# Bioinformatic Identification and Analysis of Extensins in the Plant Kingdom

**DOI:** 10.1371/journal.pone.0150177

**Published:** 2016-02-26

**Authors:** Xiao Liu, Richard Wolfe, Lonnie R. Welch, David S. Domozych, Zoë A. Popper, Allan M. Showalter

**Affiliations:** 1 Department of Environmental and Plant Biology, Ohio University, Athens, Ohio, United States of America; 2 Molecular and Cellular Biology Program, Ohio University, Athens, Ohio, United States of America; 3 Russ College of Engineering and Technology, Center for Intelligent, Distributed and Dependable Systems, Ohio University, Athens, Ohio, United States of America; 4 Department of Biology, Skidmore College, Saratoga Springs, New York, United States of America; 5 Botany and Plant Science and Ryan Institute for Environmental, Marine, and Energy Research, School of Natural Sciences, National University of Ireland Galway, Galway, Ireland; Iowa State University, UNITED STATES

## Abstract

Extensins (EXTs) are a family of plant cell wall hydroxyproline-rich glycoproteins (HRGPs) that are implicated to play important roles in plant growth, development, and defense. Structurally, EXTs are characterized by the repeated occurrence of serine (Ser) followed by three to five prolines (Pro) residues, which are hydroxylated as hydroxyproline (Hyp) and glycosylated. Some EXTs have Tyrosine (Tyr)-X-Tyr (where X can be any amino acid) motifs that are responsible for intramolecular or intermolecular cross-linkings. EXTs can be divided into several classes: classical EXTs, short EXTs, leucine-rich repeat extensins (LRXs), proline-rich extensin-like receptor kinases (PERKs), formin-homolog EXTs (FH EXTs), chimeric EXTs, and long chimeric EXTs. To guide future research on the EXTs and understand evolutionary history of EXTs in the plant kingdom, a bioinformatics study was conducted to identify and classify EXTs from 16 fully sequenced plant genomes, including *Ostreococcus lucimarinus*, *Chlamydomonas reinhardtii*, *Volvox carteri*, *Klebsormidium flaccidum*, *Physcomitrella patens*, *Selaginella moellendorffii*, *Pinus taeda*, *Picea abies*, *Brachypodium distachyon*, *Zea mays*, *Oryza sativa*, *Glycine max*, *Medicago truncatula*, *Brassica rapa*, *Solanum lycopersicum*, and *Solanum tuberosum*, to supplement data previously obtained from *Arabidopsis thaliana* and *Populus trichocarpa*. A total of 758 EXTs were newly identified, including 87 classical EXTs, 97 short EXTs, 61 LRXs, 75 PERKs, 54 FH EXTs, 38 long chimeric EXTs, and 346 other chimeric EXTs. Several notable findings were made: (1) classical EXTs were likely derived after the terrestrialization of plants; (2) LRXs, PERKs, and FHs were derived earlier than classical EXTs; (3) monocots have few classical EXTs; (4) Eudicots have the greatest number of classical EXTs and Tyr-X-Tyr cross-linking motifs are predominantly in classical EXTs; (5) green algae have no classical EXTs but have a number of long chimeric EXTs that are absent in embryophytes. Furthermore, phylogenetic analysis was conducted of LRXs, PERKs and FH EXTs, which shed light on the evolution of three EXT classes.

## Introduction

Extensins (EXTs) are a diverse family of hydroxyproline-rich glycoproteins (HRGPs) found only in the plant kingdom. They are cell wall proteins characterized by the repeated occurrence of serine (Ser) followed by several consecutive prolines (Pro) [1,2]. Some EXT molecules have Tyr-X-Tyr motifs (where X can be any amino acid) that are responsible for intramolecular or intermolecular cross-linking with other EXT molecules in forms of isodityrosine (Idt), di-Idt, and pulcherosine [3]. These cross-linking properties contribute to the extracellular matrix and play roles in plant development and defense mechanisms [4,5].

Besides cross-linking of Tyr motifs, post-translational modification of EXTs includes hydroxylation of Pro residues to hydroxyproline (Hyp), and subsequent glycosylation of Hyp and Ser residues. Peptidyl-Pro is hydroxylated by prolyl 4-hydroxylases (P4Hs). Plant P4Hs belong to a family of 2-oxoglutarate-dependent dioxygenases [6–8]. Characterization of P4Hs is reported for a number of plants, including *Arabidopsis thaliana* [6,9–11], *Nicotiana tabacum* [12], *Dianthus caryophyllus* [13], and *Chlamydomonas reinhardtii* [14].

*O*-glycosylation of EXTs predominantly occurs on Ser-Hyp_n_ motifs, with often four to five oligoarabinosides attached to Hyp residues and galactose (Gal) monosaccharides attached to Ser [1]. In Arabidopsis, the sequential addition of arabinose (Ara) residues is carried out by distinct arabinosyltransferases: hydroxyproline *O*-β-arabinosyltransferase (HPAT1-3) [15], reduced residual arabinose 1–3 (RRA1-3) [11,16], Xyloglucanase113 (XEG113) [17], and extensin arabinose deficient (ExAD) (Petersen et al., unpublished). The addition of Gal to Ser is carried out by Ser galactosyltransferase (SGT1) [18].

EXTs can be divided into several classes: classical EXTs, short EXTs, leucine-rich repeat extensins (LRXs), proline-rich extensin-like receptor kinases (PERKs), formin-homolog EXTs (FH EXTs), long chimeric EXTs and other chimeric EXTs [2]. Classical EXTs have signal peptide sequences which direct the proteins to the secretory system and ultimately the extracellular matrix. Most prominently, they have Ser-Pro_3-5_ repeated motifs throughout their sequences. Moreover, some EXTs have Tyr-X-Tyr (YXY) motifs along with the Ser-Pro_3-5_ motifs. EXTs that are less than 200 amino acids in length are referred to as “Short EXTs”. LRXs are a class of chimeric EXTs which usually have signal peptide sequences at the N terminus, followed by leucine-rich repeat (LRR) domains, and Ser-Pro_3-5_ repeated modules near the C terminus. The LRR domain is known to be involved in protein-protein interactions [19], and the EXT domain is thought to contribute to the insolubility in the cell wall. These features make LRXs candidates for regulatory functions on the cell surface. In Arabidopsis, LRXs are implicated in root hair morphogenesis [20]. PERKs represent another class of chimeric EXTs. They lack a signal peptide sequence and their SP_n_ repeated motifs are predominately located at the N terminus; they have a protein kinase catalytic domain near their C terminus. In Arabidopsis, the PERK gene family contains 15 members, and PERK1 was localized at the plasma membrane [21]. Microarray data showed that there are two major groups of PERKs: those that are specifically expressed in the pollen and those that are generally expressed throughout all plant tissues [22]. Research has shown that PERKs may affect cell expansion and normal floral organ formation [23]. In Arabidopsis, they are associated with an abscisic acid response [24]. In *Brassica napus*, BnPERK1 is reported to be involved in signal perception and response to wound and/or pathogen stimuli [21]. A third class of chimeric EXTs is the FH EXTs. FH EXTs are characterized by significant homology to formins and the presence of repeated Ser-Pro_3-5_ motifs. In eukaryotes, formins are associated with actin dynamics in that they control the assembly and elongation of unbranched actin filaments [25,26]. A fourth group of chimeric EXTs are termed “long chimeric EXT” because of their extraordinary sequence length that have more than 2,000 amino acids. Lastly, some EXTs were characterized as “other chimeric EXTs” as these EXTs have an EXT domain and one or more domain(s) not known to HRGPs or the above classes of chimeric EXTs.

Showalter et al. [2 and unpublished data] conducted the identification of the HRGP superfamily in *Arabidopsis thaliana* and *Populus trichocarpa* in which 59 and 60 EXTs were identified, respectively. In addition, Newman and Cooper [27] identified numerous proline-rich tandem repeat proteins (TRPs) including EXTs through a bioinformatics approach using EST and NCBI Non-Redundant protein sequence data of a number of plant species, but the search criteria for TRPs were not tailored for identifying EXTs. Nonetheless, knowledge about the number and distribution of EXTs in plant kingdom is still lacking.

BIO OHIO 2.0 is a newly revised and improved bioinformatics software program developed at Ohio University that was tailored to fulfill this task [2,28]. The program was designed and developed for protein identification based on amino acid signatures, such as biased amino acid composition and common HRGP amino acid motifs in the genome-encoded protein sequences (i.e., the predicted proteome). The program can also further analyze identified proteins by checking for the presence of potential signal peptide sequences and GPI anchor addition sequences and finding similar HRGPs via the Basic Local Alignment Search Tool (BLAST). Using this bioinformatics tool, Showalter et al. [2] identified and classified the HRGP superfamily in Arabidopsis (*Arabidopsis thaliana*) and poplar (*Populus trichocarpa*), two fully sequenced plant genomes ([2,28]; Showalter et al., unpublished).

Rapid advancement in the “next generation sequencing (NGS)” techniques is increasingly making genome sequences available. Thus, it is now feasible to conduct a more detailed analysis on the EXT family in the plant kingdom. Here, we analyzed 16 plant genomes: *Ostreococcus lucimarinus* [29], *Chlamydomonas reinhardtii* [30], *Volvox carteri* [31], *Klebsormidium flaccidum* [32], *Physcomitrella patens* [33], *Selaginella moellendorffii* [34], *Pinus taeda* [35], *Picea abies* [36], *Brachypodium distachyon* [37], *Zea mays* [38], *Oryza sativa* [39], *Glycine max* [40], *Medicago truncatula* [41], *Brassica rapa* [42], *Solanum lycopersicum* [43], *and Solanum tuberosum* [44]. We also integrated previously studied data on Arabidopsis and *P*. *trichocarpa* to determine the number and distribution of the EXT family members in the plant kingdom and examine the evolutionary history of this fundamental cell wall constituent [45].

## Materials and Methods

### Identification of EXTs

The predicted protein data files from 16 plant species (*O*. *lucimarinus*, *C*. *reinhardtii*, *V*. *carteri*, *K*. *flaccidum*, *P*. *patens*, *S*. *moellendorffii*, *P*. *taeda*, *P*. *abies*, *B*. *distachyon*, *Z*. *mays*, *O*. *sativa*, *G*. *max*, *M*. *truncatula*, *B*. *rapa*, *S*. *lycopersicum*, *and S*. *tuberosum*) were downloaded from the Phytozome website (www.phytozome.org). The protein database was searched for EXTs using BIO OHIO 2.0 software, which integrated more functional modules into the software compared to BIO OHIO 1.0 [2, 28]. Briefly, a regular expression of two or more SPPP repeats was used to search for candidate EXTs. Candidate EXT sequences were then analyzed for the positions of SP_n_ repeats and YXY cross-linking motifs, the presence of signal peptide sequences, the presence of GPI anchors, and for similar sequences using BLAST searches against known Arabidopsis EXTs. An EXT is determined by comparing all the above information with known features of each class of EXTs. If a sequence fails to fit in any class of EXTs, it is called a potential EXT. The BIO OHIO 2.0 program is freely available and can be downloaded from github: https://github.com/Showlaterlab/BIO-OHIO-2.0

### BLAST Analysis

The functional module of BLAST was integrated into the BIO OHIO 2.0. All candidate EXTs identified were subjected to NCBI BLASTP analysis using the current Arabidopsis protein BLAST dataset (November 2010 TAIR10 Genome Release) downloaded from The Arabidopsis Information Resource (TAIR; www.arabidopsis.org).

### Signal Peptides and GPI Anchors

The functional modules for signal peptides and GPI anchors were integrated into the BIO OHIO 2.0. All proteins were analyzed for signal peptides using SignalP (www.cbs.dtu.dk/services/SignalP/) [46] and for GPI anchor addition sequences using the big-PI plant predictor (mendel.imp.ac.at/gpi/plant_server.html) [47].

### Sequence Alignment and Phylogenetic Analysis

Amino acid sequences were aligned by use of the Geneious software program (http://www.geneious.com/) to obtain conserved domains. Aligned sequences of LRXs, PERKs, and FH EXTs were input into Mega 6 for phylogenetic analysis using the maximum likelihood and the maximum parsimony methods [48]. For LRXs, the analysis involved 78 protein sequences. There were a total of 294 amino acid positions in the final dataset. The evolutionary history inferred by the Maximum Likelihood method was based on the JTT matrix-based model [49]. The evolutionary history inferred by the Maximum Parsimony method used the Tree-Bisection-Regrafting (TBR) algorithm with search level 1 in which the initial trees were obtained by the random addition of sequences (10 replicates) [50]. The bootstrap consensus tree inferred from 1000 replicates was shown and branches corresponding to partitions reproduced in less than 50% bootstrap replicates are collapsed. For the phylogenetic analysis of PERKs and FH EXTs, the same methods were used as for analysis of the LRXs. The analysis of PERKs involved 93 protein sequences, and a total of 283 amino acid positions were present in the final dataset. The analysis of FH EXTs involved 76 protein sequences, and a total of 377 amino acid positions were present in the final dataset.

## Results

**Fig 1:**
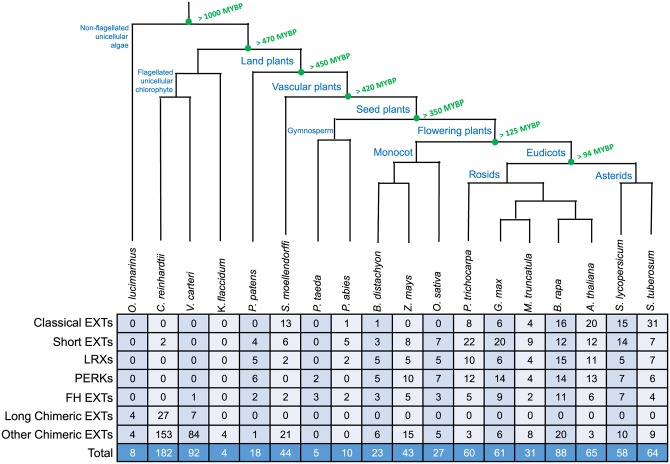
Phylogenetic distribution of EXTs in selected plant genomes. (A) Dendrogram showing the evolutionary relationships of species selected representing major plant divisions. (B) The distribution of EXTs identified in this study and in the previous literature (Showalter et al. 2010, and unpublished data). EXTs are divided into seven subclasses including classical EXTs, short EXTs, LRXs, PERKs, FH EXTs, chimeric EXTs, and long chimeric EXTs.

**Fig 2:**
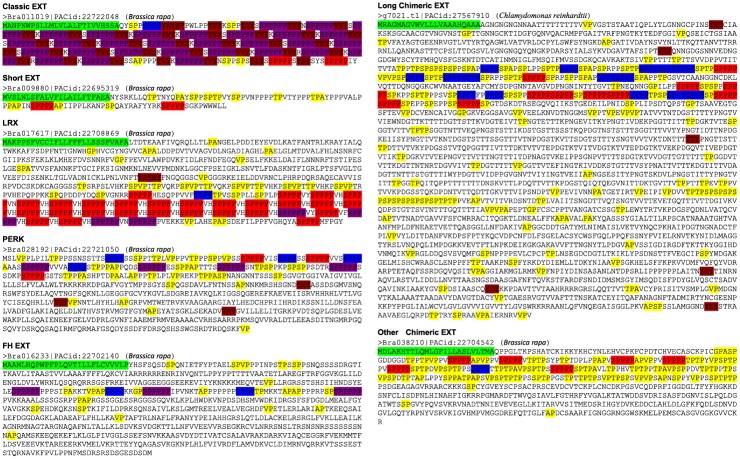
Protein sequences encoded by representative EXT gene classes in *Brassica rapa* and *Chlamydomonas reinhardtii*. Green colored sequences at the N terminus indicate predicted signal peptides. SP_3_ (blue), SP_4_ (red), SP_5_ (purple), and YXY (dark red) repeats are also indicated. Sequences typical of AGPs, AP, PA, SP, TP, VP, and GP repeats, are also indicated (yellow).

In order to identify candidate EXTs, the BIO OHIO 2.0 program searched for protein sequences with two or more SPPP repeats from 16 plant proteomes: *O*. *lucimarinus*, *C*. *reinhardtii*, *V*. *carteri*, *K*. *flaccidum*, *P*. *patens*, *S*. *moellendorffii*, *P*. *taeda*, *P*. *abies*, *B*. *distachyon*, *Z*. *mays*, *O*. *sativa*, *G*. *max*, *M*. *truncatula*, *B*. *rapa*, *S*. *lycopersicum*, *and S*. *tuberosum*. This initial screening obtained 2563 candidate EXTs, among which 758 were determined as EXTs and 1804 as potential EXTs. The EXTs include 87 classical EXTs, 97 short EXTs, 61 LRXs, 75 PERKs, 54 FH EXTs, 38 long chimeric EXTs, and 346 other chimeric EXTs (Fig 1). In addition to having at least two SPPPs, these EXTs contain a HRGP domain that is rich in Pro, Alanine (Ala), Valine (Val), Ser, Glycine (Gly), and Threonine (Thr), and most proteins (76%) have predicted signal peptide sequences that direct them into the secretory pathway and ultimately to the cell wall. A representative EXT sequence from each class is shown in Fig 2. Detailed sequence feature analysis of identified EXTs for each species is shown in S1–S16 Tables. All the identified EXT sequences are shown in S1 Fig; the sequences of potential EXTs are shown in S2 Fig. These potential EXTs have at least two SPPP repeat motifs but mostly lack a signal sequence and a HRGP domain.

### Classical and short EXTs

Classical EXTs are categorized as having EXT domains throughout the protein sequence, except at the N terminus where there is usually a signal peptide that directs the protein into the secretory pathway and ultimately to the cell wall. The EXT domains contain repeated motifs of SP_n_, where n ≥3. Moreover, most classical EXTs have cross-linking YXY motifs in addition to the SP_n_ motifs.

In this study, no classical EXTs were identified in the five earliest diverging species (*O*. *lucimarinus*, *C*. *reinhardtii*, *V*. *carteri*, *K*. *flaccidum*, and *P*. *patens*), indicating that classical EXTs are absent in these non-vascular species that are either aquatic green algae (*O*. *lucimarinus*, *C*. *reinhardtii*, *V*. *carteri*, *K*. *flaccidium*) or land plants that are dependent on water for reproduction, lacking roots, and predominantly living in humid habitats (*P*. *patens*). However, classical EXTs were found in tracheophytes (vascular plants), including early diverging members as 13 were identified in the lycophyte *S*. *moellendorffii*, 11 of which contained YXY motifs. *S*. *moellendorffii* EXT11 was found to share high similarity with all 12 of the other EXTs, indicating the likely occurrence of gene duplication events (**Data not shown**).

Despite the presence of classical EXTs in tracheophytes dating back to more than 420 million years before present (MYBP), classical EXTs were nearly absent from the genomes of the two gymnosperm species and the three monocot species examined here. No classical EXTs were identified in loblolly pine (*P*. *taeda*), and only one classical EXT (MA_74039g0010) was identified in Norway spruce (*P*. *abies*). Furthermore, while this protein contains 35 SPPP_3-5_, it lacks a signal peptide. Similarly, no classical EXTs were identified in corn (*Z*. *mays*), or rice (*O*. *sativa*), while *B*. *distachyon*, a non-crop species, only contained one apparent classical EXT, Bradi3g10280. This protein contains 11 SP_3_ and three SP_4_ repeats, along with 19 QAAA repeats, which is not known to be associated with any other EXTs or HRGPs. In addition, a BLAST search with Bradi3g10280 as query found no hits of significant similarity to other protein sequences (**data not shown**). These findings are consistent with two previous studies in monocots that found a lack of the SP_n_ repeat motif in *Z*. *mays* [51,52].

Classical EXTs, however, were ubiquitous in eudicots. In this project, five species were chosen for analysis: *G*. *max*, *M*. *truncatula*, *B*. *rapa*, *S*. *lycopersicum*, and *S*. *tuberosum*. Classical EXTs were found in all these species. In addition, previous research reported on the identification of classical EXTs in Arabidopsis and poplar, respectively [2 and unpublished data]. An overview of the number of classical EXTs identified in these plants is shown in Fig 1.

**Fig 3:**
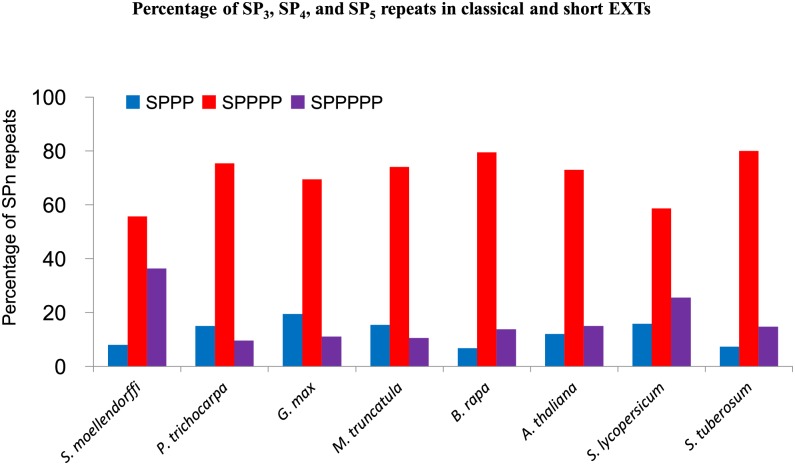
The frequency of SP_3_, SP_4_, and SP_5_ repeats in classical EXTs of selected genomes. The frequency was calculated by the total number of each type of repeat divided by the total number of SP_3_, SP_4_, and SP_5_ adding together in each species.

The frequency of SP_3_, SP_4_ and SP_5_ (or more) in classical EXTs among the above species was calculated to determine which of these repeat motifs is dominant in classical EXTs. The results showed that SP_4_ repeats universally predominated in EXT sequences, with the lowest being in the lycophyte *S*. *moellendorffii* (56%) and the highest being in *S*. *tuberosum* (80%) (Fig 3). However, the dominance of the SP_4_ repeated motif is not seen in other categories of EXTs (**data not shown**).

**Fig 4:**
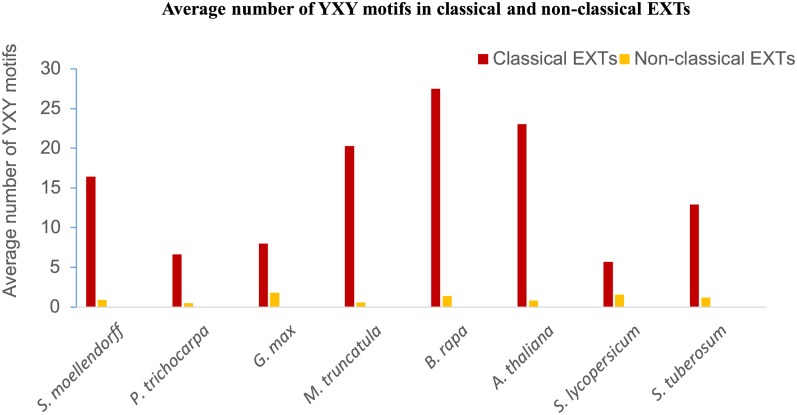
Average number of YXY motif in classical and non-classical EXTs. The frequency was calculated as the total number of YXY repeats divided by the total number of classical and non-classical EXTs in chosen species.

The average number of YXY motifs in classical EXT and non-classical EXT (i.e. all other classes of EXTs) was calculated to confirm the observation that YXY motifs are abundant exclusively in classical EXTs. As is shown in Fig 4, the average number of YXY motifs in classical EXTs ranges from 5.7 (in *S*. *lycopersicum*) to 27.5 (in *B*. *rapa*), whereas less than two occurrences of the YXY motif were found in non-classical EXTs in all species studied.

Short EXTs were found in most species in this study but no short EXTs were identified from the *O*. *lucimarinus*, *V*. *carteri*, *K*. *flaccidum*, *and P*. *taeda* genomes. Interestingly, two short EXTs were identified in the aquatic species, *C*. *reinhardtii*. Unlike classical EXTs, short EXTs were also found in *P*. *patens*. Overall, there is a slight increase in the number of short EXTs in the embryophytes, which indicates the importance of this group of proteins in plant growth, development, and defense (Fig 1).

### LRXs, PERKs, and FHs

**Fig 5:**
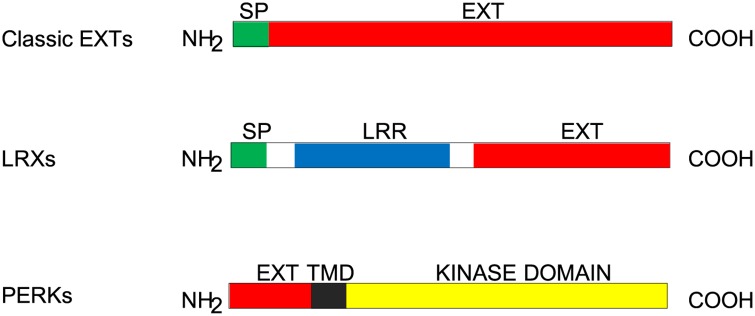
Structural schemes of classical EXTs, LRX, and PERKs. Classical EXTs have an N-terminal signal peptide (green) followed only by EXT domain (red). LRXs have an N-terminal signal peptide (green) followed by a leucine-rich region (LRR, blue). The EXT domain (red) of LRXs is located at the C terminus. The EXT domain of PERKs is located at N terminus followed by a transmembrane domain (TMD, black) and a kinase domain (yellow).

Leucine-rich repeat extensins (LRXs) are a group of chimeric EXTs. A typical LRX has an N terminal signal peptide, followed by a leucine-rich repeat (LRR) domain, and a C terminal EXT domain. A representative structure of an LRX is shown in Fig 5. In this study, LRXs were found in all but the four algal species (*O*. *lucimarinus*, *C*. *reinhardtii*, *V*. *carteri*, *K*. *flaccidum*) and one gymnosperm (*P*. *taeda*). *B*. *rapa*, found to contain sequences for 15, had the highest number of LRXs of any species. However, most species contain two to seven LRXs (Fig 1 and S1 Fig). Five LRXs were identified in *P*. *patens*, suggesting the possibility that LRXs were derived during plant terrestrialisation (and subsequently lost from some species e.g. *P*. *tadea*). A BLAST search against all the LRXs identified in this study revealed that these five LRXs share more homology among themselves than any LRXs identified in other species, indicating they are likely paralogs derived from gene duplication events. Interestingly, for the two gymnosperm species included in this study, two LRXs were identified in *P*. *abies* while none were found in *P*. *taeda*. According to evidence from chloroplast, mitochondrial and nuclear genes although they are closely related members of the Pinaceae the genera *Picea* and *Pinus* diverged ~140 million years ago and it is reasonable that differences in LRXs may exist between them [53]. LRXs were identified in all flowering plants in this study, with eudicots having more LRXs in general.

**Fig 6:**
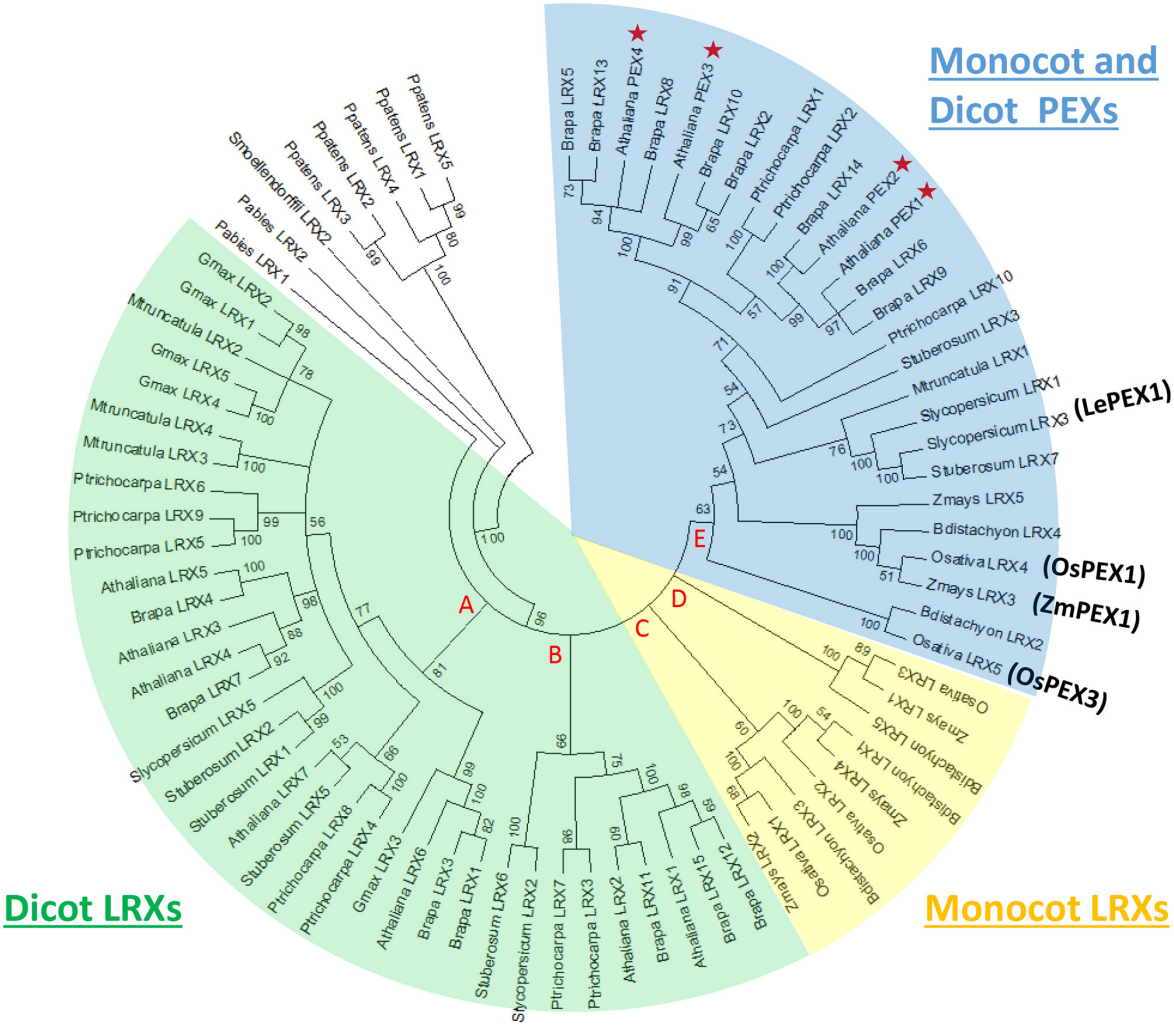
Maximum Likelihood Analysis of LRXs. The evolutionary history was inferred by using the Maximum Likelihood method based on the JTT matrix-based model. The bootstrap consensus tree inferred from 1000 replicates is taken to represent the evolutionary history of the taxa analyzed. Branches corresponding to partitions reproduced in less than 50% bootstrap replicates are collapsed. The analysis involved 78 amino acid sequences. There were a total of 294 positions in the final dataset. The green-colored fan area (clades A and B) indicates LRXs from eudicot species. The yellow-colored fan area (clades C and D) indicates LRXs from monocot species. The blue-colored fan area indicates possible PEX clade. Previously reported PEXs were either marked with red “☆” or in parentheses.

To explore the evolutionary relationship of LRXs in different species, phylogenetic analysis was conducted using the maximum likelihood method based on the JTT matrix-based model. The phylogenetic analysis showed that all LRXs in the moss *P*. *patens* were clustered together as the outgroup. The rest of the LRXs fell into five major clades. Among them, all eudicot LRXs fell into clades A and B (*G*. *max*, *M*. *truncatula*, *P*. *trichocarpa*, *A*. *thaliana*, *B*. *rapa*, *S*. *lycopersicum*, and *S*. *tuberosum*), while clades C and D contained all the monocot LRXs (*O*. *sativa*, *Z*. *mays*, and *B*. *distachyon*). This topology indicates that either LRXs in monocots and eudicots went through quite different changes, or one or more clades of LRXs were derived after the divergence of monocots and eudicots. Notably, previously reported PEXs were found only in clade E, which contained all PEXs from Arabidopsis (AtPEX1-4), two *O*. *sativa* PEXs (OsPEX1 and OsPEX3), one *Z*. *mays* PEX (ZmPEX1), and one *S*. *lycopersicum* PEX (LePEX1). Therefore, it is likely that ancestral PEX gene(s) existed before the division of monocots and eudicots, and that the rest of the LRXs in clade E may also be PEXs (Fig 6). Phylogenetic analysis was also conducted using the maximum parsimony method (S3 Fig), which showed almost identical tree topology as inferred by the maximum likelihood method. The aligned sequences of LRXs are shown in S4 Fig.

**Fig 7:**
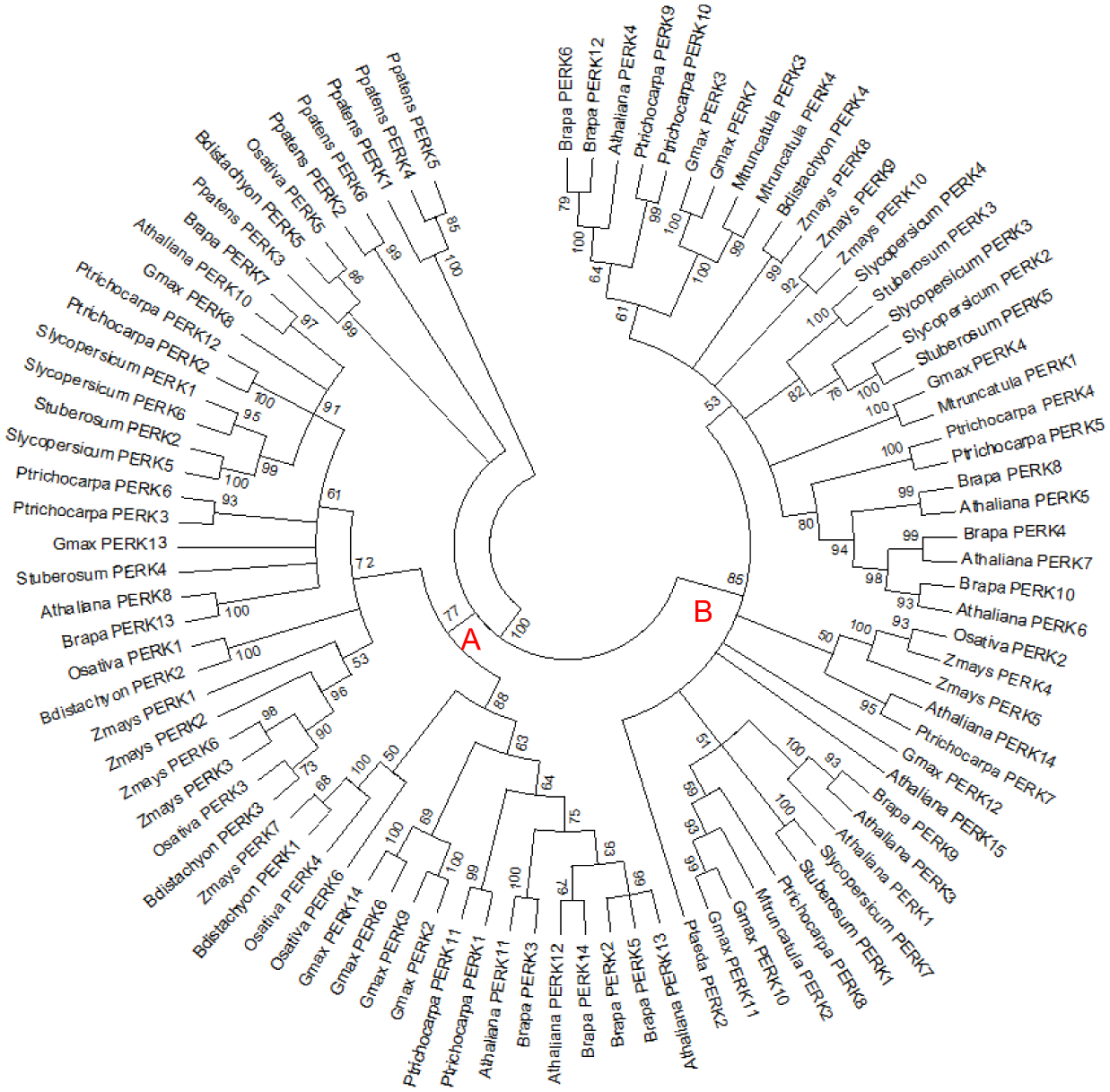
Maximum Likelihood Analysis of PERKs. The evolutionary history was inferred by using the Maximum Likelihood method based on the JTT matrix-based model. The bootstrap consensus tree inferred from 1000 replicates is taken to represent the evolutionary history of the taxa analyzed. Branches corresponding to partitions reproduced in less than 50% bootstrap replicates are collapsed. The analysis involved 93 amino acid sequences. There were a total of 283 positions in the final dataset. A and B represent two major clades.

Proline-rich extensin-like receptor kinases (PERKs) represent another group of chimeric EXTs. They have an extracellular EXT domain at the N terminus followed by a transmembrane domain and an intracellular receptor kinase domain (Fig 5). In this study, PERKs were identified in most of the species in this study but not in *O*. *lucimarinus*, *C*. *reinhardtii*, *V*. *carteri*, *K*. *flaccidum*, *S*. *moellendorffii and P*. *abies*. Notably, BLAST analysis for two *K*. *flaccidum* proteins (kfl00031_0230 and kfl00671_0010p) revealed that they were similar to Arabidopsis PERKs, but a closer look found that they differed from the general PERK structure and were thus classified as chimeric EXTs (S4 Table and Fig 5). A number of PERKs were identified in *P*. *patens*, suggesting PERKs were derived after the terrestrialization of plants. With the exception in *P*. *taeda*, at least five PERKs were identified in each of other species with *G*. *max* and *B*. *rapa* having as many as 14 PERKs. To explore the evolutionary history of PERKs in these species, phylogenetic analysis was conducted using the maximum likelihood method based on the JTT matrix-based model (Fig 7). The phylogenetic tree shows that PERKs from these species form two dominant clades (clade A and B). The expression pattern analysis of PERKs in Arabidopsis showed that some of the Arabidopsis PERK members were pollen-specific genes while others were more broadly expressed [22]. In this tree, five AtPERKs that are pollen-specific were clustered in one sub-clade under clade B, namely, PERKs 3–7. However, other pollen-specific PERKs were also seen elsewhere in the tree (AtPERKs 11 and 12). The phylogenetic tree showed that most groups at the tips were either the same or closely related species, and the tree had high confidence at the tips in general. PERKs from monocot plants did not form a single branch, but they tended to cluster together. Similar to LRXs, tree topology inferred by the maximum parsimony method was nearly identical to that inferred by the maximum likelihood method (S5 Fig). The aligned sequences of PERKs are shown in S6 Fig.

**Fig 8:**
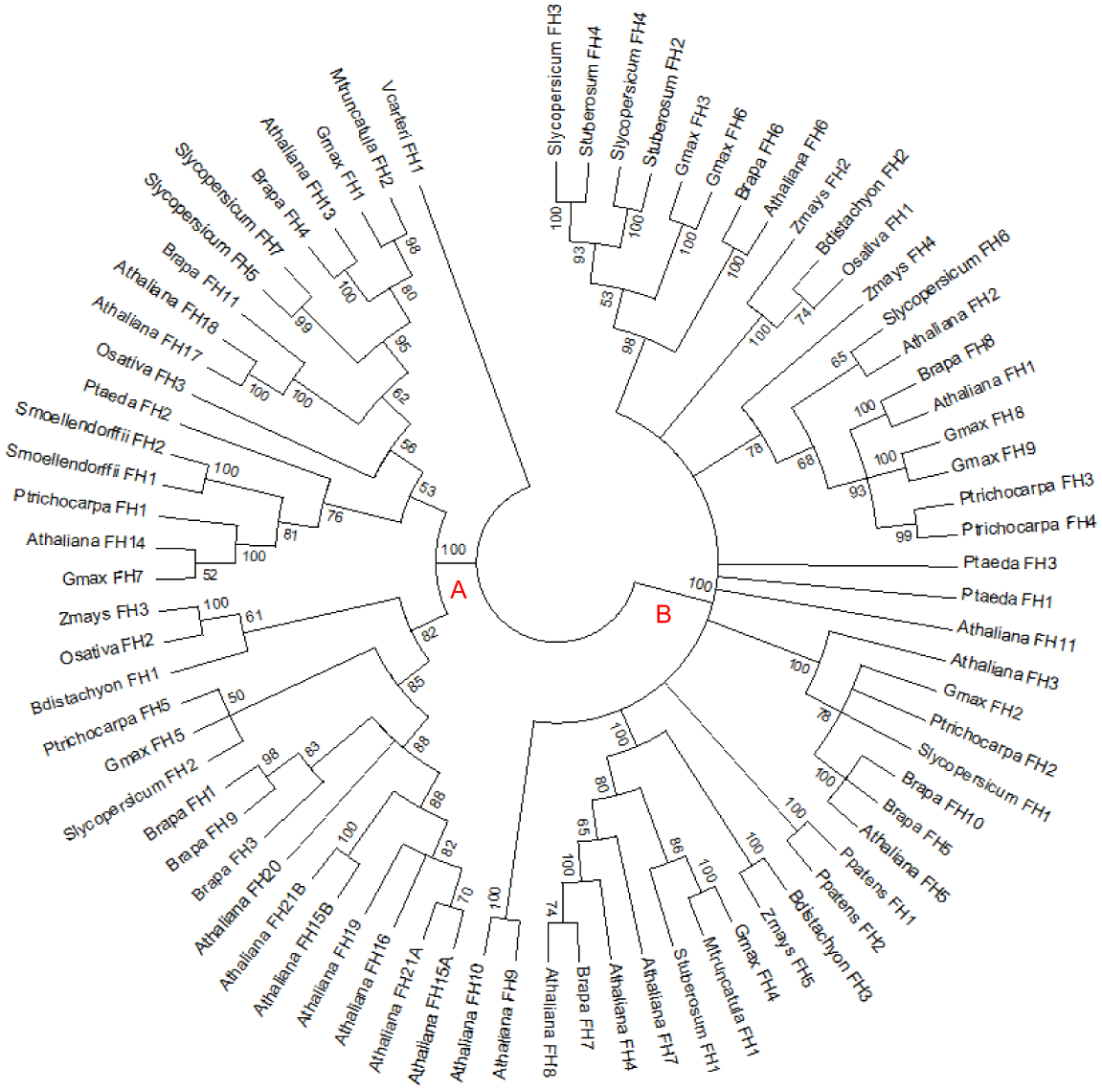
Maximum Likelihood Analysis of FHs. The evolutionary history was inferred by using the Maximum Likelihood method based on the JTT matrix-based model. The bootstrap consensus tree inferred from 1000 replicates is taken to represent the evolutionary history of the taxa analyzed. Branches corresponding to partitions reproduced in less than 50% bootstrap replicates are collapsed. The analysis involved 73 amino acid sequences. There were a total of 377 positions in the final dataset.

Formins were first found in animal cells as cytoplasmic proteins that are associated with the organization of the actin cytoskeleton. Plant formin homologs are quite different in structure and function [54]. Plant formins may play important roles in cell cortex organization, including cortical actin, microtubule cytoskeletons, and the attachment to the plasma membrane [55]. In this study, formin homolog EXTs (FH EXTs) were categorized as a third group of chimeric EXTs, as some of the formin homologs were found to have an N terminal signal peptide and contain a number of SP_n_ repeats. FH EXTs were found in all but three of the algal species investigated: *O*. *lucimarinus*, *C*. *reinhardtii*, *and K*. *flaccidum*. Interestingly, one FH EXT (Vocar20008550m) was found in *V*. *carteri*, the remaining algal species examined, suggesting that plant formin homologs were derived before divergence of the embryophytes. In general, an increase in number of FH EXTs was found in higher plants, with *B*. *rapa* having as many as 11 FH EXTs. To explore the evolutionary history of FH EXTs in these species, phylogenetic analysis was conducted using the maximum likelihood method based on the JTT matrix-based model (Fig 8). Notably, the FH EXT identified in *V*. *carteri* was placed as the outgroup given the considerable difference between this sequence and other FH EXTs upon alignment. Similar to the analysis done by Deeks et al. [54], phylogenetic analysis revealed that FH EXTs were clustered into two major clades with 100% confidence. Clade A contained Arabidopsis FH1-11, while clade B contained AtFH 12–21. In general, FH EXTs from the same or closely related species tended to cluster together, and higher confidence was shown in deeper branches (with over 80% bootstrap support). Phylogenetic analysis was also conducted using the maximum parsimony method (S7 Fig), which showed almost identical tree topology as inferred by the maximum likelihood method. The aligned sequences of FH EXTs are shown in S8 Fig.

### Long chimeric EXTs and other chimeric EXTs

A group of chimeric EXTs were characterized to contain an enormous number of amino acids, usually over 2,000 amino acids per protein. This group of EXTs was referred to as “long chimeric EXTs”. A few such EXTs were previously reported in *C*. *reinhardtii* [56,57]. In this project, 27 long chimeric EXTs were found in the *C*. *reinhardtii* genome. In addition, four and 84 long chimeric EXTs were identified in *O*. *lucimarinus* and *V*. *carteri*, respectively (Figs 1, 2 and S1 Fig; S1–S3 Tables). Long chimeric EXTs, however, were not found in any other species analyzed in this study. Other chimeric EXTs were categorized as being chimeric EXTs but did not belong to LRXs, PERKs, FH EXTs, or long chimeric EXTs. In this study, chimeric EXTs were identified in all but the two gymnosperm species; however, the number of chimeric EXTs in each species varied greatly (Fig 1). While most other species have no more than 21 chimeric EXTs, the two flagellated unicellular chlorophyte species, *C*. *reinhardtii* and *V*. *carteri*, contained 153 and 84 chimeric EXTs, respectively.

### Comparison with previously identified EXTs

The literature was searched to compare previously characterized EXTs with EXTs identified in this study. A total of 54 EXTs were obtained, of which 32 were identified in species included in this study. Among the 32 EXTs, 24 EXTs were found to be identical or similar to previously identified EXTs. The list of previously identified EXTs and their counterparts in this study is listed in S17 Table **[**1,4,20,21,51,52,56,58–89**]**.

## Discussion

### Bioinformatic identification of plant EXTs using BIO OHIO 2.0

The first comprehensive bioinformatics identification of EXTs was done in Arabidopsis in which 59 EXTs were identified [2], followed by poplar where 60 EXTs were found (Showalter et al., unpublished data). In another study, Newman and Cooper [27] identified numerous proline-rich TRPs including EXTs from proteomic databases, but they adopted more stringent search criteria which resulted in fewer EXTs identified in their study. Up to now, there is still a lack of understanding of the number and distribution of EXTs in the plant kingdom. With a rapidly increasing number of plant genomes fully sequenced, vast amounts of data are being produced. A need to process, mine and analyze genome data to provide biological meanings awaits.

The Bio OHIO 2.0 software program provides an efficient and reliable tool to identify proteins with biased amino acid compositions and known repetitive motifs [2,28]. The newly revised and improved 2.0 version integrated more functional modules that include searching for the presence of a signal peptide, GPI anchor, as well as automated BLAST searches against the Arabidopsis proteome. These improvements make the program an ideal bioinformatic tool to study cell wall components, and gain insight about evolution of protein families.

In this project, a total of 758 EXTs including 87 classical EXTs, 97 short EXTs, 61 LRXs, 75 PERKs, 54 FH EXTs, 38 long chimeric EXTs, and 346 other chimeric EXTs were identified among over half a million predicted protein sequences in 16 plant genomes ranging from primitive aquatic plants to eudicots. Moreover, the functions of searching for signal peptide, GPI anchor, and BLAST searches against the model plant Arabidopsis were incorporated in the program, making it more robust and efficient for identification.

### The origin and evolution of EXTs

The EXTs identified in this study showed that the EXT prototypes might occur early in evolution of green photosynthetic organisms, most likely in the form of chimeric EXTs, as they are found both in the early diverging species and later diverging species. This suggests that ancestral EXTs might originate as a stretch of amino acids or a small EXT domain that functioned in favor of evolution, and thus had more widely spread over time in evolution. On the other hand, long chimeric EXTs were identified in only three of the most primitive species (*O*. *lucimarinus*, *C*. *reinhardtii*, and *V*. *carteri*), but are absent in all land plants, indicating the lack of evolutionary advantage for these long molecules. LRXs, PERKs, and FH EXTs predated the evolution of classical EXTs, as they are found in the land plant *P*. *patens*, where no classical EXTs were found (Fig 1 and S5 Fig). The origin of classical EXTs was possibly associated with plant vascularization, as although they are absent from the (non-vascular) bryophyte *P*. *patens* they are present in the (vascular) lycophyte *S*. *moellendorffii*. Further analysis is needed to help address whether they are indeed associated with vascularization or alternatively with terrestrialization, since *P*. *patens* is the only bryophyte examined in this study. Interestingly, classical EXTs are absent or nearly absent in gymnosperms *P*. *taeda* (0) and *P*. *abies* (1), as well as monocot plants such as *B*. *distachyon* (1), *Z*. *mays* (0), and *O*. *sativa* (0). However, further analysis is needed to provide greater support for this conclusion, as it is possible that classical EXTs may be more abundant in other gymnosperm and monocot species. Eudicots have the greatest numbers of EXTs and the largest number of Tyr-X-Tyr cross-linking motifs, which largely occurs in the classical EXTs.

Combining previously identified EXTs in Arabidopsis and poplar, phylogenetic analysis was conducted for LRXs, PERKs and FH EXTs, but not for classical EXTs as the latter failed to align in the EXT domain due to the highly varied number of SP_n_ repeat motifs and the Tyr-X-Tyr motifs, which is necessary for a meaningful outcome. Similarly, phylogenetic analysis was not done for short EXTs, chimeric and long chimeric EXTs due the heterogeneity of their non-EXT domains.

The phylogenetic analysis of LRXs included the LRR regions of 78 LRXs from 13 plant genomes. Five *P*. *patens* LRXs were clustered in the phylogenetic trees generated by both maximum likelihood and maximum parsimony methods, indicating that one ancestral gene duplicated multiple times. LRXs were found in almost all of the more advanced species in this study, demonstrating the widespread nature of this protein family. Comparable to Baumberger et al. [90] who reported LRXs form two clades: reproductive pollen-expressed LRXs referred to as PEXs and a vegetative LRXs, analysis here showed that LRXs form three major groups, including one group of eudicot-specific LRXs, one group of monocot-specific LRXs, and one group of likely PEXs. The existence of eudicot and monocot specific clades indicates that LRXs in monocot and eudicot evolved quite distinctly from ancient LRXs. The PEX specific clade contains LRXs from both monocots and eudicots, indicating that ancestral LRXs duplicated and diversified to become pollen-specific LRXs before the division of monocot and eudicot [90].

The PERK phylogenetic analysis included the receptor kinase domain of 93 protein sequences from 12 plant genomes. Similar to LRXs, PERKs identified in *P*. *patens* were clustered at the root of the phylogenetic tree by both maximum likelihood and maximum parsimony methods. However, PERKs were not found in the lycophyte *S*. *moellendorffii* or the gymnosperm *P*. *abies*. The phylogenetic tree shows that PERKs form two major clades, both included monocots and eudicots. This indicates that ancestral PERK genes existed before the division of monocots and eudicots. Expression pattern analysis of PERKs in Arabidopsis showed that some of the AtPERK members were pollen-specific genes while others were more broadly expressed [22]. Here, pollen-specific AtPERK3-7 were clustered in clade B, but other pollen-specific PERKs were also seen in other branches. The phylogenetic tree shows most groups at the tips are either the same or closely related species, and the tree has high confidence at the tips in general. This may be due to gene duplication events that lead to gene redundancy as is seen in Arabidopsis [91,92].

Phylogenetic analysis of FH EXTs included the FH domain of 76 proteins from eleven plant species. All 23 formin homologs in Arabidopsis were included in this analysis, as it was interesting to see their distributions in the phylogenetic tree. Notably, only AtFH1, AtFH5, AtFH8, AtFH13, AtFH16, and AtFH20 contain two or more SPPP. The phylogenetic tree showed two major clades (clades A and B) with high confidence, which was consistent with the study of Deeks et al. [54] who showed that Arabidopsis FHs form two major types.

A major gap in our understanding of the evolution of EXTs in green plants is a result of the lack of significant genomic information for charophytes, i.e., the group of green algae ancestral and most closely related to modern day land plants. However, immunological based screening has revealed the presence of EXTs as well other HRGPs including arabinogalactan-proteins in many of the charophyte taxa [93–95]. A previous study on cell wall biosynthetic pathways in charophytes [96] and this report have also shown that the presence of EXT-like macromolecules in the charophyte *K*. *flaccidum* and charophyte ancestors including the prasinophyte (*Ostreococcus*) and the charophyte sister clade, the chlorophytes (*Chlamydomonas* and *Volvox*), thereby supporting the presence of EXTs in charophytes. Similarly, the lack of genomic information for bryophytes and ferns, with only one bryophyte sequence and no fern sequences available, makes pinpointing some of the EXT distribution patterns difficult to interpret. For instance, classical EXTs were found in *S*. *moellendorffii* but not in *P*. *patens*, suggesting that the origin of classical EXTs might be associated with vascularization. However, since only one bryophyte and one lycophyte genome was investigated it may be possible that *P*. *patens* is an exception and that classical EXTs are present in other bryophytes, in which case classical EXTs may be associated instead with terrestrialization. A significantly more resolved interpretation will soon be possible as more genomic data for charophytes, bryophytes, and ferns become available.

## Conclusions

A revised and newly improved bioinformatics software program BIO OHIO 2.0 was utilized to identify and classify EXTs from predicted proteomes of 16 plant species. A total of 758 EXTs were identified, including 87 classical EXTs, 97 short EXTs, 61 LRXs, 75 PERKs, 54 FH EXTs, 38 long chimeric EXTs, and 346 other chimeric EXTs. Analysis of these data revealed that: (1) classical EXTs were likely derived after the terrestrialization of plants; (2) LRXs, PERKs, and FHs were likely derived earlier than classical EXTs; (3) gymnosperms and monocots have few classical EXTs; (4) Eudicots have the greatest number of classical EXTs and Tyr-X-Tyr cross-linking motifs are predominantly in classical extensins; (5) green algae lack classical EXTs but have a number of long chimeric EXTs that are absent in embryophytes. Furthermore, phylogenetic analysis was conducted for LRXs, PERKs and FH EXTs, which shed light on the evolution of the EXTs.

## Supporting Information

S1 Fig Protein sequences encoded by the predicted EXT genes in *O*. *lucimarinus*, *C*. *reinhardtii*, *V*. *carteri*, *K*. *flaccidum*, *P*. *patens*, *S*. *moellendorffii*, *P*. *taeda*, *P*. *abies*, *B*. *distachyon*, *Z*. *mays*, *O*. *sativa*, *G*. *max*, *M*. *truncatula*, *B*. *rapa*, *S*. *lycopersicum*, *and S*. *tuberosum*. Colored sequences at the N and C terminus indicate predicted signal peptide (green) and GPI anchor addition sequences (light blue) if present. SP3 (blue), SP4 (red), SP5 (purple), and YXY (dark red) repeats are also indicated. Sequences typical of AGPs, AP, PA, SP, TP, VP, and GP repeats, are also indicated (yellow).
(PDF)

S2 Fig Protein sequences encoded by potential chimeric EXT genes in *O*. *lucimarinus*, *C*. *reinhardtii*, *V*. *carteri*, *K*. *flaccidum*, *P*. *patens*, *S*. *moellendorffii*, *P*. *taeda*, *P*. *abies*, *B*. *distachyon*, *Z*. *mays*, *O*. *sativa*, *G*. *max*, *M*. *truncatula*, *B*. *rapa*, *S*. *lycopersicum*, *and S*. *tuberosum*. Colored sequences at the N and C terminus indicate predicted signal peptide (green) and GPI anchor addition sequences (light blue) if present. SP3 (blue), SP4 (red), SP5 (purple), and YXY (dark red) repeats are also indicated. Sequences typical of AGPs, AP, PA, SP, TP, VP, and GP repeats, are also indicated (yellow).
(PDF)

S3 Fig Maximum Parsimony analysis of LRXs. The evolutionary history was inferred using the Maximum Parsimony method. The bootstrap consensus tree inferred from 1000 replicates is taken to represent the evolutionary history of the taxa analyzed. Branches corresponding to partitions reproduced in less than 50% bootstrap replicates are collapsed. The MP tree was obtained using the Tree-Bisection-Regrafting (TBR) algorithm with search level 1 in which the initial trees were obtained by the random addition of sequences (10 replicates).
(PDF)

S4 Fig Sequence alignment of LRXs. The analysis involved 78 amino acid sequences. There were a total of 294 positions in the final dataset.
(PDF)

S5 Fig Maximum Parsimony analysis of PERKs. The evolutionary history was inferred using the Maximum Parsimony method. The bootstrap consensus tree inferred from 1000 replicates is taken to represent the evolutionary history of the taxa analyzed. Branches corresponding to partitions reproduced in less than 50% bootstrap replicates are collapsed. The MP tree was obtained using the Tree-Bisection-Regrafting (TBR) algorithm with search level 1 in which the initial trees were obtained by the random addition of sequences (10 replicates).
(PDF)

S6 Fig Sequence alignment of PERKs. The analysis involved 93 amino acid sequences. There were a total of 283 positions in the final dataset.
(PDF)

S7 Fig Maximum Parsimony analysis of FHs. The evolutionary history was inferred using the Maximum Parsimony method. The bootstrap consensus tree inferred from 1000 replicates is taken to represent the evolutionary history of the taxa analyzed. Branches corresponding to partitions reproduced in less than 50% bootstrap replicates are collapsed. The MP tree was obtained using the Tree-Bisection-Regrafting (TBR) algorithm with search level 1 in which the initial trees were obtained by the random addition of sequences (10 replicates).
(PDF)

S8 Fig Sequence alignment of FH EXTs. The analysis involved 92 amino acid sequences. There were a total of 283 positions in the final dataset. The analysis involved 76 amino acid sequences. There were a total of 377 positions in the final dataset.
(PDF)

S1 Table *O*. *lucimarinus* EXTs identified in this study. (PDF)

S2 Table *C*. *reinhardtii* EXTs identified in this study. (PDF)

S3 Table *V*. *carteri* EXTs identified in this study. (PDF)

S4 Table K. flaccidum EXTs identified in this study. (PDF)

S5 Table *P*. *patens* EXTs identified in this study. (PDF)

S6 Table *S*. *moellendorffii* EXTs identified in this study. (PDF)

S7 Table *P*. *taeda* EXTs identified in this study. (PDF)

S8 Table *P*. *abies* EXTs identified in this study. (PDF)

S9 Table *B*. *distachyon* EXTs identified in this study. (PDF)

S10 Table *Z*. *mays* EXTs identified in this study. (PDF)

S11 Table *O*. *sativa* EXTs identified in this study. (PDF)

S12 Table *G*. *max* EXTs identified in this study. (PDF)

S13 Table *M*. *truncatula* EXTs identified in this study. (PDF)

S14 Table *B*. *rapa* EXTs identified in this study. (PDF)

S15 Table *S*. *lycopersicum* EXTs identified in this study. (PDF)

S16 Table *S*. *tuberosum* EXTs identified in this study. (PDF)

S17 Table Comparison with previously Reported EXTs. (PDF)
