# Supplementary material for: Bioinformatic Identification and Analysis of Extensins in the Plant Kingdom
Source: PLoS One. 2016 Feb 26;11(2):e0150177. doi: 10.1371/journal.pone.0150177 (PMC4769139; doi:10.1371/journal.pone.0150177)
Supplement: S1 Fig — Colored sequences at the N and C terminus indicate predicted signal peptide (green) and GPI anchor addition sequences (light blue) if present. SP3 (blue), SP4 (red), SP5 (purple), and YXY (dark red) repeats are also indicated. Sequences typical of AGPs, AP, PA, SP, TP, VP, and GP repeats, are also indicated (yellow). (PDF) [file pone.0150177.s001.pdf]

S1 Fig

Ostreococcus lucimarinus

>16632|PACid:27413750
MARAKKRARTIARAALVVSTALAIEGARATTFTADGRSCKWPLELAEDVFVNECVPYGRDGALWCVAEEDGKWGVCA
DLDFDASARGKVSMEAAARAATTLAREEELTYEARRQAMEVLESTSTLMLKALARESIEIEGKIVENPVVEFLAKRQ
RERPDNFNAVNDPTTSTPAPASTTSPPLQRPPPSPPPPSPPPLSPPPPNQPRCVARTDIRWESPQADGSSEFGMDIY
IVLASGESISGGWKILFKFARDGVVLYSSSAYGADARTMYAPEDGRVFELRDRGYDAYIALYSTKRIGFNVRTRSAQ
SLRLSTLAVNGEDCEIVSESSIRA

>16112|PACid:27414204
MRARARRVGARRVALARAAIAMTMMASAAAVDAPPYPPYPEYPEAPPAPAPPRPPRPPPAPRAPHPPRSPPSPTPRS
PPSPPPPPSPPPPTIARTADGAALVIFTALTLIVVSGLVYVGIGLGGARALGERRRGASGRNARGARQLDDSDSEEE
MTLRDELREFAKQAKVIVAEGARAAIVRVQRLTHGESGERDEDWRRLSDTNPFVRWVKRQLADDDNVDDGVGEPLID
ERDDDYEDLMAEPYRFSEFADYVDAVDASSLDAVEDAAFVRLPRAQPEPSWFARSPTTNTTDGTP

>16425|PACid:27419256
MCAHRVDVGSSTDPTECICTHCPTTCADVNGVTDSADSLTGTVDTCTEIYNCTEFLMGMNWHLIGNPDSYPWNETDS
YGCGCGVSVCPSPPPSPPPSPPPSPPPSPPPSPPPSPPPSPPPSPPPSPPPSPPPSPPPSPPPNPPPSPPPSPPPSP
PPSPPPSPPPSPPVPIDRIVNATSTLVGYSSSDFDASRRAFFRVGIASALSAIDSDDVGITSITDVIAKRRRRLLQS
MAVDIDYYVFVDSLTNTTLVEVSSTLNTAPLLTSLQSSGLVAVTAVSTAVRALPSPPPSPPPAPSISGSASQNISAN
ATLTVAEQDPDFSGARVAGLFAGAAAMLFTSVAYQYRQSMFNLFQTRASKPTNTAGGSRKSAFDDKSSNAAEDFITK
ACE

>24979|PACid:27419296
MTTTRNTRPHTRRNVRAVVTIGRALAVALACVTVSIAAVGTLGAYTHVDDPRVPHPGCMRYSHRGVTDRFDRCPGIM
MRENPRYANYDTQIMQPFNLPKFQSALTAYVSEHYAGDADYDACVITGADDDGNRNEVWKTYSPDYFGSLRLTYTCP
WMTESVLRPASGYGDPSDTTTFVMDMRYAAFLMINARVQRDLQAWLTRHAISDADIASDTKDYLFEFFRLYYRAGDV
SKLGKFYRKGLSISASTKKWMDRFEQLSGDKHLELQIDEQAALDDFLLSNAATGYAPSGWPIRDVVNDLEGYNAALE
SAYDAHYWNTESSRVSTWASTHSYSVTTPEARLQSNDDVTSTNAAVKERYITTKVDEWQSSQCTINTYLCDLSLDSE
AAAADVWSFNQNVNISADEVYQQHVVDTIEAWKTTNSYSFLTDLDAQLAQDDLATFNLEAEERYLEYHNAVLAPNAA
AALAAAPTPTPTPTPTPSPPPSPPPSPPPSPPPSPPPSPPPSPPPSPPPSPPPSPPPSPPSPPPSPPPSPPPSPPPS
PPPSPPPSPPPSPPPSPPPSPPPSPPPSPPPSPPPPPHFAPFVPLASVPEYDEHESFEMEPFVVFVANPYVVPVYGH
RNNHTHIPILPVPLASIVDDVADDITMPAYLSKYHRNSLSTYGAQWHFRYGRNCKATCAGSRTQLDVQGPVGALWNC
DSDDLVCPQDNDTSELEGILEALSASDHKHPSCPAADPCMVVPPPCYYVSTECIPQSVIEYTLNNTSTREAEEGVDE
RYLHVPLAVDDLTQVKLVSQRPPLGNRMRWPYSRGIAMTPITGRDGNDYYHTCTYGATLSGWSGSVAYGFALADAEG
NHVYDSGHIFGHEMWQAYYGETASPAFTSDCTDVQAGKWRNRVQYHTPNLASQNFVFGTCAGQCSAILGSVPEDQIV
APIGLSKGHLRDRLIEANDAGLGAAEKNVITAHELVRRAAAATVALGVKELGVKDVGVKDEHHEKARGDAVDDVQGK
HASLGDVRDFERPFGSFVAFAAPFAFVCVIVLAVFKSRSILDFARERRERRTPEGRAERANLVV

>31292|PACid:27415672
MSALLLALCALASLLGVARGGGSPSPSPVHCSARWIDVNKGGCPSCGPGTYSKRYQIDRDPAYDGRRCPHVNGHVSY
GHDCEAPPCPPPPPSPPPPSPPPPPPGQFVPVQVSINDPVPRVSDAYDFHILTSHPSCDSQNDCFESHIETDVSEHH
VGLRCDVTVQKKDQVCATFGRHMPCRLWNDFYPTTLTLTDEHGDIVEGDYVIEYECYFELANGELVPEPTKVKKTLH
EFSVSKGCDLKMNLNRDSKAELAAYLLSGADEFNIADCSSLKSIYAAKEAVFRIFDSNPTDGRLDHEEILIGVEAHS
MDTQIVNHWKDIVDAEGGDGLFLTLSNFMSADIAPRGCQSGAGDVVTFTEATYPTSDPGRETGSRQCGEMAGSMKTT
WSFPRSMQLGDWSCVYIDGLLYSQLLAQTSRNAVSMQNSVTDIRPVIGSFHDAEQSLAAHFTFAGVSNARLISSESP
RADGQEVVPTLSIDDTYTGSLRVFTASQHGKHGACLNSCQGCTFHSYKYIGEPFKNDFAVSMQIFIPTQLSSNSGFR
KKSEPLMSFTSDASGSMQHSIKLRLGTALSKFSLQFIHEKVNSVTGHRDELSEPIPLGAFGRWTSIGFSFHPKDGLT
LYRFSVEGGRVTHEIKVPSNNDEWKSRLRDTLHLMHRREFFKENRIEYDDLRVYTGRVKNNTFIDAYKCDALGAQCA
PRAHATPNSRRVVCVMLDVHDSSGGDARAPYSCTGALYYDGGAIEVRAKMDLTGVAFEFRDTAWHESSFEILRRTHD
PSGDNAFDTVVLVDGGLNGCASMFSSITYLDREAGQQPNSRWQYKIKTKTDDVDVSFLSLTTHFKTPWIGQIVGEVM
AGKSVVPVGEVRICADFVKPNGTFLMYRSHNHSNLALHMPVVHTSNLTISAEQQAHRATDGLHLKPDSIRVARGQYL
RVNLDYWSAIQEVEVCTATGENNFRTFVREFDPGYSVNHGHECKLRETNATQTFSASRVTCFFFTCRGSKLTTLHGQ
FVTAAVEEGDDVRMTQIRVVGQRGRCTFTAISDDDGRYAIDVLDRSGNVPVKANMLIGAYKEEVFGEEFEVPLLDTS

DSDGDDDSLNVPTRVLLVLRRKNSSLAARLGHEVPDYSSSYYDGETHQYLDQHGYSPPYGSSYYDGETHQYPDQHGY
SPPFGNTPYVHTIYDNAYDSGVPAAKVEFQSLTNLRLENTTLMKKDGVADGWNAGGSSNQSMFNVTGRFTGMDAVEG
IRVECSCDDGEYVVGLSYAEHIANFSKAATEFAIHCRGKGYEPAIISGGRSYANSNNYRMAMETRPSHHMTDLYDVY
QSTDITSSPPISTANDFSYAAAQRRLLAPTSSYPARSPTSNEYPTTSPTPSSQLLNSYPQSYDAQTNEYPQSYDATT
STYFPPYHIDDYLDRGYRSNMCMKKTFTIKLTRSGRVLFYETQGHWLYDHPVQHENFHHTQTWQTGGQSPPYGYTEQ
TPPYGYAEQTPPYSARDKSSLGAHADVSHTGQALHEYHEPAQLIVPVAYAERAHSPLFVRMVVISQNVTLSDADIRW
TVPTPSPPPSPPPSPPPPSPPPSPPPPPPPPSPPPPPPSPPPPPPSPPPSPPSPPPPPPAKAIRGNSVDWTVKKNVD
STSSGGVFLVGWNSKGAAVSARAIHSSSNVTRGISATLERGPYSMGHTCVGLTSSADRETVDSSNIDFAMCCEWGYL
GVFEKGSRKWRASGNLQDSCSQTDTLQIVVNDGDGAIEYFKNRRRVYTSQRTPQYPLHADAEVYFGRLGEVVWIERI
PSPPPSPPPPPSPPPPPSPPPPPPPSPPPLHQFTSADVDPRINTAAAIPHITREEFEDFIENITGFPNLGHAIVMDE
IWQYIDDDGDGVLSEIEFQHASYKMHTHELYVDPIIVYPSRSIKHRYGFKVSKSVRSDSSACVGDAVSYDGPSASLG
SCELTSEKEPVEITLWGNGFAGTPSTTTIYSLGASKNGDTTYIASARVSVGTWFTYNVCAVIKFRVIKKDGQCSIVV
DQVHKMWPSLVSSKCGITPPIYDRWSRYMVASVATSATNGFAGMKSSMFKSCAASCENAILLRHNKTVPYNTSEWRN
YYESLVKRDMEPTTHVDCSHREDMVVLGREEDTLAMPMVLSSMARKQWLLDLDSARRKQSTSENVTKLLGTMHSNFA
ISSDLWLPDIVHVFDKIPKPENASSGPSKEDEIHVVDNDLGNSFSNFARIEVNRLQLEHKRIGEKTFTDDTAAVVKG
AILFPQHLTGGSPNCGLFEAMIRVYDVEEDGEPDVYLTEEDGSFEMALTRGKTFRIEAFYEGHTICYAGSQPQDATS
PNGYDFEGGQQACVKTTHTLARVGDGNFLYFTDITRGNIDLGLYAGECEERYTGEDVKFRVTPVNGCHKPVIVSETD
INERWHTIEDILPEGQQVGDNARVWKFAAMDYSITLASGSDISRVTEKACPDEACPDYWDGCQVEPDDMRQFFRNRN
SLERLALMRDEFAWNSIRYKYHGYICAQIMDIPRITDNELRLETCVGNLTSGMLTEKHLLGSSDHDTISVAADKSLR
VKVFELHDLTPNAADAPFKCTKFPNTQSRTGNTKFSVRQTVTNEEDNVCHPNRGGGPLCDFEVNISTADVGKLIFPT
EEDTDQETTQMVIAAGYPNLANPYRREVTITVTRDDTPLDGGLGRSLQATMTRVLIPLGSKPRGGFDTGDDTLWATV
PIDGLVYTVVHDPPGGNSVAELQSGSEITIQWEIASARSVKVGRSIELNLGFAGKTTFDLGFNAGYTAEAATKSSVL
TLETEKDFGHKESGPHFTAKATSEEVWETTVTMDRHIRSSDDEGTPGRPGDVILGGGIELVYKVSDTLDVGTVDDCL
TTGVGITWLPRRPTSYVFNVFSIESQVLPNLYFLYTVAKSGENTAKTPSDRIVKDGSGMRYECASSPCSNSEMNQHW
ASYILRRIHTWKRTLLWSSPEVYWMPKGASYEKNYKAYDRINEPYIDSRSLFERRMSSALAQRDSKRDPSILRVAFE
LSKMWYEQSLLSVGDRGMLRSKPHIPALFLTAGLEIPRLAPFLENSRAASFASIAYPRSSKGAWSFDEVVNDQTNIG
MYSFGMNEVAARDLDAQVKKCDGVLCETSGEFDAAPHDVLYGGSLNAMGETGDLRATDPSRLMASLTGTPGPTGMLQ
KDAVASGGEEETIYLTFGGGGHALEFSFSAKESINRDAFAYSLDFDASAENTNNMKFSGSPIPTIEFNRATDIARTL
STDRIFAWNKYGTMTTVYSLADPDYGDKFVLKVGSDARFGTPLFMTMGGRSMCPGELTTMWRESGNIIERPRSLSFG
TLPLNPGERAVHEITIQNESPYREARPLYLRIVDGHSESLRSLVRAALNIIKDDESASAQHVATVVTNTAANSVASA
SLSMATVIEKVEEAAGSGDATALSVMQVVVAEVESVSKSAMTPLQDVEITVNGKKVVPVAEWLPLKHVIGDALESQR
TVHKTVFNLGFKPTETSHPIIEHIQVEIGSLCEMQISYDGAGLYRDPIAVKAQLEKMRWESKCPSVTFSKSTVIDSE
ITRANATAPPLRLVVVNPDSGNLWDPSSASRLEKVVVQYRPLSGGEWITAKEDHVKYKDDSYKKNLICEHSRTSGCT
WDWDLNNKYDKLLSGYKDGAYEVRVKNFCSGGDAFAATEVHEYVGDKTLLLFTDTKNPLVEQHVSSPAARTVTIVYA
EEIDCADPPTFEVAQTHDESCVAVAGDGTVSAAIIRESYEQTCLNTGVQGKWMMQYPNNEDAPNPGLYRVTVRDIRD
KAGNPFVGDLTINYVVGDVVDDCDILKASKDDLTPLIGLGNFADVQGNTIWGVYSKNIDCRSQRVTITKSFDQSCRS
TSTEIGTTNFTLACVNVGGTGQWIMKYPEDLESGVYTVRVQGVKDRTGHEASSFIRKFTTLHNSDGTLNECDDASAS
FAIGLGRMRRRRLAPSASATKTSTNLILSDLAIVVFAICALVMIIKKRIALPADESELREREAFDKVDIPRYGSSL

>25682|PACid:27415895
MMRGLVVASALSSLRGVHGGSSPSPPKTDCVGFWQGSCTAFCGPATYSQTFTITVKPRNGGRSCGAYEGQTRTENCE
GIANCSPPPSPPPPPSPPPPPSPPPPPSPPPPSPPPSPPPPSPPPPPGAGAAIEVRFGSTTERVAAYSYDFQVAVHH
PACAEASDCLLNVNEESVSNHALGLRCDVRVKRDDQSCSESDYADNLPCRKWNDFFPKALALTELDGTHSVGSHTIT
YNCHFVTVDGIVPGSETVGQHSFEVIKGCDLHLPLGFNAAGQASANQNSGLEELVRYILLSNDQFNNVLCDDGEIYK
TKESIFRRHDVDPSDGLLRLNELQAMLVEQSVSRYILNVWNDQFRAAHDTDLAVSIVDVMEIPVRPVKCSGAPSNSF
SIDSITYPTLDWQTENRCVSGLNGLTTAWSYSPKPTNGDYVCAYVDGILFNQINVADGTVDDVVDVRFEEYEVGGTT
LQLPISFTDIRPATSDFLDEHASLVAAFTFDEDPLHDGSLDSVAPIVDGQSSVPMRLQPKSARTISGCSGGNGFRCL
QAVDDPGTNDYSYSLDKEDTLGHDIAMSTWVRIDPSRCSQPKSSNDESLMTVAQFQGQLGLHDHRLHVYLKIRTSDG
MVELRASHQVKESVDPPSAFTSSKDVKLDFACDGEWHLIAFAVNSVNFMTLYVDPTSDAKATVIDDTTYTRDETWPL
SALESMTDVTLLGAVDFEFDDVRVYTGVIREVMFIDTVRCGHYKRCVLRQSSAPKARRIVCLSGVISDAREETYSKF
ECGGGLYYDGSTIDVNGKLDPVGVTFSFRDTAWNDVGFEILRQSMQDSLTDAPYESVVMIDSGLTYCHRSYAPLLFA
DREAAAIPGQIWKYKVVTKTSSGGSINSVPFKFITPWHGEVGGYVYAGDSAVPVPDVRICANFHDPIVIDGEVANYR
GSRPPDADENENSTITLRPSDDVESSSSNVALFKHAYSNPLNEQAYTLTDGNHIPTGESVVVSTGGYARVDLGLWMS
IGSVQVCLQPQSIKARQLAEGSLTDRLGYSGDTGDDPDVIVDPPRYENDVIRLVRADEKLWAKNTDRLDAEAGDTVL
LECWVRAVGKEAIMTFQILGYEQQTTAAGRQTVGDALVITAEPNKWRKIRLKHTLEDAAFVGFTVSAWSESTFYFTG
AHFTLNTLTERLELVQTSAISPLTASPTAPPPGFEDVVKSVDAGVSVTVHVPGESLRALTGDSVVVSFWAKSLGGER
AVSIDLRGTSATDASTESFTIHEESWRRFNATLISSQDQYITFPEISFGAESSKVEVTGFEFRQQASPTLSLPMKVH

VHEIDPEDTTNYGYACNHDPSDVSDEFADCLTFTCGGGGGDTVQAMHGQFVSVVAVRDVNLTEIRVLGGETRCPFSA
VTDSEGSFVVHITDSTGMTPIKTHVDIGAYKVEVFEKTTQPILQTSDVTDPLNVPSKVLLVLKEDDSSSPETTTSLL
GRQRWGRRLLGNIAQYIPPPPSPPPPSPPPPPPPEKAAVGNVVWVTLRRAKASNRKVWRRSPNWWNYFSAAGAVSRR
SIHGLTDSVRGISARCGSESGLKFIGLNRQSAAETVSSRMDFSIRCNFHSDAVEVYEGGKKIKGAAVAYTDSDTLQV
VINDAGAVEYFKNEIRFYTSQQTPEYPLHADVAFIMRGSFSDVKWVERMASPPPPSPPPPSPPPSPPPPSPSPPPPP
PGKAVAGEILGSVAWVSQVGVESTESGQVTRTAAVTGAWDAGAVSQRAIRNGSDSVRGISAVCAGGYNNRMIGLNSQ
STTDSTNYQSLDFAIFCTYTKRIKVYEKGKRKYTMAGTYSSSDSLQIVINDAGAVEYFKNKIRFYTSQQTPEYPLHA
DISVKAGSLTNVHWVERIASPPPSPPPPPSPPPPSPPPPSPPPPSPPPPPPGKAVADDHVAWVSQVGVESTESGQVT
RTAAVTGAWDAGAVSQRAIRNGSDSVRGISAVCAGGYNNRMIGLNSQSTTDSTNYQSLDFAIFCTHTKQIKVYEKGK
REYTMAGTYSSSDSLQIVINDAGAVEYFKNEIRFYTSQQTPEYPLHADISVKAGSLTNVHWVERIASPPPPSPPPPS
PPPSPPPPSPPPPSPPPPPSPPPPSPPPPSPPPPSPPPPSPPPPSPPPPMHDFDETDTDGSGGIDKSELTAIIEKGA
SFPSNGYAIISEELWNSSLDMDGDGLLNAQEFLSVAHRLAQRTLFVEPVLVFPPRKITYDFDLFTVQSVSAAIADES
HQDARSQVFGKACENFIVRRSTSAHVPYSSEGWNEWYDELSAESGGDVVSKIDHCRPLEDAEFDAIVVGDETSVYAT
PLLATSPGSNKIFVDLDAALLHDVVQDDPQTLFARVTRPGLDVALWSTQQLPEIVHVFNNNDVVKQAGTSDDDGDLY
ESDTVFSDTADIDVINLELTHNHQVEQKMQDDTTVVILGAVMFPIEWTELSECGLFEASIYVKDESEAGAPRQFTTD
ETGWFEFAVTNGKTYTITAEYPGHEICYSGTTIVEATRIWSCDGKPTTVTLHAVSSKKYIFFSDTTTANVDLGVYQG
ECEKLYTGATFKITPINGCHPPAIFTSAQVSGWTLPDRDKENSDVIPTYQEVPNGRRWPLAAMDYSVVIEEAPSTAN
FTAMREEKYPNALCHVGVDGIIPYFRQHPTTIERLVPLRTEHTWYSARYKYHGYLCAEFVDLSVITDDGDYCWDVDG
VKAGGIRHNHFVGISPFFDDFGGEGLYALGPKLVSAKVLELHVENGSLDECLTLPSAESGGMTVAMFRQSVTDIAEN
PCHTDRNGGPLCDFDVEIDPESDKLLFPTDEGEKTTDRLIVAGDPKLSGNYRRSVELTVGRFDGSMTVTVPLKRELI
SLGSKPRGEGIYGEESDDVYWATVPLEGLVYMTVHDPPGGNSYAELLMGTEVTMSVELSDEQAASVSSSHEGGGGVE
LEVEAKPGFGAGYGMEVLFQLGMPLFKIDFDAYHEESGPEFSVAQSSAVGWDLTATINRVIRTSTDVAIPGRQGDAI
LGGGVELVYRLADTLDLALREDDKPCLRISTAITWMPRKPTSYIFTVHSIEAKVIPNLRYLLSVVESGLIVGDDSKM
EAPNWPQYIADKINVWQRTLLWASPTVYKVPMEPTAEGAPVLYGKNYGAMERIMVPFMDEQSAFGMEAEKMETSFIS
DASGPISTPIDELKLAWKRIDRVSPYIHGVPGDLGDADGTIDTLNDFILSDETDESRLSQLISGYVSNPGTLFSDLL
SGNLGFRGDDDDNDATPDVGKVFWSRKSERMKPKFLEQFQEADELVTTMGMNSPTREDLNNTVSSCITEKCKTQALD
VSSQLLQSSDLSDLTLGEDASIYMNDTKRVDASFTGRMGPRGYSTTDSNGPADEAILLSFSGGGASTEYIFSSNENV
DGQDYAWTLSLDGSAENGYSFAGSIAIVKGHVGATTDMSKSVSKSRAFAWAKYEHMSTMYSLGDPDFGDKFVLQVSS
DKRFGSPVFITMGGRSQCPGEKWTMFREAGVTIGKESTHNTNLNPGEHALIQLLISNESPYKEVANMGLRLVDGVAD
CVGKIIAAAHNAAQLNEDNATFVKNAAYAMADSHECFASESDDIQDLKARIEDIVASYEQSMSVRAGRLLANAIARL
TQTTTAQGTLLQGMKFSINGIQMWSFGEVLPLRRLAGERMDSQSVVRESRVLLSVEPSDGVYESSYLGISLVSLCES
MIEEYMYRPIISSSVTLGAMSWSKDCPQVAFHSSTLSKEASYFEKSAASDSSVLSISVVNPNRYSLWPKETASTSEA
LVTNGNLAYVYVQYRSVSGGEWITAKDAERGAGTNKNFNLLCPESRGGDGCIFDWDLNDPYNKLLSGYKDGKYDIRL
KTMCVGGSHLAKPSVHEFVSDQNLMVKIDTKDPLVGDFKYVSSQVTQRVDFMEDIDCTKQVITAKRGNSSTGPFEAV
SNEDLRQYVLQCVNDGAGGHWLMKFPYFSQGYHKVTVTEVTDVAGNPAAEFEFVAPVRVGASTAETPQLGSSGSSPR
QKSSLASSSPQDDIDGSQKVAIFYTPFAFTLCVALFAFAFRARARPADAEMSERTSLVATTSRQVDGYSSVI

>27866|PACid:27417425
MRVGDRARDARLRTLVRVALALFALAPSPRSSASAPGSEAAGARGARARDARRALGVRTTEAAHDDALTAWNASDES
IVSPLRCQRRAVFGECVQEVGCGWCDSRDVDGHNPTCYSLLDAALCVSHLPPVMNAPRVVNAVTNEIEIPCDPRCAA
IGNAEHCAKADGCGWCAASSTCMSGSVYNGPCRTCAYNWTYSAPTPTCVGAMTGDGCATCGCENNGVCAATSKECIC
PDAFTGSLCQFNTTTNNGRDACNGHGTWDATAKDCVCDRGWVGVGDNRCKFSCSPSVDCNGNGYCSERDGTCVCDLG
FAGESCSCLSQSLVSYPYPDALLNTNKTGCCPYSYIECPSGSLNAGQCVIAPADTTKCPVESRDDGFLVFLGLLLFF
LGMYLSAAIIAAISGHPDPTCTVVVRLPTIVRLRDSLFMRNIDDVNEMQTISLTCKLTDNFQKVKRMVCRRLKHQPD
PDSVDLFYLGCLVDPGWRLGDGINVAQTCHVHLKLRGNHETHKYTSKKKALKRLRYIFSDNVIIFCLYFFFILSLSL
CASTWSWDRKNDSGIPSPPPPPSPTPFSPPPFPPPPSPPPSPAYPPPPPAAWIDLLNAQNTVNTNATFGTDSTFIGA
DQVGHIPTDILALESITVTYATQLAVTSNSSGNVVNFYTYEDGPLWQRELPLFTSRSGIPFVANIFTVCDPRKAVEA
TFQVEMNAGYGVVSYQPKPNENGVDVFTFRGTVEYTVAQALGLDTDGTVDCSPAVLALYDNSCTTDIKEARVDIAKR
NDAPVAAGGEQILSYESTADIIPMRTQVDATATGSKLSRPMTATDIDGDRLTWTIRQQPAHGTLELPAGPVAYSSTF
EYIPDINFTGYDYFIYSVDDSLDNTPDTTLYDVASVVVAVGSATGKPVGVVSHYWVDEDTTMTGRFGRTIMASEYAG
TLTYTVTNAGVQNGTVTKLCNDVEVQYWWNCSVNTDDGNLNFQYFNYTPSANFTGTDSIEFSIVSSYDSTLAASASV
NVTIRPVNDPPFLANMEVNAVMNRDRAADDLGDADLTPITFSPYDVDAGPYFRLFLTWKPIVTQSDWATRRRGRWYK
SIDRFGRPVDEITAATLDQVGLLNNGTSITLYYLAPPLLYGFFPSTTLVRQNPLEKYAIRVVDDGSLVSAEATLNIH
VGCALGYTTESASAVGDASNAKTYGTCTICPLGQVTDAINSHTCRACPVGMAGGTVQRGFCTRCSNGYYSDTPGLET
CISCPAGSTSVQGATSRDQCFCNIGWYGVPGYCFPCPEYSDSYGAPERWTYCAETNLTVPYPQPGFYVVSPETRSYK
QPISMRQCFPHKACPGVKGIAGSAQVDAIAAGGSSADAVQCAEGYVHDACETCVTGYYRLNGQCLKCGSALGHTLYG
AGVGLVMLSAILMPWLIDEQSFRYIVYCRILSLVVFLQEVGIIGRYNLGWNDYPGLTLLLQLCYILQLNPEVIGLEC

LTEYDFVARWNSLMAMPAIGLALMYGIAQLYAYIWGKFYIKTQPSVEVVRNGQRRWVLMLRSILNVIQITYVAVIIA
TFDFFIFHRSVTDKFGYIRAQPNLRFGYFSSTTGSTKWQQLFPLALIALLLYPIGFFAIMYWVIKYAMGGWEKLWKR
DLIGFATFQFTDDYVWYRVNEMARILALCLIQLLGYELRNGNGVVQALTALLVISVSFATVILRPFKTSEAKRYLGL
HLLAMVLVLMLSVISIAPVSYIINQRTKDNARNAVWQTIAVAVFVFCAGIFYEAYSSAEWFKRADWHLRRAYKNFIA
RVISIVVSEHRSSQWLRERKWVEWKPTLSLLNPSAGAILQLESAEGDQGLYFSGNFDMHLRMEFARGIRSLWRHKTV
ETILGTHDDRAKSFYKMLSRPVRQSLIRAAAVDNRDMNRQVQLFIENKMIQKRYNFYRKFYRQAQKDLRGVSGAHNP
SVERFLLRDSTLARAKSHATQEKEKEDEEKQLAEYEVLQQADNVRRAFQTDVNDAADLDQEELFFQTSEVSGRTHFV
RFQGYFGDIHPDWDAYDESLAKAKAEAQDEDGDVLPWHLDLDDVYDDFCCLCECEPGERCARHGNLYVVIQQDDEEV
SRIKQERERHFRTRETIIIQEEDPDRSRDLGWNVYVPVRLKEEVSSVHTKLATELRDMRDRGEFAGEDELPAVTGSK
FDISHKGKLLDTHNTVKLENLQWKAEVQLENRRGNLAKTTAWLILQDDPEEARDPDEDERMYARPDIEEGPVLAQPV
LQSNLVHENPEMTVLDLRRMLADSNSKPLERTILRYRNNGKELIQDAAPLTRKGISKPNHVLTSFEPEDGNYYVWSE
IRNHWTFLEGLFKTYASIPSGPSDNETRVGADDHLLMSYRQFMYLISSCKIEDKLPNSQYNQLSAVVRSVFDTVKAP
ELLAKCLTLDRETGSVEGSLCPSEGPRLELRIKHAWGMHLDEFLTSLVFMAWIIYGHRVPLTDRGVARATRWLIERI
LRPKAHHIVEAYEAQARFATAMKKQGKSSDEDIRAVSTVYKAFTDKDGMGVMEFKAATMWAVGAESPLNKFGELVFR
TYSDFSTSTPDWVYHGARHGWVHAIDEKPDGYLCCQGSRLDAKKFMLAFGDFCFRANPTVKPSEALQAQFKQAQERK
PLAESPSVHAVLEEPSDELARKNLVMPDSRCSICGMTDHEAPNCPRKKDVNCPNCGITVEPPNKIVIGPDDLEYCNE
CLREDVPEDEPFVCPNCETPCEPEEVAISDVDGKKHCPRCAPEPFVCPNCETPCEPEEVAISDVDGKKHCPRCAPEP
FVCPNCETPCEPEEVAISDVDGKKHCPRCAPEPFVCPNCETPCEPEEVAISDVDGKKHCPRCAPEPFVCPNCETPCE
PEEVAISDVDGKKHCPRCAPEPFVCPNCETPCEPEEVAISDVDGKKHCPRCAPEPFVCPNCETPCEPEEVAISDVDG
KKHCPRCAPEPFVCPNCETPCEPEDVIKVDGGQFCPKCWSPQRKRSIPTCPNCDAPCTLNDIITGPGGKDYCPRCVP
EEEPEMDEAPRQASSKAPAPFECPNCSREMEPEDVMPGPDGNVYCAECFPDEALDDDIDDEDYPSAPAFTKPKIEKA
QRQRVARVRKVTKRRVDDDAPIAKPRAFTPSSPPPPKPERERIARVRKVTTTVVDDESVARPRAFARAPSRADRERA
EAASSASRLGKFFGFGGASSSTARE

>93602|PACid:27418757
VDDEATNVDFAGVNCEGFGGGALSIDAYAWSHSTPTDSMLRVGVDAPRADDADRGARETYASVLIVNDADREVNLNG
VTVQFEFSKLIRLDDAASEFTTLGEFEFTCWGSQIVAPSGARRIVDCGDLSIQMNDVGPRVTFGAIALNAGEFLAGG
YSNFLFSFRDRESRVLDAESVRALDPTCDPSAFTTASSIVSVAPTSTFGWNPNAQIMLSACPSLLDVVFSTLESQIE
DGVARIRGIVGHRSCSTLDASKVTFAIDLGGDYEQSQLQAACEGLQIVYPISSVPMSDAAATCKAVVTERGVELSFG
DDLVLCPGCFIVGGPNNVVLDLSYADGRGFSSTEISPNGLREPICAGFPRGDGPTDAELVRPCDEEANAEYEGSTLT
TGLFLVPSPEACCLACRENPNCNVWTFCTANDGCGRSEFAYSYSSCELKYAAPEIISQDTIPGKRGPNATYISGALR
DKEVEPVPFDTSNERKECRAETNANYDGIQISIVNAVSWSACCDACLADPACAVWNFCDDVSGCDGEFAYQTCVLKL
FLVGGDPQKPPAIARGDGVPWVSGSLVDRIPEPVPSIEGCRVEENANYKGHPLNSGSDLVVDSETECCAECKKMKKC
NAWVFCASIDGCGNEYYDYKFGECWLKKLSKEDVETIPVPAWERGENVTWTSGVVGAEVMMPSPPPSPPIVQPSPSP
PPPVEVPSPSPPSPSPPVEVPSPSPPPQPPVEVPSPSPPPQPPVEVPSPSPPPQPPVEVPSPSPPPQPPVEVPSPSP
PPQPPVEVPSPPPPPPVAVPSPSPPILPTCLRLLQDLQGRCGSIFGIENDEAFDPSCCDIVRGMNVERCFCDTAIID
ELGDAIRTVQITAPTLCDSGLELVSGLSCFPSPPPSPPPPSPPPPSPLPSPPPPSPPSPSPPPPSPLPPSPSPPPPS
PPSPSPPSPPPPPPVPSPPPPSPPPPSPPPLPPPPPPPSPPPPVDECPTLRLVGGPNAVQNEVLLNSLYVDPGAVAT
DELDGDISDAIEADTSEVDTSRVGTYRVYYRVRDSGGCDVSAERLVRVIALVPTSEYSQLTSLCVSNIPRVEELCVG
FETATISGPNPASFPSCCVALSDLDEAGCFCVPEFIQSFEGIRPSVGELSAFTPLACGFRIKVGDVCEDQQLLTQFL
STPTAVPEFVIPDEKWERIVDDGGALRDDISCADTIDVTRKYCSAIQQNDVGVIPRISDFAPCCGAAARLHNESCLC
DDFTLITDDNVMFLREIVGFTPLGCGFNLTEACPALSEELGVQTIVVERPGNDTDPTIPIGTDTPPITRPLEGYGVV
VENGQVIPSDTPGRSECDPVRELKNFLVVASRPNSTDYDVLGAIGCCANSTIRVPEVTVVIGYRRREGNMNEFVDAL
SVPSVECAGLSLISARGEVLDDTLCDRATIEESIAGIKITLANLTIPPDAAITGRVQDGKLFTAKHESEATLDALAP
TIRALQCDVGGSWTDVFESPPNDSRRKLLQFFGGIPFFPDQDQFGDANRTDITPSIAPLRPLAGFNLTQYARPSFRD
GFDGETSDGGRPVDCLDVLPRVQASLSWFGERVDVGGDFRVAPFNRYDFRGSIGVNNDGGAIRLIDVNIPIVLSSWV
NDINGTWREVQNPFDEYAIECLMITVVNGESRTPSLEPCAGLQFRMASYAPDGVFNEASGRPLPKVTLLEVSLSNVT
LCAGCKLQGDNETLFRVVSLSGARFDYVGPSVGNPTCRRDIQGQLVEPIAMLPTSPPMTPTPVNGLVCNGEKGLNLK
GILLRDGSKFIVDTEEACCQACFRTRDCDTWVYCTGDCKDFAYHSCWLKRAIGGGYTAERGPTEIAAWDRGPDVPWT
SGWFPTQRSPPSPSPPPTPSPTPSPTPSPTPSPTPSPTPSPTPTPTPSPTPTPTPTPTPPTESSPTPPAPDSATYFT
LQPGVPVQIPASTITVIPGAQLPPGAPPSTPTPAPIPVDGLSRTCSPDDATICPTESAPERLRETDARLSLQLLRGE
GGSQAVVTGTAINFGRVSDQICLNGVAVMLPFERIVVAANEAPIVAPPEDFIVDCIFVGVRSRDGPPSSDDNGCGVY
VEAKVVEAGVELTFRDISLCAECWIVGGPSSALFTVRHIDGLALQLPEQNVTIANIFTEDAAFCATS

Chlamydomonas reinhardtii

>Cre07.g353837.t1.2|PACid:27563804
MDNDQVTYRSSDPRSNYMTNVAPNTRTVLSSPSLGVAYNLVLYIGAQFGSQSEFVPVYICRFITADTECDTLANVLA
NAPKSSPPPPRPPTPPPSPSPPPSPPPPPPPPSPPPPPPSPPPPPPSPSPPPPPPPSPSPPPKSSKGKPGERAPPMP
PMPETPSDPPSPGHARPPPRHRRSPPPQRRPRKHMP

>g11639.t1|PACid:27565474
MRLSRQHGGLRLAWAFAVVFVLALNTAEAQRKKSPPPAEAPPPFSSDYDSPPTAEPPGGAPPVALEASPPPGLPPSP
PPPSPRPPSPRPPSPRPFSPPPPSPQPPSPPPPSPTPPKPTPKPRPTGTPPKSRTAQPPPRQRVPYLRPRPANWAVN
KHGDN

>Cre08.g365300.t1.2|PACid:27562825
MGALALLLVASCFVGGVFSARMAKLALDDSASVSDASASSADQQQELESVVSALAHMNADSEAMRRVLASGVYPPSP
APPSPSPSPPAPPSPSPPLPPGLALEAEHYVAGFSANATIVADLDVTSQASVDAFIAAFIQLTATALNIDPANVYVT
RVTKGGASSGRRAALEQSGAAEHVWSASELSALLPGAGLSLLALKRVTPADDATVAGLRSAKLELVLGPGAKLPQA
RRSAQQHQPSSTADVIVDFTVVTLVEVALPPSPPPSPPLPPGVSAPPTAPVSPPSPPPRPPRPPPLNVAVLTSALNA
SWVNEAPSPPPSPPPSPPSPSPPARVTSVRVSRMPAQGTLSGDSSKPVVWYNDASFRSYRKPRSPLNLVFWSARRQC
TATVGVCGGCPRAWAVPSNATQTTTTLPLYFREPMQLDSITITQLQNPGVLSVELLPWPATPIPELPGVAPVSGPKG
QPVYSAASDSTPCGGDLVINVPSDRSGSSESVPPRGSQSELPPRLRRTAVGGIRITVKAQAKGAKPTFISSVRFSGR
VLYPANPAAYDGM

>Cre08.g374750.t1.3|PACid:27562835
MSSTVFWTHRVPRASSGAGGARMATSGGPHSGSVDAPVPIPPSSSHRPPAGSAHGSSPAEQQQQQQQQLHGGGGG
GAGGGWRWGWVNGLLRTAASPPTSAPNTAGTGTGSGASSSGVGAGGVRRTLQQRSSGSGRTALSREQPWPGAAAAPH
LAPAAQATSPQPHHHQQHQQHQQHQQHQQQPAPQPQATREVGGSRSGPQLRQQGRSTTAAEGEAAAPAVTVDGTGQG
EASQTQAWRRTDAVEGTRGPDCKRELSGPGHAAEGSRRVRRRPSAASASTSTTTATSGPSLLSSSLSGLRSACASSP
LLTLLLLLLASSGPLLPPSRLPNSFENPSASYTSSNPNNHPSNHPANHPANCLTRLGSLARAWGAGWGPRLAAAQSP
GSLLPPPAPGRVCPVYSRDPALWACTPLYQNCVLYMYCPNPCNTAVDRFSFVNMSSCAAAGRTYITNGTCGAITCTA
PPLVPDTSPPPPSPDPPTVSITPAAPPPPPPPPSPPPPPPPSPPPPSPPPPRPPPPSPPPPATSTSPRPPSPRPPSS
SPPPPKRPSPPPRSAEDMAPPPDSGGPDTPPEYASAPPPLDESFPPPPKTSLPPKPRLAPPLPPSPPPGATAPKPPS
PRPLSPQPPVPRPPSPRPPSPAPSASSGNEPPAKSPSPAPPSPLPPSPRPPSPPPPPPPPPSPPTPTRDFSFPSPPP
SPPTPRPPSPVPPSPPPPSPPPPRPPPPPPPPSPPPPSPPPPSPPPPSPTPPARPAPPSPSPPPSPSPPFPPPPSPP
PPSPPPPSPPPPSPPPPSPPPPYPDPPPPPSPPLPPPLPPAPPSPAPPSPAPPSPPSPSPPVLAPPRPPPIPPAPPR
PTGQPDFVRTIDVPTIPTAQQMTALQASFKSSVGTSSGDVTVGVDIISKTITVTYSMFAIGAAAGGSCPACNAALTD
ELRVKVAAAARARPANVGCACLAFFQEQQAKAVAAAAAGSTQTAATKEVLAAATAAAAAAAAAAASKSSSAGSSSQG
RRLTQTSGSSGSSSTGTGTSAAASSALTACPESAFVAQIVVAPSDDLTAIVSAILSTSVISPVVATIIPLPSAQQLS
LSSQLLLSLGGSSTAAVQVAEKVVGDSQGVAARVASALTVSPDAITVADPAITGASTTSPQPNSDTPANGAGGDGGT
DSGGGSSSSGVSTGAIIGIVLGLVGAAVLLWGVLYFARVGHKRRKMAGSYQGRAAERMDPRIIMWQNACAVRTVQRN
YGGPAKAAAAAPPPYGQAPYAAQLPYTLAAPPPATAGAAAAGGAYGNQLVARDAYMGAGGLGMGGMGMGMAGSPYGA
TATGSTVASSGATVARPAAPAGPPLPLAATQYTAATAHAAVAAAARGTGAGAYYSQAPMRVGVAGTPPPSAAAAAAA
AAAAAAGAAAGGYRGALQQPALVAAAAPLQPSGSGGGAVPGMGAYPAPVSAAAAAAATAARVSTLSSSLNSTKNRRR
MGESLVTVPTNGAVPGQR

>Cre08.g385550.t1.3|PACid:27562904
MTTGIAWFWRPDGASVVAEQGRPQSCGERLRQVHGPYAPASAAEEHRWCTGADGTEACNRTASTHDTARCAAASGPL
CPAAAGHRTTAGAPPCSCSHHQVAGCDAACSSCSSAPQHTAADTSTAPDGSPRSTAGHTTSSGSSNSGTGGDQGCQR
RHRPQSPIRRRRCHLACSPLAPLATWWLTALLAALAGGPLRGLGPAPAAAQYTAVLDLYSLPGSFDLMSDCPTLLYD
IMMQLPYRPSRATCDTLGGGAQISASMAYVQAEGLQDLMARLGSAALWRLAAGLLGPGCGWGAAVAAASTPGSSPVV
AGELRVCAWGAGGASQPFLPNSPLGALHAKTCSPDLVLAEAACPPPPPPPLSPPPSPPPPNPLSPPPPSPPPPPPPS
PLPPSPQPPSPAPPSPTPPSPAPPSPTPPSPLPPSPPPPPPPPSPEPSPPPLPPQPRPPSPPPPPPPSPPAPPPSPK
PDWLRLTVLLPAWAPAPTSAGCNALVPAVAAFAAAATGTPGVVPDSGLGCVVQLAGSGSSGSAFNAYVVQGPAYSFP
IAAAVANALTVSPSFITAAAGLPCGTALSLSAQAASASVACGALADDRAHAAALPAPGVPYTSNWLLCCQAPPLPPS
PPSPPPSPPSPPPSPVPPRPPSPQPPQPPSPEPPSPAPPSPPHPSPTPPRMPPGRPPQWPAASSPPPRPPPRSPLPA
ITETTQAPPTRRPPPPRPQPPSPSPPEELAPPPWHEDASPPPPPPSPSPSPRPPSPRPPPKKKPAKGKGGKKQKAPP
PPPAPPPGKRRGAAGRR

>g8557.t1|PACid:27563040
MGALALLLVASCFAGGVFSARMAKLALDDSASVSDASASSADQQQELESVVSALAHMNADSEAMRRVLASGVYPPSP
ASVDAFIAAFIQLTATALNIDPANVYVTRVTKGGASSGRRRAALEQSGAAEHVWSASELSALLPGAGLSLLALKRVT
PADDATVAGLRSAKLELVLGPGAKLQQARRSAQQHQPSSTADVIVDFTVVTLVEVALPPSPPPSPPLPPGVSAPPTA
PVSPPSPPPRPPRPPPLNVAVLTSALNASWVNEAPSPPPSPPPSPPSPSPPARVTSVRVSRMPAQGTLSGDSSKPVV
WYNDASFRSYRKPRSPLNLVFWSARRQCTATVGVCGGCPRAWAVPSNATQTTTTLPLYFREPMQLDSITITQLQNPG
VLSVELLPWPATPIPELPGVAPVSGPKGQPVYSAASDSTPCGGDLVISVPSDRSGSSESVAPRGSQSELPPRLRRTA
VGGIRITVKAQAKGAKPTFISSVRFSGRVLYPANPAAYDGM

>Cre08.g365150.t1.2|PACid:27563123
MGALALLLVASCFVGGVFSARMAKLALDDSASVSDASASSADQQQELESVVTALAHMNADSEAMRRVLASGVYPPSP
APPSPSPSPPAPPSPSPPLPPGLALEAEHYVAGFSANATIVADLDVTSQASVDAFIAAFIQLTATALNIDPANVYVT
RVTKGGASSGRRRAALEQSGAAERVWSASELSALLPGAGLSLLALQRVTPADDATVAGLRSAKLELVLGPGAKLQQA
RRSAQQQQPSSTADVIVDFTVVTLVEVALPPSPPPSPPLPPGVSAPPTAPVSPPSPPPRPPRPPPLNVAVLTSALNA
SWVNEAPSPPPSPPPSPPSPSPPARVTSVRVSRMPAQGTLSGDSSKPVVWYNDASFRSYRKPRSPLNLVFWSARRQC
TATVGVCGGCPRAWAVPSNATQTTTTLPLYFREPMQLDSITITQLQNPGVLSVELLPWPATPIPELPGVAPVSGPKG
QPVYSAASDSTPCGGDLVISVPSDRSGSSESVPPRGSQSELPPRLRRTAVGGIRITVKAQAKGAKPTFISSVRFSGR
VLYPANPAAYDGM

>Cre08.g381900.t1.2|PACid:27563160
MSRLALLALLLALALGPVLPVGVKGLQGGVAASGSGPAATAEAARIADAATRVLLARPLHDAAGTAAGDGASGAGGS
GAASGEGAASATTAAGSSGTAQPAAGVVPLHRSRHDRLAAAAAARRAAAPPPAPPAPPAWELELAPAVAALYPAREP
NGTLLRGPPRLAGTYKGKWLQEALGPALAAAGYLTTARGAIILQLPATDNPPPAAAPATAASTASASSPAAAAAAAT
ASLGGSSTGSGSGANSSSSSSSSGGGSSSSGFGALPGGVQELRGELVLRDGPGVTDRDVRFRLRGVYVPAAGAVHAV
LEPHWPTVVTMTEEEMAAAAAAGGGGGGATEEGAAAGHSYREALRTAARQMALSGSRWHHALVRASPPPPGARRRGP
YDTQELRVAAAVAAAAAAARRDRGGGVGLPRPPSPPPSPPLELALRKTCEFRAHFHVYYKGDPDAASHVSHLRLDVPA
PPPPAPPPRGGWLMRRASPAPALDGRGSGGSGGGSSGGSSGGSSGGGGGGGGGGGGGTQQGRGQEPGSGGAAVGH
HSSSNTLDTAAAAAAAAADTKRRSLQHNAASSSSSSSSSSSSSSSRSSGGSGRGLLADTEIFHSTQVQLHGGGTGSG
SGSDGDEQGLEGVYGLPPGMDPDDPDLDLIGAVVSPNCGLRLSFNATSVHLERYYRQVLRYSVMVTAVTIGQVVLVV
VQMEAAAAPSASARTSLYTVTLQAILDAYQCLLHLTGALVVDALFGAFASVAFLQFLLFAVFEMRQTLLVWRAQRAA
ANPAAAAATDYWSVRRDMSAVYTRFYGLLLLGLFLLFQLQSHVWVLALLLHSWWLPQIVHSAVTDTRPPLKAEYLWG
ITALRAVSPLYFWGCPANLLRITTRPGLCLALCVWLAAQAALVAAQIKWGPRVFVPKAFLPQRYDYHRAATRREMGL
DDGSSSSTGNGSSSTGNGSGSGPGAPSGAGAAALAGGRAVLHRLVAWLRPGGSSSSSSSSSSSSSSSSSSSSRAAP
RARRSDGVGGADGAGDVETGHGSKECVICMNPVALLPPRCRMVTPCGHFFHEPCLSRWIAVNSTCPTCRRPLPPP

>Cre08.g365100.t2.1|PACid:27563238
MARSSASRPTMGALALLLVASCFVGGVFSARMAKLALDDSASVSDASASSADQQQELESVVSALAHMNADSEAMRRV
LASGVYPPSPAPPSPSPSPPAPPSPSPPLPPGLALEAEHYVAGFSANATIVADLDVTSQASVDAFIAAFIQLTATAL
NIDPANVYVTRVTKGGASSGRRRAALEQSGAAERVWSASELSALLPGAGLSLLALKRVTPADDATVAGLRSAKLELV
LGPGAKLQQARRSALQQQPSYTADVIVDFTVVTLVEVALPPSPPPSPPLPPGVSAPPTAPVSPPSPPPRPPRPPPLN
VAVLTSALNASWVNEAPSPPPSPPPSPPSPSPPARVTSVRVSRMPAQGTLSGDSSKPVVWYNDASFRSYRKPRSPLN
LVFWSARRQCTATVGVCGGCPRAWAVPSNATQTTTTLPLYFREPMQLDSITITQLQNPGVLSVELLPWPATPIPELP
GVAPVSGPKGQPVYSAASDSTPCGGDLVISVPSDRSGSSESVPPRGSQSEVPPRLRRTAVGGIRITVKAQAKGAKPT
FISSVRFSGRVLYPANPAAYDGM

>g7389.t1|PACid:27563634
MATRLPLILLVIAALHAASAVHVPDTYARWALERNEDKQHRRLKSELVPDGIPSMESVKGTDLPIQGMPSRTTRRLL
QSSGSSFTAPPDLSVLTGGLDCIADPKANALYPVAVAPPGGSRVGNVLCVEVNRDVGACSAAGSACCTAMRVGGIQI
PSRSRCSAAVQSVEVDGAAWSSWGWEAGSASLRITGLNKVTGGTKTVCITLGQDSACSRPRRFCRDNGDGSGSTCMM
LVYNAGAGPSKAAAPNCCPLYDVDLRAAAVTDGVTSSPPPPSPTPPSPPPPPSPEPPSPPPPPSPQPPSPEPPSPQP
PSPLPPSPAPPAPPSPVPPSPVPPSPAPPYPPSPAPPYPPSPRPPVPPSPRPPSPRPPSPKPPSPAPPSPAPPSPAP
PSPAPPSPAPPSPAPPSPAPPSPAPPSPAPPSPVPPSPAPPSPTPPSPAPPSPTPPSPTPPSPALPSPSPLPPPLPL
PLAVPATVPYPPPYYTSVTSPPPVYGVYSAQPPMYGVYSAQPPVYGVYSPPPPYTASVYPPHYPASPPAAAVLPKPS
PRPSPFPLSPPPGSPPPSPPPPSPPPPSPAPSPSPVQAPPSPSPSLRPSPRPTAFPLSSPASPLPSASPPPSPAPPV
PPPPPSPQPPSPPPPPTPPSPVPSPPPSPPPPSPPPPSPPPPPPSPPPPDPPPPSPSPPPPDLPPPSPPPPSPPPPD
PPPPSPSPPPPDSPPPSPPPPSPPPPDPPPPSPPPPSPPPPDPPPPSPPPPDLPPPTTTKDRSPRPPIHPPQPPRPP
RPPARPADEAAPPDGMPPPDSAPPGDFAQPPPPPPPPPANFAVVPSRLATSFGGTYTTLLVNGKSTCRYQVTLSARL

LLGGGRPAGGRITGLALRLAAGANTELRFPSAKAIYRSLTVWMGAAAKRPADMTRAFDNNMVTNTRSLVHLARPFTL
EAGAFKTGASSWFWIAFSSGGYTYTGGDLVVEIVLSDVVGGAPKPVPFDAYPSGPDAAAAYAPNYKATSATGPANRD
VLAMLLPYTN

>Cre07.g332900.t1.3|PACid:27563847
MSSGLRRANSNSFEGQDSPPNCQRRCHEKIRNNCERSRSKAVTPGRALSCAAASEQATEPAPGLHTAYVSTQHTAVP
GAGGPGARVALSCGCMAGDTAACALPFLQLQQQSQPAGNGAHQSRLPVHIMPPGPARDHHNRKLSVARLPAQPSFDV
AQGAGVQGICVAGTPATQAQGLGAIAPVRNADCTSSATAQEEWAAVEPLLTASPHAGSTQAAVRGPKRPQLTTAAND
GADASGNDAGCAVSAGTAETECSADSGPLPAALRMPQQSNTCRGGHQPAQQREHELRRKRGGSLRSAASITRAGRTH
GSLGSLRPLVLLLLAAAASWLPPLLFLVLAPQPAAALDAPPYFCTPLAPQASAVGTTTCSLCDYGNTCTSLPESSLA
DMFYSDARLCTLPDVSDLVDLPVRKMGAGQICSGLQTVRSFALVNESDSSGASVGRVYVFMSHNGRLYITSRFSCGV
MFATDDTYLSEAGRQTAAAYVWRDGDAVTNTASYVDVLYGTGYYSCYTLSVDLKRVCDASRQARFNPSPNDPTLSDC
NCPMAGGASPTAPCDPVDLSTQGSVFVGLRLKLAYYGGSPLPPDGAGNLTDVCPRSSISSYVLKDDTTGNVGVQEFI
LPDCMYSPPPPPPPPRPPPPPPPPRPPPPPPSPPPPPPLPPPNRFAISINSPARLPSGLCPSLNNTFNYLLSDPFLKG
KYQALSTCAVSDSVTGPGSFVLLELYFYFPEASSYFEQVLVGMGNTTTPGSGQGVGGGANGRSRASELILFFTLNVV
CGTRMVVSSSGLSTSILPDINPPGTWPELVCKPPPAPPARPPSPPSPPPPSPPPPPPSPPPPPPSPPPLPPPNPPPL
PPQPQPPQPLPPSPPPAPYKFTVMYGNSSRLSLNTACNEVTYILEFTYKTLFARNFSAVTCSAAAGSDGPVLEATIL
FAVSNSSSDSRNMVYVFNPLNVEVLARRLQLPCGSRFLIRNEQGQINFIDGRQIPRLSCDAPPPPPTAPPPALPPPS
APPPGLPPPPDAPSGDQASAFSPPPPKKAPPRPPPKRPPSPLPPVLPEGLMPPPSEELPVLTPPRPPSPKPPRPPPR
VRPPPPTDGGTEQPQQPNESPPPPPPPPETPPPPRPPKRRPRPPSPKPPRPPRVRPPPPPPEAPPPPPPDAPPPDTP
PPPPSRSPRPPRPPPPMPSPPPPPSPPPRPPPRPPPPPDAAPPDDQASTSDAPPPMAPPPSPPPPPGPPPPSPKPPR
PPPPSPQPPPEPAPPGAPAISPSPPPPPPPNPPLPPPPPPRPPRPPLPPIPSNDTSAGRLLRMSVTTSRTDAFGPND
CRSFILIAQQSLGGRPLVSGFNCTVANPATSGAALTGLFEQVADAVSYFDTIATTSTASTLRAALGLPCGVSSVLFS
VNAPGVLPRLYNAANTANLRCDVAARKTAAPALPPLPPDWQH

>Cre07.g336600.t2.1|PACid:27563858
MGSLALLTYVWVVFVLHPARVADAAVTQGGADSGGARIGRLRLNTGGATGHDLSVSGQAASQSSRSLGATGAQASSS
GSSSSGASSGSSSGAATGGLGHLNVGYGRGLGAGLQHRGVGSAGGGVGSSASSGAVLTTSNQHLLFQTPSHPTKPQK
TAAAKASSPPPPPPDEDDVPEGPHPMRHYPYQDAKYRIWSLPKEDVSPRCRASGICDGKYDCGADGMGCITDALKRK
MKVRDAAAWTWKGYRQYAWGHDELNAGSRNSREWFNMGLTIVDSLDTLQILGLLEEYAEARYWVVNHLDLNQGDVSV
FETTIRILGGLAAAFYHSGGDEAFLMKAVEFADRLMPAFNTSSGLPQPHFSVHDKNVATYRGSAGSSCLAEIGTLSM
EFTAVSRLSGRPEFRDTAMKTWPIIKSMPSMDGLYCTMLNADLLSCNGNHYTFGASADSTYEYMLKQWVLTNGTDEM
CLDMYKKAIGGMRRHMLTELWMGPDIGEVWIAAENDAGASRNLVLEHLTCFLPGTMALGHMYGVNTGGAGEDDDLMV
AIKLMKACYELYHQTPSGVAWDSVHLVPRYSPPPSPNPPPPRPPSPPPASSPPPPPPRPSVDEVGGAGVGAGAGATY
RAGSGVTRTQRRLLEATAQEGQARKGDAYWDRDGDDGDDEDEPVEVTSTREAGVIDSDDGDDGADGVDGLGYVVLDA
EVRKQQQQQQQQQQQQQQKPHAEERRHLMAEASGGAGDGAKPPGWGKVKYEFAPRSRENFLRPEVAESLFYLWRAT
GDPIYREGGWNMFPAYERWCRVDSGGYQGAAITTTTTATTTTTTTTVINNVESVPPGPGNKMESFWIAETLKYFYL
LFSDDPNEIPLDEFVFNTEAHPLAIWGSATDRKLRKILERYHGAPPPPSPPPPSPPPPPPVTPVAGDATGGAGGAAL
GSGQQQALDHVTQVVHRARQLLAQQEAAERAQQLRSGGAGADVAAESLMDALTAARRRAAGHRLSSATLAGGGDASS
ERRRR

>g7574.t1|PACid:27563985
MQARSFFLLLAVAMAYVGMAAGQDCAASYLTPHLFTAADGSAGPQVVCSPKPNFISNSNLIDWSIGWNTAQLPAAFT
TSGFTVELPLYGPLLNSSSSSGSGSSDGSDGSSSSTAPQVQIRPGWYRLVFLPSDASVGVTVSAAILEGGPATDGGG
SSSSPGPGSSYYSSGYGRLHDGLAPTPAYAPLIYLSGGVAGLQLTVSVPPNAPPALSVAFRVESAANVVPACTAPAV
PLVIAGGTASLGSCLTITGGGGGGGRQPTSFSFSFQVFLSGLSASFKSKAVTAAMLPIPLPGGAAMPVGAYTVKLLL
TTKPAGAAAAATVTATLCDAGSGGGAAACGDGYSGRQLVYGGSSNIGTDSVGSWVAAASGIGGIGLAVVAAPGAPSE
IKVGIMIDAVSVGGGGIAWPYPPPPPPPPPPGNPSPPPPQQPTDPIPTPAPSPSRTPSPAPTPSNSSGPAGPGPGTA
DAPPGQAPQPAAPPPPQPEPATSPSPAEASSPPAVASPSPSPSPSPSPSPSPSPSPSPSPSPSPSPSPGLSTPDP
SSPAPAPSPGSSPPDVPPSPLPPQQSTAPPPDAAATPVPSPSPSPSPSPSPAGDPAAAASPPPPADVVSLPPSPADV
GSSPQPQSQPQPPSPPDSGAGGSQPSPPPAGDGAAATPPPTATDPASSPPPTGASAPPGTQAPPPLPRPLLLQPSSS
SSSAPPAPQELQGGQLQPPAGSADGTTPAAPAPPVPPLGQTGGGGGSGSGGNLVTDGGGATSGGSGGRGSSTSNTGG
GGGGSSSAALVPALATVVPISAVAAAGLALHWWRRSRVKWTMIA

>Cre07.g344000.t1.3|PACid:27564156
MRQQVESGGAETGSSEQDGSRDDGISVPRRQLQARRSRPPRTPRPPPNDIGSRSVSPPPPSPQPPSPPPPPSPTPPL
RRHRRRSPSPPPSDIEARGLDQPPPPTSPPPPPPPSPPPPAPPPPSPPPPSPPPPSPPPPLPPPPSPPPPSPPPPSP

PPPSPSPPSPPRPPRAPLPKPDVDYLGWTGPIDLTTLKTYDKTDPAIDAGLDIWIIAGQSNAVGENMQGSRPACCSP
VPGKLLTFNLGNNPTNQWRDATPCVGCISRGANPAFYDSCGPDLGFGRVLLQLGVSGRVGFVPTAAGGTNLADMWCP
GCPLYKDMAQTVVRAMRAAGPNARLRGMLWVQGESDANNDWNAAAYGARFAAFLAAVRQDFAPYMSYAGAPAGGLPV
VMAVMSTWKRADIFPYIQEVRDQQLSFNGTNVLKVDMANYGFYLQSMRNPYQPDQVWWDQAIHLTQQGACDMGGDMA
AAWAASGLQPW

>g8068.t2|PACid:27564161
MYSPKAKPQAWGLAALPAVLLLLLAATLPTSTSAAGVRGVGSWGSGVGVGVGSSWVRSLLQTDAFPFQDCKRPGLRC
DNSPWNLVGVRRTVKRPSRNVVYCLRLEAKACTSQQQCCTLLANRVEALSLSMATTGPCNATSGVVSVTAGGVQVER
SLVYGSDYVDGSAADVSLVVRGLGAAADASGGIASMEVCYEVRAACAAWTDVCRPGTPGAADNCRFAVNEADPSGDV
ACCNTCSANGLTFPAGGGTASPPPPPAVPPPDAPPGSPRLRRRPPPPPLQQQDESPPPPPSPPPPSPPPPSPPPPSP
PPPSPPPPDAPPPPPPDPPPPPPPPDPPPPFINLNSSPPPPPSPQPPPELPPAGPPPLSPPPPDPPPPPPPDAPPPSP
PPPPPPSPPPPSPPPPSPPPPDLPPPPPPEELPPPPSPTPRLPSLLPPFPPPPSPPPPSPPPPEAPPPSPPPPDTPP
PPSPPPPETPPPPSPPPPDASPPPSPPPPSPPPPSPPPPSPPPPSPPPPSPPPPSPPPPDPPPPSPPPPSPPPPSPL
PPSLPPPSPEPPSPEPLAPSPPSPPPPPPSPSPPSPPPPSPAPPSPVPPSPPPPSPLPPSPVPPSPAPPSPAPPSPM
PPSPAPPSPVPPSPAPPSPAPPSPSPPSPPPSPAPPSPVPPSPAPPSPAPPSPSPPSPPPSPAPPSPVPPSPAPPSP
APPSPSPPSPPPSPAPPSPVPPSPAPPSPAPPSPTPPSPPPSPAPPSPRPPSPPSPAPPSPRPPSPPSPAPPSPKPP
SPPSPPSPEPPSPAPPSPPSPAPPSPRPPSPPSPAPPSPKPPSPPSPPSPAPPSPQPPSPPSPAPPSPLVPSAPPPL
PPAYPPLAPAYPPSYPPSYPPSYPPSYPPAYSPAPGLSPPAYMAATPPEPPSPEPDTPPYPAGQKKPPSPPRPPSPP
RNPSPPKAPRQPPRPPRPPSPPPSPPPSPPRPPRAPRPPSPPMPDPPEPPSPPRPPRPPRPPPNPFPPPPSPKPPRP
PRPPPLPPSPAPPPQPPRPPSRPPRPPRPPSPMPPPTRPRRPLNPPRNPSPPRPPPRPPRPPSPPKPPNAPTRPPRP
PRPPSPPSPPSPAPRPPRPPSPPPQPPSPPSPEPPPLAPPNPPRPPRLPSPPPQPPRRPPKPPPMPPGGISDGGGDD
GYDSDPPSPSPMVEPPSPVTDGDEQDLEPPAGEVEPPPSPDVPAEDPDA

>Cre07.g356950.t1.3|PACid:27564209
MPLLATAKRRRHQQQQRVWALLLVAMLLIDGGGGGGRGRGRFFPTLAAAAGGDGGTPFTADVAAAAAAPAAADTPAA
AAAAAAAAAAGTAGTTAGVATPEAVAAALAAAAAGGDDYSGDGGDGGGDGGEDGAVRPMQCMSYTTDVSFPGGSPG
SVISTLLVQEQLGPTVGAVEVRDLAFTHPRVGALVIALYRLPYSHAPADTPYTAAQLASMSGGRRALLKGLQQGQGA
TGGGGGGGEGEGLGLGFSDAAGAVLAVDTPHAAGNLTGIYRPATPLSAFAAANATSAARWVLRLADLGEGWQSRTLQ
LDSWTLVLCPPPPPGTPANSTAPIALPPQLQIDMAQQAARDRIVNRGAATGAASAARLAALGGLPLSPAAGTAVNIT
ATTVFKSYSDLADDAVAAVIQRIDNIQELFGLPYTPPPGISVWLPWVELLIGGSEIILMNFVWPGKIVSYALQLEVT
VRTRMTMAAPNTTTTTTSNSGAGGSTGVPAPAAGGGTPAPAGGGTNSGAGGSTGVPAPAVGGGGTGGTGGTGDGGSG
GSGGGGGPPYNGPCSNDVSVYDRGDPFGYMPRPGAPAGTPPERVDKQAVLDALCDLSKGQSGAAMFGDSSGSSSSGS
STDMPSTRSSNDPTAAAAATTSRLTLYGAADGSLPDFEPSGRAAEMRRSLLGGAGGGGAGVLAASGTPLQPMDFTRV
DQPGLQLLGHLAGDPSDPSPSSDSGDDSGPTTRSSSASASAAPDPDAPDPGFLDSVSAMMAAALNPDEYAAALDGGL
QDLVDQADTAAAALGAAAGSPALRSAAGPDGVKAAEALQAGAQTYGHILRAGQASLKRLSIQQQKNQTRAVLQQNGP
LTAAERAFATRFVWNFNFVRINFRFAASDGLVTTLQQFVYQLSDVNTIISSARSLAALDQRQFGNASTQAAFQARDA
QLKRIQDQVSGAWLVDRIDNQTQAIKAKQQQQRQARMPRPPPRPPSPPPSPPRPSPPPPQPKPPPLPASPPPTSSPP
NNNNNNNNNKKPLHGRGGHVDDMGGSGRRSLQEVRRLLQAAEEGSSGIGSQEVAEQEQVAEQEVAVEEEVAEEVAGA
GADAGVDADSAGTGAAGAVGGGDEEEDDPLLPTGETVDAWLARITAEAEAEAALAFRRIPHDRSTGLDRVMGLRRSA
A

>Cre07.g321400.t1.3|PACid:27564322
MLGVAMGRRLFSPWLGHMLVVALVAFAFSANAARVSMEMSTSDATETTLFRRRLNQASPKASPRPPSPKPPSPSPPS
PKPPSPRPPSPPSPPSPPPPSPPPNPPVPPASFRMDKGKGGFTFFSSDDADDAVHCQGTRCGALYSNVISMAISFAP
ADADGILAVGTFKANTQARNSIEGWVASANYPVSKITYATTAAAVTNANLSSYKMLYIPSNDLNTPGGISSTLNKAL
ISIKADIINYVNKRGGSMVVLTQAGFGKDSYGFLPVPLNFTALDFQDADVTEDMMQISPVTNSDNVDHVYWHGYWTG
PIDWNGLRVLATQARLCPEPYGPNQNCRATVLCNQNTILTAENCYDGIDNDGDGLIDKLDEDCWRCGDGILDPDEQC
DDGNVLDGDGCSSTCQLQDMPPPSISPSPPPPPPTNGREVDYCYAGGLASCPPCLGKCSTVDNFGTDGIICKVPQYL
KGSYPDSDVCKNNRGVLALQTIAVTNQDTSGNTVTVGKATVFRTSSGMLYATVQMTCPYLLWSPKTDSSGVDTNSFL
LLSYVLNGQAGTAVSSLTRSPSSSWATFACYTFQFNVDALQPGGLGCTPFDIDARLTVRSVFARDSSSSSTCNMDTE
KVSTTPTVSTKESFVAVDPAPPSPPPSPPPPSPPPPRPPRPPSPLPPSPSPPDAPPPSPEPPSPEPPSPPSPQPPSP
PPPSPPPPSPPPPSPPPPNPPPPSPPPPSPPPPSPPPPSPRPPSPPPPSPPPPSPPPPSPPPPSPPPPSPPPPSPPP
PSPPPPSPPPPSPLPPSPLPPSPSPPLPPPPSPSPPPPSPRPPSPPPPPNPPVPPSPPPEPIPPSPPIPPSPPPSPT
PPSPLPPSPEPPSPEPPSPPPPSPPPPSPPPPSPPPPSPPPPSPPPPSPPPPSPSPPSPPPPSPPPPSPPPPSPPPP
PPPSPDPPSPPPPSPPPPSPPPPSPPPPSPPPPSPPPPSPPPPSPEPPSPPPPSPPPPPPPPPPPSPPPPDPPPPSP
DPPPPSPPPPSPPPSPPPPSPRPPAPPPTPNCDCSKLVRAPAGGCGSAQAMVSFKAGDRYDTTIPSVDICVPTFGFD

PVTGIYKIAFCWNTDCESLRDLQGKPIRAYINNLDTTPGTGPDGLAGTNVVLTALAPECARSTNIITIKDTGATYRF
GATELLVTVPAPIATLDVDVAINNTAQCNDHWVVTALVSVSAPLGTTPPIPPASCPCWMRTTNGTCPGIKVPILLET
IDDLGNNRGQSVMQCIDPANFDIYTGRMAYSYCWRYDCLPPVFATSRFKATCSNLERWNIAKMTGKFLLNMQHSEGI
YMSVTGHPKDPTQQPVMWTIADDQQWIDLPEGGVGNFTFEYIIPPGQDDLSAAVVMDTSPRPPPPPVPPGPPGSSCS
CPLTPEETVGGEPGVCNGTYATMTVRLESDPTVTYKQCVSWPNYDLIMQEMAWSHCWRYDPCLPKAFYGQAYRVSIN
EVSKHNIPLIPPAGYELLHFVAPFGMSIETTFKYVNGDVVVYNSTQGSLVIQGRKNVITVPTQISNITIEFKIPAVW
GIDGMAAAVVLRHIDGTDFPFPPSPPSPPPSPPAPPPLPASAARVLITTDFLVMANVPITALLKSDNVALTGYYVTI
PFKFYDYLDMPDCSDSNRDAFKAKVAAAFNVSDPSTITVSCRFNVPTSDQPILRRMMRAVSRLVQTVTTGTTPADVA
VETSVTVKLPVDFPAAVASGCAKTEGLLASDVSCDTGSVTTKPSVTVSMSKSESVIAGGADPCTGAVSESANLLSAL
ARDAGSSVNSNTLRSTGCKSQRVAGTTSDPNAPGATPVPGGAPGTAPTPGPSDSPSSASLSSGAIAGIVIGAVAGAV
LIGFVGIAVRNKMSQRSPVTMVGRDGTTRTTTPRGWARGRWMTYSMQEKAEQARSGSAPVAGVSTY

>Cre07.g346900.t1.3|PACid:27564496
MAWYLAFVALSAFALVSSVMGHGGGLHDWTFLPEPSPGREFIRHQVASCSSFAPVPVSANLGPLPAFPSPASSIQVV
SGSASIFYYLDGNCTDRATTSIDMDVNITLNGQPYAGRQVNGYGFIGNCSNPNDLYRHNASRGDDRFNMVWFKKENS
NTTEDGNLFGAAPNVFISRVPPAADALRSLVLYPSPEDSVPICCDLVYNPPPPADFFTQGTFCRTPAPPPPSPPSPP
NSPPPATRRASPPPRRTPPRKQLPK

>g7805.t1|PACid:27564561
MIGAAAYDTNRCNERQRQPWRQPYGARSGRHRSALVAALLFMVLLMRVGSSSGTMAGLPRQSRKREDVLEAVPGDMQ
RVSVVSTYGQRAQKPRIHDRRLSNSLAPPSPEPPSPGPPSPEPPSPEPPSPDPVPPSPEPPSPAPPSPPPSPEPPSP
APPSPVPPSPEPPSPGPPSPPPSPAPPSPEPPSPEPPSPAPPSPPPSPEPPSPVPPSPEPPSPAPPSPPPSPEPPSP
APPSPVPPSPEPPSPGPPSPPPSPAPPSPEPPSPEPPSPEPPSPPPSPEPPSPAPPSPPPSPEPPSPVPPSPEPPSP
APPSPPPSPEPSSPAPPSPVPPSPEPPSPGPPSPPPSPAPPSPEPPSPEPPSPAPPSPPPSPEPPSPEPPSPPPSPE
PPSPVPPSPEPPSPAPPSPPPSPEPPSPAPPSPVPPSPEPPSPGPPSPPPSPAPPSPEPPSPEPPSPAPPSPPPSPE
PPSPAPPSPEPPSPAPPSPPPSPEPPSPAPPSPVPPSPEPPSPGPPSPPPSPAPPSPEPPSPEPPSPEPPSPPPSPE
PPSPAPPSPPPSPEPPSPVPPSPEPPSPAPPSPPPSPEPSSPAPPSPVPPSPEPPSPGPPSPPPSPAPPSPEPPSPE
PPSPAPPSPPPSPEPPSPEPPSPPPSPEPPSPVPPSPEPPSPAPPSPPPSPEPPSPAPPSPVPPSPEPPSPGPPSPP
PSPAPPSPEPPSPEPPSPAPPSPPPSPEPPSPAPPSPEPPSPAPPSPPPSPEPPSPAPPSPVPPSPEPPSPGPPSPP
PSPAPPSPEPPSPEPPSPAPPSPPPSPEPPSPEPPSPPPSPEPPSPVPPSPEPPSPAPPSPPPSPEPPSPAPPSPAP
PSPEPPSPGPPSPPPSPAPPSPEPPSPEPPSPEPSRASKSRAAIPGATITRSAIPGATIACTAESCTIQPGPSKPGS
TQSGATKPGATCPFTPEPGTSEPASTVTPQPGAAIAGAAQSITTQSSTAESITAKPPTSEPCSAKP

>g11753.t1|PACid:27564902
MGRFRRAAGALLLLAFGLSLIPSGYAQDGLPVDWGQDSTAALATFPYCQCNDYRCKASPYRLRLGEVGPSGTSGKLD
KICYNIEMPGCSDATNQCCKIIGQRLDKIEFEVQEVCQKAVRQVTLNGQARTFAFLTQYNTGVLSVRALAQLNGGAN
GSQLCIHVNNTAAPTCNTPAALCQRGDGTCRYTMFESSSHSCCATCIEGLYPSPPPVTPAVRRPPPSSPPPPPSPPP
PPPRSAAAAYADDLAALTNSLDDLQVQCDKIASYAEWASLRVNHTKCATTAIWHDKSPIQXXXXXXXXXXXXXXXXX
XXXXXXXXXXXXXXXXXPPPPPPPPPPPPPPPLPPFPAKPPMSPCDCLSPLIDNHSRWRFRFQESTAIGSDNVFIFT
LSVNPDAKCSPITYRPGDCCNQALDGVSLALDPVYAGLVKSVKVYTYDGNELDVRFTQTAEFGLAMTLPTMLHTSDM
PTNTFYTFEIGFAQSDWADVTKMPCRPSQYEPTLPACDYWVNGWQTQPGNPAVVLPPDERTAGCCPEGVVSFCSEKI
KGTCNPRLSDSPFRLSYVNTSSLVGVRTSSVAFSLTSQAVTNAIGPMEGLPDCSNMAALNGVKIYITDAAAAKVAQV
ALNGAAVTYAVKKDAGGSYVDATVPGNRVGTGNWVITLNDRFNGTDVCGYKVGSYSVCNYVLNGKSGQCCTMGDMPV
ATLTTPMVKFG

>g11583.t1|PACid:27565033
MPPNCCGSSLGGRPLIAPVLLAALFLLGTHGHDHISLSDESDGTMGRSLQYESTTAAAVTDPAAAEAAGVIAAGALE
RRRLSSRSVSVSERLRLRWLQLRGAAAHSSSAAATSGAAGSAGPRRHLATGLGWVTDQFGLEPPQCSISTAWNRSPL
SLALERPDASTTCFVITVAAASNASSSAPCTGGAKACCDSSRRAVDQLSVLADPTCGDASIAGVTVDGAVWTNYHFE
NQTNVFTVSGLSSGGVSAISSPPFPAPVPSPSPPSPLPGKSGGGGRGGGGSGRGERDSGSAASPAPSPSPSGGVVSE
TRSASVGIQPPVYGSDGTDPAAAAERQVRVCITLSTRTRRCNVLRDFCRDVQGSSPTECGVMVRDEQQCCPVTRVP
YGDVPLDQPYTFLLQGRFTGSGITCEVLRLSPQAGTTILDALALAYVVGLGLPQSDDVRFLSYTCVSNGPGPQYDNL
LNAVGTVNLRGLDPRVIYRQSASILNATASRYALSITGAVPYTVPPPSPPAPPPSPPTPSPPPPPSPPPPPPPRPPM
PPLPPAPPSVPAPPILALLDFNFVSSDPGAAQALACNRVFARPRTGRTLTDSLTSSYETALDLDPELDTLTVDGLSC
ADFSNKGGKGNSTGNGNGNGNGNGNSNGNGNGNGNGNGGDTQSPPVYGGDNETETAGLPNRSKQLRLQVLLRLKLGV
NDTSVTVAARLRLIIASLNSSALASEAVLPSVLGFITERVEVQGGGRNVSIFATVVVVSVTLAAPSPPPPGPPSTVP
TSPSPAPTYAPTYPPPAYSLSPNPPAAYPPAPPPSPPPPPPPRPPRPPLSPSPSPPAPPTYPPPPPPYPPAPAPAYPP

PPYPGPPRPPMPAYLPPFPPPPSPPPPSPPPPFNSTPASPPPLPAPPPPSPSALLPPPPPTTSAPPPQSSPPPPPAT
ASASPAPPASPPPPPLYPVVPLKPPPAAPVTQPPAPPPVYVPCYLLPPSASPPPPPYYNHQGGNYGNYGNYGNYGAQ
PPPSYYGMPWGQPQAPPPYYGAAHQPPPPYYGAAHQPPPPYYGGGYGGYGGYGGYGYGGGYGYGGGYGSPPIVLCYV
PPPPYGGYSQPPSPPPYYAAPPGYYGSGPPGYGYGGYGAEPPAYGGY

>g11460.t1|PACid:27565130
MARLGRRALSVDVVTQRCSQGAFFRPLFRVVIVLLSILSLANSLPNYALNGRPCTHHPDTLVKAATGTTVHNAVVSD
LATVFTVLTGTDSNSPVAVSLCRGKLYTLQVAFPAARRALLTATEGGISGSVTPLCPNRVALGGATGTDAGVAPSYS
LLYRVPCSTAGSSVTFQITSAAPSAPTLLLGASASLPLDAACSSTLCGSSSGGSSGGDGGAVRDAAASSPPPPPPVR
GGAQGAVIPAMPGSIASWRTGQDLAYVAVRVSRQEAEAACAARGSGTRLASTAEWGAALGDMLGGPLGGLMVDVMVS
LYDFGDNGGRPWHVWTDAGSASGSTCGGAVSLDTSTMTLARTTSNAICAQTLFYMCIGPTGVYAAGTATATKSPPPP
PRTLLPPPPRRPPPPPPMTPTPTTSVSGGVSQTIGTRTYTVFGTRVTFATAQSACDQLGGARLASVSDWGDISGLIV
AGEARVLWDSLSQSMFGFGSYGDKPWNEWASGGGGPGKGGCGAARMDPMALVLTDSSLTASCSQTLMFVCVYEPGAA
TKSPPPPSPPPPAPPPPPPLPKGVPVKVSPPPPPRPPPPSPPSPKPPKKKPPPPPSPKPPPPPRIQPPPPSPPPPS
PPPPPTRPASFKSPPPSPPPQPSPPPPHPRPPNTRKQSPSPPPPRPRLPKVRSPPHPPPPSPPPRPPPPPSPPPSPA
SCDASSAGYACSMALGESMRLHWSPANLATAASAAASTPPPASFCAAVYGSGTRGSVHIMLEAAGADRLSLIFPAAA
AGGLSPGITAAITDDGSILTSTFRIKTTPGPSDTVALPYTVPAGWGARLRPPGTSLPPPPPPSPPPPVNFDPAVFGP
GPWPLGLPRAINWTEGRAAVLLPPNAADIEAAGNGVGVYRLALCFTAASGAPTGIRGLDSYLASLVPADPSVLAARL
AATAVLGRRRLQQQEEQQQEEALVTGEASGVLIAGAVVVGGQRRLAAAEDAPPPEGEVVFSPPPPPRPPPPPSPPGQ
AVFIGNAVAAQLGAGGRRVLLLRQAGIESTCETSCAALGPRWSLGPSAAPGYLLQGTEYTAAWAVLLQQIPPRPDAL
AKGLVVGAWLGKAAATYVPRNPDVADGPAATSPCTGLRWGDGSLRSTPCESYGWCMCWSA

>g11953.t1|PACid:27565165
MLALMAFSHALAQPGGKPRRACDTTPEPCHREGTVCVDTPGQQPGYRCVCDTENGYVNNAAEGSNGQACKKRDVVQQ
SMPPPSPAPPSPAPPSPAPPSPVPRSPPPSPAPPSPAPPSPAPPSPEPGSPPPSPAPPSPQPPSPAPPSPEPGSPPP
SPAPPSPQPPSPAPPSPEPPSPAPPSPEPGSPPPSPAPPSPKPPSPAPPSPEPPSPAPPSPQPPSPAPPSPKPGSPP
PSPAPPSPEPPSPSPPSPQPPSPAPPSPEPGSPPPSPAPPSPEPPSPAPPSPQPPSPAPPSPEPGSPPPSPAPPSPE
PPSPSPPSPQPPSPAPPSPEPGSPPPSPAPPSPEPPSPAPPSPQPPSPAPPSPEPGSPPPSPAPPSPQPPSPAPPSP
EPGSPPPSPAPPSPQPPSPAPPSPEPGSPPPSPAPPSPKPPSPAPPSPEPGSPPPSPAPPRPEPPSPAPPSPEPGSP
PPSPAPPSPQPPSPAPPSPEPGSPPPSPAPPRPEPPSPAPPSPQPPSPAPPSPDPGSPPPSPAPPSPQPPSPAPPSP
EPGSPPPSPAPPRPEPPSPAPPSPQPPSPAPPSPDPGSPPPSPAPPSPQPPSPAPPSPEPGSPPPSPAPPRPEPPSP
APPSPQPPSPAPPSPDPGSPPPSPVPPSPQPPSPAPPSPQPPSPAPPSPEPGSPPPSPAPPSPKPPSPAPPSPEPGS
PPPSPAPPSPKPPSPAPPGPEPPSPAPPSPKPPSPAPPSPEPGSPPPSPAPPSPQPPSPAPPSPQPPSPAPPSPEPG
SPSPSPAPPSPQPPSPAPPSPQPPSPAPPSPEPGSPPPSPAPPSPQPPSPAPPSPKPPSPAPPSPEPGSPPPSPAPP
SPQPPSPAPPSPQPPSPAPPSPAPGSPPPSPAPPSPQPPLPAPPSPQPPSPAPPSPAPGSPPPSPAPPSPKPPSPRP
PSPAPPSPAPPSPRPPSPAPPSPPSPRPPSPRPPSPSPPSPAPPTGMNAIYSLNTSTCIGYGSNAPYLLSCNTNNAG
AYNALVTPVGANNLFTIRANSDTSKCLSATDNSASAGTASFVPQMLPCVPGADSQQFFVNVTQFGLWTLSPKNNRTR
CLKPAANGTLTSMTLADCSGNPATESVGFTMSFTPAQPPACTLPALWCSYATQRLIGPLDCNGDGVLDWACYSPSTG
KRWLLLSNPSPGLTCLTTSGYAYGAANNPPNGGDPAFLSNCPAAFNLPPLPPSPSPPFPPPLPPSPGPIPPSPAPPK
PPSPQPPSPAPPVGFQRIFHANSSQCVRCNNVRTGLEACNPTFLFAYYGVLVTPVGANNLYTIQYNSDVSRCVTAEE
SAPYFQTYLTTCVPGADNQKFFVNQTQNGLWTISAKELGRRRCWQPPPANASIQNIIYMYDCEGDLAKEGFGFALPF
APAQPPVCSVPAAWCALPTQQLVGPVDCNGDGVLDWACISAGTNQRWLLLSSAANPAATCLATSATALGAAANPPTG
GTVPLSACPAAFTAPPPSPPLPPSPRPPSPPSPRPPSPPLPPSPGAATQAVAAIAAVAAVAAPTLTQATQPG

>g11998.t1|PACid:27565167
MAAAPPMFILVLCLAAVANACNPFTLETSRIGPAHRRSVIEKSLSSAVQASSYISRRSLAANPAANKAPPSPPAAVC
TCLPETSVCGCSPYRLSGPVVTPLSPNVFPFFTGTRFCFTIQSVGCGITPLPCCITAATAPLEQVLLNVGKQCTGRF
VDYTVNGQSWLDSGVDGPYPKSSVVYFNNLGLSLLTAAGTQLCVATTDQVGLGAPCEKVKTLCDDPRDGNCRYMFRE
GDNATVCPACGTVPEPLPPPSPRPPSPHPPVPPSPLPPPSPLPPSPLPPHPPSPTPPSPPPSPVPPSPTPPSPAPPS
PAPPSPAPPSPPPSPVPPSPAPPSPAPPSPAPPSPPPSPVPPSPAPPSPAPPSPAPPSPPPSPVPPSPAPPSPAPPS
PAPPSPPPSPVPPSPAPPSPAPPSPAPPSPPPSPVPPSPAPPSPAPPSPAPPSPAPSPMPPSPAPPSPAPPSPAPPS
PPPSPVPPSPAPPSPAPPSPAPPSPPPSPVPPSPAPPSPPPSPLPPSPAPPSPAPPSPAPPYPPPSPVPPSPAPPSP
APPSPAPPSPPPSPVPPSPTPPSPAPPSPAPPSPPPSPVPPSPAPPSPAPPSPAPPSPPPSPVPPSPAPPSPAPPSP
APPSPPPSPVPPYPAPPSPAPPSPEPPSPAPPYPPSPAPPYPAPSYPPSPAPFYPPSPAPSYPPSPAPSYPPSPAPS
YPPSPAPSYPPSPAPSPPAPHPPSPAPSPKPPSPLPPSPTPPLPPSLKPPSPAPPSPVLAPEPPSPEPLTPSPRAPK
PPRPPPNPPILQTHGCRTAVSGQPYSLSPLAQVASGVQPDVPADEEQSDSPPMLPPGPWATYCTTVSLRWGFTAGRC

SPGEPSSQCCGMNFEKLETLIDGACRRSIFNMTVNGVFRERIISPYSQWNAVSFKWTAMDLSPTQASSTTFCFTAVG
SCADLSVLALQGRLQAAVFDPTHTCCPTFFLYTGRHRRLG

>Cre11.g481750.t1.2|PACid:27565263
MRSQSSRALVGGALLILLSGVLVFAAPTMADSGLRGRALLQKKKASPSPARSPTPSATTTATSPAPKLVFPPLCNCE
RNPRKSPFRMSLDDAASAGGVYCFTIQNVGGCDPKQKCCDGTQGVSKVELDVVASCKDSVRNVTVDGKKWSYEFNTA
LSVIRITNLGKTASTAAGTEVCIALAPKSQCPGLAQLCSAGAGLCKYALSNQDRDCCPVSILGNAPPPPPSPQPPSP
QPPSPSPPPPPSPAPPSPPPPSPLPPSPPPPPPPSPPPPPPPPPPPPPPPPPPSPSPPPPELPPAQPVTPARKRPPP
PAPPPPPRSDFPFCQCQRNARGSRLMTTASNNITVVNGLTRICFNVALKDVCENPNSKCCEFELYKMEFEADPTCAD
ALAYTTVDGAYKAKFFQTRPYTVIKVTNIEKPINKVAGTEVCLFMRPQCNSLEKLCSFHDGTCTVALFNKPGSSATS
CCPLTTVGPL

>g11602.t1|PACid:27565407
MPVILLALLFVSLTSGADDTQYYSQITRSLSTRRRVLRAQPDLPAVNDVAGIPGGPTRARQLPKPTDPALFGVGTFE
SPTDVGTAAAVEPPSLPPPLLRGTSAPDSQQQTADATTMQTRQAAAPLVSPVKATGNTKAKTAVAARRPASRRPPRP
PPKPPAPPLPPDQPSLPDGSIAPPSSPPDTPPPDTPDQPSPPSPSPQRREPSPPRPAQVLIDGWWITGITDGNSGSD
SGSNGAGDTISPPPPSPRPPIPRRLASGGGLGIGVERGEVGSPPVAARPPPPSPQPSPPPPPSPPPPPPPPPSPQPP
PPTPPLPPRAPRPPPAPSLIDIQIILDAHNAARALAGVRPLSWDAGLAASAQSWSSQCTWQHSSGSYGENLASGAYR
TSKDPLNGIGIWYNEVCEYDFSKPGFASATGHYTQMVWADTRTVGCGYTACPDGVMGLGAKTGVLVCQYWPPGNLDS
ASKFRDNVPPPRTFPDRCPNGYGSSAGRR

>Cre11.g483250.t1.3|PACid:27565449
MAQHPLLLSAFVTTLVVASITVGYACNPFTLETSIAGRRLSEGGYVRINDARRRAQAAPCTCLPETSVCDCAPYRLS
EPVVTDLDPDVDLTTGSRFCFTLEVVGCGSSQLPCCTNLAAQPLQQVLISVANGCSGRFVDFTVNGEFYPDASVDGP
YSKSSQVIFRNLNLDLASAAGTEFCVSTSDQAGAGPACSTVSQLCDDPAGNCKYLFREADDATVCPTCGTLSLASPP
PAQLPSPPPPSPEPPSPPPPPSPEPPSPSPPPSPEPPSPPPPPSPEPLSPPPPPSPEPPSPPPPPSPEPLSPPPPPS
PEPPLPPPPPSPEPPSPPPPPSPEPPSPPPPPSPEPPSPPPPPSPEPPSPPPPPSPEPSSPPPPPSPEPPSPPPPPS
PEPSSPPPPPSPEPPSPPPPPSPEPLSPPPPPSPEPPSPPPPPSPEPSSPPPPPSPEPPSPPPPPSPEPLSPPPPPS
PEPPSPPPPPSPEPPSPPPPPSSEPPSPPPPPSPEPSSPPTPPSPVPPSPPPPPSPEPLSPPPPPSPEPLSPPPPPS
PEPLSPPPPPSPEPLSPPPPPSPEPLSPPPPPSPEPPSPPPPPSPDPPPPSPPPSPPPPSPAPPSPAPKLLRTHNCR
TSTGEDQPYLLSPLTRVASSASSAQAASAWATYCVIVSTRDAADPSGCPPSDAATPVGRCCGMTMDKLEFIVAPGCR
FKVTKLKVNGQSRATSTSHYPQFDAASFKWTSLGLTPAEASSTTFCFTAVGSCADLSVLALQGRLQASIFDASRSEC
CPTFSLLEGGRRRTRRA

>Cre11.g481600.t1.1|PACid:27565488
MIRGSPRLYGGVLLVMVAAAMLLAAPASADSEYFGLRGGGRVLHQKKAKAKAAPTPSPAATSPAPKLVFPPLCNCER
NPRKSPFRMSLDDAASAGGVYCFTIQNVGGCDPTQKCCDGTQGVSKVELDVVASCKDSVRNVTVDGKKWSYEFNTAL
SVIRITNLGKTASTAAGTEVCLALAPKSQCPGLAQLCSAGAGLCKYALFNQDRDCCPVSILGNAPPPPPSPSPPSPP
SPSPPPPPSPPPPPPPTPSPPPPELPPAQPDAPARKRPPPPASPPPPRSDFPFCQCQRNARGSRLMTTASNNITVVN
GLTRICFNVALKDVCENPNSKCCEFELYKMEIEADPTCADALAYTSVDGATRAKFFQTRPYPVIKITNIDKPANTVD
GTEVCLFMSSKCNTLEKLCSFHDGTCTVALFNKPGSSATSCCPLTTVS

>Cre14.g631150.t2.1|PACid:27565524
MIASLSFTNSWGESWGDGGYMQIVRQGSSQLAARLNKCGILNSLSYPTLKPVSPERRGQLLRQGWCAAEDVVVPADG
GGNATATTLAAKHDVAINDLIRVNTHFKADPDALIEVNTRYFVPPCSVNPPRPPVPDLECGTRAVLQLLGDGADGSR
PRIQLGQPTTPATASPPPPPSQTPADRGGAEERFTATCPSGAHMIGFRGRAWAGQPEVESCVLAALAGQQSNPASSI
LYNQLVNLRDRIVNSILEQAAVSADPAWNGLFTSVDLVCSDGSVRPTGRGTSDIGGYDLQAGDSIPVDTTARDCPGG
YDMARSRLRRAPYERFEFFLRCAASQQWTSYPAGLLAARRAPGITTSSLTIARYRFSSTDAGASTPGTAGTAGLSVT
EALLLTAIRNSCGPVLAAPPVGVPDTLLQFNESAPQTLRPPTSTESARLAVPSLLLPAYRSAEHLVYFQRQARLEVL
GGVGGGGGCPPGQVVVGLRGRRLALQPSSLFFNGSAEWKYGSPLPFRFRYAPDYTSLSAVDELVCGRTGTSTVMPKA
PQPKVASGSSGVLATAVPGGVPGSGPAAGAGLVKITPLTCGASALLTSVFAANDGVDINLLGARCSDGSAAGSAGGF
GATANGSISGRFTVDYLCPEGFDAVKLMDGSTWDGSSNGSGVGGVALRCSAAAKAQSDERYWVQLGAPNFLRATDSS
PAFTLSEAFLNQPEYSVCAAGSVITSLNVHTATVGVASVVVKLEFICGPRPPPASYASLSDIAAGYGVTVSDLARAN
PALFPSGSASAAAGSVDPALNNTEIRIPLMCGPAPVQPPATTIAAGCPKYWPLPNTTNAGTTTCGMVARSYVSGDLA
YLSRINNNICPNASTTVAKGQRLCIAPQSSVLSTSQHSFEATASTTTVTTSAGHRRRLTATAAAFASAGVGAVGGFV
INNTIVTCILSQWVAVGQSCEDIAVAYNLPISTFLAYNPDLACATLQAGAEVCVRTGPLLPETVQIVSLKACAVRPG
VCGDGTCHDDDSSPVGFTCTCAADALFIQGRCFGTDPPLPPPPPPPSPRPPPPPSPSPPPPPSPPPPSPPPRPSPPP

PPSPPPPPPPSPPPPPPPSPPPRPLPPPPPSPPPPRPPPSPPPSPPPPRPPPPPPPSPPPPPPPRPPPSPPPPIWTP
SPSTDFATSPVFCGSDSFASSYVYPPTSDESMIGLPISSMTIWSSSQLNGLQLRFGSSLQAGPQHGSASGTVVTQSL
QGVTVTQVHACCSAAAYQGVARVQLLTAAHGVFTQGVGCDVNNTWVPVPAGYRFAGVRTWTSSSLLSTLSRSSGEQF
IHRIQFLFAPTAYTPSPSPTGYTPSPTGYTPTSYTPSPTLTPALAPRATSLSPAVCGGSPSYQLTSSDVSLAQFYTL
DRIGYRWGQVLGQIRSYYDNDRQVGPLHGDSHNGGPGEFLITPDLNIVAARACCIVNTFYDNSVRYIELYTSAGRTL
VMGDNTTGCEAMQAWWSVPSGYRFAGFVTSTNPAKYVQRLQFVAAAASAVPSSPPPPSPPPPSPPPTSSPSMFSCVD
QVATCQAWSQQGYCDSQYTYNGQSVHDVVCAKSCNIIACVPQNPIGCYGDGLPRALPSLLSGNTSRPDMTVELCDTL
ARSADMPYFGVQFGGFCFGGSNLTAALDMYGPMTDCTMQCSGDDTQYCGGSWEQNLYFTYPEDVCQSNPCAEAGVTD
ATCTAVSGGVGLSAYDCNCPANSVRLDAAAACTCEEGYYLYFGSCY

>Cre14.g609900.t1.1|PACid:27565684
MLRHWSLLALLALLAWRGAEAYPRFWYDRNYLVEYGYGPENGLDWSVFPALTCTNYPDKAYNGFYPAHGTPTPDNTI
GMTFALSGGPAVTTLCPGATHTMRLTFPIRRLALVTTDPPNVAAFTSPAPPTPDCPGRLDLGNSLTSGAATSFTTTF
TVPCTAKGTSVAFHVVSAGTSGTTRRWATAQATMPVSADGSGVCAAVSAACAPVASPPPPSPPPPSPPPPSPPPPSP
SPPLPPSPPPNLSPRPPPSPPSPPSPPSPSPPPSPLPPSPPPPSPPPPSPSPPSPPPPPPPTPTTDDSTGDNGPDLS
NSDVTSPPPPPASNASAGSCTPSPLGYACMSTQGDVTLHWTVNTPSAPPNACTGTDSAGKTVLAAGDVATDGTIHMA
VQAATSGYVAIGFSPNPGTMGPSDIVLGWAGSSGSSIRAFYVTGEDMDDSNALSGTRDWAYDKGVMESGGVTTICFS
RRLREPRALASSDLRAAGGLTSAASGASGSSSRRRLAQALPQMGFNWATFPQDRLVQHLSRTVGGFSLDVASGAADV
VAVDKGYWVNVHGALMAVAWALLLPLGTFLPAHRWVLRDVRGPAGKHLWFLLHVGCQYVGISLFVAGFVIAYVKLDN
GGVVVGGKAGSAHAPIGIAVMAAAGAQMVVGHVRLDPTHRRRWLWNLVHHNLGRCTVLLAWANVYIGIYMAHTGYQA
SYAAWAAPIAAVMGVLVLADLVLRIAAPAAPATAYKAEADPGIKAGGAHMHPQEQGVSQYGNSAYPLSPNQGHTNKL
SKQKSSSNAAHHGFQGEEEDAAAAGGNGGERFTGRYSPGGSSGGGAGDTAAHVNQLYGGTNKV

>Cre14.g609700.t1.3|PACid:27565732
MARPLVAFLTALLAWRGAEAYPRFWYDRNYLVEYGYGPENGLDWSVFPALTCINYPDKAYTGFYPAHSTPYTDSTIG
MTFALSGGPAVTTLCPGATHTMQLTFPIRRLALVTTDPPNVAAFTSPAPPTPDCPGRLDLGNSDGSRAATSFTTTFT
VPCTATGQVVFAVTSAGTSGSTRRWAQAQATLAVADAASAQCAAVSTTCVAAVPDPSPVAEPSPEPAVDPAPSPAPK
PEPSPSPAPGPEPSPVPMPQPAPASQPSPSPAGAPNCTPSPLGYTCMAAAGDVTLHWTVNTPSAPPNACTGTDSAGK
TVLAAGDVATDGTLHMAVQAATSGYVAISFPVRPGTMAPADIVLGWAGSSGSSIRAFYTRYQALENGDALSGTRDWA
YDKGVMESGGVTTICFSRRLREPRALASPDLRAAGGLNSAASPLGPAPQMGFNWATNPQDRLVQHLARTRGGFSLDI
AAAAAAAAPAGGGGPVPSAPSPATTGSAPSPSPSNDGESAESPKPSPSPPPSPSPSPAPSPAPQPNQTLPSAAGPSP
PPPSPSPSPSPSPSPSPSLGPKPATTPPTGGSSSSGSPRPAAAAAARRRPPPRRSPPPRAWRLAPRKPHRTRRAAV

>Cre14.g621700.t1.3|PACid:27565742
MARGQDSDLNLERWEGTLMMMTGEMAGEEGDDHTSTDIIYLLQVTEPAFSLFELHIPHALLHSPLGSGDPVMVTGYR
QGEDAIVAVSIASRMAAFGGSSTEGVAGRHRLMMEQVDPNAALAAAAAAVAAAAADAAAAVVAAATRPRGLQAVGAA
GGDGPTGAARAAAAATALRNTRVVRDMPVLYMVVGMCGFGPSISVQDLESVLFAGPNNRGPTLEAYYAECSRGKTAM
NRGNSLVVGPVQLPCNFNSSAYRFSTSTCSYRDYWGWHTWAQQYAASALGVDLTRYRHRVLVMPRGFMYEAGCGWTG
LGTLGPVDKGPDGLQYVSSMVWIQGESSSSVMAYMHELGHNLYLHHANDSKGCEYCDYSSLMGGCCALRCFNAPNNW
QLGWGQPLAVLNSATWPQHSWLYFDLPAQHTTDAHIIWIQPDWTPDGRREVSTGVANGVATTGPASFPVSYYISYRI
DSGRYDTDMPDMYLYATSIYFFDGATQTSVRRTRHVQAVFTGETFSNATEGWMLKQLSHGVLGSRVGLCRFSEVTEK
SCGDGQDNDCDGLIDKADPDCTQPSPPPAPPPAPHPPRPPPRAEPPGIMDDSPPGEDSPPPMKRRPSHPPSPPPPPS
PPASPSPPSPPSPPARSPPPSPKPPRPPPPTPPSPKPPSPSPPSPKPPSPPPPSPSPPSPSPPSPPPPKLNPAPNTD
VSNTGPEPSQGSAKSPPPATATGGGRKRPPPPAPISTGGEIAPTKKGKKSPPPAGGAVNPRKAKPPPPSGKKSDSSP
PPSPPPPPPPGKKRLKERRLTFALDHEEDSVLESEERDL

>Cre14.g616700.t1.1|PACid:27565921
MFRRTLPLLPLLLVFIAAVQSYGAAGQATSARPGSRLPASLAPDVAYFDAGEASSLVVDPSTGRVSEQRDLLATAQS
PRPALKWQGNCRYQGGPAGANGSVYMDGWTCHAKLSAPLCNGTCLKGAYTIVVVVEYGSVYQSSILSALEPSSNPSK
RMVWRDSELLLRTIPDGGFFVGDSYDPSSAVYNATPEARAKTVLQATTFISDWTVQALAVQPSSGKKGGKATWMRQP
LGLGEKSATVASGRAPGLFAPVGSDFVIGCDYMETMPDNAGTSNATNAGLPITYRCDPRYYKLRGRLVAVLVYSKAL
KEAQVARISTFYSPRWVPASPPPPLMKSPPPKPVRGLPSDLLPDVAFFDAGDRASLTITSSQVTAQRDILATPEVPR
PSLTWSGDCRWDSAAGGSVVLDGKSCFAILDTKARLCDWDRVDGGYTVVLVAQLGPSQQERVALLTSRGYEQQSFWN
AERMLAWRPNGILTREIVTFVDPVNDNPNDEYDQLRFPDAKANSTFSFTRVPARTWTLEALVGQPNYLNALNGTARY
YRGGPDDSAGARVLYSGRAPLIRAPSGADFALGCDYMKFDIPDEDYSNYEYRCDTKSYLQGRLAAVLVYSRQLSAAD
VSRLLTYYRTTRWLAAGTPPSPPPPPPLPPSPPSTGLPAALMPDLAYFDASAATAGRLVVPQPGGKVATGGGVRALR
DVLNPGQQRLDWFGDCRYDATSGGGSIVLDGSSCYAQLNTNLCAEWQLMLNRNGGVNLTFTLVVVLELDNTRLPPPP

KPPLAPSVNGDHEIAELDTGGMGIMSYGMQDGAYYYFGNPSMHWSTKATYMTMDGSEDYNNVDFRRNSPAPVPRGVW
SMEAISSYVYDVPDVNTYGYNEVEQGYTYFRGTSAQAAGTLLAVDDDLYNFPPPDGTEFRIGCTWRDSDLNYDCQPG
SHLRGRLSAVLVYSRALEKKELQRLHAYYSGRWSAAGKGR

>Cre14.g613050.t2.1|PACid:27566143
MMANTRWLLVVVSMAAAMCAWPAMAGRLGASRAADVATQSFLQRRVLQSDCLTCSALSNCVRAVCLYSDGNATNYVS
LDFSGCKNNSLSWFCCQFGTCSTTACNGVASTSTCNNVVKATVAVPAGAASVTLQVHDGRITGNFKCTTATPCCGGD
GGACGTGSTSSVCGGVVVPISGVSGCFARPPSPRPPSPSPPSPKPPNPPPPSPPRPPSPSPPSPAPPPPPPSPPPPS
PSPPSPSPPPPPPPSPPPSPPPSPPPSPPPPPSSPEPPSPEPPSPPPPPPSPEPPSPKPPSPPPSPPPSPPPSPPPS
PPPSPPPPPPSPEPPSPEPPSPPPPPPSPEPPSPKPPSPPPSPPSPPPSPPPSPPPSPPPPPPLPPLRQHPPPSPPL
PPSPAPPSPSPPPPSPPPSPAPPLPNQASCSSSCPNGSQIRAAGFKTTCGATCLLTQPSACLVEPRPGAFDSPGSYV
PAFSGVYGCFLKSSGTYTSNSSGSSSSYPLYLSYGGANLDSFFSSAAPGAVAALACYDSPQLINLTQYLLVTVLPTP
SDNYACLTCGFYNVAPLAATSSANCDCNDTAWAFPTPAQLGAAQAGNSTSEIRLNVTDPGFGNGVFWNPRQPTSSAW
GGYFRIAPPASPNAVLTLAVCAGCGQNQPDKGYYLSTGFTITFTNFTKGNGSITIKALAPGASAEVDKLQIYLSFVA
PPNLNPGQFQTFTTTSPTVTPTNPLNAFPAGPFTAAARANVTYTTSSGSTATVPASVDPSQGLFFALHLDVGQACPA
PY

>Cre14.g625850.t1.3|PACid:27566162
MRNGVSVRNYKDELDKPAMVTRRTLGEETASGAPPGYLFCRFFGLRHILAASDRTNGGLRRCHTCTFDADSCVVLCA
YCTDTSGDPTPSEVSLELSHCRPAEGEIIADTDGYLMCRMAPNPPDTPMPPPEPPTYPPPPPPVVPVTLTGTVEVYT
SHLHNSPNRTAPYMSYAVTFANESTGYSLKASFSLGSLANTADVQTGDRLSFKVTSAPPGGSGRRHLLSNCDEVTGL
CFDPTAAEGNATASGKDFVVDGKAVNITSIVMLATVCGNRPGIDITKLRSTLFNPDQSAAVNSRNVTLQHYYATCSY
GKVQFRPENNLVTAVSLPCTALYQTSSSGVQTWYKSQTSCDAPELYGWMRDALAQAQTNLGLASTTPYKRKVLFLPN
RQACDWPGMASVGCDASCNTWINQMSNTNVDMQVLFQELGHNIGLQHANRWIDGVNQEYEDFTDPMGTGWVQNDDYQ
NKTMICLGAAEAYKAGWVLPAASFNLTEDLVPGYPVTLILPSMHATDTNFLYINVTSLPAYKPSWPANAQPLNRTNQ
LFVSYRVRQPAPGFDSGLTTDMSKKIYIHNFNATLRSPPRPDPGDAAMKTSLIAILGTRAGSVQGMDVNTAFRFDFG
PLAGGRTSVNGLHIKPLSLDAASATLTVCYFAVAKESDAEGGCSNGIDDDCDGYADEADTDCGGVRSPPPAKSPPPP
PPTKSPPPPPPVRSPPPPVKSPPPPKSPPPLKSPPPLKSPPPLKSPPPPAKSPVPLLTSPPPPLAAAVKSPPPPKPP
KKRPPPPPPGASGHHRRTQLLGQHQHQRRRIRAAV

>Cre16.g693500.t1.3|PACid:27566510
MARASAVAQAALAIVMGAMLVSANINVCVYSNTDVVESFERAGLIYETVRYLVPSHPSYSSSQPDFTGLGAFVIPGN
KGSYWATEGLAANAAKRSALVDFVTGGGVLVLADGGSTGSNQFAPILDLFLGKPSGCVVDPITLPFLAYRRYADGRL
NDLPSPIKVKPDQGVSTLVCPAAPNAKAVYTGWNPATKRSVSVATTWSFGKGVVVWIGSGYTQPNLKGYAELAAGVI
YDTEEGVSSPAPVPTPSSSPSPSPSPSPSPSPSPSPSPSPSPSPSPSPSPSPSPSPSPSPSPSPSPSPSPSPSPSPS
PSPSPSPSPSPSPSPSPSPSPTPSPSPSPPPPSPPPPSPPPPPPPPPSPPPPSPSPSPSPSPSPSPSPSPSPSPSPS
PKPSPKPSPKKSPPPAFSPPVDMPPPEDLAPPPQAQ

>g16842.t1|PACid:27566553
MAARRLFVTILVAVLGLGVRGQSSDTLPYISLQAYLNGEPALSMLLQLAIPSYSLLSDTGLKATVFLPTSDAVMSYL
YTEFGVSDLADLWSNAVLNQRLLGEFNLRVLPQMIVKDRLLTAQQMSNGMKLRTNSSEEVTLVVSKAADGTMTITGP
QNSAKVVKANQLLANGKVVAHLVDKVLLPPDIVETPPLLEDSPPPPLRSPRPSPRPSATSPRPSPASSPSPSPSPSP
SPSPSPSPSPEKRKKSPPPAGRPSPSPRPSPSPSPSPSPSPSPSPSPSPSPYPSPKKKPSKSPSPSPSPSPSPSPSP
SPSPSPSPRPSRPSPSPSPSPSPAKSPSPSPRPSPKPSPKPSPKPAPKPSPSPSPSPSPSPEEVPEFGSFPNLQAAL
SAIPQLSLLSLLYDTAVQDSLVRIPLDAIMLSPFTLWLPTNQAFEAWFADPESPWHWQNDLGQAVGANPRLLLTLLS
PHALAGNQLLLSDPNDLHDGSTFTTGLKVLGRPPPGAQFVVRRDGDTVALEGTRGANTGVVINVLRLQAALVGKAGV
VHVVDRVIVADNSTPASHRRQQRRRLGHQEPQQEQLHAR

>g15607.t1|PACid:27566600
MRLHFAALAAVLIAAHAVHSAAAFDVPLYANDKVTAGKARLDALTDNIKYLAPGSVVKLATSQPDLSMATGYVIPSN
KGSYWTQEGLSPSSPKVAALKAFVSGGGALLLLDGKVDGDNSHLPLLSALLGYDTKCSAMDIPADTNMNLRLTTGYF
AKLDGKIKVKPRQGVSAMICGTGGPIYSAYVQGRKQMSVAIYWPLGKGTVYWIGSAFSIPHLKGYQEAMGAALLTAA
AGPAPEPAPKPSPAPKPSPSPSPSPSPKPSPSPSPSPSPSPSPSPSPSPSPSPSPSPSPSPSPSPSPSPSPSPSPSP
SPSPPPPSPPPPSPPPLSPPPSPRPPSPLPKPSPSPSPSPSPSPSPSPSPSPKPSPSPSPKPAKKSPPPADAPPPDY
LAPPPGTQ

>g16843.t1|PACid:27566630

MNIRGRNWIGAILCLVVVCGAPSSAYRLAANRGLSWNSLAAGASVEAQAASELLERRIAQEGTTNSKLPPGSFDFPP
EGAPPTEHEPQAPPSPRGLQPSPANSSAAPDSSSPNPAPQPSPGEPGTLPAAGAPPPGSSPWVMPAPSYPPPPYGGE
SPEPCIGGSYGYGGYAVAYGSYGYGGYGCHTPPSPPPYGGYYGSTPPPPYGGYGGYGGYGSYPPSPEVQPSPEPSPS
SSPSPEGNPSPSPDENAPSPPPERWTTIWRYLEDVVVPDLWPFLQAAMDARQKAYLQDDQARFTLFVPLYEDMLKDE
LILSIFANPADPRNAMRLNRLMAYHQVLDVTIYPQDLQDNMTLTNMLGEQLHITQKLTEDTNEFYVDGVNIARWQVG
GRGIVYWLNGVIHEPVAPSPPVAGPNRFGTLGEALTSLPQCSIMSALFAGVSADPAYGPLVNALLANPFTLLVPTDT
AIATFLAGMGLGPSDLLTMAPEAVAAIVAPHLVVTGDAFLPAARLTPGAAVVTGLPPVLPSATLVVQAAAEPGALPV
LAYEDGGAPPLHVLVADVFVRAEGDPTEAVVHVVDAVVTAPAGGAGRRRRNRRALSSH

>Cre16.g693450.t1.3|PACid:27566907
MARKGVFPVLAVAAVLACANAVATVTIYQNSDILANPDDVLVYLLDGLNTGEGLQVATVGQQPADLLAGLTEWPSAY
IIPPQVRSDASAWGDSPYALAEGLASDGPLDALRNYVHAGGNLVLLAGGSGGGGGLGLSVAAAVLDSPIGCQRAEAL
PGLLLGQSTTSPHVPYLPGLEVIELDPQAANSQALLVCDDMAGAAPWFEGWDDVAMRPLTAALMWTYGAGRITWIGA
GLDSESSAQAWLPVIKSAVSATPVPVAGATHPKPSPGPIAMYGGYGGYGGYYGTPPPAVSYPSPAPPTYGPYGAYPS
PAPAYSSPAMSYPPPAVSYPPPAVSYPPPAVTKSPSPSPEPSPEPEPSPVPEPEPSPVPEPEPSPTPEPEPSPAPEP
EPSPEPKPSPSPAPGPSPSPPPPLPPPPTPPSPSPHPPPPPSPPPPTPPPPSPPPPRSPPPSPPPPSPRPPYPSPS
PVDPRGGKGLGAEPAGCFKTSGVVLAEGNAATAQAFDPLACAALVHGDGSYSYVGFAEGNKCYGYKKQQPPGDSAKL
TTCKACQNSKFSADMCGGSGAMSLYSLEELFPAVPPPASDYTPPPMDS

>g16193.t2|PACid:27567135
MMPTIVWWAMYVYLRVSAADNPPPSPGPPPSLGPAAPPSPAPPSSPDPKPPSPAPPTSPAPLAFGSARSVNFNTAPL
GKLVSASLESLGVDLFMPAADDAAMFDGWVIDCEAGWQGSNRINYTATGCKRALYYSITEGISTSFRPLFPRDPFWR
LTLTYRKNNPIGGVGFRSSSSSINSDCEVFLDRTDPLVSIERPPATYNFTSTPKTVTLTPVDCQWPAGSAFEYFYFF
TARSGAAYGSDTFEYLIDSFTIETGTAPSPPPPLPSPPPRTVPWPPSPEPPSPAPPSPVFPSTRSIRVDTLPLGELA
STSLEALGIRLFTLSPFPEPPIWGEVVDCAAGWDQQGTAMTAAGCTKTLRFPGSYSTGKFAPLYVPGTFHSLSLTYR
TTIPDVVLYLYDCEVWLKRTEPFVTTQQRPATFTYTTSTTVTLTPADCGRPAGSTFPFFDFYVNMDSDGSGVRELLI
DSFTMETGMAPSPSPPSPAPPSPAPPSSPDPKPPSPAPPTPPAPLAFGSTRSVDFNMMQLGLLESASLESLGVELFM
TAAGAADAAVYSGWVIDCTAGWQGSWNNIPHSATGCNRALYYSIPEGISNYFRPLFPRDPFWRLTLTYRKNHLKGGV
GFRSSSSSIRSDCEVYLDRTDPFILKKRPPATYNFTSTPKTVTLTPVDCQWPAGTAFEYFYFITDQSGAAYGSDTFD
YLIDSFTIETGTAPSPPPPLPSPPPRTVPWPPSPEPPSPAPPSPVFPSTRSIRVDTLPLGELASTSLEALGIRLFTF
LSFEPPVWGEVVDCAAGWDQQGTAMTAAGCTKTLRFPGSYMTGKFAPLYVPGTFHSLSLTYRTTIPDVVLFLYNCEV
QLNRTEPFVTTQQRPATFTYTTSTTLTLTPADCGWPAGDAFTEFYFYVNYEGYYGNGVRELLIDSFTMETGMAPSPS
PPSPAPPSPAPPSSPDPKPPSPAPPTPPAPLAFGSTRSVDFNMMQLDQSGAAYGSDTFDYLIDSFTIETGTAPSPPP
PLPSPPPRTVPWPPSPEPPSPAPPSPVFPSTRSIRVDTLPLGELASTSLEALGIRLFTFLSFEPPVWGEVVDCAAGW
DQQGTAMTAAGCTKTLRFPGSYMTGKFAPLYVPGTFHSLSLTYRTTIPDVVLFLYNCEVQLNRTEPFVTTQQRPATF
TYTTSTTLTLTPADCGWPAGDAFTEFYFYVNYEGYYGNGVRELLIDSFTMETGTAPSPSPPSPRPPTPQPPTSTTSP
PPPPPPPPAAPPPSPTSPSPLPSGLLAPTSLPPPLTPPSPPPSPPLLAPPSPSPPAPPTPSPNTQPPPSPSPALLPS
PTAATAPPSPLTPRPQQQLPPSPAAPTAAVVATFRSFFLGCFALDVAATSVAAASRALPIALVANDAAMTVAKCAG
LAQAAGLDHYGITHGHTCVGGVSRQQATQYGPLPASACNQPCSGDAAQTCGGGPTTTTTSTMSLYSFSPANPPPRPP
PRAQRKPSSAAARPPAPPAPAPPLPAPPAPSRQVETQTLSRTAAVDLPALQEDVAINSSRRVIAVPPSSARGLILLH
PAAVPVFTAATVGGGGALPLPNSTAPVIASARVGRLDGRLVAFGTEAMLTSCCGQAAVGFDASELGKVIVNAAQWAA
AAAGAATDRKATIRVSDSRLEPLARFIVSMAPATFAKATRKGHLLELSAFARGGHERCDVYVVLGADPQQRYNAKIK
GALRDFVEKGKGVLLAGPPVVAGVDAAAVMLAAAAPVPSDEWTRPYLMFAKQNGITAGGAASAVTLDRVASEQFRAA
LSRLLRIKDQQQASNASAEFASLKARVMGVRADLDASDIAEFDEVLRARIAEYDRDLGAL

>g16193.t1|PACid:27567136
MMPTIVWWAMYVYLRVSAADNPPPSPGPPPSLGPAAPPSPAPPSSPDPKPPSPAPPTSPAPLAFGSARSVNFNTAPL
GKLVSASLESLGVDLFMPAADDAAMFDGWVIDCEAGWQGSNRINYTATGCKRALYYSITEGISTSFRPLFPRDPFWR
LTLTYRKNNPIGGVGFRSSSSSINSDCEVFLDRTDPLVSIERPPATYNFTSTPKTVTLTPVDCQWPAGSAFEYFYFF
TARSGAAYGSDTFEYLIDSFTIETGTAPSPPPPLPSPPPRTVPWPPSPEPPSPAPPSPVFPSTRSIRVDTLPLGELA
STSLEALGIRLFTLSPFPEPPIWGEVVDCAAGWDQQGTAMTAAGCTKTLRFPGSYSTGKFAPLYVPGTFHSLSLTYR
TTIPDVVLYLYDCEVWLKRTEPFVTTQQRPATFTYTTSTTVTLTPADCGRPAGSTFPFFDFYVNMDSDGSGVRELLI
GEFVHHGNGHGSITLAPQPGTSQPGAAQQPRSQAAQPCAAHPAGTPGLWQHAQCGF

>g16780.t1|PACid:27567247
MTASFITSIVTLGTPCPGRQQQPAACRSSPSPSSSSSSQASARTQRRNAAGTTAPGTGRQTLLVAALLLLTAGLAAA
QPPPSFYCNTWKGTTSATCPICASGSPCTTITTADDFWRKPELCSLPDIEPLRSTVTGATLFPTTSICSADSGIAAY

PIRGLPATGAAGKTGGTAYAWIGYNGRLYLTLAFDCNFMFSSNPNWPGPKVSVALWNAANGLGRPQYVDVLYAAGFY
TCYTLSIDLRNVCNPADGAVFNPSPNAGSTCQCNGGACPSPPTNLFNEGMPLYMDVRVSLGDYASQDGLCAPGSIGN
YTLSSPSDGPAVNPLFVPNCIAPPSPPPAPPRPPPPTPPIPPRPPPSPPKPPSPPPPRPPTPPTPPSPPPVDVSVVI
TTPMILDRDTTCPSVILWLASWYAIVNATVSNPPCVVGVSPASSTMFITYSLLPNQAVPFVNIIFPAYPEPRSIQLK
VLMLDLLKLPCNSTSSVQGVGIYRVVPDNLLPSNMKDDRWPELYCAPQPSPPPSPPLPPAPKAPRAPPPSPPPPPPP
SPPPPSPPPPPPPSPPPPPPMPPPPPGILYYWQMFYPAPIDQARAKDCTYVSFIMKYSYKIAELSPGQTDPNCTLSS
TKLVATLMFYSIERGAKSMVGVFNEAAIGEFVTMYGIPCNSIIYMSYDDGSGATVGFKTFSGSNVPALKCGLPPRPP
RPPSPNPPPPTPPFAPDPPSSPDQASNFSPPPPVRQPPSPRPPSPRPPAPAPSPPFNASTIPRPPRPPSPPRPPPQP
PQTPDVVFQPPNGDQPSILDPPPPSRQPPSPKPPRPRPPPLRFPPEVPFLPLPPDITQLPPPPPPRRRSPPPPTPPS
PKPPRSPRPPPPSVSPPPPPSPPPPKSPRLRPPPPQPDTPPPPPPPKSPRLRPPPPLPSPPPPRVPRAPRPPPPAGK
PPPPTGKPPPPGKSPPPPPPVVRSPRPPPPSPPPSPPPPTGNITVNNRTVIMSVDTILTRALTNAHCTALTVITAQS
VPPSVNVTAGPNCQLNDPPTTGAKVIVVLETNTQALDFYNAYATPTRADTIVRVLSLPCNRSSVIFAAPGLAEPRVF
DQRNVPALQCASRTTTASGRRLVLAAERAMRAFEEAEAAAAQGGRRELPEEVAFGDVPEEERAGVALPLVSLPILSH
GANEADPRAAASAATAAAVSRALGEAGEENMVTVGGQAAAALVGEDGRRRTAVLQDELAGLMLVNLDDEEEDSSSSS
SSQEDALNPGSGVSAARGRQQ

>Cre16.g693200.t1.3|PACid:27567248
MLLATALSAGAAAAARIPGAYGPPAYGGYGGSYGYGGYGAAPPPGPIYGGYGYGGPSYGHYSPPYPPPAAATPPSYV
MEWPPMAEALERPPAYPESPPPELPPVWLSPPQSSPPPASPPPSPPPSPPPSPPPSPPPSPSPPLPPALLLTDIVGC
YQFLGPGSRRLFPAAELSAGHGSAGGGVGPNAGPRSFDVAACVRMVAGSPGSHFRYVGFFDGHMCFGIGGILPPSAL
AAAEPFAACEPCPLPSGRNSTGSSSKDGGGGSSAQQQQPEPQPQQLRCGSGTRMAVYDLAAWPWVRLAPSLPPMAP
WPPEPEDFGSGWRRRSRRRRRQ

>Cre16.g693300.t1.3|PACid:27567406
MQTCPRSGAVWPTLLVTLAILSCIHQPAAALRRVRRRTRAVEYPPWPPYFPEPPADGPATTGAEPPPPPLESGPAAY
LYGDQSVLLNPDSVGLLAASMLGLGLDVSPPLLQLPVEQLAAQKQWPAALVVPPLSAAARPGGPDSGGGPWHKLPEA
LAAAAAFLRSGGNVVIFGGGGGADNGLGLVEELLLAAAAPAAAGPGAGAAGARRSSGCMAAQVSGAFFVQATNEDGS
DVLPYIDGLPVILVDDSTAASVRVLACPPSSGAIPWFESRSGNTGPVAAALVWPVGRGRLVWLGSSFGNIVEAAPWV
SVVRAAVVALPRDAAPPVGGGSTPPAGARPSPPPDVEGAFPPPVYGPVSATRPPPPPYYGSGGPGNYPPYGGYWPLS
PPPYYGGYYGGGGGYYGFYSPPASRAGPDADPPVLPSFGCLPYVVNGQWDERLFDAVQVPLHDSEGGGGGAVQFDPV
KCGRAAGAAGYFLAGFVATAGGESGGRCFGLEYPPDGAADPPSSKPEVVGGGGGGGGGTCQPCAASPDWARLWCGVE
DARGEGTTGSVSYMSIYETFYFDSATEA

>Cre16.g663750.t1.3|PACid:27567478
MPRARGHSHRAPAALVPVLVLAAVLAGCTDAARLHRAAAGAAAGDIPLTDPITLLTPAISTTASSLVKHRAAMSAVL
AYDATFSATAAASAAAAAAGHAKGHAGHVASGGGAPRRQAASDTWGSHDRGVDAAISRQQVQAEPLRRQRLAAHRRA
LSGGCPDFPPPNLVMCAGGVTYPSTCDAQVKGFCAEHILPGNCVPGSRMALTCSLSFAEQQLSSGAFAPAPKPLTLV
LTAALKPSGNEDGGGCPWYLIKELVGGGGSSAVGSSGAKLSYGGKSYSLALAGQGDFNGNDNLVQLADDPLEPTSQI
LSYAGLALYDKKDEWTISITAATDPYSGGGYGGYMTLTSDGAPFDGVVLVARTASCSLDLGRSVPIPSPPPPPSPPP
PSPPPPPRPPPLPPSPSPPPRPPVPPVPPSPPPKPTPPRPMAPPNAPPPSPRPPRPPSPPSSALEVFTDNLAFADSA
GRRVVYVLRLSATPVPEAGDGGNGTTGPVFNFRVRHVEGTVTVIDPGEYATAPVVTEEVDLLPPGPDGYEGNDNLLQ
PLPSPGPGGSSNTAARLLPVFSSRGLALRGTSSGSKFLIRVEDFGSESESNSTGGSAGPGPGPRWRTVTTALVGGYN
ETEDVAEDEFVYTPNGTLSRYVSVPALCFDTNATAANTNASSSSPGADPGRTDILFLTDSTGSMGTAIADVRAQARR
IKDAIAAVAVDVWFGLAQYRDTGDVFVWHLDLAVGPHPPAVIQAAIDTWSANGGGDEPEGQLYALQQCALNPGVSWR
SDATKWLIWFGDNPGHDPSNAGVTLPASIAALTSRGIRVIALEMGKLNQWGQATAIANATGGAYLSGATVNPANLSS
AIIGAARSGLAGRLAARPAAGGCDDPRVSLSFGDAASAFALAAVDRGQRLCFDATAQVGTCSGLTTTCRWQLVDVTG
AVVHSAAAHVTVPGTCPPAPPSPPAPPPAAQPPLAGHPTGYPPAVPYPGYPPHYPPHYPPHYPPHYPPYYPPGYGEA
APPPYYGPGYGSYPPPLYRSHP

>Cre16.g652200.t1.2|PACid:27567574
MVLTRLQVSILLLATAGFAASGPTKKNIEGELVKLDRDDGDAGNWALLNRRTGLFEPLGKMGPGHKMPQKDKNGKG
LAAGQYVTIGCTVDAAADQCTFVAQQDVAIVKPAYVPVAKQVRERLLILIMDAPACGAAAPMTVSTAETLWFGPNKD
ARGGYASRAEMCSYGEFVMEPPPASKVMTVTPPCSWPTATCDSWAMANAARTQAQALLGAAAYGAFSHHILVMNFPA
ACQWAGLATLGGGVEGGGQVWLNGATYSQTFGSFQVPLHESSHNFVLYHGYRFHLEYQDKTSYMGTGTACPASTETS
LLGWSSPVEGGNLNSTTLPTGPAASGPFVLPATYLTGLGNYVRVQPDWLPYYNNAQYGFNLYMEVRVRANGDDMMDP
VYDHKLIIHEVSSLYNNDPVTYRTADPQSNYLTSMPASSRLVLQNDYYPYSLVLYTGAFTGSSGAFLPVYLCRYAVA
ESECPSLSKVLSGTNTASSSPPPASSSPPPAASPPPPASSPPPPVSSPPPPASSPPPPVSSPPPPASSSPPPASSPR

PPASSLPPPPVSSPPPPASSPPPPASSPPPPASSRPPPVSSPPPTASPSPSPKVKPGKKFQQATGRRSALGNEWQGL
DNPIDQRMPSSTTTTSDPSSSSHERSAAASGSGAKPARRLLLLPMPSDQQSISSEAP

>g6305.t1|PACid:27567926
MASKFLLLALLGALAALSVNAGRVDATGRTLLQAKPPRFPYCRCDDRLRVSPWRLKWDGYLQGVANNDWICFKVFTN
FTDTRCTGPVDLQPACCKPNLSKLEFDVAPECKGTSNRALINKDVTDLKTIAVEVLDNGRAILRISKGLDYSAATLP
AGGLSVCVNRLAGQCTISELFMSPTSVQYALFNSKNCCPTGVTGPGGPTFPTPVSAPPPPFRDRPVASPSPPPPPPP
PPPPPPPPPPSPPPPPPPPPPPPPPPPPPSPPPPSPNPPPPKGPSPTPNNFPYCNCATYGRNSFPWRVSVLNKTTTA
TSERVCLKLYVDPAVAAVCNNKFGCCKQGLQKIELEATSTVPGQCKGSITPFTLTGTTDIKSSFLWDSTRPVLKFTK
LDLSYDLGVAGAALCFSLKGPGCTRLNQLCVSGKQCPTAVFNGQTPYNTCCPSFYLDA

>Cre06.g287800.t1.2|PACid:27568046
MGPKPLLLLACALVLLKSCGGIIYDPCQPLTPIARGDPFVIGLALIPAVNATLLANLTASYGGLCNTTFQDYLITNY
KALIAIYNLRVDRLQVLRMPYPDIIFNMVNATPPTMALAAFRANVTSPAAYIASGDPAVTYGAGFVVSLALLPRFDK
GNLQYLQWYDQSCNECGGKNGALCMHSTQAGVVACSTPLDNCTCTVSGVTNSSCSLDDARFDVCSTSINTAWMGTDR
NQAVMRTGPQVQRLNAYSITSLFNTARNKFYDLKNFVYANVQSSWGAVSSDAQSQYVDFEGGLSQYTRSPPPPPPSP
PPAPPSPPSPPHPASPSPPPAPSPPPSPPPPSPHPPSPPPPPEAPAVVVAASIETEVLPDTATETAAVAPADSGTTT
DTTTSVETPAT

>Cre06.g272250.t1.1|PACid:27568053
MGRRDVLATSVALLFLAGLALGSAQLDMSDDFGMSNFGMRRLTAFYSRNFAAHFPATCFQSSYFSPFRMYFKGYNFE
TDRTTFIYQLDVSSCPFPKADCCRLPLDHMLVRTKSDASVMEVRLDGRVLGHDVSERGLTIYDLAVSPTQLKSMQLE
IVTPATGYNYPSDLCPQSRYPGSCDVISYDESKTCCAERAALPYELFFPNNLPPGSVGPDVKAPPPSPGPPSPPSPP
PPSPPPPPPSPPPPPPDGWSFCCIDDLPKSPYNLKFMGSRTSGSDTAYSFQLVVRKLNYSYPDFDGPEKGDCAQMNL
RDMGVAVYDNLLIKSVQFNGSVVPNYWTEPQGVRPDANWLYIPVMKDFADFSEENPIDFVVTVRGLVPSLCPANEFL
HSPNACEFAMHGKDEDTHCCPHGATKKGGPYDDCCVDDIEKAPYRMEYYKTTPTEAGTTYDFAVKVVNVTGIDYDLE
EEALCDRMTLDYAQIQIYKNVEVVQVQWEDRIMAFNTTPATDYSKWLNINGINRFINDFDPNRPAKFKITVKGYVNE
LCPAGWLLDAGGKFICEYALHGTQSGHTCCPHDITRPGQEQPDCGCRDDLAGTPYRLGYQLAGVAPNQTMFNFDLAK
VDPTAAADFDGAPDKNFGVDVDCGGMSIKSISFAIHQNIKVKDVIFNGFNYTWQYEPYTNTTKWLRILDLDYDPADF
PASAPIPLQVLTTGPAVTELCPAASQYNSQAACEYLIFGLSNYAECCPVGVTSWYTPNLLMTARR

>g6351.t2|PACid:27568068
MLRGAVQTCVLVLVLVLVCVSALGEVEWRGDQEEHTHQHRHLLQKKVKAKKPPSPPPSPPATGGGGKAVRNKKSPPP
APSPRQVSAPPSPLPPSPAPPSPRPGALPTVVDGATEAASSPPSPSPPSPPPPSPPPPSPPPPPPPSPPPPVPPPPS
PPPPSPPPPSPPPPSPPPDVVPSPSPPPPPPPSPPPPDSPPPSPPPPSPPPPDSPPPSPPPPVDSPPPSPPPPSPPP
PSPSPPSPGPPAPVPTGGSGVTLDGSGCPDAQAALDLHNSYRAAHGGVPALQWDAALAADAQAYAEVLAGLGCTLIH
ATQPPGFSATRFGENLYMSAGYPRTPLTCAPGITSWYNEVSYYKFTSTPYTDSYSTGRVVGHFTQIVWRATSRLGCG
MASASYSFPGFPAPGQCKVVVCRYRQAGNVVGDSNYFQNVLPKA

>g6351.t1|PACid:27568069
MLRGAVQTCVLVLVLVLVCVSALGEVEWRGDQEEHTHQHRHLLQKKVKAKKPPSPPPSPPATGGGGKAVRNKKSPPP
APSPSPSPPSPGPPAPVPTGGSGVTLDGSGCPDAQAALDLHNSYRAAHGGVPALQWDAALAADAQAYAEVLAGLGCT
LIHATQPPGFSATRFGENLYMSAGYPRTPLTCAPGITSWYNEVSYYKFTSTPYTDSYSTGRVVGHFTQIVWRATSRL
GCGMASASYSFPGFPAPGQCKVVVCRYRQAGNVVGDSNYFQNVLPKA

>g6303.t1|PACid:27568088
MALPGWRRSGDLAALLLAVWMASVTCSALSLPPSPVLVNPAPARPLGSTCPTLLPECSRSIVAPRNILCIRLSKENN
ITVNQLRAYNPELDCAARIPTGCALCVSAILPPPSPPEAPLPPSSPAPRAPRSPRAPRKASPPPRSPPLPPSPQPSP
PPPSPRKQPKPPAPKPPAPSSPPPPRRSPPSPPPRASPRPSPSFVAKTTPAERGVTSSPRPAPLPPPPPPPATTPAG
ILLEGNNDPSNPASWWYLDAGVTLARHNLYRTKHQAQPLAWSTRLQKEAQDWADNCWFAHSSRPYGENLALGHPSIT
AAMDAWYSEEKVYDYANPQFNAGHFTQMVWQRTYLVGCAIGVCPDGVSYVGGVWMGKLYVCMYWPPGNYMGQFSENV
YPPVDGSRRRQALGSGEL

>g6408.t1|PACid:27568149
MCRVASCATLLLALLFLLRGAAAQQCPDETVALVYNNGTGYAVQCYQYLQGRYSYEERQERSELSRTDFGNYYYYWK
QRQPTVRVVESGATVQCSGNVFDEYTYQYCNAGGCTGDTLTGTQSKNSSCVQYRFYAFTGNVYAWVMGDVSNLQTFR
QLVRDTDDSPNLILNWRDFTSFDSTYSSTDTILIQNLTLLGGNSSPLPGTSPSGSLSPPPAGTSPSPPVPPPFATPS

PPPPVPPPFATPSPPPPVPPPFATPSPPPPVPPPSPTPSPPPPVPPPSPTPSPPPLVPQPSPMPSPQPSPTLSSPPS
PPLVPPPAPTKSPSTSLPPRLPPPPPPSLLRPSSPPVRPRPPVRQPPPSLRMPRPLLLSKSKPPPRPLRRPRATSSG
SGASP

>g6301.t1|PACid:27568444
MGLRALPVGMAQPAEVEAALVGSDCPDRLPTCTTTVAAPRNMFCLRFSRDNNITVNLLKQYNPGIDCSLRIPEGCDI
CTSATLPPPQPPADPAQPPGEPPLQPPPPRRPRRPAWPSNPSHPPPSPPSVPALPPRPVRARASPAPPRPPPSPPRS
PSPPPSSPDRLAKSPPPSPNQSPPPSPNQSPPPSPNQSPPPGPPPPSPRPPPLRRPPPPPPRSPPPPTRRPPPPHLP
SPPPPPPPPPPPPPPSPPPPPARRSLPPRPPRPAHSPRVFSPPPPPSPSPPPPPPPPPPPPPPVPLPPSPRKRPPPP
KRSPPPSPPPPEESPSPPPPPFRPPPRSPRPPRPPPPPMIVTPRVSPPPPARLPPPPPPPPLLDASNDPADPSSPWF
LDAGVTLARHNLYRTKHQAQPLAWSTRLQKEAQDWADNCWFEHSQTSYGENLALGHPSIEAAIDGWYSEVDKYDFSN
PGFSSGTGQFTQLVWQRTYMVGCAIGVCPDGVSYAGGRWQGRVYVCMYWLPGNYPGQFAANVLPALEDASGRRQQR
RRAQQGIMQLQLHQHTSDDEL

>g6296.t1|PACid:27568494
MAGGNSFLRLFAVAALCLGITVAGGNSFLRLFAVAALCLGITVDGKYGAVTIDYRLGSALTHSSKSSVAPKRRLQDG
GCAFGGLRGVLASSGPFGGLRSCTYCVFDAGACAVACNNCAGINTNSSSVQLSLLGCQAGPLTIVVDASGQLACQEP
APALPFPPSAPSPPGPEGGLTGSVIVWHSLDENRTSPLPVTYTAVFDTGNNSTLTSQFSLGALQRGADVVTGDMVTF
KVKAPGTGRRRGLRSDAFVLDCDPDSGLCLDTDTAVLNGTSTGAKDFVINGKPVNVTSIIMLATVCGTQPLMSVSAL
RSTLFNPASGAPTRATMQEYYDTCSYGKLNYLATNNLITTVNLPCSALYQTTASGTQVFYDSVNKCGDPERMGWVRD
AVVQAQQNLGLSDISFYKRRTLILPKRKACAWAGTASVGCDTNCITMINQLNSYLDVSSTFHELGHNIGLQHANSWN
SATGDINEYGDFTDPMGTAWPANDPYQNKSFICSAAPTAFKAGWATPSYNFSIAGGGLVPGSPRTLAVPSMHLTDQN
FVYFNISTLAPFPVTWPAGAPGLPKTNQLFVSYRVRQTGVTGYDSGLPNDQNRRVWIHSYNATYRLPPRADPDALDM
GPALVAVLAPSAPAAAGSMRTARSFRVNYGSIAGSTSPVNGLNITTLSVNATHAVITVCYFRALNEDGAENGCANGF
DDDCDGLVDDADPDCASGVVRSPPPPPPPSPPSPPPPPKSPSPPKPPSPRPPRKPRKSPPPLPPPPKSPPPPPPKSP
SPPPPKSPPPPPPKSPPPPPPKSPSPPPPKSPPPPKPPSPSPPPPPPPKSPPPLPPPPKSPPPKPPSPKPPRKPRKS
PPPLPPPPKSPSPPPPKPPSPKPPRKPRKSPPPLPPPPKSPSPPPPKSPSPPPPKSPSPPPPKSPSPPPPKSPSPPP
PKSPSPPPPKSPSPPPPKSPSPPPPKSPSPPPPKSPPPPPPKSPPPLPPPPKSPPPPPPKPPSPKPPRRPRKSPPPL
PPPPKSPPPLKWPSPPKPPSPRPPRKPRAPPPKMR

>Cre06.g309900.t1.2|PACid:27568528
MRPARPAFPLALALALLAFVIRAGATLGVGITEPVLNKPLPSGFRVTCFDGQELIFTNDNYTNGVLVAADAYAYPVG
QCRRCPAGTATMDGFRCIPCPSGYWSDEGARECIPCPVGTITVSTPKGTSGPSRTYDQIQRLNAGAGSCRKCPPGYF
QPALAGTVCLPCPSGFTSTLGAEGCSPCQEGTFHGDGWQLAANGAYGARGMRNASGGTGGREASVVELADITGIVPT
AGVEYTVLPNTCIACPRNTYQPLKAQAATGSLGLGAFSACRRCEDGWWSPPGSAYCQPCPAGSYRNSYFDGTVQLSN
AAQSLTYNLANETAPNTLASCFLCPKGTFAPDPGASVCQPCPAGTHAAATGSTGCNRCGAGTNSLYGLRGQQLSWQS
NIATGPTAAYYTYTISGFDKALYVGGGAAPGNRSAWRLLRNATTEDRNFWLAGKGEPCAYNLPGYYTDVEGLPVQLP
CKPGYFSPEPYTDKTKCFTCTFGTFNEEFAQPICKACWPGSFASQRAMTKCEITLPGYFTNPNPVARNATYDLTVLT
TTAPNMTELVAGQSAPTPCGLGYYQPEYEKSYCLACANGTYADVVGLRTCKDCQAGRYQPSTGQATCFQCDMGWYSD
YGSDRCTRCPAGWITPAAGTARCSRCKAGFYADKPIGATACRSCPRGYYGPYEAAYSADGFTPEGPRGCFKCNFDTY
TNRGAMTYCNNCTDLLLSTGNAVPTCTETTGAMRCKPCSMLINRVENRTTIFVPPPPNPSPPPPLPPSPPPPSPPPP
SPRPPSPRPPSPRPPSPAPPSPFPPSPRPPSPAPPSPRPPSPAPPTPPPSPPPPGNPALPAGQGVYVAGDNPAGRRR
RLMMGGAASGEQDQEQDEMEVTDEAMLAERLRARQLHQSQQQQRQQRPLELEDLAEEAELLRLMAQLPQEERGTGGH
AGGA

>g6308.t1|PACid:27568673
MAGGRYISFLWAVLSLITVLAVAEADLASWLHRPPPASARRPPPSRHRKPPPSAVRVDPFVQLNVTVTGELSLEAVD
PDLTAGQATDPLDSVAPGEVVRRLVERVNESTSVNTQVDFDPEAVKGLVTGDVVEVPLTLTLPQSVVDLLQLDSGSG
GTGQHRRLLGDEVHSLRRQALEMHGSRRSLSDFVAFQGRLQDLIRSVGAAGGQEDIVNLVPTAAAATSTGKSNSAKD
LMIVGGKPLNVTSVTFVFTSSSCNPTRVPRTINYDWVSSRWDNSTTPATFTSLSRQHDICSYNKLNFVRVINKVYGP
IEIPCTGSQPTKGKYDLNTGCSDPEIWGLWDFAKSWLAKNDPIMSKGLFAVRRKIIVFPFSKGSGCGWAGRANVGCS
NGGDCMTWLAPGVTDTVLDMGTVFHELAHNIGLAHSGRRMCDATGTCAVREYEDRTCIMGMGFPTDNQKKYICTNAA
QSYKAGWASPLEQNGNSPFNGTFTYDAVTGDFNDNPIVLPAMGVTDKNHLRIVLDQTGIDRNNARQRALYVSYRARV
PGIEYDNGLNNNYHQRVFVHEFNETADNKPSDQDNPPLIMAVLDVKGANGRPPVIGDSWGALPDRYTYTAPPGLGRL
VIRVVSKTASTASLQLCRALVDVEQGDDLCFNGKDDDCDGLTDDEDPDCTGDVQAVASSPPPPPVRIFSSPPPPPPS
PSPPSPPPPPKRPPPAGKKKSPPPSGSTSSPPPPKKRPNKGH

>Cre06.g258950.t1.3|PACid:27568681
MQAPREGAGQRRMWPAWHAAALFLCLGFGLIVQARAAASGLAPWCAVSPLHQVTTACQTSTSCETGDSWWGDADVCR
LPGPAGTFPFTVSSFDVCGGPAAVTQLPLSDGSGRQLGTVSLYRLYDSSLLHVSVSLGTADGTVVLYRETPGEASAS
LFLSSQLLPGPLPQYSQLLPPPAAANGSSSSSSSTPPYSCFTWSVDLAAVCNPLSSYQSGVAPAGGSSSGSSSSSSS
SSSSSSSVGGAGIGGWAPVCSCLPSVGAGSCPPQDLAAAAALYFSLQLDVLLARPAADTCGLDSAAATPVTITTRFP
SAAAAPSPGAIAVGPVRTGCWEPAPPGADRPPYPPQPPQPQVSPPAPLGLYPPPTYPPPPTYPPPASPPPPPWPPA
PPGGPVATLLVRSPLRSLLQAEQCSQAVMQLYPLYATRAARFNCESRPGSLGVGSDVTFQLTFLTFDGLSAFSGGMV
QPLTWEGLLGAMTVGCGSLAHYSDSTGAAFSVCGSADQAPGCRVVLPGMSCQPPPSPPPPPPPSPLPPLPPSPPPPP
PPSPPPPTVCTASTAVFKQGVSFMYATCDALLVFNEIMFLKGVNVVGTFACTLNAARDTVSVAGVLASPADAARFVS
NAKMPVNVETLASYFNLSCVDSITFDATPCLGQEAGLVQYTGQSLPAAACRRPPPAPPTPPPPPPSPSPPKPRPPFT
PRQPWGPYPPEMPLSPDPSSPPSPPAPIAPPHPPPAEPAPTSPPEGPSPPPPDTPPPDAPPPPSPSPPNAPTPPDAP
PPPSPPPPDLPPSPPPPGLPDSPPPPEAPPPDLPPPSPPPEFPPPPPDAPSPPPPPTNPSPPPPPETPPPPPANPRL
PPRPQGPNFPQFPPRPPRPPRAPAGRRRRVAAEDVEVEQEVAPLQEAEAAASDASAHSTSGSSGRSRRRSVREQLQL
P

>Cre06.g302950.t1.3|PACid:27568867
MPAAFESRLLINSDASQGSQASADCTLTGPVAIFRAPTGDQSWLLDDESRLPSYRLVPASADVAIPDQGDFVQVTGR
CELSSRTLTYAELLVDSEGGPDPHLAALSHQPGTNAPGSEQTLDTLAGHGGATNATAAAGAIVDATIEMRLDDSEAA
RRQRPPPPQPDAGAAPPPPPIRRRERAIPPSPPPELPPSPPPRTPPKPRSFRAKPPPPTQSPSPPPSDPPSHPPPKP
RISRAKTPPPTQSPSPPPSQPPSLPPPKPRGAFARPRPLPASSPPPPPSPPPRSPPPKRPPPTSTRTRPSPPPPRSP
PPSPPPSPPPPPTSPPPPPSIIQPRGDQVPTDPAPVWPVRGNAKRLTSLVMVVRLCGQIATGVDPQFMRQRWFKSFA
TPQTTKDGTMDSLVTQCSYGKWAFNDFENLVVPVIVDLPCSGRGSLGDWSASNCRDVDRQGWAEAAMKYLTDNTDID
VKSYTHKLMIFPTQTANCNPNLAALAMQNCAWTGNCYIWSFGETSPRSLLHEIGHNMGLFHSWVWPVHDPAIDQYGD
VAALMGMGPTPVCWNAPQGQALGINSPIAQLNASSLPAGSWQTIRLPPSFVSDSNFVTVAVSWMSDPVLRRGYLYLS
YKLARLAEAGLPKELDAHVHIHSYTGDLTLAARNYSMLVGNISAGEQWPPADQAAATAAFPWRLLVLARSTPRDTST
VDGMTATLQICRFTSSPRECGTAPAPLESGASTRSAALGDMAVAAAGSDSSGAGVRSASIDTSAAGEMVAVQAAFDS
AAATGLDQLSALSDLASSLLAGVCGDGVCGTGESMLSCPSDCCGADSETAARCGDGRCDAWAGENCITCPSDCARVR
PAGSRDILPFPIDASGHFQFLSHDMPPSSGLVWWCCGGGELGDGCGEPACAALPDSAKGGSGTSGSKGAGVCRSTCA
TPHASRRRLQRKVLSGTATVAR

>g6304.t1|PACid:27569078
MAGGNSFLQLFAVAALCLGITVDGGLSAVTVDSYPRQGAALTHSSKSSVAPKRRLQDGGCAFGGLRGVLASSGPFGG
LRSCTYCVFDAGACAVACNSCAGINTNSSSVQLSLLGCQAGPLTIVVDASGQLACQEPAPAMPFPPSAPSPPGPEGG
LTGSVIVWHSHEENGTSPLSITYTAVFDTGNNSTLTSQFSLGALQQGADVVTGDMVTFKVKAPGTGRRRGLRSDAFV
LDCDPDSGLCLDTDTAVLNGTGTGAKDFVINGKPVNVTSIIMLATVCGTQPLMSVSALRSTLFNPASGAPTRATMQE
YYDTCSYGKLNYLATNNLITTVNLPCSALYQTTASGTQVFYDSVNKCGDPERLGWVRDAVVQAQQNLGLSDISFYKR
RTLILPKRNACAWAGTASVGCDTNCITLMNQRTSYLDVSSTFHELGHNIGLQHANSWNSATGDINEYGDFTDPMGTA
WPANDPYQNKSFICHAAPTAFKAGWATPSYNFSIAGGGLVPGSPRTLAVPSMHLTDQNFVYFNISTLAPFPVTWPAG
APGLPKTNQLFVSYRVRQTGVTGYDSGLPNDQNRRVWIHSYNATYRLPPRPDPDALDMGPALVAVLAPSAPAAAGSM
RTARSFRVNYGSIAGSTSPVNGLNITTLSVNATHAVITVCYFRALNEDGAENGCANGFDDDCDGLVDDADPDCASGV
VRSPPPPPPPSPPSPPPPPPPPKSPSPPNPPSPPPPRKSPPPLPPPPKPPSPRPSPKPRAPPPKLR

>g6920.t1|PACid:27569240
MGRQSGASGLRALAVVALSSFLLASGAAAAQSGLYTQFPFCKCIKSPSAYKLADVVVAKGNSQYCFRLSAKVPAGCN
NYCCNKADLKKIEFNVNTACDVFGSVVKATVNGVPTKVGTSFDQPTDGPAGSTVLRLTQLGLGLWSDGAEICITLAP
GKNAPGCTTLEQLCVPPAGLPKGVCSAALFDSQNDCCPLSTPGIPSPPPPRPPPPSPPPPSPPPPSPPPPSPPPPSP
PPPSPPPPPPPPRPPPPSPPPPPPPRPPPPSPPPPPPPVCQACISFNIDSYPVFVWSDFLKRYYFNQGRCLDSLGLI
SGDVNDAAEQMGIKLAQGFSYTPASCGSSRWTTCGSFHTEADAKKMQDWATQQANNVWMESITGPACSSYWHGWQFS
ITSANSQCFSMGASSRACALDDVPFPTHTQCKKTQFSTPFALVPYVLEAGRGRSNNTLLYCFQTYTVSGDQLVDPQN
KCAKSTNLNKVEFFADESYRRAIMGVRVKPKGGSATWLASTWGAVGQTTFKVTPLDWSIEQASQGEICFELKASVSI
DDFCLGPETDTCHGSIFDNSMDCCPTYPTTLLP

>Cre06.g306000.t1.3|PACid:27569292
MPARGLTLALLLAACLVATPFATAMELKPQQQRELQGSKAQLEEEQSLLVVVAAAGGTVSTANTAAASHQQHQQHHH
HRRVLSDGSAAGSSGGRVQWPLSQSDLGVAATAPRHLREGSSGRSSAGRSSGRSISTGGGSSVRSSGLDGWFGLPGE
LMRRALGWTSVAEEDVAKAQDLLATTGGVGGGGLEEAAMRRAAQEGQAPPPPRRRRVPVPDGGGTLLSLPPPEVLQS
PPPPPPPSPQPPSPPPPDVPPPPGAPPPPEQNNNVPEDSPLPPGLPPPPDVPPPPDVPPPPPKPPKKRGKSPPPEAK

RSPPPSPPPRPLPPRPSPPRPSPPRPSPPRSSPKPVIAPQQESSPPPSPSPPSPSPPSPSPPPPPSPRPRSPARQPP
SPPPPQAPYSPAPPTQPQWPPLSPNPPPGRVLPPNNVPEDLLVTGRYIRAGTSMIVAVHMTSVPGTDRFLFMERPSG
YHPDGSNTIAGFFDVPTGGWTHIKSPDGLFCCGHTLLDSGDVLIVGGHQANAGYPDGMKSIRTFNRSCTDLQLRKLR
EMNWRRWYPSATLLPDGRALVMGGTQGVGAGTANNPFWEIYDWATNGLQQFAMRPGYLDSANQVYYPFNYVLPSGLL
FTFCGRAGFILDWNTNTWRQPVPRLRGYATTQFPYTGTSVMLGLYPENNYQVEIMLFGGQKEAANKDLSLRANRGAN
RLKLYWDPAISNYTFDGWAEEMMTISRVMPDSVLLPNGQVVVLNGAQTGLAGDSASGGDSRADYPVLYAELYDPDAR
QGQRFTQLGWTQIPRMYHSTACLTTNGTIIVAGCDRCYRYTVNDGWDYVASPAKADYRVELFQPPFFFMDNLKPAIV
SIQSDKMAYGGTFRITYAFPSVVGGGPGGAPLTFNGQPSLPNGVGNGNNNQLRITRAVLVAPCSCTHSFNTHQRLVG
LEILSDNAATGALVVRGPPDIYTAPPGMYMLFLLNGPVYSRASWVLLQRP

>g6393.t1|PACid:27569295
MRAAGSTPRRSLPLLALAFLAMASSLQLAHGSLLDRLFGEAPAAPAASTADSVRDIIENELLRQEEDLHRRALTALS
ALAHNPAGVAWLLAAAEAALAAGQGDDLRHGGAAVKLLREVAEVDRLRRRRVAEALNIDMDISLEVRQAQQLPTRTA
SVASSQQQQAGGSGSGYPAYGQPYRSADADVAEQQTRERLLSSSGASGSGSGMHVQPRRLQRAAGKQQQQQTKQQKK
QVPSSAADPTDAESLLTDEFDAEREAAFAAQQQQLWLEAQKEDWGNDAISAEDLAAYTGGNTGSGSDAEGSNRKNLL
ANSAAAAAAAAEDDDVDALVSGFGDPAVTRWGAQMQERQQLFEAQEALRMEESEVEIAEAELAEARRSAAALAAAAAA
EADALLSAGYDAGSAAAAGAEEEGLPHAAGSQHYHEGAIRRGLGNARRTPSPSPASVPSPAGDGAAAAKPTNFQLAP
PPQVEVADLKAKAPKAKGGKGKPPPPAAKPTKAGKGKPPPPSLILPTVSAPVPGVNAPSPSPPSPSPPSPSPPPPPS
PKPPAHSPPPPRPPPPVRRPPPPKHNEAPPPPPPPPPPPPEFVGYLPDFPDSPNAPPSPPPFNGKYSPPSPPPPPSP
PLPPSPPLKVAPDAEGPPDQIGMYQQVGISYAVAVHLVHIPGTTRFLFMERPSGKHPDKKTFIAGFFDIATNKYTNV
FNTDSLFCNGAVQMANGNIAVVGGHIAKSGYLDGLKSLRIYDRTASTLITTNTMKFPRWYPSANLLPDGRIFVSGGT
QSPGAGTRNNPINEIWDPQNNPTAPPVQWTLPQNFVNKAGDIFYPNNYILPSGHMLMYCDMVGIIVDPYTGTVIANM
TNHGSIQKTVRLEYPFSGTSVMLPLTPANGYTPEFVFFGGQFGYGWTNTPAVDLAMRVKVNWDPVAKNYTYGTWEAE
KMNARRVMGDAVLLPNGQVVVLNGAMNGVAGDSATGGSSKANFPQFWPVLYDPQAPNGTRFTRMARSQIARMYHSTA
ALTPDGTIVVAGCDRCDYFNVSVPYSKSPWGLPEYRVEVFYPPMVFWDMRPTLVSVPSTAGYGTRFQVLYDTITTMV
DIDGVVLMAPSSTTHSTNFNQRAVGLRIVSDNGNGIITVESPPNINIAPPGFYMVFLLAGQAYSTAQWIQLLP

>Cre06.g261123.t1.2|PACid:27569444
MLAAAHGGKVTQGAAEEEGGQRTGGRRALPQPIADDECGARRGEASPCSPGRCLPKNAGTYVCVCPTGYATGPNEKT
CNEINECRGYDPNTVESCVNTIGSYTAVCKPGYAVYNPVTKACGVATSPSPPSPPPPAPPPSPPPSPPPSPPPSPPP
SPPPSPPPSPPPSPPPSPPPSPPPSPPPSPPPSPPPSPPPAPPPSPDPPPPSPSPDPPSPSPPPTPPSPPPSPPKPP
APSPPPPSPPPPPPNRAYTCTSWTATAGPSAANPNLNYFGNNRTPTPECTTARSAVVALFSPYLSSTYFDFVFKYQF
FGVGCNGNEVTACYSAFDDAELCNISQNLMRLDPDALINAVFGFSGQQCPVALQGYSLGVTSTVSCGISMVGQAC

>Cre06.g258800.t1.3|PACid:27569520
MGAVLHLAARMARSTALLVALLGLAALGAANAQGAYPPPARGLNAYCAIQNRTTNAWACNLGVNTPTACETTDLWST
DATECSLPQVPGFPVPANTFPYTDTCQYQVARLPLVYKGTTTVGGFLVLFKDYADNIYYTLSLNATARAANQPDGQW
LYVEPTLPGQTGPSGAVYLWDSPPDSSKPLSVQASVQDYMIEDRNSYKRWSCATYKTSLNNYCAPGYVFDAAGICQP
KAGATQGPLPSKDLSGAAGSSLFLAAEYSIVKYSVSQIGTAETGFYCGDPRTSMIAGQTLQTLALQGGVVNLAAIPG
FFLPPSCKTTSRPPSPPLPPSPPPQPPSPLPPSPAPLPPSPPPSPLPPSPKPPTPPSPLPPAPPTQQFTGSVFVYAP
NRPNGFNQDDVTKVYAMMRYGLGCGIGNNPNVCLKSQPYVTINETNNAFYPAYTMLGVKMFFDGSKDGSGQPYPTSE
QNKWAADAVTSMFAVLGSSELWKALAGPVQFGGLGLGCGAFMSFEAVVDGQYYSQPIGGGSPPLVDSYDGSTYTYQG
ARYVCPLLLGGFGGPQWPNASLPYYIPDGVAGLPGYIRLGAIPQNTNYQRRRCATILTPPDLMQPSTSCPLSPPPSP
SPPPSPPQPPSPPPVPPSPPSPPPSPPSPANPSPPPPAPPAAFCGLNFKVISRIPKNGVDRNPDCSLLATLVQSTFT
QGVQLRNPPQFRCGTTVSATEISAVADTLTTEGAQVFLRNMAGNIGAFNQFTFPAPGGLGLICGDYFNVTSTCGMNT
TAPVPVFQPNGTVVNATYPFVVGPDAIFYPQFVLPSYTCPPPPPAPSPPPKPPTPSPPPLPPQPNPPPAPPSPNPSP
PPPPPGFRFQMSVINGDQNDAINCPRYTSWMNAMMDSFERTNTIQRVNPSAPYCSRPAQETLLSPELAQPSQVNFLY
QYLSVNSTIGVFVRDGGVPCGSAVRLYNPAGGGFFTDYRCSRDVPTNPAVAVLDLCCPLPPSPPPPTPPSPPPPSPP
PPVLSPPPSPPPPSPPPPAPPPPSPPPPVPPPPSPPPPSPPPPSPPPAAASPPPSPPPPPPPSPPPPVARLPPWPPL
PVNNVPPAPTARNTTWTAPAGTTVRTYEIFAPFKGTGGRNTGVSGRTVQFWLCPALKAALQDQLGQPVSVKPNVNAS
ISCTDWYQVKKKAKGLFYRITVAAQQSAHDAVMDFILSGGDFVRQARLVCGSQIRIGEAEPPRQKLKAVFPSPFDPK
AAVDPYLYLSGKAGSPGAGVCIDKLPV

>Cre06.g306900.t1.3|PACid:27569535
MGGARPWRGSASHTVCISIAVVAALAALAPLAIADNEPPSTHVEPEPPSKSRPPHRPALIAANDDLTPIDFPPPDFH
FGDYNSPPPIESDNPPYIPPPPVASPPPPGTRVPPRPPSPPRSPSPPPRPPSPPPQPPYPTYGPLPMRLVGGTDNSS
GVVEIWNEKEQDWGALCFPAKLFSETYYTQDFMYNQDSDVAKLICRQLGQPYLGAWLLHPDAFPSVATARLRGWALS

TNPGYSCGNAATALSVLDCDDIELLRNMSDNCQSAVLTNEPDVKLRNYVPTGVVCTDTPRRRPPLPPSPPPAPPPGV
RPATDMSIPAVNYTMRIVHLRSGLTTYRNGSRVTQGRLEVLLPRVGPNGTREGEPVWGSVCQSAFVSDEVAKYACRS
NGLPYKAATLVYGVRPKQPRLDSQPVHWVLLRGCTDRPDGLACGATMSRDDVAAFQSANRAAVRAGLPKQPLPYDDQ
TGWYTGLPYAMAACDTQRHQVDVFVRCTDSYPSYSRPPPLPSPPPSPPYPPAVIRNAVQFNVTTIADNLYYIMFGVP
VPGGQPGELVWGHFCPRNTFPGGDDLYIDRTAANAVCNQITGGARPYGYYNFLQNDKPWPLLPPQDERLSRPVVLEA
IDCSMVPPPAIAYDGGHVLTQTELQYPRVENISLCEVTLAAYPPEPLQDGDSCYFRAWGARRLISCIDSPWIETPYM
TSVRLAGGDGTWGRLEIVMHRESSDYLGWGTVCAPEFTIKQAQGVCRDLGLGWTEARLLPTSAAAPLENADSVPIHM
DGIACFANRVWWARPWSEEREQPPSFLRDCRPDWRGSIIKTCDHRTDVVIACGGSAEVIHAPVPGPPPAATQPTSAP
PPYGAYGVQPPPPYGGPYGGYGSYAPPPPGYGGPYGGAYGGYGA

>g6297.t1|PACid:27569536
MPITICGLLALLPPINAAVDERWLVGTVTVQQGRAGRTVWLQTVNVTQAALWVLDLSLIGETHFSTGEDVAVHGLCD
ATRYIVVVDSYVAKTYTLSADPLHSWTGSLQGVSVARPTSPPPPSPSPPPPVVFPGDGGEDDVAALRRSPPPLVDRG
RRKSPPLLPPAPPSPSPPSPPSPPPRPPPPRRRSKMPPPPQNHPRSSTPPAPSPPPPSPRPPRPPPPRPRPQPPSQHP
PSPSPPLPSAPSPSPPAPPPAPSDATRFSLLAVVVSLCGRQPAVRPDTLQGYLLGPARTTLQSYTTAVSNGMGYLDP
SAVAVAPGVVDVPCSGFSSGGPWGTGSCGPLDQQGWSEYVVAAVAARWGLPTGKYGRLMLVLPPGNACGVVSWGTQS
CAPDFIGWPLSRCYVIWTATGNFWPYWNVMHEMLHNKGLFHSIRDNSTDPYGDGSCIMGSGYNTCLNVAQMARLNWA
TPLATLGTPALPANAWQRYSLPAHNRLLANHIRINPGWLAPSPLADPNAGALFVSYRVRSPSGCDTGLDDRFHGRVQ
LHSYIDTLPLGSPWRGNVTVLVANVGPRQLWPPAGRRNLGPSSLQLALRVVSRSPLTDAVPRVTLELCRYTRVTECV

>g10076.t1|PACid:27569719
MLIVWAFTIIPHVSAGRSLLAAAADGQQRAASSSACAQLQGGSDPCFPGKCQGKSSSEFTCTCPPGYKVAASNKKCD
ADPCASVDWESVDACVFANAQLSVTCKSGFNSYNSNSKKCRDIDECDGFDRATVASCTNSKGSYAVICKPGFHDYDT
NSKTCTVAPSPPPPPNIDECDGFDRATVASCTNSKGSYAVICKPGFHDYDTNSKTCTVAPSPPPPPSPSPPPSPPPP
PPPSPPPPSPPPPSPVAAASSPHAPPPSTTFSFRVFGGFVCMTVPPSGGPMYWGGCFSGASAWQNFMVEDTDTPNQY
VIKLAADPTLSLNYSGNLDDGPAKVVLSSDAASMRAWTMTSR

>g10076.t2|PACid:27569720
MLIVWAFTIIPHVSAGRSLLAAAADGQQRAASSSACAQLQGGSDPCFPGKCQGKSSSEFTCTCPPGYKVAASNKKCD
ADPCASVDWESVDACVFANAQLSVTCKSGFNSYNSNSKKCRDIDECDGFDRATVASCTNSKGSYAVICKPGFHDYDT
NNIDECDGFDRATVASCTNSKGSYAVICKPGFHDYDTNSKTCTVAPSPPPPPSPSPPPSPPPPPPPSPPPPSPPPPS
PVAAASSPHAPPPSTTFSFRVFGGFVCMTVPPSGGPMYWGGCFSGASAWQNFMVEDTDTPNQYVIKLAADPTLSLNY
SGNLDDGPAKVVLSSDAASMRAWTMTSR

>g9845.t1|PACid:27569788
MRQTIAPRIVAAGLLAGLWVLTAVRAQPAVTTGLLDIFSGLYIDKQDAACTGIHLKTDYVYITPQVEAQQEPAWTEV
LSGDNIIGDGTDGTFNIRTGDKVNVIYSNSTNRRMLRSDQPALLDFEILEPGSPKEVFTGEPIEVSSYIYVINTCGW
PQSTTADRIQQLYFSGNQNIENYHSTCSYNKVFFREENTRIFDNINVPCAGTVQSGVLKYNYDGSKNCGAAEQFAWR
MAGENLARSLGYGAEMDSIQRRRLIVVLPSAVKCGWAGLGSVGCSGKSCSVYIKGGYANDLLVHMHELGHTQGLSHA
GRGLDEYGDKTDIMGTAGSSPGYLCMNAGNQLRVGWNSPIITLTPPFAEFNVVGRMLDGTPTEGGRWELPAAASTDT
NHLFLNFNSLGGVPFPNTFISFRARSPTFDNILSSDMNNRVLVHFFNGSASERDYNRTLVVGVLSSGQSFASPFVDP
VGGINQYGGGWKVTVLWIQNGNSALIQLCRMYSRADCSGTIAPVPVPSPSTSSPPPPSPPPPSPPPPSPPPPAVVNS
PPPPPSPPPPSPPPPSPPPPPPSPPPPSPPPPPPSPPPPSPPPPPPPRPPPPSPPPPPSPPPPSPPPPPPPSPPPPP
PPRPPPPSPPPPSPPPPPPPSPPPPPPPRPPPPSPPPPPPPSPPPPPPPSPPPPSPPPPNPPPPPPPSTPPPPPPRP
PPPSPPPPSPPPPPPPRPPPPSPPPPSPPPPSPPPPSPPPPPPSVVNSPPLPPSPPPPPPSPPPPPPPRPPPSPPPP
PPPRPPPPSPPPPSPPPPPPPSPPPPPPPRPPPPSPPPPPPPSPPPPPPPRPPPPSPPPPSPPPPPPPRPPPPSPPP
PSPPPPSPPPPSPPPPPPPSVINSPPPPPSPPPPPPSPPPPPPSPPPPSPPPPSPPPPSPPPPSPPPPSPPPPSPPP
PSPPPPSPPPPSPPPPSPPPPSPPPPSPPPPSPPPPSPPPPSPSPPDASEPPLGAIMLSPSPRPPRPPPMRPPPPRP
PRPPPPRRMPPKPPKPSPPPRPPRPPPPRPPRPPPS

>g9215.t1|PACid:27569917
MGRHQPTRRRSAAASSLLLALAGVAVLSLCPASEAARLRGMSSVLGGGGARGYSRDVATAVLSGPGDVAAAHDKAGP
ASGGAEQHAGSRRRLRGLSSGLTGILDLNVTLPSIWNLTLTLRPASPPPPCVQWHKEFPEPCPSPPPPSPAPSPPPG
PPKPPKPSPPPPSPPPNPRPPSPRPPSPRPPSPRPPSPRPPLPPSPRPPSPRPPLPPSPRPPSPRPPHPPSPPLPCE
IWHKEFLLPCPSPPPLPRPPTLRINPGLLFP

>Cre09.g394200.t1.3|PACid:27570208

MGRLSVALAVAVALLAVLPGGVLSQSSPYDYISSRSDMTFMKKCIDNLLPKNKQPWTATSNKALTMFLVTDDGFRTT
FSGLNIRTTLGTDPERFCTVVTKKMNNTRNAIFKYHVLAANLGARTRAQLPGNCPVGPDGLPGCPYNTQAGGKNLLD
VITVAEADDVVGTDKTYLIGGVDANYADYIKFPNGTNDYDRAVKGRILHSINALLVPTRYVPIPRSPSPSPAPKPSP
KPAPTPSPASSPGPVISSSPSPPPPPSPPPPSPPPPSPPPPAPPPPSPPPPNPPPPSPPPPPGVASPSPPPSPAPSP
NPPSPAPPPSPYAGFASIYDFVSQRVADLSRMKTAIDNVKLTARLSDPALAWTCFFPEDTSISWGLNWPGTARNYAE
QGAFVASCIANPAFAPTCNTSVIEYTGVMTNVILQQCTPAALITTSNWVNSTTNYTTVAPFGGLTIFRSSNLANATF
WVASKFDPNPQPISGVLAANNVTSTGLIVIRKRDNFVGQSVVQVVTSFTGNYAAAPPPPPPPSPVPPSPAPPPIAYG
SITSAINGVAGLQITRQLISIFNLTAKVNALAGTTCYFPSNQAWALFGNNVNDLGAFVNVTANGTAVPLSSRRLLSA
MEDGDNLQFCVPSHGFCAMASTVPLRRMLINIPNRFNATQFLDLLAWNVTVEPSGVASMPVFKAQILANTILSGCYN
PPTAPGSQNVGNAVRKLVDTDNFAWETALGWSVDVGIVVQKTPYTGTCGNNVTVTGTATSGAAINLPNVDLENLGYC
TQCTTYDNDTMLYSGCSFTVYSISTGGASGFDTVNGVQQITTLGYSRGRSPVAYWVPHDIVVGTYDNPTGYVQLIDR
IILSPRLPPPPPSPLPPSPPPAPFPPPPFTGLRPLLEGNSNFNDCAAKLFAATNYYPYVEQFDTSGWTLFIPTDDGC
QAALTAYDPATYNGNLTQNVNTAISNGYARTLVKNMLVVNAYLSSGSINNLTTAVTDQGLYGNTTVGTNTLTFLKTS
VISVSQQYPGFPKMTSNITIADIPVQRNPNALGGVGLAGYVQMTTMLFTPPLAFQAQSPPPSPAPPLPPPSPPLPPP
PVAFPNGFQDYLNANPELSILNALLSCTNSTTTIQNLLNQGAQYTLLAPTDTAFSSFFSSNNIGGNWRDVLCNTTNG
QNTKTAQLLAVHVIAGANFAVNITANNNTAANLYNYAFSAGFKIQLLNLGGVYTVRQQMPITSQTFVAGKYDNAING
TAAVAHMITAVLALPVPFPPPPPPPPPPGNGPAGAAAVSVYVGLRNEPEAALFVNLVDKFNVTLPSGVSTPATAYLN
TPDTVATVLVPSVNAMNGFFSRIAFSGTPMSYADCIAPTGGANVVAAKTAVCAAIVQFAVLPGAQFLPSFIPATYNV
SGAATVGVSDAASMYAALGNVTGYGATTASKLYYKLNTTPPVNAATLAFQTTTNPPCVGIASNIKGPIPAGVPNFLS
KLQGMIYVVSQVLFPKEAYDIAVNAGATFSGCP

>g9797.t1|PACid:27570258
MSLVPQALPQAGSMTQASGEHGDSGRQALTLVEERRKATRLTSPALSGQQWSPALPEVANSVGVLNALQRQAALMPC
VPQLATDRAACSAAGQRQALPGGLGAERSRRSRSLHAGCWLLDPLPVHHREGTQLPASPSSGRDAPVSGAWGLLSGA
LGEEQEMEQAGQRELSLLGRGASRAAHGSLACQRGPEPAQQGSSGVDEVVGEEEGVGRPADRDEVRSWRHPACPRRQ
QQEMHGAAEFVPATSSTSTCSGGGQGAAARCGGWARAQRSQEEQEERRLGQQLAGCCPGGAEAEAQRNTGCVPYPNS
SPSTWSSPRSSSLPKTRCELQGGGRRRRAPTATAAGTAPASGPAHSKQHERNPRLPSLLAALLTPLLLLVAAPAALL
TPAAAQLATDPPPYMVNPLNPFGGCLSLQMDVYLYVNCQYIYLGDLPYCNIIFTCADCAGGVSKAPWNITLFNDPNV
APSPSRVSCPTFNLDDAGNAALLLNYNLGNNCPDLPDDSSIVCKAVPSPPPPPFSPSPSPKPPRPPPGPPRPPRPPS
PEPSPLPPSSPPAPPAPPSPPTAPPSPPTPPSPPVAPPRPPSPRPPSPPAPPPSPPSPPVLDGTSPSPPSPAPPSPA
PPSPAPSTPPSPAPPSPVPPSPAPPSLAPQAPSPPSPTPPSPAPPSPPGPPSPDPPSPAPPSPAPPSPAPPSPPAPP
PSPPIPPTPPGPPPTPPAPPAPPSPPPSPPPAPPRPPPTPPAPPPSPPQPPSPMSLPPSPQPPQPPQPPPPATAGVP
PAPPSPPAPPTPPSPAPSPPLPPAPPPTPPSPPGPPAPPAPPPAPPAPPPSPSPPPSPLPPTPPAPSPPPSPPPSPA
PPSPPPPSPVPPQPPSPPPSPTPPPSPPSPPSPAPPSPPHPPEPFFAPSPPPPTPPPPRPPPPPASVTEVPSGFSYT
SFYGVPMYPTQSRLKIISDNIAKELGLESDGVTTTIATTFVATVYSVLTSSLANPVTNRRALLQTNPLPPSPAPPSP
TPPRPSPPSPAPSPPRPPVPPSPPPSPLPPSPAPPSPPAPTNTTSPPPAPPAPSPRPPSPLPPSPAPPSPAPPSPQP
PLPSPPAPPPEPPSPLPPPSPTPPSPLPPPAPSPPPHPPSPLGCMGCTPQVAAGMLAAICTQLSVDDCSTIKVACVG
VGGLNASLTVFPAMPDSAYGYGDNCSARVTATFSLPASAAGQQALFVLNVITNKVAVPGYAVTSPTTQAEVAVSTTL
QNIISNSGSSKVPVSDQKVALSIATTLGLPPDQVFIVTGPSGNTTGGGSGGGGILTPLSTPPPPPASRCDSPRLGSL
CGAEAVGAIVGIIVGGLLLLGLLIGLAYSLISRGRQPVLPVDDYAWVQKYAHVAPGLATAYTPPVFRATPYSVDLNH
PTMYAGTATFKE

>Cre09.g401900.t1.2|PACid:27570285
MAPGNRWAARTGLLATLLLALQLARGVVAQSATYPPIAVYDDSDGSDGRDVSRKAALTTSVQGQGGAGVTVVKGPVA
LSLEARTHVMMANTLQSVLAGGSRTTLVNLVDGNAGMLVLTSVTGDPVPDYNALLAPFLGGASPGCGWADLTKTANN
VYGYPGMPLSLFAGGSPRPLAFSCSAGAPWFVTTDGAAPVIMFHTPGGGWVKLLGFDWSVPSRISVWEKILLYLPAS
PPPAPPSPPRPPKPPTPPPFSPPPLPPSPPPEPPLEGTVIVLTDPATPSDVTRKTKLRLSVLQMIGQGNVQLRTNPG
YAPLHTVYDNTYINLPQASKDYILDLVRRGKTLLSIVVTPELPEERLTQVLTSALGQPAKCVKRALIGAAGNTLTAA
GFSDYSNLLGVPRFPNTMIPANNTVSLVCDSGRQLVLVEGADAAEEGVMWEMSVGRGTVRLVGFDFTTDGFQTTWQN
ITLAQRLPAMASIAVANIKSMLPPPPPFSNPSPPRPPRPPPIMKPPPSPLGGRVPAVVMLTDPTPLPDADLKQKLQD
GIDALGNSAGLWPFGGGVEGALVHVAFDTTLVRFTASQLASLAALVRARKTVLSVIFTNSTTDVQLTAALAAITGAN
RLQCVTSAVAENQRVNRAVPDLIGDLKSDGWRAQLVTRSVRCNTGVILFNSNADSTQAVVHEMSTLPSNASKSGNDT
GGVVRLIGYNFFSGGRGDNRKPLTSLTIFSSNLVSQAALSNATLPDLAGGKPSPPPVQPPPPPARAPPRPPGGAIPP
RPIRPPPPPPPGADAQAVDTIVLVDAVPPATAVDADRKAKFLAGVREVKDMAIVAQTGLGAARSHVVYDNTYMSLPAL
VREDLVTRVLTLQTLLTIIVTPGTTTNNITNIAREVTALGGAVPISCTKERVGTGARIRLNPALSILDVLGPWRVDG
GTSAVSCAGATGVYLTTDRSRPVILEFLTPGGGTVRFIGYDFSTGARGAGKIALTRLAVSYTRLTGTPSPSPPPSPP
PSPRPPPRPRPPSPPFVVRPFAPPLPPDMPLPPADPSFPPSPPPAPPAPPLDTGLVLAAVITDPAGTVLPTADATRK

SNLFDAVNRFLNGAARQSPVKYMPGVSAHIMVDNVNTRTAFTASAKTDLGLAIGTGETILTVIVTPSAATPVTVLNT
IIKTFTGYTATCTKTATAVGTATVGLTAVSHTAAAAALPDTLTQAANGVVFACSGIPDGAFIPIYVTASGNTAVAEV
ITPTGGVLRLVGFDFTSTSPGLTDWAQLVSYNFRIATGAEDSQQ

>g9537.t1|PACid:27570297
MRASALLALVLAAGLACSALGQTFPPYSCTRDPQQSRFRLDMPYTVAGNRVCMTTRVVPCAKPGSPCCDKKIDLYKI
ELDINAQCKFAVTGVTVNGRPALAPTFDPYGPSNSNKAVMKLTGLNLTLATAEGAVVCMKLGGACSTMDTLCTVGNG
FCQTALVQSGTCNCCPVQMLGFMPPPPSPQPPNPPPPPPPSPPPPPPPSPPPPSPPPPSPPPPSPPPPSPRPPSPAP
PSPKPPSPQPPSPRPPSPPPPSPPPPSPPPPSPPPPSPPPPSPPPPSPPPPSPPPPSPRPPSPAPPSPKPPSPAPPS
PRPPSPQPPSPKPPSPPPPSPPPPSPPPPSPPPPSPPPPSPPPPSPRPPSPAPPSPRPPSPPPPSPRPPSPAPPSPA
PPSPEPPLPPSPEPPMNPLTPAIRRPPPPVPVSFPFCECNRTVGNVPFTFDPTPVVKKAASGPNKMYCLTLYTTDCI
DPSNKCCAQRLSKVEWWSKDACRSSVKAVYLDGVKVDQQWAPKGTFKIPALNMAAASVPSAGREVCIELNAKSACPT
LQSFCARSAQGRCYYAMFSEDKTCCPLDTFVAVSGRR

>g10156.t1|PACid:27570299
MRDYKALPLLRMGRLPLRLTKGCLLAVLFLGTLAWTGRAQDDPEAGISPILPGFEADVAALNRFPFCQCEDYRCSSS
PYRLDSIDSPTGRTDELCYRINRVGCSQANPCCKMLLTSLGKVEFSVEKSCSTQYLGSWVNGVKRNSYFDTDFATGK
IRVTPLGMTADSAANTTICLRFQPGATCGSYPALCGNTAGMCTYAVFESSNHRCCPICAQALPPPPPNAPAIRSPPP
SPSPSPPPSPPPPPPPPPPPPPPPPPMPPPPPPPPPPPPPPPPPLPLLLPLPLPRLLLPRLPLPTPTPPAPRRPSAT
ASPHS

>g9447.t1|PACid:27570337
MAGNGGLASAITAFLAVLLWLGSTTAEVGARASHRHPRHFHLNHRHRQLRSSNNNLISGPSPAPSYVGPVYPPAYDA
APPYYAAWAPHYPPSYAPPYYSTPAPYPPTYPPTYQPPPPPPPPPPSPPPPSPPPPPPSPQPPSPLSVLCAAPTSCP
DTATLAAAGIPNAACGDCSVSEQCFVESRQDQFLGAGAASSFLSVASCANAKTDGSVTDPVLSFLTFPRYPTLPVAD
PRYPYLHLYLAAADTAPGDSVAVAVACYTDASLTEVDKYLVLQRIHTATLAAAGIPNAACGNCSVSEQCFVESRQDQ
FLGAGAASSFLSVASCANAKTDGSVTDPVLSFLTFPRYPTLPVVSPRYPYLHLYLAAADTAPGDSVAVAVACYTDAS
LTEVDKYLVLQRIPTAADNYRCPSCKWFWSFEVRTPAAPSC

>Cre09.g389050.t1.3|PACid:27570366
MAWRARCTRIPACAVVVLFVATTVWVTNGVLAAPDDERRDNHADSAMMSHRVGRGLAQDSSFVFKPPSPAPTPPSPP
SPPPAPPSPVPPSPPPPSPQPPSPKPPSPPPPPSPSPPSPRPPLPPSPSPPSPRPPLPPSPEPPSPKPPSPPPPSPK
PPSPPPPSPNPPSPPRPPGLPLPPSPPPSPPLPPRPPPSPPRPPPNPPSPRPPSPSPPSPRPPSPKPPSPPSPRPPS
PSPPSPKPPTPPTPPVPPSPKPPVPPAPPPPPAPPRPPRPPSPPSPPPSPSPPRPPPGPPRPSANARSGDGKGGFVF
FSTDDADDNGHCEGLGCGRLYANVFTLAVELAPADARGILAVGDFSYGTPAKSSLESWVTAAQYPIELIDVVRTPAQ
LLAANLTSYKVLYVPSNIYNTAGGVDVDLNDALISIKDTIKNFVNVRMGSLVVLAQAGFGIYDNSLAYGFLPVPLNF
TALDFFDVEVTPDMLEISPETNGDNSDHSFWHGYWTDPIDWNGLRVLAYQARMCPVAYGALQKCRASVLCNRNTILT
AENCENGIDDDLDGLIDKNDPDCWRCGDGVVDPDEECDDRNVLNGDGCSSTCKFQDLPPPSAQPSPPPAPPKNSDAI
DYCVRSGSPSCANCGGKCVTIDNFANDGTICKLPEYLGGAVPNNEVCMNDKNFVAFKTLIVYSSVDPTLIVGSATLF
RTPDGTLHVTVQMDCPYFIWSSAGDVNMQDNNNVVLLSYPDPTNPTTGDLQYTRVPLTRKYATGDETYACYTFSTDI
NFLAPSGQQAGCYAIDIDAKAVSIRSTKASNSVTSANCTYQPEKIGQTDTFTTKTMFVSEDPAPPSPPPSPLPPSPP
PPWPPRRPPPSPVPPSPPPPSPSPPSPSPPPSPLPPSPSPNPPSPAPPKPPSPGPPLPPSPRPPSPVPPSPSPPPSP
MPPVPPSPVPPSPMPPVPPSPAPPSPSPPPSPAPPVPPSPVPPSPAPSPVPPSPPPPLPPSPTPPSPPLPPSPPDFP
IPPSPSPPPLPPSPEPPSPQPPVPPSPEPPSPQPPVPPSPEPPSPPPSPLPPSPPPSPEPPSPEPGLPPPSPEPPSP
EPSPPPPSPEPPSPSPDPPSPAPSPDPPSPLPPSPSPPPPSPSPPDPPSPPSPFPPGWPADPPSPPSPPPSSPPLLP
PPPSPAPPRPPSPPPPSPLPPAPPSVPNCNCARMQPPAADGSCPSSPRPYVKVTFKAGDRYDESLAGISICVPTLGF
DNATNIYKVAFCWNTQCSIMAPLRAASAPVRAFLTADAGPATAPANHLSGAAVVLTTLSPVCANATNTLALLGGVGT
YGWGAANRLLSIPEPGVASVDVDVKIADIGTCDDDWVVAAIVVVTTAVATTPPLPPAACPCWQRTLNGTCPGIKVPI
LLETQDNWDNRGMSVMQCIDPANFDIYTGRMAYSYCWRYACLPDVFSTRPFKATCKDLERWNIAKMQGNFTLFMQRT
EGVRMTVTGKPKDAALPNVVYDASDDGTVVTLPPGGVGNFTFEYIIPPGFDDLSAAVVMETGPRPPPPPSPPGPPGS
SCACPLRPLETVGGSATTCNSTFATIKMEVVNDTSVQPEYRCTSWPNYDLLLQDMAWSYCWRYDCLRTDFWGKAYTA
TIEQVTKHNIAQMTPARYQLIHFVAPYGMTITTTFYFADAAMAPAACNSTVGSPVVSGRVQEVTLMPLSNLTIQFYI
PAVWGIDGMAAAVVLRHIDGTDIPLPPAPPSPPPAPPAPPPLPATEASVLVTTDFLVLASVPVAALTSGSSNVVQSG
YFVTLPIKFYDFLDMPDCGDASRAAMQTKVAAAFGIANASSVTVSCRFAAPASDQPILRRLMRALADGLHRVLQAAT
GGNAAPATEKVVVTAGVSTAVDYSKALSAGCTKLDSIVSGDSACDTAGVATYPRVSITTSLPATAAAAGTGSLCAAL
TTSNAAFIQSAAAVRSTAVVSNGCTAVYQAGKAATPSGSGDGSSPSGGNAPAPTPSASGGSNGGLGTGAVVGIVVGA

IGGVILVALVAVVVRNRMVTRRSNLLFVRADGRVAAAGGAETSAAASRNWATLGGRKPWRTYSMQEQEAQARSGSAP
IAGVTRY

>g9535.t1|PACid:27570430
MRASALLALVLAACLACSALGQTFPPYSCTRDPQQSRFRLDMPYTVAGNRVCMTTRVVPCAKPGSPCCDKKIDLYKI
ELDINAQCKFAVTGVTVNGRPALAPTFDPYGPSNSNKAVMKLTGLNLTLATAEGAVVCMKLGGACPTMDTLCTVGNG
FCQTALVQSGTCNCCPVQMLGFMPPPPSPHPPPPSPPPPSPPPPSPRPPSPTPPSPKPPSPQPPSPRPPSPPPPSPP
PPTPPPPSPPPPSPPPPSPPPPSPPPPSPPPPSPRPPSPAPPSPKPPSPAPPSPRPPSPQPPSPKPPSPPPPSPPPP
SPPPPSPPPPSPPPPSPRPPSPAPPSPAPPSPEPPLPPSPEPPMNPLTPAIRRPPPPVPVSFPFCECNRTVGNVPFT
FDPTPVVKKAASGPNKMYCLTLYTTDCIDPSNKCCAQRLSKVEWWSKDACRSSVKAVYLDGVKVDQQWAPKGTFKIP
ALNMAAASVPSAGREVCIELNAKSTCPTLQSFCARSAQGRCYYAMFSEDKTCCPLDTFVAVSGRR

>Cre09.g393450.t1.3|PACid:27570620
MGHIGLLLLALALLVVAPGALSQSSPYDYVSSRTDMTFMKKCIDNLLPKNKQPWTATQNKALTMFLVTDEGFRTTFS
GLNIRTTLGTDPERFCTVVTKKMNNTRNAIFKYHVLAANLGARTRAQLPGNCPVGPDGLPGCPYNTQAGGKNLLDVI
TVAEADDVVGTDKTYLIGGVDANYADYVKFPNGTNDYDRAVKGRILHTINALLVPTRYVPIPKSPSPSPAPKPAPKP
APTPSPASSPGPVISSSPSPPPPPSPPPPSPPPPSPPPPSPPPPSPPPPNPPPPNPPPPPGIASPPPPPSPAPSPNP
PSPRPPPSPYAGFKSIYDFLSQRVADLSRVKASVDAADLFLQLSDPSLAWTCFFPDDDSADWNQLWAGVYGEGRNIL
QQTQYVNQCWDSVRAGALTNPKCPNATREYVGVMQNVYLQSCSPSAPLVSTDVWANRANLSTVAPYSGLTTWRMGPN
PANASTTLWVTTVFDPFPMPNSGLLLPNVLNAQGVVAIRKRDNLVANSMVQVISSFLGDYAAAPPPPPPPSPAPPSP
APPPQAFATISTALLGQNALSVTRQVITLYNLTQRVNALADTTCYFPSNQAWALFAANGNDQGAFISVAANGTFTPL
SRHRRSLLQSQPLPQHVTAIRFCALGFCATADLTTAGASGAAAQARRSLAMGPNPFNGSRFADTLTWNVTLEPADSY
NRAIMRNVILQSCYNPPSAAGSQNAGANAVRRLVDTDEFAWETALGWGSSTGIVVQKTPYTGPCGNNVTVSGVATSG
ALTNLPNVDLEALGYCTQCAQSDPATGLYAGCAFTVYSIATGGASGFDPNPTNTSASVITGLGYSRARSPVAYWVPH
DIVVGTYDAPKGYIQLLDRLVLSPRLPPPPPSPLPPSPPPAPFPPPPPFTGLRPLLEGNSNFNDCAAKLFAATNYYPY
VEQFDTSGWTLFIPTDDGCQLGLQTYDSTTYSGTLAQNIATAIANGYARSLVKNMFVVNAYLSSGAINNFTSAVTDQ
GLFGNSAIGTNTLIFRKTGAISLFQRYPGSSGAFPGGFPQMTADVLIADIPVQRNPNALGGVGLAGYVHMTSMLLTP
YLVAQSPPPSPAPPLPPPSPPLPPPPTAFPNGLQDYLNANPELSILNTLLSCTNSTTTIQNLLNQGAQYTLLAPTDT
AFSNYLNSAGIGGNWRDVLCNTTNGRAAQTADLLNAHFIAGVYFGVNITANNNTAANLYTYAFNASYKIQILNLNGL
YTVRQQMPITSQTFVSGKYDNAINGTAAVAHMITAVLALPVPFPPPPPPPPPPGNGPAGAAAVSVYVGLRNEPEAAL
FVNLVDKFNLTLPSGVSTPATAYLNTPDTVATVLVPSVNAVNAFLGRVSFAGTPMSYADCIAPSGTPQQVAAKLAVC
GGIVQFAVLPGTQFLPSFTAADYTLNPPIVADGTFKYILIGNNTGAGSSATKIAYRLDTSTNPIVTTSLAFQTTTSP
PCSGLAVTSKGPIPAGVPNFLTKQQGMIYVISQVLFPQEIYNSAIAGSGATYAGCPP

>g9538.t1|PACid:27570668
MRASALLALVLAAGLALGQTFPPYSCTRDPQQSRFRLDMPYTVAGNRVCMTTRVVPCAKPGSPCCDKKIDLYKIELD
INAQCKFAVTGVTVNGRPALAPTFDPYGPSNSNKAVMKLTGLNLTLATAEGAVVCMKLGGACPTMDTLCTVGNGFCQ
TALVQSGTCNCCPVQMLGFMPPPPSPQPPNPPPPPPPSPPPPPPPSPPPPSPPPPSPPPPSPPPPSPRPPSPAPPSP
KPPSPQPPSPRPPSPPPPSPPPPSPPPPSPPPPSPPPPSPPPPSPPPPSPPPPSPRPPSPAPPSPKPPSPAPPSPRP
PSPQPPSPKPPSPPPPSPPPPSPPPPSPPPPSPPPPSPPPPSPRPPSPAPPSPRPPSPPPPSPRPPSPAPPSPAPPS
PEPPLPPSPEPPMNPLTPAIRRPPPPVPVSFPFCECNRTVGNVPFTFDPTPVVKKAASGPNKMYCLTLYTTDCIDPS
NKCCAQRLSKVEWWSKDACRSSVKAVYLDGVKVDQQWAPKGTFKIPALNMAAASVPSAGREVCIELNAKSTCPTLQS
FCARSAQGRCYYAMFSEDKTCCPLDTFVAVSGRR

>g9176.t1|PACid:27570737
MGLSLSWAALVLAATLLVVADAQRQSGLYTNFPFCKCVARGRYSLDRVVDSVGAGQYCFTIRVQTPAGCNGYCCTQA
DMHKLEFHAQPQCDINNGASVSATINGVPTRVGPAYDQPPDGPPGGTILRLTQLGLTAATANGARLCIKLTAGRNGT
GCGTLEQMCVPPAGSPPGVCTAAIFDSGNGCCPQVQAGTPSPPSPLPPSPRPPSPKPPSPPPPPGPPPPSPAPPSPS
PPSPPPPSPQPPVPPSPAPPTPPGPPPPSPAPPNPPPPPSPAPPSPKPPSPEPPSPPPPPSPEPPSPKPPSPEPPSP
QPPLPPPPPSPYPPSPSPPSPPPPSPPPPSPAPPSPAPPSPPPPCEVCLQLSITAFPAPESFPPFRLPVSLCDSLLA
QLNATITASGVTVLSPFVATACSPNTVRVCGVFFSGAEASEKLQPLVGGLLDSALATALGGGCSGASAGYTLGASIF
GVNGSPDCLAGERELTCAFLPPSPLPPSPAPPSPQPPLPPPPPSPYPPSPAPPVPPSPPPPSPYPPSPAPPAPPSPP
PPPPPPPPPPPPPPPFPPPAPPSPPPPPSPPPPSPAPPSPQPPSPPLPPSPPPPSPYPPSPAPPYPPSPAPPSPAPP
SPAPPSPPPPPSPPPPSPYPPSPTPPSPAPPSPTPPSPPPPPSPPPPSPYPPSPAPPSPAPPSPEPPSPPPPPSPEP
PSPAPPSPAPPSPPPPPSPPPPSPYPPSPAPPLPPSPPPPSPYPPSPAPPSPAPPSPEPPSPPPPPSPEPPSPAPPS
PAPPSPPPPPSPPPPSPEPPSPPPPPSPEPPSPAPPSPAPPSPPPPPSPPPPSPYPPSPAPPLPPSPPPPSPYPPSP
APPSPAPPSPEPPSPPPPPSPEPPSPAPPSPAPPSPPPPPSPPPPSPYPPSPAPPLPPSPPPPSPYPPSPTPPSPPP

PPSPPPPSPYPPSPAPPSPPSPPPPSPAPPSPAPPSPPPPPSPPPPSPAPPSPAPPSPPSPPPPSPAPPSPAPPSPL
PPSPAPPAPCPAVCIYVALLPGPAVLFPYAFNADTCASIGDSIASDITEAAQAAGLNVSTPFTLSACIDNLIKVCGE
FSSIEDDVVSALQPFVEIQAAVWRELVSGSSSSGTCAPFLSGYSVVVAVAGDNGEVTPDTDVSALLTSLPPSCLLAT
DSAGCSPEDTGGNFPKCQCNTSPLSTRYAAQPTITTLPGRRPGRTNYCFQLALVQPSKPNTFCANTSSVYKVEWWAD
DTKRRAITGFGVRPGNVPASTPLRYASVSWGAVGEDTVKLTPLNWNNDQVDGGLVCLELDGAIAPTLGDFCISSTQS
GGEATGSGTCWLNIFDTSKKCCPTYNAAQP

>g9749.t1|PACid:27570824
MLGRRARRRGACRSHALSILLVLVAYVAAQYQAPVSCPQPAGYTFLRLHGSAERNLGRVARPPSTSAAAWTAYLAFV
CSGLSACTGFSSAGWLKAERAGFGQVGGRWYALSSWEVVDDPAGPCAGMYVRHGWERGGCPQASGFFFLEGKDSSGG
DLVAPQPAGRPSLAPTSAGRVAGWAAECEGRGSCLAFNSFGVLRASLTTYQNLEDIDPALVSGYGDCLGLYVRIPQP
LPAPAFRPPMSRSNTSVELPVFANANYRPLAPDFRVPADWTSGSNWLELSFEGTYLPSSAALPADLGILELAQSRNV
FTNAVPLWLTGPDGSRLVRVTWYLPNVDLSPNGLARIRIRTPYAGWSGLRFTVAFTWRSPAPPPAPTPPPLPPVPG
MPPPPPSPEPPAPPPPVPPTLPPANPPPWLNDDVEPSPPPPPGLPPPPAPPSPPSPPGLPPSPQPLQRTSRSSSAAP
VTLPPLFGSRSAVLVALPLPAGWRSDDSRLVVSWAAAAYVPTANATTSPGPGPAWGAMGAYGQFLFTEERYAPAPPP
SPAPPSPVPPSPAPPSPDPPSPQPPSPAPPPSPPGLPRPPPDVPAVVTSARRQRRARELLQGAAAVPPAGPTPPLPP
PPPSPPPSPPPSPPPPPPLPAFTVLSYTYTLPIEATSGSVLLVNPPLAAGSRLTLAVRTWNGGWNALTANLTAEWRP
AAPPDPGTAALPPAAPGGDPSAGPLQRLPNMPNFPLLPFPPSPRPPSPRPPRPPSPAPPSPRPPVPPSPQPPSPAPP
SPPPSPAPPSPPLPPSPEPSPPPLPPLPRAPQAPSPPSPSPPRVPPPAAPMPPSPPAPPTPPSPPAPPRPPSPSPPS
PSPPSPPAPPPPPPSPPPPPAASPPSGAPIINPSPEIGNAKPPPAAVPFSPSSPNEEVVGSSGGGSTNLALAVGLPA
ALAAVGIFACVAAAGFVMAARRRRREEAAAAAAALLSKSRDGSSAGGEVVGAAGPGGSGMGPGGPAATGGAGGGGLG
PRAAQARQLLSPLGVPASSPHPSSSQLPNVGSTSGSMAGSPSGAGGGGGGAGGGVGGGGGGIGGLLSPRSQVLAAKA
RRLLRTVTTLNPFGRSGRGGRLAAGAPVAAAGAGPGAGRRPHLAPLAVAAVAAEAAAAAAPSPGGGPGGTLSSGFLD
SDVEGPQGAAGAGGAFLDPPAHAAAIFTTGGALPVGRGSNTRDSPAGGEGRGSGGSAAGSAAGGGSAAGGGHYTPSP
LGNLRALSELLGVPLPPQLQPGGPSGGVLGGAATRSLHTRSLDGLPAHVAAGRGA

>Cre17.g708450.t1.3|PACid:27571083
MLRYRASGLRPLAPFGNTGRATFFAALVLIARVALSQEERPCPFKHVNQAQNMFPSGPGVAANLQTCKSCRLDTNRC
RLNCRGGCLLPDGSGRGPGVALSLRECDPDTHRIGVTDDGHMVCQDLTGEPPAAPEQASGLPSPSPSPSPSPSPRPR
RSPSPSPSPSPSDGDTVNVTITGTISVLMGHRHGEGAAREVGNWTLQEDAGAAIPLTLDFGEGENTLVSGDSVALQV
EVPASAVPPGTRRLRRLSELALPPGASLTVVGTRQTASGRGKDFIVGRKPVNLTTAIFSFSFCGWKPAVTPDVVKAR
FFNKYAADPLRSVTVERTFNTCTWGLLRTPEPLSVVLPTEEIPCSGTRPDDNKPTVQIKYDLGRLCATPEVDTLRYY
AKLLANTKYKDRVNWSSLKRIVMILPNMPACGWAGLGNTGCAPGTQCNSWLQVPDDVDIPVMFQEMMHNTGLYHSER
LLVDGFTSVPYGEVTDPMGQAINDPAIPNGLICSNAAQAWKAGWATPIERNMTDMIPGQAAEWTLPALGVSGGLEAM
LRIRLYDDGRAFDGNPLRQRAVYVSYRARTPAPGYDSGLPAQYNQRGYLHQFDGAAVPRTDPWLTPTRLLGLLDTAP
DNAIQGLEAGPLPVRRTWIYTPLSYVNATDRPDLAQAVGPLGSLNITIKAKDTTSMAVSVCRFVDTRESGSQCVDGQ
DNDCDGLADEDDPDCADEWATGDFSDKPSPPPQRIRSPPPSPPPAPPPPKSKPPPPTGRSRPPPSPPPPSPSPPSPS
PPRPPPPPKRKSPPPARSAAPPPPPKKRG

>g17376.t1|PACid:27571094
MRLTVAVLAILALAIGASAQQTGLYPSFPYCQCTKTPSAYRLSPTVTSTGAGTYCFTLSAKVPAGCTHKCCKADLKK
IEFNVNDACDVFSPSLKATINGVRTKVAPAINKAQNGPVGSTTLVLTQLGLGLGNDGAQVCITLGLNKNGKGCTTLE
ELCVPPAGMPAGVCTAALFDSQNDCCPLSQANVPSPPPPSPPPPSPPPRCEVCAYIALVDPENNAPFPYAFSADECD
SYAQTLIDDITAQAGDAGATIVTPFAKVDCQERLIKVCGEFFSNEEGALIQDWIGEQVSVWNDMVTGGQCPAYLSGY
SVVTAVGGDGSDVNSLPMSCLNAFKSTACAPETVDFPKCQCTTKAFATPFAVKPMMSEMAGPSKDTTSYCFELAVVA
PANPGSACGKTSTVNKAEFFADDTKRRQIKSIGIKPAGAAGYKWVAPSWGAVGDQTLKVTLGWSTAQAAGGRICLEL
YNTTSLDDFCMGAAMDTCWLNLFDTTRNCCPLYTSSLV

>g17407.t1|PACid:27571100
MTPAVTQSLPPVCVHNTTDFTQVFPPDATGPADTCVDQAACLDIYYDTSTCKTTGGYLYCQVCIFWNQGRYNRCPKD
VTNTLSHTCRGDELDPGITAFGGAPQAQGGDTAKLNGWPSGVANRYCQWVRWLGEDTNPVNVRFTVKDGSQSCGTAT
GNFKINGMTATCQPPRTLSSGAIAGCGRGDQANECLWTFSVPRIPVCNTKSPPPPPPRPPPPPPTALRPPNNKGGYAC
VSSDDADDAGHCQGTACGSLYINMIRKALDLAPIDADGILAVGSFTNGTSARSSLEEWVKAAGVSVSKITYADTAAK
VAAANLTAFKLLYVPSSYLQTNGGITDALNDALIAIKGKIADFVNLRGGSMIVLAQQGLLNGKSYGFFPTNFTYVET
DFYDSNNQPEMELVSPSTDNVNIDHRFWHGYFTGPQDWNGLRVVSYVANQCPTPQGRGQKCNATVLCNLKTFLTKEN
CYNNIDDDGDGLIDKADPDCIRCGDGIVDPGEDCDDGNMLDGDGCSSVCKFQAFAPPLRAPPPPFMESNDPDLDYCY
QSSNAGCATCAGACETVDGFWGSARACKLPKKLKGGLPDANVCKNDMGGLAVRVEDLYVTDNAGAVSSIVGQVHMFR

TTGGILTATVRMGCPQLLYSDASNTTDINQFMLVSYTAGGRFVSKTVPMVRPDVGKKRYACYSFEYDMNEIVPGIGC
TLVDMDVRITVRSSFTTSMVGSTTCSSAPDKLSQTTTMTERTSFLVAPQLPPDMPPSPQPPSPPPPRPPSPPPPPPP
PSPSPPSPPPPSPSPPLPPSPPPPSPPPPPPPSPPPPPPPSPPPPSPKPPSPLPPSPPPPPPPSPPPPSPPPPSPPP
PNPPPPQPPSPLPPAPSPPPPSPAPPLPPSPKPPAPPSPAPPSPGPSPPPPSPIPPSPVPPSPAPPLPPPPSPPPPS
PPPPSPPPPNPPPPPPPSPVPPSPKPPSPPPSPVPPSPAPPSPKPPLPPVPPSPAPPAPPSPGPPSPVPPSPEPPSP
APPSPKPPLPPSPRPPSPKPPSPAPPGPPPSPVPPSPEPPSPAPPFPPSPPLFPVPPSPAPPPLPPVPPSPAPPGPP
PPSPPPPSPPPPSPPPPNPPPPPPPSPEPPSPKPPSPPPPPSPEPPLPPSPPPPSPVPPSPEPPVPPSPAPPSPPPP
PPPSPEPPSPKPPSPPPPPSPQPPLPPSPPPPSPAPPSPEPPVPPSPAPPSPPPPSPPPPSPPPPSPPPPNPPPPPP
PSPEPPSPKPPSPPPPPSPEPPLPPSPKPPLPPSPAPPLPPSPDPPSPEPPSPEPPSPGPPRPPSPRPPSPEPPSPK
PPVPPVPPSPAPPSPRPPSPNPPSPRPPSPMPPSPPPPPSPEPPSPEPPSPEPPSPAPPSPEPPLPPSPAPPRPPSP
RPPSPIPPSPAPPSPEPPSPEPPFPPSPPLFPVPPSPPSPPSPPKPPVPPSPAPPSPPPPSPVPPSPEPPSPEPPLP
PSPRPPTPPSPSPPSPRPPSPRPPIPPSPAPPSPAPPSPEPPLPPSPEPPSPEPPSPTPPAPPSPEPPSPEPPSPEP
PSPRPPSPKPPSPMPPSPKPPSPAPPSPLPPSPPPPSPEPPSPEPPVPPSPEPPSPPPPSPVPPSPPPPSPPPPNPP
PPPPPSPEPPSPKPPSPPPPPSPGPPSPPSPAPPSPAPPSPEPPVPPSPAPPSPPPPPPPSPEPPSPRPPSLPPPPS
PSPPVPPSPVPPSPPPPSPEPPSPEPPTPPPPSPPPPSPPPPSPPPPNPPPPPPSPEPPLPPPPSPQPPLPPSPPLF
PVPPNPPSPPMPPAPPSPVPPSPPPVPPSPEPPAPPSPEPPSPEPPSPAPPSPAPPSPHPPSPRPPSPVPPSPPPPS
PEPPVPPSPEPPLPPSPAPPTPPSPEPPLPPSPPPPNPPPPSPPPPNPPPPPPSPEPPSPPPPSPPPPTPPLPPSPA
PPQPPSPEPSPPPLPPSPRPPSPAPPSPEPPAPPPSPPPPNPPPPSPPPPFPPPPGPPPPSPPPPSPPPPPSPTPP
SPEPPLPPSPTPPTPPSPPPPPSPAPPSPEPPSPSPPPPSPPPPSPSPPSPPDAPPPSPEPSSPPPSAPPPPSPPLP
PISSSCDCATKLFAPTNGACPDNHVILNFKAGNADRFDTTTPVRACVPRLGSNDDNTMYTFAFCWSTSCPVLSPLTE
AMSTVGLRIYVDQMSVLPAVADRLADKAVVFTNLHPTCAAPTSTLTLLNGLGTYKIEASQSLRQIPAPGLDTLDVDV
QAARNCDNEWVMVAMVPVHDIGADVPAPPPAACPCWQRTYDGTCPGIKVPITLSIEGAVDPHLFPPSTTNTTIQQCI
DPANFDVYTGRMAYSFCWRYACLPPVFASYPFRAVCKDLERWNIAKMEGEFLLQLQRTEGLEMFVEGKTKDPVLAGM
AVVNYTRNDDLTFISIPAGGVGNMTFTYVIPPGFDDLSAAVVMDSKPRPPPPPSPPSTRLTTCACPLTPPDTVGAPD
AITCNGTYATMTARIANASTPEEQAAIAYQCVPWPNYDIFMKELAWSHCWRYDCLPSIFFGKPYTLVIDRVTKHNIA
DIPAAGYELITFPAPYGMTMNVVLYGAADGAAMEYNSTQGGLVVNGRLQVIQVPKLQSLTVQLNVPNIYGVDGMAAA
VMLRYIEGSEMPLPPRPPSPPPAPPSPPPMPEGVPRVLITTDFMVTAKVPVETLINSSATITHSGYYVTVPIQYYSF
EQLTDCSDASVAAYKQLVATTLGLNDTSTITVSCRFATNTAGQAIARRLMAAATWVMESLGLRSQPGPLERVRRQLQ
TGTGAGASGDAKLDVTLTYGAPKTVNPADSATTNCGVMMTAWGASCDASKVRAATRVTIRSEKADTSQSAVASACSS
AAAEGTAALLAASPTLSLNDLETMACQTAIVTTSGTSSNGNGNGNGGNGNALPPPPPAGAANPPGASGMSPGAIAGI
VIGSVAGVVIVGFVALAAKNKIQQSRSGTQFVDPNSGRPGARRWRTYSMQERAEEAVRGSRPVAGVSVYT

>Cre17.g696700.t1.2|PACid:27571196
MARHCILLALTLCAFASVALARDLLQATSSNPSWKNLFPPYGCDRNPNHSRFKIVPGYSVQADGSVCFTVMTQTCTG
SATCCKVKTANKLEINVNPTCNRAILTATVNGRRTLAPTFDEYGTADQHALYKITGLNITSANADGSQICINFNPAG
ACKSIESLCPEGDGSCTLAIVQTDPCNCCPVNPVGLFPPPPSPSPPSPPPPSPAPPPSPPPPDAPARRQPPPPSGST
PFPFCRCNRTKDIMPFRLADQAPTVKKIGPNNAYCFKLLTGPCAEPGSFCCQQDLRKIEFWASDNCRSSVRQTYLDS
VPVSFSWSTSKQGTTFKLPNLAIKSSDVPAQGREVCIELVASSKCPTLQTFCHYHPRAPGCVYSVFSNDAKQACCPS
TSFPSV

>Cre17.g705500.t1.3|PACid:27571235
MGTSKPCLLALTLAALALAASLQLGSAQPVYGDTPSPPFDPNDPDAAYLFGFPFCRCSDYRCGTTPYKMMRFSEQTL
ANGNYQVCFNFQDVGCQSGNACCDSILRLMDKIEIQAEKTCSKAVAAVTFAGVNEIGSTHFDTEFTVGKIRITGLNA
NRATMQSAQLCITIKPPCNSFDVFFDSDKLGQPGIYQYAVFNGGHDCCPVCVWQQPSSPSSPPPATVTPGIKRPPPS
PPPPPPSPPPPPPPPPPPPPPPPPLPPPPPPPPPPPPPPPPPPSPXXXXXXXXXXXXXXXXXXXXXXXXXXXXXXXX
XXPPPPPPPPPPPPPSPPPPPPPPPPPPPPPPPPPPTPPPPPPPPPPPPPPPPPPPSPPPPPSPVQPKNESICDCISVF
EPNTRWRFRYRDSELKGDFNYFSFNIFTVPSDYCLEVNYRPGYCCDQTLEEVEVAIAPQFQDSWWKLAPRYWFFNTD
GSIFQSGAISTQHKSDLGLIFKGFNFNTSSVPPGTELVLTLALNATLWNNTDAFPCGQSHLVDEGGICDYLMYGNQI
HDGQPVMSPYGDFVPSCCPEGVYLIENPTPYCGCTDNKLESPYRLVMEPQPVTVRGATTYSFRVDTTNPVPPESNAE
VDCNGMDLDAVRLYVLPEIANSAAITSVMFNNKTIPMKNITFGNDTYQYWIEIKELGKAKPAIGTSWKLDITVAGAA
PPSICAPNALGTGECEYNFFGKFSLKDLEYQCCAHGLSEPTLVSEEPEQCGCDANILATPYRLDYASAAYTRAADAT
TLNFTLTYDQLDCEPNSACCASDLKSVFIEMDTTAFVSAAVTPAVPGGVTVEKLASGIKLTALFGSANGYDVAVRVS
GQKTLASVCGSLSAKDGCKYRLEGGYNVNQPYGCCPTDVTGV

>Cre17.g717900.t1.2|PACid:27571405
MRLTGFALAVIGLLAIGASAQQTGLYTSFPYCQCTKTPSAYRLSPTVTSTGAGTYCFTLSAKVPAGCTHKCCKADLK
KIEFNVNDACDVFSPSLKATINGVRTKVAPAINKAQNGPVGSTTLVLTQLGLGLGNDGAQVCITLGNKNGKGCTTL

EELCVPPAGMPAGVCTAALFDSQNDCCPLSQANVPSPPPPSPPPPSPPPPSPPPPSPPARCRVCAYIALLDVNNNAP
FPYSFSDDQCVEVSDVIVADITAQAESVGATIVSDFALVSCQGQQIKVCGEFFSNEEGALLQGFVSEQVAIWRDLIT
MGSCPAYLTGYTVVAAVGGDGTDVNNLPVTCLAAASSTACARETVDFPKCQCVTQPFSTPFAAKPVVTTMDGPTKGT
TAYCFETMLVTPNNPGSKCGKTDMLLKAEFYADDAKRRKITNIGVKPAGGAMKWQAPSWGAVGEFTVKATPLNWSKA
QADGGMLCLVLTNDTTLNDFCMGNTENTCWINLFDSTKNCCPLYTSSL

>Cre17.g736800.t1.3|PACid:27571553
MLLLIAVALLTMGLQQVTAQSCSGSTWTGGGSPMYDCSSNMTAYVSSNITTPSSAIGGSAGGLAGPPYWGTSSKCLS
GEPTVLAEQLTSWFTGNNGATSCNITVYFDTYAAVRHTGECCLRCQLRIPRVGILIVHKGAAKSNISPNLTLLPVWG
SPVTTSCGSEPAAKCGTSDNVFWYTCSFSGTLTYVAGITFGVQDNGYKVFDAVRLIGYRGYMPPSPPVFSPPSPAPP
SPSPPSPAPPSPNPPSPAPPSPAPPSPPPSPAPPSPKPPSPAPPSPAPPSPPPSPVPPSPAPPSPPPSPAPPSPSPP
SPPPSPAPPSPAPPSPPPSPQPPSPGPPSPAPPSPAPPNPVPPSPAPPMPPPSPTPPSPDPPSPGPPSPLPPSPPPS
PPPPSPMPPSPAPPSPTPPNPAPPSPEPPSPVPPAPPSPAPPSPPGASKPSTAQSLSSQPSPSLPGASITCTTKPST
AQPCSAQPGAAKPRTSKSGSAFTRAAQPRTAQPGATKPSAAITYSTVTPASQPWAPCSPIACTTKPKPASQPGSAEP
STTHATKPAACASFPPPSPPKPPQPPFIIPSIGVPPEYVAVNDSLPQGYLTVTQVVDVPDDLATSPEALAALFPGGA
SLAVTGKNIRVPVEITTAVQATAVLTTSLGISTQVEVGCNSAFLDAFRSELALRANLTTDIISNVSCNAGAPQQAAS
RHHRRRLYSSDSQGGIAGGGEPYSDAWDGGEQKDEAGLELDDGYGGDGDGAGAEMRRLLAAAASGTCPATGAGSLSL
LVVLSVPAAATNNSQLYKSQIAAALDAWTAESAAGSGAPGMLQLCGPSMDDIVTTTTVRVVSEVALSSMGASNLSSV
CGSADLTTTTALGGAGSSVACRVTAASTGDDGRPPAVSNPSPAPASAPVSAAGATTSYLSIIIAAAVTGGVVGAVCL
AVIGIVARRRSREKRQREAGDPGELSSSELDHGQAGTETNQFYVGAEAAASAGSGVEAGGILAVILPTGATRGSSSA
RPVAGTAGATAAGASGGGNAFGRRTADMSAAEESEIRSAFQHALQSAGPPPLPRSDDWSVHGRHADPTSSALVPLAV
SAPPAGGAAAWSGPNGPMSADQATIGTWGGSSGGPGTGSGYVGGGAAAAGAPAMRASFDRRASSPGISGRSVRVSRR
VQPLGNSNQVPLQSRGASPAVSGAHISSGGLPPVELSGPQEPQAFRSFASMSGATGAAAAASAPVAGSFRRQSLVMN
PAFESDWEPRASPAAGAAAGTSAHGGARPMSGSVIVARATAGGGAAADGASRVAGSPTGSEAISLFERFKSQLTGGL
NSASGRPASIDGSPGGSPRAGNRGLSLRNITAMFKEGGGSGAGTSVRPSLDGLPVANGGGAAAGMAAGGALGLPLRE
DVPDIEGAPPLETRGSGGAALARHQMSEAPPRLRGTLGGRSSNLRPTLASPLGPGGAAAATPASGAAAGGSGAAALA
QAMSSGGAVRRFMVSPAAGASTVGVRFAASIPEGSPEHSPSTGADDAIADGGAAVTGVPPPPPRAPNSAAPAGAGAP
VPVLGSPLAPPSAFALGARPSRLLRKAKTQTASPSLHGDELGAFDDDEPGAGLQQRQRPGAAASALSAISPANVRAG
AAAGQRSTDTGMLSSAAGASAAAAAAAAPSPGIRSSVRRAMFPTGSKSASVLGGRRATHSGVPDDVAVPGARSAKPP
SLLSPDASASRAPAPAAAKQPLREAFAPVPDAGPPGSLNA

>Cre17.g713150.t1.3|PACid:27571580
MCRIRTDITRPSAFLAALAGLSCVLCVFAQAPCSNLTSARTIEALTKRCPTDLSSFGTILAKIPKYAQWLLDCGTGA
CDNAQRTLLMPTDMALDTYLKAKGTTLTKMLAKTGGGRKALQAVLRCHVLQPSIALTSVNITAAAAPLSYKSLQGPS
LTFAESSSGGVTVECGANSVAEVVHADITTGKSVVHLIDTVLQCQAAPSPPPPPSPSPPSLPTPNSSPSPPSPETPP
PPSSPPPSPPPSSPPPPEPSLPDPPSLEPPSLNPPSPDPPSLPPTPPSAPLTPPSPPSPPLPPSPAPSPPDPPSPPA
PPSPPLQPSPPNPPPPPPWYSLGGLDSYPFCAGEVCSRPQLCTAERKCMCGTGPGCGGNTACWATPDMAGWPTPPAA
SCVVQERICSTWDGNPRLCLPDFFCYKDIYCMSRGQLRIMLESWTSVGDLDMWVTNPLNQTVWWNSDGWNADETRDP
KWLPYGFEDNGYAVLDTQSDQYGPENVYYIDKNWQDGTPAAPLPSGNFYVCARVYIQDLDPSVWLKFPVIVTTIAES
AKYGINMRTVVTLRDANFEPSTPTLESDCTASSPSLVMTIPIP

>Cre17.g714800.t1.2|PACid:27571604
MEPRTLTWLALLGLACYAATATATAPRVLLYNSSRVVASSLRTDLIMGVMGEVPGVSIHTTKAQPYLHELSAFSAYV
IPANDKMPYLDAEDAAFNLWKPLELREYVQAGGSLILLDGSTGHNPRPGSSGNSFTGMLDLLLGSEGAAHCKGYLYG
RDLPMYLRLSDQGALGNIHTPIMVKPNRGLSGMTCTGEQPGEPIYSGRADGSADAGLSAARLWHVGQGRIIWIGSSY
TMPKIRAFREVLTGAIVDSQFPDTAPTRSPPPSPPPAKASPPPPRPPPPAKASPPPSPPPPRPPPPSPPPPPARSPP
PTPPRSPPPPPPRSPPPPPPTRSPPPPPPLRSPPPSPPPPPRRPPPPPPQRSPSPPPPIKYLHTDPPPTDFVTNPPPD
WIQPPPLEDWSPPPVKKAPPPRPPPPRNAGRRLRRQQ

>g17702.t1|PACid:27571698
MRIGGWHMRAPAACKQLAPMPNAALAAPGTVSARHRRPSNSSLQPRFARASAMLPVLIAALVAASQLAAATRRPEQP
KPSPAYDGNGNQDSCLHGCGENYKPVCCNGKCEKGPEFTAYEFCPSPPSGAGAYTSPPPQQPSCNPDTYPYVNLTYY
NPRECRTYTLFDVNAGCMPHWDKAKRYCEGIGQELAPWGDSESEDSCRKLCAANRYTCWMGGDAPEGLCNLMTQEGY
GVQQACKQPVRFVCRTKDSRCPYPPPPPPQSCYDKCVYDNPWVNTDVPYCDPQGKPYRNYCDGHCKCTEEIKPCAPP
AYSPPPPSPSPPSPEPPSPEPPSPEPPSPEPPSPEPPSPSPVPIGQPPAYPPAYPPPYPPPPSPPPPPPPSPPPPPP
PSPPPPPPPSPPPPPPSPPPPRPHPPSPPPECKVCIYVYIVATPKPESSPPFRYTNESCTYISNMVTSALGDASASK
GVNLIAPFALKECGGKGVKVCATFFSAAEAQKLQAEVTALLQALNAYAAKSCSSAMLYGYTLGVKVIGSQSSEVCLT

GSVRHTCPYPSPPPPPPPSPPPPPPPSPPPPPPPPSPPPPPPPSPPPPPPSPPPPPPPSPPPPPPPSPPPPPPPSPP
PPSPAPPYPTPPSPVPPSPPPPYPPPPSPPPPPPPSPPPPPPPRPPPPPPPSPPPPPPPSPPPPPPPSPPPPPPPSP
PPPPPPRPSPPPPPPPCRVCIEIYIAATPKPESSSPFRYAQQDCTYLSPLITGPLNNASASKGLGITSNFTLTRCSD
KSIKVCASFSSASEARKLQPEVDELLKALKDLAAGSCSHETYGYTLGIRIISEGGAKEECLKGSFTHYCPYPSPPPP
PPPSPPPPPPPSPPPPPPPSPLPPPPPSPPPPPPPSPPPPPPPSPPPPPPPSPPPPSPEPPSPAPAYPPSYPPSYPP
SPLPPTPPPPSPSPPSPSPLGCYEQCIYNNPWSKDGVPYCDPQGKAYSNYCEGHCKCTEPLHPCAPPPASSPPPPSC
NPDTYPYVNLTYYNPRECRTYTLFDVNAGCMPHWDKAKRYCEGIGQELAPWGDSESEDSCRKLCAANRYTCWMGGDA
PEGLCNLMTQEGYGVQQACEQPVRFVCRTKESSCPSPPPAVPVRSPPPPPRPTGPMSCYDQCKYDNPWVNASLPYCD
PNGKPFSDYCAAKCLCKEVTSPCRPPNPNPPVTEIGTCIQRCDIKYPNWYVWVCDKDGNAYQNWCYAACHECEYYTI
CKPPQAEAPSSDHTWIALTESGYSYKKWVDTTGAGVTYAEAEAKCAELGTNWEVTPYDNAGWAACKQLCSSNQAGCW
LKRGRPLDCPIINTAGQLSLAPCGSNALQVVCRRKL

>g17926.t1|PACid:27571737
MSNCCDASRLAAAEVAGRRANKGHLADSGISGHPHAQPRLPTGSLATAVAFAQGPTSASTVAPIAVAAAVAAVAAMW
VGGALALRRLSSVWRPSRRPRRRTAPDSSSRHGMAQPETYGATTCAMAARRRPAPPRRWCGSTCATHTCSTGTLEAN
ARAPAVRCGDSGERAGRGAPAADAAARHGCSSTASYQRGWAAVGKDASASSRVGGCRRDVRGVGEGRSNGANGRSGS
RGHAGSSGVSVGVGVSGGRRLGARRAASASAVLLLQLSLLPLLVTSQVVNIATVSMSPTDKTTSAATLTNDGYYVIA
AGAAPQAVFAVSSSGASSQFNVVIWYRYPATNGNGPLKKSGSSLAICPSGDTSNPIVTGGNTQAIKLTATNETDVYD
STNAGIATGCYVVRVGLVTPGTMNFVGSPAHAGMRVGNPPNIAKCSDVVNIPPNCDSTLFTYNPTGIPGGTAVVSQP
TTGSTCANMTISVPANGCTDPEGSVMTYRWDVYKSGTTTPIQSTVYGQSVSFSTGLPGLDIPAGTWTMVLTVSDSPI
NASLTLAKVISFSNIVVQQCNINYAPTTPVLTATATCGASSIGVTASSTDANGHALSYQFVLTNSLGATVSTQAWST
SSTATYSSPTLVAGTYTVQAQARDNATSPLTSSTSTLTFNLGVACTPPPLASPPPRPPSPSPPNPSPPSPQPPSPAP
PSPQPPSPAPPSPPPPSPGPPSPAPPSPQPPSPAPPSPQPPSPPPPSPRPPSPAPPSPLPPSPRPPSPPPPSPVPPS
PAPPSPPPPSPDPPSPAPPSPPPPSPGPPSPAPPSPAPPSPAPPSSPPPSPPPSPAPPSPSPPPSPAPPSPAPPSPV
PPSPPPPSPPPSPQPPFGAPLTPPRPPSPAPSPPPPSPEPPSPAPPSPAPPSPPPSPTLPSPPPPSPEPPAPSPPLP
PPSPQPHSPEPPSPIPPSPPPPSPPPSPHPPSPEPPSPAPPSPPPPSPPPSPLPPSPRPPSPPPSPVPPSPPSPGPP
PFGTPSAPFPPPSPEPPSPEPPSPSPPSPPPPSPGPPSPEPPSPEPPSLPPSPAPLNPPSPRPYSPPPRPPRPPPPQ
QSPPDLPPPVPPSPEPPSPPPSPTPPSPEPPSPVPPSPPPPSPAPPSPHPPSPPPSPPPPSPEPPSPAPPSSPPPSP
PPSPLPPSPAPPTPPPSPSPEPPSPAPPLRPATWPADPPAPSPPPPGPPSPAPPAPPSPDPPGPPSPAPPAPPSPIP
PSPAPPSPAPPSPHPPSPAPPLPPSPAPPSPLPPSPLPPSPPPPSPPPPSPLPPSPLPPSPRPPPSPPFPAGATVLN
ISDTSTSGVLIDPPPTLDLIAIDVSDNPITGTLPTSWSQLSALQALDVSFTFVNGTIPVAYSALTALSLLAASNTSV
SGTLPALLSALSNLAILNVSSSLISGTIPPSLAAITALVVIDTSNTSIAATLPPGLSTLTRLQALDVSSTLVNGTLP
SVYSALTALTLLAASNTSLTASIPPELLASLTSLQILDLSSTHINGSIPQTASALTALTYLDVANTSTSGSLPAALS
SLSALAVLDVSSTNIGGTIPDQYSALTALQVLNVSFTNVSGSLPISTSALTSLTLLDVSSTHATGTLPGALSTLTNL
AILDVSNTDISSSIPAKYSALTALQTFSADGSQLSGSLPMALSTLSRLQELSIDGTNISSSIPAQYSALTALQTFSA
DGSQLSGSLPAALSTLSRLQELSIDGTNISSSIPAQFSALTALQTFSADGSQLSGSLPAALSTLSRLQELSIDGTNI
SSSIPAEFSALTALQTFSADGSQLSGSLPAALSTLSRLQELSIDGTNISASIPVQLSALTALQTFSADGSQLSGSLP
AALSTLSRLQELSIDGTKISSSIPAEFSTLTALQTFSADGSQLSGSLPAALSTLSRLQELSIDGTNISSSIPAEFSA
LTALQTFSADGSQLSGSLPAALSTLSRLQVFDVSGTAISSSLPEQWSTLAALQIFDGSGTGLTGPIPASWSALSSLS
HLDLSSTGVSGRLPSVLPPALVSLDISDATLTGPLTATNWTGLAALTQLDLSGNAGINGSLPALPPSIRSLDLSGCG
VSGTVPASWSALAALTSLSASSSGLSGPMPAQLPASLQRLAIANSSLSGPLAASSLTLLASLTFLDLSYNSLTGPLP
DLGSMTNLVAVNLSGNAFAGPLPASWSSLTRLTALSLGGNALSGPLPASWPTSLVALASLNLTGNSPGVCKGVGYAG
GFSSGASVSGLLDVCLYPAPPSPAPPSPRQPSPAPPIPAPPSPAPPIPLPPSPRPPSPPPPSPWPPSPLPPSPAPPS
PPPIPVPPSPTPPSPPPSPAPPAPRPPSPVPPSPAPPSPPSPRPPSPMPPSPAPLSPTPPSPAPSPQPPSPAPPAPS
PPPSPPSPPPSPSPPPPSPSPPPSPPPLPPSPPPAPSPPNPPAHSPPFPPSPRPSPLPPSPPSPPFPPIVIPNIGIP
PEYVASTGTATSGFLTVTQAVDVPDSLASDPAALAVLFPNAAEISVDARKVSMPVEVTTAVEATAVMNTGAGSSIEV
TVDCTPPFLEAFRGELVRRSNISTSVLANITCAGLSLGSGGSSSGGHRRSVLATTGSGSIFSGRDDCSGSNSSISLM
VLLRVPADSNNTNVYKQQLQDALGLWEAESQDPTSNPDGSGSMLRLCGPRPGRITTTSEVRVVTEVPLNNVGTRSLN
DVCGASGFTSTTAFGGAGSNVACQVAEVQMPSASIPGPAPAPAAASPSSSINWLPIIISAAAVGSMTTIMCAAVLFV
LVGRRRKRRREEEEERKAHRAAAGGGAAAHGSGGGDDDAPWLSGNQLGAGGGGGSPPLTSGLASPTGVRLAAATGGT
LSIVPPPGASSRSAGAPLRRPDLSPNAEDDLKRILASAFHPSQSGAAPVAVAAAAAAAATGSDRRSGSRQLDEMDGS
GGGAGTGTGTAVEFSPSAAVSRQGSRQRNSAATAGGTGTNTNTATRLSGMVGTDSALLSGGRLSPAASHADARFSLT
GADAAGGAIAPVLLGSSGYGAAGLMPPGSGATRLVTDVGLVGGGRPGSGLASPRSGARLQERLWGTGSAGGISAVAV
AGGPASGGSVMYMNAAFREDPLAEDDMEAAGMEPTAPAATSTTAAAAAFGAAATTTGIGSMPAALASAPPAPPGRAT
TGGMRATLGKFAASFFKRERPSGSGAAGGALQSASTASGRSPMSDTNLDLPEGHVSPVPAHMRDAQSLGGGGGGPAS
APHSPQRFASPLSPGARLPVPPTAALAAAGAGGAAAVTTSTRGNAWSRAVDSGAAAPADELTPVKGARVKFSHVPEN
ETDALQRRPSGLAAAAAAPGGSHSFRHMRTMTQQPDQPSDSLAMHLDDDDEDLPASDTAAARAAIAAVAGGGAGTR

ALTPTRPALGGERNGKWLSMRAAPETRLAPAAGAGPPSASVVGRSSAAGPPPRRATFSGLAAASAAANAERSGLGSA
GDAPRGSSALLLPAKPTPAKPMAWGGDAGSGAGGVGGPERLTQASEVASEPGLRSGLGAGRGGKLRPSPLSPLGR

>Cre17.g706350.t1.3|PACid:27571811
MRPGAALRALAQLATALYLTLLVRAGLLVRDAPGGQPYQYELHTWLGAGYVGAGWLGAAAFCQSRGGALTPLSSTAE
LSTVAGLVGDGCLIDSKWSDQLFWVGLSTLAHQSATQPQALGFMDRPRPGSSTTLLRNSSRIATVLGKMAATANVTL
WPDVATAATPAAAAVAAAIANASGGGWVLSDVPVQPLCAAVRLLLPIPAAAPTAPAVANTSATANATAGAANATTNA
TANATTLAAACGDAYRQYQASKNGSSASGGSGSSNSSSSGGSSTVDVSNYQMELHFQDCGGLSGLAAAGAAAAASHG
FVCRVPAGTNPSPSPSPPTPSPPSPPSPPPLPLPPPLPPPLPPVPTSPPPAARRHLMALSQIAPAAITTGAEPSQLV
RSGHGSSSGCGGGSSSGAQQWLSRGLQPHRAEAPGGDGSLALGLSRRQLLQQVGGNTTSDTTTNTTNASNSTTSTTT
NATGTNSTTAAGASSPPPTPPQPPSPSPAPPPLPSPPQQLPPSPASASPKCIPLIPLDMGAANATTSANTNTSANAN
ATRTGGVRYLACTAAAPAGGGGGPAAAAAAAASWSEAAELCEGLVRGGTLAVLESQALIDALTYLIRSRDPTAAYDL
WFGLVSGGPQPSSLFTTAAAPPLEYILTEPHIVPSYMRQSWLNGTLAVHTNCQAPPPPSPPPPTPSPPPPTPPPMPS
PPPPKPSPPQPPLPQPANITAAASNTTGNGSATGNSTTNATATAGGAGNHHRRLLLTTNTTANATNSTMPTNSTTAS
NSTTPTMDNTNTTTAGTNTTATSNSTATPTNSSSPSPSPSPAAPPQLQPHPSGCCASLYVGPTPTGSSVDSGNSSGS
SGSSNATPPRLLLRACSDRISFICQAPYVAPAPPAPPVPAATSLQPPPPLGSTPPPVGGDGSGGGGSAGGAVALTRE
ASLFLNAMASNYNGAYRTFIATVSLSLGGVPYEVITSANTTHDQFTNETLQQLSRASRLPLSSFAPTSLTPGSVVLD
LNMTSRALPRATAADWQSFLTGGLAALAPDQLLSSDYMTTWGVTDVRLVVLSATEVIPEPPPTAPDTSSSSSWWDDP
GHKAAVLVPSIIGGILLIGGIVAGAFVLYRRRHAGGRSVAAVQPTGAVMPAAAEPPPAMAAPGSVAAVGTVGAAPGY
GSYGDGAYGGYGVPSPGGYGTAGYGGVAVQGGIEVDAKALAATAYAEAAAAAAANGGMLPPGYVVPAAPTAAAYPGA
VPPPASAETTAAAAAAAAADGAILSPTSARRAHQPFPPPSPGGGGPHPGAPQYSLSGGHVSSAGGGLPANGAWWEQE
DAGAAASRAYGSGGALSPLSPIRTGSRGGSSHALLPPLSSPSGGGSGRAASRLAPLPPLSRGRSAAAQPLSPAAVAA
SATAAALAASGGSGVLTPPVDLAALAGGPMCIAARSPLPPIHRNPTPGSPGGGAAAASLASPRAAAPSPLSPTRTTP
SQSALAAASPSGGGGGGGGGGGSGSRPSSPPGAGAGAGAASAASAAAASPPPAL

>g17622.t1|PACid:27571875
MMAASGRSLALAGLALATLLAAGLPYAAARQPTDFPWCSCSTYDCDCSPYKISLTSQRKVNETITRFCFNVDSDPCD
TSRACCRRMMANVDKLSFKIAPECQRVNIQRITFNGDRWNSWDTYSHDLGNGDKGYELKFYDIQSNSSAFPGTRICV
NTISPCSSVFQICDGPNGLCEYSFADTPATTQKSLLDDIDAYSFDNNIQYADNGDLFFESSTCSSTEIAICGTFATA
ADSWLLQTWLFDNSKTYIRSLLCGRSVTDQDECTDKCTTAYQAYTVEMVVETGEGGSESCLSYSDENNCEKPRVRSS
PPPRVRPPSPRPPSPKPSPSPSPSPSPSPSPSPSPSPSPSPSPSPSPSPSPSPSPSPSPSPSPSPSPSPSPSPSPSP
SPSPSPSPSPSPSPSPSPSPSPSPSPSPSPSPSPSPSPSPSPSSSPTPTPTTSPSPPPPSPPPPHPAPPSPPPPAPP
PPSPPPPNPPPPSPPPPGPPPPKQPPPPPPSPAPPSPPPPGPPPPSPPPPQPPPPSPPPPSPTPPSPKAAASPPSPL
PPSPPPPSPPPPQPPPPSPPPPNSPPPNPPPPPSPLPPSPPNPPGVTTSPAPPHPPPVPPSPPPPSPPPPSPPPPHP
PPPSPPPPSPNPASPPPPPSPLPPSPPPPSPPPPSPPPPSPPPPNPPPPSPPPPLSPPPPPSPLPPSPPPPLPPPPS
PPPPPPPSPRPPSPPPPPQDVPGGPRCVSKNDNTPFGWSALTIANTTDIYGSAAIQICLTVEEKGCKNDKAACCDMD
FNKISIPIVDSCQPKIREMRVNGVKHEYSWLQRDGNQNIALRNMRAYLPNPIGAQICIVVLPGQCAAPAGLCKDGTC
KVTIWNSNNKCCPSTKLGLASRGGKKKAYFF

>Cre17.g718000.t1.3|PACid:27571972
MHLAGNLLLAALAATLWAASVSAQQTGLYPSFPYCQCTKTPSAYRLSPTVTSTGAGTYCFTLSANKVPAGCTHKCCK
ADLKKIEFNVNDACDVFSPSLKATINGMRTKVAPAINKAQNGPVGSTTLVLTQLGLGLGNDGAQVCITLGLNKNGKG
CTTLEELCVPPAGMPAGVCTAALFDSQNDCCPLSQANVPSPPPPSPPPPSPPPPSPPPPSPAPPSPPPPSPPPPSPP
PRCRVCTYIALISQPEVSAAYAFTADQCAQVSDVIAADITAQAESVGATIVSDFALVSCQGQQIKVCGEFFSNEEGA
LLQGFVSEQVAIWRELVAGETCPRDLAGYTVVAAVGGDGASVQDLPQSCLSASDNKACALESPPPPPPPPPPPPPPPS
PPPPSPPPPSPPPPSPPPPSPPPPSPPPPSPPPPLPPPPSPPPPRPPPPSPPPPSPPPPCESCVYVELQTPSHEPFF
KYRFDSAMCAAISEAIAADLNTAAEVAGAVLPTPFEGVDCSGQEIKVCGTWLDGEAATELMQPYVDTQVTSWLALVT
GGRCPAYLRGYSVLVAVAGSVSYELPEEYSGQEMPCLYAKDQSACAPETVDFPKCACETAELATPFAALPIIDVGPG
PSKGTTAFCFNLTLVSPTNPGSKCGRSSGLLKAEFYADDKQRRKVNSIGVQPAGGAMRYLSATWGAVGENTLKATPL
NWSKAQANGGRICLVLYDTVTLDSFCMGNELDTCWLNLFDSSKDCCPLYTSSIAVA

>Cre17.g705200.t1.3|PACid:27572079
MRARKTGSTSLLLLVALQILVWHDILWCQAQDTVTLRGHLIVIDIDIPAGERGSGVPAADNTHYILRTGVREVWPLR
FPDGTQPPDRLGSGAPMEVQVLVGDATDNGAQVVGSSARRRRALHAAAQVAEGGNGGSGSADAGEAPQHIANLEAGA
AGVGSGSMVEGSGTSQLRKPRQQQRQGSQASGVEEALAKLHDRRMGPAGSRHRAKPQATLEPGASNATLQQERHGVD
GASTGSSSGSSGTSEQQPEDAAAARLDLEWLRRHRQQRRRRQQQEVDPGATLDGGSSSSDSVGSSSTSTDGAVSGAV
EDDSFVDRLTVLSWRLLPTQTKPKYYLSGSSSTTASRSGDTSGAHQFVYNTNRLRMDDVSTVIFITNMCGYGPAMTV

EELEARLFGTAEPSSEADMSVQRYMSSCSNTQATFSRDNVLIVGPVDLDCDGSTWDLAWSTSSCFENDYVGWQWAAE
EWAYGQDIDISSYRHRVIITSPGKANWMTAGDPDCAWSGMGVLGPAYNAAGEQNMDGTAVYSQGVFSYAWVSGDHWD
RPQGWLHELGHNYYLHHATSLGCGSAYCDDSCTMGYCCTPRCFNAPHNWQVGWDDPTALLGDWDLSPGVANRYTLVS
GLGRDAAPARVRSGAVLRVEAAWAGVDDGGVGTTLWVSYRAGGSVYDELPDELTDRVLIYWYPGSDPNDATVTVRLA
ALAEGEVWRNDTLLLSVTRKAPSGAGAAAAAAAGGGAQVVLCRYSGDVETECGDGLDDDCDGLIDMDDPDCTDGVVQ
PPDAPSPVAEPSLTDSPPPTTPESPPPLESPPPPPPPPPPPPPPPSPSPQPPSPPPKARPPKTPNAPSPPEPPTPPFN
PLQPPDTPNAPPDVPPPGAPPPDAPDAPPDAPPLAPQRPPLAPAPPLPPPAGPVLPLIPLAPSVYELAPNRPPPLPA
SPARPSSPTAPPPPPSPPPFAWPGLCLTAAYNDGCSTNTVPQPPLLSCPAAAAANGSSSSCYLTALNASLLNGTLQP
GSLASCNGLTSPSGKYRLVVQDDGNLVLYNLQTSQPAAAPAVQWASNTTGVAAAAGPVVLKLWLNGTLGLVWYRRDA
KSATGWRVAAVRNTAPNGGLQGGVYGGGAAGAANGPFALNVRDDGRAYVQNKAGAVVWATSDPGGPAAFSAIGAPVC
IAPPPPPPMPPRPPPSPPSPPPAPPAPPAPPSPAPPGPAAPGTLMPPSPASPSPPSPPSPSPPPSPKPPSPKPPRRP
PSPKPPAAKPPSPSPPSPAPPSPVYGGPLPPVLSVPSPSSPSPSSSSSPSPDPSPSPAQVVPASPELSNPELSPSPE
PPSPVPPSPSPPSPEPPSPEPPSPEPPSPEPPSPEPPSPEPPSPEPPSPEPPSPEPPSPEPPSPEPPSPEPPSPEPP
SPEPPSPEPPSPEPPSPAPRTPVSPPPPRAAVAKLSAPPLVFAPQPPSPPSPSPPSPPLPVPATPSPVASPLPSPVP
SPSPLVASPLPSSLPPPVPSPPQAASPSPSPTATSSPAQLPAVPSPAISSPPLPAPSPSQTASSPAVATPSPSASPP
PPTAAVPASSSPSPTNANATGGAPAAVATSRAPSPPNSQQRRSLQSAKAQAAVASESWAAEAVGGDDEA

>Cre17.g717850.t1.3|PACid:27572149
MTSRMTSSLAFLALTLLAVGASAQPQSGLYTNFPFCKCIKTPTAYRLSPTVKSVGSSTYCFTLNVNVPAGCNNYCCN
KADLKKIEINAFQACDVFSPPVKATINGVPTKVAPAFTTPKDGPVGSATLVLTQLGLGLASDGAEICITLGLNKNGK
GCTTLEELCVPPAGMPAGVCTAALFDSQNDCCPLSQANVPSPPPPSPPPPSPPPPSPPPPSPPPPSPPPPSPPPPPP
PPSPPPPSPPPPPPPSPPPPPPPSPPPPPPPSPPPPSPPPPSPPPPSPPPPSPPPPSPPPPSPPPPSPPPPSPPPPC
EVCVYLTLVPPANDPFQYRFDAATCDDAATAISQDIELLAEDLQARISVPFTLTECTETSIKVCGEFFSNEDGALLE
PDIQNIYLDQWLQIITGAQCPAFLAGYTIVVSIASANPDVPSACLSGVLSTGCSPETVLFPKCECTTTPYNMPFAVN
PVITTTPGRKTGSTAYCFKLASVTPSNPSSFCGKTTTINKLEFFADDTKRRKVTGVGLKPAGSATTTWRDPIWDTPG
QNTLRATNVNWSSAQADGGQVCLELTVPLADFCLGSADGSSCWVTLFDGSRTCCPTALTSITAP

>g18268.t1|PACid:27572311
MINRQAGARTHLAPVVILETSLHVATSERAEILASLPSQVRVCTRSAIAQAKSISSMKVAVVAAALAALVAVAAAQP
PPPGFARFPYGSCSKSSPYRTYEATFPQGQSGNVFCWTLQYQGGRCSDPGFQACCQNDVHKFSLAFEPSCAPGFKAS
ATLDGKAIAANNVVLETPPNADKRMTLAVKNLGLAGPEVEGRQVCITVQAGNCRTLASILPVFDQSPLWQNSLWSDD
HACCGVSRGSPPSPPPSPNPPSPPPPSPKPPSPRPPSPPPPSPAPPSPPPPSPAPSPPPPSPVPPSPPPPSPAPPSP
PPPSPKPPSPAPPSPPPPSPKPPSPPPPSPPPTCDFCVDYFLFPGLTRPELPWFDYYCDEAVFNLLEIYQPFKDDGT
IISGAGEADIICKGTNVTVCGVTKNEEAGRIMAQSLREDSLFQAAAILGIDTGRCYPALSGYTFITTPAPGYDNSPC
TPQPSAVPMCGPAPPSWPEYPSCRGKSANDYFQFLVGTRYTVAYGDKTSEYCVPIEINDINAQDARGQTCYGKNLNK
VQFYLDYSRRYQITGIAIKYNNGSTFTRSQSWGPVGSNSLKITSLDWSVSDVARLEPQICLTIKSGTTLTDLSMDKT
KTLWAATFSADHYCCPIQAGSPFTTDGVPVEL

>Cre05.g243450.t2.1|PACid:27572621
MLLPLLLLLLSAPFIASAASAASAAEEASAEAVADHSRGLKALGGGHWHVQRRPQLAAAAAISAAAATSARLTAAEV
AGGAASAAAATDGGSEQLAWSADPQLQQGQTFGHRRSLLKAKKGDSNAGSSGSSISGMTIIAGAAATPPLAPKKHKK
SPPPRSPPPRSPPPPRRHKSPPPVRPLTEPPLPAPPTVLETTDAPPPRSPPPQPAPPPPPPPSPTPSPPPPSPSPPS
PPPPSPRPPSPRPPPPPSPRPPVPPPRPPPPSPPPPSPPPPSPPPTSLPPPPLPSPEESSGPSDGYDGEYGGEDGYM
GGGSGAGNATDADNSMDEPEWPPFPPPHPRDANAPSPPPPPPPAPGSRHGQYEFIGSANIIAVHLMAVPGTDNFLFM
ERPSGRHPDGSHNIAGFLNLPTRAWTHLYSPDGLFCAGHTFLDTGDLLIVGGHQANAGYPDGMKSIRTFNRTCTDLQ
LRKVVEMAWKRWYPSATLLPDGNVLVMGGTQGVGAGTANNPFWEMFYPSNGTTRTFAMKPNYLDSAEQVYYPFNYVL
PSGLLFTFCGRSGFILDYTNNTWMQNLPRLRGWATTQYPFTGTSVMLGLYPETGYTAEVVLFGGQAERATRNLSTIA
AKQAQRLTLSAYDPVTRNYSFGPTGSWVGENLGSPRLMGDSVLLPNGKVVLLNGAQTGLAGDSASGGDTRANYPVLF
AELYDPDRPQGQRVRSLGFSRIPRMYHSTACLTTNGTILVAGCDRCQRFVVDEGWTYDPSPTGKADYRLEIFHPPFV
FFPSDRPIILAPSLDASPPPPPRASPPPPPPPPPTGTKAKQPLPSQHDDLGVAVVMGYGTTHQIRYAWPAGFAGPQP
GSSIGMNATAPPAADPYDVWTPGVITRVVLSAPCSNTHSFDMNQRLVGLEILSNTPDPSTGSSSGTTGSSSGSSSTV
AGGVLTVRSPPDINIAPPGMYHMFLVNGDVYSSAVWVRLARPQ

>g5503.t1|PACid:27572654
MRSLGLLVAVLALASTARSQSDDLDVLARAAAARGDPDFPYATCTRSPSRSIYSTLPVTTNPAPSTYCWTIRVSQSQ
CTTQNACCSADVHKMELDVDAACDVPSQKVTATINGKPTPQAADIVKPPNAADYQRTLRIPGLALNAGNANNAVVCI
KLTGPCDTLAKLTPNDVWSTALWSTSHECCPISRGASPPPPSPPPPPPPSPPPPPPPSPPPPPPPSPPPPSPPPPPP

PSPPPPSPPLPSASLFSIIPPSEYRELYTFTPELCARVADRVVDDLATIASDSDARLIKDFALSECVASYDVTTNSY
PYMTICGEFFSKEDGDRMPELSDAMDSWIEEVSAASWVSRISSARLCRRRRPLLPRRRRPRLPRRRGLLLLPRLLPR
LLPRPRHPLPLLLRPRLLPRRLLPPPPPPPPPPPPPPPSPPPPPPPPPPPPPSPPPPSPPPPSPPPPSPPPPCSTVC
VTWVRYNGRTNQQVCNELASTVQALAMDKGYELATDFFCSQVNISAPRPNGRPAMRVCGDFKLESEAREFGTYLSDG
GGFRDDLAPAIGFGNYIVPVFGQRICLVSELSYVVTDRTGKICDQFTYSQGCAPPQDEFPYCTCKPGAMLSTPYNLT
YTRKFASNGNNNYCFRVNVDTQRCVGRCCSMDLDKVEWMSDLMNCRYAVAGFTVSTKPNAFIAPVWATQTDNLIDAS
GPVNVAVLKTNNLNLDIRNSNGVEICIALKATGKCPTLEDFCYDGLCKYAIFDRSAGCCANDFAEGNDFGYNRRR

>g5501.t1|PACid:27572671
MRRTAVAVALLAFVAAATALDFPYSGCTRALTKSKYSVKPVTTFPGTNTICWTVQYSDPQCANPSEPCCSADLHKLQ
LDVKQSCASGFRATATVNGQGTAAPSIYRPPNAGSDQYVLSIPGLGLDGASANGATICLTLTGGSCMTLASLTPSPV
WSVALWDTAHQCCPVTRGASPPPPPPPSPPPPPPPSPPPPCRVCSTWEAIAGPTVLVDDLNYFGDAYNCAVGKDVIS
ELYAPYMEDGTIIAGTDSITCNGNKITVCATMFSTEVGSKMASELQAMGSGLVTSVFGFGGDCPLAFAGYSTFVTST
DDDCGVFMAGPACAPPTAPFPFCVCNKQQGSTPFYLDNAVEVDDVNNEYCVSVYTAAAVGTGACSKKTTLKKAEFWF
NYAMRWRMTSITLKYATGAKATRSQSWAKAGDNTLRVYDLNWTVDQVASLKPKICFTFKAGTTLADFNADPNGFVWG
TLFDPEQDCCPTFPAQ

>Cre05.g238650.t1.3|PACid:27572820
MSISAGHRRPWTIIAFNMRHTAVAVALLAFVAAATALDFPYAGCARALSKSKYSVKPVTTFPGTNTICWTVQYSDPQ
CANPSEPCCSADLHKLQLDVKQSCASGFRATATVNGQGTPSPTIYRPPNGGSDQYVLSIPGLGLDGASANGATICLT
LTGGSCMTLASLTPSPVWSVALWDTAHQCCPVTRGASPPPPPPSPPPPPPSPPPPSPPPPSPPPPPPPSPPPPPP
PSPPPPSPPPPSPPPPSPPPPSPPPPPPPSPPPPSPPPPSPPPPCKVCATWEAIAGPNVLVDDLNYFGDAYNCAVGK
DVISELFAPYMEDGTIIAGTDSITCNGNKITACATMYSKERGAKMANELESMGNGLVTSVFGFTGDCPLALAGYSTG
VTSTDQDCGIFKAGPACAPPTTPFPFCVCNKQQGSTPFYLDNAVEVDDVNNEYCLSVYTAAAGPGKCASATIMKKAE
FYFDYNMRWKLTGITIRYNNGRTATRSQSWGPVGDNTLKVSDLNWKTGDVASLKPKICFKFKSGTTLADFSMDPDGY
VWGSLFNPDQSCCPTFPAE

>g5109.t1|PACid:27572914
MACGRPAAAVVLAIFVSLVAFFSPISAAIASVGVDSVTGLYIPGWADNLNPASLNYFNTSGLNLTGVSHMYYAFLWI
DETTYRVYDPFNNLGLLRGLKARWPATQLLLSVGGGGFSTAIWSAAATTGRAAFVDSMVAAVKAAGADGIDIDWEIP
GFADRSALTALAAALRSRLDAEAAAAGRAGHYWLTVATPAIWAESGVFDGFDLAALKNSFDLFNIMAYTMHDPCYWE
TQTAFHTAWSDCSTALSYFISQGVPASQLVLGLAFYGHSYTLIDAATYTYPAPSVESRDCSSMDTPSYRAVMQELSW
GYGGGVFTDAGQRSAYYVRGTKWVGFEIEETLVAKSQGARDFGAAGVMIWDASLDVPEGRLLRAVASRNAAPPPARP
CGGGFIGTGQCPGSTGGSGQCCSEFGYCGTGEQFCGPRCRGGPCIQYPSPPPAPPQPPPACGSGQVGGGLCADPTQC
CSLAGWCGVGADCAAGWCGVGSDYCGLGCKGGPCVVYSPPPPGTLPLCGGGAVNGGQCQYPGDCCSQWGWCGTSADH
CGAGCQGGACYLPTPPPPSTSSAPPSPKPPSPRPPSPRPPSPKPPSPAPSAKPPSPYPSPPSRTASPPPRPPSPRPP
SPRPPSPKPPAPTPRPPSPRPPSPSQQQQQSPPLPPPSPVAVVGTCGGGDVGNKLCANPEECCSGAGWCGTGTDFCY
WYCVGGPCHWPPPPPRGAGPAPPAPPPETPCGNGQVGSGRCESPGMCCSSAGWCGDSADHCLHFCAGGPCWSPPPAT
AATVAATAAPPSPPPPRGNKSPQAKGRRRQMRGGAGGGVGGDTRGRHAREERQEEAE

>Cre13.g591550.t1.3|PACid:27573397
MHDPQVISLLILLLAAIPLWPELPRATAARALVSVLPRSLAADDVASRTLTTYATAATPLGDRGLENFGLHPVNCPG
GSFLNSWRLVVSNATSGSSSTGEQGRTFHLEFACLRVPAAAQCSRGTDRYYKAGPGRTAAQYRTTDFRALLDFNPDV
GTVGRGYLQPLTGFRVFLDSSGRISAEWWGCELPDSFTPARVYAYRTSPRRPDATTGGVAELALHPAACEDNEAMTG
WFLNHYTWENPATIDMAAQCYTLAPPEPIAPLGLGLPGLYANSTSGDFTPIPPSVSSSGGAAATVTGFVFVTSSASS
IAAGALVPVCAEGVGTAALPLQLCAGLGFSRGSVLSWRRNWDGGSLPPPVVLHNVSCSSGGAAGSGGSAGVGLLAAD
AAEAACVAVPAYDGYCFEVARVTCSAPLPASPPPSPPASPPSAPPQPPPPTLPSPPPAAAPPQPPPPQPLLSPLPAS
GAAQSSPPPPSGAGPQPSPLPVPQGNESGAPARTNLTAANLLTTYTTAPTPLTERGLEDFGLHPVNCPGGSFLNSWR
LVASNATSGSSTGEQGRTFHLEFACLRVPAAAQCSRGTDRYFKADPARNIRYLTTDMRALLDYFPEIGSLSMGPLTG
FRVFLDSSRRISAEWWQCELPDSFTPARVYSYRSSARRPNLATVAMSITELALYPAACKNNEAMSGWELNLYDYASP
IYMGASCYTLAPPEPLMPLGLGLPGLYANSTSGDFTPIPPSVSRSGGAGATVTGFVFVTSSASSVAAGALVPVCAEG
VGTAALPLQLCAGLGFSRGSVLSWQRNWDGGSLPPPVVLHNVSCSSGGAAGSGGSAGVGLAADAAEAACVAVPAYDG
YCFEVARVTCSDPFQPPPPPSPQPPPAPPLPPPPPPQPPQPPSPPPRPPAPVIGPPPPRLPPSPPRPPPSPRLAPGE
QPPPGYCECAAGDNKCPGEECTPNYRYRNRVCSYPTLFCGTERYIDGYSCRFSSSDACAERLDDYSRCTGRSECTWQ
TSELPPATPELQYRLVNGKTPNEGRLEVSLDGKKWGTVCDDGFGRTSAAVVCRALGLPYAAAVALRKAPYGRGTGSY
LLDDVACRGDESSLLQCPFRKPFGTSDCALDHIEDVGVVCQDEPIVPGDSDGGDNINGGGPGAGGGGSGGGSPRPSP

AATASPSPSQQPSPPPPDTGLAPPPANSTLDPPPASPPPVPQLEQPPPVPSDTAPPAVPAPPAFSPPAGPYPPPAYG
HYGYYGGGYQYGGYPAPPYGYHHTPPGYYGYYGDHWGGGYSGPSYAAAQQPSA

>Cre13.g589400.t1.3|PACid:27573732
MHSPAILSLLLLATAPHVLGSGSLSIRRLLRHEVDASPPSPAPEPPLPTPPTPPLPPSPPPAPPNPPSPPPVPPSPP
YPPPTPPSPPSPPPAPPSPPSPPPEPPSPPPAPPADPAPRRSSSRGQRAPPSPGAPDPPALPPPSPSPEQRPGRKRV
PITVERGTTRPSPSADTTTDEADRSGSDGELGSPSPPRPPSPRPPRLPPQPPTPATSNAASFLMAGGKCQDAQMTLD
YANRYRAAHQAPALQWSSSLAAAAQSYADKLASQGCAIKMEHSGAPGELLYWETGDDMYCRFAVIDWYGESMVYDFT
PAPFTDNVALTDNDTSHFTQLVWRSTSSVGCGVQRSTSGGFSPCTYVVCRFSPAGNIANDAAFLANVLPKTSLGQN

>g14187.t1|PACid:27573811
MRSQRLTLWLLAVSAAVQALSVANGFECAPPALVGTYNAVVLIAAEALNVTEQRQQGPNGTVVVTRTTEQARGSEGV
FMNLQPTYSLKSCNKSSGSSQYGLRIRVQGCNAFSSGRTTPASPDACTASAGCSRNATACPRNCLTDIMEAVEATYS
LDVEGGLLTDSIQLTLLSSENSASGSSSSSSSSSGNLTAKVAACFRVNTTAAIDKQVGSLQRVFNMLARQSHARFGN
VSEQVPVPVNALVTPKNTNDQDEDISSQTTTKFSPPSPSPPPPSPPSPEPHPNSQPMTSDSLYVFNSESTLSEPRPD
GTQILTEKRFLVPTDEIFEAMDGGSFDIPAGGASRSLVSPSGAVTARADLTVTEFLAPASATNANMTGNQTQSSRQH
GNSTLASAFPGNTSSNSSQEAAAGSSSFPASASAVITRTRVLVTLYKLPSANVAGGADEGENEGTNPGALLSTAKDM
AASLSKLTIDAIYPGRSYSSYISGWKGQLEENLRRRRRMALQTADVSNLSDDGPSRNTTSSLARRRRLLYWNKVNMF
DQYRSTTVYGREYGVGVYVNRRHGVDRWNSREFEVGVAVDGGINVKLFGYWYADAAYFSIEGRASHRRTDSGNYYQS
DAKLRRQLDLLGYSKVNSYDYNFGYRSNCDDYTKNWRGAYYKVWEQTYKIASVGVNWGVLSATVSLMAAPSLLAAFG
GCDLYGRSTLAGGLGAAGSLYATASGGVDVVLASVSIDADITIIEPSITGSLMYTHNHLLYGYSFTPSCAAATLGLT
SLKAHVHWTKRWWSWFEWKEDTKTLAETDGVYFGTDFITCDWDLIYYSDRAAIAAFTPPPPPLPSPPPPSPSPPPPP
PSPPPPPSPPPPPPAPMANSNARLTLTTSWACPLCSTWGVKSSTGMDFDLLVSWTVGSATYAVSYGMTAGDGYSVYG
GDNSVFLSSYEYVTWPAGGPPLAYVTYHLCLRDYTGGIAGLNYFQIRAWATMNGQDFPINYGSNVGWWSTYNTNYMY
YSSVIPRPAGSACTPGMTGYVGSFKINAPPPPPLPPSPRPPSPHPPSPRPPSPRPPFPSPNPPSPRPPSPRPPSPSP
PTPPSPRPPSPKPPSPPSPKPPSPRPPSPIPPSPKPPMPPSPRPPSPMPPSPPSPRPPSPKPPAPPSPRPPSPAPPS
PKPPSPKPPSPAPPSPKPPSPKPPSPAPPSPKPPSPKPPSPLPPSPKPPSPLPPSPKPPSPKPPTPPVPPSPAPPPA
APKLSVRTEWSRDGVPQPTANFDLKMAWVTSSSAKYTINTKNSSPVRGASHSGNNTRFNRNYEGVTWPSNATVDKGI
YYVCIAWTPTEINEYNNYELLTTIRIDGALVSKATNYWYNFDSTDELATACSDYASGYLMYFNYPPAPRLRFKVEWY
AAFGDLTPGTDMDIVVRWYDKTAGTYRDIGHYNQRAGGGQYGGENSVAKVNYEEVFWPATTVTSIPDDTWFDICVVW
YTTESLYARYSVEEDGVYADGSSWDVWVDTSLSRPTEACAPGVAGYLGSYHYNAPDWLRNSGSASVASADDSADAED
ATPPPPAKRSDAPIDRVPTTTYLLNKPSISGSSSVSTPQPSPVAAQAGQLQSALSPPSPLPPAPRPPLPAPSPQATA
ATTTRTSTARQDDGTLTPSAAAPASTPQPAATGSTADVVSSQRDTPADLPQPSTGQPQAGVSDTVAARPQSDGSAGI
SSGAPSVGATAGPIPAPQQQGGAGSTQGTALRVIIPSVVLSCVVGVVVIGAVMVVWRRKSLAHSDGRVVPI

>Cre02.g118650.t1.3|PACid:27574650
MNRCSGGRGLPVLLPAAIVILYLQCALLVVDCKPTACKTSQGAFYLERNALFLGLGQYCIPLVITSTACNASTTTGC
CAAERLDSVSINIGNECRITPRRVSAYLLLGEDGGRKRAGRLLNVTLARGRGWRQLVISGFALSPSLLAVNSSSNDS
SSGVTTAGNGVSGSGSSGTGRAQLCLQFKGATAAAGSSSGGATSPGECATLAGLCATPVTGSPASLSNSSPQVTCTA
VLSAASGGCCSTSVVAVPSSQQAPPLGAPPPVPPAVVREAGEDAAGSSGSSPPVPPPVSPPSPPSPVPPQPPADGLL
LCATFSLLLLDSSGGTGGGTGAALLLSQAQCATWQAAVGSELTTYFTTPAATPPAAGLLAILPTAASCSGSGAVVCG
ALTWAAADAGGAADAAAALQDFLYLRVDGWLDAALSGTSCRTAAGGLYTATAASLVVEVEGLRVETARGVCGSDSAE
EGDSGGGEPAAAGPAAPPPSPGLPGPPPSAPPPPSPPPPAPPSPPPSPPPPPPWWSDDARWQYLWISPPPPPSPPPP
PPPTPPLPPPPPPPPSPHPDAPLPPAAAYATDVCLEVYVIPRDPAVGPAGFVLDDTVCTAAAARLALGLANVTLGSG
GVVWRPFRLDACSTDYVPSADGGSGDGGGVFPYVRVCGSVDSPLLYDTLLALYIWAAGGDPGAIDIPVPRVAAGLVT
GGRRGTGDPGCPYTANGITFRVGMYDSVYYTNGFPATDYLCGNADPPPPRPPRGLPRTPPRPPRTPPSPKPPRPLPP
SPEPPPPPPDQPPSPEPPSSPPLPPAYPPQPPLPPAYPPSPSPPPSPPPPPPPPPEVFCQLCIQLSITASSTDVFIPY
SLSEYECGAFGATIADELAFAVYDLLGPAAPGGFNIVACVPDATDAFGRHTTYVRVCGQVREPISGSDFATLQQVLE
TQVGTWQTLPGNGQCPVELAGHFITAATYSDPAARDCGFKASATGACALVSPPPPPPPPPPPPLLPSPPPPRPSPVA
SPPPPPPPPNPPPPRPPPPAPARCQLCLTLQFIGTGTSGAKSFCAALNKAAADTFQPLADAQGIRLLGSGSGGGSVW
RASGCGLTDSPGIAAVSVCADLLAAQAPALPRVLSQGLLQLFDSLFPPLASGAGSGGGCAAYPNTARSYRLRVSDGS
ASSGGASGSSGNSSAAAGGCFPAEERDCSTPVPVATSFPPDAACSKDSPAGAFRLRRDIQFKNVTILTTRYQLHCFG
IQLNNGSSANTSACAGSGRVVQLAILANATQRGALTSVVVRGSADQRRLPVSSPATSTPTSNGTAEALDAWWVRPGA
TGADRKLGNALWVRPIDWNADLTRDLVARGRAEVCLELRPGVGLGGFCLGDGGATCYVAIYSSDTCCPVAGAALP

>Cre02.g095150.t1.2|PACid:27574663

MGSSRTLLLAALPFLLALQVLGQTPALNAGRWCSLPGYNQSLACQNTTGAFAPTSCATVDQYYGRNDPRFDICMIPG
PGTQWPYGKDYYKDVCNGPAGVLKVPMKNRYAMDWVNAPVLADAYIYRTYSADPTKSLLYITVAIRGVNNNFPTQQP
TGQAPNQLFYVEPYPLEPEQDDEGLYLDMSASIYLWTDARNVTYKQYVDPMIYSGKYSCFSAVFNLQRICDPARSYK
NTAKRNLNERCTCRPGFENNCDPVDISKSDRFFIVLNVNGVPFSSSQIQIPNPTCANPPTITKPTVFSNSNGPAIIT
EYVLNPDACNLRPTAPPDPPLNPSPPNPPLPPSPAPSPPNPPLPPSPAPPPNLSVYAQVSVTTFNPLRFFSSGYDCS
MARNATSYYWRGRTVVQRVTCTITSVFQSAFDSNFDVLTLTFYFNSITNLRYFFDSVNNEPFWVDLFTVLTPGCGAV
GNYTDIVYNPFSIIPPVPLAKQNLTNPQPFCAYGGEVSSDDCWFVMDQFACPHPPSPPPAPPSPPRPPNPNSPPHPP
APPSPPPPPCGVTVQAINYRYNATSDLDACWDFIVTVKYLYTFNATSWYCDTVSLNSTFMSIYTVFATDKEAQTFWT
NFNNPYFAVVTLSSINPTCRTGLLGMGTPSCNQTDVYWDVRNSNYPVRQLGGVCPPAPPRPPPRPMPVPEPPGGPAN
MPSPSPPFPPNMVPPSPPVRPRPPPPYNDPFIMTVESPTPYADDMCERATATLVSKLAFFDRPYQGPVCKLGATIAY
FMVITVGFTDPRHSTAFGMTFHDEFNEYAATVGLPCNSSTLLQANGASWVERCPDVPALCCGVGLNSPPPRPPRPPR
PPSPRPPRPPPPDLPAGPDGPSQPNAPFVRRRPPPSPPPPRKRSPPPPPPVRRPKPPRPPPPSSPPPSPPPSPPPRN
LPTTRRPPSPPPPPPPTISTFTQNRIFAIVAPPTLAGSDVSFDLVPILCRNLMDGLRAIFTTYGWEEGTQYDLLNDD
CPNRILSSGAKQYNMTIGMTQGAAQSLVTLLNQPDNFQLFVTASDILCGSRVSLIKLVTQEVYFTKNKDNSPLALDP
NNPFSSCVHTLLAMRK

>Cre02.g090050.t1.3|PACid:27574773
MAVLTAAARTGCALLRGAVLLLVLQMIMPSTEFIPLTVVEAQSAASASDPQCVTEFNGSIPEGKVCLYNCQAPPSPP
RPPSPPPRPPRPPPPPVPSPPPSPQPPSPPSPPRPPRPRPPPSPPPPSPPPPPPPPPSVPPPPSPPPSPPGVPSPPPS
PPRPRPPPPLPPSPAPGVPPPLDASPAPPSPAPPSPRPPRPPPRPPSPPPDPVTSDPPPPAAPPPFPRPPPSPPPPP
PNPPSPPPPSPPPPSPPSPPSPPPPPPSPPPPPPSPPSPPNPPPNAPPPCFCTCYDIPNPITGRVLAVGGVSGALSS
RPARISLASDGGFVNATYTNGTAYILGAKDSSTGASFADVTLWSKLPKKYFNRTIISPLSGLASVLNDESDLDSAMS
RYARRLGVSSSYTGGLAGYDYVNVTVEAPTTKNYSQKGVAVALANYQVLGTVVNIASLFTTLYGASSRDALEAVWTG
LALMLNIDGVRLDDTLGVSTVADASLAALLGSDESDLFSNLYGGGATDLSGLLALINGLGGSTTSTSSPSGTSISTS
TSGSSGSTSTSISTSGSGGSTSATITTGSGGTFTTTTTSGGSTITTTGSGSTISINGGGGLNSISFVGGRHRAARRS
AIASTRFADAAAAEFEPEPSSEEEDGGFASRQRLLSHEIVQLAEWIAAGRRARMQGAGAEAAPHRHQHHHRRLRTAG
VLGARRQLLQNNWALQDNVTLALAVSLAQSNALITQQLALVGSSNEPLSGILTVAGQVVTVLTTSMPSAASKMGAGT
LSYSDFNATYTGDGLTQAVTQASFSTAAGTGSSDDKNTGLMIGVIIASVAAGLLLIALVAVLIVKRKRSSNAVANLR
DEKAAAEPA

>Cre02.g078800.t1.2|PACid:27574927
MAECHAPVARVAIIVALLISAASAAGVSTLSISPDLAAVRSGVGRGLLTTASGFPWCQCIDYDCACSPYKVNFESST
QSGSLTTTCFSVAYIGCNTSRACCRGMLAAVDKLTFETTAACGVKSNIAGITINGKSWPSWNPYPHPSGPGTGYELK
IYNLKGNNGTFPGSKICITTKAPCSSLKDLCSSSPSGDCTYSFADTSSTTYCPICPLNVYPPPPPPSPQPPSPGPPL
PPSPAPPSPSPPLAPQPPPTPPPPPPPPPPPSPPVPPPPPPPPLSPPPPPPPPPPPPPPPPPFPPPPPPPPPPPPPP
PPAPPPLPPPPPPPPPPPPPPPPLPPPPECPEVCATVQIIPPDSLFSNEVPANIFTFDGTEDVAWRHSPLFPCKGEPPS
LVTFDGPDGVDCASYGDFMAATFNDRAWEVGADLSAAFVVTSCSGLWNLTTGIRPNITACGTFASNATAQLFQPEVG
DVLEQLVNFITGGACPANLEGYTISAQIGGIAPSGAFGGAEPSCVVGQTSQSCKADAPDAPPPPPPPPPPPPPPPP
SPPPPPPPSPPPPSPAPPTPFPKPSPSPSLVKTHICVQSTNNIPFAISDLVLSPTVDQLGFPAVSMCVSASSATCRR
GAFCCGMDFAKMELPITTDCKPELRRIVINNEASSAYSWGFYDSFTTLKFPSLTKSLPGGPYGATLCWVVRPGACAD
PANFCLNGRCQVNIFSSDNKCCPATIVN

>Cre02.g076950.t1.3|PACid:27575028
MGRAWGLVLHVLFFVLLGELCGSGPGRPAAEASKLPRQSQKYTPIPSASASGVQEVATDVLPDRFLVKLRPKSGKFS
TRGSSNVQSRAQQIVGRLRTAKLAVRLTHEFSTAWEGFGLQADDVDSALAALDEEYEVLSIYPVTWLPAPDSNVSEV
DAAALRGVFTVADASAPTNGTTARLAAGGFKRLDGWDVKVGIVDTGVDYRHPALGGGFGPGFKVAYGRDFAGDAFKP
GGVPAPDADPMDCAGHGTHVAGIIAGSYSSADWSFSGVAPGVTLGAYKVFGCTGGTTSELVVAALDAAAADGMQVIN
LSLGDEGAWGGPVAEAAARLAGLGVLVVAAVGNAGPSGLFMPTSAASASGVLGVAAVQSRAAPVAATLTVSGVRSAR
AAGRDFAIAAAFGDVGSLANAIVRPSDAALIAGVAAVPGSTAATGSADGCSPAVLGDVRGTVALLAAGGCSASIKAA
VAAARGAVGLLLYGDTDTVAAEVSRAPLRSAPGGAGWLFPTNPYGIPLMVTDTQSGQVLYDAYRTMAESLQLLSPPP
SPPSPQPPSPSPPSPSPPSPKPPVPLPSPKPGSGNGPSNALTPTPVPATIPALSPQPSPPPSPGQGQGQTGGNGATQ
GSQGRDSSPSSAVTASSPPPSPPPPSPAPPGSPKPGNSGNTPAKSPSPPPFPPPPSPSPPTGKPPPSPPPPSPPPPA
PPPPSPPPPSPPPPSPPPPAPPIKKWPPPNQPPEEDTLPPPVEELSQLGGDSPSTSTSGSNSGSSGSSGSSGSSGSS
GNSGSISGSSNGNSGNSGKGNSGNGNSGNGNSGNGNSGNGKGGRRLRHRLMLEDGPQPPPLGRPARRRRQLMQGRPS
EPLLPTVVMVTGSLASPFSSWGPTPDLHIEPMLAAPGGAVLSTVPIVTRADGSRSTGYAYLSGTSQSAPYVAAAAAL
YLQANKARQVSAAQVEAALVLTAKPLPDLRPTAAAVDAASAASAPSPATAAAGTAAATPASVLRVGAGVVDVAAMAT
NLVDLSPSKLELGSGLLGRGNLTFNMDAVNRAAAPITFRLLHLAASTIDGGVMMTVDRSIGAGGPNNSRVAQPFSPV

ELPYGAATVAFANTRARVIDTVTLPGRGRVRFRVTLGVADWVAPDAQLFLSGYVVLEPVPPPAAAAAADPAVAAAAL
QPPLYVPYMGATAPLADVPVLLPTLPGGMSDPTGEWWWDEDAAGLSVSDNSPPAAAAPPPAEYDSALLDAEPAPMPA
PMFSYALAGPRASLPALAVFLQRPPAAQELRLHSATYGGQYLGYVSLTAQPLRRPADGSPLLLAWRGTYFDVNRKRE
VQVVPGDYYFIVRMRRPAAAPDGPLTAGSVVPVDEWTSPVFRLTRI

>Cre02.g120000.t1.3|PACid:27575167
MNLAAINRVKVLVAVCLAIAAPTHAAGTGARPESVAAFLRGGNLPSMGNIGALAAGTSSITPATQAAATSTARPLQR
LLQSDPTLPFTCTGISPPQNCKPSPLFGNSMDCSQATCPLEPADATTFEDCVCATNCRGAVMIAQCAVGYRQCDQSC
VPLMQTGCPSSCYLNLAFYTYCRCSPAEVAECVQSVVEAVKQTYLQVCAKLPRRDGSPVNALPPQTWEPAPAPIQPE
PEPFPEPEPSPEPEPSPEPEPTPEPEPSPEPVPSSSPSPTRSPLPSPAASPSPSPSPKPSPSPALRPPPPSLSTPEP
DALYGPPPAWEDASPPPPSPRPPRPRPPSPKASPPPSPQPSRTSPSPVPLLPPQSPQSPDLPSPAEPPSPEPPSPEP
LTPEPPSSEPPSPEPLLPPQSPSYGQPTPAPSYSPAPPYGGSYPPSPVYGPYGGVQPPPPAAYGSYGSPPSPVYGGY
SSSPSPSAAYGSTDTNRPPRPPRKLHSRPRPVPQ

>g1869.t1|PACid:27575192
MCSFSSRRGRPMARLLLLVTLAVALVVQQAAAQGNSKGVNRNPLFPDCKCERDRRSSPYRASLTSTEDYPGGRRYCF
TFDVVSCQVDNPCCYMNLYKIEFPFTYDCRGSLQYIEYQGGNRSIIWGPSYLNADIPRVDLKITNMRLSQTNAAGQG
ICLGIADYCKEIDQWCWPNTGKCRMAMFNADPFDCCPIQNFPTTDSPPPLTPAQRQPPPPSDVSPPPPPPPPPPPPPP
PPPPPPKTAAPPPPPGCTVCVRLKLATIPAGGTYEITDAQCDDYQATIADAANEAANTYGAMMTKPFQPDINLCYPD
EVSVCGTFFSAAEGEKLQDWIDFDGALDDVVNLITGGSCPAELSGYTVVAFLTDAVEGESCLTGSSQQACALLSPPP
PPPPPPPPPPPPPPPPKRPSPRPPSPKPPSPPPPTPPPPSPPPPKPPPPRPPPPNPPPPPPPSPPPPKPPPPSPPPL
SPSPPSPPPPSPKPPSPPPPKPPPPSPPPPKPPPPPPPSPPPPSPPPPSPPPPKPPPPSPPPPSPPPPSPKPPSPRP
PSPKPPSPRPPRPPPPTPPPPPPPSPKPPSPPPPRPPPPSPPPPRPPPPSPPPPRPPPPSPPPPAPPPPSPPPPKPP
PPSPPPPSPKPPAPPPPRPPPPNPPPPRPPPPSPPPPCQICFTLTLAAPPGGTYTFSDNECTDLQSFMADLVNTEAG
DLGAQLLTPFAPDLNNCQPDFVQVCGTFFSDAEGAKLQPWVDTNEPFELWHNTLLQGGCFNVPYLIGYSFAYEVLTP
DGSPDGCIVGYWQPPACAPSTSPPPPPPPPPPPPPPPPPPPPVRSPPPRRRAPPPPPAAEWPVCECDKRPGSSTLTFN
TTTVTRLASGMNQYCFSLGLTACLRASPCCLQDLTKIEFDVVPACVGSMVYSIQDGEQRPAQYQLAPYPALKMNRLS
KQYIDVPGTVVCVVLKPPCATLTDLCGGPSCRYALFNKRVGDQPKCCPVRTVNDPQ

>Cre02.g099350.t1.3|PACid:27575194
MQALIALVSMYSIIVVLVDAQPLTTTKPPPAPLLPPPGPPAPRPPLPSPRGYPPTAPPLPQLPQEGQVRLAEISAPG
EGRVEVFLTAQPPAPGVGEATSAWVSVCDDRWTATEAAVVCKMLGFGWVGAAALRGLTAPPEVPIGLDGVDCSRTTL
GAPDGEQLDTPDLQQPLPPAGPNVTLLECASQRRGATACLPKEAAGVRCPPPPPPPAPPLPAGEDASPPPPSPRPPR
RRDVVLPPECEGFGDKITNFRGRLMTERAVKLREEYFGGGDGSGGGGGEFGAPPGSLGGGGPVAIDPEVPDWLKHTD
LYKTTLPHGSLRLVGGPSPQSGVVLLSWCGYSGTLSMQPTQHLETDLRGGGLAAVRRPAAAANETAARVVNELRAAA
VCSALGFQYSRSKRGSKRYGLGAGLVFDAAPICMRGAYDFAAALLAPVGSDGGPSAASVAMPGGLPGVVFPGACSGP
FVGGPSAAGGGAGGSSGGGYWGDVQLSASGHDRDLSVECFNDPDEIHGPLDPSPPPEGALRLVLYVQEPGAATSWPD
STAGGTGFVQIYRDGSWGAICSTGWDDADAEVACRQLGYAAGGAAWGSSWPDRFPASDWAGRWRDIVLTDVTCPSAS
DGDGADGVPVEVPPRLDLCPASTDAASCHYTDLAGVKCNPAPPPPSPTPPLPHPPPPSPPPSPAPPPRPPAPPLPPS
PRPSPPSPPSPRPPSPAPPSPPSPRPPSPAPPAPPSPPSPPSPPLPRPPTPSPPSPPSPRPSPPAPPPSPATRSALS
SLALALGTAAPPPQLPPASQPKPPVLEGPTGCRTCVTLRIDIRLGRTMDPFPREACDRLADGVTMALATPPLDGDGV
TPPASVLYPFYCSSPPVQPSPPPPCADSGGNGEEGTAGGMAGGGGSPHWHEMTVCGAVEDGGALVAYLSDPVVSNNL
LRTNMGTALLDAMNVAAPCAALDVDYDYEVTGPALGPTLLFSVCPASADPGATPSSEGVGGQGGGAGAPPLLATVEQ
GNGADGEREVTWTPDAQCDPNAPKWTFPPAPEPAEQPGDAPPPDGDTGGDSSPPPPEGEQPPPQLPSPAAVPPSPPS
PPAPRPPPRPPRPPPLPPSPPPLPYTAPPLVACVNRSALYQTPEQLILITGSSPSTFPSLSLPNCGSSAHSKGSFFF
GSWTYWPFIGVSAFPAANTSGLGGARGASIVCLGLQPSNSSNVTAARNGSTSAGAASANGTSSGVVSVAGLLVPAWG
LLPAAACQVASCAPPPGYFWNVTTDGPIPCGGNNTDAVAVYRLEDADPSPAPPPAAAPPRPLPTASPPLPAPPGPKQ
RRALLQQELQQRWLARRQKAGVAVAEAAGARAGERRRRRA

>Cre02.g077850.t1.2|PACid:27575250
MLLLCGLAVSSAARVSLYMSEANNVLDLSWDNGPSNLESQLRSFGFDLSVAVDRQPRLTGTDAYVIPAQHGSAFYSS
AEDMSAVSKFVENGGLVVLLGGKADADRDFVAKALGYQGSWSVCKHAGCNSKSSQGVPLLAEQASSFFSSKAQDAWP
ASLEDAKTISFNTWCRHEDVEAFTIPLYTAAGDEMKVAAQAFGKAGVSGAVVVLGYSWKEGAKARWGELLEKLVSDF
AAGAYVVPAQGNADEHPEAVDSVLESAADVADEAAEVVRRFLQSAAGTYPSPAPTPSPSSSPSPSSSPAPSPSPSPS
PTVIPSPSPPSPLPPSPPPLPPGIAKVVEDFVVTYQNQTIGDQDPADLQKNQTAINSLVDDIQNTYAKALGVNATAI
VVRGFLFVYRNGTVITIMVDTTPAKRRRMALENVAKVYDRSEVADMFGSGSGVTGLIHEGSYAANEDEIMEMLDAEA
ALPALLYRKLNEMSRRDLQQDDYYIQVLVSIVQFIEVALPPSPPPQPPVPTGTVLPPSPPPTPPSPPPRPPRPPPVN

LNAIADAIGANQVERGFPPPSPPPPRPPSPPSPGPTVKFLNLTAYGTVGPNKPVVWYNDPNFSSLVSNLSPLNLINP
FKPSCPLKCEGCPYTWRASDDMTVVPLFFRDPVQISRIFIKQVRNSGVVKVQFIKWTGSPSNGNYAPDLGRVIYNVT
KDTTKCQSVLAIRVGPKKSGVNMAVPTGGSQSNIQGKLKDKVIGGVVITMQRPAGSGPNYGPFVEYVRFQGRAVYPL
DSSIYTQFGRKKL

>Cre02.g077800.t1.2|PACid:27575300
MRFNCACAGPLALALALALLAGAQASSRVALYMSESDGVLDLKWNNGPSNLQKQLVQAGFDLKVTADGQPRLTGVDA
YVIPAQNGHTFYSAAEDMSAVASFVENGGLVVMLGGNVEADREFVAKALGYQGAWSVCKHAGCNSKSSQGVPLLAEQ
ASSFFSSKAQDAWPASLEDAKTISFNTWCRHEDVEAFTIPLYTAAGDEMKVAAQAFGKAGVSGAVVVLGYSWKEGAK
ARWGELLEKLVSDFAAGAYVVPAQGNADEHPEAVDSVLESAADVADEAAEVVRRFLQSAAGTYPSPAPTPSPSSSPS
PSPSPSSSPSPSPSPSPSPSASPSPSPSPSPTAIPSPSPPSPLPPSPPPLPPGIAKVVEDFVVTYQNQTIGDQDPAD
LQKNQTAINSLVDDIQNTYAKALGVNATAIVVRGFLFVYRNGTVITITVDTTPAKRRRMALENVAKVYDRSEVADMF
GSGSGVTGLIHEGSYAANEDEIMEMLDAEAALPALLYRKLNEMSRRDLQQDDYYIQVLVSIVQFIEVALPPSPPPQP
PVPTGTVLPPSPPPTPPSPPPRPPRPPPVNLNAIADAIGADQVERGFPPPSPPPPRPPSPGKVDTINFLNMTAYGTV
GPNKPLVWFNDPNFAALDAALSPLNLINPYKTSCPLKCEGCPYSWRANDSMVTVPLFFKDATQISRIFIKQVRNSGV
VKVQAIKWTGSPSDGNYVPQLGRVLYNVSKDTTKCQSVLSIRIGPVKSGINIKVPAAGSAANIQPKRLRDRVYAGVL
ITMQRPPNAGLNYGPFVEYVRFQGRAVYPADVSIYTPFGRKKL

>Cre02.g079500.t1.3|PACid:27575707
MGAGTRTRTLQSPWLLVAALILLVVPGGLAQAPVYPPAPAPPSPAPARQPSPPSPMLAYPTFPDPPAPTPPSPPGPP
PPSPSPPSPPPPSPPPPSPPPPPPSPLPPSPPPPSPAPPPAGTPLSGSVYLLGSVAQCTGKAGPPGEMLDVSVDATG
ALSGKGTAVGYSKDLTVSVSGTTCADSFTGLPVPFALVAPWASPASFVDGVSGIVVTPATRLLAATKPTDPSLTAAY
ALVGVSVPAGTLATNAGPVSALRSAIAATRRTGVRMLFADAALASLATVASAATTSACGSATAAATAVFNALAALTT
ANGSVDLFRADLASEIVTAAATACGKDAPASAASDAATLYGKVVSELNAPADATRAAEFVSVPLEAAALAARVLAVV
QTRGVAAAANWAAELDAVSISRAAISSTLAVSPVLPSTAVNLRLNVRAAGPLSQCPVSYKDFTSPTSPRSPTTSDAG
VVSMSQVTVGIATVPGGCKDNALSSVNRTVSSKFSMRALLPPGVENAALDVVAYLASAVFTMDQARSLKEESAPPRL
INVDDYKKVYNALGVTDALPAGTDFARQNWVSTTLTGANLIARAYFLNQKLQGALGPTCGFLAGLTRGKRTVDDVAA
SFVDRLASDLIDGTLRLDDKTYLNGLMRAYLTNGANLPVTFPSAKFGSSAVGRRLLQADPTATDDLLSFDLEQLGAL
LDSVATAVADSSNKLVQLEQEVAAAAASGTPVNIAEKLQQAAQVASVQQSSLSDSLTTLAQEVAAAVAANDTAALAT
KLQSATDAIKADYAPEKLTAMIQSAQVNTAAIKTVAEAGTAVDPAPPSSDSGSGISSGAIAGAVVGAVVGSALTAIV
ALIVIKRRRDEHRTAPSNHNSPGIRAVASGALVDAAAPAAGAVPAVAGGGPGSARPAAAVAGPSSARSAAPTSHHHV
DVEKGAGGAAGSSDPDRSPPAADAAPAGRTASGRLNVVHPMPVAESRSRSPSAGFAPSPRGLAVSGGWDVAAY

>Cre02.g102050.t1.3|PACid:27575763
MARRSALAKGLGPLVALLLGVVGRCVSIGARPRVSLYMTESSSVLDLSWSKGPENLRQSLLKAGVELSVAASGQPRL
AGLDAYLIPPTHGSASYAEAENMEAVANYISSGGLVVLTLNGLHQANDADFVSRALGYTGKWKVCDVISSGSKSLYL
SAQASGLFRASSLDFPASLEVADTVTRTSWCAHEDVDSFIVPIYTSEGDESQVAAQLFGKAGTPGAVVVLGYDWKAG
PQAQWNALLNKLIADFAAGAFVVPASGNADENATADGNFASVLDSASDVADAAAEVVRRFLQTAGAAGVYPSPAATP
SPSPSPSPSPSPSPSTNTSSPSPSTTDSPPPHAPRPPRPPRPPPPSPLPPTPPPATPSPPTKPSPPMPPNPPPNLNP
RPPPSPTPPAPPPQPPSPPPPSPFPPSPPPLPPGIAKVVDDYTVTYGSDMAASIIGDNPADLKNNASAVAEIVFNIQ
GAYADKFDVDPDAILVRGFRFLFSDGTILEVLVDTTPAAKKRRMALASLAPEDMLHVYNHAEVAAKFGADSGMSGLV
HETRTSVTEDEIMEMLDASAAIPAALARRMAEVDTRRFLQATDYVLEVLVTVISFQEVPLPPSPPPRPPYAPGTVLP
PSPPPTPPSPPPRPPRPPPINVQKIAAELGADFINNGFPPPSPPPPSPPALNLPFINVDNATGTGTVGTKRAVVWRD
DSDYTSLLTPGSPLYLIDPAKPACPAKCAGCDKAWKAGTNNVLTLPIFFKEPTQVSKIYIKQIRNSGVVKVQLLKWM
GAPSTGQVTGNVLGKTVYNVTKDTSKCQSVLVVRVGPKKSGINLPVPAGGSQAKLPTKLAAKALGGILITMQRPADA
GLNYGPFVEYVRFKGRVLYPKDASKYAYAAAKKKKL

>Cre02.g145950.t1.2|PACid:27575795
MAPRALVCLGLLLGAVGYASALAGLKIDQYKDDATATATHAKWRTKTRQLLQTDEDSVCYEGCFDAPDAQTDPLPVF
LVGTTDGSPMPLLTCYTAALEANLTYFAIVDVTDCFGGNEMVQGWAPDTCPLDCVPDGAQPAICGAINKASVFSMGL
CQYLGAPCAPEEAPPPPPPLTFDYCYDGCYVAADLDSDELNTTTSVAACQVLNANGGSADLFGITNGTECLALYSPP
AEKVDNLLCDLPCDEGTPCGADGYIAVYTMSACPSPPPPPPSPFPPPPPVPPVASPPPPRPDTAISPPPPTIEELNP
PPFEESSPPPPAGPVFRCKEGIALWGVFLPNTPVKLNPTKSQAENQAHCEELCADNDACQGYYYKTTQWCFQMKEVY
SFGKEYSTAIIACKKF

>Cre02.g077750.t1.2|PACid:27575843

MRSSSACAGPLALAVLVAALACASASSRVALYMSESDGVLDLKWNNGPSNLQKQLVQAGFDLKVAADGQPRLTGVDA
YVIPAQNGHTFYSAAEDMSAVASFVENGGLVVMLGGNVEADREFVAKALGYQGSWSACKHAGCNSKSSQGVPLLAEQ
ASSFFSSKAQDAWPASLEDAKTISFNTWCRHEDVEAFTIPLYTAAGDETKVAAQAFGKAGVSGAVVVLGYSWKEGAK
ARWGELLEKLVSDFAAGAYVVPAQGNADEHPEAVDSVLESAADVADEAAEVVRRFLQSAAGTYPSPAPTPSPSPSPS
PSPSGTAPPSPPSPPLPPGIALVVEDFTITISNQALLNITDPSSTDTPAEVEKLVNTIKNLLSASVGLQPENVLIRG
IRWRYANGTVVTVAFSSGGLRRMALEMSAGKVFTHDEIVDKFGTALPIRRAVMEASSPATEDDLLQLRDEETEAKAQ
IGQALLEHRRVLQTNDGLVVEILFTLVYYTEVPLPPSPPPQPPLPPGTIVSPAPPSPPPRPPRPPPVDLVGALNNIT
NGTVNIVPYLTPLPPSPPPVPPPLFLNVKNMTGQGTLLPAGPLIWYDSPNFAANNKNLSTTQLIDPWKPECPAKCAA
CPFSWKATAGSLVVPIFFKEPMNINRIFIKQVRNSGVVKVQYIKWTGAPSNGNYAPDLGRVIYNVSKDVSKCQSVLS
IRVGPVKSGINIKVPAGGSQANLPGKLKDKAVGGVLITMQRIPSAGANYGPFVEWVRFQGRVLYPASVGVYKTVLDR
ARK

>Cre03.g156200.t3.1|PACid:27576091
MARHPSLAAAVALLLLAVCVGPALAQRKKSPPPSPLSLGICRRGKCPPPPTEGGSEDPVASPPPPAGGKKKKPPPPG
NSLGLCRRGKCPPPPAEGGEPQTASPPPPAEKASPPPPVGAGLGLCRRGKCPPPPVGEVDASPPPPTSSTLQPPSPP
PPPASTSPPPSPPPPSPPPPSPPPPGPPPPSPPPPNPPPPDPPPPSPPPPNPPPPDARPSPPPSPPAPPPSPPYISP
QPPPPPPPPPRPPRPIASTGSFAQFTMGNYTYEFLQKRLNFPDAGSMEPDPSTPSTGTKPKGNPKPPRGRGEIKRSA
FTIARARALGHSKAEAHCNSRGGNLVSLTSFEENSAVIAKIAELGLYNKMMDPGSTANLDLWIGLRTNLIANTFWTD
GSGLTYLPSLFLGGIDTYQDGLCYTMSCRADNSCKWSAVLPPAEGCVNVTRGNFICKYDTSLVKLTWQDSAFNSSYA
LFKPAPPGQPYFYSNYLCTKINGRLVTPNNEAEFRMLVNKVYDVYTTNTTFSPGELDSIGNLRLWIGLYFQTSLSDS
FWQDGTRADENPLAFKVSLQLPQTCYAMHCRTGYCDWQPIWCGTSLPGFICEVRGAYP

>g4380.t1|PACid:27576383
MARPRASGALVALGVVAALCLQAGHVFAQAPGQDKKNIEGELTYIDYHDGTGDWALLDKSNNGRKFQPLGKGKKPPK
KDKNGKDLEPGQYMTIGCTVDATTGQCSFIDATDVAILKPAFIPPATNVRERLLVVIVDAPSCGSGAAAGATPSNLA
TLYFGPNSDGKGGWADRLETCSYGEVVWEPPTSGLTQFVTVTPSCSWPTSTCDSWAMANAANTAAQTKLGATAFATF
THFHLVMAVPSACSWAGLATLGGGVGGGGQVWLNTNTWTQTFGTFQVPLQESIHNFVLYHGFSSGIEYQDKTTFMGT
GLGCPAITEKRWLGWASPVAGGDGLDAAALPAGAALGPFNLPASWSTGLGNHVRVRPTWTSFYTNTLYGMNLYFEFR
QAKMGDISLDALYANKVVVHEIMSYMDNDLATYRSSDPHSNYMSNVAPNTRTVLNSPSLGVSYNLVMYVGAQFGASS
EFVPVYVCRYATADTECDTLANVLANTPKNPPPPPRPSPPPAPPPPPPPPPAPPPPPSPAPPPPPPPRPPPPPPPSPK
PPPPPSPRPPNPSPPPKGQKPKPGLNAPPLPPFFESPAVRAPPPPRRRSPPPHRRSPPPHRKSPPPHRRPHRK

>Cre03.g192200.t1.3|PACid:27576385
MTGSALLVLAAVLCFGPAATEANTAGACPSSCAPPSFCPLSVLSAQRWAEPSGATLIRSSWRGLTNGTDVLTLGASK
GLAAVALIFSSPDSVTCFLARQLSTSSSTGLRLSLPLASSAIVSISRASDGSCPATSPHFGTASTWVADILALKLNV
LYTRLLAGVAGSAATALETAVIGSAYGMPVCECRTVGTLLRDAELYLSGDAGNDGAPPYDLCATDIVLQYGDPSCRT
SPYTNWLCYNVTLAAQPSPPPFVEPHFPPYQPYTPDAPPPGEESVPSVAAPPPRKSPPPVPAKSPPPSPVAAKSPPP
SPVLAQSPPPPTVKSPPPVGAKGPPPPRRPRGPRGPAPPRIPRPPSPPPPSPRPPVSPRKVSPPSPSSPPPFPPAPP
SPPPPSPPPPSPPPPSPPPPSPPPPSPPPPSPPPPSPPPPSPPPGRRPPSPRPPSPSPPSPPPPSPPPPSPPPPSPA
PPPGNHRAPRSPPPSPAPPSPPPPSPPPSPRPPRPVRSPPPPKGTVPKSPRPPPVERPASSPPPPKGTPVFWPPRPA
GAKNPFPFGQCLARGSTIGTPYTLTYTNESRAEEDSMYCFQVGLNQCDRPRCCGMRLEQIQFLIDDNCYGSVYGVHV
NGKPRSPSYHVYDVRGSTYTVLKLSGFGLTLQQAEGATLCFSLRSSVCPDLYRLCAGPVCQLTLANQPGRQKSSCCP
PQDLLLP

>g2892.t1|PACid:27576607
MAHHGALYKLFLVAGLCLAIQVDAASRSYLTRPPPLKRPPPHRSPPRQVSPAPVEVVTVLVNVSGQLSQSLGHGIVE
KDASSDDSAWNLVLQEEQIARLVPTSPKTPEEASVNTRIELETDDTLITGDPVETSLALDVPKSVADKLGLDVAADG
GPAASGNSTDGTDSGSRRRLTEVEHRARRMMLDFHGTRRTLAEFNDLTDVLSQLGVQNQVPKAKVPKTGSGKKNGGA
KDLMIIGGKPLNVTSITFFFTSSSCGINPVLSESQIRARWYDNGDSAPVTASLQRYHRVCSYNKLGFYPDRNLVFGP
IDVPCTGSMPTKGAYDLRKGKGNKINLDGEMYGLYDLAKAWLRANRPDVLAQLPFLRRKVLVWPWNNQAQLLKNGDL
EIAAWAGMANMGCPGNPDPSGIVPDCLTWMNTDLPSTTIDLTTLFQELGHNIGLAHSSRTVCDAAGCVKDEYGDSTD
PMGGAGPISGDKSYICLSAPQAYKAGWAVPLANLNSTNLEDGGGFTDYAVPSMHTTDTNYIRVAIDQTGIATTDRLK
PERAIYISFRVAQPLGGYDSGLPKELNARVWVHEYNETANGLTANLKTPPMVLNMLDLPVLDPKTKKVSPPVVPGWG
PLPARAVLPSAFGAGDSLTIWLKSKTATAAIVSLCRTTAASETEETCNDGLDNDCDGLTDGDDPDCGGADPADDKSP
PPPPLAVLRKSPPPSPPPPPPPAKREKSPPPPAKRADNPPPPANAASVPSPRRPKANGRRQHH

>Cre03.g155750.t1.3|PACid:27576717

MMHHNLKSAYWYSLALDMLPMRQGRLALAALGLLLLAAAGHASYLVPNGASKFPFEGCKLSAQVNRWTVSLFSYNED
PVRGSHACFQFHIKDPSECVDAGLRCCNGMTNLNKFKMFIDNVCQRADIFNITVNGGQTTAAFKEYMNDDPARSTLK
ITNMQIPFAKIDGTKLCFTLRGTANNGNCPTLTAWANPVTRQYGLLELGLYDKKVSNYECCPMFVFPVTGRVGSPPP
KGNPTDQPSPNTPPAPPDAPATPEPVEAPLSPPPPPSPRPPSPRPPSPRPSPPSPSPSVMPAPPSPPPPSPPPPSPP
PPSPPPPSPPPPSPPPPSPPPPSPPPPSPPPPSPPPPSPPPPVLSPPPVDQSPPSPPPPSPPPPSPPPPSPPPPSPP
PPSPKPPSPKPPSPKPPSPIRRVRPAKPPSPPPPPPSPPGATPCKKKKGCAPPPAGGVAPCKKRKCRPPPPEQLPDV
STPSPDPSPSSPSPTGPTAALSFTRGGFRYDIYADRSATFVTAEAACAAAAGGSGHLVSLLTAEESAALESLLRSAG
AAASVGNGSSVSLWIGLRMRLADGKFWTDDNQLEYLPVADAGNVVDGACYSLVCGAASGTCNWTPAMTSSPATGCLS
STQDGYVCKTTSA

>Cre03.g179450.t1.3|PACid:27576791
MPGGALLGALLLLGALCQVSLSAPRRLTIHKSSDWPPTDEGATAPRAPHPPPPPPPPPPPPPPPPPPRAVPLPPPLVV
RAPAREGDLRLVNGSSPLEGRLEVYYRGQWGTICDDFFTTAIASLACRQLGFAHGAPVRWAAFGQGTGPIWMDNVQC
LGGEQRIWDCDAERPNRECTHSEDVSIVCEAAAPPPPRPPTSPPSAPAAPDGTIRLLHGNSSSIGLVELSLFGHWGT
LCSATWTPQTAVVVCRQLGLTGGTLLFDAKYGTRTPPTGPVWLQGLACDGSEASLMACTYDAVLDPGCSDPYWAAAV
MCGVDDGAVRLANGTGSAGRLEVMHNGAWGTVCHDRFGDREAGVACRQLGFSSGRVIPGDRYGAGRNVVWMDEVACG
GDEARLVDCRFDGWGWTDCSHFDDVAVSCYMTSPSPPPLPPSPGPPPLPAPAPGLYPPLPPPYGTGVYTPSPPPSPA
YAPPYYGIPPYGGPGNPVGSYSPPPYSSPAYGPPAYGPPAYGPPAYGPPAAYGPPAAYGPPAYGPRGYPPPAYAATP
GYAPPMYTSPPAHASPPTYGAPTYPQGGAPGYR

>Cre03.g155300.t1.1|PACid:27577404
MGRRSLLLPYVAGLLIAGLACGFAQLDTSDDFGMSNYGLRRLSAFYSRNFAAHFPATCYQSSYFSPFRLYFKGYNFE
TERTTFIYQLDVSTCPFPKADCCRLPLDHMVVRTKSDAQVVTVRLDGKELRSDVGEMGLTIYDMAVDPTNIKSMQLE
IVTPAQGYNYPSDLCPFSRYPGSCDVISYDESETCCAERAALPYEMFFPNNLPPGSVGPDVKAPPPSPRPPSPPSPP
PPSPPPPPSPPPPPPDGWSFCCIDDLPKSPYNLNFMGSRTSGSDTAYSFQLVVRKLNYSYPDFDGPEKGDCAQMNLR
DLGVAVYDNLLIKSVQFNGSVVPNYWTEPQGVRPDAHWLYIPVMKDFADFSEENPIDFVVTVRGLVPSLCPANEFLH
SPNACEFAMHGKVDDTHCCPHGATKKGGAYDDCCVDDIEKAPYRIEYYKTTPTEASTTYDFAVKVVNVTGIDYDLEE
EALCDRMTLDYAQIQIYKQVQVVQVQWDDRIMAFNTTPATDYSVWLNINGINRFVNDFDPNRPVKFKITVKGYVNEL
CPAGWMLDAGGKFICEYALHGTQGNHTCCPHDITRPGQEQPDCGCRDDLAGTPYRLGYQLSGVAPNQTMFNFDLAKV
DPAASLDFDGAPDKNFGADVDCGAMSIKSISFAIFNNVNVKDVIFNGFNYTWQYEPYTNTTKWLRILDLDYNPADFP
ASAPIPLQVLTTGPAVTELCPASSKYSSQAACEYFIFGLSNYAECCPLGVTSWYTPNLLMTSR

>Cre01.g013100.t1.3|PACid:27577752
MRPASLILIVIVGTTLCASAAAQVKSPKPPRPPRPPPRPPTMKPPPRPPKPPRPPPPLRGSPRPPPPPRSPPPPRPP
PPPRPPPPPPRPPPPSNIVRGAALPPPPVQRSSPPPPIPTAGGGSGLLGGGGCPDAQAVFDSTNMYRGWHQAPPMTWS
PTLAAAAQAYAEVLAPKGCPLIHAGVGGENLFTTTQYPKPDNTCLRAINAWYSEEAYYDYSVANAFTYNWPRGVGHF
TAMVWKGGRSFGCGVARADVPMAAMPGGFAGCKIVVCRYSLPVNVADNTAFKQNVLPKV

>g1051.t1|PACid:27577964
MDASFAARLAIAVLVLAVTDGAVAAALEAPDLGNSTFLGHPVASPPPAPGPAYGSPSPLPSYPTIPPPYPPPGPSYP
PAYPPAYPPSPAYPPAYPPSPAYPPAYPPAYPPAYPPAYPPSPAYPPAYPPSPASYPPPPPPTYATPPPPPPPPPREP
LPIGPITLGFNASAADARCSVDMGIARQDPDFPIDPYQLQTYPCTREPSTVFTLYTLPLGDLTRTLVYLVSWRGACL
TPVDLGAAAGDRNFTSLLVWQDCEDGLLPQEEQLFELEAPPQAQGGWLLRSSVGDSTQCARVLGTEPLSEVTLAPCD
PHDPLQIINITPYTPSPSPSPTPIPGPEVSPPPAQELPSPPPSLQPSPAPSPSPSTGPVTLWGQFQMGIVAAPPTRP
CSTDPGLLDPGLAADTLVWVTYPCISPSTVFDVYRAGEDATGRPVVFIVSGRGFCLAPYSRRPRTPIVWSACPREGG
PGGPPVNAAQRFSLVEVAGTAGRRHLLQSTGTRYLLESTVRINGAASCMTLSGTAARLTPCNATDASQYVVFTAPPS
PPPLPPSPPPVYPPPPVPVSPPSPPAYPSPGFPTASPPVAPPPVVPSPPPPYPAVPTPAPPPSPSVASPSPSPSPVP
SSPPPAYPPPIYLPSPTPSPAPSPELSPSPSPAPSYPPPAYPPPAYPPPAYPPPAYPPPAYPPPAYPPPAYPPPAYP
PPAYPPPPSPAEHPTSPPPEQPSPSPPPPSPVSTPTASPPPAPSPPAPAPPPPYPPPPYPPSPAPPSPPPPSPPPPS
PPPPSPAPPYPPPPSPPPPSPSPPSPPPPSPAPPYPPPPSPPPPSPLPPSPPPPSPAPPYPPPPSPPPPSPLPPRPP
PPSPPPPSPPPPSPPPPSPTPPSPPPPSPLPPSPSPPSPPPPSPAPPSPPPPSPPPPSPAPPYPPPPSPPPPSPPPP
SPPPPSPPPPSPPPPSPAPPSPPPPSPLPPSPPPPSPPPPSPAPPSPPPPSPPPPSPPPPSPPPPSPPPPSPPPPSP
PPPSPPPPSPPPPSPPPPSPAPQSPPPLSSPPVAEPPTTTTPSPSPPVISSPSPPSPSPPVISSPSPPPDRAPKPPR
PPPPPPYGRIPFPYYTCDRSSEAVPYAVSYVPQLQDPTAHCFRINVRGCDAAGPCCHMDLYKIIINSNIHCKGSLTN
STVNGQDRGVVFDTYWYRGQQMATFRVSKLDSNASNADGLLFCFNLEGPQCTTLDELCYGGVCTASVAGYHTDRCCP
VMDVPGTEEAADSGAGTEPAGSGVDGGSSEGDAPAAGPENSDGQRRRLLDGRKQHRLRRHRR

>Cre01.g017900.t1.3|PACid:27578059
MAMQGSQQQRGRRDWSRQWSQLWAPLLVMTAYCALVAKSQTPQVCWNRDLGDYGGQQLSTTDIADGSILTSGGDSLV
CFEKTGQRRWLFRFNRKTEVVADNQTFTYSARQDGASPFIAVSSTGLVYLAAADAVLYALDVRTGDLVWHWRDSPST
FESITYGDGRVYVSTTDFKTYALDVVTGEVLWGTQFRRVSSWTTGWFKGVFVRPDVDGRVWGVDAETGDLIWMFNMG
AEIQYRWDPELHFHPSGIGFMMARPSQIWAYNVTSGDVLWKMNAREGYMSPDNDGDNRQTFEVWGDLVIAQDEGAVE
TNPNKYGILQALDVFTGVRVWIRDDVYPGKYGMVVTEGLLVYTTYIPDMSTLDVSSSLLISAMGPDHQDVWNWQRDG
ATPAGQIQALDGLVFALQGEIANTTFNGLGSSTVLVALPATPSVICPPDVKSPPITVNNNQQGTNAVIAPPPPPFVS
GKPFCWTHSFSYKPLGSPTSDGVRAYGQDNAGLVIAVYLDTGAIAWKATCGPDSNSLYYPSNVVVVDGVVYLGCSNL
DVQARDAATGHLLWRRPFQLTPDRAAYRGGIIGNPAVANGKIVISKPDGFLQALNITNGQLVWSYDTGILAADSVGI
RDGVAYAIARHNAEVRYSTIVAVDLDTGVEVFAKPLEPKWAMEPGRLNFRHDNTLVYATWWPDAQLQATTIGRDGGD
LAWNYPISDSTGVYSVLVVSGEDLGLPHDALFYNDNEGLVTALALGSDGPTEVMWTVDLTDARLWPGVENLLTPGQS
DWAQITYSNQTLYVPTTQGMFVVGATNGEALWSDLRSRLNANIHVIERPGGPQVIYGRYLSNFESNTPQCRPPKPSG
LVTKISVRGSVPADGGEEPSTPTDAPAPDSRPVPVTASPPPSVTPAPAPSPSPSPPPSPSPAPVRSPSRSSSPPPPP
VCPPCPAINCSAPAKRPSSPAAVAVALGLSGLPYNIVKADSKLRDRVAGAVEKLVSGKVTGFVRVRVLGVESTAGGT
AAVLRLSLQRSGGASATAVASAIEDAALNGKLQEVVLAAKVPAKVRSVVLKV

>Cre01.g026500.t1.3|PACid:27578078
MNRLWTSLLKAALVVCAWSSACSAIELGAEAVGSPLAPSHTRAFALPVRHHKLPDGVRRRRHLLRSSTRPVYGNVPE
LGYYYTYLTIGTPGQTVSGILDTGSTLPAFPCSGCTRCGPSKTGMFKPELSSTSSTFGCSDARCFCGANSCSCNNEQ
CGYSIRYLEGSSTSGFLAEDMLAVGDGGPAANFVFGCAQSESGLLYSQIADGVFGMGRTPASLYGQLVQQGVIDDAF
SMCFGAPREGVLLLGNVALPADAPAPVVTPVVGNTNKFNIQIEGLNFNDQQLVSGQTWFSYGLGTIWDSGTTFSYFT
RNVYNALVAAIRRHVEANPDLVSVPGAYSDDICWKGAPADDASKLGAYFPDMELLLAGGGRLTRSPLHYLYPYGAAW
CLGFFDNAYSSTVLGANLMLDTVVTYDGRLNQMRFTTYECDKLSEALGVNGQGSNNSTSPGSAPSPPLPQPPPPPPP
SPPPAAPPPAALKPKRPRRTSPPPPRPPPRSPSPRARTKKSPPPRS

>Cre01.g039950.t1.2|PACid:27578574
MRTRLYLNRWLTFVASCLMQASAGLSYSFSIYAPVLKEIWGYHETQIATVGSCFNIGGYLAIPSGALYDRLEKHKRF
GPRFVAVMGSLTLALGYLGLYAAASGLLQPHFALVCLFAVLGGNSSTWFDTACVVTNVRNFPRDRGTVVGILKAFVG
LSASIYSAIYAADFMSGSGASPPPPASPPFSPPPSSDHHVPPASALRSLLSFAAHLPPAAVPSPSLSPSPAPSPSPA
DTDARNSGALSFLLFIGTVPPLFSLLLVSLINLVPEAYHEPQPVKEEDGTGPGTGAGASAPSAPADTSRSGRRRGGS
GGGARGEPGGSDLQQPLLLPEPQERHGLGTQHPAPNAQQGPSRPQLDQWQHTPRAGASPSSSLPGASSPQRPAASRF
TTQARFTFTYCLVVVVAVYQTVAALLEGPAAHATPIERQELLAGLLGLLGVLLLVPLASGDCVHRKPRPDAVERVRR
NLLMRLARRALSTAAASAASAASAASFGIRGWSISRRGASVSVTGGAGAGGPGAGGAAAGGGAARVAAAGPAVVAVV
AEEVVVEKLAVVKVPAEAPQASTAAKTAAPLVPATGKPESLLPPPALPHIHTHRGGPIPTVHAAAATPTGESAATST
TPDTAATVALTEPTGPQGGTGGVGVRDQDLEAALERLESVPALPDLTLGQAARTPMFWLLMFQFSVGLGTGLAYLNN
LGSIVVALGGKQGGQVVFVSLFSVANATGRLMGGVLSEHVLRRYGTPRTLVLLAVSCLSLLAVGGAAASDLGDLYAV
SLVAGLAFGAHWGVIPAVTSDLFGLTHFGSNYTGLQLGPAAGGYLLATVLTGKLYDRAARQHGDSLFCQGADCYFAT
WCVLGGLNLLSLLGTRELYALTVRQYRRMVRAGGV

>Cre01.g025200.t1.2|PACid:27578767
MRLVLLAVVLLVGSGVAAAQQASPAADAPGILRKTLRGTLAQVTLDDDGGEQWAIVSESDGTIQPFARGVRPPKKDK
NGNDITAGAVVGLSCPTDSTGSCTYVSSSDTTLLAGGATPGAATSKVVQRNLIINIDYASCNYSASHNESSLRTLYL
GAAGDGTGGHALKFEQCSYGGLTMNATAFTVVTVQPNCTAAILVNCSYSGISNGADAAAKAILGTTAFSSFTHYTYI
LPPGFESICGWSGLALLPGKQTWLQTSSYGVSRWATAMQEGIHNYGLWHSWLGTAEYNDYSTSMGRGNSCPNAPELS
YMKWATPVTGGSALNASLLPVGQAVTFNLPATYLTGTGNFLRVVPDWIPNYLYNVYIAVRANKIGDSALGNGYANRI
NVHYLNASLDNQRWNGNYTYQYKDRKITVLNTTQVNVTTDLPDYNLVVYGGSWVGTDIMRMYICRYSLSRAECPASA
AVLQATASPPPSPSLPPSPSPPSPSPPSPSPPPPKPPSPSPPSPSPPSPSPPPPKPPSPSPPSPPPPPPPSPPKVTG
GGNNKNKGRRMLRRAGQQ

>Cre10.g434650.t1.3|PACid:27579290
MRGPLCIAFAVAAFVAAATAQSSCPPAPEVTRTALASACSGTSAASRAIGCSMFALCNNAALTPPIFSALCSTWRIA
SSLCSDDTDVVAAASGLCSGIRAAYVDDAQYRSCLQPYTGIRSTSGIRAGHLRACDDMAGEPMGGCDTCGTASSCPN
PLVSYADGCIDMDMGQCAAWRSFCTGDAAPTGAAAAMLCRRPNNRGGIVAPPLPPSPPPPALSPSPSPSPSPSPSPA
SSPPMHNMNMTDGGPSPPAAPCVVDGTLPECGNFSYPHMSVMADINNLCTAMDYMPGCGIWHACDAGLVSGDYCQPF
TLLATICVDMPTMAGCRAYRGLCKAGSVVRQCAIRPAIPKTPSTKQAQALIDTVCSTTPAPALCATCNQLECSDYIG
AVTDACTSAPDTEGCDELYEGWCAAASAWEGRGNALAPDASSNSSLAHYCESSAAYAALPSPAPAASPSPAPAPTPS
PSPDMHGGSSPAPAAAACVSDPTRPECATYVYPDASVAADVTSLCNMMPYMPGCIIRDACTAGLVSGDYCRPFTLLA

TICVDMPGMAGCRSYKPMCMVPASRVPQCSALPAIPKTPSTLQAQDLMDSVCAAPVGATLPECAACTPTTCPDPLTP
LARVCANAAVAADAAGAGCGAYFEDWCAAASAWQTAGSGNLSHFCRDSKAYAGLGSSPAPSASPKPAPNPRRKSPPP
RRASPPPHRASPPPRFVKAGKRQSAASL

>g11256.t1|PACid:27579408
MGQTYGLVGGLVMVIIALASIPSDVEARDFKLNVQKGQQTLWQKSPPPKHRTSSPPPKRRAVAPPPPSPEPPSAPLP
DPANSREKNGVRLVLGDGPRGRLELSSTEGWLTDYVDGAVAWLPVCQMIGFDDSKAQIMCELLGYTYGRLYYDDEVA
WRPPNDTASYTDLPIENLDCSPNYPDAPPPSSEGGDQTAGGHRRRLLAPLRADINIPKNAPYTCSFYSYATKRCDYS
GPLVGVECGDDVFPPAPPPPPRPPSPPNAPPDRNVDLKLYGVGVGTVADLESNLCDSEEQDGCRSYERAEVLIADPA
NPGSQIWAPICSVDNNDDLAILVGDTLCKQVVNYPDPGYVSYSGEVQLPLQIPEEPDAAAEGNLFRPSKYTQWVTFT
GGEPDARYPALQQLEYTVASACESAQLFAVFCSYADD

>g10543.t2|PACid:27579554
MAPAGRASAAPLSLLALVAYCAALLAAPASAGSQGFLPRRALLQTCLTCSGLTTCIKATCLYTDGTVNYVSVNLTSC
KGASISWFCCQQNSCTTTVCDSTQVNGATCNNVLTSVVTTAVGATSVMLQVHDGRLIGDYDCGAPGNSCCGGDGGAC
GSGSNVCDDVVVPIAGVDTCSSRPPSPAPPSPKPPSPAPPSPKPPSPPPSPAPPSPKPPSPPPSPAPPSPKPPSPPP
SPPPSPPPSPAPPSPEPPSPKPPSPAPPSPKPPSPAPPSPAPPTCPPGCRCTDTAWGFPLPSQFRNADLSSTAELPL
TVDPGFGGAVFWNSRQVGGAWGGWFRITAPGSGSLTLGICGGCGQNVVGKGYYFGNGFSITFNSFTT

>Cre10.g423850.t2.1|PACid:27579884
MAPTSFRLLVLALAIDVTVGGRLQKLERRSLQDGTLRLVPDAYQGRVEVLYNGVWGTICDSGFSDRTANVICRQLGY
AGGRAHWGAWWGEGSGPIQVTNLQCKGTESDIQECSSSPWGAESGCSHADDAGVVCFDEQPVRLADGTAPGTGRLEV
LSMDGIWGSVCELGWRPENTYKACTQLGFPDNLDVAVLVGGSAFGSGDLRVAMADVACSGLERYFAACSFAGWGVSG
GCDPETQTVGLDCLGTLGHSPGPDQNQPPEAAEASPPDLPPPSPSAPPMEGDAPPPRRPPFNPAGDGAAPPPGNEPI
GADMPPPPLAPPPNRPPLSPDTPAAAPPPDNAPDEWSPPSDSPPDSPPNHEPDPGTDWSPPPPPYVIPAPVPDPSPP
PYYGSSYPPPSPPPPPYYGSSYPPPSPPPPPYYGNGVPPPYYTTSPPAPVYGPPYGSAPPPYYNHTHKPPAGYPPPG
GQSGHQAPPLPPFPADDNQGSYLEAGTIGFPPPIRPGSSGALFLEVLCPPGFYATAIQVLNQETASGLGLECISIPA
CADPPPPPSRSIKIHYRAPGCGPRRSRRSLRGLGSELAAEDPDSRLSSGSQQESNGQLRSSTAAGGSARVRSSSRML
GSATDVGAIVGNAFVLSTARLTDGAGNTVDQLAVYFGDPSSPDLQTKVVIRLNSEWVDGFSVPDYRFDCPSGFSAVR
AQTGGMSQPPTRVGFRCGSDAAAAGGWTSPELGVGDYGPAVPLSEVQCPEGSRLAGLLASTRRIWDGGPLLVEAFTA
YCTKACAA

>g11098.t1|PACid:27579953
MASRAFITFAVLLFAGSAFAGELGVTLARGSARELLGATPRFPYCSCDVYDCACSPYNVTMASVTNVVGGKRFCYQI
GYLGCPTNRPCCNALKANVDKIAFTTSAACQKKNGGVTRVEISGNLWRSWETREWALGNGDYGYELRIYDLDGTNSN
NFPGTRICVTTGAQCNKLAELCDIDGGNCKYSLAESSNTKYCPICPVPPTAKPSPPPPSPPPPPPPSPPPPPPSPPP
PPPPSPPPPPPPSPPPPPPPSCALRIIPPAVTRPGQKPTWGNTGAAGLPNCLDVANIMTDTLNIAAEDADARMVWPF
EYRDCITEYGAQNPSVVVCGTFLSAEDGALLQDEINDLLELILSEVKGGESCPPRLSGYTLLATIEPVDGSPDCLSG
TSSSACAPELTPAPPPPVQLVQTHTCPQSTTNVPFILDAITQSSGTDTNGSPVQVFCTTVKSQSCSASSACCRMDFA
KVELIVNTACRSDLRYITVNGERQAYSWQFYSEFTSLKFTGLSKLSPVAGSTLCWAVRAGGCASTSSFCYKGVCQAN
AFSSDNKCCPANII

>Cre10.g421350.t1.2|PACid:27580077
MRSASASHVALGVIAVCLLATAQQAAAGLYQSTGLISVVDTSYPSGAHTSDYVLTADVGGIYTLIATATAQAGGDLF
TGLVSGGIVRVVSFYEPKLGQPWDIFGLTVISPPPPPMPPAPPPSPPPPTSRIGSRATTKSLPWVSSWNPGVILNQD
IPDPPRVVRDLPTLFILLDLCGKGGGPAATEDALSNMLYRGLTPFQDYVATCSYGKAQFDTTNTRILTVKLPCSGVS
KVSKMAWDSSSCSKDNLFNWMYEVEYYIDNVLKPKDWNHRRYRHHVIITPPNMTQWAGPDCDWSGMGSIGMAQGTWS
YAWVSGDNWQTKQVYLHEMGHNYNVMHAATMQPASEGGPDACSHCDWSSAMGFCCETRCMSAPHNYQLGWAKPAATV
NSTQLSEGNTLAFELPSQIMSDANFLQVTTDWLTAASVSAAGIDDMGGPAFYNPANATFYFSYRMDFADFDEVADGY
AGGTNVYLFTIGSQMDKKDSVHLALLTDAAPAWTDPSGSLVVRQVGAGGDTAIVTVCRPDAGGEKSEDACTDFWDND
CDGLIDADDPDCAWYYDSLPSPSPPPPAQKASPPPSPPNSPPPKRSPSPPSPPPPPKRSPPPPPSPSPPGLPPPPSL
PPPPKRLSPPPRLAEQVDASPPPSPAPPPAISDTRSPKKKRPPPPASGKSPKIPKSPKPGKRGLQAQDTSRAPTRPA
PAAIRHRRHAAT

>Cre10.g452250.t1.3|PACid:27580296
MRSLSGSVALGLGLLLGTFLLGGSARGLLAGGSPAQAAGSFDSKEGCATIIEQGMYKTFYDRGAAPDEYMSFTAAVC
SLTLTADLYPALPASEYANFQQVVAQLAPNLKSTRWSSTSAFLDYWTATNDPDVASATAMGAYAPYARVRAYVLTVC

RGLSTASAPDLTDTDFELAGTVLTTSTRSTFMSCVQLEAAGLQLLRTSAVGANPFTMRMTFDASTASNPASMIKVIE
PVLSSPSSAVSCAFTTASGSTVQWEDDRSFTLQDKATITVSCSIAPAASNNGRTTAVALGFYVDGSSVSYAATLFNG
LPFPPPPPRPPSPPPPPPSPSPPPPPAPPSPPPPPPPSCESSLRGQPCYKLYSLESAASPLLELVGAPAAALPAGGV
LAQIRALLDALHTANQTIYKQAAQIDSLQTQINDLKSKAAQVDSLQTQIKNLTTKAAQVDSLQTQIRDLTAKVPTSS
SSVDVLRLCIGEVCLQQSASNTANSDGRLFFVRKGTSLASVRISTADIDMDILQVFPYRDGGDNLPSFPTFFFVNQY
TDFGIYTGYFNSRHTYGGRLLIDKVCIGESCLRASSTSAGVLMISGKDADTRRTAHFDTRLGGRDIFRVYRDRDGNY
PYFYYNNANGFGTNDGVTNDLYA

>g13810.t1|PACid:27581269
MVLTTVACCALLRSAAGIALGSPPPPPSPPSPGSPSPAPPSPAPPSPEPPLPAPPSPTPPSPEPPSPEPPSPAPPSP
EPPLPAPPSPAPPSPEPPTPPSPPPTCRADPYLLYYHSITESISGPVRTSEPESRVLGTATLWPDYPVPGSVTLELE
AAPGGSWTPGAVVHWALMTPLQYLNDVFTVPLPDLEGPPCHEPTSLPHNRTLTANDVSFFRVFTLTYAQLGVVDDCN
PRALFPVVRIDDGETSAWLAWDWVNPATGEGANICSGSPTAWGVGSFSLQSCPCQPPSPSPPPPAPPPCGTTILSQE
SEPVASGYLSGPIRDTANGWEVGAATVHVEPSGPDAVTLSLSLPLPAGTVIKYNFFDWVGYADAVFTLRPPALEGPN
CHEPYDDSLLTAATVPAGTNEYNISVSYSSLQLSPCSRKTFFPVVKITTPEGLTSWLAWDWVTPNDPENDVCGDSPT
AWGVGYFDLYNCPCSPPPSPPPPSPPPPPPPSPPPPPGSPPPSPPPPSPPPPSPPPPPPPSPPRQSPVPTAQLGTGF
PFCACRKRKPSLSPWRLGPLTGPTIAAPNSATEPTLATWCADITATAEPSPPTGVCAGMDLFKIEFLVNHACYSSSP
KAIRRVTVNGAQAFPTWSQKYIKGVYYGVFGVSRLAFTAADGPVRLCLSFAAEAEGGCGTPAGLCYGSACTYSIFNK
KQNCCPTSSISSSVDVRPGH

>Cre12.g506750.t1.3|PACid:27581283
MPVRARFLSALLVGLVGLASAQTQPTFPFCQCVRSTSTSRYEVVDAPAVALKAGYWCVQLRVKEQCDKPKNPCCNAD
LYKIELLAQSGCVGSRMTASYGYSAASLVEVPSVPTWEVPDAAPEGAAVLKLVRLGLNTSTADGMYICYKALSGSCK
KPEQVCLPGATGQCQLSLFNTDSSCCPTETFPLPPPPSPPPPSPPPPSPPPPSPPPPSPPPPSPPPPSPPPPPSPPP
PCVVCYNLASAGFSFNNRTCVLFAGFTNGLLTQDWMFQEGLILEPFVCTQATPNFVQICATYASAEVAQEAADYISA
DDFSLVELMWDYMRLRRYLCGGQGSLQFSSESDCINPLLYNSRACALEQTPFPFCECDKTPGTMPFGVDYYYSEPAN
SKFCFTVLAVTPNDPSSSCGKITTIKKVEFNIQDSVKRQIKAVVDASGKTLRTAWGSLGDNTFKITPLSWTLPQLTA
APQQICIITTGAELSDLCVGGGCTASVFDDSQKCCPTSTSQLEPVVPDDAFRSFP

>Cre12.g487950.t1.3|PACid:27581478
MSRKVIWSRCNVARCCIALLELAILFGFTQTTCGALTGLTLPPLDLTAISYGHPWRSANTLVFTDLMKHSAPFKAGA
RGASATGDCGWLGDENIQWRTDGYPAGLSPPYGWTHCANSTIGAGGAFYPAGKYVVLYDGEGVVEFAGDAAWPPLAS
SPGRLELNVTPSSAGMRLRIVKSSFANPLVNLRIIPADLESSYNTPANTFRPEFLKQLEGVPLLRYANWMLAVGNAG
SGRAAPRNWTRRTTPTTSTQSFRDVDGVAVEHCVDLANVVGADMWVSLPRAADDNDPYAYNMLKYLAVNLAPGRKLT
LEYMTDGPGWIEQHLPNNTRLLARIAQRVFAEVGRPRAQLRVVQTAPGINFVANMMGVLDQQKQQDPKANYSAWLDA
VALPASFGGSAYSSVGADRNPTAWQDGAFFLSPNRPQSVSLEAALKLIRSSVIVADMNYNRALQVLKARGLEVLGYA
GGPELAAPQYGARANMDRILGDCGFSFTTWPCRVNNLYPPSNSGSISFDNAAARNASLLGVVLANATREAELTEVLR
RVRLHDYTYDLMLDSLWRWHYQMNGSDLVVDVTKQGSRCDYAPGLGSLRKGFAMLAPTECQPGVLTSASQPLPGAYP
TDAPSFRAFKAWIAAEAAGGSAGVGVGGLRGALPRTAAEVTAPVFPPAAACNPACVYGTCTYYGVCSCWAGASGPDC
SVLAPSSTPSSCAPRLGINLEGIADWSRSWFFVDAWKSSRAWISQSFVESAWSTGTAQELISRRDGPFGPAAYGYPA
RLAPYQKVSSIMTRDLEGHAPGGWYTVLYEGKGVIAFSLSDVKDVVYEEAGCIRVLFNPSTDFNNGMLLSIERTDPS
DPIRNVRVLMPGFERQAEAGDVLHPLQLDFLRPFGLIRFMDWMATNQATLPTSWDTRPRTTDISFAYSVGGVPLEVM
ALLVNKLGADPWFCMPHTATDDYVRGFAQAALQLLRPDVKVYVEYSNEVWGTGFPGGQYAQEMGLKMNLTEEGNRWY
GGATNEARLCFVAHRTANISRIWKEVWGAAAGRVNIVISGQAVWPITSSKLLSCGGAHKYIDALAIAPYFGSYSKTR
DTNLETFVNTTLVSQVNDSLAYVSQHAAIAANFSKPLLCYESGQGLIGDGSSMDLALQANRAPGMYARYRQYLNGLI
ARNVSRAAHFSSVGAFSRYGSWGLVEWQDQDRRDSYKYQGLMSVLNEQLRCPLPPPLDPSACPNACSDNGVCARNGA
GQPACYCNTGFGGDDCGQGQYTEVYKCGYKCTFDQGFCNNSVVTQRSIRTWTCSCKPGISGLECGINSCTDNCNGHG
ECVDQNVCACYPGYTGSTCDVDCGCNGYGRCTNTNSSSGGGAPTCICDVGWRLGPNGCEWDCSDCDDGTSCMGPGEC
GCADTCVYGDCFHGACRCWAGYGGARCDLDEQAAAAVGLTVVPRLNRGSIAGVNVDGLSYWSTEWVWTDVMKGSGQW
WTANKPDTAWENPYNTGQPLELRPDGYPARLPPNTIAHTLILRNVALHAWPGRYVVLYDGEGRLDFGFDAQIASRDR
NRLEIILTPTADLACLERGDAYCGDNGIYLAIIDTNPSNPVHNIRILPSMGGAAASAGGAGAGGAWEGRYGRAPFHP
WFLKSVARYSVIRFMNWMAVTQGEPAYQGAQDWEARRRKPDYHTQTGSEGGGAALEHIVQLCNMVGAAPWISVPYIA
GEDPGTLRAMAALLRDRLRPDVPVYVEYSNEVWNPGFDSYKWARDRGLALKLSTDGQTASYRYHARATVAIAEAFRE
VFAAAPGGGASRVKVVLGGWGFLCNGGTGCGAWHMADTLGWNGTASKVDYYGVTAYWNCGLGGNGAADAILSVGDML
AKCRTQLAADEAAAAALAAKVATYGKTVITYEAGPAIVEQAALGNGGETPAATVKYVAVNRHPDMYGLYRDYLEMYR
RVGFVSAGRPYNQFTSVGRYGKYGSWGLQEYTGQPLDTAPKYRSYMDWLDSQAGGPGPRAQCLSLAATGGAAGGVSG
TAGGGGVALAEGLLQGAPAVHLPATGSVWVQGQNVTLKWATVGWPSGPSRPLTITLWSDADCVDGSNQSQQKPIDQA

LAVLARDAVLNPPGRLEWTVPLGAEGPAALAVAVAAAEAAAAAGGGRRRVPRFFIRISDGVSTNYSEPFDIRLPYTY
SAGAWGGCDCVAAASRQRRTVSCRATRPPGSAAAGLGDAVPERLPRCDPFSDWNITRPDPACSISATGCREYRTSNV
GQWAWSNFKPAVDCTAQQSPWPAAGDAVSTEAADSACVAVGAVPPPTSRTCSDASVRLCPNIFKPPSPVPPSPSPPS
PAPSPKPPSPRPPSPRPPSPRPPSPPKPPRPPSPSPPSPRPPAPPPKPPGPPSPSPPSPRPPAPPPRPPSPPPKPPT
PPPKPPGPPSPSPPSPRPPSPPPKPPSPPPKPPGPPSPSPPSPRPPSPPPKPPSPPPKPPGPPSPSPPSPRPPSPPP
KPPSPPPKPPGPPSPNPPSPRPPSPPPKPPTPPPKPPGPPSPNPPSPRPPSPPPKPPSPPPKPPGPPSPSPPSPRPP
SPPPKPPTPPPKPPGPPSPNPPSPRPPSPPPKPPTPPSPKPPSPKPPSLSPPKPPSPSPPSPEPAA

>g13811.t1|PACid:27581651
MLAITGQLVAYSGAGSDPAFGTATLALDTTSMVLSLTLAPGPGRTWPADPATVSYATHTSGAAWLAAGACSAPLAGD
PNGAPLAGGLAGAADPLTLAVPLDGLGLAPGDVCGGRRAAVYVLAVVRAQSQCIWLQWYDGARACPNSAGSKPFGFG
AFGLSCSAPCPESPPPSPPSPPASQPPPPPDPPPPPSPPPPRPPPPSPPPPSPPPPSPPPPSPAPPQPPPPRLAVPD
MPPPPPPSPPPPSPPPPSPPPPSPPPPSPPPPVPSPMPAPPPPSPSPPTIPSQFPYRTSCRKQRGPGLSPWRLGPSA
AVGPGTAGSSSLSGSSAGQLAGALAALDPEALRWFCMPLTWQLLGRVVRPYTSPCAGMDLDRVDLLVRGECMSQVPK
ALRRVALGDTLVSHHFTSHAWPQGGGAKAWVLSVTGLRELVPVPEPTAGQAQPQQQAQPQQLCLGFARDAAATNASA
CATPERLCYGSGADCTYALFDSGAACCPTTSTATASATAASASVATSSDGGSSG

>Cre12.g487700.t1.2|PACid:27581682
MPRLRGAAVAAPLLLLIGALSALQLGAAIEANAQPPVVVYSDPDPNNSNDVTRKAALVASVLGVGSALGAQVVSGRP
VPLSLDARTHVMMTNTLNDMSDLNRRALVNLVDRSGSLLVLAANQIGGQDYNALLAPFLGGASPGCGWESQSRAANQ
VFDLPGMPLSLFAASRGANILAFRCTAGAPWYATSDGAAPVMMFYTPNGGWVKLLGFDWGTPSRVADWETILLYVPP
GPPSAPFPPPPPPGVPPPPSPPPRPPSPSPPSPPSPPQMPPMPPSPPPDPSPDARAILLTDEALPFDTLRKTQLLLT
VLQLVGQGNVLDRPNPGIAPVHVSYDNTLVKLSNGSKAFLNEKLSLGKTMLSIVVTEEDSAPDLSALVSSIMGFAVN
CSKRVMVRGSLTAVPESNFTGVLGIPRYPAAMSLVANTVALTCNSGRRVVLVDGSDTEAVLWELSVGRGVVRLIGYD
FTNGGYDGTAPANRELPLAQRLSATSSIAAVLTTRMLPGDTSSPGAVRSPPPAKRPPLPPPTGRPPPAPFGGKTPDV
VLLSDPEVQPDSANKEKLRDGINALGSTPLVWPNAVDGAPVHVAFDTTLAALSNDTLAGLTSLISAKRTVLSIVFTS
GLTDAARNTLLQSLTGYSDLSCMTGAVAAKLRVNRVVSAIAELRSSGWRAQQVTRYIKCNIGTTVFAANVVGSKQAV
IQELTTMSGGTVRLIGYNFASGGRGSDRKPLTSIAVFAKPLVPAAAGR

>g13210.t1|PACid:27581768
MLCAGCATIGCGLETLPPIRGTFSPASAWEDVSHLRLDDASCLLLLPLYHPLTLRHTLPPPPSPTSPPPASPPPPPPP
LPPSPRPPPPPPPTPSCRLPSSSSSSASRPTATPSLPPPSPPLPTSPPPPPLPPIPPASPPLTPPPPSPPALPLQPL
TVSTTAFPSASSDVIHSPFLLAAVSPGSSSHATNCPNRCLAAFMTPSTIPSLRPTSSHSASSPPPLPPPLLRLRPLP
LSLLRLLSLNHRLPLRFLLHPLCHLLLPCAHLRTLRLLLLLDLRQPFPSSAATTAASSTSTDSSPPPAPPPPPPSLL
PSAPSSSSTPPAPPPLPFRLPLAFPPPSPPPPPPSPSAAASSPLLLLSNTHPPPPPLHLHCLHLRPRPRLRPPLRPP
ITFPPPPPHLSATSPYPSPRHLYTCHLSVCHQIPVPASSSTAAVSPAVPAYIYCGVLSPLFSSPPGPSSPPPATSMA
HRPLPPPTLSIPAETSSASSASFRAASSSPAAAASSSSPAASSSSSSPATTAGASSRAASSASSSSLAAARAAASSP
AAAASFSFSYPAVAAPCSFPAAAASSSSSPPPPPPPPRPPPPPPPPPPPPPPPPSPPPPPPPPPPPPPPPPPPPPPPA
PLPPLSTPLPLRATPLSPHSSASFPPPSASTSSPSASFSPSASSYSSASSSPFVSSSSHSSTSFSHPAASSSHSSAA
SSHSSGWLSGAGLPYGA

>g13652.t1|PACid:27581909
MRASRPPLLLPLLLAAALLLAGLLQPRGAAAYPDYWVATPAGQQDCAAQPTRAVIGSPHGTPIQDSTITITLRPLDA
GSSGDEALQLCPGQAYTVQVGFPQKRRALISATSGAFSGVPSGVLTSPDCPNRLIIGPSQPFTTNAAMPLGLTPGAT
LTLPCDLSATSAAGGQPPAALVLRVTTASPISTRWRAASRSYSLSSNCPVAPQCGGASGSSSTGLGGSGSSSTGLGG
SSSNTTETGTSTGGGSGSGSGSATTDGTGSSSTDNSGSSADSSTPGPGGSSADDSADTGTSSLVPLATPSASSGPAV
VAAAGPSDPAGASPVPSPPPTTASPPPATPTSTSTSTAATPPSPPLQFRGDAAGANGTATTTSTDSNTNGTGTGSGS
GSGTGITPAATAGAAPSSSSTAASPPPTTSDANPPPSSPSQQVPPPTASAPPPPPLPPSPSASPSPSSSSVSTSPSA
AAAAAASPLPPAYPPSPPPPRQSSAPTVQAASGRQDEGDFRESNQDASGASSSAAAAGVDPTATAGSSSGAGGSSSP
VVPGGGSQQGGKQAPDVGSSSTTGGPDTSPHNPVSVGKQLPVAGAAAPAAATAEPSQQATVPAVAPSAPGTAGAVGA
AGGAAGVIRAAAGPLVAFSVTCFVALALALGI

>Cre12.g546500.t1.2|PACid:27582082
MAAFSLSIALLALAATRSVVAYPELIEEYPSCSAHPERNDVFPHHWFVVPDPTGTAWTAYLDGELLDGRLCPGHTHS
VLVSYQDRTGGPDKRRALLTSSIGQLRAVGGVETAPGVFKDPSCENRLYLAPHSSSVPGYIELDAALPTSYSVTLTL
PCSASGATVELKSSSATHECLTNCGYSNLMVGTWTVPVDPECAAPACRPPESPPPPPSPAPPPSPPPSPRPPSPPPP
SPEPPIDLSAPRRPPSPPPPPSPPSPAPELPPSPPAPPPSPPAPRPPRPSPPPRPPPSGALQGDGIFVDPPFPPGAP

QPPAPPASCEPSSLSYACSKMLGQVRLHWTIGGAAPPANDCTGPAPRALSSAGEALVHFGVEGPGVSGYVSLGFPEN
PDLMYDADMVLGWVSADGRGVVETYHVTSYEMSATDVVSQDWALGSGVVEKRGADGSPTTIMCFSRRVAEPLARSSP
LLDMNDGTIKYSWAVSPEDALVEHPPNGYGAGLVNLRSGTSSAITVKDNSKIIIAHGVLMAVAWVLLLPLGAMAPAH
RWLFRGRMWGSKAAWFWVHFVGQLGGFGIFCAGFILAMVAFDRPQGGTLTSSHAIMGYVVAGMAGLQMVVAFMRPDP
GTKLRVMLWNPLHMNLGRATTLLAWATCLVGAAVHSESICKAPIVPWVATLGSAMGLILLADWALRDARSRKADKEL
QDMKSRRYPTDAVPVNVAVAADVVAKEKAAPVRPIVDPGNPVVTVLAGDSDSGIGPRAGVKGALPTTPSSGSGSAVA
DSDVQIVVQTQRTG

>Cre12.g559750.t1.2|PACid:27582112
MASVRRRARGLLSLLVVLAVMAATSTTAATRVTAHISNRHNLRGLRKAVGRQPSARRLMVSGGLTRLDELGSTPPGD
TEAAAGPIPVTTRAGTWDEEWEAVIANQVSPKPKTGSAKRAQPPPSLPPVSEEEAASPPLAPTQQDASPPPPKPTQA
ILRLARMPRPPPAPPAPPAPPSPPSPPAPPPAPPSPPSPPAPPPAPPAPPAPPSPRKPPMPARKPFAGNVERGEENP
TPSKDAASPPPSPRPPSPPRPPPPRPPSPRPSPPPSPSPPGAPDGPSASNNPLIDKALILESHNWLRRATGVPDLVW
SDDLAASAQAWSNQCVFEHTRSGYGENLAMGGFRTAADVARGVALWTGEVCEYDWSKPGFGMDTGHFTQVVWRNTQR
VGCGFRTCEGGVAGYGSRSAGAGVLVCQYDPPGNYLSSSQFTNNVLRPNPMPACDKRAIGRR

>Cre12.g520450.t1.3|PACid:27582572
MAMLQLLPALAAGGGHGVHIAYLTDCTMYSDWQTVGMVFSYKRSRQPRDSQITRIMCCTEEERKRYNEQLLSIVNTH
VAPSFAKNEKTGDHYAAYNKPGAVYDWLKHVDPPEDWLLVLDSDMYLRRPFYPQFFNATRGWCVSADYTYMVGVNNE
LATRHIPEIEPRHDTLAGPYGRRGDQVGGFFFMHRDDMKRVAPLWLSYTEDVREDPESWRLTGDQYVEKGGKPWISE
MYGYAFGAAKANVWHKWDKKTMMYPTYRPTDCQMYSDWQSVGAAFSFKMSGQPGSVIRVMCCSEEQAKNYNKGLLGM
VDTWVAPDATHSKRTGDRYAAYNKPEAVIDWLDHNVPKHDYVLVLDSDMVLRRPFFVENMGPRKGLAVGARYTYMIG
VANELAVRHIPHVPPRNDTLAGPFGRRADQVGGFFFIHKDDLKAMSHDWLKFSEDVRVDDQAYRLSGDVYAIHPGDR
PWISEMYGYAFGAANHNVWHKWDTFSMIYPGYEPREGIPKLMHYGLLFEIGKNYSFDKHWHYDFDVTVCPPWDLKDP
KRRTHGIFPEPPRPSSLRKVFPKNGDMASMDDFIGYYKELLAIETLATLNAAFCDYHISHCPPSEQLVSVCKEVFSL
YNEAREFIQEAEASYDCQDFHPKCEEWKESGECTKNENYMTENCRKTCDKCNKIEKFFPETTTKELEEKLAKMSKEL
QPLSEDPDNKAGAGSAPKTESPLVIPKQEQPVAIIPRNEVPPKQEVRASSPAMQSSPPPSPPPASPPPVDSPPPMSP
PPESPSPDKPPPKVVTRKALADPKKVTQKALMVRCYKLSLGIDEVKDCVKAAKEGKEYEVPKRTKATDEEEEAPKAK
HAESHLTLDGEGATTESANDEVAKTKAPDASAEGNEGKKNIRVVQRDLEDLSLTQGDGKKGKAPVIDAPVVGPSLHS
LLGRLNTWQALVLWLVVVVAFLALVPRIAKLRRRQRSGMRTE

>Cre12.g549000.t1.2|PACid:27582720
MRSRLLWACGLAALLALAAMPAALGQKILPKFPYCRCVKKGPYALAKNVDEIDGYYCFEIEQVPCGKDKCCKMDLRK
VEFDANVACRDTTKVTAMVNGLLPKQQPTWTAPQDTPAGKAVLKLSGLGLNITTAPGSLVCLKLDGQCNTLEKLCAV
PKGQMPGICAISMFNTKDTCCPISQTGWPFPPPPPPPSPRPSPPPRPPPSPPPPRKPSPPPSPPASCPICITLNTTD
VSLTREVCDNLADYVNSVWVDRSNATLLQPFSCSLATPSQVKICAVAEGKPDTVDFAGIAEDTYEIQLMLEELGLSC
AFPSMMGTSMVYLVEYGTCRAVFTYVQDCKQPGPQPFPFCVCNRRPKATPFALDPTIRVKPGSSPRSTNYCWTATTV
APYDPTSPCGKTNELYKAEFYVRNNTRGAVTGLTAAGKSWSPVWDSDGKVFRLTNLMWTVDFVNTNKPQICVELTGI
TLGQFCVTANCQYALFDASKSCCPMGTASLG

>g8540.t1|PACid:27562763
MEPSITRLTASARDRVGDRVQHIDVSFILSAAGPRVEVDLDVGALRLSRSVECDVACAGKEVTFEGVEVETEAVTSV
VVLARDGRGRTSMAYTSFEQIGVAGVTVSHGVAEHRRALDATTPTYSPTPSPSPMYGPSPSPSYGSPSPSPYGTYSP
SPSPSYGSPSPAYSYGSPSPSRTPGPTASPSPSVKESPAPSPEPEASPSPAASPSPSPAVEESPSPSPAVEESPSPS
PAVEESPSPSPAVEESPSPSPAVEESPSPSPAVEESPSPSPAVEESPSPSPDVEESPSPSPAVEESP
SPSPAVEESPSPSPAVEESPSPSPAVEESPSPSPAIEESPAPSPGPSPAVEESPSPSPAIEESPSPSPAVEESPSPS
PAVEESPSPRPSPSPAVEESPSPSPAVEESPSPSPAVEESPSPSPAVEESPSPSPAVEESPSPSPAVEESPSPSPAV
EESPAPSPAVEESPSPSPAVEESPSPSPAVEESPSPSPDVEESPSPSPAVEESPSPSPAVEESPSPSPAVEESPSPS
PAVEESPSPSPAIEESPAPSPEPSPAVEESPSPSPAIEESPSPSPAVEESPSPSPAVEESPSPSPSVEESPAPSPEP
EASPSPAASPSPSPAVEESPSPSPAVEESPSPSPAVEESPVPSPAVEESPSPSPAVEASPSPSPQPSPALSPSPSPF
PSPSPSPNPSPVPEPSPVPSPQPSPSLTPKPSPSPSPEPSPEPEPEPAPSPAASPSPSPAVESPSPSPVVEESPSPS
PAVEESPSPSPAVEESPAPSPGPSPAVEESPSPSPAVEESPSPSPAVEESPSPSPAVEESPSPSPAVEESPSPSPAV
EESPSPSPAVEESPSPSPAVEESPSPSPAVEESPAPSPAVEESPSPSPAIEESPSPSPAVEESPSPSPEPSPAVGES
PAPSPGPEASPSPTASPSPSPAVVESPSPSPHPPSPAPPSPAPPSPAPPSPAPPSPVPPSPAPPSPPPPSPRPPSPA
PPSPPPPSPGPPLPPSPPPPGPPRPPPRPPRPPPPTPPSPPPPSPTPPSPRPPSPPPPSPSPPSPEPPTPEPPSPEP
PSPEPPSPEPPSPSPPMPPSPAPSPPQPPSPPPPPPPFPPGVSLDLSLLKLTFAGNVSELNATDIYNLRKQIADFYG
IPIELVIFDMGASSANGSGRRRLISGSDDMPLAIYSRGQLGSQFDVHAGHALVEMKLVGKLDMGSHEFEMMRAAMAA

MTGEQLAGARRRQLLQNSGYTLGFVVGSISVAPSPPPAPPSPPLPPSPEPPSPQPPLLPGQFAPPPSPPTPPLPPSP
EPPSPEPPSPPPSPPAPPPSPPKSPLTDALNATSAEVLQFPPPTPPSPVPPSPPPSPAPPSPRPPSPRPPSPRPPSP
RPPSPRPPSPRPPSPAPPAPPSPAPPSPSPPSPAPSPPPNCDVHEGFTFYPMQNYDCTETGAFNCTIYTSFNGDPSA
VDLAAYTCGFGSDTVDAQKCGAFDSDGNVLSTYEQGVASYPADPVEVTNLSGQCAGVYVKNEYRYFPPPPPSPPTPP
SPAPPSPAPPSLAPPSPSPSPSPGHPIAPSPPVPVPTYSPSPSPVASPAPSPAYGSPSPSPSPEASPSPSSEPSPSP
SPEPSASPSPAYGSPSLSPSPVASPSPSPEPSPSSSPSPSPEPSPSPSPESSPSPSPEASPSPSPELSPSPSPETSP
SPSPVASPSPAASPAPAPSPSPVYGSPSPSPSPDFGPSPSAAPPSPSPESSPSPFASPSPSPSPVPEPSPVPSPAPS
PTPAPIPSPSPEPSPSPSAEPSPSPSPEPSPSPSPEPSPSPSPEPSPSPSPEPSPSPSPEPSPSPSPEPSPSPSPEA
SPSPSPEASPSPSPEPSPSPSLEPSPSPSPEPSPSPSPEPSPSPSPEASPSPSPEASPSPSPEPSPSPSPEPSPSPS
PEPEPSPSPEPSPSPSPEPSPSPSPEPSPSPSPEASPSPSPEASPSPSPEPSPSPSPEPSPSPSPEASPSPSPEPSP
SPSPEPSPSPNPEASPSPSPEPSPSPSSEPSPSPSPEPSPSPSPEPSPSPSPEPSPSPSPETSPSPSPEPSPSPSAE
PAPSPSPEPSPSPSPEPSPSPSAEPSPSPSPEPSPSPSSETSPSPSPEAFPSPSPEPSPSPSPEPSPSPSPEASPSP
SPEPSPSPSSEPSPSPSSEPSPSPSLEPSPSPSPEPSPSPSPEPFPSPGPEPSPSPSSEPSPSPSPEASPSPSPETS
PSPSSEPSPSPSPEPSPSPSPELSPSPSPEASPSPSPEASPSPSPEPSPSPSSEPSPSPSSEPSPSPSPEPSPSPSP
EPSPSPSPEPSPSPGPEPSPSPSSEPSPSSSPETSPSPSPEPSPSPSPRPIPSPQPSPSPSPLPRPSPSPSPSPSPS
PSPSPTPSMSPPPSPAPPSPPPPSPRPPSPPPPSPAPPSPRPPAPPRPVRRPPSPPSPAPSPPPPSPMPPSPPAPPA
PPSPPPPPPPLPEGLGVEVSKYELFFQDSNMTASEMTQLCLDIRDEMAAAFGVPIDQVIIPSCSAYLVSSGGSNRRR
MVELGHSAAPIKEWSGEQLTELLGSDSAVAASVEQLVGMVLVSRRFASERQWNMDRVAANAFASARLRRRGRLLSQS
SNTFAVEVIIAQIVEMPSPPPSPLPPSPTPPSPPSPPLNPGQFAPPPSPPAMPLPPSPPPPPPVYPPPPRPSPPPPV
NLDVIGAKLGANVSQIYDPPSPPLSPPPSPRPPSPPPPSPVPPSPAPSPPPVCEREGYVYFPNAAYDCAAASSSCLV
LESWGDSPGDAEYNCNYGPEDDHYTPADCGAFDSNGNVLSHPSGGRYAADPTTVQGLSGDCPGTWVKEEYRYVPPPP
PSPPVPPSPAPPSPSPSPSPSPSPSTPVAPSPPAPVPAYSPTPVPAYSPTPSPSASPSPSPSHPVSPSPPAPVPTYS
PSPSPSGSPSPSPSTPVAPSPPAPVPTYSPSPSPSHPVSPSPPAPVPTYSPSPSHSPSPSPTYSPSPVPAYSPSPAP
TYSPSPSPVYPLSPTPSPVASPSPSPMASPSPSPVASPTPSPSPTTSPSPEPSPMPSPEPSPSPSPGSSPRPESSPS
PSPEPSPSPSSEPSPSPSPEPSPSPSSEASPSPSPEPSPSPGASPSPSPTPEPSPSPSPEPSPSPAPVPSPSPEASP
SPSPSPSPSPAVGETVTTTLRLVGADYGIVSGSEDSMIAFESAACISLTSTTATSSCSLVSATSGSIIATFDLVSNV
GVTAAQLTSEIDSKAASNTLWTPDFTTEYSVTSTEVLPNPSPAPVPTPSPSASPSPSLSPAPSPSPSPSPSPSNETS
PSPSPSPSLSPSPSPSPSPSNETSPSPSPSPSPSPSNGTSPSRSPSPSPSPSPSPSPSPSPSPSPSPSPSPSPSPSPSP
SPGPSPSPSPTPSPSPSPSPSPSPSPSPSPSPSPSPSPSPSPSPSPSPSPSPSPVPVVPVSSPPPSPAPRLPSPPPPAP
GAKTIFGQAYDGYLSGCTVFLDISSDGLFGADEPSGRTTYDRSAASSGWTFSVNASLTPLLARRQIRLLPADVAELA
NGNRTSDGKGCHDVATLTKLAVPLAAPGSCSTPVITPLSSVLSEMDLLTTGQFTVAALRQRLVGALGLNRTTTPTLC
EYDATSDALVDLQSPGGRLYAQEQAMMASVTTLCSYVTPALKDLPTCARLMVQQVAARVLAGTSARRRVQAARAGRT
LLQGNSGNTPAANNSASNSNSNSNSNSTLQLADPLGGLLNLQDPATLAALLTGVAAQADKLPAGSYNPAALNDTARI
NATVNSVSQMLAIGANATSLQTLLSSSLVAQDYLAAQIASLAAANSSAAAAALNSFANSTSSAALSQQVATTPVPGD
YRPFNNTNQSGGDNAPPPPPSDSGGGDDNNNLAIGLGVGLGVGLGLLAIGGVGAGVYYKKKKADSRRDAYNVSGAAE
GADPEHRGGVAPVHDVRPQSPPPPAGLVTAQLAPPGAAGGPQLTGTAV

>g8431.t1|PACid:27563294
MALCLFSLWQWGDASGDWIDTPYRAAVGVYRRAAALAASVSAPAPQTAVQQARLWAAVALEGMSVAAAPMALQRLGG
AEGWSLQCRWQPAAGAATGSAAGRAEPGTAAQGDAAVAEAAASGERWQPLAAGSLELWRCAGRDGGCSSLHRSEDQA
AANPVDGAGDVVALLALLSFVAPAETPLQQLWQQLSGPGPAPAVDCSISLADTPLQGLALPAPTGVKAATGAAVAAA
ADADGSTRELLLRQLQQLGWALADSLGLDGSSSHSHSHSHGSSRRLAQATEDAELRDFTWPDLPPDTPLAPLPPLPP
RPPPPPPRPPRPPPPAPLPPAFPLPPDPPAPAAPGFPQFPQFPPRAPRPAQPASPSSAGAPPPQAPPPGPDTPMPPGA
LSTVQAPPPASPHGQPVAPSQPPPPPASPGPSVPCSACVVQTIVPPVSGTPGDIFTFVPELCEEQGAFIADSLNNAA
ADVGAALATPFATASCSAAWDPAAQPPVLPVLRVCGAVATAADAALLQAWLESAAAPLDDWLSHTAGGDDCPARLSG
YTLAIEFDPATIQEGACLTGRSQKACAQLSPPDQPPSYPPGAPAPPAVYPSPAPAVPAYPPPAPVGQPLQSPPSPPS
SPPPSPAMAYPPGQPTQPPPYPPSPPSPPPEQQPSAYPPSPRSPAPSYPPSPAPPSPEPPSYPPAYPPTPAPPSPEP
PSYLPAYPPTPVPPSPEPLSYPPAYPPSPAPPSPEPPSYPPAYPPSPAPPSPEPPSYPPAYPPSPAPPSPEPPSYPP
AYPTSPAPPSPEPPSYPPAYPPSPAPPSPEPPSYPPAYPPSPAPPSPEPPSYPPAYPPSPAPPSPEPPSYPPAYPPS
PAPPSPEPPSYPPAYPPSPAPPSPEPPSYPPAYPPPEPPSYPPAYPPSPAPPSPEPPSYPPAYPPSPAPPSPEPPSY
PPAYPPTPAPPSPEPPSYPPAYPPSPAPPSPEPPSYPPAYPPTPAPPSPEPPSYPPAYPPTPAPPRPELPSYPPAYP
PSPAPPSPEPPSYPPAYPPSPAPPSPEPPSYPPAYPPSPAPPSPEPPSYPPAYPPSPAPPSPEPPSYPPAYPPSPAP
PSPEPPSYPPAYPPTPAPPSPEPPSYPPAYPPSPAPPSPEPPSYPPAYPPSPAPPSPEPPSYPPAYPPSPAPPSLEP
PSYPPAYPPTPAPPSPEPPSYPPAYPSTPAPPSPEPPSYPPAYPPSPAPPSPEPPSYPPAYPPSPAPPSPEPPSYPP
GYPPSPAPPSPEPPSYPPAYPPSPAPPSPEPPSYPPAYPPSPAPPSPEPPSYPPAYPPSPAPPSPEPPSYPPAYPPS
PAPPSPEPPSYPPAYPPSPAPPSPEPPSYPPAYPPSPPPPSPEPPSYPPAYPPSPAPPSPEPPSYPPAYPPSPAPPS
PEPPSYPPAYPPSPAPPSPEPPSYPPAYPPSPPPPSPEPPSYPPAYPPSPAPPSPEPPSYPPAYPPSPAPPSPEPPS

YPPAYPPSPAPPSPEPPSYPPAYPPSPAPPSPEPPSYPPAYPPSPPPPSPEPPSYPPAYPPSPAPPSPEPPSYPPAY
PPSPAPPSPEPPSYPPAYPPSPAPPSPEPPSYPPAYPPSPAPPSPEPPSYPPAYPPSPPPPSPEPPSYPPAYPPSPA
PPSPEPPSYPPAYPPSPAPPSPEPPSYPPAYPPSPAPPSPEPPSYPPAYPPSPAPPSPEPPSYPPAYPPSPAPPSPE
PPSYPPAYPPSPAPPSPEPPSYPPAYPPSPPPPSPEPPSYPPAYPPSPAPPSPEPPSYPPAYPPSPAPPSPEPPSYP
PAYPPSPAPPSPEPPSYPPAYPPSPPPPSPEPPSYPPAYPPSPAPPSPEPPSYPPAYPPSPAPPSPEPPSYPPAYPP
SPAPPSPEPPSYPPAYPPSPAPPSPEPPSYPPAYPPSPPPPSPEPPSYPPAYPPSPAPPSPEPPSYPPAYPPSPAPP
SPEPPSYPPAYPPSPAPPSPEPPSYPPAYPPSPAPPSPEPPSYPPAYPPSPPPPSPEPPSYPPAYPPSPAPPSPEPP
SYPPAYPPSPAPPSPEPPSYPPAYPPSPAPPSPEPPSYPPAYPSSPAPPSPEPPSYPPAYPPSPAPPSPEPPSYPPA
YPPSPAPPSPEPSTPICPKAEYSAAVTLRCTYSYVDEEPCLLLAWCTTCSSPPSAVYTPIDMTACAQARALFIDVSC
NGTAFCAASPASPPAPIPPSPVPPSPAPQHPPSPEPPSPVPPSPAPPSPEPPSPAPLYPPSPAPPSPDPPSPDPPSP
EPPSLVPPSPAPPSPEPPSPAPLYPPSPAPSSPQPPSYPQPSPPAPAPPTPAAPTGVCPALTYSPAFASRCAKSYPD
EEPCALDAWCYPCENLKSPGFSVVDVAACAQAGALYIDVNCNGTAFCTSMPASPPAPLPPTPRPPAPTSPSPAPPSP
QPPAYPPQQLHPPPSPEPPSPAPQYPPSPAPPSPQPPAYPPQLPPSPPPPSPKPPSPAPLYPPSPVPPSPVPPSPLP
PSPPMNPLPPTPPPSPRPPSPPAPPSPPPLPPAPPSPPPPPPPLPPGLAIELSDYALTYTIDESQLSNSTAVEEFLN
KVRADMAKFFGVPVSQIVLTGIKLPAAPAAAPAVNEAGGAGSNGGGKGGRRRLGDGGDSDFALEPLEVWAGAELAPH
FGGSAGLFGLRASSLVQTSRRRPADEAALHELRARAMLADVVGALARVAVVEPGSQAQAQEPAGDAVPSSTHMRRRL
QTGSSIIVETTVVNQFEVPLPPSPPPMQPPSPPSPAPSSPPPPPPGVMLPPYPPPLPPSPPPMPPSPPSPPEPPSPP
PPVDPPPRPPPAPPRPPPAPPINLTALAEATNADSAQVIYHPPPSPPSPAPPSPEPPSPEPPSPAPPSPAPPSPSPP
PPAPPLVCDMAGYVFYPFSDYACAPADPAQPWCIVLEDTGDADGAAYACEWGGAGALAPDAPRYPVPPESLEVVLG
PAASSGCQGVYVKLQLQYLPSPPPSPEPPSPAPPGPSPPPPSPRPPCPPPRPPPNPRPPRLPPRPPSPAPLVPSSPP
SPSNPPPSPEPPSPEPPSPAPLYPPSPAPASPQPPSPEPPPVGQPLPPSPLPPSPEPAGQPLSPPPLPPSPEPPRPE
PPSYPPAYPPSPAPPSPEPPSYPPAYPPSPAPPSPEPPSYPPAYPPTPAPPSPEPPSYPPAYPPSPAPPSPEPSSYP
PAYPPSPAPPSPEPPSYPPAYPPSPAPPSPEPPSYPPAYPPSPAPPSPEPPSYPPAYPPSPAPPSPEPPSYPPAYPP
SPAPPSPEPPSYPPAYPPSPAPPSPEPPSYPPAYPPSPAPAYPPTPAPPSPELPSYPPAYPPSPAPAYPPTPAPPTP
EPPSYPPAYPPSPAPAYPPTPAPPSPEPPSYPPAYPPSPAPPSPEPPSYPPAYPPSPAPPSPEPPSYPPAYPPSPAP
PSPEPPSYPPAYPPSPAPPSPEPPSYPPAYPPSPALPSPEPPSYPPAYPPSPAPPSPEPPSYPPAYPPSPAPPSPEP
PSYPPAYPPSPAPPSPEPPSYPPAYPPSPAPPSPEPPSYPPAYSPSPAPPSPAPPSYPPAYPPSPAPPSPEPPSYPP
AYPPSPAPPSPEPPSYPPAYPPSPAPPNPEPPSYPPAYPPSPAPPSPEPPSYPPAYPPSPAPPSPEPPSYPPAYPPS
PAPAYPPTPAPPTPEPPSYPPAYPPSPAPPSPEPPSYPPAYPPSPAPPSPEPPSYPPAYPPSPAPPSPEPPSYPPAY
PPSPAPPSPEPPSYPPAYPPSPAPPSPEPPSYPPAYPPSPAPPSPEPPSYPPAYPPSPAPPSPEPPSYPPAYPPSPA
PPSPEPPSYPPAYPPSPAPPSPEPPSYPPAYPPSPAPPSPEPPSYPPAYPPSPAPPSPEPPSYPPAYPPSPALPSPA
PPSYPPAYPPSPALPSPEPPSYPPAYPPSPAPPSPEPPSYPPAYPPSPAPPSLEPPSYPPTYPPSPAPPSPEPPSYP
PAYPPSPAPPSPEPPSYPPAYPPSPALPSPAPPSYPPAYPPSPVPPSPEPPSYPPAYPPSPPLPPSPEPPSYPPAYP
PSPVPPSPEPPSYPPAYPPSPAPPSPEPPSYPPAYPPTPAPPSPEPPSYPPAYPPSPPLPPSPEPPSYPPAYPPSPA
PPSPEPPSYPPAYPPSPALPSPAPPSYPPAYPPSPALPSPEPPSYPPAYPPSPAPPSPEPPSYPPAYPPSPAPPSPE
PPSYPPAYPPSPAPPSPEPPSDPPAYPPSPAPPSPEPPSPELPSPAIPSPAPSSLAPPSRQPPSPEPPNPAPPVGQP
LPPPPFPPSPAPPSPAPTYPPSPAPPIPAPPAPAPPSPLSPSPPEEGPECPPPLTLSPQVGGRCTNSYDPALPPPCL
LLAWCNSCATPPVMKYSVLDVAECTAANRTYVQVDCNGFVECVVHPPTPSPEPALPPPPSPPASPAPTPSPATSPSP
SPAPAPVYPSPVPSPELSPSPPSEGQPPPSPAVSPPPTPEPSPEPSPAEQPSPSPAPEPSPEPSPTPEPSPPPVPEP
PPAPVPSAGPSPSLEPSPEPSPGPSPEVSPSPAPAPEPSPAPEPSPSPPVPEPATTLPPSPQPPSPEPASPMPPPPA
PPLPPSRPPAPPRPPPRPPRPPPRPPRNPPLPPSPPAVPPPLVPLAPSPPSPPAPPSPPPLPPAPPSPPPPPPPLPP
GLAIELSDYALTYTIDESQLSNSTAVEEFLNKVREDMAKFFGVPVSQIVLTGIRRPAAPSASPTGGAGSNGGGSSGR
RRAAMEAGAAGEALETYGSVELAPHFGGSAGLFGLRASSLVQTSRRRPADEAALHELRARAMLADVVGALARLATGA
PYHAGPGVRSHAHLHRRRLQTGSSIIVDTTVVNQFEVPLPPSPPPAPPPTPPSPAPPSLPPPPPGVMTPPTPPPSPP
LPPPSPPSPSPPPPVDPPPQPPPAPPRPPPSPPVNLTALAEATNATRAEVVIRPPSPPPPPPSPRPPSPRPPHPPGL
PPPSPPPACDVDGYAFYPGDTYSCGGSGTGGQQGWCTVLEDTGDASAAAYACEWGGDSYVGPDCGAYDTLGRVFSVY
GYDSSTATVLQRYPGPPAPDQDIADDSLSAGPCRGVYVKQGARYIPPPPPPRSPVPPSPPPPAPSSPPLPPATDQPP
LPLPPSPAQPTPSPTTASPPAGQPTALPPLPPVPPSPSPAAYPPSPAPLPAAYPPSPSPAAYPPSPAPIGQPAQPPP
QPPAPASPLPAPSPPPSYSLSPSPAAYPPSPAPESPAPVPGPSPSPSPAAYPPLPAPTPAPSPEPSPVPSPSYSPSP
SPSPAAYPPSPAPSGQPAQPPPQPPAPAPPASEPSPALAPSPSPSPAYPSPLPSPIPSPPPAPSPAPSPSPSPAAYP
PSPAPIGQPSPSPSPAYPGSLPSPAPIGQPAQPPPAPPAPAPPSPPEEGVVCPPPLTLSPLVQARCNPDYDPTKRPC
LLQSFCTTCDSPPASVYSALFVEECTLTNRTYVVVGCGGRLECVLPPPARSPSPSPSPGASPSPSPSPAPVYPSPAP
SPEPPPSPEPAPVPSPSPEPPLAVSPSPAPAAYPPSPVPTPAPSPEPSPVPSPSYSPSPSPSPAAYPPSSAPSGQPA
QPPPQPPMPPTPASSSPAPTPSPAPESPSPIPGPSPSPSPAAYPPLPAPTPAPSPEPSPVPLPAPSPAAYPPSPSPI
GQPAQPPPQPPAPASPGPAPSPVAPSPSPAPTSSPSPAAYPPSPDPTGHPSQPPPQPPSPGSPAPAPAPSPSPTYGA
PSPSPSPVPSPEPSPGPSPGPSPEPAPVPPPEPTASPPAPAYGTPSPSPESASPPAAQLQPPSPSPDQALPPSPPSL
PPPRPMTTRPPPSPPLPPPPSPPLPEPFNVPRPPPLPPALPAPPVAPPSPPAPPPSPPEFPNTPNTPNPPPSPAPPT

PLAAPPPPPPALLPPSPPPPPPPAPPLPAPPPQPPPPRFLPVTRSGRALDGYLDNCVVYADVNGNGGLSTAQGEPFN
TSTFQNGWTLTLSDAAAVSAPLRVVPAPPSTYAKSRIAVSPQDDCHDATSLLKLYLPLAAPSAACAVISPLSTLLLA
MQERGYGQAEAKIKAAFGISAAANISICQTDPIKALYVDLDPAARPYMAAEQQVMAAVVQSTGYLLVNDTDLPTLGR
FTWQGLARAVENTFRSSPAASPAASPAAAGRHRRRLDSGAGGASSISSEASAVDAAASNSASRALLQTSSSNTTTPL
LFDLTSPAVLGSILDATVAELAAAAAAANSSSSNATAPPPANTLGVTDPARREAALNSMSYMQALVAGAVASGNLTT
VAAAGLVAQSNMTDGLTQLAQPAANVSAFIDATTPAALAAAVAATRITGPTLPDNISGNPEIGTLPPPPPPGTVPAP
RGGTGSWIDNNTGLFVFLVAVIPVLIIATCGTGYYVYRRRQRRTAEFGRMHVTGGEGGPPVQPLAVASTPAGSRRMQ
SDDGGGAGGGGGGGGAAAARGTSTTSSAGAPPSHSGPLPAAAPMVTSYNVLYEGESRDVAGSASLAAAVLATRGAAG
GGAPGVSPHGSVPTSLPAAAAPGDVVHNYTNVMYGEQPSLAAALNDGLNVPPPAAAMAALAGGAAGSMHRSGGAGQP
QGADDVLNEFGWAADGRGSRGSGGANPVAARAAAGMQPVSAAALGAAAAAAATAAAHQQHRHRQAARLGPEVEPFAG
RAPSSQGSNADSELRRPETEYASAEFHTGKLSFTSRQHTGEVPPLSGAGPRSRASGLGAFAGAAGASAAAAAEAGGA
LAAPPQAALPGPGGGGYLGQSTASGRLPAFSLDRSYSLARLSPTAAATAEAAAVASGSTGGSLAEPSLSSPYDHGEP
VYSTAVAATASDARGGGGGDESGPSSGRNLNPLFEPVTMFANPLHGAAASSATVTPTASRRHTTDAAPATPPSVTSA
AVAATASPRRPGDLSMGGGGLHGTVTPPQHLQGAPKPWPPPPPRRGTGGGAAEV

>g8527.t1|PACid:27563512
MPEAPPAQGHQQQHQEKCGVYGMHGDRRRQGAAASRAGAHATAAPGLSAAAEPQGTGCQDRRQLQQHQHQQHLQGQQ
HRQEQRQRQRQHLRTSARSSGVGASRLRANLLRPSHGALLLVVLLLLSLMLGGGWPGTGGGVRLVAAAVTAAAAGDI
VLAPPPFEDYAPPPQTDFPPSPPTLPPSPPSPPPLPPRPPAPPPTAPIEAPPPVDEPPMAPHPPPSPPPSPPKPPRS
PKPPPRPPSPPPSPPSPPRPPKPPRPPKPAPSPPSPRPPHPSPPVPSRAFRQAPPSPQPPKPPKPPRRPRPPPPHPP
SPQPPVPPMPPHPPSPLPPSPAPPPHPPSPIPPSPAPPIPPAPPSPAPPSPAPPSPEPPSPKPPVPPMPPSPAPPSP
EPPSPPLPPSPTPPSPQPPSPEPPSPEPPSPPLPPSPTPPSPQPPSPEPPSPEPPSPEPPSPPAPPSPVPPSPSPLV
PSAPPPAPPSPYPPTPPPAYPPPYPPSPAPPPSPYPPSPAPPSPAPAYPPSPAPPSPAPPSPAPAYPPSPAPPSPAP
PSRAPAYPPSPAPPSPAPPSPAPAYPPSPAPPSPAPPSPAPAYPPSPAPPSPAPPSPAPAYPPSPAPPSPAPPSPAP
AYPPSPAPPSPAPPSPAPAYPPSPAPPSPAPPSPAPAYPPSPAPPSPAPPSPAPAYPPSPAPPSPAPPSPAPAYPPS
PAPPSPAPPSPAPAYPPSPAPPSPAPPSPAPAYPPSPAPPSPAPPSPAPAYPPSPAPPSPAPPSPAPAYPPSPAPPS
PAPPSPAPAYPPSPAPPSPAPPSPAPAYPPSPAPPSPAPPSPAPAYPPSPAPPSPAPPSPAPAYPPSPAPPSPAPPS
PAPAYPPSPAPPSPAPPSPAPAYPPSPAPPSPAPPSPAPAYPPSPAPPSPAPPSPAPAYPPSPAPPSPAPPSPAPAY
PPSPAPPSPAPPSPAPAYPPSPAPPSPAPPSPAPAYPPSPAPPSPAPPSPAPAYPPSPAPPSPAPPSPAPAYPPSPA
PPSPAPPSPAPAYPPNPAPPSPLPPSPAPAYPPSPAPPSPAPPSPAPAYPPSPAPPSPAPPSPAPAYPPSPAPPSPA
PPSPAPAYPPSPPPPSPAPPSPAPAYPPSPAPPSPAPPSPAPAYPPSPAPPSPAPPSPAPAYPPSPAPPSPAPPSPA
PAYPPSPAPPSPAPPSPAPAYPPSPAPPSPAPPSPAPAYPPSPAPPSPAPPSPAPAYPPSPAPPSPAPPSPAPAYPP
SPAPPSPAPPSPAPAYPPSPAPPSPAPPSPAPAYPPSPAPPSPAPPSPAPAYPPSPAPPSPAPPSPAPAYPPSPAPP
SPAPPSPAPAYPPSPAPPLPEPPSPTPPSPAPPSPAPPSPQPPSPEPPSPEPPSPPPPPPWWAGMSPPPPPPPPPSD
TPLTPPAPPSPPGAPPPPPSPPPPPPPPPQTPLASSPPPPPVTQLSPPPRMPVDPSPPPSPTRSVMETRTVRTRFPV
EGQVVLGDVDTRLSFLTSVQGIIAKAAGVDASDVIVTGAYPGSVVVEARVTFNPFFTSAADVDAGLAALSADPSAFF
DSNFLQRFAIDTSSVEVIVMAPPPPPDAPPPPAAPPVPPALPPPPPPRPRRVRRQQPPSPSIPGIPQDPPQSPVALP
PSPRPPASPPPSPPSPRPPSPPFPPLPPSIARMLQLNLSMPYDQLRDRTVREAFSWDMRLAAAAFFGLEPQQAVVVA
LLPLPNTSLPYTTRVNVLLFFTQSTLDPATVNAAFARAAASPMSIVDPVKSKYGITAAQAYELAQFNYPPPPPEPPE
PPSPPPPAPPPSPEPPFPPELPGPNAPPSPPPLPSPPRSPRSPRPPRPPSPPPRPPRPPSPAPTPPPPPPSPPRLPP
PSPRPPSPPPDPPAAPPPPPPPPPPPPPSPVPPSPPPLAPNSNPLPPSPPPSPPPAPLPPSPPPLPPQPPGVVPRYL
AIDFNTSLAGIATSDFSYESTAFGTGVRTALGKAWRLDIRRIEQIAVYRLPVTVPSLDPGAAEPYVVRMNVLVQFTG
ISENFANSRVNDFVDEPMPYFTEGFVSTWRITSITAARVATTAYRVPPPPPPPPSPPAPPTPPSPPPLPPHPPPPPR
PPRPPPPSPLRPPRPPRPPTPPSPPAPPLLPPPPPSPGVPPPPQEPAPSPATPSPPTPKSDDYDYLYPPPGGFDPTD
SPPPPSPPALVSRSVQLTFDVVPDLILGDIDTRDDFGSIVAAAVASAARLPSTAGAVAVALKAPAGGATAPLVAEAF
VTFRPGGDAPSVADVDAGLAALAADPRAYFGPDFLSRFSITRINGVDVWAPPPPPPSPRPPQPSPPPLPRPPSPKPP
SPRPPPRPRPPPPRPPSPAPPSPPSPAPPSPPSPRPPSPPFPPLPPSIARMLQLNLSMPYDQLRDRTVREAFSWDMR
LAAAAFFGLEPQQAVVVALLPLPNTSLPYTTRVNVLLFFTQSTLDPASVNAELSRATADPFGVVRPIADKYGITAAQ
AYELAQFNFPPPPPPDPPEPPSPPPPLPPSPEPPSYPPSPAPPSPVPPAYPPSVPPSPPVSPSPPPSPPSPRPPRPPR
PPPRPPPPRPRPPSPAPHPPSPPPPPPPSPAPSPRPPSPPPDPPAAPPPPPPPPPPPPPSPVPPSPPPLAPNSNPLPP
SPPPSPPPAPLPPSPPPLPPQPPGVVPRYLAIDFNTSLAGIATSDFSYESTAFGTGVRTALGKAWRLDIRRIEQIAV
YRLPVTVPSLDPGAAEPYVVRMNVLVQFTGISENFANSRVNDFVDEPMPYFTEGFVSTWRITSISAARFPGSIYRVP
PPPPPPPSPPAPPTPPSPPPLPPHPPPPPRPPRPPPPSPPRPPRPPRPPTPPSPPAPPPLPPPPPSPPPPPVPSPPE
VLQPPLIEEEPPSPPPGPQLPPPVPSPPPPSDEYEYEYIYPPSGEVYLGPSPPPLPPPPRSRRPPLPPRVRKPPPSP
PAAPPTPPPPSYPPSYHPAYPPPAYTPAYPPPAYPPSYTPAYPPPVYPPPAYPPPAYPPPAYPPPYPPSPAPPYPPS
PAPPYPPSPAPPYPPSPAPPYPPSPAPPYPPSPAPPYPPSPAPPYPPSPAPPSPEPPMPHPPSPPPSPRPPRPPPPV
PPSPMPPRPPRPPRPPPKPRPPPRPPRPPPPSPPLPPSPAPSPPPSPAPPPPPPRAPPAAPGTYAPPLYPPSPPAPP

TYPVASPPPPPAAPPQPPTSPPATPPPPPEPLYLSVVFTAVLPPQSSAAFAEEVRQALASHFGVPVDQIRILSYGTT
PLSAAAPPPPPASSVASRSASLSLTGGALRPLGGSSSSSSSGVVFGSSTTGSAVASRAGPAGSYVTSISLALDPSAA
APPAGGDVADAVDGFVVDPFSAFDNAFLDAYAVEDVVAAVVSATDVFVPPAPPGVPPPAAPAVEEPAAPPPHEDNGS
PPPDMPPPMPDAPPSPAPPSSPSPPVPSPPAPSLPPPPSTPPTSPPPPSPPIPSTDRGNIDRTAVPPTAPSPPSPPP
PLPSAGGDPDLQLGEVVSPPPPSGSGIIAGGSSPPPFSAAAASGGGGGGGTNVGPIVAAVVVPVVVIVLLAVVVYKW
QRRKHMDNAFNSIMTPLEVDDGQDDGMPLPSPPDTAAAGGYMTIAFARSSGSAGSGGAAAAAGSPAGGRSPSNLSGG
ALPPALGSAASAHGSTSGGAGGGAAAGASRGLTPSVSRVVVVNSDLPLDDIMKIRFSPAFYAASDAAEGPNSSRRTT
SNGAAAAAGPSSTTSAGGAGGAGAEGMRPQQRSSFLRR

>g11926.t1|PACid:27565054
MAWRWRAWTFTLLVLFATWPTARGQGASTPSDEYDAALPARSTSSFPWCQCVAYDCGCSPYQLQVLPPTRDGINVRS
CFRVTYVGCDTTRKCCRGMLAAVDKLALETSAACSERGRVLGVTLNGRVHESWNPYVHGTGAELKIYNLQRSNATFA
GVLVCVTTAPPCGWLNDLCVHGSGGPGGAGADGTSGGGGGMQNSCRYSLSDTPEATYCPVCAVRLPPAPPPPSPPGP
NAPGRRRARPPPPEPTPPPSPTPSPAPTPCEVCLTLTLLPPPGAAAATQGGGLPFALTDPLCTDLESPMLSGLADAA
RAAGATISIPFARTACSTSYVPTATPMVLPYLRLCGAFRTASDGLRLQSWLAGPTTGLSAWLLQLTGGEACPASPGL
SGYVITSLASSPAGGDTCLSAAAVQTCVAAQAPPPPPPPKARMPPPRASAAARAPPLPPLPDVPQVGGPQCELYLHI
QVLPAAASAASASSSLAAGAFGRRRMQQAWGPGVEGEEVVGKGGSAAAGEAAAMAVTPQAVALRLPGGSGSLSGSGS
GSRAASNGWRRQQKDITTGEQAFSFDVASCAELADRIISGFAVQAPLVGSEITVPFTQFACAPQQLTLYGAFATAYD
GHLLYDWLNGPDGMQAWVHAVTGGDACSQRLSGYTIRSLVTGPGGELAASCMSGFAQQQCPSTPPPFLPPLPPESPS
SPSPPLPPPDSPPPPSPWPPPDTPPPSPPPPPPPDAPPPPPPNSPPQSTPPPPPDSPPPSPPPPPPTPPVAPEPPSP
QPPGLPSPSPPQIPGPQVPSPPLPSPLPPSPPPSPAPPSPQPPLPPPSPSPPPCVVCITTTIQPPVSASASTVYNLT
DAQCLDLSSTIIDNMQMLAFDTGAVIVTPFTQDACSPSYNATSVPAKYPFARVCGAFLSAEDGALVADPLKELMKEW
VESLTGGAACSSASLSGHLISSKATDPSGAASCLDASFRQACSIRRPPSPRPPQPRPPSPAPPSPAPPSPGPPSPQP
PSPEPPSPEPPSPEPPTPVPPSPAPPSPAPPSPAPPSPAPPLPPPPSPVPPSPAPPSPVPPSPMPPSPAPPSPTPPS
PTPPSPVPPSPQPPSPAPPSPAPPSPTPPSPAPPTPAPPSPIPPSPPPPAPPPPCITCVSVFVLPDSLISGVPFRPN
ASLCSSMAAMVSAGFTAAAADAGAVVVQPFAVTSCVRGGGPLAGVLTPLMKICGGLLSADDGAKLQLRIPAATAQPS
PPVSATTFATAAFATAAFPTAAASATTQPATTEPATPEPVATVSTATQPSTALAVAALAAAAGPSASFTAATLAAAA
PSPAAPTAATVAAATGSASTKPFAFDDSECGKLAKRIDDGFKQQAALLGANIQVPFRLDHCTPTYLKLMGTFGSARD
GEMLQEWLNGPLGMLDWVKSVTGECPDRLSGYAVRSAVQPPNNDTNSCLVGDFKLECFYQPPPPQPPLPPPSPPPPC
VVCITTTIQPPVSASASTVYSLTDAQCLDLSSTIIDNMQMLAFDTGAVIVTPFAQDACSLSYNATSVPAKYPFARVC
GAFLSADDGALVADPLKELMKEWVESLTGGAACSSASLSGHLISSKTPLSPAPFAFTPASCADLAAVITDDVSQQAA
DMGSVMLAPFALTECSDNSLRLCGTFQSAADGAALGPWLEDQSQFWLEALGGGDCSAELAGYSMVFEAVAGPGGGGV
CLQVNAPPLPRIPCEVLVHILLQPPDGLPAVAEAITDGVGSATSTSRHRERLLLQQLTVTATAADASGSAAFSFDAA
SCAALSTRIATGFAVQAPLVGSDITVPFTQFACAPQQLVLYGSFATAYDGFLLYDWLNGPDGMQAWVHSVTGGDACS
PELQGYLIRSVVTGPSGELAPSCMAGGFKQQCEGGPALPPPPPPPPSPPPPLLPPSPSPPSPGPPPRQATPGLRQPS
MPPPPPPPSPSPPPPPPSRPDLVQTHECVASTNDVPYSVGPLYVRQSLDQFGAPAVAMCTRVSGRQACRQAAFCCDMD
FAKIEVPINPACRNELRRIAVNRRLKDWSWGSYPSGLLTVKFESLITDLPDGPDGAELCWLVRPGGVCADPSVFCLQ
RSCQVNIFSTNNKCCPSAFIS

>g11990.t1|PACid:27565431
MHIQARGSVQLLVVVLLLLAHAHSSAADFAGYPYCTTSPTGTCQPCEVSSSGSSDVPCTTGDAFGDDPAICTAYFSP
NYPNNFGGADKCATTGGGSSTAWRAEVFAADPAGGGAPDIRVGELQAWEDTSGYMVVGFMPQCPYVLDLSAGAGGPL
TISITDTAISGGATQGSWSTSIGSVAAAAAAVAGGGGVPTDGGGNVPYLSCAGWRFQIRGTGAGQLAGAACQPYKRT
VTLTAKLVTVAPAGTGVCGVLSSVSSNISLSLTYVYSAGLSPPPPTPPSPAPPSPAPPRPPGTPPPLAPPPPDLPPS
LPPPPSPPPRPSPPPSPPPAPPRPPSPPPLPPSPSPPPPLPPSPPLPPVPPSPPPPLPPSPMPPSPPPQPPSPPPTP
PRPPSPPNPFPPPPPSPKPPSPPPNPFPPPKPPRPPPPTPPSPQPPSPEPPSPAPPSPPPSPAPPSPMPPSPFPPSP
PPSPSPPPSPPPKPPSPAPPSPAPPQPPSPPPSPRPPSPSPPSPRPPSPNPPSPKPPSPFPPSPKPPSPAPPSPSPP
SPFPPSPPPSPPPLPPSPHPPSPAPPSPAPPSPAPPSPFPPSPPPSPEPPSPAPPSPAPPSPAPPLPPSPPPSPRPP
SPNPPSPKPPSPLPPSPRPPSPFPPSPNPPSPTPPSPPPLPPSPGPPLPPSPAPPSPAPPSPAPPSPPPSPEPPSPA
PPRPPSPVPPSPAPPSPSPPSPPPSPVPPSPAPPSPAPPSPAPPSPPPSPRPPSPSPPSPPPSPAPPSPAPPSPSPP
SPAPPRPPPSPVPPSPAPPSPAPPSPSPPSPPPSPAPPSPAPPSPAPPSPAPPRPPPSPEPPSPSPPSPPPSPEPPS
PSPPAPPSPAPPSPAPPSPAPPSPVPPQPPSPPPPLPPSPAPPSPSPPSPFPPSPPPPSPTPPSPSPPSPLPPSPAP
PSPHPPSPQPPSPSPPSPPPSPEPPSPAPPSPAPPSPAPPSPAPPSPHPPVPPSPSPPSPAPPSPAPPSPAPPSPDP
PSPNPPSPAPPSPTPPSPAPPSPHPPSPHPPSPDPPSPAPPSPAPPSPAPPSPAPPSPAPPSPAPPSPEPPSPEPPS
PAPPSPDPPSPAPPSPAPPSPAPPSPEPPSPAPPSPAPPSPAPPSPAPPSPAPPSPVPPAPPSPSPPPPSPSPPPPS
PPFPPSPPPLPPSPEPPSPAPPRPPPLPPLPSPPPPSPPPPPRPPPPPTPPRPPPLVSVLGIPDSLLGGSSGITASN
STSSAGGSTGDNATQQQPPTTALLLLTQTTTVSDADLEAAAAAAAATGNGSSSDSSSSTGAVSSAALAALFPGASNV

TVTGRSVTFPIGLVQLAVLPVSISTGGSGGGTIDVNGGTSTTTPGGANGGTSTPTTGGANDLCPGELAALARSIRSE
LAARLGLNDSQVSRISCTLDVSPPSARRSLQQQPQGAPPPSVGSAAQQLCDAGGGGGAGGGGNNRTAVAINVELTLP
PDTNVTRLRTSIQEVVAALSAAPQNATAYTGAASSWVVCSPPPSADSIRAVAGVAVTYAVPLTAEGAAAYAAQCGGD
TSSGTTSSSTTSSSTAAAVTPLQLSSSLTCALVPVAGGPTVPSDRDLMAAPPGVGGNTYAGGPPPAGDAGSGGGRSG
IDRSLAVILAGMAVGGAILITCSVTALVLVLRRRERRLEEEARRKRLREEGGEAAAAAAAAAAGGGRGLQAKSPGGL
EGSGGAMTPDRHTPGGGGGGGGAAPWTTAGGGLVTAGAAGAMHYGAVPYSGSSDSSRLDGGGGVGGGSNQLAVQGGA
AAAAAGQQGRTGVSRSGAAGKSVSLSGVVLQPAEPSIHAAALDAALAALVGGGRGLQWPGGPDGPASAAAVLVSSSN
QQQPQPPAQPSPPPRRSPNPFHLASQGPRLSGDGRSSLVNGGGHAQLASPSFGAGPGLPPGVGANGGARSAAHLPWG
ANGGAGAAVPPGGDAGLGGSGSGSGGRSGGIGRWSSSGVMPAGAGAAAGAGAAMHPSSQQPSRLRLASGSDALVGGG
PALLLPTGSGSLGVMAGGAAGGGAVRSYRPALASPAATSNPWRSMPIPSDPTADVSDVETLSQQGGSSGDEAGSLPI
RLGSPSLAGAAGATAAAAAAAGVRRQRTSGGLASPAAAASTPAGGGRSAVRRVLSSASRMMLGRSAGGGAAADGLPA
SGPQSASGASRHVLTTNVAFEPGPQQPQPQATAAVGAVAPGSAARAVPGSADVAAGTRRTKRVTLAPAADPPAGGSA
GTHGGSLPRRLHSHSGTSAAGAGPRSATVVGGMGVGPVTFSPGARAGAGAAGVAPGRTGPGATGRAATDTGLLATGG
QALPRPRPPASGLVEVGGLPGSPRRASLVMYEQAPSPSR

>g15825.t1|PACid:27567088
MIRASRLLVLAAVLLVAACGTGAQEPTDPQQPVDDGDYIAGGHGYEYSSVDKTYTELDFLDNGATLAASPSPTIVEN
RDGFTSRSQFPYCRCGRGTSNSPYRLVLSRVTQLSNGASSRACYRIVAAADCGALDASSRTGMCCNEVERQLHKIHV
EADPRCRGSVRSVSLNGWSGKAWQWEDSLGPSASLKISNLGLNNTQALATEVCFTLAAPCASIQDLCFNEPSCRYAL
FSAAGVNADGGYCCMIGDLAINGTSSGGGSPPPPGASGSTPAPPSPPPPSPPPPSQPPPSPPPPSSPPSPPPPSPPP
PSPPPSPPPPSPPPPSPPPPSPPPPSPPPPSPPPPPPPPSPPPPSPPPPSPPPPSPSPPSPPPPSPPPPSPPPPPPP
SPPPPSPSPPSPPPPSPPPPSPPPPSGDCSEVTDIIVGGIDSVLYMGYVVTQPFEALVCSPTLLKVCGVLEQSTTGG
RRRSLLVEMVQGAAQDALRAELQRQLESLVDIVTGTNCPTDTDVTALLAGDGNDLQDLPPSPLIGEPSPRVARQCAP
TASPPPDFVIPTEGAPPPPPGGSTGSPSPEPSVSPAPGGSPAPSPGASPAPGGSPAPSPEPSVSPAPGGSPAPSPGA
SPAPGGSPAPSPEPSVSPAPGGSPAPSPGASPAPGGSPAPSPEPSVSPAPGGSPAPSPGASPAPGGSPAPSPEPSVS
PAPGGSPAPSPGASPAPGGSPAPSPEPSVSPAPGGSPAPSPGASPAPGGSPAPSPEPSVSPAPGGSPAPSPGASPAP
GGSPAPSPEPSVSPAPGGSPAPSPGASPAPGGSPAPSPEPSVSPAPGGSPAPSPGASPAPGGSPAPSPEPSVSPAPG
GSPAPSPGASPSPGGSPAPSPEPSVSPAPGGSPAPSPGASPAPGGSPAPSPEPSVSPAPGGSPAPSPGASPAPGGSP
APSPEPSVSPAPGGSPAPSPGANPAPGGSPAPSPEPSVSPAPGGSPAPSPGASPAPGGSPAPSPEPSVSPAPGGSPA
PSPGASPAPGGSPAPSPEPSVSPAPGGSPAPSPGASPAPGGSPAPSPEPSVSPAPGGSPAPSPGASPAPGGSPAPSP
EPSVSPAPGGSPAPSPGASPAPGGSPAPSPEPSVSPAPGGSPAPSPGASPAPGGLPAPSPEPSVSPAPGGSPAPSPG
ANPAPGGSPAPSPEPSVSPAPGGSPAPSPGANPAPGGSPAPSPEPSVSPAPGGSPAPSPGASPAPGGSPAPSPEPSV
SPAPGGSPAPSPGASPAPGGSPAPSPEPSVSPAPGGSPAPSPGASPAPGGSPAPSPEPSVSPAPGGSPAPSPAPSAS
PSPGPSGSLAPPTAELPPPPEPSREVFFPPSAFNRSPPPPASGASPAPSPSPGSSPGPGLPPGPSPGASPAPGGSPA
PSPEPSVSPAPGGSPAPSPGASPAPGGSPAPSPEPSVSPAPGGSPAPSPGASPAPGGSPAPSPEPSVSPAPGGSPAS
SPGASPAPGGSPAPSPEPSVSPAPGGSPAPSPAPSASPSPGPSGSPAPPTAGLPPPPEPSREVFFPPGAFNRSPPPP
ASGASPAPSPSPGSSPGPGLPPGPSPGASPAPGGSPAPSPEPSVSPAPGGSPAPSPGASPAPGGSPAPSPEPSVSPA
PGGSPAPSPGASPSPGDSPAPSPEPSVAPAPGGSPAPSPAPSASPSPGPSGSPAPPTAELPPPPEPSREVFFPPGAF
NRSPPPPASGASPAPSPSPGSSPAPGASPAPGGSPAPSPAPGTSPLPAPSSPTPGPSVSPSVSPSPSNAPPPAPEQP
PPMYGAYPPPPYPAAYPPSYPPPYGGAPPPPYYTPAYPPAYPPSPTYGPVPSPSVGGSPPVQAPKPPSPDPPSPVPP
SPAPPSPVPPSPEPPSPVPPSPEPPSPVPPSPEPPSPAPPSPEPPSPAPPSPEPPSPAPPSPAPPSPVPPSPTPPSP
APPSPAPPSYPPSPAPPYPPAPSPPLAPPPYYGVPAYPPPPPPYYSGAQPPPPFYTGAPPPPPYYSGAQPPPPYYTGA
LPPPPPYYTGAIQPPPPYYVSSPPLYARPPPRPATVLPPPYPPAGPPPVEVCATEVLIPPSELAPGREPFTLTEAQC
ADLILLLADDLSFASDDVGAARLTDWGAGRTACFLSAEAAAAASPPAAGPGVQLCAVFASAAEALQLQPQLDNDYMD
QLLEHGASVTGDGSGAACSAALSGYTPLITIAATQPLPDGVTNADVPFSERTGGACAPLSPPPEAPVYGGSPPLAAL
PPPYGPLPPAPTLPSGAVTVCASAGAVPPAQLSPGQAEFTLNDTSCADLISKEAALLSQITAATGLELLQDWASSPY
LYTCYRTADASRDPADRGVGVRVCAVFLTPADTKALQAVVEDSPVLDTLIEILASAAGGSGADGGGGCAAAALSGYT
LAATLAVPTPLPDGISEGDVMTPLFRQNACALLQPPAAPAPPDGSFPTVLAPPPVLVQSPPDPSREVFFPPGAFSRS
PPPPSPDPPSETGAPPSPSPSASPSPSTAASPSPAVAAPPPSPAPPAPSTSPSPGSPPPPLGAVLPVPVPSTSVPWA
DTGYGWSMYDTSENATYYVTSYLCVVPSSSQEMRGWSVVVDSSLLGSDLQVSGVTGTRTDIDGAVSAVQVIPYTSSS
VPTVTVVDNMTASTSVIYTLYWPKTFTIASRHLQQQHRRLAQSTASPSPVSGSGTTTRFNNLTIIAWTPDVISSVTL
APGLTGPNSAALSLGAAACVAAGSTSPLPWPVVGSDGCLVGTMSRTYIGTSGRTVVTLRVAPNGALDATVAANCTTW
FASLSQPVFVRMPLTNSTAAAAADLFVNRTAASAAGSVLPVGVYVKDGALVLPRAAVGNAASSSYFKMDVQLALPSA
LALSDVCEQAMLSGQWPNTCALQVVDRNLCTQGVAVESANGEAGPVGQAPGVAQAPSPPPTQGSLQGESGDGGSSDR
GSGGSGSGLSNSEIAIVVSLVVGCFLLALIVVALFVRYRRRKWAPAKEVFSGGTGAVGSSGVAVDINAVATSGALPA
GSGMRSLPAAFGAADAAAGDTAGAAGAASGVGMALAPRIESSVSNDGTFYTAMESGLMQADGANLSSTLGAAGPGGS

GAAGPAVGADTSAHRLAAVRAALARSGVGSATGHAGPGPSRLSGSHVPAAVYGGPGPSTSTGAGPSTSGGAGLGDAG
AGPSTSTGGGAVGLGPEGAVNWAMTQPSTAP

>g15955.t1|PACid:27567211
MVPSSRRSCVTLVLLLVAFTTTSAQQSAEGSNGQACKKRGEASFPSPPSPEPPSPPPPSPRPPSPAPGVPPPSPAPP
SPAPGVPPPSPEPPSPAPGVPPPSPAPPSPAPGVPPPSPAPPSPAPGVPPPSPAPPSPAPGVPPPSPAPPSPAPGVP
PPSPAPPSPAPGVPPPSPAPPSPAPGVPPPSPAPPSPAPGVPPPSPAPPSPAPGVPPPSPAPPSPAPGVPPPSPAPP
SPAPGVPPPSPAPPSPAPGVPPPSPAPPSPAPGMPPPSPAPPSPAPGVPPPSPAPPSPAPGVPPPSPAPPSPAPGVP
PPSPAPPSPAPGVPPPSPAPPSPAPGVPPPSPAPPSPAPGVPPPSPAPPSPAPGVPPPSPAPPSPAPGVPPPSPAPP
SPAPGVPPPSPAPPSPAPGVPPPSPAPPSPAPGVPPPSPVPPSPAPGVPPPSPAPPSPAPGVPPPSPAPPSPAPGVP
PPSPAPPSPAPGDPPPSPVPPSPSPGSPPPSPAPPSPTPGVPPPSPAPPSPAPGMPPPSPVPPSPAPGVPPPSPAPP
SPAPGVPPPSPAPPSPAPGVPPPSPVPPSPAPESPPPSPVPPSPSPESPPPSPVPPSPAPGVPPPSPAPPSPAPGVP
PPSPGPPSPAPGVPPPSPAPPSPSPGSPPPSPVPPSPTPGAPPPSPAPPSPAPASPPPSPAPPSPSPGSPPPSPAPP
SPSPGSPPPSPAPPSPSPGSPPPSPAPPSPSPGSPPPSPAPPSPFPGSPPPSPAPPSPAPPSPAPPSPAPGSPPPSP
APPSPAPPSPSPGSPPPSPAPPSPTPRSPPPSPAPPSPSPGSPPPSPAPPSPSPGSPPPSPAPPSPSPGSPPPSPAP
PSPSPGSPPPSPAPPSPSPGSPPPSPAPPSPAPPSPAPPSPAPGSPPPSPAPPSPAPPSPAPPSPSPGSPPPSPAPP
SPAPRSPPPSPAPPSPSPGSPPPSPAPPSPSPGSPPPSPAPPSPAPRSPPPSPAPPSPRPPSPRPPVPTPPSPIPPS
PAPPAPGLNLLASEEYQYNCLSIALPVASPSFVANSIYTENCATVPEDKYMQLQPVGVDNLYVLRQDSDTSRCMAAT
NTTPTTFTPQVLPCDPASDGQKFFINRTTGGAWTLSPRARGSYVLAAADYSRASNTTLSALDTFTSIGFRWAFKLPF
TPTNSPVCARPEAWCSSTSATMYGPIDCDGDGILDWACRRLRTDGTEQRWALRSAEDCNFQGILASGAAFLNGGSGV
ADSVCPAAFSTTPPQPPSPRPPSPRPPSPAPPSPKPPSPSPPSPRPPAPKPPSPAPPSPVPPSPAPPSPVPPAPPAP
LPPSPAPPTPQPPSPQPPSPVPPSPSPPSPAASPPSPPTGFQVIGIPANNKCVRFGTTAFQADCSFIFATMYALLQP
VGANNLHVIRTNVDTTQCVTAVEVATDVFRPQIQACVAGADNQQFFVNQTQNGLWTLSPKIRKTRCWQGSTINSDIY
LDDCTGTNAAEQIGLTLPFVPPQARVCSLPARWCALASQQLIGPLDCNGDGVLDWACVSYSTGQRWLLQSTADPTTT
CSATSATALGAATINDFSTPTGGTGLPVSACPVAFNSPPPSPPLPPSPAPKPFPPPSPAPPKPPSPAPPSPFDGYSP
VCYDNMDAVGLEMANLTASSESTCRELCKQDSRCEYYVYCATTSRCRLRQDIASPGGSASVIPAGMYFNGYISYGDI
YTRGCRTCLLRTTNFGFSCSLESTVWSDATNSSGRVLSRVPGVSTYAACLALCDENPACMFAVYDAPPAAGTTCTLA
TAWLDGNARYGGSDPSISCFKANYGPAPPRPPPPSPPVPPSPSPRPPSPQPPSPRPPSPSPPLPPRPPSPPPPPVGN
ACLQDADLRGYQLDFAIRTADTCRTACIANAGCTYYVTKPDGYCSLRYRFLEPSGSPEAGTVAVGNTQPFQTNVTSC
FVRSTVGRYLCSIPGTSNAVPLNTVTSLTGYTYNNCVTVCEGDPQGCRAFTWDEQVVGSGLGECSPARSWDYSSLYL
SVASGGDDTTCVKVYN

>Cre16.g654700.t1.3|PACid:27567296
MTTALVALAAAAAFIVTASANTNADWSPEDYVNPNEEVHALDTAPVVRTRLGEVVTRAQFPYCRCGRGYANNPYDLV
LSSQKQLKDGRSVTCYKVAQIRTCNGLTPGSRAEGCCKAMLGDFHKIEFDADPTCAGSVKGVSINGKAGKAWAWVTD
LGPSAVFKIANLQWNASLAVGSEICYTLGGPCASMDRLCYNEGGCNFAIFTAGLYSQTVGECCLLGSMELGANGTAA
SPSPAGTGTPSGVSPTPSPGPASSPAPTPSPVVAKPPPVASKPPPPTAKPPPPPNSSSGSLVNVSITVSRRVAASAP
PPPATRTRSTKRGLLQTSTNGIFDAASCSLLADVVNRRLRALRNPVGWDVPYSCSSVSADRLVAGGSWPAGTTSADL
AWVYTELRSAALLAELASALGLSCEDSVAVADSLGGVQVQGACGGSSTAGPSPSPSGTSVSPSNGGASPSPSTGGAS
PSPKPSPSPSTGGASPSPSSGGASPSPSASGASPSPTPSPSPSTGGASPSPSSGGASPSPSTGGASPSPKPSPSPST
GGASPSPSSGGASPSPSAGGASPSPKPSPSPSNGGTSPSPTPSPSPLAGGASPSPTPSPSPSTGGTSPSPSSGGASP
SPSTSSASPSPTPSPSPSTGGASPSPSTGGASPSPSTGGASPSPSTGGASPNPSPSAGGSSPSPSAGGASPSPSPST
GASPPASSPAPSPPLDAPILPTQDNSPPPPHDSGSGSSPVPSGSSPSPTSGGVPSPEGASPSPAPDGTGSSPSPDTG
SSPTPAGTGSSPSPASGSSPAPAGTGSSPSPAGTGSSPAPAGAGSSPSPAGTGSSPAPAGTGSSPSPTGTGSSPAPA
GTGSSPSPTGTGSSPAPAGTGSSPSPAGTGSSPAPADPGSSPSPAGTGSSPAPAGTGSSPSPTGTGSSPSPAGTGSS
PTPAGTGSSPLPTGNGSSPTPAGTGSSPSPTGNGSSPAPAGTGSSPSPAGTGSSPAPAGTGGSPSPTGNGSSPAPAG
TGSSPDPAGTGSSPTPAGTGSSPAPTGTGSSPAPDGTGSSPAPAGTGSSPSPTGNGSSPAPAGTGSSPSPAGTGSSP
APAGTGSSPAPAGTGSSPSPANTGSSPTPAGTGSSPSPTGTGSSPAPDSTGTSPSPTDTGSSPAPSGTGSSPAPDST
GTSPSPTGTGSSPAPAGTGSSPLPAGNGSSPAPAGTGSSPLPAGNGSSPAPAGMGSSPSPTGTGSSPAPAGTGSSPS
PTGTGSSPAPDSTGTSPSPTDTGSSPAPAGTGSSPSPTDNGSSPSPAGTGSSPAPAGTGSSPSPTGTGSSPAPAGTG
SSPLPTGNGSSPTPAGTGSSPSPTGNGSSPAPAGTGSSPLPAGNGSSPAPAGTGSSPSPTGTGSSPAPAGTGSSPDQ
AGTGSSPAPAGTGSSPSPTGTGSSPAPDGTGSSPAPAGTGSSPSPTGTASSPAPAGTDSSPSPNGTGSSPSPTGTGS
SPSPTGTGSSPSPTGTGSSPAPAGTGSSPSPGSSPSPTGTGNSPAPDGTGSSPSPAGTGSSPSPATGSSPVPAGTGS
SPSPSGTGSSPSPSSSGSSPSPSSSGTCEISVSVSVSRTSNTPIASYIDCSSFVDGLQRYIADLTGADAPVLACDSE
SDPGISLTAIGATPSTTADALFFALSGNGYSDLVAQLQLPDTGGCVAGSQPFLSFASSCTDPFRPGACAVDTCAMDA
LVKHVLPTSGAGASGPTDAELDSQCTALTGEMNALLASVAADLGTSLAPEITFQCTYSIETLVMGLVDGAAVADGTQ
AVVVKASVSQLPSDVSAALRAAFVTIGYNSLASSLSFPALTPAGASSCMATPAAALATAAALDVAGADTCGGSYVPP

ACPDTCTLSAVVQHPAPGSGGSGAMTADDVAAECDTLVKVMDILMVGAADELGVDSTALFFECSGAAVATAAAGATT
SASATLPFALAAALQAQLSEPLGYNALASTLGMAELTPAGCLAYGTDLTTTTVASFATDCAAPFTPSVCPPAPPSPR
PPMPRPPSPPSPRPPKPSPPQPPSPRPPKPSPPAARPPSPAPPSPAPPSPRPPSPAPPSPAPPSPAPPGPRPPSPTP
PSPTPPSPTPPLPAPPLPEPAGTTPSTTPSSASPSPSPNLSPSAQPSPSPVANTTTPSPSPSTSTTSPSPSPSFSPS
PDQAPSPAPTNGDSLPPSPSASSPSPSPLPAAESPSPSPSTSPSPSPTDAGSSPPPSDSSAGAMPPPAPSRDIDINQ
PPSPAAGGSTPSSSPPPSPGPEMASPSPSPSISPDMGSPSPSPSSTSSPSPSPDAGAAYPSPPSPAPPLPSPPVPPP
SPPSLSPPPVPPSPPSPPSPPPQPPSPPSPPPLPPGPPSPPPVPPSPPSPPPAPPSPPIAGGAVLPVAVPSNVVPWS
DTGYDMSVYDTSENATHYTVQYLVLVSDSAQPMSGWSVVVDPSILGTDLDTSAVTGTRTRDDGTTASAIVIPVTDAA
VSTVTVIDQLAPGSSAFFTLYWPKTTAANHRRRLAQTSTDPATASRGVNVTIIAWSNSTLSTIVLPPEVTGPNSPAL
SAGAALCAAASAAGVTTLPTDTSVNLLGGSASSCITTSLSNIFTSASNHTTFNTTVATVGSNTACTSYLTTLSRPAF
VRLPLTPTASAALAAALANRSTTGGASVLPLGAFISGGSLVLSRSLASTSAIALRFSLPSALAPSDVCEQGVLADQM
PNTCAVQLIWRSGCAQGILGNASALAAGGGVGQVPSVQAQPPAPAGNLQAEVIGDRDVNASSSDSNSLTASQIAIVV
CLVVGFVILAIIVLVVVMHHRRKKWTPAKGEVNSAPGPLILRPSAGGARHVININASAVSGGGAPPATSAGGASATY
TAAGGLDTPRTPAGMGRSTGGGSTFAVSGGGAAAAAAARGPGVIAMQLDGEGEDPMALPPPPVQASGRRAHFVNPLS
VMPPVVSTESATESAAEVGAGFGAAGSMAAAGSGAAATEGAVPVGGLSSGGPSGSHTGGVGAASVDDQKGAVGWAQS

>g7021.t1|PACid:27567910
MRAGMAGVWVLLLVAAAHQAAAAGNGNGNGNNAATTTTTTTTTVVPVGSTSTAATIQPLYTLGNNGCPINSYNYCIA
KSKSGCAACGTVNGVNSTGPTTGNNGCKTIDGFGQNVQACRMPFGAITVRFPNGTKYTEDFGGIVPQSEICTGSIAA
TPVFAYAGTGEENRVGTLVTYRTQAGWLVATVRLDCPYLSWFSYNGKDAPGATIVDAYNSVYNLTTKQTTANVTTRR
RNLLQANKASTTTCPSLTTDGSVLYGNGNGQYQNGKPDARSYIALSYKVDGAVTAFSIPTYRYQNNGDGSNNVEDNG
GDGWYSCYTFMHQVSFGSKNTDTCATINMNIGLFMSQIFMVSSDGKQCAVKAKTSIVTPPTTTTPTTSVVDPPVTIT
TVTATPPTPSPSPSPSPPSPAPSPPPSPAPSPLPPSPTPSPPPSPAPSPRPPSPTPSPPPSPPPSPPPSPTPSPPPP
VPVPSPSPPPSPTPSPPPSPPPSPRPPSPTPSPPPPSPRPSPPSPAPSPPPSPPPSPAPPTPGSVTCAANGGNCDKL
VQTTDQNGQGKCWVNAATGEYAFCMYSDPGNNTAVGVDPNGNINTYTGVTTPNEQNNGGPILPPSPPPPSPSPPSPP
PPSPKPPSPTPPPSVSPSPPPFPSPPPSPSPPSPPPPSPPPPSPSPPPPSPPPPSPPPTPSPSPPPSPPPSPSPPPP
SPPPPSPEPPSPEPPSPSPPPPSPGTDSKDTRSEGQVQIIKSTGEDEILPNIDSPPLIPSDTQPSTGPTSAAVEGTG
SFTEVVPDCVENCAIVGGPGKVEGGSAVGVPVEELKNVDTNGGMSVPPTVPEVPVPTATVVTNTDGSTTTTYPNGTV
VIEWNDGTTKTTTTDGTTSTTKVDGTEIVTTPNGTETTTKPNGEVITKYPNGTIVDEKPGGTTITTTPDGKTVTESP
GGTVTTVTPTPSGGTNTTVEQPNGSTSTTIKDSTGSTTSSSTKQPDGTNSTSTTTSGGTTTVVTYPNGTIDTTNPDG
KEIISYPNGTTVTKNTDGSTSTTTSSGTTTVTNPDGSTKVTTSDGTSTTTYSNGTVVTVKTDNTTTYTYPNGTTSTT
TPDTTVTVKYPDGSSTVTQPGGTTIVTTTDKTVTVTEPDGTKTVITVDGTKTVTEPDGTKTVEKPDGQVTIVEPDGT
VTIKTPDGKVTVTEPNGVTTVTTTNGTTTITDPNAGTVKEIPPSGNPVTTTTSDGTKTVVDGTTGTTTTTPGGDVT
VTTKDGTTNTNRTDGVVIVTTPDGTTTTTVPGGTTVTEFINGTKIVDDKSTGTTTVSYPNATTVTTTKDGTITTVDA
DGTTNTTTSTGTTTIWYPDGTTVIDPPGVGNVTVIYINGTVEIIAPNGSESITYPNGTSKDVLPNGSTITTYPNGTV
ITTTPGGTPQITQPPTTTTTTTDNSKVTTLPDQTTVTTKPDGSTQIQTQEGVNITTDKTGVTTVTPPTTTPEVTPPV
TVVKPDGSSTQKDPDNTVTTQDTTGKVTTVIPSNDPTVPPEVIVNNPATGETIRTVPEIPIVPDVVTPTPSPSPSPS
PSPSPSPSPSPSPTPTPPVEAPVTVITVRDSNTDTPLVAIITSNGDTTVQTSNGTVVTTEVETGQTTTVTTDGIVKV
QDPSTGTTSVTNTTTGQTTTTIAVPVPAPEPTGPFCSCPPTVANGATCSSGTPLEWYTRTVKPDGSFQDNLVATQCV
DAPTVTNADTGAVVFSFCWRRACITQGKTLDNEALFFKAPAVALPAKYQASSSYGIALTILRPSCATNAAATGHTVA
ANFMPLDFNEAETVETTQASGGLLNFDAKIVAPGGCDDTGATYMLNIMAISLVETVYNQCPKHAIPDGTNGNADCTF
PLVPVYLDITDANFVKDPSFPTYKQCVDPCNFDLFSGELACSYCWSFGQLPPQFSSNYFVIELRNVTKYNIDLMAGG
TYRSLVNQPQLIMPDGKKVEVTFTLNPKDEIKGKGAKAVVFTTNGNYTLPDGVAPVSSIGDIRGDFTKTTKYGGIHD
VNMQIKVPRGLDDQSVSVQMFGSPNPPPPEVLCPCPTPLNSEGDCDGTLTKIKLYPSDNSTTFVEGCIPSPYWDGAK
GELMISHCWRASCLENPPFFTPTAAKPNSPFYRKDYDIEMLLTDKYNIAAVPSGKHELSFFKPKVVDMFVQFQYVDR
ARPTETAQFSSRDGQVQSITVPAGGIAKMGLRMKVPFNPIYDINSASANLNTDPSRLIPPPPPPPLAITNYSYTINRN
AQVLRSTVLIDDLLNVTTAASNGSDAASGAFNFTQVKSTFARDFSNDIDMSMSGYIMSQDISFPDYGLVQSCTSADV
QAVINKIAAKYGVSPDSIAASCQYDYKKPDAVTTVTAAHRRRRGRSLQQTTVTTPAVCYPRISITVSMPLGVPMDGN
VTKALAAATTAADAVYDAVGTDACYVPTGAESRVGTVVAFTTTNSKATCEYITQAFANAGNFTADMIRTYNCGEENP
KKPYPPGLIALWCVLGVLGVVVIGVVTYLGVSWRKRRAANLQFVNQEGAISGGLPKVAPSPSSLAMGGNDYKYSTKA
AAVAEGLQRPDTPTRYSPRAAVPAIERNRVVPV

>Cre06.g309950.t1.3|PACid:27568709
MLAILCIAQPAGGQPPSPEPPSPEPPSPAPPSPEPPSPAPPSPEPPSPAPPSPEPPSPAPPSPAPPSPAPPSPPPSP
EPPSPAPPSPEPPSPAPPSPEPPSPAPPSPAPPSPAPPSPPPSPEPPSPAPPSPAPPSPAPPSPEPPSPAPPSPAPP
SPAPPSPPPSPEPPSPAPPSPEPPSPAPPSPEPPSPAPPSPAPPSPAPPSPPPSPEPPSPEPPSPEPPSPAPQSPEP
PSPAPPSPAPPSPSPPSPPPSPEPPSPAPPSPEPPSPAPPSPEPPSPAPPSPAPPSPAPPSPTPPSPEPPSPAPPSP

EPPSPAPPSPAPPSPAPPSPEPPSPAPPSPPPSPEPPSPAPPSPEPPSPAPPSPAPPSPAPPSPAPPSPSPPSPPPS
PEPPSPEPPSPEPPSPAPPSPAPPSPVPPSPEPPSPEPPSPPPSPAPPSPLPPSPAPPAYSATGIYGDSPWFTTRNG
FRWALFKDAATFSAAEEACRDLGGYLANPVSLAVYQDVLDAYSVPFTYLSDTRVWLGISDQDTEGWFYFTSGRGTAT
VEQVDLITWEDAWDSSHWTQDGSGLDCVAAQLGTATAALTTGAWLEMDCSTPLPFFCSGPTATLTNAPPPGAVAPAP
PPPYAAVAYPATSVVVSYATYRLVSPTASTWLAAQQACVGLGGHLASFASAAELTAVRDALVAALAAYSPPYTTSWA
PLWIGLYERSLSYGEDWRWSDRSPLTFSGFGQYELNAASNPPTTPNCGALHLDRAGQWSVQGCGTQLQYLCKLANVA
NAPPPSPPPAAPATARQLAFRFAVGDLTYTLVMQPRSYAAANRYCGSLDPGGTLAVPADAREYMDVMSQVYLYLSSD
PAAAGGSALAPPPAASPPPWPPAQVVSALPSQAGEAPFWIGIRWVGAASALGDFTQVDGTSPPTFTQWAAGVYTGPR
PVLACASVDPTAGFQYVPAGCGVQLPALCRAATADNAIAPPPSPPSPAPPPPAATAVQTRVGLYLYSLYNEAALTYP
EAAAYCRAGGGYLASFHSLAEFNAVWSYMTTTATALAAGDASVWIGYDEIGRNETLLRWLDGGPAAWTHFGLPYTDT
EGNDCAAVDQLWGGQWVMDDCLEPRPFICKRLLGISADPAPPPAAVAASPPPLASLPALRNVTVAGVLYQYVASPAL
SYEAADAACTQYYGVLAWFASASEFNAVMLALQADVAGLYDPSVWFGLYRSYANSADWFWYGNTTLNGTAMADPAQF
LFNPTSAACAALGTSLNYQPDNWGGDQRCVAASVLDAGGGSYCYRWDDRACDQRLGFICRVDDRMPLAPSEPQQPPS
PPGLPPLPPSPAPPGPDPDPPSPPAPSDPHAPPFPPAPFVPGEPAAPPQKPPTPPPAPPGSDFWSAPWEPPSRPATP
DPFPPPPPPPPPPPALPPPGLPPPPPPPPPPPPPPPQHPDLPFAPGEPFYPDPPEAPPPPSFESGVPPPPPPPPPPP
PPHAPPLPPSQPGTPSAPAAPSQPGVPSVQPDAPPPGLPGAPPPPPPPPPPPPPGVPPTASAPGAPPPPPPPPPPPG
APDQPAAPLAPRPDTQSPGAPPPALPGAPPPPPPPPPPPPPGVPPTAGAPGAPPPPPPPPPPPGSPDQPSAPAAPSV
PAAPQPSPPAEDSTGAVPPPPPPPPPPPPPQTPPAPPAGTAASPPPPPPPPPPPGSPDQPSAPATPAAPAVPSPPVP
APDTYTAPPPPPPPPPPPPPGTPPSGQPTEPQAPPPPPPPPPPPGTPSAPAAPSQPSTPAAKVDSPPPGLPDAPPPP
PPPPPPPPPGVPPAASAPGAPPPPPPPPPPPGAPDQPATPLAPRPDTQTPGAPPPALPGAPPPPPPPPPPPPPGMPP
TAGAPGAPPPPPPPPPPPGSPDQPSAPAAPSVPAAPQPSPPAEDSTGAVPPPPPPPPPPPPPQTPPAQPAGTAASPP
PPPPPPPPPGSPDQPSAPATPAAPAVPSPPVPAPDTYTAPPPPPPPPPPPPPGTPPSGQPTEPQAPPPPPPPPPPPG
TPSAPAAPSQPSTPAAKVDSPPPGLPDAPPPPPPPPPPPPPGVPPTATAPGAPPPPPPPPPPPGAPDQPAAPARPGP
DAPAAPPPAQPGAPPPPPPPPPPPPPSVPPPTPDAAAAPPPPPPPPPPPGSPDQPAHPATPSVPAAPQPPPVDKEDA
APPPPPPPPPPPPPGVPDAPVTTQAPAAPPPPPPPPPPPGTPTMPGAPTKPGAPLAPEQPAEHAPPGAPPPPPPPPP
PPPPDAPLPPPARAPDAPPPPPPPPPPPGAPDAPAAPAQPTASTQPDAPPPALPDAPPPPPPPPPPPPPGVPPKPAA
VDAPPPPPPPPPPPGSPDQPAAPMSPGAPGPTAPPPAHPDAPPPPPPPPPPPPPGVPPPAPGTTPPPPPPPPPPPPP
QTPAMPDVPSEGQPAAPIAPSPPVQEDASAPPPPPPPPPPPPPGTPAAPPQVSAPQAPPPPPPPPPPPGTPDAPAMP
RLPDAPLTPGAVQQPPAAPGLPPPPPPPPPPPPPQAPPVPGSPNAAPPPPPPPPPPPGAPDQPAAPARPGPDAPSAP
PPHLPDAPPPPPPPPPPPPPGVPPPIPDAAAAPPPPPPPPPPPGSPDRPAAPASPDAPGPTAPPPAHPDTPPPPPPP
PPPPPPDAPPSPRTHPGKKGDDDAFAPPPPPPPPPPPGMPDMPAVPSQPGAPMPPAAEQVPASPPRLPPPPPPPPPP
PPPTTPPPEPDHTAPASPPPPPPPPPPPGTPEMPSVPATPEDVSVLRPDAPPPSLPGAPPPPPPPPPPPPPGAPPAL
PPQDASPPPPPPPPPPPPGTPDAPADIKDWSQPDAPAAPPPALPDAPPPPPPPPPPPPPGVPPPTPVDAPPPPPPPPP
PPPPSVPPPPLLDVGVPDLPPSPPPPDQPGAPPPPPPPPPPPPPGVPPPLESPPPDAPPPPPPPPPPPGAPAMPTVP
TQPGAPQPPDAADAPAVPPDLPPPPPPPPPPPPPAIPPPTPDAALSPLPPPPPPPPPPPPGTPFTPDTPADWSQPDA
PAAPPPGEPNAPPPPPPPPPPPPPGAPPAQPPQDASPPPPPPPPPPPGTPDAPADIKDWSQPDAPATPPPALPDAPP
PPPPPPPPPPPGVPPPTPVDAPPPPPPPPPPPPPGAPPPPVLDVGVPDLPPSPPPPDQPDMPMVPRAPNAPNVPSPP
ALPASPPDLPPPPPPPPPPPPPGSPASPIPLAVSPPPPPPPPPPPPPSTPDTPSLPNLPPSSPMPPEIIPPDAPPPP
PPPPPPPPPDVPPPGSPPLESPPPPPPPPPPPPATPDAPADIKDWSQPDAPAAPPPAFPDAPPPPPPPPPPPPPGAP
PPVPKDSPPPPPPPPPPPPPPGVPPTPEFDPGVPELPPSPPPPDQPSAPPPPPPPPPPPPPGSPPGVPGLPDTPPPP
PPPPPPPGTPDMPTLPGAPGPPPPPPPPPPPGAPARDPPPPPPPPPPPPPGMPPGSPSSPLPPPPPPPPPPPPGAPP
PPNMPQQPPPLEPDMPPPDFPSTAPPPPPPPPPPPPPGAPPPPSPPPDAPPPPPPPPPPPPEPPPPSLGVPLAPLAP
GLPPPPPRPRPPPPKPRPPPPPPPDLPPYAPDPPIPQSPPPPPPPPPPPIPPPPPPPPPPPPPPLAPSPPPPPPPPP
EPTAQVDQILGAQPPPPPPLNLTDVSLANITGGRTTTGDAVPDAVTSQPGNTFDVDAYRLVPVVFANVTVVVDAWMQ
PQLYEKLRRREFADVMRQVLPFSQYLIRVRVNKLTWRIGDYLTEQRNRTLTARGFPPPSPPAPPRAPGSRALSPPPP
PPPPPPSNDTSYPRMTLFIQMYAEMTTEADVRAGFGSAALVSSFQFSRFPVYALSLGATSITSYTVYTRRLNVTDNQ
PPQLTLRGPPEVFVPLYGSYKELGAAAYDLLDGTINDDLVIINLKRWNESEYCCETTFPIDTSQVTEPDKPFIVTYD
VFDFVWNSAPTVQRKVHVYDPCAQASNGVERTCPDTRLCSVLGGVCGSATLFSDIRDLATPLTEPDPNNPGITLGIH
TLSSDPVYDTIIPNLQLLPLDGLFFVIPPNLDGTPGLQGTLTYLTVGDPWVEPGYIANDDRDDSVQQHVVRRYAELV
DTTGPTPPNTPFAITYDVKDVSGNAAITQVRLVFVLCPATLPVLCPPDERVLARPVWYCAAEVCGVPDFFTNALGGA
AAVPAVTRNTAPVVRLRDNKYLVTIFDNATYARCPDRGDPTGPCDSGVTATDAEDGDISYKVHAFCADVSTAGVDTM
WEAVGLAACQFGNVLKAGRRVITYTVTDTAGATSQISRTLLVMQRCEDGEVQCPDGTCTDFNCFDPSTVITLTLFGN
GTDDTTGSGLNVTDGSSFVDSSNFTTSTVNDITGGGSSILTAFASGAPVVSINEPPVIALRNSTLLSQTVQVPQNFV
WRNCRASEGPNPATLCELGAQAFDPEEGNLTNTIYVCPPVACLATCSHLNCAGHEFTSKGVRGCVDTGADPGNIFNV
IFLVCDNGVPRQSAQVTRTVLIARKCAAGLYECDGVCETVPCAAKAKVARDQVVNNTLPALRLLPPGSSAENAPVSA
AGVPLSLTQILTYGVPAPISFSPCASYDANAACAAIAMDVEDGDLTSFINVVDATDCSGKTPNTCLRCRPEQLSLGK
CLPGTYRLRYFVEDNSGGISEAFWNFIVESVAVNRVWFLMPPSFNISAPASNSTNATRRFMDQIQVNATRSRGFLVG

QLQRFGVDLKDVRSANVSSLALVNTNTDEGQYAINADFCICMINVTLEFTTGLQVNYTVPEVFEIFEPDNFPPPPGR
LRPPPVPPSPPPSPAPRPPSPPAPPPPSPGPSPPPAPPDPPSPPPTPPSPPQPPSPPPPPSPAPTPPSPPAPPPVPP
SPAPPRPPAPPTPPSPPAPPSPPLPPPSPGPPPGNGNAGRRLIDGTPAVAPAIAPANRVWSWGDDYEMGAVWSPPPP
LQRLFNQLRRSMSEPGAAAGAGADVSDYDDDGDGEADWAPVPPADYEEEDPEQDVMVVSAAGTHGPGDSDDNPLPWG
LSAAAWGGPAVAGVARKASGAAGGGSGGAQSAPAPAPSVPVPSYLHTALMHASAPSEATGPIDPLLSGAGGPGSAGA
GASAAGAPLANTLAAVLGAGEITAASSLSIGARIWQLHVKPALQKQQAAAALAAQAGPHQGLLPVATALYEPFGGFG
GGADTGASGLLGAAAAHPAAGSNGAGGGLVGQVVRGDAAKLRASVGVLGKVFTREGEQWDLPGSGALRRAALEEASR
AETAAAASDPAASAAASVAAAAIGPRKGQGKGSVGHAATVEAAVAMFSPAALSLVTADSHLDPRTIKGGPVGGLADD
FNLHELMGSAKPQPAAKTDDADAGADEPSRRTVVRTLLSEQLVPALAEISRRLEQSQRELDAIRLSPTALGVVEQVG
RHGRSRRRLLQTTSGSVANQTASCAPAINATNLATNCTAPPPTTTDMYLAVTLSLIADIDNVNRALSANMTNSTDTI
SVLDEKFTATDVKYESQYVAYKNLATTYLSALENRTNTVILNLAALAAADATNSMLLSALAGLLKGAVTSAAEEADR
LGITTAMLVDGLGLSEENWFFESYQQCLIQRSGSGTTYNFTINREQQAALRYDLETGRVAAPPPPPPARAGFTLLQA
TQMGRGRSLLGWGSELLSAAAAGAGLGWAGLGPADPQDDSAASAATGSGYHAVRDRRLQTGTVPSTVSAELPQGLTP
RLQYGGYELEVAAGRPPAGLTVTYDSRGIPERRRTAGARGNRVVGGLMLHQVRRRLDQVVLGRDSEACQSKFGPLRA
ECVRSKYSTKNLQDLGGIGEDPVFDRSSSIYSPDVAGRAWAYYNFSEGNSEISTLKLPFGFTHYPVPGLPDGYPVFF
DTRLGADRARKLLTYLEDGRYLSPIFSSELRLRLLVYNPDAHVFGYLRAAWDWGGDGVVRLRQWIAAIPATDYGKFI
SEHQYYMFIPDWIMVLFILLHLASTLLDCVRALQHQRLLQRATEGEPLAGLTADELFRRKRRVKKRYTWRMTPFWVA
WEVASCLIQIAALVVLYWFVFVASPGIPTATTYDVYDADVNAPARFFMINKDERGWNPYLASVLQAFQTRSDLKPTN
DTIDLAATAAAMAPPSMPPRGGDYRWLLPDDVSGLHALGDMYSYLDNMEYLWNLYFVLQSLVLLMLMVRLMHYVSFQ
PHLAVIGGAIARAIPMLLYWAVIAGMVVVMLLMLNIIAFGDRLEQLSTISGSAYAVARWLLIKHSDRGNRATITEML
NDNTVILNGGESLVAALVRGLGAIFILFVLYAFVFAILLRQQRMLRRWRHAAPTVGEDLRHMFRWWVQRSWRDAPSN
KRLDLTIDWVTRPEVRYTWAYSVLYSAVVRGVAAVAGGTALAKRHPTQHQAVTERAVLLPGGPPGFNSPDEPDPRYC
PRYNLRAMMMVFEELLLVNKELRRELFFFMQTNNEDGRTKRMKAMGAPRRAAAAAAVNDASARAMADSLASLLLERF
GKRVPRRAARSFDEMMHQLRKRLGTPAGGSGSADGSEFSGSLDGGSARGGDSENSDDDMLPTGVRVGSLKHRGYGVH
NQQTVHDLQLQAAVQTAMRNIHTIRENMGRELGDDPQLLATLFVAQFAGQIPEPERRPPGVRPSLYRQRVQQAGSGN
SLVNVGAGSAPAPAAPGGPPRPGRSRLGGAAMMGQSPQAAPAPTQGPVAAVLDTNTPEGMLTKALQAMLAPPSTTAN
AAVSADGTVPAEAEASSASVTDGAPRARPRPKGAKSRARFAADLEIPGNTTSTAGEEAPASPGVASPGAVGSPSGRH
NSVSGQLGGSRTARSMLPRHLQPEPRAASFAHPAPATLGSLGQLGSPSSVRSSRSMVMPAGSGSAYFTPSASMGGAA
GAATAPGGPTASTGGTDSGAAPQPEPLTIRRPQIHKMRSLGAAAAVAGRMRSFVSGAPGEITPASPTGATSMSAAAA
AASLITSPGSYGRSGGSTSMARSGVATPRGDLVSAQISALPLDGTIIFATSAAVVQRLTSHVLQLLRELRGVQADAD
AMLRSTLVLIERLSAGSVPFPRRAARMVPGVMGSPASSPPVSPQNEKPPGWEDAAVARVAAANAAAAQRAAVGTAS
PRLGSSLRVRVEDGAGGAAHGHGAAGANVDDGEDMLPLPPPPALRGVDSYTASPRSLAALQAARGAAGPLSGLLTPP
TPTPEASPEKALPPAAAKSAEPAQAEEAPAGESRVVSRAASQAGMKPVDEMREVEPEPELVTFMVQSGMKSRAAGDA
AALAAAALAAEEDERREREALAASEGLDSLAPPPGTSSSSGQVHVPAPYGPVEYGAAPHIARLEMAGMRPSSGASQS
SDPAIKVLSPASEAHRRASTPKSSHGSRVGSPLLPTEAIRRLSPETSNLSRPGSAAVVDAAPAREAPEEASARRNLP
LPSPQGGSRPASVQRDEQGVEVLALPDTVPEGQEAEAGGPMDEPVQGEGEEGEHGALEASRSLGRQPSRIAFSESSM
RVRAEDQEEDDELDEPPSATAAAGAGPAQASEPQAPPRAGYIPPPSREPAMQSEPLEGTRPMRPSRSVRLVGVPPPP
PPGGVPAPVRRRALASQASLAGTTSQASLGAEASTGEGLVAPPQVLTPEEALSVISQALGTSAPRPIGKPATRHVQL
PPDVERRQSQLAKQQELLAAAQAAVAMRRMASNANSRVGSRATSFSAAAAPGAAGLGLQSMDSTGSGAATDAPGGGA
GPSTSSAIPPPPGRAAASPLRPGRRALLASAQSGAGFGLDAAPGEEPSNAVQMAAAALERKQAEEAASKAYVDELMN
RMRSGMSATGERLALSGRLGSAAGPSGAASPALGAAGGGSRQDSLAGEVSRGSAGLLQQDSLSGTMQLRPSGSIVAP
RGAPPMAAFGSLRKPGTGRSSLNLPPMSPLGATPAATGSGEGAGSGLGPIVPPPPLFTNAPGRPQRVSQLMGQGSQG
SFGAGSPSAARSAAAQAQLSAFTGDLDVEDFDLDSFGSMRRAPAGPTPKRRQAGDDE

>g5732.t1|PACid:27569118
MLRKSTGKAGRHDLRRVAMALAFATLFFLPDLASTQTTYGPWNATEDLNEQHKGLLAFILSGDTSFWSRPEVATRLG
FGTAPWRCISNCQTQIFTSDQQCAPDCESRTYCEPGGAALGSENTTCCALSLLDQTYASAQPPSSTQPAWCSTYPGW
GARPRPSVCDFNVFAARGTTPPAGADDPDLAVSCSSGTVPTYSISGTRYRQNDTVYRIMHNGAVTNPANVTRNQVKS
IKLRHSAMWHHPSVNITSPPEFSLVSELACLPLEEIFLEDVALRADYSVLMAQPNTTLDNGKVFNLYDPSTYPFNVT
GLYNNVFSMLIEPSFMLHSSVSVKASSFNPGLALRNAALRTCNFAPFTELHAMRWTGQAQLDWSAEYAQALSYVRDG
SQLRPRGWLPPVIEPVSARLLLPDTLRRLTIRRTRDEQHGAVQWTSRPLITGPLPGEWALLRNLEYLDLSDEMETGA
IVGPIPSTWLMMSHLRVINMTGHHNFCRDWHKIISWQIRMYYRAATHEPNLNVPHYYGPWGGNNGNMTRYNISVYDL
SGHGWQWYDEVTTEAGFVEVIAPHGQCCWDKWSQTIKDNNYEILYPDGSRFGNNVVQDEIYGGFYQDEEWCEPTSPP
QPPPPPAPPSPPSPPTTPDVPPMPPSSPPAPVMPPAPPPQPPIPPASPLTPAAPPRPPLPPTWPGKWEGAWPFRPPI
PPRPPRPPPPPLPPSPPLPPVTPSPPPRPPPPKSPPPPKPSPPPRPSPPPRPPPRPLPPSPPPPPPLPPNPPSPAPP
SPPPPPSPSIPPPSPGPPSPEPPSPAPPSAAPPSPMPPSPAPPSPDPPSPKPPSPVPPSPLPPSPEPPSPVPPSPPP
ASPEPTSPAPPSPPPPSPEPPSPAPPSPPPPSPEPPSPAPPSPPLPSPEPPSPAPPLPPPPSPEPPSPAPPSPPPPS

PEPPSPAPSPPPPSPEPPSPAPPSPPPPSPEPPSPAPPLPPPPSPEPPSPAPPSPPPPSPEPPSPAPLPPPPSPEPP
SPAPPSPPPPSPEPPSPAPPSPPPPSPEPPSPAPPSPPPPSPEPPSPAPPSPAPPSPEPPSPAPPSPAPPSPEPPSP
APPSPAPPSPEPPSPAPPSPRPPSPEPPSPVPPSPPPPSPEPPSPAPPSPPPPSPEPPSPAPPSPPPPSPEPPSPAP
SPPPPSPEPPSPVPPSPPPPSPEPPSPAPPSPAPPLPLPPSPHTQSPPSPVPPSPAPSAPSPPSPQPPSPLAPSPPS
PAPQAPSSPFPPPQPTAPTAPPPPFPPSPAPPSPTPPSPEPPAPQPPSPTPHAPPSPEPPSPTPPSPLPPSPEPPSP
SPPSPAPSVPSPPSPAPPSPMPPSPAPLAPQPPSPTPPSPAPPVPPSPEPPVPPGPDPPLPPSPTPPSPQPPVPPSP
TPPSPHPPSPAPPSPAPSAPLQPSPDPPSPQPPSPAPGPPPSPPSPPSTPSPPSPAPLAPAPPVPPMAPQPPSPPLP
SPPFPPQPSPTITPASPQPAPAAAVLDCSAAATRTSFAVASSSRGAFYIAVAVPASSPSYCQVCGCELSYAVLDPGA
SQQYVIPSSGSSSTAGGSPTVAVTSSVSTPAGAGGLGNGTHGSTARRRALVVEATASSSGPAAGVGARHLLLAATAN
STTLEGLLATGRSRSAAGAGMGMSRLQVVDVQTGGLDPVTAAPPTGTSPNTTSGAGEAGGSGTVRYSSMGAGGSGGL
DAAWRLTPGATGDYLLRLKVADQEWWRWVSVDIDPPRAAGQLLLARRTGGSSSSSNSTSGSGALAAAEDEVQVEVNA
AHAAAGATAVSAQAAVVRLMMAVIAMSEPVQPFSLTSALRLSGGARLLSTQCFASATAAAEVAAAAGTVDASTPPGS
DASSSAATVAPAAIAPVSGSTSGTSNSSTSGSAYVQSCVAVLFAEQDATPELLLPPGTLTDMHGNINAEPLILSVNL
TASADSLSTVERAGAPVAAAVAGGVFASAAFTSASASFLSAFSSRSSLLQSGYHIQMLAMSSSLASPGISPAFRRIS
RYLRWSLLGIQGNIPLLDGAFSSGSAAAGSGSGSSSSNSSSGGLGDVDVAAVALDRLQLSVPPPLPAAGDAASQAQP
PANLSPPPSASQLVADGSTALAGRRSRSLVQAAAPVAPSPPFTQAPPTPAPFGTTGAAPAPPPAPPRQPSTPPPAPP
PMPALSLSGDRDVLVSWLQQVGAIGSGSNSSGGGSATSDSASVGAYSLGGAAASPRGDVVVLPDGQLGLLGPGGSAP
PPMQPGSGSGGQQSSGASVATDTTIHTNVQDLLYTLAIAALLMVALVAAHLLVIGLWRLAVMYDVCGAAESGVEGLH
PVLRFPRAEMVLGGLLLVALTFYSALTLSGAASPRWGDNTAAGRLIAVLVLAVLVVPYGLLLWWLTVCRWYLQEEEV
DHYMLGPHWQAFDGVIPGGGAGAGGDGGGHGASALPAVAGFGGTGGGGGVAFAACQAEPGSGDGGSGGGEDGYGLGP
HWALAPAGAKTLDYESIASPAAGAGSAAGATRTDAAGSTVGGAQSGKGRPRTLRSTDVTAGGAAAGTAAAAATAAAA
AAGAPAGAAGGDVGAAFQRRYGEADDNHGDSYDGEDGEGDLDMAPWRRRGPPADGVDAAAPATAARKMAGSDDGAAG
ERRRANPHARKGSADGADGGAGDPQAAGRPLLRRGVSYGDNLLTAGANTRGSALRSTPGPVADDDARSRFRRNVTYA
AGDNALTSGAASAGAAPSSPTRTQPDSPQAPLSPRRGAAMGSNAYTAGASRFGGPRTRADSPPRAASPPPEPASPLS
GDAGAISEVGFNAMVSGGSRPPRSDSPTMEDEAAAIRIQQDAADPAVEANRSRRKQAGSVEEQQVAINPMAVPRASV
LERPDVAASLAAAVAASGAPPQGTVPLPPITEATGPNGLGSSPRDGRVLTTASVSHGRDTSRLLHPMARPRSLGASL
VPASATTNGGSGNSSGNGRESGSGSTSGAPAAMPRPRVGGAPSNVSAAAAAAAQPAAHQDLFGELDTTVVTPKMAAD
AGAASPPPNLPSPMMRLGSRLGLVRGGSNSGAAQVAPASPLPAKAPSPPAAAARPRPPGAVTTNAAVPMPPPQKVFR
SPSEPANARAQGAAPPAAAAVANGGGRELKQQPAVQTVNSTTSSISANSSGSGNPLARALRYMSVPKPGAQPATASN
AAPAAAEAEPAGPPSRHTAWGGSLSQPPAAAAAAAGAPPSPPIAPQSPMRLPMPPPGAGGAGGAGAKAGGGFTRSPQ
ASIVMESSPAAGPVWPARRRNNRAAIMDEDDLVAEGGGAAGPVAEGAEAPDSAGHYFGGWEDEDPRLLLREAPRSPV
MPPPPPLRAGGTARGSSTGGMSAAAVSIHSHSPSSSARGGGSTAAGHYMTYNPLSLPSAVSAAAEAAAVTPSALASP
PLPLSLLPPRGRSQQAAGSPRSVLLLQRSSTFAPNPTAMAILGTRGAGGVGGCSTRSLQSAVGEGVEGPLLPSAGAL
AVVGSPKSAGGAISVGGGGGGRKSVADLDAEEAEVMRQKQLAALDDWSEVVDPGPGGPAGAIAAPPGVDKRLLPLYP
GKELPMYRLGYRPGCWPLRLRLTPPVYLLARFEFLFEDAIGEGPREQHGRETPLILCVTAVNFSHKVLCAAVFGGFG
LLERSVPQLGFLMALQGAMLVYLAAVRPFREALLQAVELTCHALEAGLFACALALVNAQPNNGAATTYVMVACFFGV
ALLVIVYELRRLALLVMALWRMWRERGTQARRASHAIMDEMEGGGVSAAAAAAAAASRTPSALAPPVSITARRSSAL
KEGAVRTGVASSLSDGGGSTPTSPLGAQPQPSAAAPTRRSRRSSAAGGSSSSDGGTAGRQAAAAIAELAQGQDGPGG
DAVGEAATAQPETSTAAGEIAMRGATATGAGAAR

>Cre09.g404250.t1.3|PACid:27569887
MKTLLFGVALVVQAMILEVNSQRFDVKQKGLSFPPSNADFIQNAAFTQRYGAVKVPQMMDWRAMQGILSPPADQNPC
GACWSFAVSHAIESHVNIAVQQAGGMGVRLAPMQPVMCSTEVTGNASQPRCKDGWVGKAYRYAAQRGLVPDAFWQKW
TGNAYVEAQTCPAQVLLNSTAFSDAAYLIHDWETAPSTPTALKMVVANQPAVALVHASPDWEVYTGGLFNGSCSGDL
ADANHAVLVVGWTEDAWIVKNSWGPDWGEGGYMRLPQGSGCGVLNKVTYPVFDSVSADRRGEVLREGFCAGVGKVES
TGADAAVTLRSLATSLNVTLDELIRVNSHIKGGADTPIESQTNYFIPPCTREVPPPPVPQAACGTTYSIFAASEPLD
AQQGKPAQDPVSGRRRLAAMAARAAEQTHVVYVQDGFQVQPDGMSSQQHHLHQRRRQQEEALLERLAAARHRGDTSR
RSLLGISVNGGAVGASGSASKAATRQRTPPPHQQQRMPPPRRGIPSPRQETTVISLSPPHLPPPSLPPPSPPPSPPP
SPPPSPPPPSPQLLLDTHLEAPPPWEYEDSPPPPARRPPPPALSPPPPSTQSGSSPPTDPPVIGMRAQCGNAGSTPK
FSSNGVCAGISWTCPWFQASGSMDVTYRVIRQAPDGTRSPGGSGTVTLRCASSSYRLPLLYVELSSPVVLSGLGADA
LVLQQRNASCGAASLTWVDVAAVTEAGPRSFLYFTPAPEAPCAGNEVVSAVARSPPIQAPPAPPSPTPPLPPSPTPS
PPPSPTPSPPPSPTPSPPPSPKPLPPSLPRPPPSPTPPSPRPSPPSPPPSPPVPPSPPPPGDIDAMSRQFPTEYSIL
TTGIPDHRFPALVNASDRQPTRLLLLSASFPVLSGPDGSPLVAAALQGQGKVLVIGASSVLSDVGDSGWAWSSRRSF
TLVNNALGWLAGFDRPLARGDAFRNVAFWSTGLTTSQASSIRNYLRDNGWDPKGTAALTTIQTRYATQALPTASGPL
SLERLASFKVLIMTSAELLSSKFSADIRTYVQQGGSLLVLHTIQIKAFPSVRPGITFANVYSCNRLLGPMGLVITGA
TAEAVPTWRPAVPVPATATPDMLLAANSEYAAGVLSRYYAGQERRLPVEAYEQAVKSMELAKRLLTPSRQLFPSFFS
LLSQVRGPPPPPQLPQITASSVLGSAAGGGAGSTRLGPLYTYWLTSPALERIEEGLEEFSTVCPAGSHVVGFEGMAW

STRDARPWGATRGGMFTTEFKFQCSDGSLQDTGYATAPKKYKSSVVPLRFPGHQSYEMPHLVANPMFGVPWVEPACP
SGYDAVRARAHSNDEDNYVKPIQMMFRCRATGYWTSYTAGVGMVNVFRGEQANARFLGYGPGSLSRRSWYMDPEDPV
PYRGSQHLQQIQDMVQLEDTPGAVECPEGQVVAGITGLRWVPPITDFSSADESDPYLYPRLTDITDHVTSTSTGWYT
TRFFRPELSDDIRMNPGYTVVLSAHDVVCRPAPTVAASDNMASRSNVAAVAEHAEEAILATPYDGTYLSSVFHAGAK
PAAGDDSSANTTLLTCGEENMVIAIMAASDTSSDLNYIAARCTDGTKTAVGEYLPSGSTGGSVVDNPCRLGYDAVKA
IGGTVAGGSGESVGGFALRCASPLTSDSSSRSGSSWTRFGAPILSDVGPNNDQYALTEASTNAGVEECPVGQRISTL
RVMQYEGVVTGIDFYCAPMPPPPKDHSFYDIAVRYGITLSDLFGANPQLDRTKPVLAYNGTVINIPQLCGLPPIQPP
VSTIAASCPRWWPLPNITVTGAETCGMVSQSFKLTLPYLNTVNGNRCPNASTTLTRGTRLCIAPPASVMSAAGRRRL
QQYTNTDADCTTQSTIGVGDSCESIAAVSGVEVADLLEWNRGMRCSSLTIGSVLCTKKASALGGIDVSGLVAAPPSP
PSPPSPPPQPPSPPPRPPALPVNITFYASGYYCNCTSGFVSAEDLQTCLPLANVALRQPTYASSVMGGSPSYRFDSR
WAVDGSVISTDPDISGYFSSGRGDPSPFLSIDLGSFYTLSHVVLTLRSGYSHRLSGAAIRVGFTPIRGAEDREDLAR
NPVCWQQQGNATSDRLQAACAASGQWVTIINTGGLYLQLAEVEVFSFRQLDACPEGTVPAGNGTCATNPCGGAPNNC
TNIPGGGYACSCGANFSKSIVQPPTCDSLTRNVARGRTFYTSSSAGRYDQGWVEESCRQPSLIYWTTTFGVSAGLTD
GKTDAGSVTEMWIPSDSDRDGPWASIDLGTPFAVSKVVLYVGSKNMRCVNTYTGYGSLNATYGGLAYLQNAVVRVGM
LPVLWGDSLNKEFPDLRGLEGNRVCSVQLDAVPAGARVELSCNSPVVGRFVSIQQNTFTGNELLSLTEVEVYGQPAV
QCSKGYVTSADGTSCVDEDECVTSAPCGRFPNQCVNLPGNYTCVCAPGWVYYSQPSGPICLAMGQVPYPNDEHQITS
WGADFGPKGKMAFASSVYTMPKEAAFFNDGDGSAWAYGPQQAFDGASNGASWASAYVQWSGYTFQDSWRSAEDDRQP
WLSVFLTRPYVVTHVKVYNVPGLMGEYLSNVEVRVGTVGVGSVNVTRWIGQNELCGTITAAADPGEVVTVVCKTPLV
GRFVSIQKVAPTVRTASSSAAFNGTLSRTLVVAELEVYGYRAPQCPAGHTFDGVSACTPTKARPIRYLDIRSLELRP
SGHVWFRTNDNVWTGNSREWTKNDRYDWVYNDPYSPVPVCGTAWNDVTAAAVCRGAGFSGGKARSVGAPNDQAFGRA
IWYAVDNITCANATMPYGFCTRIPVDGSCTTWAGVSCNSDKLPAGPETWTEPPEPQCPKGFNRLPDGSCQDEDECAF
VPCGSANNTCTNLQGSYNCSCGAGYFWVAQASGPSCAAIAEVPYANSRRYITSWGADYGLFGKMVFASSSVQSPALA
FNGITYVGSGSTTAYTDYYRSAAANDTQPWLSVFFTRNYVVTQVRIYNVPGDLSDYMSDLEVRVGTVGMGSLNATGL
IAQNELCGTVLGPVRSGEVVTVTCAKPLVGRWATLQKVAPTQRSAASSGAFSDNLANTLAVAEFEVYGFQPPVCPMG
QTYDGVRACTPTKPRPIRYLDIRSLELRPSGHVWFRTNDNVYTGNSREMTKNDRYDWLSNDPNAPIPVCGAGWTDVA
AAAVCRGAGYTGGTARILGTPTDANFGTWIWYGINNVTCASSSTVYTDCTFIPVEDPKGVDICGKVFAGVSCTSATV
G

>Cre09.g400850.t1.3|PACid:27570373
MRRHWSFSIWLLLVLLVALPASGSTFRDFTTFTDASVGGQDLGGVSTSAGQASWQACVTLCQSYSECVGFLYRSSPS
ASCVLKRTRGRVAYGALPAVGAVAGVRGFEGAGAQQPQVVTVSLGSTGNFTFALGATYRRPPLVLPSIKGYQQADYG
TYAMHVTDVNTTHFTIENQHNSALGLNVLVLDGLTDDMTYILPAPSCDSNGACNTTGCRVLSQPLSFPWPLPSSHVP
DAFVVVPEVAGPGAGAPADSGSTVAVSGSPPNAVSMRLCPSSGGGSSLTSFTAGFLLTDHPLTRAGSYRYDCPLGSR
TSDSACPVAYNGPTGTDCALREISTYITFSRPMPADPTLVLSIIASDGDPSTDKNFDVYVPSVSIYGFTLNTRAYCT
TRLWSLEVSWLAVSPTSSPPKPFPSVGRSRYGYFYRGCYTNDYQAVMMYTMSSNDMGMTIERCAALARASNYAFFAI
KDGRDCTAGNDGGWGLRSYEGTDACAVPCTGSASAGCGDTNRISVYQLVDRFYKLDAVTDMSAAEGLCSSSGGVLAM
PLSGEENAAVAAAAADLLNATSGYVRLGMRRTSNTPSTDREAGWDVASGRQLTGNYLNWRNPADVSYPAQPSGSTDD
LCVSGFSTDLLWEDTSCTIGSVAMCERVFSLPEQTTWVGCYSDSSGSPLLPTYLGWVGSIAGCAARARLEGFSLFAV
RAQTSCYGGNDASILRGASRGTCDLDCNYYNPTQPHNGQLLKCGGSSASAVFTAGNQFCRSPLMATGNVTTTNMPSS
GTVLTASFNRNFRAAPVLLYALHNWRRPSQTLSVSVTLNDVTWDRLTMWVEINAMTYAGNPGTLEYMEANWMALDGL
PQGMRIKREIGCSAGGCPSPSNCWIYTYPPLPPGAALPFVAVTATHTTDGGSTATLSGNTLSICPVSGGAINAFRAD
LVLADTAVVQTGWVSWACPAGQETNATACPLTAVGGCADRDQQFSGSFEPSFPSGSDTEVLLSLVAVEAVCDGGCYG
VDVRLAGQYHDTDQLLFRVVAGCSTSIRSFTVNWMAVLRSRPGDSSGVCTGSPRRSGIRRLGCYGNATTVPNAVLPL
TLPQNHPSMTIRRCAEAARLYGSDMFAIVNGTSCFGGSLSSVGGLPPPVPASQCSTGCSGSGSQKCGGPDSMSVFGI
YDRYNTIGSSPLTSNPCGSGRVPATVASAEDDVIIQNAMTVANVYDAWIGLRLTGATSGAPPMLWDTRWTWSSGATY
RDPVYNIGAPLTSALPGCTVKESTQYGAANSQFSTLTVASWAVCCTACAANYACYYWSFRPSDGSCQLKGDQGPSSW
SYDVNYVSGASPEVQATPDDRPSLALWHSSPERRYTSPGCVAYATPSGLSDHPPPPRSGRAMRGWVHDDCTSYHTAV
CERLMWIPEYLGQDVQFQGCWWHVAASDMVLPTVISSFSADMNIARCAKLASDARFPLFGLHRGGECWGGTGTAASV
TAGRAPAQCNIPCPLQDEGETQPCGGLTGLLLFSQSLGPYNMAQGRPAYASSVTRPGGSAGWGYAQGTALLTDGRPM
ANETLSINMCITTAKQARPWLGVLLPALSDVSAVRVLAPPADCYLTNAAEVNAADPSLGPGNCTSSGLADFEVRIGY
YDLLQTPFPDWASTVLCDTFTGVAAPGTWVTLNCTTPQLGRSVIVTRRAGFTQPLGLALCEIQAIGQPNLSFGAPPP
SPAPSNMGCFPISDAVRGCTSAGPGSDVGNPCTLAYDHNATTYWSRSASGPGQELRLDLKTAYSVCSIAVDWWCGAP
GNTPCNMTIATSLNSSFVKPYGHFSATSSRMTHTVWPSTDAMQVKIVIDANGGVLAGVYEVTLMLREAAGYYNIIPT
IPAGNRFVSSVKDSNPATYGDAKAVDGIWTASLPTGFCWSSAAVAAEPSWISFGMGGGAVFAGVQFLLPDDITPFSG
TFNLRTGWTGVSSTTYLTYNSITTTVSDTFYPGQVVDIPCSWAGSFGVLSLELQTTNTELQICELALIGRAQVYPPP
SPPPLPPGSGGGFGNYALNAPVFASSVVRKGFDSAFSPAPFQRILTDGRPMLNDTLTSGFCVTTAKMRQPWFGIALP
TLLDVSSVTLQAPPDCWFYDVWNTNSLATPAPSGSCSSVGLHDLEVRVAHSELDLGTPFADLFSTSVSCGSYTGAAA

PGALVTINCTTVVTGRTVIIVRLNGAAPAGLALCEVQVMGSPPAPGPPPPPSPAYNPMCAVSNYLINSCNSQGPGVT
GNCSAAYDGNGGTYWSRLSDQADQQLSLNLHTVLRVCQIQVDWSCSMAASCNMSVSAGLLTSSILVFNTTAASASPY
TVYMYPNADANHLIFRVDGLSAGAYTYIYEVRVFLAEAKGAYNFISLAYPPGSLLVASSQKNNDPIYTPANLVDNVW
NAGLSSGWCWASDSTSSDAPWLAFNLPAQHTLRGVQLLLPDYITAYNADITVRAGNSTVNTTAGLSANPTCYVDARS
GGSTWTSAEVVTLYCNSPSARVITLQANLPYWEWDLCDVALWGRQLLQGPGPSPGGGGSYAEQPFPTGYPSFPPSGM
HKRVASYEPDLAGAMSSPAMVQCPGAAPDLLTAVRIKKTSSGPWVQAVQLVSCGSNATVNSGLNDGGVTGWEVDDAC
ASGYDQLRGYERNSTSDPLWGVWGLSFHCSGSGVWTTPVGSGPGGYTGRLVTLQCPRDWRMSGLVLWGNTSQVGVVA
PFCTPASGVPYDPLATCPFIKGGTLLPGYVSPNPITADLGTSIAANTAVSTVISMCQATASCAAAVLSPTGAGASGD
NYNAVLTNVPLSDLQSPGYLDGLTGCTGTVMLNPKGRPSWYCMAGFTPPPRYVTAYSPITVTGAEECAWQAAAYVVD
AFLTSASGTQCTMLSYSDTVAASDTGGVAVNSSANAGAFCWRQSAPWNCTDVGNVPGNGYDSFQAATPEVCKWACDK
DFSCTHFFYNATDFGCYLRTLAPSATSGTSQYASGPGQQRACMKTPYHSDYSYGNPPASYGSMCWVGVHLGNSVPSW
SSPYGNALSATECAKFCKQYGCGHWRLHNDGKCDVYSPNTYLGLYGTSDMYGPDVDTAMSCLATVRAEYECLPAGFT
VQYSLASSVTTNVSRDACADACSGSSSTCTGFTMIDTGSGFTCDLHARSFVGAYGVNTYDPSAGAVSVCLRTFAARA
LRLANATAGGGYSPSPSYSPGFSPTPTPSGAAASVLMVSLPRGYPGTGWATGVASPSYANMDCITGYATSVTLGGLS
SPYLSYMSLDSCSGGGSRTGASYVGNITVMSVGDQASFSCPNGFSAIAAIQSDPPSLAALSPFEFRLWCTDGSGGQW
TAPGIGSGPGATNFKDTVVTCPIGAVISGFKVHYTSSVVARVVIECKPRAGGSTWIDPRGQCPWPAGYDFLPTYFDS
TVATLFTYTNANATGGITGIRAAVPDCESQAACVAIAHKVLNEDAADVESRLQADTGAAAGLDMWQGAAVGQPVMPH
CDGSFWKTPRFPRQYFCVHRMQLSGTVLTTSNSASLPHSCSQLCDATSSCRGWYLDEAFTTCTLFDTPFVSNAAYSV
VTNTAGLGMSCWLANDPWYCLPPGLATAGNADSMAGLFAIPEKRASPDECRADCDANPACSHFVFSGAYNGQFNCYL
RGAAFTGPTSEFANGPSPDAHRTCFKTLMHRQLSASYINNYALTHAQLCFPGIRFGPTGAWRLLGDVWDSASCSAYC
HSQPGCAWWEIKIDGSCMLHDDWGWFQNGAFGPDSSVVMSCLSAVTGTFVCLPSGATFSFNLVTSLTNVFSTTDCAN
SCKSSSTCSAYHYDSVNNVCELGDQPFTGATGVSAPTGGVAGDGFCLRTLNYGALFSAGPPAIPQSPPPPLSPAAAS
AGFQMAPIPLRYTPSSYLAYLAGGMSVSCVSPAAINTITLDDAGDIFLTHLNAINCTDDTAPSGGGFGSPGGSASTF
SCPDGFDAVRGEEAYKPPGGSPAIAHPLVQVSFRCSTTGVWTSPGRGSGSGLAGGNRTLTVITCPATTAITSIQLSG
DSTYIGVVSIVCGAAAAPPSGTDDCPAVNGFYSLPGYTAAGATNVGSVNTGANAELAFQDCVTSGSCNAVAWGFSGG
SAFGQGLSADLAALQGVLPGDGGGYCDGTFAEAPPLPPQYYCARGSVLTSGSSGLPVATASSVAECAYECSSFGCTG
FVFDFAEATPCNLFSSVFTVADTFTVSTSTDIIGCWPDAAAPWYCLPSGLQFTASSPIAPVYRRATPEQCRYDCDSY
PSCFYYRYDPVTFECGLYAEILYPGAGVVAGTNPVRTCFKTPQHPDLSASGLGRAQGSLCFPGVDLGGVKSNTLPGY
FNPTSCAVQCAMYGCGHWSLRTDSTCELFAPYTYLGMGAGAAIPYGVDNTTVMSCLARVTGSFECLAPNRTIAYESA
GAVTAATLADCGASCYSSGSCIGFTYTTAATSNCFLMADKLFFGTSGSNGLASGTDDVTTCLRTTMVRQLFGDPPPV
ASPPPPEFPPSTPMPPTGGGMTYPPPPGISAFTVEMAAIPIRYTPVGAWTTTHSYYCNDDATMNGVTVDDAGGDYLT
HLAVTSCTNALGPYLGGAGAFGSASGSATAFSCTDGFDAVRAEEAYTPPGGSPATPHPPVYVALRCSTTGLWTSPPY
GSGSGLSNNRTLTTVTCPATTAIQTLDFASNGGYITILRITCASATAPTASDTMDACPAVSGFSLLPGYTDPAATNT
STYTTFKMDAFSDCNVDSTCTAVAWGQNGGGLFSETQIADLVSLQGVRAGDGGGSCLGTFVEVPQAPPQYYCAPGIF
VGVVPGAMASMPAATAAHCAYECEAAGNCKGFVVDVSAGTGCDLWGMEPLPSEYPFTVSSSTTVIGCWPQANAPYFC
LPDGVSFSGVSVSTPINRATPEQCRFDCDQAPGCYAYSFDPVTFSCALFADLLAVATSVIATANPVRTCFKTPQHPD
LSASGLGRAQGSLCFPGVDLGGLKTDTVTSIATPTACAKLCADYGCGHWRLRTDGACELFAPYTYLGMGAGAGIPYG
VDNTTAMSCLARVTGSFECLAPNRTIAYESAGAVTAATLADCGASCYSSGSCIGFTYTTAATSNCFLMADKLFFGTS
GSNGLASGTDDVTTCLRTTMVRQLFGDPPPASPPPPSPDPPSPEPPSPAPTAPPPSPEPPSPEPPTPAPPSPTPPSP
VPPSPAPPSPAPDAPPPSPEPPSPEPPSPEPPSPAPMAPSPPSPDPPSPAPPSPDPPSPAPPSPAPPSPLPPAPFPP
SPAPPSPEPPSPEPPSPEPPSPAPLAPSPPSPDPPSPAPPSPAPPSPFPPAPFPPSPAPPSPEPPSPEPPSPEPPSP
APLAPSPPSPDPPSPAPPSPAPPSPLPPAPFPPSPNPPSPAPPSPEPPNPAPPSPAPLAPSPPSPDPPSPAPPSPEP
PSPAPPAPLPPSPAPPSPDPPSPAPPSPAPPSPAPNAPSPPSPEPPSPDPPSPDPPSPPPPSPPAPSPPPPSPEPPS
PEPPAPSPPSPNPPSPAPPSPPPSPLSPPAPTPPFPEPPRPTSPSPAPPSPPPPSPGPPPAPFPPPMPPSPPPSPPA
PPPVPPPSPPSPPPNPLPPSPGPPAPAPPSPAPPAPPAPLPPSPQPPSPVPPSPPPPMPPPSPAPPFPSPAPPPPLV
FLDSLSPSPPPPIILVAELSLEASRPPSPMPVPDTEPPALSLMGSDSLELEVFVDAYVERGATALDTYNGQTYNVRV
VVESSNVNMSAASPPGAPYQVIYAATDAANNTARAVRRVVVVDSCADAGEFRCAATKACSVFKSCTAGLGGAGSNST
NSNTTSVQKVLERPPDTIAPVITVLGDGIRYFTASGVQGMISVAYVTAGGAFTDPGASATDEVPSAGGAPSTTMSVP
VYNYIMDPTGEDVSVIPLDSPTGNDTESAVPYLITYTARDFYMNEAALVRRRVYVLCRPPEFACPAYDPEDPLTCSS
SRICGVAMGGSDGAGVMFSTDSTLSSNGGDATNTTSASGTTTTTTSSSSSGTNETLTRLVPRFKLNMDPVISISQGA
MYGPCTGGMAGTCEPGATASLVTTGDLSGQLRACATGVRNPQPYDAVGLQYCGVDTRVPGNYTISFHLTWPSVGELI
VYRTLVVEEQCVGERTCSDGRCSVDGTCANEMTVTAASSSNNNSSSNIGSSGSSSNSNSPSLSSSTSTATINAAPKL
TLNMSDIVGMRVNVKRGTAYQRCTPGQAPTADRPCETLGSASDTEDGDLTSAIALCPPSDCGKVQCRSHWADRKAPS
ECGIDTVSAPIGTSYRLVIAVFDSRGANATAERTIAVVSPCNATQDYCEEAVPGSAAGATRWVCSDVSCAERSRLAT
LAAAAGNGSASGVLSPRLFLLPGYQTTNLSLAEADQAVFLAYRTPAPFSLAPCAAFADGAPPAPPTCAAIANDTLNG
DLTAAITTVVVPRCAANTSVEEGTCTGCSVAALTTGTCLPGRYSITYTVAATDGNGGSASASRRLDVAVEQLTTTII
ELNLFPNRTAGGSTNVTVAEAFAASLLSNSTARAALLAPVMQVFGITPSTIRNLFYITPPTVVPVYPSGATAANGTA

GNNTAAVNGTAAGNTTAPGNATAAGNTTAALALTNTTVTPSYYVVRMVLNITTGSADAAGMSAFSGMVQAGGLGRRR
RALSLPSSLPDLPRAEGVSAAQTASSQRAKLRLKPVGPEQDDVIYLADASANSTNSASDSSSGATSQSATGEPLGAP
EPVSVREMVLHSILTGLLGGVRPDQLVHAYEARGSSSGGGSRRRSALSVAALRSSSTGGGASSGGLPLVMSALGDVR
AQLDMAAGLVHLDVVEQRQPAAAAAAGDRRRALAEVPQLRFALGDDQDQWDESELEEEEDMDAPDEGEGAVPNLRRF
LSIATRTTSGNSGGSGHVDGYGSLSAVRGVVRRVLQTSSCGTAATAPNASSTSAASVGLASVGGVSSACASPAVDAT
GVALGVLAGAVSDLDRLGKQMADQQSNMVALLGTLDDKFSAKDEVYRTSIALLASEADVAFNTTAAKAQRLLELVEQ
TLAQQNANADALVATLNLLQSTLNEAEAATSRAVVTAAAILEGYGANGPAAWLSTEDDLSTYSACLYSRGASRAVAF
RTSWAQAATGNATAYYSAAASTLASATAAAAAKAAGAAAAAAAAEAGARRRRAMEAGAAGAGSVADAGGCVWPAWLQL
LPASLQRGIITFAQTLGPWVSEALGVQHCAAAGCLAGAEYGVPVTGTAAQGTSVAAAAHATGRNPPSPPAAEALAGP
DGSGTSTFDLLLPKWNRRYSPTASHLPDPGEGAPAPAATATERLLLTSLGGSSSDSGDGSGSGDGSSTGADDAYYAD
FMGYSLPTGDGYDYSLWDVRNSDRARLLGLNNRVLVGLLLHQVRRSEAEVRASSGVDGVICKSTSFDSLVVGCDADS
TNDDRYPASGTLGGIGNDPVFNRHSTLYEAAASISDYYNTSTNSTDLLPTGLPYGFFHEPLSGLPAGYPLLLDTQLS
AQRAEQAITFIRDGSYLSATLTKSMRAQLVSYNPDAQVFGYWRLDLTWLDSGVIQASAKLLGLPAISYGQSIKNLQV
SQFLPDFFLVLLVIGYFVMTAIDIYRQLQSQKRRRRLLWRRAANQLSRAVAAAGFRRHTAVAAAAAQAGKGGVGGNA
FAPDRAQGGDGGSDSDSEGEDDDGDGDPRGIKAALGIKKYRPKMSVKWICYEALICALMAASIAVFYTYAVRLSVRD
DFTARFDVYDALTFARARYFLLARDPAAAEAAAAAAAAAVAAGLASNTTAGVTMSTGGTATSATTSGSSGSNATGAEA
GSAGRWKLPADNGPLSDAGAMYARVDDMYNTFVLYGFLQGLVLVFIIVRWLHYISFQPRLSIIPGTLALALPDLLHF
AVVVLTCVVMFASAAALTYGPGVSQLSSPGSAVYLMLRYVLLRNDDFVFRDIVRSTSERTAAEDALAWLLYLIGPLF
FVFLLSNFIMAFLAWPFGLLKFAVEGQPGVPKDLSRILGWYWQRLRHRAPKNKHILQWTEEWLAGDPRGIMYTISKS
VANLRARLGGKSHIGGPPGAASGPSTGFGAAARAFRSVTAGHGWPGKGGVGFGSPGATASGVPPAAAAASRSAISVS
GGLGAAGGARPAVIRVARGAPPLDGTALAAALHAVSRAHITAVGVGSERDMPPPPPHSCD

>Cre17.g732500.t1.3|PACid:27571497
MLLLGLLVMLPMGFGQPLSCMSNVYDCKSNTTAFGAGPATVTNTMGGTTTAACASNLTGPPILGTCSNGPPKAPGDA
STLCFSKSNSNATIQISFQTPLYGYSLGVYIVDCQAGFISGDVWVQTVGGATVKVPCNPDLSSGDSAFWWTCTLPTP
YLYIAGMTFMVTGTGEKVMDAVRLQGYPAFPESPPSPPSPAPPNPPPSPVPPSPQPPSPAPPSPAPPSPPPSPAPPS
PQPPSPNPPSPAPPSPPPSPAPPSPQPPSPAPPSPAPPSPPPSPAPPSPAPPSPVPPSPAPPNPPPSPAPPSPQPPS
PPPSPVPPSPAPPSPPRSPAPPIPGPPSPEPPSPAPPSPPPSPTPPTPEPPSPAPPSPAAPSPQPPSPRPPSPAPPS
PPPSPAPPSPAPPSPEPPSPAPPSPPPSPMPPSPAPPSPVPPSPAPPSPPPSPTPPSPGPPSPAPPSPPPPSPTPPS
PTPPSPTPPSPTPPSPPPSPAPPSPSPPSPSPPSPAPPSPPPSPAPPSPSPPSPSPPSPAPPSPPPSPEPPSPAPSP
PLPSPAPPSPTPPPPPRPPPPSPEPSTPPPPPSPRRSPPLEPSSPAPPAPAPAAPSPPRSPPPSPPGTPAAPSPAPP
SPDPSMPPMPPSAPPPTSPPGPQPPLPIFTPFTLPSEYVNATDPVGILTVSRQVLINGSELTNSSALNYFFPQATSV
QLVAVVLGIPLVLAFGELASGGAASQQITCDSPFVVQLASAFPSLAGLPPGAAVDSSSCTQLVQAAALPGSSRELLL
PSTSTITTGAAHRRQRALQAASSGSSSSTTGSSTTGSSSSSSCGSSSTASSSVSLAVSLSVPVGSSSGSSGGSSSSS
SSRAAAYDSVQAWQSGNTPSVIPSGVSVCGVSAVKQISVATKVRATYEVPLSSLGVEELASTCSGSGTAGSSSAASL
GLSGTECQVSSSTTVRSSEDGMPTPTAAAASSTSGSSSSGVPTVLIAAVAAGGVAAAVSLGIAGVVVRRRRRLRVAR
IEAERARQAEEMHRDNSFISLEGNISVAPAPLTRALRPSRSRRSFGGGVSSGGGAPGSAAGSVGAAGLHPPQQLRAG
ASRAGALRRLSTADRVYHSYDGAVVADGSGPASGYPSRRFHCVSLGQQDAGAASEGEYLPRSTGGAAGMLGGARSQR
VVRFGDGVEPPVDFATAAAAGLASGGGGATRISLMSRLREGGASSGGASRRASAAFGDGISRSGEARNGAGGGSPSR
SQSRRHSHGGGLSSTHSVRFRFDDDGGDEESGAGGGGYASGTVATLGRTSVATTIGADRTSAGSHAWLTSDERGDGG
GDYSDYAAESAAAATGGGGGGDGLARNSAPQARAARLHHYMNIIDGGHGASSAGHSGPIARRHSGAAAAGSGGGGIA
TNSLRRSHNFDTLALALASSSGMRQNSRGSRLLSYRNVGGAAAAAVANANARARRCSVAAANASEDEQLADEDVAAP
WVDVQQATVLTEAQLARVLAWQQAAEAAVLADGPPSPPSPVGSCASSECAPVWPVRQQLLQRLQQQQQQLHSQRGE
PSGAAPPAPLSASAAAAAFAAPPATGRGDLSFLSPPSIQVLPPPPAAYLQPRAPASAGSGGASPALRTAATAREAAA
AAAAAAAAAEPSVPPSRPYELSNLVPMKRVHTATDVPAIDRDAEAGEAGSSGLAGMEAAYETAGAGVAEDDSGEELL
TLQLQRPAPTAAATTPFASMAAAVAEPQLAQSPSLLRRLTAAAVPLVIPASVSGRLRVSVTPGSDSPAGGGSAAAAA
AAGCSDLAGVHSSMVAPQAHSPSGGTQHTFRVSSNGNCGAGGSGGSGAPLTDVLSRRPWMSPRVRPFTDNGVPPPPN
GPARAATGVGSISDTNAALTVSAVAAAAAASAERGATTVSGDGTAGDVAGGGGGGSHRRPLLHSEPNGTPRTSQDFN
GLTRMGSHGSTASTTTGSVRRTGSRTAWDRSHVGGAAASSGGGGAVIVASASAPVTGVVGASGDIGCGDAAATGRGN
VVGVAVRSLAAVSPARSAILNASGKSPSQPLLHLGPTTAVGAGVAGSVGAAAAAGSKGSSCSAGGAASSSPRRLHSR
PPTPTLPAYLPSSSQPYAQQHQQQRPRMRAASMPDADAGMLGSRLAREAPSEAVMDMKMYDVDNDEDAFYHGRSAAT
VAAADGESVPGEVYSVPHVALLPQPWRFSRGSNRTAGSMGSNGGITIGVVRGVNRGTDASDAGPVVGRLGQVPPPRA
SLPATTAPPMQLNQSVFSAAAAAAAAVSGVGGAATGAVAPPGGQRATTPGGGSSGGAAEAAQQLLALDWAVEDFEMG
KEETQGGRAVTDAPVGIPRRSMQRQTQPQAQVQARRGGSPSSRLASSSSAVDAQLARGVL

>g17844.t2|PACid:27571620

MCGPAAPSTATTETEVFTLYLQPMFADPSSTCSTTITSSWTGLVCNGTAWYGVGCTGDGSAVTNSTGNVNSIALPNC
FPGGVGVAASLPSEVSVLTALTRLDLTGNKLSPNSGLPADYSALTGLRLVGLASMDLEAGPVPSSGFPQEWSSLVDL
QQLDLSNNRLLGGTIPASWDILASVTALNIANTAACGDIPPTLSNSTTPSTLDSYCELVNCRRYLAETLQPMIDSQV
QPGSACNLTSAWPSSGRIDSWAGITATATTVTTGTPINDVMSFVLNDCYANNSNSLNVVLPSELTCFSSLTELQFMR
NNAAGSLPDVWTQLTQLQALWLADNILSGSLPASYSALTGLTILKVHNNLFTSSLPPEWQAMSLLEEITISDNYDLN
GTVPSAWGNLTSLTSFWAWGVGAGGQLPPSFSALSLLSELTFVSDTLSGSLPSEWSTLTALTLLNIASDRATSTMTG
TIPDVWFGFGGAPAMFALQDITISNTGVSGGLPTALWLVTSLTKVDFSFNRLYGGASPTGFPMDYSMLTGLVSLNLA
DNLFLAVHAGGNAWAHVRGCDWAVTPGSALTYLNLQNTGVCEPFPAGLSSALTPGTSVNSYCDDLSSAYNLFTNFKP
IFDSTTPGVGCSLASWDLGANPLCSNTWSGARCQVSPADSSGVSLGSLTSLELLNCNAGGSPVHFLPRSLTDLGGSL
SRLAISNSAVTGTLPSEMVYLGQLTDLDLSGNSFTGTLPGDWSSFVNLYYLKLNNNPQINGSIPDSWGFGLYSLIQL
NLGSMGLLTGPLPDSLAYLYSLEDLTIQSNSLSGTLPASWSSIYRLSSLVLGAGGNAGPGPSINGAIPQEWFLGAPL
PYLWNLRISNTKVTGPLPDPGSAFTAGQLGLTSLDLSSNLLSGTLPDSYQGFSILQRIILSDNTQVSGSIPTSYSAL
ASVGLVSLDVTATSVCGSIPSSINGAVTPNPLPTCPSPPPAPPTPPEPPSPPPSPDPPSPEPPSPAPPSPEPPSPVP
PSPPPSPEPPSPAPPSPEPPSPAPSSPPPSPEPPSPAPPSPEPPSPAPSSPPPSPEPPSPAPPSPEPPSPAPPSPPP
SPEPPSPVPPSPEPPSPAPPSPPPSPEPPSPAPPSPEPPSPAPSSPPPSPEPPSPAPPSPEPPSPAPSSPPPSPEPP
SPAPPSPEPPSPAPPSPPPSPEPPSPAPPSPEPPSPAPPSPPPSPEPPSPAPPSPEPPSPAPSSPPPSPEPPSPAPP
SPEPPSPAPPSPPPSPEPPSPAPPSPEPPSPAPPSPPPSPEPPSPAPPSPEPPSPAPSSPPPSPEPPSPAPPSPEPP
SPAPSSPPPSPEPPSPVPPSPEPPSPAPSSPPPSPEPPSPAPPSPEPPSPAPPSPPPSPEPPSPAPPSPEPPSPAPS
SPPPSPEPPSPAPPSPEPPSPAPPSPPPSPEPPSPAPPSPEPPSPAPPSPPPSPEPPSPVPPSPEPPSPAPPSPRPS
PEPPSPVPPSPEPPSPAPPSPPPSPEPPSPAPPSPEPPSPAPSSPPPSPEPPSPAPPSPEPPSPAPPSPPPSPEPPS
PAPPSPEPPSPAPSSPPPSPEPPSPAPPSPEPPSPAPSSPPPSPEPPSPAPPSPEPPSPAPSSPPPSPEPPSPAPPS
PEPPSPAPSSPPPSPEPPSPVPPSPEPPSPAPSSPPPSPEPPSPAPPSPEPPSPAPSSPPPSPEPPSPAPPSPEPPS
PAPPSPPPSPEPPSPAPPSPEPPSPAPSSPPPSPEPPSPAPPSPEPRSPAPSSPPPSPEPPSPAPPSPEPPSPAPSS
PPPSPEPPSPAPPSPEPPSPAPSSPPPSPEPPSPAPPSPEPPSPAPSSPPPSPEPPSPAPPSPEPPSPAPSSPPPSP
EPPSPAPPSPEPPSPAPPSPPPSPEPPSPAPPSPEPPSPAPPSPPPSPEPPSPAPPSPEPPSPAPSSPPPSPEPPSP
APPSPEPPSPAPSSPPPSPEPPSPAPPSPEPPSPAPSSPPPSPEPPSPAPPSPEPPSPAPPSPPPSPEPPSPAPPSP
EPPSPAPSSPPPSPEPPSPAPPSPDPPSPAPSSPPPSPEPPSPAPPSPEPPSPAPSSPPPSPEPPSPAPPSPEPPSP
APSSPPPSPEPPSPAPPSPEPPSPAPSSPPPSPEPPSPAPPSPEPPSPAPSSPPPSPEPPSPAPPSPEPPSPAPPRP
PPSPEPPSPAPPSPEPPSPAPSSPPPSPEPPSPAPPSPEPPSPAPPSPPPSPEPPSPAPPSPEPPSPAPSSPPPSPE
PPSPAPPSPEPPSPAPSSPPPSPEPPSPAPPSPEPPSPAPSSPPPSPEPPSPAPPSPEPPSPAPSSPPPSPEPPSPA
PPSPEPPSPAPSSPPPSPEPPSPAPPSPEPPSPAPSSPPPSPEPPSPAPPSPEPPSPAPSSPPPSPEPPSPAPPSPE
PPSPAPSSPPPSPEPPSPAPPSPEPPSPAPSSPPPSPEPPSPAPPSPEPPSPAPSSPPPSPEPPSPAPPSPEPPSPA
PSSPPPSPEPPSPAPPSSEPPSPAPSSPPPSPEPPSPAPPSSEPPSPAPSSPPPSPEPPSPAPPSPEPPSPAPSIPP
PSPEPPSPVPPSPEPPSPAPPSHAAPLLNPPSPAPPSPEPPSPVPLLPLPNPPSPEPPAPPSSPPTPNPSLPSPNPA
PPDPATPAPRSPEPPIPVPPSPPSPLPPSPSPFAAAGSDVFIDVNFQGVNLPDVNQTKFAQQSCTELQTSTAAFNCV
NNKLVSGSIIGGYVLTAPPGTPLDVFYTVVQTAVTTDPASLFSGSFLSAYGISSVSGTVLPGAAVPPSPPTPSPPPL
PPSPPPPPPPSPPPPPPPPPPSLPPPDLSPSPPPPSPAPPPLTPPSPPPPSPVPPSPVPLPPPPSPQPPVPPPLVPP
TSPSPRPPSPSPPPPPAPFPPPSPPPPPGQFVSGYEVTYTPTFCAGLASDTGFNVFDPSALDQFGRITAITFVHDAA
IPGVVVAVKLVYGSGSATHTDVLAGSVYDPSSPTLSLSTVRLVSSGGSALQTLSVCCTATGSVAQLAVGFSDGTAAS
TGLCPAGARRRVLLQARVGAGGGSAGVPSGAVVGGVRGGRGAGVNSIGFALAVKKPYPPPRTVPFPPSPAPPSPEPS
PPPGRRRPPPPNAPSPPPPPPPSPPSPPQPVAPGAPARPPLPPRPPSPPPPPPFLPSVPPLLPPPVGTPAAPPSPPP
PPAPTTQSVQVAFQVSAKVDYTRIVANVPPQVPNTVPASTLVLLGPAVPIVRNSLADPLIASTLYGRGRVAAFGGEK
MITGCCKPRGKPTPSDPSLDTIIVNAADWAAWYGKKVGKAIIRVADTRYMPMAKYVVKKRPDSFDTLKSAKIKSYYL
SLKAFLKDGHKACDLYVIGSYDIQYLEPRVSHFLQEFVNYGKGLIVVGPDVMPSIFYPGSLAAPSLAPSTAGRRSML
LQRAAVLLGDAFEPDVTDAGDNLSDPDAGVDLRHQLHREMAAALVRRRELQQSGSSSTGSSAPGNPLFNSTSIPVNQ
VSGPMGLVFSGYVSDPGGNLTVTSPSELQNAELAASQYVEYLQGRMQLTIPELSLAVSTITKARASVPRGTPGASRF
WDLSDEATVLSGKAPALPPLFGAPPPPPQLRPPPPLPPPRPPPPSFLPPLTAYVGCFFEAASRRALNTTLLTGTGVS
VDSCLGVATNVSNPNMVGTTFLGLQRTACFGGRELSASFVAAQIAEAACPVSCPAASNQKCGGNANATGIPVSVYRI
LSAAPPPPPAALSPPPSVTGAIPPVPQPRQQPPSPAPRPPAVSQQPPPSPAGAKKRKRVLMRAY

>g17813.t1|PACid:27571782
MSRRLHARPLLAPCVLLLAVLLTLGWGLDGASAQTYCDGSGNWNGTGPALYNCGLGPTVQYALNTSLDAKSAAPLKG
DASGPPDWSAGLGCLMSGASTNALQTTTLYSQTQGNPSTPSATVLAMFAAPMYPLSVGILIMTCQQGYVQPNLELRL
PNGSYAATTCPADPCTSATSCLGNQTYWYTCSCSGTAISQMPVTGLRVVLSTNAVGQNVWKVIDAFRMTGYVGYSPP
APPSPAPPSPSPPSPAPPRPPPSPAPPSPAPPSPRPPSPRPPSPSPPSPAPPRPTPPSPVPPSPAPPSPPPPSPHPS
PAPPSPAPPSPPPSPAPPSPAPPRPPPSPAPPSPAPPSPHPSPAPPSPAPPSPPPSPAPPSPPPPSPHPSPAPPSPA
PPSPPPSPAPPSPAPPRPPPNPSPPSPAPPSPPPSHAPPSPGPPNPPPSPAPPSPAPPSPPPNPEPPSPPPPSPPPS

PAPPSPAPPSPPPSPAPPSPAPPSPPPSPAPPSPAPLSPPPNPEPPSPAPPSPPPSPEPPSPAPPSPAPSPEPPSPV
PPSPAPPSPPPPSPPPSPAPPSPAPPRPTPPSPVPPSPSPPSPAPPSPPPSPAPPSPVPPSPPPNPAPPSPAPPSPP
PSPEPPLPVPPSPQPPSPAPPSPAPYPEHTSPVPPSPQPPSPAPPSPAPSPKPLSLAPPSPPPSPTPPAPAPPSPPP
SPAPPSPAPPSPPPSPAPPSPAPLSPPPNPAPPSPPPPSPPPSPAPPSPAPPSPAPSAEPPSPAPSSPQPPGPAPPS
PAPYPEPPSPVPPSPQPPSPAPPSPAPSAEPPSPAPSSPQPPSPAPPSPAPYAEPPSPAPPSPQPPSPAPPSPAPYP
EPPSPVPPSPQPPSPAPSSPAPSAEPPSPVSPSPEPPSPGPPSPAPSPEPPSPAPPSPEPPSPAPPSPSPPLPAPAA
SQPPNPAPAAPLLPSPEPPSPAPPSPAPSFLAPAVPQPPSPALGVPAPPSQASAAPVPPTPGPSSAELPSPAPPSPD
LPSQAPAAPEPPPSPAPLSPEPPSPAPPSPGPSSQAPPSPAPALLQPPSPTPAVPALPSQAPAAPVPPSPEPPSPAP
PSPGPSSQAPSSLAPAVLQPPSPAPAVPVPPSSEPPSPASPSPGLPSPAQPSPAPAAPQPPSPAPAVSVPPSSEPPS
PAPPSPGLPSPAPPSPAPATPQPPSSVPGVPAPPSLAPAAPVPPSPEPRSPEPPSPQPPSPGPPSPSPPLPAPAVPR
PPSPAPPIPAPPSPVPPSSGPPSPPPPSLAHAAPQPPSPASGVPAPHSPAPAAPVPPSSEPPSPAQPSPAPPSPAPS
QLNQLPSPAVSTPLAAVPLPPMPQSPLRPLTPSTPPPPPSPPSPNPAPPPLSLLPVPPPPPLLLAAPRPTYALPVVQ
VPPQYIASDPSTTNGLLTVSQGVAIPDSYLSAVAFGGSSSSSGGSSTVSVRDLLALFPDASDVRIVARTMSVPVVVT
ATAAVDAITTASFVSSRTGQVVDVVVGCNSGFLEAFRSELVARADISDEVLANVTCSSSGSSSGSSSGSSSGSGGSD
SSNGRSSISGGSRGSAAARRALLAGMCPAAGSSSAGGSSTTSSMSLVLQLRVPVDAPSAAGWLPSPTPFMSATAAPN
TTADGASASPSSSPSSNSPADVASPLVGTNSTDALVAEYRAQVLKALSDWEAESAAAAAAAAAAAAAAAAAGSNNAT
NAPVPPPASFPLLVCGPSSEDDVLLETQVNVMRALPLNAAGTAALAAVCASSTPAASSTTDPGGLGLSPTTALGGEG
SHVACRVTTQSALSSYGAPPPLFVDAHVAANRNGALLPLIITGAAVGTVVCVLCAAAAALLAVRRRKRQRQDAEDED
AASAANPQQNEQRRRQQQPSADGGEQQAGQPPFRVRGAAATDAAVGDAAGGLDPGGVLCILPPAGFSQHVAPGRAAA
TAAAARAATARGGRVSAWSSTGAVDSGGPRVAAAQAADRIHDAVPKRASSPVVGFAEATAAAPLGAAGGGAAYDATR
AASASILPAGGAAWALALPADGRQRQPQQQQQQLRVSSPLSSPRSPASWPDELPLSPTVAVAAAQPPATAASALLSA
AGPSAVASSHSRGTSSNRSGMMLQHNLMFEDDARDRALLAFATAAAREAAVARGVGASAHASQTSSFKSAAPHHGGR
ATPAAAEGGGGAGSPGSRTGSPLTRAAIVSNPQRQGQRVLQPSVAAVGVSSSQGVVALRVAHGGPHATADEPEGPPP
WLARWHRQQGALAAAVMAGGAGNSGPLARVTWDGGPPHSGGASSPLVVSSPRARRGVAAVATAHSSSAANGMLQLAS
QAGLGQAGVKDGSSSAAATASGTAAAVLRSGSSSSAGGSSKGSRNAAGHASSTGGGSRFLASNLSARIMRVFRGPSA
APASTDAAADVGAGIGVPAQPPAARASPFPVAWGGAADGASGIMIPALRSAPAAVDMAMQPAAATQPSAWAMGSSGF
GAQGRRAQSRRQLLDRPRYPTLDPQAPAAASASAGAVASGQAAAVAHSRVQLQLPPHRPAAWGVLASTSEATAAAGG
LEGGDGTRSLAAGGVPRAAAAAGEVRPPGSADVVSPRVGPRRPPRVRRASTTLGLMAPPAPPAAVAATAVAAAFTNG
GGARGTGASYGAGAAATVSLRP

>g5228.t2|PACid:27572559
MAASREVGAWRAFLAFGLLLVAIKSNAQTWFDDRDAAVICKTLGLPTSRAKAVFKAWNVYTRGGPWENGKPASAEEI
VAYLNSVDPPIAMTNLRCRGDEASITACERDVTNLGTCTRAQSVGVQCFENDYAVRLVNSGSNPYLGNSLRGGWVQV
WMGSGWSYVSAASFDVRDAYVVCKQLKMPFGGARVFRGVVEGTALFGPANLTSSTRTLLPGLQCTGSEASLGECPID
PAAVDAANDPTAPPQGGASMLCEDAEFDVRLRGGTHVSEGRVEVYSKYGVWQTVCWSTFDASALDVVCRQLGFQTTP
LNPPSYLNFSSSGIPDARPAAGISSIMNCPPGANAISQCGNLTSYAAINSYTSFPLGCTAALDVVIKCYQRNPLYTQ
VEYRSICTDPWSTQAGLSPEAGVTAMLVGEFDDQSQGERSQDVLLLKYGSDGKLIGHAAFGRPWASTFSAEADIDFG
STPKPEVLLLADMTGDGRDDLVVMTSSQVQLSCRLSAFMPIVAVASGARTFTQLSPWLDLAGSPFYIDTSNTTTRAY
IVTDVTDDRAADLVFFDQPSLRLGLMPSNRVNKLLDDTDGDGTTWFELSTVSGRCASLGEDCFLLTTDYNRDGLNDV
AVVYLDRVTDTEEFYLTMVATSPPAPLTWHLSAAAIFPRGACTSPMAVALGQFVGADGSMDGTISGTVNPQLVCLSS
YDQRIYIGGLGVWGSLPGPVTSLHVRDVDVDGRDDLLIFTEVGSFYLISTGAAFEPALSTAAFLSPASVSLAVPNTP
SLGQTDQTASTAVASADVVAAAPVEHRCGPPRRLVAYFTNRRPDKSPACVASVADVVATPLQRATAATHLIFAHVRP
NAETLGIDLINDRDGPVLANLDGNLLAVNPDIKVLVSVGGPGGNDAEFTRLVLNQQSVTGFANATLAFLNTYNLDGL
ELSWPSLQAEQVFGFTALVELLSSSLRPAGKLLSLAVPPREVYLSLAWGRLGSLVDMINFQGFDLEGDEVLGAAPYV
ETPLFDCLEAEGLSVNTLIDLILAAGAPPQLVNVVASSMGRSFVLDGDGYVGGPGSPGPCMGLEGLLDQSEIKLLLP
PGAAKLDPEALANTGPYASNQFAHWEDGYTIVNKFCFARAHCLGGVGVWDVDGDSYGELLAAVTRTMQGDPAVCEAY
SPPECTNAVSTRGSEDLGSPELVATLGDAEYLLYQVRKTWTDARDHCQQIGGDLVSVTSRGEAGVVYSLISSWASSG
QLGQDDIYSGRDVYLWLGGTDAAQEGRFVWAATGAELTYTAWAGGQPDSRYGSEDCLAASVRLGGSGGAGLREVLSP
EALWNDLGCSAVLPFVCQRNRDLARSFLQGAKEVPWLTATYHVMAPVPGSGDPGLMLTQPEANKLCRTMGGELPTLT
DPWVREDLTSQYHRDLPSHTWLGLRSYGDGQLFWNDGTFTTDGMLNAWEPGEFGDAACGLIIGPNGANVTVSVGALY
SFWNATAFQGAIRVASPPPPPPSPPSPAPPGPTPPRPPPRPANVTPPAPPPSIPSAPFPPDFDTVLIYNLSTYFTDE
PELVTVFLPQGVYSLSCNERMPTTCQTGAPTVSLNPNFYCLSRANGRSYVVPGKELTGMPLYLTTERSCASACMLNI
RCVYYTWLPRFRASFLPSDEMLGRPNQLPGQGSCYLMGRPWASNADRLPKLLSEITDNDRVCFRSGAVFGGDSIPVN
DTSLIQPPASIGRLHGNPTPTTPTPAAPPPSAPFSLLCGGDGSAAPLLSSLTFLVDNATRGIQDVGTTCAGTYTAGV
IGYLSAEGYEMRLRQLQRPIIQSYTARCGPGGVTGLLGSYDNRGMCQVSLMCTGGGVEPVLPPGASRNCSIGASAFD
FECPRGHYAVGLQGVFLNPATYTAADNILATLRMVCAAVPVAQVAPPPPSPPNPPPPPPPAQRRRRAALESSAAWRR
SAAAAMASAGRSSKDGQSAASGSFFEEALSNQQGRRLQQILPPSPSPPSPKPPSPSPPLPPKPPLPPSPPKPPSPSP

PSPSPPSPKPPSPKPPSPSPPSPKPPNPPLPPSPGPKPPSPPLPPSPPAPPPAPPRPPRPPPAPKPPRPPAPRPPSP
QPTPPIMSPPPPPSPPPAPRPPPAPTPTAPGVPNPPTPPPRPPSPPSPPPKPPTPSPPPAPPPRPPSPPPPPPSPPP
PPSPSPPPPPPPSNATSDVIPCRTCSPIFGETAGSNSPFANTSRPGALRCPQGHLLTQLFQPVVINQLLAQQVCCTN
FSQPITGLGGVCGPLAPSAPGADDTMRAGLPATPSQPLSATTPFLTTVVNGSTCSGGIAQVTGLFLSFRPPSPGVGT
PSYIMGLTARCRNVATPLPLPAPASIFQRPFSYTCPPGTVLSEVLWNMQDWPAQPGTARSQPVGVIANIVFKCSNAA
FPTPATPMPSPVPFTQVESSNMALVSINCPAGSYVTSVYGSYDLSPANMGGVAFVRQVGITCSGATTITREITTSLG
ATASATPFTSAVCPGGVGALTARALPAPGAPTTSPPALLSLDAHCYDTQAHTRRLSFLPPSLPPSISGSVFELTDRC
MGLTLVSGVTMLRSMFSATPALITVGIPAASFLHGFKLSCTDVPEPSAPALPVSAQLPPNQLPTAPAVAFKYECPAG
SKVVSVLSRVDADEDLLNLRIECDNAPLGDAEPLAAVAARLTPFFAPPVVFTANCSNCTTAANISSMGLGQRRYISP
DVAIAAPRSSYVIERTCPLGVAGVVNSATTSRLAPELGGLARPVSTADMPNAPTGSQVALFDVLLDWSNAAAFCTAQ
GGALMTFEDELQRVVASSVVAKWAAENTGDFLMGVWIAARRSGNGLYDFSYLDGTSMANYVPPWARGEPNDMSGVED
CVELVIDVTAVQQASWNDRVCTGLVRRPLCKLLPKAAFDGVATADAAPPNVQIAATNNDELSFYNISVTWSEAATFC
QRRGGDLISFRDQDEIRRFGAAMSDWTRFIYTGRPSPLQVRTWTGLRRQVPASGTPAEALRVATATFQYQNREFLFI
PNMAVPEDASAACRGFGGELASFLSLVEYNTVFGQFLSTIIAYGITSTASTVTSHIGLIRNNFGTFNWRDGNTWTGF
TAVQAPQNLPSLCGVVLTRCTAGNATAGFSGCSWAISTTSCSFQTNNGFYPSSNSYICSRNTPAAASIVSGSRQYLA
FAERRTWPDAAVACTSIGARLAAFHRPEDYTAFQTAASRSQAFLSATGITNITAGFAHIGMSHNAAGLYTWSDGRPL
LYSVWAAGMPDVLSANTNCSAFTVSCTNTTGLWGSCSSPQLRDVACNTPAPYVCSYDFDDEWAYSDSTPLVYGRDTP
AGLGQPAATFAGNYQWDRVLNAAVCTNANASLDYTGTAIMSFSTPDTYKCCSACSATPGCAWWTHVNNTFVCTLKRG
NGAAVTSVNANMWSGASGRPAPSLANATALLDDSGLNCVYLTQQMSGGSTYQMPPLQCQQRMGVACRRSSAREAVPE
DERRAITNYVQPMGRVFTNRGESELSLYGTFQDFPSAQKLCELRGGTLVEPVNMEWQDAVFRLGTDVIYRGASNIII
EYLVGLSERAQEGQYVFNSGTRLLTPFWSAGQPANLSNPLYVEDQDCARARHSVNSNPNSTWISWYCRFAPSNAMCQ
RPAILTPEYATIRSMAPAAQTVVGRAHFMFYPRLMDWYQAKTLCEANGGDLAYFDSAQQYEEVMDALLQWLRTRPFV
SRSIPANRAAVGTWLGLNNRLNMNNWRWSARPYVLASATNPSWFARRWNGANITVGGRLGRAYDNPAMGLASPGIDT
PFTLCPNITDSDAFVIGFRAQVDGRSTSSTSAAPNTTHNLLGLMGLRMRCEVIDPDLNSFAMPETAVNSINNVPGVG
AWGATEFTCPSWSPASKIYMVGIQLRAAPRRDQPIMQQVKNCIPSGSNPCATYTISWQATNTTWGDDVGITSMRGIC
TNTVSSPSSWAEIVNPAPAADAVWLPAITCATGYAICGISTRVDYAGANYTVRAANTSSNDDSGISGITLKCCPLPN
PFNTAVMGFECVEARVFGNNGTNTWIQRPCASRNSLISSSNVLCRRELPDYNANAALNVQNTTASVRAADTQTASIP
TLPDPLLQQVAQSVEPLGTERVLYAGSTRANRNIITQKYEVLLYDLVMPWGPAVSFCKANNAELMAIDDQNTANAVT
RLVARVMPAAAFAYFNTTSLQFWLGASNRGGNTAWTWSTGGSVTYYEWSTGAAQPQRPSCAKLTYTINSQSGAAYVK
SGWGAEPCGSHMLVLCRRTTTSGSGRSRAAPTNTKAPAASLTIRDVRTTLFREAVTWDQAKDYCQRRTGVLASINSA
NEGDVIATMVREWARTSTAGEVKVWLGGSWHPDAMRYAWEDEMSFDYVRGGGSLSAPERPTQGCLSWRLRPDGTDVW
QADDCAEYALPLCQSELQPFICNTGPNSTRPIDSNPNDAKAKVFCPPGTVLTGITGSISNASLSGASLTLADGLQFS
GICGNPAAPAVLSTRDNQELPMCRYRWSLPRIEGPDVFIDGTNATTALDSCFRMCSVGDAPLQQVFRTPMLAVLYQV
NGVFSCRCGRALVGRQLLNDDAIKVAEATGAANFIYGRSYDIYAICTISEELMTAGYFKQISFQLTLSDISAPPKLT
SLVRGAKYRVFTIPQAVDWNAAQRICMLNGGHLATLESTTDIEDLVKLLRENTNVAVDFPRGSGVWLGLYAAQNNMY
RWVDGTPLRYPLGPVLEDWGYAPANCGFLDLWLNSTRDRRIITGDVRAGSCDNINAFVCETSTMVDDSLSDITPGIV
RTYDTFGQGTRGWGKWELKHFAAHRVTWHDASRICRYNNMDLLWFFSPSEEDFVRTQMLAPAGSLTPSTSWLGATPG
PDGRISWRTRAESASALRSSIWSLPAIWNASAAQYLAENDYGQLCGLLDPRARLSNPARGSFLLAHCSQQAHPFVCK
SRVIPWRDLPLKNQVQPSPPPPPASPPPPPGIGNVLLRVDRPAAQYVLLDQVTSRAEALRMCRAYNGRLPEFTSLSE
YDLVSENVRRYWTGKTSESFVSYWLGGSKKVVGNTTSWLYDSGNVASPTAWGLDEPNNYGGNENCIHVIIYLVDTPT
GASRYGAVGDKRNERTYWNDVKCDAGLPVFCQLSPRTGGPLTPPASDLAAIEYVGTLNGIRYVMYNQLLGWTDAKAY
CEANGQVLADFIAQDELDGVVRGVFHYVYSLPQFQDTLLSLWTGYNSFTSDFRGNSILTANGFAGPS

>g14575.t1|PACid:27573829
MRQTRSASSTQGRRRNCDLVCIVLVVAALLAPATHAGTRDRPWCEGGAAVCRGSTTPESRYEHVSLTSGNGLVYTFG
GRKGAWPADVAPSSTIEEMDMRSRSLRTQALSGWQNVSGRAGHTMIEYQDAAGRRHLVVFGGVMYDASAASSSSTS
SATDTSGGGGGGGGAVSTANPAANETLLNDVASVGLQDFVWVAHRPTGTAPSPRKYASAVLWGTEVMLVYGGVLADG
STSSQLWAYNITERRWLGQVTPQTASYGDSPPALRSAALAVSGSTLLVYGGYKTAVLTVYAPSPPSPPPSPPAQTGP
GGGGGGASTGGNGSSTAGGSTGPSGGATGGAAGRRRGLAAADSPPPPSPPSPPPSPSPPSPKPPSPPQPPPTVSLSQ
FQLSRALWAVDVRLAPWSPPSYVATWVRAGLSMTSPQPASSSSSAAASDDVLLAGFEASLSVSEAGPDTLVVYAAGG
CKPSGLETQSTWRSWYARYATDPAFNLTSALTAFTEQVTAAAAAAQAGTAVPSLSLSLTLSRSRLFVHPDLMTSSSS
SSGGGGGSSSTAWVNADGSSVDLTTGQVSALPTSLSPAFVRSQAWVTSADGSGMVTGGYRNADAGTDVVVTSRRLQL
RWGSGGTWLPDGASLKHAFFETSTASTVMTSMLPLAVPEQPVTYDTAGGGGVGGGASTKTGLIFLGKYDTTSSDQPE
VSVGLVADSSSYSLTPLCTPDPASECSSVSAAFTGPLRPPRREWYAAAPLYGTFNGSAYDGSGSGAATSYSISAPLV
LVYGGVAVVGGPPPGKRPEMSSGGGASILRTALVGCDTRGQVPASDWSSYSTCAWNKISSSVTLKTATSASPTASSA
GSTTGGVTPALTPGRRRRQLAAAAAAGASGVAADEADRLAAAAAAEAVLAAGAAGTPSGSSLRPRQVGISSDVDVDV
DYGVAFNDYDDTGLSAHLDQTCSCAGGSGTAEPGGGTRAQQQQQQSWRRRRVVRRALQQASSSPTRTYYDSATVLAY

QVEGSLPLAVRAKRGTPALVYGDVLNLRTGGSSHSPGPRVGASLSALSALGGGDGRTVLLFGGATFNQTAALANDNA
VTTDELTLAADVHFLRYYVGGDAVQPLPPSGVRDCPQDYPAAASASGSSSSGSGSSACVSAAPFVWDTGSAALPATA
PIAIPTIPDLPTSLAATFSCATSRVVRVEVVASSSQMGAGQAGVRITTWNGTVLLQADGFGSSSGGPAMPSPPGSVT
VVGTVELPTGIMQVWMTAADGLGWTRDEDGMGPAGACCGAANKTCCPQLRLYSVGCSAYLSGFGAGEAVWDVGAANR
RSPDYGVFRKLFAVAVSPTAATLPSGSSASYLQYPLAQRGSYLHSFQLRPAADSPLPPPRHRHAAVFVNDSSLASNF
GPLGVLLVYGGASSYSAAASDPGTRLSDTWAFCLTNNSWVQLRPAGSPLTGGANIYNTSSSSHGAYSMAYVQGANQV
AFSTPTDLLMIPVTVRSTVDSRGAGSSSSSSSGSSTDAANAQLATMASVTTKSLSPLLSSMYDGDTALLTSIDLVNV
TYNQPLAVTAAILVQGRFAKPAKSSASAKSTRLGARLLRELGHAGVMGVRAAGGDNGEDDDAGADDGTDGFGALDKL
AAIEQSLVSRGGGGGGGRPDVPEEQAAVGGAAAAAGGRRRLTLVYDGELDDFTLHHHEEVASTADSVDEMAAPDSNR
RRRLQQTDPPSPSPPPSPPSNPAPPPSPAPAPPPAPPPLPAPPSPSPPPPPLVTLSPWAAATAAAAWGAVSNDQLLA
TYLDGIALTVVDCGGNDNAMIVQQPGAVFQNLLFRNCAATAVVVDSGSTAATVRFANCVFLNNNGTRGGALRVAAGS
NVTLADCLFLGNEAALGGAVYVEAGGVLAAVSGVLLMRNGNGPTSQAGGALYAAAGSCITSLRSSAFDGNGNIHGLA
NGTGTGGGLMLQQPACATAISRCAFARNAAGTGAGVSITNVDSILTLDISSSIFGSNNATTYGGGGLALQVVYGAVI
LSNVTFDDNVAARRGGGVFSSNVRQVNASDCAFTRNGVRLGTGGGWCAEKEGAIHIDRAAVRANGALYGGGFGFSRD
LTVNLTDSVFEGNTATHGGAMEASACTWLQLDRCSFRNNSALSAGGVSIMQTSSGVRLNNCTFTTNWLLNSSAGISA
GETCGRHGVGGGGGACFDVAADVVVSGGHWANNTATNGGAMWVAQKCNPSVDDTCGYVRLYGVSLYGNLADGGGGGA
AFLYEADDLIASCDQFPNSTAVPDLLTTVDVFALAVPVSTVASADALSVNSTSTSSTSTNSSSSSGSSGGALGSNAT
VLSVCGGAWYGNRAQYGGLLATTAAAVRVVEPAGAVKTVYRSNDLLPVLVEVVDAFGQRITGGSTEAAATLAAVAAS
TQPLGVTTQSATSGLAAFASLRVREVPGNYSFNISVSGTFHALAPEPLAVDVRRCWVGEVEKEADLCVPCTSGTFSF
HTANSTCDACPEHAECAPNSTLADSGFIVLHDDGYWRSSPFSPQVLECPNSDACAYDGTSSVAAVPWVNGSRQEVLL
AALRQINSRGPFAVSPNTSAPALALWRAVQCSEGYVGNVCGGCLEGYGITGDATCSKCPNKSLNSLYFFLVSLVNVF
MIMITIRAQLVRSGDEKAARARAKAESRAASMTVAAAASAMARSISAAASKQQQQQQGQQQQLQQLQQLQQLQGRRS
SSDVGDGDDDDSVGGMGGTGKAGGGLPPARKEDSVLPLTAAAAAAGRGPVAEGIQEEEGGATEDASSHGTAHGAHKN
TVGFAVGTADAGAGGAGGKTVAAAAAADVLLEEPVKDEELMDGTHSIVIKILVSYLQVAAIVKDVAVVWPSPVAWLL
NLGNQVSSGVTTMVSLDCSLPSNAIPKSVQKVLITCFVPFILMALSVPTWLAIYGWKAQRKQRQGQSPPPAAPYLRT
RLIVTGITIVFFTYPDVTDALLGIFACPLLDDSPAGGTPYADQLRAAGRWWSSDYNLQCYTGSHLLLLLVFGLPGVL
LFSCGVPAFSAWFLRRNTQLLRDRKFYRAYGFLYTDYEDRCYFWESVVMLRKLCMVVVVVFLGVISVQVQLLVSLGI
ILTALGLQVAYDPYRCPRMDLLERLSLIGTTLIIYVALFFLLNLSDATSVALSVTLVGLNGLILAWFILILVREYAH
GIMHALDADGDGVLSAADVGQALDGLSSKQPWLARTLVRLDAALRRRPLLAGVISRAFPYGETNDPWVAAHLVSEDY
DVDEHAEEEAARMGGKDGDKGKGKGGGRGGKGVADADEGMVTAVADSASQPGAWSKIPKQQQPQAALGVREAAALGG
GGGTSGGGIATVAAAAAAPTPAAVAAAAAHRGSMDRPIRASSSSSRSRVAPEPMPAATAATAATAATAAAAGVT
GGSGNSRPFSGGAGAASSSRRSSAWEHGDIGGGAATAVTTAANTIDDGAANGSSPPASRVASTRLGSPPAAAGGGGG
GDAIAASPIPQPAARAGSGSVNGGGSRPMSRQRPLAAAAHGTTAAAGSSAAIASFGSAGSYTAAALPGLDLDSDLPV
EPLGPAVGPGQE

>g14346.t2|PACid:27574185
MTYESRRGGRKCGRARRQHPGALRLALVLVAALALWAGGAAGQDNPPSPEPPSPAPPSPRPPSPAPPLPPWPPPSPP
TSAATLLDLKAALGNPSGLSTWAGGDPCVGTWARVTCAGGSVTELDLSSLPGLAAGQAFPGGLSFMSSLRRLNLASN
ALQSALPPLPTGLTWLNAGSNNLWGPLPPSWSRLRDLQTLSLAYNRLYGSIPASWPGGMTAMATLFLTGNGALCGAL
PGTWATSGVVEGTGTGLGAACGANPAPPLPAGGAPRAALFAVRLSTNSTWPAGLDSSWQFDTDPCGSTAWAGVTCSG
GSVVRLDLSLYNMQGTLAADLAYLGTALTFMSFALNKYSGPLPAAWSALQGLSRLELVGNSLTGTLPLQWSQMTALT
YLDLSNTRLSSTVPDVWGAGMGSLVTLDIRGVSGICGAFPTGWDSKVLRDATYATSCAPPPMPPSPLPPSPAPPSPA
PPSPAPPSPIPPSPAPPSPAPPSPMPPSPEPPSPPPSPAPPSPAPPSPAPPSPVPPSPAPPSPVPPSPPPSPEPPSP
APPSPPPSPAPPSPEPPSPRPPSPAPPSPAPPLPPAPPAPPPPPLGSGSALALVYNELGQPGSMSSWSGLPATDPCT
LAEVTCGGGGSVTGIDLSYTYGLTGTLPPSMRYVTSLRSLNLGGYVGMTGPLPASWSVLAQLTELVLAGNQHTGFLP
ASWSALTGLVTLDLYGNKLEGLVPSGWPSGLSTGTMTRLTLSGNAGLCGTLPGWFGSSSKVTTAGTNVSQACGITTN
TAGGLLSLKAAAGASWPSGLSGWVAGTDPCVSGAVWQGLTCVAQTQPYTDRVDRVDLRGLGVAGTLPAAASTLGTLV
TALLLGNGNAWSGTLPGAWAAAGGLRGLRWLDLSFQTALTGTLPDAWSQLAALTRLGLAGASGLQGSVPASWDTGMS
AMGSNGIVITSASGLCGPLAGSLTTKVLRDAGYLTSCSPPPPVPSPPPVPPSPAPPSPAPPSPAPPSPVPPSPEPPS
PPPSPAPPSPVPPSPAPPSPVPPSPAPPSPAPPSPPPSPEPPSPAPPSPPPSPAPPSPEPPSPRPPSPAPPSPAPPL
PPAPPAPPPPPLGSGSALALVYNELGQPGSMSSWSGLPATDPCTLAEVTCGGGGSVTGIDLSYAYGLTGTLPPSMRY
VTSLRSLNLGGYVGMTGPLPASWSVLTQLTELVLAGNQHTGFLPASWSALTGLVTLDLYGNKLEGLVPSGWPSGLST
GTMTRLTLSGNAGLCGTLPGWFGSSSKVTTAGTNVSQACGITTNTAGGLLSLKAAAGASWPSGLSGWVAGTDPCVSG
AVWQGLTCVAQTQPYTDRVDRVDLRGLGVAGTLPAAASTLGTLVTALLLGNGNAWSGTLPGAWAAAGGLRGLRWLDL
SFQTALTGTLPDAWSQLAALTRLGLAGASGLQGSVPASWDTGMSAMGSNGIVITSASGLCGPLAGSLTTKVLRDAGY
LTSCSPPPPVPSPPPVPPSPAPPSPAPPSPAPPSPVPPSPEPPSPPPSPAPPSPVPPSPAPPSPVPPSPAPPSPAPP
SPPPSPEPPSPAPPSPPPSPAPPSPEPPSPRPPSPAPPSPAPPLPPAPPAPPPPPLGSGSALALVYNELGQPGSMSS

WSGLPATDPCTLAEVTCGGGGSVTGIDLSYAYGLTGTLPPSMRYVTSLRSLNLGGYVGMTGPLPASWSVLTQLTELV
LAGNQHTGFLPASWSALTGLVTLDLYGNKLEGLVPSGWPSGLSTGTMTRLTLSGNAGLCGTLPGWFGSSSKVTTAGT
NVSQACGITTNTAGGLLSLKAAAGASWPSGLSGWVAGTDPCVSGAVWQGLTCVAQTQPYTDRVDRVDLRGLGVAGTL
PAAASTLGTLVTALLLGNGNAWSGTLPGAWAAAGGLRGLRWLDLSFQTALTGTLPDAWSQLAALTRLGLAGASGLQG
SVPASWDTGMSAMGSNGIVITSASGLCGPLAGSLTTKVLRDAGYLASCSPPPPVPSPPPVPPSPAPPSPAPPSPAPP
SPVPPSPEPPSPPPSPAPPSPVPPSPAPPSPVPPSPAPPSPAPPSPPPSPEPPSPAPPSPPPSPAPPSPEPPSPAPP
SPEPPSPRPPSPEPPSPAPPSPAPPSPLPPSPAPPSPSPPSPPPSPAPPSPAPPSPPPSPEPPSPAPPSPPPSPEPP
SPSPPSPPPSPIPPSPDPPSPAPPSPSPPSPPPSPVPPSPLPPSPPPSPEPPSPSPPSPPPSPDPPSPDPPSPAPPS
PSPPSPPPSPAPPSPLPPSPPPSPEPPSPSPPSPPPSPIPPSPDPPSPAPPSPSPPSPPSPVPPSPLPPSPPPSPEP
PSPSPPSPPPSPIPPSPDPPSPAPPSPSPPSPPPSPVPPSPAPPSPAPPSPGPPSPAPPSPAPPSPEPPSPSPPSPP
PSPAPPSPSPPSPVPPSPDPPSPDPPSPAPPSPMPPSPSPPSPPPSPAPPSPTPPSPAPPSPAPPSPPPSPAPPSPA
PPSPAPPSPDPPSPPPPSPAPPSPDPPSPAPPSPAPPSPAPPSPRPPSPAPPSPAPPSPAPPMPPSAANTLQVIKFQ
LGNPAALSSWASADACDGTYYGVSCILGAVTSLDLSYTQLSRAAPPEDIQWLRALKVLNFEKSNISGTLPLSWAALT
AITELNLARNGLYGTLPDAWSAMSRLGTLNLGFNALVSTVPASWGSGMTNLTRIILTNNTALCGLLPFAPGHVTVTL
LNTGLNASCPGPPPPPPPPPSMASGLVALKDAATQWPLTDWGTGDYCSWTGVVCDGAAVAVDVSDSSIQSRRTGSSA
AEMREQSSQVFTRLLALLAITFTTSATQLDVLNSLKASLYDPGNQLATWTGSDPCTGWRGVACNGSDITTLDISYLG
LSGSLPDGLTGLTALRVLDATGNKGLNGTLPAAWGTVGAWPFFQELYLGSNALTGTLPNSWSNWGSSIQQVYLLQNR
ISGTLPPSWQSWQSVRYVSLTNCSLTGTIPEVWATGSPAMGSLVRLYVGSNSLCGPLPTGWGMKVTYTNSSITQACP
SPPPPPPSTNSGLLALKAGVTADPSAVLDSWSAATLGTVCSTWIGVTCSGGTLVDIVEIQDNQLQGTLAADLKYLTT
LQRLRIASNSFSGTLPPEWSVLTQLSMLDASNNAGLTGTLPVAYSALTALRNLNLANGLGFSGTIPSAWSSIGLTKL
VVEGTGVCGAIPSGIPSANVVPNPLPACAPPPAPPFPPSPAPPSPDPPSPRPPSPTPPSPVPPSPVPPSPAPPSPTP
PSPAPPSPVPPSPPPQPPSPQPPVPPSPAPPSPSPPSPPPSPVPPSPAPPSPAPPSPAPPPLPPSPDPPTPPSPAPP
SPAPPSPRPPSPAPPSPAPLAPPPSPAPPLPPSPPAAAPSLSLVLGELSNPALLAANWTGSDPCVAKWIGVTCTGSV
VTALDIRAPYVSGSPLPPSVSLLTGLTSAQFQSSSYSSTLPAGWSTLTRLTNLNLQSNSITGTLPASWSNMVAMRTL
LLGSNQLVGSIPLSWPAGMSAMSRLSVVSNLAMCGFLPPSWEHGTVVSVSNTNLNMSCPSPPPSPPAPPSPAPPPPS
ASASLYAVRLAISNWPGLAGWTLGSDPCTSNAGGPWEGVACTGGVPTTLDLSGYGFSSVSLPADLAYVDSLRVVNLG
SNVDLRGTLPASWSALSLLQELDVSGNKLTGALPDAYSHLSLLTALRLSSNQFVGTLPSAWRPGGGAGMASLSTLSI
GSSGLCGPAAANYSGVALVGTLPNCAPPPSPPSPAPPSPAPPSPAPPSPAPPSPAPPSPVPPSPAPPSPAPPSPDPP
SPAPPPVPPSPEPPVPPSPRPPSPAPPSPAPPSPTPPSPDPPGPRPPSPAPPRPAPPAPPTAANALLQVKYELGNST
ALSTWDDVLHPNPCTSWYGVSCDGSGNVVDLSLPSTSPRLAGPLPASLVQVRTLRSVTLSGNTLTGTLPDSYSQMTQ
LEVFNVAGNQLTGLLPASWAALQLLQELDLSGNNFASFIPPAWPGGMTALVYISFAGNSLLCGFLPAPWTEGNPAYS
SASLSTPCPSPPSPPPRPPSPAPPSPAPPSFGNALAGLRLTISPWPASILGWNTSSDPCTAAWTGVSCTAGQPSGVL
LLQRGLTGSLPSSWLYVTSLTSIRMNNNSLSGTLPRQWAGLIGLARLELLGNAFSGQLPAEWSTLTNLRTLDLGGNG
LTGPVPSTWSALTQTTRIQLSDNTMCGALPATTAANFVYNGTTIPSPCPSPPPPPSPPLPPVPPPPLPSHPPSPQPP
SPNPPSPIPPSPSPPVPPSPVPPVPPTPHPPSPMPPSPAPPSPEPPAPLPPPSPAPPSPDPPSPSPPAPPVPPSPMP
PSPTPPSPDPPQPPSPAPPVPPSPVPPSPTPPSPVPPLPPSPAPPQPPSPAPPSPVPPSPVPPSPAPPSPDPPSPLP
PPSPAPPSPSPPSPAPPSPNPPAPEPPSPVPPASPVPPSPRPPSPAPPSPAPPAPPTAANALLQVKYELGNSTALST
WDDVMHPNPCTSWYGVSCDGSGNVVDLSLPSTSPRLAGPLPASLVQVRTLRSVTLSGNTLTGTLPDSYSQMTQLEYV
SVANNGPGLGGLLPVSWSALTRLTELDLSGNGFYSTIPAAWAGPGGMGALQLLWLDNNGALCGSIPTPWAAGSPVTS
SGTQIGLACPSPPSPPPVPPSPAPPSPAPPSPAPPSPPPPSFGYALADLRVTASPLPAAILGWNTSSDPCTAAWTGV
SCTAGQPTTVVLNQRNLTGSLPESWAFVSSLSTIRATNNSLTGTLPRSWSVLTSLTRLELLGNRYSGQLPAEWSTLS
NLRTLDLSGNLLTGPVPSGWSVLTGLFRVDLSGNSLMCGPVPSAVYVYNGTQIPATCPSPPPPPTPPSPPPSPVPPS
PAPPSPAPPVPPSPAPPVPPSPEPPSPAPPSPAPPVPPSPAPPAPPSPAPPSPEPPSPAPPVPPSPAPPVPPSPAPP
SPAPPSPEPPAPSPPPSPAPPSPVPPSPAPPVPPSPAPPSPTPPSPDPPSPRPPSPAPPSPAPPAPPTAANALLQVK
YELGNSTALSTWDDVMHPNPCTSWYGVSCDGSGNVVDLSLPSTSPRLAGPLPASLVQVRTLRSVTLSGNTLTGTLPD
SYSQMTQLEYVSVANNGPGLGGLLPVSWSALTRLTELDLSGNGFYSTIPAAWAGPGGMGALQLLWLDNNGALCGSIP
TPWAAGSPVTSSGTQVGLACPSPPSPPPVPPSPAPPSPAPPSPAPPSPPPPSFGYALADLRVTASPLPAAILGWNTS
SDPCTAAWTGVSCTAGQPTTVVLNQRNLTGSLPESWAFVSSLSTIRATNNSLTGTLPRSWSVLTSLTRLELLGNRYS
GQLPAEWSALSNLRTLDLSGNLLTGPVPSGWSVLTGLFRVDLSGNSLMCGPVPSAVYVYNGTQIPATCPSPPPPPTP
PSPPPSPVPPSPAPPSPAPPVPPSPAPPVPPSPEPPSPAPPSPAPPVPPSPAPPAPPSPAPPSPEPPSPAPPVPPSP
APPVPPSPAPPSPAPPSPEPPSPMPPSPEPPAPSPPPSPAPPSPVPPSPAPPVPPSPAPPSPTPPRPDPPSPRPPSP
APPSPAPPAPPTAANALLQVKYELGNSTALSTWDDVMHPNPCTSWYGVSCDGSGNVVDLSLPSTSPRLAGPLPASLV
QVRTLRSVTLSGNTLTGTLPDSYSQMTQLEYVSVANNGPGLGGLLPVSWSALTRLTELDLSGNGFYSTIPAAWAGPG
GMGALQLLWLDNNGALCGSIPTPWAAGSPVTSSGTQVGLACPSPPSPPPVPPSPAPPSPAPPSPAPPSPPPPSFGYA
LADLRVTASPLPAAILGWNTSSDPCTAAWTGVSCTAGQPTTVVLNQRNLTGSLPESWAFVSSLSTIRATNNSLTGTL
PRSWSVLTSLTRLELLGNRYSGQLPAEWSALSNLRTLDLSGNLLTGPVPSGWSVLTGLFRVDLSGNSLMCGAVPSAV
YVYNGTQIPATCPSPPPPPTPPSPPPSPVPPSPAPPSPAPPVPPSPAPPAPPSPAPPSPEPPSPAPPVPPSPAPPVP

PSPAPPSPAPPSPEPPSPMPPSPEPPAPSPPPSPAPPSPVPPSPAPPVPPSPAPPSPTPPSPDPPSPRPPSPAPPSP
APPAPPTAANALLQVKYELGNSTALSTWDDVMHPNPCTSWYGVSCDGSGNVVDLSLPSTSPRLAGPLPASLVQVRTL
RSVTLSGNTLTGTLPDSYSQMTQLEYVSVANNGPGLGGLLPVSWSALTRLTELDLSGNGFYSTIPAAWAGPGGMGAL
QLLWLDNNGALCGSIPTPWAAGSPVTSSGTQVGLACPSPPSPPPVPPSPAPPSPAPPSPAPPSPPPPSFGYALADLR
VTASPLPAAILGWNTSSDPCTAAWTGVSCTAGQPTTVVLNQRNLTGSLPESWAFVSSLSTIRATNNSLTGTLPRSWS
VLTSLTRLELLGNRYSGQLPAEWSALSNLRTLDLSGNLLTGPVPSGWSVLTGLFRVDLSGNSLMCGAVPSAVYVYNG
TQIPATCPSPPPPPTPPSPPPSPVPPSPEPPSPAPPSPEPPSPRPPSPSPPPSPAPPSPVPPSPAPPVPPSPAPPSP
TPPSPDPPSPRPPSPAPPSPAPPAPPTAANALLQVKYELGNSTALSTWDDVLHPNPCTSWYGVSCDGSGNVVDLSLP
STSPRLAGPLPASLVQVRTLKSIDLSGNTLTGTLLDVYSQMTQLEAISVASNLLSGVLPLSWSELRSLRSLDLGTNP
QLVSTVPVAWPAGMTALTLLVLRNNPNLCGSLPGTWVVNNPVVTTGSSLGTPCPSPPSPPPAPPSPFPPSPTPPSPE
PPSPPPPSSGNALADLRLTVSAWPATMAGWNSTSDPCIAAWTGVTCTAGQPTSVQLPFQGLNGSLPSTWSFVTSLQS
VVLGGGTNTLRGTLHAAWSSLLALTRLDVVGQSFTGPLPPEWSVLTALRSLGLSANQLTSSVPAAWSVLTALTRVEL
SNNTLMCGPVPSAAYIYNNTLIPGPCPSPPPPPSPPQPPSPVPPSPPPPPSPAPPSPEPPSPVPPSPVPPSPAPPVP
PSPAPPLPPSPVPPSPVPPSPEPPSPRPPAPPSFADALYAVKNELSNHTVLANWTVGTHPCIDSWTGVQCTGTDVTG
ISLQYVSPSFNAPVPRLLYQVPTLRVINFVGSGLTGTLPPEWSILTNLVSIQLTSPNSIQGPLPVGYSALTLLSSLN
LAGNQLTTTIPPQYSVLTALTRLNVASNAAMCGPLPPVISVKVVATGTNVGLPCPAPPLPPSPPPQPPLPPSPIPPL
PPAPPSAQPVLLLVKQAVTSPWPYADWVAGTDPCAVGANWNGVTCPVGLVEQLDLSYLNLVGSLAQDLRFLTSLKSL
DFSVNQFSGALPWEYANLNQLSVLRLGNNSFSGYMPPEWSSLTNLMELDLKGNQLISTIPPAWSTLTKLTRLDLSGN
PLACGAAPPAIAAVVVPNLLANCPPPPFPPSPTPPSPEPPSPSPPAPPSPEPPMPPSPVPPSPEPPSPVPPSPAPPS
PHPPSPVPPSPMPPSPAPPSPDPPSPMPPSPLPPSPSPPMPPSPVPPSPPPPTPPSPPPSPEPPSPTPPSPMPPSPV
PPDPPAPPPAPPSPPSPAPPDPPSPPPPPSPNPPSPPPRTPPNAPPPPPPSPPPFPPEAVRPTISMFVSFPSMDCNE
LSDDPFLEQQVNNEVRAALGASLGVDSSSVAILQMVCGASGGGGVTATGTTVRRRQLLTAALEAAATAALEASASEA
PPPRPNSGAPAPDEDRLLLVSSAADGSSSSSSTGAAALYEDYVYTEMEALAQTPEPRVLMAQQAYASAAGITSDPAS
ARDGQAPAPYAAATGIAVFDECSDEPPGQCGHRQPSSEADGAMGPQPSPAPGTDPDHWQRAGRPAPGGAAAGAHGGR
RWRRRMAAVANPSGVTLELAAMLPKTATQASIDLLMAKINAAPEDVTWLFSASSMLVQYGTPRVSAVAFDVRASLFS
DSRPCDKRPALAGYAPAFVSRYEFAVLLRFAAPFNFTACGANYGCVLGVSKRGLVAEGSLRPLDSQGSTYRLKVTVL
SEGTLELVLRDDPCDPTTRAAYLIVTVDFTNPQAVLTLATAPTMSNATFLVHADFTEPVQPILPSDIAAIDCRVASV
QLLTPTRMLLVVEGQTGATAKVQLSSFAFADRSGNPGVPSNRLEVVVPSQAVALVADISHYATMAVMGASAVSCGAT
ALLAPGWRSIACVGGLLRSLGHLQVLSYTESLAFRTPDAYKKAASASEWSQLAWLPASATSWSESFSKQLGRLTSAV
PPPPPPRLQSFSGTLETYIVDSGLVSATPYLTYAAVAAPDATDVQAVSSLTRYADGLVYNVTQLPDMRPPVREAWSD
IVATAIVSGAVLAAAVLVHLVLVCSTDRDMRLLGYSFYAAWPKLFLIGLVAVTPPLVYGSFRVLSYETVGGAAPPIA
VFLLLALAVGAALLVVAWLMVVALTRAPKKLPVQPEKPDPQWAEGAPRSRGAAAATPRTPRNQVAPAPMSPPPPQNG
AQNILVNQQQPQLQPGMAPQDPHQLFMPRGTPIAAGRPMAVQAAPLPLMEMDPAPPAMPGAMLPPPPMPWHMPIRPP
QLMGYVPQYATGPRPAPIVMPAPPYAPMPPPPAPPPAAPPPLLASLFGRWRGQPVAGAASTGAAGPLAEAHEQPEMH
RQQEKHPQGTSAQEGGGRRQLLRNRSKSWSSRSPRSPATAGPVGEVAESGEPAAGRTSPAEPAVGSKRRPCPLEEHD
AGGASDDAGGDNSPLQGLRTDSARRAAAIERLSPRGLLARQARSSCPGPLLLGAASVPVEEQAAVPAAAVAVGAATQ
SSGGDTGSPRSPRMRQPVLAMARPGSSAGEISPASGSAPAAAYMPNAPMLLGGAAARALASSRARVSLSQEVGAGSG
SGPASRLAEILATPPRRSGAGGRGSDAGGSAGPSRLRAEALSQPAPRGRFSMMPPVPPSHGRNASSARSEASIDDAM
TSARVGTARLGAGPAGASGAGGMEGTSGGGARSSAGNTTWFDIASDDELVRRRTTDIDMGSGSDNGTGGGERGSGGN
RNAARRAGPGPVAEGREGNLFALDRDPEGRAKRVSKIRQARRAPEAPLVNRPITGYIPGVRGGGGGAGPGGAGNAAA
MAAAAAAGQGEWPGLAPRGETADGGLANWLRPPPGAHRDRGQARGRRSRSRGRDDDDGGGGGGYDDRLSTAASGDYG
RGRTAGRLNTAGSGNYEPGPSGAPTYDGRSAFCASYGWLVADVVGNTDESALNRGGQRRILVANVVSLLRTLLLAGL
LGGWAARTASGSMLQVIAVIAVTAGWLQYLLVVRPYVSILGLVAEAVVSGFECVLLVMAALAQGRVRMSPAPATLAI
VALFIAVGVVAVLEVVRTVLVTIYIVKRMQQAADDADEASAGGAGKGGRARSSRVGAFGASTSGSRRGGEP

>Cre02.g116100.t1.3|PACid:27574329
MSSSRAWQRCLAALLLLLTAVASSHSEAISPLEHVLPFYTPKITHAGRGVGYSRWADATSSTRRKLGVNAEYSYCLT
SPTKRCYPCENGYDDDPCATDDLYGQDDTKCSMPYYYYGNVDDESICGDEVRPMNVGALELKALVPSLLPGGTTLY
SLGQMYAFMQVNGNLTVTLRLDCPWLAVVDAGRAGAAIQLSVAHPNGTVWNKWINLTDTGSSFVSCASFTVPAKRPN
PALPGDNGCDPLTYTVSARFLAETHQADPAVGTEPATCSVNDEATANTRLYYSMDYTPIQFRPPSPPPSPPPSPGPP
PSPPRPFIPDSPPDAPPPNPQPPSPEPPMPPVIPPSPPPGPMVFSQGNGAGCAHSDPGNYTTVSGTAASGPAVTCRD
QAACLDVFYTDDCSYNIPVNGVLYTYCQVCLLWQDAATNGRGCPKAGTGTLSHVCAGDEFSAVLSGSGATPVVGATA
KRNTWYQGRAQRYCTWARWTDSQSTVPVSFTVKDGSGACLPAADSPPLSVVLNGIPATCQNPRTVGGVLAGCGTGDQ
PNECLWTLQVSKPGAPGYKCPFRPPSPQPPSPAPPSPRPPSPAPPASPSPPPSPPAPPGPPSPPPSPPSPPAPSPPP
SPVPPSPAPPSPEPPSPEPPMLPLPPSPLPPSPAPPSPVPPSPAPPMPPAPPYPPSPQPPSPPPSPQPPSPPPQPPS
PAPPYPPSPAPPLPPSPPPLPPSPPPSPPEPPSPSPPPSPEPPSPSPPRPPSPEPPSPSPPRPPSPQPPSPPPSPKP
PQPPAPPSPPSPPPAPAPPSPEPAPPSPVSPPLPPLAPPSPPSPPQPPSPAPSPPAPPSPEPPSPPSPPSPPAPPAP

SPPPPSPRPPSPPRRPPRPPSPPPSPAPPSPAPPAPPSPPPGPGCLQVSARSGKLYGTSDFLDFGSITVRPHNATAI
SLDLQLATVAPLPGFGVPAFSPSPSSIGVELRSFFTSSSCPNAPFLGNTAAHRHRHRQLLQAAGTASSAGTRTERPC
TSAAGGGVGEVLLDVAPLLGCTVDTPPTTLTLQLWLALDSVAADSCAAGPQAPATTLWAGTPFSQVLPAPDNGNTAC
AYMNVTLACGFTYECHNVPFPPPPSPPAPPLPPSPAPPSPVPPSPAPPSPRPPHPPPRPPSPAPNPPVPPSPPPRPP
SPPPSPPLLPPSPPSPPVPPSPPSPTPPSPSPPLPPSPAPSPPSPPLPPSPPPSPPQPPSPSPPPSPEPPSPPSPPQ
PPVGGGSGSGSASPPPGSSGSGSAPPAPPSPPPPPPSPPPPPTPPSPPPEPPSPPSPPPAPPSPPQPPSPPPSTTGG
SGSPGSEPGSSGSTPGAGPSGSSPGSGPGSSPGSSGSGSDVPPSPAPAGEFVLPPSPAPPAPANSTPVLSALVLLNT
TLAPGDPLPVVPGAVVSLDYNYLTATARFQGDSPYASCDPAVQGALVTVLAGSLNFPAANVSTACAPDSGGAAHHRL
SRRQLLGRDLMETTCLPTVKSDITFKVNPSVPMDGFKAAVYGALGNGGIAGVCPLGLLAGSWAVTGAALRAFMLPGG
SVPNCAAFANVLTTALAASGGTAAGGATVSGCAVAQMAPGDAAAMLGAGATTSSTAAAGSSSSSGGGVPIVPIVAAV
VGAAVLAAAALALYMYKKKTGFNVSAQAEPGKADAAGGPAARAGAPPGGPEGEPAGPDGGAGGPGGAGGAAGGEGAG
AVNWATPAAGAAAGGAAAVGGGALLRKLFRNLSTAAAVTFGRDASVAAAAAAALAAAGAAGAAGAATPAGGPLSGQA
NAARAMKGPNAVPGEWEDNIISEVPDIETLEADGAGGAGGFGASAGGAGGVGIGLGAAAAGAAAVGAGAAAAHRTGQ
GMSGSDATAGAGAGAGAGLGVEGSGVGDANAAGLPVGAAAAGAPPDLLGDGGAAAAAAAAAAAAAAGAGAAGAHKAV
LPPIRAPMNKLPQLDPSLAGHAPAFGAGSAADKLVLPTHAGYAGSAADMAAAAAAAAALGLKGALPAIAARKSALAP
LQRPSVGAIPEVGTSPGAGGAGAGAVVGGAGGEGAGAFGASAGAAAAAAAAAAAAAADPFAKPVLPGLRNGSTAGGAA
AAGAGGATAGVASAAALMPGTLPTASLDHAAARALEEQLRAGKIGGGGMEALAEEASAGDVRSSNINLFVRNPDAAA
AMAAAGAAGAAAGGAAGGAGAGGPLLGSGYAAASMRLPTLPDAGSSFTEAAGAAMAAGAVGAAGAAGAAAGAGGGRG
AMSRFFGGGGSSGADVQALKPGETGKSMRFGGGAGEGGAAGGAGGAAADAAAGSAAAGGAAGAAGGGAAAAGGGGFL
SRMFGGLFGGNKSAAAAAGGAAGAAAAGAAAGAAAGGAGGAAAGGTGAAAGAAGAAVQWASPAGSPALKAGAGVGGS
AMARTGGAAGAGGASAGETLPEMDEEAAFGASAGALHAALGAAAGAGAAGAAAGFGLGMSPGARRGTSSNGMAMGGA
ASSPAGRMSANGAGSAGPVPRTSTSGIEAAAAAVAAAAAKLFSAGGSGAGGGSGSGRGSPSSNVVRRGSGSGLPSIF
MHNNAVYAEPREAASTAAFGVGAGGAAGGFSTNSDPDIRGGGAAGGGEFASTLGAAAGAGAGGTAAPRMSQSGPQRF
GRTSVDMDPELGVGAGGAAGAAAGAGAGGNRTVRFNKPGGGLSGDFDPEMGAVGGAAAAAGAAAGGNRTVRFNRPGG
ATDGSFDPESGAAAGGAAATDAAAGGNRTVRFNRPGGATDGSFDPDAAAAGGAAAGAAAAAGGNRTVRFNRPNAGGH
GDLDEDEAAAAAAAMALGGAAASGNRTVRFNRPNAGGHGGDFDAPPADLPADAAATAAVRNGMPANLRDFQRPGGGA
SDGAFDAEAAAAAAAAAAAAAGGAAAATGRAAREVKFAPGLGNLPEDEVADGEDGGRGAGDGDAGGRGGNAGTGHRTT
RQFRFADMRAGRSIARDVDMEELDEGVPGNGARAEEPPPENATEEERRAYEMRRARMGTYGDGKIGPVDNIQLQREM
RRKAMLEQMKNNAWSSFNPRTAEK

>Cre02.g075950.t1.3|PACid:27575893
MGNNCSVQRRRSSRQQTCCRPRSAGIPSLPLLLLALACGLLPPAWGPLRGAYAQACNSTVADCSGGELTQYGVSATA
APPPKDSIAAPYSALLGPPDYTATPLCISYAPNTTQQQTAWSQASNDPSAATRFSSVTAGFSTPVYARQVGVLIVS
SATPNITDNLFLVTADSGSIRLSCPENTGRTCLGANASETPYWFTCNTSSLVGPIVGMRIRLNATVVVDAVRLTGFV
AWPSPPPPSAKPPSPNPPSPNPPSPNPPSPNPPSPNPPSPAPPSPHPPSPDPPSPNPPSPKAPSPEPPSPKPPSPKP
PSPGPPSPEPPSPPPSPEPPSPGPPSPPPGPDPPSPQPPSPEPPSPPPSPDPPSPPPSPEPPSPPPSPDPPSPPPSP
EPPSPGPPSPEPPSPEPPSRPPSPDPRSPQPPSPEPPSPPPSPEPPSPPPSPEPPSPEPPSPPPSLDPPSPQPPSPE
PPSPPPSPEPPSPPPSPEPPSPEPPSPPPPSPPPSPEPPSPEPPSPQPPSAPPSPNPPSPEPPSPPPSPQPPSPEPA
SPPPPNPPPSPEPPSPEPPSPQPPSAPPSPNPPSPEPPSPPPSPQPPSPEPPSPPPPSPPPSPGPPSPEPPSAPPPS
PGPSPPPSPQPPPSPPPSTPPSPPTPPRPSAPAPSPPVAPSPPAPEPASPSPPSPSPPPAPPASPLAATLGLPADMV
DTTGKPVAVLTLARESTLSGVELTDPGALMVLFPGAKTVQAVGFQLGVPLQVVPRGASGSGVDVCSGTYLATLQEEF
YARLPFSTTTATSVTCTRTAAVAPAPAPTSRRLLQLLLGRHQQQRHQQQQQRRQHRRLLAASRPDGGLEAHMLRG
LVQQAASEAGGRRLQQQLFPSSSSGSSSGRGAEERNAVGACAGESAGAVNFSMQVPIDSSGSAGGSSASGSITSVRS
ELYDSMRSWQVAQEASGAGSLLVCSPSVDEISVSTKVRAVYEVGLSAAGARALSTACTGAGAGSGLLGGSSDVSCNL
APGPGAAPVEVSPAPGDTNSVASPAPVDIAAPSPSSSGNNGGSSAVTLLAAVAGGAGAAAVLAAAAVVLVVRRRRRL
AREAELKNADCVQPVSDLSYADRHSSYLPREVAGGRGSHHSSRRSGRSGAMRVSAAGSAASSVRSARASSARRSALG
QSVVTANSSSSTGSGSGAGRVSASGSHSTRLGASSVGNASSSNNGGSASNTPSARSSTSGIGSPGGRASGTGMGGPE
QAHPHPPHHHHGNMDIEAGGPDAQAHAATASEAAATSGPSAQHGLPAEQYQQPNPPLRGRALRMYERARGGVAGPSV
TSSSASRNLRALVAVRAAWRAAANAGGQAASREPNAPGAGGDIPAVTGAAAVAAATGAVPGSRPRSPGSLPPAVPPG
SVDVGDAEAAAVVAAAEAEAADPAFALEDDETAAGFIVEDGDDSFSSVRSHESGVSEAARDGAESASATAAASASAA
QAGAPPVFQRRTTSGNSNGRPSRPASPGSALSRPSSPGHVVAPGSRPLSPGLLAATKAAAAAIGALAAAVEARKSSN
GGASAPSSNRTSAGRSSAAARGVSAEGAASASSRKSSSRSSGSRKTSPRLLTRMPSPRVHPSPAGTSERGDSPPEGY
NPRALGRAATTGHGAGAAASASAGLSDWARPLAAAAGALTAATSRFANFMLGRPLPRSVSAGNTPTSQRGSSGGGGT
SGGGGISTRPSSGIDGSRNGSPESTASGSTAVTAKSNFVAAVDEHIPPPVTLAAGMCAEPEALAATPAEATAAAVAA
AAAARASAAPPQAATWMRSPASSTSGVEGADTTTTVIAVQPAPSLSNRSSSSGGPSSGDVSFGMLPLPPRVPSVIQP
PSQLRSPSQLPERPQPELHSPRQQRSPSYTRARSNLGSPQGPRSPSVAEPQPMLVPVQLRQSPPRLTRSAPGAHAAS
VDATRGAGSSGAAQRALHASHSTLPPRPHHAPTLSSLGRTGSLGAAAGAAPASGPGGPLAVPRVLRRTVSEAPQGSV

ARAAAAPPPAPPAPNPSRLAWSSSMAGGSHEATAEADQATASAAAPSPAPALAQLADFARISMLPAQAPGSALREVL
KQLPPTLDATYNAAAAAAAAAAAASAAIAASEASDSSGSSMWSSTSSEGSLAAAAAAAAAAPVPSRRGSGSGTPAGVA
AALSRAARSRRRLQGMLVAQAQQHPQQQMQLLPPPAAAAAAAPARDALTIDDVTIGTSPHLPGFSLLFRVRWKQAPG
RFGHMPYSAWVPYDQLPAADGQEAMRRFMQSGRWRQFKSTSQYATFARENRDRVPRLLTITEDSRAEIGSGHLPVEA
AAEAETGADTSGRPSITGIDADSERTSGSVRGG

>Cre10.g421150.t1.3|PACid:27579278
MRALRHSAAPGASWRPRTSSICCALLAALALHVSTRQLAAAQSFIRLAGGGECLQAAPVRRELVGVRACNTSEPDQV
FVLKAAEGGTAGVYLIMHAQQPGFTMDFYVTNNTVGMWSLRNSTNQQWLLSEEAVAAGGGTALQTARYPDSPNCVE
PAPAAEGQPDLRLAPCDRSDSQLFFLVAPEDMAGWQMPASPPPAPPPACPQVPGYTLSAGFDQFASDLGQARFDNYG
GVNLLDAAKACDALCECVALNTDGSMKRASDNLVDKRAEEDRCWGLYVRTSNANRTCAPIKVKPTEPVLPDRHMGCV
YDTIPRVMDYIMSDEGLMTRRLCALLARSKGYKYYGLEYGQECYGSDQDESAIRRVGGSYECWRPCTGDGAEGCGGG
WSIDLYLVDGDKYLPAAPPAAPLPACPQVPGYTFQRFTDQFGGDIIQTGSNLADMAKVCDKLCACVAFHSGGYLKSR
SDNLVALKQGADLCVGLYVRDVAPSATRLCETHTGAPFAEDGPLGCIWDTLPRVMPVRLANDPARMSRRLCSLLARS
LGFKYYGLEYGQECYGSNDTLATITARGRSYFCDRPCVADGTDGCGGGWSISLYEVDGLKNLNSPPPSPPSPPPRPP
RPPTPPSRDLSYSSWFCGTQGQQYEMVSDEELARHYRLQGVRARYGLVLQEVQSVFDDGAEEGPVHGGYGSGPTTFT
SVAPRWDSERVVAVRACCATGGAWGNGVQAISLYRANGREDAVGDPKWCFGFQDWQFVPEGFEFNGFATMQAIGIPG
TVWVSRVAFVYTAIPGFYRPPSPPPPPSPSPPVPPSPPKPAYPQSTLRTVRSRDSGLGLYLTAASAAAGAAVGYQTA
LLGSAAAGGAAWPADTQLWDVEPAPGQQDPENIKYNLMLKSPSNGTVRLCLTMKPESEPYLDTCASNSTFQRVTLSP
GDGVANTDFGAPVGWIERVVLGDRARGEDQYGCLRMPQTPEAGSRAFSRAVTWQGWCGTLLEQIYVEQMSVDMAVFD
ESSRPPELLASPPPPASVIPNATMPTAAPPTAPPPPSISSVVPITLRSRANGLGLYLTAVSATAGSSVGFGPGMDLT
AQLLAGGNAAVAAEPSAKQLWVLEKSDLQPSSGKPGHLLVLWATAFAAERLCLALRPDAAAPAPSLAPCSRNASLQL
VTFEAGAARAVVSSDAGSWVGHLQLGAGAGATSGAAFGCLAPFWKGAYEPERINAVSALASACGVSSSAQQNISAVA
FDVNPYVNAAAQGAQSAALPVTSSDFFCGYNTGSAPYQYQTATDEPVAAEGYQLSGVRSRGGALLEELQSLFVADGA
AGLKTGPVHGGYVSTSSSALTNLGGDAGAHVTEVSACCTGGSWSNGVQAVRLRTAGGRLIYLGSPTSCNQPQPWVPV
PEGSTFAGVATQYTPSAAGTTYPQRIAFLFASAMLQPLAAPPPSTVVPSPPGLAPVYMPNRPPPSYGGYGGYGGYGG
ASPMPSGPAPYPGHVSAPPPVYPSPPPPVQPPSPPLPPPGPPGSGCPAIPGYTYVPAASVDGKLIQMAYVISYENML
VLAKACDSLCECVGFKGASPAYATAGTLYTQRVSGAQWSDDDLDCRAGAYVRNDTERAPGTCEPLKGGTLPDRPEGC
FKDMSRYSQLMDKVLASDDSRMTPRLCGLLARAAGYAHFSLTEGSRCFGTNANISAITRYGAASTPDCSAPCTGDIA
AVCGSIYYEYMFTRFFNLWTVNGTVNEPVYPPPGTPPPASPTACPAAPGYTFTRVYQYPSYLANLGPRVAGLDINDF
SDAAAICDRTCECEGFDHVGMLQWKLSSQTTAFPVDDPSSTCQGTYVRNNAKARSTCESLKALDQPFGDVPLGCFRD
VYYSQATVMGGMYVQDYQKMSPRECSVMARAQGHRYFALKRGSTCLLPSGPAVGAGVDILTQNGPSSECSYPCPGDS
SQLCGGFYEFNLWSVDYSVRSPSPPPPSPAPSNPAVPPELPPAPPSPEPATPPSSPPMPPPSPPAPPLSPPGSRCPS
IPGYRYVPFSSMSVQIVRASYGFAYESMLVVAKACDSVCECAGFGGWGYAGPSGVMFTTFTEGAEWTADDEACSSGA
YVRTAGERAPGTCEPLEGGALPDRPEGCFRDGTPYGTLMDKNLVSDSAAMTPRLCGLVARAAGYKYFSLSSGGTCEG
SNAEDVSVLTRYGAGSAADCRSRCTGDSSKWCGGSVAGGRFFNLYRVGAGASGEPPITPPTAPPPSPPPRSPPPPLV
CPDVPGYAFIRVDSWDADLGVQMSTLNATDFGIACDGICECQAFSNYGYLRQSVGSQIPLGAGSPPCNGLYVRNTTR
SAACESLQPPSAGAVSPLTDKHLGCFKDDYVSVMGIGSFAYGFTTMTPRVCSLLARSGGYKYYGLKNGYECVVPRNF
AAGADADALTRLGPSTGCIRPCPGDDSAMCGGGLLNVNIWEVDYTVRRPSSPSPAAPPPSPEPPSPELPPSPPAVPP
PPAVTCPQVPGYRLERMQADPAAAFVTFYTTKDLATLVKSCNAMCDCVGVSTQASLMSSVSGLFDPGASYPWSGSIA
SSPECNGVYVKEASAAAGCAPLRATDAGATPLPDTALGCYRENTASPLLTSYATSATLNSPRLCGLLARAAGYQFFA
VRGSSCQVSNSAQSTLTALGTSTTCTAACAGDSAAKCGARTPPVSFNLYRVGA

>Cre04.g216050.t1.3|PACid:27580547
MTLRQKRTWGPSGSLPVAILWLLGVLSLFKAEAQSPPQTPEFLQPPPPPPRRLPFDGIPAAPRQDTAQPPGDTQGTV
DAPPPPTAPPPGLPLPPTALLPPLAPSPPPDLPPPNAPPPPAQPDQPPTIPTTPPVSPDVPSLPQQPPPPDMPPPGT
PSPPPDLPPPAAPPPDLPLPLNPPPSMPPPVAPSPPPETPVGELPPPPDAPPPATPPPDVPPPATPMTEQPPPPDVP
PQATPPPDVPPPAIPVAEQPPPPDVPPLVAPPPATPVTEQPPPPDAPPPATPPPDVPPPATPMTEQPPPPDVPPPAT
PPPDVPPPAIPVAEQPPPPDVPPPVAPPPATPVTEQLPPPDVPPPATPPPATPPPAAPLPPPETPVGEQPPPPDVPP
PAMPPPELPPPAAPLQPPETPVGEQPPPPDVPPPAMPPPELPPPAAPSPPPETPVGEQPPPPDVPPPATPPPETPVG
EQPPPPDVPPPQAAPPPLAPPPETPVADQPPQDALSPPALEPPLPPTVPPSPEPSLPILPSPESLPTVDAPPPPSVS
FLPPTPAPADPTPSSPEPPSPAPPSPAPPLPAPSPHPPSPHPPSPQPPSPQPPPPPPSPRPPSPAPPKPPPPTPPAP
PSPRPPSPDPPSPPPPSPQPPLLPSPPPPVPEQQLVFNAPLLLPLSLSQPNATVDPFARLVSLKLMQVAAPAGTYLC
EFLPASAPPPGLGYMNLTEANSFGSRLWYALPPGFTALLAPVNSSPYIFLLLITGGGSSGGSRFVVQQRCTLTAAYR
AAAGEEQGNATSAATALLPGGAYTSTYTADAASSWGALVYCHVLYGGGLGGRDAVSALLLPDPAVRPLLTNTSFFGP
ALPQALSQPLNHKAFFFWTQTTSPPCCGNETAPTATCDPLSFLGPLPLYDAGGGGQQLNTTSGEVPLFPPCDRNWGL
HRSFTCQVPRVWARAPAASPNPATGRPREVVMFAAAVPYWTAVQRCALLDGEGGAGPGRLMSLPDDSSGVWGAEALA

SWPGEPGQAVWAVDAGVNCSTAALPLSLSGGNGGSDVTSAGQVLCDDLLFFVCVRDLDFPSPSPPSPAPPTPPGPQP
PRPLSPPPRVLLQPSPPDVPPPPPDGPDQASAFMPPPPAGSGGSSLQAQAEALLRIASTFDSGPGSQCDLSSWRVPG
TTPCDWSPRVVCGTTAPNSTTGGTDGTTAAPPAVDGGFSETVVLLNLTDCWGPYNASANSVGGMQMPSSADLGTMLG
LRTLLLTNTGVQGPLPMSVTGMGGLEVLSLGLDRAALAADFAAANGGASAPKVAGRGVTGSIPAGYSSLSALSRLDL
QGAALSGPLPAGFSTLTGLVSLDLSAEDGLSSGGDGSIPSSSLTGPLPAAWSTLSQLTLLSLSGQQAVGGSVPAEWG
AALAQLQWIELSGCGLTGPIPPSFSGLQDLRYLVLSTNSLTGQPPEALSALSDLRELSLEDNTFSGTIPEAWSALSN
SLTLLDVSSNAGLGGPLPDGLSSLSILQELNLQACNLTGPLPASYSALSNLASLLLSTNALTGPLPDEWSALTVLEE
MAIAGNGIRGTLPDSWADGLQSLTTFDAAYNRLTGPLPASWSGLAALQLLDVSHNALSSSLPPAWSALLALSSLKAD
SMRSEDGSTSVGLDGPLPDSWSALTVLAHLQLADGAFTGTLPASWSALTALSGLDLGASELPGGPAGELEGPLPPQW
SALQRLVHLSIPRQPGINGSLPADWSALTGLTHLDLQGDSLSGPLPPAYSTLTRLATLDLDAPADAQGALTGPLPAA
WSTLGQLTLLSLSGQESVGGSLPRSWGNGLSQLECLDVSGCSLTGGIPGTYSRLTGMTRLDLSTNSLTGQPPASFSA
LTALEELSLTDNLLAGSLPDSWSTLTTLSVLDAAMNEGLTGPLPPAYSTMTGLRQLDLQACGLTGPLPGSYSALVSL
VGLSLALNFLSGTLPASYSALTDLEDASLAGNSLTGPLPPSWGGAMAGLFSFDASHNGLTGPLPASWSGLGSLQLMD
VSQNDVAGPLPPSWSVLTQLAVLDTSGSSGAAPSESRGPLPDSWSALTALQSLGVQGASLSGSLPSSWSALQGLTWL
ELGNGGAGSPAAAGSLSGSLPPEWQALSGLQHLGLGGQPGLSGQLPPTWSVLTALTALDLDGSGLSGPLPPSYSALT
RLVVLDLSAPTQPSTGAPGGGGLTGPLPAAWSTLGRITLLSVSGHAGIGGTLPANWALGSNGLGSSVRWLELAGCGL
TGPLPPEWAQMIALQVLDVSDNTLTSSLPPAWSSLTTLREAWLFDNALTGSLPEALSGFSSLEVLDASGNAALGGGL
PEGLSGLSALRDLNLQACNLTGPLPASYSTLTGLVSLVLSTNALTGPLPTEWSALTALEDLSAFGNRLDGPLPVAWG
ASLGANVATVDLSHNRLTGPLPASWSGLAALQLLDVSHNALSSSLPPAWSALLALSSLEASSNAVPANVTAGAARGI
SGPLPPSWSTLTALARLGLEGERVNGTLPSAWSTLTQLSWLALGAPSSALAAGAGGGGSLGSSSGSDGALTGSLPPS
WSNLVALQRLAVPGHPSLGGSLSAGWSALTALTALDLEHSGLSGPLPPSYSTLTGLVVLDLAAAAQAGAGTNTSSLT
GPLPEAWSTLGRLTLLSLSGHGDIGGSLPPSWGSSLTALRFFDMAGTGVTGSIPSTYSSLGLLQHLDLSANGLTGQV
PDSWGTTFTSVQELLLFDNSFSGTLPDSWQLLRSLEVLDASSNPGLSGRLPDGLSGLSALRELNLQACNLTGSLPAS
YSALSSLASLLLSVNNINGTLPVEYSTLSSLEELAVAGNQLGRQLPPGWATGPLTTSLLTLDLSHNSLTGPLPASWS
GLAALQLLDVSHNALSSSLPPAWSALLALTSLVASRNPNIAGSLPPEWAAGLPSLQQLLVAGCFLDGSLPASWAPAG
AAGGGAGSGSGLGFALARLELGSNNLEGSLPVGWSGLTRLSYLDLSDNQIRGSVPAPWLRAGSGMPSLQVLLLGTNL
LDQPLPDVAALFLASQSGNANAAAIAAAAAAIGVAPLLQLNVSFNSISGPLPGPSQPYMSYLVGLDLTSNGLSGQLP
PAWSLLTSLTALRLAQNGLSGPLPTVWAALGGSSAATAAVGGRRRISLEAMGAGARGHRSLMGHVHSWPLERKGDNE
ATAGLSQAARRLHSKSNGGLLSWLAGLHQQRLQQATAILGPSHRRLLQQSVGSGRNSTGAGSTGAGAISGAVPGSGL
AVLQLSDNSLTGSLPPSWSSLRALTQLTLARNSLQGPIPTAWPVGMTSLQELTTAGNDPLPGICGTNPGGSTWPLGG
AGELASLSACTGLSSPPPPPPSPPSPVPPRPRPPSPMPPGEPPLTPDQGSVFSPPPAPKSPRPPRPPPPSPPPAPPS
PPQPPSPPPSPSPPEPPFFPDEPVVRPPPPPPPPPPRPPRPPPPPPPSPPRPPAPTPPQGVGIAPSPESGNQGEGNGGA
QASPPPPGGSSSSGSGFQPWWIAAIVGSIVGALLILLALVAIFRARRRRSSGSAYAVRSAGSSPLQPPPAPAQLPPG
FFPGAPAAQPALLTAPAPPPAGLDRPSLNARWHNPVFDTRENGEGAADWMDVMDAFYLVESEGAPPAPATVPPGAAN
PLFMSSRRGAVMGAEGAVQQPASLYGGYGTERSAGGMVAGASGGGAGVRPAGEVVENTGAVQAEELHIYQPQPPRQS
RASPYGYGTAARGGGARASNRRYTADV

>Cre04.g229550.t1.3|PACid:27580819
MSSRDVLLLLALALLGVVAVASQGPAAPSPSRRPPRPPRPPPPLPSPPSPPPAPPDNQNFRLVATALELYVRGSSGW
LDYTAGAVGYVWIYSESAGDYFPFCLPDGGNTTWDAGAASYACRAAGYDRGVQVATGNGTANRRVGFGSPLPQVQDL
TCPEGAATAQLRDCRAAVLTEPTACGTMAAVVCNNESPPPAPPSPPPAPPSPPSPPPLPPAPPSPPPPPPPPSFPAL
PSPPSPSLPPAPPAPPSPPPGDGAVRLVGGPSLYEGRLEVFGNGSFGSVCDDSFSDAAARVVCRQLGMTGGEALCCA
AFGPAPASAPIMLDEVTCRGEEPALLLGGCMTGAWGSHDCDPGEAVAVRCDSGGAQVDAFAAGQFFIPSPAGVGGLI
TADQALVSGRNLSVTPPLRCIASGSHTTGTGCDPRFEPTYFFANNMFNDVVYRIVYVYLDGAHSCPSPLTVRAVSKL
YGNSRVQLDMVLPPGGDPVYLDPRVPGRLMLQRKGRISTGYQISPSWVDGVWLDASRYILDDGKTAVTWALDDAKGW
LADRGVMQPAGPAVAGWRWKHGFVGNTDGYSPATFPELITTDGGAGGGGGGINATYQLVYELDGGTHVVYSYPSDR
RLVVSHPRSTAPLAVYIQLEFLPHPTGARGLVVQRMEGDSLWNVAFLAVSSPAVQRRAFVVMDTTSRDTWGCPQALA
PPGAPAPSPPPPDAAPPDAAGEEGDANGQQPAPPPPTDAGTVATPLQICRFRLLGCGSGAAASGQAAAAAAQQNDTA
TAAAAPVCVREAELHCWMEGNDGSSSNSSSGTSSSTGTSSSNSGSGVVVEVGSALLPHLNYSGVQVVSVPALPAAVD
GSSNTTSGSSNTTSGSSSSSSAAPAQQPPHWGITITGASRLLLNSSVIADLPLSPGGPLLSCRGCMEVTLRRVQLRN
LSAAAIAAGSAAMSQPPPEPPLMSYGAVLLHGARAATLEDVACTDVRGGTNSAGWACVLVGVPAPAAAAPPVEARVQ
IIRSTFAGNAVAAAASPAVLNAAAAGNSTAAGAAWEAAGQLLAATAKGSSGAAGGAPPPQGAGAVAVIILLNTPAAA
AAAGSSIGGSSTGGSIQQAVFVNIADSRFDRNEGVLGAVLFTGPNMTTWLSASNSSFNSNHAAFRGGVVALAGPAAE
VHFTSGSMVVNNSATQGGGVVNSVTDIQAFRLTGGCSVLGNTPGEGGYGGVLNAETYLDIVDFINVTVSGNGGSSIS
SYGSVIKADPTLASSVPWDYDRLGGQHTGANITILNSRVTNNGGAWVFLFGLFNMIEVRASDMSRNNGLWFSPWRGD
QSSNPRAALLGFHLQEGTRVCGNIAPAYQGLAPVLYTAVPVGRVEINNTTVCSNYGRDASAFGSVASIKSVVITNGS
HIYNNSIGARASMKPTILATGESDEIIIEGGSTITDNDGGQIFMVVRHLGSLIVRGAGTAVRNSTSYIGDGSLLRSD

SLGSLELSDGASITQGAALNGNGGAVHVANDVGSISILGGATLSHNRAARGKGGAISVGGQVTGDVRVAGGSSAEHN
EAGAGAGGFLFVNGAILGGLFVEGGSSVRQNTAATSGGAVHAGGGIFGGVRVAGVGSRLCNNSATTSNGGAVSAGPA
MASLEVADGAAVTGNSAGGAGGVAWVQGDVSSVRAVNGSTVSGNTATLEGGVLLASLLGGLELLSGAVMSRNVAGGN
GGAVFADRIRMLGVSDSIVEGNQAGGSGGFVAAGTLIDTVSFARARVSRNVAEQGPGGVLSLSIPPPGSQLLVQAAP
GGRASLTVADGCVFSRNRAYESGGAIHVAAASDRTSNAPRITLQVDVSGSSFLNNYAGGAGGALSLSAPAAGAIDLA
VAVRDCAFAGNSAGSELFLLGSSLYSGYGGSIALVSLPKLRAGAVMAAQQQAEEDSSSNTTSSSSNSSSSSSSSQGS
RAITGGNLARPGSLGAACALVLERSTFVNSSALQSGGALSVVSCGTVIRSSTFTGNRAQFRGGAVTGMIEAANPLSS
TATASNGSSSSVAAMSGGDGNGAGGRRRSLLLKRRGEEERGSGSSRSGTQQQLLQRLEQQQQLQHRRRRAIAGTVPT
SSSSSSSSSSSSGADLAHAILQLQRARSVWLDVSGSTFTNNLAEADCGGALHAETAHAAGVRLHDVTVRGNQAAEGG
GLCLSARGSAAAALVSGSRLSDNAAAGGGGAVLAKLSGGANNTLELVDCTVYGNRATSGAGAAVVSGGRGSQLVVSG
SSVEANLATVSGGGAHVQCDATAAAAAATAGEARCGPEPLLVLRNASLSHNAAASASRSYEGRGGGVFVGPGASASL
QGLAMTSNVAGEAGGAVAAEECEALVLEGCNVTDGQAARFGAGLFVRSCGSTVAHGVRLVNNTALTGGACFFAGPTA
DAGILRLVSASGGTTATTTAVVDGTVSTASRGGSVVLRGCVLAGNAAASSAREDSSSLGLQRTYFRYSGHGGAVFVL
GNVSVLAAGCDLAAPAAGRGGASGGGGNSGAVGPSVASTQQCASSSGSSSGGAASLAAMQTAVTALAAAADSAAVQR
AIADAAALLAKAGQEEGCWRLALSDLRLPSSQAYPLWMQDERARSLQAGCSLGDASASDSSADTTSSTGTILALTSA
ACNSSSAAVPPSLQHACEQMATLRGCSARVAAAAARSGSSSGNSTLSLSEGPGGSAAAAAAAATAGLLGGLLEVPPS
RMELVAAVAAGSAVATGSSTSTSTSSSSSSGGGGVSGSGSRSAAPLRLRPGTPMRLAVRLMDGLGQPAKRDDLRWAV
TLFILPAQPPTAAAAATAAATAAAAAATAATATGGGSSNSSSSLVPAAAFPARPQRPWHDPRLAVLDPDAASGGSLS
VEVVEGVAVWPRLTVRGWVGAYVLVFRAVSQEDAALYQISSLEVAVEVEPCAPGEALDLSWARQSWGRPSWVACAAC
APGQFTVWRDARPPQWRVDGSDYVDVMKALSEAAAAGEAKCMACPDRATCPGGAVVVPKPGYWHSGPDSPKLHRCPF
QPACGNPAGGSGTGDRWPADAAAAAAMAPAAVVVLNSSSTQTLLPLSGSSSSGSSSLFGVLAQSSLESPRSQWLARC
QLLGYLQVSGGANSNSSTDANSTAAAMLKGCTEWRQQQVAAEAAAGGAEAVGSSGSSSGAAVAPYQQLQCAEGYTGN
LCGACSPGYFLNSEQQCMECPPLVRTIGVGLLALFASVALVAYTSFVTFEEHFETTAAAAAAAAADDANSGPATPRT
PSGVVGAFGGSGASPTAHAASSRKLALLSRRDELCAADILKVLIVHVQYYVIITRLPVSYPGSINALAAILNAITGA
SSAIVFSYSCLVPGQDSWEQALSQLLGSLITPLVIIAVSMSIWAARYAAVRMRAAAAHKRLHARLAAVQLQQQGQQG
GAGAGGADASRAVTPHSQHALSNVVAMLTALSADAAAGGGAGGQGGGGGGSGAAQVVDLAGVDDDDDVAAPALAPEQ
GVGRTTAPPPPPPVLSSGGASGNASADVALQSRDFLLTSAPSPPSPPPALPVPPPAVTSPSVGGAPEGVQHHFSSRY
PEILAHGAHNTHSTGGHSLVSSSDANHAQQAAATTQQSPKDSPQSPSQPPSQPSSDATSQPYSQPPSQPPSQPSSQP
PSQPAPKPQQCQQQQQRQQPLRSPLLQKLIGGGSRAGEAATQPASCTGGGVAPDPFARLPPAFSLATLQSVTSSSGV
HASVGNLPDDTAAAAAAAAAAGVRPPPPQQPLQPAGSLLSMASSTHSIASSTVTITASGAAYSYGTGGGTAHGASSK
RRISAVNSSSRHHQPTLLPSRLSNATRLPSKALSRVQSSVSSRYAASALRRTLTQVDGAVSLPEQLWVVALVGVFIM
YPGWANASLSVFTCYHIDPQPAAAQRAAEPFWDRYRATWQYGYWIRNMQQECYSGEHLAAAVPIGVVAVCLFLIFPP
LLSFLLLWRHRRALGEPDVAKLFGFMYNRYKPLFWWWGSALMLQQLLLVAVEAFGRALGNVMQQVLVMLVAFLALAV
MNAACSPARSRLATLLDFFSLSVLSLTLTLSLFFTGDSEAQLSASAQDAVGGIIIAINLALLAAFVALLLRRTWVWA
RGRLDDRMAALRQRYAAVRRRHLLPDTEASHHVNVHEDGTEGAVDAAEQQQQPFKQ

>Cre04.g219050.t1.3|PACid:27580944
MHSENIHPGWFFFALVCSATLCTLPTHVIAASDAPPPFSDAVAPADEPPPLVRRNSDPPPPLPSLPLPPSPSPPVSP
PPRASRPPPSPRPPAPPSVVYTIAGARLSLYPFAYIRPNATYPNGVLVPLTNATAGGLGYIIAAVAPSNETGAPLPA
GNPTGIPVCLPGFDNIAAALACRAAGLAGGRQVATGSSSGSGSSGGGVGGGNGTGSNATYPNLSFGPGRVGNFRIVR
NVACPYWATSLAYCQAELVDWAGAGDCASVTAVVCDKGGSPPPSPPLPSPPELDAPPPGEAPPDLGLLDLDIVVGWT
YDGVDYELGYDHTSVRGGLYGGNNLDNRTSWERVYWPRGSTPDATTYHVCVRWFTGSRSLQLDLELEVTVAGALAYS
ETAVWGTWLTRNSQCTPDANGYIGSYSYTAAGSGSSSSVGGATGSSSGGPPALQFLVAWGERSSPPPNPPPQLPPTP
PPPPPPPPPPPPVVPPGMEPVCLDTTYVAGRVLEVLGATDGPAASSVGSCLQACLNRQPRDCQFIVYDGMLGTCYLK
SDFLVTNATSEDAVSSDEYGESGHNPDTTVCITPPVVSQVLDDAGMPVVSSRGNYVCTHGWDLRGRVLGVGDRFATQ
EDCAAACDLNAQCQYYVYDTSESSPCALLYDVLAGHPKYKFSYDSEAAACVKALRPNAPRRLELPALSGLGMDLAAL
AGPDTDGSWPAISVRNTGGGAIVVYAWLVSFDSGLGTGGGASSSGGGGSDPKLYDPITGIAAVPAVPLCAGDGSSGW
GDAQAARLCRDVGFAGGYVAAGGADSAVEALHLPGGAVTGLSCPDRGGSDVPLLQCRGVVEEDRGCERVAAAFCTPA
PPPPPQPPSPPPVVSPPPPPPPPQPPRPKSATRTARLSDLAQTVLPYAHGVLGVSADLSAYAGGMPPLVSVDKPRGF
VWAATSHGPVLPVCAAGGGSGGDGWNDAAAALACRAAGFPGGRAGRAVVNLRKQLTAMITNVTCGGESNGIGDCSGV
FVEYGRCLRLASVECNWEPPPGLAPQSALFDAYEGVRTSSQGVTAWRDPRTQDQTQPLQLTMSGTCGYDNSSTEAGG
GYGSVVFDGRSCLGRLGVNLPAWSSARTAPFTLLVVAKLDVPVSARPTDSYSLIQISRTPTDYDFEAAFQVATRGNT
SSSFLYDNQAGQAFGKLVMPASYDEWAMYVFVRQPGGTAAAVYRSSQPRNGGVGTFPGGVELIASQSVPPPGVSVQP
DNLVLGADWRDNNTFVKGRLAVAAVYTTALDLGQLNDLYNLYAPRFGWARAPRPGPPSPPSPAPNVPYPPQGPAPPD
HASGDAPPPWDEGVGSVTISSPPPFPSGFFRPNWPIAILTRDTYVGCFDGDPRYPAATDPAGDSGGGAILPYCLMYE
VDNMTAQLCAEAARSGGFKAYGLADGTTCLAAFDPQPAAALLGVAPQGECSTRCGGDQQQACGGDGSFDLFLVGSAG
SSLPGTDPSSGLGSVSAAYLGVRLSGGFSSAAGRVEVQLEAGGQWGTVCGLPAFGDVEAELVCRTLGFSTGIAVFQP

APRSAALILMSDVSCNVSIHAQFSECASSRGAAVDRTCTHTNDMGVRCFSTETRPLPEIATSMIPHTWSLDTQSWGG
QLAEDDGSVGYVWSYVPSAAAWLPVCHPGAFDDTAATLACRQAGFGSGTALPYDEWGVASLGLLSQTQVLRDVRCVA
GASSLRGCTGSLAPVAMCRSLVTVNCTYDPPELSVALVDGPDGDPNTGRVEVTIDGDGKPICFDNEMLEPTSPTVDL
LCHTLGYRGGGRASRGPQELNATNLVGWLTGLACPPGAEWLSQCSFGRVTSAGSPSAVLSECPSKRPLQLRCFDTAP
PPAPPAPPQRPPATPRQPPLGNRQKADTCVYIRITTRPPFEERRSSLLRLPSSECVPFASDVAREIDLAAFELGVKL
RRPFSDTEGGCDGLGMQVCGVFEVPNRDRIWFARGGRPVEDLPKLAPFFVAALPRWIDRVFGGPGCNDTSLGYGVSA
FLQDKNGQVQFKSTRTCAEVSSPPPPPPPPAPLAPALPPGAVPPPSPPEAEPPAGEPPAVDYFCTGCVEIVMTPREG
TLGQSPPMEMPPAAPPNDGLEPPPSPSRPPPPTPQTLYGNGWPIDPDFCRTAAAEISTVINRAANDFRAVWSQPFAL
AECDPNPYRPVLRVCGVLDFVPVQLEKLLQDEGQGPRTWLQPYLMPYNPDTYDYSNSPDEELCLPRFRSLDTNEHIY
DISVRMVSNDTFGMQCLGNASYDVSCAIPDTDYSSPPPPSRTPPPAIASLAPPPPPDRPDFPPPPRRPPTPPRPPPP
SPPPPSPPPPPPSPPPPPPPSPPPPPPPTCQICATLDYAAGNPRFAFLRPTHCAAYAAAVSSGMAQIATGRVDFATA
WPTAVTAPAVTCDTTRITVCNTLTSRGSGIRIRDQLNARLESIARSANGICTASPGIVFTAAVQDGRAFPPPPASMS
CFPIVPVSFNCSLPANRDFPFRPCVTARNATAFVLDKVVPDTSRANSHCFYFRNNTPISPGGVCASAARLNGVLFRT
NSTVRPRVRTVFVENGADRSARTALGLVWTNNDTVLNVAVPDWGVASVSNPAAPDPTRPRVCIQLDPGVALSQFCGS
GAGGAAGCALSILTDVADTCCPVQPYTPPALATAGTAGRH

>Cre12.g513400.t1.3|PACid:27581085
MVFTNADVESPNATLLTTAALRSRPPTNAWWTSWLQYTGADTTPINMNPYMVKVLPTGLWVASPRDYTQLDGTCLGG
ACIRFDFDYKLQITTTTGVSVVEVESYDDLSVTFVWRAVAGGGVVMRSTLLQGLPYVTVEFVAVKPRVVVITGAILA
ANPSTWAAATKFKVPLNNQLTWVIYTSSSVAATSTSTNFEATTTFTGRWRLATVTHAMAQPYGWRKADLAANAAAFE
TLLDSCAAAVPTGARVTLGIQPAAQSPTGADRAIQQIDYTVVSLDGTASTNLLMFTMPHHREALVSPSPNPAAYLIV
KSPRGNLKTVVGTKWVLAFELPSITWAAPGGAGSNSAYLQTIANQITATDVNAAGGTDVLPNAAPQLADMARLYQIA
TELSPTYTALSASAVTLRSNLLTMLNQWLTSSDLSNTRVLSYDSKWGGIIAYGVAKWATGSGGNRDGWGKNNAYTNH
LEDYGPLLYAAAVLAKANVTWGNDVTPSVMALVRDLANPRADLSDPYFPFARHMDWYEGHSWSTGLLSRTSDGGMVN
WGKYQERSGSAVAAYYSIGLFGAAIGNADLQKWGQVLAGIEAAGARNYFQILATGTEAYPAGTFYVCNDAGTETSYP
GYPTNGKHVPGKVYQSYVWYWTEAGGVSQDAAGYTALQLIPFLPGASELTQRKPWIQEAYDSIAGSSTATPWTAFRA
LLGSATSAAQQGTSWTEVQVNVSADPTMVDITGGSFSVKHSKASLLYWVASRSFGAVPVPVPSPSPPSPAPSPAPPS
PASLSPPPSPAPPSPKPPSPPPSPAPPSPAPPSPPPSPAPPSPAPPGPPPSPAPPSPKPPSPPPSPAPPSPAPPSPP
PFPAPPSPAPPSPPPSPEAPLNAPPSPPPSPAPPSPAPPSPAPPSPPPSPEPPSPAPPSPPPSPAPPSPAPPSPPPS
PAPPSPAPPSPPPSPVPPSPAPPSPPPSPAPPSPAPSPPASPSPVSVSPVGWTPLDVSIVPTSAPPAAFSRYTNTWY
KGNYPAWGDWTNPSFDNVTYAPQVDLFATGASAFPYPTNSWWSSWSHNRTIGSTRGVGDEPVQMHPWRARVMPSYLE
LVPPGVQWDVKPGYIVPAYDRNISITAAEGFVSRRIVDFNEMGVTFEWLTATGVSGGPGSMRVTMLQGTPFLTARFV
GVTPVVTQHVASPLVVGTGSGQVGKSFKVTSNGRISFKYYFSQTVTADINDTRVALTAPFTGVMRIAVLNSTIAPLG
ILSSVDSVMQEAAYDANADIYPTSATMTPGYQTPAQSGTGSERGIVRIKFTTASMSGTPTGQLMMMTMPHHRTHLLY
PAVPGAGAARVRMDDMRGELVSVLGDEWFLGYDLAAVSAVGWGARNTIADSTRRQSIISTLLAEATSWPGLTPRDDS
YYGASDMAAIGRMAIIADEMAALESASASSLAAAATSLRTKLKAALDARLNAAGTGNNASLVYDTTWGGLIVYKDAR
YSLEHNFGNRVYNDHHYHYGYYLMGAALLGKADPTWLTANLPALTTLVRDFANPNKADSYFPLARMMDWWEGHSWAG
GMQVFGDGKNQESTSESVNGYYAVALLGRALAQAGVAGAADLTRWGQLLMAVEVSGAQHYYQMTEAATAFPVVYPKP
FRDNKAVGILWNSKVDYSTWFGSTPIYIHAIQYIPFTPASEVLLRREWMVESYPVASFNLSNLAVTPCWKQFGDAAL
AMLNSTGTATAWSRTLALPYTAADGSPNFFGGWASHPKTALLYWIASREGAPMPSPPPLPPAAPPPPSPSPAPPSPP
QPSPAPPSPPPAPAPPSPAPSPVPPSPAPPSPPSVPAPPSPAPSPPSPPFNSPVSWTPLDVSIVPTSAPPAAFSRYT
NTWYKGNYPAWGDWTNPSFDNVTYAPQVDLFATGASAFPYPTNSWWSSWSHNRTIGSTRGVGDEPVQMHPWRARVMP
SYLELVPPGVQWDVKPGYIVPAYDRNISITAAEGFVSRRIVDFNEMGVTFEWLTATGVSGGPGSMRVTMLQGTPFLT
ARFVGVTPVVTQHVASPLVVGTGSGQVGKSFKVTSNGRISFKYYFSQTVTADINDTRVALTAPFTGVMRIAVLNSTI
APLGILSSVDSVMQEAAYDANADIYPTGATMTPGYQTPAQSGTGSERGIVRIKFTTASMSGTPTGQLMMMTMPHHRT
HLLYPAVPGAGATRVRMDDMRGELVSVLGDEWFLGYDLAAVSAVGWGARNTIADSTRRQSIISTLLAEATSWPGLTP
RDDSYYGASDMAAIGRMAIIADEMAALESASASSLAAAATSLRTKLKAALDARLNAAGTGNNASLVYDTTWGGLIVY
KDARYSLEHNFGNRVYNDHHYHYGYYLMGAALLGKADPTWLTANLPALTTLVRDFANPSKADAYFPLARMMDWWEGH
SWAGGMQVFGDGKNQESTSESVNGYYAVALLGRALAQAGVAGAADLTRWGQLLMAVEVSGAQHYYQMTEAATAFPVV
YPKPFRDNKAVGILWNSKVDYATWFGSTPIYIHAIQYIPFTPASEVLLRREWMVESYPVASFNLSNLAVTPCWKQFG
DAALAMLNSTGTATAWSRTLALPYTAADGSPNFFGGWASHPKTALLYWIASREGAPMTSPPPLPPTAPPPPSPSPAP
PSPSPAPPSPAPSPVPPSPAPPSPPSVPAPPSPAPSPLAPPLPPSPPTPPDTQDSPVPYTPLVVGSVAPDAPPGSIA
TVSNNTQYIQGGVVRSHQPTLPASFFPYPTNTWWSPLSHLNLDNVDFKTMLHPWHVKVQDSQLEMVAPGAYWSVVGD
TISSAYSREVGLGAVEALGRRSIVSGNEMGATVEWLTDAGVSGGDGSMRVTLLQGCPFITARYVGLTPIIRHKVTEA
PLIVGTGLGKIGTSFKVENNAGITYKYYFSQRVTVDITSGSVVVTSPLPFTGVLRVAVLKSSMADAIALQPLLNVDV
ATLAAVEQVYDANSDIYPVGASVKYGYQTPAQSGTGSERGVLRIKFTTASMSGTPTGQLMMLSLPHQRPRLLYPPAP
TGQLVRVNDFRGELVHRLGDDWVLAYDLPPVSFTSLNGVTNTARRDAIATTLINDISGWSGMEPRNNGYLGTSDLAA

IGRMLSIAEEIAPQLPSGTTKDWLLSQVVWARTRLTDSLDARLRTSTINSSFVYDSTWGGLILLGDAKDGQLSNYEH
RTYNSHHSSGGYLLAAAAALAKSNSTWLAARQANVMALLRDFANPNKADPYFPFARHMDWWEGHSWGTGTQVFVDGK
NQDSSSEAVNAYYAVAQLGAAAGDADLRRWGQLLTAIEIAGAQHYFQSPSNGAPASPYASPFKDNKVVGRLWNGQVD
RGTTRWGDAVLTNQASHYAPFTAVSPLLLRTDWIGESYAVAAADTTTATAAGTVGWSFIANMARALTTDVSGAWNAI
SGVSPTDARFFGNWPSAPKSVQLYTIASCDTPPTPSPSPPSPAPPSPAPPSPPPSPAPPSPAPPSPPPSPEPPSPAP
PSPPPSPAPPSPAPPSPPPSPAPPSPAPPSPPPSPAPPSPAPPSPPPSPEPPSPAPPSPPPSPAPPSPAPPSPPPSP
APPSPAPPSPPPSPVPPSPAPPGPPPSPAPPSPAPPSPPPSPAPPSPAPPSPPPSPAPPSPAPPSPPPSPAPPSPAP
PSPPPSPAPPSPAPPSPPPSPAPPSPAPPSPVPPSPPPSPSPPSPKPPSPSPPSPKPPSPSPPSPKPPSPSPPSPKP
PRPSPPSPRPPSPSPPSPKPPSPRPPSPKPPSPSPPKKTPGGTGRRRQLQRESRNFKRHSRRLAM

>g12152.t2|PACid:27581525
MISDALSLTVAFLFLLCAAGFAQDQRIASFTSLPVGALTGSSLSAQGIELSAGSNPVSPAWGEVVDCAAGWSYGGGT
IMTAAGCTQALRFPYDDFSESFRPASPPSMFWSLSLTYRTTLPYVTLYLYPTSGQGNANACKVRLNRTEPIASPRPP
AFVYSASNSVTVTASECGWGAGSTFFSFSFYVGVEDQRIATFTSLPVGALTGSSLSAQGIELSAGSNPVSPAWGEVV
DCAAGWSYGGGTIMTAAGCTQALRFPYDDFSESFRPASPPSMFWSLSLTYRTTLPYVTLYLYPSTGQGNANACKVRL
NRTEPIASPRPPAFVYSASHSVTVTASECGWSAGSTFFSFSFYVGVEGSAAGIDVFELLLESFTLEAGAAPSPSPPS
PEPPSPEPPSPAPPSPQPLVFGSSRTINTSMLPLGDLTEASLEASGVGLVRLEGHIDTWGTVVDCAAGWLGLTTSYS
GMTIHVAATGCSRALSFDTEGTVSLFRPVSPTSPFWRLTLTYRTAAPNVGVGFYQTGDTNYNYRCEKTLDRTEPIVT
APAPASFTYTAPPKTVTMTPADCVGGWPPGSTFDNFYIFTTLSDAGAVLWNLMIESFTIETGMAPSPSPMPPTPEPR
TVPWPPSPEPPMPSPALPSTRRVSFDSFPLGDLTSTSLQAIGIGLGDPTFPTTPVWGEVVDCVAGWSYGLVSNTAMT
AAGCVKTLRFPYALDTGRFAIASSPVDRFYSLTLTYRTTMPYADLVLQPVDSQYSPCTVKLNRTDPIDRRQPDVFTY
AASPVTVTVTASECGYAAGSAFWSFQFYISYGDDDSELINVFELLIDSFVMETEAPVLASPPSPAPPSPAPPSPEPP
SPAPPSPEPPSPAQPSPAPLVFGTSRAVAFTSLPVGALTSSSLSAQGIELSAGSNPVSPAWGEVVDCAAGWRYGGGT
TMTAAGCTQALRFPYDDFSESFRPASPPIMFWSLSLTYRTTLPYVTLYLYPTSGQGNANACKVRLNRTEPIASPRPS
AFVYSASNSVTVTASECGWSAGSTFFSFSFYVGVEGSSAGIDVFELLLDSLTMETGMAPLSSPSPPSPAPPSPEPQS
PAPPSPQPPSPMPPSPEPPSLAPPSPEPPSPAPPSPAPLAFGSTRTASFDALPLGSLTLTSLEAQDIRLAHQNESNA
LPGWGQVVDCAAGWTNNGSWAMTATGCNKTLRLTNQLSDFFLPLSPPSTFWKLSITYRTFAFTGLVLYGTGDVNKFC
EWTLQRTGDVKYNYRCEVVLKPTETIAGPEPAEFTYTASPNTVTMTPADCGWAAGSTFNSFFLFMYVEGDSFTIETG
IAPSPPPSPAPPPPRTIPWPPSPEPPSPAPPIPLLASTRVVSIEVLPLGNLASTSLLGLGIGLDADMYTSSPAWGEV
VDCAGGWSYEGTTMTAAGCNQTLRFPYQGSTGKFSPLWAPNDGFYSLSLTYRTTLPSVQLELFNWQDCTLRLNGTES
IVRPRPAAFTYAVSKTVTVTPEACGWAAGSTFINFYFYVPPNEYIPGGIDVYELLIDSFTMETRMAPLPSPSPPSPA
PLSPEPQNPAPPSPEPPSPMPPSPEPPSPAPPSLEPPSPAPPSPAPLAFGSTLTVSFDALPLGSLTLTSLEAQDIRL
AHQNESNALPGWGQVVDCAAGWTTDDSWAMTATGCNKTLRLTNELSDFFLPLSPPSTFWKLSITYRTTFAYTGLVLY
DTGDVNNFCERTLERSDPTPLPNPTTFTYIAAPITFTVTPADCGWDAGSTFDRFAIFIYTFMAVGRDRVELLLDSFT
MKTGSAPSPPPPPPSPPMPPPRPPSPPPLPPSPPSPEHMVFGSTRTVSFDTLEIGLLTETSLEAQGLALSDYPFGTG
HFKMTWGQVVDCAAGWYLAAAAVSMNAAGCKRSLQYKDEGFSSAFRPLSRVSEPPPFWRLTLTYRTTSPNVRVGFYK
TGDVEYSNFCQVELKPTETIAGPAPAAFTYTAGPNTVTMTPADCDWDAGSTFDSFFFYIAMADDDAPVSELLTDSFT
METGMAPSPSPSPPSPAPPSPEPQSPAPPSPEPPTPMPPSPEPPSPVPPSPEPPSPAPPSPAPPNPPSPPPLPPPSP
PPVSPPPPPPPPPPSPPPPDPLPPISSPLPPSPSPASSPPSPSPASPSPAPPSPAPVVAPSTLPSPAPPTPSPTGSP
ATPPPSPSPSPSPPSPIASALPSPPAPLQQSSSPPASPMIVATFRSFSLGCFAFDVVSASPSLRALPVTLATNDAAM
TVAKCAGLTQAAGLDHYGLTNGHTCVGGVNRQQATQYGSLPESACDRPCPGDSRQTCGGGPIDSADSAGTQLSALSL
YSFNPASPPPRPPPRAPRTRPARPGSAAKPPSPAPPQPTPPSPSRRAEAQALTRTAATDLPALQKDVSPTINGIPSA
RSLLLLSPTAVPVFTATLLGASAAELRAPVLASARVGMGRLVAFGSEAMLTSCCNQPGAAAAANGTESAAEINKIIA
NAANWARAAAGAAGRKANIRVADPRLDAVARFIVSMLPDIFAKSRQDYYMSLTTFAKGGHERCDVYLVLGADPQQRY
DTKVRDALRSFLENGKGVLLAGPVVADAGISGAAAPVPSDEWTLPDIMFTKQNGVTAVASATAVAPSGVAAEHFREL
LSRLLQIKARSSSPEFISLKARVMRARSDIAVTDMSQFDEVLKAKIAEFDRDIAEL

Volvox carteri

>Vocar20008550m|PACid:23124792
MAKYVYWMGLPFGKSAGTTLLPHIRVLTSLDVHRPFMQSRMCCSPLAANRSFAAGKLCAQRICEFCTLDKVEDERHV
LELLATFHCAFKSLICCQDNFYVWRKHYFIIFSGGEPSIGLYFQFKTDASPDKSKPLVGCRVLVRAVIVWRDSQAGV
AQNTSTSFKSEALGLGAKHGKRTQDILEQQGRFRFTIEFKDKQRWYLASHSVDDRKEWLTAVVAHYHALLRRSGGFS
FRHGHDGALAGARRHHQGGHGHAFQAPRSTASSPPGTPTRRQASPTASLRTSVGDLTAEDGDRDGLPSASTSELGTP
IRAMPSGNTTPQSVTGSMTSMAGLQQQMEQQTHSRRPSGDSSAGFQAHGQGNIFRGGDGSTRGRSATLAGLAPAANS
APPGGFLPSFSSQSALLTAASHAPSSASKASGYAGAAASGPAPAAVKGSFFTKFLWWDAPVTTTARPALSAGAGGQQ
QQQQALAAVRPRPYEVTPLGPDLGQLDLISGKFDGSLVTSSVCEQFERARTYVDKHPEHFSERQRGALQNWASLVEG
QAATPDVLMACKALYVLEVTRVRPDWSAADEGDTSFAGPEAVIVQVRRMPTAISLTTLKECLDYCSKDWLDMFCRLD
GATLLLDVLRSHEGPARQGIPEALEALMVTLQCVQSLTSKPGGMAAVLAVRGFTRAVAALLRPVDSDTTRVALELLT
KMLLFHDQSYRQARSLVASGEYCCSMTVVREIAIPFHSLTSESSATVPCSVSGNAALTQPSYLICGHVYIGDSVHIL
TDTERNGMRMCRVRACCTGCLGAAVSRLKPFEHVSGVGRAAATPPPPPPGPPPPPPVTGVARAAATPPPPPPPGPPP
PPPVVVTPRGAPAPPPPPGPPPPPPAVVRPPPPPPPPAPPPPPPPPPPCLQMSGGWTYLPGATVKVNESTPNFHSSD
GVEETTWDYGTRLPDEWTRALVANTPGTQHVPLFRWVDAGMPPPPPAPLSPPPPPPPPPPGPPRPIVVIPKSPTSY
VSSPNDGNDQGPFSFSTPHRTGDEDDESEGTPLLGSAGIASRSGFSTRTAAQPPAPSSQPVNPDPRSQTSGASATEG
STSARQSSGGAPVCSAGVGPAAVSGLSMQPGGPGSTCADGGAMALGVAGARSDGDQHLAAEVAEEYVSALLDLLDME
NDKFDQDLVLHVVKFISVVLVSPEAEANGPLMQRFVDALMSRRLLSLFADLTGMDNTFLDKEVMAIKDTIMQIVAPL
PPPPPPPPEPEPAASVVKPLQHSASFKPQQQHPLAPLPRTPLPGSPPPQVVIPGLSPADSSTPPPPPPPAAATPPPPP
PPGIFARPASVPPPPMATPQRYTAKPVPQPSKKMKQLFWDTIPYARLHGTFWVEQDLDEECKGPSDAAAATGSQQAG
EVPEGEGEEEGGEGPPSLDWSLMEEMFAQVIKARATPAKTSNNSKQQQSVIDSKRAYNIGILMGSKIKIPVDALRAR
VVQLDPRAFDSEEAVGALLQCVPSQDDAAALAAYRDSGKPLEDLGEAEKVCLQLMSVPAVEQRLRVYSYKFQTPHKL
KSAKQVFAANLRAIEAMRTSPMFHRALRLARDAGNFMNFGTRLGNAIGFRLRALPKLQDTKSADAKHTLLSALALEV
MRVGGRSTGESVLADEMGTLSDSMLKVSHAEAGEMLAIAEQQLDEIRGFLKTYTPITDAGWEAADLAMTGAVTCAGV
EDTFATTMAAELEAMEAQYSEVRGMQTCMKEQYEAMLKYFGENLNSTPSDTEFWAGIAAFVDKFSATQKALLAVRRP
LGGEQGTERQLGEWLGDGTSSMLWLHEAKDVKPTSSLGIVTLSLHRPGRGWRKGFLPMPSRVEYDVPHPGVSKVTKG
INGMPLTQKRLETLEARERLLSSGRTPSKTMSLAGAPNGTTNALAAAASAVLHTHPGTPSHDGSSNGAAGQTGSPGF
LSPLTLLTCQQGDTPRATIRPTSTLRLDSSRDDESGWPLSSPIRGQSFAARLSEVSFASSSHVVSTTTLGSPDAAAA
PAPVQLPPPRLSASQAGHGGGNHAPGLAHGRGEAAMAGLAAAAAGLAAATASVSSTAAAAAVCAAVHTRSVSTGSAG
QLASLSTSTTQQASAASALSDATLLLQPPPSSMAEAAAQKAAQEEREIQAAKQVSNRRLADILMVESRRRKTAEGML
AAAESAAPGTATCSVSSGSLTPKMIGR

>Vocar20013465m|PACid:23124129
MARASSGSFLLVLITVLVTLSGCLGIKFDPCQPLSEIARGDAFPLGLVLIPSINASLLANLTAANGGMCNTSFQNYL
VTKFNARVAIYNMRVDRLQVLKMPFPDIIFSMVNATTPLMALAAFRANVTSAPIYIASGYTPETYGAGFMVSTALLL
RFDLGMLQYLQWYDMTCNECGGIRSDLCLHCTQAGIRACATPLNSCTCNTTINTDAASNSSSSNSSSSSSSCSLADE
RFDVCSTSINAAWLGTDRNSAVLRTGPQVQRLNAYSITGLFNTARDKFTQLKNFAYTNVLSSWNAVSSDAQSQYVDF
EGGITQYDRKSPPPPPPSPPRPPAPPPSPSPPPAPPPAPPSPPTPPAPPLSPTPAAAPASGRRLTQDEAGTAAAAAA
AVQDAAAKEQEPEEAKL

>Vocar20011773m|PACid:23124285
MSMPGHKLTTLLALSVLAFSCLVEGHGGGLHDWSFLPVPTPGKEFIRHHVASCSLWTTIDPSLGVLGPLPMFPPSGS
KLTATGSASIFYFLDGNCTDRATTSIDMNVNLTLNGQPYAFKQVNGYGFIGSCSNPNDLYRHNASLGDNRFNLMWFR
RENTNTTEEGHLSGAAPNVMISRAPPATDALRSLVLFPSPADSDPLCCDLVYNPPPPVDFFTQGSFCRVPAPPPPAP
PSPLTPSPNPPSPPPPSPKPPSPPPPSPKPPSPPPPSPKPHGDSPRPRPSPRPSPRPSPRPSSRPSPRPSPRTSPRP
SPRSSPRPSPR

>Vocar20009362m|PACid:23124791
MNSMSSAQMLPLALVITLMSFNAVNGQPGGGGGGGGGGGGGSGGGGGTSTPCPRGDGQVVRLVSKTSTEYTVFFS
GCPFYSPYNQTTPNTPTFQNKTVTFRITPVLSSTVTYVGIYASGTTSNSRKIMSTIGYTVNGMGMYSDADALGRDAY
VYEGQSFDGCRGHADQSGLYHFHTEPGSGCVYNETAGTHSPLLGIMYDSIPIYGALGDNGVPPTNLDECGGHTDTTY
PFYHYHTTYNLKSPYIVKCLRGCIFNANGNPSIQQYNLVKTDATCVKAATQYDYSSFVNPLLSSSPVTTSSPPPPPQ
TGPSSPPPPPSALRSPPPQLGKPPKAQAGSPPPPPPPKMVMQPKPAKPIQGKPALGRRHL

>Vocar20008839m|PACid:23125087

MPSSFPRHLISLLAFLSSTSLPDPTLPSGRNLVCSLANASLMACPMLDWSARGSFYPQQQCLDDPLGVSCWNKDDPA
ATAILTSPPGYYQCTSEGAVRLRDTDTVDWGRVEYCANGEWGSICHDGWDDLDATVVCRQLGYPYGTALKGRLGGGL
SPPGPEGMAIWLQQVNCSGSESALADCPILTLPGTPLACSHRADAGVACHDSKPSTPGVPYFSGPICNDPGALRIVG
VSDAAGGALGRLEVCYAAQWGAICESGFGYNEAAVSCRQLGYTTGRAVPAVSMPGRPANLDEPGAVRPAHEFAAEMA
AALDHLRLLGNGRCKLPVRRCGGCAVHQRHEICALSAALQLGFQTGNLLKGGLTPAGDPDSPVWSGALDCLGYEDRL
QECPPGDDSALPGDYSAACDTHSDDVGLQCRNSLTQYEVPRDTAVSGAFACENQGQLRLMDGIGRTAYQNGLVQICL
NGRWGTMSLGLMAMSGARVCNRGFGKEEARVACRHFGFVSGMVTTSSLPINYVILAFSQPINMANVDCTGTEERLTQ
CKYDMYDGACTHRMDVSVRCVWGELPPSSPPLTLRWPSPPPKAPPPLPRPRSPSPPAALLRPPPPAPRSLLMHPPVP
SPPPPRPRPSPRPPPQFPPSPPIPQPPPPPPPPPPPPPLPPPPPPPLPPPPPPPPPTLPPPPPPPPPPPLPPPPPPL
PPLPPPPPPPLPPPPPQHEVPSPSLIRTPAPSLTAALPSPPRRSPPSTTPPLEAAFEACDYEGDIRLAGGNLPGSGM
LQFCGDDGTGSFKWGSVCGAAPLREDAVNMICRLLGYRDGFAVVDPNNPSYGYDGQFYTFQPNPYNMPTWLYDIECA
ANPSVMGDCWSTSQYCAPQSDIAVICLRTGRATRRYGCGGKPGALRLVGGRSPNEGTVQVCKNVVWGTICDTEWDDV
DASVTCRMLGFNWGAAVSAEGRFSNGTAAGTTFPQGPSDMRVQYANLACDENYAETLDDCERASLRVFPPECKARGF
RRDAGLICWD

>Vocar20008475m|PACid:23125583
MAGALVFSAWISVLLLLAADAPQTFAAIDCPVVPGYTFYPRVQEVPGSLNLSSPTSNASSMTPASIAAACDQTQFCN
GFHTSGSLKLLPMAPVLSPVMNGTSTGNNESSCVGIYYSNRTLSGLSLPDGLSFETVRQTGEQKVQGMMATIAVAET
VASMFKDQGVDDLTKLPESILLQAVASTVAAISREGNSSNSNSSDSITSDGLLSALLYPVWDSRSVNGSNYISPVKD
QGGCGSCVAFAVTGAAEAAVAAARKTTVNSNDYSEQWLFFCNGMSSAAFPNCDSGWFATAAAKVVVTKNIPYELNYP
YTGSRGCALGSPPERRAEGGFKDTAYTDITQAKQHIRMYGAVTSYFAVYGDFFRWRASSPPYAWDGISALAGYHQVL
VVGYNDIGSYWIVKNSWGTRWGDNGFIRISYSANVGFMSGYVGSIIGLRWEAAPPPPWPLPSPPLVSPPPPSPSPPS
SPPPPPPPTCQKSASYYWNEATNPLGPNRCNPAGRGCECDGLRTCSSYGWCQGISRPSPPLRSPPPSPPPPAACQKS
ASYYWNEATNPLGPNRCNPAGRGCECDGLRTCSSYGWCQGISRPTSPPPPAACQKSASYYWNEAKNPLGPNRCNPAG
RGCECDGLRTCSQYGWCQGTARTRRALRSFLSNLSNGNRRVN

>Vocar20008683m|PACid:23125635
MVALVAFLIGFLGALGPAWANPKHVALYMSEAGGVLDLSWDRSAMNLEQQLRQLGLNVMLATTGQPRLEGSAAPKAY
VIPPQNAYNYYSSAEDMGAISSYLASGGLVIILDATHGQGDALSTFVAKAFDYTGKWIVCDQLYTNDEQALGELQLS
THASAFIQVKTSAQGSWPSTLEDAQTTSLYTSCLHEDLRASIVPLYFAQDSDIKVAAQAFSKAGVPGAIVWLGYDWR
GGPQTQWGNLLKSLISDFKPTSPNWQHLGTFGKMLFSASDVNLAGFLQGEFHFTSRKSADSEAVTVEMEAYDIDAEL
LDADLLEDADLLYSVLESASDMSDPKHASEALRRFLQNAPTGLYPMPPQTYPLPTYPPPSSPSQRSPPRPRPKPPSP
PPPPPSPPPPSPSPPSPPPPSPSPPSPPPPSPSPPSPPPPSPVPPSPPPPGLRPPSTPKPLSSPPATAAPSPPVTFP
PRSRNPRPPFIRRPPPPPPPKVSAPPPPPPKASPPPPPPLASPSPPPPQSSPPPPRSPPPSPPPSPPPPSPPPPSSP
PPRPPPPSPPPPSSPPPSPPPPSSPPPSPPPPTTSDAYIMVNNFTAQGVIGSDGLVFYGSPFFRGQLKPRTAAQLVD
PNKKPCTPPGSAVCYACPRAWMATATERSVALFFKDPTQLSRINIKQIKNPGVITPHQLQRDIRDSRLGAGIPGAPN
GATHGDDDACSSLHANRACQRHRMSVGSEGVIEGNLFTTVYNISSPSQTCQSVLSIRVTPSQSGIYEDVPEDGSSAE
LPENLARTAAGGVLITAYPFPHNAASNFGPFLEWVRFSGRVLYPSDLESYQARMQK

>Vocar20008459m|PACid:23125714
MKNILLAALCVVAVFSASHALSSPLSPPPTRYQAFPYCAKCVALESIYRLKPDFSGLGNRTFCFTLEVNPLNADFMC
KNAPIYKMDMLLKKGCQRDVVIKASVNGIFTYSFLEVPRYASPGTVILKLNDLTARHLGTNSNGARICLTILNGGKG
NCTTLRSLCADPKTTSFRKINNGNYRNTNSQECMVALFAGSDNANDRTCCSDPPLHPPSAPPPPPPPPPPPPPSPPP
PPPPPPPPPPPSPPPPPPPPPPPPPPSPPPPPPPPPPPPPPSPPPPPPPPPPPPPPSPPPPPPPPPPPPPPPPPLPPP
PPPPPPPPPPPPPLPPPPPPPPPPPPPPPPPPPPPPPPPPPPRYCTEEKVCVYLTFGFRESNATFPNGTCDRLAEQI
QSDLLNSSASYILRPPVVPQITCTSDFIKVCLNLDLYANATKLHPVVGENAGRWLNYALTSTCAASRSKFNLTIAVG
GDGDWKNGNVSFRCFNASDVLPCQGSDQNVTCNCNMTRNITPFAALPTMSGPFTGRKNTKLYCFNITLVKPIEPNST
CGKSNLLDKVIIWADDTIYRSSIKSIALYAAGDTSAKYVVPNWNKFGSQQVKATGIGWTKDKANGGAICLELDQDVD
LSDFCLWRFPQQKNSCIRSSNIACATADGSPRNEAEQPKPLLRDCRFRQTCTFPTTTRCSTTRRIFSCDSNPSRDSA
KILALRFVAITVPESPSEKE

>Vocar20008317m|PACid:23125917
MRWANRLGVGLLFLSYFATAATSQSNPTSPLWICDDCPDEISPVCVNGITFRHRCLAECQGHRLGTDGACTGDDLLP
LAPWASTAAPASAPPAASTAALASSKSSTSPPPPPRSPKLRKPRASDMKRFESDGFVYVGSVELHSGPSGGGDPSNL
GGRMRASVGGAGSGSMAEAAGAVGASSLDNLQRSYNTQGKPVREHLAQVEGKPSSCSSYANTFRHASASYAAIHPAQ
QASAQSTHGPLAFYPAPTISPLRVTLVVQVRGMRYSPDTGDFFLEATPRPIRQHRVIKRALMAGEDDASRGGGGSGG
GSRRVLGDNRWGVLDAKVYPFSALAHFIFSDPSATAKFQCSGAFITPWDVLTAAHCVWSFSTGTGYKDWRISPALDK

KPNYDSPATYISADYATFYRTETTNRSYADQRGAVNYFDIAVIRVMSPHTAWLGIKYDCARASYPKTMACGYTDPIY
NPQCNQCFLTTSQCRPMWMMYNYCYSRRGQSGSSIVDLDDLRVLGVLSGGPPNDWDYSYWTPIDAFHFSNIVRWMWK
PPPPPSAFRSPSPLPTTASPAVRQSQPSSSSPSRQSPPPPPPRVSLSPPPPPPPPLVRMPPPRPRFPPPLVIRTAAS
LPQVLSRNAGLPETSQDSTSDLDANPPELVPVLATALSPPPPTTARSSVLSTVLLSAAQAAAASVPTPASSGCTNGQ
LRLMDGPNAWSGRLEICRNNVWGTICDMGWGWDDARVACRQLGFPAGGEAVEGGWFPNATESSPIHYSNVTCLGYET
ALASCLLPNRSTSCNHKYDAGVICANPAVASPPPSSGFTNGSNSWQPGSYPCSQDGVLRLVPKDGMILHSNRPVTSN
SFIIGRVEVCSGGQWGSVCDDNWNNNDALVVCKQFRFPSGRALTRAITSVTATPAAPPPPDSNMLDLWTPGPLNMSI
WLSNVDCTGAESSLLACRRRTIASGFRLPCTHQEDAGVVCFNTPAPVAPPQPPPPECSEDGALRLVPIAGRQGAGRL
EVCYSGRYGLVCDNGFGMPEARVACRQLGYLYGRPMGPSNSAVAGDPGPGAFFWMSDVDCVMPYTYNGGWLLRLTQC
MFLGWGGSTCNPRRQAVGLVCSNDPSTVAPSPRPPPSPYLCECEEACGSALTLASKRKGVPFARRAQPPPGFKTTRP
PHCAAASGPTRPFHQCALLTRAAAGSTPGTVRLAGGSSTSGRLEVCSNAGLWGTVCDSGWSDVDASVACRQLGFARG
VAAAPNSFPSGAADQLVLLSDVACTGSETLLSSCNSRNASAIQGCNRHERDAGVVCSNSQPPSPQQRLRPNITCNVE
GAVRVVATSGVIYTSVPAVGRVDVCYNNEWGALCNLYDDPDIGWDSISATVVCRQLNGGSDSGVAVLALNNADGGVP
PLPSYMRFWKYGILCTPEDDGTPVEARVADCPGSGWGKVLDSCKASNVAGVSCKL

>Vocar20009430m|PACid:23125934
MLSSLLVLLALMTTGAGAAPVKKLPPPPSLRSPPPPVPTPSQTSVALALQGKLQYRTTRPAGTWLLTGVVVNKVTTN
YQLPGQPIDGTTGMPIPPGRTISLTCILSGPKSTVCTSVAAAKVTVAAAPVQSTNLTLSVLVMVVSLTDSASCASRP
GANVTDVRNAFLEPDGYADFFGNCSYGRMVFNRQALTVVSTAVSCSLPIMRCAEDAIASAAQQQATASGIKIGSYAR
FLYVLPVDFAVTCGWVGLAELPGAQSWFTADSQGIFSKGTVMQEMLHNVGLYHGWQDGVEYNDESTAMGYGDSCPSA
PELMRLGWAFPLVELNSSSFPVATFRTFTLPATYLGPTGVMIKIRLDWLDFYTKNVYLALRVKAAGDISLLDKFNGK
ISIHEVNKDIDNDFTAPGDPRVSIIGVVGANSPSSYFNYKLYLLTGAFNPVTSSMSVKICRFVDGPNECVDMPPPPP
PPPPSPPPPPPPTQSAPSVLLNQPFPPSPEDREARPPPPGSSSPPPSPDDSLTVRPPPKDRSPPRTPSPLRTDRSPP
RTPSPLRTDRSPPRTPSPRRSNDD

>Vocar20008988m|PACid:23126293
MSVGTRCLLLVVAALLLMPTSGPALLAKAASDTSSSSTLTASHQVTQALTAAGSSLGSAAEGARLRGSLGRAGAAGE
RRAAAETAADIIFPFFRPPPPPCFIRIFRGDIPIWIPCPKPPPKPPSPRPPPPRPPPPPCFFTIVLGGIPVRFPCPS
PPPRPPPPPPRPPRPPSPPPPPPPPRPPRPPPPLCFITIVINGRIIRIPCDLLNRSPPPSSSPCFVNGLGSDGTTLPI
PCNLLPPLAQES

>Vocar20009617m|PACid:23126309
MLPFRMIPGRSVVALAALLGLLAVHVNAGSLNCTAVMFLRSNYGGETFTISLAPQGQTGKTVIPDLAALPKPATYRS
WDDSINSALLECVCTGSDCSIDYAQVTLRLFAEPYWRKNGGGDKIDVRCAGMPNMPSCAGLLPVMPKGWGSRTSAVA
LLYNLTSYVLPVKLPSSPPPATQPSPSPSPSSSPTPTPSPSPSSSPSPSQSPSPRSSPSPTPSPRSLKPPSPTKPAT
RSPPPRA

>Vocar20009279m|PACid:23126591
MNAEGSLQMLLAGMLAINLLSLSVVTAQPGGTTTPCPKGNGQVVRLVSKTSTEYTVFFSGCPFYSPYNQTTPNSPTF
QNKTVTFRITPVLSSTVTYVGIYASGTTSNSQKIMSTIGYTVTGIAMFSDADATGSDAYVNEGHTFDGCRGHAEMNG
LYHYHSEPGSGCVYNETAGTHSPLLGIMYDSIPIYGALGDNGVPPADLDECGGHTDTTYPFYHYHTTYNLKSPYLVK
CFRGCIFNANGNPSIQQYNLVKTDATCVKAATQYDYSSFVNPLLSSSPVTTSSPPPPPQTGSSSPPPPPSALRSPPP
VLGKPPKAQAGSPPPPPPPTSRSPPPVLGKPPKAQAGSPPPPPPTSRSPPPAMTRSPPPSLPSKSPPPPPPPAVAGS
PPPAGMRPKPPKAVVGRRL

>Vocar20003715m|PACid:23126790
GVGSPAWVAVNAQTGRYPFFPFCQCTKTPSPYKLSPSVKAMGKGRFCFTLNVNPPACSFPCCKADLRKIELNVNPEC
DAFGVTIRATVDGKFTPVAPSFDEPDQGPIGAKILRIPQLGLGLNSDGAEICITLSKPKGGAGCTTLESLCVPPGNM
TTGVCSAALFDSSNDCCPISSVNVPSPPPPSPPPPSPPPPPPPPPPPPSPPPPPPPSPPPPPPPPPPPPSPPPPPPP
SPPPPPPPPPPPPSPPPPPPPSPPPPPPPPPPPPSPPPPPPPSPPPECVLCTSVIYSSAGSIFSYSVPKSTCDQLSS
EISTSITAQANAIGAAIIPTPAAITCESDKITVCTTFLSAADAQKLKEDNWLDSQVAIWVSRATQNCPATLDGYSVS
VFADSDCVSAGATASCFLSNLEFPKCECNTGRLSTPFVVLPSFTRLPGRDRTTNLFCFKIDVRTPKNKVGLARSGRG
RPCPSPNPLYTTCFAHSCKRTTAHEPWRPSSRTLHVHLPPLP

>Vocar20003702m|PACid:23126828
MTERLVLLLSCWLMVILGCLRTTASAERSLLQAYLETFPPYGCNRDPQQSRFRLDPVYTVSGNRVCMTARVVDCAKP
GSDCCDPKIDFYKIELDVNKQCKGAVTGVTVNGKPALAPTFDPYATNDTKAVIKLTGLNLDLATADGAVVCMTLGGS

CPSMETLCAEGNGFCKYAVVQSGTPSPPPPPPPPPPPPPSPPPPLAPFTPVKIRSPFPFCAAEDVFPTTHPLPTRSC
NRTLPGLVPFTFDKTPSLSLNGGNRRYCLTLRTVPCADPSSPCCDQALSKVEWWSRESCRGSVRAVYLDGIKIDSQW
GVNGTFKIPQLNMAPSSVPLQGRQGGLGACTYSMFSEDKDCCPIGSFVTLGRRR

>Vocar20003588m|PACid:23126887
AQLVPPPPSVLLPPNPFLPPFPSPPPPVHPLPPPPPLSPAPPPKPPPECSYCVQVGVTPPNALAANQNPFTLDCDGA
ARRLASYLEYRFRLSDLSLAKQFNLDSCQGTNDSANSSAVGEQFVRVCGMYSAPLPTDKLPFTDISRYLIESSNMPM
RLVTGADNTSSCTYDARNYTFTLRSFFVKGNSLLCSGNVGYANGTDHICIPKSSPPPPKPRAPPQPPPSPAPPAVPR
LPPSPPPRPRPPPPPPAPPPQPPMPPTPPSPPPKPPGPPGPPPSCKVCVTVEIIPPPDASSNPALYFNEGLCNRTAS
NISRTLGELFGSNQVTLLQPFVVTVCRPPSPGGKADGRSSVEVCGALPDGDSGVFPLNATVLGDALLAFARDIINPE
ECGYDVVATILGSGSANSSYCDVNDSKRQPSCALRSPPPPPPPPPPPSPPPSLSPRPPSPPPSPRPPPPPSPPPPPP
LPSPPPPPVATCKICFSISSDEPNTCSSLSSATSAGTNYLQTRGVSLMTQDWRQSCIGRETRICIDYAANSISGLSS
ATDIVARVGDTMYRALSPQPRCGERKRIWSEIPISGSCPSVSRDVDICSAPQPVLRCVGNNPRNANSPFRMLAPAED
KITMFGTRNTYTCFQLQPSGGTNDCADDALVGIIFTGNSALLRSTNVLLRTTVDEPVAGSDLYPSPANTKVANTLTV
KLTRSWTPALVTDWANRGKGKVCLDMSLSSLRDFCLGGTCSVNLVYSFDDCCPKQDFSIG

>Vocar20002258m|PACid:23127240
MGSLTMRAALVALLVIIATSGGDAQLRNYRSKNGVRLAGGSGPKGRLELSSVDAWFSNSSAPTKPAWNAVCDEDVDE
DVALIMCQMLGYSYGRKAYDARFSYRKPPTSTARIGRITCDQKDSSRRALRGLPTQTTATKNAGGAADQPLLEVNVG
SASDAHDPRVDRSLYTPRMATLRSPPSAPYTCMFGMGRCDTQGPLAGLECSHTPLGDASPPPPSPPSPPPAPPQHSS
FIKLVGGRPADQPWNKVESNLACISDYYPCTNFGRVEALVDSLNGDGTMTWAPVCAIDDPDLADRVRSVICQQIVDW
MPNRDGMSLTVNADVSFEVPLDPVTSSSDFDPNKYDTWFTVLGYDGALARLQDFMYMASQTPCESLLGVQCQVFRST

>Vocar20014516m|PACid:23127568
MISKLILRHLTTALLAVLVSSAEGSRGRGAPSSECPLVTGYEFHRKQEAASVLILGTSAASSPDSIAEECGNTRFCN
AFTAAGDLLSVPMFPVFTDMEGDGSAADVKPCDGTYLSKRTLSGLRLPADMKLEALRRVGAEKARNLRAAVAAAKRV
AAKLRMKNRNPRTAYRLPAEETALDQSSVTAGSYMYDDVLDALSYPEWDSRFSGPFNFVSPVKDQGGCGACVAFAAA
GMAEALIATVNNASAYDIDLSEHWLFFCSTSSGPLCEEGWYASSAVTVIANQSIPFEFNYPYRPDAPSCKLLSPPER
RPGGVFKYIYITDLTAAKQHIRTYGSVTTFFAVHADLYTWSPASGASRVYVLSAALTSFVAHALTFLNPYVWDGVSP
QTGYHQMAVVGYNDTGSYWIAKNSWGTGWGDKGFLLMSYNANLRPSPPSAPRPQKGVRDPPSPPPTFPPSPSYPKPP
SPPPSRDCGVCRNCNRNFLCEPHLGEKCVIGGTGCLDCGNCKTIFCGDGKCTSNSPTGGYKETCSSCPMDCGRC

>Vocar20014578m|PACid:23127645
MARLLLLCFCCVSIFIIHAKGQNTKAQNANPLFPDCNCERDVRASPYRAYFTGMVPYTGGSKFCFTFDIVPCQIDNP
CCYMNFYKLEFPFMLGCRDSLQFIEYQGSNKSITWGPSYALANPPRIDLKITNMRLSQTNTRGQSICIGIADKCAEL
HSWCWPASGNCKLAIFNADPFDCCPVQPFTRIEPAYRASPPPPLVKHAPPPPPPPPPPPPPPPPPPPPKKRAPRPPPT
CSVCVRTYLSYVPPGGKFNFSQTACQDLHFFLVDELNAQVQCGGSGLRESSKVHCVLAGKAVEADALDARMLVPFTP
EPCTATEIRVCGSFFSAEDGQLMQDWMDSGALAEFNSFLLPPNDPCPSSLQGYYLTTEVTESSCLTGPPPPKPPPPS
PPPPSPRPPSPPPPRPPPPSPRPPSPPPPCEVCITITITITPPGKQFTFDEGLCTRMQTLIAKDVREQAMWVGARIL
TPFAPDLYECYPGEIRVCGVFFSDADGQLLQPWISSGAPFINWYDALFPGSCSSHPEIAGYTFTLKAANYDGVPGCL
TGNYVSPACSSFPPPPPPPPPPPPPPPPPPPKPKLPPPPPPRTRPPPRPPVATFPQCECDKRPGSSSLFFSQSNVTD
LDTGMRMYCFVVGITPCLRQSPCCTQDLHKIEFSVLPACVGSVAYSATDGELRPAQFQLKPYPAIKFNNFAKAFSDA
DGTEVCLLLRPPCDTLEALCGGPTCLYSLFNNRVSNQPKCCPVRNVLDPLLE

>Vocar20006332m|PACid:23128305
MSPLVLLLISGALAASVVVSSPVITKAPQPRLSFGPPKSPAPGPPSPKPPSPKPPSPKPPSPKPPSPKPPSPKPPSP
RPFPSPVQATPPPMPRPPPPSPRPSPPPPRRPPPQPSPRPPPRPLVSPSPPSICADQEPLASSCPIWKSSGFCTPMH
FAIDQSVVDYWCRKTSCFSGARTSKLYRLILATSNDIMVLSYCVELKRSGYCDPKYTSGGLSIRDYWCRPTCEVCTE
CVDQLSYCKVWKNSGYCGLTYSADGLSVRDYWCRQSCGVCTAPPNAPSPPPLPGDAIIGGCADPAAALLLHNQYRAR
HGSPALGWNNTLAQQAKNWAANLALNNSCSSYYEGIGNENLYTMSTSSQTSPLNCSRAVEAWYDEISLYKFTSTPYT
DNTFSSIACFSQLVWASTVQVGCAAARGMNCYVVSCRYAPLGNYDTDSDFLANVLPPVTTRRSLEEYVAAVDHVLPT
SGDRDGRVVITSGA

>Vocar20007485m|PACid:23128677
MASKRLLLAALLAAVCAVIPHALSQSAPDGGDNLNVTVFVEGMVQVYADSVFTLDGEIGPAPPIQPEYTYTLVDKAV
NDEPPNEVPVAFGEEEGASLTTGDIVQTALTLTLTAEQASELGFGFSDSSNGRRLLSEEHEKARRMVLDFHNTKRSL
QEFISLTNVLGVLSSISLSSKPKAENPVLVSKEDSKDLFVTNGASLDVSSLTFVFRSTSCGLYPSINSSVVRKYWFN

EESLNPKVTATLQRYHSACTYGRLTFTPINNNIYDVDIPCKGTLRGGIAYDLEDGTGNIGMLDGLMALVDLAKDYLR
ENDRDAYRTWKQYRRKIYIFPFGWKRVYNADFIGRASHGCVSNGNCFAFINSAPYGLATEQVTVPLVFHELGHNIGL
THAAAFDCTDPRCKVVEYGDLTDPMGLGAPYDLEKNLVCMSAPQAYKAGWASPIPGGHIAAGRDLPPGIAKTFTLPS
MSLGSENMLRIITDAANTALNGSATTTRREAQRALFVSFRIRGTDPASYDSGLGRDNNIYFDLNNYVWVHEFNETAN
GMPGVRHSLLLAKLDNKAGKDSFTQVLSTTLGGVTVRVKSKTAKAATVTVCRFLRTNESGSSCSDGLDNDCDGLVDM
DDPDCNPALRPSPPPPSPPPKKQVTQPPPAQQATSGGGPAKKPSPSPPPAHKLKKPPTKAKSPPPKTKKARPPPKKN
TNRG

>Vocar20007569m|PACid:23128768
MMLPAYSPRFVPLVLLLLLLLTSITCNASLASPRRQVHLASSLATSDESSADPTAALSPSRQRQFRDLVSPGGRRLL
EAAAYALDNARSVTAAPEDRRQIAPSRLLQRSSALDLNLRMVDRDETASVLGDITNAGMLARTYLQLPPSPPRPPRP
PPPSPKPPPPSPRPMPPSPVPPSPFPSPQPPSPAPSPPKPPASPPYNSSSDDYATDASDVFVPFRNDGPVSLPLLMD
NYPEANGQWVLKAVGNVVAVHMSLIPGTDKFFFMERPSGRHPDRSSNIVGYYDYLTNRFTNINYTDSVFCAGHTVTQ
DGHVMIVGGHISKSGYGDGLKAVRILSRRTATLYRITNMSYPRWYPTATLLPSGKVTIMGGTVLPGAGSAKNPIYEI
WDPANPTQLDVRRQSAGLVSQTKDIYYPNTYVLPTGDLLIMCAAYGEITEPLSGTLRTVLPSWSNVAGDLQLEYPYA
GTSVMLPLTPYNNYTPEVVVFGGQYDKARINTTASRLALRLKVSYNATTNLYSFGGGWTAEKMPLPRVMGDAVVLPN
GKVVVLNGAVLGVPLLFIMLCYTTYHMGEVRYWWTTPGGISKSPTSFAEYRIEVFRPPCWFNVTAKPQIISMDAATW
DEYDSVNVMQYGEPFALQYSMFYANDTVTSAVLVSPGSTTHSTNMNQRVVGLEILAQDVDARRLVLNGPPDINIAPP
GWYMLFLLNGDVYGQSAWVRLPVSDFCSTCLIGASHLSATCRTIHIMARSNRVTPGASHKPPSRPPPRKPSPPPPKP
PPPSSPPPNSPPSPRPPSPQPSPPKPPASPPYNATSDDEGPGPSDGNSSTPGDGNVNLPIVPDNQPDAYGQWILKAV
GNVVAVHLCMVPGTDKFFFMERPSGRHPDGGNNIAGYYDYLTNRFTNVNYTDSVFCAGHTVTQDGHVMVVGGHIAKS
GYADGLKGVRIFSRRTLTFKRITSMSYPRWYPTATLLPSGKVTIMGGTVLPGAGTGKNPIYEIWDPANPTVLITRNQ
SNGLVTKTNDIYYPNTYVLPTGDLFIFCNRYGEITEPMTGTVRTTLPSWSTVAKGIFTEYPFTGTSVMLPLTPDNGY
TPEVVYFGGQFSYGWINTTASRLALRIKVVYDPATRNYTFGDGWTAEKMPLPRVMGDAVVLPNGKVVVLNGAVKGLA
GDSASGGVAKANEPNLWPVLYDPDAPSGSRMRLMSRSMIPRLYHSTVSLTTDGSLLVAGCDRCDKYWWTTPGGISKS
PTSFAEYRIEVFRPPCWFNVTAKPQIISMDDATWDEYDGVNVMQYGEPFALQYSMFYATDSVTSAVLVSPSSTTHST
NMNQRVVGLEILAQDVDARRLVLNGPPDINIAPGWYMLFLLNGDVYGQSAWVRLPGTAPRLDDFFATLK

>Vocar20007606m|PACid:23128782
MYRFCNNQTLTPAIFPQLCTTVKIASSLCNDDTAVASIGLCTQLRAAYSSITEFQRCLPPYPITSTSSIKALHLTAC
DQMEGFWEPMAGCDTCTRQSCPDPLASLSAGCIGMPMDQCGGLDALCALGASALCGKNLRTAPRPPSPPPEPLAIA
GAPPPSLPCVANSSLPACASYEYPLANMTADLDSLCTSMPYMPGCTIRFACQAGQVTGDFCRPMTLLATICVDMPGM
SGCRPYMQLCRNTSVVRQCAQFPAIPGLPNTMDAKKAVVIDICGSSQVANISQSTKLCNDCNQLRCADYLDPLSTLC
SLSPSLPGCTTYLTWCRASSAWEAATPGSNSSLGYYCRRGQSPPPSPSPPRGQSPPPPRPGNPSPRPSPPLRPPPSR
SPPPSPPPAKSPPPSAKPPAKSPPVQPPSPPPRKPPPPSPSPPAKRPPPPPLRR

>Vocar20007701m|PACid:23128931
MASLLLVLLPALLLLASTSASASAHVHSLTTAADTAPADLHGAAGVGSKAGRDRLRQTAGRWTGRQLLAPETNDNSH
LTKSSNGNGYSVESSNSKMTDLASTLLQTGRRVLATPKTPPSPRPSPPPQKPSSPPKKPSSPPKLPPSPPKPSPPPR
KPSSPPPKPLSPSPPPPNSPPSPRPPSPQPSPPKPPASPPYNASVEDESSGPSDANASTPGDGLISLPIIPSDDREA
YGEWALKIMGNVVAVHLCMVPGTDKFFFMERPSGRHPDKGSNIVGYYDYIANRFTNVNYTDSVFCSGHTVTQDGHVM
VVGGHIAKSGYADGLKGVRIFSRRTLTFKRITNMSYPRWYPTATLLPSGKVTIMGGTVLPGAGTGKNPIYEIWDPAN
PTALAKQNHSNGLVTKTNDIYYPNTYVLPTGDLFIFCNRYGEITEPMTGTVRTTLPSWSTVAKGIFTEYPFTGTSVM
LPLTPDNGYTPEVVFFGGQFSYGWINTTASRLALRIKVVYDPATRNYTFGDGWTAEKMPLPRVMGDAVLLPNGKVVV
LNGAVKGLAGDSASGGVAKANEPNLWPVLYDPDEPSGSRMRLMSRSMIPRLYHSTVSLTTDGSLLVAGCDRCDKYWY
TTPGGISKSPSGLPEYRIEVFRPPCWFNVTAKPQIISMDDATWDEYDSVNVMQYGEPFALQYSMFYANDTVTSAVLV
SPSSTTHSTNMNQRVVGLEILSQDVDARRLVLNGPPDINIAPPGWYMLFLLNGDVYGQSAWPQSFNPSLESRARRLF
FGLSWLKREKETQVRVWY

>Vocar20007457m|PACid:23128944
MLRCHVYSIVFALLVLYAGAECPAPPIVYDSTLRAVCSKEPARGAIGCSMYRLCTDNTISPPIFSKICGAWRIASSL
CSDDQNIAESTGICGRLQASYANVTEFRSCMKPYNFTSTSDIRAKHLAACDEMYDPSFMLGCESCTATSCPDPLVSY
SLGCAYMDMNQCAAFHGFCGGEATPADIRTTYGHGAAVLCLYIKPGPGVVVAPRPPSPPPEPPSSSPLVAPGATPA
AGPPRAGLSPSLLLPPKSPPPPSPARQSPSPSPSAKRPPPPSPSKRSPPPSPFAKRSPPPSSAKSSPPPSSAKRSPP
PSPLKRPSPPPPSSSRKPPPPMRKSPPLTGTRSPPSNRKSPPPMKTRSPPPSTRRPPPTSHRQ

>Vocar20010940m|PACid:23129183

MTASESPMAAGGTLFPAARLKMLWLTSAVALHFMGIIQSAVGQVTPVPSCIHSDPNNYTDVSSGLLSSCNDQARCLE
FLAIETLRPHILHIHVHRCHPVSTSTPYRHTHSKRVAHGKGDQSQDSSSCRITNTSTGSWLYCQFCIFWSDARNCSK
PSNETFQYACQADDSWTPGVITMLGAEPTIGATKTINSWGSSVKSESREFILEGLRTSMSATFSPHSAAFTSQSAAF
TSYSAAFIPPSPPSPPPRPPPSPPTPPPSPPIPLPSPPRPPPPPRPANTLCPCPLLPEQTIGGSPGLCNSTYATMVA
TLANTTDDNTVFYQCVPWPNYDILMREMAWSHCWVRDCLPEPFRGKPYNLSINQVTKHNIALIPPASYELILFPAPF
GMQIYVTLYMTNGSVATYNSTVGSPVIGGTLQRLSLSPLSNLTIVYYIPATFDVDGLAAATILRMIKGSQAPNPPLP
PSPPPAPPPGPPRAIPIPRVQISTFFLVAANVSLTSPGAGLTDAGTYVTVPINFYDYSLLADCSDPSVRAYMARVQA
LGLKETSVNCRYAAATYGESLLRRLRRTLISASSWLLDKAGLQSASADVKRRFLQTTTTSGAPELLNVFLTYMETQR
DIAASASSTCAKLTAFFGGSCDPSAFQTARRVRYTTAKVASSFANGAAACQAGSNEGFNRLLSTARISVYDVKLMNC
TIRRF

>Vocar20010621m|PACid:23129221
MGTSRQRLLAWSIAAILLAAFLQLGSAQDYDSSSFDPNDPDAAYLFGFPFCKCSDYRCGSSPYEPVKSSEELLASGT
YRVCFKFVDSGCQSGNACCENILQLLTKVEFMADKACNKSVVGVTFGNVSKLTSTFFDTKYAVGKVRVTNLGATRTM
VQNSRLCFTVKPPCASFDAFFQSSKLGGSGLYQYAVFDIDHQCCPTCAKTMPPPPSPAPGPTHPPAPLSPSPPPPPP
PPPPPPPPPPPPSPPPPPPPPPPPPPPPPPPPPAPPPPPPPPPPPPPPPPPPPSPPPPPPPPPPPPPPPPPPPPMSPSPP
PPPPPPPPPPPPPHPPPPPSPIPVPKPEQCDCVSVFEPNSRWRLRYRGSEIKGPYNYFSFNIFTVPSSDCLEVTYRP
GHCCDATLDGIEMALTPAYSNNWWKLVPKFWLYDTNGSIIQSGSVTTQHKSELGLAFTGFERNTSQVLPGDQLVLTL
ALNATLWNYTDAFPCGKSLLVEEAHICDYMLSGQQVVNGVYVSVPGGRFVPGCCPRGVYLLGDPTLTCGCTDNKLDS
PYRLYARPEPQPANGGAMVYSYSVVTTVPTPSAVNDDSSCNDMDLDRLRLYIRPEVVNRLVAASINNRSIPLSNVVY
GQDSAQVWMELRELKLTMPAVGTTWSLLLVIQDVSPPSICAPNALGSAECEYVMFGKFSIKDLDFLCCAHGLSETTV
VKPAQLQCSCDHNMLHTPYRLDYAGGDYRYATDVSTLNFTVTYGELDCDASDTANAGCCSTDLKSIYVELNTTAVVA
VMVSPPVPGGVFQEPLGSGVRISGIFGSGAGYDLSLVVKGKQGTSFKYRFEGGFNLQQPYGCCPTAFT

>Vocar20010802m|PACid:23129224
MRLPILGTLILAASLLAVSEAQTFGSGLFPNFPFVACQRSQSAYKVASTVTSGGSNTYCFTLRVSVPDNCASYCCQD
ADLRKFELNVYRKCDISGIKLRATLDGAPAKTVPTIVEALDGKNDSTILRLPALGLNQSTADGVELCITLGPNSNGE
GCLTLEELCVPPEGFPASTCTTALFDTQNDCCPLSRVTVPPPSPPPSPPPPQADCDSFAATVSSGMDGLSIVAVVRP
FELQLCSDSVVKVYLLSATLENLASFWFEQALGGGTCPAYLLGQTIRVNIGGDGDPINPPPSCGVSVTKAAACSLPT
AADGPACDCNSRQRATPFAMVPVYTEKKGRFANTNMYCFDFLTIAPEFPNGPCGSTTTLAKVEFWVDDAYRRNLTSV
GVQPSDATRMTWLAPSFATSGDLTLKVSNLNWSLSQARGARICMDLDKSVPMADFCLGKVPGHCWAALFDTSLKCCP
LYIAST

>Vocar20010920m|PACid:23129231
MSLLLAVDIFAPALTPCGAPHRVDVSGIHLPAPRQLHAQPDMHMPLRGLALQQPAFVHVHFETWRAQSSLGHRQGSD
SDVRTYLDVNPACNVTPTADIRATINGVPTNIAPAIRSAENVPGVNVLRISRLGLNLSTAADAVLCITLKPNRLGRG
CTSLEDLCVRPDGYPSGTCRAAFFNTQRNCCPIDVFPSPPTTTPSPPPLSPSPPSNPLLPSPPPSPSPPPPPSPPPS
PPPPPSPPSPPSPPSPRPPPLPRRPPRPPRLPPTRPLAPPPSPPPSPPPPSPPPPSPPPPSPPPPSAPPPPPPPPP
PPSPPPPSPPPPSPPPPPPPSQPSQPSQSPKPPAPADPEDITRETPPPPLSPPPVTPFDCPVCLTVQLIPPAVNLYP
YSFDMSECSQGLLRLVDGLNGLALAYGAQMTGQFRPVDCSDNAMVACGAFAFPAEAAKVPAGALEELSYTLLDDIAG
GGAECHPELQGYSIQAYTSASSCLQLSVSKDCVISPLPNNCTCNTTAGVMPYKVSPRYNVTAVPARKSNLYCFTIEA
LPDGVSPLNAACGPVDILSKVEFFAGLRRRRVTQLQKHLNWSFRAAHMTHGYDTIRSLVRGFELIPSVGTSRTVSPT
WGPKGLNTVKASQMKWNATQAIGGRVCLELRDTTSLDQICLDGSGNPTSRCLAKAPASTDRVAAADAQACVRDTSLP
ASHPDGAAPRSPSSLRGEELLKAAPATPIILAQSLDDKVAEIIARYRLMEDRLAAVERQIVDMVRELRAAPPGAVLA
AAVAGPVTAAAAPGADGGAGSSSTSPSVAVPPGQQLHGSGGAVSSTSGQQAAADLLSGGGGSQQGKARLFEINRIQS
AITEVVNSGYLESVKGLRETLLVQEWGLQAKNWARRRGVSAFEEWCAVNAPEEVSYDIISALYDDPDALHVLPTPRL
RMMARAMIEQHTLQAYDEVFRRCTSTAEGLARVGLGDLLPHTAGVGQVLRGLLPGGLPFLSGGFWSRGLSPHYGVWE
GTEGMWGKRGAGSRCGVLEG

>Vocar20010865m|PACid:23129550
MAAAGAFLPAAGLMLLLMSSVSVASRSSEGAWSTVKSINTGRKLAQVISSFPTVPSCVHSDPTNYTYVDTDISGPAD
SCYDQAGCLDFLADSTTCRTISTSTGSWLYCQFCIFWSDVRNCPKDAVDTISHVCSADEFWTPGVITSAGAEPTIGA
TNKLNTWASYSDDKYCQWVRWNNSAGASAWADLAFTVKDGTQACLSTGTPPLNVVINGIAASCQGPRTVNGVRAGCG
QGDQANECLWTFRVPRPGTPSFKCSNVSPPPSPPRPPSPPPPPPPAPPPPPRPPPPRPPPLPPLPPGAVLPPPPPLQ
LSNVGGYTCVSTDDADDANHCQGDACGKLYANMIKKALDLAPADASGILAFGSFSSTSNARKSLESWVTSAGYSTSI
ITYATSSSTYSSYNLTRYKMIYVPSDNGNTDGGITSTQNQALIAIRPKIVDFVNKRGGSMVVLTQSSFGTSAFGFLP
VSFTFTALDFVDVSITREMQLFSPESNNSNLDHVYYHGYFTRPIDWNGMRVMAYQTGFCPVTSGPNQDCRATVLCNT

KTILTAENCYDKIDNDNDGLIDKQDPDCWRCGDFVVDPGEQCDDGNILDGDGCSATCQFQDFPPPPPVVFAPPPPPT
NQPDVNYCSYDGVTGCATCQGSCETEDKWWSNGRICKLPQALGGAMPDDYLCSNDFGAVATKEFDFYTVGSDGSVTS
VGMVNTFRTTRGVLHATMRTTCPNLLFTNTNTTNTTDINHFLLISVAFSGNLTTVSLPMKRNKTGIVLYSCYTFTFD
LNEVFPDIGCRQINLDLRITARTSMVTYSTTSQTCITQPDKHTQTDNIIEKYNYYNATPSAPPPPLIPPSPPPPPPP
RPPSPPPPPPPSPPPSPMPPPPPSPPPTPPPPSPPPPPPSPDPPSPEPPSPLPPTPPLPPSPPPPPPPSPPSPPPSP
PPSPPPPSPLPPSPPPPSPEPPSPPPPSPPSPEPPSPAPPSKALGDRQSSLNMLNISCPRSCDCSKLPALDATTKTC
PDNNIILRFRSGTNRYDTSVPEIQVCVPRFGHDNVTDIYKFAFCWSTNCEVLSSLAGKPFRVYFDNMDLAPALPSNR
LAETPVVVTHLNPECAKPTTIVDLVNNVGTYKIGTNQQLLRIPAPGLDNLDMDVAMEAGCNDDWIVFAVVPVHDAGG
LTPPIPPAACPCWDTPENNTCQGYKVPIYLSTLDISEGQSYAQCIDPANYDSRTGRMAYSFCWRYACLPPVFSTTRF
TATCPHLERWNIAKMGGNFSIDFQGPEGVNMVAIGHAKNGTIGNITYTNIDDGSTVQLPDGGFGNMTFTYTIPPGFD
DLSAAVVMDAVGPRPPPPPRPANTLCPCPLLPENTTGGSFGLCNSTYATMVATLANTTEDNTEFYQCVPWPNYDILM
REMAWSHCWVRDCLPEKFRGKPYNLSINQVTKHNIALIPPASYELILFPAPFGMQIYVTLYMTNGSVATYNSTVGSP
VIGGTLQRLSLSPLSNLTIMYYIPATFDVDGLAAATILRMIKGSQAPNPPLPPSPPPAPPAPPPGPASVPRVQITTD
FMITANISLASLTGSGLADAGTYVTVPIKFADYSLLADCSDASILAYTQKVQKALNLSDTTGINVNCRYAAATYGES
LLRRLRRTLISASSWLLDKAGLQSASADVKRRFLQTTTTSGAPELLNVFLTYPSPQGTSATTPSSTCTTLNTIGGTC
DASGAQTAMRVQYTTEKVASSVSSESGACEAGTAEGLNKLLSTGQISIYDVMAKGCVVKQLPSAPSAQSVPSTPSAQ
SSSDKGLSKGAIAGIVIGSVGGAVVLAVVGLTIKNKLDQSQTVNVGNVRESGQAPSGRRWRTYSMQQRAEEATAGRR
PVAGVSVYT

>Vocar20012678m|PACid:23129750
TITSLELGEPVYYLRQQLDQQRLGSDPGGGGGGSGGVIHRLVFCAGMSPSDIPLETMVAVSYDKVVDGVMYSCKRPI
PQPSSTEEEVKGQQQHDDGLRRRLTRRHQQHQQQHQHQERPQQRQRQRQRALLGESITTPTSLRILVYIASFCGYDT
PASVTPQQVTNLLTIADSSDGNRSLTNYLMTCSYGQVSLLPENIRVLGPVTVPCNGTLREPQPFSSGGKFTTRSCNE
LDNAFKWHYWLDAWAAENYKVDALDYHHRVLLLPSGYVGRNDTGCGGFSGMGTTGRWSILRTAVNKWGSGLVWWSGD
AYGDLEVLLHEFGHNYGLTHASVPGGCDLGDQCDHTCTMGATGGQGIRCFNAPHNWRLGWGQPALQLSYDNLPYGEA
TAVRIPPQLSTAQSSVLVWGLPYGPTADSAGDIQQLQRQRLFISARLNVYMYDLPWAPDVGMPFLLMHTYNGTDSKP
TIQTNQVGEIAAGEMWRDSNSGLVVRFDSWNSSTGAAIRICRRRAATEVNCTDSLDDDCDFLPDSLDPDCIPSAPIS
LGKRMSPPPKSQKQSLPPPERLPPPSPPSRQRSPPPKLPPQQPPSPRPSPQPPPSNAPPKQPRSPKRRNNPPPPEQP
SVNLWPPPAAGSPPPAAGRPPPDAGSPPPEQPFVNLWPPPTAGSPQPAAGRPPPDAGSPPPEQPFVNLWPPPAAGSP
PPEQPSAELWPPPAAGSPPPEQPSAELWPPPAAGSPPPDQPSAELWPPPAAGSPPPEQPSAELWPPPAAGSPPPDQP
SAELWPPPAAGSPPPEQPSAELWPPPAAGSPPPEQPSAELWPPPAAGSPPPEQPSAELWPPPAAGSPPPEQPSADPP
PEQPSAELWPPPAAGSPPPEQPSAELWPPPAGGSPPREQPSAELWPPPAAGSPPPEQPSAELWPPPAAGSPSLQQRH
ESTPPPPPPPPPPPPPRPQQP

>Vocar20012997m|PACid:23129879
MVLFLLAFALLLNSTPSCRGLAVATVTDPTTNPNTRFSLLSTASASASASEIAAAGSLAVAAVNGGAPRFAAADVAA
ADDDDDDGEKPDNDMAITGSAAAADVAVRELQETGWLTSDASPPDYLNWPIAPPSQPRPNAPPPRPPKAPKKKRPPP
PPRPPKSKPPPSSARVCITTPPSPSPPPSSPSSPPPSPPASPPPSPPPSPPPSPPPSPPPSPPPSPPPSPPPSPPPS
PSPSPPSPPPSPPPTPSSSPPPPPRRPPTSPSPPPFPRPPLPSPRSPPPPRPSPPIRPPPPFKSPPPPIRRPPPPSP
QRAPPPPPFRPPPPQRSPIPTTTETGDAYPPAPPTAPNFPPVAPSPPPISRPPKAPARDVRDVGRYDQIGIGMIVAV
HLVQIPGTDRYLFMERPSGYHPDSSRSIAGFFDLNTRKFTHVFSPDGLFCCGHTLLDTGDVVIVGGHQANAGYPDGM
KSIRTFNRSCTDLQLRKIREMGWRRWYPTPTLLPDGRVLIMGGTQGVGAGTANNPFWEMYDPATNSTRPYAMRSMYL
DQSEQIYYPFNYVLPEGLLFTFCGRSGWIMDWRNNNWLQDVPRLRGYGSTQFPFTGSSVMLGLYPENNYQVEIMTFG
GQREAAVKDLSFIGNRGSGRLALTYNRTSGNYSFRGWELDLLSIGRVMPDSVLLPNGRVIILNGAWTGLAGDSANGG
ESRANYPLLFAEEYNPNAPLGSRFRRMATTLIARMYHSTAGLTTNGTVIVAGCDRCYKYQVQDGYDFDPSPTSKAEY
RVEIYSPPYFFMDELKPLIVNTSSTSMAYQGLFTITYTFPAGWGNNALTRVVLVAPSSTTHSYNTHQRLLGLEIVSN
SVGDVNGVAIVRGPPNINIAPPGMYMLFLLNGDVYSRAVWVTLIRPRPGEPGYMT

>Vocar20012894m|PACid:23129936
MRSPWLPSLLALVYCSVLTHRAYAGLATTAPDSVTGLYVPGWAENTGSVLNFFNTSAINLEGVSHVYYAFLWIYGGN
STVYDPFGNLKLLVSLKSRWPATKLILSIGGGGFSTTIWTKAATTGLTAFVNSAIKAMKSANADGIDLDWEYPVAST
RDTFTALCAAMRLRLDAEGAAATPARHYWLTAATQSIVSGDSFDGYDLPVLKNHLDLFNIMTYTMHDPCFWETETHF
HTAWAECATALDYYLSKGVPRTQLVLGLAFYGHVYRLTDPSVYKYPAPSVEGRDCSTMTTVSYRAILLELKNSNGRG
GVFVDVAQRSAYFVYDTRWIGFDIPETMALKINASRAYGVGGVMIWDASLDTPDGSLLRAVASRNMSTTRPCGGGFV
GNGACGDASLCCSEFGYCGLGETFCGPKCRGGPCIQYPSPPPRPPAPPPNCGNGVVGGGVCAVISECCSVAGWCGTG
EAWCGVNCVGGPCWYKPRSPPPPPRPPPKPSPPPPPPLSYGCGEGIIGNGTCPFPYECCSAFGYCGTSDAHCGYGCQ
GGPCTPFPPPPPGDIGPTCGNGNVGNGRCGFPGDCCSKFGYCGNTVDHCGPGSCTGGACWVPPPPPPGGTNNDKPPP

PYPSPKQPPPSAKPPPPSSPKPPPPSSPKPPPPSSPKPPPPSSPKPPPPPSPKPPPPPSPKPPPPPPSPKPPSPKPP
PPSPKPPSPTPPGATRSPPPPPLLPPPRPSASTFCGNGKVGNGTCRDPTQCCSAAGFCAVSADHCYWYCVGGPCWVP
PPPPPAPPGMLASPSPPPPNPPGVVTVYCGDGVVGNKQCRLKNLCCSAAGFCAYSADHCLHYCVGGPCWAPPPSAVQ
KMMNATSSP

>Vocar20012648m|PACid:23129972
MLCRMLIVTAFLLILPAFSSTSPAEYDLMGMPALRNEDYSGKGDNTLTSAESEEIEALEQPQGYSGEQRLGTHAKLP
SEAYLSTGVARPWLDRAHLSGAYVDYGHTHDPTVRPPSTIHHTSAPDLSEHPRTDHQDLITLAHSLDGEHSYCLTSA
SGQCFPCQVENPDDENPCASDDNFFRDYDKCSVPYYYFGNVTDESICGDANFLHAGVYVAIQVRPRNIGYQNLTTAQ
KAKVGQMIAFMQISGLLTFTLQLDCPWLAVVDPSRPGAFIRMSIRHPNGTVWNRTISQTSSGSLYVSCISFAVQAKT
LNPNNSGDNGCDPITYTVTVSFLTETYQPPSPSPPPPLLPSVPRRPLLPPSPLPPPPAPPAPPGCLRVTARTGKLYS
EYVLTDSGNLTVGSYDSRTIKLDLSMTQFFAADDSIVMDLYRQTLPLPTDCPAAEDVGYSGTVAYKISPNCSTKSTR
LLVDVSSVMPCYADNPAVVGTFIIWLAVYTGPTVAPPSPPPSAPSPPPSPPSPPSPPQPPSPPTYPLSPQPKPPTLP
TPTPARPTLLPAVNYSPMSRKQPPAPVPPAPSAVPPMAPPAPPPLVGTLETYVNLSNAVDPNTTLPEIPGAVVYVDF
NYLTATARFQGDSPYASCASSVQSSLLTALSGLLNFPAGNMTSSCTVESTSSSSSSSATGRRLNADSSSSCTSMVKT
YITFKIDPAVPMTSFKSSVYKILNNGGVSGVCPLGLMDGTWATEGSTVRAISTGNSSYTDCGSFSDAVQSALSSSGA
TSAAASNCRTLQFSDGLGSPPPPAPAAAVILAAAAAKKGGFPIMAIAGAAGGIIAGLLALAAFLYYRRKKKKGNAFT
VTSGAEPGVADVFEAKTEAVPGADVGSAAAAGGGGGGGGDGVPGGATAWVGVAGGAVPGSAFGKGQGGGGDAVAGKD
AAAAAAVAAAAGTLDPGTMSQTSSGNGGVGGAGGTFLAGPSASISGASAVTLGMESSAAALAASGGGGGPVETGGAL
FRKTSKALGEPGSGGGEAGSGGGSATASPRRTTDADGSGGGGGGGGALGALLLRNVAAKAKVLPPLQSAAGAHLPEL
DPNLAPPALAGKAVLPTVTGGAAAAAAAGTTGGVGGSTSSAGGAAAADVAALLVPKGRKSALPALGVPVSQSLAVPS
ASNPLVKPLLPALHTAEHSPSAAAAAGETGGGEVDSAFAGSSAAGAAAAAAAAAAATPTRASATIPGSLHSSSMNGA
AGRALEAKYGGNIGDGLDGLVEETQSSGPSVASTHGLFVRTGEASGGTSLKVPTLAEVSGELQPLDSTEAGAAGAAA
AAGAAGAAAATGVGVGSGRRSSAGGSGKEWVVPVTPTAGDGHAAAAGGAATPSGKGFFGKMFGGMFGGGGSGGGGGS
GGGGGAAKEPAGSFVSPRGGSATTSELPTLAELDAAAATAAAAAAATGAAAGSDFVRTAHAGALSRGPSVSGFGVPT
LGMELGSAVSGAVAAGPGPRVTGMGTTWQNSAAAAVPTEGAASAGTAPSIRTGAILTSGGAAAGAAAAPDVSTDAAP
GGAAAAASSDVPADRPRNALPSIHLKSTTQLRAAANIGIDVAEDGSGGGNWATAAAAAAGGASAFQGRRGSRTRGLFG
DGAGSGGGASDGRSPPGSGSSRRSVHLKRAGAAGDVGGDIDGEDAARSPSSPRRGVPGGQRSRTSRSAIGGDVGDDG
DAAATAITGAGARAGFREKPSGVRLRLGDGGDVGDVDGDDDDAAAGGVSKGMAKFKRGGDGPGGEFDEDDAAAAAAG
GAAGGKWGQGMAKFKRGGDGPGGEFDEDDAAAAAAGGAAGGKWGKGTAKFKRGGDGPGGEFDEDAAAAASAGGAAGG
KWGQGMAKFKRGGDGPGGEFDEDDAAAAAAGSAAGGKWGKGMAKFKRGGDGPGGEFDEDDAAAAAAGSAAGGKWGQG
TAKFKRGGDGPGGEFDEDDAAAAAAAAASGGKAGPAGHRSVRFNRPGDAQGGGMDSEHLDGGSPSIAKKASSGQRSVR
FNRPGDGTNGDLEDDDAVAAAAAAKLAGGAKAGGGRSVGRFNRPDGGQSAGDVDEEAAAAAAAAAAGHPAGLPPNLRN
FNRPGLGDHDGSIDPEAAAAAAAAAAAAGAPPPVTGFGKPSRKMKFAPGTGEGADDVGDDGGSSPQNPAPGPTRSGRS
VGGGRSVRQFKYADINGVDEDDVEEELLDDAPQLSANATAEERWMHEREMRRRALLEQMKGNAWGGSVKHRERC

>Vocar20012568m|PACid:23130014
MSKFLCFGIFLAVFANNVSVTISLRLMQDSAQEALHDFDAEDDSVLFVLGKRAPQNQQTVQQKDNPNAYWEVWHSKR
VAEGPAPVYGPQMPQPPSPPYVESPLPSIPLPPPPYGDRSPPFPMPVEPPSPFPGPGPRESEAPTAPRPSQQPETSP
SPPPPSPWASPPTLTMPSRGQPIRESEPPTAQQPLQQPETSPPPSPPSPLASLPTSTTTSPGPAPPQPDSLSPPYCV
LAPAACLLVNATSGTTLDAFELLAPGWQSRSAVYPELLYEFGLLVNSGGGIVRKPWQPYADATIVIFAGLPEGTNSL
YVCVRAARDLLTATGPRACASVKVQVSAQVSAAQIAREMSAIQSIVLSASTVPTSVVLTMARRLGAIAASANGSFAD
STGTLASTLLAELVASAGNKTSSMSSVLTVFGGISTLWLLARADGRAAAVDGVNSLAEKLALQPLAFEDAQPIAGFF
DVIIAGAMDIVAASSSGGNGSVAARSKAARGMLASVIHGTDSLVRGLLNSAKCDGSSTRLATSRLSIAAGCMNMELA
AAAGSAMSVAALGTAVARHRRMLASELSATPPVTVVLDPAVAPLCVADAACASTGLRLAVSIFTDLSLLMTALGGSA
VPLAMNVSDYRMGGLVEIVSPIVRFSAPGLPVSAYGLSKLVTFDLMLNATAIMARGANTKRALVRLQDSGAAASDPT
SGIYDGSSRTQTLQSEMINNVVTISGYSNFLGDFVVLQYENTANPTSGASPSLPEVSKVGNTAGDGLQPSLLLTAAL
VSVAMQSINAFLQRC

>Vocar20012618m|PACid:23130037
MATCNATDARQKFGLFLGHPADGPLYFVVPSFAINATGVSPRCLRQNSASFVVLMPCTDDDLDQRFEIRSFTAGRPP
RMLPPSPPPPTPSPNAPPQQWYPYSLVFRTDWYPIGGSVNLNDYDYMAGHNGYGDYSYGVPLDDLDIMASWTWNGAT
YTMGRWAQNADNISATTGAEYGGDNTKYGTQYEIVYFPAGVSPPPTDFYVCVAWTRTQTSTTRVVLTMYKGYAVAST
TKVFNTTRQSDYSCTPSSAGYVGSFNPSAPSPPPPPPSPTIVQQSYPYPLLLRTDWTIISGVRGRSSKPYDFDILVS
WTEGNTTFMVGSVALTAGRAVYGGDNLRNNGTNYEYVYWPAGASLPATNTLHVCVRWNGNPKPLARVMLSVYHGTRF
TTSTTMVDTTFDWSETCVPGAIGYIDSYWLGQARSLLDDLGYSNCYTPPVPSSATSLYFLVQWTPANSTSPGPTLFD

WDLVVTWSYGGAAYEISPYLRFVASAVHGGDNMRVSVPGNSEAVYWPTGLTGMEPSPTQYDVCVRWYTRGRLNVTLT
VFLYTNPVLRVTKTLESVAVNSKTCSQYAVGYVGSYNYTGGSAPARQKVVFQAQKTWRI

>Vocar20001324m|PACid:23130651
MNRAALWIIILALLADCVASVTTSVVPDCAAAERVLLPSKQPSTSGPLTDIGFSGSPGLATIYVNSPGSGDITVKVN
FAQTHDPGQRASPPALPQAYWGLFTEEQYMQKSYVRTAGGASNSSWSCNEPTLSSSQPLSLGSSTFNVTYGRDQLRK
IGLKSCVESKFVFVAVVESYGLVRDADGTVLYDRNEAVQMSDPFLRDDVMISSAAQLSFKADAPGVASLDLFLDLPA
GTEVYWQLYNEELYDQAVWVTDYLTAEEYCMGNPYYSIEARQVLDAPASRVSIPISASDLLALGLSPCSDFAFILAV
RIHLPSGGDYYVAWDWGGCNRDGSDQLWPLARLGLHHCPCPSPPDGTAPNHGPMQSPASGSPSPPQAMKDCSLAEKV
SLPGDATFIAGNLTDLQIRGSSPGVATLYTNTPNLGDITFTISFAQTAPSGNRTTAAPAPPKASWGLFSSEEFMRLS
LQFAATNRSAEGLGSAGPSTTCTEPSLPWSRTLDDQTSSFNVTMTSSDLRLAGLQTCSETQLVFMALVESYGLVQSD
LRWELYRQYVAWQTLQGCVDGGNVSYAAFSLSYCPCESPPPPSPAPLAPPSPPPPPSPPPQPPLPPRPPQPPLPPLS
CGSGFTFLSNGVPVKGITSDVLLDVQGAINNFDFSSTDYLNASSAARLSVKPNAPGIATLDLFLDLPAGTEVYWQLY
NNATYEQAVWVTDYTIGQEYCLGSPYNAMFSPRAVLDAPTAKFSIPISASDLLALGLSPCSDFAFILAVRIHLPSGG
DYYVAWDWGGCNRDGSDQLWPLARLGLHYCPCPPPPSPPPKPPSPPSPSPPPRPPTPPSPSPRPLPPSPPSPPRPKP
SPPPAPPTPPTPPPRLPPPSPRPPASPPPPPRPPRPPMPPSPPNPASSTTTPA

>Vocar20002518m|PACid:23130844
MARSSVLLVALMGLAALQAAVAQGPARGVSKWCAISNRATAAWACNLAVGTSTSCETSDLWATDSGECAVPNFGQPL
AAETFKYQDTCRYQVAQVPLVFNGTSTLGAYLLIFKDYSDNIYFTVQLEGTSRVSGQVDGQWLYTEPVITGANSPSA
AIYFWDSPPNTAVQIAQQAQLVNLMTEDRNSYKRWSCFTYSAPTTNFCAPGSQYNGSTCVASSGSPSSKNLALTPSS
NLYISVVVNVVKFPVFGLGAPDTGFYCGDPRGDLSLRVGTLVLNTTGNVLRLADIARFDLPTNCANTQRPPSPPLPP
SPPPLPPSPAPPSPRPPSPLPPSPPPPSPGPPPSPPVPFPPSPPTQQFSVSVFVYHPNRPTPFADADVAKVIRVMRF
ALGCGYNGDPDKCLRSKPYATFNTTNNAAFPLFTMLGVKMFFDGYYDGDIIAAETERPGLARASVTSLYNNMNANLV
WAQLAAADTGLNLFCGAYMSLEAITDGAFYSQPGFTDGASGTNLGALYVCPIIPNVGVPTALPADVVGLPGFVRLGN
ITSNTNYVPRLCSPSLAQGPPMMCPMPPPPPPSPPPMPPSPPSPPPSPPLPPSPPSPPPSPASPSPPPPSPPAQFCS
IRFSIMSSTIRYNSRAADCSALASQANIVARSAGVVVRQPGSFACNTSSPVELAVTADLLDATAAQTYLIALSNNGA
LYGNIGLMINLLCGDYFNISASCIGTTVPSVSVTLPGSSTPTTVSYPLVIKAGDPLYPVGFAFCPPAPPSPPTPPSP
PPPSPPPAPPSPPSPAPPPRPPPALPPPPPGFTLQLSIINGDINDATTNCDRYKKWLNAMLTSYEIAGIINRVGVIQ
CDRPAAETILKQELLRPSEVNILYTALLVPGVIGAFARDSGVPCGSAVRLYNPTGSVFTDYACSTNSSAPTYVQDLC
CSPPPSPMPLPPSPPPPRPPGPLASPPPPNPPPPQLGVAVSFKPHPQGIACSDYYTLSRAKGIYYRITVAAQTAAHD
ALLDFMSTSGDFVFQANLVCGGQIRVGMGEAARSKIAAIPPTPFDPKSTSDPYEYLVGGKPTLGFCLNTL

>Vocar20002859m|PACid:23131336
DNGCAKDIYSKQDRKIQFIGATESMSQMVLDAYNLIVYAGASFVTDILRVHLCRYVTSPLECPSLSMLEGIAVKTSP
PFVPPPPSPLLTSPRPPSPRPPRPSPPSPPPPPSPPPPPSPPPPPSPPPPPSPPPPPSPPPPPSPPPPPSPPPPPSP
PPPPSPPPPPSPRHPPSPPPRPRPPRPQPPSPPSPSPPSPRTDNVVAWITQPDTGRIVQFSNSGVAGDLLLAPVDTQ
RSNYSQMWLIKVVDTDAAVHTVHPSHNTSLCWGVRGGSYDDTAVVELQPCLDGGSLAKHRKWIIQQSCTPGQVHLFA
QHSGSCLDIYMAQSRVIQWGCHWGLNERFMIRGWNQQGLVTSTPWYATAQSPWFTCDGNTPGDPPAWVLPLGGGGIV
QALAAAGVTGSGLIARGGSQTQSPSEMWLVRTEDAPAAIHTFRLSNNMSLCWGVRGGSMDNGAAVELQSCLGGDGGD
NLLPDHRKWIVLDSCIVGQVYMVAKHSGKCITMDSGSGNQLVQLDCNWGNAQLFGIRGWIQTGLPIAPSTVEYSSAG
NPRWFTCNGTTAIGESPSSCFQYTNIAGYNIRSVSTADMKECRAACNTEPSCVFAVRNDAPLCYLKSAPLYGPDGVN
EEDGAIEQACWIRANYGRFYCIDNWDVNGDPAAVSSSSSTDYVDCSMASYQQGYSLDQCMNECLGNPYCQFFVLSTN
GDCYLKTHPMRGTCGVTEYRPDIARSCFQVY

>Vocar20006778m|PACid:23131841
MGMQWLLHSSLLLLALFKGSAAAEVLGPPGSSPPQDPPDVRAPPAFPSSPPDVKTPETIMQLSGATRLSLCADLQKL
FSDFLDSANIFSRNQLCYIQLQISEGQTLGQNRSQSPNQQPPPDLYWFILSAVLPRSDVARSAVLTMGRPQGPDIGK
FLAGAGVPCDDQLIATTQTEEQDAASVYIYSCNGMLSLPGSSEPLTLPALCCSERPTQPGGGRSSSPPPPQPALPRA
DSSSPPPPRDPPPPPLEQPPPRPVASPPRASPPPPGSTAAAQLTLFVMAPLERLSCYKLRLACETLAQPYGLVEGNY
SCHASETAAPIEARYGAMAAVGSPSAVESTAYVIVKAEWSARAYARSFRRLQAALASSIDVFASLGAVPCGTEIQSK
RLSKEVVAVVAVLAAAAAVVASIVAAGYVLYRGRRRLGQPPAGFGLKGAAGMDGLPLITGYGHDNGSGPGGGGGAGH
PCHHPHQRQQPAPLQQQQRNGSSSSWVVKEGLYAVVVKVVQSGLCTAGAAVPGGVSGVGGSLAGSVSGCSSLTERSS
ALATATGLLLDVSATATATAAGGGAREGGVAYGSASLDIHDHDSVAVVRTLLHASSPSRPPVVPVIQQQQTRGAAAT
AAVSKPPAVTAAAAARQASNFAATAGSASWGAGELAPDGSAFGFMDVFRGAAANHICLSTSTSAAPSASGVLTAAGV
PRNNPLYAAACVHGANGGGAGFGVGGITPRIADDLLLRLRRSCGGGLAPSQPSWSGQHRYVTMQDGRRAQGDDGGSN
DHPAWGRAAAPGDDDAYSFQMGHSAAVRDRGLRRTQQPQQQGVGGGYPSGGCGAGWVAQRPREVTLLELFNQPPLAG

AMLSTPSTSSGGAPPSPLPSGAIPRLSSDGITTAAVDTAGPGAEAARQPSLSGLRQRSSPAIALERLSVQLAWNIAR
LSCSVAAGQGDEGGPDGDEAGGSGPAGEPLVGQLLPAAPGDSHGGSSSRTSGGDGSRSSGSGPGTATSASASDGRFT
VRSGTVDTSSASAAPSSDWELAGLSARSSAAGPAAAITASCPPPPPNGVVARVPPPEAALGRRAAGAVAGPQQQQQQ
GGGMAAVAGPPLRTTPAAAGPAEATGALDVPAKRHAAGPAAAGGAGPPGGGGGGGSLPCIGGQRGGTDLDISPKELR
IHTDGLLGAGAFGSVYRGSYRGQPVAIKVLHHLHFAAAAGRGGPGAGGGAAAGGVLEQKDVASFRQEIAILRMLAHP
NIVRVLGGCAHAGHPFLVMELMPRCLHNVIHGAAGLALPDALRIATDVARGLAHLHPAIVHRDLKPANILLDAEGTA
KISDFGLARYHLKPYISTQQPDAGSVAYMAPEGFDPAIGRLSAKCDVYSFGVLLWELITQEHPWAGESNVSIIYRVA
VHRMRLPVPTDPRVCPPRLAALLHACMSYVPSDRPDMRHVLGELEGMSGAQQQQPEGGLAAPLECAAEAAVLPLTPL
PAPSPPNKDDDAAAAAAFARGPLGARQTG

>Vocar20001807m|PACid:23131880
MAGEKCLTFCAAVAFLTIYHGMITGSLAQDGGLSGDVSTGVAAVCGTCTIDYTPVCGEDNNTYSNNCYAECLLGQGS
WTPGRCPHELNPVAGCIGTAKCANNPCEQRWPSTCQNRLPADAVCFLNACSGIEYQGQLLVPCAPLWVDPATKRVIR
CNDTETQPCKCSREYNPVCGSDRVTYANNCTAACALGQDGIWTEGECKASCICTKEYRPVCGSDGRTYGNKCMAACE
LGTNGTWTEGACRSPPPPSCKCSREYNPVCGSDGVTYANNCTAACALGQGGTWTEGECKAPCICTEEYRPVCGSDGR
TYGNKCMAACELGTNGTWTEGACRSPPPPSCKCSREYNPVCGSDGVTYANNCTAACALGQGGTWTEGECKASCICTK
EYRPVCGSDGRTYGNKCMAACELGTNGTWTEGACQVLSPFAGCLGNVKCKRNTCSDVCANVPSGAVCFQNTCTGQSL
NGEPVDPCAAVWVDPDTGKAVVCPFEAIKPNISCFSNETVQCFVDPCAVSSCPADPTAGCIANYCAIGTIGDRLIGP
CEAVYVDSDGNPVDCTAVDSEPAITCPKGKTVQCFADPCAVSSCPANPSARCLASYCSEGTFQGQPVGPCEAVYVDS
EGNRVNCTAPFEDCICTMQYDPVCGTNNRTYGNACEAACAKQEVAYNGPCMSLPSCPKGKTVQCFADPCAVSSCPAN
PSARCLASYCSEGTFQGQPVGPCEAVYVDSEGNRVNCTAPFEDCICTMQYDPVCGTNNRTYGNACEAACAKQEVAYT
GPCMSLPSCPKGKTVECFADPCAVSSCPANPSARCLASYCAEGTFQGQPVGPCEAVYVDSEGNRVNCTAPFESIYSC
SPNSPRFLCKSPSPCANATCPAFANAECIVKPCTSTYHGVDLPACSAIWYDPIMDKLVDADSCEGAAFKDCQCPYTY
DPVCGKFNRTYRTFPSSCAATCAGVTTIQKTGKCQHCNGQECTIGKLNDYCMLQAPSETQCWSVKYECQSIRGGPAD
GFIGSCLPKANISSPAAKKVAPTVATPSNRRRHRHLLEASEDV

>Vocar20004103m|PACid:23132302
MPLASLLLLLLFLLFTITLIITLMNVFSDVCGGPGGVLAVPLKNRAAANVDSTPVLGTAYIYRTYSTDITKQLIYIT
VALNGSNNSGTGQLFYQEPFPLDANADVTEDLRIDVSSSIYIWTDAANVTLKQYVNPMMFTGIYSCFTYVFNLRRIC
DPVRSYKNPAKRNLNEVCTCRPEYAGNCDPIDISAAPSIFIVLNVNGVPYSASILQNASNATCPSPVSSTAPIVLTN
QNYVTQYSLSPSACASRPPQPPIPPPPPSPPSPPSPPRPPPPSPPLPPAVPPPPRPPPDLTYSATISLMTFDRNRIF
SSGYDCFVGRNATAAYYRGRVTVQRIQCAIVSVYQSALDNDFDMIQWTYYFNSVDNLRYFFQSVNIETFWSDLYDAL
VPGCGAVGNYTDSFFNRNNAPPVPLAQQNLANPQPFCSFGGVFSRDDCCTNLPVGTTAASACIALRLALNVYTMNTT
TWYCDLDRYTSNYVAVYTTFANFTDANMFWVNFNNTFFAVATLNRLNPDCRTFLLATGSPSCNTSDVYWDVRNSYYP
VLSDSCGPAPPRPPPRASSPPPPVPPVISPAPPNSWPPGGAPPIPPPPPSSPPPPYAAPMVLTISANQPYVTGICAA
AIKSLSAKLAFFDRPFQGPVCTLGQTIAYFMVVTIGFSDYRHAISFAMTFNSEFNEFASTIGLACGTTGTIQANGAL
YNLACPNVTTFCCNSPPRPPFPPSPRPSPRPPNVRSPPPSPMPPSPGSPTEPGLPFAPRIRKPPPPPRPPSPRPPSP
MPLEPAMPGVPPSPPPPDIPIPPESPLTPRMPAPVRRSPRPPPPKRPSPPPPPRRLRPPPPPPQQPPSLPPPPPPPP
RRSPPPARQPPPSPPPPPSSPPPPNPAVNPQLTQYRTIAITTPSVLAGNPVTTDLLPYLCTNLKAGLRSIFTVYGWE
SNLQYRIPLEYCPTRNLPNGSKQYNVTIQLTPSAATTLITNLNSPSNFQLFVTATDILCASKVSVLKVATQEELFTK
SKDVAPNALDPDDSFSSCVHSLLLMRK

>Vocar20015274m|PACid:23132601
MARSGASPGAVLMLALVAGMAVFVRASYLTGTMFPYENCVQNTKYSRFSASFSSYSVDTIRNTSTFCIRLHVGPTCV
SGPYRCCNPTAYINKIKLSPSLGCRGSVGSVNVLTPSGKNYSVSSIYFERHMGQDVMKVTPLMQWLKSPDAIRDVRV
CFNLKRPCWNLNMFSYNTRKIEYSLYDKKVDDYECCPVGVIGLPQPPSPPPRPSRPFGVSDPPMPMEPPTLPSAPSP
PNEPNPPAEPRMPRPPSPLPPGPRKPPSPRPPPPPPRPLPPSPRPPPPPRRSPPSPRPSPPPRRSAKGVCFLKRSSTEL
LRVLPTLAGCHIILVLRRFYRLSYAHALSMLQSYDVIGGRRRPQADIPRGNGQQCRHVGFKDLGIDAPADKAARHIV
QCEGCTRRFRTLQCDDNYRSNVVVKMLQYVLRVLLR

>Vocar20000600m|PACid:23132769
MCKQMLFYVFLVVSVALASGQLFSKPNFLVIVTHSSDLTSTTSTTSITSSPPPPPSPPPPHSSEVPSGETNNQRIPQ
DDQDYMFNSTHPYYMPMLNQHIVEQGLQLRNFLISTAACCPSRSILMTGRYTHNNNVTSNIEPHGSFWKFMSQRLDD
DYLPVWLQKAGYRTMHVGKFLNAMDPTDPRFRCPKGWDTWDALVEPYVYLYYNPAFSYNCGPIEAEGMERLYGPYVG
TVRMEMFASPYRNLIVNSTSQVLPNQYSTDVISDKADAYIRKAVAEGMANGAANYKPFYLQVTPVAPHTQCDYINNE
GGCVFPVPAQRHKLLFPDALLPMNPNFNVPPPPELGLINEMKSSSGVQKHYLARLRTLRAVDEMIGRLATTLASVGQ
LSNTYVIFTSDNGFQLGNHAQSSGKQFHWEEVVRVPFYIRGPGIPPGYVTDWQGNMVDIPATVMDLSGAGVPDIADG

EPIPLRTLLPSFSYPYTPAPATAQLTKGRRRLSGSSGSSGSMLYVESDFEGPDVLAVDSDAVDPDLDLELEDQHGGG
RRLAQSTEYNRGMREMMPIEMWGHAWDKRITMKDYRALRICTGWLAFGSAADYRSALINGTLGWGDTFYGSIGRIFA
GPGLNGGINAYDDSFVDDNSTDDSVPDMRRRLLQGGSVVVPGSLLDNPDPSSSSSMPSSQIEAQLEAYVVEVIEAVS
KRLKATAVESAADTAAAAPSVAAKVAVLPARPYTLRLSFIYGEPLDTDSWSKQLDSACVYITAATGAESCTANAATT
FLATSGLSSVFDAVLVRGSGLPLGDFNSAVSDFARDPSLFLPLPYRSAFNISSISAINCGIASSAAGAVNAAGPCGS
AATPPPLSAIHGHRHRFLLFAADIADGSDNLTIPDNTPPSAPDDPTPRNPPPKPSKPPRPPPPRPPRPPRPSPSPRPP
SPPPNPPKPPSPPGPPSPLPPPPMVRVVMMSMNYRRAFDTDRFRQQLDSTCLFLRIFSTAVTCTPGEMPQGAWLADD
GLSSWMLLTLGQNSRPSDFEFTSSWTLLTNWPYPAKSTAQAPPGGTKAPGPRCNYHHATPVRRRYHHERALTLAGVP
LTPHTPLRRFQVANLQNNTAFYFGDLYRRVYDVIDIKISLVMPPLPPSRPSSPPSPPSPPSPPPSVPLPPPSPAPSP
PQPAPPSPVPPRPLSPSPPPLPYPPVPLPPPPYPKPPSPPLPAVYSPPPSQTPPPYVARSPPLKSPPPKPRPPPPFR
SPPTAIANVQDPAEPSSASLPPPYPSVPYPPALPPSPPTTSTCLFIRIATRAASCSTASVYFADYGVGSIFKVVLTQ
GPGITAEDFAREVDVFASDPMRYLGESYLSLYNIKTMSAQWPSPPPAPPPSPPPPSPPSPPPSPPPRPPVPPSPEPP
LPPSPSPPPPPPPSPPPPSPPPPGPPPPSPPPPNPPPPSPAPPNLPPPPKPPPPPSPPPPRRPPKPPPPRTPPPNTP
QTPPPPPNPPPPRPPDERVKIGTCYKYVEWCMNRFREIHDLVLDPYELNNRKPRLSRKSIRVIVPYRLIQHCFPPRI
PTAPAALLDRLDALMTAVGYCRGGDMCSNPYQLLHPDGSVNSFEEAMDPQYDGLYASLRKFAFGRCSTGYFPNTESS
WLLGDAKPSPPPRKKGQGN

>Vocar20000673m|PACid:23132772
MEATIWRTSLRLCLALMYCLLCAQFGVAQQHNFSQVLALSYRFYEAQMSGDVPTWSRASQAAGGWRNKSHLLDGTGP
NGIGVDLSGGWYDAGDHLKLHLPLGVSASLLSYGVLTWEAAYRAAGQWDIAVRNLDWVASYYLKCHYQASDTPSANA
FVAQVGDVDTDHNTWWGRPEQQAQGGSQGSPGWRPVHVITAAGGRGADIVGEAVATLAGVSLLLKRPGAYSNPTRAA
TLLSRAKQLFEFAKTIKNTWGPTSGSNAYPSSSYNDDLAWAAAWLCRADVDGGAMSLTASAACNAAPSLWDATKYAN
LDLSWDNVAAAAALLLRDTGAGGATYVASYDDFVNRLLTRWTGSGACSTGATPCMTPGGLAWYSDWGSARYAANVAL
VALAAAARSDGGGGAALTSAARASRICWAKNQVSYMLGTNPQSQSFVVGYKPTTSHKAPEKPHHRSSSCNPSYAITCD
WTALDAAGPNPSVLAGALVGGPGRDDSYVDNRRDYMKNEVALDFNAGFTAALAGLTDLEQGLQRSGPSPPPSPSPSP
SPPPSSPPKPSSPPSQSPPPAPLASCSPTDYACQQCSQSTYTAPDACRSCVGAMRSIGQDPYRCFSCSNGVTDPTIQ
GICFTECVPPTALKGIDWSCNSYCADPNFVGTDAAKSRECVSCLTGVSNAWDCYNCMAVTASLSDSAAARATCFKCV
TTTTLGGWSCGDCASRATPAERDSCIKSRGG

>Vocar20000622m|PACid:23132804
MPRPTSFLVVALALFLQVALFGSVVEGQRRRPPPTAPPPPPLVRRKMPPPRSPSPVRPTLPSPPPPSPQPPSPPPPI
PRPPIPRPPSPVPPSPAPPRSQGTKPNFVLIMVDDQDYLLNATHPAYMPKLHKYIGDQGLHLNNFIVTSSLCCPSRV
SLLTGKFTHNHNVTSNQAPQGGWGRFQAQKLDAEWLPLWLKPLGYNTYFTGKFINLFDVPPGDVKNCPKGWDMFDPL
TDKSVYDYINYDFVPQCARMDSFRNAYQTDTISTRALGYIDEAVDKGQPFYVQINPAACHTACPKGAEEGGACVPPV
PSPKYSGRFSGVSVPRLSNWNVYLSNVLNIGPDGSTNPAGIDKHYQRRLETMLSVDDMIEDVVNKLEERGVLNNTYV
IYVSDNGYHLGNHGLPKGKTLPYEEDIRVPFYIRGPGIPAGVVHPYMATMVDLTTTLITLGGGAVPDQMDGVPLPLD
RIVQEAPGAAAPETPMPPSATVTNGTMRQITTNCRALPPADRLPVEMWINSLSKDLDKKDYRSVRICTNYTIANDGR
TETCWKYIIWCVWPTTTNFKELFDLGADPGEINNLMLTLDSNPNPNYKRLVSRLDALLQMMGYCKGASCRQPWQRLH
PEGGVDSFEGAMNPIYDGKYEAYTKFAWRSCQAYYDPINNEVGDRSLGALSRWVSG

>Vocar20000651m|PACid:23132816
MRRGLLPAIVLVTVLLALQTSTGHAARKKRSPPPPPLPPPPPSPPPPLTPASPLSTGCARRKCTSPPPSVSSPPPPP
PPSPPPPGAPPPPSRPPPQNPLTPALIFQSPNPPPPSPPPPSVSSVFNFTFGNSSYSVYNGSRTFGQAEMFCNTNGG
SLLSVLSAEENAAVGQSLKNINVYGIIGNGGGGTVTLWMGLRINQTSKRYWTDGNDLTYLPPLFNGGIDVFADGGCY
SLRCAPGSDCIWSLAVPPTLGCVNVTRDGFICKIDNNRYKGVYYLTRAGQPLGTVYALVRPPSPVSWRQADDNCKTM
NGRLVSINTADEYWLLINSTSNLVLRNQFPPSDLDNFGNVRAWIGLQFITSLENSFWVDGTRADRGYNPQGIVLTHY
YACYAIYCRIGFCDWQSTWCGDLLPAYICELRNEWSRR

>Vocar20000570m|PACid:23132872
MALKVLRPSLLVCLITALCFHVSNVVAQQHNFSQILGLSYKFYEAQMSGDVPTWSRASQAAGGWRNKSHLLDGTGPN
GIGVDLSGGWYDAGDHLKLHLPLGVSASLLSYGVLTWEAAYRAAGQWDIAVRNLDWVASYYLKCHYQASDTPSANAF
VAQVGDVDTDHNTWWGRPEQQAQGGSQGSPGWRPVHVITAAGGRGADIVGEAVATLAGVSLLLKRPGAYSNPTRAAT
LLSRAKQLFEFAKTIKNTWGPTSGSNAYPSSSYNDDLAWAAAWLCRADVDGGAMSLTASAACNAAPSLWDATKYANL
DLSWDNVAAAAALLLRDTGAGGATYVASYDDFVNRLLTRWTGSGACSTGATPCMTPGGLAWYSDWGSARYAANVALV
ALAAAARSDGGGGAALTSAARASRICWAKNQVSYMLGTNPQSQSFVVGYKPTTSHKAPEKPHHRSSSCNPSYAITCDW
TALDAAGPNPSVLAGALVGGPGRDDSYVDNRRDYMKNEVALDFNAGFTAALAGLTDLEQGLQRSGPFPPPSPSPSLS
PPPSPPPRPSSPPSQSPPPPPLASCSPTDYACQQCSQSTYTAPDACRSCVGAMRSIGQDPYRCFSCSNGVTDPTIQG

ICFTECVPPTALKGADWSCSQYCSAPALVANDPTRARDCISCVKGVSNPWDCYNCMAVTASLSDSAAARATCFKCVT
TTTLGGWSCGDCASRATPAERDSCIKSRGGRRLLAGENF

>Vocar20004196m|PACid:23133079
MGTSKPRTAACYLGTVLLVSLLRLARAQGTAVPGDAQYLFGFPFCQCDDYRCTSSPYKPTVASQENLANGQYRVCFS
FQDVGCTSGNVCCEAILQQMSKVEFQADKVCNKSIVAATFGGVSFTGSTYFELDNAVGKVRVAGLAATRTMVRNSQL
CFTIRPPCASFDIFFQSAKLGLTGLFQYAIFDQTHNCCPTCALRPPSPPRPPPVSSPPPMPTPPKSPPPPPPPPPPP
PPPSPPPPPPPPPPPPPPPPPPSPPPPPPPPPPPPPPPPPPPPSPPPPPPPPPPPPPPPPPPSPPPPPPPPPPPPPPP
PPPPHPPPPPPPPPPPPPPPPPPPVPLAPSSPYCDCLSPLVNTNSRWAVSYVASNITNGELLYNFKISLNDGTHCRA
INYRNGSCCNATLEGVSLPILDQYRTAFLKAVVALGDERLKARLYKVEYEYGLRLQFNERLFVTSLPAGASLAMTLF
VDPAIWSDASKIPCPPSRLDPYGSACDYWLHGLQTAPGKPEQELVNVQTAASCCPEGVISFCPADNAGACMSDLTAS
PYTLSFASRVQAGSKTIFSFQLAHRNLVSTCSAMAIDSVLLFVSRSYALPSAAATLGSISTTVAPGSIGPQSYLNIS
MSSYASVQPNTLTLELEGNLELSDICVTPAGSSRVCNYVLVGAQRSGGTSQACCPAGNIPATLPSGRRLLALAEDVH
GVLAAGGSICSKYDAFSSCFSVAPVEISETFDEGMTFSYKLTRTSVEANCPSEFQVMVSSAAMATFKQGRGHIWAGG
IPAGDHFSWMLPAAGSAVDSAIGSTHIVSFTLRGHSMDRLSRVCTLQGQDSCIFRLLGESGCYQDTTFTVAGPRDHY
RQQTLGGGFNIERAPIYNNFFSELPHYPPSTLRLKNGDRLPAFIDRNYHQQNRDQTDLQQRSDAMAVSGNQRPKYFR
RPLLAAAEIHVKQAPLPPLPQYQDLNATAPAAMGGPEPRSKTIGTQSDYRENEAQTAPWEPDYVLPEHPTIKQQALS
QRHHCDGPEVLHLRDMAFADGLPAGLQEVIRIDKMRAKRAFEATLPPIDDAARLPLRQKMIEEWEAREWEEREQEIL
DIQERRLALLDNALQVREEELDEEHRQRVDDRKKATLAARAGRFADVQATRIKTMRQLIENRKYVEKHRKLHKPTIV
ERYANFGSGTYAPLQREGRFPESKPMGQEIETEGYEPATLKGVLELEAFLPARLLNPKMSAPKRPDRLDYGQRKEAA
TQRDLKTINDLLDVSKATAGRGHGDCWPAPLRDDSLGSPGLGNPGSLKRASSSSLSKSGAQQPPASAGGMGAGQLPP
SAAAGSGTVTAAAASGGGGGAAAAAASGGGSTSPSRRVLRAMERPATPELPVPPALSQPQHAALVLLQRLLRGRAAQ
NIMYEGRLRRQELIDELRVSERLSGDGARLDGKPIRRPEERDAATLRLDALLGSTVAEVCAVLSEVDPTKREVLLAA
LETSRAHATAAAFAAAASELASADLTAAAAERTSRSSIPSALQSAPLSSLSQKSLHTAAVNLEALGITREQAEEAAT
RIQAAFHGHKARKEVAAMRERGALLRNIMANGEEGKVVKCQAAVRGYLDRKRVHQMRHAQAEAQAEDSGAKATAGAE
GEEMGERSEAGFGFDPSSYSPEQVEAAKRIQAVQRGRLARRRMAAMRAARDVAAEEPPVTGETGGDEHTAVATEREL
NPEYTVEQQVAVIKIQAAGRGYLARRRVAAMRVEQEQLLQEEEGTGSRRPTVIGRQGDPEMYVAAAAAAAEAAAAAA
AGGDTGAEDEGLWPKMQNLSGFVLTPAAPPGGQEAVSLLPISPEAEAERASSQRPAEGEAEAEASLAGGGFGSMEDA
GAVEAEAPPEGEPSLAVGSSGAEGGEGDEPRPAPSGIADEGSAAEAAGEEAEEAGGEAYKGRDGDEGQENGLGEES
QAGPEEGEGGYEEGGEEGEEGEGEEGDWAEGEGEDGEPQAE

>Vocar20013094m|PACid:23133852
MAWSLGALLLHLALLTICPCLADQHGTTRVALYMDATSDVLDFNWNEGPNNLLEQLEQLGFHVQASHAHQPNLLGTD
KPSAYVVPVQNGHLFYSSVEDMGAVAAYVQSGGLVIILDANHGEGEALRNFVGSSLGYEEDARAITYTTWCRHEDEF
AVTLPLYTSQLDSTRVAVQAFSKVNNPGAVVWLGYDWTDGPQDQWGALLKKLVTDFAQGVYYAPLFGNADAHPLFAV
DTVLETASDVVADAAEIVRRFLAVPGMYPSTPANPPPSSPPELTGFPPLPLGKAMEVAKYTAVYQEPAGEEFDFMGL
INRLKISVAAANDVPTGNVIVNFIIFPNGTGLRGHWRRRAIETSERIFTDAEIAEMYAKTDMRPLELYLVSKAPITD
EMMLQLAQEETEFIRRAMLDGTADNAAQGGLLQIGYLVIKIISVAYPPSPPPSPPLEPGQIAPPMMPPSPPERPSFP
PPSPPVNFTRIELETGAFGVKDGWSPPSPSPPQQPSPPPMPPSSPLSPSHPSPSALPSQPPPVFPVQSIPLPSMPLQ
LPPVPSPPSPSRPSLSPPSPSRPSQSPPAPPSQVSLSPPLPSPSLPSPLLPSPKPPSPKPSSPKPPAPSPLPKPRSP
KAPRKPRTPKPPSPAAPPPSLFIKVTNVSAHGARGSSRSLVWNDDGDFKSQLTKQSPLQLVYPAKPSCPTTCTACQY
AWKATADQLSVAVFFKEPMQISRIYLKQIRNSGVITVQFIKWVYPPRGVVEGNLGRTVWNVTDDTSMCQSVLAINVG
PAKSGMNLAVPAGGSPANLPTKLRATATGGVLVTMERPRNAGLNYGPFLEWIRFSGRALYPSGLVPYN

>Vocar20013200m|PACid:23133935
MPPFKPVAPGAMSHFLLLSLVCGLCFYHATSTTVSVYMSEAKDVLNHNWDGSTYKLAQQLEALGFNVERSEDEQPNL
SGDKTPSAYVIPAQNGPAPYSSAEDMDAIASFVANGGLVVLLDSSSNRGESVPQFVAKAMGYTGEWESCLSMGSNDM
VAIGQPVLGSAALQFLPETEDAWPATLENARVISSYSACQHQDESSTSIPLYTVEGDNGKVVAQAFGKAGISGAVVW
LGYSWQDGEQPLWSSLLAKLIRDFANGAYTAPSQGSSLSLLDQQHTLDNVLQAATDAAAGTEEIVRRFLQTATVTYP
PPARSPPKPPQPGRRPSSPPRAPKQSPSPPKSPRSSPKPPNSPPSPPPSPPPPSPPPSPPPPSPPPPSLPSPPPPSP
PPPSPPPPSPPPPAQPNPLTPSLTRPPVPSPPLPVAPITYFESILPMGARGKTRSLVWYDDDDFRDQLTPKSALQLV
FRRKAPCPDRCANCNRAWKATANQLSVALFFKDPVQLTALTIKQVKNPNIQLVQLIKWTGKPTGSFPESAIGPVIYN
VTSNRVPCGSELRIDIPAVKSGINQNPPPTADVNNLPEALKVSQGILITANRLETAGKGYGPFVEIVKCWGRVLYAE
NPRAYQ

>Vocar20013164m|PACid:23133995

MGRLSAALAVAVALLTLVPGGRSQFATPFEYISSQPDLSITKACIENIILRNQAPWTSQKDQDFTIYVPTDDGWRVI
FDSFGLGDPERFCKETRKVKLNQTRNSVMRFHLLPGARTREQLPGNCQGGQGCKYATLGGGNNLVDELTIVIDASVG
TYVFGANPDDYGDYQYTTLANGTRVPRFNIRINGRILHVLDAVMIQARFIPAPSPSPPPPKQSPPPRPPPPTTQPPP
PPPPRQSPPPPVLSPPPVSNSPPPPAPPPPSPPPPGPPPPSPPPPSPPPPAPPPPSPPPPAPPPPSPPPPPGASPPP
SPAPSPRPPSPAPPPSPYIGFYSIYSFLSQRVVDMTRLKTAVDAAGLASYFDDPSLQLTCFLPNDTAATWDAPVFWT
STMTNVRVKDQTVFVAACRANMSSSAYCTSALVTEYSALVRDLLLRHCISPRVLTTNWTNAVYTTVQSFVSLSTAVS
APTYSVSLVGGPVRTLLPDTPSARNRDNIVAQSVIQIISDFIVGGPFAPPPPPPPSPPPPSPSPPPPAYTSITAAIN
GVDQLSITRQLISLLNLSPLIDALNGTTCFFPSNAAWYRFSVNDNNLLVNVVASPSPPSPSPPSPPPPSPPPLPPFP
ASTTGRRRLSSVEEDSKTVKFNIAPGVVAHGVITDRRMLAQVDNVFNAADFYDRTIWTVSLESGITPYLTQMLRNSI
LSSCVNPANATRSFNAGQNTVFKLQDANTELPLETALGWGEDVAAYVKRIAFSGTCSDSVTINGVANVPLETLGYCS
QCPKYETSDDTYPNCNFNVYYLSYGGASAFDTISTDNTVRIPFVYWVPRDIVIGSPAQPRGYVQIVNRVYQPPNVPP
PPPSPAPPRPPPAPSPPPPVPGGLPGLLNNSFSLAHQLFTAFNYYPTVAQFDTSGWTLFVPTDAALTAAMTARGLTI
STVISSGIGLSIVRNMFVPNTILFTSNITNSTFVTTNVGIFGSGTSSNAVTFLVLGSSITLNQTFPGSASPQTATIG
ILRDLQVLRGAGALAGVVQQLDSVLWPDLALPSPPPPPPTPPTPPSPPPPPPPPTFQDGIATYLATFPELSIFRQIL
TCTNFTTLFNDALRQNPTTIFPPIDSAFNSYFATSGRTLSYFCNPDNLGDAINLIKAHVISGAYSSATFAANTGAST
PVTSYPYFYVAEMNSQTLTVSGNGTVTNVTARYPPAAGTFVPNRYDIILFNTTPPPTPTSVGYVYAHMINAVLPPAN
FAPPSPPPPPPPNVYNAISIWDGITKEPLLATYAGLASVLKIPGTNTTFRAYFENTSTIATVLAPTTDALFAMFPRV
TITLASGSSRALTNSDCLGNYSAAVDFCSRILAFTVIPDVEFFPSYNAAEYFLSFPFGDASRYIFMPVTLVGPSVSA
NFGYTMSLASNPPDTTTLAFATVPCSGKAVNLKGPVPVGVPNATTQKQSALYVVNDVLFPPGGMPNIGTTPSSACP

>Vocar20013899m|PACid:23134121
MQVRSNAAIWERQTAQNVLVSVLAVLAFSSLATSLSFLPLNTTFSPNGAPPQTFKVNPAVENVSRMFPVGQPAPPTN
AWWASWVQANGYDGDTTAIDMQPYLVKVTSSGLSMAAPHDMTWDPECSGSRCIKNEYKRYVIITTGSGLTVVEVESF
DELSVTFRWRPASGAAVAMRATLLQGSPYVTVEFLSSAPVFRTSTDPWLGPAPSSITGDSYNKFKLPCGSGLTWLIY
STSAQALSITATLTDFSATGPVFSGRWRVAMLLPRFAIPYGWTRASLDGSSAAFEAQLDKYANVYATGGQVQLGVET
GVAQPRAILQHLFNTSTMWGPAATGLLMWPPATSDTGQLTCKGIRGNLIAVEGSTWTLAYPMPDITWSAPGGIKDPA
YVAPIIDQLLQTDINSNLYGAQVFKVSQALADMGRIAEIAAELVPYDSRLVNAAWALRQRIASHLAVWLAPSNPNSM
GLVYDAKWGGIVSAYPIWINTVNPAAGRNEEDKNNVYWHHLAHYGPFIYAAAVVAKGDPVWAAANRDAVLALVRDVA
NPRSDRSDPYFPFARYMDWWAGHAWATGLANAQIDGAVWTWGKWQEVRYLAAVAYLPYSLRRPALCCAFVHCSGTAV
TAYYSIALFGAATGDPSLQRWGQVLAAIEAGSVRTYWQIPAAGSDAYPLGTFVAADAGGIDRLYNGYGNDGKRVPGK
VYQARIAFMTEVGLVFPEPLVLYGLQWAPFLPGASDFLQQQGWMAEAYAAVANATLSPSNTVAWEAFRAMARGAAGD
RAGAWDVAMNAVPNGPPYAVDATDSQGLRHSKTSILYWIATRESGSSPTNSPPPLSPSPLPPSPRPPPPPPPLPSPP
PPPLPSPPPPPLPSPPPPPLPSPPPQLSPPPPFRPPPSPPPSPPPRPPASPPPTPPFRPPPPTPPFRPPPPTPASRP
PSPPSRPPPPPPPRPPSPVPASPPPPVATSPRPPPSSPPSPPPVSTYIAYRALNPAVGIAAPPGLTNFTNTYHTMGT
TEVRLDRVPSLPGRFFPFPTNSWWSTWTHVSQYSLRMGEEPVAMHPWRLKALSDRMVVVPPGVQWGIAANAIVPAYD
LYLGISVAEGLAGRSVADGNEMGVTFEWLRQGGVSGGGPGSMRATMLQGCPFLTIRYNSLTPLVEQFANAPLVEGLG
TFTGRTFKVANNAGLRFKYYFSSSVTASINATSLRLSAPFTGVMRIAVLYSSRLPLMAATSAGDAERLYDANADIYP
TGATASFGYQSATESGGPGERGILRMTFTTASMSGTNQGQLLMLAMPHHRTQLLYPSAQPVSAAGSVTMDDLRGPLT
PVLGSDWYLGYNLSDIGWNAPSGISNATMLSGVIAALKADAASWQGRAPAADPYFGSSDLAALGRMAVIADELAAYS
STPLADASALRNAASSLRSLLTTHVNARITTNPAAEASLVYDTTWGGLITYRDATEHKSLYFGNSEYNDHHYHYGYL
LYAAAALGKGNPSWLTSKRESLLALVRDYANPRRDDPCFPLARMMDWWGGHSWASGILAFGDSKNQESTSEAVNSYY
AVSLLGRVLGDPDLTRWGQLLTAIEVSGAQHYWQIPSSSPVYPSPFRDNKVVGILWNGKVDYATWFGNNPAQIHGIQ
YIPFTPISEVLLRANWVAESYPVAVSQLVSQLSPAWLQFPAMARAVLNPDAAWSTITSQTTFYGAWAAHPKSVQLYW
IASRKAPAATTQG

>Vocar20014446m|PACid:23134624
MRRPKQPRVRIESLLVLVLGLASFGHRVLSAHNVIVDPISGRRGYAPQRAEGGFGLRRQLKAGCGSFVACPSATDAA
KTILGNNPNLTIADAAFTKGTCTSTMAQWGVVPAGGWSPGFQLASWFPNGSLVLSSGDASAANCAVNTLDYYTLSVG
GGGDANLNALIPAYTTYDAVALEFTVTALKDGLLVFKYAFGSDEYTEWVGTAFNDVFGFFIAPISQPITSGHNVAIV
KGTASTQVSINNVNHKSHQDLYNNNRIGAADGYTNLLNTQGFQVTANQQYRVKLAIADAGDHILDSWVWLGGDSLII
DNRPIANGTIDTPVDTTNSTCTKQIITLNANASYDPDSNDVLSYNWTISAPCYPTITLSGAVVTGLAFLTTLTAFRH
PARAPLATGRAPPHVLQLDLSKLAQNVSYVVSLSVSDDSDLEDILTTTLFVPPGCGSALTKTCTSTKPPPPPASNKP
SPPPVIYVVNPPSPPPPPPLPPPPPPPPPPIIGDYVIAGNAGVVPCGSPGVTIDVCAADDEMLAQIAKAMDGDPYTL
YYIWAVFDMYDNDIPVFNKTLTSDQGCITSFTATELHQGRSLKKYKILVDVTNVDTWDKGDGGILGTETFIQVLQCP
ITPSWPLPVVLFDSPESFTLACGTTVQLDASTALAWATAKYSKVPSTQVLRWSLHNAFDVVVWATDSAPPYYNLDGT
ALVKNGAILAGATYTLRLLIILDTHDFDNDAWETKVSVEPPSPPPPPPPSPPPKSPPPSPPPPSPPPKPPPPSPPPP

PPPPSPPPKPPPPSPLTKSPPPSPPPKSPPPSPLTKSPPPSPPPKSPPPSPLTKSPSPPPPKATSPPPPPLATPPPV
VNSPPPTSTAQKSPPPLKAPPPSPPPSPPPPKPSSPPPPLLKPPPLKAPPSPGKLKSPKLEPPPTPDSPELGD

>Vocar20014418m|PACid:23134633
MRRPPCRQLRLVSALALLLLLPTAILADIFETVTDPLSGRRGHVPSRARRSRRGLNAVSSCGGFISCPSASAAAQMI
LGNNPNLTISNAVFSKGTCIAGGSVQWGIVQNTGWAPGHVMASWFPKGALVLSSGDATAGNCAVNTLDYYTGLVGGG
GDANLNALIPAYTTYDAVALEFTVTALADGLLVFKYAFGSDEYTEWVGTAFNDVFGFFIAPISQPITSGHNVAIVKG
TIDTQVSINNVNGNSNSNLWNNNRAFEVSPTKPIEADGYTNLLNTQGFQVTANQQYRFKLAIADAGDQILDSWVWIG
GETLLVDQKPVANTTNPTTNCATKTATLNASNSYDPDKGDILSYTWVLTANCYPSVTLTGQTAKVMLGAMLRVAAFS
NITGLSLFDSGRRLPVLVCSYCYRCGSVKPRVLSSQLPYICTASSTADDSALALIASNYDPGDFYTVFYTWRVFDIT
DKGYDNPIISVTVPQDPSYPDPTITFTAADLQQSTALKKYRVALDVTDVATWDQGNGVILGVETFIQLLACPIQPAD
PPTNIIFDGLKSPEAFTLACYASVVLDASAPLAAAAAKYGKPITTQIFRWSLLDINDVVVWTTDSSTALTLFDGTAL
LSSYVILPDTFYTLSLAVILDDKDFDDDNWVTKFAVENCGFVLEPLPPQSFNPPPPPSPPPPPPPSPPPPPPPSPPP
PPPSPPPP

>Vocar20005909m|PACid:23134750
MALHGGTRRSPVGPKLVMAYLLVIFLSLLGPGAQSQTVQPTKIEGKLVYKDAHVRKIWSLVTMSTGVVYTLPSQPID
ASLGSAIPAGSTVSLNCIAAASQPTNCTATTAARITTSAAPVPTTDLKIKLLVMVLSLTGTCTKTGATVTSVQTAYT
SPTGYANFLRNCSYGKVVYDTVTVVATPVACSSTILNNCNEDAMATAAKASVTAQRGASFLNSFTHFSYVLPNGMVN
TCGWVGLAELPGTQTWYTPDQDGIFDKGTVMQENIHNLNIYHGWKAGVEYNDYSTSMGMGQSCPSAPELWRLGWATV
LDLLNSNNFPANAFKTYTLPATSASSMGNMIKLQTDWMPSYTKNVYLALRTIGGGDQDLKAEFDRKVNIHELNKDID
NNFMARGDPRVTIIGTVAASSSMDLPAYKLFIRTGVLLESGTKIVVKVCRYTASSSACQDAAQASPPPPSPPPPTVV
KKSPPPPPPVKKSPPPPPPPPPPSPPPPSSPNKSPPPPPNKFKKSPPPPPPPTPPPAPPPPASKKSPPPPPPGTRK
SPPPPPKRKSSPPPPPDAPPPIEEAPPPDYSSPPSGGGDAPPPYDAPPQSWA

>Vocar20006105m|PACid:23134803
MRRQQMVASAICRPAHFVLATLLALTNVARAEVGVAQVMSALEEGYFDSPPGVPNFHLNNNNKDYLYLADIENPSGS
AWRLVAEGPNLIDKYNVLTGDYVRVNYKNSSTASGSRRLLSDEPLPEIDSIDVISESEGHEIYTGTQIRVKSMIYII
STCGWAPSATVEQIKGTFFNNTNNIAGYHDTCSYGKVAFDPSENFVFGPVEIPCKGTVTNGVLKYPYDASVACGAAE
QFAWRQYSEAAARAAGYGAFMASSQRRIIHILPREVRCPWAGLGNVGCSGSSCIVYIKGAYAQDLTVNMHELGHTQG
LSHAGRGYDEYGDFSDVMGTAPSEGYLCMNVGNQYRVGWNKPLATLLPDNTTNAPGNRGVAGRWVLPPTAATDTNAL
YLRYNINGTPFPNIFVSFRSRTSRYDNALATYMNNRVFVHNFNGSATERDYNRTLLVAVMAAGDVYTSPFLGNTTFD
DSGLGVKIRVISIAAGTSATVEVCRLTEKVETSCFDGDDNDCNGLTDELDPACAMASSPPPSSPNVPPSPSKSPPPS
PPPPISKPPSPRPPPRPRSPPRSPLPPPRPPTSPPPPPQLKASKAPRFPTPPQKPRPPRPPPPPRPPPRRPSSPRRP
RMPTAPTRH

>Vocar20006106m|PACid:23134804
MRRQQMVASAICRPAHFVLATLLALTNVARAEVGVAQVMSALEEGYFDSPPGVPNFHLNNNNKDYLYLADIENPSGS
AWRLVAEGPNLIDKYNVLTGDYVRVNYKNSSTASGSRRLLSDEPLPEIDSIDVISESEGHEIYTGTQIRVKSMIYII
STCGWAPSATVEQIKGTFFNNTNNIAGYHDTCSYGKVAFDPSENFVFGPVEIPCKGTVTNGVLKYPYDASVACGAAE
QFAWRQYSEAAARAAGYGAFMASSQRRIIHILPREVRCPWAGLGNVGCSGSSCIVYIKGAYAQDLTVNMHELGHTQG
LSHAGRGYDEVFVHNFNGSATERDYNRTLLVAVMAAGDVYTSPFLGNTTFDDSGLGVKIRVISIAAGTSATVEVCRL
TEKVETSCFDGDDNDCNGLTDELDPACAMASSPPPSSPNVPPSPSKSPPPSPPPPISKPPSPRPPPRPRSPPRSPLP
PPRPPTSPPPPPQLKASKAPRFPTPPQKPRPPRPPPPPRPPPRRPSSPRRPRMPTAPTRH

>Vocar20006101m|PACid:23134811
MWCVLWCVLWCVLWCVLWCGVLSIYASYTRLHADPQPEGYTARTLPSASPSSPPRPEIRSGCCISCGSVNHNWGYTS
PDGRYSCHTCGFSNRRANGFYGPLGSRSQEEADGAGGSACGECGTVRTHAWHGHKERLQVMVCNACHMRYQNCGSYS
LRAGADREAWLNLGNANRGEKEGSSSGGEAAAAVAAAAAAAAQQVRHRLPRITSAPAAAAAEPTAAGQTTADSHESR
RTKRTSQTPGASNSAGEKFGNAGKHQAQGGASKTRGSGASCRESESGVMSKERRSGERKGGFGDTDRGSGARERSAG
ALEGPCCVCGATLSVRWYLHKLDPGKVTCQACYDRHRRRGAYLSSGNRTEAGDPGAPASDDQRAEAAAAAAGGPVPP
AKRTAAAANHVNGAGPSPKAVATVAHPGGDALLTGGSMEAVAVAAASPLPLPELACELAPDGARGSGSGFGGKRGKR
TLEEDDRGCAVCGLRHYDNAGVDAANADSGCHLDDDGAAGRDGGATRRRRGNKKLAYTFTCAACTTAQVAAAPLQGM
QPGGRHCVSPPTASSLDVQDGRLTAASNGGGAPTGVLQQGYCSDEKALAAQVQAIEAGSGAKEIAAAAGTPPPPLVK
RVAAAALEFTGKVEAAGNAGAVAVTAAAAAATATEAREANMTECNPRPGGRRRTCPGLVTCRRCYDRFRVRRSYGRG
DALTAADAAMRAPGATAAAPMASVAGTAVDGAEKSQPKGNRCRTSRSRKSNPDGTDSVCAGDWVHVRQTATPAGTAR
SPKRIDAAAAADEDDEDAAPSLRTVFRVRRMRRWSYNASQGAEGDDRDEDYMYDGDGDGGDDVSNRDGDDDEGEGDE

DDGDEGDEGDDGDDGHDMEEDVEKGREEQVERMSEDEGLELVSDEMEMGQVQDQVQNQNQNREREAEDGVREPPQAA
AAAAQGGEVARIRIPAMAAAAAAAEMQQAMLRYGTNRQGAVAAAATAAGVALVPGTPEPERIPPPPPRYTPPTMPLR
REDSVAPQGGSLSTPKSPTRSLVQALAVALAHPQPPQRQPLPMPPPHDRSPPYHHHQSHQCQYGSPGPFLNAAFQG
PLQQQQQAQQQQHAVPFPALHELLPLPPQPPSIGRINQRPLQHMAPRTKRRRRSRTCSRTRRTRLEQLTLEFGDL
LGKACEALVEMEDKDGIRLLESGNGASLPSPPPPTGHWDLAANGGGHDGGGEGSRTQLPPSPSPMRLRSPLRVAYEE
LCVADPFLRARLLGDLAVRWLEKNAWRSPLEALLGRGVRQRSMDRIGPFECLDEQQQVVLGGCGLWANWDRNRRTLY
SHVVVVARAAMETTAAVAAAAAPMVPKVEHNDHKYTRGSGGSDGAGDGDGDGSSGTAAATSIATSFGRASGDGNPAV
PAPEPRASLASIRRFLQVTDYLAEGLAAHLLGQPPPPPLPAPNQSIQALTTPSLQPPPAPPPAPAQQHQELPPPPPP
LPPPSPRPSSLPAPPSPPPMPLTASPTPTLAASPLPPVAVRPSPGAGPAADPLAPRGAEAVLAAANCADGEGGHGSG
AVSAVVAVDGAMQTSDGLAADGDGVSRNGGRGGSAATAEDGGDGSAGADGNGVISSIGADAGGNGGIVSSIGADAGG
NGGIVSSIDADAGGNGGIVSSIGADAGGNGGGVDSIDADAGGNGGGVDSTGADAGGNGGIVSSIDADAGGNGGGVDS
IFSDAGGNGGGVDSTGADAGDSGNRDGSAGVNGSGLQAMDEHTARKAATRLLRTAVQAAVHLALQVGASPSGRLALR
VPTPGDPFVLEPLKPPPPQPLLLQPSAHSSAQSQAPCSSFINLTAMRANRLAVAVDWVPSRPGLAQVPAGRTAAAGA
TNGDGNQLNLAMPGATAGHAAGCQARSPPAAAANINAADGPAAVPLKRPPPPSYDGDVIRQDHDVPYAGELQPRRVR
QRRQEEERPSQDQHSEDRAQLASSVDAAMTGMTYQADVATAAAAAAAVPQGPIPTPQPSPPPPPPPLPLAGSAAPPP
PSHPTPVPLRDRYPPSHHVIPRAPNTRGLNNSTSFPGSGDSGGGGDGGQRREDGNGGGSGGGSGSFQVGDMVGSVLW
VISPAIIRRDALWRELQDGGPSRELHSAFGRPNGDRQHMGEPPVAAAKTAAATATATLAATVTAAAASATAAAADAV
TGAAVASRAAATEAVLNLNGDDGSSSGGGCGCGSGGYTGPLVPSRRRLFVAEATVVPARVVGCGLPDA

>Vocar20006081m|PACid:23134918
MHQMAWSLVLQFVLLPALAMTRARAGALPTDILTLGTARYVLKAFNYKEGDGTFVKLDKSMEVAVGYVFAVPFPISA
AYPAVPVCLPGGWDDIGAAAACRQAGFSTGRTFATGGNTGYPKVQLTGTVSVRNITCPNGATSLSQCTATRLLSGVC
KALAAVVCKGAVWLPPKTPRPPKPPPPQPPPSPPRPTRRPPPPPPPTPLPRPRALRPPPPKSRSSPPPPPPERSLPP
PYEYESPPPPPENSPENTPPPQYEYESPPPPPENTNDNTPPTQYEYESPPPPPENNLPPPYEYDSAPPPPKHYPPPP
FKDNTQPSGGDVRLIGGLQVWEGRVEIFFRGQWGVVCGRWTSVEAAMVCRQLGFNSTSVRVTRWTPPPNLPIWLSDI
NCSKIDARSPYKGLAACNSNGIGVITCQAGSDVAGVRCFA

>Vocar20005948m|PACid:23134966
MASAMSRLIQFVLAVQLTLTNVVRAEIGFAQILSGLIIDKRGYNIHTGTDYLYLSPNTTNPTQSAWKLLAEGSNLID
KYNVLTGDLVRVNYKNSSSASGSRHLLSDAPPEIASIDVISESEGHEIYTGTQIRVKSMIYIISTCGWAPSATVEQI
KGTFFNNTNNIAGYHDTCSYGKVAFDPSENFVFGPVEIPCKGNVTNGVLRYPYDASVACGAAEQFAWRQYSEAAARA
AGYGTFFDTTQRRRIVHILPREVLCPWAGLGSVGCSGSSCIVYIKGAYAQDLTVNMHELGHTQGLSHAGQGYDEYGD
YSDVMGTAPSEGYLCMNVGNQYRIGWNKPLATLLPDNTTNTPGNTGVAGRWVLPATAATDTNALYLRYNINGTPFPN
IFVSFRSRTSRYDNALASYLNNRVFVHNFNGTATERDYNRTLLVAVMAAGGVYTSPFLDSTTFDDSGVGVKIRVISI
AAGTSATVEVCRLTEKVETSCDDGDDNDCNGLADDADPACWKPPPPNPPLPPLRPPSPPSPPKKPSSPSPPPSPPPP
SPAKPPKPPSPRPPPSPRPPPPSPPPPRPPPSPPPSPPPPPPSPSPPPPLSPLQPPPNNPPPSPPPPFTPPASPPVS
SPPKNPSPPPSPRPPRPPPPPPRPPPKPPSPPRSPPSPRVPPPSVRH

>Vocar20005650m|PACid:23135121
MAARPKGLAPRALIGLCLGGLLVLLCNGADAAAEGQRTLPRPAAKQTLKLHRPPRSPLEPSSPNAPTSPPASPPMLR
SMEGELNVIVLESDGYLTYLRHDDGGVTNVLLDDEEAEKYSGQRVRIEALPDTPSDYDNLEATNGEEIPARSYRRRM
QATAVRARVAESFGFAGGADQQATVKDVTTGQQVLNITSVVFLLKFCNYNQETLSSLRNLYANGPNTAPNEGTVQNY
FNYCSRGAARLSAANQLFFEVNEIYAMHDLAEAYVQDTLRVNISGIKQRVMVLPPLAFCGRSWGGLANLGCLGTKCF
TWIAYFHRKIFFHEMGHNYGLHHSNVEGGGEYSDYSCAMGYGYTCLNAPNSWRMGWLQPIAGGDLNGSTLPFGQWKK
FTLPVQVKSLQSYLRISPDWKLGPNQAFTSWSTPVPIFFVSYRVREDPFEYFNDNNPLANTSGKVWLYKFNGTQAAN
SWVKSEQLGSYWAGEVYRDDNWSGLVVKVLDAKRTLDGYAIVSVCRSGGPKEAQGGNSCSDGRDNDCDGLVDQADPD
CNGVMNSPSPPPSPPPPPSPPPSPPPSPPPSPRPPPTPPPTPKPPRPPPPRPPPRPPPPSPPRPPQYNGYLQARRPR
PRRGRRHRSRRHRSRHGRRAHLQCGPHHARRRDHA

>Vocar20005613m|PACid:23135371
MKMHYQRTMLLCAFITNILAMAVGAHPLRTATTVAVYSSTTSGVLDQTWDPNGLANLQLQLRQLGLYVAGVDSFPEI
SGTRAAAFVIPAASGDNPYKDVEDARTLSTYVKSGGLVIVHGSARHQNDAQDLVAASLDYQGSWQYCELVADSTRSA
IGISSVSPYAEQFLSVAWPSTLEDAPYQQVRSSSAASRAPSHLTGSSNFPQETQEIHQPTVYQVHSWCKHEDPDALS
YPLYTLGGDSMKVVAQAFTRVGSSGAVVWLGYSWQDGPQQGWGEMLGRVIDGFHQGTPVRHLDTICMQLYDRRYRTR
HGTLRSPFQGLYQRPVDASIMDSYPLRLDAVLDAAASMLDDTAEAVRRLLYGVMLSYSFGSGYYSAGGYYGIGYYGF
GGGYYGGYGGYYGGYGGYATPGGNICLFGRTQQVRQPVIPARWSQRRRFPDVPAHRHLQYDTVPHHLLYSPLQRRQH
LRSPSRRPLVLQCRKRLPYHSCQLLPYHKAGLRLRSPPPYHSCQFLPYHKARLRLLLPPPYHSCQLLPYHKAGLRLR

SPPPYHSCQLLPYHKARLHLRLPPPYHSCQLLPYHKARLRLRLPPPYHGCQLLPYQKARLRLRLPPPYHSCQLLPYQ
KAGLRLRLLPCTPAGLLPLRRHRHRQLRNRFQGPSAPDPVLLRQGSRRIHRHRCDRCRQSCRRRRRREYGRGRRRHP
FGFRRHGTIAGWVCTDAAER

>Vocar20014733m|PACid:23135488
MSTLLGFLLICPATVFGGNQYMHRAVSGPLRLLQQQQQQQQQRQQQQQQQPAVAGRYRCNVTGTVRFRDDGTVRLI
DTNGKSWVLSWSRNPPPPSPSSSPSGAGAVMPAAAAAAAAGRGPLQTGQKVYVRGLCGITSRPCWVRVELLTHIRIA
KNGGGGQSSGGGGSDGGGSEVGKDGSWLAAPTNASLTSSESVYGSSEGAGGTEGGGGDGGVLPPGVGIFRDRPSPSP
PPPVPGYFPPPSPPPSMQEESPPPPPNTPPPSAPPLPPHPTPPAPSPSLSALPPQPSPQRNPQPPRPRDQLPPRPQD
SKPQASPPLPAYPRNSSKTPSSQPGTLPGSPPKVLKPPNPRPPPSPRRSPPPRPPPPLSPPPRQIPPPNLPRPSSPP
HRTPRPPRPNNPPRPSPLPPRPSPMPPRPARSPPRPSLPPRGSPLLRRPSPPPPTRSSPLPSRPSPPQPSPSPLPPS
NPNPRPPPPPDHSPPQPSPSPLPPSNPNPSPPPSRPSPPQPSPSPLPPSNPNPSPPPSRPSPPQPSPSPLPPSNPNPS
PPPPDHSPPQPRPSPFPPNPRLPGHPSSPLHPSPSPHPPSPSPPYPKNPPPQPRKPPPPPMRLSPSPRPPTVRFPPL
PRRPARPSPPPYTPAMRNQFPTFQEAVEGFSEISFRHPPTPPWGIVPDMMRIMVLAVDICGIESSANMTFMQSTTLP
SPVYAHRLWASAQEVWATDIRPFFEKGSFGDLSMPANVNFTRVVPFHVSIPCSGTSSRGVRWSVTSTPDEGILSGMA
EAADSAAKALGIPADRYDTRVYLLPDVIGSSSWGTINCPYSPLPVRVWTGAVSGSNAQLRLLVHEMGHNLGLMHSRG
YDENGVLLEYGDESCPMGFVQVPTHYNAAQSVWLGWTVPQEVLGADNLLPGTWTTFALRGLADSHFSSLQILPGTWM
SSGWGASDKLYVSFRRQTATNTDAGLAAPFRNKVQVHMHGYGSQGCTSPVLLATLDVPANDVSWGSLWPRPGSTLPQ
DAISPLLVVRVMRIDLVVGVARVALCRAEQFEPESGSQCFDNVDNDCDGLVALVDTGFFLSFTSFNRVGMAGVWEWE
WE

>Vocar20014804m|PACid:23135617
MASSSSSSSWRGAMLLLLLPALLLLGVTTAEIQGRGGGPSSGGVAIGTITSITHLKPTYYLHEADSGNVHRLIFCDE
MPPSAIPIGNPGVTVMYDKTVNGVMYSCNAPTASEEQQGSQQLRRQLAETGGSITVPQEPRILVYMTTLCGYPYGGL
PPEIVYDLLVIGNKQFVGKTLADYMNTCSYGQIKLLPSNVKVLGPVEISHNYGLTHADIPGGCDSGDQCDHTCPMGG
GGGQGIRCYNAPHNWQVGLGRPFLRLNDTNLSYGKITNLTIPSQISRPNSSVMVTGLRMPTGLSLFISSRQNTYTYD
LPYRATQDRDTFILMHTFNGSKTILTGQINVGGIWSDPASEFTIKFESWDDVTGANVRVCRRRFNITTELNNCKNGL
DDDCNFLTDSQDPMCFSPPPPSPSPPPRPPPSPPLPPSPPPPSSPPPPTPPAPRRPPRLPPSPRRSPPVPSSPPSPR
LPPPYRAPRGPSTPRPPPNPRPPTARSKP

>Vocar20006909m|PACid:23135743
MVPTSQRHSLHLLGVVLVISISGSSAARSIKQLGSPFLGCYNDYQNDGLPYNFEISLTNMTVDRCSRIAQLNGIPFF
GLKAGRGCYAGYDIQRVLSNGYSDQCTSPCTGNQGQICGGEWALSLYATPPLPRPSPPPPYISSGCFVGCFRDRVDM
RTLPNMYVESPDMTVDRCRQLARSQGHPYFGVEAGTQCFAGYDLYMAYYVNGRGAACDWQCGGDASQICGGDLAISV
YHTIEPRPPPRPPAPPPSLRKSDLLGCYRDSETFRALPYRLSVSENMTVAECQSAAKLQGYSLYGLEAGRECWAGYD
SYRATSLGPSISCDWPCTGDDCEMCGGDWAILVFSVTAEMDQQSSPPPFRPYSPHWPAWPQEVPSYPPIAPPMGPAS
PPWAVVYEPRWPNVPPPSPPPPPPPVYGTQPPNAAADAFVLPEGKRPEVAYFDAKVGVSAYTDAVYEDIVLQWKDVF
LSERPDNQLYFLGECHTAPATPSSSPAIRFDGWSCWARMADPLPNWDSSETGAFTAVWIGRFTAHDYYGFGYSEQAY
ATLSRTENDFDRELSWTSQRIMVYSHANGMAADIYTATPPSEQWTMQVLSRSPDGNGGVTISYFWYGISGYLNKWSL
HAGASSIDPDNFALGADSRDSDKFFSGDVAVVMLYNRTLSEGAVRGLIDFYSPRFGWPNPNA

>Vocar20007352m|PACid:23136164
MTSTSWRGASRATAVRRLLWCSVFLQVIALQQLAWASIGVIETMDKGGFVCASMDAAERLCRGTDCGALYPNMIKKA
LQVAPEEARGILAFGAYTPGSAARTALDGWVKAAGYSPSIITYVTNPAAVKSYNISTYKLLFVPSGERVVQGGISAD
MVNALVAIKGGIADFVNLNGGSLIALAQPVMFDELEASYPFFPAPLKTTLIYDQESWFSDVSVTNEMSLISPSTQKN
TISDHQWLGFFYGPVDWSGLRVVAYQTGMCPTPQGKNQDCQATVLCNTKATLTAENCYDGVDNDGDTWVDKEDPDCE
RCGDGVVDTNEECDDGNLLDGDDCSSQCRIPPPPSPPRPPSPPSPPPRPPAPPSPPSPPPPPLPPPPPPPPPPPPPP
GPPPPPSPPSPPPMPPTPPNPPKALAPPKKSVTAQFLQTTLSFRRISPYFALCDATSMERMRGQLATALNLPTENVT
VTCEWPKNGAGSRRLLQDTDGQDSTDTGDSNLADVCGANSPSYTVNIKPEGRHRLVYVARDPTVPYYEFKVNVWNSV
NSKLAGLCPLGSFDGDWAQNGYTVGGQKGVVVPFQVVIRVSRSILWVPMGCWEKTWSMVGVTDIDPLRMFDTCLERP
PVAPLLHGEAKYAESAEEKEVKKKANIVAPVVGVIVGVFGVGAVVGITVFLKKRKSQLYESDSAQSDETAASRGDAA
GDKAIVITNEIFSTTSGATNIGDRRSEGAHHHRGK

>Vocar20007184m|PACid:23136303
MRCGQVEMSSRGTYWLMAASLLLFCLLSLASAVSAEALEETAGTVGLSAASHTAATANRRALLGVTSPPRKSPSPPR
SPPPPRPPPPVRPPPPPPPRSPSPPPKSKPTPKSPPPKQAPPPPSPPVFSFEVFMYFSFSNSGPLTPKPNRQFGRDVN

CSKLAGTISTKLADFATKAELSFTAPLQVTRCNGTTFSMTGSVLYNESACAAMDRQIDFSVPYRWMNEAMYENKNCK
AYPYIEVAVIIQSIGTVGETKDVPCVSRFFGFVFCADRLQPSLPPPSFPPV

>Vocar20007419m|PACid:23136364
MACRSTRPASLAFALLCLGTLVVPHCQVQGRRPAPSRSPPPSPPPSPPPSPPPSPPPSPPPSSPPSPPPSPPSPSPP
SVLRNPLVPRWPRQPYRQRPPPRPWGPPSAPTSTSSSAVTATPSQPPAAPPPLLRMQKSAEAPFLTVEGEFDVLTSH
TSSPSVYLREPGGQVTAITNMGDTEALRYSGYLVLGLADEGAGKDGPGAGIGSGTVSSVVFLLSFCNWRPALDTQAF
RSMWLNDDNAATGPGGSRTMEDYWNYCSRGAARMSASSQLIFEVPIPCNGKWYDREYDLRTQCGTSDLYLMMSLRQD
ISGVKQRIAVLPAEVDNSCSWAGMGSVGCSGSRCFSWINGAYARDLTTYMHEMGHNFGLQHSGRAGDPDEYADPSCT
MGRGRTCFNSPNQWRLGWISPLPGADLNGTNLPSRSPQSLIRIDPTWVPLAASETRNDIQTPAPVYYVSLRMHQPPF
DDFELPINRVYVHTSRVTQAATSYIRSSLQAAINPGGTYKATMPYGIIVRVESITPAAGSCVVTVCRASGTSEAQGG
DSCGDGLDNDCDGLVDDLDPDCDGPQLISTPQQQQQPPPPPPQSSPPPSPRPPRPLPPPPRPPGPSSPPPRPPRPS
PPPRPPPSPRRPPRPLPPPRRPPLPRPPPPK

>Vocar20007116m|PACid:23136376
MQRCEALALFAALELGLLVAVQSCGHHFVHQDIKDLVARHVAHQAHMASASWGPRTTASYTAKFSDNAHRLTSELGT
HSHDHGPGGGHRHARTPDDAAASSGGLDEEPAAATTDQLPQWPEGSQRDGAVGSEGVAATRGLDGEGAASRGDQQTN
KEEEEEAKEDLHDSNPAGVEEEEEEEVKAVSGLRGRSLQQTTTTTTTAPVPAPIRLSVTYQQLGALNLMQQQRLTRV
VEAVRRILQKYINVKQPSRNGMLVDPLCDVETWSCFPSFATSSTDADRMCGLAVIQPAHIVNPVSCATGVTRGQFTL
QMLGRTAPFSAAGNGSSAYGTNCKSYAGTKGEETDMYMYITAVQNNDCEAGAAAWAKPCLMDIGINRPLLGAANVCP
GALELLDEERLTAVLTHEIIHALGFTDSMYNVTRRPDGTQRPGSEMVAVADVGGKAVKLLTSPNVREAARAQFGCPS
LLGAQLEDEGNAGSAGSHWEYTHYQGEVMVASTIFAADGSPARVSNLTLAYLDDTGWYVTNRSAAGLLSWGRGAGCD
LPTKSCSAYMAAAPGQKLFCDPTAASSASLPTLLCSADYKSTGVCRSLNFTGGCGVLLTSSPQTCMGPDAANDRQEV
FGWGTGSPSGRCLPVVYRFQASIGSIRYTYPTNGSDGDSGAACFDTACSANGSVAYVKLLGQQFPCPAATDLCPSQT
CSACNSAGGQCQNGVCYCYLSYNGSDCGYSLISEQYVTDSGWGWVVGNGSQPSPPPPPPLTIWTQLVQLTLSLNNSV
VDVKSRQGKLQSTIAEWAGLSGSAVTIVIVSAGSNSNSSSYVGTGNSSVAGESYGGATDTSIAVSGSRRRSDSRRRR
RARVLQQSTTSTTTARVGAVTTVMLTPSGTRPATTLLGWLKSNAPERTALVAKLANTSFFVVPNGISTESIITQAMD
TSSPPPVIAAVAPASANPARTRAIIGVGIAVAAAAVMGIIAAFIVVLVLRHRQEQRRSRTSFYQVPAAVAMDESPYG
SGGGAPYDSGFGMGGGGGGPFGRGPPGPQPFDCRGPQGGGGPWPSARYGTLAASGPLGSFGAAAAAAGQPQLPLPPS
SMPEFSAAQGRFSPLYCGAEARGYM

>Vocar20014359m|PACid:23136619
MAMLRLIPYLLLLLLALPVGTFTQTVQGCGSRAFIYHNGVPANSIDVMFRDSETGASVGWGYIAMNLSALTLPEPIA
TDQTVPGTINFAVKLEAFVGSISADVLTESMFQYGYPGGGADCSSDPDTNGYLSFPMDVPQNVVFASTLDYSRLITN
LLVHPLLGLPKCQPIVLYMRAKFTDKNNSSRSAYLATDWVSAGNASDPPPPPPRPLSPPPLPPSPSPPPPPSPSPPPP
PRPPPPAPKAPKSPRAPKPPKAPKAPQPPPRPANNLLVNKFPFSSCSASDVSLTPYRMTSPMGPFNVTATAVTYCFR
ISASATVDSTSPCARMVINELQIVIDPSCVESFPNSFRVTTVNGVTVSPSYTYKTWREQTYGVMTITSLASTFPITP
AGGLYVCLAMSRNSRCGTPAGLCYGNSCVYSLLNADNSCCPASQVPY

>Vocar20003361m|PACid:23136736
MFARKMLANVWRILVVAAVFAFLAPAFGSPTAELLRGQNDTSTTRPPRTASTQSDVSTRLKGAANKLGLNFLVSFAE
SVMPALADVFEDAPVAAEYIARLLSRGLDPNLADPEGPVAESLYDILQTMFDKIATTRTGNIAMERMMFQLDFNITN
LAAARDFREMLHILKKSDWSYYPWWHTGTVTGANNIVYVAATTVQALVAESLGLHYWSYSTVLTPYTVAADSADFLL
AVAESTLSHADTLRTSADASLLMTVEEYDAFSRLRASAAELQRSLEGITAELDGRESKARAMVKPPPPSPPPWWWQY
NQPSPPPWWSNYPSNPPQTPPQPPSPPSIRADKVAVRILNSLGLQYLLRFAEDTYVVALDIAYYVPNAAKYIYRLVD
VGPGSLLDVDGGVAYALHRIVQTSVNSWTNSTTGSIIADFLLQSHNVDLYALGAASDLLEVVDIMKRSFWNPYHNEA
DYGPTDVVNTVAGIIRTLVMEATTPNGRVGPIEIALDAVDFAKAFVGSVVDNAYTLRYSADGSLRMTDDEYEAFYMI
RTSLDDLRGALGRLRDGITNGGGGADNGEYDYGYRRPRSSPSPPPPWWSGYDTVPPPPSGSSRPPWPPQPLRPPWGW
SDYRSSPPPPSGPSWPPWPWWSEYDTVSPPSESPQPPWRWSDYRSSPPPPWWYSSPPPPSGPSWPPWRPQQPSGPSW
PPWPPQQPSGSSASPPPWWWQYYSSPPPPSGSSASPPPWWWQYYSSPPPPSGSSASPPPWWWQYYSSPPPPSGSSAS
PPPWWWQYYSSPPPPSGSSASPPPWWWQYYSSPPPPSGSSASPPPWWWYSSPPPPLASPGGTEGPLAALERLVRTLT
YNANKMGLHFLLRFVETAYPPAAEIGKDLPTAAWYVYKLLQQGLGPFLSDPSSQAAYSLYRILQAAFANLASTPSGS
IIADVALRNHDIDLVALASARDFSDLISSFKSASWGSDGSWEVEGIALVAQSITDSVQELLWNAVSPYGRMGPVQLA
RDVVSFVRAVVGATLDNADELRNNADAQMFMSNMEYDAFLRLQHNLDELNQNLSYLAEELNGIVGDYGTAEHVRHPP
TIRHPPRRLSPPAESSGDVADLGGIILRAANNLGLDFLVAFGQSAYPAVNNALLQAPLVAQYIRRLLRHGLDPSFTD
PSGPTAEAVFNILHPTFSRLLETDTGSIVADYLVGHELDLQRIATARNFTELIGELRHAYWWQRVYDFYFRQYDTSA

SLAQEGAASIVDTVTNIVRHIVRDAVLPYGRISPQVLASDIVEVTLTLTRATIRNMAQLRGAAEHSLYLSDLEMNAF
YALQYHLTILRESLINLAASLNQ

>Vocar20004320m|PACid:23136990
MSRLFGVLALGALLAMLQCAQQTAAQSGLSMLAPIAIYNNANSTDAADVARKTALVNAIRNLVPNVTVISGPFTNFS
STARTHILMTNTLPSDGNISPATVSAIDRPGAMLVVLAIPELGTIFNSQLTPFLSANPNCNVTILKAAANTVYNFNG
LPVSIFTGQNKVQTYRCSFGTGWYMAGDTSPVIAFYTPRGGLVKLQGYDFSFGPATRFSDWATVTVFSPPSPPPPPP
RPPPPPVPPSPPALPPSPRPRSPPPPPPEPGAATQHTLITEVSNPDGNDALRKAQLKVGVINLLGAGNVSSTTVPPA
SATSMSIFDNTFDALSPANKQLLVRQLYSSPRKLLSVIVTQETRNELLSSIVSLITNTTAVCSKRVLPSGNATSLLA
APFSDYSNIMGVDLFGSAMTAPPVTLSISCSIGRQVGYDFTTGGYTKGFFNPNPSPGQRLIATATLAVVYNKPMLPP
PPPFSTPSPPRDRSQQQQQQQQQQQQQQQQQQQQQQQQQQQQQQQQQQQQQQQQQQQQQQQQQQQQQQQQQQQRQRQH
LELPTSPPPLPFGGRIPATILLSDPVDLPDSKEKGLLMDGISDLGTRATPWPVAAQGVLVHIAFDTTLARMSDADLA
GLTSLVRAKKTILTVIFTANVADSRLTSLLSTITGAVNLQLYSGSVPETQRINRLVDTLGDLKTDGWRALNSTRYIR
ANLGAPLFGVATDTRQAVVIEAPTLGGGVVRLIGYNFASGGRGDNRKPLTSLVVFAAPLLNMTALTTRPLPPLPRPA
PSPPPQSPDAPLEPVFYRPPRPPPAALPPRPLRAPPPPIGNRYDAIFLTDNITRGVVDTSRKALYLLGLGELSDLNI
VNQTTLGNAPVHVVFDNVYLSLPPLVREDLLTRILIGQTLLTVIVTPETTATNLSSIVTQTTTYTNVQCLKGPFANG
TRFPPAANILNDLVDAWKSDGGTTSVGCTNSAPVYVSADNGRPIAAEFTAPGGGNVRFVGYDMSSGARGAGKIAITR
LTVYPLPLVPAPAPPPPPSPAPRPPGPSPPRPPSPPRPSPPPAPSPPPSPPLPPSPPLSAGSKVALVITDGPANTLN
AADQARKKALADALEAFAPGYIAYQPLQYVAGISATIVYDNTYTRSNFALSDKIVLADVVNNGTTILTVMVTTPNVV
PASGLAVLLTRITGYPEVTCTKTVIVNSPSDSVTLFKANMDPYGETQVPATLSHSANVMTLSCTGIPAASWKPIYVT
SSGGQVVVEVITPSYGVVRLVGADFSRANSPNQWATLLTYNFRLSAGAPDV

>Vocar20004321m|PACid:23136991
MSRLFGVLALGALLAMLQCAQQTAAQSGLSMLAPIAIYNNANSTDAADVARKTALVNAIRNLVPNVTVISGPFTNFS
STARTHILMTNTLPSDGNISPATVSAIDRPGAMLVVLAIPELGTIFNSQLTPFLSANPNCNVTILKAAANTVYNFNG
LPVSIFTGQNKVQTYRCSFGTGWYMAGDTSPVIAFYTPRGGLVKLQGYDFSFGPATRFSDWATVTVFSPPSPPPPPP
RPPPPPVPPSPPALPPSPRPRSPPPPPPEPGAATQHTLITEVSNPDGNDALRKAQLKVGVINLLGAGNVSSTTVPPA
SATSMSIFDNTFDALSPANKQLLVRQLYSSPRKLLSVIVTQETRNELLSSIVSLITNTTAVCSKRVLPSGNATSLLA
APFSDYSNIMGVDLFGSAMTAPPVTLSISCSIGRQVGYDFTTGGYTKGFFNPNPSPGQRLIATATLAVVYNKPMLPP
PPPFSTPSPPSPTSPPPLPFGGRIPATILLSDPVDLPDSKEKGLLMDGISDLGTRATPWPVAAQGVLVHIAFDTTLA
RMSDADLAGLTSLVRAKKTILTVIFTANVADSRLTSLLSTITGAVNLQLYSGSVPETQRINRLVDTLGDLKTDGWRA
LNSTRYIRANLGAPLFGVATDTRQAVVIEAPTLGGGVVRLIGYNFASGGRGDNRKPLTSLVVFAAPLLNMTALTTRP
LPPLPRPAPSPPPQSPDAPLEPVFYRPPRPPPAALPPRPLRAPPPPIGNRYDAIFLTDNITRGVVDTSRKALYLLGL
GELSDLNIVNQTTLGNAPVHVVFDNVYLSLPPLVREDLLTRILIGQTLLTVIVTPETTATNLSSIVTQTTTYTNVQC
LKGPFANGTRFPPAANILNDLVDAWKSDGGTTSVGCTNSAPVYVSADNGRPIAAEFTAPGGGNVRFVGYDMSSGARG
AGKIAITRLTVYPLPLVPAPAPPPPPSPAPRPPGPSPPRPPSPPRPSPPPAPSPPPSPPLPPSPPLSAGSKVALVIT
DGPANTLNAADQARKKALADALEAFAPGYIAYQPLQYVAGISATIVYDNTYTRSNFALSDKIVLADVVNNGTTILTV
MVTTPNVVPASGLAVLLTRITGYPEVTCTKTVIVNSPSDSVTLFKANMDPYGETQVPATLSHSANVMTLSCTGIPAA
SWKPIYVTSSGGQVVVEVITPSYGVVRLVGADFSRANSPNQWATLLTYNFRLSAGAPDV

>Vocar20004293m|PACid:23137161
MERTFPTNLTSPGPHHQVHRQRMQRRRRAAVSPYSGKATLLVAAAAALMCLSFVGVRTVLASSSGVDTTATSEEASA
AGAEVTAVADGGAAIVVAEGEVVPASRRHLATACTAYTGYIAYRDIDRDGDDIGNYGSASSALAACNRNPSCVAFNS
QGWAKRSATPAMPYGGLCLWVKRSSMGAYSGQYQQGSPTGDGARIIHTRACAGGPPAQPTQFYPGSAIQALTDLVQD
YCYSNSGAKWSGTTGKGHTDDPEGSGIETCQGGKSFRCDEIDFMGTTNWYQEYFYDFPSLSNNTCNTQMVLTPASDA
PRAASRSVLTCRPNPTSEGVCTFSPRLETASSVAVTQARGYSDQFKAGVVTKVTATFGAMEATMESTFDFAFSSTAS
ESTARIIGQSTGATASVTTSPNDTYCSSLTISLGRTAAVGKLEVYLEFSGGVGTRQNDWRAWKNINNGRTDFKYLYH
PLWYIVNTAKAAQIARANAFTVIGTTVRVTTPITLSMQLPAYGYVNPQRYSLNSLTCTSASARTADPAPNENAKPSP
PPPLRSSPPPPPLRSSPPPPPLRSSPPPPPLRSSPPPPPLRSSLPPPPLRSSPPPPPLRSSPPPPPLRSSPPPPPLR
STVSSRMASVDTAIDAVSTATAAYEKSSAAVTTTTTTTTTAPYMLDESSVNIAARAAQQQTSSYSDQQTAAKLDTRG
ASVSAFEGSISPVFMQRLKALMSAYCRAQSGGDDCEVDWDGNTDWWRDWFLRLQPAQIATGLDEPVWNRTASGLDNV
TSMLVVCPYRPFNYRCQIDAHSYQSTIITRSRTSTQSFGVTNRCCSSSVQIFHVHCTSCTDARAAAGCDMTGAKERL
RQIATCQLVLRHPRYAWFIRSVIAPYADCLGLLQPCATASSSFSWSDGTAVANTETATTVQSYSVQMNPATNPTQKC
AHLLTRMTVANVRGIQSAWLTINGYVGFHHDDASPNPLCLPARSIDRSIDRSAAAAAASSYSIKSSRVWKKWADSNP
HYYRAQPFSSVIENAKSFGLKEAAEWTMVNGMARIQVPIDVVVMTGVSGSTGAVYYDAGSPQCNLPEPA

>Vocar20004451m|PACid:23137292

MLYALPRTRNGLKVAVSRRNHGRFRRAASGTLKNIVRFGKAWSLDGNRAARKRFGQLGGPAMACATRTFTALLMSRV
DGLDSSRFYLTVGEGRMFRLEFCANVTVHDEVAPNSIVRVDYVNITNGNMLSCRLPQPVDRQRRSLFGDTIDTPTRP
TFLIYIVSFCGFSEPPAASPQLKGTGHQTMMPVRRKLDWEVSVSTVSIAAFGASGCCRGHFLRHFTSTPAVGNPVWR
AIPSATHGSDRLVALSVPEISAGCGRGLAVMNFYNGTGYQGRSIMEYYRTCSYGQVLPSQLDVVGPIDIPCQGTLNV
PFTFPTGNSFNTSTCGNDNMLKWHYYLDSIVTDPKRGYNIVPTNYHHKVILMPRYFSSRIRGRFWVLPKAPGGQQED
CNGFAGSGSIGPWIRQLSSVNRYGTGLIWWSGDVFNSIEFLFHEVGHTLSMAHADIAGGCDLGDQCDHTCPMGATGG
QGIRCPNAPHLYQLGWGRPYRWLQDSDLRYGMYQPIDLPPQMSGTSTSVAVEIGSDRFFFSARINTQLFDLPYRSWE
NGPYVLLHSYKGTAVTPYARTILLASTSLRGIALDPISGITMNFTSWDPRLGARVIVCRRSKSKEQTCGDGLDDDCD
FLPDELDPDCAGRMPEAVGAGTDEEYQGSNDRSPLPRQPPSPRPPSPPPPSPLPPSPPPPSPPPPSPLPPSPPPPSP
PPPSPIRAQPPSSRPPSPLPPSPPPPSPLPPSPLPPSPPPPSPIRAQPPSPRPPSPLPPSPPPPSPLPPSPPPPSPQ
PLSSLRAQSPSPRPPSPPPPSPPPPSPPPPSPPPPAPEPPELPLPPGAWMTPPNLPETPTPPNEPSTPPLPAAAKPP
RPRSPPPPRPVETGKSPADLEPRPRARQSQTARGAIARLGSFCFCTRTRAVQMRAFGVRRVSAGGQAFAASKYLGRR
RN

>Vocar20003888m|PACid:23137326
MLWIMQARFWFTVPTVYLLFSTLSRYVHAADGNGVHIAYLTDCTMYSDWQTVGMVFSYKRSRQPLDSQLTRIMCCTD
EERKRYNEQLLSIVQTHVAPSFAHNEKTDDWYAAYNKPGAVYDWLKHVTPKEDWVLVLDSDMYLRKPFYPQFFNATR
GWCVSADYTYMIGVNNELAVRHIPEIEPRNDELAGPVGRRGDQVGGFFFMHRDDLSRVAPLWLKYTEDVREDPEAWR
LSGDQYVEKGGKPWISEMYGYAFGAAKANVWHKWDKRTMMYPTYRPTVVSSARKMMRFLLGVFLLLVFQRGATAASE
HQPVHVAFLTDCAMYSDWQSVGMAFSFKMSGQPGSVIRVMCCSEKDRKNYNKGLLTMVDTWVAPDMSRSPRNGDRYA
AYNKPEAVLDWLDHQVPKHEYVLVLDSDMVLRRPFFIEELNPKRGLAIGARYTYMIGVANELAVRHIPHVPPRNDTL
AGPYGRRADQVGGFFFIHKDDLKAMSHDWLKFSEDVRMDDQAYRLSGDVYAVNPGDRPWISEMYGYAFGAANHNVWH
KWDTFSMIYPGYEPREGIPKLMHYGLLFEVGKNYSFDKHWHYDFDVTKCPPWDLKDPKRRSQGIFPEPPRPSSLPKG
DFLGFYRDLLAIETLATLNAAFCDYHISHCPPSEQLVTVCKEVFSLYNEAREYIKEAESHYDCQNFHPKCEEWAKTG
ECDKNQGYMTETCRKACNRCSQQNMYFPETSTKDLEDKLAKMAKELQPLAEDPDQQKGATGVSPADSSSPVVVPKQE
QPVVVVPRNMKPTEQASPLPSPSPVHSPPPSPPPHSPPPSRPPHSPPPSPPPSSPPPNPPPPSPPKVQSEKQLLVRC
YRLSLPLAEVKDCVKAAKQGIAYEPKQTTAGGSQASSGESADGAEGVGQKDLTRVGRVIERDLEDLTLTQQDVKKGA
APVLDPPVVGPSLHALLGRLNKWQALLIWLLVVAAFLAIMPRIARLRRRARSGMRTE

>Vocar20003835m|PACid:23137341
MSSAQSLQLMAVLCITVLLLLLPAGAEMHSSYQHYGGFMGGTGGNDIETALSAAAAGHSEDSVGSNPRRSLVDTAKQ
NRTCILGKYECTVDLRYFASLNQRPATDIQKLLARSIADQCYSEDTAGAVTALGCPYNSGGIFHASLLRNFVYCPGS
LLDQAITCLETSSETSCSSNTACTWTNRYLAVWSDYRQLNASTVMMNAIDAAMINLGWLKGPHDEDLTDNYDTNSGG
IMPFKYCAARWTINQTFLMSLYNTYTDWQRAAFISGPAYQGPIALVGTAGQTLTGSCPAGQKLTSMAAACTNNVTTK
AKCQALNANGIYCSWNLTTGVCTPYALPSLGDFLTSEYAADPWIADLYKISLACSGYSSTNATACAAGGSLFTYNTS
QLGAFSVVVPSWYLPPEASSPPSPPPAAGTAPPAGLLGTPPAGAGGSGTPPRASPPPPKAGGPPPPADGSSSALRMG
RLTALLQAVVAVLAVWLGGGVLIVAL

>Vocar20003932m|PACid:23137612
MRWAFGSLLSMVFLNSFICSHSQPCEQVSCDGLGAYMNDDNCTDAQFYRDWAAASGLKGAMQEGYAVGLLEFRLVIS
SQAFENFILFIILCNCVTLALSSPRQDFGSTRLGETLAIFDYVYIGVFTVEMLLKVIALGFVFGKGSYLRDGWNLLD
FLVVIMGYVSLLSPSNLTAIRAFRAMRPLRTINRVKGMKVLVNTMLGSLPMLFDVFLLCAFTFFMFSLIAVQLFEGV
LRNRCGLPDFSGAYNVSLAGIAGGGGHGGGGYGGVVVANVSYLVPDDQTEDMCSGPLSSEVVWHLGNDDLVPAANAG
KPYAGRACDSGMYCTWYGNPYNGFVSYDNILWSWLTIFQHITLSGWSDVMYLVMDAVNYWVWIFYVGLIIFGAFFMV
NLALAVLAVNFSAEGQQATDEKEVEDRQQDKEEDRKRLKRQEEEEGEQQQEEEGERGKGLRQQQQQQGEAYRRSST
SRRFEAVLAAVGLANGAYGEGISTTSRLVAALKVRRRALLANDEDAVLAGSSPPPDLGRLRRLTWRVAVSRGMELTT
AALILVNTAVMCVNWYGMPYKVEQATNYINYGLTCYFAVDVAIRLSAFGFARFFRSGMNIFDFLVVALSLAEMVLDL
LPGVAGLGPLSVLRAFRLLRIFRLARNWRELNALLTGMFKSVQASIMLVCVMFLFLFVAALVGMQLFGYRFMFCDYV
SGSRPVCPPGHRVWGQCPNHFHCYLPCAEDQQGSWVEAPGSFYNGLAYCERFCPDWGELAIRNDIMIDAMRTVSPWA
SLYFVFVILVGNYLVFNLFIAILLDNLNSKVVEAVAAVAAVAAATSGNSNNSKFGKGRSSSSRPSATTAVSSSSSPP
PPPHPPPSPSPPMHQVQLLDWEDVAQEDAALTRAAGGGSRSSGGGAGNRACGAEKSGGGGFGNSVCGGSGGGGAGAG
GTNGVSRGDGQAVGVAGLPQDGVVVEAQSPRLASTTPRRSVREVSGRWEELEEGKSPEPSQETAAGSAAAPPLAGVT
RMGSASYRENTRHQREEQLRQRSWLSCRRVPSSSGGGGAAAGEDSMHGRAYSGSGGGTALTVSARCSAGGSPGACSI
ASTQSSGFRMPLPPVAIADTAAAAAAAAAAAATQATSAATSGGAGVRVQVQAGAELAARRLLLLLLRLPRRRWRRRA
RRRCPKSTGAAAAPVSVKGVQEEDESSCWADVAAPISASFASKAAVGATPPEAMGKTTTWSPSGSNSGSSQNVVTPL
SHAPGVGGRMARVEPSLLNFPQLSSPPSSPRHHQQQQQQRLRLQPARQDHQQQQELTTPAGQPPVSLFALSSKSAL
PQLQRLQSNHQSSSQLCRLPSTHQNHHQQQQQVRQYQLGFIQSSQRSFRAAANVFRNVLYGFSSFRTTPVNQLSRA

ASNASALSSAQPAGEGGGPPAWLDRLEGRSLLVLPSESRLRRGAAAIAHNSWFEAVILLLILASCVCLVLDAPSLNP
ESRLALALRVLDYIFTGAFTLEAVLKIVTFGFAFTGRHAYIRNGWNVLDLVIVMLGWALIVVELVGRDGVKDNVKML
RVLRALRALRPLRAVQRFPGLRVVVNTLFAVLPAMLNVALVCVFFYLIFAILAVNLFKGKLYNCVDAASGERIDPYY
VLPYGQQLTRAWCEAGNQAISQSAYYSALNVSMPEYSIATQWVNPKANFDNVGVAMLTLFQTATLSLWVDITFTAVD
SVAVDKQPVWNHNPWIILFFVAFLLVCSFFVLNLFIGVTLDKFAELQAEQDAAGGAFLTPQQQSWVQVQRLLLRSKV
PYVPPRPAGRLRGAVYSAVAGRRFELSMLLVILSNVVFIALVHTDMSATWQAVMSYSNLVFTCIFVVEAVLKVAAFG
PRPYFRDGWNCFDCFVAAVSAVSVALDFSGTRDLSFMPVMRVLRVVRVVRLVRQATGMRKLLHTLITSLPALANVGG
VMLLFFFIFAIIGVNLFAGIKRGDSMNDHANFDTLPNAMLLLFRMITGEGWDAIMQDCMNTDDCVLVLQDWNVTSSQ
PTSQPSSAVSATPAPANSTPADPATTIFLSAGTYLDPSDPLLAALPSDATDNQCPLSPFAAVVYFPVFVVLCTFILL
QLVIAVLRENLLESDDGGDEAEAARPVPRTAIESFTKAWCASVAEAAGPGGGGGGGGGGSGGVGGLLHASRLAGVL
AVSHPPLGALGSDHPRQEVQRVLLRLRVPLYEGNKVSFMETLHALAGSVCATPLPPPEEDKLHRKLSKRLPKAQPHA
KYTSAHVRRGTAMMGCGGVCAGRDEEARTRRRRRRGRKGFKGGESEGLGTRRVLVVRKWGVWRMGSCACAGSSITLL
PGLGGRQRITALHLQLLVSVSREEGAEEEEG

>Vocar20000905m|PACid:23137682
MTTRAGPAREYHCRSRNGSLYGTVLQTIKLQPPLSNKAAVAECQKLCDQAADDCSGYYYKTTGFCTLMTGHVVFSKP
YPADFYACIFHGYSPAAPEEPVLSPPPLHPPPPAPGLPQLPPSTPPPLQPSPARTPPSPPSPRTRSPPRTPKSPPPS
PKAPVTKPPPPSNPGVPGVPSTPPSLPPPEKPSLPPPPQAMPPPSPLSKPPPRPRPARSPPPSSSPPPSPPPSLPPR
LPPSPLPPALPVRSPPPTPSRRPPFPVLTRTPPSPPPRSPPAPGSSRVYRCKNETSVYSSLPVLRVLKLSPPLRSSA
AVKECRRRCDTFAGGACSGFFYKNTGYCTLIAGDARFSGKLYRSDIIACVLQRQPSTPRSSSSPRPPPPLTPMNAPS
LGFPSAVPCHVNVSIGHDGHPSERDTNVSVLTWL

>Vocar20001045m|PACid:23137811
MTRPLSQMFPPMRLLTVSVFLCIIQSSAIPPAEAIGLGQELGDNSGSSEFVLSDGGTARRQLHQTTCNKNLGYKVYP
DMDHVGDTITTSNRPATQCTNDANCVAYNSNGQLKRAWSPMTKSTGTCLYIKEAFAPTACASYEGYTASADVDVRGN
NISNLALGKAATRCLADPKCKGFNSAGWLKSALAPLVQSRGICFYTKIDESSPPPPSLSPPPPKSPPPPPPPSPPPP
APPPPRSSPSPRPSPSPSPSPSPSSVISTAGCPDAAAVLAQHNAYRANHSSPAMVWDETLAKQAQVWAQGLTSPTCR
LEHATSGGQFGENLYLTFGSLKCNDAVKTWYGEIRSYKFTDNPWTDNQANFGNIGHFTQVVWKSSTTLGCGAATDAG
GSCAVVVCRYKPAGNVAANSFFKANVLPRI

>Vocar20001970m|PACid:23138061
MPFFSARMLATTAFTVVILCFLPQLNSVLAQDPPDYPQYNCSTSPAVTYTAVREDSLYYVSMKFNASVSQIQLDNRL
DNYTAKIREGQRLVVTCPERSSAWSPPFGIPDMSVNLWSGMASAPTVVKCPPNEYVVSFFISANREWYSWTGTLGIR
CSGGRNFSFNAQPANTNATIDGETSQPSGLKVIYLTTATISIESVLGVGLYKEGNTEVFECPQGMLIAGFSAGAYPL
MWQAPRRSSWICNLQFLCTGLTFPPPPPPPPPKASTQPPPPPPSPPPPRRGFPPPLPAFPDFDCGNSRSLSYTAAATD
SFYDLANQFQIQLSITQMMYDNGLDYRTGLADGLELIINCPVGVWSKPYGTYNSSDVAIYDGMAAASGVWLCPPGEY
AVKFSVKSSLAWSGWVGTLTMQCSGGRSFTADAQPATSSFAPMDGAAWQPKGFQIAAIEVGPVDSAIYSVFGVGASA
TKGGAQEWFTCPTDSLITGFSAVVSYAPPPAGSPPSSKPPPPPLALASPPPSPSLSPPSTRPSPPPPPLVAVPPSPA
TAVPPPPTSAVPPGEAPGSSPGTVISWSPRPPSRFAPRPPPPAFGTLYAEQRAGGRCKGVYCLHSNTATAYGILTAI
ICGGICFISLLIIAALYIRKKKKVAKYLARRTQPDEPNAEPEPHDITDVASISRRQGAAASPKDKRLITAAAAAAAA
ADKTNDEFALTMDPDMVLEGSVSSMDRARHQHGEGDAGYAAAAAAATPARGGDPAFEVSKVIWKLTIGPSAAARSTI
ERTTTITSVISNKCTGIPSAVVIWNIIISIQTIAAIIKSESLKARTVGAAAIARTIASKVAVKGITAKCSVAIVSPG
DAPAAAVETGGHCKRENDNDKGISVKDVIGVEDVITINV

>Vocar20001955m|PACid:23138146
MRCLAVLVLLVLQAGVGLGSTDGELRGAGENLIVNADSTSIKPTQPSSLLPPPPPPPPPPPPPPRKFYSFGPLVWCY
FNFPQWPDMPEMPPSPAQPSSPAGFAGRDTAAFESPSQPPSPRKTATKAVQQKAPSTKPPPSPKMRPPPRLPSPPPP
PSPPPPSPKPPSPSRSPPRPRSSQPTSRPATGSPAPKASRRLLRL

>Vocar20008139m|PACid:23138937
MAQKLRLGAFGGFAFTALLFVGIATAADLASVYNTCRNLAKSNQAVHGCVTKNSDGSWAVVDPGHGMLGSDGCAASG
TFTDSSQTASNFGKLRIVTSSSYSDLDQAYAAGFLEGYLTAARIYDHHYNLKQYFVVMLNESESLELSLNWLERQEE
WANAQVRSPPPGEEEPYWQLLGLVQAQFEGLVGGYQARAKQEQQQRQQQQRQEARRLAGGDGGRSGSGEGGYGDDDV
RVGWLERRDLMFMNSNGDVYDIIDALEAGFGGEVENGTGEGLLPGAVRQGRGRGRRNPKWADVDPDPVRMSLKLGLQ
GKCSALIKVTGDLGELLLGHSTHDSFTAMTRIYKHYDFSGLSNTAVAAPRMSFSSYPGELFSDDDFYLLSSGLAVME
TTNHIYVGDVYAPLSYRCVLSWQRIRLANWMASSGGEWVDVFGRHNSGTYNNQYMIVDTKRFIPHSELQPGLLWVVE
QLPGLFKTADVTAELSRGYWPSYNVAYFPEVYVAAGYPDMVGAGGHQGRIERLTAMGAKKYAFSIRLLKYQIAPRAA

IFRRDQGVVDSLEAMKHIMRYNEWSKDPGQPPFTWSDARWEKLPHRGMPDTFNFTFERMSPRDLPTYEECAAAAKAV
AAGAGAGAGAGTTRGAQLRRKTPIMSPPPLPLSPSLYMLLRRRMFPMYGGVVCRRSGELRGAAG

>Vocar20007052m|PACid:23139095
MHRPLAGPTPAFSASRACGRNLLVVLALLLGAAATLAQTPSYYCNTYTVGTGQCPICAAGSSCSQITTADNFYRNPS
ICSLPYYGSLTSIPGANLIPTTAICNAASGINTYDIRGAAGSSYANTVVGTAYVFVGYNARLYVTLQFNCDFMFSTN
PNMPKNFVSIATLNSWNFVVQGPYTTPGCSVGLSAGASSLFITYAFLPASAADFINRIFPPFPEPASVQLRYLLDDI
LALPCDSFVTAQGVGVLRQVPDKTLGFQDPRWSILYCLPSPSPPPSPPPPPRPTAPPNPPPSPSPPPPPLPPSPPPP
SPPPPSPSPPPPNPPPPPAIMFFWQLFYPAQTSRPGDCAAVSFMLRYSYRLADLAPATADPNCTVTSTKLVATLVFY
TVDRGAKSMVGLFNEGAIGELVLQALTNAHCTALVVITDQSRPPGVNVTSGPNCQLNDPPTTGAKIIIVLASSAQAL
LFYDAYATSTRADTIVRILALPCNRSSVIFAAPGLTEPRVFDQRNVPALQCSTTTTTSTTTGRRALQQSAPTPSPSP
STAVVTDSVVERSVVLSVDTLMTTPLTVSHCTALITLATLAIPAGVTPTSGPTCQLNNPPTTGAKVIMVTRTMFDAM
GLFNSYATANRANTISRILSLPCERSSLIFAAPGLSDPVVFDQLNVPALLCNISNAITISGRRRRTAQDMAVLEVPR
LDERTTTEGADNGPNGNGRSNDDDDDDAAEGQYWWLNSYTGGGRPLALPLALTAADGGGVGGGIAASTFLPGGAADL
LLQQAGSVAAAAAAAADEEGEGKGKGEEEAD

>Vocar20011363m|PACid:23124253
MFALGLAILLCIASLLQVAVGQPVISRIDPAIGSLAGGARLVIYGNNFGDMYTTSRSVYIGPYPCNIISHYSSNEVL
TCETSPGSAALKAVLPRAGPPGTEVFIYGDRTAYMMYRDCVESNWGDMNCVGSILFGDYLCRQDPADPDSPIIMTTN
PRLGPYLYRIGCTMPAQNSNNTQPALGKAASLNATIHFEASMRGGAPTALPSSFLYNVDSVPYQFQLYPEVTGVSPA
TGSNAGGTLVKVTGRGFPDLRLGMNDTIAISIAGVPCDIVNSTYDTLFCVTGPKPAATVSPIGGLYPAMRGIEYEFY
NISRLSYGNLWQLNNTIKVQPLNGSYRAVLTGSWEGNEYSTPYYCSRSKAFFTAPRAGNYSFYMSADDYAQLNGTWK
QSNGALVSTTLINLQSWTYIDNFFQSPSQRSLEVTLNAGQSILLEMNHCNGASVGFTQLAVRMPVPEPRLNSLPEIQ
KISISATFFPRSVAIKYLYGDGAAVTAFNITISALNDTALDDPQIGLYVQINGGWDIVLPLGATTGAMDDLVEASLG
GNFTKNDTLFGIRKTRAYGTLTLQMAALDGSMGSFNVTAARLVLLPPPPPPMDNTCPSNSSAAINVTSMYPPPAPQA
LQSAGTGFTVNVSRAQAVPAVVPTGDFLLGLYGNATTSRVSATANSTAMVAAIQNVTGYNSNYYSVNVTNAREGAYF
ARIWNITFTYSWLDNVPALVVGPSSTTPPGVLLTMESRSASPRLKGSFKVAFGSSCASVKINLGDSSETIEAALGSL
PGLAPPKQVDVSGDGTSGYVYTITFDPINNPGDQPELRIADASDLQGVNPTGSITTVWNGTTDAFYAPIPTEFLRLA
VSQPGTINLQVNGVPSACADASGICGFLYSDAATPRITGVSPTRLAFSSQASLPLTITGSGFSSGPSVEVRVGTALC
NVTSVTATQITCNVPDTAPAGVRLVSVNVAGLGFADGAPNVTLETLYVTGSAPSPAVLSSAGISVLNFTGKGFDYVD
CANNKLTIGGVRCSIIKCQPNMLTVIYPGNNGANAAAASVTAEVYEKGQLIDSDTPSGLTVSVSSSAPSISTVSPAR
LPGAGGVITWSLTGATASQVISASMVPFISSFTNYTEASVAFQEPIPCTDVTAGSSGTLTCISPALRNGRYHILAVL
SSGLQLLSSDTILFELFITSVSPNLGSIGGGTTLTITGSGFSQVVSENVVFITVPVSTTFLNGVVQCIATSVTSTTL
TCKTLPNLAADASANDPFAKQVMPVDTTPGRAVSVVICDSSYNSSFLQEYCWSLPETARARCDAAGAATCNFGYSTE
LTPAVDYLYPSSGSLGTRITVVGSYLNMVTEVQFLQGGVLKGAGSNIISSRTNVSCDAPDLPPGVYTILLLKANGEK
SVDPYGKGTFTFTASIDRLTRNVGSLAGGLPLVLSVGGAGLAVGPDNLTANAITIADLPCPILSVKNRTEVTCRAPG
MNGYVFAEYWNLGVSTSSMPDIFSFTKPHISRIEKGTSFSWGTMSPEPGVINFDYWAARFTFYYQISVTENVTFYMY
ADDDARLYIDDVLVGGRNTPLDTILYPGLRKLVVTHVEYNGGAYLSVVISRRQPNGARGPFAYLEWQKVTPVPPGTP
LPVKLTVNGIDSVTQCPVTNLTLVLPGQPKAPFEPSWVQTPNRTCSYVYSVYRTPSLSALSGITLSGIMQPTFNSTN
TTLSIFGDFLTDPSLPAASQISVTLANRLCSIVGVAPVTVNGTNTTNINCTAPALPAGQWPVSIMVEGLGLTRPTAT
AATDLPIVTYTVRVLSYTFNNPNSKCYFSLFGGGTFNITGYGFVKDLVDTTLQVNMTAAWETVFCGSGFSNSPCQA
VNSSRLNLVPLASDGFTASFGLTRFKVPNPEAFAGNNSISGVNLRYKPRVYYTSNGTYAATASIELLYFCGGRTPAL
SGVTPSSAPPDAGATLSLTWYLSGVGTLNNIITAPAAKNGASNATVEFEVGPTVLPCRNPVVTDFNITSTTYREAIS
CTMPAYMPAATYTLWLCIQPFGCGLLPAYTIPLTISAMSATTGGSAGGITVVITGKGFDTNTTLVSVRFGNSTCKVI
SSTATSLTCVTGPLDTKPVSPVTWPLSITPTAGTNETTLSNFTFTFDPALESTVSGIVPARGSTEGGTPVTITGADF
RTGVSTTVSIGDVACQSVVVVNSTAITCTTGKPPNSILRVPLPVTVLQQGRGYARSSAQYQYIDVWSRNSTWGGGPL
PGYEDSVVIPAGVTVLLDISPPKLYVIVLEGNLVFDDTKDYINLQAHYIIVKGGNFTIGSAEKPYPGRANITMHGAP
NSRDLPMYGAKALAVRQGVVTFFGQPKIPHYTKLNRTADPGDTSIVVNGAINWQVGDRIVIASSSFYGNEVDEATIT
ALDNRTVSGCTVITLDTPLQYVHLGELHSVQGSTPLDMRAEVAVLTRNILLQGDYTSAKNMYGVQVMVNSPSYLTNK
GLVRFDNIEITQSGQAFRLGRYSMHWHLMGDVAWQTWVRGCAIHHTYNRAITVHGTHRAIIQNVTAYHTMGHTFFLE
DGIESGNLIEGNLAIHVKVSDALLNTDTSPAAFWITNPNNTVRNNVAAGSAAYGYWYRMLDNPEGPSYTTTICPKFT
PLLEFTNNTAHSSMFYGLRIHPEFYPRNVPCNGFSGTFEQVPAVFNGLVAYKNGMKGAVGTQVGMVQLVNVIAGDNG
GGPQQHVVNGKDHGGDLELSWVVDDRSRVGVQITEMAGIVNATIYSRTSVGKRGSAGQWPSCRRVAGIITQSPVKGD
PKHSALMSLINVTFVDFKGWDDCTLFSMLEACGKCKDHQGGSTTFTANISIITSDNSSSMMSYWSWGHQGVYLDTDG
TLLNNKSLPASLLPNVGTNVTLGPGATWHSSVESELFDPNECKYVRNTRTSNNGAYCSPGLSFRRVMLHDHTPQSIG
IKDLILTSVATNRSSLVHFTKYNEFGYQFTIATARDYWVQWDMPFRVDPETYELHKMDLMNTSDYVYITTKHIQVKD
HFSINNNDQWNNLTAMPPPTNSTHGMFYYNKTKAPNSWWWSNLSYYDTKFTVFVDGRLDTALDMKAFTCPNTGCTNV

PDAVVDTRNNTLYWSNTSTWNTSAVNNFRKPVAGDNVTIPYGWDLVIDESTPPLLALTVQGNLRFDTTRDINLTATY
IIVMGQGVLSAGSATEPHPAKATIRLKGARDTPDYGIDNNFNLGSKVLAALAGGTINLYGKPARKRWIKLGSAAQIG
NNAINVSDPNHGWSVGDKILITSTSFNWNQSEFRIITGIRNNGSQLLLDSPLAHPHGARVKAYPGGPTVDMRAEVGL
VSSNVLITADDGESTHSYNGEMFGARVVVSGNSTGRFDSMGMAYCGQAGFDDRACIYFDRMAPVTVMRTDNVTKITT
VSAVPNPSFVKGSVLLYGMASNVLIYGNPSVASPVTLADNIMYEAFDKHSVDVQTRDNVIRSNLVIGTIKNMTERSG
NDVRMPSSFRIVYASNWVTDNVAAGSERYGFAYYGLPCSNFTNGSFRNNTAHSSLAGLWFQASPEALLQGCAQLTNF
TTYMNWDFGIISTHGVSTDIVLKDVNILDTKHAGIHLMRYGEMTDKGWMRWDGGLLVGQSSNDVCSACTKRGDPGCH
PKMSLQSFNQYDPFTPAVGLQSSQFALGFAVGPEKKPFDKPMGYSLIHGMFNISGITLADFLGPAGCGGSQSGTYAL
ANQPKAPEAFYPHYFSKINVQNVSTGVNQGMFYHTPPDPDWRNEADCGEAVYTRPDGSQIKLNCAGPAHAYWRDLDG
SLTGTVSTTAGIFTSKRVFPMDQGSPVLPGACTYSEYQDSYQCVVNSTSYIAEAGLDLRYKPDPVPADGIWGDPQML
VLESRDKDTEDRNFGPVFFNVSGSIDLVTAAMDHGWCFAYTCQKRLSTFWTYVPTGQTVYINFTRTPSQSFRIWFPY
ADPDKEIVLVINMLYTLNRRFTWLPPGNGNTTGRVMPLDKPIRIGDGTPHGSYFWDQDNALLYVKIKGGMTVETRTE
SAVMVSQSFAVTIDNFYAQQAVFLKNLAAVMGIDMNRLYVAKIVAGSVSATIGLEPDKSIPVDPLLDNTVSAAVDPT
DPVVTDPAITAANSSSASDTSSPSTPPPSTPPPSTPPPSSAVAVDLTAAYNAFVTAVTDPNATSTLGIQPIGVPTAD
WSTAGITFPPPPPDSPTVPSPPSPSPPDTPPASSPPPPPASSPPPPPASSPPPPPASSPPPPPASSPPPPPASSPPP
PPASSPPPPPASSPPPPPASSPPPPPVSSPPPPPVSSPPPPPASSPPPPPASSPPPPPAASSPPPPPAASSPPPPPA
ASSPPPPPAASSPPPPPAASSPPPPPAASSPPPPPAASSPPPPPAASSPPPPPAASSPPPPPAASSPPPPPAASSPP
PPPAASSPPPPPAASSPPPPPAASSPPPPPAASSPPPPDQIVLSPNLPPPPSIIWGSTEVPANGESSPSRGGGSSTN
TGGSSPSIGGSSTGSSGSSASSGGSSTVTGESSSNSGTGSGNGSKGATPMYIVGIVVVVAVFVVRRRAKGPLPVLPS
GSPSTSEVSSSMSSPPAAGRPGSASTRQAFVATNPAYETPGERAAALPASVAAVPVQGPEENPTSPRASVLAAGPTP
LSPAQFIGPLASTARMPHSSLPLVPPRTPVAAVDAMEGEAMLAPGASVSVAAAASGAIATTASGSVGGSRSPAQLTP
GQPTTNHPSDTTVTSPSNMATGSTGSKVDSAEIEQVPDHDQTKAAAAEGAPAAVHVVAPPGTSPAPVTGDATDAATT
PKARMPASLQ

>Vocar20008751m|PACid:23124883
MRYIYLLWKQLGYLEKTRVELPGCVVVAFLLIHWAILSQGQDADRLLEFSDSCSTSSQFVGWVAGSDACSFSGVTCT
NSAVTDVELNGLDLNLCQIPTTWNIGDPSTGLEQLTRLTFRNCNNFLISSDLMFVTQLKKLELADTTEIDPLPALAD
FSQMVNLVSLRLPSVGFTDPFPAEFSALTALTELRLGDNAMTGSLPTVLSTLSKLAILLRLYFFITPGNCIFARRSL
WEFRRSLSAAEPLSLSTPSNIQPATKSSTTAAKPDTTAPKPDTAAAKPDTAAPKPDTAAAKPDTTAPKPDTAAAKPD
TAAANPSPPPPEPPSPIPPSPEPPSPSPPPPGPPSPIPPSPEPPSPSPPPPEPPSPIPPSPEPPSPSPPPPEPPSPI
PPSPEPPSPSPPPPEPPSPIPPSPGLASPSPPPPEPPSPIPPSPEPPSPSPPPPEPPSPIPPSPGLASPSPPPPEPP
SPIPPSPGLASPSPPPPEPPSPIPPPEPPSPSPLPPGPPSPIPPSPEPPINNTTPPPRNWDRLLDTFLETPLQRVGR
PLVVLLRSHDSLDQANTQNWTAAISGPGNYMSVQSLGPMSGPTPVAAEVQWNVTVPGGQLQEAGNYTLFVYYNGNLS
MAPAVQGNGSTVSIYPGLPHPSNCSLQVKPFTNTTAVGDYVAVDLRLHDAYGNPALAARNVSVSLLGSVSKHKIVSG
ANFTSLEPAGSGLFRYVHVLEAREQLTVWLNVSGTAVANVTLNVSAISPAAVAFNESMSSAVMQLYGSTTVQHLPPQ
AALRLPVLVVHRLEVPVLRSPSTWSSTSPSFGSDPGLVASLELMAQNGTQLMFSGSWHGGNHSYVIFFRVLAVGRYT
GTVRLQHPTNQTAQASLTLPLNATLSPVASFSLVVAAANQSRVGDWVYITPTLLGTLGEPAAAPTGLTLTARGTYSG
AQSLNLSIDGIRFSPVRYTFQIQETVTFVLAVNQVAVTNATVIVQGVAPSQLDLPRSLAAACFLDVVGGAAAGASGS
CSTTASSSAAVRANTTFMVQQGRQATVEMPVYTVDGQVWQSDPGLSVVLTLVPSALVEYIDNAYPGSLSEARRRMLI
LAVEQNSGHSVEELSAWGGSLRAGRSLLQEYGSNYGGAQPYDYPVYYGEYANINAAGAGYDPDSYDLGEEPLSPWPA
SGNWSLPGAASLNIDSPDGNSTLQELLAEEPQVISIRGSFSTYKQSYQVPVLLNDSDTYRALLSVRSPALNGSLRLS
RFWVTAMDVAKLNTLVSSNTTSSASSALDPAVNPVVSLNLTLSAASITSYPGGPETMLADYRALLASRAGLTSTDNV
TVSITAGTIGINSGSSGTVMLSFQVYFDADWANTSSSFTSMTSLEWFYFRIMKLPETVLQDTATYPALNASTVQVTA
VSLDEATSSALASKATASDVAGSTAETSITILSEDLDSDTSAATTSSSTSVATLPASSVSSPPVITLIGDPYMEVQE
ADTFSDPGVYVYDSIDGFAVNARVTVRICARTPNLEAMMYALDAAAAAASTVGTAAGSGMSVAAAKAAAQAAPPSV
FTCVGGTAGSNSTTGQLTIYPNGGLTLDNSFINAVTQMYLLSYSAVNSRATAATPRYRAVAVKPRCRTADGEFWCPS
LSACSIRGVCNSTLVALSTALQSVAGGSTSLSADTAVSAASNAIPGVDPSTLTVTYTPAGDMTVVSPDGSLIIVDPL
SYARSSLASPDSESFGILGAIMSGGIGASTASAAASQQQPVYIKDTLPPVITLLGSGKLALTPSGAAVMIDSVLVGS
EWTDPGATVWDAVDGDLTAMLQTFGAAAVDTSRPTKQGSLYSYMVEYQATDLTGNVAQLARRLIKVVCPSNESYCTD
KDDLPSCTSQGICGDTLATVSSGATSGSYSTGLPQITIVGPSTIRVQQGTKYDRCTSLIPQNQPCDPGAVALDARDG
SLDLRVQVCGIPLRAARAGQTLLPLLIACNVSVSRPGNYTITYSVANSAGRSASVSRVLIVQPVCPSGKILCSDGIS
CSEDGGVCRSNVENASVTISSTAASQTAAGMSVALKPRLTLITATAAPARVLLPRGSTYGPCTIGADLAAASTPFCE
PGATAVDSAGTVNLTDRVVVCPPSSCLYGGGCSADLLRRHTLASKGLAGCGINTLAPPGAVFHINFWVWDYARPPNN
ATVRRTIVIVDPCANKSAPYFCSDGSGGYLCSPLPCTASASLLPPKVPGPNITLLPPADVAYVEYGSVSPVYLGPCT
SMFDTRSCGAVATGVLLPTSPAANTTTVDLTAQLSVINTTPCNTTSGGSTTCQSCSLEALAMGSSNCLPGVYTFQYS
VTDDQGVTATANRTVVVYQQAIISVNLTWLADKLTDAEAAAQLSINLLNSSHPDHASAVQDLATRMAKYGVQASDVE
ILSATVSPTSPPPTNSSPASMLVDVAIHIYNPRRVHRSATGLTLSTPTSSSAWRRRQLHNDDENSTPSSYLILHGSS

SNQRYHWDVGKTNGMVKQPLEEVWEVLLAARMQDLQHRHQRLLLQLQQPDAMDRACRHVETAGFRQGDFSQPASALK
NDMAMNLDQRRHGLRSLLQAASNTSANSTSALASLATSAASSLNATSVIITVPSAPDLTAGYLESLVGLVQALLQTS
DAAYTKASTLNSTLPSILGDTAATADEERGTAIKTAVGALAVEAADAQNRTLVVSAQVEAGFDAQLAAEQQLLEQSS
ELDLELRELSALTRAQTDRALYMAMVAAEAAKDAAEIVMDQDCYRLQLTGFQAAFTLSRFSLPSSAGSPNAGSNATG
SSSMATGTRRRLYRGTEDESSSSSGSVVPWLGYYLLTQGTSALDTLLAESELDVGADLRVAVARRYAGTRAENRVVG
GLLLHTTRRTLRSPAEAVGRNVSSETDSCSGVFSGLDASCHWFDVAFLRDVASGSSYLQRLYAGTNNSVAPYGVDPV
FLRSSPLYREDLTSQLSWYYNTSDPAQVSVTGTPYGFTSRALSGRSPGFPVLLESGLSASRAAQMMRYLADGNYLDH
RQTVEMTAELLVYNPGLHAFAYFRGDFQWSEAGAIIGHLSTVGFPAMAYLEEGEALTRKALPRIFARELLPLWVLTV
FFSVVTIVSTVSATIRAARFAAAAAAAAARERALTNALKAFEGPLQRTSGGGGFEKGASIAIIAASGIAESTEGTAG
ELPASRPGGFAALVAGTTGTVPWAQTGSSGGQAASTAIGGRGGAGIASAFGGRGGVAGLVGAFGSGGGGAAASHIRI
LRDEVAARREERLELLGASNFSARRVWFREFVRRDGGLLYDIPLALLLIAVAAYWTVYVERHLVLYNARSYYRVYDA
SASTSARWLLAARQDPGSVSNSASVYAEAASMGIDLPTGAGDPGRWLLPTEDSDWDALNDVLRNAHTLVDMWTTYGM
LQAVVIVGLVAKLITVMSFQARLGIICRTLMTMFTPVLHLVIIIVVVAVMLAAAANTVMGDRMAALSSLGGAISDTM
STILGPASMDSVDLLSRGLVMPGVLHFAVAAVLVTKVLLMLFMMFNFFVAAMGHIFMKQKHSIDWARAASVPQDLIQ
VVVPDLMRRIMGAIPGSESRRWRRRGEGATAAITASKDGRTSGGGDGDAEAAAVAVTGCTRLYRTVDTPTNTQVLHA
LASGGIHELMCAAELPKTWNGRVRAAKVSGGRLLIDKATMRQLIGLVASSPSAAAARLPLNDDDESEGDAKRRAAER
TVAQRELAMSVARRVMERVGRTYGSKAPEYLILEKAAEAEAGARRRSKAAPTVVGTGLFRAQAPVSQQEEGSDEQLS
SESASRPRRRLSSEIQIHLSIYDALRAAMESIVRWQSGVHRWQVRTWKQMASAYLFNQQLLAGLGVAPGPAFVERPP
DLQDDDLPHVARVSLQRQLSPAQSMQVPPLPLQGSMRSWMGDDANGILSPSQLSVADISRRCSRTSDSPMQLGTRRR
TSRSPASVWPGLTPKPMTPPSPRVQSPSQRQSALLEAPTGAQGFEEPAVEVAALTGSRPSFPPSSTEAPCLAAAAPS
PGSASSSVHNLSAAEVPAVSSRSYSIGGVPNTATAVPTPFRASWSPGTVTGDGAAAATVIGAGTGGSLPSLPSGRFL
ALRAPSRWMHAPTAADGAVNAGLSRGQSYSYGFAMVPEEEPSSPELDEDSGNLESLRAASDVVARAAIQPKGLPSAR
DRTRWGGVAPMMDEMRERLMALMNRRNSRTATSAGAGSGGRRRRNYSGSGSGPSSPISPVSAAHSSSGAIGPSEQRQ
SGSGGMQDDPNLVYDIAPSRNRPGMRDEESAVDSDLVQERGGRRPSA

>Vocar20011956m|PACid:23132921
MTSPKSRRRRGIQGLRAEHQRLVQRLIAKRQQQAQQRSTGVSKLLADQWVAAAFAEVQQIRARIHQIEAMLAAIVRP
EGSVELETGSAAAAGPSQHLPFMVQPPGVAGAAAAGSSPSNNGLPLPWQQPVHVPLAPGAFPGVPPFGIMPPMMMPV
VPFGMPMGTTTATISNSAAAVIGPAVATVQVPLHHHQIAAAAAAAAASAAAAAMFGSGLLPNGGGMLFVPQPLQPHP
QPAVQPAAAAATAEEGPSTSAGNRGSAADVEPASTAAEAAAIAAAVPAVDAQPAAEATIEVSAAHNGSAAVAAAEAG
AATAAAPDVSTRIAQDQHSVTTSGDALPCSASATAAASEASGDDASASSALTPSADCPVTATCRGMEGRQSWERMKL
QIGPCSVPDSARRTDLSGPCFPKQRRGALHCVQVSRPCSQEAASFFLNAVTHRREENKDCARGVPLKRCDCLARAMQ
SFRNASFYVHKEHILAAAKRTATVLTRIPWDLKLQVHRCRFQAVGNSPDAFAYALTIPCTGVEDVDAQATSQLRAAL
DAFDEGVFEYHNRAAAQAAGGPQADAGFKGAGGRGQRRTRREQAYASRIPEDRQFFRPVEVQSAEWAVFKPHLWLRG
RQVPATEPAPHSHPYAHMHTPPPSPPPLSTGSPGGEHRPAAAGVSSSSGGAAAAAAKGGSRAVTAAAATTASGELAV
FGSKIRLTPPPAGLSGTQEEVLASDGTFEETFVVQHDEWEAHVPAHLRRRRERPRDEKAVAIGLPPVEPKSAVLYDI
AETLLPELWTRLAPLLAPFLSRLETERRNQPDDGGGGVRASSAFRGAGGGGGEFEIITFSSESPSDNAKLAADSTTA
ASGGGGRTPPPGPPPVLPRDLLPLDSALAPPPLPRGALLQQRPSVPPVAPLILGGAPSLSPLTSPSHSHSHSRSTSV
SQAPLRCSYSGEFAAAAAAIAAAAAQPQLAPLTQPPLAPLHVPNATGFGSSSPQTSQKTLVGATAAPFQLTHGSHGS
PAAAPPPLAASCSSGSPYLVAHQLLGSSSAGQSPSPSHGQQNPYQHPHSHSYTHTSQVHTATSLPAGSTVAAAGDAL
MLVGLVPLRATASTGTGAPALHHHQHQHQHQSQHYPSPQPSQLQHQNQQQQQQLQQPLQSAAIPPVNQQQPLPRRVP
SLSVMSVQPPYAALSASSSSPGAANSGGTQASSSSGPYGEVPWYMYSTINHHHHHHHHHHHNNNNPAGPSPVAVLAQ
HSRFIAVQGTVVPGAGGGCGGSPPRTRQTSLGGGLTPAAGAAAAAAASTASPAGPAAAGGVSSTSGGTAAIAGGGGGR
GGGGASAVARSRAPSTVANAAVGAVAHHSVHLAPLAAAAATVGSAAAPQLVRQQQQQHQVSHVQPPGLASRKTSSDL
AAPPQQQFGTSATAGTAAAAAGVVYPSESSVESASVISRNRFVFMQSSGCNEPWPPWGPVQETTTSFSSPPPPPASV
TTNVAFGKPAFSSSVYMGQDAYGPSRAVDGVADHTSMYISRFGDVNPWLSVDLGEAFIVSRIVLSLREDCCGNETCR
AEITYNSLAYKMPGSGVTGGFLDITVSPPLIGRFVTVQNFNPAVNTSRLAVSELLVYGYPAEPQNDIKAKTQNEKQR
GKLASSQLTQSHTHVAAMPPEGAADFDRVWEGGLRRCIMDETNLLTNAVRLKMPYGRVAHLPPGGTAGVPFGVIEAF
AGWSANSAALHGNCLGFGVRASCSILQYGAGYGDDIRALKTLILPDEGACCEACYTAPDCMFWDYQHSSKTCRLKDN
EGALVPPGHIYPGFWRDDGRVAGAKRVVSTFKRHNGLVFTPDNITKWIWSRPDALFNPQPTDAVYYFARSLYLPAAL
SGVQVRIVVDDNVTVIVNDQVWPPSAFWPNIANITVNFAAGYNLVLLRCYNRLSYAAGAAAFWAPNGTVLLRTDERW
AFVEALPSPPPPNPPPPSPPPSLPPLRPHAPSSPPPHYPPPPPPPPPHPAPPAPPPPPSPSPPSPPPPPVPPLPPPS
NPPSPPPPPPPPHPPPPTPPPPPPPTPPPPPPPPSPPPPPPPHPPPPPPPPPPPPSPPPPSPPPPPSPSPPHPPP
PPPPPPPPPSPPPPSPPPPPSPPPPHPPPPPPPPPPPPIPPPPPPPPPPPPPSPPPPPTPPPPAPPPPPPPPYPPPP
PPPSPPPPPSPKPPVPPPPHLKSDCYVGCYMDSADPRVLPIVLLTADNMTIDLCRQLALEAAQLLVGGLSYYGLEAG
RQCFGGNDLSQAVSLGASSSCTWSCAGNRRQVCGGDWAVSVYRTIFPPPSPPVPPSPPPHALPLGDFVGCFSDSDPV

RSLPALLEVSPANMTVDRCRQLAKEGGFEYFGLESGVECYGGDSLEAAWVNGRSSSCDYACTGNDCQICGGNWAVSI
FRTEL

>Vocar20014913m|PACid:23135477
MSPLGLLVATLWLVLLVPGQTSPLDICFRNVVATLATGLQVHNRCNKVNAATNSSNSLPWNETEVFLNLVGQCFMEE
RRNITLMAPNMSFTSYVATMNSTVAVLSQRLVDGSDLEAHSLDFMTVPMAAWGRTLSLIGGVAPTILGMSSDTSPPG
EVDGWMVDPIALADLYDSGQTLDLSGLIQEMPYRQQLEWFGIHRLFRGNVVVDEGTVASIPLGGLVYQMYYRRDVLQ
KRGLSPPVTWDDFLLVAAAVNGTDMNGDGHPDYGVCLQRPRYCFNGFSLAAIWSSFIQSEGTHQGAFFDPDSLQPLV
NNSAMRRAMEIFADLRAFGPPDETTAPCLPYNARFVRGECALTLSWGYQLKANLFLDGSRVRDNVGVALLPGSSEVL
NRMTGELVNCTSRTVCPFAEELVTASNAVAVQLVNRAPYLAVSTASAGISAKANVWHQTALLNFFSYLASRNVSWEL
VTSRYTELAPYRYEHFDPSYVGEWVRRGYPEPIIREFLDVMKRQHCTGLPREKKKRNVFESIIFMTRGVGTGLKVLS
YVQWCAPCKPLLDVYATHLSNQEMTVTTAMTKLAEELYGVYGPHNPRFDTIRRLYRYSIGLSDFHSTSSSDAGTQGT
GNQAVVYFAVVIPVVVVVLLAAAVAAAAAVYYTVLRRRRRLQHRRRHGPTEHPGLGDDTTLVVSDVEGSTVLWEAIP
PGVMDVVFNLHHDCMRRTAVRFGGYESQTEGDSFIIAFFSANDAVQFALAVQQALLDEPWPRELLAHEKCRPLWLAR
SGVDVPLDLPLEPAAAQQHRNSMPYSTNEGSRHGALQPGTQQGSFASTAAHGGGGTGGLAGFSAPTCDGGNGAAAAA
AAAAAAEPEVAASEPVAVQSKFNKLLATRWGSRRRVYCTNGGSGGGAVAAVTHTASTDPGSTSRIASGGNGEGLFAH
LRRSASGGPGATAAASSPVPPTAMMSAVAAAAAVAPPPPPPLLPLRIETVGEALRALWREVPAHIADPALSGTSGMH
TGVRHRSDLVYSDVAKRMKYSGEVLAAAKAISDAANGGDVLIGTATLDRLDPDVLTKRCRLLYTGRHVLHGGDADAA
AAAPPTLTHPPPPPQPGAVADLYALYSPRLFPRVWHYRPPRTQLSLGLSVMEAPLGRVSYGLLVCHGLCNMAAAGHD
TAVVSEAHTALTAVSEDQLRACHGVPAAPPSAVGGAGAVVGAFRNPYHAILWALQAQEDLLRFPWSRELLAHEQFEA
IVVPAAAAKALIGGSGEGGGGGAEGLTDVRSSHAMTAAGPTPSHAAAQAAAAAAAPFAQRRGHGPGSLPPPSSVEDA
DFGCTASGQGSTKLSMASGSLTGGGGGFVAVMEAATEAAAAAAAVAVSPAPPAPQVSSARRTFRNLVVKGHSGNSPS
AGCNSSPAGGATSSGMGPGGGGGGGGWAVGWNGSGASTRLRQSAPSHMPPPLQRQATPAVSSLAMAAAANAARSETG
ATSAPLGMVNYGSTLSTMPYDVSYAVSTTDGEPDYRNAMTWTSGVCFSRAHTHTPPSPPPPTRTATTPPPSPFPARP
PGKHVGTTTTWAISEESDAQRSQPAAAAAAALSATAATAPPPAAASVAAAAAASGAEGDMVLYRGPRIKAAISFGP
LKASLDPLTGRVRYEGKAASYAAKMVSYASVGMVVASVEAVEAGHAEESEGCGAAGGEFPREHRQTSTGCDIRGTPP
VDTTATDGDPCSTTLRPSATLTAVGRQESGHEDPEGPSAAMAAAAAAAAAAALLNLAPGADGDGGAVLHRSSSLLDT
VFLTDPSIQPGAGGGGGVGHGGPCSAGTTTEAGSVWTGGRSSVLLQPPPGLNGCSDSSLPPMAAVMWRRSLPGTAVA
VGTVTFAAAAAAAGVSMSEDDPADAVTECTDGSRRQAPPPPPPPLPIYAEPTLLLRRLETLGDMDGGAAEAATEEE
PDGGREDEDQKEGEEEADVALKLPPQKQLAVAVTAGAPQHANDPPPSVHDSEATRGFAAVRRVRESELWRQLSGDAV
AGGGGGGGGRTENSGAGVARAANDIDDGGGVAVAAVTEGHGSASPLRNQDPVSRRFPDLHTGGGSGFPAACPTESAF
GGAAGRSRAGGGANADAFVVMAVKAPPLTRIQASSAAAAAAAAAAPSFASSGFAAGRASSLRHGDGRTAEAEAVIAA
GLSSSLRYGGGGGGGEGVAAAAFAAGRASSLRHGGYDGLAPPLPPADLLRQRQQQHLLLLQRDSQRVFMGGAAAPAS
GSRASTALPSVQVISLGGGGSGGGGGGSGGSGGGSGGSGGGGPASSGHLQVQVPSLHPITTPHNQFHKHQHQHQHQH
QHQHQHQHQHQHQHQHQHQHQHQHQHQHQHQHQHQHQHQHQHQHQHQHQHQHQHQQFVRAVDAAPLTAPLPTTVAIG
ATGPPANNNSKSSPDLELSPAGGGGGGAAGGSPGTAPPRFPVLINAPANVAAAGAAAVAAVPQPVQQLNHLSSWRRS
TSSSFASGARGGAAAGGGGGRSSLLGTSSRRYTQGVPPGGVGVGGGVGGSSYDGGAAASLLSSQAHSRCEDHPSAVH
ISVDGGGSGFAGGSGGGGGGGSSQMVSGGSGGPYFPQLDNGGYRGIAAAAAALAAADAAAGGGGYCAPWLAATEVPP
PRSQALTAVGLDEVSMMPVALKKRGHSQPLVLYLCRFSSSARTFEMQQRLMKLGHGDPTAAAAVVASAAAAAGAPLE
EAGAAAAAAAAAVVASDYQPGAGSCTEPQTTAGGATAAAPRDTAPGSEASLAQLASGAAAGHVTGTDLAAAAAAGGS
GGTSFLSERLDKTLKSSVVSILGVSRFGGDVSAALSPPPPPPPPRLRAGMLGTAAARRSSLTGLHSTPPAAPPPLLA
RRSLGGMLQNDPTFAAINAVPHAAQRGIFGTYGRAVSCAGAAPSPVGLGSGGLRSTGGGGGTGGFMRRFRRTSFDVV
PQKRRSERTATAAAAAAAGMLGTYGIAQPMGEVRTMSVKRVQRGTTSGPIITLTLVGGGGVSLAAAPPLMATATASA
TLPTFHVHHVYGAASSSPGIAVGGSAGGGGGGGVSPYDVASLVYGAPQQQHQQPYRTAHGATGSLLSVGAAAAAAAA
AAAGGGGGGMMMQRPLYRSTAAAAAIGAVNHGGSSDDSPRAVAAPGTASDVPLARYGGGGGGGGGGAGGGAGVDGIF
VGNSAWEYGGRSGGLLNWAPALVGPTEEGHETDEEGEGEGNGEGEGNGEGEGV

>Vocar20004500m|PACid:23136854
MGRTPLQLLKCSVAQLLLAAALAWTVSADTDADPELGVAATLPGAADVEALNKFPFCQCQDYRCSSAPYRLKSIPSP
TKRSDELCYLIERVGCSQANPCCKILLDRLGKVEFAVNKTCKVDYVAAFVNGARFNSLFDTNFPDGKIRITPLAMNV
TTATNTSLCLRFKPGSACASYSSLCNTADGACTYAVFESSAHQCCPICSQSLPPPPPGMPPPTPPTLPAVPSGPSQP
PPPPPPPPPPPPPSPPPPPPPPPPPPPPPPPPSPPPPPPPPPPPPPPPPPPPSPPPPPPPPPPPPPPPPPPSPPPPP
PPPPPPPPPPPRPPPPSPPPPPPPPPPPPPPFPDAVRAEVQKCDCISSLITTTSRFRLDYVRSIPTPNQVIYHFNLY
VVSPEDCWEPGYRYGSCCNQTLVNIGLSLVSSIAADKDVGNVTLTTDLGFEMTVSKQRTDWGIKLYPNDITQGRNYT
PSLFVTNFTSGKPWLLSVPIRVDATPAPKAGMWPCKRSAFLPNDDRVCDVEINGYQTLTDEPTVFLGIEAFDVPNCC
PLGVVRFGHDDTCCVDNLAENPYSLSYNGSRHAAAPNQDDTVFTFQVSYNGATAVNVPGVSNGTRPNCSVSEVDQVT
LFVAPSVLDSVYKVTVDGAAVDWTRSSNSYQSWVRAINLYKTNRTPSLMSVYVAADVTAEQLCPTRVAGSPLCEYVF

KGAYDDTAREFTCCPYGVAPASAASCPVPGAWLPVMKLGSDGAREIHTMKWGLVPSFTRLAPGERPDHYRMFNARCE
GLTSKPVFSRLLSGKRCVVLLDGFYEWHSQGGGGGAASRKQPYYITTADEPQQPAMYMAGLYDVCHDPDGEPLHTFT
IITTDSSEPLTWLHDRMPVILTNPEEISAWLGEEGDGGLKCLAQAPQNRTALKTEPSVRILMKSEYEHPFSSEQPHA
KTEEPQEEQRGEAGDKTGNDGRGGGGGARSCTPSSAGMRTCAIAGSETQMVVKSEARPSSVHPRGAADLLGTGEGDG
DGKGGEDGGGGGGGGGGESGQVEEGNDGGSQGKVVEGGDDNSSGARNTRASGGGGGGSGSGMGGGGGGGSQGKGGLR
AVEPNRQRCEAMVEKVRRRKRRRKRRRKGREMAKVNVMPSKMYLHTPPGPPDTHNTPSFPQSPQICKPYGGPLLRWF
PVTPEMSKPGYDKPDCCKHPSVFPNSHADSHPHGRSIAEPAGHPSWSQGLAAATRIAGGPSPGGSILDQLGRTSIPN
EGALRAILQALEARLDQASLSPSSRTTSLDLKTAATAAAAAAATAAAAKRSAAAAAAGSLRRMPKRVSADDGEGTDK
LRTEGGGTRRHPMREVVTMLRFGGAADVAGGGGGGGGCSDVWPGAVAPAAGMSPWVGAMSVPAGCYPAAGGGGIGGG
GGEVGVGGGACSQPPLQTLFDPQAGLSRSALVARLKEAEVQLHFTSVAFGRFLGSSSFSLELMGYQGKQKAPDALRG
GRCEYLAESVRAGRSDRGAAARQAEAARKLTAQHGTAREERRLLQRRAENLSVFLRKEKEAREVVDRELATERQRSS
SLAVQLAGAHQQMKHMQDLLDEARQQYRHFLDVNKQDRLRRSGSALTSARQEAAACRLVRDRAEVAVAGMRRLVEEM
YTSRASGQLEQALQVLPAGLAAILAAAGGGGGGGDGRQRTGSVDAKKGRLQAGDRDDDKRVRGETPAGFRRVCRRLQ
AHAALMVDAATRVGGGAASTRTGRYRAQILSSSTSPCVSSSCGGRGGGGGGGGGSVNSPTGSTISAATTPLSSSSGG
GCTSGSGSGASAWANMVLVAGRRRRSSAGGAPAGGGGGGCGGRGDAPGRVPLGRISRGNGIEDGGSGSGRTGLDSSD
SESDDSSGGATVPLALGGHNMQQKGRPRRLRGRVLSGGGGGAFAAATITTGAARTVAFDLSPTPTPAAGAPDGGVGG
GVGGAQRQRSRSRPPQRPGSGTDTDDTAPSDFGFTPRPDERGRDDVTIGKGAAEGGGGGGNGGGRVHRVAGGGSSHF
AVGVRDLERLRIALLGAAASAAPSGLTGDGGQRAAVAAAVSPPQLSVAPSLASPPAQGHAPISRGGAGEAHSGVVRG
LKAAAAVATVAASREEYNDDDDDDEDEDEDEDGVVSIAGQSARVGVGVGVGGVGSTGEAEWGRDAEGDGSSGDDEGA
RGHRRGGGDPGYVYGGKHSPGPSSSVGTTAATPAGPAVFEKAASGQATAVAAAAAAAAKGGSRLRAAAGSGDQDPNL
DLDLDLDLGFGWRPSWDPDPSHRKLPPLTSLPLLPLPPPLPAVLVLVVPYLRQGGPTGQSFAPEAAAYPWMQGRPAA
AAAAAAATGMAPEEPPHRQPSAARGRRRPDSVAAAAAAIVASAAAALGPESRRAALRRWSARITGTAVPASPHRSAS
TGYYALPQLAAAATAAAAAAGRAAAGLGTNSKNGRLHVRPRTAQTCRAFLGGPCVGVGVCVWGGIRGRGSALLEGAG
DG

>Vocar20004391m|PACid:23137042
PPPPPSSPPPLTPSPSPPPQPPSPPSTPPPPSSPPPLPPSPSPPLQPPSPPSPPPPPPPSSPPPPQSSPPPLPPPPT
SPPPPPSPLSPPPPTSSPPPPLPSSPPPPPSPAPPSPAPPSPAPPSPAPPSPAPPSPAPPSPAPPSPAPPSPAPPSP
VQPSPAPPSPAPPSPSPPSPAPPSPAPPSPAQPSPTPPSPAPPSPAPPSPVQPSPAPPSPAPPSPAPPSPVQPSPAP
PSPAPPLPPSPSPTSPPTPFPSPPPPATPSPLPPSPPPLPPSPSPSPSPSPPSPLPPPLSPPPSPPFRPASPSPPAP
PSPPAPPSPRPSPPPSPPLPLAPPSPPAPPSPPAPPSPPAPPSPPPTEDAPLPPTPPPKLPPSPPGLPPPNPPSPSP
PPSPSPWTAQWASVLPSPPPPPPPPPASFTLIRQPPSPPPPPPEVTVAAVALEQSVNIVATTTVDVTAPEIVLLGSS
EVVLQVYDIYTELGAQAIDNQDGVLPVNVLGGPVNTSSTTADRPIIVVYEAIDRAGNVARTERRVLVRDNCMLQRER
RCPNTLRCSVFGSCNLNSQLESMSTTTSTESSPQASKTGADHMLRAPRQRIRLGLFQTNDALVKSSPKGFQVPVDNI
PPTIQIRGTGQLYVTPAGARGMINVVLAGSAYVDAGATAADVVPVAASRSGSGTTTGSTTKTVNLTSTILTRVFSPS
GLQVGTVSTDEPTGDEAAGGVPYLITYDVVDDAGNRASTVYRRVYVVCPAPEYECPREDEDSPRACSKGGMCGVTGL
VSNQAASTTIATAGSLSGSIVTASSGSTSSGSTSSGSASASSSSSPGGSSGSTYQATRVPRLVLNGESVITITAGTS
YLPCRGATATDCEPGSTATLDTFGDLNHQVRACATGVPNPLPYNFIGLKYCNLDTRTPGIYKISFHLVWPDVPEMVI
YRTVVVQDLCEGERACSDGKCSVDGVCSNELKIEVQAAASWQPAGKTTSSATSTAPAITAITNTPPRITLSTSEIIG
QQVSIFSGVAYSLCTAGQRPTQALPCEPLGIAEDVEDGNLTALAIVQGANVGPMWQLAIYDSTGANATAERLIIVIS
PCPADQFTCDDSMAASDSSVPAGVVCSPVPCALRQGLQGLDANAGARPRLFLLPSLQIDHLSKAELNQSLVLTYRQP
APFSLAPCAAFTDAVPATPTQQTAAGLCGAVANSSTGDDLTPYITTAVKALCPSGGSSSCYGCSVTGLSMGTCLPGR
YRIVYSVKDGDAVSGLQKAARLDVSVEQLKAQTFTFSMYYNGTAARSPDRMAAEAFAATLRSANVSTKVEMLSIALK
ALGVATGSVRRLDLLAEPGVQPCNVSVVSYCVAVAINVTTGSADFWDTSDMFFGALYSDATASEDGTCVNVTLTELT
AILASADSAVASLEPYSTTCTEKRPEGASMAQSLISGAVDGVGGASQTILSNQAALPWLIARVEDKLSETDEPPAGR
SLPPPDPSVRNHKGYRRASTGSMQAAATALASETQQAQELLAKMTGKEAERGRAGTLGLTASLLQIAVNEAESAALL
ANITAVLLPEASAVALDADLMNAYLDPAYIACVSSKVSVASFAFHVSRYTTAAPPDPQPPAQAASPSHRRRLFRSPD
EDGWARWPSPGSGQIATTARLPWLSQAYSRMSSLVCASPQGYHGHLLTSVQGVFCDTRGGNRPNADTTEGASTGQRG
RMYGSRRRSTRGTSNSNSGRSQFTGYTLPDGSQYNYSLLDDSDLRRRRAGGRNDVMLGLLLHQERRTVDGLADSNRR
VCKQSSYSYFYGGCDARTFKVVLSGNLGGIGNDPVFSRVSSLYDATLRSSDWYNMTEGSPEINPLGLPYGFFHEPLE
SFSPGYPLLLDTRLSAKRAGQALTYLRDGGYLSATLTKSLRAELVTYNPDAAVFGYWRVDFTWLDSGDIRATSYLLG
LPAVSYGEYIKNLQVARFLPDFFLVLLVLAYCCMTVYDVVMQLRAQREQRNQLRGGGPQRPWRAAASASASSFRRSF
TQLGKVAPCDRTIPDGDDNFEEIDGASGPRVRGHVGSNRKYTPRMSAGWVMYETAICALMVAAIAVLYTYTVRLSVR
EDFIARFDVYDADAFAPARYFLLRRSTSLNSSEGASSAATNTTRTAGSPDRWRMQADPRALNAAGAVLTRLDSMYNT
IVLYAFFQGLVLCALEVRWLHYISFQPHLSIVFGTLVLALPDLLHFAVVLVISIVMFAATACVVLGPSVAQFHNLSS
SLTIMFQYSLLRNDGGTFKKVLSSPLERPALEHMLVGVLYFVAPLFFVFTMLQFILSFLVLPFQELKRAVDGLPGVP
QDVLRILHWYWERFIKAAPKNKLLLHWLEDSLAKGPSRSFLYRMRTMLSARGASMMARVRSVRLDSRRINWSMSMQG

SISANGQRSAPRSPPEGPSAALTATRTTDSAAGAQNCSSIGGGSHGTGLLDLQQSFGSKLGSRPSKRALVKAVSFRS
GSQQLTWQAAPLAPVAANTSAVTGDIWVNRLAAEGEARKVVIMDPPPSDLTSGGRARTLHRFFSTGFESAALSRPRP
QGVPAAALRSDAMGGATARSRMRLVLLAKRLSNPRQQGDGPAAGGSSSREAGVTVVEDAAIHLADKVLANLVMRLGD
GTDLSELAEAAAMTPAPLSRAASNVGREVSPSPPPSTPTSAAAQPSMLWRRPSAPVLASDLHADTPPVSVGSGCASS
VHEKSRNRAGDGGADGASGPSSLNTAMDGAREGMINTVSWADCRQAALQRFMSSAQLEAPCESVAACNVVSRRSELG
NNDINEMLPPSFIMTSAMMRLPTPPALPTAHGAALPLSPPSSSSRLAARSPLATTRGSFQRRASPKPQISDIACWRQ
PSLQADSDSTAISPNTNTGSGDGRISEGPSNPPAGGSNTDKVYSSCRDVLDALLELIRYLSWMQRQVSAATMEIEAM
ATFLSRLISEVEALPSSSTRDRKAVRAALHAGLQAVLAQGGGQEANSDGRVLASRRYFGAHDPQRETSRWKQASYGN
QPPNWDLLRQRVAEMGSAADNGGSAVQLSHIAASACEVLQNAVTGPTGYSVVPLAYYSTRRLDRDTAARQRRGWRPR
RLSLLAAEKSRVSGPGVIIPVSSKRNDIMGAEVGYNNRDSLPDSGHARMAVRPTADRGEKM

>Vocar20008165m|PACid:23138784
MESDWWVVADPGGRSRSPPAEPGTPPPVAPLPPWPFDPPPPPPPPPPPPPGAPPPSPNEPDISPPPPPPPPPPPATP
DMPAIPRSPSVPQAPLPPFNPNMPPELPPPPPPPPPPPPPQLPPPPPPPPPLPPPPPPPPPPPYLPDMPGFPRAPLY
PPPFAPWSPAPNQPPPPPPPPPPPPGLPPPVPESPPPPPPPPPPPPPPPPDPPPPKWIFPSVPMEPGYPPSPEPPLP
PPPSPRPPPPPPPDFPPYTPDPPSPSPPPPPPPPPPPPIPPPPPPPPPPPPPPNFPGPPPPPPPPPPPPLQVVAAALG
FPPPPPPLSSVNLSLSDIKGNNNNTDFEADATTSTPGSSFDPSEFTLVNVVFVNLTVVADAWMQPLLYDPAQRFIFE
NTIRSALPFMQFSARVRINKLTWQYGDWIGEMRNYTAARRGFPPPSPPTPIPPPFAPNARFLPPPPPPPPPPDGNTT
WPRMTFFLQLYIVHSTELDVRKSLGNQMLVDLLRFNLFPVFATSLLSTKPTTFNVYTRRLNTSDTTPPELTLLGGTD
VYVPLYGTYTEPGAVATDNLDGVIDSDLIIMQLIQPNESQYCCQTTFPIDTSAVTRSPFIISYDVYDYVWNKAPTLY
RRVHVIDPCAANSSNVERMCPDQTCSVLGGKCGEVLAFPELVALATALLNQSQATGGGDVRPDSVVAMPLGSTISTL
TSNPVVDTQPPYIQLLPLEGDFFYIPAGEPGILAGTPELSGIITRLNLGDTFVDPGYKASDNLDATVQWRVQRRYAE
LVDTSVPTPSNGPYAITYDVKDVSGNKATTQIRLVFVDCPSALPLLCPPDERVTSPARWYCASTVCGVPDSISGVLT
IAPPVSSVVTNTPPTLTLNDRSSLVTMYTNQTYAKCPMPGQPRCDTGVTATDAEDGDISYKVRACANEAGENVDSLW
DAVGLSACNLTFKRTPGYKNITYTVTDSSGATASIVRQLLVLEICKEGEIQCPDGTCSDEGCFVYDGSYVDATNSST
GTFGASGTAALYVNQAPTITLVTSNLIPETVSVPQGYGWRNCRASDGNPPGRLCEPGAIATDPEDGNITSKVYVCPP
ELCLATCSSTACSGHELSIKDSRGCVNTSASVGSAFLVKFLSCDSGYPVLSAVTFRTIVIANPCSADQYYCNGVCKN
VPCSAAEIVANPPAVSNTAPAIRLLPPGTTLETAPVAAAGAVLNVTQFLTYGVPAPISFSPCPSYNVTSACVAVAWD
AEDGDLTTRITVVDTTVCYDKNPNNCLRCKAEQLTLGKCFPGNYQLKYSVTDSGGATSLAYMTFVVESVAANVIWFL
MPPSFNLSAPASNMRNETVLFRDALLSRRPRPHPAFPFLKFLCGGNTTLRRGFITAQLQRFAVNLRSVRTANITINI
INSNTDDGIYDPNYCICMLNVTIDYTTGLQNNFKIPQVLPYYEPDNFPPPPGAKRVVASPPSPPDSLSFRRRLTADI
DSGVGPTITGVIGASATLPMTLTATTATTPSRTADPHSAAFDIDEEDLHLFEPLDDATPKTTRGTSGRAVTRSPQHG
HHALPPTSSPPPATPQPPIVPSPLRRMPTAPKKPDPHAAALDIDEEDQHLYDPEKLPPATVVLGEKRRRRQAAAAAA
APAPAASVPSYLHAAMLGASVPDSAAAAGEISRASALALGAKIWQMHVLPARQRGAGAAAPAPAPGSFTSLSTVVDD
VDDEDVVAAADAAAAEEEAQLLMQSVEVLGRVYTRNRDPPRDLPISKELRQAAYDRAAIAAAAAAPAASAADRSNVG
PRDGRRRVPAAGTAAIAAADVDVDVDGVDATAAAAVIDESTEAPSRQSLMHDIMVNHLLPGLASISGHLDKVERDLA
ELAGGFGGGAGVERDRRRWLQGTSSTSVCAGTNNATNSVSTCVAAPPTELETSLAVMASLIMDLTVVSSSMLSNVNN
TVIIMNNLPEKFTAVDDTYSTSYTEYKPSSSTTATTLPILNCVVRTGYCAVQGYALSYLTNITTRTDQLINSALLAE
LQSQDAVNAGLIGAVAALLSGSVASISELADWVGLTTAVIIDGLGLAEDEWFYEDYKQCLIQRSGGGLRVNFTINRE
SQEAVLAAIAAGVYAAPPPPPGSPVSYNLISAIQLSAISRRLMSYTFPWGSNAASDSSNGEGSGSKAGSWFGFRSGT
WGGSGGSYSSSSRDRRLQAGSGAPPPSFLPVISSRLQYGGYEIEVAAGKPPPGLTVQYDSRGIPERVRVAGIRGNRV
VAGLLLQQVRHKLSDVILSPDAAACRSRFGFLRSECNRVSRYNSTGLRDLGGIGEDPVFQRGSPLYFGDVAGRPWAY
YNFSEGNSEISATGLPYGFTHYPVPHMPDGYPVFIDTRLGMARASKLLSYIKEGGYLSEVLTKQVLGRLLVYNADEK
VFGYWRATLNWAGDGVIKLRQKLSIIPALDYRESLSPPDSVKRFYSATTPASVPIQSTRVGHFVVDWIMAVFVCLYG
ANALLDVVRALQHQRLMQRAKEGEGLQHLTPEELHRRKRKAKRRWAWRVTPFWVLWEVVAWSLMMAAMVILAWFVFY
LCRRAPTANTYDVYDADISARARFFMLRKDEGGWNPYLANQLMSFRTRTDTSPSNDTVGLATTAAGMPNPGMPAPGD
DYRWLLPDDPSGLQALGNMYDYMDRMDFYWYVYFLIQWGPEGDRGGMRNTSTAVTYGNEPTRTAYNPQAVVLLMLMV
RLLVYLAFQPQLAIIGGAVVRSVPALLYWLAITGIVFAMCLVLLTTCFGYRLADMATLGASAYALMRYIIVINRNFG
TRASITELLDMRFLEHHGGEVMIAGFVRALGPLFLAFVLYAFVFAILLRQQRLLRALRRRAPTVAKDLRRMFKWLLQ
RTTGNAPSNKDMDATIDWVTRPEVRYSWAYSMLYNAVVRSVAAASGGAALARRHPKQHQATTERAILLSYGGGRFGS
PDEPDPRVCPRYNLRALHKVFEGMLGVNKELRRELYFFFNTNAKDSRAQRLKVLLQARGRAANSDPSADTMADSLAR

LLLERFGQRVPRPQARDFADLMRKMKKRMGAAGDGSLDGSDDTQSATVDDSDDDILPTGARVGDLRHGGYGAGAAAT
LHELQLQNAVRTAMRNVHHIRENMGKELGDDPQLLATLFVAQFAGQIPEPQSRPFASRPSLYRQRVQLGNVTGVGSA
SRASSMQRESSAASMPVPTAASGPLAPPPLPRSSGPVAAVGDTNTPEGMLTRALQIMLAPQATTAAPTQQQQQLAPE
GDDGTDEQPRAGRSRPQGAKTRARFASMETPFEGDTGAAAATTAAAAAADTAGGGGGGGVPSRLTGSRTARSMLPHH
LSQSHLRGGMSFNAVPSTLGSFGGLLGAPHAPVGAAAQEYPGVTPPAVQLQGSGGESVNFALSPPVTAAAAPTDATA
TAASEIIPAAAASMSPSGTMTSTAPELPGIRRPQIHKMRSMGAAATAAARLHTLVSGAPGEIMPATASSLAAASLAS
TPTGSLSRNRSGIPTPRGELVAEQINSLPPDRVIIFATATGVVQRLTSHVLQLLRELRGVQADTDGMLRSTMIMIER
LAAGKRPFTRRAARMMPGVLLSPAASPPVSPQTEKPPGWEDAAAARVAPANAALAAAAAAGRGGCAGGGSAAAGGGG
GSGGAAAAGTMGSGSRFGSLRVRGPEEPEDMLPLPPHPVLRGADHYSNSPRKVPALQSPGPLLPPARSPSGSAARLP
PIQGMSPLPPLEGPLAPAPTEATSVPLPLPPMAEEAPASPTKLVFRPPPLPVDEVREVAREPEPVTLVVHAKTPKGT
PELPVGDAAAQAAAAFADQEADQAQREALAEEEGLPAPPPGAAGTPRNPSLTLSSMASPLRQSLTPTRPSSGASQVS
EPAMRLLTPPRLAAAGINGTLRTVVVLFSCAHWLGAICTAMVAAAVGDGFGVLLLLGCRIEEPKPFRTSPRTSRVSS
PLLPAEAIVRLSPEPSSGSSRPGTALVDPRPNPDLDAAPAREAMEEPGANRNLPPAPRGSDLDGTVRRDEYGVEILR
LPETETVVPEASELAGEEGEAGSPRSGRVPMRQPSRIAFSEAVTPEEEYDEDEDLPRGAAAAGPSQTVAPPPPPPPP
VGRSSVYVPPPSREPAAFSEPLTGTEPPSPARPARMSSLASILTGLPPPPSGVPAPIRRRGLSRNASIAPPGTPGTN
AERAAAAAAAAVPPPQVLTPEAALQVINQALLPQPSGQPSGIRQSPSRRVQLPPDLERRQTQLARQQELLAAAQAAV
AARRMAGAASPSRGASRATSRAASPSRTSRAVSRGRAAAQQRPGEQFGLPPPAPGTDPSNAVQLATAALERRQQEEA
ANKAYVDQLMERMRTGLISTDERMTLAGRQPSTRPQQQPAASSGAPARRGSDASEESAAIGLADLPTTSLGRAATMG
AAGNVTMGQSFKRPQPAAAADPAAASPQQRAGSPSRPRAAGRQRPPMPPPQSGSSMLSPPGPFLGPALGPIVPPPPL
FTTSPSRRARPGSGEAGSGAGGIAPPQTATASSRPAQPRAEFEVEELEDLDFTVGRELPQPRRRQAGDD

Klebsormidium flaccidum

>kfl00031_0230
MHLSASFLLCALFAAGLVAGQVERVCQGVSDESSLQTALAAATSGSVVTICSNVNLTATLVVKAPLTVQGNCSAPGC
KISAALPRTVNQMFSVAPSTGPVFFRNLQLDSGVALSSQTGTLLTGGAVLLSTNSVTTFVNVLFTNNAADEGGAVFS
NGANATFVGCTFTGNTATASGGAFQAAGGSASILSSSFFANSAPLGGAVYLQQTAAPAVVRGSLFVGNRATVQQGGA
IFVGVRTVNASIGGNTFAGNTATVSGGAVHDDGASQSGAVVSTAFTVLSGNAFSQNTAPQGGAVSNSVAQTSFCPAE
LFVNNSATTGTSLFAAGSTNSEATRFCPVAPTDLAFISDVGGVFATDCAYCNCGPNNPCDANAVCTPGSRTANLTCA
CRNGFSGNGYVCLGASNSSLLLGNTGASTSLAALASLTAVSSPAAGAPAPGPATILVAPGPAPPAVSRNSTLTNTTA
GNVTSLANGTALNGTVPLNGTGTLNGTAINGTVPLPLNVTVPAPAPSANRTNVTAAGPNAANVTLPLPVNATANVTG
PVSAPAPGPIFNGTLPLPTNGTVNVTSPPPPLLAPPPPLATNASSPPPLATNASSPPPPPPLLAPPPPLATNASSPP
PLNPPPPAFPPPRPNVTFPVATNATAPPPIETNASAPPPLLLPPPPPPNPPPPGAPPPPLPTVPVIVTPDVRPPPPI
ITTLPPAFPPPEVATLPPAPPPPAATPPAPAPTGGLLGGPASPPPPVCTCPTGQAPGIAATALACSCNYPLTASYRM
VNRNLADWTTIFSSSFSNDLAGLIGVQPSQILYTNVAQGSILLDTNVVPLSGPTLDNATVSRISGVLQQGQPLNLST
AQYGEVLVTSVGQPAQLTSGTPPSGSGVSVTPSGPTPSNAPGTTNTSSGGGGSSIGVIIGAVVGGVVGLALVLAVIA
LLIRRKKRKPRLPPPITPPELAQPPKALERPLSARAASSRGGDPALPTPGREDIADKALFGTFRRFGYPEIQEATDD
FAAANLLGEGGFGLVYRGTFADGTMLAVKHLKNTQQGQAESEFLSELGTLGRVRHRNLVALKGFCVTAADCFLILDF
AAGGNLDQALRPATRPPLTWPQRMNIAVGAARGLSYLHNDCRPTIIHRDIKSANILLDSEGEAEVADFGLSKLLDSG
QTHHSTRVRGTYGYVRTLYLVAPEYAMYGQVTKKTDVYSFGVVLLEIITGLKALDVAQSGERRTLTDWVLKRHQQPL
TIMDPRLNGEYFREQAEKFLQLALACINPSADRRPPMLDVLRTLETLNPSGSVPAPALGPAERSGDVHDVYPTTSSD
WSLGAGSGTRRTPVRPSSAGSGSSPAATHFSWSQGNSSTVLHNTTQYDITGINEGR

>kfl00047_0130
MGGGHWPGVKVWTMWIFLAGLAAGVTQAAPVTDCNNLAAEISAAGAGGTVEIQGILPCSPAITIGSSVTLAGVSSNA
ANDGFDGQAMTQLFVVQGSGTQVNFNFLTLTNAKTNAQGGAVLVNSAASASFSNFVISNCVVNTGFAGAAVFIGGSA
SFSDGLITGSSSDYAGGAIFADTGSSLSLDNVQFTNNVARTGAGGAIFSNSGLQASFTSVTFDTNMAATDGGCVFLG
GSIKSTFAGCTFTNCTSTNGRGGALFMGGDNALTSIIDTSFMGNQALGNGGAVFVSSSTSVTTLTNVQFTNNKATRP
ASGGGSAGGGMLADGDVSMQQVTMTGNTAGGNPNDGSRDVFVNEGGFAYFCDELPWPGNTNPHTVSGPYSLGCPGYN
NTTPIPANKDCVSFLTALQNAPNDGTGVVNFTDVIRFDALTCGTFSLPFVISKSVTIQGASDNPLYNTLDAYNNNAL
FRVTGTGVRVFFNRVQLTRGVGGNGPAISVESGAFVQTNNAVFSYHQATGNGGAIYLAPGTSIEMLSGTTLTNNIAK
KVAGGGGGNGGAVYGDDGSKVYLYTVTMQFNKAEQIKAVIARHVQGGAMYIQDAQVTSLFTGSFNNTISGPDNSRSV
FITPGGLLRICSDQFGFNFATIVGPYETICPLNIPDVASTCPELLMRIANPVVYSYDGGNRDPAVPTIRFTGFITLP
AESCPAGTTFPIVIDSDVAGLRFLGDDANPQFNQITGNGSNALFKTTNPNGIRFERLWLTRALRASGNGACLEHTAG
PLTILNVNFQDCIATNGNGGAVYLGPGTSASTTSFAALRNYGQFGGAMYTESNVPVIASGSGFFQGGAYQNGGGIYV
QGPFSASRTSITSNMADAFGAGLYMTGPANVQWNTGSFDSNTAGTEGGGAFFGASTIFKGVFFTQANNQRRGKSRTP
PLGGSISDAGYLDTGAMVTLCQYTGQNNDQGNNGTTPYQYFAGPGKDGLVIFDNQDCTNRGPPPPANVNPAPVATSA
NPPPVSITPPAPGVISPAPSDVPPPPFPTNVACDPAPAFKFTCYSSGGDSSICCDTPCPETPGPGPAPPFPTNVACD
LDPANKYTCYSSTGDSSICCNTPCPAIPTAVPYCEGQPIPTARRPRPPPRGVLTLLAPALHVLRQHWLQLHLLRRTL
SRNPRAYSRLHRAANHRHSPAGAGLPAPPNAVRPRVESLQHVLQLRGEHLRVLRLAVPRNAVGRSVLLRAAHRGTLG
AFRAERAGPGPTRGHRVVPAPSGGAREPPTSAAHRGTLGAFRTERAGPAPLAVTLSSPPPAEVLASPPPVETTAAPA
PAPDTPTPTPTATVASSPPPAETTAAPAPAPDTPTPTPTATVASSPPPLETIGAPAPAPETPTPTPTPTPTPTATIP
SSPPPVETTAAPVNPPPPPPPNNPPPPLETPTPTPTPTATASAVAPTAEGPAP

>kfl00209_0190
MAAPGRLPALVMLMALLAVALFPGADARIFPERRSLAQTGVTAVFFDEIHWDNNGTDVAEAVEIFGPVGTDVTGYKL
TRYAGAGGLCSTSVCPLPSGPTLTFPAGSILRGDGSSGSTTSGTIVVVFPVDGLTNSGAGMALSDPASQLLDFISWS
STSNTFTVTASANTGPASGETSTPVPARETSSTPVGYSVQRTGSTTWSSPAANSFGPAYAGQAVPSSEGPRIIGAGN
LPSQLVVDGLLTSTGYRGTAPTGGNTPAPTPVGSTVSSPPPALSTSNPPPVDTTVSSPPPAGGATGTCGSAGETPIY
AINGNGQAQAIADKQVVTSGIVTGEFDEANRLNGIFIQTPDAQADPITAGTSNGLFVYFVGTSRATFPATAPGDYIQ
VSGTIVNYNGLPEMNAVTALTICSSGNPPPTIPAVTLPTDLRQYLSMQVNFPQTLTVTDNYNDDFYGEIELSVNGRR
YVPTQVADPGAPANAVAASNDAQAILVDDGSSLTSRSPAPLKPPYPYPGLSDSNTIRVGYTLAGPLNGDLSYGSSFG
YLVEPIGNPSSAALTFDPSTNPRPTAGPAVAGSLKVVGMNLENFFTQLQANGCSSSDCRGAKTQQQFDTQLAKEVLA
ITALNGDIYAFQELQNNGFGPSSAIATLTNAVNNAVGAGTYAFIRVNQPDGGPASIISTDPNCARQCVGGDAITVGM
MYRVSAVTPVGAARAITKNVDPTFPSDNTRPSVAQTFADNQGGKITVVANHLKSKGSACSGDADQKDGQGNCNLTRQ
RAAAAIVRWLASDPTGSNDPDFLLLGDMNAYAKEDPIKTFEAGGFVNMIAAKIGPLAYSYVFGGESGYLDHALASSA
LAPQVQDVAEFHINADEPIVLHYDPATGLAPATSMYRCSDHDPVILGMTLTPSSSALPPPAANPPPIAANPPPASVN

PPPAAADSQPPPVVNANPPPVENANPPPVAIPPAPTAEIASPPPVEVASPPPVQNASPPPVQISSPPPVQIASPPPV
QIASPPPVQIASPPPLAKSPAPAVATPTPVPTNPKGGCGNACNCPNENACTNGNKPCAWFNNACHFMG

>kfl00671_0010p
PATMEDIGNLVAGPLWSQTRLSLSLKGLLGPIPPQLGGLTNLTYLDLSQNNLFGPIPAELGNLTSLTYLDLWDNKNL
SGPIPPELSTLTRLTYLDLSFNNLSGPIPSKLSSLPNITTLLLQVNNLSGPIPPELGRLTNLSILGLAANNLSGPIP
PPLFRQSNSSFYPGNSGLCCIGCTFSLPCTGNPPPAILPAPPRSPPPLPPLLPPAPIEPPTSLPPVLPPIYPPSMAP
AFILPAMQPPPLPQAPAPSPPPSVGPIVGGVLAGISFMLALATLGFCLHLRKKRMSNQGPPTGLEEGGKDELQNQAA
LKQPAPIKRQERITFKQLQKATGNFNSANVIGRGSFGTVYKGVLADGSMLAVKRLETRPGRRDVEERSWKTEVEALG
KVRHINLVPLLGVCAERGERLLFFDFFIRGSLDRRLHHSAAGGGVLTWGDRMNIARGVCQGLTYLHSHIRPPMVHRD
IKAANILLTDGDPTDACIADFGLAHLVQDTGGKTSTMVKGTVPYMAPEYLHGGARFLPAKCDVYSFGMLSLELISGR
PVVRQLESGVVERLLKVATDLVIEGRGLELVDPALRTAYNAEEARNYIHVALACMRSDPSARPTMQDVGSLLARASV
VDASFIKEEIWESDESSAYLGSLGSSTAERSAYESTVQVASRFLIPR

Physcomitrella patens

>Pp1s66_120V6.1|PACid:18038303
MTGGNGSRSSRPSLLLIFMLIPVFAATAHDAVHGDDRSSLPRQSPRAPDSAHEISGEPGTNFKYGKMTFDEALHHGS
DSDEPTTCTCTCYRDVPESPKTPLQSSPMHRDRSPPPAPYRYSPSPAYSTPAPPTGSTTSPVTFNSPPPTPYYDIPA
YNKPSPPVVAAPETFVPPPPPSPVY

>Pp1s469_11V6.1|PACid:18055055
MKSVQAMVVLLLLAALLECVQHSEAGRTMLTVGSEKTLSSLLPSCSPPPAPVDVPYTPTTPDTTSPSTHDTYTPTTP
TTPSEPSPVYSSPPPADGY

>Pp1s304_18V6.1|PACid:18057161
MASSSLPLAVVLLLLVSACSSQALPGYRVLPHRFLEAGSDSDNSRSSGALKKNLREMLSSVVSSPPPHKSPHKQHSS
PPPACPPPPPTPPPTPVAPTSI

>Pp1s8_132V6.1|PACid:18074183
MTAAPAMTVALLLVALLSCALPSEAGRALLTLGSQKNLKSLLPGCSPPPAPVDTPSYSPPTYTPESPPYTPSYSPSP
VYESPPVDQSPSYSPGSPSYSPPSTGYQTPPTYSPPLYLSPPTYSPSPVYSSPPPYSPSPVYSSPSPVYKSPPVYQS
PPAGGY

>Pp1s287_63V6.1|PACid:18046460
MGTSMKILLMASVFVSLLAAVSATDENPFFGNRPIGNHPPGVGHHPPGVGRHGPAWSKWRAYIALQAWKKAIVDDPN
GILDTWVGKDVCEYKGVFCSPPEDPNLSYLEVVSGIDLNEADLKGPLVPELGLLREIGLFHLNSNRFYGTVPDSFRY
MKTLFELDLSNNHLTGEFPQVLLDIPLLEYLDIRFNKFYGKLPRELFSKRLDALFVNNNNFNGEIPDNLGESTVSAL
VLANNYLHGSIPKSIGDMKNLNEIVALNNDIDGQLPDNIGNLKNVTLFDYSDNHITGGLPKSIKGMSSLETFDMSKN
KLGGIVTAELCELDNISAINLDNNYFTGVAPSCAALGDILSLDGNCVPGSQGGQKDAATCAAFYSPPSGGTPNPTPPS
PVTPSPPPPSPSPPPPSPSPPPPTPVTPPPPHPEPECPEGYAPGYKSGTCFMIVKEPKTWSQAESYCREHSDGNLAA
AADWAELEDLGVLCFKSNVTVFGDASNPLGLGCFLGGRRPYSLNPATKGWNYPLSPCVEFNQTWWNYGEPNNLGGYE
GCLTVKFESEEQARNPAKLPYMLNDLSCDVRLPFICALTRCEDVGCDLPETCKTFGDKQAKCYSDHRNSVGYGCDCS
AGYYPDGSGACVPRSS

>Pp1s35_305V6.1|PACid:18055256
MTRFTLPRMEASATTMLVTALLVSLFMLQAAAISAPVAPATSAVPAHPFSGTQTIENHPLGYGRRLQATGRNGFAWS
QWRAYIALRAWKKAITDDPKNVTRSWKGKYVCRYEGVFCQHPPGQKYSSIKVVAAIDLNGAHLKGTLVPELGLLREI
AVFHLNSNRFCGGVPDSFRNMKLLYELDLSNNQFSGRFPMVTLAIPKLVFLDLRFNKFYGRLPSELFAKRTLKIIFV
NNNKFEGTIPSNFGQSRVAAITLANNKFRGTIPRSISDMSNTLFEILALGNKFEGRIPSDIGKLKNILLFDFSTNKI
KGDLPESIRNLTDLEVFNMSNNLLGGTVTAELCRLKNLATLDLSDNFFTKLESACRALNRRVLDISGNCLLTRSYYN
QKSEATCASFYGWSPPPPLSSPPPPSPPPPPTPSPPPPPTPTPSPPPPPTPTPSPPPPPTLSPPPPPTPSTYTPPPP
TSR

>Pp1s35_242V6.1|PACid:18055307
MTRFHLQGMEATATTMVVTALLVSLFMVQAAATSAPATSPAPSHPFFGNRTTGNHPPGHGGHPQGTGRHGYAWSKWR
AYIALQCWKKAITEDPNNILASWNGKDVCSYKGVYCAPPPDPKYSYLTVVAAIDLNGAQLKGTLVPQLGELREIALF
HLNSNRFYGGVPDSFRYMKLLTELDLSNNQLGGDFPKVVLAIPKLAFLDLRFNTFYGKLPSELFSKRTLQVIFVNNN
NFEGTMPSNFAQSEVAALVLANNKFQGNIPKTINNMSNTLYEILALGNEFDGGIPDGIGNLKNLLLFDYSSNKINGG
LPDSLQNLQALEIFNMSKNYMGGAVTAEICQLKNLSALALSDNYFNSLASACKQINQTVLNVTGNCLFADKIPDQKD
KDTCARFYGWSPPPPPPSPPPPSPPPPSPPPPSPPPPSPPPPSPPPPPPCPYGYQPGKYSGTCFMLVTHCKTWEYAE
HYCNQQSGGHLAAVADWDELKDVGELCAQSNVTVQGDSSDPKGLGCYVGGRRPCNLSPATNGWNYPGSPCIDVPSLW
NMGEPNNYGGQEACLGVKYESDAQAYDNSYLPFMLNDLRCDIQLPFICTLTRCSDYGCGMSKTCKTLGDHNSTCSWD
DSAAVGYKCQCSEGYFSDDSGTCSPK

>Pp1s96_30V6.1|PACid:18068597
MGATINALLLTALLVSLFSLQAVATDPFFGDRPIGNHPPGVGRHPPGIGRHGPAWSKWRAYIALKAWKKAITDDPKG
ILKTWVGKDVCKYEGVFCSPPEDPDLQYLEVVAGIDLNDADLKGTLVPELGLLREIGIFHLNSNRFSGEVPDSFRYM
KTLFELDLSNNQLSGSFPLVVLDIPNLVYLDIRFNEFFGKLPRELFSKPTLDAIFVNNCNFEGDIPDNFGESPVSAV
VLANNRFDGSIPSSIRNMSNTLNEIVALGNNFHGTLPSEIGSLKNVNLFDYSANYISGGLPTSIKNMRDLEVFDMSR
NYLAGTVTAELCGLNNLTAVALDYNYFKGVDPTCARLGDILSLVGNCVPGAPGQKDEATCAQFYGLTPPRTPSNPTP

SNPPRSSSPPPPTPSNPPRSPSPPPPSPSSPPPSPSQPPPSPPPPSPSPPPPSPSPPPPSPSPPPPPVVSPPPPPVI
SPPPPPPYISPAPQPDTECPEGYEPGKKSGTCFMLVKEPQTWAEAEYYCRQHSDGHLAAPADWDELRDLGKLCYKSN
RTVFRDENNPWGLGCYLGGRRPYTLEPETNGWNYPGYPCIEFNQSFWNHGEPNNLGGQEGCLTIKFESKEQARDKSK
LPYMLNDLRCDVQLPFICSLTRCEDGCDNSNTCKTLGDDGAECHSDASAAVGYSCDCSNGYSWDESGTCVPE

>Pp1s118_151V6.1|PACid:18069169
MAPTKAGALTCFQLGRMGTTMKTLWLVSMLVCVRAAVAATEHPFFGTRPIGNHPPGVGNHPPGVGRHGPAWSKWRAY
IALQAWKKAIVDDPKGILDTWVGLDVCEYEGVFCSPPEDPNLSYLEVVSGIDLNEAYLKGPLAPELGLLREIGIFHV
NSNRFYGTVPDSFRYMKTLFELDLSNNKLSGKFPEVVLDIPRLEYLDIRFNNFYGKLPRELFSKPLDALFVNNNNFD
GEIPENLGESKVSAIVLANNNFEGGIPESIGQLENVEEIVALNNNFKGGLPHGIGNLTGVTLFDCSENKITGALPKS
IEGMASLEVFDMSDNMLGGMVTAELCELDNITSIVLDNNFFTGIAPSCAALGDILSLDGNCVPGSQGQKDAATCAAF
YNGGGTPSPTPPTPTPVTPSPPTPTPSPPTPTPVTPSPPPAPSPPPPAVLPPAPEAEGECPEGYEPGRKSGTCFMLV
KEPQTWAQAEYYCRRQSDGHLAAAADWDELKDLGMLCYQSNETVFGDANNPLGLGCYIGGRRAYKLMPTTRGWSYPM
SPCVEFNQSWWNNDEPNNLGGYEGCLTIKFESEEQSRDPEKLPYMFNDLSCDVRLPFICALTRCEDVGCDLPDTCQT
EGDKDAKCYTEASKAVGYGCDCSDGHYADESGTCIPSDS

>Pp1s145_144V6.1|PACid:18049923
MVRAFATTSGYMKAAANGPVAAPGVAPSGQGLPPTALTPPAPPPLGNGVSPPAPGIVSPPAPGIVSPPAPGIVSPPA
PGIVSPPAPGIVPPPAPGATSPPAPVPSSPLLTSPPPALSPPLLPPVYAPPPLPTPPPAIPPAVPPPVPVPVATPPP
ILPPPPVQAPPPILPPVAPPIQSPPPTATPPPSNASPPSKAPPPPWSTPPSVSPPAPPPKNAPPPTVPPPKAPPSQA
PTLTIPTIPDLSPPPPVFITQGSPPAPGPPPSSEGTSSTLVVGGAVGAVAVLFVAICVVVCCCRKKRKSAISDDGFS
YKGSSIATMPLTGAQFKSSDAPSDAWGIHSASSTGTGSDIPPPPSGSDKMGNSRSYFTYNELAVATDNFSKDNLLGE
GGFGRVYKGILPNGTVVAVKQLTVGGGQGEREFRAEVEVISRVHHRHLVSLVGYCVADRQRLLVYEFVPNGTLENNL
HNTDMPIMEWSTRLKIGLGCARGLAYLHEDCHPKIIHRDIKSSNILLEENFEAKVADFGLAKLSSDTNTHVSTRVMG
TFGYLAPEYAASGKLTDRSDVFSFGVVLLELVTGRRPIDMSQEAGFESLVEWARPVAMRILEDGHLEDLVDPNLDGN
YDRDEMFRVIETAAACVRHSAVKRPRMAQVVRALESEDRAGLYQGMKPGQSMDSDSQYGSEYGGTSRYGGDSGEFDQ
NDHSSNSGPRPFHKQGANHLPRIESAEHSIAEEPDDQTMEYVRSESSGIHAFKPPVFGTQQFHPPKFTSGEYDPDKE
SSDFPGAYRSSSKKVSSYAPPRSGLPNNGMSGFMGAPPPPPGRYSKPVNLGGEDYVPIGKTVSFAPNVEDKKF

>Pp1s134_145V6.1|PACid:18054792
MSAPAPTGGITPPVNSTTPPVAVVPPVAATPPVVSPPPAIVPPPVIAPPPVELPPPPPAPVISPPPAIPPAVVPPPP
DPTPPAATPPVPVTPDVPPPTAVAPPPPTPTNSPPAPTPPTDSPPAPPPPTGTNPSPPGLSPPSAVRGPPPAVLSPP
STPSAGGTPPSSSGSSSLSTGAVVGIAAGGGILALFFLFALIAFCRKRKYKKDSLPYTAAGGGGGGAATAAAYEVG
GMDDPKGYAAGGYAAGGNGRLPPGSHGGSVPLPPDGTSSVGNSRSWFTYDELHAATNGFAIENILGEGGFGRVYKGE
LPNGKVVAVKQLTLGGGQGDKEFRAEVEIISRVHHRHLVSLVGYCIADKQRLLVYDFVPNGTLDVNLYGNGRPIMNW
EMRMRVAVGAARGLAYLHEDCHPRIIHRDIKSSNILLDDKYEAQVADFGLAKLASDTHTHVSTRVMGTFGYLAPEYA
QSGKLTEKSDVYSFGVVLLELITGRKPIDTRNPAGQESLVEWTRPLLGEALAGNMEELVDPRLDGRYNYKEMFRMIE
VAASCVRHTASKRPKMGQVVRVLESEEENAGLYHDLRPGHSSEHEPSFDRYGGGSDYDTQEYNSDVLRRKRRDTNKS
HGSEYTSEYSSSLYPTGTVDSSNEFESGNSRPGDTRSNIVEQPPRRPPVSVRTGRASLATIPVPPNVTHTSLSGLPP
TRAAPPRPGQLGNSSVASSDTSGSVSSSTFAPPPPPPNFSDEYDPIDINPKGIRKSYDDLRDGR

>Pp1s115_198V6.1|PACid:18060166
MAELMWRAWLIGSYGVFCYVLLLEAWLVNGLRQNDADKLQYRGRYLMQAGPIPTPLTIPLPPPPPSEPPTSLSEQRS
PPPSETSLPPAITSHSPLAPPLETIPPPVSPPPSSVEPPPSPPKESLPPVVGPELPAQTPTESPPPPSITLSPPSPA
HSDVPPPSVPPLTPDSPPPPAVDAVPPSIPDSPPPLLEAPPPPPISLPPLSPTPSVTATPDVPAPSTLPPTSDSPPP
PAEGAASPSIPDSPPPPLIIIPPPSPAPTATATPDVPPPSTPPPTLDSPPPPVEGAAPPSILDSPPPQIESPPPPPI
TISPPSTPPPTSDSPPPPDVGAVPPSIPDTPPPASNPPPLSIPNAPPPSLEVPSPPSLGAPPPTTPDSPPPPNVLPP
TTPDSPPPPNASPPTTPDSPPPPNASPPTTPDSPPPPNASPPTTPDSPPPPNVPPPTTPDSPPPSISPPPTLTPPSI
PVDPPSSQPPAVSPPPPVSIPPSEPPTNSPSPPAPIPFAPNPSPTTSPSPPSTVFPPPSPSPPSPPPSSPDSPPSPI
PPNSSPPDASPPPPRSPSPPDVVSPPSEVPQPAAPPSTQLSPPAARSPTTVSPPLPSTQPPPPRDHLSPPAGAPPPN
SILAPPPPSIFAPPPYALAPGPGVQGCCAPNMMLQPGSLGTIQCRCVYPVTVKLQFINASSDTPNLQEIFQYELASQ
LKLLDVQVFVNYFKFGENSSFSVKAGKVDGPMNVESDIGPISGISFSVAEISRINQTIWSGKVKFNETYFGDYSVIS
VTPEFIPPTIPVAPPPVITSQPSHEIAPTPSKSSSTALYAGIGSGVGAVLLCLVIAFCIWNSLHSRKRNEENDTVSS
SKVSALQSHQLAGALPSRWVPVDSKSFPRPKQTREFTYEELSEATNGFAPSAFIGEGGFGKVYKGILRDGTEVAIKK
LTTGGHQGDREFLVEVEMLSRLHHRNLVKLLGYFCCREPLVQLLCYELIPNGSVDSWLHGTLCATFGPLDWPTRMKI
AIGSARGLQYLHEDSQPCVIHRDFKASNILLQNNFHAKVADFGLARLAPEGQGNYVSTRVMGTFGYVAPEYAMTGHL
LVKSDVYSYGVVLLELLSGRRPIDHAQEAFENITAWARPLLTDSNRIHELADPLLDGKYPTEDFEQVAALAKSCIEP

EWRARPTMGEVVASLNQICWSGEYNTSSDVERASSEHETGNLQSVGLPRISNSTSTSSNSSSAFSMPWPGDRRPPFS
SYAAGIGPSAFTPNSSNQFTSVSGLVDDSFSKTNIVSEDLQEGR

>Pp1s160_128V6.1|PACid:18068326
MVRAFASTRFYSPSAGAVPLASTNVPTASESKNHDDEGDYGIQAEVNDSNIENDSGSPVLAPWEGTSPPPPPPEDTT
PPALPPVPDSASAPPSVESNETSPPALPPIPDPTSPPPPSVQPDATAPPSIPPLPDTVSPPPDPAAQSPPSPPPETP
PPDPAAQSPPSPPPETPPPDVFLPPPNPTVGPPPLPAPELAPPVESPPPLAAPPPVIIPPPQPVETSPPPIVESPPP
QALPPQLVPPAAPPTIVSPPAIPPVEAPPPSIASPPPASPPLPGAPPPEPTIAPPPAEALPPPPSGSPPPSVSSPPS
LPPSESPPPPPSESPPPPPLPPLSAPASAPKSPTAPPPLLSPLAPPPTSLAPALSIPKITQPDLDTPPPPGRSSGPP
STSPSPPSSGVSSRALVVGGAVGGIVALAVVVCLLIFCCRKRRRIDGDGFFSQKGSSAAMPLTGAHYKSDNPDAWG
IHSGSVNGSDIPPPPSGSDAMGNSRSYFLFSELQEATGNFSKDNLLGEGGFGRVYKGTLQNGTVVAVKQLNLSGAQG
EREFRAEVEVISRVHHRHLVSLVGYCVSNQQRLLVYEFVPNGTLENNLHNPDMPVMEWSTRLKIALGCARGLAYLHE
DCHPKIIHRDIKSSNILLDENFEAQVADFGLAKLSNDTNTHVSTRVMGTFGYLAPEYAASGKLTDRSDVFSFGVILL
ELVTGRRPIDTTQEAGFESLVEWARPVVMRILEDGRLEDLVDPNLDGDYDPDEMFRVIETAAACVRHSALKRPRMAQ
VVRALENDSDRAGLYQGVRPQQNADTDSQYGSESQFGTNRYGGDSGQFDSDDHSTASGLQSFHKQNTNVSRQPNNLA
AIQSADHSIIPEESGEFDIENPSNHLPVPDFKPPEFKSPVLGTQQFTSPTIRSGEYDLDNEASDFPGAYRSTSTKQR
PGASVPNNGMSGFLGAPPPPPGRYQQPLSYSGEYVPIGKTVHFALSVDEKKF

>Pp1s118_23V6.1|PACid:18069168
MVRAFLSTRSYFEAPATSPPAPTDSSFGYVVKKKQDGEKIKPPKGPKAFIGNPAQAPFDGNNPAFPPAWTDSPPAPP
PDTTAPPDPAGQPPPLLPPESSPPPNPSPPPLDPTSPPPDPFQPPPFVPPPVESPPPILPPPPQPAPVAPPPIIVTP
PPQVPPPVLPPPAPSTAPPPSISPPPANSPPPWSPPPASPPQSSPPPPPPPSGSPPPSRESSPPPPPASAPAPPPTA
VPPASAPRGSPPISLAPAVIPRITQPDFTPPPSTNHSGPSSALPSPPSSGVSNNSLVVGGAAGGIAALVVIICLLIC
CCRKRRRHINPDGLSFKGSSAAMPLTSSQYKSDAPDAWGIHSGSVNGSDIPPPPNGSDKMGNSRSYFTYSELQTATD
NFSKDNLLGEGGFGRVYKGTLPNGTVVAVKQLNLSGGQGEREFRAEVEVISRVHHRHLVSLVGYCVSNQQRLLVYEF
VPNGTLENNLHNPDMPIMDWNTRLKIGLGCARGLAYLHEDCHPKIIHRDIKSSNILLDEKFEAQVADFGLAKLSSDT
NTHVSTRVMGTFGYLAPEYAASGKLTDRSDVFSYGVILLELVTGRRPIDMNQEAGFESLVEWARPVVMRILEDGHLE
DIVDPNLNGNYDPDEMFRVIETAAACVRHSALKRPRMAQVVRALESDSDRAGLYQGMRPGQGSDTDSQYGSESQYGT
NRYGGDSGQFDSGDHSTNSGLRSFHKQNTNVSRQQNNLGVIQSAEHPPIPEGSGEFDLGNSSGQMSVHDLKPPVLTQ
PTFQSGEYDPDKESSDFRPTNRPRPSVPNNGMSGFIGAPPPPGRYNQPLSYSGEYVPIGKTVHFAASVEEKKY

>Pp1s83_193V6.1|PACid:18073418
MSATPPTSGGVPPVVSPPTPNAVPPVAVNPPVVPPPVATPPTIPPATPVVNPPTATVPPAATPPVPVAVPPTATPPV
PVAVPPVVSPPPPVAVPPVVSPPPPVAVPPVVSPPPPVAVPPVVSPPPPVAVPPVVSPPPPVAVSPALSPPAPVAVP
PTLSPPPPDALPPSVSTPPPPALSPPSPTSSSPPQPTPVPPSAPGGTPPSPILLSPPPAVNRTSPPAALTPPASTSS
NSSSTAIIAGVVGGGALLALVALVLLFVCCRKKRSRKDTLPYITPHGGGIHGAGKIASAYNATGTSDLKGYAVDGNP
TVYPPGSVPLPPEGVASVGNSRIFFTYDELHKATNGFDHGNLLGEGGFGRVYKGELPNGKLVAVKQLTVGGGQGDRE
FRAEVEIISRVHHRHLVSLVGYCISDKQRLLVYDFVPNGTLDVNLYGRGKPVMTWDLRVRVALGAARGLAYLHEDCH
PRIIHRDIKSSNILLDDKYEAQVADFGLARPASDTNTHVSTRVMGTFGYLAPEYAQSGKLTEKSDVYSFGVMLLELI
TGRKPVDTRDPNGAVSLVELARPLMTKAMEDGDLDELVDPRLGDNYDPKELFRMIEVAASCVRQTANKRPKMGQVVR
ALESEEENAGLYQNLKPGHSSEYESEFDRYEGSNYDTQAYLADLKRGKKSNPSDISYESDDTSEYPTESVPSSSGEY
ESMIRPKTEPMGRANMGKERGPISMRTDRGFSMTGRGSRSENYSVLPSMPPTRPAPPRPDSSSDQSGSLRSDTVFKP
PPPPPNYNEDYVSIDFNSIESKKEDRRRFME

>Pp1s71_197V6.1|PACid:18039683
MSNSTVVAPAKAIVNVSAAPPATTPVLSPPPAYLVPSASSPEGTLVIAKTQSSDISWIAIVGVLVAFLVVAVITLIW
FWYKKREYKKALQEHRDLYEENEANFNREGEVDEEQGIPSKTGQKILSDFERALASQSASKKFDGGDDGDDATSGAR
IVEQGIPVYTRDKHVVEGFRKKDSGGFRNKVLPGDFNWKQEENFSDELFYKGEKMGVRYSRAELGNIDFLTKIPSSE
KVASKVGRSESESASLLGTLNRSHTIAASPAREEERSKIGWKSRTLSLPAFHHRSLQGSRDTSVNMKGENRAEAPRT
VIFPLTPHPHALPSSPSHRYSSTAFGDNLAPAPLARVSCWKASRLSVQSNAALNNPTFIVPPSTPPPLLPLPATLGE
ADEHGRTLKGSKKAESKWAIASYCPSTPPPPIPVPFLSPLAPPSQEHTQSLLLATSLTLSTSPSSPSSTLGADGSPS
SGLSHANFNEGGDLSSQPPPPPPPPPPPLPTLSCRKFINLPAPDDPMKLTPPPSPPSNPHEERKLTPPVSPECPQDG
RKITPPPSPIPPFPTLPRKSVTPPPSPPPPVMPSSASKPKLAALILPGRPDLGADNVEGFRINRRSPKQPQKMRPLH
WEKLKPESHKSMVWDNITNSMELDEEMIEHMFGVTTRASEDEGPKQSASTVTIERAEMLYPRKAHNIAIQLRARGLS
RIEVRNALLEGEGLSQEILELLVKMAPTDDEMTKFQGYHGDPTLLGPADRFVQGILQIPSAFERLNSMLYRASFSEE
LTQLQCTITTLEMACKELKSSRTFTKLLEAVLKTGNRLNTGTFRGDAKAFKLDTLLKLADVKGVDGKTTLLHFLIKE
IVKAEAFRAARLAGFHDSSTPSSTWSSLCPSPTCTSPSSPLSHFARGMEAELNRCQSEISNSSSLGGFRRIGMDVVR

GIPNELDHVRKAGGMDISTIRLGVSRLQTGLQDMKTTLEKLHEPSEGVGIGIRTTTYDLTDDVFSDRMAGFVEDAEA
KLSVIQKDLEIVLASAKDISVYFYGEADTAKSTQPLKVFMVMRDFLAMLEQACEDVMKGNAPLPPPSTGPSRRNSLT
LPM

>Pp1s17_57V6.1|PACid:18058763
MSNSTVEVYADGPVKAPARAPALVPAPVPVQTPGHFGPPAPLPVGNLPPQSAIGISWVGIVGILAAFLVLAVIALIF
FWYKKNAYKKALQDQRNFYVANAVSQIREEWSDDDVNRSKRGQTTLADFEHALVAQTRSKRFNWGNNAAFGDSPSGE
RIVEQDIPICTRAKPSSRGSKDWVLSGSFSDKHMTGSFGSSQCDDLVGEVFYTGVEGSEQMGVRYSRAELGDIEILT
KIPPNPKTPKTPKKWKSESESASLLGTLSRSHTIASSPAREEHNKPGGWSVRSFSLPTYHHTSSSPPGSLVTSSGIK
ADNRLEALKSLSPLTPHPSSLPASPTAHNYFPPPFGNNLTPASWDHVPSRLSVQTNAAGHTNGALHANAALHNPTFV
APPSTPPPPVPLPMALEEGEGRDSPREKTLKELRRDMKQAESKWTIAHYCPSPPPPPLPVPFQPLPSPPYPLRAPSR
LGSQLDVESPSFRALCKSALTIAASPSPSSPSARRPATLSNVATSPPPRSPLGKGSEDNTSPPSPPPPPSPPPPPPA
PPPPPFSTNLPSKKPAPPPSPPPPPPPSLSSRKFTPPPSPPPSADEGRKLTPPPSQPPATLPCKELTPPPSPPPPPM
LSSSESRSRSIPKLAPLILPARPDLGENKEIFRAEQRSIKSNQKMRPLHWDKLKPESRTRMVWDNMSNSMELDEEMI
ENLFGVAPSTSAGSITRQSSKLSVSAKSEILDPRKAHNIAIQLRARGLSKMEVCDALVEGEGLDQEILEILVKMTPT
DEEITKFKQFQGDTTILGPADRFILGLLQIPNAFERLQAMLYRASFEEELRHIQDTITTLQMACKELKSSRTFTKLL
EAVLKTGNRLNMGTFRGDAKAFKLDTLLKLADVKGVDGKTTLLHFVITEIIKAEGARAARLAGFDDGSTPTSQMSSA
CTTPCATTPSSPLSHFARSMEAELERCQGEFSNSDGRDDFKRIGMDVVRGIPNELVHVRKAGSLELVALKLAVSRLQ
IGLQNTKTTLEELRVFTSDAVETAGYDLGDDVFKEKMMDFVVDAEAEVRMVQRDLEAVLASVKDISIYFYGEADTAK
SKQPLKVFMVMREFLAMLEQACKDVMKTNASLTVSPSRRPSLSLPNKTN

>Pp1s38_64V6.1|PACid:18058026
MAEQLWFAFVLLVMICSTGWTGVHSARPLPEELPTLPEPTLGKLLAWPQDPSTTPNLPWNPPPPPLPFLRNSPPPPL
TFPWNTPSPPNPVTPWTPWNTQLPPPSPLSTTPSLPISVTPWYTPSPPPSPLSITLAPPSIVTPWNPWSAMSPPPPS
PPTPTLSPPMSWTQPWQTPPPPVDSTTPSFPGNKPPSPPSPMWTNPLFTPPPPFDTTPDSPPPPWNRPWVITPSPPP
SPTTSSPPPPSLFTLFASSPPPPPAGQIFPWTSSPPPPVGPIFPWTPSPPPKVGPSLPPFGLPPGGPLLPSGIQFTF
TNFTQAWLPQAGVKVTWQSLITMELSLQLLVEAVVKPIMVLIQQFDPPPGKGLGKWGWRNVLEDSKLAAKVGATCTT
SNESGYPERGMVIVQTPINNTTSNAVAWFTVLFNGWSQTTPFHATLDSTPGTFGGFVTDSTLPDLNTFCLHPSSLAR
RANRQGLHYVMG

Selaginella moellendorffii

>440537|PACid:15405643
MGPSPTLVVFFVGVTLAALAGADEPPVYKYASPPPPTYYYKSPPPPVYKYKSPPPPVYSPPPPVYKYKSPPPPVYSP
PPPVYKYKSPPPPVHSPPPPVYKYKSPPPPVYSPPPPVYKYKSPPPPVHSPPPPVYKYKSPPPPVYSPPPPVYKYKS
PPPPVHSPPPPVYKYKSPPPPVHSPPPPVYKYKSPPPPVYSPPPPVYKYKSPPPPVYSPPPPVYKYKSPPPPYHY

>402661|PACid:15405846
MKGVILGFAIESAMIFFKSCSMLLLLLVYTVTCDGSQEVLDRNTGSFGDAKNFGFGIPKSTIDFFCWRPWLRRPWWF
PNRLCPPNAPPYCYKSPPPPMELPTPPVESQPPPVESPSPPHHQKSPPPPVESPPPPYQYKSPPPPPPPPKCGKTPP
PPPPPPKCEKSSPPPPPKSPPPPPPKREKCPPPYEYKSPPPPPPKCEKSPPPPAPYEYKSPPPPPSSPPSPLYKYES
PPPPAYEYKCAPPPPPPPHHQHVKSPPPPSLPIYWYQSPPPELVKK

>448871|PACid:15409144
GKVKITCGESYYYGYTNKHGIFRIELPHQSWEDASSCKAKIIKSSYIKSSCNVITDYRSGATGAKLKFKSKTEKELV
LTAGPFIYATPEPSSVCFYTSPPYEYKSPPPPTPVYHYASPPPPVYYYKSPPPPPYYYKSPPPPPPNYYYKSPPPPS
PYYYKSPPPPPYYYKSPPPPPYKYESPPPPPYKYESPPPPPYYYKSPPPPPYKYESPPPPVYKYETPPPPVYKYKSP
PPPYKYESPPPPPYKYESPPPPPYYYKSPPPPPYKYESPPPPPYKYESPPPPVYKYKSPP

>405021|PACid:15410012
MGPPRRTRMPMEATAALSLVVIALAFAAQVEASPYEYKSPPPPVYETPAPVYKYKSPPPPPPVYKYASPPPPVYHAP
APVYKYKSPPPPVYHYSSPPPPVYKYKSPPPPVYHAPAPVYNSPPPPVYKYKSPPPPVYHAPAPVYKYKSPPPPVYH
YTSPPPPVYKYKSPPPPVYHAPAPVYKYKSPPPPVYHYSSPPPPVYKYKSPPPPVYHAPAPVYKYKSPPPPVYHYSS
PPPPVYKYKSPPPPVYHAPAPVYKYKSPPPPVYHYSSPPPPVYEYKSPPPPVYHAPAPVYKYKSPPPPVYKYQSPPP
PVYHSPAPVEDDNKCCRIFLEIRTRVAAVETELLHRKREM

>405114|PACid:15410799
MRTSQVRGLGLVPLLLMVLVANAYDVDPPSPDYKSPPPPKYEYKSPPPPEYDPSPVYEYKSPPPPKYDPSPVYEYKS
PPPPKYEYKSPPPPLKYDPSPVYEYKSPPPPKYEYKSPPPPLKYDPSPVYEYKSPPPPKYDPSPVYEYKSPPPPKYE
YKSPPPPPKYDPSPVYEYKSPPPPKYEYKSPPPPKYDPSPVYEYKSPPPPKYEYKSPPPPKYDPSPVYEYKSPPPPK
YEYKSPPPPVKYDPSPVYEYKSPPPPKYEYKSPPPPKYDPSPVYQYKSPPPPKYEYKSPPPPPKYDPSPVYQYKSPP
PPKYEYKSPPPPPKYDPSPVYQYKSPPPPKYEYKSPPPPKYDPSPVYKYTSPPPPKYEYKSPPPPKYDPSPVYKYTS
PPPPKYEYKSPPPPKYDPSPVYKYKSPPPPLYYYKSPPPPAKYKSPPPPKYDPSPVYEYKSPPPPKYDPSPVYEYKS
PPPPKYDPSPVYEYKSPPPPKYDPSPVYEYKSPPPPKYDPSPVYEYKSPPPPKYDPSPVYLYKSPPPPKYDPSPEYK
SPPPPAY

>413006|PACid:15411341
MIFLKSCSMLLLLLLVSTVSCDGSQEVLDRNAGAFGDAKNFGFRIPKSTVDFFCWRPWLRRPWWFPNRLCPPNAPPY
CYKSPPPPVESPPPPVESPPHHHKSPPPPVESPPPPYHYKSPPPPPYEYKSPPPCEKSPPPPTSPATQSPPPPKSPP
PPLPPKCEKSPPPPLPKCEKSPPPPPPKCEKCTPSPASYKYTSPPPPQSPPPSPPYKCTPSPAPHKYTSPPPPLSPP
PLPPYKYESPPPPPPKCERCTPLPAPYKYTSPPPPQSPPPSPPYKYESPPPPPYEYKSPPPCEKSPPPHHHHRKSPP
PPSPPLYWYQSPPPPSPTYRSCNEILKRSSIKAAWMEHTFLRQIFEDVAAKSMFAA

>412461|PACid:15411617
MIFLKSCSMLLLLLLVSTVSCDGSQEVLDQNAGAFGDAKNFGFRIPKSTIDFFCWRPWLRRPWWFPNRLCPPNAPPY
CYKSPPPPTPPPVESPPPPVESPPHHHKSPPPPVESPPPPYHYKSPPPPPYEYKSPPPCEKSPPPHHHHRKSPPPPS
PPLYWYQSPPPPSPTYISCNEILKRSSIKAPWMEHTFLRQIFEDVAAKSIFAA

>229952|PACid:15414583
MGTRLMASHSSIALGWALALVTIAALGEINVVSANYDYSSPPPPYVYKSPPPPSPSPPPPYIYKSPPPPSPSPPPPY
VYISPPPPSPSPPPPYIYKSPPPPSPSPPPPYVYKSPPPPSPSPPPPYVYKSPPPPSPSYVYQSPPPPSPSRPPPYV
YSSPPPPSPSPPPPYVYKSPPPPSPSPPPPYVYKSPPPPSPSPPPPYVYKSPPPPSPSPPPPYVYKSPPPPSPSPPP
PYVYQSPPPPSPSPPPPYYYKSPPPPSPSPPPPYIYKSPPPPSPSPPPPYVYMSPPPPSPSPPPPYVYSSPPPPPVY
YYSSPPPPSYY

>420737|PACid:15415204
MESFSMIRLLLLVLCVTSSVAGSAARLTHLVKDDPPPPPPPSNSSPPPPPGYSPPPSPSYEPPPSEPPTPPPSYSSPP
LPPEYPPPPPPSLSYEPPSPPPSYSSPPPPPSVSYEPPTPPPSYSSPPPPPEYPPPSPSSPIPPPGYSSPPPPPALSL

TPPPSFSSPPPPPTLSLTPPPSYSSPPPPPGYSPPPPLSYEPPTSPPSYSSPPPPLEYPPPPPSPSYEPPTPPPSYS
PPPPLSPGYSSPPPPLEYPPPPSLSYEPPTLPPSYSSPPPPPSPGYSSPPPPPEYPPPASPSYEPPTPPPSYSSPPP
PPSPGYSSPPPPPEYPPPASPSYEPPTPPPSYSSPPPPPSPGYSSPPPPPEYPPPASPSYGPPTPPPGYSSPPPPPE
YPPPPSPSYEPPTPPSNYSSPPPPPEYSPPPTRYAPPPSYSSPPPPEYPPPPSPSYESPPPPAPSPSYEPSSPPPPP
SYELPPPSYNPLGGRTTSCRRLETPTSLSDTDTRLLNSLLKMEYILSQFYVTVANGGTFHMSNHNSSQTSSLVHKLM
NEFAVHQLDHISVLSQFLTNRAVARPRMNVGRQAFSGILQAAIGQKLPPEFDAFGSPERVLLASFVMSPMAPSLAEA
ILPQLENEASKAIVAGMLRALTSEDAAVRTMLISMSENKVVPFPMTVGAFSSKITELRHLLALLSDECAGKPFASPS
LSQDNSMVVEPVKMVARQGTMGVFNVLSDLGILA

>429269|PACid:15415476
MGSCHFAMALLAAALVLLRSSQVQAFDFSFPPMPDFYKYLSPPPPPYYKYESPAPPYYKYVSPPPPYYYKSSPPPYY
KYESPPYKHESPPPPHYYYKSPPPPYYKYESPPYKYESPPPPPYKYESPPPPPYKYESPPPPPYKYESPPPPPYKYE
SPPPPPYKYESPPPPPYKYESPPPPPYKYESPPPPPYKYESPPPPPYKYESPPPPPYKYESPPPPPSNMSLCHHIRV
STTSLLEVQVLPPPPYYKYESPPPPYYKYKAPPPARPQRLLEKLMFIYNYN

>422106|PACid:15416343
MGSCHFAMALLAAALVLLKSSQVQAFDFSFPPMPDFTPYYKYLSPPPPSYYKYESPAPPYYKYVSPPPPYYYYKSPP
PPYYKYESPPYKYESPPSPPYKYESPPPPPYKYESPPPPPYKYESPPPPPYKYESPPPPPYKYESPPPPPYKYESPP
PPPYKYQSPPPPPYKYESPPPPPYKYESPPPPPYKYESPPPPPYKYESPPPPPYKYESPPPPPYKYQSPPPPPYKYE
SPPPPPYKYESPPPPPYKYESPPPPPYKYESPPPPPYKYESPPPPPYKYESPLPPPYKYESPPPPPYKYEPPPPPPY
KYQSPPPPPYNPPPPPYKYESPPPPPYKYQSPPPPSYKYESPPPPPYKYESPPLPPYKYESPPPPPYKYESSPPPPY
Y

>113758|PACid:15418587
MPPPKPSPRRARGAPPPHSSAHSQAPSPAPAEQHHGAAKQHSHGGAPPPKQDSHGAPPSPPTSHGAAKQHQHSPPPP
PASHGAAKQHQHSPPPPPPGHGAAKQHQHSPPPPPASHGAAKQHQHSPPPPPPGHGAAKQHQHSPPPPPTGHGAAKQ
HQHSPPPPPVGHGAAKQHQHSPPPTSHGAAKKHQHSPPPPPASHGAAKQHQHSPPPP

>422873|PACid:15421761
MEKYRRNSVCVLVCRCKWDFFVAISSLISSKETPQATGYRFVNCKYAFRTRLEAEAVRANVRPIRVDCFSQLTRSPP
FSLRGALKANTKHTKGRKETPSPGSDKLRALELGLDRLEKKERWWRQEIQGSISVWDFDQYESPPYKYESPPPPHYY
YKSPPPPHYYYKSPPPPYYKYESPPYKYESPPPPPYKYESPPPPPYKYESPPPPPYKYESPPPPPYKYESPPPPPYK
YESPPPPPYKYESPPPPPYKYESPPPPPYKYKSPPPPPYKYESPPPPPYKYESPPPPPYKYESPPPPPYKYESPPPP
PYKYESPPPPPYKYESPPPPPYKYESPPPPPYYYKSPPPPYYKYESPPPPYYKYESPPPPYYYYKSPPPPYYKYESP
PPPYYKYVSPPPPAYKK

>404120|PACid:15407734
MASSSSSHWCCLLLFTVILTQSTAGSTLKNQNACSTEGLLREWCGKDDGTTVNKGANRHELFKNNVCRLMILGKSMV
SSNSLGGSTQSPSRAPVPSPSPSAPPPSPSPSPPHPSSPPSPPPPSPPTASPPPPSPPPPPPPPPSPSPPPPPPPPP
SPLCPPCGYGQKCCCMFPSCLFLFKCCPITTVCGYGPHCY

>405687|PACid:15414821
MEISVKLAFVMLAAPMCLLNCAAPSNADATIDCQMGDFQDVGTFPIGICKCSEGWTNILGSPYLPCGIPKCDFKMSC
DGVDPGSPPPTPNISFSPCLIPSICGEGGDCISQGLSLAYTCVCRNGYRNLLNLTSGVCFRDCQLGEGCQNLVLGIN
PTIEPLSPPPPVPFQNSEDKSGLGKAF

>406865|PACid:15419161
MFLLLLLVSTVRPWLRRPWWFPNRLCPPNAPPNAPPYCYKSPPPPPPPVESPPHQLLTVTSLHHFHQSVRNLHLRHH
PSLHHHHHHQNARSLHLHTSTNLHHHEKSPPPYEYKSPPPESPPPLPPYKYESPPPPPPYEYKSPLPPPSPPIYW

>430475|PACid:15420154
IYPSPPPPSPSPPPPVAHIYPSPPPPSPPLVYPSPPPPSPSPPVYHSPPPPSPPCDNEWMPTPAPPGKSPPPPAPQY
HYESPPPPSPHYYFSPPPPSPPPPYHSPPPPPPPPPPPSSPDNYYQYPSPPPFAPPPPEYKK

>408211|PACid:15420277

MLLLLLVYTVTCDGSQEVLDRNAGSFGDAKNFGFGISKSTIDFFCWRPSLRRPWWFPNRLCPPNAPPYCYKSPPPPM
ELPTPPVESPPPPVELPPPPHHHKSPPPPPYQYKSPPPPPPPPKCEKSPPPPAPYEYKSHYKLNISSFAQNLRAYDF
YLIFYKDDDRNLYRNLSQTLSSEVDSRTPNSPHNCSLT

>431330|PACid:15422079
MNFFKSCSMLLLLLLVSAVRGDAKNFGFGIPKSTIDFFCWRPWLRRPWWFPNRLCPPNAPPYCYKSPPPPPPPPKCE
KSPPPYEYKSPRPPPKFEKSPPPPPPYECKSPPPPPKCEKSPPPYEYKFPSPPPLPPYKYESPPPFRYKYESPPPPH
NSPPLPPPPIYWYQSPPPLRELVTFCPGL

>81544|PACid:15409022
MRFQRQRRLEGASEASIRSLWDAYTALQAWKAAITKDPLNITATWIGDEVCSYKGVFCSAAPDDSCERVVTGIDLNH
AYLSGKLVEELGLLSYLVLFHINTNFFSGTVPPSFCKLAHLYELDLSNNRFSGPFPNVTLDLPSLRYLDVRFNRFRG
GIPPRLFDRGLDAIFVNDNDFQCAAPATLANSTASVLVLANNMIQGEIPSSIGALNGSVEEIILLGNMLTGCIPDSI
ANLTQVTVLDLSGNQLGGYVPDAIAAMKSLEQLNLAGNLLSGTLPEGICELPKLQNLTLEDNFLTDIAHQCLELPAS
NGTIVDTDRNCIPYQPNQRPHDQCADFLSQPTSCQPEPLVMAPPVLPPPPPSPPPPSPPPPSPPPPPPEYHYESPPP
PSYHYELPPPPPY

>54019|PACid:15414762
FAAIQAWKRAITSDPGNVTGSWIGPDVCSYKGIFCSPSPDNAYERAVTGIDLNHARLSGRLVPELANLKYLALFHIN
TNFFRGTVPSSFRRLAFLFELDLSNNLFSGVFPSVVLEIPSLAYLDLRYNLFMGSVPQELFQRPLDAIFINNNRFQC
EIPESFGSSPASVIVLANNNLQGKIPASIGNMSRTLEELIILNNEFSGCLPFGTGLLSSATVFDVSFNLLGGELPTS
ISRMGRVEELILAHNLFTGPVPAAICDLPSLRNLSIADNFFTSVSSHCLRSRRGIVFDDSGNCIAGQPAQRPADECR
FYKEEPLSDCSSIAATIHPPSPPPVQSPPPPPPVYSPPPPPPPPPPPPVYSPPPPPPPPPPPPPPVYSPPPPPPPPPPV
HSPPPPPPHFYSPPPPHIYSSPPPPP

>74599|PACid:15410807
MSLFRRFFYRRPPDGLLEISERVFVFDSCFSTDVFEEETYKLYLRQIAMQIHEQFPDSSFLVFNFREGERKSQLTEM
LSQYDMTVMDYPRQYEGCPILPMEMIHHFLRSSDSWLSLEGQQNIVLMHCERGGWPLLAFILASFLIYRKMYTGEFK
TLDMLHREAPKGLMQLLTPLNPMPSQLRYLQYVARRNNSPEWPPPDRSLSLDCLILRVVPAFDTEDGCRPLVRIYGR
DPRSKAGNRTTRMLFALGKKSKSVRHYRPTDCDVMKIDVQCAVQGDVVLECIHLDLESDREEMMFRVMFNTAFIRSN
ILMLNRDDIDILWNGKERFSKDFRAEVLFGETDGFSSPVAPVPSLMEEKGGLPMEAFAKVQELFSSGDWLDGGGDAA
LKFLQQLTTVGNGFEDRRLPMDRAALAAVDDEWTLISSSTTSSTIATATPLTDDQNGTSPTSSSNPSFAPGTPSPPP
PPPPPPPPRLAISRSPPPAPPPPPPPPVVSVAGRPPPPPPPPPPPPFKLPSSTAKLPPPPPPPPPPPPLPRGNSTAPPPP
PPPPLPRGNSAAPPPPPPPPPRGNTAAPPPPPPPLPQAARPLASSSSPPPPPPPPLPPPGMRGTPLPPPPPFKGPPPP
PPPPPPGSAAPPPPPPPLGVKSPLPGVPPPPPPALGRGRGSSPLTPSPPGGRGRGQVAGASGLASATPKKTSLKPYH
WVKVTRAMQGSLWAESQKQEEQSRQPEFDMNELESLFSAAVPNAAAGGDRAGGRRASLVPKQEKVLLIEHRRAYNCE
IMLTKVKMPLPEVVKAILALDGAVLDVDQVDNLIKFCPTKEEMETLKNYTGDKECLGKCEQYFLEMMKVPRVESKLR
VFSFKLQFTSQVSDLRENLVVVNEASAEVKESPKLKRVMQTVLSLGNALNQGTARGAAIGFRLDSLLKLTETRARNS
RTTLLHYLCKIVSEKMPEILDFDKELPHLEAATKIQLKALAEEMQAVSKGLEKVEQELTASENDGAVSDGFRKSLKS
FLDTAEAEVRTLASLYSEVGHNADSLARYFNEDPARCPFEQAVSIIFNFIVMFKRALEENSKLAEMERKKAEKEADK
DKVLPLRRELDGLLSPRRSRIA

>77222|PACid:15417080
MSLFRRFFYRRPPDGLLEISERVFVFDSCFSTDVFEEETYKLYLRQIAMQIHEQFPDSSFLVFNFREGERKSQLTEM
LSQYEMTVMDYPRQYEGCPILPMEMIHHFLRSSDSWLSLEGQQNIVLMHCERGGWPLLAFILASFLIYRKMYTGEFK
TLDMLHREAPKGLMQLLTPLNPMPSQLRYLQYVARRNNSPEWPPPDRSLSLDCLILRVVPTFDAEGGCRPLVRIYGR
DPRSKAGNRTTRMLFALGKKNKSVRHYRQTDCDVVKIDVQCAVQGDVVLECIHLDLESDREEMMFRVMFNTAFIRSN
ILMLNRDDIDILWNGKERFSKDFRAEVLFGETDGFSSPVAPVPSLIEDNSGLPMEAFAKVQELFSSGDWLDGGGDAA
LKFLQQLTTVGNDRRLTMDRAALTTADNEWTIIPSTTTTSAAATLPLTDDDHNGTSPTSSSNSSFAPGTPSPPPPPP
LPSPPRLAISRSPPPAPPPPVSVTGRPPPPPPPPPPPPFKLPSSTAGATAKPPPPPPPPPPPPFPRGNLGAPPGNSAAPPP
PPPPPLPRWNSAAPPPPPPPLPRGNSAAPPPPPPPPPPPLPRGDSAAPPPPPPPPPLPRGNAAPPPPRPVPTAPPPPP
LPQAARPLASSSSPPPPPPPPPLPGMRGTPPPPPPPLKGPPPPPPPPLGTKSPLPGAPPPPPALGRGRGSSPLTPS
PPGGRGRGQNTLESATPKKTSLKPYHWVKVTRAMQGSLWAEQKQSRQPEFDMNELENLFSNAVPNAAVGGERAGGRR
ASLVPKQEKVLLIDLRRSYNCEIMLTKVKMPLPEVVKAILALDGTVLDVDQVDNLIKFCPTKEEMETLKNYTGDKEC
LGKCEQYFLEMMKVPRVESKLRVFSFKLQFTSQVLDLRENLVVVNEASAEVKESAKLKRVMQTVLSLGNALNQGTAR
GAAIGFRLDSLLKLTETRARNSKTTLLHYLCKIVSEKMPEILDFDKELLHLEAATKIQLKALAEEMQAVSKGLEKVE

QELTASENDGAVSDGFRKSLKSFLDTAEADVRTLASLYSEVGRNADSLARYFNEDPARCPFEQAVSIIFNFIVMFKR
ALEENSKQAEMDRKKAEKEEKDKILPLRMELDGLLSPRRSRAA

>416549|PACid:15401636
MRSILVLFLLVSCVSILRLWLERSPPPPPRQRGSIFEQNLSAASAASASASASATPSPSPSPSPPPSPSASGFWIGT
GSSVASFLASVPVVLIAYDYPRRPPASAGLAPGCFVLLCSASLGFLNFLATTEKWIFTMAEWSAFCRWSATFTALAY
GISAAQLAITRKSLPKFVRGIATPILYIIACVVPSIFGRAFRHDPHGWAYNALAVSYGIFWGLPFLMRSFASWARWY
VGVWGEGAELGFTTEPRFMVSSMLNTIAALLWVFYSDTVEHHDGHFWISIVASIGGACVFLGQLVTLLEGKKC

>438958|PACid:15402604
MELANISELRLLLLLLLMASCFATDDPATVGDNDLVVGRVFCDRCIEGRFSEYAIFVKGAKVALECTQQDGGIHVVE
AVTDSAGAFAIPVDEPGSSCAVKLLSTPTPQCNIAAKHAQTLNANMRTKDNVIYVGLLSFRPEEPLPICSQRAYYAS
PPLPPEYCYAPPAYSSPAPVSRPPSPPPAYSSPAPISRPPSPPPAYSSPAPISRPSSPPPYASPAPISRPPSPPPYS
SPAPVSRPPSPPPYSSPAPVSRPPSPPPYSSSAPPAYSSPAPISRPPSPPLYSSPAPIVRSSPPPLYSSPAPTTRAS
PPPLYSSPAPTVRSSPPPLYGSPGPIVRGPPPPLYSSPAPTARPSPPPLYGSPAPTVRPAPPPVYSSPAPIARASPP
PY

>416970|PACid:15404683
MAKWLLLFFVLVFGANGKRSPLYGGTDGENFSDGQSQGIVRVKIWSGYIVDGIQVQSDQGGEHVWSDPHGTSKGEAK
EVVLAYPDEYIYKISGRTGPNPWTSNGLSRLYLHVNNRRTNAQTTFGPFGTHDYSNQTTFDSPEGTVVGFFGYASST
TYFRGIGVYFQDPCDCPTPSPPPSPPPQPLPSPRRPCKGYQWSTTGSSCCCVERESKKHTCCQSSTRECPPLRYGEA
LPPASPPTEELCPVLGLEDVIRKYYELNMNLL

>426017|PACid:15404870
MACKIITPILFSPLCFADPISRMGVTVPYYIGGDGSSSIGVLLVTTLSSTGKNTGKHTGNRIEDPSPRGDSCRNSGQ
ESRGLPSTALAARGSDRQQWPSIKRMTSMSVPEYDPPPLPVLEGDNHDSQELSSNRTFPLVQLPSRAEIHLEVEELK
KLIGERTWKLPTTLQSIKEEMWSRLLNKFAKAAQQDGEEQPVSVFWQRLQGNGTSTVRKHRRPPDKNSDEQELVAAM
AEESSYKVLKSHAAAGSRKLRQVDVSSTPVWQSLRGDGTSTVGMHRRFPYKNSEEHAAAMAEESSYKICQESDVLKS
HAATGIPSQKLLASGTGDTKEASSNASPPPASPPPFMWRWFPRSLGEDEFSVCGVSNGSSIASEFIQLFEGEGISSS
DAILNSLLEGEGGCSSSNADLRLASSNDLNLQAMAVAATPLLLPIHAVVLVALLFLFLLSFLLVIKRRWGRRNRGLL
SSTFRCKCIRMCSGRDQVVASMARFSSAGSNSYIANSFVSQLMPKDEKGCMQSLFSCKPGTKGRTPASDQDCLLFIA
LIPLQRSSEHRFSHSSGTTSPESLNMKFAAMPCKSMYKNVPNGVTFTEKENIRNVTRSVENARAARPRPLALQGQVV
P

>410458|PACid:15405653
MLLRALALFALCLLVSATPSSPPPPGSCSSDADCPEAYPCCSPWGNCGNEVPWCSSPPPPPPPCKSPLYGTTSGGTP
FSGSLTSTPLEGLRIISGCVVDAIQWKPSGSSWTQMYGTNSCSGCLPKPPPQAGLRGTLYEISVSSDEYIYKLSGTT
GTDKTCGAGGAGEGVVSLTVHVKNMKTGVTKTYGPFGDSKSGTFFETAPGNIVSFFGYASTRSYMKGIGVIYSTCPS
GSVRITPMGAFSII

>441276|PACid:15406378
MLLRALSLFALCLLVSAAPASPPPPGSCSSDADCPEAYPCCSSWGDCGNKAPWCSSPPPPPPPCKSPLYGTSADGTA
FSGSLTSTPLEGLRIISGCVVDAIQWKSSGSSWTQMYGTNSCSGCLPQPPPQAGLRSTSYEISLGSDEYIYKVSGTT
GTDKTCGAGWAGEGVVSLTVHVKNMKTGKTKPYGPFGTSTSGTSFETAPGNIVSFFGYASPKSYMKGIGVIYSTCPS
SSVQTTPMGAFSII

>410639|PACid:15407085
MMKVFGANGKRSPLYGGTDGKSFSDGQSQGIVRVKIWSEYIVDGIQVQSDQGGEHVWSDPHGTSRGEAKEVVLAYPD
EYIYKISGRTGPNPWTSNGLSRLYLHVNDRRTNAQTTFGPFGTHDYSNQTKFDSPEGTVVGFFGYASSTTYFRGIGV
YFQDPCDCPTPSPPPSPPPPPPPSPPPPSPPPPSLPPPLPSPPPPSLPPPPPPRPCKGYQWCPAGSSCCCVDRACNK
DTCCISSTQGCNPLGYCEASPALPPPSPLPSPPPPSPRPPPPPPSPPPPSPPPPSPPPRPSPPPPSTPSPPPPPSPP
PPSPQPQPTLGRCKNFDRHCQGRYGRKFCCCVPNSAGKCGSTWSCCCDSCNPRKNCVTFGLGGCNQKIL

>403585|PACid:15407999
MRSSSVMLLLLGFLVLVSTQELGKNELEEAIVLYSGSSDDPVERLSFSSFASFPTLPPFPPSPPPPPPPPIAPDDHH
HSRPAPAPAPAPSDGDDDDDDQGSSGTPPPPPPPPPPPSPPPPPHHLPKALLAGLNVTNGGKLSPDFSPRTYNYSSS

VSSGVRKIRIVATVSQEEERDEYTVTVNSIPLKSGVPSPQLQVGKAGEDTLFEIAVTALEHEPSTYYLLVHRGKSKW
DRFGAKFLLALAIVLVVAVVLFLCYGCYVCIQSGRMPSIWPFRSRDADEYSLLPGPSSGRP

>449338|PACid:15408044
MENKTFLYLILLLAIILKNENFCVATTSIKVDGYVYCTNCGSLSQSGIFNAKVNLVCPGDSDSSFTNSKGYFNIFEL
VKTGTDIGSCRVSVDTSRAPRGCSFLTDINGGSSGQSLSTCTNNGNNKFTCGPYYFTPNACSPPPPPNSPPPPPPPP
KASPPPPRKPPPPPPPRRPPPPPPPRTPPPPPPRTPPPPPPRTPPPPPPRTPPPPPPRTRPPPPPRKPPPPPPRTPPPP
PPRTQPPPPPPPPPPPSPTPAPSLSPTPPPESPASAPGPAPTP

>411518|PACid:15409314
MLLRALSLFALCLLVSAAPASPPPPGSCSSDADCPEAYPCCSSWGDCGNKAPWCSSPPPPPPPCKSPLYGTSADGTA
FSGSLTSTPLEGLRIISGCVVDAIQWKSSGSSWTQMYGTNSCSGCLPQPPPQAGLRSTSYEISLGSDEYIYKVSGTT
GTDKTCGAGWAGEGVVSLTVHVKNMKTGKTKPYGPFGTSTSGTSFETAPGNIVSFFGYASPKSYMKGIGVIYSTCPS
SSVQTTPMGAFSII

>419497|PACid:15409790
MVLHSLLLAFILAGLSSATRKDTITVTGVVFCKRCVHGTAVGTPLPGVEVSLQYTGNPAILALTDSNGVFKLPVKSA
FFADPGAKSRQLFVKIVRLPDSTCQIPTTTEKNAGILSKAARRTGIFTVGAFTFRPQKTLCSELPPSLSNTSIIPPL
PPAIFPFPHPGYGGAPPIGSSPFLPFSPPPYEELPPPPNAEPPEIVIPPQPTSPPPPHSLPPAPVRRNNGPPPPLHA
LRPPLPPPAPRMKSPHPSPPAPYVRDKSPPPLPKPPARSKSPPRPKLSPPPPARNKPPPPLPKLTPPPPRNKSPPPL
PKLSPPLGPPKHAPPSRAPSPGRKPLPSPSPSPTPPPFPSPSPSPPRKESPRAPYLPSPPHCQQKP

>441903|PACid:15411640
SKTFLYLILLLAVILKNENFCAATTSIXVEGYVYCTNCGSLSQSGIFNAKVNLICPRDSDSSFTNSRGYFNIFELVK
TGTDIGSCQVSVDTSRAPRGCSFLTDINGGSSGQSLRTCINNGNNKFTCGPYYFTPNACSPPPPPSPPPPPPPRRPP
PPRPRTPLPPRPPQPPPKTPPPPPPRTQPPPPTPPLSPTPTPSLSPIPPPLSPTPPPLAPPLLSPPAPTPPALAPES
PASAPGPTPTPSSLL

>413143|PACid:15412174
MATWWMILLVLQLWWCSWLEARPIKNIVLQYPSPPPPVTYVYSSPPPDGYSSPSPPQTSCPPPPSPPASPVYPPPAS
PPPDGYSSPSPPSSCPPPPSPPVYPPPASPPPDGYSSPSPPSSYPPPPSPPVYPPPSDGYSSPSPPPSPPVYSSPSP
PSSYPPPPSPPVYPPPASPPSDGYSSPSPPSSCPPPPSPPASPPPTSTPSPVYPPPTSPVCPPPPSPAYPPPSPPPC
DSKPPPMQEYPPPPCEDHHAPTAPEYPPPPCECHTPTPPEYPPPPPPKDCHTPPPPAPSPCNESAEYPPPSCDDCSS
PPTEPPPCEKCEYPPCDDCGKLPASTKVTDARILAEILSAELLLSDQYSRAARFKDPSVKMLTSEFQSQLKSHISVL
QGLLKDAAGKPQIDAGKGVFTKIMNAAFGKDLDPAFDAYNTSTNFLLAASVTAPLASSLSVAGLSQLQGADAKKAVA
GISSTLAGQSSVLGVLLKLRKLEKVQPYGLTVGEFSSQLQELQKKLELLHHGQVAEAASSSRNSTNVSSSKAKVSPS
TARDPKQHPEEIAAILMELGFYAPGQQ

>412728|PACid:15413899
MEQHKQAPLWALLLILASGLILTAVGLTKNLTLLLNNYPNFKRFNTLLTTSGVVDQVNPMLTATLFIPENNTLLNDF
IYDMGKHPSEEKLADLVRYHISDFYIESEFLAVRRNFSSPIKTLFRERPYEETLHFRWLQLNVDNHGVATLSRPPHQ
SPPLATILRNVVQEPFSITVYAIDRVLEPEGFLPPPPPPPPSPPPPSPPPPPPPPPSPPPPPPSPPPPSPPPPPSPP
PPSPPPPPSPPPPSPPPPPSPPPPSPPPPPSPPPPPPPSPPPPHPPPPPAFLPESEDGIPPPQSLAMQRFHRSVLAS
TLVSLLAMLLLKF

>420661|PACid:15414560
MASIIRRSVPPSSPPHRAPPPAAHRSVHPPRPGHRSVTPPKTRAPPPGRSMPPPKPSPRRARGAPPPHSSAHSQAPS
PAPAEQHHGAAKQHSHGGAPPPKQDSHGAPPSPPTSHGAAKQHQHSPPPPPASHGAAKQHQHSPPPPPPGHGAAKQH
QHSPPPPPASHGAAKQHQHSPPPPPPGHGAAKQHQHSPPPPPASHGAAKQHQHSPPPPPPGHGAAKQHQHSPPPTSH
GAAKKHQHSPPPPPASHGAAKQHQHSPPPPPAGHGAAKQHQHSPPPPPAGHGAAKQHQHSPPPPPAGHGAAKQHQHS
PPPPPASHGAAKQHQHSPPPPPVGHEAAKQHQHSPPPPPTSHGSKQHSHGGGAKQHPPPPAKQHTGPPPSSGTITW
RPRRFGPPPPPPAKEHTGPGTTTWWPRPAHDTITWQPGSPVFHTFSSPPPPGPLSIAPPLGSSSSTAAAAAAAFATP
GLLAAGIVLFLLAMVAIVTGIWILVKGRRKKANDILVRYVGSSHLPGSGSSSSSQREHLVPLSKIEDAELRDKLDSF
VQERSKQQLRPSQLNASTKVASPPLARPPRRLPPSAFLKMNPIYGAATDSVDDRLTPPLGRDPPAAIHGVDERKKVP
RASLPEDPFLSSAARRSGNDRRAQEQAVDPVSRRGTVFDPGDAFVVRLAAAAEKRAEMEKHMQRIIEFGMRRPGNKH
SHWIREVENLEKPVVEKPVVEANHSANSGGSHLAARVHDSDAKPSTSEVKLELDGA

>420679|PACid:15414600
MASIIRRSVPPSSPPRRAPPPAAHRSVHPPRPGHRSATPPKTRVPPPGSMPPPKPSPRRARGAPPPHSSAHSQAPPP
APAEQHHGAAKQHSHGGAPPPKQDSHGAPPPPPISHGAAKKHQHSPPPTSHGAAKKHQHSPPPPPASHGAAKKHQHS
PPPPPASHGAAKQHQHSPPPTSHGAAKQHQHSPPPPPASHGAAKQHQHSPPPTSHGAAKQHQHSPPPPPASHGAAKK
HQHSPPPPPASHGAAKQHQHSPPPPPASHGAAKQHSHGGGAKQHPPPPAKQHTGPPPSSGTTTWRPRRFGPPPPPPA
KEHTGPPPSSGTTTWWPRPAHDTITWQPGSPVFHTFSSPPPPGPLSIAPPLGSSSSTAAAAAAAFATPGLLVAGIVL
FLLAMVAIITGIWILVKGRRKKANDILVRYVGSSHLPGGGSSSSGSQREYLVPLSKIEDAELRDKLNSFVQERSKQH
LRPSQLNPSTKVASPPLARPPRRLPPSAFLKMNPIYGAATDSVDDRLTPPLGRDPPAAIHGVDEHKKVPRASLPEDP
FLSSAARRSGNDRRAQEQALDPVSHRGTVFDPGDAFVVRLAAAAEKRAEMEKHMQRITGFGMRRPGNKHSHRIREVE
NLEKPVVEANHSANSGGSHLAACVHDSDAKAQNEDS

>422030|PACid:15415694
MESFSMIRLLLLVLCVTSSVAGSAARLTHLVKDDPPPPPPPSNSSPPPPPGYSPPPSPSYEPPPSEPPTPPPSYSSPP
LPPEYPPPPPPSLSYEPPSPPPSYSSPPPPPSVSYEPPTPPPSYSSPPPPPEYPPPSPSSPTPPPGYSSPPPPPALSL
SPPPSFSSPPPPPALSLTPPPRYSSPPPPPGYSPPPSLSYEPPTPPPSSSPPPPPEYPPPTASTRYGPPPPPSPSYG
PPTPPPNYSCPPATTVTNPPPPPEYSPPPTSAPPPSYSSPPPPPYATPPPSYSSPPLPEYPPPPSPSYESPPPPAPS
PSYEPSSPPPPPSYELPSPPPPSYNPLGGRTTSCRRLETPTSLSDTDTRLLNSLLKMEYILSQFYMTVANGGTFHMS
NHNSSQTSSLVHKLMNEFAVHQLDHISVLSQFLKTRAVARPRMNVGRQAFSGILQAAIGQKLPPEFDAFGSPERVLL
ASFVMSPMAPSLAEAILPQLENEASKAIVAGMLRALTSEDAAVRTILISMSENKVVPFPMTVGAFSSKITELRQLLA
LLSDECAGKPFASPSLSQDNSMVVEPVKMVARQGTMGVFNVLGNLGILA

>421413|PACid:15415772
MEFHGRLPALLAILLLLCIFGAAQSSRKAGSGDANGDVDRGGGQDQDHQSPPPPPSPSPPPPPPPPPSKIESKEDFN
ATAGSSKNQTKEEDPPPSTGAQPPPEAEEEDPGHCCGLKMRACLSSPGDSSDGMLSLVIKSHEVVKVKVITPSFLSA
DPSELTTTADGKEIQVKIEMVDPEAAKLQSSIQITVKSDTESCKLPVPSHNLGYSDQKKTLLGGFRYSALRTPVVGL
SMLAAALVLVMGSWLYCRYRVKKTGQSSAGEHRYVELEMGAPDRDGAGTALEVEDDGSWDKVWDEEWDDGEEAVGSS
SFKLTESLSAKGLAPRRTSKDGRVD

>444340|PACid:15415930
MATWSTFITLFLAISTGLAAASTYEIYGDPTCTTVLTNFSTPAQCFPVLPVFGYNFIFTEEPGITFFGAACSMSING
STILTKNTTVCGPSTVLSFGILPLVDCNLPPRRSPPPPPPRRSPPPPPPRRSLPPPPPPPPPPPRRSPPPPPPRRSPP
PPPPRRSPPPPPPPCKKVIYGDARNFEKQIYPSAIFLIHFLILVIVILKH

>413730|PACid:15416712
MASIRLVVAFLCLLQAASVALSREELVYVRGSVECKTCSSSEEPTPLSDVTLRLSCDGDTYYQNSHYRGRYVFDVYA
RKKMKCRVSVLTSTLPYSCSRAKTSLHRDVEISAISGTTFVVKRFVFGPPACVTHSSKSSHHHPAHSHHHHGHHHWY
HHPPPPHHHAPQSPPPPPPHHHHHHQHHQHHHRYRFDHPSKGFYPPSKGSHHHSPRSSSPGSHDQHPAPPGHRHGHG
RGHQQHLAPPPSKVPAPSFQAPAPSPPSFQPRQLPPAPTATALPPPHGQVLSPPPEKPIVEANVWPPPGAKAPISST
FIRQVTAPPPQPEERVGTPTLSPPPVKSAETPASPPLPEQLLLSPPLPSAVLPPKSSEAALSPASDSTPPKDILLVP
PEGSVSVVRPPSFIP

>415476|PACid:15420378
MAWLAFLVFVLTASVAGGQQSPPPPRSPPPPPPPHPPPPTPPPPSPPPPTPPPPSPPPPTPPPPTPPPPSPPPPPPC
PPPPTPPPPPPPTPPPPPPPCPPPPTAPPPPPPPCPPPPTRPPPPPPPPPPTPPPPPPPCPPPPTPPPPPPPPPCPPPP
PPTRPPRTPPPSKTPPPPPCPPPDTPPPRPPCPPPSPPRHTCPSLPSCPSHRQTPPPPSHCSPCCGKAHHPKLPLTQ
IFTVMNHLQRLYVTAASKKSANFSGEEALTMDIISEFGIQSALQTRMIQKHLGDGVEAPEIASTKLAFQRIVHAAFG
EVLSPAFDPFSTPLSALVASSTVLPLATSLSVGILPELHSKSSKALVAGVVGALAGQDAAVRALLYRHRKEIVAPYE
HSVAHFHGKVLGLTRSLELLSGAHPAKYQSSLFSLPVDDSGVSPALSAEQARLVLQRLGFLQ

Pinus taeda

>PITA_000081371.RA protein AED:0.11 eAED:0.12 QI:0|0.66|0.57|1|1|1|7|870|686
MASLAPSPAALPGGGSAAAPPVGNNGTTSPPTAPPATPPVSSPPPTSPPPPPASTPPVSAPPPSPVTVSPPPATAPP
PTPAVPPTVKSPPPSSTSSPPPASSPPPSASPPPPPSNSSPPPASSPPPSASPPPPPTAKSPPTSHSPPPSPTPSPP
PSKLSPPPAPQPSRSLSSPPPPNVPTSKGSPSAPVSSGSPPSPTPPSTTPPTPNSSTNSSSTPNTPSPPTASGGGNT
SSIVIGVAVAGLVVLALCAVLFCIRRKKKRDGYLGDHYGPPPPGGASFKSDGMYAAVSRTSEQYLVKVSPQPSTPGH
GSSGTAAFGVPPSSVDTPGLGNSSWFNYEELAVATNGFSPQNVLGEGGFGCVYKGYLPDGQVVAVKQLKVGSGQGER
EFRAEVEIISRVHHRHLVSLVGYCIADSQRLLSFLQLPSAGHPRIIHRDIKGSNILLDDRFEAQVSDFGLAKLASDT
NTHVTTRVMGTFGYMAPEYASSGKLTIRSDARPLLNRALEEGNFEELADPRLEKNFDEGEMFRMVEAAAACVRHSAR
RRPGMGQVVRALESNAAIGDLNNGVKPGESVMYNSAQYSADIRRFRRMAFGSRDNASDFSEVTSENDTNLSQYSNEF
RKSKELEAKQTRLWKSELAMSCACGGESPSSGESETLPINRSHIYRDETNEEKSSGEAPIPINCKPLTGR

>PITA_000023436.RA protein AED:0.16 eAED:0.17
QI:290|0.85|0.62|1|1|1|8|370|657
MSSPTTAPAPVSPTPSKNNTSPPPPVNLTPPPTSPTPTSPPASPPPSSSPPPPKAPSPASSTSPPPPKSLLSPPPPT
PHSSSSPPPPPKTSHGSNTPSSTGSSNSSSSFSTTAIVGVAVAGVVGVLLLCILIACCCRKKRKRRPEFIPGQYYGA
PPQPSKDDYYGGRPQYLQNNAPQPGNNAVKIRPPSSPPSHPVIPKPPPSPPDVMTPPPPPPPVFSSGDDSSSGFSGS
KQPLPPPSPRIGLGISRTFTYEDLEAATNGFSRTNLLGQGGFGYVYKGILPGNKTIAVKQLKVGGSQGEREFQAEVE
IISRVHHRHLVSLVGYCIAGSQRLLVYEFVPNDTLEHHLHGKGQPTMDWPTRLKIATGAARGLAYLHEDCYPKIIHR
DIKASNILLDSNFEAKVADFGLAKLASEGFTHVSTRVMGTFGYLAPEYASSGKLTDKSDVFSFGVMLLELITGRRPV
DTTPSFSEDSLVDWARPLLARAMEDGNVDALADIRIQHNYNVNEMMRVVACAASCVRHSARKRPRMGQIVRVLEGDV
SLDDLDEGIRPGHSTVYGSIGSSDYDTNQYSEDMKKFRKMALGSQEHGSDYSGTTSEYGPNQSVSSSEYLQSKEIGS
SVGRSQVPRTVGHYRTPSSGTSQEYVPRKHTNRDFPSVFRD

>PITA_000045997.RA protein AED:0.07 eAED:0.07 QI:0|0.5|0.4|1|0.75|1|5|0|1032
MGMRRITRGFPGLLLLFSLLLFSALSDTQEIISLTAEDNSREGGSTDRYWLRGFKGSQFGIQNRRIDEIEQLETNCE
LAQLEGKDVDRGDLYYFSDRGRCVDAVGQIHNTDSRFQHTLAKTISNLPPKKSSFLPVSLHKHGAITSSVDPVYGNH
DKFKIRAVSGEDENSNMLWPLDRLRVLLGFHTDEKILSHSPSLEISAMHALAPAEPPTTAVSPSPKATPPDKYDGPS
PSPSLLTVLPPVRHSPFPLPTHISNSPPIEQSGKKSGNSKTVVVAVVITAISTFALAGVFFYFCQKCRRHGSNEHKD
ERPLLFPSMDNGSVNHFYGESNGFKWGTVENSTGNSHKVASLETGKAGGMQLSSFNEGNHLHISASRTDISSTQPSQ
EVSQIHPTFIHPSSSDSPVPFHALQNTASQPIRPALSSHPHLQSTDTQIPSSCEPPSLLLPLDTPSAHSPVKSTSPP
PPPPPPPINKSPPPPPPPPMKNSPPPPPSTYKSPSSVPVAPAKGKSNVPMPPPTPAKSSKIPPPLPPRSKAGQPMSS
AAQSAFGTGVSAGHLNAEGEDGNLQRKLKPFHWDKVQANPDHSMVWDKIRAGSFQFNEEMIETLFGYNSTKTNSGVA
KQSVIPPSQNLHLLDPKKSQNLAILLRALNVTKEEVCHALFEGEGLSPQLLETLVRMAPTKDEERKLKDYNGDISKL
GPAERFLKALVNIPFAFERFDAMLYRENFREEVSYIRESYETLEAGCKELRCSRLFLKLLEAVLKTGNRMNVGTYRG
GAQAFKLDTLLKLADVKGTDGKTTLLHFVVQEIVRAEGMRAARAGEQSVSDTTSNIVDGQTYHKSDENESSEDREDY
YRKLGLLVLANLSTDLCNVKKAAAIDSHSLTSSGSKLATGLAKMKSFLRREFSSQKGDENASNDMPEGIFHKSMALF
LQQAEEEISQIQSEERRVLSRVKEITEYFHGDAAKEEAHPFRIFVIVRDFTNMLDHVCKEVGSIPKRNPPSSSQKGP
SSISDDNLSRALFPTVFDRNVYSADDESSTA

>PITA_000042657.RA protein AED:0.15 eAED:0.15 QI:0|0|0|1|0.93|1|16|0|1504
MDYPKQYEGCPLLSLEMIHHFLRSSESWLTLDQKHNIVLLHCERGGWPVLAFMLAGLLIYGKQYTGEQKTLDMVHKQ
APKELLQMLCPLNPIPSQLRYLQYISRRNNGIEWPPLDRALTLDCLILRIIPNFDGEGGCRPIFRIYGQNPFSPDDR
TSELLFSMGKKSKNVRYNRQADCDVVKIDIHCHVQGDVVVECINLDAELEREEMMFRVMFNTAFIRSNILMLNRDDI
DTLWNAKDRFPKDFRAEVLFSDMDMDAASALLNVSTLGGEEKQGLPIEAFVKVQEIFSNPDWVGKDDTTIHIIQQFT
ASNTLQEKQEKQQENQEKQQENQEKQNGASNTLHEKQENHIIASNALHEKQGKQIISSQEKQVIASNAVVQNQEKKI
ISSQEKRVTASNALHQKQEKQIVVANALHEKQEKQVIASNALHEKHITDTNSLHEKWDKQVIASKPLHEEQEKRIIA
SNTLHEKLERQVVASNALHEKLEKQITASNALHEKQEKQIMASVGLQEKWDDQNTVSNSPHEKQEKQIIASVGLQEK
WEDQNNTSNSPHEKQEKQIITSSALHEKQEKQVIPSTTLHEKQIIESNVSHEKQEKQDITSKALYEKQEKQIIASKA
LRKKQIMALNALQERQEEPKTASKTSQEKQEEQIIALNSLHKKQIMILNAPEDKQEEPNTVSNTSQEKQEKQISAPN
ALQKKQEENTTSNILKEEQLEQIIASNILQENHKEKQDKTLSSDNSNTLLFPPTETKGAPPFPDDEEINWKELQSSR
LLPGQPKALSPWTQPYFLLNDVAGATAPPPSLPPSRYHSAPAAIGIRAPSQSRSAKKSSVEMSSISAQQAVQGVSSY
LQPSQSVFDVPPSSLPSAGTDVLLPSPSLAPLLTEMTYRPPPPPPPPSSPKPRSSNASAPPPSPPSIHSGVNAAAVWP
SPPSPPPPSIHSGAPTPPPPPPPPPPPPPTIRFGVPASSPPPPVCSIATVPPPPPYPPSPPYFGGPALPFSLVLAPSP
PPPPPLPRSGPPAPPQPPPLCPGASGIHNLSPVPPLPPSMPSRGAPPPPPPPFGGRNIPPPPPPLRGHGVPPPPPLL
GGNVPNGTPPPPPQGRGRGFVSRGGFASPSTPRRASLKPLHWIKVTRAVKGSLWAEAQMYDEAIKPPEFDMSELENL

FSAAMPTSGHGGSGERPGGRRTARGAKSDRVHLSAVLALDDTVLDIDQVENLIKFCPTKEEMDLLRGYNGERENLGK
CEQVFLEMMKVPRVESKLTVFAFKSQFISQVSDLRKSLNIVKYASQEVRESIKLRRIMQTILSLGNALNQGTARGSA
IGFRLDSLLKLIDTRARNNKMTLMHYLCKVLAEKLPELLDFNKDLAHLEAASKRQLKTLAEEMQAVSKGIEKVEQEL
TASENDGPVSDGFRMGFLVDAEADVKSLTHLYSEVGRSADALALFFGEDPARCPFEQVVSTLLNFVTMFRRAHEENV
KQAELERKKAEKDAEKERMKLSPLRIDAGEQLPSPLRRKLK

>PITA_000026470.RA protein AED:0.02 eAED:0.02 QI:0|1|0.75|1|1|1|4|607|1096
MEVAGLWHCRSINPKPQLRFATLLLLLLLLLLVFVGSSEAKASGRFDRATSGKKRVRSRIQIAKRVLHEPSFFPLPP
SPAPSPSTGFESPPEPGNRPRIPFTNEPFFPLSPSFSPPSPPTGISIPANVGSPHVGNKSDRSKKIVIGTLVSLSAI
VALCALLFVLHKRRDRDRWGSDKFADDTPLTSVGTPGNPRVFQEPGPVTKVATNGGSDVLYLGTLEPFGNRTSNGDG
DTHMDNAHTHAQNMDETHNPNPNTKFSKPNGEEIEDRFLRRSPEIEPLPPLAKRLRPDNSTDSSDEESFYSPRCLST
SSSRSHSGSPRSSSVCKRLFSPPSQQPAAVKSPQSTGSYPSSASSSTPSSPSPLDSPPPPITPTVLTPPIRGHFHSP
PPQVSRHVLPSTTPPPNSKPQPPAAPHPTHQSKIHPPPPPPPPPPPPPASLFQNPPSAKHESNQQLLHSIAREKTSL
SSPTSSPAKGNANPPAPSSLPTEKKSSLQSAEQNPNKLSAPSPPSKDKPDPQSPHSPPSSKSSRSPLPPLPSRPPPP
FLSSRQGKLSLSPSSSTKFPPPPPPPPNQNKPSPPDKPLPPPPSQPASRPQSALYSARTGKSLLSVTSTSGHSPSEN
STEQSNSSGTDGTPKPKLKPLHWDKVRASPDRAMVWDQLKSGSFQINEEMIETLFVYNTPTQVKGEGTRQSVHPPLG
QNQRILDSKKSQNIAILLRALNVTKEEVCDALMEGAFESLGTDLLETLAKMAPSKEEEIRLKEYNGDISKLGPAERF
LKGLLDIPFAFKRVDAMLYRANFKEEVMYVRKSFKTLEAASEELRSSRLFLKLLEAVLKTGNRMNVGTIRGDAQAFK
LDTLLKLVDVKGTDGKTTLLHFVVQEIIKAEGARVASTSEQPLTNSTSDNINGKFCPQGAEDKSQNDREEEYRKLGL
QVVAGLSTELSNVKKAAGMDSDGLSNSVSNLARGFGKLRDILQMIGGGKANESSTQGNFFKSMSSFLQEAEEDIARI
QSEENRAFSLVRETTEYFHGDAAKEEGRPLRFFVVVKDFLGVLDQVCREIGRTRTRMAQSSPRPPQVVVHPISMPLF
PKFQQRQPDSSDDESSSP

Picea abies

>MA_74039g0010 high_confidence
MDSFKGSSIREVMEADNMRIWGYYPSSVIKFLLPLLLSCLLYFNSLMVVDASYYESPPSHYKSPPPPPVYKYKSPP
PPPYHYKSPPPPPPVYKYKSPPPPYHYKSPSPPPPVYKYKSPPPPYHYKSPPPPPPVYKYKSPPPPLYKYKSPPPPI
SHVIPGTPYHYKSPPPPPYHYKSPPPPSLYKYKSPPPPPYHYKSPPPPPPLYKYKSPPPPPYHYKSPPRPPLYKYKS
PPPPPYHYKSPPPRPPLYHYKSPPPPPPLYKYKSPPPPPYHYKSPPPPLPMYKYKSPPPPYHYKSPPPPPPVYKYKS
PPPPPYHYKSPSPPPSVYKYKSPPPPYHHKSAPPPPPVYKYKSPPPPPKYHYKYPPPPHTVYKYKSPPPYHYKSPPP
PPPHLVYKFPPPPYHYKSPPPPPHHVYKYKSPPPPYHYKSLPPPPPHPVYKYKSPPPPPYHYKSPPPPPPHPVYKYK
SPPPPPYHYKSPPPPPHVYKYKSPPPLVYKYKSPPPPLY

>MA_108099g0010 high_confidence
MGILARMSFVSKLLVVLIAGCICSANPSAASYYKSPPPKPYSYKSPPPPSHVPSPVHYKPPPAPTPVPKSPVVEKPP
PVQSKPPPKPVPVSPPYKYKSPPPPKPTPAPYAKPPPVQKP

>MA_5375790g0010 high_confidence
MAFLLALLCLIASSTAVFADYYSPPPTPYSYKSPPPPPYSYKSPPPYNYNSPPTPSYYYKSPPPPPPPSYKSPPPYY
YKSPPPPPYYYNSPPPYYY

>MA_618974g0010 high_confidence
MAFLLALLCLIASSTAVFADYYSPPPTPYSYKSPPPPPYSYKSPPPYNYNSPPTPSYYYKSPPPPPPPSYKSPPPYY
YKSPPPPPYYYNSPPPYYYKS

>MA_7447685g0010 high_confidence
MVVLAGMSFICKLLLVLFAICICAAIPSSASYYKSPPPKPYPYKSPPPPSHVPSPVHYKPPPAPTPVPKPPVVEKPP
PVHNKPPPKAAPVSPPYKYKSPPPPKPTPAPYAKPPPVHKPLPPPSPPKSWSTPLPPHHNPSPVTKPP

>MA_7862055g0010 high_confidence
SPPPYNYKPPYYSTPSPPPYDYKPPYYYTTPSPPPYNYKPPYYSTPSPPPYDYKPPYYYTTPSPPPYYYKPPYYYST
PSPPPYYYKPPYYYTTPSPPPYSYKSPPY

>MA_100985g0010 high_confidence
MLHLGIVGWVSWHLGLLLLLVLGSQSSVSAAHGGRGLLSDSEIIRRQLLALERDNDLEFLGAKVKVDPSLSFPNPRL
MNAYIALQAWKAAIFSDPSNVTGSWVGANVCNYNGVFCAPALNDSGINVVAGIDLNHADIAGYLPEELGLLTDLALF
HINTNRFCGIVPRSFRKLRLLFELDISNNRFVGAFPNVVLSLPSLKYLDIRFNEFEGGLPSQLFDKDLDAIFVNNNR
FHFEIPSNLGNSPVSVIVFANNNLRGCLPSSIGKMAGTLNEIIVLNNNLTACLPPEIGKLKNLTVFDASFNKFVGSL
PNSIGGMVSLEQLDVADNMFSGSIPRTICALPSLMNFTYSFNFFTGEAPNCLALPNRGAVFNDRANCIPGRPAQRSF
RQCASFLSRHVSCSSFKCNPVVVSPRPALPPPTSPSPAAPFKPSPEQRPPSPPLPPQPLPSPTPPSPIHPSPPAVPV
SPAPSPQEGVSPVHVFPPVTSPTLYYSAPPLPSSSPPPVHYTPPPPPYSSYPQPGKYTAPPPPVYDTPPLPSPEPVH
YNAPPPPSPPPPPVHYTAPPPPSPSPEPVHYTAPPPPQPVSSPPPCADEMPPTPPAPSSLPLPSPTNPLPPIFDNTP
LPPTLGFTYKSPPPPSIY

>MA_7188g0010 high_confidence
MLVAVVMFTQEKMFIKIGLISMLVLVVGVLHVSAIATAGNTLGEMAEGRRVVESSSSGIGWGGGGGGLGFGGGGGG
GVGFGGGFGGGGHGGGGGFGFGGGIGGGFGGIDRKSGGGFGGDVCGSRLYNAYVALQAWKTAISDDPLGITKTWIG
RDVCNYTGVFCAPALDRPCDKVVAGIDLNHGFLKGFLVEELSLLTDIALFHLNTNHFSGSIPLGFKDLKLLAELDVS
NNQLSGVFPSVVLSLQSLIYLDLRFNYFTGAIPAELFNKKHLDAIFVNNNYFEDKIPDNMGNSPVSVVSFANNKLTG
SIPSSVGNMAQSLNEVLFLNNALSGCLPEETGMLDQLNVLDVSFNRLGGQVPLSLGCLGNIEQLILAHNEFCGSFPD
VICSIRSLQNLSVSYNYFTGEGASCPSLPTRGVPFDDSHNCIPGRPLQRSQQDCAAFRTQPKTCTEMTFSHVQCGLP
PPIISPPPPPIPFRYKSPPPPPLQVPAIRYASPPPPAPLAVPAFRYASPPPPTNLVPPPTQNCQPPLPIH

>MA_10432176g0020 high_confidence
MDIRRVNGGFSGLLLLFSLLLFSASSDTQEIISSAAEDNSRDGGSTDLYWLRGFKGTQFGIQKRRIDQIEQLETECE
LTQLEGKDVDRGDLYYFSDKGRCVNGVGQIQDTDSRFQHTLAKTISNLLPKQNNFVLVSLHKHSAISSSINPAYDNH
GEFKIKAVSGEDENSNMLWPLERLRVLLGFHSDERLLSQSPPPEISAMHASAPSEPPTTAVSPSPTAIHPDKYDGPS
PLPSLITVFPPDQSSSILSPTPFSNSPPLGQLGKKSGNSKAVVTAVVITAISTLALAGVFFYLYQKCATGRHGSNEQ
KDDRPLLFPSMDNGSVNHFYGESNVFNWGTLENSSGNSHKVASSETGKAGGMQLSSSNQGNHLYISPSRRDISSTQP

SQEVLQIPSTFMHPSSADSPVPLHASQNTVSRPINPALLSHPYSQSSHAQIPPSCESPSLLLPPNTPAVDSPVKSTT
PAPPPLPINKSSPPPPPPPMKKSPPPPPPTYKSPSSVPVAPTNGKSNLPMPPPTPAKSSKLPPPLPPRPKAGQPMSS
VAQSAFATGVSAGHLNAVGEDGNLRPKLKPFHWDKVQANPDHSMVWDKIRAGSFQFNEEMIETLFGYNSANTSSGVA
KQSVIPPSQNLHLLDPKKSQNLAILLRALNVTKEEVCDALFEG

>MA_10436302g0010 high_confidence
MALFKKFFFKKPPDGLLEISERLYVFDSCFTTDVLEEEDYKIYIKNIVIQLRDQFPDASFLVFNFREGERKNEVTDI
LSQYDMMTVMEYPRQFEGCPLLPMEMVHHFLRSGESWLTLEGQHNVVLMHCERGGWAVLAFMVAALLIYRKQNTCEQ
KTLEMIHKQAPKELLHLLSPLSPIPSQIRYLQYISRRITGSEWPPPERALTLDCVILRXXXXWPPPERALTLDCVIL
RIIPNFDGEGGCRPIFRIFGQDPFSTEDRTPKMLFSTPKKSKNVRHYRQADCDVAKIDIQCNIQGDVVLECINVDSD
LAREEMMFRIMFNTSFVRSNILMLNRDDIDILWNAKDCFPKEFRAEVLFSDMDASSSPLLNVATLGGEEKGGLPIEE
FAKVQEMFNNVNWGDEAAIHFIQKISKSNVLQEKSDILILDTANNWSPLPAKGQLSPFSTNGEDENDAKKLLSPRLS
SQLNSPSSMMLKPSLLTTNSHALPPPITQQSRFHSAPGVLGLPVTSQETAEYQSKHQNATVVSAVSATIPQSAEISG
FEHPSTCSAVASPQPNHAQAPPAPPPTLPPGIPQTPGRQGMPPPPPPPPPSLPFSGMGAPPSPPPPPPPPPGVKGAP
PPPPPPPPVGVPPPSPPPPPPPPPGVKGAPPPPPPPPVGVRGTPPPPPPPPVGVRGTPPPPPPPPGGKGAPPP
PPPGGKGTPPPPPPPPSGRGAPPPLGGRGAPPPPPSLGGREGQPAQLMLSGGKAPVNASTPPTLSIQGKGTTTASSP
GVKSLGSATRAGSTLATTPRRAALKPLHWVKVTRAMSGSLWADAQKYDEANRAPEFDMSELECLFSTALPTSDHSGS
GDKAGGRRASLGSKVDKVHLVDLRRAHTCEIMLTKVKMPLPDVMSAVLALDDSILDVDQVENLIKFCPVKEEMELLK
GYAGDKDNLGRCEQVWTSLILFNKWICQMDLPFSLVSS

Brachypodium distachyon

>Bradi3g10280.1|PACid:21831068
MRLLLVAVLPLVILIISFPISRAYDRGSSAGHDNSNVHKPNGVGPVKNPHCPKPGRGPNPHEKGEPGNNGWLPHRKE
CDGDDPSPTTPSSPPPPSPLYQANEPPPPPPSLSPPTIAPSSPSMTPPNSNPLATPPTYQSNGNPPPPQYSSPPTFA
PSTFSPPPSPPATPPPYRGNDQPPPPPPSFSPQPLPPSSSLPNTPPALSSPSEIAPYNSPPPPSSPLATPTYQDNDQ
PPPPPPSSSGPSPLWSSPPATPPTYQVNQPPPPTSSLLAQSAPPPPPPFSPHATPPTDQGNGQTPPSPPLPPPFSPP
ATPPTDQGNGQPPPSSTSPPPFSPPATPPIDQGYEQPPPSSPSPPPFSPTTTPPTDQHNGQPPPSSSPPFSPPATPP
TDQGNGQPPPSATPPINQGNGQPPPFSPPATPPTDQGNGQPPPSLPSPPPFSPPATPPTDQGNGQPPPSSPSPPPFS
PPATPPADQGNGQPPPSPPSPPPFSPPATPPTNQGNGQPPPSPPSPPPFSPPATPPTDQGNGQPPPSATPPINEGNG
QPPPSSPSPPPFSPPATPPTDQGNGQPPPSSPSPPPFSPPATPPSDQGNGQPPPSSPSPPSFSPPATPPTDQGNGQP
PPSSQSPPPFSPPATPPADQGSGQPPPSSPSPPPFRAPATPPTDQGNEQPPPTSPPPPTPLATTPTYQPPSQPMPPP
GPPTGTNGWVEVHNFRDTLYWQIARFAVLIFKLVNKKEYTLVDVLYVSMQPAGTGNNYFLEIKVADENKKVGKYHVL
VWGVPGSTTQTWKLLMFQFVGN

>Bradi2g11770.1|PACid:21808457
MAPFPPVLLLPLPLLLILSSLLLPNAARADTGPTVVLKDGTTCTLCASCDNPCNPTYYPSPPPPVVNPTPQCPPPPS
YSSAGGGGGGGGPVIYSSPPPSNGGSGTGGGYFYPPPTGGSSGGGSQGGGGGVYPTPPPPNPFLPYFPFYYYSPPP
KFVDKSSAAGAISSSPATLLLLALSGLLLWRD

>Bradi2g45060.1|PACid:21808745
MGRAMSPVLVLAVLAVMAATAAAADGDVKCADCPIVYPSPPPPALPPPPPYYYYSPPPPASYPGVSNCPPPPGGYIE
IGGSPPGQGRMYPQDPGFMPSSALSTHGSRALPFTVCALAILRLLL

>Bradi4g11290.1|PACid:21813278
MASSVSALALKVAAIAAALAMLAVPSLGRCPSLGPAPPPPMQASTPPPPPAYASPPPPAYASPPPRPLPTSPPPTPL
PPAAQPAPAPGPGPMISCNDCHIQCSSPCIGTISSKCSYYCDYK

>Bradi2g05080.1|PACid:21806530
MRGGALLLLLLVALAALCCGAAADFSGDGGGEGVMASAAAVEVDPSWRFPSRRIREAYVALQTWKQRAIFSDPRNL
TADWSGPEVCNYTGVFCASPPSDPSAGLAVAGIDLNHGDIAGYLPPELGLLSDLALLHLNSNRFCGVLPRALARLRL
LHELDLSNNRFVGPFPEVVLSMPALRFLDLRFNEFEGVVPSKLFDRPLDAIFLNHNRFRFNLPDNIGNSPVSVVVLA
HNTFGGCLPASVANMSGTLNEILLINNGLDSCLPPEIGRLRELTVLDVSHNRLAGPLPPEVAGLRKVEQLNVAHNLL
SGPIPQAVCALPRLKNFTFAYNFFTGKPPSCARVVPRDGDRRNCLPNRPAQRPPQQCSAFYARPPVNCAAFQCKPFV
PPVPPPPPPSPSPPPPSPPPPSPPLPSPPPPSPSPPPPSPSPPPPSPPPPSPPPSPPPPSPPPPSPPPPAPVHHSPP
PPAPVHHHSPPPPAPVHHHSPPPPAPVHHHSPPPPAPHPHPHPTCPPPPPCACPTPALPPPPPYYPGPLPPVSRAEY
GSPPPPLRQ

>Bradi2g12760.1|PACid:21807326
MGTLPPFIAVLLLLLSSASGLTADEAAARRRQLLLGPAPEPHVDRVHIDINIQIKISNPRLQSAHTALHALKRALYS
DPGNFTGNWDGPDVCAYNGVFCVPSPDDPSASVVATVDMNGADVAGYLPKEIGLMSDLAVLHLNSNRFCGVIPEEIK
NMSQLYELDASNNRFVGPFPDVVLRVPKLSYLDIRFNDFDGPIPPELFLRPYDAILLNNNRFTAGIPDTIGKSKASV
IVLANNELGGCIPRTIGDAAATLDEFIFVNNSLTGCLPVETGMLHTTTVVDVSGNALTGPIPPTLSGLAKVEQLDLS
GNRFTGELFKAVCELPALANLSVSYNFLAKEDGVCSAAGNPPEDKYSFEDQGNCMGEARPFQRSAAECGPVVSSPVD
CTKLQRCGWPAPPPPPVFPPPPPAPVPSPPPPPPVFSPPPPAPVPSPPPPPPVFSPPPPAPVPSPPPPPPVFSPPPP
ALVPSPPPPPVFPPPPPYHKPVFLPPVRGTKYQSPPPPQFAGY

>Bradi2g42477.1|PACid:21807550
MTKPLLFLSLLLLAAAAADAAAGQQRRTLLAGDDNAYAFPNPRLRDAYVALQSWKRAILSDPRNVTGSWSGPDVCAY
YGVFCAPSESDHYLTVVAGVDLNHADLAGHLPEELGLLSDLSVFHVNSNRFCGVVPRSFHNLGLLHELDLSNNRFVG
AFPDVVLRMPSLKYLDLRYNEFEGAVPPELFDRPLDAIFINSNRFRFQIPDNVGNSPASVLVLANNDFGGCLPASVA
NMSSTLNEIILMNTGLKSCVPPELGMLAELTVLDLSHNQLMGSIPAELAKLRNIEQLDLGYNRLTGDVPEGICRLPR
LQNFTYSYNYITGEPQTCLHVKALDDRRNCIPYRPDQRSPDQCHFASNYQHVNCDAFRCKKFVLPSPPPPPPSPPPP
TPSPPPPSPPPPSPPPPTPSPPPPPSPPPPTPSPPPPSPSPPPPSPYYEVSPEERYLSPPPPAYVEPTTPPHYDIPS
PPYYEVSPEDRYNSPPPPATGVPKYDYASPPPPAYSEVSPEERHPSPPTPTTMWKLPAYDYASPPPPAAGQP

>Bradi4g11130.1|PACid:21810435
MLLLLLFAAGGMSVARALPLSSAEASYIARRQLLAMKEAEASASVNVGGGAVGEGAGLSVGGEASDDLRGVGGEGGE
QGAPASGPAAGGGEVASGPAEGGGDVAGGPAAGGEVGAEVASGPAVGGGEVGGVASGPAVGGEEVGAPVSGEVGSDG
GIGGGGAADPDAWRRAFYSDLKGYTNNWVGTDVCAYNGVICTNALDEPNTTVVAGIDLNGADIAGYLPPELGLLTDL
AFFHINTNRFCGIIPRTMSRLTILHEFDVSNNRFVGVFPYVCLDMVSLKYLDIRFNDFEGDLPPKLFNKDLDAVFVN
SNRFVGPIPETLGNSTASVVVFANNKLVGCIPKSIGRMVKTLDEIIFLNNKLDGCLPLEIGLLKNTTVVDVSGNGFV
GTLPKEIANIDKLEQLDVSRNMFSGILHESICQLPALVNFSFAFNFFNSESAPCMPSDKAEVNLDDRDNCLGALRPA
QKTTLQCAPVLARPVDCSKHACAGYPTSAHPPIVGPYMPGGSPKLAPVVPGPIISPYMPENPTPPPVPLPKTSPPAG
HAAPPPPSADWLPGTPERERSPPPEVQPPPVVLSPPPAPVKTYSPIFASPPPAPVTYPPPPVKLPPPPAPQKPPPQ
EKTPPTPTPKISLPPPPAEKKTLPPPAPVTSPPPAEKKSLPPPAPVEKKPLPPPAPVSSPPPEEKKTLPPPVEEKKT
LPPPVEEKKTLPPPVEEKKTPPPPVEEKKTRPPPVEEKKTRPPPVEEKKTRPPPVEEKKTLPPPVEEKKTLPPPVEE
KKTLPPPVEEKKTLPPPGPVQVKPPPTPAPVSLPRSARHLLR

>Bradi3g03370.1|PACid:21832433
MTPLAAPPRRLLLQPFLLLLLLVATAAQEGDSSSPSTARFAAPSSWPFPSPQLRAAYAALQQWKQTAIFSDPSNFTA
NWLGPNVCAYNGVYCAASPTTPGYPLVVAGIDLNHADIAGYIPSSLPLGVPDLALLHLNSNRFCGIIPSTFRALTLL
HELDLSNNRFVGAFPDPVLALPSLKYLDLRFNDFEGAIPGKLFSVPLDAIFLNSNRLRHGIPGNLGDSPASVINLAH
NDLGGCIPPSIGKMAGTLEQIVLIDDALQGCVPVEVGLLKKVTVFDVSGNKLQGGIPAAVGGMAAVEQLDFAGNLFQ
GAVPIGVCGLASLKNFTYADNFITGRPGCAQAMADGAWNCIPGAPAQRPAAQCAAVMARGVFDCSKAQCQSGAGGGG
GPSSGPVLPPSGGTTSPSYPAPPKGGSATPSYPTPPSSASTPSYHAPPQGPTTTPSSPPKGSAPPSSSSTPSSPPKG
SSTPPSPPKSSTTPSYPTPPSSSSTPSSPPTPSYPTPPSSSSTPSYHSPPTPAGGAPPSPTSSNKPGSHSAAPPGS
SYGPTPSTPPSSSSSGSTPPGSYGPNPSTSTPPSSSSSSSPPPSSGGQHQPSPPTGYPGYALPPHAPGTPGAAGSPPS
HTHPSTPSSPEHCAPPSKGGATGRHPPSSGGNLPFPPVYGVAYGSPPPPVKPYN

>Bradi2g44830.1|PACid:21805961
MPRVRLLESAERLASAPAGGPPPPPPKSSTSPPPPPPSSPPPPIPSPPPPSPSGQNSSSATFEQFLVKDPPKTSGSP
PPGKSPTPSPPPPVSPPPSPPQTSRSPSPSSPPPSSPPQRSSPPPASPTPPEASTSASPPPSQPEIPAPATPTPPSP
SGAASASPGTRFSLPATTRGQTPPAPGTPSPPSTSQLLPSGVTVIMPASGPPAGTWQSPPHTKPTAASPPPPPLAPP
SGGSAITVPNGVLVGVAVGGFLMALASLFMVACVTNRENNDKRRPPMPMRKRTVVVPARGVASPEYVSSGPAAPSPS
ETGSYDFSGSKSWFTYDELAGITGGFSAENVIGEGGFGKVYMGALGDGRRVAVKQLKVGGGQGEKEFRAEVEIISRI
HHRHLVTLVGYCVTENHRLLVYEFVCNNTLEHHLHGKGRPVMDWPKRMKIAIGSARGLTYLHQDCHPRIIHRDIKSA
NILMDDAFEAKVADFGLAKLTNDSMTHVSTRVMGTFGYMAPEYASSGKLTDRSDVFSFGVVLLELITGRKPVDSSQP
LGEESLVEWARPVLVDALETDDFRELADPALECRYSKTEMRRMVESAAACIRHSGTKRPKMVQVWRSLDVDSSSTDL
TNGVKLGHSTAYESGQYSEDIELFRRMAFGNDLNTAEYGFSEEDEQQRTAAGR

>Bradi2g49240.1|PACid:21807984
MGGYGYGGEADPFLPPQPGSSAYVAPPGDHGQPQMQPGPRPPGCPYSSNASAPPASTSYHSMPPAASPPPVSSPPPA
SPPPEPLPSPPPALPPSLPPPALSPPPPDAPPPSTPPPSPSPSPPSIEVQAPPPPTADDQPRVQPRVYPSPPPPSLP
PPPPATVSPPSPPPSPTPSPSPAPAAQAPSPAPVAPYRVPPPPRIVSPPPPHHHNKPHYAPPRSPARHSNSTHAPSG
VGKNIEISRETATTIVAIAGLAMLSFIGATIWFVKKKRRRTEPPSALPTQQQAPPPPPPNYIPSSAGSSLASDGFYL
RSPGYPFMRSSTGSHGFPYSPADSGIGYSRMLFTPENLSAISNDFAEENLLGEGGFGCVYKGILPDGRPVAIKKLKI
GNGQGEREFRAEVDTISRVHHRHLVSLVGYCTAEGQRMLVYDFVPNNTLYYHLHVNEVALDWQTRVKIAAGAARGIA
YLHEDCHPRIIHRDIKSSNILLDNNFEAQVSDFGLARLAADSNTHVSTRVMGTFGYLAPEYALSGKLTAKSDLYSFG
VVLLELITGRKPVDSSQPLGDESLVEWARPFLMQAIEHRDFGDLPDPRMEQKFDENEMYHMIGAAAACIRHSAVMRP
RMGQVVRALDSLADSNLNNGLQPGRSEVFLEPRTEEIRLFQLREFGSRECSDELSQASWRSRRDL

>Bradi2g00900.1|PACid:21808541
MASSPSAAVAPTGTPSPAAATTPASPAPVKLPNATPADPPASPPAAAAVPPPQPTSPPPPTPTPPPATVPPPPVVVA
SPPPTPSAALPPPSPPTAVPPPPAGAAPPKPSPLRPPAAASPPPSQSNLPPPNPPDSSTTPPVAQSPPPPHRRSPRP
PTTEPQAPPPSASVITPPTSPSPASGDPIMPNSTSTSPPTPSAPGSAPSDTADKPTTQPTFGISNPAPNKPWSPSVQ
DSSPSPSGDGVSYGAKVGIGVVVAILVLSLVGAAFWYKKKRRRMTGYHAGFVMPSPSPSSSPQVLLGHSEKTKTNHT
AGSHDFKDAMSEYSMGNCRFFTYEELHKITNGFSAQNLLGEGGFGSVYKGCLAEGRLVAIKKLKDGSGQGEREFQAE
VEIISRVHHRHLVSLVGYCISGDQRLLVYDFVPNDTLDYHLHGRGVPVLEWSARVKISAGSARGIAYLHEDCHPRII
HRDIKSSNILVDNNFEAQVADFGLARLAMDFATHVTTRVMGTFGYMAPEYASSGKLTEKSDVFSFGVVLLELITGRK
PVDASNPLGDESLVEWARPLLTEALGTGNVGELLDPRLDNNFNEVEMFRMIEAAAACIRHSASRRPRMSQVVRALDN
LADVDLTNGVQPGKSEMFNAPDTAEIRLFQRMAFGSQDFTTDFTQSSWNSHQSRDVDASALRHSQS

>Bradi1g07010.1|PACid:21815588
MSSNSSAPPPSAAPPPSPDLSPPPSPPTANSSSPAPTNSDGPSPPPAPSNSSSHGAPSLPAGNPSPPSESSRGSSRP
SSSQASTRRVGSNTTAEIIFAVAGAAALLALLVTACVCCSRRTAPRRRRRKPQNPMHLYADSSAYKGNSSYYTSEPQ
PQWQSDANAGAPSMSAPGLGSPGCSSWHVPPPDTSSGTHGQPMPGLGKGKGTFTYEELALATGDFSAANLLGQGGFG
HVHKGVLPCGRAVAVKQLKSGSGQGEREFRAEVDIIGRVHHRHLVSLVGHCVAGDRRMLVYEFVPNKTLEFHLHGKG
LPAMAWATRLRIALGAAKGLAYLHEDCHPRIIHRDIKSANILLDDNFEAMVADFGLAKLTSGDSDSETHVSTRVMGT
FGYLAPEYASSGKLTEKSDVFSYGVTLLELVTGRRPVDARRPLLEEDDGLVDWARPAMARALASGDYGGVSDPRLEG
GYEPAEMARVVACAAACVRQSAKKRPKMGQVVRALEGDMSLEDLNEGVRPGRSGVFDAAAESSGYRKEAPAATGS

>Bradi3g31967.1|PACid:21833106
MASPPPSRKLSPPASRPRPVAKSPPRRLSHPPPHPRPRPPPQRQHLHGQQHKQQGRATTSSAWSVGSMSARLSQRTP
VLGLRAWVLVAAGGAAVALAVLLLAVCLCRCRRRRRRCPRVAPSLHHTSARSLKHRARQAMAEEHAAADVEEPPVRW
HPASPPPPFQPPFQPPIEVIKAEQEAPLIAVESARTSGETASSSGGGSAREWSTDSGGCGGDDAEPEAARRGWGRRY
TRRELEEATDGLAAYNVLGEGGYGVVYKGVLRDSTAVAIKNLHNNRGQAEKDFRVEVATIGRVRHKNLVSLLGYCSE
GACRMLVYEYMENSNLDKWLHHDDSEVSQLNWDTRMHILLGTAKGLAYLHEGLEPKIVHRDVKSSNILLDGQWNARV
SDFGLAKLLCSERSYVTTRVMGTFGYVAPEYARTGMLNERSDVYSFGVLVMEMITGRTPIDYTRPTAEVNLVEWLKR
MVAERRVEEVVDPKLPEPWPPSKVLKRAVLAALRCVDPDGGQRPTMGHVVHMLEDDIRFRDELQLARDLSPHASSSG
SYEREE

>Bradi4g03720.1|PACid:21812481
MALFRRLFYRKPPDRLLEIADRVYVFDCCFSTETMDQFKYKNYLDGIVLQLREQFADSPLMVLNFRDEGKSLVSGLF
SLYRITVKDYPCQYLGCPLLPLDIIIHFLRLSERWLMLEGKQNILLMHCERGGWPVLAFMLAGLLLYRKQYNGEQRT
LDMVYKQAPKELLQMLTTLNPQSSHIRYLQYICRMDYELGTQPIPFTMDCVILRGVPNFDGVGGCRPIVRVYGQDIL
TADKGRNVLATPFKAKKHVRRYRQADNIPVKLNVGSCVQGDVVLECLHVDDGLENERLMFRVMFNTFFIQSHILQLN
FEDIDVSWDADHRFAKNFKAEVLFSEFDAESDVSTEIASDDDDDDCGDEIEVGSTDEFFEAEEIFSNPDSHDVHKDA
DTLSVASTDSTLSAEVRKVSPFSNLELMNDIDGSQESKTDDMSLSFEILNDEKACTSVDTNIMHEDITRVVNSSLET
TTYGSRDSSNSSSATYRDKDDGCSVENSNSEKDSTVDPKQDLSHTDNVLVKEVIILETNSPKDIQMIKEVIISEVTT
PKQLLEGDTMEIESGDAVHNSESIASAEVDNIEGLDIALKQGEGDSPGEGCVVYDNGTKQEDNSNTEQASISGTNVP
VIEHTDENNRVDLPSLEKSHPQSTSATLVLSSSEQKIKQSDASNSNETREQTVGMEASISNSTGQPSNTSSTVNILP
EGCSLAANGARTCAGTSIVTADSSRLVLKKKGFLPLSTYSIFAPLSPRRNPLRSASTDLSFLSPLQTESNQNSVPST
STEHKWHPTPSSSTSLRSSKVPSLVNPPLRPIKTVSSLPSSSLETYLEMSTSYSPASHANHQQHINPHPPWIPPPRQ
LHPAKTQGKDLHPFGLSFPAFKRYGIPPPPPPPHTHSTQKNSSSLIADHEQRRVEGSCSSSPFIQTVLNLGFSSPIL
PSKSSIDMPEFLLGASSFLDAELTNRRNIPSGMDVPTTSEDPKYFFSVPHSLSPKISHHNTPQPPPLPPLLPPLPLP
ITCNETVPLVCSKSPSDPSYKEPPMPPERQPPSPPPPPGTNDPSISNFVNEQLDPMETCKEFLRSEMSTESSPSTVH
RKEHGGIPTPPHPPICRWPRGVSSVQSLTPRPSTPPPPPPFPPHLSSLQSPQHLPSLSPSPSTRSLLVPSRTNVPSP
HLPQPPPIHINQPPPPPPMLPHRPSLPRKHATPAPPPLPREHQVIFPLPPISPTRHVLPSPPPPPPFGRHQAPLPPA
LPSFVRHIAPPPLLYPHFSEDLSTPPLTTDARIPLPPPPPPKDTAGNFAPLPTEGSPRPALPECQVEAPIPLPPLEG
LERIPPALSAEGDKTILLPSLVGGLEKTPLQDGFGGGAPPPPPPPPLGAYGGAPLPPPPPGGYVGAPTPPPPPLGVD
GVPPPSVLIPGGYKRAPPPPLLPPPLPEGYEGAPPPPLPPGAYGGAAVTRLPPPGGYGGGAPPPPPPPGEIGAPPP
PPPPGGIGAPPPPPPPGGIGAPPPPPIGGLGGTPSPPPPAGFRGGGPSPPPPGGYGGTPPPPPQRGHGGVGGPPPP
PGAPAPPMPPGVPRVPPPPPGVPGGPPPPPGVPGGPPPPPGGRGGPPPPGGRGPGLARASTAVRRSSLKPLHWVKVT
RAMQGSLWAELQKQADADSNSEFDVNELASLFTIAPKTKGGSKSEGAGKSLGSKTDKIHLIDIRRANNTEIMLTKIK
MPLSEMMSAALALDDSVLDSDMVENLIKFCPTKEEMELLKNYTGDKEGLGKCEQFFLELMKVPRVESKFRIFSFKIQ
FQSQIRDVRKNLQTVASACEELRGSEKLKVIMKNILLIGNTLNEGTPRGQAVGFRLDSILKLVETRATSSRTTLMHF
LCKSLAGKSPELLDFHEDLGSLEAASKLFKQLQLKALAEEQQAVVKGLEKVEQELTASESDGPVSEVFRKTLKEFLD
ASGADVRSLSALYVEVGRSADALSLYFGEDPAKYPFEQVASTLLTFVGLFRKAHEENLKQIEAERKKALKEAEKEAS
QDRTPVKSKDGGADRSPRSPFK

>Bradi1g22980.1|PACid:21817600
MGTRGGFVIAVVLRELLSSAAAAADDGSLRRRSLHQPFFPIEWSPPPPASGSVVLPTPPPASAAAASTTSPARSAPS
FTNTIAIALTAGLVALAVVSYSCVLLWRRVSDGGGEDDGRATTATSTKPVAVGAVPARVPSDVGSSSRRHRSPPPSS
TASDAIYLDPLTSLMEVHQHRASPDLRPLQLPKQPSLSPDLRPLPPLKRPVSHPPPPPASTPPMTATTEYSSEEDDQ
ATYYTARKTAMSSFSRSTSQRSTLEQTAPPPAPAPVPPPPPPQVNHLRPPRPPPPPPPLPRQRLLRPLPSESPPPAAL
ANLALTSPPEPSIQNRGIENSDVLGGSTRPPNLKPLHWDKLRAISGRTTVWDQVKNSDSFRVDEAAMESLFPRSGVP
AAGNSDQAVARGAPGKQQSLLLDPKRLQNVAIMLKALNVTADEVIGALMHGNLEEKPEFYETLAKMAPTKEEELKLK
HYSGDLSKIDPAERFLKDVLDVPFAFKRVDAMLYRTNFDTEVNYLRKSFGTLEAACSDLRSSNLFLKLLDAVLKTGN
RMNDGTNRGEAKAFKLDTLLKLADIKSTDGKTTLLHFVVQEIIRSEGFDSDQTASNPGSASKERFKKDGLKVLAGLS

SELSNVKKAATLEMDTLSGNLSRLDTDLEKVKLVLQLKESCADQGSSVKFFEAMDVFLRRALTEIGSIKIAERSALQ
RVRETTQYFHGDATVEEPHPLRVFMVVCEFLLILDRVCRDVGRTPERVMMGSGKSFRVTAGTSLPPRRQEQQRQLSS
SDEDSSSS

>Bradi3g59780.1|PACid:21827983
MAPPPPLLGIPLLLLLAAVVCPLAAAQSPQRNIQTRFPSTSTPAFDAAPPPPIVSPSPPPTTAPFLPPQSSSSSSSS
SSTKRSDIAVAVVSTALSSFAVSGLAFFLFLRHGKKKEKDFAHAAGGGGRGNGYHQNGGFAAGKQPERAPKQPLRGG
GGGIGMVDENGLDAIYWREFEKEGDSGGGRGRKLPAGSGWRPPRQPSERRQVSSSLPSPSPPRPRKGKVDQEPLIPR
GSLDSSSAVFDDAPSASSSSPFSVAAAEAYSRPAPAIAVSSVPRPSPPPSASSSSLAPPPGRASPPPPPPIASVSPP
RAAPPPPPAPRGPPPPPPPKGPPPPPAPKGPPPPPPPGGKKGGPPPPPAWANSSRPPAAPGVPTGAEDQQAKLKPLH
WDKVNVAATDHSMVWDKITGGSFNLDEGTIEALFGTAAANRKPKSADSKESSSGLGRSNSEDQIFLLEPRKSHNISI
ILRSLTVGRDEIIDALRDGHTELSTEVLEKLSRINISKEEESTILKFSGNPGRLAPAEAFLLRLLLDVPNPFARVNA
LLFKINYSSEVAQLKQSLQTLEMASQELRTKGLFFKLLEAVLKAGNRMNAGTARGNAQAFNLTALRKLSDVKSADGG
TTLLHFVIEEVVRAEGKRLAINKNYSIRRSGSLAKTSTDGGISAAGTTIQGPSREERQNEYMNLGLPIVGGLSSEFG
NVKKAATVDYDVVVNESAILSRRLTEIKKLLETCGDDGFARGLRGFVKAAEQELKAITGEQEKVLDLVQRTTEYYHA
GATKDRNAHPLQLFIIVRDFLGMVDQACVDIKRRLQQKKPPPSSSQPTTAAAAAAAKEAADGAAASAPLPAPAPAQK
PPEEVDSKRKRVMPRFPNLPAHFMKDGADSDSSSDEE

>Bradi2g57740.1|PACid:21806590
MKMSVGGQPKGHLWLVLAVVIAMFAMHGEAARSLSPPSLSLSPAYAPVIKVVGKVYCYRCFNEAHPEESHGKEHLQG
AMIKVTCQANDQALVRFGYTESNGKYSVGITDLPLSSAYGADSCKVELHAAPGGSDCNVPMELNLSGVNVYSKSNKE
VVFQANQVMAFGSKKTFAGCSKPHVLPPVHPYNSSPLPYQYPSPPSSYKFPPLPYQYSPPPSHQYTSPPMNFYSSPP
PHQLSTPPNNYQAPPTSSNYPSPPQGYKYPTPTHKYLPPPYYYNSPPPQHQYSSPPNNHVAPLVPYQKSPPPTYNYK
SPLLPPSPAPVYHYNAPPPYHYSPPPLNYGSSPPPYQYSPLLPPRHLQPNLPNAKSPPASTSPQPLRNSYQSPPPPN
KLS

>Bradi4g11260.1|PACid:21810624
MALSVATLTLKVAALIAALAMLAVPSLGRCPSLGPSPPPPSSPPPPSYASPSPPPAYASPPPPPLATPPPMQASSPP
PPPLSTPPPPPMQASPSPLPPTYASPPPPVPTPSPPPLALPPSPAPAPGPGPMISCDDCFTQCSTPCRATIHSNCSQ
YCAVDSSCDYCKAEIAKNCKAGGTCADGSCDCDKLNTAGSCASACGTRYCDACIYALESHCRDDCRSECSAPRCVPW
KPS

>Bradi4g07580.1|PACid:21811380
MAKPMSFLVLQVLLVPVLLFLLPGAECQSSHGGGANLTVTGTVFCDACSRSSFSNHSYFLPGVRVRIDCTFGANSPS
KEEIKITAEKTTNSYGAYQLDIPAIDGVGCTTAAAAVSFCRAAVLDNPSALCNVPALTTTVGHISFSTQAQEPNSNA
NVCLYNLNSLYYRPRPNKGPGLCDDDGGGPLQPAALNDSLFYCPRWPWPPIPFCTPRPWFPPIPFFTPPPPAFPFPL
PPFPPPFPFFTPPSPPPPAFPFNLPPWPWTPPAPAPAPPSFPFPRLPPIFSAPSPPPPPPPPAFPFPNLPPFPHLPPF
AFPPLPSFYSPPPPPPPPPPPPPPPPSFHWPFPPLPFFPPTSPSAPSPPPLKRSRKDPTTWSSSKTTPAP

>Bradi4g11250.1|PACid:21812666
MASGAATLALKVAAIAAALAMLVVPSLGRCPSLGPAPPPPMQASPPPPSKAYASPPPPPLPTPPPPPVAAQPAPAPG
PGPMISCNDCFTQCSTACYATIRANCSSPCNSKEYSCGNNCKTDVIKSCKAGSTCADGRCDCDNDVDTYSSGYCDRE
CAYWYWFCESCGRGLSKGCGENCARQCHADGCVEP

>Bradi4g11240.1|PACid:21814077
MASVVATLALKFAALSAALAMLLAPSLGRCPSLGPAPPPPSHEASPPPAAAPASPPPPPPPAQPVPAPGPGAMTSCN
DCITQCSTPCYASIPWDCSRSCQVCDQCRAEAIKSCKASSSNCADGSCDCDNMNTSASCGNDCKSWSCESCKNGRTR
GCGIYSAHGCYAHGCVEP

>Bradi3g12902.1|PACid:21830894
MAPAAKRGAKGQKKWTRDPQLGDLVLAKIKGFPAWPAKVSRPEDWDHVPTPRKFFVVFFGTKEIAFVALPDLVPFTE
EVKNDLVDRAREKRFPKRHMKSMDEAVVQICKAYDELPKSSGPANGLLPDETLDLIEKATEHLVKLPDDGETPKLGQ
MEGYSSVGNLNTVVHGSGTEGDMKGGHDRKDSFPTASKRKKPAGKDSDHPKKKKSITSKPAINMRLEQERSPTSLRP
EGEAEDQQDGKECHPTEALILDPTVDVICALEVPKKYNSEKQLKNSERNELKRVDVAGMSGMTSPKALTDTVPSNSA
DKESKGFKKSKMVMKQSLTNKSERRDYDKIMLDKPNKRLSGKSPDAFSSDKKPLPGSVQCKPEGSMDMRLAKKPKLM
DKANETVKTGAKNEHKLPVDNEKDKAVKSEKSTSMGAGNSTVPEIVTGDDRKRKSGIVLSPLPRSHSDGLEHAPGSA

TKLSVLGAAKKGSSMREDGLRGRPLAKPRRRACRFDEDEEEEQRTPPHKTVAKTISTHVAPTEKTGIRRIPSSQVGN
VSARMSGLARKENPRSVGMSPVKHEPLYSPSQEKVHIRPQMMGRKSDTSSLDSIAALGNKINLADRKSSGQVRMPAS
SEVKKMPASSEVKKPQVSSSKVLHQTSGNSHSQSQAALEKDVLLSKPENAKAKPKPSTQVATSVENRLSASLSGERI
GKLDHSNEDRSNFDDRADFAEPNPDSVKSMKHLIAAAQARRNLLASAQGNSDGTSADNAVLASTPYGLRGLSPSPVF
NTRSASGIVISESDQFQDSICEPGQRVDMEKPAEIDREHGKSPKPKQSSTSGLLSGGTDAAIARDALEGMIETLSRT
KDSIGRATRHAIECAKRGIADEIVELLIRKLESEANLHRRVDLLFLLDSITQCSHAQKGVAGSSYVPIVQAALPRIL
SAAAPPLAGARENRRQCLKVLRLWLQRKIMPEDVLRRYMVDIEVPNDDTSTGVLLRRPSRAERSVDDPIREMEDMLV
DEYGSNTTFEFSGILSATVFETHEDLPRVNGSSPLISLQVERSGMPENKEIIAPDSVEEHMMVLESVTSDAVMQDAF
VLPSNMQQTDGAVFIEHDLRQEVCSEEALTNHHELPPLPEGPPPLPFDSPPPPPLPEGPPPLPLESPPPPPPLPPSP
PPATPPPPPLPLSPSSPPPPPPLPLPSGLPPHPAPPHPHPCIPPPVPPSPSSLGYQPAVSEYFRTPNGNQLTQVVGN
SSIQGIGSTANFIPGGPVNAQASVNFVPSMPPDYGSNNGFMAQQASNGNYQFRPGVPFHQGTFSAFPSTQTPPVHPH
NHHTHMNPMGQQPVPPSCNSYVVQSFPNSQSQYTSEEQWRMTSGNFSPDDHHNAWLPGGRPLSCSDGSFMQDGYPRS
NIERSSMDPMSHQYPVLNHLPSGAPLPGHVVPQMLPARPDIHVLNCWRPSG

Zea mays

>GRMZM2G076029_T01|PACid:20825479
MARRPRPCAHLTATLLLLAVAAAVARAADGDEKCGSPCGNPCGEPCVYASPPPPPVYYPPPPPPVYSPPPAPEYYPP
ATPTPTTPYCPPPPSGGGYEPSPGYTPYTPGYNPTPPSGSGWYTPSYNSPPGTLYPQDPGFRPNAAPARVAAPWRAA
LFLAAAASAALARACLDL

>GRMZM2G370193_T01|PACid:20828984
MGGRRSSSVAPAAVLALAAVTIAIAAAAAAEGEVKCGGCSPCGGADCPVLYPSPPPPALPPPPPYYYYSPPPPATTY
PGDSSYCPPPPGAYIQIGSTPPGKGPLYPQDPGFMPSGAPARAAPLALAVALAALAFFL

>GRMZM2G065259_T01|PACid:20837083
MVRPWVLVVLPLMLATLLIATTNAQNYIEPAPSPLPDSPSPPPPPPPEPSTAPPPASTPTPAGSSPPPPAPPASSPP
PPPSPASFPSPPPPPARASNWTPVANANDPKIQQVAQFAVRIYAISKKELKMQFQNVVSGETQPCDGGYNYRLVITV
RGGKKAQYDAFVWGIQRTTSWKLLSFTPKY

>GRMZM2G065199_T01|PACid:20838013
MARPSVLVVLSLTLATLLATTANAQDYTAPTASPLPNSPSPPPPPPPESSTAPLPASQPPPDQASSPPPPSPLPPAS
SPPLPPASSPPPPSPAASSPPPPSPPPPPPPASNWTPVVNVKDPKIQQVAQFAVRIDALSTRELNMQLLNVVSGETQ
PSNGGYNYRLVVTVSGGKYTQYEAFVWGILGTTSWQLLSFTPMY

>GRMZM2G079638_T01|PACid:20849189
MAALLLLPLVLAASLGLGNADPAPATIVHKDGTTCTLCASCDNPCNPSYYYPPPSPPPAPVTTPCPPTTPSYPSPSG
GGGGPVIYSSPPPPAASGGGGGLGGGGFYYPPPTGGGGGSGGGASQQGGGVYPTAPPPPNPFLPYFPFYFYSPPPPL
HSGARAVTAASLLPVLLLTSMLLLLCQTP

>GRMZM2G396541_T01|PACid:20851039
MARRPQPMPNLVPTLLLLLLVVAAAVVRADDDEKCSNPCGNPCGEPCVYASPPPPPVYSPPPPPPTPEYYPPTTPTP
TTPYCPPPPLGGGGGYEPSPGYTPSTPYTPGYNPTPSGSGWYAPPYTPPSAGTLYPQDPGFRPNAAPRRAATLFSLA
AAVACACFGLL

>GRMZM2G310158_T01|PACid:20851097
MRSPIRGGMLPLHVAAAVVVTMLAFPAAVVLGIATDEDCKCFMCVCDLDPHPLPPEVPTHHPPPAQPEPVPSPPPPP
PSPSPPPPAPALPVYYPPQTGYYYYPPQPYGYPAGEMYPRDDNRRESKSDAARRHGSCGRASLALAAALACGVLALL
LRSAAA

>GRMZM5G894582_T01|PACid:20852841
MMGGRTVAPPLVLALVTIIAIAAAEGDEVKCGGCSPCGGADCPVLYPSPPPPPYYYYSPPPPATYPGESSSYYQPPP
PGAYIQIGSAPPGKGPLYPQDPGFIPAGAPGRAAPLAVVAALAPALACAWALL

>GRMZM2G082823_T01|PACid:20822009
MIPAPPASSAGGSITSGWRNLLLLLLLLTLTLARPAAAQPPTSPALQAAYAALQAWKRTAIFSDPSNFTSNWAGPNV
CAYNGIYCAPRPSDGALAVAGIDLNHADIAGHIPADLPLGVPDLALLHLNSNRFCGVLPDTLLHLRLLHELDLSNNR
FVGAFPAVVLALPSLRYLDLRFNDFEGPIPPPLFDRPLDAILLNSNRLRGPIPANLGNSPASVLVLAHNRLGGCIPP
SIGRMAATLNEIVLIDDDLTGCVPPQVGLLTKLTVFDVSGNHLQGPLPPAVGGMAALQQLNVAGNLLRGPVPPAVCG
LQGTLRNFTYEDNFFTSRPGCAVATADGRWNCIPGAPAQRPPPQCAAAAAPFDCRTAQCQPPPTSGPPGSGPGGPSQ
PLSPPGSSNTPSHPSPPGSSNTPSYPSPPGSSNTPAYPSPPGSSTTPSHPSPPGSSTTPSPPGSSTTPSHPSPPGSS
TTPSPPGSSTTPSHPSPPGSSNTPSYPSPPGSSNTPAYPSPPGSSTTPSHPSPPGSSTTPSPPGSSTTPSHPSPPGS
STTPSHPSPPGSSTTPSYPSPPADGNSPKPSMPPSSHGGSAPPSGYQPPPWSQWAPSGQPVGVPPPTEHPGGVWPPH
TPTAPGTPGSAYPPGTPGSPSTPTTPGTPGSTFPPTTPGAPGSLPSTPTTPGTPGSTFPPTTPGAPGSLPPTPTTPG
STFPPTTPGAPGSLPPTPGYHPPSPGDHGSSQEHTPPSAPGSSGGVLPFPPVHGVAYSSPPPPPSDPAAGNPPSFSP
VHGVSYSSPPPPLPPVYPVSYASPPPPYKSN

>GRMZM2G333811_T01|PACid:20827274
MTTPPRNATLPVLLVLLLLAPHLLATAPAFASRGLSASDAAHIRRRQLLQYHGGGAGNGSGVVVDPSYAFPNPRLRD
AYVALQAWRRAILSDPHNVTGSWTGPDVCAYAGVYCAPSPQDPDLTVVASVDLNHADLAGHLPEELGLLADLAVLHL

NSNRFCGLVPRSLDKLHALHELDLSNNRLVGAFPDVVLRLPSLRYLDLRFNDFEGPVPSELFDRPLDAIFINSNRFR
FQIPDNFGNSPASVLVLANNDFGGCLPASVANMSSTLNEIILMNTGLKSCVPPELGALMGLTVLDLSFNKLMGAIPD
ELARLHSIEQLDLGHNRLVGDVPEEICRLPHLQNFTYSYNFITGEPPVCLHVKSLDDRRNCIPYRPDQRSPEQCNFF
RNHHHVNCDAFKCKKFILPSPPLPPPPSPPPPSPPPPSPSPPPPSPPPPSPPPPSPSPPPPSPPPPSPPPPSPPPPS
PPPPSPSPPPPSPPPPSPPPPSPSPPPPSPQPPSPPPPSPSPPPLYYEESPEERYLSPPYPDTLRCRCRSTTRCHGS
TVTSRRIHGVQVSRHL

>GRMZM5G841015_T01|PACid:20846217
MDPPLRLLPDGRLMAALLLLAACLSACSVQAVTSAEASYIAHRQLLAMKEAGGGEAGDLPADFEFDDRVGAANFPNP
RLRRAYIALQAWHRAFYSDPKGYTANWVGEDVCKYNGVICTEALDDPKITVVAGIDLNGADIAGYLPPELGLLTDLA
FFHINTNRFCGIIPKSMSRLSLLHEFDVSNNRFVGVFPYVCLEMVSLKYLDLRFNDFEGELPPALFDKDLDAIFVNT
NRFVGPIPENLGNSTASVIVFANNAFVGCIPKSIGRMVKTLDEIIFLNNKLDGCLPLEMGLLVNTTVIDVSGNMLVG
TIPEQLSNIAKLEQLDVSRNVFTGIVHESICELPALVNFSFAFNFFNSEAAVCMPSDKALVNLDDRDNCLGALRPAQ
KTALQCAPVLARPVDCSKHVCAGYPTPGGGPPSSPVPGKPAASAPMPSPHTPPDVSPEPLPEPSPVPAPAPMPMPTP
HSPPADDYVPPTPPVPGKSPPATSPSPQVQPPAASTPPPSLVKLSPPQAPVASPPPPVKTTSPPAPIASPSPPPPAP
VVSPPPPVKSPPPPAPVASPPPPEKSPPPPAPVASPPPPVKSPPPPTLVASPPPPVKSPPPPAPVASPPPPVKSPPP
PTPVASPPPPAPVASSPPPMKSPPPPTPVSSPPPPEKSPPPPPPAKSTPPPEEYPTPPTSVKSSPPPEKSLPPPTLI
PSPPPQEKPTPPSTPSKPPSSPEKPSPPKEPVSSPPQTPKSSPPPAPVSSPPPTPVSSPPALAPVSSPPSVKSSPPP
APLSSPPPAPQVKSSPPPVQVSSPPPAPKSSPPLAPVSSPPQVEKTSPPPAPLSSPPLAPKSSPPHVVVSSPPPVVK
SSPPPAPVSSPPLTPKPASPPAHVSSPPEVVKPSTPPAPTTVISPPSEPKSSPPPTPVSLPPPIVKSSPPPAMVSSP
PMTPKSSPPPVVVSSPPPTVKSSPPPAPVSSPPATPKSSPPPAPVNLPPPEVKSSPPPTPVSSPPPAPKSSPPPAPM
SSPPPPEVKSPPPPAPVSSPPPPVKSPPPPAPVSSPPPPVKSPPPPAPVSSPPPPVKSPPPPAPISSPPPPVKSPPP
PAPVSSPPPPVKSPPPPAPVSSPPPPIKSPPPPAPVSSPPPAPVKPPSLPPPAPVSSPPPVVTPAPPKKEEQSLPPP
AESQPPPSFNDIILPPIMANKYASPPPPQFQGY

>GRMZM2G169669_T01|PACid:20848767
MRGSVILALLAAAASSFFGAVTAASEGRPTVAVEVDPSWRFPSRRLWDAKAAGHLPRPQPHCRLGRPGGVQLHGCLL
RAAPLRPRPPQRARGRRGRPQPWRHRGLPPAGARPPPRPRPAPPQLQPLLRRPPPRASPPPPPPRARPQQQLLRRPI
LGRRPRPPDAALPGRPLQRLRGDEGTVLPRLFDRPLDAIFLNHNRLRFQLPDNFGNSPTSVVVLTHNAFGGCLPASV
ATMSGTLKEILLINNGLSSCFPPEIGLLRELTVLDVSFNQLAGLLPPELALMRKLEQLDVAHNLLTGAVPPGICDLP
RLKNFMLAYNFFTCEPPSCARIVARDGDRSNCLPNRSSQRTPQQCAAFYARPLVNCAAFQCKPFVPPMPPPRRLPSP
PPPSPPPPSLPPPSPPPPLPSPPPPQLLPSPPLSHSPPPPSPPPPAPVYHPPCPIMPPPLPCTPTHLWPSPPPYYPG
PLPPTNPVRYASPPPPQQHHPWPLVHPVQYGSPLPLCCIRRNQSQTQGRKAGSALAQPPPPPPPL

>GRMZM2G300969_T01|PACid:20873201
MEAAVVRLRRCRNGSLVLVYLAAILLAATAAAALSDAEASDIARRQLLSLREGDGDLPDDFEFDIHVDVTFANERLR
RAYVALQAWRRAMYSDPKNFTGGWVGADVCSYFGVTCVPALDDANTTVVAGIDLNGGDIAGYLPAELGMLTDIAFFH
INSNRFCGIIPKSFSRLTLLHELDVSNNRFVGGFPDVVLQIPVLKYLDLRFNDFDGTLPPHLFDKDLDAIFVNSNRF
VGFIPENFGNSTATVVVLANNAFIGCIPRSVGRMADTLDELMLLNNRLDGCIPPELAELVNSTVVDVSGNALVGTLP
EGLVNMIRLEQLDVSRNQLAGAVAENVCKLPALANFSFAHNFFSVEAEACVPSEDSPVALDDSGNCLDGGRPGQKPL
PECALVLAHPVDCRTNVCSKMPAPPAKKVPRPPDVSPHPLPPAPSPENQPPKKSPAPQPVSPQPSPPPSVKSPPAPA
PAPAPISSPPPLIKSPPPPVPVSSPPPLLKSPPPPVSMSLPPPSVKSPPPLPPRSPPAPVKSPPPPPPVSSPPPPVP
VRSPPPPPPSPISSPPPSMKSPPPPAPISSPPPPTKFPPPPVSVSSPPLVKSPPSPAYVSEPPPTVKSPSPPSPPPM
IKSPPPPVPVRSPPSSVKSPPPSPLLSSPPPPAPVRSPPPLTKSSPPPPPIRSPPPPQANSPPPSALISSPPPPMQS
PPPPAPVSSPPPPIKSPPPAPVSSPPPLAQSPPPPAPVSSLPPPVKSPPPPAPVSSPPPPVKYSSPPTPVNSPPPPV
KLPPPLAPMVSPSSPMKSPPPTAPVSSPPPPVKSPPPPAPVISPSPPVKILPPPAPVSSPPPPVPVSSAPPFAKSPP
PQFKSPAPPTSRRSPPPLVKPPPPPPLVSSPPLPPMTSPPPPTLASSPPPPVRSPPPPAPINSPPPPVKSPPPPAPV
SSPPPPAVPSPPPPAPSSPPPSAPEEFILPPIMAQQYASPPPPLFQGY

>GRMZM2G028731_T01|PACid:20820176
MATLVQPPAGAVVGSVANTLTTPGSASGPPPSSSSPTSASTPPPTDVPSSSTPTPPPPSPASPTPPLPSPSTPPAAS
APPPPSPDDSAPPPPEASSPPPPAALPPPPAPAALPPPPPAPVPTATPPPPPPPDIATQPPSSSQASPPPSAPPPRQ
SHATPPPSPPPTASPVSATPPPPPVHSPPWGGASANYSATSAASASGDLSVGNTKAFAFDELYGITGGFARENVLGE
GGFGCVFKGTLGDGKVVAVKQLKGGGGQGEREFQAEVEIISRVHHRHLVSLVGYCIAEDHRLLVYDYVSNNTLHHHL
HGRGRPVMDWPTRVKIAAGSARGLAYLHEDCHPRIIHRDIKSSNILLDDQFEAQVADFGLARLAENDVTHISTRVMG
TFGYLAPEYASTGKLTEKSDVFSFGVVLLELITGRKPVDSSRPLGDESLVEWSRPLLNRAIETQEFDELVDVRLEGN

FDDVEMFRVIEATAACIRHSAARRPKMGQIVRVLDDSLTDVDLSNGVQPGKSQMFNVANTADIRQFQRMAFGSQDFS
SDYSQSRPSIGSSRRDF

>GRMZM2G458548_T01|PACid:20820499
MASAPTTASSPLPASSRPTATPPDTAATPPPLTSPAPAAPPPSSHSHSHSQPSPGPPQHTASPPPRAAAAPPRPPLL
SPPPTSPTPSAAPTPSPSPPASPAPAPPPFSAPPPSARPLPTPAIAVDPARGKNPSWNKSSSPGAPSRSPSAPFGGL
GNGVVAIAAAVVALLLLSLLVTAVWFNRKRKRKQMSGYRAGLMSPFSSQQPSGESANVGWSADPSVHTNYSAGSPRL
KQCLSDISMGNSRFFSYDELYQITDGFSAQRLLGEGGFGSVYKGRLPDYKDVAVKRLKDGGGQGEREFQAEVEIISR
VHHRHLVSLVGYCISNDQRLLVYDFVSNNTLHYHLHGHGMPVLEWSARVKIAAGAARGIAYLHEDCHPRIIHRDIKS
SNILLDNNFEAKVADFGLARLALDAVTHVTTRVMGTFGYMAPEYASSGKLTERSDVFSFGVVLLELITGRKPVDASR
PMGDESLVEWARPLLSRALDTGDLEGLVDPRLEMKLDEVEMFRMVEAAAACIRHSASRRPRMSQVVRVLESSADIDL
TNGVQPGQSQLFNVANAAEIRMFRRMVAGAQDDDSSDLTQYDWSRGSDAAPGSRIL

>GRMZM2G355636_T01|PACid:20827980
MASSPSPSPGTIRAPLPPSSATPSPATTTPTPASPAPVTQPNATPADPPSSPVAPPPVPSASTPPPQLTSPPPSLPP
PPPDAVPPPPLVASPPPAPAAVVPPPSPPAPVAVPPPPTPAAPPKASPIHIRPPAAASPPPTILPTPNPPADPTPPT
LAQPPPPRRRPPRTPQTEPPPLAPPPSGIPVKPSTSSPSPTSADPLIPTPSSPSPPGTTTPGSGSTPSVPAPAMAAD
PVRPVTTGGSNKSSSPASQSSGSPVDSGGMSSGAKAGIGAVVAILVLISFVGAAFWYRKKRRRVHGYHAGFLMPSPA
STTTQVLAKTNFSAGSPESKDSMPEFSMGNCRFFTYEELYQVTNGFSAQNLLGEGGFGSVYKGCLADGEFAVKKLKD
GGGQGEREFHAEVDIISRVHHRHLVSLVGYCISDEQRLLVYDFVPNNTLHYHLHGLGVPVLEWPSRVKIAAGSARGI
AYLHEDCHPRIIHRDIKSSNILLDNNFEALVADFGLARIAMDACTHVTTRVMGTFGYLAPEYASSGKLTERSDVFSF
GVVLLELITGRKPVDASKPLGDESLVEWARPLLTQALETGNAGELVDARLNRNYNEVEMFRMIEAAAACIRHSASRR
PRMSQVVRVLDSLADVDLTNGVQPGKSEMFNVANTAEIRMFQRMAFGSQDFRTDFSQSSWSSQSRGLDASGSRPL

>GRMZM2G358830_T01|PACid:20830300
MSSPAPTSPATPSPPPPAPVPPTPSPQPPTPAPVPPTPAPVPPTPAPVPPTPAPIPPTPAPVPPTPTPSPPPPSPSP
PPPVTPSPPPPATVPTPSPPPPASPPPPTVPTPSPTPPASPPPPATAPTPTPPPPVLRPPPPSAVPASPPPPDSVPS
PPPSPNGVPASPASPPSHQLLAPPPPASSTGTSSTSSSPSKSSSNATVVGIGVAVGAVVLIGLAVGLIYCFAGRKRR
RRSPLPSQGFPGEFYDPRRGPMTPLHMSHVQSTTPSSTPPLMHSWQQSSHGLSESPLPPLNPSPAITGGTFAYDDLA
AATDGFSDANLLGQGGFGHVYRGTVAGQEVAIKKLRAGSGQGDREFRAEVEIISRVHHKNLVSLVGYCLHGEQRLLV
YEYVPNKTLEFQLHGSGRPTLDWPRRWKIAVGSAKGLAYLHEDCHPKIIHRDIKAANILLDYNYEPKVADFGLAKYQ
AAEVTSVSTRVMGTFGYLAPEYAATGKVNDRSDVFSFGVMLLELITGRKPIMTSSEYQPETLVSWARPLLTRAVEEE
NYDELIDPRLETNYDAYDMARLVACAAAAVRQTARSRPRMSQVRLLLQNVSHTLGAAVAASVTLVSPFPNGQVVRYL
EGELSVEDLNAGVMPGQSAMQRSSGGTTDQINRLRRMAFGPGAGGSTGTGAITEYTSSEFSDPTTEYGLNPSSEYTA
SSAGDTGEVAAGAQGQQQWHGRRAPGDTERMSRRTTGRRTQS

>GRMZM2G459003_T01|PACid:20843584
MSAPPPSNTSPPSTSTPAAAAAALSPPSPSTPEPSNSTTSAAAPSLPPPQAPVPPPPPPPTPVAPPAVSPPGTPPAG
PPATVSPPPDRPAPSPPAPPLLSPPVQPPAPPSAPPALSPPPLAPSTPPPGPTPSAPPAPPAPLPQSPVTPAAPPPA
ASPPTPQPPVTPSAPPASPAPIPQPPAIPVPAAPPASPSPIPQPPIPSPPAPVMPPPLPAPSVISPPSPPAIPAPVT
PTPSQPTITPPSPSTPTPAPPMPPVPAPTPTPPSPPVTPATPLTPLPPPSPAVPAPVTPSPPTVAPVPPTPTPPAPP
LPSPPSPVTPVPPVPSPPSPVMPSPSIPVPPLPSPARPSPPSPGSNGTVIGVSVAVATVVMLGLIVGLIYCLCCSKK
SRQRRRSSPPGTYVCQQRSYTPIRLSDTLWPWPVQAHHSAVVLDTHAVSARAPTRAPAAAQEPPSPMLGQSWQTSSG
SSSGVLLGPLRPPPPPPPPLFTGGTVSYAELVAATRGFSDANLLGQGGFGHVYRGTLERGGGGGEVAIKRLRPGSGQ
GDREFRAEVEIISRVHHRHLVSLVGYCIHGDQRLLVYEYVPNKTLELHLHGSGRPVLDWQQRWRIALGSAKGLAYLH
EDCDPKIIHRDIKAANILLDYNFEPKASDFSSRRVSDFGLAKIQPADDTHVSTRVMGTFGYLAPEYATTGKVTDRSD
VYSFGVVLLELITGMTPVLSPEPDNDETLVSWSKPRLARALGEDALDELTDPILGTNYDGVDMRRLIACAAAAVRST
ARTRPRMGQIVRFLEGQMSAEALNGGVAPGQSGTVDDGAADQLRRMRRLAFVPAASSTGGFLTEDTSRSYVSEATSE
YGLHPSSSSSSDDGATSEAASAGRDGQRSGEIGVADGMTRRTRSGRADE

>GRMZM5G872442_T02|PACid:20850060
MASSPSPSPITIRATSPPSTATPSPATTTPKPDSPAPVTQPNATPADPPSSPAAPPPLPPPASTPPPQLPSPPPSLP
PPPPDAVPPPPVVVATPPPAPAAVVPPPSSPAAVPPPPTPAAPPKASPIRPPAAASPPPSNLPAPNPPADPTPPTVA
QPPPPRRRPPRSPQTEPPPLAPPPSGIPVEPSPTSPSPTSADPSIPTPSSPSSPGTTPSTPGSDGTPSVPAPATPGD
SVSPVTTGGQGSNKSSSPASQSSSSSVDSGGLSSGAKAGIGVVVAIIVLSLVGAAFWYKKKRRRVHGYHAGFVMPSP
ASTPTQVLGYSAKTNFSAGSPESKDSMPEFSMSNCRFFTYEELYQITNGFSSQNLLGEGGFGSVYKGCLADGREVAV
KKLKDGGGQGEREFHAEVDIISRVHHRHLVSLVGYCISDDQRLLVYDFVPNDTLHYHLHGRGVPVLEWPARVKIAAG

SARGIAYLHEDCQPRIIHRDIKSSNILLDNNFEALVADFGLARLAMDACTHVTTRVMGTFGYLAPEYASSGKLTERS
DVFSFGVVLLELITGRKPVDASKPLGDESLVEWARPLLTQALETGNAGELVDARLNKNYNEVEMFRMIEAAAACIRH
SASRRPRMSQVVRVLDSLADVDLTNGVQPGKSEMFNVANTAEIRLFQRMAFGSQDFTTDFSQSSWNNSQSRGLDASG
SRPL

>GRMZM2G004330_T01|PACid:20852655
MASTEQLASSPAGGPPGPKARAAPPESSSSPPPPPKASSSSPPPPTPPPPPSKQSSPPPPAHNSTTSPNTPATPPE
KSPAASPPPPASPPPPSRSSPPPSPSSPPPSPPPQSSPPPPAASTSQQSPPAPEPTKPPTPPSSEKPGAAGTTASS
PPPNAGQPPPRETPSTPPDTAGHQPPPPAPTQMLPPPMALTVIMPGGVSGGPPAGGMWRSPPGPPAGGGGSGSNSNM
KNEAVVIGISVAGLVLALASLLIIACATGKQGNRGRKRERHASRRHSIVVPERQCGAAAGVVSADVYQPSNGPAPSP
SPSGTSSSYDLSGANKSWFTYDELAGITGGFSAANVIGEGGFGKVYMGALGDGRRVAVKQLKLGSGQGEKEFRAEVD
IISRIHHRHLVTLVGYCVTENHRLLVYEFVANKTLEHHLHGKGLPVMDWPKRMRIAIGAARGLTYLHEDCHPRIIHR
DIKSANILLDDAFEAKVADFGLAKLTNDSLTHISTRVMGTFGYMAPEYAQSGKLTDRSDVFSFGVVLLELITGRKPV
DASQPLGEESLVEWARLLLVDALETDDFREVADPALECRFSKTEMRRMVEAAAACVRHSAAKRPRMVQVWRSLDVDE
CSSDLTNGVKLGQSMAYDSTRYSADIELFRRMAFANDLSTAELGVVDEDDHRYHNSSKASSSSRRYK

>GRMZM2G065214_T01|PACid:20866099
MSSNSSTGPRPRPSPSPPPPKSSPSPSHSTAPPPSADSSSPGPSLPSDSSPPPAASRSHGAQASSPAKPHSPREQPS
RGSADDGGRPPPAPAHRGGPTMVEIVFAAAGAAALLVILIAACVCCSRKTAPRRRKRPHNPMHYYADFPSSACKATG
NSSTYYPNGPQPQWQNDTVAAATRSTFGPPGGGWHAPPPDMTSGPHGARPPPPTTPHEALLGLGKGTFSYEELAAAT
GDFSAANLLGQGGFGYVHKGVLPGGMVVAVKQLKSDSGQGEREFQAEVDIISRVHHRHLVSLVGHCIAGARRVLVYQ
FVPNKTLEFHLHGKGQPVMEWSTRLRIALGSAKGLAYLHEDCHPRIIHRDIKSANILLDNNFQAKVADFGLAKLTSD
SNTHVSTRVMGTFGYLAPEYASSGKLTDKSDVFSYGVVLLELLTGRRPIDAGDARSFLDVDDSLVDWARPALSRALA
DGDYDGVADPRLRGNYDTMEMARMAASAAAAVRHSAKKRPKMSQIVRALEGDISLEDLNDNEGVRPGQSMALGTAAA
ASYKAKAPGPYTHGVERIRQAPTVYSGAVGRPSTIGNDGDESREPHQ

>GRMZM2G172081_T01|PACid:20869796
MASSPQSSPSSSKKSPPPQEASALAPKSSSKSPSPPKGESNSSPSPPSKSSLSPPPRPKKSASTSSSKDGKTKKSSS
SSSSSDNTAAVITGVVLGVVGFALLLSIVACVCCAKKKKKRPPPMNMPFYTDEKGNVYYPNAGLPPMWQQYGSNGSI
PPPGWHHHGGGNPLSPSLGSMAAPLSGEMYSSGPHGPPLPPPSPNVALGFSKSSFSYEELAAATSGFSSANLLGQGG
FGYVYKGVLAGSGKEVAVKQLKSGSGQGEREFQAEVEIISRVHHRHLVSLVGYCIAGNQRMLVYEFVANNTLEHHLY
AKDGPVMDWSTRMKIALGSAKGLAYLHEDCHPRIIHRDIKAANILLDNNFEAMVADFGLAKLTTDTNTHVSTRVMGT
FGYLAPEYASSGKLTDRSDVFSFGVMLLELLTGRRPIDTTNYMEDSLVDWARPLLSAALAGETGFAELVDPRLGGEY
SVVEVERLAACAAASTRHSAKRRPKMSQIVRALEGDASLEDLHQDGGKPGQSVLFSGGGSDNISRLRQLAFDSGDYS
DYSTDSSTGGGGRPPRRP

>GRMZM2G072292_T01|PACid:20871335
MATPPDGAPAGPDDTSPPPSSNSSTPPPSDSSTPPPKSSGSGSPSPSPPAPSLSPPPPASPQDSTAPAARGSPAAPS
RDSPSPPAPKRSDSDSDNSGSSKSGSGSGSRSRSSGSTQVDVILAGVVIGVLAFSLLMCIAACVCCAKKKRRKKPPH
MNMPYYTDEHGNVFYANSMPKWQSSAMDHGWHAPYSPASGDMSGSHGPGLGQMPPSPGMPSLGFSKSSFSYEELAAA
TGGFSSTNLLGQGGFGYVYKGVLAGSGKEVAVKQLKAGSGQGEREFQAEVEIISRVHHRHLVSLVGYCIAGSSQRLL
VYEFVPNNTLEHHLHGKGVPVMAWPARLAIALGSAKGLAYLHEDCHPRIIHRDIKAANILLDENFEAKVADFGLAKL
TTDTNTHVSTRVMGTFGYLAPEYASSGKLTDKSDVFSFGVMLLELITGRRPVDPTNYMEDSLVDWARPLLARALSED
NFDELLDPRLENRVDRLELERMCSSAAAAVRHSAKRRPKMKQIVRALEGDASLDDLNEGVKPGQSMMFSSGSEYDGA
NYAANISKFRKVAFESSEFSNDYSGTSEYGADSGEAATQRQQHR

>GRMZM2G410951_T01|PACid:20830169
MALFRRLFYRKPPDRLLEIADRVYGTVPHNVDYHSVETAAVVLWHLAYYFLPPIVFDCCFSTETMDQYRYKNYLDNI
ILQLREQFADSSLMVLNFRDEGKSLISGIFSKYNITAKDYPCKYLGCPLLPLDIILHFLRLSERWLMLEGQQNILLM
HCEKDGWPVLAFMLAGLLLYRKQYNGEQRTLDMVYKQAPKELLQMLTTLNPQPSHLRYLGYICRMDDGIGWPTQPIP
FTLDCVILREIPNFDGVGGCRPIVRVYGQDIPTTDRSHSVISPPSKAKKHIRRYRQADNAPVKLNVGSCVQGDVVLE
CLHVDDGQGNERLMFRVMFNTFFIQSHILPLNFEDIDVPWDADHQFTKNFKAEVLFSEFDAESDASTEVAPDYDYDY
DYDNDMDVASADEFFEAEEIFSNAESQDGNKDADTLSMVSTDYIRTPSAECWKNSPFSNFDLNIDIDGSQDNKADNL
GLLLETVNDGKTCTSTEANILLNNETAVVKSTFTATIACTSTEANILLNDETAVVKSTFTATIDGDTDSGISSSTYK
EDGCMFEKGSSKQDIRMGSNQDLSQIDNVLVKEVIILETNSTDDIQMIKEVIISEVTTPKQVVEGNMMETELDEAVH
SSESTALGEDEDTKRPNTFFKKDEGQSARDEYAAYDNGIVIEREESRNKEKFTITGTDSLVAEPKDLPHLDSSNTSL
ELSSANENIDQLRACSSNVTAERRAGIDTSFSSSRSQSSNISCANILPEESNLIINHASTSVNANTDTTDSSRLVLK

KKPFLPLATSSLFSPSSPRRNLLRAASTDLSFFSPSQTESKQNSVTSTSGRGDSDTSSVLPPFQLYTSLGSSSKMSL
VHPTLRPIRTISSLPSLSFGTFIEMSMASSRSPKHQEHVRSPSIPQDQHLHPPMTEENDLHSGSLTLPASNQYGPQT
PHPPPLPPPHDSCTQSYSSTIIYEPEQIRADGSCSYSPDYRQSVLNLGDSSLTSLSNSSIAATECPLGASDFVDEEV
TSRPNIVTAMDVPFTNEDTNSLLHMTCSSPPKTLQRAEPPTLPPPPLPLVPPPSQLPLPTICSDSGSVPLVYSNPSS
DCPYKEPTMLPEHKSDVPATSLEGHEASEVQFLQSDTAVELSPSEHSEYKVQHVVGSAEDTISSMSSYIPAAPPYPM
FHIVTDNSSCSISAEQVNCEQPLDQTTLSIHLRPFEVEISQGETINGVLASIIDDKEHGGIPILQLPQKLPHSGEHM
KSPPPPPPPPQPPPCHATVIPSLCLSSKPPSPTEHYENPPSSPPPFSRESCVVLPPLPAPLLNHPSLPGKHINLYPP
PPPPPPTLHHIAPASPCSYQPSFYADIATQPTFSEDIVIVPPPHSKGVNIIPLPPPPPRTSETLSQPLDGGQSTGPA
SLLEVTKENPPPPLLPPEGQMGSPLSTLCGEIVNIPIPPPSLPGGHGEVPLSTPPRGCGAIPPPPPFTKGIGGITLP
IGFHSGDLSFLQSTRESKGPSCLPPPPPPPPPPPPPPLSSNGHIGDLPPPPTSVFVEASIPLPPQTGCGGDPPPPPP
TGGHVGPPPPPPPLGECAWDLQPPKACVRAPPPPPPPGGYAEVLMPPPPPRGYVGAPAPPPPPGGYAGAPPPPPPPG
GYAGAPPPPPPPGGYAGAPPPPPPPGGYAGAPPPPPPPGGYAGAPPPPPSYGGIGGVPPPPPPFGGLGGTPPPPPPA
GFRGGAPSPPPPPGGHGGPPPPPPRGHGSGPPPPPGAPSPPMPPGMSGGPPPPPGGRGMPTPPGGRGHGLARTLGPT
LQSAMRKSSLKPLHWVKVTRAMQGSLWAELQKQVEANSHAEFDVNELESLFTIAPKAKAGSKSEGRGKSLGTKSDKV
QLVIY

>GRMZM2G057247_T01|PACid:20831838
MTRGTEVVVRALLVAAAVAGLLLSAAAEDGVVIQRRSLHQPFFPVDPTPPPGFDDSIVPPPPPPAAAAGAAAASAAS
KGGGRSVTNAVAIALATGLVVLAVAFYSCFLLWRRRSDGGGGGVGGDGLRAVKSSRPGAVAVRVASDVGSSARHQRS
PPPSSTASDAIYLDPLTTMVEVSRHRPQSPDLRPLALVKQPSPDLRPLPPLKRPAPQPPPPPASTPPMTTTTGDSSD
EEDQATYYTAPKTAKSSFSRSTSQRSTVEPTVAQPPAPAPASTPTPLPPQANAPRPGRPPPPPPPPRQRLLRPMPEE
SPPPAVLASLALTNPPEPSGQDRGGENPDGHGGRARPPKPPSLKPLHWDKLRAISGRTTVWDQVNNSDSFRVDEAAI
ESLFLNNSGGVGNSDQAARRGGAGKQESRLLDPKRLQNVAIMLKVLNVTSSDVIGALMHGNGDLGSEFYETLAKMAP
TKEEELKLKGYNGDISKLDPAERFLKDVLDVPFAFKRVDAMLYRANFGAEVNYLKKSFGTLEAACTDLRSSKLFLKL
LDAVLKTGNRMNDGTNRGEARAFKLDTLLKLADIKSTDGKTTVLHFVVQEIIRSEGLGSDQAAAAAANPGSTSKEQF
KKDGLQVLAGLSSELSNVKSAAALEMDTLVGSVSRLETDLEKVTLVSRLRQTCPGDDQVSSEKFFEANDAFLGRAHA
EIEAVKAAGERALQRVKETTEYFHGDAVKEEPHPLRIFMVVRDFLATLDRVCRAVGRTPERVMMGSGKSFRVSAGTT
SLPQRRNDQRRELSSSDEDSSSS

>GRMZM2G414002_T01|PACid:20846116
MALFRKFFLKKTPDRLLEISENVYVFDCCFSTDSMGEDEYRDYLSGIVAQLQEFFPDTSLMASNFWSGDKRSRISDI
LSEYDMTVMDYPQQYEGCPLLQLEMIHHFLKSCENWLSVEGQHNMLLMHCERGGWPVLAFMLAGLLLYRKIYTGEQR
TLEMVYKQARRDFIQQFFPLNPQPSHLRYLHYITRQGGGSEWPPISRPLILDSIVLHVVPRFDTDGGCRPYLIVHGQ
DSSPGNKSAKVLYEMPKTKKHLQRYGQAEVPIKLSVSCHVHGDVVLEFIHIGDNIENKETMFRVMFNTAFVQSNILE
LNRDDIDVAWNVNNQFPRDFRAEVHFSDPVSFKPAATIEEVADDGDETDVASVDTGEEFYEAEEYWHDARKDPATHS
IDGGISLNDIAEFDGGATTEERSSLEKHRSDEGVKIAVSQNFGSMNEKVAIAPTSSFENQEGLQQPREVLPNAKLSK
NNDQENSDVHDIQVVATSVDSEGHKFGSTGQEDTKGVIAQTLVTTVDLSGSDGIHCQTDEQTKMSEYPDLDYTAFGS
PRSLSRTDGDAHMRTKKNEGLQNGDIKIITENTISVDNELVIYEEKTIVVNGNAIQVVKNVVNEESIAAKIGKPITE
SRTSLDNDNGKIQTAKPSDIANGKLDGCRLESCLETVPTKKTTVHDRIVVLPATGVATEIKAKQEVLVDKQDFGIVL
PQSRTVARARSPRLGSDDRGQIPDKAVLSVLKEMVVGNAKMEDQPKLAKPKIMRRWISPKKESDATSVRRPSHPPSR
YYSSPAALGIRSISTDGKISVVNDAPLLSLKQPYGSNLTREATSAQSAPSPPQKSLLLGAQSASRSQVSPPPPPPPP
PATNYSSSSTPMHMGLASTGLSNLPQGASPPRSPVAQKHAFAPPAPPPPPPPPPRSGVGGNTPPPPPPRLNNSAIAP
PPPPSTITRSSAPPPPPPPQSMSRSAPPPPPPPPPSLCSNVPLPPLSCTLSAPPPPPPPPLPSMARSAAPSPPPPPP
SRGAPPPLPLGRGAPPPPPPPPPPGRGAPPPPPPPGRGASLPPPPPPSRGAPPPPPPPVSRAGPPPPPPPPGARCGP
PPPPPPPGASAPPPPPPPGARPGAPPPPPPLGARPGAPPPPPPPGGGGRGPLPPRAPGVPTPPRAPGVPLPPGSNPS
LGRGRGAVRPMGSAIGAAASRKSTLKPLHWVKVTRALQGSLWEELQRNTDSQSVSEFDVSELESLFPAAVPKSDDSS
KSERRKSLGSKPEKVHLIELRRANNTEIMLTKVKMPLSDLVSAALTLDQSTLDVDQVENLIKFCPTKEEMELLKNYT
GDKQILGKCEQFFLELMKVPRMESKLRVFSFKIQFGSQVADLRRNLDIIDSSCNEIRTSLKLKEIMKKILLLGNTLN
QGTARGAAVGFRLDSLLKLTDTRATNNKMTLMHYLCKVLAARSPQLLNFYADLVSLDAASKIQLKMLAEEMQAVSKG
LEKVQLEYDASERDGPVSKIFREKLKEFTDNAGSDVQSLSSLFSEVGKKADALIKYFGEDPVRCPFEQVISTLLTFV
TTFRKAHEENLKQAELEKKKAEKEAEAEKAKNAQLTGKNHSKSSNPSRQAKQAIERTRSVSRRGRDAG

>AC155376.2_FGT004|PACid:20853194
PAPAVGPPPPPPPSPPPPPPPPVGYWESRVRKPDTSKETRSPALSPPPQAANFRSVPAPTDAFSSRPPESSDQGDKS
EDTTPRPKLKPLHWDKVRASSDRVMVWDQLKSSSFQVNEEMIETLFICNPANAPAKEATRRPVLPTPRAENKVLDPK
KAQNIAILLRALNVTKEEVCDALCEGNTENFGAELLETLLKMAPTKEEEIKLREFEEETSPVKLNPAEKFLKAVLDV
PFAFKRVDAMLYIANFDSEVNYLKKSFETLEAACDELRSSRLFLKLLEAVLKTGNRMNVGTNRGDAHAFKLDTLLKL

VDVKGTDGHTTLLHFVVQEIIRTEGSRQSASAQTTPRTQANPLREELECKKLGLQAVAGLANELSSVKKAAGMDSDV
LSSYVTKLAGGIEKVTEVVLRLKNEGTESRDGAWRFHDRMQRFLKKADDEIIRIQCQESVALSLVKEITEYFHGDSA
KEEAHPFRIFMVVRDFLAVLDQVCREVGRVNERTIASSVRHFPVPVNPTTPQLFPRLHALRAGFSDDDDESSAASVS
SP

>GRMZM2G142779_T01|PACid:20881670
MAPRPCPLSLLLLAVAVAVALSPLAAAQPQRNILTTFPSTRTPAFATPPPPTVSPPPSPVTVPPSSPPPPPSSVKRS
DIAVAVVSTALSSFAVSGLAFFLFLRHGRRKELTAGDGNGYPGRPLDGAFAGKRSEREPTRPSRGGGFGMVDENGL
DAIYWREFEKDGEAGGRGREKKPPTTGTGSRRPPQPPPLRQQQRAEMWPEPQPSSSPPPSPPRRSRRNRIDQEPLI
PAGSLDSASAVFDDSLHPPSAGSSSSFSVSARPPPPTPAVAVSSVPRPTPRPAPAASPGLPLPPGRASPPPAPPMVA
ASVASPPPPPPKPAAASPPPPPPLAKGPPPPPPPKGGPPPPPPPRGGPPPPPPPKGPSPPPPPPPGGKKGGPPPPPP
KGGASTSSSRPPTAPGMPSGAGEQQAKLKPLHWDKVNVQATDHSMVWDKITGGSFNSVREKSRRFTRVGMKKRIPQN
LQRQSLDEGIIEALFGTAAANRKPKPSDKESGESSASLGRSNTPEQIFLLEPRKSHNISIILKSLTVGRDEIIDALR
DGQTELGTDVLEKLSRLHISKEEESTILRFSGDPDRLAPTEAFLLRLLLDVPNPIARVNALLFKVNYGAEVAQLKHS
LRTLELASQELRTKGLFFKLLEAVLKAGNRMNAGTARGNAQAFNLTALRKLSDVKSTDGSTTLLHFVVEEVVRSEGK
RLAINRNYSIRRSGSLARSGHEGGSSAAGFASQGPSREERQSEYMNLGLPIVGGLSTEFANVKRAALVDYDAVVSEC
AILDGRLNEIKRLLETCSDDGFARGLRGFVRAAEQELKALRGEQERVLELVQKTTEYYHAGATKERNAHPLQLFIVV
RDFLGMVDQACVDIKRKVQQKKPAPSSSQPNTAAAPTVAAVAAAAATTAATASATDGQTAPAQKPPEADSKRKRVMP
RFPNLPAHFMKDSADSDSSSDEE

>GRMZM2G138401_T01|PACid:20827111
MASAAAASSAVKEALVVVAVCIVLLLHSSAGHQPPKSPPPPHCHYTGQQPPPSPVPASLYSPPPPPPVPAPMPSPSP
PPPPPPVQAPMPPPPPPAPTPTPAPTPTPSPSPAPVNNCSYMYCAMQCSPVCQANHDAGIAKCESDLATNYNGCYDS
CTSHVCPGDSCAGSGCGFGHCPCDNANATSCCQSCGNVLYPEEQRCRNYYDRAVEYCMIDCQDTCYKNCTQGA

>GRMZM2G129935_T01|PACid:20833272
MATKLAVLATLVLALLGPVSCQQQQAGYGYGAGSPTPTSSPPPPAAYPPMTTSPTPSSPTAYPPPPPPPTPSPPPPAA
GAGLSVGYYKDKCAEAETIVQEAVRAADAGTKAGLVRLFFHDCFVQGCDASVLLKPDNDTNPQPEMLGVPNLSLRGF
EVIDAAKAAVEARCPGVVSCADIVAFAGRDASAFLSGGAINFTMPAGRYDGTVSLANETLPNLPPPFADVRRLKAMF
AAKGLDTVDMVALSGAHSIGRSHCSSFSSDRLPPSNTSDMDPAFAATLQASCASSANGAADNTVVQDYRTPDQLDNQ
YYRNVISHKVLFASDAALLKSSDTLGLVYVAAFSQKLWQDKFGQAMVKMGGVQVKTAANGEIRRMCGYVNKP

>GRMZM2G032145_T01|PACid:20836344
MARGIILLLLVTPLAISMALPSLSTRIEGSVIGERSNLFSQLHSDNSSPTENKLQPGEKSFASEAYAVNWNEPPDQQ
SQDSLEQLEPYRVIWKQPVQGEKSFVSEAYPAKWNKPPSPSKQSQDSFEPEAYRVKWNKSPPPHQQSQDSFESEAYR
VKWNKSPPPHQQSQDSFEPEAYRVKWNKSPPPHQQSQDSLEQEAYRVKWNKSPPPQQQTQDSFEQEAYRVKWNKSPP
PHQQSQDSFEPEAYRVKWNKSPPPHQQSQDSFEPKAYWVGWKKHAQGATDVADETTPARGHGLHVATGMLFTRKSLL
PGTALPEGTKFCGHGFLDPERFELRADADAIPFSYSQLDTILRMFRIPRGSKKAEQVAATLRTCEAESPEAHACATS
EQAAADFAASSLGVRASKLVALVTTVHGGKDATRYVVAPNGITRIGKAAGAAAVVPCHPMPYPYMVHYCHQPADVEA
LRVELTGLGDGGVTTAIAMCHANTMNWDDRYFQMLNVTRGEEICHFMPRNYVLWLPAAELLGN

>GRMZM2G085246_T02|PACid:20838293
MQRAPERRRGAGPLLLLALLVALAAAPRLVRAVTDATDVSAINGLYMSLGSPLLPGWTGNGGDPCGESWQGVVCTGS
SITGITMNAANLGGQLGSLGNFTAITSLELNNNNIGGTIPEDLPVTLQSLFLSANQLTGSIPSSLSKLENLTAMSVN
GNHLNGDLPDTFDSLNRLVNLDISSNNLSGVLPSSMKNLASLTSLHMQDNQLSGTLNVLQDLPLKDLNIENNLFSGP
VPASLLNIPNFKKDGNPFNTSIVPSASPPSTGPAPTPAASKPAPTPAPTTSNSTPPAPAPSFPSRSPPPPKTTSNSS
EGSTTRDSTSPSKKHSTSTLKIVGFVLAGVVLFIIIVLLVLFCLSKYQERRSRYDHNRSQQARVHHRVEPQIKPPPV
QQSNDLKKGSGEVLDRRGHELSSSTAALAKKSPENQKEHVINFDRTDSDLFPVSLPPPPPPPPLPPIERVIANPIVP
PEKRYSPPPKTSSSTSATSFSIASLQQYTNSFREENVIRESRLGKVYLAELPDGKLLEVLKIDNANGRISVDDFLEE
VECILDIKHPNILELVGYCAEYGQRLLVYNHFSRTTLDDTLHDGEDTESALSWNARLQVALGSGKALEYLHASFQPP
IVHQNFEPANVLLDKKFSVCVAECGLAELMPPSSVTQLSGRMRALLNYEAPELQDSGVVSERGDVYSFGVVMLELLT
GRKPYDSSRPRHEQHLVRWASSQLHDIESLSKMVDPSIRGQCSEKALSRFADIISRCIQQQPEFRPPMSEIVQDLAR
LVNATGEESE

>GRMZM2G373578_T01|PACid:20839575
MATPPVLAFLLLLLVSSFSHASVAAVLPAHSNPNPFPTTTPLHLQARRQQRVRQPNSPADATKLAAAAADDTGTTAG
PFTVHYFQQELDHFSFTPNASTVFYQKYLVNDTFWRRPGGGGTAGPLLVYVGGEADIECIAHNVGFMFDIAPTFGAL

LVFVEHIEYIFGDLNIGPQKDMARVVWWSKRKHRFYGESLPFGNNSAQALGYLTSTQALADLAILITDLKRNLSAET
SPVVIFGGSYGGMLASWFRLKYPHVTIGALASSAPILQFDYITPWSSFYDVVSQDYKSESLNCFSVIKAAWDVLEER
GSNGNGLVELSKLFRACKTVKYADSIRRWLRTAFVSIAMMDYPTPASFLENLPAYPVKEMCKIVDGFPADADILEKV
FAAASLYYNYTGDQTCNQIEDEGNPRCLNLNYWGWQACTELMMPMSSANDSMFPPHTFSYEDKANYCLQTFGVRPRP
HWITTEYGGYRIDEVLKRFGSNIIFSNGMRDPWSRGGVLKNISSSIVALVTEKGAHHLDLRSATKDDPDWVTEQRRQ
EVEIIHGWIDQYYRDMAQGFNLAASSIRKTSPPPGLPYAVVPRRRGCIVARDLTSPPPGLRYPVVPRRRASIVARVL
TSPPPGLPYPPVPRFSRKGFHLAAARSSARLHSAVAFQPSIPFFPDVVLRIRSRGVSCLSLCCITESPLQRSLGTGK
MDSADSIADKAIGRMQEISDKIFSVARETIHPGRGLTSFDATKLRVALEEIACMLEQDSLVLQEHLDIVNNFNIPVD
SSYEPSTLSSPPPSDDDLTPHWDFAEHFQNFWGVEYDSDQMPSFSDNDMDKSGKVIAKWDSRATKIYTEICVEEVNA
RNRPQQFLNAEGYANLIRKFKERTGRTYTRDQMKNRWDSLKRMFTQWKTLNERATGLGRDPHTGSISAPDEWWAKQN
EDYELVQAMPGCIMFKTAPLENEDELKVMFGPIVCTNERTLVPGVEDANSSSDDHLEYTLGGGENSTPDPCTNATGK
RKMRHDSPKPKKKKDSREEYMKRLVEAFESRSLTTNKSITSSDNDPVRLEI

>GRMZM2G160966_T01|PACid:20844614
METRQLLLVFCLLGAGMAVVVDAQAQSAPAPPPRQTPSAPPQQTQFGRTMSTFITVAISVFFFLLFVCAYINQCRLA
DPGAAAAAAAAAAAAGGGGGGPSRRGKRGLDPAVVATFPIVSYREVVEHKIGKGVLECAVCLTAFEDDDDLRLLPHC
SHAFHPECIDPWLQSRVTCPLCRANLEKPAPAPAVAPPSPSPRLAPRQPSPPPEAVAIPVSDDGSEGGSSSCEDDRK
EEAVELETLRSARRAARMPRSHSTGHSLFAAAAAAAAAEEGDHERFTLRLPEHVREQVLRSRRLRHATSLLNLSDMS
SEGSSRGGRGVAGAGSFGNGGGSSHGGRRWQSFLARTVSWARGGGDGSVRRGWDGSTRRGRDGGESSWKGVASPPPT
GRP

>GRMZM2G434363_T01|PACid:20853802
MAMPSLLRFLVLLTAGAAVSQVVAAWVDCGDASPSPPPYSSSPSPSPSSTPANGSSNAPFRANLMTLLDALPRAAAP
TGFASLSLGAGRDRAFVRGLCRGDFDPPRCLAELQEAVRTLGGSCPSSSRRGAVWLDVYVAYADTNVSTPREDDVRM
VLYDTRLVAHPAAYLQAYSALMGTLVARAVGGGGGEAGTSRQFFATGEANYASDDPNGTMYGLVQCMRDITAADCVH
CLQASVPQLPCCKGNQGGVVLAYNCYLRIQVYTYYDLALDAPPPAAEPAPPVPSPPPAGETSGTGTRPRNTIVLLAV
VVPLGTLLLLAFVVAGVYLRRRRGVVVEERGRERGCTAVEDEPSMTYVHPEKFTLAALRVATGNFAAGNKLGEGGFG
EVFKGRLEDGHPVAVKRLSKGSSQGFDELKNELILAAKLKHRNLVQLLGVCLEESEKLVVYEYVPNRSLDTVLFDAG
RRPQQALEWSERYKIIRGIARGLLYLHEESRLRIIHRDLKPSNVLLDSDMTPKISDFGLARAFWGDESRGVTRRPVG
TLGYMAPEYAYYGHVSTKSDMFSFGVIVLEIMTGQRNSSPSVEDGSSNRNLLSYVWDQWRRGSVAEVVDASPCAGGQ
CAGTEALSCVQIGLLCVQKDPRSRPDASEVVLMLEGRCAIQQKPSRPAFYSGHISVASARRAARGSAVSYGRLSAIG
TVSENGVTVSELDPR

>GRMZM2G120839_T01|PACid:20854508
MTPRPRLLLLLLAIALPAASAASTLAVSASSTSPTVCGVAEPNGTVYCAPLQGPSSSNSASPVAPSAIAFAELSAGR
GFVCGLQAGGAALFCWPSTPAPQWGQLRRLYNGPAPLADLAVGGGHVAAYDASGKVVLWWRGGGRFPARADGAFRSL
VSGDGFSCAVQANASAAVRCWGPQGSAVQAGLANASAAPYLAAGGARACAVLASGAALCSGSDASAGALPRDLFGYG
LAVGDSHACALRRPDHTAVCWTLGGPTTTLYEPALGISFAFLVAGGNFTCGVASSDFSVYCWSAGAVAAPVPLPRIR
PGVCVSDDSSCRGCGFMSQSQKFCGGSGGICDALCDDSPPPPAPAPPSPPPSSSRRVSKAWIAFCVVGAVGGFAGLC
SIVYCLVFGFCSNKRVHNSVQPNISAAAAGTGADNNGGGGGAAASGSPYGSPNGSRARGLFRRQLSRAMTRQRSGPS
SFKDPAEEFTFAQLEAATKGFALEAKIGEGSFGTVYRGKLPDGREVAIKRGESGPRARRFQEKESAFRSELVFLSRL
HHKHLVGLVGYCEEAEERLLVYEYMKNGALYDHLHPKASAAPSPVASSWKLRIKILLDASRGIEYLHSYAVPPIIHR
DIKSSNILLDGGWTARVSDFGLSLTGSPEPSSEESRSQQLAVTTKAAGTVGYMDPEYYGLHHLTVKSDVYGFGVVML
ESLTGRRAIFKEAEGGSPVSVVDYAVPSIMGGELGKVLDPRAPEPAAHEAEAVELVAYTAVHCVQLEGKDRPAMADI
VANLETAFALCEGSDRDRGTGAFGNSSSSASLSVTSMDRSGALA

>GRMZM2G435592_T02|PACid:20858509
MGARSSSTWTQHLLLLFFLVLGVATNSSHSSTINITNRCSYTVWPAATPVGGGMQLEPGKTWVLQVPGNTQSGRVWA
RTGCSFDGPGKKSCQTGDCGGVLACTTSGQPPMTMAEFTLSDSNNMDDYFDITVVDGFNLPMDFLPVPSSKGSSGCS
RGPRCAADITSQCPDELKVPGGCRSACNGSSCDASTVNSNTVFYARMCPDAYTYATDNGPVTYSCPSGTDYQIIFCP
PVDLVSLSPPPTSPLSPPPTSPTATNGTSSITSSSKSKRPRIFGYVLGGSIGGFILIASLVFFFVLHRRRLHRRQEM
QEEEEAEFGRLPGMPRRFTFEQLQEATDQFREKLGEGGFGSVFKGRFGEQAIAVKRLDRAGQGKREFLAEVQTIGSI
HHINLVRVIGFCAEKTHRLLVYEYMPNGSLDQWIFCRQGQGDDDAPRLHWQTRHKIIAHVAKGLAYLHEECMKRVAH
LDVKPQNILLDDNFDAKLSDFGLCKLIDRDKSQVVTRMRGTPGYLAPEWLTSHITEKADVYSFGVVVMEIISGRKNL
DTSRSEKSFHLITLLEEKLRSDRLVDLIDMCITSDSQAQEQEAIQMMKLAMWCLQIDCKRRPKMSEVVKVLEGSISV
ETDIDHNFVVTNPPSFSAPRIVVMSAPPLASEVSGPR

>GRMZM2G002555_T01|PACid:20858754
MGARRSSTWPHHLPLLFFLVLVNTSHTTTINITNRCPYTVWPAATPVGGGMQLEPGKSWVLQVPGNTQSGLVWARTG
CSFDGHGNMSCETGDCGGVLACASSGQPPFTRAEFSLGGLNNTDFFDMNLIGGFNVPMDFLPVPSNGSSGCSRGPRC
PADITSQCPGELKVPGGCRGACQHCNGSTVNSNTVFYVRMCPDAYSYSLDQGPIMYVCPSGTDYQIIFCPPVDLVSL
SPPPITPLSPPPTPTSPTANGTSSITSSSKSKNRSLFGFVLGGSLAGFIFIASLALFFILHRRRLQRRQEMQEEEEA
EFGRLPGMPRRFTFEQLQDATDQFREKLGEGGFGSVFKGRFGEEAIAVKRLDRSGQGKREFLAEVQTIGSIHHINLV
RVIGFCAEKTHRLLVYEYMPKGSLDQWTFHRQGDDETPRLHWQTRRKIIAHIAKGLSYLHEECMKRVAHLDVKPQNI
LLDDNFDAKLSDFGLCKLIDREKSQVVTRMRGTPGYLAPEWLTSHITEKADVYSFGVVVMEIVSGRKNLDTSRSEKS
IHLITLLEENLKNDRLVDLIDMCSSSDSQAQEQEAIQMIKLAMWCLQIDCKRRPKMSEVVKVLEGTISAETDIDHNF
VVTHRPSFDAPGIAGMSAAPLASEVSGPR

>GRMZM2G020986_T01|PACid:20861449
MGAAPVLVLLLLVAAAALPRRASAATDAGDVSAINGLYVALGSPKLPGWSASGGDPCGESWQGVTCTGSSITSIIFN
AANLGGQLGSLGNFTSITEINLSNNNIGGSIPEDLPVTLQNIFLSDNQLTGSIPVSLSKLHSLTAMSLNDNHLDGKL
PDTFDSLTELVNLDISSNNFSGPLPPSLGSLTSLTTLHMQDNQLSGTLDVLQDLSLKDLNVENNMFSGPVPPKLLNI
PNFKNDGNPFNTSIAPSTSPSSPTGSPTQTPSSSSSPSGSPSPSNAASNSSSGSTARNSNSTSSKKKKPSTLRTVGY
VLLAIVLFIVLALLVVFCLSKYQERQTRRDYSMAQLGRHQRAEEPKSKQASVQSRHDAKKGLSEVPERKKPREINLA
VPVAIEKPPEKRKEHVINLERTESEIFAAAPPPPPPPPPPPPPPPPPPSTPPPPSPPPRLPSPPPAEKVTVNPIVRP
VKRVNTPPRTGPSTSATSFSVASLQQYTNSFQEQNLIRESRLGKVYLAELPEGKLLEVMKIDNANGRIPVDDFLELV
ACISDIRHPNILELVGYCAEYEQRLLVYNHFSRKTLLDVLHEGEDIDNPLSWNARLQIALHAAKALE

>GRMZM2G048175_T01|PACid:20872145
MRRRCNQLGLPLALLFAALLGGAQCQSSHGGGGAAANLTVVGTVFCDACSTSSFSKNSYFLPGVRVRLDCMIKVNSN
SKEEIKITAEKVTNSDGAYQLDIPAIDGFECAAPGATAGESFCRAAVLDNPSALCGVPAVTTTVGHISLPSQDPSAC
FYSLNSLYYRPSKPGPAQCGGAAGLSPPAAALNTSLFYCPPWPWPPIPFCTPRPWFPPIPFFTPPPPAFPFPLPPLP
FFTPPSPPPPAFPFPLPPLPWTTPPSPPPSPSFPFPHLPPIFTTPSPPPPPPPQFPFPMPPLPHLPPLPHFPPLPSL
YSPPPPPPPPPPPPPSFPWTFPPLPFFPPGPGSSGPSPPPLMSGSRKDPTTWSPSNKQP

>GRMZM2G062390_T01|PACid:20875015
MGRGRGGAFSCSMLDLLVLSALLPFAASQSPSSLLSPAQSASRPPTAPSARPSFPSPAAPAPRLSRPPAPPAAKPSS
PPPAAPAGKPSPSPRPAPTRSPAVATPAPRASPAASPAPKPSSPPPKTAPAPKPSPSVAPAPRPSPPPVTPVPPAPK
PPPPPPPPPAPLQPPSNSTPTTSSALGQLSPSFYAQSCPDVELAVRDVVRSASTLDPSIPGKLLRLVFHDCFVEGCD
ASVLIQGNGTERTDPANLSLGGFNVIDAAKRLLEAVCPATVSCSDIVVLAARDAVVFTGGPAVPVALGRRDGLVSLA
SNVRRNIIDTGFSVDAMAASFTAKGLTLDDLVTLSGGHTIGSAHCNTFRERFQQVANGSMTPVDGSMNADYANELIQ
ACSANGTVPAGTAAVGCDSGSASVFDNTYFANLLGGRGLLRTDAALVQNATTRAKVAEFAQSQDGFFASWASSYARL
TSLGVKVGADGEVRRTCSSVNG

>GRMZM2G335638_T01|PACid:20876250
MGAAGGGRCVRLGAAPVLVLLLIAAATLPRLARAVTDAGDVSAINGLYVALGSPKLPGWSASAGDPCGESWQGVTCT
GSSITSIVFNAANLGGQLGSLGNFTSITEINLSNNNIGGTIPEDLPVTLQNLFLSDNQLTGSIPMSLSELHSLTAMS
LNDNHLDGKLPDAFDSLTGLVNLDISSNNFSGPLPPSLGSLTSLTTLHMQDNQLSGTLNVLQDLPLKDLNVENNMFS
GPVPPKLLNIPNFKNDGNPFNTSIAPSTSPSSTPTGSRPTQTPSSSSSPSGSPPPSSAASNSSSGSTARNSNSPSSK
KNKSSPLRTVGYVLLAIVLFIVLVLLVIFCLSKYQERQSRRDYSTSQLGRVHQRVEEPKSKQASVQSKHEAKKGSSE
VPERKKPREINLAVPVAIEKPPEKRKERVINLERTESEIFAAAPPPPPPPPPPPPPPTPPPPPPPPRLPSPPPVEKV
TVNPIVLPEKRVSTPPRTGPSTSATSFSVASLQQYTNSFQEQNLIRESRLGKVYLAELPEGKLLEVMKIDNANGRIP
VDDFLELVARISDIRHPNILELVGYCAEYEQRLLVYNYFSRKTLHDVLHEGEDLDEPLSWNARLHIVLHAAKALEYL
HDTCEPPVVHQNFEPANVLLDNRCSVRVAECGLAQLMASGSVTQLSGRMRALLNYEAPEIHESEPFTRQSDVYSLGV
VMLELLTGRKPYDSSRPRDEQHLVRWANSQLHDMESLSKMVDPSIRGECSVILLSRFADIISQCIQPGPEFRPAMSQ
IVQDLARIVGASGAVSE

>GRMZM2G167578_T01|PACid:20876646
MANSSNYGLGLACFFAIAAAVAGGTQFMVGGANGWSVPTAGAEPFNTWAERTRFQIGDSLVFVYPKDQDSVLLVEPA
DYNACDTSSYVRKFDDGDTVVTLDRSGPLFFISGVEANCRANEKLIVMVLAARSNGNGTGGGAQAPSTAPQPASPAA
SPPPASSTPPPPSSPAPKGASPPPASAPTTTPGTPPPPAPSASSPAPASSTPPPPSAPQAPPPPASSASSPAPALT
TPPPPSATANAPQAPPPPSASSPSPSAHGATASSTGTPSSPPAGAEVKNGAALTVATGLASSFGACILGYAMLAL

Oryza sativa

>LOC_Os05g01040.4|PACid:21941426
MANKSKSAAASPSSSSSPSVALGLAATTPTTSPLPLAPAAAASSSNPNATPADTTPTSPPPASPPLPSATPPLAASP
PPPPPPPPPPRNSPSPPKPPSQAAELFNVANTAEVRMFQRMVLGNHDDSSDMSQYGWSSSRQ

>LOC_Os11g05935.1|PACid:21944221
MVGGGAVVGPAVMLAVSVIVLLSAACSGAGEGPDASLLCVSECGTCPTICTSPPPPPPAPPSSLSPSTPSVLPLYS
APPPPYLTLLLPPPSPADESDMFPPPEAPTTTNPPPSPPPPPSSPPPPTTKSSSGSTASPPSSSSHFSSPPSPPSSS
NPYYYFYLSGSGRRCGGGGAASVYTALILAALLPIATFLT

>LOC_Os01g45700.1|PACid:21904983
MMRRPAPVLVLAILAVAASTAAAAAIEGEVKCGGCSPCGGADCPVLYPSPPPPALPPPPPYYYYSPPPPAYYPGSYC
PPPPAAYVQFGGGAQSGRGPLYPQDPGFMPSSAPSSHGSRAVRLFTACAAFASLWFLW

>LOC_Os01g02150.1|PACid:21907107
MARWLQLPCSLLLVIMLVAATAAAADVVVRDDEKCAACGLPSPCGTTCTYASPPPPDVLPTPVYYPPPPPVYYPPPS
PPPVAYPPPTTPSTNCPPPPYGGGGYNPTPSYNPTPGYNPTPSGWFTPPNMPSYLTPPGTLYPQDPGFRPNAAAGGA
ASWRAVVLAAAAVAGALAL

>LOC_Os01g20780.1|PACid:21909948
MALHLFLLLLLAASLALANAAPPPNDEGATTIVLKDGTTCKLCASCDNPCNPSYYPPPPPPPVVTPTPQCPPPPSYPS
GGGGGGGGGTVMYTSPPPPYSGGGGGSSTGGGGIYYPPPTGGGGGGGGGWQQGGGGGGAYPTPPPPNPFLPYFPFYY
YSPPPPFYSSGSSVAGVSAISSAAAATFTLLLTGLLLW

>LOC_Os06g08190.1|PACid:21932252
MHLRPLLSLAWLLVAALFLLQSSPTSSSSSPPPPPPPPPPSGAAAGEDNGGGSAIWAGVVAADTGGGGGEKAAQRRS
LRRVGLHRTPPPPTSNDGGIESISPPPPPEQDGQFFSSTGYPTRPPPAS

>LOC_Os01g07060.1|PACid:21908199
MAAFLHLAILVSLLAAGATANNGYTTPSPPPPPQHTPPPSPPPPAPAAHQSSDKVLVRVEGKVYCQSCEHRNSWSLD
GARPLRGAEVSVTCRDAKNRAAWWRLAVADESGYFLAEFGVTRASDFLGADPRGACYARLLSSPDRKCDGLTNINAG
MVGAPLRDEGKRWPGQGYDNVVYAAGPLAFRPANCPPKHY

>LOC_Os01g41120.1|PACid:21909835
MTTTTTTTNHSAKSILLLALLLVPHLAAASSGDALISQRRRLLDYHGGGGGHGGGDVYVDPSYTFPNARLRDAYVAM
QAWKRAVLSDPHNVTGTWIGPDVCAYEGVFCAAARDDPHLVVVASVDLNHADMAGHLPDELGLLADLAVLHANSNRF
CGAVPSTLERLHLLHELDLSNNRLVGAFPDVVLRLPSLRYLDLRFNDFEGPVPAELFDRPLDAIFLNSNRLRFRIPD
NVGNSPASVLVLANNDFGGCLPASVANMSGTLDEIILMNTGLKSCIPPELGMLTGLAVLDVSHNSLMGAIPGELARL
ENIEQLDLGHNRLTGDVPEGICHLPHLQNFTYSYNFITGEPPVCMHVKAFDDRRNCIPGRPDQRPAEQCQFQNTHHV
NCDAFRCKKFVLPSPPPPPPSPPPPSPPPPSPSPPPPSPPPPSPPPPSPSPPPPSPPPPSPPPPSPSPPPPSPPPPS
PPPPSPVYYSSPPPPYYEVSPEDRYLSPPPPPAYHEAPPPPYYEVSPEDRYLSPPPPPAYQETPPPPPQYEVSPEDR
YLSPPPPSPVKWKLPVYEYSSPPPPAATWKP

>LOC_Os01g08470.1|PACid:21910710
MRGAGFLLLVVFAVAVAGGAVAAAADLDGGDGGGGVAAEWRFPSSRMRDAYVALQTWRREAIFSDPGNLTADWVGPD
VCNYTGVFCAPLPWDRREVAVAGVDLNHGDIAGYLPPELGLLADLALLHLNSNRFCGVLPATLRRLRLLHELDLSNN
RFVGRFPEVVLDMPALRFLDLRFNDFEGGVPRQLFDRPLDAIFLNHNRFRFDLPDNFGNSPVSVIVLAHNSFGGCLP
ASLGNMSGTLNEILLINTGLSSCLPPEVGMLREVTVFDVSFNRLAGPLPSAVAGMRKVEQLDVAHNLLTGAIPQAVC
ELPRLKNFTFAYNFFTGEPPSCAHAVPRYGDRRNCLPNRPAQRTLRQCAAFFARPPVNCAAFQCKPFVPALPPPSPP
PPSPPPPSPPPPSPPPPSTSPPPPSPPPPSPPPPSPPPPAPIFHPPQPPPPPPPPAPQPHPPCPELPPPPPPPPPCG
GATPALSPPPPPPYYPGPWPPVHGVPYGSPPPPPLQHHSSWPPIHSVPYGSPPPPPLH

>LOC_Os06g49100.1|PACid:21930799
MAGRAALLALLLALAAPAPAASQPAAVDPPPSWAFPNARLRAAYVALQAWRRTAIFSDPANFTANWSGPDVCGYNGV
FCAAHPTDGRVRVVAGLDLNHADIAGYIPASLPEGLPDLALLHLNSNRFCGVLPDTFSHLRLLHELDISNNRFVGGF
PEVVLSLPSLRYLDLRFNDFEGAIPPKLFDRPLDAIFLNSNRLTRPIPPNLGSSPASVVVLAHNRLGGCIPPSIGRM

AETLNEIVLIDDELTGCIPPQVGLLRKVTVFDVSGNHLQGPLPGSVAGLAAVEQLDVAGNLFEGPVPATICSLQSLK
NFTYEDNFFSSRPGCPAATADGRWNCIPGAPAQRPPAQCAAAAAHPFDCSKAQCQATPPTTRRPGGRTPLAPHRSPL
PHHMPPRRTPPTPPPPSSPTPSHLPPPPPTYSESPKSSMPPSTSPPSSHGASPPSSSSSPPTEHPGYVLPPLTPPPP
TTTPPGHHAPVPGTPSSPPSSSWSPPQGGGGKLPFPPVHGVAYSSPPPPPSGDKLPFPPVYGVAYSSPPPPSKPYN

>LOC_Os11g43640.1|PACid:21946250
MDLRLLLAPGGRRAAMALILAVLAACLSSAANVGAVTSAEVSYIAHRQLLAMKEAGVSEEGDLPSDDFDFDDRVGVA
VGDFPNPRLRKAYIALQAWRRAFYSDPKGYTNNWTGNDVCSYNGVICYAAIDDPKIMVVAGIDLNGADIAGYLPPEL
GLLTDLAFFHINTNRFCGIIPKSMSRLSLLHEFDVSHNRFVGVFPHVCLEMAVLKYLDIRFNDFEGELPPALFDKEL
DAIFVNSNRFVGYIPGNLGNSTASVIVFANNAFVGCIPKSIGCMAKTLDEISFMNNKLDGCVPMEMGYLQNTYVIDI
SGNVLVGTLPTSLSNCSKLEQLDVSRNVFTGIVHESICELPVLVNFSFAYNFFNSESAPCMPSESSKVNLDDKDNCL
GALRPAQKTTLQCAPVLARPVDCSKHVCPGHPTPGKPSEPPEKPPLIPVPVGPPEKSPAYEEPPAAPSTPTSHGPPP
PEEESPEEPPEEPTPSPTPSSPESPAKMAPPPAPAIKGVTSPPAEYGAPPPPSSGWLPKSPERKKAPPPQAEPPTEY
SPPATPESSPPPEGKSPPTPTASHSPPPVPEGHTPSPPKSGPPAGESPPTPESKASPPPTPEEYTPSPPKSTPPAEK
SPPTPESKASSPPPPAPEGHTPSPPESTPPSEKSPPTPESKASSPPPPTPEGHTPSPPKSTPPTEKSPPTPESESSS
PPPPAPEGHMPSPPKSTPPVEKSPPTPESEASSPPPPAPEGHTPSPPKSSPPEEKSPPIPPTSHTSPPTPEEYTPSP
PKSSPPEEKSPPPHSPEKSPPSEAHPTSPPPSEKSPPTPAEESSPPTPEKSPSPPSGHEGTPPSPVKSSSPPPEAHV
SSPPPEKSSSPPPEAHVSSPPPPEKSPPPPETKSPPTLTPEISPPPEGKSPPSHTPESSSPPSKESEPPPTPTPKSS
PPSHEEREVPTITYSGELIPTI

>LOC_Os01g25460.1|PACid:21905341
MGTLPPFPPLLLLVAVIFSFLSSSCFAVTSHEAAAIARRQLLNFEKHGDHVHIDIDIEIKVSNPRLAAAHRALHALK
RALYSDPGNFTGDWAGPDVCAYNGVLCAPSPDNASASAVASLDMNAADVAGYLPREIGLLSDLAVLHLNSNRFCGVI
PEEVANMTRLYELDVSNNRLVGAFPGAVLRVPELSYLDIRFNDFDGPIPPELFLRPYDAIFLNNNRFTSGIPDTIGR
STASVIVLANNDLGGCIPPTIGQAAATLDQFVFLNNSLTGCLPLESGLLANATVFDVSHNLLTGAIPATMGGLAKVE
QLDLSHNTFTGVVPGDVCGLPALTNLSVSYNFIAGEDAQCSSALLDAKLDKSLEDEANCMGNVRPMQRSAGECAPVV
SHPVDCSKTKPCGWPAPAKKPAPESSKHSPPPPPPPAPVQSPPPPAPVVSPPSPVFSPPPAFSPPPPPKTSPPPPVS
SPPPPPPPTMSPPPPIQEPVILPPILSAKYQSPPPPFFEGY

>LOC_Os02g04540.1|PACid:21923130
MISRGHAAAAWALLLLLLGAAVAQHEGNVSEEYAASFASRYDAPPSWAFPNPRLRAAYAALQAWKQTAIFSDPSNFT
ASWVGANVCAYNGVYCAPSPGYGGGGGGGLVVAGIDLNHADIAGYLPASLPLGLPDLALIHLNSNRFCGVVPDTFRR
LRLLHELDLSNNRFVGAFPEVVLALPSLRYLDLRFNDFEGSIPPALFDRPLDAIFLNSNRLRNPIPANLGNSPASVV
VLAHNNLGGCIPPSIGKMADTLNEIVLIADELTGCVPTQVGLLRKVTVFDVSDNHLQGPIPASVAGMAAVEQLDVAR
NLFEGAVPAGVCGLASLKNFTYTDNFITSRPGCGVATADGVWNCIPGAPGQRPPSQCAAAAAHPFDCSKAQCQAASY
TPTPGGSPGRGGQGPPAPVSSPPRRRGPYPQPPSSSPTPSYPSPSSSYPAPPGSNTPSYPSPPSSATTPSSHSPPGG
SSSTTPSYPSPNGGKPSTPSHPSPPGSTTPSYPSPPSSSTTPSYHSPPQGHTTPSHPSPPSSSTAPPSHSPPQSTPT
HPSYPSPPVTYTPPPPTSADRPDVRFAPPPGSYGPIPSTPPSSGSPPSPSSGYQPPSGQPGASPPTQHVPGAGTPTT
TPPSHSHPSTPPSPSGPSFHPPPTPHNCSPPSHGSSTGGGHGGGHPPSTSTPPGGKLPFPPVYGVSYASPPPPVKPY
N

>LOC_Os01g53640.1|PACid:21907106
MGMVGAYADPFLPPKMALSAYAAPMGEYGQPQPQPAPRPPACPYSSSSSSPPPVSASYHSWPPATSASPVSSPPPVS
SPPESFPSSPPPALSPPPPDAPPPSLPPSPPPSPPPSPPHVEVQAPPPPMTTDQPHVQPRVYPSPPPPSLPPPPPQT
FSPPSPPPFHPPSSSPAPAPVPAAVVYPPPPPPPRIASPPPPRNHIKPHYVPRSSARSHSNSTRASSGAGKNIEISRE
AATTIVALAGLAMLSFVGATIWFVKKKRRIEPPASLPTQQPAPPPPPNYFPSSGGSSLTSDAFFISPGYHPVRLFS
AGSHGYPYSPADSAIGYSRMLFTPENLAEFTNGFAEQNLLGEGGFGCVYKGILPDNRLVAVKKLKIGNGQGEREFKA
EVDTISRVHHRHLVSLVGYCIADGQRMLVYDFVPNNTLYYHLHVSEAAVLDWRTRVKISAGAARGIAYLHEDCHPRI
IHRDIKSSNILLDDNFEAQVSDFGLARLAADSNTHVTTRVMGTFGYLAPEYALSGKLTAKSDVYSFGVVLLELITGR
KPVDASQPLGDESLVEWARPLLLKAIEHREFGDLPDPRMENRFDENEMYHMIGAAAACIRHSAAMRPRMGQVVRALD
SLADSNLNNGLQPGRSEVFLEPQSEEIRLFQLREFGSRDCSDEMSQASWRSRRDL

>LOC_Os01g54700.1|PACid:21903658
MSSQAPSAPVTPAAPPPQTPPVTPPPVTAPPPVSPPPVTPPPVTPPPVSPPPVSPPPVTPPPVSPPPVTPPPVTPPT
PVAPPPVPPSPPPPTPTPTPVTPSPPPPVTPSPPPPVASPPPPDVPTAPPPSNNPPSPPPSPSNVPASPPPPRISLS
PPPPPSTPTQSGASSGSKSSNNGTVVAVGVAVAAVVVLGLAAGLIYFFVSKRRRRQHPPPPHHPGYPPFPAEFYDP
HRPPSQQQSHALSPSPSSTPPLLLQPHSFVSSGGASEAASAVPGIAMMGGAFGYDELAAAADGFSESNLLGQGGFGQ

VYKGTVRGQEVAIKKLRSGSGQGEREFQAEVEIISRVHHKNLVSLVGYCIYGEQRLLVYEYVPNKTLEFHLHGSGRP
ALDWPRRWKIAVGSAKGLAYLHEDCHPKIIHRDIKAANILLDYTFEPKVADFGLAKYQATEQTAVSTRVMGTFGYLA
PEYAATGKVNDRSDVFSFGVMLLELITGKKPIMVSHGDQPDTLVSWARPLLVRAVEEENFEELVDPRLENNYDAYDM
GRLIACAAAAVRHTARSRPRMSQIVRYLEGELAAEDLNAGVTPGQSAMQRTSGGTTDQMKRLRKMAFGSATGTGTVS
EYTSSEFSEPTSEYGLNPSTEYTTSAAGGDTGEVTVDVQMTAGASGEAAGTERLSRRTTARRGGRV

>LOC_Os01g02040.1|PACid:21907573
MASSPSPSPGTATPATAAAAPSTATPTPATQPNATPADPSITPPAASPPLPSAATPPPQPDSPPPSPPPAPPVAVPP
PATVPPPPVPVASPPPTPSATLPPPSPPASVPVPPTPATTPPKPSPVQQPPVAASPPPSSPADLPPPNPPARSDTPP
VVQSPPPPHRRSPRTPWAPPMAPSPSGSPTKPSPASPSPIAGDPIIPTPNNPSSPLATPSAPGSGTPVVTPSAPVSG
PPSPGTAPATAADRSNKSLSPNTQDGSVSSSDGGMSSSAKAGIGVVVAILVLSLVGAAFWYKKKRRRATGYHAGFVM
PSPASSPQVLGYSGKTNYSAGSPDYKETMSEFSMGNCRFFTYEELHQITNGFAAKNLLGEGGFGSVYKGCLADGREV
AVKKLKGGGGQGEREFQAEVEIISRVHHRHLVSLVGYCISGDQRLLVYDFVPNDTLHHHLHGRGMPVLEWSARVKIA
AGSARGIAYLHEDCHPRIIHRDIKSSNILLDNNFEAQVADFGLARLAMDAVTHVTTRVMGTFGYLAPEYASSGKLTE
RSDVFSFGVVLLELITGRKPVDASKPLGDESLVEWARPLLTEAIETGNVGELIDSRLDKNFNEAEMFRMIEAAAACI
RHSASRRPRMSQVVRVLDSLADVDLSNGIQPGKSEMFNVANTAEIRLFQRMAFGSQDFTTDFTQSSWDSRSRDVDAS
GSRPL

>LOC_Os03g37120.1|PACid:21912609
MMPRVLMASTEDTATAPAGGPPEPPPQSSSASPSPSPPPPPPTPSSPQRPPPPPPPATPPPPPPASPGKNQSPASPS
QDSPPPVASPSVSSPPPAPTTPPSPPPPSKSPPPPSPPPTTSSTPPSHQSPPEEGTSPPPSPSSGATTPSPPPNAQS
SSSSSTPPAGAGTSPPAPREMPSPGTPPSPPTTLITTQAPPIQPPPPPGGNSMIMPSSLTTAGTSQSPPDATTAGAP
PPPAPSVGAWGGNVPSGLLIGVAFAGFLLALASMFLFLCIKNRWKRRRRPAQVMNLARRRTLVVPERVASPEVYQPS
NGPTASPSGTSSYEFSGTTSWFTYDELAAVTGGFAEEKVIGEGGFGKVYMGALGDGRCVAVKQLKVGSGQGEKEFRA
EVDTISRVHHRHLVTLVGYSVTEHHHLLVYEFVSNKTLDHHLHGGGLPVMDWPKRMKIAIGSARGLTYLHEDCHPRI
IHRDIKSANILLDDAFEAKVADFGLAKFTNDSVTHVSTRVMGTFGYLAPEYASSGKLTDRSDVFSFGVVLLELITGR
KPVDSSQPLGEESLVEWARPLLVDALETDDFRELADPALERRYSKSEMRRMVEAAAACIRYSVTKRPRMVQVWRSLD
VEGSSPDLTNGVKLGQSMAYDSNQYSADIELFRRMAFANDLSTAELGYSGKDDVRRPPR

>LOC_Os02g26160.1|PACid:21921289
MPPRCRRLPLLFILLLAVRPLSAAAASSIAAAPASSYRRISWASNLTLLGSASLLPGAAGVALTTPSRDGVGAGRAL
FSEPVRLLLPQDAAASASASRAATPASFSTRFTFRITPSPTYGDGLAFLLTSSRTFLGASNGFLGLFPSSSASDEGE
LRDVSTVAVEIDTHLDVALHDPDGNHVALDAGSIFSVASAQPGVDLKAGVPITAWVEYRAPRRRLNVWLSYSPSRRP
EKPALSADVDLSGLLRTYMYAGFSASNGNGAALHVVERWTFRTFGFPNSSYAPPPTKYIGPMPPNNQPLPPPPSPSP
SPPPPSPPPPPHPNHRRRHLFYKVLGGVLGGMVLLGLVVVGSAVLLGRSVRRKNQEHAVASEDMGEATLSMEVARAA
TKGFDSGNVIGVGGSGATVYEGVLPSGSRVAVKRFQAIGSCTKAFDSELKAMLNCPHHPNLVPLAGWCRSKDELVLV
YEFMPNGNLDSALHTLGGATLPWEARFRAVYGVASALAYLHDECENRIIHRDVKSSNVMLDAEFNARLGDFGLARTV
SHGGLPLTTQPAGTLGYLAPEYVHTGVATERSDVYSFGVLALEVATGRRPAERGISVVNWVWTLWGRRRLVDAADRR
LQGRFVADEMRRVLLVGLCCVHPDCRKRPGMRRVVSMLDGTAPLILVPDKMPPVLLQPVPNASSMNSADTANTAFFS
CR

>LOC_Os06g29080.1|PACid:21928576
MSSESDQAPSPSSPSSSSSSSSGNGSNKAPPPEESDNSSSNGSSSSSPTPPSSQSSDSDSGGGSSSPSQGSSSPSPP
PPSGSSSESHSSPPPAPPQSSSSSSSSSSSGGGSKSSPEAPSPPSESSGNGGGGGGGGRSSPPPNWSPPPQQQQQHQ
SGGSTPSPPPSPSSNQPPPSSGSSASSSEPSPPRSSPPPSPPQSSGGNNGQPPKPSGGQQQAPPQSPPSAANQSVVF
IPVPVASNSPPGMLPPPQVIDATPSGAISSTNFPGGRNSTAGSSNTSLSQQQHTTVSSTAQASSSGHIAAAIAGAAV
TGLLCAIVAIYLIVSSRRKKKMDGLVYHYDGNNYFVPSSQFGGSSRNHHPPPSAIMLNSGGASADGGGYYNSGTFSG
GEGTGPAGSKSRFSYEELTGITSNFSRDNVIGEGGFGCVYKGWLSDGKCVAVKQLKAGSGQGEREFQAEVEIISRVH
HRHLVSLVGYCIAAHHRMLIYEFVPNGTLEHHLHGRGMPVMDWPTRLRIAIGAAKGLAYLHEDCHPRIIHRDIKTAN
ILLDYSWEAQVADFGLAKLANDTHTHVSTRIMGTFGYLAPEYASSGKLTDRSDVFSFGVVLLELITGRKPVDQTQPL
GEESLVEWARPVLADAVETGDLSELVDPRLEGAYNRNEMMTMVEAAAACVRHSAPKRPRMVQVMRVLDEGSMTDLSN
GIKVGQSQVFTGGSDAADIQQLRRIAFASEEFTGEFEQRTTNSNSESRPMNRIPE

>LOC_Os06g29340.1|PACid:21928806
MRTLLQVQPGGSGGGSVPLPPTPTVTSPLTGGSPPPPATSSSSTPPPPSSSSSSAPPPPAPSSASPPPPSPSEPPPS
LPPPPPPVVTSPPPPVAEPPPPPTALPPPPPSPVPTPSPPPPTAPPPADNAAPPPAPTQAPPPPAPPPRAHATPPSS
SAAPPPVAAAPRASPADAPPPPPPPPRHSAMDSATPPPPPARTSAATTTRSPAEAGSGAGVPAPPVSGGLSSGTTAA

VVVVVVVAFLGLAGMFACLSQRRRRRQAERYYPGFAVPSYTPQHMSGEAPFLRPPSASGSMNFSAGQSQGVSPMMSS
GQAYGQSTSYGQQQRLTSANYSTGSQGGGAARSVAASGELSVGNTKAFTFDELYDITAGFARDKLLGEGGFGCVFQG
TLADGKAVAVKQLKGGGGQGEREFQAEVEIISRVHHRHLVSLVGYCIAEDHRLLVYDFVSNDTLHHHLHGHPRIIHR
DIKSSNILLDEHFEAQVADFGLARLAENDVTHVSTRVMGTFGYLAPEYASTGKLTEKSDVFSFGVVLLELITGRKPV
DSSRPLGDESLVEWDKETGGLDPLLTLIDTSRQNQPTSRPLLNRAIENQEFDELVDPRLDGEYDDVEMFRVIEAAAA
CIRHSAARRPKMGQVVRVLDSLTDVDLSNGVQPGKSQMFNVANTADIRQFQRMAFGSQDFSSEYSQSRRQKGFREVS
LELVGNERQDRCRVNSQRCVAHFGLAKLAKNDVTNVSTRVMGTFGYLAPKYAWKLAEKSDMFSFGVVLMELITGWKP
VDSSRPLGNESLIEWESSNTSAPSDCKYLLRDAQFYHVMLVFGDMFFLTILILTLKIYVFKQFIIQHIH

>LOC_Os07g39920.1|PACid:21901643
MRRRVALSTAIALLVGAQLCVAAEVEVAGAGGGVVRRRSLHQPFFPIEWSPPPPMSGSEAVPPPPPAAAASATTGGG
RSTTTVMNTVAIALSAGLVALAVASYSCCLLLRRRRREEEDDGDRAAKRAVGAAAAVAARVPSDVGSSSRQHRSPPP
SSTASDAIYLDPLTTLVEVRQHEKSPDLRPLPLLKQPSPDLRPLPPLKRPESQPPPPPPSTPPLTTTGYSTDEEDQA
TYYTAPKTAMSSFSRSTSQHSTLEQTAMPPMAAPAPPQTNPPRPVRPPPPPPPPRQRLLRPLPAESPPPAALANLEL
TGSPVKPAVEDRGGENSGAARPPKPPHLKPLHWDKLRAISGRTTVWDQVKNSDTFRVDEEAMESLFLNSGGGGAGSS
DPAARRGGSGKQERRLLDPKRLQNVAIMLKSLNVAADEVIGALVRGNPEDLGSEFYETLAKMAPTKEEELKLKGYSG
DLSKIDPAERFLKDVLGVPFAFERVDAMLYRANFDNEVNYLRKSFGTLEAACEELRSSKLFLKLLDAVLKTGNRMND
GTNRGEARAFKLDTLLKLADIKSTDGRTTLLHFVVKEIIRSEGFDSDQSAVNPGSGSKEQFKRDGLKLLAGLSSELS
NVKRAATLEMDTLSGNILRLEADLEKVKLVLQLKETCSDQGASENFFQAMVVFLRRAEAEIKNMKTAEENALRLVKE
TTEYFHGDATKEEPHPLRIFVVVDEFLLILDRVCRDVGRTPERVMMGSGKSFRVPAGTSLPPHRNENRRVLSSSDED
SSSS

>LOC_Os07g40510.1|PACid:21901985
MSFGAQTRTFVPPPPPPPPPPRSGVGGNTPPAPPPPPLRSTVPAISPPPPPPPPPLKPSSGAPCPPPPPPPPPPPPP
SAPSSRAFSSAPPPPPPPPLLRSVPPPPPPPPISHSNAPPPPPLPAARFNAPPPPPPPPTTHFNAPPPPPPPPITRS
GAPPSPPPPPSPPPPPPPPGARPGPPPPPPPPGARPGPPPPPPPPGGRPSAPPLPPPGGRASAPPPPPPPSTRLGAP
PPPPPPGAGGRAPPPPPAPGGRLGGPPPPPPPGGRAPPPPRGPGAPPPPGGNPSSLIGRGRGVVRASGSGFGAAAAR
KSTLKPLHWIKVTRALQGSLWEELQRNDDSQSVSEFDLSELESLFPAAVPKPNDSSKSDSRRKSLGSKPEKVHLIEL
RRANNTEIMLTKVKMPLPDLVSAALALDQSTLDVDQVENLIKFCPTKEEMELLKNYTGDKENLGKCEQFFLELMKVP
RMESKLRVFSFKIQFGSQVADLRKSLNTIDSSCDEIRSSLKLKEIMKKILLLGNTLNQGTARGAAVGFRLDSLLKLT
DTRATNNKMTLMHYLCKVLAAKSSQLLDFYMDLVSLEATSKIQLKMLAEEMQAVSKGLEKVQLEYNASESDGPVSEI
FREKLKEFTDNAGADVQSLSSLFSEVGKKADALIKYFGEDPVRCPFEQVISTLLTFVTMFRKAHEENRKQAELDKKR
AEKEAEAEKSKAQLASKNDSKPSNPSRQVKQTPDTKTRAASRRGKDVG

>LOC_Os08g17820.1|PACid:21887535
MISLVHEEITQVVDINTGCLSSLDMTVPSTMNSSRPVLIDQNSKLDDQFGSLQSSSPTMIMSQQFPVSRSSSVLSSD
FSPRLLSACPRFHSAPSALGITALLEDHAAFGDTKNSVKVSSAVVKIPSKQSSQQHPITVTPVVTKCTPSPPPLLPP
LAPVVPVPSDDQMISQEKDMSQQAQKHPDLSSFPSLSPTQQKQSTSKLCQTILPTNHQLSSSNITKEPLQISPAPTP
PPLPTPSTSSSSSCHCLPPDSMLSTTTALFRPPAPPPPPLQSPSTPRCSPVRTLASPPPPPAPTSSPVRMSGPPPPP
PPPAPNSCPSRPAPPPPPPPPPLASTSSPPRPAAPSPCQLHTSTSSPARPVPPPPPTLSTIRSSAPTPPLLPGATSAP
SPPPPPPPCSSSNQLSAPPPPPPSFSKNNGSIAPPPAPPGGNAKLPGMRGRGPAPPSGPMSRSLQSGQAASRRSNLK
PLHWVKVTRAMQGSLWEESQKTDEASKPPVFDMSELEHLFSAVLPSSDGKRSDKSGSRASGSKPEKIHLIDLRRANN
CGIMLTKVKMPLPDLMSAILTLDDTILDADQVENLIKFTPTKEEAELLKGYKGDKQVLGECEQFFMELMKLPRVDSK
LRVFLFKIQFPSQVSDLKRSLNIVNSSAEEIRGSAKLKRIMQTILSLGNALNQGTARGSAVGFRLDSLLKLSDTRAR
NNKMTLMHYLSKVLSEKLPELLDFPKDLASLELAAKVQLKSLAEEMQAINKGLEKVEQELTTSENDGPVSEIFRKTL
KDFLSGAEAEVRSLTSLYSNVGRNADALALYFGEDPARCPFEQVVITLQNFVRLFVRSHDENCKQLDLEKKKALKEA
EAEKTKKEPENAQKTKEPGNDKAKHNNSIKELDISLQSPAQTASAK

>LOC_Os05g47940.1|PACid:21940036
MAGPARWALLLLLAVALLVPAALAAGGGGNGGASASTPNNGNGGNNGNNGNNGNNGNNGGGNEKHEKSPPPPHHDSP
PPPRASPPPPVYSPPPPPPRSSPPPPPVYSPPPPVSSPPPPVPSPPPPVSSPPPPVPSPPPPVPTSPPPSPLPSPPP
PVPSSPPPPVSSPPPPVPSSPPPPVVPSPPPPVLSSPPPPVVASPPPPVVPSPPPPGASDVVYCTNKTRYPTCTSPA
YCPSRCPKSCHMDCATCKTVCDCNLPGAVCQDPRFIGGDGNTFYFHGRRDRDFCLLSDANLHINGHFVGNHVPGLKR
DPTWVQAIAVQFSGGHRLYVGARRTAVWDDDSDRLAVVFDGETVQLQRVAHARWESGSGLSVTRTKAANGVLVELDG
VFKITANVVPITKEDSRIHRYGVTDDDCLAHLDLAFKFYALTDDVHGVLGQTYRSSYVNRLDVSAKMPVMGGEKQFT
SSGLFAADCAVARFGRAGDAGAVAVASEELVDVKCSTGLDGVGVVCKK

>LOC_Os03g14615.1|PACid:21914352
MVASMASAAFVLVAIVLPISPFAHAQRTTQPPIVAPTMPPTPPPTQPPSPGPMAPMPSPPPPTQPPSPGPMPPMPPP
PAPMPSPPPMQPPSPGPMPPMPPPPAPMPSPPPPTQPPSPGPMPPMPSPPPMQPPSPGPMPPMPPPPAPMPSPPPPT
QPPSPGPMPPMPPPLAPMPSPPPMQPPSPGPMPSPPPPTQPPSPGPMPPMPPPPAPMPSPPPMQPPSPGPMPPMPSP
PPPTQPPSPGPMPPMPSPPPMQPPSPGPMPSPPPPTQPPSPGPMPPMPPPPAPMPSPPPPTQPPSPGPMPPMPPPPA
LMPSPPPPTQPPSPGPMPPTPPPPAPMPSPPPQNQPPPPGRCPLIKIVILKECTKLSLFDLVLNPSKARQQCCPPLE
DLSSSGATDCLCRALKGPIGVLPPPIRVILGLCGKTVELNLFCH

>LOC_Os01g67390.1|PACid:21907520
MKFSMRGQPKGIPWLVLAMLIATFAMLTEGATSLSPPSLSLTPTYAPVIKVIGKVYCYRCFNEAHPEESHGKEHLKG
AMVKVTCQANDQALVGFGYTQDNGKYSVSITGLPLSSTYGADSCKVELHSAPGGSDCNVPIELNLSGLSVYSKSNEE
VVLQANQVMAFASQKTFGFCSKPHIQPPIFPYNSPPPSPYQYPSPPFNYKSPPLPNQFSPPPFNKFPPPSHQYPSPP
QSSYHSPPPYQYTPPNSYQAPPTSYNHPPPPYGYNSPIPPTNKYLPPPYYFNSPPPQYQHSPPANSYVSPPLAHQYP
PPPYKSPPIPPYYFNSPPANHYSPPPYNFGSSPPTYQYSPPLLPKTPKYLPPKVPLEMSPPAHATSPQPLVHYSPPP
PLQHAGISSTTPSVNSYQSPPPVNQLS

>LOC_Os03g07640.1|PACid:21910977
MKPSLLLLALLATFVVAIANADDYTTAAPAPSPEAEASPPSPPTEASPPPLAPPPSVTSSPPPPAAGPLMPPPPPPP
SVTSSPPPPPLPPPPPPPAASPPPPPPSPPPPSPVKSSPPPPPAWSPVTNVNDYTIQQVGKFAVQSYCLNTGAKLVY
VNVVSGQTQPCSGGGSNYQLVINVAAGVRTAQYSVFVWGILGTTTWKLWSFTPKN

>LOC_Os06g07220.1|PACid:21930757
MAAALATVVSFLLVVLVSVAHGWNKDCPPPGSGSSGGGHHGKPPGSGSGGGGHHGKPPEHHHHHKPPPSPRCPSCHP
PYTPPTPRPPPTPPYVPSPPPYVPPYIPPPTPPYVPPYIPPPTPPYVPPPTPPSPPPYVPPPTPPSPPPYVPPPSPP
ATKTCPIDALKLNACVDVLGGLIHLVIGQKARAKCCPLVQGVADLDAALCLCTTIRARLLNINIYLPVALELLITCG
KHPPPGFKCPPLYGA

Glycine max

>Glyma02g01850.1|PACid:26289743
MAPFPLMCFVILLLSSTVSVTATATQTTLTVGKLDQVPHIMCKECELPPPPPKECPPPPIPLAPPPPLPPSPPPPLP
PSPPPPLPPSPPPPLPPPIPLSPPPPLPPSPPPPLPPSPSPPNLPSPPPLAPPPPLPPLAPPPPPLPPLPIPPNGPG
EYLVPPPRDPNLPDLDDLNGAKIINPLLCFSNTSYFSLHSFTLLVLLCLPYYFFM

>Glyma08g47751.1|PACid:26309653
LIHPTTTTTLFLQSPPPPSPKPRSPYYYNSPPPPVVYSPYIPYPHPHEYSHPLISKPKPSPTPYHKPYYYNSPPPPS
LSPHPYPPYYYKSPLPPPPSYYYKSLAPPSYYCKSSPPPSPSSPPCYYISPPPPPPPPLLKEPFHPLYYYKSPPPPS
PSPLLHYYKSQPPPSPSSLSSYYYNHLHHQNPSPPPSYYYKSSLPPS`LSPPPSYYYKSPSPPPPYYYKSPLPPSPT
SHPPYYYKSPPPPMSSAPAPYHYVSPPPPTKY

>Glyma01g45440.1|PACid:26325275
METHLVLLLALIFTVVAGVGAQGPSTSPAPPTPQSSPPPIQSSPPPVPSSPPPAQSPPPASTPPPAPLSSPPPASPP
PSSPPPASPPPASPPPASPPPASPPPASPPPASPPPATPPPASPPPFSPPPATPPPATPPPALTPTPLSSPPATSPA
PAPAKVVAPALSPSLAPGPSLSTISPSGDDSGAEKLWSFQKMIGSLVFGCALLSLLF

>Glyma15g03856.1|PACid:26331582
MGTSAGPRHWPQLIYTLAFCLIAVSVVAGHDNPYYASPPFNEELPPCHHLPPPPPFPSPPPPYIYKFPPPTSPSPPP
PCVYKSLPPPSPSPPPPYIYKFPPLTSPSTPPPSVYKSPPPPSPSPPPSNLHKSPPPPSPSPQPPYVYKSPPPPSLS
PPPPYYNKSSPPPSPLPLPPYVYKSPPSQFPSPPSPYTYKSHPLPSPSPPPPYIYKSPPSPSPSPPAPYIYKSPPSP
SPSPPAPYIYKSPPSPPAPYIYKSPPSPPPYIYKSPPSPSSSPPAPYIYKSPPSPFLSPPAPYIYKSASPPPPYIYK
SPSSPSPSPPAPYIYKSPTPPSSSPPPPYIYKSPPSPSPLPRAPYIYKSPPLTSPSTPPPYIYKSPPPTPPATIY

>Glyma20g01150.1|PACid:26339393
GKGNDNGSTLSPSPVPKPPKSNPPKLETPKAQPPPTPKVEPPPTPKVVHSPPPPVSSPPPPIHSPPPPIHSPPPPKL
VLSPPPPAYSPPPPINSPPPPINSPPPPVHSPPPQTSVLSPPPLIHFPSPPVHSPPMPLHSSPPPVHSPPPPINSPP
PPVHSPPPPTQVLSPPPLIHFPSPPVHSPPPPVHFPSPPVHSPPPPVHSPPPPVHSPPIPVHSSPPPVHSPPPPPIN
SPPPPINSPPPPVHSPPPPTPGLSPPPPVHFPSPPVHPPPPPVHSPPLPVHSPPLPVHTPPPPVYSPPPPIHSPPPP
VYSPPPPTWDDVVLPPHFGSAYSSPPPPTIDGY

>Glyma16g28605.1|PACid:26352944
MGSLMASLTLTLVLAIVSLSLSSQASADKYDYSSPPPPVYKYKSPPPPYKYSSPPPPPKKPYKYPSPPPPVYKYKSP
PPPYKYPSPPPPPKKPYKYPSPPPPVYKYKSPPPPVYKYKSPPPPPKKPYKYPSPPPPVYKYKSPPPPYKYPSPPPP
PKKPYKYPSPPPPVYKYKSPPPPYKYSSPPPPPYKYPSPPPPAYYYKSPPPPPKKPYKYPSPPPPHYVYASPPPPYH
Y

>Glyma16g28590.1|PACid:26353896
MGSLMAYATLTLVLAIIVSLSLPSQTSADDKYDYSSPPPPEKPYKYKSPPPPVYKYKSPPPPPPEKPYKYPSPPPPP
VYKYKSPPPPYKYPSPPPPPKKPYKYPSPPPPIYKYKSPPPPYKYPSPPPPPYKYPSPPPPVYKYKSPPPPDYKYKS
PPPPYKYPSPPPTPKKPYKYSSPPPPYHY

>Glyma02g09201.1|PACid:26289570
MGSLMAYATLTLVLAIIVTLSLPSQTSADKYDYSSPPPPEKPYKYKSPPPPPYKYPSPPPPPKKPYKYPSPPPPIYN
THLPPPPPYKYPSPPPPVYKSPPPPYKYPSPPPPPYKYPSPPPPVYKYKSPPPPYKYPSPPPPPKKPYKYSSPPPPY
HY

>Glyma02g09220.1|PACid:26290460
HSPPPPYYYHSPPPPKHSPPPPYYYHSPPAPKHSPPPPYYYQSPPSPKHSPPPPYYYQSPPPPNTSPPPPYYY

>Glyma02g41410.2|PACid:26288537
MSRHHILHAKVVLLLFCITISSPVYANTFSSKLDQSLSSQPTHTEIKCGSCPCGSPCDDQLLSPPPPSPPPLFLPDI
SSPTPPESCDDSSPLPPPPPPPSSSRPPPTPPPPRFIYVTGVPGDAYAYYYSAAQNRLVGLLVLAGLGALSVTMLFG

>Glyma12g06111.1|PACid:26293356
MSMSVAADDKPYYGQPSNYYPHPTPPTYRQINPPYYYKSPPYYYKSPPPPSPSPPPPYVYKSPPPPSPSPPPPYIYK
SPPPPSPSPPPPY

>Glyma12g06101.1|PACid:26294643
MGTRKWPPLIIALAFCLMAMSVSAEDKPYYGQPSNYYPHPMPPPYQQVNPPYYYKSPPYYYKSSPPPSPSPPPPYVS
KIPPYYYKSPPPPSPSPPPPYVYKSPPPPSPSPPPPY

>Glyma11g14131.1|PACid:26294991
MAISVGADDKPYYGQPSNYYPHPTPTPYHQANGPPYYYNSPPYYYKSPPPPSPSPPPPYIYHSPPPYYYKSPPPPYY
YKSSLHPSSSPPPSYIYKSPPPPSPSPPPPYVYNSPPPPSPSPPPPYVYKSPPLPLLSPPPPYVYKSP

>Glyma11g33894.1|PACid:26296372
MASPTPSLWPQKPTIVLLLICISFSHSYSVEEAITSSKKLDQPVFPPPAYTEIKCGSCPCGDTCGEQLPPPPPQPSP
APPPPCLSPPPPPPSPPPPKIPSCPQNCNPLPPPPPRFVYVPLPGVPKPYTWVYYYSAAENRGVGLIVLAGLGGLSM
ATLLLDDILKLKLYF

>Glyma17g03400.1|PACid:26311866
MRLCLCLTLLLVLASVAMGEEDADHNKHSLKAKSHAQIQCTMCSSCNNPCNQVPSPPPPSPSSNNCPPPPSPPSSGG
GGEGAPYYYSPPPPSQYTYSSPPPPASTGGGMYYYTPPSNRNYPRPPPPNPIVPYFPFYYYSPLPPGSTAAPPPSTA
SSILCALAFSSFLLLLL

>Glyma15g15500.1|PACid:26331718
MGTRVWCCFVMFLSLNALAIALEDHNLKTTSKDDIKCTPCGQIPSPPPPSPPPPAPTTTYCPPPPSPPSSGGGGAYY
YSPPPPSQYTYSSPPPPASTSGGGGGGAYYYPPPRGYYPTPPPPNPIVPYFPFYYHTPPLSSTAAPPPVKGSLVCAS
AISLLTLFIAFL

>Glyma15g13785.1|PACid:26332924
MGSKGVSLLLFLNLLFFSMISSSSSYKSPPPCNVPSPGTPPPPKHSGPRSPPPTGHFHPPPKPSSPTPSPPNSSSSY
KPPPPSPSDIPVHVTPPPPPPPPPRCPNLRICPLLSGLTDLEAAVCVCAQIRAELLGIPININLFLDLVLNRCGKRL
PANYRCN

>Glyma03g08478.1|PACid:26336472
PGQPSPTPLLKSIHSLPPPPRNRYKYPSLPPPVYPHPHPHPHPHLHPHPHLLYVYHSPPPPPKKKPYYYHSPPPPPP
KKAQTN

>Glyma20g32470.1|PACid:26337040
TPLLHHPRSLIIIHLHPPYKYHSLPPPPKKPYKYPSPPPPKKPYKYTSPPSPPPVQKYSAPHPHQVYHSPPPPKKKT
YKLSTHLHHHQCTSTLPHTQSITHHHYLKRNPTSTHLHHHHIRNHTNTHLLHLQCTKSTLPLPLTSLPQ

>Glyma20g01700.2|PACid:26338771
MLIKGHQIKAPILVLNLFLVLICSTASSVNVVESRKLDETTPVPATNGTEEKCGSCGGTTYPSPPPPLLPPPSPPPP
SPKKTPPSQYCPPPPPPSSFIYITGPPGNLYPVDENFSGATSHHRSFAAVLMPLLVGLFSTLAFL

>Glyma09g04450.1|PACid:26340568
MGTRVWCCFMMFFLSLNALAIALEDHNLKTTSRDDIKCTPCGQVPSPPPPSPPPPAAPTTTYCPPPPSPPSSGGGGA
YYYSPPPPSQYIYSSPPPPASTGGGGGGVYYYPPPRGYYPTPPPPNPIVPYFPFYYHTPPPPSTAAPPPVKGSLVCA
ISFLTLFIAFL

>Glyma10g35080.1|PACid:26346115
LPSEISANNYIYSSPPPPKKPYHYPSPPPPVPSPPPPKAPYHYPSPPPPPKKPYKYPSPPPPGKQPYPDPHPH

>Glyma07g22970.1|PACid:26348037
MPTKLSHQIKAPMILNLILVLICVTTPIKGEKSRKLDEVTNGNEKCTPCEGGYTPSPPIEYLSPPPPIEYFSPPPPI
VYPSPPPPSPKKPPTKYCPPPPSSAYLYMTGPPGNLYPVDENFSGASSNRHHSFAVFLPLLVGLLCMMAFW

>Glyma07g34050.2|PACid:26349625
MLIKGNQIKAPILVLNFFLVLICSTSSSVNGVESRKLDETPVPATNGTEEKCGSCGGTTYPSPPPPVLPPPSPPPPS
PKKQPPSQYCPPPPPPSSFIYITGPPGNLYPVDENFSGAISHRHRRSFATMLLPLLVGLFSTLAFW

>Glyma07g37200.1|PACid:26350443
MRLLCLCLTLLLFLASVAMAEEDSDHNKHSLKATSHAQIQCTMCSSCNNPCNQVPSPPPPSPPPPSPSTNNCPPPPS
PPSSGGGGGGAPYYYSPPPPSQYTYSSPPPPASTGGGGGGGIYYYPPPSNGNYPRPPPPNPIVPYFPFYYYSPPPPG
STAAPPPSTASSLLCALAFSSFLLLLL

>Glyma18g04391.1|PACid:26354157
MASPTLSLWPQKPTIVLFLICVSFSYSVEEAITSSKKLDQPVFPPPAYTEIKCASCPCGDTCGEQLPPPPPPSPPPP
CQSPPPLLPSPPPPPPQSPPPPKFPSCPQNCNPLPPPPPPPRFVYVPVPGVPKPYTWVYYYSAAENRGVGFLVLAGLG
GLSMATLLLDDIMKLKLYF

>Glyma08g04390.1|PACid:26307290
MMMMMKKKDAAYSSILTLLLIATLLSLTVSASSLSDAEAMYVKQRQLLYYRDEFGDRGENVIVDPSLAFENDRLRNA
YIALQAWKQAILSDPRNQTADWVGSDVCSYTGVYCAPALDIPKIRTVAGIDLNHCDIAGYLPEELGLLLDLALFHVN
SNRFCGTVPHKFERLKLLYELDLSNNRFAGNFPEVVLRLPSLKFLDLRFNEFEGTVPRELFDKDLDAIFINDNRFVF
DLPDNFGNSPVSVIVLANNRFHGCIPASLGNMSNLNEIILMNNAFRSCLPSEIGLLKNLTVFDVSFNQLLGPIPNAV
GNAVSLEQLNVAHNLLSGQIPASICMLPHLQNFTYSFNFFTGEPPACLALPAFDDRVNCLPARPLQRPPAQCKSFLS
KPVDCNSFKCKHFVPSPPSPSPFPSPTAPVTPSPSPSTPSPFPSPTTPVTPPSPSHSPPSPSTPSPTTPVTPSPSHS
PPSPATPSPFPSPTTPVTPSPSHSPPSPATPSPFPSPTTPVTPSPSHSPPSPATPTTPVTPSPSHSPPSPATPSPFP
SPTTPVTPSPSHSPPSPATPSPFPSPTTPVTPSPPSPSTPSPFPSPTTPVTPSPVHSPPSPVYLPPPHSSPTPPAHN
YGSPPPHHSPAQPVYPYLSPPPPPSVHSSPPPPPPPPCIELPPPPPQSTPSPTPYLPPPSPSPPPPPPPVYHSPPPA
SPPPCETPPSVSPPPPPAPVYEGPLPPIVGLPYASPPPPPFY

>Glyma05g35330.1|PACid:26319865
MVMKKEATYSSILTLLIATLSLALSDEFGDRGENVTVDPSLVFQNDRLRNAYIALQAWKQAILSDPRNQTTDWVGSQ
VCSYTGVYCAPALDNPNIRTVAGIDLNHGDIAGYLPQELGLLVDLALFHINSNRFCGTVPHKFDRLKLLYELDLSNN
RFAGKFPEVVLRLPSLKFLDLRFNEFEGTVPRELFDKDLDAIFINDNRFVFDLPDNLGNSPVSVIVLANNRFHGCIP
ASLGNMSNLNEIILMNNAFRSCLPSEIGLLKNLTVFDVSFNQLLGPLPNAVGNAVSMEQLNVAHNLLSGQIPASICM
LPHLQNFTYSFNFFTSEPPACLVLPAFDDRLNCLPARPAQRPPPQCKSFFSKPVDCNSFKCKPFVPSPTPSPATPVT
PSPPSPSTPSPTTPVTPSPTTPVTPSPPSPSTPSPFPSPTTPSPFPSPTTPVTPSPPSPSTPSPFPSPTTPVTPSPP
SPFPSPTPPVTPSPPSPSTPSPTTPSPFPSLRHLYAFTTTPNFLHPLPKSTPSPTPYLPPPSPSPPPPPVYVSPPPP
PPVQYSSPPPPVYHSPPPCETPPSVSPPPPPAPVYEGPLPPIVGLPYASPPPPPFY

>Glyma20g28790.1|PACid:26336660
MASHSPNREGHYLNIVLFLAFLISIINPSQQDPSSPLPPLNPRLTKAYTALQAWKYKITSDPKNFTLNWCGPNVCNY
TGIYCAPALDDPHIYTVAGVDLNHATISGSLPEELGLLTDLSLFHINSNRFCGSLPNSFDKLHLLHELDISNNQFSG
PFPEVVLCIPNLKYLDIRFNNFHGNVPSRLFDVKLDALFINNNNFQFSLPENLGNSPVSVVVFANNDLKGCLPLSLV
KMKGTLNEIIITNSGLTGCLPSEIGDLDKVTVFDVSFNKLVGELPESLGRMKRLEQLNVAHNMLSGEIPESVCMLPR
LENFTYSYNYFCSESPVCLKLKEKDDTKNCIPYRPLQKSPDECKAFYAHPVHCSAFGCASPPPPPPSPPPPPPPPPP
PPPPPPPASYHHYP

>Glyma09g32080.1|PACid:26340754
MRKKTYSDLSLFFLPLLLILVSHSLCASAFSHNGPLTDSQVSFIKQRQLLYYKDYFGDRGEKVTLDPSFVFENDRLR
NAYIALQAWKEAILSDPKNLTQDWVGPNVCNYSYVFCAPAPDNHKIRTVAGIDLNHGDIAGYLPEELSLLTDLALLH
INTNRFCGTLPHNNNRFAGKFPDVVLRLPLLKFLDLRFNEFEGTVPKELFDKDLDAIFINDNRFVFDLPENFGNSPV
SVIVLANNRFHGCVPAGIGKMKGLNEIILMNNAFKSCFPAEIGLLQNLTVFDVSFNQLLGPLPDAIGGAVSLEQLNV
AHNLLSGKIPESICKLPNLQNFTYSYNFFTGEPPRCLALPAFDDRRNCLPARPAQRSAGQCKSFLSHPVDCNSFRCK
PFVPSLPPPPPPSPPLPSPPPPSPVYIPHSPPPPPPPVYSPPPPPPVYSPPPPPPPHSPPPPPHSPPPPPPVYPYLS
PPPPPPVHSPPPPVYSPPPPPPSPPPCIESPPPPPPPPPCEEHSPPPPSPHSAPYHPPPSPSPPPPPIQYNSPPPPS
PPPPTPVYHYNSPPPPSSPPPAPVYEGPLPPVIGVSYASPPPPPFY

>Glyma07g09730.1|PACid:26348106
MRKKTYSDLSLLLILVAQSLSASSFSHNGPLTDAQVSFIKQRQLLYYKDEFGDRGEKVTLDPSFVFENDRLKSAYIA
LQAWKEAILSDPKNLTQDWLGPNVCNYSYVFCAPAPDNHKIRTVAGIDLNHGDIAGYLPEELGLLTDLALLHINTNR

FCGTLPHKFDRLKLLFELDLSNNRFAGKFPDVVLRLPQLKFLDLRFNEFEGTVPKELFDKDLDAIFINDNRFVFDLP
ENFGNSPVSVIVLANNRFHGCVPAGIGNMKRLNEIILMNNAFRSCFPAEIGLLQNLTVFDVSFNQLLGPLPDAIGGA
VSLEQLNVAHNLLSGKIPESICKLPNLQNFTYSYNFFTGEPPRCLALPAFDDRRNCLPARPAQRSAGQCKSFLSHPV
DCNSFRCKPFVPSLPPPPPPSPPLPSPPPPPSPVYIPHSPPPPPPPVYSPPPPPPPPPVYSPPPPPPVYSPPPPPPV
YSPPPPPPVYSPPPPPPPVEEVVTVD

>Glyma07g21210.1|PACid:26350617
MAYPFGAIPPTFEVQNDAPLVNERLKKAYVALQAWKEAIYSDPLNTTGNWVGEDVCSYNGVFCAPAIDDPTLNVVAG
VDLNNADIAGHLPEELGNLSDIALFHINSNRFCGIVPETLENLTLLHEFDISNNHFVGGFPSVVLKWPNLKYLDIRY
NDFEGPLPPELFEKDLDAIFLNNNRFTSIIPDTLGKSKVSVVTFANNKFTGCIPKSIVNMKNMNEIVFMGNDLGGCL
PQEIGLLESITVLDASHNGFVGTLPNLSGLKNVEVIDIEHNKLSGYVSNIVCQLPLLKNFTFSNNYFNGEAQTFNSP
PPPVSSPPPPVYSPPPSPINSPPPPTPVLSPSPLIHSPPPPVHSPPPPIYSPPPPVNSPPPPVSSPPPPVYSPPPPL
INSPPPPTLVLSPSPPIHSPPPPVHSPPPPVYSPPPPVYSPPPTWDDVVLPPHFGSSYQSPPPPTIAGY

>Glyma02g14310.1|PACid:26288980
MSAVSPSPSTSTAAPPPQTSPSANTTSPPPSTVTTPPPQQTPSSTSSPQQPASPPPTTPSPANPPPSPPSPPPSSPP
PSMSGTPPPLVPPPSPPPSPPSSPPPSSPAAPPPVPPSSPPPPSPSAPVPPNPPNNTSPPPSQPPQSPAPPPQPPAP
PPQPPAPPPQPPAPPPNNTSPSPPRNSPPSPAATPPRASPPQNSPSPPAVPPPRNSTRSPPSANSPPPPVNAPPPRS
SAPPAPEPSTPPSRTSTPPTPSSQPAPTSNSTPRSSPPSPPSTTNLAPPPPSRVLSSPLPSPAQNGTKNPSPDGGGD
GIGTGGVVAISVVAGFLLLGFIGVLIWCMRRQKRKLPVSGGYVMPSTLASSPESDSSFFKTHSSAPLVQSGSGSDVV
YTPSDPGGLGNSRSWFSYEELIKVTNGFSTQNLLGEGGFGCVYKGCLPDGRDIAVKQLKIGGGQGEREFKAEVEIIG
RIHHRHLVSLVGYCIEDSRRLLVYDYVPNNNLYFHLHGEGQPVLEWANRVKIAAGAARGLAYLHEDCNPRIIHRDIK
SSNILLDFNFEAKVSDFGLAKLALDANTHITTRVMGTFGYMAPEYASSGKLTEKSDVYSFGVVLLELITGRKPVDAS
QPLGDESLVEMGSTFTESCTRH

>Glyma02g04010.1|PACid:26289527
MANDTSSNESSSSDSDKDKASPPSPSSSSNDSESPKSAPSKSPPPSPPPSPSPPPPEKSSPPPTESLPAPENSPPPT
PSQSPPPTPSQSPPPSPSPPTPPSPPPSSTPPPSKDSAPPPSPSEPPPSPPQSSPPPPQNSPPAPQSQAPPPHSLSP
PTAIQTPLSPGSHVTPPPAPTGSGPNHPSPPRSPPSTQPKPSGALPKNDPPSTSPSSGNNTGETVGLALAGVVMIAF
LALVIFFIFRRKQKRAGVYAMPPPRKSHMKGGDVHYFVEEPGFGSGAQGAINLRCPSEPAQHMNTGQLVFTYEKIAE
ITNGFASENIIGEGGFGYVYKASMPDGRVGALKMLKAGSGQGEREFRAEVDIISRIHHRHLVSLIGYCISEQQRVLI
YEFVPNGNLSQHLHGSERPILDWPKRMKIAIGSARGLAYLHDGCNPKIIHRDIKSANILLDNAYEAQVADFGLARLT
DDSNTHVSTRVMGTFGYMAPEYATSGKLTDRSDVFSFGVVLLELITGRKPVDPMQPIGEESLVEWARPLLLRAVETG
DFGELVDPRLERQYADTEMFRMIETAAACVRHSAPKRPRMVQVARSLDSGDQQYDLSNGVKYGQSTIYDSGQYNEDI
TIFKRMVNGSFDDSEFDMDSTTDYRSTVSREMSGSRHARMLYSEGGDSDYRVFHGQRSNTQNMS

>Glyma11g07175.1|PACid:26295349
MSSPHNSSPDDSSPDNATPDNSTPDDSDNSSPPSQSPPPSSPPPSSPPPSSPPPSSPPPSSPPPSSPPPSSPPPLSP
PPSSSSSPPPSPSPPTSNSPSPPSPNGSSQSPSPPGSSHSPPSFESPSPPHKSLDSPPSSRNSGSGSGSSNGGGNDS
GGDGSSKAIVGAVLGVGSVLLILVIVCVVCNRKKKKNTNTWNHNNYYNSGQHPSYYGGPHGDHVVRMQQNGMGPGG
GGGWGAPPPPPMMMNSADFSSNYSTGPPPLPPPSPTLALGLKGGTFSYEELAAATNGFNDANLIGQGGFGYVHKGVL
PSGKEVAVKSLKAGSGQGEREFQAEIDIISRVHHRHLVSLVGYSISGGQRMLVYEFIPNNTLEYHLHGKGRPTMDWA
TRMRIAIGSAKGLAYLHEDCHPRIIHRDIKAANVLIDDSFEAKVADFGLAKLTTDNNTHVSTRVMGTFGYLAPEYAS
SGKLTEKSDVFSFGVMLLELITGKRPVDHTNAMDDSLVDWARPLLTRGLEEDGNFGELVDAFLEGNYDAQELSRMAA
CAAGSIRHSAKKRPKMSQIVRILEGDVSLDDLRDGIKPGQNVVYNSSPSSNQYDTMQYNADMQKFRKAVFSNSDEFG
TSSGSSGEVSQKQQRL

>Glyma04g01480.2|PACid:26304506
MASNSDKDSPTPPSPDSSSSQSPPSSPPPSQDSSSPPPSSQDNSSPPPSSPPPSSPPPSSSPPPSPPPPSSNDQKSP
PPPSDSSSSPPSPPPPPPPDKDSGSHRSPPPPPHSRHEFSPPPPPNHRLSPPKSSSSGSSDGLKDSESHSLSMPVII
GIAAGAGILLLLLLVVVCACSRRKKRSQPSVSYYANQPSDDGYYKRPRGGQNQYGYNNNGEHVLNIPPPPGAGWGAA
PQPPQMISSDMSNSSFSGSHGPVLPPPHPTVALGFNQSSFTYDELSAATGGFSQRNLLGQGGFGYVHKGVLPNGKEI
AVKSLKSTGGQGDREFQAEVDIISRVHHRHLVSLVGYCMSESKKLLVYEFVPKGTLEFHLHGKGRPVMDWNTRLKIA
IGSAKGLAYLHEDCHPRIIHRDIKGANILLENNFEAKVADFGLAKISQDTNTHVSTRVMGTFGYMAPEYASSGKLTD
KSDVFSFGIMLLELITGRRPVNNTGEYEDTLVDWARPLCTKAMENGTFEGLVDPRLEDNYDKQQMASMVACAAFSVR
HSAKRRPRMSQIVRVLEGDVSLDALNHEGVKPGQSSMFSSASREYGAEAYGADMMRFRKLALDSGVGSSEYGGTSEY
GLNPSSSSSEQSSAEYARRTTGGGRMHTP

>Glyma08g28596.1|PACid:26307659
MTSTNPSPNASPVAVPPALGGILSPPPSSSSPTNSSKSPSPPNSPQPNQTQTPNSPAPSSPSAPPPSPPSPPQAVPV
TPPPSISLSPPPTLPPPSPPDSPPPLPPASPPSPPATTAPPPAETPPSLPNLSPPSPPDGSPPPQSPPTTIPPPSRP
VSPSPPPPANVPRPPSTSTPPQKQSPPKTTPSHASPPSVSESPPKPPSSDVHPPSTLPSDTSGSSPPATLPDPPTNT
TAAQGPTVSLPSLPTEKPTARPTNDGTNSMSSNNTPSHSGGLSTGGSVAIGIVVGFTVLSLVMAVWFVQKKKKKGTG
SRGGYAAASPFTSSHNSGENRPVLDWPTRVKVAAGAARGIAYLHEDCRKPVDASQPIGDESLVEWVVRALDSLDEFT
DLNNGMKPGQSSVFDSAQQSAQIRMFRRMAFGSQDSSSFFNESQSSWRSRDHNSNTMFSQNKTGPWNV

>Glyma08g39480.1|PACid:26308813
MSTKHTEEDNDSSYLTIPNKDKRKQKSSHDNDKESAPSPKPPDLDTQESAPSNSPPPSPPPPPPQLKLPILSPPRLK
PSSISLLSPPSKDLSGSPSWSPPPPPSRSPSPPSSLSLLSSSSPPPSPSSPPPPPSSPPPPPPPSSPPPPPSSPAPP
QSVPLRNNGNPSPPPPYYPRKQSPPQFHAPPPPSSLPRNAPSNDSTPRTSDKPRSGHHGKHGGATSSHTNSTGSSPN
QTSSDDNSEKYIGYTLIGIFAVALVALAVSLVFVFKKKKSRGDAHVTPYMPPLNIHVKSGVNGHYYVQQPIPSPPLA
NNYGNGNASMHHLGASFDSAQFKSAQIVFTYEMVMEMTNAFSTQNVIGEGGFGCVYKGWLPDGKAVAVKQLKAGGRQ
GEREFKAEVEIISRVHHRHLVSLVGYCICEQQRILIYEYVPNGTLHHHLHASGMPVLNWDKRLKIAIGAAKGLAYLH
EDCCQKIIHRDIKSANILLDNAYEAQVADFGLARLADASNTHVSTRVMGTFGYMAPEYATSGKLTDRSDVFSFGVVL
LELVTGRKPVDQTQPLGDESLVEWARPLLLRAIETRDFSDLIDPRLKKHFVENEMLRMVEVAAACVRHSAPRRPRMV
QVVRSLDCGDESSDLSNGVKYGHSTVYDSGQYDKEIMLFRRLANGTFVDSDFEIYSREYSLSRDTSMTSRGSHQELI
RHSSSGSLNLELSTCIGPVLNENRD

>Glyma01g38105.1|PACid:26323849
MSSPHNSSPDNETPDNSTPDDADNSSSPSSQSPPPSSPPPSSPPPSSPPPSSPPPSSPPPSSSSSPPPSSPPPSSSS
SPPSPSPPTSNNPSPPSPNGSSQSPSPPGSSRSPPSFESPSPPHKSLDSPPSSRNSGSGSDSGSRDSNGGGDDSSK
AIVGAVLGIGSVLLILVIVCVVCSRKKKKNRMYYYAGEQSMGKGNNNNYYNSGQHPNYYGGPHGDHVVRMQQNGMGP
GGGGWGAPPPPPPMMMSSAEFSSNYSTGPAPLPPPSPNLALGLKGGTFTYEELAAATNGFNDANLIGQGGFGYVHKG
VLPSGKEVAVKSLKAGSGQGEREFQAEIDIISRVHHRHLVSLVGYSISGGQRMLVYEFIPNNTLEYHLHGKGRPTMD
WPTRMRIAIGSAKGLAYLHEDCHPRIIHRDIKAANVLIDDSFEAKVADFGLAKLTTDNNTHVSTRVMGTFGYLAPEY
ASSGKLTEKSDVFSFGVMLLELITGKRPVDHTNAMDDSLVDWARPLLTRGLEEDGNFGELVDAFLEGNYDPQELSRM
AACAAGSIRHSAKKRPKMSQIVRILEGDVSLDDLKDGIKPGQNVAYNSSSSSDQYDTMQYNADMQKFRKAVFSNSEE
FGTSSGSSGEVSQKQQRL

>Glyma01g23180.1|PACid:26325636
MSAASPSPATSTAAPPLQTPPSSNNTSPSPSTATTPPLQQTPPSTSPPQQPASPPPSTPSPANPPPSSPPSSPPPPM
SGTPPPLVPPPSPPPSPPPSTPAAQPAVPPSSPPPPPSPSPSVPSSPPPSNPRNNTSPPPSPPPQPPAPPPQPPAPP
ANNTSPPPQRNSPPPPAATPPRTSPPQTSPSPPAAPPPRNSTRLPPPVNSPPPPVDAPPPRSSSPPAPAPSTPPSRT
SPPPTPSSQPAPPSNSTPRSSPPSPPSITNLAPSPPSRILSSPPPPPAQNGTENSSPDGGGDGIGIGGVVAISVVAG
FLLLGFIGVLIWCMRRKKRKVLVSGDYVMPSTLASSPESDSSFFKTHSSAPLVQSGSGSDVVYTPSEPGGLGHSRSW
FSYEELIKATNGFSTQNLLGEGGFGCVYKGCLPDGREIAVKQLKIGGGQGEREFKAEVEIISRIHHRHLVSLVGYCI
EDNKRLLVYDYVPNNTLYFHLHGEGQPVLEWANRVKIAAGAARGLTYLHEDCNPRIIHRDIKSSNILLDFNYEAKVS
DFGLAKLALDANTHITTRVMGTFGYMAPEYASSGKLTEKSDVYSFGVVLLELITGRKPVDASQPLGDESLVEWARPL
LSHALDTEEFDSLADPRLEKNYVESELYCMIEVAAACVRHSAAKRPRMGQVVRAFDSLGGSDLTNGMRLGESEVFDA
QQSEEIRLFRRMAFGNQDYSTDFFSRASLNP

>Glyma01g03690.1|PACid:26325784
MAKNTSSNESSSLNSDKDKASAPLPSSDDSESPKSTPPKSPPPSPPLPPSPPKKSAPPPTESPPAPAHSSPPTPSQS
PPPTPPHSPPPSPSPPTPPPEKSPPSPSNSPPPPSTPPPSKDSAPPPSPSEPPPSPPPSPPPPPPQNSPPSPQSLTP
PPHSLSPPTAISTPPSPASHVTPPPAPLGSGPNHPSPPRPPPSTQPKPRGTPPKSDPPSSSPSSGNNTGEIVGLALA
GVFIIAFLALVIFFMFGRKQKRASVYAMPPPRKSHMKGGDVHYYVEEPGFGSGALGAMNLRTPSETTQHMNTGQLVF
TYEKVAEITNGFASENIIGEGGFGYVYKASMPDGRVGALKLLKAGSGQGEREFRAEVDIISRIHHRHLVSLIGYCIS
EQQRVLIYEFVPNGNLSQHLHGSKWPILDWPKRMKIAIGSARGLAYLHDGCNPKIIHRDIKSANILLDNAYEAQVAD
FGLARLTDDANTHVSTRVMGTFGYMAPEYATSGKLTDRSDVFSFGVVLLELITGRKPVDPMQPIGEESLVEWARPLL
LRAVETGDYGKLVDPRLERQYVDSEMFRMIETAAACVRHSAPKRPRMVQVARSLDSGNQLYDLSNGVKYGQSTVYDS
GQYNEDIEIFKRMVNGSFDDSEFDMNSMEYRSTVSREMSGSRHSRMPYSEGSDSDFRVFHGQRSNTQNMS

>Glyma09g32390.1|PACid:26343321
MSTAPAPSSPPANNGTAPPPSTPATPSAPPPATPSAPPPSTPSSPPPATPSSPPPATPSSPPPATPSSPPPATPSSP

PPSTPSASPPPSTPTTPSTSPPSPPSTSPPSPPSGGSTPSPPSRSSPSPPSGSSPTTPSPPSSSSSSISTGVVVGIA
VGAVAVLLVLSILCICCRKKKRRRDEEYHAPPPQQQQPPRGPKDGAYGGPPGQWQHNVPPPQDHVVSMMPPKPSPPP
APPAYAGQPPPPPPPPFISSSGGSGSNNSGGEFLPPPSPGISLGFSKSTFTYEELARATDGFSDANLLGQGGFGYVHR
GILPNGKEVAVKQLKAGSGQGEREFQAEVEIISRVHHKHLVSLVGYCITGSQRLLVYEFVPNNTLEFHLHGKGRPTM
DWPTRLRIALGSAKGLAYLHEDCHPKIIHRDIKSANILLDFKFEAKVADFGLAKFSSDVNTHVSTRVMGTFGYLAPE
YASSGKLTDKSDVFSYGIMLLELITGRRPVDKNQTYMEDSLVDWARPLLTRALEEDDFDSIIDPRLQNDYDPHEMAR
MVASAAACIRHSAKRRPRMSQVVRALEGDVSLADLNEGIRPGHSTMYSSHESSDYDTAQYKEDMKKFRKMALGTQEY
GASSEYSAATSEYGLNPSGSSSEAQSRQTTREMEMRKMKNNQGFSGSS

>Glyma07g09420.1|PACid:26348580
MSTAPAPSSPPPTNGTAPPPSTPSAPPPATPSAPPPSTPSSPPPATPSSPPPATPSSPPPATPSSPPPSTPSSPPPA
TPSASPPSTPSASPPPSTPTTPSTSPPSTSPPSPPSHSPPSPPSGGGGGSTPSPPSRSSPSPPSGSRPTTPSSSSSS
ISTGVVVGIAVGAVAVLLVLSILCICCRKKKRRRDEEYYAPPPQPPRGPKDDAYGGPPRQWQHNVPPPQDHVVSMMP
PKPSPPPAPPAYAAQPPPPPPPPFIISSGGSGSNYSGGEFLPPPSPGIALGFSKSTFTYEELARATDGFSDANLLGQG
GFGYVHRGILPNGKEVAVKQLKAGSGQGEREFQAEVEIISRVHHKHLVSLVGYCITGSQRLLVYEFVPNNTLEFHLH
GRGRPTMDWPTRLRIALGSAKGLAYLHEDCHPKIIHRDIKAANILLDFKFEAKVADFGLAKFSSDVNTHVSTRVMGT
FGYLAPEYASSGKLTDKSDVFSYGVMLLELITGRRPVDKNQTFMEDSLVDWARPLLTRALEEDDFDSIIDPRLQNDY
DPNEMARMVASAAACIRHSAKRRPRMSQVVRALEGDVSLADLNEGIRPGHSTMYSSHESSDYDTAQYKEDMKKFRKM
ALGTQEYGASSEYSAATSEYGLNPSGSSSEAQSRQTTREMEMRKMKNSQGFSGSS

>Glyma07g00680.1|PACid:26349072
MSAPAEAPSFTASPPSETTPSPNSTALSPPPPSTINATVSPPPPEAASPPTSTVNSGLSTGTVSGIVIGAVLGSVGM
LIIGGIFFCFYRNWKRKKNHSQPPQPKADIAGGTLQNWQDSVPPTTDGKVGFSPKPPPGGLVNQQQSSAALLTLVVN
SSNTSSSLGSEKAKSYISPSPGTSLALSQSTFTYDELSMATDGFSRSNLLGQGGFGYVHKGVLPNGKIVAVKQLKSE
SRQGEREFHAEVDVISRVHHRHLVSLVGYCVSDSQKMLVYEYVENDTLEFHLHGKDRLPMDWSTRMKIAIGSAKGLA
YLHEDCNPKIIHRDIKASNILLDESFEAKVADFGLAKFSSDTDTHVSTRVMGTFGYMAPEYAASGKLTEKSDVFSFG
VVLLELITGRKPVDKTQTFIDDSMVEWARPLLSQALENGNLNGLVDPRLQTNYNLDEMIRMTTCAATCVRYSARLRP
RMSQVVRALEGNISLEDLNDGIAPGHSRVFGSFESSSYDSVQYREDLKNFKKLALESQEQGISEYSGPSSEYGRHPS
VSTSSDQQNTQEMEMGNKKGSNHDSGIQVLD

>Glyma18g51520.2|PACid:26355274
MASANPSPNASPVAVPPALGAILSPPPSSSSPTNTSTPPSPPSSSQPNQTQTQTQTPNSPAPSSPSDPSAPPPSPPQ
AVPLTPPPSISLSPPPSSTPPPTLPPPSPPDSPPPLPPASPTPPVTTSPPSPPVTTSPPSPPATTSPPPAETPPSLP
NLSPPSPPAGSPPPQSPPTTIPPPSRPISPSPPPPANFPRPPTTRTPPEKENPPKTTPSHASPPSVSETPPKPPSSD
VPPPSTLPSTPPSDPSGSSPPASLPDPPTNKTVVGGPKVSLPSLPTEKPTARPTNDGTNSMSSNNTPSHSGGLSTGG
SVAIGIVVGFIVLSLLVMAVWFAQKKKKKGTGSRGSYAAPSPFTSSHNSGTLFLRPQSPANFLGSGSGSDFVYSPSE
PGGVSSSRSWFTYEELIQATNGFSAQNLLGEGGFGCVYKGLLIDGREVAVKQLKIGGGQGEREFRAEVEIISRVHHR
HLVSLVGYCISEHQRLLVYDYVPNDTLHYHLHGENRPVLDWPTRVKVAAGAARGIAYLHEDCHPRIIHRDIKSSNIL
LDLNYEAQVSDFGLAKLALDSNTHVTTRVMGTFGYMAPEYATSGKLTEKSDVYSFGVVLLELITGRKPVDASQPIGD
ESLVEWARPLLTEALDNEDFEILVDPRLGKNYDRNEMFRMIEAAAACVRHSSVKRPRMSQVVRALDSLDEFTDLNNG
MKPGQSSVFDSAQQSAQIRMFRRMAFGSQDSSGFFNESQSSWRSRDHDPTTVFSQNKTGHGNV

>Glyma18g19100.2|PACid:26355442
MSPPPSKYLSPPPSPRPPPLPLPSPSLRPPSPLPPSPPPPSLPPPSSPPPPQSQSVPLRNGNPSPPPPPPYYPRKQW
PPPLQPLSSPPQFRAPPPPDRPSSRRNAPSNDSTQGTSEKSRSGHHKHGVASHTNSTGSSPNQTSSGDNSGKYVGYT
LAGIFAVALVAVAVALVFVFKKKKSRRDAYTTPYMPPFKIHVKSGANGHYYVQQPIPSPPLANNYGNGNMSMQHLGA
SFDSAQFKSVQIVFTYEMVMEMTNAFSTQNVIGEGGFGCVYKGWLPDGKTVAVKQLKAGSGQGEREFKAEVEIISRV
HHRHLVALVGYCICEQQRILIYEYVPNGTLHHHLHESGMPVLDWAKRLKIAIGAAKGLAYLHEDCSQKIIHRDIKSA
NILLDNAYEAQVADFGLARLADAANTHVSTRVMGTFGYMAPEYATSGKLTDRSDVFSFGVVLLELVTGRKPVDQTQP
LGDESLVEWARPLLLRAIETRDFSDLTDPRLKKHFVESEMFRMIEAAAACVRHSALRRPRMVQVVRALDCGDESSDI
SNGMKYGHSTVYDSGQYDKAIMLFRRMANGNFDDSDFDMVSREYSLSRDTSMTSRGSQQELIQHSSSGEFDSRDINM
HRSSIE

>Glyma02g36446.1|PACid:26288403
MAMLRKLFFRKPPDGLLEICERVYVFDCCFTTDAWNEENYRVYMDGIVGQLRENLPDASILIFNFREEDTKSQMANI
MSEHDITIMDYPRHYEGVPVLKMELIHHFLRSGESWLSLSQHNVLLMHCERGGWPVLAFMLAALLIYRKVYTGEQRT
LDMVYKQAPHELLHLLSPLNPTPSQLRYLLYVSRRNVALDWPPLDRALMLDCIILRFFPNFDGEGGCHPIFRIYGQD

PFSADKNPKMLYSTPKRSKSVRAYKQGECELIKIDINCHIQGDVVIESINLNGNMDREKMMFRVMFNTAFVRSNILM
LNRDEIDILWDAKDHFPKDFRAEILFSEMDAAAAVIADGTSCFEEKEGLPIEAFAKVQEIFSHVDWMNPKADAALNV
LQQMSASAMNDRLDTVSDQCMENGTLLHETSPRIPQGNLSEARQSLSSTKRSPDNDMSRKEDKANKVEGIPQQPSTS
NIIYQETAISSERTTESNKCPTGPTNVDIKLQAPHPALSSSVDTSFSPRTPPLRPQSTSAKEVHDSPRQTESPPSYL
LPLQSKHQTQDRSSIPTPGTQLSSTFHSKSPADTISHPSASAITSTQPSPLLSSKNVNEIPPIRTRLESSPSRPPTP
PPPPTPPLKDHRLVRAKPPPPPHPPPPPPKKELHVKAEPLPSPLSPMNVEPQVRGGPSPPPPPPPKEEQPVTFNAPP
PPCLSGKVAGSTIAPPPPPPPSSDHINSSLQKSPPPPAAPAPPPPPGAPAPPPPPGAPAAPPPPAKGGLKSGSPFPL
SLSVSGDGNNVSGPTSSKGRILSRTISSKNNTKKLKPLHWLKLSRAVQGSLWAETQKSGEVSKAPEIDMSELESLFS
AAVPSGPAKKSNVQSSAGPKSDKVQLIEHRRAYNCEIMLSKVKVPLHDLMSSVLALEESALDTDQVENLIKFCPTKE
EMELLKGYNGEKEKLGRCEQFLMELMKVPRVESKLRVFSFKIQFNSQVSDLRNSLSVVNAASEEIRNSVKLKRIMQT
ILSLGNALNQGTAKGSAIGFRLDSLLKLTETRARDKKMTLMHYLCKVLDDQLPEVLDFSKDLANLEPAAKIQLKFLA
EEMQAINKGLEKVVQELSTSENDGPISETFRKKLKDFLGSAEADVRSLASLYSSVGRNVDKLILYFGEDPARCPFEQ
VVSTLLNFTRMFNKAHEENHKQLELEMKKTAENEKKKCESERILPTAIRTGNAK

>Glyma12g34350.1|PACid:26293662
MGSLSSRRYLGQVFPQHVSEISIKEDESPKKNKPKEKDSNKKDKKKSKSSGSKDKNKNTSMAITIIVTILVAALLFL
CCGSGRVRQNDERPLLSMSMNDYSVGSSSNNNPYKNSMKEEKIGFQSSSNTLVDDRKNSMMETQLAGVAASAATRPS
FELKPPPGRVANNGVLPLKPPPGRPDLLPHESCSIKPFDNSIVPPPSPPPPSPPPPPPPTPPKPNSAGPPPPPPPSN
GPRAPPPPPLRGKPGPRPSPPPRVGGGPPRTTPPFGSKVAKTQEEVGVNSEGEVNATNKAKLKPFFWDKVQANSDQT
MVWNQLKAGSFQFNEEMMETLFCYNTTPVEKSKGQQKKEASSPSASPQYIQIINSKKSQNLSILLKALNVTIEEVSE
ALLEGNELPTEFLQTLLKMAPTSEEELKLRLFNGNLAQLGPADRFLKALVDIPFAFKRMEALLYMGILQEELTGTRE
SFAILEVACKTLRSSRLFLKLLEAVLKTGNRMNDGTFRGGAQAFKLDTLLKLSDVKGVDGKTTLLHFVVQEIMRTEG
IRAARMAKENHSFSSIKSEDLLEDISYESEDQYRELGLQVVSRLSSELENVKKAAALDADGLIGTTSRLGHGLIKTR
DFVNKDLSNIDDDKGFHETVKSFVEKAEADVTSLLEEEKKIMALVKNTGDYFHGDSGKDEGLRLFVIVRDFLVMLDK
VCKEIQNGPKKPVAKNVKREASNHSRKSSSSEIHPLPPDIRQRIFPAVVANRRMDGFSSDDESP

>Glyma11g05220.1|PACid:26295856
MKARHLSFFYLHIIVFTLSLSLPIQVNTAKGINNTITNESGNNRRILHQPLFPASSAPPPAGTASPPPSTPVVETPP
SPDGGNNIPFFHEYPAGPPADQNQHAAPSSTVNSTIANPTATQPTKGTKKVAIAISVGIVTLGMLSALAFFLYKHRA
KHPVETQKLVAAGSGNNNNSNRNSNEVANTTSAPSSFLYIGTVEPTDSRDNRNAIKPNRSPYHKLKRSDRYRPSPEL
QPMPPLTKPPDGNYPPAVSSSSDSDEESRDTAFHSPQNSSVDGYYTPASRHSSLVNGSPAKKETNSTPTPVAVPFSK
RTSPKSRVSAPSPEIRHVIIPSIKQPPPQSPPPPKHSRKPKFSAPPPPPNLKRLQSTKTTDTALHVSRTSLNPPPPP
PPPPPPPPPLQRKSVSPAVSASSTTCASVKRQSWSPIQCSVTNVSEEVEQSQSVSSSERHEANDTDGAKPKLKALHW
DKVRATSDRATVWDQIKSSSFQLNEDMMESLFGCKATNSAPKEPPRKKSVLPFVDQENRVLDPKKSQNIAILLRALN
VTKDEVSEALLDGNPEGLGTELLETLVKMAPTKEEEIKLKNYDGDLSKLGSAERFLKAVLDIPFAFKRVEAMLYRAN
FDAEVNYLRKSFQTMEAASEELKNSRLFLKLLEAVLRTGNRMNVGTNRGDAKAFKLDTLLKLVDIKGTDGKTTLLHF
VVQEIIRSEGAGAESANDNVKMDSKFNEDEFKKQGLQVVAGLSRDLSDVKKAAGMDSDVLSSYLSKLETGLDKVRLV
FQYEKPDMQGNFFNSTKLFLKYAEDEIVRIKADERKALYLVKEVTEYFHGNATKEEAHPLRIFMIVRDFLNILDLVC
KEVEKMHDRIVGGSGRSFRIPPNASLPVVNRYNHRKQDRSSDEESSSP

>Glyma08g40360.1|PACid:26307339
MALNTMILQSWPFMSLVFVLLLHSLSITPTCHCQTSTNSPTQNIETFYPNGTSATAPATQQPPPPVPTGNIAPSRGS
SNRNIATAVAATAASTIVVCGLIFFVVQRCLRKRRRRREEIRSNTAGGGGGGGDTRVVPQGNLFERIDGNVRGLIVD
EDGLDVIYWRKLEGKNSNKDLLHKEVVRSSRNNEKEDEKHGHDENQVKKSKYIQEVPLLRGKSSTSHLNVSPEEDEP
YRFSPLPSPPPSASVSAPNSSSVPASVGIVIKGAQKPDSPTQPSTPPPPVSPSTPSSSTSFAAVPKTNIPAPSPPPP
PIPARRNPTPPPPPPSSKPPPAPIEMQAIKQRNSSGKGMLETSNDQVKLKPLHWDKVNTNNADHSMVWDKVDRGSFR
VDQDLMEALFGYVATNRRSPKGKSHSAIPSKDASAQSAKTNFLLDPRKSQNIAIVLKSLAVSQGEILDALTDGKGLN
ADTLEKLARVSPTEEEQSLILQYKGDPARLAAAESFLFSILKAVPSAFKHLNAMLFRLNYNSEIQEIKESLQTIELG
CNELKSKGLFLKLLEAVLKAGNRMNAGTARGNAQAFNLASLRKLSDVKSTNGRTTLLHFVVEEVVRLEGKRVALNRN
GSLSSSSSRSSSNSNGNYENNIASNELVEREYVTLGLPIVGGISSELSNVKKAAQIDHNNLVGSISALSTQLVEIQQ
LVSSCGNGEGGNFVKEMDHFLGNAEEELKLVREKQTSVFQLIKKTAQYYQGGSSKETAENNLQLFVIVKDFLGMVDQ
TCIEIARDMQKRKPPKALFR

>Glyma17g11100.2|PACid:26312273
MALFRRFFYRKPPDRLLEISERVYVFDCCFSPDVLEEDEYRVYMGGIVAQLQDHFPDASFMVFNFREGERRSQISDI
FSQYDMTVMEYPRQYEGCPLLPLEMIHHFLRSSESWLSLEGQQNVLLMHCERGGWPVLAFMLAGLLLYRKQYSGDQK
TLEMVYKQAPRELLHLLSPLNPQPSHLRYLQYISRRHLGSEWPPSETPLYLDCLILRVLPLFDDGKGCRPVVRVYGQ

DPSIPANRSSKLLFSTSISKKHVRHYVQAECMLVKIDIRCRVQGDVVLECIHLNEDFVREDMMFRVMFHTAFVRSNI
LMLNRDEIDILWEAKDLFPKDFKAEVLFLDADAVIPELTTVMVSEDANETESAETESASPEEFYEVEEIFSNVIDAQ
EGKTEYDSQAFHENAVDVETHKEVWRQESDPHTFEDCTPDDRIPKQVDKMNSGINAVKDISIDDVHYKFGGSMDSDP
HAVKDIAVDDGEMKSTSTTFVSDMVKPPLETKEVTMDVHPELAVMQNEYDEDKELDSKAGHHMSDLSEQKSGKLLPS
TAKKQPTSNSKPVGDTIAAKPKIKQQDAHGFQAKQAKPNAVTRWIPSNKGSYTNSMHVYYPPSRNNSAPAALTNFSS
SKEKMEDAKTRSLSAPVVSAVVSIDKINDLKSRKVATSRSSGYIAAEVDEKCPPSPLLPIKETSFQSDTQAQQQSSE
QVLQPPPPPPPPPPLPYQTSTDRTPPPPPPPPPFASHKASPPPAPPPPSPSLPPLLPPLANSYIYLAPPPVPPPPPP
PPFGKASLPPSPPPPPPPPFRQAPPPPPPTLHRAPPTPPPPPPPPLSRAPPPPPPPLPPSSGAPPPPPPPLRPSSGA
PPPPPPPMYGAPPPPPPPPSGRGPPPPPPPMYGAPPPPGGRAPLLPPPPPPPPGGRGPPPPPPPMYGAPPPPPPPGG
QGPPPPGGRGPPPPPPPVGRGPPPPPPPGVPGAPSPPGGGPPPPPPLGAKGANVGADPRGRGRGYARPAGGGAMAPR
RSSLKPLHWSKVTRALQGSLWEELQRHGEPQIAPEFDVSELEKLFSANVPKPTDSGKSGGRRKSVGAKTDRITLVDL
RRANNTEIMLTKVKMPLPDMMAAVLALDESVLDVDQVENLIKFCPTKEEMDLLKGYTGDKEILGKCEQFFLELMKVP
RVESKLRVFAFKIQFGSQVTEFKKSLNTVNSACEEVRNSVKLKEIMKKILYLGNTLNQGTARGSAVGFKLDSLLKLT
DTRASNSKMTLMHYLCKVLADKSPRLLDFHLDLVSLEASTKIQLKSLAEEMQAIIKGLEKVKQEFAASANDGPVSEV
FHKTLKEFIAVSESEVASLTNLYSVVGRSADALALYFGEDPARCPMEQVTTTLLNFIRLFRKAHEENIKQAELEKKK
AEKEAEMEKAKGINLTKKGAKDS

>Glyma01g40080.1|PACid:26324205
MKALHLSFYFYIIVFTLSLFLSFQVNTVKGVNNTSANESGNRRILHQPLFPASSAPPPAGTASPPPPPAVITPPSPD
GGNIPFFHEYPADQNQQAAPSSTVNSSIANPTATQPTKGTKKVAIAISVGIVTLGMLSALAFFLYKHRAKHPVETQK
FVAAGSGNNNNNNPPNRNSNSNEDANTTSAPSSFLYIGTVEPTVSRDANKPNRSPYHKLKRSDRYRPSPELQPMPPL
TKPPGGNYPPALSSSPESDEESRETAFHSPQNSSIDGYYTPASRHSSLVNGSPAKRETNSTPAPVVPFSKRTSPNSR
VSAPSPEIRHVIIPSIKQPPPPSPPPPKHSLKPEFSAPPPPPNLKRLQSTTTTTDTALHVSRTSLNPPPPPPPPPPP
PPPPLQQKSVSPAVSASSNSASVKRQSWSPVQCSVTKGSDGVEQSQSVSSSERHEANDTDGAKPKLKALHWDKVRAT
SDRATVWDQIKSSSFQLNEDMMESLFGCKATNFTPKEPPRKKSVLPSVDQENRVLDPKKSQNIAILLRALNVTRDEV
SEALLDGNPEGLGTELLETLVKMAPTKEEEIKLKNYDGDLSKLGAAERFLKAVLDIPFAFKRVEAMLYRANFDAEVN
YLRKSFQTMEAASEEIKNSRLFLKLLEAVLRTGNRMNVGTNRGDAKAFKLDTLLKLVDIKGTDGKTTLLHFVVQEII
RSEGAGAESANDNVKMDSKFNEDEFKKQGLRVVAGLSRDLSDVKKAAGMDSDVLSSYLSKLETGLDKVRLVLQYEKP
DMQGNFFNSTKLFLKYAEDEIVRIKADERKALYLVKEVTEYFHGNATKEEAHPLRIFMIVRDFLNILDLVCKEVEKM
HDRIVGGSGRSFRIPPNASLPVLNRYNHPKQDRSSDEESSSP

>Glyma09g34830.1|PACid:26340274
MSLLSRFFYKRPPDGLLEFSDRVYVFDSCFSTEALPDGMYSLYLRQILSDLHEDFPESSFVALNFRDGDHRSRFSRV
LCDLDATFVVDYPKQYEGCPLLPLSLLHHFLRLCDSCLADPGALILLHCERGGWPLLAFLLAAFLVFRKAAQTGERR
ILEMVHKEAPKGLLQLLSALNPLPSQLRYVKYVARRNIGAEWPPPERALSLDCVILRGVPGFDGGNGCRPLFRIFGR
NLLSKGGLSTQMIYNMHKKKKTLRHYRQADCDVIKIDIQCLVKGDVVLECVHLDLDPEREVMMFRVMFNTAFIRSNI
LMLNSENLDILWDSKERYPKGFRAEVLFGEVESISPPRAQTSILDGEVKGGLPIEAFSRVQELFSGVEWVESGDAAA
AWLLKQLTVLNDAKELSRFQGRGSWYSSPADSDSENNESSTADSSDEAFDVIPKSYADPSKLLISDNPDLVHLAFEN
NGGDYVNLTSRTPDQSLTDNVSLPHHAPRKEYSPCSSPPSPSPVPATQPFPTPAPPLPTSMPIQPDLAPPLPPHQID
QNVNSASTLPSPPPPIVSNAPSQSPPPPPPPPLPPRSVQTGSAPPPPGPVRTGSVPPPPPPPGSVQTGSAPPPPPPP
GPVRTGSVPPPPPPPGSVKTGSAPPPPPPPSPVRTGSAPPPPPPPPCPVRAGSAPSPPPPPPPPPGPVRTGSALPPP
PPPPPPPGPVRTGSAPPPPPPPPLGPVPTGPALPPPPPPPPGPVRTGSAPPPPPPPSVRTGSTPPPPPPAPKPPGVL
PPPPGRGASPMPPPPPAARAPGVAPPPGKASTANVGRGRGTGGTVNAPKKTLLKPLHWVKVARAAKGSLWADSQKQD
SGTRAPEIDISELESLFSAASTSDGSSTKGGGRRGPNINKPEKVQLVDLRRAYNCEIMLSKIKIPLPDMLKAVLALD
SAILDIDQVENLIKFCPTKEEMEMLKNYTGNKEMLGKCEQFFMELMKVPRVESKLRVFAFKITFSSQVNDLKLNLNT
INNAAREVKESGKLRQIMQTILTLGNALNQGTTRGSAVGFKLDSLLKLSDTRARNNKMTLMHYLCKLLAEKMPELLD
FDKDLVHLEAASRIQLKALAEEMQAVSKGLEKVEQELAASENDGAISTGFRKVLKNFLDIAEADVRSLISLYSEVGR
SADSLSQYFGEDPARCPFEQVTQILVVFVKMFNKSREENERQADAEKKKLEKEALKERTANNLHSKKDGVR

>Glyma07g06440.2|PACid:26349843
SNNRRILHQPLLPQNGSPPPSQPPSPPPSSPPNPKYPFSTTPNTNASSSTPFFPTYPSPPPPPSPSAFASFPANISS
LILPQTQKFKSSSKKLLAVAIAAVACAAAVVALSAFVYCRRRRKNYSADDKTLRSDSSIRLFPREASTTSGAASRKA
RNTSSTSSEFLYLGTIVNSRGGVDELSDPRAAALNPRKMDSPELQPLPPLARQTSRLREESTATVEDDEEEFYSPRG
SLNNGREGSAGAGSGSRRVFNAIAGENLVGRSSSESSTSSYSSSSSASPDRSHSISLSPPVSISPRKSLPKSPGNTI
NPPIRPPQEATAIRSSASLSKNESGSPRLSNASSNGKSSAFSLPSPVIGMNLHHELDQSPTISDVSDRYRHSPLSSL
HLSPTLLSSPERELNSQPQPPPSRKHWEIPDLLTPIGEAPNFSVPQRKQWEIPVLSVPIAPSSSVLAPPPPPPPPPP
PLAVPRQRKQWEVPSPVTPVDQQISRPAPPLTPPSRPFVLQTPNTKVSPVELPPASSQNFEEGSEETSKPKLKPLHW

DKVRASSDREMVWDQLRSSSFKLNEEMIETLFVVNTPNPKPKDTTPRSVLAPQNQEDRVLDPKKSQNIAILLRALNV
TIEEVCEALLEGITDTLGTELLESLLKMAPSKEEERKLKEHKDDSPTKLGPAEKFLKAVLDVPFAFKRVEAMLYIAN
FESEVEYLRKSFQTLEAACEELRNSRMFLKLLEAVLKTGNRMNVGTNRGDAHAFKLDTLLKLVDVKGADGKTTLLHF
VVQEIIRTEGARPSSTNQTPSTNLNDDAKCRRLGLQVVSSLSSDLASVKKAAAMDSEVLSSEVSKLSKGIAHIAEVV
QLDEAAGSDESSQKFRESMNKFMRMAEEEILKVQAQESVALSLVKEITEYFHGNLSKEEAHPFRIFMVVRDFLTVLD
RVCKEVGMINERTMVSSAHRFPVPVNPMLPQPLPGLVGKRQYNSSDDDDHSPSP

>Glyma16g03050.2|PACid:26351725
MCSTALTLTLFLLIAPLLSTSTPTTNRRILHQPFLPQDGSPPPSQPPKPPPPQQSTSPSPPNPKYPFSTTPNTNASS
SAPFFPTYPSPPPPPSPSAFASFPANISSLILPQTQKSKSSSKKLLAVAIAAVACAAAVVALSAFIYCRRRRKNYSA
DEKTLRSDSSIRLFPREATTGGGSAPARKVRNTSSTSSEFLYLGTIVNSRGGGVDELSDPRASALNPRKMDSPELQP
LPPLARQASRLREESTPTLEDDEEEFYSPRGSLNNGREGSAGTGSGSRRVFNAIAGENLVGRSSRSESSTSSFSSSS
SASPDRSHSISLSPPVSISPRRSLPKSPENTITHHSSPPEEAAIRSSASSSTLSSPSPVFGQHVPSSPSMSSTPERR
ECQSPSLSPLSLSPKKSQTPDGESVPGLVVLEKTQSFGSSKSKNESGSPRLSNASSIGKSSAFSLPSPDKGMTLHHG
LDQSPTISDVSDRYRHSPLSSLHLSPTLLSSPERELNSQPQPQPPPSRKNWEIPDLLTPIGEAPNFSAPQRKQWEIP
VLSVPIAPSSSVLAPPPPPPPPPPPPPLAVPRQRKQWEMPSPLTPVDQPVSRPPPLTPPSRPFVLQTPNTKVSPVEL
PPASSQNFEEGSEETSKPKLKPLHWDKVRASSDREMVWDQLRSSSFKLNEEMIETLFVVNTPNPKPKDTTPRSVLAP
QNQEDRVLDPKKSQNIAILLRALNVTIEEVCEALLEGVTDTLGTELLESLLKMAPSKEEERKLKEHKDDSPTKLGPA
EKFLKAVLDVPFAFKRVEAMLYIANFESEVEYLRKSFQTLETACEELRNSRMFLKLLEAVLKTGNRMNVGTNRGDAH
AFKLDTLLKLVDVKGADGKTTLLHFVVQEIIRTEGARPSSTNPTPSANSNDDAKCRRLGLQVVSSLSSDLANVKKAA
AMDSEVLSSEVSKLSKGIAHIAEVVQLDEAGGSDESSQKFRESMNKFMRMAEEEILKVQAQESVALSLVKEITEYFH
GNLSKEEAHPFRIFMVVRDFLTVLDRVCKEVGMINERTMVSSAHRFPVPVNPMLPQPLPGLVGKRQYNSSDDDDSSS
P

>Glyma13g25550.2|PACid:26317970
MHIAYLVVLAIPPLFSLVVPSLLAQAPSPSQHILLILHHLQKIHLLLSTSTPPSSKHSPPPPSSPPPSPKHAPPPLP
PSSPPPPSPKHAPPPPSSSPPPPSPKHAPPPPPPPPPPPPSSPPPPSPKHAPPPPFSPPPSQTHVPPPPPSPSSPK
HAPPPPTPSPPSSTHSPPPPPPSPTPPPARAPSPPPPPPPSSPSSACKSTLYPKLCRSILSSIRSSPSDPYNLGKFS
IKQSLKQAKKLVKVFKNFLTKHKSSSSLNTAEIAALEDCSELNKLSIDYLESVSIELKSIDSNNTELVEKIETYLSA
VATNHYTCYDGLVVIKSNIANAIAVPLKNVTQLYSVSLGLFTQALKKNLKKHKTRKHGLPTKDYKVRQPLRKLIKLL
HTKYSCTGSSNCSTRSERILQESENKGVLLKEFAIVSLDGTENFTSIGDAIAAAPDNLRPEDGYFLIYAREGNYEEY
VTVPIQKKNILLIGDGINKTCMTGNHSVVDGWTTFNSSTFAVSGERFVAVDVTFRNTAGPQKHQAVALRNNADLSTF
YRCSFEGYQDTLYVHSLRQFYRECDIYGTVDFIFGNAAVVFQSCNIYARKPMPNQKNAVTAQGRTDPNQNTGISIQN
CKIDAAPDLAADLNSTENYLGRPWKVYSRTVFMQSYIGELIQSAGWLEWNGTDGLSTLFYGEFQNFGPGSDTSKRVQ
WSGYNLLSATQARNFTVHNFTLGYTWLPDTDIPYSEGL

>Glyma13g06860.1|PACid:26319359
MSWLLIIILFLSLTHGSFSETSHDKKLPYAVVVGTVYCDTCSQQEFSIGSHFISGASVVAECKDGNSIQSFKKEVKTN
EHGEFKVQLPFRVRKHVRRIKGCTFKLLSSSEPHCSVASVSTSSVSLKTRKQGEHTFSAGLFSFKPLQKPNVCNQNQ
NEFSPNTYPSKPTPDKTTVFKTNPTTKSSDKSKSANKNLAEDFFFPPNPFFPPPLVPNPFQPPPLIPNPFQPPPLIP
NPLQPPSPPLIPNPFQPPSPPPLIPNPFQPPPSPPTLFPNPFQPPPSPPPLIPNPFQPPPSPPPSLFPPFPPIVIPG
LTPSPPPPPPPKPIFPPLPPLFPPLFPPPHTPGTPPASASKNHPYP

>Glyma19g04410.1|PACid:26325961
MSWFLVILFLSITHGSASETSHDKKLPSAVVVGTVFCDTCSQQEFTIGSHFISGASVAVECKDGNSIPRFKKEVKTD
EYGEFKLQLPFKVRKHVRRIKGCTFKLLSSTDPHCSVASISTFSTVSLKTRKQGELIFSAGFFSFKPIEKPNFCNKK
QDEFFPNTYPSKPTPEKTKVFKTNPTTKISDKSQSDNKFLAEDFFFPPNPFFPPPLVPNPFQPPPLIPNPFQPPPLI
PNPFQPPSPPLIPNPFQPPSPPPLIPNPFQPPPSPPTLFPNPFQPPPSPPPSLFPPFPPLVIPGLTPSPPPPPPTKP
IFPPFPPLFPPLFPPPHSPGTPPASASMNTSP

>Glyma19g04540.1|PACid:26328309
MRIMYWFLVLFLNLTYGSVLDASHGKTLPSTVVVGTVYCDTCFQHTFSTRSHFISGALVAVECKVGDSVPSFNKEVK
TNEHGEFKVEVPLKVWKHAKRIKGCTFKLISSSEPHCSVASVATSSSVSLKTREQGELIFSAGLFSFKPTKKPNFCN
HKQSVPNSKAHVKNEFPPNTNPSTLDKKSAEDTFFPFPPIPFLPPIPFLPPLPFPFPPFPFPFPPLSPPPTPTPVLP
PTPLPAPLNPPLPAPLSPPPLPPLSPPPYTSPWPQLSSPLRIPNR

>Glyma15g35290.2|PACid:26331978
MSTPPMPFLFLFLFSLIVPLLLAQAPSPSPTHSPPPPSPSPSPKHTPPPPPSTTTPPSPKHVPPPPPSSPSPSPKHA
PPPPHSSPLPSPKHAPSSPPPSQAHAPPLPSSQSPPPTHSPPPSSPSPPSPTHAPPPSPTPPPARAPSPPPPPPPSS
PSSACKSTLYPKLCRSIVSSIRSSPSDPYNLGKFSIKQSLKQAKKLVLVFKDFLTKYKSSSSLNAAEIAALEDCSEL
NQLNVNYLESVSEELKSADSSNDTELVEKIETYLSAVATNHYTCYDGLVVIKSNIANAIAVPLKNVTQLYSVSLGLV
TQALKKNLKTHKTRKHGLPTKDYKVRQPLKKLIKLLHTKYSCTASSNCSTRSERILKESENQGVLLKEFAIVSLDGT
ENFTSIGDAIAAAPDNLRAEDGYFLIYVREGNYEEYVTVPIQKKNILLIGDGINKTCITGNHSVVDGWTTYNSSTFA
VSGERFVAVDVTFRNTAGPQKHQAVALRNNADLSTFYRCSFEGYQDTLYVHSLRQFYRECDIYGTVDFIFGNAAVVF
QSCNIYARKPMPNQKNAVTAQGRTDPNQNTGISIQNCKIDAAPDLAEDLKSTNSYLGRPWKVYSRTVFMQSYIGELI
QSAGWLEWNGTDGLNTLFYGEFKNFGPGSDTSKRVQWSGYNLLSATQARNFTVHNFTLGYTWLPDTDIPYSEGL

>Glyma18g03040.2|PACid:26355061
MKTPPSSSVNLCLAVAVAAVLILSLVPSSHGFGSGATLALSDSSSTVCAVVASESTRRIECYRQGQVVPIAPNVSFS
SISGGRNYFCGIRSSNSNLLCWNTSSSFERRRLYNDSSVPLENLAVGDTHVCATAVGDGTVRCWRTGNTFQIVSGSD
QFASISSGSGFSCGILKNGSRVRCWGDTNVSEQIENSFRNISMLSLVAGGSHVCGLNLTGFLVCRGSNGSGQFDIPQ
GGAFEYSGLALGAEHGCAIRESNGSVVCWGGNGQFSVSNVTEGVSFEVIVSGSNFVCGLTTNNLTVVCWGPGWSNGS
RFELPLPRVLPGPCVQSSCGECGSYLNSEFLCSGSGNICKPMTCRPQTTVPPPLLPSPPPPSPSPPPPPSLTSPSPS
PSRSKTLTRGLLAFAIIGSVGAFAGICTIVYCLWSGVCFGKKKVHNSVQPTITRGSSGSNGGGASNNSNSSISSMIM
RQTSIIMRRQRSGTSSMKHPDRAEEFTLAELAAATDNFSHENKIGAGSFGVVYKGKLTDGREVAIKRGETGSKMKKF
QEKESAFESELAFLSRLHHKHLVGLVGFCEEKDERLLVYEYMKNGALYDHLHDKNNVEKESSVLNNWKMRIKIALDA
SRGIEYLHNYAVPSIIHRDIKSSNILLDATWTARVSDFGLSLMSPEPDRDHRPMKAAGTVGYIDPEYYGLNVLTAKS
DVYGLGVVLLELLTGKRAIFKYGEDGGTPLSVVDFAVPSILAGELVKLLDPRVEPPNVTESEAVELVAYTAIHCVNL
EGKDRPTMADIVSNLERALAICESSHDSISSGTISVVSE

Medicago truncatula

>Medtr2g098060.1|PACid:23014732
MGTPLIYAIAFCLIAISISINVAAYKPYYASQPNNYSPPSTKHVEYPRSQKTPYYYAKHPHLNHKLTPLYKHKSPLS
TKHAEYPQSKYTPKHAKHSHPYPKLTPLYAYKSPPPPKYAKHPQSQYTLYHAKHSHLYPKLTPLYKHKSPLPTKHVE
YPQSKYTPEHAKHSHPYPKLTPIYTYKSPPPPKYAKYPQSKYTSHHAKHSHLYPKLTQLYTYKSPPPPKYAKYPQSQ
YTPHHAKHSHLYPKLTPLYTYKSPPPPKHAKYPQSQYTPHHAKHAHLYPKLAPLYTYKSPPPPSPSPPPPYIYKSPP
PPSPSPPPPYVYKSPPPPSPSPQPKYVYKSPPPPSASLPLSYIYKSPPPPSPSPPPPYIYKSPPPPSPSLPPPYVYK
SPPPPSPSPPPPYVYKSPPPPSASLPLSYIYKSPPPPSPSPPPPYVHKSPPHPSPSPPPPYVYKSPPPPSPSPPPPY
VYKSPPPPYASLPLSYVYNSPPPPSPSPPPPYVYKSPPPPSPSPPPPYVYKSPPPPSPSPPPPYAYKSPPPPSPSPP
PPYVYKSPPPPSSSPPPPYIHKSPSPSRHVLKHPAYHLPYLYNSPPPPPRVY

>Medtr8g078670.1|PACid:23035907
MGTSIEPRNLPRLMYTIAFCMIAISTFATADDKPYYGGQQPYYNYPQPTTPPSYYYKPPSYNNYQSPPLYEYKSPPP
YDHKFPPYNYYKSPPPSPPPPFAYKFPPYNYQSPPPYEHKFPPYNYKSPPPYDHKFPPYNYYKPPPPSPPPPYDYKL
PPYYYKSPPPPSPSPPPPYVYKSPPPPPYVYKSPPPPPYVYPSPPPPSPPPPYVYKSPPPPPYVYKSPPPPPYVYPS
PPPPSPPPPYVYKSPPPPPYVYKSPPPPPYVYKSPPPPPYVYPSPPPPSPPPPYVYKSPPPPPYVYKSPPPPPYVYP
SPPPPSPPPPYVYKSPPPPPYVYPSPPPPSPPPPYIYKSPPPPPYVYKSPPPPPYVYPSPPPPSPPPPYIYKSLLLH
HLHHHPTICIQVATSTTICLQVTATSSICLRITSSSTICLQIPSTTTFMFYESPPPPSPSPPPPYYYKPIPPPYVYE
SPPYIYKSPPPAPYVYKSPPYVYKSPPTPYKPYQYSSPPPPVY

>Medtr1g099150.1|PACid:23045086
MRSPMASVYPPVSPPYHYPSPPPPKVYPPHSPPPKVYPPVSPPYHYSSPPPPKKYPPHSPPPKVYPPVSPPYHYSSP
PPPKVYPPHSPPPKVYPPVKPPYHYSSPPPPKKYPPHSPPPEVYPPVSPPYHYSSPPPPKVYPPHSPPPGVYPPVKP
PYHYSSPPPPKPYKYSSPPPPVHHVSPKPYYHSPPPPKKPYKYSSPPPPVHPPYTPKPVYHSPPVHPPYTPHPVYHS
PPPPVHSYIYASPPPPYHS

>AC231338_17.1|PACid:23053389
MASITLTIAFTIISLSLPSQISANNYVYSSPPPPSKPYKYASPPPPVYKYKSPPPPVYSPPPPVYSPPPPVYKYKSP
PPPVYSPPPPVYKYKSPPPPVYSPPPPVYKYKSPPPPVYSPPPPVYKYKSPPPPVYSPPPPVYKYKSPPPPVYSPPP
PVYKYKSPPPPVYSPPPPVYKYKSPPPPVYSPPPPVYKYKSPPPPVYSPPPPVYKYKSPPPPVYSPPPPVYKYKSPP
PPVYSPPPPVYKYKSPPPPVYSPPPPVYKYKSPPPPAYSPPPPHYIYSSPPPPYHH

>Medtr3g049390.1|PACid:23010035
MARIMRSSFLNLLLLVVLFTNLLHCFDAQPVPTPAPSPNSPSPAPNLRPPSPTPAPVPPPNSPPPAPVPSPISSPPP
NPNAPEPEPTAAGQGGSSGLSGGQKAGIVIGTLLGAAILGFIGMVCWKRRVNIRRNRYSDAARNIEL

>Medtr3g015420.1|PACid:23011094
MAYLKKVILNLTLLFVIILSMEVATTAARLPLWKIEPHHKINGRLLYIVDDVPRGHHYKSPPPPRPSPPPPPPRP

>Medtr3g061500.1|PACid:23012681
MAPYLFLNRFIIILNLTIQVLDFSRHQSPPPSSTNKAPSTKPPLPKKDFSRHQSPPPSSTNKAPSTKPPLPKKDFSR
HQSPPPSSTNKAPSHHSLRNDGGGTTPYYGSITCNSNIPSTQCNTNKGNLTNTQCNIDKGNSTNTRKETPPKGFADD
GGIGRE

>Medtr2g034910.1|PACid:23018654
MVTLQTTTSMCCFMIMLLFLSVTKSFGDLVTDHKNLTTVTKDEVKCTPCGQVSSPPPPSPPPPSPPPASTNNCPPPP
SPPSSGGGSGSTYYSPPPPSVYYYSSPPPPASTGGGGGGGLYYPPPTGGSGSGSGGNYGTPPPPNPIVPYFPFYYHT
PPPGAAAPPSLKGSVMLCAVSVLPIFMALF

>Medtr5g020350.1|PACid:23028696
MSYLFLTLIAADFGMMPRGPVKGSGPNPVFPDSPPPPRTSTNLNFGMLPKGHVPWSGPSRGSSDSPPPPRTPANLNF
GMLPKGHVPWSGPSRGSSDSPPPPRTSTNLNFGMLPKGHVPWSGPSRGSSDYPPPPRTPTNLNFGMLPKGHVPWSGP
SRGSSNSPPPPRTPTNLNFGMLPKGNVP

>Medtr8g020530.1|PACid:23038239

MASTFYSHLGFLIFGISICFYAFVVQSQIQDPNGFQSQQNQVVSSPPPPPPPPPPPPPPPPPPPPSTPPPPQDFNSPP
PPTLPDIPPQNQNPSPISSPPPPHLRRWHDHVQLPPPLAPPPQHSMNAGKKVGLLFVGIAAIMQVGFVGFLVIKRRQ
LLKTNDNDRYENCS

>Medtr1g012690.1|PACid:23041870
MASNKLSALIILFSLLSYSTFSHACGSCKPTPTSPPPPSTTPKASPPPKASTPPPSTTPKSSPPTPSTSQKCPSDTL
KLGVCADVLGLVNVIVGSPASSKCCTLIQGLADLDAAVCLCTAIKANVLGINLNVPVTLSLLLSACEKSVPNGFQCS

>Medtr1g012700.1|PACid:23042689
MASNKHSALIILFSLLAYSTFSNACGSCKPTPPSPPPPSKTPKACPPPPSTTPKASPPPTAITPPSTTPKSSPPTPS
TAQKCPSDTLKLGVCADVLGLVNVIVGNPASSKCCTLIQGLADLDAAVCLCTAIKANVLGINLNVPVTLSLLLSACQ
KSVPNGFQCS

>Medtr4g039330.1|PACid:23051859
MLHLPTESPPPHTTKPPPHTTKPQPHTQPETCIISLPIHQNSITTSTITTSPPSPPPITTTSPPSPPPTTTTTTQPS
PPPPTTTTTQPSPPPTT

>Medtr3g100950.1|PACid:23009334
MQVQGWFLLFSFFLFSPTSFFSHAISNEEAASIARRQMLHLQENEDLPENYVDTYKTDLKFPNPNLKSAYIALQAWK
KAIYSDPTNFTSNWEGPNVCSYNGIFCAASLNDSKIQVVAGIDLNQADIAGYIPAEFGLLADIALLHINSNRFCGVL
PKSFSKLKLLHELDISNNRFVGKFPCHVLSIPDIKFIDIRFNEFEGEIPPELFNKTLDAIFINNNRFTSTIPENMGN
SPASVIVFANNGISGCIPSSIGQMKNLNEFVVIGNNLTGCLPEDIGKLQQLAVFDVSENLFVGALPKTLQGLSEVEV
ISIAHNKLTGSVPKSICSLPNLANFTFSYNYFNVEEEGCVPPGKEIELEDMDNCIPNRPKQKTTNDCNVVISKPVDC
TKGLCSSKPSQSNSPSNPPTEKPTPSVPKPQPQPTPSQTPTPSTPKPTPSPQPKPTPSSPPPTPSTPEAEPPIEDDP
HNEAPKRRTRSPPPPVQSSPPPVHSPSPPVHSPPPPVHSPPPPVHSPPPPVHSPPPPVHSPPPPVHSPPPPVHSPPP
PVHSPPPPPVHSPPPPVHSPPPPVHSPPPPVHSPPPPPVHSPPPPVHSPPPPVHSPPPPVHSPPPPVHSPPPPVHSP
PPPVHSPPPPVHSPPPPVFSPPPPVHSPPPPTASPPPPVNAPPPEDDIILPPHFGSSYASPPPPIIAGY

>Medtr6g086120.1|PACid:23025461
MRKKQHYSSPSYVIVLFSFFLSLTTTLSSSIVSDGSHLTEAETQYIKHRQLLYYRDEFNDRGENVTVDPTLVFSNNR
IKNAYVALQAFKQAILSDPRNCTVDWVGSDVCSYTNVFCAPALDNPKINTVAGIDLNHCDIAGYLPEELGLLTDLAL
FHVNTNRFCGTVPHKFEKLKILFELDLSNNRFAGKFPEVVLRLPELKFLDLRFNEFEGTVPKELFDKDLDAIFINHN
RFVFNLPENFGNSPVSVIVLANNRFHGCLPAGIGNMTRLNEIIAMNNGFQACLPEEVGLLKNLTVFDVSFNKFLGPL
PEKYGNAVGLEVLNVAHNYLSGQIPASICALPNLVNFTYSYNFFTGEPPQCLVLPAADDRQNCLPARPRQRPAKTCK
AFASHHVNCNAFRCKAFVPSLPSPPPSPVFPPPVVTPPTPVFNPPPSPVLSPPPPSPPPPVFSPPPPVYSPPPPPPP
VYSPPPPPPSPPPPSPPPPPPPVYSPPPTPPSPPPTFSPHHPPTWFIYLLPPPLIPSPPPPTSPPSPLSLPLPTPPP
SPSITPSNHPLLPTRSQSTQTSQPLPSPPANMRLIDSHDHSPFLLSPFQPTSLHPHPPLPHRPRPSPPPILHISFPR
SNRPLTACPHPPLRMTTIFQTPSISLNTSASASQYVKTHSRPYIVSPPTRTPWLSIDSTPLAVIFITLLLACHRLDS
RGSPPPPPHSPPPPVYPYLSPPPPPPVHSPPPPVYSPPPPSPPPCVEPPPPPPPPPCVEPPPPSSPAPHQTPYHPPPS
PSPPPSPVYAYPSPPPPVYTSPPPSPVYAYPSPPPPVYSSPPPPPVYEGPIPPVFGISYASPPPPPFY

>Medtr8g103700.1|PACid:23038523
MMKKSQTLLIVTLLLTINTLSLSAQTIDNPSLVFENNRIQNAYIALQALKQAILSDPLNFTTNWVGSNVCSYTGIYC
AQALDNPNIRTVAGIDLNHADIAGYLPDELGLLTDLALFHINSNRFCGTVPHTFQKLKLLFELDLSNNRFAGKFPKV
VLSLPVLKFLDIRFNEFEGTVPKELFDKDLDAIFINDNRFAFDLPENLGNSPVSVVVFANNKFKGCIPSSIGNMSNL
NEINFLNNLFKSCLPSEIGLLKNLTVFDVGFNQLVGPLPAAIGGAVSLEELNVAHNMFSGKIPSSICMLPNLKNFTF
SDNFFTGEPPACFGLPASDDRRNCLPARPFQKTLVECASFLSKPVDCKSFGCKAFNPSLPLPSPPGVSPSSPPSPGG
SPTSPSPPGTTPITPSPPAVSPSTPSPPGTTPISPTPPGTTPTSPEPPALPPSTPSPPGTTPISPSPPGTTPSIPPP
THSSPPGTPNSPSPPGMTPSSPPPTHSSPPGYTPSPITPVVSPPSPAPHYGSSPPPHSPFFSPPPGTSPSSPPTPGT
VPSFPPPTHSSPPAHPTPPSTTPTPPVVSPPSHTPMPPSHSLYPPPPTGPHYGRQSPPPPSESPPSIGPPPSLSPPP
CEEHPSPPGPPSSSTPTPPVHVSPHPPVQHTPPSPSHPSPIQYNSPPPPPPLVHYIYNSPPPPTLPPPPPPSPAYGG
PLPPIVGLPYASPPPPPFY

>Medtr8g083560.1|PACid:23039342
MMKKSQTLLIVTLLLTINTLSLSAQTIDNPSLVFENNRIQNAYIALQALKQAILSDPLNFTTNWVGSNVCSYTGIYC
AQALDNPNIRTVAGIDLNHADIAGYLPDELGLLTDLALFHINSNRFCGTVPHTFQKLKLLFELDLSNNRFAGKFPKV
VLSLPVLKFLDIRFNEFEGTVPKELFDKDLDAIFINDNRFAFDLPENLGCIPSSIGNMSNLNEINFLNNLFKSCLPS

EIGLLKNLTVFDVGFNQLVGPLPAAIGGAVSLEELNVAHNMFSGKIPSSICMLPNLKNFTFSDNFFTGEPPACFGLP
ASDDRRNCLPARPFQKTLVECASFLSKPVDCKSFGCKAFNPSLPLPSPPGVSPSSPPSPGGSPTSPSPPGTTPITPS
PPAVSPSTPSPPGTTPISPTPPGTTPTSPEPPALPPSTPSPPGTTPISPSPPGTTPSIPPPTHSSPPGTPNSPSPPG
MTPSSPPPTHSSPPGYTPSPITPVVSPPSPAPHYGSSPPPHSLFFHHHRERPLPLHQRRERCLPFHHQHTLLRLRPH
YGRQSPPPPSESPPSIGPPPSLSPPPCEEHPSPPGPPSSSTPTPPVHVSPHPPVQHTPPSPSHPSPIQYNSPPPPPP
LVHYIYNSPPPPTLPPPPPPSPAYGGPLPPIVGLPYASPPPPPFY

>Medtr3g116450.1|PACid:23008156
MSSISQESSPGPDAQNSSPPDSSSSPPPSLSPPPSSPPPSLSPPPSSPPPSSPPPQSPPPSSPSPSSPPPSSPSPTP
PSNTSPSQPSQSPPPPPQPQSSQAPPNNSPLPPNQGVFSPPPPPPPTSHHHHKSSPSNNGNNTNDNNGNQLSVGGII
AVAGAACIILLVIILLCVICTRRKKRSQPHHIHYYPTPAHGANQYMYNNGEHVLNIPPPAQGGGWTPTPPKKTPPKM
TPSKKTPPHQVSSSELTNSSYSGPVDPVLPPPHPTVALGFNQSSFSYEELSTATGGFSKQNLLGQGGFGYVHKGILP
NGKEIAVKSLKSTGGQGDREFQAEVDTISRVHHRYLVSLVGYCISESKKLLVYEFVPNKTLDYHLHGKGRPVMDWAT
RLKIAVGSAKGLAYLHEDCHPRIIHRDIKGANILIENNFEAKVADFGLAKFTQDTNTHVSTRVMGTFGYMAPEYASS
GKLTDKSDVFSYGVMLLELITGRRPVGTAGSDYEEDSLVDWARPLCSKALEYGIYLGLVDPRLEENYEKQDMTRMVA
CASACVRHSGRRRPRMSQIVRVLEGDASLEVLINQDGVKPGHSAMYSSASGDYDAGTYSADMKKFRKLALESSTGSS
EYGATSEYGLNLSASSSDQSSVDYTRRTVTGTGGGSKFHEK

>Medtr6g088610.1|PACid:23025389
MSTAPAPSSPPAGNATAPPPATPTAPPPATPSSPPPATPSSPPPATPSSPPPSTPANPPPVTPSAPPPSTPATPSSP
PPSTTPSAPPPSTPSNSPPSPPTTPAISPPSGGGTTPSPPSRTPPSSDDSPSPPSSKTPPPPSPPSSSSISTGTVIG
IAVGAVVVLVFFSICCICFRKKKRRRRDEEYYGQQNYQQPPPAQRPKVEPYGGPPQQWQNNAHPPSDHVVSKPPPPA
PIPPRPPSHVAPPPPPPAFISSSGGSGSNYSGGELLPPPSPGIAFSSGKSTFTYEELARATDGFSDANLLGQGGFGY
VHRGILPNGKEVAVKQLKAGSGQGEREFQAEVEIISRVHHKHLVSLVGYCSTGFQRLLVYEFVPNNTLEFHLHGKGR
PTMDWSTRLRIALGSAKGLAYLHEDCHPKIIHRDIKAANILLDFKFEAKVADFGLAKIASDLNTHVSTRVMGTFGYL
APEYAASGKLTDKSDVFSYGVMLLELLTGRRPVDKDQTYMDDSLVEWARPLLMRALEEDNLDSLIDPRLQNDFDPNE
MTRMVACAAACTRHSAKRRPKMSQVVRALEGDVSLADLNEGVRPGHSSVYNSHESSDYDTQQYKEDMIKFRKMALGT
QEYAGSSEYSGATSEYGLNPSGSSSENQSRQTTREMGQTTREMEMRKMKNGQGFSGSS

>Medtr5g019940.1|PACid:23029656
MSSPPDSDNSSSTPSDSPPEESPQSPPPSPPSDSQPSPSGSPPPPPPSGSQPSPPPPTSNKPSPPSPNGSGNSPTPV
AGRPPPASNRSGNSQSPPHKSPTTPTGHSNGNHNNNNNNNNNDDATKAIVGVVIGVAVALIILVIACFVCCRKKKRK
YYYGERPPPGKGNSNYYTSGHHSNYYGDREHVVRVQNGMGPNGGGGGWGAPPPPPMGMTSTDMSSNYSGGPPPLPPP
SPSLALGLKGGTFTYEELAAATDGFIDSNLIGQGGFGYVHKGVLPSGKEIAVKSLKSGSGQGEREFQAEIDIISRVH
HRHLVSLVGYCISGGQRMLVYEFISNNTLEYHLHGKGRPTMDWPTRMRIAIGSAKGLAYLHEDCHPRIIHRDIKAAN
VLIDDSFEAKVADFGLAKLTSDNNTHVSTRVMGTFGYLAPEYASSGKLTEKSDVFSFGVMLLELVTGKRPVDASITM
DDSLVDWARPLLTRGLEEDGNFSELVDPFLEGNYDPQELARMAACAAASIRHSARKRSKMSQIVRTLEGDVSLDDLK
EAIKPGHTTVNTSSGSEYDTVQYNSDMQKMRKTVFSSHESNTSSFTSSGEMGQTPPKLKTPQQLP

>Medtr1g025070.1|PACid:23045508
MSSPPDSDNSSSTPSDSPPEESPQSPPPSPPSDSQPSPSGSPPPPPPSGSQPSPPPPTSNKPSPPSPNGSGNSPTPV
AGRPPPASNRSGNSQSPPHKSPTTPTGHSNGNHNNNNNNNNNDDATKAIVGVVIGVAVALIILVIACFVCCRKKKRK
YYYGERPPPGKGNSNYYTSGHHSNYYGDREHVVRVQNGMGPNGGGGGWGAPPPPPMGMTSTDMSSNYSGGPPPLPPP
SPSLALGLKGGTFTYEELAAATDGFIDSNLIGQGGFGYVHKGVLPSGKEIAVKSLKSGSGQGEREFQAEIDIISRVH
HRHLVSLVGYCISGGQRMLVYEFISNNTLEYHLHGKGRPTMDWPTRMRIAIGSAKGLAYLHEDCHPRIIHRDIKAAN
VLIDDSFEAKVADFGLAKLTSDNNTHVSTRVMGTFGYLAPEYASSGKLTEKSDVFSFGVMLLELVTGKRPVDASITM
DDSLVDWARPLLTRGLEEDGNFSELVDPFLEGNYDPQELARMAACAAASIRHSARKRSKMSQVERLALSKCISDQ

>AC225458_54.1|PACid:23046114
MSSPPDSDNSSSTPSDSPPEESPQSPPPSPPSDSQPSPSGSPPPPPPSGSQPSPPPPTSNKPSPPSPNGSGNSPTPV
AGRPPPASNRSGNSQSPPHKSPTTPTGHSNGNHNNNNNNNNNDDATKAIVGVVIGVAVALIILVIACFVCCRKKKRK
YYYGERPPPGKGNSNYYTSGHHSNYYGDREHVVRVQNGMGPNGGGGGWGAPPPPPMGMTSTDMSSNYSGGPPPLPPP
SPSLALGLKGGTFTYEELAAATDGFIDSNLIGQGGFGYVHKGVLPSGKEIAVKSLKSGSGQGEREFQAEIDIISRVH
HRHLVSLVGYCISGGQRMLVYEFISNNTLEYHLHGKGRPTMDWPTRMRIAIGSAKGLAYLHEDCHPRIIHRDIKAAN
VLIDDSFEAKVADFGLAKLTSDNNTHVSTRVMGTFGYLAPEYASSGKLTEKSDVFSFGVMLLELVTGKRPVDASITM
DDSLVDWARPLLTRGLEEDGNFSELVDPFLEGNYDPQELARMAACAAASIRHSARKRSKMSQIVRTLEGDVSLDDLK
EAIKPGHTTVNTSSGSEYDTVQYNSDMQKMRKTVFSSHESNTSSFTSSGEMGQTPPKLKTPQQLP

>Medtr5g036540.1|PACid:23031773
MLYGKCQLPKPIAQDKEENKTCNDHQKEKKHKSISYFRNLIVIVNLCSSSFMSLNMMFLLLSHVLILQTFLIPTFYC
QTNSPQNIETLYPIEIPEQATITQPPNPQESPPPSPSSSQGPISSTSSTNSSTNSKIVTAVAATAAGTLVLSALIFF
FVNKCCRAKRRNVIVNNNNTSSRPSLEHRNAVPQAKAFERIEGNIKGLIVDEDGLDVVYWRKLQDQNINKDLQKGVV
LDSPRNNEGYDHEENQEEKSESIQEMHLLRGKSSTSHMNIFPQEESYTIMKITPPAPPPIVPTTQPFSLNLLPSSPK
PPSTSFSSIPQISSPLVSSIPSISGDRKSQKPPPPPPPPPVPDRKISAPPPPPPPIPNRKSPSPPPPPPPKTGGLK
LKSSSKPPPTPIETTSMKNKQGSSSSEVKLKPLHWDKVNTNLDHSMVWDKIDRGSFRVDDDLMEALFGYVAAKPKSN
TPKGKESTSPSRDASTNAFILDPRKSQNTAIVLKSLAVSRKEIIDALVDGQGLNADTIEKLSRIAPTEEEQSNILEY
EGDTEKLAAAESFLYHILKAVPSAFKRLNAILFRLNYDAEIVEIKEFLQTLELGCKELRNQGVFVKLLEAVLKAGNR
MNAGTNRGNAQAFNLVSLRKLSDVKSTDGKTTLLHFVVEEVVRSEGKRAVLNRNHSLSRSSSNRSSSSSSSSGDSKN
SATSNEQKQREYTTLGLAIVGGVSSEFSNVKKVALTDYNSFVGSISALSARIVDIRQLVLQCGNNGKGGKFVREMNH
FLENAEKELQLVREEQTRIMQLVKRTTEYYQGGASKDGAGEQTLYLFVIVKDFLGMVDQACIEIARNMQKKKTPKTK
F

>Medtr4g131020.1|PACid:23052955
MAMLRKLFYRKPPDGLLEICDRVYVFDCCFTTEAWNEEKYKVYMDGIVGQLRENVPDASILVFNFREEETKSLMANI
ISEYDITIMDYPRHYEGCPVLKMELIHHFLRSSESWLSLGQHNVLLMHCERGGWPVMAFMLAALLIYRKVYSGEHRT
LDMIYRQSPHQLLHLLTPLNPIPSQLRYLQYVSRRNVALDWPPLDRALMLDCIILRFFPNFDGEGGCHPLFRIYGQD
PFSADKSPKMLYSMPKRSKNVRAYKQGECELIKIDINCHIQGDVVIEGINLNDDMEHEMMMFRVMFNTAFVRSNILM
LNRDEIDVLWDAKDHFPKDFRAEILFSEIDAAAAVILDNTSFFEEKDGLPIEAFAKVQEIFSHVDWMNPKADAALNV
LQQISTSAIMNDKLDKVSDQHVETATSLGETGPKTPQRNSYAAIRSLSSTKRTPNNDMSRKEEKTNKVDSIPQRANT
SDIIGQEKICSSEKSLESSKCPTGSTNFDIKPQESNLASSSSADSSLSPGTPPPRPPLTTRSKEVHDSPPHTELPPH
HILPSQSGPQSQDRSYSPISSSTPETYHSLAPDSSIEPSPPLSSRKPLNDIPPVKTRPKSPSSQPPTPPPPPTPPLK
DQKVVRAGPLSPPPPPPSPKKDIHVKAGPPSPPVPSHMNEKPHVRDGLSPPPPPLPPSPPLKAEQPTRFQPPPPPP
PPRLSVEAASPITAPPAPPPPPSATLSSGNPNASLQKSSPAPPPPIPFGKGLKPGSAFPMSLSVGVDGNKVSGPQSS
SLAGSKGRVLPRAIGSKNDSKKLKPLHWMKLSRAVQGSLWDETQKSGEASKAPEIDMSELESLFSAAAPSSGPAKKS
NVQSSVKPKSEKVQLIDHRRAYNCEIMLSKVKVPLHDLMSSVLALEESALDTDTVENLIKFCPTKEEMEIIKNYNGE
KEKLGRCEQFFMELMKVPRVEAKLRVFSFRIQFYSQVSDLKNSLKVVNSSAEEIRNSVKLKRIMQTILTLGNALNQG
TARGSAIGFRLDSLLKLTETRARNNKMTLMHYLCKVLDDKLPEVLDFSKDLANLEPAAKIQLKFLAEEMQAVNKGLE
KVVQELSTSENDGPISETFRKKLKGFLCSAEAEVRTLASLYSGVGKNVDALILYFGEDPSRCQFEQVVTTLLNFTRM
FNKAHEENRKQLELEMKKTAESDKKKCESERILPTTIRTGNVK

>Medtr3g010770.1|PACid:23009928
MSPSLSLLLFILLAPLLLLAQSPPPSPSPPPPSSSSAACKTTLYPKLCRSMLSAIRSSPSDPYNYGKFSIKQNLKVA
RKLEKVFIDFLNRHQSSSSLNHEEVGALVDCKDLNSLNVDYLESISDELKSASSSSSSSDTELVDKIESYLSAVATN
HYTCYDGLVVTKSNIANALAVPLKDATQFYSVSLGLVTEALSKNMKRNKTRKHGLPNKSFKVRQPLEKLIKLLRTKY
SCQKTSSNCTSTRTERILKESESHGILLNDFVLVSPYGIANHTSIGDAIAAAPNNTKPEDGYYLIYVREGYYEEYVI
VPKHKNNILLVGDGINNTIITGNHSVIDGWTTFNSSTFAVSGERFIAVDITFRNTAGPEKHQAVAVRNNADLSTFYR
CSFEGYQDTLYVHSLRQFYRDCKIYGTVDFIFGNAAVVFQNCNIYARKPLPNQKNAVTAQGRTDPNQNTGISIQNCT
IDAAQDLANDLNSTMSYLGRPWKIYSRTVYMQSYIGDFVQPSGWLEWNGTVGLDTIFYGEFNNYGPGSVTNNRVQWP
GHFLLNDTQAWNFTVLNFTLGNTWLPDTDIPYTEGLLN

>Medtr3g117760.1|PACid:23010081
MELPEKLLFAIFLILSVAIDGTKADTMLTGTVICDQCKDGQRSLYDYPVNGAKVTLSCSDSNGQVTMSREETTNWFG
SYTMRFDGAPDLGGCSAHVSGSNNRQGGPMSCGEGAGPAQNPRLMFRMFDMEYAVDPLLAQPSQPMQYCSTSSNPS
PPPPISSPPPHFNLPPMIFPEASACPSQKWTMPEYECYWRGVNRDTKVGVAFGMVAARRYGTDMTLWYGLKGRGDPY
RTLLREGITALLNSYNSIQFSYHPLGVITHMNYALMGSTRDVLVTAYHFMRANSGAGNVSCKFTSCN

>Medtr7g072980.1|PACid:23022961
MASSMLVKVTCLAMICLVLGIPLANAAASCPEIEQTLTPCLEYVTHPGSPPPPEPCCNAVKAIHGQTHTPQDRRDCC
SCVKSMIGDIPGLNLPAAASMAKDCGVDLGYEISPDMDCSATHYSTIPCLGYLRNPGPSVPAPYCNGIKGLNNQVKT
THDRQSVCRCLKSTVTCLAMICLVLGIPLANAAPSCPEVQQTLAPCVPYVTHPGPPISPPPPCCNAVKTLNGQSKTT
QDRRDVCGCLKSMMGGIPGLNLPAIASLPKDCGVDIGYIISPNMDCNKVN

>Medtr5g020530.1|PACid:23031304

MSAAKLTSCLAAATAAAAASISSQSNPAYSESFFRFPFFSSSPSNSPPPTDQSSDKKSDSPPPAEEPNKSGFDPESL
ERAAKALREINSSPHAKKVFDLMRKQEQSRLAELDAEKVNYELIQTQGDIDRLRKMAEEQRNLIQEQNQRQAQVLRF
EDELARKRMQTDHEDQRRHNVELVQMQEKSFVRKEQARKDSEEQMQAQKLLTEQKKAEIDKETIRAKEKANAEKRIR
LKVLTEEQNRRELKDKLQGETDKWIAAINATFSHIEGGLRILLTDRDKLLMTVGGATALAAGVYTTREGAKVTWGYI
NRILGQPSLIRESSMAKFPGSRMMSQAKNKVLNYSTLAREKKSVGIQNGLGNVILHPSLQRRIVHLARATSNTKAHQ
APFRNMLFYGPPGTGKTMVAREIARKSGLDYAMMTGGDVAPLGPQAVTKIHEIFDWAKKSKRGLLLFIDEADAFLCE
RNSSHMSEAQRSALNALLFRTGDQSRDIVLVLATNRPGDLDSAITDRIDEVIEFPLPGEEERLKLLNLYLNKYLCDE
SNGSKGGLFMKKQPQQITIKDLSEDVLKEAAKKTEGFSGREIAKLMASVQAAVYGRPDCALDSKLFREIVDYKVVEH
HQRLKLAAEGGLPA

>Medtr8g066950.1|PACid:23035986
MDSPLSEIPGSVPEWSLHRRSSLERRTKEKGSMKDCAMKGTMMIFLVTFIWDNDDLKHIKVKQKYIKKNEQSYRYYL
DTTPSLFPICPPSRHYPPRIGPTIGSIPTMPTTGSPPPPGSPPPDPTTGSPPPKGSPPPNPTTGSPPPPGSPPDPTT
GSPPPPGSPPPKGSPPPDPTTGSPPPPGSPPLDPTTGSPPPKGSPPPDPTTGSPPPPGSPPPDPTTGSPPPTGSPPP
DPTLGSPRPPGSPPPGSPPPPNSPLDPPWESFLVAEHMLKPNSTKIAMLRTFEVAI

>Medtr1g083260.1|PACid:23042476
MSCGSELLTMIFIIIITLLVPLSSSQRTHILSVGALINAAGSFHVHTLQDNYYMKEQENEKKQVQKISGLDENEEKQ
GFIVEKFRSLLGLKSFHKRVPSKSNGDSDSDSDQFLTPSPSPSQNIEAEVEAPAPAPTPSQVMHFHPHSYHQKHHFH
WNQPPKKLHHDDRGRTKRILVAVFVSVGVAAFVISLGLILFCRKKFTNHKKKKPKRTMPLCSSNTKGKTKGKVSLNP
GLDLFYLDALGEDVEQHACTLTKTSDNNVSSSFTKEIVSVHEEELVIKNEHECVDKIVHEDCDSSEDESFHSFVDSQ
SNTRLSNASAGSLSDTQSLLLSPQNSFSLLPNQLPSSPQNTNDSHQPPYSPKQKDQDIENETFVQCPQTSNSSPPPP
PPPPPTPPLKMPLFTLHSLTTSSRVSSHSPLSLTSHTLSSPVNSETSSRSNLSPEKDSFSPSSSNPTKSPPPPPCPP
PFPRGNSNKNAKTPPPPPYQFPQSPLGKDGTPLAKLKPLHWDKVRAAPNRTMVWDKLRSSSFELDEEMIESLFGYNL
QSSINNDESKSKTPSPSKHVLDPKRLQNITILSKALNVTAEQVCDALMQGKGLSLQQLEALVKMVPTKEEEGKLFNY
KGNINELGSAEKFVRAVLCVPFAFQRVETMLYKETFDDEVVHLRNSFSMLEEACKELRSSRLFLKLLEAVLKTGNRM
NVGTIRGGARAFKLDALLKLADVKGTDGKTTLLHFVVQEIVRSEGIRVSDSIMGKISQKSNKNRTEEEKEEDYRKMG
LELVSGLSTELYNVKKTATIDLDVLASSVSNLYDGMVRLKQLVENELHEDEMCHNFVMSMKLFLQYVDGNLKELRGD
EDRVIARVKEITEYFHGDVSKEDNPLRIFVIVRDFMGMLDNVCKELRRSKTPRTPNPLAPFR

>Medtr1g021620.1|PACid:23042625
MAIPSLLCSLLIIIFTSQTKADVVYDEINLYHYVCDQDNNRGNYTTNSTYDKNLNTLLSTLTSNTEINYGFYNFSHG
ENSDKVYAIGLCRGDIMPNSCRSCLSSARTNLTRNCPNRKEAIFWSEDEKCMLRYSDRLILGVMEDVPMFSSKNENN
SSDVDLSNEVVTTLLNNLTSRAVKGDSMIKYAAGSQPGPQYEVIYGLVQCTPDLSETDCNSCLVENLQQIPSCCNNK
IGGRVVRPSCNMRFETSYLFYEPRASPPPPPSSPPPPATTNGTTLSQGDDIFSIGYANASKVFNEMLLFQKQNHEQI
MWYRLPTIHSC

>Medtr1g012630.1|PACid:23044392
MASNKLSALIIIFSLFAHSTFSHDCASCKPTPISPPPPSKTPKACPPPPSTTPKASPPPPSTTASPPPKASTPPPST
PKASPPPPSMPTASPPPKASTPPPSTPTASPPPPSTTPTASPPTPSTAQKCPSDTLKLGVCADVLGLVNVIVGSPAS
SNCCTLIQGLADLDAAVCLCTAIKANVLGINLNVPVTLSLLLSACQKSVPNGFQCS

Brassica rapa

>Bra004749|PACid:22685694
MATPAWSQARGQWVVAMLALLVGSAIATEPYYYSSPPPPYEYKSPPPPVKSPPPPYEYKSPPPPVKSPPPPYYYHSP
PPPVKSPPPPYVYHSPPPPVKSPPPPYYYHSPPPPVKSPPPPYVYHSPPPPIKSPPPPYYYHSPPPPVKSPPPPYYY
HSPPPPFKSPPPPYYYHSPPPPFKSPPPPYYYHSPPPPVKSPTPPYYYHSPPPPVNSPPPPYYYHSPPPPYYYHSPP
PPCYDWTYPKKSHDKKHLKGAVVEVTCKAGDKIVKAYGKTKINGKYAITVKGYNYRKYGGEVCTAKLHAPPKGSPCN
IPTSYHMGNKGAKLHVKSKTKYEVVLYAKSFAYAPKKPYGECHKPAPYHPPYYYKSPPPPSPVYYYKSPPPPAPTYV
YKSPPPPTPTYVYKSPPPPTPTYVYKSPPPPTPTYVYKSPPPPTPTYVYKSPPPPTPTYVYKSPPPPTPTYVYKSPP
PPTPTYVYKSPPPPTPTYIYKSPPPPTPTYVYKSPPPPTPTYVYKSPPPPTPTYVYKSPPPPTHTPTPYYYHSPPPP
VKSPPPPYYYHSPPPPVKSPPPPYYYHSPPPLVKSPPPPYYYHSPPPPVKSPPPPYYYHSPPPPVKSPPPPYYYHSP
PPPVKSPPPPYYYHSPPPPVKSPPPPYYYHSPPPPVKSPPPPYYYHSPPPPVKSPPPPYYYHSPPPPVKSPPPPYYY
HSPPPPVKSLPPPYYYHSPPPPVKSPPPPYYYHSPPPPVKSPPPPYYYHSPPPPVKSPPPPYYYHSPPPPVKSPPPP
YYYHSPPPPVKSPPPPYYYHSPPPPVKSPPPPYYYHSPPPPVKSPPPPYYYHSPPPPVKSPPPPYYYHSPPPPVKSP
PPPYYYNSPPPPVKSPPPPVYIYASPPPPTHY

>Bra015238|PACid:22692069
MHRDHFCNLHVLHADADLPLDHQRVSPPPPYYYHSPPPPVKSPPPPYYYHSPPPPVKSPPSPYYYQSPPPPVKSPPP
PYYYHSPPPPVKSPPPPYYYHSPPPVKSPPPPYYYHSPPPPVKSPPPPYYYHSPPPPVKSPPPPYYYHSPPPPVKSP
PPPYYYSSPPPPKSYPPPYYYSSPPPPPKSYSPYYYSSPPPPVYKKYEKKRTQR

>Bra025889|PACid:22695898
MGSPVASLVATLLVLTISLTFVSQSTANYFYSSPPPPVKHYQYKSPPPPVKHYSPSPVYHSPPPPKKHYEYKSPPPP
VKHYSPPPVYHSPPPPKKDYVYKSPLPPVKHYSPPPVYHSPPPPKKHYVYKSPPPPVKHYSPPPVYHSPPPPKKHYV
YKSPPPPVKHYSPPPVNHSPPPPKKHYVYKSPPPPVKHYPPPPFYHSPPPPKKHYVYKSPPPPVKHYSPPPVYHSPP
PPKKHYVYKSPPPPVKHYSPPPVYHSPPPPKKHYVYKSPPPPVKHYSPPPVYHSPPPPKKHYVYKSPPPPVKHYSPP
PVYHSPPPPKKHYVYKSPPPPVKHYSPPPVYHSPPPPKKHYVYKSPPPPVKHYSPPPVYHSPPPPKKKYVYKSPPPP
VHHYSPPHHPYLYKSPPPPYHY

>Bra003189|PACid:22700292
MRSSSRMGSSTHLIYALGVIIMATMVAAYEPVTLPPLPSYSPSPKVEYNTPPLPYISNSPPPPTYYSPSPKVDYKSP
PPPYVYSSPPPPPYYSPSPKVEYNTPPLPYISNSPPPPTYYSPSPKVDYKSPPPPYVYSSPPPPPYYSPSPKIDYKS
PPPPYVYSSPPPPPYYSPSPNVEYKSPPPPYVYSSPPPPPYYSPSPKVDYKSPPPPYVYNSPPPPPYYSPSPNVEYK
SPPPPYVYSSPPPPPYYSPSPKVDYKSPPPPYVYNSPPPPPYYSPSPNVEYKSPPPPYVYNSPPPPYYSPSPKVDYK
SPPPPYVYNSPPPPYYSPSPKVDYKSPPPPYVYSSPPPPYYSPSPKVDYKSPPPPYVYSSPPPPSYYSPSPKVDYKS
PPPPYVYNSPPPPYYAPSPKVYYKSPPPPYVYSSPPPPYYSPSPKVVYKSPPPPYVYSSPPPPYYSPSPKVHYKSPP
PPYVYSSPPPPYYSPSPKAHYKSPPPPYVYSSPPPPYYAPSPKVYYKSPPPPYVYSSPPPPPYYSPSPKAHYKSPPP
PYVYSSPPPPYYSPPPKVHYKSPPPPYVYSSPPPPYYSPSPKVVYKSPPPPYVYSSPPPPYYSPSPKVHYKSPPPPY
VYSSPPPPYYSPPPKVHYKSPPPPYVYSSPPPPYYSPSPKVPYKAPKHPHDLPTPPHFFPPPPPPPYYSPSPKVEYK
SPPPPYVYSSPPPPPYYSPSPKVDYKSPPPPYVYSSPPLPYYSPSPKVDYKSPPPPYVYSSPPPPYYSPSPKVYYKS
PPPPYAYSSPPPPYYSPSPKVYYKSPPPPYAYSSPPPPDYSPSPKVYYKSPPPPYVYSSPPPPYYSPSPKVEYKSPP
PPYVYSSPPPPYYSPSPKVEYKSPPPPYVYSSPPPPPYYSPSPKVDYKSPPPPYVYSSPPPPYVSPSPKVEYKSPPP
PSYY

>Bra003699|PACid:22700295
MASLAATLLVLALSLGFVSETTANYYYSSPPPPVKHYTPPVYKSPPPLIKHYPAPPVYKSPPPPKKQYEYKSPPPPV
KHYSPRPVYKSPPPPKKHYEYKSPPPPVYKSPPPPVYHSPPPPKKHYEYKSPPPPVYQSPPPPVYHSPPPPKKHYEY
KSPPPPVYQSPPPPVYHSPPPPKKHYEYKSPPPPVYQSPPPPVYHSPPPPKKHYEYKSPPPPVYQSPPPPVYHSPPP
PKHYEYKSPPPPVHSPPPPVHYSPPHQPYLYKSPPPPYHY

>Bra012274|PACid:22700304
MGSPMAYLAATLLVLTVSLTFVSQSTANYFYSSPPPPVKHYEYKSPPPPVKHYPPPPVYHSPPPPKKHYEYKSPPPP
VKHYSPPPVYHSPPPPKKHYVYKSPPPPVKHYSPPPVYHSPPPPKKHYVYKSPPPPVKHYSPPPVYHSPPPPKKDYV
YKSPPPPVKHYSPPPVYHSPPPPKKHYVYKSPPPPVKHYTPPPVYHSPPPPKKHYVYKSPPPPVMHYSPPPSPPPPK
KHYVYKSPPPPVKHYSPPPVYHSPPPPKKHYVYKSPPPPVKHYSPPPVYHAPPPPKEKYVYKSPPPPPVHHYSPPHH
PYLYKSPPPPYHY

>Bra015937|PACid:22702258
MGSPVASFAASLLVLTISPLTSVYQSTANYFYSSPPPPVKHYEYKSPPPPVKHYSSPPKHYVYKSPPPPLKHYSPPL
VYRSPPPPRKDYVYKSPPPPVKHYSSPPPNKYYVYQSPPPPVKHYSPPSVYHSPPPPKNHYVYKSPPPPVKHYTPPV
YHSPPPPKKHYMYKSPPPPVMHYSLPQVYHSPPPPNKHYVYKSPPPPVKHYSPRLVYHSPPPPKKKYVYKSPPPPVR
HYFPPHHLYLYKSPPPPYHY

>Bra000292|PACid:22710639
MATPAWSHARGQWVIAMLALLVGSAIATEPYYYSSPPPPYEYKSPPPPVKSPPPPYEYKSPPPPVKSPPPPYYYHSP
PPPVKSPPPPYVYQSPPPPVKSPPPPYYYHSPPPPVKSPPPPYVYNSPPPPVKSPPPPYYYHSPPPPVKSPPPPYYY
HSPPPPVKSPPPPYYYHSPPPPVKSPPPPYYYHSPPPPVKSPPPPYYYHSPPPPLKSPPPPYYYHSPPPPAKSPPPP
YYYYYSPPPPVKSPPPPYYYHSPPPPVKSPPTPCYDWTYPKKSHDKKHLKGAVVEVTCKAGDKTVKAYGKTKINGKY
AITVKGYNYRKYGGEVCTAKLHSPPKGSPCNIPTSYHLGNKGAKLHVKSKTKYEVVLYAKSFAYAPKKPYGECHKPA
PYHPPYYYKSPPPPSPVYYYKSPPPPTPTYVYKSPPPPTPAYVYKSPPPPTPTYVYKSPPPPTPTYVYKSPPPPTPT
YVYKSPPPPTPTYVYKSPPPPTPTYVYKSPPPPTPTYVYKSPPPPTPTYVYKSPPPPTHTPTPYYYHSPPPPVKSPP
PPYYYHSPPPPVKSPPPPYYYHSPPPPVKSPPPPSYYHSPPPPVKSPPPPYYYQSPPPPVKSPPPPYYYHSPPPPVK
SPPPPYYYHSPPPPMKSPPPPYYYHSPPPPVKSPPPPYYYHSPPLPVKSPPPPYYYHSPPPPVKFPPPPYYYHSPPP
PVKSPPPPYYYHSPPPPVKSPPPPYYYHSPPPPVKSPPPPYYYHSPPPPVKSPPPPYYYHSPPPPVKSPPPPYYYHS
PPPPVKSPPPPYYYHSPPPPVKSPPPPYYYHSPPPPMKSPPPPYVYHSPPPPMKSPPPPYYYHSPPPPVKSPPPPPY
YYHSPPPPVKSPPPPYYYHSPPPPVKSPPPPYHYNSPPPPVKSPPPPVYIYASPPPPTHY

>Bra024612|PACid:22716064
MAMNEGVEYKSPPPPYVYSSPPPPPYYSPSPKVEYKSPPPPYVYSSPPPPPYYSPSPKVEYKSPPPPYVYSSPPPPP
YYSPSPKVEYKSPPPPYVYSSPPPPPYYSPSPKVEYKSPPPPYVYSSPPPPSYYSPSPKVEYKSPPPPYVYISPPPP
PYYSPSPKVEYKAPPPPYVYSSPPPPPYYSPSPKVEYKSPPPPYVYSSPPPPPYYSPSPKVEYKSPQPPYVYISPPP
PPYYSPSPKVEYKSSPPPYVYSSPPPPAYYSPSPKVDYKSPPPPYVYTSPPPPAYSPSPKAEYKSPPPPSYY

>Bra007440|PACid:22717742
MATPAWSLAGKTHLVVAVLAMLVGLTLATEPYYYSSPPPPYEYKSPPPPVKSTPPPYEYKSPPPPVKSPPPPYYYHS
PPPPVKSPPPPYYYNSPLPPVKSPPPPYYYHSPPPPYYYHSPPPPVKSPPPPYYYHSPSPPVKSPPPPYYYHSPPPP
VKSPPPPYYYHSPPPPVKSPPPPYYYHSPPPPVKSPPPPYYYHSPPPPVKSPPPPYYYHSPPPPMKSPPPPYYYHSP
PPPVKSPPPPYYYHSPPPPVKSPPPPYYYHSPPPLVKSPPPPYYYHSPPPPMKSPPPPYYYNSPPPPVKSPPPPYYY
KSPPPVKSPPPPYYYQSPPPPPKAYSPPYYYTSPPPPVSYPHPHPHPHPKPLVFKVVGKVYCYRCYDWTYPKKSHDK
KHLKGAVVEVTCKAGDKTVKAYGKTKINGKYAITVEGYNYRKYGGEECTAKLHAPPKGSPCNIPTSYHMGNEGAKLH
VKSKTKYEVVLYAKSFAYAPKKPYEECRKPAPYHPPYYYKSPPPPTPVYYYKSPPPPAPTYVYKSPPPPTPKYVYKS
PPPTTPTYVYKSPPPPTHFPTPYYYHSPPPPVKSPPPPYYYHSPPPPVKSPPPPYYYQSPPPPVKSPPPPYYYKSPP
PPVKSPPPPYYYHSPPPPVKSPPPPYYYHSPPPPVKSPPPPYYYQSPPPPEKSPPPPYYYHSPPPPVKSPPPPYYYH
CPPPPVKSPPPPYYYHSPPPPVKSPPPPYYYHSPPPPVKSPPPPYYYHSPPPPMKSPPPPYYYHSPPPPVKSPPPPY
YYHSPPPPVKSPPPPYYYQSPPPPVKSPPPPYYYNSPPPPVKSPPPPYHYNSPPPPLKSPPPVYIYASPPPPIHY

>Bra024614|PACid:22718260
MTSSPGMGRSAHLVYALGFVIMATMVAASYEPYTYSSPPPPMYNSPAPKVDYNSPPPPYVIMATMVAASYEPYTYSS
PPPPMYNSPAPKVDYKSPPPPYVYNSPPPPPYVYNSPPPPPYYSPSPKVDYKSPPPPYVYTSPPPPYSSPSPKPAYK
SPPPPPYYSPSPKIDYKSPPPPPYYSPSPKPIYKSPPPPYVYSSPPPPYYPPSPKPIYKSPPPPYVYSSPPPPYYSP
SPKPSYKSPPPPYVYNSPPPPYYSPSFKPAYKSPPPPYVYSSPPPPYYSPSPKPVYKSPPPPYVYSSPPPPYYSPSP
KHVYKSPPPPYVYSSPPPPYYSPSPKPAYKSPPPPYVYSSPPPPYYSPSPKPAYKSPPPPYVYSSPPPPYYSPSPKP
AYKSPPPPYVYSSPPPPYYSPSPKPVYKSPPPPYVYNSPPPPYVYSSPPPPYYSPSPKPVNKSPPPPYVYSSPPPPY
VYSSPPPPYYSPSPKPVNKSPPPPYVYSSPPPPYYSPSPKVVYKSPPRPHVCVCPPPPPCYSHSPKIEYKSPPPPYV
YSSPPPPPYYSPSPKHAYKSPPPPYVYSSPPPPYYSPTPKHAYKSPPPPYVYSSPPPPYYSPSPKPTYKSPPPPYVY
SSPPPPPYYSPSPKVEYKSPPPPYVYSSPPPPPYYSPSPKVEYKSPPPPYVYSSPPPPPYYSPSPKVEYKSPPPPYV
YSSPPPPPYYSPSPKVEYKSPPPPYVYNSPPPPPYYSPSPKVEYKSPPPPYVYTSPPPPPAYSPSPKAEYKPPPPPS
YY

>Bra014810|PACid:22719988
MRSSSRMGTSAHLIYALGVIIMANMVAAYETSTYSLPPLPSYSPSPKVEYNTPPLPNVYNSPSPPPTYYSPSPKVDY
KSPPPPYVYTSPPPPPHYSPSPKVEYKSPPPPYVYISPPPPYYSPSPKVEYKSPPPPYVYSSPPPPPYYSPSPKVDY
KSPPPPYVYSSPPPPPYYSPSPKVEYKSSPPPYVYSSPPPPPYYSPSPKVEYKSPPPPYVYSSPPPPPYYSPSPKVE
YKSPPPPYVYSSPPPPPYYSPSPKVEYKSPPPPYVYSSPPPPPYYSPSPKVDYKSPPPPYVYSSPPPPPYYSPSPKV

EYKSPPPPYVYSSPPPPPYYSPSPKVEYKSPPPPYVYSSPPPPPYYSPSPIVDYKSPPPPYVYNSPPPPYYSPSPKV
EYKSPPPPYVYSSPPPPYYSPSPKVEYKSPPPPYVYSSPPPPYYSPSPKVEYKSPPPPYVYSSPPPPYYSPSPKVVY
KSPPSPYVYSSPPPPYYSPSPKIHYKSPPPPYVYSSPPPPYYSPSPKVYYKSPPPPYVYSSPPQPYYSPSPKVHYKS
PPHPHVCVCPPPPPCYAPSPKIIYKSPPPPYVYSSPPPPYYSPSPKVYYKSPPPPYVYNSPPPPPYYSPSPKVDYKS
PPPPSYY

>Bra037731|PACid:22720029
MATPAWSHARGQWVVAILALLVGPAIATEPYIYSSPPPPYEYKSPPPPVKSPPPPYEYKSPPPPVKSPPPPYYYHSP
PPPVKSPPPPYVYHSPPPPVKSPPPPYYYHSPPPPVKSPPPPYVYHSPPPPVKSPPPPYYYHSPPPPVKSPPPPYVY
HSPPPPVKSPPPPYYYHSPPPPVKSPPPPYYYHSPPPPVKSPPPPYYYHSPPPPVKSPPPPYYYHSPPPPVKSPPPP
YYYSSPPPPKSYPPPYYYSSPPPPPKSYSPPYYYSSPPPPVSYPHPHPHTHPLVFKVVGKVYCYRCYDWTYPKKSHD
KKHLKGAVVEVTCKAGDKTVKAYGKTKINGKYAITVKGYNYRKYGGEVCTAKLHAPPKGSPCNIPTSYHMGNKGAKL
HVKSKTKYEVVLYAKSFAYAPKKPYGECHKPAPYHPPYYYKSPPPPSPVYYYKSPPPPAPTYVYKSPPPPTPTYVYK
SPPPPTPTYVYKSPPPPTPTYVYKSPPPPTPTYVYKSPPPPTPTYVYKSPPPPTPTYVYKSPPPPTPTYVYKSPPPP
TPTYVYKSPPPPTPTYIYKSPPPPTPTYVYKSPPPPTPTYVYKSPPPPTHTPTPYYYHSPPPPVKSPPPPYYYHSPP
PPVKSPPPPYYYHSPPPPVKSPPPPYYYHSPPPPVKSPPPPYYYHSPPPPVKSPPPPYYYHSPPPPVKSPPPPYSYH
SPPPPSPPPPNYYHSPPPPVKSPPPPNYYHSQPPPVKSPPPPYYYHSPPPPVKSPPPPYYYHSPPPPVKSPPPPYYY
HSPPPPVKSPPPPYYYHSPPPPVKSPPPPYYYHSPPPPVKSPPPPYHYNSPPPPVKSPPPPYYYHSPPPPVKSPPPP
YHYNSPPPPVKSPPPPVYIYASPPPPTHY

>Bra014807|PACid:22720680
MRSSSRMGPSAHPIYALGVIIMATMVAAYEPLTYSLPPLPSYSPSPKVEYNTPPLPNVYNSPSPPPYYSPSPKVDYK
SPPPPYVYTSPPPPPYYSPAPKAEYKSPPPPYVYSSPPPPPYYSPSPKVEYKSPPPPYVYSSPPPPPYYSPSPKVDY
KSPPPPYVYSSPPPPPYYSPSPKVEYKSPPPPYVYSSPPPPPYYSPSPKVEYKSPPPPYVYSSPPPPPYYSPLPKVE
YKSPPPPYVYSPPPPPHYSPSPKVEYKSPPPPYVYSPPPPPPYYSPPPKVETKPPPPPYAYSSPPPPPYYSPPPKVD
YKSPPPPYYSPPPKVDYKSPPPPYVYRSPPPPYYSPSPKVDYKSPPPPYVYSSPPPPPYYSPSPKVEYKSPPPPYVY
SSPPPPYYSPSPKVVYKSPPPPYVYSSPPPPYYSPSPKVHYKSPPPPYVYSSPPPPPYYSPSPKVEYKSPPPPYVYS
SPPPPYYSPSPKVVYKSPPPPYVYNSPPPPYYSPSPNVHYKSPPPPYVYSSPPPPYHSPSPKVHYKSPPPPYVYSSP
PPPYYSPSPKVHYKSPPHPHVCVCPPPPPCYSPSPKIIYKSPPPPYVYNSPPPPYYSPSPKVYYKSPPPPYVYSSPP
PPPYYSPSPKVEYKSPPPPYVYSSPPPPPYYSPSPKVEYKSPPPPYVYSSPPPPYVYTPPPPPHYSPSPKVEYKSTP
PPYVYTPPPPPHYSPSPKVEYKSPPPPPYYSPSPKVSPPPHYYSPSPKVEYKSPPPPYVYSSPPPPPYYSPSPKVEY
KSPPPPYAYSSPPPPYYSPSPKVEYKSPPPSPSYY

>Bra011019|PACid:22722048
MANPNWPSLLMLVLALFTIVVHSSAQYSPPSPPPYAYSYPWLPPYVYKSPPYAYSSPPPPPYVYNSPPPPPYVYSSP
PPPPYVYKSPPPPPYVYSSPPPPPYVYKSPPPPPYVYKSPPPPPYVYSSPPPPPYVYKSPPPPPYVYKSPPPPPYVY
SSPPPPPYVYKSPPPPPYVYKSPPPPPYVYSSPPPPPYVYKSPPPPPYVYKSPPPPPYVYSSPPPPPYVYKSPPPPP
YVYSSPPPPPYVYKSPPPPPYVYSSPPPPPYVYKSPPPPPYVYSSPPPPPYVYKSPPPPPYVYRSPPPPPYVYKSPP
PPPYVYRSPPPPPYVYKSPPPPPYVYSSAPPPPYVYKSPPPPPYVYNSPPSPSYIYSSPPPPSYSYSYSSPPPPIY

>Bra016333|PACid:22724258
MTSSPGMGHSAHLVYALGFVIMATMVAASYEPYTYSSPPPPSPVYNSPAPKVDYKSPPPPYVYTSPPLPPHYSPSPK
VDYKSLPPPYVYSSPPPPPYYAPSPKVDYKSPPPPYVYNSPPPPSYSPSPKVDYKSPPPPYVYSSPPPPPYLYNSPP
PPPYYSPSPKVDYKSPPPPYVYNSPPPPYYSPSPKVDYKSPPPPYVYSSPPPPPYVYNSPPPPPYYSPSPKVNYKSP
PPPYVYNSPPPPYYSLSPKPTYKSPPPSYIYNSPPPPYYSPSPKVDYKSPPPPYYSPSPKPTYKSPPPPYVYNSPPP
PYYSPSPKPTYKSPPPPYVYSSPPPPYYSPSPKPAYKSPPPPYVYSSPPPPYYTPFPKHAYKSPPPPYVYNSSPPPY
YSPSPKHVYKSPPPPYVYSSPPPPYYSPSPKTTYKSPPPPYVYSSPPPPYYSPSPKPAYKSPPPPYVYSSPPPPYYS
PPPKHAYKSPPPPYVYNSPPPPYYSPSPKVVYKSPPHPHVCVCPPPPPCYSPSPKIEYKSPPTPYVYHSPPPPSYYS
PSPKPAYKSPPPPYVYSSPPPPYYSPSPKPAYKSPPPPYVYSSPPPPYYSPSPKTTYKSPPPPYVYNSPPPPYYSPS
PKVEYKSPPPPYVYSSPPPPPYYSPSPKVEYKSPPPPYVYSSPPPPPYYSPSPKVEYKSPPPPYVYSSPPPPPYYSP
SPKVEYKSPPPPNVYSSPPPPPYYSPSPKLEYKSPPPPYVYSSPPPPPYYSPSPNVEYKSPPPPNVYSSPPPPPYYS
PSPKLEYKSPPPPYVYSSPPPPPYYSPSPNVEYKSPPPPYVYSSPPPPPYYSPSPKVQYKSPPPPYVYSSSPPPPYY
SPSPKVEYKSPPPPYGYTSPPPPTYSPSPKVEYKSPPPPSYY

>Bra027383|PACid:22683961

MGFLNIVLLVAMILSFHAFLLAQSQQQDQDQDRRQSQSSPPPPHSFWESQSPPPPTPQFNTPPPPQSTVGIISPPPP
PPSPSPPPPPPPPSELASPPQSHRNKPRRLRPPPPPPPPPVRTFNQSEKSGRLNTGKIVGLVFAGIAALLQICVVAFL
VFKRNQLLRMTHTY

>Bra029602|PACid:22686984
MEAFSNVSALLILLLLSLSLCVTSKDQTVSCTMCTSCDNPCNPVRSSSPPPTPPSGGGSYYYSPPPPTPQSSSGGGG
GSYYYPPPSTSGGGKYPPPYGGYGDGGQSYYYPPASYGNYPMPPPPNPIVPYFPFYYHIPPPGDSGSERLMSSFLFV
LFSVLLCFG

>Bra039829|PACid:22687433
MGRQFVIVAICIVLVAGVGGQAPSSPPTATPAPPTPTTPPPAATPPPVSSPPPVTTSPPPATTAPPPATPPPVATPP
PATPPPVATPPPAPLASPPAQVPALAPTTKPDAPSSSPLSSPPSPATDAPGPSIESLSPGPSTDSNDQNGATKTVSS
LVLGSVLVWFMI

>Bra023357|PACid:22688841
MGLIIMVMVMGILVASSPSDQTDVLTPLCISECSTCPTICSPPPSKPSPSTSPPPSPSLPSSSPLPPPPPSPSLPSS
SPPPPRKHSPPAVSPPPLITVIQPPPPRFYYFQSTPPPSPLSSDVKGSPPSPPSPKGQPKGQQDPNPYPYFYFYTAS
NATSLFSSSFLTSLFYFLYLFCLTIR

>Bra009880|PACid:22695319
MVSLNLSFALVFILAILFTFAEANYSRKLLQTPTNYQPAYSPPSPTPVYSPPVNPPPPTPVTYPPPTPAYPPPVALP
PPAPINSPPPPAPIIPPLKANPSPQAYRAFYYRKSPPPPSGKPWWWLL

>Bra036401|PACid:22701512
MARQFAVVAICIVLIASVGGQAPSSPPTTTPAPPTTTTPPPAATPPPVSAPPPVTTSPPPATPPPVSTPPPVASPPP
ATPPPVASPPPVATPPPAPLASPPAQVPAAAPTTKPDAPSSSPLSSPPSPATDAPGPSTTSLSPGPSTDSNDQNGAS
KMVSSLVLGSVLVWFMI

>Bra011767|PACid:22702685
MERNVLLTVTLICIVVAGVGGQSPASSPTKSPDAPSTPTTSPSSPPVEAPKSPSPVTSSPPPASVPESAPPSPPPKA
SAPVRSPPASVPEAATPPAPVADAPAPSKGKKHQNATAPAPELDSPPSPPMEAPGPSSDAVSPGPATSANEKSGAES
TSVLRNLAAVGAAATAWAVLVMAF

>Bra026249|PACid:22705845
MHFVNYKSPPPPYVYSSPPPPPFYSPSPKAEYKSPPPPYVYSSPPPPPYYSPSPKVEYKSPPPPYVYNSPPPPPYYS
PSPKVYYKSPPPPYVYSSPPPPPYYSPSPKVDYKSPPPPYVYSSPPYYSPSPKVSYKSPPPPPYVYKMPYY

>Bra013116|PACid:22712242
MARQFAIVAICIVLIAGVGGQAPSSPPTTTPAPPTTTTPPPAATPPPVSAPPPVTTSPPPATTAPPPATPPPVASPP
PATPPPVATPPPATPPPVASPPPATPPPAPLASPPAQVPALAPTTPDAPSTSPSSSPPLPATDGPGPSAEGPGPSTD
SNDQNGASKTVSSLVLGSVLVWFMI

>Bra001213|PACid:22712632
MEAIRFSNLSALLILLLLSLSLCVTSKDQTVSCTMCSSCDNPCNPVPTSSPPPPPTPPSSSGGSGGGSYYYSPPPPP
SISGGGKCPPPYGGYGDGGQSYYYPPASYGNFPTPPPPNPIVPYFPFYYHIPPPEMAKSKNHTAHNQSAKAHKNGIK
KPRRHRHTPTRGMDPKFLRNQRYARKHNVKSGENASVEG

>Bra024560|PACid:22716131
MPGSYHRRGSDVSVIIFTVMITLLTCPVIINASSSSEAANTRKLDEVDPIKCSPSCVQNPPPPSPPPPSPPPPACPP
PPALPPPPKKVSPNCPPPPSADFLYITGPPGNLYPVDEQFGAAAGKGFTVVKLSGLIGFGLMGLLVL

>Bra032604|PACid:22717649
MPEILRLMDLTASFLMAVVFLASLVMIDAARSPVEKLNEDPIKCTPCIQNPPPPPSPPPPSCPSPPPPSPPPPSSPP
PPKMSYCPPPPKQEVYFSPPPPPPPPPGDLYPVDHDFGGAAAGEMFTTVKLIALLFIGFMVL

>Bra033914|PACid:22685598

MREETFLFQWRFLASCFIFFLILPQAFTYNTPPINPCSPHAPNWFPPIANPRLLKAYAALQAWKFTMTSDPNGFTSN
WCGPHVCNYTGVFCAPTLDNPYLLTVAGIDLNHANIAGYLPEELGLLTDLALFHINSNRFKGQLPMSLNCLKLLHEL
DVSNNKLSGEFPSVVFTLPSLKFLDIRFNGFYGDIPSQLFDLNLDALFINNNNFRFRLPGNIGNSTVSVLVLANNDL
QGSCVPPSFYNMGKTLHEVILTNSQISGCLNREIGLLTQLTVFDVSFNNLVGSLPETMGDMKSLEQLNIAHNKFSGY
IPEGICRLPNLENFTYSYNFFSAEPPVCLRLQEFDDRRNCLPMRPIQRSPAECRSFSSYPINCASFGCSPPSPPPPP
PPPPPPPPPPPPPPPPPSPPPYVYSSPPPPLYVYPSPPPPPYVYASPPPPPCVYPSPPPPSYVYPTPPSTLSPPVHD

>Bra015245|PACid:22691761
MTKPPPFSLPAFDCFLLFFFFFSFSSVVLALTDAEASFIAQRQLLTLPEDGDLPENIEYKVDIKLTFPNQRLKRAYI
ALQAWKKAVYSDPFNTTGNWHGPHVCDYTGVVCAPALDDPNIAVVAGVDLNGADIAGHLPAELGLLTDVAMFHLNSN
RFCGIIPKSVSRLKLMHEFDVSNNRFVGPFPSVVLSWPAVKFIDVRFNNFEGKVPQELFKKDLDAIFLNSNRFTSTI
PESLGESSASVVTFAHNNFNGCIPKSIGNMKNLNEIIFKDNKLGGCFPSEIGKLANVNVFDASMNSFTGVLPQSFVG
LIGVEEIDISGNKLTGFVPENICKLPKLNNLTYAYNYFNGQGDMCVPGSQKDIAFDDTRNCLRDRPKQRSAKECAVV
ISRPVDCSKDKCAGGGGSSPTTPSKWTPPSRVPTRPVRKPRPSKESPKPNAPQNQSPVNFKRSPPPPIVLSPPPSPL
VHSPLPPVHSPPPPVHSPPPPPVHSPPPPVHSPPPPVHSPPPPPPPPVPSPPPPEPSPPPPVHSPPPPVHSPPPPEP
SPPPPVHSPPPPVHSPPPPEPSPPPPVHSPPPPVHSPPPPVYSPPPPVYSPPPPVHSPPPPVHSPPPPVHSPPPPPV
HSPPPPVHSPPPPEPSPPPPVHSPPPPVHSPPPPVHSPPPPPVHSPPPPVHSPPPPPPVHSPPPPVQSPPPPVYSPP
PPHVYSPPPPVQSPPPPVYSPLLPLVHSPPPLVHKPQTPNESPEPNDPFDQSPVKFRRNPPPPQQSHPVDSSPSPFH
SPPPPIYSPPPAPVQSPPETSVNSPHPRAPTRTVEAPLPSEEFIMPTIIGHQYASPPPPMFPGY

>Bra023805|PACid:22703815
MREEPFFFHCRFLASWFIFFLALQEKAPHADSHLCRVVLLPQAFTYNTLPINPCSAHGPYGFPPIVNPRLLKAYTAL
QAWKHTMTSDPNGFTSNWCSPNVCNYTGVFCAQALDNSYVLTVAGIDLNRANIAGYLPLELGLLTDLALFHINSNRF
EGQLPKSLNCLKLLHELDVSNNKLSGEFPSVVFSLPSLKFLDIRFNELYGDVPSQLFDLNLDALFINNNKFRFRLPK
NIGNSQVSVLVLANNDLQGSCLPPSFYKMGKTLHEVILTNSQIGGCLNREVGLLNQLTVFDVSFNNLVGSLPETMGD
MKSLEQLNIANNKFSGHIPESICRLPNLENFTYSYNFFSGEPPVCLRLQEFDDRKNCLPLRPMQRSPAECKSFSSYP
INCASFGCSPPSPPPPPPPSPPPPPYVYPSPPPPPYVYPSPPCTLPTPVHY

>Bra013339|PACid:22703831
MKTKKMAQIYALFVLHFTFLFSTGLSHSYSLASSNSDLSDKEVHLIRQRQLLYYRDDFGDRGENVVVDPSLVFENPR
LRSAYVALQAWKQAILSDPNNFTTNWIGSDVCSYTGVYCAPAPDNPRIRTVAGIDLNHADIAGYLPQELGLLTDLAL
FHVNSNRFCGTVPHRFNRLKLLFELDLSNNRFAGIFPAVILQLPSLKFLDLRFNEFEGPVPRELFSKDLDAIFINHN
RFRFELPDNLGDSPVSVIVVANNQFHGCIPTSLGDMKNLEEIIFMNNGFNSCLPSEIGRLKNVTVFDFSFNELVGSL
PASTGGMVSLEQLNVAHNRFSGKIPASICQLPRLENFNFSYNFFTGEPPVCIGLPGVDDRRNCIPARPAQRSPGQCA
AFLSLPPVNCASFGCGRSVTPSPRPPVVVPSPPTTPSPGGSPPSPSISPASPPMMVPPSPISPIPAPVSPSSPPSIG
PSPPSTPPSPPGSPSPPGVVPFPPPSPVYSPPSPPPSTGHSPPSPSPPTKFSPPSPPPSAGHPPPSPPPSTGPSPPP
SPPSTGYSPPPPPPSTGYSPPPPPPSTGYSPPSPPPPSTGYSPPSPPPSAGHCPPSPAPPTYSPSPPPPPPTYYPPQ
PPQPSPSQPPQFSPPPTPYYYSSPPPPPHYWLPPPPHSPPPPVYHYPSPPPPPTPVYSPPPPCIDHSPPPPPPPTVH
YSPPPSPVYYNSPPPPPSVHYSPPPSSPPPPVIHHSPPPPPPGYEGPLPPIPGVSYASPPPPPYY

>Bra011483|PACid:22703864
MAKPPSFGCFFFLLFFFFISSSFAAYAISDSEAAFLVRRQLLTLPENGDGELPDDIEYEVDLKATFANTRLKKAYIA
LQAWKKAIYSDPFNTTANWHGPHVCAYTGVVCAPALDDPNVTVVAGVDLNGADIAGHLPVELGLMTDVAMFHLNSNR
FCGIIPKSFEKMKLMHEFDVSSNCFVGPFPNVILSWPSAKYFDLRFNDFEGQVPPELFKKELDAIFLNNNRFTSVIP
ETLGESTASVVTFANNKFTGCIPKSVGNMKNLNEIVFMDNGLGGCFPSEIGKLSNVTVFDASKNSFVGRLPTSFVGL
TGVEELDISGNKLTGLVGDEICKLPNLVNFTYSYNYFNGQGGSCIPGGGRKEIVLDDTRNCLTDRPDQRSAQECAVV
INRPVDCSKDKCAGGGSSTPSRSSPVHKPSPVPTPVVDKPSPVHKPQPPKESPQQDDPYDQSPVKNRRSPPPPHQSQ
PPVVSSPPLPSPPPPQVHSPPPPVHSPPPPVHSPPPPVYSPPPPPVFSPPPPVYSPPPPVHSPPPPPPVNSPPPPVH
SPPPPVQSPPPPVFSPPAHPPQTPVQPSPSPAVILPPPSQSPPVVYSPPPTISSPPAPAPVEEKQTPPAQAPAPVEK
EQAPPAQAPAPVEEKETPPAQAPGPVEKEQAPPAQAPAPVEEKETPTAQAPAPSDEFIIPPFIGHQYASPPPPMFEG
Y

>Bra037543|PACid:22704893
MTRRTMGKPYGCFLLLFLFIIFVIVHSAAGLTNEETSFLTKRQLLALSENEDLSDDMEYEVDLNLKFANTRLKRAYI
ALQAWKKAIYSDPFNTSANWIGQNVCSYKGVFCAPALDDQSIMVVAGIDINHADIAGYLPPELGLLTDVALFHVNSN
RFCGVIPKSLSKLTLMYEFDVSNNRFVGSFPMVALSWPSLKFLDIRYNDFEGKLPADIFNKDLDAIFLNNNRFESII
PETIGESTASVVTFAHNKFSGCIPKTIGHMKNLNEIVFIGNKLSGCFPNEIGLLNNVTVFDASNNEFVGSLPLSLSS

LTNVEQMDFSFNKLTGFVTDDVCNLPKLSNFTFSYNFFNGEAQSCVPGTSQDKQFDDTNNCLQNRPKQKLANECLPV
ITRPVDCSKDKCAGGGGGGGGGSNPSPKPTPNPQKLEEPSKPKPEEAPEPQQPSPKLETPKTSEPSKPKEPKPDAPK
QQSPKPESPKHESPKSEQPNPKPESPKQESPKQQPPKTETPQMGSPPVKPPVSNDPYEASPIKKRRPQPPSTEEIPS
SQSPPPHVFSPPPPVHSPPPPPAYSPPPPVYPPPPPVYSPPPPVYSPPPPVHSPPPPVHSPPPPPPVHSPPPPVHSP
PPPVHSPPPPVHSPPPPPIFSPPPPTPIYSPPPPPVHSPPPPVHSPPPPPVHSPPPPVNSPPPPVHSPPPPVHSPPP
PVHSPPPPSPVYSPPPPVFSPPPKPTTIISPATSPMANAPTPSSETDEDSSPVQAPTSESETVEGPTESDQSPVFSS
SPTLTPTPLSQPDIAPLPSGEQVEAPSPSTQPPVTTAPPSKKDDDNDSFILPPNIGFQYASPPPPMFPGY

>Bra001950|PACid:22707988
MKNTTQSLLLLFFFFFFSVHSLSISSNAPLTDNEVRFIQRRQLLYYRDEFGDRGENVTVDPSLVFENPRLRSAYIAL
QAWKQAILSDPNNITLNWIGSNVCTYTGVFCSRAPDNRRIRTVAGIDLNHADIAGYLPEELGLLTDLALFHVNTNRF
CGTVPHKFKHLKLLFELDLSNNRFAGKFPAVVLDLPSLKFLDLRFNEFEGTVPKELFSKPLDAIFINHNRFRFELPD
NFGDSPVSVVVLANNRFHGCIPSSFVEMKNLNEIIFMNNGLNSCLPADIGRLKNVTVFDVSFNELVGPLPESVGGMV
SVEQLNVAHNRLSGKIPASICQLPKLENFTYSYNFFTGEAPVCLRLPEFDDRRNCLPSRPAQRSSEQCAAFLSRPPV
DCESFKCGRSVTPLPPIVPQLPPPPPPSPPPPSPVYSPPPPPPVYSPPPPPPVYSPPPPPPPPPPPPPVYSPPPPPPP
PPPPPPPPPPPPPPVYSPPPPPPPPPPPPPVYSPPPPPPPPPPPVYSPPPPPPVYSPPPPPPPPPPPPVYSSPPPPPVYS
SPPPPAPIYCTRPPPPPCPSPPPPPHYSPPPPPHHSSPPPYYYSSPPPPPPHHSSPPPYYYNSPPPPHHSPPPPYYY
NSPPPPHHSSPPPYYYNSPPPPHHSPPPPPMYPHSPPPPPPHSPPPPPRYSPPPPPPCIEPPPPPPCIEYSPPPPVY
SSPPPPSVHYSSPPPPSVHYSSPPPPTVYYSSPPPPPPSPEYEGPLPPVIGVSYASPPPPPFY

>Bra017617|PACid:22708869
MAKPPSFVCCIFLLFFFLLSSSFVAFALTDTEAAFIVQRQLLTLPANGELPDDIEYEVDLKATFANTRLKRAYIALQ
TWKKAFFSDPFNTTGNWHGPHVCGYNGVVCAPALDDPDVTVVAGVDLNGADIAGHLPAELGLMTDVALFHLNSNRFC
GIIPKSFEKLKLMHEFDVSNNRFVGPFPEVVLAWPDVKFIDLRFNDFEGQVPSELFKKELDAIFLNNNRFTSTIPES
LGESPATVVSFANNKFTGCIPKSIGNMKNLNEVVFMDNKLGGCFPSEIGKLSNVTLFDASKNTFIGRLPTSFVGLTG
VEEFDISENKLTGLVADNICKLPNLVNFTYSYNYFNGQGGSCVPGGGRKEIELDDVRNCLPHRPDQRSAQECAVVIS
RPVDCSKDKCAGGGSSTPSRPSLVHKPSPVPTTPVQKPSPVPTTPVQKPSPVPTTPVHKPSPVPTTPVHKPSPVPTR
PVHEPQPPKKSPQPDDPYDQSPVGNRRSPPPPHESQPPVVFSPPPTPVSSPPLLSPPLPSPPPPVYSPPPPVHSPPP
PVNSPPPPVHSPPPPVHSPPPPVHSPPPPVHSPPPPVHSPPPPVHSPPPPVHSPPPPVHSPPPPVHSPPPPVHSPPP
PVHSPPPPVHSPPPPVHSPPPPVHSPPPPVHSPPPPVHSPPPPVHSPPPPVHSPPPPVHSPPPPPVYSPPPPVFSPP
PPPVHSPPPPVHSPPPPVHSPPPPPVNSPPPPPPVEKKEVPLAHEPAPSDEFILPPFVGHQYASPPPPMFPGY

>Bra001730|PACid:22709312
MNRRTMEKLYGCFLLLFFFIIFTIIHSTTALTDEEASFLTKRQLLTLPENDNLLDDIEYEVDADLKFANNRLKRAYI
ALQAWKKAIYSDPFNTSSNWVGTNVCSYKGVFCAPALDDPNMMVVAGIDMNHADIAGYLPHELGLLTDVALFHINSN
RFCGIIPKSLSKLTLMYEFDVSNNRFVGPFPIVALSWPSLKFLDVRYNDFEGKLPPEIFDKDLDAIFLNNNRFESII
PETIGKSTASVVTFAHNKFSGCIPKTIGKMKNLNEIMFIGNNLSGCFPNEIGSLNNVTVFDASKNGFVGSLPSSLSL
LDNVEQMDFSYNKLTGFVTDNICKLPKLSNFTFSYNFFNGEAQSCVPGTSQDKQFDDTSNCLQNRPQQKSAKECLPV
VSRPVDCSKDKCAGGGGGSNPTPNPPKTPEHKPNTPKLEEPSKPNPKESPKPQQPSPEPETEPSKPKEPKPETPKHD
SPKPEQPKPKHESPKQESPKKQPPKQEESPEPDMTKPEEPPKHEQSPKTETPKIRSPPMEPPVTDDPYDASPIKKRR
PQPPSTEETPQSSPVHSPPPPLVFSPPPPVHSPPPPPVHSPPPPVFSPPPPPVYPPPPPVYSPPPPPPVLSPPPPVH
SPPPPVHSPPPPVHSPPPPVHSPPPPVHSPPPPVHSPPPPPPVFSPPPPAPIYSPPPPPPVHSPPPPPVHSPPPPMY
SPPPPVHSPPPPVYIPPPSSPINSPPPAFSPPPEPATPLPPATSPMINSPTPSSEVKEESSPVQAPTPDSETVEAPS
ESDHSPVFKSSPAPAPSSEIEEESSPVQAPTPYSKTIEAPSESDHSPVFKSSPAPAPSLKIEEESSPVQAPTPDSET
VEAPSESDHSPVFKSSPAPTPSSEIEEHSSPVQAPTPDSETVEAPSESDHSPVFKSSPAPTPSTEIEEDSSPVQAPT
PNSKTVEAPSEYDQSPVLNSAPAPTPISQPNHTPTPLSQPDISPVPSEEQIEAPSPSTQEVKPPVTTSTPPKNNDAG
DDDFILPPNIGFQYASPPPPMFPGDHHDVPCDTKKRTEETVKTLFDFPIKEKIKVKRDEVNPVGYYDGEHTKNVRDW
NKDPSALWKWKWTGSDASDNLLVWRLVFRFVRQSPLWRRCAQPVTFLYMRSGLNRCNGWSGDSLLIPLFCLLLLR

>Bra013092|PACid:22712347
MTKPPPFSLPAFDCFLLFFFFFSFSSVVLALTDAEASFIAQRQLLTLPENGDLPDDIEYEVDLKETFANNRLKRAYI
ALQAWKKAVYSDPFNTTGNWHGPHVCGYTGVFCAPALDDPSVAVVAGVDLNGADIAGHLPVELGLMTDVAMFHLNSN
RFCGIIPKSLDRLKLMYEFDASNNRFVGPFPSVVLSWPAVKFIDLRFNDFEGQVPPQLFKKDLDAIFLNNNRFTSTI
PESLGESSASVVTFAHNKFNGCVPKSIGNMKNLNEIIFKDNRLGGCFPSEIGKLANVNVFDASMNLFTGVLPQSFVG
LTGVEEIDISGNKLTGFVPENICKLPKLVNLTYAYNYFNGQGDSCVPGSQKEIALDDTRNCLPDRPKQRSAKECAVV
ISRPVDCSKDKCAGGGYSPSTPSKSKQPSPVPSQPVHNPQPPKESPKPNDPSDQSPVKFRRNPPPPQPHHPVVSPPS
HIQSPPPPVYSPPPPPIHSPPPPVHSPPPPVHSPPPPPPVHSPPPPVHSPPPPPVHSPPPPVHSPPPPVHSPPPPVY

SPPPPVHSPPPPTPVYSPPSPVQKPQAPKESPQPNDPYDQSPVKFRRSPPPPHQSHPPTVSPPLSVHSPPAPVHSPP
TVYSSPQPPKESPQPNDPYDQSPVKFRRSPPPPHQSHPPTVSPPVPVHSPPPPVHSPPPPIHSPPPPVHSPPPPVHS
PPPPVHSPPPPVYSPPPPPVHSPPPPVHSPPPPVHSPPPPVYSPPPPPPVHSPPPPVHSPPPPVHSPPPPVHSPPPP
PVHSPPPPVHSPPPPVYSPPPPVHSPPPTVPSPQPQSPTPIQSPPQTPVNSPPPGTPTQTTEAPPPSDEFIIPPFVG
HQYASPPPPMFPGY

>Bra027044|PACid:22714046
MLLLPSLRPFFFLFILFSACFLHTRAQEGQGDISSDNIKVDPSLKFENPSLRQAYIALQSWKQAIFSDPFNFTANWN
GSDVCSYNGVYCAPSPSRPKTRVVAGIDLNHADMAGYLPPELGLLTDLALFHLNSNRFCGTVPTTFKRMKLLYELDL
SNNRFVGKFPIVVLSLPSLKFLDLRYNEFEGVIPSKLFDKELDAIFLNHNRFRFGIPENMGNSPVSALVLADNDLGG
CIPGSIGLMGKTLNEIILSNDNLTGCLPPQIGNLKNVTVFDVSFNRLSGPLPSSVGNMKSLEQLNVANNRFTGVIPS
SICQLSNLENFTYSSNFFTGDAPRCAALSGDNVAVNGSMNCIAGKERQRSAKECSSPASRPVDCNKFGCNNIFSPPP
SFRMSPTVRVLPPPPPSSKMSPTFRATPPPPPSSKMSPTFRATPPPPSSKMSPSVKAYPPPPPKYEPSPPPPPSSGM
SPTVRAYPPPPPPSPPPPYIYSSPPPPPPSPPPPSPPPPYIYSSPPPPPPSPTYVYSSPPPPPVEYYPPTTQSPPPQ
YWQTPSPSEPYPSPSPPYYQYNSPPPPPTSYTVQSPPPPPPADCPPVTNSPPPPPVYYTPVIKSPPPPPPVYYTPVT
HTPPPPPPMYYPPVTQSPPPPPPVYYPPVTQSPPPPPPVYYPPVTQSPPPPPPVEYHPPATPNHSRPPPPHKGCKDG
PSNEHHYQTPTPPSPPPPSYDDTPLPPIHGVSYASPPPPSIPYY

>Bra026996|PACid:22714316
MLIAPLRLVFFLLLSSICLSQIKADESDLGDHIKVDPKLKFENPKLREAYIALQSWKQTIFSDPFNFTATWNGSDVC
SYNGIYCAPSPNSYPKTRVVAGIDLNHADMAGYLPAELGLLSDLALFHLNSNRFCGEVPLTFNRMKLLHELDLSNNR
FVGKFPSVVLSLPSLKFLDLRFNDFEGMIPSKLFDRKLDAIFLNHNRFRFGIPDNMGNSPVSALVLADNDLGGCIPG
SIGQMGKTLNELILSNDNLTGCLPPQIGNLKNVTVFDISSNTLRGLLPSSIGNMKSLEELHVANNGFSGLIPPSICQ
LPNLENFTYASNFFTGRAPICAALSVTEALVNGSMNCIAGMASQRSVKECLSLLARPVDCSTFGCFNIFSPPPPTFK
MAPAVRMLPPPIYVYTSPPRPSSKMSPTVRAYSPPPPASPSPPPPYVYSSPPPPPYIYSSPPPPSPPPPCPESSPPP
PAVFYAPVTPSPPPPSPVYYAPETQSPPSPIYYPWETSSPPPPSPVYYPWETPSPPPPTEYYYSPSQSPPPAKGCKD
SHPPQASPSYEPAPEYSYTTPPPPSYPDTTLPQIPSVSYASPPYY

>Bra034594|PACid:22722317
MASVLAMAKTPSLGCCVFLLSFFFLSSSFVAYAISETEAAFLVRRQLLTLPENGELPNDIEYEVDLKATFANSRLKK
AYIALQAWKKAIYSDPFNTTGNWHGPHVCNYTGVICAPALDDPNVTVVAGVDLNGADIAGHLPAELGLMTDVAMFHL
NSNRFCGIIPNSFSKLTLMHEFDVSNNCFVGSFPCVILTWPDVKYFDVRFNDFEGQVPPELFKKELDAIFLNNNRFT
STIPESLGDSTASVVTFANNKFTGCIPKSIGNMKSLNEIVFMDNGLGGCFPSEIGMLSNVTVFDASKNSFIGRLPTS
FAGLTGVEELDISGNKLTGLLADSICKLPNLVNFTYSYNYFNGQDGSCFPGGGRKETVLDDTRNCLPDRPEQRSAQE
CAVVINRPVDCSKDKCAGGGSSTPSRPSLVPTVPVQKPSPVPSLPVPEPSPVHKPQPPKESQQPDDPYDQSPVKNRR
SPPPPAPVNSPSIPLPSPPLPPPVHSPPPPVNSPPPPVHSPPPPVHSPPPPPPPPVYSPPPPVFSPPPPPVNSPPPP
VLSPPPPVHSPPPPVNSPPPPVFSPPAHPPKSSSPPQTPSEPSPSPMIFSPQPSQSPPAVSSPPPGPPKVDCPPAAQ
APAPSDELITPTPAPVENKQTPSAQAPAPSDELIIPPFVGHQYASPPPPMFEGY

>Bra014193|PACid:22723452
MDKPLGTFFILLLISPIVVATINEETSFPENAHLTNNLDQKCVDIIKVDPSLKFENDRLKRAYIALQAWKKAIYSDP
FKTTKNWVGPDVCSYNGVYCAEALDDPSLKVVAGVDLNYADIAGHLPAELALITDLAMFHINSNRFCGIIPKSLSKL
ALMYEFDVSNNRFVGPFPEVSLSWPSLKFLDLRYNEFEGCLPSEIFDKNLDAIFLNNNRFESVIPDTIGKSAASVVT
FANNKFSGCIPRSIGQMKNLNEVIFTGNNLTGCFPNEIGSLNNVTVFDASNNGFIGSLPLTLSSLSRVEQLDLSNNK
LTGSVVDTFCKLPNLERFKFSYNYFNGEAESCVHGKNNGKQFDDRSNCLKNRPDQKSVNQCVPVVSRPVDCSKDKCS
GGSQGGSPPSIKTPEIIPPKPKELVIPKPEESPKPEPQNPLRPETPTTNVQQPIPEHEPPKHESPKPENPTNKPELP
KPEETPKPQPPKSEESPKPEPPKTSETPEPVSPPKEDPYNASPVKNRRPPPPPPPPKVKEIQVPPPQPPMPSSPPPPV
YSSPPPPAPINSPPPPVASPPPPSPPPPVNSPPPPLIFSPPPPSPVYSPPPPIHSPPPPAHIIIQPPIQAPTPVQAP
SSESDQSPVSSPVQSPTPIQSPTPSSVLDQPTIDAQSPGQTPTPLNEPASSPKEAEDRDAPEPSLSTPSPSPSPSEN
VAPPPENNNHSGFNLPPHIGFGYGSPPPPMFPGY

>Bra016796|PACid:22724373
MLFHPLRIFLFLFLSSLCLLQIKAAESGLGVHIKVDPKLKFENPKLREAYIALQSWKLAIFSDPFNFTANWNGSDVC
SYNGIYCAPLPGAYNKTRVVAGIGLNHADMAGYLPSELGLLCDLALFHLNSNRFCGEVPLSFNRMKLLYELDLSNNR
FVGKFPKVVLSLPSLKFLDLRYNEFEGKIPWKLFDKKLDAIFLNHNRFRFGIPKNMGNSPVSALVLADNDLGGCIPG
SIGQMGKTLNELILSNDNSTGCLPPQIGNLKKVTVFDVSSNRLRGPLPASVGNMKSLEELHVANNGFTGVIPPSICQ
LPNLENFTYSSNFFTGRAPICAALSVADAIVNGSMNCLTGVARQRSVKECLSLLARPVDCSKFGCYNIFSPPPPTFK

MSPVVRKLPPPVYVYKSPPPPSSKMSPTVRAYSPPPPPSSKMSPTVRAYSPPPPPLSKMSPTVRAYPPPPPPSPSPP
PPYVYSSPPPPPPYVYSSPPPPPYVYSSPPPPPYVYSSPPPPPPSPPPPYIYSSPPPPPPSPPPPYVYSSPPPPYVY
SSPPPPPYVYSSPPPSPYVYSSPPPPPPSPPPPCPESSPPPPVVYYAPVTQSPPPPSPVYYAPEIQSPPPPTPVYYA
PETQSPPPPAPVYYAPEQSPPPPTPVYYAPETQSPPPPTPVYYAPEQSPPPPSPVYYPSETPSSPPPSPVYYPSETP
SPPPPTEYYYPPAQSPPPAKGCTESHPPPTQAPPTYEPTPEYSYTSSPPPLPSYPDTSLPPIPSVSYASSPPPPSYY

>Bra018431|PACid:22685365
MDKVQEQAELIEKKLTPFVASQPTTVVIGGGLADQKTTGGESTDQKTTGGGLADQKTTGGSQTTQPPTTSPPSPPSP
DSGGGGGGGSQSSPPPVLTLSPPPSNQSVNTTSPPTPPSSPPPSITPPPSPPQPQTPPQSTPAGNSPVVIPSPTPQI
QLPLTPPTFVTQQPTVNGLALPNNPIFNPSPPSTPLSPPSPENSGSQGSPPSPPVVPLTPNAPRNPSQPLDSPPAAG
GSNHVPSSSPPSLSGSDNNSGGSNRHNANGNGNGGSGQQKNDSNYTEKTMIGIGIAGVLVIIFVAAIFFIRRKQNKS
SSPRSNQNYLPPANVSVNTDGFVNYRQKPGNGNISAQNSSPDTNNSLGNQKPGRSTTPDSAVLGNSKTIFTFEELSQ
ITEGFCKRYVIGEGGFGYVYKGIICDGKSIAIKQLKSISAEGYREFKAEVEIISRVHHRHLVSLVGYCISEQHRFLI
YEFVPNNTLDHHLHGKDLPVLEWTKRVKIAVGAAKGLAYLHEDYFGLARLNDTAQSHISTRVMGTFGYLAPEYASSG
KLTDRSDVFSFGVVLLELITGRKPVDTSQPLGEESLVEWARPRFIEAIEKGDISEVVDPRLEKHYIEGEVYRMIETA
ASCVRHSALKRPRMVQVVRALDTRDDMSDLSNGVKVGQSTVYNSGQYSNEIRMFRRASEDSTDYGYGSSNGYYTSQD
FTSRESERAFNTSRRTNY

>Bra007915|PACid:22691065
MSDSPTSSPPAPSADSVPPPDNSTGGSAPPPTDSSPPPSPPADSAPPPSTPSPPPASPPPESNPPPDSSPPPPPDAP
PPSDPLPPVDSGSPPPEPTNSPPPPPEEFESPPPPPPNEDNTSPPSPPEQPPPPPPQAPSPKGGPKNPKKSLPPANS
PPAPPNAPSHALPPKSTGAGGPLKSPSRGVPSFPPPPPTSNDGGYQGKTMAGMAVAGFAIIALIAVVFLVRRKKKKN
VDVYTDSQYLPPSNFSIKSDGFLYGQNTTKGGYSGPGGYNTKQQSNNSFGSQRGSTPDSAVMGSGQTHFAYEELMEI
TQGFAKQNILGEGGFGCVYKGKLHDGKLVAVKQLKVGSGQGDREFKAEVEIISRVHHRHLVSLVGYCISDVQRLLIY
EYVPNQTLEHHLHGKGRPVLDWARRVRIAIGSAKGLAYLHEDCHPKIIHRDIKSANILLDDEFEAQAIKKTILVADF
GLAKLSDSTQTHVSTRVMGTFGYLAPEYAQSGKLTDRSDVFSFGVVLLELITGRKPVDQYMPLGEESLVEWARPLLH
KAIETGDFSELVDRRLEKHYVENEVFRMIETAAACVRHSGPKRPRMAQVVRALDSEGDMGDISNGSKVGQNSSAYDS
GQYNSDDRKMAFGFDDSSDSGMHSGDYSVQTSRKGSNGASTEFTRNESENRNFNNRRF

>Bra019913|PACid:22698262
MDKVQEQADFIGKKIAPFVTSHQPNLPGFTDQKILGGSQTTQPPATSPPSPPSPNSGGGGSQSSPPPVTVSPPPSNQ
PPITTPPPKPPSSPPPSITPPPSPPQPQTPPQSTPSGDSPVVIPSPKPQLPLPALPPPTLVTQQPEAKPNDNGQEQP
NNPTSPPSPPLNPLSPPSGSQGSPPFSSPSPPVISLNPNLPRNPSQPLDSPPAEGSNHVPSSSSVPSPPSLSGSDDN
SGGSNRHNGGNGQQNNEPNFTEKALIGIGVAGVLVIIIIAVIFFFRRKQKKSSSPRSNQQYLPPANVSVHTEGLIHY
RQNPGNGPASAQNSLPDTNNSLGNPKPGRGTPDSAVIGTSKIPFTFEELSEITEGFSKRFVIGEGGFGCVFKGILSE
GKPIAIKQLKSISAEGYREFKAEVEIISRVHHRHLVSLVGYCICEQHRFLIYEFVPNNTLDYHLHGKDLPVLEWTRR
VKIAIGAAKGLAYLHEDCHPKIIHRDIKSSNILLDDEFEAQANYVQVADFGLARLNDTAQSHISTRVMGTFGYLAPE
YASSGKLTDRSDVFSFGVVLLELITGRKPVDTSQPLGEESLVEWARPRLIEAIEKGDISEVVDPRLEKHYIEEEVYR
MIETAASCVRHSALKRPRMVQVVRALDTRDNLSDLSNGVKVGQSTVYNSGQYSNEIRMFRRASEDSSDLGTSNGYYT
SQDFTSRELESRGFNTSHQTNN

>Bra018782|PACid:22698690
MAEGQSPENSPPAPPPPSPSSPSSNDQTTSPPPTSDSQTTSSPPPSPTLAPPPEQSPPPPENSSSPPPPPYESSTPS
SQSQSPPLPSQSPPQQSDNKGNNQGNDGGGNNASPPPPPPSKTSEHSSHSPPKSLAPPTSNGGSNSSSNDRPNMGVV
IGLVAAAGLLFLVMILLCVCCLRKKKKKSKIEQMPYYGSNAFAPDGGDQYFNNAATQQQQHYNQNDNTANLPPPPGT
MGTNWVNSPPPPPPGNWQPMPSPPAPVSGGANAIHSNEMSSNYSSGPYAPSLPPPHPSVALGFNKSTFTYEELAAAT
QGFSKDHLLGQGGFGYVHKGVLPNGKEIAVKSLKAGSGQGDREFQAEVDIISRVHHRHLVSLVGYCSNSGGQRLLVY
EFLPNDTLEFHLHGKTGTVMDWPTRLKIALGSAKGLAYLHEDCHPKIIHRDIKASNILLDLNFEAKVADFGLAKLSQ
DNNTHVSTRVMGTFGYLAPEYAASGKLTDKSDVFSFGIMLLELITGRRPVDLSGEMEDSLVDWARPLCMNAAQDGEY
GELVDPFLENQYEPYEMARMVACAAAAVRHSGRRRPKMSQIVRILEGDASLDDLNDGVKPGQGSYMGSSGGDGSSDY
ETGTYGAEMKKFRKVTLDSRDYGASSEYGGTSEYGLDPSSSSSEEMNIGGGSNKTTTTSTRRV

>Bra016218|PACid:22699541
MSDSPSSSPPAPSSDSTPPPDNSSGGGSAPPPTDSAPPPSPPADSTPPPDNSTGGSAPPPADSAPPPTPPSDSSSPP
PATPPPVSEPPPDSPPSPPPDAPPPADPTPVDSGSPPPEPTNSPPPPEESESPPPPPNEEDSSPLPQPPPEKSSPPP
SQKSSPPPSEKSSPPPPEKSSPPPSEKSSPPPPEKSSPPPPASSPKSAPKKKKKSPPPPPPGAPTKSPSNAPPPLKA
PHALPPKSTAAGGPLKSPSTGVPSFPPPPPNGNDNGYQGKTMAGMAVAGFAIIAVVAVLFFVRRKKKRNVDAYSDSQ

YMPPPNFSIQSDGLLHGQNTTKGYSVPGGYNTQQQSYNTQQQSDNTRTSFGSQRGSQRGSQRGYPPDSAVMGSGQTH
FTYEELMDITEGFAQRNILGEGGFGCVYKGKLHDGKLVAVKQLKVGSGQGDREFKAEVEIISRVHHRHLVSLVGYCI
SDVERLLIYEYVPNQTLEHHLHGKGRPVLEWAKRVRIAIGSAKGLAYLHEDCHPKIIHRDIKSANILLDDDFEAQVA
DFGLAKLNDSTQTHVSTRVMGTFGYLAPEYAQSGKLTDRSDVFSFGVVLLELVTGRKPVDQYQPLGEESLVEWARPL
LHKAIETGDFSDLVDRRLQNHYVENEVFRMIETAAACIRHSGPKRPRMAQVVRALDSEGDMGDISNGSKVGQTSSYD
SGQYNSDAMKFRKMAFGFDDSSDSGDYSVRSSSRGSYGASTEFTRNESENRKFNNRQY

>Bra039607|PACid:22700350
MASSPESSPPPSNSSSTSSTPPSPPSPTQGDSSSPPPDSSSPPAPQAPSPPSSSNNSPPSPASQGGGGNGGGNQSPP
SRGSPPSRGGDNSGSRSSQSGNNGGSRSDNSPSGGSGGGGGGGGNNTNTAIIIGVLVGAGLLMIVLIIVCLRRKKKR
KDSFYPESMKGNQYQYYGNNNNNNNNNSSQNYPNWHLNSQGQNQQPPNSWGGGGPSLPPPQQMPTSGDASSLYSGPA
RPVLPPPPPTLALGFNKSTFTYQELAAATGGFADSNLLGQGGFGYVHKGVLSSGKEVAVKSLKSGSGQGEREFQAEV
DIISRVHHRYLVSLVGYCIADAQRMLVYEFVPNNTLEYHLHGKNLPVMDFSTRMRIALGAGKGLAYLHEDCHPRIIH
RDIKSANILLDFNFDAMVADFGLAKLTSDNYTHVSTRVMGTFGYLAPEYASSGKLTEKSDVFSYGVMLLELITGKRP
VDSSGTMDDTLVDWARPIMARALEDGNFNELADARLEGNYNPQEMARMVTCAAASIRHSGRKRPKMSQIVRALEGEM
SLDALNESVKPGNSKVYGTSGTSTDYSQTSYNADMKKFRHVALSSQEFQSSEAEGSCSTDSRETKSPAAPK

>Bra004331|PACid:22701814
MATSPAQPPVSNSPPTPPVASPPLGNSLPNNNATSLPPSQSPPLATPPPVTSPPLGNSLPSNNATSPPPPSLSSPPA
TPPPVTSLPLGDSLPSNNATSPPPVTPPPSPSPPPPNGAPPPVTTSPPNGSPPPPPLPKPPETSPPPPPQPVISSPP
PAIPPPPPSVQPPQASPPPPPSAPPLPSSPPPPSSVPPLPPTSQRSPPPPPTERPVQSPPPPSPPSERPIQSPPPFS
PPSRRPPSSPPSHSPPSDRPIQSPPSPPENTSPPPSDSLPPPTFSSPPVPGPNNPPQNNPTPSSPDTSNPTHSTSGI
GAGPILGITVAVALLLFSLIGLLVWCIRRREKRLSAVSGGYVTPSPRSDSAFFRTQSSAPGSHHHTYFSQSESGGLG
NSKALFSYQELVKATNGFSEENLLGEGGFGCVYKGVLPDGRVVAVKQLKVGGGQGDREFKAEVETLSRIHHRHLVSI
VGHCISDDRRLLIYDFVSNNDLYFHLHVSKEVLDWATRVRIAAGAARGLAYLHEDCHPRIIHRDIKSSNILLEDNFD
SRVSDFGLARLALDCNTHITTRVMGTFGYLAPEYASSGKLTEKSDVYSFGVVLLELITGRKPVDTSQPLGEESLVEW
ARPLISHAIETEEFGSLADPKLGGNYVDSEMFRMIEAAGACVRHSAAKRPRMGQIVRALESLSAEDLTNGMRLGESE
VFDSAQQSAEIRLFRRMAFVSNAPRTMEQDCGVFTADCVVLCCCCECFILQFFIFVFYKMPSKVAQKMKRFVIRRLL
RGKKRRLLPAKNKDCREEERLPGDDVSRVSCMEDIEEMLHELSMEGEFVFGSFWRQGETTNDLDFGNSQYEIEETND
HSFVIAHCTRCLKRTQSGI

>Bra011534|PACid:22704632
MADSPVDSPPAPEAPNAGTGSPPNETSPPVAPVSSPPAPDSAPPPTNNSSASSPPPAPPSQETLPPPSPPPSPPAAG
NPPPKTPENPSPPSPEVKTPVTPPAPPQAPANQSPPSQRPTPPSPGANDDRNKTNGGNNNGNNRDGSTPSPPSGNNR
NSSGGDSPSPPRSISPPRSSGGSDSSSSSPGESHHQPNVGLIVGVLVGAGLLLLLLVLICICCKKKRKKRDPQVNHL
HYYNNSPFGAPNGNGGYYNNGTPQDHVVNMGGGNWVPQQPVSGPHSDTSNLAGPTPSPQAATLGHNQSTFTYDELSI
ATEGFAQSNLLGQGGFGYVHKGVLPGGKEVAVKSLKLGSGQGEREFQAEVEIISRVHHRHLVSLVGYCISGGQRLLV
YEFLPNNTLEFHLYGKGRPVLEWSIRLKIALGSARGLAYLHEDCHPKIIHRDIKAANILLDFSFETKVADFGLAKLS
QDNYTHVSTRVMGTFGYLAPEYASSGKLSDKSDVFSFGVMLLELITGRPPVDLTGEMEDSLVDWARPLCMKAAQDGD
YSQLADPRLETNYNQQEMAQMASCAAAAIRHSARRRPKMSQIVRALEGDMSMEDLNEGGRPGQNSYLSPGGMTSEYD
ASSYSADMKKIRKLALETKEYQSSEYGATSEYGLHPSASSSEEMPRGSSMRRNSQL

>Bra028371|PACid:22706472
MSSAPSPSATPSPPLPPTVIPPVPPLTPPPPTTPVIPPPPPTADPPTTPLTPPPPPPTAIPPTAPVTPPPPLPSTDL
TPSPPSSPLPSSPPPPSPATPTPLTPSPPPPFTDPTSPPPPSSPSPPSPPSPSTPPPPRPVTRSPPPRPSGGGSPRT
PSITPGFSPPPPASSPGGLSSGVVVGIAIGGVALLVALLTLICFFFFCKKKRRRDEEAPPGPNGPYGAQKQQNASQP
SDHGLTSPQQPNPPSAPPRPRGMSSSSSGGYDSNYSDNAVHPPPSPGLALGTYQGTFTYEELARATNGFSEANWLGQ
GGFGYVYRGILRNGKEIAVKQLKAGSAQGEREFQAEVGIISRVHHRHLVALVGYCIADAQRLLVYEFAPNNTLEFHL
HGKGRPPLAWSSRLKISVGSAKGLSYLHENCNPKIIHRDIKAANILLDFKFEAKVADFGLAKIALDTNTHVSTRVMG
TFGYLAPEYAASGKLTEKSDVFSFGVVLLELITGRRPVDVNNAYADNSLVDWARPLLTRALEERNFEGLVDPKLNNE
YDREEMARMVACAATCVRHSARRRPRMDQAKGNISPSDLNQGIKPGDSNVYSSYGGSTDYDMSEENEGINKLRRKAL
GAQEYSESSEYSNPTSEYDLYSTGWSTEGRTTREMETRTIKRTG

>Bra001723|PACid:22707669
MAEGQSPENSPPSPTPPSPSSPDNTQQQSPPSPDSSSSSSSPSSSPPAPPPPDNSSSSPPPPSSDSQHQPSPQQENN
NNNNNNNGGNNDNNNNGNNNNGGNNDNNNNGNTNNGGNNDNNNNGNNNNNGNNGNNSNNGNNGNNNNGNNNNGNNSG
GSNNHSPAPPSRNSDRNSPSPPRSLAPPRSSGGGDAPNTGAIIGIAAGAGLLLLVMILFCVCCCRKKKKKNPMPYYA

SNGYATGKGDQYQQQQQHYNNQSEHVMNLSHQHPGLNSNNNWMNSPPPPPPQPVSGGLNNGNSSEIYSGPYGPALPP
PHPSVALGFNQSTFTYDELAAATQNFSQARLLGQGGFGYVHKGILPNGKEIAVKSLKAGSGQGEREFQAEVDIISRV
HHRFLVSLVGYCIAEGQKMLLVYEFLPNDTLEFHLHGKSGNVLDWPTRLRIALGSAKGLAYLHEDCHPKIIHRDIKA
SNILLDESFEAKVADFGLAKLSQDNVTHVSTRIMGTFGYLAPEYASSGKLTDRSDVFSFGVMLLELITGRRPVDLTG
EMEDSLVDWARPVCLNAAQDGDYSELVDPRLEGQYEPYEMARMVACAAAAVRHSARRRPKMSQIVRALEGDASLEDL
NEVKPGQSLGRGSSSDYDSSTYSADMRKFRKVAMDSHEYGATSEYGNTSEYGLDPSSSSSEEIHTGGGSGGANKNNK
TIPSREL

>Bra024725|PACid:22715278
MATPVQPPTAEVPPSPPLSPGGNAVSPTREPIDGNSPEIPNPPPLSTPPPAAPLSSPPPEPSSSPPPPTGAPPPTPT
LPADPPPPPPPPSPDTSPPPISPPSFPPPETSPPPTPSSLDPPLNPPPPETSPPPPPPLHEPAVSPPSFPPPEVSTPP
PPSSPESSPPPPSLPESSPPPPSSPDRPAISPPLFPPPKSSPPPPASDPPTNPPPLVPPLHSSPPPPSLPPPRPPPS
PPGSLRPALSPPPRDPEHPQQPPPPDSKRPPPPEKIHPPSKPSPAPLPSNSSSSPSPPLVPSPPSPQKSVPDSGHPP
QENNLTPPVTNNSSNSGFSTVAVVGVSIGLVLVLLGLIGVIVWCVRRQKKKLPAIGGGYVMPSPLVSSPRSVYVYGY
RYAHLFITSLCCSDSTPSKTNSSAPLVGNRSSNRTFFSQPEPGGFGHSKELFSYEELVKATNEFSDENLLGEGGFGC
VYKGALPDGRVVAVKQLKIGGGQGDREFKAEVETISRVHHRHLLSLVGYCISENRRLLIYDYVPNNNLYFHLHGHPR
IIHRDIKSSNILLDNNFHPLVSDFGLAKLALDCNTHITTRVMGTFGYMAPEYASSGKLTEKSDVFSFGVVLLELVTG
RKPVDTSQPLGDESLVEWARPLLSHAVETEEFEALVDPKLGRNYVGAEMFRMIEAAAACIRHSAAKRPRMSQIVRAF
DSLAEEDLTNGMRLGQSEVIDSAEQSAEIRLFRRMAFGSQNYITTFVFFKVPCKLGQKIKKFAKKRCGRTRTEEDVV
KEERWYGNGVVFEEGSSRSNCIEVTEGMLSMSKEFGFGSFWRHEDLPTL

>Bra037220|PACid:22716788
MASSPESAPPPSNSSSSPSPPSPSPPPPTQEGSSPPPPDSTSPPAPQAPSPPDSSNTSPPPPASQGGGGNGGGNESP
PSRGSPPSPPSRGGDNGGSRTSPSGDNGGSRSNNSPSSGGGSSTSGGGSGTSGGGGINTNTAIIIGVLAGAGLLMIV
LIIVCLRRRRKSKDSFDAESMKGNQYQYYGNNNNNSNNASQNYPNWHLNSQGQNQQPSGGWGGGGPSPPPPPRMPTS
GDNSSLYSGPARPVLPPPPPTLALGFNKSTFTYQELADATGGFVDSNLLGQGGFGYVHKGVLPNGKEVAVKSLKSGS
GQGEREFQAEVDIISRVHHRYLVSLVGYCIADGQRMLVYEFVPNNTLEYHLHGKNLPVMDFSTRMRIALGSAKGLAY
LHEDCHPRIIHRDIKSANILLDFNFDAMVADFGLAKLTSDNYTHVSTRVMGTFGYLAPEYASSGKLTEKSDVFSYGV
MLLELITGKRPVDSSSTMDDTLVDWARPIMVRALEEGNFNELADVRLEGNYNPQEMARMVTCAAASIRHSGRKRPKM
RQIVRALEGEVSLDVLYEGVKPGHSNIYGTSGTSSDYSQTSYNADMKKFRQIALSSQDFQSSEEAVTEPEFISGPGY
TGPVKNLFYFIPWPFSHPLSVPLLPQILKLHKNPQIGDFSISLLFPNLTMLRLRTLLSLKSSFSSPSAATKTILAPF
HSTSVLSEKSRKHFGSDRASKGGGGTTSESSKVCLKIRDWSDDLFITAKWNEWNIGPNPLIRDRHMKKKSPPGRGKK
PRDKKTKRWHREGNTDDDFEADATNKFENKWREAWTNQSQKSSYSYSRDSGFQWREGWSWTTQSQRSKAWNNDHCDE
PLVVGSQSDRKALGLPLTGPLKLEDVKNAYRSSAKKWHPDTNQGTSKAEAQEKFKLCVEAYNSLCSALS

>Bra028192|PACid:22721050
MSLVPPLPILTPPPSSNSSTTSSPPPSSSPPTTPLVPPPVTPPPSPPVPSSSPPPPVISSPPPSSSPPPPVVSSPPP
AAASSPPPPPVVVASPPPSTPPPAPPQDSSPPPPPESSPPAPTTTSSPPPPPSNTPSPPKPSPSPPSDTRSPPPPPS
SDKPSPPPPGSTSTPPPASHPTDPAALAPPPTPLPLVPREKPTPPASPNANGNNTSSSSPSGVGTGGIVAIGVIVGL
LLLSLFVLALWLTRKRKRKDPGAFVGYTMPPSGYSSPQGSDAVLFNTNSSAPNNKMRSHSGNDYMYASSDSGMVSNQ
RSWFSYDELAQVTNGFSQKNLLGEGGFGCVYKGVLSDGREIAVKQLKIGGSQGEREFKAEVEIISRVHHRHLVTLVG
YCISEQHRLLVYDYVPNNTLHYHLHAPGRPVMTWETRVKVAAGAARGIAYLHEDCHPRIIHRDIKSSNILLDNSFEA
LVADFGLAKIAQELDLNTHVSTRVMGTFGYMAPEYATSGKLSEKADVYSYGVILLELITGRKPVDTSQPLGDESLVE
WAKPLLSQAIENEEFGELVDPRLGVNFIAAEMFRMVEAAAACVRHSAARRPKMSQVVRALDTLEEASDITNGMRPGQ
SQVYDSRQQSAQIRMFQRMAFGSQDYSSDFFDRSQSHSSWGSRDTRDQSKFVP

>Bra016342|PACid:22723179
MSDSGGSPASSPPAPPADSAPPPETSPPPPSDSSPPPPSDPQPSPPPPDSLLPPLPSLLPPLTATPPPASNPPPADS
TNSPPPTSTETPSPPPPANSDAPPNESNNPPPSPDVQSPPPPPSSPSTNPETPPPESPPAPPSDPTNSPPIQPSPPA
YSPPPSSLLAPHKPNGGSGRPVVSPSVPSRETPPTNSSNGNQTMVGMAVAGVAIMALIAIVFFVRRKKKKNIDAYAH
SHYLPHPNFSVKSDGFSYGQDPSKVYSGPGGSMYNSQQQYSSMGNSYGSQRGQSNDSAILGTGQTHFSYEELADITQ
GFARHNILGEGGFGCVYKGTLHDGKVVAVKQLKAGSGQGDREFKAEVEIISRVHHRHLVSLVGYCISDQHRLLIYEY
VSNQTLEHHLHGRGRPVLEWSKRVRIAIGSAKGLAYLHEDCHPKIIHRDIKSANILLDDEYEAQVADFGLARLNDTT
QTHVSTRVMGTFGYLAPEYASSGKLTDRSDVFSFGVVLLELVTGRKPVDQNQPLGEESLVEWARPLLLKAIETGDYS
ELIDRRLEKHYVEHEVFRMIETAAACVRHSGPKRPRMVQVVRALDCDEDSGDISNGIKVGQSTTYNSGQYNQDITKF
RKMAFGSGDSGEAGMYSGDHSVISSSDFSGNESETRPFNNRRF

>Bra028684|PACid:22688369
MALFRRFFYKKPPDHLLEISERLYVFDCCFSSDVMGEDEYKLYLGGIVAQLQDHFPDASFMVFNFREGEQRSQISDV
LSQYDMTVMDYPRQYETCPLLPLEMIHHFLKSSESWLSLEGQQNVLLMHCEKGGWPVLAFMLSGLLLYRKQYQGEQK
TLEMVHKQAPKELLHILSPLNPQPSQLRYLQYISRRNLTSDWPPSDTPLLLDCLILRDLPHFEGLKGCRPIIRVYGQ
DPKSKANRSSVVLFSTPKTNKHTRLYQQEECILVKLDIQCRVQRDVVLECIHLHDDLVREEMVFRIMFHTAFVRGNI
LIVERDEMDILWDAKDQFPKEFKAEVLFSGADTVVPAIATAPVSDDDDENDFDMASPEEFYEVEEIFSDAIDGHDLK
REDSDSFVVVDSASDDSEGKEVWKGDVEPNAFLDCASDDSNHKHEASVDPVKDITVDDVQYRSDGKADTNDSVKDIG
IDDSDEQRKRTVEAKENDSRTEESQQNGDGEESTAQKANADLNISVSDKTKAAPRKVVGANAKPAGDSVKPKSKQQE
TQGGNVRMAKPNAVSRWIPSNKGSYKDSMHVAYPPTRTNSAPASITTSLKDGKRATSPDGVVTKDAKTKYLRATVSS
SDIMRSRTPIWLSPDSSPKDKPPSLPVSPHHAPPTPQHPPATPSLPSLTSEATSTSQIPEPPPFPSERLNSGTTLPP
PPPPPPSAPPPPPFASEKPNNRTVLQPSLPWKSVYTSTLATSTACSTSQPPPPPPPPPWKPGYASILETHEEGSTS
YNSPPPPPPPPPPPPPPFSSSNTTKSSGGHIPTPSPLPYMSIAPSPSPKTSLINGFSAAPPPPPPPPPPLPPFSKA
HSVPIPPPPPPSYGSPTAPPFGHVSSVPPPPQPPSHGAPPPPPPPPPPFGHVGSIHQPPSHGAPPPPPPPPPPPS
PFGHVGSIPQPPSHGAPPPPPPPPPPPFGYTRHPPPPPPPPFGSAGPPPPPPPFGKTSPPPPPPPPFGSTRPPPP
PPPPPFGKTSPPPPPPPPFGSSRPPPPPPPPFGSNGPPPPPPPFRSGGPPPPPPPFGSTGPPPPPPPFRSGGPP
PPPPPPFGTSGPPPPPPPFRSGGPPPPPPPGGAPPPPPPMRGGAPPPPPPMRGGAPPPRPPMRGGAPPPPP
PGGRAPGPPPPPPPGGRAPGPPPPPGPRPPGGPPPPPGPRPPGAPPDLKGAGRGRGLARPGLGSSAPKKSSLKPLHW
VKVTRALQGSLWDELQRQGQTAPEFDVSEIETLFSAIVPKPVDKAGGRRKSVGAKPEKIQLIDLRRANNTEIMLTKV
KMPLPDMMAAVLAMDDTVLDIDQIENLIKFCPTKEEMELLKNYTGDKATLGKCEQYFLELMKVPRVESKMRVFSFKI
QFGTQIKEFKKSLNAVNSACDEVRTSQKLKEIMKKILYLGNTLNQGTARGAAVGFKLDSLLKLSDTRAANSKMTLMH
YLCKVLASKGSDLLDFHKDLGSLESASKIQLKSLAEEMQAIIKGLEKLNQELTASESDGPVSEVFRKTLKDFISVAT
TEVATVSSLYSVVGNNADALAYYFGEDPKRCPFEQVTATLLNFIRLFKKAHEENIKQEELEKKKAAKEAEMEKAKGV
SLTKKPVDDS

>Bra020386|PACid:22689500
MDLLRKLFNRKTPDGLHEICDQVFVFGSCLSTDSLEEEKYKTYLTGAVNQLQEHFPDASSLVFNFRDVVDTRSVVAD
VLSEHGLSVMDYPRHYQGCSLLPVKVMKKFLCYCDSWLSLGPSELILLHCERGAWPLLAFMLAALLIYRKQYSDEYK
TLDMICKKAPVELMHLFSPLNPIPSQLRYLQYVSRRTMVSEWPPTERALTVDCVMLRCLPDVSGQGSFLRPVFRVYG
QDPLFVDDDKKPELLYSSAKKGKHLKIYKQLFSNKVELVKIDDIKCHVQGDIVIECLSNDMEVMMFRAVFNTAFIRS
NILTLNRDEVDTLWDIKEQFPKGFGVEILFSDMDDDSSVDLTEKVGLPVEAFSNVHEFFNQVAPNDVPSPLGQPVRS
PPPVSKSDKTTALPPPPPPPPPPPPPPPPPMQHSDQKTASRVFPPIPHPELATAASPPPPPPPPPVHNKSP

>Bra009306|PACid:22692783
MVWSCRVVSEPSLALYLKSTTFNRFASKILGFHVAVENNHRRFGSHRMAMFRRLFYKKPPDHLLEISERVYVFDCCF
SGNVMGEDEYKLYLGGIVAQLLKHFPNASFMVFNFKEGEQPSQISEVLSHYNMTVMEYPRQYESCPLLPLEMIHHFL
RSSESWLSLEGQQNFLLMHCERGGWPILAFMLAGLLLYRKQYHSEQKTLEMVHKQAPKELLQILSPLNPQPSQLRYL
QYISRVDWPPSDTPLLLDCLILSDLPHFEGSKGCRPVLRVYGQHPKARADKSSIVLFSTPKTNTQTRLYQQEECTLV
KLDIQCHVQGDVVLECTHLHDDLVREEMVFRIMFHTAFVRSDALVVRRDEMDVLWEAKDQFPKEFKAEVLFSGADAV
VPTIATAPMSEDEKEDITSPEKFYEVEETFSHVIDDHKIDSDSFGVVDTASDDSEGKEVWKGDVEPHAFLDCASDGS
NHKHDVHGEASTDPVKDITVDDVQYRPDGKADTNIDSVKDIEIDDGDEQRERRTLEAKENDVEGSDSPNDIEPTTQE
TNTKINNPISEKTQDGAAASPPPPSQNDSFFQTPQIPPSPPFAFERPNSGIMLPPPPPFASEKPNSGIMLPPPPPPP
TPPPPFASSRRKSETMLPPPPPPPPPWKSVDTSSFETHETCSTSYGSPPPPQPPPPPPFSKAHSVPPPPPPPPPLPL
TKAHSIIPPPPPPPSYEPPVPPPPPPFCKTSPPPPPPPPPFSSGGPPPPPPPPFRSGGPPPPPPPPTPMSAPPPPP
PMRGGAPLPPAPPPMRGGAPPPPPGPRPPGVGPPPPPGPRPLGGGRGRGLLRPGVGPSIQKKSSLKPLYWVKLARAL
QGSLWYEFERRGVGKIAQEFDVSEIETLFPATVPKPADKSGGRRKPIEAKPEKIQLIDLRRANNTEIMLTKVKMPLP
DMMAAVLAMDDTVLDIDQIENLVKFCPTNEEMELLKNYTGDKEMLGKCEQYFLELMKVPRVESKMRVFSFKIQFSTQ
ITEFKKSLNVVISACVEIRTSEKLKEIIQRILYLGNIMNQGTARGAAVAFKLDSLLKLNDTRAVNSNTTLMHYLCKV
IATKASDLLDFHKDLESLETASKIQLKSLAEEMQAISKGLEILKQELTASESDVPLSEVFRKTLKEFISFAEDEVET
VKSLYSDVGKNADLLSCYFGENPKNCTFEQVTATLLNFIRLFKKAHEENVKQVELEKKKAAKEAGMVKAKGVSLRKK
VVDDS

>Bra002668|PACid:22692848
MALFRKLFYRKPPDGLLEICDRVFVFDCCFSSDSWEEENYKVYMAGVVNQLQEHFPDASSLVFNFREVGTRSVMADV
LSEHGLTIMDYPRHYEGCSLLPVEVMHHFLRSSESWLSLGPSNLLLMHCERGAWPVLAFMLAALLIYRKQYSGELKT
LDMIYKQAPRELLQLFSPLNPMPSQCRYLQYVSRRNLVSEWPPLDRALTMDCVILRFVPDVSGQGGFRPIFRVYGQD
PLFVDDKKPKLLYSTPKKGKHLRVYKQAECELVKIDINCHVQGDIVIECLSLNDDLVREVMLFRVVFNTAFIRSNIL
MLNRDEVDTLWHIKDQFPKGFRVELLFSDMDAASSVDVMDFSCLEEKDGLPIEVFSKVQELFNQVDWADQTDATRNM

LQHLAIANAVHERPEGGNSSPRLQGLSPRSIHDLVKQAAMDSNAKIKLSSVSEVETVDSPEKQPTGTAKKFIAEDTH
SVLQISSQDATKLSGQESPSLKLVHHSATVKPFVDGSEFSEKAEENNGKLVSFASSTPSLPHPTIASLPPVAAPPPP
SPPPVPTVASQPSEKLQHSVVQPAETVSQGNSWVSLAGSTFNQTTPNEKHPVTLPPTSLASDVSPQESSRITKSSSV
LPSATLSADPTKPPIGAPNDVASPLGQPARLPPPVSKSDKTHELPQRPPPPPPPPPPPPPPMQYSDQNTVAAVSPPI
PSPAPAITASPVSNSDKTHGLPRPPPPPPPPPMQHSDENTVARVSPPVPPPPPAIAASPPPPPPPAPPIPQSHGNSA
MKSSPPAPPRPPAPPAPPNAPPPPGQMKAPSAPPPPPLGQMRSPSAPPPPPKLGTKPSPSSGTGVPPTPALPGGSLS
SGKGRMLRVNSKNTAKKLKPYHWLKLTRAVNGSLWAETQMSSEASKAPEIDMTELESLFSSAPEEAGKSRLNSSRGP
KPEKVQLIEHRRAYNCEIMLSKVKVPLQDLMHSVLNLEESALDADQVENLIKFCPTREEMELLKGYTGDVDKLGKCE
LFFLEMMKVPRVETKLRVFSFKIQFRSQISELKNSLSVVNSAAEQIKNSEKFKRIMQTVLSLGNALNQGTARGAAVG
FKLDSLPKLSETRARNNRMTLMHYLCKILAEKMPEVLDFTKDLSSLEPSTKIQLKFLAEEMQAINKGLEKVVQELSL
SENDGPISHNFNKILKEFLLYAEAEVRSLASLYSGVGRNVDGLILYFGEDPAKCPFEQVVSTLLNFVRLFNRAHEEN
GKQLEAEAKKKAEEEKSKTSEVDKESRKPLSLEEQVKKEKTKISGFDKETKEPLDEGTPA

>Bra002969|PACid:22693168
MGNQNRGGLLLWFILISGFLVISSLEVNLDKDEPFLTPFVAPSTGMVNEPVVESSWAKSCWQDSDCVKEAVAVFNLC
LPASRELFGFKHSHLKQTLLGCIQEQAKLNGHNLKYLKLLPYLLDTPRRNLASRPVSLSPSPSPSPPPKRSRVPPTR
SRSPSPSNSFFPPSRSPPPAKKTASSSAKRKEEHEKTIIIAVVSTAVSTFLLAALLFLCCTRVCGKGSGGRKNDERP
LLSLSSSDHSVGSSINYGGSIKGGNQSFNIYSNQGKMSSFDGSNSDTSDSLEERLSHEGMRTHGLPPLKPPPGRTSS
AHLGKPPSGKVEPLPHEPPKFLKVSSNKGSHHTQPPVPPPPMPSSAGPPRPPPPAPPPGSGGPKPPPPPGPKGPPPP
PGPKGPRPPPPMSLGPKAPRPSSGPAKSPSDDDGAPKTKLKPFFWDKVQANPEHSMVWNDIRSGSFQFNEEMIESLF
GYAAADKNKNDKKGAAGQAAPQFVQILEPKKGQNLSILLRALNATTEEVCDALREGNELPVEFIQTLLKMAPTPEEE
LKLRLYCGEIAQLGTAERFLKAVVDIPFAFKRLEALLFMCTLYEEIAFVKESFQTLEVACQELRGSRLFLKLLEAVL
KTGNRMNDGTFRGGAQAFKLDTLLKLADVKGTDGKTTLLQFVIQEIIRTEGRRAARTIRESQSFSSVKTEDLMAEEA
SEEMEDSYRNLGLQKVSGLSSELEHVKKSANIDADGLTGTVLKMGHALSKARDFVNSEMSSGEVSGFREALEDFIQN
AEGSIVSILGEEKRIMALVKSTGDYFHGKAGKDEGLRLFVIVRDFLIILDKICKEVKGKPVKMARKQGSTASASSET
PRQAPSLDPKQKLFPAITERRMDQSSSDSD

>Bra024447|PACid:22696956
MSFLHSRFFFFLLFFSSFSVSSFSVGEVNEIHRRILHQPLFPDASPPPPPVFQSTPPPPDAPDQPFFPENPSTPDQS
QLPPPPPPPPASSDANGGLPIPTATTQQAKPGKKFAIVISVGVVTLGMLSALAFFLYRHKAKHASDTQKLVSRGGDG
GSRRFQEDPLPPTSSTFLYMGTVEPSREPASESIGPVNTSPYRKLNSVTRSDRYRPSPELQPLPPLAKPPQPSENSP
SALSPSTSSSDEECRGTAFYTPHGSAISSDDGYYTAFPRSANSNGHSSGSIPHSKRTSPKSKFGSRSPEMKHVIIPS
IKQKPPPIQSPPLRGLETDPYPQNKPKFSQPPPPPNRAAFQAITQDKSPLSTRVSPRRSPPPLHTPHPPPPPPLYTP
PPPPPPPPRPPPPPQIRPRDFQIPRKLSNPEATKPDDQSRKLAFKTPSPQSKAVEEAKSVSADGDTDPSKPKLKPLH
WDKVRASSDRATVWDQLKSSSFQVNEDRMEHLFGCSSASSAPKEPVRRSVMPPAENENRVLDPKKSQNIAILLRALN
VTREEVSEALLDGNPESLGAELLETLVKMAPTKEEEIKLREYSGDVSKLGTAERFLKTILDIPFAFKRVEAMLYRAN
FDAEVKYLRNSFQTLEEASLELKASRLFLKLLEAVLMTGNRMNVGTNRGEAKAFKLDTLLKLVDIKGVDGKTTLLHF
VVQEITKSEATTTTIDETILHENKDGFRKQGLQVVAGLSRDLANVKKSAGMDSDVLSGYVTKLETGLEKLRAFVKTE
TTTTPGKFFDSMKTFLKDAEEEIRKIKGGERKALSMVKEVTEYFHGDAAKEEAHPLRIFMVVRDFLGVLDNVCKEVK
TMQEMSSAMGSASARSFRISATASLPVLHRYKSRQEDTSSDNEHSSNSST

>Bra016233|PACid:22702140
MAAMLHQPWPFPLQVTILLFLCVVVLPYHSFSQSDSPQNIETFYPTAELSPVPPPINPSTPSSPPSNQSSSSDRGTI
TKAVLITAASTVVLAAAFFFFLQKCVIARRRRRRENRINVQNTLPPYPPTTTTVAATETLAREGFTRFGGVKGLILD
ENGLDVLYWRNLQSQRQRRSGSFRKEIVVAGGEEGGSEEKEVIYYKNKKKMEQVTEVPLLRGRSSTSHSIIHNDSYE
LESPPPPPPPPSIPAKKTVPAPSPPPKKGPSPPPPPIPVNKTAPPPPPPPSPPPTPMKKTAPAPPPPRPSPSPPPPP
PVKKAAALSSSGSRPPPAPRGSSGGESSRGGGSGQVKLKPLHWDKVNPDSDHSMVWDKIDRGSFSFDGDLMEALFGY
VAVGKKSPEHDKQKSNSPTKIFILDPRKSQNSAIVLKSLGMTREELVEALVEGHDFVPETLERLARIAPTKEEQSAI
LEFDGGDAGKLADAEAFLFHLLKAVPTAFTRLNAFLFRANYYPEIAHHGRSLQTLDLACKELRSRGLFVKLLEAILK
AGNRMNAGTARGNAQAFNLTALLKLSDVKSVDGKTTLLNFVVEEVVRSEGKRCVLNRRSNSLTRSNSNRSSSSSSSN
NAPQAMSKEEQEKEFLKLGLPIVGGLSSEFSNVKKAASVDYDTIVATCSALAVRAKDARKVIAQCEGGRFVEKMMTF
LDSVEEEVKTAREEERKVMELVKRTTEYYQAGASVKGKNPLHLFVIVRDFLAMVDKVCLDIMRNVQRRKVTSSSEST
STQRNAVKFPVLPPNFMSDRSRSDSGESDSDM

>Bra013217|PACid:22707193
MLFLLFFYFLFSSSALADRRVLHEPFFPVDSPPPSPPQSPPLPKLPFSSTTPPDATSPPFFPTYPSPPPPPSPASFA
SFPANISSLIVPHATKSPPSSKKLLIAAISAVTSAALVAALIALLYWRRRRGRSSQESNAPDDSKTWTTDSSRRVYP

PPPPTTAAPSARRNAEGRASKQRTTTTTARSSANTSSEFLYLGTMVNPRGVEDHQSVSNRSNNGSSSRKLESPDLQP
LPPLMKRSFRLNPEVGSIGEEDEEEFYSPRGSASGRETLTRVGSESRRVFSSVPNQNVRSVNNDTISCSSSSSGSPG
RSTFISISPSMSPKRSEPKETVISAPEPPPPKTSPGSSESSPSKITDFRLVRSPSLSLASLSPRLCPGKNAEGGGLG
QISRSPTVTSLTTSPERENKEENSLQNLDDDSPRSSSRSSASTSPERRPNDTPEAYLRSPVHSSASTSPYRCFQKSP
EVLPAFMSNLRQGLQSQLLSSPGQGFLNELDALRSEKTSHKSPPEKGSHDPQSASSSSLSSSPDRDFSHSLDVSPRI
SNISSQILQPPVSAARVPPPPPPPPPPFPTWARRNQATPKVSRPPPLTPPSNPFVIPSENLPKTSPEETHKETFSAN
EPAEETPKPKLKALHWDKVRASSDREMVWDQLRSSSFKLDEEMIETLFVAKSLDSKPNQSQTTPRCVLPSPNQENRV
LDPKKAQNIAILLRALNVTIEEVCTICSWGRALNVTIEEVCEALLEGNAETLGTELLESLLKMAPTKEEERKLKEYK
DDSPVKLGHAEKFLKAMLDIPFAFKRVDAMLYVANFESEVDYLKNSFETLEAACEELRNSRMFLKLLEAVLKTGNRM
NVGTNRGDAHAFKLDTLLKLVDVKGADGKTTLLHFVVQEIIRAEGTRLSGDNNTQTDDIKCRKLGLQVVSSLSSELS
NVKKAAAMDSEVLSSYVSKLSQGIAKINEAIQIQSTVTEESGSERFLESMNTFLKRAEEDIIRVQAQESVALSLVKE
ITEYFHGNSAKEEAHPFRIFLVVRDFLGVVDRVCKEVGMINERTMVSSAHKFPVPVNPMLPQPLPGLFGRKQSSSSS
SSSSSDDEHNSSH

>Bra005952|PACid:22709355
MALFRRFFYKKPPDHLLEISERLYVFDCCFSSDVMGEDEYKLYLGGIVAQLQDHFPDASFMVFNFREGDQRSQISDV
LSQYDMTVMDYPRHYESCPLLPLEMIHHFLKSSESWLSLEGQQNVLLMHCERGGWPVLAFMLSGLLLYRKQYQGEVK
TLEMVHKQAPKELLHLLSPLNPQPSQLRYLQYISRRDLGSDWPPSETPLLLDCLILRDLPRFEGREGCRPIVRVYGQ
DPKARANRSSVVLFSTPKTKKHTRLYQQEECILVKLDIQCRVQGDVVLECIHLHDDLVREEIIFRIMFHTAFVRANI
LIVQREEMDILWDAKDQFPKEFKAEVLFSGADSVVPTIATAPVSDDDESDFDMASPDEFYEVEEIFSDAVDGHELKR
GDSDGFVVVDSASDDSEGKEVWKGDVEPNAFLDCASDDSNHKHEASADPVKDITVDDVQYRSDGTSIDSVKDIGIDD
GDEQRKRRTMEAKENDSRTAEKAQAKPRKQVGANAKLAGDALKPKSNVRMAKPNAVSRWIPSNKGSYKDSMHVAYPH
TRINSAPASITTSLKDGKRATSPDGVVTKEAKSKNLRASVSSPDVRSRAPSCLSPEFSPKEKPPSLPASPHYAPPPP
QHSPSPPEQPSLSSEAASPPPPPPPPPTYSSPSQNDSYSQTSQIPPPPPPPPPFASKRPNSGSMLPPPPPPPPLASEK
PKTGTVLPPLPLSPPWKSVFASALATPTTCSTSQPPPPPQTSSQSQTSQLPLPPSPPPPPPFASVRRNSETMLPPPP
PPPPWKSVYTSTLETHEACSTSNNFPPPPPPPLPPFSSSKENGDHVTPSSLPYLSNAPSPSPKSSPINGFSAPPPPF
SKPHSVPPPPPPSYGSPTPPPPQPPSYGTPPPPPPPFGKTSPPPPPPPPPFGSSCPPPPPPPPFRSGGPPPPPPPPM
GAPPPPPPPMHRGAPPPPPPPMRGGAPPPPPPPMLGGAPPPPPPPMRGGAPPPPPPPMLGGAPPPPPPPMRGGAPPP
PPPPGGRAPGGPPPPPPPGGRAPGPPPPPGPRPPGGGPPPPPGPRPPGGGPPDLKGAGRGRGLSRPGLGSSAQKKSS
LKPLHWVKVTRALQGSLWDELQRQGQTAPEFDVSEIETLFSAIVPKPVDKSRKKSVGAKPEKIQLIDLRRANNTEIM
LTKVKMPLPDMMAAVLAMDDTVLDSDQIEILIKFCPTKEEMELLKNYTGDKAVLGKCEQYFLELMKVPRVESKMRVF
SFKIQFGTQITEFKKSLTAVNSACEEVRTSPKLKEIMKKILYLGNTLNQGTARGAAVGFKLDSLSKLSDTRAANSKM
TLMHYLCKVLASQGSDLLDFPKDLESLESASKIQLKSLAEEMQAIIKGLEKLNQELNASESDGPVSEVFRKTLKDFI
SVATTETAAVTSLYSVVGNNADALAHYFGEDPKRCPFEQVTATLLNFIRLFKKAHEENIKQEELEKKKAAKEAEMEK
EKAKGVNLTKTADDDDS

>Bra029012|PACid:22709967
MILGGGMCNQNWARLVLLLVLLSGFFLVIALAESSEKDETFISQFMAPSTEQANEQMVETLWAHRCWQDPDCVKEAV
TVFNLCFPGSKDNNLELFGFTPSHLKQTLLMCIQKQGELNGHNLNYLKLLPSILDNAPRRNLASTPSPSPPPKRSSR
RPPPAASKKSVSEKLTSKPAPAAKGKEDHQKTIIIAVVTTAVSTFLLAACLFLCCTKVCGKGSGGRINDERPLLSLS
SNEYSLGSSNNYGGSGKGHQSFNGNNSDNFVTLEERMSMDGMFNNINNSHGIPPLKAPPGRKSSKISWKPPSGKVEP
LPPEPPKFLKVSSSKKSSSAPSPSPPPPPMPSSAGPPRPPPPAPPPGSGGPKPPPPPGPKGPRPPPPMSLGKKAPPA
SPGPASSGDDDAPKTKLKPFFWDKVQANPEQSMVWNDIRSGSFQFNEEMIESLFGYAAADKNKNDKKGGSGQAALPQ
FIQILEPKKGQNLSILLRALNATTEEVCDALLEGNELPVEFIQTLLKMAPTPEEELKLRLYSGEIAQLGTAERFLKA
VVDIPFAFKRLEALLFMCTLHEEMAFVKESFQTLEVACQELRGSRLFLKLLEAVLKTGNRMNDGTFRGAAQAFKLDT
LLKLADVKGTDGKTTLLHFVVQEIIRTEGRRAARTIRESQSFSSVKTEDLMAEETSEEMEENYRGLGLQKVSGLSSE
LEHVKKSANVDADSLTGTVLKMGHALAKAREFVNSEMKSSDEESGFREALEDFIQNAEGSVVEILEEEKRIMALVKA
TGDYFHGKAGKDEGLRLFVIVRDFLIILDKSCKEVREAKGKQVKMARKQGSTASSVASEIPRAPSLDPREKLFPAIT
ERRVDQSSSDSD

>Bra007822|PACid:22714932
MALFRKFFYKKPPEGLVEISERVYVFDCCLTTDMLEEEEYRVYVGRIMSQLREQFPGASFMVFNFRDGGESTSLMES
VLSEHDMTTIMDYPRHYEGCPLLTMETVHHFLKSSENWLLLSPQNILLAHCERGGWPVLAFMLASLLLYRKQFSGEE
KTLEMMYKYAPRELLQLMSPLNPLPSQLRFLRYVSSRSVGGHSQWPPLDRAVTLDCINLKLVPDFDGEGGCRPIFRI
YGQDPFMASDRTSKVLFSMPKRSKAVKHYKQADCEVVKIDINCHILGDVVLECITLDSDHEREEMMFRVVFNTAFLR
SNALLLNRDDIDVLWNTTDRFPKGFRAEVIFSEMGAGNNHVSVDLTDMEENDGLPMEAFAKVQEIFSDGEWLDPNSD
VAVTVFNQITAANILQESLDSGSPRSPDSRSLLESALEKVREKTKLMISENVAVSPDAFSPVWRERDSDSCHRSYAD

PNSLIKKVDEPQGLRVSVQRQAHSKIISPRLLQSSVTSPVLNRSPTQGSSPASVSRFHSPSSLGITSILHDHGPGK
GEEATSSSSPSITFQPALHPLILKASPSNGSPPAEAVVKPPTLPLLKPLKILSPPPPPPPPPPPASSSSLRSTATQG
PPPPPPPPPPPQRSAQPFFPSPPPLPPPKKVVTTSNAPPPPPPPLRSKPLSGAAAPPVPPPPAPSALSRSQNGGCNG
NVPPVPGPPLGLKGRGMLQTSLKGQGQTRKANLKPYHWLKLTRALQGSLWAEAQQTPDEAATAPEFDISELEKLFSA
AIPSSDNETKGGKSGRRGRPKVQKVQLIELRRAYNCEIMLSKVKIPLPDLMSSVLALDESVIDVDQVDNLIKFCPTK
EEAELLKGYTGNKENLGRCEQFFLELLKVPRVETKLRVFSYKIQFHSQVRGSAKLKRIMQTILSLGNALNHGTARGS
AIGFRLDSLLKLTDTRSRNSKMTLMHYLCKVLAEKLPELLDFPKDLVSLEAATKIQLKYLAEEMQAIRKGLEKVVLE
FTTSETDGPVSKHFRMNLKEFLSFAEGEVRSLASLYSTVGGSADALALYFGEDPARVPFEQVVSTLQNFVRIFVRSH
EENCKQVEFEKRRVQKEAENEKLKKVLGTQKLHVPAPTIL

>Bra022423|PACid:22684428
MALATRVQILGCILLASLALTMADTPPGIAKNPSHATCKIKKYKHCYNLEHVCPKFCPDSCHVECASCKPICGPPSP
GSDDDDGEDDGGYTPPAPVPPVSPPPPTPTPAVPSPTPPVSPPPPPPPTPTPAVPSPPPPVSPPPPPPPTPTPAGAS
PTPPVSPPPPSPTPAVPSPTPSSSPPPPTPTPAVPTPTPPTPSVPSPPGTPTAPVPPYSPPATPTPSIPSPTPTSPG
STPPYVPPSSPTPTPPSDGEAGAGVRRARCKKKGSPCYGVEYSCPADCPRSCEVDCVTCKPLCNCDKPGSVCQDPRF
IGGDGLTFYFHGKKDSNFCLISDSNLHINAHFIGKRRPGMARDFTWVQSIAILFGPHRLYVGALKTSTWDDSVDRIS
ASFDGHVISLPQLDGATWTSSSLGVYPQVSVKRVNADTNNLEVEVEGMLKITARVVPITVEDSRIHGYNVTEDDCLA
HLDLGFKFQDLSDNVDGVLGQTYRSNYVSRVKIGVHMPVMGGDREFQTSGLFEPDCSAARFTGNRGSNGGRSKMELP
EMSCASGVGGKGVVCKR

>Bra037084|PACid:22684501
MGFLHKQYHSFVILLLFGFLAISYACECSDPPKPSTPPPYTPCPPTAKPPPPPTPSPPPPYVKPPPPPTPSPPPPYV
KPPPPPTPSPPPPYVKPPPPPTPSPPPPYGKPPPPPTPSPPPPHIQPPPPPTPSPPPPYVKPPPPPTPSPPPPPTPC
PPPPPAPTPKPKTCSINVLKLGACVDVLGGLIHVGLGKGYAKTKCCPVLDGLVGLDAAVCLCSSIRAKLLNIDLVIP
IALELLIVCGKTPPRDFKCPAPQQRNPLLG

>Bra040669|PACid:22685898
MASIYSLLFLSLLQLHCTLSTGNNHIIHRKSLEIISGSGVGGIPPPLPSPQPKPEECPPPCPPRPLPPQPEECPPPP
PPPCPPPRPPPPQPEECPPPPPPPCPPPPQPKPEECPPPPPLPPSPPPPPPPSPPPTPSPKTPPLPPPESIHSPPKA
PPLSPSEPSRSPPKPPKSPPPPQLTFASPLLKKVYPVLQAFKKLVEVDSKNILASWNGSDICGKYRGLECATFPGTK
YQAVASVQFNGFNFSGKNLRLDNFLDKLDTVTIFHANSNNFLGSVPKVSNLKYLFELDLSNNKLTGEFPASVLKATN
LTFLDLRFNTFSGSVPRQVFNLDLDVLFINNNNLVQKLPHNLGSITALYLTFANNRFTGPIPASIGNIKYLQEVLFL
NNQLTGCLPYQIGKLNRATVFDVEYNNLTGPIPYSFGCLDKMEQLNLARNKFFGTIPEIVCEISSLKNLSLSYNFFT
QVGPKCRNLIKRNILDVRMNCILDLPNQKTPLECANFFMRKHTCPNSKSMFRIPCGKNPNRIRLDQEQLKDEQAQTS
SPVSYWALNPDRIQNR

>Bra004715|PACid:22686484
MRGMKSLTIWAIFAAVLSQQLFASVASIKFEDEKTYYSPPDPNAGSPPSGTPPSHGGYTPTPSTPSHTPPSNCGSPP
YDPSPPSHTPTPSTPSHTPSTPSHTPSTPSHTPTPSTPSHTPTPSHTSPPCHCGTPPSHPSTPSRPSRPSHPSRPSR
PSTPSNPPSGGYYSSPPPSTPVVVTPPTPIVDPGTPSTGGTPPSSGGYYSSPPPSTPVVETPPTPIVDPGTPIVGET
PPTPSSGGYYSSPPPSTPVIETPPTPIVDPGTPIIGGTPPTPFIDPGTPGTPFLPAPFPPITGTCDYWRNHPTLIWG
LLGWWGTVGGAFGAVSIPSSIPGFDPHMNLLQALSNTRTDAIGSLYREGTASWLNSMVNNQFPFTTSQVRDQFLAGL
SSTKAATKQAQTFKLANEGRLKPRI

>Bra035635|PACid:22688850
MEILMFLVRIYLVSSVLVAASSSGLDLLSPSSSPPPPLPETSKGFGEVPISSPESHKPGNAPPPKASLPSSPPLADV
AAPPPSYSSGTKAPNREPIVSVPSAPGPVSSPVSDIPPFPSVALPQPTPSIVPPRNASNKKPVAPVASPPTISVDIS
PPVIPKLPHSRSPDSPTSTAPSPPKFNGHSHHTSSSPPLNHLHHQEPKKIKDSPPPPPPKMSNRPISSSMHPISIAP
SPSPTQGLLPPLLKLFPTTHRQNLIAKLSFISPKAFPLRSSSKPRKLPPLQALPPPPPNSDCSSTVCLDPYTNTPPG
SPCGCVWPIQVELRLTMPLYDFFPMVSEFAREISAGVFMKQSQVRIMGANAATEQPDKTILLIDLVPLGDKFDNMTA
MLTYQRFYRKKVYIDATTFGQYEVVYVRYPGLPVSPPSGGMTVIDHEPFSRNNNNNGMVKKPFGVDVPKKMRRKEIN
GGSIAVIVLSAAAFIGLCFVVVWFLAFRRGRARRRLSTRASLPSLTKPPGSVRSLTGSRFSSTSLSFESSIAPLTLS
AKTFTASEIVKATSNFAESRVLGEGGFGKVYEGLFDDGTKVAVKVLKRDDQQGGREFLAEVEMLSRLHHRNLVNLIG
ICIEDRNRSLVYELIPNGSVESHLHGVDKESLPLDWEARLKIALGAARGLAYLHEDSNPRVIHRDFKSSNILLEQDF
TPKVSDFGLARNALDDEDNRHISTRVMGTFGYVAPEYAMTGHLLVKSDVYSYGVVLLELLTGRKPVDMSQPPGQENL
VSWTRSFLTSREGLEAIIDQSLGQPEIPFDSIAKVAAIASMCVQPEVSHRPFMGEVVQALKLVCNECDEAKELNSVT

SLTHDDLGDDNGAESSCGGEGSRRMVRYPLLPSYDSEPGTERGLSVSEMFTGSGRLERVSNSGPLASGGGKRFWQKM
RRLSTGSLSEHGSSSLMVRSGSR

>Bra002639|PACid:22692658
MESPMRFNLRTAFSIIFLTFLPLNLKSQEVFDPSQDHSLIQSEASWNRRSLVETPPLPGKGPAVGASPPSPDQAFEG
STKPPPAPETQTPPGGDGTPSPPPSSVRTAQTPNPPSEPPPQLLSPPPRTKKTHNISMIVGIVVGVFTVSVALIIFF
LIHTRKIPIKPWTNSGQLQNALITGNHTLFNVPRMQLSELRAACEDFSNIIGSFSDGTIYKGTLSTGAEIAVVSIAA
GSRANWSTDMETQLLQKIRKLSKVDHKNFLNVIGYCHENEPFHRMLVFEYAPNGTLSEHLHSQHTEHLDWPTRLRIF
MGIAYCLEHMHNLNPPILHTNLDSSCIYLTEDNAAKVSDFSVLNFISPSKESSSSKNLLEHSTLDPQTNVLNFGALV
FEIITGRLPDPDSLFLEPKPARDLVDPTLKTFQEDVAERLLGVVRQCMNPYSAQRPTMRKVVVKLREIIGIEADAAL
PRLSPRWWSEMEIITTDGN

>Bra002357|PACid:22694518
MGVWWIVVVALLAHTASAAVREYHWEVEYKFGFPDCKEGMVMAVNGQFPGPTIHALAGDTIVVHLTNKLATEGLVIH
WHGIRQLGSPWADGAAGVTQCAITPGETFTYKFTVDKPGTHFYHGHYGMQRSAGLYGSLIIDAAKGKIEPLRYDGEF
NLLLSDWWHESVLSQELGLSAKPMRWIGEAQSILINGRGQFKCSLAAQFSNATAQFSNTSLPMCKFKKGDQCAPQRL
HVEPNKTYRIRLASTTGLASLNFAVQGHKLVVVEADGNYITPFATSDVDIYSGESYSVLLTTDQDPSQNYWISVGVR
GRKPKTPPALTVLHYVTAPSSQPPSSPPPETPRWNDFDRSRNFSKRIFSAMGSPPPPRKFKKRLILLNTQNMIDGAT
KWALNNVSLVVPATPYLGSVKYKLRRGFDRKSPPTTFPMDYDIMNPPRNRNTTKGNGIYVFPFNVTVDVILQNANGL
DANASEIHPWHLHGHDFWVLGYGEGKFRPGIDEKTYNLKNPPLRNTVALYPYGWTALRFVTDNPGVWFFHCHIEPHL
HMGMGVVFAEGLNRIGKVPDEALGCGLTKQFLMNRNNP

>Bra019737|PACid:22695369
MDSLWWWETICSGAPDSLEATIDLLVFEREKCEPKRTKETHAPPLSSHFANTWPQIRSLLIKADGVLGSRIKVDPKL
KFENPKLRQAYIALQSWKQAIFSDPFNFTANWNGSDVCSYNGIYCAPFPGSYNKTRVVAGIDLNHADMAGYLPSELG
LLRDLALFHLNSNRFCGEFPNVVLTLPSLKFLDLRFNEFEGKIPSKLFDRELDAIFLNHNRFRFGIPKNMGSSPVSA
LVLADNDLGGCIPGSIGQMGKTLNELILSNDNLTGCLPPQIGNLKKVTVFDVSSNRLRGPLPSSVGNMKSLEELHVA
NNAFTGVIPPSICQLPSLENFTFSSNFFRGRAPICGAVSVVNGSMNCLTLMARQRSAKECLSLLARPVDCSKFGCYN
IFSPPPPTFKMAPIFRKLPPPPSSKMSPTVRAYSPPPPPSSKMSSTVRAYSPPSPPSSKMSPTVRANPPPPPPPPP
SPSPPPPYVYSSPPPPPYVYSSPSPPPPPPYVHSSPPPPPYVYSSPPAPSLSPPPCPESSPPPPYVYSSPPPPPPSP
PPPCCPESSPPPPVVYYAPATQSPPPPSPVYYPSETQSPPPPPSPVYFPPVTQSPPAPPPTPVYYPSETQSPPPTTE
YNYSPSQSPPPAKGCNDVHPPQTPPSYEPTPEYSYTSSPPSPPSYDDTSLPPIPSVSYDSSPPPPSNY

>Bra030020|PACid:22703356
MKSSIVLVAAAILCIVAFPTATVGKNLRFGLKPTQGWPHPSEASTNQMFMSTSQKFNYGDSKVWRCTYSNGSAPAIS
ISISPPTPTMPSPSTPTTPSPSPPTPKTSPPPPTPSPPPPTSKKAPSPSPPPPPPTPSLPPPTPTKVPSPPPPTLSP
PPPTPTKASSPPLPKPSLPPPTPKKTPSPPPPTPSLPPPTPKKSPSPSPPSDDESSSPSQPSNPPQEHHHHHEFPLE
HIGRCYRNMGQVGFCRGQMAISFYTRLFKVSKYCCNLIVNMKNECDDVIWGYFYDPHFVPLVRCTCHVSF

>Bra039563|PACid:22703663
MGILHKQNLSFLILLLLGFCAVSYACDCSDPPKSSPHPVKPPKHPIKPPKPPTVKPPQHTPKPPAVKPPHHTPKPPH
HTPKPPTVKPPHHTPKLPPHTPKPPTVKPPHHTPKPPTVKPPAPYTPSPPPYTPKPPPVKPPPPYTPSPTPSPPPPT
PTPPVVTPPPPPTPTPEKQCPQPPPPPTPCPPPPPAPTPEPETCSIDALKLGACVDVLGGLIHIGLGKSYAKATCCP
VLGGLVGLDAAVCICTTIRAKLLNIDLIIPIALELLVDCGKTPPRDFKCPAPQKKTSLLA

>Bra038210|PACid:22704542
MDLAKHTTLQMLGFILLASLVLTMAQPPGLTKPSHATCKIKKYKHCYNLEHVCPKFCPDTCHVECASCKPICGPASP
GDDGGDTPPTPVPPVSPPPPAPVPPVSPPPPVTPTPSYPTPTDPLPPAPVSPPPPAPVPPVSPPPPTPTPYVPSPTP
PVSPPPPSPTPDVPSPTPPSSPPPTTPTPAVPSPTPSSPPPPSPTPAVLTPPHVTPTPPTPAVPSPPDVTPTPPTPS
VPSPDTPTAPLPPYSPPATPAPSVPSPTPTPPSSPTPPGSTPTTPTPSVPTPSPSVPVPSAPNSPPYVPPSSPTPTP
PSDGEAGAGVRRARCKKKGSPCYGVEYSCPSACPRSCEVDCVTCKPLCNCDKPGSVCQDPRFIGGDGLTFYFHGKKD
SNFCLISDPNLHINAHFIGKRRPGMARDFTWVQSIAVLFGTHRFYVGALKTATWDDSVDRISASFDGNVISLPQLDG
ATWTSSPGVYPQVSVKRVNADTNNIEVEVEGLLKITARVVSITMEDSRIHGYDVKEDDCLAHLDLGFKFQDLSDNVD
GVLGQTYRPNYVSRVKIGVHMPVMGGDREFQTTGLFAPDCSAARFIGNGGRNGGWSKMELPEMSCASGVGGKGVVCK
R

>Bra013345|PACid:22704845

MKLPLPLLLLLLLLSPTTSAAPSLSPTPSPTISPVPRTSPTPPRTSSSSSPLDPKQLKALESLNIPTLRNPCDHHPS
SSSKPPTTVVTCDTGSPFRLVTSLSFTNCSSDLSISSTALKALSPSLPSLSFHNCPSLSPPPHLPDSLHSFSAVSSF
PRLSGLSLARLVNLTDITVSSVPVSTSGLFVILGNMHDIVSLTISHANLSGNIPKSFHSNLTFIDLSDNLIKGPIPT
SITLLSNLKALNLSSNSISGEIPDSIGDLISLKNLSLSSNKLSGPIPDSISSIPDLTHLDLSGNQLNGTVPRFISKM
KSLKYLNLANNAFHGVLPFNASFLKKLEVFKVGGNSDLCYNHTVLSSKMKLGIAPCDKHGLPLSPPPRKEDSSSDDD
YSEDDSSEKKKEEHHGPNKAVLGVAIGLSSLVFLIIFMILLAKWCG

>Bra000517|PACid:22709442
MASVECSIGDSISTLHPDIIQTQILTRLDGPTLASTASTSSYLQTLCYEQKLWREISTATWPCINDSRVVQAISSFP
SGYRSFFADSYSSTEYTWESEKQDTPTTGLISAVDLYYRGELIYSKVQEMETENGKAGWFLSAPFRVDLLDEKESVQ
TQILYPGGDYEAWVRNMEESMELNWIVIDPVKKRAANISSRKAVSAKRNWLTGDLEIRFSTVVAEAAGKKNAAEVAV
VVSCGSAETWKEVDEEVGGEVHVRDVRLQLEDIEGKCLKGRDSLVILQGMLEGKRCCKDGGGGGRGKGRYEEYMAMK
THMREKKERREKAQDTICMIFGFSLPSIRPPKSTSLLPKKSNRYEFEICVNQSCSELTRNQKNPIMDLVAPTKNEAD
VHLQSDSSQAFYFLIAILALLVGSAIATEPYIYSSPPPPPVASPPPPYKYKSPPPPVKSPPPPYEYKSPPPPSPPSP
YYYQSPPPPVKSPPPPYYYHSPPPPVKSPPPPYYYHSPPPVKSPPPPYYYHSPPPPVKSPPPPYYYHSPPPPVKSPP
PPYYYHSPPPPVKSPPPPYYYSSPPPPKSYPPPYYYSSPPPPPKSYSPYYYSSPPPPVYKKYEKKRTQR

>Bra012849|PACid:22710594
MGRSWVSSLLLLLLLILFFSKASSRGLADEQPPKDTSNTTTTQSPPLPPNVGDGKANATSSLPASNSTSPDPKEPDS
ASPPPPPPPPPPQQLQAEGEKNVTVVSNTTEAVSPPPANQTESVDKGKLPKKMAPPPKSLESGEKGKVPETGNPPPG
KDPEKVDDAKGSSESVIVETCAGKSKICRTENSLVACTLSIEKDSSKWSILVQNEGEESLKAKIVLPVSSSPDLTLP
KHQSQRVNISISVDTNKIILDAGKGECVLHMYPPKENTLSIHLPSYEKLVTPINGAYFLIVSVVIFGGAWGFCLCRK
NRRADDNGVPYRELELSGGGTGLESVHDVETADWDEGWDDDWDENNAVKSPGGAANSARISANGLTARASNRDGWDN
DWDD

>Bra012706|PACid:22712622
MKQRSSLLILCSVLIVSFVASVSAQTCVHNGKNFIPNSTYDANRRLILSSLPSNAAAQDGFYSGSIGQEPSRVYAAG
MCIPGAEANDCSACIKGASDWLVQDCTNQTDAYYWALDPTLCLVRYSNNSFSGSAGYWEILPRQKSRYKADVAALTP
FQNIYALMQCTPDVSSGDCDNCLRQSVIDYQSCCGEKTGGYVMRPICFFRWQLFTFSKAFGNITLAPPSPPPSPPPL
LQRQPSSASDQAKKTDTGSRNISLRTIIAIVVAVIVFIIIIIIIVVVFARRSTRGGKSDQQVEFNQTGITTVRSLQ
FDFKTIEVATNNFSERNKIGRGGFGDVFKGTLMPGGREVAVKRLSRTSEQGEKEFKNEVIVIAKLQHGNLVRLLGYS
VKGEERVLVYEFVPNKSLDSFLSDPLKQAQLSWTKRFNIIKGIARGILYLHQDSRPKIIHRDLKADNILLEADMNPK
ISDFGTARIFGIGQTSSITERIFGTEGYMPPEYRCEGQFSVKTDVYSFGVLVLEIICGKKNRSFCFPGPDLVTYAWR
LWREETPLELVDQTILENFPAEEVTRCIHIALLCVQHEPTDRPHVSTIVSMLTSTTIISHVPQTPGSLDSLY

>Bra026744|PACid:22713371
MKGLIIICVVTFLLISVSITESIENQHRHKGKDKQGLVRRHRRAKSRRRSSSRNGEAFATQCDVFFRCVFGTCGQWN
FPIVPCPQNPFLPPPPPVVTPVVIPPPTPCVNCVQPSPPPPIPVPCPPPPSPPPPIPVPCPPPPSPPPPAPCITCVT
APAPLPPVPCVTCVTAPAPPPPQPCIVCTTAPAQPPPVACPPPPLPPVIPFVPTPIILPPLPPLFPVMSPPATPTPV
PILPPPTPVLPLPPPSAPLPPPLSSSIPSPSLPLVLSPPPPLPSGSVSQPPFMMTPTPVLGGGTPGFIGIPPPVQDL
PPILPPPVQDFPSILPPPVQDLPSMLPPPVQEFPPILPPPAQDLPSMFPPPAQELPPPVQDFPQILPPPAQEFPPVL
PPPVQNFPQILPPPVQEFPPILPPPVQDFPPVFSTPPILQDPPTEPIFSTPPALGEFPPQAPVFTTPPEVTNPWLPP
EQPPVTSIPTIPENPYPNPDMGSNQLPPPSWDSPPFNR

>Bra026743|PACid:22715247
MRTKQLVVVGFLFSLLLLVDSHTTESISDHEENANAKAVTVKDHKQINRGRRSGSGQNRGRRSCDPLFQYLFGICGR
WPFPTTPSPDNPFLPFQPPRQPPRPRPRPPPLAPSPPPPRAPSRPRPRPRPTPPPLVPSPPPPPPTPLVPSPPPPSP
PPIFIFPSPPPPVLAFPPPLVPSPPPPLWLPPPVFTLPPPLDEFPPMPPIIWVPPLDVPGQSSPAEDFDLITP

>Bra020993|PACid:22721222
MKPLSPSLPLLLLLLLSSTTFAAPSLSPTPSPTISPIPRTSPRTSSSPLDPKPLKALESLNIPTAKNPCDHRPTSKP
PSTVVTCDAGSPFRLVTSLSFTNCSSDLSISSAALRALSPSLASLSFLNCPSLSPPPRLPTSLRSFTATSSFLRRRK
GLSGVYLARLVNLTDLTVSSVPVSTSGLFVILGNMDKIVSLTVSHANLSGNIPKSLHSNLTFIDLSDNLIKGSIPTS
ITQLSNLKSLNLSSNSISGEIPDSIGDLISLKNMSLSSNKLSGPIPDSISSLPDLTHLDLSGNQLNGTVPRFITKMK
SLKHLNLANNNFRGVLPFNASFLKKLEVFKVGGNSDLCYNRTVLSSKMKLGIAQCDKHGLPLSPPPQKEDSSSDYDY
GSEDETSVKKKEESRGPNKVVLGVSIGLASLVFLIIFLILCAKWCG

>Bra038089|PACid:22721399
MGLLHKQNLSFVILLLGFLVVSYACDCGDPPKPSPHPVKPPKHPVKPPKPPTVKPPPHTPRPPTVKPPHTPSPPHSF
PPYTPKPPTVTPPPPYTPSPPPYTPKPPTVKPPPQPTPTPSPPPPYVKPPPVPTPETPCPPPPPPSPPPTPCPPTPP
APTPEPETCSIDALKLGACVDVLGGLIHIGLGKSYAKATCCPVLGGLVGLDAAVCLCTTIRAKLLNIDLIIPIALEL
LVDCGKTPPRDFKCPAPQRKSPLLG

>Bra014023|PACid:22723120
MKSLIILIVAHFCIIVSPTTTMGGWPKPSEVSNEEKLVNTGQAQPHLYAGKFNFGDSKVWKCTYNNGSGVAISISYP
SPPQPPSQKPPTPSSPPTPKMAPPLPKPSPPRPSPKKSPPPPKPSSPPPTPKKSPPPPKPSPPPPTPKKSPPPPKPS
PPPTPKMSPPSPTPSPPRPTPKKSPPNPSSLTPNESPPPAKTSILIIHSPPPPHEPIPAQSPPKEPTTPSTQWPPYR
NWNPLGL

Solanum lycopersicum

>Solyc04g071080.1.1|PACid:27276990
MASLVATLLVVLVSLSLPSESSANYQYTSPPPPKESYHPSPTPYHPVPLYKSPPLPTPVYNSPPPPRETYYPPHTPV
YMPSPPSTQIYKSPPPPNESHTTLHTPQPTSHHLHQLLLTSLYRCQFQFTILHHHPRSHTTLHTPQPTSRHLHQLLL
TSLHRHQLRFTILHHHPRSHTTLHIPQPTSRHLHQLPIYKSPPSPTPVYDSPPPPKELYYPPHTSTYKSPPPPNEPY
YPPHTPTYKSPPPPIPIYKSPPLPTPIYKSPPPPKEPYYPPHTPLLPSSVYKSPPPPTHVYKSPPPPIEPYYPPHTP
IYKSPPPPIPVYNSPPPLTPVYKSPPPPKEPHYPPHTPVYKSPSPPKEPYYSPQNPVYKSPPPPTPSYKSPTPPKEP
YYPPHTPTYKSPPPPTPVYKSPPPPKEPYYPPHTPTYKSPPPPKESYYPPHTPTYKSPPPPTPVSNSPPPPTPVYKS
PPPTDYVYTSPPPPYYY

>Solyc04g071070.2.1|PACid:27279171
MGKMASLVATLLVVLVSLSLASESSANYQYSSPPPPVHVYPSPPHYPVYKSPPPHHHHPIYKSPPPSEKPHYPPHTP
VYKSPPPHHHHPVYKSPPPPTPVYKSPPPPKTPHYPPHTPVYKSPPPRHHHPVYKSPPPPTPVYKSPPPPKDPHYPP
HTPIYKSPPPPKEPHYPPHTPVYKSPPPPKSVYKSPPPPVKPYHPTPVYKSPPPPTPVYKSPPPPVKPYHPTPVYKS
PPPPTPVYKSPPPPVKPYHPSPTPYHPTPAYKSPPPPTPVYKSPPPTHYVYSSPPPPYHY

>Solyc06g076420.1.1|PACid:27284841
MKLITFFALFFLLQSTAIFSFVVADYSIATSQYGGNTPPPVTSQPPPSHGHQPPSAPGQAPPQHSHGHVPPSVGSPP
PPSPMISSPNPPPSHGHGPPSKGHAPPGGYHPPSPPSHHGHPPPSTPTPTYSPPPQVQPPYSQPSAPPQSPPSHGHA
PPPKRHTPPGRHNPPSHHQPSPPSHHGHPPPKHLQPPPTPIYSPPPQVQPSPPSYSPHPPTYSPPSRSPPSHGHAPP
PKGHKPPGGHHPPSPPSHHGHPPPTYSPPLPTYSPPPPPLTYSSPPAPTYSSPPPTHSPSPPPPTYSSPPPAQSSPP
PPTYSPPPPPPPSYTPPPPAQSPPPPPSYSPPPPVQSPPPPPSYSPPPPSQSPPPPPAQSPPPPPSQSPPPPPIYSP
PPPVQSPPPPPTYSQPPPPSPPPVYSPPPPSSPPPSTYSPPPPTSHPPPPTHSPPPPAYSPPPPPTTYSPPSPPYGL
SRSTP

>Solyc02g030220.1.1|PACid:27287299
MISFGNLGQRSLLAFALVICFVASTVVADYSYEYTSHSPSPYYKKREKHVEHSPSHHYYKSHAPSKYYKTHVVAKYY
ESHAPSKHYYKSPVVAKYYKSHAPSKHYYKSPIVVKYYKSHAPSKHYYKAPVVVKYKSPTPSKKYYKSPTPSKYYYK
SPSPTKYYKSSSPAKYYKSPTPSTHYYYKSPSPSKYYKSPTLSKYYKSPPPPKYYKSPIYYKSPPPPTYYKESTPSY
KSPPPSPYYKESTPSYKSPPPQPYYKESTPSYKSPPPPPYYKESTPSYKSPPPLPYYKESKPSYKSPPPPPKYYEQS
PTTYNSPPPPPQKYEQSVTYASPPPPPVYY

>Solyc03g082770.1.1|PACid:27292317
MRSFGNLGQWSLLAFALALCFVASTVVADYSYGYSSPSPSPYYKKPEKHVEHSPSHYYYKSRAPSKHYYKAPVVAKY
YKSHAPSKHYYKTPVVAKYYKSPAPSKHYYKTPVVAKYYKSPAPSKHYYKAPVVVKYYKSPAPSKKYYKAPTPSKYY
YKSPSPAKYYYKSPLPAKYYKSPAPSKHYYYKSPSPSKYYKSPAPSKYYKSPAPKKYYKSSVYYESPSPPPTYYEKS
PSYYKSPPPPPYYKESTPSYKSPPPPPYYKESNPFYKSSPPPPKYNEKSPTTYNSPPPPTYSSPPPPQEYEQSVIYA
SPPPPPASPPPTYY

>Solyc11g065910.1.1|PACid:27297197
MASSLPHLVWFVAILSISNSVEASHNPYSWRKYYSYKPAPTFTPNPDKSLGQSSPSPQPSSSDYYISSPPSPLGWFS
PQPSLVPSPTPTSSPLSPSPPSPSKWAWEPSPSYTTSPQLSPVLAPSNPDQSPPSPIEWFSRRPSQVPSPAISPSPS
PSHLRWAPSPYSYSWEPSPSYTTSPQSSPVLAPFDPDQSPPSPIEWFSPRPSPVPSPATSPLSPSPYDNSSPPSPSE
WAPSPQSSVVPVPAPAPSYSPPPYYYKSPPPPSPSPPPPYYYNSPPPPSPSPPPPYVYKSPSSPPPPPYYYNSPPPP
SPSPPPPYYYNSPPPPSPLPPPPPSY

>Solyc01g005890.1.1|PACid:27300227
MRSFGNLGQWSLLAFALSHAPSKHYYKAPVVAKYYKSHAPSRNYYKAPVVTKYYKSHAPSKHYYKAPVVVKYYKSPA
PSKQYYKASVVVKYYKSPAPSKKYYKAPTLSKYYYKSPSPAKYYKSPSPTKYYKSPTPSKYYKSPSPAKYYKSPVYY
KSPPQPPPTYYEKSPSYYNSPPPPPYYKESTPSYKSPPPPSYYEKSTPSFKSPLPPPYYEESTPSYKSPPPPPYYEE
STPSYKSPPPPPKSYEQSPSYYSPPPPPIVY

>Solyc01g097690.2.1|PACid:27300243
MESLGKLGQWSFLTVALAICLVASTVVADYSYEYTSPSPSHNSNKYYKSPSPSKYHVPTPYYKKPYPSHYYYKSPAP
SKHAYYKSPSPAKYYKSHVPSKHYYYKSPIVTKYYKSHVPSKHYYKSPVATKYYYKFPTPSKHYYKSPVPSKYYYKS
PSPAKYYKSPSPTKYYKSPTPSTNYYYKSPSPTKYYKSPTPSKYYKSPSPTKYYKSPVYYKSPPPPPKYYEQPPTYY

NSPPPPYYQESTPSYKSSPPPPKTYEQSPTYYSSPPPPYYKETPTYASPPPPVKYEEPVTYASPPPPEKYEVPPTYA
SPPPPPPTYY

>Solyc01g097700.1.1|PACid:27300589
MNSLQKLKQWSFLTIALAICLIASTVVADYSYEYTSPSPTHNSNKYYKSPSPSNYHVPTPYYKKPYPSHYYYKSPVP
SKHTYYKSPSPAKYYKSHVPSKHYYYKSPIVTKYYKSHVPSKHYYKSPVTTKYYYTSPTPSKHYYKSPVPSKYYYKF
PSPTKYYKSPSPTKYYKSPTPSINYYYKSPSPTKYYKSPTPSKYFKSPSPTKYYKSPVYYKSPPSPPKYYEKPPTYY
NSPPPPYYKESTPSYKSSPPPPKTYDQSPTYYSSPPSPYYKETPTYASPPPPEKYEVSITYASPPPPEKYEVPPTYA
SPPPPPPTYY

>Solyc01g097720.1.1|PACid:27301141
MESLGKLGQWSFLTVALAICLVASTVVADYSYEYTSPSPSHNSNKYYKSPSPSNYHMPTPYYKKPYLSHYYYKSPAP
SKHTYYKSPSPAKYYKSHVPSKHYYYKSPIVTKYYKSYVPSKHYYKSPITTKYYYKFPTPSKHYYKSPAPSKYYYKS
PSPAKYYKSPSPTKYYKSPTPSTNYYYKSPSPTKYYKSPVPSKYYYKSPSPAKYYKSPTPSTNYHYKSPSPTKYYKS
PTSSKYYKSPSPTKYYKSPVYYKSPPPPPKYYEKPPTYYNSPPPPYYQESTPSYKSSPPPPKTYEQSPTYYSPPPPP
YYKETPTYASPPPPVKYEEPVTYASPPPPEKYEVPPTYASPPPPSPSPPPPTYY

>Solyc01g107220.2.1|PACid:27301967
MISLGKLGQLPLLAAALAICLAASTVFADYSYGYTSPTPYSSNKYYKSPSPSKYHVPTPYYKKPVEYSPSHKYYKAP
VPSKHNYYKSPSPIKYYKSPTPSEHYNKSPIAKKYYKSHTPSKHYYKYPVPSKHYYKSPSPVKYYYKSPSPIKHYYK
SPTPSKYYKSPSPSKYYYKSPTLVKYYKSPTPSKHYYKSPAPVKNYYKSPTPAKHYYKSPTPKYYSTPSPTKYYKSP
VYYKSPPPPAYYEKSPSYYKSPPPLPPKYYEESPINYKSPPPPYYESPPPPPKTYEQPPSYYSPPPPTKYEPSTTYA
SPPPPSPSPTPTYN

>Solyc01g097680.2.1|PACid:27302043
MESLGKLGQWSFLTVALAICLVASTVVADYSYEYTSPSPSHNSNKYYKSPSPSKYHVPTPYYKKPYPSHYYYKSPTP
SKHAYYKSPSPAKYYKSHVPSKHYYKSPVATKYYYKFPTPSKHYYYKSPIVTKYYKSHVPSKHYYKSPVATKYYYKS
PTPSKHYYKSPVPSKYYYKSPSPAKYYKSPTPSTNYYYKSPSPTKYYKSPAPSKYYKSPSPTKYYKSSPPPPKYYEQ
PPTYYNSPPPPYYEESTPSYKSSPPPPKTYEQSPTYYSPPPPPYYKETPTYASPPPPVKYEEPVTYASPPPPTFY

>Solyc01g005880.1.1|PACid:27302838
MRSFGNLGQWSLIAFALVICFVASTVVADYSYDYTSHSPSPYYKKPEKHVEHSPSHYYYKSHAPSKHYYKTPVVTKY
YKSHAPSKHYYKTPIVAKYYKSHAPSKNYYKTPVVTKYYKSHAPSKHYYRAPVVVKYYKSPAPSKQYYKAPVVVKYY
KSPASSKKYYKAPTPSKYYYKSPSPAKYYKSPAPSKHYYYKSPSPSEYYKSPAPSKYYKSPAPQKYYKSPVYYKSPP
PSPTYYEKSPSYYKSPPPPLYYKESTPSYKSPPPPLYYKESTPSYKSPPPPPYYEESAPSYKSPPPPPKYYEKLPTT
HDSPPPPTYSSPPPPQEYEQSVIYASPPPPPASPPPTYY

>Solyc01g097710.1.1|PACid:27303832
MENLGKLGKWSFLTVALAICLVASTVVADYSYEYTSPSPSHNSNKYYKSPSPSNYHVPTPYYKKPYPSHYYNKSSAP
SKHAYYKSPSPAKYYKSHVPSKHYYKSPVATKYYYKFPTPSKYYYKSPASSKYYYKSPTPSKYYYKSPVATKYYKSP
TPSNHYYKSPSPSKYYYKSPTPSKRYYKSPSPTKYYKSPTPSTRYYYKSPSPTKYYKSPVYYKSPPPPPTYYEKSPS
YYKSPPPPPKYYEQSPSYYNSPPPPPKYYEQSPSSYKSPPPPPKYYEESATYHKSPPPPYYPESSPSYKSPPPPPKS
YKQSPLTYNSPRPLKTYEQSPYYEQIPTYSSPPPPFEKYEQSVTYASPPPPSPTY

>Solyc12g038700.1.1|PACid:27307667
MSHGKKHLEGVVVEVTCKDGDKKTVSYGTTKINGKFSITVEGFEYQCQKPKPTPAPCYYKSPLPPSPTYVYKSPPAP
SPKYVYKSPPPPTLTYVYKSPPPPAYYYKSPPPPTKSTPPHYYYKSPPSPSPKPTPAYYYKSPPPPTKSPPPSYYYI
SPPPPSPKPAPIYYYKSPPPPSPSPPQPYYYKSPPSPSPPPTYYYKSPHSPSPSPPSPYYYNSPPPPSPSPPPHYYY
KSPHPPSTSLPPPHY

>Solyc04g071100.1.1|PACid:27277702
MSPPPPVKPYHPTPVYNSPPPPTPVYMSPPPPVKPYHPTPVYKSPPPPTPVYKSPPPPVKPYHPSPTPHHPTPVYKS
SPPHYIYSSPPPPHHY

>Solyc04g007670.1.1|PACid:27278290
MSKLPIIFLIFVIYSTQITNGLLNNPRKLDEANIVNKCGSCPCNNPCNTPSPPPPPPPVPSPPPPPPKKPPSSVYCP
PPPFEGGAGGGNGGSGGGYSPNPPSDAQYIYMNGPPGNLYPVDQYFNGAKSCFSSGFSLLISGFFLGIIALI

>Solyc10g084190.1.1|PACid:27280595
MITKSPPPPPPKVFSPPPPMVTKSPPPPPPPKVFSPPPPPSPFLISDSPPPPPPPHPPVPISDPFLPVFGP

>Solyc06g009760.1.1|PACid:27284219
MGTLSPPHSQFIFHIFTLFFLISLNHYDSQAMSTITAMAKDQLSCTMCSSCDNTCQPISPPPPPPQLYPPSSSGYIC
PPPPPVYVSPPNNSGGNNGGEGDANGSGNYPPPTNNYPTPPPPNPIVPYFPFYFHSPPPPTSKSIHLERHPLVTCLI
FGVAIFFLL

>Solyc06g068530.1.1|PACid:27284338
MTKKIIERPSISSPPPPKPSPNSKQISTYDFANNNELAVYYWDAPNKGNSLGRSSKSPPPPPRPAPSTGQLFSS

>Solyc03g116200.1.1|PACid:27290285
MKETLIISVLCVVTLQYLFLVSADRLSLPHNETFGMPLSSPPPHEPSPPPPX

>Solyc05g008110.2.1|PACid:27299082
MSRFVIFLLFFVVGENKCGGCPCNKPCIPPSPPPPPPSSPPPPPPKKPPSGYDCPPPPYSGGGGGGGGGGSNIPYPP
NSQYIYMTGPPGNLYPVDHDFGGAKRSFSSGFSLLIGGFFLGLFYFW

>Solyc01g006390.2.1|PACid:27299718
MKMFIMLMMATVLFCSLHQVVMAREVVVNVNNNINDLIPCWPIEWPWCSSPPPPPTPSPPPSPTSCLASDQEKIKTC
MFNTTSIDECCPIFKSTLGTSCPCYNYAEDLDNQVLITLDTYCDIDSPCKSVQVIKLSKE

>Solyc01g065510.2.1|PACid:27301370
MGVMLAFSILIITMMITGMQNEGNLVNANETTWAGSKYQIECTMCAACDNPCATPSPPPPQPPSPPPPPSTSYNCPP
PPSPPSSGGNYYYSPPPPPPSSSGGGGGGNYYYSPPPPYQNYPSGPTPPPPNPIVPYFPFYFYNPPPPSQSAAIKLT
DFSPKSSIFFTVFIALLIFH

>Solyc08g078020.1.1|PACid:27304210
MALSHPMTIFSLFLTFLALTAAQSPMMAPTMPPSTMSMPPTTSTTTPPPMSSMSPPPSAMSPTPSTMSPPPMSPMTP
SMSPMGPMTPTMSPMDSPPAPAGPGMAPGMSTPGPAPGPMGGESMASPPPSSGFVHGISISMAMVAIIGSVALFF

>Solyc12g038710.1.1|PACid:27306542
MYKSTPPTSPSPPPPYVYKSPPPPSPSLPPPYVYKSPPPPSPSRPPPYVYKSPPPPSPSPPPPYVYKSPPPPSPSPP
PPCV

>Solyc12g038860.1.1|PACid:27307567
MRIQGGGVAKGRQYLPQILTALAILVIANAVSADPYVFSSPPPPLYDYMSTPLPSPSLPPPYVYKSPPPPSPSPAPP
YVYNHLLPLSITTCTTCV

>Solyc12g098800.1.1|PACid:27308244
MRLNNGGPAKGRQYLPQILVALAILAVANVVSAEPYVYSSPPPPVYEYKSPPPPX

>Solyc09g075580.1.1|PACid:27311307
MFAPNSQFQFFLLLFFSFSLNNVQITQAMSTITAISKDQIACEMCTTCENPCQPIFPPPPPPPSPSPPLLCPPPPSP
PPPPPPSPPPLPPVNYCPPPPPPPARRSCPEDCSLQPRSPPYSIYPYFSPPSTYPNKSTRYKNQPIVTYLIIAIACL
FKTFGLM

>Solyc10g050470.1.1|PACid:27281819
MTVPPNIKALGCFFLLILLNLVSSSYALTNDEVASIARRQLISGTGDLPNIVEYEKNIDLKFENPRLKRAYIALQAW
KKAIYSDPSKFTSNWEGANVCAYNGVFCDKALDDPNINVVAGIDLNNADIAGHLPVEIGFLADVSLVHINSNRFCGI
IPKSINNLKLLDEIDFSNNRFVGPFPKIVLELPKLNFLDIRFNDFEGPVPSELFDKNLDAIFLNNNRFVSTIPPNIG
NSNASVVVLANNRFHGCIPHSIGKMENTLDELVFTNNNLSGCVTEEISKLKSLKVFDISKNKFVGSLPRGLELMQKV
EVLDIASNMFRGEIPKAICTLPNLANFTFSYNYFENLDEGCAPPLPRNPVLDDKENCLKGKPGQRSEKDCQSVLSKI
VDCSKDKCRSNGEGPAPKDPKTPMPPKKTPSTPKPPKTNPPKHVPSPKPKSPPVQSPPTPVASPPPPVPSPKPKPSP
VQPPPTPVPSPKPMPPPVHSPPLPVSSPPPSVPSPKPMPPPVHSPPPPVSSPPPPVPSPKPMPPPVHSPPPPASSPP
PPVHSPPPPVSSPPPPVHSPPPPTSSPPPPVHSPPPPVPSPPPPVHSPPPPTSSPPPPVHSPPPPISSPPPPVPSPK

PMPPPVHSPPPPVSSPPPPVPSPKPVPPPVHSPPPPVSSPPPPPVFSPPPPKPVQSPPPAPLASPPPPVFEDVVLPP
NLGSIYASPPPPIFPGY

>Solyc11g005150.1.1|PACid:27295065
MFSDPFNFTANWTGPNVCSYGGVFCAPSLMDDSIRVVAGIDLNHADIAGSLVAELGLLTDLVLFHLNSNRFCGVVPK
TFSHLKLLRELDLSNNRFVGGFPKVVLSLPSLKFLDLRFNDFEGPVPSGLFDKDLDALFLNDNRFRFGIPENLGNSP
VSVLVFANNDLGGCIPASIGKMGKTLNELILMNDNLTGCLPMEIGLLNKLTVFDVSFNKIQGSLPSTVSKMRSVEEL
NVAHNKLTGVIPASICQLPRLQNFTYSFNYFTGEAPVCAATRSGDGQENCIVGKKNQRSAKECSSDDAKPYDCRKSK
CYSPFATSPSTKPKPKPKPRGPPPPTWKSSGSHNKRSPPPPKSTPLPPPAPYKKSPTYQHRSPPPPTHKISPVTHHS
PPPPSPVYYHPSPSSPPPPVYYSPPPPVYHEPPPTYKPKSPPPPPTPSYEHPKTPSPLPPTPSYEHPKTPPSHEHPK
TPSPPTPSYEHPKTPSPPTPSYEHPKTPSPPPPTPSYEHPQPQSPPPPPTPSYEHPKTPSHPTPPTPPCNEPPPPPP
NSHWEPKPSPPYTYSSPPPPSPSPPPPTYYYSSPPPPSPSPPPPTYSSPPPPPPFYENIPLPPVIGVSYASPPPPVI
PYY

>Solyc01g108900.2.1|PACid:27301164
MVALGCFLFFLVSFCSFSPSYFALSDIEAASIARRQLLSNNGQLSNTYESEMTINMKFENARLKKAYVALQAWKKSI
YSDPTNFTANWEGSNVCAYNGVFCDNALDDPNISVVAGIDLNHADIAGHLPVELGLLADVSLIHINSNRFCGIIPKS
ITNLTLLDEIDFSNNRFVGPFPDVVLELPKLNYLDLRFNDFEGQVPSALFEKNLDAILINNNRFHSTIPESLGNSNA
SVVVLANNKFYGCIPSSIGKMGNSLDELVFTNNELSGCLPEEITKLTSLTLLDISGNKFVGSLPQDLKSMQKVEIFD
IASNKFMGNVPKNLCTLPSLKNFTFSKNYFESMDETCRPSESKQVKIDGNENCLGGRSEQRTEKECFPVVSKPVDCS
KGHCGVSREGQSPKDPPKTVTPSKPSTPTTPKPNPSPPPPKTLPPPPPKTSPPPPVHSPPPPPVASPPPPVHSPPPP
VASPPPPVHSPPPPPVASPPPPVHSPPPPVASPPPPVHSPPPPVHSPPPPVPPPVHSPPPPVHSPPPPVHSPPPPVA
SPPPPVHSPPPPVASPPPPVHSPPPPVHSPPPPVHSPPPPVASPPPPVHSPPPPPPVASPPPPVHSPPPPVASPPPP
VHSPPPPVASPPPPVHSPPPPVHSPPPPVHSPPPPVASPPPALVFSPPPPVHSPPPPAPVMSPPPPTFEDVALPPTL
GSLYASPPPPIFQGY

>Solyc12g088950.1.1|PACid:27306494
MQVYRCFLSLLLISAVLFSSSCALSDHEASLLARRQLSTLPENGNLPDNYELEVNVEYTFPNSTLRRAYIALKSWKE
AIYSDPSEFTSNWKGPDVCNYKGVFCSPALDDPNVTVVAGIDMNHADIAGYFPVELGLLTDVALFHLNSNRFCGIVP
ESFSKLTLMHEFDVSCLHSCLIKIFDALFLNNNRFVSTIPETLGNSSASVIVFANNKFHGCIPSSIGKMSNLDEIVF
MNNDIGGCLPVEVGLLKNGKVFNVAGNLLSGILPKTLDGLSHVEELDISHNTLTGFVPENLCSSLSLKKFVFSFNYF
NGEAKGCEARSRKDMSLDDTNNCLPGRDLQVRNEVPPKPAPVPKPRPNPKSPPTPKKVSPPPTQAVSSPPLVHSPPP
PVHSPPPPLAHSPPVSSPPPLVHSPPPPPPHSIQSPPPPLAHSPPPPVHSPPPPPVHSSLPPQVHSPPPPVHSPPPP
PVHSPPPPQVHSPPPLVHSPPPPPVHSPPPPQVHSPPPPVHSPPPPPVHSSPPPQVHSPPPPVHSPPPPPVHSPPPP
QVHSPPPPVHSPPPPPVHSPPPPQVHSPPPPVHSPPPPPVHSPPPPQVHSPPPPVHSPPPPPVHSPPPVSSPPPPIV
HSPPPLAHSPPPPVHSPPPTFEHVVLPPNIGSIYASPPPPIFQGY

>Solyc12g006980.1.1|PACid:27306904
MKEKTQTPICSLFFLVLCFLCCSVFTEHTNAEFTVSENGPLTDSEAQFIKHRQLLYYRDEFGDRGENVKIDPSMVFE
NDRIKNAYIALQAWKQAIISDPFNITMNWVGPNVCSYTGVFCAPALDNPKIRTVAGIDLNHGDIAGYLPEELGLLTD
LGIFHINSNRFCGTIPRKLNKLKILFELDLSNNRFAGKFPYVVLSLPKLIFLDIRFNEFEGNVPSQLFDKPLDAIFI
NHNRFAFELPENFGNSPVSVIVLASNSFHGCLPASIGNMSNLNEAILMNNGLRSCLPAEIGLLKNLTVFDVSFNELM
GPLPENFGGLVNLEQLNVAHNMLSGTIPKSICQLPKLENFTYSYNFFTGEPPVCLALPEFHDQRNCLPNRPVQRSPG
QCKAFLSKKIHCSAFKCHKFVPVLPPPPPLSPPLPAPPPPPVYSPPPPVYNPPSPPPPPPPSPPPPSPPPPVYSPPP
PSPPPPPPVYSPPPPPPSPPPPSPPPPPPPSPPPPSPPPPVYSPPPPPPPPPPPVYSPPPLPYCVRSPPPPPPPPNS
PPPNSPPPPLAHSPPPPSPYYYNSPPPPPPNSPPPPPNSPPPPPPTYIYSSPPPPNSPPPPPPPYVYSSPPPPHSPP
PPSPLPCIEPPPPPPPPCIEPPPPPPSPSPSPPPPPMYHYNSPPPPSPSPPPPPVYHYNSPPPPSPSPPPPPVYYYNSP
PPPPPSPPPTPVYEGPLPPVIGVQYASPPPPPFY

>Solyc04g006930.2.1|PACid:27278738
MDSSLLYEGSAPPTDNAPPTSSPPPTPSSPPVSSPPPPSSSPPPSSPPPASSSPPPSSPPPESTSPPPSSPPPKSSS
PPPASPPPAPTPPPPVSSPPPTSSPPPALSPPPHVQNPEPPPAPQSHGNSSPPQSPEPKKGSSSSSPPSPPSSNSPG
DGGRGGDTPPSPTSKSSENSPPSPALGPSKDSPPSPATLSPSNSPSSDSPSNSSTPTSVIQWSPPPPSSNGAQSSLS
PPSSQVPSNHNSGNRSTESPKPAGGNSSGTAESASIGIVIVLLLVGIIGAVVWCIRKRKKKNSVHCSGYVKPISTCS
SPKSADPGGFNNAKTWFTYQELVEATNDFSAKNELGKGGFGSVYKGYLADGRYVAVKQLNIGGSQGEREFRAEVEII
SRIHHRHLVSLVGYCISENKRLLVYDYVSNNTLYFHLHAQGRPVMNWPTRVKIAVGAARGIAYLHEDCYPRIIHRDI
KSSNILLDDNFEAHVADFGLAKLAQDAESHITTRVVGTFGYMAPEYASTGKLTEKSDVYSFGVVLLELITGRKSVDT

SQPSGQENLVEWARPLLSRALQKEEFDLLADPCLEKNYVGTEMFRMIEAAASCVRHSSAKRPAMGQIMRAFDGMAIH
DLSNGMKVGESAIHSAALQSAEISWFRKMLKDIFMRFDLNGDGSLTQLELAALLRSLGLKPGCGDQLHVLLSKIDHN
GNGSVEFDELVDAIMPDMNEDILINQDQLMELFQSFDRDGNGYITAAELAGQMSKMGHPLTYRELSNLMQEADTNGD
GVISFNEFANILGKSATDFLGLNTVSESVA

>Solyc02g062790.2.1|PACid:27285769
MDSSPGTASSNDTDSKGTPSSNGTPPSSSSSDSKNDSPSSSSSSPSPKNESPPSSSPPPQSPPPSSNASPPPKNDSP
PPSSPPPSSPGSDASPSKDNNNDNNNNNDNNNNNNNNNNTNNQSPNPSSNTPSQSPPPSSTSNNPSTPNSPKNPPSF
LSFSPPPPVLTPAPPHAHLSPPPRSGSNEPSPANNDSSSTVGIVAGIAVGGIVIVALIILCVWCSRRKKKQRYYMAP
GPKGGDPYYNNNNQWNRRQSMDHVMKVPQSEMGTQSGWAGATPHQQGANSSGEFGSGYSGQAAPLPSQSPNMGLGGF
SQSQFTYEELAKATDGFSQANLLGQGGFGYVHKGVLNDGRVVAIKSLKAGSGQGEREFQAEVEIISRVHHRHLVSLV
GYCIANGQRMLVYEYVDNKTLEFHLHGKGQPVMDWETRLKIALGSAKGLAYLHEDCQHRIIHRDIKAANILLDLNYE
ALVADFGLAKLTSDNNTHVSTRVMGTFGYLAPEYASSGKLTEKSDVFSYGVMLLELITAKKPVDPSNMMEDSLVDWA
RPLLTRALEEEKYDGLVDARLEGNYDTDELHRMIGCAASSIRHSAKRRPKMSQIVRALEGNSSLEDLNDNPKPSKVA
SFAGAPTQSYDTGMYNADMMKFRKMIMPTQEFSSSPSE

>Solyc02g085430.2.1|PACid:27287295
MQSPPSTASSDKDETTSSSPSNSSPSPPPPAPPTSPPPESPPPVNSPPPSKESSPPSSPPPSASESPPPPSDSTTTS
LNAPEASPPTSSKFNPPPPVSSGSPSLSAGKLSPPPPSEKSAPFEKPDGAQHSGKPAAGSSGNNNGSPSSNSGSSDM
FVVTGIAVAGLMIFATIIVCLIYWRRKKKEHYYTNPPPGPSRRPPKGAGSTDPYYKGPKPMEHIIKVPTTVYTSNDS
TFAVNGQALVPSPSLGGFSKSQFTYEELARATSGFSKANLLGQGGFGYVHKGVLVDGTVVAVKSLKSGSGQGEREFQ
AEVDIISRVHHRHLVSLVGYCIADRQRMLVYEYVPNKTLEFHLHGKGHRVMDWGTRLKIALGSAKGLAYLHEDCHPK
IIHRDIKAANILLEDNFEAKVADFGLAKLSSDNLTHVSTRVMGTFGYMAPEYASSGKLTDRSDVFSFGIVLLELITG
KRPIDPSNIMEDSLVEWARPLLKTALEEGKYDELVGAPFEGNNVPKELHRLVVCAAASTRYSAKRRPKMTQIMRALD
GDSSLEDMSDGAKTDTYDTSAYNADMIKFREMISNQEFNSSEYGGTSDYGLNPSSSSGDSSELDHHQRSRG

>Solyc03g034060.2.1|PACid:27290982
MEDSPSSRSSNSSSSTPPSSSSSPPPPNSSPPPSPPESSPPPLKSDSSSPPPPKSDSSSPPPPPSKSDSPSPPPPES
SPPPKSESSPPPPSPNQSPPPKSESSPPPASPPPTESSPPPASPPPTESSPRKPSDSSPSSDNDKPNTQSPPPSSNS
NSSSSSSSSSPSPTTNTKPSPLKNSSFNEPNSPPSSTFSPPAPISPESSQFPPGKALPTSPPSRDASSNKSPGSLGT
INSPTHEQSSSSTVAIVAAAAVAGLLIFAIIIVCLLCNRKKKKQPYYVDPAHPPKGGDPYYNTGNYSSPHTDHIVTL
APPPGVMGTPQEGGRGWTPPPPPPGANTSSEFSSGYSSHVPGGGPATIPSPNYGGLSKIQFTYADLATATGGFSDAN
VLGQGGFGFVHKGVLTDGNVVAVKSLKSGSGQGEREFQAEVEIISRVHHRHLVSLVGYCIADGQRMLVYEFVPNGTL
EYHLHGKGRPVMDWGLRLKIALGSAKGLAYLHEDCHPRIIHRDIKGANILLDNNYEAMVADFGLARLTEDNNTHVST
RVMGTFGYLAPEYASSGKLSEKSDVFSFGVMLLELITGRRPLDTTNKLMDDSLVDWARPFLTKALEENNYDELVDPR
LEGNYDPDELQRMVACAAASVRHSARRRPKMSQIVRALDGDSSLEDLNEKAGKNNTANFGGASGPASDIYDTRAYNA
DMVKFRQMVMTNQDMNSSEYGNTSDYGLHPSDTSSDFSSDNNHSGPNKQSK

>Solyc05g010140.2.1|PACid:27299318
MSVVVSPPPSLAPIPVPPIVLSPPPQLSSPPPASQPNATAPPVSSLPPTLPPQSSPPPALPTLPQSPTPSNVTSPPP
SNVTSPPPMASPPTESAPPTVSPPPIPPASSPPPVSSPPPSSSPPPQSSPPPASAPPPTSSPPVSSSPPPEVEPPPV
SPPPQPTVPTSSPPPPPKDDPPPASPPPQPTTPPSSPSPPPKVDSPPFSPPPPAQNPDPTAPPQSPEPPKGSPPVNS
PPSPASVPPRGSPPTPASDPPANTPPSPAFTPPQGSPPTPSLEPPKNTPPSPSVPSGGTTTDRPSDNAAGSANSSSS
SGIGTGGTVAIGVIVAVLLLGIVGLVGWCLWKRKKKAFRPSGGNVMPTPSGSTPNSDSVLLKIQESTPDTRNGTGNK
FLNSPGGSGGFGNPKIWFTYEELVKATGDFSAENLLGAGGFGSVYKGCLPDGRDVAVKQLDIGGRQGDREFRAEVEI
ISRVHHRHLVSLVGYCISENRRLLVYEYVPNNTLYFHLHAEGRPVMDWTTRVKIAVGAARGIAYLHEDCNPRIIHRD
IKSSNILLDINFEARVSDFGLAKLAQDAKTHVTTRVVGTFGYMAPEYASTGKLTEKSDIYSFGVVLLELITGRKPVD
TSQPLGDESLVEWARPLLSHALEKVEFDQLVDPRLERNYVIPEMFQLIEAAAACVRHSAAKRPGMGQIMRAFDNMSA
SDLTNGMKVGESTIYNSADQSAEIRLFRRMAFGSPDFSSDFFSQGTQHSGESAEDRV

>Solyc01g010030.2.1|PACid:27300075
MSIVSPPLSSSTPFAAPPIALNSPAPIYQPNENSSSIASPQSAPLVATSPLPESPPPPTNITLPPPASSPQPQPPIS
TPTPPAESAPPIISPPPTPSGPPASSLTPPPSSSPPASPTESPSSSTPSSPPPLPPQPEPTRPASSPPPPSTHPPAL
SPPHSSIRPPALSPPPPVQNPRTPPAPRSPGNPSPEPPKRSPPSSKPPANAPPSKPPRNTPDSPALVPPKNSPPSPD
ALPPINSASSSSPPTNSSPPQSTNGAQPSLTPPSPSFPSGNTTTSSNSSAVKSKDIGDGGIGIGGSVAIGIILVFLL
LGIVGAAGWCICKRKKKDSGLSGGYILPTTLISSPKSGSASLKFRESKPEIGNGAGSNIANTTGNTGGLGQSKPWFT
YQELLEATNEFSEHNLLGEGGFGSVYKGCLANGRDIAVKKLNIYGSQGEREFRAEVEIISRIHHRHLVSLVGYCIYE

NGRLLVYDYVSNDTLYFHLHEQGRPVMDWATRVKIAVGAARGIAYLHEDCCPRIIHRDIKSSNILLDNNFDARVADF
GLAKLAQDAKSHVTTRVMGTFGYMAPEYASSGKLTEKSDVFSFGVVLLELITGRKSVDASQPLGEESLVEWARPLLG
NALEKEEFDQLTDPRLGTNYIDSEMFQMIEIAAACVRHSAAKRPGMGQIMRAFDSMLMSDLTNGMKVGESAIYNSAE
QSAQIRLFRRMAFPSQDVNSDFSSQSTNYSRELSEHV

>Solyc12g007110.1.1|PACid:27307399
MSTPTPVTALAPSNATNSPPPSTPATTPPPASPTPPATAPPPASPTPPATAPPPSTPAQAPPPARTPTPPAATPPPD
ASASPPPPTTPAPSSNPPPSTTPAPSGSPSPPSTTPSTPSPPARGNSPPSPSGGSPSPPSGGRPSPPAVSSPPSDEG
SSGISTGVVVGIAIGGVLILAILSLLFIFCKKKKRRNHEPVNYYVPPPPPPLGVKADPHGGQMHHWQQNAPPSADHF
VAMPPKPSPPPVGGLLPSHAPPRAPSPQPQPYMNSSGASSNYSGSEIALPPPSPPMSLGFSQSTFTYEELVRATDGF
SDANLLGQGGFGYVHKGILPNGKEVAVKQLKAGSGQGEREFQAEVEIISRVHHKHLVSLVGYCITGAQRLLVYEFVP
NNTLEFHLHGKGRPPLDWPIRLKIALGSAKGLAYLHEDCQPKIIHRDIKAANILVDFNFEAKVADFGLAKLTSDVNT
HVSTRVMGTFGYLAPEYASSGKLTEKSDVFSFGVMLLELITGRRPVDSTQSYIEDSLVDWARPLLTRALEDEKFDGL
VDRRLENDYNHNEMARMVACCAACVRHSAKRRPRMTQVLRALEGDVSLSDLNEGIKPGHSTVYSSYTSSDYDTLQYN
EDMKKFRKMALATSQEYASSDQYSNPTSEYGLNPSGSSSEGHQTAEMETGRMRKDSRGFSGSKGFSGTS

>Solyc10g006540.2.1|PACid:27281441
MGVRGVAQLSVFIIILFTIVAADPASNGLEDHLLANQISSVGINQELAEELWLNCRLELVHSNEAVEDLEFSHPGEE
ANGIFTNRRSLTKNKEKNANLLTKEALMGCLVKKNLLFLISGEEKQSPTWYTRCMDFLFSWYGEPRRRELVQVGDAP
APAPAPVTSSSETPNSPPPARPPTLPFFPRDYNDSSKTSGPSDQSSTSQNSTSDGQSNKKKSNTKTVLVAVLVTAAV
TFIVVALFFICYCKVCGVGYRKGKNDERPLLSLSISDYSVASRHGNAGSHSVSDDSHNKMGKTFYMESNTLNGSKSE
IPLGTVTGIAVAAAGVSQQIPPERMGMQGQPPLKPPPGRVNPFEVPTSPAPPPLPPAKITNLAAPSPPPPPPPPKPS
AGSPRPPAPGPPPPPPIPVRAKAGPRPPPPPGPGATPPRPPPTGLKPPRPSPLGSNASSSASVEGSESDPSKTKLKP
FFWDKVLANPDHSMVWHQIKSGSFQFDEDMIESLFGYAHADKDKNGPKKDSMSQDASKQYVQIIDQKKAQNLAILLK
ALNVTTEEVCDALKQGNELPSELVQTLLKMAPTADEELKLRLYNGDLSRLGPAERFLKVLVDIPFAFKRLESLLFMC
SLEEEASMAKESFATLEAACTELRKSRLFHKLLEAVLKTGNRMNDGTFRGGAQAFKLDTLLKLSDVKGIDGKTTLLH
FVVQEIIRSEGIRAARARDRGSVSSIKSDDLPEDQSQDSEEYYRSTGLQVVSGLSSELENVKKAAILDADSLTGTVS
KLGRALKESRDFLNSEMKNVDDENRFHQTLKSFVQNAELDITWLFEEEKRIMALVKSTGDYFHGNAGKNEGLRLFVI
VRDFLIILDKVCIEVKNAQRKLNGTPKKENVASKTSESSNPPSLDLRQKLFPAITDRRIDDSSSDDDGSESFSSSSM
VNRETRFSF

>Solyc06g069670.2.1|PACid:27283148
MQDDDNIETWKEELEHHAFQDCSFVEGHRKLEENMDSTQQASKTNGLGDRDHLINSEAVISDVSSRKDSVAQTSGAN
VMSQGDNKQDIEGPLSQERQGSQFTADINKGKNEKAPSSLKKQLASSSKLAVDGIGPKNKSKQKESQGTLSRLAKPN
AVSRWIPSNKGSYTNSMHVAYPASRYKSAPPALAVTKDSQSGFKSKSPSPRASSEAKILAGAGRIPKKRSSCPASLD
MSTTVKEAPVAMTYSSESVESQALDLQASPSSPLPSFQYPSTLSLELPPIQEASTNMPASTMLPESSQKDHIITASL
THHSSPPPPPPPPPPPPSQIPSHNQNVKSLSPPPPPPPPTNSLRKTQIGRPPPPPPPPGGAPPPPPPPGGGAPGPPP
PPPPGGRAPGPPPPPPPGGSAPGPPPPPGAPRAPGGGPPPPPPFGAKGPGAAGRGLPAGRAPALSRSRSNLKPLHWS
KVTRALQGSLWEELQRHGGPQIAPEFDVSEIEILFSATPKASSAGKAGAKKKSADSKPDKVQLVDLRRANNTEIMLT
KVKMPLPDMMAAALAMDESILDADQVENLIKFCPTKEEMDLLKGYTGDKELLGKCEQFFLELMKVPRVESKLRVFLF
KIQFKAQVTDFKKSLTTVNSACGEVRSSLKLKEILKKILFLGNTLNQGTARGSAIGFKLDSLLKLADTRATNNKMTL
MHYLCQVLASKTPELLDFHESLVSMEAASKIQLKSLAEEMQAIIKGLEKVKKELESSVNDGPVSEIFCKTLKEFIGA
SEAEVGSLRDLYSVAGRNADALALYFNEDPAKCPFEQVTATLLNFVRLFRKAHEENLKQAEQDKKKVQKEAEMENAE
KKQLKKEGTLVLSQTRSLQYRSIGFGFLWAQERTSNIKAREPGNSRSALQRETEKDTFLSGLLECFAERSSRKHTVV

>Solyc02g092470.2.1|PACid:27286697
MRAAGLSIFFILSLLSSFTCQFQDLVVKEKNRRILHQPLFPVSSTPPPDSEISPPPPAEPVNSQPFFPEVPTGTTPD
QTHQPQVTPANGTPVSNSVATQTAKPVKKVAIAISVGIVTLGMLSALAFYLYKHRVKHPDETQKLVRRNSDQRINEE
SRTPPSTFLYIGTVEPPAKTSAMTDSNDATGSPYRKLSSVKRMDSRYRPSPDLQPLPPLSKPQPPPSINSPTAMSSS
DEESHDTAFHTPQGSTVSNEEGYYTPSLRESYSSNKNYVPYSKRTSPRSRLSDSSAEVKHTMIPSIKQAPVPPLPPR
QPQGGLIEQLPPEPPLQYTRPELYVPKRANFSSPPPPPDMTRLQLISNQAQQISKAPPPPPPPPPPLPPPPPPLPFST
PHKPEGSQRNVPSAAYQQMVKTESRSPTPKSTPGSEKTSTSEEQNGGASSLERHDSSDIDPSKPKLKPLHWDKVRAT
SDRATVWDQLKSSSFQLNEDMMESLFGCNSANSVPKEATRKSVLPPAEKDNKVLDPKKSQNIAIILRALNVTKDEVS
EALLNGNPEGLGPELLETLVKMAPTKEEEIKLREYSEDASKLGSAERFLKTVLDIPFAFKRVEIMLYRANFDGEVKD
LRKSFQTLEVASEELKNSRLFLKLLEAVLRTGNRMNVGTNRGDARAFKLDTLLKLVDIKGTDGKTTLLHFVVQEIIR
SEELDSEPPGEDLSNKANIKFKEEDFKKQGLQVVSGLSRELGNVKKAAAMDSDVLGSYVLKLAVGLDKARSVLQYEK

QGMQGNFFESMKVFLKEAEDGIVRIRAEERKALSMVKQVTEYFHGDAAKEEAHPLRIFVIVRDFLSILDNVCKDVRR
MQDQTVVGGARSFRIVATASLPVLSRYNVKQERSWDDNSLSP

>Solyc03g044060.2.1|PACid:27290584
MRAHPICNFLSTLLIFITITVAEHTQIQHLIDINPLTTRRILHQPLFPSTSSSPPPPPPQINSEPIFPTPDHPFFPE
VPTVQTPDQPQQQQIQPNGTPVSRNNSIAPQQTSPTKKYAIAVSVGIVTLGMLSALGFYIYKHKTKHPDETQKLVG
NNSFQEESRMPPSNFLYIGTVEPSAASQTQTNHSIVASPYRKLSPPKKSDRYRPSPELQPLPSLRNNPTFFNPPAII
NSSEDDDETKIYKPYNITSASSTSIMTRSNNSIPHSKRTSPRLSLSCSSPDIKRAIIVPSVKQTSPRSPPPPSQLQQ
HKKPTLTYVPQRVKFSQPPPPPDMSRLKSPNNQLQVTSKASAPPPPPPPPPPPLSTSRALGGKVGSRITNSIERSRP
QRSFSSVKPQSSSPTPRSGYVVSEKINHLEEQNGGAMLDTETIDESKPRLKPLHWDKVRATSERATVWDQLKSSSFQ
LNEDMMESLFGCNSVNSVTNEVIKKPVRPTVEKENRVLDPKKSQNIAIMLRALNVTKDEVSEALLDGSAEGLGPELL
ESLVKMAPTKEEEIKLRDYNGDTSKLGSAERFLKAILDIPFAFKRVEAMLYRANFDAEVKDLRKSFQTLEEASQELK
NSRLFFKLLEAVLRAGNRMNVGTNRGDARAFKLETLLKLIDIKGTDGKTTLLHFVVQEIIRSEGLSSDDNNLANLSS
NIKFKEQGLQVVAGLSRELCNVKKAAGMDSDVLSGYVSKLEAGLVKVRSVLQFEKTGMEGKFFESMKVFLKEAEDGI
VRIKAEERKALSMVKEVTEYFHGDAAKEEAHPLRIFLIVRDFLCILDSVCKDVGMMQDRTMVGAARSFRIATTASLP
ILNRYNARQDRSWDDDSSSP

>Solyc07g005480.2.1|PACid:27292844
MKNLLRLNLYLMHLLLLHPKPKDEKPTLSQPDTYLLPLAAQKPDHKGEPASSPHLTGLSLSPSPLVPSPHTPPVKDK
LVTGTIPSASQATPPRTQCSPIVSLKDDRPAMSQSDAPLLPRTLQQPAYEGESTSPPRTAQQLGDEAAKKEPTRPLH
PSKSCPPSLAPPNSSSPSAAIPFIKPIEQQFVQSSNFSPPPPPPPSQHPIPLLNENAGSVGGSPQSPAPPTPPLKEH
SVFRGVPSLPPPPLPPPPPRDSYPLSTPPVLSKNSVCTSEPLGPVPTLKESSAFRDKSPPLPPPPPPPPCQLNTTQI
LSPNSASISEPPPPPPPPTPPLKEKVVPLTREPPPPPLPPPLPVQSMKENSSFNGGPAPPPPPPLPASQASKPANVS
VMPPPPPPPALGSRQSVPSAPPPPALGSRQTVPSAPPPPVPSLKPDLKAGSGMIQSASKGSNLPTSPSPPPPSAPPP
GLKGRGPLSRTMNSRSQSSKKLKPLHWLKISRAVSGSLWAEAQKCSDAPKAPEIDISELESLFSAAVPTSGQGSSGG
KRNSGTSMGQKLEKVQLVDHRRAYNCEIMLSKVKIPLHEMLSSVLALEDSALDVDQVENLIKFCPTKEEMETLKGYK
GEKEKLGRCEQFMLELMQVPRTESKLRVFSFKIQFESQVSELRKSLNIVNSAADQIKGSSKLKRIMQTILSLGNALN
QGTARGSAVGFRLDSLLKLTETRARNNKMTLMHYLCKVLADKLPELLDFSNDLSSLEPCAKIQLKFLAEEMQAISKG
LEKVVQELSMSENDGAVSENFRKALKEFLCYAEGEVRSLAQLYSGVGRNVDTLILYFGEDPARCPFEQVITTLLNFR
RMFNQALEENRKQVEFERKKAEKEAMEKQKMSHSEKT

>Solyc12g019480.1.1|PACid:27307679
MLSSLCIINLVVFTFFSFTTTTAGAGVYYRHRRILHQPFFPVVDSLPPSSLPSITHPPISSPQPQPKFPFSTLSPPE
TPLTQNPFFPVFSSPPPPPPPPPIRSDSYATFPANISSLILPQTSSHSSAKPISGKLIAIIISVSVLSAAFLTSLVA
YFLHYYRQGKVEEKMYYQRTDSLRLVPPNATPSDGVVIKKHLPSPPPPPPAMEVQRHTPTSNSSEFLNLGALVSSRE
VESPEVQPADGVAVNFQRLGSPELLPLPPLPRQHYQQTRKNGAGYSGEDDENDDEFFSPRGSSGDKGSPSQTVSSSH
ATPYEVPLQTQNRFLYSNSNSPSESSLLNSPSLEFNLSPKISDSSARISESSLRNLGGFRSYVSMKVPPPPPPAPPP
RFWEAPQVPKSVEAENGGPPVLVAPSMPVLGHHVNGNIKSSEAVERRNDEIIKPKLKPLHWDKVRATSDRAMVWDQL
KSSSFQLNEEMIETLFTANCSNSNPKDGITRLVQPVLNQENRVLDPKKSQNIAILLRALNVTNEEVCEALLEGNADT
LGSELLESLLKMAPTKEEERKLHEFKDESPFKLGPAEKFLKAVLYIPFAFNRVEAMLYIANFDSEIEYLKRSFETLE
TACEELRNSRMFLKLLEAVLKTGNRMNVGTNRGDALAFKLDTLLKLVDIKGAEGKTTLLHFVVQEIIRAEGSRLSGA
DDQNPIVEKTLQDEVEFRKIGLQVVSRLSGELTNVKKAAAMDSDIISNEVAKLAAGIAKITNVLKLNEELVSSENSR
KFSESMNGFLKTAEQEIINIQAQEGVALSMVKEVTVYFHGDSAKEEARPLRIFMVVRDFLSILDQVCKDVGRMTDRT
IISSGRQFPLTVDAGLPQVFPGYNVRQHDSSSEEETTPPTS

>Solyc12g010110.1.1|PACid:27308455
MALFRKLFYRKPPDGLLEIGGDRVYVFDRCFSTDVWEEKNYKGYVASVITQLRDHYPDVSILAFNFREGESESLIAN
DLSEHDVTIMDYPRHYEGCPLLSMEMINHFLRSSESWLSLGQQNVLLLHCEWGGWPVLAFMSAALLIYRRHSNGEQK
TLDMIYKQAPHDLLQMQPLNPIPSQLRYLQYVARRNSNTQWPPLDRALTLDCIIIRMIPNCNGKGGCRPIFRIYGQD
PFIVSDRSPKSLFSTQKKSNVVHYYKQEECELVKIDINCHIQGDVVLECISLHDDREKMMFRTMFNTSFIQSNILIL
DRDEVDTLWDAKDQFPKDFRTEVLFSDMDVAASVVPVDLSCFDKKDGIPEEAFAKVQEIINSTDWLNQKGDAASKTL
EQITESNLIPEKLGSPPDTIATTKLIDQATLENPQERQELAALVNNTKGLAQSTLEQQVGSSSEAYRSNKQEAMFQL
VETKESSASVTSTPVSPHESKTVEHPSLHGKERSKLKEVSSLSEIKDRPLMTNVLMSPTPPPLRTKDQGIVTGKPLS
PALTPPALFTPLKDKLDVPSPSQPTPPRDQSTNSIYLKDDATTVSKPDTSLLPLGMPLSPCENDIIAKMEPSLPTFP
SGSPSPSGPIPPNIKHLEEKLVSKNGTSPSPPPLPHLVPVLEENSAYVCGTPQTPAPPTPTLKQNLTFSGGLSPPPP
PPPPSLPPPTPSSSPCLLPTTPGFTKNCSSISGPPQPPSPPTPPLNERLVSKGGMLPTPPPPPPPPPPLPEQPVKEG
FSLIEKPCPPPPPPPPLPEKHVKEDFSLTEKLCPPPPPPLPSSQTSKPTELYAVPPSPPPPSMVSPLKDNNNLPKSV

PSVPPPPVPFPKVNNMPASPSPTPPIAPPPSKNYRRMLSSTMTSRSNSTKKLKPLHWLKISRAVQGSFWAEIEKCSY
ASKSSVIDMPELVYFFSVQNLDQVGSGRNGNSKTKFGQKIQKVQLVDHRRAYNCEIMLSKVKIPLHDMLTSVLALED
SALDIDQVENLIKFCPTKEEIEVLKGYKGEKEKLGRCEQFMLELMQVPRIESKLRVFSFTIQFQSQVSELRNNLNIV
NSATDQIRGSSKLKGVLQTILYLGNALNQGTARGSAAGFKLDSLLKLTDTRSWSNKMTLMHYLCKILTDKLPELLDF
SKDLSSLEPALKIQLKYLAEEMQAITKGMEKVVDELSMSENDGPMSENFCKALTEFLSCAEGQVSSLAQLFSDVGKN
VDSLIIYFGEDPARCPFEQVVSTLMSFQRMFNQALDENRKQLEFERKKAEKEAKEKQSTSASNHKKT

>Solyc10g084200.1.1|PACid:27279494
MDKNILFLFFLCITLHLASTVSAQLQVGFYNTKTRCPSAETIVRDTVRTRFSSDRSITAALLRMYFHDCFVRGCDAS
ILIDSKNTKNKKSEKNAGANGSVRGYELIDQIKSKLEAKCSNTVSCSDIIALATRDAVALSGGPSYSIPTGRRDGLV
SDPSQVNLPGPSITVPQAIQSFKSKGFNVNEMVTLLGGHTVGITHCNFIQGDRLSRADGSMDSKLFSSLRKTCSSNG
GSPVFLDQNTSFTVDNSFYKQLRLKKGILKIDQLLASDRSTSGIVANFASNPKAFQQAFANALIKLGNTQVLVGKSG
EIRKNCRAFNPPPPKITKSPPPPPPKIIKSPPPPPPKITKSPPPPPPKVFSPPPPMITKSPPPPPX

>Solyc02g078050.2.1|PACid:27288463
MAFRSSKAMVLIQVLSLVVASFSELSFGEVAENSSLDNDREDNEIISTKGLGIGRVPKKSPSTPAPAKRPSPPAKSS
PPPSPPAKSPPPPPPTPTKSPSPSPPPPTKSPSQSSPPPPTKSPPPPTKSPSPPTQPPINRPPQPSPPATQVPIRRT
PPPSPPTSKPPIRSSPPPPTDYDEPPDIEPPVDQEPPPVSHEPPPINEPPIIPFPPTLSPPVKLPPPTIHPTGKPLI
VVGRVNCKSCSNRGLPSLFKAFPLHGASVKLVCHNNGRKAHVQTALTDKNGDFSITPISLTRADVHKCRVYLVKSPK
SICNVPTNFNNGKSGAQLKPILPPGNHGPGHGLMFDFFGVGPFIFEAPNKFPCRK

>Solyc02g089250.2.1|PACid:27288610
MMNIRISWVWILVLFLGFTFHNLSTSQAENTRHRHHSTAVIEGTVFCDTCFQQHFSGASHFISGATVAVECADSVRR
SSFYKEVKTNEHGKFSVDLPISVSKHVKKIKGCSVKLIKSSEPYCAVASTATSSSLHLKSRTQGTHIFSAGFFTFKP
LNQPDLCSQKPSIQKSKKKLTDPQKSAISNPNDPTFYPPIQDPPAPGTLLPPLPRLPPLPLLPPLPDLPGLPISPVP
KDSSKYYSQSETAAKPNFFNPIGGLPLPPNPLLPPPSILPPNPFLPPPSIIPPIIPSPPPSIFPPLFPSPPPSIFPP
LIPSPPRSPPSLFPPLIPPFIPGLTPSPPPPPPSLFPPIIPGFPGVPPASTSSSLQKKNPSP

>Solyc03g116230.2.1|PACid:27289195
MKETAISVLGLLSLFLLEVVSANEIINGTFVLEGINKNASGGVFANGDECGMQANHRSKCPSGMCCSIWGWCGTTSE
YCGSGFCQNQCTGPSPHGSCGMQGGGTKCPSGQCCSLLGWCGTGSDFCKPEICQSQSDGRLCPRGQCCSVDGWCGTT
TDYCASGLCQSQCPFTPPPSPPPPSPPPSQYQCGMQNGGTKCNRTGECCGISGMCGNTYEYCFPGYCQMQCPGPYPE
GRCGWQADGKSCPTGQCCGNAGWCGIGPGFCDPIFCQSQCSGAPISTAKRDGGIRSFLLNAALV

>Solyc03g121230.2.1|PACid:27290033
MTRDLIFTFLICISLFALEANAQIDQSGFISIDCGIPRGSNYTDVATGLQYVSDSAFVDTGSNATISSEFHSDDLEQ
QLYTLTSFPQGKRNCYTIRVAEGKGKKYLIRASFLYGNYDGKSQLLINFDLHLGVDFWTTIQIVNASVPLYEEIIHI
LSSDFVQVCLVNKDRGTPFVSALELRLLNSTIYKTTSGSLQTFVRLDLGSTATQIVRYQDDIYDRLWWPYNNDENTI
SLSTTSTIDNTNSYLPPTKVLSTAIAADNDTDGISLWWEPANSTDEYYIYLHFAEIEANHVNRQFNIYVDGELYQGS
FAPDYMSVTTIFSTSALKPKDRHQISLNKTGNSIRHPTINAIELYKVVKQIINPQTNDLDVEAIMNVKSTYEVKKNW
QGDPCGPVADIWKGVTCNFNGDLPTVISLDLSSSELQGAISPYIISLTKLETLNMSNNQLTGEVPPNLSQLAFLQEL
DLSNNLLTGKVPANLAKLPYLKKLYLKGNSFSEKIPEELLEKSRNGSLDLRYDEFSPPPPPKDEISPPPKDEFLPPK
DGNNLSAGALAGIIASVVVLGVLALLLLLWFIIRRMKNKKGKITETETTVQSPKIALELKNRQFTYSQVLHMTNNFQ
RVLGKGGFGTVYLGYVDNRDVAVKMLSPSSVQGFKEFQAEASLLMSIHHKNLISIVGYCVEGNHIGIIYEYMANRSL
DMQLSDRNPNALTWEERLHIALDAAQGLEYLHHGCQPSIIHRDIKSSNILLDDKFQAKLADFGLSRTLPTGEGSHVT
TIIAGSPGYLDPDYYRTNKLTEKSDVYSFGVVLLEIITGRHLLGKHDKIYVITWVNGMINDNGDVSKVIDPRLGGQV
DLNSAKRIVALAMACVSLEPTNRPAISLVVTIIKQCLRQMIENYDSN

>Solyc05g026060.1.1|PACid:27299350
MAITLIYLFVFFHVCFFFVSSSLSDFSILDYNFDSDPFKADYSPPSPPPPSPPPHPPSLTCHELQGIGSLNTTCQLN
YNLNFTRDVYLEGTGNLFILQGVLLSCHVPSCSLTINITGSLELKANAKILAGSVYIVAGNASFISGSVINVTGLAG
DPPEQSTGTPKEYQGGGGGHGGRGASCVMDNKKLPEDVWGGDTYSWKSLEQPFSYGSKGQSTNKEDNYGGNGGGKIW
LDVKDIFDACGTLLADGGDAGIKGGGGSGGSIYIKSKKMIGGGKISASGGNGFAGGGGGRVSVEVFSRHDDSEFFVH
ERCRSHAYLQEDQL

>Solyc01g006400.2.1|PACid:27303568

MATMKMFMMLVVAAIMFCSHHQVIVAREVVVDVVEDGNKLNLWPWEIPCYLPWPFPFPRPYPCPPPRPRPRPRPCPP
PPPPRSPPPPPRSPPPPPPSPSPPPPAPATTCSPGDKARVKTCMFNTTSIDECCPTFQSILGTSCPCYKYAEDLDNQ
VLITLEAYCDVSTPCRSPPPKPSCPASDQEKVKTCMFNTTSIDECCPTFNSILGTSCPCYKYAEDLDNQVLITLESY
CDVNNPCNGAQVIKLSKDDE

>Solyc01g107710.2.1|PACid:27303647
MSRSYVVGLIVILLVIVVEATPPGIADHPSHSHCSDDEIKKCKNLPHVCPKFCPNGCITECRSCKPICIDGPSPSPP
PPTPYHPHPPQSPPPTPGHPHPPKSPKKVKCKSKDKKHSKCYNQEHTCPSTCPGTCKVDCVSCKPVCSCDKPGAVCQ
DPRFIGADGITFYFHGKKDKDFCLVSDSNLHINGHFIGKRNENMKRDFTWVQAIGILYDTHNISVGALKTATWDDAI
DRLYLNFDGESILLPENEGARWQSETRPTTSITRTSDTNEIIIEVENILKITAKVVPITEEESRVHNYGITEDDCFA
HLELGFKFFALSDEVSGVLGQTYRRNYVSRVKMGVLMPVMGGDKEFSASGLFNADCSVAKFQAVNEGLLNNLEIPNL
KCSSGMSGRGVVCRR

>Solyc12g098780.1.1|PACid:27308148
MRLQGGGPTKGRQCLPRILTVLVILVVANVVSADPYVYSSPPPPTYEYKSPPPPSPSPPPPYVYKSPPPPSPSPPPP
YVYKSPPPPSPSPPPPYVYTSPPPPSPSPPPPYVYKSPPPPSPSPPPPYVYKSPPPPSPSPPPPYYYKSPPPPSPSP
PPPYYYKSPPPPSPSPPPPYYYKSPPPPSPSPPPPYYYKSPPPPSPSPPPPYYYKSPPPPSPSPPPPYYYKSPPPPS
PSPPPPYYYKSPPPPVKSPPPPYYYSSPPPPKKSPPPPYLYTSPPPPVKSPPPPYYYSSPPPPKKSPPPPYHYTSPP
PPVKSPPPPYYYNSPPPPKKSPPPPYHYTSPPPPVKSPPTPYYYKSPPPSTHYYPPHHQFVVKVVGKVYCFRCYDWK
HPKMSHDKKHLKGAVVEVTCKAGDKKIVSYGTTKNNGKFSITVKGFEYRKYGAKACKAKLHYAPKGSKCSIPTNLHW
GIKGANLKVKSKNKYEVVLYAKPFAYGSKTPYAECKKPKPTPAPYYYKSPPPPSPTYVYKSPPPPTPTYVYKSPPPP
AYYYKSPPPPSPKPX

>Solyc09g098510.2.1|PACid:27309150
MRLFAGGPGRGRHYLPHILVAILALVDIVSADPYIYASPPPPYVYKSPPPPSPSPPPPYVYKSPPPPSPSPPPPYVY
KSPPPPSPSPPPPPISPTTILLQVSSTTITITPTSILLQITTTTIPITSTSLLLQVSSTTIAISSSTILLQVPTTTI
TVSSSTILLQITTTPITITTPSLLLSLSTSTREVSSSSILLQLTSPTRKITSSSSIHLWFSATTSPLLSSWKSLAIF
HNHSNFSFSHKISDGFLRRRLIVPCSNAINKMFNFAIERLIPSDLDHFASASLVSLLHIVLCVSSRGKWLK

Solanum tuberosum

>PGSC0003DMP400004216|PACid:24377933
MRLQGGGLAKGCQYLPQILTALAILVVANVVSAGPYVYSSPPPPMYEYKSTPPPSPSPPPPYVYKSPPPPSPFSPPP
YVYKSPPSPSPSPPPPYVYKSPPPPSPSPPPPYVYKSPPPPSPSPPLPYVYKSPPPPSPSPPPPYYYKSPPPPSPSP
PPPYYYKSPPPPSLSPPPPYYYKSPPPPSPSPPPPYYYKSPPPPSPSPPPPYYYKSPPPPSPSPPPPYYYKSPPPPS
PSPPPPYYYKSPPPPSPSPPPQYYYKSPPPPTKSPPPPYYYNSPPPPMKSPPPPYYYSSTPPEKKSPPPPYHYTSPP
PPVKSPPPTYYYSSPPPPKKSPSPPYHYTSPPPPIKSPPTTPYYYKSPPPPTHYYPPHHQLVVKVVGKVYCFRCYDW
KHPKMSHGKKHLKGAVVEVTCKAGDKKIVSYGTTKINGKFSITVEGFEYRKYGAKACKAKLHNAPKDSKCRIPTNLH
WGIKGANLKVKSKNNYEVVLYAKPFAYGSKTPYAECKKPKPTPAPYYYKSPPPPSPTYVYKSPPPPSPKYVYKSPPP
PTPTYVYKSPPPPAYYYKSPPQPTKSPPPHYYYKSPPPPSLKPTLVYFYKSPPPPTKSPPPLYFYKSPPPPSPKPAP
IYYYKSPPPYYYKSPPPPSPSPPPPYYYKSPPPPSPSPPPPYYYKSPPPPSSSPPPPYYYRSPPPPSPSPSPPYYYK
SPPPPSPSPPPTYYYKSPPPPSPSPPPPYYYKSPPPPSPSPPPPYYYKSPPPPSPSPPPPYYYKSPPPLSPSPPPPY
YHKSPPPPSPSPPPPYYYKSPPPPSPSPPPPFPSPPPPYYYKSPPPPSPSPPPHYYYKSPPPPSPSPPPPYYYHSPP
PPVKSLPPPYYYSSPPPPVKSPPPPAYIYASPPPPTHY

>PGSC0003DMP400008279|PACid:24379593
MKFNGGGPTRGRRLRLILVALAVLVVANVVSADPYVYSSPPPPAYEYKSPPPPSPSPPPPYVYKSPLPPSPSPPPPY
VYTSPPPPSPSPPPPYVYKSPPPPSPSPPPPYVYKSPPPPSPSPPPPYYYKSPPPPSPSPPPPYYYKSPPPPSPSPP
PPYYYKSPPPPSPSPPPPYYYKSPPPPSPSPPPPYYYKSPPPPSPSSPPPYYYKSPPPPKSSPPPPYYYNSPPPPKK
SPPPPYYYSSPPPPKKSPPPPYYYNSPPPPKKSPPPPYHYTSPPPPAKSPPPPYYYSSPPPPKKSPPPPYHYTSPPP
PAKSSPSPYYYTSPPPPKKSSPPPYYYTSPPPPTHYYPPHHHFVVKVVGKVYCFRCYDKEYPEKSHAKKHLKGVVVE
VTCKAGDKEIVSYGTTKINGKFSITVEGFEYSKYGAKACKAKLHNAPKDSKCSIPTNLHWGIKGANLKVKSKNKYEV
VLYAKPFAYGSKTPYAECKKPNPTPAPYYYKSPPPPSPTYVYKSPPPPSPTYSPPPPSPSPPPPYYYKSPPPPSPKP
APVYYYKSPPPPSPSPPPPYYYKSPPPPSPKPAPVYYYKSPPPPSPSPPPPYYYKSPPPPSPSPPPPYYYKSPPPPS
PSPPPPYYYKSPPPPSSSPPPPYYYMSPPPPSPSPPPPYYYKSPPPPSPSPPPPYYYKSPPPPSPSPPPPYYYKSPP
PPSPKPAPVYYYKSPPPPSPSPPPPYYYKSPPPPSPSPPPPYYYKSPPPPSPSPPPPYYYKSPPPPSSSPPPPYYYM
SPPPPSPSPPPPYYYKSPPPPSPSPPPPYYYKSPPPPSPSPPPPYYYHSPPPPVKSPPPPYYYNSPPPPVKSPPPPV
YIYASPPPPTHY

>PGSC0003DMP400008280|PACid:24379594
MKFNGGGPTRGRRLRLILVALAVLVVANVVSADPYVYSSPPPPAYEYKSPPPPSPSPPPPYVYKSPLPPSPSPPPPY
VYTSPPPPSPSPPPPYVYKSPPPPSPSPPPPYVYKSPPPPSPSPPPPYYYKSPPPPSPSPPPPYYYKSPPPPSPSPP
PPYYYKSPPPPSPSPPPPYYYKSPPPPSPSPPPPYYYKSPPPPSPSSPPPYYYKSPPPPKSSPPPPYYYNSPPPPKK
SPPPPYYYSSPPPPKKSPPPPYYYNSPPPPKKSPPPPYHYTSPPPPAKSPPPPYYYSSPPPPKKSPPPPYHYTSPPP
PAKSSPSPYYYTSPPPPKKSSPPPYYYTSPPPPTHYYPPHHHFVVKVVGKVYCFRCYDKEYPEKSHAKKHLKGVVVE
VTCKAGDKEIVSYGTTKINGKFSITVEGFEYSKYGAKACKAKLHNAPKDSKCSIPTNLHWGIKGANLKVKSKNKYEV
VLYAKPFAYGSKTPYAECKKPNPTPAPYYYKSPPPPSPTYVYKSPPPPSPTYVYKSPPPPTPTYVYKSILL

>PGSC0003DMP400008281|PACid:24380792
MRLQGGGPAKGRQYLPQILTVLVILVVANVVSADPYVYSSPPPPPYEYKSPPPPSPSQPPPYVYKSPPPPSPSPPPP
YVYKSPPPPSPSPPPPYVYTSPPPPSPSPPPPYVYKSPPPPSPFPPPPYYYKSPPPPSPSPPPPYYYKSPPPPSQSP
PPPYYYKSPPPPSPSPPPPYYYKSPPPPSPSPPPPYYYKSPLPPSPSPPPPYYYKSPPPPSPSPPPPPPYYYKSPPP
PSPSPPPPYYYKSPPPPSPSPPPPYYYNSPPPPVKSPPPPYYYSSPPPPKKSPPPPYHYTSPPPPVKSSPPPYYYSS
PPPPKKSPPPPYHYSSPPPPVKSPPTPYYYKSPPPPPKKSSPPPYYYTSPPPPTHYYPPHHQFVVKVVGKVYCFRCY
DWKHPEMSHGKKHLKGAVVEVTCKAGDKEIVSYGTTKINGKFSITVEGFEYRKYGAKACKAKLHNAPKDSKCSIPTN
LHWGIKGANLKVKSKNKYEVVLYAKPFAYGSKTPYAKCQKPKPTPAPYYYKSPPPPSPTYVYKSPPPPSPTYVYKSP
PPPTPAYVYKSPPPPTYYYKSPPPPSPKPSPIYYYKSPPPPSPSPPPPYYYKSPPPPTKSPPHPYYYKSPPPPSPKP
APVYYYKSPPPPSPSPPPPYYYKSPPPPSPSPPPPYYYKSPLPPSPSPPPPYYYKSPPPPSPSPPPPYYYKSPPPPS
PSPPPPYYYKSPPPPSPSPPPPYYYKSPPPPSPSPPPPYYYKSPPPPSPSPPPPYYYKSPPPPSPSPPPPYYYKSPP
PPSPSPPPPYYYKSPPPPSPSPPPPYYYHSPPPPVKSPPPPYYYSSPPPPVYIYASPPPPIHY

>PGSC0003DMP400008282|PACid:24381417
MRLQGGGPAKGRQYLPQILTVLAILVVANVVSADPYVYSSPPPPTYEYKSPPPPSPSPPPPYVYKSPPPPSPSPPPP
YVYKSPPPPSPSPPPPSPSPPPPYVYTSPPPPSPSPPPPYVYKSPPPPSPSPPPPYVYKSPPPPSPSPPPPYYYKSP
PPPSPSPPPPYYYKSPPPPSPPPPSPSPPPPYYYKSPPPPSPSPPPPYYYKSPPPPSPSPPPPYYYKSPPPPSPSPP
PPYYYKSPPPPSPSPPPPYYYKSPPPPSKSPPPPYYYNSPPPPVKSPPPPYYYSSPPPPKKSPPSPYHYTSPPPPVK

SPPPPYYYSSPPPPKKSPPPPYHYTSPPPPVKSPPPPYYYNSPPPPTHYYPPHHQFVVKVVGKVYCFRCFDWKHPKM
SHGKKHLKGAVVEVTCKAGDKKIVSYGTTKNNGKFSITVEGFEYSKYGAKACKAKLHNAPKDSKCSIPTNLHWGIKG
ANLKVKSKNKYEVVLYAQPFAYGSKTPYAECKKSKPTPAPYYYKSPPPPSPTYVYKSPPPPSPKYVYKSPPPPAYYY
KSPPPPTKSPPPHYYYKSPPPPSPKPTPVYYYKSPPPPTKSPPPPYYYKSPPPPSPKPEPVYYYKSPPPPTKSPPPP
YYYKSPPPPSPKPAPVYYYKSPPPPSPSPPPPYYYKSPPPPSPSPPPPYYYKSPPPPSPSPPPPYYYKSPPPPSPSP
PPPYYYKSPPPPSPSPPPPYYYKSPPPPSTTLLLQVSSSTFPTTSLTLLLQIPSSTISVTTSTILLQVTTTTFPISS
TTLLLQVSSSTFPTTSLTLLLQIPSSTIAFSSTTLLLPLSTSTGKVSTSTLLLQLTSSTSEITSSPSIRLCFSTTSN
SLLSISRSSTILPNQSSE

>PGSC0003DMP400001496|PACid:24381922
MAKIAYLLTTLLVALVSLSFPSECKANYYYTSPPPPTPVYKYKSPPPPVYKYKSPPPPPPVYKSPPPPVYKYKSPPP
PVYKYKSPPPPPPVYKYKSPPPPVYKYKSPPPPPPVYKYKSPPPPVYKYKSPPPPPPVYKYKSPPPPVYKYKSPPPP
PPVYKYKSPPPPVYKYKSPPPPPPVYKYKSPPPPVYKYKSPPPPTPVYKYKSPPPPVYKYKSPPPPTPVYKYKSPPP
PVYKYKSPPPPTPVYKYKSPPPPVYKYKSPPPPTPVYKYKSPPPPVYKYKSPPPPTPVYKYKSPPPPVYKYKSPPPP
VYKYKSPPPPVYKYKSPPPPVYKYKSPPPPVYKYKSPPPPVYKYKSPPPPVYKYKSPPPPVYKYKSPPPPVYKYKSP
PPPPPVYKYKSPPPPVYKYKSPPPPVYKYKSPPPPVHKSPAPYYYTSPPPPSHY

>PGSC0003DMP400001493|PACid:24382126
MGKMASLVATLLVVLVSLSLASESSANYQYSSPSPPNESYHPSPTPYNPAPVYKSPPPPTPVYNSPPPPKETYYPPH
TPLPPPPTPIYKSPPPPTPIYKSPPPPKEPYYSLHTPVYKSPPPPTPVYKSPPPPTPIYKSPPPPKEPYYPPHTPVY
KSPPPPTPVYNSPPPPKEPYYPPHTPIYKSPPPPTPVYNSPPPPTPVYNSPPPPTPVYNSPPPPTPVYNSPPPPTPV
YNSPPPPTPVYNSPPPPTPVYNSPPPPTPVYNSPPPPTPVYNSPPPPTPVYKSPPPPKEPYYPPHITSRHLHQLLFT
SRHHHLMSHTTLHTPQSISRHLHQLPYTIHHLHQLQSISHHHHPKSHTTLHTSYKSPPPPTPVYKSPPPPNEPYYPP
HTPTYKSPPPPTPVYNSPPPPKEPYYPPHTPTYKSPPPPTPIYKSQPPPKEPYYPPHTPTYKLSPPPTPVYNSPPPP
TPVYKSPPPPHYLYTSPPPPYHY

>PGSC0003DMP400001489|PACid:24383537
MGKMASLVATLLVVLVSLSLASESSANYQYSSPPPPVHVYPSPPHHPVYKSPPPHHHHPVYKSPPPSEKPHYPPHTP
VYKSPPPHHIHHPVYKSPPPPTPIYKSPPPPKTPHYPPHTPVYKSPPPHHHHPVYKSPPPPTPVYKSPSPPKDPHYP
PHTPVYKSPPPPTPVYKSPPPPKEPHYPPHTPVYKSPPPPTPVYKSPPPPKRPYHPPPTPYHPTPVYKSPPPPTPVY
KSPPKPYHPAPVYKSPPPPTPIYKSPPPPVKPYYPAPVYKSPPPPTPVYKSPPPPVKPYHPSPTPYHPTPVYKSPPP
PTPVYKSPPPPVKPYHPSPTPYHPTPVYKSPPPPTPVYKSPPPPVKPYHPSPTPYHPTPVYKSPPPPTPVYKSPPPP
VKPYHPSPTPYHPTPVYKSPPPPTPVYKSPPPPVKPYHPSPTPYHPRPVYKSPPPPTPVYKSPPPTHYVYSSPPPPY
HY

>PGSC0003DMP400001492|PACid:24385089
MGKMASLVATLLVVLVSLSLASESSANYQYSSPPPPKRPYHPSPTPYHPAPVYKSPPPPTPVYKSPPPPEEPYYPPH
TPVYKSPPPPTPVYKSPPPLVKPYHPAPVYKSPPPPTLVYKSPPPLVKPYHPAPVYKSPPPPTPVYKSPPPPTPVYK
SPPPLVKPYHPAPVYKSPPPPTPVYKSPPPPTSVYKSPPPPVKPYHPAPVYKSPPPPTPVYKSPPPPTSVYKSPPPP
VKPYHPAPVYKSPPPPTPVYKSPPPPTSVYKSPPPPVKPYHPAPVYKSPPPPTPVYKSPPPPTSVYKSPPPPVKPYH
PAPVYKSPPPPTPVYKSPPPHYIYSSPPPPHHY

>PGSC0003DMP400001491|PACid:24385299
MGEMASLVATLLVVLVSLSLASESSANYQYSSPPPPKEPYHPSPTPYYPAPVYKSPPPPTPIYNSPPPPKETYYPPH
TPVYKSPPPPTPIYKSPPPPKEPYNPPHTPTYKSPPPPTPVYKSPPPPTPVYNSPPPPKEPYYPPHTPMYNSPPPPK
EPYYPPHTPTDKSPPPPTPIYKSPPPPKEPYYPPHTPIYKSPPPPTPVYKSPPPPTPVYKSPPPPKEPYYPPHTPIY
KSPPPPTPVYKSPPPPKEPYYPPHSPVYKLPPPPTPIYKSPPPPNEPYYPPHTPVYKSPPPPTPVYKSPPPPKEPYY
PPHTPVYKSPPPPTPVYNSPPPPTPVYKSPPPPKEPYYPPHTPIYKSPPPPTPVYNSPPPPTPVYKSPPPPKEPYYP
PHTPAYKSPPPPTPIYNSPPPPTPVYKSPPPPKEPYYPPHTPTYKSPPPPKEPYYPPHTPTYKSPPPPTPVYKSPPP
PHYLYTSPPPPYHY

>PGSC0003DMP400001495|PACid:24386311
MGKMASLVVTLLVVLVSLSLASESSANYQYSSPPPPKEPCHPSPTPYHPAPIYKSPPPPTPIYSSPPPPKKRNYPPH
IPVYKSPPPPKEPYYPPHTPTYKSPPPPTLVYKSPPPPIPVYNSPPPPTEPYYPPHTLVYKSPPPPTPIYKSPPPPK
EPYYPPHTLTYKSPSPPTPVYKSPPPPTSVYNSPPPPRETYYPLHTPLYKSPPPPTLIYKSPPSPKEPYYPPHTPTY
KSPPPLTLIYKSPPPPNEPYYPPHTPVYKSPPLPTPVYKSPHPPTPVYKSPPPPKEPYYPPRTPIYQSPPPPTPIYN
SPPPPTLVYKSPPPPKEPYYPPHTPVYKSPPPPTLIYKSPPPPNEPYYPPHTPVYKPPPPPTPVYKSPPPPKEPYYP

PHTPTYKSAPPPTPVYKSPPPPKELGYPSHTPTYKSPPPPKEPYYPPHTPTYKSLPPPSPVYNSPLPPTPVYKSPPP
PHYLYTSPPPPYHY

>PGSC0003DMP400001490|PACid:24386342
MASLVATLLVVLVSLSLASESLANYQYSSPPPPKEPYHPSPTPYHPAPVYKSPPPPTPVYKSPPPPKEPYYPPHTPL
YKSPPPPKEPYYPPHTPVYKSPPPPTPVYKSPPPPKEPYYPPHTPTYKSPPPPTPVYKSPPPPKEPYYPPHTPTYKS
PPPPNEPYYPSHTPTYKSPPPPTPIYKSPPPPKEPYYPPHTPTYKSPPPPNEPYYPSHTPTYKSPPPPTPIYKSPPP
PKEPYYPPHTPTYKSPPPPNEPYYPSHTPTYKSPPPPTPIYKSPPPPKEPYYPPHTPTYKSPPPPKEPYYPPHTPTY
KSPPPPTLVYKSPPPSKEPYYPPHTPTYKSPPPPTLVYKSPPPSKEPYYPPHTPTYKSPPPPTLVYKSPPPSKEPYY
PPHTPTYKSPPPPTLVYKSPPPSKEPYYPPHTPTYKSPPPPTLVYKSPPPSKEPYYPPHTPTYKSPPPPTLVYKSPP
PSKEPYYPPHTPTYKSPPPPTLVYKSPPPSKDPYYPPHTPTYKSPPPPTLVYKSPPPSKEPYYPPHTPVYKSPPPPT
PVYNSPPPPYYLYTPPPPYHY

>PGSC0003DMP400020659|PACid:24387859
MGSFGKLGHWPLLAFTLAICLVASTAVADYSYGYTSPSPSKYYKSPSPSKHQVPSSYYKKPIKPVEHYPSHYYKSPT
PSKYHSHSYKHSPSHYYKSPSPSKYHSHVTVTYKHSPSHYYKSPAPSKHNYKSPSPIKYYKSHVPSVPYYKPSPSYY
KSSPPPPPKYIEKSPTYYKSPPAAYYKPSPSYKSPPPPPKYIEKSPTYYKSPPPPPYYKPSPSYKSPPPPPKYIEKS
PTYYKSPPPPYYKPSPSYYKSSPPPPKYDEKTPVYYKSPPPPYYKPSPSYYKSSPPPPKYDEKTPVYYKSPPPPYYK
PSPSYYKSSPPPPKYDEKTPVYYKSPPPPYYKPSPSYYKSSPPPPKYDEKTPVYYKSPPPPYYKPSPSYYKSSPPPP
KYDEKTPVYYKSPPPPYYKPSPSYYKSSPPPPKYDEKTPVYYKSPPPPYYKPSPLYYKSSPPPPKYVEKSPAYYKSP
PPPYYKPSPSYYKSPPPPPKYIEKSPANYKSPPPPYYKSSPSYYKSPPPPPKYVEKSPTYYKSPPPPYYKSSPSYYK
SPPPPPKYIKKSPTYYKSPPPPYYKPSPSYYKSPPPPPKYIEKSLVYYKSPPPPYYKPSPSYYKSPPPPPKYIEKSL
VYYKSPPPPYYKPSPSYYKSPPPPPKYIEKSLVYYKSPPPPYYKPSPSYYKSPPPPPKYIEKSPVYYKSPPPTYYSS
PPPPTNYYKQTPTYASPPLPAKYQQSPTYSSPPPPPTYY

>PGSC0003DMP400052946|PACid:24396861
MKLITFFALFFLLQSTTILFFVAADYSVATSQYGGNTPPPIISQPPPSHVYQPPSSPSTPPLRHAPPHHAHPPPSHG
HVPPFVGSPPPPSPVISPPNPPPSYGSPPPSHGGGHPPSQGHKPHSPSYGHAPPPTEHTPPPGGYHPPSHHQPSPPS
HHGHHPPPTPTYSPPPSQMKPPLPPYSQPSPPPHSPHSYGHAPPPKGHTPPKGHHQPSPPSHHAHSPPPTYSSPPPP
YSQPSPSPHSPPSHGYSPPPKGHTPPRRHNSPRHHQPSPPSHHVHPPLTHAQPPPTPIYSPPPQVQPSPPTYSPLPP
TYSPPSHSPPSHGHVPPPKGHKPSRGHHTHSPLSHHAHPPPTYSPPPSLPTYSPPSPAQSPPSPPTYSSPPPAQLPP
PPPSAKSPPHLPTYSPSPPAQSPPPPPTHSLLPPTYPPPPPPPPPTYSPHPAQSPPPSSPIYSLPPPSTPLPPTYS
PPPLTSPPPPTHSPPPSAYSPPPPSTTYSPLSPPYGLPLSTP

>PGSC0003DMP400037451|PACid:24399233
MRLLAGGPGRGRHYLPHILVALLAILAVVDIVSADPYIYASPPPPYEYKSPPPPSPSPPPPYVYKSPPPPSPSPPPP
YLYKSPPPPPPPPPPPYVYKSPPPPSPSPPPPYLYKSPPPPPPPPPPPYVYKSPPPPSPSPPPPYLYKSPPPPPPPP
PPPYVYKSPPPPSPSPPPPYLYKSPPPPPPPPPPPYVYKSPPPPSPSPPPPYLYKSPPPPPPPPPPPYVYKSPPPPS
PSPPPPYLYKSPPPPPPPPPPPYVYKSPPPPSPSPPPPYLYKSPPPPPPPPPPPYVYKSPPPPSPSPPPPYLYKSPP
PPPPPPPPPYVYKSPPPPSPSPPPPYLYKSPPPPPPPPPPPYVYKSPPPPSPSPPPPYLYKSPPPPPPPPPPPYVYK
SPPPPSPSPPPPYLYKSPPPPPPPPPPPYVYKSPPPPSPSPPPPYLYKSPPPPPPPPPPPYVYKSPPPPSPSPPPPY
LYKSPPPPPPPPPPPYVYKSPPPPSPSPPPPYLYKSPPPPPPPPPPPYVYKSPPPPSPSPPPPYLYKSPPPPPPPPP
PPYVYKSPPPPSPSPPPPYLYKSPPPPSPSPPPPYVYKSPPPPSPSPPPPYYYKSPPPPSPSPPPPYYYKSPPPPSP
SPPPPYYYKSPPPPSPSPPPPYYYKSPPPPSPSPPPPYYYKSPPPPDPSPPPPYYYKSPPPPSPSPPPPYYYKSPPP
PSPSPPPPYYYSSPPPPKKSPPPPYYYSSPPPPKKSPPPPYYYSSPPPPVKSPPPPYYYSSPPPPKKSPPPPYYYSS
PPPPKKSPPPPYYYSSPPPPVKSPPPPYYYSSPPPPVKSPPSPYYYTSPPPPTSYYPPHHPLIVKVVGKVYCYRCYD
WKYPKKSHDKKHLKGAVVEVTCKVGDKKIKSYGTTKINGKYSITVEGFDYAKYGADACKAKLHKAPKDSKCNIPTDL
HWGIKGANLKVKSKNHYEVVLSAKPFAYAPKTPYEECMKPKPTPTPYYYKSPPPPSPKYVYKSPPPPSPKYVYKSPP
PPSPKYVYKSPPPPSPKYVYKSPPPPSPKYVYKSPPPPSPKYVYKSPPPPSPKYVYKSPPPPSPKYVYKSPPPPSPK
YVYKSPPPPSPKYVYKSPPPPSPKYVYKSPPPPSPKYVYKSPPPPSPKYVYKSPPPPSPKYVYKSPPPPSPKYVYKS
PPPPSPKYVYKSPPPPPYYYKSPPPPSPSPPPPYYYKSPPPPSPSPPPPYYYKSPPPPSPSPPPPYYYKSPPPPSPS
PPPPYYYKSPPPPSPSPPPPYYYKSPPPPDPSPPPPYYYKSPPPPSPSPPPPYYYKSPPPPSPSPPPPYYYKSPPPP
SPSPPPPYYYKSPPPPSPSPPPPYYYKSPPPPSPSPPPPYYYKSPPPPSPSPPPPYYYKSPPPPSPSPPPPYYYKSP
PPPSPSPPPPYYYKSPPPPSPSPPPPYYYKSPPPPSPSPPPPYYYKSPPPPSPSPPPPYYYKSPPPPSPSPPPPYYY
KSPPPPHHHTTTSLLHHHRHHPHLHTTTNHHHHHPRHLHLPTTTSLLHHHRHLLLHHTTTSPHHHHHRLLLHHTTTN
HHHPHLHHHHLPTTTTLLLHL

>PGSC0003DMP400024837|PACid:24404277
MKINIVILSLIALLLISSTLLVEARSGINEEEKRRSRGGVGRSRRSSGGRRSRGRSSSSSRNCDPLFSYLFGSCGQW
PFPRNSQNNPFTQPMSPSPSPRRPATPFRPPILPSPPPAVIRPPVIPSPPPLIPSPPLVTPSPPPLVTPSPPPVVAP
SPPPVVASPPPASPPPVFPPPPLVPYPPPPSPPPPIPPPPLVPSPPPPESPPFFVFPPPLVASPPPPAEPVFPWLSP
PDPDDTPNFPLFSPPPLVPDFPFPTPQTPDFPPETPLVPIFSPPQQDFPPPTPLFPIFSPPPIFTLPPPEQNLPPAT
PLVPIFPPADDQPVLPDQPPNSFLPSPLIPEQPPTNFLPSPVFPDQPPNNFLPSPVFPDQPPNNFLPSPVFPDQPPN
NFLPSPVFPDQPPNNFLPSPVFPDQPPNNFLPSPVFPDQPPNNFLPSPVFPDQPPNNFLPSPVFPDQPPTNFMPSPV
FPDQPPVNFLPSPVIPEQPPETFLLSPLVPDQAPPVTVIPPFELPPQPDSPPFA

>PGSC0003DMP400059595|PACid:24408179
MTSFLPHLVWFVAILLISNSVEARHKPYSWRKYYSYKPAPTSTPNPDELPGQSPPSPQPSSSDYYISSPPSPLGWFS
PQPSLVPSPTPTSSPLSPSPNDFSSPPSPSRWAWEPSPSYTTSPQSSPVLAPSSPNQSPPSPIEWFSPRPSPVPSPA
TSPLSPSPSPSRWTPSPHSYSWKLSPSYTTSPQSSPVLAPSNPNQSPPSPIEWFSPRPSPVPSPATSPLSPSPSPSR
WTPSPHSYSWKLSPSYTTSPQSSPVLAPSNPNQSPPSPIEWFSPRLSPVHSPTTSPLSPLSYDNGSPPSPSEWAPSP
ESSVVPVPAPAPSYSDKPSQSPSPSPLPNYYYNSPPPPYVNNSPPPPSPSPSPPPPYYYKSPPPPSPSPPPPYYYNS
PPPPSPSPPPPYVYKSPPPPPSSPPPPPYYYNSPPPPSPSPPPPYYYNSPPPPSPPPPPAYYYYNYPPPPPSY

>PGSC0003DMP400011030|PACid:24412419
MPPPVHSPPPPVPSPKPMPPPVHSPPPPVPSPKPMPPPVHSPPPPVPSPKPMSPPVHSPPPPVSSPPPPVPSPKPMP
PPVHSPPPPTSSPPPPVHSPPPPVSSPPPPVPSPTPMPPPVHSPPPPTSSPPPPVHSPPPPVSSPPPPVHSPPPPTS
SPPPPVPSPKPMPPPVHSPPPPVSSPPPPVPSPKPMPPPVHSPPPPMSSPPPPPVFSPPPPKPVQSPPPAHLASPPP
PVFEDVVLPPNLGSIYASPPPPIFPGY

>PGSC0003DMP400041618|PACid:24418635
MPTKHYYKSPSPAKYYKSPAPSTHYYYKSPSPSKYYKSHTPSKYYKSPSPLKYYKSAVYYKSPPPPPTYYEKSPSYY
KSPPPPPYYKESTPSYKSPPPPYYKESTTSYKSPPPPPYYIESTPSYKSPPPPPYYKESTTSYKSPPPPPYYIESTP
SYKSPPPPPYYKESTTSYKSPPPPPYYIESTPSYKSPPPPPYYKESTTSYKSPPPPPYYIESTPSYKSPPPPPYYKE
STTSYKSPPPPPYYIESTPSYKSPPPPPYYKESTTSYKSPPPPPYYIESTPSYKSPPPPPYYKESTPSYKSSPPPPE
YNEQSPKTYNSPPPLKSYEHSPSYYSPPPPSSVY

>PGSC0003DMP400055428|PACid:24420089
MRSFGNLGLWHLLAFALAFCFVASTVVADYSYDYTSHSPSPYYKKPEKHVEHSPSHYYYKSHAPLKHYYKTPVVAKY
YKSHAPSKHYYKAPIVAKYYKSHAPSKHYYKAPVVVKYYVVTKYYKSHAPSKHYYKAPVVVKYYKSPVPSKNYHKAP
VVVKYYKSPTPSKHYYKAPVVVKYYKSPAPLKKYYKVPTPSKYYYKSPSPAKYYKSPTPSKYYKSPTPSKYYKSPSP
TKYYRSPVYYKSPPPPPPTYYEKSPSYYNSPPPPPYYKESTPSYKSPPPPPYYKESTPSYKSPPPPPYYKESTPSYK
SPPPPPYYKESTPSYKSPPPPPYYKESTPSYKSPPPPPYYKESTPSYKSPPPPPYYKESTPSYKSPPPPPYYKESTP
SYKSPPPPPYYKESTPSYKSPPPPPYYKESTPSYKSPPPPPYYKESTPSYKSPPPPPYYKESTPSYKSPPPPPYYKE
STPSYKSPPPPPYYKESTPSYKSPPPPPYYEESTPSYKSPPPPPYYKESTPSYKSPPPPPKYYEQSPTTYNSPPPPP
PAYYKQTPTYASPPPPQKYEQSVTYASPPPPPASPPPTYY

>PGSC0003DMP400039038|PACid:24420144
MKSLGKLGQWSFLTVALAICLIASTVVADYSYEYTSPSPSHNSNKYYKSPSPSNYHVPTPYYKKPYPSHYYYKSPAP
SKHTYYKSPSPAKYYKSHVPSKHYYYKSPIVTKYYKSHVPSKHYYKSPVATKYYYKSPTPSKHYYKSPAPSKYDYKS
PSPAKYYKSPSPAKYYKSPTPSTHYYYKSPSPTKNYKSPTPSKYYKSPSPTKYYKSPVYYKSPPPPPKYYEQPPTYY
NSPPPPYYQESTPSYKSSPPPPKTYEQSPTYYSPPPPPYYIETPTYASPPPPEKYEEPVTYASPPPPEKYEEPATYS
SPPPPSPSPPPPTYY

>PGSC0003DMP400045001|PACid:24420564
MRSLGKLGQLPLLAAALAICLAASTVFADYSYGYTSPSPSYSSNKYYKSPSPSKYNVPTPYYKKPVEYSPSHKYYKA
PVPSKHNYYKSPSPAKYYKSPTPSKHYYKSPIAKKYYKSHTPSKHHYKYPVPSKHYYKSPSPVKYYYKSPSPVKHYY
KSPTPSKYYYKSPSPVKYYKSPTPSKHYYKSPAPVKYYYKSPTPSKHYYKSPTPKYYNSPSPTKYYKSPVYYKSPPP
PAYYEKSPSYYKSPPPPPPKYYEESPTNYKSPPPPYYESPPPPPKTYEQPPSYYSPPPPTKYEPSTTYASPPPPTNY
EEQAPTYASPPPPTEYEPSTTYASPPPPSPSPPPTYN

>PGSC0003DMP400055427|PACid:24421042
MRSFGNLGQWPLLAFALAICFVASTVVADYSYDYTSHSPSYNSKKDYKSPSPYYKKPEKHVEHSPSHYYYKSHAPSK
HYYKAPVIAKYYQSHAPSKHYYKAPIVTKYYKSHAPSKHYYKAPVVTKYYKSPAPSKHYYKAPVVVKYYKSPAPSKN

YYKAPVPSKEYYKSPAPSKYYYKSPSPAKYYKSPAPSKHYYYKSPSPSKYYNSPAPSKYYKSPAPQKYYKSPVYYKS
PPPPPTYYEKSPSYYKSPPPPPYYKESTPYYKSPPPPPYYKESTPYYKSPPPPPYYKESTPYYKSPPPPPYYKESTP
YYKSPPPPPYYKESTPSYKSPPPPPYYEESTPSYKSPPPPPYYKESTPSYKSPPPPPKYYEQSPTTYNSPPPPPPAY
YKQTPTYASPPPPQKYEQSVTYASPPPPPASPPPTYY

>PGSC0003DMP400033774|PACid:24421327
MKSLGKLGQWFFLTVALAICLVASTVVADYSYEYTSPSPSHNSNKYYKSPSPSNYHVPTPYYKKPYPSHYYYKSPAP
SKHIYYKSPSPAKYYKSHVPSKHYYYKAPIVTKYYKSHVPSKHYYYKAPIVTKYYKSHVPSKHYYYKAPIVTKYYKS
HVPSKHYYYKAPIVTKYYKSHVPSKHYYKSPVATKYYYKFPTPSKHYYKSPAPSKYYYKSPTPSKNYYKSPSPAKYY
KSPSPAKYYKSPTPSTHYYYKSPSPTKYYKSPAPSKYYKSPSPTKYYKSPVYYNSPPPPPTYYEKSPSYYKSPPPPP
KYYEQSPKYYKSPPPPPKYYEQSPKYYKSPPPPPKYYEESPSAYKSPPPPPKYYEQPPTYYNSPPPPYYEESTPSYK
SPPPPPKSYEQSPSTYNSPPPPKTYEQSPTYYSPPPPPYYKETPTYASPPPPEKYEEPITYASPPPPSPSPPPPTYY

>PGSC0003DMP400039036|PACid:24421405
MKSLGKLGQWSFLTVALAICLIASTVVADYSYEYTSPSPSHNSNKYYKSPSPSNYHVPTPYYKKPYPSHYYYKTPAP
SKHTYYKSPSPAKYYKSHVPSMHYYKSPIVTKYYKSHVPSKHYYKSPVATKYYYKSPTPSKHYYNSPVATKYYKSPT
PSNHYYKSPSPSKYYYKSPTPSKSYYKSPSPAKYYKSPTPSTHYYYKSPSPTKYYKSPVYYKSPPPPPTYYEKSPSY
YKSPPPPPKYYEQSPSYYNSPPPPPKYYEQSPLSYKSPPPPPKYYEQSPTYYKSPPPPYYSESTPSYKSPPPPPKSY
EQSPSTYNSPPPPKTYEQSPSYYSPPPPPPPYYEQTPTYASPPPPEKYEQSVTYASPPPPSPTY

>PGSC0003DMP400039037|PACid:24422457
MFPSPVAGRRWSMLPRLLRQWLLPYYSYYKSPSPAKYYKSHFPTKYYKSHVPSKHYYKSPAPSKHFYKSPTPSEHYY
KSPSPVKYYKSPAPAKYYKSPSPTKYYKSPSPTKYYKSPTPSTRYYYKSPSLTKYYKSPTPFKYYKSPSPTKYYKSP
VYYKSTPPPYEKSPSYYKSPPPPPKYYEQSPKYYKSPPPPPKYYEQSPSSYKSPPPPPKYYEQSPTYYSSPPPPYYQ
ESTPSYKSPPPPPKSYEQSPATYTSPPPPKTYEQSPSYYSPPPPPYYKQTPTYASPPPPEKYEPSVTYASPPPPSPS
PPPPTY

>PGSC0003DMP400031352|PACid:24422667
MGTPSKLGQWPLLVYAFAFCLIAISVAADYKPHIDDSSSLSYRYRPRSPPPPPPRRRYYYKSPPPPPPRRHYYYKSP
PPPPPPPPPRRRYYYKSPPPPPPRRHHYYKSPPPPPPRRHHYYKSPPRPPPRRHHYYKSPPPPSPSPPPPRRHHYYKS
PPPPPPRRHHYYKSPPRPPPRRHHYYKSPPPPPPRRHHYYKSPPPPSPSPPPPHYYKSPPPPSPPPPPNYYHHPPPP
PPILSPPTTSPPPPHYYKSPPPPSPPPPPNYYHHPPLPPPPPPPSPSPPPPYYHNPPPPPSSSPPPPYYHNPPPPPY
YHNPPPPPTPSPPPPYYYNPPPPPPIHSPPPPPPPSPSPLPSPPLYYTCR

>PGSC0003DMP400039034|PACid:24422873
MESLGKLGKWSFLTVALAICLVASTVVADYSSEYTSPSPSHNSNKYYKSPSPSNYHVPTPYYKKPYPSHYYYKSPAP
SKHAYYKSPSPTKYYKSHVPSKHYYKSPVATKYYYKSPTPSKHYYKSPAPSKYYYKSPSPAKYYKSPSPAKYYKSPT
PSTHYYYKSPSPTKYYKSPTPSKYYKSPSPTKYYKSPVYYKSPPPPPKYYEQPPTYYNSPPPPKTYEQSPTYYSPPP
PPYYKETPTYASPPPPEKYEEPVTYASPPPPEKYEEPATYASPPPPSPSPPPPTYY

>PGSC0003DMP400039035|PACid:24423799
MCPLLITRNLIPSHYYYKSPAPSKHAYYKSPSPTKYYKSHVPSKHYYKSPIVTKYYKSRVPSKHYYKSPVATKYYYK
SPTPSKHYYKSPTPSKYYYKSPSPAKYYKSPSPAKYYKSPTPSTHYYYKSPSPTKYYKSPTPSKYYKSPSPTKYYKS
PVYYKSPPPPPKYYEQPPTYYNSPPPPYYQESTPSYKSSPPPPKTYEQSPTYYSPPPPPYYKETPTYASPPPPEKYK
EPVTYASPPPPEKYEEPATYASPPPPSPSPPPPTYY

>PGSC0003DMP400055409|PACid:24423929
MATMKMFMMLVVAAIMFCSHHQVVVAREVVVDVDVVEDGNKLQLWPWEIPCYLPWPFPFPRPYPCPPPKPRPCPPPP
PPRSPPPPPPSPPPPPPAPATTCSPGDKAKVKKCMFNTTSIDECCPTFQSILGTSCPCYKYAEDLDNQVLITLEAYC
DVVTPCRSPPPKPSCPASDQEKVKTCMFNTTSIDECCPTFNSILGTSCPCYKYAEDLDNQVLITLESYCDVNNPCNS
VQVIKLSKDDE

>PGSC0003DMP400051731|PACid:24425945
MVVISGKSLLIHYLLVLLVSSFTKLTHVKGFDLGDLFDFPPDPFPYNYDYEQSPVPRVIEPRLQVWPPPPPSPPPPP
PPSPPSPPPPKQSPPPPSTDSFFIPPFVIPPIDIPPLIPPLLPVISSIINPITSSPPLLKSPSQCKPDKSIKLTPPP
PAKQPPVKAPPPPPHAPSPSPARQPTPPPVKAPFPSPARQPTPPPVKPPSPSPPPPPPPLSKKSPPPAPPPSIANPP
IMAPSPSPAPHPPVIIAPFPSSPVAKPPVIPRRPVKPILPPSPAVKTLTVNGIVYCKPCNSYGVPNLLNATPLQGAS

ARLVCYNGKNAIVQSAITDKNGEFRLTPKSLIGADIGKCKVYLVKSPNPTCNVPTNFNGGKTGALLQHVVPPKPPIV
TPAVIVPVQPPMSDLYGVGPFIFEASSKVPCAMHRKL

>PGSC0003DMP400010630|PACid:24383996
MGIMSKLPIIFLIFVVYSTQITNGLLNNPRKLDEANIVNKCGSCPCNNPCNTPSPPPPPPPVPSPPPPKKPPSSGYC
PPPPLPPSEGGGGGDGGGGYSPNPPSNSQYIYMNGPPGNLYPVDQYFNGAKRDFTSGFSLLISGFFLGIIALI

>PGSC0003DMP400053159|PACid:24386628
MSRLVFFLLFFVVATPITNGLNPRKLDEGENKCGGCPCNKPCIPPSPPPPPPSPPPPSPPPPKKPPSGYDCPPPPYS
GGDGGGGGENIPYPPNSQYIYMTGPPGNLYPVDHDFGGAKRSFSSGFPLLIGGFFLGLLYFW

>PGSC0003DMP400045517|PACid:24391134
MALSHPMIIFSLFLTFIALSAAQSPMMAPTMPPSTMSMPPTTSTTTPPPMSAMSPPPSAMSPPPSTMSPPPMSPMTP
SMSPMGPMTPTMSPMDSPPAPATPGPAPGPMGGMPMAPPAPSTGFVHGSSNSMAMVAILGSVAFFF

>PGSC0003DMP400008622|PACid:24395558
MDPSLLILTFLGVCFCFLALAVQSQQNVSPPPPPPPSKESPPPPPPPPPPPPPPPPPSPRPPPPSQQKHHSPPPTKSV
HSAPEMTTSESKHSNHDKKHHNSYEKSHQLAKKKKPNLGKKLGSQSCKNDISLRLSILWNCLGFVLENIMLRLLCPL
PGTWYLLLVGGGWYLVELVEVRKSWPGHHGH

>PGSC0003DMP400008623|PACid:24395559
MDPSLLILTFLGVCFCFLALAVQSQQNVSPPPPPPPSKESPPPPPPPPPPPPPPPPPSPRPPPPSQQKHHSPPPTKSV
HSAPEMTTSESKHSNHDKKHHNSYEKSHQLAKKKKPNLGKKLGLVFVGVAGMLQVCVVAFLLIKRRQLLKAGSRF

>PGSC0003DMP400055120|PACid:24399389
MSTITAISKDQIACEMCTTCENPCQPILPPSPPPPSPSPPLLCPPPPSPPPPPSPPPPPPVNYCPPPPPPPARRSCP
EDCSLQPRSPPYSIYPYFSPPLAYSNKSTRYKNQPFVTCLIIAIACLFKTFGLM

>PGSC0003DMP400044408|PACid:24423807
MGVKLVFSILIVTMMITSMENEGNLVNANGTTWAGSKYQIECTMCAACDNPCTTPSPPPPQPPSPPPPSTSYNCPPP
PSHPSSGGNYYYYSPPPPPPSSLGGGGGNYYYSPPPPYQNYPSGPTPPPPNPIVPYFPFYFYNPPPPSQSAAFKLTV
FSPKSSIFVTVFIALLIFH

>PGSC0003DMP400000762|PACid:24378261
MVFENDRIKNAYIALQAWKQAIISDPFNITLNWVGPNVCSYTGIFCAPALDNPKIRTVAGIDLNHGDIAGYLPEELG
LLTDLGIFHINSNRFCGTIPRKFSKLKILFELDLSNNRFAGKFPYVVLSLPKLIFLDIRFNEFEGNVPSQLFDKPLD
AIFINHNRFAFELPENFGNSPVSVIVLASNSFHGCLPASIGNMSNLNEAILMNNGLRSCLPAEIGVLKNLTVFDVSF
NQLMGPLPENFGDLVNLEQLNVAHNMLSGTIPKSICQLPKLENFTYSYNFFTGEPPVCLGLPEFHDERNCLPNRPVQ
RSPGQCKAFLSKKIHCSAFRCHKFVPVLPPPPPLSPPLPAPPPPVYSPPPPVYTPPSPSPPPPPPPVYSPPPPPPSP
PPPSPPPPPPPVYSPPPPPPSPPPPSPPPPPPPSPPPPPVYSSPPPPPSPPPPSPPPPPPPVYSPPPSPPPPSPLPY
CIRSPPPPNSPPPNSPPPPLAHSPPPPSPYYYNSPAPPPPNSPPPPPNSPPPPPVCIYSSPPPPNSPPPPPTYVYSS
PPPPPSPLPCIEPPPPPPPCIEPPPPPSPSPSPPPPPVYHYNSPPPPSPSPPPPVYYYNSPPPPSPPPTPVYEGPLP
PIIGVQYASPPPPPFY

>PGSC0003DMP400002002|PACid:24379130
MKEKTQTPICSLFFLVLCFLCSVFTEHTNAEFTVSENGPLTDSEAQFIKHRQLLYYRDEFGDRGENVKIDPSMVFEN
DRIKNAYIALQAWKQAIISDPFNITLNWVGPNVCSYTGIFCAPALDNPKIRTVAGIDLNHGDIAGYLPEELGLLTDL
GIFHINSNRFCGTIPRKFSKLKILFELDLSNNRFAGKFPYVVLSLPKLIFLDIRFNEFEGNVPSQLFDKPLDAIFIN
HNRFAFELPENFGNSPVSVIVLASNSFHGCLPASIGNMSNLNEAILMNNGLRSCLPAEIGVLKNLTVFDVSFNQLMG
PLPENFGDLVNLEQLNVAHNMLSGTIPKSICQLPKLENFTYSYNFFTGEPPVCLGLPEFHDERNCLPNRPVQRSPGQ
CKAFLSKKIHCSAFRCHKFVPVLPPPPPLSPPLPAPPPPVYSPPPPVYTPPSPSPPPPPPPVYSPPPPPPSPPPPSP
PPPPPPVYSPPPPPPSPPPPSPPPPPPPSPPPPPVYSSPPPPPSPPPPSPPPPPPPVYSPPPSPPPPSPLPYCIRSP
PPPNSPPPNSPPPPLAHSPPPPSPYYYNSPPPPPPNSPPPPPVYIYSSPPPPNSPPPPPPTYVYSSPPPPPSPLPCI
EPPPPPPPCIEPPPPPSPSPSPPPPPVYHYNSPPPPSPSPPPPVYYHYNSPPPPSPSPPPPVYYYNSPPPPSPPPTP
VYEGPLPPVIGVQYASPPPPPFY

>PGSC0003DMP400027068|PACid:24381357

MQVYRCFFSLLLISAVLFSFSCALSDHEASLLARRQLSTLLENGNLPDKYEFEVNVEYTFPNSRLRRAYIALKAWKE
AIYSDPSEFTSNWKGPDVCNYKGVFCSPALDDPNVTVVAGIDLNHADIAGYFPVELGLLTDVALFHLNSNRFCGIVP
ESFSKLTLMHEFDVSNNRLVGPFPKVVLNMSSLKYLDLRFNNFEGELPSQLFDKDLDALFLNDNRFVSTIPETLGNS
SASVIVFANNKFHGCIPSSIGKMLNLDEIVFMNNDLGGCLPVEVGLLKNVTMFNAAGNLLSGILPKTLDGLSHVEEL
DISHNTLTGFVPENLCSSLSLKKFVFSFNYFNGEAKGCEARTRKDMSFDDRNNCLPGRAKQKSQKECQPIVSKPIDC
SKAKCGGPLTKSPPSKKNEVPPKPTPTPAPKPRPTPKSPPTPKKASPPPTQVVSSPPLVHSPPPPVHSPPPPLVHSP
PPRVHSPPVSSPPLPVHSPPSLPVNSPPPPIVHSPPPRVHSPPVSSPPPPLVQSPPPLHINSPPLPIVHAPPPHVHS
PPVSSPPPPHVQSPPPPLVHSPPPPVHSPSPHVSSPPPPIQSPPPPPVHSPPPPVHSPPPPISSPPPPVHSPPPPPV
HSPPPPVSSPPPPVYSPPPPPVHSPPPPPPVHSPPPVSSPPPPVHSPPPPVHSPPPPFEHVVLPPNIGSIYASPPPP
IFQGY

>PGSC0003DMP400027067|PACid:24381358
MHEFDVSNNRLVGPFPKVVLNMSSLKYLDLRFNNFEGELPSQLFDKDLDALFLNDNRFVSTIPETLGNSSASVIVFA
NNKFHGCIPSSIGKMLNLDEIVFMNNDLGGCLPVEVGLLKNVTMFNAAGNLLSGILPKTLDGLSHVEELDISHNTLT
GFVPENLCSSLSLKKFVFSFNYFNGEAKGCEARTRKDMSFDDRNNCLPGRAKQKSQKECQPIVSKPIDCSKAKCGGP
LTKSPPSKKNEVPPKPTPTPAPKPRPTPKSPPTPKKASPPPTQVVSSPPLVHSPPPPVHSPPPPLVHSPPPRVHSPP
VSSPPLPVHSPPSLPVNSPPPPIVHSPPPRVHSPPVSSPPPPLVQSPPPLHINSPPLPIVHAPPPHVHSPPVSSPPP
PHVQSPPPPLVHSPPPPVHSPSPHVSSPPPPIQSPPPPPVHSPPPPVHSPPPPISSPPPPVHSPPPPPVHSPPPPVS
SPPPPVYSPPPPPVHSPPPPPPVHSPPPVSSPPPPVHSPPPPVHSPPPPFEHVVLPPNIGSIYASPPPPIFQGY

>PGSC0003DMP400010509|PACid:24397485
MHYHFSVHSCCLNTLFYSIIFLILFDAASTTNQISNYIDHPFGGIAIETSNSRKLLSYKGDVLAINPSLSIENFRLK
NAYIALQSWKEVILSDPHNITQNWVGSNVCNYTGVFCSPSLDQPSELTVSGIDINHGDIAGKLPHELGLLFDIALIH
INSNRFCGTIPESFLNLKLLFELDLSNNRFVGKFPDVVVQMPNLKFLDLRFNEFEGALPKQLFERDLDALFINNNRF
SFELPDNFGNSPVSVMILANNNFVGCVPVSIGKMVRLNELLLLNNKLHSCLPNDIGMLKNVTVFDISYNEMMGTLPD
SIGGMMSLEQLNLGHNMFSGMISSNICTLPNLENFVYEHNYFSEESPACLNLNAFADQQNCLRGRPMQRSALDCQRF
LSNEVDCTTFKCALPPPSPPPTTPPQNNCCVCLSPPSPSPLPSTEPPSSPLPLPAPSASPSPPSISPSPSSPQPPDL
PSPSFVEPPLPSPPPCENQRIPSSQDMSSPSP

>PGSC0003DMP400010700|PACid:24409847
MRPPSRSVFISFLVISLLSYQISNVVGQEGDNDIDLDDIKANASKLSFENPKIRDAYIALQYWKTAMFSDPFNFTAN
WTGPDVCSYGGVFCAPSLNDDSIRVVAGIDLNHADIAGSLVAELGLLTDLVLFHLNSNRFCGVVPKTFSHLKLLREL
DLSNNRFVGGFPKVVLSLPSLKFLDLRFNDFEGPVPSELFDKDLDALFLNDNRFRFGIPENLGNSPVSVLVFANNDL
GGCIPASIGKMGKTLNELILMNDNLTGCLPMEIGLLKKLTVFDVSFNKIQGSLPLTVSKMRSVEELNVAHNKLTGVI
PASICQLPRLQNFTYSFNYFTGEAPVCAATRSGDGQENCIVGKKNQRSAKECSSDDAKPYDCKKSKCYSPYATSPST
KPKPTPTPKPRGPPPPTWKSSGSHNKRSPPPPKSTPLPPPAPYKKSPAYRHRSPPPPTHKISPVTHHSPPPPSPVYY
HPSPSSPPPPVYYSPPPPVYYEPPTYKPKSPPPPTPSYEHPKTPSPPPPTPSYEHPKTASPPTPSYEHPKTPPPTPS
YEHPKTPSPPPPTPSYEHPKTPPPTPSYEHPKPQSPPPPYEHPKTASPPTPSYEHPKPQSPPPPTPSYEHPKPQSPP
PPTPSYEHPTPPTPPCNEPPPPPPNSHWEPKPSPPYTYSSPPPPSPSPPPPTYYYSSPPPPPPSQSPPPPTYYYSSP
PPPPPFYENIPLPPVIGVSYASPPPPVIPYY

>PGSC0003DMP400044985|PACid:24422116
MVALGCFLFFLVSFCSFSPSYFALSDIEAASIARRQLLGNNGELPNTYESEMTINMKFENARLKKAYVALQAWKKAI
YSDPTNFTANWEGNNVCAYNGVFCDNALDDPNISVVAGIDLNHADIAGHLPVELGLLADVSLIHINSNRFCGIIPKS
IKNLTLLDEIDFSNNRFVGPFPDVVLELPKLNYLDLRFNNFEGQVPSALFEKNLDAILINNNRFHSTIPESLGNSNA
SVVVLANNKFYGCIPSSIGKMGNSLDELVFTNNELSGCLPEEITKLTSLTLFDISGNKFVGSLPQDLKSMQKVEIFD
IASNKFMGNVPKNLCTLPSLTNFTFSKNYFESMDETCTPSQTKQVKIDGNENCLGGRSEQRTEKECFPVVSKPVDCS
KGHCGVSREGQSPKDPPKTLTPSKPATPTTPKPKPSPPPPVASPPPPPPVASPSPPIHSPPPPVHSPPPPPVASPPP
PVHSPPPPPVVSPPPPVHSPPPPPVVSPPPPVHSPPPPPVVSPPPPVHSPPPPPPPVHSSPPPVASPPPPVHSPPPP
VHSPPPPVQSPPPPVHSPPPPVASPPPPVHSPPPPVASPPPPVHSPPPPVASPPPPVHSPPPPVASPPPPVASPPPP
VASPPPAPVFSPPPPVHSPPPPPAPVMSPPPPTFEDVALPPTLGSLYASPPPPIFQGY

>PGSC0003DMP400000755|PACid:24377931
MSTPTPVTAPSPTSPPSNATSPPPSTPATAPPASPTPPATAPPASPTPPATTPPPSTPAQAPPARTPTPPAATP
PPAASASPPPPTTPSPSSNPPPSTTPTTPAPSGSPSPPSTTPSTPSPPGSTPSPPAGGNSPPSPSGGSPSPPSGGRP
SPPALSPPPSDEGSSGISTGVVVGIAIGGVLILAILSLLFIFCKKKKRRNHEPVNYYVPPPPPQGIKADPHGGQMQH
WQQNPPPPADHFVTMPPKPSPPPAGGLLPSHAPPRAPSPQPQPYMNSSGASSNYSGSEIALPPPSPAMSLGFSQSTF

TYEELVRATDGFSDANLLGQGGFGYVHKGILPNGKEVAVKQLKAGSGQGEREFQAEVEIISRVHHKHLVSLVGYCIT
GAQRLLVYEFVPNNTLEFHLHGKGRPPLDWRIRLKIALGSAKGLAYLHEDCQPKIIHRDIKAANILIDFNFEAKVAD
FGLAKLTSDVNTHVSTRVMGTFGYLAPEYASSGKLTDKSDVFSFGVMLLELITGRRPVDSTQSYIEDSLVDWARPLL
TRALEDEKFDTLVDRRLENDYNHNEMARMVACCAACVRHSAKRRPRMTQVLRALEGDVSLSDLNEGIKPGHSTVYSS
YTSSDYDTLQYNEDMKKFRKMALATSQEYASSDQYSNPTSEYGLNPSGSSSEGHQTAEMETGRMKKDSRGFSGSKGF
SGTS

>PGSC0003DMP400043552|PACid:24389342
MSVVVSPPPSLAPFPVPPVVLSPPPQLSSPVPASQPNATAPPVSSLPPTLPPQSSPPPASPTLPQSPPPSNVTLPPP
MASPPTESAPPTVSPPPIPPASSPPPISSPPPSSSPPPQSSPPPASSPPTTPSPPVSSSPPPEVEPPPVSPPPQPTV
PTSSPPPPPKDDPPPTSPPPQPTTPPSSPSPPPKVDSPPFSPPPPAQNPDPTPPPQSPEPPKGSPPSPPVNSPPSPA
SVPPKGSPPTPASDPPTNSPPSPAFTPPQGSPPTPSLEPPKNTPSSPSVPSGGTTTNSSSDDAAGSSNSPSSSGLGT
GGTVAIGVIVAVLLLSIVGLVGWCVWKRKKKAFRPSGGNVMPTPSGSTPNSDSVLLKIQESTPDTRNGTGNKFLNSP
GGSGGFGNPKIWFTYEELVKATGDFSAENLLGAGGFGSVYKGCLPDGRDVAVKQLDIGGSQGDREFRAEVEIISRVH
HRHLVSLVGYCISENRRLLVYEYVPNNTLYFHLHAEGRPVMDWTTRVKIAVGAARGIAYLHEDCHPRIIHRDIKSSN
ILLDINFEARVSDFGLAKLAQDAKTHVTTRVVGTFGYMAPEYASSGKLTEKSDIYSFGVVLLELITGRKPVDTSQPL
GDESLVEWARPLLSHALEKLEFDQLADPRLERNYVIPEMFQLIEAAAACVRHSAAKRPGMGQIMRAFDNMSVSDLTN
GMKVGESTIYNSAEQSAEIRLFRRMAFGSPDFSSDFFSQGTQHSVESAEDRV

>PGSC0003DMP400031066|PACid:24406621
MEDSSPSPSNSSSSSPPLDSSPPPSPPESSPPPPKSDSSSPPPPKSDSSSPSPPPSKSDSPSPPPPESSPPPKSDSS
PPPPSPNQSPPPKSESSPPPASPPPTESSPPPPSSTNFSPPSPKSSDSSPSDDNDNPNTQSPPPSLNSSSSSPPPTT
NTKPSPSKNTFFNEPNSPPSSTFSPPTPESSLFPSEKALPTFPPSRDNASSNKSPGNSLGSNNSPPHEQSSSSTVAI
VAAVSVTGLLILAVVIVCLLCNRKKKKQPYYVDPAHPPKGGDPYYNTGNYSSPHTDHIVTLAPPPGVMGTPQEGGRG
WTPPPPPPAANTSSEFSSGYSSHVPGGGPATIPSPNYGGLSKIQFTYADLATATGGFSEANVLGQGGFGFVHKGVLT
DGSVVAVKSLKSGSGQGEREFQAEVEIISRVHHRHLVSLVGYCIADGQRMLVYEFVSNGTLEYHLHGKGRPVMDWGL
RLKIALGSAKGLAYLHEDCHPRIIHRDIKGANILLDNNYEAMVADFGLAKLTEDNNTHVSTRVMGTFGYLAPEYASS
GKLSEKSDVFSFGVMLLELITGRRPLDTTNKLMDDSLVDWARPFLTKALEENNYDELVDPRLEGNYDPDELQRMVAC
AAASVRHSARRRPKMSQILRALDGDSSLEDLNEKAGKNNTANFGGASGPASDLYDTCAYNADMVKFRQMVMTSQDMN
SSEYGNTSDYGLHPSDTSSEFSSDYNHSGAHKQAK

>PGSC0003DMP400017893|PACid:24427542
MASPDSPPSFFPFPTFPTATPSNSTSDSPPAPPPDSSSPPPPPPDSSAPPPSPPAPDSPPPSESKSPPPAESPPPPP
PTAAAPPPSAPPPKPSVSPPPPSPKAPPPANSPPPASSPPPPSKDSPPPAPPPSPPPPPPAVSPSPPPPVKNQPPPP
DSPPPAPVANPPQNSPPPPALAPPPASLPSAPPPNLLTSPPPSISPPAPPNNTSPAGAPPPLPVTRLPTEKPTAIPK
PAITADSSARNGGGNKTGSVAAIGVVAGFLALSLVIVAVWFTRRRKKRESAFNLNYLGPSPFASSPNSDTSFLRSRS
QHSTYLAPTGSQSNFMYSPDHGGIGNSRSWFTYEELSEATNGFSPDSVLGEGGFGCVYKGVLNDGREVAVKQLKSGS
GQGEREFRAEVEIISRVHHRHLVSLVGYCISEQQRLLVYDYVPNDTLDYHLHGKGMQTMDWATRVKVAAGAARGLAY
LHEDCHPRIIHRDIKTSNILLDINFEAQVADFGLARLAGDASSTHVTTRVMGTFGYLAPEYASSGKLTEKSDVYSYG
VVLLELITGRKPVDQSQPLGDESLVEWARPLLAQALETENFENVVDPRLGNNFVAGEMFRMIEAAAACVRHSGSKRP
RMSQVVRALDSMDELSDLSNGVKPGQSGIFESREQSAQIRMFQKMAFGSQEYSSDFFNYSQGSYKS

>PGSC0003DMP400027121|PACid:24427808
MDSSPGTASSNDTDSKETPSSNGTPPSSSDSKNDSPSSSSSSSSSSPPPKNESPPSSPPPQSPPPSPNASPPPKNDS
PPPSSPPPSSSESNSSPSKDNNNNNQSPNPSSNTPSQSPPPSSTYNTPSTPNSPKNPPSFLLFSPPPPASSRESPAP
PHAHLSPPPRSGSNQPSPANDDSSSTVGIVAGIAVGGIVIIALIILCVWCSKRKKKQRYYMAPGPKGGDPYYNNNNQ
WNRRQSMDHVMKVPQSEMGTQSGWAGATPHQQGASSSGEFGSGYSGQAVPLPSQSPNMGLGGFSQSQFSYEELAKAT
DGFSQANLLGQGGFGYVHKGVLNDGRVVAIKSLKAGSGQGEREFQAEVEIISRVHHRHLVSLVGYCIANGQRMLVYE
YVDNKTLEFHLHGKGQPVMDWETRLKIALGSAKGLAYLHEDCQYRIIHRDIKAANILLDLNYEALVADFGLAKLTSD
NNTHVSTRVMGTFGYLAPEYASSGKLTEKSDVFSYGVMLLELITAKKPVDPSNMMEDSLVDWARPLLTRALEEEKYD
GLVDPRLEGNFDTDELHRMIGCAASSIRHSAKRRPKMSQIVRALEGNSSLEDLNDNPKPSKVPSFAASNGAPTQSYD
TGMYNADMMKFRKMIMPTQEFSSSPSE

>PGSC0003DMP400026200|PACid:24429195
MQSPPGTASSDKNDTTSSSPSNSSPSPPPPAPPTSPPPESPPPAKESSPPSSPPPSASESPPPPSEYTTTSPNAPDE
ASPPTSSKFNPPPPVSSGPPSLSAGKLSPPPPSEKSAPFEKPGGAQRSGKPAAGSSGNNNGSPSSNSGSSDMFIVAG
IAVAGLMIFATIIVCLIYWRRKKKEHYYTNGPPGTPGPPRRPPKGAGSTDPYYKGPKPTEHIVKVPTIVYTSSDSTF

AVNGQALVPSPSLGGFSKSQFTYEELARATSGFSKANLLGQGGFGYVHKGVLVDGTVVAVKSLKSGSGQGEREFQAE
VDIISRVHHRHLVSLVGYCIADSQRMLVYEYVPNKTLEFHLHGKGHRVMDWGTRLKIALGSARGLAYLHEDCHPKII
HRDIKAANILLEDNFEAKVADFGLAKLSSDNFTHVSTRVMGTFGYMAPEYASSGKLTDRSDVFSFGIVLLELITGKR
PIDPSNIMEDSLVDWARPLLKTPWKRENMTNW

>PGSC0003DMP400042537|PACid:24402639
MNTPGPPPTNSSQNQVVLKAVIAAVVSTLVVAGILFYFFYRYYVIRRWKKNKLSSSFRREMPHQEFQQTGALKGLIV
DENGLDVIYLRRFEGSQLGSCFSKIWVNHMDEDEDEDEEEEKRIDSGQEQPVSSDHIQEMPLLQNCSNVGSYENKEA
VTASQQTTNSLFAASRSSKQPSTSLQFQQKEIIPPQPETPSQLALVSSQNPPQPPIPPPAPPPPPPQLPIPPPAPPP
PPPPLPMKRIAKAPTPPNAGKIKSSPPPPPKGGGLSSLIKPPIAPRGKGSRKDKAEAQNEEKSNENGETQVKLRPLH
WDKVVANADHSMVWDEINNGSFRFEDDLMEVLFGYNVTSNRTPEGNRATTSSGTSKLARPTQMFILDPRKSQNTAIV
LKSLSVSRKEILDALTEGQGLSVDTLEKLTKIRPTEEETSKILQFDGNTRKLADAESFLYQMLKAVPSAFKRFNAML
FRSNYDPEILNLKENLQTIELGCKELRTSRIFIRLLEAILKAGNRMNAGTARGNAQGFNLSALQKLSYVKSNDGKTT
LLHFVVEQVICSEGKRCLINKGHKDDSDRKTEKVEKDTEHLTLGLPVLQGLSSEFSNVKKAATIDYDSFINTCSTLT
TRVNDVQQLITCCGIAEKDQFVKETKGFLEECEEELKVIREEQTRVMELVKRTTEYYQAGTSKEKGTQSLQLFAFVK
DFLDMVDKVCVDITKKVQKKSAATVESSPPPRSPRPITPVRFHNLRTYFTPEIVSSESENDF

>PGSC0003DMP400026177|PACid:24405503
MRAHPIYNFLLVLIVITASSITAAAEHTQIQHLIDINPLTRRILHQPLFPSTSSSPPPPPQINSEPIFPTPDHPFFP
EVPTVQTPDQPQQQIQPNGTPISRNNSIAPQPTNPTKKFAIAVSVGIVTLGMLSALGFYIYKHKTKHPDETQKLVSN
HNTNSFQEESRMPPSNFLYIGTVEPSSSATAAAAASQTQINHSIVANNTSPYRKLSPAKKSDRYRPSPELQPLPSLR
NNPTFFTPPAIINSSDDDETKIYKPYNISSSSTGIMTRSNNSIPHSKRTSPRLSLSCSSPDIKRAIIVPSVKQTSPP
SPPPPSQEVTLQQHKKPTLTYVPQRVKFSQPPPPPDMSRLKSPNNQLQETSKPSAPPPPPPPPPPPLSTSRVLGGKV
ASRVTNSIEPPRSQRSSLSVKPQSSSPTPRSGYVVSEKRSHLEEQRGEAIHDTDTRDESKPRLKPLHWDKVRATSER
ATVWDQLKSSSFQLNENMMESLFGCNSVNSVPNEVAKKSVRPTVEKENRVLDPKKSQNIAIMLRALNVTKDEVSESL
LDGSAEGLGPELLESLVKMAPTKEEEIKLRDYNGDTSKLGSAERFLKAILDIPFAFKRVEAMLYRANFDAEVKDLRK
SFQTLEEASQELKNSRLFFKLLEAVLRAGNRMNVGTNRGDARAFKLETLLKLIDIKGTDGKTTLLHFVVQEIIRSEG
LSSDDNNLSNIKFKEEEFKKQGLQVVGGLSRELCNVKKAAGMDSDVLSGYVSKLEAGLVKVRSVLQYEKTCMEGNFF
ESMKVFLKEAEDGIVRIKAEERKALSMVKEVTEYFHGDAAKEEAHPLRIFLIVRDFLCILDSVCKDVGMMQDRTMVG
AARSFRIATTASLPVLNRYNARQDRSWDDDSSSP

>PGSC0003DMP400025365|PACid:24411742
MGVRGVSQLSVFIIILFTIVAANPVSNRPEDHLLANQISSVGINQELAEELWLNCRLELVHSNEAVEDLEFSDPAEE
ANGIFTNRRSLTKNKEKNANLLTKEALMGCLVKKNLMFLISGEEKHSPTWYTRCMDFLFSWYGAPRRRELLQVGDAP
APAPAPATSSSETPNSPPPARPPTTPFFPRDYNDSSKTSGPSDQSSTSQNSTSDGQSNKKKSSTKTVLVAVLVTAAV
TFIVVALFFICYCKVCGVGYRKGKNDERPLLSLSISDYSVASRHGNAGNHSVSDDSHNKIGKTFYMEPNALNGSKSE
IPLGTVTGIAVAAAGVSEQIPPGRMGMQVQPPLKPPPGRVNPFEDPTSPAPPPLPPAKITNLAAPSPPPPPPKPSAG
GPHPPAPGPPPPPPIPIRAKAGPRPPPPPGPGVTPPRPPPTGLKPPRPSPLGSNASSSASVEGSENDPSKTKLKPFF
WDKVLANPDHSMVWHQIKSGSFQFDDDMIESLFGYAHADKDKNGTKEGLYVPRCF

>PGSC0003DMP400037176|PACid:24425905
MRAAGLSIFFILSLLSSFTSQFQDLVVKEKTRRILHQPLFPVSSTPPPDSEISPPPPAEPVNSQPFFPEVPTGTTPD
QTHQPPVTPANGTPVSNSVATQPAKPVKKVAIAISVGIVTLGMLSALAFYLYKHRVKHPDETQKLVRGNSDQRINEE
SRTPPSTFLYIGTVEPPAKTSAVTESNGATGSPYRKLSSVKRMDTRYRPSPDLQPLPPLSKPQPPPSINSPTAMSSS
DEESHDTAFHTPQGSTVSNEEGYYTPSLRESYSSNKNYVPYSKRTSPRSRLSDSSPEVKHSIIPSIKQAPAPPLPPR
QPQGGLIEQLPPEPPLQYTRPELPYVTKRAKFSSPPPPPDMTRLQLISNQAQQILKTTPPPPPPPPPPPLPLSTPHK
PEGSQRNVPSAAYQQMVKTESRSPTPKSTPGSEKTSTSEEQNGGASSLERHDSSDTDPSKPKLKPLHWDKVRATSDR
ATVWDQLKSSSFQLNEDMMESLFGCNSANSVPKEATRKSVLPPAEKDNKVLDPKKSQNIAIMLRALNVTKDEVTEAL
LNGNPEGLGPELLETLVKMAPTKEEEIKLREYSEDASKLGSAERFLKTVLDLPFAFKRVEVMLYRANFDGEVKDLRK
SFQTLEVASEELKNSRLFLKLLEAVLRTGNRMNVGTNRGDARAFKLDTLLKLVDIKGTDGKTTLLHFVVQEIIRSEE
LDSEPPGEDLSNKANIKFKEEDFKKQGLQVVSGLSRELGNVKKAAAMDSDVLGSYVLKLAVGLDKARSVLQYEKQGL
QGNFFESMKVFLKEAEDGIVRIRAEERKALSMVKQVTEYFHGDAAKEEAHPLRIFVIVRDFLSILDNVCKDVRRMQD
QTVVGGARSFRIAATASLPVLSRYNVKQKRSWDDNSSSP

>PGSC0003DMP400030108|PACid:24400695
MAISHSHLCLLLGFVLLFTPTLSLRRDYFFRNGPNVNDDHLDILHDKKFPINFLSAFDDLSITPPPPDESPDVASPV
VSPSPEETSGSAPPPEETLGSAPPPEETSGSPPPTEETPVTSPPPEETPGTPQPPEESPGTAPPPEETPGTPQPPEE

APGTPQPPEEAPGTAPPPEETPGTPPPPEEAPGSSPPPVDAPGSSPPPAQTPSTLPSPAQTPSSSPSPAQTPSSSPS
PTQTPSISPSPAQTPSSSPSPAQTPSSSPSPTQTPSISPSPAQTPSSSPSPAQTPSSSPSPTQTPSSSPSPAQTPSS
SPSPAEPPTIPPPPEEPPIIPPPPVEPPTIPPPPPPTPPHHHHHHHHHHHHHHHHHHHHHHHHHPPPSTEVSIQDPDQ
SMISDNIN

>PGSC0003DMP400033948|PACid:24406407
MKETTISVLALLTLFLLEVISANELSLPFHLPINGTFGLEVFQGISNASAGVYLNELGESGMQADGAKCPTGMCCSI
WGWCGTTSDYCGSGFCQNQCIGHGPSPHGRCGMQAGGGVKCPTGQCCNILGWCGTESDYCNPEYCQSQCSGPFPKGR
CGWQAAGGLCPNGLCCSVDGWCGTTWDYCASGICQSQCPSTPPPPPPSPPPPPPPPPSPPPPPPPPPSPPPPPPPPP
SPPPPPPPPPSPPPPPPPPPSPPPPPPPPPSPPPPPPPPPSPPPPPPPPPSPPPPPPPPPPSPPPPSPPSPTPPPQY
RCGMQKGGRKCNITGDCCSIWDSHIRVLFSWILSNAMSRSIPRGTMRMAS

>PGSC0003DMP400047498|PACid:24408586
MIAATAVLESPPPPFSSRRHRRSRVAATAVLESPPPPFSSRRHRRSRVAATAVLESPPPPFSSRRHRRSRVAATAVL
ESPPPPFSSRRHRRSRVAATAVLESPPPPFSSRRHRRSRVAATAVLESPPPPFSSRRHRRSRVAATAVLESPPPPFS
SRRHRRSRVAATAMYRSRSARSPSLREPETRLSESDKIEGAGSWDALEWTKIDVNFFTSLRPVARSIPIGVKQFLLE
AEQVIVEGYGVVLVNTDDAGTLYVTNFRLLFLSEGSRDIITIGTIPLATIEKFQKIAVKLPSGPRQPEKTRSQRLLQ
IIGKDMRIIVFGFRARTKQVNS

>PGSC0003DMP400027948|PACid:24414440
MLGVLLMPPLLGDLVNLEQLNVAHYMLSRTISKSICQLPKLQNFTYPYNFFTGELPVYLGLPEFHDERNCLPKIPVQ
LSPGQYKAFLSKKIHCSAFRCHKFVPILPSPSPLPPVYSPPPPTPSSPPPPPPPSPPPPPPVYSPPPPPPSPPPHVY
SPPPPPPPPSPPPPSPLPYCVRSPPPHLQAFDHSLAGLLFSSFTFFLSSNETHKSNSFLCFSFLFFLLVLNSTKPQF
ICLRPLLLDLTNLIAS

>PGSC0003DMP400055411|PACid:24420108
MATMKMFMMLVVAAIMFCSHHQVVVAREVVVNVDVVEDGNKLKLWPWEIPCYLPWPFPFPRPYPCPPPKPRPCPPPP
PPRSPPPPPPSPPPPPPAPATTCSPGDKAKVKKCMFNTTSIDECCPTFQSILGTSCPCYKYAEDLDNQVLITLEAYC
DVVTPCRSPPPKPSCPASDQEKVKTCMFNTTSIDECCPTFNSILGTSCPCYKYAEDLDNQVLITLEILL

>PGSC0003DMP400051732|PACid:24424989
MAFTSVKAIVLIQVLALVVASFSELSFGEVTENSSLDNHEDNEIISTKGFGFGRLPKRSPSTPTPVKRPSPSPSPPA
KSSPPPSPPAKSPPPPSPSPPPPSPPPPPVKSPPPPSPPPPVQSPPPPSPPPPLAQSPPPPPTIPPPAPPPPAQSQK
QTPPPPPTIPPPAPPPPAQSQKQTPPPPPTIPPPAPPPPAQSQKQTPPPPPTIPPPAPPPPAQSQKQTPPPPPTIPP
PAPPPPVQSQKQTPPPSPIAPPPAQSQKQTPPPPPTIPPPAPPPPAQSQKQTPPPPPTIPPPAPPPPVQSQKQTPPP
SPIAPPPAIQRAPPATQPPISRPPQPSPPTQPPINRPPQPSPPTQLPIRRPPPSPPVTQLPIRRTPPPSPPPTNKPP
IRSSPPPPTDYDEPPNIEPPVDQEPPPVSHEPPFVEPPPTNQPPIEPFPPTLSPPFIPRITPPVKLPPPTIHPAGKP
LIVVGHVNCKSCSSRGLPSLFKASPLHGASVKLVCHNNGRKANVQTALTDKNGDFSITPISLARADVHKCRVYLVKS
PKPICNVATNYNNGKSGAVLKPILPPGNHGPMFDFFGVGPFIFEAPNKFPCRK

>PGSC0003DMP400018095|PACid:24425367
MKNVKGTYLLFFLFLILAALSSQVYSISSDFSILDRSNDFISDESVFQLFQEWKQKHGKVYKDEKEEEMRLEKFRWN
VKYIVEKNSERKSASEHFVGLTNFADMSNEEFREVHGSKIKIPFNKRKIIQMKNVEEKSTSISCDAPPSRDWRKHGA
VTEVKNQEQCGACWAFSACGAVEGINAIITGELISLSVQELVNCDNSTNTGCCGGYMDPAFEWVINNGGIASEIDYP
YTASQGACKITKVNHKVVTIDGYRDVPQEETALLCAVAHQPVSVGIDGTSADFQLYRGGIYDGSCSSSPDDLSHGVL
IVGYGSEGDNDYWIIKNSWGTSWGVEGYGYIRRNGDLPYGVCAINSLASYPTKELSFVLSPYPSPAVQPPPPPFPLP
TPPSSAIPPPPPSPSPPPPPSAPPPSTSPPPPPSAPSPSPPPPPPSEPSPYPSPAIPPPTPPSPPPPPPPSAPFPYP
SPIVPPPPPPSPPPSPDVPLFPPPPPPPSPQPPFAPYPYPSPTVPPPHPPSPPPPLPYPSPVVPPPPSPFPPPPPSP
SPLPPPPPPPPSPPPPPPSPPPPSPSPPPPPPSPPPPSPSPPPPPPSPPPPPPPSPPPPPPPSPPPPPPPSPPPPPP
SPPPPSPSPPPPPPCPPPPSPSPPPPPPPSPPPPPPSPPPPSPSPPPPPPSPPPPSPPPPPPPSPPPPPPSPPPPSP
SPPPPPPSPPPPSPSPPPPPPSPPPPPPPSPPPPPPPCPPPPSPSPPPPPPPSPSPPPPFPPAPSPSPPPPPPCPPP
PFPPAPSPSPPPPPPSPPPPPPPCPPPPPFPPAPSPSPPPPPPCPPPPPFPPAPSPSPPPPPPSPPPPPPPCPPPPSPS
PPPPPPPSPPPPPPSPPPPSPSPPPPPPSPPPPSPPPPPPPSPSPPPPFPPAPSPSPPPPPPSPPPPPPPSPPPPPP
PYPKPSECGGWYYCPEYQTCCCDLYFFGICLRHKCCPYENGVCCHGSDYCCPTEYPICDVYEGVCLKRFDDTVGVAA
KKRRMAQYKLPWSTSTKETEEMGQTLKWKRKHVAPMF

>PGSC0003DMP400002485|PACid:24428704

MMNIRISWVWIVVLFLGFTFHNLSTSRAKSTRHRHHSTAVVEGTVFCDTCFQQQFSGASHFISGATVAVECADSVRR
SSFYKEVKTNEHGKFSVDLPISVSKHVKKIKGCSVKLIKSSEPYCAVASTATSSSLHLKSRKQGTHIFSAGFFTFKP
LNQPDLCSQKPSIQNSKKKLTDPQKSAISNPNDPTFYPPIQDPPAPGTLLPPLPRLPPLPLLPPLPDLPGLPISPVP
KDSSKYYSQSEAAAKPNFFNPIGGGLPLPPNPLLPPPSILPPNPFLPPPSIIPPVIPSPPPSIFPPLFPSPPPSIFP
PIIPSPPRSPPSLFPPFIPPLIPGLTPSPPPPPPSLFPPLPPLFPPIIPGFPGVPPASTSSSLQNKNPSP

>PGSC0003DMP400053338|PACid:24428810
MRLLFLLLLVMHFTDFSAGKQPRLPEYQALLALKTAITDDPQLTLASWNISTSHCTWNGVTCDTHRHVTSLDISGFN
LTGTLPPEVGNLRFLQNLSVAVNQFTGPIPVEISFIPNLGYLNLSNNIFGMEFPPQLTRLRNLQVLDLYNNNMTGEL
PLEVYQMTNLRHLHLGGNFFGGRIPPEYGRFPSLEYLAVSGNALVGEIPPEIGNITTLQQLYVGYYNTFTGGIPPAI
GNLSQLLRFDAANCGLSGEIPPEIGKLQNLDTLFLQVNSLSGSLTPEIGYLKSLKSLDLSNNMFSGEIPPTFAELKN
ITLVNLFRNKLYGSIPEFIEDLPELEVLQLWENNFTGSIPQGLGTKSKLKNVDLSSNKLTGNLPPNMCSGNNLQTII
TLGNFLFGPIPESLGRCESLNRIRMGENYLNGSIPKGLLSLPRLSQVELQNNILTGTFPDISSKSNSLGQIILSNNR
LTGPLPPSIGNFAVAQKLLLDGNKFSGRIPAEIGKLQQLSKIDFSHNNFSGPMAPEISQCKLLTYVDLSRNQLSGEI
PSEITGMRILNYLNLSRNHLVGSIPSPISSMQSLTSVDFSYNNFSGLVPGTGQFSYFNYTSFLGNPDLCGPYLGPCK
EGVVDGVSQPHQRGALSPSMKLLLVIGLLVCSIVFAVAAIIKARSLKKASEARAWKLTAFQRLDFTCDDILDSLKED
NIIGKGGAGIVYKGVMPSGEHVAVKRLPAMSRGSSHDHGFNAEIQTLGRIRHRHIVRLLGFCSNHETNLLVYEYMPN
GSLGEMLHGKKGGHLHWDTRYKIAVESAKGLCYLHHDCSPLILHRDVKSNNILLDSSFEAHVADFGLAKFLQDSGTS
ECMSAIAGSYGYIAPEYAYTLKVDEKSDVYSFGVVLLELVSGKKPVGEFGDGVDIVQWVRKMTDGKKDGVLKILDPR
LSTVPLNEVMHVFYVALLCVEEQAVERPTMREVVQILTELPKSPGAKSDDSTVTDQSPPPSDSALESPTSIPEETKD
HHQPTPQSPPPDLLSI
