# Supplementary figures and images for: Bioinformatic Identification and Analysis of Extensins in the Plant Kingdom
Source: PLoS One. 2016 Feb 26;11(2):e0150177. doi: 10.1371/journal.pone.0150177 (PMC4769139; doi:10.1371/journal.pone.0150177)

S3 Fig

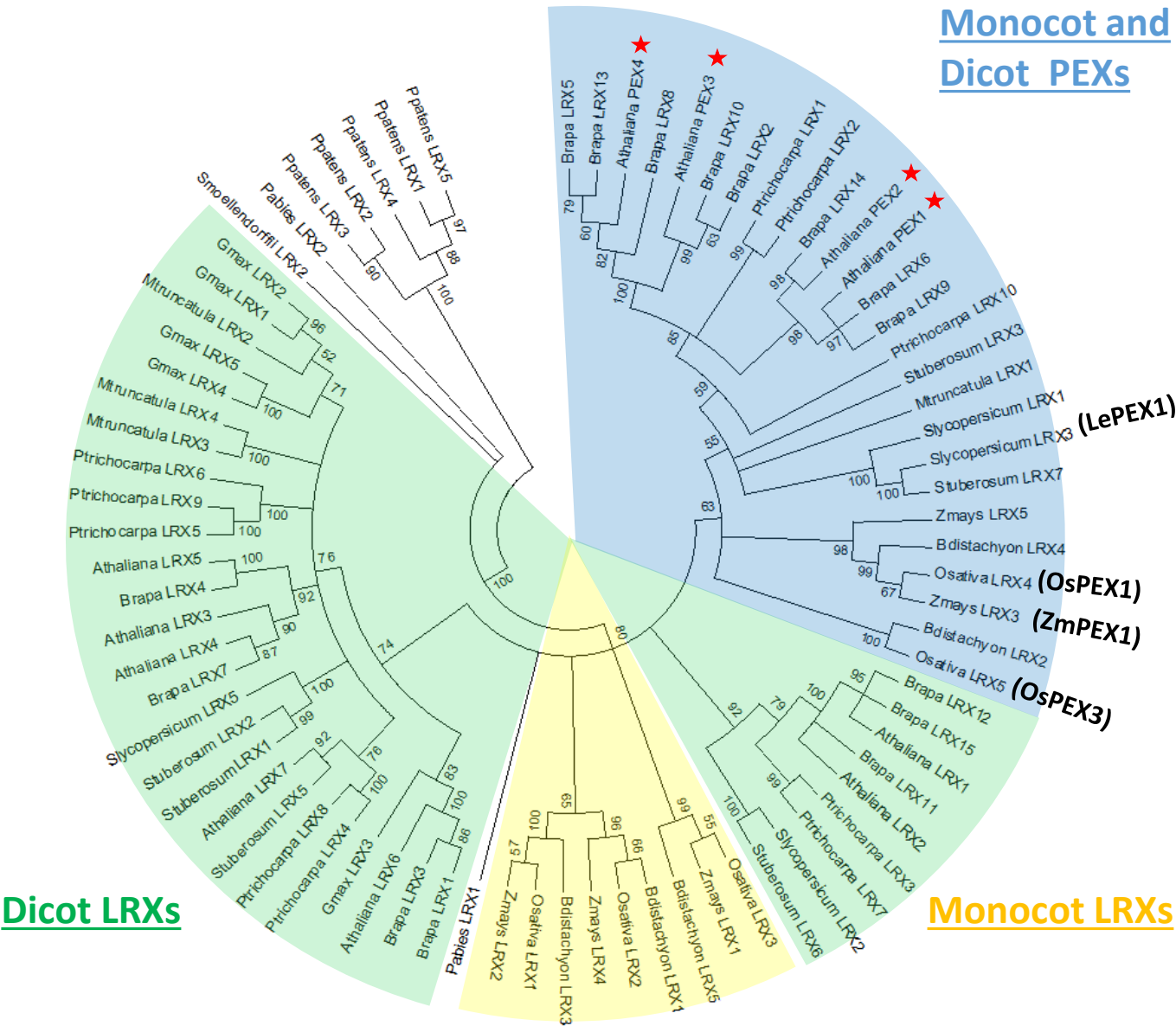

Supplement: S3 Fig — The evolutionary history was inferred using the Maximum Parsimony method. The bootstrap consensus tree inferred from 1000 replicates is taken to represent the evolutionary history of the taxa analyzed. Branches corresponding to partitions reproduced in less than 50% bootstrap replicates are collapsed. The MP tree was obtained using the Tree-Bisection-Regrafting (TBR) algorithm with search level 1 in which the initial trees were obtained by the random addition of sequences (10 replicates). (PDF) [file pone.0150177.s003.pdf]

**S5 Fig**

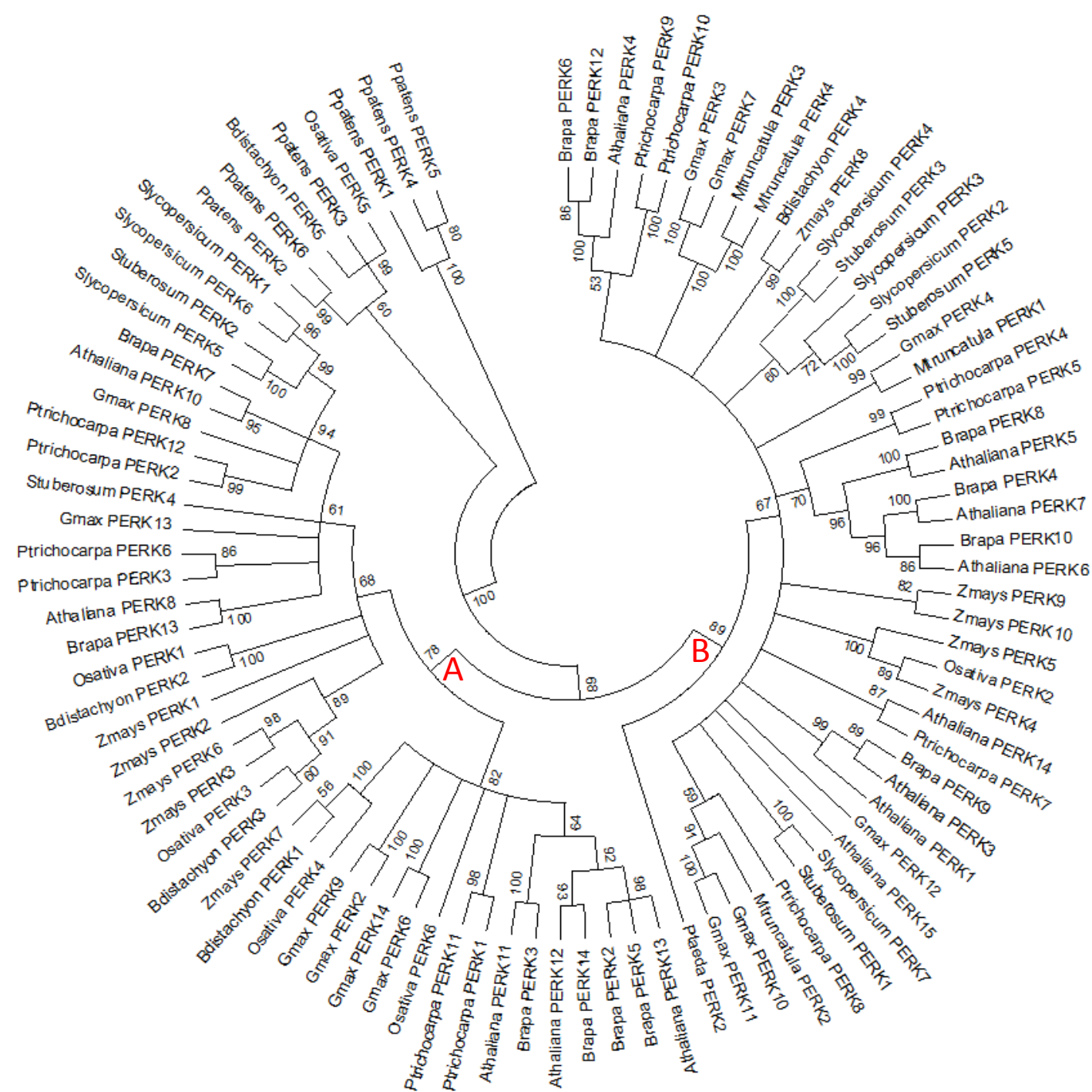

Supplement: S5 Fig — The evolutionary history was inferred using the Maximum Parsimony method. The bootstrap consensus tree inferred from 1000 replicates is taken to represent the evolutionary history of the taxa analyzed. Branches corresponding to partitions reproduced in less than 50% bootstrap replicates are collapsed. The MP tree was obtained using the Tree-Bisection-Regrafting (TBR) algorithm with search level 1 in which the initial trees were obtained by the random addition of sequences (10 replicates). (PDF) [file pone.0150177.s005.pdf]

S7 Fig

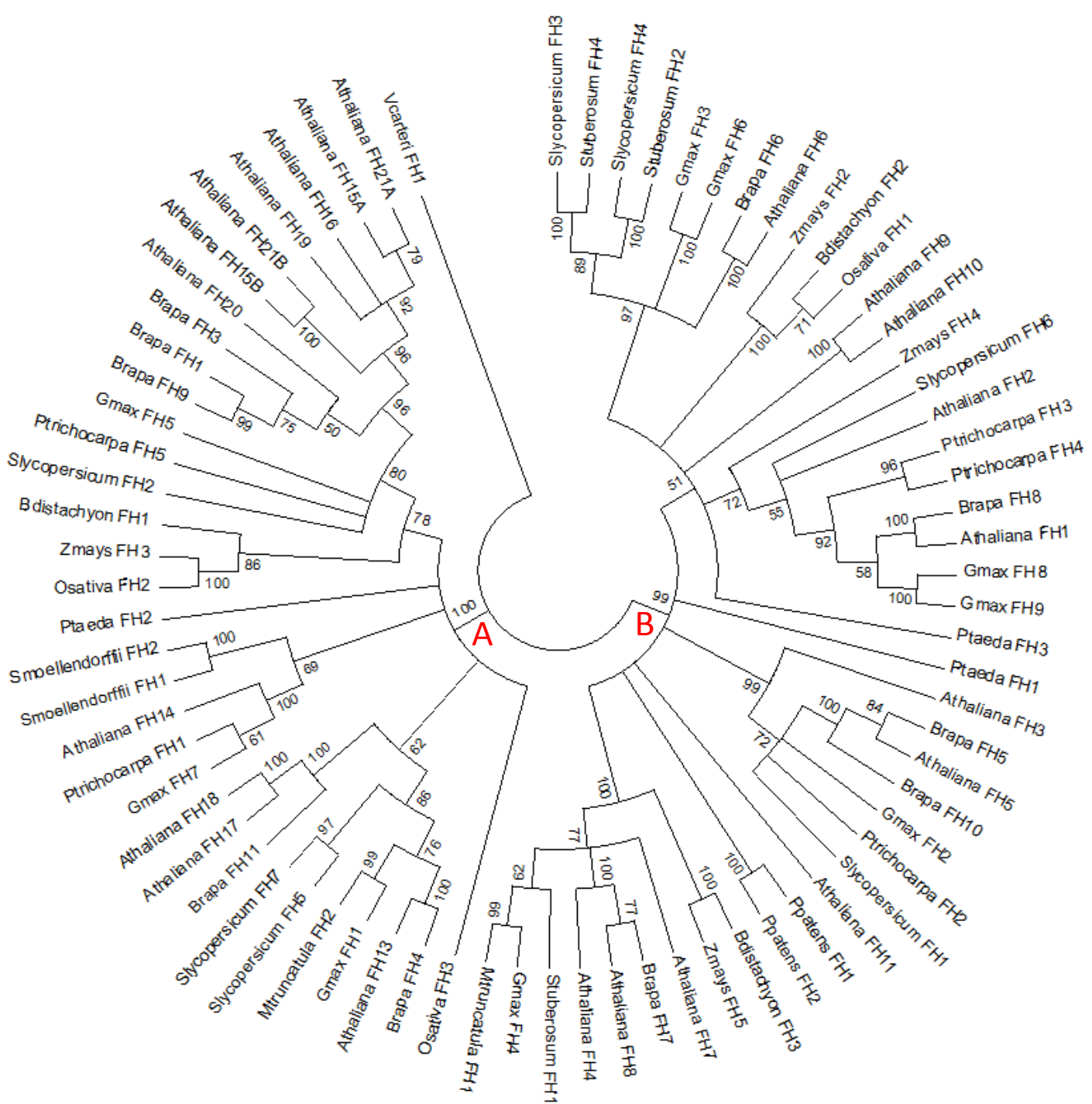

Supplement: S7 Fig — The evolutionary history was inferred using the Maximum Parsimony method. The bootstrap consensus tree inferred from 1000 replicates is taken to represent the evolutionary history of the taxa analyzed. Branches corresponding to partitions reproduced in less than 50% bootstrap replicates are collapsed. The MP tree was obtained using the Tree-Bisection-Regrafting (TBR) algorithm with search level 1 in which the initial trees were obtained by the random addition of sequences (10 replicates). (PDF) [file pone.0150177.s007.pdf]
