# Supplementary material for: Bioinformatic Identification and Analysis of Extensins in the Plant Kingdom
Source: PLoS One. 2016 Feb 26;11(2):e0150177. doi: 10.1371/journal.pone.0150177 (PMC4769139; doi:10.1371/journal.pone.0150177)
Supplement: S6 Fig — The analysis involved 93 amino acid sequences. There were a total of 283 positions in the final dataset. (PDF) [file pone.0150177.s006.pdf]

S6 Fig

```
                        1        10        20        30        40        50        60
                        |        |         |         |         |         |         |
   Bdistachyon_PERK2    FTPENLSAISNDFAEENLLGEGGFGCVYKGILPDGRPVAIKKLKIGNGQGEREFRAEVDT
       Osativa_PERK1    FTPENLAEFTNGFAEQNLLGEGGFGCVYKGILPDNRLVAVKKLKIGNGQGEREFKAEVDT
   Bdistachyon_PERK3    FTYEELHKITNGFSAQNLLGEGGFGSVYKGCLAEGRLVAIKKLKDGSGQGEREFQAEVEI
       Osativa_PERK3    FTYEELHQITNGFAAKNLLGEGGFGSVYKGCLADGREVAVKKLKGGGGQGEREFQAEVEI
         Zmays_PERK3    FTYEELYQVTNGFSAQNLLGEGGFGSVYKGCLADG-EFAVKKLKDGGGQGEREFHAEVDI
         Zmays_PERK6    FTYEELYQITNGFSSQNLLGEGGFGSVYKGCLADGREVAVKKLKDGGGQGEREFHAEVDI
         Zmays_PERK2    FSYDELYQITDGFSAQRLLGEGGFGSVYKGRLPDYKDVAVKRLKDGGGQGEREFQAEVEI
         Zmays_PERK1    FAFDELYGITGGFARENVLGEGGFGCVFKGTLGDGKVVAVKQLKGGGGQGEREFQAEVEI
    Athaliana_PERK10    FSYEELVIATNGFSDENLLGEGGFGRVYKGVLPDERVVAVKQLKIGGGQGDREFKAEVDT
         Brapa_PERK7    FSYQELVKATNGFSEENLLGEGGFGCVYKGVLPDGRVVAVKQLKVGGGQGDREFKAEVET
          Gmax_PERK8    FSYEELIKATNGFSTQNLLGEGGFGCVYKGCLPDGREIAVKQLKIGGGQGEREFKAEVEI
  Ptrichocarpa_PERK2    FAFEELVKATNGFSSQNLLGEGGFGSVYKGYLPDGRDVAVKQLKIGGGQGEREFKAEVEI
 Ptrichocarpa_PERK12    FAFEELVKATNGFSSQNLLGEGGFGTVYKGYLPDGRDVAVKQLKIGGGQGEREFKAEVEI
        Brapa_PERK13    FSYDELAQVTNGFSQKNLLGEGGFGCVYKGVLSDGREIAVKQLKIGGSQGEREFKAEVEI
     Athaliana_PERK8    FSYDELSQVTSGFSEKNLLGEGGFGCVYKGVLSDGREVAVKQLKIGGSQGEREFKAEVEI
         Gmax_PERK13    FTYEELIQATNGFSAQNLLGEGGFGCVYKGLLIDGREVAVKQLKIGGGQGEREFRAEVEI
  Ptrichocarpa_PERK3    FTYEELVQATNGFSAQNRLGEGGFGCVYKGVLVDGREVAVKQLKIGGSQGEREFRAEVEI
  Ptrichocarpa_PERK6    FTYEELVQATNGFSAQNRLGEGGFGCVYKGVLVDGRDVAVKQLKIGGSQGEREFRAEVEI
   Stuberosum_PERK4     FTYEELSEATNGFSPDSVLGEGGFGCVYKGVLNDGREVAVKQLKSGSGQGEREFRAEVEI
 Slycopersicum_PERK5    FTYEELVKATGDFSAENLLGAGGFGSVYKGCLPDGRDVAVKQLDIGGRQGDREFRAEVEI
    Stuberosum_PERK2    FTYEELVKATGDFSAENLLGAGGFGSVYKGCLPDGRDVAVKQLDIGGSQGDREFRAEVEI
 Slycopersicum_PERK6    FTYQELLEATNEFSEHNLLGEGGFGSVYKGCLANGRDIAVKKLNIYGSQGEREFRAEVEI
 Slycopersicum_PERK1    FTYQELVEATNDFSAKNELGKGGFGSVYKGYLADGRYVAVKQLNIGGSQGEREFRAEVEI
   Bdistachyon_PERK1    FTYDELAGITGGFSAENVIGEGGFGKVYMGALGDGRRVAVKQLKVGGGQGEKEFRAEVEI
       Osativa_PERK4    FTYDELAAVTGGFAEEKVIGEGGFGKVYMGALGDGRCVAVKQLKVGSGQGEKEFRAEVDT
         Zmays_PERK7    FTYDELAGITGGFSAANVIGEGGFGKVYMGALGDGRRVAVKQLKLGSGQGEKEFRAEVDI
       Osativa_PERK6    FSYEELTGITSNFSRDNVIGEGGFGCVYKGWLSDGKCVAVKQLKAGSGQGEREFQAEVEI
         Brapa_PERK3    FTFEELSEITEGFSKRFVIGEGGFGCVFKGILSEGKPIAIKQLKSISAEGYREFKAEVEI
    Athaliana_PERK11    FTYEELSQITEGFCKSFVVGEGGFGCVYKGILFEGKPVAIKQLKSVSAEGYREFKAEVEI
         Brapa_PERK2    FAYEELMEITQGFAKQNILGEGGFGCVYKGKLHDGKLVAVKQLKVGSGQGDREFKAEVEI
    Athaliana_PERK13    FTYEELTDITEGFSKHNILGEGGFGCVYKGKLNDGKLVAVKQLKVGSGQGDREFKAEVEI
         Brapa_PERK5    FTYEELMDITEGFAQRNILGEGGFGCVYKGKLHDGKLVAVKQLKVGSGQGDREFKAEVEI
        Brapa_PERK14    FSYEELADITQGFARHNILGEGGFGCVYKGTLHDGKVVAVKQLKAGSGQGDREFKAEVEI
    Athaliana_PERK12    FSYEELAEITQGFARKNILGEGGFGCVYKGTLQDGKVVAVKQLKAGSGQGDREFKAEVEI
  Ptrichocarpa_PERK1    FSYHELMEITNGFARQNIIGEGGFGCVFKGCMTDGKAVAVKQLKAGSGQGEREFKAEVEI
 Ptrichocarpa_PERK11    FSYHELMEITSGFARQNIIGEGGFGCVYKGCMADGKVVAVKQLKAGSGQGDREFKAEVEI
          Gmax_PERK2    FTYEKIAEITNGFASENIIGEGGFGYVYKASMPDGRVGALKMLKAGSGQGEREFRAEVDI
          Gmax_PERK9    FTYEKVAEITNGFASENIIGEGGFGYVYKASMPDGRVGALKLLKAGSGQGEREFRAEVDI
          Gmax_PERK6    FTYEMVMEMTNAFSTQNVIGEGGFGCVYKGWLPDGKAVAVKQLKAGGRQGEREFKAEVEI
         Gmax_PERK14    FTYEMVMEMTNAFSTQNVIGEGGFGCVYKGWLPDGKTVAVKQLKAGSGQGEREFKAEVEI
          Gmax_PERK4    FTYDELSAATGGFSQRNLLGQGGFGYVHKGVLPNGKEIAVKSLKSTGGQGDREFQAEVDI
   Mtruncatula_PERK1    FSYEELSTATGGFSKQNLLGQGGFGYVHKGILPNGKEIAVKSLKSTGGQGDREFQAEVDT
         Brapa_PERK4    FTYEELAAATQGFSKDHLLGQGGFGYVHKGVLPNGKEIAVKSLKAGSGQGEREFQAEVDI
     Athaliana_PERK7    FTYEELASATQGFSKDRLLGQGGFGYVHKGILPNGKEIAVKSLKAGSGQGEREFQAEVEI
        Brapa_PERK10    FTYDELAAATQNFSQARLLGQGGFGYVHKGILPNGKEIAVKSLKAGSGQGEREFQAEVDI
     Athaliana_PERK6    FTYDELAAATQGFSQSRLLGQGGFGYVHKGILPNGKEIAVKSLKAGSGQGEREFQAEVDI
  Ptrichocarpa_PERK4    FSYEELAAATEGFSQAKLLGQGGFGYVHKGVLPNGKEIAVKSLKAGSGQGDREFQAEVEI
  Ptrichocarpa_PERK5    FSYGELAAATAGFSQANLLGQGGFGYVHKGVLPNGKEIAVKSLKTGSGQGDREFQAEVEI
         Brapa_PERK8    FTYDELSIATEGFAQSNLLGQGGFGYVHKGVLPGGKEVAVKSLKLGSGQGEREFQAEVEI
     Athaliana_PERK5    FTYDELSIATEGFAQSNLLGQGGFGYVHKGVLPSGKEVAVKSLKLGSGQGEREFQAEVDI
         Zmays_PERK9    FSYEELAAATSGFSSANLLGQGGFGYVYKGVLASGKEVAVKQLKSGSGQGEREFQAEVEI
        Zmays_PERK10    FSYEELAAATGGFSSTNLLGQGGFGYVYKGVLASGKEVAVKQLKAGSGQGEREFQAEVEI
         Brapa_PERK6    FTYQELAAATGGFADSNLLGQGGFGYVHKGVLSSGKEVAVKSLKSGSGQGEREFQAEVDI
        Brapa_PERK12    FTYQELADATGGFVDSNLLGQGGFGYVHKGVLPNGKEVAVKSLKSGSGQGEREFQAEVDI
     Athaliana_PERK4    FTYQELAAATGGFTDANLLGQGGFGYVHKGVLPSGKEVAVKSLKAGSGQGEREFQAEVDI
  Ptrichocarpa_PERK9    FTYDELAAATGGFDQANLLGQGGFGYVHKGVLPNGKEIAVKSLKAGTGQGEREFQAEVDI
 Ptrichocarpa_PERK10    FTYDELAAATNGFDQANLLGQGGFGYVHKGVLPNGKDIAVKSLKLGSGQGEREFQAEVDI
          Gmax_PERK3    FSYEELAAATNGFNDANLIGQGGFGYVHKGVLPSGKEVAVKSLKAGSGQGEREFQAEIDI
          Gmax_PERK7    FTYEELAAATNGFNDANLIGQGGFGYVHKGVLPSGKEVAVKSLKAGSGQGEREFQAEIDI
   Mtruncatula_PERK3    FTYEELAAATDGFIDSNLIGQGGFGYVHKGVLPSGKEIAVKSLKSGSGQGEREFQAEIDI
   Mtruncatula_PERK4    FTYEELAAATDGFIDSNLIGQGGFGYVHKGVLPSGKEIAVKSLKSGSGQGEREFQAEIDI
 Slycopersicum_PERK2    FTYEELAKATDGFSQANLLGQGGFGYVHKGVLNDGRVVAIKSLKAGSGQGEREFQAEVEI
    Stuberosum_PERK5    FSYEELAKATDGFSQANLLGQGGFGYVHKGVLNDGRVVAIKSLKAGSGQGEREFQAEVEI
 Slycopersicum_PERK4    FTYADLATATGGFSDANVLGQGGFGFVHKGVLTDGNVVAVKSLKSGSGQGEREFQAEVEI
    Stuberosum_PERK3    FTYADLATATGGFSEANVLGQGGFGFVHKGVLTDGSVVAVKSLKSGSGQGEREFQAEVEI
 Slycopersicum_PERK3    FTYEELARATSGFSKANLLGQGGFGYVHKGVLVDGTVVAVKSLKSGSGQGEREFQAEVDI
   Bdistachyon_PERK4    FTYEELALATGDFSAANLLGQGGFGHVHKGVLPCGRAVAVKQLKSGSGQGEREFRAEVDI
         Zmays_PERK8    FSYEELAAATGDFSAANLLGQGGFGYVHKGVLPGGMVVAVKQLKSDSGQGEREFQAEVDI
         Brapa_PERK9    FTYEELARATNGFSEANWLGQGGFGYVYRGILRNGKEIAVKQLKAGSAQGEREFQAEVGI
     Athaliana_PERK3    FTYGELARATNKFSEANLLGEGGFGFVYKGILNNGNEVAVKQLKVGSAQGEKEFQAEVNI
         Gmax_PERK10    FTYEELARATDGFSDANLLGQGGFGYVHRGILPNGKEVAVKQLKAGSGQGEREFQAEVEI
         Gmax_PERK11    FTYEELARATDGFSDANLLGQGGFGYVHRGILPNGKEVAVKQLKAGSGQGEREFQAEVEI
   Mtruncatula_PERK2    FTYEELARATDGFSDANLLGQGGFGYVHRGILPNGKEVAVKQLKAGSGQGEREFQAEVEI
 Slycopersicum_PERK7    FTYEELVRATDGFSDANLLGQGGFGYVHKGILPNGKEVAVKQLKAGSGQGEREFQAEVEI
    Stuberosum_PERK1    FTYEELVRATDGFSDANLLGQGGFGYVHKGILPNGKEVAVKQLKAGSGQGEREFQAEVEI
  Ptrichocarpa_PERK8    FSYEELARATDGFTDANLLGQGGFGYVHRGVLPNGKEVAVKQLKAGSGQGEREFQAEVEI
     Athaliana_PERK1    FTYEELSRATNGFSEANLLGQGGFGYVHKGILPSGKEVAVKQLKAGSGQGEREFQAEVEI
         Gmax_PERK12    FTYDELSMATDGFSRSNLLGQGGFGYVHKGVLPNGKIVAVKQLKSESRQGEREFHAEVDV
    Athaliana_PERK15    FTYEDLSKATSNFSNTNLLGQGGFGYVHRGVLVDGTLVAIKQLKSGSGQGEREFQAEIQT
         Ptaeda_PERK2   FTYEDLEAATNGFSRTNLLGQGGFGYVYKGILPGNKTIAVKQLKVGGSQGEREFQAEVEI
       Osativa_PERK2    FGYDELAAAADGFSESNLLGQGGFGQVYKGTVR-GQEVAIKKLRSGSGQGEREFQAEVEI
         Zmays_PERK4    FAYDDLAAATDGFSDANLLGQGGFGHVYRGTVA-GQEVAIKKLRAGSGQGDREFRAEVEI
         Zmays_PERK5    VSYAELVAATRGFSDANLLGQGGFGHVYRGTLEGGGEVAIKRLRPGSGQGDREFRAEVEI
    Athaliana_PERK14    FSYEELSKATGGFSEENLLGEGGFGYVHKGVLKNGTEVAVKQLKIGSYQGEREFQAEVDT
  Ptrichocarpa_PERK7    CTYDELVVATNGFSDANLLGQGGFGYVHKGFFPCGKEIAVKQLKEGSNQGEREFQAEVEI
        Ppatens_PERK4   FLFSELQEATGNFSKDNLLGEGGFGRVYKGTLQNGTVVAVKQLNLSGAQGEREFRAEVEV
        Ppatens_PERK5   FTYSELQTATDNFSKDNLLGEGGFGRVYKGTLPNGTVVAVKQLNLSGGQGEREFRAEVEV
        Ppatens_PERK1   FTYNELAVATDNFSKDNLLGEGGFGRVYKGILPNGTVVAVKQLTVGGGQGEREFRAEVEV
        Ppatens_PERK2   FTYDELHAATNGFAIENILGEGGFGRVYKGELPNGKVVAVKQLTLGGGQDKEFRAEVEI
        Ppatens_PERK6   FTYDELHKATNGFDHGNLLGEGGFGRVYKGELPNGKLVAVKQLTVGGGQGDREFRAEVEI
        Ppatens_PERK3   FTYEELSEATNGFAPSAFIGEGGFGKVYKGILRDGTEVAIKKLTTGGHQGDREFLVEVEM
   Bdistachyon_PERK5    YTRRELEEATDGLAAYNVLGEGGYGVVYKGVLRDSTAVAIKNLHNNRGQAEKDFRVEVAT
       Osativa_PERK5    LSMEVARAATKGFDSGNVIGVGGSGAVYEGVLPSGSRVAVKRFQAIGSCTKAFDSELKAM

   Bdistachyon_PERK2    ISRVHHRHLVSLVGYCTAEGQRMLVYDFVPNNTLYYHLHVNEVA-LDWQTRVKIAAGAAR
       Osativa_PERK1    ISRVHHRHLVSLVGYCIADGQRMLVYDFVPNNTLYYHLHVSEAAVLDWRTRVKISAGAAR
   Bdistachyon_PERK3    ISRVHHRHLVSLVGYCISGDQRLLVYDFVPNDTLDYHLHGRGVPVLEWSARVKISAGSAR
       Osativa_PERK3    ISRVHHRHLVSLVGYCISGDQRLLVYDFVPNDTLHHHLHGRGMPVLEWSARVKIAAGSAR
         Zmays_PERK3    ISRVHHRHLVSLVGYCISDEQRLLVYDFVPNNTLHYHLHGLGVPVLEWPSRVKIAAGSAR
         Zmays_PERK6    ISRVHHRHLVSLVGYCISDDQRLLVYDFVPNDTLHYHLHGRGVPVLEWPARVKIAAGSAR
         Zmays_PERK2    ISRVHHRHLVSLVGYCISNDQRLLVYDFVSNNTLHYHLHGHGMPVLEWSARVKIAAGAAR
         Zmays_PERK1    ISRVHHRHLVSLVGYCIAEDHRLLVYDYVSNNTLHHHLHGRGRPVMDWPTRVKIAAGSAR
    Athaliana_PERK10    ISRVHHRNLLSMVGYCISENRRLLIYDYVPNNNLYFHLHGT--PGLDWATRVKIAAGAAR
         Brapa_PERK7    LSRIHHRHLVSIVGHCISDDRRLLIYDFVSNNDLYFHLHVSK-EVLDWATRVRIAAGAAR
          Gmax_PERK8    ISRIHHRHLVSLVGYCIEDNKRLLVYDYVPNNTLYFHLHGEGQPVLEWANRVKIAAGAAR
  Ptrichocarpa_PERK2    ISRVHHRHLVSLVGYCICETRRLLVYDYVPNNTLYFHLHGVGGLALDWATRVKIAAGAAR
 Ptrichocarpa_PERK12    ISRIHHRHLVSLVGYCISETRRLLVYDYVPNNTLHFHLHEVGRPALDWATRVKIAAGAAR
        Brapa_PERK13    ISRVHHRHLVTLVGYCISEQHRLLVYDYVPNNTLHYHLHAPGRPVMTWETRVKVAAGAAR
     Athaliana_PERK8    ISRVHHRHLVTLVGYCISEQHRLLVYDYVPNNTLHYHLHAPGRPVMTWETRVRVAAGAAR
         Gmax_PERK13    ISRVHHRHLVSLVGYCISEHQRLLVYDYVPNDTLHYHLHGENRPVLDWPTRVKVAAGAAR
  Ptrichocarpa_PERK3    ISRVHHRHLVSLVGYCISEHQRLLVYDYLPNDTLYYHLHGEGRPHMDWATRVKVAVGAAR
  Ptrichocarpa_PERK6    ISRVHHRHLVSLVGYCISEHQRLLVYDYLPNDTLYHHLHGEGRPFMDWATRVRVAAGAAR
    Stuberosum_PERK4    ISRVHHRHLVSLVGYCISEQQRLLVYDYVPNDTLDYHLHGKGMQTMDWATRVKVAAGAAR
 Slycopersicum_PERK5    ISRVHHRHLVSLVGYCISENRRLLVYEYVPNNTLYFHLHAEGRPVMDWTTRVKIAVGAAR
    Stuberosum_PERK2    ISRVHHRHLVSLVGYCISENRRLLVYEYVPNNTLYFHLHAEGRPVMDWTTRVKIAVGAAR
 Slycopersicum_PERK6    ISRIHHRHLVSLVGYCIYENGRLLVYDYVSNDTLYFHLHEQGRPVMDWATRVKIAVGAAR
 Slycopersicum_PERK1    ISRIHHRHLVSLVGYCISENKRLLVYDYVSNNTLYFHLHAQGRPVMNWPTRVKIAVGAAR
   Bdistachyon_PERK1    ISRIHHRHLVTLVGYCVTENHRLLVYEFVCNNTLEHHLHGKGRPVMDWPKRMKIAIGSAR
       Osativa_PERK4    ISRVHHRHLVTLVGYSVTEHHHLLVYEFVSNKTLDHHLHGGGLPVMDWPKRMKIAIGSAR
         Zmays_PERK7    ISRIHHRHLVTLVGYCVTENHRLLVYEFVANKTLEHHLHGKGLPVMDWPKRMRIAIGAAR
       Osativa_PERK6    ISRVHHRHLVSLVGYCIAAHHRMLIYEFVPNGTLEHHLHGRGMPVMDWPTRLRIAIGAAK
         Brapa_PERK3    ISRVHHRHLVSLVGYCICEQHRFLIYEFVPNNTLDYHLHGKDLPVLEWTRRVKIAIGAAK
    Athaliana_PERK11    ISRVHHRHLVSLVGYCISEQHRFLIYEFVPNNTLDYHLHGKNLPVLEWSRRVRIAIGAAK
         Brapa_PERK2    ISRVHHRHLVSLVGYCISDVQRLLIYEYVPNQTLEHHLHGKGRPVLDWARRVRIAIGSAK
    Athaliana_PERK13    ISRVHHRHLVSLVGYCIADSERLLIYEYVPNQTLEHHLHGKGRPVLEWARRVRIAIGSAK
         Brapa_PERK5    ISRVHHRHLVSLVGYCISDVERLLIYEYVPNQTLEHHLHGKGRPVLEWAKRVRIAIGSAK
        Brapa_PERK14    ISRVHHRHLVSLVGYCISDQHRLLIYEYVSNQTLEHHLHGRGRPVLEWSKRVRIAIGSAK
    Athaliana_PERK12    ISRVHHRHLVSLVGYCISDQHRLLIYEYVSNQTLEHHLHGKGLPVLEWSKRVRIAIGSAK
  Ptrichocarpa_PERK1    ISRVHHRHLVSLVGYCISDNQRLLIYEFVPNKTLEHHLHAKDMPVLDWPKRLKIAIGSAK
 Ptrichocarpa_PERK11    ISRVHHRHLVSLVGYCISDNQRLLIYEFVPNKTLENHLHGKELPVLDWPKRLKIAIGSAK
          Gmax_PERK2    ISRIHHRHLVSLIGYCISEQQRVLIYEFVPNGNLSQHLHGSERPILDWPKRMKIAIGSAR
          Gmax_PERK9    ISRIHHRHLVSLIGYCISEQQRVLIYEFVPNGNLSQHLHGSKWPILDWPKRMKIAIGSAR
          Gmax_PERK6    ISRVHHRHLVSLVGYCICEQQRILIYEYVPNGTLHHHLHASGMPVLNWDKRLKIAIGAAK
         Gmax_PERK14    ISRVHHRHLVALVGYCICEQQRILIYEYVPNGTLHHHLHESGMPVLDWAKRLKIAIGAAK
          Gmax_PERK4    ISRVHHRHLVSLVGYCMSESKKLLVYEFVPKGTLEFHLHGKGRPVMDWNTRLKIAIGSAK
   Mtruncatula_PERK1    ISRVHHRYLVSLVGYCISESKKLLVYEFVPNKTLDYHLHGKGRPVMDWATRLKIAVGSAK
         Brapa_PERK4    ISRVHHRHLVSLVGYCSNSGGRLLVYEFLPNDTLEFHLHGKTGTVMDWPTRLKIALGSAK
     Athaliana_PERK7    ISRVHHRHLVSLVGYCSNAGGRLLVYEFLPNDTLEFHLHGKSGTVMDWPTRLKIALGSAK
        Brapa_PERK10    ISRVHHRFLVSLVGYCIAEGQMLLVYEFLPNDTLEFHLHGKSGNVLDWPTRLRIALGSAK
     Athaliana_PERK6    ISRVHHRFLVSLVGYCIAGGQRMLVYEFLPNDTLEFHLHGKSGKVLDWPTRLKIALGSAK
  Ptrichocarpa_PERK4    ISRVHHRHLVSLVGYCIAGDKKLLVYEFVPNSTLEFHLHGKGRPTMDWPTRLKIALGSAK
  Ptrichocarpa_PERK5    ISRVHHRHLVSLVGYCIEGGQRLLVYEFVPNSTLEFHLHGKGHPPMGWPTRLKIALGSAR
         Brapa_PERK8    ISRVHHRHLVSLVGYCISGGQRLLVYEFLPNNTLEFHLYGKGRPVLEWSIRLKIALGSAR
     Athaliana_PERK5    ISRVHHRHLVSLVGYCISGGQRLLVYEFIPNNTLEFHLHGKGRPVLDWPTRVKIALGSAR
         Zmays_PERK9    ISRVHHRHLVSLVGYCIAGN-RMLVYEFVANNTLEHHLYAKDGPVMDWSTRMKIALGSAK
        Zmays_PERK10    ISRVHHRHLVSLVGYCIAGSSRLLVYEFVPNNTLEHHLHGKGVPVMAWPARLAIALGSAK
         Brapa_PERK6    ISRVHHRYLVSLVGYCIADAQRMLVYEFVPNNTLEYHLHGKNLPVMDFSTRMRIALGAGK
        Brapa_PERK12    ISRVHHRYLVSLVGYCIADGQRMLVYEFVPNNTLEYHLHGKNLPVMDFSTRMRIALGSAK
     Athaliana_PERK4    ISRVHHRYLVSLVGYCIADGQRMLVYEFVPNKTLEYHLHGKNLPVMEFSTRLRIALGAAK
  Ptrichocarpa_PERK9    ISRVHHRHLVSLVGYCIAGGQRMLVYEFVPNKTLEHHLHGKGLPVMEWPTRLRIALGSAK
 Ptrichocarpa_PERK10    ISRVHHRHLVSLVGYCIAGGQRMLVYEFVPNKTLEHHLHGKGLPVMDWPTRLRIALGSAK
          Gmax_PERK3    ISRVHHRHLVSLVGYSISGGQRMLVYEFIPNNTLEYHLHGKGRPTMDWATRMRIAIGSAK
          Gmax_PERK7    ISRVHHRHLVSLVGYSISGGQRMLVYEFIPNNTLEYHLHGKGRPTMDWATRMRIAIGSAK
   Mtruncatula_PERK3    ISRVHHRHLVSLVGYCISGGQRMLVYEFISNNTLEYHLHGKGRPTMDWPTRMRIAIGSAK
   Mtruncatula_PERK4    ISRVHHRHLVSLVGYCISGGQRMLVYEFISNNTLEYHLHGKGRPTMDWPTRMRIAIGSAK
 Slycopersicum_PERK2    ISRVHHRHLVSLVGYCIANGQRMLVYEYVDNKTLEFHLHGKGQPVMDWETRLKIALGSAK
    Stuberosum_PERK5    ISRVHHRHLVSLVGYCIANGQRMLVYEYVDNKTLEFHLHGKGQPVMDWETRLKIALGSAK
 Slycopersicum_PERK4    ISRVHHRHLVSLVGYCIADGQRMLVYEFVPNGTLEYHLHGKGRPVMDWGLRLKIALGSAK
    Stuberosum_PERK3    ISRVHHRHLVSLVGYCIADGQRMLVYEFVSNGTLEYHLHGKGRPVMDWGLRLKIALGSAK
 Slycopersicum_PERK3    ISRVHHRHLVSLVGYCIADRQRMLVYEYVPNKTLEFHLHGKGHRVMDWGTRLKIALGSAK
   Bdistachyon_PERK4    IGRVHHRHLVSLVGHCVAGDRRMLVYEFVPNKTLEFHLHGKGLPAMAWATRLRIALGAAK
         Zmays_PERK8    ISRVHHRHLVSLVGHCIAGARRVLVYQFVPNKTLEFHLHGKGQPVMEWSTRLRIALGSAK
         Brapa_PERK9    ISRVHHRHLVALVGYCIADAQRLLVYEFAPNNTLEFHLHGKGRPPLAWSSRLKISVGSAK
     Athaliana_PERK3    ISQIHHRNLVSLVGYCIAGAQRLLVYEFVPNNTLEFHLHGKGRPTMEWSLRLKIAVSSSK
         Gmax_PERK10    ISRVHHKHLVSLVGYCITGSQRLLVYEFVPNNTLEFHLHGKGRPTMDWPTRLRIALGSAK
         Gmax_PERK11    ISRVHHKHLVSLVGYCITGSQRLLVYEFVPNNTLEFHLHGRGRPTMDWPTRLRIALGSAK
   Mtruncatula_PERK2    ISRVHHKHLVSLVGYCSTGFQRLLVYEFVPNNTLEFHLHGKGRPTMDWSTRLRIALGSAK
 Slycopersicum_PERK7    ISRVHHKHLVSLVGYCITGAQRLLVYEFVPNNTLEFHLHGKGRPPLDWPIRLKIALGSAK
    Stuberosum_PERK1    ISRVHHKHLVSLVGYCITGAQRLLVYEFVPNNTLEFHLHGKGRPPLDWRIRLKIALGSAK
  Ptrichocarpa_PERK8    ISRVHHKHLVSLVGYCITGAHRLLVYEFVPNNTLEFHLHGKGRPTMDWPTRLKIALGSAK
     Athaliana_PERK1    ISRVHHRHLVSLIGYCMAGVQRLLVYEFVPNNNLEFHLHGKGRPTMEWSTRLKIALGSAK
         Gmax_PERK12    ISRVHHRHLVSLVGYCVSDSQKMLVYEYVENDTLEFHLHGKDRLPMDWSTRMKIAIGSAK
    Athaliana_PERK15    ISRVHHRHLVSLLGYCITGAQRLLVYEFVPNKTLEFHLHEKERPVMEWSKRMKIALGAAK
         Ptaeda_PERK2   ISRVHHRHLVSLVGYCIAGSQRLLVYEFVPNDTLEHHLHGKGQPTMDWPTRLKIATGAAR
       Osativa_PERK2    ISRVHHKNLVSLVGYCIYGEQRLLVYEYVPNKTLEFHLHGSGRPALDWPRRWKIAVGSAK
         Zmays_PERK4    ISRVHHKNLVSLVGYCLHGEQRLLVYEYVPNKTLEFQLHGSGRPTLDWPRRWKIAVGSAK
         Zmays_PERK5    ISRVHHRHLVSLVGYCIHGDQRLLVYEYVPNKTLELHLHGSGRPVLDWQQRWRIALGSAK
    Athaliana_PERK14    ISRVHHKHLVSLVGYCVNGDKRLLVYEFVPKDTLEFHLHENRGSVLEWEMRLRIAVGAAK
  Ptrichocarpa_PERK7    ISRVHHKHLVSLVGYCINGSARLLVYEFVSNNTLEFHLHGTGQPVLEWETRLKIAIGSAK
        Ppatens_PERK4   ISRVHHRHLVSLVGYCVSNQQRLLVYEFVPNGTLENNLHNPDMPVMEWSTRLKIALGCAR
        Ppatens_PERK5   ISRVHHRHLVSLVGYCVSNQQRLLVYEFVPNGTLENNLHNPDMPIMDWNTRLKIGLGCAR
        Ppatens_PERK1   ISRVHHRHLVSLVGYCVADRQRLLVYEFVPNGTLENNLHNTDMPIMEWSTRLKIGLGCAR
        Ppatens_PERK2   ISRVHHRHLVSLVGYCIADKQRLLVYDFVPNGTLDVNLYGNGRPIMNWEMRMRVAVGAAR
        Ppatens_PERK6   ISRVHHRHLVSLVGYCISDKQRLLVYDFVPNGTLDVNLYGRGKPVMTWDLRVRVALGAAR
        Ppatens_PERK3   LSRLHHRNLVKLLGYFCCREPQLLCYELIPNGSVDSWLHGTLCATFDWPTRMKIAIGSAR
   Bdistachyon_PERK5    IGRVRHKNLVSLLGYCSEGACRMLVYEYMENSNLDKWLHHDDSEVSNWDTRMHILLGTAK
       Osativa_PERK5    LNCPHHPNLVPLAGWCRSKDELVLVYEFMPNGNLDSALHTLGGATL-WEARFRAVYGVAS

   Bdistachyon_PERK2    GIAYLHEDCHPRIIHRDIKSSNILLDNNFEAQVSDFGLARLANTHVSTRVMGTFGYLAPE
       Osativa_PERK1    GIAYLHEDCHPRIIHRDIKSSNILLDDNFEAQVSDFGLARLANTHVTTRVMGTFGYLAPE
   Bdistachyon_PERK3    GIAYLHEDCHPRIIHRDIKSSNILVDNNFEAQVADFGLARLAATHVTTRVMGTFGYMAPE
       Osativa_PERK3    GIAYLHEDCHPRIIHRDIKSSNILLDNNFEAQVADFGLARLAVTHVTTRVMGTFGYLAPE
         Zmays_PERK3    GIAYLHEDCHPRIIHRDIKSSNILLDNNFEALVADFGLARIACTHVTTRVMGTFGYLAPE
         Zmays_PERK6    GIAYLHEDCQPRIIHRDIKSSNILLDNNFEALVADFGLARLACTHVTTRVMGTFGYLAPE
         Zmays_PERK2    GIAYLHEDCHPRIIHRDIKSSNILLDNNFEAKVADFGLARLAVTHVTTRVMGTFGYMAPE
         Zmays_PERK1    GLAYLHEDCHPRIIHRDIKSSNILLDDQFEAQVADFGLARLAVTHISTRVMGTFGYLAPE
    Athaliana_PERK10    GLAYLHEDCHPRIIHRDIKSSNILLENNFHALVSDFGLAKLANTHITTRVMGTFGYMAPE
         Brapa_PERK7    GLAYLHEDCHPRIIHRDIKSSNILLEDNFDSRVSDFGLARLANTHITTRVMGTFGYLAPE
          Gmax_PERK8    GLTYLHEDCNPRIIHRDIKSSNILLDFNYEAKVSDFGLAKLANTHITTRVMGTFGYMAPE
  Ptrichocarpa_PERK2    GIAYLHEDCHPRIIHRDIKSSNILLDNNYEAKVSDFGLAKLANTHVTTRVMGTFGYMAPE
 Ptrichocarpa_PERK12    GLAYLHEDCHPRIIHRDIKSSNILLDINFEAKVSDFGLAKLANTHVTTRVMGTFGYMAPE
        Brapa_PERK13    GIAYLHEDCHPRIIHRDIKSSNILLDNSFEALVADFGLAKIADTHVSTRVMGTFGYMAPE
     Athaliana_PERK8    GIAYLHEDCHPRIIHRDIKSSNILLDNSFEALVADFGLAKIADTHVSTRVMGTFGYMAPE
         Gmax_PERK13    GIAYLHEDCHPRIIHRDIKSSNILLDLNYEAQVSDFGLAKLAN-HVTTRVMGTFGYMAPE
  Ptrichocarpa_PERK3    GIAYLHEDCHPRIIHRDIKSSNILLDDNFEAQVSDFGLAKIADTHVSTRVMGTFGYMAPE
  Ptrichocarpa_PERK6    GIAYLHEDCHPRIIHRDIKSSNILLDENFEAQVSDFGLAKIADTHVSTRVMGTFGYMAPE
    Stuberosum_PERK4    GLAYLHEDCHPRIIHRDIKTSNILLDINFEAQVADFGLARLASTHVTTRVMGTFGYLAPE
 Slycopersicum_PERK5    GIAYLHEDCNPRIIHRDIKSSNILLDINFEARVSDFGLAKLAKTHVTTRVVGTFGYMAPE
    Stuberosum_PERK2    GIAYLHEDCHPRIIHRDIKSSNILLDINFEARVSDFGLAKLAKTHVTTRVVGTFGYMAPE
 Slycopersicum_PERK6    GIAYLHEDCCPRIIHRDIKSSNILLDNNFDARVADFGLAKLAKSHVTTRVMGTFGYMAPE
 Slycopersicum_PERK1    GIAYLHEDCYPRIIHRDIKSSNILLDNNFEAHVADFGLAKLAESHITTRVVGTFGYMAPE
   Bdistachyon_PERK1    GLTYLHQDCHPRIIHRDIKSANILMDDAFEA-VADFGLAKLTDTHVSTRVMGTFGYMAPE
       Osativa_PERK4    GLTYLHEDCHPRIIHRDIKSANILLDDAFEA-VADFGLAKFTDTHVSTRVMGTFGYLAPE
         Zmays_PERK7    GLTYLHEDCHPRIIHRDIKSANILLDDAFEA-VADFGLAKLTDTHISTRVMGTFGYMAPE
       Osativa_PERK6    GLAYLHEDCHPRIIHRDIKTANILLDYSWEA-VADFGLAKLADTHVSTRIMGTFGYLAPE
         Brapa_PERK3    GLAYLHEDCHPKIIHRDIKSSNILLDDEFEAQVADFGLARLNTSHISTRVMGTFGYLAPE
    Athaliana_PERK11    GLAYLHEDCHPKIIHRDIKSSNILLDDEFEAQVADFGLARLNTSHISTRVMGTFGYLAPE
         Brapa_PERK2    GLAYLHEDCHPKIIHRDIKSANILLDDEFEA-VADFGLAKLSSTHVSTRVMGTFGYLAPE
    Athaliana_PERK13    GLAYLHEDCHPKIIHRDIKSANILLDDEFEA-VADFGLAKLNSTHVSTRVMGTFGYLAPE
         Brapa_PERK5    GLAYLHEDCHPKIIHRDIKSANILLDDDFEA-VADFGLAKLNSTHVSTRVMGTFGYLAPE
        Brapa_PERK14    GLAYLHEDCHPKIIHRDIKSANILLDDEYEA-VADFGLARLNTTHVSTRVMGTFGYLAPE
    Athaliana_PERK12    GLAYLHEDCHPKIIHRDIKSANILLDDEYEA-VADFGLARLNTTHVSTRVMGTFGYLAPE
  Ptrichocarpa_PERK1    GLAYLHEDCHPKIIHRDIKSSNILLDDAFET-VADFGLARLNTTHVSTRVMGTFGYLAPE
 Ptrichocarpa_PERK11    GLAYLHEDCHPKIIHRDIKSANILLDDAFEANVADFGLARLNTTHVSTRVMGTFGYLAPE
          Gmax_PERK2    GLAYLHDGCNPKIIHRDIKSANILLDNAYEA-VADFGLARLTDTHVSTRVMGTFGYMAPE
          Gmax_PERK9    GLAYLHDGCNPKIIHRDIKSANILLDNAYEA-VADFGLARLTDTHVSTRVMGTFGYMAPE
          Gmax_PERK6    GLAYLHEDCCQKIIHRDIKSANILLDNAYEA-VADFGLARLAATHVSTRVMGTFGYMAPE
         Gmax_PERK14    GLAYLHEDCSQKIIHRDIKSANILLDNAYEA-VADFGLARLAATHVSTRVMGTFGYMAPE
          Gmax_PERK4    GLAYLHEDCHPRIIHRDIKGANILLENNFEAKVADFGLAKISDTHVSTRVMGTFGYMAPE
   Mtruncatula_PERK1    GLAYLHEDCHPRIIHRDIKGANILIENNFEAKVADFGLAKFTDTHVSTRVMGTFGYMAPE
         Brapa_PERK4    GLAYLHEDCHPKIIHRDIKASNILLDLNFEAKVADFGLAKLSDTHVSTRVMGTFGYLAPE
     Athaliana_PERK7    GLAYLHEDCHPKIIHRDIKASNILLDHNFEAKVADFGLAKLSDTHVSTRVMGTFGYLAPE
        Brapa_PERK10    GLAYLHEDCHPKIIHRDIKASNILLDESFEAKVADFGLAKLSDTHVSTRIMGTFGYLAPE
     Athaliana_PERK6    GLAYLHEDCHPRIIHRDIKASNILLDESFEAKVADFGLAKLSDTHVSTRIMGTFGYLAPE
  Ptrichocarpa_PERK4    GLAYLHEDCHPRIIHRDIKAANILLDYSFEAMVADFGLAKLSDTHVSTRVMGTFGYLAPE
  Ptrichocarpa_PERK5    GLAYLHEDCHPRIIHRDIKAANILLDYSFEAMVADFGLAKLSDTHVSTRIMGTFGYLAPE
         Brapa_PERK8    GLAYLHEDCHPKIIHRDIKAANILLDFSFETKVADFGLAKLSDTHVSTRVMGTFGYLAPE
     Athaliana_PERK5    GLAYLHEDCHPRIIHRDIKAANILLDFSFETKVADFGLAKLSDTHVSTRVMGTFGYLAPE
         Zmays_PERK9    GLAYLHEDCHPRIIHRDIKAANILLDNNFEAMVADFGLAKLTDTHVSTRVMGTFGYLAPE
        Zmays_PERK10    GLAYLHEDCHPRIIHRDIKAANILLDENFEAKVADFGLAKLTDTHVSTRVMGTFGYLAPE
         Brapa_PERK6    GLAYLHEDCHPRIIHRDIKSANILLDFNFDAMVADFGLAKLTDTHVSTRVMGTFGYLAPE
        Brapa_PERK12    GLAYLHEDCHPRIIHRDIKSANILLDFNFDAMVADFGLAKLTDTHVSTRVMGTFGYLAPE
     Athaliana_PERK4    GLAYLHEDCHPRIIHRDIKSANILLDFNFDAMVADFGLAKLTDTHVSTRVMGTFGYLAPE
  Ptrichocarpa_PERK9    GLAYLHEDCHPRIIHRDIKAANILIDNNFEAMVADFGLAKLSDTHVSTRVMGTFGYLAPE
 Ptrichocarpa_PERK10    GLAYLHEDCHPRIIHRDIKAANILIDNNFEAMVADFGLAKLSDTHVSTRVMGTFGYLAPE
          Gmax_PERK3    GLAYLHEDCHPRIIHRDIKAANVLIDDSFEAKVADFGLAKLTDTHVSTRVMGTFGYLAPE
          Gmax_PERK7    GLAYLHEDCHPRIIHRDIKAANVLIDDSFEAKVADFGLAKLTDTHVSTRVMGTFGYLAPE
   Mtruncatula_PERK3    GLAYLHEDCHPRIIHRDIKAANVLIDDSFEAKVADFGLAKLTDTHVSTRVMGTFGYLAPE
   Mtruncatula_PERK4    GLAYLHEDCHPRIIHRDIKAANVLIDDSFEAKVADFGLAKLTDTHVSTRVMGTFGYLAPE
 Slycopersicum_PERK2    GLAYLHEDCQHRIIHRDIKAANILLDLNYEALVADFGLAKLTDTHVSTRVMGTFGYLAPE
    Stuberosum_PERK5    GLAYLHEDCQYRIIHRDIKAANILLDLNYEALVADFGLAKLTDTHVSTRVMGTFGYLAPE
 Slycopersicum_PERK4    GLAYLHEDCHPRIIHRDIKGANILLDNNYEAMVADFGLARLTDTHVSTRVMGTFGYLAPE
    Stuberosum_PERK3    GLAYLHEDCHPRIIHRDIKGANILLDNNYEAMVADFGLAKLTDTHVSTRVMGTFGYLAPE
 Slycopersicum_PERK3    GLAYLHEDCHPKIIHRDIKAANILLEDNFEAKVADFGLAKLSDTHVSTRVMGTFGYMAPE
   Bdistachyon_PERK4    GLAYLHEDCHPRIIHRDIKSANILLDDNFEAMVADFGLAKLTDTHVSTRVMGTFGYLAPE
         Zmays_PERK8    GLAYLHEDCHPRIIHRDIKSANILLDNNFQAKVADFGLAKLTDTHVSTRVMGTFGYLAPE
         Brapa_PERK9    GLSYLHENCNPKIIHRDIKAANILLDFKFEAKVADFGLAKIADTHVSTRVMGTFGYLAPE
     Athaliana_PERK3    GLSYLHENCNPKIIHRDIKAANILIDFKFEAKVADFGLAKIADTHVSTRVMGTFGYLAPE
         Gmax_PERK10    GLAYLHEDCHPKIIHRDIKSANILLDFKFEAKVADFGLAKFSDTHVSTRVMGTFGYLAPE
         Gmax_PERK11    GLAYLHEDCHPKIIHRDIKAANILLDFKFEAKVADFGLAKFSDTHVSTRVMGTFGYLAPE
   Mtruncatula_PERK2    GLAYLHEDCHPKIIHRDIKAANILLDFKFEAKVADFGLAKIADTHVSTRVMGTFGYLAPE
 Slycopersicum_PERK7    GLAYLHEDCQPKIIHRDIKAANILVDFNFEAKVADFGLAKLTDTHVSTRVMGTFGYLAPE
    Stuberosum_PERK1    GLAYLHEDCQPKIIHRDIKAANILIDFNFEAKVADFGLAKLTDTHVSTRVMGTFGYLAPE
  Ptrichocarpa_PERK8    GLAYLHEDCHPKIIHRDIKASNILLDFKFEAKVADFGLAKISDTHVSTRVMGTFGYLAPE
     Athaliana_PERK1    GLSYLHEDCNPKIIHRDIKASNILIDFKFEAKVADFGLAKIADTHVSTRVMGTFGYLAPE
         Gmax_PERK12    GLAYLHEDCNPKIIHRDIKASNILLDESFEAKVADFGLAKFSDTHVSTRVMGTFGYMAPE
    Athaliana_PERK15    GLAYLHEDCNPKTIHRDVKAANILIDDSYEAKLADFGLARSSDTHVSTRIMGTFGYLAPE
         Ptaeda_PERK2   GLAYLHEDCYPKIIHRDIKASNILLDSNFEAKVADFGLAKLAETHVSTRVMGTFGYLAPE
       Osativa_PERK2    GLAYLHEDCHPKIIHRDIKAANILLDYTFEPKVADFGLAKYQTTAVSTRVMGTFGYLAPE
         Zmays_PERK4    GLAYLHEDCHPKIIHRDIKAANILLDYNYEPKVADFGLAKYQATSVSTRVMGTFGYLAPE
         Zmays_PERK5    GLAYLHEDCDPKIIHRDIKAANILLDYNFEPRVSDFGLAKIQATHVSTRVMGTFGYLAPE
    Athaliana_PERK14    GLAYLHEDCSPTIIHRDIKAANILLDSKFEAKVSDFGLAKFFSTHISTRVVGTFGYMAPE
  Ptrichocarpa_PERK7    GLAYLHEDCHPKIIHRDIKASNILLDHNFEAKVSDFGLAKSFATHISTRVVGTFGYMAPE
        Ppatens_PERK4   GLAYLHEDCHPKIIHRDIKSSNILLDENFEAQVADFGLAKLSDTHVSTRVMGTFGYLAPE
        Ppatens_PERK5   GLAYLHEDCHPKIIHRDIKSSNILLDEKFEAQVADFGLAKLSDTHVSTRVMGTFGYLAPE
        Ppatens_PERK1   GLAYLHEDCHPKIIHRDIKSSNILLEENFEAKVADFGLAKLSDTHVSTRVMGTFGYLAPE
        Ppatens_PERK2   GLAYLHEDCHPRIIHRDIKSSNILLDDKYEAQVADFGLAKLADTHVSTRVMGTFGYLAPE
        Ppatens_PERK6   GLAYLHEDCHPRIIHRDIKSSNILLDDKYEAQVADFGLARPADTHVSTRVMGTFGYLAPE
        Ppatens_PERK3   GLQYLHEDSQPCVIHRDFKASNILLQNNFHAKVADFGLARLAGNYVSTRVMGTFGYVAPE
   Bdistachyon_PERK5    GLAYLHEGLEPKIVHRDVKSSNILLDGQWNA-VSDFGLAKLL-SYVTTRVMGTFGYVAPE
       Osativa_PERK5    ALAYLHDECENRIIHRDVKSSNVMLDAEFNA-LGDFGLARTVHLPLTTQPAGTLGYLAPE

   Bdistachyon_PERK2    YALSGKLTAKSDLYSFGVVLLELITGRKPVDSSQLGDESLVEW-RPFLMQAIEHRDFGDL
       Osativa_PERK1    YALSGKLTAKSDVYSFGVVLLELITGRKPVDASQLGDESLVEW-RPLLLKAIEHREFGDL
   Bdistachyon_PERK3    YASSGKLTEKSDVFSFGVVLLELITGRKPVDASNLGDESLVEW-RPLLTEALGTGNVGEL
       Osativa_PERK3    YASSGKLTERSDVFSFGVVLLELITGRKPVDASKLGDESLVEW-RPLLTEAIETGNVGEL
         Zmays_PERK3    YASSGKLTERSDVFSFGVVLLELITGRKPVDASKLGDESLVEW-RPLLTQALETGNAGEL
         Zmays_PERK6    YASSGKLTERSDVFSFGVVLLELITGRKPVDASKLGDESLVEW-RPLLTQALETGNAGEL
         Zmays_PERK2    YASSGKLTERSDVFSFGVVLLELITGRKPVDASRMGDESLVEW-RPLLSRALDTGDLEGL
         Zmays_PERK1    YASTGKLTEKSDVFSFGVVLLELITGRKPVDSSRLGDESLVEW-RPLLNRAIETQEFDEL
    Athaliana_PERK10    YASSGKLTEKSDVFSFGVVLLELITGRKPVDASQLGDESLVEWARPLLSNATETEEFTAL
         Brapa_PERK7    YASSGKLTEKSDVYSFGVVLLELITGRKPVDTSQLGEESLVEWARPLISHAIETEEFGSL
          Gmax_PERK8    YASSGKLTEKSDVYSFGVVLLELITGRKPVDASQLGDESLVEWARPLLSHALDTEEFDSL
  Ptrichocarpa_PERK2    YASSGKLTEKSDVFSYGVVLLELITGRKPVDASQMGEESLVEWARPLLNHALENEELESL
 Ptrichocarpa_PERK12    YASSGKLTDKSDVFSYGVVLLELITGRKPVDASQVGDESLVEWARPLLNHALENEEFESL
        Brapa_PERK13    YATSGKLSEKADVYSYGVILLELITGRKPVDTSQLGDESLVEWAKPLLSQAIENEEFGEL
     Athaliana_PERK8    YATSGKLSEKADVYSYGVILLELITGRKPVDTSQLGDESLVEWARPLLGQAIENEEFDEL
         Gmax_PERK13    YATSGKLTEKSDVYSFGVVLLELITGRKPVDASQIGDESLVEWARPLLTEALDNEDFEIL
  Ptrichocarpa_PERK3    YATSGKLTEKSDVYSFGVVLLELITSRKPVDASQIGDESLVEWARPLLTTALENEDFEAL
  Ptrichocarpa_PERK6    YATSGKLTEKSDVYSYGVVLLELITGRKPVDASQLGDESLVEWARPLLTDAIENEDFEAL
    Stuberosum_PERK4    YASSGKLTEKSDVYSYGVVLLELITGRKPVDQSQLGDESLVEWARPLLAQALETENFENV
 Slycopersicum_PERK5    YASTGKLTEKSDIYSFGVVLLELITGRKPVDTSQLGDESLVEWARPLLSHALEKVEFDQL
    Stuberosum_PERK2    YASSGKLTEKSDIYSFGVVLLELITGRKPVDTSQLGDESLVEWARPLLSHALEKLEFDQL
 Slycopersicum_PERK6    YASSGKLTEKSDVFSFGVVLLELITGRKSVDASQLGEESLVEWARPLLGNALEKEEFDQL
 Slycopersicum_PERK1    YASTGKLTEKSDVYSFGVVLLELITGRKSVDTSQSGQENLVEWARPLLSRALQKEEFDLL
   Bdistachyon_PERK1    YASSGKLTDRSDVFSFGVVLLELITGRKPVDSSQLGEESLVEWARPVLVDALETDDFREL
       Osativa_PERK4    YASSGKLTDRSDVFSFGVVLLELITGRKPVDSSQLGEESLVEWARPLLVDALETDDFREL
         Zmays_PERK7    YAQSGKLTDRSDVFSFGVVLLELITGRKPVDASQLGEESLVEWARLLLVDALETDDFREV
       Osativa_PERK6    YASSGKLTDRSDVFSFGVVLLELITGRKPVDQTQLGEESLVEWARPVLADAVETGDLSEL
         Brapa_PERK3    YASSGKLTDRSDVFSFGVVLLELITGRKPVDTSQLGEESLVEWARPRLIEAIEKGDISEV
    Athaliana_PERK11    YASSGKLTDRSDVFSFGVVLLELITGRKPVDTSQLGEESLVEWARPRLIEAIEKGDISEV
         Brapa_PERK2    YAQSGKLTDRSDVFSFGVVLLELITGRKPVDQYMLGEESLVEWARPLLHKAIETGDFSEL
    Athaliana_PERK13    YAQSGKLTDRSDVFSFGVVLLELITGRKPVDQYQLGEESLVEWARPLLHKAIETGDFSEL
         Brapa_PERK5    YAQSGKLTDRSDVFSFGVVLLELVTGRKPVDQYQLGEESLVEWARPLLHKAIETGDFSDL
        Brapa_PERK14    YASSGKLTDRSDVFSFGVVLLELVTGRKPVDQNQLGEESLVEWARPLLLKAIETGDYSEL
    Athaliana_PERK12    YASSGKLTDRSDVFSFGVVLLELVTGRKPVDQTQLGEESLVEWARPLLLKAIETGDLSEL
  Ptrichocarpa_PERK1    YASSGKLTDRSDVFSFGVVLLELITGRKPVDASQLGDESLVEWARPLLIHALETGEFGEL
 Ptrichocarpa_PERK11    YASSGKLTDRSDVFSFGVVLLELITGRKPVDASQLGDESLVEWARPLLIHALETGELGEL
          Gmax_PERK2    YATSGKLTDRSDVFSFGVVLLELITGRKPVDPMQIGEESLVEWARPLLLRAVETGDFGEL
          Gmax_PERK9    YATSGKLTDRSDVFSFGVVLLELITGRKPVDPMQIGEESLVEWARPLLLRAVETGDYGKL
          Gmax_PERK6    YATSGKLTDRSDVFSFGVVLLELVTGRKPVDQTQLGDESLVEWARPLLLRAIETRDFSDL
         Gmax_PERK14    YATSGKLTDRSDVFSFGVVLLELVTGRKPVDQTQLGDESLVEWARPLLLRAIETRDFSDL
          Gmax_PERK4    YASSGKLTDKSDVFSFGIMLLELITGRRPVNNTGY-EDTLVDWARPLCTKAMENGTFEGL
   Mtruncatula_PERK1    YASSGKLTDKSDVFSYGVMLLELITGRRPVGTAGYEEDSLVDWARPLCSKALEYGIYLGL
         Brapa_PERK4    YAASGKLTDKSDVFSFGIMLLELITGRRPVDLSGEMEDSLVDWARPLCMNAAQDGEYGEL
     Athaliana_PERK7    YASSGKLTEKSDVFSFGVMLLELITGRGPVDLSGDMEDSLVDWARPLCMRVAQDGEYGEL
        Brapa_PERK10    YASSGKLTDRSDVFSFGVMLLELITGRRPVDLTGEMEDSLVDWARPVCLNAAQDGDYSEL
     Athaliana_PERK6    YASSGKLTDRSDVFSFGVMLLELVTGRRPVDLTGEMEDSLVDWARPICLNAAQDGDYSEL
  Ptrichocarpa_PERK4    YASSGKLTDKSDVFSFGVMLLELITGRLPVDLSGEMDDSLVEWATPLCAKALEDGNYDEL
  Ptrichocarpa_PERK5    YASSGKLTDKSDVFSFGVMLLELITGRRPVDLSGDMDDSLVDWARPFCAKALEDGNYDEL
         Brapa_PERK8    YASSGKLSDKSDVFSFGVMLLELITGRPPVDLTGEMEDSLVDWARPLCMKAAQDGDYSQL
     Athaliana_PERK5    YASSGKLSDKSDVFSFGVMLLELITGRPPLDLTGEMEDSLVDWARPLCLKAAQDGDYNQL
         Zmays_PERK9    YASSGKLTDKSDVFSFGVMLLELLTGRRPIDTTNYMEDSLVDWARPLLSAALAETGFAEL
        Zmays_PERK10    YASSGKLTDKSDVFSFGVMLLELITGRRPVDPTNYMEDSLVDWARPLLARALSEDNFDEL
         Brapa_PERK6    YASSGKLTEKSDVFSYGVMLLELITGKRPVDSSSGTMDDTLVDWARPIMARALEDGNFNEL
        Brapa_PERK12    YASSGKLTEKSDVFSYGVMLLELITGKRPVDSSSTMDDTLVDWARPIMVRALEDGNFNEL
     Athaliana_PERK4    YASSGKLTEKSDVFSYGVMLLELITGKRPVDNSITMDDTLVDWARPLMARALEDGNFNEL
  Ptrichocarpa_PERK9    YAASGKLTEKSDVFSYGVMLLELITGKKPVDPSSAMEDSLVDWARPLTISALETGNFIEL
 Ptrichocarpa_PERK10    YASSGKLTDKSDVFSYGVMLLELITGKKPVDPSSAMEDSLVDWARPLMITSLDTGNYNEL
          Gmax_PERK3    YASSGKLTEKSDVFSFGVMLLELITGKRPVDHTNAMDDSLVDWARPLLTRGLEDGNFGEL
          Gmax_PERK7    YASSGKLTEKSDVFSFGVMLLELITGKRPVDHTNAMDDSLVDWARPLLTRGLEDGNFGEL
   Mtruncatula_PERK3    YASSGKLTEKSDVFSFGVMLLELVTGKRPVDASITMDDSLVDWARPLLTRGLEDGNFSEL
   Mtruncatula_PERK4    YASSGKLTEKSDVFSFGVMLLELVTGKRPVDASITMDDSLVDWARPLLTRGLEDGNFSEL
 Slycopersicum_PERK2    YASSGKLTEKSDVFSYGVMLLELITAKKPVDPSNMMEDSLVDWARPLLTRALEEEKYDGL
    Stuberosum_PERK5    YASSGKLTEKSDVFSYGVMLLELITAKKPVDPSNMMEDSLVDWARPLLTRALEEEKYDGL
 Slycopersicum_PERK4    YASSGKLSEKSDVFSFGVMLLELITGRRPLDTTNLMDDSLVDWARPFLTKALEENNYDEL
    Stuberosum_PERK3    YASSGKLSEKSDVFSFGVMLLELITGRRPLDTTNLMDDSLVDWARPFLTKALEENNYDEL
 Slycopersicum_PERK3    YASSGKLTDRSDVFSFGIVLLELITGKRPIDPSNIMEDSLVEWARPLLKTALEEGKYDEL
   Bdistachyon_PERK4    YASSGKLTEKSDVFSYGVTLLELVTGRRPVDAR-EEDDGLVDWARPAMARALASGDYGGV
         Zmays_PERK8    YASSGKLTDKSDVFSYGVVLLELLTGRRPIDAGDDVDDSLVDWARPALSRALADGDYDGV
         Brapa_PERK9    YAASGKLTEKSDVFSFGVVLLELITGRRPVDVNNADN-SLVDWARPLLTRALEERNFEGL
     Athaliana_PERK3    YAASGKLTEKSDVYSFGVVLLELITGRRPVDANNADD-SLVDWARPLLVQALEESNFEGL
         Gmax_PERK10    YASSGKLTDKSDVFSYGIMLLELITGRRPVDKNQMED-SLVDWARPLLTRALEEDDFDSI
         Gmax_PERK11    YASSGKLTDKSDVFSYGVMLLELITGRRPVDKNQMED-SLVDWARPLLTRALEEDDFDSI
   Mtruncatula_PERK2    YAASGKLTDKSDVFSYGVMLLELLTGRRPVDKDQMDD-SLVEWARPLLMRALEEDNLDSL
 Slycopersicum_PERK7    YASSGKLTEKSDVFSFGVMLLELITGRRPVDSTQIED-SLVDWARPLLTRALEDEKFDGL
    Stuberosum_PERK1    YASSGKLTDKSDVFSFGVMLLELITGRRPVDSTQIED-SLVDWARPLLTRALEDEKFDTL
  Ptrichocarpa_PERK8    YASSGKLTDKSDVFSYGVMLLELITGRRPVDSTQMED-SLVDWARPLLTRALEDGNFDTL
     Athaliana_PERK1    YAASGKLTEKSDVFSFGVVLLELITGRRPVDANNVDD-SLVDWARPLLNRASEEGDFEGL
         Gmax_PERK12    YAASGKLTEKSDVFSFGVVLLELITGRKPVDKTQIDD-SMVEWARPLLSQALENGNLNGL
    Athaliana_PERK15    YASSGKLTEKSDVFSIGVVLLELITGRRPVDKSQADDDSIVDWAKPLMIQALNDGNFDGL
         Ptaeda_PERK2   YASSGKLTDKSDVFSFGVMLLELITGRRPVDTTPSED-SLVDWARPLLARAMEDGNVDAL
       Osativa_PERK2    YAATGKVNDRSDVFSFGVMLLELITGKKPIMVSHQPD-TLVSWARPLLVRAVEEENFEEL
         Zmays_PERK4    YAATGKVNDRSDVFSFGVMLLELITGRKPIMTSSQPE-TLVSWARPLLTRAVEEENYDEL
         Zmays_PERK5    YATTGKVTDRSDVYSFGVVLLELITGMTPVLSPENDE-TLVSWSKPRLARALGEDALDEL
    Athaliana_PERK14    YASSGKVTDKSDVYSFGVVLLELITGRPSIFAKDTNQ-SLVDWARPLLTKAISGESFDFL
  Ptrichocarpa_PERK7    YALSGKLTDKSDVYSYGVVLLELITGHPPISPAEMNE-SLVAWARPLLTQALEDGNFEAL
        Ppatens_PERK4   YAASGKLTDRSDVFSFGVILLELVTGRRPIDTTQAGFESLVEWARPVVMRILEDGRLEDL
        Ppatens_PERK5   YAASGKLTDRSDVFSYGVILLELVTGRRPIDMNQAGFESLVEWARPVVMRILEDGHLEDI
        Ppatens_PERK1   YAASGKLTDRSDVFSFGVVLLELVTGRRPIDMSQAGFESLVEWARPVAMRILEDGHLEDL
        Ppatens_PERK2   YAQSGKLTEKSDVYSFGVVLLELITGRKPIDTRNAGQESLVEWTRPLLGEALA-GNMEEL
        Ppatens_PERK6   YAQSGKLTEKSDVYSFGVMLLELITGRKPVDTRDNGAVSLVELARPLMTKAMEDGDLDEL
        Ppatens_PERK3   YAMTGHLLVKSDVYSYGVVLLELLSGRRPIDHAQEAFENITAWARPLLTDSNR---IHEL
   Bdistachyon_PERK5    YARTGMLNERSDVYSFGVLVMEMITGRTPIDYTRTAEVNLVEWL-----KRMVERRVEEV
       Osativa_PERK5    YVHTGVATERSDVYSFGVLALEVATGRRPAERGI----SVVNWV-----WTLWRRRLVDA

   Bdistachyon_PERK2    PDPRMEQKFDENEMYHMIGAAAACIRHSAVMRPRMGQVVRALD
       Osativa_PERK1    PDPRMENRFDENEMYHMIGAAAACIRHSAAMRPRMGQVVRALD
   Bdistachyon_PERK3    LDPRLDNNFNEVEMFRMIEAAAACIRHSASRRPRMSQVVRALD
       Osativa_PERK3    IDSRLDKNFNEAEMFRMIEAAAACIRHSASRRPRMSQVVRVLD
         Zmays_PERK3    VDARLNRNYNEVEMFRMIEAAAACIRHSASRRPRMSQVVRVLD
         Zmays_PERK6    VDARLNKNYNEVEMFRMIEAAAACIRHSASRRPRMSQVVRVLD
         Zmays_PERK2    VDPRLEMKLDEVEMFRMVEAAAACIRHSASRRPRMSQVVRVLE
         Zmays_PERK1    VDVRLEGNFDDVEMFRVIEATAACIRHSAARRPKMGQIVRVLD
    Athaliana_PERK10    ADPKLGRNYVGVEMFRMIEAAAACIRHSATKRPRMSQIVRAFD
         Brapa_PERK7    ADPKLGGNYVDSEMFRMIEAAGACVRHSAAKRPRMGQIVRALE
          Gmax_PERK8    ADPRLEKNYVESELYCMIEVAAACVRHSAAKRPRMGQVVRAFD
  Ptrichocarpa_PERK2    ADPRLEKNYIESEMFRMIEAAAACVRHSASKRPRMGQVVRAFD
 Ptrichocarpa_PERK12    ADPRLEKNYIESEMFQMIEAAAVCVRHSATKRPRMGQVVRAFH
        Brapa_PERK13    VDPRLGVNFIAAEMFRMVEAAAACVRHSAARRPKMSQVVRALD
     Athaliana_PERK8    VDPRLGKNFIPGEMFRMVEAAAACVRHSAAKRPKMSQVVRALD
         Gmax_PERK13    VDPRLGKNYDRNEMFRMIEAAAACVRHSSVKRPRMSQVVRALD
  Ptrichocarpa_PERK3    VDPELEKNYVPSEMFRMIEAAAACVRHSAAKRPRMSQVVVALD
  Ptrichocarpa_PERK6    ADSGLEKNYVPSEMFRMIEAAAACVRHSAAKRPRMSQVVRALD
    Stuberosum_PERK4    VDPRLGNNFVAGEMFRMIEAAAACVRHSGSKRPRMSQVVRALD
 Slycopersicum_PERK5    VDPRLERNYVIPEMFQLIEAAAACVRHSAAKRPGMGQIMRAFD
    Stuberosum_PERK2    ADPRLERNYVIPEMFQLIEAAAACVRHSAAKRPGMGQIMRAFD
 Slycopersicum_PERK6    TDPRLGTNYIDSEMFQMIEIAAACVRHSAAKRPGMGQIMRAFD
 Slycopersicum_PERK1    ADPCLEKNYVGTEMFRMIEAAASCVRHSSAKRPAMGQIMRAFD
   Bdistachyon_PERK1    ADPALECRYSKTEMRRMVESAAACIRHSGTKRPKMVQVWRSLD
       Osativa_PERK4    ADPALERRYSKSEMRRMVEAAAACIRYSVTKRPRMVQVWRSLD
         Zmays_PERK7    ADPALECRFSKTEMRRMVEAAAACVRHSAAKRPRMVQVWRSLD
       Osativa_PERK6    VDPRLEGAYNRNEMMTMVEAAAACVRHSAPKRPRMVQVMRVLD
         Brapa_PERK3    VDPRLEKHYIEEEVYRMIETAASCVRHSALKRPRMVQVVRALD
    Athaliana_PERK11    VDPRLENDYVESEVYKMIETAASCVRHSALKRPRMVQVVRALD
         Brapa_PERK2    VDRRLEKHYVENEVFRMIETAAACVRHSGPKRPRMAQVVRALD
    Athaliana_PERK13    VDRRLEKHYVENEVFRMIETAAACVRHSGPKRPRMVQVVRALD
         Brapa_PERK5    VDRRLQNHYVENEVFRMIETAAACIRHSGPKRPRMAQVVRALD
        Brapa_PERK14    IDRRLEKHYVEHEVFRMIETAAACVRHSGPKRPRMVQVVRALD
    Athaliana_PERK12    IDTRLEKRYVEHEVFRMIETAAACVRHSGPKRPRMVQVVRALD
  Ptrichocarpa_PERK1    VDTRLEKHYVESELFRMVETAAACVRHSAPKRPRMVQVVRALD
 Ptrichocarpa_PERK11    VDTRLEKHYVESELFRMVETAAACVRHLAPKRPRMMQVVRALD
          Gmax_PERK2    VDPRLERQYADTEMFRMIETAAACVRHSAPKRPRMVQVARSLD
          Gmax_PERK9    VDPRLERQYVDSEMFRMIETAAACVRHSAPKRPRMVQVARSLD
          Gmax_PERK6    IDPRLKKHFVENEMLRMVEVAAACVRHSAPRRPRMVQVVRSLD
         Gmax_PERK14    TDPRLKKHFVESEMFRMIEAAAACVRHSALRRPRMVQVVRALD
          Gmax_PERK4    VDPRLEDNYDKQQMASMVACAAFSVRHSAKRRPRMSQIVRVLE
   Mtruncatula_PERK1    VDPRLEENYEKQDMTRMVACASACVRHSGRRRPRMSQIVRVLE
         Brapa_PERK4    VDPFLENQYEPYEMARMVACAAAAVRHSGRRRPKMSQIVRILE
     Athaliana_PERK7    VDPFLEHQYEPYEMARMVACAAAAVRHSGRRRPKMSQIVRTLE
        Brapa_PERK10    VDPRLEGQYEPYEMARMVACAAAAVRHSARRRPKMSQIVRALE
     Athaliana_PERK6    VDPRLENQYEPHEMAQMVACAAAAVRHSARRRPKMSQIVRALE
  Ptrichocarpa_PERK4    IDPALEGNYNPHEVACMIACAGASVSYSAKRRPKMSQIVRALE
  Ptrichocarpa_PERK5    VDPALEGNYDLQEMACMVACAGASVSHSAKRRPKMSRIVRALE
         Brapa_PERK8    ADPRLETNYNQQEMAQMASCAAAAIRHSARRRPKMSQIVRALE
     Athaliana_PERK5    ADPRLELNYSHQEMVQMASCAAAAIRHSARRRPKMSQIVRALE
         Zmays_PERK9    VDPRLGGEYSVVEVERLAACAAASTRHSAKRRPKMSQIVRALE
        Zmays_PERK10    LDPRLENRVDRLELERMCSSAAAAVRHSAKRRPKMKQIVRALE
         Brapa_PERK6    ADARLEGNYNPQEMARMVTCAAASIRHSGRKRPKMSQIVRALE
        Brapa_PERK12    ADVRLEGNYNPQEMARMVTCAAASIRHSGRKRPKMRQIVRALE
     Athaliana_PERK4    ADARLEGNYNPQEMARMVTCAAASIRHSGRKRPKMSQIVRALE
  Ptrichocarpa_PERK9    VDPMLESKYNHQEMQRMIACAAASIRHSARKRPKMSQIVRALE
 Ptrichocarpa_PERK10    VDPMLENNYNHQEMQRMIACAAASIRHSARKRPKMSQVARALE
          Gmax_PERK3    VDAFLEGNYDAQELSRMAACAAGSIRHSAKKRPKMSQIVRILE
          Gmax_PERK7    VDAFLEGNYDPQELSRMAACAAGSIRHSAKKRPKMSQIVRILE
   Mtruncatula_PERK3    VDPFLEGNYDPQELARMAACAAASIRHSARKRSKMSQIVRTLE
   Mtruncatula_PERK4    VDPFLEGNYDPQELARMAACAAASIRHSARKRSKMSQVERLAL
 Slycopersicum_PERK2    VDARLEGNYDTDELHRMIGCAASSIRHSAKRRPKMSQIVRALE
    Stuberosum_PERK5    VDPRLEGNFDTDELHRMIGCAASSIRHSAKRRPKMSQIVRALE
 Slycopersicum_PERK4    VDPRLEGNYDPDELQRMVACAAASVRHSARRRPKMSQIVRALD
    Stuberosum_PERK3    VDPRLEGNYDPDELQRMVACAAASVRHSARRRPKMSQILRALD
 Slycopersicum_PERK3    VGAPFEGNNVPKELHRLVVCAAASTRYSAKRRPKMTQIMRALD
   Bdistachyon_PERK4    SDPRLEGGYEPAEMARVVACAAACVRQSAKKRPKMGQVVRALE
         Zmays_PERK8    ADPRLRGNYDTMEMARMAASAAAAVRHSAKKRPKMSQIVRALE
         Brapa_PERK9    VDPKLNNEYDREEMARMVACAATCVRHSARRRPRMDQAK----
     Athaliana_PERK3    ADIKLNNEYDREEMARMVACAAACVRYTARRRPRMDQVVRVLE
         Gmax_PERK10    IDPRLQNDYDPHEMARMVASAAACIRHSAKRRPRMSQVVRALE
         Gmax_PERK11    IDPRLQNDYDPNEMARMVASAAACIRHSAKRRPRMSQVVRALE
   Mtruncatula_PERK2    IDPRLQNDFDPNEMTRMVACAAACTRHSAKRRPKMSQVVRALE
 Slycopersicum_PERK7    VDRRLENDYNHNEMARMVACCAACVRHSAKRRPRMTQVLRALE
    Stuberosum_PERK1    VDRRLENDYNHNEMARMVACCAACVRHSAKRRPRMTQVLRALE
  Ptrichocarpa_PERK8    VDQKLQNNYDQNEMARMVACAAACVRHSARRRPRTSQVVRALE
     Athaliana_PERK1    ADSKMGNEYDREEMARMVACAAACVRHSARRRPRMSQIVRALE
         Gmax_PERK12    VDPRLQTNYNLDEMIRMTTCAATCVRYSARLRPRMSQVVRALE
    Athaliana_PERK15    VDPRLENDFDINEMTRMVACAAASVRHSAKRRPKMSQIVRAFE
         Ptaeda_PERK2   ADIRIQHNYNVNEMMRVVACAASCVRHSARKRPRMGQIVRVLE
       Osativa_PERK2    VDPRLENNYDAYDMGRLIACAAAAVRHTARSRPRMSQIVRYLE
         Zmays_PERK4    IDPRLETNYDAYDMARLVACAAAAVRQTARSRPRMSQVVRYLE
         Zmays_PERK5    TDPILGTNYDGVDMRRLIACAAAAVRSTARTRPRMGQIVRFLE
    Athaliana_PERK14    VDSRLEKNYDTTQMANMAACAAACIRQSAWLRPRMSQVVRALE
  Ptrichocarpa_PERK7    LDPRLGTRYNNSEMASMVACAAACVHPSSWIRPRMSQIVHALE
        Ppatens_PERK4   VDPNLDGDYDPDEMFRVIETAAACVRHSALKRPRMAQVVRALE
        Ppatens_PERK5   VDPNLNGNYDPDEMFRVIETAAACVRHSALKRPRMAQVVRALE
        Ppatens_PERK1   VDPNLDGNYDRDEMFRVIETAAACVRHSAVKRPRMAQVVRALE
        Ppatens_PERK2   VDPRLDGRYNYKEMFRMIEVAASCVRHTASKRPKMGQVVRVLE
        Ppatens_PERK6   VDPRLGDNYDPKELFRMIEVAASCVRQTANKRPKMGQVVRALE
        Ppatens_PERK3   ADPLLDGKYPTEDFEQVAALAKSCIEPEWRARPTMGQICWEYN
   Bdistachyon_PERK5    VDPKLPEPWPPSKVLKAVLAALRCVDPDGGQRPTMGHVVHMLE
       Osativa_PERK5    ADRRLQGRFVADEMRRVLLVGLCCVHPDCRKRPGMRRVVSMLD
```
