# Supplementary material for: Bioinformatic Identification and Analysis of Extensins in the Plant Kingdom
Source: PLoS One. 2016 Feb 26;11(2):e0150177. doi: 10.1371/journal.pone.0150177 (PMC4769139; doi:10.1371/journal.pone.0150177)
Supplement: S1 Table — (PDF) [file pone.0150177.s009.pdf]

S1 Table. ***O. lucimarinus*** **EXTs identified in this study.**

Gene Identifier	Class	$SP_3/SP_4/SP_5$/YXY Repeats	Amino Acids	SP	GPI	Top Five BLAST Hit in Arabidopsis HRGPs
16632	Chimeric EXT	1/2/0/0	332	Yes	No	None
16112	Chimeric EXT	0/1/1/0	296	Yes	No	PERK13, FH13
16425	Chimeric EXT	20/0/0/1	388	No	No	FH3
24979	Chimeric EXT	22/0/1/0	1065	No	No	FH3, PEX4
31292	Long Chimeric EXT	6/2/8/4	4003	Yes	No	PERK5
25682	Long Chimeric EXT	5/19/9/7	4076	Yes	No	FH3
27866	Long Chimeric EXT	2/1/1/5	3182	Yes	No	EXT18, EXT22
93602	Long Chimeric EXT	9/9/2/2	2146	No	No	PRP2, PEX4, FH3, PERK5
