# Supplementary material for: Bioinformatic Identification and Analysis of Extensins in the Plant Kingdom
Source: PLoS One. 2016 Feb 26;11(2):e0150177. doi: 10.1371/journal.pone.0150177 (PMC4769139; doi:10.1371/journal.pone.0150177)
Supplement: S2 Table — (PDF) [file pone.0150177.s010.pdf]

S2 Table. *C. reinhardtii* EXTs identified in this study.

Gene Identifier	Class	$SP_3/SP_4/SP_5$/YXY Repeats	Amino Acids	SP	GPI	Top Five BLAST Hit in Arabidopsis HRGPs
Cre07.g353837.t1.2	Short EXT	3/1/4/0	190	No	No	PERK5
g11639.t1	Short EXT	2/3/0/0	159	Yes	No	PRP2
Cre08.g365300.t1.2	Chimeric EXT	4/0/0/0	552	Yes	No	None
Cre08.g374750.t1.3	Chimeric EXT	4/15/2/1	1481	No	No	PRP2, PERK1, PERK5
Cre08.g385550.t1.3	Chimeric EXT	5/2/4/0	787	No	No	PEX4
g8557.t1	Chimeric EXT	4/0/0/0	503	Yes	No	None
Cre08.g365150.t1.2	Chimeric EXT	4/0/0/0	552	Yes	No	FH6
Cre08.g381900.t1.2	Chimeric EXT	1/1/0/1	1076	Yes	No	None
Cre08.g365100.t2.1	Chimeric EXT	4/0/0/0	562	Yes	No	FH6
g7389.t1	Chimeric EXT	6/16/4/1	1011	Yes	No	PEX4
Cre07.g330750.t1.3	Chimeric EXT	1/1/5/1	950	Yes	No	PEX4, FH3
Cre07.g332900.t1.3	Chimeric EXT	3/2/8/1	1428	No	No	PRP2
Cre07.g336600.t2.1	Chimeric EXT	2/1/3/3	1006	Yes	No	None
g7574.t1	Chimeric EXT	2/2/0/0	814	Yes	No	None
Cre07.g344000.t1.3	Chimeric EXT	1/8/2/0	473	No	No	FH3, FH18
g8068.t2	Chimeric EXT	9/25/5/0	1204	Yes	No	None
Cre07.g356950.t1.3	Chimeric EXT	2/1/0/0	1156	Yes	No	None
Cre07.g321400.t1.3	Chimeric EXT	6/41/4/3	1914	Yes	No	PERK5, FH3
Cre07.g346900.t1.3	Chimeric EXT	2/0/0/0	256	Yes	No	None
g7805.t1	Chimeric EXT	31/0/0/0	990	No	No	PRP10
g11753.t1	Chimeric EXT	1/0/2/1	704	Yes	No	None
g11583.t1	Chimeric EXT	2/3/6/3	1125	Yes	No	PRP2
g11460.t1	Chimeric EXT	4/6/5/0	1138	No	No	PRP2
g11953.t1	Chimeric EXT	26/0/0/2	1535	No	No	None
g11998.t1	Chimeric EXT	13/0/0/0	964	Yes	No	FH3, PAG17
Cre11.g481750.t1.2	Chimeric EXT	0/2/3/0	472	Yes	No	FH3, PERK5
g11602.t1	Chimeric EXT	0/1/2/0	491	Yes	No	None
Cre11.g483250.t1.3	Chimeric EXT	3/2/28/0	787	Yes	No	PEX4
Cre11.g481600.t1.1	Chimeric EXT	0/2/2/0	433	Yes	No	FLA10, PERK5, PERK3
Cre14.g631150.t2.1	Chimeric EXT	4/6/6/3	1740	No	No	None
Cre14.g609900.t1.1	Chimeric EXT	2/6/2/1	833	Yes	No	PERK5, FH3
Cre14.g609700.t1.3	Chimeric EXT	2/1/0/1	615	Yes	No	FLA10, FH3
Cre14.g621700.t1.3	Chimeric EXT	6/2/2/1	809	No	No	PERK5
Cre14.g616700.t1.1	Chimeric EXT	1/1/1/5	964	Yes	No	None
Cre14.g613050.t2.1	Chimeric EXT	13/2/6/0	772	Yes	No	PEX1, FH3
Cre14.g625850.t1.3	Chimeric EXT	4/5/2/0	804	No	No	None
Cre16.g693500.t1.3	Chimeric EXT	1/3/1/0	421	Yes	No	FH3, FLA10, PEX4
g16842.t1	Chimeric EXT	1/1/0/1	578	Yes	No	FH3, PEX4, FLA10
g15607.t1	Chimeric EXT	3/2/0/0	393	Yes	No	PEX4, FH3, PAG17, FLA10
g16843.t1	Chimeric EXT	2/0/0/2	597	Yes	No	None
Cre16.g693450.t1.3	Chimeric EXT	1/4/0/2	587	Yes	No	PEX4, PERK5, PERK1
g16193.t2	Chimeric EXT	4/3/1/6	1831	Yes	No	PRP2
g16193.t1	Chimeric EXT	1/1/0/3	518	Yes	No	None
g16780.t1	Chimeric EXT	4/7/5/1	1253	No	No	None
Cre16.g693200.t1.3	Chimeric EXT	7/0/0/2	330	Yes	No	FH18, FH3, PEX3, PERK5
Cre16.g693300.t1.3	Chimeric EXT	2/0/0/1	567	Yes	No	None
Cre16.g663750.t1.3	Chimeric EXT	2/1/2/0	1023	Yes	No	PERK5, PEX4
Cre16.g652200.t1.2	Chimeric EXT	4/8/0/1	673	Yes	No	EXT9
g6305.t1	Chimeric EXT	0/1/2/0	443	Yes	No	PERK5, FH3
Cre06.g287800.t1.2	Chimeric EXT	3/1/2/0	396	Yes	No	PERK5, FH3
Cre06.g272250.t1.1	Chimeric EXT	0/1/2/2	758	Yes	No	None
g6351.t2	Chimeric EXT	6/12/2/0	429	Yes	No	PEX4
g6351.t1	Chimeric EXT	2/0/0/0	278	Yes	No	None
g6303.t1	Chimeric EXT	2/2/0/1	403	Yes	No	None
g6408.t1	Chimeric EXT	2/4/0/4	390	Yes	No	PEX4
g6301.t1	Chimeric EXT	6/3/5/0	560	No	No	None
g6296.t1	Chimeric EXT	8/13/8/0	1036	No	No	None
Cre06.g309900.t1.2	Chimeric EXT	0/4/0/2	928	Yes	No	None

g6308.t1	Chimeric EXT	1/1/3/1	735	Yes	No	LRX5
Cre06.g258950.t1.3	Chimeric EXT	1/5/5/0	925	Yes	No	None
Cre06.g302950.t1.3	Chimeric EXT	8/1/3/1	946	No	No	None
g6304.t1	Chimeric EXT	1/1/2/0	759	Yes	No	PERK5, FH6, FH3, PERK3
g6920.t1	Chimeric EXT	0/6/3/0	572	Yes	No	PERK5
Cre06.g306000.t1.3	Chimeric EXT	4/2/2/1	974	Yes	No	None
g6393.t1	Chimeric EXT	1/1/2/1	1151	Yes	No	None
Cre06.g261123.t1.2	Chimeric EXT	16/2/1/0	383	No	No	None
Cre06.g258800.t1.3	Chimeric EXT	11/10/2/3	1259	Yes	No	PERK5
Cre06.g306900.t1.3	Chimeric EXT	5/1/0/1	891	Yes	No	None
g6297.t1	Chimeric EXT	2/3/0/0	616	Yes	No	PERK5
g10076.t1	Chimeric EXT	1/2/3/0	350	Yes	No	FH13
g10076.t2	Chimeric EXT	1/2/2/0	336	Yes	No	FH13, PERK5
g9845.t1	Chimeric EXT	1/36/25/3	1035	Yes	No	None
g9215.t1	Chimeric EXT	3/3/0/0	262	Yes	No	None
Cre09.g394200.t1.3	Chimeric EXT	3/4/2/3	1496	Yes	No	FH3, FLA10, PERK5
g9797.t1	Chimeric EXT	9/2/1/3	1398	No	No	None
Cre09.g401900.t1.2	Chimeric EXT	7/0/0/1	1276	Yes	No	None
g9537.t1	Chimeric EXT	0/19/1/0	576	Yes	No	None
g10156.t1	Chimeric EXT	2/0/1/0	313	Yes	No	None
g9447.t1	Chimeric EXT	0/1/1/2	426	Yes	No	FH6
Cre09.g389050.t1.3	Chimeric EXT	13/9/1/1	2086	Yes	No	None
g9535.t1	Chimeric EXT	0/14/0/0	527	Yes	No	None
Cre09.g393450.t1.3	Chimeric EXT	2/5/2/3	1520	Yes	No	FLA10, FH3, PERK5
g9538.t1	Chimeric EXT	0/19/1/0	573	Yes	No	None
g9176.t1	Chimeric EXT	0/20/16/0	1339	Yes	No	PEX4
g9749.t1	Chimeric EXT	4/0/3/1	1287	Yes	No	PERK5, PRP2
Cre17.g708450.t1.3	Chimeric EXT	4/1/0/0	798	Yes	No	None
g17376.t1	Chimeric EXT	1/2/0/0	500	Yes	No	FH21a
g17407.t1	Chimeric EXT	6/40/14/2	2996	No	No	None
Cre17.g696700.t1.2	Chimeric EXT	0/2/0/0	391	Yes	No	FH3, PERK5
Cre17.g705500.t1.3	Chimeric EXT	1/0/4/2	889	Yes	No	None
Cre17.g717900.t1.2	Chimeric EXT	0/4/0/0	510	Yes	No	None
Cre17.g736800.t1.3	Chimeric EXT	6/1/0/0	1734	Yes	No	PRP2, PEX4
Cre17.g713150.t1.3	Chimeric EXT	2/1/1/0	582	Yes	No	None
Cre17.g714800.t1.2	Chimeric EXT	6/3/5/1	422	Yes	No	None
g17702.t1	Chimeric EXT	3/6/26/6	1345	No	No	None
g17926.t1	Chimeric EXT	21/18/0/1	3540	No	No	None
Cre17.g706350.t1.3	Chimeric EXT	4/3/0/1	1516	Yes	No	None
g17622.t1	Chimeric EXT	2/21/4/0	878	Yes	No	PAG17
Cre17.g718000.t1.3	Chimeric EXT	1/16/1/0	749	Yes	No	None
Cre17.g705200.t1.3	Chimeric EXT	6/2/1/1	1609	Yes	No	PRP2, PEX1
Cre17.g717850.t1.3	Chimeric EXT	0/16/4/0	603	Yes	No	FH3
g18268.t1	Chimeric EXT	2/8/0/0	648	No	No	None
Cre05.g243450.t2.1	Chimeric EXT	5/5/3/0	967	Yes	No	PRP2, PEX2
g5503.t1	Chimeric EXT	0/7/5/1	768	Yes	No	FH3, PERK5
g5501.t1	Chimeric EXT	0/1/2/0	478	Yes	No	None
Cre05.g238650.t1.3	Chimeric EXT	0/9/5/0	558	No	No	None
g5109.t1	Chimeric EXT	3/2/0/2	827	Yes	No	None
Cre13.g591550.t1.3	Chimeric EXT	4/2/0/8	1285	Yes	No	None
Cre13.g589400.t1.3	Chimeric EXT	5/0/0/0	384	Yes	No	None
g14187.t1	Chimeric EXT	0/2/3/7	1765	Yes	No	PERK5
Cre02.g118650.t1.3	Chimeric EXT	2/2/6/0	1384	Yes	No	PERK5
Cre02.g095150.t1.2	Chimeric EXT	5/1/3/3	1094	Yes	No	None
Cre02.g090050.t1.3	Chimeric EXT	5/3/4/1	856	Yes	No	None
Cre02.g078800.t1.2	Chimeric EXT	0/1/2/0	721	Yes	No	None
Cre02.g076950.t1.3	Chimeric EXT	4/6/0/1	1355	Yes	No	None
Cre02.g120000.t1.3	Chimeric EXT	1/1/0/1	496	Yes	No	None
g1869.t1	Chimeric EXT	3/18/3/0	980	Yes	No	FH18
Cre02.g099350.t1.3	Chimeric EXT	3/3/0/1	1350	Yes	No	PRP2, PERK5, PEX4
Cre02.g077850.t1.2	Chimeric EXT	4/1/0/0	783	Yes	No	PERK5

Cre02.g077800.t1.2	Chimeric EXT	4/1/0/0	813	Yes	No	FH3, FLA10, PEX4, PERK5
Cre02.g079500.t1.3	Chimeric EXT	0/3/1/0	997	Yes	No	PERK1, PERK5
Cre02.g102050.t1.3	Chimeric EXT	5/2/0/1	883	Yes	No	PEX4
Cre02.g145950.t1.2	Chimeric EXT	0/3/1/2	401	Yes	No	PEX4
Cre02.g077750.t1.2	Chimeric EXT	3/0/0/0	773	Yes	No	None
Cre03.g156200.t3.1	Chimeric EXT	3/9/1/2	597	Yes	No	PERK5
g4380.t1	Chimeric EXT	5/0/0/0	612	Yes	No	PERK5
Cre03.g192200.t1.3	Chimeric EXT	9/16/0/0	700	Yes	No	FH3, FLA10, PEX4, PERK5
g2892.t1	Chimeric EXT	1/1/2/0	756	Yes	No	None
Cre03.g155750.t1.3	Chimeric EXT	2/16/2/1	629	No	No	None
Cre03.g179450.t1.3	Chimeric EXT	3/0/0/0	572	Yes	No	PRP1
Cre03.g155300.t1.1	Chimeric EXT	0/1/2/2	756	Yes	No	None
Cre01.g013100.t1.3	Chimeric EXT	0/2/0/1	290	Yes	No	FH6, PEX4
g1051.t1	Chimeric EXT	10/35/0/1	1294	Yes	No	PRP1, PAG17
Cre01.g017900.t1.3	Chimeric EXT	2/0/1/0	1053	Yes	No	FH3
Cre01.g026500.t1.3	Chimeric EXT	2/1/0/4	508	Yes	No	None
Cre01.g039950.t1.2	Chimeric EXT	1/1/0/0	882	Yes	No	None
Cre01.g025200.t1.2	Chimeric EXT	1/2/1/3	557	Yes	No	None
Cre10.g434650.t1.3	Chimeric EXT	3/1/0/1	721	Yes	No	None
g11256.t1	Chimeric EXT	2/0/0/2	422	Yes	No	None
g10543.t2	Chimeric EXT	5/0/0/0	375	Yes	No	None
Cre10.g423850.t2.1	Chimeric EXT	1/0/3/0	778	Yes	No	None
g11098.t1	Chimeric EXT	0/1/5/1	553	Yes	No	None
Cre10.g421350.t1.2	Chimeric EXT	4/2/3/0	705	Yes	No	None
Cre10.g452250.t1.3	Chimeric EXT	0/0/3/2	639	Yes	No	None
g13810.t1	Chimeric EXT	3/4/4/0	713	Yes	No	None
Cre12.g506750.t1.3	Chimeric EXT	0/7/1/2	517	Yes	No	None
Cre12.g487950.t1.3	Chimeric EXT	12/0/0/6	2607	No	No	None
g13811.t1	Chimeric EXT	1/9/0/0	516	No	No	None
Cre12.g487700.t1.2	Chimeric EXT	3/0/0/0	741	Yes	No	None
g13210.t1	Chimeric EXT	4/0/5/1	710	No	No	None
g13652.t1	Chimeric EXT	3/1/0/0	648	Yes	No	None
Cre12.g546500.t1.2	Chimeric EXT	2/1/2/0	784	Yes	No	None
Cre12.g559750.t1.2	Chimeric EXT	2/1/0/0	447	Yes	No	None
Cre12.g520450.t1.3	Chimeric EXT	5/0/0/4	966	Yes	No	None
Cre12.g549000.t1.2	Chimeric EXT	2/1/0/0	493	Yes	No	None
g8540.t1|PACid:27562763	Long Chimeric EXT	9/12/3/4	3975	No	No	None
g8431.t1	Long Chimeric EXT	14/14/5/3	6907	Yes	No	PRP10
g8527.t1	Long Chimeric EXT	28/8/7/3	3734	No	No	None
g11926.t1	Long Chimeric EXT	3/4/3/0	2100	Yes	No	None
g11990.t1	Long Chimeric EXT	27/8/1/2	2349	Yes	No	None
g15825.t1	Long Chimeric EXT	4/25/2/2	3188	Yes	No	PEX4
g15955.t1	Long Chimeric EXT	25/1/1/3	2018	Yes	No	PEX4
Cre16.g654700.t1.3	Long Chimeric EXT	7/1/0/0	3003	Yes	No	None
g7021.t1	Long Chimeric EXT	15/11/0/6	2574	Yes	No	None
Cre06.g309950.t1.3	Long Chimeric EXT	18/4/12/5	7988	No	No	None
g5732.t1	Long Chimeric EXT	7/15/2/0	3884	Yes	No	None
Cre09.g404250.t1.3	Long Chimeric EXT	9/4/0/4	3235	Yes	No	None
Cre09.g400850.t1.3	Long Chimeric EXT	4/8/0/14	8145	Yes	No	None
Cre17.g732500.t1.3	Long Chimeric EXT	12/1/0/2	2370	Yes	No	None
g17844.t2	Long Chimeric EXT	67/2/7/1	3760	No	No	None
g17813.t1	Long Chimeric EXT	19/6/0/1	2640	Yes	No	None
g5228.t2	Long Chimeric EXT	3/0/7/1	5226	Yes	No	None
g14575.t1	Long Chimeric EXT	4/1/1/0	3554	Yes	No	None
g14346.t2	Long Chimeric EXT	52/11/9/9	9388	Yes	No	None
Cre02.g116100.t1.3	Long Chimeric EXT	23/1/2/5	3027	Yes	No	None
Cre02.g075950.t1.3	Long Chimeric EXT	18/4/0/0	2343	Yes	No	None
Cre10.g421150.t1.3	Long Chimeric EXT	4/4/1/9	2671	Yes	No	None
Cre04.g216050.t1.3	Long Chimeric EXT	8/3/1/1	3723	Yes	No	None
Cre04.g229550.t1.3	Long Chimeric EXT	6/1/1/4	4137	Yes	No	None
Cre04.g219050.t1.3	Long Chimeric EXT	5/2/5/3	2658	Yes	No	None

Cre12.g513400.t1.3	Long Chimeric EXT	30/0/0/4	3761	No	No	None
g12152.t2	Long Chimeric EXT	5/1/2/6	2674	Yes	No	None
