# Supplementary material for: Bioinformatic Identification and Analysis of Extensins in the Plant Kingdom
Source: PLoS One. 2016 Feb 26;11(2):e0150177. doi: 10.1371/journal.pone.0150177 (PMC4769139; doi:10.1371/journal.pone.0150177)
Supplement: S3 Table — (PDF) [file pone.0150177.s011.pdf]

S3 Table. *V. carteri* EXTs identified in this study.

Gene Identifier	Name	Class	$SP_3/SP_4/SP_5$/YXY Repeats	Amino Acids	SP	GPI	Top Five BLAST Hit in Arabidopsis HRGPs
Vocar20008550m	Vcarteri_FH1	FH	1/0/1/2	2183	No	No	FH13, FH18, FH16, FH14, FH21A
Vocar20013465m		chimeric EXT	1/0/1/0	402	Yes	No	PERK5, FLA10, PERK3, FH3, PERK6
Vocar20011773m		chimeric EXT	0/3/0/0	319	Yes	No	PEX1
Vocar20009362m		chimeric EXT	1/0/3/3	368	Yes	No	None
Vocar20008839m		chimeric EXT	1/1/0/2	1011	Yes	No	PRP2
Vocar20008475m		chimeric EXT	1/3/1/1	658	Yes	No	EXT9
Vocar20008683m		chimeric EXT	5/12/2/1	826	Yes	No	FH3
Vocar20008459m		chimeric EXT	1/0/5/0	713	Yes	No	FH3, PERK5
Vocar20008317m		chimeric EXT	1/1/3/5	1441	Yes	No	PRP2, EXT18
Vocar20009430m		chimeric EXT	1/1/1/0	563	Yes	No	None
Vocar20008988m		chimeric EXT	2/0/1/0	243	Yes	No	FH21A
Vocar20009617m		chimeric EXT	2/0/0/0	238	Yes	No	PEX4
Vocar20009279m		chimeric EXT	5/0/5/3	404	Yes	No	None
Vocar20003715m		chimeric EXT	1/2/8/0	504	No	No	FH3
Vocar20003702m		chimeric EXT	0/1/1/0	362	Yes	No	None
Vocar20003588m		chimeric EXT	4/2/3/0	753	No	No	None
Vocar20002258m		chimeric EXT	1/1/0/1	385	Yes	No	None
Vocar20014516m		chimeric EXT	2/0/0/3	536	Yes	No	None
Vocar20014578m		chimeric EXT	0/4/0/0	745	Yes	No	None
Vocar20006332m		chimeric EXT	1/1/0/0	476	Yes	No	PEX4
Vocar20007485m		chimeric EXT	3/1/0/1	697	Yes	No	None
Vocar20007569m		chimeric EXT	1/1/0/3	1379	Yes	No	PEX4, PERK5
Vocar20007606m		chimeric EXT	6/1/0/1	439	No	No	None
Vocar20007701m		chimeric EXT	3/1/0/2	788	Yes	No	None
Vocar20007457m		chimeric EXT	7/2/0/0	371	Yes	No	PAG17
Vocar20010940m		chimeric EXT	2/0/0/1	698	Yes	No	None
Vocar20010621m		chimeric EXT	0/0/4/4	838	Yes	No	FH3, PERK5
Vocar20010802m		chimeric EXT	1/1/0/0	468	Yes	No	None
Vocar20010920m		chimeric EXT	5/6/3/1	1021	No	No	PRP2
Vocar20010865m		chimeric EXT	6/3/5/2	1857	Yes	No	PRP2, PERK1, FH3, FH21A, PEX4
Vocar20012678m		chimeric EXT	16/0/0/1	944	No	No	AGP51C
Vocar20012997m		chimeric EXT	15/2/1/1	902	Yes	No	PEX4, FH3
Vocar20012894m		chimeric EXT	1/1/3/2	856	Yes	No	None
Vocar20012648m		chimeric EXT	2/2/0/1	2230	Yes	No	PRP1, EXT18
Vocar20012568m		chimeric EXT	1/1/0/0	785	Yes	No	None
Vocar20012618m		chimeric EXT	1/1/1/7	598	No	No	None
Vocar20001324m		chimeric EXT	4/1/2/0	900	Yes	No	PEX4
Vocar20002518m		chimeric EXT	6/5/0/1	1071	Yes	No	PERK5
Vocar20002859m		chimeric EXT	1/0/10/0	724	No	No	FH3, PERK5
Vocar20006778m		chimeric EXT	0/3/0/1	1492	Yes	No	PERK7, PERK8, PERK12, PERK4
Vocar20001807m		chimeric EXT	0/2/0/1	1044	Yes	No	None
Vocar20004103m		chimeric EXT	2/4/2/1	1028	No	No	PRP2
Vocar20015274m		chimeric EXT	2/0/0/0	421	Yes	No	None
Vocar20000600m		chimeric EXT	9/6/2/4	1636	Yes	No	PERK7, FH3
Vocar20000673m		chimeric EXT	3/0/0/0	724	Yes	No	None
Vocar20000622m		chimeric EXT	0/2/0/1	672	Yes	No	None
Vocar20000651m		chimeric EXT	1/3/2/0	423	Yes	No	None
Vocar20000570m		chimeric EXT	2/0/1/0	732	Yes	No	None
Vocar20004196m		chimeric EXT	1/0/5/1	1966	Yes	No	PRP2, EXT22, PRP3, FH3
Vocar20013094m		chimeric EXT	2/0/0/0	838	Yes	No	PEX4
Vocar20013200m		chimeric EXT	2/7/0/0	622	Yes	No	PEX4
Vocar20013164m		chimeric EXT	4/11/4/4	1462	Yes	No	PRP2, FH3
Vocar20013899m		chimeric EXT	6/2/3/2	1707	No	No	PRP2
Vocar20014446m		chimeric EXT	14/4/4/0	920	Yes	No	None
Vocar20014418m		chimeric EXT	0/1/3/1	701	Yes	No	PERK3
Vocar20005909m		chimeric EXT	0/3/7/0	591	Yes	No	PRP2, EXT22
Vocar20006105m		chimeric EXT	2/1/1/2	625	Yes	No	PEX4
Vocar20006106m		chimeric EXT	2/1/1/2	522	Yes	No	PEX4
Vocar20006101m		chimeric EXT	1/1/1/1	2224	Yes	No	PEX4, EXT18
Vocar20006081m		chimeric EXT	0/0/4/4	425	Yes	No	None
Vocar20005948m		chimeric EXT	3/4/1/2	662	Yes	No	None

Vocar20005650m		chimeric EXT	4/0/1/1	651	Yes	No	PERK5, FH3
Vocar20005613m		chimeric EXT	2/0/0/0	713	Yes	No	None
Vocar20014733m		chimeric EXT	6/3/1/0	1080	Yes	No	PRP2, PEX4
Vocar20014804m		chimeric EXT	1/3/0/2	491	Yes	No	None
Vocar20006909m		chimeric EXT	1/1/1/0	678	Yes	No	AGP30
Vocar20007352m		chimeric EXT	2/0/1/0	728	Yes	No	None
Vocar20007184m		chimeric EXT	2/1/0/1	282	Yes	No	None
Vocar20007419m		chimeric EXT	10/0/0/0	647	Yes	No	FH3
Vocar20007116m		chimeric EXT	1/0/1/3	1103	Yes	No	None
Vocar20014359m		chimeric EXT	1/0/2/2	432	Yes	No	None
Vocar20003361m		chimeric EXT	8/10/0/4	1332	Yes	No	None
Vocar20004320m		chimeric EXT	4/0/2/0	1283	Yes	No	PERK5
Vocar20004321m		chimeric EXT	4/0/2/0	1214	Yes	No	PERK5
Vocar20004293m		chimeric EXT	0/1/7/4	1070	No	No	EXT9
Vocar20004451m		chimeric EXT	0/14/0/0	926	No	No	PEX4
Vocar20003888m		chimeric EXT	6/0/0/4	981	Yes	No	FH3, AGP2, AGP17
Vocar20003835m		chimeric EXT	1/1/0/0	488	Yes	No	None
Vocar20003932m		chimeric EXT	1/0/1/1	2341	Yes	No	None
Vocar20000905m		chimeric EXT	7/0/0/1	419	No	No	None
Vocar20001045m		chimeric EXT	0/3/1/0	415	Yes	No	None
Vocar20001970m		chimeric EXT	1/1/1/0	886	Yes	No	None
Vocar20001955m		chimeric EXT	0/1/1/0	199	Yes	No	None
Vocar20008139m		chimeric EXT	2/0/0/0	680	Yes	No	None
Vocar20007052m		chimeric EXT	1/3/2/1	801	Yes	No	FH3
Vocar20011363m		long chimeric EXT	0/1/27/10	4784	Yes	No	PRP2, FH3
Vocar20008751m		long chimeric EXT	1/9/0/5	4282	No	No	PRP2, EXT18, PERK5, PERK1
Vocar20011956m		long chimeric EXT	4/4/8/2	2392	No	No	PRP2, FH3, PERK5
Vocar20014913m		long chimeric EXT	0/1/1/2	3210	Yes	No	None
Vocar20004500m		long chimeric EXT	0/0/5/2	2466	Yes	No	PRP2, EXT22, EXT18
Vocar20004391m		long chimeric EXT	10/4/5/4	3141	No	No	PRP2, PEX4, PERK5
Vocar20008165m		long chimeric EXT	1/0/3/4	4535	No	No	PRP2, FH3
