# Supplementary material for: Bioinformatic Identification and Analysis of Extensins in the Plant Kingdom
Source: PLoS One. 2016 Feb 26;11(2):e0150177. doi: 10.1371/journal.pone.0150177 (PMC4769139; doi:10.1371/journal.pone.0150177)
Supplement: S4 Table — (PDF) [file pone.0150177.s012.pdf]

S4 Table. *K. flaccidum* EXTs identified in this study.

Gene Identifier	Class	$SP_3/SP_4/SP_5$/YXY Repeats	Amino Acids	SP	GPI	Top Five BLAST Hit in Arabidopsis HRGPs
kfl00031_0230	Chimeric EXT	2/2/1/1	1365	Yes	No	PERK11, PERK13, PERK12, PERK3, PERK8
kfl00047_0130	Chimeric EXT	5/0/0/3	1438	Yes	No	EXT18, PEX4
kfl00209_0190	Chimeric EXT	9/0/0/2	1069	Yes	No	PRP2
kfl00671_0010p	Chimeric EXT	2/0/0/0	663	No	No	PERK15, PERK13, PERK3, PERK12, PERK8
