# Supplementary material for: Bioinformatic Identification and Analysis of Extensins in the Plant Kingdom
Source: PLoS One. 2016 Feb 26;11(2):e0150177. doi: 10.1371/journal.pone.0150177 (PMC4769139; doi:10.1371/journal.pone.0150177)
Supplement: S5 Table — (PDF) [file pone.0150177.s013.pdf]

S5 Table. *P. patens* EXTs identified in this study.

Gene Identifier	Name	Class	SP3/SP4/SP5/YXY Repeats	Amino Acids	SP	GPI	Top Five BLAST Hit in Arabidopsis HRGPs
Pp1s66_120V6.1		SHORT EXT	2/0/0/1	179	Yes	No	None
Pp1s469_11V6.1		SHORT EXT	2/0/0/0	96	Yes	No	None
Pp1s304_18V6.1		SHORT EXT	2/0/0/0	99	Yes	No	None
Pp1s8_132V6.1		SHORT EXT	2/0/0/0	160	Yes	No	PRP1
Pp1s287_63V6.1	Ppatens_LRX1	LRX	0/3/0/0	632	Yes	No	LRX4, LRX3, LRX5, PEX4, LRX2
Pp1s35_305V6.1	Ppatens_LRX2	LRX	0/2/5/0	465	Yes	No	PEX4, LRX4, PEX2, LRX3, PEX3
Pp1s35_242V6.1	Ppatens_LRX3	LRX	0/5/2/2	642	Yes	No	LRX1, LRX2, PEX2, LRX4, PEX4
Pp1s96_30V6.1	Ppatens_LRX4	LRX	1/5/3/1	688	Yes	No	LRX3, LRX4, LRX5, PEX4, LRX1
Pp1s118_151V6.1	Ppatens_LRX5	LRX	1/1/0/0	655	Yes	No	LRX4, LRX3, LRX5, PEX4, LRX2
Pp1s145_144V6.1	Ppatens_PERK1	PERK	2/1/0/0	843	No	No	PERK10, PERK9, PERK8, PERI5, PERK11
Pp1s134_145V6.1	Ppatens_PERK2	PERK	2/0/0/1	757	No	No	PERK8, PERK13, PERK1, PERK12, PERK9
Pp1s115_198V6.1	Ppatens_PERK3	PERK	7/13/1/1	1276	No	No	PERK1, PERK10, PERK3, PERK5, PERK8
Pp1s160_128V6.1	Ppatens_PERK4	PERK	8/1/3/0	976	No	No	PERK12, PERK13, PERK1, PERK11, PERK3
Pp1s118_23V6.1	Ppatens_PERK5	PERK	8/0/2/0	844	No	No	PERK1, PERK13, PERK12, PERK10, PERK9
Pp1s83_193V6.1	Ppatens_PERK6	PERK	1/6/0/0	801	No	No	PERK8, PERK13, PERK1, PERK12, PERK3
Pp1s71_197V6.1	Ppatens_FH1	FH	1/1/0/1	1081	No	No	FH6, FH2, FH1, FH5, FH3
Pp1s17_57V6.1	Ppatens_FH2	FH	2/0/5/1	1127	No	No	FH2, FH6, FH1, FH11, FH5
Pp1s38_64V6.1		Chimeric EXT	3/5/1/0	474	Yes	No	None
