# Supplementary material for: Bioinformatic Identification and Analysis of Extensins in the Plant Kingdom
Source: PLoS One. 2016 Feb 26;11(2):e0150177. doi: 10.1371/journal.pone.0150177 (PMC4769139; doi:10.1371/journal.pone.0150177)
Supplement: S6 Table — (PDF) [file pone.0150177.s014.pdf]

S6 Table. *S. moellendorffii* EXTs identified in this study.

Gene Identifier	Name	Class	SP3/SP4/SP5/YXY Repeats	Amino Acids	SP	GPI	Top Five BLAST Hit in Arabidopsis HRGPs
440537	Smoellendorffii_EXT1	EXT SP4 YXY+	0/23/0/14	229	Yes	No	EXT3, PRP1
402661	Smoellendorffii_EXT2	EXT SP4 YXY+	1/6/5/7	277	Yes	No	EXT22
448871	Smoellendorffii_EXT3	EXT SP5 YXY+	0/6/11/21	291	No	No	EXT22, EXT3, HAE3
405021	Smoellendorffii_EXT4	EXT SP4 YXY+	0/21/1/21	348	Yes	No	EXT3, EXT18
405114	Smoellendorffii_EXT5	EXT SP4 YXY+	0/35/3/35	546	Yes	No	EXT3, EXT22, EXT13
413006	Smoellendorffii_EXT6	EXT SP4 YXY+	6/13/4/9	364	Yes	No	None
412461	Smoellendorffii_EXT7	EXT SP4 YXY+	2/6/1/4	207	Yes	No	PEX3
229952	Smoellendorffii_EXT8	EXT SP4 YXY+	0/32/1/19	319	Yes	No	EXT3
420737	Smoellendorffii_EXT9	EXT SP5	3/6/15/0	727	Yes	No	None
429269	Smoellendorffii_EXT10	EXT SP5 YXY+	1/4/12/21	282	Yes	No	EXT22
422106	Smoellendorffii_EXT11	EXT SP5 YXY+	0/5/23/35	386	Yes	No	EXT22
113758	Smoellendorffii_EXT12	EXT SP5	1/1/7/0	211	No	No	None
422873	Smoellendorffii_EXT13	EXT SP5 YXY+	0/9/16/27	402	No	No	EXT22
404120		Short EXT	0/2/2/0	194	Yes	No	PEX4, FH3
405687		Short EXT	1/1/0/0	181	Yes	No	None
406865		Short EXT	3/0/2/4	152	Yes	No	FH3
430475		Short EXT	1/9/1/2	139	No	No	None
408211		Short EXT	0/3/2/3	192	Yes	No	EXT22, PEX3
431330		Short EXT	5/1/3/6	183	Yes	No	EXT22
81544	Smoellendorffii_LRX1	LRX	0/3/1/2	398	No	No	LRX4, LRX3, LRX2, LRX1, LRX5
54019	Smoellendorffii_LRX2	LRX	1/1/6/0	411	No	No	LRX4, LRX3, LRX5, PEX1, LRX1
74599	Smoellendorffii_FH1	FH	1/0/2/0	1100	No	No	FH14, FH18, FH13, FH16, FH21A
77222	Smoellendorffii_FH2	FH	1/0/2/0	1121	No	No	FH14, FH18, FH13, FH16, FH21A
416549		CHIMERIC EXT	1/0/1/1	304	Yes	No	FH13
438958		CHIMERIC EXT	11/0/0/1	387	Yes	No	PRP2, PRP1
416970		CHIMERIC EXT	2/0/0/1	263	Yes	No	None
426017		CHIMERIC EXT	2/0/0/0	617	Yes	No	None
410458		CHIMERIC EXT	0/1/1/1	245	Yes	No	None
441276		CHIMERIC EXT	0/1/1/1	245	Yes	No	None
410639		CHIMERIC EXT	2/8/2/1	377	No	No	PEX4, FH18
403585		CHIMERIC EXT	0/0/2/1	292	Yes	No	None
449338		CHIMERIC EXT	0/1/2/1	273	Yes	No	PRP1, PRP3, PEX1
411518		CHIMERIC EXT	0/1/1/1	245	Yes	No	None
419497		CHIMERIC EXT	3/2/0/0	374	Yes	No	AGP31
441903		CHIMERIC EXT	0/0/2/1	246	Yes	No	PRP1, PRP3
413143		CHIMERIC EXT	6/1/0/1	566	Yes	No	None
412728		CHIMERIC EXT	0/6/7/0	321	No	No	FLA5, FLA4, PEX4, FLA3, FH3
420661		CHIMERIC EXT	1/1/13/0	749	No	No	None
420679		CHIMERIC EXT	3/1/6/0	652	No	No	None
422030		CHIMERIC EXT	5/2/9/0	588	Yes	No	None
421413		CHIMERIC EXT	0/0/2/0	333	Yes	No	None
444340		CHIMERIC EXT	0/0/5/0	204	Yes	No	FH3, PERK6, FH21A, FH13
413730		CHIMERIC EXT	2/0/1/0	400	Yes	No	AGP31
415476		CHIMERIC EXT	0/3/2/0	447	Yes	No	None
