# Supplementary material for: Bioinformatic Identification and Analysis of Extensins in the Plant Kingdom
Source: PLoS One. 2016 Feb 26;11(2):e0150177. doi: 10.1371/journal.pone.0150177 (PMC4769139; doi:10.1371/journal.pone.0150177)
Supplement: S7 Table — (PDF) [file pone.0150177.s015.pdf]

S7 Table. *P. taeda* EXTs identified in this study.

Gene Identifier	Name	Class	$SP_3/SP_4/SP_5$/YXY Repeats	Amino Acids	SP	GPI	Top Five BLAST Hit in Arabidopsis HRGPs
PITA_000081371.RA	Ptaeda_PERK1	PERK	10/1/3/0	686	No	No	PERK13, PERK12, PERK8, PERK10, PERK4
PITA_000023436.RA	Ptaeda_PERK2	PERK	1/4/1/1	657	No	No	PERK1, PERK4, PERK5, PERK15, PERK7
PITA_000045997.RA	Ptaeda_FH1	FH	0/0/3/0	1032	Yes	No	FH6, FH1, FH2, FH5, FH3
PITA_000042657.RA	Ptaeda_FH2	FH	0/2/1/0	1504	No	No	FH17, FH20, FH13, FH14, FH16
PITA_000026470.RA	Ptaeda_FH3	FH	1/1/0/0	1096	Yes	No	FH6, FH1, FH2, FH5, FH3
