# Supplementary material for: Bioinformatic Identification and Analysis of Extensins in the Plant Kingdom
Source: PLoS One. 2016 Feb 26;11(2):e0150177. doi: 10.1371/journal.pone.0150177 (PMC4769139; doi:10.1371/journal.pone.0150177)
Supplement: S8 Table — (PDF) [file pone.0150177.s016.pdf]

S8 Table. ***P. abies*** **EXTs identified in this study.**

Gene Identifier	Name	Class	$SP_3/SP_4/SP_5$/YXY Repeats	Amino Acids	SP	GPI	Top Five BLAST Hit in Arabidopsis HRGPs
MA_74039g0010		Classic EXT	4/11/20/38	501	No	No	EXT3/5, EXT22
MA_108099g0010		Short EXT	1/2/0/2	118	Yes	No	PEX4, EXT22
MA_5375790g0010		Short EXT	4/0/3/7	96	Yes	No	EXT22, EXT21, EXT3/5
MA_618974g0010		Short EXT	4/0/3/7	98	Yes	No	EXT22, EXT21, EXT3/5
MA_7447685g0010		Short EXT	1/2/0/2	145	Yes	No	None
MA_7862055g0010		Short EXT	7/0/0/11	106	No	No	None
MA_100985g0010	Pabies_LRX1	LRX	2/1/1/0	634	Yes	No	LRX3, LRX5, LRX2, PEX4, PEX1
MA_7188g0010	Pabies_LRX2	LRX	0/2/2/1	532	Yes	No	LRX3, LRX6, LRX5, PEX4, LRX2
MA_10432176g0020	Pabies_FH1	FH EXT	1/0/2/1	659	Yes	No	FH5, FH6, FH1, FH2, FH3
MA_10436302g0010	Pabies_FH2	FH EXT	0/0/2/0	962	No	No	FH20, FH13, FH14, FH17, FH16
