# Supplementary material for: Bioinformatic Identification and Analysis of Extensins in the Plant Kingdom
Source: PLoS One. 2016 Feb 26;11(2):e0150177. doi: 10.1371/journal.pone.0150177 (PMC4769139; doi:10.1371/journal.pone.0150177)
Supplement: S9 Table — (PDF) [file pone.0150177.s017.pdf]

S9 Table. *B. distachyon* EXTs identified in this study.

Gene Identifier	Name	Class	SP3/SP4/SP5/YXY Repeats	Amino Acids	SP	GPI	Top Five BLAST Hit in Arabidopsis HRGPs
Bradi3g10280.1|PACid:21831068	Bdistachyon_EXT1	Classical EXT	11/3/0/0	792	Yes	No	None
Bradi2g11770.1|PACid:21808457		Short EXT	2/1/0/2	186	Yes	Yes	EXT37
Bradi2g45060.1|PACid:21808745		Short EXT	0/2/0/2	123	Yes	Yes	FH21a, EXT33
Bradi4g11290.1|PACid:21813278		Short EXT	2/1/0/0	121	Yes	No	EXT18
Bradi2g05080.1|PACid:21806530	Bdistachyon_LRX1	LRX	1/14/0/0	548	Yes	No	LRX3, LRX4, LRX5, LRX1, LRX2
Bradi2g12760.1|PACid:21807326	Bdistachyon_LRX2	LRX	0/4/4/0	505	Yes	No	PEX4, PEX1, PEX3, PEX2, LRX2
Bradi2g42477.1|PACid:21807550	Bdistachyon_LRX3	LRX	0/11/2/4	534	Yes	No	LRX4, LRX3, LRX5, PEX4, LRX2
Bradi4g11130.1|PACid:21810435	Bdistachyon_LRX4	LRX	5/0/0/0	812	Yes	No	PEX1, PEX2, PEX4, PEX3, LRX4
Bradi3g03370.1|PACid:21832433	Bdistachyon_LRX5	LRX	1/1/0/0	670	Yes	No	LRX1, LRX2, LRX3, LRX5, PEX1
Bradi2g44830.1|PACid:21805961	Bdistachyon_PERK1	PERK	5/3/2/0	669	No	No	PERK12, PERK9, PERK10, PERK5, PERK1
Bradi2g49240.1|PACid:21807984	Bdistachyon_PERK2	PERK	5/3/0/1	681	No	No	PERK8, PERK9, PERK10, PERK12, PERK13
Bradi2g00900.1|PACid:21808541	Bdistachyon_PERK3	PERK	2/2/0/0	682	No	No	PERK9, PERK10, PERK11, PERK12, PERK1
Bradi1g07010.1|PACid:21815588	Bdistachyon_PERK4	PERK	2/0/0/0	537	No	No	PERK4, PERK1, PERK5, PERK15, PERK3
Bradi3g31967.1|PACid:21833106	Bdistachyon_PERK5	PERK	1/1/0/1	545	No	No	PERK12, PERK13, PERK9, PERK11, PERK1
Bradi4g03720.1|PACid:21812481	Bdistachyon_FH1	FH	0/2/2/0	2024	No	No	FH18, FH14, FH16, FH13, FH2
Bradi1g22980.1|PACid:21817600	Bdistachyon_FH2	FH	2/1/0/0	778	Yes	No	FH6, FH2, FH1, FH11, FH5
Bradi3g59780.1|PACid:21827983	Bdistachyon_FH3	FH	2/0/1/0	884	Yes	No	FH8, FH7, FH4, FH6, FH1
Bradi2g57740.1|PACid:21806590		Chimeric EXT	6/1/0/9	388	Yes	No	None
Bradi4g11260.1|PACid:21810624		Chimeric EXT	2/3/2/0	234	Yes	No	FH3
Bradi4g07580.1|PACid:21811380		Chimeric EXT	1/1/2/0	377	Yes	No	AGP31, FH3
Bradi4g11250.1|PACid:21812666		Chimeric EXT	0/1/1/1	189	Yes	No	FH3, FH6, EXT9, EXT22, PERK6
Bradi4g11240.1|PACid:21814077		Chimeric EXT	1/0/1/0	172	Yes	No	FH3, FH6, LRX5, FH21a, FH13
Bradi3g12902.1|PACid:21830894		Chimeric EXT	1/0/3/0	1437	No	No	None
