# Supplementary material for: Bioinformatic Identification and Analysis of Extensins in the Plant Kingdom
Source: PLoS One. 2016 Feb 26;11(2):e0150177. doi: 10.1371/journal.pone.0150177 (PMC4769139; doi:10.1371/journal.pone.0150177)
Supplement: S10 Table — (PDF) [file pone.0150177.s018.pdf]

S10 Table. *Z. mays* EXTs identified in this study.

Gene Identifier	Name	Class	SP3/SP4/SP5/YXY Repeats	Amino Acids	SP	GPI	Top Five BLAST Hit in Arabidopsis HRGPs
GRMZM2G076029_T01		SHORT EXT	1/0/1/0	172	Yes	No	EXT18
GRMZM2G370193_T01		SHORT EXT	0/2/0/2	136	Yes	Yes	EXT31, EXT33
GRMZM2G065259_T01		SHORT EXT	0/1/3/1	184	Yes	No	EXT9, FH18
GRMZM2G065199_T01		SHORT EXT	0/3/2/1	198	Yes	No	None
GRMZM2G079638_T01		SHORT EXT	1/2/0/2	183	Yes	Yes	EXT41
GRMZM2G396541_T01		SHORT EXT	0/0/2/0	165	Yes	No	PERK13, FH3
GRMZM2G310158_T01		SHORT EXT	0/1/1/3	160	Yes	No	FH21A, FH6, FH3
GRMZM5G894582_T01		SHORT EXT	0/1/1/2	130	Yes	Yes	FH21A, LRX5, LRX4, EXT12
GRMZM2G082823_T01	Zmays_LRX1	LRX	0/2/1/0	801	Yes	No	LRX1, LRX2, LRX4, LRX7, LRX5
GRMZM2G333811_T01	Zmays_LRX2	LRX	1/15/0/1	555	Yes	No	LRX4, LRX3, LRX5, PEX4, LRX2
GRMZM5G841015_T01	Zmays_LRX3 (ZmPEX1)	LRX	23/30/1/0	1188	Yes	No	PEX1, PEX4, LRX4, LRX3, LRX7
GRMZM2G169669_T01	Zmays_LRX4	LRX	1/7/1/0	527	Yes	No	LRX4, LRX3, LRX5, PEX6, LRX1
GRMZM2G300969_T01	Zmays_LRX5	LRX	15/26/3/0	972	Yes	No	PEX4, PEX3, LRX4, LRX3, LRX7
GRMZM2G028731_T01	Zmays_PERK1	PERK	2/1/0/1	556	No	No	PERK8, PERK9, PERK12, PERK13, PERK10
GRMZM2G458548_T01	Zmays_PERK2	PERK	2/0/0/0	595	No	No	PERK8, PERK10, PERK9, PERK13, PERK11
GRMZM2G355636_T01	Zmays_PERK3	PERK	3/0/0/0	691	No	No	PERK8, PERK10, PERK9, PERK4, PERK1
GRMZM2G358830_T01	Zmays_PERK4	PERK	1/8/0/2	735	No	No	PERK1, PERK4, PERK3, PERK15, PERK12
GRMZM2G459003_T01	Zmays_PERK5	PERK	2/0/0/1	896	No	No	PERK1, PERK3, PERK5, PERK15, PERK10
GRMZM5G872442_T02	Zmays_PERK6	PERK	2/0/0/0	697	No	No	PERK8, PERK9, PERK10, PERK13, PERK4
GRMZM2G004330_T01	Zmays_PERK7	PERK	4/5/1/0	683	No	No	PERK12, PERK11, PERK8, PERK10, PERK9
GRMZM2G065214_T01	Zmays_PERK8	PERK	1/1/0/0	586	No	No	PERK4, PERK5, PERK1, PERK15, PERK3
GRMZM2G172081_T01	Zmays_PERK9	PERK	2/0/0/1	557	No	No	PERK4, PERK7, PERK1, PERK5, PERK6
GRMZM2G072292_T01	Zmays_PERK10	PERK	1/1/0/1	583	No	No	PERK5, PERK4, PERK1, PERK7, PERK6
GRMZM2G410951_T01	Zmays_FH1	FH	1/0/2/4	1853	No	No	FH13, FH18, FH14, FH16, FH21A
GRMZM2G057247_T01	Zmays_FH2	FH	2/0/0/0	793	Yes	No	FH6, FH1, FH2, FH11, FH3
GRMZM2G414002_T01	Zmays_FH3	FH	0/0/2/0	1608	No	No	FH18, FH14, FH13, FH16, FH21A
AC155376.2_FGT004	Zmays_FH4	FH	1/0/1/0	541	No	No	FH1, FH6, FH2, FH5, FH11
GRMZM2G142779_T01	Zmays_FH5	FH	3/0/4/0	947	Yes	No	FH8, FH7, FH4, FH6, FH5
GRMZM2G138401_T01		CHIMERIC EXT	0/1/2/1	227	Yes	No	FH3, FH18, FLA10, FH7, FH6
GRMZM2G129935_T01		CHIMERIC EXT	0/2/0/1	380	Yes	No	None
GRMZM2G032145_T01		CHIMERIC EXT	6/0/0/1	525	Yes	No	None
GRMZM2G085246_T02		CHIMERIC EXT	1/1/0/0	780	Yes	No	PERK13, PERK12, PERK8, PERK10, PERK11
GRMZM2G373578_T01		CHIMERIC EXT	4/0/0/1	1052	Yes	No	None
GRMZM2G160966_T01		CHIMERIC EXT	2/0/0/0	388	Yes	No	None
GRMZM2G434363_T01		CHIMERIC EXT	2/0/0/3	708	Yes	No	None
GRMZM2G120839_T01		CHIMERIC EXT	1/1/0/1	814	Yes	No	PERK12, PERK8, PERK7, PERK9, PERK5
GRMZM2G435592_T02		CHIMERIC EXT	2/0/0/2	653	Yes	No	PERK3, PERK12, PERK13, PERK15, PERK8
GRMZM2G002555_T01		CHIMERIC EXT	2/0/0/2	645	Yes	No	PERK3, PERK1, PERK8, PERK15, PERK13
GRMZM2G020986_T01		CHIMERIC EXT	2/0/0/0	606	Yes	No	None
GRMZM2G048175_T01		CHIMERIC EXT	2/1/2/0	367	Yes	No	AGP31
GRMZM2G062390_T01		CHIMERIC EXT	3/0/0/0	484	Yes	No	None
GRMZM2G335638_T01		CHIMERIC EXT	2/0/0/1	787	Yes	No	PERK12, PERK8, PERK9, PERK3, PERK15
GRMZM2G167578_T01		CHIMERIC EXT	2/0/0/0	306	Yes	No	None
