# Supplementary material for: Bioinformatic Identification and Analysis of Extensins in the Plant Kingdom
Source: PLoS One. 2016 Feb 26;11(2):e0150177. doi: 10.1371/journal.pone.0150177 (PMC4769139; doi:10.1371/journal.pone.0150177)
Supplement: S11 Table — (PDF) [file pone.0150177.s019.pdf]

S11 Table. *O. sativa* EXTs identified in this study.

Gene Identifier	Name	Class	SP3/SP4/SP5/YXY Repeats	Amino Acids	SP	GPI	Top Five BLAST Hit in Arabidopsis HRGPs
Os05g01040.4		Short EXT	1/0/1/0	138	Yes	No	PERK8, PERK10
Os11g05935.1		Short EXT	0/1/2/2	194	Yes	Yes	EXT38
Os01g45700.1		Short EXT	0/2/0/2	135	Yes	No	FH2, EXT33
Os01g02150.1		Short EXT	1/1/0/0	173	Yes	Yes	FH3
Os01g20780.1		Short EXT	0/2/0/1	192	Yes	Yes	EXT37
Os06g08190.1		Short EXT	0/0/2/0	126	Yes	No	None
Os01g07060.1		Short EXT	0/1/1/0	194	Yes	No	AGP30I, AGP31I, PRP3, PRP7, PRP1
Os01g41120.1	Osativa_LRX1 (OSLRX5)	LRX	0/14/3/2	570	Yes	No	LRX4, LRX3, LRX5, PEX4, LRX2
Os01g08470.1	Osativa_LRX2 (OSLRX4)	LRX	0/8/3/0	520	Yes	No	LRX4, LRX3, LRX5, LRX1, LRX2
Os06g49100.1	Osativa_LRX3 (OSLRX2)	LRX	0/1/1/0	538	Yes	No	LRX1, LRX2, PEX4, LRX3, LRX7
Os11g43640.1	Osativa_LRX4 (OSPEX1)	LRX	9/6/0/0	946	Yes	No	PEX1, PEX2, PEX4, PEX3, LRX7
Os01g25460.1	Osativa_LRX5 (OSPEX3)	LRX	1/4/3/0	503	Yes	No	PEX1, PEX4, PEX3, PEX2, LRX2
Os01g53640.1	Osativa_PERK1	PERK	6/3/0/1	671	No	No	PERK8, PERK10, PERK13, PERK12, PERK11
Os01g54700.1	Osativa_PERK2	PERK	5/6/0/1	682	No	No	PERK5, PERK15, PERK3, PERK4, PERK12
Os01g02040.1	Osativa_PERK3	PERK	4/1/0/0	698	Yes	No	PERK8, PERK10, PERK12, PERK13, PERK11
Os03g37120.1	Osativa_PERK4	PERK	5/2/1/0	675	No	No	PERK10, PERK4, PERK1, PERK14
Os02g26160.1	Osativa_PERK5	PERK	0/1/1/0	695	Yes	No	PERK1, PERK4, PERK10, PERK14
Os06g29080.1	Osativa_PERK6	PERK	5/1/0/1	748	No	No	PERK4, PERK1
Os06g29340.1	Osativa_PERK7	PERK	0/4/0/0	839	No	No	PERK8, PERK13, PERK12, PERK10, PERK11
Os07g39920.1	Osativa_FH1	FH	2/1/0/0	774	Yes	No	FH6, FH1, FH11, FH5, FH 3
Os07g40510.1	Osativa_FH2	FH	0/0/3/0	741	No	No	FH18, FH2, FH16, FH13, FH19
Os08g17820.1	Osativa_FH3	FH	1/0/2/0	893	No	No	FH18, FH14, FH16, FH19, FH12
Os05g47940.1		Chimeric EXT	1/16/2/0	510	Yes	No	EXT51, PRP2, EXT9
Os03g14615.1		Chimeric EXT	6/8/0/0	429	Yes	No	None
Os01g67390.1		Chimeric EXT	6/1/0/5	412	Yes	No	EXT18
Os03g07640.1		Chimeric EXT	1/2/3/0	209	Yes	No	FH3, FH2
Os06g07220.1		Chimeric EXT	3/0/0/0	246	Yes	No	PRP16, FH3
