# Supplementary material for: Bioinformatic Identification and Analysis of Extensins in the Plant Kingdom
Source: PLoS One. 2016 Feb 26;11(2):e0150177. doi: 10.1371/journal.pone.0150177 (PMC4769139; doi:10.1371/journal.pone.0150177)
Supplement: S12 Table — (PDF) [file pone.0150177.s020.pdf]

S12 Table. *G. max* EXTs identified in this study.

Gene Identifier	Name	Class	SP3/SP4/SP5/YXY Repeats	Amino Acids	SP	GPI	Top Five BLAST Hit in Arabidopsis HRGPs
Glyma02g01850.1|PACid:26289743	Gmax_EXT1	EXT SP4;	1/5/0/0	209	Yes	No	PAG10, PAG30I, EXT22
Glyma08g47751.1|PACid:26309653	Gmax_EXT2	EXT SP4; YXY+	3/7/1/11	263	No	No	None
Glyma01g45440.1|PACid:26325275	Gmax_EXT3	EXT SP3	15/0/0/0	211	Yes	Yes	PRP1, AGP17K,
Glyma15g03856.1|PACid:26331582	Gmax_EXT4	EXT SP4; YXY+	4/10/0/17	383	Yes	No	None
Glyma20g01150.1|PACid:26339393	Gmax_EXT5	EXT SP4	5/22/1/0	341	No	No	None
Glyma16g28605.1|PACid:26352944	Gmax_EXT6	EXT SP4; YXY+	0/13/6/20	232	Yes	No	EXT3, EXT22
Glyma16g28590.1|PACid:26353896		SHORT EXT	1/9/4/15	183	Yes	No	EXT3, EXT22
Glyma02g09201.1|PACid:26289570		SHORT EXT	0/7/4/11	156	Yes	No	EXT3/5, EXT22
Glyma02g09220.1|PACid:26290460		SHORT EXT	0/7/0/5	73	No	No	None
Glyma02g41410.2|PACid:26288537		SHORT EXT	1/1/0/2	153	Yes	Yes	FH21A, EXT31, EXT33, PERK6, FH3
Glyma12g06111.1|PACid:26293356		SHORT EXT; YXY+	0/6/0/4	90	No	No	EXT22
Glyma12g06101.1|PACid:26294643		SHORT EXT; YXY+	1/5/0/4	114	Yes	No	EXT21
Glyma11g14131.1|PACid:26294991		SHORT EXT; YXY+	2/8/0/9	145	No	No	EXT22, EXT18
Glyma11g33894.1|PACid:26296372		SHORT EXT	0/1/1/1	169	Yes	No	FH6, FH21A
Glyma17g03400.1|PACid:26311866		SHORT EXT	0/3/0/4	171	Yes	Yes	EXT37, FH18
Glyma15g15500.1|PACid:26331718		SHORT EXT	0/4/0/3	166	Yes	Yes	EXT37, EXT34
Glyma15g13785.1|PACid:26332924		SHORT EXT	2/0/0/0	161	Yes	No	None
Glyma03g08478.1|PACid:26336472		SHORT EXT; YXY+	0/0/2/3	83	No	No	None
Glyma20g32470.1|PACid:26337040		SHORT EXT; YXY+	1/2/0/3	146	No	No	FH6, EXT22, AGP45P, PERK3
Glyma20g01700.2|PACid:26338771		SHORT EXT	0/2/0/0	141	Yes	Yes	EXT31, EXT33
Glyma09g04450.1|PACid:26340568		SHORT EXT; YXY+	0/4/0/3	165	Yes	No	EXT37, FH6, FH18, EXT41, PERK13
Glyma10g35080.1|PACid:26346115		SHORT EXT; YXY+	0/4/1/4	73	No	No	EXT3/5
Glyma07g22970.1|PACid:26348037		SHORT EXT	0/3/0/1	148	Yes	Yes	EXT31, EXT33, EXT18, FH6, EXT30
Glyma07g34050.2|PACid:26349625		SHORT EXT	0/2/0/0	142	Yes	No	EXT31, EXT33
Glyma07g37200.1|PACid:26350443		SHORT EXT	0/5/0/4	181	Yes	Yes	EXT37
Glyma18g04391.1|PACid:26354157		SHORT EXT	1/2/1/1	173	Yes	No	FH6
Glyma08g04390.1|PACid:26307290	Gmax_LRX1	LRX	3/0/5/1	735	Yes	No	LRX4, LRX3, LRX2, LRX7, PEX4
Glyma05g35330.1|PACid:26319865	Gmax_LRX2	LRX	1/1/4/0	595	Yes	No	LRX4, LRX5, LRX3, LRX7, LRX2
Glyma20g28790.1|PACid:26336660	Gmax_LRX3	LRX	0/0/2/2	399	Yes	No	LRX6, LRX4, LRX3, LRX5, LRX1
Glyma09g32080.1|PACid:26340754	Gmax_LRX4	LRX	2/6/10/4	585	Yes	No	LRX4, LRX5, LRX3, PEX4, LRX6
Glyma07g09730.1|PACid:26348106	Gmax_LRX5	LRX	0/0/7/2	488	Yes	No	LRX4, LRX3, LRX5, PEX4, LRX2
Glyma07g21210.1|PACid:26350617	Gmax_LRX6	LRX	2/14/0/0	454	No	No	PEX2, PEX1, PEX4, PEX3, LRX4
Glyma02g14310.1|PACid:26288980	Gmax_PERK1	PERK	7/2/0/1	638	No	No	PERK13, PERK1, PERK3, PERK4, PERK15
Glyma02g04010.1|PACid:26289527	Gmax_PERK2	PERK	7/2/0/1	680	No	No	PERK13, PERK12, PERK9, PERK8, PERK1
Glyma11g07175.1|PACid:26295349	Gmax_PERK3	PERK	10/0/0/0	632	No	No	PERK4, PERK5, PERK1, PERK7, PERK15
Glyma04g01480.2|PACid:26304506	Gmax_PERK4	PERK	6/2/3/1	645	No	No	PERK5, PERK7, PERK1, PERK4, PERK6
Glyma08g28596.1|PACid:26307659	Gmax_PERK5	PERK	4/1/0/0	453	No	No	PERK8, PERK9, PERK10, PERK5, PERK11
Glyma08g39480.1|PACid:26308813	Gmax_PERK6	PERK	2/1/5/1	718	No	No	PERK2, PERK8, PERK10, PERK9, PERK1
Glyma01g38105.1|PACid:26323849	Gmax_PERK7	PERK	8/0/0/1	634	No	No	PERK5, PERK1, PERK7, PERK15, PERK3
Glyma01g23180.1|PACid:26325636	Gmax_PERK8	PERK	8/3/2/1	724	No	No	PERK10, PERK13, PERK12, PERK1, PERK15
Glyma01g03690.1|PACid:26325784	Gmax_PERK9	PERK	4/1/1/1	686	No	No	PERK13, PERK12, PERK11, PERK9, PERK10
Glyma09g32390.1|PACid:26343321	Gmax_PERK10	PERK	7/0/0/0	664	No	No	PERK1, PERK5, PERK4, PERK15, PERK6
Glyma07g09420.1|PACid:26348580	Gmax_PERK11	PERK	8/0/0/0	671	No	No	PERK1, PERK5, PERK4, PERK15, PERK7
Glyma07g00680.1|PACid:26349072	Gmax_PERK12	PERK	0/2/0/1	570	No	No	PERK1, PERK5, PERK15, PERK4, PERK6
Glyma18g51520.2|PACid:26355274	Gmax_PERK13	PERK	5/1/0/1	756	No	No	PERK8, PERK9, PERK10, PERK13, PERK1
Glyma18g19100.2|PACid:26355442	Gmax_PERK14	PERK	2/2/1/1	622	No	No	PERK13, PERK12, PERK11, PERK8, PERK10
Glyma02g36446.1|PACid:26288403	Gmax_FH1	FH	0/1/1/0	1209	No	No	FH13, FH18, FH14, FH16, FH21A
Glyma12g34350.1|PACid:26293662	Gmax_FH2	FH	1/1/1/0	757	No	No	FH5, FH1, FH11, FH6, FH2
Glyma11g05220.1|PACid:26295856	Gmax_FH3	FH	1/1/0/0	895	Yes	No	FH6, FH1, FH2, FH11, FH5
Glyma08g40360.1|PACid:26307339	Gmax_FH4	FH	1/0/1/0	790	Yes	No	FH8, FH7, FH4, FH11, FH6
Glyma17g11100.2|PACid:26312273	Gmax_FH5	FH	1/0/1/1	1409	No	No	FH16, FH13, FH14, FH18, FH21A
Glyma01g40080.1|PACid:26324205	Gmax_FH6	FH	0/1/1/1	889	Yes	No	FH6, FH1, FH2, FH11, FH5
Glyma09g34830.1|PACid:26340274	Gmax_FH7	FH	0/1/2/0	1226	No	No	FH14, FH18, FH16, FH21A, FH12
Glyma07g06440.2|PACid:26349843	Gmax_FH8	FH	2/0/1/0	978	No	No	FH1, FH2, FH6, FH11, FH5
Glyma16g03050.2|PACid:26351725	Gmax_FH9	FH	1/0/1/0	1079	Yes	No	FH1, FH2, FH6, FH11, FH5
Glyma13g25550.2|PACid:26317970		Chimeric EXT	2/4/2/0	731	Yes	No	PEX4
Glyma13g06860.1|PACid:26319359		Chimeric EXT	3/0/1/0	354	Yes	No	PRP11, AGP31I
Glyma19g04410.1|PACid:26325961		Chimeric EXT	2/0/1/0	340	Yes	No	PRP11, AGP30I
Glyma19g04540.1|PACid:26328309		Chimeric EXT	3/0/0/0	276	Yes	No	AGP31I, AGP30I, PRP2
Glyma15g35290.2|PACid:26331978		Chimeric EXT	3/1/1/0	690	Yes	No	PEX4
Glyma18g03040.2|PACid:26355061		Chimeric EXT	0/1/1/1	809	Yes	No	PERK15, PERK9, PERK12, PERK3, PERK1
