# Supplementary material for: Bioinformatic Identification and Analysis of Extensins in the Plant Kingdom
Source: PLoS One. 2016 Feb 26;11(2):e0150177. doi: 10.1371/journal.pone.0150177 (PMC4769139; doi:10.1371/journal.pone.0150177)
Supplement: S13 Table — (PDF) [file pone.0150177.s021.pdf]

S13 Table. ***M. truncatula*** **EXTs identified in this study.**

Gene Identifier	Name	Class	SP3/SP4/SP5/YXY Repeats	Amino Acids	SP	GPI	Top Five BLAST Hit in Arabidopsis HRGPs
Medtr2g098060.1	Mtruncatula_EXT1	EXT SP4 YXY+	0/32/1/22	591	Yes	No	EXT22, EXT3
Medtr8g078670.1	Mtruncatula_EXT2	EXT SP4 YXY+	5/19/12/34	505	Yes	No	EXT22, EXT3, EXT21
Medtr1g099150.1	Mtruncatula_EXT3	EXT SP4 YXY+	5/11/0/9	250	No	No	None
AC231338_17.1	Mtruncatula_EXT4	EXT SP4 YXY+	0/30/0/16	287	Yes	No	EXT3, PRP1, PRP2
Medtr3g049390.1		SHORT EXT	2/0/0/0	144	Yes	No	PERK15, AGP54C, PEX4, FH14
Medtr3g015420.1		SHORT EXT	0/1/1/0	75	Yes	No	PERK6
Medtr3g061500.1		SHORT EXT	3/0/0/0	160	Yes	No	None
Medtr2g034910.1		SHORT EXT	1/4/0/1	184	Yes	No	EXT37, EXT34
Medtr5g020350.1		SHORT EXT	0/4/0/0	182	No	No	None
Medtr8g020530.1		SHORT EXT	0/2/1/0	168	No	No	FH6, EXT32, FH3, FH21A
Medtr1g012690.1		SHORT EXT	1/1/0/0	154	Yes	No	PERK6, PERK3
Medtr1g012700.1		SHORT EXT	1/1/0/0	164	Yes	No	FH3
Medtr4g039330.1		SHORT EXT	4/1/0/0	94	No	No	None
Medtr3g100950.1	Mtruncatula_LRX1	LRX	2/23/2/1	685	Yes	No	PEX1, PEX3, LRX4, LRX3, PEX4
Medtr6g086120.1	Mtruncatula_LRX2	LRX	7/10/7/4	838	Yes	No	LRX4, LRX3, LRX2, PEX4, LRX7
Medtr8g103700.1	Mtruncatula_LRX3	LRX	4/2/2/1	712	Yes	No	LRX4, LRX5, LRX3, LRX7, LRX2
Medtr8g083560.1	Mtruncatula_LRX4	LRX	3/2/2/1	661	Yes	No	LRX4, LRX5, LRX3, LRX2, LRX6
Medtr3g116450.1	Mtruncatula_PERK1	PERK	8/0/2/1	657	No	No	PERK7, PERK5, PERK6, PERK15, PERK3
Medtr6g088610.1	Mtruncatula_PERK2	PERK	4/0/0/0	674	No	No	PERK1, PERK5, PERK15, PERK4, PERK7
Medtr5g019940.1	Mtruncatula_PERK3	PERK	1/1/1/1	604	No	No	PERK4, PERK5, PERK1, PERK7, PERK6
Medtr1g025070.1	Mtruncatula_PERK4	PERK	1/1/1/1	537	No	No	PERK4, PERK5, PERK7, PERK1, PERK6
AC225458_54.1	Mtruncatula_PERK5	PERK	1/1/1/1	604	No	No	PERK4, PERK5, PERK1, PERK7, PERK6
Medtr5g036540.1	Mtruncatula_FH1	FH	1/0/1/0	848	No	No	FH8, FH7, FH4, FH5, FH6
Medtr4g131020.1	Mtruncatula_FH2	FH	0/0/2/0	1198	No	No	FH13, FH18, FH14, FH16, FH21A
Medtr3g010770.1		CHIMERIC EXT	1/1/0/1	577	Yes	No	None
Medtr3g117760.1		CHIMERIC EXT	1/1/0/1	298	Yes	No	PRP11, AGP54C, PRP3
Medtr7g072980.1		CHIMERIC EXT	0/2/0/0	281	Yes	No	None
Medtr5g020530.1		CHIMERIC EXT	2/0/0/0	630	Yes	No	None
Medtr8g066950.1		CHIMERIC EXT	11/6/0/1	287	No	No	None
Medtr1g083260.1		CHIMERIC EXT	0/0/2/0	909	Yes	No	FH11, FH6, FH1, FH5, FH2
Medtr1g021620.1		CHIMERIC EXT	0/1/1/1	319	Yes	No	None
Medtr1g012630.1		CHIMERIC EXT	2/4/0/0	210	Yes	No	FH18
