# Supplementary material for: Bioinformatic Identification and Analysis of Extensins in the Plant Kingdom
Source: PLoS One. 2016 Feb 26;11(2):e0150177. doi: 10.1371/journal.pone.0150177 (PMC4769139; doi:10.1371/journal.pone.0150177)
Supplement: S14 Table — (PDF) [file pone.0150177.s022.pdf]

S14 Table. *B. rapa* identified in this study.

Gene Identifier	Name	Class	SP3/SP4/SP5/YXY Repeats	Amino Acids	SP	GPI	Top Five BLAST Hit in Arabidopsis HRGPs
Bra004749	Brapa_EXT1	EXT SP4 YXY+	1/79/0/51	879	Yes	No	EXT8, EXT3, EXT22, EXT18
Bra015238	Brapa_EXT2	EXT SP4 YXY+	1/17/1/11	208	No	No	None
Bra025889	Brapa_EXT3	EXT SP4 YXY+	10/26/0/16	407	Yes	No	EXT3, EXT4
Bra003189	Brapa_EXT4	EXT SP4 YXY+	2/54/10/31	928	Yes	No	EXT18, EXT2, EXT15, EXT12, EXT14
Bra003699	Brapa_EXT5	EXT SP4 YXY+	1/22/0/10	271	Yes	No	EXT3, EXT4, EXT22
Bra012274	Brapa_EXT6	EXT SP4 YXY+	7/19/1/13	321	Yes	No	EXT3, EXT4
Bra015937	Brapa_EXT7	EXT SP4 YXY+	1/15/0/11	251	Yes	No	EXT3, EXT4
Bra000292	Brapa_EXT8	EXT SP4 YXY+	0/83/1/52	907	Yes	No	EXT8, EXT3, EXT22, EXT18
Bra024612	Brapa_EXT9	EXT SP4 YXY+	1/12/8/11	303	No	No	EXT18, EXT15, EXT12, EXT9, EXT14
Bra007440	Brapa_EXT10	EXT SP4 YXY+	4/69/1/46	845	Yes	No	EXT3, EXT22
Bra024614	Brapa_EXT11	EXT SP4 YXY+	0/43/11/28	772	Yes	No	EXT18, EXT9, EXT16, EXT14, EXT6
Bra014810	Brapa_EXT12	EXT SP4 YXY+	2/27/11/20	623	Yes	No	EXT18, EXT10, EXT15, EXT12, EXT14
Bra037731	Brapa_EXT13	EXT SP4 YXY+	0/75/1/48	876	Yes	No	EXT3, EXT8, EXT22
Bra014807	Brapa_EXT14	EXT SP4 YXY+	6/34/15/26	805	No	No	EXT18, EXT2, EXT15, EXT12, EXT14
Bra011019	Brapa_EXT15	EXT SP5 YXY+	1/2/29/37	384	Yes	No	EXT22, EXT3, EXT17, EXT21
Bra016333	Brapa_EXT16	EXT SP4 YXY+	3/47/13/31	889	Yes	No	EXT18, EXT2, EXT15, EXT14, EXT12
Bra027383		Short EXT	0/2/2/0	168	Yes	No	EXT32, FH21A, PERK7
Bra029602		Short EXT	1/1/0/3	163	Yes	Yes	EXT41, EXT34, EXT37
Bra039829		Short EXT	2/0/0/0	166	Yes	No	PRP1, AGP18, AGP9, AGP6
Bra023357		Short EXT	3/1/0/3	180	Yes	No	EXT38
Bra009880		Short EXT	0/2/0/0	125	Yes	No	EXT39, EXT35, FH3, PRP1, FH4
Bra036401		Short EXT	3/0/0/0	171	Yes	No	AGP9, PRP1, AGP17, AGP18
Bra011767		Short EXT	2/0/0/0	178	Yes	No	AGP18, AGP17
Bra026249		Short EXT	0/5/5/6	148	No	No	EXT18, EXT12, EXT9, EXT15, EXT10
Bra013116		Short EXT	3/0/0/0	179	Yes	No	PRP1, AGP9, AGP18
Bra001213		Short EXT	0/0/2/2	193	Yes	No	EXT34, EXT37, EXT41
Bra024560		Short EXT	0/2/0/0	144	No	Yes	EXT33, EXT30, EXT31, FH18, PERK5
Bra032604		Short EXT	0/4/1/0	139	Yes	Yes	EXT33, EXT31, EXT30, PERK1, FH18
Bra033914	Brapa_LRX1	LRX	1/2/3/5	461	Yes	No	LRX6, LRX3, LRX4, LRX5, LRX2
Bra015245	Brapa_LRX2	LRX	3/31/5/2	834	Yes	No	PEX3, PEX1, PEX4, LRX3, LRX4
Bra023805	Brapa_LRX3	LRX	0/0/3/3	436	Yes	No	LRX6, LRX4, LRX3, LRX5, LRX1
Bra013339	Brapa_LRX4	LRX	8/4/9/2	758	No	No	LRX5, LRX4, LRX3, LRX2, LRX1
Bra011483	Brapa_LRX5	LRX	1/11/2/2	694	Yes	No	PEX4, PEX3, PEX1, LRX3, LRX5
Bra037543	Brapa_LRX6	LRX	2/17/5/0	840	Yes	No	PEX1, PEX3, LRX4, LRX3, LRX5
Bra001950	Brapa_LRX7	LRX	3/10/18/5	756	Yes	No	LRX4, LRX3, LRX5, LRX7, PEX1
Bra017617	Brapa_LRX8	LRX	1/27/4/2	766	Yes	No	PEX4, PEX3, PEX1, LRX4, LRX3
Bra001730	Brapa_LRX9	LRX	2/14/6/0	1153	Yes	No	PEX1, PEX3, PEX3, LRX4, LRX3
Bra013092	Brapa_LRX10	LRX	2/27/6/2	861	Yes	No	PEX4, LRX3, LRX1, LRX2, LRX7
Bra027044	Brapa_LRX11	LRX	2/5/11/4	737	Yes	No	LRX2, LRX1, LRX4, LRX3, LRX5
Bra026996	Brapa_LRX12	LRX	1/9/1/5	584	Yes	No	LRX1, LRX2, LRX4, LRX3, LRX5
Bra034594	Brapa_LRX13	LRX	1/10/2/2	670	No	No	PEX4, PEX3, PEX1, LRX4, LRX3
Bra014193	Brapa_LRX14	LRX	0/10/0/1	727	Yes	No	PEX1, PEX3, LRX5, LRX3, LRX4
Bra016796	Brapa_LRX15	LRX	4/16/9/12	770	Yes	No	LRX1, LRX4, PEX1, LRX7, PEX4
Bra018431	Brapa_PERK1	PERK	3/0/0/2	711	No	No	PERK11, PERK12, PERK13, PERK8, PERK9
Bra007915	Brapa_PERK2	PERK	4/0/3/1	674	No	No	PERK13, PERK12, PERK11, PERK9, PERK10
Bra019913	Brapa_PERK3	PERK	3/0/0/0	715	No	No	PERK11, PERK13, PERK12, PERK8, PERK9
Bra018782	Brapa_PERK4	PERK	2/1/3/0	679	No	No	PERK7, PERK6, PERK5, PERK15, PERK3
Bra016218	Brapa_PERK5	PERK	6/4/2/1	751	No	No	PERK12, PERK1, PERK10, PERK5, PERK15
Bra039607	Brapa_PERK6	PERK	2/0/0/1	610	No	No	PERK4, PERK5, PERK1, PERK7, PERK6
Bra004331	Brapa_PERK7	PERK	4/4/4/0	866	No	No	PERK9, PERK12, PERK13, PERK11, PERK1
Bra011534	Brapa_PERK8	PERK	2/0/0/0	672	No	No	PERK5, PERK7, PERK4, PERK15, PERK3
Bra028371	Brapa_PERK9	PERK	1/4/0/1	660	No	No	PERK1, PERK3, PERK15, PERK13, PERK5
Bra001723	Brapa_PERK10	PERK	0/1/1/0	700	No	No	PERK6, PERK7, PERK5, PERK1, PERK15
Bra024725	Brapa_PERK11	PERK	4/5/1/4	819	No	No	PERK12, PERK13, PERK11, PERK1, PERK1
Bra037220	Brapa_PERK12	PERK	0/3/1/2	916	No	No	PERK4, PERK5, PERK1, PERK7, PERK6
Bra028192	Brapa_PERK13	PERK	4/3/4/3	669	No	No	PERK8, PERK9, PERK10, PERK12, PERK5
Bra016342	Brapa_PERK14	PERK	2/4/1/1	659	No	No	PERK12, PERK13, PERK11, PERK8, PERK9
Bra028684	Brapa_FH1	FH	0/0/3/0	1627	No	No	FH15, FH16, FH18, FH14, FH21A
Bra020386	Brapa_FH2	FH	1/0/1/0	456	No	No	FH13, FH18, FH14, LRX5
Bra009306	Brapa_FH3	FH	0/2/1/0	1237	No	No	FH18, FH16, FH13, FH21A, FH14

Bra002668	Brapa_FH4	FH	1/0/1/0	1292	No	No	FH13, FH18, FH14, FH16, FH21A
Bra002969	Brapa_FH5	FH	2/0/0/0	877	Yes	No	FH5, FH3, FH11, FH6, FH1
Bra024447	Brapa_FH6	FH	1/0/1/0	897	Yes	No	FH6, FH1, FH2, FH11, FH5
Bra016233	Brapa_FH7	FH	2/0/3/0	802	Yes	No	FH8, FH4, FH7, FH5, FH11
Bra013217	Brapa_FH8	FH	1/0/1/0	1091	Yes	No	FH1, FH2, FH6, FH11, FH5
Bra005952	Brapa_FH9	FH	0/0/3/0	1480	No	No	FH16, FH18, FH14, FH13, FH21A
Bra029012	Brapa_FH10	FH	1/0/1/0	859	Yes	No	FH5, FH11, FH1, FH6, FH2
Bra007822	Brapa_FH11	FH	1/0/1/1	1118	No	No	FH18, FH13, FH14, FH16, FH21A
Bra022423		Chimeric EXT	0/4/2/0	556	Yes	No	EXT51, FH3, PEX4
Bra037084		Chimeric EXT	0/6/1/0	261	Yes	No	None
Bra040669		Chimeric EXT	1/1/1/0	555	Yes	No	LRX3, LRX4, LRX5, LRX2, PEX1
Bra004715		Chimeric EXT	3/0/0/0	410	Yes	No	None
Bra035635		Chimeric EXT	0/1/1/1	947	Yes	No	PERK8, PERK9, PERK10, PERK1, PERK12
Bra002639		Chimeric EXT	2/0/0/0	481	Yes	No	PERK3, PERK6, PERK7, PERK12, PERK4
Bra002357		Chimeric EXT	1/1/0/1	577	Yes	No	None
Bra019737		Chimeric EXT	3/7/6/6	684	No	No	LRX1, LRX5, PEX1, LRX7, LRX4
Bra030020		Chimeric EXT	0/5/1/1	301	Yes	No	PRP8, PEX4
Bra039563		Chimeric EXT	1/1/0/0	291	Yes	No	FH3
Bra038210		Chimeric EXT	1/6/0/0	617	Yes	No	EXT51C
Bra013345		Chimeric EXT	2/0/0/0	431	Yes	No	None
Bra000517		Chimeric EXT	1/17/2/12	608	No	No	None
Bra012849		Chimeric EXT	1/0/1/0	389	Yes	No	None
Bra012706		Chimeric EXT	2/0/0/0	611	Yes	No	None
Bra026744		Chimeric EXT	0/4/0/0	500	Yes	No	PRP5, PRP1
Bra026743		Chimeric EXT	1/4/1/0	227	Yes	No	None
Bra020993		Chimeric EXT	2/0/0/1	431	Yes	Yes	None
Bra038089		Chimeric EXT	2/1/0/0	256	Yes	No	None
Bra014023		Chimeric EXT	3/5/0/0	238	Yes	No	PRP8
