# Supplementary material for: Bioinformatic Identification and Analysis of Extensins in the Plant Kingdom
Source: PLoS One. 2016 Feb 26;11(2):e0150177. doi: 10.1371/journal.pone.0150177 (PMC4769139; doi:10.1371/journal.pone.0150177)
Supplement: S15 Table — (PDF) [file pone.0150177.s023.pdf]

S15 Table. ***S. lycopersicum*** **EXTs identified in this study.**

Gene Identifier	Name	Class	SP3/SP4/SP5/YXY Repeats	Amino Acids	SP	GPI	Top Five BLAST Hit in Arabidopsis HRGPs
Solyc04g071080.1.1	Slycopersicum_EXT1	EXT SP4 YXY+	2/18/0/3	479	Yes	No	EXT3, EXT4
Solyc04g071070.2.1	Slycopersicum_EXT2	EXT SP4 YXY+	5/14/0/3	291	Yes	No	EXT3, EXT4, EXT22
Solyc06g076420.1.1	Slycopersicum_EXT3	EXT SP4/SP5	6/9/9/0	467	Yes	No	None
Solyc02g030220.1.1	Slycopersicum_EXT4	EXT SP5 YXY+	3/2/4/4	338	Yes	No	None
Solyc03g082770.1.1	Slycopersicum_EXT5	EXT SP4/SP5 YXY+	2/3/3/6	322	Yes	No	None
Solyc11g065910.1.1	Slycopersicum_EXT6	EXT SP4 YXY+	1/7/1/7	334	Yes	No	None
Solyc01g005890.1.1	Slycopersicum_EXT7	EXT SP5 YXY+	0/1/4/1	262	No	No	None
Solyc01g097690.2.1	Slycopersicum_EXT8	EXT SP4 YXY+	0/5/2/7	318	Yes	No	None
Solyc01g097700.1.1	Slycopersicum_EXT9	EXT SP4 YXY+	0/4/1/7	318	Yes	No	None
Solyc01g097720.1.1	Slycopersicum_EXT10	EXT SP4 YXY+	0/6/2/9	362	Yes	No	None
Solyc01g107220.2.1	Slycopersicum_EXT11	EXT SP4 YXY+	1/4/1/5	322	Yes	No	None
Solyc01g097680.2.1	Slycopersicum_EXT12	EXT SP4 YXY+	0/5/1/8	306	Yes	No	None
Solyc01g005880.1.1	Slycopersicum_EXT13	EXT SP4 YXY+	2/4/3/5	347	Yes	No	None
Solyc01g097710.1.1	Slycopersicum_EXT14	EXT SP5 YXY+	0/3/5/8	363	Yes	No	None
Solyc12g038700.1.1	Slycopersicum_EXT15	EXT SP4 YXY+	3/7/0/12	246	No	No	EXT22
Solyc04g071100.1.1		SHORT EXT	0/6/0/1	93	No	No	None
Solyc04g007670.1.1		SHORT EXT	0/0/2/1	149	Yes	Yes	EXT33, EXT31
Solyc10g084190.1.1		SHORT EXT	0/1/4/0	71	No	No	EXT21
Solyc06g009760.1.1		SHORT EXT	0/1/1/0	163	Yes	No	FH6, EXT34, EXT37
Solyc06g068530.1.1		SHORT EXT	0/1/1/0	74	No	No	None
Solyc03g116200.1.1		SHORT EXT	1/1/0/0	52	No	No	None
Solyc05g008110.2.1		SHORT EXT	0/0/2/1	124	Yes	No	EXT31, EXT33
Solyc01g006390.2.1		SHORT EXT	1/0/1/1	137	Yes	No	AGP51C
Solyc01g065510.2.1		SHORT EXT	0/2/2/3	174	Yes	Yes	FH6, FH3
Solyc08g078020.1.1		SHORT EXT	3/0/0/0	152	Yes	No	AGP7
Solyc12g038710.1.1		SHORT EXT	0/7/0/4	81	No	No	EXT21, EXT3
Solyc12g038860.1.1		SHORT EXT	0/2/0/3	95	Yes	Yes	FH21A, FH6
Solyc12g098800.1.1		SHORT EXT	0/2/0/2	55	No	No	EXT22, EXT9, AGP45, EXT4, EXT3
Solyc09g075580.1.1		SHORT EXT	1/0/1/1	161	Yes	No	FH3, EXT34, FH6, EXT41
Solyc10g050470.1.1	Slycopersicum_LRX1	LRX	2/21/1/1	710	Yes	No	LRX1, PEX4, PEX3, LRX3, LRX4
Solyc11g005150.1.1	Slycopersicum_LRX2	LRX	0/11/3/2	619	No	No	LRX1, PEX1, LRX5, PEX3, LRX7
Solyc01g108900.2.1	Slycopersicum_LRX3 (L	LRX	1/32/3/0	708	Yes	No	PEX1, PEX4, LRX4, LRX1, LRX3
Solyc12g088950.1.1	Slycopersicum_LRX4	LRX	7/18/9/0	661	Yes	No	PEX1, PEX4, LRX4, LRX5, LRX6
Solyc12g006980.1.1	Slycopersicum_LRX5	LRX	3/14/15/7	727	Yes	No	LRX4, LRX3, LRX5, PEX1, LRX2
Solyc04g006930.2.1	Slycopersicum_PERK1	PERK	12/2/0/1	800	No	No	PERK9, PERK10, PERK8, PERK13, PERK12
Solyc02g062790.2.1	Slycopersicum_PERK2	PERK	7/1/0/1	654	No	No	PERK1, PERK7, PERK15, PERK6, PERK12
Solyc02g085430.2.1	Slycopersicum_PERK3	PERK	4/3/0/1	610	No	No	PERK5, PERK4, PERK1, PERK6, PERK7
Solyc03g034060.2.1	Slycopersicum_PERK4	PERK	9/4/1/0	744	No	No	PERK4, PERK5, PERK1, PERK3, PERK8
Solyc05g010140.2.1	Slycopersicum_PERK5	PERK	15/1/1/1	750	No	No	PERK8, PERK13, PERK1, PERK12, PERK15
Solyc01g010030.2.1	Slycopersicum_PERK6	PERK	2/3/0/1	730	No	No	PERK9, PERK10, PERK13, PERK12, PERK11
Solyc12g007110.1.1	Slycopersicum_PERK7	PERK	2/1/0/0	685	No	No	PERK5, PERK4, PERK15, PERK3, PERK6
Solyc10g006540.2.1	Slycopersicum_FH1	FH	1/0/1/0	933	Yes	No	FH5, FH6, FH1, FH2, FH11
Solyc06g069670.2.1	Slycopersicum_FH2	FH	0/0/2/0	924	No	No	FH14, FH16, FH13, FH21A, FH12
Solyc02g092470.2.1	Slycopersicum_FH3	FH	0/1/1/1	889	Yes	No	FH6, FH1, FH2, FH5, FH11
Solyc03g044060.2.1	Slycopersicum_FH4	FH	0/1/1/1	867	Yes	No	FH6, FH1, FH2, FH5, FH11
Solyc07g005480.2.1	Slycopersicum_FH5	FH	0/1/1/0	884	Yes	No	FH18, FH13, FH14, FH16, FH21A
Solyc12g019480.1.1	Slycopersicum_FH6	FH	0/0/2/0	888	No	No	FH1, FH2, FH6, FH11, FH5
Solyc12g010110.1.1	Slycopersicum_FH7	FH	1/1/1/0	1299	No	No	FH13, FH18, FH14, FH16, FH12
Solyc10g084200.1.1		CHIMERIC EXT	0/1/4/0	374	Yes	No	None
Solyc02g078050.2.1		CHIMERIC EXT	1/4/1/0	363	Yes	No	AGP30, PRP1, PRP7, PRP3, PEX4
Solyc02g089250.2.1		CHIMERIC EXT	2/0/1/0	370	Yes	No	PRP11, AGP31
Solyc03g116230.2.1		CHIMERIC EXT	1/1/0/1	295	Yes	No	None
Solyc03g121230.2.1		CHIMERIC EXT	1/0/1/2	894	Yes	No	PERK3, PERK15, PERK1, PERK11
Solyc05g026060.1.1		CHIMERIC EXT	1/1/0/0	322	Yes	No	None
Solyc01g006400.2.1		CHIMERIC EXT	1/1/2/2	251	Yes	No	FH3, FH18, FH6
Solyc01g107710.2.1		CHIMERIC EXT	1/1/0/0	400	Yes	No	LRX1
Solyc12g098780.1.1		CHIMERIC EXT	1/42/0/25	554	Yes	No	AGP30, HAE3, EXT22
Solyc09g098510.2.1		CHIMERIC EXT	0/6/1/4	302	Yes	No	EXT22, EXT21
