# Supplementary material for: Bioinformatic Identification and Analysis of Extensins in the Plant Kingdom
Source: PLoS One. 2016 Feb 26;11(2):e0150177. doi: 10.1371/journal.pone.0150177 (PMC4769139; doi:10.1371/journal.pone.0150177)
Supplement: S16 Table — (PDF) [file pone.0150177.s024.pdf]

S16 Table. S. tuberosum EXTs identified in this study.

Gene Identifier	Name	Class	SP3/SP4/SP5/YXY Repeats	Amino Acids	SP	GPI	Top Five BLAST Hit in Arabidopsis HRGPs
PGSC0003DMP400004216	Stuberosum_EXT1	EXT SP4 YXY+	9/65/0/45	885	No	No	AGP30, PRP7, EXT22, EXT3
PGSC0003DMP400008279	Stuberosum_EXT2	EXT SP4 YXY+	2/76/0/43	859	Yes	No	AGP30, EXT3
PGSC0003DMP400008280	Stuberosum_EXT3	EXT SP4 YXY+	2/37/0/24	533	Yes	No	AGP30, HAE3, EXT3
PGSC0003DMP400008281	Stuberosum_EXT4	EXT SP4 YXY+	2/65/3/43	833	Yes	No	AGP30, EXT22, EXT3
PGSC0003DMP400008282	Stuberosum_EXT5	EXT SP4 YXY+	1/61/0/35	865	Yes	No	AGP30
PGSC0003DMP400001496	Stuberosum_EXT6	EXT SP4 YXY+	0/31/7/37	439	Yes	No	EXT3, EXT22
PGSC0003DMP400001493	Stuberosum_EXT7	EXT SP4 YXY+	1/28/0/3	485	Yes	No	EXT3, EXT22, PRP2
PGSC0003DMP400001489	Stuberosum_EXT8	EXT SP4 YXY+	5/23/0/3	464	Yes	No	EXT3, EXT22
PGSC0003DMP400001492	Stuberosum_EXT9	EXT SP4 YXY+	4/21/0/2	341	Yes	No	EXT3, EXT22
PGSC0003DMP400001491	Stuberosum_EXT10	EXT SP4 YXY+	0/32/0/3	476	Yes	No	EXT3, EXT4, EXT22
PGSC0003DMP400001495	Stuberosum_EXT11	EXT SP4 YXY+	1/24/0/3	476	Yes	No	EXT3,EXT4, EXT22
PGSC0003DMP400001490	Stuberosum_EXT12	EXT SP4 YXY+	8/28/0/3	560	Yes	No	EXT3, EXT4
PGSC0003DMP400020659	Stuberosum_EXT13	EXT SP4 YXY+	1/22/12/2	732	Yes	No	None
PGSC0003DMP400052946	Stuberosum_EXT14	EXT SP3	11/4/1/0	504	Yes	No	PRP5
PGSC0003DMP400037451	Stuberosum_EXT15	EXT SP4 YXY+	0/120/16/87	1561	Yes	No	EXT3, AGP30, EXT22
PGSC0003DMP400024837	Stuberosum_EXT16	EXT SP3	9/3/0/0	516	Yes	No	None
PGSC0003DMP400059595	Stuberosum_EXT17	EXT SP4 YXY+	0/10/3/10	458	Yes	No	None
PGSC0003DMP400011030	Stuberosum_EXT18	EXT SP4	1/21/1/0	258	No	No	None
PGSC0003DMP400041618	Stuberosum_EXT19	EXT SP5 YXY+	1/3/14/1	342	No	No	None
PGSC0003DMP400055428	Stuberosum_EXT20	EXT SP5 YXY+	1/1/20/4	579	Yes	No	None
PGSC0003DMP400039038	Stuberosum_EXT21	EXT SP4 YXY+	0/6/2/7	323	Yes	No	None
PGSC0003DMP400045001	Stuberosum_EXT22	EXT SP4 YXY+	1/6/2/6	345	Yes	No	None
PGSC0003DMP400055427	Stuberosum_EXT23	EXT SP5 YXY+	1/1/11/5	422	Yes	No	None
PGSC0003DMP400033774	Stuberosum_EXT24	EXT SP5 YXY+	0/5/7/10	462	Yes	No	None
PGSC0003DMP400039036	Stuberosum_EXT25	EXT SP5 YXY+	0/4/6/6	372	Yes	No	None
PGSC0003DMP400039037	Stuberosum_EXT26	EXT SP4/SP5 YXY+	0/5/5/2	314	No	No	None
PGSC0003DMP400031352	Stuberosum_EXT27	EXT SP4/SP5 YXY+	0/10/10/5	358	Yes	No	EXT3
PGSC0003DMP400039034	Stuberosum_EXT28	EXT SP4 YXY+	0/5/2/4	287	Yes	No	None
PGSC0003DMP400039035	Stuberosum_EXT29	EXT SP4 YXY+	0/6/2/4	267	No	No	None
PGSC0003DMP400055409	Stuberosum_EXT30	EXT SP5 YXY+	1/0/2/2	242	Yes	No	FH3, FH6
PGSC0003DMP400051731	Stuberosum_EXT31	EXT SP4/SP5 YXY+	1/2/2/2	422	Yes	No	AGP30, PRP1, PRP3, PEX1
PGSC0003DMP400010630		SHORT EXT	0/1/1/1	150	Yes	Yes	EXT33, PERK3, EXT31, FH21A
PGSC0003DMP400053159		SHORT EXT	0/2/1/1	139	Yes	No	EXT33, EXT31, PERK13
PGSC0003DMP400045517		SHORT EXT	3/0/0/0	143	Yes	No	None
PGSC0003DMP400008622		SHORT EXT	1/0/2/0	185	Yes	No	FH3, PERK6, FH21A
PGSC0003DMP400008623		SHORT EXT	1/0/2/0	152	Yes	No	EXT32, PERK6
PGSC0003DMP400055120		SHORT EXT	0/1/2/1	131	No	No	EXT34
PGSC0003DMP400044408		SHORT EXT	0/3/1/4	173	Yes	No	FH6
PGSC0003DMP400000762	Stuberosum_LRX1	LRX	3/12/14/5	632	No	No	LRX3, LRX4, LRX5, LRX6, LRX2
PGSC0003DMP400002002	Stuberosum_LRX2	LRX	3/14/14/7	716	Yes	No	LRX4, LRX3, LRX5, LRX2, PEX1
PGSC0003DMP400027068	Stuberosum_LRX3	LRX	5/17/4/0	698	Yes	No	PEX1, PEX3, LRX3, LRX5, LRX1
PGSC0003DMP400027067	Stuberosum_LRX4	LRX	5/17/4/0	536	No	No	PEX1, PEX3, PEX4, LRX3, LRX5
PGSC0003DMP400010509	Stuberosum_LRX5	LRX	2/0/0/0	494	Yes	No	LRX3, LRX4, LRX5, LRX7, LRX6
PGSC0003DMP400010700	Stuberosum_LRX6	LRX	0/15/2/3	724	Yes	No	LRX1, LRX2, LRX4, LRX5, PEX1
PGSC0003DMP400044985	Stuberosum_LRX7	LRX	2/22/7/0	674	Yes	No	PEX1, PEX3, LRX1, LRX4, LRX2
PGSC0003DMP400000755	Stuberosum_PERK1	PERK	3/1/0/0	697	No	No	PERK5, PERK4, PERK15, PERK3, PERK13
PGSC0003DMP400043552	Stuberosum_PERK2	PERK	13/1/1/1	745	No	No	PERK13, PERK12, PERK1, PERK15, PERK4
PGSC0003DMP400031066	Stuberosum_PERK3	PERK	8/5/0/0	728	No	No	PERK4, PERK1, PERK15, PERK12, PERK3
PGSC0003DMP400017893	Stuberosum_PERK4	PERK	6/4/3/2	682	No	No	PERK9, PERK10, PERK12, PERK13, PERK1
PGSC0003DMP400027121	Stuberosum_PERK5	PERK	8/1/0/1	643	No	No	PERK7, PERK15, PERK6, PERK13, PERK12
PGSC0003DMP400026200	Stuberosum_PERK6	PERK	3/3/0/1	494	No	No	PERK5, PERK6, PERK7, PERK1, PERK13
PGSC0003DMP400042537	Stuberosum_FH1	FH	1/0/1/1	755	No	No	FH8, FH7, FH4, FH3, FH11
PGSC0003DMP400026177	Stuberosum_FH2	FH	0/1/1/1	881	Yes	No	FH6,FH1, FH2, FH11, FH5
PGSC0003DMP400025365	Stuberosum_FH3	FH	1/0/1/0	517	Yes	No	FH5, FH11, FH6, FH8, FH4
PGSC0003DMP400037176	Stuberosum_FH4	FH	0/1/1/1	886	Yes	No	FH6, FH1, FH2, FH11, FH5
PGSC0003DMP400030108		CHIMERIC EXT	4/0/0/0	393	Yes	No	None
PGSC0003DMP400033948		CHIMERIC EXT	0/1/9/0	358	Yes	No	PEX4, PERK5
PGSC0003DMP400047498		CHIMERIC EXT	0/7/0/0	330	No	No	None
PGSC0003DMP400027948		CHIMERIC EXT	2/2/4/1	247	No	No	LRX1, LRX2, LRX3, LRX4, PEX1
PGSC0003DMP400055411		CHIMERIC EXT	1/0/2/2	223	Yes	No	FH3, FH6
PGSC0003DMP400051732		CHIMERIC EXT	2/7/2/0	592	Yes	No	AGP30, PRP1, PRP11
PGSC0003DMP400018095		CHIMERIC EXT	1/12/35/3	1038	Yes	No	None
PGSC0003DMP400002485		CHIMERIC EXT	2/0/1/0	378	Yes	No	PRP11, AGP31
PGSC0003DMP400053338		CHIMERIC EXT	2/0/0/3	1017	Yes	No	PERK8, PERK15, PERK13
