# Supplementary material for: Bioinformatic Identification and Analysis of Extensins in the Plant Kingdom
Source: PLoS One. 2016 Feb 26;11(2):e0150177. doi: 10.1371/journal.pone.0150177 (PMC4769139; doi:10.1371/journal.pone.0150177)
Supplement: S17 Table — (PDF) [file pone.0150177.s025.pdf]

S17 Table. Comparison with previously reported EXTs.

Name	Species	Amino Acids	Repeated Motif	Sequence	Reference	Best Match	Identity	E value
pDC5A1	Carrot	306	SPPPP	Full	[58]	NA[a]	NA	NA
pDC11	Carrot	43	KPP; TPP; SPPPP	Partial	[59]	NA	NA	NA
P1	Tomato	NA	SPPPPTPVYK; SPPPPVKPYHPTPVYK	Partial	[60]	Solyc04g071080[b]; Solyc04g071070; Solyc04g071100	NA	NA
P2	Tomato	NA	YK; SPPPPVYK; SPPPPVYKYK	Partial	[60]	none	NA	NA
P3	Tomato	NA	SPPPPSPSPPPPYYYK	Partial	[61]	Solyc12g098780	NA	NA
The class IV gene	*Chlamydomonas reínhardtíí*	202	SPPP 2	Full	[56]	None	NA	NA
THRGP	*Zea mays*	NA	TPSPKPPTPKPTPPTY; TPSPPPPY	Partial	[52]	None	NA	NA
A sugar beet EXT	Sugar beet	NA	SPPXPPTPVYK, where [X] is [VHE/KYP]	Partial	[62]	NA	NA	NA
ISG	*Volvox carteri*	464	SP(3-5)	Full	[63]	none	NA	NA
class I	Tomato	NA	SPPPPSPSPPPPYYYK	Partial	[64]	Solyc12g098780	NA	NA
class II	Tomato	NA	SPPPPSPSPPPPTY(1-3)S	Partial	[64]	Solyc11g005150	NA	NA
class IV	Tomato	NA	SPPPPSPSPPPPYYYK; G(2-6)YP; G(2-6)R	Partial	[64]	Solyc12g098780	NA	NA
NA	*Prunus amygdalus*	278	PYHYK, SPPPP, and SPSPPKH	Full	[65]	NA	NA	NA
SP2	Douglas fir	NA	SPPPP	Partial	[66]	NA	NA	NA
Tom J-10	Tomato	388	SPPPPSPKYVYK; SPPPPYYYKSPPPPSP	partial	[67]	Solyc12g098780	NA	NA
Tom L-4	Tomato	322	KP; SPPPP; SP(2-5)TPSYEHPKTP; SSPPPPSPSPPPPTY(1-3)	Partial	[68]	Solyc11g005150	NA	NA
NaPRP3	*Nicotiana alata*	NA	SPPPP	Partial	[68]	NA	NA	NA
NA	Tobacco	NA	SPPPP	Partial	[69]	NA	NA	NA
PTEL15	*Solanum tuberosum*	290	SPPP; SPPPP	Full	[70]	PGSC0003DMP400001489	82%	1E-70
Potato lectin	*Solanom tuberosum*	NA	SPPPP	Partial	[1]	None	NA	NA
pCNT1	Tobacco	318	SPPPP(K2); PYYPPH;TPVYTK	Full	[71]	NA	NA	NA
NA	tomato (hybrid of *L. esculentum* and *L. peruvianum*)	NA	SPPPP	Partial	[72]	NA	NA	NA
6PExt 1.2	*Nicotiana sylvestris*	139	SPPPP, YXY	Full	[73]	NA	NA	NA
PEX1	*Zea mays*	1188	SP(2-5)	Full	[74]	GRMZM5G841015_T01	99%	0
SbHRGP-1	*Glycine max*	NA	SPPPPSPSPPPPYVYK	Partial	[75]	Glyma12g06111; Glyma12g06101; Glyma11g14131	NA	NA
SbHRGP-2	*Glycine max*	NA	SPPPPSPSPPPPYYYK/H	Partial	[75]	None	NA	NA
SbHRGP-3	*Glycine max*	NA	SPPPPYKYP, SPPPPPYKYP, SPPPPVYKYK	Partial	[75]	Glyma16g28605; Glyma16g28590; Glyma02g09201	NA	NA
Ext26G	rhizobia (*V. unguiculata*)	489	SPPPP	Full	[76]	NA	NA	NA
SbHRGP3	*Glycine max*	432	SPPPPKHSPPPPYYYH; SPPPPVYKYKSPPPPYKYPSPPPPPYKYPSPPPPVYKYK	Full	[77]	NONE	NA	NA
Dif10	Tomato	396	SPn	Full	[78]	Solyc02g030220	72%	1E-105
Dif54	Tomato	438	SPn	Full	[78]	Solyc01g005880	69%	4E-80
Ext 1.4	*Nicotiana tabacum*	224	SPPPP	Full	[79]	NA	NA	NA
Hvex1	Barley grains (*Hordeum vulgare L.*)	NA	APP; SPP; KPP; TPP	Partial	[80]	NA	NA	NA
GAGP	gum arabic	NA	SPPPTLSPSPTPTPPLGPH	Partial	[81]	NA	NA	NA
PERK1	*Brassica napus*	647	SP3	Full	[21]	NA	NA	NA
NA	Pea (*Pisum sativum cv.*)	NA	SPPPP; SPPPPP	Partial	[82]	NA	NA	NA
LSG1	*Volvox carteri*	415	SPn	Full	[83]	Vocar20001045m	100%	0
LSG2	*Volvox carteri*	625	SP(3-5)	Full	[83]	Vocar20006105m	99%	0
S9	*Volvox carteri*	568	SPn	Full	[83]	Vocar20005537m	43%	7E-127
AtExt1	*Arabidopsis thaliana*	373	SPPP; SPPPP; YXYK; VYK	Full	[4]	At1g76930-EXT1/4	98%	1E-86
AtExt2	*Arabidopsis thaliana*	300	SPn	Full	[84]	At3g54590-EXT2	100%	0
AtExt3	*Arabidopsis thaliana*	325	SPn	Full	[84]	At1g21310-EXT3/5	99%	1E-175
AtExt4	*Arabidopsis thaliana*	246	SPn	Full	[84]	At1g76930-EXT1/4	100%	3E-88
AtExt5	*Arabidopsis thaliana*	203	SPn	Full	[84]	At1g21310-EXT3/5	91%	2E-58
LRX1	*Arabidopsis thaliana*	744	SPPPP	Full	[20]	At1g12040-LRX1	100%	0
mPex2	Maize	1016	SP(2-4)	Full	[85]	GRMZM5G841015_T01	72%	0
tPex	Tomato	711	SPn	Full	[78]	Solyc01g108900	99%	0
NpLRX1	*Nicotiana plumbaginifolia*	725	SPn	Full	[86]	NA	NA	NA
NA	Sugar beet (*Beta vulgaris*)	222	SP2	Partial	[87]	NA	NA	NA
Ext 1.2A	*Nicotiana sylvestris*	311	SPn	Full	[88]	NA	NA	NA
MC56	Zea mays	267	PPTYTP; SPPPP 1	Full	[51]	None because only one SPPPP	NA	NA
Hyp3.6	Bean (*Phaseolus vulgaris*)	163	YYYKSPPPPSPSPPPP	Partial	[89]	NA	NA	NA
Hyp2.13	Bean (*Phaseolus vulgaris*)	367	YYYKSPPPPSPSPPPP	Partial	[89]	NA	NA	NA
Hyp4.1	Bean (*Phaseolus vulgaris*)	230	YYYKSPPPPSPSPPPP	Partial	[89]	NA	NA	NA

a: NA indicates not available or not applicable.

b: Green highlight indicates EXTs identified in this study.
