# Supplementary material for: Bioinformatic Identification and Analysis of Extensins in the Plant Kingdom
Source: PLoS One. 2016 Feb 26;11(2):e0150177. doi: 10.1371/journal.pone.0150177 (PMC4769139; doi:10.1371/journal.pone.0150177)
Supplement: S2 Fig — Colored sequences at the N and C terminus indicate predicted signal peptide (green) and GPI anchor addition sequences (light blue) if present. SP3 (blue), SP4 (red), SP5 (purple), and YXY (dark red) repeats are also indicated. Sequences typical of AGPs, AP, PA, SP, TP, VP, and GP repeats, are also indicated (yellow). (PDF) [file pone.0150177.s002.pdf]

S2 Fig

Ostreococcus lucimarinus

>93986|PACid:27412924
VDRAAKPSQRLSSPRERCPTTPSTPLASNAARGAIDPRVRERFRRGDDRRARTTLGAWRHRPSSNERTISRSPPRPP
DPSRLTAFFAFRRPAQPVAHDSTLKRVTVANDDGDVYVESVGTDAEIVLKTQKVRVDGDVYCGASSSVGISSRVDTV
ESTHAALESRVNALNATEVELSAVMTSRLDALNATNAELKASDVAMTSRLDALNATNAELKANETALLSRINELLAR
VDALSNSTLPPKCIPPGGEKLQYDGTQWTCVCAGSWSGETCETPPSPPPSPPPSPPPPSPNQVSQTTQSGRPSSSDV
WYRSPSYSGSSRPSETVVKERVFSFSATCAASTWVSDYTQCGEGAWREVGMAYSKTSGSIGLGWETIDGQPCLEKDE
PDRTFNTGFCNHWHCWIDACGPSGDSFYGTNNNWITISGWYALASGSYTMSLIFDDSCDVYLESSTSSWTKIFAHGS
DASSVVFSVSNGGEYRFVFVLGNGAYSAVVAITSFESSVDGTYW

>94768|PACid:27413781
MRRDPRWASNAAAEYDANVGAKGREVNKLLVELKESQDKMAQKIRDAEEATAWVDTIEGAKATMDKLPEYTLKAREA
SRRMQELRQRLEKVEARASDTFVAECAAERLEALVSACDANSEATFDRNECCVRLRELDSRDCFCNVNLGAVDFELR
RRALPEVLRAERLCDARLKFGNECAPLPRFPPLNVQPPPQAPSQAPQQPPRAPSTPPPPPAIVSPAIISPIQATCSV
DGLVTLIQAGCASIVDSTDSVLRQRVTSECCRALVKLNDERCFCSEGDMIQELLREFPANFGGMFKAAPNVCGSTIV
GGWQCLPFVDRTPPPSPPPPASPPPSRAIQRAPLPPFIGRTFGLERDVVETITRDVQTRIRERRALIPYFNDARERP
VYVGDAARAKIAARKVEDFLALPREETSDALRALRAIVEDGWATHEREGCGHARQWPEIFVAASALIREQFPQFGVA
SEILPGTGWGTESEAIDWVIRRPEKHIIASKIHFATRLHRDPDSWTNPATTQRPRDGWPELSMPDRSRYQYRFINVH
LTTSALDPTGNAWQSPLVVMLPRDGGAREWAFVKRKLEMTSEGAKDENNFDDKLNIFDPKTWIIRGNRLTRGAYTKE
SKVKDDTDTRGADGMTDAERDFEFPEVVSQDDQEFVSNGAIMFDSFDCWHGAAKWQEDKAFKKTLTDVDPKGRQPFH
SARCSIEMRFRVRIDMNAAGAEKLQWGPFTSAVRDGKFLDEPLRDSEARYDLARGRIVRATR

>32639|PACid:27414247
MAPSTPAARVPTMRRAREDEPVTNLSVAALGALFLANIALVILGLILIVVGRRAGGVGSVFAHGGLDDPLFENPGTS
ASVIGTLLCALASYGLAGLYHRNKTYLLSYHVLSIFMLVGIIYACAVMNIYKSASADMVRNYWHTFQQNTNITGITR
VGRPTRLDHAVWIMRVEEARAEARLFLRTVASCMGASIFFILTALGCTAYIMGLKYTGVYVGITTNVAGVAFALTLF
VLCYYVAKSTYNVPDAVSMKFDVAFMHDVGVSVPPEATTLFNTLRKSPDLIFQTADPSNGRRRLLQSGDNSTQSPPT
PPSPSPPPPPSPSPPPPPDADWQAQSVWVFEERHVRVDNVQYNLTFKLALAGISGDDLDESFSAWAAKKIADFGEIT
PDKVYVSELKAGAEHKIGGVWAPRFLAATAFIIAFFNVYGLVAIYKDDRFLMATHFCFSFTGILLMIIAAALVSKHA
DATSRLISNNWHSIQNKVIGAGVEPVDAGAFARAHFKAAAALGITVIVLQVTSIVATLLNFFSNEPSYTLLTPGKSG
SNSNGATIGRFAGVELDSFDEEYGKRGSLSQQRGHTRRGSDGLVPLGNLATPPSKHFSID

>31000|PACid:27415503
MDDDAGTTGGAGRAFEDAADGGLIDSQYEDADVPRDARYGDMGVIEREAARRTRRGRRGRGRGGEAEGALGHTYIRV
YEDGNDARGGGASTRNGGAQRYRTVTVPIPTRVRVPMEPASVGPAPRAQRRGHSPLPPIETTGGDLFGLGTGYSYYI
DPADWAYPPTVVVELPEGVEFADAELFARGASEHPPNEAGLVMRVNGQRTSPNTLVKNLDVLEIDAQAHNVLNDVRT
VVLRYTWRDPGAGGARKQRRGLIRLNTNGDARTGETQPHRWPIVEHPDTPSVPAPNVPAPTPGTYVPAPNVPAPTPG
TYVPAPDVPAPDVPAPDVPVPAQAPNVPAPNVPAPTPAYFAPPIAWPPPPAPAIVSIEEEDILAANELVIPVPPNML
IGIPTDGHFTVSGLPDFDSDNTPHRQMIMFSFGWTGNNGAEAELGASDSQFVDPPVMDTSSTPPDWISIRVNGQLHA
LPYAAQNGDRIVLRVRAPSEPNSSRMAQMLIGDLHPKVVLTTLDIAAPPSLEETMDAMEPPAPPMVSPPPPREFTSP
PPPRVLTAAPPSPPMPPSPISVVADDDASYYDTGTPSSTPAPAADDASYYDTGAPTPTDDDASYYDTSTPSATPTPT
PTPTPTDDDASYYDTSTPTTPTPTPAPTDDDASYYDTSTPTTPTPTPAPTDDDASYYDTSTPTTPTPTPAPTDDDAS
TDDTASSSPTPAPTDDTASPSLTPAPTDDTASSSPTPAPTDDTASPSPTPAPQQSPVDAFEPTPGAPTESLDDAPPE
SDDSYADFAPASAYPSSVNGTSGYPSSVNTTTPDATSSASEPALPTTPPSPPPPPSPPQPPSPSPPPLPPAPPGIVN
VHNVTMQAGSKAVMFHPPKTVETLQSPNQPLTLHFMENGNDAQLASSGLPLWTEVYVNGDLQDDADITLSNGDRLQL
FVKASPAPLVRRAAYLSYGHKQFVLDVTTAQTSPPPPLPPAAPPPPNDVILAHVAANHPTQCTSAQAYKWSTLTQND
RSTVPMNAWCSRNTTFGEFQAPLCSHQTGRDIIILVDASEAIGSEVFYGKMIDMLHDLYCTIEGTGSQVGVLLLPGA
GNPYVCDAYTSYIPLQQHTSEDFHASLERMRADEGACCGRSVPLAQGLKAANGLFRDFGIFPPNERSVIFVTASHPS
TPVEFETCLSISVPEFRDLTRDHPFNVLPSGEEMTTCEYKLRSVPVAAAELKTAGVRVSVVTVPGADGAVPSSAYYS
GSPWPSKCTADGQCEFSAPYGGDRGHWGSWYTDANGDLTRDYNPGPELACQMRVHPGTPVVSRPVLANALRVNAWNP
RGYADTLAVAMCPAPQCLPSSLFGSTFADNCVGDDASNWAQTLSCVRGESYSMCDRLIPRTERCHAEVRSDPVAVGT
VNNVTAVSVRIHCEQHCSGERTDTHAKSIRVVCPRGASCDGEVIGDDVITAVEAQSATPPTSSNQ

>29265|PACid:27417724
MRQLFKDKQSFNVDISAWNVGQVTDMFNMFSNAISFNQNIGGWDVSSVTQMYQMFHNATVFNQDISGWTVAQVQDMA
YMFSEAKAFDKDITSWSNYTGSGSPVYVQMFVGATAWNAAWGRPGGLQNFDGPASVWAVDGTYTPPPPPPSPPPPSP
PPPPSPPPPSPPPADVPTYGIGIMMGGGLLCVFFGYKYYRRRQKARAPAVSIKDRMDEDEEAMTKSTVEFFDKLDSG
RIKSP

>28656|PACid:27417945
MTTRAPDDGDARRGRSTTGASSDAFVEGDAFEYARRTTANTTGQLTWPAARALAAFLDASDALRRAREVLELGSGNG
WLGLAVLRARGARDVRVTMTETTEGGALRWLEARARANAAWGRIEGDDGARARCRALNWNAFSSDAAASDAGDAGDG
EEGANADARGSARTEDETPLTADSPELADVDVVLGADLVYDDAGVVALPRVVKALLSRARSGAVFYYAHTKHRYDGM
DLDFFANVSANGLTCEEVRPRGAPSPPPSPPPFESLFPDQRIAIYRIALA

>32063|PACid:27418250
MGLCAFVLLGNCATQACYVGFGAAWPRNPWQADALRYLGFGRLDGIIDYVLFFVPYFGIALAGGSKLYVDGASERAS
ALDGGDAEGAFATTVSDKETVMRVTVCTAALAAVTVPSLSSMMYLLFVVSYFATEMRRGVGGKFQGARESLLNLPRR
ATVACLCYTAIHFMAIYMYQLPELQDAARESSAQLLGLYSMDRTEGPVARLLRLTQIIAIGGLFMALCAVESHDSDY
DTADGEGIRAPLMEGESASDADVVDTYDDAGTVARLLARFGGVLAGFTLLATSLLVVNVIAYPMLLLGLILVVNPTR
HTTFANVHGPVIFAYVALWSVGEYALLCIPPVMMSSMNGKVRVLLQIFGASPAHNGEQSGINACYMGSLVATLTFVG
WLTKNAKSTPTDDVLEQRVQDGVIVDFVPLDDLEIEHNPAVDDSRLMWDLPLVQLISHLLVPFALWIVALSRADLLH
AALLLAFLFNILKPGDRTVLHLTAQTLAGFVAALYLWAMNEHVNIFNVYHTKWATTLRVLGLEQSNMIHTTGPFACV
LVVICIVLYLQKVLNALTPETEEDSAPGQGTLNSLVSLGKAVVYECSLVLMKLMTRFGAYLVLLVGLVTLETSQCSL
INLTLLVLLGLNCIAKRHSEETESGRWRAVLLFCLFNLASRYAFGAFPLDEIFGFSTEFKAFLSNAVGLAQNVEPGK
LMAELIGPSTLLVVTYFHEIGLLTMSNAVDARRSLPLAATELGFLPLVKRAIILHTNKIVTLCAILFSLERRDLLGL
LMLAALIAATLSMKTSDGPTELVGTVALISAIIDYAYKIQYTQAGQHWGILTLLGAHKWPESPSDFIPENESMLRYP
VLVILSVELTKATRFWLRKLPPALKSGCSPEPCHLFWPQRDARAMLVQRKTENTDASKEMDDLDDKAMSEISESTSD
IADEGESAASDEARREFTKRVRENAQTNLGQMIMLLSIVPEMIEGMMANLMPGLLYVVFAIVGIASTNMLSLVYLGV
TFKLINSKLGSTPRDRLLLWRFTTAICALIVLWQYVVSIDIVQNRVDDVPPSPPPSPSFARALLSTASEQMRLSRET
RQWLAVYPCSKMMLFGYFSAFFIAACQLRMDAILAALEERARMTIEATQAEGAELDEAMISELIATMVPRQTPTQVA
LLPPETQETSYFDILFAPLTWEASTNWTILEWMRYFYLRYAVDWALVMVIFFGTFSRDLMHVGYLILPFYFLRTRDS
LSMDSLMGLRILQLYNVSVIGAILLYQAPLEGLLGDWYVSVESECSLLQGFGFYKLPTVLGWQSGQLSPDLVIFAIV
HLLRSIITQPTYKAAMIMRRQSIEAERIVDDALKRGWIHESLATCLTKVSEKENRRQRVEDIRNEITSLSREILAVE
SRGTRRKPTGDRKPPSDTIRRRRGVRDSDDDENNEEWIREDEDNAERQDLRAAQLRDHELKMRTSVDDPKKDLTALL
HTWLGDRSSTIGLLFGAFLKAWPWLTAPRYSMCYFFMILAFAIDFSIAALVFPTSIIAYGLMTYPVVPAGFWRLILL
YAELSLAVAYVVTIPCSLQCSGMTACAHTGVVFGIQDASSAFVPSMIGAFMAYIAITVHHSAQQTPRDEIHRREERD
EGNNGDLFAANALFSRFKNVHFAEFEMNFARQDDSESSPFHIEVSRGLLAEMDNFFQRILTPEAEYGPSFVALSVLA
QEDAEPDWDEIVARVNDALINHQTISEGGSKLPLSYYTLEFVQVDTDSLRTSPGHRAAIFRVIAPVSVLRPSAEASK
ALEAMQLREAAGGSDVAPLGLTSVRGFHQEGQDFYTATLCFDILATFFVAICYQNTVDSDAEALVETYYQGLFKIDY
VVSVFICLVLIVVDGVIYMKKAKIAKVFYHYLTYAGFVGFLLMIFHQNTTARHSYLQFFFLLKSLSFTLGARQIRCG
FPAEVFTNSLIFQRSDIISFGAFMAYFSIPFMYELRVLLEYACTDSALDLYDWLKLENISRDLFRINVRNNTYRKYH
PFGQPQPWWKKVFLEGGGLFTIIFSLLVVPFFVFSTSNPQVDINPVYDIRFNMSLIGAENTFKYPIFEGGFRKTIET
MPWSTLRTDGPIPMINKPGQIQEACLAKDSDTVWSPPPPSIEALKKSLSSGAKLTTGWMFKRHLPLDNPILYSEGVD
YTFAEDEAAALIRVLNGEQNNVFLENIYPRVWHLHGKGSPVPNYSSQQSISCTLALNGYNTTSPWWSVDCGTYVVPL
AASEENRQSLDWWRPCSAFENGPEIVLISSEVPTGVFATFSQFVGGLTGVYVVYILTLASIVRGFTNNLVGSIQHVD
LPSTHRLISLCSDIYSARQVRDFVLEERLYWLLIRIYRSPAVLFEFTRTSDGSAMPLRPKTS

>93102|PACid:27419381
MRARVVGDAATRNRRATRAEAAESSPTMRRANRRRRRDGATTALTLTLTLALLDAATAMTMRVRGTLETLTRACATS
ESASFEFTRRDGGATTRCVFAPYDVVREAERASANATAMKAIFVERDANAEIFAQNMVGIDDDGVAAALAANASASA
DYERAKAVNASAHAAVEAALAIADSGTATQVSASGSEATYGCSIPAGAGGVYAVVAAADPAGGVTDVTKPDSSKTIR
RTSLRDYSPKSGPSTGGTVVTFSGDGFNDVAGSTMNCSISDGNSVVVQRATHLPDGTVTCEMPAQSGVIDVKLVYPD
SCYETAQFLYYQNPEVLLVIPRHAPRFGRNNFSAYATNTYYASQYGPGGSDATHAGKVNVTCALGMGTSSNQTASAI
AVVPGVVSASGQAITCMVPDITIPMGTHDVRVSYNGQQYMLPNLVLTDPSKVSIVARGPVLRTKHYYSTAQNGNEDA
RVIAVPVELEGVNRRLVSVGVNVTYGGISQMPGTSGDQALVQVTHASARDNATDARGDYDFSVSPTTLHWMAGESGE
NFVYYVRIRNDRVHENALEALTLTLVDAMNADIETDFKNKSAVVTIADDDPAPLFGVRARNVAFPPLYRYSQETVKIP
IDIIGGGNFALPAVIDYEIVTSGAASSAVAGVHYAAATGRLTWMPGDYGKTQHAEVVLDWSNIPSEASLRLHVNLTA
VSESRVAAIPSSFVESEDAALFIFGVPPGSCPPGTRRTDPTTYVAPPPVPQALSPPPPSPPPPSVSVDSAILSLSVV

ANTTVAMSSVFPYVPDGIAIQPAFDPTRHTYTATVPHGVTGVQVFYQFRQSTTSITTTLSARRRLLAASLVNMKYFT
LNQGDNVIRLITTSPNGQSSETYRLTIERLAMVANPPSPPPPPPTPPSPPPPPPWTHPSPPPPEGALQAPPAPPAPP
APTPRPTEPRDNATCTYCSAGSFASDMNSLQCQLCPPGSASPSVLTKACVQCPQGMYMAESGATQCLQCPRDAFANV
TGARLCTPCPRQATTSTAGSTMCNVSTAPVDRAHPDVFYVDVVFGVSFHQAGGSLANMAANIGVDADANSAFVRAME
IDVATKFNVTRGSIRLDVAQPGATASTPAGRRRLLQSGSSVSVTCSASQNCIRAVVVTVTMQATEMLRFGVVRDYET
TKAAIDATRAQAAAILLTLRSDPVAFFSTTTAGIGGVVQGQLYEDPSGSLIVEKEPTPPRGVPNVFGDVNVALIVVS
CICAMFAAFVAYTLLRRTRARRMLTKIENEVIESEKRRVGVRKVKDSSKVTATVNKATWKKVLAQKDAPDDAEKGQV
KKQIQMREQSQITLAHVSRFKQSRESSAIEKMWQARNERAAIGVGIQRVKR

>19151|PACid:27420266
MTRATGRARASEDDATTTRGTYGATRTSRGSASDDARDDARRRGTTRARRWRAAGACACLALAIGTCGTGEGGAIER
WGEATRALGARAWTKGVGAALGEGSVSSTVTSSVTSTVNGGTPSPPPPSPPPNPPPNPPPPSPPPNPPPNPPPNPPP
TATPDAHPSSTPVESPSPAPEPVSSPPALTPDQAAKLKRDIRRLTRKIGRRKPDHPSMPGLQQQLAELQRELNGEAS
PTSSPSPASEPVSSPPALTPDQAAKLKRDIRRLTRKIGRRKPDHPSMPGLQQQLAELQRELNGEASPTSSVSARLGS
KASIVVSTRDLDVSRASLFCVVAVTIVGIVIRRLRRAPEFETDEIARLL

Chlamydomonas reinhardtii

>g8674.t1|PACid:27562805
MDRLYDIFSAAAQAQQQKQEPRSQLVAAASSAPAVMGSGSGGSGSGGSGSGGGAQPPAPTAAPPESADLEGNGPGGV
PTTAALGLAAAAADCAGAAQRDVMGRAARCGSGAVAGGANPPGGWAGGPSSSHVSTLGQLLLPAATAASSLATASSL
ATAGSSFTGSSCGTGRSGLGGGQQAGGRSGLGGSGDPGGSGSGGCGSSSGGGGSGGTAEGSTGRGCSFLTVSGGSPE
AAADAGPGLEPTGALENLNELQPAACSVQHTWRLLGAARVVSAPSDSSSVGECSLPKGLIQIPAAAPWRPPARLDGA
VATACAPAPGLAAGAAPAAGALQKDQQEGLLQALVYMGVISDTGQTPGRQGPTANVRAAAPGTTGSGSRSRDGGFRR
ANTAAAAAQDGCEPAGAFAPLAATLSSPAAADHVEQAGIQSLREAVAAEESHGAAACATAVDACAAAVSLLGSAQRQ
AVTDCASRSSSCSRYGKHGEQPGPLSTTEARPLPLLPPPQGLLLLRGGCGSSGGDKNSSNSSSSSKHRADGRSALLA
ASTGLTAAAPLTGHRGLSGAPALAAPPASPPSYTFAPASDMEHGAGAPDSPPAAASKRLTCVPGKCDAPATAAGPPS
TLPALAPPPPAPPPALAPAPLSAPTLQLRRLLLQPPAPAVMAATPAAAAATAAGALPSASVGWARTGGAGSSSSSPS
ALYRSPARHVTVSMKFDTPPGMSFEETAAALSASAHRTVEQVLRRSSAYARSQTANVGTDSCDVASGSAGTPGTGVA
GLPSSASGTGGGGGGAISSDTSSISGIPSSSGTSSLFAATPSSEAAASAAGLAPGSTTPAAAAAGAPGAAAAIRGAG
AADPSARVAAAAEASEPAAAASDGGDGAVVAAAAAAATPVPGGGGLRWHSYATQVQYLRGCIEVVLRLQFDAPDGPD
EAAASDALLHAAMEALHDELHGQLLQLPEAAHAELLLPLHLLHPAGGNAVGADGEYGEPPLPPQPPPPQMVPLLVYM
DPPVLIAAASPPRAPLHRPLQQQQVPPQQPQGQPQAPVVAGSSRATAPGDPRTAVRAAVAALSPASRWGSADAAAES
EAPGGGAEPQAATATTTLGAPQQGSERRSALAEPLQEPGRQAVAAALVPSSSPSPPPPESPAASARARRAARRRAPP
RRGLTCAAEYFGTRSSREWHGLAGGGGGVDDTASEKGAAGGRRLRHGISGEVGGAGVGEDLMAGDSASELSEYGFND
GGGGGGGEGWVAVQERQQERQQERQQERDWERKQPAGVRWPATGQLTVAAEEGKGQEAAESSGVVQLGADLQGSMAT
PFAAAAAAAAMEAAAVPAGIAGASGSTGNSSSRSSSRYHNVLDLSLVPPHHIATTAVPVTEQGRRPRHARAHAPAGT
AEASAAAPDSPDIGSGDSAGVGEAGAAAAVTPFLHPSLAAVPPLPMATASEPAALLPHQQVVMELLTASDSESLDQD
RFAPAANARGSLGGNGGGGTPDSEAVNVTAQGWLQHCGAEAASHSTSPSAAATSMIFAGQVRDLGGAAGGSSSGVG
AGGRVSWGGDAGSSGGGAVSPFAGALMQRQPSGHIHGPDQAAAAGRDAATGADTVPPARAGSHRSLRPSPFVGAAAA
PESTGTGGGGGGGGGGAVRAPSLDRSFSLSAAARLHQHRMEHQPQQQQQQQHQWWRQQQVQGENTGGLATAGEAG
ARAHGGVAVAAPAVNPVAEAAAVAAFSPPPWLLLARRSNSGPAGGRFSPLRAPAVASGPLRAPAVASGAAAVAAPVA
LVAAGGSSGQLASTTAFSDFAAGRARGPPAVPPAIAAQRPVPRQRAAVPEAEAAAVASAAVAAAPTQTVCFTLHLDM
SSDAGGGTAGTAAAATAVPPPLLVRVVVTQAGAVVAEVPQVEVGPRGASVSLDLPAALAEGSAALLVLPYRGDGHGQ
EAEPAAAGGAAAAAAAPAALAALHYSPIAVVPPEVAAELCGLMGRMEQELAAEAEAAVAAAGEGGGSSSTSSAMVRA
RAFTHSFSPFVSDMMSLLMGGNRFAAARSAAAATAVATASGGAAAPPDAAAQDLQEAVRHLQQLQMLVNALLSYMRQ
THLPYTQQYMAAGLAGAGVLPASSFGSASPPPTVARRDTARQRLQEQQQQQQHSQERHGYEQQAEVASQLLPQECGD
HVPASAPAATAESAADSAAEAEVGPAPPLAEARCRGFAADAAWAVGRAAAVARRRLLWGLAASGQRLHGPLCQAGLS
PAAVSAAAAAAVAVAGALVLRRLQLRAAGLPPMDGGPGGGGGGGGAGRLPGRGTDALQQTHGGQRMGQDRQGQQGHG
QPWSVGLHSGFEVVAMWATAMATVMAVVLAAVAWVTQRLLRRRRGVCAASTAAAGQQQ

>Cre08.g379800.t1.2|PACid:27562877
MVNSSEPVRLYNRQAIQVVFSRPVIALGSDFGDGAAAKQVPFRLSCGVPGSFRWVSTTAGRWDPALDWPPDLSCSLR
WNTSLRSYDGARLMLDGTPPRRALASSSLYMYGGGATSEAAARLTGGAWSSDSGVAGDKLPEVPPDGKLLLQFSYPV
NLLMLKSAFVVLQGRDESVEEGAPSLPYNLQPCWSPAPAAPLPGGAAGSGTPSNTALDVNTTCAAVEVGAALQPDTW
YTLRLPAGTRYAALAGPTTYTQQVVFAGLRRFRFPLAQDAWRASSPSSGISYRRLDLWLPHGLQPGTPLDALARVLS
VCELQPAAAVTGAPAAVAGGEQASPPPPDVCVPGAAPLPFTLSLPSSSRLRMTVTDLAPGKKYRVTAGADPTVIDGF
GMSLEASNSTDFYMTQIAYRFEGADTTGGVYSFSNAPLAVLEPEPADNLTQWPYITIAPPPQPALGVDVPPYTSRTV
RSFVLDPANRNHVIKLLQVFQRNFPNASSSGDPTAHLGLEPVSVVPVPEPLTTPGSGAGWTRVSAPLQPGAALQLLD
ACCTPPSYVGGPPSRRTQVLVRSGLSMSVLASRSNTVFTAWVTDASEGSGGGGVAGATVSFYVLPTSGYYREGSELL
PFLAASCVTDAAGTCSVDIPPNESGQSPQSGDRLSVMALAPPSPTPTASLTSSTPSASTASPTASTASRVALVLDEL
WLGSRPQPPSGTLASLVADRRLVRPGESIKITGFIAQASLAGMQLPPATWGILEVSSGWDVSASGSVTGRRRRRAAA
AAAVATISSSARFTKLPGTAGDAAAAGTTVQSRQEYELSSSAVEAADSSGGRDIGSSSSGRRQRALRQAAPPDDGVG
SPPAEEPYTPPPRVLVAVNASSGSVHGEIQVPAGARQQPYNVMLYLPAAVGTVGTAPLDGPPPDLSAPDLLFGWNYV
RSFEFTVADPRPPTAELQIVAPPWVSPIGTFTVTATAVSYIGAAVGGAPMTLTWRHARASGVLRITTDANGVATATV
DLGALPEANRTTVYDAISVSVEWIGPTRERIVESASIAAADAEYALSYSLSLTPDLPGVPFGVTAILTSNTDGSDLS
GIPVTATLSPATPGTAGGAACDSVATCSTSSGAGFVGPSACRLALPCLGRFSLRVCAVLPPAVAGATAVGGGSSTEV
VTVCGRFEMILGRNASEWQQYAFKLRGMPMPALSLDKNVYSIGETPRLRFESPWPHARLLLLWGNDFATQSKAIDTL
PAGAMADVELGPLSYDTCAGGCAVTAVLHVPRLGPSDMPLPPPDAVPVSVVCDPRAPLTYSSKLRIAVADDNGLEVV
VAVGPVAGEAAGGGRVVDAGGEELLAIEPGQKAEVSVRVSGAGPGVEVTVYGVDQAVLDLMPYDLPAPQLDLVLRLA
ADVQVYGMAAYRLAPGAVRAVFDTLVRRLSQLDPWLPPDTAVRAAAAAYWSGGVQAADMSDEEYLDQFTTGITLLAG
GYVDNNRAPLTPAFRRPPPQRRAERAPPVGGPSDPESGAGGADVSGELRSSDDFVTTPLFTTVLTGPDGVARVNFTA

PANLGTFVLRAYAAATGAAAARRYGSGEGRLVVRRALSLTPSAPRFVRVGDGFEAGCVVTVGSAPARVSVTLTVEGG
ASSPLQLSDGGGVGGASTMTKVVTFQAGADLQEEVRFPLSGRGIGTARLTLRAVDFQDGGGSDGLQVSMPVYGQQGD
VLVATTFSLTANGTTGGGWAEGVELPAAVPGSGGLTLTAGVGNLPAITAMYANLLQQERSVAEAGDRYPYAERALTW
AVLPPLLAAYGQPNTSSAAQDQQVTRGFADLGALTEPGLGLVWSDFRRWDGWRPLRADTRLNAWALFLTGLHGPALT
SPPPPLPASGLGRYLGPWSDRLEAQLLPVWRRALAGQLVADAQEARANNGTYSDWHTLAWTRLALGADWDPADSSTG
GGARGRAAADLSLAALIGANGKNASRPRLDRDTRVVLSLVLLSMGQSNPEPRLVNATLVELSSAVRLQGRTAYIAAG
PGNPGPASLEDQALALLLMVRARSLSPLLPRLAAYVAAGGSSSSPALSRRRVYYPESWFVQALAAAALMSYDVSRSS
AQPQLQLEADVNGLTVLEASFRPGRTQPVTTTTPWESLPPPPAVGQPGELNFEVSGRGEVSVSAALQFVPVTLPAFP
TYRGLYVEAAVQVVDPATGQGAGPRVSGVPLGSVVALTIQVTSPDELGAVTLAVMMPAGLEPLDPHVVTDLGSGCDL
GDWSGGGFGFGFGGGGGMRSRWWYWWPVCPAQETTPALVTFSYASLRSGTSTVTIKAVAASAGTFVLPPIRAWADTQ
PELMGMTAGSAFAVCGDCSEATPAAPPAALVPCPALDCSGNGVCNLANGACECDEGWGGRDCGKALA

>Cre08.g364400.t1.3|PACid:27562900
MEPSPSEGPPPGPPPGMLGATTPAAGQQASAGGPLPGSTTMPGPGQPGQLGQPAAPGQQAPPAFAVPGVPAPGLVYG
APRPGMPAVQPNPLAANPAALQQQTAAAAAIYSTLLRPPGTAGTAPALTPAQLAQSLPPNVLAAMQGLQAAQAQLQA
TAQANAAAQAAHAQAQAQLQAAEAAKAAQQGASTNSRAASPEGNPNQPAAQAPPPGVVPGAMPALPRPPMPGAVPPA
PAPPSTVPPSPAAAMQALVSAQAALQAAQAQMAAAAAAAAAVTAAGQAQAGAAAPGGVPPGQVPTPAQLQAQAQANF
QAQMAAAQQLRNAAAAAAARPPFPGAPMPGQPAPGAPAAAPAPLGTPPSAPPGSAPAAGLPQPAGSLPPPGQPGGIR
PPAVSPAAASQAVAAAAAAAAAAAAAVAGVRPPAPGGPLAPPPAPSPSPAPAPAQAPVPMQHHQHQQPGPGAVSQS
PASPPAPAAPTLPNPSTSPPAPPAPSPPPPPAGLRPTEQSPPPGAIPPGGGLISSLPPPPLPPPGMALPPGSDGAPP
PPDLSGMPPGLTLPLLGPDGQPIDPDAPLTEQQQLQLSEAMQAQMEQQMQMMAAQQEMMAQAGMQFPPGMQFPPFPP
GMPFPPFPPGMQLPPGMQMGMPPLPMPPMQPPMQMMQEEELEPESSDSDNDMTSPRKRREKKKAPRGGGGGGGGGGG
GDDVVSKGKNKTYRGVRQRPWGKWAAEIRDPTVGARRWLGTFDTAEEAARAYDAAARAIRGPAARCNFPLSSSHDDH
YHHHVQQQQQQQQQQLEAQAMAAAAGGHMGGPNGMGGPPMGLMHGGPMGSVPEDEPLPGGPGGMPPPMMEQPKVKL
PAAAGLKLRGLKQKVGPKKGGMDEPLIQSSMPGGLGAADIHGAMSVSDAMAAAAAAGGAAGGAMPGLNGSPGNPFQM
RIKTGLTPNFSGWHGPLPPGVAGSLGGSAAMMATSLGNSSGGLLANFPPLPEWPPAGSMGSAASMMMLGMSPGAGME
AYGTSYGNQYGKSVDMMDMCAQLLEAGGEVPPSLHDADGSDVAKAGGAGSAGARLHLHSMWREWQAGSLGSGANVVD
PLATLGSLRNELVIPPDFKSEGPNAGAGDGDLDGVHEMDEMMILGTTPDFGRPSNDFFKNLPNSFGTGASSIGKGAA
AGKAVAAAAAAAAAAAGAGGAGGAGTGASSGTLTLAPPGSVAAAAGAGAAPLPFGSRGNSPPSDVPDPGGAGGSPLT
HMLLHGRHGLAADPALLGMGQLSSDGAALGVGGNDEDDYLMGMSPDLPTGSGKGKQPPGIREPGFESFMKAHAAQAA
AGAATPKGAVAAAAGGGGSSGAPSLELPVAAAAGGSPNRDTAGTGDLLSPTISFAAGVGGLDLPPPDQI

>g8883.t1|PACid:27562934
MSGRAGKRARPDSEAEEAEAAGEEAGGSPMSVGEDAEEAAAPDTEGTEVDDEAGAAPEAQLDAGGAEAADEAAEAAE
AAAQAEDAAGDDAGDGGGAAAGGTSRSRSRARGRGRSKSMGAAGGGAAAPAAAAAGGGGGGGGGASRSRARKSVDP
ALADVSPEAAAAIAAAVAAGTETGRPGRAVRQKVVKYRENKIEYDWEREGFKEAAPKEERPGRGGGGGGKSGGGKGG
KGGSRSGRGKGKGRGGGEDSDDEDGAGDAPPDGEDGWDTHCKVCNSDADIEKLLCCDGASSLGCTAVYHMYCLDPPL
SRLPPGDWFCPECAHKYTYQDIERVLDYRDVPLSGDEPTTERRRHHHHHHHHHDHHHHSTGPLRREFHVKWKGESYI
HCTWEPEEDIAKMYKMFPAIKQKVQRFWRAREAGLLAGGGGGGGGDTAAAADLREAEEAGEVVHGVHAAWQEVERVL
AEQPVAAGGGEKKHGARKAAGTSAGGSPAGDGGSGSGLGGGSGGHMPMEYLVKWRELGYEQCTWEQESDIAGYEDAI
RRFRESRSVIEDARARKAAAAAAGPARKKARVEDTGAADDAAAAAAAAAASTEAPGGEVQVQGQGSRKWHATPDFLSP
AAGSLHPYQLEGLNWLFHKWCAREAVILADEMGLGKTVQTIAFLAALHAECCELPHLVVVPLSTMRNWEREFAAWAP
QLNVVSLAGNAEARAIILEYELFVQPHSGNKQLKRAAGGHRLKNKDSKLFQLLTQFDVKHKVLLTGTPLQNHLGELF
MLLHFLEPDKFPSRSAFEARFASLSHNEEVSQLHSLLQPHMLRRLKADVMKQLPPKQEQLVRVELSRRQKDIYKALL
LRHSQVLVGNRAAVAAAGGGQAALKNLVMELRKTCNHPYLTGSPAEQAEAREDDAADSLAALISASGKLQLVDAMAQ
RLKAAGHRLIIFSQFTRTLDLLEEWLNGRGLGYMRIDGTVSGSERQRRIDRFNQQPDAYFAFLLSTRAGGLGINLAT
ADTVIIFDSDWNPHNDLQAQARAHRLGQDKPVMIYRLVTRRTIEERMMQVSRKKMMLEHLVVRKMSYAGGGGPGGGG
GGGELKQSELDDILRYGAQELFAEDEDEGSPAGKEGGEAAEGGEAAAAAEGKAEGGEVKAEGGEDKDASKPAAGTAP
GTAPGTTTGAASSSSSKRIVWDDAAIERLLDRSGLNALRNYGESVAADEAGGGGGGGGGGGGGADIFSAFKVANFDM
VEVLPDPEPEPEPEPEAAAAAGGSGAPDEALVAGLCPEQGGEAGPAFWQSLLAGRTDLAAGEAEVMAAAVAAAQARMGK
GHRQRKKISYRDVAGENTSSSSDSDDRLSGSSSDEEEDGEPPGAGGAGGAGGPGGAGGGGVPGHVSKQMLAAMMGQG
VRSAVEAFAAARPDVWRQLVAAPLAPGSSAAAAAAAAAAAAALVGAAGAQAAAPPAAAAAAGAAGAAGAATLPPLKSG
NGTTLQVYGLNPRDRSTFIAALMRFGLQIADGQPDEMYRAFAARLPHRPPAAVREYTNLLVAALSEPVGAGGAWSNG
LRPEDLLGPHRATDVLERIGFLHLVRRKMAEYRNRTALVDEPGQPDFKLKEAGRVRALSAPGRVWRPHHDGYLLRGL
LNYGYGQWQRIIKDPGLGVREALRGELGVPVDYDTLVAAPAGDKEAGGKEGGKEGGKEGKADKEEGGKEDGAKAEGA
AVKTEPGQEDGTDGGVEMADKAEKEEAAGTQPEGTQPDGTQGDAGTATQPDAMDVDGDAAGASSPAAAGVVKSEGGG
GGGETSPPPTGATATAITKEKARAWSSNESRWITRRLAAISEALNLEYLAVPAAVAKAAAGAAAAGRIVPAPSSLVA

ATAAGAAGLHRAAAAMQLLHQNAAAKAAAAAAATAAAARGAANKPKAAAAAGGAAGAPPAAAAASVTDAFLTNLLPV
VDFPTAPAAERALKRQLIEQYNALVRTVATIRHETATAAQRAATEGTAALGQAAERFRLQVGVLEAYATQMLVMLPP
AGQTMAQAAPTLLARLQQAAAQQRAQQVAAVAAAQQRVVQHVAAAAAARQQLQQRQSAGAAAAAAGPAGAAGVGKAS
ASPPPPEAPAGAGAVAKPGAGEVQTDVEVVGEAAGTQGEAAAGVKQEAMQE

>g8541.t1|PACid:27562982
MVELGHSAAPIKEWSGEQLTELLGSDSAVAASVEQLVGMVLVSRRFASERQWNMDRVAANAFASARLRRRGRLLSQS
SNTFAVEVIIAQIVEMPSPPPSPLPPSPTPPSPPSPPLNPGQFAPPPSPPAMPLPPSPPPPPPVYPPPRPSPPPPVN
LDVIGAKLGANVSQIYDPPSPPLSPPPSPRPPSPPPPSPVPPSPAPSPPPVCEREGYVYFPNAAYDCAAASSSCLVL
ESWGDSPGDAEYNCNYGPEDDHYTPADCGAFDSNGNVLSHPSGGRYAADPTTVQGLSGDCPGTWVKEEYRYVPPPPP
SPPVPPSPAPPSPSPSPSPSPSPSTPVAPSPPAPVPAYSPTPVPAYSPTPSPSASPSPSPSHPVSPSPPAPVPTYSP
SPSPSGSPSPSPSTPVAPSPPAPVPTYSPSPSPSHPVSPSPPAPVPTYSPSPSHSPSPSPTYSPSPVPAYSPSPAPT
YSPSPSPVYPLSPTPSPVASPSPSPMASPSPSPVASPTPSPSPTTSPSPEPSPMPSPEPSPSPSPGSSPRPESSPSP
SPEPSPSPSSEPSPSPSPEPSPSPSSEASPSPSPEPSPSPGASPSPSPTPEPSPSPSPEPSPSPAPVPSPSPEASPS
PSPSPSPSPAVGETVTTTLRLVGADYGIVSGSEDSMIAFESAACISLTSTTATSSCSLVSATSGSIIATFDLVSNVG
VTAAQLTSEIDSKAASNTLWTPDFTTEYSVTSTEVLPNPSPAPVPTPSPSASPSPSLSPAPSPSPSPSPSPSNETSP
SPSPSPSLSPSPSPSPSPSPSPSPSPSPSPSPSPSPVPVVPVSSPPPSPAPRLPSPPPPAPGAKTIFGQAYDGYLSG
CTVFLDISSDGLFGADEPSGRTTYDRSAASSGWTFSVNASLTPLLARRQIRLLPADVAELANGNRTSDGKGCHDVAT
LTKLAVPLAAPGSCSTPVITPLSSVLSEMDLLTTGQFTVAALRQRLVGALGLNRTTTPTLCEYDATSDALVDLQSPG
GRLYAQEQAMMASVTTLCSYVTPALKDLPTCARLMVQQVAARVLAGTSARRRVQAARAGRTLLQGNSGNTPAANNSA
SNSNSNSNSNSTLQLADPLGGLLNLQDPATLAALLTGVAAQADKLPAGSYNPAALNDTARINATVNSVSQMLAIGAN
ATSLQTLLSSSLVAQDYLAAQIASLAAANSSAAAAALNSFANSTSSAALSQQVATTPVPGDYRPFNNTNQSGGDNAP
PPPPSDSGGGDDNNNLAIGLGVGLGVGLGLLAIGGVGAGVYYKKKKADSRRDAYNVSGAAEGADPEHRGGVAPVHDV
RPQSPPPPAGLVTAQLAPPGAAGGPQLTGTAV

>Cre08.g374250.t1.3|PACid:27563071
MVRASVARNRPLQTGSLAWLVLLTFSLAWVTIPVSDALQFPVNVSVTSSAATDAPSAIAYIGALLDVLWNVPWQPNC
INDTRRSYDAPWPSRCALPAVQYGYYDETYIDATVYSGNSLRPFYSTCRYPASSDPYAFLEPYSMPWVDDFGIYQPV
LDGFDMWWGFTVNTVDSNLKWIDPAWTAQGAWLGGQDFRDAVWNFGTHYCSWPFVECSSCENYDIADPYDPDKIANG
IVPAVITALDFRNASLYIYYLDFGMFPAGSLDNVWYLNLAYNFIGGPLPANLPTLLPSLQHLALDHCRATPDVRGTA
SLQYGWQQYPSTGQPYEYCSDGDVTGSDGTEYVISGMIPDEWGDAVAAAASDSSLPWANLRTVRLSNQALYGPIPEG
LRSASSSISSWRLQGNTELCGPLPEFAAPINSLLYLGTKLGTHTWVWHDPADHTQGGECLAPPPPPSPPSPRPPRPP
PLPPSPPPPLLPPSPPVPPPSPPSPPSPPPSPPEPPSPPPLPPSPPSPTPVARCIQVGGICDSPSPMPPSPRPPQPP
SPPPPPPRPPPPRAPRPSPPFHPPSPDSPPASSVPPSPEPPSPKPPSPAPPSPAPPSPPPPSPAPPSPAPPSPAPPSP
QPPSPVPPQPPSPVPPSPKPPSPAPPSPPLISPVQGLLLGGPGLWAALLSIPDAANSVFVVSAGLPTSVAPSTPPEL
LASFCTVCSCQLTATAISLVGGSSRGNNTNGSSSGGNYYSNGGDAAIQRIPAGNSTDRGGSSSGSGGSSSWGPGTET
AAEWAVDAVQDGTYQLQLSIGGVTYTRTVVVDRTPPSVSGNVTLSANRIKQEPSAVGEASLNALGSKQAMLLTISFS
EPVPAFDPAASLIVTGALVAEWVAAADKMTFYVLAMTLPAELVATAAGSSSSGTSRSGNGNGTAAAAAAAAPPPPA
GTTGRRRALQQQAAAPPPPASGSSSSLSGAATANQQQRHVHFLLPATAYADAARNPGRNDLSLSVELTDNAVASPAV
GEALATTARVTAATYPAVAATTTLVAAASSSFAQAIRAKGSLLQGSYHIQMLTMSLYLASRGVGREYGEYAVEFKYA
VLGVKGNLGPAEAAMPTNEKEVTAAEQARQVGGDLWPIGNDLLGGSSNTTASGSSSGSSSSSNSPPRRPPPPPAAGS
TGLLFSNADASPPPLAVATPALPAPLPSTIAAAATAAPPRLPSPPPPAVGSSTGVLPRRHLMQQMLQPPAAAVAAPP
PPPASSSALVLQPSPPPPPPPSQLLIQQASATYVSDMQDLLYTLVVAAMLITAVAAGRLIAAVLYRLLVSPEPHPFL
AFPRLETTIAGLILVALTFYSCMALGGPAADWHGSRTAAYCVLTIAVVPYAAFLWWLALARAWMVPQFTLVEPMTTS
SYASPRPSAFERADRTSPRPSTVSIATAADIGGTATGGGGGAASGACADVGAMAAPATVAAGADVPSEDDRYARGPH
WKQFDGVLPPTVASGVGGSGGGVVPLPPLVALPGSGRHLPLPPLLPAAGAATAAGGGAATAPGSPRGAAAGEDTHQF
GPHWRKFSVPDDGAATAGVGVDAAAGGAGGGGGRGSRGGSTDGVRFANGTRTPSDDGAKGRSSSHGGGNGGNAMSSG
AASFGASAGGGGAAAASRLGSRSGSPGDGDYNAMSSGAAMRGGGAGASRFGPAGSRAGSPPEGAGGYAGGANAMFAG
TAGRAGAISPAHDGSSRPGSGSNEGGSRQMSYGSNNAMTAGAVGVGGGVRRPAGSLRNASDGDAPPPLPAPGTVPLP
FANLLTSGADVRSRRALNAEAVAAAAAGGGGGGGTSRSGSGNSNGSRAIGLASVPGTADRQSLNVLPEEAAAVASQM
PGFQKEQLVASGGSGGGSVAGSVVGGSVGGASRRVLAASASRPGSLSGLAGGGIAAAGPVNGGAVRPRVGGMSLGGG
GAADVNDAPGSPRQPRPRVGSTPTNVPAAVAASAGAPRPPRITEVDEMGIAVVSAAEVAATAAASSSPPSPPLSPPM
AGKRGFFLPTPPPASLQAAAAAATAAANASAAAAERQAAGTVGMAPLPSTASARAAAGAGGGASLQRSRTSLAPAAS
SEPVYGDGLGGAAGMLARRARVRRMVTLGDAASPGGDGSTAAAAAADRRGLRFGSVPVATPSADAAAAEAKAQAAAA
AAAAAAAKPPQKQARSNGWLNNDAGGSAAAGPVAEATEEDPAAAGGGAAAGAAGAWRTRYASQLAAAGGAATDTTDS
DGEGTQPWNRDSFRRRDRDAAAAAAARRRGKAPLPPGADPHQFSGGAAIISTAAGEDLFVLPDADADAAARRKFSSH
EDIVLQTDGGGGAAAAGGGGRRSVALVTGYRYSRGYGRDGGGGCSRLLLPSPRLMAMFEFLFEDMLGSEPEQQLTRA

QRPARLVATALNFTHKAGCAAALGYWGLREQSWAQLGTLLGLQVVMLVYLLAVWPYAEWQLAAMEVVCHAAELGIFV
AATAVANRFVHSSAATFAMIALFFLTLAAVLLYEVRRLYLLLKEVWAVLRTKIASCRGSGAGSGGGGDGGGGCFGRF
GGRGGGAAAAQEQGGKNAFADVHPPAAILSAPQPVLLPQLQAAATAAGGDAAAAVASGPMGGMEAAAAAAAAQTRHP
SVGNATTGAPASATAAPAASATAAPAASATSATAAAVAATATHAQAATVGGRASTSNV

>Cre08.g359600.t1.2|PACid:27563086
MSLHNHRRTSARLQQAIFVAAVFVTPCVLGFAPRVAVYQNEEVTGNGWSNGPDNVRAQLDSADAGAAELVDASAKPR
LAGLDALLIPGQHSAFYSGVEVMSDVQAFVANGGLVLLLSSSSHGAAENDFVSQALDYQGDWVRCKQTASSSSSTGS
WGEPVLVRHALPALFSASAASAASWPSSLEAARSTSFFSWCRHEDADAFSLPLYTAADHTDKVAVQLFGKAGVPGAV
VVLGYDWRQGPQRRWGRLLTQLLSSFASSAFARPAQGNGFEHPPLDELDAGDDMISNDMSSSADGGKALGGLLQLSE
VDDAAVRLADETAEVIRRFLQAGSAAGTYPGPSPTPSPTPSPTPLPSPSPDPRPRPPSPPPAPPSPQPPLPPGLALV
IEDYTVTFENTTALGINSPADIDTPAKVATVIDHIKAMFASALGLSKSEVLVRGLRFIYKNGTIIEVLTPTAANRRR
AARLMVELDNKSSSSSSTESYDLVDPAVHKKYDGSELTQMFVPASGVVGLVRERRSAVQQEQVMELLDVEAAELLHL
YDNLPAAVRRVLADKQGDATIEVLITLVYYVEVPLPPSPPPQPPVPPGTVLPPSPPPAPPSPPPRPPRPPPITLGSV
INATGAAGVLMGISPLPPSPPPRPPAAPPTTLKVVNMTGQGTLLPNGRAIYYDSPNFQTALKAGSPLHLVDPLKEAC
PNKCVACPQAWKVVEGNTSINNANRTELVSVPIFFKEPTQISRMFFKQVRNSGVVRVQLIKWSGRPVNGTWSAALNA
SSLGKVVYNVSKDSTMCLSVLVVRIGPKKSGVNLPVPTNGTQANLPAKLKAKSLGGVLVTMLRPPGAGPNYGPFLEW
VRFQGRVLYPSSVSVYKNLLDPVSRRK

>g8807.t1|PACid:27563125
MAPPPSPYPAIPNAPAPPAPQTYPPSPEPPSPQPPSPSPPSPTPPSPEPPSPAPPSPEPPSPEPPSPEPPSPEPPSP
YPPSPAPPSPEPPSPAPPYPAPPSPVPPSPAPPSPAPPSPYPPSPAPPSPEPPSPAPPYPAPPSPAPPSPAPTSPAP
PSPYPPSPAPPSPRPPSPAYPPPTYPPSPEPPSPQPPSPAYPPPAYPPSPEPPSPQPPSPAYPPPTYPPSPEPPSPG
PPSPTYPPPAYPPSPEPPSPQPPSPTYPPPAYPPSPEPPSPQPPSPTYPPPAYPPSPEPPSPGPPSPAYPPPAYPPS
PEPPSPQPPSPAYPPPTYPPSPEPPSPQPPSPAYPPPTYPPSPEPPSPQPPSPAYPPPAYPPSPEPPSPQPPSPAYP
PLTYPPSPEPPSPQPRSPAYPPPAYPPSPEPPSPQPPSPAYPPPAYPPSPEPPSPQPPSPAYPPPAYPPSPEPPSPQ
PPSPAYPPPAYPPSPEPPSPQPPSPAYPPPAYPPSPEPPSPQPPSPAYPPPAYPPSPEPPSPQPPSPAYPPPAYPPS
PEPPSPQPPSPAYPPPGYPPSPEPPSPQPPSPAYPPPAYPPSPEPPSPEPPSPGGDRTSPPPPYYGGGYGSGDGGGY
GGGYGGYGGGYGGGYGWGGGWSPGDGDDDFQNGEGAGTPPASLAPPQAQPPQQAQPPLPPLPPRPPPAPGIPFRDGQ
QQSQFQPQPPAAGDGGIGSSGDGSPPSSPPPPAPPGGGDEASGTPSPPAHFSSLEPPPAPASDSSLEPPPAPASGSG
SGAPPVVGGSSSLPPTPTPPPAPPAPPAPPPPLFASTPPPSPAPSGGDGGGDGFNSSGGGGGGGGDRGVNVNGQALD
EVPSGGAAAAGDSSNGGAAGPAAAGNSSSGGGGGGGSGAGGALATDNVVTAGGGGGGGGGSGGMSAAGVGALAGVVG
GVGLITVAGAVFAAAVAAKRRRFAPAPDVMAQLMNTEPASIAGGYGGGGGGGGGGGGGSGYAASRYAASGGSIAGFS
AFGGGGGGGGGGGGGGGFSSSQPSGASVPSQGVPEPAVATSQAGARPWAAAAATTAASPASAAATTSSAGPVQDVAP
AAPVAEAVDAARAAAATNGNANAMTTPAAPCLDPAALSYIHQHGSETAIATAAFAAATAAATAAAATAAAASPATLP
PSLSPVAAPPAWSSTRSSIRASVRSATAAAVAAGAAVSAATAATPPPPPPPLSAPLSLAGLWTSRRHRRQAFTFNTA
AAAAASSSNGNTGGGAWAPPPSPSPLSVSSQPQGQQGATAAAATAGATAPAANATATAGGASLSLLHRWRYSLTASL
RTSISNSTRTSAESSFGSMLLPPPPANAAATAAAGARGLQPLQPPVAAAASSVRSSRMLDPIPEPDHPDPADLLDVE
TPPAAAAAAEAAAYRDDDEDDDAAFSVAEAEVDAASEAEEEEEEEQEEQEEEVDAVAAEGAWEAAAEFTGQQQGSPG
DEQAEADEAEALRRIVLAAAEAAADADAAQAAMPPLLPSPPLPYATATASGEKSAAVAVVVAEAVEEEDTADEEETA
PPPPAAPVATASATAPQGQQGATAAAATAGATAPAANATATAGGASLSLLHRWRYSLTASLRTSISNSTRTSAESSF
GSMLLPPPPANAAATAAAGARGLQPLQPPVAAAASSVRSSRMLDPIPEPDHPDPADLLDVETPPAAAAAAEAAAYRD
DDEDDDAAFSVAEAEVDAASEAEEEEEEEQEEQEEEVDAVAAEGAWEAAAEFTGQQQGSPGDEQAEADEAEALRRIV
LAAAQAFGWRRSWSSPPPPSSGIAGFAATAATASGEKSAAVAVVVAEAVEEEDTADEEETAPPPPAAPVATASATAV
GASAAELQGKQCV

>g8874.t1|PACid:27563146
MEDWVAQCPAGAHVVAFRGRAWSTEDYRPWGATEPGLLTTDFVLICSDGTQLPIGRPTAPYTYEHLTAYPVVKDGWI
PSRSGIEKLIRQTGDPNTPPKRFTSFGLDWTEPKCAGGYTAARAQAHGGDAGNGIGKPLQLFFRCNGTGAYTTFTAG
IGLTTFNPFLGPDNYNPNWWHFTLQPGVPTTTSRTIPGLTQMQSKAQLEDMPGYRTCPPGQVLSGLRGNRLAPPLVD
WVKATTEIRDFMSPEGYLINPGYTVLLSVLDVYCTDAPAAAAAGPKPVAPAPAGSPGGELARIPSVPPSARTLEHTV
LNTTSTRLACGKAAVISGIAAAQAGPYGLDMSIIGIRCSDGTSEFQGTLAADRASASTSAASTSEPSSLFVDNPCIT
GFDAVRPIGGQGIGLDKYEGGQPVLGVGVGAVAVRCSRSISTDAGGNRRGSPWTRMGPQVLTNTTYGQPGLVYSLTE
ASVNTPDAACPGGQVITAINVTVATVRVTFLRPELNLTLDDYQYKNESRVVTVVTDLAFFCGPVPTPDPDRSLYDIT
VRYGISLNDLLAANPKAKLDVSLPIAAYNGTTLVIPQLCGAPAVKPPVSTIAAGCPRWWPFPNTTVTGAETCGSVSM
SMKIPLPTLNSMNDGKCPNAATVINRGTRLCVAPPASTASASNFFGTASVGRRRRQLSQEGEGDGTADGTGGATNVP
AEGDGLAVEGPEQQSPAVDVVPVEAPVPSPAAETPAAQGPEVEGPSPVERGGDPAATVSPQPSEGQAHAPTSAGDQA

PAADHSPAVEPAPSGDDTPSSGSEPAASGGGASVQQGDTQDEEGSVGADAPPPPAPEGAAPHAHTPAAYGPAGSAPT
TLGAAAGDVTENAPPPDAYPAGDGISGSSGGGGGGLGGGASPATADEVDTRCLQNYTVALGDTCELIGAVFDLSFKQ
IMALNKGFRCSNLAVGMELCVERKSLQGALSPPPPAPPHLSPPVAPPHPPLPPAPPPRPPSPPAPPPKPPAPQSPRP
PSPRPSPPPPLPPRPPRPPPPAPRTPLRKPSPPPRPSPPPPSPPRLPPSPRPPPPNPTPLPPRPPAFDDASDCHSVA
DRNGGRSPCGPEPVNHCIDTPPSGYTCSCGERFWPSVDGRACVERTNVAFLAPTYSSSQLRGATDTDVDARFSPRWV
ADGATVSVSKYVASGYFSSSRGDAQPWVSIGLRGTYLVTQVRVVIRPGPGVRRLRNAEVRVGATPITSPQDTPLLPR
NALCGPPLLPSSQYDGKAADMPERHWLDCTRSGSRPPLAGSWVTVQNLHPDGEMLQLMEVEVYADPAWRALPPSPPA
SPPPLAAPDVLPTQELPRPRKSPGHPPPPGVSPPPPWPPGERLVFEPLPLYRVTERGKRLPLVYVYSSAASDRVADC
AVDGRRVAPLKPKGKLGGGCLFDTITNGSNPAFLTIELGYDTPRNVSHVRLFNRVGGNPRVALESGLEGAEVWVLRT
GAVTGAQVNQLLDDPDAYGAVRCGKTVRAADAGEAVTVSCVGEPGVGATGLPGSYVLVVKRPAVGSSGAKAVTTQLA
FAEVEVIEGCVTECPPK

>g8359.t1|PACid:27563163
MASSSSSSSAPASAGGCPLWPMPLPAWQPHLLAQLDHAAAAAAPPAAAAGAPPAALAGGSGGVARMGMAAAHLAAAA
PANGAPLSSAGAAWPSAAAMLRPARPIPSWSQTGRQQYDGERAGRARFMVDAHGYTEEQAACADINSPYAQDPRLIA
RALINGASVPESRAWVWNNEELDGGQRTLERHRGQWVAVGGSLVDWRGQAFIDADGTVRVPHVAHFWLAEFLAAMHT
PASFGPSPLFGFTLGATEAATAAAQRFLSLHRADTALCHKDMLGKVFSEGVMRTAVTQPWRDLFIMNLTREQKALPE
GQVLSVTLVANCLAEAQAAFRGIAAPTPAAAVHVTAASSAAASPAPAAAPAAPAAPAAPPAPAAAAPAAAAHAH
QVNQLPVVSQADLMRRENLVPDTTVNQRKPGDAQWCLYHQTSHHAADQCRAYSALVSSNRAREAARRIATGEGARAF
HKAPAPAPAQAHAHVTAALPAETAQPASAAALCPPHRAARRELADDLSVATVRSLYDNGGHSERPPLPSATAAVSHS
QVAGASGMELGDCWDAEFGPAHIFHLAARPPSSGPMQSALPLSFQPLPAAAARRCAPAPADGAATPTASSSTTSAAC
PSATTGSDAAASTSTRPAAAIPAATAASSDGAPSGADNRVRAHLNDAKFAALHAVLRDQGLQITRLEGDVRRYARLL
RRLDAQAAAGCTHHSAMKLGRGVGPLLLVDGLSSQGTPYAAALPDPASDVTLTVTEAASSRGLPLQYDTGIYVSSVG
GVTSELAQIRGGLPLTVLAGTEQQHTTRVVCFVVPGPQVFDILLGTNWLIDAYRSVWDAGRNMLGLRLPHQPDASPP
AVELPLRQRNRAAGACHAVSSGRAPDTSTSRTVIAAALACVAAPTARSHTDAVGVPPLTGTGAGTGGCTSGGRNGPE
SDEESSDYESGEESSDYESGEESSGYESGEESSGYESGEESSGHESDEESSDDDSGGGGSPSASAGSAPIAPGPPTA
RPTDVLCERCDSWDHHARSCPWRCDQLPCIRSFNKAQRRCAEAAVACGPADVAAERARDPARSPCRAFPTASPSPPP
PPPGSAASSPPPPPQPPSSGLPPPLQLLAAAAPSPAFAWAPPSPGSAAPSPPPPPQPPGSASPPPLQLVATAAPSPA
YATAPPSPGSAASSPPPPPQPPGSVWPPPLQLLAAAAPSPAFAWAPPSPGSAAPSPPPPPQPPGSASPPPLQLVATA
APSPAYATAPPSPGSAAPSPPPPPQPPGPASPPPLQLLAAAAPSPAYAWAPPSPPPPPQHPGSASPPPLQPPGSVLP
PPLQLLAAAAPSPADAWAPPSPGSAASSPPRPPQPPGSVLPPPLQLLAATAPSPAYAWAPPSPGSAASSPPPPPQSP
GSASPPPLQLLAAAAPSPAHATAPPSPGSAASSQPRPPQPPGSAPRPPAYTSALPLPGFADLLPPPRRSPGSPPPLL
APSPQRPGSAADPWRSKLPSETWWQFLGRMRAAGIHLTAMPESEWAGQGRPPPEADPPPLSASRSASPVHQVALGFA
APPSPAVASSAAASAAPSPAAGRTPERMTFDALLLRHAAAEAAASSTYPGAPPPGRPAPLSPAAPHTAAAASLLPPN
ASCAPAAPLVAPELGLPRLRPIMTRGGSSSAAPLPSGGCDLAPCSPAMPPPVAWPPQSASGPGLPPLPTAALPDQAW
SAPPSQPSLGAPSGLAAGPPPPSPSLLAEAEDLVAAFLHRVPPHARAWARLAVMTAAAGGTCDRRAAGRRAVMSAHG
AGGEFGGSATYDPEDSEDDEVPPLNPPSDDEEDFDDAGARGGPSSHMTTGQLPPADSLRVLPTAIPLEAGPALPSCN
DPEGRREPLPHDLHTKQPGGWMLYQHPDATDADVNRLLRLVAQFDTGGSDGLFARSLDDLTGYRGGCDPVEINLSHD
RSIYECPRKHTPAEYAIMDAKCEQLRQAGIIVRAPPEAKYAMNSTMPAKRALDGTMTDTRFCQDARRLNEATIPDSH
QLPLPEDLFQLMANAKVWAKADARAAFNQLPIKLEDQPKTAFWWRRELWMYTRLLYGLRNATSNFQRVLDTHLRDWG
LAHRVVAFVDDLAAWAETVPELIDVLCGLFRMLTSIGIKLHPEKTVLLADGIEFLGHYVSPRGLQPTAAKIAAFMAL
QPATCLLECQRLLGSFGYYRGYVDHYSVKMAPITALTAKAVEWGPDTWTAEQQRALDAIKHEFAEGDLVLRPLDPQR
ELILHTDWCEVGISGVLGQLDDDALEYMVACVSRTCNVHERRYGSYKGELLAAVWAVQTLRPYLHGRRFTLVTDHGP
LEWLMTQRDLTGQAARWALILQQYVFSVVHRPGKLHQNADALSRCPLPTDVDAAGARLDQADEPPPPPPPVVLYPGR
RLPAYAQPPTATTLTTIAEAALPSADGWLPRLAAHLASSTGAAWPSTTVAGARASAVCLATLHASRAAGPGACVVDG
PDLLAGHNGTTSDVREMAPAADTAVRTAVALVNRHGASLRQLRLTPRLRLRLCTARGSVVGLDTSLQQHSFFDAAQG
DGVAVLEAPGGLAAGLQACLRLGIKVRRYVSMEPQPAVLGAVEKLVRALAAANPLCLPLAAWRQAGAPRQTALSGLP
LRDGGQWLVFASWGSTNGWSDADALSFVGGLQLALRKQHCAPPAYLLHGPTTTRAEQEQLGFPFGLPATMDTVQAGV
AAHRLVTLYTNLAAAEHLAAVLERLPPEPDRTLAQQLQPGRRPMVALRVEPLPYRPAHVPGQRLVALPAGSDAPPLR
TVPGDEPCHLSVTDWEAVLGHRPGTADTLPPDLAIPALRNPISPRTAATALAACLALQRSYLTPHDTHAPPPLRVAP
APSPLGGDEPAGEDDQATVIARGLLQRQPAPDLAFTFSTLAAVAADAQEQATATAENQSWTDVWEDAEVLAALRAGG
AAAGGAAPRRVTRRMADYRWVNGQLLRNMSDGLTRVCPPPDQRVALTQAVHQRTGHLGVRRTLALLQTGHWWYGMKN
TVRQVLRACRACDMSNARGEALPSRLSPLPVMGLFYRLGVDLAGPLPPSEPDGNRYVMVVIEHFSKHVELLPLRDKT
AARTARALADVFARFGAPAEVLTDNGSEFEGEFAELLERSYVDHRRTSPGHPQADGAAERVVQVLKQALRKTCYLTA
DASAWEQALPELLLGYRCSPQASTRYSPYELLFGGVAPVIPPAVRERISVPLDFEDPEAATASLLARMEWVRQAYPA
AASNLLIAQHRDTLRYSAIRTGRYLAKPITYAPGDYVYVRRVDAAQRPTLHFGQHDTILRVECIGPLGVAVLVGRDG
ARVRRRAEHLRPCHLDIDPAIDPQLFRPQRDLQCEVCGSPHQEAKMVLCDGCNTGWHTHCLRLPRVPRGQWLCPRCK

ELDAEEEVLRAGWAAEETGDAAAPAAAAAGLAGTAAGEAGAAAAAPQCGAAGRRERRGVPAAAAESEELGRLLFPRA
RTRRLDEAASDLGGRRVRRAEEGPDGVLEYLGALARPRYFRVQWSDGEEQQLTLAEARRLLAD

>Cre08.g363950.t1.3|PACid:27563201
MSQTIKTGAREVAASLAVTMALLVSCFLYLRNDIMVSQQPCEMTYMYAQYEPVDMPAELLISAGTRARSAGASSRSS
NASGWHRGDSVAQQEVPAWGRDMTAAAARVAEVAAELAGDRHRLWRYTGESSHGPGTDPRAPPALPILFIPGNGGAH
EMGRSLASETARRLGRLGPGARLAGAGADWYLMDLEGEWSAFDGDTLERQVSFMLAAMRHLARTYNITTACSSDSSS
SSSSSSSSSSSGSSCRPRTGATAHPGMVVVGHSMGGVVAAEAIARAAAEPDLGPSLVALLLTLGSPHARPAVPGHPS
LLRFYQRAAAGAAGAGAGAGASGAAGVGARGRPVVPVVSIGGGAADVQVAAALTDPSSLAPAGLSRGLSLPAASPGA
WCPAAHNSLVWCNQLVAALGQALVDMAQDAFPSLDAARKPVPTAATAAAANKPPRPGPSGPGGELATAELDAALREG
HRPDSADGGSGSGSSSSYSSDGSSSSSSGSSSPPPAVCGPEGLGRWSWALRRVEARLGHPAQPYLATALMAATAAEA
RRGPDATAATATASEAAAQAEATSELLRRAKPALAPAAAGGAACGGANLAWYLQYQSPAAQPRQGRTALRLLPYDLV
GAPGTGRTFSWRLGRWQGAGDASRLPRGWSGWLSGAQPCTAFRVWLSVVVEQPASPATATAAAATAGSSSGSSSSSS
GGSGGEGREALEELEAEVRRAAAAPARTLRHDVEVTALAAPLPAVVPEGRWHVRNRSAWGEIQGGRDYQSRAAWLLL
LPPALVEPLLQLQPGAPPLRPAGALGGGSSSKPTVTLTLTLWLAAAPSYEPAFRVVAAPLLPPHEPADRPLPLRPSP
GSPEVVLQRQPRASSLSAAATAAKKKGAGAPPPLLLPLQLPHLTPPLLLPIPAGGAAASSSSRGSSAGSSVSSSGEA
GSSRTYTLEDGSAIRVERALAGEAAAASPLRAALTAAAVRWLGLWRYAALRITALDSRGRPLLQPPNAGSAAKGGGR
SQRRRPSVAGKGGDEGEEEEAEEEEEGGAGAGVGSCLYPVVMLVGGSSSGGSGGGSSASDWQAATLLQVSANHTYHS
LEPCAAAGPAGCAAAAALLDPACAHRVEVVPFLAARLQQLLGSQLGRLWLGAQLAALAAAGGLAAAAAARRRAAAAA
AADAAAAMVDGGDGGVSAGAGEEGGGGGGGRWRRRGGGVGFGGWPLLPLDALDVLAEVMWRRPVAAAAVLLATAATT
ALMAHASSAWAAAAAAAAAAAEAGAEAAAAAGGDVAAAVEAGLAAVAKSCGALAAAWPLVRRGAVALGLCPLPHLAG
CGWGASVSEWPGGFSAWLAAATNATASTTAAAATATAAAPVVLAAPPLDGETLAALDWALLAAAAALTAWLLAAPLQ
LAAAAATAAEAALGAAWWLAAVLPYRIVTAPMAAARRRRQAGSSGSGSTGPAAPGGGGGSGDVVLGPAARAGLAAAA
RCSLRAAALAAAVVLAGHAAAAPGAVLLAAVLSLAQALSSPAAVLPSPPPLPKPPRRPAAVAAGKRAGATGAGAAAA
AAAAAGSAGPPPDGDNAAAEVAAGEDVPQEWWRDAWWDEPWREDMNEPCSRDGDGNMAVGGVAMAGGQRRRRLTTAT
AAGRRRRAALQAQHAAHEAAVAVRHCRLAWLLYYAAYGACAAPAVVAVLRRLVLRWAAVGRLAGAVGEAALAAVSAA
SGGQVLGVAALAAGGLRDAVVGALAAACGGAAAWVGDLAAGGGVGQAGYVLAALHCAALAVAAAAEAAKGHAAAAAE
ASGSDGSAASGRSGGAGKAGGGGGAVAGGVQASSSGAAVAQGRGSGRWAVSAAIRGLAVVAGCVVLAMAWWLGAAEA
AHLVAAGVALAQLVLVLQAAS

>Cre08.g373800.t1.3|PACid:27563214
MLGINFELWLPASTRCSRSSLGRRGLCRPGQQHTDVLPRATAAAASTASPISFGASPPPNFVPSSTGVGACPRPLRE
AQQRRRFNQPNVLQGGWALPGRPHLASPLSATAANPGQPGDITGGGSSSGSGGSKSGGRSSGGRGPSSAAAGSGATG
SGAEGGFKRSPGAAGSSSGSSTNMSTNMSSRSGGGAADRTPESGTSRSDTAGTSSRSSSRSGGFSSGAGAGVGASRG
PGAAGGGSARNPPIRGGTGSSTGGSSGGSTGGSSGGTAASGSATRGSSSSGGGGDGSGGSGGSATRNGSRIPGAKSN
SRSDAAASGGTAAGGSGSRTATGTNTTGFSSFSFSASPSSSSSSPSSSSSSPRLQRLIREAMAEAIRTRGATASEAV
VVDPESRNGKGRILGKVHWVVDPVAALREILDGVDPGSAEGMLEGLEGAVGLHPALDVAQGRRWLAEDPWLGTGVGA
SGGAGKAGAAAAATAEGKAAAAAASAAGTGGQQGRFLHDFRGHLEAERAAGAATPQYRFLVVPTAPAAATAATAAIA
ATAATAAAAATAAGASAAERPAGSQAAAARPPAPAADMPAASSTAASRQSGPAAAPAAAPPGAATAVAAGAATSASA
PAVPTSAAAATAAATAAAAAATQAPRPLAAAAAEPATPAVVPEAHAPPLPLPPPLPLPLPLPPPLPPPLPLPLPLPPR
QRYDYRVRLDAAAAGAGGAAGSGSPVRAMAGLLKKALTRHEVVLTEAPSRHDLHAAIKAVATARAQLQAQQLQAAAR
AAAAAGVAEAAEAADAAAAGEEGVGEAAEEEELEARQGQPRQQKQVVPQRQGRQAKRQPPPPSQQSPQRLSREEEEA
AAEALWAGSDIALQLLLPKQPYLRPPPSPPPPPSPPAQQQQQKQKQQQQQQSSSPSKSLSNAGPGGGSSGRVWRI
YSHRVPLPPPSPPLPPPSPPPPPPSTTPTASSLPSSAPPPPPPPPPPPPPPPPPPPPPPPQQARPSAPSLPPLPLPP
LLAPPPPLQRLVARPGVSAAALAEALAAAVLRDGAAELAAPRGRDDLAAAAAALVMARGWLARGGGGGGGGVEGEAV
AAAAAAAGEAVAAGEAAAVVPASETDLAAFPSWETFGPWEVAEEGGSGEVDDGGDGRGGEEEGDEEEAGKEEGSDGA
GDAGMGHGGGGVAAAAAG

>Cre08.g363100.t1.3|PACid:27563220
MLAPTLLALLMLMSRSAAAIQEDVAMDMNAAAASGLLAQDLELAKTATDLVATRRSAQDLTQVVSAIKAVITSVPSV
LSKSPGLPSSFSADFGLFMNAAGTDGKFFITAATSTGVWKNDIIAAIGQLVPWLDIMDNAPAADEPGWLFRATIAVT
DTSMGMRLATTAPLTIAGAISDKLGLNGLGIDPQEESGNALTSASIEIVVARGTGKIQKFAFAMGGGSFSIASLAAK
LGLSVSGAATGITLSNPSLSIEVPDDATEPTNVEVALTVSGIPFLPGSTFDAVLVVQGSTSISIDITGDVVLFDGTC
TANAFNLAVGSAARTLSASLTISCPDAFINGATASLTYDNGAISVVADSIFSYLDGALTINRITFSSGRNATYIAAS
ASGGIANYVATVAFNVSRIPVNGKRPLSLSLSLPEANVGAVVRAIGPTADDLLPDFVKNVNLPSLSLSTWDKVTGTG
NRTNLTSLYRVTVGPQSSTGIYADFVLDKLGILIANVTMTKSLDLGAAVTAALPGVTLPFDVSGIIIIKEPFISVVG
ARAPSRGVVPAGTKLGTVMGFGLQVPALLGDSMVYANMMLMDANKTRVGIFEIGWVGDFTPVQYVTLRGALLRIQSG

SGVIAIANGTLVTVDVAVVLQISPNKQFNLTAEARNVQVAQVLKTLFPSTPDLIVKVLDPVRFSYIAIGYDSVKKFT
CEASPDLTGVPGLRELIQFLGFKQEDIKIRPKLNELAIEIAIIKTWELNLGSPFVGKSLLSFALALSQSGANTQFAA
SADFSAQLQLSFLEPEVIWFSLGATVAYDSLAPPPSVSFSIRAAVAATGNPFYIKGFSWIKIAYLGGEIGLTPMTVF
PFVSLYKINFNAKGIVLSTNVEVAVLWDQPKADFGILFAISNFDLQGMLNEMGIRASLGPFNIQISSAKFSFARQTI
ALPSGTVIPQGLKLSANMTFLSIFVNFDVSVDTTGFELQLEVRDVLKTPIFDAIFTGAQAVLDALKINIKLREQIRI
DLIRVGLVLKTTRVAFRFGVKAYIFGVTIDFDINLDTAAAPWDVVVKTLQDKFIEPITAACTKDSYVRGSGFVDRCP
AGRERQGDFCYNNCPTDWQYGATGKLNICYQNCPPGYRDDGVGGTGLTCSQVACNADQIWDGALLCWNGCAGGYSYK
ATRCYKNCPSGWGDDAVGLSCVRNSCDGGDEDNGAGLCYPQCRSGYTSNKLTMCIQSSCPSGYRNDPLSCWRDVDIY
GKGCCCVYFFGWRCCGCNGGYTDDGCTCRRNADLIWKSTYDRGVGYPKYRTYIKDSYLNSAVSAVRSFSKRTQWRDA
YPMVCAPDKDYDGAALCYDKCQAGYTGAAFVCWQNCPSNKGLIDCGTYCAPAAAGSCANFIGATYQNCKNKYPTRRM
VLDMMLSGNYTYEQMQAAADGDTTVFKGSAWDPKSVEIIHHTSHSGRDAASHAGLLHPETTRVPKTTIKVDAAEAVV
VEEKTQEEEWEEVEVDHPHYAGTTSADSWQHVRIMHERLNNIDTMFNDGSSSEGGDTGVHVVRMLQSGASPPPPGAG
CKPAATTPQCWCVGKAYGIYADPAGRKSRYLICAGDSGLLQTCPGTDVFSPSLKLCVSPSEAAAPSPPPADPNQKPP
LPAKNCTGRDDGIYFQDSRDNTTAYKCDSGQLILDFCPDDYVTDETTGDCKIPDYKPPGQLAPCQDPDCFCSDKKKG
NYTDVLRGNIKRYINCRGNKGRWRKCGKDSVFDSKTLQCGFMPPNATMPIKDCKDVDCFCVGRADGVYVNPWSNQTG
INCGFQMPAIIPCSDGYDFVITKQPYCQPKDVENPLNLPPLPPSSNDTTSSTAPSAGGYRRNRRRGG

>g8806.t1|PACid:27563229
MVTHFAAGSLALALVLLASVLTDGQEQTVVVNVQELHSALLNGLTNELLLAASLRPTQPAEPYIIKGHLRLVGTAVP
GNAGTPLPTLDLSLVQGWLQLARNASLTLRNLRLVSSQLPPGAVNATAAAATAAGSSSVSPADLLLPYRPLLRALLA
PPPTPAPSASTTSTAAASDGDSGGPQLLAVSLEGCVDSTDVGLAQALAREATASIPRVPAAVAAAAVSAAAASSGAG
AWAALTPSLAAAWPPVTQAVSDELCLAQAASPSGSPLAAAARRVLRRQGQWGAGRSLASWLGAAAGRWAGGGSGGDV
GRADCGGDRAGSSSSSGGARGAGVGLEWREVLVGRASGGVVGSVGSSVGGSGASALRRHGRRQSWVDVGQAHHQQQD
KLQELEQQEQPQEWLPEQLLHRDQEGPDDCPESTVGEGALREEGKVEVGQRAQGQLPAAPRRQTAEASPCLAGTVYV
DSAVVALELATARSSAAASGSSSSTSTSSSSSSMPYLREGSPLAASFGNSSSSSSSSSSSSSSGGGSSSSAAEGSAA
LLLLVRGSYSASAAQVDEACLSYLRPLTCVRQALDAAAAAVGPAAAVEALPAASPPLPAAPSASPPPPPAAVGAAPA
GGGSDHKTAFVATLAVLLPAAVGLLLAFLVLMYMYRRLRQRHRRERGAKQAPGVGPLTTLLITDIANSTALWESLPE
EVMDLALKLHHACLRSLLARFNGYESATEGDSFLLAFHNPVDAAKFALTAQVQLMDVMWPPEVLAHPDGQEVWVVPK
ASEPPPSGREPSGSLTPFMMNTGVSPYVEASGASISVYNAGGGGAAMYRSSGSAVPLALGSMPSGLLLTARMSSTDV
GMAAAAAAGYAGYAGGAGSVPGGVGLGGGGGGIAGGRSARRASWAVLTKSFRSNAASAGGMGGSGAAAAATGAMQQG
MARALDLEHLGAGLDLDYDDIMGGGGGSHVAYYGGHGGHGHGGGHARPRTLLRSFTSHIASALGRTSGGASGGAASG
GDGGGGGGLYASGAVITAVAAASGMEEGGQPVSSRVRRFIRSSISGISRAAAAAVGADVSGHSGGSARPSHYTDSHS
TQHHDHVQRQPHHHQPHRQQHPHHYGEYGDSAGLTAAAASAAAQPPAPASVRLPSGSRRRLSSSLVAEREALEHMRR
SKSFAAVHQLHQQQPQPGGGRQAGGSVGAGTGTGGDMRVLGRGAAAPAAAVAAVAAAGVVPSSGSFTARSLLKQALS
FNRRQHSFRGASRAAVASGSVAAAAAAATAALNSGSGAGWASAGDSGGTPASPLSVGAGTNGSRRALVIHPSATTSS
ALLRKSLTSVAATAGVAVAAGAAPASGASAADHLSTGGGGGGMTSTDEDQPPHPQPGRPHGSGPLPSAAAHALGRAL
GRNSRSMNGNVAVGSTGTRPGAPGFRGRGLAPLAGAGAGAGAGAGTGTGTGTGAGTGAGTGAGTGTGAGTGEAAP
SGFGTSASARRASVGIGYAHLCNDVVLPGPPPDPAASGGHDDDDDDPAAVAAVQMYGFAGGSSVGDGGGGGGGSLEL
GGLHSGVGMEGCSGVLGRESTYLRVGMESITHIPINSNSILGGGSSTATGTGGASRGTRDGIDSMGQRQPRGSIDDP
RASNASASAVMDALDSLARSGSVTAAAMAAPSPPTVPAAAPSMGAVARDAAVAAAARDAVASAAGMHALTAAQRRRA
SAPSEAALLPGGGGGGGGEYLPLQPPVGSSARAVGSGGQPTTALEAFAMIGDGTGTSGAPSRAGSIASGGGSASALA
SAHVQLLSAAPRRGSNLGTSMADPAAAGGGGAAGRRTSNSQHTYSTSSSILNPAFDTRHDDLVAAEGARSGLATSGE
PTLQLLLLGAGASVGGPARVATSPGDVGPLALGGAGGGGSFRPPPVARRGMSRSAEQVPDRVTAELVNAGTAMLGPG
GAGGGGRGGAGTGGAGGAGGAGTSGWGSSHTDGSVMGGTGTGGGGTCSGGAMLVTEDGSSFASATHPALAIAAQLGS
SAASRPISGSHGAGGGAGGGMLQARPASGSYASGAAGTDGGGAGGGGGGGGGLLTAAMARMMMMRTKSGRRDGRVHS
SPAAPRFDPGDAAGSFFAGTATAGTASATAVAVAGSPAALAMPAHPHPQSRTIGQPLASIPEPITTGPSRASLDAGG
GLGVSRAGTGGTEGSLGEGADDKDYDRGGAGGGGTQADADDLLGSLRDDLYPPGSGPSSPAVASLLSGSGGGRLLGG
ALSPLKAVVAAAAAAAAALSPAGGGGGGGGGGGGGGGDSPVASPPVSRASSVSNSLARSLTRGASRANLLMRQITRD
RKPSSFTSRAARSASAVTEGGGGGDGGGGARGAGGDSPGGGGGGGGAGARGAGGDSPGGGGVGVMSMTALAVLDSDV
GGGSGGSGDGAGGRGGGGGGRARSGAHLPLLSDDGEEVTSTSMAVEPSALGMVSRLLDGGSGSSSRARTRSVTAGDG
GPRRAIATPLAGPRYAVVEPDEAGEPGAQVHVFTGGSGGRPPVPSSPQLGHLSQRALRWQGPGVSVRASSGPQPAML
LTQQQQQQQQQQRSALQGLLRHQAAQAAQEEADAAAAAAAGVVTAGGRQRNPPPRPKTLSSLMAFGNGSSNSGGGG
GGPGRRNSTHDESQLPATPRSTGLFANLSMGLGLSLGLGSNTASSNAAAAAAAAAAASGSNNHSGRLSGSQQPPLLDL
GGGGGGGGGGGTSSAGASGALPPGVLMGGAGAPPPPPPAVMMLSGGASRRMSFLGLGLGLGLGLVRSRTERRASLSD
LPLWAGAGLPPGGAAGAATAGAAATAGGAGSVAGGELQVLGGWAGGDGGSMTHQHGGASSAQLLQIQMQLLECRSYK
EKCKDEWPAALRPAPPARCAYRGLRVRMGLHTGISTATDVTFNPTTSHMAYGGVAMKVAKAVGDAAAGGMILLSQSA
FLALAAQLSALPGTPQAVFSGESDLGLGPGGDRYSLYQLVHRELVQRIGYAAPLRNIPLTQLGTEAAPTGFCAISFM

HVASASMLVAELGQPGVEALQQFKAIVTELCSRCGGYVVEASEGLCLAAFKHAAAALVWALSSREALAEHVWSPEVR
RWYRFTVAPPSTSAAPPGSHHHHHHHHHHHHHHGHQGHHGHHGRHTQTSTSGAGGRGPRPRTGVHVGSVNVEVNAAT
GRMTYRGKVMNRTSRIAHKAASEQILCSREAWEAVCASLSATVQRIQEEEEEALRQQAAAEAEAAAQAAAATSANGE
EEDEEEDGDGDGYGSDEGDLLPELATGGRYTPGVSGPGGAGTNSSGGGSGGGGGGAGGLSSIRKLSIRNLLSGGGGG
GGGGGGGGGGGGGGGGSDWAGGVVSSKSGGGTPPTSSRSARGAGPLSAGGTAAGHTSGPGTLREVASERIARFRGLS
IASGVTTAGASGGNIGGAASSAVQHITTAKNMSAAVSSAVDKVTTVAAALGRRVASFSTKVSGGGGSGSGAGGGAAT
NAGRRGSSGTGMAGAAGGGAAGDLSTAVDDRRAKSLTSGRKRRDSGAAAISAGSGGAQPQSPSRLAAVSQLLLWPRA
SAASARGSPTSPAGLAAPGSGVAGAAAAAFGGMMLPSPRASAPNACMTPLAISAIAAAAGANASAAAGQATAVSNPS
SGGGASGGVSGGVSGGGGGPAGGIRSAPASGSWPRASPRAAQLELDTGGASGAESSGGRARATATSPPSPGSPQPAA
RSPRHTSTSLAPDGGFGGGDGSDGGGRGGRGGGTGRSPPTLTPAALSSNPTDTPPDALAAAACGPSTGSPRAAAAGR
STGSPRAAGGDTAAGRSAREGEGEGGGGHEGGGGESSSSSGSSSSGGSGDDDGMPPPPSVRWQALSRLAGGGVELPE
GQRLAARFLGSYSLKGVREEMRLFEVSWAPEGDDDTAGADSIVSPRRDGGGGGGGGGGGGGGGGGGDSGTEEALASS
RGAAGSMVREQLAGASRRARRDSAAAPGWAGGPPASRGASMTEVTAALPGMSAAAVGRGDRFQQRGRSAEGSQGGRR
PSPQGMPALGGPSAAAAAAAMAAAVAARVGGGTEGVQQVASASRGKVPAAATATGMAFGGSPGGGHSGAASGVGFGG
SAGASGNASRAESRVNSRRRLLSLPAQPMTVGSAATDAGGGAATTGGHGGGGGTRLLSSGQMEMGLSIGRGGGGGGG
YGGGYGHQVSGALSGASGWAYGMTTTGESTYDDDAPPVSDSCNAFATGGMVTLAGNAFRASANANANAFRDGAAGGG
GGREGSSSHFDISPAARRVTPSLATPSLMGAAPAVAGGQGPPAPHGGAPWSHANMSRPGTAPVAATDALAAPHRHDS
PIQAVGIRAALAATAAAAAAAGGGGGGGGGQHPLAGALPSQPPSPPAGRIISPLGAPSAPRGRPDAGVVAAAAAAAA
AAAAAAAAAAGAPSSRDPASDQLSFLRASTLDDPQMPVSAAATTGGAATGSHTAGANYNHVASGGTAASAAADATQL
LAHRQPAAVTAAVAVSPAGADAGFVALSAAAGGSGSAAAGGLHAAATLTGGGGGGGGGGALVGGSASGARRRLMGVT
FADDALPPAAAATAGSSSGMGVGGGSPAHQLSGGSGGSSSHTLMPLLTGSPLPAPGLPSLNQWGDATSGAPGSGGVN
TLLVDYHGGTAHQQYYSHHGGLGSDVGTAAAGRPVSGGAAAAAVAVAAGTQQSFASALRAHLMGPAGDGLAAAPAAA
ASSVAQLLLRGGAGAGGGGQGGSGGPSTASTGGRMAATATTTATTSIQEEREQEELLVGQEEEEQEQERQRRQQGSP
DHVHMRRRNSAAHQMARLRDSPSPPPPPPQPPGT

>Cre08.g376800.t1.3|PACid:27563240
MRAPGLLARGGPNLICHQASAAVDVLCPGIAAVAVDGSGAHHAETRSAEALRLNFTYSYVELQTCCVASQRAPPLGR
TNDAVVPHNTVTSDSFLSASSTGSGSASTSYERDARSGRATGVLAQASRSAGVGVAQLRALHTASGPPATTRASASA
AAPIQHHHTTSLQSSLASSHHRREVLGMSSFGGQYAACGASRAQLHSSASPSGAQPELAREAAEAASHSRLSTHGYN
HRSSSAAGASSSSGAGRVAAGGVTGSSSSGGALRGAGSSSSVSGSSSASSSGGDGRAVPDSQYLASLFGGEGELATH
HAADEAAEVAEAAGRINTATGTAARELSPPPQAGHPASASAAASRSHAASPDAASGLSQEVLVDQLVGRHLRRPFLD
TAPGAHRTHPHAGTQPSPHPATARLGQALPAAQGGQPQWPAAPPPPPAATRDQASLRDLVLPRSRQGSTPHPVRPGL
QHVAALSRLTQRLPQVAAAAAAAAASQQAMLRPDGMRQQQQQQQQQQRPKEPAAPPGAVAETVEQAAAGPVVVVPTM
PGAAVAAPVPAALQPAMAAVPVPLQPAAAEHPAAAELPAVLQQQPPVLQQQQPVLQQLAEQMSVASLADAAAAAVTP
SVAPHGAEAVPPEAAAGRADPAVAPALDSERQAGTAQKTPPLSPPPAAAAAGTAASGSGGAFDMYPQTSFSASYQSP
APQVSPPDAMLPAAVAAAPKLSGLAQRLLNNAAPPQHAQQGQWEAVAAPAPPTALRSGGRALPTAGSSSVTWLLGSI
EEMRALAEKRAAEQKLASAEAEPAAAAAAAGGRAVTVAAASQYDDQLLYSLSRRHAEALRRQNYEERRQEQQRQLR
LERMREQAVATGGAAAANMAAAATAAAAGQARLAAEVAAAAAAASDPAAALAAARQAAAALAAEAEVAVGAGPSVAVA
TAPQTSGTVLHSTAAVAQAGAQPSEPALAAQPQQTTTTTTTTAAPATATPTSPAAAEGAAEAAAAGRYCDSQLTHAP
PDDEPYLGDRDAAQAQALKQRRNSLLGWAMASLETLRSWRPSSGLFSGSSSSSGSSRPATETASTAVGTDAPSAAAA
AAPVSAPAAAAAPEVAAGVTASELQPSHAQLQPQPQAQLQPDPVYDELMASLKAASGRGKQVTAEAEAGAATAATAA
TAEAAAAAAKAEAVVAAEATPAGGTLEAAAAAAEAAAVAAEAAAAAAAAAAEQAQVALTAAAGAAEAQMAAAAAAAA
EAEAAAAALHSAVPDAAAAAAAAAAKPAAGSLSLLDLIRSPVAVQAVTKFAEVREAATRRRQEGARARAEARAAAAA
AAAAAAAAEAAAGGRAVAAASQESLAALASKVAATAAAQSQQPQLQPTSGVDAGDSYGYGYGYGNGGWRQQQWQQHQ
GQQQWQPRGKPYPIKPATDWAKDMAKTLAGSGPAAAAGELGGYTDEVAAALQAELQERGGGGAAGAAAFGEEAEAAA
LAGAGSASGRRTLFGEPPQVLIPDGITLRGLAAALGVPAARLEAFLRDQLGEVVRSDLEEVAPESAELAALEFGRIG
IRAAAAEAAAAAADTSGLAPPPAPPRQPVVTILGHVDHGKTSLLDALRSTNVAAGEAGGITQHIGAFEVRLSTSLSA
PAPTAPAASTSSGSPAASTAEAASAAGGEEPGGGGAGGGGGAITFVDTPGHEAFSAMRARGAAVTDVAVLVVAADEG
VKPQTREALAHARSAGCPVVVALTKCDKPTARPDYVKVVHTSATTGEGLRELEEALLLQAELMELRAPVEGRAAAAV
IEARLERGLGPVATVIVKRGTLRVGDPVVVGTEFGRIRALRSDAGGGGGDAAAGGVRPGQYALVSGLEGLPQAGDEL
LVVPDEARARRLADARRLRAEEFRRGRLAAALHAQRSRETAALADEYNRRRAMKADVMRLLAERKKHTAQKLKERAA
ALRAAATATSADGAAAAEGAAAAAPPVVQPGAVAAAAAAAAPATAAAAGAGAGAGAATASPVPVAQLNLVVKADVQG
TVEAVLAMVESLAAEQQQLAASGSSSGAAATAGGGGGGHGYVSVKVVHAGVALQPSDVHLAVAAGAHVVLFNAPLG
NKVEQLLRDAQKSSGVRLLSHSVVYHLMDAIRGEMERAAAAAAAGGGAAGGGAAEVLAEVGDAEVLATFALSATKLV
KDGRIAGVRVLSGELAASSADHVWRVVRGGQLVWEGPCTSLRRHKAVVAAVAAGSECGVVLGGGQFSDFQVGDRLVC
LQRRPAAAAGDSS

>Cre08.g366600.t1.3|PACid:27563243
MAAERARQAQQLEAFVNQNAARPCTPSTVLREARVRHGYDVSYLLRATAPPSGPALPPSRRSPSRHQFGRLNIAAAA
ASGCSGSGPAASGTGHAAFGREEEVDGEDLDEADLRMLDAGFAGEGDGWRGDPRQRRGPRLPATITIKAPAPHRFGS
VLLAPPGAPGATAVAASAAVDSGRGSSISNGSIGTCAAGSLVQLVSARSTRLGPHIDSPPPAARPTPFASGTKRISF
ACVPDATAAGLSGPGGPSGTGSSGSCGGSKVTRAPASVAVLMDRVSEDQVAAVVNAGVAAALSPRARTADPHSHHLL
GGGYGPRGGGGSGGGSGVVGGGGVLQPSCSPRLSARGRDGACRQAAVMPGAAGTALGSRGGGGTWTRRFSLECQQPT
LAMSSLAGGSPPGENASSTGGAGHASVRGPCVPGSSCSGATQQGQHAAAVVAPPQQLTDGALLPRACSPCCLHSTPR
SVVGGRAATASTAAGKRGAVAGGSPPRQAAIRRGPSGPAAAASPCYAQCGGGPRPATTTCCSSLASDTAAAAATPFS
ATASSCSSPQSHRASPLPSACSSPVPPSAAFAIARRRSIDPQLSVVGGRANSRAGSGSLAVGAISSCCPATGSTAAC
GGLLDAAAGSSSGGEPAGPASVPALACARGSGQADGLEMAGCATGSGSCTGTDKAAAGIAAVPVAAATPAPPGVAGL
GSQSAPLPNVASSPPVGDACLQHHHQQFQHQHQHPWPPSRAAQLPLSAEDVAGSHHGSTTTAVSTAGIHPYPHWHGG
SHVMFPELAAADTATATAMAGRATTSALQLESLPLPRLASCGSGGGGETGGGVHAGAVTCRISELSSSGRSMGEYQR
ATSRARRYSAPYPESVSGTSAGASTRICHGSVNGYSSNLPSQLQLSSRATGTADVHRFPAGWNGSAAVEPALASERA
SPAAGGGGIPQEPHLHYSYQPQQQQQQQQQQRRRPSDHDGSDGGALPPPQQRHSQSGAAQQLHVHVIGPVASPAAAA
ATEAAAVSRMTRVMRAESRKGPADQDWTLDPEAVAAHAARVLGAASADQVPVAAAGTAAFGRGHCVRLHGHAHEPTA
APAPRVSGSGDASGGGATGAAACAFPEEARRRSSHTGEGGTDAAAAAPSAGAAPPAAGLGSRRELGLNGSVGPAVLA
SAPAARVAGHGHGYATLPHLPQPQPQRLPFLPGVSRLQSKLTPGGPQQQPAAGGLPAAAVSGGGTGGAHVPPTSAGS
WSASAAAAATTCAAATRGPISPAGVMRQQQMASSRPVTSGARLNATPSNAQGVSPPPSPPLTAKSSGRSHKRAGTA
TAAAAATAAGHAMPPGAATAAGHAASCRGSAAPFGTGREGPTADCASSGRGADAAAPVAAAATAQSVEMEERASILL
RIKNALSFLHRDE

>g8760.t1|PACid:27563262
MRLLCTGAPAPPPSPGGGLAVGASAPPSPPPVLLQSAAAVAPAAVTALPSAATSKSAPGPPARCSASAPPPSPGGGL
AVGASAPPSPPPVLLQSAAVVAPAAVTALPSAATSKSAPGPPARCSASAPPPSPGGGLAVGASAPPSPPPVLLQSAA
VVAPAAVTALPSAATSKSAPGPPARCSASAPPPSPEGGLAVGASAPPSPPPVLLQSAAVVAPAAVTALPSAATSKSA
PGPPARCSASAPPPSPGGGLAVSASAPPSPPPVLLQSAAVVAPAAVTALPSAATCKSAPGPPARCSASAPPPSPGGG
LAVGASAPPSPPPVLLQSAAAVAPAAVTALPSAATSKSAPGPPARCSASAPPPNPPPAQSTGFCAHVLQLQRPIGTA
VRGDSWQFT

>g8553.t1|PACid:27563280
MIQPADDVFPLDHNDSAIDSEAIQQVVSALAHMNQDSEVMRRVLASGVYPPSPAPPSPEPNSPSPAPPSPSPPQQPP
SPPSPPSPPPSPIDPPSPPSPAPSPHPPSPPAPPSPSPPLPPGLALEAEHYVAGFSANATIVANLDVTSQASVDAFI
AAFIQLTATALNIDPANVYVTRVTKGGASSGRRRAALEQSGAAEHVWSASELSALLPGAGLSLLALKRVTPADDATV
AGLRSAKLELVLGPGAKLQQARRSAQQQQPSSTADVIVDFTVVTLVEVALPPSPPPSPPLPPGVSAPPTAPVSPPSP
PPRPPRPPPLNVAVLTSALNASWVNEAPSPPPSPPPSPPSPSPPARVTSARVSRMPAQGTLSGDSSKPVVWYNDASF
RSYRKPRSPLNLVFWSARRQCTATVGVCGGCPRAWAVPSNATQTTTTLPLYFREPMQLDSITITQLQNPGVLSVELL
PWPATPIPELPDVAPVSGPKGQPVYSAASDSTPCGGDLVISVPSDRSGSSESVPPRGSQSELPPRLRRTAVGGIRIT
VKAQAKGAKPTFISSVRFSGRVLYPANPAAYDGM

>Cre08.g359200.t1.3|PACid:27563338
MSSSTSSSAAYSMLMYSICKDYESYEYSSYSSERAALDSAINEHAGTDASYFASSAFAEQASSYCNSDARSFEVAAS
FKGFGVQVDASTSTGSSLAQCGSQSAASSYTGRNGGQNSYFYGYNSKLQEAKFGEAKANVKQLRSTVCGSDSSVNSN
SDSVGYLQQVLEPAVVGAYTGCLELFKAGLQVKQNTGIGSHSFSVDLRFTPGLPGATAFLEGVAVSPYGTAVCRMLG
CRNSTRTSTSSSTMTRSAAVVVVAPAADGNVVPAAGALATLAAVEEVEEDEGGSSGSGFDGSTATAGGGGGGRRRQQ
QRSLLATASPPARQQRSPPPPPPPAGAAVASPPPAATPAECPAAEVSMNLELEPNAVYSLLCELAPGAPADGGVTDV
YLSTSVGGTYKGVLFRNGNQTRLAIDQAAADAAAAAAAAAAQEHAVLRAQLQMQTALLNNLMSGVAAVQSVTSLTLS
TLQAAAVTQAALLRNLTAAQQLQEQQPAPPAAATPQQQGGPRVQVACGGYHTCVAVAGALKCWGRNDYGQLGQGLSL
SATGGQQQQQLISANVPPVALPNGNVVAAASGGDHTCVLLGGSGAISCWGSNAYGQLGFGDKTDRPSPPVSLAEGQL
TLSVVDLGPEARAVQLAVGGFHVCALLSDSRIKCWGHNDYGGLGLGDVSRRGDQAGEMGANLPAVDLGLSSLPGVGV
ASITAGCYHSCALLTNGRVKCWGNNVYGQLLGLGSGNNRGDQAGEMGTGLPFVNLGTGVVATSVTAGCQHTCALLAN
GTVKCWGFNFYGQLGLGHNTTKQGNTPADMDAALPAVDLGPGELATAVVAGGFHTCALLANSSAVKCWGRNDYGQLG
YGDKKNRGERAGQMGAALPVVSLGPAGTQVQDLSAGLLHTCALLAGSNAVKCWGANDSGQLGLGDTDNRGDSVNEMG
VNLQVANYG

>Cre08.g363250.t1.3|PACid:27563376
MPSALGFIIEETGPNSVEVFALSHGDGSRPSLVTLVEALPHTASHVRFRGLECMPQDALHALLGRCLHLAALALPAR
ELPSSCPLGAAEQRAPAELVAARLSRLPGAWALQQLPCSLTELSLTHCGWIHPNSLVHLRGLTALTRLSLRGVRVPP

PPAAEHPQRLGAAGLPGVQVSGLDASGEEVCRSAASGSGSQAGPASAAATAAEAATAGASSNPHELGATCPVAAAME
HWTRLSRLQDLELIGEAGGTAAPESTGCGSGGSGGAWDALLPAFLPCLESLTRLAFHPPSLDSAAHALAPLSLCVSL
RRLELSSCASGGSSGSASGGGSSRSGGSSSSGNGSSRGLVLDEHAWRALAAAPVLEELLVGQCEGQDVGRALGLASR
GFGAGDNTAASVGAAAAALALWNAGSARAGVGRAGRTGSDGGGGAGFWGAMGAAAGAEGGAGAWSGSRVWSNQWADS
EWGDEGGGGAAAGSAWGGGQAAGGLEGGGWRAAAGMAGASRPLSVTPEGSPRRRDGGCPTTAAGAGWATGAGGSSGA
GSGNWAQAAAALATAAPAIPANATWAAAGAGLTSHALVEEVEEATARPHGLVAGRRGSAGDDENDNSYPLSTPGCGQ
LPSRAHAPGAVLGARPATSHAASDRCSSDGEEGSSLGRPPSSAVTSSLHRDGDKDRGRDRGDPPEASAAASIMHSSY
SPPSSSSPCSTSRAATTVLTRVSAGGEAMLSAALALARGGEHAVAKPGARSAGGGASVSDARRLESEDGGGGGGCSW
CGSLAAPDEHEAHSEGHELDGHSVHGEEAQQEEAADGPAASCQSDGQEATASDGEEVDVTQASAARPIVRPLQRGG
ANSTGGMPLLPRSAGSSAGLQAQVGAMRQRFGSSSGLCCSPGGDAAVVAAALSAAAAFRASLQVSGAASPVSGGAAA
AASAAGVNSGSGGNGSGGSSGATAAAPADLLAASTAAATAAAVAAAWDAADAAAAAAGWVRSTGCGVPGRRAPTIAA
APASPATAVQGTPAHAPCVAPARVPDPSVSIPAGAGLGGTQSGALQRCLLDAGSCGDDSGTAAATNHLEQEEEQEAD
AAELGPAVDEEDALCRQLLDLQQQGGEDDSAMGGCGDDAHGMGRHAGAAAAEERGYEAAMRAISLGDQGRSSLAGWR
MPAGPEEAEDARLAAAAAAACRFADAASEGTRSAAGACPSGSFPGQADQLPGLGAGLARRTHAGNLTASAGAKSAGA
DVPAPLPPLDFTPFAALTRLSLLRLADGCAVALSGCWQLQLRLRTLSVADCSGLGDVGLKALPKLRHLHQLSLRGLA
HTHDGVFSTWTGPRLLGAAGVGGVGGGADGGGLDELRSLELGMSRAAALALAAAADAAAANAAANAAANAGTNAAWN
AEQGHVCADATDGMGLGAVFGSGFGCRNPEPVAPGVWTPAAAGSPVSAGAASGTASGAGPGTVVSAKDVQAPTAATA
ASLGAKRLRRRTPPDPFGGPSCWGGLLGGSRDALPPPPPSPPPPARSSSPPPPPLQTPPQHLAEHDAAGAAAAAAAA
TNDDDDIAAAIAAVAAAGGFSSDDHDDGGNVFRRRRGAAAAGPVRGGHSSASSYSGAAMYSGSSRRSSLCGALSDLG
EEVRHQGRDADHMRPAGGGGHGQELGLGEEGEGLEEWDGLEEEGLSFWDFEAAQGGHDGEQASAAVGGDIVAAGDVV
DAELQRPGAAAGPQEATEQQQCQQQQNQLHSVAAPAPAAVAGVTGLTAKPFATAANDYAVGALALSLGEQLMAQLAN
RKPKQQKPLLQPLQQQTLLQPPPQPSAALLRMAAGGGQLWGRSRGGCSTGGLGGCSPDGDMPDLLPPDATAAAGPGV
GAAVQPTAPAHASGWSTAVAAAAAAAAATAVCMRSGAGQGASSSGGGGSSVPVAAAAATAATAGQGRGRGSIYGALL
HTSSAASGSHGAASLPAGTSIASTGGKAPPAPAAASAHPVTPTAGAASRVDARPVQDSSSGGGGDGGGSSLRLAPRH
CWQLDSQEDSAAVRWASPMALAPGRRCSGGGAGGGAGGSGAGGGGAGSGSGAGGGGGALLGARNFRAVVTQASAAAA
AAAGAGAGSQAGSSDADDEEETEMRGEGGAGVAAAGEGGRVHGGRRGVAALQSAATATVDAASRDGVLGGRRRGSAL
SEDWTMIELEPPELAAPAGPVEPAMCDTGLAVLLQTLPRLTELHLHHMPHLTPATVEQVLSRPRLAALVVDGCRQLT
SDVCDLLAVSALDLTSGGGGGTGGAAAGGEGGLKLRLVPWVPPAPPRPSAPAVSVPRVGTRF

>Cre08.g364950.t1.3|PACid:27563388
MNQDSEVMRRVLASGVYPPSPAPPSPEPNSPSPAPPSPSPPQQPPSPPSPPSPPPSPIDPPSPPSPAPSPHPPSPPA
PPSPSPPLPPGLALEAEHYVAGFSANATIVANLDVTSQASVDAFIAAFIQLTATALNIDPANVYVTRVTKGGASSGR
RRAALEQSGAAEHVWSASELSALLPGAGLSLLALKRVTPADDATVAGLRSAKLELVLGPGAKLQQARRSAQQQQPSS
TADVIVDFTVVTLVEVALPPSPPPSPPLPPGVSAPPTAPVSPPSPPPRPPRPPPLNVAVLTSALNASWVNEAPSPPP
SPPPSPPPSPPSPSPPARVTSARVSRMPAQGTLSGDSSKPVVWYNDASFRSYRKPRSPLNLVFWSARRQCTATVGVCGGCP
RAWAVPSNATQTTTTLPLYFREPMQLDSITITQLQNPGVLSVELLPWPATPIPELPDVAPVSGPKGQPVYSAASDST
PCGGDLVISVPSDRSGSSESVPPRGSQSELPPRLRRTAVGGIRITVKAQAKGAKPTFISSVRFSGRVLYPANPAAYD
GM

>Cre08.g370050.t1.3|PACid:27563441
MNRLYETFSAAAQQQQQQEPRSQLVAAASSARAVMGSGRGGECRGAQPPAHTAAPPEPADLEGNGPGGVPTTAALGL
AAAAADCADAAQRDVSRAVRCGSGAVAGGANLPGGWARGPSGSHVGTLGQLMLPAATAASSLATASSLATAGSSFTG
SSCGTGRSGLGGGQQAGGRSGSGDPGGSGSGGCGSSSGGGGSGGTAEGGTGCGCSFLTVSGGSPEAAADAGPGLEPT
GALETLNELQPAACSVQQTWRPLGAARVVSAPSDSSSNVGECSLPKGLIQIPAAAPWRPPARVDGAVATACASAPGL
AAGAAPAAGALQKDQQEGLLQALVHMGVISDAGQTPGRQGPAAIVRAAAPRTTASGSRSRDGGFRRANTAAAAAQDG
CEPAGAFAPLAAALSSPAAADHVEQASIQSLREAVAAEESHRATACATAVDACAAAVSLLGAAQRQAVTDGASRSSS
CTRYGKHGEQPGPLSTTQALPPLLPPPQGLLLLRGGCGSSGGDGNSSNSSSSSSSKHRADGRSALLAASTGLMTAAP
VTGHRGLSGAPALAAPPASPPSYTFAPASDMEHGAGAPDSPPAAASKRLTFVPGKRDAPATAAGPPSTLPAMAPPPP
PPPALASAPLSAPTSQLRRLLLQPPAPAMTAATPAAAAATAAGALPSASVGSARAGGAGTSSLSSPSALYRSPARHV
TVSMKFDTPPGMSFEETAAALSASAHRTVEQVLRRSSAYARSQTANVGTDSCDVASGSAGTAGTGVAGLPSSASGIG
GGGGGAIGSDTSSISGIPSSSGTSSLFAATPSSEAAASAAGLAPGSTAPAAAAAGAPGAAAAIRGAGAAAPSARVAA
AAEASEPAAAASDGGDGAVVAAAAAAATPVPGGGGLRWHSYATQVQCLRGCIEVVLRLQFDAPDGPDEAATSDALLH
AAMEALHDELHGQLLQLPEAAHAELLLPLHLLHPAGGNAVGADGEYGEPPLPPQPPPPQVVPLLVYMDPPVLIAAAS
PPRAPLHRPLQQQQLPPQQPQGQPQAPVVAGSSRATAPGDPRRAVRAAVAALSPESRWGSDDAAAEAEAAGGGATPQ
ASTATTTLGAPQQGSERRSALAEPLQEPGRQAVAAALVPSSSPSPPPPESPAASARARRAARRAPPRCGTTSAAEY
FASRSRSWPGLACDGGSVEDTASEKGAAGGRRLRHGISGEVGGAGVREDLMAGDSASELSEYELNEGGGGAGGGGGE
GWVAEQERQQERDWEREQPAGALRWPAIGQMVAAADEGERQEGEGQERAGAEAGEAGGAAAQAAGAVQLGADRQGSM

ATPFATAAAAAAMEAEAAPAGIADASGSTGSSSSRYHNVLDLSLVPPHHIATTAVPVTEQGRRPRHARAHAPAGTAE
ASAAAPDSPDIGSGDSAGVGEAGAAAAVTPFLHPFLAAVPPLPMATASEPAALLSHHQVGMELRTASDSESLDQDRF
APAANARGSLGGNGGGGGAPDSEVLNVTAQSWLQHCAAEAASHSTATSATTTSMVFAGHVRDLGGAAGGSSSGVGAG
GRVSWGGDAGSSGGGAGVSPFAGAPMQRQPSGHAHGPDQAAAAGRDAGTGADTVRPARAGSHRSPRPSPFAGAAAAP
QSTGTGGGGGGGGGGGGGGAVRAPSLDCSFSLSAAARLHQHRMEHQPQQQQQHQWWRQQQVQGENTGGLADAAART
RMPGGVAGAAPASPAAEAAAVAAASPPPGLLLTARQRDAGGRPSRSATAVASAAVAAAPTQTVCFTLHLDMSSDAGG
GTAGTAAAATAVPPPLLVRVVVTQAGAVVAEVQRLEVGPRGASVSLDMSGLAEGSAALLVLPYRGGGNGQEAEGELA
GGAAAAAAGAEAATAPSAALAALHYSPIAVVPPEVAAELCGLMGRMEQELVAAEAEAAVAAAGEGGGSSSTSSAMAR
ARAFTHSFSPLVSDMMSLLMGGSCFAAARSAAAATAVATASGDAAAPPDAAAQDLQEAVRHLQQLQMLANALLSYMR
QTHLPYTQQYMAAGLAGAGVLPASSFGSASPPPTLARRDTARQSLQQAAAQ

>g8396.t1|PACid:27563467
MQASLPAGGVPGPSLYLPGPVAALPGQAIPLVVTASFGGPATAVNVTMVAMGSAPVAVLTGPAGDVPDDTPVIQMQQ
IQRSAAQGDSQSAPGAWLLPAGLLATGKTHTFTVTVSQQPVAAAGSTATAQSASQSLVLRPRPASGSGGGLGGEQQR
FDLRVRDPDCCLRGPRRAVNAAVGRHTFRYAGGVAPGRVTAPGSYVVAQGPAMPSPPPGVVAAPPGTSPSPPPPLAP
GQATADTQTSVTAAGPTSQEQGSTAGGSTAVIAGAVGGAVGAAALAAAVAAVVLARRRRAKQQQVRQGGTSQPQPD
RLPVEEYAPAAVGRGQRPAPSQAPHPGEEERRQNGEATPATPRRQPVRSVAAGVVPVSTQLMTEGRGPGGSQRAAHS
PLSGTPRALSRTSAIGGWASGDLERPASVSAAAVAPDAVAPSVGGAAAGVLGSEGMRSPSGNQLSMPSRSRLGQGVA
HQSDAQRSYTRRQASGT

>Cre08.g367100.t1.1|PACid:27563481
MPPPIARVASFSVDIEDWHSCTPSDDDAYQEALERNSNASFFTCRTHAPSVVGNSAAPNEPESPTRLQDADAALAGL
VPESLSEAKLQHVAPALELCDTPAAKKTKSAALPPQVPPAGGAQPAARPKPALTRRTTPLRAASPRTTSSPSPALAA
AAADADADADAAPAGADSSNIAAAEATLVRVHVIPRPDDSGVQLQILPSVLCLFGSVPFPAPSASSLDFAAALLLQA
GGRSEVVSCAGATALTPAASAGGAGEEVYAGASPAPGLRSAVPAHSEGGASAVSEDPAVQKDQSSKARRGAAGKAAT
AGASDKQATLDPRKSQAAFPGSLLLKRIFSGFGNSSNSGNVSDAAASPPPASVQELGAELLAACGCGAADTQLTAAA
AASTTMVAGYVQAASAPVRLAPAGEGSAASASSVPPSPGGDIAFGADDAAAAEAEAAAAVHRMSTDIFRNVLRASGG
GGGDVSSPGKAQRSPRDTAFLSSAAATKAAATASPRQRDQAPTAAFAADTGAAADSINSKMPTRASATAIGASAAEG
SPCKDEQSAPGVAAMALLAGLAAPPAAAAQPPAAIRTGGGLAAAGSLEMLAPGQSGEMSEGGGLPMPDLEKCESPVM
AKAAAGSGKVPGGSPPPRPGVPLATAASEEVVAEEEEEVTLSVAPEQEAEPVPEPVAAVQAAVVPPVPTAAAEAVAE
AGATASPRTVSKKAYGTVANIVRKMVPGFRDSALMAVSASAEGAAAPAPAPPRPPVAPRAPKAAAGAKPPTLLAATT
AVAAAAGVEHAVPEVQESQKEQADAAAQSPGAPMFIFGQEQPKTAKTPVIRPRPISAKPAATPAAGAAGEPTTPADA
AGRKRKSELEDLPIAKMPVSSGKNAIAGGAGPNTPGASTEPGAPSPAPAAAKPAAQELVVAPAPATDAAAPREAIAE
AGRKAQAAVEAAPSAAAEPVCAPQSLHNSANPEIVPTQDEGAVVQQQAAVVAAAPAAEVPVPPQQQQREPVKPAAAG
AGKKQGGGGLFACFGCFKA

>Cre08.g383400.t1.3|PACid:27563492
MALTVRGRWRLSLTLRSLLAAVVAACLLGAFYTADAALQQQLPKGTRFAVTAATSQLPSRYGDLWGRAEVTLPDGRR
GSVCARGWGNDLASDLCAQRSSSWRFGVPTNGSVFGQPSQLLPVLVADIRCPGLGSDAAGAAAAGGKLGSNCTATVY
DSAAEAAADGCGLDQAAGVTCFLYDYLTPPGSPPLPLRLVSSGNQTALDAAARAGLVAAGRLEVQWGGVWGGVCSNP
YYDFWWDDARAQVVCRQLGYPADGHAYSAGTQTPLLPPMPSRDQPQFITFAQCNGTEPGLGACRGRPINSTYKSCGP
DFTAQVACYSQKRLASALDGRTIVVLNVETGGAVRPLPPEVDYGDWEGHPRITLTDSRYVSVCDRGWDDAAATDYCR
RLTDQRSPLGVAFRGSYFGSRTSATAWIADLRCPAGDETNIGSCLGTFYNSSAEAAAAGCDNTTIAGVRCFTYNYLT
PPGSPPLPLRLVSSGNQTALDAAARAGLVAAGRLEVQWRGVWGGVCSSTYGQWNWWRDHHAQVVCRQLGYADGHAYR
VGSRKALLPGPPPADQPQFITYVQCNGSEPGLGACRGLPINQTRIAPSNGGCRPEDAVQVACYSQKRLNTALGRQLT
LRGLVNASRPPGGYGETQGRPEGWLAGSVALCDRGWGEAAASAFCGMYTRNTGSVQPYGVPLRGSYFGSTASAVAWL
ADLRCPAGDEENLGSCLGTFYNGSAEAAAAGCDNTTIAGVRCFAYDYLTPPGSPPLPLRLVSSGNQTALDAAARAGL
VAAGRLEVQWDGVWGGVYSLPPAWSSRSWWSDTHAQVVCRQLGYPDGHAFVVGTNTSSLLPGPPPADQPQFITYLQC
NGTEPGLGACRGPPLNSSTGATYGPDNAVHVACYSQPRLLEMFGQPPTLTWQFGNNSLPEFGYGEIQGRAEVSTGYL
NPPVAVCDRGWDDAAASALCRQLQYGSWRSRLLYGVAYRGSYFGSTAAATRWVADVRCPAGDETNIGYCLGTVYNSS
AEAAAAGCDNTTIAGVRCFAYDYLTPPGSSPLPLRLVSSGNQTALDAAARAGLVAAGRLEVQWGGVWGGVCSTTALA
TFDWWRDTHAQVVCRQLGYPDGHAYRVRTVNALLPGPPPADQPQFITYLQCNGSEPGLGACLGSAPNGTGRGTLPAA
CPPEAVVQVACYSQKRLVTQLLAASAVTGSRPGTGSGTVLNGIALLNTSSPPYSYGYTQGRVAVRLPNGRAVTLCDR
GWNDAAATFVCLELSYTLRNAYGVAIRGSAFGADPERPPQIADVRCPAGDETDLSMCVGTFYASAAEAAAAGCGNDS
VAGVRCFAFDYVLPPKAPPLPTRLVELNTSYSGGGSSGGGGGGGGGGGSGGGGATVLGRVEVQWAGVWGGVCSSRT
YPNQAWWNNATARVACRAAGYWGGAAFQPRSPEQLLPPDVPDAAEQRLLQPQWLRYAQCSGEEASLGSCRGPPLNST
GNAGSNSNTACTGADAVFAICSLTPPPDTLPLPTVGSPSPAGEPQTRPLPMRPPSPPPPTPTPRTPSQPAVGADGGG

GGLDAAPPSPPRPPLPSGEVLQNSTGGVFATSAAEDYPELVSDVPQTLPNLYPATTLALLGPGLALLLSPQQQVVAA
ATRYGGGRAAVFGAEKMVTRCCSSGSSNGSSSYSGGSSTSTGAMTNGASDPAMDRLLVNVVGWAATASRNSSSSSSS
SGGLLCVSRPDFATAAAYVVSQAPRVFDTHYGSSNSSNNSSSSSGWLEVPLWAFARPLPHDTALANGTNGTTTDATS
DAVLLPLPRPYNCSVYIIGTHDTLYLQPYIQRRLLSYVAGGGGLVVAGPDLMPAAFYTEEEEEEDASSTTAVAAAAA
GVGSGGQRRRLQQAAGSGGSNDSGSRPQIPPGSFEVNAVTGPLGLVLTGFVSDPGGNLTLASPSVAHNAELAAAQLV
GYLQGRLPLATPDLLMLLATTTRTRAVLTSLASAAAGAVSSSTMDAVAQALERVDSLIRQIDGLVATAPANLLAAFA
SPPPPPPSVRRPPPSPPPRPPPRPPPLPRPQQRQPPPPPAPPLPPAPLPPAPPSPPSPLPDTGTGGGTPLPRRAPSG
RSPSGGGGRSRGETTRRPPPRMVVVSGDVAEAVLMNGKQQSKQQQQP

>Cre08.g376400.t1.3|PACid:27563498
MGNNQSAAGLQPGGSLSLDVDASLAQRAQSEEAIEAPAAPETSFAPYNPFDRRGTDFNFPPLADALRFDPTTTDQEA
SRPSDIETLANLYPVEFPQGESPPTQVPTQLPDLPLRGSNDSERSSVHELRAAPPPPPISEPSTTNLSVMRLQSVLP
GDSVADPDASPSRKLLSSLGHGHGIGHGESPPTQVPTQLPDLPLRGSNDSERSSVHELRAAPPPPPISEPSTTNLSV
MRLQSVLPGDSVADPDASPSRKLLSSLGHGHGIGHVMGGPSSRLSSFRTHGGSGIVSADQLGTRRPSSRDGAVSTSL
ASAGAGAAGAVGSGDAGTASRPGQQRPSRHSSYIQAFSERGSERFSLESVGAGSSRRPVQLLQPQAQAMLQPPRAAA
AGALKRYDTFEEDSLSEVLLQSERTSASLGFNAATASGGAAAAAPIPAVIPATLPAAITAAIPLAAPAAVPGGAAGG
RAAAAAVVVGTVASGQQKQVQRPEWEQEQGQWQTGMLARAGGGSTAHGSVASMRSAGRSEGLGGAVSGGTGWQRREG
SIHLRGGGSSHGGPAGHSGDGRSSRGPGSSAGGGGGGDGRSSRGGGGSRGGGGSGSGGISTMDHLDLPGMSGGVHAV
AGASGRDMAVQSFTSFSTDAPEPPTAPQQQPVQQPAALQPHKVQEQQQEKREEEQREQRAYEQTQEEEEPPPPPRDV
AELMVLGSDDSFCFREDLDPMIGTSAELDSDSEPSAAPDAAAVDKMDASGEMGRPVGRESAPGSSPSPSPSPSTSPS
PSPSARPPSSGLVGDFGLQGGTGGSHLGSRGGSSRGNSSHGGGSSGGHDGGGSDGGGGGDSDAQGVRRPRRSPAAAV
WLRPGLTQELATDSLPPSVSGRSTGGYTTDDVGRGDGSDGDGDNDLGDDGHGDGGSSRGRPGSDDGSNRSAGGDGSG
GAGIRGPGSENGSGSGSGASLGAAAGAVTDNSADLFASFRQRSAAAASDAAALTATGGTVGAAADDTVADPDAFLAG
GAGRPPARVTLAGWGHQDEWALASGSAQTQPGEGGDAKGRGGSRGGGSGSGGSDAASVRGSGGSAGAARAMGGEDAA
KQEAAQLEVFEEEEEEEVQDLREGPASGSRRRLLVAELQSSRRASGRDLQPAGDLQEAGHARAGATSEEEEDTDADV
EAAGLGRDTSVPPSRVASQRPPSQDSSQRWKRSEPTAARATSTAAEEDDETADHVTAAGLQPTRSSRALQGTAGGVA
SRTSSRSRSRSQSDRHLQRRVPGSAAASASTSFSTRGGVAAERSGGWLGGFAEHYHQHHDGDRGRGGEAAYAAAVKD
NTRDGGADDYDDEEAEHDDEGGDEDAGLRAFLRTASYHSGGIATGAAVRAAGFSLAQPQRLQQQPAAAVSARQPHRT
LPVEEQQQQQPLQQPPPLPPSAMDFPPLPRQPPVTGPWGSLHLESMRPRATAAAAAAAVAAAPAASSAADTPAATAA
GPSSDALPQPLRQASGWLLPPQPQARVRMQHHPHRHHQQQQQQQQQSLFAESSLASSITSASEDGTWPTAAAAAAMK
AAAAGLTWADLAQAAAAEQWLLEQLEAATGAAADGGDRSSLGGDGASTAEGTGYGDSRASGMPGAWPDVPGAGPGVG
EAGRGVRRVRSPPRPGEVAASPPPQRRQGTWPHVAAGPLSGLEPEPAAAAAAAARTAAGAEVASVLEAVAGPPAWSG
ERAEAVVAAAMVAAPPAPAPAPAPQVLPAQVQLSLQLDESALQQVLRTCLEAALPQLLTRALGPLAAGPAGAAAEPA
HEAALRTLLASQATAHRDPQPRAGGEVAAPGGHLGLEAVGPKGSAGQGHNRPGQQPQLQQQPPQQQQVCNAGTQTAS
PPREQAVQTDEPAALLLLPPASEPPLQPMLTFQSSQAAVTAVSAAPAHPSSPSGRSERSALHSESLATPANAPAVPA
AATYIPTTHGAIASTPSGTGTNTNVGLGHTTITVSTGAATATSPATARTTQGPSRPEPSRQVPAGYNVPSPQDDAAL
FCSRVAADLLADLRRDIHKRAAAGPPGGGMGAGPGSGFGLGAGVLGGRGGGGGGGLSASGIELPSALRPLDKRPRLG
SPPPARAGPAPLHGAYSHQPQHQLQHQPQQHQLQRSAARSGAAAALGTNPGLLRFAPPSVSSRHSAATSSRLPATAT
AAATAASIASLTSTVGAASYAAAAAGAAWAPPVLPSEQLFAASVAAPPATTSTTADAGGALLVHRGWGGARAGGGTY
GLPGAYGVGGVGTTAGDTSAAEQLDSLLRSMARGSGSGGDYGAAGPGGDAVAAGWHVGARASEGPLERAAAYADAFG
TALEQGPGLSAEPSFGAELRGLASGLGPVEEWRRLQAAADGSEWTAAAAAAVAATARRVSDAEGPGHGARGGPAGGG
GAAAGEKLHGSPPVDLERRSEMLLRFLSDLQQQQQLGAEAYRSPPRF

>Cre08.g383600.t1.3|PACid:27563562
MQSSGLCGAAALAWLYLTCLLNTSCTYAATQLPAGTQLRIIFGGPPPDRYGDYEGRVEVNLPDGRSGSVCSRGWDVE
LSTGVCNRWNGYWRYGIPINGSAFGPLVQLAPVLLADIICDGGQRLATTLEFPSSANVSCTATVYDSAAEAAADGCG
LDQAAGVTCFLYDYLTPPGSPPLPLRLVSSGNQTALDAAARAGLVAVGRLEVQWGGVWGGVCSNTGIATYSWWRDAH
AQVVCRQLGYPADGHAYAIGTRNSLLPASAPADQPQFVNYLQCNGSEPGLGWCRGYPINGSVTPGACNSSDFAVQVA
CYSQPRLQAFTGFGPRINVENRTVPPLGYGESQGRPEAYGNTARAVISICDREWNDTGATAFCKLYGAYTSGVAFRG
SYFGTNDPRVPVTGWVTGLRCPAGDETNIGSCLGTAYGTAAEAAAAGCDNTTVAGVRCFAYDYLTPPGSPPLPLRLV
SSGNQTALDAAARAGLVAAGRVEVQWGGVWGGVCSSNNYGQWNWWRDQHAQVVCRQLGYPADGHAYTVGTVNPLLPA
SAPADQPQFINYLQCNGTEPGLGACRGRATNGTSSTCGPDLAVQVACYSQPRLLVLMGQESGLSVQLPNNSVPGLGY
GETQGRGELLVQYNRPPVAVCDRGWDDAAATVFCRQMSYGGSRGRLQYGLSIRGSYFGSTAAATTWVADVRCPAGDE
TNMGYCLGTVYNSSAEAAATGCDNTTVAGVRCFVYDYLTPPGSPPLPLRLVSSGNQTALDAAARAGLVAAGRLEVQW
GGVWGGVCSTTRTGTWDWWADQHAQVVCRQLGYADGHAYRVGSRNALIPGPPPADQPQFITFAQCNGSEPGLGACRG
VPINGSAPPGACNSPDFAVHVACYSQPRLQPQLLPGPAVAAYLGALQLSNSSTPPNNYGAAQGRPELRLRDGRLVAL
CDRGFDDAAATSACRYVTASGMYGWPYGVSIRGSYFGSTAGAAAWIADLRCPAGDEANIGACLGTVYNSSAEAAAAG

CDNTTNAGVRCFAQDYLTPPGSPPLPLRLVSSGNQTALDAAARAGLVAAGRLEVQWGGVWGGVCSSPGSTFSNWWRD
THAQVVCRQLGYPDGHAFVVGTNTSSLLPDPPPADQPQFITYVQCNGSEPGLGACRGLPINGTYSSCGPANAVSVAC
YSQPRLVAQVLAAANLTGARPANPLLIAVLLNDSTAAGGYGLRQGQAVVSFTNGRRVSVCDRGWTDDAATLFCRQYY
RPSTDAQARVGQVYGQAVRGSYFGSDPTRPALVADLRCPAGDETNLVWCLGTLFASDAEAAAGGCGAADTAGVRCFN
FDSILPPKAPPLPTRLVELNTSYRGSAGGGGGGGGAGKDGGGRGGAGGGGGGGSGGGGATVLGRVEVQWAGVWGGV
CSSRAYPDQAWWNNATARVACRAAGYWGGAAFQPPSPEQLLPPNVPDAAQQRLLQPQWLRYAQCSGEEESLGSCRGP
PLNSTGIAGSNSSTACTGADAVFAICSLTPPAAEQPLPMRPPSPPPPTPTPRTPSQPAIGADGGGGSLDAAPPPPPR
PPLPSSEVLQNSTGGVFATSAAEDYPELVLDVPQTLPNLYPATTLALLGPSLALLLSPQQQVVAAATRYGGGRAAVF
GAEKMVTRCCSSGSSTGVMTDGASDPAMDRLLVNVVRWAATASRNRSSSSSSGGVLCVSRPDFATAAAYVVSQAPRV
FDTHYGSSNSSSSSGWLEVPLWAFARPLPHDTALANGTNGTTTDAVSDAVLLPLPRPYNCSVYIIGTHDTLYLQPYI
QRRLLSYVAGGGGLVVAGPDLMPAAFYTEEEGSSTAAVAAAAVGVGSGGQRRRLQQASGSGGSSGSSNGSGSGASTA
NRPQIPLGSFEVNAVTGPLGLVLTGFVSDPGGNLTLASPSVAHNAELAAAQLVGYLQGRLPLTTPDLLMLLATTTRT
RAVLTSLASAAAGAVSSSTMDAVGLALERVDSLIRQIDGLVATAPANLLSAFASPPPPPPSVRRPPPSPPPRPPPRL
PPAQPLPQPPRRESPLPPAPLPPAQPSPPSPLPDTGTGGGTQAPRRAPSGRSPSGGSGRSRGETTRRPPPRMVVVSG
DAAEAVLDDRQQSQQQP

>g18235.t1|PACid:27563628
MARQKSKAAKIRHGLHAANNNLSALFDANAVEDLEEEAVGVIAGMLSLTLEHARKRLAAAKLLRSRVDKQPEGSGRA
VQTALSRHTTKKSRGGKEGHGREERRGDDVSRGDDVSHGGEASSSGEASSSDEEGGGEEGSGGEESSSGEESSSSEE
SHGCSDVDEDAPCKKCGGADATVFDMVFCDDCNDCYHVNCHEPPLTAVPMGRWSCSTCAQKPVGCAGGGLGGGAEAL
QRAGGPGADLEVAALGRAETAAGATAAVDCSSTGGGDGGALEPTASPPPGLGGLAEALQRAGGPGADLEVAALGRAE
TAAGATAAADCSSTGGGDGGALAPTASPPPGLGGLAEALQRAGGPGADLEVAALGRAETAAGATAAADCSSTGGGDG
GALAPTASPPPGLGGLAEALQRAGGPGADLEVAALGRAETAAGATAAADCSSTGGGDGGALAPTASPPPGLGGLAEA
LQRAGGPGADLEVAALGRAETAAGATAAADCSSTGGGDGGALAPTASPPPGLGGLAEALQRAGGPGADLEVAALGRA
ETAAGATAAADCSSTGGGDGGALAPTASPPPGLGGLAEALQRAGGPGADLEVAALGRAETAAGATAAADCSSTGGGD
GGALAPTASPPPGLGGLAEALQRAGGPGADLEVAALGRAETAAGATAAADCSSTGGGDGGALAPTASPPPGLGGLAG
APVHYKRIAVDELPRRNKGAKLQSIPEMLKK

>Cre07.g325850.t1.3|PACid:27563673
MTRPPAPPSSLPASLPSPSPSPPPPPPGPSPPSPAAPPRPPPLPPYPPEELNVLPSGPFPCAWAELGGEDTRVRRFL
FPPSPDPSLANIPGLQYSVEAAGAQPSTPVAVCATAAACATPQAAAAAASSGAALILPSPAPITSSALPPLANGTRA
LVAAADHPDGVYALAASDGAGTAYSVDVCTRVPADTRCPDFFGVMSVMRPATALPAATNTSAGAPYGAIRSPVTWTA
AAAQAAAAAADSPLAVLTSACTPPPGVDASRKLVYSWGWDFSGLPPSLAASGFVAVYRRYPLTRMAPGSYNVQMLKP
AAVRLAVIVRLLDANPDVAGTPLGVREVVTVGDSPSFVVPGGNVTDLTFVFVVPPVAQLDNVAAMSAAISMAWRPAP
PSPPPAPPLPPSPAPPSPQPPSPEPPSPSPPPPSPPPLPPTPPLPPSPPPSPPQPPSPPPEPPAPPSPPPGNVQRGQ
EGAATCRGGGAPGWGLSLSLLLAAAVASWARAGL

>Cre07.g353600.t2.1|PACid:27563677
MHKSAGSRRVSALLAASVLIIQVLGLLAQPGQDKKSFEGELTYVDYHDGTGDWALLDKAVRKFEPLGKGKKPPKKDK
NNKDLQPGPGEALWPPLARPVHDHSDAQSAHCDCQWSASLAEGCTVDPTTGQCSFIDSADVTVLKPAYIPPATNVRQ
SLLVVIVDAPACGAAAASGATVSNLQSLYFGPNLDGKGGWAVRLENCSYGEVVWEPPTSGLTQFVTVTPSCSWPTAT
CDAYAMSNAANAAARAKLGDLVFYDFTHFHLVMAVPSACSWAGLATLGGGVGGGGQVWLNTNTWTQTFGTFQVPLQE
SIHNFVLYHGFASGIEYQDKTTFMGTGQACPSVTEKRWLGWASPITGADSLDEFALTPSAASGPFNLPASWTTGLGN
HVRVKPTWASWYNNTNYGMNLYFEFRQAMLGDASMDVTYANKVVVHEILSYMDNDQVTYRSSDPRSNYITNVAPNTR
TVLSSPSLGVPYSLVLYVGAQFGSQSEFVPMYICRFITADTECDTLANVLANAPKSSPPPPRPLTPPSPPSPPPPGA
SPPLPPTKPPSPSPPSPRPPSPRPPSPSPPKPPAPKPPSPRPPNPSPPPKNQKSPSAKPGERAPPLPPVRTRPRRRS
PPPSRHPRTRSSDAADVPAPASSARR

>Cre07.g353600.t1.2|PACid:27563678
MHKSAGSRRVSALLAASVLIIQVLGLLAQPGQDKKSFEGELTYVDYHDGTGDWALLDKAVRKFEPLGKGKKPPKKDK
NNKDLQPGPGEALWPPLARPVHDHWLHGGPHHGPSLLVVIVDAPACGAAAASGATVSNLQSLYFGPNLDGKGGWAVR
LENCSYGEVVWEPPTSGLTQFVTVTPSCSWPTATCDAYAMSNAANAAARAKLGDLVFYDFTHFHLVMAVPSACSWAG
LATLGGGVGGGGQVWLNTNTWTQTFGTFQVPLQESIHNFVLYHGFASGIEYQDKTTFMGTGQACPSVTEKRWLGWAS
PITGADSLDEFALTPSAASGPFNLPASWTTGLGNHVRVKPTWASWYNNTNYGMNLYFEFRQAMLGDASMDVTYANKV
VVHEILSYMDNDQVTYRSSDPRSNYITNVAPNTRTVLSSPSLGVPYSLVLYVGAQFGSQSEFVPMYICRFITADTEC
DTLANVLANAPKSSPPPPRPLTPPSPPSPPPPGASPPLPPTKPPSPSPPSPRPPSPRPPSPSPPKPPAPKPPSPRPP
NPSPPPKNQKSPSAKPGERAPPLPPVRTRPRRRSPPPSRHPRTRSSDAADVPAPASSARR

>Cre07.g328250.t1.2|PACid:27563705
MLEAGIAFGLGSSCCSGSEETPARETSRCCHGGAAAVAAVTKQTASTPPTAVAAAAVQAAAAAESDAAAYVCACTVT
ANSSGSDGDQTNGGSSPRKRLAADGSSSANNNNNNNGSAGSAPDAARALRHLLLPLAAQLELPPSAGSATAATITTM
APPPQSPTAGPDPPQPQQQPVAERLQRLPSPPSPVPTPTRAPPSPPRPQTPPVSPAPGERTAATNSCSGEDGGGGGS
SYSGVQQAPPSPSDSAAPLALAQQLQPPAGPATAHQQQPQQQPGAAVASAQPAGEGCSGRSDGGSSSGATAAAVSQS
EGEVPVPAEPAVPSANLDVNAHTNAHTAVAKAAAGAATPPEAAAAGAAPATPPGGITAAAAALLAPGQFQRYYSRMC
DAHAEALAAELAAAAAAAAAADMSPPPPPPPLMLPQQQLQLLSPHAAGYPPFPAAAPQQHAPPFQSHTLPQPQPPPP
PHHLHHPLHYGGSPLLARGGHNGYNGNPNHHNHLGGGGGGPCWTSLDEWRQRHLLEIEAMQGIVPGPVADNRSTNRH
NHHHHNHLQPPTNLFGGYSGAGGGGGVGGESYSRPPPWQQQQQQQQHYLLQQHPLSPDPRHLSLPPPPPPPPPPPPQH
YAAEALPAAGRHPNHPNHHHQSGDQQPNNRGVDNGGTGGGAAGLSPAGRMLTRFFSQRTGAGGDGFGGPAPEEEEAA
TATAAITADGGVGGGATAGTASELGSGDNGGGGSGGSASRRSDEGPLPCPDAFATAAAAAAAAAAAAADAAAETAAD
VADQPPPRSHSPPPSGLLRFDGSQREHMSPQPHTQPPSVIGSNPMSSSVYGGGYGGGGHAALPYQMSYDSLVLEGGS
YGGGEGGFGGFLPAFASVPEGLGLFGAGGGYSRTGAGGASATAGGAAGGGGDLLLPMGLGGGGPYDISPYGTPPMSY
TSGGGGVGGGGGGFQAFGATPPSPPSRLLAWGNSAPAGGGGTIGPFGSLGGADGGGGFGGGGGGGGRSGNGHSLYTG
GGVKGISGGGGGGFGVSGGGSCGSGGAGGGMGVGGGGGGCVRLESLFGRLTALSCDQQGSRVVQRLLGSAASEQVGR
CLDELLPSVYDTSCHPYGNYVLQQLMAVGGARHKLRLCGALSGRIRELSFDPYGCRVVQKLLEVVPEPQAAAAVAEL
DGTVMRCVRDKCAHHVLRAALHHVPHSRQTFLVDTLQSALPALARHPYGCRLVQSLLQTTSEPERWEVIARDLLQDA
PQLGGHEYGNYVMQTLAQVAAPHVRSALAAALAPHAVRLACCRHGSPVLEAVLKYGSEGDREVLLAALLGPTTAGGG
GGCSVGSGSPYGSNGGAGGWPFEIGGGEGGGGAAAGAIAAAAAGVGSGGGGGGVSGGGSGSGSGSSSCSRALRLACD
PFGNYVLQRCFQVCSPGQAARLAAALLPAIPELSARCAASHHPGMAALLARLQEMTPDPGQLHLQQQLQRQQQDPNH
QQDEHYQQLQLQLQQHLGSSSYPLSPHVHLQHLQPPATPPHPPSPVFGGGALPPPPYAQHSFGGGGGGGVSPFGMPG
AGAAMFGSSMMGATADNRYFGGVASDGGSGSLDLSVLLSKLSQEEQRQLLLQLQPPSSLFGSSGSDAAAALYGYDSV
ASVGGVAATAASAAAAGNLADGGSSSGVTALEVGSRRYCRNSTSSFGSSSTAAAERASGAAATATITAGDASPPPSD
QASSGAPANAAATAAAGPAKVPESEAAVAAAAAVAVAAAARALSLEEWALVAAIGGSSNGGPDASGGALAAATAASG
LRSPMPLLPAAVWAHHIPGAELAGGGDSSTSAKRPHGQHPTTAEAAAATAAVAAGGDGWSRLLLRTLSRVASGGGGS
GGNKAVGGAAGGGEAAVDELSEYPEDAVLTEDSVAAALRALGLAPVGSGRQR

>Cre07.g339100.t1.3|PACid:27563712
MADRPERPSSAPEARRPASAQALGSAAAAGLIVAPGVQPSTLRPGAGAGGAGHGGSILAAVVRGDGSSGAAHGVGSS
AGGFSAGHGGGRIVLGRTTGLASRASASRGSQLGGGSGGGGGGGGGIAAGVGAGGGLTASVAAPSPSGAGSHGHAH
QSSTQAPGPGAGLGGAGLGLGLGPGLAAESSGGAWGLCAVGMSVRGSALSLASPAAAGGNAPPDGLGVYLSGVGGPE
AGGETARRRKPSLPPEARAAAAAAHKPSTAPAGPSSGAPGAGRPARKSSMDGGGGSGGSGGAGVSGSTAAAARVYGL
EAAPRRASVGTTGAGGGGGGGVGGGGAAGARPSSAVKRQAAAGSAGAGAAPAPGKAVGAGAGKATAGAAAGAAAAAD
AGAAKPKKTKKTAQAATGTEAGAGRAQGEMKAPKASAAGAGAVPAKAAGAKGTGKGSKGSKAAAGGAGGKAVASSRG
GSAKGSAGFVGPDGGAAAAAAAGGDVGLALSVIPEEGGSGAASASATGVMGSFSLAAGGGAGGGSMPPTPSAAAVAA
AAAAAAAGGGGAAAAVRASAASAGGGGKKPSRPVPRELDAAQFELGRVLGSGSFATVYLARQLDAGGAKVVVKRLDR
RPATATLGHNPMAPSSEVDNEVAVMTRAGFHPNLVEFRGWYRDPRDGVMCLVMGFCSGGTLAQLIKNPPAVEGGAAG
GAGPGRNPYNAGGGPIADPNAFTTFGSPMPAGGGGGGGGGGGGAMGDGGTIVGGAGGGGVAAARTPFYFPEELVMFW
FVQLLMALHHLHGRKIMHRDLKPDNIFLAAARRVIKLGDLGVAKHMSGTFELAITCLGTPYYMSPEVLASRPYTYAS
DIWSLGCVLYEMAARRTAFEAFGLPQLMFKIIRVAYDPLPPAYSRPFQQLVNGMLRADPDDRPTTGDLLAQPYVRRH
LARLLTLGAPAGAPALQGPSPSMPPMVKPSAARSAVKRLEKAAAAAEGAAAAGGGAAGTGARRRRRPAAAAAEGGAA
GGGEDAEVASGLMSMMAGARGKNVHQWDSGAKFDVRRQQQAEMRFRAREFEQRITREQVRQQRRERDPAVRAREAEL
ELLRQELLARRERKAAQVYEQRILADKLGDDEALPGGGGGGAEPGELLAAAAFRTRPKLARTSQLAPDLAGVDDYRA
ALGPHADDADMCALAARRMLGGDSGDLEEEEGVEEEDEEAEEGGNRLGAATVAAAARVRHAVADGEEGEAEPDTAGR
RHQRHQHQRAQQGHASAAAAASQIAGGARQRYGAAPGGGGAAAAAAAAAAAAAVHSDDLYDGEEEDEGELYDVDVDE
DAEYDEYDDDDDAFEYDDDGEGEEGEEVEDGEEEGGTVAQARRVAAVTAAVEEELVDGEDAEEAALLEDMRRELLEQ
ALADGAAEEERQRELLGGSSEWQAAVASLGDAPATGRRRGGGAAAAIASKAGTRSSVARRSDPAASARVRQQQRRR
QGIEAAAGPGQGPGEEEEDDEEEEEEEEEDVERFDGLATADAVSATSLRASRLGGAGGAAAAHHHGQEQQQDEELG
EELEERQGTQRRRGGAAKSGSRGRREVSAAPDAAAGAAAAALEAQGSFAEDMALSSVPDDDHDDEEDEDDEDEDEED
EEDEGEGEDGDEEDDQGDEEDEEDDSEDEGEEDEDEDGGEDEDDSGDGEDEDDESVGLESQEPSGIYDDDAASDGGA
ADGPGSAAHDHDGEEEPQRATRSRLGRPHQRHPAHARATALSHPHPHPHREQPCGTASPQREPQLEEDELAPQTGAG
LERGPDCASALEMASLAAVSGRRRQEVHAAAAAAVAAAAAAAAAAAAPSRLDPQELERSFKDLEQQLTAVWQSRHVS
ASASVSASEHASVRASVRDSVRDSVRDGVRDSVRDSVRDGFKADGDAGGAEQRQSQGSGGSGGGSGASGGGGTGPRT
RAEQDDGGDLGDGGGAAAVVGLLERSGGGDRLGCAGSGRDGRAAAAGAGLLAAGPEPEPEPDPQVTGAMKELLGKLW
TLTVEEKAAGAGKAAGGKSLGGGGGGAAGLRRSGGADGGGNGGGAAPARRSVVGRAGAPASVHRAPPLAQQAQGGRQ
QHRHARPAAAAEPSAGVSSTRASLTAWAEPGAPAAASAAASAPAPAELAAAADSPVLNGRARADGGARGARVASAGS

TALGSSARGSTARSLSFGSAVDGGMSAASTLRAAVAAAATTDSGEGPERTGPDQDLGGGGGGARDAAGESPVRFSSG
PHPHPHPLLAPQRPSSSPQPHQQPHQQPHQQPHQQHTQQQQPPQLSRPPLRDGPLPPPRVPRASGGSDLGNPYSPPP
SPPALLPPVGPRASPDPNRRRQHAAAGLLPPSAPPQLYPQHLPEVRPPPDWAHHSSSGAAGGGASAGGGDDCAGGGV
DGLFDSLMSGDSFNAGASSGIRPATRELAPLRTPRRSSGLNPQLAPPGPDGPLQPSYSTDGGVLSPPPLPPGGAATM
FSQALGGGGGGSPGRGGAMLLSGPGGGGGGSSGALGPRLSGAAGPLAPLRTDSWPRASVHTSISLLAAGGGAGGGGG
GTGSGYATPPSPSRPHSSGAAGGEGPPPPSSSSLRPLNHAAHHGHDLHADHGERATQHTGGSGTGTGAGAGAGPRAL
PRSTSRRGSQGALLDVNSAAVGADGAGGGGGLEGGAGGGGGGGSGWAGIPPPSATRTVSGTGLLPALHGTAGGAAAG
AGGGARAYDSYEGAASPARSASPAPLWSEEEAEEEEAVVSGHADASGGGERRAGQVAGGGSSRRSLVPRSPPAGVME
SEFAPSTQGSGVAGVAGAGVGAAGLLPPLPTGPSGPPSAEASGAGGGGPAGASAAIGSGAGVAGGTRPVVSPLAVG
GVAPPAATREGLAAAGVAAGVELGGLMADIRRWKVDRNRHRAGPAGAAASAGAK

>Cre07.g330750.t1.3|PACid:27563717
MGTSRRSLLAWAVAAVFLVATLQSARAQDSPPSAPPFDPNDPDAAYLFGFPFCRCSDYRCGAAPYKMLKASEETLPN
GNYKVCFNFNDVGCPTNNACCTSLLQSVGKVEVQADKACKATIAASTFGNVSKAGSTFFDDEFSVGKVRITALNANR
QMMDNSQLCLTIRPPCNSFDVFFQSSKVGGSGLYQYALFNSKADCCPTCVFAPSPPPPDVPAIRRPPPSPPPSPPPP
PPPPPPPPPPPPPMPPPPPPPPPPPPPPPPPPSPPPPPPPPPPPPPPPPPPPMPPPPPPPPPPPPPPPPPPSPPPPP
LISVGSFIAVSFAVAKASWTDTSLFPCPASKWDPSRPHLRLLAARLPDXXXXXXXXXXXXXXXXXXXXXXXXXXXXX
XXXXXPPPPSPPPPPLPASPPPPPPPPPTPPPPPPPPPPPPPPPPPPPFPAAPTVVPCDCLSPLVNTNSRWSVVFQG
SEDRPQNIWYNFRIYLNAGTGCRPVPYRNGTCCEASLEGISLPVIPDLKAAVVDATAKVGGDLLTATLFHSFEYGVR
LQFKDPVYTDSISVGSFIAVSFAVAKASWTDTSLFPCPASKWDPSGPTCDYWLHGYQTAPGNPQQPLIDPEVIPACC
PEGVVHMCPPGTPGSCLASLDASPYSLTFAGRARSGADTVFTFSLAHRNVRSACSAMAIDNLLLYVAKPYATSAATA
TLGGVSTKVVAGAEGPTNFLNISMSSYASVAPGTVTVTVKGDVALADLCVTPAAGGSRVCGYVLKGAQRTSGSTQSC
CPAGNIAATLPSGRRLLAVTEETQAAVAAGGSICAKADPASSCFALAAVEISETFEEGTTYAFKLTRTSALASCPTE
FQVQLSSAAMATFKEGTGHIWNSGVPAGDHFSWVLPEASGDGSLGTTTILSFTLKGRGMDRLSRVCTLGRNQDACVF
RLVGSSGCYTGTVATDFQYAGNAHHH

>Cre07.g356200.t1.3|PACid:27563746
MMQSYTSAQPPAEAGYAQAPASQLHRDVSNDQRLRRTVSACARADPAVVTDLRAGFNVGLGMMDRPRSEALPPELQL
GLGPTGATGAHAGSAAPAGFSSASGHMTMGPGQPSAYAAHVEAGGADWPSEGSQAQRLRAHHYNMYLGYEATGSATP
LHGGSGGGLAGMDTASTPPPYGGAAGDQSMAPSGAAANTASPGPASADAARHAEADAFHNAFGARLPRLAVIHAAGG
SFGGGAGSSGGAYAANDWGSGSPLRRVWASSLHEAGGSTGGTGRPSAGPNSPSARAAGSPIAVRGTPTKHVAGSQGG
SILSPRGGPLSAGSPGFAAASAQLAHLGLGSGPGSGSAGASRHLRSSSGGGGLGGIAAACLSPQFGATASAGGGGNA
SSTSSEQRRLALSSVSNLAQLRETVAADGALPPREKQNLLQLIEGAMAEQQQSQQLLQTRDAAGSSASGSGEAPYSN
QGAGMTLQQLQMLTQAQPRSQSTPSGPMSAPYGLARPQQQPSPPPRPAGPHRLSLDAASRAAAVTTAATATGRLQSM
PTPFGGATSVAATAASAGVGVAPGGSQPSDPGLCGISGSGALSVSSGSVPASGPMSGGPPAPGGVAGAPAGFGRQSF
GMDTSRSQAASLSLPESMGSMGRGAGGILSGGGGYGAAMHGVGLASSISAASGSRLRRCSGGLSPPPHTAFTEIPGL
SGGHVHGHQQLHQQQYHLQQQQQQQQHAKTEDPQQQHAKQEDQQQLLREVYTAFAGDAAQREEHQQHQQPYPMRTS
AGGGDGSGTGLIMPSRLDSNQSSRSLLTPGGNRVATSGGSGSGSGSGGASSGGAAPHGMEGVLHHGVCGGDGVTAAV
GADTAAGAGNAAGRATSGSRSGSEGPRAPRPSRAAGSEGGSQISSATGCSGEPASVAGVVGAMHVQPGPPPSSAAAT
PEGPRSAAGESMLPHSAQNPATAPWPETAAMGPAAVGRPQQLAMDADHMAALPRSHSPRALPSPSWQAAPPPRPASA
IASRASYNDLPRLLPQQQQELQPQQQQQQQELQPQQQQQALQPGAAAVLHGVSVSPLARQMLAMPFDVSGRSGQANS
TGSGGGGAGAGGGGGSAVTGLLPMDVRARMLAGASMPALLPSYQQQQHQSRLQPRPPPNFASGSSGWVPELHGGDAA
APAHADTSTWAARNDSVAEFPSLSHPPTNGSGGGLGGRGGGPDVPQLLDSPQHRHLQQQLQQSRFHRSHQQLLVSSG
PASSTGELTAAAAAQPAWHHMDARAHAPYVDDAGGSSLSSRSRPASSTFGGAGGGLPGTGFAANVRAGAPPGYNTEP
GFPQSPLPHHPASTPQSGFGFSSPHSRPQQQQQQEQQHQQQRPSGYSTPTMQAATAQQGSLQQPGATAAAAFGSPGV
FTGQQQQPQERQWQVPREMQRAAAAAAAAAAQARAQALAQVAHGFTGARLSGSVADLGPAPPPLQAFEEAMASEPGR
APVAGRGGTAAVAGVAGADQDGDLLMLGGLGVAGGDDGHTGLLRDMWNTTGGAAAPIAAPGGVGSSAAAAGGGPPRA
ASGDGALLSPLPPMLEDGDVDALDCMLDELLGVGDMGDVVGPGSGGDGDEDMDNLHLHFSALQDGSGGRRAAVRRGS
SGGRRAGEGAAGAAGPAGADPVVQQQSQLRAGGPAAGAEAGAGGLGGLGGSFGHLAARGSSEMMGVVELGADGGGGS
EVDILALGLGDGTDTDMGFMSGF

>Cre07.g338550.t1.2|PACid:27563756
MRPERLGCPTARPAEPRWGATRSWSKLTQLLVPLALFVVGFLHGGARAGKSRPWCTSGVGLCRDAPATPRFDHSMVA
VDDKVYVYGGRRSGSAFTGILAGDSVDVMDVRTLSVMQLETPGSPYARAGHGMAAWGSGLLVFGGATNSFAPDSTAA
GAGGAATGANAAAPLLNDVALLDRGTLEWRSLAPYLPAAGGGGGGGGAVAPQPRARAAVAVSGDVLVVVGGNVAPSA
DTPRLLSDVWRFNLTRRSWSGPLQLLRSSDAAAAMAAEDAALTAVPGLPGVLLLYGGRAWDNSSSSGSSSSSRGWRS
QNRLLLLDTRTSPAAAFTLGADLPPPGGNAAAPAGNGTTGVNGTMTGLGGNTGGRPPPPAHDVRLDWQGGDLLISCA

GGWAAAGPLAAYSADAESDLKRWFAVYRIQGLLNQIQAELYEVQGGGAYPSAAAVVVEAATTAAVAAAATAAAGAAR
SAALARNSSAAAAAAAGAVAAAAVTAADVSAADVAAAAADLGLVLLPDLWRGLVREHIYVSGLASDTMARLMAAHSV
YFSALASIPAAAGVTPLNGSTNGTSSYTNGTSLNSTNSSIVSSSDDAAAAAIRAGALANATAAMPAGLGPLWNAALG
GGVLDLGPAAGRVADGSALGDSDLSFVKQARWVLLGGRILMHGGVQGPLGSAKVSRRTHTLDLVRRQVAWLDISSKP
PYYESGIQTASLRTMLLVSPPAPVVDPVMATAAGLLPPLLPAAAATAATAATAGGGNRSYVSGAWGAATASAVAAAA
AAAGSPTLRFAVGGANSSYSLTINTTATTANAAALLLAAADTAAAGVVDQHLVFLGLMDAFSGQEPEFSVMYRASVA
RQDITYTCRVPRSGAPSAADANCFIVSAAAEKQLPSRRDLYGAAGLMYIYEGNTTEYDFTLTTDLVLLYGGQIATGA
SRGGGGGTAAGTSGGGGGEGDADGSFEAVLQSPPPPAPSAAPRPPPPVPPTPAAPTASADGNSTGGGSAGSDVNGTQ
PGSPTPTPVSINGNQSSTNTSRSTTASPTPPMFPPPAAARRQLHTARALQQASQPNGAGAGAGVDTGAMTAAQPPAQ
PPPPAPSAPWGSQSDGATGSSSGGAGQDTTIWNGTAATTPPPVALATPPPAAPTSPAPASPFTSTAASPSPPQAPPA
SPSPSPSPPPVPVPTTSPASSASPAPTTSQPTASPTASTPFLTNGSTAGTYIYGGAYVATWQPAAGPRISASLPPAT
AGYVFEAVRNANGSGDVHSPGPRSGAALVGAGNTEMLLFGGLSLDERGNVVLMNDVHYLRYTPNLDPGRVSTDPTLL
SPVPEPCPPGERCVSCQRSRAACTAAAVAAAAAAATAATPPPPQRSNGTAGGAAPGSGRRRLRESQRDASVDAQHSS
AAAQQQPQPQLQLQLQQGLPRRYNAPQPGEEPRQRKPQPRKPRQRKPRPQPWLRRRKAQQQQQPQSQPQQQGPTAAT
PSGSALSSSYYDMGGDGPFGTTNTGPNARADAAPFPTARALPPGYAYVVASTGKRARASSQYDAAEAPARGAAGNAV
DGNLADRRYEQGSCAHLDLRADSLSPRNASWWYVDLGRATPVDYVALWSRADCCGSADTGYKDRNRDIEVRVGDVEP
GSAADGPAGAGAANRLCAGGVWVPQGGRGVADCGGALGRFVVVRKPYPDYLALCEVQVWAHTKPFPYDQGHYLTSLE
LLPAAPPRTPAFPPPRHRAAAAMLRPGAAARYPAGALVVFGGTSDPDDADPAALLGDLWVMDLAGHTWGRVTAQGVA
PAPRSTAAMTSAASQIYLSGGLSYAGGRWIQSSDIYGLDLGQPTLVWTVQQASTTSSGSTRQDGTGSGAASQRAGGS
SSAVVSNLVLTRQPSRLATSTQADLVLVPTPGTVSVSTPGPTELLQAVAAMSDGDLLSVQAPRGHVVRLRSTLVVSS
AAIIAFEPTDNGGSSSGGAGRRRLQQAAASSSSSGGGGSSSGGSSSGVGGGDAGAVLDEGSLLVLDCGGLEVGVLVE
SASVVLQGLAVVGCSQAGVLVRRPVSASAALADEPSLTVTDSQFANNSGSGIVMEAGSLSLVNVMLSNNSRSGLLVR
AGAVVRDVVASTFRGNRNLMPGDGAAGPTPEGSSNSSATPSSSSSSSGSGSSSSSSSSSSSSSSSSSSSSSGSSSSS
SGSSLAVLTSESLAAAAAGRGGGLYAEAGSQVLAMTGVLFVNNTASTGGGFSLQSLDQASALFTDLVLAGNAAARDG
GGAELSNMRTAAFAFVGGRVEANSAAGTGGGISLSDCILPISFTGTAFAGNAAGGTGGGLAADLVRRLDLSRCRVSR
NVAAGGAGGGLAADTSGSITVSRCRLVANAALQGGALFGGDNVGVGVSNSVFIGNMAVAAVGGAAAVTAAGGAGAGG
ALLCARCARLALTGSLFASNAAWQRGGAVALMQARGASRVSQCTFTNNTIVTGLPAAEEARLATAATAWVLGTRPAS
ARAIMAAAAAPPPPQRQQAASAPTAASDAAPSSAPNSTTLPTAAGGSASAAASSDESLATDSTGINADAGGAMALLL
SVELEVADCDFVSNTARSGGGLYAAWDTGVQPYGALTLRCATFGGNAARRGAGGAVFTHQPAAMALLCGAGANPGSG
AGASANGVGGGGAGGNGSSGNSDGGGMLAKCGGVNGTATAAAAAAAALQDAGNVDGSSSESGSSTAAAAAAVAAAEA
EAAAFAQALVDTAAAAVYGYVVNATARLNASSGSASNANATPPLSLLSPPPPPSLIPDSCVPTWVGNVAVEGTGPVL
ASSAFSMFLDGASASSSSNAGSGSSTGPGGSSNGSAGGGAPAVAVLAADGALELNNMQSDSQLPVTMAFLDFYGQRV
SGGVEDSRVAVSVTSTPPVVSGQTQLLTEAGVGHFTDLKVRGTPGAYNISFRAAVRDRTLPPLVMQVRLRPCLLGEV
RTLDNDKCIRCAAATFSLDPTNTSCDLCPNNADCPMAGQGRVLLPEDGYWHSSPYSPQVHYCPWSEACSYGNRSARL
LAFQMALPRLPVINASSSLFRLPPEALAYLNSVRRAGGGANGSGGGGGGDGWQWALGGDWDAGVWVLRAFNATSGYS
DIQCAPNYGGNLCGRCNKGYGRTDFGSCIKCQPAALNHFLYGLKIGLTVFMIFISVYSIQTDLREVRQLWTLQRLNE
SMLQDNKDGKDSGLDDAHSKDNTHRTDTRTVSGAPGSSCNGDGTGNGGDGGGGGGGGGGAGGGGDQGGGGGGGAAAG
LAVLSSGAAGGDGQCNPLESGPLFVLVEEEDAAAAAAGGPEQRVLLDTAAPSATASAGAVAGAAAAPSATAAAAGAV
AGAAAAPAAAAAAAGAVAGAAAAPAAAGAAAGAVAGAAAAAAGIAAARLPTRPQSPRLLLDTMDDEEGAQHRRVGEA
EAEADASSADAPQRSSRPAESFRLMRLQGPSAAEPQGSGPVTSPSRNGNHLFLGPSAVTVDGVADGLPCGSDGSDGD
AASVFELPPVDTPGGVGRGDGGGGRRVAPWLLDTPSQASICSLAAGMPSFQTASAAGLPPAAVVGAESGLLAGGAGR
GGGAGGGGRGGGVAIGIRSYSLGRCGSPDGADSVAGAEAAPATGSSAGLLVTLPWRQSEGSERSPGGFGGAQPRLVP
ATSRLLAARAADAAASPPAADSGGRLAGGTSRRALVRFASSGHRAERAAAAAAAAAAATAVTYRAGGGGGGVGAGGG
GSSRLSRAMSAASVCRSRSTEELLEPRAEALTAAPPGMHSQVEDLGRLRLAGGNVGGTIVEDADEEAGLPACAAAEG
DIATAAAAAATAGPRRTLMRFYSSGRRAERAAAAAEAFMHHLDALGANGGGGDGGGTVGLRRSARLARGTSAAGVEV
NASGWVGEMVAPDGEPRAAATAAMAGGQLAALGGGGGVAARVHTGSGERLQLLPRGLGAVLAETRAAGMLQRAYSAG
RVEPSTSGGMDAATGHGATAGSVYGAVRLVGGADAGLEVRAADAAGVAAAAGALEDPGCPIRHLPRSLELGAAEWAA
AAEEDGAGPAPPSSDSSTGRGLPRRSRVSDPAAAAGGSSSSTGRGGRGSGDVPEQHAYYGHPPNSGSSTNAMRPGTP
TFSRSYTGTHTPLTTQQSLTPDSCPDTETAVAAGAPGAVAAVARAAGGGGGIGNLASLSTGASLLADGPSIGDQSFA
SSTAAAGAGPGGVAAVAAAANAVAAAAAGTGRVAAEPHIVYLDGGGGGGGGLQPALATASVEVGVRTGAASREFAGA
AAGADASSRFIRRSNAPRALLTPPPRPPPGVAQAAAAAGNGDVDDDAVAFICSDAEEEGGEQGHMVGMQGHERLMQQ
QQQQQQQQQQQQQQQQQQRWQRPGDVFAEPLLLSSGAGLLAETPTTSARQPRAPDSSHFLSSSGGTNATSPRQEGGP
AAGGTGGVPLRAPWEVLADDRPAAVPAACAAVVGGAAAGGVGAARVSVAQPVPAAASGEMSRELMLWHQQLQLEQQQ
QQQQQQQQQQQQLELEQQQQQQQQQQQLQQQWSVAAEQSAPDHDLTRGPERPSPPARRKRPAAQIYGVLLKGSADDK
NETQASDTGGGHAYAAAGSRSTGGGAAALSSGRGPVIHSEGGRRQLPDHSHGAPPVPVRAQTLAATTAAAPAPAPPA
PTAWSPRSAAAAPAPATAATAAAASASASTAGTPRRLFVPAAPAAEPFSAAAAGPTTTTSTTSTTTTTTTTSNGTAP
GQPFLSLSTILARSPSLSPGAHGAAASTGAAAAPPSRLLLAATPAAGMPGLSPEGRASAGGGGGGGGNSGGGGGGSS

GGGGSSSLPERLRATLHTLSSLREGIGTSRGGGGRVVVVSHNPLFGEEQAEAPPQLQPTGRRGRMPAPPAAPLPPAP
APPPVAVPAPAPAPAAASASAWPLAILTSARTQLNAAVAAVTAVTGARNTASNGNGGGGGGALPPLLGRAGETGAST
LPASTAAQDGSSRILQPATADATAGATATASAAAASTDATAGGGPQGGRVMRAPPPPPLRMPTLEHSGNGSSRRGSV
RASSPGSGLSGLSGLASPAALSRSQSPGGAMAAAAAAAPVPSPTGSRRRVSFRLRPASPAAADSGAHANAGQQSAEG
AAAATAATAATAATAADVGSSRRMPPPLPPLPPSLSLSRKAGSRRQSSTTAVGDGGAAAVRPPSPSSPPAEVRRGFR
RPPPLQKVPSSSVTPSSGPSPAGGARTPPPPPRARSAHRADSMRRGQQQQQPGDQRRSHASQLQPPEQPQKRRRSRE
PLPGQSPPALGAVGPSPRRFDSASLKTPGGGPAAIGGGGGHVRLLSPARSVRRKPGCVPTYSTVIKIFVSYTQVLSI
TRKVSLQWPAAVREFLAVTNSLSYSVSSFISLDCSLDHTSAVPVSVQRTIISLSVPLMLALLAWLLWTALYFRLKAR
KAVVPSYGRYMNTRFVITLIVITFYCYPGVTDMLLSIFSCPRLDTGGAATPYGAAARAVGTFWAEDYDLECFKGVHN
FLAMALALPLVVVFSLGVPAASALFLWYNRDRLENDPAFAAKYGFVFGEYRPQYFYWESIIMLRKLVMVVVIVYVGY
IGVGVQVLVALGVLMLATVMQLLCRPFREPAMNDLERLSLYSTCSTLYLSLFLYVEGVSEAGRIVLSCMLLGGNSLV
AAFFTYCLGREVRRHTIAEYDMDGDGRLSWAEWEVMVLTKLGENWFGHGVLGLARAVRAGVHLARRVLGRSVRSSAR
AVARVASGRPLFPQDSSRGSRGFGGDASLASTSRTSVLRRASTRSSTTTTTTTTATATSSGGASRARHSLLLHHQQP
SPSAASARTAVVTTAPSHSSSAGGGGGGGGSSLRLSGLLPFRVSGLSGREQKPQPLAQPPAPQSGDSSQQQQPQPLL
RTATPPPAASTGSVASGSGMSSRAASGRPGVPREPV

>Cre07.g350300.t1.2|PACid:27563759
MQLAAGGCGCRRGSSRSSSRTSLLAAVLLGVVVIMAAHGGPLGASAQQQDDELEALDGAQVRGLFGGLNNINQNNKH
NNNQNNNKNKNNNKNNNKNNNKQNPPPPSSPPPPSRSPPPPSPPPPSPSPARKPPPVRRPPTPRPPRPSPPRSPSPP
TPPPPARSPPPARSPRPSPRARPSPPRKGGFKVFSYKKKLVYIQSDEQYNYNEGVNFCQSKGLFYVSYNDKVKKAPL
RPLCYSNGKGCWVGGQVQNMCAYIDPHGNGGPYPEPCNFRHYVVCWGDLKKQRNNPAIFGNQQQQG

>Cre07.g331450.t1.3|PACid:27563760
MGNKTSSPQAAPAGGDPTAGTWVTTNGEQVERSVYRGNAAPARPALKAAGAEPSVRRGVAFSGPVDGEADHHNHHNH
HQTESHPLDLDAAYDDEALDRSVSYYDHEYDSATEDEDGVRQYGDEPYDPDDPLRREYGPDGGPGPWGEAGEGGEYG
PMSRTTTGATHVSTKSNWSSRVVTALRGLLPSGVATDSKLAEARRNGLTRRPSVLVYTNRRPPAHARAEERNWWFKV
KAAANGAAGVMSGRWGGVSGKFSTAGGGGGGGASSGTASAAASSRFRGGGGGASINSRANRSFRVGGSVKGAPAVAA
STSRLAGPSPSSRAVSLSRAAAAAAAAAAAAEDSDSEAGSEAAAALPPADRSVRGAGGGGAASRHGSRGPGQAGTGGL
EAGSRSMRGTMRQHAAAAAAAASAVADVDGQEEAARVGRPRPVAAAAAAAAVPRPSKAAAAASDARARVGGVAAGESD
GEPDCGTEAEDVSGSGRATASGKSASSALEGEDGSGSTGNRSGRGRQPQDHGQPPEQEREREREQALRLKHQQQLPQ
LLQPQTSAPTAAAAAPAAPRALQAPQTPQAARSSSLLLGTSGGTCRPAATAATVLAAAAATNAAVRSSLLLGGGAAA
AAAASASVSGGSGGAGNHRPVTIAAAAADASFVVAGQQGQAAEEGEVGDEWGGGADLDAVERALTQSGVAADQAVRR
ASIGLLRAHTGLPEATASAAPPPTPSGRDRGGGSGGSGGSAAGTAAAASPPPSPPPSPGARGGTVRTSETEAWLQAQ
VLQVHGGGGGGGGSSDAAGWRGWEQVAAAAAAASRPMAVAPAAGTGTALGVSPFSGVTAAGDVSAAAAAAAAAAGLLP
STSSLSAGRATPPALLQPSASMASMPSALSLASPSAISLHTQPSLRSPRTVAAPAGAPAAAQPSPRLRTQPSGLSYC
TSPVSPLPSAAAATSDGSSSMRGAIRLFGPSPSVGGGAAAAAASQQPPSRPMSPMTTLIAAACGTAGSGSRTGLSSC
QQLAAAPPTGASLRRHASTGGLEPVAAATAATSSQAGGAGPAVAAAALMDPDAEFLAAFELSMRPPAAGGPGGGGAW
RPGGGYGAATGAEDDDDDEDTALIQLAPPAPPAQLAAAPLRRPTLSIHTSDTAAASAGALAAPPPGSPRGDASASGN
SALRRLVLPASPTPSPLPSPAQPGGAAAAATAAGAPSGTSSGAASGESTPTGGSRSLYQQLHQQLRAKLMRQQLSHH
SPPASHRQKQHQRHPQQPSPSPSPSPSPSQQLQQPNTRDARDGTSTRHHQQHIDHSYHGVGTNYASAVRTDAGLGPN
SAGADALGLYRFGRSSDAAFPDHRSRGYTSEAGNGLAAAASGQQLQPPEAMEAAVHGPLAQYRSQPHDYMLLLPPDR
AASPFAAAATADAGGGPCSSGSGVDSVGDSSNRLASPRMRPNASMPLPVVGGAAAAAAATAAAAAGAAAMPPPSYSN
SGGVIAAVASAWAAPPDHLAAGTAISKEGMGAATAAAGAAAAAVEASEVDAWLGAGSPDTPGAGLGAAALRDAVWRR
QQHPHQHQLPQPSHLAVQHQRQQQQRQQQRQDVLQPASSTPNPAATAAAGTATADTYTELRRPSRPYVDLRDEERLS
RSLLLQPPQASASFAGHGSSGAAAGAFAATAGGQGGLGSSAASSGRPSNGSDSLFRQSSAPVTNTLQQQQQAAAVA
ATAAAGGHGGIGGGGNATAGIALAGRQQQQQQQLQQQGAGAVAALFAHAASAAALDYVRPAAVATRAAANTVVTIPG
ALDRASVAAPGVLA

>g8123.t2|PACid:27563777
MELEDAVCATEADTHLASVLGGDSQDLLQDLLNVFNEEPPFGGSFVQEQPDAPTSTRTENAGSGQPASSSEGQAASG
GTARMPSPQLGTPPVVEAQLVHPNLLGPAKPEHPTAVALAAPAAPTAWPVPGPAAALSAMTFGSPTQPSDAGVPPFA
TNASLAAGMCFAPATSAMAASNAGPQQQHPALAMPTPVVATAATHPAALLAGPPAAGTSPQPQLQLQAQTPSQQAQQ
LLLQQHLQHLHLQQQQALHQMLRAASLPAATGDRAGTSGLGAATDAHAAVASAAAYAAAAAAAVARNSLEASAAAAA
GSSRSAPPAASNHSLAAFAAAAAAAAGASLASPRSGGNERGLATTAAGAAGVSHSAVEKQRRDRLNSLIDELRCLVPP
QAEPAGGAAAAAAAAAAAAEGGAAAERNRPKHAVLADTVALVRALKQQLLLGSVTDVLGPSAGEGRGRTGGSPPPPL
SISGPTPTRDAPSPAPSPPPGAAANQLAAGQSHVIVKRGPDCYYVQIECRDRRGLLSDIVGALKELPLQIRNASVTT
GADGTVHDVFELAPTTPTTTAATTTSSAAAPPAAAAVAAALSYEPAALKAWVQAALAQRELQQQAARQQQEQQAAAA

AMQMQMLPPGGGHSFPPAAGATGMALTSGGSLATAAPVALATSFAAPAAQHAAAAAGATDAELMFANFNMEAEQLMQ
KRPRAH

>Cre07.g346950.t1.3|PACid:27563792
MGHGGGLHDWTFLPEPSPGREFIRHEVASCSRFVPVPDSANLGPLPAFPSPASSIQVVSGSASIFYFLDGNCTDRAT
TSIDMDVNITLNGQPYAGRQINGYGFIGNCSNPIDLYRHNASRGDDRFNMVWFKKENSNTTEDGNLFGAAPNVFISR
VPPAADALRSLVLYPSPADSVPICCDLVYNPPPPADFFTQGTFCRTPAPPPPSPPSPPNSPPPATRRASPPPRRTPP
RKQLPK

>Cre07.g324100.t1.3|PACid:27563833
MEPHGNANVGVLGPQPTGLLGQPGTRPGSGPDRPGPPLPMGTLSSSSQGPGSSSPRALDDDSAAEWETPAGQPWRCS
ASGAQPQPEQLEAASPASPAGQRGSACEKVVRQLFSPDVSHAEPSPSQTPAAALEYSWPQPGADAVQRRATSTPRTS
QHHQLHQLPRRPQSQPRSLRHCAQGPKAVAPLRDAQELLCMAGHADDPSRIWTTDVVQHMSRFLPHNDLPATLRLVN
RTLAQQLKQPQHTTIRLSHACPTHAFERHWAAPGATAGLRLHDRRRLLRLTARSDVIENLDIAIAAADCCLAVEVLA
AAAGANRLAVAEHLRARHGVPLGGEVLVAAAGAGRAAMVDWSLVQGCTSDKAAAAAAKHGHRAVLDRLLGRTAAEGG
AVLPASPPPGRVDVGALLEGAARGMPLAVLQQLHTEHSVRAAERRAAGGGAEGNGAAAGGDGAGAAAAANADGGAGG
GAVVRMPLLSTREQVKIVEAAAASPAVLAASISAPSAVAAAYVAASSAADEEPGDAAGSAERTGAAAEVPLVAWVAS
ASAAVSAPHVSVPLTDDGDEYATAAQQQSATADRPAGGIADPHSAAVEAAREDAMEAEAAADDVAGSAAAEPSPAVA
PSPVPTTEVALTLPAEAEPAWRAKLKWLESQQYPKPAEAWSAVAALSPPPCGSSSNSSNSHGGGSCRHFLPRSAGCC
LARVSCQGAYAVAASSNCTSGSTSGSGSSSLGQGGDWRERFEYLRSAGYPLNAPEAVRRAASAGHEAALQYLLATQE
GRWLVRGMRGAQPRRSDGGAARPPNGGEEAGWEDEDVGWLGLGPGNGEEDEDDNGPGHRGAAASRAGALHAVSRAAR
AAARGGHIGVLRVLAAHGLDLQQVPLLLPAAVRKRQLECAAWLLEHVFGLPSPSASSSGSSPGDTNTGKYTGRGGIN
CAMGSARGAASVGDIEAGKLVMDASAAVEACLESTSAKPRTSPVPQSGTALTTAAVVPSRNQPLPPGLLTRTLLLSA
AGSGCPRMVAFVHERLRVGGISTSTSTSTSISASELASGSADVADGPGCGVSPPAASGAASSGPGSEAGGSQSGDVS
AGPAGAGHTEPPPPQSEPAQPPVWPHVQSHGEGNGAAGSADAADKAGAAPGGPLAAPNVQAQSVSAPGGAAGEGGVP
AGVPEALASAAATGHTAAVEAAEVVAPSPEADTEGHKEPQPTWDEAAWAAAAKAGCVQVLELLASYGCPMGTNGDPY
VAAARNGDLATLRCLRRLGCSWGPPVPPQPQHDSDDSFTFSIGSTVFARAVTATARREWSSLPVLQLLLDLGCPVDW
DEAASRARDFRGSKPETFLWVVEQQAKAEEEGGGDRGGQGGRRQGCGRARGSGAGTGAGARRRDGAVGAAGGGAGGV
AGSGWKRCVALAVLGCCAFAARSLLHRRASGRG

>Cre07.g354650.t1.2|PACid:27563916
MDFQLPAVTRYLADTGGDGSGAVAVGSLRESLPRSKTAEVTTMLSEQMYATSPPTRLPKALEAYFTGPAARGYSPPD
LEAQYVVPFNKPPPPYRPPGGGAGQGEQEEPDPNGTHTARAVPPGSAPLALGPPVHSSVQTGHVAAAPTSSPAGAVA
GADTVAGGWGAPMDWSHLFGTPAPAVVPSSAAAGGGAGAGASGDKGGAPPGLPVPRSLARMPSQAGSIGGASAAMTA
VTGGGVSVLSRRTHNAVTAALPGTAEGKFCGAVVGVMDRLAATSALQAGGGGGGKYRSLRVQTAEHKESMVSAISLP
PPPQTGAFSSRAAAARMAAVLDRSGASRDDMADIVRQIKQFAGLPPALRDAVSGRFQLFCYPPGSYLYEAGCRCDEA
FVLVSGAVMLHVEGRPPQRLQPGSVLGAELLMRRGAVPLSATTDPHFEAQLAVLCWVDYEEAVRGWAAERIAELVPD
LQASQVARLRSGLQRGVQYVQRQAGEPLWVGGQPADAVALLVQGQVLLASPWEANALVTENQIVSQLRVLMGPLADT
MVLNEMPRPATHAAARAGGLRGGLKGGGGGSMRGSIKVALGGEAAGGGGLSASTSFAVRGRGGDGDEEEDGAGGRGT
GSGSGGGKPVGFSLAEKLERAATGSGAGSADSTPRRSARIAEPPPPTTTTASLGKEDEDEEDASPLRFRLAGGGGGG
GSSKKGSRKVALEGPAGPRFVEDSPEPQPEPEAGKAPDPQAAGAAGGEGGAASGSSDRPASGQPRRARQGQPPGRNS
RNATGSGTSGEAREEAGPEVDASEATAEAGGDTGPAEAAADAGPAEGEATQAEAGPPPAESAAAASSRAARVSLKFS
DEPDAEAHGAANVAAATAADGATAAAAPQPHPESQPQPQHEASAESAAAPPSVRSAGMSAEASLLPAVSVWDLHDGA
HPPADLLTPVIGLRAAMDAAEASRAAGGHGSDVGDGASSVGGPLSAEASLTSPPMDTRGMGFAFGGAAGPGVGGPAD
AAGPTASASFLAAVGVAPGPGSGGGGGGGAALASGMMTPALVLRPAAPAAVKDAGAVPAAAQEQQDQEQQPGDAAPT
GNEAAADEAKAAGDASSLPPRPAPAPRSLISKIGGGSQAGSAAGSKAVSRTGSRTASAHVSPSSSRRVTGSGDMLPG
VTSTAPASAASAAAASVPTGLPPRPPASSGGGSARSAGPTMAEAAATAAAGGLSSVDSTAAAAGCSSARERPSTPMS
EAPSLDDGRSEQGEAVAGGDELLLGDCSHPVLPPRSMPSPPPGQDEARCAAAPPVGTQPAAADPAAAAGMREGTGSG
AGDGATSSLATQDGSVDAREAAEAEAAARRPHARVLLGAAAAAPALASIEDSFAPSSEASGAVVAGAEAADVGRKPS
KLRMRIAEGPVGVSALEGERGDVKPSSLRPKLRMNDDSNDADDNDGAASVAASNTMRFMPARASVGGLKPTENGGGG
GAMSARGSVAGHVSGHGGGGGGVTGPCQPLANALATQFKFHRQRVAAVYSAPVVFGEEALGDPVPAAVEAGATAGKK
GAAEPTKPPMVHSAIATARCELILVPAALLRALLPPPAQVPGLLGKVAAAAVAAQRRLLQATGHNTNDSGHAQWPAA
ADRVREVLTSTQPGQRQPWQVELLAAAFSGMPAFARLTWQARLRVFQELEYRRLPVGSSLDLARLTVTEKELPDPAA
AAAAAAARNGLLSTRMSQAVISPPPPPDPSSAPPPSEAAGLQQGQEQHQHQGQGQGHSLSVSPVAGLAAGAAVSASP
PAPSAGAAGATDAIPPVLPSAAGQGAAVAGSAPGCPAATPPVGAPAAAAAAAADIAASAVATHASAPAAAQRQSSTA
SAASTDGSNVPGQASGSGAQQQQQQHQPDHQLQPQLQPQQQPHQHHTVEAVFDPSAEEPLIPSLALLQRASASTITG
DGGIGDGSTPRGGGVAPTESALAAAAAADMLRLTTMYTAESSAPSVGTPFTPATPHDPAAAAAMSAKYANYIIGEAK

RGPMTPPTPVATLPAAQLTAPVEHFPPKPEAPPAAAPPPQPLPDPTQRPAPAREPPAPPPATRQKQREAVALRLDDK
AYWCIWGDIALEAAPAPPAPARSEGGEDGDDTSAPASRRASGVGPYGPAHASITGGGGGGFTSAAAAAAAAVATAAA
SGDISARAATAGGHKLQLVGLGDAFSGSALADATAAALNTPVMLSSTPRMSTTGGPGDIQLQPSIAGAPTAEGSFAQ
GSRRQPSPTSPQRRNTGSPSSPKRQQQREQPPAPPPPQVPLLCLDPHTAENAALLHESLPLGDNILRARVLTAGGDH
PPPPSEGGEVAHSAGGAGGGYAEVLCMSKPMFELIAGGMGRSVPPAVLPVLARHVDWLAGAGVDLAAMAAGMEGRYK
LEEHKQGTELLRAGEAAPAVFLVAAGECVMRGPVVGLRPGATAGGGSLDLATLREGSTCGELSLLAGRPLWYSLVVA
SPTAKVIALDLQALRGWLLGAPAGMAAAAEVVAAVAEVDIALYGRAVKQIAIQVLNEKLELSMFQSNMKSMIRRVIE
DNASELNAILAQQLQQEAAASAAAAGGKAAAAAAAAAAAASRPRTIVLEGWDQVEAIAASVARPYEPGVQSWELIAQA
RFNAARRAKARYLDSLYTATALATQEDAGGHAAGGQRVALPAPGPVAAGLAAPGTVRQPHLHDIRVVPKDLRGGEVE
VRRRGGDAVLHMPPHELSAMMGAAVHLVAPTAVAAAVGAVAAGASSAAAAARAAGAAGPGTEGSGKGAGTDGGLSAT
GGASASAASVTAGASASLLARKPVNYAATAVSGGFGSDLGSPSRNAKRPAGAPVEGLGRALTAARRTYADYYGAPGT
TDDDGVESPAAGAAAGAGAEDDNTGGSKPGLGATAGGGVAGFRSRLRVSAPVLDAPTVVQLSPGPVAPWDSKGRLPP
WNLNGEAAGAGEPRRFPWDAPTAGAGAGGASRLGGGAAADHLARQRELLASGGAASTAWSTAPLEGHRGRLVVALSA
APSMASVALDPETRALIERQMEEEEAAEAAEAEVAEQEAVRMALGDAAARKLLQAGLQPAAAAGSGPVAADAMAAAE
RAASLSSTTATSLSGSLGFSRPGTSLTDDPHGSLNGSQPDSRSASRAGQLLQPPHGHHPHGHRASSGITAAEVRGLT
ELPVHVLASIGEEDSMEVLAALEGAQQHMAAVAAARAAAGLPPAPSALASASMTALPPPPARASAGAWISKSQEISA
GGGGKASTLPPLRAAQRPTSTPPAAVGSVSSLLLGGGASAVSAPGPLADASSSALLHGLTAAAATASDARSTASGAA
ASTQSGANAANAHAAAAAARRSRIQARNAAPAAAGGEVAEAPDRAFWVRDRLHDPQGLASSVMADAAARAVVQAVEG
GGAPPDPLSMTAPAALGGVGGGGTSRVGPSTSTSANVLLASAGRPAIPTAAAGSSTASGRSPRLQPLRRNASSATAA
GKSAATTPRQRPSGGGGVAGGLAAAAPAAAELAEQGMLYVTLVVPPAPKLLPEFPSPAQAREAAEAAEAAERAAREA
RVDAAINNPATRPLSIDHRGEMRYVPNYLRQRQRPPLAPPPVRPVLLDGEDEGSSWVVPSYTSGIGFTDGFYDEDSQ
VIYFD

>Cre07.g326550.t1.3|PACid:27564021
MASLRRSLSATASGANALDSRAIKATQSIVEHARVGPGLDSPSSFASLRAQTTDASAVASGAHTTQTHHAICQGPVC
GAVPECLGSAAAGAASSATTTDTAALPAATTRTTASPNSSGDGTSGPCLPAADYGFDHMFDQLGGIGDISGGGEGGS
YSGYGMTELPLSPLGGGGGGGFWDDDNDELQLPLPQLAPEPQHQQRQQHMQHMRHAHDAQQQRLVSQAAAAAGQGHQ
LAASGNPNQQVTQTTLPAAAAYMANAAGSATGTPDERQLLPLMPSPSSPPPPPRPQHHTAAGARPDPLGFSVLSRRP
APSAPAVSANAAADTAQMQHQQHLQRQQHQLQHAPVAGAASVAAGPLGLYSLGSFTDSSGCHAGVAAAAGLVTSPAG
SAAAAGTAATHHVQLSSTSPAWPIAAGATLACAPAAAPPARGVLVAASAPPLAGVASGTAPATPWQLDGCGGDDEDN
AVDAVHSLMRRWSSEPRVSGMSGGRPGAAQPPLPQLQSQPQQAAVQQQQAQQQAQTQANTMQQQAAHLFCHQQMQHK
LHQRPADVVTPVKPLPAMPAAATPPPAGSNMARPQQSAGGPGPQPSGSAMTDGGGPCTSDGGAASPAPSAPLPPPHV
QHMGARPYGTPSAAWAHSPPPPHMSPYGHSPYGHHPYSRAPAYYAHLQPPHQYGHVPPYAADVPAAGVSPVRRVRWV
YPAAPPPPLPGLPPPLPSAVPRPVTQQTPQQQQQQQQQQVVPHMQAAPACSEAAAPAAAASGYYYPMTPTTPRGKV
PYFGAPPPAVPPMPQLSCMGAPAAPPPPAAAQLLASPYPVTTYAPAQARR

>Cre07.g346850.t1.2|PACid:27564095
MGHGGGLHDWTFLPEPSPGQEFIRYQVASCSSFAPVPDSANLGPLPAFPSPASPIQVVSGSASIFYYLDGNCTDRAT
TCIDMDVNITLNGQPYAGRQINGYGFIGNCSNPNDLYRHNASRGDDRFNMIWFNKENSNTTEDGNLFGAAPNVFVSR
VPPAADALRSLVLYPSPEDSVPICCDLVYNPPPPADFFTQGTFCRTPAPPPPSPPSPPNSPPPAIRRASPPPRRTPP
RKQLPK

>Cre07.g335250.t1.2|PACid:27564103
MPSPPLPPSPGPPSPAPPQPPSPAPLAPSPPSPVPPSPAPPAPPSPLPPSPAPPGPPAPPAPPSPVPPSPAPLPPRP
SAPSPPSPAPPNPGPPSPAPAPPQPPSPNPPLAPPPAPPSPPKPPSPNPPSPSPPSPEPSPPSPPAPPPAPATPLAL
GPAPPSPAPPRPPSPAARSPEPPSPAPPSPAPPTPQPPIPAPQPPSSPPAPNPLPPRPPSPPPSPVPPSPEPPTPRP
PSPHPSPPLPPLPPMPPIPLNSPPPVLPQPSPQPPLPSPPPPLPMSQVPIGSQFTTDTGVPTYVPTPARLLAVSYNV
AKSVGVENDAVTTTVEGAFITTVYSVSGTSARRLLERLVVQQEEAEQEEPQRLEQAKQHLQQALVQPQDPQDLQQQQ
QARSRALLQTDSPPPPRVCLTCARAVESALAAGLCISLKLNNCSILTVSCLNESVIAVAAFPAVPTDAAQAALANSC
DAALLATFAMPNTTSAQQAAMTQALLSTPIIAPGYGVVPPPASTSSVAPSALLRTVVVNTGKPNPGSNSSEASFSIS
GAMVAESVAATFGLPQGQVVLVTDPSSRPSGGQTVPAPAPGSIPTPNLGPDGSPSPPSNAACTENPKFGSLCGAAAV
GAIVGVAVGGTLVVGLAALAIFSAATRRRQPVSPMYGDRSTMPYSTAAWPPAPGPTTVAAVPGPWSALGPGASLGAA
AARSAGGAAPGAPSAPWHDRDVGVGAAAAAAAAAAGAVLTKSPGASARAGKYYSGGGVAPRWSSAGGSAAGGAYGTS
GGGGNGAYGSFLDINQAPPPPPGMMLGGNPMFYDLAGNPIPAPGVDSNMAAAAMAAAAPGGYGGGGSAGGAGGSPSR
LRPPALIIPPQGAGAYTEGLNTLGSLASPVPASAGPAYGYRLGTGVGSPGVYSKYPGRGPQSLAAAAASPASVAMAA
RYSNRAAGYSNDPAAMAALRSSMAAESAAAEEIRRSRGAATMAGLASAAYSSSPYVGRTGAPPAASSLGGAGGEPLP

RFYPQPQQVARPSPLGADAAPVRGPHESPRGSYGQPAHYGVGGLGSPSGMYLPGAYGKTGPAGPLPSPGAGRPASSM
LGAGGVAVPMGLYGEPAVQQAYSPSAPDAQQYFQSPGGATGPYPQSGLGPY

>Cre07.g354500.t1.3|PACid:27564105
MTGEGKGGGNQGQPAPFLRKTYELVDDATTNHVISWGPSGKSFIVWKPSEFAANLLPLYFKHNNFSSFVRQLNTYGF
RKVDPDRWEFANEYFQQHNRDLLLTIHRRKPASTVTAPGGPGGVGPGAPGGSGSSTGPGTGSGSGSAQQKSAPGPPV
SPLQQFTSQVSSEAGQPGPPGPPAPAGAPSAVLPIPLPPGVPPAAAAAAVAAAASAPFSLSPPFPLDLGIAATLPLF
TAPPPTAGAGAAPGLTFPALTIPPVGVMVGPGGPLATGPGPILSTGPGPGVTPSAGSAFSRPLLPASGSGAGSGSVP
SLKTELPPPHALGPLLSPQAAAAAAAAVAAAGGPSPPPAQLQQVLAALASQSGTPGSAAAGQLARDGSIEVGSGWPA
GSGSGMTDGAAHAQGTAGNTNAATSNSPADAPGGASAAAANGGGPRPSHSHSSNNAPSASSSDAQQHQQQQSDPMLA
EDEYGGQGGAGSAGKAAKRSRADMYTSARTAGGGGGGGGSVEAALAAVAAGGLPSASGLPSASGLASGALPSGGGGM
GHLTSAGLPSGGGGLVGLNSGGGGPGAVGLHSAALQLLGTSPPPGGLAGLSGVTGAAAATAAATSGPASLTSFDAAG
NPLPAGITRPLPQHHAMRGTTAALLNEMAGQLGRLSALAREVEGLKEENTALKRSMEALTHAYTGTGTGGGGGSGSA
GAAGPSAGSGGMGAAGLRSSGAGAVAGGCGGIGLASGPQGVSLAALAGELGMGMDEEAGTGNGVTAMDAAASGDGGA
AAGGGGGASDGSGGVPSDLGAGGSSSGAPAVNKDGTTATPLTQAVEEQGKRLWDQKLRHDELEKQLLAQKELSARQA
AKTAALELALAANSAKAVHDAAFLGRVLEERVSAESGNMRGRLEAQERANKAAHDVNSRLEARVAELEAQLRALGQD
TAPGSSNRMSQHDNTNTNNQQQPHLQQPTGAEALGSRTFAGTAAAGDVGSQHGQQGQPALLDGARNGSASGGAAAAA
ACSPPPPGTGSAGFVAFMQRLGSGAAGLGSGQAGQALGLGAAGGPLGSGAAGLGSGLGGGLSGGLSGGLSGALPLEA
DLYAKTIEDRLWGTVEQIVNSAPDPAHGARLVLQVAKARQDAYAAAAVVGAAAASTSTGGRSTSSGGNGGAVSGAGN
GTSAGCGTGDSNTSGPSGSNTPGGAVAAAGQRGAASLAAAANGVAGSRGAAKEDGQSTEGSGSCPPPSVPVAGTAAA
ATAAAAAGGDMMDTGA

>Cre07.g327150.t1.3|PACid:27564171
MASQELSANGNKGLDSTGAMELSADAKLREEGLGSLGVLPDHLLSYIMYLLDVEDMVRVSCVSRLLRVLGCEEPLWL
ALSLKLQNGLVEYKANWRQTALASYRRPRAGPVPPLAVAAAARPPAPLPGLASLFLYKRWYRCHVDLRGFLPPPDAA
TVPRVVAAGGPAAAAGAGMSPAEFRRLYDGPGLPVLLQGLQDSWPAACWSLPALCAAFPAAAFKVTKPHGGRALMTL
PAYVDYMARQADEEPLYIFDPDFVEAAPGLRRMYDIPAVFEQDYMGVLPGGGRPHHRWLVLGPARAGASWHVDPSLT
SAWNALLSGRKRWALYPPHSPPPGVALSDDGEDARPDDADGLTSLQWWLEVYPSLPPERRPIEFVQEPGEVVYIPGG
WWHAVLNLETAVAVTQNYVGDANLPRVVAYMAAGSSAYFRQPLSYYSREEVAAWRAAYPGPMAAADAAAAADAAARA
AAAARDSASGGGDTSGSEDFSGSEVEVEVEGEGAGDKAGRVGDGGSDSSDVEGSGSESGSDPAASCLDENGVEGGGG
GGGGGGGGVELAAAAAVVGAGDGGGGDDSGGCSIRPLASRWLLGPARPSREGGVAAATGAGSEAEVGAEVEAGAVAL
LPLGPRSAAAAAAAAATATAGGGAHGTRTRTRTITTTVTTTSAASKQEQEEEAEAATSSGKADEAVQPEEEDDEGGP
WEGPDAPPALRCWRRERILGRWLRRLWEQRPQLRPALGCCLAASTAAPVWAAVLAAVARQHNTLLRRAAAAAAAAAG
AAGGSSAEEGAHSGGGPCNGHAGTGSEQGQAQALQGGAAESQSLQSQSLLALQALVGAPEGRGLLGLELMPLVGADA
LVFLTGAPAAAAAGSTAAAGSSGPIAGSKGAHGVAVKVFTHELPAMRGLMCALEAHVPLLLRGRLTATAAHTTGLGP
TALGLGGGSDDSHGVAAAAAAAAALEAADQVPLPASCGLLSVRFPQAGAEAHGPGDADEPRHVSGGRRRHCHNGSSNP
VKEQAPAGGEQQEEQEQQGAQAAAGQGRQGAKRSAAAADLSPREAGRRRTKAAPPPPAGTANTAPSQHPKPMEVEAV
KPLGEGGAEEHEEPPQPPQPSQRARAAPEHRLLYLVEQRMQGSHVLPELWDELSHHQRQQLATALGHLIGRMHRAAA
AAAAVAASGTAAAAAPQAQPQMEVEVKAETAAPPRAALAGVGGSSWESLLRSRGVWHDRDGRIWVSGLGRVCSPHSA
DDSDSDSDGEGDGEEEAAARDGGGSSADGKAGGGPSADRGEGAAAAAAAAAAPAPSAAPQRPRRRCHVPLDSPWWPF
VSHLRRRQRRLLRRLAVAAGPAAAAQPLQPAARGSGPVGGAGAAEGGAAAQSPPPPPPYARPAAGDADEELPVWVRQ
QLPAYLPEDPAQLLGFQLQPPEGERREGAGGEEAVAAGGGGAAASGPGPGVPPLLLHGDVTSSNVVLQPPPQAAADC
ATANGGAAGADVSTAAPAAATAATAWSPVPRLVDFSDAGHGDPLAELVPVLASCLGCHVGHMRAFWAAYTAHVDPRA
PNGSGTSGRGDGGGSAGATGESAAGTAGAAAARGVWPLRGSSNGGGDGGGGREALSYAAMCYCLIHEEVDVLLERAW
DSLGAVAAVAPAAADTAAGPTLERLATALWGFLDEAEEGGRPAA

>g7710.t1|PACid:27564300
MGICATKPVSSSSTPDSLVTGVAPRRSRGGGGATDPRHQHTDSFDPQQDFSTDPDLNDDDDDAGGWRAPSGDSDDL
APVSAAAALGEDGSTAAAAASLIIFTKRRPPLAKRSVQSKWWKSFKSAAGSAVGAVSGRWGGAASSKSLFGDSGGGS
GGSGGAGGGGGASSKKWLGWSGKWGWGKGAESTKTTGGVSTTSLRKATVAAGGGYSVGRSSSRRFFSFSNITDDSDL
PGDSPDGEAEDIGVEPEGLPAAAGEQEWQHGGQQGGAGAMSAEDAAAAIAARAAALTGPPRAVPPPPSSSSRSTGMK
APAPAAAAAAQQQQRRDASSGMRSINGKQGIGSGAAAVAALARDPSRKRVAGGGGGTSLAARDAAGTAAAASAVAA
ASALLAGGSVGNARRRSSVKLTTKTFKYHHASTSNNGGGGGAAAAGVTGIGATKNFRSFGALVGSSAATSTAASSGG
GSGSGGAAGYAAPGGAAAGGFNLRAASLGRAAALSVAAAALAGNSNSGAPILGLLRKVASLEPAGGGVGSVSSYSSS
KHNSTGGGAGSFGGGSGDSMASGGVGGSYRISGGGGGLQRASGPIGSGGLQRASGNIGSGGCVAAGGNYRTSGGGAA
AVDAQGASAAACSRLKAVSWQRSDAVCVYSEAAAVVAASSASCTTPAADGGGGDGDGDGDGRGAGGSDEGPASCPPY
MAGVLEATSARQQHAAADSAGKQRADPSATPAAAAAAAAAAAAAAAAAATDESMGASGPFATAAALAAASASASGAT

AASVTPVNSGALNTAAAATHGWPPSTAATTTRVTTAASAAAVATAAAAAAAAAAAAGSGSSSSGKGMTGSAAASSAAG
SRAAATRGFIASLTPLEAVPSPQYPHRPQHQHHHHQLQHHHTAAAACDSPVSCSSLSLMGLASPSGINLMPSTSFTA
GGLLLPIDELLPMVDSLHDHTAVAAATATAEAAAALAAAAAAAAAAQAAPAPVQQAEHLPPPPSPPPATSPSPSPPS
TAAARALSQPLTTSNSAPLFRLRSPAGTSSIYTAVDAATSPFGAAAMAAAAAPMSPSPSGRLLLSRHSAAVAVDGRV
SRSSTGGHTPAGGSGDLGAHGAHGSGGSGGGAPLGRTMSRADSSGGGGGGGGRPQRLRLAPAAAEGEDAAAVVAEGH
GGDDGSARQLLSPPGFVRGSDLGGLLTPPPNHNDEEVSSSSTPDSLVTGVAPRRSRGGGGATDPRHQQHTDSFDPQQ
DFSTDPDLNDDDDDAGGWRAPSGDSDDLAPVSAAAALGEDGSTAAAAASLIIFTKRRPPLAKRSVQSKWWKSFKSAA
VAAPAAKNDSDLPGDSPDGEAEDIGVEPEGLPAAAGEQEWQHGGQQGGAGAMSAEDAAAAIAARAAALTGPPRAVPP
PPSSSSRSTGMKAPAPAAAAAAQQQQRRDASSGMRSINGKQGIGSGAAAVAALARDPSRKRVAGGGGGTSLAARDA
AGTAAAASAVAAASALLAGGSVGNARRRSSVKLTTKTFKYHHASTSNNGGGGGAAAAGVTGIGATKNFRSFGALVGS
SAATSTAASSGGGSGSGGAAGYAAPGGAAAGGFNLRAASLGRAAALSVAAAALAGNSNSGAPILGLLRKVASLEPAG
GGVGSVSSYSSSKHNSTGGGAGSFGGGSGDSMASGGVGGSYRISGGGGGLQRASGPIGSGGLQRASGNIGSGGCVAA
GGNYRTSGGGAAAVDAQGASAAACSRLKAVSWQRSDAVCVYSEAAAVVAASSASCTTPAADGGGGDGDGDGDGRGAG
GSDEGPASCPPYMAGVLEATSARQQHAAADSAGKQRADPSATPAAAAAAAAAAAAAAAAAAATDESMGASGPFATAAA
LAAASASASGATAASVTPVNSGALNTAAAATHGWPPSTAATTTRVTTAASAAAVATAAAAAAAAAAAAGSGSSSSGKG
MTGSAAASSAAGSRAAATRGFIASLTPLEAVPSPQYPHRPQHQHHHHQLQHHHTAAAACDSPVSCSSLSLMGLASPS
GINLMPSTSFTAGGLLLPIDELLPMVDSLHDHTAVAAATATAEAAAALAAAAAAAAAAQAAPAPVQQAEHLPPPPSP
PPATSPSPSPPSTAAARALSQPLTTSNSAPLFRLRSPAGTSSIYTAVDAATSPFGAAAMAAAAAPMSPSPSGRLLLS
RHSAAVAVDGRVSRSSTGGHTPAGGSGDLGAHGAHGSGGSGGGAPLGRTMSRADSSGGGGGGGGRPQRLRLAPAAAE
GEDAAAVVAEGHGGDDGSARQLLSPPGFVRGSDLGGLLTPPPNHNDEEVIGVFAASASPFASPSSLGRVGSGAAGPA
HAQHSTSPSPPSRSGGGGGGGGGGGGFAAPNDSAAAEHQPAVMSAAAAAAAAAAAAAAAAASYPPQQLQAATSGRHVGN
GNSNSRLRRVTVGSLHAAHGGGSGTTAGPTPAHAATTAATTASRTAHDHNHNDTLSHESGGLIRVGNGMMTHAGGCS
TAGASAAGGTGGHGGGAPGGGDGLPDDLEGELEGLSPYPHCFGGFVRGASDGEVYGEVGGGAVQGGGGAGGWGVGLG
AADGGGGGGGARDLVRGASDGIAYSGTDSHHGHHGGQGHPMNHGHHMLSHTAATAAAVAAAAATAGHSGVYSGGFVG
SAADSGGSSAADATITGWSPPLPPAPLAAAAADSPAGAIHLLQPQQKQQQQHASHGQHPQQHPQHPQHPQQSHTHDG
QHHPHQQHQQHQQHPQHQQHQLRAQLMRHHLQQMAVQLPRSLNGSRRPSVGAAGGGGGGGGGDASAGPDSADAAGAGS
SAGPSSPLLPPGSPRTPTAAAPAGALSPVGSRGAGGLWRTGSGRVYPTSPLPH

>Cre07.g338950.t1.2|PACid:27564308
MSASGGPKPKGPERLQLAFQEIYAADLGPWPSYDTKSSPLGARGTQPPGAASRPSPSYANTPSALVRRSLQQQQQQ
QQQQQPQQSQQQQQQQLCSTVEQVEELLAGGSRAAAAAATTPGRKAAGAGGYASRHTQRAAYAAAAGSPGAGPAGGL
PPTASSPAGSKAAASPGGRAGAAAAAGAARAAGKAAAGTAAGGADAKAAGASTGKAAGQSRGKGAAAGGRTAGAATS
AVIAALALDASLSDLDPSALALLEAIMSPADAAAAAAANSKGAAAPAAAAATGATAAVPVAAAAAGILTSPVTPAGTA
ALPPRPPQVRASLDGSFAAAADAPRSGCAADARSQAASAAESPAAGSLTAAMAQRAVAAAAAGDSRRNSPRELASVG
AAGGGGGGGGNGGASTLPSDVSLHLPHSSAPSATGSATSAATAPPLAPPPRAGSGKPGSCSYPSGSARAPLPQSSMS
AGGLVPTGAAGAGALPADADEVAEEDCGAAASDVEDGGCVSDGEGASSRGGRNTAGQTQAQRGTGAGAGHSSANNLN
AAAVGAQGGGGGARELIKRLCRSHATRTLSNRSLRVAAGRPLTYAELQGSSRLQGCASAATDPAAPASGATSRRTTG
ERPSAGDRPSTASAGAGLLRGMADNNLSDRTIPEESGGQSAPNSVSSVAGAARAAEATAALAAAAAAGDAAPAAAGQ
SGRLSQRVSQGRQQTPPAAQPPKQAEVKKRGFFARLFSCFGGGGGEKGSQLCNTSSGSASAGAAVASVPAAMPTAAA
VEQPVLSDGGGLLLKRSLSVGTGAPASFPINIDAAAAGGEPGKTRGAAPGIPMPAEFDPAFATATGGLSGAAPLGSD
ASVASVPTSPRSPLGTTAAAAAAPAPVAAGSSRLAAMASVGSSSLLPPAQSMSQPQQRSPLPGGAVASRRGGYALAS
PAGGGASYSGNGPQPLRASTDTGPSSLIMRSFDGNPAAAAMGPATAGGAAPASAAASPLGSSRGSHRQVARSASRPG
SALAAAAAEAGAGRGGQFRTGTSTGTGSAAPVSTHGHSAERLQRAISDGTAIGAHPPAARSRLSPGGARDRRATVEA
AMAYEDAAPGTGAGATAAAGGGSHSASPNHVTAGDATAAPVPGAGLPPLLFSDAPSLRPAAAAAGSAPSEAPLAGTV
ATAVAALTGGRYSRKRTRATHPTGGSLAHLDAKKLAALPLEEMLETIQAAIEADDAARGRQSQQAPQQHLSADGAVA
ARVASIRRRRSAAGARGNGAAAAGAGAGGASTAAVQMTPGGKGGAADGGGRRRRRRRVKNPKRATGASNAPPLAAVA
SQPQVSPGVPALLLQAQRSSPAFGATAAAGAGATAAAAGSAAKPEWRHGGRALAFNGIGIAGAPALPSSIGIVISPR
EPHTAGAASAAGSASASAAARASVDSYFGLVRQPPYKKPLLSRNSPSPPRPSVSPSLSSPPPLAAMAAASSSPAAPM
AAPAAGPPAGHTTLEVGPAVAAVLRASAPVHPTASLAAAAAAASPPLGTTGRAVSRLLLQSDPAAAAPVTSSLDLTS
LISSAGAGIGYSRAGTPTSMPPGAASPAPDAASPSRRPALASSCKDAGAVAAVTMGKDKAGKDVSIAAASGPSEAQL
FSQLMDLISRGMEVEQNVLGRGGDSGDAAKALAQAPKPQVLATQRPLPPPPAVVVPVMDVHVFEEQHPHHRQAHQQQ
QASRGYQLLLKRPATPPAATAISLAELLSRAPPAAAAAAAAGAQEVSAGAAEAGAAEAEAAAAGAVVAVKPLEAEAV
PEDESFHCSGEADEEVAEEEEQHEADGGEEQQEEAEAVDDSSLPQPYCATTASATATPTAAATEAMASQAAAPGPAV
KVQVKMSTLESAISEALSADVPQPPAAAAASDVVLHCSGSTAVSEFDREQQAASGLLSRTSTAASDDNRAAGGDGAG
SISGASASEPLKATLSSEAPAMTKLDSRSVSYVRLSLSQQAHAQQAAAAPPPPHYAAPTVASAAASGQRASSSSGGG
GGAAAPSEGGRRVSLSGGGGGGARDVGRSPLRQRAHSPPPGSYSALAAARLSGAGINPSPSATTTPTKAPPLPPPLP
TAAAPAASPARAVAPFTLPLVESGAAVAAVLHSTPSVGELSSGTVAGRVSGGLVDEQCTSGATQEVVVAPGCGIPPA

EAVAEAEAVVEAVTARGDDGRDRMSSSFKAPASQRDLGGSGICSGAFESVLTAEEVEEVAAEAAAEEPSAFRDEEVV
MAVAVAGVAAPVVGAAAGAALLVYEAALSPRTSAGAGWDATVAFEDIPASSHAPTPAAAAASAAVQQAVAAPAAPAA
ADPPAAAVAAVAAISVAGGSGSGIGCARGSHHDSGSIDLDAYEKSLAEFDPYLNSGGPAPAVVKQPVP

>Cre07.g354750.t1.2|PACid:27564334
MTAGNIIKVDPNTQAWKVDRSGHPKPSSRRNPPSQRSPTAGGRRGLHPPPPRAASPSPTSSPPSTSNDRDIPSLLTS
PPPPPALPPTLKVVFSLDWNFTGDVDIVVSPPPPASTTISFVQKGCAACPEAGYGRLTTPKTNARSGPETVEFETPL
LGTYHVCALYMAAASITGHVSIPTHGVSLAFTGTTTGRALNYRACDAAQAGYIFSYDLK

>g7578.t1|PACid:27564346
MRNAVALAVLSLLLWGASAKASQAPPPSPLSSSPPGLPTAVVASPPSPAPSHPSPPQQPAPSPPSSSPPSPPQPKLP
SPTSPLAPSPLPPSPATPTPPSPLPPNPAPPAPSLRPPSPAPPSPQPPSPAPSTPAPSPMPPGPSPPLPPGPPTPTP
PSPVPPSPAPPAPSPRPPSPAPPSPAPPVPAATSPSPPSPRPPSPAPFILAPSPLPSAPPLPQPPQPPSPLPPSPAP
SILAPSPLPPGPSPPQPPSPRPPQPGPPVPFSPSPPLPPPPQPPPSPPTPPSPQPPRQSPPSPQPPSPHPPSPAPPS
PPSPPSPPPPSPAPPSPAPPAPAPPSPAPHSPTPPLPPPPSPSLPPFPPGPSPPSPPPPPRPPPLPPYPPEELNVLP
SGPFPCAWAELGGEDTRVRRFLFPPSPDPSLANIPGLQYSVEAAGAQPSTPVAVCATAAACATPQAAAAAASSGAAL
ILPSPAPITSSALPPLANGTRALVAAADHPDGVYALAASDGAGNAYSVDMCTRVPADTRCPDFFGVMSVMRPATALP
AAANTSAGAPYGAVRSPVTWTAAAAQAAAAAADSPLAVLTSACTPPPGVDASRKLVYSWGWDFSGLPPSLAASGFVA
VYRRYPLTRMAPGSYNVQMLKPAAVRLAVIVRVLDANPDVAGTPLGVREVVTVGDSPSFVVPGGNVTDLTFVFVVPP
VAQLDNVAAMSAAISMAWRPAPPSPPPAPPLPPSPAPPSPQPPSPEPPSPSPPPPSPPPLPPTPPLPPSPPPSPPQP
PSPPPEPPAPPSPPPGNVQRGQEGAATCRGGGAPGWGLSLMLLLAAAVASWALAGL

>Cre07.g342500.t1.3|PACid:27564439
MGAGWSSAYDITALVLQLYGFLLADDSIDQAVGRAIERQREPLPRPEQEQQPQEQPQEQVEQEQRRHQRLGPPADGG
GTAAAGQPQQLRVAYGLTCSCGFHDRPRLRLASSPPPGVVAVPVAAATGAGVEDAAMSDAAAAADPAGLGRNGGGGG
GEVVAGGKEAAADGNVAACRLPDLPMEIIHKIFTHADSGTVSKAAAAAAAATTTAAAAGDQPPPPAKRARIRWSPPP
STSPAPPAASAAAAAAPDAAAAGPALGAAPAGAAAHLMLLSDAALRAKQEAELICFFRKAPPAAATWVSRRQPPGAA
AGVGGNDSKANGGDGGSGGAAGAASSRQPGGSRPAGGGAGGGAAASIDPLDDVFGGGYDRRGSGGGGGSGGGAIPPE
LDVLGVGVSVELHDRSASLRRLSMTHDTLSLAAFAADGVRRSAWNQPFEAFLPLAVNAPHGRAALRVLRHSVAAMLR
LPHARHVRAEHLLDVVARLMNSLVVDICTPPKKKEGGGEPDLDPEMADDPFLDWQGRRQQERPVPRHMSDAALQSFC
HLHHLLLTLVVEGGSSGEGGSSPGSGCSGGGGGGALGRAVLEAAARDVSEFITRPEARRKSSCPDLGLLLVKYLLVP
RDAAPWAAFAPPFLRELLARQVRWVVKGLAEELPQPPQAVGGGRSGASAREAAREAAREAAAAAAAAFERPWEGGGD
LRYAQRLLARGGGEGAAAAAAAVAADATRLRQHFEHARTSLSLVALQAFFANHLARPSSSAAAMQQLAAIKASYDAR
NGQPPPPVFDAFNAHARSVIAQRGWGDFLSAVRMGVSAEAVAAVAAVAAVGGGAGAGMGGRAAAASGGAGSKRAAPT
MAVPSATATVMVGDVARVMADTLRQAVVEAHRAGYQQVPDGRGSGGGGGRDGRGGYGGGGGGNRWDRKPAVPSRPPR
HVQFVDWDGPTPPLVVVGSEW

>Cre07.g329550.t1.3|PACid:27564525
MIQPGKLAPAPETGDHDFDYMFAEGVKPRGDLLRSFEQLRSRRTVPRGLYGDTRVSAFGPPPPGAPDVFRQPPRRKW
HVAGASAEMPLASAAPASAWAAPAEAPPQHAAYSHQQLQPQPMTTAQHRPQLLTAAGPPQSAGASPAPLNNVGIGHG
VRVPMAPGAAQPGPSPMAPPPARTGAARSGKRAVNHAGPRSKLAPSEANIERARRIAAAAAAAAARAAKLAAVQQHA
AAAVSAAVSRGRGRSASAGGTGPAGDQHGAEAAAGASSHAGPAAGRGRVAGPVVGRPGSPPPQHYPQGVRARPSASP
GRGIPPAPAVSPPRQHGAASSPGRSRIPSPPPGYQLNDHRHASPHRSPHRPPHQESPTRSSRSRSPGSPYLQQPPQP
HLHHHHGQQQHRDHHNHSERHQHQQHQYDQHQHMQERARAPVSYSSLDTVSHERDHRRPSPPRGYLGGRSPPRAAPA
DSTSTAYDATSYSSITGPPSSTGSPPVPLQDAGYARRQRLQALKDRRSRGAGAVLPGVLPAEASDLVNMVGYADQAA
AADPAWVRSASPSPMKRKAAASPPNQRRGASPQPRRSPGHARSPPARDEQGEAWPRPGPRSPEKGTQTEEAWPVGGR
GAHQGGEDDTRDPTDQGAAEGADTRRMRMADMVRRFREWDPEVESRRDAATEGEATLARGLGNAQAPQAAHSDTAGT
SSLNDEVGVAGPYCTGRAILPSLPSPGLYQPGFTPEGMFYMQGRGRSAPAEEGGVESLAAAVLQRSRNLLKERDVIQ
QRIQAALAKYSSVTGGSGAGAGTSSSGANAPGTGRRQSPSPEKPAVVREPGAMAAGLDRFGARLGAEVRLAGQLEGE
RPAIASPRGAERTPPPARASGGGDGPAGYGSQERPKSSGISVRQAPWRERLRALLAQAGVEPGPATSPGPAVAAVAT
GAGTAGANAATGSPSLLRQADAGAPEDPLEAWRKRRQRQQQPENAEPAGSGEVGAHLPGLSGARGALADWLAQVSG
SAPPRIIEGKAPSSFPRSSAQFDGAKPASSAAPAAAPAAPRASAVPAASAAPDAQAGDVPSVDDLLARLAQRFGLAP
GSLPGARSGATEVGPARLSDFQSLAAVSQGSGAELAAASAGGSSFSDRPVPQRETSLGQSASPPPRANRPPGSEHSH
SSVAHEHSSAGGKAPLATAARQASQASLSSSYELSDGVLGSPQSPQSLDGQHAGQHHAAEPQSQTQPQAAAAGAQAS
SFAPAAEREPSSRELAQEAEGESVGEAEGELVEQADGEPVEEVVVEDYEVDFESEHSDQSAASQGLEGSGGRQEAAG
AEDDDGVRMGLSGVLELQLSGGSVSMRSSGGAAAELQQEQSLGTGSSGGDVSGDVSYGGSSAHGPAAPQPGMQGGDG
ELLHSAGSAAAPQQLERLPSSRGSFRTCDNATPQQPGQASVTEAPAQQPSTEYVAFSRENSDQAASEQVVEEEVDYR

LDDSNDQLNMTLAPGRGLGLGRGLGLGGAAAGGAWPDWLSALGIPRPVDLAALPAEFRGSSESGGSGGARSSGGSGG
GGAGGNAEAGTGVGGAGGHDDAGADMHAQAFTATEPSGDAAAGESAWSRRSAAATRTSGGGVDVASGGSALRPDLIG
GALFSGNPDVGSSADLAVAGTAAGARRRWAVPDRAAAASSSYPAPGAAVDPLTASAFGSLLDQAVAGSLFADTPSPA
RIASGAGVGGTRAHTAISPASATDGATVAAAASAWSPEAPQYSGGGGYGVGHVDSSLESVGSGVPWALTSVELPFSD
LSPLRGQGRGGGGAHRGARLRGSGELTSSLASSGGGRLTAILQHTVTAALFGDSPSASMDGLEGLGSARSHGSQATA
DSPARRVGVSSGGGGVAPHPARTPSPLRNTGASPSEGGASPAAEWPRQQQLQQQQQGHQAHMQVDDSSSLTERVTRF
GRRRTTSDGGAQSPPLTATRIQATGEAASSNGAHDTGRENERATTDSGAASARRPDADVDAPDADQQHSEGELQPAT
QTPAVLSEDAEAGSAHQGPELDEDGQAEMEDDEEEEAEEVYEEVYEEDFAEPSPPSSEGSAASAALAALRPPGTGLS
ALGSAGSLSGRQSAGQREVGRPPSPSHRHHTGSTPTSTVQRHDPGTDQGPGSPFAAVGSTSGGAASRYAAAFNTADL
SGDVLLGLSGLGGGATDTGSRGDASTGDASYASSAAAGTDADGSERDGSRPAAAGARGSSRLGRQHPHRPSGAGGGP
GGGRRELQRQDSDTDPNMNSRMMDSGLPEALLDLLATGSGGVLLAGAGSGSGGGTGSGRASGQRRGEGTLGGAASAA
AVLAAGGAPEDGADAGDSGSLERVGELVVRTSPPSSGSQSEVSGEADEGPPPPVCRRPRGSQGSRRTPGSSEDGGGG
DHGSSQGQGAGYNDSYGEQEVEEDDMGTGGLPSVGFGIALDEAGVPYVVPLPYSLDLEDVLLRSSSPQPPPGAAAAA
AAASSSDPLFRSPAAMAAAAAAVLSGSSGSIPVAAMSGGGGRGGSGEGLVFVMEDPLEALLFGNSAASSGGYSGLLG
RRTPAVTITEINTSSDDSGDGGGDGDGDDGLSSKGNGDGAAHTAQPEASGSQPASAPSPASSGEVSEERSATAGGGA
YVGAGTAAPGAAPAASNPSFRRWTADRHPAATAAAAAFPGTAAPDSSVTLAPHHASTVAAAGGETGSAVAGAQPTAP
ASSTTGSIFAPRAEVHVADAGAGQGAGPSPLGLQAGRLPALKGRLPPISPADSAGLGSTSIPSVDGAGLSIATSMHH
APASAADMVARRSSSAPAPSPTAAAESEAAEVEASGSRAPTRRTLFSEGEARAAPSAAAQSETPARPAAPTGPTPMA
AFAGAAATDPAAAAVVTQPTAARASAPLVYTAAPAPRQVTPAGAAAQPLADAPAVAAAATGAHPAANTSPLPGSSAP
PMSTWTGHGAAAAAALPDNSTRPQPHLPATEQAAASRPARYVPAPVYPASFAMPATATSLAAPLPGLAPTVPAASGT
GMPGLHQHPASAAQVPSSWAHMPYTAAAAAGAHHAPRASAGVSEGLMVRPLATSGGSSDGLSRGLYGAGAGTYGSGT
SRAAGGAVVLQEEAGDDVIVAMLKRKMDACMDKLRSLERNLKSPPPRV

>Cre07.g333400.t1.3|PACid:27564586
MLLRSLNVLLCLALLGLQSRSNGARAASDDQRPCPFASWRAVIGVLSASTSCRGCDFDAAECVLRCKRCAFGWPAEV
DLRTCPTHGTLRINLTPLGLVCTTKGVKVSNSTRVSSPPPPLRPPSPPLSRPPPSPSPLPPVVASQPPFPPPPPPSP
RPPLPPSPRPSPPLLPPPPVQSLPPSPPPPSQAPPSPAPSPALVFGPSPPITEVFATIAQAEVAIAYGHMRPADDAW
SGSAADEAAETDVIALVQRVDNSTDFVQETDADPADPSLDLRTGDVVDVTVTQAVDPSTPIRRRVLLEAAAGTRRSL
QDLSSGIVTPGGRGRLSITSFKKTSSNGGKDFIVGDQPYNLTSLTFLMHMCGKNLTYTRDTVNRMFFNRFATTNNYT
LQRHHEVCSYNKLRFPPDTNLVFGPVSVPCSGTVAVTSNTDNRLTGVQYNYDFSTRCGNDERYGLWELAKRWLQTNQ
PSIYANIRFYKRKLLLMSEAPACGFRGMANSGCGSSHCISWIRSTPSLTDDIWSKQGLPDATTIFQELGHNIGLNHA
GRLYPAAGSDGSSFTVGAYDDMTDPMGGGYPDASRMTAGPVCLSAPQAYKAGWASPVAGDDTGVLPGDFELWDMDEG
VNYYFSLPSMHLGDKNLIRIRMSAAFSLTANAAPNNRLLQQAYFISLRQRQSTRGSFDHALRPAFDSRVAVHEYNGT
ASAKTDNPLVVTGLRQLLTAGIPTTTQVFGSAAFNISRVWTSPALPSKYSDPNIKGGWLTVGVERFTTATGIARVRL
CRTTRTLATITVEDCTAGDFDYNCDGLPDSQQDFCDNLINGAGSVRRRELIAAAEL

>Cre07.g324150.t1.3|PACid:27564598
MPQGRITAEEQTKEPRERCVELLRLLAAVDTFPRGGLCSGYYAVCAALRYLQVWRPSLVHLNQQDALPPLDVAWAWL
VARQQPIPYRALGSGNGPYATAFLFGVTSDRDRRCWAEAVAKAAAAAAADAEGVGSRAGLAGQAAWPPPPPVPGEQP
WAGISTELLQEAVELAEDMQRFSTLLRSWLRPQFLDTDFLVRAQFRYSQFLELHALNPHTVLVPTADIALMWHTHIA
LHGEYARSCGELFPAAPAPAPATITTTTTTTTMASATASAASDHAAPSQPPLPDSGTGGKCQGGEGSHSEGSHGEG
SHKVGYLFSPDYLRLRGIQLEAAFAETARLYEEQYGEPYLSYDVAWIPPGEVYPAADPAGPLSALLDVFNAVPAAAS
DAPATHLAQQVPSTRLQSPNAPKWHAAAAPRAGGHALFTLWLIARTLAEGPPFPRLSMRVGQTGVTGAAEADAAAAT
CCGCFGAPRRALPAERVLGLASVAQTGGSEAVGDVSAPKLSVLSDLFALAIGERIGLLYHMRLLPAWTEHPFLACIR
ERPGLWVPPRTVVVSPSQTSVDSTKRDGSRFAAVVVAAANDARPGGVGTTPAAPDRLVLRKEVQFEAADSEPDSPPP
AGGTAPLSPPPPPQDATLNRVWEPAPSACQGGRYAVTDCERFMDSTAPLGPQLAGLAEALGGPLSVEALPSSQSGRE
LVAGSRRLLGRRSETASRRPRSSSMRRARDAGGGGGSSTTDAGEATRPIQDLLRKQQWLVEVRRVVRGRAEEPFDPH
ARGLRYTPGSATLTEGAGLGLELPVNRSRDADGSVGAGGAETAGDRVADRRAVRSIADVYDLEVELQLVTAVAVVLP
AAVDTSGGRRDP

>Cre07.g346000.t1.2|PACid:27564654
MRPSGVRPRTGPYLGTLLLCIICWSSVAAGDSGGAAATVLDTNDTLVAGACTNNSLEALRLGVLSLNGSSDAGQLGA
LLQASWAGLSSIPVDIVPVGFGTAGWEAAVAGQGFTAWTSTPATAAAAAAASLDLPPAMLLSDPELVWLDLLPMLRS
GAVLYDGRLVALPYAVEAPLLLYRRDALERLGSGPAATWPELVALLQRLKQLDTQTQTQAQAQTGNSSSSSSSSLGS
NGTTAATTEGAAAASAVAAPPTWGFCNALPSGCDRDADLLLSIWASAALPALPPPTSRAELQRLLDSQLRDGLAAPA
LAWALGVYRQLLAAAPPGSGHGRCTRPRVEAALARCAVVLGPSSGIKAALWRLSQGSSSSSGAGNGSSSSAGNGTST
SGGGGGGGARRRLRHDGAPLELEDGEGGAGVALTTMLPGSVLAADPVTGALRPCAGVGQAVGSSSGSSSGSSSSSSG

SGVYLACRHLVVLPGLQATEPDRVVMGVSPQPLAFTAVPDSPALPLAAAATATAAAAAAYTNSTSTGRRLLAAEAPA
AAASSAAAAAPPGVAPSVAVYLGNEVLALHINASAPPPQQYAAWRLLAAALSPGAVWPAVVAPTEGGATVTRRSQLR
DVDRWEAAGYSRADITAYLQALSATLDGNPLTFTALRDLPPATRDALYGAFAEAANRAAGFATPAASASASGMPAAA
AAAASGADATSDGGGLDDTTAIAKETLRAIAALYTARRAEVTQGFIRSLHLSFPPAAASDGGDAGGGGGSAGVLRGA
ALAGLIVALTVVGVVAVTLLLLRARRWALLQDLRKKLRFLDRASAAPGYGPDTTLMLTDVQDSTQLWELLPASVMDA
ALSLHNGAFRQLLPHYRGYECHTEGDSFVLAFHTAVEAAAFALEIQIILISLDWPQELLDTELCGMVKNTAATAAAA
VAAAAAAASTAPPGAGGGGYLLSTAGGQRTYDQYGLASVGHGSGGDGGAAVAANSALASSLVAMHFPGVVLGTGAGGG
GGGGSAFASNDGDRRMGGGSSNGGGGGACGMDGDGGGGAFLDSNGGLLSSHSAAVVARAGGGGGGGGYAELDGGEAA
VAAAAAAAALDEPWGLQAVDEERADSDEHLADVFLASSRVTGGGGGDGGGIPGSTGATAAAGAAAAAGTFGMSRRLV
RRSRTFSTMQALMAAVQERPLWSGGGGAAAVTGGGGTGGHPMQGIRRSGGIARAPPPSADVTAWGGGGGGGGGFLMS
ADGYRAGGGGAATTDGAWPLHSPPGNWRQRGPSPPLALMGPRPHPHQQHYHQVVQPGPGGVGSPSAGLPHGPLGFRA
PRSTGGGSSDHGNGSRHVNGIEMGHLPASPTSPHRMLGGSGRGSGVGSGASPRGATAALLPEARSSLTSAAAPAPAP
APAPAGPGPGSGLLPPGAVRQLSFVKPPPLPPLRVPGSGSVTGWESGLPRSGGGAHAVTPRSLGSGSEGPGTTVAAV
AVAALGSGASASPTPRASAPGSSSTGAFRPVRSTGSLAAAVVAGAHSDAAAAPGDFGGGGGGGGYERPSADSSRSSA
NRALEEASLRLYPVPMHAFGSVTSPASAAASSRGGGAAAAGGAAGAGGSSGANARGVGSLRSLPSGGAAPAGSSSMD
TSSARLLQLGGRSTDISGSGASRSLRLLAAGRASRRSSTGAATPPFTGLLATVRVSNTSAAAAEDDNGWDCDIAGGA
TAGVGLRGGVEFMLGMGGGAGLRGGALGTIVDMQLEEEAAGRVADESNAAAMDTAAATAAALVRGRSGRSPPPPRGM
GSGGGSEGAPVATAAATIMRPLLADSPLIVVGDPAKEARRRRRRRALKAARSDDGSGGDVDGDGSSGAGDGTDGGFC
DASTRSSTFDGGANGAAASGAAGGVSSGGGLSGGGGGGGGHALLAAAHRIILGGALDGWQGPGAAVTRSAAAAMQHA
ATGSTGSDSITTDGTASTASMDLPFSSTALDAVQARRSSRLTDWQPPQQASTAAAAAAAAAAAAAAQQSPKSGGRRS
LHLGDGGADGPAVMGSAGAATSRRAASGSAIAVPRFLTAASGLPAGGGGAGGTGGVRRSMTGTRTESGGGMVGSMHG
GGTASPSASNHPLVTLGSNVVHISAATNEGWGSSAGNAAAAAGGGGSSSLRTGRSSLTGFGGFTCGGGGAALYPHLE
GSTPGSSVRGYDTGQSSQVQGWDAAEAPAADGHGGGDLRGGRSAGGRSATAGAGASPAGAAGGGLWGTDARRRLLHV
VPPPLLLSMDTDSIGVCADDTEGLEDTGAEDLEAGGSADTARHGSGAAPAAAVAADAAVPSAITAPLASDGDVGGNF
GGDGNGDGDGDGGESGDAGAVLSPFVRSAGSDHGAESDSGEGGGRAAAAATAGLRSSPSWRRGVSRSASVNNIPSGA
AAAAVAAGGAAAASSGGAGTGSGFTGFASVSVSGAPALAAPPAALRGRSNTSLLPRRQRALTEYVQSPLGRSASKGP
TAPWEQGPEEGESSEPYHFGFGGGGGGGGSNACGGGGGGDRGSAGVSSAASGNLASRVTSVAGTGKILRAGSIDGSG
GVDSSSSRLKRLGSGSHGMRSGPARALAIAAGIFGFGESKSTGAAAGAGAAGTGAAALANLRATLEQQVGNDAAGSS
FPGVAVRPLSRSGSGLQYSGRIPALARVGNSPGLRGGVDIDGRRHSVGSASRLASPSAAAAVAAALPRHSSSGAPST
PAGDRPSSSSNISNSVRVRLLQATVGIDGGSGGGGGGSPRRFSRRRSSTHSDPGGPVPLPAGSGAATDGGPGTSGGT
SQVGGPVGLADAGGGGAAAAATAAAAAFAQANRYRTPASLAGADAGFTGPGGGRYDRSSAAAAVAARRHRLPARSAT
PPPRLPGTAGGVGPFGAAGLGSVAGAGGLNYANPTYASTSRAGIAGATTSHLPTIAAGSAYGGMSSTGYGGVDGAGA
PWGSGVVVGSGRMLAGLDALAEAYSTAAAQQAAAAAAVNGGGGGSAAAGSQPASGRTTPAGGSRWSLAARAARTSVS
QSLAAALHGAAGSAAGSAGQHDSRMLLDGRCSDGRVFNPLYNAGGTNPGGPGYGYGYGSVHGAGPGSTAAGTSTALL
TQYGRDSTAYDAMGASAALLSLHGIEVGNASNGNNPNGPGGGGGGGSQQLIGGSNTGALAPPLVQTVAAPSSGSRPRD
SGLLAPYPGVSRDLPGAGRDSNTGRLLTALGTAFRSKRASRGGWAGQDSGGVGASSRRLFGSRAGSGALGGGGGGGG
PESMGASSRDGGGAAAAAAAAAAAAGPATFLEAMLQMATGAVGSPAGSKASYATNAHISPSYGPQHVQPQQLLSACV
LPSTDEAAGVRVMSGGTAAAASNTVGGASGGENAAAGAALANRRSTSGGRNRLRRSITPLGLLLGTFGTGTSGASGV
DGSGGSIITGGGGTCAGGLGRSAVGGFRSVRGLHRFIPWGSGASRDGGHRAESACSNDVAEHTVPSGGGKPRQSLAS
PLQYALSGNSVIDAPRLRGSTAAVSSPRSRHALLAAAAQRAAGGGGVVSVGGFPSVFTAHGVGPMSTPGQDSATGEA
GSSVLGDTRTDSHPSPMPRLAPFSGAATIHGAATTHGTSTDTETLGGTPRDGQYGAASIQPAPSAPMPLTSASAAGQ
SPQRSPYAPGGAFIPGQRFGTGIVTPTGGYARSASVDAPPPWLTHPSAPSAAAAAAVPSAAAAAAAAAGAASDARTPR
TSAPARSYTFTYGQPPQRQSLSLLRQGLVSGFPLNGGGGSGGGSGGGSGSGGTVLHLSPRLSSRLELEPVEEGVPAA
AASLARGGGAAAGSASVSGGIAASAGAAATVVTHLMLVAALQSTSPCGDSHDGRSSGFASSGAAVAAAATAATTARS
SAHQTAAGAVLPMYVEPDLGHSPPADADGRGGSDAAAGSAAAAARSSTPLDTAPASFLHNATTDHELATAAGANRRA
GNQAAVTAAAAAAAAAGDGDGGGGGRVGSPASSIYGEPAAVPQLGPAPGGGAFTPVSGVGGRRGRVSWAGAGGGALR
TSMLGRSPSQSPPGTAEPSLASPGVSAAAAGVAAGAAGAAAADTGGMLYLLGVGVKMAAPSSLTSDVDLTPRPSGGG
DGGGGSESALQLSCDDGTPPHPQPPQPRRSVSGIPATAAQLPIAAAVSAGGTAEPGPASPAVAPVRSATSVHLQVLR
EGGAVVALPDAAASRGGSGVSGGVYGGGGGVPSARPVHMSSLMNRAMAAPGSASGGAAGSAIRSLGGGGGGCGGGSS
RTGGSGAATSTSGGLITADSLEGAPRAGSARSGSMLLPSHGPSGRRSSSPIVLPSRAAAQTHSAGLPRQAALSVLAA
AAAGGGAGTGTATATLSTGSSAIPWPASYSGFPTGPSPPPLQQQQQQLLTSRAPGPAVSVSLMPLSQLPQHLPNAL
QPLQAQQGARGFTHRSGASSQSRPRVSPQSAQARLGGSARGPGVLSDVATTPAAAAAAAAAAAGGGGATWDVDLGGV
WSGVPASEDPLLDEADVLSSMGAAGAAPLTHSYHTISATATAAAAAARSGHTSFTVMGAGGGGGAGIAVGTGSGVIS
GAGGGGYGGGYGGDGYGGGGSTAGGCLLASGLSISVSRVALPAALTLTAPSPLATATAAVLEACASGAPAHGGPGTP
RGGLTSRRTSLQSVRRGVTQDNLLAGLGRRLDSVAAVSGSGAGSLLGGGGGGGGGDMPAWDAAARESYRLRGAGAGV
GIAGGAGGGGGGGGAGARVAGMVHRLAGGSKKGLYGPAGAVSDFPICFRGFRVRCGLHSGLEPGRDVFYTKVTGRRG
YAGPAMALAKTVSDLVPGGMVVMTAAAFARLQPWPSPDALPGAIVWSRGRYRVNVGEGVGAASVEVDLFQLLCPQLL

ARQPHLEARPWRGAEQVLPGVLAAPLGRLALVQVQAAGVQVLSAWDVDVARASLQVFAGVATEVLPAWGGFPAALDM
QAGSLLAAFREPALALGFLHALIDVARDAAWPEELLQHELGEPLAIPPPQASGGATDGRMSPLPPGWGSSQVSPPPA
FRGLRLRCAAEVANVRVDLGCATAAALYEARDAKAFKSLRRMTRLAHMGEVLCSGRLLAEVLAGPPAALAALHELLA
FSPITPAAGGVPERGGGFGGGGGKDVDTSTRGLAVPVSPLPQRYSVYAAAAQQSGRLSPQPAPHALLRTPQPSGRLT
SPPSATLMGLAAAAAASGHGMAAAAADGDVNGDGGLHGLASPLFGAGSGAVTPPLSPPAPTPTPAGGGAGATATTGY
GMAAAAAAVAPAPHRLTGKEASRAAVYLCTRRRQVRRSGMGDAAFAMVGSVGGVYGNGMYGASAALYGGQSNASGGG
AAALAAAAGSNAGMGVHGAAFASNYSAGASSHAGGR

>Cre07.g324500.t1.2|PACid:27564666
MSSSAEEGSDVTDRYLQTDDGAFYRVDFCAGLELSEIPSGARATVFYTSLANGVLSSCQAPRMEGAGGRRSMFGEDI
TTPTEPKFLVYLVTLCNVPVPAAATPQAVLDMFNGGGSYTGASLSGYYDTCSRRQVRVLPSNVKVVGPIRIPCSGVL
DNPIRFASGNAFDTGSCGNDNQLKWQFYLDQLMSAAPYNINPLEYHHKIMVLPTGFSNYLKNCNNIVVGTATVGPWF
RGSPTSGSNTWGTGLVWLDGGAFDIGALFHEVGHTLGLAHANVPGGCGGFDQCDSSCPMGSASKQCLNAPHAWQLGW
LQPARSLADADVFARPTQVALPLQQASNNSYAMVSLTSTGSTPNNQLFVAARTNSAPYDVMFDSKLDGKTMVLVHSY
NGTAVSSYQRTFALATLDVGQRYVHPDSNVVVSYTSGGSDGGAVVTLCRRSGATEAVCGDGLDDDCDGLPDESDPDC
QGRMNGNSRSPPPPRVLGGGLNVQNGAVRPPVPRASPRPPPGPPPGPPRPRPSPPPSPRRRPSPPPAPLPPSPAPPAL
SNTNDDFWWMGGVGSNDPKTSSPSSNKAPPKGVALDTAPPPPPPPPPPPPPDAPPPRKPGKRRQVLLPNAPDSPLSP
PNLPPDAPSNPPPPAKRVARPPPFPDIVRGSGGTWDDWTSSGRRLLRRR

>Cre07.g356500.t1.3|PACid:27564753
MRSNATAWRHALLAVLLSLNALVVAGEAQCGNASAAVGICGCVGLTNSSTAPHILVRAGLRPSLPLLATLAREHSAR
ALQPNPDERLAVVDVHARAGKGPGSSGGGSSSGLQEELAELLQDVGWAAAAAAAAGGTNVDGSQQGSGAAGASTWAG
AALDDSWALDAALAPTAQTPLPAAAARRQAIGLESSTHAAGRQEQAPAAGASARRSAAEAVGAAGTGSGGGTAATVP
AGWGWSSRVLGTAEYAVRPLLRSHAAGNTSWLQQLNASAAAGAELPAELLTPRLGSADGPAAAPLLLLYRRDWWQRL
VATGGVGSNSTGSNGPDATAAEQLLPPTWDLLVQLLSALRNSDLDGDDKADHVLCADLQPGCKGWALLAAVYASLAQ
THGTQQGIWFNATDLSPAVGGPAMQAALQTYAALAASNAAPFTPGGAAVSLSNVSVSTEELLRGGGGELPTDGSAPA
CGAVNPLFAAGRCLFTIDWATAVLRLTRAHTQGMSGQLGVAPLPGSAYVVPADTASSKQAGGAAASSSALLPCNASL
CPHAAAMPDAALQLVTALTAPPSGSASPGPPQPQPQPAQPPPLVNRAPLLGEASNVWVLAAWQAADDLIRLDDYAGL
VAYQLGLKYASLLQPLRRPSGNNSTSSSGSGTANSGGASGGTGLAALTISLEADAVVAAGPSGLAPGPAGGSQLVAL
DLEAEDVAAALRAVDACLRHPNAAVDVQLPFSSQYRLALDDLAAAWLAQSSAAAAVPAATASGSSRALAQAAGSGSG
GTDVLLEVVVEAFANVTNAFPYPAILLRLYWQATGFTPRSRAGDSGSSSSDRAMVVPLAASLGAVGGAVVVCAAVTA
LLLWRRRDWRRRQAAELQQGKGLQPPGPGPATTLAVTDVQNSTLLWEVLSAELMDECLATHHRIMREAIELYCGYEV
FTEGDSFAVAFHGPDDGVGFALYVQMALLEADWPAELLEQPDGCEVWARRNTHLLQPPPHLHLAPLLAPTGTPAPGS
PSVTAGLHTGGGGGRGHSRAPSASGYENPFAARLTAGSKRLRQLLTGAASRSHSYLVPSSAPGTGLGLGFGGSPRAG
GSAFFSPVASMLLLDGSGAGGSGHSSTAAGAAAPPAAAEAAAALAAGRPPLPGPFGSSRLARQQRNSYSDYWPTQAA
HGLGSPRLATHTGAAAGAAGGAAVVLGGGGWPDGGGGGISASGGAWGNAGAGGGAGQMGRVWSRAHTARRSFDVGAS
SLHEAAGGLEWQSRRNGSPAHGSARLLSEAGLGLGLEQAGRHRSASTQALPNLAHGWRGLLAAADAQAAAAAGTGDG
NNGGGTRAGPVALDSPAASVAVRSYRPHGAVSSPARAGWPGSGASAHSAIAAPAFPPGGAASASHLLTTNALAPARP
GPLVPFGRSVSFGHYDGDGSGASEVVIHNDLFEGREGELEAGQPGETCAAACEDAALPARRGACSMAVRGAGRPSVV
MEACEEEGGAADAAGAGAGAGANAPAALPGTAAGKSWVGRKGALVGDRRGSGSWRQVVQRVMSPVLLAAGASARSIG
SPSSSRSLGSEAAAAPAQQQKQQLQQQYPVSAGGSAAEAGAAAPGLLARQQRQGPSQGAGGTTVQGAGAGSMGSGGG
GTGGSSYSMPLPPQMLLPSLSHAAAAAGILAPGGPGARTHLANPNPVGYVRTPLSRTSTLDTPRPGGAGAGAGVGSA
GQQLPPSGSLLRTGSSGAWGGGIMVSGGAGGVSGSRWAHGGMLHSGSLGTFGGGAPGAASGGAAGMGGSSSLGSFGP
QYVHRAPYAGAPAGVPPRPRPLHALQAPTSAPGFGLNSLERAGNPVSSMGRGGSLEPPLQAVSGPDGGGGSSGQVSS
LNTPSSPGAVEYSAQAAPGASGLLLPPWHDPRHRGGGGSSSALLHHQQKLQQQHRSVRSGADGSVGLVSSERDSPGS
IGCYIGVSSAAGLSPGGADGGCNSTSQLLMCSSAGSSARVTPSWGLVMGPAAAGNGLVAAATAAADHSHSSSSALVL
LGPSATRLLSGDLGSVLEGNEGEAGDGGGGAEPRNERLVSPAAMASAPAPSAIAAGAAGTGAAGAVARAPGGGNTGA
APAAACPSPIHGVLVEEARSEVEGSMARIGYASPGEVTAELSASSEQALSEGAFEVSLRRQPWRQGSGSNSNTASTN
NSTRYPASSMHSPPPGQLQVHMQTPGSEDTVVAAIASAGPAGEEAAHGDADSQFSLGRGLRLNLRALVEQAEAEQAA
AADGGGAGQQARTSSDGGGAGGGSGSGALAADGADTPHLTTVPAWSRLMAGGADLDADEAAAVWKGAAEEAVGEAPA
QSPHTAAPSATAVAEINVGEMAVKAPYKPGPAAAAAAAAVEVQVQALGPAASAPASPTVPGERSPSLPGLPPPPPPG
LVNALRLSTAAAPQPQPQPQPQLLQRSRLSSAGGRADARSGGHAAGSVPARMSLPDQAVQVPQRHSGARMPPQLRRS
SSSLESSLSARQLLAAQAAAAAAAAATAAAATAAAADAAPSPPPSRQSATGGQTRAVLRVSPKGAAYSAVQSASAS
PAGSPSRQRAAGLPPPAPITSQRAADPGVLPEPQPVELPPQGTGADDGVDLQGGDAEAAAAAHDCQLGGTLSGSGSS
GPREIVTSDRTPRVKRLGSLINASLAEAAGAVPSSAPPLGSWSPHPGAQPQHSLEALCGGAGDAANGATAQPAHSPQ
MDDNGDCGTSPWDVTAAQMGLVSGHDGGGYVPRAGAQAPAPQVASRSRRWQTRASALPPLPPITSGAEFLPSRPSSN
LAPLDMSPPVPQQPQPQPQPLSRQINTSARAARDCKAMGVDGHQGPEVPRAEAAAERTEADEAPGIDSDLLRWRDGEPQ

SRVGRDSTGGFLGDLEPGPVEGASNSRGRRGSSGSGAGAVTAAGGGGPTAPLQPLPPSAAGAVAPDAAAVNVPAPPP
ARDEAAVASPQAAKKQAASHRAATAAAAATPQLRASPSGRRQSDAASCPPARREALPRSLSSAAQQATQQLAEVERR
RQLRQQHEPQCQAHGKEQGAGTSGSRSGRASRDIASKAEAQSLEGKAAAAAAAADITDGRRFLPPALTPPPAVATAT
AATAAAALVQASAASGSSRGPASSSSSRYSGLAGLAGLEGLEDALGPLMEWRHVVEWCVDADDMAEEEEDEAEVDEE
EDAMVRSEAGDGFGVEQADHSGLGGRGGGSTKLSTLQLHVQGLRHRRNAAGVHGSGSHNAVFGPTSTRGGGAARRTM
DGGPPGRVGARSTVITGAGGGRHPPPGQPPPPPPGQQPQQPARRTTLDGQPLHMPLPLPLLPAFGLSGLLSGGAGSG
LSALKRAATTITDKLGVGQAARGTTGAAAVGGASPLASTGADNNASSGDNAIAPGAAAAPAAAAAGGRFGLLRGQLQ
RHLTLTLPDGEEPPTAVRSPGSGGGAGGLPLRSPAGAVAAPMLRSSGSTPGGGTPGGAVVGRAGGSATDGVGHWAMG
GGEEDGPPSNRSGGIGGGALTLESRRGTVAAAGQLLSRLGAAVTAAVKQLTPTHAQAPPQTPADPTPQQHGISATDP
FKRTTSTGLATAEERPSGGSHRGTAAAAAAVLRRVLGGGGSTPPASPQGNTLPSSSPLGSVTRSSLSSHTKESKRGQ
QPGDASAAAHADANGGPGTGWSLGVADIETGLLADGGPPGDMGAAVSLGVRAATATAAFGRRIPRDTEPAAVALAPA
PAPPPPSAASYASTTGAEAAAMAAVRASAAGHLPESGRVAGSLCLTPMWMRLAPYTVPAAAQHDDRGREWAQAGQVA
PRPDVAEEAAAVALAAQGGVTVGHSRRFPKMDTDADADADADAYGEADPQVLLPPTGRAFGSVVMLPVAGSSSAWER
VTSSTRRSTSLAGMAPPALLAMGSGRANTNTLSQSGGLSRSSMPRVSGMGGATAAGSGADGDGGGAAGGAAGLLLG
TTGHGTGSRLGFSALSINPGGPGWSRLSRHPTSNLDTTTNNLTAAAAAALATTTEPPSPALQGGYTMLSGYSQAGYS
LAGYSNAGYSHGGGLVGGGGGLMLGDGRESNARQGLFPQVHTSNLAGMLRRNSVTGALAGLPPGVSAGGLGLGGLRS
SFADLGGLMGGVGGVAGAEGVFGFLWRDLNAATSHMAAAAATYAADGALSAGMGVRAASGWLSSVATTVASALQLLY
DRLSPGEVLLAHVAAGPRRSLGAGVGAAGEGSGHPAAPGTAAGTPSHAGGAGAAGASCGGGGVEPELVLRGLRVRVG
LHSGPGEAEVVMEVVEGALVARYVGDFLAVAKEVSDSAIGGMVVLSGAAFRAYQQQLRRRERHGGGGGMGLREGVMV
LHVGDHVIKEPHGGAAAGHDSTIANAFADAESSPVVSGARNISRTQRQSLSRTHSGCLQASPIIAAAPRAASMSRLG
LSDAGCARELYAAVCPAMVARLALLPSPVRTHEEVALGCLSAPAGLVAPVFCNVHGVETLLAWEKVLQERFMRSTTP
HIATPAAVTLQGYEGGADGRASAGGASSLAAARGGVHSSAAGPAAARACVVQVALAMLRDAATAAARRHGGYVVASS
ADGGHWVLVFGCAEAAVGWGLDMLQAMLTAEWPDGFLEHELTEEAWEGGRLVKRGLRLRIGVDFGRAMVRLVPRSGR
LDYVGRPMNRAARIAAKAKAASLLVSDAAWSAARPTLGSGVAATNLGTMQLKGVKEQLELWALRACSAAEQPATHSG
LAGAETQ

>Cre07.g343350.t1.3|PACid:27564764
MGGKRKQPAEPLPGQQKLTSFFGVTASNGRSAGGTASATLAVSGTAASPAPSISAAHTAQAAQVSGVAAPSPSRQHR
PGMSAAASAAPGGSAAAPQAVHSPGRAAHPGVVAVSPGRGAQAASSLTAAHRPAGAAPAPAHTPPPPKQPIAQLPLH
HPPHDGAIPMTSAAAPPASPQRSAMQQQQQQRPARSGLPPGACPAQQLLPGAPPPKKPPAGTAPSTQSGRGKAKTAA
TGAATATTTALDSSGGAAAQQPQQAVPAPSQSARRRGAASSGGVAGGGSSSAAAARAAASAAAGAGAGGVWGTAGAA
AAAVAAVHPYHIKYEVGAAAARRVRVLYGIADAGDPETAHNRYQRLTSQLAPPAVDAGGDVAGGGRGRGGGGGGGGG
GGGGGGGGVGAKYAPGGPTGPAGHHQQQQQQHHHHNHQHQHQHQHQQHHHHQQQQQDHYHHQQQQQQQPSSSAATHA
ASGSAPALRSHAERSPFGGDTASSPPRWARHQQDAAMLGAVLPRMTPGTGPGTGVGWGVGGSANAAWRTAQSGCGSV
EPPHCGGGGGGVVATSTAAPPDGEEALWAQPPPSRTPPPPQLQHQLLHQPRGPAHHQQPAASGQPHHHQLTPPSQWQ
QQQPRRQQQQQQDWQQQQQQQQQPPQQQDWQHHHQLWNRQQQPTQQPPSQQHWPQPQPQPQPKSGQGPRQGAWQQQA
PGGQGLQPAPGQQGQAPGQGQGQGSWSQQLQQPGTLRPLQQAWHGHGPTPPPLQGHQQAWQEHGPSPPQQQPWHGHG
PSPPPPQPQQQQAWQGQGPSPPPPQMQPQQQQAWQGHGPSPPPPPQPQQQQTWQGHGPSPPPPPPPPQQQQAWQGQG
RGPTPPPPQQQQAWQGQGRGPTPPPPQQQQPWQGQEQGRGQHRVPMGAKAAQAGPWQAPSPQHHPTAAGQGQGQGLV
QGLVQGQGAAAWPGQQQGQRWQQPAMQGPAAVAAAAAAAGSAPPQGQGTWPQQGPAGQGPHPGAWQPQQHHHHNHQQQ
LQQPWPGAAAPGPRPAEWQQPQPRFQQPPRNGPPAAAVAAAAAAAAAGPSSSSWQAPAAVATAAAAGTAAAATAAPGT
AAVGTAAAGRGAGAGAGAGSVGCGGGASGGAGAAANATGGGGGAGGIAPWRRDLNESQLQAVTGDLERAQLIIAGAG
TGKTTTLIKRILYLIQEKRVPPRHILCITFTNKAAAEVRDRLDRAGVDLAGLIAATFHSWAYGLLRTYHRAAGFAQC
PTVWKDADLLRAVALAIRLSSLERGRGDMAAWLGLDGGAEPATWAQVLAAAQAQHPDICSSCAEQARQLEAKQQKRS
KKKKRDAAEDAAGAAAAGGGPDASPAGAEAAACAGAAESEGTGAGAGRGGRGKGRGGGGRGRGRGGRGKAGGREPDG
GGRAGGGGSETAGAAGASYGEASPGGGGRAGAGGGKRQRTASAAAATSTAATPAAAWHHQQPLSQQQHQHQQTPTKP
VGPEGHERHGAAAGATADAAGAEAQPPQPQPPQQPQPPQQQQPPQQQPLEERLLYKTEFISGLYARLQAIHAPTATE
KLGAKPKPATVKEELGWIDFFKRQTVDNISQYPGKQMLFETYTRILRDANAIDFNDMMLLLVQLIRERPGVLQRLQD
RHRFLLVDEYQDCSLMQVALVSEVAGTHGHVTAVGDDAQSIYRFRGAAPGVFGLFKDIAERLALSAGRPREAALQQV
VLELNYRSTPRILSVGQFVLQAEGGAPAATAKTLRATKPDTGNKVVHVDAGLPEEEAEWIAAEVERLNKEELVPYGG
MAVLYRCFKLAGAKTHAPLQEALRHRGIPFRLVRDTSVFERREVQDLLAYLRLALNPADDGAFLRVYNTPPRRLGDA
KAAFITRLTELQETLDRRRKELQRRRGQPQPQRQPQPQRHGEHHAGRGEPGPAAEPPAAAAAAASSVRPPEHSLYGL
CAVLLSGGPAAVAAAAGADGGGGGGGREGGAEAAVWEALGGGGAAAALTAAHRRALEGFVEVVESLRRDIWQLGPAG
ALVRVVELTRYVKWWSELKTNKEKAAADTAMVAAASPGGGRAARTAAAAVHRAVAAQGSDLEYTDDDEEDEVQQAGP
ADAAQPPPTPGSPGGTPGTPRRQQRTARRRPPAAEGAGGADGGDGDEDDDDGDGDENDEDNDEGNEDDDEEEEEDAF
GQGEANCEPLPRGLAPEPRALPHPEWRRVLGLRVLFKEAQVFGRRWRLHNEAPFPPTAADSGDDNTEQQPGPAQEEQ
QQQQQQAQGHPMPPGPQQPQQWPPPLVPLHAPRPQAPPQPFGATPAHVPGQGQGQGPVGAAAAAPTEESGDGGGEGE

GLQVPDLFRIATRALLNSYTGEEVQAALGCVCPHTSSPRVMPFVLEQVFGEHGRGPLALRDFLAHVALSDKDVESGG
HDGAAAGAAAAKRSAVTVSTIHAAKGLEFPAVFVARWSQGYLPTLYRPDKKRGKNMSEADRAAFEEQDAQEHTEEER
RLAHVAVTRAMERLYITSIRVGQMFGEPILVPPSSISLPADPAVVERRVLPVSNKERKLAERAEEIAERRVNGLG

>Cre07.g343350.t2.1|PACid:27564765
MGGKRKQPAEPLPGQQKLTSFFGVTASNGRSAGGTASATLAVSGTAASPAPSISAAHTAQAAQVSGVAAPSPSRQHR
PGMSAAASAAPGGSAAAPQAVHSPGRAAHPGVVAVSPGRGAQAASSLTAAHRPAGAAPAPAHTPPPPKQPIAQLPLH
HPPHDGAIPMTSAAAPPASPQRSAMQQQQQQRPARSGLPPGACPAQQLLPGAPPPKKPPAGTAPSTQSGRGKAKTAA
TGAATATTTALDSSGGAAAQQPQQAVPAPSQSARRRGAASSGGVAGGGSSSAAAARAAASAAAGAGAGGVWGTAGAA
AAAVAAVHPYHIKYEVGAAAARRVRVLYGIADAGDPETAHNRYQRLTSQLAPPAVDAGGDVAGGGRGRGGGGGGGGG
GGGGGGGGVGAKYAPGGPTGPAGHHQQQQQHHHHNHQHQHQHQHQQHHHHQQQQQDHYHHQQQQQQQPSSSAATHA
ASGSAPALRSHAERSPFGGDTASSPPRWARHQQDAAMLGAVLPRMTPGTGPGTGVGWGVGGSANAAWRTAQSGCGSV
EPPHCGGGGGGVVATSTAAPPDGEEALWAQPPPSRTPPPPQLQHQLLHQPRGPAHHQQPAASGQPHHHQLTPPSQWQ
QQQPRRQQQQQQDWQQQQQQQQQPPQQQDWQHHHQLWNRQQQPTQQPPSQQHWPQPQPQPQPKSGQGPRQGAWQQQA
PGGQGLQPAPGQQGQAPGQGQGQGSWSQQLQQPGTLRPLQQAWHGHGPTPPPLQGHQQAWQEHGPSPPQQQPWHGHG
PSPPPPQPQQQQAWQGQGPSPPPPQMQPQQQQAWQGHGPSPPPPPQPQQQQTWQGHGPSPPPPPPPPQQQQAWQGQG
RGPTPPPPQQQQAWQGQGRGPTPPPPQQQQPWQGQEQGRGQHRVPMGAKAAQAGPWQAPSPQHHPTAAGQGQGQGLV
QGLVQGQGAAAWPGQQQGQRWQQPAMQGPAAVAAAAAAGSAPPQGQGTWPQQGPAGQGPHPGAWQPQQHHHHNHQQQ
LQQPWPGAAAPGPRPAEWQQPQPRFQQPPRNGPPAAAVAAAAAAAAGPSSSSWQAPAAVATAAAAGTAAAATAAPGT
AAVGTAAAGRGAGAGAGAGSVGCGGGASGGAGAAANATGGGGGAGGIAPWRRDLNESQLQAVTGDLERAQLIIAGAG
TGKTTTLIKRILYLIQEKRVPPRHILCITFTNKAAAEVRDRLDRAGVDLAGLIAATFHSWAYGLLRTYHRAAGFAQC
PTVWKDADLLRAVALAIRLSSLERGRGDMAAWLGLDGGAEPATWAQVLAAAQAQHPDICSSCAEQARQLEAKQQKRS
KKKKRDAAEDAAGAAAAGGGPDASPAGAEAAACAGAAESEGTGAGAGRGGRGKGRGGGGRGRGRGGRGKAGGREPDG
GGRAGGGGSETAGAAGASYGEASPGGGGRAGAGGGKRQRTASAAAATSTAATPAAAWHHQQPLSQQQHQHQQTPTKP
VGPEGHERHGAAAGATADAAGAEAQPPQPQPPQQPQPPQQQQPPQQQPLEERLLYKTEFISGLYARLQAIHAPTATE
KLGAKPKPATVKEELGWIDFFKRQTVDNISQYPGKQMLFETYTRILRDANAIDFNDMMLLLVQLIRERPGVLQRLQD
RHRFLLVDEYQDCSLMQVALVSEVAGTHGHVTAVGDDAQSIYRFRGAAPGVFGLFKDIAERLALSAGRPREAALQQV
VLELNYRSTPRILSVGQFVLQAEGGAPAATAKTLRATKPDTGNKVVHVDAGLPEEEAEWIAAEVERLNKEELVPYGG
MAVLYRCFKLAGAKTHAPLQEALRHRGIPFRLVRDTSVFERREVQDLLAYLRLALNPADDGAFLRVYNTPPRRLGDA
KAAFITRLTELQETLDRRRKELQRRRGQPQPQRQPQPQRHGEHHAGRGEPGPAAEPPAAAAAAASSVRPPEHSLYGL
CAVLLSGGPAAVAAAAGADGGGGGGGREGGAEAAVWEALGGGGAAAALTAAHRRALEGFVEVVESLRRDIWQLGPAG
ALVRVVELTRYVKWWSELKTNKEKAAADTAMVAAASPGGGRAARTAAAAVHRAVAAQGSDLEYTDDDEEDEVQAGPA
DAAQPPPTPGSPGGTPGTPRRQQRTARRRPPAAEGAGGADGGDGDEDDDDGDGDENDEDNDEGNEDDDEEEEEDAFG
QGEANCEPLPRGLAPEPRALPHPEWRRVLGLRVLFKEAQVFGRRWRLHNEAPFPPTAADSGDDNTEQQPGPAQEEQQ
QQQQQAQGHPMPPGPQQPQQWPPPLVPLHAPRPQAPPQPFGATPAHVPGQGQGQGPVGAAAAAPTEESGDGGGEGEG
LQVPDLFRIATRALLNSYTGEEVQAALGCVCPHTSSPRVMPFVLEQVFGEHGRGPLALRDFLAHVALSDKDVESGGH
DGAAAGAAAAKRSAVTVSTIHAAKGLEFPAVFVARWSQGYLPTLYRPDKKRGKNMSEADRAAFEEQDAQEHTEEERR
LAHVAVTRAMERLYITSIRVGQMFGEPILVPPSSISLPADPAVVERRVLPVSNKERKLAERAEEIAERRVNGLG

>g11650.t1|PACid:27564996
MYAPRPPPPWPPPPSPPPRQLPPRPPPRPSPPVRLNTSGGAIGGVILTPTGSPYGTYAPPGGYYGGPVPPVPHNASG
DGDGGSGGGSGQYGASGEGAGGSGGGGSGADPDHNEENEQPEGGGAWDNEPPSDMPPAVYGGEPPPPPPDLASLAGA
GRRRRRRVAQETGNGSAASPSSSDSSSSGAAGGGAEVDAEADLASALLPLLPNLRLGAGASLHLSFSAVAVRCSLLQ
TLWARQQAYGWGSSSSSSSSSSGSSSSGAVVGLNVTDDDNLIRFAFPRAYVRTWRRGRLTAEELTLTCERSDDFSAA
ACAADRAATDRTVVWDAADGTALLAALAAISTGATPTATAATAATAGAVSSQAPDCRSHAAHVIRLAAGGVVALPAA
AWPQQGVVLHVNVTIQGRQQLGAAGGSSSTSSSTTTTTSSSGSSNSSSSSNATAVGTVLDLRGLEAVAAVRCGARVT
LAGLGLVGLARHPAAYLAASVGVVMADRGANWTTNAAAASAPLLLSGVTLLLPTPELQALAYWGALAANPTVGSNGG
AGSGTGGGSGGGAAAAAALLGWRRSLLQRIQGAEFAADRLRLRSGGLMGTALADVTLTDDPAVAYDGVPLLLTSAAA
AEAAAAAAAAAGANSNTTGANSSAIGSGSSASGVSSSGSLLQLALQAAADSGAGAGAAGAWAAAAAELLAAPPPAWL
VAAGDTAALLDPALTSPTLLLAAGSAGQLQEAVAVAADLGQLKRSAAAAGIPTGTVANGRVLLQLLAPVALNRTQWP
ADGLALALPTTLASHPPGSVHSLDMGLGLGYLAAPAAASGGSGGTAAAAAAQLTLRSVQVLRSGWLRNVTGQLGGGS
AWPVPPPPPPSPPPPPPLPPLASGGGEPATILGKGSGGAANVFSLEATQSQPPSPRPPRPTPTPAPPPAPPATTTLL
AESLQLRLWSLVPGSSTSSFSSSSSSSSTSTGAPLVLDGAVVTLSREEVLLLLACAVRGGAALPHTSAPGTSGAALS
AASVDDLSSPAAGGSGGGSGTDAGALAILSPAARRRSALAQVVVFAPGDVGHGGMGTDAGVDGPHAVNPDEAAAVAA
LVEEAARPQAQVPRWRRRLRRSTSSRQLAQASAAPPPPPPPAPASAESVLAAWQAATAEAATLLTDAACAGLEDLGL
KPPAWPEPWTWASAGGGEVELAVATDAAVAVLGGSSSGGGSSSNSPTPQLLLRNVTLRSSAGIIRFDMQKLLFLVAP
ASPTIEPAAPVPSLPPPSADGSGGGGSLSGGAIAGVVVGAVVGAALLAVVAFLFVSRRRRHSGYAAGLDKANMSQDD

SALVRGMSPRNQAALGSILAAASAAANRSVGGGSAASAALEAAPPRDSDDLEQGGGGGSRRGGRAAAVSADGSSGSP
DQQAAGGRRPAAGRGRLSTGSVAAAGGGAAGGGGGGGGSLQRRLTLGVTPEEAAAGGLAAPARAASAAGAHMRPLPL
AVGTAAGSAMASPAGGGAGSGTAPRGSLSWLNRVMDRLRAGEPLDSGSLVPGGGAAAAAAAGGAAGGGGARSRSSHG
TLLSALGGAGGGGDGSPEGRVASSQADAATSATILTTTASGGGGGGERISPAASKASSGLRERDTAFTAHGGGVTAA
DAAATATTESSLTDKLEGRATGSGDGTTAALTGSGGSSNNNTHNTHNTLRGSRALVNAAALTANASNAAAAAAGAGG
GGGAGTHSAFNALSNTVASRVAVKKVVHRTQSAVLDELHSLSVDLSNEIDDHQLLVLDVVASGGYGTVYRGSWQGLS
VAVKTILFQEASYSRKVALQEAALSKSISHPNIVATYAVDARPIGVVPHRRPTNHNHNGGGDGGVTAASAASTASSG
GERSMADIRDWRLYIIQEFCDGGTLLRALKGRFFCTTTNTGTSGTATGAGFAGSDTSAPSQQPSAAHSHVAVACSAG
MDGAVGDASSAGGGGRESGGGGGGGGGPNMLMVLELSLGIAAAIRHLHSKQVVHGDLNPRNVLLKRDSAALAGYVPK
VADFGMSVKLSAAQSHVSNVWQGTQFYAAPEQIEHGILTKFSDAYAFGVMLWELAWSQPVWLAVQPPPQPPGGARHH
RAQPPAPSYVAHPNFPKLPPSVPPEYAALVGACLRPMHTQRPAFTAIHRALRALQHQLRAAMAARYCPELEPLDSPA
TSVISTLTTPASTASGAGGGAGGVGGGNGGRSHTSYGIGPAAAAAATAMAAAAAAAAGGSVGGGGALSPAGGVAATLA
ERRRANSMAAQRHVVGGVGAAATAAGVAASVPPTPPTPQQPQQQSQVEPMQLQRGSGSGSGTSALAGLGNGGGPAAA
AAAASANGPGAATAAGQVVVLGAAAGTAAAAAPPPPPPVLAVGGGAAAPSPVPSPNSPLGMAQMVLARHHAHMQELH
AALGGRGATGGRLGAMRRVSSARAWAGGVNHLAVGGGSAAASSTAASSTAGSDAHAALAAAAGMPVARFDASSAGSS
SMAGSASGATASAGGVPAAAASFLAAQQRLLFRKSLSSNSNHTQSSVGAGAGAGNMTVGGSNAPYIAYPPHLGGGGG
GGTAGVTVTTGAGGGTDGMESPFTPASNASSANVPYHLANYHNHYYGGMPLHPATAAALGLPPLPGGGGSAGGSGHP
LLLGSNATGAFTGGGNLMLSSLTTAGGGTGTGPATERDSNMSERLSAERASGGSRGSAGSSSIPLHTYLYSPIGPAA
GGNGFSGMPGVGVVVGGTGGAAGPAPPGAATLSPGSSAAGSAACLPPAGLSVGGLLGLPRLEEGDEAESHSQTLGVS
SPPSATAEAAAAAAAAAAASVTMLGRSGPVPPPAPGGGVAAVVATDWTGNGMARSGSNRRLSRMAPPSVAAAAAPATP
MATVVATSAAAATAAFGGAGGGRGGGGGAAAASRLPVDSDVANRQDVPPGIPPTLMRCATGGGSDTGGGTGSASLSM
GATAGDASTSGVSGGGSGVRGRLQVQDSAEGVARLDTACAQYLGGPLAESAGADVASTCVDLACKALAPSGADTTIS
TATYTSAAATPAACAVTRFERAHEAAAAAAAEASALASAAAAADRRPAPGPGHRASAGLSMTDFEAAAIAAAAAAAA
ASPAGAGAGIIAGESHAVAPPAALSLAGGLAPGSPGVPPPSQLQSPCGGRPSRHGSFFGSLASSLRYASANLQVGFS
RASSLVRVGSHTTTASHASAASTSHGGAPTPGTGTETGTGTAPGTGTTGTGTGSGALPRALSRGGGGGGALSPFEAT
GAPHGAGAGAAAPSAAMATDAGGSPCLPQPPAGALEGRPDAGGGAAGSAADAVVDSPYADATAAAAAARRRDSAPLV
REAFGRELNRVLSNSRHVSGNGGASPGRTGGGGGGGGAGSGQRRNPLLSTPRHVSVQDELPSPGAAASAPSGGAAAA
AAAAAAAVATATATPPR

>g11650.t2|PACid:27564997
MYAPRPPPPWPPPPSPPPRQLPPRPPPRPSPPVRLNTSGGAIGGVILTPTGSPYGTYAPPGGYYGGPVPPVPHNASG
DGDGGSGGGSGQYGASGEGAGGSGGGGSGADPDHNEENEQPEGGGAWDNEPPSDMPPAVYGGEPPPPPPDLASLAGA
GRRRRRRVAQETGNGSAASPSSSDSSSSGAAGGGAEVDAEADLASALLPLLPNLRLGAGASLHLSFSAVAVRCSLLQ
TLWARQQAYGWGSSSSSSSSSSGSSSSGAVVGLNVTDDDNLIRFAFPRAYVRTWRRGRLTAEELTLTCERSDDFSAA
ACAADRAATDRTVVWDAADGTALLAALAAISTGATPTATAATAATAGAVSSQAPDCRSHAAHVIRLAAGGVVALPAA
AWPQQGVVLHVNVTIQGRQQLGAAGGSSSTSSSTTTTTSSSGSSNSSSSSNATAVGTVLDLRGLEAVAAVRCGARVT
LAGLGLVGLARHPAAYLAASVGVVMADRGANWTTNAAAASAPLLLSGVTLLLPTPELQALAYWGALAANPTVGSNGG
AGSGTGGGSGGGAAAAAALLGWRRSLLQRIQGAEFAADRLRLRSGGLMGTALADVTLTDDPAVAYDGVPLLLTSAAA
AEAAAAAAAAAGANSNTTGANSSAIGSGSSASGVSSSGSLLQLALQAAADSGAGAGAAGAWAAAAAAELLAAPPPAWL
VAAGDTAALLDPALTSPTLLLAAGSAGQLQEAVAVAADLGQLKRSAAAAGIPTGTVANGRVLLQLLAPVALNRTQWP
ADGLALALPTTLASHPPGSVHSLDMGLGLGYLAAPAAASGGSGGTAAAAAAQLTLRSVQVLRSGWLRNVTGQLGGGS
AWPVPPPPPPSPPPPPPLPPLASGGGEPATILGKGSGGAANVFSLEATQSQPPSPRPPRPTPTPAPPPAPPATTTLL
AESLQLRLWSLVPGSSTSSFSSSSSSSSTSTGAPLVLDGAVVTLSREEVLLLLACAVRGGAALPHTSAPGTSGAALS
AASVDDLSSPAAGGSGGGSGTDAGALAILSPAARRRSALAQVVVFAPGDVGHGGMGTDAGVDGPHAVNPDEAAAVAA
LVEEAARPQAQVPRWRRRLRRSTSSRQLAQASAAPPPPPPPAPASAESVLAAWQAATAEAATLLTDAACAGLEDLGL
KPPAWPEPWTWASAGGGEVELAVATDAAVAVLGGSSSGGGSSSNSPTPQLLLRNVTLRSSAGIIRFDMQKLLFLVAP
ASPTIEPAAPVPSLPPPSADGSGGGGSLSGGAIAGVVVGAVVGAALLAVVAFLFVSRRRRHSGYAAGLDKANMSQDD
SALVRGMSPRNQAALGSILAAASAAANRSVGGGSAASAALEAAPPRDSDDLEQGGGGGSRRGGRAAAVSADGSSGSP
DQQAAGGRRPAAGRGRLSTGSVAAAGGGAAGGGGGGGGSLQRRLTLGVTPEEAAAGGLAAPARAASAAGAHMRPLPL
AVGTAAGSAMASPAGGGAGSGTAPRGSLSWLNRVMDRLRAGEPLDSGSLVPGGGAAAAAAAGGAAGGGGARSRSSHG
TLLSALGGAGGGGDGSPEGRVASSQADAATSATILTTTASGGGGGGERISPAASKASSGLRERDTAFTAHGGGVTAA
DAAATATTESSLTDKLEGRATGSGDGTTAALTGSGGSSNNNTHNTHNTLRGSRALVNAAALTANASNAAAAAAGAGG
GGGAGTHSAFNALSNTVASRVAVKKVVHRTQSAVLDELHSLSVDLSNEIDDHQLLVLDVVASGGYGTVYRGSWQGLS
VAVKTILFQEASYSRKVALQEAALSKSISHPNIVATYAVDARPIGVVPHRRPTNHNHNGGGDGGVTAASAASTASSG
GERSMADIRDWRLYIIQEFCDGGTLLRALKGRFFCTTTNTGTSGTATGAGFAGSDTSAPSQQPSAAHSHVAVACSAG
MDGAVGDASSAGGGGRESGGGGGGGGGPNMLMVLELSLGIAAAIRHLHSKQVVHGDLNPRNVLLKRDSAALAGYVPK
VADFGMSVKLSAAQSHVSNVWQGTQFYAAPEQIEHGILTKFSDAYAFGVMLWELAWSQPVWLAVQPPPQPPGGARHH

RAQPPAPSYVAHPNFPKLPPSVPPEYAALVGACLRPMHTQRPAFTAIHRALRALQHQLRAAMAARYCPELEPLDSPA
TSVISTLTTPASTASGAGGGAGGVGGGNGGRSHTSYGIGPAAAAAATAMAAAAAAAGGSVGGGGALSPAGGVAATLA
ERRRANSMAAQRHVVGGVGAAATAAGVAASVPPTPPTPQQPQQQSQVEPMQLQRGSGSGSGTSALAGLGNGGGPAAA
AAAASANGPGAATAAGQVVVLGAAAGTAAAAAPPPPPPVLAVGGGAAAPSPVPSPNSPLGMAQMVLARHHAHMQELH
AALGGRGATGGRLGAMRRVSSARAWAGGVNHLAVGGGSAAASSTAASSTAGSDAHAALAAAAGMPVARFDASSAGSS
SMAGSASGATASAGGVPAAAASFLAAQQRLLFRKSLSSNSNHTQSSVGAGAGAGNMTVGGSNAPYIAYPPHLGGGGG
GGTAGVTVTTGAGGGTDGMESPFTPASNASSANVPYHLANYHNHYYGGMPLHPATAAALGLPPLPGGGGSAGGSGHP
LLLGSNATGAFTGGGNLMLSSLTTAGGGTGTGPATERDSNMSERLSAERASGGSRGSAGSSSIPLHTYLYSPIGPAA
GGNGFSGMPGVGVVVGGTGGAAGPAPPGAATLSPGSSAAGSAACLPPAGLSVGGLLGLPRLEEGDEAESHSQTLGVS
SPPSATAEAAAAAAAAAAASVTMLGRSGPVPPPAPGGGVAAVVATDWTGNGMARSGSNRRLSRMAPPSVAAAAAPATP
MATVVATSAAAATAAFGGAGGGRGGGGGAAAASRLPVDSDVANRQDVPPGIPPTLMRCATGGGSDTGGGTGSASLSM
GATAGDASTSGVSGGGSGVRGRLQVQDSAEGVARLDTACAQYLGGPLAESAGADVASTCVDLACKALAPSGADTTIS
TATYTSAAATPAACAVTRFERAHEAAAAAAAEASALASAAAAADRRPAPGPGHRASAGLSMTDFEAAAIAAAAAAAA
ASPAGAGAGIIAGESHAVAPPAALSLAGGLAPGSPGVPPPSQLQSPCGGRPSRHGSFFGSLASSLRYASANLQVGFS
RASSLVRVGSHTTTASHASAASTSHGGAPTPGTGTETGTGTAPGTGTTGTGTGSGALPRALSRGGGGGGALSPFEAT
GAPHGAGAGAAAPSAAMATDAGGSPCLPQPPAGALEGRPDAGGGAAGSAADAVVDSPYADATAAAAAARRRDSAPLV
REAFGRELNRVLSNSRHVSGNGGASPGRTGGGGGGGGAGSGQRRNPLLSTPRHVSVQDELPSPGAAASAPSGGAAAA
AAAAAAAVATATATPPR

>g11472.t1|PACid:27565006
MQDHPFLAELVCERLPWRDALRLGATCRALHSVALSSLARGGGSGSSSSSIGGTCCTGGSSDGGWSRHLTTPVHALP
ALGQKPGGGGSGGGSSSGGGGYDVAAAMLLRGLHGLTLTVSAAGAAAIAGGGASEGGDGYLGGGRDRLLPPPLGCHG
GGHTACGGVDCGGGGSVKSSARMEAAGRGLAAGADVGDEEDEEAVELEMGAAAESDTLCESEAAEAEAAEALLKWLA
SATAAAAAPAPAHKPALAQSPPQQLRPPPPLLPAVRVLELQLDVGMAAAAALLAACPQLRRLTCRCLDAERHCRGAP
REQLQELEGEEEEEEEEAGEQEEEEELEDGEDGELAMQEEDGAEQDEAAGGSSRPAAAAAAAAAAASGDTDVEMVAG
EGSEADDAEDEEDEEDGVGDEAGGSGSEAGGDAGEAARRRLWRALAAGPVRLALSCPACLDAWLGELQRRRRLWQGR
RQDLQMDQQRQRRTAAPLPCRVKALEARPHPRSAAAAAAAAGPPLALPRAAAGDLAAAAAASAAAAADAGALLAAVC
KARRLCLPPPPPPNDTCPKAPSPSTALPSAARPPVASASTTVSFYVAPSASPSASPSASPSASPYLALTHLQLTGVA
LAPRLARAALPPLACLRSLVLTDVCCRPPHPAGWDWLGDCRGLTRLGLAGECPLTPGRLGLAHPAIGRLTGLVELRA
PAWSLPREAPGVLAALSRLTCLEAGGAASLPYVVPQALPTIRALSVRCFYGGTPLGGHLAALLPALTALHTASPAPS
ASAASVVTAFAAGTHRLPLQPPHVGTAGAGGRHRRRGTSTSIGNGNGIAASAAAAATGTSAFRQRLLQLHLQGGAAA
GPAAAQQGRRRQPLAGAAGGGAAGQPHGPDGGHEAADADEGGEDEEEDEDEDEEDDEGSRGPSAPTASAARSHVCWA
LLPLDVHTAVRLPTYGGLRRLELVGGPVAALLELSRPLRGLTSLTLRWASLPDGDEDEEGDEAEGEEEEVGTALPAL
RLALAGQPQLSAGGCGGAGGCRSGAATPTAHSGFHTSGSAGGATVVVAAAAAAATPACSQPHTSCSGGGAAAAAAGT
AADTDTDTAEGAGDVDGVVGPGPAAAGGAGGVLAVNPGVSAAAAAPSGGATAAVPAATATTAAHSGRSLRAVHWADE
EVGGGAAPLGNGSLARPSSSRASGGSSAGGSAGAGTGGVAGGGSGGGSRPPPAQGRARGVRFVSPPRLLRDADSGPS
GDEGVGPAPGAAAGTQGGARGLLSRRHTDNSGSDAAATVAAAAATVAAAAAAVVGSCEPPPRPAARVRRVGWACGES
EAEAAAAPAAGCGAADAAASASRGDSRQVKTEEVQECADECVGEASSPPPPSLLKGASGTGRALRTGVRFASPPPPP
RPSNSRGGGEGGRGEGGRASGGRARSSNSSGGGAGGSAGTGAGGAGRTGGSARRKQPAARHGDEAEHHDPEEGDKQP
GIVGAAGPSLSLSRGQPRARTRTRCRSTGASAGADPRTAADAGAAADAGGGGFASGLEDEDVAGGSGAATPATSHHS
VAGLGRVASGGSGTTSTSTSGSQPSSSSSSGHSGGGRRARCGGGAGSGGSSSTDSGARRAALAWAVRLSGLLPGLTE
LGLHWVAGLGGGTGHDVLDVLVGVGGGAGPVGGAESGGGLSRLTELRLSGFSGLQAERLGRLMRGRGAAGGRTGGTG
EAGARAESGAAEGGARGGDGLEAGVAVVGGPGGTGGCGLRRLVLQRCRGGDGEAACQAAVAAAAGSVWAELVWLP

>g11685.t1|PACid:27565058
MPRRLAFHTSSMYTAVTTAALLLAAGAAFLPLLAAAAAPTSATRFEGRLVTFSHVGDAGHGEAGNAPGLAEAREWAL
VHPQTNTLVHKFARGFAPPGRDRNQRRITPGAQVSMSCVSFDPVTGLCTAVAPGDAYLISGPTPPAAAARKVENILL
MRLDFTACGYKMTSLTDTDIKTMYLGPNMDGNGGHAQAFEDCSYGALRLNASAFRVVTVVVPNCVATCDFYGITVPS
DTAAKALLGVDIYTAYTNLVYLLPDNYFSLCGWSGMATIPGQQVMIHSAPNYTGIYNRMTVLQESLHNFLAWHSYQF
DMVYQDPSTCMAQTPVCPSAADLHRLGWGSPASGGDALSAATLPPGTVKSFNLPASYLTGTGSFIRIKTDWTSFYNS
TTYGKNIYVSVRVPKRSDAALKPEQASKVLVHHVRAAINNAFPADTSDPYLNYLMAVSPNSAGNATDYNLVVIAGNW
VGTDILSVGVCRYLASPAECPTSIPAAVPPRPPKPPSPPSPSPPRPRPPSPSPPTPLPPLPPSPPPRPSPPPSPLPR
PPSPRPPSPSPPSPPPPPKSKKGRRLLQ

>g11595.t1|PACid:27565062
SPIAGLYPGMRGVEYEFYPLTSPPTSELWRLNTTIKAANLTGGYSGVLMDVVETRDIDAPVPWACTRLRGFFTAPRT
ATYTFFYSADDYGEVNGTWIQNGVEVRQRLVALGQGTYLDWYYWLPSQRSAAVNLTAGQPLLLEANSCNNAGRGFQQ

VAVRVTDDRVRPNTVERTIQLRVFGPSMPRSVALKWLYVPSPTVANSTAVQVTVSADSAARFNDALLALNLTLEWVN
GTGVAGSGSAPPAGPVRQLNVTLPLAVPSAEMTARLGAALGPPFNRTTGGVGGAGLIGVRRVLSGAVLLLEIGASTA
AMPHLAITAAALVALPSPPPTIVSNLSNCSFANPLMAPVPGSAAEAAYLAPPAPPATVVPTASLATVLPALNVSYGP
APPPPEPASNPSGTWALRLLSGTDTRNLPYDISAAALLSVINDLTSNGPECAVVRTTVREGQYLANVWNITWVNANY
IIVYAFTVTPGPDAPLGSVLATEARAASPFTTGTFRIAANSECEYVDVNALDDYVPSIGAKLASLPGLSNSSNVARG
YGSDSATYVSVTLDAYAHSPTANQPSLRLVDSRGLSYQDATTLFVPAWAAPPEQAFERLPVDFFQLPVAGPGSIRLI
VNDAAAACVHPSGICSFRYDSTATPTVAAVQPQSLTWPTDTPAVTAAAGNAAAELTINGRGLNTTTAAAAAGEQVWV
MVSGANCTITAVNDTQIVCNVPWAALATGQRQVVVYVPGRGYAAGTPPLLVSAFAVRAVVPAALPLAAGDGSSSSNG
GMALLQISGQGLVDAADVAAAAAAGGNPCAGLGVSVGGVACGLVACGPSQLLALYPGGGAAVADGMSMPLELQAIVN
GTAAVRFTVPVAASPLGSTTAPAVVSLTPTATGASVLPASGGANVTIGLRGSFNPSDVLGMYLVALPQNWTNATTSG
SSTGANSSSSSSSGSTGSCSGSGSGAVGMTPLAAALAAAVPCAAVTWQQQPSSSSSASASVAVCTTGPMPPGSYQAM
VVLNGSSTASGGSSAAASATAGGSSSTSSSAYNAQGLTLLSSGSVSVDLSVTSVSPAAGSVGGGTLLTLTGFGFVAA
GAINSTDAIRIFVTVPVSGTFPTGLLPCDVVSATATQLMCRTRPHLAATADLDDPLAAKRLPKVTPAAAVVVMLCRA
VSNNASANGGSGTGSTSAGPSASLPSTCPFGTFPVATSRCTVSNGCRYAYTTAATPVLGYVLPSAGGAGTRITIVGR
GLTGATTARFFNAASSTGTGAASSSSSSSGAASSSGSSAVAQASCSLDAATPPAVASGSAGGVLTAVACDVPAALRA
GRYSLVVELPSGSMSLDPYQGAVFSYQPTLASVSPRAVSLAGGLPVTLSLATAGSGPLLQPDTPAAHAVTVGAGMLG
PAGLPCPVVNVSSTTTLTCTAPPLTGWILAEIWNLAPFSGFRPLVESFNVTPTATVLVPSANFDHTVGTLPPIFPPG
ILQPEYYGIRFTFYVQFTRAENVTFQIAADDGGRLLIDDAWVADTGRTLTLPLGAGVHKFVWAFYQAGGSSVSRVYM
AVSPSAATSFPWTKATPVPPGTLLPLTLSVNGAPAAAACAGAQVSLRTAPGQQDLSFEPSNITLPSNASACGLLYTA
YATPTLVAAVTAAPAGVEVPALSYPLDTNTSAWTANVYGSWLTPSPARDVTVTVGGWPCVPTATGPVSTVALDAASS
AALSGNGGVNVTRMSCIVPKLPAGDWPLNVSVAGYGLARPPATPRAELPVLTYPLRVLRQQPLSGTANQQDCQLSIF
GGSTVTLAGVGFVSGLTNATLGRTMFLEWDSPANGICVATASASCAWLGAPPPPLVAAAAGALPLQLLSSDGATAVF
RIGRYSLADLPASAVAAAAAAVAGGGGSISTGALAPPATYLTGNATLRSVLRWRLRVFNSGVVPPTATEVTGTLAFC
GGRTPAVTSVAPATVAAGLQPSGGVALVISWQVSLAGSSNGTWNASAAGSSSAATVELVAVARGGSSTAGAGPLLLQ
PLVLNCSDARVNASSLGSTSYNETLRCQLPAYVPAGTYTLWLCAPPLGCGYAAASWTAALQVSGFAPGSAMQGPASG
GTEVTVVSSGHSLDPAAPPAARFGGSACAVLAMDASSFRCRTGAVALPAGAASATLPLSVTPAPGADEVTFSNVTFT
FAAAGGMWVTAVSPTQGPVAGGTALRLSGSGFDVTAAAQMNVTIGGVPCGNVNVLSSDAITCVTGPLLAPVAAGGAA
AAAAAVVVPGQWLDVVVSTPGRVSGALSAGPNGSTSGTSTLSPPAASFRYLDAWSSPSSWPSGRLPQAGDDVAVAAG
RALLLDVSPPPLGALLVLGWLVFDDTQPALELQARSILIDGGGRLSVGSADQPYASAATITLIGNPPAVSPDDSGGV
SGCVDDGASGAAGGLALYGTKVIAVRQGVLEMYGTPRSPPYTTLNITANPGDGAILVNGAVDWKAGDRIVIASSSFF
ADEVDEATVVSVTTTNSSTSGDIVSRVALDAPLSYTHLGVVVAPPATAAGLTRPAQPLDMRATVALLSRNVVLQGDA
ASANSMFGARVLVHTPPSLLAQGPKAAVRLSHVAMRRMGQAFRTGAYSLHWLQQGDVGSSSSSSSSSSSSSSSSSSS
SSTAAAGSFGASWVRGCVLRDAYARGFVVTNTSGLVLERNVLYEVYGHGFVLEEGTEYGNVLDGNVGILTNANPALP
PTAAWDAGNPATFYVANPNNTLRNNVAAGSDHTGFLYRLPDAPEGSGSTLATAAFYSTCPRFTAMPTVPIGSSNGSS
SNGSSSNGSVSSGGFASNTAHSALLYGLRVHPEYFPRASPCSRYAAPLQQAAAVFDGLTAFKCGLKGAAASVVGLVQ
FRGMALGDNGGGPVTPNTTLIKETGAQAEIAWVHDDRSRITAGLTNLAGVFSTTLFGRTAAGAVGTAGRWPNATRRI
AGVATQSTPQGSSYHQCGLALVNVTFANFTVAVPLSIAGAVAPPPPQLFALEACARCLSLQGGCTTQTAGLQFVELA
DAVGTGAGGAAAGTDGSNASSSARSGTRRPALAAWSWLQQGIYEDTDGSLLSAAQLSTYLSYPAAFPAAANTTTAAP
GSTAAVAPPPWAGSTFHSAINNTLFEPSECVYLRGAAAWPSSNTAAVCAPRVGPFRRLHVAVAPATIGSTEMVVVNA
ATNRSSVVPYVAVNEASYMVTLPTGGREFWIHWRAPTGIRVDATSYNLYRLDLLNASNHFSYITTRYTINGTFTVNS
TTVTASARPAASVSTAHGATSYNRTFTTNGWRWLGTGNGTVARFNDTALTTFLAGGSNALLRIQSGSCPSVGCPTQF
VEEQEPFLRWSRDTTWANVTGGKPGPGANVTVPVGWALLLDESPPALGSLVVLGSLRFDPLQDITLTAAAVMVLAGG
SLSAGAPGAPHPRAARILLTGFRGDPGWRVPNASGAPFAPGADLGTKVLAVLGGGTLDLHGRAQARRWTRLARPAAP
GDTVLRLDVPLSDLDGSWQPGDRVLIGSSSYNQYQSEVRYIVAVLPYSALAAVDGGGGGGAASADGNLTAVVLDSPL
RHPHAARGRSYPVSGNGSSSSGSGGSSSSGNATLSVDLRAEVALLSSNVGIAAAEAYSMAAGSLPPSDLYGSPPSAA
SGSNSSNSSTTAAWVTYGARLLVAGNSTARLVGAAVEYCGQLDGSLLRPCVLFQDLLQTAVAGAAATNGSSSNNSSS
SNGAANSTFSSAAGAQLYIRGSAIAFGVGGSIAIAGASTAPASADIRNTTNTTAVSPANSSAAAVAAVALEDNALYG
TYGASNVLVLSSSNLIKNNLALGAVWDGGVAFGALTAGGAAATLLPQQVPHPLNASNSGDIVASTGNSSKSTAYRAR
VLSNFYIRDPGNWVEGNVAAGSDAFGFAVQGTPCIDLTAIEALAGIFENSSSTSLTSGGQGSSSANDSSSSGSAGVW
ALGSFRNNTAHGCLVGLWLRSSAASRAAAPGGGGGRCTALSNFTAHTNWDYGLLTLRGIDTDVRMYDVALLDNRHAG
AALLRQGGFTERAGVSWRGGLLAGRTSPVLCALCASPSDAGCHQRLTPQSYNRALPYTPSVGFVAAAFALGFSVGPD
RSPWDAVASYPTVNGMALIDGVVVADFEGASGCGGGSFAFANNPTAPDASHPHFFRRITLANTNTDGSAQGLFLMRG
PDPAWRNPLDCGTANYTAGATPLPLNCAGPSRALFNDLDGSLGVSPAGGPVTVLGALPDGPRPQPLAQAPAMPSGSG
CIYRGDLGTYVCAGGPAGSGATGVFDPQLFVLASRDTDAEVRGPEAVAFNVSGSVDVVVPAMDQGWCLGSASCKRRL
AAFWSVLPTGGSVDVSFTGSVPDSFQLWMPYASPSAELVMAFDFPTNCTAARRFAWVPPLVANTVFGASGSSSAGGR
GQRLSYGPPAPYDGTPHGSYWWDVDGGRLVVKMVGGGRSLEVRTEAAVPVRLNATVTSVPMTLAARRALASALATAA
GLDPLRIEVMPLPQQLYPGDASAATAATSGGQATLPVAVHIYPPAADSNISRPDAFPQPARCSTAGGLDAGTQGALL

ATIWSGLVNANEEQQAAAVAAALANATAAAAAAALSTDDDYSWAQQLAMAAAAAGGGLLASWTPPPPAPPAPALAVQ
LTSAYLADLYRAWVMLAWPPTPAVNGLAISGGPVLAGVCGGADGAWLANTGTRGALDATLRAAIVAANTSPPPMPPP
PAPPLPPPSPPPSPAPPSPAPPSPAPPSPGPPSPAPPSPGPPSPHPPSPRPPSPFPPSPMPPSPAPPSPAPGPPSPA
PPSPAPPSPAPPSPAPPSPVPPNPKPPSSPPPSPVPPSPAPPSPAPPSPGPPLPEPPSPAPPSPAPPSPVPPSPDPP
SPAPPSPLPPSPPPSPAPPSPAPPSPEPPQPPSPPPAPSPPPPDPFLRWSRDTTWANVTGGKPGPGANVTVPAGWAL
LLDESPPALGSLVVLGSLRFDPLQDITLTAAAVMVLAGGLLAAGSTASPHPTVARVLLTGSPSDQGFILPPLEAAMA
GPGYPAGSNIGSKVLLVAAGGRLSLHGRSVTRRWMRLARAAAPGDTSVRISGSPSGELRGIQVGDRVMIASTSYNQY
QTEVRRITGVVAQPADSTAGAGNLTALVLDSPLRHPHAARGRSYPVSGNGSSSSGSGGSSSSGNATLSVDLRAEVAL
LSSNVGIAAAEAYATITAANGTQPLASEPSLDLNRLYGSPGANDGPSTAAVPYGARLLVVGTGSAQLTNVVVERCGQ
LAAVGQEQRPCVLFQSLSPRPAPANGDDSDGSSRCSDGSSDGSSTSCESDISSSQSYMYDSAIVAGVGGNVAVLGSQ
SPTVRVELYGNVLYGSYAVSNVQLLTTGNHLYNNLALGAIAATNRTANSNSTAAGATSGPASVSPANFLLSQSANWV
EGNVAAGSDRFGFVYQGMPCTQPSAPPSASPPASRPPIRTPPTTSPSPPRAPSVPAPGPLPPAIPPPSAAPAPVPTR
LPPPAPPPQSPPSEPWDVGPVAWTRGSFQNNTAHSCLVGAWLRASVASSGVGCTALSNFTAHTNWDYGLLTLRGIDT
DVRMYDVALLDNRHAGAALLRQGGFTERAGVSWRGGLLAGRTSPVLCALCASPSDAGCHQRLTPQSYNRALPYTPSV
GFVAAAFALGFSVGPDGGSTWDAVRGYPTVFGHANITGVTVADFEGASGCGGGSFAFANNPTAPDASHPHFLRRITL
ANTRTDGSSQGLFLMRGPDPAWRNPLDCGTANYTAGGTPLPLNCAGPSRALFKDLDGSLGVSPAGGPVTVLGALPDG
PRPQPLAQAPAMPSGSGCIYRGDLGTYVCAGGPAGSGATGVFDPQLFVLASRDTDAEVRGPEAVAFNVSGSVDVVVP
AMDQSWCFPAAGSSSCSQRLASFWTSLPTRRTTHISFYPESSAPRSFQMWMPYADPGSETILVIRFDAASLSRRFVW
LTDASDRATAMTASPPALYDGTAHGSYFWNATTTTLTLKMVGGLTLEVRAEPAVMMGTALAIPVDSFGPSAQAAFVS
KLTNASGLQPSQIEVLRVLAGSLRLNMLIYPDPRLFAAPRPYYWPPEMRSASDLTGNVAVDAGGLDSQTQAALWAQV
VATASNSTTGTSTGASIAAVHLLEAAIAAARERVIAALTATASADDGGTTTSEHPPAPSAPPDAPVQTFSDISSSFF
TAMAKVAAVLSLPSAAESEVVLGARVMDKPTLLSLYGLGGGDLLLEDPAIAAAAAAASLQAAVTAAESLRPPPPPPPP
PPAAGGQPSAPGAAGGSSGSPVGAGGTPVSSGGGGSGGAPGVSDPTGATGGGGDRGTTGTGTGTGVNEIGSYSPSPS
LQTDGGSGTSGGAFSPSANAGSAPRQVGGNGADGGADGSTGSNGTGNGTSSSSGSGGSSTAGASNGSGSSGGGSSIH
LAVLVPAVVGGILLVALAAAAFGYAAAVQRRPRRHSAGGADAARGHGDAAVAASGSAAVAASSWRRRLGEGGPQVAS
SGGSWAFRLRQGGEDYEVASRASTIVFENLMAVALVLDDLEEPTHVSGTVAVGGAGAVTALGGSGWVAGGRNRGTHD
HQPPPSPPPPLMVPDERDEDAMSQHMPELASGAATPLAVAGSRSTSPQRWALMAAAAAATAAANPAGSTATAADAAI
AAGSLRDGRLCLTPRASSLRAAAPPLSRCGSAFNLHECVRAEDPAERLMRTAGSPPLSCRTLAALDKGTAAGGSHGV
SLLRIGGNVAVTTPGEEASGAAAAGVAGVGTASTPGATPGVLAAALGLLRRQGFAAAVASATVNAAGVDAERSRVLS
RGGSFSVVSRKNSVVAVAPEPTPADAASAATAAGASGTATALSIGAAATAAASAMAAARRRGGLPDLATVLSSGVML
AAPPSSPTAAGISGSFGGGRTSPFSHGPGSARSRLGQHSRSTIVPFMAEYGTAGGPPPPIVAAPVVGSSRAQFISGP
GISSGAPPPAQPLPPPRTSDGPPSAGLDGGVASRQRPSRFMPDSRATSAGGVTPVTLATTTAATSRRSSTGGAVVCG
AAGAASPPQGESLRTTPASARLRDAVVVEGEPFEMADNGAGAVGAQAVLQPAAAAAQAAAAGPAMSAKAAAVAVAKA
PSLVGRFVAWARGLFGKASSPSPPTTAAVTPEPMSPPQSAAAADSRVVGSEVAEPVTPFAHASTVAASAAGTAGVGA
PEGSETAAVSSSPAPAAAARRSSAPGGADSVSHALSPPQHSEHVQPGAQPGAHPAPGAAEYPATSTPAQHLSMAEVW
DAASPAPKELKLCGDAAPPPAAAAAATQAACGAGLGPSISAPSEVLHGRGADAQASDSTQPQALAAPAISAPQPSAA
TGADAGTAGMSTNAAGIPLTPAAPSASLPLPAAALALVESHPHGLPPKAPASPAAAGPLLSPAHQAPGSLPPLSRGR
HTYKPRRLPPIEAAAPAAAAAATTQTESSGLLGAGTCESVPAAPAGATAQASSKAARAEMPQAGDMSCASQPSSPPA
SPTRATPGAPVSMPCGGDAGSLMSEAAELQPDSGAESVQAPQPEPSHPPSVSGGATAQVQLLQILAAQQQQLSAAVA
SHALLPPSPLLLPSSQQQSGAGLPSPASVQLPQQPHSSRPQSAAQQDPASPRVRALASALSIARRDRRGLLNPLQL
PNDPRAGAEGEGDATATAAETMAALQARSVSARRLAPVVSFRRASITDAASIFPAAAASDAAAAAAAAAAPGSSRHR
VGGVQRDFGSQSSVGSGTGSHVQDDADNDDACEWDDDWGAGGSVIEAGGRVSARLKSSTRFGAASTSRTRLHPLASA
TSGLEAPPPRGVSPAAALLASRSFTAAAAAAAAAAGVAASSPRGQLRFHMPGALQPAAADGSEPVSPKPQPSSAVLP
TSQTEARSGAAVGTSSMPEPTEAGVAAAVGKPETGTVVAAVGPITPEIMRPDALLAWSSDPHAVASAVDAPAPREVV
DGEQLGEWHGAGAGVAADAVQHNATHARRLALEDAASPPPIVDTVAVPPLTAAVEAASAAIADGLDPEQPMPPRLQ
QLRGRAGLQELGAGQEQERQHGARSYHQAGTAPAAEAVHPAPPPAPPSLPPPVSQQLPPAGLQPLKRARPQALEPEP
EPEAVPAAEAHLELQLEMPAPDSAAPEPSSADEIESDAAGGVAVGSRGPGLNVPTEGSNTFRAEGGTGVQPLEQHEQ
HEEFLGMEPEPEPQRHMITRAEKAAKLAERAERVAKREAKAAAKMAREERKRQLAVAADAARSLPSASMGGY

>g11593.t1|PACid:27565087
MSVSIGPYPCAVAPTLSSAQQVTCEAAPGLSGTYWVTVRRVSAGGAGPNSTTATTVTARLWGYTYTAGRSSSLRVVR
PSAGPPGAELSVIGDPTGVVRSDCTPSVTGGDSECLGDVRVGLLQCKTQEGDLGAALDFSPRLRWSCCSPYRIRCTT
AQPLRNNPRLPSLEPGAGTAGFFNVTARFEASSRGALPLINGNAWLYTADGTPYLYSVYPEVVSISPATGSAAGGTR
VTLRGRGFPDLRRQLQGGSGGSGGWGQRERVSVWLGGAPCEVVDSTYSSLTCVTAPQPAAPPLNASATPIAGLYPGM
RGVEYEFYPLGEAAVPPYNELWRLNASGSRGNNSNSSTNGSSSASSSGSGSSRAIRVEDVPGAYRVVLDGRMEGRDD
DFNRSYACSRMRGFFVAPRTAMYQFIVGSDDYAQLNGTWINGTTGTSNSSGSNTGAGANGWEVTRLLARNTGAYYVD
WWTQALSSTVHPPVPLAAGQPLLLDVAHCNRQGPGIAQVGVRILGDTPQANSLSETQRVVVAGSRLGRSLAVKYLYA

AAANTSALALVQVTLATDSAARVANDTQLGLQLTLEVGSSNGSSAGGSSGSSPAAVRVNVTLPVTAVAGEMTRLVRN
ALLAAGAIQLLPPATAANGSSRGSNNSGSGAVGPRMRSNATAVGVRKVVNGNILTLEIGVETWALQPPPSPLPAPSN
GSSSSSGAGAAAVVPPTFTVLAAAVVVVPPTRNTSSNPSSVAIVPDLTSPTCRFANLLMAPVPGSAAWAAYHAPPRT
APGPSPPNLTAVYSTFNISVDLNPVRPAADVDPTGSWILSATNPGAAAGVSFTFSWNSPPEALAAALLNLTSTTMDV
RMSGILGGASSSGNGTSSSNITGITAGTGGSGSVTSDPSASAAASAPLPPAAPFFGGDDVFSPPPPPPPPSGPASGP
AVVGDGPVREGAYLAIVWNVTVPSWSLFVPAVSNLTLSAVPGDGFPTAAPTVVLPVTGGGSAPPLLPATAGNASSGG
TDSTSGVAAAAATPPPVAMMAVETRPVAAPPTGVIRLAYDNECEYSAEIDVYWETSDVIGAKLAALPGLTKPRRSGR
FYANDTSLHITFQMDPFLHPGDQPRIRIADTRGLSGEATAFYATSDYAGSNDTLYFPLPVDFMRLTVGAPNSGQLTV
NGVSAACAHPSGVCGFAYDGSATGAGGSNGTGSNSLPLTPAVVSVEPIRVTFQPPAVLSKQLVITGSGFTPSTQANA
SIASSAAAAAASGAAAGLLPADTLEVRVGGAPCVVTAATDTTITCNLTLSAPAQSQQQLAGQAPPPPPVPSLAAGLQ
PLTVYVPGKGRAAGAPVLVVDALVVTSVWPQQVSMYGATLIRIGGHGFVSSASISILSSAPSTAPGATEAAAGAGAA
LVGPCAGLRVRVGGVGCSLVECAPDHLTALYPGPAVPSAAVAGDGVMEVELQVLAANGSVITSSYLTQQAAAAVGMP
PAPSTILSTMAPAITAIDPRPSIQQTQQPQQAQAQAPLSAAGGDSVRVTLSAAARPSDVLGVFLVPSAGSSSSTGAN
SSNSSGSGSGSTGSCSGSGSGAVGMTPLAAALAAAVPCAAVTWQQQPSSSSSASASVAVCTTRPMPPGSYQAMVVLN
GSSTASGGSSAAASANAGGSSSTSSGAYYAQGLTLLSSGSMSVDLSVTSVSPAAGSVGGGTLLTLTGFGFVAAGAIN
STDAIRVFVTVPVSDTFPTGLLPCDVTSATATQLVCRTRPHLAATADLDDPLATKRLPKVTPAATVVVSICDPAASG
GGGGGTSGISTARLSGALACSESRPTPVVSRCTGQCEFSYSADATPVVGYLDSPAVPAGVADVQVLVFGRNLSAVTT
ARLLLLPASFGTDGSSSGVSLGAGTSSGAGASISPAAAAATLAATEAAILAAEVVATPVAAAAAAAAGPAVEAVGFG
VPGDLPVGRYAVQLVDTYGGVSVDPFQAGILIVQLQLGSVSPAALSLAGGLPLTLTLSGPGLLLPGNPAAHTVTLGG
NSITGGNSSGAAPPPLGPAGLSCPVLNVSSDGRQLTCRAPPLTGLLLAEFWNLPEGLSGSVLPPLEIYMNPDIVTTQ
PGGVAFNYLTGQSPLPGDIRTRFWGARFSWYMQLTRDENVTFSLAADDLCRLLVNGVPIIPSAGSGGRTAAATLRAG
INSFVLVFLQYGGTAWLRFLVAPHPGTGTPVAPDWSRVTPVPPGTPLPVTLVLNGASAAAGCDATQLVLRLPLAAGA
RQQELPFEPDQVHLANGTCAVVYSTYRTPTLVGVIASNGTLNPAASGVDLPALNATRPSFTTGGGGGAASSNANSSS
TATAAWTLYVYGSWLADQPLDAVRVTVGGRPCVYLPSAASAIANDSNTVTGSGSDGADGSSANTTETSSSAPSVTAA
TLVPLDPPAVAAASGNGGTMLTRLACLAPPLPWGTWPVAATVDGLGAVRPPPSSSSAVAAVSYGLRVTEVLSPVTGA
RAYCQVSLFGGGTVSLAGVGFVPGLSDASLGRSMNVSWWSSTSSSCNSSSSVTAAGAACSWFGVATPPDAAAATGQL
RLALLASDGATATFTMQRYSLRSVAPRLNATILAGSSNRSSNGSSGSSSSNGILLDDWRYLGNVSVRSALRWRVQVA
ATAVAGGAAAGATTEVTGLVGMCGGRTPRVESFEVLPAAADPTSTGGGNGTVMPTSLRLSWVLPYAANAGGGGGSTG
SWPDLLQSPPQPGNPSAASVELEAVAVAADGSSSSSAISSGGNGSGGGSSGWVLSCANATVVTSAADEAAGSYSETL
ECALPADLPAGVNYTIWACVQPFGCGYHPFDFPVALQLNGLDPASPTAGPTTGGTAVVIRLRGHSQDPGQVAARFGS
SICRITAVDARSITCITGLLPYDATQSDPTVARVLPLSVTPRWGAAEVTDPGTTFTFLPDQDVYVTSVAPAYGSTAG
GTRITITGRGFTPRQAGGNTTSSGSSSSSSSAAAAVVAIGGALSCGSVVILNDTAVQCTVPVLPFNMTSGVWMTVTV
TPAGRTPSAPRTIRTAASANQTVPPQLLPPPAASFRYLDAWSSPASWPSGRLPQAGDDVAVAAGRALLLDVSPPPLG
ALLVLGWLVFDDTQPALELQARSILIDGGGRLSVGSADQPYASAATITLLGNPPAVSPDDSGGVSGCVDDGASGAAG
GLPLYGTKMIAVRQGVLEMYGTPRSPPYTTLNATANPGDGAILVNGAVDWKAGDRIVIASSSFFADEVDEATVVSVT
TTTTSSPSGDVVSRVALDAPLSYTHLGVVVAPPAAAAAGLTRPAQPLDMRATVALLSRNVVLQGDAASANSMFGARL
VAHPPPSPLASVLRLSHISMRRMGQPLALQPLQPSPVAPPAVAPAGFSMLWHSLGDLPTNADRSINANNSSGGSTST
GASSSTTTGSTSSGGQASIPASGGSSSSNSSSSSGTSSGTSSSISSNSLPSPWLRECVLQDAYARGLVAIGTRGGVI
ERTVTYNTYGHSFALDTGAETGILFDSNLGILAREARALTTRDTQMPSVFWVSNPNNTLRNNVAAGSDHTGFLYRLP
DNPEGAAALTSSTSICPRFTAMPSNVLATGGSSNGSSSNGSSSNGSVSGGFASNTAHSALLYGLRVHPEYFPRANPC
SRYAAPLQQAAAVFDGLTAFKCGLKGAAASVVGLVQFRGMALGDNGGGPVTPNTTLIKETGAQAEIAWVVDDRSRVT
PLLIALAGVFSTTLYGRTAAGAVGTAGRWPNATRRIAGVATQSSVPGSSYHQSGLALVNVTFARAYLTGRLAVTPRA
PWPQHYALEPCARCAPSTGGATAFLAGAQFVDGAAGDEAAALAALAAAGAAAAGDLTDAGVPGGSVVARLVRRPALA
FWSWPFQGVYEDTDGSLLSAAALSPLLPLPPSVLQALGGASSNSSGSISSTSTAGGSAGNVTATSTAAAAVAWAGAT
FHSGVSFSRSLSYGGTGSSNSSSNSSSAAALPGIFDPESCVLLRGLRGQPSSNEGVVCAPRAGPFRRLIISDVGPTP
DLAGQPLVVVDVATNRSAWVQPSTSNDNGGYVLVLPVRREFWIHWRTDTWERLDPATYRMWRLDNLANSTQNHLYIT
TKYMDVYDSFTANTALITGAAATPRPPALTSTAHGATSYNVTLTPNSWRWSIPPPPGTSTTQSLATVSFSDTAVTTY
LSGASTSSVRIIAATCPAAGCDSPPAKFSALREPYLRWSRVSTWASRPGGKPAPGANVTVPAGWALLLDESPPALGS
LVVLGSLRFDPLQDITLTAAAVMVLAGGSISAGTPSAPHPRIARILLTGSRGGAGWRLPSAAGPGYAAGTDLGNKVL
AVLGGGTLDLHGRAQARRWTRLARPAAPGDTVLRLDVPLSDLDGSWQPGDRVLVGSSSYNQYQSEVRYIVAVLPYSA
LTAVDGGGGGAASADGNLTAVVLDSPLRHPHAARGRSYPVSANGNSSSGSSSNGNATLSVDLRAEVALLSSNVGIAA
AEAFDSSSSGLVGPGSGLDLADLYNTDTGSSSSGSSSAGSGPIGSSSITGSFGSGASGIIHGVRLLVSGSSNARLSN
VAIERCGQEAAVAASDTLASGAGSSSGNSSSSSSSSSSSGLRPCVLFDSLSAPRAGSSITVDGRSYSRYLRDCAVVA
GVGGNVAVTGNQTSPYGALVSGSVLYGTYGGANVLLRTAGNTLRQTLATGAVQPSIAGSGTGSSSSSTGSSNGSSNG
AYVANIQVVQAGNWVDGNVAAGSDRFGFIASGVPCATTIISNTSSSLPPSSSNSSVNSSANTTTTAANSTTGAALDG
DGDPGLPTWALGSFRNNTAHGCLVGLWLRSSAASRAAAPGGGGGRCTALSNFTAHTNWDYGLLTLRGIDTDVRMYDV
ALLDNRHAGAALLRQGGFTERAGVSWRGGLLAGRTSPVLCALCASPSDAGCHQRLTPQSYNRALPYTPSIGFVAAAF

ALGFANSAGSGPWEAVTSYPTVFGHANITGITVADFEGASGCGGGSFAFANNPTAPDASHPHFFRRITLANTRTDGS
SQGLFLMRGPDPAWRNPLDCGTANYTAGATPLPLNCAGPSRTLFKDLDGSLGLSPAGSPVTVLGALPDGPRPQPLAQ
APPVPSGSGCIYRGDLGTYVCAGPAGGEATGVFDPQLFVLASRDSDNQVRGPEAVAFNVSGSVDVVVPAMDQSWCFD
GVMHASSGGGGNASSVTSCRRRLSAFWSYVPTGGEVGVELIGNAPRSFQLWLPYAPSASEVVLTITFPARQRASRRY
VLVPPTAFSTANSTSSNTTNTSAFNTTSTGNATASSTNNTSTDPLEPSVRANALPYGPPVAYDGTPHGSYWWDEDGG
RLVVKMVGGSKSLEVRTEAAAMVGLGFELPISDWTPGIQAAVVAALSVASGINDTRRLQVSRVTPGSVLVDLLLYED
PAAAAAVAAAAAAAAATRPNSTAGSSVWPSAATAGGLGGLDPATQAALSAQISTQNPTQQQTLATARSAATAAATAA
AASSDDPAAAAATNSNGTSSTDGATAPDAFPASLLADLLKLVSMLGAPTASAKVGVSVLPGDIKLIAAYGTDGSNLF
VTAPETAAAISAAVDAAVGAVTAPPPSPPPAPPPSPNPAPLLPPSPPQPPSPPPLPPALPPPPSPPPSPPSPPSPPP
LAPSPPSSPGVRDLVSPPLPVLPRPPTPSPATLIPQIQLEDAPVEPAMSGFQDPPPPPSSIAFSDPGNAPSPPSSLA
SGSSSGVAIGVIVPAAVGAAVAVGVAAAAFMFFRRRQAAAAAAAAAGAGGPTSPGGGKTRSSALDRWNFRRVQGRGG
KRGDDDGDVVEYDLEHEGEEGDGSVAAAGELSPPRSPRRIAMNAAMAAAAAAAAAAAASATAGGAGGDAGGGSSRTSP
ADKPSGLLSRPASGRSGAATPRHHSQSAVTPDPFGTSLPLPPSLGAGNGSAASAIKLLQPPLLPSWRTAAAASAKLL
PPIVKLKDLPRRLPAISTKLYDAGGAAAADAWSPKGGAGGGGGVGYEGATSDRTPRFEGGLATAPLPQATPTRTYTP
RTMARPPLAPSSRTPRAHSAPRERGISDTGSGGADGFFNGGGGDKDSGAFTPRLDAVASLPGGMDGSGGESDGCSSP
RRAGLRPVSSAFGAAAEAAAGDSTPTYAARPGAGLAGGLAAAYAAAAAAVTTAISARGFGRSTSTTGSARAGAAGGS
PSDPSILLWHNAIAAPSGAPFHDAPAAAGAVGLQPPPANARTLSMPRMGPGHGPGWSRFAPSGGAGGGPGGATVASA
TGGRSDSGEANRPWSARMAAGSTAAPAKVGGGGPPASGVWRDGPRSAAVMPGGDGHRGVPSGGGGYAAGGNGYNLAT
VTGATATDGATSALLPLRACSSTGNMAALDHGPGSVSPPRPLPPRVVLEGEGPRTAHVGAVGYAAAGGALAARPPLP
PSSSIGRRASFGAADAAGSMGGVL

>g11754.t1|PACid:27565150
MSSPITVVFAGGDMTTWCAAVETLSLATCDAKSECCARGAWPVARLEVLADRACTASNIAGVTVDGQPWSSFSFNAA
AGVLSISNLYDPAVPALTSPQPNSQVVCVSLKGTGTCAKSAGLCRRITVGGDCAVLAADAAGACCPVAEAPTYQAAD
GSVPLDRETSQAFNVTLQLNRKSATYTCAKLGASRADRRALFKRVAATYAAALGLPGKSVAVEDSVCGRRSNVLSLL
VRMSGGRLPHWAAVEGRSAAILKALSADTGGVLVAATPVVPSSRGKFVKGPAASTGSGSGAVPTPSDPSPSPSPSPP
VPSPAPSSPQPPSPGPQPPSPPPVPAPPSPRPPRPPSPRPPNPAFLTVAISSLDMLFSPDLPPPPSNPSPPPEPPAL
PPAPLPPWAPGTNVTYDENGNVISISIGEVYACEENCCECCCVRITPPVFSTGGWPGGGHGGGWTGGGDDGGFDDGG
FDTGSTGGGVMIQSPPPAYPSPPAPPSYPSPPSPPPAPPSPPSPPPEPPSPPSPDPPSPDPPSPEPPSPEPPSPKPP
SPEPPSPEPPSPEPPSPNPPSPNPPLPPPPPPPPPPPEGEEGIEKDPWG

>g11469.t1|PACid:27565206
MVLSQSLCGRCRTTPFNARPALALLASVAVLCAALLLVGTPAPACASRDLADVVRNYAVAHGFDLGSTTEHPEGAST
AGEEVTIGAIGRSLLAKKSPPPLRSSPPPKPPPSPKRSPPPRPPPPSPPSPRPPPPPLAADSTDEGPGPADGNPNTP
GDGKVGLPSVPNNDPDNYGQWLLKAVGNVVAVHMCMVPGTEKFFFMERPSGRHPDGKNNIVGYYDYQTNKFVNVNYT
DSVFCAGHTVTQDGHVLVVGGHIAKSGYGDGLKAVRVFSRKTLSFHRIANMTYPRWYPTATLLPSGMVTIMGGTVLP
GAGTGKNPIYEIWDPANPTQLMRRNQSAAMVARTNDIYYPNTYVLPTGHLLLSCNRYGEIMDPMAAKVITAMPSWMA
VAKGVFTEYPFTGTSAMLSLKPENNYTPEVVYFGGQFSYGWINTTASRLALRLKVEWDEAAGNYTFGEGWVAEKMPL
PRVMGDALVLPNGKVIVLNGAVKGLAGDNAAGGAAKANEPALWPVLYDPDAPLGSRMTVLARSNIPRMYHSTVSITT
DGSLLVAGCDRCDKYWWTSASISKSPTSFAEYRIEVFRPPMWFNVTAKPNIVSIDEDTWDDEDGVNVMQYGAPFALT
YSMFYESDKVTSAVLVAPSSTTHSTNMNQRVVGLQVLQHDAATRRLIVSGPPHINIAPPGWYMLFLLNGDVYGQSEW
VRLPGDAPRLDPFMQQYASGSTKK

>g11927.t1|PACid:27565211
MHENIRVPRRASWAGLVPWALVVVLLHLAAGGHAPSSLATEDASSLGDLDGAITSTNNGGRGLLGLTSRFPFCECLS
YDCACSPYKVLMVSRNQTGDTARLCFRVAYVGCDTNTDCCRGMLAAVDKLTFETTAACANRSNLLGVTINGRPHASM
LPYWHGKYTEVKIYSLRRNNATLSGSEICITTRAPCSTLQDFCSSTTFDGCRYSFADVPSATFCPICAMDKPLPPAP
PSPPPPPPSPPPSPAPPSPEPPSPEPPSPVPPSPEPPSPEPPFPPPPSPEPPSPVPPSPMPPSPVPPRPPPPSPPPP
CEVCFFLTIIPPTGYGASLPPSPLLSVSPPAARLPPGAINGTEVTGGGGSGGGGPGFSPSPPPAYGAGSPTAASTPP
GPAASMVPPPSPFLPAPSPSLPNLLPPEACAILAAAIQDTLNRTAMATGALITRPFVLGACSGTYNASALPPAAGYP
YVRVCGAFLSSQVGSRLTAALAQGGAFRQWARLIANGGNSSSSGGGPGGPGGGGGGGGSGDDDECSPLFQGFNVTSQ
ALGPGGETSCLTGQYSRYCPYPSPPPSPVPPGPLPPSPSPPSPAPPSPSPPSPAPPSPQPPSPLPPSPLPPSPEPPS
PEPPSPLPPSPAPPSPAPPSPLPPSPLPPSPLPPSPEPPSPEPPSPEPPSPAPPSPAPPSPLPPSPLPPSLLPPSPL
PPSPEPPSPLPPSPAPPSPAPPSPLPPSPLPPSPLPPSPLPPSPEPPSPLPPSPAPPSPLPPLPPSPPPPPRACVVC
VYIYLHPPGTAGGALGRRLSSEPFVFDDSECGRLAKLIDDGFKQQATLLGANIQVPFRLDHCTPTYLKLMGTFGSAR
DGEMLQEWLNGPLGMLGWLTSITGECPDKLSGYSVESVVQPPDSDEESCLVGSFQIDCFYRPPSPEPPSPEPPSPLP
PSPLPPSPLPPAPLPPSPEPPSPLPPSPLPPSPLPPSPLPPSPEPPSPAPPTPAPPSPVPPSPLPPSPLPPSPEPPS

PEPPSPLPPPPLPPSPLPPSPLPPSPVPPSPAPPSPLPPSPVPPSPEPASPVPPSPAPPSPTPPSPVPPSPEPPSPL
PPSPQPPSPLPPSPLPPSPAPPSPKPPSPVPPSPAPPSPEPPSPEPPSPSPPSPAPPSPKPPSPVPPSPAPPSPEPP
SPEPPSPTPPSPAPPSPKPPSPVPPSPAPPSPEPPSPEPPSPTPPSPAPPSPKPPSPVPPSPAPPSPEPPSPEPPSP
TPPSPAPPSPKPPSPVPPSPAPPSPEPPSPEPPSPTPPSPAPPSPAPPSPEPPSPEPPSPTPPSPAPPSPTPPSPAP
PSPAPPSPEPPSPEPPSPTPPSPAPPSPKPPSPVPPSPAPPSPEPPSPEPPSPTPPSPAPPSPKPPSPVPPSPAPSS
PEPPSPEPPSPTPPSPAPPSPKPPSPVPPSPAPPSPEPPSPEPPSPTPPSPAPPSPKPPSPVPPSPAPPSPEPPSPE
PPSPTPPSPAPPSPKPPSPVPPSPAPPSPEPPSPEPPSPTPPSPAPPSPKPPSPVPPSPAPPSPEPPSPEPPSPTPP
SPAPPSPKPPSPVPPSPAPPSPEPPSPEPPSPTPPSPAPPSPKPPSPVPPSPAPPSLEPPSPEPPSPTPPSPAPPSP
KPPSPVPPSPAPPSPEPPSPEPPSPTPPSPAPPSPKPPSPVPPSPAPPSPEPPSPEPPSPKPPSPVPPSPKPPSPVP
PSPAPPSPEPPSPEPPSPTPPSPAPPSPKPPSPVPPNPAPPSPEPPSPEPPSPTPPSPAPPSPKPPSPEPPSPEPPS
PEPPSPEPPSPLPPSPMPPSPLPPSPAPPSPAPPSPAPPSPKPPSPLPPSPAPPSPLPPSPAPPSPAPPSPAPPSPA
PLSPKPPSPLPPSPAPPSPAPPSPLPPSPAPPSPAPPSPKPPSPLPPSPAPPSPLPPSPGPPSPTPPSPKPPSPAPP
SPAPPSPLPPSPAPPHPPPSPPPPCDVCIMVTITPPIFADPSSVFTFTNDTCSDTADTVIANMQAIAADAGSVIVTP
FSLTACEPAYNLSTSTYPYLLVCGAFLSAYDGARLEATLKLAMSFWIEDLFGGDAQDCAASHPGYSISNEATDPGGA
TSCIQARQLQACNIRKPPSPRPPSPRPPSPAPPSPKPPSPKPPSPAPPSPAPPSPAPPSPAPPSPAPPSPVPPSPAP
PSPAPPSPHPPSPAPPSPQPPSPIPPSPAPPSPRPPSPLPPSPAPPSPAPPSPKPPSPAPPSPVPPSPAPPSPAPPQ
PPPSPPPPCEVCVTLTIIPPVLSDGTSVFTFDNDSCADSATTFIASMEFIASDAGSLIVAPFVLTTCSPAYNVSESP
IVYPYLRVCGAFLSASDGAMIESALQDFMQLWIQDLVGGDPEACMLTHPGYTITSTAADPSGAASCLHAEYRQACAI
PRPPSPHPPAPPSPLPPSPIPPSPAPPSPAPPSPQPPSPEPPSPAPPSPAPPSPKPPSPKPPSPTPPAPTPPSPAPP
SPAPPSPVPPSPRPPSPMPPSPAPPSPKPPSPAPPSPAPPSPVPPSPRPPSPAPPSPIPPSPVPPSPLPPSPAPPSP
EPPSPAPPSPLPPSPAPPSPAPPSPSPPSPEPPSPSPPVPPSPEPPSPTPPSPPPRPPPPCEVCITLTIRPPDILPG
PLFTFDRDTCTSVSDMMIINLANIAADVGALLITPFTLDICSGDFSATESPPVYPYLRVCGALLSAEQGARMQPGVL
AAMQIWIMSLAGGPAEDCSMAVGGYSITSVATNPAGAPSCLDAGWSQQCAFRSPPSPPPPTPPPPPPPPPPDELPDA
PPSPPPPPPPPGLPPPPRGQGGSRAPPLPPLPKPACEVFIHIFALPPNSPATLAGAFSSSFSTSTIFSSGTRVGHHH
HRRRRHLSQLANDFDAGTAAALPAGAFSFDAASCAELADRILSGFAVQAPLVGSDITVPFTQFACAPQQLVLYGSFA
TAYDGYLLYDWLNGPDGMQAWVHAVTGGDACSPELEGYLIRSVVTGPSGELAPSCMSGGFKQQCEGGPAPPPPPSLP
SPPPPLLPPSPSPPSPGRPPRQATPGLRQPSMPPPPPPSPSPPPPPPSRPDLVQTHECVASTNDVPYSVGPLYVRHS
LDQFGAPAVAMCTRVSGRQACRQAAYCCDMDFAKIEVPINPACRNELRRIAVNRRLEDWSWGSYPSGLLTVKFESLL
TDLPVPDGGELCWLVRPNNLCADPAIFCRGGFCQVNIYSSSNKCCPSAWVT

>g11760.t1|PACid:27565230
MAEAGGGSKKGTGRLHSAEPGSPSAGDGGAGPSGSTSPSPPRTGGGSGSSRAPPGTTRKCPECLRNYKGKWYRHKH
IDGEYTCDGCYRFYLKNDHYKPASSPGTAGGTGGTGGGDAVAGGGGDEEEESVAAGGGGGGGGGTAAAASGGGKRGV
KRKADTSPPPDQGSGGSGADGGGGGGGGGTRGGASCKKRGRLNEDADDPAGGGAGGGEGAVAESQHGPGDVKTFARR
RSSQDGAAAAGAAASGRGRASRGRGRTSASGSEDIGGGGGGAAQAGRVAAGSATATAAAAAAAAGLSTITEEEGTAD
AAAPANSAAAPVGPEPDGEESRADAGADAGAAAAGADLDADAEGARRRPAHRGLGHHGPAHPHAQAPPPPPPQPPPQ
PPLGQGHQGHQGHQGHQGHHGHPHHALGGAAAAAMARRSALLARAEEHGGVRCAECGCTESRRWVKQRAAGGGGGGG
TAAARQEGEDGCAPLVCERCEDYHRRHGSYPAAAAAAAAEPGAAAAAAADGGAGDGGLQAAAVKGEAPPDEAEAGAG
GGGGGGSSAGPWPGTAAAAGAAAAAAAAAAAGSDDEAQFQTASERESQEFSPLKQKQPQHPQPQQPQPQSPPAALPP
PSSRSTPSRRSAAAGVAAMLRAAAAADNNDSDHDEEENGDAADGDDGDDDEEFAPPGASRGGGGGGGGGGGGGAGGQ
RKQAAGQSAAKPAARQPRVTPTGRGRGTTAAAAAAAAAAGSTGDGAAAMEADGGGAATPGGGGAAAAPGGSMAGRRC
AACGSTSTQSGWRRHQGGLPGVVVCKRCYDWHYRHGTYERGSGSVAASPRGGGGSGGGFGGGGSPSKSGAAAAAAAA
AAAAGGGGGGGGGSSGGQPRRASARQAAAGAAAAVAAVAAVEADMAAESPTASIRRQRTPLSPPPALSPPPPAPQEQ
EQQQFFSPPPPPQQLFSPPPQHAQMLKQQQQSQYMAAGSGGFAAPPLHTPAAAAAAAAVAGTAVAGTAACGAAPVPP
PHLSPAAFMAAAQHQQHHQHPYQHPHPQQQYPYQQQQQAPLQTPSAVTSPHLQPQPPPPLPPPSPLPLPTAPFAAAY
APPPPPPPSSAAAATPLPHPHQRLAAEPDLDVSELRGQVAAFAAALSRCCQALVELDGSHGGSLLLLGPTPTPTPAP
TPAPTPTPAPAPTPAPAPPSHTPSALALNPPPPPPSESSQSPPQSARRRVYAAVCGGEPRLRCRFLGDALMRWFQAR
AWPCPLALLMGPSAELGLGREAPLPPASPGQQALWSASRRTAAANWAAVAAACSTPCYSAATAAGAVPSLVGAAAPH
AHLCAASFRRVVEQLAAPLAASLLAAPPPAVQPSAAAAAAATALPYVRDAVAAALTLALQVCAASSRLQLRLAAPGD
TVAAAAAAAATAAVEWVSLPVEAPAEAAQQLAAGLRAAAAAAAATDGGGGGADGDAGSAFDGLPALVPAAAPESAAQ
PPPPSPSASALASGGAASDGVSTAVWVTAPGLTLREVRPQYPLQSPASVAAAAVTAATVTAAEAGAVAAAMEAAEAV
RAAVRAALAKGDQDELHCSAEAAAAVVLHLVQALTAAAAASTANGIGGGGAGGGGGGGFAEPGQVVMVVSQPAAKSG
GGGGGGGGGGIRQLPAVLAALARPVPPPPSPPVVVEVPLVPVSVVALRQLGS

>g11902.t1|PACid:27565253
MEASTSSIVCVESISGSGRGERAKWQPRLEHAQSRQPWRHNGPASTWRRCKIAFRWRRGFLSALSHLALLHVIVTLL
WGIGQRVAAANSPLEYWSPDPPSPEPPLLRPPSPEPPSPEPPNPVPRSPEPPSPEPPSPEPPLLRPPSPEPPSSEPP

SPEPPSPEPPSPEPPVPPSPAPPSPAPPQPPSPGPPQPPSPAPPVPPSPEPPSPAPSPSPPVPPSPEPPVPPSPAPP
SPAPPQPPSPGPPQPPSPAPPVPPSPEPPSPAPSPSPPVPPSPEPPVPPSPAPPSPAPPQPPSPGPPQPPSPAPPVP
PSPEPPSPAPSPSPPVPPSPEPPVPPSPAPPSPAPPQPPSPGPPQPPSPAPPVPPSPEPPSPAPSPSPPVPPSPEPP
VPPSPAPPSPAPPQPPSPGPPQPPSPAPPVPPSPEPPSPAPSPSPPVPPSPEPPVPPSPAPPSPAPPQPPSPGPPQP
PSPAPPVPPSPEPPSPAPSPSPPVPPSPEPPVPPSPAPPSPAPPQPPSPGPPQPPSPAPPVPPSPEPPSPAPSPSPP
VPPSPEPPVPPSPAPPSPAPPQPPSPGPPQPPSPAPPVPPSPEPPSPAPSPSPPVPPSPEPPVPPSPAPPSPAPPQP
PSPGPPQPPSPAPPVPPSPEPPSPAPSPSPPVPPSPEPPVPPSPAPPSPAPPQPPSPGPPQPPSPAPPVPPSPEPPS
PAPSPSPPVPPSPEPPVPPSPAPPSPAPPQPPSPGPPQPPSPAPPVPPSPEPPSPAPSPSPPVPPSPEPPVPPSPAP
PSPALPQPPSPGPPQPPSPAPPVPPSPEPPSPAPSPSPPVPPSPEPPVPPSTAPPSPAPPQPPSPGPPQPPSPAPPV
PPSPEPPSPAPSPSPPVPPSPEPPVPPSPAPPSPAPPQPPSPGPPQPPSPAPPVPPSPEPPSPAPSPSPPVPPSPEP
PVPPSPAPPSPAPPQPPSPGPPQPPSPAPPVPPSPEPPSPAPSPSPPVPPSPEPPVPPSTAPPSPAPPQPPSPGPPQ
PPSPAPPVPPSPEPPSPAPSPSPPVPPSPEPPVPPSPAPPSPAPPQPPSPGPPQPPSPAPPVPPSPEPPSPAPSPSP
PVPPSPEPPVPPSPAPPSPAPPQPPSPGPPLPPSPAPPVPPSPEPPSPAPSPSPPPPSPGPPQPPSPAPPVPPSPEP
PSPAPSPSPPVPPSPEPPVPPSPAPPSPAPPQPPSPGPPLPPSPAPPVPPSPEPPSPAPSPSPPVPPSPEPPVPPSP
APPSPAPPQPPSPGPPLPPSPAPPVPPSPEPPSPAPSPSPPVPPSPEPPVPPSPAPPSPAPPQPPSPGPPQPPSPAP
PVPPSPEPPSPAPSPSPPVPPSPEPPVPPSPAPPSPAPPQPPSPGPPLPPSPAPPVPPSPEPPSPAPSPSPPVPPSP
EPPVPPSPAPPSPAPPQPPSPGPPQPPSPAPPVPPSPEPPSPAPSPSPPVPPSPEPPVPPSPAPPSPAPPQPPSPGP
PQPPSPAPPVPPSPEPPSPAPSPSPPVPPSPEPPVPPSPAPPSPAPPQPPSPGPPLPPSPAPPVPPSPEPPSPAPSP
SPPVPPSPEPPVPPSPAPPSPAPPQPPSPGPPQPPSPEPPFAGATVPHTPPPLPMPAPVPAPGASSPTPPSPHPREA
PSPEPSAGGPPPSPEPPSPEPPTNTSSVNVTVSLSGVDYALVTASSTELEAFKTNTCTELLSATKAGSCVVTSVMAG
SVTVTAALAPAVAGAGSSPVAMLAALSSAVDALVSAPASAFSQVYFTAYGVTSVRVIKGGTGGGAGGGEPGGSGQQ
PAGSGGGNGPSPVAPLLPPPTVPGEPPSLSPSLSPFPIPAVLDVPPSPLQPSSPLPESPSPSPVQPPNPLPRVPPSP
EPPSLPASPSPPSPAPLQPQPQPPSSLPAFPPLMPPPRPFTPPPSPPSPVPGDIQLSGLASPPPLPPSPRPPRPPAP
PPSPRPPPPLSLQQLLAAPPPLTPPPPPPAIAVPASWLLTAAAAAAGGTDGDAGAARNPDITPPTVVLVGSSTVAVV
IYEPFQDPGAYAVDDRDGRVAVAASAVGNPVNTNRLTAGPDQAYLIMYTARDAANNRGVAFRRVAVYDNCTAPEFRC
PDTRGCSVFGRCSAQLGALSSLLGSSYDGRDGIGADGSPSPGAVVTDGDVPVARQRAPDSIPPELTLRAGEDSARGS
VRSTLFVDPTSSSTNSEGGALRLVTYVPVGVKFVDPGATAKDWQDGFPGGPPTSTDISDRVLVSVSPSSGVDTSHPS
RPDAPFRLTYTVRDLALLAAAPAIRLVVVECPGGADPCPDPNDPDGPYMGCSVQGVCAAGLQPLMNTEDNSVSGGSG
STDSSGISSGGGGSSSDSATALLLLNGQESVTVPQYTPYVYGCFDAVGCDFGATASSSTSVFGDLNTEVLACAESVK
NPEPYLNVGLQYCGINTSVVTTYYISYTLPPAKYPGAGAVYRKVVVQASCAGRTCADGTCADEDDSCPGDLLGIISS
SSGAGSGAGGSSSGDASAGNTASSGSGIAQAAAQATTPPPLPPSLTLRAVVLRDGSVSLKQFTAYKWCAPGQPPTES
IPCEPGAEATDASGNNLTSQVVLCPPTDCSASGCTGHLPSVKAVSDCGVVTETAAVGKVFRLILAVFDSRGLNATAE
RIITIVPPCSTGGSYCDGACYSITCEQRAALLALQGPGADLSAPPQVYLLPTAADAAALDLLSYGGGSGRSLTLVYG
TPAPLSFEPCTSLRAAMAASAVGSSGSGGGGGAGGGAGGGPSCAAVALDAQDGDLSPEITVTDVSPCPAAGNGTALG
ASDREAGADGASSNSVGSSSSCYRCTASDLSLGSCLPGKYVLRYAVVDNDGIQGRADLQVAVEQRLAVTLGAELRFD
ASPDPALGASGKASAAAVGAAQAAAAGYAASLAANATEAFAAALRLVPLYGIAVDSVRQVALLGVEVFVAPAAVAIA
DATGGGSVAAALVRVSFNVTVASTERPALLAGSSSSSSSSQGTLTSKNRPTPKSTEPPAQATSTPNYAA

>g11855.t1|PACid:27565317
MKWSSAPARDGGGQRLCVDLQQFSLLDGRVPGNQLDAVGFQQLMGTDFVVHDPSHFLPALTALLLDTEEQAPAPATA
TAADAPATPATPAAAAANRGQHAAAGPSALGSSASGRAVTLRGCRLLVPLPPPPRLLLDGRPHSGHGAPGAGHAAGT
HKQGVSANDGAGAGGGDVGGGNGGGGQWDRCRQAARAALGAHVPTEEVAPAGQPAVRPRHLGQDQRQRQGDTAGGVA
VLRVLPLSLQLPLPLPLPGPVPEAGSLPGAGPSLGQERPHPNVAAAAVAAGGAVRAAAAVAALELRLPEGGLEVVCG
TAEALDGLAPPPAADRAADGAVAPAGLAAMAARATAPSGSDNTKAAVAVAAAAAPAAAAAVAPAPGMAAALATTATG
ASGVWHRQRSAMAVPGGNVVNGAYGAGGATGGSSGGSAGLPAAAVGALCAAAAAAAVLFGCGWWRRRRRAGPYAQEA
GGVELGSTTAATAAVHSQPAVLRLAETPQAQQGQQALLEREPVKALPGGEGLRGGGADGARAGGGGGDVAAPEAIEL
DVLAGPMSDSAMTAAAAASAAAAASGQRLAKAPGGFVSDTGAPGWLSTRNGGGGGGGGGGGGGGRPGSSGGAAAAA
AAAGVGDAAGGGASDLEASNCFTEGGADRPRSLQLISTTGGGSTARSGGGGGGGGGALGGSRLLADVMRALRNGIAG
GGSGSGGGSAGDVSRGADVRVAGRRPGTMAGAVVAPPDGSGDGASPSQPHFATLSAAGPDSAAAAAAVQPGGGGSLT
GGVGGVGGGSGGGGVGGGGGSGGGGGGVGGAAVAAGSGSAGGSGNPLPSLFSSGGRLGGAKLPMPLTLGGGGGGGGG
GAAALAAGGGGGGGGGAAALAAGGSVGGGVGVLGTWGAGSVPAGGSGSSGVLFAVGLGAGGGGGGGGGLDVTPTFHR
MSPPEVLFSTQDIPECPEEEEEEVAAVSAGGRLAGGGGGGGGAGTGGSRRRNAISSSGANGNGNGSGAEDLTANASS
ASAARAPTCLYGSKPYGAGGGGGGGSAVHPEVSAARERVAAMLESLMSSPALEASGGLVAARDVIIDTNAELGRGTA
GATYRGRWRGADVAVKIIAYDRAANRKLLAELLPVSAVSHPNVLCVLSHFYVQAPLSSRMSPWTLHASSAQQQPQQQ
QQPSQVLMSRGVMAAPPSAPHSGISAAASLSGSVTAAWLGHDAATSALGAPVPGTAAAAAAAAAAAAQPGVAAAAAA
QGFQVSKLDPPRPLPEAPSVDVTPPASAFTSTANFSNYLLPSYQLTPNQLSTASSRGGPLAMSAASPPGPAGANGNS
SMPIQLLTTANHHNRHPYNRGLGTVTGVPAAAPAALTRSDSNGGGGGGGSGGGGGGSCGGAGRTDGGSAAGMMMEYP

SAAIPSNTLLSPTPPPGSLQPSFGAGGSGNRGETNLLTGPVAATGLGPGLGLGTGPVEVSPLPLSPRATGAGGPPAT
GYKLSAAALLQLQQQQLRRRRLQQQQELLAKHLQDAQQRRQAQAQEGPGGGGAGGSLAGPQLAGAAASPEGSIAIL
PTELPTHAHLRDTGDVSEGTAGGGGGSAASGAADIGRDGGGGGDLNSSHGLPPMAMASAALRGPSTAPNDPMHQSYG
KDLHGTSMSSLLAAEAAAAAPSSSYEHLYGASVDGVGGCGGGGCGADSGGGGGGGGCSGGGGQHQPSASVAGLLSEG
SWGTTPGASGLVSELLNQLNATTSAAAPKVISPQTAQERSSVGAAGSAASGSGGGTNANSGNGGGAAAAAAVTGSPA
KAAAEGPTRSQLYGLIDIAPSPTGSSKAISDIHCGWDGRRTPSGEAPEARSGELGTAVAMAQAAAVAATGGDDARDG
GGSGGRDGAAAAAGTRASEGRVMAFELLAGSGGRGRGAARSFRSGGRGPVAPGGAGGGGGSGGNAPPYSGADTPQQM
SGGTGTYSGAFSSLQRLGSAQTDGEDDWSGRPKRDPALLQAVAWIAASGNSGAAGAADPIRGIAAPRNEKSAALRAA
AAVASTVATAAQAAGAPVAAEEAAAAEEAAAVTSLNPGTTSSGYSVTGGPFRSGVLRVLSPVNETGSSAGEGGSGGA
TSIAFSVPAASPFSLPPSPGRDASTAAAANRREVGGGGGDEGRDAPRSNRAAASGSAATTGPTGPSEPEHQLHDTPP
NGTAQAVGDGGSAAAAGTAAGAAATAAVSSFARVSDADSHSSRSAGHSRKVRPAAVASATNNDKLSAISSPLRKLVV
PPEGMLSAAATMAAERRLHSGVEPQQPPASLLASAAAAAVAAAARVSSGAGAGVDMGGAGPSSGSRMFAPHPQAPAP
APTHAPAVQLPLPPSPFADAGLEHRPSMGLLNATPPEPPRQAQPAQPAQQPPPPSRGAGLEGTERWPRDAGGTGRLR
TDGGGGALGDDAAGPASTGVVALGGGPAGSSTGGGHSAGGGAGSAGSAGTGATTVTAGSALIFAVGSGAGAGAGTNS
SSLTAQLPPPTAVGTQTAAAAAAAAYNAAAAVEPAVATAFALARGTASTSADGSTGTGAGRPLRVLLSESAVAVAAA
AAAEHGGGSHAAPPFALPSLGPVTPASGPGGSPGPHPDRDGTPAASSVPDIARGSSDDAEELMPTLAPLRRPPADGD
DVAAAAGASLPPPPPPHGAGGSPVIRSLPLRPLDGADTPRLSHESDRGGGGAGGGGGGRIGSDWAVCFDSAIVAGAG
AAATTLRLLGARGSAGASSAAAGRGSGGAATSSSYGPASSGGGAAAAAGAKASPGDASGGPQDSPVDAASSGPGSIS
AAVDANTRYNTLQQLSLPAPDLGGSVYGPAGGIPSTATSKLGLDDLLERLNTQSSASSLHTPPAASTPNGGAAAAAA
AAAAGHSATRPVAAPGGGFAAGGFQPVPLLSQPAPNPAAAASPGGFTGALMGVSRYGDSGYGASRYGDSGYGTSKYG
DSGYGTSKWADSGYGTSKYADSKYGDSGYGGSKYGDSGYGAGGAAAAMGTGNVSGIFPMSSAASNLPRVPTPAGSSA
AGTNSNTASGSAGRSDRGNNNGRASGAVPGGFTPTTHLAAVAEQDPGSASGMGRVAAAALALADAAAAATGGALAAP
LLTPTALPSAAARPALSGVTKLSFPLQLALPSRSAAAATAAAATAAAAATAGTTAAAAAAASEAAPDQAGSASAPPP
PPLAAQPHRALLPASLRKPASAAGVAAAATNAAGAAVDFTFAPSPPVSGGAGGGGSGGGVNSNSAGGGRGRTFAVQP
LELLPPPPVAAVVSASAAAGPVPTASRYTTAAVGGASRPGRLLGASGRVGGGGSGHSGGVPRARVPVIPIAPTLDPP
MPGPPTAPFAGAANWARILAAGSGDARRRTSLDGDRDGGGGGGGRGGGRGTARHGDDGASDGDRPSSDRDAGRTDET
DELFRGLVGSTGGRGSTRSQLSPPPPQPPQPPAPPALPLARLAAPPPALPPGLATSFSAGLSRSSTARSVPQVPSRL
SIGGSSDGLGSGAAGCGTPRPRSSLAGGAVAGLDRRVASAAASPGGGGGGGGAGGDQRSSGRSSLGSGLRALMLDGS
EGSGGVDRSGSSDLAVGFTSGEAAAAAAAAGQRGGGGGDMLQLSAPALPRPLAALIPPAAFPTAVDVGQVACANWSA
TGDEATAALAKEEEAESVTSAGGGGDASSTSWQQLAAVAPPTGAGPSRMVATRLELAAPVILRSPVPTDGHPNFLVP
PNRGLAAAAARRRSSSAGAGVSGGAFRAAAAAALAAAGGGGGGGGGGIADVGAGAGATGAAAAAALASRGLAMYSRR
SRRQLHQLRSMPMVRARSRLGLQTVGEVLLTQAAAVAAAAAAAASAGAGSDSGAAGALLQGLRGGAAGGGSAGVGGG
GAGGRRRWSAVFPTYELGTAGSAYDDVLDLYDADETGSTAADEDEQIDRTSAGASSDARSTVEGGRSSREFAGAGAG
GGDGMTPNGAGDSRRSAGCGGRGRSRRRARGYQSEPALVPAPRRRKSTDGGLDGVGGVGGVAAAVPEGRSSEVSVTG
GAAGASADMHWAIAQGLGSASGSGWGGGGREAGPAGATVGRMVVVTLLPASQRVGLGVGVGVGAGTVSIAESGGSVL
RADAGGSSAGSHSHSHGGSGSAASGCSGDVFETAADAGEGAAAAEAMAVAAGAEELRLVEQAAPSAVAAAAAHFLAP
SPVSHANAHAQAHSHMLGAPPLGADFTEAMSLSGSPSHTSAGHAAADPAEPPAPAPGLSRFGGGDGGDGVVAQGSLS
TGGSVALAGPIGSRTSSTSQSGTPPPSLGGGGGPLLQPAMLPSPLLQPSAVDTGLAARVGLPPDVSGSGQLSAERLR
KLTVVTVLPPSAAASAAAAAAAAAASERRQSAAAATGAPPAATEAAARTGAASAGGAFVGVLPVAAATAGADGAAGS
GAAAAAAEGAPLLPPPPSGLTLKLGHGRSSSSTTAVGGPLLTSLTGTGTGSGSRSPGTLGRGSNAATPMQARLSGGG
AGGGGGGTGTTAILSSTASVQSPSLAALANATAAGGAGGGGGMSQHALLTSSSLGSEVLTLAGYGTPISFEPWRNSH
ASLTRNSAASHAAGLIGGGGGAAVGSQALSLLHNGGSSSTHHDLSERQNATGGGSGPEAAAAAAAALATEPPGGSAT
GGGAGSAAVMAAGRFAVSSLVSSDSGDGAGRSDAPPRTAAPFGLPPPPPSRYLDRLPEVGESGSADVGSCAVGGGSS
SGGGGAGLIGRGSGGSGGGGGGGLIGRGSGGSGGGGGGGGDGLMDGLAFTPPPPEEPAALNGTVSEAEEGGSGPLPP
PVTLGDGVLGDPQQQQQQQQQQQQQQQQQQQQPQQGAADVSRSGHGSAAAGPAGGGGGSGPSRRTPFRSAAGGAAAA
AANAPAESALMPIDVLGHSGSLPSAGGGGGGGARAAEAADAAGRPLAAMLRGRARSAVCPTTVAAASAAPAPPPPLT
GQRGVAAAGSSVSSSVSDVNGAGASSASRGAARRLLLQPPPALGGAASLQPPVPRAASLPAAATGSAVTVMHARAAA
GAAIERDAGGAAAAVASGGGGGAGSGAVHGPSSVGHGLSTLEGVSISDSIFDRASLPNSELLADQSTMTEPITPRFQ
ATTSIGGAGGPGTGAASASASASVARTPYSGGHGSAAGAAAAAAASAQQQSQSRSQSQQGSIEEDADARVRVPTSSA
ATSRTGPAFFTERLPVRPPAPASSSATLGRTARSAAAAAATPVAAQPTAYGGSGPLAMAAAAAAAAAFTSPAGVPVA
LPASGVGAGVAAPPPQQLPRSLGLAAPPLGRPPSGRHSTSGAPGDTFLVPMPTSLGAHSGQSAGGAATGGGLPPPPR
RNVGVHSISNLRGVPEAAPFLVPDLDSPLSPGVGAFGAGSAAALFCGGGGGGAETAPPSPGLTRSFDIELLPLPLHR
TTSSRRGGGGGGGGGGANRTRTANIDALLAARSPPPSTRTHGGGGGGAVWPHRAASSSQFVPSSAMATALARQKASS
ASGLHPMRMPMPMPAVGASSPTHPLQLQQQQQQQQQQQQQHSGGLAVAAPAGSGATAPPLLPTSKSAEGPSGSEAPG
VWRAGNLPAAAAAAAAAAAAATAAASPAAAVGIPQPLLLPIAVSAVPAAAGGPSPSPSPPPPPHPLGAPSGGALVPQ
HCLSAPPTPAASAGSNFTGLYASGGAGAAGGAGAGTIVIGAGGGALSGGAGGGRTFGGGYGYGYMPPASRSGPQSHI
SGGFSGLPISSSGTPNLLGSGVYDINNNNNSSTTQIPTGGLGLDELLVPNGCLSAQAWLQSAIAAACGALAAGQPPA

AAAAAAAAAGPPAGPVSGGAVSGGGGGGVCCTLAVVVMELVPGGNLRLLRSRLGPPGAKVDLLSLLHLARGVAAGMS
ALHSRRPPLPHGDLRPENVLLAPDPAAPLGLRPVVADYGFSRLMLTGRSAALTRPHGAATHLAPEVWADGSPSRPAD
VFAFGVLLWELAMGRPPWEGLSAGQVLAKVVLEDRRPEWPPCAAAAAASLAAAAGSATGSTAGSKSGSKSGSKSGSA
SRAAAAAAAAEQQQQQHGEGGQQLRAGRRVPVRTLDGTRGGAGWLPRGYTQLAQACWSRQPASRPDFAAVLQWLDEL
IMEQYMLQPPPPPLQPPPPPVLQPQLLQPAQQIQLRLLQASERSRRLLQQHAFPHPHPSPNPHQMWLQPPMAAAGEP
LRAAVTWSRGGAGGGATAAAGAGGSSSGAGAAAFGGRPAPLASSAAGAARPGSRGAVGLLPSAGGSGSSIQTALATE
ESAAAAVDRLLRRVAAAGGAAGGMARAAIRDATGGRRTGGRGAGAAGAGGGGGGGGGPHPPPFISIEM

>g11757.t1|PACid:27565363
MADSRQVAGGSRPQRGAAAKASQAISSMYRGASQPDGAPAEGAAAESGRPSLSPDRPSLTRAGRPAAALPSPRAADG
AAAPAATARKLRSNAPGLATAPGLATGRANAASNTSGATTQQLPSATNSQQHPQQHHQAGPEPSASAEMGPAAGTAL
NANAEKFGGCCTCGSTNRNWGYIHADGKYSCHGCCYRNKAENGFYGPLGTRSRAELDATCGSRACAECGTRNAHSWR
GHKMTPGQMVCNACHMRWKRTGTYDRPAAAPRSKPGGGSGGGGRRRTAGGGRGAGGAAGGGGRGRARAKHRQQQQE
EEEEEEEEEEAKRGGGGSTDGSTASSSGSSSSSSDDDGNGDSSSSGDEEEDGSGSSSSDGSASSGGGSDTGTDDDGE
DDAGTSDSGTRAAAAAAPPPARRRPPPAATRAAARAEAAAGAAGGSGGRAPAPTPPTPTAGAAALEGTEHAATASAR
PPRTPSPRAAAAAAAPNEPSPSLQPAIISSPDAAVAAADAPAAAAGPSGGGDGAGGSGAGSAPPPRVPTPTTTPAKP
PPTAITAAATHDAALLSTPTPPPPQPQAAAQLAAPAAPAAAVPQPFVGSCCGACGATRSVRWYAHKADATGLLKTCQ
GCYDRQRRHGAYPPPPNEEAAAAAAAERRAAAAAAAAGAVAGGAVGTAPAGAAGTAARQGLGNGGGGSGGGLVQVGR
ARPAKSTMTPAAATAGAAFGKSAAGQGNGALPVVPLLAPGPSRPAVFRTALMAGGGGGGAAAASKVPASTAAYLTPT
KTTATAAPPAASAAAGRSGGAAATLNAAASAAEVAAEAAADAAAAAAAGGGGGGRRRCSVCGSAGTSKGWRTHKLDP
GLGPTCRSCYDAFRVHQSYNVRRDAAAATARRSSGGGSGGGGGSSGGGGALAAPSTPTPLAAALRRQHLASPLASPL
GSPPSLLSAPPGGGGAAGGTAGAAAAPQQPVLSGRMPPLESRVCAVCGATGCSAWRQHVFRPGQVTCRGCHERFRSH
GFYVPPLSAPRQQQQQQQQHLPQTNGAGSSGGGSGNGGGGGAAGGAALGAKRGRAEPQPAATAAGAAAGGAKRPRVT
AADISAAAAEAAAAVAAAAAAAAAARLRPGDYVVPLRHAPKAVAAIASATGGAAAAAQQPQPQLQPQPPMLLRVQTVG
RRGVAVVLGRDGSRIRVRADHLAPWQPPPPSAAAPAAPPPPAYIRDPLSERRAAARAMAAATGGDGAAAAASVTCCE
LLLLLQPRQQQQQQQPSAARRAQQGAGRRAAAALPPAEEEEWWDEEEDGERMEVGEEGDMDLEDYDGWEWDWEEEEE
EEGAGEDGCGEQREEPVPLPSAGQAQRQQQQQQQHRQQLLQERVWERPQRLLQQSQPQPLPLPLPQAGGARSAAPQQ
QQQPAGAALAPMWVAAAVAGAAAAPAGVPPRAVAAGSGAPSGSGAAAAVAAGAPPPPPPPLLVGRRAKLLQQQQTQQ
QQQAQQQQAQVTSPAGVRPTHPHLHAPPLQPQSPPHNPHYHRQQQQQQQRPLAPHSPKQAPPPPLHQVLQPPPPHHP
PLQPQQQQQQQQLQPPLLQPPAAALLPEGQAVSGGVTAGEVERLGREAAALGAALQEACRRLAELEDVHGLRLLAPW
DPQQQQQQAAVAAQQDRGSAAATAAAEGGGGGGGQPSQSPQSQPLPLSQSPLRAAYEALCGREPELRARLLADVALR
WIEAELWSSPLALVLGTYAVGVLGIDRIGPLPELLTGREADARRQWALDMARRGVYERHLAVADVAVADVAAAAAGG
AGGAAAAAAAAAAATADVSAAAVARFHQVVAHCAESLAAHMLGQPPPPPLPPPNQQAPPLAAPPPPPPLALLPPPPL
PPPGHYYTPYGGPPPPPHLALPAPPAAVPPPPPPHHYQQQQQQQQPYPAAGGYGWSAGPAGGQPPAQALGPVGGQQQ
QQHPHPQPHSTAFAYQYPHPHPHQQQQQHAVAMRRSITSSAGAGGGGMAAAAAAEPHHHSPPPPPPPPGPPHVHLEP
PYAPPRHHHQNHPHMQHPQPLLQPQPPPQQQQQQPPHPAGSGPWSHWMPPPSRPPQQPPPHPHEGDQTPEPWWRQQQ
PHPPPPQQQPQQPQQPQQLYRPQPTYARPPTLPPTLPPQQQPPQPQPQPPQQQQQPLPLLPLPPPPPPPASAGPDVP
AASPPPLLPPPPPPPPQQAPAAALVAAAPAVAPAAAPAPAAPAAGPAVAAAPATAAAPPSSLATPSQQAPAPPPPASA
AVAALASPRSTQRLMGGSTYLLLGSGAGGGGGGGGGGGGGGGGGGGGGGGGAGGGGEGAGSLAVSGSAAADALLQA
RSAREEGPQAPPRMGQLQPPPNLAVWEDVGGAVWEDEAAGGETTTEGRQAAGRVVRQVARQGEQGAEAAAVAEAAEA
EAEVAKAEAEVEAAEAKAGAADAEDETAEAAAVASGGTSAAAELSPARSQPLALALPLPSPLCPQHQLLQEQQEQTP
PALDSPPAQEEKADVSAVHAGPPAAAAGQQQLQPVQQAQQQLLPMPPPLRLHLQPSPPPHQQPREQEHAEQQHKQQQ
QQQQQQPQFTAGGGLAAILAPLTRSLAAAATAATAAPAVAAAAAVPAAGPGGGTGPAGQADGAAEAEAAVEAAEAEA
TVKVEAAEGMETAEAAEAVAQAGVVVPPPPRPLPPPPSPRPPPPLPPPPLAATSTAAAVGLGTVSVNDGAAPDPVAA
TATAGGSGGGGGSPQQQGTPEIHRSSSSSSSHGRVPSPGGSAVAAALVMRPSSDQRRDPRSSSSSGGSSGGSGGGAG
GTSGGGGCGVIVAVEATGAANGMELPLRAGSSSMMSEQQQLLPPPPPQPHPGMQQASGGQPPPAVAPAAAPETEPAA
AAAAAVGSSAAVAAATQMPPSSPEPQQQQPQPERPQQQQQQALLLPPHQPPPLPPFTAPPPPLPPPVAVSSDMGGIP
VVAAPPRGATGGRGAAAVGAAGISRGGGWSAFGTPFAAAARGGGGGGGDAGAVSMAPAFPPPPLQSGSTAPGSYGDS
WRDGGSFSQQGPDQQHRREAQPAGPPPPHAAVPTFSAAGPEQQQQLQWQQLQRQQSGTAVAGGFTRTGSSSAAAAAA
TPPPSYPHSYPYPPHGPQPPPPLHPHPHPHPHPQPPPPQAGSSSYSSYSSHPHHQPPPPQQQQLLPPPRPLQLLPPP
LPPPPQPPPPPSSSRDAAAGQQPYGGSPHHHPQAPGGATGAMGSYGGGPATGTPSQQPGSAAGGGYGPAASGTTPQQH
HNGGSGPYGPGAAGAGAGAASYGPPGGYGTNIVAAGPSYGPGYGGPSSISYHHPAHPHPHPHHPYPYPHPYPHPSAA
AAADTAPSHTPQFHHHASLHHHAAAAAAAASRASHVRAAAAAALRAVTAAALRLAFLAGAAGNGRLALRLAPPGHSLP
PLPPRELASLFASATLQAMAAREGRGGKEGGEGRGGATGVGGGAAANTPHLVRVGGGAAAAAAAAGGGSSGSSSSSS
APPQPQPLFRASEWLLPGAAVAAATAAAETEMEARLELRRLGYDDNAGSGCGAGSGSAGGGGGDGGRKRALPAADED
GGDGDGRQRRWAPRPRLDPPPVQTQGGADGDEGAAADAAEEAAAAADAAARVAEGGGGTGGGGGGQRAGTTAAGRAG

GSGGSGSGGISGSLRVGGGGGCAVLWVLAPGVVSREARYRVPPQQQQQQQPHPAASAAAAMGLQPQQQPQGGGGGSW
SVEEVPLVPVDLVGSLL

>g11466.t1|PACid:27565464
MRSPEARGASSGTSCTKQHFELCSSSCGSAAFWPALVRFFAGGSLASLSESRGGSGVRTAGAASQNYAKARRRGYTR
CWALARLLSVCIAFCCLAAPTRALTNSYCSSFKGTGACPPCRSPFDLCQPVDSYWDSPDFCVLPDTPAGSGGVGSCG
PIVTLDMVSASDRTAVAGQLIAFQTPDDYLYLTFRLACPFALRPGFSTDVPYNLGVYLWNNTDMLGNPQYADGLTLS
SGVYTCLSVVVDLRRVCNPARSVYMSNTPSAQANFNCGCFPGSRPCPPSDMSAAQLLVAQPAFYTLPSPANCKPAAG
TALNKYIANTLDGSGLLQWDPTACGPVGTPKRSPNPSARPSEPPTGVRNDQLPDAPPDMSPPPSLLSPPPSVSPVPA
SSPPRGALPPPPRLQEEADSPSPPPLPTPSQLQQSPPPAVAPIPASPNTSQPSFGPTPPPTPATPTPATPAPAPSSA
ISNGDTGGDVGGKNTTTSNTTSPDPPQLQPAPSSPLPAQSSAGGGDGSSSFLSGAGIGIIVGCAVIGVAVVACCCIT
IYVYCRRRAPARSASSVDGDSLARHSTKSALGLGLGRHSSRSDRSLTNFSFAMVPHAYEYESGGGGGKEGSLRGGMV
VLGGGPGDSSRHGGGRGSSGRDMERGGSGGGGPGNGNGSGGIYTYALDGAAASGGSATESHFSYLSRLSGHDNNLGS
RSRPLSSHGDGFVPQGFTELDGPVPLPVAQPVSSSALPGAGGADPRGLLQAHAVAAAALRGTARQVAGRAGADGNGA
GSSAIELPPVGPSGNSTSGKASRGSKGRKYFLFGGGSSAGAGNKSTGTLASAADIAANAALPLRRLSAHDAADDRHR
HDHDHASEVASDEPGGGGACGSSFSADTTGTLLPRASGLSTSSLLSAFSGASGRLGRLGGSRSPGGTGSRSPVLSGS
HAHTAAAAATTVDLLPELPMPPLPPPLLQPTTSLEGSLASVSVARAGSSVSPMAGATSVAAPVTPSACAAAAVTQVD
ASEPSLSGRSSIRSPSTTTVLASAGSLGGHALLLPAQGSLGPNHHHTGGLLHACVSGAMGAPSGTSAVAVTIAAAAA
APFSTSSRAESSAGASPLLSGRPSLAAPPLAMGGPLLAPAQSLGGHSGAVLSSHPSGGLLGSRGSLRTAPSATGGVP
TLAPSSSGHNYPSAVAVMTTSTAGPTASYSSVSSHTAITTIGGNRYPSLFNDAHPTAAPSMPLVAVSAPATTCASPG
TTTPATGLPTSRRPPPPLASIPSLGTLPDHLPESSFAIHAPLNLDDDDDDATLSGTMLRAGATGEGAGALAPYGSAA
PSPSLTPSGGIEAHSSATTTSVSVGSAVTLYSASLRRAGSSSHGGVGAAGTAAAAGSPFGSMARAATTGGSGSSSAS
GPSSLATASSNVASSNASSAAARLPGQASAATAGGSRSGPNSQGGAAGTPPSSINPAGSMVLPAEASPFAGATMLQQ
QLALAFLQQRASLSPPASPPPHSAGGMSPAPMSAPAPGMRGAAAGGAVASSISSAIAASGHRDSGKFTFNNNGTGPG
EKLLVSTGVGTALGLLRTAASAAMMDAYVEKQRASAANSPLHPGRASAAAIPGTSPLPSPTSATAAAAAVVVASSSP
QPGSPRSASPAPLPHVQSVQSGPHGSMPQPVSPQPHSLPMTPPASLLTSSRSGSGSPLPVPPLLAGAPSLASASSAA
LGSPSSSLLQPAPSLRTTHSVATDGSSGGASLRSTESVPAPGGCGGPGGGGAASMDLAAIRRYCAEGSRDGEERSLG
GVGGGDVLTAVYNAVCKLERGTLFCNKYKVRDSPLLGRHSVVLLAKNVYSKARVAIKFHATRQQYEREKAALRALSS
TYVPALEEPLEGPELAAMGLPPALVMEAGRCTLTEWLRGPGGVGGAGRGPGRDLLALKSALHQVLGALVHLHSRRLV
HRDLKPANIMWFDGAHRFKLVDLAEAVPVGEVAPPCCTPLYSPPEQLRAALEGQACASAVTPAADMWAFGVVAFEVL
SGSRFYGPSPSLQAVLEAVFGHSPLPCEGGEASALAGVELAQARRLIAHLVVRPPAQRWSAATCHRNALFASGDDTD
QRARAWDARTQSELRPGGVGAGAGPGRRGSQLQPSLAGGGAGPQAAQASASPPERG

>Cre14.g613250.t1.3|PACid:27565512
MPGVMGGTPPGYPHDGPHGFYHDVPQGGMDGAPPHMRHQPPGPGPAGPGPGRGLGLGLYGGGGGGPVSGSGACPPTQ
HAPPPTYQYGAPQPQLPYVLPQPPQQPPQYDYSSYYAHARPQGPYGAASGPGYPPPLAVSGLASPLAPIGSPPAPPP
GISPTGRMPPPLAPPLPPEEPPQPPPPGEGSGPGAATPPPLPPSDDGEDGECDDGGVGTTAAVAGRGRGGGQAAPQL
SPPQQPSAGGANGSGKGAADGAGTGTQGSAEAASAGADVGRAAVEVVAGAGAVQAFTIKAAKPPAVISHNTLVGSRR
GLPVGVTAAAAAAKGSKAGTRAKSTTPQPSFGGGDVVDYDFDGTAADKGGGGGAAGGAAAGAALGAATTSTADSAAA
AGGDAGKDTAAVQAVAAAAAVEAAVAAAAAAAATAARLTSPASRATTPEPCIAAVTATRATTTITTTTSAGTRSPLP
QRSPGPHRWGCAEPLAATAAAAPLAALAGSPLHARIGTTAATAAAAVAAPALAPAIVAAAAQLPPQLQQKRNRWDKK
TAQEGEAGRGADTSAATSTVATAAAAAASEGAILMEDIMAQLECMKAARRGSHPPQDPPPVAAAAAAAAAAAAAAGA
AAAGAAAGAVTAAGAAAGAITAAGAVTAAAAAAGAAAAGAAAGTVTAAGSVGGAAAEPPGVPAGHRSGGATPLSPPH
AVAGAAEQHRVLARSPGRRDSRHGGNRSRSSSRSRSRSSSGSSSSSSGDGSSRSSSTTRSHSGSRGSRDRHRRRRES
RPSTGAGAGAAAKAPEQTAPAAAARTAAAATSRLVSPAPMGAAAGGARESGAGRSRDRSRDRSKGKERDRDRNTDRE
RDRRRDSSRERERDKGREREREPRGRERSGGGSERRSSRDKERSGAGDKAGRQERDRSRDRRRDRSRDRARGASAAA
TAAAPANGRPGSRSPLRRPPPRSPSPSGATGRHTHSRGGGRPGDAHLPGLGPYGSHGSHGLHGLHGLHGLHGPHGSH
GRERRLHEPEAHGRDRDRYGRDGRDGWERRGPERGERGTYHAGEEGRRRPGSREGSPRGRPPPASPGGRLGATYSER
ERERERLRGPRVGLGGEGHRSRPGSSPPRSPPPPSYEPHRDAAHRRGGGAAAAAAAPAAAPAAGHSSRLDAATGATT
APVPGHAAVDGALGHSPLPPLPPGPPPPDAQTVTVAAAAPMPNYSPFTSDDEDAMILSSDEEGAVPPPPPPGAPPAP
APHGSQRPPQLPPHLQPQYGGTAAPPQGHHQPQAHSHSHSHHRHGHSHHSHHSHHQAHAPHHSPHPHPHPHGPHTPA
PPSSPKPMVPPGVTDEHLAAMREAYRSEVGRLLRLYGRAGGEQGEQQAQPGSGPRRPPAPPAGVQALQLISEGYHAT
VDAVELGKTDSEDVVPVPMPAAVSAPGGHQGGRGGGDDDDEGASPGPENAEQLPLTDETRVFPWAVDRGAEPADLR
VPPRADQPPLAPASPVFLLAVEFPSDDTAFQPWHLLTWPQPHQQNQQQRAGGGDAAAAAAAVAGGGAAGSYGLEYAG
SAGETVLAAAAAAAATASDPEIGVYRYPVTVPNKTDAKLLCTERRAEPLAKLRQWAARGGAEGSAAEHFLPVWGQSV
QRVADGLWLPLTQELITPEQLRWEARREDAVAALAAANTQPTSATALLAAAFAAATASAAAAATGSAAVRPTSAASG
PGPLAAAGASRVSRVAGSASPGALPARASGESDLLELLERSRGPDCWLWPRGSGGSGRPGQGGAKRTQPLANGRSGS

ASLSARATSASPPPPAAAAAAAAGAKAGAGTGSALAALHAALTSAARSRGGPRPGGSGRGDGGDGGAGNGGGGGGGA
RGPSASPCSPNAPSSLFFSVAASQREPATSPPPPRPLAAALPDWLAALPYTRVYGDAVSGPGGRGGTAKEQQQQAGG
QQPGQGQQQGQQRPGEKSRALWGPPPPLQVLADLRFQLFEDAHKRLPLLCYNLVKEVLEEIRSGKRTSASQRPPAAG
PAGRGAPAPAPPHPPSAPPAPPPPAPPPPPPAPSPAAPPPPAGSKRPHGDDERDQQADGRVGAPPVPPPPPPPPPPS
PPPPEAGAAPQPPGETTAPAARASDTTSPAAASDKAAGAAAADAAAAAAAAAPRPAKRSRWDVPSKAGNAAPPQPSD
AGATAPAAETAAPAPAVAPAAPAVAAPVAAIPAAVSPAAAAEPAPATAAPAMAAAAAAATPTAEGVADALVVGDVEM
EDAGAQPNSAAAAAAGEAHAGAGAVAEAQAGAGTVAEAQAGVGAEAEAQAGAGAVAEAQAGAGAVAEAQAGAGAVAE
AQAGAGAEARGAATGRPADDQSAPEADMRQSAAEPAPVPASDANAAAAASSAVEVVEAEAPAAMECDAPVQQAAEAA
SGVAAEAEAVEAEAPVVEGGHAAAMDVDEPGAAPAPEAVGPHPAQAQPQQQPQEQPQEQPQVQPQVQPLQQPQEQAQ
EQQPEPQAEPGTEQQPLQTGTAAEALRAGEDATPLPGASTAPHKPAPGAATPGRGAAMSPGGSDPGTRAACAALLAA
DLLLAAAPPLVAVVEEEPEGAAGQPKAVAAAEEQKADAPEQAVEVVAAVQAAAAAVPQAVPEEVPEAAEAEALPPAA
AAADAAAASQVVQHAEAMDVDEPVTAPAAVAAAVPPGVPAATVATEVPPVVTTEAVERCLLEAMRLMSEGIDQAALL
QDTDEEEEEEDEEEGEAGEEAGPGAAQQRRPRPLRALRTRMQRYGLLPSVQSDPGAAAAATADAAAAQGLLPQAATV
DKATAAAREDAAAAAGAAGAAAGDAGGAAAAAAAAAAGAALAALQAEWEAAAARAAARGEEGLQWVAGMMEDLQASAA
DSGVPLEETSDLQNVEARKERFTRMVALGPQLAERARAVFGRLRAQLAQAQAPGPGPG

>Cre14.g631150.t1.3|PACid:27565525
MQIVRQGSSQLAARLNKCGILNSLSYPTLKPVSPERRGQLLRQGWCAAEDVVVPADGGGNATATTLAAKHDVAINDL
IRVNTHFKADPDALIEVNTRYFVPPCSVNPPRPPVPDLECGTRAVLQLLGDGADGSRPRIQLGQPTTPATASPPPPP
SQTPADRGGAEERFTATCPSGAHMIGFRGRAWAGQPEVESCVLAALAGQQSNPASSILYNQLVNLRDRIVNSILEQA
AVSADPAWNGLFTSVDLVCSDGSVRPTGRGTSDIGGYDLQAGDSIPVDTTARDCPGGYDMARSRLRRAPYERFEFFL
RCAASQQWTSYPAGLLAARRAPGITTSSLTIARYRFSSTDAGASTPGTAGTAGLSVTEALLLTAIRNSCGPVLAAPP
VGVPDTLLQFNESAPQTLRPPTSTESARLAVPSLLLPAYRSAEHLVYFQRQARLEVLGGVGGGGGCPPGQVVVGLRG
RRLALQPSSLFFNGSAEWKYGSPLPFRFRYAPDYTSLSAVDELVCGRTGTSTVMPKAPQPKVASGSSGVLATAVPGG
VPGSGPAAGAGLVKITPLTCGASALLTSVFAANDGVDINLLGARCSDGSAAGSAGGFGATANGSISGRFTVDYLCPE
GFDAVKLMDGSTWDGSSNGSGVGGVALRCSAAAKAQSDERYWVQLGAPNFLRATDSSPAFTLSEAFLNQPEYSVCAA
GSVITSLNVHTATVGVASVVVKLEFICGPRPPPASYASLSDIAAGYGVTVSDLARANPALFPSGSASAAAGSVDPAL
NNTEIRIPLMCGPAPVQPPATTIAAGCPKYWPLPNTTNAGTTTCGMVARSYVSGDLAYLSRINNNICPNASTTVAKG
QRLCIAPQSSVLSTSQHSFEATASTTTVTTSAGHRRRLTATAAAFASAGVGAVGGFVINNTIVTCILSQWVAVGQSC
EDIAVAYNLPISTFLAYNPDLACATLQAGAEVCVRTGPLLPETVQIVSLKACAVRPGVCGDGTCHDDDSSPVGFTCT
CAADALFIQGRCFGTDPPLPPPPPPPSPRPPPPPSPSPPPPPSPPPPSPPPRPSPPPPPSPPPPPPPSPPPPPPPSP
PPRPLPPPPPSPPPPRPPPSPPPSPPPPRPPPPPPPSPPPPPPPRPPPSPPPPIWTPSPSTDFATSPVFCGSDSFAS
SYVYPPTSDESMIGLPISSMTIWSSSQLNGLQLRFGSSLQAGPQHGSASGTVVTQSLQGVTVTQVHACCSAAAYQGV
ARVQLLTAAHGVFTQGVGCDVNNTWVPVPAGYRFAGVRTWTSSSLLSTLSRSSGEQFIHRIQFLFAPTAYTPSPSPT
GYTPSPTGYTPTSYTPSPTLTPALAPRATSLSPAVCGGSPSYQLTSSDVSLAQFYTLDRIGYRWGQVLGQIRSYYDN
DRQVGPLHGDSHNGGPGEFLITPDLNIVAARACCIVNTFYDNSVRYIELYTSAGRTLVMGDNTTGCEAMQAWWSVPS
GYRFAGFVTSTNPAKYVQRLQFVAAAASAVPSSPPPPSPPPPSPPPTSSPSMFSCVDQVATCQAWSQQGYCDSQYTY
NGQSVHDVVCAKSCNIIACVPQNPIGCYGDGLPRALPSLLSGNTSRPDMTVELCDTLARSADMPYFGVQFGGFCFGG
SNLTAALDMYGPMTDCTMQCSGDDTQYCGGSWEQNLYFTYPEDVCQSNPCAEAGVTDATCTAVSGGVGLSAYDCNCP
ANSVRLDAAAACTCEEGYYLYFGSCY

>Cre14.g610200.t1.3|PACid:27565550
MKPQRPADSGEARRPIGYWAAFNGWWRGKLREQGCRPSQTELAEWFEANAPYCWQPDEAPSLKELKTHAKCLRSTEQ
VRLYFRKYRAARKAGSTNSARGASDDGGGDDSDSDGGATAPSGPRAHSAQQQRPSTSYGSGAGGGRASAGAAWQGVT
SWPGLGAGADGAAEGMYGFGSGMPLPLPPLGPFGDGTVDVAAAQGPLAGFVPFAFGQPGAGAGLGLGGGAAGLFTAG
GAGGALAGLAMHQPSEQPYPPIQQQHKQQNRQQRQQTPAVTTTTSAGGGRGRTPYAKYTARCLPAGTATTASGGLPP
RHQPPASASAGLATPPLPPAPAASTGAAVSFSAAGAPQHCTNAMQRGIGGGIMLQLPSAGLATADATASAAYMQQPQ
QLQPLYRTGSAGARMQHPAHPAPLLLQGAHSDPTSPSGGADGGLGAAAAAGGGISGAAAAAGGGGGQRPAKRQAVGP
AHRSSSYSGPYGAFGGVDGSSSAAAGELEVGGYGFLTAALPVTTGGPFLPSASQVSHSVSQPGMHCGQAAAQPHERR
VSAPNPTTHNAAVAAGANATAAAAGGAPATPTTATMVAAAVAAAAAASSALSPTLAFFRAWQDADQPAAASAAAQLAG
GAMAVGDVGVVADVCRDGNVDEDGNVEDLLLDLPAFEDLLPTAGRSSGGGAAARAQRNSGKAPAVPLAAGFEGLSAA
ELSSALSLPLTPGGGVGGGLLALLQEDGGSPLLSSPALLRAVAAEQEQVEAAWRPGAPQQQQLRRQGSGDAMDVDAE
PAPEQQQQQHRPAIAPAAVMPAASCAATEATMLQLAAAAAAGAAAGAPHQRHASVGSGRVAAAAPAAAAPAAASTPP
PPGGDMAWWLQAPGGAAAGITLPPLRTSASQIPSYSGSGPCPVASSTAVTEEETGAAGQQPQAAAATTAAAGVAVGD
GGGDGDGGGTFQGFRLSALVTPTAAQLASAGAGPTPEHAAGGGPGAHGVCGTPPGPGSGSVGGGGGGGGGGGGGGGA
PRGSPGVAGLSPASTQRIMSLLGMAAPLGDRDGARDCSGHCQHEQLHKHQLQEMEKLQQQEMEMEQEGAMEEADRLA
LAPVSLPVAQPAVGPADAEMAPAVADGARLPAPAAGGAGGARAAQPAWSEVTLAADSMALTLAVTSGGGLEAAGGA

GADQQQQQQQASQQVATSAFASRQAQAAVGPMSACGAEAPFAIATPFNPHTQSLPYHPHHQDHQPPQPQHSEELSL
PAGTYPAIYSQHPHHHHHHQHHSAACAPWPPTAVASTYAYGMPPPPLQGSHSAPAGAECAAADPATAAHMSQLQPHP
YGCSSGYPPYPTPHYSYCGGGAPPHPAYGMQPPHTPAYYGYPPPPPPHYHPDMYGAWHQHHHQQQQAAAYSMMPPPG
PHGQFGYPPATPYAVPYTPHTSSYPGAPPPHPFSVHHHPHHPTMPPPAAAAAAGPHHHPTAGATPASCAAAAAAAAA
QMPPPATIPQPRQQLQQQLTKAAQPPIQQQPIFTAAAAAAAGGPPEASSAAVRAAAAAAAAARKLAGGSLTLLGAGAG
AALGVAAAGGRGAGGALAPLRPTGAEPLMPGRYVWQATAKPGRGADSCAPAATGEGGGEYGDACGGSGGGGAQPACS
GGGPQATATTLVGASHCSAGHACYSESVGVAQATPQAQALTATPCKGGGGRGGGGDEEEVSCGRGRCGGGDGDGGED
SGAQAGGAAGGAPAASDAQLMPPPPPRLPPQSRLPALGAPAGSAAAWACAADEEPAAVKGGDGEGDQEESEAEPEAS
EVSGESDGGMEDDGLQQAQEAVSGGQAAADMDAAGPEDRPDQVRGQGRGCQPVGDDGHASDAEVEDRSSDEEEAEEE
EEGEEEEEEEPLLDVSAITAGLFSPPLSSQYNPYAHSGGGGAGSAPPAATATTTATATTCTTADDEAAGAGGSGSVL
ASPPPVSRRRRSACLPGSSHSHPHPQGLQSRPVRDGRGLAAVVSADHEQQHQQEQQLQQQAADATPKAGAGLGSTFV
FAAATPVQTVSAFDVGPRRHPFSSYTAPAPAPGPAPALPGAAAARRGAQLGPTETPHGAAGGASAAGPFAAPTPGSA
TAGAAAAGAATAAELLAASASPLSGSLLASWLTSPAASLEASLAGSRSLGLLSPPPPRSAQAAAQAAAQLQAPYSCG
RALGGGAGSSALRLRLRRHWGGEGAVAVEARTPSAAGGDAGAEVFALEPRELLAGTPCGGGTVGQPHAAQAAVAAVM
VMGRTAQQDHAQEDVEEVGDVDVTAQIRISYMPPPAAAAVAAVASATGGLPCGAAGGGKGAVAGAIGAAVAPLALLA
GSKQQQGQSPVLGQNPVL

>g14763.t1|PACid:27565559
MTFDTTYSQACQPNTPFSGCKHEGAWSAVTTALSPDAVSCDDQFACMDLQIYSGECLDVVESGIVNKYCKVCLTWGT
WSEPDGQFSDRPLCGKTSSSMEYICAADRLSNWWKAPSTYVNNTRLLTKDTGIMYGWNEGRDGAYCQWARWGPGDTA
NKVVSFTIKDAYGSCLPFGVNTKSYTVQGMAATCQRKRSSGGCSSSLPDDNQCLWTFNVPHPVNMNCLLSPPPSPRP
PSPRPPSPKPPSPSPPSPAPASGGSPRPPVPRPPSPSPPSPSPPSPAPPPSPLVQGQPPSGCVFSDPTNFTAVTPGK
GPLDTCVDQASCMSVYYSDACSMVTLADGTTAQDCQVCLYWDNSRNCPKAYPADSVSHICSGDQFYGVIPSAGASAV
TGATAKRDTWWGGLSQRYCQYVRFSTSTLAPATVYFTVKDGSGSCLAAGAPPLSVTLNGLAATCQGPRTVNGQLAGC
GIGDQPNECLWTFTVPHPDSPDFKCATRPPSPAPPIPAPPSPRPSSPRPPSPSPPSPVPPSPAPSPAPPSPRPPSPA
PPNPPPSPAPPTLTPPSPAPRSSSPPPSPQPPSPRPPSPAPPSPPPSPAPPSPAPPSPAPPSPPPSPRPPSPSPPSP
SPPSPAPPPNPLVQGQPPSGCVFSDPTNFTAVTPGSKGPLDTCVDQASCMSVYYSDACSMVTLANGTTAQDCQVCLY
WDNSRNCPKAYPADSVSHICSGDQFYGVIPSAGASAVTGATAKRDTWWGGLSQRYCQYVRFSTSTLAPATVYFTVKD
GSGSCLAAGAPPLSVTLNGLAATCQGPRTVNGQLAGCGIGDQPNECLWTFTVPHPDSPDFKCATRPPSPAPPNPAPP
SPRPSSPRPPSPSPPSPVPPSPAPSPAPPSPRPPSPAPPNPPPSPAPPTLTPPSPAPRSSSPPPSPQPPSPRPPSPA
PPSPPPSPAPPSPAPPSPAPPSPPPSPRPPSPSPPSPSPPSPAPPPNPLVQGQPPSGCVFSDPTNFTAVTPGSKGPL
DTCVDQASCMSVYYSDACSMVTLANGTTAQDCQVCLYWDNSRNCPKAYPADSVSHICSGDQFYGVIPSAGASAVTGA
TAKRDTWWGGLSQRYCQYVRFSTSTLAPATVYFTVKDGSGSCLAAGAPPLSVTLNGLAATCQGPRTVNGQLAGCGTG
DQPNECLWTFTVPHPDSPDFKCATRPPSPAPPSPAPPSPRPPSPRPPSPAPPSPTPPSPAPSPQPPSPAPPSPVPPS
PAPSPAPPSPRPPSPAPPSPPPSPAPPSPAPPSPAPPSPTPPSPPPSPQPPSPLPPSPAPPSPTPPSPAPSPQPPSP
APPSPVPPSPAPSPAPPSPRPPSPAPPSPVPPSPAPSPAPPSPRPPSPAPPSPPPSPAPPSPAPPSPAPPSPTPPSP
PPSPQPPSPLPPSPAPPSPTPPSPAPSPQPPSPAPPSPVPPSPAPSPAPPSPRPPSPAPPSPPPSPAPPSPAPPSPA
PPSPTPPSPPPSPQPPSPLPPSPAPPSPTPPSPAPSPQPPSPAPPSPVPPSPAPSPAPPSPRPPSPAPPSPPPSPAP
PSPAPPSPAPPSPTPPSPPPSPQPPSPLPPSPAPPNPTPPSPAPSPQPPSPAPPSPAPPSPAPPSPAPPSPAPPSPA
PPSPAPPSPAPPSPAPPSPAPPSPAPPPVCSPCPHSFISEFHYVDTSGQASAQFVELIVPTMLDLSTLSLATYTYSA
AGGSLVVDIPLGSPPPASSGVTLAVSELPGTGFSVVTVRATAGGPLASAGGLLPMVPAGAALMGGIALVSHCAEADT
LKVLDYVAYGNDSANPLPAGSVSLVPLDGPAKGAYFVVLPEVTGVAESGLTTAPGSSIERTGSGNTGNQEPGAAGMS
WSTTPSVLDYVAYGNDSANPLPAGSVSLVPLDGPAKGAYFVVLPEVTGVAESGLTTAPGSSIERTGSGNTGNQEPGA
AGMSWSTTPSASPGSVDPTSLEVPLAPPDSRCGLLPPPSPAPPSPAPPSPAPPSPAPPSPAPPSPAPPSPAPPSPAP
PSPAPPSPAPPSPAPPSPAPPSPAPPSPAPPPVCSPCPHSFISEFHYVDTSGQASAQFVELIVPTMLDLSTLSLATY
TYSAAGGSLVVDIPLGSPPPASSGVTLAVSELPGTGFSVVTVRATAGGPLASAGGLLPVVPAGAALMGGIALVSHCA
EADTLKVLDYVAYGNDSANPLPAGSVSLVPLDGPAKGAYFVVLPEVTGVAESGLTTAPGSSIERTGSGNTGNQEPGA
AGMSWSTTPSASPGSVDPTSLEVPLAPPDSRCGLLPPPSPAPPSPAPPSPAPPSPAPPSPAPPSPAPPSPAPPSPAP
PSPAPPSPAPPSPAPPSPAPPPVCSPCPHSFISEFHYVDTSGQASAQFVELIVPTMLDLSTLSLATYTYSAAGGSLV
VDIPLGSPPPASSGVTLAVSELPGTGFSVVTVRATAGGPLASAGGLLPVVPAGAALMGGIALVSHCAEADTLKVLDY
VAYGNDSANPLPAGSVSLVPLDGPAKGAYFVVLPEVTGVAESGLTTAPGSSIERTGSGNTGNQEPGAAGMSWSTTPS
ASPGSVDPTSLEVPLAPPDSRCGLLPPPSPAPPSPAPPSPAPPSPAPPSPAPPSPAPPSPAPPSPAPPSPAPPSPAP
PSPAPPSPAPPPVCSPCPHSFISEFHYVDTSGQASAQFVELIVPTMLDLSTLSLATYTYSAAGGSLVVDIPLGSPPP
ASSGVTLAVSELPGTGFSVVTVRATAGGPLASAGGLLPVVPAGAALMGGIALVSHCAEADTLKVLDYVAYGNDSANP
LPAGSVSLVPLDGPAKGAYFVVLPEVTGVAESGLTTAPGSSIERTGSGNTGNQEPGAAGMSWSTTPSASPGSVDPTS
LEVPLAPPDSRCGLLPPPSPAPPSPAPPSPAPPSPVPPSPAPPSPAPPSPAPPSPAPPSPAPPSPAPPSPAPPSPAP
PSPAPPSPAPPPVCSPCPHSFISEFHYVDTSGQASAQFVELIVPTMLDLSTLSLATYTYSAAGGSLVVDIPLGSPPP

ASSGVTLAVSELPGTGFSVVTVRATAGGPLASAGGLLPVVPAGAALMGGIALVSHCAEADTLKVLDYVAYGNDSANP
LPAGSVSLVPLDGPAKGAYFVVLPEVTGVAESGLTTAPGSSIERTGSGNTGNQEPGAAGMSWSTTPSASPGSVDPTS
LEVPLAPPDSRCGLLPPPSPAPPSPAPPSPAPPSPAPPSPAPPSPAPPSPAPPSPAPPSPAPPSPAPPSPAPPSPAP
PPVCSPCPHSFISEFHYVDTSGQASAQFVELIVPTMLDLSTLSLATYTYSAAGGSLVVDIPLGSPPPASSGVTLAVS
ELPGTGFSVVTVRATAGGPLASAGGLLPVVPAGAALMGGIALVSHCAEADTLKVLDYVAYGNDSANPLPAGSVSLVP
LDGPAKGAYFVVLPEVTGVAESGLTTAPGSSIERTGSGNTGNQEPGAAGMSWSTTPSASPGSVDPTSLEVPLAPPDS
RCGLLPPPSPAPPSPAPPSPAPPSPAPPSPAPPSPAPPSPAPPSPAPPSPAPPSPAPPSPAPPSPAPPSPAPPSPAP
PPVCSPCPHSFISEFHYVDTSGQASAQFVELIVPTMLDLSTLSLATYTYSAAGGSLVVDIPLGSPPPASSGVTLAVS
ELPGTGFSVVTVRATAGGPLASAGGLLPVVPAGAALMGGIALVSHCAEADTLKVLDYVAYGNDSANPLPAGSVSLVP
LDGPAKGAYFVVLPEVTGVAESGLTTAPGSSIERTGSGNTGNQEPGAAGMSWSTTPSASPGSVDPTSLEVPLAPPDS
RCGLLPPPSPAPPSPAPPSPAPPSPAPPSPAPPSPAPPSPAPPSPAPPSPAPPSPAPPSPAPPSPAPPPVCSPCPHS
FISEFHYVDTSGQASAQFVELIVPTMLDLSTLSLATYTYSAAGGSLVVDIPLGSPPPASSGVTLAVSELPGTGFSVV
TVRATAGGPLASAGGLLPVVPAGAALMGGIALVSHCAEADTLKVLDYVAYGNDSANPLPAGSVSLVPLDGPAKGAYF
VVLPEVTGVAESGLTTAPGSSIERTGSGNTGNQEPGAAGMSWSTTPSASPGSVDPTSLEVPLAPPDSRCGLLPPPSP
APPSPAPPSPAPPSPAPPSPAPPSPAPPSPAPPSPAPPSPAPPSPAPPSPATPSPAPPPVCSPCPHSFISEFHYVDT
SGQASAQFVELIVPTMLDLSTLSLATYTYSAAGGSLVVDIPLGSPPPASSGVTLAVSELPGTGFSVVTVRATAGGPL
ASAGGLLPVVPAGAALMGGIALVSHCAEADTLKVLDYVAYGNDSANPLPAGSVSLVPLDGPAKGAYFVVLPEVTGVA
ESGLTTAPGSSIERTGSGNTGNQEPGAAGMSWSTTPSASPGSVDPTSLEVPLAPPDSRCGLLPPPSPAPPSPAPPSP
APPSPAPPSPAPPSPAPPSPAPPSPAPPSPAPPSPAPPSPAPPSPAPPPVCSPCPHSFISEFHYVDTSGQASAQFVE
LIVPTMLDLSTLSLATYTYSAAGGSLVVDIPLGSPPPASSGVTLAVSELPGTGFSVVTVRATAGGPLASAGGLLPVV
PAGAALMGGIALVSHCAEADTLKVLDYVAYGNDSANPLPAGSVSLVPLDGPAKGAYFVVLPEVTGVAESGLTTAPGS
SIERTGSGNTGNQEPGAAGMSWSTTPSASPGSVDPTSLEVPLAPPDSRCGLLPPPSPAPPSPAPPSPAPPSPAPPSP
APPSPAPPSPAPPSPAPPSPAPPSPAPPSPAPPSPAPPPVCSPCPHSFISEFHYVDTSGQASAQFVELIVPTMLDLS
TLSLATYTYSAAGGSLVVDIPLGSPPPASSGVTLAVSELPGTGFSVVTVRATAGGPLASAGGLLPVVPAGAALMGGI
ALVSHCAEADTLKVLDYVAYGNDSANPLPAGSVSLVPLDGPAKGAYFVVLPEVTGVTESGLTTAPGSSIERTGSGNT
GNQEPGAAGMSWSTTPSASPGSVDPTSLEVPLAPPDSRCGLLPPPSPAPPSPAPPSPAPPSPAPPSPAPPSPAPPSP
APPSPAPPSPAPPSPAPPSPAPPSPAPPPVCSPCPHSFISEFHYVDTSGQASAQFVELIVPTMLDLSTLSLATYTYS
AAGGSLVVDIPLGSPPPASSGVTLAVSELPGTGFSVVTVRATAGGPLASAGGLLPVVPAGAALMGGIALVSHCAEAD
TLKVLDYVAYGNDSANPLPAGSVSLVPLDGPAKGAYFVVLPEVTGVAESGLTTAPGSSIERTGSGNTGNQEPGAAGM
SWSTTPSASPGSVDPTSLEVPLAPPDSRCGLLPPPSPAPPSPAPPSPAPPSPAPPSPAPPSPAPPSPAPPSPAPPSP
APPSPAPPSPAPGTPPPSPEPPSPGPGVPPPSPAPPSPAPGTPPPSPEPPSPAPGVPPPSPAPPSPAPPSPEPPSPA
PGVPPPSPAPPSPAPPSPEPPSPAPGVPPPSPAPPSPAPPSPAPPSPEPPSPAPGVPPPSPAPPSPAPGTPPPSPEP
PSPAPGVPPPSPAPPSPAPGTPPPSPEPPSPAPGVPPPSPAPPSPAPGTPPPSPEPPSPAPGVPPPSPAPPSPAPGT
PPPSPEPPSPAPGVPPPSPAPPSPAPGAPPPSPEPPSPGPGVPPPSPAPPSPAPGAPPPSPEPPSPAPGVPPPSPAP
PSPAPGTPPPSPEPPSPGPGVPPPSPAPPSPAPGTPPPSPEPPSPAPGVPPPSPAPPSPAPGTPPPSPEPPSPAPGV
PPPSPAPPSPAPGTPPPSPEPPSPAPGVPPPSPAPPSPAPGTPPPSPEPPSPAPGVPPPSPAPPSPAPGTPPPSPEP
PSPGPGVPPPSHAPPSPAPGTPPPSPEPPSPAPGVPPPSPAPPSPAPGTPPPSPGTPPTSPEPPSPAPGVPPPSPAP
PSPAPGTPPPSPEPPSPAPGVPPPSPSPPSPAPGTPPPSPEPPSPAPPSPAPPSPEPPSPAPGVPPPSPAPPSPAPG
TPPPSPEPPSPGPGVPPPSPAPPSPAPGTPPPSPEPPSPAPGVPPPSPAPPSPAPGTPPPSPEPPSPAPGVPPPSPA
PPSPAPGTPPPSPEPPSPAPGVPPPSPAPPSPAPGTPPPSPEPPSPAPGVPPPSPAPPSPAPGTPPPSPEPPSPGPG
VPPPSPAPPSPAPGTPPPSPEPPSPAPGVPPPSPAPPSPAPGTPPPSPEPPSPGPGVPPPSPAPPSPAPGTPPPSPE
PPSPAPGVPPPSPAPPSPAPPSPAPPSPEPPSPAPGVPPPSPAPPSPAPGTPPPSPEPPSPAPGVPPPSPAPPSPAP
GTPPPSPEPPSPAAGVPPPSPAPPSPAPGTPPPSPEPPSSAPGVPPPSPAPPSPAPGTPPPSPEPPSPAPGVPPPSP
APPSPAPGTPPPSPEPPSPAPGVPPPSPAPPSPAPGTPPPSPEPPSPAPGVPPPSPAPPSPAPGTPPPSPEPPSPGP
GVPPPSPSPPSPAPPSPAPPSPEPPSPAPGVPPPSPVPPSPAPPSPAPPSPEPPSPAPGVPPPSPAPPSPAPGTPPP
SPEPPSPAPGVPPPSPAPPSPAPPTPAPPSPEPPSPGPGVPPPSPSPPSPAPGTPPPSPEPPSPAPGVPPPSPAPPS
PAPGTPPPSPEPPSPAPGVPPPSPAPPSPAPGTPPPSPEPPSPAPGVPPPSPAPPSPAPGTPPPSPEPPSPAPGVPP
PSPAPPSPAPGTPPPSPEPPSPAPGVPPPSPAPPSPAPAPGTPPPSPEPPSPGPGVPPPSPAPPSPAPGTPPPSPEP
PSPAPGVPPPSPAPPSPAPGTPPPSPEPPSPAPGVPPPSPAPPSPAPGTPPPSPEPPSPAPGVPPPSPAPPSPAPGT
PPPSPEPPSPAPGVPPPSPAPPSPAPGTPPPSPEPPSPAPGVPPPSPAPPSPAPGAPPPSPEPPSPAPGVPPPSPAP
PSPAPGTPPPSPQPPSPAPGVPPPSPAPPSPAPGTPPPSPEPPSPGPGVPPPSPAPPSPAPGTPPPSPEPPSPAPGV
PPPSPAPPSPAPGTPPPSPEPPSPAPGVPPPSPAPPSPAPGTPPPSPEPPSPAPGVPPPSPAPPSPAPGTPPPSPQP
PSPAPGVPPPSPAPPSPAPGTPPPSPEPPSPAPGVPPPSPAPPSPAPGTPPPSPEPPSPAPGVPPPSPAPPSPAPGT
PPPSPEPPSPAPGVPPPSPAPPSPAPGTPPPSPEPPSPAPGVPPPSPAPPSPAPGTPPPSPEPPSPAPGVPPPSPAP
PSPAPGTPPPSPEPPSPAPGVPPPSPAPPSPAPGTPPPSPEPPSPAPGVPPPSPAPPSPAPPSPAPPSPEPPSPAPG
VPPPSPAPPSPAPGTPPPSPEPPSPAPGVPPPSPAPPSPAPGAPPPSPEPPSPAPGVPPPSPAPPSPAPGTPPPSPE
PPSPGPGVPPPSPAPPSPAPGTPPPSPEPPSPAPGVPPPSPAPPSPAPGTPPPSPEPPSPAPGVPPPSPAPPSPAPG

TPPPSPEPPSPAPGVPPPSPAPPSPAPGTPPPSPEPPSPGPGVPPPSPAPPSPAPGTPPPSPEPPSPAPGVPPPSPA
PPSPAPGAPPPSPEPPSPAPGVPPPSPAPPSPAPGTPPPSPEPPSPAPGVPPPSPAPPSPAPGAPPPSPEPPSPAPG
VPPPSPAPPSPAPGTPPPSPEPPSPAPGVPPPSPAPPSPAPPSPAPPSPEPPSPAPGVPPPSPAPPSPAPGTPPPSP
EPPSPGPGVPPPSPAPPSPAPGTPPPSPEPPSPAPGVPPPSPAPPSPAPGTPPPSPEPPSPAPGVPPPSPAPPSPAP
GTPPPSPEPPSPGPGVPPPSPAPPSPAPPSPAPPSPEPPSPAPGVPPPSPAPPSPAPGTPPPSPEPPSPAPGVPPPS
PAPPSPAPPSPAPPSPEPPSPAPGVPPPSPAPPSPAPGTPPPSPEPPSPGPGVPPPSPAPPSPAPGTPPPSPEPPSP
APGVPPPSPAPPSPAPGTPPPSPEPPSPAPGVPPPSPAPPSPAPGTPPPSPEPPSPAPGVPPPSPAPPSPAPGTPPP
SPEPPSPAPGVPPPSPAPPSPAPGTPPPSPEPPSPGPGVPPPSPAPPSPAPGTPPPSPEPPSPAPGVPPPSPAPPSP
APGAPPPSPEPPSPGPGVPPPSPAPPSPAPGTPPPSPEPPSPAPGVPPPSPAPPSPAPPSPAPPSPEPPSPAPGVPP
PSPAPPSPAPGTPPPSPEPPSPAPGVPPPSPAPPSPAPGTPPPSPEPPSPAPGVPPPSPAPPSPAPGTPPPSPEPPS
PGPGVPPPSPAPPSPAPGTPPPSPEPPSPAPGVPPPSPAPPSPAPGTPPPSPEPPSPAPGVPPPSPCASQPCAWHAA
AFAGAAQPRAGPAPGTPPPSPEPPSPGPGVPPPSPAPPSPAPGTPPPSPEPPSPAPGVPPPSPAPPSPAPGTPPPSP
EPPSPAPGVPPPSPAPPSPAPGTPPPSPEPPSPAPGVPPPSPAPPSPAPGTPPPSPEPPSPGPGVPPPSPAPPSPAP
GTPPPSPEPPSPAPGVPPPSPAPPSPAPGTPPPSPEPPSPAPGVPPPSPSPPSPAPGTPPPSPEPPSPAPPSPAPPS
PEPPSPAPGVPPPSPAPPSPAPGTPPPSPEPPSPGPGVPPPSPAPPSPAPGTPPPSPEPPSPAPGVPPPSPAPPSPA
PGTPPPSPEPPSPAPGVPPPSPAPPSPAPGTPPPSPEPPSPAPGVPPPSPAPPSPAPGTPPPSPEPPSPAPGVPPPS
PAPPSPAPGTPPPSPEPPSPGPGVPPPSPAPPSPAPGTPPPSPEPPSPAPGVPPPSPAPPSPAPGTPPPSPEPPSPG
PGVPPPSPAPPSPAPGTPPPSPEPPSPTPGVPPPSPAPPSPAPGTPPPSPEPPSPGPGVPPPSPAPPSPAPGTPPPS
PEPPSPAPGVPPPSPAPPSPAPGTPPPSPEPPSPAPGVPPPSPAPPSPAPDTPPPSPEPPSPAPGVPPPSPAPPSPA
PGTPPPSPEPPSPAPGVPPPSPAPPSPAPGTPPPSPEPPSPAPGVPPPSPAPPSPAPGTPPPSPEPPSPPTPPPPVP
LCRGCITYQLIPPIFPVGPVISINSSTCLDLVDIVNMDVEQLALDGGATVFIPFSTTSCDDDKVTVCGAFGPGVDLD
LSVQLEFWLQDVILAGRACDGYSVQGSTDTLATSDRLSCLSGQAQKSCAISDPPSPEPPSPEPPSPPPPPPDAPPPS
PEPPSPPPPPPPDAPPPSPVPPSPPPPPPPDAPPPSPEPPSPPPPPPPDAPPPSPVPPSPPPPPPPDAPPPSPEPPS
PPPPPPPDAPPPSPEPPSPPPPPPPDAPPPSPEPPSPPPPPPPDAPPPSPEPPSPPPPPPPDAPPPSPEPPSPPPPP
PPDAPPPSPVPPSPPPPPPPDAPPPSPEPPSPPPPPPPDAPPPSPEPPSPPPPPPPDAPPPSPEPPSPPPPPPPDAP
PPSPMPPSPPPPPPPDAPPPSPVPPSPPPPPPPDAPPPSPEPPSPPPPPPPDAPPPSPEPPSPPPPPPDASPPSPVP
PSPPPPPPPDAPPPSPVPPSPPPPPPPDAPPPSPEPPSPPPPPPPGAPPPSPMPPSPPPPPPPDAPPPSPVPPSPPP
PPPPDAPPPSPVPPSPPPPPPPDAPPPSPEPPSPPPPPPPDALPPSPVPPSPPPPPPPDAPPPSPVPPSPPPPPPPD
APPPSPEPPSPPPPPPPDALPPSPVPPSPPPPPPPDAPPPSPVPPSPPPPPPPDAPPPSPEPPSPPPPPPPDAPPPS
PVPPSPPPPPPPDAPPPSPVPPSPPPPPPPDAPPPSPEPPSPPPPPPPDALPPSPVPPSPPPPPPPDAPPPSPVPPS
PPPPPPPDAPPPSPVPPSPPPPSPPPPPPPPPDGPIPPPPSPPPPPPPPGMPPPSPEPPSPSPPSRPPPSPSPPLPP
PPSPPPPPPPDGPSFPPPSPPPPDTPPPFPPPPSPPPPPPPNLPPPSPPPPSPPPPPPPDGPIPPPPSPPPPPPPGA
PPPPPPGLPSPPPPGTPLPPSPDPPPPPPPAVFFGSPPPPPPPPPPDSSPPPPPPPPPDLPPPPPPDAPSPPPSPPP
SPPPPSPPPPDAPLPPPPPSPRPPAQAGERAPPLPPLPAIPAVPAAPPPPPSECYNQGDTTRLDTPPPRACGDQATC
VDATLDAKKCVYRVAGGRRYLYCQSCLEWPREGSSCALLRASPPPPATRGRTGAGATTAATSTGVDTICASDRLGTA
IEGAGGVPVPGGINQVSPWLPGGNSTKYCQWVRWADGDFTTQPVLFSVRSGDKTCVAGSSGAEIMVTLRGLSATCTG
PRFSPSGTVAGCASNDGTVYNECLWTVMVPRPDGPEWNGPTDCIPPEEPTLPPPPKRPGAPNLRRNPPLPPFPATPS
ADPTCKHTGPNTQLPGNSGLSCNSQGACIDLDYDSSECYWRSGFLYCQACLVWPRAGSSCALNAASPIDYVCAGDEL
SSVLTPDGSSTSGGTSLLQPWQPQSRYCQLIRWAADDMEPTEVLFSIKSGSARCTRRTNVTMTLEGLDAVCAAPRDN
ASGGAAGCQGNDNVDNECLWSMRLPRPGAQGWNGKNCLPAPPLASPPPPRVRRPATPGIRAPSVPPPAPSEPLTPAK
GRPPPPKRRTPAAPGKRAPNAPPTPQPPIEGFYGFPFCACKRRTLKPTPYRLVYDSSTPLPALADGKDRVRHCFHIQ
VVGCDSKSQCCGMGIKKVELSVENECRTSVKLALLAGRSYPWSFTQNDYNGETYTTFKLTSLGLEKPDVPDGMPLCI
ILTEPCSSLADFCYGGGDWCRFTFFSTDENCCPTGDLLASSDPTQVEVVDTAGGDVEVVELRRRGLLSGDAA

>g15257.t1|PACid:27565579
MEDTEPSSELRPQQPSPVAADPADLAGPSSQQPWKYDIATEHASLTTTGGRIWDAARRLASYLEAMSGPLGLQRPGL
RVLELGAGLGWLGMTLARNIRVDPGQGGMVVLTEQEAGGGLDWLRHNLRLNAAALGLQGEGLLEGGLQQGGPQAAAS
VGQQEGQGGEGGQQQPAVRAEACDWFLYCNPDLTTARAGAGAGAGDRGDQGSQGGGGGGGGGGGAIGAAAGSATGQA
TASADAGTAAASRRYAEPLAPGASLAAAGLAAAATTTAATTAATAVAATVATTAATAAAAGAAGGTGADGCSGSGGS
GTGAGSGAGGGGGGGGGEWLSGVAWDLIIGSDLVYNEVGAVYLPRVLRALSRPGHTRVLYAHTKHRFDTWDMRFYEE
LAAQGLSLAEVWETGAPSPPPSPPPLTELFPDMRVAVFQIGQQPPPS

>Cre14.g620600.t1.2|PACid:27565597
MRCSILVSVLFAAAIAAAQGAQSGLYTSFPYCQCKKSPSAYKLGDVVVAKGNNQYCFKLSAKVPAGCTDYCCSKADL
KKIEFNVYEACDVFGEVVKATINGVPTKVGVSFHEPTDGPVGSTTLVLTQLGLGLASDGAEICITLSMNKNGKGCTT
LEELCIPPPGLPKGVCSAALFDSQNDCCPISTPGIPSPPPPSPPPPCQVCVYISLTVSDASRVLFPYSISNDECQGY
IATIQDDVATQASDLGVTVTPGSTSCAGNLVMVCATFFSNEEGAKLQSFVDTQINFWLDMVTQGCRPALAGYTVVVS

VGGMGDAVAGLPQSCINAQASAACALQTPDFPKCQCVTTPGATPFAIAPAMSSMAGRTKDTTAYCFSITLVTPNSAT
SPCGRSTSLLKAEFYADDKKRRAITGVYVQPKTGTGKWLSATWGAVDEQTVKATPLNWSKDQANGGKICLELKNDTP
LSDFCLPANDGVCWGNVFDDTKNCCPLFAAAQP

>g14958.t1|PACid:27565601
MRSSYSLVAVLVAAAFAAAQGAQSGLYTSFPYCQCKKSPSAYKLGDVVVAKGNNQYCFKLSAKVPAGCTDYCCTKAD
LKKIEFNVYEACDVFGEVVKATINGVPTKVGVSFHEPIDGPVGSTTLVLTQLGLGLASDGAEICITLSLNKNGKGCT
SLEELCIPPPGLPKGVCSAALFDSQNDCCPISTPGIPSPPPPSPPPPSPPPPCTACVYLSILETGNVVFPYEFSQQD
CDDYAAMIGDELQTEAENLGVAINFAPAVCSGDVIKLCATFFSSEQGAELQPFIDERLPFWLQLITGVQGCPAFLTG
YSAYAAVGGIGGSAQAPDASCLSGFQSQACAPEPVDFPKCACVTTPGATPFAFTPVISTMAGRTKDTTSYCFSITLV
TPNSATSPCGRSTSLLKAEFYADDKKRRAITGVYVQPKTGTGKWLSATWGAVDEQTVKATPLNWSKDQANGGTICLE
LKNDTPLSDFCLPAPGGSCYGNLFDDSKNCCPLYQASLP

>Cre14.g631100.t1.3|PACid:27565620
MSVGGQRNARHRLLLLVSLLCLLALSSTLVAEANRDFGPAFTRRLQQILGSNGTIAGGRPPPRPPPLPSPPATATLV
SGISIRLVAVQFFAATNTTPALFRPFNISSGTAGGLGYAVARVTRTNATALTVPVCLPGWSDTFAAIACQGAGMPSG
RAVRTDGSPGSYPNLNSPESTILTDLSCPPGVMSLAACTAVLRQYSAGQCKSLGAALCDGRPPAPPSPPPPAPPSRW
PAQAAPPLSVDPAQSPPMGDEPALPPGAGFEPPGGPADGDVRLVGPEAPLRGRLEVYYQGVWGTVCDDNWDKKGNLA
AQVVCEQLGFIGGAWSTITTPTAYGPPGPVLLDTVFCGANATRLDECTHDPWYQTDCDNTEDVVVACDLPPSPPASP
PAPPPRPPNAGRSPPEPPGTPPLEPPGAPPPAPPAIPNLARFALTYASSVLDTFCEVGGCYPFYAVDGDTTDETKML
HTAAGDTLPWLSIDLTNVSRITSVRIYHRPGYLGRTRNSELRVGNASVTSTSDSLYGNTLVWKQPAALSENVSVLVF
DPPVVGRWVTFQNRHDNATSGEQVLQVLELEVYGNEVAATQRRFPDVLLGCVDEVLDIPLLLYDNTASTPDLCAQAA
RALGAEYYAVQGGTCWGFPSNDSLATQLVDNIAGGAYYYAFCNAPCPGDATQRCGGGAAFDLYQANLQVPPPPPPPP
PPPPPPPPPRDVPPPQCCACPAPPPPSPPQPPLPPPRQEQTGLVLPETSALLVRKDESSSRRYSAVVPPDTVDWRSSG
VLPPVTTQGECGSCYAHAPAACIEALAVMEHAYTNLTSLLGAGGARALLGPEQVVQCKPGGCAGGWVGDTYIQAMRG
LLYQEEWAKAVALTTGAVSGAPSANGTCPVATIQRLWTDLNSTALRYSPTGLRIEAWETIPASEFALIQALANQPVV
ALLHASEDWFDYWGRGRVYDGNCSSDPSKSTHAVVIVGFTPDTFIIRNSWGESWGDGGYMQIVRQGSSQLAARLNKC
GILNSLSYPTLKPVSPERRGQLLRQGWCAAEDVVVPADGGGNATATTLAAKHDVAINDLIRVNTHFKADPDAIIEVN
TRYFVPPCSVNPPRPPVPDLECGTRAVLQLLGNDADGSRPRIQLGQPTTPAAASPPPPPSQSPADRGGTEERFTATC
PSGAHVIGFRGRAWAGQPEVESCVLAALAGQQSNPASSILYNQLANLRDRIVNSVLEQAAVSADLADPAWNGFFTSV
DLVCSDGSVRPTGRGTGTSGGYQLLPTAAANASATGSYSILDWQEPDCPGGYDMARSRLRRAPYERLELFLRCAASQ
EWTSYPAGLLAARRAPGITSSSLTIARYRFSRTDAGAFTPGTAETAGLSVAEAQLLTAIRDSCGPVLAAPPAGVPDA
LLQFNESAPQTLRPPTSTESAQLAVPSLQLPAYRSAEHLVYFQRQARLEVLGGVGGGGGCPPGQVVVGLRGRRLALQ
PSSLFFNGSAEWKYGSPLPFRFRYAPDYTSLSAVEGLICANATAASAANATVAAPYDPQPNVMQGSSGLLVAAVPGA
AGTGNNSDVLTTTTVTCDDSLMTSVFAANDGVDINLLGARCSDGSAAGTAGGFGATANDTISGRFTVDYLCPEGFDA
VKLMDGTTWDGSSNGSGVGGVALRCSAAAKAQSDERYWVQLGAPNLLRATDNSPAFTLSEAFLNQPEYYVCAPGSVI
TSLNVHTTAGQEPSGDVPVMAKLEFICGPRPPPATYASLSDIAAGYGVTVSDLARANPAIFPRGSASAAAGSVDPAL
NNTEIRIPLMCGPAPVQPPATTIAAGCPRYWPLPNTTNAGTTTCGMVARSYVSGDLAYLSRINNNICPNASTIVARG
VRLCIAPRSSVLSVPQASSTTTVPRGRRHLLADEVFGNPIATGGFVINNTIVTCILSQWVAVGQSCEDIAVTYNLPI
STFLAYNPDLECVRLQAGYEVCVRTGPLRADSLSIVSLEEARKTTCELLPGVCGPGTCVNTNATAQGYTCTCPNTTV
FTGATCTFLPQPSQVPPPAPSPQQPTPRPPPPQSPPPRPRPPSTSPPPPPPPPPPPPPPQGSFTNAGFFNGRSIFVYR
PSLTNYSRCSSICTQELGGSGGNLLSIRNVADNSVIQQIFAFYREEIFAGSGSFPSYLITGGIRRANGTWYWDDGTA
PSFGLAAGYSNWPWDVGEPNDATPGENRVAMYADGSWNDVNETRDGMCACWRPYIPGDTPTPVTGVVPATAPLLSTA
EQFAYYFTTPFRQSFLYSMLYRGRQYFSTASMPLTSCNTTCRNGLVGGGHMLSIHSAEENDVIAQLFRATRRRLYDA
GSGIVWLLMTGGSDAAEEGTWVWDDGTPFDYGLPLGSMPPWIFSDKSANGAQPDNAAAGPGEDCLNMWVNGSWNDIG
CGTLQGMCMCYVESV

>g14660.t1|PACid:27565630
MQGWPRERRHATAASLGAAGGTGAPAAVHPDMLRRLRRVLLLHPGGLAPSALPAAWYDAYGAPLLPELAGPFTSLEQ
LLAACPSAAAVGTPGRPRVARSQQQGQQPLLVTAVVTAAAVEEVADMVWTELAHRAAAAAAAAAGIPGGGGTGSAAA
AHVSYEAVCAGVRYWLGGQGVEELRPPLEPPPSLQRLRQLDRRVGATMEAYCAARCVVTLWELERHVCASEGVEAFG
ELRLGPLTAHPVVRRTFFASLPPAQEEMWRRQQQQQQQQWWPAGGNYGGDAGAAALPPPPGADPCPVTAADVAAAV
RRLVVSRWAAAAELRAAAAAGVASWAGAELCWDVLTEVAAVAEAQHQHQQHQHQQYQQQQYRQQHQECPLPWRLGVL
VNDLLLYVEVAAAAVGAEQQAAAGGGVAAGGGTAMLLGGEGGSDGAAAAALAVAVAGTQLQQGQGGPAGEERQLSRK
QRKALARAQQHQQAQQKGQGAAVAAGAAAAVPSPPPPQLPPPPQSRHYDFLSPQQQAALSRRQLKDLFLPGGGPAAQ
RSAAERQQAAAAARQRGALALEALRAGRQAAEAEAEEAEEESAAGSSSGEDEGAAGASSENESSDGDSSSSSSSGSS
SGSSDSSGSSSSSLGDGAQGKQRQRGAGRMRRRKGSGAAAAAAGGRRREAAAGGRDAAEDWGLPAGLATLSERGGME

AGGRVRAALAEAHGLPPDFSEEQLQACFNRTLRQALGGGRVQPRPAPGFYGAVVGLLLDGFAAPVLQAQAEAARAAA
AAALAAANTGDGGGGGKNSGNKASAKLAAQLARYSPAAVRRRLSGLGRAAARNALLLALVRAAHAAMRGQLAVSGAQ
AAALRAQAQGQAQGQEQEENAAAGGGGGGGRGGGGGRSVSGSSSSSSSDSEGADEPYVRGPAQRAALRAAAVGARAP
KAAAGRGGGGRGGGARRAAGAGVGPAEEEAEAEEGITTAAVRGVVRSLGAVEAALTAARPPAGWSAAALSRLRAGLF
GGGGGGGGELEEAPDAAVKRGAHFPIQKLQALATFVDFLLQRGPRPAAAAATAAAAAAAARHQQQPQPTGEAAAAPP
SEPAPATATATATPAAVVQLPAPPPSGPSAATLEALRSAVLQLRGGYSLSALAALEQQVAARLAAQLLPPPLVPPHN
NTHTAAAAAGVYCALGLGGSLLSALEVAGEPELAAALAGDAEGPAAAPLHVVLGLVAEVMRSWRLAAAAAGSAAGPG
PDSAGGGGGDGSGSRESGGGGPGVVAAVSAALCRHFGVAAVECLGHGPAARLVALAAASHTAPPPGWHSAVALALAP
PAAQLLLAADGGGDNTNSNGTISGTRGTGGGDARVGALGVVGRAAALAALRAAPALCDLRLATCWGAVFEPQLGPLE
AFLREEAAAAGVAVMELPQPPVGGPLLELAAGGRLVKLPPPPPAAGPAAHVVARAVARDGRGLAAALLALVAGCGGL
AGCPCGGVAAALGGALADVAAQAAGRSELAGEVGPLVVAAPLGRHPAGAGGGGGGGGAGGGGGGGGGGGGVDSPGAA
AACRLVLSALPHLPLPLLAPLAEAVLLPALAAALSGAVAGQGGAAGGQAAGMAAGGGGGGAGVGYGAGSAALHAALL
RQAAALAVSSPAGGGGGGAQCVLSCLHHLGFSLGVTAWREDWSSRHSAEEEEEGEGEEEEAGAAGGVQRVQGSVAAA
AATAATAASTAATSVGAAQPALVGVTPGCTPPAVPTTEQGQGGRPDAVAAPAPVPGATAAAAAPPAAPAPAPGPGSP
ATAAAAPAPEAVVASVPPPPPESECRALVEAIRREEFGLGLALGGEAGELSGRLAARTGRALQRLAAELYGSDSHFV
MELVQNADDNAYPAGVTPCLEFVLGRGHITAANNELGFSAAHLRALCDVGASTKAVGGGAGGGGGGGGGGYIGQKGI
GFKSVFRVSAAPQVHSRNFHVGFDLRRHGCLGYCLPDWCPLPPPATAAAAAAATAASEEGCAAVAALRRMGCPLPPP
PPPSPPPPSDGSGPGSGSAPSTCPFPVYGTAIVLPLRPDMDPAAAGGGGGGGGGEMAAAGDAGGLGGGERGEVLRRR
FADLSPTLLLFLQRLRRLVVRDETWGQPQQQQLGGAGGAGAVGPAGEVGPSGSGSGPCVARVLEMERRAVAGSPHLL
QLVVRTAVLRRQLAAVAVAEPAAGAAATVAAGGALTGPGLVQEEAAVDVAGASTAISTWAVVTSALQPQTSRAGVVV
AQTRVSLAFCLGPPVAAEESAAVRSGGPPPPPLAAPHWREGGRPPQQPVFAFLPLRGYGLRFVVQADWVLPSSREAL
DADSPWNQELRQQLPGLFLRALDVFRSLPPPATSTSTASSTSTASSTPTAASCLFWTDQWLRCVPLEGEAQGFFAGL
PHSIAALLRAAPCIPVAGGGWATPGETAVVAGGEVGSTHLQPHRPHAASELMALLAAAVDPAAAAPGSPVGGGQQIK
PPPPRPHASQHPPHPQHPQQRLQQSGVLSVLGLKLVAPEARCLYASRPLRALLGVRDLGPTHLLEAVKTLVRGGALA
GGRVSLAWLARALALLHTATATATPAPGGGGGHDWAHGPGPGQPHLTRTALLRELRGLHLLPLQGGGWAAAEELCGG
PAAAAVAVEVQAEVSAAGGTQPQEVVEEEEEERLALVRQLAAELGDGAGGGSGGGAAAGCVFFPLPPAAASTAARGR
SGTDAAGAGAGAGARGGGVVGLQAEAHGSPATAAECVRLLQRCGLPTDMPQLLELFGGAGGRGGSAVTAAVAAPRPQ
PPLLRVLDVRLLEAAEAAEEAEGVEPDLEVPVAGDDAAAAAASARQVLAGQQRRAVLMSCLQDLGVRHLAPRDLVLR
LAAPALITAPAPGQHGAGLSGHGPHNTHTRAGQEQEQEEGGQAHRQRKGSKRDKRDGKRKQPHDGAVGGGGGGTEAA
TPPARLLTACLALPLLAGLLQPDGSSSSSSSTGPPGNLGNRLLQLLRRRCLVLTAAGGARLLRGGELRPPPPPPAHS
APTTQPDPAQSGGRHKRGRKENPEAGVTEAAATDGATSPAAPLPPRQQQLYLPAAYLAAAGGGGGPAVGLDDVLALL
PRGGAAAGSSGIHCTMTTAGSGEQWPLVSGDYLRLCPELLPQWRWLFGQLGAAALPRPQPVVTAVRTRQQLQQALQH
AAAAAAAAPGGGLQISPEALAAVPLPCRLRDWVCPPLERLVALAAAAGGAGGAGGGGAGTGGGQADVAVQACSRLLS
LMAAHWEAAGLGGAMRLQLLPAEVPTNGPTTPTATTPTTSSSVQPPQPHQGLSSLALGPSRLGAALQLTAWLPCTAT
AAEQQAAAEQQQQQKAAGGGLRGQRQQAPLQAGGFVVLRRPVDAFAPLPPLRRVLGDLVPYVAAGGAQTGQPGTGQE
LVTGDGAAAANAVATRLLQQLGVCCELSGRRLLALLRQWSAGGGGGCGFTSSLGAMCGVYGALEAALRRAEQQQAEA
EAGVEAAGVEVAGGPSQRGNEEGLHSATATTAAAAPDLAGAGSDLTQAELVAAFSSEPLIWLPTAAPHQAPAGVPAP
SSGAAAADVGGVADEGLGSTRQLPGRFYCAGEVVWCDPAGVVEPLATAAAAAVAGGATAAALLPLLRVLPPHYTHDG
GDTRPVVDARWLQSFAPHRQRPLQGGMAGALFARLAAAAATTCSSGAAPVDSVAEEQPHSQQQQQQRLQPNGGRLTR
SAAPAAGAAAAAAARVKSEPHDDNVIDLDITSDDDGSSSSSSSSSSSSSSKNEEPVGTEVQQAQHAGAQAAGGAARG
VISLEPSPRQYTDCLRACAALALQQPRPRAPTTTAAAATRNMGAAVAAAPEAPVRWALRIWSHWARQHLSGAAGLGP
GGSELAALRRALWTEPLLPCASGCWRSLAGDAAAPPQRQTGPPAKTVAPQQVWVLINDDPLAAEALQVAATAAAAAA
ASASAASRGGRRGAGPQSPPPQLDFLVLPGTGHAGEEGDSDGVAGGDGGGGGGGQAAGDGGGRSDLQLQLVPPAAEA
LAEALGAARLSDCVSYRVHLAPAAAPQPGTQVEAAVRTAVAAAEAVLAARLAPPQPAASPAVDQFLQLQASFAKVLP
DFKVLPVSDFAASLVFSPPPPAAASGAPPGLQHGAGGSTGGASVGLLPGMEVRVAPPPSCLLVGAQLYVRLAGGGAS
DFAGLTDQMCRLFLGGRPWPGLSSGLLPVLMAAAAGGEDAVRQLLRRQGVPPLPALELGAGPQRQGASRGHLWRLPP
PLPMPPPPSPPPVALHCEDAAAGGGAGNAKSESKKRRRDAAAAVDALEGLAAGGAAAACGGAAGQVGQQDGGAVGAA
PNTAEAAAGPAQRRRLDGSAAATLIKGEGACSDSSSSSSSDESSGDSETKRAEHEQSVVKSKHSSGSEEEGRSSSGS
EETSNSSSSSDASSDVDEEDDDGNKAGADARNLEAGAAAGAAAAQVSSRGPQAPLPPPPPQPLGSGPLLPAAAVAGA
VQQYPHRQERSAAERMRSASDWWARKEQREEARRARLARAGITGETEEDRRMQSIGRWGEELVFRTLVAEAEERNRR
RAAAQRQLMAGGGGSHGGPGAWQQPATGAQAGGGAAGGGGGFERVVWVNRQSESHLPYDLYITHADESVTYVEVKSS
AAAAKDLFEISVPELQFAAAQGERYLLVRVRGAGGPAPTLERLTDPVALLRQHRLSLCLAV

>g15168.t1|PACid:27565660
MIAVLLALVIALPLLLGLAAAVLGWLLWRQCRRTRTLAGSVRPPGAGPQTTLLVTDIMNSTVLWEEVAAFSMDKAIR
LHHACLRKLLRPYNGYESATEGDCFILAFWSPADAARYALAAQQELLDQSWPPELLEHPDGCEVWVSCPPARHNPFE
AHHKDRERQERLDRAAEDRAAAVAAAAVAAAVAATAGGGGGGEMLGGDRDPSSHRGGAGGRDSSYHSYGGGGGGGRT

GGGGGAYGSGGYGAVYGGYVGGYGNDTDGSVQAGSIGGGGGEGSHRPLMQRLGSLHRRMSRLTLNIGGGGGGGGGT
HSSSRGGGGGVGGGMPSSPTTAAAVAAAFPPLLAAPPPAAASSRHGDGSVGRGGGGGGGGGGGHSVGVSAASRADSP
VPAAPPLPSLPIPIPEYSSSPNARSLGQLKPSVNNSGLAAAVAAGRGGGGAAASSPASHGFAAAASSFAGSFGGGGG
GGGLTPRAVISAMASSWRLQSRRGGGGGGVMHGSGGAAAKDDSAVQLVQRERFSVSGVPAAATYRTTYCGGGGGGGA
GAVGGGGGSPATLGPGASGGGGNRKSPTSQSWIHQHQSHQHQHLQSHQQEHSDSSSLQQRTRAGGGGGGAGGGGGGG
GGGPRRARQGSICLAYSGSTFLLPMSLMIPEPPRYGSDHDGGGSAAAGRDGAAAAAAASDNDDTDYGGNTTNDDERP
SPATETDYEGEWPAAGSPVWREAAPTTSLGPPSASIDAPPYYAGRAATPPPLAAAEAEAVAAAAGGGGGSSSGPGVA
TAAGGTTGPSTAAAAAAAAVLAPAALAERPFLELPPPPLTFPSVEELRAMRQRHRSRHFSNGSAAASIAAAEQARRE
AAAVPGGVYRSNHSEPLPLLTAVGVQAVGPNDRRLLSVDGGGVAAAAANVDGGGGGGGGALRFGLYATSSSRQGLQQ
SLSRDCWPRAAVPHAPPLAAAAAPPGALPGPLGGQLGPGGLLGGPLRQWAGGGVSSAAAGGTLQGGSSSTAGTRAKP
PHGSSSNSSSSGSSRNRRRRSPLLLLPPPPGLAVLLDEAGVVVGGGGGGGGSRQRRRRNFGAGGGDHLGSGSESDSA
DFAQIGSGFSGSDEPASPRPTARVVPASRLQQQQQQPMLLPSPSSAAIDVPSAGAAASSRRRLSVSDDVGDVGHAGA
GGAFGELPMVGAADGGGAGGGGGGGGGGGGGGADVGSGAVAMAPPLPPSLMLPSSALGGGGGAATAAGTAGSAAGPA
ADTGLAAADAAAAAAAAAAGQQQVTAAAGTLALTPPTPLTVLGEALASGANSLQGSRHGSRNNSGRWVNATGAASGPA
GGAHQSPLSQNGGGGGGGGVASGSNTASGVQQQQQATADLPQPPSPLWCVPAGGAGSSSNHARRQHLRNAHLKNVLH
AAIGSGSSFTSAGSSSRPASSCNSGVAGGVMLHGGGADGGGGGGGGGRRSGSGLLSQALQQLHINASGGGGGGAMLL
PGSGQSAASATAGISASATACLVGSHSGTGGDFGNACRASFADALLPDSPPSPPPSPPPPPPPAATAAAAATAASIP
AALQDLDFEQPRSSRSNVPAVQFQIHHVPSPQLQHQHQMSPLRPAATAAAASSGAPTAAAAAPKQLQPPSPPHLARS
GPLPPPLAVAASPAAGQPRARMSMDDARQLQPPPGTTASTAVARNRTSSQQSDLMRQLSNTLLIKRLSFAGGGGGGG
SGGGGSGGDGGGREIGGGAGLFWTTPPSPIDNDRLLPQPQPLLQLQLQLQPHSPLQPAAMTAPLLQPPAVAAAAAAN
GPALTAEAGVAGNVDATAAVAAGTEGELSCMAVATEGSGGSRSPSESGDSGRGRLQQQLQPSGSGSTKRRRAADLLA
GVSVTTAAEAAVADSSSAAASGGGRSRGQPAAGDNATAAVLAAAGGGTETIDSPPSLHLPIIIHTYAAAEAVAEAPH
QLQPTAAVAPAGGGGPTEDRSSLHVDVSVRGGNANNSRDLAIPPVTVAVAAAVTGAGAGGGGGGGGSSAPLPVPVAGA
AATARPRRLAAAGTLAAAALEGGGGGGGGIGSSSRGGAAVAAAAGAAVAVGSAGAGVGAGRGGAVMLEAAGMVGLPP
SQAALAAQQQQQQQQQQQLLRAGTLSAGAGQLGRMLAHLGNGSSGHVTVGAAAEAVAGGGGAAGQLMQQLQPPPPPE
ECRPLTWREQLRDLFPVINPNGSAFNMSNRGSSHAVHDGYGFGGGFGGGGGGAHGSVYGSVYGGLAAVAAAATAAAA
AVGGATTGGGQAVYGTHLMYSNPTAATNVAGDASGRGGGGYGGGYGGDVSVGGAGGLYGASRRGGRSSRYNGSGPAL
VFRGLRVRMGLHTGISNPADCCFNPTTGAVAYTGAPLKIAKAVSDAAAGAMVLASERTLHQLLPLMPLLDSRPPMAL
YCGEVELEGLLLPPPPPAAVASAVAADALDATAVAGYRGGGGGGTMLLAPPPPLQPPGYAVSHYVMSAAAAADVSAH
SSKASIYPARLLLEAPSGDPAAASAAGGPAGFGFGAAAAAATAAGDESVRSGPASPTSAGALFMPPAALTGASGGGG
ARGHYGAAAVSPLPSAAAAAAAAAAAGLLRRGVALYALVGYRLRPRIPHWAPLRGVNFLQLGNVDAPVGRSVAITFLT
VPAAQALLTDLEEVGARALELLRATAVALCARRGGYLIEAAEGLVLAAFHDPAAAAAWALDSLEALQRLDWAPPVLS
HPQGREVYGGSSNGFGFGVGFGIGVGGGWGTVAAEYGGGPDLTSVSHQRPHSQHPHGSASPPPSQPPPPPQPPQQQL
LLLHRGPRVRAGIHVGEVTCDVHSSTGRLSYRGRVMNRAARIAYKAGDSQVLCSREAWGLVRRTLPPAATHEVAAAA
AAAGVQQQQQQQQPAAALQPRHMSASGAGGGGGAGWAAGGMRSLLSSIRGNDTAGGGGGGELAASNDAGGLMDLATA
VGGTGSSCGDFRRPSSSQAPNVPSPHPPTSPAASFLSSSTTPMPASGVGHFAPSHHTHAHAHSHSHSPFGGHSYPPP
ASHHHQPHQQQPMQHQHQQHSPTLPQLPEAEAAVAAAAVIPAAGGGSSTYATATAGAGLLQLLQPPPARRSPSQVSP
VSQTMTLSAALYNAAVQTAAAGGGGGGGGGGSWSTAPAAAAVGAAAAGGGAGGGGGGTGSGASRLLRAAPAALPYDQG
PAVAVPPPPGQQLAEARASGGAGGGGGAVAAVWSSPLLIRQRTDGSSNSGILPTAVAAQTELTAVGAASTAAAAAAA
AAAATAAAVLYRRSSDGLSSLRTSRTSGPDGATIGGGGSRGGGSRGGSRGGSRGGGSRMFAYESLEALQHQTQSGHC
SENGTGPMTGVGGYGIGGGAKAAGGSGRGLHVGGGGEDAAAAGAAGTSGRLSPSQQYQSKGLGAAFGHLQHPLIAFS
HPHHYHLSEEEDEEEGSGAQASEPSPSRKERQLQPSAAAGGQRGEGRQPRSPGSSSSSSSSGEAEGAQPHSTGGRVL
LACSLGLASLKGIAEPIELMELRWSAKMVRART

>Cre14.g619400.t1.2|PACid:27565709
MAADLQGSLMISGCTFTANGGAPQADLAHATPPVGVLCTGDDAATCGVSVTDSNFLRNYGSVASGMLLQTMGSATVT
LSGVDFTDNTASWTSLRTASIAVRTAVPASAFLHLIQSAGPAIVQIRASDVSFSNSSSSHGMSVLRVDDVNDKATVT
AIELTRATFTRQTSGASALLLVGLRQITATGLTVEGSTCSLNGVDGQRFLSAGSCVCIGVEEDEGRTSSVDLTDVAI
LNNTAENSAALFITTYYQVMPRLAAAATAGRLSVSGLRVEDQTSTVLASAASFSYLSSVTVTGGSIARNLAGGGLLI
EGAASEQAADVTVSGVTFTGNTRQLQKDAQGRTQSSLSRGGGLYITNAVTVSLTDVAFFDNVAELSGGAAIFESLAS
LAVANVTAGNNSVGESTAAEGGDGGAFFISGVSGLTTFGGGTFTRNSAFRNGGALAIQESGQLSLINVTFNSNDAGG
SGGAVALQQLSGEDDPSGFLQCNFTSNTARVQLNVKDSETITAPSTGGGGAIAAADVSNGMRLESCVLVGNSATHAA
GGAVNIQTSRYLLVSFTAAKRNSAGTNGGAFFISNMASETSSWMPKNSFEQNTAGLATGGNSGLGGMGGAIFAETST
IATTCSSFIGNAAIRGGAIYARLQARIAMLGEQDLSDCTARATLAAVSRATSASAPTLYPPQGFTAAAEPASYGSVF
ANNTALSGGAVFAQDSTVWVFENPEPKWFTYGRKGVLFANNTASSGGAVLAYSALAVTVRADFYNNSADAAAATQAV
AARLGVPASSLAAATEPGAGGALAIIGSEQSSLTLTDSAFVGNSAVDGGGLYLAASEGCSKPDSCYVASVQGVIMER
NVALGGGGGGVFWEYPGLLSMTCAAPAELAPPPSPLGDGSDGSDGGGFVDPGLGGAEAPPTQPPPLTPTAPPSPSP

PPPPSPPPQPYPPSPPLPPPPAEYHRAPPVVLPDAQLRACDSWSGNAVEGGGYGPDVASTGFDLVSSPHYLDFYTSS
APVQANLSVLDYYGQRMSGGNRSVVIRAVSVQVFGQTYMAADGGLAEFDDLKLRAQAGWHDVDFAAETAYRQLSASG
LSLYVRPCTIGEYLSPTLDQCLACSPGYYNFNHTANLCNACDTQAALCSDPEFPGMIIPQDGYWHSNFFSEQVMDCP
NGDACTYDNRTQALSDVQQRVMAMALAIQESLVTRPNATSTPTTSPAASGAVSSADGSSSSSSSSSSNGSSSIAQRV
LLQERYQELRRLAGDIVASLELGSGGRGRDSSSPGLPSSSSSSSLGAVAIRVDTDSAHSASRSAADVRRWRAELGSA
EARPLLPAPLLALRAAMRELLAAAGGDASSSSSSSAYGPAADDDDYGLLLQAVSVLYSNYTAALCAPGYVGVLCGSC
DVGYGSTGPATCRLCPSAAANAVYYTLAIMLTMVILAWTIRSLLTQSMIAANRARNTRKWEVMGEMRIMGRPVVDAQ
LMSVLDDDELPDMEPPAGPIYGRALPANLAGGLAQLRLAYRAGGLHDDGSQHTASGPYNAGGALGGGGGGGASGPFG
RGSTAAYQDSGYLSEQAVPIAHAHSALSAAGSAALRVTRSNHPTSYSYSGWGAPAAPAGGAFSATNMTTYAGLQQQL
QMQHSAMSQQQHHHHQQHQQPHLTAQRISLQAQQLSQSQSQQPLAAQASAPVISERPGLPPLPPGRGWSLRRSQASA
SVLPVATGGGAAALGYGTGTSGSISPAVGGGRHFSGSQFPNPLASSSGAPLSSSPPAAAGPHFSPFASYQAAAAAGA
VASARSTALLSTGGGADPDPDGYAFGAASSSAATAAAVGLLTMPSRGVSNQGGLDPSPLSAAVQLTTAAGEVQELAA
VDEEEQLTSSHGSRETQVYAAAPSVGAPSAAALSTGPPSAGAPSAGAGAGAGVGMAAGAGGAASGGVLTYRVSLGSQ
HEQEGAGELRRTSLAGPSMHRLSRSRGPSHNGVAEDREGCARLPRPQLQVQLPSPAQAQQHREAEMEGAGPEQEQQQ
AQQEQDPEQQQDRGEGEGINPRDSAQDRPGHATALEAAQAALKGRKRGGGDGEPNGKNGGDGGKGGGGKDRQHPQQG
EGDEEQQQQQQQQQMPQSYPAPQQGQGQAGYGPEPPYLRPHASVPYPAAPPPPPAVPAPRAARARRASAGVYGPCGS
PFGMPPPGYPHPYPQSPGAGAGVASPFYPPVMAAGGGFAGLGGGGGAAAAHVSMAEALLSPRMASATAGVRRHPFFG
GVDRDGNDVITNVVPRAAGSFLATNRRGSLLNLMAAAFSGGAASGSGMAAAAAAPAPPPAPYITPVGRFAAGAYGPI
AAGASPVLIPAHGSGGGGSLVVSPRMASATGMPSAFMAEVLRRRTREDARRVGRAARSGSGAGNPSAEPTPTPGALV
LENAAAAGVSGTAAGLVAALNVGRRSVTSLGAVLRKRLAGSALLSAGGVSSGAGSGGPNSAEIAAASRAASGSRGPL
PLGPAGLGSAGPGAASGPGGISGGGAPLSGSIIRGLGLEGAGVVDEFGVDDDYCYYGMSSGSPWSGAMSQQQQMQT
QQQQQYAPMQAFGISGGGGGGGMPPPDYPGTGGGGGGPGGYGEFRDIGGAGGYPRDSAPHPSGSGSGKVVNSIAANP
APKPLQDAAVIKIFISYVQILALTRAVPIPNLPRALLGYMRFYDQITAIPGSLVSLDCSLPDATGVPKAMQRIILAA
LAPLYVSVIVGLVWVGLMLPVYRQERRAALRAADVPTLRGVALRYLPPRLLMSFATVMFYFYPNTVRALLSIFSCVS
VDDGYTDNALAVAAGLHAGKYWTLDYDVRCYVGQHLALAMGLGVTGLLLLAVGWPLGVALWLYDRAGRRDQQLDFSD
FSLVVYSSYRELYLLKYLYWESIIMLRQLCVAVVVVLLAHKGVAVQMLVVIAVVAGALVLQLCVQPFKTPTMNRLEE
TSLVVIMITIYLNLYYISPDISQASRVALEIVIVLLNAVMLAVFVYFITRTSWDKQLDKMGLDRRRVYEMEAGEIQA
SLRRKYGRRTAALLTRAVTFAQRFRSVQAQLRHTSSTMRQQLAQLPLRALTGSSRGKADSAKSLDPVDGEGKNHKNA
LEVDGTGAEAQGQSGAEPHNGKQTERGSRGYESGGLGKGASGGGAAVKDRDSAAAADGRVRKELEAVAAADGDGGRR
AGGGGGEAVLGRQPEVAMELEEEGLDVGAAARCGNKRH

>g15189.t1|PACid:27565754
MGANTRTLRSVWTRWAIALCLFLLGSEQFQFANGAGSAFAETATTVGQLLSTQGQLQQGVLAIVPDASSGAGGAITL
PPATGASLTLTGKGGAVPPGLLNSSFPAASVPPFTALTWGRLADQLDLQGPALALTTGQTLSLASLSLLLADVIQPL
GRGPLLRELGLPALVALGGGGVLELRNVTLVLADVDLRAALGSMCRVDSWPYSPGVSITNGQIQISDFNSTPPGGGR
VHWRDVVITAAASPGGLPPPWPCAAAGVTHGTQLGGGAGGLLPGLLGSSTGAVFVSLARDLALPSDGSWSPVELPAG
RLLVLLGDPDRSAAGSPTVLDLGGIAAAWTAPRRQLVLTGLSEDGSAVSVSPDSGIPGADKLYGSDPGLPVAAAPYA
VAEYGVARLYDLLLVNLPPARRPAAEWALMAAAVNSFGVSWDVEGEGPAAAAPALRLSRCTLVAPDGEVAFLTRAAR
NATEAALVPQYLALAEAPRTVVSADLAGRLQLTSLAFYGQEGTQVVVMANATLLPATTYAGLAGAAPLLPDTRVWPP
LQLQPSRRVANQYGPLGLAVAPGLAFAQERIAECGALPGGVTQILITSPDDRSVWPLARAQPFTAVPPPADDSGSGG
GGGAAAEGCVVAGYPPGLSSGRRVFVDLKGTLRRLGLSAPLTLRGLVLYNLAPGGAYPLVRPADAAAGDAAAVLPPA
PPPPYAADAAWVNSSLPLWYFDVPRSEADLRSTPDTATTTTTSTTTTSPPRLVLEDVTLVLPNEAEWRALAAAMLLV
HQPDALGGRHHHHHRSSRRLLAELGQGQGQGQERQLQGRGSRRSLLQTGTSPKPPSPAPPPSPLLAAEPPAAAEQPL
TPPPPPLPPSPPASPPPAPSPPPPPPAPPLNVATLNAMLAFANMSTPGAYNYAARTLTLAAARHYGWYGTNITITCR
LPPDAPAAAAASVAALPYAPLSLPYQELADMDVDVVVGWGVPPPLPAPPPPAAPPSPPRQPNSTAPGGGGAAAFSPP
PSTLSESSGGGADASGGGGGGKRAWVLPVVATVVTFFGVLAIVAVITAVLVVRMRRKRKQEEAEAESKEAAAAAAVV
VAAAAAANGADGGRRKRRSDGGSLAGNGSTAGSKAASRQPSKRTLDLAAGGATGGAATAAAVVAGAGGEDSAAGHSA
RIAIVLDTDLDAVPDGAGDSFISRAANSFRATRDLILGRGSGCNSRLGSPLPPGSGEATPTRGGGKRASGDGTPGGA
GGGGGTAALLAALASSTRFGSGRRSPAYRGSANGMVPASGGGGAAGGGISVAAAAPGSPLTGAPSRFAAGPTSGRML
ASSSGAVGAGSGKISIDGYAAVAATVGGAAATAAGGASRKSLADTALLGEPSAAAVAKVYRAYVGQVIVRAQQQSLR
DKASRAAGAKDTTAKEADNNNENSKNGDAAKAEERKAQPPPLETASSKDSAAAATAPPAPSAPVAVSVAAAAAADAA
AGTSGVLSQAMATGASLQFDGPNSGAFATAGLRDAAAAATSGAASPAGALSPAPSAKAMSTLPSTSALLATASSAGV
AAAAAAAARFATAPLGAEDEAGFLREMDAYLDGFRSAVTRTLPPLGDLGEDEGSDEDQEDKEKEKEKEKGKNTKDKA
KKDKKAAKEKADKADGKADKDGKTGKSKSSTDSENDDAGNNDNNDENDGLGALRPAAVAFVPAAVSASSRRRSGAAA
AAGGGGGGGGRSAADKWDLPRLMRSLQAELRCSGVLASIQIQDIIDVGRPGPAFGSGSGSGLAAGGSSKSLMLLPAS
RGSKSKSKSREPGRAVYRGLWRGQQVAVKTRVEALAGGREARARLRAVLESATSTALSHPHIVATYLYDLRVLGPLE
LPTGGGGGGGGGGGSASAAASRRSLMHSESGASASAAAATAAVNGGGAPGASVSAAAAAAAAAREVSKLYVVQELCS

GGSLRAALQEGVAGCVLAGGLFRLLALRLALDAALGMRQLHACRLVHGDLRPENVLLVAGPRAGTPGGPSAVGPHGH
HTPAPLLLPTPAMQRPSHFLESPLTATASGGGGGGGGGGGGGGGGDGGDSNMGSPLMHAASARNFGGDRPSPFGLRQ
MNSPVAGAPRNLDSPGQLAIRNLDSPGLPGSGAASPAPERASLQRGGEQADGPAAAAAAAVAATAAATVRAAAARAV
AARAAAEHEDPVPGGPPPPPPPGAGLAEVVAKAVIPGRRNPDSPTLGLPPAAGLTGGLATPLRSPPAAIPAAQLDGS
DGIATPDAAAAAAAAAAAALGSPGGRGGGGGGGAAATTGGGADGVGGGGGGGGAARLGGGPSFRASANGRLNSSVLG
SAFGGGGAAAAAAKTMASPSPLGDPSAQLHVTAKIADFGLAAPPVPAAAAAMSEAAKRAAAAAITSYTAPEVAAAAS
SSSSHSAASPALTQRGDVWSFGILLLELYYGVGLDDCRRLYNSLQLEAGARTGKPLEALLIEDMLSRSETRPYAQLA
AECLSRDPRRRPDFVQVVARLQAIYATASVLALAKATRQSSAMSAAPGGAGGAGGGDGRSSSNGTMRAPNNADAAPF
ASRAGGAAAAAASLNVRTSSVKQISMSGMAGGGGGGGGASPGLANGSPGVSRASSSFRIRPVPMPAAAATGPAAAAA
GLPAPSRLSGAGVSLETRTSIGGAPPDGGGGGGRRRTSLGGLMGGDGADDAGGPPYRGAVAAAGFDVAEEERGVASD
GDAGGWGRHHSHAAAASGALGGLAGGRSRHRLSTLGDLGTLEEQRSSAGSAAGGGQEPPLGSSNGGGGGIGRHGRPV
TSGGAAAAAAELLLPPERASWGGGRSNAAALAATLVSGGGGSGGGGGRSQSGGGMLQRADSNGGIGRFKLSLSGQAS
PSAAAVAPLPFAGGGGDGSGGGGSRRSIAISNGGAAAATDGHTASTSRLSIGSAAAAVTLTAGGEEDARDSQGGRAG
PPPPPPPSAHALGSTGSGSAHNLPAAAALAAGDRRRVSGGGGGLVSRRSSGSNLAVVGYADGSVQVAPGSAFSRAQI

>Cre14.g612350.t1.3|PACid:27565760
MARQKSKAAKIRHGLHAANNNLSALFDANAVEDLEGSQLKQLPGALYIVADPTSSSIEVHAYGSMDDPMLVPSITHL
VKCAYAVHQLRQPPPNITIVKAKATGTTAGPAPKPESYLVRETLRFVYQNAVLPGLRELYDTGFTVHMCNGGKWSKD
DGVSAGKIASWIPADGGSCTCDGSTCMDARVKLGFPNVPLVDYMKHELRKEEAKKAISWGVRRSTFVAQKVAELEAE
WKEERADHKASQARSGAAAARVQQSAESDASDSDEEVVNPVVARKVRKQQGATALSQQLLQTHFERFNMDGKGAGEG
SLLQVLKEEGYGLSKAALARALRDASLRWGKLTPTQEEALGHFQEVKEEYGEEEAVGVIAGMLSLTLEHARKRLAAA
KLLRSRVDKQPEGSGRAVQTALSRHTTKKSRGGKEGHGREERRGDDVSRGDDVSHGGEASSSGEASSSDEEGGGEEG
SGGEESSSGEESSSSEESHGCSDVDEDAPCKKCGGADATVFDMVFCDDCNDCYHVNCHEPPLTAVPMGRWSCSTCAQ
KPVGCAGGGLGGGAEALQRAGGPGADLEVAALGRAETAAGATAAVDCSSTGGGDGGALEPTASPPPGLGGLAEALQR
TGGPGADLEVAALGRAETAAGATAAADCSSTGGGDGGALAPTASPPPGLGGLAEALQRAGGPGADLEVAALGRAETA
AGATAAADCSSTGGSDGGALAPTASPPPGLGGLAEALQRAGGPGADLEVAALGRAETAAGATAAADCSSTGGGDGGA
LAPTASPPPGLGGLAEALQRAGGPGADLEVAALGRAETAAGATAAADCSSTGGGDGGALAPTASPPPGLGGLAEALQ
RAGGPGADLEVAALGRAETAAGATAAADCSSTGGGDGGALEPTASPPPGLGGLAEALQRTGGPGADLEVAALGRAET
AAGATAAADCSSTGGGDGGALAPTASPPPGLGGLAEALQRAGGPGADLEVAALGRAETAAGATAAADCSSTGGGDGG
ALAPTASPPPGLGGLAGAPVHNKRIAVDELPRRNKGAKLQSILEMLKK

>g14681.t1|PACid:27565765
MKSQPPGAAQTGFTSPVARFRRCSLLSITFKRDEEDANDDADEDDADEDDADEDDADEDAVEDTDEDVDSEHGDAIQ
HPALLASLCVAGVSADVARNITELRLKLPLDLPSLVTVAVVLAGTLRHVRILALGTQPCGIGRYGFYNMHALHTRLR
AAFPALEGLNLPARPCLRDLEAFAGSSLHTVRVMADSPGILRMSHVRSLVQLPQLRQLDLDGEDWGTEWGGDDYIWD
DEDDHVGYDKAANSPAGAALRDASDDEAFHGMFDEDVEELLTVRRLLVSPPPALERLRWPGRAWLEVGFTGGRITCV
DLHEDDLHHAALVLLPVLAATGRRLPLLKVKRMTEHSDDVISQLQPHTLFSRLLAATDRVELGVLRLIIWRQPSAEV
AARLQAVVRAVGGMPEKLQFGRYWVSYLSLQTRPRYSGGAGGHRQAAGSAATPAAPAAVTEVTAQQVLERAAARMWD
TAEAEAAATVARVEAAIAAYEADGDDRRPRTRLDNMYVLLRGPLVWQLTCGPDGAASGGGAARLKEWLESLVARSPP
PATAATAAAAAVAPGSPAGTAAAAEAPGEVKVRRCSFAACGFGSAVAVVWCDDPIAALQLYRAAAAAAGEAPGCPQV
SACKADDGHLATLDSQCWAPAINEVIRELWDDHLRSAPVPAAPTAEVGQEQAALGARDAGKAVTRPVGMAAWEGDLQ
FIEHLLLLVEQAVDIVSCAW

>g14678.t1|PACid:27565768
MKLRPRPSFTSPLARFRRCSCLSIIFWEGEEAGAHDVIQHPALLASLYMAGVGADVARNITELRLEGPLELPTLVTV
ALVLAGTLRHVRILALDTQLCGMGSYSFNNMHALHTTLRAAFPALEELNLPARACLRGLEAFAGSHLHTVRVMAESP
GSLRMSHVRSLVQLPQLLQLDLDGGDWDACWSGDSDDDVWDDEKADSPAGAAELPLGDASDEETFREMEEEQVWDLW
ALRRLLVSPPPALERLRWLAEPRQEVGFAGGRIACVDLHGGADGPDLHRAAATLLPALAATGRRLPLLKVNYLVGYP
ADVISQLQPHTPFSRLLAATDRVELGHLSMVDGTAAEAAAALQAVVRAVGGMPEELQYGRHGHWRLSLQTQPRYSGG
AGGHSQAAGSAATAAAPAAVMLAEVTAQQVLERAAARMWDTAEAEAAAAMARAEAAEAAYVAENSWHRREELARMYI
LLRGPFVWQRTCGPDGAASGGGAARLKEWLESLVARSPPPATAAAAAVAPGSPAGTAAAAEAPGEVKVRRCSFAACG
SGSAVAVVWCDDPIAALQLYRAAAAAAAGEAPGCLQVSVCKEGADEDWTPVIHAVIRELWDDHLGSAPVMAMPTGTA
AAAGQGLAARGAPEAGGVSAGQGGVAARRDGAAVGQCRAAVGRDEAMALECDLQVLQRLLLLAEQALKAVGKLQMDS
SQLAGLWACEA

>Cre14.g608350.t1.2|PACid:27565776

MSSQPPWARTGFTSPVARFRRCSRLSVELELDADEDVIQHPALLASLCVAGVGADVAHNIAELRLKGALELPSLVTV
ALMLAGTLRHVRILALDNRPYGMGSLDSNKLHALHTTLHGAFPALEELILPARACLRGLEAFAGSRLHTVRVMADSP
GSLRMSHVRSLVQLPQLRQLDLDGEDWIADWSSDSDDEEEEADEDEDDCPACAAYLHLGDASDEETFRDMNDQEVGE
LWALRRLLVSPPPALERLRWPQWPQQEVGFAGGRITCVELRGHCAADLHRGAAVLLPVLAATGRRLPLLKAGHLGDY
PERIISQLQPHTPVSRLLAVTDRVKLMGLKLMERTSRHQPSAEAAATALQAVVRAVGGLPEELRFGGYGRPARLELQ
TRPRYSAGAGGDRQAAGSAATPATPAAVAEVTAQQVLERAAARMWDTAQAEAAATVAEAEAAEAAYVADNSWYRREK
LVRMHVLLRGPFMWQLTCGPDGAASGSGAARLKEWLESLVARSPPPATASVAGATPGIQARIAAEAPGEVKVRRCSF
AACGSGSAMALAECGDPIAALQLYRAAVAAAAGEAPGCLQVSVCKAGAAWTVEISKVIRELWEDHRRSAPVLAMPTG
TAAAAGQGLAARGPPEAGAERPGQAAAGQVGAAAGQGEAVAGRAGVAAWECDLQVLQRLLLLTEQAHEVVGSGE

>Cre14.g613400.t1.2|PACid:27565804
MSLAPEWQHPFVNIFKLCDVDTMREFETKGDVTEHIDKIIGKKVFKIRGMIPAGNYLRVPRTKLQTLGLTGRLLYIQ
LKVTPIKVFAIHIEVVTEDHNIHRISISNMYNPESMKRKSNGVQLPFPKPSHRWCVLAVDLREALRGYTSSPFASVK
AVQTCSWMTVRTMFASDYKFSLQSLPGDMALSHALDSSLFEMVWLPAEPQDAPTMDFMPPPKRYGTRARRPGTAETA
DTAGAAGRKSLSGASAGGAGPAKGGAKAGAAAGGKRAVSSAGATRGTPSQRGSTAGAATPGSGAGGGGHDGEEDFED
DLSEDLDNGAPLPPSPAVPPPLSPSVAARIAGRATSANIGAATAAGAIVGAAAAHPRPGESDASARPFPRPTTALLA
GAATTASLRPPIASANVAKPEPGQPAAQPLLPDPALTLTRVNAYSGEFVRCLAWLPGTDEVVFAAASVVVIMGVADP
GAAAQQQAAVTPGRQRYCLGHTAFVCALAVASDGRLLASCQEGKEAIVRLWDTANCACLAILNAHASGLSCVDLSPD
LRALAAVGLDVQGRQTIALWNIAELRAAGGPKVELVTRHATEYNIKVLKFSAYQEDHLLTAGRDSIRIYRLKAGQLR
GTSVRLVPQDKRVTSFAGGVSAAVGPNIFTDIGFEAGVGVYGVDAFKVYVSSASGALFVVDYTTRQLDAIFQLHAAA
INCLVVSDGLVLTGGDDRLLRAWPLDFRDYLLEAEHEGAVTGLAMSTDALRLAVGTENGTLGVLAIPTHAYTTLLRS
HCGAVNAVAVDPNRDQYCTVSSDGTLRIWHLATHQQLYEFDAPGEAVSAIAYHPHPSHHELAAGFANGRLRVFDVPS
TTLLQEHHQHRAGITELLFSPGGDRLFSGGADGALVVYDTARMYAPAQYLSAGVRDIKVCCAVSSNGAFFASLVRDP
YRRTTSLLVFHGRTLDPFMRIETDAAAYVKLAFSADCNELWALTSGRRLDRYELKDGQLVQQIHEVSPLELTALCLD
PAGRYAATAGADGLLRLWGCVPLPALRGLKGLPPNSAFLGHPSAILGAAFHRSGYLVTVGDADCVCVWRVNADHMQQ
ERGHLAAAAHALRGAAAGGGAAGASPLLLQGAAHHTQATGALDPTDPRSRAHPSTALGPVASARDHQLTTRAAALGT
PLPAGVLGLVPAPSAFTGTYTQGMAAPSLGSTVASPTRVTAATANSMAALSLVANSPAVAATAPGLPSHALVVPKPS
PPPPSAAPASPGRSPNRALAGAGPARSRLPVPLPPSPQPLPPPPPPRCSWLLGYSPTGSSNVAWNATTGLFCYVVED
MVVMEKLATRQQRYLRGHNRPLSCLAASPDGALVAAGPAAAEPAFTEPAAGARAAQLQQQGVAVGPGGASPTAPFAD
VVVWEAASGRELWRLRYHPLGVQALAFSPDGRWLVSLGRDPERGVVVWDVAAGLLVAAGRTEQSPRAAAWLWGGHNP
AFATAGADGLLLWTLQDNFLEQRTVPLSPTASPRDPRPVTALAVDPHGALLAAEAPAAPGSQVPVWQVQVELQPPQE
PGGEAAVAPGGGGGMSAAVVQVAALPSGCVVTAMAAGLDFALLATDGGSVVRYRRSTLSGTWLESSAVRLDGRVAAL
SAEPNMCDAVAATDTATIWFVGMQDASCVPIVCGQAAPVTGLVPAPHSGRVMASTAADGLLRVWRLGPEDAEPALEL
HSTSPATAAAFLLRQPTDAPAHTLRHPPSAAPSSAASSSPLDPLDPLPAADAAPARPVLLPAPHLLGGYGDGSLRLF
ALEPAVQLSWALARHPAPVVGVAPHPRRGLVLSASSDGSLAVTDLSSTRLVSYITAFTAAGPTPHSPGGTGRRSPRG
AASPPPAPPRPGTGPLQAMAVSSGPGAPLAALAWRDRLVVFAAPWDDPACSPLAEYDVTPSDGRTDAADSHGLLSPD
VPACLAFVNSSSGSSSSGTSAASPGLSAHLGGSSSGAPSSKLLAYASPLLPGTVLLYDVRLAAVARRVRLPQMVRSL
AVAPDGGAMVVGCMGRSVFLVHLASGNTEELGGHAGAVAAASYTGDGGHAVTASGSVLMVWDAAQLLKGVTPPPPRM
LREF

>g14698.t1|PACid:27565832
MATARATAAFTAPLFALLALLAWRGAEAYPRFWYDRNYLVDFGYGPDNGLDWSVFPALTCSNYPDKAYTGFFPAHGT
PTPDSSIGMTFALSGGAAVTTLCPGATHTMQLTFPIRRLALVTTDPPNVAAFTSPAPPTPDCPGRLDLGNSANSDAA
TSFTTSFTVPCTAKGTSVAFHVVSAGTSGSTRRWATAQATMPVSADGSGVCAAVSAACAPAVASQSPPSPLPPSPPP
SPRPPSPSPPSPLPPSPPPPSPRPPSPSPSPSPSPSPSPSPSPSPSPSPSPSPSPSPSPSPSPSPSPSPSPSPSPSP
SPSPSPSPSPSPSPSPAPTSSNATTTTGGPASAPSPSPAGAPRCTPSPLGYTCMAAAGGVTLHWTVNTPSAPPNACT
GTDSAGKTVLAAGDVATDGTLHMAVQAATSGYVAIGFAAKAGTMGPSDIVLGWAGSSGSSIRAFYVTGHVMVDSNAL
SGTHDWAYDKGVVESSGVTTICFSRRLHEPRALASPDLRAAGGLTSAGTSVLGRRRLAQAPLQMGFNWATFPQDRLA
QHPSRAMGGFHLNIAAAAPGAVGTGGAGGAGGPVPVSATPAPSSGESENEHEDENEDEHEHSEGHVVSPKPSPSSKP
SPSPKPKPSPKPSPAPKTSPGPKPPTSGTRRPGSRRPGSRRPGHR

>Cre14.g631400.t1.3|PACid:27565933
MQRQRGASSGIGAPTTIRLVFWLLVAAAIAGSHARDLPYSSRHAREQLPSPPPPVLRLLHTERRLQQYTAQPPQLQP
QLQPRSWRRVQQQLLPDPGVSSYSYCTTSSSGECAPCAQIPYYGDPCASDDGYGGNGFACVLPDRPEEYEATWGVGL
CGWTSGGTYVSSVWAQMPNGTQVPAGELFGFEDAAGVFTVTLRLDCPWMMQLPAAAAGGGEGGGPRLSLNLTQFDGT
HDWAELSPVLRYNLSSTPQYTADAQYVTCASWRTAVATPSTACGWPFRRLVGLNATVLTTNALTLDVCSVLDPATQN
STISTTVLFGQVPEPPPPSPAPPSLPSPPVAPIAPPLPPDAPPPPPPPPPPPLVPSPPLPAPPLAPPPTPPAPPPAP

PAPPPQPPSPPRPPSPPPNPSFPTPAIAAAQPSGSPAATAAAATCPVPAAAAVAAASLSKAATSPFPSSKAALALAA
IPTATITPPAKPSPAPAAVTRAAVSVATLTSTTPPLGDPYWTPSIPPPRPPVPPLPPAASLVTSELLLPAYVAEVSE
LQPAMKLLGSSWVATVKTYNVMLQLRVPSSRSAIRASPSQPAACDVSDVASLQSAIARDMPYNASLLRVACSITLPA
DPATAAGGSSSSGRRQLLLGAGAGGRAESHACGQQSLCNAVSDPGAVESPYPAAVATAVAAVGSSSGQGSWTASRRL
LQATTTAAPPAAAATASSLADELAAATAADGGSSGSPPPPPPPGTALGCGAELQLNVTLVVEVAGGATSASAAAAAA
AGGGQQELLQPLHDLADRLLRKYALLSDGDGSSSGSTPPPASCLPTWWASRNDFAPQLPTAVASSIQLTTVSGLPLD
PTGGGSNNSNSTAAAAAACQAVQAAAVSAFQAALGLLASTSVSETWGSGGGGACLIEAATPTRIMELFMAAPVGAPG
SSGGGGGDDGSSGVPLPPPPASTQFRRETPAAPGSGSGRGTDLASLAVPPSPPASGAQPKATVGMPNWAVAVVAVVG
VLLLTGVGVAVMLLIKSAGLTMLGGATAASATATTASRVAAAAAAGGGNAFATGASASAPGAVVMNSSPLFEADAAA
GNAAPGVVGEGVGTNVAEIRPVVL

>g15254.t1|PACid:27565978
MEDTEPSSELRPQQPSPVAADPADLAGPSSQQPWKYDIATEHASLTTTGGRIWDAARRLASYLEAMSGPLGLQRPGL
RVLELGAGLGWLGMTLARNIRVDPGQGGMVVLTEQEAGGGLDWLRHNLRLNAAALGLQGEGLLEGGLQQGGPQAAAS
VGQQEGQGGEGGQQQPAVRAEACDWFLYCNPDLTTARAGAGAGAGDRGDQGSQGGGGGGGGGGGAIGAAAGSATGQA
TASADAGTAAASRRYAEPLAPGASLAAAGLAAAATTTAATTAATAVAATVATTAATAAAAGAAGGTGADGCSGSGGS
GTGAGSGAGGGGGGGGGEWLSGVAWDLIIGSDLVYNEVGAVYLPRVLRALSRPGHTRVLYAHTKHRFDTWDMRFYEE
LAAQGLSLAEVWETGAPSPPPSPPPLTELFPDMRVAVFQIGQQPPPS

>Cre14.g616650.t1.2|PACid:27566018
MKLLRLVLFLGACHSIKAANHAPTIKQGSRLLPASLAPDVAYFDAGDASSLVVDPSTGRVSEQRDLLATAQSPRPAL
KWQGNCRYQGGPAGANGSVYLDGWTCHAKLSAPLCNGTCLKGAYTIVVVLGFGHGYYSTILSALAPSSQPRQRLVWR
DSELFLRSIPDSGFYVGQEFVPPFSAAFNATPEDFYKGLLRPVSYYGNNGWSVQALAVQPATRRKDSRATWYAQPRA
PNAKSTTVASGRTPGLFAPVGSDFVIGCDYMETMPDDAARINATDIRLPITYRCDPRNTGLRGRLVAVLVYSKALKE
AQVARISTFYSPRWVPASPPPPLMKSPPPKPVRGLPSDLLPDVAFFDAGDRASLTINRTSSQVTAQRDILATPEVPR
PSLTWSGDCRWDSAAGGSVVLDGKSCFAILDTKSRLCDWDRGDGGFTVVLVAQLGPSQQERVALLTSRGYEADIWNG
ERMLAWRPDEVLTRKLVKFVDPVNDDPNNEYDNARFPDAVANSTFTFTRVPARTWTLEAFVGRPNHTNELNGTARYY
RGGLDDSAGARVLYSGPAPLLKAPSGVDFALGCDYTMFDIPDEDYGNYELRCNTNSYLQGRLAAVLVYTRQLSAADV
SRLLTYYRTTRWLAAGTPPSPPPFPPPPPSPPSTGLPAALMPDLAYFDASAATAGRLVVPQPGAKVATGGGVRALRD
VLNPGQQRLDWFGDCRYDATSGGGSIVLDGSSCYAQLNTNLCAEWQLILNSNGGENLNFTLVAVLELDNTRLPPPPK
PPLAPTVNSDHEIAELDTGGMGIMSYGMQDGAYYYFGNPSMHWSTKATYMTLDGGEDYNNVDYRRNFPSPVPRGLWS
MEAINSYSYDVPAPGNTGSTIHNQGYTYVRGTAAQAAGNVTQVAGDEYSFGPPDGTELRIGCTWRNTDMNYDCQPGT
FLRGRLSAVLVYSRALEKKELQRLHAYYSGRWSAAGKGR

>Cre14.g624900.t1.2|PACid:27566056
MPPKAPAPPPPPRLDLPSALAILANPPDAASGGDPLLLPQALSFLGGLNFTTIPQDIELQTTLVTAVIPYVCHADGA
VSGVAMKLLAKMAAEPRLQPVWSEPACVQALWAIVSGFHANYDEYMARLESMAAEAAEAAAGGGGGTGRGGKDGGKG
GKAAAPLTPVDPESTLPGPSKFALDAAMAALSAAVVHSGAAGVEVVGGLLAADVAPLLSLLRHNSSRIQVLALRLLR
LLLRSDAMAAALTAVDAAAALTELLATSPYGAVREEALAGLGHLAGASATSIQQMLEAGLPLLLLAHTMDPTTGALP
PPPPSVADALPEEDSEVEAGLAAAATAPPPPPTPIDFGRPMVSSAARLQELSSRLLLTLARHHQGVPQALLRAGCLQ
LLLAALPAPQLPELPPPPPTKRPSSAADTIFAGVVPGLKKAAPAAVASEPEAAAPVSKAEWAAALEGPMPLPAPAVP
VTEPRLQAVLLQLVATLLSEPGVQDYWWSVHSTRRPHLQLAYLLQPDRLPPPPPPEPTAAEAAAAAAAAAAAPPSAP
AAPAQRPSTQGKAAPPAVAAAPAASPPPPPQPPQLPTEPPFAPEVRAAAVQCLLQLSKHPQWLQLLPFLTSLQKAVR
AAASGAAELDGDGDVIDVGGAATSPAVIQPMADLLQKLLASRAPPPPRAAAARAAAGSSEGGPELSATGTVPRRPNA
VNNRLPLDANGLTEEELPTRDITETLKALARLARRGNDHWALSAVAAALMLLPDEALYAPPWPPLPSPPPTPPPPPL
PTSQFLWDALGRPVMTDGIKLVHL

>Cre14.g631300.t1.2|PACid:27566057
MIVICSSFCAKALARAEVTQREAADRRLRDLQQLILQAQESAGLRANHVEREGAAAARPHRGLRGESLAVSAPSPPE
GGGQQYAVVNLDLGLSSRGGAVNISRLEPRSHTPPGGSRIQPGSNATANGSIGTWGPYSPAGAMGYLWARVDYSSGS
SSGGGSSDAAWYPVCFRTENEPALKAANLMYAVTACVDAGYSLGYSLPPSYTAAYGGSSTTGASTPPPHFPGGGGGN
GTVGQGEQQQQQQLFISNMGCILYSGPDGSSTECTGDLGPQCLGAPAVLSCIPNATDPGSVLDLDVVVDWRAPPAT
AVTSGGGTAPTYEGDNVLYGRNYERYVWPAGSTPYETVYDVCVGHFAVVDPPTPLRLELTVWDAGSLALHQSVTWAY
PEYATGSPQQLLADLLRGAFGSDDGNGGPASPPSPPPPGPGSGGACAPGMRGYLGSYMHTGRNSSSDSGSGGNGNGS
GSGAEGGQPELEIMVSWSPAPVRFPPLLDANSSFVFINNTNDVDIITSWPAVQFPPPPKAASPPPQVSGQPAPSPSP
SPSPSPSPSPLPSPYPAPAASGGANPSSPSSGGDGVPPPALVVGANVTVTNLMPRTYIEASRLWTLYRPAGAVAFLW
ASVGDPTGVSSYLPVCTKRGEAIVDYYAAMACWNDPGRAGGYVLGYGLPPWYLAHRPGTTLAPAGGGALLGPAAAGP

PRFLTDLECGGGGADSPQQQCAATVADSCEDYALLSCIPHSSDYLVLFDLDAVVDWAAPASAALSVGSPPTFMGDNV
ATGLDYERYMWPPADRTDVRRPYLTVYHVCVDNFQVVEPAGPVRLDLTIRNGGAVVLSMSVVWNSSALGHGGLFGSF
GGPPAGPPIPGRAVACNPSKRGYLASYNYTGRVSGGRYSTTWTTGSHDALPPELEVVVGWSWAGESPPPRLPPPSPP
APPGDAAAAGAPLVSFSSLLPWQYQYPNRNWAFYRNPGALTFTLATVRGSADEALRYPVCGGAVAKVVTLLAATTCR
AAGYIFGYALPYWYVPRADLDTGSLGHPEDRRFANNVRCNISMQALLKQDPLPPYECTGTLSDTCDGGYALASCIPN
AAAATNAVFDLEAMVDWMPPGGTEAAVAAAAAAAGTGPIFMGDNVATTSNYERYVWQAGSSPYKTAYAVCVEYLAAV
VPGGPIRVDLTVRSGGVTVMDQTVTFPNTTLVGGRLLEVVPAAGRGVACNAGSRGYLGSYTYTGRNGSASGGSSGGV
SGSGGSGAGAVPAGSHDLAPPELEIVVAWSLAGVRPLPPPAPPPGTTPPAAASPPSPSPSPSPSPSPSPAPSPSPAQ
SSSGTPPPSQSSGSPQGPPPNCTQQLQNLARGAAAYSSSYLTWWCLGGCRATYVVDGDTATDKQMLHTAPFDFDTWL
SLDLGAPSIISWVRIWHRSGFLGRTQMSELRVGNASVMAATASDTANLQKNTLVWKQPAALSEDVTVLVFDPPVVGR
WVTFQNLVPSPRMDDEHALQVRELEVFGVRVPATPPRLYQNVYVGCVSQEALLSPAASAPPPAASLLAIYRNNTAAT
SDLCAQAARRLGARYYAVQGGDCWHLSNFSTAAVSVVTQQCDAACPGDAQQMCGGSRAYGIYEIAPPGASASPPPPA
SRLAPPPPPPPGGADARECCACPAPWPPSPPPPPPKPEHTAGSPAPRKLPVRSPRPLRRPPPRAVGQERRSAGAGPA
P

>g15144.t1|PACid:27566068
MTTSPRALRAYGLQPVDLLSALEDQERQHQQRQQQQQQQSRHARSTDNSRHAVNIWRPDSSYDDIDGVGSPAAGRGA
LLQVPLLHPVGVVGTIGGAADAIASSTRLPPSPPPHSRDLTAAAPPQQQHTGCSGGGSGSGSGSGAGGGGGGGLSFGF
GPMAAAAAVLRRLTPRLVIPRLTTIMPQLPNAATAPAAAAPAAAPPPPGQQAAAAAPPQRGPRATALQAAVAAAMME
EQQRERQRRGASRSPSPSLQSSPTAVATVASSGTPTPAQRSPFAAAAAGTTAGVAAAVAPAPPPPLAALRLVARPPP
PPSPVAAAAPAALPTPPPPPSPSPPPPPPADAGGAVAEAREEHSPPHSRDSLLPPAAATAATAATAASPSASNPAST
SPNQPQPLPPSAAAADAWASRTAARGYSAAAAAAAAPPPPYRSPLLASPFAAAATSGAAGTSAAADAAGLGGGQKQQ
QQQQQQQQQQQQQQQQQQHLRAPAGIHEGGGGGGGGGGGVVSSSSSGGSSTWQLPPSAFATPRAGSAASSSTPGTPQ
HQLQPQPQQQQHQQQQQHSGVPLLPSQTQQYLQHHHQHQHGQQQQQMQQQQQLQLPQGPGDAGCSAAAAAATAGGGG
GGGRAGGSHMWGGGGGGSSSSSRSSCYSFLASYLVDWLLAAGVFAIAMTVPLKAIPVINRHAAPGDPALSYPYRHDT
VPTAALAALVVAAPAAVFAAAAAWEWSLALLHHGLLSLAEALSLFLLFKQSINIVGVYRPDWYAGLASGDAAAPSDG
RRAYPSGHAGLSAAAAALTAAFLVGQWRLVAEGGGGGGGRKPALFARALLCVVLPAGAAAFVGASRVADYRHASADV
NAGAGLGLLCGLGCYLLNFHSPLGRRAGEPRLHDGEDGCSSGYRGCWHDGSSSCWAGGSSRGVAGSRRHYRHHHPDH
LPGNWQRPPPQLSLQQQQQQREEEEQEEEAAGCRVGLEDLDLDLEGGAVGGVAGRAVGERGAVCSNGRGPGASEGQ
EEAGVGARRRGVGGGGGGDWRALGRWWWQWRPHV

>Cre14.g629050.t1.2|PACid:27566097
MRAFIAFVLALAVFAAVQVQGAQYNVTWADDGSPIDLSVNCSDVVQFSWLPRHELEQSRGYGCGKPTIRNFGEGVNI
VAPLVFANPGTYTFACNIEDHCTVGKQLAKVHVGACSTPKNTINWAYRRDNASYAALQLTCGETLTFSWTSGRHDVA
EVEKPDCTSPTIAYYGNGTRFDNGTYFPPASTGSVSFTYTKAGRHHYKCDVPSHCSVGRMLLTVDVTCPTKSASPPP
RRRSPPPRKWVHWG

>Cre14.g619800.t1.2|PACid:27566118
MGRKKAEKGTARAPHGDELLRILHSPATHGKLRTLVKSGMDPNCSVETACFDSTTWILPGVAKPCIPLLVFACFNGV
PAATVELLIKLGANPNSVTDCGLPCPLAVLCAKITPEEQRALFQVLKQHRCTTFNSRLRSARNEEPCHITCKVLDLS
DWTAPRKVEGVEMLIQFGTFLLPDPPGGCGGCGGAGGANKTGGGGGTDGTAQQQQQEQLDEQALYDFVVTAVRQPCG
HKLLDSLWRHYPQRLLPRLPLRSSLFLSALSESWMAGAWRHSEERQHTVTQAVLLRLLFEHPAMSAPPQRKALVAWL
RANMTALLYRPELVAAVLDRFKGAGAGDEDVIHVDDTNLVLPVGLLAGPAALQGEVPLAAGGGGGGEGGDAEGGDGE
GGGGEGGGGGGGRGGGGLSLMLAYAVDPDEQLARRGVEMLLARGGRVDVPCRLGPEVLGAAGSTAAAATATAAAAAA
GKEGAGGAEGAGGEEGSEGGEGGGAGGGGGATTDGLVTPLVAAFMPYQRNQALVELLWQRFAETHPADATHDAVARL
RAAAQSLFLALPGWRGALDRAARAAAAAAAAATAAAAAGGAGGSAAEGGGGAAGLPGGAEGGAGGAAAAAAAAAGGG
VFGLGVLDAVPLVCSRERLRDLLDSNVLDFVDGRNLEQVEAALMDLVGALSALPDQQEPDQEAEDQDQDDKAGATAP
SSPSSSRLEAQLQLVEARLAADKVWLESLAAAGRPESELVDDPEVWRAEAAVEAAAAIRACLLRSAAGVEPEAAPSA
AAAAAYAAAVAAAAGAPYAPPVRGYFSSAGFAMDRDAVVTPAAATAAAAEDGGSSSSSRARELPPGVAFVEVVAARV
MSQPEWGFSRGLLLQLGVCQMRRMMRQLHGEDDVDGVRGMLGQIQVLRYHYEELLRSMAPPERRLLADLVAALEVAD
AARAAEEAGGAGGAAPPPEAAVAAEPSPQAPQAPAAPLMPRHSTDLSALRALLPGESLGGDSDEEDDLGMLGEEEEE
DEDEEDDMDGLGGFGLRGLRVGMSPVVAAPPAAAAAAPPPPAVAALARRQLHGCAATGLDVMVGSAPPSGTAGGASAA
ASARATGGGCVSDSYDGSYLDEAIDAALAVGGSGGGVPHSLPAHVRKLREANSSVDSESLASAYGRTGSYELPPYEY
LGAAVMRRKDPNGCNDGADLDFGLELESDVPDPAPRAGGAAAAAAAVGKAGGGASAVRGRRATVGKGRGGAGRAAAL
AGSGGDEGDSGSDRFDTPSGDDAGAAGAVPASAAAAAAGSIAFAARATGSARCIATPPAAAPAATVPAPQTARSGSV
GASAAGSKKQERQGLQQKQQQALRSQRRSGDGSSGAESSGSAGGGGGGGMYGVYGASHAGGGGYGGYGPEYGMESVG
GDMGALELPHGGGGADVLGLGGGTAAGGAGGGGGAAAAAVARGGGVSLSAAAEATVAGLRSGSHSRKAVAAGGSTPH

DGGQGNAAAPPLLSFGGSRRRLEREDSRRADQVAAAAAEIAAAAALLPEEPEALDSGSDGGSAAAAHARRARPPPPP
PLPHHHHHHRNGRCESGSDADGNGNDSDGSSVNFDGVPGSQQKKQQQRQQQQQQQQQQQQQQQLQPAAPPRLSGGN
ASAPVSRTSSAASLDSTRAYIVHRRRPGGGGGGALGGGGAADPDSRDGGGGGGGHGVSDFYAGGDEGWEPMPGMRMS
DQGAAGGGGAAAAAWAAAGARGESARRLRAAAAVAAAGAAGAAGAGGHGQYDDDADSACSGGGGGGGSWGADADGED
EGAAQDEEDEEEEGGWGGWRGGRAAAPPPPPPGPGSAAGRQRRRLQVRRAHAAAAAATAAEPAAAGAEPPLQPASHL
LQPDLTVDSEGDVAAAAARGARLAPPPAPLPRRGRASVSDDGAARLRHRGGDGDGSGCARACGSSSSSGGGGGGGGS
SSSRLSYGSEGSVTGGARGRATSAAAAAAGSGSINGGGGGGGVNGGASGAESDANSDSTQEWPPRPRQSQPSAAKAA
AAAAAPGDWTPPPAARRRHAGAAAKGVLGRDASESPGSSIGFSGRRRQHLDSDRSDEGGGVDGCGSAGNGNGRRASG
GGGEDSANGGASSDGTVEWGGAEVGVVQRRRGGGGGSRGSSGGSSGGGGGGRGNRRAVRSPSGSVDANVGAGGGGGG
GGDSDGGSSDSSVTSRPAPLAASAAATAAKARAAAAAAATPAAAGGRGAAIADAVATLSTPCSSHRTTHPSAKTGKT
NPAAPPPGAAAISDDDAPGMSGRGGGGESDAASPLAAPVAAAAALATARSQPAPMAAVAADDAIAAAAAAAAAGAAA
PAQLAAAVAGTSAAPAAATAAGALPAGGADKALTSLLAALAAANVHPRTDAISPGGGGGGGANGSASGAAAQAGSAN
VSSSSNAGAGGGAASGDGGGGSSSNGSVQQCYVLQLSGAAPEAGFSAPPPAVMQVLAATPASADPLHGWLAGLRGAL
AAASGCEQQRLYVVADCPARAWPAWLPALRAAVAAAVLGAVPHDLEQEAADAAADAAAAAARRSRRRRLQLRRRRHL
AEGGGGGGGGGRGRSGRADGEEGDDEDGSQDDEDGSDDEEGGGGAAAVAAAARRRRRRRHCPEWLRQAVALLPLCHV
VFPFGGMFLTQATARALQATTDPARHAAYASPTASPPPPPPRLWLDAACLPAAVAFLEAHPGNLYGRALLMQLAGKQ
VSRALLRSPRYGLTGAARTAESLQELLYAGGDVGYSALLGGGGGSSSGVGGPGAATAAAAAAAAALLLDEGEEEGE
EGLALRGAGMTISVLNTALCAARQLQAVWRSTWPGGHSLRLAAYSYLAAAGYPRAVVSALHACVPLVLEGALDASEL
LAELRLLAAAPGTALQVAEEILAPQPDDDPPSPGAADPHSPHSPHGGGGGGGAVAFRHLVSALRAALDPNQPHTLHT
HLAADAAARRRQQQQQQRQQAASPPPPPPGGGGAAAAGKPAAAAKPGQPPLAPWSALPPPLREQLVEEMLTISDLI
MIVHAVLLALRTALTHAADVLLAAAEGGAGAAAGDGSGGAPAAVAAAAAALRREVAEVQAMRAAASRMLVERMEAQE
RAPACAAAAASISTNLSHSHSLVSNHLHHGGAAAADAGGGGVGGGGGAAAAVEDEAATAETAATAAANDPVTWRHIN
GLYEHGKAVLQCAADAAAALLDPRTRAEEVERCEVLVGWLASDSQLLTGLAALRQLPPAAGLALPPHHYAHTHPYTA
PGGTADDDGEEGDEEEGAVAWGEAGRGGVPAPPPHHQCNEVLYRMMVPNWRDEPPAPASPAPASAASSSPASAPAAS
PSSSLASEFERSSWSLPTLMRGEFRPVGQARLRRLFDEVRALACECLDQLSRIAPPPPPPPQPAAAAGPAAGSFPNF
HYQHQHQHEHFGGRGGGDEEEQADDDAEYLTSEAAAAATAATAASAAAGGSGSLGAADAADAIAAVCAFLRQAHQAQ
AQAQAHEAHQSHQAQAQAHQTQAQAQAQAQSHQSQAQAHQAHQAHQAQAHHDGELGGQGQQGRGHDGYGGYGDEDGE
DAGEGAGGECGGADGAEGCEAAGAQFVYLPGLPPHLQVPLQPHHIQQLQARLHAQAQHQQQAQHEGLGGQQQQQPGE
GEGEGEGEDGGSNHFHFIGQEEWEGEEGEEEEQQEGGGLPWEALGGLAGFVFADGSGFGFAAGDGGAAVGVGSDGVQ
YYEAGRGRGARVVVEEVEDDDEEEEEEGEGEGLDDPL

>Cre14.g626800.t1.3|PACid:27566132
MTSFSSAALANVGLGTSKPTSFARQSTAITPVPGDLPAPPSVRSAGSLPLWHLSPSQGPAHLRPSAAASRRGSVAAA
AHRLVGTLAAAAATLAGPPQTRCAAPAPTAPQRRLLGSHGEQLHHQTSQRQHQHQRQVRGGQDQQEQEAQEEQEEQE
EQEEVCAGRHQPDGLNGPCRPQLDAAPRSAAGSRSAGGRGGAAASIRPNPDLDSRSTVWRSGGSPRLDPNPDPDADV
PPAVAPDNGSGSAQPVPLDPHHGQQHHEHHHHHAHVHVHVHPPPRQALLRRGGSYTGAVAPAAAAAAAAALPLPLYG
EHSERQRQQQEHMQQPHHMQQQHTKHPAHSQHTLQHTQGPHLHAQPAPRSLSLLTAPAGGQRGGYRHSLGSMGLDCG
GSGSGSTSASSTSSRGSLALWSGAGFTSRARVAAAAGASGGGAAGSGGVGGGGGGGGGGGGGGSGFGGGGASGQC
SGLGPREGAVGGLRRFVGPEPHAVGGSRSSSTSRSIASSTGGTARSSSSNGSSSSSDRSNSGSSRSSGSSSSTSARG
ASTPGPFQAAPTSPSTTPRPPVTRTSSSSSSPLYGSHLLAALRQAGTVSEVAQLMAPYCHGGGGGPVSGVGGANAAA
GATGGSAVALGGGRCGAAHVAAALWALAEAAERAVQAAVAVPDVAASGWAESAAVAERLLEMAAALFEEDGSSSGGS
SGSRSRSSSGRADAASQGSASGGLPGLAGTSAGLDLALAVALVPHRLTLLQPSPPPQPRPQPPRSLSPPSLLQPRWL
LPALAALGPRLHRLPPARLVELLECLAAPAMWPYAPASGTLGRVLLLRAVQSLVRGGLQQLAPPAAARALGALAALA
PAGPGRYFCVHAARKGPAAAEWAAGTPGQLLALARSEATAVAVAAGGAAVEVAAEVSVGGVARQPLSVDAAARQLDL
AFATSFMSYIRAALAAPQGNKRGSSDGGGREGEAPGSGNGDSSSSSSSGGGSSSGTDGACGVGGGGIVAADAVAFLH
AIAKFRNIGRAQQHAGASSSGSSTGRGSSGSSSSSSTSSGWLFDWPGAEALRGHQLTEEEMGDTRGASGSSSSSSSD
SSRNGCSSHVYHIDSSISISSGAEEAAATSAAACNEDSSSNSSNSSRTSWSCSNDVTTSSSSSSDSHRTSGQSRRAQ
ELSLPAQAEATARQLRRRPRPGDVSPLARPSPPPPPQAQTPSSSPSRARGTRSSSNGRSSRASSRDRRVAAARNSTA
QAAAQDAAAPAAVGAVADPDAATLLQLIARLPPTPPRPTSQGPAPLQAASTAVAVAAAVAPAVAGLCDSDLLQLTRA
FWAMARLGVRPPAGWMQRFLAASAGSSGSSSAPGSSSLNGSGSSSLNGCGSSSLNGGGSSSLNGGGSSSSGHLARLP
PPLLARLCWAVAEVRWAPGGVWLGAAAAAVSAAAEQLQPDEVCMVLVALGRWGVRPRPEWLWPFMQRAVQPRLATFH
ANSCSTLLVALARLLQPVPPQLLQPASGSDAASVHPCAEPHPWPLPHAPAAATVSSSSRQRANAVAGTAAAPLGPAV
STQSTPAARQQRLQRRIEQHGRRREVAAAAAAAVAEQEAARRRGVRRRAAAALLLPRGLAAALVSQAVAPQLRYMSG
RELVHALGALGRLGLTADRIPRLMLVAGSAASSESSSSPASLASPAASLSLASPASAGAAASSAERGAASMEATAGV
LDWRQQLLAASLAFLEPSAGAGAGIVAGGHMAAASSSVVTSATPGGSAGGPAGSEGGASPRAAVARPPLTPMQLALL
LLYTARARLAPGPGWRAAYWAATAGGPPAAAAAAAALPAWRSGNGYSHSRPGSYSHSKRGSNGYSRDQAGSYSHSHS
HGPGGQRAALSLMTPLALAALVEGLRSLRVVPPAWWAAAFFAATEASLPALPPSCVYQLARLLLYSPLRPRGAWVRA

FRARLDLVAAALTGHQLRTCQRMVRAVSRRRRYALAPGWLPNAAAAAAAARRRRIGRRRAARRAAVRAMGAQPDMDA
GTGVAGV

>g15141.t1|PACid:27566137
MRTYSYASTGAGSARTAEPAGTRPRRVPSPELLRRSSKHKWSSACPPGRAAAALLILLGALLQRSCVAAQGLAPPPP
PPLLSSTTPASSGFGVAGSRNSTSNASSINANSISGGSGHNDTPTNHVTVGCGLRAVLPALTSYCREATRRALQAEA
SHNPRLAIVDIVSDDGGVTPPAAAVNADVRVLSAAESALQPLLYEHARGNTSYLSQLDANVSAGARLPSSFLSVPGG
AAGGPLHAPLLLFYRRDWWASMLARTSWRNGGGGSSSDGGGEAAAPPPPTWQALVEVLEANVNTNLEPDVSEPDHVL
CVDMMEGCKGWAVLAGIYASMTQTKGGWQGAYFNRSAAGGLSPSVGNVAMAAALRLYSRMAQVNAAPYTPGGAATSV
DRVPVDPKHYMVAGGDVDPATGLPACGVVNSLFAAGRCLFTIDWAPAALAMTAGDAVGVSGNLGVGLLPGSSEALDP
TAGNTLQLCTRASCPLAEALPKPFNTVMSAYQIATEMSAQPPQPPQPPSQPPPPNMPPPPSPPPSYPPPPSPPLPPS
PPPPSPPPLPPGLGSTVEMMNNRAPLMAAVTHVYVQPADQSALQAARVNEVMRIVGYQGGLKYSLLTGLAPSNATVD
TPADGGSNSTSTSNSNSPSGETCAAQMALGSGGAGSAAAVAASSILDLAPEDQDALVVAILASTRHSNEYFDFKLPF
SNVYRSWLDAAATRAALQIKHNLTVDAAAIAAMAQMISRGGSSDSSGSSSGGSDPAAPASGGGGTASVAAAVSMTGA
ATAADGAAGGAGAGGGAGQGSAHTVARHRHLLQPQGQGPPAVADGPQLPLLVVPVAQSGGGSDSGGAGSNGSSSSNS
SGNGVSVVAVAVLDPDPVVQQLVMELQRNFTETMSRFPYTNILRFLYEYYLPAKPVEAPPPPAPIDNPALRLGLPIA
LTVGCTLIILGVVAAVAVWMVRSRRFRSKALQPPGPGPATTLVMTDVQNSTLLWELLPLGVMDVCLSIHHRIMRQAI
SDHCGYEVFTEGDAFCVAFWSPEEALGFAFQVQEELLMADWPDVLLQQEDGCEVWAQRCHVGPSLGSNGGGGGYVAA
AAASSSMAAAVTNRGLRMLRTSTAQQPLALPPPPSFSATPSAAGAGPVGMGAGVEGGNPRLAPTLASALAARWSASS
SPRQPLPLPPPPPPPRAPTAPAPPEQAPASKAPSRLRMLVAKTDALNRFQRRNSAPLAAAAATLLSSGSGSRSGGDG
GGGGGEQQATVAATGTHRVVADGGHGAPLGRGPAGAWPISAGPPAALLGTGSSSTTSPAAATATATARSPAEEAGQG
PGPGQGPGLGKVRSPTGQVPSPSEPLHKLQLPGSCGVREPAVGAAAATAATTAASTPTGRRSGEGRGADVSIRRLAQ
VREHDDGARAPAAAATAGGGGGGRGGSRGGGGGLEAGLVVLQPAGIAVSQDGSSSSSSGAGSSDDDRDARERPSAAA
AGPSTSATARERPSAAAAAARAAAAGGDGDSNLVGGDMEFAIDDFFKGDADWRNEVTWEPTDQVLEAGAKGSRKAVR
LSSGNQRGGGAAGAGSRNRAGTASADRASGGGAAAMISPLLQPYLARDRGKAPGSGSITAAAAAAFGRRRGGGVLES
AMSDGANDRDSDVAIRTASIRGGGGGGGGSSGGARNLFDSGGTGVAPRRKSVLGLPRLHLSAFYGASADRGSGGGVA
ATPSPMAVARRHFDTVSQASGSPAPAGLTSTATGGAVVAIDLERSPLASPASHRNGSARHGTAGGAAATSGDGYGQL
HVPDLESAPTHPPPQPVHTGVAGGGAGGGGVLAKLSSLRLALLRLGRQGSAAAGGGGGSGSGGLGTANTSPSATPPA
AAAAAACTPRRCATSPLVVLDVADVAEAAAAATAAANGAAAGTHHTPPHPYAHDHAHGHGQGHVHVQGISPHGLGSR
SSTAAAAAAATAAAAAAATAAAAAALPPPPGLDDTQPIIVSAGPFIASETTTMGTGPSRWGGKFMATMGPIDRRYLT
GALPGGTGAAAGAGPSGGSAAAGAGIFDKLWYDINTAAVAPAAADGAAPPAMAPVSETTAAPSEGMADTPVGTRKGS
VSHWRSMTAAVSMATAGARSVLPGGAGGGGGGGGGGASGAVMEEVAGQGSGSGAAAPMPPASRAPSASGGAAAAQAA
AVAAAATAAAANAAGPAALVTVGAVLRRLWVNDPLGPLHFFRATAAGGAAHLLDSPATCAIAEERGGGGGGGGSATA
AATAASAAAGPSVLQLGTPRARDNDLNHYASRQANNHQPPVLVFRGLRVRIGMNSGLEESEYMVTQSGLGVQAVAYC
GDFFATAKEVGDVAMGGMVVLSASAFAAYHSTQTRTRKTGGGGGGGGGGGRIAASDMLILHLGEHAFDKAAPPAPPT
PPPLQEQQQPSGPQKLEHEETLLLRPPERAATGIPTAAAGTTAITAVTAATPEVPVRSLYGALSPAMAPRLAVLPDL
VRNTRVVAPGCLAAPAGVVVPVFCNVVGLEVLVAWERVIQQRAARELSAASTHATAASGGGGTGGMSSDDGGGGGGG
GGAGGSVPGSSALTGGAGVVQAALRLLRAVGRALVARHGGYVVASSWDGAHWVLVFPSPESALLWGLDMLEAMLTAD
WPPGFTEHELTEEVFNDGVLVRRGLRLRIGVDCGRAMVRVVPRSGRLDYVGRPMNRAARIAAKAKAAWMLASDAVWQ
AARPAVAHAAVAHSLGSLSLKGVREDMHLWALRPRRCFPASPPPSQTQEEQERLLARVPPPGAAAAAAAAAVRDASA
TAQGYGSVGQGQGQQQWGPPLAAQLRPGSEAAAAAAFAGRTMSAAAVVSAAVADGSGI

>Cre14.g608200.t1.3|PACid:27566141
MADAENTTKAPPRAPEKSKPAKNRTSELMRPDPVPQGAVWPPVAAGFGVTPWPLGGPFWPPVTPLRLGAVSPGMPFW
RPPMPMQPFNPFLAHAHGMPPGGMRPHPLLGPPGALLKPPPLHVPSPRPHAPPHTPPPPAPGPAATPPLTPAPPSAP
PPAPPPAPPPPPPAASPAPPAVDPRFGLDLQTALLAATGPLPLSDLGALWLRQFGCPLPADHLGFSSLLELLRALPH
AVAIGRPPHYTLSLPLPAGPPTAAPAVAAAAGMAAAPGTPPASSGAAAPLTIQIRKSVVPIPTGSGAGASAVSGQRY
KTSTPQAPRPALPVFAATAAPAKPGPGGPTAGTPARKAPSTAAATTATPSRWGPLPTPTPMPLEPGRAAAAVVAMSP
EARQQAVAAGTVGVLATRAAVLALSETAAARALALSKALRAEPKEPSQPSQPARAALPAPAPERHRPLVSVALKQVA
AQPSPPDRERRSSRHTHQRRRRSQDRSHAAAAAATAAASGHPPVWGRSPLPEGYHLDSRSSTSSSSSSSSSSSSSSS
ESSGSESSSDSSSSDSESSDLGSLRRRRRQQASPRRDEPQEQAAAPAAEAAAAGGDGDAAVERRVPVVPVVLEGQDR
QATGAGVADAAAPAAGPTTEMSGGSQKEQQQQLQQRLLLASAGGGGCGDAMDVEVDVDVDGVRAAEQPPTPPVAPLL
PDLLPAAADAHETCASLRLSLPSHQPPGEHQQQPEQPEQQVEEGGGPCAGTGYLPAGPVDPPGQSGPSPGPGTAAAL
APLAASRTAGAHEGVAGEDAEMEEVEVEVAGEAMEAAVEVEVEVVDLTRSDSSEPGDGREDEHELADIGTAAVPCAG
TGAGSSADAGTGVPEGEEGEAKEQEKEEELGLQPGSSPHAPGVAAGNAGVIAAVMAGAAGDNTCGAAPRGHHGTAGG
AAGGADVDGGADPGPADLRAAGSPPPQLPPAPPPPAPPPPAWYDVRQDAALAAEAAEAEAEAECPGPSAGESGPAAG
GEAVAVAPAEEPAAAVAVAVAMAPPEEAAAAASAAAPALEEEADMTNMAEPHAVAASGDVAAAAAEGEGVDEEEEAS

GEVAAVPRVLVEAGVTAADDTSACEGPEARPAPGAAAEAEAGIGAEEAAARGVGEGLALGVPAAVAPAAAVGPSAGA
EAAVSQSEVEVWVREEVESGPGAGQELWREVDTSTPPPPPPPPPPPPPPPPPAAAPPLAPQQLQQAAVEVATEGTGS
DMELDEEEEEGGKVDGGWGAWRGRQALVAVGTGGDDDMDMCSACEECEGWEVEAEAGAAVAQSLPSVSPAVASSVQH
CLAVAAVAAGDCPQLMQQEVLPPPPPLPPPPPLLLPPPPPLPPPPPLLPQSLQSPHFMPPPQHQAPQQPELSHQFAP
IFTPHQLPLDLSPQQLPLDLPPPQQQQPQQPAVLLPPPPFQCLQPLSPPPQLHLPAPQLSPQPPPPPPTWGPSPASA
PPPPLHQQPQPAFQELQPNPFQEPQEQPFQLPVLQEPQPLQDPPQQQQQQPQQQQDSQQQPASEAPPRFLAPGGVDL
LSAAAPLFQPPLAPPPPPPLQPPLEARFAAFVRLLRRLLLLHRDWPRGGLPVSLLPGAWRSAYGCPLVPAAWGGGGC
GELHQLVGRAVWAGAAALAPADLPGEPQEQQQQPPVRERGREPQQPQRPGRGAGRDQVLIGVVDTGGAEELVVAVWE
DMLRHKHNLNMGHHPQHQHPQHPRPPGDPLGAAWPPPQLGVQVQPAAAPEAGPAAHVSYEAVCAGVRYWLGGQGVEE
LRPPLEPPPSLQRLRQLDRRVGATMEAYCAARCVVTLWELERHVCASEGVEAFGELRLGPLTAHPVVRRTFFAGLVK
GGRRRRLFTGAEAGPPADYGFPPLSAFDVAAALRRVAAARGLAWCRAAASGNVASSQPHTALVAAVLEELDRGLRAL
AVHEERIAAHVHAANQPAGAAARGRKGTGGAGKGDGGGGSGGGGGARPPPIVESSTWPDWWLPSGCGQLSRYPAEVR
ATALALRLGVLVCDLQLYVEVAVAALREEQWGALRGAVARPAEEVRRLAAEVPARYRAAFEAGRQPEAAGAAAAAAA
LPTATKAAATAATAAPVKAVAQAKAQRPPVKEGKEEGEEESEDEEEEEQLQEEEEVCEQEEEAEEGTQTKSGKPQAA
KQPDKPPQPPQPRAQPPASYATPAQQLLTRRIWIQMDTQSQLHKATAKAAAGEAALPTLQKPAAKPQPPPLPPLPPP
PATTTATTTATTTTKAKAKLPKHKRQALKAVEVQAAQARRLSWSDPALPRLPPTPPAPELLVRQGHQLKSAITRSAV
LCALRREPPLPPSLPAGGYGFLQRLLEDFPHSVARGKQPHPLYMAGSVHVPRSALLPADLPAFALRVEDCLAALAAA
EEAAAPEACREEGRKEGRQPPQQQPVAAAATAAAAAATLKLAPAAAQAARGPPPRLPLPDEGAVACGVPRVLRAPEA
WLGSVLRNLEELPGGGPDPGKYPVAPPEWDGGSFLAVANSVCVRRELMRVFPDLTSPDFTAHDLASAFGARVRQLLT
GAHTEVRAAAEVGPRDSFYSQALQRLAKDLLGALCRGEAAALVTGADSRRLGRTGQLAPIKYGRQKHRADTLAPEAV
TTRVNAVVGRLCASPLCHLLIRCLHTALTSQQQLQQQQQQQQQQQQQEGPGQGPGREPGAAAAADADKEQPQPAASE
QVECSSGGSQQFWSCCEEGEGEAVGMQVEVAAVAGAGPTTSSSGSGSYYTACEPAAEAESAGVQAVQGAAVAAVAAA
AGEVDVSSASEADPPPEADPPPEADPPPGPASMPAARDTVAGVGAGVEPGLSPAANRGAITLCGTAPARGGPLQEGQ
GVGTAGAAAAAGEGGGGGGGGAAAELSPGVEAQRPPWLDAAEGGRRQRQRVGKRGRDGAEETAATTAAAEAGPLPLL
GLGAAAPVGRREPESAAKRHKRPDGAGHVAGVTDREEEHCVPPPPPPPPSPTPPPPPTAPPPAPPSAPPPQPHQSTQ
PQHPRPQLAQPHQSVPQPPQPHQPPPQPPPPAAGGCWPGRPAGPPTAATPPAAASATADLAAPARDSADNLLTGRWG
EELVYRDIRRQLQVQTQQQPQQQQQQQQQQQHRTYHHPHTPQQQQQQQQPVWDVEWVNAAGESHLPYDVVVRRGSEV
VSYIEVKASTSLSKKQFEIGAGQLLRAAQELGPRWELALVRGARGPCPNIVRIPHALRALRQRRIQLFVGLAGAEE

>Cre14.g613050.t1.3|PACid:27566144
MRSDCRGGTECCESPAAGVPGTTGAAGGRREGGRRIEYVSLDFSGCKNNSLSWFCCQFGTCSTTACNGVASTSTCNN
VVKATVAVPAGAASVTLQVHDGRITGNFKCTTATPCCGGDGGACGTGSTSSVCGGVVVPISGVSGCFARPPSPRPPS
PSPPSPKPPNPPPPSPPRPPSPSPPSPAPPPPPPSPPPPSPSPPSPSPPPPPPPSPPPSPPPSPPPSPPPPPSSPEP
PSPEPPSPPPPPPSPEPPSPKPPSPPPSPPPSPPPSPPPSPPPSPPPPPPSPEPPSPEPPSPPPPPPSPEPPSPKPP
SPPPSPPSPPPSPPPSPPPSPPPPPPLPPLRQHPPPSPPLPPSPAPPSPSPPPPSPPPSPAPPLPNQASCSSSCPNG
SQIRAAGFKTTCGATCLLTQPSACLVEPRPGAFDSPGSYVPAFSGVYGCFLKSSGTYTSNSSGSSSSYPLYLSYGGA
NLDSFFSSAAPGAVAALACYDSPQLINLTQYLLVTVLPTPSDNYACLTCGFYNVAPLAATSSANCDCNDTAWAFPTP
AQLGAAQAGNSTSEIRLNVTDPGFGNGVFWNPRQPTSSAWGGYFRIAPPASPNAVLTLAVCAGCGQNQPDKGYYLST
GFTITFTNFTKGNGSITIKALAPGASAEVDKLQIYLSFVAPPNLNPGQFQTFTTTSPTVTPTNPLNAFPAGPFTAAA
RANVTYTTSSGSTATVPASVDPSQGLFFALHLDVGQACPAPY

>g15268.t1|PACid:27566160
MRRNGARAAGARWPLRTLCFAAILLICIGQDGTSTGGGPSSGAVADCGSECGGWAAAGECGANPGYMLVNCAAECEQ
CADCPDLDPSCGGWAAAGECGANPGYMLVNCRKSCGVCGPPTPPPPPPPPPPPPPDVPPPPPSPPDPPTTPPPGPQPP
PPPPTRPFVLPPATACRLVVDVTAVAPDLLSSGSSSGSGSSSSSGNTSSNGSSSGRLAGAVVRLDCLPPPAQQQPPD
SQPGPAPAPGPTQSSTSSSASTAAPTRVRVVLGSRLLPYSDDFTGVTVVTAAEAAAAVAAATAAVAPAGNTTAAAAA
AAAAAAGALPVAVSEWGVAFLDVTHLRLRDSLVAGPPLSPAGPHLATHRVPHLALTGTTLRTMFGPARNWQYQDGYD
PSIPPVYGAAVCDELVTLRLDGFECSSVRQGAGWACLLALWAGRRGARALYGIDTDAYGAYGAGYGRDPELGPPVSP
PWLFVTSSRITNNRVQRGGPLGVHQRAPAAAGPSSGCSSSSSGSSSSGSSSSSSGGGGVVQAVVSYNEGFGAVVLAP
AASQQAESAAWRASVLDSSDPGAQAAVPLLATLAAGTTATSNSGGRGAVLAAMYGVNLLHLALTSGTQLLDNYVDLG
GGAVYGQPPGGGGAAATVTLRVSRGSRVAGNRAAGGGGGGLLLGGGVALLEVSDGGALGPGNVAEDGAGGAVAVADE
SGLSASPPILGRLLLTRGGAVVGNSAGAARAAPGGGRGGGVFVAGSCGGVRVESGGVAEGNSASAQGGFLACVAYGG
SDAAIGAIEIADGGGGGGAVVANNTAGVSGGAFAALGRGVGLLNISSLVYDADAASPSRRPVLVTGNTAAGGRGGLL
LAAGAVGAVELSSSGGIASSGPDGSDGAGSSSSSSSSGGSDGDGEGQSALAPALRVERNRARSDGGVVAAEGGCGSV
AVRGPGTHVWGNWADEGDGGFIFAAAPGTAGSSGSAGSSSGARRRSALSGPQQQQQQQQAVLVIRREVPAAAAAAT
AFGPTPHTNTSSTSNMRIGAAAVAAVATAAATTAGTWSPAATLSATAGAPSATAPGDAGRQQWLATSRRRHRALSAQ
GGGSSPGERSGRGSGNSSNSRRRQLQTASASPAIGRLLLEDGVQVSYNAAGGGGGFAAAWGDVGQVVLRGGAVAEVN

SAAAGAGGFLLVARGDLGPPAPAAAAAWVSVAAAPWAGGDGPSPSGSPSGSPSASPSGSPSGSGSGSDDSVPTPEQQ
APPPAAAEEQTDTGGGAGGGAGGGAGGGAGGGAGGGAGGGGAAGTVTVTVTVTGAGVVWSWSWGVVVSGGGTALRWN
RAAEEGGGVAVVGGRLRGGVLVEGGAALWGNQGSSGGAVYVSGPAGPLVLHGAGTAAASNGAAQDGGVLAAAAVAQG
VCVQAGAQLIDNAAGRSGGAVFAAQLQAYTGGGEAAGAAAAAAAAAAAAAGTGCRYGGGVWGVAVAGAGTRVSGNSA
GQHGGVVAVAAVPVAVSITDGAQVVGNAAVGGDGGVLWLREGFRSLTVKGAGTRVSGNRADEGGVVWGDAASGALAL
GPGGEGEGREVEVAGNTARMNGGAFRVNTLEQFSISGPGTRLADNAAGASGGALAVLGAVTTASSAVAAGARVSGNS
AGRSGGFLFVQGRSSGSATGDAPSGSSENSGSSSGSGSSSGSSSYADGLPGLAVVSGAVVSDNRAERGGGGAVAVVG
RLGRLTVSGGARLSGNSAGADGGAVAVAAGGGGSGLLEALEVSAGAEVSDNQAQGSGGAVWAVWGRAVELSGGAAIS
NNRASLDGGALAFASCAAFGTAAGVSNDVADGLDAAKGSSSSRDGGLRVLLTGGSSMIGNTAGRDGGALHAAESGSS
SSDGGSSSGGSSSGGGGGGYFPYPANGCCMLARLELRGGSSIFGCKAQAAGGAVRADSVGAVLLYGGSSISYNSAAT
GNGGALSVAYRLGRLVVAGGSGMYGNAARSASGGALAAAAIAAVEVAGEGSAMSGNTAALDGGAIATQALGALSLSA
GGAASQNVADGGSGGFLLFAGDGGSGGGSSRCGPPPSSREQVPRLRLRLRDCVMSQNWATRRGGALAIANPYTPVSS
LTATNCTFERNSANYAGGAIWLLLQDSVVLLPPPPPPPPAPPPPPTPPPGPRPRPLSLVLLGRAPPPAPPLSPLPD
SSDSGSTSDGSSGSSSSTDSRSGPAAASPPEVFVGVEVRADAPPPPPPDGQQPLQPSPPGLPPPPQRPPGVAQAAAP
PEGAFPDEGGPAEHSSLPPQQQQPPAELPGATPPPPPPPPPPPPDATAGGGALPPPTAVLQLLFDGCSLNGNSAVLG
SGGAVALEREVGYVDMTIHDPLRRVIGSWIQPPAAAAEPSSRGGDGSGSDGSSSSSSGGGGARLELPPAVRLVFRNC
RLIENRASEAGPGGAVSAVSRSSLGTVTAADIVMRMEYAASQQPPPPPPPPPPPPPPPSAPAEGPGGSPTAADGGDG
TGDGSSSDSSSNGDGSSDGSSSGTDGSTRGSSSSSGSSSSPFEQLNSRGRRPMLRLLTLVQAPELQVVFEGGDTRVA
DNSAGASSLFASLTAAGAEPHGGAVSVRTELAVQQLVLLQVGIPLYYGQPDGAYEPGGSQSYVQRDTSGRTPTAVLA
DALAATAAANAAGDEAGRWGVSGVLPAPCRVILRPGSSGSSSSGGSGGSSGSDGSSSGGGMQWVNNTAKGRGGALHL
AACSLEVIPSGLSSSNDNGSSTGSSNSNGSSSSSGQVGPVIVFAGNEAGVDGGAVSVELEAAGSSSSSSAGAAAVGL
LRLLLDEARNASRQAAADAAAVVAGSSSGRRRRRLLQQQDSGTAATAAAGAATAASDPGGGEVAALERALAAVVVRG
QPRLVVWPPAAAAAEAAAAAAANAATAAGSAVGSGTANATAAAAVLFLGNVARYGQGGAVRVELHPLLAPPRLQLQL
RQLLQPGSGTGAGTGAASPPPGPAVPVAGSSTSANSGGTSGSSSSSPGPQQQQQQAAALVWMEGVELRANEAGFGGG
GLAVVSLSGNSDTAGSSSGSSSSSGDGSSSGTGGSSSGSSSGGSGSSNTGSSSAGSSNIVEAAVLRHTQEASAFLNS
LNGSSSSSVNGSSSGGSAAVLLRRCSFVGNQVTGVGAAAAAGSATTCGGGGLQLVGGLAAAALEGVALTGNAAAPTG
RPRACGGGGAYVGEGGAAMLSGCRLERNTASANGGGLLCSSCSALHVYGGWLRGCSSEAAGGGLALQADGSSSGGSS
SSDGSSGGGGGWRAALVVVEIWGNRAGTGGGVSAWALASGSSSSSGSSGGSSSGSWLLLQQCRLANNSATGVAGGGA
AAVASLGQPAASYGGAGGGVFVGGSVAAVVSRCDLSAGNWAGGAGASIASAQRCPGGLAATPAAAATAVAAAAAPQQ
QLLLLPAGGLPVGSSWVSPPPAVPSLLHRALARLARFAPSPSSPSSSSCWPLALADVALLTAPQALALVQQANATTN
NNTSTSSAAATATATASRAALWFEDTYAEALAVGCSGGGAQALAGMQTAAAAAAEGGNSGGWLKGEAAGGDGGAEQD
VTAAALLIDSSGSSSGNGSATATTTSERLRAQQQRVGLGLATCVARQVEAAAGPQAAADFANATSSSSNGGSSSSSG
GSSSPLSRLAAALLYLPPARLRLVGVALAATGQGQGQQGAAAAGASSLVMTVPDTEAGAEGLGSGLLLLAGGAATAE
AAAAAAAAATAAAAAALRFGVEPAAVRVAPSLQLPTGQPFALALELVDGAGQRVADHSQVVVELTVSPAARAALTAS
SSFAAPVLAGAAQWYGVAARGWPGPVALHFTAVSRTGDYSIPPLSVPAALLPCGLGEQLAAGRDAAPPPWTACAACA
ADEFGLWTDTRGSSSSSSTHIGSSTSIGSSSNGSSSGNVSGDGVVALIDGGAGAGAVAAAMLAALAGAAPCYNCPPN
AVCPGGPLLLPRPGFWHSAANSSAMQRCPQPDACVGDGAAGGVGDPVSALLARLAAQAQQQQQGQQGQAPLAKKRAL
LQAASPSQAVAGAAATGAADSGCSSGGGSSSALPRQWQAPSLRQCQEAWYASNPPGAAVLAALAAYQADMRLQLIQP
AAGASGNGSSGSSSSSTGCLPASNATAIGAAATAAAGELAGEGGLELLRPCVLWGLADTHPLSYMAAQCAEGYAGNL
CAACRPGYFIDAEFECQRCPGAAQTAVLGVVAVLLSSLTVLYTAFATFAEDQTNTASVNASEIFKVLVVHLQFFVIV
TRLNISWPQIIQTMRAVMATVTGAENYVTFSPSCLLPAARYDPPHTHTGTGTGGGDDGGSSGSSGSSGSGGSSGSGG
YSAASAEAQLAGGLLLPLAVVGVSCAVWTVRHQLFGQALLKRLSNRSGGGGGGPGSGRGASVLGLGGGGGGSSTGGG
GGGGGGSSSSGLWKALTLATRRGGGSLWRRSDTSSTGGGGGMGSGGGGGGGGGGSSAVMRRAGRRAAIAAEILATMAV
RQDSIFGSSAGSGGSGGSYSLSPCEEEQEDELREMDGEEEAGGQAVGKAGGEVGEVGKEREEKQAKEGREPAGNGHS
NDGGKWSGEAAADDGSSAAAPAVTAPAAAAAAGFAAAVEPAETLLGTGCSGSSSSSSSPRQLQPRQLQPQPQLQPQV
WLGNPLAEDCGGGSSAGAPPGGRRRARSTSGGWWRRRRAVAEGASRPEPGPCAAAAAAAATAAAIARAGAGSWRPLA
PAAAAAACSRGGPPSAFAAAHLDLLEDEEEEGEEEEEEGVGLREQAGGAMSGREGGAGQRQQRHCPLPTTTFSVLRQ
SHTKHQQQQQQQQRPPPLQRCVRFSDPGASAGAGGSEGGGGGGGGGGMAAQVGPNSGSASASGSGQLSSQASLLAA
ACSAGHPSLPPPPHLALAIDTVMKPAAAAAAAAERGVEGGGGSGGAGATDAAAAGAAAAAAGGGGERAEQPLRAQQS
APGCLSPSAGGARVPLAVQLLVLVLIALFILYPALAQVSLSVFSCYRIDTGGGPYPEMQRAGFTVISLVSMAAAPLR
SRLLVVLEFLSLGVLSKPQNTNGSGGGAASKAKGAGGSSKGAGSSKAGAGGGAPLRSGTRSSTASSSRRSGSGSRSG
SLPKRGSSSSGVGGSSSGVGSSGGVKLAASRAGTLMSRARAAVRPLVVEVVPLRVAKRVRDMNARCYTGTHLALYVP
LGVAAVCLFCLAPPAASAAVMWVHRRRLDEYHTAQTYVTIMLGLFGMDRAASMNFSQGSDLALGVVILALNAAFLLG
LLTALAAHSASLLRPLLQPPAQAAAAAAARAVAVRLLPAAAGWRCWATCVEEQGVAEGEQGEQGEEEEEGQGGGGGPV
GQVGLVLAEGEAVGVPVGRRQGKA

>Cre16.g693950.t1.3|PACid:27566220

MAGARYHGVAASVPVSSKHLRLRHRRSSQAQRACRGANPVSLSPLLLLLLVLLQATAPPLAAAAGLCAAAVATGPRT
LGALAAACPEQLSTSLALLAAAAAGPLGDHAGWLLGCAPGERSCPRTVFLPVDSAWQKHFQATGSSLATATAALTSA
PAGSSSSSSPQQQQLLPDVARVLLLHVLGAGEGKLRARQFPKVPGARSYGTRLGGARLTVSRSAGAANTLQLRGPVV
AGGAAATSLTSNLPAGKVFVYLVDVVLLAPPPASPSPSPPLPQATATSPSPPSPPPPSPRPPSPPPPSPPPPSPPPP
SPSSTPTPTPDTTAPSPPLPPPPPPPSRNRRPRPPPGPPPRAPASPRPPLSVSGLAGYLDLITDMRAPVISVLMVAVP
GTRKYVLVERPTDVFYVNGGVIDMDTRKTLRFLPAENRFCSGVMVLPSGMPIFPGGFGPTGLLKDGRANIGGFFVGN
NSLINVGNMRALRWYATPAVMPDGKVLIAGGSPQYNAGAPPPWAELWDPAQPWAPTRTVAQPPAFESVEHRWYPLLW
LLPRGDVFWWADRAGAITDSDTFRVLRNVPRFPDNYRYRMLYPFTAAGTMLALRPQDNYQPSFVVFGGGDMSGRPFI
NDDDVTLAASSQAARLDLTACSSDPSGYCTSGWQLEDMLGLRRLMGDAVVLPNGRVILHGGAASGLTGLPKGGPNQA
ADPQLVPLAYDPYAPPGRRFSRLEPGPVVRLYHSAVCLDPSGLLLVAGCETCSGYRGGTPFISPSPDGMFEYRLEWI
TPAEVAPGVPRPVWVDEGAAADGGGGGGGGGGGVRPPSVVQRGSTFNVTYDYDYELGAAPGSSSPLLAAASLAAPC
AATHSVAMNSRVVLMTVLSDVEVPGGGGGGGGPSEPPRRRVATIAAPPASLPGLAPAGPYLLFLVGQAGSYSRGVWV
TVT

>Cre16.g691150.t1.2|PACid:27566259
MLTVHYATPSVVLRVLEAEGPRNRHAAFFSTRAGNGSTLLHAALFGTTAPVLGGIITNNNGSDTLNDAPAGNPTLGF
AWVTAMPPVAAANTTSTGEFLTDPALFDSLTPGEPRQPDQLGFLALTGGRALYVVNATDGSLLYKQEFGSRVLGPGP
GNFAVPPVNFRGLAVFPTANNTLLAFNLATRTLAWEAAFPVSAFSSLQVSTERLLVRLTKPSTTPGYPYYSVSVAGE
VRGFEIDPALSSLSSPRTTNPLSPLTPEGIHFTFLDANWSAPTSPSRPPPSPLVANAPLPPTPPPSPPPNRPQRIGM
TDLVTLESRKLTLNFTIRYPPVPPAIAYDSCRLVVYGNGFVYEPAAGEDSILTSVHVIDVASGKLIWSKNLPRLDEF
GDSFIQHPYIDPVSGSIYLLQNEMLWIFDPTPDKLPPPPPSPPSPPRPPAPPPAPPSPPSPPSPPPSPPAPPSPPSP
PPEPDYGGAASVSHASGHVMALSLVLTVLLLPLGLLW

>Cre16.g679050.t1.2|PACid:27566277
MARQKSKAAKIKHGLHAANNNLSALFDANAVEDLEGSQLKQLPGALYIVADPTSSSIEVHAYGSMDDPMLVPSITHL
VKCAYAVHQLRQPPPNITIVKAKATGTTAGPAPKPESYLVRETLRFVYQNAVLPGLRELYDTGFTVHMCNGGKWSKD
DGVSAGKIASWIPADGGSCTCDGSTCMDARVKLGFPNVLLVDYMKHELRKEEAKKAISWGVRRSTFVAQKVAELEAE
WKEERADHKASQARSGAAAARVQQSAESDASDSDEEVVNPVVARKVRKQQGATALSQQLLQTHFERFNMDGKGAGEG
SLLQVLKEEGYGLSKAALARALRDASLRWGKLTPTQEEALGHFQEVKEEYGEEEAVGVIAGMLSLTLEHARKRLAAA
KLLRSRVDKQPEGSGRAVQTALSRHTTKKSRGGKEGHSREERRGDDVSRGDDVSHGGEASSSGEASSSDEEGGGEEG
SGGEESSSGEESSSSSEESHGCSDVDEDAPCKKCGGADATVFDMVFCDDCNDCYHVNCHEPPLTAVPMGRWSCSTCAQ
KPVGCAGGGLGGGAEALQRAGGPGADLEVAALGRAETAAGATAAADCSSTGGGDGGALAPTGSPPPGLGGLAEALQR
AGGPGADLEVAALGRAETAAGATAAADCSSTGGGDGGALAPTGSPPPGLGGLAEALQRAGGPGADLEVAALGRAETA
AGATAAADCSSTGGGDGGALAPTASPPPGLGGLAEALQRAGGPGADLEVAALGRAETAAGATAAADCSSTGGGDGGA
LAPTASPPPGLGGLAEALQRAGGPGADLEVAALGRAETAAGATAAADCSSTGGGDGGALAPTASPPPGLGGLAEALQ
RAGGPGADLEVAALGRAETAAGATAAADCSSTGGGDGGALAPTASPPPGLGGLAEALQRAGGPGADLEVAALGRAET
AAGATAAADCSSTGGGDGGALAPTASPPPGLGGLAEALQRAGGPGADLEVAALGRAETAAGATAAADCSSTGGGDGG
ALAPTASPPPGLGGLAGAPVHNKRIAVDELPRRNKGAKLQSILEMLKK

>Cre16.g679050.t2.1|PACid:27566278
MARQKSKAAKIKHGLHAANNNLSALFDANAVEDLEGSQLKQLPGALYIVADPTSSSIEVHAYGSMDDPMLVPSITHL
VKCAYAVHQLRQPPPNITIVKAKATGTTAGPAPKPESYLVRETLRFVYQNAVLPGLRELYDTGFTVHMCNGGKWSKD
DGVSAGKIASWIPADGGSCTCDGSTCMDARVKLGFPNVLLVDYMKHELRKEEAKKAISWGVRRSTFVAQKVAELEAE
WKEERADHKASQARSGAAAARVQQSAESDASDSDEEVVNPVVARKVRKQQGATALSQQLLQTHFERFNMDGKGAGEG
SLLQVLKEEGYGLSKAALARALRDASLRWGKLTPTQEEALGHFQEVKEEYGEEEAVGVIAGMLSLTLEHARKRLAAA
KLLRSRVDKQPEGSGRAVQTALSRHTTKKSRGGKEGHSREERRGDDVSRGDDVSHGGEASSSGEASSSDEEGGGEEG
SGGEESSSGEESSSSSEESHGCSDVDEDAPCKKCGGADATVFDMVFCDDCNDCYHVNCHEPPLTAVPMGRWSCSTCAQ
KPVGCAGGGLGGGAEALQRAGGPGADLEVAALGRAETAAGATAAADCSSTGGGDGGALAPTGSPPPGLGGLAEALQR
AGGPGADLEVAALGRAETAAGATAAADCSSTGGGDGGALAPTGSPPPGLGGLAEALQRAGGPGADLEVAALGRAETA
AGATAAADCSSTGGGDGGALAPTASPPPGLGGLAEALQRAGGPGADLEVAALGRAETAAGATAAADCSSTGGGDGGA
LAPTASPPPGLGGLAEALQRAGGPGADLEVAALGRAETAAGATAAADCSSTGGGDGGALAPTASPPPGLGGLAEALQ
RAGGPGADLEVAALGRAETAAGATAAADCSSTGGGDGGALAPTASPPPGLGGLAEALQRAGGPGADLEVAALGRAET
AAGATAAADCSSTGGGDGGALAPTASPPPGLGGLAEALQRAGGPGADLEVAALGRAETAAGATAAADCSSTGGGDGG
ALAPTASPPPGLGGLAGAPVHNKRIAVDELPRRNKGAKLQSILEMLKK

>g15951.t2|PACid:27566287

MRTTYQHTRRQPHPSQHRSIVWTLLAYLPQALIVSALCFIAVYAGWALQTISVHGGDSGAVVKTSGSAVGAASPAVR
ILRTADPSPSPRATRKVSKPPAPGPPILNSPPPPSLPPPSPPPPKESPPPPSPPPPSPPPPSPAPPSPPPPSPPPPS
PQPPASPAKQVEDKPAVMSTEAKKQTGTVAGSDKGPEDEPYDDKVPKDADAEYDNDADKPKQESSSSSSSSKKKEDG
KLEAAAAGGSGAVARELLPTSVSGRTGRSSGAQLTRPSPGGTLFSWGLKEWRLGRNKGDNAIPEPALKDLTVISVAA
SRHSAIVSGEGVVWTMGHNDSRGGGGHGSPPLDASGQLGRGGNTQPGPVGGPLQGKFAVQAIVGRYHTMAVTEDGEL
WSWGLNDWGQLGRAAQGAASEDDPSPCNSGPSCHSGIPNKVEFPAGVKIRGAAAGRYVSMAVDDDGVLYTWGHDGCS
NGGKLPAQAEAWRPRKVEGELAGKRVVAFDAGYVFWLAATEDGAVYTCNSQDDGYAGTLSNKHQPNNAGELGREGDP
LVPGRVGGVLAGHKVEAVAAGREHAVVSTSEGKVFTWGGRDLLLGRSGSPKEPGQALGDLVDDNVRYVMAGEYHSGA
ASHTHIYGWGSNDYLCTGVGRHNPVQRMKKGRDRGDVFRPARAVGPVADGGWQVQALVGGFQHALAIAANKGGSWET
KPSSWLDSTNPNSTSHGAASSRLQHARSEKAVAIATAAEVSTLRSRGPSSRSSGPVASAKARVASKRLVVPHGRVGA
AHGAVRPAARTTSSVAAAPQPSALKPQEQKKRGGLGETDAGAAVKAASPSPSPSPKELSAADVAAAAEEQAAAGADA
TQVKAADTTTTATSATAAEAEAKPAAEDAAATALQTAAGAYSTTADGAAAGATHTDTSAPDYHTRYLAPQLHPIRYN
VSDYVHRYRRSDRFRKIEAESPDVWEVLPRNYDRRYKNPCWVTKDSKFRCAPYFHILGVSKCGTTDLYHRLSQHPQL
FESRNKGPHWWDECPYPPKGACTAPPNGDFDGYVELFADAAAKIRSKDPDGITGEASSNTFTAAAGVYLRGPSWDRN
TTVTMPELMREAAPFLRNIIIFRNPVDRYYSAFYYYRWWVKDTPPPTPDDFHKQATEEVATWRACVEAHGQKHCVRH
YHPQQLVKGMYSEFIMDWLNHWPRDQLLFLRNEDYSAATREHMQTVIDFLAMRQPADKEWDVIMAMKTRNKNSDKYK
PMLPETRAMLEAFYRPYNERLAHVLNDDRWLWRDPPKSPKPAGVQSDAAGTATARRLLLQPSV

>g15951.t1|PACid:27566288
MRTTYQHTRRQPHPSQHRSIVWTLLAYLPQALIVSALCFIAVYAGWALQTISVHGGDSGAVVKTSGSAVGAASPAVR
ILRTADPSPSPRATRKVSKPPAPGPPILNSPPPPSLPPPSPPPPKESPPPPSPPPPSPPPPSPAPPSPPPPSPPPPS
PQPPASPAKQVEDKPAVMSTEAKKQTGTVAGSDKGPEDEPYDDKVPKDADAEYDNDADKPKQESSSSSSSSKKKEDG
KLEAAAAGGSGAVARELLPTSVSGRTGRSSGAQLTRPSPGGTLFSWGLKEWRLGRNKGDNAIPEPALKDLTVISVAA
SRHSAIVSGEGVVWTMGHNDSRGGGGHGSPPLDASGQLGRGGNTQPGPVGGPLQGKFAVQAIVGRYHTMAVTEDGEL
WSWGLNDWGQLGRAAQGAASEDDPSPCNSGPSCHSGIPNKVEFPAGVKIRGAAAGRYVSMAVDDDGVLYTWGHDGCS
NGGKLPAQAEAWRPRKVEGELAGKRVVAFDAGYVFWLAATEDGAVYTCNSQDDGYAGTLSNKHQPNNAGELGREGDP
LVPGRVGGVLAGHKVEAVAAGREHAVVSTSEGKVFTWGGRDLLLGRSGSPKEPGQALGDLVDDNVRYVMAGEYHSGA
ASHTHIYGWGSNDYLCTGVGRHNPVQRMKKGRDRGDVFRPARAVGPVADGGWQVQALVGGFQHALAIAANKGGSWET
KPRGLGETDAGAAVKAASPSPSPSPKELSAADVAAAAEEQAAAGADATQVKAADTTTTATSATAAEAEAKPAAEDAA
ATALQTAAGAYSTTADGAAAGATHTDTSAPDYHTRYLAPQLHPIRYNVSDYVHRYRRSDRFRKIEAESPDVWEVLPR
NYDRRYKNPCWVTKDSKFRCAPYFHILGVSKCGTTDLYHRLSQHPQLFESRNKGPHWWDECPYPPKGACTAPPNGDF
DGYVELFADAAAKIRSKDPDGITGEASSNTFTAAAGVYLRGPSWDRNTTVTMPELMREAAPFLRNIIIFRNPVDRYY
SAFYYYRWWVKDTPPPTPDDFHKQATEEVATWRACVEAHGQKHCVRHYHPQQLVKGMYSEFIMDWLNHWPRDQLLFL
RNEDYSAATREHMQTVIDFLAMRQPADKEWDVIMAMKTRNKNSDKYKPMLPETRAMLEAFYRPYNERLAHVLNDDRW
LWRDPPKSPKPAGVQSDAAGTATARRLLLQPSV

>Cre16.g668750.t1.3|PACid:27566307
MVPGDYRPGSYAAAYLQQQAESAARRPSSTERQRARREEAAWDGGHGPERQDSSDAAAEATEPVANQGSYAAIAAAA
AAAANAAAAPATGSPGRPASARGHGTRSPSPSPPRASWDPSVSRPATTVLAQATQMRMARLRRLAQRQAQIQQPPPA
RGGSLTSSGPLQAGLRASSARASASSRLGRAPVLLSAVLQQQPQLYDRQRLSAVERFIDVLDELPSRYRDAQVLYLS
KNSLTSVSGLEQFGSLRVLGLADNLLSEPGQVEALAAACPGLEALSLEGNPLSMVPHYRSRVILALPGLKALDGRPV
TEEERAAAPGELAAEEATVAVLMRNACEVHKMASVVQRAQLHMELLHTMYSRGAVMARVEALGDAAAAAAEGRGGAR
SLTRLVELWDYEGGMGAEERRKIRGALLREVGRMYRQSRAAQGAGRSPSWEDAFAQVMMAQQQTTAALLDLLTQCQA
ALDELLRSMAATAGPHTSSWSAAAAAVGGSGVQGAGGVGGEGAGRARLLREAEEDAERQLALRMEREGAMADMRRAA
AARPALKSALKRTAHQRQSADGAAAEAAAARRSGSGAGGSQHGGDEEEDAAMMGNLSDDEGLLVRRRRKSPSPQRWQ
SARSSPPPPEAVERLLRARRGVVASPVRAGSTPAAGLRPATGRTAESRDAGTASAAKAPQSARATLRQQLPWSAGPG
LASATTAGGTGSSRSGSTDRTRPWLQRRSRSVDAATLRRGGSSARGAAAGGPKATATPVAVGEGADDRRRASSVSPT
KRLQSPPLNHPAAWRPPGAAPPGWSSPAGAASPSPDYKVAAQAVERAVRAQWNEEQVDAGVRPHAALTAAAAGGLAD
GASPMRQSGQWADLPERGVSASPEVLGRRQGHSQQWHGAAHSLPAHHARRSLQGALSGSSAPSGATVGSSAPPSAQA
RRNPAPADARAAQLRASSPAAPLAAGGARTRNAAGNALGGAASWSQIDDLRRQVEESVAETARLRHIADLAAQPIPA
RSPLPSTGLDARPNAQLRASWQLQAEVAPLAPSSRPHPATDAGPPYAQLAPPPWAMAPPQAQAWGVSSTPPPGGLGG
APTFSPQPQPQAPPPSLQPHRNSSARAPDDASLTGAAAPQGEPFPSLPRIFAPPTAAPSGASWPHAQPQPTFQQQQQ
QPLADTEASFTVAPRTGEQAPTQPAVHLRLQPPPGAATAAANQAQSAYTGQQRSAPAAPEYAQPSAPPLPAQLQTEY
AAAAIAAAAAAAGTPGGSWTPQQHQPPLQDPQEPTSLGGMSVMRASLDVLTPGSVQTSANFPSVSSTPSFSRRLGEA
ASGAGGGGSILDAYGAPVGQAQQQPQPPRGYAPQEAPAETPTQPQMTGTWGVVDTAGPMQRDSVDFPRPPTATSILA
TPAFTLFAPPAGGAAGMGPASRAGGGVSFINAGGATAAAATPGGTIVSFNDAAGGTARVEVAEMITEDSAGYKDQHI
HLHIHPTGSQAPDTGGTDASTPQLFMQTGPDRRRAGGVATGAPPTQPTLFGADTSSAAGTLAVIAPPGSPLHASLAS

GKVVYEKIERIKPAGGGQGAAGASAGRVAKSGGETRRSKRRVPGGVGADGRAAVPGFGYTYEDDEEDLSGTEAEEQG
KVSLRRRARGPLAHSAEEGGDAPHDEEGQDQEDREGYDDDIARDGDWRQRRSTSSTSGRSGQVPEGRRAGLRSPHPS
RRAAGSSRSPAASMRQRDQDAREQAQYAELQRQLSEQAVLSSQLEQQLAQMQTQGLTAGAAGGVPAPAEVLEPGAAT
FVYGTQQHASPQASMASAQRGPLAAVVAADAPAGMTGAGAGERALLPSGSQRERTQLPSVRRPATSDMLPTFQLAPT
RDTAAAASPEPRGQQQQQHSTTSFQASPSRLGATASTAAASATGTTGAQLLPGASSTPTKHAPSSPPPPGTTRQQSG
QQLHSEQQPSRPLQGAEDLPPALRGISDQIQAQAARNRSLQEQIRSLHAQLEVEPAADAALLQAPAEAAAAGVALRS
QLPTATAAAAGLSGPTTDPLPAGSLRALEQQLVSLTLEKEAVERSLLGEQGEVRLLQQELGLMGQVADALEESQAQL
QETRLAVAHLQQEVVERDQQLDAGRAREAELLEQLADAREELVTETRRLEGEAASATVRHQQQMEEARVDAAERLAS
HEAASVAALAAAQAERAAAEAALLEQHTAQMQELADAHAAELATLRSAYAAEVAAVSAEHAEAVQRDAEAAAAAQAQ
LEERRVAEAAAAAEAAAVAAVALEEAVRVGRERLEATEAAHKGQAEEAAELHKQQLQDAVVRGRAEVEVARQEGEER
EAATRAAAQAAAEAAAAEHAAEVAALHDRIRYTAEDAAAARRQGEERAAALQAQLEEALVALGNERMAHSELRVQHQ
AVEEVAADTRRQLEQLLDRLEVERRREGAAELLARRTLLRRALWHWYGNGQLCAEEARGLHRAAEHFRRRACRAALR
RWHVVALRTRLLKRMLRERRTKVQRLVLASLKRLVLERRSEARSDTIAAAHHGMWLLGRCLSAWRRLWRAHARAIAH
HDEDRAAAATTHHRHHVLRTAIAVWHRQVHEHCLPKTQKKRRAAAAHRRRLLRRGLQGFRQLLERCAAKRNSMARSV
RRHRFSLLGRALGYWLDFMETVRRKRSAVAAAELQFGHSLVRAWHRAAGRSAAIRRGVEGMQASSGTRRLRTCLARW
RAFLICRRQSALAKRLAEEHRRGAVLWRCLSAWLVLAQQAAARQLQVKGMREYQRMMQAAIRWQRMVEQSRRQAAVV
ERAVRLFRHRVLAATQRAVLRGWRDLVEERRALMRSAAIAFVQAFLVIRTRRLVTEWQGLARRGAIARGRAAAEELR
SRLEASESSLRAAFARLEAAGADCDALQQQLREAQSDVLHLKQQLQDTERQLAEACAALEEARTGMEERDVHLAARA
EQLATLQEERDLLQQKTQTLRQQIAAGEVSITSLHEQASSLQRSLAAAQAQSRELGLQMKQQAAQYEEQLLAARDAT
ALVRREANSLAAAAARAEQAAAEGEAQLRTVRTGHESLVASLRQELRTREAHILQLSQRLSSMPCCSGGAPAAATSA
AARPATAPRPPSPLPSASPLVDRSLISGLAARTAAGARALRPTGGLLSGRNVAGALASAALHDDPSSSVDTTTLSTT
TVTTTQAQQHEEVAAQWRERERLLAAAIAARERGPLFEPSTAAAIAATATAVAAAVRPPSPEVVLRSYEARGPDTMP
AAGSEATGLLRTSLVSQPLLQPTAQRASPAAPAADVWIRSPDPPASSSPQPYLHLHPQPVAGRSGSHHVTFAGATSY
LANAAGGGEHDGDGDDSSSDSSAALQSAALEEAVAAAAERLRRSAAEAQHAERSRQVGEADFLRRRAGILQQRQEEL
QHLSGGHTPGAAAQGRPANGSVAAGGHVGLAGAAGSTSAPVDRVVILAGGTAPGPAEASAATTHRASRNVANPRVSS
SAAQVGARPSTAAAEMDRSLDFAAQQLQVLGAQQQAYQRQLLLQPPPPLPPSQPEARAQQPQPQPRQQPQAPVSSSS
AANVDVMGEGPPSELPALTAGIVSTLTNLRDAIRFGQPVSHDSSRAPGGVAASSAGQQEPLQSAAVAAASTGSLSSQ
GSDALAPAAEATLRELLTMAPDRGQPGAAGGSASSRRPPSAPSSQTHHFSWWPAAAGAGGGTSPGMSGESGDSRGAG
DGAAVPSLHSVPASYRAASPAGSTSGRVATGSDGVGAASAFAGAASATHGARASGAWLRRSADGADALSSSMGSMSA
GTAPAALGRSAGSASGAATALQQLRQLASGHARPQLPPLPQQQQDQHPLPRYLQDQGLSTQLLVEGGVDASVEAERL
PDAGAAFPEQGHELYMLAGAHVAAAVVRRRLLGDTVEAEVSMALMAAGTGMAAQGGVGKIGYVSDDDEEDEALCLVD
ESTPSSTARSMTHDV

>Cre16.g653900.t1.2|PACid:27566330
MSKNGADNATQRSPAEELNHVLHWVAYLLMACSAGLLLTSAVALFRWPYPSCLCYNTPFSDSAMCLGSPCCACANIR
QGNVTLIFAWTMLLLCRMAIGFELSYLYPYLGFCLLHAVTASLVYVMVRGVQRQQAEEEAAVAASRGRMLLLHELGV
PGALGREGQQPAPLPPIQTMSAELVLVLQPDYCSACGGVGGGSSCEEKGEGADGDGKPRASCCEAWGAEGRATGAAA
DAAAGAAAHAAGLGKAAGALPPGVAWALRYTRVEPPHAAPHGEDQASPKAAGLVRGGSGASPLAAPTPATATGGAGN
VGLVGISAFAGAPAAAAAVLPEAEPSGCEGPGAAEAPGAGCGAVSSAAGGCFGRLGCETGAGADCNGTGAQQSALWR
SVSEALTPAHALQQQRLQARLSSARSHMAGSSPALVLAHGVSLPLPELANRTPVASPTASVPTHARAWSGSRAQISA
TSLPVMVATYRGGSPPPSALRGGSPPPSALRGGSPPVPRQQQRAVLGHRPASTDAAGHTDCPPLTPVTLAADAAAA
AAAAPAAPQAADTQGNTAGP

>Cre16.g650625.t1.3|PACid:27566434
MSVLGKDQPLGGTSAQCNQTPVRRPRPPSPPPARPHLLTAPDGSTRIDPYHWLRDDEARDPEVLSYLRRENDYARAV
LAGYQQTQSELLEAMTARVPPTEHTPPVPGPVVFTASATRAAGSSSSSSSSSSSGHGYSQLPGRSGDGDDDSGGDD
GSGGDGRWCWYWYGTTRGPRQRHWVHRRWRVPLPAAAPHDAGGSAGPLAYGPLAAAPAEGAAEVVAAAEGEVVLDLN
RLAAEVAAGADWEAGAQAGEEGAREEAGPEEGAASRSGGSGSEERGSAGSSTSSTTSTSSSSSSSTGGGGRRGSSYC
DVGCVKPSPDGTHVAFTLDVLGDEQYSLYLQRVGSRSSSGSGNSGGGGGSSIGGRGSGGSSSGNTGGGPRPRLLLRG
CGPAVEWSADGRTVLALRLTRRTHEAYQLVQLDLGPDLRGGTQEPRVGAGETEEPQPEQIMGLTEKPQLEPETRTTR
GEPQPESGAAAAGGGSIGAGAAVQVLLEDSQPGSSLELAGHTSCGGWLLVGRKGPGGRTLLAMQQEHPLPHARQPSS
ETESVQAAQQPQPQPQPQPQPQPQQQLQLPAGLSLGSPQSDASSQPHRLPNCCWAVVAPASDECDVLRADYMYGGGS
GGSSASSSSSSSSNGAHAATAGAATAGAATAGAATAGAVTAEAATFAVLMDTDTYPDGHVCAVTGGDLRSRVVLLPG
SPGRQVVDLVVVEWEEAQQRGQPEEDEDEEEGQQQQQGCSTGVGGVSSSRSSCSPRNHVRRYLVVHTLSNMQAAVR
VYDVSDVRQRVEAARAAAMAEQQKGRIAQEAGVGSRSSSSSGLAESEEGEGMETEEEEDEPPCVSPLPLLWTYLPG
DEVAGGTSTRNNSGPDTCTGGGGGEQASPSAGGGGATAASPAAAPGHGASPCMPAACPSPPGAAACGFDSNPATTCH
TTDGPASAPNPDPGATCHACCLGAVSSLVLQHGVWTPHVRLPYVRLSYSSLTTPTTTVDLDLERRVAWRRQVEEVAG

GFAPGAYLSATLWATNPADGTRVPASLACRADAWPPATGRPLPAVVQVYGAYGRKLLPDFNPADLAILDGVSVGVGV
SQSGDSCAAGGTCDNHGSGSAAQQELGARTEPSSSSSSGGGGSGGGGGSGGGGGSSGGGGGGGGSGGGSGAGGGGGG
GDSADTRRQPCLLVIAHVRGGGELGAAWHRAGKRRCKVNSAADLAAVAGALVAGGYSRAGKMAVWGRSAGGLAAGMA
LCGVHHRPPPGTGSPPPPHASPSPIPPPPPPLPSPAPGALFSAAVLDVPFLDVLGAMCDSRLRLTAQERPEWGDPEK
CQADYAYIRSYSPYEHVAALAAASPPAPALLLTCALHDSRVPYWGPAKFVARLRSELRGRMALDEHGPRETEEEADA
GVLAMGCKCGPAIESAAVGGGCVRFEGGVEGEGREVVDSIRLTGVLLLTETGAGGHFASGALGERAMRSAFLLQRVA
SVEADARASTRRGLNI

>g16452.t1|PACid:27566659
MVPGKALGWIFCCFKPEQDTGRTQRQGSEATLLAACRTADAAPPPLNNYFALMLQHISDPVALFTRNGTLLANNAAS
EALFDRMRAAAGSCRPAPPSLRLLFTADPDALQEAMADIAAGRTWRGLIQIQAMDSRPPPAPAETSKKEAANMLLTA
ASGRLSQQCSLASSCAMPSLLQPPPPTAGAVAPGGSGSGAPWTAHDMASLRLQLQLLQPERAGHGSDAYGNPRDCYS
HHCPSSSGALPAYHVLHGPPLLAPQQQQHLMQTSMRITHSLSLPCASRTAGAAATDAAGSSSAAAEVLAPDRRVSSE
TGAAPGRGQRRDAAGPARHYSAAAVAPRSGEVGCCPSERPGELLVDVLTSGGGGGQSVVCGGGSQPANSAVAPVQAA
TVMEADTAAGSGASAADLELHQHLFSRGSVGPAGRAGGQASCDHSVGSSTWASAAAATAAAMAGSSSAAAPPSVPLE
GGRRRGDAGSQQKQQQQKQMPQTQERQRQQQQQQQQQQQQQQQQQQQQQQQQQQQQQLLLLLPQTQAQERDQEQGSQH
HRPSRFGTSYAAGTSSPTSPAAGVPQARTVAEEPKGPVSVSALWDWKAARSALRSRGRRASVEVPSSAAASYTTGGN
ATATAAAANSSPSAGVLQSPRRASLDEDASFRPRWAALDTTTSHHNSQQTHISRRVQAVAPAAGSTTSKASRAGSHR
MSMDVPAGSAVVKLAVPGFGRTSLPRTCGAGGASGSSGLVGSRGSNSGVSGATTAGRTAPLIDVRSFLRQAEKDYAG
ATAAVAKPSAAAAQAAVGGAGVSAATAAPLMGSSGAVGVRTRGPSRLPPQQQPQQTPQRKEPPPPPPPPSQEWQAATR
TGGLRQPDTHSGVSQARAAAAAGGSPAAAAAPPPVHNADGQRARSQSPAADRSRDALLASVVSGDAGNEAGVTTAGP
QHVLLRLPSLTGGPSAVSAGSFRAGATGGAAIAAAGCGGSPSSASGRRSLSPLSRQSRSAHCGTVGEEPPLPQDQQH
HEQEKQLEQQEQQHGMDGLGSRAPAAVLGEADATTAARAAVAARERELQEQEMFAAERRLLAASAPTCEAINSSAGT
AQLQHVAQDGATGESQSHKDELPGDVGGSPSQCRPAADIGLLSNRTLPQLRANPTHAAVPIRADPPDLAPAWGLPSV
CVVLEAAAPMLQVDSRYSLHVGSVAGGSGGGPIALAAAAATARGSAAARRRSSWCAGAAAAAGDAAGDAAGDAAAGA
ATAGAEQGLARHHVRSSGSPHPPHHVTATARRMLFPLGPDQQIGACQQPCDSHPSLPSAEGPHTQPTAAAASAGGGA
AAANALGAAVAAAAAAASIDFQCDKLSYASWADHGTGAHTSGNTISAVLGNSNSLEMPFGGVAHSATSASGNALTAE
SLALLVSCRARSAAAERSNSSLANARAPAAGGVSGGVASGEAQPQAAAVGQVSSHGGRRKLEVPAAAAGTGGQSGEA
ALTAAVDLQEAASTARGLAVTGAVDGARTSPAVRQDASPRGPEDGCGSDDSRARRMSCNRGSACRPSLLSAALAGND
GGVWGSPAAPPDTTGGGGTAAQAGGRPAAANAATGAPGRSASLAVSSAFALISSANLPDSVAPLSASTRRGQRYYAA
GANADAAVGVPTAASASSDASLARSPSGGQQPRSLRLPPSPHAHLLSAAEAPHPAGGVACGSEVHSSSSGRVQHVAA
ARTPLSRSKSGVGNSGHFVTTYASGGAAAARAAAVAGGVSGSPPAVFGWTASGAGGLADTDPAILASLEQQQLMQLM
QHQQQYIQQLHQRQQLQQLQRQQHQEALMQGLSTSAFGAARRGARAAAANRASCAGSVASGGAAAGGTVAMARAGGV
AAAVSAAHAGASARDIDVSVGGSVARRQSTCIGVGAAAYQHQQQAAAGAVASGMFGSRALHHSGVGGLPAGSLYATA
AASIGGGGGGGGRRTRSVENLMLVSNGSDDFCSMESTAAAASARSKPLPPPQPGSHTVHFPMSRAAAAGAGGASADA
PAASSRAAAWIPVGALSPTSGTAREALSMNGAGPASPRSSRGKNFLARSLARLTSLAKMGGGASGSTGPGAISGMGD
GGTSSSNQQQRSGLHRSPALSDGQVMLSNELLSVMAGVTSVDQAKEASRTFGGNSVGAAAAAGAAAGTGLKVVAAAA
AAAAGMALEPAHRHVSVSWNHLTRAASGPLGAATLEVPQQPPPELWTFTPPAAKNSGSSGNMPMSSPRTAASGKGAA
ARNFGNSFNASTGHGVGSVSDLTAVVSGKLSVPVSALSAAGRQRPVLNTAPLMAAAAVDPFAGVALDAVDGPVADVA
SKSPRVIASPLPQHLSIATAAACPSPAAMFRGSSGGAGGGGGGGHTSPPPLHLGSHPSPPPPTPPAAGLAVPRIVQL
TMRSMARAGGAGVTGGTSALGGARWAASNGTGGGNSTNHASGPLSSLAAAALLESVGDRMPSALMHTYATGLCDDAL
GVGSATGGLAEAAAAAAGGEEDAAAVADLVADVSIRGGIHGSVGASPRAGASAGLSGSFGSALHGHAGSSAVLMPPV
IASSAGILTTTASCNSNQHVMLQHQSGHSALGALSTTAAGLGRLERSSAATLMQQQDLQQHQQQAADGANSRSCSPQ
KDGRDAETQLLAGHSGSWRNSCRAGGGGGGGGMGEATGGQDSGAQQTGGTMRSFASTPEAVGGADGSPAHAVDVRGA
QDGRASRRRRQVDHGSGGGGGGGGGTLAVSSYASAGAGAGAAAAAAASGLLSAEMLTAGAAAGNGRGGGKMAGMLVVT
RCGSHSATSTASAAMSTVALPPPPGLQLATDTAAASFSVDTAAGGGGAADATAAVGSVLGGGAGGLGTEGGSWYELT
VSAVPHPTSPGEFLIMVVQHDVSARVWAERQLARVVEAEHALLEAIFPAHVLEHIAVATMDAALTGGGAARTGPATQ
QQGSGADSVGAPVAAVSGPLHITGDTFIHLATNHSMITVLFCDIQGFTGMCNSVRPGVVMAFLNDLFTRLDALLDAF
GVYKVETIGDCYMVAGGLMKVDEETGAVTVRSDDVDPLHAYRTVQFAKALLQAAGAVRLPTTGEPVRLRVGIHSGSA
MSGVVGTRMPRFCLFGDTVNTASRMESTGEAGAIHVSKAVFDLVPGEEWEPTGGVQAKGKGQLETYLLRPAMEDHGD
AEATVPKL

>g16708.t1|PACid:27566710
MMPDPAEAETETRPAARRWWWPWAVADEPEERTSASAPVRNFSDLPSAQEEVVGPDAEVPRPIARASLFQWRGSVRS
SVDRLKGSWRTSFAPRFTLYNIERPEEPERERCCPRPGLALVLTALVICTVVVVVVPVCTKYDCGVSGSHGGGNNAA
VQPSQQRPSSFDIVAFQALLQMSWTAAGGGSVLVPAGSTQQQQQQAACAQLAGVNSSSNTTGRGNSSISGNGLTLN
ASITQGVQETLGQLGAPGLPVRVVRVTCTQQQPLAPTTTSVTQRATASAIMSATGSNSNGSSSSGSGSGSSSATHAT

AAGRRQARRLLLLRDGGSALQTPGVGSDSRSSRRLAATATAVVTLEIVFEVQVPTASGMAGLVTAVRNTMQSTDGAA
ALEQQLADSLQPLTGSSTTVRLLQPLAATGPQLEVPGGGTPSPAAPNSSVPSPSPRPVASAAPPPPPPPAQSAPPPP
DAGTAPVPANAPSPSPQSAASPQPEAALPPPAQQAGSQPPPPSAPAQLTPPPPPRTPASPPPAVQGVPLAPFPPWPP
LPVGTVAPAPSPPALPADSLSPPPPPPPETEPPVAEGSPQQSPPPVAGGDGGGGTEQPPPPPTYPPLLDLSVNVLLS
RDGAGRR

>Cre16.g671600.t1.3|PACid:27566742
MDVQPADSQSAEGGAKGSCGNPAGTALAPTAAPTAAPSAAPTAPPTAAPSAAAPAVDFANVSSLALLRRHRKNKFAI
PRQAPVPEAAALSPAKTVSTGVAAGAAGSPVEPSAGQQSEPRDQPTQQPQQQLPVQVVGLSDVGAAVGAAADAGTPR
AGVVGGGPALGSDTAPAAANVGAAAGGGTAGAEAGSHEVQPVPAAVAAVAPPAAAPEAATAAVHASGCGLQPAPGPK
QPAASAFAVSPPSLQTVLLAAMGPEAAPPPAGSAAVEVLPSAAPPQVAAAVATAPSPAATAPIAAPQAQQALVEAHM
ADAGGEPAAATAPAAGSDPAAAPTATAAAPADAAEATLVAAATQPAVGAAAAAAAAATAPQPATAGQPPHDGNQLDA
LRAGHCVRAFTIHFTVTVLPSRDPAAPRRKLLALKPADLRAAGLTCVAELGPLVLDSGAGAGAQDMVPVSDGDGAGA
VLFTVLGLSAADTLRPLQEVTGGGAAAAAAGAPAGAAAAGRGAGGKAKGKGKDAAWRIRLNGPSRWLTADGGVLDPT
TPEAAGLQLIREPVEGVPRGRYTLLRANGSCASGPADPPRPARPYEIVALLRPAGEEADAAAAGAGAGGSAGVCVHA
GAVAGAAGLGGAEQGSRHPALGFGAAPAAPPPLAELSGLAQVGSSGNVGGLGGLGGGGGGARSVAAPGSAGGGGDRY
SGETVGAVITTSGGRGPTSAAPGDAGAGAQGSRASRDGGEEEGEEASGSQWERQQGRVPSGSAGAVVAAAAPAAEGA
SPALRKPTLEKVAARPSPSDSKGAEEGTAAARAAVPRRPPPPPPQTTPDLSEERAIRSKPAPLQLQATPQGPAGSVS
PTPRSRPRALPQPPPLLMGGGGRGGGAGASLDRLCSKQLSNSEVDSVKGGPICFTSAMTGEDGVFHGLHADNGEHPF
CGPVILHDGAGGGSWAMRVRGNGNSAQRLHDTKAMLDHYKPTVGDALVFARCLHPPAPWASTAAATAAAVSPAQAHE
NASAAAARMAADWSVPPVHFTVWCVRKADLAAGGVALLQPRADQQQPLQPQRRPQARSPAAVPDAGEADAHMAAAEE
GEQQKEAEQEGQGVDGMERAESNPAAGASPAVGGVPAPGAVAGGFPAFNISCMKELTKTDCAQKVKFPVAWLKAGGV
MAGTTRGQALCLHARGSAEFTQANVGLSGALGVRVGKALKDALRMERGRVLVLAPCGTDGRPLRPGDAPLTLRHFCL
EVLGTRTDGAGLHGRSGRAAGNAGAAARGAAGAVTDEDEDEDEEPDEDEVLQSEDEAVLDGEDEVLVEALPQSGRLA
AAAAAAQHGGDRAAAAVAAEAGAALRKVLSKTDADGCIIIPQAWCCPGGGFAGLGIGSSVVLHIGRTKETFTRKLCS
RRANLYMTIPADMRKRLGLAVGKELVLEACDNVGRPLAAGAARRAPPHYAVRCLWRWPRGAAVADEQSGQEQGAYEG
LDEGEEGSEDDEQEGERAGAAAGGLARRAAVAVAHGARGVTDPGVAAAAPAAPATTSREAPSPRSADGGAAARSAGS
KGEAAAKDGAPPRPRAPPPAPVAPARPKPSVSPPPLARDAATGQQQQQQQQRKPPQEPQPLQQPGLGTAAAAGAAGA
AACVHDPDGWVPAGADPASFTPPAAADAQWEQVAGTITLEQMRWRRLLPGAAEAAGAGGGAPQAAAREVPTEAEDDG
SLRLCGVVFAVRRDGGAAAAAAFARAGRADWLRRHAGNEQTPAVEIAPLPGGGSRKGDKGHGQQAALPASAQPRPWH
ALFRGGEGGGSSSGTRTGVLQRRLASLLNLKDLYDATTPLPRGPVTLPCVVACRDARRGGLGACVTTEVRKFHPLGV
MPGWVLQGTAGGSEDAYNFAYAGHPRAASSHRPASGDPPLLLELRRRGETGGVWVFLSLSYRLPYPPELTETPGAAA
AAKAAAAAAAADRATAAAAAGAGTGTAPPDSAQSMHSQLPAPRQQEFTLFSMGAARSVLALVNDPGQRRHATDDLELP
EPPEEERQANCVVVPVAVRGVVLPVLVAVRDLRPGEQLLLDRGDDWWAPVADRWEVAEDRLPQAAAAEEALDDAQAS
LARHEALLHLPGEVQAFPLPQEAAAWHQAAQGAAAGAAAAAAAAAAVQAGSAGGEAGVAAAVPLQPPQPQQGLPPPQ
LSLPCSPQHGASQRDDAGGRGAVTTPSGTAPAATPDNGAAQPTRPPAATTSTADATPDRRRAVDSTAAGGGDGRCDA
ARDRDRERKREREWERGDTRDRDRGRDREGGRQKPEQERSRRRDGAQGLSANKEHGDGRLKDRERERDGDRDRDRDR
DRDRDRDRDRDRAVSGDRQRGMDADKVRQQQKDKDRDKERGKEKERERDRDREKEREREKRKQQERAEGKESQQSLR
GKERGAEPSKSDRADGHGGSEPSAAAAAAAAITAATGPSPAAPTMAAPAAAAAPQQLPPSPRPASLGAFGHNTGTPK
AADAAAAAAAAKPAARDRSRDGDRDRERQGKRDRDRTDMARRQDSSKERERERERDKQASRDAETGRDRDQPQSQQQ
ALPPPPPLAQGQLQQMQMQQQQESLRPLSPSLLPPHLAGAAAPTASNTPRSVPDTNEQRQESRPREERGGTDSQRHR
SKTAGQAGAALQPDVGLRSPQAAQQGQTGKDKAGGMQHQQQEGLDASGTMRRRSSTGCTPIVWSPSAVQRMSPPPQP
QPAEQPLVSPPAAAPPRPLEPQQPQPPLSPQPQLQPSQQPPLPSPREQPPGQAGSLSALLSAALPLPQAQAVDREPA
VEREQGLGVEQQPNDKKRGRDEQPQGNSSDCDQPQARSSGRERPRDADRKRDRDRSPERKRTAEPGRERDRQRDRDK
EGGRGRDRERGPGPEQTRERGSEREHDQQRKHDEKESTRGREQERERERDSDRGQKRRHEGEQERQHDLEGRARGGV
SGNEPKPVPEPKRDPGRSSGKASDRGRDRGQGRERDPEPGPRGRSPGQSGSQAQDGERAAGSSPESGKRRRAEAEGG
RCAGGGGEGDAGAQGREARRAAGSGPGRDVAAKESPASAAAGSGAGNSRLGGRGGDCSSGRYEPFSDPYRHQLPPLD
EVVGGRLHYPPDMGCEERMKRILRRMERIRLLRSNIFDPDSEPGGPGFNPNAPRSSKEWPAAWLAATPLGWRFPAGS
ATAGGSGSAR

>Cre16.g671200.t1.3|PACid:27566794
MFAKASFSEFIEAALDAGDDLGNGDLSLDSGGAAMLLGSSPPILLRSSLGPVTVSPAGSMHGLMQPAFGGFCIDGSG
NNLSFSQYPQQPMQQQQQQQQQQQQQQQPSSVIGSGGAFAQRAQQQQQQQQLQQLQQLNMQQAQMQAHFSGMVPVA
VSAHASATGGGQAMGVGPGGPQQQLQPPPQGSLQQGFPCGLAAPSPPPPAPSPAAACTPMDTTGTTSASAAAAAASP
GLPPGRTSTAGTGAGGAGGDAVGSSGSAAAGGGGGSNSSSLAPSLPPGGGGGGSRASTLSLSLPAQMQAQMQQLQAL
QAQVQAQQLQLQQLATQQAQLQRHLPMSTLAGAAATNSGGGAATTTATSAGATQKLAGSSASLSTEPAPGTSPFLV
AGALSATAAATTATAAAASELAVRVMVMDPMAPGPVPASAAASGGSSSGTAGLLQPAAYSGWQQQQQQQQQQQQPSN

MGAMSPPPPLPLLPPTSPPLTSHKHPHPPDHDAHGMAGGGGGGGGGGGGGGGGGGGGGGGGGGYSAAQPVPKKRTSY
TSEYSGFNGTPLASPRGGLSPSSSALAGQLSLHEGNSTGAGGAAIATAAAALAAASAGPNVGSFGALGAGTPVSYSP
QLSPQLSSQLYQSAEGPMLSQLLRTATSSSGLAAVPGAPPPGGGGLAGGGSLLPMASGQLPPLPAFMQQQQQHFQQQ
QQQQQQQQQQLQPLGMSSALLPLELQQAYHPLNAGYYNSGGGTGGDGSNSDGIGGERSSSRLHHLHHPHSQQVTRDK
NREAQRRYRERQRGALATLQAKTEEQARVIEGLTREKKMVEQHNRMLLERVWKLEGLVETLQRSAIGGGGGGGGRGG
AGSMDGGPGSAGSLPPHQQHHQQHQQHQQQHQHLQAGAGPHGGAGGGDDGAAAAAAAAAAADMQQGGGAAGGMGGL
AVPMSTSPTQQHHQQQQDLQQAWQQQQQQEQQQQQQVAWKQEPAFSAGSGVDSMGVGS

>Cre16.g694200.t1.3|PACid:27566859
MERVSSVAARISGCWRQLLLLCIYLVAAIGAVDTSSLVPFNVVIYGQLNVFIAHPSGLLDAGLPRGAPPPRGGAQAG
KRAPPFPPISLEDASPPPASTKAPHASSSLPPPRRRSPPPRRLLAAPPARGSHRHRELLATSDGSDSASSSYSVTAR
LQEPVNATHWLYVSIDLGEQGRYLVTGSWLQVPATFFLNPAVAQQLGLAPPGSGGDSSPPLSEAEATAAAAALAALN
NTSERRPLGDLAAQGLLRLLDGLSVAERRARPFSGPQAAWPATGMPGAVASGPVREVVRVEPKQETWGPELGQPLEV
SALVLLFEDGAGAGAAAGGCGLAPLSAAAVRAALLGAEGPSLQRTLQACSHDMYRLRSEPDGSSSSSSSSSGAAAGS
LVLGPVPLGGAGAFPCSGRTAGGGGGGGGGGADYDLASGCGQPEAAALAEAAQAWLQRERPDVWGALPAYRRALLL
AASAACFAGSGADGSGGGGGGGGGGGGGAQQAFHGLVSAGCPDGGPCTSWLFVPPGSSAPDPAHLLHALGHNEGVPH
AAGLLVAGSTDQAAQPDPTDPMGSAGAGVCFAAPNAYKLGWSGPLELQGVDPGTGAAATSRADMPLRELPAGVFTEF
QLPAMALAAHCLLRLVVEQAGLPAQAAAAAAAAGGLGWQRALFLSYRVRQRAGGGGGGGGGGGYDSGLPEEYDMRVW
VHESNETATGAPAAPGSHTLIRAMLQPADADADATASPPAAATDPSTNTDASSSSSSPPPPAAAPWPVGSAFRYLSS
DPVATGGHLAVEVRRRDGQSATVALCRFTAEVAEGAEDGEQCYNGLDDDCNGLQDSEDPACQ

>g16697.t1|PACid:27566937
MPVAPTSAPSAPPTAAPSAAAPAVDFANVSSLALLRRHRKNKFAIPRQAPVPEAAALSPANTVSTGAAAGAAGSPME
PSAGQQSEPRDQPTQQPQQQLLVRITDVGAAVGAAADAGTPRAGVVGGGHALGSDTAPAAANVGAAASGGTAGAEAG
SQPVPAAVAATAPPAAAPEAATAAVHASGCGLQPAPGPKQPAASAFAVSPLSLQAVLLAAMGPEVAPPPAGSTAVEV
LPSAAPPQVAAAVATAPSPAATAPTAAPQAQQALVEADMADAGGEPAAATAPAAGTDTAAAPSATGAAPADAAEATQ
AGAATQPAVGTAAAAAATAPQPATAGQPPHDGNQLDALRAGHCVRAFTIHFTVTVLPSRDPAAPRRKLLALKPADLR
AAGLACVAELGPVLDSGAGAGAQDMVPVSDGDGAGAVLFTVLGLSAADTLRPLQEVTGGGGAAAAAGAPAGAAAAGR
GAGGKAKGKGKDAAWRIRLNGPSRWLTADGGVLDPTTPEVAGLQLIREPVEGVPRGRYTLLRANGSCASGPADPPRP
ARPYEIVALLRPAGEEADAAAAGAGAGGSAGVCVHAGAVAGAAGLGGAEQGSRHPALGFGAAPAAPPPLAELSGLAQ
VGSSGNVGGLGGLGGGGGGARSVAAPGSAGGGGDRYSGETVGAVSTTSGGRGPTSAAPGDAGAGAQGSRSSRDGGQE
EGEEASGSQWERQQGRVPGGAVVAAAAPAAEGASPALRKPTLEKVQVAARPSPSDSKGAEAGTAAARAAVPRRPPPP
PQTTPDSSEEPAIRSEPAPLQLQATPQGPAGSVSPTPRSRPRAQPQPPPLLMGGGGRGGGAGASLDRHCSKQLANSE
VGSVKGGPICFTSAMTGEDGVFHGLHADNEEEPFCGPVVLHDGAGGGSWAMRVRGNGNSAQRLHDTKAMLDHYKPTA
GDALVFARCLHPPAPWASTAAATAAAVSPAQAHENASAAAARMAADWKVPPVHFTVWCVRKADLAAGCAALLQPRAD
QQQPLQPQRRPQARSPAAVPDAGEADAHMAAAEEDEQQQEAEPGEQEGQGVDGMERAESNPAAGAGPAVGSVPAPGA
VAGGFPTFNISCMKELTKTDCAQKVKFPVAWLKAGGVMAGTTRGQVLCLHVRGSAEFTQANVGLSGALGVRIGKALK
DALRMERGRVLALAPCGADGRPLQPGDAPLTPRHVCLEVLGTGTDGAGLHGRSGRAAGNAGAAARGAAGAVTDEDED
EDEEPDEDEVLQSEDEAVLDGEDEVIVEALPQSGRLAAAAAAAAAQHGGDRAAAAVAAEAGAALPACCKVLSKTDAD
GCIIIPRAWCCRGGGFAGLGIGSSVVLHIGRTKETFTRTLRSGRTNLYMTIPADMRKRLGLAVGKELVLEACDNAGR
PLAAGAVRLAPPHYAVRCVSRRPGGAAVADEQSGQEQGAYEGLDEGEEGSEDDEQEGERAGAAAGGLARRGAVAVAH
GARGVTDPGVAAAAPAAPATTSREAPSPRSADGGAAARSAGSKGEAAAKDGAPPRARAPPPAPVAPARPKPSVSPPP
LARDVATGQQQQQQQQRKSPQEPQPLQQPGLGTAAAAGAAGAAACVHDPDGWVPAGADPASFTPPAAADAQWEQVAG
TITLEQMRWRRLLPGASEAAGAGGGAPQAAAREVPAEDADDGSLRLCGVVFAVRRDGGNAAAAAFARAGRADWLRRH
AGNEQTPAVEIVPLPGGGSRKGDKGQGQQAALPASAQPRPWHALFRGGEGGGSSSGTRTGVLQRRLASLLNLKDLYD
ATTPLPRGPVTLPCVVACRDARRGGLGACVTTEVRKFHPLGVMPGWLLQGTAGGSEDAYKFAYVGHPRAASSHRPAR
GDPALLLELRRRGETGEVWRFLSLSYRLPYPPELTETPGAAAAAKAAAAAAADLATAAAAAGAGTGIAPPDSAHCMH
SQLPAPRQQEFTLFSMGAARSVLALVNDPGQRRHATDDLELPEPPEEERQANCVVVPVAVRGVVLPVLVAVRDLRPG
EQLLLDRGDDWWAPVADGWEVAENGLPQAAAAEEALDDAQASLARHEALLHLPGEVQAFPLPQEAAAWHQAAQGAAA
GAAAAAAVQAGSAGGEAGVAAAVPLQPPQLQQGLPPPQLSLPRSPQHGASQRDDAGGRGAVTTPSGTAPAATPDNGA
AQPTRPPAATTSTADATPDRRRAVDSTAAGGGDGHCDAARDRDRERKRERERERSDTRGRDRGRDREGGRQKPEQER
SRRRDEAQGLSADKEHGDGRPKDRERERDRDRDRDRDRDRDRDRDRDRAVSGDRQRGMDADKVRQQQKDKDRDKERG
KEKERDRDREKEKEREKRKQQERAEGKDSQQSLRGKERGAEPSKSDRADGHGSSEPSAAAAAAATGPSPAAPTMAAA
AAAAAPQQLPPSPRPASLGAFGHNTGTPKAADAAAAAAAAKPAARDRSRDGDRDRERQGKRDRDRTDSARRQDSSKE
RERDKQASRDAETGRQPQSQQQALPPPPPLAQGQLQQMQMQQQQESLRPLSPSLLPPHLAGAAAPTASNTPRSVPDT
NEQRQESRPREERGGTDSQRHRSKAAGQVGAALQPDVGVGSPQAAQQGQTGKDKAGGMQHQQQEGLDASGTMRRRSS
TGCTPIVLSPSAVQSMSPPPQPQPAEQPLVSPPVAAPLQPLEPQQPQPPLSPQPQLQPSQQPPLPSPHEQPPGQAGS

LSALLSAALPLPQAQALDREPAVEREQGLGVEQQPNDKKRGRDEQPQGNSSDYDQPQAHSSGRERPRDADRKRDRDR
SREKKRTAEPGRERDRQRDRDKEGGRGGDRKRGPELEQTRERGSEREHDQQRKHDEKESTRGREQERERERDSDRGQ
KRRREGEQERQHDLEGRARGGGSGNEPKPGPEPKRDPGRSSGKASDRGRDRGQGRERDPEPGPRGRSPGQSGSQAQD
GERAAGSSPESGKRRRAEAEGGRCAGGGGEGDGAGMAPEAQAEARGDAVGPVGWVGGSVYRSSAGTSDVAAAATSAQ
VRGTAGGKGAAAADAPEPHQAGGAEPSAATAAEASGGWGDGDGHGGAATATAEWEDLRTHSSTAGASATATAAAAAA
AAPAGGAADAVPLAEEAAGAQGREARRAAGSGPGRDVPAKESPASAAAGSGAANSRLGGGGGDCSSGRYEPFSDPYR
HQLPPLDEVVGGRLHYLPDMGCEELILRRMKRIRLRSNIFDPDSEPGGPGPNPNAPRSFMEWRAARLAATPLGWRFP
AGSAPAGGSGSAR

>Cre16.g662350.t1.3|PACid:27567015
MLQPAARAAPAAEGVQGSDSPFGANAPAGATAGDPRREGGGARESVLSVGCVTGRVCAAAPSALLEASALPAGTDDT
GVGMGTGRDVGTGVGMGMARRRAASSGCLQAQLEAQMPLWQPAAVAAAVAAAATAVAGPGGSSAPGAAAGSTAGASG
GSGSGSAEVRGLDALLQLRPPPSDGGGVEDAGGGAGDGYGAGDAVYGTGGGHMPEPPQPPLGPFGRARRHSSGHYES
YGGGAAIAAAAAAAATAGVVDVGVAWLFPSFSTPPPQPPPPLMNSGIRSGGTCSGSSGGSSSSPSGQGGRSQGPEVE
AAAEAEAAGGAGAGAGAGPGALAVGPTALTPPAEAGWAAPACSAGGAGGSPALLLPPASPPPPPRHALLLPHLQHLL
LPADPAGGPLAGAAAATAGGRSGSGSGMRSSSAVGGGGGGGGSIAAPSVAASDMPTLTRPTPTRHPPYLHPHPHPHP
HLLLPALDPVPECALEADSSAASLGSSSAAATAGLTALPPPPRHASEGGSSPEAAAAPAPTVAAPAIAAPAVAAPTV
AAPAVAAPAVVAAGRAALVSAFSRVAHGAAAAAAASGPPPQQQPLTPVLPDNGAPTGAAAAAAATAGFGSIAEVAPAD
AAAGFGPGVGPASSSGSRYVVGRQAVAPPPPAAAAPPAPACGPAAATQDPWAAAAAARPGANGAAAATTAAAVAAAA
AGPEPPAAGSGRVLPPPQQQPQPQRQTHMQTQLQQAKQPQRQAPRRTQAPTQLVVAAHRLPVTCVREPQEGGGGWSV
QASPCLLSSAMCGIQQLGLEGPEGPEGGGGGPEGGGGGGYRVTWVGWPGLWLEPGPERRRLEAALAAGGYTPVWLQE
DLLDQHYNGFCNGVLWQLFHYVPPHYSLMSGWRQQAMMQRGGGSSSGGGGGGGGGGAGGGGGGGGGGGSSSSGGHS
GGQGPQWAAYQAVNDLFADAVMAAVAGSSSNCGGDGGSSGNGGGGGNGPAGSGAGADSAAATSNGGGVAAGGGCGGG
ASSQQGGGGGCAGEEVVAVWVQDYHLMLLPEVIRRRWAAAEAAADQGAPGGGGGGKVRQQRQQRPRRRRLRLGFFLH
TPFPSSEVYRTLPVREQVLRGVLGSDLVGFHTYDYARHFVSGCARLLGLEGTPQGVEHASGALTRVATTPTGIDPRP
FTLLLRGGPGGGPDPGVAAHLTALRHRFAGRKLVLGVDRLDPIKGLPHKLLAFEKLLEDRPEWRERVVLVQIAVPPH
TEVPEYQQLRSRVHEIVGRLNGRYGSLTHVPVHHLDTCLSRQQLAALYAAADVALVTSLRDGMNLVSYEYVACQLDD
AEGGEGAAEQQAEQRQGTEIGGSGGHVPGVLVLSEFTGAAQSLGSGALLVNPWDPADVAAALHAALVMGAEERRERH
RQNAAHVRTHTAQHWARTFISELTAPPQGGHQGQGAAATDPLRALRPSPPPPLDVKAVLRAYRATAAAAISSSSSSN
GSSSINGSSSGGGGRAQQCVRRRLLVFGYSGLLSSFGGGGGGGGGGGGGAGAPAAPVVGNGSSSGSSRSPAPAVAVA
AAGGGGGQRESRDHGGGSAVALTGSGDAAAAAAAAQAAAGSSSSGGGGSSSRRELHGGSRALLARVDPRVMAALRQL
AADPANVVVLVSGSDTARMDALFGGLLQPDLLQPPAADLLQPPGRQQQQQQLQPQPQQPSQRPTGEVWLMAENGVFA
RRVGRRGGVRAVITPARPGAAAAAAAAGGVRGGPGGGGAATAPATEDTAAAADGDDDDVWCRWHSLFDVQSLPALAAG
GGTAAYLPPQQQQRGGSSRRHPQHPQHQQQQQQQQQQQQQQLQQSVQTTTQSGATPAPAPAPASAASSSSHVARRHP
ATGPHHQPPPSPPPSPQPPSPQPSPSPPPPPSPPPLSWMGAVRLVLDYFAERTPGSFVEARATSLAWNYRHTDPDFG
RLQARDLLQHLRAGPISNAPLEVATCGRTVEVRPAGVTKGRAMQRLLGLIRASSSAPPDPDPAASAAATTAIAAATA
IAAAATASAEGPGVSSPAAVPAPDSHSQLAARDGGAAISGAPATASSQPPPPPASDYSFVLCVCGHLSPHDEDIYSS
LLQPPPAAQPPPPSSSAAGTTAQTPHQPQPLPPPPPPPQPPHQPHVFTVTAGGGRDASTKAAYSLQAGGGEVGGLLL
RLAALSSGSSNSGAGDGSSSNGAGDGSSSSSRPQRGHEGGADRSVGAPAGGGQPPPAGDTRSSERRSFEFPTCAGGP
VQPLASAGAPPPAPTAAPPTPAPAAAAAVQGDDVFVTCHSPVRLRQARQQTSYSSDGGPGGPGGGGGGAAAAAAARG
TSGGSSTRTSGGGGGGGSSSDTPSSPSPWRRASIRAWPSAAALAAAVAAATATAAAGAAAPAAVAGGAGGGSGPNSGT
LPGIGSSTAGGVGSSSGVWSASAGAAAPAAADGGAGSSIWAGYGGGMLQPAAAAAGTVSPTSLLQPPLPLRRGLPGS
SGGMLRGSSGALSNMSGLTSEQLQSGGGGGGGGTGGGMGVCRLSSVYSTDALMLDQLLLPLGLSSYSPGGGGGGGAG
GGMLLPAAAASPAALEGALSGTAAAAGASAASGAGAGAGATASGGGLPSLPAWRWVAPSPRAAAAGGGHQEAAGVGG
VAPLGAAAGAGGRGGGSAGSMCGGGGDEGQQVEGEGEGEGGGGLGSSGAGGPAGGLWSPFTAVRPRRPQQQPQQQQP
QQQQQQQQMLRLQQQQQQQQQQQQQQMLRLQLQARQWMSEGGEAAWQPAGGSVGAGDVGRGGGGIQGGEGGGGARLMS
VGGCREDGGEQQAPWQWQHPQWQQQPARRLWQQQEERAGDSVAAVAAVAAGWEQGGELARGTQPLQRGRGSRGRHED
AGDGGEAAGRQAAQEEAPLPLPPTPPRRQPEAFSPRGPRLNDGSSPVVAAVAAAAAAAAAAREGQGGGGREGGEGGGG
AGGGPRPE

>Cre16.g694500.t1.3|PACid:27567070
MAAMWSQKGLAAGLHAKRQRDIFVPSVHRNVACAAFRTHYDVLRVHPRASQDEIRAAFRKLAKTLHPDVSASYDSEE
EFMRVKEAYDTLADAGSRAQYDRELHQMGRPQPTAQPKPPHTPGQSVLGRRMGRARPGSSGATRAHHQHQRPVTSGR
GRTVRNFSPPGSPSASFDEDSWLSPPPPSPPPTTPVGATSMRAAAAASAAAAAGAAAGATAGGSSSSSGGAAGTSFG
ERAGSSSSGRFVSFGEDARASVWESAPSVSASSYFGAEPRGNGSYYWDNVATAAAAAAAAAATAGSGSEDEMEAVLRG
AGGEAAGGEAWGAPATEAATAAAAAASGGATGGTAGAAAEYADAPAYTAQPQYTADVGVVGSSFASDNQSGFWGSGS
SSSWAGAAAAATAESESAAGVSFGGGGATAWDDVPSTPPPPATPPPPPPPRPVNPWSSYMSAFSMPATDVGSVGSSF

SSEYAAASAAYSGSSEPAAAAPEPPLEVSEPASDSPAGEVAAAAGIAATAAETAPAPAPAAATATGKEAAVAMARAT
AAQASAAAAASAAEAAAAALATASSSAAAALAAATEAATAAAGAMAEAAEMRQRLAEAEVRAAAAEMKAAAAEARVE
AAEARAAAEQARASAAESKAAVAEGRAAAAEEAVAAVGRKATLAESRVAAAVERAAAAEAKAVAAEGKASELRSRLQ
AVGAELAEARAAEARSRAAEEAEARAATEAEAAAALVEQRAAAAAAELAAVSARAAAAESRASAAEKRVGEMRMQLA
EAAAQMAEMQQAAAPAAPPRPRPPRPSARPPASPASPATPATAPGATSTSPPPPTTAAAAATAATAAAAAAAATRQP
DVDSGATGRPGVKQGPTAAAAQTVAAARSDGSSSSTSTRGASDSSSNGSSNGSGSGSSNGSGARAGHAHGSGSVATR
SQRFEKAAAAATAAAAAAQGAPQARPETAAAGASGGGGAAAASVAAEAAQVAEVMARMAEAHTLFGGGSSSSGPSSS
TATSTTTDGSSSSSPDAVTPAQAVAAPAPAEVAAPPPPPVAQQQQVPEPAPAPEEEEQVEVFSSISGSWDDSADVGD
WVGDWGWDSSGADDDDGERVAALARVEAALAAAAAAAAAAAAAEQQQPQEQRLAVDAAAEDLVAAVGAGDGATDADP
PAGAAAASRDDEAQQQQQQLQPELQPVRAGMATASSSGEAGAAGISFHGPGAPASSTGSSTNGGSTTGSSSGGAVSS
GTGRGRSGSPIQSAGVYYTSSYEGLLRSDSPSGSSSNGGSSGSSSSGSGSGSSTAARATASSSNAGPAGAAARQHPG
PSGSSGSSSSTGGGSSSIFSRPHGGAAQPRRTLRVRHDPAARRRAVSIFAAKAMTGGAQAPLRFALGPRGGGSSGAG
GSAGAGPNAGA

>Cre16.g694500.t2.1|PACid:27567071
MAAMWSQKGLAAGLHAKRQRDIFVPSVHRNVACAAFRTHYDVLRVHPRASQDEIRAAFRKLAKTLHPDVSASYDSEE
EFMRVKEAYDTLADAGSRAQYDRELHQMGRPQPTAQPKPPHTPGQSVLGRRMGRARPGSSGATRAHHQHQRPVTSGR
GRTVRNFSPPGSPSASFDEDSWLSPPPPSPPPTTPVGATSMRAAAAASAAAAAGAAAGATAGGSSSSSGGAAGTSFG
ERAGSSSSGRFVSFGEDARASVWESAPSVSASSYFGAEPRGNGSYYWDNVATAAAAAAAAATAGSGSEDEMEAVLRG
AGGEAAGGEAWGAPATEAATAAAAAASGGATGGTAGAAAEYADAPAYTAQPQYTADVGVVGSSFASDNQSGFWGSGS
SSSWAGAAAAATAESESAAGVSFGGGGATAWDDVPSTPPPPATPPPPPPPRPVNPWSSYMSAFSMPATDVGSVGSSF
SSEYAAASAAYSGSSEPAAAAPEPPLEVSEPASDSPAGEVAAAAGIAATAAETAPAPAPAAATATGKEAAVAMARAT
AAQASAAAAASAAEAAAAALATASSSAAAALAAATEAATAAAGAMAEAAEMRQRLAEAEVRAAAAEMKAAAAEARVE
AAEARAAAEQARASAAESKAAVAEGRAAAAEEAVAAVGRKATLAESRVAAAVERAAAAEAKAVAAEGKASELRSRLQ
AVGAELAEARAAEARSRAAEEAEARAATEAEAAAALVEQRAAAAAAELAAVSARAAAAESRASAAEKRVGEMRMQLA
EAAAQMAEMQQAAAPAAPPRPRPPRPSARPPASPASPATPATAPGATSTSPPPPTTAAAAATAATAAAAAAAATRQP
DVDSGATGRPGVKQGPTAAAAQTVAAARSDGSSSSTSTRGASDSSSNGSSNGSGSGSSNGSGARAGHAHGSGSVATR
SQRFEKAAAAATAAAAAAQGAPQARPETAAAGASGGGGAAAASVAAEAAQVAEVMARMAEAHTLFGGGSSSSGPSSS
TATSTTTDGSSSSSPDAVTPAQAVAAPAPAEVAAPPPPPVAQQQQVPEPAPAPEEEEQVEVFSSISGSWDDSADVGD
WVGDWGWDSSGADDDDGERVAALARVEAALAAAAAAAAAAAAAEQQQPQEQRLAVDAAAEDLVAAVGAGDGATDADP
PAGAAAASRDDEAQQQQQQLQPELQPVRAGMATASSSGEAGAAGISFHGPGAPASSTGSSTNGGSTTGSSSGGAVSS
GTGRGRSGSPIQSAGVYYTSSYEGLLRSDSPSGSSSNGGSSGSSSSGSGSGSSTAARATASSSNAGPAGAAARQHPG
PSGSSGSSSSTGGGSSSIFSRPHGGAAQPRRTLRVRHDPAARRRAVSIFAAKAMTGGAQAPLRFALGPRGGGSSGAG
GSAGAGPNAGA

>g15616.t1|PACid:27567077
MTGTDCLLFTCAQSDVRCPASYMSKCPGYTPTSCGKAPGGNGPFWMHHCLPGTYPAPGKALKLSCKLTRGPDGAFQC
YITQEGSYVASLGMRCLTGTCIYRMPSPPPPSPPPPPPDVPPSPPYSPGPHDDPPPPPFVPPSPPPPPLPPPPSPPP
PPPPPPSYIRTHTEVALLIAIGITVAGLMTAAAVLSYRDNAIATAWQSAAANDQPSSGGGWGGGGGGGGGGGGGGR
GFGGRGFGGGTPRAGEAFVPLLNSAHAPSPLDSGVVYDDPYDGEDEGGGAGAGGVAGGAPVVLTWTNIHYRVQRASG
GKLHILRGISGVAGSPSALQGPAAAAAVQGLGAAAAAAGAGRRSGGGGMQAVMGPSGAGKTTLLDVLSGRRTGPGRS
GQIRINGNLVSAHQIRALSGYVLQDDVLPGTSSVFEYLAFNALLRLPPHRHSQRKRDARVWGLVRRLGLTKVVHNLI
GDAHVRGLSGGEKRRVSIAVELLTRPGLLLLDEPTTGLDSTNAARVVEVLGGLAAGGVNVLLSIHQPRPDVLRAMDR
LLLLSGDGRVVYAGEVAAAAAHFAALPALPGLPPDVPLSPPDAASGINIADWLLDVVIKSPREAVAAMADAFGASPA
AAADAATAAALADSPRPLPPAKYSPPYLLQLRALSVRLLRNTYRHPFSVALNFAATLAVAVCLGLIFRNSGTETSGI
QNRLGVLFFMLLYLSLMSLSSLPIWRDEKLLFMRERASGVYGTPAYYAAVVLFDLLPLRVLPPSFFALATYWMALAI
NEFHHFPLDFTFTAPINTSALPPLRISGDGVLKEFGFNVDLFYLDVSMLALLGAACCGLTYVFLYFSGHTLLDEFED
LTGRTVAWLLLRAQAAGEAIAAVARRVGAATRRSAAVAADRFRNHRRRGSGGAAAGSSTAAATAHSAAADQAHAALD
PLLLPEPGSGSNSDDEDREVDEARSVMSPAASLAAPLPSPGGLDLGGGAGTAAAGLPLPPPGTMGAEAAAAARGGAA
AAGGGGFVAVTLPGVGSGLSTGTGPMVLSWENLSVRVRLGGGRVRYVLQSVSGISGPTPPPLPPPARASLGGSSSPP
GGRKAGSGTSGGGGGAAPAVVHVVGWPALAPASAAATIGPATDGIGAAVDVSGSSHTSKTNGSSSSRHGSAAALTAD
PAGAHDGGSGGGATAGASVAAAALQPLSMAPPPPPPSGTGSGASGIFSRLGLTPSAALSRLRPHRPSGDGSAHAHTH
THPHPHHGMWSTDSDTAAAAALQAADAVGGRCCLFAIVGPSGAGKTTLMDVLSGRRHDAAGGVSGEIRINGHCVGAA
QLRKVCGYVAQEIVLPGTSTVTEYLSFHAALRLPAALVRASAGSSSSSKHMFGFGAGGGGGGGSADAGVAAAALTPV
QARVAAVVAELGLTRVAKSLIGDDFVRGLSGGEKRRVSIAVELLTRPGLLLLDEPTTGLDSTNAARVVEVLGGLAAG
GVNVLLSIHQPRPDVLRAMDRLLLLSGDGQVVYTGPTERMHAHFSSLGYSLPQDSAAVADAVLDLVIRAPPSESAAL
VEGWRSSAVAAEDAGWAGRLQLGDALLHDQRVKALSGLRKYESSFRHQVAVLSGRRATGLWRHPLLLGLHFAATGLM

ALGVGAIYWHTGRDTGGIQDRFGALFFMLLFLALLSLSSLPVWRDEALLFMRERASGVYGTAAYFTSVVLWDVLPLR
VLPPGLFTAVSYGMIGLRASGGAVAGHWLVLVVANITAAAANMSIGAAVGSVALANMVGSLCVLTSTLFGGFLLSRA
RMPRLVAWLADLSYVRYAFEALLIGEFGGAAGFRFTGYHQPGTPPDQVPYVDVTGDEVLQTFGFRTDAWLGDVCGLL
ALMAAFLTSTFLLLRYRGRP

>Cre16.g695650.t1.3|PACid:27567083
MTRVSVQPGGSCVDVYTGLAPSYAVTAALPVGAGAAVAGMVGVASLAAMSYLELVAASDTGLGAAAAAANRSSSVSG
VGRRLTQQGVAAQQQALPVEAGLDASSVGASATRAQVPQLIPRWLQATGANSTSGTNATATNITSGSNSTETTIVTG
GDTNSTNTTTSTNSTTPIPSPSPAAGNITATNTTHSNTTASGGTGTNSTSPPLSASPPLPPSPQPPSSPLISTLGGL
PLDGTATQLLSNVTTVRLLLSRLYGTLNVSLAPYGVDALALQPLALLRSGLRAERQLGVAVLAADAQLGSVVGLGAA
LLRAAGGVSTAGNSSRLAALVAAQLVHQLALHEDAAAKMKAMATNRTGISSMNGNSSDTVQPSPSLSPSPLPPSPLP
PSPPLPPGSSADLVGPAAVFTPLPYMPGLYSTVLVREVYITAAARAGVPLTSAAVNATVAVAASVAAYAEVVKQVAL
AELRGVVMGLNPLNAAMGLARLAVVQAEAEGVVGQVVSGANAAALDTYVLDALPERISKAAVDVAAVHSSLGLAVPA
AAVAAVGVAHLVGPLRGCSGQANDLWTPAYEAFTTGSAGGISMRRKGLGSVTVSPYRLCYDALTQLPLNLSVSNLSP
SMGIASVSPVAMLAQGLYTVQATQQLMTVTAAQQDAAFAAMGLDRAAYNALMVAAANASAASASNSSSSSSSTSSST
SSRALSSQGFLLNQPLMGAVLAANAQLLGLFYAARAMFTSSCYYTYGLDSWENAHTFTVTLTLYAAAQQALASGSGK
LDLSSPTTLNSMWAMLVDACDFNSAQLLVSPSPPPPPSTADPGSALANVSGAAGSSGPTITPTGTLLAGGDGTAVGA
ATTSTAALTGITTVLGHRRQLLTSRQRTELLLGGDGDGGSSAWSEVVSAGWQQGALDAEGVLRHAEGSPATAAAVAL
HRRLRAHLLADLGLVGLEALLPEGSPARRHLLQQPRNGGERADEPRTVRGDLLGQAATAVATATGPGTGGRRLSVYA
DDPDMGNVYTKVSEILSGINAQLRSIQAQSADAALLPGAAASLYNLTGLVLNATQLGHVAQYGLYNRLVAVLRLRYS
DGLSLVTALQKVAADYTGSSLALRMTEAAVAVDTVCSEPGSQCSVYISNGGVGYSPPPSQSGAGSSSSSSSNMGAVI
GIAVGCALGGLALLVGGFMAYKYVRRRQIKQTVVNTFKTGDQGGLNLRVASTLHGNGFSPLNNAGLGTGALAPLRPG
GEAYSSTVPAALGHNPAVAHDYPLYPTPSYGAAAYGSPAGPGGEVMMTPAGAGGAQYLHHGGAAGAMGPGAAGAGGM
ASARGEAGLVDRNSPAGGPGAALLGATSGYKTYPNSAYVPDQRTAAADSTALRDTGLPSPRHQAAQPYPYPYSYLDT
AGGTASPRAYRPTTSSSAPPQMLAPTLSAPVTPTHANAANTNLNGTTGRRGGQLPPLAAYTSVQNPAAPTSSLALSG
GLNSSIRAIPVNSNFNSSLRPPTASAHNSDGGSGGHNSGPNSYVALRSPALAGTGVAASGQLHGPSTLGPSSGVNAR
RSWGPGQDYAIASGLRESSFYTPQ

>g15694.t1|PACid:27567111
MRSAVGRLPPLRPQPRAAHFNAPLRSLTRALLLIIIITCGSPLLLPAQLAAAQPPLLTPALQQGGNAGTGTPPSAVA
PRPPEHLTHLEPSPPTPTSSPPAASPPQPPPRVDSSQSYVGNSTGPSSSNTSSSPGVSNSSNVTALPPPPQHLPPQL
LAASTPPTRPQPNPVQAQASPPPLASPHSPSPPPSIQPPQLQPQPPPLAAPPPLSSPVPVAAEPAAAEPPSPALSQP
PRPSPAFLVAAPPPSTGGGGGLPLPLLQEPRSPPRPPRPPPPPPEGTEEPTGPPMLPLPRAEPPPHPPQPHPQPMPP
RPEPPSPHPSPTPEPPSPHPPPTPEPPFPHPPPTPEAPFPHPPPQPEPPFPHPPPQPEPPRPPQSPQPEPPRPLPAS
APPSPRPPTAPSPPVPPAPTPPMEPPSQPLPPLTPDPPGSPAPPPPLAPPSPERRRPVRSPPAQPPFPPLPSPPMDA
TSPQCALVPDAEGGGGGALSMLLSGGVFPSGFRFAVGGIAALLGNSSSSGSGGGTSGSSSTGEPGMADCGCSGSPYS
ALLGADVTVADTTYYAFRLEARPYGDTGSTPYGTGSTAPPAWRLPLQAVVVAMDPTRATSCYGVADSDLVQPTSGGA
GGSLGRPFPSGAWFLPAGADTPWRGAGAGGGGGGGGVWPGIPLQAFIGVSAMGSRAIELTLNLTDLRAQLATLRPPP
PAPRPPPPAQPPPAPRPSPPPPAASPTIPMAPPDAPVGQEPPLAGEEPPELQMPPPPLPPPRPRPPAPSPAGSGTNP
DQYWMGGGRRRLQQQAAVSPDTVDSGSFDGSSSFGGGSWQQQARRRLGRALHQQQQDAGSGRDAATDTVPPSPSSNL
ADPITAGTFLLGRCGVLPMRRLCRARAPDGSNTCLFAFVMDPGPASGGGDGGGGDDGQAPAAPPAEPPPASPVAPLS
PTPSPSPPSPSPPPPRPPVPSPAPAPPPPRPPSPSPSPPHLPPTPSPGPPSPTPPFPAPPAPAPPAPHPPAPAPPLP
APPSPAPPYPPAPSPAPQPLPISSPPTPVVAPPPAPPPAPAPAPPTPAAGTTPPPTADPPPLVYAPPPPTGSPSPSP
SAIPSPTPVPAPAPAPPPAPASPRPPVPLPAPAPLPAPLSLPPGQLPPPAAPPPAPITTTNNNTTSPASSASPPLPA
PAVHPPPVTSLNPPPSPTTASSPPPLPAPSPAPLSSLPPRPPQPGPPQPAQQAHSPSPPPSPGLAPPPQPAPSSIPT
LSPPAAAAPPHPSPPAPQPPPAIAQPQPVPAAPSPPPPGPPASPPSAQSPPPAPHLPPPPPVPSPPLPSPPPQLVPP
PLPRPLASAPPPLAATRRLLQIAGSPAITSINTSSTTSSGTPPTGSNATQATSPPSPSPSSSPSPSAVVFDTDGQPN
PPPRPPRPPRPPPPEADEDPPSSALPPGPGPGSSPPQATGPPPPLPNPRPPLPQPITSPAPAPAGQQPFPPSPPLPA
PPALLPPPPLPSPMAQLQPPSPQLQPPSPPSSAPPHPAPPSPAPAPAPLPAAAPPPPPQPQPPASPRPPPAPVLTPS
ASPSPSNASSPSPPPALAPMPSPAPPSPRPDGPISTQQPPSAQPPSSAPGSGNTTATTPPPQPSALAPKPPVPPPQT
PPHPPSTPPPTAHPPPAATNTTNTTSTSAVPPPATVNSPQPSPLPPSKPTPPTPQPQPAPPQPQLQPAPPRPQPQSQ
EHPAGSPPLPPAGPGQLLHQPPPLPVSMPPPYPAPAPASAPAPPPAPASGPSYAFPPQPQPYPRSQPQPQPQPQSVS
YPPSQQPGSRPPVMAYPPSPASSPAYPPPAPPSPRPGPPSPAPTPPPPSPSPPRPPPPPPPSPAPPPVRPAPPPPPP
PPPPAPPPPAATAAPPVVAVCYSRVEQDTQLLPPASPPPSPPPTPPSAPPPAPQPPAPPPPLKVRPPPPAPHAAFPP
GYVAPPPPPMDISVECRKTKSDVMITDGEVFPFGQFRVATTCGCDTAPYQLVFGKDITYNGDVYHTFRLLPKPCNES
IKLGKAPICNLTRYEVNKIAIRLTMESAPCFNTSDSPLQYLDKARNYYGVRFWNQSKPDRPDSWLPSRVGMTFSEKS
IELYVFGLKYAYTDGFFMMLRCRFQPSFAAMCKRTSANMCLYAFVDTPGHAYVSICKVQGLG

>g16863.t1|PACid:27567221
MTGLALPPLDLGAAQYGGAWAAASSGVFTDLMRHSGSPMGCGQRSAAAGIAPNGTWSYLNPQLITWRPDGYPAGLSP
PAGTLWSATAVIAPGPGPGHYSPGRYVLLWDGSGTVVADGDAAYPPLESAPGRQLLDLLPGRGNGTGLRLRLVASAA
SDPLVRLRLVPAALEAGAAYEEAPFTPTFLQLLRGIPLLRLTAWMRAGTDPNSPRTAPRDWATRTTLAHASQVRDAD
GVAVELLVGLANAVNASVWLSLPRAAAADDAYVAGMVGAVAGGLRPGLGVVVEYGAEGPGWLGPHLPNATRLLAAVA
RRVWAAAGRPAAQLRVIQAVPAADVLANEVEGAGGDLSHLDALAVPATFGGRSHWVWPGLDCPTGWADPAFLAAPQR
AAGAYGLEEALGYIRSSVLHADVAYHRVRQQLAARGLGLLGYAAGPELTGPSYGARANYDRVLQACGGSTPTWPCTF
TGPYPPWGGPANTTWSDAAALAASLRGALAANASRETDMEAVTRAARQHSSMYDMTADWLWRWHGQLGGGDLVLDLG
RTAGSCDTAPGQRAGVYAPEECRTPSLVVASPADSPVYRALQDWIAATGRGGEGAAPADPTSLPGALPRSAATAPPA
VPPAAACPAACVYGSCVGGRCRCWVGAGGPTCGQLAAANATPAACNPAVGINLEGISDWSRAWTFVDVFKAARDWIH
QSLTPGPWSLAPPLPVISSPDGPGGAAALGYPAAPLPPDSRASTLVMRDLQGHIPGGVYTVLWEGRGALDFSMSDVR
ALQLVEAGHARVTFAPSSDMNNGILVSIERSDPADPVRNIRVLTPGYEADALSGAAPFHPALLQWLRPFGTLRFMAW
SRTNDADQPTSWPQRPTPDTAWSYAAAPGGVPLELQLLLANTLGAQPWLCLPAAADDDYVAGMAAAVAAGLRPDVGV
VVELGNELWHTGFPGGQYAQAQGLEMNLTEQGDRWPGGLNNEARLCWVAHRTANISRMWRQAWGRDRAAARLRVVVS
AQAVWPLTTAKLLSCGGPAVTELIDAVAIAPYFGAYDPARDTNLTTFMTVTLPAQIDALLVQVRQHAAAAAAAGKPL
WAYEAGQGLTGDGSSADLAIQANRHPAMSGLYSRYLSGLVSAGVSRIVHFSSLGAASRFGSWGLAEWQDQDPATAPK
QQALLSFLHDNLQCPLPPPPDPAACPAGPDPTSSPSSSGAPPLLLPCSGRGACLAGGRCACWSGYSGEACGEATYTE
VYDCGYRCTFDQGVCNTTSVSVSGSGSIRTWSCACGGPPGLIGGRHCSIVSCPNDCSWNGECVDVGVCSCYPGYSGP
DCSADCGCGGHGTCDGAGGCVCDVGWRLAAGGGCEWDCSGCAPGTACVGPGECGCADTCVYGDCFHGACRCWAGYGG
AGCDLDEVAAAAAGRPAVPRLNRGSGAGVNLAGPAPWSTEWVWTDLMKGSSGWMTANTPDTAAENPFDTGAALELWP
GSGYPARLPPNTYAHVLLQRDLQLHAWPGRHVLLHEGEGRLDLGMDATVVSRSKGRLEFEFRPSADLACAATGAAYC
GDNGISLRLTATNPGNPLRGIRILPSAPPGAAASAGGPGAGGAWEGRWRRAPFHPWFLKSLARYRVLRFMTWQATNT
ADWAPADGRTAAASTYSGPGATQWGARVTPDFHTQAVSSLGGVAAEHVAQLANLLGADVWLAAHHTAANDTVAAAAG
LLAARLRPDVAVYVEHSNEVWNPLFPAYGYAVDRGRALGLAPPSAPNTTAAYRYHALRTMQIGEIFKAAFASAPGGG
SGRVRVVLGGWGYLCDGGAGCGRVHMNETLGWRDTAQQVDYYGVAAYWDCGGLGGNGPADVLLSVEQMIARCNASLP
AAAAAARALVSAASPWGVPLITYEAGPSLVEAAALQSGTTTTPGLAAKLTAASRHPAMYGLYRSYLAAAVAAGLVSG
GGEGRPYMQFVSTALPSQYGSWGLQEYTGQPVAEAHKYRALMDWLDEQSDASGRALPPRRPLCLDLTAAGGAGAGVS
GSAGGGGVGLGEGLLSGPPAVWLPAAGSVWLLGLTAPVRWATVGFPTTNTSASNPTSSTLDITLWRESDCVPAAAVG
AAVGVGVGVGSAAPMSAQQRPSSQALLVLASGLSAAAAASGRLDVSLPLPGEAPAALLAALAAAAPGGAGGRFFVR
LSDGLSANYSEPFDIAPALTYTADADWGACDCAAAPAPAPTQRRNLTCTPHPRAPAGLAAALTAAASTSTGAAAAAS
LLPQRELAPMPQPRCGTFYVPWNLTQQGVPGDTRCSFNADTGCRTFRTSNTGMWAWSVFKPVEDCTAGDSPWPAAAA
AASGASAALPLSLCAAWDPAPPPAASQPCPADACPSPPPAPPSPTPPSPTPPAAAPSPPPSPPQPPPSPRASPAPPP
FCTTSDTAALLSQLNWGAADCDPTAPFPACGEGELCVAVVGCSQWGCDAAAAAQGSLTQRPCVGLCRPLALGPLAAR
LTPDGRAVRLSLSAPAAPLAAVAAAGGSAASAASGVGCDQVFAADTAALLGGAGAVCWLEADSSSGSSGSSGSSTSG
SSGRVLVARLAANSMALPGLPLQLRGAGGALVWVRDRTQPFTGEVTLDACDPCAPPRVVISGPAVLTAAACDSPLQP
TSASASSSSAAAVAVWDASASSDPAGRALAFTAVRWSVPEGAGSPGGRAVLAAAALRTNFAPERLVLALSAQEVQAL
EEGLEYRIQVELTSWLGSSAAATASFRRAPTAALPPPPVSIAGGTQQSLRLGAGLRLAAEVAGGGCRGRSLGWRWSL
APSSPPLLRAGGAWGGLVALAAAALSGQSLYMPGAALRASGLSHGDRLVLRLTAAYTATAAGNSSSGSSSSSGSSAA
AVTEVDVSLAVVGSEPVAALSLSGWGSGGDVWDNVTLALDAAAGSYDPDLLLGSSNTGSSSSARAGLSYSWSCLRED
LAPCFDAGGGGAGGSFSADGGVWSVPPGLLAAGKSHTFTVTVTKGGGSGPAAAAAAAGAPLTASASLTLRPRSVAGA
VFPRAVLSRQCAAAACAAPHPADRDLVVLLRLLPPHDTAATAAAVNVSWSSPDLAVAAAVASLGPSTAAAAGDPATQ
LLTVPAAALPAGRSSLTLVASLMLAQPQQGALSGTASLTVPLNSAPYCGRDRAAPAQCLALALTGSFPTAAAALRAQ
DWVDDAAYDAAAPAPDTELAAAAALRYEFGLIRRTATTTAATAAGASTVRQLYEVRQVGAAATAVLAGLPVGEVTFY
CCAVDVAGARACAALNATVPAPAAVGSGGGGSGFDAGAALAALDVSSLSAAGDRHTLLAAAHQAAALLSYVSSVRGG
SSSASSSSNSSSSAPVLSAELVATQTAALVSAILGTGSSVTDSNDNSSTTAAALLLADADEARQMVSALAALAAAAA
SLLPDSAKEPLMMAARAAMAALTSYSSSSSSVSSGGIATSGTLSADASSPWSSAEATEGFVTELCRLVAAAMPASAA
AAVVAEAGATNTSARRAVLHEVYPARHLAADTSPTSASAAGPPASLAVSRLADVALLAAAAGGALGRQAVPGGGPGY
EAAGDAGLYVSAGTALLSSGTSASSSAGSQLLLSAGPEAVEAAAAAAAQGGSSSMGRHRRRELLTTASATATASADAY
LVLAGAVATGAGGYSLSLTYAPSASAAVASVVAAAASAAGLAPVTPLGGLAVVGWSPLTSAAAAAGSPPALDGTSSY
LQLRLPVTSTSFNASRTAACLAYDPASGRLSGSLAGVALPAGSGAAAATTPPPCALERYDAASSRVVCRCAAVGAYV
AVQGPEQPRPPSPAPGSPGVSPPPPPSPPLPPPPAGQEQLGGTSAPVATSSDAATPPDAAAGSDTGAIVGGVVGGVL
GAAALAAVAAVFVLRRRRLGSGSGATVAAVEDGSRSGTGQNGVSFTGSSDEVASDSGDTAPDSGGLAPHPTSPPAGK
RAAGTGYAKGAALGATPRTASASAPPTPRAPSSPRSAARLFGACFGGGGGGGGGGTGGGGGGRIGSASRAAGGTL
EEQWANLLQAPPAGSQRRHSINLSFGMGAANVRVHSATDPPAAGPSGLHGGSGPAGVHPGMGLESRLLRGAAGGVGA
GGGSGAGGSGAGGSGAGGSRPASRMGSATPLGPGLNGGVTGAPGYGRSSGGPDLAAGGGGGARSPAGAVAWGGGVGG

AGHGVGGDGAAAATPRSRLSSVHTAVAATVAAAMGGGSPDPRRAAAAATAASAGGGDDSSGSSSANHSVAPLTARSG
GGGGSGAAEAPPQPLLPLPRDIAKSGSQQQLLPGGSGGQQQQQQQQQQEEEEEEEEEKEEDGCEARGPGEVP

>Cre16.g693200.t2.1|PACid:27567249
MLLATALSAGAAAAARIPGAYGPPAYGGYGGSYGYGGYGAAPPPGPIYGGYGYGGPSYGHYSPPYPPPAAATPPSYV
MEWPPMAEALERPPAYPESPPPELPPVWLSPPQSSPPPASPPPSPPPSPPPSPPPSPPPSPSPPLPPALLLTDIVGC
YQFLGPGSRRLFPAAELSAGHGSAGGGVGPNAGPRSFDVAACVRMVAGSPGSHFRYVGFFDGHMCFGIGGILPPSAL
AAAEPFAACEPCPLPSGRNSTGSSKDGGGGSSAQQQQPEPQPQQLRCGSGTRMAVYDLAAWPWVRLAPSLPPMAPW
PPEPEDFGSGWRRRSRRRRRQ

>Cre16.g674250.t1.2|PACid:27567260
MSERPDRPLFIGNFEYEAEERDIVRLMEKYGPVEKIDMKQGYAFVCMRYKEDGDVAIRKLDRTEWGYKKRMLKVEWA
QKSEADRKRDTKPSRTLFVVNFDVRRTTERDIERYFGRFGRLTRVQIKKSYSFVQFQNVEDAIKAMERANGAQMEGR
TLAVEYVQNEDPNVRLVEYGRGGSGDRDGGRGRDERGGGGRYRSRSRSRSRDRYRRSPERSPPRYERRASPPPRRDS
RSPPPRERSPPPRRGVSRSPPPRERSPPPRRGASRSPPAAERRASPPPPRRSSRSPPPRERSPSYDR

>g16792.t1|PACid:27567277
MNGRLTCPDATFRQLSQYLPTPGRVRLSEDPSSRPLVLHALSPDWEDAQHPSGPGATTSCRRSRQDEETRSAFHVQL
QRQGNGDWVVHPARLLVAAVLGDSASSGTLELLGRFGKLLLVAGEQEGALGGASQAITASAAAPQRRRRRQETSAAA
TAPRVHDLQQAQSAPRAGTAGPSARVPRTKPAAPPAGAATSPEAIPIDAAAAGKQPRQKQPEPDVGRGTFPADLLLG
AANDATALVACAASCACAPLAAGAASGSDAPSSERALAARSPAAGEQEAQQPRRDSGDVGHVGQAQQPKAVSAARAT
TASTSSAPSSAAAASTAHHVAAASSGGSAPKSDGSGGGQQQAASAEDTCSTEIGIHAQPHTEDDEDDLLRLDGQCCR
AYNKKRLCALYPKWARTPLPGGTRHAYKALLLRVITPPELGFGPELEQTFPAFLHKERRSTGFIHLYLDSLWVVARE
LDWQGGALFTLDRELHPTTATAAATAAAAAAAGGYGDTTKHPEELVVRVAAAGTGQMDEKTARMVDTRQRAEEERTR
RRLAVPRDYVVLRLTSSMLRQRSESLEFTAPAAAASWLGRGARWDVAGDGALLPVRWTESDDTAGGESADLQRTSIT
LSARHQGVSQQCVLRALHPQLHKAQAVPGDVLVLSPLWRSVAAAALPATATDSIGPSRRCSSSGAVRSGGSSSGNAD
RAAGHGAVCGNGPATKAAAAVAGGGAATAGVPDGSGATAGPPAAAVHGQQQDQQSPAGLSNHPTVRYVSAVKAHLRR
CSEWDAGGVVRAQHDGISSARRRRWLLSWTRTRTATAAACLAPAVTDAESAAGTSAGSAAASSGVTRTTGTPHTAAA
SSKSAGSPQRTTSASVQRRRLRRSSISVQQQPAPRAPQQPARPTASSPSKAPLHHHPVAAGTAVDPAAAAPPAEAPL
SSNEATAEPVGPAAATPAAATPGKESGEVDNQHHAAAAEGSKQQQEQVTTEPRQPLRAVQPQQEVLQPPRAPVSPLA
SRALSQQLTLSPGRLTPLRLLSPPTSPGSATGVFRAAAAKSSYGSASGAAWRPSAVPRTLARFGAKPASGVPKTPLA
APSTPEPSLAAGAGAVPQPHQAVTTPHQNSAVICALAMTEASVAQAHFKFAAGNSMPVGASGGVSSSEAQAVEPVDE
GCKGGSRSSADTPSQQPLPPVFGEPQQYQQQELRRRLLLARRSLHPLQQHAPDTAAVEGPSDAPLQSEAAGRIVSPP
LPRPLPLMARRLLLKGWPPATSCVNAGPTAAAAGDSASQAADTFLVAAAQDLVLTTASQQQSSFAFIGDEPLLRCER
PAEAQEDPCGLPRVAGSAAAGIDEAGHQLPSPNGVASIHLLHGAKPAGLVLHSSDGVLQQGMAVNGSQLSTAARAEE
TGPPAAASPPDLPSLQQPEAAAGESHLNQNQTGEDAFTREPEELGQRVQQQRAGGTGDQQVEGQVAAAQQHIAPTER
APEKPCHTLYTLRLKLSQKARLGSAKAAAAERPLGSKRTAVDAGCEEEQPQQKRQRGPTRWDHPLQPLEQQQHQQQS
NPLPLMTAVPEPVGSAVQRRPAPSAAVASSLLTAPQLQQVTPQPPQPAVRDVQREPFSPPPSPPPARRHQLSDGGLV
ECQPAAQHLAAASSWGTLLPLTPSRCDPHSACAAAEPLQAACEHSPAIPCKRLLRALLAELSSKGPEMRRLCSGPAG
DVVTERLQWLGMAAVIAAKELSKRGALQPQQLCGGKAVEMRFQNCPLPELLVQVWLPQLGSPAAAADVDWTQASALA
ACGQIVGAIKKASQQGDSAALARWFQALVVGDFNKICLDLLEEESGRNDSTRGAARLVWASLLPEAMGPRYYCHVQI
LEAHS

>Cre16.g650600.t1.2|PACid:27567288
MAMPRHGRRPARCSSNRASQWLLLVLGVALAASPRFLLLVEAATYTLTLDKLGPTVNPTTSDAVTFTATVASPDSTT
AVFFTLDYGDGVTAETTRTTTGALSTTPTANLVSAGTYTVTYASIGTKFVTLRLYDSAVAPGVLLASKTVPIYVEDS
TLTATLLQSGVPRLNLAFSGFKGRVSSSTANRADMWATIQLDTAPGVFESSRIFIGIAPTASTNYDFVIPDQVYNLE
GAKTTVLRIYDAPVGGTLLRTFTPAAANAVYVVDPSKYVLTLTVGPTSVTTADQVTFTQTTTEVSYSASATSPILQW
RFNWDDPSVVETPLAYPDALTAASNFPTTATAVSSAAASTFRYTSTGSKNARLRLYDGANNVIAEKIVVITVSNAGY
TLALAKTTADPVTTDDTIAFSAGAKHLSSTSQVWWTIDYGAGESSPRTALTMTNVGAAAPNAIASLSNQYTSGGTKL
ATLRIYDRDGVGANTGLLLASTTVTFTVTPVLYALESAVEPFSPIATVAAKWSFRIQRSKATPAGVTESIKCAFFGA
DTGTAPADLAAWLTAANGAGGLTATILPSSIPSDIISFTRTYAAAAASLQGKLQCFIGSTPLWDPYYPTPVFQVLAA
APTYTLSASVTPAVVPVDTATLWTYNIIRSVPVPAGGPSLPILCSFWDGKTGAAPTTDAGWAALAGSANGKGTSMAP
GSTTATCSFTPSYSTTGTATPTLQLIQNSFALDAATTVGFLSPVYTAPAFATVTAASYTISSYLNPVTPVAGGAAAV
WRIVITRNAAVTASAKTLTCQMPDNGQGGSPADVTADIAVGGTTTVCVFSIAGYTTATPGPYFATVNVVDGAVTTSH
ITKNFTVLASGTTAPTYAVTSVVSPATPVKVSTPVTYTFTITRTTAVPAGGIPQPIICEFFNGEGTAPASAAAYWRV
STTIPDADTVVAVMAPGETTTTCTFTTYYTTVSAGGFTAKLMVFGESATAAPLLTSLSVTPSQLLAAVHSFATPMVV

AAAVVAVESTTISPNYNPTTPYTNIPTYFTFTLLRDPPVPPSASSGVQFACALYTGQNVNPASAPSAITDAVYKTFT
DVTTAVATDANYFADQQLRVVTMAPGTGRVSCTFPTLYAAAGPFSPKFFVFEYASSTVGANALAVADTVTSLTSFTT
QAAPTFITGPTNVPQRVPLPKGFRTTCFDGYELIFSNDNYTNGVRVAVDAYPYPVGQCRKCPGGTATMDGYRCIPCP
SGYWSNEGARECTACPAGTIAKPAALTARAKYSIDPTTYHFVTHLAMGPESCKKCPKGYFQPNIAGTVCLPCPSGFV
STSGATGCTACSEGTYHTDGVGTTTPGEATSLDTTDTFGSIYPIIPNTCRQCPANTYLPLRGQAAIASMNLAAVSSA
TPCRPCEDGTWSKAGAAGCQKCPPGTYRNTWFSGQLGSPFITADGVPVATTLTELGSGCSQCPPGTYAPTFGMSVCL
PCPAGTFASAPGATACQQCKPGTNSLMGDRTQQMALVVTNAANDFPALRAYTISGMVAGPAYAKPIVTGPDTNFFMA
GKSETCSTNLPGYYTDVDGLPIQLPCKPGTFMPFDTATANLLDTGLTVDGTQCYTCQTGTFNDEFSQPVCKACWSGS
FASKRGLPTCEIAQPGTFTNVAAAANATFNTATLIPTGLVKGAQAPTPCGMGYFQSSAETTTCTACAVGTYADQAGL
AACKPCQPGRYQNSIGQRVCKPCDMGTYSRYGGELCTKCPAGTVASKTGSSQCTPCAAGFYANAPDSATSCRACPRG
YYGPYSGAYADNLGDEFEGPRGCYKCPYDFFADRPGVRQCTACPPLDLGGGNLVEQCTEDLGSQRCKPCSLLSKPKT
ARTEQSPPPPSPSPPPPPPPSPRPPSPNPPSPRPPSPAPPSPNPPPTSPPPSPPPSPPPPRPPPPPPPPPSPPPPNR
SPPPPPPASSAINPGGGVNQNGDPVGHRRAILSLMEDEDAEAAQEEQAIVDVDAEMQPQDDE

>g16528.t1|PACid:27567335
MARQKSKAAKIKHGLHAANNNLSALFDANAVEDLEGSQLKQLPGALYIVADPTSSSIEVHAYGSMDDPMLVPSITHL
VKCAYAVHQLRQPPPNITIVKAKATGTTAGPAPKPESYLVRETLRFVYQNAVLPGLRELYDTGFTVHMCNGGKWSKD
DGVSAGKIASWIPADGGSCTCDGSTCMDARVKLGFPNVPLVDYMKHELRKEEAKKAISWGVRRSTFVAQKVAELEAE
WKEERADHKASQARSGAAAARVQQSAESDASDSDEEVVNPVVARKVRKQQGATALSQQLLQTHFERFNMDGKGAGEG
SLLQVLKEEGYGLSKAALARALRDASLRWGKLTPTQEEALGHFQEVKEEYGEEEAVGVIAGMLSLTLEHARKRLAAA
KLLRSRVDKQPEGSGRAVQTALSRHTTKKSRGGKEGHSREERRGDDVSRGDDVSHGGEASSSGEASSSDEEGGGEEG
SGGEESSSGEESSSSEESHGCSDVDEDAPCKKCGGADATVFDMVFCDDCNDCYHVNCHEPPLTAVPMGRWSCSTCAQ
KPVGCAGGGLGGGAEALQRAGGPGADLEVAALGRAETAAGATAAADCSSTGGGDGGALAPTGSPPPGLGGLAEALQR
AGGPGADLEVAALGRAETAAGATAAADCSSTGGGDGGALAPTGSPPPGLGGLAEALQRAGGPGADLEVAALGRAETA
AGATAAADCSSTGGGDGGALAPTASPPPGLGGLAEALQRAGGPGADLEVAALGRAETAAGATAAADCSSTGGGDGGA
LAPTASPPPGLGGLAGAPVHNKRIAVDELPRRNKGAKLQSILEMLKK

>Cre16.g666300.t1.3|PACid:27567379
MSFICKVLRSALLLLLLAGSLLDAAWAQPAASPPPGGGWPLSAPPPQSVSDVAKTPETIMQLRGLTGIRSCAELQPM
FLELLDSGNIFSRNQLCYVGREVLTDGSEDLSDPGLQQLSPPSLPLSLTAPSPPSLSSFVLSAVLPSLEMAQRAIAS
LGTDTGADVSTFMQVAGMPCGGALLATTLTEDQDTATVYTFSCDGTLSVPGSVASLVLPAMCCATNVPRPPKRHTPP
RPPPVLPPWRALPDPPSPEPPSPQPHPQLPTRPPRPPTRRYPPPAAVPGAPQLTLFVMADPQRLLCAKLLAACETLA
QPYGLLNGKYGCYASNELSSSGSTTPMPRYGSAAPLPSAYTASNESLAVNGTGANSTDLVLAVPYMIVTATWPARLY
SRSFRRMQAALPSSVEVFASLGAVPCNTEVRAVLLSTTGMVHREEAYGCSNALPAVYTALLEQDGLKVVPRVLLCCR
PPPAPVPPNAPPPGGPALPPPVHPPPGPFTPPSPPGAPPPGLIIELAPPPQPPSPPRSPSPPPPPPPPPPPPPPPFPG
YEDHVSTHPRPPPGRVQNGQEWFPPPPPSISYTSANLSTGAIVVIAALASAAFVSTLVVVFVLYRRRSLGGAGANKS
GPAPGPGPAPAAPASSEGYQAQHESGAAADTAWVGISLTDGDSTYHQLAHPLQQQDKQHVEQLLQQAQKKQLSTKRA
DGGLLAYQSSLESQVSVAGTGVALGGTTSIGTEAAASGGVASPQWVVLYPGGGEAGLDPHRVSVLLPEGDSVAVIRA
SPHRALPAPVWQQQEAAAVEGGVAGDTLAAAAAALEGCGGDRAPGASSFGVMDVFREAAQNICLSNATSAAPSAAGL
LAANGLSAVPSSLPPLSPGALHGAAASASRQPTGVAATPFQRTPRVGGVSAGSGCPQPAALVGPVQPMASRAFSSGS
PGTPGSVPPATSAFAPSPARHPASSSMRRREAPCELTLQDLFMQSPLTGAYLATPPTSSGGAPPSPPRHAASEPQTP
QRPVPAAAAPTATLERLSVQLAMNIARLSRATPSGDLGMRSSALSGLPTVAHGGSQASSVARLQRWRTYTEGLGRGV
AAALSGLACGSAALHDVVSGGDGAPEQREDGLANVNHSSAGRRNYSSGSQPVSDSASGAPSGLESGSISGNGTTLSM
RSSQGTAGAVAVLSDAEGAAGHPPAKTGGVAGLSPTHDSALPPLPPTRSRGIPAAADTSASAATATTAGAAGSSGQA
VLLPAPADPHLDLDISPRDLKLQADGLLGAGAFGSVYRGRFRDQPVAIKVLHHLHLQQPMPGGSPGGPGGALPYKDK
EVESFRQEIAILASLRHQNIVRVLGGCAHAGRPFLVMELLPRCLHNVIHGANSRLPLSEVLRIATDVARGLRYLHPA
IVHRDLKPANILLDATGTAKISDFGLARYHLKPYISTQQPDAGSVAYTAPEGFDPAIGRLSSKCDVYSFGVLLWEMI
TQEHPWSGDSNVAIIYRVAVHRMRLPVPADLAVCPPRLATLLEACMAYRPADRPDMRHVLGELEAMSTVAVRH

>Cre16.g677300.t1.3|PACid:27567517
MRGIPMCCNMQRRGAGGSAVGCSVATAVLVIAIFTAMPHSAQARTPLTLADANPYAGRSLLQTSLPPSPKPPGLPKP
PPKPPGPPKPPPKPPGPPKPPPKPPGPPKPPPKPPSPPPSPPKPPSPFPPSPPPSPPPPLPPFPPLAPGTAAPAGYF
EALPFTAKVVSLSLANIPRTRKYMQGWRRVSSTWTPMAGSLDLETNTSTGYAPGIAIGCSGPTILEEGSIIYMGGEW
GNSGNKPANVDGRFSATRYDAATGKYIQVGTLSVPRWYPTALRLNDGKVLVVGGTANSDSGPAYTFSELWDSNNPSA
PTTPVPHPAAFSASMGLNYYPFMALLPNKEILWWGDRGGSITDEHFNDILSPPLPTNYGPWHTMYPYTATIAMHAL
RPNAATGVYDTFSFTIFGGQNPYRVSPGTPASNVSARLDFAYCGPTNTDICVVNGGWQIELMPDRRLLADAIVLPNE
RILVHGGATTGRAGVSATGLKAANGAPVSFVYNPSKPEGGRYQITAPVLIMRSYHSTACLDITGHIFSSGCDECALP

VPSGYEGLIDPNPTGDYEYRLTLGTPAEIRDVDRPVITSAPDLIHRGDVFTVSYTYTGVHITGVTLTAPCAATHCIN
MNQRAVVLPFTVDAATSTITVTAPPTSQPGVAPRGEYVLWLLGDEVGDFGRTYSEGHWMTLKD

>Cre16.g682550.t3.1|PACid:27567544
MHGQRRSAAVVTAAAVSWHQLARDPCRRRPRWRQRQASAVRRLAATPALVRLLLLLVPPLLLLWATPTLAQAPPAYP
PSQPPSSAPSSPYPAAGSMPPPSPPSAPPGTPAVASSPPPGLPPLLPVSAPPSPAPTHPQTPPPPAPSPPAPSLPAP
SSPPPAPLPPPAPPPVPTGPWAQLAALLDVQAAMAVPYTDWNNTQPCGPPVWSHVTCDGSGNVIGLVRQGPAAAGPA
GCPPASLTALTGLQSLVWAVPPLNSTYTCDWPAIASLTALTRLEMHGLFSPAAASASLANLHSYATLTNLVDLDLSN
TPIYEGLITTSASHAFSALVGLTRLALANTCFGRVDPLDWPTVSQLTNLKVLNLTNAMGRNWDGVAPHNASCGRISD
LWIGLADHPSLEVLSFDNNGPRVDGAFPSVYFSSTKALAARLRRLYVHKNNMMNTIPATSIDTVSVPHLEALTLWGN
PLLCGALPSGGATSLCLDYSGTKLGMSCTVAGQVLGFPVPGTDCTPVNATAALVGIDMPQDLPAVQAAAVALQVFRN
GSSANAAALSSWGAGGNPCLWRGVQCDTNLQVAVLELAGLNLTGPVPAGGPLTSLRALSRLDLSRNNYTGTIPAANL
ASFTALTYLSLAGNQLGGTLPAGLTALPNLRILHLSHNSFAGSLPAVWFSGTNKLGNGTLLRVACGHCNLTGNLPAI
DAGFALPSLQLPYNNFAGGIPAYNLTLNQELDFSHNQLTGPAPNFGANIQRLCLSGNMLTGPLPPLNNMQDVRLSTN
NFSGPLWPSLNPGVFILDLSYNQLSGTLPAAWSTATNLSALYLQANALTGALPASWGDMQLVASPAVGTSTLAFHLK
VLHLSGNQLSGPIPAEWASFSPWGPNAFGCLDLTGNTNLCGPVPEGMVCPRTAATSIGYDCANTTKPLGPSLCRNFP
FCQAPAYSQAPADIYASIPPPADAAVNQALVSLQTGQIAPPGNVFATWRTSLDTCLWDGVYCGNNGSRVDGLSLPRR
GFTWSSPGPYLTAIAGLKFLNLAHNKIAGTLPDWDPASPTAAGLADIDLTDNLMTGTVPSNWTALISLTSLRLASNA
LGGGGTPLPAALGSRLRALKVLDLSDNGWNTTLPDAWCYTGGAGGSMTSLESLKCNFCGLGGGGAGAPLPRLDYCTN
LRQLQLIGNNIAGALTAASFPLPQLTALELGMNALTAITVNGSDLAAPAALPALQKLDLSYNNISGTLPCALGSWTN
LRQLLLRSNNFSGALPAEPGGLVISNTCANCSRLTLKVVDLSYNNLEGPLPEGWWPMPSLTGLYLQHNRFSGPLPSI
WGSNWPAYPGGASGGGPGLVSLQLSSNALTGSIPASWTRFLPNITLAVGVNGTNFTSGTGAGALQCLGLRYNRALCG
ARLSPMTWQDDCASVAGTLLDKNCSTLAPLPLPACPTLLLPGACDPLPPLYKDLPTPTPRQGLVEAAPRANAGPDVQ
PLQDLKANILASNNTFVSSTNTPLVSELFNSWDANMEPCSTHTCVPCRLGGGGPPCGMSYATWANMTGNSNAASSNA
TTCNYMYVSCEAGRVVGIHLNLNGDTEVASIAASPPLLGCVALPYSLSTMTPLKYLELGRVAMSGTMPPSYSSLTAL
VNLLLTNGPTASGGGTGDLSGPVPVEWGTGITLLENLMLHVATTSIAAWAHDWQIYGLPYLKSLSLRGEGDNSMQVA
DVFFIVRLASLTRLELSGRAVSGGLDQMAWLGTKSSLSVLRIKDCAVLGGSIPIEWQSANFTLTEMTLSGLPAVSGT
LPAWLTQHMASGSVLKLDHCGFTGVLPAAWGMANATGSPPRAAYFKTLVLTGNHLSGVLPDTWADLVYRSTSISMCC
QTPAVAGSGQELRGPIPTSWLAASTTFAPAPPSLTSLNLSGLNCLCGDVAGSWLTTTAGLALSLPATMGTSCDDVTC
SGAGEGPMLMRLKSELQAATTAANASDWSLAAAAIFDSWSNVTGAPPPCIDAPCAVSLAGQTAGATDPNGSGLPLCV
WRHLSCTADQRVSGIFLGWESLPGNSSNLTALPLPLPPALLLTSVPSYLSALTALTTLDLEALKVTSGTLPDGWSVM
TGLKQLRLAGLGPGVTGPLPNAWGSMTVLERLSIRGGLGLTGTIPGSPWGVVPTLQSLRIEDAPGISGPVPNYYLQT
AALTELRLLRVGAVALKTGWPATLSAEPNTGLTSVRISSVPGAVPPGCPADHAGAKAPDTGTWYCYGPLGGLVRLRT
ATGGTSVQALAPAPAGGSPPAAGTAVQQLVQNDTDVGQRWHIEPNGAFPFSTIREASSGLCLGVAAPGSGAYGGLDG
PYMGASLELQVCGSNGTTGAVYDTQRWTFYRHPNPSLADGFILSPAVDPTKCAVVANGSISTGGAGLAVDGVGSAAT
GAGVFLTRCSSASPVDQAYLQPARVGFNPQPSDCMADTTYWCTAVPYQNSTLVDCDGDGLLDAVCTGTGTNTSGRGV
RLSTRGCSTAAPDTGWPSAPNSLCQPLWGASSPPAGSPPAAPPPSSGSFRRPCPLPNYVIAAAYGNDGYSCLGPFAS
PVVILTSTGQDLALDVPYNYPQPGVAIWQYARGQALTIPDPAQLWIIEHAGNDTYTIRDNGGGFCLAAKDAATADGT
VVELQICGSGYGSPAAPHQRWRLRATSAWIRYSTFYISPVHAPHQCLNIVNNSSTNQVPLQTLTCNASSQYFELQSP
PQQTKDAPATQWSTLTGLLQGFSVAAKNLRELEISGQPWLGGGSSGGANGTAASGLGGLAGGGSQPLSALTVASGNG
FAFPVLESLTLRGLGLTGTLPGPAWLSGAGAAGALTWLDLSGNSLTGNLPPGFNLMFNASTAGDDNVLLLGGNRLSG
TLPGAWVTNGYAGAFNTLDLSDNQLTGLFPYEWASLIYSSRAVKLANNLLRGAISPRWQTYTSTYGPALALLDIRNN
NCTCSAPVLNGTWIAARVAAGSLSVLTTGSFAADCLNTPCASPDEVQSALIDLKAAITNATGASEADVINTFDTWLP
NVRPCGNATANGGTCVLCRRNQTCGAPANATSHYCGYRYITCAGGAVTGIYLSHNLTGDPPPSTVAGGDRFLIGAAD
GGYPASLAVLTALQDLVLDDVKPASAGSTFFQTSWSSLAALRRLSLNVDAGNALPPEWSALTSLRALRLYGGGGQAA
ALPTAWSTWTALTSFEMQSTPAGMAAYGNYWVFDGVTPLPAAWSAWTGLQVFRLDGTSGLTGDIPSAWGGAWTALKE
LSLSYNPGMVTSATSVAALLQPPGSPALQLVSLSLAYTPAGSDTLDHIPLAAMTTLTSLQLNGRGCSGTIPDAWAAA
ADVPNITATVTASNVTVGSGPKMTALSVLDLSGNLISATIPAWLNGLFPRNLPAGLAVLSLDTNQLTGTLPAEWATN
GGQASFAELSVSDNFVTGVMPPEWAPLVWGTASLDLCCNHHVGGSWNGLQGPIPVEWASYGVIINGVNASSNVYMGS
LLSKLDLSGNDCTCDAAVPAGSWLDVKLNASLLAYNYTGSFPGACTAVACAAYTYPPPPPSPDPPAPLPPSPEPPVP
SPPSPGPPSPVPPSPAPPRPDPPSPAPPSPAPPSPLPPSPAPPLPPSPAPPSPAPPSPIPPSPAPWPPPPSPLPPSP
TPPSPLPPSPAPPSPAPPIPPSPDPPSPMPPAPPPPVRLSPPPPPPASLPPALVFDPQFNPSVAFTAILPGLSASTY
PGGATSLLANYRSIIARTASLPSVDYVVASLSSGSAGTAVARRRLMQQTATADPVSLTTLVWFPSNWASQPGLFPNQ
TALDAFVFNLRHLPAAALSAPTAFPAFNASSPLVSGLTSTVTTPSLLDSSDLANSGSDAATTVVAVVNVDLSGAGAE
VAALTASSGFNLTALAAADITPPSISVKGDTVSLVPASVTTPYADPPATASDALDGLGRLGLITTYGLCRLPPAGLT
GLVMASEEPGDLDLQASGLDCGGGGGGGGGGDLLLPFVNVSAPNAPGEVWLTSWGVVNSRGIPAVPRYHVTLVTDP
CAASNEVWCAAFGRCSVGRQCVAVNLGSSLASSGSLSSLGGGGSSTGSSSSAAGTGSSSSSSDEGDDGSSVDSGALS

ILAGGGSTTGQPILFTDAVADVSAGGSGRTVVSVPRDTSPPRIMLRGSGKPGVSPLGEPVMMEEVVYGSGDWTDPGA
SATDVNAYGVTVDVTDAIRRYGAAKVDTTSATPLDAPYGFAVLYTVEDVAGNAAVPAWRLIRVVCIMPETFCTTADG
DRQCTVNGVCALGAQAASLFGSSSITASFSGGSASSATSSSTSSNATSSSSSSSGAGSSSASSSSASTGSSSASSST
GSDSAGDDGLPYEPAMVMGGPRLALRGPRYVEAAQYGTYDRCASSASFRGADCDAGVAAWDARDGRLDALVRVCGSA
PLRPPTGASPVPILIACGISTAAPGAYNITYVVTNSRGVAGSTWRQLTVRAGCLPGEALCPDRVTCSVDLVCPSSLK
LPGTAGANTTGNATADSSTDSSGSSTGTSASNSTGGSGFNTVLTTPTAAAASSSGVPANVPPNITLIVSEAAPVHVL
IKRGIDYVFCNGAEPSADVPCEPGALAFDPDGLPGSSNLTGTVVACPPTACLTTRGCSQDDLLNYALQKVGLAGCNF
RPLAPVGTVFLVDLWVWDAGKANASVVRYVEITDPCPSNGSDSARYEFCRDAQGRYFCSPLPCEQAVALRPPARDPP
VLALLPEVAYIEYGTVPPYYLGPCTALLRPNTFNSSSQAGSASSINSGRGSGGSAVSCSAVAAARTVAADGTQALLD
LTPSISVQPVSTCSAAADGTNVACATCTLEQLHVPGRCPPGAYSYMFWVTDGSNTVSLTRTVIVYHRSSVRGLVAPF
PPTTNFSAALETAAAINTTVALLARAPASKVAALLIANTTAPYRSAVSYMTARLQGPLGVNASDVALRGAAVVPLPP
VPAAGKSGAGNRTNSSSNASTTAGPASYVVQVDAEVFTFLPSPSGGSSSSSSGSQPQQGGVAQLLHEAELRAWRLYA
SALTSSADFLAQQLATALGLVGDGTSGSPSPSLLPRSAIAGLEAMPPAAPPPPEPPSLPPPSPPSPPSPAPPAPPSP
HPQPPIPPVPPAPAVPAGAGGGSGRRRRLGQAARRSMHLDNDGTSGDVLPQSDKARTTPQQQPRRTPRPQQEGAAAC
PGGPACGSSSDAADELDDPITSGYADYDSIIRGGDDAATATATPPIGGGVDADGVVGHGSGESTEAGQGRPESLVVW
GGWRLQSHPLIRGDHAALPPGGSRPALSAHVQVALEALVKELVEAEASGAEAKSGPLARWRRRLRTPVPPAATSMAQ
PAEDSARRHLQQQTAANFTSGANSAIAALLSSSVNMTNTSATAYTSAAVPAIGSVDVMLASLAAQAAVVAVQVSTLL
GDTGLLSARLNSTGSGSDTNSSSSGGSVSTAAASGSAGALSAAGINATEDAYQQRASAAFATLLAKQTAASDATTNN
ADVVSALLDAQLAAAAVQSAALSDTAAQLSDLTAATEAAARRTAYDTVLMEQATGEWEYVWANCNATLLNEFQPQAD
LVWSFTLDRYGRAAANSAGAIGRRLRRQMRVVSTSAGTVAVTRQQQSAAASSNTSSSGSGRWQGYVPVATEEVVDVQ
VAGSPDAPQAPQRDRVLFPSQSLYMVGGVLLHATRRAIDELGRCTDVRAARFKQLNFECIRHTVGSSSAPLSAATRD
RLFATELHPLHPYGVDPVFLPSSALYDSHLSDSTVQYYNTTAGSGEVSATGAPYAFYHRPLQGYADGFPVVLPVELS
STRMADLLRYLQDANYLDRYSSSLGVRLLLFSADLRAFGAAALTFKWAPDGSIGLRFRFSGLPALTYLRDGGQDGTS
SSASSNASSNASSSSASSSGGGATDWQTVVTREALGLWILAGVFGVVVAVQAARAAASAFSRDLTLRQKWDRFGNLL
IDLAVACLLLASAVVLAWYMAAHAATFSARQHYTLYDAIANARARWLLPPKADPATLLDGSSATTGASNITTGAGLG
AGGGGASAAMDRATLKQVAAELNISEPLQAGDPGRYLLPDAAVAAGQMEALADVMGSAQELSDLWAAYGLIQAITLI
LLIGRLLSTLSFQGRLGLIVRTLYHAVPALGHLVLVMVVIEVAFGFFAHMVLGARQETMSTLSGSLYDTFSLIIGES
ELLSVDSILPPEQEFMWGEHFAASLVMVSQVLLMSMVLVNFFFAILGATFMKLKHSWGFRHGTSVTTDFANVLLPDA
AAALARWSRKLTCGRLHYRERHTAPLTNRQLARLVRERALAGCDVRRSERMSLKAITWVLPMVKVADRGSGASGASS
QGVAAAGSAAAAAAATTCRVVPTEVYIDKLTLEQMLLACAASAGQAGRASSPLPAADAGKNRTAALAEVPSARSVDE
EHTQQLQQGRSPVGCGSWMRRGAWWRGKGASSGGGGEGVGTRVRAFLGSSRSHRALPAEVVLDDAARATEAARSAAS
VVEPPARWPALAAGPTAARERPGATAAAPPEERRWSNEVVAAVAAKRLMSHYGHRVHVWPDADPRRSLSSAVVASDA
GIGLQHPQGQSGHKGGSGNGKALCSGNAAGGLGSWGGSGSSRPGSSSGSAHVLGAHVKSVEQQSGSGSSAAKQAAAL
AVPTAMTRGRQQSEPQLGLLTQQQQLGRLYAAMRDMASAIEKSQAGVYRWQLKTWRQMMAMAEENAAMISQRTGQPE
PLPPMPPQPCVTALADAGGAATPALGDASRHNLAARAAGAAGSNRPLSAASTGSVAAAHPNAIGAVAAAPWSGVGRS
AAGGAGSNLSGNSRVRRSTDLQASGPSVASVAGDSIRRGRSTTGEGAEGVSATNRALLQVAFGRGSVDATGSGGVAR
PESPQWAELTSSASMLGSSLTATVAAVMSTSGAATSEHAARHPHPHQPPGKRASSGMHMQVLGNLGAGRSTSAIVPL
PSASDESLAAGADAPAGSRQPQSPPASAAAGGPRSPAAMIPAAAAPDDASPHARSASINAPAAGGMSPPTPSGMRRP
SGLQRFDSVASTMDVTRTVSGSNISVHFNPSRRASLMSPGGWGPSITGGAGGGVASARQLLLPGGGGSGAASPSPSS
QRALGGGPRRSSVLGDPEEDAAVSSAAAAMATARMAAAGQRPSRRSMEMLLAGAAGGGGGRGGRRGSALGEEAPLAK
DGAECEDEEDDDGEWRAGMASLRAGGARNRSAASWSMPMPEGGLQGLASPVGSRANSFIATAIGGAATSSPAAAHVM
PSLGAPSPAGHRSRGVMRKSRLHVAPGLGGGPSGNGGEEEPQAAGAEAMPPHGMMASNPAFDPVSPR

>Cre16.g665350.t1.2|PACid:27567590
MGSCLAKPAESAEQKKASSVPSPPPAPAPQPVQQAAAPAPKDSGDVKPGQQATPVPPPSPPPQPKPAPPAQPAPAPA
AKVVEAIPAVADEKSPQGKQPRRSQEHDNGVKAEEIELVKQKSTEPQPSASATAAVATPKAIEPAMEPKVAAPSAPA
AAPAPAQAPAAAPTPARPGSKLRTDVKLRDLYKLGKTLGTGGFSVVKLATDRGTGVEYACKIMALPPVGQEVGENEN
TREDIFKEIDLLCGMNHENVIFLKEYFEEGNKVYLITELLTGGELLEAVLKRGSYTEAEARLCFVQVLRGIEYLHSK
NVVHRDLKLENLLLAKQDDISLVKIADFGLAKHAVNGMQTICGTPQYVAPEVIVGAKGHVYGPGVDMWSAGVVLYIL
LGGYPPFWSDSEPQLFDMIRKGKYSFGDPVWNKVSEGAKDLIRKLLVVDPTKRLSATEALQHQFILEGNFNPPSTPK
GPR

>g16758.t1|PACid:27567606
MLQRGAAGRGTARGVAATTHAARPYVPRWSRAAAARVVRAAAPSPPPAQDTARGTEAAGPGPSSSSAGGGGGGGAGG
PLLSLSALFGRGGGQQQQGPTPEVRVPLNNVGKVPIFQLLYELYSSHGGVFRMRLGPKSFLVLSDPGAVRQVLVGAV
DKYSKGILAEILEFVMGNGLLAADGEHWIARRRVVAPALQRKFVSSQVALFGAATAHGLPQLEAAAAAAAAAGDSR
GGGAASVDMESFFSRLSLDIIGKSVFDYDFDSLRHDDPVIQAVYSVLRESTVRSTAPFPYWKLPGISLLVPRLRESD

AALAIVNDTLDRLIARCKSMLEAEGSIPMPASPSSPSSSTATSSSAPSSPSAPLEESSAPTVLHFLLGSGEALNSRQ
LRDDLMTLLIAGHETTAAALTWALHLLVAHPEVMKRVRDEVDWVLGDRLPGSDDLPLLRYTTRVVNEALRLYPQPPV
LIRRAMQDDVLPGGHVVAAGTDLFISVWNLHHSPQLWERPEAFDPDRFGPLDSPPPTEFSTDFRFLPFGGGRRKCVG
DMFAIAECVVALAVVLRRYDFAPDTSFGPVGFKSGATINTSNGLHMLISRRDLTGVPPPAPRAPAAAAGAAAGSCPH
AAAAAATAAAAAAVGCPHAAAAATSGAPAGVTPQ

>g16805.t1|PACid:27567672
MRGRDTRGAGLPFKWLLLALVVVHAVHGQDPGQGQDAPPAPPPDPSPSPPPSPAPAPTYSCDRMDRVPRAERCTFVR
EQCESDSRLPYSEWYYCSVAPHGAFASFLFTLFLVALLPLLFTLLGDTAEIYFSPIMTHVAQCIPKMRPRFAGVTFV
AIGNGAPDLSSNISAIRNGDWLLSTGALTGAAMFVQCVVAGEVMRVSGGPVKCRGATLRDVAIYSASVLLVLASFAA
GRITHWFIAGACVLYLTYALWVFAGDEWHERGRPHARTLWRSLRDTWDVWFGACQEPQGLPWAAVDVGGPLSPRVSG
TELLLPLVAAGRSDSLGWAAAPGAATGAGAGPRTTLPEPRVRSPGARPHQTHHHHHLMSAKAYQTMVWADLSPENPS
GSSSSRSLAAAGGAGGGEDEEQGLLSRRRGGRKGRRQGGGGGGGGAASRGPGARGPAGSTLELGPAAGYAPPALSPS
PPPMSSATAAPPSATAAAAARLGSSGGGSGLLTLSSLDGSALAAPAPVDAAPHWGSAAGLAAAGSITHNTRSNSAGG
GAGPGSAAGADGGVARKASLAPSGGSGRLAGSGSGFAADPWSSGLTPAGSGLGPGAGRGGAGSEEGSYVGSEVEEDD
EEGADAYDREAATVAAREARRRRSRQGDEEDEEGDGASSSEAGGDSGVSSREAGPPRELWARVQYELTVGNSIEWSD
LPPESLQRRWRELTFPLLLPVYLALRLTIPFADPASYSRRWLLATCAAAPLLAAVYLAPSAAAALPMMIAAAVAGGA
LAAAVGYFTAGEEDALPDWDVLGTGFAFGPAAFAVFGFFMGVIWIDTLASEAVGVVGLLAGLLRVPASVMGLTLMAW
GNSLGDFFGNPAMARRGQPTMALTACFAGPLFNMLASLALGFGSYFARKHVSSAEVMLRPEVALGCGFLLLYNAAVA
AAGLLNGGKLPERFYVFARGWYGLYFVAACVLGLAAGQGDA

>g18202.t1|PACid:27567683
MLYAQKAGVGTYCASVPRPARPALAVGLLSALAPRRHRHLCCCSPATAAVAPAGVTALPSAATSKSAPGPPARCSAS
ALPPSYGGGLAVGASAPPSPPPVLLQSTAAVAPAAVTALPSTATSKSAPGPPARCSASAPPPSPGGGLAVGSSAPPS
PPPVLLQSAAAVAPAAVTALPSAATSKSAPGPPARCSASAPPPSYGGGLAVGSSAPPSPPPVLLQSTAAVAPAGVTA
LPSAATSKSAPGPPARCSASALPPSYGGGLAVGASAPPSPPPVLLQSTAAVAPAGVTALPSACCDLQVRAGPACALQ
CFGTPAQPAARTANGLLSSGCLSTPAS

>g6294.t1|PACid:27567840
MASPYVQGTASYATTSSPWSRERPGPAVPGVAQQVAVDGVSLAAAGAPGTALSSAASAGGLLSRPNSFKVLHASYAG
APEAVSSELYSSAASAGSSVPPPAFGRGAVAGGAGGSAAPLFSTSSGRSLLSPDHATAHAAAAASAALSYARGPSLD
RPSPGGPNSSSLSGGVGCSTGTSTSGGVVSAFNHTLALATGNASSRRGTGFVPRAASPNPSPLLAARPAAPPPSPPP
PPAPAPVAAPPAAPAPLSTSHGLVINLSLSPALHSATSTDVPLASPASPPPVTLASAFLQARTHDGAFGGASGAAGG
ASGGLTSSPSASNFLGAGSGAVASAGFNAGVGVAGSNLALGLTGASSSGNLGLRTASERRIQRLLNKIDSVCSNHSN
HSNHSNHSNHSNDSRGGGDGGGGANSAHGAMADVGGASRSDSLQPWQGRPGEERERDRVGRDRRTSAPAAQDLATLL
QLLAPHDESGADSRGTSPGGGRARRSTAASGGSRLQGGPNEAVATAGGGAVATAGGGAASVTAAGPGGVGAAAAARP
QQLGARGADGRNASESSSRSVSRSGSSSSGSSRRSSGSGSSSSDWDGEGEGPGARLRQPLPAAAAGTMAPPGRDVVD
VTLRRRTSSPVGRHTFTPAASPFAAKPRPQSPGQARGGPSGGSAAPAGAGRGPHAALSPLPPPPAPAEGLSGLVAPL
PFMRLSSSAVGSEVGQRDGEGDVDDDDEGMQREPSARQLVRPHGRRAAGKEETEDSDEGDRAAAAEAGQQRQLRDGH
HQHRQHQGELDHRQEAERDRERPRQRGRADRDGGGGGGGGGGGGGGGGGPAPPSRLPSAASGGLPSSPSSAVTLSPS
GVHGTHGDGGAADGSPSGGIAPAAVAGGGGGGCVAATVGPAAGAAEARPRPSIVIPTKSYGLGPGEGDKCARPPSQR
SSVDGFARRSVDGVAAAVAAPADAAGAAFAAAAGGVAARRPGAVAPVARPMEERLTAAAAAPAAPAAPAVVGALPVG
ADVPLSLPGSQQAGAQGGGYGDADDDASAADSSTGAPPPRQRSAPQQPSPFAAPLPASVAPGRSLRVRGPRGGDPAD
SSSASSGPDLAARPGAGGACGADQPVATSPVMAAAPQAALTLAPAPAVAAVTAPAAAAVPVAAANAAALARTPSGRA
PPRPPGGAASPGMYASPTTPPHPAAAAAGLSGLSLGLDRAQRHDGGGGDDSDDSGCVESTVSDSDLTIVAYSTGLSA
RIDARLQQRPPGPSVAAVATAVAAAATASRGAAAAAGHTQFGGGGGSQRYLAMPPHADALPSVGAGMGVAAGGLPAS
SSSAALATAATLVALIDEDLHRGGGGSVGTGMRGGGGGSGRRAESAGRRRSSGYPPLLQTPGAAGSPAVATAAALQR
QLSQQRLSARVGLLGEPSAGDVGASATAAAAAGSSSSWRWAPGTDALAVARTSLSGVAGQPLLQQLPARRDSSQGRQ
PSINPSQSQQQQQLQMLLSSQPNPHHHRHHHQHHQHHRHHRSHRRGSGASPLSDGDGGSSDDDGGALAAGRVGAGYP
LASYKSMRDSLSLPGAQPSQMSGAAMSAAALPAHLHPPFTAPAPSHVSSQHRLLTAGLVAAAASGGGCVGGSATASA
AVSGAGGGAALGSIMRRLDGQLAEVQVATVPGAPAGAGAGGSAATTASGAAPLALAAGGAGAAPQLLGRQLSGRVAF
GGLVTGRAVGSTSGGLLLGMGGVPGVDEVRGGSAGAVDIDMLKRDLERVLGK

>Cre06.g302900.t1.3|PACid:27567856
MAQAQQQTAKAIRALAQKPENKRCFNCDSLGCAYVVPAFNVFVCTECSGIHMKVGHRVKSISMGTFTAEEARALEAG
GNAVAARSWLANWRPEGDARKPVDKNARKLDAWVRTVFLDKRYFGAAPPQPAGGAAAGVGGVGSPGQASRSGDTDNI
PVRPLTDVLGNGSGSVKLTVSPTPSLGGSGATSTSTTSPPPATHPAATAAPKPAAPLAFTDNAFGDAPAFPAAAAPR

ATPAHAVAPPAFDPFGSYVPAAASPATAAPAAAPAPQHAAPVAAASGWPPEQAPAWPNSAPAAHASTGAAVSAPAAQ
LPPPPHHHAHAHPHSHTQNHLHQAAPQAAAPTLAAAQAAPAAAPSSANGGFADDADWGAFSGAPAPAAATSHAAHAA
TLSTSSGGAAAQAHAPAPAHAPAALHTPAAAAPAHTAAPPPPPPPPSPPPPPAAPRAELPSDLFSEPVMQHPVALAG
PAVAVVAYGIAPGGLVPGGYGVGVPQQAPYGAVAAAAAPAQPHPGAMPLQGGAPAGYPFPGAGAAQAEFPGLGVSYG
VPGMAAAAAPPAAAQGYGPYTAAPGAFNFAQVGQALPTAAAPAAPPGAANGNPFA

>Cre06.g254100.t1.3|PACid:27567865
MPPTRLWLLGRLFAKDSQPATQLRSQQPSACAAAPQVASAPVGPSQQSAKQHQQSSQQHTQTAQPPGSSGCRPAPPR
RSNSPPWQLAMLPLLLLALALAPCAVRAELEATLVFRSINRPFTLNDCSVLDDYLTDALIAKNVPIDFANPRLYRPT
CNVSNSNPPAAGGVRGTMKYDVVFDGLPFLENLQAFALAMRDELLWRSLFLVTYTGCGGEALYSDVANDFGLTQEVN
VCTAAPGTTRLPPTASKDCTYLLPQQVCSPPPPPPPPPQPGTPPGVAPPSPAPAPLTPPPSPAPPQPNPPDPPRPPR
PPRPPFPPGLGPCILVTKAIRPANWSSSGLECNMLVQSMQGFFTVGAAMVPDRGFKCLDDGSSNGVMLVVGTAASRE
DAQTVANNFASKPLATATIKLLRGMNCGSALSLEGAACGVAVRYTSGAQPEMFDCWPSPPPSPMPPSPPPSPAPPSP
PSPRPPSPRPPSPPPALPSPPASPPPPTFLQIRITGANMLKMNCTALAGATTLSILTSGLQPLSDTSCNVDYILREV
QLRTRMADAQQAAQAGIALGDFLGTFIAFGQLPCNSILEMSVSTSWWYNALTCPIKPALCCPSPPPPPPLPPSPPPP
SPPPPSPPPPSPPPPSPPPPAPPPPSPPPPRPPPPPRLPPKPPSPMPPDWPADPQAPNAPSRRKRPPPSPPRPPPPP
PPKRKRRPAKRGLREN

>Cre06.g255200.t1.1|PACid:27567929
MPPSPLPPPPRPPPPSPGPPDPPAPPSPPSPPPAPPFVPPAPPRPPANDTTYTFMTNTATYALYRNRASWTTALEVC
RARGYTLASWAASNQELYDIFNGGWDYFYAAEFWVGVVRRANKTVYEFADGGAVPAIDGTPVDDYIVLADPDCLTDI
RGCCATLTDRMVGPFLGGLQTPRSPKLVVRNCLTQFMYLCKGPLPPSPPPPSPPARPLAGNVVLRFTMDYVQLTTVA
GRTQQFRQAVADKVAAEYSEAGTTVNVYELYQDTATDGAVVVKLLVGLPSSINENSAANYLFRLVNTPSIIFDNTFR
SEYLISTPIMASLANTMELNSPPPSGVQQLASGGASEQDKRKVGLIAGLCAGLGLALAMGGVAAYVVLKKRRASMRK
IHEIH

>g6878.t1|PACid:27568087
MHSSVEQCKIILARPGSDRSGFSGNPFSHYNLCRIRLRLSGLTLDAHAMARAHLQEVEANATAAAGSAGASGGGGGG
GAQATGQGTAGQQAAEQAVIAASSPPPPPSPNPPPPSPPPPSPPPPSPPPPSPPPPSPAAAQDATQQQQAAAGAGGS
TTTTTTGAGAGTATGAGAARVEQAAWHEYDVSNLISTEISKTATDMHGDGVEDWSDFQLPRTQDDIRRLQGLPHVDE
CFYCKMCVPTTAMNLDTSQHRRRQQQHRRRGLLSEEELASEAYRANPAEAEGLIYGLPPLEGPWPTAGSSSSGSSSC
SGGGCGSSSSSGSSSALYRRRLQASGEDPAAVEAELGRELLGLGGEDEEGDEEGEEQEWSEEDMTAAAAAAAEALVG
VGRRGGIGAGGGGSSSSSSSSSSSSSSRRLRRQQREERAEQRRRRLLEEEEEEEEEEEKYNALDTTESGPDYHIGGR
VYNISDIAPGRRIFQAKQHLPNCTVCRGCDHTLEMMASGSVLLGSYFAHEPGGSPSWLFRVRTDRTQSGWAVVKVWC
IPVLKAKQRGLGMCDPKKVYAQVKLLLAQQKGEGEESAPGSEEEEEEEEEEEEEEEEEEEEEEEEEEEEEEVTSEDEE
EEEEGEGEGGNSGSSRGQSPVPDPRARSHAGAGGTGGPRQPVPRSAPHGRSVSVGGGGKAAASVAAARIAAKAAARR
RGAGGAVTGV

>Cre06.g264150.t1.3|PACid:27568101
SQGAIASDGRSNAGGNGHKQAGSVNSTTTCTSWNSAGASGTFAANATTNCTVDPLTTNTTLTNSTTTQTNSTAPTNS
TAVVPGMDGGASGSTNSTDAANATASTTNIASNSTGASNSTASNSTASNSTGASNSTGASNSTGASNSTGASNSTPS
ITSGSNSTTSAGSTGTGGSSTGVSGVANTTTSSTVGSGGSSGSGTGSDTTATSGGAANNATTSSGSGSGTGSTGNDG
TMGDTTTPESTTTGSSSSSSSSSSSSNTNSSNSVNHGGNSNGSGNGHGNNGNGGDGAAASSSSPPPPASVAPSQPQS
QQQQQAACARDGVTAASVSGDCGKHRVPVTFSSSSSSSSSSSAAAAPASSVTMCAEPAAFAADTGTLQLALCWRDSD
LPAWLLLPSGGSSNSSNAGATLRVTLPRLQRYGIASMAAGSYSLSVDGGGVEAVGGTVHAAAAAAAAAAGGAADVPF
GPLPGGSTADALAVPAGGVDALTLWFELPAVASAAASGAASSSVPATKSSSSSNGNGEGNGNSSGNNGNNGNGNGNG
SSGNNGNNGNGNGNSNGNGNSSGNGNGNGNGKVDNSTSGNSGNGNGQGNGNSGGNGNSSSSSGNSNGADSGNGTNST
GGNGNSGSSASELLLPARTISVTLTGPPNPPSPAVTCKCSPAASAYPGGACPAGTAPLTATLTSASSPSTESPSYAV
CVAAPSYDRVSRRLVFSACWRYACLPDAFARQPYTLTLGPLAAAAAGTPPAGHYRLHGSVPAGVDMSLQWLPANASS
ATASATAVTSADGGGAAAAAGWRSAAALGGAGLDEVAVAASAIAVVLHVPFDMGVDDSAGSLELKYLHSTCNFDGSN
STSNHSVVIEAADGGGAVMLPQLPAAAADSAANATLTVVSHTWVPRRVVRTDAAAGSSGGSSHTVAELEASVRSSLE
AAAGSGGGVSVSVSVQVSGYRVSIPIGLQDFSAVPDCGAESLALLRSLAAAASLPGGAALLGVSCAFAAVDASASAT
GSGARRRSLMATTTATGTSTASTCAPRISVPVTLDSSSTASAIPTTTSAPDLDTLLSASVAALSSSYSGSAGAVCPL
DPASPAIEVVSKLSVSVTMPAARMVAAAAAAGGDAVASACTSGPAALAASLGLGTGEAQVVGCELSAAVAGPITVVT
DPPSSSGSGPAGAAGSDVTAASPQPTGAAASSGHSGGSGGTSSVLVGVAVAAGVAAAVVAAAAAVLVTRRRQAARAA
AAENGEPLSGRSSKYDCRRPSLSGGGVEVFEPDSDDSAGQAMRDAALGAKRQRRVSYGGGGVMGGRGDSVNSGGGGG
FFGRVSKTVSAKNGMRGVSNSGAKTPSARPSLFGTDSPGAGLEAAFLSIVPDAYTIGTNDNSRTVSDVGPKSAPPTL

HHAAQVNTWTPTAMGGGCSAVAAAKSRTSRASRGSASGRGAGAGAGAGVLGSFTQGSQSWYPTPYDEEDDDDGEGPG
DYCSNGGKGAAGGRVTRASQSHTEGVSRRTARSSGALLGAVLCARVSYPGCTPDLDDDGDEGAHASEDEEMQSASAS
DSDNGSDPAAEAMEVEVQVGAVRASGLSLTGRFRRLQPNRHGTGSGSGRGAPRRARNAGGAGTRAHHSDGCAVYGGG
GGAGFHDFFGLEELVGAEDLAGVMCSLPTAPPASAAERLAARRSSASSSQTPLTAAAAAAAARRRSRTEHPAVVAGV
GCAGGAASGSSPFLRRSHNAGLLGSNPSHPDVEEVDDEGEGAMRAAGDPAERLFTRGSYGAQQGAAAGGPSRTASVA
SASAAGSSGSAASQRVWICAPPLPPAAATVAFILPGAGDAVQATTAAELAAAAAGCDKDRDLLRVQSRRPGFVDRSE
GGCLAAAVASSVCSAAAAASGFSHQGSGADTDGAAAAYRLEDLDDCGGGVSAASSGGATAASQRRHSALNAAARVEP
RRGGFTRARRHNLVAPYLGPGGHAPPAYIPHMPPGAAAVGGVVGGGSGAASIGNSFTAWSAPATAVNSPGAGGRLFA
DASAWAGVVGAGRRSSAGAAFAVASGGDNNDEDDSCALYGDDGRWEAHRRLAPLSGSGAAAGMPRCAAGLNLSRGPS
SRSRDRARDSRNSGPGDEGDDEAEAEAAAELGGAAVLAPRRPRRCSGGPDDAADGPVFNASASTAATVSAYRGAGPD
SRELDSGGKCTIRRAPFVLGSGALDDAEAAAATAAERERERHDVGGDGDGAMTPLVDVMIAEAGVADALVGVDRYHR
MQRLVGPHTRPPPPHARQPLVSHAALAAASGLVPATPAADAFASPAANWSSGASGGAAEADASLPEPLSSAALAAVA
ALEGAAVVLPCGTGAAAADEAPSAVVGGRAHFRLRRSNASSVLASRPPSPPPEEVLLPAGEVQPAVAAGAIAAFGDG
VVCAADFVLTERRPAPVPASRRKTWDDEEEDMGPSATRYLPALPPSPSPPPLLLAELPDASDRGSAGSCTMDAAAAE
AVAAAAAAAVGAAGGDPGATGATGAGLLASIHSADTAASMEEVAPAFELLRARQPNRHPHHSLAGLTHDGTSAASSA
ASSMNGRGGLVDAGVNANAEGGTARLAALRRLAVGPSRPAWGLVHAGVGPFKRSNANVHAAAASVNNRSAAAAAAAA
AALAAAGAQPLSPRSCALHEAEVARAAHAARGASAGAAAVLAAPGAVSDGGGFSPRGGGPRGPLPRLLSRNSTGGLS
IQASDASGPFIHLVRDVGVLSPRPSGAHRASLDM

>Cre06.g259950.t1.3|PACid:27568145
MAADGTPRRGSTIAEKIALFRSPSAAANAARPSQRDDEFWWQQQQQQQQQQQQKSAQRRPDAPGSQTSPTTREGSPT
RPRQPSPPVLASSDALYRRKDQGLVTTPGVAGHGNATSGLSAATAAALQRLAGEHRSLYNAIMSLERGPDGGLSDSA
MRMVESALARSRAAAAVTDAQVAAASNAAQAAAAAGGSSYGAGRAAGTSPPPGNGRGHTTTTFVAQPAPVSGRAVQA
MQRQSKQQQQEVLLQQVRMQQAQTHMHAQAQVAAQVQAQAQAQLQAQAAVRMQAQGQAQLQAQMQAQAQMQAQAQMQ
AQAQMQAQAQMQAQAQMQAQAQMQAQAQMQAQAQMQAQAQALAAAAGAARLRSSSGGSGVAAENSVESLLNRYRPSA
QARNVASTAPSAAGAIVSGSAPASKIPAPEPPPPAAAAAVPAAAPGPSSDAAAAASAAAAAGTNINNNSRSEDSRQR
DSSGGSGPDSRSRSRSRSGSPERRPGSAAADKLEKIKRLANIAARIAGSASGSGGEGPPSARGDGSGGGAAAEVAAA
ASAAAAAAADAAAAALAPPHAAAADAAADEASEASSPDGLVSEPSLSAFHAQTAAVIAASVESSVVFAPTQPPPPPP
APAPPAPAPPPPEPTPSALAPAAAAVAAGRRVQFSSGQPEVRLFSQGVIAGLSPTSSVNPAATPAAPGVAAPGNVGT
APAPGVTPAPASTDANVGQAAAATSAAGVAAQAAAGEAGAAAAGPSTSAAGISAAPQQQQQQQQQQQQLALAPPPVV
LLPSPIPPGSPGSPKPTHIFIPADPKLPPSLVAVPGFQPLPRSSVSPTRLRRSQSGSPGQGTSPPRRQGDSRQSSPA
KDGAAAAAASKPTAESKPDASAGAGSSEAAGSAAPAAGAAGTPAGAGRKSEIDRMLDSDSDDEVPGVKGAAAARVVN
EMEVEKNARAQAAAQMEREAASYSRNRLTRLLDEVANWKRVEGERLLREGERLKMLDELKKMNITQLGVLLGPDSAL
LKEITRLQDMYTGNLEAVKGQADQHTRAVLDALQAAGETALAVKEAVVEAAIKQQDDVLELVERVAPPVPPPPPLHS
PLLSLPNQTLYGGAPPASDLLSPTWWTSLLDPGRRQQVEADVTAALHGGGPDGAAAATLPAAGGVAAELLAHQVAGV
ASAIRGSLDTPGTREASVSAITQAARQVSAKLDMAVRDYLDRTSGVATAPGTGADVGAGGTSIPVSDATLAGGLASP
GFGLDPLRPGAMPATLAGMSLLPQLGGFSGGLYGAGTGGAMPAAAPGDPWIARPVQLPPVMLSDGTLLQQPLTAAAV
LQPPAASTLGSALPGTASMSGASLPHGTASVVALGTPAAPTAGTGSLLAGFPGLAPPAPPTLVAVQGALPAGGASGT
ALQNLPTCNPAYLAAPAASYAGQGVGAWTAAGQPMPGTAGGMAVANGGPMPGEGLPCRLSVHMTEVYPGSADLARSA
AQAAAAHHATTSVYRDAVGATAAAAAATAAAAAAASPRSDGSHGAVPAQELGDYERARRSSGGGRPMLSGRDGDGD
GGSVLDVAVSALTEDLLRLRGAAADAVADLAAAAAVTAAASPPPSPPRSGRSGRPPAASSGASRRLHFDTQSINSDR
RAQPGSSRVSGAGSAHGGGGGGGGGGGGGGGGSRRVSSSSRRADDPTEAAGSQDRRVAREEERVRGERSGRDRGHRD
HEGRPGRDRAREADEEAGQRSVRSSIAGSVAGSMGRRESDHHGRRQEQWEQQQQQQQQSGRARGGGEHKRPGYLDAG
SRRGDAASADGSEASLNAGLAELTFSPHRQPPAGAAGSAAAVRPQHAPPQHQQLAPAGQEHGAAANRLFPRPSTAPA
VTAAAAGAASYGPAGTELAAPPLVAQHPQSMPAPQPYQGYHPQSHPASPGAPAYPGASAGGLSVLPHSPSHGAHQTQ
HQHQPRSPGHPPPRHHHPVPPYSPPYGGGSAAVHAPAYAPPAGVSQPMSPYAPHAPLMQPPQQQQQQLQPQQLQPQQ
QVSGHYLYSAPQQFFQLPPTGANGASPPAMAPPGYAPAAAAGPGMQLMSPAGSVAPGAWPYGGGHMPAPHAPQHAP
PAPQLQLQAAPQQAASLSPYRQQQQQQPLSFPGMMPAFGSGGVGGGSSTVGAPAVALAAGSQGDAVQRAYQATLAQL
QAVYQGSAPPNL

>Cre06.g259950.t2.1|PACid:27568146
MAADGTPRRGSTIAEKIALFRSPSAAANAARPSQRDDEFWWQQQQQQQQQQQQKSAQRRPDAPGSQTSPTTREGSPT
RPRQPSPPVLASSDALYRRKDQGLVTTPGHGNATSGLSAATAAALQRLAGEHRSLYNAIMSLERGPDGGLSDSAMRM
VESALARSRAAAAVTDAQVAAASNAAQAAAAAGGSSYGAGRAAGTSPPPGNGRGHTTTTFVAQPAPVSGRAVQAMQR
QSKQQQQEVLLQQVRMQQAQTHMHAQAQVAAQVQAQAQAQLQAQAAVRMQAQGQAQLQAQMQAQAQMQAQAQMQAQA
QMQAQAQMQAQAQMQAQAQMQAQAQMQAQAQMQAQAQALAAAAGAARLRSSSGGSGVAAENSVESLLNRYRPSAQAR
NVASTAPSAAGAIVSGSAPASKIPAPEPPPPAAAAAVPAAAPGPSSDAAAAASAAAAAGTNINNNSRSEDSRQRDSS

GGSGPDSRSRSRSRSGSPERRPGSAAADKLEKIKRLANIAARIAGSASGSGGEGPPSARGDGSGGGAAAEVAAAASA
AAAAAADAAAAALAPPHAAAADAAADEASEASSPDGLVSEPSLSAFHAQTAAVIAASVESSVVFAPTQPPPPPPAPA
PPAPAPPPPEPTPSALAPAAAAVAAGRRVQFSSGQPEVRLFSQGVIAGLSPTSSVNPAATPAAPGVAAPGNVGTAPA
PGVTPAPASTDANVGQAAAATSAAGVAAQAAAGEAGAAAAGPSTSAAGISAAPQQQQQQQQQQQQLALAPPPVVLLP
SPIPPGSPGSPKPTHIFIPADPKLPPSLVAVPGFQPLPRSSVSPTRLRRSQSGSPGQGTSPPRRQGDSRQSSPAKDG
AAAAAASKPTAESKPDASAGAGSSEAAGSAAPAAGAAGTPAGAGRKSEIDRMLDSDSDDEVPGVKGAAAARVVNEME
VEKNARAQAAAQMEREAASYSRNRLTRLLDEVANWKRVEGERLLREGERLKMLDELKKMNITQLGVLLGPDSALLKE
ITRLQDMYTGNLEAVKGQADQHTRAVLDALQAAGETALAVKEAVVEAAIKQQDDVLELVERVAPPVPPPPPLHSPLL
SLPNQTLYGGAPPASDLLSPTWWTSLLDPGRRQQVEADVTAALHGGGPDGAAAATLPAAGGVAAELLAHQVAGVASA
IRGSLDTPGTREASVSAITQAARQVSAKLDMAVRDYLDRTSGVATAPGTGADVGAGGTSIPVSDATLAGGLASPGFG
LDPLRPGAMPATLAGMSLLPQLGGFSGGLYGAGTGGAMPAAAPGDPWIARPVQLPPVMLSDGTLLQQPLTAAAVLQP
PAASTLGSALPGTASMSGASLPHGTASVVALGTPAAPTAGTGSLLAGFPGLAPPAPPTLVAVQGALPAGGASGTALQ
NLPTCNPAYLAAPAASYAGQGVGAWTAAGQPMPGTAGGMAVANGGPMPGEGLPCRLSVHMTEVYPGSADLARSAAQA
AAAHHATTSVYRDAVGATAAAAAATAAAAAAASPRSDGSHGAVPAQELGDYERARRSSGGGRPMLSGRDGDGDGGS
VLDVAVSALTEDLLRLRGAAADAVADLAAAAAVTAAASPPPSPPRSGRSGRPPAASSGASRRLHFDTQSINSDRRAQ
PGSSRVSGAGSAHGGGGGGGGGGGGGGGGSRRVSSSSRRADDPTEAAGSQDRRVAREEERVRGERSGRDRGHRDHEG
RPGRDRAREADEEAGQRSVRSSIAGSVAGSMGRRESDHHGRRQEQWEQQQQQQQQSGRARGGGEHKRPGYLDAGSRR
GDAASADGSEASLNAGLAELTFSPHRQPPAGAAGSAAAVRPQHAPPQHQQLAPAGQEHGAAANRLFPRPSTAPAVTA
AAAGAASYGPAGTELAAPPLVAQHPQSMPAPQPYQGYHPQSHPASPGAPAYPGASAGGLSVLPHSPSHGAHQTQHQH
QPRSPGHPPPRHHHPVPPYSPPYGGGSAAVHAPAYAPPAGVSQPMSPYAPHAPLMQPPQQQQQQLQPQQLQPQQQVS
GHYLYSAPQQFFQLPPTGANGASPPAMAPPGYAPAAAAAGPGMQLMSPAGSVAPGAWPYGGGHMPAPHAPQHAPPAP
QLQLQAAPQQAASLSPYRQQQQQQPLSFPGMMPAFGSGGVGGGSSTVGAPAVALAAGSQGDAVQRAYQATLAQLQAV
YQGSAPPNL

>Cre06.g250700.t3.1|PACid:27568212
MIDRIKSKPKQSSLGVGAASASRTQLAASPELPGAEIEAAAADVKAATGAAAEEAGAEAAGLAAAAAAAAGTSPEVG
QLPLPSAAGDDDAAACEPAEPAGCDGTGCEAESPPPASPLPPLPIASRTQLAASPELPGAEIEAAAADVKAATGAAA
EEAGAEAAGLAAAAAAAAGTSPEVGQLPLPSAAGDDDAAACEPAEPAGCDGTGSEPESPPPASPLPPLPIASRTQLA
ASPELPGAEIEAAAADVKAATGAAAEEAGAEAAGLAAAAAAAAGTSPEVGQLPLPSAAGDDDAAACEPAEPAGCDGT
GCEAESPPPASPLPPLPIASRTQLAASPELPGAEIEAAAADVKAATGAAAEEAGAEAAGLAAAAAAAAGTSPEVGQL
PLPSAAGDDDAAACEPAEPAGCDGTGSEPESPPPASPLPPLPIASRTQLAASPELPGAEIEAAAADVKAATGAAAEE
AGAEAAGLAAAAAAAAGTSPEVGQLPLPSAAGDDDAAACEPAEPAGCDGTGSEPESPPPASPLPPLPIASRTQLAAS
PELPGAEIEAAAADVKAATGAAAEEAGAEAAGLAAAAAAAAGTSPEVGQLPLPSTAGDDDAAACEPAEPAGCDGTGC
EAESPPPASPLPPLPIASRTQLAASPELPGAEIEAAAADVKAATGAAAEEAGAEAAGLAAAAAAAAGTSPEVGQLPL
PSAAGDDDAAACEPAEPAGCDGTGCEAESPPPASPLPPLPIASRTQLAASPELPGAEIEAAAADVKAATGAAAEEAG
AEAAGLAAAAAAAAGTSPEVGQLPLPSAAGDDDAAACEPAEPAGCDGTGSEPESPPPASPLPPLPIASRTQLAASPE
LPGAEIEAAAADVKAATGAAAEEAGAEAAGLAAAAAAAAGTSPEVGQLPLPSTAGDDDAAACEPAEPAGCDGTGCEA
ESPPPASPLPPLPIASRTQLAASPELPGAEIEAAAADVKAATGAAAEEAGAEAAGLAAAAAAAAGTSPEVGQLPLPS
TAGDDDAAACEPAEPAGCVGTGSEPESPPPASPLPPLPSAAGGIRWGQQPAGTARQPLLTQRQSNYLWGLGYTVVDP
RMSVGEAASRIRFQLELRAAIMERYNFPPDAPVKPRPPCPEHLSQLQADPELCALLDRAQRPLLLQGQRLPVLAFSD
AARLARVSRQRPLDLGALGRALVAMDADMRARKDGPPSQEQLEELELLRQALKQKGKALPKVAEGTPATYGDAMVMI
AFVIPP

>Cre06.g250700.t2.1|PACid:27568213
MIDRIKSKPKQSSLGVGAASASRTQLAASPELPGAEIEAAAADVKAATGAAAEEAGAEAAGLAAAAAAAAGTSPEVG
QLPLPSAAGDDDAAACEPAEPAGCDGTGCEAESPPPASPLPPLPIASRTQLAASPELPGAEIEAAAADVKAATGAAA
EEAGAEAAGLAAAAAAAAGTSPEVGQLPLPSAAGDDDAAACEPAEPAGCDGTGSEPESPPPASPLPPLPIASRTQLA
ASPELPGAEIEAAAADVKAATGAAAEEAGAEAAGLAAAAAAAAGTSPEVGQLPLPSAAGDDDAAACEPAEPAGCDGT
GCEAESPPPASPLPPLPIASRTQLAASPELPGAEIEAAAADVKAATGAAAEEAGAEAAGLAAAAAAAAGTSPEVGQL
PLPSAAGDDDAAACEPAEPAGCDGTGSEPESPPPASPLPPLPIASRTQLAASPELPGAEIEAAAADVKAATGAAAEE
AGAEAAGLAAAAAAAAGTSPEVGQLPLPSAAGDDDAAACEPAEPAGCDGTGSEPESPPPASPLPPLPIASRTQLAAS
PELPGAEIEAAAADVKAATGAAAEEAGAEAAGLAAAAAAAAGTSPEVGQLPLPSTAGDDDAAACEPAEPAGCDGTGC
EAESPPPASPLPPLPIASRTQLAASPELPGAEIEAAAADVKAATGAAAEEAGAEAAGLAAAAAAAAGTSPEVGQLPL
PSAAGDDDAAACEPAEPAGCDGTGCEAESPPPASPLPPLPIASRTQLAASPELPGAEIEAAAADVKAATGAAAEEAG
AEAAGLAAAAAAAAGTSPEVGQLPLPSAAGDDDAAACEPAEPAGCDGTGSEPESPPPASPLPPLPIASRTQLAASPE
LPGAEIEAAAADVKAATGAAAEEAGAEAAGLAAAAAAAAGTSPEVGQLPLPSTAGDDDAAACEPAEPAGCDGTGCEA
ESPPPASPLPPLPIASRTQLAASPELPGAEIEAAAADVKAATGAAAEEAGAEAAGLAAAAAAAAGTSPEVGQLPLPS

TAGDDDAAACEPAEPAGCVGTGSEPESPPPASPLPPLPSAAGGIRWGQQPAGTARQPLLTQRQSNYLWGLGYTVVDP
RMSVGEAASRIRFQLELRAAIMERYNFPPDAPVKPRPPCPEHLSQLQADPELCALLDRAQRPLLLQGQRLPVLAFSD
AARLARVSRQRPLDLGALGRALVAMDADMRARKDGPPSQEQLEELELLRQALKQKGKALPKVAEGTPATYGDAMVMI
AFVIPP

>Cre06.g250700.t1.3|PACid:27568214
MIDRIKSKPKQSSLGVGAASASRTQLAASPELPGAEIEAAAADVKAATGAAAEEAGAEAAGLAAAAAAAAGTSPEVG
QLPLPSAAGDDDAAACEPAEPAGCDGTGCEAESPPPASPLPPLPIASRTQLAASPELPGAEIEAAAADVKAATGAAA
EEAGAEAAGLAAAAAAAAGTSPEVGQLPLPSAAGDDDAAACEPAEPAGCDGTGSEPESPPPASPLPPLPIASRTQLA
ASPELPGAEIEAAAADVKAATGAAAEEAGAEAAGLAAAAAAAAGTSPEVGQLPLPSAAGDDDAAACEPAEPAGCDGT
GCEAESPPPASPLPPLPIASRTQLAASPELPGAEIEAAAADVKAATGAAAEEAGAEAAGLAAAAAAAAGTSPEVGQL
PLPSAAGDDDAAACEPAEPAGCDGTGSEPESPPPASPLPPLPIASRTQLAASPELPGAEIEAAAADVKAATGAAAEE
AGAEAAGLAAAAAAAAGTSPEVGQLPLPSAAGDDDAAACEPAEPAGCDGTGSEPESPPPASPLPPLPIASRTQLAAS
PELPGAEIEAAAADVKAATGAAAEEAGAEAAGLAAAAAAAAGTSPEVGQLPLPSTAGDDDAAACEPAEPAGCDGTGC
EAESPPPASPLPPLPIASRTQLAASPELPGAEIEAAAADVKAATGAAAEEAGAEAAGLAAAAAAAAGTSPEVGQLPL
PSAAGDDDAAACEPAEPAGCDGTGCEAESPPPASPLPPLPIASRTQLAASPELPGAEIEAAAADVKAATGAAAEEAG
AEAAGLAAAAAAAAGTSPEVGQLPLPSAAGDDDAAACEPAEPAGCDGTGSEPESPPPASPLPPLPIASRTQLAASPE
LPGAEIEAAAADVKAATGAAAEEAGAEAAGLAAAAAAAAGTSPEVGQLPLPSTAGDDDAAACEPAEPAGCDGTGCEA
ESPPPASPLPPLPIASRTQLAASPELPGAEIEAAAADVKAATGAAAEEAGAEAAGLAAAAAAAAGTSPEVGQLPLPS
TAGDDDAAACEPAEPAGCVGTGSEPESPPPASPLPPLPSGGIRWGQQPAGTARQPLLTQRQSNYLWGLGYTVVDPRM
SVGEAASRIRFQLELRAAIMERYNFPPDAPVKPRPPCPEHLSQLQADPELCALLDRAQRPLLLQGQRLPVLAFSDAA
RLARVSRQRPLDLGALGRALVAMDADMRARKDGPPSQEQLEELELLRQALKQKGKALPKVAEGTPATYGDAMVMIAF
VIPP

>Cre06.g290800.t1.3|PACid:27568239
MSRGVLAEKWRAAKERGSLSTVLDLSLSDTTTYRFGRLVITSVRLHDVGLVGAKEPAPAASEPASDTEASSIVSELA
SLGLPTAFGKKGGGPRDAAKRGGAGTSSRFAAGERRPRGANDSNGGGRGPQGGSTGGEDETEGSARAKDTPWVQSFD
ERWGCHYYHNSLTQESVWEEPAEGYVPWTGHTQPPQQRHGDTAAAATSSAPKAAAAGGSRPAPRMLTEEELLKSMGL
WQGGSSGAGGATGAEAGGSSATAQPAGDTGGSLLRGLIGGGGAKGAALGAAPSAASASAHLPQRVVPATAMSEEQLL
ARMAAAASMPAPPPAPLPPAPSPLPPPPPLPIPPPPSPPPPPAPEAAVDAAPPSAQQRPQKPPGRMLTEEDILRSMG
LLEGGGRTGTEAAAGASNAGPAAAQDSEARAAAGQSSRSAAPPPGFGGHAAAGPGAAPPQSGPGHAYPHTQGPPHGG
PYGPPGSHGPHGHPPPPHHGHPPAMAGPPPPGFHPHGPPGMPRPPPPGFGPQHPMPPPPGMPHPANGGWPPHGVPMP
PPFHGPGMPPPGMPMPPGAPPGFGPPPPPYGMHGPPYGPPYGHALPPGYGLPMGPPGFGGPPPPPGFQPPHPHGPPH
PHNQHLHHGPPHPHSHGPFPHGPHPGQHPPPLDLPHGHPHGHGLGHGHPHGRPPLPSPPPPPTPAGGDPAGQASANG
NQQRQQPDGTGQPQSHPQSHPQQQPAGGSGDSGRRGAMWTLEELERQLEMLAVEGEVEDGAAGGGAHTHRGGRGRQH
GDDSAGGAGAAGGRRSRGGNAGSGGARSSLHGQAAARNQQQQQQQQARQQKQQPHPGAAVRHGKRGGAGSGVNTEGG
EDTENDDAAFYDSFAAPARRPAPVTNMPAVGDDDGTRRITLDPNTAAYVRTLLPRSAAKYWMQRYSLFSRFDEGVQL
DTEGWWSVTPEVLASHQALRSRDLAAGGRGRVALDACCGCGGNVIQMAAGVFPVVYGVEISERRVEMARHNAAVYEV
SPRCQFVCADFFQVAPNLKVDVLFMSPPWGGPKYQHVNTFDVFYPLVGFNQSLVRLLDVAMDCVRDQDGVVAAFLPR
NTDLSQLAAVVPEAAVWEVERAYVNNKLKGITLYCHPGRGKRGSGGEPNVRAKAEVEHAAAVEALHAAGAVEAAAAA
AEAVGGASALEPGSGSGRESSSGRRRGGGGGGGRGKGAGGGAGDAGAATSVLLGGELNDAVMLQELERALAGL

>Cre06.g277800.t1.3|PACid:27568299
MSTAKVPKHAFRARDLPWAELVRELEFISTLKRKAARLHWFLSTRIVRQPSPPGYPRAALAHDLHGLLRLLLPRLDV
ARGAFGVKEKRLASLLVLALGASERDARAVKGWERRACELRMRFPDMMQQLLLDKYCAESLPPDLAGMTLGELNAQL
DALAAVAARAEASAVRPNTLVAGAAAAAAAAAATAPPPATAAAGAATAGCASVASGGLEADAAAAGTTAGSAGAGAG
VGVVGAGVVGAAGGKKAVAAAAVVVDERARLRALCGPEATAQQAALLRWLLRRCTPAAGRWLLCVLLRGEMPVGLSE
RSLLGCLHRDAWEAFSSCCSLEEVAASLGPAGAVYKRTAVQPGLCVSPQLAAKPPPGGLEEVVRRHSGRPFRVDTKF
DGWRIQLHVVDPKPKPAAAGAAGSAAGAAGAAPAPVVHYYSRRFLDHGPRSGFNVLDGAVLSFLRPPPPGPAAPPGS
LPGPQGAAAGVDVDGGAGRQGMEGKGEEAEAVAAARPPQVVLDGEMLVWNKDRRAWEPFGCLAAAVHAAAAGWAPEE
LVKGWEVMVPPAAAQQQQQPPAPPCVWQLEVVYVVYDILYCSDTSTLHMPLQERQELLAQVVAAQAPGGGVRGGVPV
GRVGAVTFRMEALMPGRPLQQDGRLHSWLCSNVEQLRRLHDQQTREQREEGVVIKSLTAPYRPAARSESWVKLKPDY
LTLGEFDCVVLGWRAGTAGGDRTSFGSWLLGLPDPQPQPQPQPVDTTTTTTPTTPTTSTAAAAASSPPPPLQFVTFA
VVSALVREERREVLEARLLSSGLMAPPAEVAAAVGRSNSGNSSGGGGSGGGGGGSGGGGGGKSGNGGGGAGSGGGGS
GRLSSRAERLARGCPAWLRATGDPEEWPDLMVTDPHKSIVVQVNADVRLHASHVFAAGCTLRFPRITRLREDRSPAS
ATSLAELRNRLAAAAAAGAAAAAAAAGVAGSAGGDSEGLGDGDGEDGDTADEKEGERRRAADAAGAAAAAAAQTGRKR
RRLTVPDAVVDEEEAEAAGRHRPHQPGGMVVDGPPPPAAWTPPATAPPPRPAAGGRGRRRRAQQQQQQQQQQQQQGS

LPGGGRSRCGGAPAAVPLALRPPAGQGEQPTHFDLLHGAWVCVEGVYGTAAAVRRRRAELGALVGLLGGRLLATYTG
RGSCAASYVVLDGWDPQRAAADTRRRLLLRPAWLEDCYNERRLILPPRPAHAWRLDLTPTTTSATAASATTAAASAS
ASAVDMYGVPLYERLDDEDVRMLLERHVLATPAPLLLQAPPPAPPPPLHHHQQRRQHQHQHPSEKPRLPYRRATVA
PPAAVAAAGNGGGGGRVESYAGLKQRLAVAAALGPRQLFTLRQQQQQLRRCPDGIGDGIGAVTGGGSGLRDCVTMR
DVVVWVQRQLEAGGAPVGAPPPPPPVEVEEEEEAAEEAGGDGGGRKCGGGGGGGGEWWYGSGLGRVFAGAHVLLLDV
SQQQLAAAEAEAKADLNVEVEAAAGEGQQPAPAPVTSAAAAPQLNPPLELMAGVGAVMGLVGKWCTPAGPGEAAATA
SAAAAGAATAGAATAAAAAAGAPGAADAASAAAPSWCCWRPEAAAAAAAAAAAAMPAGAAAADAALQRLRLEVVRGGG
RLAGGWSEGVSHVVLFDGREHPPAVSGGSGGGGSGGGGSGGGNSGGGSREASDRLGLVPGRVVRGAPPLVAAEAVVA
ALRSMCSGQQWERAAAAAAQMYREAAAAAAAAAAAAGVAPAAASAITTTAAAALTAANAGAGAGAGAGAAATVAAGHQ
HQLQHLEDHSPRPQPQCRQRCREVRRGGAPGAAPVSPPPPPPPPPSPPQPQSPPPPPPAAVASSLSVIRRQAAGPSS
RAGPEPSELARLLAWLGVGMGGGGGGAGKRMRTDADADVDTRNAGRVGGGGGGGGGGHCCVGTARLPLPAGGVRVVD
WRWVPDSVQRCEQLQLQWHRHAVAAPAAVPHRRTPAAAAAPAAGGGTSGLLLRTCLRGGGEGGSSGTPSAAAGGASG
PAAPAPAAVGRDGGSGSGGWESECDWGSSAGSEAPAGSEAPVGGCNTTEEQADEEEEEEEEEEGEEREEGEEREGTA
QGGTLEARRQRRRRRRRLHVVAARPHALSGFSGARLELSPLPEDWYPPRIAPPKQPPPPPPQLPSASASHPPPPPQS
PPAAGGAGTAEAARLPASHPAAAAAAATAATPVDVSVVMASAAAAAGKADPWLLLAERLREAPPPPRGSGAAAAAAA
GNQARARDRDRGRGRAGECAAAVPTDTAAARAAAARAAVGDTAVGAAAGADGDGGDVVCALWRRLLHGSSASGADEE
EEEKEEEGAGVTQQPAKAGADVDQPGDACDACGACGASTGGVDTVEGSTSRRLQPLQPLPLLPSEGSDRDRPPSQLL
LTPPQRPRSDHQLQHHNDPQPEQLKPVEHEQQPQPQQQPPQQQPQQPQPQPQGRRLVEGLPTPACDHPAATTAQASS
DASPKPAGCSKGGASTATATATAAGMDAAGLGMPPAPAPPTAATDGGPSTPAPRSTTVLRGASSSQSSSALDHLDRL
PPPPALPLPLTAAALPLPLSLAPLPLPRLPALALLDGQVDGAAAVAVGTMPATEPAAVAATLESGAASAAAGVKSGT
AAADGEVAAAAAALPMAAASSRGCCVEAGGAPAGRPSDRRHHHQQRRRRRPATPPLRLMLQLLMSEPQLADDSPPPL
AVPALPETRAQTRQPDADGDGNGAAGTATASRAAAVAPLAAGSDAADVVAGTDAGAGAGAAEVCVGGASGADKEEER
AGGCRPDDECASSADRERERCSASAANVQQNTASEDPAGPKQAVQDLESREHSAEAAREAGRKRSRSRGGDSEERRD
AARRRKAARRRDSRGRLLLTRLAMVGLDGGSDDGGDGGLDGVKD

>Cre06.g307350.t1.3|PACid:27568417
MEAAGWLRDYVTAIKHGRVHPFTAVERKVRAATRNEPWGPTGTMLNELAELSYGMDQVQIMFAVLDYRIRSPEHKWR
CTYKALQVLEFLLKRGSPACLTAAAELVVPLAALANFQYVGPDGRDYGLNVRLRAEAVKAAILDSEGLAQQRAALAA
RADAMRFAGYSRADMAATAGAGAPGAAAAAGPGGQLQLPGAAGLPGAAEAAAGGGYGSAPLGPTAGSQAAAGGYRPP
NALGTTQPAGGAAGLRNAGETKGVTFEQNKRQLEQLRQLTMAEGNRTCADCASAAAAARPTWASINLGVFICMKCAG
IHRGLGVHVSKVRSTTLDTWLPEQVATMARLGNRRANAHFEAGLDPAMRPQRDNALELEKFIRLKYADKVWAAHGTW
PPPPEDGPTAAAAAAAPGSTQPGADGQRYPAVAASAGGGFGDAPPAWGSLGMSPPGTAQPAAAAAAEHAHTNGVAHS
QQQQQPRRLLVPPPPGSGQKQHQQRHQQQQQQQAAEQQLLLMALDSDTEEDGDESGEGSGGGAGGATAGGGRRGGSG
GRDRAGGAGGPQSASTSAASAGGNAALWELLDPTLDVMTWLDRVEGGGAAAGGAGGAGGGWGAAAAGGGAAAAAAGA
AAGPAGVGPGAYGAAPSPLPQQQQQRTAMASPAPAPAHAGAATPLFDFGSSAFPPPGSAAPAAAQPAPLAPAPAPRP
SPVPAPAFAPVPTAAAHARPPATAAAPPPQRTGASLGSASSGVIHTSEFPVYDLLTPNPTGGGFGPPSAGGGVAGAA
QRATPHFASPPPHYPSHHFQQAQQQGGAGGGTAPGMSAISSPPPPQAIGYAAPLHAPQPLQQRPAHNHHFPSNGSAG
GNAGGAFVAGGNPGAASAAVTSVVAGSGAGAAGGFPLAAGVWPPAGPQGPGAVAGRGGVGMRAMAAGNAGGGGMAAA
GKPAAAPAGATAGTAATAAAATAAAAPAGPPVNLDSLLAMQLDGLGSSLAGGGGAAGRQANGAAQAPWTVSPAGVRG
RH

>g6870.t1|PACid:27568439
MSDAIAAAAAAATSSQQQQQQKTAAPTSNAVIHSAASKPSCIGARSWCHLPESCTYCGGGDSEAEGRRLLVLCSCCF
AAGAHVGCYEDVNGAALPREVTHGDADYFCTPECQHVHDTLKAASHGSSSSSGGGGGGGGGRGCRIRDEPEMFTFEL
LPCAEYRHGDRRLCREIDQALRLFKSSFSPLLMDNGRDLLDMVCTGWETPDEQLTETEPGHNFSGFHLAVLRQRGAV
VTAATLRVFGRRFAELPFVATREGYRRAGNCRRLVKAVEDLLLSAGVGQLVMPSIKPLLPMWAAKFGFTPLTEQEVA
AIEDWVVDTDRETSTLVRKPVLPPPQRPAQLAAAAQAAVAAGIGAKKRGARSGAGAPAPAAVAAGGGVAGGGGGGAS
GGRLVDLRRRRRPVKIVRDEDLSSDGDSSSSEESGSGSGSDGRHGGRRSRKAAGRGGGGGGGGRRRRRARSAGSGSD
ESDFRPEAEGGGGGGGGSDEEDESEDAADLEPEAEVLGGRRAAAAAAAAAAAAPAIVKGRKKAAVAAAAPAAAAGPTA
NGSGGGTTAAAAAAPAAPKRKRARRTPAPRPAAAAGPPPVQQRYDPANELVHPIPGLYTHLEMNTVLLPELRPLPPP
LPPDVEAARPPLSDTFELTAAIQSTVVRKVERAVERSARTGTPLLTLPPGLLPPCAIHCAAAMGRAPPAALEALAAA
EAAAAAEKAAAAAKEAPAAAAVQQPQQQQPPKAPKPPKGPKPPRPRYPKQPHLLQGQEQAAQLAAEDARLRHSDGGG
SGGGAGGPPAARTPEQEEARRRRQRLRLAAARRRQSYRELMMGFVLADTLQRLLTSEPQLLLGPELPSLGPRLGPHT
STPMPASHAPQQKPSRSLWLPQQQQQPQPQPQSQQQPQPQPQPQSQQPAAAAVSALARAPALEPLPVPSGPRASPSP
AASAATATPAGTATAISAAAAELVRLRSENEALQRRLAEAEAALQSSRQQQPQALVLQPPQPPLLQPSLRQGQKRAY
EQQQQQQQQQQQQQQQQQQQQSPRSPAPAISPLPFLLQPSPLYAEAGATACGGGSGGSSTPAATATTPLPTGDIVAL
AVRAQRHASSVAGFGTGGCGRDAGGVGGGDVVDGIHGAGSSGGGGDSDGDGGADTCACDEPVTYPDDDYGSTAVEEV

DDGDGPYTTAGADPRAAAATAAMAAAAADGMWDVAQATAAEGRVNSIAAAAPAAFGFWAARQAAEEVAGAVAAAEAL
PAAAAPGLLQRLPVLQLGAPALPLPPPVNASAAIAAFEVMAGGAAAACGHTNEAPTTETGTEPAAAGAAAVPVRYVH
SAFQQPQPQLQPQLQPPVSQPPAAALPPLHTFGSPLELGLFPPSQPSQQQLSQPLSQQLSQPPSQAQPQPEAAALAT
LTAAPLVSMETAEGALRQPRSLPGSPLPLPQQQANSHTAATPAAAAAAAAATVVPHTDSVAGGGDTTPVRAAGARGA
DSITPGGSAGGAAAAAAAAAAAAAGTATAAADYCDDEGARSPPFSLLAEVARLAAAMGRRDGSSTGVPGTGVGVGQDQ
QQEMASISSPLTPPAGRSPPSPRPALLGSPAGGGGGGGGHGVCHRSAADTPHPVAVAGAGSSVGAGGGGSGSRSKVR
ATRRRIVMLASSSPGSSGGGSGSGGSGSGGSTPRGWGSIDGDLAHAAATDGVESAGEASDAAAGACDDIPAGGSSYY
GGGGGGGGSSSSCGGGLQMEMEWSRMDMEAAEALALGPLLAAAAAAQKEQQQREKQQQQLQQQLLQPAFYGRAAMAP
LWRSLAAGGSAGVADSVPRPPRAPPSPSPSPSPPPTGSLFAAAAAAAAAAAAAAAPSATAAPSPATAAAASSPATAA
IAAAQTVAGTAASTPAAATGTTLTTTTTTGAAGRISGSPFASPTSLLRFERACLQPLLPCVVRVLLEDSPPTSAPRP
RPRASTQKEEEAEAGAGAGAEAEAGAGAEAGRGSPPSSCKRRRLALEAESEGLQQAAAAVTDMEVGTEAEAEAKAGR
AAAAAAAQPSVAAAILADGDKGCGYPAGAAAAGAAAAGAACAAGAACVPEACLEGTAGTEAAVPAAALGGGSPAAPA
AAATHALLGHQQQQQQQQQQQKVAVVVVPPGAGTHVHASPPPASGQDLIAAAGLARSGSALCSIGDTSNSRTAAAKA
PALSPAPTPDPSSSSGGNGGTAGRKRRLPQRFADPVGLEGATSVWGGGSGDDTVAVATASPGQDDQAAVPPPPLLPP
VPQPDPAVATAAAATAVAIAVATAALPAAVLHTSGSSAGGEGVAADAAAAATPLPPLLAAAGGVYMSPIAFAAAAAP
ATSPAPRSAAIPAAVAAPPVAPKRARLVQLGALLQPPLSPLPSSTAAAAAGGAAGVSPAPAATPTAAATAVAAGAAA
VVPAVVEADPYAYQPDEPVRLAAQEQASAAAADANASAPASTAAAAAAPAVATRPPPAVARPQQRLAKRQKAAASLF
GEEVVLTSATSTLADGSGSGSGDSSSSPGASPLASQGGGKASAAGPGVGVGGGEGGGAGGATTPHSQLRAAAAAAAA
AAVAEAQEADGGSPGSSDGAAGTDAVTAALVAAVAGADVAAGAVPPPVPPVTQTVPGSSAPTAPPPPVPVQRAPASS
TASPQPAAAPAATAALTGSGSGTGTGTGSLFMPLASPGPRGRPVASAAAAAAAAGGGAAQRRTPQPSQSQSQSQSQPQ
SQPQSQQPLPSLTSPPSSLSAGVAVSRRGAAGAGGGAAASQPQPALGPMRLSPSSLRPLPNTQSGASLQAPGNGSGV
CGGGGGGGSGRPAFPSPRRPQFSAASGGAASGAAGGRGRGGGAIAVAAAGPVVINKSKLLSSIIAGDEDEDDEDADLD
EGVLSIMRAIDRDLAQDRAAAAGAGGGGSGGGGGGRLGDELGGLGALLGEMSGGLGLLTAAARGPMTGRGGVASVSG
AAPVGASSGWGGSAASRRGVVAAGRLAHAGPGPSGDRRMAASAGAAAAAAATTAAATAAAAAAAARGVNALCDRLCV
DDGMDEDDE

>Cre06.g279200.t1.3|PACid:27568462
MGRLVPKQPAKPPAVPIPGFESDIHKIVDEFTLKVQEGAPLNLSTFKEIWRSRGFSFVHQAVPLNVPPWDFLQLLYG
AALNRLADPSPFLAYQPDPAELLQQRERRQKLIQEHQARLAQEQNKPAAVPRPPRVLRRGPDGRFVMKEAGAEQEQ
EQEQGQEQLEQAPGAEALRQLLLEQFPSLEAALASAQGTMVDAGLFSGAVAGAPPGLGSGAFGLEAGAGAGMAATAF
PPGLADGARMLAAAKASLAAAAAGGHGQGLGQVHGHGQAAVDPHQQEFLDQLDEFIGHMQVVEAGQQALAAAAGQQG
PLAGPSRGAATSGLGHGGQDQQHPQQQQQPQPAAAAAAGDDSGLDLIGSGFGSGFGSGFGSGFGLGSGLHELSRQA
SGRPGNGAERPPEQPQGTGLVAMALVAAAGGMSVAQEDDYDALEGEAAPIPPVGGMASGAGAAGSEIAPYGAEVTAA
QGPGPVGPEAQAGVAAAAPTMPFALHDTPVGFSPADSLPSALGSPTRWGALLGAPAGTERAGASSLAAVAGGSLLGS
PLLRGAMSPLMRGLGDGGGAGGGEAGGLAMPSPMRGPLPSPLRDLGFSPLQSPGMTPIRSGQALGGGDGSGALPQLP
PLPSLMQLPPPAVPLEPAPTGAAAPGPLAVAPWVGASGQPQQQQQQEPAMVDAVLPALRELAEVHAGRAAGALHAP
AQPLALPAPQALQPGLPTAPVHGLQPPHGGDGAPLALTLHAPGDGAGPLGPGPQAGAAPPLGPPPLLSMAPSVSPPP
HTTNGVAQQPVTVPGRAAASHSPGMLAAAEVVRDVAGTAGESERGPSPPMPGAGPLPALARVPSAVMAPGPAPGPVS
PPLPRQLSMAPGPSPPPTLASGPSPPPGASSQTPLLPQPAQPAAAQGPLPHPSMAPGPSPPALPLPGPPPGGSSRGP
SPTHAMAPGPSPPPPAIMALGPSPPLPAGPLIANPLRLNGPAAFEAAYAAAAASGILPAFGAGQGVEAGGGGAVGVM
LDPQSVASVAAAAAAAAAAAMDAAPTAAGTGATGSGLSGLTLGSAAQAAGAAGALALAGQAGPLTAPLALHAPGATG
SGAAVAAGPGPQTQAAPATIVELPPPAPLPVALPPPGPDVPLAARVGAVYAVYCLYGTQLCSPRVRVYVPLPLLGSL
AATVREAADKGVKDAVAAMRAMWRQGVLVPGGVLRYPGATTPRPRRAPPAPPPPRELTPAQRAARAAARAANPVPEH
ERLRPGAPFQSVLVSNPVLRQALLHLRMALPGVTDTAALQRLCDTYEQQREAIFAEPERRRGGRAAGMGVAAGAASG
AGRSAAGAAAAPATGGAEAGGAGGVRDRRQRRHVPEVLFEASIGRQVADALQTSFTRVIELLRQPPLSRRKAEEKKR
RKEELQAAIAEEEARAAAEAAAAPRTVADAVAEVAARPAGVTGRRVAGELGALMRQRAGGPTSGSGPTGPSYTVRVI
RHRVELQSAGLLPDMAEAGAAAGRAGQAHEEDEDVAVAARQLHLPGVPARLLQKEAERRQRVASVADRWLSEQDRQM
RREAGDVSSAAGAGSAARGDRPGSQPGSSGRPAGTASASGATAKASARPARGRGSSRANAAARTARAESEPEDDLAD
VNVDIDLDLEAALEAEFSRIEAHQQAGTSARTVPADAAANIAATGQPRQQKAKPAAAKPYGTAPATAKAQPGPRAAA
AADAASGAGGTKSPKPPQRLLHEAYPGLDVVCGGTRGRLDTRARTVTCACTACEHAGASSGAVVDVAEWPGHLAAGE
GRKNWQTAVQVLVQAEEGGGAGDGGGGGKDAVRRPTLAAFLRRNGLLLGRRVAAAANAAAAAGGTVAKGKRRQEAAS
TAGAKKQRRRAGSPGSESDWESDGGGSSNEEDGGTGVGVPATATRTLPPRQRRASAAARTADGVMEDVNDASDSETQ
LPGGSSRGLGQGRGLGRGRGRGGGRGRGAAADGAIPGPSAAGGGPGAGRLAVNADEESDEELAELLRSTQEAMAAAQ
RVTAGLVDGGGGGGVASAAAVAAAVGGAAGAEAARKRQTAAAARPQLGRGGGDRSETSGRGRGRSRGRGAAAAGGAA
AGDDAAPAAKRGRKASNSPAAPTGVEGAVPAASGAGRKRRVSEAPLAADARISDGTPSAAPMPAAKRQRVGMAAVSA
GARAPVSGSALPPRPPLVPGRAPAVSPAAAAATQAPVPTRQAPVAQGLGSTVVMRQDLDEDAEVVVSDVEEGDDDS
GAEDAPGTGGRVNGTAASANAARAPGVGAVSGAAPDDEDEDDDLEDFNRFFAAVKDLQ

>Cre06.g265700.t1.2|PACid:27568530
MTLRIRAKRCNTWALVVCVLAVSVAAREGVTAPQQQQLPAWTHACRVVIRGSSPDYTSSWELVEASLECWAAQQVDA
GAVTATAATPPLQVAVGRRLLPYLNLQQQQQQGPQLNASSTDSDTAAGSTITGSSGAGGGYVVVSVRSSAPPPPPPA
TPPPPPSPPSPPPHPSGDAEPPAAGHAFSGPTIPNPDLSDDWGITFLGPMGGTLRIVDSILTNVPLSPVGPLLYCAN
CSYVSLENVTFSRLLGYSLPADQPFDMAWQMYVVRLARAPYPVYGAFAATGLRGAEVVGSRCANVRRGHGWSCLLLQ
YEGAPDSGIVPSSAAVPSAADAAEGNSSDSSSGGSNGGRSASLIAAAAAAPYMLLLTDTTIFNNSISAGGVPGTEVG
FGAVVIAGPGAGPLVALDEATAAWAAAAEPDGPVAAYAAAASVAAAALSAAFNAQDSDSTGVLEAKRLVAMGAVMRN
STVHVVARGCVLSHNMGAHGAALAAVLGANLGNVSLLEDCEVASNTAYLSGGWLFATGSVGALSIGPNVTVTANSAS
TAGGAVYVQGNITGAFRVGGGSRVTGNSVLRYGGFLTVNGSMGSLELAEASAVDYNTAGRTYDDGSYIGGKGGVGYV
RGGIGAVLLAGGSSMSYNVVSPGTDWSSGGGGGALAVQGPVGTLALLDGSSMVNNSASNAGGAIYIWSGLDRLLVSG
GSRLSDNACVDDSGGAVAVEGPLRSAVLEGGSAMNGNSGWQGGVRRCRVAVVVVAVVVVVVVVVALWVGGPMQELVV
RGRSSMSGNRAATTSGGAALVSGDIVAVRVEGGSRVCGNRAGTAGGALRARRIQTLSVTDSELSGNTALGTVAPAVV
GWSFSGSGGAVYASGGVHDLSLLRSNVSHNRGVSGAGGFLFQGAENAVETGAASLPAGSIDPSPGVHWLESSAVVGN
EAGLGGGAMWYSRLPLQVVVTGGSRLEGNVAHGGSGGALWAGWEPGAMSQPRPTELSSDTPLCRQGARITVNGSSTL
TANRATMDGAAICVSLDIFAAYGNCTKTGNSSLTLELYGGAAFDSNSAAGGGGAIFVLQPYNFQAARMDVRINASGV
RFANNSAGADAIGLLRGSGLGLGDGSSSLASSAVGGSRRVSAFGGHGGAVLVWSERRGTLVEAVAVPVSSTGSSSTG
SSNTSSHAAGGGGDRGGGSSGAGVGPAALAEAIAAEFGGGSLAQQQPQPRAGGSCLVTMRGVELVGNQGTGGSGGAL
LLGACPTSISGSSLQGNSADVSGGAVALWDQTLESVARAGGSSSSSSSSNSGGGSSLQQPQALLLPVLSLINCSFA
GNTALGKDGGGALFVDVSSAGHSAVMSNCSLTGNRAVLGPGGAIAAVLRGVDLGPADSISSEGNSSGGSGGAGMQAA
VAAVEVRGCAMLGNLAGGVGGGAMYARLYGGSGVWVTDSRMVGNAATSSAVGSSNSSGGGGSSSGGALLLDGRRLGG
GSRSGSNGSSSDGWILARVQSCELSRNRGPDGGALAAFLDSDAPATAASAAPAASGATAPAAPAAAPTAWLLVRDSV
LSENSAMGSEGAQPHQALQQALLTLPAAAATVAATAAAAAAASSSPDTAEGRHSYGGCGGAVLVQTRSAAATAALAE
LASTSPSAPSARVLGPPPAVFSMQNCRLMSNAAASWGGAVFVPPGSSATLLNSTFESNTAGASGGAVAGYACGSLVL
SDCRVTGGGAVRAGGGLSAAACAAVRVERTELIRNNAAGGGTAAAAAAGAAGGGTGGTTEAAGLGGAVWLVGVAETQ
LLGCRIEGGGAQVAGGGVYATAVAAASVGAAPTTLVVADSGITNNTAAQGGGLHLGAGLRAVLLRSQVLGNAAVAPQ
TATAATAATSGSGSGSSSGSSSLAAADTAEAMSDSSPYAGYGGGMFVERGAAVLLSGCSFWRPNATAAVAAGGGASN
GAAAGAAIATLQSRGGDIGGQVAVASRAQVLDGLVAALTAASAGMVAGGTVPSAAATAAGAAGPTPAPAWTTTPSTS
LLTALGLGGAACLGGSTGGDAAAAAAASAWAAAGSSLVLLSTALPPASTQHNTSQVTALWAEDATLSAIYARCGNGM
TTSAGSPSADPSAGSKPSSTSARAAALADLSGCVGWDTDGAAARAGGNGSGSGAGSGTESWEDQQQLQLDGAVRSGA
AVLLPPSLLGLVQFCTVDERSGHQERCVVGGTGAAAVAAAGALYFTQQQQSPPTPAPTPALALLAAGVGLQQHLEMQ
PERPFTLAVQLYDDLGQPWTADWPPMRVGLAVLPVPPAPANASAPSQQSSAAHGGADSNGTLLTPAAQLAAACAAAA
ADATAAAAAAAGGGVNATGGARVAPPLPLWCEPKAVSLATLDGSSAVTAGTELLVEVRSGAASWPWLLVRGWPGRYV
LTISTNGAYAVAPLALALTLRRCAAGQTLDTSEAAAAAALGLDLDLMAAPTWTACRSCDMFQVGLWSDDRPELSALD
GPDQLAAVTAAAQDAGACLNCPAHAVCPSGPVLLPAPGYWHSSPNSPLVLRCPNSDACTYDDGNGLGDGGGGSGGSG
SALSSSSTSAAAAGRRRLLQLGTATGSSNGSSNSIGAWDVAGAVARMGFNDSRSLRLALCQQLWYSTNPPGQAPELL
QLLQPAALSTTPPQQPPLQPPALQQPPSSPSPSASSPAPQPQPQPLSQAQALQACVLWGLDPGSPLSYMSAQCAPGY
AGHLCATCQPGFALSLDFQCSACPTTAQTVGLGLLAFAASVALVLYTAYTNSGEPSGDGDDKGGGSGNGGSAGGGSP
PEIGDVIKVVVVHMQMLIIITRLPIDYPNAVLKFRGALNAVTGTASYVAFSPSCLAPDQSPAGQARAGLLGALAVPL
LVAAFALGLWALRYQFFSQARMKRSSALPRWRLTPHASGGSTPNTATPRQQSRGADGTQPQGPQGQQQQGQQHGTGS
GRQRTNSGGLLPFRLSLGRWWLGGGGGSTHTPFNFSFRTVQEGEGQDDDVHEQGQGQGQGAVADEECGKVYGSGGGA
AHDGGGSGGGGDEEGLTGDRDTGRTGGADVGTTAAGRQQQHGQEQQLQQLLMGKEKLLRGGSGSGGGDGDGSPLRDE
GPPGDVEQQGGGGRRGADSTTLLEAAAGAAAVQETRRRHRVSWAGSGNPTALLSSSQPHGTASLDAQSSGRTQHRTS
FNGSGAAGGAQGSLPPLLPSTSSASVSDAFPTRPSPSMLLPAAPRSHSGHAGPAVEGHGGVPLRAHGVDSNGSGSGS
GSGTSACANPILRSALRSASASREAATAVAPAMAAGAPPPVTPPAAAHSPASTAGSPPPRSSSCHAPSVASSATGGG
SGNGSPSTRPLLPPTGGPGNAAASSGDAGSSQQQQKKMLKSSATGMLGADRSSGGRGAAGSGAGSPLHALADEAGGG
VAGGGVGIMGPPGTQRSSWASGWSSHKSILRTAATSSSGIGQQQQAGSGTSSSNDPSSSGALKVPLAAASSPSASSA
AHRNPTPAASRQQSYIRHGSRGSCASGASGGGGQAHRHSGKASGGGGGGGGGSGQSSGRYGDGRSSGGSQQRYSHGH
LSGLSSRLLRSVSTAVRRLPAPLVKQLGSLHSVGSHLQHYDQALSLPRQLGVVLLAGVFVLYPGLVSASLSVFACRI
LDTPGAEYGGVYDSNLAATWRYGYWIRDMGQQCYSGTHAALYVPVGVVAVLLFCIGPPAASFVIVWRVRDRLNDHHT
RRTYGFLYSRYRSTRWWWESVLQVETLSLVAVDVFARALTVTQQALLLLAALTLILTANMLASPLRHRLLVALEFVS
LAALSLTITLSLYFTADTDTLSPAAATAVGVIILVINVGIISLFMAVILRRHQPQLSKTWAEWRQGLQAGAARCLGS
RSCCGRQLLPIAWRSLALPGHQAHSQHAPQQQKVPSARLEGVDEEEDADEAGDGKEAGHVGHGGARGNSDSSSGGGP
NELGCSLHGSSSGRGRGGFSRLLAAPHVPVDSPDLDLPVVDETGGEQDESDAEHERKLQALVELREEEEEGKRQEQR
QEVGRGMGLEGQGGTSVTAAVPRGLTWGRWLLP

>Cre06.g270050.t1.3|PACid:27568581

MEARDDDDMAGAAATAGGMDVGEEEVGTFPTTPVAEPPKLLPPYAHRTFPNPSKYDETARKRLAKVHPRLAECYRGL
TVVSLGTGTKVKSLFSLRDPSSADNATEVKLGYGKACKWLEEFHARLLLLASSAGSGAGASGGGDAAGRTASARAKP
QLPAPKRQNPSDQPTAALVQPDPDHSPAPPDKKRQRKLSAAARVRAPSSVPGRSSGGAGPSVAAARHSPATCAGGEG
AQPVEQRVPLVGSLGDDWLPPPHPQQSPPPSPQRSPLHRKASASPPPSPHHRPPQAQAKPQRHQQPRAPPPLQQPHQ
DVPQQRTRQQGTPQRPAAGGAAGGVAKQPPSSSSPAFRKQQHTQQPSTTTTAEPPAAAGACRLPPAPALPPAAAGAA
PAGSSPAPAPLGPMHGTTPPTKATAGSSSGKGRPGEVSKSGGSGGGRAPLAVAAPEVLDFAFVGAPTAMPRLLPSLS
NRPSLSNTSNSAAREATTNPGASGPAQQPSTAAAAAAAAAAAAAAAVAPAVGEANAVTSRARSDGGGGGISGFSGPA
AEGAAGANGRRVSSNGGSGGGSASGDHQHHHQHRQQHHHHHHQQHAPPPSPPRGSTGGATPPQQQPQHRHQHQLQPQ
AQEVSPLHPAFSVLGAPTSPRWCPPPSPGTGPPSSPLRWTPPPSPRAELPPSPRAEPPPSPRAEPLPSSRSAEPPPP
SPLRHGGGGAPTGGELRRSLSNPCLHGGSSSAPPAAAAVAGSSGSGDANGGGVVVVLDESLANTRDLRRLQQLVTSL
QCASNDRAAAAASATPTPSPPPRARIIASYSLLQKLRAAFPQQHGGSKDDLPVIASAPLLALNDVLAPAASMSAAPA
AAAAAAASADCRFTSAKAAYARFYDDLQRRGCTSGPAALGPSVVPLASSLYLARLEADAASPVQAHVGSTGPAGARS
SWQLPATALIVVVDELDGFAGAAAGSRNGSAGCGALVPVESLPAHPVTGEAPGPTTKRWTTVVTLAQAVRAAGPAVA
RHGGQRVVAALRGLAAQAAVRQCLRVVAAPGAAMRNELRLWCEVLGADE

>g6771.t1|PACid:27568590
MSPSTTTLVLLLLVAARISSSGAAPAGEAAWEHDVSRSDATSTLQSWSNVANRCLSLNTYADSCDTRTDGSVYCKTC
LTWRHEAGEAGCQRALARVLHVCAADRLMQWYSGPGKRSVGGSTDVHVLPWAHGKTEVCQWARWNTSEQDTRVVFTM
LSKGLDCAEDATPISVDGATFAACGRSARGCRKSGAAPGEVSITLGVRRTTGVTPLPLPATGNGATDVAADAAVLPA
HARSLLWYQTQPCTSSVLYNSSGSPVTFIDGPVYAGADKQLVGTATLYPNTPPGFLTFTLARIYNSDVSNSYFWDNS
DLLWRLYPDLGSLNNAIDGPACSRPNQLGRNFNHVSMDGNIGIISFVIPNSNLSLTEGSCDRKRFFIMIKAGVAGET
AFLSWRYMTKNINEFPELCTSVSNSWFGLGDFYVGCQPCATPPSPPLPPRPPSPSPPSPSPPSPSPTTVSSPSPPSP
APPIPAPPSPRPPSPAPPSPRPPSPAPPSPAPPSPAPPSPRPPSPAPPSPAPPSPSPASPSPPSPAPTSTLPPSPAP
PSPAPRPPEPPCAPCPDTALYANATSSKVSFITGPVYAGADKQLVGIATLYVNTPVAGSLTVTLVRTSNNGFNSPFW
STDDALWQLYTDTGSLGSAIDAPGCSRPNQLGRDFNHVPLNGNVGLFSFVIPYTSLGLTPESCERKRFFIIVKSGVE
GDTAFLSWRYMTKNINEFPELCTSVSNSWFGLGDFYLSCKPCTPPSPPSPPSTVALSPPSPAPPSPPAPPSPAPPSP
APPTPAPPSPAPPSPAPPSPAPPSPAPPTPTLRPPSPAPPSPAPPSPAPPSPTPPSPAPPSPAPPSPAPPSPAPPSP
APPPPCDDCPSLFISEFHYDQNPRGGNSDKREWIEIIVPAGFDWRQDLWLAQYTFNGNPQTASGSLHGNVFPFTSPQ
ITVISETRLQTPNFDWTALVIDLQGNLQNSNGGWDGFALLTKCTDGVRWSVADFICYGANSQGFNSIAANGPAAGYS
CTNVGAGVIENNNTPQGTSIQRLNTGDWSTVATWNWQANLPQTWGSLFNPSIALGTIPQVEACGVPPAAKPPSPAPP
SPAPPSPAPPSPAPPSPKPPSPAPPTPTLSPPSPAPPSPAPPSPAPPSPTPPSPVPPSPAPPSPAPPSPAPPSPAPP
PPCDDCPSLFISEFHYDQDPRGGNSDKREWIEIIVPAGFDWRQDLWLAQYTFNGNPQTASGSLHGNVFPFTSPQITV
ISETRLQTPNFDWTALVIDLQGNLQNSNGGWDGFALLTKCTDGVRWSVADFICYGANSQGFNSIAANGPAAGYSCTN
VGAGVIENNNTPQGTSIQRLTTGDWTTVATWNWQANLPQTWGSLFNPSVPLGDIPQVEACGVPPAAKPPSPAPPSPV
PPSPAPPSPAPPSPAPPTPKPPSPAPPSPAPPSPKPPSPAPPTPTLRPPSPAPPSPAPPSPAPPTPAPPSPAPPSPA
PPSPAPPSPAPPTPTLRPPSPAPPSPAPPSPAPPSPTPPSPAPPSPAPPSPAPPSPAPPSPAPPPPCDDCPSLFISE
FHYDQNPRGGNSDKREWIEIIVPAGFDWRQDLWLAQYTFNGNPQTASGSLHGNVFPFTSPQITVISETRLQTPNFDW
TALVIDLQGNLQNSNGGWDGFALLTKCTDGVRWSVADFICYGANSQGFNSIAANGPAAGHSCTNVGAGVIENNNTPQ
GTSIQRLNIGDWSTVATWNWQANLPQTWGSLFNPSVPLGDIPQVEACGVPPAAKPPSPAPPSPVPPSPAPPSPAPPS
PAPPSPEPPSPAPPSPAPPSPEPPSPAPPTPELSPPSPEPPSPAPPSPAPPSPAPPSPEPPSPAPPSPAPPSPAPPS
PAPPSPAPPSPEPPSPAPPSPAPPSPEPPSPAPPTPELSPPSPEPPSPAPPSPAPPSPAPPSPEPPSPAPPSPAPPS
PAPPSPAPPSPAPPSPEPPSPAPPSPAPPSPEPPSPAPPTPELSPPSPEPPSPAPPSPAPPSPAPPSPEPPSPAPPS
PAPPSPAPPSPAPPSPAPPSPEPPSPAPPSPAPPSPEPPSPAPPTPELSPPSPEPPSPAPPSPAPPSPAPPSPEPPS
PAPPSPAPPSPAPPSPAPPSPAPPSPAPPSPAPPSPDPTPPTPELSPPSPEPPSPAPPSPAPPSPEPPSPEP
PSPAPPSPEPPSPAPPTPELSPPSPEPPSPAPPSPAPPSPEPPSPEPPSPAPPSPEPPSPAPPTPELSPPSPEPPSP
APPSPAPPSPEPPSPEPPSPAPPSPEPPSPAPPTPELSPPSPEPPSPAPPSPAPPSPEPPSPEPPSPAPPSPEPPSP
APPTPELSPPSPEPPSPAPPSPAPPSPEPPSPEPPSPAPPSPEPPSPAPPTPELSPPSPEPPLPAPPSPAPPRPEPP
SPEPPSPAPPSPEPPSPAPPTPELSPPSPEPPSPAPPSPAPPSPEPPSPAPPSPAPPSPEPPSPAPPTPELSPPSPE
PPSPAPPSPAPPSPEPPSPEPPSPAPPSPVPPSPAPPTPELSPPSPEPPSPAPPSPAPPSPAPPSPAPPSPEPPASP
PIVPGEVPKLQFDGAVCGATGETIADSIGQLSADQGTALKESFQLRTCSVGNLSAVPPEFPYMTVCGIFGSLSDAAS
IQAGIQALMPTWVSKLAAELMNAFNGGGSAGHRHRQLLELPNMLTQQQLQHHSRSLLQSILCTETLIGYGFSATLEP
EPGNPQCLVANYNETCPYFPPSPPQPPPGPPQLPPSPAPQPPVAPPQPPPLPAPPSPTPEPCRLCLTLVLLPGPTFV
GPLTFTDDDMRSYGEQFATFFNQEVLAGLPSPGNVFEVPISAYNATWIPGTGVTPFSTVCGNFTTDGINALAGLDDV
VWSQWFNILYQGDNCPADLAGISSAIQFAGTPQTCFLHTDIVRDACAPEVPPSPEPPSPGPPAGPPLPPFSSISPPP
PPPPPENPPPSPDPPPPPSRRRPTSPPENSRACGDQATCMDTNIDSSQCQYRATCMDTNIDSSQCQYRVQGNQLFLY
CPVTFTYPRPGSICALLNMPPPAPPPARGGGGGSGGDKSSPYICSSDKLGTVVEGSGGVPVPGGINQQPWQPGNTTV
YTQWVRWAEADMQPRTVEFSVRSGSKACSLGSRGTAITVNGMAATCGGPRFLDPAGTVAAGCQANDGTIFNECLWSV

NVPRPGGEGWNGDVCVENNVPPAAPPSPNPTLATPPSPPSPRPPRPGGNPNINAPLPPINDCLLSGVSSSKTPGGT
ECVNQANCLDVYYDSAKCEWRASSDGNLYLYCPVCLRYPRDSSMCPLTSSIEYLCAGDEVSTVLSGDTGEPVPGGIA
TLPGWAPYSNYCQWVRWQQDDFAQQPIYYSIKDGDGACVRRAGNSVNVTIQGLDAMCTAPRQNAFGKPAGCQGNDNI
DNECLWQFLVPRPGGPGWEGQVCTSPPPPSAPAPNTPSRQRPPRPPAKPGKRAPNVPPAPAPPPPRRNIPFPFCACK
KRNVKNTPYRLTYASSNPMSPLPGGIPRIKHCFEIDTVDCDPSTSCCGMGMKKIEMFVRNECRQSVKLALLGDQSQS
QSISWSFTQDTHDGSTYTTFKFPNLHLSRDEIRNATSLCIVLSDSCAKLENFCYDGKDNQCRVTFFSEDESCCPTGL
TSVGNQATAPEVEVDNSPPEAVVTGDLGRHRSSFRR

>Cre06.g281450.t1.3|PACid:27568646
MCKRSDQTVTAATRASLLFAAVLVGLTAYTAQGATPQELQLRLTGGGDPFNGTLEIYHAGKWGLVCAQYFDDVELRV
VCDALGYPGAWRHYTSTATRGSTTRIWLDWISCPSRHTPLSDCYHSDWGSSISCDTSDVVGIMCDTQANDAQPPAHG
GDEAPPSADNGSFYYDPPPPPLAEPPPEGDAPPGFPPPSPPLTEPPPEGDAPPGFPPPSPSPPSPTPPRKRPPPPPL
AKSPPSPLPPSPPPPPPPPVKPPPPPPSPPPRKRPPPPSPYPSSPPPPPPPPPPPPPSPPPPPSPPPPRPRSPPPRP
PPPRPPPPPPPPAPKPPAPELSPPPPPPPPPPPPPPRPPPRKRPPPSPTTLRPPRPLQPPSAPPPPPPPPPPPPSPL
SPRASGASWYAQTRQGCASIAQTGVYSLLYQAAAAQQTGSASSSSQVAQSAFVSFMDVVCHLTDSLGGAAAAGGATG
EGAYLLQLDTPAAQRAFGVAASVLTTDYYGAWDAAAYEEQWGWQGESPARAAVNLFVLAACVDLTPDAARAAALSGL
DFGTMARTSSVADFLAMYTDCVDGFAGKYVELARVSDVGSSPLVLRVVYSPPAADGGAAVSASSSAAAVFEPPELDA
EGVTCTLEELPAATTAGAAAGGDGGGAVLGGRVLGTWTSANWSAAAADPSSAAGGVDAAGGGLELRPQQPYRVACYL
DETATGLTISSGLRVLSSLAGEQARTSFFPLVLFPDGGGGSGGGGGDSGGPAGGPEPAMQGPPAVTAYASPPPSLPF
YGGGQYGYGTLSYPPPPSPYYGAVPPPYPPPHDYASSAPPAQSPPWSRPAAHADEPPLSEQQQPSQPPLQQQPPSQP
DQVSEQPSQQPPLEESPSPPPPPRRPAPHPPARRPPPSPRPPPLKRPPPPPSEPASQQPPDSGGFGNLPME

>Cre06.g305450.t1.3|PACid:27568693
MTRLQAECCANAGASGPLTLTLTDAALTRGPCWRQAGWSRAPDSSGGIVASLPCSRRLARPNPALWPLLFLLAFTAS
FTTAEAFTLQSLSVTGAPTAIGCGFYTLNYALWCDPLPVYAYVAFQSECTLWLATRPSPPPASPPPPPSLPSSPLAT
PPATSPPPPSPLPPAPAAAANASLLSQWVSSGSFSGSFLYTGELITAECPPARPYAAVMSCINKTTCSQDWRGAETN
SKELCLVLIASVYGSYGAPRQPWGNSTLCDVRITGRLEPYVPPPAAPASNSTELLWWQILLMVVAGVVALAICAGCL
MLSWWWCCRSGGRGHAAVAPRGDSAAAHMAQALPKELAMVPPAMLLKAYSTRLTDNKWDNPDMYAPNDKEQAAPIPG
TVPQPMQAPMPPQQANLAGMAVAAAGVGAATGAAAAAAAGAVAAHNSHPPPSDVNHAPSMRAGAPHLEPLSHPSMPP
AAPPAAHLQQAPAGVGLEMARTVSSRPPPIRTSPSFGPAPGQGGPLGPGQTPGGHLGALPPMAPGVTPHGLDGAPGG
YPGAQATPPAAAAAPPPPPPPPPQPMDPQYETAYQVSSAPPPGAVLAAAGVAAAAAGAAAAAAAGGQHNDPHNPQT
FPPQHQQPPLQQSQPQSYQAPGPADTFPPHDPNHPYPPPPPGMQPQYAPQLGPDGRPLPPHLQPPPPPPPPGMGPQG
QLLPPAQGAGPEAGAAAPLPQKKSMFSRFFKKAAPAPAPEAAAAAAPHQMPPGPPPPGPSGYPPGAGSYPGAPGPAA
AGAAGPYGNDRQHPPPPPPPPPPPGPYPYQPQPGQHGQPQAPPGFPGSAPAPHGAPPPPAYAQQPPAAGAPPPPASA
LPPQAHAAPPPPPPLVLAAPGEELMQPVRSAAPPPAPASPPAPASAHAPPPASAPAPVPLAYVPPPQVPPPPPPAVV
HTPTVHAAPPPPAPPPAPPAHQPASVMDAPPLAPKRGGLELLGGGGGGGGAAPLPPPAGGLPPLSRPQLAPLGAPAA
PPPLAPAALPPTAFAAPAPLAPLAGPRVFARAAPKDSDEDSLLGFRQDLKQETSAAAAPLPPPQVSRPPAGGREDLE
DLDEDFAPPPPKKGGFF

>Cre06.g294250.t1.2|PACid:27568714
MNQRTEEWVDLLTAELVAGASAGHHRRSLLEAVQCDQALAGYSFAADITPAPGSGICLHGNYSRTCLYLPPSPMPPS
PLPPSPAPPSPAPPSPAPPVPGIPPPSPAPPSPEPPSPTPPSPAPPSPLPGIPPPSPEPPSPAPGMPPPSPAPPLPG
IPPPSPVPPSPPPSPEPPQPSPEPPSPAPPPPKCRMCATLRMIPNVNIVTPPLAYTDADLFDFGAQFAAIINEQLIN
DGAPFDELFTNVPIAYNATYDPSTGVAPFSSLCGNFSAATMALIANVPDDVWDAWFILVDPNADCPVELAGISLIVE
FDSTPSGCFDKNPRSHPECAPESPPPSPDPPSPPPPIASMLPPPPPPPPPPENPPPSPEPPSPPPPSPELPPPSPLP
PAPLAPTPPSPEPPSPEPQSPSPPSPRPPAQGKQNAPPLPPFPPEAPVPLECVNKGPSTDMTTPPDTRACADQATCI
DVNIDSEQCQYRRQAGQTWLYCPVTFTYPRPGSICALLNIPSPPPPSARRGTGARADAATANSPYVCSSDKLGTVVE
GLNGVPVPGGINQQPWLPGNTTVYTQWVRWAETDLKASTVEFSVRSGTKACQLGSSPIDLFVNGMSAKCTGPRFTDP
AGTVAAGCQSNDGTIFNECLWSVDVPRPGGEGWNGDVCVDNGGPAPNTPPSPRPPRPGGAPGLRAPPLPPFNPCLLS
GVSSDKTPGGTECVNQANCLDVYYDSAKCEWRASSDGNLYLYCPVCLRYPRDSTMCPLTSSIEYLCAGDEVSTVLSG
DTGEPVPGGIAVQSNWGPSSRYCQWVRWQQDDFEPKEIFYSIKDGTGACVRRAGNSVNVTIQGLDAMCTGPRTNPFN
GPAGCQGNDNVDNECLWSIKAPRPGGPGWAGEVCTKPPPPAPPPMQPNAPSKKRPPPRPAKPGQRAPNVPPSPSPPP
QQYIPFPFCACKKRNIKNTPYRFDFISSTPLPTLSDGKPRVRHCFNIDTVACDATHSCCNMGLKKIEIFANNDCRSS
VKLALLAGQSISWAFTQDTYNGNTYTTFKFPNLMLSRADVGKGMSLCFILTDTCSKLENFCYDGKNNSCRVTFFSVD
ESCCPTGPASLEASTPEFETAAPPPDAVTVDPTGGHR

>g6692.t1|PACid:27568783

MGAGSTPSAGPHQGCVTRCPAERYIRTRHGDGNSGHGPVQDSSPLGRSRGQAHTPASGHSSSTEAAADLVTSTSGSY
SCSDFGGAGSSCSSSSSSSSSSSSSTRCRSSPACGKTGGRNGEGSRAHLCPGPCDSSPTAAAGPALCPGQVELYRRS
RCGGRVQYSGPALLLLAALQLLLGAVSEAVWAALALTAAQAAELGSAAHGVLQLGLRTCSGSADAADETGRRSGMSR
ARCTEAAGGASAGARVFQGTYRHSDGGCQSIPRRSSWLAAASVTQQDGAAAATAAAAVAEAVGARAPQRQRRRYRAQ
LLSGAPPLRGLLPAALVVLVLLLGTWPRASEAQVVNIPSLVMSPDSKSTTAAGLTTDGYYVVAAGAAPQAVFSVTSS
GASSQFNLVIWYRYPDTDGNGPLKKSGGNLIISAAGDTSGPIVTGGNQQAVPLTVTNETNVLDTVTNGMLAGCYVVR
ISLVPPGTISYVGSPAYAGIRAGNPPWNKCSDVVNIPPNCNSALFTYTPSGTSVPGGTAVITQPTTGPSCAFLGFNV
PNGACTDAEGTALTYRWEAYGNGSTTPWIIKYGQNITFSTGLPGLELPAGTWTVNLVVVDTPLNTSLTRNKTISFTN
IVINACKINYPPTAPVLTGSTCGVDPVTVTANSTDANWDPMTFAFTLRNGLGVVMASSGGYTSNTTVSYTSTNTAGG
TLAPGTYSLTVAVKDGNAVPAVGPNTTVSIGVGCPPPLPPPPSPMPPAPPSPPPSPPSASSPPPLPPSPPPAPPLSP
PMPSPPLLPPPPSPPPPPARSPPNPPRPPRPPAPPFPPIIIPNIGVPPEYIATGGGSSTTTTTTTTSNSTTSTSGSG
SGSGSPSANGPTTSNGPTTSNGAPDGSTGTAALAAPPPAAATAAGGGPAQSFLTVTQQVDVPEALASSAEALAALFP
TATEISISGSKVEIPVEITTAVSAVAVISAPISTASGGSGSSGSGSSGGTQVTVDCSSTFLNAFKAELVFRSDVSAS
VLANITCNSGGGNGGSSSKSGGTSAPAAAHHHHHRMLQQRHRQLMQTPDSGSGGGVSSCVSNSSMTLVLVLRVPADD
SLGADVYRQRLQSALEQWQAESDAGAADGVANWLRLCAPTAERIRTSSEVRVVQQVPLNAAGTAAVSAVCGRAGNPT
STTAFGGAGSTSVTCQLDVPAAGQPSDTGPPGAFASNTDRSAAAPAPAGSSISWLPIIVTAAGVGVVGGGLCCIIIG
VVAVRRRRRKREEEEERRQRPEEEEQQAAKRQALDAEDTAPDSGGDGSGWGAAAAPPPAPPGNAFAAADVAAAAAAS
AFAAGASRRDSFMTGAAAAAAAAPNNDAAMNATPVGGMLSILAASRHTAAPADNMAASGRMRRASVTLEHTDAIRKA
LADVLKQGNGGGGGAAAATGVAAAADALGSAGAPGPAVNSGMMISTSGVSPQTSVTGRQPGPSASSPVASRVLGRIS
DSDAGDVIVGSGELPNLATGGCLGATTSSGYTKTAAAAAAAAAAGGGAASVTEKRSGFLARLTARTVSRTSVLPDRS
PSSTATGFARPEPEGAAAGAGADRISPAAAAAAATATALLAGVRAGSAPASPASQRSAKRSHTDLGHMPSRLQLVTT
PAGAAPLSVYSNLTFAPDGPTDGTERRSGSGSSSARTSRMSGNAASTLLRVGAPATPPTGLHRLSGSGGPAAPASPS
GAAAAPVKRGTTLRNLASAFFGGGRLSGNGAPSESAARAPPLSAPTDVGAGFAPASPGASARLGLVSPPSRTRGALT
RSAVSMKEVFGRSRGAMTPPAASEAKSGSASYPWQQAPSSPSMATGAAAGAAGPQMTPVRGMRVAFVSSADGPGAGA
GVGREGGSGTAVPRSHSLRGTASATRTFGLGLEPDHTDLELDVDIGASDGPASGMVAAAGSITSGGNGSAAAAAAAA
IAGAPKWLAVRAGDVAGGRTNTLAAGQQARSMPQRRATFHAGRTGGGSTGGAAGSAPGSPRAAAAITSTGYPLLPSR
SAALQPPPPALPSASPTAGDGGTGGGGTGGGGLEGKSAIGRMLASGSPSGVVSPGSATRSGLVVMPTRGYSGSGLIP
ISTAPASRSRSPAGGSGGGSLGGALGSAAASAMATTSAAAVASRASPNASGGRQGVDRSGSPGPAAAAAKGAPGSSG
ARRQQVLSPLGR

>Cre06.g309450.t1.2|PACid:27568798
MQSSTAALTRAAAVDATASATPAVGRRKRPPPPQPDSPPPPLLPPPSPAPPTPIASPKKAAPGTAGTPKKKRPPPAF
PLSPSPDPPSPPPTPPPLKGGRKKSPPPATPPSPPSTPSPPKKQRRIRPPPPDMPTAPSPPPPPPPSPPPPPLQMEA
DVDWSRYPTFAQLSEYLALRVAGSLGRCSLASAGKSVQGRDLWAVTIGDPAGVYYPDPTNPDVPFPKARAAYIGVMH
GDEKGHISAVLRLVGELCDPLSEPKFAPGGVLDSNVTDLLGSTVLYVLPLMNPDGYTATQRYNANGVDLNRNFYTSA
FPFAMPTAADGYALQPGTSNALYNAAADWTDNGGGGAHEPETQAVMSWLASVRPHVSADLHGGALVGSYALDACDSR
GALLDCPSPEAPLPGYLANVYSMNHPSMRFSWGEVQASRQVQFFNGTTQGATWYPAIGTIADWLHHTYRRHMLTLEL
HYYKYAMFLNDKLNMYATNRPSMLRLAGVGAHMGLRALLRDAASGAALAATVTPQQPAGAWAPEVESGGLVWKMAMP
GVTYSGTIYPYDPADSSIAYEPIPYSFVVPYTWDDVLSKVPRANLSIARVFRAVRVGGSQASTATAPPPQQPPPPNA
PQPPLQQQQQRRRRR

>Cre06.g250300.t1.2|PACid:27568849
MSSDSRKTFVVTTIACAIAPAGQQDQYAAYLGQDENGAIASFLDSTQNTLQAGVSGLGTGQLQLKLSNAAEFPEGCE
FSVVLSKLRAGPIRTEDVPAGVAVATVAHSPLSSLYHTLKDVYSPLIKSQTAEGVPVLDKRLSELLAQVQAGLGTAV
RKGVPASAQEVADPNQAPLQEVVTPLDEINFWAELTNSPNAGPIKSSALQVSSALEPLRQSFEQLSSDAPEGEGGSI
GWEGAKELVDLTTNALRAAWPVKLPIGGWAMGQKRADNFLRVVGAALATYVQRRVDRLAAAGRGDLWSAPFGEVRAV
LVGASGLMTRWVQESTALVEDWRLGVDTGGHDWEGAAFADAYVRSFQERLEEIYNMREMVDELAKLVGREEAGSLGV
QQVFAPFQGLQALQVSDFNTHVWKAAHEDFERRMGPIEQRISQKLKELFASVIIPSLTSAIGPGRGGDRAAAGGSLI
QPQQVFAEIKRYSSLMGRKNIASALQSEKETLAKQVDRHLDSVQAEFDAHRDSATGGAKVAPVGRVTASIVERIMWC
MQTLQKLGKVSEVLKHMLRGGVAGDEGSGGAALRNTLGVVSELQKEVEIFRKEQYSAWEEWMSEELGEMANWKNSKL
MTFDSQNSHVKTHFNDQLVLLLREVRQLQSLGFGVRKDILNEVEIANKFYRYGMVLKQRANFYNNIATEMVQCQKPM
MLKDALDFEKVLMNPKDAQGKEITWRNAAALDGYVRRLNEVADRLAEKNRTLRKWHSVLSDKVVTLAGTDLVRHKDK
WAAGVKEMREIFGRLEAEGYSRESQQVWRQHWDFQLYKALEVQYLSGLEMINKTLPEVEVKMVFRQHRLQYDPPLEE
LRIRHVKDLLNTFLGLPLRMKGVSDLSERPGFFRPIVDANPTGIARVYAAAESLFTQLADELKKYQDWMVLGTLDLD
EFADANLLEVSDWELNFRMLKAASRDAEKLPNEIRIECYKVSLAPVKGSIDEHMKKLQDTLVASLRRKTVAEKDQIE
DFMKNGRELFTRQANTVEDIGLAGQEAKGLTAKLAEVQAARRRIDEKNKLLRQMAGGGRDAAFAVVDLTEVNNSWDA
FTNQLQQFDAHLEEQKGNLAVQISRQLEEFKGKVAGMNSRWQELKPKSGPSGNPAVVLAKIQEYANAIKELREESAK

LYKEAEAFKIDVPDFELMTEMETDVMATKAHWDRYADFLRERDEMANRDWLSMRDQVWKIEDFLAKWTKATAGKSDD
PIAVILTQEIDNYTLCLPHLKSCLRGAGWEDTHWNQLFGLLGMKTSGPAAVSKETVTLTHFLEKADLVVKHADVIKS
LDAQAQGEAVIRKALTELKMWGMAREFTFTESTQSVAGRQRRTPLIKEWRDAMTEVGDNQSLVASLKQSSYYNMFKD
EVSSWENKLSFLQEGLTLLNQIQRKWVYLEPIFGRGALPSQQQRFRNVDEEFRRTMTSLESTKKVVTFADIPGIRDK
LPQMAQQLDVCQRALADFLEEKRSQFPRFYFLGDDDLLEILGQARNPAVIQSHLKKLFAGIQKVKFSQDQSTIQAMQ
SMEGEVVDLAPTVRITEQIETWLGDLTRSMKNTLQQQNEVLCAGRMNDEFRAAASQCLQLKEAVAFTEKAEVALKAG
SSGLAKLVTEMRAQLMKLTGSDFTGHHLLQLKKQALVLDFIHYCDVAEYLAKDKIGGTTEWGWTRQLRYYHRAEGSV
KVAMAEATFDYTWEYQGNAAKLVYTPLTDKCYLTLTQGMALGYGGNPYGPAGTGKTESVKALGQALARQVLVFNCDE
EFDFKSMGRIFVGLVKCGAWGCFDEFNRLDEEVLSAVSQQIQTIQLALKEGAKTMMFMDKTVEVDKNAGIFVTLNPA
GKGYGGRSKLPDNLKQLFRSIAMTVPNNELIAEVLLLSEGFNHAKDLARKLVSLFSLSRELLSPQQHYDWGLRALKT
VLGIAGRELRDARKAGQNVDAEIEAEIIIRSVAATKLPTLTFDDNSRFKALINDLFPGAKLTDARNEALEKALAEAA
AACKMELTQQQIDRMLQLHLACEQRIGVIIVGPSGSGKSTLWELLEKAYERLGRKPIVYKMNPKAMPRQQLLGSMNM
DTREWSDGVLTAAARKVVKEPLEQRSWIICDGDVDPEWIESLNSVLDDNRLLTMPNGERIQFANNVNFIFECHSLEF
ASPATVSRCGMLFMSDEAMEVERMLQRWLKVQATDNGDPGQMQSWMNDFFDKAFQWALSHPRAVETTKGGILDSGLS
HLKLGPGSKQEFMAGLCRGLGSNMNPDIRNQFYNDMARMSGEGGIMDVGVATDPLIVLGDELRERGMDEADGGLVVT
PEVTQNLLMMAPWFKNRDPFLVVGPEGCGKGALLDYCFKRIMGVQVAVVNCSAQTSAANVVQKLVQVCGKPVTTTSG
KALRPPDNTRVILYLKDLNLPRPDKYNTCQLISFLQQLIAHHGYYDENLDFIRVERVQIVGSMTPPGSVGRHALSTR
FTALVRIVTMGYPDRENLATIYTNMAQRVLANSKTASSVSPAALSKAMLEVYSSVRERFTPNDYPHYEFNARELSDW
INGIQRYSLEGGLTLVQAIAHEGLRVFRDRLVGDHQEQFTSMLYGTLTSLLGYKPDATPWYTSTLGASAEERISGDL
TKIKMLRWEQDTFAELVAEKLKGYEREHKELNLLLFPEVLERVSRFDRVLSQQGGSLLLCGNSGVGRRSLMLLLAYM
HNMDFITPKMTKNYDLKSFRNDLKEVLRRAGVEAKPVMLFLEDHQLVNNAFLELVNSLLSGGEVPGLFTPEELAKEL
APLDKARDEDPLYTGPSNSYAFFSYRIRRNLHIVVSMDPSNEMFRSRCEANPALFTRCSVQWLEGWSVKGLQQIAAA
RLTELVESSPELMKLGRDKLINHMIHIHASSGSQTTREYLALVSLYGQIYNRKRTQVLEQQNFLKGGLGKLAEAAVT
VDTLSAEAEKQRVVLKAKQAEADEALVHIQDSMLKAADRRKEVEVLKKRTAIEEVEMKERRVKVEEELSEVQPLIDA
ARKAVGNIKKDNIAEIRSLKMPPDAIRDVLEGVLMVLGQQDTSWNNMKTFLGKGSVKDDIINYDAHKITPEIRARCA
KLLAAKGNSFEDAVIRRVSVAAAPMAQWFKANLEFSKVLERVSPLESELHRLQSSLEESQRLIKQYEEELVQLDAAV
ASLKAEFSKKTSEAETLKISVDKAESVLSSARQLLDGLRGEKVRWEITVGTLGEQLKELPLSSLLAAAFITYLPSHP
EEHRLKVTKDWCAYLGAAEFDVTRFLSSESEMLKWKAEGLPADGLSAQNAVVILNSTSRSPLIIDPSTQASEWLKSH
LRVTGQNVEVTTMADQRFTTTLELAVRFGKTLVVAEVDKVEPILYPLLRMDLDRQGPRFVVQIGDKATDYNDTFRLF
LVTRNPDPYLPPDARSLLAVTNFTVTRSGLEGQLLGLTLQKERPELEEQKSTMLRQEDECKVALAELERNLLQTLAT
STGNILENKDLLDKLNETKTRSATVEKALTESKTLQASLDQQREVYRPIAARGSVMYFLLADLQALNQMYTFSLSVF
LNLFKKALDRDTPPGGDVTARIALLAESLLELVFAYVSRSLFNADRLTFGMHMARHLQPSLFPEAQWAFFLGKPVPD
SASPPPKPSWVREEQAGAFSALAAAFPQLVAAAELADSALWAQWASGATDALPGKIAGGKVNPFQQLLLVKAFRPDR
LQSAMSSFICGTLNIKSVSPPPFSLKALIEGETRPDEPVLFITTPGADPSQELSEYAAQTMGKERYFEVAMGQGQAE
KAVTLLRECAKNGDWLVLKNVHLAVSWLPSLEKELLMLQKHDNFRIFLTSEPHPKFPSTLLEMSLKVTFEAPPGMKK
NLQRTYEAWSAEYLASGPPIRAQLLFVLAWFHAVVQERRTYIPQGWTKFYEFSFADLRSGMDVITLATRAGTAPQWP
LLLGLLDDAIYGGRLDNPFDSQVLLTFLRRLFSAETVGAAGGKVRPLPGSKVVVPTTNHRADYVSIISALPDVDTPG
LFCMPDNIDRTAQQVNSARVISQLKAMSLRADAAGGFNRAQWQAQLGPLLRLWDQLMSGASALKAAMKDIRARGTTD
KGGAPIENFVALERYKGASLVALIDRTLGAIARVLKGTDTLSSGVQSSGAALLADVIPGSWDAAWEGPEAPMDYCRA
VVAKALAIEGHWARCQQPGGGGLLDGSGGAGPLELSSVFHPGTFLNALRQQSARTLGCSMDMLKAVTSWETAKLKAA
AGGAPVALLGGLIMQGATFDGSRLSPVAAEAPAFRAVPAMSMAWLHKDSPMAYASYMEAPLYMTSDRSKLLARVQLP
VSGPEEMDGWVLAGLSLFLSV

>Cre06.g301802.t1.2|PACid:27568910
MPPNPNTRPAIYGPDSTHEATGYYGGPPPAAALTYPPRAPTYSPHSLPPAAYPLGGGGGSGAGGSYPPPGYVSYVAP
PPAPRPPLYQPQPPGGGAGRYGSPLPGTYPPGGGGDAGGAGAGEYGGTGSGRYGGGGDNRPPLPAADAHPAPRPPLR
MPAPAPAPTPAPAPAHPRPSFPSTQAASPSPPPPPSPPSPMPPPLPLDPAPPPAAPGAPAAPQPPWPPTQSRRLEGV
LLSTPSDRGTAYLLQVNATYLVGITAPSGQVVEAMLAAQLRPGAPVWLQCAFEAVTRVCFSLEALGRAPGSAAGGAV
GSGAASPPPPPPAALLTAGLAGSLRVMAASVSLRCGPGADWPPGGSLSEVRAQLHTAVDALSGCSRGAFSPQLDLVD
LHLSCSRDDDRQLFTGRCDELRMSQALTDRLQQQLQPLGNGSSSGDDSSGSSSSYSGSGDVAAWLFVLPLGVGSLCS
WRGGAGAWCCCRRSRGAAFT

>Cre06.g270000.t1.3|PACid:27568998
MPSVAAPRPAAPLPLIPLYNRKVTEAEYDTKNREQLGPALAECYVGSAAGAPGSKAKTRYTLRDPRTGRLLPELLYG
AACRYLMEFQAFLDPAAAAAAAAAAAAAAAAAAPAAGAVGATGAAGAAVPPAAAGAAAAAAHMATGTAPKRPPPQQH
ATPGPKRARPSFTPGIGVGVGGSCDPAAAATLPSANSVGSDTTQSLGAPPAAPPPVIIESLGDQWLPQSRSQRPSPP
PSPLRRQPSAPPSPLRQQSYPPPSPQRQQHSHAPPASPLRQPQPQPQPQPQPQPQPQPQPQPQPQPQPQPQPQPQPQ

PQPQPQPQPQPQPAPPKQQRQQPAQQQPAPAVKQEWEVMEVEGPPAPAPAPVPLQPQPQRQQKQKKQPAAPAAAGWK
PIVTTAIAGGAQPPPARSSPAATTAAGMPACTTTTAAAARRVAEQPPAPNDTVRRQGGGGGVPPASSDFAFVTPSAT
TAAAAAAPAPARTDASSARTAAAASPAAPSTGSGGLARQPSSVAAAVPMATASTAPTVAATRAPSPPPPAVANAASV
GRHSGGGSGTGGLSRPQASAAPGGLDNSGGSTAVRMGSAGGSGSGVSANRAAAEAAARPQWTHTEQQQHQQQAQQVA
PRSPPRAPAGGAAGAADHSTPSLKPQRPQPQPHPQPQPQPQPHPQQHQQHTAYTRAASPAAAEAPTPLHAPPSPNSS
SRWYPPSSPRWCPPPSPGMEPPSSPLRWNPPPSPRTEPPPSPRAEPPPSPLRHGGGGGGEEQGGGLRRSLSTPTMCG
GGSSSGGAGSSSTPGGGVVVVLDESLAYTRDLGRLQQLVASLRRACDDDCAGAGAASSSTSPSTSSSSSSSSSSPPP
VCVTATFPLLDKLAAAFPHAHPHLQQQQGGGGAAGGAAAGRVPATIPSASLYTLLDALTPPTAYAASGAAPGAAAGA
AAAAATAAAARRCHGSAKAAHAALYDDLQRRGCMKVVGSTEVSLASSLFLAYGELAKGPAAAATPAAGSRGAAETGS
GGGSWQPPPKTVIVVVDDLEGLMIAATAGSRNGSAGGAAVVPVESLPAHPVTGEAPGPTTKRWTTVVTLAQAVRAAG
PAVARHGGQRVVAALRGLAAQAAVRQCLRVVAAPGAAERNELRLWCEVLGADE

>g5732.t2|PACid:27569119
MLRKSTGKAGRHDLRRVAMALAFATLFFLPDLASTQTTYGPWNATEDLNEQHKGLLAFILSGDTSFWSRPEVATRLG
FGTAPWRCISNCQTQIFTSDQQCAPDCESRTYCEPGGAALGSENTTCCALSLLDQTYASAQPPSSTQPAWCSTYPGW
GARPRPSVCDFNVFAARGTTPPAGADDPDLAVSCSSGTVPTYSISGTRYRQNDTVYRIMHNGAVTNPANVTRNQVKS
IKLRHSAMWHHPSVNITSPPEFSLVSELACLPLEEIFLEDVALRADYSVLMAQPNTTLDNGKVFNLYDPSTYPFNVT
GLYNNVFSMLIEPSFMLHSSVSVKASSFNPGLALRNAALRTCNFAPFTELHAMRWTGQAQLDWSAEYAQALSYVRDG
SQLRPRGWLPPVIEPVSARLLLPDTLRRLTIRRTRDEQHGAVQWTSRPLITGPLPGEWALLRNLEYLDLSDEMETGA
IVGPIPSTWLMMSHLRVINMTGHHNFCRDWHKIISWQIRMYYRAATHEPNLNVPHYYGPWGGNNGNMTRYNISVYDL
SGHGWQWYDEVTTEAGFVEVIAPHGQCCWDKWSQTIKDNNYEILYPDGSRFGNNVVQDEIYGGFYQDEEWCEPTSPP
QPPPPPAPPSPPSPPTTPDVPPMPPSSPPAPVMPPAPPPQPPIPPASPLTPAAPPRPPLPPTWPGKWEGAWPFRPPI
PPRPPRPPPPPLPPSPPLPPVTPSPPPRPPPPKSPPPPKPSPPPRPSPPPRPPPRPLPPSPPPPPPLPPNPPSPAPP
SPPPPPSPSIPPPSPGPPSPEPPSPAPPSAAPPSPMPPSPAPPSPDPPSPKPPSPVPPSPLPPSPEPPSPVPPSPPP
ASPEPTSPAPPSPPPPSPEPPSPAPPSPPPPSPEPPSPAPPSPPLPSPEPPSPAPPLPPPPSPEPPSPAPPSPPPPS
PEPPSPAPSPPPPSPEPPSPAPPSPPPPSPEPPSPAPPLPPPPSPEPPSPAPPSPPPPSPEPPSPAPLPPPPSPEPP
SPAPPSPPPPSPEPPSPAPPSPPPPSPEPPSPAPPSPPPPSPEPPSPAPPSPAPPSPEPPSPAPPSPAPPSPEPPSP
APPSPAPPSPEPPSPAPPSPRPPSPEPPSPVPPSPPPPSPEPPSPAPPSPPPPSPEPPSPAPPSPPPPSPEPPSPAP
SPPPPSPEPPSPVPPSPPPPSPEPPSPAPPSPAPPLPLPPSPHTQSPPSPVPPSPAPSAPSPPSPQPPSPLAPSPPS
PAPQAPSSPFPPPQPTAPTAPPPPFPPSPAPPSPTPPSPEPPAPQPPSPTPHAPPSPEPPSPTPPSPLPPSPEPPSP
SPPSPAPSVPSPPSPAPPSPMPPSPAPLAPQPPSPTPPSPAPPVPPSPEPPVPPGPDPPLPPSPTPPSPQPPVPPSP
TPPSPHPPSPAPPSPAPSAPLQPSPDPPSPQPPSPAPGPPPSPPSPPSTPSPPSPAPLAPAPPVPPMAPQPPSPPLP
SPPFPPQPSPTITPASPQPAPAAAVLDCSAAATRTSFAVASSSRGAFYIAVAVPASSPSYCQVCGCELSYAVLDPGA
SQQYVIPSSGSSSTAGGSPTVAVTSSVSTPAGAGGLGNGTHGSTARRRALVVEATASSSGPAAGVGARHLLLAATAN
STTLEGLLATGRSRSAAGAGMGMSRLQVVDVQTGGLDPVTAAPPTGTSPNTTSGAGEAGGSGTVRYSSMGAGGSGGL
DAAWRLTPGATGDYLLRLKVADQEWWRWVSVDIDPPRAAGQLLLARRTGGSSSSSNSTSGSGALAAAEDEVQVEVNA
AHAAAGATAVSAQAAVVRLMMAVIAMSEPVQPFSLTSALRLSGGARLLSTQCFASATAAAEVAAAAGTVDASTPPGS
DASSSAATVAPAAIAPVSGSTSGTSNSSTSGSAYVQSCVAVLFAEQDATPELLLPPGTLTDMHGNINAEPLILSVNL
TASADSLSTVERAGAPVAAAVAGGVFASAAFTSASASFLSAFSSRSSLLQSGYHIQMLAMSSSLASPGISPAFRRIS
RYLRWSLLGIQGNIPLLDGAFSSGSAAAGSGSGSSSSNSSSGGLGDVDVAAVALDRLQLSVPPPLPAAGDAASQAQP
PANLSPPPSASQLVADGSTALAGRRSRSLVQAAAPVAPSPPFTQAPPTPAPFGTTGAAPAPPPAPPRQPSTPPPAPP
PMPALSLSGDRDVLVSWLQQVGAIGSGSNSSGGGSATSDSASVGAYSLGGAAASPRGDVVVLPDGQLGLLGPGGSAP
PPMQPGSGSGGQQSSGASVATDTTIHTNVQDLLYTLAIAALLMVALVAAHLLVIGLWRLAVMYDVCGAAESGVEGLH
PVLRFPRAEMVLGGLLLVALTFYSALTLSGAASPRWGDNTAAGRLIAVLVLAVLVVPYGLLLWWLTVCRWYLQEEEV
DHYMLGPHWQAFDGVIPGGGAGAGGDGGGHGASALPAVAGFGGTGGGGGVAFAACQAEPGSGDGGSGGGEDGYGLGP
HWALAPAGAKTLDYESIASPAAGAGSAAGATRTDAAGSTVGGAQSGKGRPRTLRSTDVTAGGAAAGTAAAAATAAAA
AAGAPAGAAGGDVGAAFQRRYGEADDNHGDSYDGEDGEGDLDMAPWRRRGPPADGVDAAAPATAARKMAGSDDGAAG
ERRRANPHARKGSADGADGGAGDPQAAGRPLLRRGVSYGDNLLTAGANTRGSALRSTPGPVADDDARSRFRRNVTYA
AGDNALTSGAASAGAAPSSPTRTQPDSPQAPLSPRRGAAMGSNAYTAGASRFGGPRTRADSPPRAASPPPEPASPLS
GDAGAISEVGFNAMVSGGSRPPRSDSPTMEDEAAAIRIQQDAADPAVEANRSRRKQAGSVEEQQVAINPMAVPRASV
LERPDVAASLAAAVAASGAPPQGTVPLPPITEATGPNGLGSSPRDGRVLTTASVSHGRDTSRLLHPMARPRSLGASL
VPASATTNGGSGNSSGNGRESGSGSTSGAPAAMPRPRVGGAPSNVSAAAAAAAQPAAHQDLFGELDTTVVTPKMAAD
AGAASPPPNLPSPMMRLGSRLGLVRGGSNSGAAQVAPASPLPAKAPSPPAAAARPRPPGAVTTNAAVPMPPPQKVFR
SPSEPANARAQGAAPPAAAAVANGGGRELKQQPAVQTVNSTTSSISANSSGSGNPLARALRYMSVPKPGAQPATASN
AAPAAAEAEPAGPPSRHTAWGGSLSQPPAAAAAAAGAPPSPPIAPQSPMRLPMPPPGAGGAGGAGAKAGGGFTRSPQ
ASIVMESSPAAGPVWPARRRNNRAAIMDEDDLVAEGGGAAGPVAEGAEAPDSAGHYFGGWEDEDPRLLLREAPRSPV
MPPPPPLRAGGTARGSSTGGMSAAAVSIHSHSPSSSARGGGSTAAGHYMTYNPLSLPSAVSAAAEAAAVTPSALASP

PLPLSLLPPRGRSQQAAGSPRSVLLLQRSSTFAPNPTAMAILGTRGAGGVGGCSTRSLQSAVGEGVEGPLLPSAGAL
AVVGSPKSAGGAISVGGGGGGRKSVADLDAEEAEVMRQKQLAALDDWSEVVDPGPGGPAGAIAAPPGVDKRLLPLYP
GKELPMYRLGYRPGCWPLRLRLTPPVYLLARFEFLFEDAIGEGPREQHGRETPLILCVTAVNFSHKVLCAAVFGGFG
LLERSVPQLGFLMALQGAMLVYLAAVRPFREALLQAVELTCHALEAGLFACALALVNAQPNNGAATTYVMVACFFGV
ALLVIVYELRRLALLVMALWRMWRERGTQARRASHAIMDEMEGGGVSAAAAAAAAASRTPSALAPPVSITARRSSAL
KEGAVRTGVASSLSDGGGSTPTSPLGAQPQPSAAAPTRRSRRSSAAGGSSSSDGGTAGRQAAAAIAELAQGQDGPGG
DAVGEAATAQPETSTAAGEIAMRGATATGAGAAR

>g6503.t1|PACid:27569154
MLSGGSRFRRITVPEGSQLVIDDAPLNLTLTTLLVQGNFSMGSASCRLQSAISITFAPAAGVPTNMMALKGMNTSTV
DMHGVLFTPTWTRLTQTAAPGTRWVTVRDAVNWRVGQLVAVPTSVWKDECRNQNEVRQIQSVSADGKNITFTTALQF
LHYGGPEYQTEVVLLSRSILLQGSPESAAELAGGHVRIESKRGRIRGVLGYRMGQQFQMGSYPFHFHMAGEVPSDSY
MTDNAVYASYWRAFTIHGTHALTVASNTAFHVAGSAFYIEDGAEERNRITGNFAGFVHPLGRVDTCDAVGSVFGAPT
IYQSATLLQPADWAAAGFYLSNAYNYIDNNAASGGTSGFMFLNLPLPIGLNRNVPLEPYKRPPLSFSGNTAHSSGYH
WDNAGAIYCGGNLQYDTDNVTLIYKIERSTFDPTDPANSSLPVKFVLRNTKVWLGGFSVASYGDRFLVDGFESHDGT
MGAAVRGDPNELLNADLNLNSAHAGWLTANIPNDLQQGYRVGLQFYDTWMRHLMSNVTIRNVVYSTTEWLTQAALVA
LTFSDQFKPFHVASIRGIRLVNVTKAAVLSNPVVRTGSWRFYNFLDADGSFTGTKRPTLVASYSATNNTGPCDAAAP
TPCTSSFNWWRTDTACTATPPNVPATSNVLACDWYPWRTQARLRLEVPSYDLTTEEAGWLWPSVNNINVYDAGVVAQ
FGQTGANRRATIITRAEGVTGLTGRGGWYAHWSLGAPKYVKVHLETVPRGTSVIFATRYPAGTTFSITRVASSWLVP
DGQLLPATSLAAVLGSDSGEFYFFDGKLLYLKLVDLWEEPYQGRLPARSGGLMVPGSRWEEYYEVTASIPGCSTSPY
PSQNGQDFSGQFCAMNGTVDSFIPPAITAAYNEWTIPYCAEVAPPLSVCSALNVSAGNCTCATLQQQGRCADMGTGG
VWLGNTSDANLRANVAKTLGYCSVTCGRCSEGQEWCRDIPIMPLSLSKNQTCSQMVPLGRCYEVAPKGYCHDTCGVC
SGAGLPCVDLRWTDQYTCADLKSWGSCNDTWVTDNNFCRKTCGACTGMPRPPSPRPPSPRPPSPSPPSPRPPSPPSP
PSPSPPSPKPPSPPSPPSPSPPSPKPPTPPSPVPPSPKPPVPPSPVPPSPKPPAPPSPSPPSPKPPAPSPPPVPPSP
KPPSPSPPTPPKPPSPSPPSPSNSPWRCTGNTAGIAGCVDPASYPAPASSANAATCGVLPSCTTIGCPHCDASLKRW
DSAATWTNYANQVPVSGADKDVTLPPGTRVLLSGCMLSGGSRFRRITVPEGSQLVIDDAPLNLTLTTLLVQGNFSMG
SASCRLQSAISITFAPAAGVPTNMMALKGMNTSTVDMHGVLFTPTWTRLTQTAAPGTRWVTVRDAVNWRVGQLVAVP
TSVWKDECRNQNEVRQIQSISADGKNITFTTALQFLHYGGPEYQTEVVLLSRSILLQGSPESAAELAGGHVRIESKR
GRIRGVLGYRMGQQFQMGSYPFHFHMAGEVPSDSYMTDNAVYASYWRAFTIHGTHALTVASNTAFHVAGSAFYIEDG
AEERNRISGNFAGFVHPLGRVDTCDAVGSVFGAPTIYQSATLLQPADWAAAGFYLSNAYNYIDNNAASGGTSGFMFL
NLPLPIGLNRNVPLEPYKRPPLSFSGNTAHSSGYHWDNAGAIYCGGNLQYDTDNVTLIYKIERSTFDPTDTANSSLP
VKFVLRNTKVWLGGFSVASYGDRFLVDGFESHDGTMGAAVRGDPNELLNADLNLNSAHAGWLTANIPNDLQQGYRFG
LQFYDTWMRHLMSNVTIRNVVYSSTEWLTQAALVALTFSDQFKPFHVASIRGIRLVNVTKAAVLSNPVVRTGSWRFY
NFLDADGSFTGTKRPTLVASYSATNNTGPCDAAAPTPCTSSFNWWRTDTACTATPPNVYPWRTQGRLRLEVPSYDLT
TEEAGWLWPSVNNINVYDAGVVAQFGQTGANRRATIITRAEGVTGLTGRGGWYAHWSLGAPKYVKVHLETVPRGTSI
IFATRYPAGTTFSITRVASSWLVPDGQLLPATSLAAVLGSDSGEFYFFDGKLLYLKLVDLWEEPYQGRLPARSGGLM
VPGSRWEEYYEVTASIPGCSTSPYPSQNGQDFSGQFCAMNGTVDSFIPPAITAAYNEWTIPYCAEVAPPLSVCSALN
VSAGNCTCATLQQQGRCADMGTGGVWLGNTSDANLRANVAKTLGYCSVTCGRCSEGQEWCRDIPIMPLSLSKNQTCS
QMVPLGRCYEVAPKGYCHDTCGVCSGAGLPCVDLRWTDQYTCADLKSWGSCNDTWVTDNNFCRKTCGACTGMPRPPS
PRPPSPRPPSPSPPSPRPPSPPSPPSPSPPSPKPPSPPSPPSPSPPSPKPPTPPSPVPPSPKPPVPPSPVPPSPKPP
APPSPSPPSPKPPAPSPPPVPPSPKPPSPSPPTPPKPPSPSPPSPSNSPWRCTGNTAGIAGCVDPASYPAPASSANA
ATCGVLPSCTTIGCPHCDASLKRWDSAATWTNYANQVPVSGADKDVTLPPGTRVLLSGCMLSGGSRFRRITVPEGSQ
LVIDDAPLNLTLTTLLVQGNFSMGSASCRLQSAISITFAPAAGVPTNMMALKGMNTSTVDMHGVLFTPTWTRLTQTA
APGTRWVTVRDAVNWRVGQLVAVPTSVWKDECRNQNEVRQIQSISADGKNITFTTALQFLHYGGPEYQTEVVLLSRS
ILLQGSPESAAELAGGHVRIESKRGRIRGVLGYRMGQQFQMGSYPFHFHMAGEVPSDSYMTDNAVYASYWRAFTIHG
THALTVASNTAFHVAGSAFYIEDGAEERNRISGNFAGFVHPLGRVDTCDAVGSVFGAPTIYQSATLLQPADWAAAGF
YLSNAYNYIDNNAASGGTSGFMFLNLPLPIGLNRNVPLEPYKRPPLSFSGNTAHSSGYHWDNAGAIYCGGNLQYDTD
NVTLIYKIERSTFDPTDTANSSLPVKFVLRNTKVWLGGFSVASYGDRFLVDGFESHDGTMGAAVRGDPNELLNADLN
LNSAHAGWLTANIPNDLQQGYRFGLQFYDTWMRHLMSNVTIRNVVYSSTEWLTQAALVALTFSDQFKPFHVASIRGI
RLVNVTKAAVLSNPVVRTGSWRFYNFLDADGSFTGTKRPTLVASYSATNNTGPCDAAAPTPCTSSFNWWRTDTACTA
TPPNVPATSNVLACDWYPWRTQGRLRLEVPSYDLTTEEAGWLWPSVNNINVYDAGVVAQFGQTGASRRATIITRAEG
VTGLTGRGGWYAHWSLGAPKYVKVHLETVPRGTSIIFATRYPAGTTFSITRVASSWLVPDGQLLPATSLAAVLGSDS
GEFYFFDGKLLYLKLVDLWEEPYQGRLPARSGGLMVPGSRWEEYYEVTASIPGCSTSPYPSQNGQDFSGQFCAMNGT
VDSFIPPAITAAYNEWTIPYCAEVAPPLSVCSALNVSAGNCTCATLQQQGRCADMGTGGVWLGNTSDANLRANVAKT
LGYCSVTCGRCSEGQEWCRDIPIMPLSLSKNQTCSQMVPLGRCYEVAPKGYCHDTCGVCSGAGLPCVDLRWTDQYTC
ADLKSWGSCNDTWVTDNNFCRKTCGACTGMPRPPSPRPPSPRPPSPSPPLPPAPPSPSPPSPPKPPSPVPPSPSPPS
PVPPGSSAVLRTATTQDTTAPADAALDASPVALESTTSGSTSQQQQSTDSSSAGRIGAGPDSSDAQQGQVAAAGAHG

QLQPDSLADGASGGGGGSGARGPPAAVVAGAAAAGVLVAVLAAVAATVAVLRSRRQSAGGAPAMGTGRVVPLEVISE
GGMEAPVPPAPKKAPSLQKRRSNRTMSIGKNAVHPAPAVLHGRRSSSGFGNDTEDAED

>Cre06.g311250.t1.3|PACid:27569203
MYPPQQHQQLQKSVQQVPSASQLLGAHVSNQSGLSLILKKERQRGQNGDPRPPWRPAGRVEKTAEELALLENGGPSY
VTSPVGSPRSSVPHYFAGPPSVGGPDSGGAPRLFYPADFSSAVPRPDGGFPPAPALAGPERRSISPTWMAAAASSRQ
PAGAGAGAGLQNSMSTVAGPTGVGLIHQPPGGTGLLGTAAAAVLAGPRGRGGGPGASSLAAAGGSSRPGQGQPSLFT
ADGAEVSGRSEDMYVRPRAAGGSGSGGTAQPVASQPQQQQQQQQHQQPTRQRSPTRDAASGGGTLPYQASLEELRRM
IAAKAASGAAAAGAAAGPVGTRTSQQQAIPWEQQPPLSQQAPPQQPLGRSQQPNLPLGATQAHNRPAAGAGSLLEHQ
HQRQSLAAHGQQQQQQDQRPLMQQQQHQQHVQFSHGGISGIKPASAAAAATVSAVTAGAKGPGGPARNGRDGLAADA
VQQHQQQQAAGAWAQPAAHGRAQHTAQQAAIAAAAASLRATATAAAGPSAPVAAAASGAGAGARHSSAPRVTVRREL
SNPRQLPHRPSAVGGYASAAAIAAALAAAQGQGQGRASAAGPSAPLGNSGDSGENSRDRVAPRAGSLGRVRTQALRF
SAGGGVMSKPAGPGVAGAHPRPHGGAIELAELLQQHTTHGQAARTRAGSYPLFAGVRGSGSGANVGRAHGAAAAKGP
GVGPGGLLSVLAHMPHAAVERALVSPLSASRHSHQRQHQHHQQDEEEPAAGFHAQQPPYASTCYMYSPPSVLTEGPH
VVAAPNQEGGEAAEAPASTAAGPAPAPTPVWTTEAVIEAVIADTYVPAPAPEEPVAPVDEEVRGRLEGLFDQQAVSK
ATGAPPQLQLQTLPQHPPAGAPSAATQPLQGKVGAAAPAAAPADCSPAGVRPSPGAVSVASLADSRLSLPAAGPAPN
LAYLCESSPEASWQSFPRNSHPVAAAAAAGPAAGRSPPALPVPVAAGSAGVFAAASPPARSVPVGGLEGGGFAFEVP
PQHQQAKPSPSASRLVLAPDARRIDTIPATAPVIVAEPAAGEVGSRSTTTAKADVEAEAGDATRGHAETEAAGMQPS
FASEVSRETDEMAELLQLMAEEAVQLLLTPNRQQQQQQQQQQQLQEAPRERGGQDHVNEAGTESGENSGERKPAAKE
AELALAAAAAAPAAQPAPAASPAAASPASSCGSVQLRAPQPQAPGRYGHAAAPPPTSAAAGAPAAPAASPQAPDTCC
SGTRRPAPLSENGDLGAAVLNPSHEHAGTGVPAGAALGAGADLDGAGPAAAASAAAGELPGELSGELPGELVGEEGV
GSEVEPELEDEDEDEEEALRANLAEIMSQAIRIVPLATDLGGANSAAPQLSGASCGSSGLRLQAADPTTCQSAQDEA
AAYSPSRSPSHSSSRSMSPAGGRGVASGAPSPFNPHGPSPAAEVSFASTLSEGVSVPPSPLPAVSVELEDEGEAGQA
GQGQGQGLAGQQLQQAADGSADFELESPPRASTQHLYSLGRGAVEFGGQAPPAAVAVPPPADHCIVAEPAAAAAAAA
VPVTASSPVASTTSPSSAANNETLQRASVVPARSQAAAGADDMATGDSLAGPRRTLLATYVEPADEAARGPLPQQEE
VPSLSLGPDAASTAHTSASGAATGLEVSHPEHPSKRQPQRHQPSEVAAGARRGNRLQLRLAPPSPLAVAEFSRQQQP
HLPDQNGGVSGPQEQQLHLHQHQQQHQDARGPPPGRQHVGRLSTDRLQFLASGGGSAGTGMEGLPDLSVSVAAQRRR
FEQARAEARGSDTGSARTAGSGSGLRGSLTGRWPPSATASGAAAHGMQPDPLAPPPPSGPNSVRAPAAAAAAAVGAA
VVQLAVEIQATHAQPQSNLGDKAVASAASEAAPASSPPPPAAAAANCWAVLCSFTPARMPAASNHAAASELLASPQL
PATDTPSASSASSATAATAATASATRSSGRVAGSGGRAVSGIPRAPHLHLHQQDPGSHSLAAAAVRPGTSASAAATQ
PGGGAEGKASAASSGSGSGRSQPASSSGNDSQHAAPSPSLLEPLFEAASSPWAAAPAQKSIGSGASSSIGGGGSHAA
GARLGSSAEVAAYGLEDLLRQEAEAGGLAGLGEGGSDSAEDGGDLLAWSTRRVMTASGGDQHTPTSRLEPQRGGSGL
GSAGAVATLTSFAASGLQSQSPGGASTRAVSLSTPKAAAASSAAVSLGAGGVEGLYSAAMRTPGPAPWSQAPGAAPA
APPSSAAGSSASSGGISHHRSAAAFQTAGNTAMPDPWGSAGAAGTSAASGGGGGGGGGASSFTSTPSVDSPAGLPRPG
GATRPLPSALSPLRPLEEAAVQAAVAQSRAQAAAPAAAAAGGDPGSGSGLGSSWLPAFDIDSFLGSGAAAGAGPDPT
APSGSARGPGSRRSSHSHVALPPSPPGPAAATASAGTGATATAGAAPGGRWQRSGLVVAIPDTAMAPGAVSGLGAGG
GAGYGGGSAAGSPGSGAYGHLPPGSPLMSPSVLQRERLLQQAGAGAGAAAGPGLSEHERTRQALWDNPFMRSDWVPR
RPTASQPTTPIATSRPASPLPQPSGALTLGVASTAGTITTTTAAPTGSIFNDPVFRSHMLGLGLGVDTSDQVQEHTA
AGGSTASAAGPVLRQAAAASARPAIAAAAHATPAAPAAGMTAVTAAATPASALAAALQPATRTPPSLSFAVYAMEPG
TAAAAAAAAAGDRAAVMYAARSTAAGYGAGGGLDAPAGGAMAATGTTAAAASAMITAAAPPQATGPSSPSPSSARKS
FGFPGLAGPASSPGGIIVGRGWGRQGSQQQAAAAAGAGVGGAAGAAVMDSPTRSTAAAVTMSAALGLLGGVEASPGA
ARTPQQQQPHQGHHDQGNAEEEEEEEDQQEAEERCSPPGSATSAASGGSSCSFGDDLVAAGPEAGPEAGPEAGPEAG
PEAELGADLGPAALGPIGEAVEAEEVEAESQQQALEGAHVGQDQSPAALAAVPGPAAPAADPLAAHPLASELPPLRR
FPSVCSSGLGGSGGGLTGGLSAANLRYSGEDEDGEAEAEGDEEEEGEGEEDEELGGEDGLVDAALEEDQREATGAAD
EEAMEAEAMQGVGEAEEEEAMVAEVEDLVLFQSLRLRQEELQTAGAEGGEGGEAETDTAAAGEELQAEADNAGVDMEE
TEGSPLSPLGWHQQASAAAAAAAAAAAHSGSAIIQVCSGLEGVPATPGPAGSSQRQVERRLVFDDPPAPDHHQHHDY
QQHQHHQKGAEGEVPPAGQLPPVPVPVVGAPGSTGPAPQGWVPLAAQRTSDGGSAGGAGELELLLPAGVSLDELLDA
AELAAAEAALEQAQAAAVAAEQAQAAEAPPAARAEAGQAAASASPELKVEAEEVASSCGSAPGPMTRAARLRQLDAD
LSGRGAGPQAADAGAVGCMAECPGLGGLLHGLSFLSPRPAGPPRPGPGGYMCSAATPLPLPRSASSAGSAGSGAAAA
TPTAHSVFVSRVRRTAAATAAATGAAAGGGAAGGGVGFGTPLLLTSASPARPQGGQRQPGGRRREAGGLPADHPWRP
LAGAVADTAAADDQDARTPAAAAVPGGRARGSGNDTDSGCSSSSSPRVDSRAFGSGQGGADSGGSGGGGGLSMGVSR
RADHGGDSSLRGSGALVSEVRQRHLLDELRATFLRRSLQSVALAGTPPHPHQQELTATDTHGYGPALSAGGGSSVLH
HTTSSSGSSGDQGGDRGAGAEVASARGGAVVPLSVSVSVGTSPLQGGRWTPHHPQHHRTGGATATTGTSTSPPPVQL
PASARPAVGPAPPPPPPPPGTRAPPPPPPPPRGPPKGFKLVTPTREQQLRLKQLHWVKLAEAAPGSFWESVQADGAG
GLRPELRDQLRAVFALSDNAAALRRLAGGGAAAAGPSAAPNGPGSVRLVENNRAHNISIVLKGIRRPPSALAADILT
LDATGLEGEQLRALLKAAPRSAELAELAAYVQGAHPAYPGLSDVALLGPVEQYFLEAGRVPWLCERLSAMALMTGFE
VGVRQVADLVAQVEAAIGGIRHSEQLKTLLGLLLEVGNTLNEGTARGGARGYKLDVLTHVAGIKGADKRSLLRWLVE

ALHREANSGGTGGSTGGTGGPVPLHQVLPLQLAGPVRAAANVQLSSVSVLLEELSGGVELMEGQVQALRALLDSRQQ
QQQGDKQQQDKYQDDIGEPAAGTAATGSDSPAAVAAETLLRSRADGLFLERCAGFLVGARPAVAGLQRRYAGLTEGL
RRLAEFFQDDFDARDPARPLLQLRDFLAALDKTVAELEAERRREAAQAAAAARVAALRLRTTTSATAATGAAAAGVG
PTSSSSASADVAAAAGTTGAAHAEAVDVEVESVVDRAGVVLPRGAAPVAAGVGAGTPPGAQAAVVVPQAAA

>g6307.t1|PACid:27569212
MAEGPVFPTFQAAGAATRCELSIQTFHTPTDPNADPYQLPPLVDNSYRLVDKTEDGSKYDTAVDLEDHTGLITGDKL
TTGLTLELPQSIADLVNAELGGGNGTRRRLSEQEHSLRRMVLEYHNTERSLKEFVQLQSLIRDVADTHVERGRRLQV
NLKDLIGAVSGAGGTQSFVNLTPKAKKTTRTGAGGAKDLMIVGGKPLNITSVTFVFTSSCLKPPQYINTAWVSNNWD
NAKAASPTAPTLQRYYDVCSYNKLRFENSINKVYGPIDFPCQGSWPTKGKYDITKSCADPELYGAWDAAKDYLKKVD
PDTFKILDGIRRKIFILPFRPVCTFGGRANVGCPGGGDCLTWMTYAAENRFIDMSTVFHELAHNIGLSHSARNVCDG
KGSCEVQDYGDKTCFMGSGYPVDPTKQILCSSAPQGYKAGWISPMVDPFAGADQTPTTGEFKYSDVPMGQNSTVQLP
AMGATDKNFLRIILDQKGITPVNGRRRERAVFVSYRSVLPSTHPNYLYDSGLSPDFNRRIFIHEFNETADNNPTDDS
YPPLIRNVLDIIPATGKPNTIGDGWGALPNKWEYAGSQGRLVLRVWSKSQTNATISLCFATVTTETGDACYNQVDDD
CDGLMDDDDPDCAGVEQPQDKNDQPPPPAPIFKARSPPPSPPPPSPPPPKGKRGRRQA

>Cre06.g308650.t1.3|PACid:27569242
MDPEVAPPPTLEGADPTEGATEERQADDAGMAPGDDYVEPGAGADTEAEPEAAEPEAAEPEPEAAEPEAAEPEAAEP
EPEAAEPEAAEPEAAEPEPEAAEPEAAEPEAAEDAGAGDDSYADDAFHEHDEAADDTEAAEPAAAEPEPEPEPEPEA
EAEAEAEAGNTEPRGVGEEPTADAAEGPEGGGEEEVPEHSGKILTYISPSFERAASAVYRIQADPEQLAAAKEAVLA
SIASAAPSRNASSVARASSGSASGSASGAAAATAASGAASKRESFSSQTASASAAAAPPAVSESAPGPGSTAGSKPA
SKAGSFSSSAAPPALEPSASAGTGASRGASFSSAAPAAAPEASAVSAASSSLRKQQSGGSAAAATDRPQASAPSASG
APSGAASRATSFVGAGQLSQSQSQSQSQRQGSRPGSTEPLEPSAPSTSSAAAQPPLLPRGLSFASNTSGPVRRAPST
STSSSAGQPVAEPSLAPAALPSSSLATAVAAAPPPPLPPLPSSAAGSRPRSAASAAASASSPAATSDPAAPASPPPP
PEPSSPPPPPPPPHHLSTTRVQPKGPLDACAAQSASEHVRTVWSTANICGIRSLPNALNPDFRERLYQELVAAAQRE
RAGDTSGDRGRRADLSYGLEGGPLAMPLSVGQKQVARIHPLSGMFTQFEYLPSEYDRVKLMGKFDRLKHKLSQTTPS
EFVVRAPPAPPRGAPAFSEFAYVTDPAEAYDSSVLADNDVGRSRVVAGPFYPAGRPKSATSLKGRLDECMMGLCRQL
SEDWPTGFLQIFEDSNGSVVVSFDKSRAVSEGDLTSYMNTLAKRHHLVAAFHLLKDATQWGLVDADTGAVFYVLWPP
WVRHKYLGPHAAAAFGEAANGRPGTRGSDSSDEEGGGGGGGGSGGAYDGEEGPRSGGGLLESYGDGASMGLGSTATG
MGGDGYGRMGSAMRAG

>Cre06.g269700.t1.2|PACid:27569245
MRVRTLPAVDANGAATRTPYARWCCRVNLRASPEPSPSADHPAAAGMGTASSRRTPPLAPGSPEFQFSRHFTTVYDD
PQHLKLAIQAAQAVLLNPASVPLSRASGFAPELVKLVAPPAEVTAAVTAEMTAAEAAAAQSEQPYGAGPREAPAAVL
PTDPAVLAQALSAAVAAALDPAKAKATAAAAAAAGTRPPAGLQVKPHHVADFLLQHRQRRRGHNHHPNHPNHHINHQ
PDNHHQHTRPQPHAAPAVHAPEASPAPPSPPPPPPPPLEVEAAQQVWEARHCDALLLRLGLFPDVYEATASRGAVVL
EVDVLAGGDGAVPKMDVLEIITAAAEPGDPRAEAPDASAPPHRITLLAPAATATSIAVSGMGIAATAAAAASGAASP
PPAAAAEQAGKPRTRPDKHKPLLRCCGHVEVLLLDLLRAATRRLAEELLTASSPGSSTGGGITSRTSSSTATTTSTT
DASPAAASLPSPLSVPPMPVPAVVPPPPSAAGSDLRRRIAAAARELRAAQADCLPCFVVGDQDVGALPELEAAAGLG
RKGVRRMSLQEVQVLLARHHSHYGSSSSSSSSGLARGGGGGGGDTVPYHALQQALNECFNEALWCAGLAAHAEQVAG
AVREAAGRAVAAVVDGVRYPETQRLLSACLAAHVDELAGAVLRRRLYELLRVERIMVYTRGGGGAFRRERASFMGRL
VAALQGPDDDQPVDEEALMLMATCLAYFKVLHAGVQAAVPRALAAHLLQPLAQPAKLQPLLWQRVLAADDITWHQVS
KELLLTHAAAGGGHGAATAAAAAAAGAAAADAPSKRR

>Cre06.g291950.t1.2|PACid:27569311
MTWPSLLQWVLRSGRPLSTRRSRRRDAAQRTSTGGTAGGAGAAAGPPGASADEGAYVSGQPGAEAFWPPAPSSSPPA
AAAAQQQQQQQQYRRLDRERSRAEAAAEARGRDRGVLREPLLPRAPAAEERRHPQVQPSLPQPAEDYVDEEEEEEKE
EQEQQRLLKEERTHAVQQALRPAGLVRRGERAGGVAGAGGAAGAGGARAGSGGGVRLHGPATAYPPPPAQPRPHTSA
SRGMSLPAGWACWGRGWREGPPMQPEQQPGGGGPAGRYGGGGSAADAAAVAEAAGAGMVKGGLPGPSWWDPHPDLDE
DEEEEDQEEDVTWEGGADQRQRRGGSGGPGDSTERTGLRRRRQASGGGSGGGCGGCVGGGGGGSGGGGREGAKGGGK
WSSKVAAAAAAAASATPFDPRNPRRVVHISRDGIPSIVRVDRHQLVADMGIAYRDLRAIDPALPIPTPTSLLIRERAL
VVNLETVRMIIGADQVYVLSVQHPLERDGRLPGGYGGSGMYDVVYGSGGGPAGDHQTTRGYPHGRQYQQQYDERQYY
GVLSSGSGGGWLGGRVADCVLGRPLHCPFIAALCARLSAATDPVVLAQHGHEHGHEHGGKGHQQSGSGGGGGGGGG
GAGGGGGGGGKRSPAGTGAGSHGQAAGKNGGSGGGGGGDGDGGGGAVLAAQHAASAPPGTHLPVAARSRRHSRRHGR
RHSPAVDTQRPSALPPPPPPQQQPSEQLARRTPPPRHGVTAPAELLTPAAAAAPAGPQQEEEAAAPDSGAGAFGGGG
GGGGPESPFQTRSTAPDHTQQQPRRRQEAQVAPSSPPPPPQLAPAAEPPSQHQSLHPLAHSHRSAQHRQPQHQQHQ
QQQQQQALHGHLTLLPRGAHDGSQTALRLPTPGDNGVGGVGGSSSTGGGGGGGGGGIGPSLEPPGAGGGASGTGGGG

GGTGGGGRGGGGGGGGGGGGGGGGGGGGGGGGGGYSVRSWLQQQGSGVVGSSAGGGTQQSMQLQPPSLPARYLARRQS
TLTQRRSTALAAAWQAEQERRRLQQGQKGEEEERERAEGQVGVAAETADAAAKAEAEQEAEMGAVAPEAAAAGAQHG
SRPVASAAMPTAGRVGGPGGDIVPAAAVAAAVAAAGGQAAAAGGGGAGAAVEPTTAAVARPTAPAGRPRQQDGGGGG
ATAATGTVISPNAGGGGTGGGGGGGPRGSRPPAAQPQRPPPSAPTNRQPPPYRAVDSGSLGDLELPYELRALEAGLA
EAVRLLDIQVRWLETHVPRAADELAQGVSPARLERVREAKRAIGAAGGRARRLAAALGGVLDDDDDMLDMYLGRRLA
LEGEGDPWHLPPELQPRPLPPGSSTDEGEAAEAEAEAAGGNAAVRAAGAGAGAGAGGEGDDSNTVMGAGSGAGAGGG
GTAGGGVAAAGPAAAGPAAARGSGIAGGSAGSPTGAAAASGPGVDDQSGDLGASAGVSGGAPAATTGGGGRRPPPTS
SPVLWRRQALSGVTEEPIERMTSSSGGGGGGDGGGGGSDDGSVAGDIEAPPPPPPALASSSAPASAPAALMTPFGTS
RVQLAAGAPPLPRPSMPRLPSPSPPPALQSARDRGSARVAAGGVGAGGGVSRGPRGGGGGDGDDDAGWWHVPAAGSA
GGGVRSSSSTGAAVSGGGGSSSSGVGGAVGRAVAPPRARRPPTPPPPVRVVDVDVEREWAARTTSAEASSALAEASA
ALAAAADRKASAERRRQRSGRRRGEAERRAGGGGGLRSHGSSSNGSSSGSSSSSSSSSSSSSGRSGSSTGGGSSSSP
CSISSVATTEWALSQGAAAAATAAGASQPPPPHGYNLPWDLAAGAAATAGRADPHDVEDCENLLEFYYVQAEALLGR
LEALSERIDDTEDLVNIDLDNRRNQIVGVDLVVTSISLMFTFITSVAGIFGMNMRNTLEDSHTAFLVTTVASTLVGL
ALCGAFLAFVASRRLLLLGPGGGAGAGGGGWMGGGGGTGGWMGGGGGGGGGGGGGSW

>Cre06.g305251.t1.2|PACid:27569391
MTTASEDAAGPWSPTLRPRTAKPAARPGTASLSLPPDPSLDARAFPPPRRSPSGASLSHVGPSSPYAPSSKRVSGGA
SSTPASPDVGSGHAATAGSRGSAPNSGALQQPAYRRLAPNSITLQARNLLASTAPSSPMAGPAGTSAAASPSHGGSR
SRVGSAHASVPSSPITRGRASPGGGATPSTPVSRGLLGAAALAPPTPQSRSGHNGHHGQAQGDVPDVFTVQMAAVTE
HLARTQGQTYGDRRASSSLGHYVQSQSGVMAGGGGPAAAGGVRASTTGGGGAFRQARPSDQLGHGHGPHHQAHVHGS
SSHNHGPVHTFTPGPGPAGPGSSTGMHPASSAAAPSVAASVATMQQRLQGKFGEEAAAFMLHDLHQVPHHLHVLFYT
IIHQLRELPSPLPSTLGQAYHAVFSQHYARTVTSVEKRHAEYKAKMATAQAQAQAQLAALQQKVAALTPSGMRQTPL
QQQAQAQLLQQQKQLQAAAAARQAEADALGAQVSKSRELHAKVMRLLCVLVAAQEPLTRQQLAGMGLEVVLPHLPAW
EVLFRMEPPAGHAAVSAMHSLHGHAHAHFGSGSRRMSINDRSSFSGNSPAAAAAAAYASASQLPAATLAVVDPSLED
WLVDAKEAGPFACDPRVGHAALGQHYRQLILSNVEASPPAGGAGASASAAGGGQGGHGGHGGGGRGPHGTTAAAALD
GYGLRYVVLHLLLSEREVTAGERLLMDADYLEQVFRAQQEGAMYLCLVRLASKTEIVSEALRWLRCNMGALRAHPRA
VVPLWQAAPHRTLTARLVSASSSGAAPPPPEPEHVAAGAAPAGKVPPSGGAGGEAGPDSAPPTPRDPSDVPFTSPRP
AAAAVWRRAGRPQCLPLSSPPFWPVGITQLPGHTGGVTGLVFDRDNRLLLAATAGGVIHAWDHATARRVGTLGGYSP
VVPTQDLHPSGELLAVGSSVDSTVRVRAIESGSGPLVTFTEPDQGGPDGGSLVAVLRGPVAGSAYGSVTPTARHVSY
SPDGNLLAAVYTDGSAAVWDWAEGVKSTHMPLLSKSSAARHACFSPDGQLLALACANGTVRTWHLKQLDPLHVSVLR
VSPAHAPGVAVRLVQYSPSGLLLATVVEGGCEVALWDVGSGAQQQLLEGAGGAAGSPVRALAFSPSGLLLATAGDDG
DVCLWNTNTRGQWTQTACLQGHAYAVNALAFSPCGQVLASGGNESAVRLWDVGSVMHRSEALRADAGLLAVSGPQPL
PPGAVIRDDEATISGRRPAPGVISCLAHSPSGSQLAAGLADGGLCVWDAAAPHRWARLQGEHRRPIGRVLYSADGGL
LASASDDGRVCIWSVPRPPPPPPSLASTSSGGSPVPGGRLGSAASSKSLLGGAGRSSTTMLAPPAAPSPPPHPNTISL
IVTILADAHLHDPAGDASTATGASPPGGASRPGRGAGGWVTLSCSMAFSPDGSFLATAHDNGTVLLWSLPSGLLAAR
LSGGHSKLAYGLMFSPPPTAPTASATSAGLQDALVGPGLLASSSDDGTVCIWDPAAAAKATEDALRVAPLSILRHDD
HVYGVDFAPDGLTVATVSRDGTLATWEPRSGTRRVWVSHGHSTATKACVDGVRYSPSGLLLATGAADGSGVRLWDAR
SGALLREVVGLRLLGWPGLGPLPWAAVFKADYAPFVVRLPERALILEAAAVCGGSDVTVEREEAAAAAADEAAEGLA
AACIGLAPAQQTGALAARRREQLNRCRLASFPAPPPPPQPHDRASSTADGDGGSGYGGAIDRPQPLLNDFTATPLRP
AVRIFSGVRSLPHVALRGRSLVLADSGAVAFYQVTVA

>g6740.t1|PACid:27569498
MCSLWSFEIPRPGTAGWKCPSPPPSPPKPPSPPRRPGRPPPSPPLEPSPPSPQPPRPPRPPRRPPAPPGTTISSEAD
DPPSPPPAPPSPPSRPPFPSPDPPSPPDVPMPPGLPPPAPPPSPPSPEPPSPPSPPDAPPPDMPNPPSPTPPSPPSP
PAPPDAPPPDMPNPPSPTPPSPPTPPEPPSPPDLPPPNLPNPPSPEPPSPPTPPEPPSPPDLPPPDFPAPPSPKPPR
PPRPPSPQPPHAPPPPPPPPPPPPDAPPPSPEPPTPPSPNPPLPPSPPQPPSPSPPYICVACPYLFLDEIHYQDAST
GTSAQKVELLAATNFDANRITLAIYTFTSSGDTNTAVIALNGGAASVSKIQLSGDYVSWSAITVSLPAGTLPTTEGA
NLLGAIAVVVTCDNGASTVTEFRAFGSTSGIGLTATTGLASGYTASLITTNDQPPAQLYESAQTAAGTSIQLTGPGR
TPDDAWTGWLSGNLATWGSINDWTINPLEPSSPAIEQCGAIVPKPKCEPCPSIFVSEFHYADSGSSSGSTAQRLEVM
VPTVLDHSKLAIVPYKFLVDAASGTNGGLALGSPVLLSTPDGAARILTNYLLQSNVAWRVMTIDMTGLMYDPSTAPG
TLGGVYLMMYCDNGLKTPVDSFTYGSLTGVPANLQYIAVTGIAMGFAVQFGHVNQVEASGVSGTSPASSIQRAGEGV
YPWDFPAWTVASVNYGTIDTSTLKPYVPPYEQCGGPAPPQSPDPPSPMPPSPPPSPIPPSPRPPSPPPSPQPPSPDP
PSPAPPSPAPPSPVPPSPEPPSPAPPVPPSPAPPSPEPPSPAPPSPEPPSPAPPVPAPPVPPSPAPPSPEPPSPAPP
SPEPPSPAPPVPPSPAPPSPAPPSPVPPSPEPPSPAPPVPPSPAPPSPEPPSPAPPSPEPPSPAPPVPPSPAPPSPE
PPSPAPPSPEPPSPAPPVPPSPAPPSPEPPSPAPPSPEPPSPAPPVPPSPAPPSPEPPSPAPPSPEPPSPAPPVPPS
PAPPSPEPPSPAPPSPEPPSPAPPVPPSPAPPSPEPPSPAPPSPEPPSPAPPVPPSPAPPSPEPPSPAPPSPEPPSP
APPVPPSPAPPSPEPPSPAPPSPEPPSPAPPVPPSPAPPSPAPPSPAPPSPEPPSPAPPVPPSPAPPSPEPPSPAPP

SPEPPSPAPPVPPSPAPPSPEPPSPAPPSPEPPSPAPPVPPSPAPPSPAPPSPAPPSPEPPSPAPPVPPSPAPPSPE
PPSPAPPSPEPPSPAPPVPPSPAPPSPEPPSPAPPSPEPPSPAPPVPPSPAPPSPEPPSPAPPSPEPPSPAPPVPPS
PAPPSPEPPSPAPPSPEPPSPAPPVPPSPAPPSPEPPSPAPPSPEPPSPAPPVPPSPAPPSPAPPSPAPPSPEPPSP
APPVPPRPAPPSPEPPSPAPPSPEPPSPAPPVPPSPAPPSPEPPSPAPPSPEPPSPAPPVPPSPAPPSPAPPSPAPP
SPEPPSPAPPVPPSPAPPSPAPPSPAPPSPEPPSPAPPVPPSPAPPSPEPPSPAPPSPEPPSPAPPVPPSPAPPSPE
PPSPAPPSPEPPSPAPPVPPSPAPPSPEPPSPAPPSPEPPSPAPPVPPSPAPPSPEPPSPAPPSPEPPSPAPPVPPS
PAPPSPEPPSPAPPSPEPPSPAPPVPPSPAPPSPEPPSPAPPSPEPPSPAPPVPPSPAPPSPAPPSPAPPSPEPPSP
APPVPPSPAPPSPEPPSPAPPSPEPPSPAPPVPPSPAPPSPEPPSPAPPSPEPPSPAPPVPPSPAPPSPAPPSPAPP
SPEPPSPAPPVPPSPAPPSPEPPSPAPPSPEPPSPAPPVPPSPAPPSPAPPSPAPPSPEPPSPAPPVPPSPAPPSPA
PPSPAPPSPEPPSPAPPVPPSPAPPSPEPPSPAPPSPEPPSPAPPVPPSPAPPSPEPPSPAPPSPEPPSPAPPVPPS
PAPPSPEPPSPAPPSPEPPSPAPPVPPSPAPPSPEPPSPAPPSPEPPSPAPPVPPSPAPPSPEPPSPAPPSPEPPSP
APPVPPSPAPPSPEPPSPAPPSPEPPSPAPPVPPSPAPPSPEPPSPAPPSPEPPSPAPPVPPSPAPPSPEPPSPAPP
SPEPPSPAPPVPPSPAPPSPEPPSPAPPSPEPPSPAPPVPPSPAPPSPEPPSPAPPSPEPPSPAPPVPPSPAPPSPE
PPSPAPPSPEPPSPAPPVPPSPAPPSPEPPSPAPPSPEPPSPAPPVPPSPAPPSPEPPSPAPPSPEPPSPAPPVPPS
PAPPSPEPPSPAPPSPEPPSPAPPVPPSPAPPSPEPPSPAPPSPEPPSPAPPVPPSPAPPSPEPPSPAPPSPEPPSP
APPVPPSPAPPSPEPPSPAPPSPEPPSPAPPVPPSPAPPSPEPPSPAPPSPEPPSPAPPVPPSPAPPSPEPPSPAPP
SPEPPSPAPPSPAPPSPAPGVPPPSPEPPSPAPPSPAPPSPAPGVPPPSPEPPSPAPPSPAPPSPAPGVPPPSPEPP
SPAPPSPAPPSPAPGVPPPSPEPPSPAPPSPAPPSPAPGVPPPSPEPPSPAPPSPAPPSPAPGVPPPSPEPPSPAPP
SPAPPSPAPGVPPPSPEPPSPAPPSPAPPSPAPGVPPPSPEPPSPAPPSPEPPSPAPGVPPRSPEPPSPAPPSPAPP
SPAPGVPPPSPEPPSPAPPSPAPPSPAPGVPPPSPEPPSPAPPSPAPPSPAPGVPPPSPEPPSPAPPSPALPSPAPG
VPPPSPEPPSPAPPSPALPSPAPGMPPPSPEPPSPAPPSPAPPSPAPGVPPPSPEPPSPAPPSPAPPSPAPGVPPPS
PEPPSPAPPSPALPSPAPGMPPPSPEPPSPAPPSPAPPSPAPGVPPPSPEPPSPAPPSPAPPSPAPGVPPPSPEPPS
PAPPSPAPPSPAPGLPPPSPAPPSPAPGVPPPSPEPPSPAPGVPPPSPSPPSPAPGVPPPSPEPPSPAPGVPPPSPS
PPSPAPGLPPPSPAPPSPAPGVPPPSPSPPSPAPGVPPPSPAPPSPAPGVPPPSPSPPSPAPGVPPPSPAPPSPAPG
VPPPSPSPPSPAPGVPPPSPSPPSPAPGVPPPSPSPPSPAPGVPPPSPSPPSPAPGVPPPSPLPPSPAPGVPPPSPE
PPSPAPGVPPPSPSPPSPAPGVPPPSPAPPSPAPGVPPPSPSPPSPAPGVPPPSPAPPSPAPGVPPPSPSPPSPAPG
VPPPSPSPPSPAPGVPPPSPLPPSPAPGVPPPSPEPPSPAPGVPPPSPSPPSPAPGVPPPSPAPPSPAPGVPPPSPS
PPSPAPGVPPPSPAPPSPAPGVPPPSPSPPSPAPGVPPPSPSPPSPAPGVPPPSPSPPSPAPGVPPPSPEPPSPAPG
VPPPSPSPPSPAPGVPPPSPSPPSPAPGVPPPSPSPPSPAPGVPPLSPEPPSPAPGVPPPSPSPPSPAPGVPPPSPE
PPSPAPGVPPPSPEPPSPAPGVPPPSPSPPSPAPGVPPPSPSPPSPAPGVPPPSPAPPSPAPGVPPPSPSPPSPAPG
VPPPSPSSPSPAPGVPPPSPAPPSPSPGVPPPSPSPPSPAPGVPPPSPSPPSPAPGVPPPSPAPPSPPPPCTVCATL
VLKPDPNVYTDLDTLSFIISDVKMQEYGMDYAAYLNDVVVSDPQYGFNSAVIFNFDSSSAIWDDDLQIYPRSTVCGS
LSQNDAAILRDMVPNDRVSGWAYWFTILFNTADTACIVSGLAADIEFSPPSCFDKSPVPYSQCTAASPPEPPSPEPP
SPEPPSPPPPSPEPGVPPPSPEPPSPPPPSPEPGVPPPSPVPPSPPPPPPPDAPPPSPVPPSPPPPPPPDAPPPSPV
PPSPPPPPPPDAPPPSPVPPSPPPPPPPDAPPPSPVPPSPPPPPPPDAPPPSPVPPSPPPPPPPDAPPPSPVPPSPP
PPPPPDAPPPSPVPPSPPPPPPPDTPPPSPLPPSPPPPSPEPGVPPPSPVPPSPPPPPPPDAPPPSPVPPSPPPPPP
PDAPPPSPVPPSPPPPPPPDAPPPSPVPPSPPPPPPPDAPPPSPVPPSPPPPPPPDAPPPSPVPPSPPPPSPEPGVP
PPSPVPPSPPPPPPPDAPPPSPVPPSPPPPPPPDAPPPSPVPPSPPPRPPPDAPPPSPVPPSPPPPPPPDAPPPSPV
PPSPPPPPPPDAPPPSPVPPSPPPPPPPDAPPPSPVPPSPPPPPPPDAPPPSPVPPSPPPPPPPDAPPPSPVPPSPP
PPPPPDAPPPSPVPPSPPPPPPPDAPPPSPVPPSPPPPPPPDAPPPSPVPPSPPPPPPPDAPPPSPLPPSPPPPSPE
PGVPPPSPVPPSPPPPPPPDAPPPSPVPPSPPPPPPPDAPPPSPVPPSPPPPPPPQLPPPPSPVPPSPPPPGAPPPS
PVPPSPPPPPPPGAPPPSPLPPSPPPPPPPGAPPPPPPDAPPPSPLPPSPPPPPPPGAPPPPPPGAPLPPPPGAPPP
PPPGAPPPPPPGSPAPPSPDPPSPPPPDSPPPPPPFFTTTSPPPPPPPPPESPPPPPPPPPDLPPSPPPSPPPPPPP
DMPPPPSPPPPDPPPPPPPPPPNLPPPKAIAAKVAPPLPPLPPDTPPPAAPPEPVCRTVGPTTQLDSPPPGSRACGDQ
ATCLDVRLDSQECQYMTVGGVTYLYCPVCVQWPRDGSNCALANIVPSSPPPSARRGVAGSTIGGITGGVDKVCSGDR
LASVIEGAGGVPVPGGINEVTPWSSGNSSRYCQWVRWKDGDMSPQPVLYSVRSGSKSCSTLDMSTMISVNGMSATCS
APRFDATTGAVAGCQSNDGTIFNECLWTITVPRPGGVGWTSDVCVDPPNAAGAPPPPPKRDGGTSSKRAPPLPPQPP
SPPDPCLLTGPSTESAGGGNTACADQASCLDLLFNSGECEWRDDPQGGTKLYLYCPVCLTWPRSGSACALSAASTID
YVCAGDEMSTVLDPGSGIPVPGGVSVETLWSPQTKYCQWVRWASDDTGLQTVYFSIKDGTGRCARRSPVDVTVRGVS
ASCNVPRTNPFNGYAGCAGNDDVDNECLWSFQVPRPGGPGWTGGEVCVDPPAPAVAPGPPPPRRRAPSSTSKAAPPL
PPDPPPPSPPPPPKRSAGPGQRMPNPPAPPSKSPPAPSINKIPFPFCACKKRNVKNTPYRLVYQNRTNLQTLSDGKQ
RVRHCFNVDLVDCDSTTACCGMSIKKIELFAKGTCRSSVRLALFGGQSVTWTFTQNEYKGVTYTTFKLPTLNIQRNQ
VNRSGIDLCLVFTEPCATLDDFCWGNPGSCRATFFSIDENCCPTGDISTTKTGDQMEQIVGAPPVVEFDFHHH

>g6739.t1|PACid:27569570
MSQTGAWSGCRYRGTSRTVNTTLTGSDTTPVTGQIISCLDQYKCVDVHYYDGECQTVGTSLFCKVCLTFNTWSSATD
SQGSTMTYCNSQSNMAQICAADRFSSYYSGPSTNSTYSINATTSGTNPTRPWQGSRSNAYCSWAYFADRTGTQYSTA

SFSLRDGSSSSANCGSAFPVPIEIAATQSHAAVTHTTVACPVAGTAKSKAAEPSTTVAAAVSHAAVAGTAKSKAAKP
STTVVSHAAVAGTAKSKAAKPSTSVAATVTHTTVACPVACTAKPKTAEPSTTVAAAVTHAAVACTVACTAKPKAAKP
STTVAAAVTHTTVASTVACTAKSKAPYPAPPSPTPPMPKPPRPPPPPPPPPSPKPPPPTPPSPKPPAPSPPAPPPAP
PAPPPKPNPPSPPRPPRPPPPSPRAGGVIIGSQCLYTDNNNYGFTDGTDLPAGDNVNGMNTCVDQARCLDPYYDDRL
CQVLGDFTYCPVCLYWSGRAGCPKSPTDSVSHICTGDEITPIIPSTITPAVGTAPQLLDTNKLDGWKSGSANRYCQW
VRFSDYETFSTISFTVKDGSGSCGASAIQVKLQGLVSTCQAPQPNPAQGPGAIAGCGTGDQPNECLWTIKVPKPGTP
DYKCLGQAPPLPPGPPPRPPRPNDPPPRPPKPPGPSPPPSPSPPSPRPPSPPRPPVPPSDMPLPPTPPPQPPLPPSP
PRATDVVSCVYRDDTYANVPTGPFQGGINSCYEQANCIDVYMDSSGCSNSGDWLYCEVCLLWGQRSGCPKSTSDTIS
HICLADEITPFIRGASGAPAVQSTAKVDGWRPFAANRVCQFARWGVDDTSRYRLYFTVKDASGYCLDGEDSISMTLG
GIPASCKGPNTTPAGLIAGCGIGDQPNECFWSFDVPRPGGPDWRCGATPPRPPRPPSPPKPPRPPPPSPGPPPQPPS
PAPRPPSPPRRPPSPPTVPDQPVSPPSPPPSPPMPPSPPRATLPNVDCVFTDPGNDFTSLNYPELASTNGPANSCVN
QANCLDLWFDGKTCSQSGGYLYCQVCIYWSNRRGTCRKSVTDTISHTCLADELFPIIPGIGAEPAVGSTQKLNGWGS
GLNNQKCQWVRWASSDDQPAAVRFTVKDGSGYCQGNLPYFPLSVTLGGVPAVCQAPRPNPAQVSGLAGCGIGDQPNE
CLWTFTVPKPGTPDFKCGARPPSPPVPPKPRPPPKPPSPAPSPPSPPPSPPSPAPKPPRPPVSPPSPPTRPGQPPSP
PGAPPSPPSPPSPPRAPIPGGACVFADPRNDFSTANDPTLVGNSKGGADTCVDQANCLDIFFDSGTCFSGPGTFDCQ
VCIYWSNRRGSCPKSVTDTISHTCMADELIPVIPSAGAAAAAGNTYKVDGWGSGLGNRYCQWVRWSASDLNPMDVFF
TVKDGSGYCQGDYPYGMPLTVTLGGLSASCQPPRPNFAQKSGIAGCGIGDQDNECLWRITVPRPGTPEFKCAIQPPS
PPMPPKPRPPPKPPSPAPSPPAPPPSPPSPAPKPPRPPVSPPSPPTRPGQPPSPPGAPPSPPSPPSPPSAPIPGGAC
VFADPRNDFSTANDPTLVGNSKGGADTCVDQANCLDIFFDSGTCFSGPGTFDCQVCIYWSNRRGSCPKSVTDTISHT
CMADELIPVIPNAGAAAAAGNTYKVDGWGSGLGNRYCQWVRWSASDLNPMDVFFTVKDGSGYCQGDYPYGMPLTVTL
GGLSASCQPPRPNPAQKSGIAGCGIGDQDNECLWRITVPRPGTPEFKCAIQPPSPPMPPKPRPPPKPPSPRPSPPTP
PPSPPSPAPRPPSPPLRPPSPPSRPDAPVSPPSPPPSPPSPPSPPNAPIPGGPCIFTDPENNQNLLPPSKSGPADSC
NDQSQCLDIFFDSSTCSYGGSRLYCQVCIYWSNRRGSCPKSVTDTISHTCLADEWYGVIPRVGADPVTGATDKLDGW
RSGLNNRKCQWVRWENGDDSAATVHFTVKDGSGSCGTDTPYMPMAVTLGGIRASCDSPRKNTTTGLLAGCGIGDQDN
ECLWTFTVPKPGTPEFTCNAKPPMPPSPPKPPNPPSPRPPPPSPRPSPPSPAPRPPRPPPDPPSPPLAPDRNIVLEF
QPPSPPPLPPTPPAAPIAGGSCVFSDWSNDTILTGGSDWTGDNVNGAETCIDQASCLGLHLDSGMCTNVGSDLYCKV
CLYWDNSRGTCVKSVGDSISHVCLADEFYETIPAPGYTPVKGLTNKLDGWNSGSANEYCQWAHWSSSDMSATTLRFT
VKDGSGYCRSRKSTSEA

>Cre09.g388350.t1.3|PACid:27569654
MYRRACRSGWRAPSHVSPAATCRVVSMVGRDVRPRVATLLLLLAAAVVIPGVAAQGNSGDNNGNGNGNGNGNGNTTS
GGGTTTAPGQLRKLQGQLVFVDYHDGGEGEYALQLVGGGTDDTQQPQSPGNSGGAASSNSNGAGGGPPRQYFKLGGR
QKVPRRDKNGQDVEVGQILTIACTVDPTSGQCSSLAPNDVSYLGTAAPPPAVAVHQRVLVLILDAPACGMPAPASVP
SVKSVYMGPAFDGKGGLAIRIENCSYGELTWNATAFTVLTVTLPTCTWDTTTCDPFGMANAARTAAYNLLGPAAYGV
FTNFHLILSTGLCGWAGLATLGGDQVWLQAQYVSYWPVTLQETVHNFFLYHSYRANIEYQDTSTFMGRGTACPNSPE
LALLGWASPAGAGGALNAASIPAGAPGVVLPRFQLPATSLTGRGAYVRVLPNWQGSYSNAAAAHNLYLEARVPLGGD
AQMSGELQYKLLVHEVNATMDNNLQLYRNYNRRINYLSSFSASNRSVIPSYQLVIYSSNWTGSQGAILPIALCRYVT
SDAECPPLSGSIFPRPPPSPPPRPPPAPPRPPQPPSPPPRPPRPPTPPPQPPRPPAPPRPPSPPPPRPPPTGSAGSN
APPLPPFDPLPPFAFEVVPSPPSVSPPKRQHAKPPRHQPPPLHRPPPHHRLPPHHRPPPHHRPPPHHRPPPQRSAAP
GLRAPPLYNQPPSYLQEAPLQQPPTYGGPYGGGYGA

>Cre09.g407120.t1.2|PACid:27569662
MADGGGRFRSAAADGAAGAGGGGSGEAAGGSSSTGEMWLRRVRPTTAMGAEAADDAGGGRARQRRRLDVSRDGAAEV
SAAGSGGGGGGASPGGSSSSDDGSSSSGYGGACAAGALESAARAVARASGAASATAADTDAAAARVTQLAAATAPEV
TKPVTATAGSASAVGGSGRPVNNSDPKMPLPEQANQPPAQQQEGRRSSSGGGGGGGGGGAVTTAAGGGGGGGGGAV
TTAAAASAAAARAAAAPAAATAATADAAAADGPCGADAVCHCAPTPPASSSAATSTATATATTATATTGTTATAT
ASAAGAAAAAAAAAAAGVLGGASACCCCCEREGGDNESRPAAGGGTICRTAVAGSVEAAPAAPAAAAARRTSSGCATA
AAVVATAAAAPASPVAAAGARPAEALAAEALTGATATAAASPHATTAATGDGDAAAAAADGAAGCYSQSLVLGSPAG
GGGAEGGEEATAEVRRQRRLLSLGQGLSRRLRRVQVLRSQLGCLESQLGCQLHACLEALHDVRAAAARAALSAAASA
NAAAGLPLPLQQQQQQQRLLAGRRGDQQHHQGTAAQAHAQALAQAQAQAARAAAAAAAAVAAADGGLREISAAAAEL
GLLGLGLGLSGGGGSGSDGYGPVPLLTAPPASAASAAAAALGGSAVVGPAGGGSSGSRRASASGHGEVEDTHPPLPQ
HQQQHQHLPNLERRTALLPPAAAAKAAGVGASGSSRDCSPPPPPPPSQQGLLPRGSSCSEALAALEQQLGLQPPPPH
LLLQQQQQQPQRDAAGDMSDAFIWRRRSSSTNGLVGTGGAGAGGGVGGGGGVGGGGGACSQLGTPKPPSHTLSQDG
GAAAAAAAAAGVTASASPSKPGLGITAGALGACDEEPALPAPAHSAADASGGDVAAVCGLRGGSGGGAAVSGTCRGG
LGVPAGGGGFDTAAGHARQSALMELLAAALRERAASASAATSDGAAGVVIGSTGGDVYVRPGAGLGGAGPVAAAAAA
AAGVAVSGGSGGGVGGGGSSAQQEWSELTWQQQQQEHQRQRRQLQQQRQLQLPLLRPVKPTVEPYHQQRQYQQYRQ
QQPLPSGLPFSSINGGGGGADNQQQHQQQKQWYLYQQYQQQQSGRKRASEGACLHPNNAAAAAAAGFAESLEVKGPS

AARRRWSDSGASGGGGGGDDELLAAARQPGPFMMQAAIARRPPQPQPMTPPAAAAATAATAATAATAACMGRGLGGA
DGGGAMAAGPQLPAGLHSSSSATRSSAADRGGGGGGGGGTRRYSDTGIGVAGCFAIDGGVGGGGGVSGGGGVSGVSG
GVSGGVSGVRRAAGTAGHAGASATGVKRRCSEATAAAAAAADPQGGGLADGDAARSAAAAARRLHTAAVQASAGVLS
GGSWVAAASPPPSPRPQQQQQQQQPSVLQHMQLLMQRSRPPLPHAATAVTAATSAGVPAATAAATAAPPAHPRPPPA
AACHAHSSWRPHLQPHQQQLQPYRGQQQHHHHHHHHRQLQQLQQRGQQLQQQGQLQHFAQQQAREWQQQAKRPRLLE
SATAAGDASSAGSAGAVRAHGDDNGARAAVTASAAAAAAAAHLLRLQQRQSQQQQQQLQHHQQQLQQLQRLQQQQQR
QQAAQEQERERDPRWLPNTPSQRASDTVDDNARQQQLRLPHQLQPAGQDDRVAVQCPPPSPPIHTNHAGDLGAGGMA
GGARRNPRMAAIAMAAAAAAAAAASAASAGRLQAQAAPPPLTGGISPPLTGGISPPSPPQPVTADAKAGVRQVLTPSA
DAGGGMGAAAETAVARAAAAAPAAASPVLGVDSGSAAAAAAVAAAAEAVLGLLKLQQHPADGV

>g10013.t1|PACid:27569663
MIRQNWRPPGEGEVELADINLTDASNRQAGDVGGVGGYRVSGSGGGQWGAGGPAGAGPSGGSGYGDAYGSGAGGGAV
YGKVSGTGMYGAPMGSSSGGGVGYSYAPAVGGDGAGGGDPYGDADASRLLNSGVLGISSTYPRGAGGYFQRLSDKLA
ARWKLVLLAWLAIAIVGIVIAVPVSLTAGSPARSPPPPMPPAPPSPDPPSPAPSPPAPPSPAPPSPEPPAPPSPEPP
SPAPPSPEPLVPPPPPPASLRDLCTYNGTALPLDPSAAPTHYALRLSLPDAAAFSAPNSTAANGVAASPANPSGVGA
AVGAPFTGVATISLDVTGDTSCLVFNAAGLNVSRVDWTLVAAAPPPSPPSPAPPSPAPPSPEPPLPEPPAAGGGTGG
GGIRRRAQSDGGAGAGGAGANATCVCGCGDGRDCSGSVIAASSAALVLDLGGAVLAAGSAVQLTLTYSGVVGGAEAG
VGLFASEPWVADLPASSTSNPAATPSSVGVLLTTQLEGASAGLLLPCFDHPSYKAIFTLSVEAPDGLTALSNMPLST
STPAATPGRTLHTFQPTPRMSGYLLALAVGNMSAISRTVNVSINGVASSMPVSVWGPAGMDVEGRLRGSLDIAAAAY
SFYAAYTGYALPLPKLDLVAVPGKTYAMENWGLLLMDPARFLYDGGAAATGTVPAAAAAATSSSWDLFQAADVICHE
MAHQWFGNWVTCRDWDNLMVNEGVASYVEYDCVAGVLSYIMAGGGSRSTAAAAADPAGAAAAEAVPLPGYPAPLRGL
TSQLRRFVSPPLGQMHGVHDGPITLTRWVDEDPNARPVLGGGGAMAGLSLLTYSKGATVLAAASGLAGGPGGLRTAL
QALLAPTGPYLYGNATVADLVGLIADVAVGADRNATAPAEVAALGGRQAFIDGVMSWLTTAGVPVVAVTDVTPAPPP
PPSPPPAPPLLPPRPDTPPPSPAPPSPEPMPPVHPSPPSPSPSAPPAPPSPEPSPPAPPSPEPSPPTHPSPPSPEPA
PLPPSPEPPSPAPPSPEPAPPASPSTGGLSGGGSGGSSSRRRLLLRSVSSAASAAGAAGGRRLLQTDPSPPSPEPPS
PAPPSPAPPSPEPPSPAPPSPAPPSPAPPAAAAPVALTTQLQVTQRRMCGYGLWPEGNTNASSFPQLVCPGGDGDNA
SAPGWPRRWWLPLAVSAAGGGAGAGANSTLAVVTASGTYPTTITLSTPLPSDGWLLRRPDFTTMYLANYNTPAGVGP
VGAVAGGNATMTHVVSLLSKIKAGFGLAIPLHNTNTTNTTNTTNTNSTNTTGHAYDVPGEAGDTGLADRLAAVLDAQ
TVTHDALLEAFSPFVAASTTLLPAGAVPGLSAAGNAAPVPLVLAAIDAGLAGPLSRTGAGLHSIAQLSLYALEMLQG
LLATSSASPATPTCNDDLTSWVLARLGPLAAAVIAADGGSSSGSSNSSSNATAGISATTATEAYLLRLSQSNLITEA
ALWGEPAATAFACRLAAAALNASTTTTASTAHVDPDWLAAALAVPLGAPNGCGANGPDAGTAFNATLAALAAAADPD
TVSTRLFALAYARGLYPHRAHLLGMLRAGVLAAAGNWSAVQNLSSYYVRPLPRAEALRVLSRMYGTAQRELFFAVRA
AAAANAAAAANGNATIDLTTVGTAPGLPPGNDLLSLVMLNSTNFGVARMISGDGLAALSMLEGAVARGLDTPAQLAT
LMAALHDPAFLGPGLDDTGLSFERSRILGRAQSRLQWLASYQQPVCAYLAAERAALAAAGNTTAPGGR

>g10013.t2|PACid:27569664
MIRQNWRPPGEGEVELADINLTDASNRQAGDVGGVGGYRVSGSGGGQWGAGGPAGAGPSGGSGYGDAYGSGAGGGAV
YGKVSGTGMYGAPMGSSSGGGVGYSYAPAVGGDGAGGGDPYGDADASRLLNSGVLGISSTYPRGAGGYFQRLSDKLA
ARWKLVLLAWLAIAIVGIVIAVPVSLTAGSPARSPPPPMPPAPPSPDPPSPAPSPPAPPSPAPPSPEPPAPPSPEPP
SPAPPSPEPLVPPPPPPASLRDLCTYNGTALPLDPSAAPTHYALRLSLPDAAAFSAPNSTAANGVAASPANPSGVGA
AVGAPFTGVATISLDVTGDTSCLVFNAAGLNVSRVDWTLVAAAPPPSPPSPAPPSPAPPSPEPPLPEPPAAGGGTGG
GGIRRRAQSDGGAGAGGAGANATCVCGCGDGRDCSGSVIAASSAALVLDLGGAVLAAGSAVQLTLTYSGVVGGAEAG
VGLFASEPWVADLPASSTSNPAATPSSVGVLLTTQLEGASAGLLLPCFDHPSYKAIFTLSVEAPDGLTALSNMPLST
STPAATPGRTLHTFQPTPRMSGYLLALAVGNMSAISRTVNVSINGVASSMPVSVWGPAGMDVEGRLRGSLDIAAAAY
SFYAAYTGYALPLPKLDLVAVPGKTYAMENWGLLLMDPARFLYDGGAAATGTVPAAAAAATSSSWDLFQAADVICHE
MAHQWFGNWVTCRDWDNLMVNEGVASYVEYDCVAGVLSYIMAGGGSRSTAAAAADPAGAAAAEAVPLPGYPAPLRGL
TSQLRRFVSPPLGQMHGVHDGPITLTRWVDEDPNARPVLGGGGAMAGLSLLTYSKGATVLAAASGLAGGPGGLRTAL
QALLAPTGPYLYGNATVADLVGLIADVAVGADRNATAPAEVAALGGRQAFIDGVMSWLTTAGVPVVAVTDVTPAPPP
PPSPPPAPPLLPPRPDTPPPSPAPPSPEPMPPVHPSPPSPSPSAPPAPPSPEPSPPAPPSPEPSPPTHPSPPSPEPA
PLPPSPEPPSPAPPSPEPAPPASPSTGGLSGGGSGGSSSRRRLLLRSVSSAASAAGAAGGRRLLQTDPSPPSPEPPS
PAPPSPAPPSPEPPSPAPPSPAPPSPAPPAAAAPVALTTQLQVTQRRMCGYGLWPEGNTNASSFPQLVCPGGDGDNA
SAPGWPRRWWLPLAVSAAGGGAGAGANSTLAVVTASGTYPTTITLSTPLPSDGWLLRRPDFTTMYLANYNTPAGVGP
VGAVAGGNATMTHVVSLLSKIKAGFGLAIPLHNTNTTNTTNTTNTNSTNTTGHAYDVPGEAGDTGLADRLAAVLDAQ
TVTHDALLEAFSPFVAASTTLLPAGAVPGLSAAGNAAPVPLVLAAIDAGLAGPLSRTGAGLHSIAQLSLYALEMLQG
LLATSSASPATPTCNDDLTSWVLARLGPLAAAVIAADGGSSSGSSNSSSNATAGISATTATEAYLLRLSQSNLITEA
ALWGEPAATAFACRLAAAALNASTTTTASTAHVDPDWLAAALAVPLGAPNGCGANGPDAGTAFNATLAALAAAADPD
TVSTRLFALAYARGLYPHRAHLLGMLRAGVLAAAGNWSAVQNLSSYYVRPLPRAEALRVLSRMYGTAQRELFFAVRA

AAAANAAAAANGNATIDLTTVGTAPGLPPGNDLLSLVMLNSTNFGVARMISGDGLAALSMLEGAVARGLDTPAQLAT
LMAALHDPAFLGPGLDDTGLSFERSRILGRAQSRLQWLASYQQPVCAYLAAERAALAAAGNTTAPGGR

>g10076.t3|PACid:27569721
MLGVFGNLSVTCKSGFNSYNSNSKKCRDIDECDGFDRATVASCTNSKGSYAVICKPGFHDYDTNSKTCTVAPSPPPP
PNIDECDGFDRATVASCTNSKGSYAVICKPGFHDYDTNSKTCTVAPSPPPPPSPSPPPSPPPPPPPSPPPPSPPPPS
PVAAASSPHAPPPSTTFSFRVFGGFVCMTVPPSGGPMYWGGCFSGASAWQNFMVEDTDTPNQYVIKLAADPTLSLNY
SGNLDDGPAKVVLSSDAASMRAWTMTSR

>Cre09.g391150.t1.3|PACid:27569746
MMVHCGVDCCGAGTGDYAVTDTKNGKGKFEALGKGKKPPKKDKNNKDIQIGDYVTIGCTVDTTTGQCSAISSTDVAI
VKPSYIPPATNVFPKLLVLLVNAPACGSSLPSTASVSSISQLYFGPNLDGKGGWAFKAENCSYGEVQVDVPNSMVMT
VSPTCTWPTSSCDAWAVANAANAAAKASLGDAVFNTYTHFHTLMAMPSACSWAGLATLGGGQSGGQVWLNLNTFSQT
FASWGQVPLQEMIHNFVIYHGYSSGIEYQDKTSWMGTGTACPSTTEMNWLGWASPVQGGEGLDAVALAPGAAMGPYT
IPATWVTGLGNFVRVKPTWTSWYTNTLYGMNLYFELRQAVNSDANLDPLYAGKIVVHEILSYMDNDIPTYRSSDPHS
NYMTAVLPNTRTVLSSPSLGVAYNLVLYAGPLSGSQSQFVPLYLCRFTTADTECPTLTVALSNTPKSPPPPPSPPAP
PPAPSPPPSPSPPSPPPSPPPRPPPSPPPPSPPPSPPPSPPPPSPPPKGGKAKPGERAPPMPPMPVEFEGSPPPRTR
NPPPRRNPPPRRMPPRHRHGKHGGKHM

>g9311.t1|PACid:27569759
MGQTTRQQCQCGHTNKWTLAGVAQGVALVIVLFSLAPSPSEAAWIRDGRCDYINTCNTGGNCYGRLTTYWDGCPSGQ
REFNRWQEEDCIWTDCTLGSSCPSGRSYSGCDCSDCLLCGGLVDRNCNNGGRSKKQCCKIRDRVQCAVSYSCTYIAE
GRCGPIAPDGVDSETFTSNGYSTTTCNTANNRASCPSGETQCGCDTGGISVNGVTYGRACCCWTRRSPPPPPPSPPP
PPPPSPPPPPPPAPPPYASPRSSGAILSADNVQQPRSSSGSGGGGISGGAVAGIVIAILAAVIALAAGGFFVYRRRR
AAAESPPTASAAAGSAGSGHTDGAGKQQQPFHNAIAKARTAMAGLGDKLRDMPPGASPKAGQGEVGSNAPCAEKGCT
DCNSGGSEAPAGAVGGTAGAAAAVAGGAVTWSSRMSGLQSRFAAMGDQFRGSMMRAAGKGGATAAGSVTAQAPGFGA
QPQPITSPPAAQPLYGGQPMADMPQQQYQQQASVPGGGNPLFPQMPMQQQNVPPAAGGNPLYPQVPMQQQLAVPYGG
NPLFPQPGPQQSQPQPQMVMQMMPDGTHQPVMVMPGSDGGMLMPAPVPVQGQPQQPQQPQPQQQ

>Cre09.g395850.t1.2|PACid:27569798
MPRPLFAAVPLGLFALSLVLLAAAELSSDGKSTGTDGLLPDDSNSGNLASGGAARQLAAAARSRGQVLSSSNGSPAP
QCVSYSGGDSVQLLSASTGEVVGWASLGAAAPAFTAADDPMLPNVTAPSYSSQARDLAQRNASYSGVFRNPRMRFNI
SLLPGQLFAGAAAPAAAAASGRGALLSNNNNNGHAGVHQLTAAVRDPGTANLRAYTFTDQPSSCPIAEMHFAQEAAL
PCAATAAAVDVDLPALFGCNAGANGGQPFSMGLFLQLSVRTAASANRACAASAASLASTSPSAWSWAFGQEAPVEVA
GSSGVAVEVEEGLEQRPECAVFEVVVTCNPAACGDASPPPPSPEPRPPRPRHNMPVADGATRSPPPAPPLPAPLAPA
PAPLAPSPPSPRSPPRRVPGASGGARAPPMPPFPVVDLDSSSPPQYPPLSVADGPLAADVRAPPPSLAQTGHSLPAP
APAPAPVPQPAPSTPATTSSSTQPPAAPSPESPKVIVTFMPPPPHRTTEGGGFGGGSAGTNVGGGFGGSRTATVSGT
TDGGAAASTTTDSSAGSGSGSGTIKPTAIAGIAAGAAVAGVAVIGVAALVMSGKVAALFAATGSSALAAITAGCGIT
GASAAVAAGTGGGANAAAAAAGGDGWYFAFGRRSTVDSEASSEADPHSTRVSMNGAYDGAAAAGSPGGASAATASSG
HTACSGQEPMVAMLMASISLPLANDGRQELW

>Cre09.g405650.t1.2|PACid:27569817
MDIEEEHEGYGEELGEGEEYEGEEEAEGEMDTEDEEYNLTHFGISQALPVPEGPPDFSAGPPQTAEEYLQWVRYEAA
QCPRVTRKDVDPEKLASSKRQAQDQNPDSADAAGDGTSEPGSSGAAASGDGAGGGGGGGGSGGGASRGRRRGTAFAA
QVPPPVDAECQDWARPNPKWLTVFLQDFARLRQALAAMRRSHVQELSATELPHLDNVRAWDRLCFEQWQSLEQQEQG
QGQQQTGQTAGGAGSGTGGGGDGRGTAAGGVGGNGEEEQEEEEDAGPPPPPPTSSPPPGAGGQSGAGGHGGGGGGE
TDRLCGPVVRTVLAMDQVAVGCLLQRHVAQLSALPLPSPPPRPPAPAPAPAPADGSSSNSGTPSGAANGSSSRGSSR
SSSSTALSYMRCQWLFALAAVLERPVPPDVSAALRELVRRCCALRAGLSRSDDPSLPRLNVLLAVAGGYFGQDEHLS
GAAAAAGLADLA

>Cre09.g411700.t1.3|PACid:27569819
MAAVVRALLWERRAASSPPRVPRTAMACSSPLCIIYLLGLACFLRLADSRGLPGATAAGAERRRMQLTFPNYPPLPP
DAPYPPDLSPPPPGAPQPPPPGRPDAPLPPPPPEAPEPPPPGLPPPEAPLPPPPGLPPPDEPLPPPPGLPPPPGLPP
PGEPLPPPPGLPPPGLPPPGSPATPPPDNPPPVAPSLSPPDNPPPEAPVPPPTSPKPPPTAPAPPPEPPTPSRPPRP
PRAPRAPRAPPSPPQSPGPPPRSLVESLPPPGPAPTYPPTYSSATPPVTSETMAPPAGIISQPPPGPAAASLPPPAP
APDSPTPPPTNGTLAEAEGAGKSSKTGVIVAATVTPIVVLALVGVGVWWYLRRRGRTYTWKEKLGGAAGPGWRSGGA
AGGGNAYMAAPPGPGGRASGKASAFDGKQDTGAPGKKAGGKSPTDPLLPKSAVPPEFESYNAGAAKSARGATRASLG

VVLGAPMVAAAGGKGGFDKANSGRAGAAGAMVGTVGGAGLRKAANDHPNAVDVRHNLLYDVRYDTDVGAGGERSRSG
ARGQAPRTSGSGATSGRQPGAAAGGGPGNRYPSAGRAARAQLDEESEQDSASEDDVGWGRRRQSPATAAAGARPQPG
RSSRSSAGRPGLAGGAAARGGARGGALPPPPASKGNGVFYRPDLAARGGADSDSDGEEQEGVGRWGRPSASSSGRG
SGGSGGARAATAAKRGGLSAPPAVGRVSGGSSAKRLSSSNGVDSEDEEDSRAPAPRRSLPGKSSGPAPAAGGKGRS
SPAAPVKGSAAALPPKAPARSQQSPAGAGGGPRRTVSIRAVTHMDLLPSSAEVSESEDSEEQDDSEEEPQRGRSSGR
GSGGRDAAPPPPPSPPPASGGGIFARLLGRSSAQQQQPQQPLVQPPTAVTSGRGSLQLTSGVAGARTAAGAAHKGAP
VLGRGFGGARGRSEDNKESEEEEAEEAYPLMRRVAARTSNPAAPVAASAGASSRATAAAAAAGRPPLPTSSRTASAA
GAHVAALGDSRAQPGRLSASSGRVGQQQPARGAYASSGVSGGTGRPRQAAVSSESDSDSDDVEAPPARRRPPAAAAS
ATRGTRGGR

>g9804.t1|PACid:27569880
MALCVGPSSMTALVARRSQPSTSHLGISESQMGHHAACGDAQPHSGGRVTEGLNYYAAQRALTPPAGDEPTTSSACQ
QHHHLGRRASHSEATTSEAGASTSAPAPHRRQSLCHCEAGAAHTAASNSSSGLGLTAWRSGTGSPREQQAACSRACS
AGQAHGLSQPAPGHGRAAAQAALMTGRPHSGSVLGMAAAPSCSEAAATSSCPAQEAATALIGTAGERPACQNTGRCN
GSRGTCATTADASGTCRCSNHAAAGHDAAAATNTGAARSCNARLHNSSTTSRSRGGGGASAARLGGLASGLSSLGGA
WRRLLLLALLALTALAPPPAAAQCPPAPQLSQRILTTCTYEYRTSICLFVTWCPKCTSPAQLAYTSLDMTICAAAGN
TFIDVDCSGFSNCITPNPSPPPFPPSPPFPPSPPSPIPPPNSPTAPPSPPPRPPRPPPSPFPPSPPPNTNPSPPPTP
PSPSPPPPPPSPPPPPPPPSPPPSPPPPPPPPPPPASASPPPPPPPPPPASPPPPPPPPPASSPPLAVIRPPPPPPPPPSPPP
APGATLYEVPQTMQLTIGYAIPLYVDATVLANIENGVSNDFNMPVADVDATIYARYLTSVYILTPNVPPAVCDQALE
IAFEATLCTQLAMPNCSYVIAECVKPGAPASMTRVPFFPTSTTPLIVNVSTAGTGTTSTTLIAATVKAAGGSPALAE
ATAEAVAAAAAKEQQPTQSQGEQHPAGGSAGASRRLWGLEGGANEAAGLERQAVRRGAQAVVSPDSVAMAVRFLMDN
ETDNAQFILSIFQKQINLLNWQVANPAPGNVSQVSSVDIVLRNPDGSARPILYVLDTRLSNDLALAVGLPVSKLYIE
RPGVVSYIPSAPKKCDNPVLGVLCGADAVGAIIGIILGGLVLIGLALFGILWMRRGRMTKVVVMDDFAWARKYAHIV
PANVIASPYLTQYGTTSTDPHAYGGMPQ

>Cre09.g403600.t1.3|PACid:27569910
MLVAGQGPFRGITWNPTAPVPDPSQPYTMLLLSDFTDSAPFVVPAGASLELRDLALLLPPPPTATASASSLLSHVFS
LRAGARLTLRRCRLFTASCSNLYDFTTALCNPSTWSFSPDLQILDAEVMYGAGIMGVGALAADNGSSSAATPFATLS
DANFTCSGGGPVTCSAAVVSDAASLRGVASLFMNTTKDTVLLSLAPSSISLAAGGPDDTWKQLTVRPGVTLGLYGSK
TRRTTLDFANAEGGLTLNAFSGARLFLADMDLVGLPYPARLLRPAHFVAAFGHGVYVNTTRDASVSPSARFPPALNI
VGCTLVLPDAEVAWWRYVSSDIADGAAVPDNWNVRRHTLPAPSSSYVRLAVLVDVAGGPRISVTNALFQSVRVYAGS
LSAAAAAAAAAWPLAGGLDSSTASTTIKLGSVSFLNTIAPAADIEFCDNGRTLLVPTDPATPPTAPGFLPAAALAAG
YGSGGSTTAGVLAVEQDGLDQLFYMMNERVNCTLAATPYVQARRRTFYDLKNVTMGLLIPATSTLTLRDMVIYNLGP
ATADYAQAAAAAARRRLLGSHRRRQLLQDGTNNTTAPVPSPPVPSPSPPAVPPSPPQVPAPPSPPLQPPSPPVPLAP
PSPPLPPLPPSPEPSPPPPPPGPPEPPSPPPSPPTPPAPPPAPPRPPAPPRPPRPPPQPPAPPRPPPPPPSPPPTPPS
PPPPPPAPPSPPPPPAPPTPPSPPPPPPPPTLVTTMRDLATPLWYFEFDRLNGSRIQLINVTLVVPQAEIDLLTRML
TRAGKLPSGASSNGGHRHRALLQDDGSSGGADIINITAIPSSVMLSCPLAQLVSFAADSQVASVSSTAIVYNSVRHL
GWYGSNVTITSVLPADAPSRLFAGTSRAADPMFDAACPPAPSDEDFPPPPRRRPPPSTLLADGGAASSGGGSGSKNL
AWILPVAICVPIVLLALFGFVIVRRRRAAAAAEAERQREALKEAGMLGLGYRSYVKSTTAGHGHEEGVPYEQMPRGG
RAGTASMSVASPYGAGGAPYGHLSGAQRSHQSAMGAMGGYEPGGSMLYSPRGGAAAGRRGGYAGGSHTVPSTPAGNA
AARRSSLLLQAHVEARAADPKPAGRMASITAALMSITHRGQSAAHPNPQHPKTPRTPRTPHTPGGGGSRSAPWRTNT
TEQMENERVSPTRPSPVKRLSILFGMGSGAEGAAGSISAGGAGPSGGGGAGGAPPHFGSVPPSPAALTHGGGNGGNN
VFSAAAAAAAASAAAASVAARAAAAAAAAGGHDRVSPTNPRVGSGGFARHSGATELALPQPEERGSGASVGGGLPTVS
GGVATISGAVSAATPSGGGAVRASDAGPPGVRSSASFAAAAQPHSAASFAEAANAAALAAQVRLPAAGSDGGRLGLA
ARESTVIDADDVELDMYGQAGSASAAIPHLAAGTAVGGGGAYSSGESLGLGSGPRALPETAAGPVPVKSAIEMTVAM
RTATSAAGVVSGVASPSLLPPDAATGASGRGASPSRGMSPSRWQQQSGQLTASPSATRSPSRSGLGMHTAPRAPALV
IPPPRLSGSGSAAALMAVGGSAGSPRGGAAMGGLPQSPSMLSGMRRANTPGGGRSPRVSGSGEPEGSGRGGDAAAVA
AAAAAAASPRGSLSGTGGGAGRGAGSVWPPPLPAPGGSAAVKAKKAAFKRGGAAGGNLE

>Cre09.g399400.t1.3|PACid:27569919
MPQNGHVVVAVAQPPHGIHRRYGGGSGGLRTCGLSTGRGRKQLLLVLTVAVMACGLPRVSHAWWSQNWACSGGCDYN
GNCKSSSSSRFSGTSSCPSGYTDGSSNWYWSGTCSYTGCYYICSDECSSGNYYTGEYSGSDCRWWGQSKCKCCPRTY
YVDCKQWLRCTWVPDPSPPPPPPPSPPPPGDRGGCIYDNYNAYVGSSQVDFSCGVSSPRAIDVSARTTSNDDLAIVV
RVGSCGGSTVSSASTSRTPSLSYGSASYNTYCVSIVCKNWVWSCGYATVSINYVQPRSPPPPPPPRSPPPPPPGDRG
GCIYDNYYVPSINYFGQSDFTCDVNGAKALDVSARSSTNDNLSIKVRSGTSCSGSTLGSTSKSSNPSLAYGISSSAT
YCVSIVCENLLSGCSGAWVSINYGTPRSPPPPRLPPPPPPGDRGGCTYDNYFVPSINYFGQRDFTCDVNGAKALDVS
ARSSTNDNLSIKVRSGTSCSGSTLGSTSKSSNPSLAYGISSSATYCVSIVCENLLSGCSGAWVSINYGTPRSPPPPP

PPRLPPPPPPGDRGGCIYDNYFVPIINYFGQRDFTCDVNGAKALDVSARSSTNDNLSIKVRSGTSCSGSTLGSTSKS
SNPSLAYGISSSATYCVSIVCENLLSGCSGAWVSINYGTPTPRSPPPGASSSGRSLAKYWSTNAGDARNAYLAALLS
SLAYNDSTTFCKKAASWGVSGTCEMVYGKFDTEYGLLDLGTSCLLAFRGSETQWVGGSADRGTDMASALIASGPLRG
VVGLAEGFAVAADNNYDTIVSRLQSSSFRASKSKPVWVMGHSLGGALSLITAQWLQSSGLFNVAGVYTFGAPPAGNG
TFKDSYSLKSKTYSYAYAKRTGCEVLDVVANLDSTLLTGHKHVVDLIPVYDNCARSGRRLLAAEAAAEAPAPAPAPS
PAAVTGPAVDAPAPPVPSTGASVADAHQGTQDTAELSEALDATPPPSCLVSVRYTPRSTMVRGRGSAPPPAEIGTLS
PALPQQSLEDGVFTSVTSEVMLSTADGASKAYTRTVQVSDVRTCSTAGWPAGLWAELTGMGIDLLELGLKEPPTPSP
PPPATAANSGNREAAATPAPSTPSPPSPPLLPPMPAVATGRSGPTPAGDSTAEAVAVEAASVVDDLVNGVYEWLSCR
PATEGCWLCHSINDYYVPGLWACLSSTDKARLPLPADLKA

>Cre09.g399400.t2.1|PACid:27569920
MPQNGHVVVAVAQPPHGIHRRYGGGSGGLRTCGLSTGRGRKQLLLVLTVAVMACGLPRVSHAWWSQNWACSGGCDYN
GNCKSSSSSRFSGTSSCPSGYTDGSSNWYWSGTCSYTGCYYICSDECSSGNYYTGEYSGSDCRWWGQSKCKCCPRTY
YVDCKQWLRCTWVPDPSPPPPPPPSPPPPGDRGGCIYDNYNAYVGSSQVDFSCGVSSPRAIDVSARTTSNDDLAIVV
RVGSCGGSTVSSASTSRTPSLSYGSASYNTYCVSIVCKNWVWSCGYATVSINYVQPRSPPPPPPPRSPPPPPPGDRG
GCIYDNYYVPSINYFGQSDFTCDVNGAKALDVSARSSTNDNLSIKVRSGTSCSGSTLGSTSKSSNPSLAYGISSSAT
YCVSIVCENLLSGCSGAWVSINYGTPRSPPPPRLPPPPPPGDRGGCTYDNYFVPSINYFGQRDFTCDVNGAKALDVS
ARSSTNDNLSIKVRSGTSCSGSTLGSTSKSSNPSLAYGISSSATYCVSIVCENLLSGCSGAWVSINYGTPRSPPPPP
PPRLPPPPPPGDRGGCIYDNYFVPIINYFGQRDFTCDVNGAKALDVSARSSTNDNLSIKVRSGTSCSGSTLGSTSKS
SNPSLAYGISSSATYCVSIVCENLLSGCSGAWVSINYGTPTPRSPPPGASSSGRSLAKYWSTNAGDARNAYLAALLS
SLAYNDSTTFCKKAASWGVSGTCEMVYGKFDTEYGLLDLGTSCLLAFRGSETQWVGGSADRGTDMASALIASGPLRG
VVGLAEGFAVAADNNYDTIVSRLQSSSFRASKSKPVWVMGHSLGGALSLITAQWLQSSGLFNVAGVYTFGAPPAGNG
TFKDSYSLKSKTYSYAYAKRTGCEVLDVVANLDSTLLTGHKHVVDLIPVYDNCARSGRRLLAAEAAAEAPAPAPAPS
PAAVTGPAVDAPAPPVPSTGASVADAHQGTQDTAELSEALDATPPPSCLVSVRYTPRSTMVRGRGSAPPPAEIGTLS
PALPQQSLEDGVFTSVTSEVMLSTADGASKAYTRTVQVSDVRTCSTAGWPAGLWAELTGMGIDLLELGLKEPPTPSP
PPPATAANSGNREAAATPAPSTPSPPSPPLLPPMPAVATGRSGPTPAGDSTAEAVAVEAASVVDDLVNGVYEWLSCR
PATEGCWLCHSINDYYVPGLWACLSSTDKARLPLPADLKA

>g10035.t1|PACid:27569998
MLAMLASNTATAMVAPRRLLSPADAARAPAAAPTPAYGTYPAVPVAPPPAYPDTLFPPSPSPAPAYGTYGPSMQPPP
AAPVYGGPYGGYGSPVSPPPAMNPEYGHPVSPPPAAYGAYAQPPPPTAYGIYTQQPPAPAYGPYGRQPSAPAYGN

>Cre09.g398050.t1.3|PACid:27570011
MAAPTSSGDYIANVIVVGAGPAGLCALNAFRAAGFNTVCFEASPGVGGWALNNEGSLQGCDSSALQFNDAAFAVALP
LAPTAQHVRSYLAAYAAFHNLLDNVRCSCRVTHVEPLLAVGTSPVPTAPPQELSSDAPRRALSDVIPSDADPPPVPA
ADCYADATVATASAAAGAAAAIPQVPAPRRKRTLDGMDGLAADGGGNGGSMAHQQQRWLVTYEDGSRGARGRMQTML
ADFVVLAVGPAGGRHDAAALRSLLAQAGVESEDCSRSSGASSHQQLQQSQPPRLLHAREVASASWLQRDGCRSVTVL
GEGSSATDAAAAVALLRHGQGVRLVNPLHLMSHALGPAPCKDAGNTLPGTLGSQELMSSARRRALAASLQPHYTAPQ
PGPLGKALRAPAKRRFWALVKDKLALGRGGRGSSSGSSSGGTGEHLVLWAPSLHDPRFMPFLSAPLRQALLGGAGGA
EGDGDMVLYRGMVHPDVPGLAFMGLEAHAGSSLLLLELQAQWLAAHLAGRLALPPGAAMRADVAAQRVWRSGALAHP
LMSVGGSLARRHEQWAVQQLLKDLHGVVAVEKRPVLAADSVFTDALVAADFDATMDRPAKKRGSHADCEVTTAAPAP
EGETAKDAELATVPPSAAAAMSVVGARAAAAVAGAGNRSPTALRRVSHLILEDEQGEAGDACPSDLLPRRTHDGCHS
GTGSVRRPPTRRATNDSAMLQLQQQQSALRRAPPRRSTVGSLMLPGQGGAASALRSAHTATSGSSGADYRMVLSVTS
PYMAPYLHEKLRRASYAAYSAGQRGGAGTGGIGTAEAEAGLLPEAASAAAAAASAATARSDDYALESVFTVAAADAA
SAMARLRTGHLGSITEGGPPQPVAPHLVGEQLSSAMTAAPPPRRRALHGMYGDSLSGGLAASGTYGGAGVFNARQYS
EPYTNGAAQAASFGAAIPAHASAITGIGHSAAQSGRAASGGMDAARGPTAAGFGPAGTVFGATPSQMLSGPGFGLSS
RNVNVSTGGHQAYITRRSSSSLASSLSIQHHMAHAALAQAVAAGMAGPCTSNPGGSRGGTFTGATSGGPSPAAASAV
PTSTSHAAYGGNGSIRPRRGSSSGGPLGPGMGGFSGFIARLSSRHSISNTNTAAAIASPLPAAGSAELSSPPLLQLQ
PQPQQGAPQLPSAALNRRLSSFGGVAGAGASTAAGGEQMPSQLRRMSQTTGVDLSILVDLITDEGGSGGLPIGILTA
TSVVHGEAAGFSGSIAPGSPNQAHSASARGGGGRSGHCLPAAAGLHRLSSAVVQDQGSNAVPAFPAVAGGLEGAAGS
CHSSAGSAAARAAAAAAAAAALRDPFGAATSSPAAAAQLLAGHPPMGMLQRDVYQHGGFGDDSMSSSEYGDAEGDVP
LGFRRGHGRDHELSHARHQSFDTAILRDESGGGGGGGTGGGPSQQPLVGLEEPQHRGAQRSAAAVVAATAVDGSDRG
RKVPAGAAVASASLLGVAVCNPTFESQPGGVACEVEGMAIVDAGVEAASALAGGLANGTTAVGPAAPEGLGRTASAA
AAEPTPAHLRSAVDAATAPAATVPASTGGEGDSLAAAGGITRNAISTAAVPFADGLQKHGDPQVANCPQPAARQPSK
SQPGARATPCPSPALAGSPTSLSPPPPAPKVSDSAKAKSALRLLPLLPSALLHWGRPQEPAPQLPSELAQTKPQPDQ
YLQQFTSPTQREHTSLQQSTPITAARSPAAAAVAPAAAEPDRLSRISTRPAAALAALFTRRSEQYPPYSPPPRKPA
SPAPAQSAAPTEAGSDIALDVMRSQLAALKAHLGNPQLVPFPALASPPPSSARSMTASPLASPAAGGSRFSRAGPGL

FAAGGGSAALPRRSRPVGTSANSSAAGLEVLPEIEAAAPPPRTSAPGSMLNSSSHASAAPVFGSTVSAAAISSGAAG
AGVSSGSGATAGRRAPPRRSATESQIGKGGIGSELRSAAPTSAIPRNSAAGAFVAPPSAAAAMPASAFAAYSAVPAD
ELAGGAAVAVAGDVAPPVISHQPGRFDRLTRNRSVTSVGGAAGVPAASSFMRRSVTAGGARSITGAGVFSDGTSAAS
SPRANSAASAMPAAAAARAAIAQAMAATQAITNSRRIYSPMMMGAAASSGQGSGQGSGFNRRSCSSAGWPVAGACGP
DSNSMAGSAPGTPLAPGHYGRQVPAGSRAGSTVGSAGTRATSASPAAAAWPLAATSPKASPVKKESGAGVVATAAAA
LLSALSPQRGTQPSAQQRSPLGRKARRLLPFGWWPGGSGAQQQGGKERSAASSSGSKQSAAGSDPRCRGPSIDADGG
ELALVASAPLPVAAATPQGEDAERSSAGSAGAVGRRGQIIASAGSKRLIQRRMSLANLLGGGSSNSNSRGTPAPSGK
PGANGDGSGGALGLLGEAADNALVPYSKRLGAVSARYGRSSRNAMPAAAAGPAGTNSGSVIGNSSGSMAFLAEAGTS
ATRLASMAGPATDLLGPPPPSAARAWCDSTAAEPAAAIGGGADNSGNSFAYTHTGSALPAEPKPTARARILRPPVGG
ADAQALATAAAGGAVAARPSEVASAAAAGGELRGAHASTADIVAALPLLPFEPAVPQHPRLRPEDAAVACAAAGSAA
APGAFIPSPFSTVGVVAGSLPTAATSAPAAQSPRQPSPLQPQEQQQRLAAAVAATGSRLPAACGQVVMVVEPVELRG
GAAPAGTAAAAAAQAATADDVTVRMQYISLASVVSTPLDRIPTVVEAEAGPMAVQAAAMGVGAGPTVSLPVPPVAAG
MHKPTAAGSPAAALPLPGEQLPLPALSAAPTSSPAPVAAVPASLASPAHLASPGAPPDEDPHASYFDLDLMLTLRRS
TIYRSTSNALPSHIGGLGTARASGPAHSQSSVSGAPSPGGAAAAAAVAAAAAAASSVQFSRGSAETPFPGRRTAEGR
DSSEGNLPAHGGSRAAARRRSSVASIEADEDSGLLRLLEQFSSEQRNSRQQQQQRQQQQQQQQVRGSRPSVDAPVDL
VRAGTALSSSATSSTNAGSGVNAGSSTLVLGAPSVPSRLRPPWSPPPTVGPQPGAATTSSGGGGATAASGSGLTFPM
PLEPRSRRDDRSSAPAGGAAAAAAAAAASAAASFRISQGDAASDAEPGYFGVSPNSSFITFQTAATPPAASSLWSQT
TRGSASPLVKQPSQLQPQPPQLQPHALSLPLTPLPEHMEVHEAEPRMTSAAAAPPPLPPAASAAPGPLPQPHSLLVP
PSLHQGQLDFRVWPGAAHVVAQLLPAGIVVGDPVSPPGAALAAQRGVGGRVGSRTLPSPPSSRLSWTVGENVQGASP
EGSAVTRQRTLGSLHRNFPTAATSAAATAGAAAAAFAAGAGLVQPTDPVLRSVHSLASPPGAASSSGTIEVEEEERS
TGRLFGRRGNSGPIRKAMSSGLDAPVFSTVLGLFGVGGGLGSGTSRPSRRSLFNAAPAGAHQSSPFRVAASSSAGVA
GAARSPRLSMGNRSSSGQAFTSHSGAHHAQIAALAAAVSSPGSTYGAVASTAGAAAAAAGGSGGGGGGTNTGTGYRP
RLSSPNFTALDDELLAAARWSAARLEQQHHNQQQHGQHHLQQGRLSPAYGGGAAPHHPSPLSAGASSHHHPPGGAGH
SPAHSQTTVAAHPHGNSPASPLLQHSGGGNYFSRIASARRSGACSSASLTPAGSMGLGPPSAALGSPPAGQQAAEGL
GETMAPERSMCVISGGVLVVRGSSCGPIGLAAAAGGDSLPEGGGSGWRESGAVTAAGGTVSEAFELGMGEAAGVAGA
PGHAPAGASAAAGVMASARDADASCSTRVDSSCSTVGGADERRGGPAWEVRQASQARQEQLVSEVSSWLRVALARKL
AGGAGPGSSGGAGSFSAAGGASSRTGSITIGHEDGGAAAGMLGGRERVTETKFQRISDVDDDTMESEAASEASHD

>g9880.t1|PACid:27570022
MNNEPAASCPGHKRRRSSSEEGEVPLPVPKKVSNAATEGAELASSLQVREQRRAEVKAIYANYGDLLNGGDTGAEAL
AFQGLLECRRGSLGARRLAARLVPRFLPRFPSHTEQALRLLLALYSDGAPAPAPPATNPGNTLRGGNAASLPNGVFK
PGPGASPSPPPQGAAAVDGDGGRAAAGGREATSGLDGSDLAAAMRRDALLGLGNVLETAVRQQQQAGGAGGAPAGAV
AAERTVPVVTELVQFVLRQLQMHCVGAGTHADGGSGGAGDGKKADVNGGGGGAGLGPHYGQSHSHSHRPSDSHKRPR
HGSDSSRGRPGPGPGGGGGGSTSGARQDAAEAAPAAAAAAMAAAAAGPLPPLPAALLDAEPDSEVGVLRGLLLRAFAL
FPRVVLAACLAPCRLPPRNRAHEDLAGSALALGLLLLQQQAPPRQAQAKATAQGKAGGGSDAAAAVGTPRAGARAPA
TPAAATGVKTEAAAAAAGGGDDCEASAMDATGPGCNGGADGGGAPGAGNASSAGAVGGNNGSSSNNGGGGGGRHPRP
SCVAQQVLGRLPETQEWVRQLVGALLRGKNAPPVPAELRSQLERLLSLCPAPKRPAAAAAADAAAGADRGAGAGGGG
GGGGGATCSPPCSPRGAAGELPPLPPLPTPTLTPSKAAAAAAAAAAAGGGGGPALSHLGSEDGSRSVPMDLDQSISIK
HEGGGGGGGGGGIGSGGAAGGGGGGHGGPADTAATSPPPPPLQPPPATTATTPAAQGASGGAAGGGGGLRGGLVCRA
EACLWLGGLPPGLTEAALVSELGRSGAVESVHAVEGAPPPPAAAAAGGAAAANNSGSHSHSLVGEEVYVVFSSLRDA
AICFEAVARTAPFGGARPLAVEFCAAYPPESAAARRRAAAAAGPSQFVWVSSSGHGAAPPTATPEAVVAALQEAGVA
VPQQILAVQGRSPGMLLHMAAAAAVPAVVACLAAAFAPPPAAAAAPPVPQLPPPPPGGGGAGAAAAAMAGTAAGGGT
PGAGTAACLTPPPPPSHPGVGGGGSGQTPAAAAAAAQLPLPPPPPQPHHQQQQQQAGGQGQGQGQGVSDPLPPPNIR
TLWFGQIHESLRDEDLLAACRQHGGEVVAWRFLRGSHCAFVDFATQAGADAARRALLSLRLGPSHQHVRVEWKLDSN
MGPRGGMGMGMGGGPGGMRGGPGGGGGGMYGTPGGGMGGGMGGQIGMGPGGGTPGGGMGLGMGQMGGGRMMMMGPGG
GGGGGGMGTPLHPSQQQQQQMLQMAGRQTPLQQQMMQMQQQQQQHHHQMQAAAAAAAQMGGHGHGHGHAGASPAHPS
LPGYGGSSTGNPQQQQQAHHHHSHPRKGGQQPGPGGGGGGSGGSGDWSGQLPPAPGSAAAAAAAPPPPPPPLPESGL
PPPPLPPPPPGPPGPPTAAASPAATAVSPARATAASPAAAAAATAAAVAGGGSSPATAPTWQGALAKSGSHVCTLQC
NTGGASAASGASPGEREPVTWPSCLDVRMRVELGYVVHSLYAHTPPAARALRRLVVSGSGGGGGSERSKLADFLGYL
AEKKRAGVIKLEAAAGLPARTLYLVPPNEQVCSALGTEWTPREPFLLALVVPTAGAAGGK

>Cre09.g404750.t1.3|PACid:27570052
MVGCYVNANYLFAALPAPAADSSKVIARVLAQECLRRGRGSQPCPIDFFQGSLPVPADLLRNTLVCRGSLLDKLLTC
ADLAGPDRPSGGADACMAAPGCVWTTRSSVEPIPEYLDLIEGGGANLRVNTSTPGLNTTAGNATYLYDAVFDADNMA
SILQGLMHYLTGAPWPSYQDTAPTTLCTARWATDRDRLAQVYKQYYKQTDMTLDGSDTDEEWALKFFGSPSAAYVFG
TCAASRTFLSAALKCHTSTSNKDACTAQPYCDYVRVDPTYQTCELGRLEPLVSRDAAIFTSDPWGAALASLTAKCMP
LLDSNTGRRYGSRDCREYRQSPEGRQGLTAQFDAVTQLQAPGAPLNPANLRPSFYVAPDTTPDPADQTASGAAKTVR

LVGGTSASNGRVEILHNGVWGTVCDDSFDVWQATVVCRQLGLGVYGTPYTHGGGSGTIWLDEVYCPSYVTRLEDCSS
GGWAVHDCSHSEDVGVSCTSFRPVRLVGGTRLDNGRVEIFNENQWGTVCDDYFGPNEADAVCRQLGYMVGVYYTHGS
ADINVPIWMDDVKCPASASRLEDCSFLGWRVNNCGHREDVGVVCYGQLPSPSPAPPSRPPRPPKRPPPPRPRLSPPP
VPRPSPAFETSLSGPAGFSLLAVRYTPRASNTPPRWSSFDVAALAPGTGMLAYVWAHVPAPDPAGPPRAPDYPACLP
DQGWDDAAADLACRLTGFASGRVALTGPESQYPAVGLVSVPTMVMNVKCPLNRATRRSELQDCTASLRRQATGMCTS
MLAALCTTARVISPSPPPSPRSSPSPGRSPSPAARPSPKPPPATSSPGVRRPPSPRPPPPPEDLLSPPDAPDIPPPP
SPSPPKRPSPRPSPSGSQQGGSGSSTGLTLRLVRPDGASSTSKSGRLEVLNSGEWGTICSDGFTNAAATLACRALGL
GISGRTIPEYTSFYDAGTGPIWLANVDCSAVSLIDPSASLADCAHDDWGVTPDCTHDQDVGVKCT

>g9613.t1|PACid:27570088
MKRGRGASKKAAADSGSDGEPEREPLDPIAAAMEAKRLRLLEAAGRGAAAWDEDRYLRAPRVDSAPDLQAAPQPSAS
MAPAPGPFTVVTTITRIPRRPPPAAAAVAAAAGAPSASAAGAAAAHAAQAGGRGSAEPEGEGGLAALLGYGDVAASD
DDDEELAEEQEGAAGAGRAAAGDSAVPASNTPTGSGPVEDAAAVGVTEDQAAPAAQQQGDGMDTELASFMQELEASG
LLEDGGDEPPPLPPQPHREAAEAAPGATANGGEAGATASADPAAVDAVAGADASAGAGDVPAHDSAGELAAAGLSAG
AGVGASGSSEEQQLLGYLVGAPEWCKCLDGGSGRTYFWNLTSGEVAWEPPNGADPDQLLPPPVGPGAAEAEAAEAEA
AQAEKENGEEDQEDGDAILNDAVRAAGSEEPADAGEAGKAGEAGPSPAAEGGMEVDDRGPGAEEEEEEVQPSVPPNS
ALAAAQERAPAVRAGDGSEQHTGEAADVDAGAGRRPAAAAPAASSGAAAAAPDAADAASGQGEAAAAAAAMELLQAV
TDMVLRPPQPHIHQVMAALETEAEVAARGYMESLSPLVRLAVEAQVRAADWRVLAAQQAAAAAAGQPADAVTWPALE
RHMVGAVRLLLEALPQARVDAERSRELPGAAATAGAAASAGAGACGRPAAAASADAAPIKGAVSPAPAPEALDADPV
AGRPNGPAGARSITVGGESQAGKATAEVADEEQAGAEEVQAGAAGSEPGADGDVDMEEGELLEEAEEAVAGPQQPPD
ARSTAPGSPETAVATSTQAEQSAADPPPPSPAHPPPPGSPPPPPPDVGLPPLPGSPPPLPPEAPPPPPHAADTEDGS
DDMAIDDDDEAAGAGTAAAAPAAVAPASTAANAAAATAAVAAHYYPHYSHDAGAGLPGAAPGAALPPGFEAAAAAYA
SGAAYGASAYDTYMSQAYGYAYGMPPPPASTPAGYEALQYVNSYLGYGAAGAAGVDAEGAAARPEVKSAKEYRAEPI
RNPAAAGAEGTPEPQSGRASASPTPPPLDLLNMHAATAPDAAAALAAARRKATAAGAVLVGGASAAGGGGAAAVGSK
KVKAGGGGGGGLGGKMGAMVQKWSAAQRELEAEEEKAARKAAEAEDPNALERKRKAELERWKWEQQVTGAATSNANF
APLGDWRSRFPKMAEGADGAATKKDKKEKKEKKEKKEKKEKKEKKEKLLTTAGAVDAAAAGSSGAGGGGAAGGAGVL
NCNSRPDLDALSAGLPSGWRAMWDKSTGDVYYGNLATKETRWVRPT

>g9811.t1|PACid:27570153
MPLPSPSPFPPPSPRPPSPAPSSAPSPRPSRAPSPPSPPPPSPAPSPPSPPPPSPAPSPPSPPPPTPAPSPPTPPPP
TPAPAPPSPPPPSAAPAPPSPPPPSPAPSPPSPPPASPAPSPPSPPPPSPAPSPPSPPPPSPAPSPPSQPPPSPAPS
PPSPPPPSPASSPPSPAPPSPAPSSSPLIIGITLKCRILSIRAGSIVVEAELGAAPGVDAADLQADLSAAASTKTLL
QPAFVESYAITESVVSIIPSPPPAPPFLPSSPAPSPLSQQPPPSPPQPPSSPAAPPSPPQQPPPAFCPVCIGWEVQA
GPVFNGTYFFDNPDNCQQATTYVSNLLHTYTIPQQGQADGGFIALTQPVSCRGNRVDVCYTSFSHGIAAKLRTDFEL
LNPLNYLGSNNLITPVFGFQDFFACPAHLAGMMLDFTTSNSQCGPIPSGWYCDPLPPPPSPAPSSPPAAPSPTPPPS
PPPLPCSVCIGWQTDARPIFMGDYFFHAYDNCGQALDFMESLLAPYLEEGVLVPAQAQVRCSGTQLQYCYTAYSSER
AAQVKAGLEALGVAGLIPRAFGFSDVSSCPEDLAGSTLQFTSSNSECGPVSTQLTCSYLPDPLPSPASPAPAPEAPS
PPQPPPSPPQPPSLPPLEPSPPSPPGPPEPPPPCAGCLIWHLNGGPEVFFDDTKNCDRATGFIKQLLAPYYLQDGSI
ISLTSPISCFANVIYVCYAAPSTESVAQMKADLEALGFSGLVTPAFGFVGNECTSEFAGTTLEFTCNTFNNVACGPS
STKLTCAGLPSPPAPLQSPLQPPPSPAPSSPPPCSVCIGWQTDARPIFMGDYFFHAYDNCGQALDFMESLLAPYWEE
GVLVPAQAQVRCSGTQLQYCYTAYSSERAAQVKAGLEALGVAGLIPRAFGFSDVSSCPEDLAGSTLQFTSSNSECGP
VSTQLTCSYLPDPLPSPASPAPAPEAPSPPQPPPSPPQPPSSPAAIPLPAPSTPAPPSSPPPPCSVCIAWQTDAGPI
FEGMYYFEIPDNCYRAIRFVATQLLPPYFFRVLPDGSAGEIRGWSSVTCRRDKVDYCYEATTRGIAANFSAGLQALG
ARGLLSPVFGGSEDGTCPGVFPAGTALEFTSSDSQQCGPPTIRTVCDGFQPPKPPRPPSPAPSAPSPPWPQPPASPG
APAPSPPPLPPLEPPPPIAPAPPSCSVCIGWETDAGPIFDTGVHFFDVPDNCQRATDVVNGLIGGYTAAGEGNAGYI
VLTQPIQCKGDRIDLCYDALSPATAAALKADFDARITLFYNGLISPVFGIPDFFSCPVVFAGKALRFTSSNIGCGPI
NPSAGSASTSAGTSASTSAAVPKPSDGSTTISPIITALAYPTQPVSSCWTRVHRVAVSSTTSILLKAAGMACNS

>Cre09.g406500.t1.3|PACid:27570213
MKTFARKRKSGGADELANSQRSASPSAAKSQRTNNLSQQNSDGRHAALRTSPRRAAGEVPRGLSPLTRAAGKRKAEN
ATEEPQKAKQLRGASPGGRQEAPERKRQRNAADAHAAGPARPATRKLASPTRATAPAPASPTAAAGAALGAAAAAAE
APSPATNTAGAAAATPTRRSTRSSASAAAAASPLTTTTTTAAPAAATTAAPASPPPVVSSPRGARAAASQAAAGTPA
AGGAGGGGADAAVTGRAGGRGAAASSPAAGGCGAANGNAEGGEEAGGDGRRGVDDGPPAEAQAAAAAGAARGGAAAA
AWHKAAAAAGGEGAAAAGGEGAARRRGVKRGAAGAEAGLVAGTAARAAPADPAEEEPVPPPPPPPQQHPPQQQARSP
RDRRTAARAAAAAAEVPPPPPQQQQPQQQPQQQEALSPRERRTAARVAEAAAKAAVASGPACAPATAVTTTATADVQ
EPGGCDGQGVSAAAPSAAAAAAPAAAAPVAATAAAAAATTAEEPASPPPAATREGRPRRGAAAAAAATASGGSQSPA
AVGLSPRGGRRARGAGQRAEEPAAAAEEPAAAAAVKEAAAAEEAVKEVAAEEVAAEAATEEAVGAGKSDGGAGGRLG

RRRPAPTRGKAAGGGGGGGGATAVAHEPAGPIQALEVANEAVEKGMAATAAAAAAAAAAKAAPAAPAAPAAPAAAAA
GSPSKGTSLFARRKAAGAAPPVAAVPVAVAAAAAAAAASAAAEPQERAAAGSGALDATPTQPDQARKVPLPPLRAAH
AAPRHGAAELKAPAAAAAATTTAAPAAAAGAIPAPAANGLPPPLPSQPPPPPPSRSASVPVLAASAPALGGSAPQPS
LPSLLGPPGAAVLPPPRSFSQPTNSGGGAAAAVAAAAAVAAVAAVAASNGAVRASRPIGRGRLPGSGGAAAVAAGMV
SAVAAGGKPPLASSASAPLPAAAGCGGGGAATTGGGGGGAGPSRMGAATVTFGRAGSAAAAANGGGIVATAGLATAA
GSAAAAAVRGAAASASERGLGLASAPGGSGSGVGGAGGAGGAGGAGGGGSSSQQQQQQLQQQQQLQGAAVSLGGARV
GATGSQGEAGEAAAAAAAVARSIVEAQESGEALQAQDDAQYALDGLRQGPAAAPGAVREAAAALARLLATARGRAVL
SRPQNLAQQALTCLARTNAAAADAATATAAALLLLSLCGSRDAAPAYGASYAAAVLWRQVLQQAESDAPPPVGSCPP
APTSAWALLSSAREALAATAAPATAATAVPATAAPPATAEGAAAGAGADEEEARGAGSSTSAGAGGSGSGSSTGPEA
VAMQRLLRLMSEGALSRQIGRNDGSGGPAVAAAGAATGAGGGGSGGGAGGDAAGAGGGGGGGGGLGCVVGPLPLALL
ALQAATHADEKRHQFAARLKDNMAKVGLLPLLARLLAARRDAVAAHLAATARWWRQQQQQQQQQQEEEEEEEEEEPQ
EAATADPAAGPAAVAVSPDTQPEAAAGSQPPSSSLAASEPSAALLPPPAATATAAATAAANVAATATAGSDPYAFGG
SLPCPGSGPSTQQEAAVAAGPPGGTAVAGAGAEREQAKREVAAAAAAAGEPAPPAAAAALPRWLVWEISLLLQVLEN
ATFTHVRNENELLALRLAVRQPLAAAAAAAAVAAAHGGGSGGGGGQAVAEGAAEPAVDGGGAGPAVAAAVAVADAGE
DGKVPFVAVLVDLVRGLSELAAAAAAAAAADGSSSSGGAGSSSAAAGASVDPVYGTHHQQQQQLLQPHPIAAAAAPA
SAAALYDACQSAAAVLMNLTHEHPDGVAAVAAAGGLAAAGSVVWRCCRPSPCLAPSTPPALGSAGAAAGPAGAGLAS
GVGVAAATGGTDAQQGHHHQHQHQHQQQEEEGLRGGGGGGGSGGGGGGARRWRVSRSDLVCRVELLSVVLGLLINLT
SNSPDNRAQLRAAQLLAGPGAEAREAAEAGQQQHKDGGGGSGGGSGGGGEMVPLLCAIVNTMMPQQLPPPQQQQQGR
QPPPQGGKQPRAGVTAGAGAAAGAGAAAGATAAKTGASAAPVVPVDVMEVDAQDGEEQGREGEEGDGEGERERAQQR
PALRLAPSPLGGQQQAAARRVGAAGAVDGSDGGGGGGGGGGSSGSSGGGGGGGGLQGPAESARQEVGSSGGGGGGRR
SGGGVGPGFEEVTEAALHEDQQDGQAASAQFYSAVLLGFLVAPAGEQQQQQQQQQHGDNRGGEIRGADQPQQQQAAA
QTGSQGGGQREVAAAARAEARQLLTGGSLTPVTSALLRGLAFYCQSGAITEATRDMLAGLAQRLTRLEAEENPPPPP
PPQQGI

>Cre09.g406500.t2.1|PACid:27570214
MKTFARKRKSGGADELANSQRSASPSAAKSQRTNNLSQQNSDGRHAALRTSPRRAAGEVPRGLSPLTRAAGKRKAEN
ATEEPQKAKQLRGASPGGRQEAPERKRQRNAADAHAAGPARPATRKLASPTRATAPAPASPTAAAGAALGAAAAAAE
APSPATNTAGAAAATPTRRSTRSSASAAAAASPLTTTTTTAAPAAATTAAPASPPPVVSSPRGARAAASQAAAGTPA
AGGAGGGGADAAVTGRAGGRGAAASSPAAGGCGAANGNAEGGEEAGGDGRRGVDDGPPAEAQAAAAAGAARGGAAAA
AWHKAAAAAGGEGAAAAGGEGAARRRGVKRGAAGAEAGLVAGTAARAAPADPAEEEPVPPPPPPPQQHPPQQQARSP
RDRRTAARAAAAAAEVPPPPPQQQQPQQQPQQQEALSPRERRTAARVAEAAAKAAVASGPACAPATAVTTTATADVQ
EPGGCDGQGVSAAAPSAAAAAAPAAAAPVAATAAAAAATTAEEPASPPPAATREGRPRRGAAAAAAATASGGSQSPA
AVGLSPRGGRRARGAGQRAEEPAAAEEPAAAAVKEAAAAEEAVKEVAAEEVAAEAATEEAVGAGKSDGGAGGRLG
RRRPAPTRGKAAGGGGGGGGATAVAHEPAGPIQALEVANEAVEKGMAATAAAAAAAAAAKAAPAAPAAPAAPAAAAA
GSPSKGTSLFARRKAAGAAPPVAAVPVAVAAAAAAAAASAAAEPQERAAAGSGALDATPTQPDQARKVPLPPLRAAH
AAPRHGAAELKAPAAAAAATTTAAPAAAAGAIPAPAANGLPPPLPSQPPPPPPSRSASVPVLAASAPALGGSAPQPS
LPSLLGPPGAAVLPPPRSFSQPTNSGGGAAAAVAAAAAVAAVAAVAASNGAVRASRPIGRGRLPGSGGAAAVAAGMV
SAVAAGGKPPLASSASAPLPAAAGCGGGGAATTGGGGGGAGPSRMGAATVTFGRAGSAAAAANGGGIVATAGLATAA
GSAAAAAVRGAAASASERGLGLASAPGGSGSGVGGAGGAGGAGGAGGGGSSSQQQQQQLQQQQQLQGAAVSLGGARV
GATGSQGEAGEAAAAAAAVARSIVEAQESGEALQAQDDAQYALDGLRQGPAAAPGAVREAAAALARLLATARGRAVL
SRPQNLAQQALTCLARTNAAAADAATATAAALLLLSLCGSRDAAPAYGASYAAAVLWRQVLQQAESDAPPPVGSCPP
APTSAWALLSSAREALAATAAPATAATAVPATAAPPATAEGAAAGAGADEEEARGAGSSTSAGAGGSGSGSSTGPEA
VAMQRLLRLMSEGALSRQIGRNDGSGGPAVAAAGAATGAGGGGSGGGAGGDAAGAGGGGGGGGGLGCVVGPLPLALL
ALQAATHADEKRHQFAARLKDNMAKVGLLPLLARLLAARRDAVAAHLAATARWWRQQQQQQQQQQEEEEEEEEEEPQ
EAATADPAAGPAAVAVSPDTQPEAAAGSQPPSSSLAASEPSAALLPPPAATATAAATAAANVAATATAGSDPYAFGG
SLPCPGSGPSTQQEAAVAAGPPGGTAVAGAGAEREQAKREVAAAAAAAGEPAPPAAAAALPRWLVWEISLLLQVLEN
ATFTHVRNENELLALRLAVRQPLAAAAAAAAVAAAHGGGSGGGGGQAVAEGAAEPAVDGGGAGPAVAAAVAVADAGE
DGKVPFVAVLVDLVRGLSELAAAAAAAAAADGSSSSGGAGSSSAAAGASVDPVYGTHHQQQQQLLQPHPIAAAAAPA
SAAALYDACQSAAAVLMNLTHEHPDGVAAVAAAGGLAAAGSVVWRCCRPSPCLAPSTPPALGSAGAAAGPAGAGLAS
GVGVAAATGGTDAQQGHHHQHQHQHQQQEEEGLRGGGGGGGSGGGGGGARRWRVSRSDLVCRVELLSVVLGLLINLT
SNSPDNRAQLRAAQLLAGPGAEAREAAEAGQQQHKDGGGGSGGGSGGGGEMVPLLCAIVNTMMPQQLPPPQQQQQGR
QPPPQGGKQPRAGVTAGAGAAAGAGAAAGATAAKTGASAAPVVPVDVMEVDAQDGEEQGREGEEGDGEGERERAQQR
PALRLAPSPLGGQQQAAARRVGAAGAVDGSDGGGGGGGGGGSSGSSGGGGGGGGLQGPAESARQEVGSSGGGGGGRR
SGGGVGPGFEEVTEAALHEDQQDGQAASAQFYSAVLLGFLVAPAGEQQQQQQQQQHGDNRGGEIRGADQPQQQQAAA
QTGSQGGGQREVAAAARAEARQLLTGGSLTPVTSALLRGLAFYCQSGAITEATRDMLAGLAQRLTRLEAEENPPPPP
PPQQGI

>Cre09.g391600.t1.3|PACid:27570223
MQREGAPGCSAAVPSLPPPVQAGAWAHPPPPPRAAARAAAAKMQMQTQAGSRHTGGSEGGGAAAAPKDPPNPASQAA
AAAVVVGDNRQPPAKRTRGSSSMLRRGPAPGGQVDAIRAAASATDGGNHGGGAGLPSASPAADTKAPAKRQSARLNS
AGLQETQPQAEQQQQPRRQQQGQQRQQQQGSLWRAARSAEQQQQQKQQAEEQQLQRQVEEGQQGTATATGARRQAPP
QRAGQQPLKRLRAAAAAAAAAPAPAGGGDRAGSEAVGGNEGNEEGDASHGKTERAEPPRPGAGEAGAGESAQGAGAG
CRGAETAAAAAAAAPQAASDGAGPGSGNGKRRRGASTADERPPTAPPATRRRSAPTARNAAAAPAASPPQSRPASPP
PGAPLAPAAVAPRTAAIATAAAAAPATAPATAATTGAAAAAAAAAAVSYACVTCGSPYLLRKNKQHGFFTHRTRAYT
CDTCCRRNVSSHGFYGPTGTRSLEEAGGRECANCGKGDGNSRWRTHPTRLGLYLCRTCSERGLWRSGEGSAPEPTGT
GAAATASGGAAAAAAAAVAAVARTTTPEEAGAAAAAVAQAGERTRAGEGEATDSAGAAAVAGAAPVSADAAEAPSAE
ATSVQASGQQHAERVAAAAAPRPGRRRGPTREARRAEAVAAAGEDAAPQGVEPPMALAVKPAADVQTGGTAQAQVAG
DAAGDAAGDAISQAAAAAAEAMAVAAMAAEEAAAAAAVPHRGTAAVLLEAAALAAAEVPADVAQAAPADVACDVPVV
AAAATAQPVAAPGPAAQHAREEPLLGMQGPGQPPQPPQPQPPQPQPPQQPQWEPQPQQWEPQPQPLQPQQPQWEPQP
LPPQQPQQPQQPQPQPPQPQPPQQPQWEPQPQQWEPQPQPLQPQQPQWEPQPLPPQQPQQPQQPQPLPQPQPPQQPQ
WEPQPQQWEPQPQPLQPQQPQWEPQPLPPQQPQQPQQPQPLPQPLPPQQPQWEPQPQQPQQPQPPQPLPQPQPPQQP
QWEPQPQQPQQPQQPQPLPQPLPPQQPQWEPQPQRLPPQPQWEPQPLPPQPQWEPQPQRSPQPQQPQWEPQWEPQPQ
QPQQPQQPQWEPQPQPQQPQWEPQPQQPQQPQQPQWEPQPLPPQQPRSPQWEPQPQQPQPPQQPQWEPQPQPPQQPQ
WEPQWEPQPQQPQQPQQPQWEPQQEPAPAEPHPQHQSPPDGANTAEAEWAAVEGAEAEGAEAKGAEAEAEAEAEAEA
EAEAEAGVEAGAGAEAEAEVEAVAEVGVGTGAEAEVEAEAVSVAVADAMAPVPRGAAQATAWLAMPSPPPPPPAVSP
AALRPPRQPRTRRLAELAEEQDPAAAVAGPSGHLDEPGGGREAGGPAPPQAMSAGNAGDAAAVGNGCGSGGAGGRGS
GAAEVVAGRAVPPTAAYGGPGPMAAGAAAADVSAAAAPTAFMTAAELPCGVPCGVLAAVPAAAAVAQSSVGGQGLRA
QAGAWGQLRQDHEAARPMPTMSAGQGAEANAGAGAKPGAGAGAGVEAAPDAVQAGALATPGNHAAALEVLPLLLDFL
PLRLRQLHMQRQTLLGLQPLLEQADRSDMQQQQQQQQQQQQQQQLQQQQRQQQQELQQQELQQQELQPQRGRLGRRP
LKMVELDFRWCAVCGAKCGGAAGGGGGGGGGAGAGGGGGGGGGGGGNAADAWVAWFECGDPSRIVCAPCHERKAASE
GVAAAGGKRRKGARVPTTAAAGAPAASSQRMPPAAAAAAAAAAATAPAPAPPAGTRAGTPGAGTPGAGMPGACMPL
RPSSAAGEGGAAGRHASGAAAGQAQPVEASAGAERACANHPDGPTPSAATSAPASGRPHLSLSRRLHLASAGARAAA
AAGIAASLAAASKQRDTAVPGPSVKAMAAFMAAAQRAGTPPQRQPQAQPQAQQQAQQHRASDGGARDAAPCLLSGEP
QPQRQQPQRQPQPQRQPQRQPHPNPPNPPRLMQDLAPLSTQAATLTSAGTLAAPAQAAGVLPAYAGAAAAAYGLGAG
AATAASLATAAVAAPASVRTAGAAALLMPAQRHMLLMRQHQQQQQQQQQQQQQQQQQQQQQQPHMQAPNQQQVEAQL
QDAQLQQLAQGTLASMQQLRLQHHQNMQRRQQQQQQQQQQQQQQQQQQQQQQQQQQQQQQQQQQQQQQQRQQQQLQQ
QQQQQLQQQQQRQHQPLQLVQYQTPGSFSPPGKLLANSTLACAHASSHASSHASEARDAVVSLTAAALPQAPPLPPP
RSAPTPLQPPPTPTPTTPPEAISPRAAAAAAAAAVAAVAEVAATGTAAAAAEAATAAAAAAKVAAAAAAAAAAAAPA
YRGHTAAGSLQQPLVASVAMPGVIYHRRSGPGAVTGGSSGPVSAGGPVSGGGAGGAGGGGAGGGGASGGGEGGDGGP
GAMHVVDNVPEEVRQRHQHFLQQQQQQQAELQRQQQQQQQPHVLEPRWQPRLLLQVLQPEEQQQQRLQPEPPEQQRL
AQAYARLEAALSSPPGGQGQEKGNGQHGQEQGRGLGQPTMPAQTLMPPLMTSQRGAGSAAAGGGGFAPHTAAGSSG
ATGSVTLGGGASDSAMLEWVLLGESRPPPQPPAQPPAQPPPQPPPSAPAPAAVPAPAPAALAPLAPLGSGLHRIGAP
TRGPAATAAALVGTGTGTGTGTGIFAPTAATAAGYVAHGGAAGSVAAATQAAAPAAAAVASSSAAGGAAPMRVRA
PTVPHASAPPPQHTQLQQHQQHQQHQQQPKEPQRRASTGAMPAAWAYPTLAPPPALWGGDGGHDLQPPTTQAAGHRV
PSHARTSSPPRAQPQPQPQPQPQPQPQPQPQPQPQAQAQPQPTQQQTLQQPTQQTLQQPQSEPQIPQLQQQQLLQLQ
VRSQQPPHAAAAWGLSGAAPQATAGVPAAASPAAAVRPADTTVRPADTTVRPADTTVRPADTTVRPADTTVRPHAEP
WTATGAAPQQQPQQQQPQQQQQQPLQQHATQEQRQDSHPAAHAGRHASQAPQPTRPGPLSPPTRVLLPPEPHSEAG
GAQAPGAATHCAACGCSLANKKVYRHKSTPGLKTCKSCYCRWCRTHSYNDAGKTRAAATAAAGAGASAAAAAAPATA
TTATPAAGASSLQPGRRVAATRRRAAAAVLREDTGGGSDASAGLKSEDGEQEVAEVEAEVEAGRGTKHAAGPEAAAG
HGAWRLMPPVKRPAAVANDEPCARGTGSPLPDSQSPPARERVLQQTALVVAAAQGQGETLQGGPSPDAAEQRMEEER
EKMEGGDEEFGQREAAVRAPQHPRPQQSRLQPQSHSLQDPAEGQLAGVASSGEESAAEQRAAKRAGGRAGGRAREHA
AAVLLAESGGHGQVEEEAEEEEEEQEGEGEEEMEEEVRDKDLERRGLELGQWPDVGGGGLEPQSAGRLARWLRQYCG
ALEATVEALDTHQQAQRQHQAQQQQQQAQQHQHALRTCGRGGLPGPATAAGDDVVGYSCAGGLGTAAAHPRSSDLAV
ATNPNGRGLAPHRGHAPDLDMQRRLADGINRWMEAHVWGRPLALMLGQEAEALGLDLEQPPLGLAPEEQTGWQCSRA
RVCAAYARVADAAMNVAQPQQQEGKEQEAKSGHQQQQQRQQKEMEEAEEYQQQRPEEARSPPSAAAVASFRGVTYML
AHQLAAELAWVGPHAPTTTSITTTTSSVGAIGASGVDGRGVEGGAPLQPEWRHQGPRPAPEQPPLGLTAPPLAAPAP
AHPEGRQQQVAVPAAVAAAGGTVAPHGAAGGEEGREAVAARERAADRLRPLLLRAVCEGLRLALRVGSQLTTRRQQP
QQQTQAQEQAQEQPAAVPGSGAGGMVGAGGVPASAGRLLLRLSAVGGAGAGAGAGAGAGAGAGAGGGGRAPAGAA
APAPGGAASSTALVLVGAPRPAAEAAAGPPWQSQQRESAALCLAPGVYLPAPASAPAAGGSGAMVAAADAGTGEPGY
LRVIAPLVVTRAHH

>g9649.t1|PACid:27570235
MAAAAVAPPDEKFLKLREENERRRKKKEEEEKRRKQDEELKTKEALERRRLERVEQDERMRKPWQQPPAPKKPILRK
GAKSKPSQPPTPSAADPMRKSQQEEKRKELRQGLQDFIRQQRQAAVAPKNPAGAGGGGGGGGNDGAPVVPLTWEELR

LAAEAGFDELVRQQEEDAEQRAEDPVAARATRHRSPYKSPFRGAPAPPQETVQVALVPTQQHAPIAAAAHVPDTPTT
AVPATADRMAALRTSYKFSDAPGWTGQHPAALQQHQPQRVGLDAGTGGGPMATTPAAVLYPPGDGPEAATGSGDVGA
ASSVASSGGLRRGVTAAARYRVAPLDAETLDSAAEDPWGRDNAAAGGAVVDAGSGGAGEGVGVGVGDSGPPGSPGGS
RSPRGSMGQRPPLPGPYDSPSQQVWPPPSPQHSQQQQQQQAVGTPRRPAAVAVSGPPADPLEASLLAGGTLAALQQ
ADGFRLTSPRPAGTTPQQSWDQPAASAHVQEPLSLQPSQSADYARTQQQQQQQQPSAQKTARRGWGAPPGQAQAPL
QQPGSPGAHYGDDPPMPAWAQAGSGAHGGGAAPEHSGAWAGGEAGGGLGESGADFGGEVGLDMGASGGLAGAGVQTA
VRHSIGVGDSGGPAMWQRESVTLPRASQAQVQRAPADFGTPALQPRHEDSEPQFQAVSPAEVQQSVPLVPAVSPAHG
PEPGARLSYNDVPLQGSPALTASVASSTTSTPARAPSEAARSVSLAASSPHQAPAPGVVSTERSSAVGAEASRGLQS
AVDASEEAVRASTGGHKQGLEDAVLGDVAEAEHEPWVPPMLSALPPPVNISTDGLPAAAPVVLRHEPLQGSATGAAT
AALTHLAAMAAGTALPPVTAAASAAPGVATSQNDKAVLPPVITSRPLSGSARLPPLPSGFHSGGSMSGSLPPVAASA
GSMTGTPPPKSQPLSASGSAATTSTSISTPTTPPQQQAHALHQPHPPPMRAPSPPGSPPRNGTPQRFHRQLSSSGGS
RPQSADLAERSAASMTAVASAASSTPVSPPQAQLTQPQSALAPQPPPQAPPAHPSRPRSADLVAAASAALPASQAAP
TAGAARASPPPALNTTPLPLETPAVAAAAVPASPRVPGVVVTTPASGTSRPHRQLQQSAPVAQAVQAVGAGGRLDGG
AVSASRDSALPVEENSAVVVIEDDKDSDIQADVEVYMDDSGLASPSPAHTSLGAPAQPVGAATVTNMPAAVKPVGTA
ASLSSKAEASPGPDEAETARLQQQQQHLLHLTDASVLPSRPPGTAGSGMGSVMSGSHISAAPLSEAGSSSSAGAGGA
GARPGSAATAGMMGSGYQASLLGMLDDSVSSFDFRVPRSAGGNSGGAGTGSRPSSAAWAAPASPAAPVHATIAAAAA
AAAAAGVPSAATARKGDSVPGKAGPAGNGVVTVAVKAGAPVPLGCSAAAPKAGHAGALPSANSFQVLQPSKAAAAAR
AVSGDLMYGGADDGEDDGGRGFDEEPVGMEEPVLPQPASYHLAAIPPPAVAPPLQLPATAAATGAPATAAPAAAAPA
GAATAGAPAAAVPAPGEPDMDDLMREIQALSALRKALKAEFEGMGHGEAVAGLQTLSANFTAAAGSNGNGASATASP
AHYAGPTQGNSASSAPEAVGIAGAAAAAAQPAPAPLLQWGFGGGAQPPPPQQQQELYGTAPAYTVPPPMPMQQQQQP
FEQHMQMPAWAALVMPPHPVAEPQPVQALPQQHPHQPVAQPSTAQAPYGAPWPQADSAQRHTQQAAPPLATTLPSAA
ATLAPGGVAGAPFPADVMAAAAAAAAGPNAVAAQGSVPQYYEEDPHGRSFAFAAPQQTAAGNGFYQQQAALPEPLPL
APPVMPAPAPAAPPQPAAAPSPPQPALQWTIPAAPGSSSPGAAPQPQVSPQRPARVSGGGAATPTKSTPSVAAAAGS
TKRALGGHAGGAGYAALDSPGSVSELRHGTRSGTGTASAPKPAMAAAERPLINDGSYARAAVEARERAERERAAREA
LAAETAAAKAAALQRSKSEHRTGGGGGTPSGGASTSPTAGTGSGATKAPSAIGSYVRQPSPLPARPTAPFGRSTSTG
RDTGARGGVAAEPSSSGTTPTGTRAASRIPTPPRPRDAGAAGVGATGGKQLAVDESYDSDLDFLERALDASDTETDT
PSPPRQGARREAFASPSSATAAAAAAANAAAYRERLAEARPKPLRIPTSGAATPMGGASTASTVASHAVPTGGGRAA
GAGSRAGGAPMAAAPQRSGHSGTSGKPVTPFTAAIRELMGLPGSAPLEQSRAAAGAAVGRGGQLLDARGQWPSRLTD
EEDRLLASLKRLDAEVLKRGLAAAGLVNGPSVTGGKGGKAAGSKGGKAMAASGAAGGGGGGGSNKLTNSLQQDQLRE
SLDRLDSQLGALRRKMEDRACMLGMPSSAAPTPSAAAAITPGKPAASTRPRDITPTKTPEERRGPPATLAKSRTPTP
NKAKAIRDQRQQQLTSHQGATPPAARTASGTASAPPKPASAPVMGAMGGVPAVPAAAQYQQGPIAASSISPPPASAS
AVPSAGYSGIPQGPANGVASGVSGAQPHGMPPRAPPARAPASAPTGPAATNAAAQQQHHHQQQQQQGPPDAGMMML
PNGMMVPYPAPAYGAMPGGGAHVMNPYGTPSSPGGGGSGAPQGMLMPPPPQHQNLQQPQQQPQVAYVPTMLPNGQMA
YVPQYVMPGGGGSYMQSNMGFPQMMMQPTGMQSPQLVMAWGASPPPSAQAVPMSPAGPSLYSFDRSSSNGTPPQVQQ
QPQNGGGPGSHDLAYQHQQYQHQQQQYHHQQHQAQHTPPFVSHSPPPSGPMPHYHSTVMRSRSIGSRDGEAMTAAAA
AAGLVQQPQQRVPHSAPQVASGGGVQGGGAELAAPQSTREPSVQFQSFGPNGGSRGLGPGAAWGGGAAGAGAGPADG
GVIKAGNLGLLFS

>Cre09.g400500.t1.3|PACid:27570260
MSTPHASVSVEGIGLLEKDAIVLELKQANADVLAELKKANEELKKANKKEPKPVPTGIKPRGLLMVVLIGLVMTPIL
IGIALPVFYTAKTARAKETSANVFMCSLYADDRQAELVAKYNTFARLDDAVYSQSALLTPPPPPQPSPPPPPSPRPP
PQPQTRRPPMPPRPPGDPPSPPSPPPPVPFAIQYLDAYNSLTQAEARFYSDLEKSCSIVGQSLWGQMDDDTFSQTPA
GNTWLQAVVNIAPVFTAARPPPPPSPASASNASSPQAAGSDAPPDYGSEYEGTFNGTGFDYPPEAPTEPPQAPQAPA
PPRPRRPAATPTGSPSSTSSTGNLTMFEKRVAQFPQAVAAWSTINATITSVDVATNAMTISFEMHLDSLVLQTAKNT
RIAKRSAKSVANVKFPALLDRTGRIKGKDTLAQTDVVSAAGADGTLSLKTGDRVTSKKMELPLVPTTSLYLYPFDEY
TATVTLVSSMTVSNVNCPTALFVKQKNAVVGWVFTAKLVPSVTDNRNVLNFQNPTDPDSAITLTYEIYLKRTTFVQF
FAVFVLMGLWLVSACSFCYAMDLLYIRPRNVTNGDATLFTAQLFAVVGTRNIMPGVPAVGVGVDFFGIIWIMIICLL
TGGLVYARIVSQYVHKEPTLYDRLAKNDVWLDDWLKQQEDAEKMAKEKKEKEEERKKKIEKYGEPGKAKIAPATAAA
AEGKATV

>Cre09.g387900.t1.3|PACid:27570272
MVACPARVWQRARSGGAFLLFCVALLAAGVCVGGQAQASTTGTAGPPPPLRVLATLASAPRPPSPSPPPPAPEPPRQ
TVLLSRLDAVTRESICALPGNASAGCVDTLSWVIDLDPFPLTVGGSAPAVGSSGAGLRCIAGVDLGLGCRWPSVSAY
LVSGEADTYTYRPLELAVRAPDISQAFQLGYTDQAAALLRQLEGLSVRCTAPAPPAVYAAFLLEPLIMALAAAGVDT
GTNVTDILATIAAASGRPPSPPLQRSPPPPPPLNGSAGAVHNSTMPSSPPPRPVASPSPPPPPPAGSSLAAATTAGG
GGTAANVSALNATASLNFTYAGIRNASDPASYFSRLYTAPTTPPSRLRRLSINDCGLSGQMPDDATWLQLPFLERLD
LSRNLLSGTLPPTFGPTPPMAHVNLSYNRITGPLPSTLNRYGGCGRVLDLSYNDLRGRIPRAWLDGACSASGKDAGS

ITPRTAATAAAAAAPTAASYLGTLDTSNPAAQSLAAAAAEAAAGGIFGAAALAGSGNTVVSAASLVEDPTAGAAAAA
TITLSGSGAFWMSYSHSYGFAGDGCPPDGVSWGSDPDLHAAVITTSPAFAALASAATGSSAGSSYTRPYPFSMLLYG
NPRLQTPGDRGVTFAAAFTRSLQFGMHDNACTAFQPLPELLWLWGSFGAAGVAVLFAAIFYAAWQWKTNRSKVTPSN
AAAQSRRPSMAAGAAPPPATAPGTAAPPTHGGRLGSMSASVPTAAASRVALSVAPSGLSRADGAAVAPSGPSGWTAP
TPGQVSGPSRTGGSMSAAAAAGANRVAVSGPSRVLAGAVAESVTPSGPSSRAAAALAVAPSGPSQRRQPPPQPLQAE
GYEEAYEGTGGAGAEDQDEEEREGGAEEEISDASGAQQNGNEQQVSHAAGYEAYQPQPEAAAVAMGASGGMARIIIV
AHARAYSRRAVARLSIYGRNAYDVTGTVTRWVLHEIPWGPMARAAAAVAAHAAAGSMTYVLSLESRRTYCSTLVSCL
PWLWVVPPALAVLAVGGTLYLMRATRELAATMSKQVGQAVADQVEGSAHGGGSGGAAAEGAAGGGGLSRGRSKAATE
LEAELEAEEAEEAAAAAGGASRREVHRSSSGGNSGETGSGSSGTEGTVSTDGVTPSVPVLAATAAASTSLGSRRHPP
PRPSPSAGVGEAQERWVSGQRRGPGTGTGMAREGDGAAIGGSVMPRSPTCVSSPSIAGPAGPSSALRSSVSRAPSQR
RVTLGGVMPTPTPIRINGSNDPSGGDVPPNRSGSMHGFSLVANTNSFANGLGSASPKSSPKASGTGAFGTPGPHDLA
AEGGGSQPGSPRSPASVRGSAMPGSPLGGGAGAGAPRSPLGRGLSAVGGASGNAVRGRSALAVGSALKRNSVDEDAV
AVAMANGGGGSSRTGTGTGMSPGGAAAGVSHRISSPISGAGECMSPSGARGLRGPAGSTSKRVLMASAGGGGPRAVA
EALESSSPPLSDDQTAADHSGSPRETVLGPQTPAPSRPSPSHADNTASGQGALTPAQTCSVSARRLVVRAVGGTASP
RLMRSHNHNHSHSPHSSPRNCRVSAQLSIASTEDTARYWEDTDGDGGDVDGGSQLDGVLPQAAAEQPVAAEQHAWRL
HAQIGVEGDAGDGKTAGAEQVHLEQQQEEAVGYWQTPYACSDDGGRVPRRSVTAAAQVASWDEQEDWYGEAAALVMR
AAAHEALAARWLPRSWCELLPPPLARSLSFWLPGGGGPALPHRELLLAAPAGVPVAEGHDVGGAGGVRFAKGTGGST
AGGAEADGGGTDRSPQHQSRSMGGRPAALTVPPGMEGSIEYAHARHVQLGAAAAAAAPYKHSHVYDPAALSAAGGGA
QLSPGSAASAAVAHATAAAAAHGGRRSMSRHPSRGAASKGASPLTSPPSNVTAHPWGVMPAPPMPGQQWHSMPPAQQ
QSPGLMHMATASVLAQMAQGRAPYGGPISPGSHAAANGPNSAQALSGSASQHWQPQLPSPQPGGARSTARSHSRLAQ
NAAAAALSASTAASMAASVAAGGVSRRSLAAAAAANAAEPPCAHAHGHLLVPERRVASMPSQRHSPNGGPASAMEPE
SSSMGFLLAGVPGTGTDGDGGGGGGGGGGGLPGAIGSTHNSPDPYSRVASRQRSSRWLEHQLTATSQRSMVTQRPSM
AAAAAVAAANAQSAASMTAAAMAASGPSAAAAMAAQRSRLPGYGAEGSAPADSAAAGAGAPADLPAAGGAPGVQMGG
ATRVRRLRPAALLVVHEYLRDSTLGRPGSGAAARLRAIALSVLGVLGWAVFGTPLALALAAPTALVILVDATLHVLA
ASGRLIAAGVSLLFRGPDRRSALARLGSYGSGAASSAAAAPVVSSVVVVGGYSGWAASVRLAAALFLGLHTHVAAWA
LCIPLAVFTGALYGLGYEWGAGAVPLPWVFLAANAGCLAGALLLLVELAFDMPAWEHGVCRYPYAMAVRAAAESRRA
AAKAAEKRETPQEVPA

>Cre09.g400312.t1.3|PACid:27570318
MGSGVSCARGEAVAVSVPAAQANPPPRAVAVSDSGAAIPGTARTGSATAGGVANPKPHARPPSAPVRPTGSLQAADA
PPPPPPPSPPPPSIPESPTPAPSPTLATVPVGSASEPAHSGSEPAHSGSGSGPGSRRVPHIGGLAVSLSFLLEFAEA
HVPPDMPTWMVVDKLVKPHTLHDTIRYVDLPHVTAHVLPPAATHYYFISHAWGRPFWEMVGLTAHYLRGAVASSVYV
WCDIFIINQHPGQQQADDLEQLRLAVADAAATLVCTDAAGSPFERIWCLYEMWTTLTVAAEGAGAAPGEQRRIKDGE
DKGADTDTSSGGGGGGGGGGGGGGGGGGGGGGGGGGGGGGGGGGGRSRSQALVVLTTGLSAAQRARLNGSIDAAAKA
TVIADKERILADIAAAPGGVTSFNAAMRCALLLRPLDFESELARGWAAAAGGVAGAVGGGGGAWDWSQLDDWLAPSP
PLPHNTNPLNNTSTSDNTSPNTSSSHQGPRAALLLGAAGSGKSTAAAAWAAHAMAGGGGGGGSGGHEGEGKGEVVAA
AARRQVAPVAVAAHFCRASDLQALDPLAALRTLAYQLALAFPQQLQGYYAFELAEAAAKQDADGGGCGGGSGKESFA
LQVISGLTTGGSAAGAGSGSGGGGPFGGGGGGSGGAADPIAAAFEVLLLRPLQLVWEAYTAAAAAAAEGVEEGQEGK
AASEGLPTPPPPPPPVVVLLLDAVDEGDSGGGGGGGHENAMLLMLQEHFPRLPPCVRLLITARPLTHIVSLLEGAFA
PLRVLPSPQVLRAPAADCVVALAARLQARPALAAALRAEALGEAPVLAKRLWEAARGWMLWITLAEDILTAAAAAIT
TAFAATAATQPTPAAPQAPLAPPALSVPEVLEQLLAAAAAAPAAAPLYRLYVRTVVAPAVKSGAGTGGRQGATAEDL
AFLLEVLGAAREPLYLSQLLDLRAEGLGLTPAVLAALEAGGGSGAAAAAPLGSLVMVRGYKLLGLHRSLYEWLAGDG
DALLPALRGTAPPASATSAPAGSATVSETVSIASAAVFPGRPRAHAALAALALRDVASSRASRAPGGGGTRGARPPD
GYSLQHGVAHVLAAAAARAAAGEEEAAVRLLGSLLLDLYFWRCVYDDGAATAATALAALLRHEAVFAGCSSTSIGGG
GNGGVTAADLAADVARWLQPYHSALLRFPEALLTSALALPACSLVRAAAEAALPAPSAGPSSLSPSPVRPGDHNTAA
ASAAAVTNATAATAPTAAAAVATTPAPEAAAVAPTPAPKAAAVAVQLAGAVWPAPPYRLVNPAAGWGDCLLTAEVLG
SGRRTPRGLGWAAAAGVLLAGCDDGRVRLVSPLSGAQVGLFSGVHQDCVYCLQLRPQRQHQQRQQQQQQQQPALQAA
GPPSPPPLVVATGSADGVVALWDGDTRVRLAELIPGLPPQPEGDVGEGGEQEAEAQTVTFGFDLSEQRATTSAAAAA
AGHKSSSGAVQVLAFSPDGAWMAVATGDKSITVWHLAPSGPAATGPTGGAGAGAGAGAGAGAGAGGGARKVVVMP
GAHTEDIRGLAFMDTTWGGSSSDGGSDSSSSSGGSSSSTGWGARCGLPPLVLMSASEDECIHVWDPTGGSSAAGAAA
GEGATAAATAAAAAAAAAAPAVVAAPLRTLRCDSEAQQAYEAHGLEPIPPPAAASGSAPHSDPWFLHGGAAATAPAP
AATAATAADADAAATAATQPPPLLSGLAEDSEGPVSRASYKLPQWYCLMPLQPLQPPPPLAGQAGMVPPPLPPGCLL
VGSGAGPVAVWDVAAGLMRERLRGHHDAVTCMAAAPAPAAGAGAAGAGTGPHFATGATRGDCSVRLWRHTPSADPAG
WAAALRAARRQAEEDASAAAAAAAERKAARAAERAAAREQALANGGDGDGAAPAAEGGEEDVEDSEEEEQEVQLLRC
RRPCQWQCVSVLRGHTAHVTSVVFLPPASPVPVVAVPGGAGLAEAFRGMRVASGSVDNTIRIWRADALAPPPPPAL
AALTAGANEPEPPPPAEAAAAAAAAGPGGREAAPPGHEGYQVLALAVSPDGKYVLTGAGDFTACLWAAATGRHLLTF
RGHHGVVGAVAFSADSSKCATGAGDGEVRVFSVPDGGWVGSCRGHKKAVTGTAFSPDGWNIATSSEDGTARIWTVRG

QQTSLLEGHPNGATAVAWSLDGGVVATGAARGNPGVQLWGARNGMRYNTLRYHVSDVLLLRFGEALEEPPLPPPLAA
TATATASGADGDAATAGGREEGGRGTAVAAAPPPEPPRKHTLLLSHDARGLTCVWNVWGNAGLVLDFYGQGADLVGS
KLLWLKDRALQYADLQELWDPKVKGTKEEAARKEQAAAAAAAASEGGCESGGQKQMPKQQQGGGSGDGGEKVKEGEA
EEEAPAPARPPSFPALLTTRALAALSPPGETVELLGGGPGLGFVAAMTDKQRLYLFALS

>Cre09.g401050.t1.3|PACid:27570346
MQPNCSIPLFILAVCAFLTPYVGAAVNAVTLLTGKVLPGDFEPYNGTVETGISTQYAAPYNNFQGVTRWWAAPNGDG
ETGELLPLQEARAGPDSGSDDKFRLLLKFRDVHRYVPAGAYVTSANLTLTFESWQDEVTVHICFLGKRWDYELGGAE
EYTSTGWINAYWDGARSLPWSQPGAAADCAAAYSAYTLFNVTPNGVYGTVSRTVALPPLLVQGWLANGGAANYGLLL
RPTTPGYIKYYTSQSSAGMLRRPALSLAWSSSSADVPPPGPQPWRLGLVPREWWVAPAPEGNDDTGTGFPDSPFATL
SKAIFEAFPLDIINLKSGVYAGAVQLTRPNITLRSAPGGHWAVLAAPRNDPVNAGVVVDIRPTAHGGVLRDLEITGG
FYYGIMFFTSWENYGTMEERVAQGAGARDWLITNVRIHDTGSNAVKLVMKAGNVTFSNCEMYNTGARLRTAGNGIDA
VQVYDITVQDCYFHDIPGAAVHLAGGTTGARLLRNYVARTTFGFNLGFDTDPEYMDTINNPRLYECIDCTAANNIIT
AVRMAGVNLWACKGGVVAHNTIWQAQEEAQASILVAALVRADTPDPADPSGATPLTSTCANATVWGNVLVRSPSARP
GPLFQIHTAGLDPSSKLVMGHNVYYDQAALGPADGSFVWGLGAMLEDERPLGPSGGGFIGTARGWADYCAGQLGQGY
CDVGSVEADPKLDSSFSPLDCSRVRRRAPAAPPPAATGLFGSPFVTDDFHGRPRPTSGSATPYDAGAVQFGASGPVK
TLSPAPLPAGLVGKPAPYMGPGLPPVYDRTWPFDYWRNRTCTDIYVDAASGSDKQDHSYESSYKKPFKTVAAAVAAS
NQCDRILLRGGQVHAGPVGIFTPNLTLMTDPRDAPKRAMLLCTGTGTTPCLRTGEGLYGGAASLTFSNFDVAMASRP
GAQGGGGTFAACVFLNEGAGGGSSAYWDYYVTQSGRSARPHTLLQNLGLFNCSSHGIKFSTFIRGVVMEGLYINNTG
GAGLEVRGGGDLVLRRSIIANTGGPGVRLGGGARNVLVERNTMWSVAERCILLGSDVTEAEYMDVDWAAYGGSRGAG
GGQQQQQQDDWHDCINATVRNNLVFDCQGAGVAFYSARDAVVVQNTFVGVATRMQAGVLLNVSPKDIGPGLEVGPPN
INITFKNNIVSLRMPIAPREDPPRTLLVEARVLPGTQPAGGSPLPDNLPAGATCAARRHRNRHRRRLSVSAVAAGAD
ADTVAGGGRAAEQQLRRALLQAPHSSGTSFAAAALNAPGEQGRNADGSCPLFPRDNAWRLDVSSLPVHPLSEQIKRR
VGIASVHTDFGGHDRINGRNVPYGIPITYVDSRKTPLVQVDIGPDGYGDESDMNPNAFPFPPDTPVEGTYPGCSDDD
CGGDRHILVVDNATCLLYEAWRAMPPAITPSRRWQVDSLARFNLSRNAQPNRRLGWTSADAAGLAILPGLVRFDELV
NKGVIDHAIRFTGPNSRPAFSPPASHFAAAGDQSDPQTLPWMGMRVRLAADFNCGGLGSGLARVFCVALQRYGGIFA
DNGSPWYFTGEATMSWESHLDDLSAVGDIPSSAFEVLDTGCLCLDSLCVQADCGGGGAGGSGPVAAASLPVYAGVND
TAQLHFDGNVYSTGDAAAAGRYVDRRLLPAGAPNPGGGAEGYDGGLAGWRAFLAAGGAGGAGAEAGSVEVADVALDP
VRDWAPAAGSAARGVAVVLSSALEDYYGRPRGVVGDRTDAGAVLSDGAVRPVPSPPPSPLASPSPSPSPKPSPSPKP
SPSPSPSRSPSPAPSPKPSPRRKKSPPPASSSPGLSPAPCPSPAKGGRKKSPCPSGPGSAPTPAPSPSPKKSPRPKG
R

>Cre09.g411750.t1.3|PACid:27570385
MALALAMLLGALLPSATGLETAGKSGRKLQQVRYPPWAPFPPEAPVSTTSPPPPPSPNPPTPPRPSRPPRAPPSPPS
PPSPPQRPPSPNPSPPQPPPRPPSPTNPPPPAPPSPPLPPPAPPSPPAPPPSPAPPSPPTRPPSPPAPSPLLAIPPS
PPAPPAPPPAPPSPPAPPSPAPAPPSPPPSPPPTPPRAPWPPLPPPRPPIYARSTSQTDNALIGVMVPLGIGVLAAA
GYVCYKIVTILRAGGGGGFGGASKPRVDIEMGVKGGKKAGKPTVTAEATSGGGNVFNPLFAWRKKKDAGAVAGGASG
PNGVASGRGAAGAGGAAGRAQRPARRPVDSDSEKGSDASGSESGSEPEESGATQDALLRKVRAGPGTSTTPPKRPGA
PAAPATGRGRGAAPPSTAGRRPVPQDSEDESGSGNGSDSGSEASAPAAPVGRGRGAAGRGGAAPAAPGRGAPAAARG
RGGAAAGRGAGRQVAASSGSEESGSGSEDEPPPRRPAARAPAARGRR

>Cre09.g388600.t1.3|PACid:27570398
MDEVRCLACYDTEGQHDLVACDGCGAVFHWTCIEGPTVPRPSSAAAAPPRPAAAAAAAAEYPGAQPQLVSTPACDWY
CPACRCSRCGAGCCPPPTAAEAAAASAAAAGASLPPSGRGKGLGGGGGGGGVLVSALDALARGHGMPEPRTGQKRW
DAAQWALWRAMVQRAVDYLLHKQPGDHGPGAVAAATGLTLTAVKHHAASARSAEELEAQLDNQLAPNPGRTAAGNAL
LRSVQAALWKHEPWTGRRRALRLLAAHLYDHHANRALAAPPPAKRPRITVAPPATPATPLATRLLASLMGGARSLEE
GDGLLVLEAVLKTLSPTALSGVDLGSLTLLRQLSAAQRQQQARTQEGAATAAGAATPGADRGPCPAGPSTLGQPPH
SSGGPGVGGGPAGVATAAAGAPQGSALHRLGSQSEPDVASAIAHLVALVQKSSPSPTQRRQQQQDAGQGAGTAAGG
AAGAGPGPGGAVPTAGAVGNAGETLVELALRLQRKRRPVDGPSDVDADAGPPDAAGDRPDRSPQDRAAPAMNPPLGG
PRGAQPHPPPRSVSPPPVLPGRATAADGRARSRTGSPEHRPPPPPASAATPGQDATAGAAAGGAAAAGGGAAVGGGG
GTEPKAVLSAMERYRLKQQQRKAAVGSVSATAVTPGPAAATPAAASAAVAPAAAAAAGPSAAGGAAAPAGLASRLLL
ASKRNKPSRTTTVNLPVLSQLYDDGDANAPGDVAGSQVLAGPGPGTDVGGTQTSGDAATGTPQQPQTQTQTQTQTQT
QTQTFEASTGAAEGGAPTRERGPPAQLPTANGVSAAARPGSPFHAAQRAGASGSSSSGGTDGGAARASPPAVEVSPD
DVVYCVCCGRPVHVACLSDDERQQVLLPPPPSPPSPPRHPPAQTAAPVRSRFGELMQPEAVKGGFRCSAECNKLVGE
LQKRCNMGVRPVQTAHSSACVGASLMDMNLEWQWLEVWPSLETGPPDSADTAGAGGKGTGEGAGGEGAAGGAGGGGC
GGGGGSNAAGALLARAMAAAAADGDRPPPNAEATARILADINACLNPTGSGEHTTTDNNSDRRHHHPNHHHPNHHHP
NHHQLHLTGGEDRGAGGSNGSGSTGGVPPGSAAVQALAAATAGVSSQFQALDVSEWLSEDGFVGGLPWVPPPQLGGT

ERGAAAVAAALARAAGGGAGGAATAAATAPGVVPPDGLAAAPGGVAAASGSARSVWPWCMQRAAVLWVDGRVGALML
VQLYGPSLAMLHVPSVHPRHRGVDLAPALLASAEASLAAAGVGHALLALAPAAAHDLALRRSDLLPKRRGRPARLMP
NEAAARAGASITFAAATWLYRLGYKAAGRDVVAARATFRLALPPPAPLLANVDPRAAPGGSSSTSEPTYPIATCPVG
HVLFAKALMPPPPRMRVRLSGAAAVAAAGGGGAAGGGTLLPADLPPSHPYHTCPHVMLLPSGDVVEEGGNGPGAGGL
PPATTGRGRGRGRGRGRGRGRGRGRYVATHYNSSSTESDDGDEDMDGGEGPGGGSSLPPRLGFVGRGKGIYLYGPNG
PPGMRYASSPARVPPAAGGGAGGGGGGQTLSGTTVSISHSRGGGGGGGGNGGEVMGGSGAGAGAANMAAAGTGGHAT
ATGPATVTMLAAPMAVMGVTGAVGTAAAAAGIGGQATPALTDSAAALALPVPTMSDNLLIDSFGGGAAGLHDLNVAD
SYGTSFGGFGSDFASIMKDPVVADAGGATSLSAGHGGAGPLTSHDVHTGLLGSTAGALAPGAAAAAGAAPVPGPLVG
MAQAQHMQSHQHQHHHPHQHQPHGATHVPQGTSHLAPRPVQHTTVPQQTAQPPPPPMALRLPSLATAAVHEARPGAG
PASGDLLASSAVREALYDMALEEAHRPNPSAAGGPPPLPPRPAAAAAAPAAPTRIRLVSNGGRSLAGGAAREQEHGQ
ERQAPEQPDPTLRAQALAVIAQAGAAAALAAAAAAMPDAGIPTVHRAAAPATQGRVAPPESAAAAAAAAAAAAAALHA
DVAMQSARMATPPYAAGGGSGSTGSVGARTPQLPPPADAFVGAAAAAATPAAAAAVAAPQTGQRSATAPADGTAVAA
GLAAANGVMHAPGTARASVAPASSIVVGQGQSGAEREAAALHRTGSTAGTDTAADLAPQICLFASVAPVAGPAAAAC
AVADADASPFPTRTPPDASTQAPPPPQPPQGQSAPAAAPPPAVTAAAHSDLAHPFGAAGMHDTGPTPGAAAAPAASD
AVARQPPLPSPVLTAAPASATPHTAAPQEPAPLPAASHARAGAVAAALAAEAATPMASPRKRATAAAAAAAAAAAAA
AAAAAAPSPAPPHAHAAAPASPLQARMSPHTSAAAGHTRRAPAAAAAATAATATPALAAGHSATRESRHGNAASHHD
TGAPATAVPRPAPSQLLGSFGTGGGGGRSSSRRSGKPSRWGAEKSEAELAAELAAAHAREGGEAAAAGQDGGQAADT
GTPRRRHHRHHSSHQSSHRHSQPQHTPQQQQLQPLQELQAAPMPPVPQHLQRQAAPHAQPQARHLEHQPHDDEQYRP
PYPHLQDQQQPQQQPQHQPQQQRRAAAQRPPWHEGPPGPAAYAQPPAPHASYASGPSLSYYAAGAGAPSQLTMSVPD
ATSSGSILEGKPGGQVRFRRKPQVLHDGSLPLVADVGTEEEEQDGAADGYEGMDPHHHHHQQQQQQQQQQHSRPGD
GGVWPAPGRPLQPPRQEQWLGAAGGAGGGWHWQRPHDAEAEAEQQQAEEEAEEYGGDARYPGQQWHTWGAQHPQPPA
VPQRRVLAEAAGADQPYAHAAPGDTWGRPAAAGARTQRRRALTPLPSVLDEVTDRLAHTDTEADPGVRPGRCHSGSL
GGGAEMGYVSEGGAGPLRRRPPLQQQQCASFVPPHLARWPAAAAGDAPYQDGDEEAEAAAWGRRARRSQPPGWAGSD
EGPGSAEGARGRRRSEDDDGYGDDEFWGRHGGAALPPPHFMPRQAPAGSRRSRAQQQQHGEDADAYACADGYHGRAP
PRQSGPPRVWPAGPELLALPPHMRRPRQALPAHDAYDQSERLLRGPADATADARLPARQRAGGAWDPHGSDWEGGDA
DGESDYDAAARLPRRRPRGPPASFGPGLGPEYDDVEAEDGLSPPRRRAPPRGRRVRDSDWEHDADRPEEEGHEGGWG
AARWRGGRPVGGAPGVQRPPPEWADTERPPHWGPPSSRGGGGRDSEQGGALRPPWHAGPPHARGGGAPLAHCSPPPW
LRGGAAEAADRQYGGLPPGSAGRKPGAWRGEAAMDGYGRPPPQQQHGTAQGEWEGAERGGYMSAGHADGRPGRPPPA
WGHVWPTHGPQAAEEENGYGPRSAAPAPGLRGPHSQPSPGSGLADAPSSGHRGQPPPSASVASFRHPPQHSRHEHSH
YSQDHPPHALPLSQQAQPLQPRLQPHTGQGQSQMHSGQLHDGAPPPAASKPTASAAVGVTAGSAPAAPHEEEQNWQL
PPRRRRRPLQPELHAQAAAPAPSAAAAAAVVAAAPAGASEGAATDRDGRKQPTGKRGRSRKRRERDRSRSRSRSRSCG
SLRSKSSSRSRSNSRSRSRSGTPSGSRRSRSASGSRSRSRSRSGSESSRSRSRSRSRSRSSGSRSTRTRSRSRSQSR
SRSRSRSRSRSRSPSVPRSPPPEEANPPARLLAAMDAAGGGGGGGGGAAGAGVDGAANGLPRRPRRSGSRERIPPIQ
SQPRPQDNELVARGGGGGSGAFANGGGGGWSAQKRGRDWDSGGTFGQYQRQQGAYASGGSEGPMRDGSGPGLNSSEQ
QRGYYGPRGGGGGGGGCGRGNGDAGGAGGGCGGGSWQSYPNGRQQQTASGWRSGADGGGGGGWAGGAQADHYGGGED
WRQQQGGAPGGPGGGGGPNARDNKRMRLSGAQADAPPYLGANGNGSGLDVFGSLAGSMPSLSVAHSRLRHGAAAAGA
GGAASAAAAAPAGPMPDMPPVVRGSGGAPPTAAEAAAAMASLTGPGSASGRAMGMGSMGRALVPGGIGAPGGAAPAA
SAAAHPAAGMVAAMGATTATAAAAPAVAAAAAVAAAAAAEHPLGAMVPQDPNVIWDLACLGMATLRGGGNPLLAGAG
GGLPAALAALQQQQQRGVAAQQAAQHQQAAAQQAQYYQAAAAAAAMQRHPGQPHPHTAAAAAAFVRGAAAAVGYGAP
GLYPGYGPAGYGVGGYVYPADGGAAGGDWAGGPGQGWGGGGGRGWGGGGGQGGMGAHVWGGAGGGGASGRGRGGGEF
SGRRGSAGALEGGGWGGGGGSRHGGHFGSRGGGGAGRGGSRSMGALVEGHAAGPAASTVAGGAARATRAVSAPAPPP
GASQHIAGVALQ

>Cre09.g413350.t1.3|PACid:27570399
MSQPPPPRPPLPPLPPINWSSIPLAGPATNATNRTQLIGNCATSNPGNCNGHGILGPGNCFCICDAGYASDYSNLLA
PKWCVPAGSVSTARNRTVDIITAANANNDGSGSQNVVWVWLGTTTGMSVTIALSVGVLVASLCFLFYCYRKYKRRCA
AKAGGHSKGGGGGGSSKARASHYYHVYHNGAAAGAAGAVNGAAAGVLAAGGPGGIPGGGWPPVMPKPPPSNFFKSWW
MHKGDKAKSKANSAAATGYGIYGSGGGRGGYSNGRDAYGNGDAGGGGYGNGGHGFGSNRMLDFAALGSLGADASPAM
AAQLATMGRSPSYSHSRSPSYSQGQGHNGYSHGYSPRAQQGPLPSSPVAGGKSQAGGARSFVNPLAHMPQEQLPPPP
YDYRLDSRNGPTGGGTSGRHHPTNYQPSTTHDSSPPPPSSSRQRPPGNDYGGGYDERQGYSHRAGPSPHDAYAGSYE
AYSQQQHQYAPRPREQQGEGIGGHRNSSMRRGLSYSGHNGGARGGYGGGYGVGDAGGGGEPRMALSHSRSPGAEDGA
QAGGEHSPRPGRGARAAADGVPRASSSSTSSRRLPVLTSSPPPSASAAAYVAPAPGGRGGPPPPPPPGAPPPPPRPQ
QYHQPRSPKHG

>g10014.t1|PACid:27570591
MSPPPMSPPPHLPAFSPIFPLLSPPSPLAASSPSRLLLTLSPPPHPFIHHASAVFAEVYGWLNAARPHPATSLRTSP
VSSSP

>Cre09.g395050.t1.3|PACid:27570600
MHATNTSHGAAGSSSSGGAAAPSAPGSSGSRAASSSQQQLCQQMYSSNEVQKEREPAGQALARDPEEEEEEEEDYPM
TEDVLAGSAAVLGGGACGQGQPALVSLLGRACRVPACLSAFGGGGSSRSRHRTAHGGGGGEGGSGGGGVGGGGSSGG
EGLFRRLVRTLKGGKSDADAAAASGPDADGGAGGVGAGGLQVVPLFAAAAASMRPRPPPTCTSSTSSPLTASAAAAE
EPAGTESFLAAHASGGSGAAAATSPPPPPAAAAAGEPSTVGRQYCSPDPCTQLYDQHWYGRPRHRQRRVEREAAVAA
AAPEPPVLVTAAAVAAAAVAAAATAAGDASAMRGSALDVTARGGSDPAQHLPPPPPADNPLAGRPTPQASSGATPPV
ADDVLMHPLRCAWDAPGNPDIPDTAEASTVPQRAYDQHSQHISGSSTPSSPATRSRRTSLHGDGREEADDDTTAPGT
PFSDWSGAAAVAATAVDGGLLQLTPDGPGRAPRSTAQHETNDLPDQHSCVLSPAATAAASAAAAAPAPAAASTTAGT
SGANAAPLHAATHGFYGSPGATFSTPDSPPAFRPNFATSTMAAPGPLGSPLRLLQPASAPASAGGWDGSGASSSTVW
GAFNPEAYASPPARASAAPLQLQLLPPPPQSPRSPRSGAGGAGRARRGRRHGPAAAAAAAATAAATAAEQHQRYESE
GLLLPLPLPPSPPAQLPPLPNLLLPSQPSPPPMQVQAAAAAALQLPGPVLQLPPLPGSPLASSLAAAARSPRAPAAA
HTPPSPVEAALATHGAAGAVVAVVQSSRAAVAAARGAAHLALQSDTDTRHHAPDQVSAAAMAPGPLLPVVGSSRFTT
AATAATAAAAAAAAAATAGATGAGGGRGGGPASPVRSQGATAPPWAVGGFRTEAAGAAGEAAGGSASSSGDSSAGGS
PQRGEEEEQDEEPRRRPQRTSSADGSVGGHTRGGSASGASGDGGAGQTRPRHRRARSVGSDDEMNAVLYGRGSGSNS
PPPVYEQLQLQLLLPPDVAGGPGGAADGAAAGTAVGADAGGHAVSCASADSPAGGAAAGSSSSSARRTPARAPRGRP
PRPPLPPQAAASPPSLQPPTSALLQPLPPLPRVPAATAAEPAGPESAASSAAATPLQPTTAATEAARRRSSSSVLSR
ASTWGSVGAVEPAPPGPTPRTPADQQWQQQHPHHCQQAQQAQQLQEDGTGLAGPSGDSSPSSATATAASPSNTFETA
AAAASAFLFHQVPGAAGYSRDGEAAAVTGRRLASAPSWGSEASFDALLQLSPAPVLQPQPQPQLDREPRSQPRRLRS
HRRQREQLLHPGLPMPSLFSSGSSTSSAGSADVLEDMLQPPYLPPPPPPLLQPTPLQRQWRDSSPPSTPSATQPLTA
GRGSNIGSSSTAGSEPPAPTMSPGWRRQQQEGSGRPLLPASPLQLDQVGVGAGAGTAACSAPGGSAAGAVAGAAAPP
ALPALRPEAAPAAVHVQLRLAPQTADGGCGVAVRLLVLDGGGDPPATAQLLPAPRQRHSHAGVRASTTTTNSNSTST
TNSNSNSNSNSSVPALHPRAPGAEPQTGAAPGTAGASLLGVHAGLTEVLDAKGIDEQGPNSDSDLDQDEQLDRLLEK
LVLAATAAGDGGGGAGGGDSEGRLAGHATRRGVGGAADVAGGERVSASPALQQRLLLRLALARSTTGSPLAGAPAQQ
QQQGRHYQPAQAGEEQVQEEQEQGDEAAALQEAAQHRILAWVGQQQRLTERGAALQLAPSPGPPPLPLPTPPPGSIF
ADPSYGSGSWEAAAATLGQCGGGGYRRGGGWREPPPPSYPSLMRVAEPGDTPTRSVPSGVAAVAVPGAAGNATAVAA
AAERRRIGGWRPRHGETGWSAIAAAQALTCRGPTQLFRSGSNSAAAAAAAGTAAAGAGAGGFAAVSDSSPTSRRIRA
LGRLRRRHSAAEAMRGGDVGSALPPPAAAPEQQRRPQHRHHSHSSSGSSPLLLPPAVNPGATAGIAPNLGLGCGREE
HIDAAAVNEDADGGAGTAGTDASTPASRRRRRALLGWALRELDSLEALLRGSGLGADDAAAVQQVVLTELLDRVNAA
MLLQPPSEQQQPHAATAAGRADGGSAGVGEAPGGGDEAERLLQLLLPPGSCSVVADVWAVLSHCLEPGGDSIAGGLG
GGGGAGVGKHCVISSKAGGRLLAGSMGDHDVSSSLLALAASDALLQPHPQLQPLSSVQPPPSALSPEAGSPAEGGWA
HRPSPEQWRRQTGPEHHRRRRKQPLGIGSAAAVGADDRLPPHMPARPPPYPVSLLPPGPQPHQQHQQQRQYQLVQQP
QQQLAGATALSSAEWSYLAGLVLAAADSSTAGSQVEGEVEEWAEEGLGHGEECGPAAAGVVDAEAPGGVGLAAAGQS
VAAAAGQSVAPVARASSPAATAAAADVHAAAAQLGLRQRQLRSLPRSAAAGGDAWGEGGAGPPGGGGSGGGGGGAGG
GSWGCDEERRELLDRWLEEVVAMSPPLMTRDADAEEEVVEEEEAQLEDAEKRPEQGERQQQQKRQQRLRLLPPPPIS
PGTAVVFRHFYQTSHPAAARAHAWQTSGTAGAAGAFGVEAAAAVHAPAAAAAVPAAAPRSASSSEHWYGEEHSERAQ
QAQQAQQAQQAQPSKPGAAETQAGVNGAGPAAAAAAAPGAAAAPDARPAASSVPPSPPPAAGRAIGSRAAVSGCAND
DNSQAAGAGAAAGAGGPGVGPGGGGEAGPTAAAAASGVYLFPGLRPRPRGEVTASAAPGPVAGSSSAAGPLSPPPVT
ALLAPSEQSAAGTAGTAAPKALPPPPPPPPGPPPPPPLLGVGVKGLTAGSGAPPPPPLGPPPLPPSEVSGVAKAPGR
AGGPPPPPPPPPPPPLPFGGVRSGAPPPPGRRGGGHTAGVQRYPQVIALHQELRRALTAGSGAGAGRKAGAGGRGWGG
GRYLPLLRGWAARVSDLQPGCMSEVVGFVAEMDAALAVLSDEAAVIRRAAAAGSLSWPERRADTLRWAASLHTELTA
LADRAAGWPYHQPSQQWEPSRSGPGGGSGGTAAGAGTGTRPGAQAQAAAQAALRGVDGVWEELSRRVEALGRDERM
EARFKEHGVPWEPGLLPRLQRSALRLLAVHLRLVLDCTNTTTDTSSSTTTDTSSGTCAAASAATATGCSTAATGEGD
GSQRERQGQQAAGQQPERQEKLAGPIAAHRDPAATAAAAVPAVPAVPAVPAVPAVPAAGTASTAGAAAADRRHAAEA
VAAAERRLLDGGVAFAFRVHQLVGGFDAECIGLLEALGPRWRAARRRAEQEEQGEREEGQQERQGGVSGAVVVGGEP
AGATQA

>Cre09.g397700.t1.3|PACid:27570643
MATLSLHTEQALNVEVVIVGYGSAGAAAANLVACGVARTRRCLVFEEAPQSERQPYGPHMSGSEPLDIAASLKRTQR
CIQGDASDQGLHVQAQWGASDAPLHGAQPLVYRHHRLIQLEWLPCSSQPRAGSVPDTHSASQDAHGGGWRAIFRDTL
NGGTSAVTASFVVLACGQDLAPPAGASLALRHQPGMQQQQVGPDDHSSCRGFGAQQPQPSGQARMLPVGVATAAQVA
AASSVRVVGAGLHAWDVAAAVALLRHGEDVVLQQPAGSVVYSDAHTEHGGAQLLSSARRRALAASLQPHYTAPQPGP
LGKALRAPAKRRFWALVKDKLTLGRGVGGSSSGSGSGGAGEHLVLWAPSLHDPRFMPFLSAPLRHALLGGSDGAEGA
GDMVLYRGMVHPDVPGLAFMGLEAHAGSNLLLLELQAQWLAAHLAGRLALPPAVAMRADVAAQRAWRSGALAHPLMS
VGGSLARRHEQCYLEQLRQDLRSASIATEPEAAVAIQQQDIGAAASPAFQPLPGPPPVVPISTDAGLRVSGGASDAG
LRTPVGPLGLASLSLQLARHGGDCRLSASHRAEESSGVVLTGSQSQRASLSQLPANGSGDTDVDVAATAQGHATAEA
PLTLAADRLQASGTSPAAQHFIIARYRTSSSHGLIAASAPTSPSRRDQAAGAAARRSASRPVSSRFRHHIDGAVSEA

EEADKAAGCCAAEPPAQAATGSQSHSAVNAGWLVNAAMRHSQSGPLHGADQPGATGPSRRAPRRSITTGTIGHVLSR
ALAAAAAAAAAAAGAAGGWTAASAATSPGHRAHAPTAVTAAAWRRERRSTTSVLDGDDQSHGAGAGSTRPPRRSAGL
GPGISTFSNSTVRNARAASMSGLGRQAAAAADSAASAPGQPVAPGSPLAERFARMVAASGLPASVLVEIVKEQHPSR
DRGTTSGSISVGASPAQLGPPQPQLVSRLQSSTLGCASASGRMETCIPLAGPRARGRMPPRRASSEVGPSPGASGAA
TSTAAADSAYRLVICTPLSDAESDADASVRGGVQRTRDGAKSSAASAVYNKIPAAMAAAPNWALYISSRAVPIAAAL
MGDVNSPPPMRMSLQGSDRLAAMDSAAFAVAQQPSPPPPRGSSQRFGMPAAHAPAGLLGLSFAALRPRSTASPPAST
SRSRGGMASYLSPSGAGASVRGGAKEQQPTMEVPSMSLLSSQANGISNGGDAGGETSVTPRAGRFHLGSFSVGGGSH
FNGSERISAGGGDVVQELEEQMRALRTHLQNPAAFPFPSIGLASTSAASRGSHTPRHLNSTAGGKSTGKSSFITAGA
ISPSKARRSAPKYRASEPNLALLENDGVVTPGRKTDVQSCIPEETESAAAGSFLVWRRSTRPRDSSSSQQLAADVTA
AAAAGSEQRQSLTGSLERPARLSRNHLHGNQPPSLLERTRSGAVAAATALASASKRQLHRLASVGSGARPSVSEAPS
VIPSQAQSPPPAPLPLPLQLRQPSHVLGTSPPRHRDPPSGAAISSHDVINHLPAPTAAHESGGGGSLPSRFSGAAAG
NGAGDVMAFSSSQTAGRTSGHDSVLGSHAFPPKVAPLGSRPRAVRSTHPSDREGYSPATAPIGGGSLPSPMAGAIFA
AAASPHGLTDPLAHLALPMTPLRHAHGLLPQPLSSAIASPLSYQQSPSGACPLAHLPLPTPALASPAHSVATAPTPF
GRCSASDVMSPRGSSPDGAAWLPSRSMSEHPDDARTPGPKSRGGATGTSAISAIPEERRSGSRHKSLRRQISAALLV
LHRAATGAVGSSAKGTPAGGKESGGGGLFALASEAALLPYSKRIPRSLSRGGANSSSMGELSNAIALGVADSIAASP
RVAVADGGREAPVRSAPPALQWPEHTEPLDKRFAQDAVKAGAASSAVLPAVLSAARRSQDSALPTATAACLPSRSAA
SATAAMLMRTAPPRTPTVNDAGHTVLPSRLAVVSVAGLAARQRIRASAPPQFGYACGPEVDVESQATRLPCLPGDRT
SAAAGSDAAAESADVAGLASGPFEASAAGLVATLAVLQEATVEEDVEDELPLRLVSNILPSAISEAEALDIALLGGC
GQRVGGAGSSSGSSSSSVPCGTPAPSGATQLALLQSRLQQHDRTGNEGQDVDELDVTVGPVGATMFGVAMLGAAHSA
LAGSSSRPQLEFVLSPPSQQAELLRRTHAGSRGSSGSGSIVLQLPSVRGGVVADLMAAVNTTRRTPHGGSDSEVDDA
EEYDIFGLSHLLPPSRRTSPAGRIAAELAGQDAISLPLLQRAPQVPGNSSRSQELNLVSGCAIPPATAQHQSRPFPM
QLSPSDLPLQQPLSADKLTDDFELLDPDADTALEDRLSPVGICARFTPGASPFRPAYASSRRLSLAPRCPNTVVGLA
DSLISTADGHGTSTGPPSAGWSTAAPGRQSMNAALLLSSVHSASFGHGGGSHGGTGAAAGGGSMASRMLGTLKKMSS
LVRTPTATPQQTAAPPRRSATGLSNGRGSIGGVLPPAFSHLASLHALAPGSVRDAYGGQGRGRGGDGRDGAASNAFT
LLSQTHSQRSSGGSGAGSGCGSGQPTASPGASAGSPAASVPASRRTLVGGTSMLRQNPAHSHLQQCLRSGPRMSSPN
LQDMHDDLLEAARWSAAQRQERDMKRHVTQRAQVRDRLCMVGMAVADASAANSHAPQQQEQQQQHDKALAGAVSSSQ
RPQLSEPLPSPLPSPLASPRSSRAPSPLLQVRAQLHAAPTLPLNWFTRHSMKVKTPGDAQTPSAGGTPPAAGSSRAF
RRSCTSPRSPLSPAGREHSRMRNIATAEGSGPSSPLRSGSAHVLTAHGGSSNALHVVASGEVSLGIARAVGRLARHS
SASGPGGLDPRALAAVSGRAADVEAVCAGLEAEAGPTEDHSGQGQAWLDAAAAGISEAWEHAHSNHGSVSSCGTTGA
LLRADAADVFEDGVRRGGPAWEIIQASRARQDQMVNQVSAWLREALAKKAAAAQATVAAQAAQ

>Cre09.g396750.t1.3|PACid:27570649
MELRCWPRRRRELGRPASAHMAGRRAAVFGLLLLFSCVACGVGLSVAAAGDSASTGGAVGPAQASMASTVDAAASDA
HSSSVSGAGSSAVCEVVELVDPLSAHFEGRLGHRLMCTDGGGGDGSSANDRIGNVHSSGSSDVGSSRRLGERRWLQA
AGSCSGFTACVSATTATQMLLGAAGGGATPDVVVSNAAFTEGLCEGAAAGAAGRRQWGLITNTSWPPGHVMKPWFPR
GAVVLSSGAAPAAACNYNSLGSYSLPAGGDGDADLDSLVPGQLSYDAAVLEFDVTPRKSGLLTLRYVFGSDEYNEWV
GAPFNDVFAVLVSPAAGQLQQGSAGAASRQTALVRGSAAQPVSIATVNAGSNADLWNNNQPGEVAQLKATEADGYTY
RLNTRGYPVQANSNYHVKIAIADIGDQILDSWLWLAGESLVINSPPVAVAAGNNINSATCGVTQKTVTFNASSSSDP
DKDPLSYLWVISAPCRPTITRRTAVARVNLTNLADGATYTARLTVTDDRDAEAVAERNVTVSVGCGSTAISNTCTQP
PSPAPPSPAPPLPTGFIPRVVWGAPAAAVVPCGSPGVVLDPGSAAADILRTQIWPDGEPADPGSEKSAYFVWALYDS
FAPEPAGAPTSFRVTAINSSADDPALTLDAATDLRQGPVSRKWMVRLWVSNSPVWDEGGGGYVSGQVFVQVLHCPLL
PSAAGAPLPEVPLGGQGQLPDPLSLGCGAAVAVNASAARSVALARAGLNTSSGPEPVVKFRWSLTHEASGRVWRRDV
VGDPLFTLNTTQVLQYAGMPLSEFSVFSLELQVELSGGQLLSDDKWRVRAGSEPCYQPPSPAPPSPSPPPSPPPEPP
SPPSPPPTPPSPPSPEPAPPANPDPPSPPQPSPLPPSPAPPSPAPPSPTPPRPPSPKPPSPTPPSPAPPSPLPPLPS
PPFDSPPSPQPPSPAPPQPRPPPSPAPPSPAPPAPPPSPEPPSPEPPGPPPSPAPPSPAPPSPEPPSPEPPSPPPSP
VPPPPPPSPQPPGINVVTHDLVAYYDTPASLPRVLLCCGSTDPGSITPQAVASFVAEFKANMSRALGIPNNQVVIRL
LVVMNKTLIQQQTDSTSPTSRRHRSLLSSLSMDFNKHHQQQQQQQQHLLPLGLPADVSTHRDQDAGIDADSAQRRTM
QQGAATTNSGAGAAIGVLTFSLIEVQNTNTPNSPPPIDLGMLPAAVMASRLYLSAPSGGGGGGGSGGGGGVEDDAPP
SPPPAAPATAPLFVKVAGMMAHGAVGQTRNPVWYDDADFADQRADGGALNLVDWATGNRSACASSSRCGQCGHAWAA
FFGPFQVTLYFREPVQLSRIDIREIANPAIAQLQLLPWPAVAFGPDLPARRGVLGRPLNVPAAAAASTACPRTTSVV
LPAKRAGMSIKVSSAGSQEELPRALRGTAVGGVTLLLKAPVTAGDVGRTWVESVRFTGRVLYPRDAAAYAAVKAPSG
AG

>Cre09.g396750.t3.1|PACid:27570650
MELRCWPRRRRELGRPASAHMAGRRAAVFGLLLLFSCVACGVGLSVAAAGDSASTGGAVGPAQASMASTVDAAASDA
HSSSVSGAGSSAVCEVVELVDPLSAHFEGRLGHRLMCTDGGGGDGSSANDRIGNVHSSGSSDVGSSRRLGERRWLQA
AGSCSGFTACVSATTATQMLLGAAGGGATPDVVVSNAAFTEGLCEGAAAGAAGRRQWGLITNTSWPPGHVMKPWFPR

GAVVLSSGAAPAAACNYNSLGSYSLPAGGDGDADLDSLVPGQLSYDAAVLEFDVTPRKSGLLTLRYVFGSDEYNEWV
GAPFNDVFAVLVSPAAGQLQQGSAGAASRQTALVRGSAAQPVSIATVNAGSNADLWNNNQPGEVAQLKATEADGYTY
RLNTRGYPVQANSNYHVKIAIADIGDQKTVTFNASSSSDPDKDPLSYLWVISAPCRPTITRRTAVARVNLTNLADGA
TYTARLTVTDDRDAEAVAERNVTVSVGCGSTAISNTCTQPPSPAPPSPAPPLPTGFIPRVVWGAPAAAVVPCGSPGV
VLDPGSAAADILRTQIWPDGEPADPGSEKSAYFVWALYDSFAPEPAGAPTSFRVTAINSSADDPALTLDAATDLRQG
PVSRKWMVRLWVSNSPVWDEGGGGYVSGQVFVQVLHCPLLPSAAGAPLPEVPLGGQGQLPDPLSLGCGAAVAVNASA
ARSVALARAGLNTSSGPEPVVKFRWSLTHEASGRVWRRDVVGDPLFTLNTTQVLQYAGMPLSEFSVFSLELQVELSG
GQLLSDDKWRVRAGSEPCYQPPSPAPPSPSPPPSPPPEPPSPPSPPPTPPSPPSPEPAPPANPDPPSPPQPSPLPPS
PAPPSPAPPSPTPPRPPSPKPPSPTPPSPAPPSPLPPLPSPPFDSPPSPQPPSPAPPQPRPPPSPAPPSPAPPAPPP
SPEPPSPEPPGPPPSPAPPSPAPPSPEPPSPEPPSPPPSPVPPPPPPSPQPPGINVVTHDLVAYYDTPASLPRVLLC
CGSTDPGSITPQAVASFVAEFKANMSRALGIPNNQVVIRLLVVMNKTLIQQQTDSTSPTSRRHRSLLSSLSMDFNKH
HQQQQQQQHLLPLGLPADVSTHRDQDAGIDADSAQRRTMQQGAATTNSGAGAAIGVLTFSLIEVQNTNTPNSPPPI
DLGMLPAAVMASRLYLSAPSGGGGGGGSGGGGGVEDDAPPSPPPAAPATAPLFVKVAGMMAHGAVGQTRNPVWYDDA
DFADQRADGGALNLVDWATGNRSACASSSRCGQCGHAWAAFFGPFQVTLYFREPVQLSRIDIREIANPAIAQLQLLP
WPAVAFGPDLPARRGVLGRPLNVPAAAAASTACPRTTSVVLPAKRAGMSIKVSSAGSQEELPRALRGTAVGGVTLLL
KAPVTAGDVGRTWVESVRFTGRVLYPRDAAAYAAVKAPSGAG

>Cre09.g396750.t2.1|PACid:27570651
MELRCWPRRRRELGRPASAHMAGRRAAVFGLLLLFSCVACGVGLSVAAAGDSASTGGAVGPAQASMASTVDAAASDA
HSSSVSGAGSSAVCEVVELVDPLSAHFEGRLGHRLMCTDGGGGDGSSANDRIGNVHSSGSSDVGSSRRLGERRWLQA
AGSCSGFTACVSATTATQMLLGAAGGGATPDVVVSNAAFTEGLCEGAAAGAAGRRQWGLITNTSWPPGHVMKPWFPR
GAVVLSSGAAPAAACNYNSLGSYSLPAGGDGDADLDSLVPGQLSYDAAVLEFDVTPRKSGLLTLRYVFGSDEYNEWV
GAPFNDVFAVLVSPAAGQLQQGSAGAASRQTALVRGSAAQPVSIATVNAGSNADLWNNNQPGEVAQLKATEADGYTY
RLNTRGYPVQANSNYHVKIAIADIGDQILDSWLWLAGESLVINSPPVAVAAGNNINSATCGVTQKTVTFNASSSSDP
DKDPLSYLWVISAPCRPTITRRTAVARVNLTNLADGATYTARLTVTDDRDAEAVAERNVTVSVGCGSTAISNTCTQP
PSPAPPSPAPPLHPEDPDLAGWRASGSWIRENFAPEPAGAPTSFRVTAINSSADDPALTLDAATDLRQGPVSRKWMV
RLWVSNSPVWDEGGGGYVSGQVFVQVLHCPLLPSAAGAPLPEVPLGGQGQLPDPLSLGCGAAVAVNASAARSVALAR
AGLNTSSGPEPVVKFRWSLTHEASGRVWRRDVVGDPLFTLNTTQVLQYAGMPLSEFSVFSLELQVELSGGQLLSDDK
WRVRAGSEPCYQPPSPAPPSPSPPPSPPPEPPSPPSPPPTPPSPPSPEPAPPANPDPPSPPQPSPLPPSPAPPSPAP
PSPTPPRPPSPKPPSPTPPSPAPPSPLPPLPSPPFDSPPSPQPPSPAPPQPRPPPSPAPPSPAPPAPPPSPEPPSPE
PPGPPPSPAPPSPAPPSPEPPSPEPPSPPPSPVPPPPPPSPQPPGINVVTHDLVAYYDTPASLPRVLLCCGSTDPGS
ITPQAVASFVAEFKANMSRALGIPNNQVVIRLLVVMNKTLIQQQTDSTSPTSRRHRSLLSSLSMDFNKHHQQQQQQ
QHLLPLGLPADVSTHRDQDAGIDADSAQRRTMQQGAATTNSGAGAAIGVLTFSLIEVQNTNTPNSPPPIDLGMLPAA
VMASRLYLSAPSGGGGGGGSGGGGGVEDDAPPSPPPAAPATAPLFVKVAGMMAHGAVGQTRNPVWYDDADFADQRAD
GGALNLVDWATGNRSACASSSRCGQCGHAWAAFFGPFQVTLYFREPVQLSRIDIREIANPAIAQLQLLPWPAVAFGP
DLPARRGVLGRPLNVPAAAAASTACPRTTSVVLPAKRAGMSIKVSSAGSQEELPRALRGTAVGGVTLLLKAPVTAGD
VGRTWVESVRFTGRVLYPRDAAAYAAVKAPSGAG

>Cre09.g410500.t2.1|PACid:27570708
MKQEGGGQPAPASARGDRTTQPASTATGAVDAHVYATGDQTMREAVAGHAPGGSSGGAAAAAATAGSSGAAVAGAGA
GVPAAMADDDDDPRPWLLFDLNGVLVEESKYAERETAREGGGGGGGGRGWGGHAGSSRFRRRNFRARPGVAALLELR
RSYRLGVYTSATHWTAARALASLGSRLVTELRAAGRSQELAALQGADRSQKLAALQEESRALAAAQEAAAMEAEAAA
AAARAVAEATAAAAEATAAADPSEIDLDGGDGGGGGGGGDADMAEAEAEAGGGAQARGGKAAGAVATGAGGQAAATS
AAAAAEGGAAPTNNNTSSSSGHGSYSRTHGNQHQHHHSYSQQHHPSYSYSQPGYSHHGSYSHHGSYNPGSHAYVDVR
VLLTRVLHRDHCAPDPGWQQREGGKEWDTVKPLAAHGLALGRVLLLDNEARKSAPGEVANLLCLPTWESAADEGVGV
WLALVRELMALPVTQDRTLDLRDHSAAISGRVCALHRPRPEANNTGTRGVHSDVVTPEPEGGFGPGGGSLLDAVAAA
ALVRAEPSIRKKQKAKAELAAAMTAGVAAAAAGGRGGAEGEGGGGAGGAVGAGAGVLDEGVAPDGLPRWAAAALATV
DGAGGATRQHQHQHQQQQDQQQQDQQQQGAAGAGELLRLVAGYALRPGAAASCDRWEAKNLREAAERWREEEAMADP
RVRLAVVACVAAADEVLRGSACHTQVCQLMTARWATFGGFDAFTLRRECSHEAIHGALLAGLIAPLPRPGPNFMLRY
VPTAAAAAAVADVDQTRPEPAVVVEADQVAQQPAAGAAAAAAEGAEAEAEQAAGSGRRPEDCFPAGLRAYIAARGPR
PNRRDRRHQKQRRREGQDQDPEHHCQQGNQQEGGAPAAADQAEAAAVAAAVEKEGGSGGGGSGGPGSEAAAPAAAAE
QTPQEQQQVPPPQQRPAWEPRTASGVPLALVGVLQDLAAQCVRDLVAAGGVNVQEVTPLVQDLTPALYTCLAAHELL
LRRLSGGGGGGGGGGGSGHGGEGAAGEGPAGAGDAGAAEAQGEGEGAGAAAGCEAQGEGGGGVQAQGLLRPHFCRQA
QVRYSAAQLPPLHLAREAFRSLAAGEALDAEGWAAATSAHPQWAAPQEEEEEEEGEGEGEEEEEELEYDVEVAAAMPS
GAAAAAAAAAAAAAMPAVENDGREEEERGRKRSRRGEREQPKAGAEAAQGAGGAAAGDTEQRHQQRQERQERKQQEQ
AQQLQQQERKQQQQQQQQLDQERPAVRGQSGDKHAGGAQRSAPAPAPAPTPAPAPAPDAAATASGHKRSRSEALGTA
AAAGTGANPIPTQQTRTHTPGPPGAAPLYGRPWRLTPAQPEPAPTSTSPPPTSTASPQPHPHRQQHPHQHGRRPYSA

LHEEVAAFAAACCPGASELAALSDAVAAVQAAAAALWPGARVVLFGSQAAGLALPGSDLDLVVLGAQLPSFTGAAEG
FGKGKGKGGKHGGKAAATRALDALARRLRASGAIRSHSLIKAKVPIIKTELLLSGPSGRGPPTRLPADISIGAANGA
AAVALVQRAVAALPALRPLCLVAKALLKECGLNEVFTGGLSSYCLVNMALAHLQCEGFDASAALVTGGSRRGEAAVG
GRGAGPLAKRRRRRAADVGGEAGGEEEDKEEYASAEEVWAEEAEAGALQRQEQQAKEEEAAAQEQEEEEWVCGDEDG
EGMDDEELDLDLHFEIEEDEDVEVGGGGGGGHAAAGGSPVAVAAGAPPPQKEQDQQGRRRRGGAGSGSPAYGADHDD
GDDDKQEEEEGQDNVRGGSPAEWPTADPDANLDPEHRGQQQHAEEDELVLIAEAAAAAVSAAADIVSPPPRRGGGGG
GGAAGGSRSAPFGKVAASLAAAAAVGGLCTAQLSYLRALPRCDPGSGADLGELLVGFLSRYGQVLDTRCEAVSILRG
GVCVKPSKWRRQRYFGRGTAAARHNDNEDEYEDDECEDEYDDHHFRGGGGGGGGQGALLSVEDPQQPGRDVCAGSFN
IRGVLGAFSRAAAQLQRAAAGAANAIATSGAAAGGGGGGGSGGGVRARPQTAGAAAGSRQIVFPDSEDGDGGGDGDG
GGEEGESADLLALEYNLPPEGSQQRRRLPEAALIAMRLALAGRRRRQTQPGAGGRGGGVAAVFPLLGCVLDTELALC
RSAEMGQARSAHAQHMSNAAQRGTGHSRSRSRDYPEGQEDWGELAEEEEEEYDDVGRGWDSGAGRGGRGRAAGGGVR
VRGKIFPTRGEKRKLVREEKARAAERQERRRARQAAGAEEREQQEEQEWEREQRQREWEWEQMDEQERAQQLQQGQG
PGKGGNKQQQAQKQQKQAEKKKKAKKAKAEKKTARQLKIDAARERRRDNKAATRARQLQEASDRRQQQAQDRGAGGG
GGAPGAAGGGAAAAAAAAAAGGAGEQQQLLPPPPSPEPYPGHRFRYQQVEPPGELQARDQAQAPARLASPRAGGSGR
KRQQRGSAVVVEGDGDGDGAAAGGKELPQWFQSLQGQDQRPHQHPHQHPHQRPHQHPHQFEGQGQGEHYQQHQYQYQ
HQRHYPEEGMEEAAGAGRPRGSGGRGSRRGASSGRGGGGGAGGGAGGGGGGKQKGKAAKQPKKQQQQQQAAPGGKAR
GGGRAGGGRGGGGGRGAPPVQGGGVSKKKAAGSKGAGGKGAGGGGRGVGGGRGGRGGGRGGVGGRGGRGKGGGSGGG
GSGGGGRGGWIMG

>Cre09.g410500.t1.3|PACid:27570709
MKQEGGGQPAPASARGDRTTQPASTATGAVDAHVYATGDQTMREAVAGHAPGGSSGGAAAAAATAGSSGAAVAGAGA
GVPAAMADDDDDPRPWLLFDLNGVLVEESKYAERETAREGGGGGGGGRGWGGHAGSSRFRRRNFRARPGVAALLELR
RSYRLGVYTSATHWTAARALASLGSRLVTELRAAGRSQELAALQEESRALAAAQEAAAMEAEAAAAAARAVAEATAA
AAEATAAADPSEIDLDGGDGGGGGGGGDADMAEAEAEAGGGAQARGGKAAGAVATGAGGQAAATSAAAAAEGGAAPT
NNNTSSSSGHGSYSRTHGNQHQHHHSYSQQHHPSYSYSQPGYSHHGSYSHHGSYNPGSHAYVDVRVLLTRVLHRDHC
APDPGWQQREGGKEWDTVKPLAAHGLALGRVLLLDNEARKSAPGEVANLLCLPTWESAADEGVGVWLALVRELMALP
VTQDRTLDLRDHSAAISGRVCALHRPRPEANNTGTRGVHSDVVTPEPEGGFGPGGGSLLDAVAAAALVRAEPSIRKK
QKAKAELAAMTAGVAAAAAGGRGGAEGEGGGGAGGAVGAGAGVLDEGVAPDGLPRWAAAALATVDGAGGATRQHQH
QHQQQQDQQQQDQQQQGAAGAGELLRLVAGYALRPGAAASCDRWEAKNLREAAERWREEEAMADPRVRLAVVACVAA
ADEVLRGSACHTQVCQLMTARWATFGGFDAFTLRRECSHEAIHGALLAGLIAPLPRPGPNFMLRYVPTAAAAAAVAD
VDQTRPEPAVVVEADQVAQQPAAGAAAAAAEGAEAEAEQAAGSGRRPEDCFPAGLRAYIAARGPRPNRRDRRHQKQR
RREGQDQDPEHHCQQGNQQEGGAPAAADQAEAAAVAAAVEKEGGSGGGGSGGPGSEAAAPAAAAEQTPQEQQQVPPP
QQRPAWEPRTASGVPLALVGVLQDLAAQCVRDLVAAGGVNVQEVTPLVQDLTPALYTCLAAHELLLRRLSGGGGGGG
GGGGSGHGGEGAAGEGPAGAGDAGAAEAQGEGEGAGAAAGCEAQGEGGGGVQAQGLLRPHFCRQAQVRYSAAQLPPL
HLAREAFRSLAAGEALDAEGWAAATSAHPQWAAPQEEEEEEEGEGEGEGEEEEEELEYDVEVAAAMPSGAAAAAAAAAAA
AAMPAVENDGREEEERGRKRSRRGEREQPKAGAEAAQGAGGAAAGDTEQRHQQRQERQERKQQEQAQQLQQQERKQQ
QQQQQQLDQERPAVRGQSGDKHAGGAQRSAPAPAPAPTPAPAPAPDAAATASGHKRSRSEALGTAAAAGTGANPIPT
QQTRTHTPGPPGAAPLYGRPWRLTPAQPEPAPTSTSPPPTSTASPQPHPHRQQHPHQHGRRPYSALHEEVAAFAAAC
CPGASELAALSDAVAAVQAAAAALWPGARVVLFGSQAAGLALPGSDLDLVVLGAQLPSFTGAAEGFGKGKGKGGKHG
GKAAATRALDALARRLRASGAIRSHSLIKAKVPIIKTELLLSGPSGRGPPTRLPADISIGAANGAAAVALVQRAVAA
LPALRPLCLVAKALLKECGLNEVFTGGLSSYCLVNMALAHLQCEGFDASAALVTGGSRRGEAAVGGRGAGPLAKRRR
RRAADVGGEAGGEEEDKEEYASAEEVWAEEAEAGALQRQEQQAKEEEAAAQEQEEEEWVCGDEDGEGMDDEELDLDL
HFEIEEDEDVEVGGGGGGGHAAAGGSPVAVAAGAPPPQKEQDQQGRRRRGGAGSGSPAYGADHDDGDDDKQEEEEGQ
DNVRGGSPAEWPTADPDANLDPEHRGQQQHAEEDELVLIAEAAAAAVSAAADIVSPPPRRGGGGGGGAAGGSRSAPF
GKVAASLAAAAAVGGLCTAQLSYLRALPRCDPGSGADLGELLVGFLSRYGQVLDTRCEAVSILRGGVCVKPSKWRRQ
RYFGRGTAAARHNDNEDEYEDDECEDEYDDHHFRGGGGGGGGQGALLSVEDPQQPGRDVCAGSFNIRGVLGAFSRAA
AQLQRAAAGAANAIATSGAAAGGGGGGGSGGGVRARPQTAGAAAGSRQIVFPDSEDGDGGGDGDGGGEEGESADLLA
LEYNLPPEGSQQRRRLPEAALIAMRLALAGRRRRQTQPGAGGRGGGVAAVFPLLGCVLDTELALCRSAEMGQARSAH
AQHMSNAAQRGTGHSRSRSRDYPEGQEDWGELAEEEEEEYDDVGRGWDSGAGRGGRGRAAGGGVRVRGKIFPTRGEK
RKLVREEKARAAERQERRRARQAAGAEEREQQEEQEWEREQRQREWEWEQMDEQERAQQLQQGQGPGKGGNKQQQAQ
KQQKQAEKKKKAKKAKAEKKTARQLKIDAARERRRDNKAATRARQLQEASDRRQQQAQDRGAGGGGGAPGAAGGGAA
AAAAAAAAGGAGEQQQLLPPPPSPEPYPGHRFRYQQVEPPGELQARDQAQAPARLASPRAGGSGRKRQQRGSAVVVE
GDGDGDGAAAGGKELPQWFQSLQGQDQRPHQHPHQHPHQRPHQHPHQFEGQGQGEHYQQHQYQYQHQRHYPEEGMEE
AAGAGRPRGSGGRGSRRGASSGRGGGGGAGGGAGGGGGGKQKGKAAKQPKKQQQQQQAAPGGKARGGGRAGGGRGGG
GGRGAPPVQGGGVSKKKAAGSKGAGGKGAGGGGRGVGGGRGGRGGGRGGVGGRGGRGKGGGSGGGGSGGGGRGGWIM
G

>Cre09.g412950.t1.3|PACid:27570739
MDKEAQGYEPGDRVDDGAPAAAAAAGAATHTRGGWPSSVALAAAPHAAGPMAGGPPAGTAAAFHQQANPWLGGAATA
GGAPIQQQQAALNEQRRREMQPQRPQQPLPQPEPAFGQHQQTPSISSDEVRLAHAQSQHQQQQLQLQLQSNEMQQRL
SAVAAAVRQRPQQPLSFHQPQQHQQDQYELELQHQHELQHQQHQQQNYPHQQHHHHQQWHQHQQQHHHHPHWHQSQH
FYPQQQQQQQQQQQQQQQQQQQPLQPVAEAEDPQVDANPCAPTRMSSSGSGSGGSGGEDAAAGGRRGGDRSGGGGPG
GQHRAAAGAVGGGGGGPLQPQQQQPLLQPRQSQAQPQSQQPSQQPSQQPIRPHPFGDVAAAAPATAPAVEVVWNAPS
SSAAVTAAALIRLAAAAAATPPPAAGTPLPPPSWPPITAAAAATAPNLYGGYIHASSSGTAPAGAAGMAAGGGSSSG
GGGMLLPGSRLGAGFGINPGVGGGGAGAGAGGGGAGGEGAGSSISISLAGGTSSSSGRLGGGGGGGGGGGERAAGLL
APGALLAAAAAGGKAAGGAAPHMAAHGQLFAPLPHTAAAADSAGAGAAGGGPGPGAGVGGSGDAAAAATYAAAAAPA
AAWFAARSNALAAVVAKPPPPPSHPPSAFAASPPPPAAHPPSQQPPPQHSSPQQPPVHSAAPPLPSSLPAVATAGST
KRRTAPADDPTTAAAGGYWATPTRVAAPRLGLVGEGASPSAVAAAAASASAPFSPFAVAAATGVLPAGPSSLLGRPA
AAGFAAAMHAATGPSSAAAPEAGAVQQRVRPGAAAAESGSGSGSASGSAAAAPPPPPQQPRQQQQQQFWAPPPPPPP
PQLRSPPPQPPPPPQRLQSQVGAAATAAAGAGAPRLNPFGEMAAAAAALSPWPAAPLLLRSARSEAATHDGRFQTDM
DALIAQQQHLQQQQQQQQQQQQQQQQQQQERQQQHHQSHSQPLPSLAIAGAAASPRSAGGGGGGGGGGGGDGSSNNS
NSRGTGGGGALLWEPNATGGAPGEALGAVAAMHAHWSADALVAAGGGDGGGDGGGTGTGTISNNAAGIVVTDAIHGL
QQQQQQRQQQQRQQPLQYQVPAAGPQTAPPPLAAQPPPPPPQQQQQQQQQQQQQQQPQVWQAWQLQQQPMQMVQQPP
QPQQQQLPHSAAAAATAHGAHATAATAAAGGWSTSPSPPTSASQLQLQQLQHLQQLLSAQPPPPALPPLAPPPPALP
PPQPQAPPPLHVQPASSHPPPYPLLHQYRMPPPPPPPRTSPFPLDGSSSSANAATSSSAGTTSGSAVGSTTAATAAL
SLAPAAATPAGAATPATAATPAAATNPASASLAAAFQAPPFQQQQQQLQVAALPAAVAAAGCVNLMAVAAGAGAGAA
TATATATAVATAARAVAVTLSVPPPGPLQLLPLNPFAAVAAAASELAATSTTATATMGAAGAGPDTTASATAATSAA
ATAAAIATTAAPLPVPMVAEALQRLQQALAAAATGGGGGGGGGGGGGGGGGGGGGGGGGGNSAGAGGSGGPVAAPM
AGAVTEGSQQWRAALLLSSGERPAAGEEPQQQQQQRR

>Cre09.g402550.t1.3|PACid:27570766
MDHLRLQHPEQRRAERGPTASSLDSRGGAQEVIRHDASTQAHRRDVSAPAAARVLPAAQQPRPQPHSQLPQRQEEAA
RASASPPPESTVQYGRVRWLRLPYGRDVAYNKPLLEAVFPAARAAALLAPTRQLAMRVTVYVEVAAAAGGAEDGVRG
GGGGGGGGAGDVGVAGDSSSATPVGGATGRDAGGSGSGNGSGGGGAAVAAAVPAAAAAPGGVQLRPYHVGLHMDCR
QGRGVKPRPRLYRLEPLSREMGWQAGAQLSLSLEPLPRPQPSRPAAAAVAAAAAADRDSANEPPFPQQGQGLSPHQA
QQHQNEQQRRQRHQQRQKPQLVPVLRLLSNGDGQRLPSALKCLATKRRRQAECLARQLGLPSTHVAMQLPHAALAAD
GREDVPVTAYTAAAAQWLRGAHLLPVQWLEEEEEEEQAEGRGAEEEDEEEKGREVGAGGATRRRGRLTAARIVSTKP
LCHEAASGGRAGGGGGTGPGVPAEGEVYVVQSLLPLLRHAGAAPGDVVVLSPLLAPQVEAVAAAAIAGGDEVMGAGG
GSSRISGLGRSGGAAAAGGGDTGGGGGGAAGACRVIAVCVMLKRQRQPQQQVEGEEEEVVAEEGGRQAAATAATSIR
RRYLFPPVRAHLRGRPRQRPQASGVGALAARAVPASARDATATTRAATATAAPRAAAPIAAAGDGGVRVAPSEADEA
AVAAVRCQQRQRQQPHAATETARPPQPQQLQPQQQQLHPQQQHPQQQHPQQPQHRLLLGVKRSAAEANLGPGARPRP
GPSQQTPPPSLRQPCVPPRPPAAAEAASGSGGRGSHLQPHQAAAAAAPVSCDTAAPSLARPVVAVSAAAPYPTADAA
PPGSPATSRRQPQPQPRPSAFQSSSGEGVGSLLLARAASQPVPAAAAVAVAVAALGGQHPHPTPGRLLRQLSAQPAG
LRRPPPAPLPSSLQPQPRRQVQLFDHRHRHHHHHHQHQHQQQQQHQPQQLPPPCPLGAPQAQRRADTQPPPLLRRQQ
SHPQSSLSRAEHSHQNQPHNPNQIQPQNQNQTLQSAPRTERLNSQPFSPLPSPQLQPQPPPQLRARQEALCRAASGP
AVVRLPPASGRQLLKPSAFLPAAAGAATAGAAVAGAPVAGRREVSQRPLPQAPAQPAGSRRLEQLQADARRDPAPLA
DAGAGRRSSSQQLGRAAVAADADQRAALAAAAEGQATARAATAPAPWAAVKPEPAEAAPTGCQPGGVRARAPPPAAC
ASSEPAQPPAVAAAGAAGARPAGLSAQQTAAAASPSGGGRADGVAPVAAADTGGSVSGGGSDRRFWRLGAHAPGSTG
VGMGVGSGLGVGAVAVASSKRVKLEVAAPANPPPPLSSVKSEPTLLAGLSSGLGPARSAPVGSAQQVGAPGGGGVSS
GPLPPSRLGPAGPRTSNGQCQQRQDLRQKQQQQTQQAQEPPTLAGSAGPAGGRSSNFTAGSQGGSGGFKSCFATAAT
AAAVIDDLEDPCKSKGHATASHLGMGMGIYTGGGPWLKAEAEEAGAVALAGGGDVGAAAPAKDLLPRATSDAALGAQ
ETAISWRALSAQVPAAVPPLPRTAAVTDQKPVPPSAAHFPPSGGGSAAAGAGTAAAHLCAPPLAPPPAASACAAAAT
ITATATAVPATAIAAPNLAAITTATADARNSRCEYTYPTSFLQLPSPATTATTSLTTRHTASAPEHQQLPLPPLQPS
ELRLCGLTFHPALAPAVTAAMAHWQAVALGGCPLSAADPTTRQLRIVGLPSPPPACFGPHRLKRNILLSKLAHLLGL
AGEAGRSDAPLPAGRLELLHVRPRGSSCSRRPASEAVGPGLGPGLGQGLGLEAAAAIKHSAVLGVLGGYVLPTDEAA
SMALNGFRQLPPASQAAVREAAREAAGAEGGQEREGAGGGRGEGGGGGGGCCLDPADAWKLLVAAHTVPLAADTETA
PPPGAVATAAAPAVAPAPGDTAVTAGGSSGSSWALCTLGGYGNLGALIADPRRQPRAWVEGNDVEDVGGAAAGRANV
TVVPVCVRGLVLPVLVALRDVAPGEPLLRDRGAAWWQALGPAWEVLEEGDGLCVPTLLAACGPAGQRQGPEQEPERE
QGQEQGVARQEQGQEQEQGQAQEGAHFEGPPERLHLLDPPPPPSVDTLQPQPPLQEQPQEQPHVQPQPQEQPQVHPQ
EQEPRMLLPELVPLPAVPVGQLPSRRLLRSGQQQSAGYHDAHGGDWRDRGAAAAAAAAAAPPPQQGSCAVAATAELA
AAGSYEQLPPPPPPPGPPPASPPRCRSPVGPSREAAGRAARLSSSSSRSAAVATGRGSSERSAGAEGGRKRGAEEVR
SPGGNGDRRRRRSKERKRGRGGEGGEVGDSGDMDQEERRGSRRREASRTSGSSRREGGGDVCDPGSRARHATGDSAR
RDGSTTAVAAEPLLQQPHQQLPQQQQQQQPGAAACAGLGLGRENSTSSGGAAAPARSSPPVAPTYMLTAADAAPADV
WMPLPHAASGDGGPQHQQHQQVLLSMVEALCATGTFNPQQQQQHSHPQLPQPALPPPPPQPPLPPPPPPLPPPLPPP

PPPQPPLLPGLRELCGPPPPGLGLLGAMLHERLDRLGMAAVLAARELVQRGVLRRRDVHEAAAAAVAAPGCRGGGGG
LALRPRCCPVPGLLLWVTGGQQPAGGGGGGGWRGGDRLPWRALQVRCSHQDLLRNVARADRVTRVLLLDWFRDLAGP
APTVRMELHTEAGAEAETGREEREAPLLAPAPERRGRPGGRGGGGGGGGGAVHGRHGGSGVVELPLTLELWRMYERE
VMRM

>Cre09.g398900.t1.3|PACid:27570782
MMRRQHAAPLVGAVNVLMVVLAFVASANAQCVPGGIFNCPPSPAPPSPAPPSPAPPSPAPPSPAPPSPGPPSPAPPS
PPSPAPPSPAPPSPAPPSPAPPSPAPPSPAPPSPAPPSPAPPSPPSPAPPSPSPPAPPSPSPPSPAPPLPPSPAPPS
PSPPVPPSPSPPVPPSPAPPSPTPPSPSPPVPPSPAPPSPAPPVPPSPAPPSPAPPVPPSPAPPSPPSPAPPSPPSP
APPSPSPPAPPSPVPPSPAPPSPAPPSPKPPAPPPPPSPPPPPPPRPPFPANTPMPPSPPSPPPSPAPPTPPTPPSP
SPPSPVPPSPAPVPPSPAPPSPAPSPPPSPAPPTPSPSPSPSPSPSPSPSPSPSPSPSPSPIPSPSPKPSPSPVAVK
LVWADDAIAFDDLNGTSTRPGSASRMVGEPDIAGTKCKGNLKGWMPKPSRNPRWGQAVFSGGRTVGSVANVTIRVAF
ATEKPALIYSSIELVVYNTGATLIRVPIAANVTRSQIRCPGFLTYGTTPIAGYPTGIDATTWPNWKIAGVRINMGAG
NKKPKTSIDAVGLNLK

>g10129.t1|PACid:27570790
MEPTVGVERPSKTPAAEAAAGKPAVKPAGAGPALQQVCLPPPECAAATPPATAAASDLPPKCVPLLPPPHWPAAACA
AAGTAGAGWPLLPLLPPLPLLRPLLQPVPTALARLVRHSAQLLLVPLAVAAVAAAGLRCFRGGVSALAAAAAAATNA
TAPTAGGGARPALLLLLLLPAAAYATAAIALAALLSPLLTRAALLLQPAAPAPAAPSAAAGHRDEPPPNMLAATEQT
AAGQQYSGQQQQQQQQQQQQQQQQQAERRRSAALGAVRRAWGLRDGTGGGGGGDGGDGRGDGDDPAGCCDGGDMAAA
AAAAAAAAAAAAAAAAARRTAAAVGLCPAAPSAAAAAKQLPSGPQQCQESAAAVAAVAGPRASIGEEEGAAGAANYAN
QGHVDDYKDSSGSDSSAIYSTSSSSHAISRVHSSTAAVAGGLSRTAGSTSIRSSSIPRSGSSATAASAAAAAATTAT
AAATPATAGAAMSALPAGTAPAAAAAAAEETAVAAAASPAAAVPSALVQGIAGALLGVLDGGGGGGGGGGGGGGGG
DGSGGGGGPPCYDKVAAGSGGAAPPAAAAATGPPPSTPPPLGAVAPLPPGAEVDPQQQHQQQQFQAAAQSQEQAQAR
AQVQAQAQAQAQAQAQAQAQAQAQAQAQAQAQAQAQAQAQAQAQAQAQAQAQAQARAQAQQVRGKAQGKAQQRPRRQEEGG
DDVLAGQSPAGSDRSPPRRWLHCRRHGRVGGADEGEALASSRMEASSSSAPPAAGVAATQLLFLTGDDIAAADDTSH
GAEEEEEEEEAGAVARAATAAMPAAGAAAAVGPAAEAATPPPPPPPPPSAAATAAADAGLVDESAAAHIPRSAAAAA
GAAAAAAAAAAAAAAGDAEDAFLVPSSYTSSPCGAPPPPPPEPEPPALAPPLAESQPQQWPATSPAASHSSGALAVT
VGKGSSSGSSGGGGGSSSGTCLAVAEAAAPSKPAADAADTAATHTTRPLSPKPSPASSCLYVSPRRHAYVSLKLRDA
PPPPPPLSSPPSLTATRLLQQRLAAVAQLRRQQHFRPHTFAGMTAGGGGSGGRSGSCGRAGGGSGGISGSARRALTA
QGAAASFPDTAAVAAMGSSISASEPASPHHHGMAYPGFATPPAAAAAAAAAVAAARSAGPSSGAASPLIVTRPQPQA
RSAATAAAGTATAATAAATAATATATRGDLQAVTGSPGHGLLLQGRGSSSSAASGGGTSGGASGRSAAATGAGGSWS
PIPEPVTAMALPPPPPSPPPSPLQPSTAPAAAAAAPPPLSFEQLTAAAVEATEAALRHSREMYGDCRAGMGPELADW
LAVPGCIQLVLSVWWRRRHPHHHQQQQQQQQQQQQQQQQQQQQQQPTRLQMPPTRLEMPPTPAGQHRARGGARGSRS
SGAAGPGSSGHGDEAVPVAAPVAALPVTGAAAAAAGAAGAAGEGAAGVAGVAADVAAAVEGVDGEPPAVAMLLQPPG
MPERCAPAVAAGVPGLEPPPSPFLTHATVAAAVAAVATAAGPAASGGGAGAAAVAAPLALTLRQREQSAAAVAAAAV
ADGSSRSTSGGYRLRINRRCSTEPPLLLPQPHPPEPSSLLAATATAIEVSSSSSGGGSFYSCSTPVGGGSSSYSPVA
SSCSATADGGGGFSTAAQPQQQQQQQQQQLGGTAAASDGTVATDAAPEAVAVLAAAAPAPAPPPSLPTATSTAEPPL
HQPPEWLWGFPPTAAEAAELPSPPPPPPRRSSLTASDGPADTVVVVRTASVPVPAPDVTAVVGASCASSASSTINNT
LAAACYAFAEASQHRDATHRTAAFGANSSSGGGSDGGSGGGSVGSDDGGGAGGAGAERHNVAAAAATAAGVVFAAAA
AEQDQRRRARNSLSVVAAARVGATLMLLDPTASTTTTTTISSSSTGSSGSTAAATTASRRLCRACSATAGVAAAGGA
LPPLPLPQLPLAPPLSRELLLQPTCALLQPPPPPLQHELSTSDAANAAAAAVTSATYATTAHVTLLLTPCHRPRADP
AAAAAAAAAACCSPPPLPPPPPAQQQPVRIIVTQLGRVLCDTCVIARCPSGGGSDAGAGVSAVGGDGDAEASSGGGS
GGGGGGGGGGAAFPLTLHGATPGVATVYVFGLAPAPPPSLPAPPPPPPRACFLAMAPLALMPAGACGELTSLYGRMV
HEQLRVMTAMAGAAGTAAGTAAAAAVGTAAGAAAAAVGSPLPCSDEGDCAGVAAAEAEVPETRQAAAAVETQQAAAA
VEARRAAERAVLDECWLPLLMDLDLALTCVPARAAADTAHHTAPPPPLVIPLPPPPQLRPPPPPHPHPHPRQPLLLP
PLQAPPPEPYCAADGSARCGRYDSGGPVTEAPSGPATESPPPPPPAATAATAPMGADGSSSRVTGGGSSISAAGSSS
SSSPMLLGSHTCVPLPAWHETLACLSRFLLAQGIHGVLLHVMEAQQAALLQPQPPLPRLQLQAAASATDGAVLLPQQ
QQQQQQQLSPSATQRLIRLLRARVAEAERFAGSGGAGGRAGAGNGRGAGGGAGSEGETVAAEAAEAVAAPHHTATAA
TAAAGSDTAGGMEAALEAALAELASVATAGAFLPAAAVADMTDAAADADSVVDTAAAAAAATTAATTAINSGRRDSS
SSSSRRSSSSSNQSSSSSGILPLPPSTLTAALPLLLGRVAAGVNTAAITAAAAACHSDRMQPSSPSPPPSPPPPPPP
PPPAAPVHRSGRSSWEAAPTAIATATAAAAAAAATAAAASPRRPSTCQYDGGEPPHPALLLPPPQLQQRLLLLQQAQ
QQAPESALLGLGSGRSRLRGGGGGVTWPPAVTGSSSPGRSRSSGSGGGGGSTPGSESP

>g9842.t1|PACid:27570816
MEHFEVELPERTLEPWLFDDEGEDDDDEYKVDSDVEADDSDSEEAEEHENALPPDEGTLELLELADDVLDSIEEKVL
SDSECVESDAEDEEEDTLNAATASQQQGTPSGHASGSAGATPSTVALQRREAAHRQHLPTIAEEATPCGGARPPGLP

GSLAHSAAAHQAAYGPGQQWQQPGFQHLTHTPDPGAAQQLPPNQDCALQPQLPGDDTDAYWQQQQQQQLQGQQQQP
AQQLDRQEYEPQEHPQGPDGSGAVGGGGGAGGSIEVQPSSGICRRTRAHVNLRDVRMEELEQQLPEHVDHEEIEAAE
DDEEYSRFLQALFAEPLPEVSSDEDPDFEEVLGLELGSGDAGGGWMALLNSPSREEGAGVSSGDDIVDGAAPGSPAR
RTRGFFRARKLLQDQQERELLEQARQRGAEVVDAEMMTPEQMSTLHAQLHCHTQMLVQMYVMMARDPDPQAQEVARQ
TKLLMDQLELAREVHSLMRKGVGLHPYTADIICRPGPPILLSDLVTQIEREGGGEAGAATDAGGAAGGAAGPSHAHA
AVAPAATQRAAVWMGPPAAGAGAAGGTAGQPGADEAAALMAAAAAAVAEAQATLSGMPSMAVAALAALQPQTGMEDG
AYGDTDADGGADLDGDECGSASAYAIQAQKAATAARSAATGAAVRAFRPPWLPPQPDNCVWTVYDVLPLRLFPQIVA
ILQTLPAYKSSYLAAHRRLKASGAQRRRRHRRRQKSDPLAFAIKWAAPALRCLQGYLCAALCFIPSPMMPDAYWTDA
EDDLLWHGISKLGTMYNAIAALYLPNRTFKQVTYRHKNLRGRGRAGDHALLAVAMQAAMRAQTEQEKQIAGAAAAAV
FAALPGAPVPPDATAAAAAAAAAAAAAATVAANADQTTANAMARRYPPKAAATPTAATAVAERPKRSRAAKQPPAAA
APVASVGEGTEAAQQAALAAAVAAAAAAATAAAIGQVPEQPQAAPAAVPPGHETISTGVYGADGGEEAPTADATPAA
TTAGAAEDGSVPAPGAVVAEGTRTAREAAAALRAASSNSLLAHDVLAMVTAARMMALPADVTAAQVMQVQHCRLAKV
QQLVQQQQQQGKTKRNVIGPLAYAPLAAMQQAAVDRATPIPLGVVTALAVGIPGPVQVAGVAVLNQILAGDPDTQT
MQDIRQLMVVALQHRAAREGYYSPRGAQAYAMATQLQDRANTAYAREALQRISEGFQRQFRMVPAVAAATGPAAAAG
VAGAAAVAPGEQSHQHAQQALQALQAAMAAAVSGGQPWNGQPPYGAAAAGMAAGPAAAPAGPLAGASQAQPVAATAG
AVHMQQAQGDAVAAATMPPAMDGGMMGAWPAMADGGAPAAVLAAATAAAAAAAAAAAQNPAVGLQQLQQLQQLLQIA
KAMPAVLQAGTAAGPNALGGGIPGVGLPVDAIAAAQAAAAAATVAALASSSMAAAAQHQQPQQPHAGFSGMLQAHAP
WMPTAAAAGPPVYAHGTVELAGTSAGAPLPQTAPGDAHGLQHSHEHGHGAVDEQHAAATPGAFHDLAEMDLDVDDVE
VGGEGPSDSLEPWHGLSRGPWSPPPLASPLGSPTPQAQAYHPPHPHAQQQQQQQQQQLQQQQPMAAAGTGAAEQAQM
AGHGAAHAAEGAGAGPGPFATAHQTPGPPHAAVAAGASVLPTDPRLTFGRFLHNRSSHTQLPAPVEGQLSGQAHLGE
PAVGHAGFLGARQPAGTAEAGPAMGSPPLLHLPEGEARPTVSDGFTLGGLSDILGASLGGFRSGAATPAVCRSPHAM
SQGHTISHLLGSGLGLTTLLQSEDGAGGTPSRLGGLTEILAGCSPGTATPTKNAAAGPSRPHTGAVTAAGPAAPGAD
AGAAGGRRRSESPPLAAVAGAAAAVDGNGGAAAQTPPEAAGAGGGGGKAAGAAGVAQPPRVGPTPTRQLFSPTSPTS
GGVGRRGQLGSMQRVASQLRAAVDVTPSSDPKAKCRSRIGALAEASRQEDTQPSPFEGTAQNDSGGPGSGERSGSQP
PGSQQRQGRGTTSRYRPRPPLFTDDAQEDSSGPGPRRTPSAGNAPNSTGRAPAGTGGGAAGAAARAQAGTAGGPAGR
LEAVAEVDEERSEEADEEGEQGLLRARKRLRLDMDDEPPAGKANATGMPGTGADATGAATSAAASPDGGAASTLTAA
TEPAGRSPVGAQPPVGRSRLGKQLPPRDQLRGKAMARALQAEATAPVKTAAAVSKEGSPASPRPAAGQHAAGAPAAA
AQTATDDQTDGLVHGVGSCGGGGSGSKPSSRQPSSTAATTSATRSNSGAAASSGETSNGGVATGSDPRKDGGPPDAA
GGGSSGGAGAGGGTIKRRSTASAPAADTTGTKRARSGRGAGAGSEQTRSRGGKEPPTAAPAADPGTGAETAAVALAR
AAPAAAKVAATSGQKRTRASAAKIAAAAAAATAAAPLAVVDANVQAAPAANCALDRPTTAAGVCPAGGPEAAAATAA
AAAGPLGSRADGMGAAAAAEPATAARGGAGSGRPPRAPTTRRVTAAAAAAAKDAAVSPSGANAAVPASAHGTKTADA
TATTSPPPAGAPGSAAAPVSPPDAVGDRAGQAPGSAGPALSAGDFSLGGLSLHSMLGMDMSTLGGALDIVSMLPAIS
TRSMHAVEDGSPPRTAAQLSGGAGPAGAAPVAAGVGAAGSGAGSDPVTARAGTAAATTAAAGDAHGGAVPMPQSATA
SAAGCFGGLRAGAGLAPGCVTGAGVAAMNTGAAPGAGGGAGAGCLVSPPRGQLLQVTPYAPSTCTLNTLDILPLFSN
SNSLWAALQLGSGGGSVGDLALGLGGGGSGGLPRTGAAGRPVVAATTTASDGGAGSAGRAVLGSAGEAAAGARADGP
EAQQLLAGCTGVSPADPWTRFMTADHSPPAGRPPAARPHGSTNAAAGAASGTAAAVADSTAAAAQGLRADSAAAEGA
VGSGHRLHAAPLATAAASTAGPVLGRSRNAANAGDTGPLAVGKAAAAPARTEAVLGFTMGAAAAGTGTVAKPQRPTL
MSPPPPPPTMVSKQRMTAGGSRAPGSETVRLFGRTVEKPAPVSIMPVPVALPRPPSGASLVGAAGGGGGVAPDATPA
FASRRSRQDDADMATDNAPRRAAPAVAPQATPIHGRAVGFEGVSRRGAPADDAAAGVASPAEGVRLPAGGQEKAVGL
PAATPAVTRTRLQPLPEAMPTPAAGAGPALAVATVVQETPAPAVAVGAPLTLTPIAIEPNEAAEAAAPASVAHAVTG
GARLASSRTATSRSAAGAPSSGDPAPSASAWGSHEPRPEPRYQGGASRAGDGEPGAATSRGGDADTPGGTCISAAVR
RAPRVAFADHQPERVAATPSASVSGLGACALGSGGAEASAAATGEDGALSNGGDARPTAGGAQAPPVAPQLPLQAEP
LAAAEPGGWSKEQDKLILRSLFEHGDCPGTWDGIREQLGMQFNTEAIEQRGRQLMARMMALA

>Cre09.g387400.t1.3|PACid:27570817
MVTLRDVPFTIRIVSLDYYMAPPIPHLDFCFSSLEGTTVDQVPVIRIFGSTPAGQKACVHVHRAFPYFYVPYDESFP
TTPNEAAVCLRRMALAIEAAVRASYMPGRDQHQHQHQQTEAAGAGAAGGPGDTGAGTAGGGGGGGGGKGPQAPRQVV
HALQLVRGRPFYGYHLQEQLFIKVVLYNPQDVGRVSGLLAGGAIMGVRLQPHESHLPFLLQFKIDFNLLGMGLLRLD
RVLFRGDLPARARPPPPGWERVLMRPPRAGGGGSGGGSIAGGGGGDATGAAVGAGGAGGVYGAGVELEYEQPLEEEL
AGAGPGRRGSGAGRGSGSGKGSGGGPGASGSAGPPTETPPDPVAMAAAADAANAATAFAAVAAAAMSGGGTQGNARR
VEGMLWTRHTTPPDWTWAGAVLPSRFRPPERQTTCLLEADAVVHDIGNRREAVRVRLEDAGADQQLLESLVPMWEEE
RARGGGRLPERPRSPPRSPGPLCPAVEAVRQEFRVVAEQHRRQLLEQRRREAATAAAAETAAAQGAAGAIGGGGGGG
GEACAPHEPPRGREEGHDAGGELADAMGYAGAAAAAGVPAVAAAAEGIAAEGTAAAWAGPGQPDRQPEGPQRWGSDA
GGADGMDASPTQAWRQQHQRQQHQQQQQQQQQQQQHGPSALRHGGDTTPQGAQGSALTPPGADQAAATQATPAAAR
PLRSPLHATMSGAAAAGGGGGGGGGWPSASQQPAGRGTPGGLFGGGQRESLFLASQAANLVLGTGRVARPDGGRTSR
AASGGSQHRTPGAEAAGMGSSQVAGGPDLTPPPPAPQHQPQQRRLHPLPPLPPPSSQRPPLQPPSRGLSQQQQQQQ
QREFIRPSPLVPPPMPSLSPPQLGGSQPPVAGPLGGSRGGGGSHAACALGADGDVGAAGGGFEPLEWVASQLHPTPA

GARRRRRHHSGPPSATASQQLAPSLTPLQDAGVLTQRPPPAAAPGRAPQQLHAMQTPAPFSNSRGAAAGLAAALVTP
RHRSATPPPAGGLAELLSQPPAHSDHPHPHPHPHPGPLSQPLYSQRQPQPDTGSQPRGSQLSGRADGDTAVAAAA
VAAGGAGGGGGGGGAVLEEEAVPLLQLDLHLRPHVHDDGYGDDVGELELDEDHLEGDDVNERAAASQLVDADALARV
REERRQRRRQRRRQRVQLSQWGSQGLRRSQPAASQDAARDAGGRAADAAREAGGFGSSRRASDSGGGSNSGSGSSGM
DSGDEWFCRQGAAGFGRHSGAEDEGEGEAAEADPLLDLADELERDDDRGANGDGGRDSGLGGAASRDIDMEDVLPQP
HQSQRQRQRITNGNEEPQSQPPQQAHPPALPQQSTPRAPAAAAPAAAPAPAAAAAAVVDAMEDDEWDALAAELASAV
EANWAAGAAGHGLGPEAGGEQEGRPGLEAALGPGGHPAAGGSDQLMALAEEMEQGEGEEQAEEQAEGDEQPVRGAEA
GRLANSAGHQQQRQQQPYAQQTEQGLGAGAEAADAEEEHYPGYHRHYHGPVYDVEGGAAGRTAALAPHGAAAAALRD
LEDLVPPPPPPRGQQQRSLQQFAPQPVQPAAAGAAAAAAAAAPAVAVAACVGDDPISGSSSDEGAGTGGANHGGADD
VEEHDLDLDGGGAAGAHVGAAAGSRGHGRSVWDPGNERTPKRQRRYPANTQAAASAVADRPQAPELAEGPQASPPAR
GGLQTGPGPPLGPAAAVTPNSVHVQRRWAGAAAAAAMAPAGQTLHAAQRPSPAVAGAVTGAATVAMAGEGGQQQQQQ
PQQQPQQRHYRSPELMSPLPLAQSSPPTARGRRPGGLLLTPSRAVTPPGASAGAIVGAAAPGMQEQTRLPVLPLQSS
WQAGASGAGGTLAGAPEWGPDGAGAAAPFDAGAWPPADGGVLRVGGAGAAGAGQAAGAAAASPVVSAWELLQQQSPQ
LQPAPPPPHHYAQAGGQSPRSAGHGAHPLQLQQWHDVQRPLLPLGPALAWRGGAPQQQQQQQEPMWQPRLWDDASDD
EEHGHTGADDNLSAGRGAAGAGTGAGNDAGGATGGGGEAQAGGRGGVIPQVDGAGDSDGEDGGRRRRKKAKKQSKSS
KGRKSSRTAAADDAAAAASCAAPVDGSPGTACAGAAVAAEAAPAGEDAAAADAAEANLAPQLQAPMADAAVVVAVAG
RSAAASTAVHTAPTRAAVVLATASGGGAVAGMGVRRGFQPPRRIATPATTPQGSPSRAHSTASPVPSVAAVAAVTAA
TAAAAAVSATTTNTVQAAFAAVTAAAAAAARPDAALPQLLAGTSRRLVSGLFRPGSARRRAAGAAGAAGTAVPGAGG
GGGSSGSRANAQPGHACGGAGHAREQLTHCQQQPPNDTQQEHGQEEQEQENEDPGQSLHTDSRPPSPALPHGERLQP
GSDAVGADDEAVGANTQATPYDEETAVGEAAVEEAAGAEGPPGAMAWESEHEDEGTAPEDIAADVSQQQEAGAASAQ
VAAALPEDRDLDRGNDQTTAMGVAEHTGECAAEPQQAWQGAEEEEEEEAWQDDMRAGGTGGHRRQEQQPPAWEDTGCH
EQHVVHSGTLPLAGHTEAAVDAAAAAAAAAAVAAVYGANEAAEGAGEDAAAEQGLGPPAMRQTEASQAADGTAEGATC
DTAGDANRSAEAGTQTQPTVHLQSQSTHESNLKIATNATLGQRQDAVAMHGCTQQEQDDRFGEDVLGLGGAGGGYAP
EAEAAAAAGPAAGSAAHVEPRGLPEAGVTPGAAAVHEHDDASPGGGAAGSGGCSSSAEEGAIAAGANAAADGRALVG
DVGSACAGPRAGSGPEPELELEPGPEPEPEPDLAAKVELGPVVLAEAGVTACRAPARTAAADSWGSAMPTAADAGAV
VAEQAADAPEGQQGCQQQDRRQEAQQEAQQEAQGGRGSFKGREEEHLPGYPLSPGPQDSGQRASLHGTAAAAAGAAS
LQTPLPQQKEQLPREAQQGSAPAGADSASAASPLWAVLFPQEEDAEPELQPEDGDEEVGEAEKAGDLEEEGVGEAED
LHAQMAQEPQPAPQQVDMVPATELQPDLDLLLTAAPQQQQSEGHEAVGAPVADGWRVAEEAGHEAQWAASVVGVTPA
RALPRAAPSARVAQAHPAATPSVAAADGGYNPSPAAAQGPARASGPEPLQARSLSYSVSHSDVDSPPLAQQPASGGP
STGRGQLPTPASHSQALPLQQPSPLPPSPLPSQAPEQAMRAFVLPPMTSPGWSTGGGSGGTGAPSCGDGTPADADLG
TPAFGLFAAASPTAGQGAATGGARGRTTGGDSGGGRGLCHGAALGGAGPPGLVLWSPGVPPLRVSPAAAASAGRAEV
GAAAGGAAAVGAVGFGAAEGAAEASVEEEPAVAGPASAARVAQEPPSRTPDRGPGASPGILPGDGADLSPGGSRSCY
LPTQPLSETPGDWQLPGRHGRLRVPMAQQQQQAQQEQLAAHAQPQHPQPSPLHHGPSPHGRHKPDFDFDVPATLPLS
LQQPSPLPVRGPRSLLALGPALPPSPPLLGGARLLIRGGGAAAEAAGRAGASAAGAPAGLQSTGTGGSQLVPTQELR
LLAEPLAASGGGTSSSMPPPSWNPLRATLMASSSHSGGPSGSPPPPQASQRHTQAALLGAALTPTQPLLHIRVGQDT
LPGTQPSSWEASLPRPQPQPPEPSRVAASPLVAPQLASRSAPADTPQQSQPMPCTERPAAVLSAADAVATTEPDAPQ
RAYQQDSIRYSSHQEHHHPHPLHKRHNDTTATVRVGSQHAPSGASPSIPPSETSAQMVPATADPRGSYSHGGGHGGA
SQPLRLQLPGAAMPQDADQRGTHAVPPPGPDAAKATEAEADAGAEADPVGTLRGLRDETPSLAPTPLDTAAQVATPP
SSGLGNSQLGTAVVPGTAPAIGTAATTQATVSAAAAAAAAAASNDITASLPATACTGAGGDVASCIGGASLFAEYSE
ADAAPAAGDVAGTLLESPPSAAPGSAAPGSAVPGSAAHAAAAGEADQGRSQDVPRTASQAAHGAACVTPPPPLKEVT
GDATSLHARDGSAGTRTTSSGGERTAAVTGATGGDRPAMSPPGTVVVTNANKTAAATSLGPSPSGWTSPQCGQRTPA
AAAAAAADVAAAVRATGAAAARGSTGGGIGTQGWGSTPVGALAPVPFPPLATPRSPPPRATGTVAAGDGTGAGPSSG
TKTAGEGGRGTQAGLGAAGALAASVALAGPTAAVQDGGGGDAEEGAGGAERKRRRAIEAGGSTGARKPGGGDGAGGS
GSGNSDHGRPGGGGGNGSGSGSGGAGNPGGPGDPDGGDDDGEEEEPSRHAGLICLRPRGRPPPPSQLLADADWYGRQ
LPPIVHVDPFYGDPKDVPPRPVILASREYKIPAWADVPPFHRGPPAAQPLARLRASAAAAKRAATAAGAATTNGPAP
EEVNRLGWGRVRGYALVPALPPPPPPSEVHAWAERQARARKAAAAAAAAARARRRRGAATAGPAAAIAGAAGAGSAAV
AATPAAVAMAMDPNTGALVPVRATAAATTPAGAPGRISTDTGLRQRHHRRRAPPRMLAGSQGPASGGVAADGVTPLK
LSADPAQSLLGTPSLGGSRHSADSMGGGHGDGGGGGSAGGSQQPGPDDEDLEEEEDDEEEKEDADEDGGDDEIGDGE
DEDSEDEPGIADAPPSPKYDERTAFLGVVALGAAAAATGGMTAARRRRRRRQRRERERKRLALDAAQPEAAAKIGSG
AAVGMAARGPGLGAGAEAAGAGASQAAVPPRRRHRRRLATQRPGVDAPDFVLGGGSGASEDSDDEDLSEPDAFAAAY
DVDGVPISGTAAPLRHDSPGGAGAARKRRRVRRRLHQSQAPQLGALAGSAPRSQEPVAAAAAAAAGSPGSATPAVAP
AAAMAATPAAAADAGSSPPSAGQEAAPQSLKPPKPPSARTQPPAPASMAATPAARMPLRPFAGAAAGAGTGSGGGPA
AAVAAAAAAAVAAATLPPPPSVFHGGTATLRTPGGPATAAPPSLLSPLRPAGQAPSGPTPPSQRGFKRKGSGGDRRE
WVTVAAVEIAAACRGQLLPDPKHDAVTAVCITVMEAGEAAPPAWAYPTRVLILDPRAVAAPAAAPAPMPTPAAAPAA
AHVAAPAAAPAATPVLTGTAVPGPAAISTAAATAVPVQLHHPHPHPYDAGEPHAAAGGAARAGPAPFATASPTAPTA
LPQPHPVAGPGAAAGPAANAAAVAATGTAAEAGAAAAGAGAGAAAGSPPLSPAERGVLTGPGGVEIGPVRGTAVTSD
GLRGVHVEAFPDEPSLLRAFAAALSSLDPDVVCGWDVQRHSLGYLAERAAALGLAPPLLRAAGRTPGTSSVKERQND

EWGALHASGFHVTGRVLLNLWRVLRGELKLGSYTFESCAAAVLGLRVPRVREQQLALWFAAGPSAGRWRTLSWLCRR
CRLQLAMAEQLDLVGRTAEMARTFGIDFFSVLSRGSQYRVESLLVRLAHTNNYIMPTPSREQVGGQPAMEALPLVME
PESRMYASPVVVLDFQSLYPSQVIAYNLCYSTCVGRPMHARAGGTPVRLGVTQYSYPVGAVLPAGANNNNSHQNHQN
HQPQHGGSQLAGRGAAGAAGGAGHQQEDQGAAGQGQGQGQGQGNDDAANNDAGEEAPAAGTATPEDLVISPNGVAYV
SPQVRPGVLPRMLREILDTRVMIKGAMKKVPPEDKVLLRVLNARQFGLKMIANVSYGYTAAGFSGRMPFAELADAIV
QSGRQTLENAIAMVESHPEWRARVVYGDTDSMFVLLQGRSRKEAFRIGAEIARAVTAANPSPVTLKMEKVYHPCLLQ
TKKRYVGFSYESPTQASPTFDAKGIETVRRDSCPAVAKLLEQSIRLLFGSKDLSGVKSYLQRQWTKILSNRVSVQDF
IFAKEVRLGTYSANAATMPPAAEVAARAMAADPRAEPKYAERVPYVVVHAEPGARLVDSAVSPWALVEAGGSLRLNG
TYYITKQIIPALERVLSLVGADVRAWFAEMPRPQRMLPQKRPAGALPLATGGGGGFGGGAAGLQAAGVAGMLAGAA
AGGTIDRFYLSRHCVVCDQLTRAAQPLCDVCKRDPQASLALLAAQVARLERQHTHMVRMCLHCGGGGGRVVPLPLQT
QHGAAAAAGPGAAAAPGGSGGGGWGGGQRGGAAATFGRGGCHGAGLGGRGARGAGVAACDLNSYNAGCGGVVCDSLD
CGVYFERRKLAAELGALGGLLRSVRMLWTDEDRSST

>g9235.t1|PACid:27570818
MTARETAAAAAAAACAAPGTCSGPPGEDCRILATPPATTAAMPPLNTDTTTTTSSASTSSCTSCTAPAAPLTPPRSP
CSTPMPPPSPPPLLPPASPLTASTSCIPSTTTATCASTSTTTTSQGVHCLITGGCGYVGLHLAWALAAEGHHVTLFD
VAPPQPAALTAAAAKPPPCSSPPCSSGSCSRSSRMGSSGGGGGGGGADEDGSSARCCYCRRLSLLHSAAAAATAAAF
NNDTGAHSPAPPQAQPQPQPQLLLPPRCRLFQGDLRRPADVLAACRGEEPPAPATPPTAAAPATAAAATAASAPAAT
AAAVHSIFHVASYGMSGAESLRADLIRQVNVDGTRNLIAAAAAAGCRRLVAVSTYNAVWRRQRVSGGCEEDEEGEQQ
QRERAEREQQQQQEEQQVENVGGGGGGTIRPPSASWAPAPPLRLLDCYSATKAEAERLVLRANGTPLLQPQLQPQL
QPPPQSPPQQLQRQTSAATAEEAAECPAGAGAGCAHGSSSGSSCGSGPCGLPNGSSSGADLSGLCGISSSGASSVGE
DSDTDAGTPPAPPRTPSPQPSQLPSLPSSLGHSPHPNQPRTQPQPADSQKPLPPLSPLLSPPPPPPPPVLLTTAVRP
PAIYGPGEVRHTPRILELAARGWLRFRFGPPDALADWIHVRNLTSALLAAWRGLDAARGHVAAGRAYFVSDGAPANT
FDFWAPLLSGLGHTPPAASLPVPLVLAGARAMVAAHAAAAAASGAALAAADATDRLLIAPTAAAVAVAVEAAGAWGV
VPGRRAAASRSAVGGSSAPGVEGASAGGGTTGGGCGRGRVGSSVSGPLVTWTRRGTAVVLALTEPLITPAEVS

>Cre09.g400450.t3.1|PACid:27570858
MVRTRAGPLLVALLVAVFLYDSSAQVVTTTLPALLGAVGTLSLQPATYSFSSTESANITSSLVVAGQGNVEGAATAS
TLDLSTYPALANSTAQRPIIIQPGVTLSLVNLTLRLPPLLASLAAAPVSLLQHVVGVAGSGARLTLDRVTLVAANCS
SPSTRQYSSLGAAHSHSELQAFARVLCRPTGTNLRFYPSNVMAIVNGVVQYGNGTMQLAATDGSSAAASAAAVAGAS
LVGSAATCAAAPVPPATAPAGPAAPYVCSATSVGNAVQLRTAAAALLNNTAEVVVISVTGNVSINASVWRGALALPR
GLVLSLLGNPTAGQGVQIDFGGVAPAFTASSYGSSGAVRLRDLNLVGLPYSRTVNSPMDFFSPWLHPFRVFRGGSGG
ATSTPQLIATSCVLWLPDQEASWWNTALLAASSVNALEARPWLARLFTLPVTASSSSSSTSGGVRGTRVTSLNDAGG
VARFTDCTFASLYDSPANAPRSEFATIAWWPLMEGVPAAVSYQAALGEYAVINSSLAAASIGAQSSWASSIFLATAN
TSAGTSAIFASAEPAPRHNPAAGDFGPQYPLAGRAVRGPPGNQTGQVAFWDLQGVTGELYGSTSATLQDLVLYNLAP
GGALPYGGGFAVPGSDLYRKLLLYGSSNWNATVNETTGEPNPLPALGSVAYEDTLANLTLPLWYFARPANSDNGLMS
LYRVVLVVPTAELQLWLYLLAQAKKLPASYGPPSNATTSTSALQRRLLLGRGSSSGGGSSRRTLQQTAGSGNSSSSS
NSSVVVPGKAAETLRAAALAATVSWYTNDTIALSYLGLPRNWVAYDTVITAVLPPGAPTPLLSYEPTYDPVSYPPPS
PPSPSPPPNPAPPLAPSPPPATPTPPRSTPDPPTSLPSSPSPSPLRPSPAPPGLSSSTGNPGSPGAGIALEGNASPS
PPPAAADSAGGGGGGSSAVAIAVGVSVGVVAASALVILGVLFCRRRHQRRAAEAAAAARKAAGGPVGGSIELAPWGG
SSSDESGLANGHGANGNGNGSGMGGKVAVGAATAKGNGNGVTVAAGASPAATPRGAAAAAAAAAAAAASCVQEQQEQ
AFERLHQQAAAAPTAAETMALLAAAAANASAAASFLAGGGGGSGAPPPAAAALAAMPSVQLVIVDDAAQNRIPVGAR
PVAVGAVGTPPPATGGTGADSGALVRPAGGSPAAAAGGAAAAVAAMGKPLSRLGDKSLLQTVDIESALLAYSSRSMV
DDREPAAAAAAAAQQQARGSLMPAAGSPPPQQQQQPAPAGGATAPAGEPTPGGEVSSAAEAPLHLPAPAAPPRAGAD
LAAAISSLQAELSGRQEQLRITTVLGKGSFGVVYLGTWRGLRVAVKTLVVHDALLGTEGRRRQRAILEAAISTSLHH
PNVVATYAFEVKPLGVVASSPPQMMLAQGSDAGGSTSGGAGGAGAGGAAGERSVWESAGDVYKLYIIQEYCDVGTLR
DALTNGVVGSVSAGGAAALCALTLALDVACGMFHIHSKNIVHGDLSSANILLTTIKVAPLPGALGALGALAGGGGGG
GQPNVFGNLADTMAAGRSAGARSLAAAVSRLRGLWRPPVIAKVADFGLSVRMGEGQTHASNKFQGTPLYSAPEVLTR
GRLTKAADVFSYGVMLLEFFYGSYIADITTNCALAAAPGAESTEPGGSSTAAGEGSSAAGGGGAETIAPIAGGRFGV
AVPASCPPHLRNLMVACIAPEPAARPTFEQVVDSLVDMLLTEHQEELSAQQQQRQRQQPQRPSLSGHHNHHQPPGL
PGPPAGGLDGGAGAGAAAGVAGGGGRLGAGAGGGGLLGGGLMTDDDLGGIVIRRLPAPGGDQPQPSAEQGGYGRLES
SGGGGGGGGLGAGLPFQPLMLPGDVENGGGDGWRGGGGDGEIPGLRVVRL

>Cre09.g400450.t2.1|PACid:27570859
MVRTRAGPLLVALLVAVFLYDSSAQVVTTTLPALLGAVGTLSLQPATYSFSSTESANITSSLVVAGQGNVEGAATAS
TLDLSTYPALANSTAQRPIIIQPGVTLSLVNLTLRLPPLLASLAAAPVSLLQHVVGVAGSGARLTLDRVTLVAANCS
ELQAFARVLCRPTGTNLRFYPSNVMAIVNGVVQYGNGTMQLAATDGSSAAASAAAVAGASLVGSAATCAAAPVPPAT

APAGPAAPYVCSATSVGNAVQLRTAAAALLNNTAEVVVISVTGNVSINASVWRGALALPRGLVLSLLGNPTAGQGVQ
IDFGGVAPAFTASSYGSSGAVRLRDLNLVGLPYSRTVNSPMDFFSPWLHPFRVFRGGSGGATSTPQLIATSCVLWLP
DQEASWWNTALLAASSVNALEARPWLARLFTLPVTASSSSSSTSGGVRGTRVTSLNDAGGVARFTDCTFASLYDSPA
NAPRSEFATIAWWPLMEGVPAAVSYQAALGEYAVINSSLAAASIGAQSSWASSIFLATANTSAGTSAIFASAEPAPR
HNPAAGDFGPQYPLAGRAVRGPPGNQTGQVAFWDLQGVTGELYGSTSATLQDLVLYNLAPGGALPYGGGFAVPGSDL
YRKLLLYGSSNWNATVNETTGEPNPLPALGSVAYEDTLANLTLPLWYFARPANSDNGLMSLYRVVLVVPTAELQLWL
YLLAQAKKLPASYGPPSNATTSTSALQRRLLLGRGSSSGGGSSRRTLQQTAGSGNSSSSSNSSVVVPGKAAETLRAA
ALAATVSWYTNDTIALSYLGLPRNWVAYDTVITAVLPPGAPTPLLSYEPTYDPVSYPPPSPPSPSPPPNPAPPLAPS
PPPATPTPPRSTPDPPTSLPSSPSPSPLRPSPAPPGLSSSTGNPGSPGAGIALEGNASPSPPPAAADSAGGGGGGSS
AVAIAVGVSVGVVAASALVILGVLFCRRRHQRRAAEAAAAARKAAGGPVGGSIELAPWGGSSSDESGLANGHGANGN
GNGSGMGGKVAVGAATAKGNGNGVTVAAGASPAATPRGAAAAAAAAAAAAAASCVQEQQEQAFERLHQQAAAAPTAAE
TMALLAAAAANASAAASFLAGGGGGSGAPPPAAAALAAMPSVQLVIVDDAAQNRIPVGARPVAVGAVGTPPPATGGT
GADSGALVRPAGGSPAAAAGGAAAAVAAMGKPLSRLGDKSLLQTVDIESALLAYSSRSMVDDREPAAAAAAAAQQQA
RGSLMPAAGSPPPQQQQQPAPAGGATAPAGEPTPGGEVSSAAEAPLHLPAPAAPPRAGADLAAAISSLQAELSGRQE
QLRITTVLGKGSFGVVYLGTWRGLRVAVKTLVVHDALLGTEGRRRQRAILEAAISTSLHHPNVVATYAFEVKPLGVV
ASSPPQMMLAQGSDAGGSTSGGAGGAGAGGAAGERSVWESAGDVYKLYIIQEYCDVGTLRDALTNGVVGSVSAGGAA
ALCALTLALDVACGMFHIHSKNIVHGDLSSANILLTTIKVAPLPGALGALGALAGGGGGGGQPNVFGNLADTMAAGR
SAGARSLAAAVSRLRGLWRPPVIAKVADFGLSVRMGEGQTHASNKFQGTPLYSAPEVLTRGRLTKAADVFSYGVMLL
EFFYGSYIADITTNCALAAAPGAESTEPGGSSTAAGEGSSAAGGGGAETIAPIAGGRFGVAVPASCPPHLRNLMVAC
IAPEPAARPTFEQVVDSLVDMLLTEHQEELSAQQQQRQRQQPQRPSLSGHHNHHQPPGLPGPPAGGLDGGAGAGAA
AGVAGGGGRLGAGAGGGGLLGGGLMTDDDLGGIVIRRLPAPGGDQPQPSAEQGGYGRLESSGGGGGGGGLGAGLPFQ
PLMLPGDVENGGGDGWRGGGGDGEIPGLRVVRL

>Cre09.g400450.t1.3|PACid:27570860
MVRTRAGPLLVALLVAVFLYDSSAQVVTTTLPALLGAVGTLSLQPATYSFSSTESANITSSLVVAGQGNVEGAATAS
TLDLSTYPALANSTAQRPIIIQPGVTLSLVNLTLRLPPLLASLAAAPVSLLQHVVGVAGSGARLTLDRVTLVAANCS
ELQAFARVLCRPTGTNLRFYPSNVMAIVNGVVQYGNGTMQLAATDGSSAAASAAAVAGASLVGSAATCAAAPVPPAT
APAGPAAPYVCSATSVGNAVQLRTAAAALLNNTAEVVVISVTGNVSINASVWRGALALPRGLVLSLLGNPTAGQGVQ
IDFGGVAPAFTASSYGSSGAVRLRDLNLVGLPYSRTVNSPMDFFSPWLHPFRVFRGGSGGATSTPQLIATSCVLWLP
DQEASWWNTALLAASSVNALEARPWLARLFTLPVTASSSSSSTSGGVRGTRVTSLNDAGGVARFTDCTFASLYDSPA
NAPRSEFATIAWWPLMEGVPAAVSYQAALGEYAVINSSLAAASIGAQSSWASSIFLATANTSAGTSAIFASAEPAPR
HNPAAGDFGPQYPLAGRAVRGPPGNQTGQVAFWDLQGVTGELYGSTSATLQDLVLYNLAPGGALPYGGGFAVPGSDL
YRKLLLYGSSNWNATVNETTGEPNPLPALGSVAYEDTLANLTLPLWYFARPANSDNGLMSLYRVVLVVPTAELQLWL
YLLAQAKKLPASYGPPSNATTSTSALQRRLLLGRGSSSGGGSSRRTLQQTAGSGNSSSSSNSSVVVPGKAAETLRAA
ALAATVSWYTNDTIALSYLGLPRNWVAYDTVITAVLPPGAPTPLLSYEPTYDPVSYPPPSPPSPSPPPNPAPPLAPS
PPPATPTPPRSTPDPPTSLPSSPSPSPLRPSPAPPGLSSSTGNPGSPGAGIALEGNASPSPPPAAADSAGGGGGGSS
AVAIAVGVSVGVVAASALVILGVLFCRRRHQRRAAEAAAAARKAAGGPVGGSIELAPWGGSSSDESGLANGHGANGN
GNGSGMGGKVAVGAATAKGNGNGVTVAAGASPAATPRGAAAAAAAAAAAAAASCVQEQQEQAFERLHQQAAAAPTAAE
TMALLAAAAANASAAASFLAGGGGGSGAPPPAAAALAAMPSVQLVIVDDAAQNRIPVGARPVAVGAVGTPPPATGGT
GADSGALVRPAGGSPAAAAGGAAAAVAAMGKPLSRLGDKSLLQTVDIESALLAYSSRSMVDDREPAAAAAAAAQQQA
RGSLMPAAGSPPPQQQQQPAPAGGATAPAGEPTPGGEVSSAAEAPLHLPAPAAPPRAGADLAAAISSLQAELSGRQEQ
LRITTVLGKGSFGVVYLGTWRGLRVAVKTLVVHDALLGTEGRRRQRAILEAAISTSLHHPNVVATYAFEVKPLGVVA
SSPPQMMLAQGSDAGGSTSGGAGGAGAGGAAGERSVWESAGDVYKLYIIQEYCDVGTLRDALTNGVVGSVSAGGAAA
LCALTLALDVACGMFHIHSKNIVHGDLSSANILLTTIKVAPLPGALGALGALAGGGGGGGQPNVFGNLADTMAAGRS
AGARSLAAAVSRLRGLWRPPVIAKVADFGLSVRMGEGQTHASNKFQGTPLYSAPEVLTRGRLTKAADVFSYGVMLLE
FFYGSYIADITTNCALAAAPGAESTEPGGSSTAAGEGSSAAGGGGAETIAPIAGGRFGVAVPASCPPHLRNLMVACI
APEPAARPTFEQVVDSLVDMLLTEHQEELSAQQQQRQRQQPQRPSLSGHHNHHQPPGLPGPPAGGLDGGAGAGAAA
GVAGGGGRLGAGAGGGGLLGGGLMTDDDLGGIVIRRLPAPGGDQPQPSAEQGGYGRLESSGGGGGGGGLGAGLPFQP
LMLPGDVENGGGDGWRGGGGDGEIPGLRVVRL

>g9873.t1|PACid:27570878
MVSGQARASGANPPPSATTGQARPTAAARAWPPGPATGSFLPPPARSGGGLRAASATSLRLWPLPLPRASGQSSSSG
NCGGGGGRHAHVAGVSGASAALASASASASAAAASASAPPQAPPLRRLLLLYGCGGGARAVGAATAAAGAAGPGPLG
AASSSSSWARGAGAGAGAGGGAASCGGSAPFVTPAPLGSRLQPHRGMARSPPLQLLPPGACAAVASRGRGGGGGGAG
GRSSSRSRQGGGGGRQSSGSSSSGSGRGGGGGGGSGGGRSGGGGGGGRASSSGRGGRTGYAGRGRGRGRGHYSHYQH
HHHQHREAEAAADDFNMNYLEGGRRAGKVGQASGGFVSFGSAASQTSRAGGGGGRGAAAGRATARSLMAWWDGYEQE
VEAASREEDERRAAAAAAAEAEAAAGLAAEAAEAADAAEGAEGAEPASSDAFHGPGLEGPEAADVATDVTSGAAAAD

ASEGAGTYKEAAAAAAAAAAAARAKEEAEYEDPSALPDYVDPDQERDRLPLARLKSLYYIRSKVIARVTATADLRQL
AEALDWCVPLMDLVATKEALRMIRTAGMSTAAYPRNRQLMSRPRFLAAFHFVAARLLGLLALYGPGVCDWAFLVTAV
GGLAEGRIRNRELLRLLHDRGLELVRAHVEAAEAAAVAAAVAAAEAAAGAEAARAEAMGAEAAGVSAEPAEAATAPE
LDVSGTAAAGQEEAAVVGVERPSAAVAEIAAEAAAAAAAAADGASGSTSAGASYTQRHGAARGAAAGPAVRRRQEGG
QGGSSSSSGSGSGSGSSSAAAADGGGGGGGITLRDLVSRLLLPLLQLDQPLHPDFFGLVERLRLLQPQLQPHPEAGS
SSSSSSSSSTGGGSSSGGNGSSMPLWQLRDILELHFRAVTPPPPALMVALQSSCEEAAAELRARARELPGGSLGSSI
SNGSSHGSSRGPGGADQQEAALAGTVASTLYLLGRLTAALRSQAAGPATAAAAAPTATAATTAGVSATATAALDVGW
THGVLEVLQPWLPVLTPLQLSGLLATAVRLGATRRLLLPPLQPPVAAAPNSSSSTSSSSGSGSGSGSSSRARSFARA
YEDAVCDVISRQPTAYQRGRNHPNRRNQTNLCNHPNCRSIITINGGAADLPAKLVSEAMRQSCRLLPARPANALAAV
ADAAAVIRWVAPPGGWVDELYEAVAVRLHAAAAAAAAVTEGPPTAAPTLAAATVDEGVEGVAATVAAATTESVLDAE
DSFGAAAAPDFGVPTAAAAYPTAAPPLSYVRPPPPPHPDRMSAAALLALLTAGRHLARVASPAPTSATATESATVSI
TATALPSSTQAGVAAAAAAAQPPRLPPPAPPPPLPPPALVGVMSEQLAARLAAQLGEVLAAAAPSSALAAAALPPQA
VPVGLARTGACDSWSTYVAALADLAAAAPPPPMAAAPEQALAATAASGGGGGSGLAAAATAGARGAAMELVDRLLGQ
PPPAAAAAAAAAAAAAAPAAAAPAATAAAAAAASRPGFRAGVFGVRGGWGSRNGSSSNGSSNNGSSSNGSSGSGIS
SSNGISSSNGISSSNGSSSSGSSSSSSNGSRPSNHHGGKAKAPSAAELAAARRCRQVVLAAAEHALALQLQAYAAAA
TAATTTRPPAAASPAAPTAGHSTGSTHGALDAGPASGPDADPEAAASRPIPDAGVTADVDATASLSQHQTISRQPPP
PGQPIPLDPDVAAADGGLMALAAALRRLLGRPPAAAWWAAAGAAAAARTAAAAAGFQASCSELHAAEEAERRRGAGW
RPRGGAGAGAGAGAAGGGWGRNGSGVAGAVPAWAPESRALHSLRQSRSAAAVRLLRCATDQLDLAEAAAAAAAAAVA
EVEADVSAWHVPAAAPQPHLQLLHPPQQLQPEWWRAMSWAAQLSLRAASASAATSSTAAAASSTGAAASRHSAAATA
ATATAAPAGLSVALGPPAAAATAAALVTGHDGNAAPAPPGQPQPPPAADAREAVADVAVRYAQHLARWLDQRRRRQQ
QQQLQLQQQQAAVHAMRPAVAGEEPGSSTHAAASGLEEAAREEAAQEAEAAQEAAVGVIQAQAAVAQEAAAAQVAAA
AAQEVAAAQAEPEAADPQPPSQPHVAAEAAEAAEAAVAAEATVAEAAVAAPPSQPPELSHHVGFLICLCEVLQPALA
APQRQPAVAGAAASPPPPKPWRLSAVAVVRLAEVLALSRFQPLRFAVLATWPDAYLDRVSELLLQQQQQQQPLQPLQ
PAAAAALLAPADACMLLRCVSSQGWRPGAELMDGLAEAVQANGGLGSLRAAYGPAALVQLASWLSYARFLPYDQWVD
ELLSQLEPCLQPQLQPQPRPPAVAAAPEAAGTAAGAAEGAGAGAPPPLPPLELPELCVLGHALGELGVAPPPAWLER
ALAAAGDAAVAAAAAAAGLVLLPPDGGGSSGPADAEGLVASSAASAASAAAAAAVAASQPGAAADGGATAGRRSLSD
QLSGMLWRWSAVAAAAADAAAAASSAAAVAAAADGGGGSAAAPASEAAAAAAAAAVAAAEEAAVVAAAALRFAGQMA
AATAPLLRRMEPLQLVRCAEGMAALVQSAATAAAAAAAAAGGGAATPTIATIGSRRGGGETTQAAGPAAFARVPFGS
DTAAPGSATAGASSAATATAAAAATPLPPPPPLVGSYSLSMPELALSQEAATAAAAATAAATAAATAAAGPVAEGHA
TAVAAFEYAFRRGTPAAPAPGDSSSSDSGSGSSSSSNSSGAAGQLLEKPAPRVAAAAAAAAPGAGTDAAAPAAEAAA
VTVRRPATAGSPAAAAATAAAGGLQGRRRQRGADLELDLGPWWREFESATAAVLLTTPPPPPQPEPEHGSPPPLAAG
DNTAASGTWPNTHWQHQQLHQQQQQQQQQRRPRPRQPPPPPPPRGGRRAAPHPPPQLSGYSLSVTLRSMAAAGHVPS
RHWLARYCHHTQPLLLLQPGGGGAAAVGAGVGAGAGAGVTAGFKPIALTSTLWALARMGISFQSGSSYSASSKSSGG
SSSSSGGGGGGGGGGRGGSTDPGSSADDAATAPPAALRPWLETALEALGAVLPSCSGTDLQYATWALAALDCPPPAG
WLLRYERCTAARLPYIGAPALLYSLWAYGKFGHAPSGQYMELALQVLRPSLSGNPAPALAPASGPAAPPPAAATAGA
TAAATAALDGHGLCLLLHCLALLDYLPPDDWMEEYLQALLRELPRITPTGLTIALRAAANLQLPLAPAAAEAWLAAA
ARLLPPPGAYAAPLMTSAGGAGSSGTGSSGTGSSRCFTEQQLETLMSALVDLPPGVLQPLQPLQPDDDTEDGTASDG
TARDGTAGGAGELLRQFLYAVVAAAPDNFKIANFYRMLTSGSSSTDISSSSSGGSSRGSSRGSSRGSVSGAGRGSDD
TSSHGSSSNGVAGHGGSSSGRRGAVLPVGGGAAAASAAAAAVAAVAAVAAAPVGAGAGGQRAGTRGAGGARRLVVRD
GPGASTNGGSSNVGGSGSSNVGGSGSSSSSYAEGQRVGRRRGPHRDVPAAGGGVVVVARPGLRGRGEQRAAL

>g9144.t1|PACid:27570909
MPDGYATNMTICGAAVQLLSAAGAVKASPFSACLLAADTLTLTLTSSGTYAPGDVVNVVAGQSTLKNGATSYTKSAA
GSVIRPTLTTVSLATTTTILIGASAPVSLPANASADVCNSIFTVALKSGTALPTALSACMAGPAVTAITATFANGTT
YSDGLTVTIKANQTLLRTGDQSASSPLFLPPASALVIAPTVLSASLTSATSITVQLPVSSTASGTLDAAGCNGAFEL
RAAGSSASKSSPFSACALASNNTALVLTLASASTYTAGDIFNVKSGQSLLQVGSTAYVPQAVSVLPTLSTAILVAAS
VVRVGLPVVSSIPAPYSADDCARSVIIAAANSTAAKAISGCVLSADGKALLVTLGAAYAAGDTVNVRTGQWQLRAGA
SVTLGPNYIAASTPVAIVPGFSSAVLTSATQVTVQLPLASALPATISASECGDAFDLLDASGTTSRVSPWTGCSIGS
NNTLVLNMTAGIYVVGDQVTPKSGQTKLTFANTTAYGVLLTPINPILTAATTAMLTASSTIVVALPYAANLPASTNN
GTVCNAAFDLVSAAGASRTAPFSACSISGGTTLTLTIASTASYTAGDRINVKSGNSAFLGSLSASGPAFVHAGTAIV
ITPTVVSTALISNTNVFVSLSAPTSASAFNQSICNGAFDVSGLATPFTNCTLSADGTGISFLLASAGSVTSGVTTIN
VKSSQLFLLAAGSGSSDSPAFVPRGSALTISEAKLAGAYLTAANTIIVKLSVAAAAVDGFSSSCNNVFTLFNSSGTA
RASPFSACTLSTDGLTVTLTTSSWVSTDKLNIRSDQTLLKVLGAANGPSYPALSSATEVSPAITSAHITSPTTIIVP
LPVASDLTGSSCSFLVLTAATSNCALSNNGTLLTVTLSGSYTIGDTININALNSALRGTSSTGALYQPVSGATAATI
QPTIGAVQVTTSTRLLVTLPAAMSSPVPNPLTADACNAALDLSGKSSPFAACTASGTTLTLDLASAFVPGDRLNVKS
TNTALRLGTGASALFFQPLATSPANILNPTFVSAKATSTSVVVVSLPAASTFVKSGSATAGLVKADCDTVLAMSSGS
LVGSGNACDLNTTSSSQLIVTLAGTTYAPGQTINVLTTNTMLLAGSSSGPAYQPRTITINPAYLSSDVVATAPDTVV

VTLPVTSGLFAADGSSLGSTLTAAQCATVLEVKAGTTAKGLASCSLAGTTLTVKLLGNNSDVYTGGDTFNFKDTNAL
LLAGSANTAPAYKALATAAVIVPNLYKAVASAGDTILITLPAASTFVVSGSAVLSVSDTVCNTILTFTNSKTVKSGT
NCVITGAVLSLTLNSAITDQTTVTINSGGQTTLVSGTGTTGPAYKAGTAAPISPAYLTSAAARSATSIVVTLPFTST
VNGATLTKATCDTIVEVLNGGDATKPRTLDSGTPCTLATTLLTVNLAATESFAPGDTVRIKSGNTVLLIGSAGGNAP
YVQATTATPIALGYVTSSIATKATEIKVTLPVPITLIKAGVSVASLDKTDCDTLLTVASGTLAATGSCALSGTVLTI
TTTSTYTPGTTTVQFTAVASSASVKILGGAGTTGTIIAALSAASSVLPGYLTSAVISGANTFTLTLPYAVTSGGNPT
CSDIIEIKAANGAMKTFNGVCSVASATTVTGTTNEALLASDTVTLKGAQNALTTTTGSKIYAATAAINIQPAYLTGA
YAIDDKKFVVVLPYASTLAAGTCSSIVSVTDNNNGPAESGCSLALDGTGIYLTVTVSASLTATAWKVDFKAAQTALT
VGSTAWAPMATGTAKSLNAGATTWGSSIMTATAVATNVLEVTLPMSSYMADISSGCSPVTFSTANTAASCKLIGTTS
SPNSILQITLTAGYVAGAVSLANAAGLIKAGSASGTAIGAASTVTIKPTFVSAVATGPRTIVVALPIQSKIIKGAPT
CTGGAVTCSSGTATCQSGSPACSAAGTYTCTATDPTVGLTCAAPTVTCPASTTAACITGGSPGSIATAGNGPGCAAA
TTDGGTAACGAGISAVCYSSSLTTAARCASGSAFSAANGNNCDDSASSIDLDPTCYTSSTMTATAVSDAALCTGWIS
GMPTCSSSGKFACVSATGPTCTASTAPTCKSPFTGTANCAITFSCGGTGLVVPTCTGSFTGCSKGTTGDTCDDDIAS
PADCSTIFSLTGGTATIAGCSNPGSDFTAPYTTTVTLATANWVPGTTLSVAADQAAAAATNYLKPTAAITLLYTTKP
SGAVVIYPSISSATLTGPKSLQVTLPVAASVPSSGLSPADCNNIFQLYATGTTTPKTAAVFSACYLGADKQTFYLTL
AAAAASTTNANTYAVKDLINIKAGQSLLKADSATAANRLAFAPLPDALLIRPAITSAVATTIASAAVIKMQLPLTSN
LGTVNCATTGIKVRVDDATDKTQANTGACSISSDANGAVLTLTLNQALGASDFTTGKGVSVVVVATSSADATKLQLF
GGSDNTGPLYVTGSIPVYDVVTPGQSIVSAKAIASNQVEIKLPAPSTITTGNTCANFLAFTPTRTISSCVYDAETQL
VTATVDQFAAGDAVNIAGAPNLLKYATAAATLTDYAALAAAATVSPALVSAYTVDSTTIAVVLPATSSFYSGATNSA
TKVASLTDVECADVLTVLLAGKTNSAGVSACATPSNCRTLFSGAACLLGTTSAGDTLLTIKLGASYAAGDAVDVWDK
NAGLNGATAAAKPLRVGTNVQALYVPRRMPVVIEPRIVSASATGARTIAVTLPVTSQLIDSTGTVISAPTQQQCASL
VALPAGKSVASCAISSDFLTLTLTLAADFAPGDTVNIASGQTLLRAWKSDVGPGYELSGPLYTPSASAVTVTLSFFV
SATSTAANTIVVTLPFPVKMYDLAASPAEILAADGATPTKDNCDSVIRVIPSGSTIARTLVAPGAGVAPCVLSGSGT
TITLTLDTTVSSYATGDRIDVGFGNSANLRGAATAAAVANTSPKYTTVATLNAAASAAVVTIGPSIVSAAAASPTQV
KVTLSATGTITTTPATVDDCNKIVTFTPAKTLAATSPCSVAGTTLTVNLDKATPYAGTDAVNIAAANSLAATTNPLK
AGSLAFVAKATAVTIDPNLYTATAIDSLVYEVALPAASSVGSTALTAAQCGAILSLTGGRTISSTVTAPCVLDTTKT
LLTVSLGTAYADGDTVTLQSTQATPWLRAASDTGPAYKVGSTLIVKPTIASAKATTATTIEVTLPVTSVIWSTSGGA
AVSTALTATECGKILSVKRGSGTVALSASGACSLSSSGSTTLTVTLASDQVFQAGDKINILASNTDSANTDKYLVAT
TSSSGQAYIARSSDVVIAPGFINAAYAVGPNTLSVALPVVSSIATDNDCSTVVTVRASATPTTTRTVSGACTVASDA
TGYYLVVTLAAGTPFVSGDEVLIKSGNTALVGSIAYASGAAGATKPIYANFDDAILTAPTTVLVTMAAVTTVSFTSK
ADCDAVFVFSGAGNTTKTTSPIASCALAPDGLSVTITLSSADAYVPGDAINVVKDQTSAKVGGVGGTNLVPYPTATT
ISPRPFGAKATLVAPTSVAFSLPAVSSLPAATAAADCNAAVRYTDVAGVDAVNPFTSCALTPDTKGVVLNTASPIYK
AGDVMNIKSGNAAVRWGPQASGAAYYPAAADVPVFATVATATLVDPSTVVLNLPTVSAIPDNFTVPDCLRAIEFKTA
AGVTKNGTVASCMLMPDRLGLQVKLLSAGNFSAGDTVNIKPEQAELRSSALATGPSYVPKLAAQVVNPALFANANLT
SATAITVRLPFASALATGADCKAVLALLGAGGVAKNVSSCSLGADGVTLAVTIPAASFVGGDVLNIVPGQRALTLAD
GTTAYVPSKAGVLVTPNIVSATLTNATIVTVALPTASVLTSTAAADCNAAVVIARNGSAVASPLSACAVSADGLSLT
LTAAATYKPMAGDTVDVAVSQTVLRAGSATGPAYVPRPSPALITVPSPPPSPPPSPAPSPPPSPPLSTANYSARGLA
TGPLSCNVLIGDLTVTTTAGNFTGMASYAGKDASYKGSCKDAVTGAVYTDDTVASTLPAGLTGLVLSPVTALASVWD
LKSVADLANTNKDYLRLFGVPVNASAYGTAVQLLAYDYYVKGFVALETPAVAVLNVEGMVAANLLMYTKFFDGLNTS
VAGRDITAAQALAAGQYALAAVLEDSIAPINTTDPASILALLNVTYSVLTANATSAAGRRLLQTAPAPFTQLQAQAT
ALAAAAAGSNALVAAQQARLITAINAGTSISAAELTNIINEASKVIVAQSTVIATAATGLGSGAISPASFTSSYTGS
ALSTLVAQQQLAATPGSDVTAGPAPSPPSDKKKSNTGLIVGLVVGLVGGAIVITLIVVFIVMRRRKQNVAAAGQATA

>Cre09.g407050.t1.2|PACid:27570928
MWLVGSFIAVCAAIMTLQGRVINAQGDNNLVPVTVIVKGQVVAFTSHKSPQRTPPPPPPRRASPPPRRRAPPPAHRA
FRGLASLSAEDATTTNNDPLPPGVLPPSKKQIRLEDAGNATFRAYAKVDLGEQAQLLRTGDNVEAPLTFQLDPAVAE
ELGLTDSGSGGSGRRRLSEAQHALRRAILQVNNTRRSLQELHDLGVLARLKGGRGARVPSAVAAGRAEVKSSGGAKD
LMFTGKPLNVSSLTFLLTAPQCGLVPSLSLDTIRGVWVPAAANATYGLASSTLQYMHKTCSYDKLRVYDNADNQVIG
PIDVGACSGTTQYGDAFDFAASCSDPELDAMYDAVMRWVQANRPEWIASGYFDTFMMMPLPSCGSVFGYGAVGCPGD
GSPCRTWLFPNKGETKLDASTIFHEMGHNQGVTHSAGQFKYGPRLITDEYADPVDPMGSAGPMTCYSAPNSFKAGWS
APIDDMGAKGVAMADLADGWNDYYVPAAAVSDKNLLRIILEQTGISEEAAAAGQERALFVSYRVAQPAPGFDSGIAT
RDGGQWNKRVWVHEFNDTATGETATVDYSLMKAMLDVPLPPLTSDKAVQAPLPPGPRVPSATWAAQGTNWTYVSTSF
EELGNLRIRVYSKNDTVAYVGLCRFKTNTEGEPGADSCYDTIDNDCDGLADDEDPDCNPPEVSPPPPPRKKPPPPAV
KKPSGSSKGRRLA

>g10206.t1|PACid:27570943

MAGVHSMSGGGGGGGGGGFNWAGAAGPAGAAPPGGGTCAAGPAAAAGAPVPHIAAMSCQARHSHQQHHHHQHQYHQS
HHHQPNPQQHSQRGQSMGAAAAAAGPRSAQQQQAPPPHHHTHWAQQHPHQHPHQHQHHPPHQQPPWRGGPAAATAAA
AAAPAPSPLHAYGPGPQGRQGKGQGQGPHQGQGPRGPRGPQGPQVQEPPPASGSFGSWAQPPHAAAAAAASTAAPGG
GGGGGGGGGGVKAGPQSQPPPQPQPVPPHAVRGLQHLHHIPQQPQRQYPLDPQRHQWPPPPPPPQPPAAGSTIYAPQ
PQQPHKYTYQPPSQQQHQHQQHQHQQRAAVLPPAAKVMTVQLGPAATLPGPPPPVMPPPSLPQQASGCSRRPVLCSG
PFPSTAIRASSGGAMAAPQHQHHKHQQQQQQQQQQQHQRLLQRLGLRVAVVGGSRCLVAAAPFRCVGRDGAPLLLTV
RLSAEDAAAVAAEEDAEAEAGQEEAAAAAMAAAAETAAVVAAARGVQDDAAERAQEEAEAAAVAEADAAQALEAAEQ
AQEEAEAVAVAEAEEKLQAAAAEAAAACGALQQPMAAAAAAGAAAGAPTHPPLLVMAEADVEVSAAAGGLQRPLPPS
AVTAVDHAAVSTDAAAFTQAAAPAAASAPVQPPPSPPPPPLPPPPQPPQQPTDSPIGTGGVQQRRRGSGCRRRMLGP
SSAAAAESLRQQLRQLQHHHHAEGEEEGGREPVGPTASDPVAAAVPAAIAAAAAAAAASAAGTRSPAAANHGVSDKA
AVAQARGRQQLLLQPVPLPSRPPLLTVPTTGAAATAATAPEAPQSQQPKSLFAKRAASSSGVTTGASSSKRSRTGGG
GGGGRGRCGQAIEEAGAADGGGAGGAGEGAPFGSTGPAEPEPTAAVAPAAAPAATPAAPAAPAAPPKVDEAVVLLEP
LPLTPLEVSQQQQQPPSQQHPSQSQSVSEQLPLNQLQLLQEQEQSQQQEPPPPEPSPQHQEQEQDQERETAQPPLLG
RMAAPQPPPLLVSFPVPLPSAPPQPSPPPQMQPMQPPPHQAPPPQQPPAALLQPPPAPCPAPPTAVGVAMAAVGWQQ
QHQALPHLRPPPPPPQLQPPQPRRCATCCPRAYYYVASFLTDDLSLYSSGSSTSGGGGGGGGCNGAAASGCRCGRRG
GGAPPPPPPPPPPPPVNLPASHPAARAALLRVVGVLVQEAAHFGAASRRRAADRYWRSLAARAAVDGTPPPALQEWAA
ATGGLEPPQPPPPPAAAAHPLAPLLTGTITAAGAVGAAGAAGAAGAAGAAGSGVASGFSSAAGRKRRREPQGDGGGG
GGGGEHQAGAAAGTGAAACVAAAAAGGGMSIAEAMAVRMAARRKAVAATGAEGGAAGGGGGGRTGGGGGVGEFRAPG
PLGVARQQQLHPQPHTRQQQLQPPPQQRPDLVERPAATAAVAKPQDLTSLGNSASAPAPAAAVEPPAVAAAAAAQPA
GKRPRPVGASAAAGAAFVNPRRQQQRVAPRHEALAVAAAAAAVGGTASAVKAPPTAATAATNAATAANANAVVVAAM
SGAVPSPAPAPSPAPAPSPANTTAAPALELASPFPPLLLLGSAACSPLGAAAPAAAALGSSAAGGAAGAAGAGSTAA
AAAAVGVSSKHAPPPFAHGFAAPTTVKFVAPGDTPTAAVGVVARGSNEEGAVLAAPAVAAVAAAAAVAAAPGAVAGR
PAAAAATEEASMDGLTASQAEALEALESKYMGGGGGGVGWVGGR

>Cre17.g739050.t1.3|PACid:27571017
MQEPPVQAFWAPTPPAHAVTCVAAHGALLYSGAADGAIVRWQLQPDQPPRAVVMLCGHACAIRHLAPGLACDEHGDC
TSRSGSTTGSSSTGSSGTGSSGTGSSTTARYLLSVDASGLACLWLEPAGLCVARRHVPELVGAAWADMPQQQQQQQQ
RPAGAEPLTARWLLAALPPAGPGDSSADGEQGGAGGGSGSNSNAARRISHDFSFHTSAGARVGGGGGGGTDAVAAAA
AAAVVAAPPPVAVVLEWRTLTVTQLLPMAPLLQLAAVTLPGAAAVAGVRGGCSSSRVARGRVVGSDDGGEDGGDAGE
VDGAGAGAAADTGRLVAVRVSYWELTQPAAAGERLAVAAAVGAGGTLYGCVVPYDPYRPPGWTLVGGDAATSDTGAG
TLPTATSPPPLAAATAREVDEFHEPLLAAALSYDLGWAVLGAADAWVVKRSRRGVVAAPPVNGGGYGGGYGALVLSY
QRLYRISLMHQQQPLMGLAAAPMPMSPLLAAGLHEPRSTPPRSSSPRPQTQPLSATLHSPDHHQLQSQLQPQLQPQL
HTSYVIRAATHMLLLEPPPPPPPPPPPQVASATAGSGRALEAAVTGLGVGEAGGVEGGEGPEAALELLCWDGSGRQQ
LVSLRRHPVPALPPPPPPPAPEVTAAAAAAANGRLEASSSFSFARLSGGGAATAAAGSGPSVSLRPAAAAVVSPFAA
LSWPGHASGGEAEHSSASEALAVGESGCSSGRAAAAALTATPSGSRNGHVLRVRELLAGEAAAAAAAAAAAGGNLAL
AHPQHPQHGRHRPLHRRLACGVCWVHWPEPTATAAAAAAAATAVDASLVSSGGSDSGGGGGGGRDEAERHRAPGKAS
GSAGWSWSVLSVHSHTAAAAAAAAAAAAAAVAGGRRWRLAGGSDAAAAAAAVPFGIGADASGGGAAAAGGPTDTTAY
QWRIRPDVSLPGGCGPDGCGPHGSSEAVLPASAPPPASSVQLPPPALPPPPAGRRGHDVLPLVGSLGAGWTATAGGV
AATAAGAATAVAAAEGAATAGNGMAPGASGAGEGGRRGQNSKAVAAVAADSGSSSACSDSSSNGSGNGTGTSSGCSS
SSESDGAGSSTSEDEQGPCFPDCCYSPASVLQPRLQQPAAGAMPAAAAANGSPGAPAAATLATAAGLAATAGDGGGG
RRARRRLLDALGLPYCLYERWAAVHWRSSRRRSQLLAAAAAAAEAEVGAGGSSMAAAPPPLLHPVVMSPGRPWLPGD
GDEADGDADGDDDNGVAAAASAGWRRAGGGGGGGRWGAAVAALTAMYRMGGLAAAAARKLQLSGIGGGSGGGWGGSG
LATGGAAAALGGTVVSASLVVCDNAGAVAAAPNGSSSSAGGGVLVDEAMLVQCTAAGAMRYCHAAAYAVPAATAACG
GGGGGGGAQPARCGWMLGHLGPVTCQLEVVYRPPPAVTATPVATATAAAAAATAAAAAAAPLSTAPSSPALHTYARA
PAPAAPSRPRSLTTAPAPPTSSASAQPHPNQPHPNQPHTNQPQTNQLHQQQPQQPQQQQLQQPHLQPQQQQQHEKLQ
QAFSGGAGGERPPSSGASSPGSGSASTSLGTFSSCPLPHPQSLPPHPHQHPAAARLSSTTAAAGSSSCRSVDLNGGG
ANGCSTRGTGSSTSGNGFTGLGGLPPGHPVRVLVTGSADGSIRAWHTHRAAFGRPIFAVHPHGAPVRRLLLPRPDPA
ATAAAAAAAHGPPLTADPVAVAAAMWTGSAAAAASSSRSSAFASSFASSFASSFASSAAAAAAAAAAAGSPGLWPGG
PAATGVFGAAPAEFRHCVVSAADDGSVALVCLARRAVVRVCRGFPYGAPEAVSWSFRRGFLACGGRDRAGRWAVAVF
DLAPATPTLDRLVSGTAARAILDGVDSAAAATSCAPAVAMRTVAAVGTHHAHTGAAAAPPPPGGRQSGSGSYGGGAG
GWIHSSAAAAGSGQRASAAEAHPSHSVLPPPPPPPPATSAQALLAAHPHPPLPHHHHHHQQPSALPPQAAVAAAES
SSPRQAAPPAGGGGGGGGAWSYGGGLHALPYGPRRGRPEVPLAAVDGAALAAVVAAAEAASSAAIAAAAAAAASKAA
AAHSAAGAAAAGGAGAGGGGGGRSTPLTVPRRPSTDAARVGGGVASPSPSNSVVSIGGIATVGGGGTGALSPTHSYP
SSVASPMGSLRPSMDGASAAAAAAAAAAALSAGGGAAATPASTVPPPPPPPGRLNGAHLPPPPQQQPQPPLPPPPPL
PGAAAVSGTPLGAAAALSAILHSWGRDAHTDAALAAALTQMGVLLPPPPPQQQPPRRSRSRRQGHRGPAVAAGGRGR
HKATCSSSRAALHGGDEGAAAACCLAPRATTAASAEQPSQRTPAAAEGAAAAAAPAQPNNSFNGLVQPAVVSACGAV
AMMLPQRSAAAAAAAAAAAASTAASAAIAGASWQNAITLAAPAQPMAAAASAAAAAWVGDGAAAASRLLHLTALSGG

LLAAASAASAAAGADAAGLARAAVRAASGLRQHYLVAERDATAAAAATAAATAAATATSATATAAAVGAAAPPPDLS
VFARRWSCENEAVREAAQLLLAAYTEPLPLPPPPPAGGPAVLAEQQEQQLRRLPPQATEILKLMAPAAANSSTPHGK
SAALPPPPPPPHRLYPAALIAAAACLAHPTAAPPALFAAAAAALSELMCLPLMPPPPPTASATAGGGYGHGVWAPDGA
LLPALAASVLTTALGGSGAAAAVRWRRALQPWDVFVDRLLAVVHAMPTDAADPTSSSASYLHPHHLALYTCSSTASA
SSTASSTSRHPPAFPGPVPGPAHAHGAASSPPALTSFVSGSSGGGSGHHGHSASGSGHGSHHHHHQQVSAAALAALR
RVSEAAAALLAAAAAADLPGLLAGLRRFSAGDHLPLHPHVAVALRALAAAGPGGRAALARSCPQLAAYLCGSCLAAA
AGRLRPDLQRLGYMAAADVAAALGPGGCAALHAGSMQFAAASPLPLPPPTPLPGTKVPLPGPTSSSTGSGSHRNPDP
NLGRLPRFVTVYDMEHGAATWQLPLQPGPEALLPTPPALAAAAVAPPPHPLWSPPPPPASCSVAEQLRAVARGGVVA
MAFSPQGDSLAVLVHGVPPSPSPSSTPQPPYDAASGAPAAAAGGVPPPAAPRAAPAAVPLAVLVFSLRLPWSARLGL
PLLGGGGGGGGGSQPLVVQPCHCWLLPPTAFGAPAVAAAAPPPAASAAAARVLVPVEARVELLDGVARGPVAVAYGP
EVGEEEEARVAQAEAQVRAAAWEAEEARAVSAVVGRVYGLRWSSASALDLLSRGRAVVRLAF

>g17612.t1|PACid:27571018
MAELTEGDGRGLTKIKISGASPANSPGRRRATASLASGSTLHPLSPRTVSILDHPGSASAAAAGLSITGSGLGSSLR
PATTSGGGAAVLLSGTSSFRTMGGAMEEAAAGIQRLVSPTDAAGMGPGGVSTPPRKVTSAPDSQSMQAQLRGSNPWR
PSGGGGGTDLHSGLMPQPPSQPPPAASPPRASLMAANGGFASAAAAGAAAAAVASSAMALGSSPGRGGGVPPAPPQQ
LVPIPPMPFGASSLGLHVNSSPPPHGLPPAGPGQAAMLAAGGGSGRERRNSASSTDSPGVYDSPMGGPAPPHLASLR
VATMGRTGSPLSPQPSTPGSTSGPGGGGGGAGSLPHTPTAAGCSSLKPRPPGEAPLGSRASRKFSHLANSPAAGGAL
TFGPASARHLHLHTGSGIPEEFLTAQRSNSGGGGGGGLTSTHSFAANLLYNNFMAGNGSQPGTPVSSSRPGSGRNSS
ARSRPVAVLVDAVRTKDDDKLREAIEAQRGPQGNILGLNDRHHVTCRTPLHEAAMSSCTSMVALLLAAGADPNIPHP
TQGPPLLHCAAFGEVDTMQLLLLEGADVNAGDVEQQTALHFACAGGHVDAARLLIKFGADVRARNEDGQAPFDLAQP
EMLRLVLAGDVVPSPDRRPGSSASTSRPSSRSAAPTAERGLGGGAEGTPPSASGLGGGGGSRRSLGGRPPLGSTGGG
MPSSPGMVPLDLASLTAAAAAVHGAAFGSGGGGGGGSGGGSLNNSIRPAPPLVDMLGAAAVASAAGGGGGGGPRSRR
NSGNFTAAVMGSAAPPSGPPPPPPSQPVHSPPPAPGQSPLRHPGELAPAGSGTLSSAAATPAGGAAAAGGGGGRAPA
SPLLSDGETVPPASPPTLTLPGGVRRQLSRGAGLGDILHAFQQDDAEMVKRSQPPDATAHVSSAQGHAAHGHAPLPP
SGSHHPPGIPAGPSAAAAKFKDTPPRPPSGGGAAAAPAAPVGFMPSAAAFAAFAGKAAPGGGASNSTAKGKDEKKEE
PQQPLLPFSMPETSMIRFGIPLQVHAADEAEASAGGGGGSDGGAADADGLRRSSGDGAGTASRQSSAAQPMPSFSAQ
SRVTAAPSFVSFGSPAAPPPPAPGGGGAAAAVGFHPPTLTVPSAAGASIGFSAVGFMPVPTTGFTPRISNSGAGGPT
GPGSRVSTSGDDFGGPTPPLVRDSSPVRERGGGAAAAGSSGAAAGGGAAAAGGAGAAGGAAGRAAGGGGGAAGSAGS
HGSAAGGMGVSGSGGGRFMPTRTSQLAAQALKRMSNTNVSTDAIEMRAAAMFRQSTEMTAGEMAWTRGELLGEGAYG
KVYAGLNQQTGELMAVKVLQLLSKHGNKEAVFAQLEALEHEMSLYKKLKHKHVVGYIDARFDTKTSAFYIFLEYVPG
GSITSMLNRFGRFSEDLVRNYTRQLLMGLEYLHGCKIVHRDLKGGNALVSRDGIVKLADFGASKAYRDHTITDCMKS
VRGSVFWMAPEVIRGTGYGRRADIWSLGCTVIEMLTGAHPWPHLDNQWTAMFTIAKTEEGPPRPKGIGEEAKRFLDK
CLQFDPAKRPTAAELLQDPFVARVGAGPAAAGNNNEDRLQHSF

>Cre17.g713600.t1.3|PACid:27571034
MPTPWPLRPTQDTSRTPPRQPAQAVAAGSETIAAVDPTAGVATAAVNVWDEAPCCSGTLGAALHQALPSSTTQQRTT
PSGQSAAPFSPSTTLGQRPSVDHSSGEALTFHPPTLPAHLAAQPRLPSTSAVQQHPHQQQQHWTLRSCINPVAPSTG
QQSESNSIVHIEMAPGSPTHSHSHSQGHTASGAGALAAPAAASVSAAPAGTEGGGVSGGGAAHEGITHGSVDRHSRT
RNSDPQQRPSGGGQQRGEPHSHSHGHHRHSRNAARHADPLRAFLPAHMRQHVEDVEGGVLGASGHGHEHPELNGHVH
PDSAGEDGQGGGAGSKSRRYSRRSAPRETSWQKWKRHHPATLVCGGFLLVAAALVLVVVAPVCTTLGCPPKSQDTSA
IAPEEQLFTLRLLARSEDPAGRPVLPDLLPADLAAGVRGTGLLLGSGLEALDCEDLWVLRRVREELGGEGRKGRVAF
VVADFPQAVPADLTGPARAAVLSALLQPGGPIVPSQAASPNAPSELRDLVALLPTLLLASASSSSSADSAAALTSLQ
QLLQAAGLGAAAQRVGLTRVMAAGGAGGSSSIQELLKLLPNATASNPDAAAIGSSLGLGFGSSGSATVPTTSSAARG
ADSSSAGAAAALAALLSQPPLDVLNSIEDVAKQTQLGGSLLSALTTGSTDGSSAAATAASGSSSATAASGMGHDALV
AAARLMSSGDPGELVSGSAGGGVLAAAGIGLTQSVTAGGWAVPVGVDARQWYMRQAGVDDAWAITRDADLPTVVVAV
VDGGFDTRHPDLAGALWVNPGEIPGNGIDDDGNGFVDDVNGWDFGGNCSSGAWRPAPAPPPPPPVASAYPPPSSSSS
PPPIKNITIPRPYSGVVPATSALELRSLASCTGDGNVSPEAGDGGHGTHVAGVIAAVRKDLAGTSGVAPHVRLMLLK
VVDGYGATYGSRVAAALEYAGRMGAHVVVTSVGPSAPLPSAPRPYQLAANAAAAAVYAAAVGSLRDKGVLVVAPAGD
DGIDLDAAKAAGLEYLPCTLAAPPYSLGNVLCVGAADASDRRIRIAVPGLDPALGSNYGSSSVSIAAPGVDIYSTLP
DRWAQVVDPVTGGGYGNMSGSSAAAPVVAGVAALVVGVIGGGAAASGVAAPRASPPPPWQAPPAPPGSVRGAAASPP
PRSGSNGGSGGSNGGSGGSNGAGGQVLTATGPFFQAELVRSVLLATSDTAPAGSSGVSGDRRINAGRAVRAAYALLG
NLHLLTPSPAYYSPGGSGASVLFPGLAEEYFTVLGQRANGSSTDVAAAVIDSRPAQVSMRVGSDREPLARLRTFRRS
GPGVLLRLRGLLRLPAAGMYRLRLQLGPGTAPESVQLAVGGRQLAFGSADLSARVMAEAAGYYDLELLLLSPTAPAN
ASLAAVELLSAVDSSSSSSPATSTTSSNTSGGGATLAAAQYSLLPDALLLSAGYAPPLPPSYSPNVDLGGARSAAA
PGYHVMWSTRGASPGDDPSAPPAPLTPALLSRISSAAAVPPGSGAAYAPFEGSALVPDLFPGPGDLANLIARTSNTS
GVPPSDWNTTGVYGVAVGHVRPPSNGQLRLQVTCTSCQVYLQGVLLLDAAQVQPLPQGPTPADTVANTPVTRSTGCV

AFPGAAASDPGSSSGGSSSGLGGSSIRGLGEVYGLEVRFVAGYVPRAIVSLRWSTCTTGSGSSSSGSSSGGSSSSSG
SGSSAALLDLASDTGQWGSLAGLLTSATMWAPTAAALPTLPRGLQCDLWLAGRNERPTSVPSPARLPPTASIRLPRR
AAAAVAAAGSAPSNSINNSTAALLAGLTALGASGGACASITAANNCTLSSNFTVTVLLLAALAANSTTGTTGTGSSL
RPPSPPIPPSPPPPAGSSAAGGAATYHVRCWTFWSARGFTDNRLAVRLPATPSRVFLGSQRVYLNDPDEPPGVSYNR
LVQLPRSSGLGPGYHQLLAYEFAGLSGDAVFGLLDGVDQFVANASLVIDRRMVLPPL

>g17376.t2|PACid:27571095
MRLTVAVLAILALAIGASAQQTGLYPSFPYCQCTKTPSAYRLSPTVTSTGAGTYCFTLSAKVPAGCTHKCCKADLKK
IEFNVNDACDVFSPSLKATINGVRTKVAPAINKAQNGPVGSTTLVLTQLGLGLGNDGAQVCITLGLNKNGKGCTTLE
ELCVPPAGMPAGVCTAALFDSQNDCCPLSQANVPSPPPPSPPPPSPPPRCEVCAYIALVDPENNAPFPYAFSADECD
SYAQTLIDDITAQAGDAGATIVTPFAKVDCQERLIKVCGEFFSNEEGALIQDWIGEQVSVWNDMVTGGQCPAYLSGY
SVVTAVGGDGSDVNSLPMSCLNAFKSTACAPETVDFPKCQCTTKAFATPFAVKPMMSEMAGPSKDTTSYCFELAVVA
PANPGSACGKTSTVNKAEFFADDTKRRQIKSIGIKPAGAAGYKWVAPSWGAVGDQTLKVTLGWSTAQAAGGRICLEL
YNTTSLDDFCMGAAMDTCWLNLFDTTRNCCPLYTSSLV

>Cre17.g699750.t1.3|PACid:27571101
MASPSLDLRKAETPACGHNGNECIAGQRCMGDAAARSVSPGSGCREQQQWSAVATAESLTAEPLARPPVAAHHSSSS
SSSSRSGGTDAPLVQEDAVADGSASSNAGPAGSVGACAQRARGCCVSDGSRLGCPPAVPSGPLSGWQQQQQCNARVS
RSSPRLVISPGSPASADNERSATCCISRPHPAGQLTTAKRKAMGLGADDAGEGRGLAWRSCGGSNRAAGSGVMGALL
RLLLPLLALWGAGRPAAASDLTGTAFPFENCKQDQRNSPYYATLYSYRENPEKQTSTVCIQIRTLDVCLPGKWRCCN
TSINKVKFFPALGCRGSVASALINGNSIMSYYWEEHQGLDILKITPLAQWLSSPAKSDGAIVCLNLRAPCWTVQSFS
YNAALMEYALYDKKVNNYECCPVGTIVLPGGSLEPNFPSRPPPGRQPPMPPGLPPPDLPTTPPPRGKSPPPKGKRSP
PPPPPKAPKAPKVQKPPPAMPNVPPSPPPPQPPPPPRVQRPPPRPPPSPPPGRKRPPPPSVPSPPPPSPPPSPPPPP
TGRRSPPPPPPTGTQNRRPPPPPVAVDQTCTFTAAIRRTAAANQGFAQSVCQLFGALIMLPLYDGSDSGSNPTFTCA
ISTPSYMVLTGSDVPAASVRALFDAYGDGGQVDNAVILLGLGACGTDSALLRTDCPGVAVNTRVLTCGDPNAALTSP
PPPPPPVRRSPPPSPPPPPPVRRRPPPPPPPPADVATPKILVFQDSAYLIFYTPTDWNSGQSTCQGLGGNLAAIDSQ
DEYNLLADAMLEDLAPEGFINLWFGLTVTTAGGAAIPGFNADGSRMSYMPINFQYDRTRAAVRYYYMTCDAMTRVCS
WNYSPGGAGDIQSGYICEFMTAALTG

>Cre17.g697300.t1.3|PACid:27571126
MLGCVRSQDFATGLKFPASGALLANPAFFARYGNVIPPDELDWRPTGFIRAPVNQLGCGTCYVHAVVHGVEALHAIK
NGSSSPANTSAAVLPEDQVIRCGGAAVVAAACDGGWVGESYIYTAITGLLSSPDWGAALTDPNLNNGGDGQQCPFKP
PSLLPPADSTTPLSLPEYFRNRGIRIYGWETVPATEGSLLQALSNQPVVALVHASRDWFSYRGGLFAGSCSSAPNSA
NHAVLVVGYTASAWIIKNSWGTGWGEGGYMYLPRGGRRGNPCGLLNMATYPVMEQTPAARRGELLQEGFCSGLLSVD
AAGAAAGVTLRVLADQQKVSVEEMVRVNGHLKNADLPLEAGTRYYLPPCSRNTPPPPVPTLECGTLYRVSLVGSPNS
SAGVVSRNLFAESGAHGRRLQRQHFHAHRHHHSRQGHHHHGQHHRSPHQHPNPLLQDAASNSVMDQQLPLGVSRRSL
AITGPGSDWVYFTVLSTANSVDSSVQQQVLELQLPDFSGGNVAFNVKYGENNQRQLWRLMQPEGSDRTLLQVQATGK
CARYEFTNSVQSGNIVGIWWACDPTDARQWVELRDAATVAAPTPATTSYMLAFVTLNMCLYGNYINYLGLRYMPYLG
DCSAIGANQKVVLATPPAPVDFTPAWLTGDPIGAQGSVADSALRAVRLANGAFLTRLENGNSFLQVGFGNVGPEGLL
PSQLWRLFKGPVDNQGRQTFMLGANVTVTGSDGKNTSILLCVAPSQYSAYDRLFMYANCEPGTGGWVGLHHFGVASG
GSGGSGGGVRLDAGGYPAGRALLWGFLRNEGNCVFTPEADKPFDPVTTGFLQRPYTEPCLPDNRFQQYDFVDPASPE
DDNPRPPRPPVDIMPDSSLRYVLDTRGTYLSSPAVRVGAPVGFLAGASSAADLSPDSSQLWYLRPVADGPAGAKRYQ
LALAASGQQLCATVAVSMDSLTLQECDAGWDRQAFAFPGADNVAPGGTGSNALPVGRLQYLVYTSDPGVGGISSSKS
YCVTGDTYQPDFAYTRARVASVAQCDADSSYTSGLFVGKPTQAYIGLAPPLTPLPATIPPRSRAGYVAIYDVRGAAL
SAAASGAVTLRYTESGSGGGSGTAAKQALQDSQLWRVRPATTPGADWFTATNYVFGDSLCLSTAAAAGSPDAYGPLT
LRPCDASDTRQRLVFWTADTAQRVTTRTVPLGRTQLGFADAGSPCLASVTPSLMIAYRAPYEVQAVSKCGPRTDETQ
LFEVADPIGTVTYQHLRWWNGGPAQTSSVAGLKPKEASCRPGTYVTGLEGVLWTGLPALEACVFAALLAESPLGGML
YSNLADELNTWDVRRQQLGFALSFNLLCNDGSKVQVNSPPDWSVSAATSYSWSESCPGGYDRVRAGTRAFLYEGRTG
YFSSQDIQKLDFSFRCRSDRRWTLYTVNGAGVSKRLATYNSTSGDGSRLLPNAALVARLKQTCGPAGSIKTATDNTV
TFFDSPQTPVDMPTADQVAGLNLAPLSAMIHRSLPQLLDLQSFPAHRLENMAGAATCPDNQVLTGVYMRSGMPALGS
TAPYNGWQAGRVIDLPVSLFLYAINPLLLSLDGVICRAVIGAEALPPATSGGPGVVVPPPAGMVFLVSAEPAAGAGG
SGRRTKDLGCAGDGAFITGVYAADDQIDLNVLGVRCSDGSSAFIGTQRREAGAPLPAYIDVPCAEGFDAVKKPWGTP
SDGRSTSRGLGSFALRCSSAAGQYDSFAGSAGDGRFWTQLGAYKAVLARGDLYAASEAFVNSEDVYGCADAGAVITQ
ITVVYDDAAKAVNTLQFFCGERPRLSADSSFYDIAFRYGITLSDLFGANPGVNSSLPLSAYNNTVLAVPQLCGSISQ
QPPITTIASSCPRYWPLPNTTVTGTETCGSIAISFCNRDLRFLNSVNNGICPNATVRISRGVRLCIPPRASVISVGH
RRQLIGALAAGGAGATQAPRPRLQLQHEALLVPLGHSQRGLLQSSDAAPRAGAVGAVCTLKTYVSVGASCGSVAAAY

NLTDTQMYDYNTDLRCEALAAGSVLCVERSDSGEEDTLEPLLVEDAPPPPPPASPPPSPKPSPSPKPSPSASPKPSS
KKSPPPPGAAKPPRAPRKPPVRKSPRPKKPTAQSPPRQG

>Cre17.g727800.t1.3|PACid:27571176
MDGRSVVLFTIPASAATTAASGQQAAGTASAERRGWQRRRSHKSQASITGVPTTLIALAFLTLVALPGAWGQVNIAS
VAVTLNKVEQSKTADGYYVVASGAKPSLLFAVSSSGASSQYNVVAWYNYPKSQGNGPLRKSGSATEICPAGSTSNPL
ITGNAATTLDFNPTNETSIVASGGGLLPGCYTVRVALVLPGTMTFSGSAASAGLRVGSPTGVGNCSSVVNIPPNCDT
TLFQYDPPTIASGNVVVAQPISGSACQTLSFKVPPAGCSDLEGTALTYKWTVFTPATDVVTLTKFGPNATFTSGLPG
LELPPGPWNINLTVTDSPANASMAQVKFVSYYFINISSCAINYAPEAPVLTGPTCGAAIAINATSTDANWDRLQYSF
VLYGAAGAGGGVVRDSGYTAATSASYSTSGAAALAAGTYTLSVTARDNATTPLTSPATTITLILGACTYLPPPLPPV
PPLPSPPSPAPPSPPPPSPDPPSPPPPSPAPPSPRPPSPVPPSPLPPSPVPPSPRPPSPAPPSPPPPSPAPPSPWPP
SPAPAPPSPAPPSPKPPSPTPPLPPSPVPPSSQPPSSQPPSPGPPSPRPPSSPVPSPAPPSPLPPSPPPPSPEPPLP
PSPKPPTAPVPPLPPSPSPPQPPAPAPPVPPSPAPPLPPSPLPPDPPSPHPPLPPSPVPPLPPSPEPPAPPLPPSPH
PPEPPAPPSPNPPAPPSAAQLAPSPPAPPPSPAPPLPAPPSPVPPAPPSPEPPAPPSPAPPSPPPAPPPAAPAPPSP
APSPPPPSPAPQSPASPPLPPAPLPPSPLPPSPDPPTSPVPPSPQPPSPTPPLPPSPDPPAPPSPVSPTPPSPEPPA
RPVPPSPHPSAPPTPVSPAPPAPPSPAPLAPAPPHPPVPPSPEPPLSPSPAPPQPPAPTPTPPPLPEPPSPEPPQPP
SPAPPTPPSPEPPSLPSPTPPSPRPPRPPSPLPPLPPSPEPPAPPLPPSPAPPAPPSPNPPAPPSPAPLAPSPPSPP
PSPAPPSPAPPSPVPPVPPSPEPPAPPSPAPPSPSPPPTPTPPSPLAPPAPAPAAPLPPSPAPSPPTPSPGPSSPPP
GPPHGSPATPVPPPAPAPPSPGPRPPSPPPARNVPSSPDAPALPAAPPPPAPPPSPPPPPRPPPAPPLPPTAAVVSS
LVTLDSYVSSSEDLKLLIDALGPEYKVSIAGQRVTFSLRVPVGLDMLDSGCQSANVRGVLAQVPAAVGVSPEDVSAE
CAPVQPAAELAALIQSAAQGPAGSSSSSSSSSSSSGARRAVLQQAVVARSGGALPRHLLNTDSGSAARQRRALQQQ
QQLQAQQPPAANTTPPTTPPPNPQPPPLPPAVDPACAGSLALNMSLLLGASSDAPQLTEKARALLKQIAFARAACAS
SSSGGGGSASAAADDSAVVVSTSTSTVLVISRVLNDGTDATASCAAVESAVEAAAAAASVTFSRGVGNATSASSAGG
GDSGCQVQAATPDLLASLTADEGAPAEAAAGFNSGRRNPTTSTEAVPLPGAAPPAPGPAQQTAIGMPVWAIAMVCAV
GGFLVIGSVIGVRMIITRGATTAAAAAIPTATVNNNAFSPESNMPTVRQLDRRPDEFTRLAEQQQGHFQNLAAASG
AGAGVAAGAGAGAALAGGSGAGRVSAPGVNSRGGMLSGGSGEHAPYMRVSANPMFEPDGAEFIAAAAGTGGAAAAVG
VGAAVTSRVPPPPPPPPPGTTWRAAVAAAAAEHAAVVPAASAAAGAGGVAAAAAAASRADEVVALQPGEVRDVTPQM
RSPPAARMAPPSTLQTAAPRRASILADLVPNVVRRASVLIMNAAHGGRGREYAV

>g17846.t1|PACid:27571208
MRGTSPPAPAAVGDRTNYEASTTETDVLTVFLKPMFGDPSSACSTDVTNTWTGTVCNGTAWYGVGCTGDGSAVINNT
GFVESLLLPNCFPGGVGVAASLPSQVSVLTALTRLDLTGNKLSPNSGLPADYSALTGLRLVGLASMDLEAGPVPSSG
FPQEWSSLVNLQQLDLSNNRYLGGELPQSWTNMAAIVSIDVSNTGACGTIPGGLSSQVTPSGLPSSCELVNCRRYLV
ETLQPMIDSQVQPGSACNLTSAWPSSLSIDSWAGITAMITVVGTTPVSDVIGFVLNGCYASNGNSFNVVLPDGLSCF
GSLTQLLFNGNNAAGPLPDAWTQLNQLQELWVTDNILTGSLPASYSALTGLTTLKLHQNQFTNTLPPEWQAMSLLAD
ISISDNTQLSGAVPSAWGNLTSLTTFRARTVGVGGQLPPSFSALSLLTELTFVSDTLSGSLPSEWSTLSLLQQLTIT
SNRATSTMTGTIPNAWFGPGGITPSMSSLQAITISNTAISGPLPTDMSQIFSLTKLDFSANKLTGGPTPSSGFLFEY
NTLQNLQLISLASNQYLGGTVPSAWAVTMSSLTFLDLQDTGVCAPFPTGLNSALTPGTFVNSYCDDLPAALNLATNF
KPLFDSGNVSLACDLSSWDISNPLCSNTWSGALCSVSPPDSSGFSIGQLTSLQLIGCSAPAAGSAAARQLPASITQL
RGSLQDLVISTSYLTGTLPFELGFMNQLTRMDLSWNLVSGTLPSLWSGLGAMQQLDLSANRNINGSIPSDWSGMNSL
RTLNLGGMDKLTGPLPETLISIPGLADVTIESNSLTGTLPAVWDNLFALTSLVIGAGGNSAPGPSVNGTFPQNWFTS
GNLNNLWNLRIANTKVSGPLPDPGSTFQAGFMGLTSLDLSSNMLSGTLPDSYQGFSILQRIILNDNTQITGSIPTSY
STLATVGLVTLNVASTGVCGSIPADISGAVTPNPLPACPSPPSPPTPPPMPPSPAPPSPLPPSPAPPSPAPPSPAPP
SPPPSPEPPSPAPPSPLPPSPVPPSPAPPSPAPPSPPPSPEPPSPVPPSPAPPSPPPSPKPPSPAPPSPAPPLPEPP
SPVPPSPAPSPVPPLPVPPSPQPPSPVPPSPPSPLPPSPSPFAAAGSDVFFDVTFQGVNLPDVNQTKFAQQSCTELQ
TSTAAFNCVNNKLVSGSIIGGYVLTAPPGTPLDVFYSAVQTAVTTDPASLFSGSFLSAYGILSVSGTMLPGAAVPPS
PPTPSPPPLPPSPPPPPPPSPPPPPPPPPPSPPPPALSPSPPPPSPAPPPLTPPSPPPPSPVPPSPVPLPPPPSPQP
PVPPPLVPPPSPSPRPPSPSPPHPPAPFPPPSPPPPPGQFVSGYEVTYTPTFCAGLASDTGFNVFDPSALDQFGRIT
AITFVHDAAIPGVVVAVKLVYGSGSATHTDVLAGSVYDPRSPTLSQSTVRLVSSGGSALQTLSVCCTATGSVAQLAV
GFADGTAASTGLCPVGARRRVLLQAGVGAGGGSAGVPTGAVVGGVRGGRGAGVNSIGFALAVKKPYPPPRTVPFPPS
PAPPSPEPLPPPGRRRPPPPKAPSPPPPPPPSPPSPPQPAAPGAPARPPLPPRPPSPPPPPPFLPSVPPLLPPPVGT
PAAPPSPPPPPAPTTQSVQVAFQVSAKVDYTQIVANVPPQVPNTVPASTLVLLGPAVPIVRNSLADPLIASTLYGRG
RVAAFGGEKMITGCCKPRGKPTPSDPSLDTIIVNAADWAAWYGKKVGKAIIRVADTRYVPMAKYVVKKRPDSFDTLK
SAKIKSYYLSLKAFLKDGHKACDLYVIGSYDIQYLEPRVSHFLQEFVNYGKGLIVVGPDVMPSIFYPGSLAAPSLAP
STAGRRSMLLQRAAVLLGDAFEPDVTDAGDNLSDPDAGVDLRHQLHREMAAALVRRRELQQSGSSSTGSSAPGNPLF
NSTSIPVNQVSGPMGLVFSGYVSDPGGNLTVTSPSELQNAELAASQYVEYLQGRMALTVPELSLAVSTITKARASVP
RSTPGASRFWDLSDEATVLSGKAPALPPLFGAPPPPPQLRPPPPLPPPRPPPPSFLPPLTAYVGCFFEAASRRALNT

TLLTGTGVSVDSCLGVATNVSNPNMVGMTFLGLQRTACFGGRELSASFVAAQIAEAACPVSCPAASNQKCGGNANAT
GIPVSVYRILSAAPPPPVAAVMPPSPAPVVSGAVPPSPRPSPPVPQPRQQPPSPAPRPPVRPPPPVAKKKLSRRAG

>Cre17.g736150.t1.3|PACid:27571314
MSADLRIASIRFTRALQAAWQARCVAPSSSTAPTRGALLGARHGLLPVLGCTRHHGTIAAFASGASGGATSQAQTSE
HGNAVPSPSLSSAPGPSASNSGLRSAGAGTATRGARVLLVDVPSLLTSVGTHTQTQQQTAPQRGGAGSNRRHIPAAP
VTRPEPQVGEPLSATATSAALLSISKAVRLARPDALLLVSHQAHAVPGGSSGAAGQQQGDEQQQQAPQWSPPAAAGA
AAAANTWRQLFSRLGPQPPGSNSSSSRSSGSSNGSGSSSESSRSSGSSNKSGSSVARGGGRRAGATAPGSATSPQPP
PPPQQQQQQGGTWAVVAAAVQAGRLDLGLAAQELLEQLQQRELELLGGSSSSSGGSSSNSSSSSPPAALAASPPPPAT
AATAAGSGGGLAGNEQEHLQQLLLREVLTSQLGPEAAAAAAPPPPPQPLPPVAASAARAPSTASHTSLHQRDASEEQ
LEHFQRSLLAMAGEQAPLPVLSSRWLSSACLTSLLANSSSSRMNSNIYGGSSSMMGSSSSSSSSGGDGSSNGLFGVA
ADAGAGGEQLQQQPQIGRVSILTADERLLQLAGAAAAVEAAAAAAPADGGASAAAASRRDGTIEASGIVMRNGNSAS
GDAVTVLYVAGGAAPASASPAAPPAGNRPLQVITHAAAACAALTEPPARPLLASARLPDYWALAGHRPTGLSAVSGF
RNAAACDLVAGLTAAEAAEAAEFKAGAAEVEAGAAGGKAPPPRQPAVMRLGGVAAAGPAAVAARVSTKLSHDNKFAL
ASFNGVMARLPGSGPGAPQLRQLLVQVAAGAAAEAEAAAVDGKPLAPAADKVSLLLPLGTTTAVATTPATAATPAAA
SPAAVAAAASRRTESATSQPPAAAAAAAAAVVLEGAEAWGWLWERAAVRDPEALAGALEARAREVLLLVQDTGDLSG
GGGSGGLLQHQLKEARSLAGVQPDVIQQQAEAGGRALELAAAGQRTAAAAAVAAETVSGGGTSAQAQAQTAPAPAPA
PAPAPAPAPAPAPAPAPAPAPAPAPAPSAAGAACVAVGGGPAVGPPPAALAATAAPAPAPATVAAAPPAAVLPRRVF
MPGPPPAAKPRTSTAAAAAAAFAAPPVAAAAASDAAAAAAAAAGLPRCVMLLDEPVVVPFWVRDETEVKAAAVVAAA
APPEHAAAAAAAAAVAAAAPTSRQALVVSYSPIDQPAELSDLVSYLTSRLTAPATSSGGGGGGAAGGGGGGNAPVPV
PVFVHWEPPPRAKKRGGGGGKRAGKDGRPPPLRTPSQAGDEDPTAAPVGLSLAWPAVDSAGAALKAAYLAASNPTPA
AAGGGTAAAAAAAGGRSPDAAAGAGSIAPAARPFVPALHTAHVKLSAAAAAAAASGGGAPRLTAQAAAELLRPLLCHT
GPAGPASFDNGERSNTSNTSNTSSATTTSAATDPAMAAAAAAAPPPPLPAWCVVTHDLKRFAITCAGLGCRLPLPPP
PGAYFDTLAAAYVFSAERFRPQENSTLKPDSVVVMSDLTRIAQYQDVRQPLQAGLGGGGGGGAAAAAHNARIDAAAE
AVHNALAYVRLQQMMAEAAAEAEADAAAATEAAARAAARVAAAPPSRPPPPLPPLTTPAAPGPLGGRAAPPPPPPPP
PPPPPPPATDQHPQQLAPAAAAGAATGDTGGGGQQQQQPGVQQQQPQPRINKDLRQLITDVEVPVQVVLAEMEMAGF
GVEVGAMQRLHEESVRRRAQVLDEVSALLGGHRAWPLSTPAEKRQLLCDTLGLDAKAEAAAEAAEAAGAPRGSVPRL
VRTKQKADGSPGEISMSLQNLEIMAPYDSTGRVVHLVMEHARLDKLIQSYTGLFLQHVDRRTGRVYPTLNQVGTATG
RLSMSGANLMALPHDKEEFGEHLRNFRRALVARPGWLLLAADYSQVELRVIAHITQDPTLASAFRERRDVHAQTAAR
VFRVPLGAVTPAQRQAGKQVNFGLVYGMGAAKLARDIGLPDNEASDFIHTFRDVFATLYDQMDRTKAQAFVSGYATS
LRGRRRGFRGAWSGKGLAWLDWQARFEPWRRQQLAAEVLGKLKADEGLAGWRGMDRDDRTTLRELGNMSYQGSTSDI
VKAAMVRCTAALGPGPGGINSRAQLLLQIHDELVFEVPEEELAAVAAVVGRVMEGTAQLSVPLEVEMEVGPNMRDLQ
DYDWRNPPPPAGLPSPPPPQTATAQDAPPRVA

>Cre17.g732350.t1.3|PACid:27571328
MGTVTTRCRERGVPNPDDDAVAGGQQAAFGVSPCSTSGRDRTNNHNDAARAHTSELQQALGAGSIRAALTTDSLPSC
RGQAGMPTADREHASGGIAVGAMSCNNYRRSHVVAPCPAVGASTSASCGSSECPQRRQQQEQRQQGPRCKRVRGAA
ADGGSRSGYSHQAQAKRPRRMGWAALLTLLALSLMSCLGLAAGQQASPSPSPAPAPTATAPRPPSPAPVAPRPPLPR
PPIGGASNATAVPRPPPLLATPPPSPLTVATPGPVPAPMPGDSTDSADAPTAALIGGGNPSPRPPRPPPKPPSPPKK
KKKKVKVKKRPPPPGPPLPPLPPRPQTPPPPPPAQSPMSPSPPRPPPSPPPPRPPPPVVTPASRPADSSPPLPPRPP
PPPPSPSPPSPSPPSPSPPSPSPPSPSPPSPRPPSPRPPSPSPPSPSPPSPRPPSPRPPSPPPPSPPPPSPPPPSPP
PPSPPPPSPPPPSPSPPTPSPPPPPSTVLTSPSPPPSPPPPRPPSPPPPRPPPPRPPLPGTGGPKPSGDAFPPTPPD
APLFPPLSPSPPALMRPPSPPLADPTVIGRYEQVGRGMIVAVHMAAIPGTDRYLFMERPSGYHPDGSHNIAGFFDLA
ARSYTHIYSPDGLFCCGHTMLDTGDVLIVGGHQANAGYPDGMKSVRTFNKSCTDLNLRKVNEMRWRRWYPTPTLLPS
GKVMIMGGTQGVGAGTANNPFWELYDPPTNNVTQFGMYPYYLDKSEQIYYPFNYVLPSGYMFTFCGRSGYILDYTTN
KWRQDVPRLRGYATTQFPYTGTSVMLGLYPENGYEVDVVLFGGQKEAANKDLSLIANRGVNRIKLTYDAPNQNYTFT
EGWAYENMVMGRVMPDSVLLPNGKVVILNGANTGLAGDSASGGDSRANYPVLFAELYDPDKPLGDRIRRLAPTKIAR
MYHSTACLTTNGTIIVAGCDRCYRFTVTPGVDFEPSPTSKAEYRVEIMSPPFFYFDSLKPTITSLQSDVVPYAQPFT
LTYSFPTPGQRLSRVVLVAPCSCTHSFNTHQRLIGLEIMGKSDADGVVIVRGPPNINVAPPGMYMIFLLNGDVYGAA
KWVTLVRPNPGEPGFVGVGIPYPDPVPAQPI

>Cre17.g732350.t2.1|PACid:27571329
MGTVTTRCRERGVPNPDDDAVAGGQQAAFGVSPCSTSGRDRTNNHNDAARAHTSELQQALGAGSIRAALTTDSLPSC
RGQAGMPTADREHASGGIAVGAMSCNNYRRSHVVAPCPAVGASTSASCGSSECPQRRQQQEQRQQGPRCKRVRGAA
ADGGSRSGYSHQAQAKRPRRMGWAALLTLLALSLMSCLGLAAGQQASPSPSPAPAPTATAPRPPSPAPVAPRPPLPR
PPIGGASNATAVPRPPPLLATPPPSPLTVATPGPVPAPMPGDSTDSADAPTAALIGGGNPSPRPPRPPPKPPSPPKK
KKKKVKVKKRPPPPGPPLPPLPPRPQTPPPPPPAQSPMSPSPPRPPPSPPPPRPPPPVVTPASRPADSSPPLPPRPP

PPPPSPSPPSPSPPSPSPPSPSPPSPSPPSPRPPSPRPPSPSPPSPSPPSPRPPSPRPPSPPPPSPPPPSPPPPSPP
PPSPPPPSPPPPSPSPPTPSPPPPPSTVLTSPSPPPSPPPPRPPSPPPPRPPPPRPPLPGTGGPKPSGDAFPPTPPD
APLFPPLSPSPPALMRPPSPPLADPTVIGRYEQVGRGMIVAVHMAAIPGTDRYLFMERPSGYHPDGSHNIAGFFDLA
ARSYTHIYSPDGLFCCGHTMLDTGDVLIVGGHQANAGYPDGMKSVRTFNKSCTDLNLRKVNEMRWRRWYPTPTLLPS
GKVMIMGGTQGVGAGTANNPFWELYDPPTNNVTQFGMYPYYLDKSEQIYYPFNYVLPSGYMFTFCGRSGYILDYTTN
KWRQDVPRLRGYATTQFPYTGTSVMLGLYPENGYEVDVVLFGGQKEAANKDLSLIANRGVNRIKLTYDAPNQNYTFT
EGWAYENMVMGRVMPDSVLLPNGKVVILNGANTGLAGDSASGGDSRANYPVLFAELYDPDKPLGDRIRRLAPTKIAR
MYHSTACLTTNGTIIVAGCDRCYRFTVTPGVDFEPSPTSKAEYRVEIMSPPFFYFDSLKPTITSLQSDVVPYAQPFT
LTYSFPTPGQRLSRVVLVAPCSCTHSFNTHQRLIGLEIMGKSDADGVVIVRGPPNINVAPPGMYMIFLLNGDVYGAA
KWVTLVRPNPGEPGFVGVGIPYPDPVPAQPI

>Cre17.g716100.t1.3|PACid:27571335
MELVSVDVPDVRIALQRGVPLLLGRGNATGITSVAVSRSHCHVLWVAEPKRSARLASALGADPGVSHLETHSRQPDL
GSAPQQQHPGNREYEDVVLTVLHASGAAVVRRPHATHTFATSPTMPPPPVAGAAAAAAAAAAGPQLVLLPAGASCQL
YPGDVVYLERRDARLATGFRLQQLQQPRSGGGGGGGEGVAATAAATPTPQSQTWSPQHQQQQRQQQLPPWAQQSPRA
LVFTAHDPPAVSAAAVAARAAAGAAPATAATAVPAVTAAAPSAPVAVLSPTPARAVFPACAAVVGSGGPASATVRPG
DGGAASTRPVRAAAGDGSARPTAAGLPPGQQPPMKQAPQPPAASFGPTGGQPTRGHVRDAVGAGAAASHALPQPLVD
PHAQPRQDSMTRHSPPHKRPRLESPSLHMSQQSQPHPDSAAAREAADTDAAAAEAVEIDAEEGEVDLRGLCVDVGTA
SGAGWAARRRRQDADAAAPPTGDGGAVVPEPTPSLAVLAAAAAAASAAASAAAAAPTVSGLLAAAPLEPSGSGAPVK
GDGTAAEAGAAAAAGAGQVTSGATAPEVLAPVAQANKAPAAAAAAEGEVEMSPDVGLGAEVEAAADRAAAPEPASVSE
AAAAQESPEPAVAEGAAAEPAGPLAAEHVQAERGAAYEGAVEQAPSEDCSKPADAPPLLRPGPAAMAEPPAGPSTSP
PHQALPSPPPPPSQSAPQPPPAQQPPQPGEATAAAFGDGSGAVAAATEGDTAAAGGAGGPRASGGGNGDGRVAVVEG
GSAVRAGGEGPAPVPATVSNAPPVSSTTAAVPAPAVAIAAPGLAATPGRAFLSGCRFVLWERGAVRDILTKVKRVGG
TEQYSVCGSTTHVVARRDLPAAEAARLLAASDLAWNRDGGGGGGGGDIASVGQHSLPLPPGVHFVTPAFLRDCLAAG
RLLDPDPAAGHVVTQLTSQQQQPLSPPPPPPQQQQQQQQQQPDDTVPAAGQPPPLQQPLQQPAEAAPEPRPQPQTAA
TEAMGVLPPQGRAAVAAGAPQPLQPQSLQPRSSQRVLPGPEDVCRGPEDWGVGDRWIEPWDDAAARRTCSIVLAHWA
KHSFHTDDGHALDYRDSLREAAAAAGAGAGAAGGAWVAAGALGAGAAGAETTPVHTPVRRTRSGGSAGRGGGRGGSR
AGSDGGGPRDTDVEDNDGDDNDDDLGLGAKAAPGSAAAASPGGGRGAGAAAGSAGGGGGGSGAAGGGGAGRRLSVGE
GGSSPAAAVSTPLSGSTTPGGGGGRGRRGGWWWRGGGAGGGGGGGGGWGRGGGWSGGKGSGSGGPRRRRVGGGGGSGD
DTEHSDDGSGREEGRKRQQQQQQQRLQPPLAAQHSGGYDTADEGETGAGRQEALARQRGSGGGGAGGEAADDDGTAG
GVGGGAVNVVCRHVCCSARPFCILEELKHTKSLYMGRDDQFRIKAIDRALAVCARLTQPLQSPDQVQRLGLGAKTAK
KLEEILATGRLQRNAVWEGSELRATMKLFTQVWGCAESTARKWYVGGARSLSDVAARSDLTAQQRLGLTHFHDFSRR
LARAEVAEAAAGVRRVTLEVLQDLTGLPQHVVADRLHVRPMGSYARGAPSSSDVDFIIAPGPEADTRLGPPVLLAAL
VARLRAAGYADPASAHMLHTRTSWKPRGAEPVRHPGAAAAYGQPRPQAHHRHHHEHQHRRRSSTAGASQQGQAQGGG
HDDDLSDGDDMAAAADAPLDPSSPAVLRLAERLASASSGSGSMEGGGGEGDGAGGGGSGAPGAGGRLAVALTAEERE
LVAASARVEDSCTWLGVWRQPVSGRYCRVDVKVYRRAQLPFAVNYFASGTDFNRAIRYYCNTPLPATRELARSYHPQ
AEAFRLSDRELAVMRRERVAAGGGPMGPDGRPGVVEHLLALVPCRCETDIFRAIGLDYVPLHMRDILAVE

>g17781.t1|PACid:27571340
MHAAAAGETDNLMGAREVRLKNIRLNHSIRLYAPRRHPIAGASLSEPPAPPAPSPRPQLTAARHPHAQQQGAAAAAV
AVAPPDPEVAAEPATPAGGLATPPFGGSSSSRHVPFYAGGSGSGSGGGGSGIGGSGSSSGIGGGGGGSGSSGSGSGS
ASRRGGGGGGGGGGSLVAAGAGGGDGDGGGGGGFGAGAAGGAGGGGEAHGGGGGAAAAGLTAPPPPPPGAVAPEAPP
SGGGHDGGGGGGGGVTSGRRQQRPLPRRLAAALLRPLVAWEVQQRRRGQRRGATEVVAAAAAAAAAGDSSRIANAGQ
ARPVSLQERRPAIAGGGGGAGDSDADGHDGGDGSSSRSHQQQQQQQQQQEHGDAATATTTTTTAAAATEGSAATAGG
DGGAAAAPPESAAAAAGGAATSPADTATADTATAAVASAGSRTSPAAPAPAAPAAARHPSPAPATTSPSSRRALWRP
PLPVAPAATPAVAAAAAPAVAATAAPAVAPAEAEAVAPPACQAAPPHPSPATPTGTPRPMVPSPTPPTLPLPSPPPA
APSSPPSQQQQQQQQQQQQQQQQQQQQQPYQIPSPPPSSPVLLPLPHPPFPPAPPSPPPPRPSTPPQPSPPSPRPPSW
NALPHAVLVDIAARATAPEAPQLLPQRLLRGPPPHMLQPLPVPLPAAAAALDAALLAVMPPSGGDSSSSGSTSGSGS
GSSNSSGGSSSSRSSRSRAQAVAAAAAVAAAAAPPPPAAATLAAVRLVCRRWRRAADEGVTALCPGQWPHRLSGLQR
FPRLTRLDLGALGPDPAPLSRAAALLRQLAMPPTPPPPLPHAPQPQPPQPLQQAASPPAPSPSPRQPQSLLTPPSAS
SPPGGSGSHKPQPPPLQPAPQLQLLQPPLAATLQHLTLSKECLVPTGRLLYDRVPLLSGLSGSSSSSAAAGGSGSII
RATLLVPPGLGQLTQLRSLAILRGSSSTAFGSSSGSSSSGGGGGGSSSSRRTHYTGGAYRTWRPMVGGEWRAAVAAA
ARHACTEFGSGIADSGGWAGTGISGDNDSGSSSGSSSSSSGSDSSSGSIDQEGGNGVRSGWWPPQREWRRQSENAGM
SAPAVAGARAAVAAAPAGPGGADEHGGGRGAGVVGPAAGRSAAAAQTEGVEGAAAGEDFGEEEWRRLQRRRRRRQR
RDVRRAAAVAAAQAEMLRASWHPVVVPPAPAVADLGEEDVAGVAAAGWLLPPTTAVGMEEQQRRRSFQQKRLPAADG
SSSYSSCALVQPSSAGDWAGSGGAARHPDGVGGSSSSASASAAWVVAGRTPGLPPLTPAATAAAGVGGGGAPAAAGA
TATAVLCGEDLVVRLNRDVSSLVQLQELELWDVEEPLCHFTATATDPVPAAAPAAAAAPATATAVAAAVTANASAGT

GRHSFSGRGQRNGGSSNSSSAKGAGSNPTRTSTPPMAPLPHLRRLALHGPTSTASAGRRGSSNHAGSGSGSSSSSMG
GVADTAAALQLLQGLRELVVGEARQEHLAAVVAQNVHQWLPASSAPDAIRTHAGRPHGLSRAAP

>g17781.t2|PACid:27571341
MHAAAAGETDNLMGARETQEHQIEPFYPPVSIAGASLSEPPAPPAPSPRPQLTAARHPHAQQQGAAAAAVAVAPPDP
EVAAEPATPAGGLATPPFGGSSSSRHVPFYAGGSGSGSGGGGSGIGGSGSSSGIGGGGGGSGSSGSGSGSASRRGGG
GGGGGGGSLVAAGAGGGDGDGGGGGGFGAGAAGGAGGGGEAHGGGGGAAAAGLTAPPPPPPGAVAPEAPPSGGGHDG
GGGGGGGVTSGRRQQRPLPRRLAAALLRPLVAWEVQQRRRGQRRGATEVVAAAAAAAAAAGDSSRIANAGQARPVSLQ
ERRPAIAGGGGGAGDSDADGHDGGDGSSSRSHQQQQQQQQQQEHGDAATATTTTTTTAAAATEGSAATAGGDGGAAAA
PPESAAAAAGGAATSPADTATADTATAAVASAGSRTSPAAPAPAAPAAARHPSPAPATTSPSSRRALWRPPLPVAPA
ATPAVAAAAAPAVAATAAPAVAPAEAEAVAPPACQAAPPHPSPATPTGTPRPMVPSPTPPTLPLPSPPPAAPSSPPS
QQQQQQQQQQQQQQQQQQQQQPYQIPSPPPSSPVLLPLPHPPFPPAPPSPPPPRPSTPPQPSPPSPRPPSWNALPHAV
LVDIAARATAPEAPQLLPQRLLRGPPPHMLQPLPVPLPAAAAALDAALLAVMPPSGGDSSSSGSTSGSGSGSSNSSG
GSSSSRSSRSRAQAVAAAAAVAAAAPPPPAAATLAAVRLVCRRWRRAADEGVTALCPGQWPHRLSGLQRFPRLTRL
DLGALGPDPAPLSRAAALLRQLAMPPTPPPPLPHAPQPQPPQPLQQAASPPAPSPSPRQPQSLLTPPSASSPPGGSG
SHKPQPPPLQPAPQLQLLQPPLAATLQHLTLSKECLVPTGRLLYDRVPLLSGLSGSSSSSAAAGGSGSIIRATLLVP
PGLGQLTQLRSLAILRGSSSTAFGSSSGSSSSGGGGGGSSSSRRTHYTGGAYRTWRPMVGGEWRAAVAAAARHACTE
FGSGIADSGGWAGTGISGDNDSGSSSGSSSSSSGSDSSSGSIDQEGGNGVRSGWWPPQREWRRQSENAGMSAPAVAG
ARAAVAAAPAGPGGADEHGGGRGAGVVGPAAGRSAAAAQTEGVEGAAAGEDFGEEEWRRLQRRRRRRQRRDVRRAA
AVAAAQAEMLRASWHPVVVPPAPAVADLGEEDVAGVAAAGWLLPPTTAVGMEEQQRRRSFQQKRLPAADGSSSYSSC
ALVQPSSAGDWAGSGGAARHPDGVGGSSSSASASAAWVVAGRTPGLPPLTPAATAAAGVGGGGAPAAAGATATAVLC
GEDLVVRLNRDVSSLVQLQELELWDVEEPLCHFTATATDPVPAAAPAAAAAPATATAVAAAVTANASAGTGRHSFSG
RGQRNGGSSNSSSAKGAGSNPTRTSTPPMAPLPHLRRLALHGPTSTASAGRRGSSNHAGSGSGSSSSSMGGVADTAA
ALQLLQGLRELVVGEARQEHLAAVVAQNVHQWLPASSAPDAIRTHAGRPHGLSRAAP

>Cre17.g700700.t1.3|PACid:27571348
MAQSRRRKRLADALAWPGASAAALATLLLISVGLARAAGTPRGPSVAFFVGTPDSSDPWPRAYLASALEARYRVTYL
QKDDAFSYKKEEYDALVIPAPDKNLGGAYPANTAAAAAFLEAGGNIILTKWASDSALAYSYITKVLTDPSGCCKNSN
LDTSKCLPQIYSPSSIASTGGYVSSQSFHTAVPLRADSKTTVGYPPYVNQLYGGSGIATLGCSGKTGGFQSLYEIIA
EQPLVEGVTRGVGQLYYVAYNWDDEGGNPKQGWTDVVVAMVQDAYNRPGALALAPVAAPQTAPSVQPGDLTSINMGE
SYLPVSRGRMSWSSGGGLTGLGAALKGPVVSTAAASGVYNARRWNAAEWGCHDVAQAGFIKGAFWVAFDLGLARPVA
RAALQYFEYWDSSTLKLNPGAVLQMEVRVDDKPFSGVADTGTVLCSKAPPVFVTSGSKGPTPTIACPSIAAADGGAV
VGRYVAVRLLDVSAGAWGSQVYKAAMCGVDVWALVRSTAGPVALASRHRPVCTGSLYKDSGFTAAFGRRPPDNATAG
LAVDGLYHDNREGAGAGMCGLSFAQDGPWISVDLGSVQQVMWVELTKSADPNYDDQLNTVFVIVYGTSANPYSCNSF
GGLQYDKVVGLSTCGGLSEAGGKGLALTAGMTFLADCGTAGVSGHIVTVYRPPTTRSPTLGYWGVLRLCEIDVYTLQ
PTYGLQQVSMHRYVDFGPAKSGVSMLPDSTNVVGAATYPYTAKGLVVDGIKPAAGAALESYMCPRVYDTSTAPSANS
YGAGDGVFMVVDLGVSGGVDLMDAVYGHAVEAVVVHFASSLVSRTNFQAEEGPYIDVRVGNTASGNRLAISPGATGS
ENPLCAYHVGHMLGLDPHDDRWWLADGTVKRRYACSGLPGRYVVLSTPRRWQFESDQPANSLFYSPAVCEIEVYARV
ASGSASSLALVSSSRTATQSGDATAIVGGASLAGRNPNTLVRENSSATLAMEQGVCAYAGALGFNNYYVLDLGADYA
VAFVRTLASLNSQLGASARVLSSAWDGKTHPINVTAGFTDCTVTSDGVATYLQGSKSIRDATAEAACIDTKTSAMAV
GRYVALWQADATLTAEPLVLCRVYVYVNGSSIPEQGDVAGSAGQPFAASGFGINATSLSGAASSSSSSGSSTYYLGG
GIVLGTKSSRKVTGRTPASLGEAPLEFILPGGGIVKGGVRRRDLLAARPAADLVDALAVVTVDGGGRTTVDDEETGG
GGSSSDPGCVRQPTAAGSDCEGEDGVRRVVGCCSSTAADALDADGSSSGTDATPAADAGTASLADYGADGAYEDYAT
TAAAGTAEAAATTGGRRGARWRRRRGLLHQDDTAQPLAVVTSGAGAASSGTSRQLLAEMAEAEAQRGGLRRLLFTAP
SQNTSVWSVDLGRSQRVDGVVLVVTVVTSSYFEYGQIQITVGDDASGATGAVVLSGGVINLLTERWYFAAGSALGRY
VTVTRTVAFSLPLNSVEVYVIPDNNEGQVVSGRPTNQAGGVVTGAGGSSNAVNGYYSQDAVIAAGNANLFWGSATPA
VVNPWWAVDLGVAMKFLFVDITPSTAALAGYELRLTNSSVSTGLEGVVAVARTATPVVALGTTARVPMPPGADAARQ
LIVRLVTDPNTASLALGQVDVVVDATVLAQTQNSGVSYPPSRTYDSAATTSGGALSAPLATDGTSTPAAAASCAFVA
APGSGGSATAVWLTADLGSTVSIRRVELTMPLDAAYAAYSTALEVRLGNTEPAAAGEGADNGVVVAGPFSLTAGEAV
IKYLATATSGRYLTVRSTNASPIALCEMAVYGPSDFDVLLIAPSPPPPSPPRPPSPTPPVPPSPAPPSPTPPAPPSP
EPPSPKPPTPPPAPPRPPAPPLPPSPSPSPPPSPPLPPAPPPSPPPSPSPPPPLPPSPEPPSPEPPSPEPPSPAPPS
PSPPPPNLTPRPPPSPHPPTPPPPAPIVYQTGAVASSSQTVSAVAATTVAVAAVSSVTASVAGSVAASVAASVASST
AASAAAATAGGIAGGMGSSTMGAASGSLSAGGAVAASFFMSHLQFFAMTDNAAANSTTSYKSVNGELSWLNLGFSTF
ALKNVPPLQRKAYNQIINCVLLVLIVLAVHLFAVVVLRCLLKTWTVPAWLVFPYLEIFMFFFSLVSLAGAAAILLSS
AATTPGGGGAVIAPAVVGAVTALLLICFVAAATFIVHRLRKAARQLGLLYEHRPPTLGKARFFAKKPAPAAQHAGSS
SDRTPPAPIAEALEREGAGASSRTASGAPSAAAIPPWERGSAISSPQWAGGSSGHDSGSSSAAGAHLRLAAAGGSAG
GSRRLSISNAAPAKSIAVAPAPVPLATAAAGTTPLTLRPGGGGGPGSHSSDDASDELGSGRETGRAGSKSVGVQDKF

HLHHHHKNPNTEHAGGDGKEKQQQHELDGKVAGEHKEAEEKKSWLERFEVGHWNRPGEEALLGKMEPWRSYKYKQDG
MSVRAAVRCLYPVDFAAEAARLRAEYEAKRRARRRQRRDKRRDAATAAAAGAGTVGSSVAAPPSRGGSRNAWGSFVG
SVASMTGRRASAVAATGGGSGGDAAAAHWHMARVKIDAAQAMERQRQRVEKIDEAADGIRSIRGMLQAITRAASRVY
SRRVPAESPETGGGAATGGVAGGGGGGARSSVEEDWRVRTNPLSVPGEGEEGPGGGDGDGRASTGAEEGVVTAVSFA
VPPARSGASAMSVMSVDEAAFGQPGGLSMSHGIAGGPGGFGSAIGSSAIGSKSGGPASASGGRPTASVSAGRHVGFA
AVGTAVTAAQAISNAERPPWRPGGGKARRGATAADSDGIPSPPPAPLPPAPAPLPPLPPVRGARPAAIAVAAAGPHA
IGSAEFDQASESRRMTWAGTGAEHLDPNAMHRPGSAKQRQHAEHGADGGVSSPGGGSPTAEAHAGAAARGRALLWGP
SADLASPSGSDAAAAAAAAVAAGAPPNAILSPHASEQPQHGGGGGGFQGFQLPDGAVRRGCVAAAPLPLMSFGHNLK
HATAAAADEALAAAAAAGGAPPDYDGPRPYTPVPQALPLPPRAETPPLARPTPPAGAQQAQLLSLQSFAPRASDSGT
SIWANAADPKLLHVGAAAAAQPPQPPSAGSAGSAAGSRQSTPPTDAEGLTLQASTSGNHLNAAYGSAAPAVPQMLPP
LKEEPRSGAVEGQESERSSPVRPDRFKAPPINLISFGVNHDSDTLITGGGPKVLTGSVTAGGGAAGSLAARPPSARR
STSGQNSPHATVAGMEEEFGGAEDGAAGGPAGRRLAVRRHGSRPGSPLAVTATVATVAAQPPPVPSWPRKAPGTTAG
GDHTDDCDYSSDDDSEPLSDEEEWKRTNPEPPDEATLRVIARAEIYERFGMVFAENQGHRMLAITFWVAVVINAIVP
AVLLGLQSGGGLAAGSAAALSANIALLCVKGGYALYMTVVLPYINVILIAAEVICAWLETAVVACIVVITLDSANDK
LPSAPPPPPGVTVVAAPLQAAPPPPGASATQSHAAAIRMAGDFMMVAELSVLGVQILAMLLTAIIPAVIALVGVARI
KLRMWRHYHTHGNLQRALTAERANAAGREAAVAKAMAELSAEEQAAAQQQQQQGGGARGLVRAGAAAGGVRRSQDG
DAGGSEQPLAAEGVVVQPQSQPQSHKLHGGGWGGSGSASSTASGRGVKSAPKLESTGSLRERRGL

>Cre17.g735450.t1.3|PACid:27571447
MSKTPKGRVHFGEGLPPGDASPSEVSNPLFNDAYGALPDASAEAVIVQIDGVSQASPRSAPATPSAVRKDAAGDEPS
DASASDDREGESGHSQMSPHEMAMLKYNTSARRLPRIADCYVEPQPAAGPAAAASPWAGRICLGLLVLLALGVCAAV
VLPAVCLTELLDFDDLPILKEVKQLLQEQHADKLDLLINDFVAGSWVQQLEGLNGLPQPPTDYSANDLTAAGGDPAS
YTAAAASAAASAAAAAAAAAAQDAAADQQRRQAQSSSSSPSPSSSPSPSLSPSPFPSSSPSLSPSSTALGGGGAVAA
DPLQASQWHLDAVGAAEAWRVTSGVREVIVAVLDTGVDLAHPDLAPNLWVNTGEVPNNGIDDDGNGFVDDAFGYDFA
GPCSTDWRAVPPPPPSPPPRPPAPPSPPPSPPPPPSPPPRPRPPSPPPPPKRAPPPPARKTSGRRLVSGAAATHGGR
RLLSALLGSVWGAGSSEALEATESLQHAQQAATAAAAATKEGASGGSSSSQGQGRRRLLDSAPVDQVYLPGRCGSDP
DPRPESGDTGHGTHVAGIIGAVRGNGVGGSGIAPGVRVMPLKVSDPQGNFWASNIVAAYDYAHRMGAHVVSCSFGPY
QPTLNPDAIDLAQAAAETRLYERAVRPLADKGVLLVAAAGNEDTNLDRARAVGMPYLPCTLDLPNVLCVAASNTQGG
IWREIVANRWVGSNTGPTTVDLAAPGSRIVSTLPGATYGDKTGSSMATPLVSGSAALVLSALGAVDGNYFQAVRARS
LLLEGGDPQPGLPVASGRTLNTARAVQAAAALAGTGATLLTPMPLDVGAASGVLLRGLSDDYYRLSTEQLAAAAAAE
AAAAGGGAAAGSGSSSGGGGALTTLGLTPFGSSVRTASTRLTSFKYGGAGVLVAVRGHLRLPVPGVYGVRLATSAPP
ARLAVTVGQRRLDWRLATATAAAAATAANASSSPGATADVVREAVLQAPTAGWYDFEVLYLLAPPNTTSTSATSNSN
SSTTATTTTPTAPPALELNWALPSSPRAFGPLPDGYLGRGSLARPIAVPHAPATLPESGAAPGGFQILWRPASMDVA
VADDDSDIGSSSSGNATAAAAAALSVAAGWDPDFLLSRVTEDTNRGGLNPYYPYSSFVDDLSFTAPGSLSAALAGAF
ATGDPAPAPANATATATNGTSSSGSSNATAVANVTAVGDRTDDFGLAAAAVAGPVYGIALAWIRAPPPVGADGSSSS
TSSSSGAPQLQFRITCSFCSLHLGGMLVAEVVDGSSSSSSSSSGSSSPALSGTVSTRLSPCISLAALRAIPTPSSGS
STSTSSGSGSSNATAAPPQLTEPATYALQLRFAVRSVAAAALVVQWGACIPTNPTSSTASTITSAASALAPVAAPPP
PPPPSGPVVYQRLSPLLAGTLWYRIPPGGAAAAAGAAGNSNTTSTTNSTTNTTSTTNTTNTTNSTLLLPASMRCAVW
RSNAVSLAGPGQVQELPRPQDPSSGPPPLSFSLPHPRASYLPCPGPVGTFCPSGYNFTLRLSDIFPAELPSSALGTQ
LYVRCWGWRAAGLAAGLVAVRSAGSLNTAVYLGGQRVFYSLSLSYGDPLNRIPRAPNATSLTPPPAASAPTSASNGG
SNTTAAAAASSSSSQYRQLLVVEWFGVTAGARLSVVEGSLNTSSLYTPNLSVQGLVA

>Cre17.g709000.t1.2|PACid:27571459
MEPKSLLLAALALIAALTGSRAASVCEVVLEADPADGITLLPEGKRIFCTGDPVAFTTGSNFIPDNNDNSTWQLTGP
FGSASVQFDDVLMFPAAYTVLTGASDVVFTNITIRNVDYGGDQAIFGLSSCTNCTIQNLYLEGMRAQVQSTNVFLID
ALSTVTIENAVVTGLGQVPANGGVGSPCSALHVRDSEVVLQNAVFTNLAVNVFATVMVSADPFTAGSSSKLTVRNTT
WRDLTLSTVDPGLISTYQPGRIAALVGGGAVLELGDGVIVANVQLAGADQSTGKPADSLFSFDTAGATVTISPSVQL
DAAAVNAGAVFTVPVASGETIDSSSAWFPYMMYGATFTANDITLSLSDVALQAGLQLHGTGATLNLRNVTMGQLTLD
TPGLTPLSLRNLTLSGLELPSFNNAVVDISDVTITSQQQQRRPLITAENDSRITLTDVTISRVSVAPDNIQVDRYSA
VLLHADTGTVATVTRLTVSGVDWAPPNDNGYFSETQLFRSGNGSSLAVLDSSISGIRSCGTVFSAVDGGALRLVRVN
ISDVDSSGAVEKRAVVRVEGGGSSLVAIDTALRRINASDSTNGIALSVLQPGAVTLSGFMLDGVYGPKSSAIQLLLK
NSTAPITLSGLNIHSATSASALKVSEPGSQITVQHSLFEDCVSSAGGTFALTGCNSDNSTMQLQLLNVTFNRNTGGN
EGGGVQTVMCHVLVRDSVFTRNAAANQGAAISLRQGSLTLENTSFVSNSLSSTTGGTNGGAVYAEQCAVSITASTFT
RNSALGAGGRGGGLFMTGSRASITRSTFFANSASGQGGAMMAYQTWLTMNDSVVAGNIAPLGGGVAMYDAVPKVTLL
QLTAPASPLLAASPPPPPSLPPLPPSPPPAPAGNLTSGYVAQTTLSDSFFGEFVNTTFTQNSCSDGLNMGGGGLYVR
EQVERWNVLVYKCTFVSNSCPRGGGMLSGYLQRTYIVRSEFRLNRATIEGGAVTMTPSNDRGSYLLIQENQFVNNTA
KILGGAVQAGSAMVEVAYNNFTGNGDGTVSLYGGAFYAEACQLVPGMPPTTANLDFRFNTWDSNQALGGGAISFRLC

NVTMYRELFVNNYAHAGGGAVEAINTLQTPLTTTQLNEVTMRNNTARQNGGAMSVLGTGVMLLHSIFEANNASIAGG
GIAALDASPSALLVLKRKTVPFYTPDEEYFLRVYMDNSTFNSNVADQGGGALYTDSCGLVVQNSRFDGNIAGDGFPN
DDWSDSGGALTAITAWSNMYIGSSNFTNNQAFGGPAGAILLVGTTAIIDACNFHNSIASTSGGAIAAADRLRNDSNA
YNVPNLVVRTSTFTNNIARRQNGGALLATGMNATTFTSTFKRNVAGGNGGAVAFLNNLDAGFMANWFEANSADQYGG
ATYVVNTPIVSVLTTTFLNNRANRTGGALSMISCNCTLTAQSTFEGNWVGGRGGAVHLQPVDNDDGNTNTLAESADC
VGHFLSTIRRMLTPEGLALLGTVDTRVETYNATIVYDGASLQVMTIGQFGRGGLRLPTPVLSGPGQITGFDTQVNLA
AGSSVKLASGSAFAFADWDRFTVQMDGSSTRLVYLDGAITTPLSPGEVVVLAPGSALTALSDSMSLLPTNTSMALTN
GGTVWKLLNGTLLSADNTTAALSVVPGARTRIGLLDGSITVSDWNRYVLWTYGSGSYLYDVVTGSARALPYADVSVA
NGSPLMRHSAADAELTAPLGITTSADPASGGEAWGLSNGTTLSINGATQIAVSALLANGSTMQIRRGSSLAPRKWAG
YTVTLSSDSPATLLSLPSIVSSAEVSSALLMGSVVSLPEGSAVSRLTSSMAMLPAGTLINPDGGSWVLNNGSALAAT
GGNVAALLVLPAGTTVALSDADVFTVGPSEWNRYSLWTGTMGTLLHNTSSGEVFVLSDSAAVSLTRAAPLTWHSDKA
VVGRQATFTVSGNTWTLGGSLSGSYTRGGSVVGCSSVLSRDSRIAMPNGTTLASSEWYRYLVWMAADGTYLFDSLTL
SNAKLPDGTLVTLTGNSVIFPHDSGTTMVAAGGQTLTVQPGAWQQSGGGSLSPSGDVTSVVVVPAEGSSISVGGPAG
ASGALPLVPRVGSSIVWLSRNGSQLVDSALSVTYRLVTGSSVQLAAGSLLLNHGSSTSIMSDAVNVTLQQAGGSQAW
SFTDGRTLSVGSGVTVLALAPAPLSSVVFNNPTTIPIAVSAKNGVWAGRYSTLLYSLEVEGSYTLAEGSEVTVGANT
SLSMYINKRITIAYWDAPDTFIVRGREWVLTNGTRISVGDGWNDVDDSQVITHVPSITEMPDEISRSCSADFTNGVY
QKDAYAYDYYALRALLNPWLQPDFETNVAPQVAAAATGLSLADTAGFNATMLIPGYTLPTFDNAGRPIGAIVTCTAT
SAPPATSRSRGLSTLLVDVVACGNVADMKGGVMVVDGGHGRVLLDRLVFYNNSVTRGDGGALALYQDDDQLNMQLEV
FITSSNFSNNAAMSGTGGALSLIADNMHQQVSVTNTTIDGNQAAMGGGISLSHNISAVFNSSVISNNVGNASAMQQG
ASANVANDRNVGSGGGIMAYRCNELLLTHGTRVENNTASARGGGVGVVSCALATVNSTLLQGNRAQSAGAVYLYNSL
PVSGTYKPEDPVGAMIAVVAKSNFTDNVAVPAPDAVMLTEGFGGGIYLRSHVSTLLADSYFKGNNASLQGGSIFIES
TCDLNDQSWFLERNRSAVTAFTSSSVSFANTVQALQVVRPKGCWGTVVYLPQFYNNEALLSGGALFSSHPEALSLSC
NDANATHASSNLPIDYADFSEPYLAGRVAETMKMASFQVLDNKVVATCYRQELESLDVNATDLTGNTLSTLVVNDNR
AKGYGYLIAIVPSKLRIINVNWTGNTSNMAGMDSSSSAISNVWPGATGSLDGLYGTVSTDAGVMALDGGSGSAGGNS
ITAGTLAEAPFFRSWPDSVFDNDTQTDRRTVSYHITTLSNKPLDLSILLLDALDQVASENTTLRQAQVRARYLNSSN
GCFAELLGGIEGVARNGTAVMSAMRLRALKGDNYTIQFKVEDSIFSGTVEPLTINISVPACSIGEVPRDNGFLCQKC
DPRSFSLWQDTEPLVNCTYPAISDITCQPCPDGAECPGGAVVVPMQGYWQSAANSTFMNACPNPEACRDGDDDMQNM
LVTCQEWWYSRPADFNYTAYINLVLNLTDAASNTFGGFSALTLSGEAFNASNLDDLCVLWGLPYNHPASYARKQCAT
GYSGNLCAVCISVDGRHYTGPGDFSCNECFDRTISVLVAVAGFVANVVTVLVTVILTFMADYTEDEDMAIGDLVKVL
IVHIQFFCIVTRLNINWPTSVSGFTGLLSALTGAVTEVYSPSCLLDESATPAEMARIDQLAAIITPLVVVIVVAALW
LVRFGLFNFRQIAGGQTGQEHIKDMRTIEEYKQDRASRVAQRRLAEHLGEEAMGTFGEEEFNNAMEDGSQQGEDGLD
LQAGDMDGQRPSHMVREMRRRMMAEHQAEPAAAAAGEDNKEEKEEEEQQEQEGGQQKPLAGVSSRESSPDSAVVEAA
TVGLLMDNESDEDSKGVMMPPPPAAPLPPPMAPPPVGPPTQDQSPANSEADAQPNTPGAADAISAAAAASVAMSDPA
LPQAAAPGLPPLPPRGSGTSPTPASGATAAPGEAAPTGGVGIPTRRPSVGGLKTRVSIKTGRSRVSFDDQALSGGTP
SSETGTPSHRTIVLANASTDRGGLVPPPPASTQRTSFRAAAAAGLSLGAIHNAAYNSDSGFSTPTGRDRRPPSLPTI
PSLGRAKVAVAAAVAKAAYDADSASASIEGADVTAEGGDIETGNGRHGASCRASPLAPKSVAGPSRLNAHARNHGSG
GSGRAVPAPDSSGSGDPLVHGSESQRGSFGPKAPGSFGGKAAGSFGSKPPALTERRTSSMLAMKQRLTASTVWVRNF
FKYDEEAVEEGEIRWASLANIDRTMTWWRQVLLVVMIACFILYPAWAQATLMIFACYYLDDGSGAYPEYQLAQWGKG
YWLLNMNQECYAGEHAALWIPIGVVCIIVICLGIPLLTFMVTFMHRTTLDTVHVVQTYGFLYRRYNYDRYFWWEAVM
QLQTLLLVIVDVFGRVLVVYQQAVLLMIVLMLIMWSNMFYQPLKHEILDQMSSMSLAVLSFTVALGLFFVPPTDRKD
PVTPVAGAAIGGLILAMNIALVVYFVYVMLTHGREAITRGVTRAREHMLVATSKMHMGLTVVRDRVSNGAHKLRALT
SRSLTSVQSKMRHVSDRRRSGSNSGLVPTPPTSNVTPDT

>Cre17.g705250.t1.3|PACid:27571536
MRLEPALGVQSFERRKLGGTEVLLAYTDAICGGYPQQLANPFTAPTIEDVIFGGIRDFTALGSVKGIDLAFTDAYVD
KTSQTYYPRGVYELALYYNDYPSPPGARFALLNRVAGRRNALTTLPTTAAKLDTSQLGKFNDKGPGSLQSATVCCSD
DNMFESMRAVRQIIGDTKIGLPVCTGYPVEVKRPAAFPLWAGVRLTVQQPIGSMEYVIASIQFIWASDTPTPVALPP
PSPPSSPVAVPAVNPPFLPKGASPDWLAIATGVVSFQLVEPTVSIIMTKRSFPIANSDIDQTVIAATRVNRGAAVAL
ASENMLQHCCGGALGASATQLNQTAKGLDLLVLQGALWAAWYWVKTGAVRVRVSHERYLPLARWLSAQRPGLFLTPA
EQDMPSYVTSPDLFIRNIKGTVGQGKRPPEHLYIVGPEDPAYTDPAMANAMRDYLNAAGGVMFLGPNPTAVRADTGG
SDGGGGTSSGTGGTGTSGGSTSGNGSPDVEVVTLDDPSPPPPATGRRKNKPPPPAATPTDTTTTTQKRKKPPPPSSR
RSAAEVASIVPVGPSWWRADADADAMANSDVGGNAVSGIASLLRGLGMPQRSQRRRLLDVAAANSSLTSIYPINAVS
GPFGIFFAPPATPSVQPGTISVVTEPRSDINNAELAAGAYVQFLQGQKPKEFDLDVVSLTIAKARASIPRGTPGSDG
FYTLVDALFALGGSLPEIKPSPPPPPPTQRSSPPRSPPPPSPQPPKSSGTPSGGNNTGVGSGPWSFLGCYIDNVGMR
ALPWRLSVSDKAISIERCAGLALKNSLGYFGMQGSVCFGGNNGTQATMYGAVAAAQCNMACAGDRSQMCGFVSAARQ
LLSLYQVSLVINIGGNRALRAGH

>g17703.t1|PACid:27571550
MARLQSILALIAVALLASGAAATQNDCLGGCASSHPSFEPVCCDGRMWYNACYMLCQAPNITLYERCPSPSPSPAPS
PSPSRPPCDPLTYPYVNQTYHCQSDCRTYTLVDVNAGCMPNWAGARAYCESRGLELAPWDTDASNGALHTLCAANRY
TCWAAGSMPDGLCPVMSQEGDMHFQGCNQPVRFVCRTTTSSCASPLPPSPNPPSPPPPSPDPPSPSPPSPNPRSPPP
SPAPPSPLPPSPSPSYPPSPAPPLPPSPPVCNPYTAPYKNTSYFCENDCRTYTLYDIDAICMVDYYTADKFCEWKGG
ELVPYDEVPALGALYKLCRDNRYTCWTGGRDDSTDLCPLMTQEGYIVQQGCKQSVRWVCRTKGPLCHPPPPSPLPPS
PAPLYPPPPSPAPPSLPLPPSPAPPYPPSPAPPSPLPPSPVPPSPRPPSPAPPSPRPPSPAPPSPRPPSPAPPSPRP
PSPAPPSPRPPSPAPPSPRPPSPAPPSPRPPSPEPPSPAPPSPEPPSPRPPSPAPLYPPSPAPPSPRPPSPAPPSPR
PPSPAPPSPRPPSPAPPSPRPPSPEPPSPEPPSPAPPSPRPPSPAPPVYPPSPAPPSPVPPSPRPPSPAPPVYPPSP
APPSPLPPSPSPSYPPSPAPPLPPSPPVCNPYTAPYKNTSYYCENDCRTYTLYDIDAICMVDYYTACKFCEWKGGEL
VPYDEVPALGALYKLCRDNRYTCWTGGRDDSTDLCPLMTQEGYIVQQGCEQSVRWVCRTKGPGCSSLPPSPAPPSPP
TPPSPAPPSPAPPSPQPPSPLPPSPQPPSPQPPRRQPPSPAPPSPLPPSPAPPSPLPPSPAPPSRQPPSPAPPSPLP
PSPAPPSPLPPSPAPPSRQPPSPAPPSPLPPSPAPPISCEANCDVRYPNWYVWVCGKDGQAYRNWCYAACHGCYLYT
PCSAPGDSNEPVIGDNVLSLTDGGYLYRMWTVDAKQRRTYAGADAFCTGLGSGWGLVPYTDLAGYGAVRRLCANNAF
TCWLKRGERDDNYPLMAADGTLQMQGPNQEVRFVCRQQQ

>g17726.t1|PACid:27571569
MTSPPQSDQGRGLRRSLRVQGRATAPAAAGPSAAERVQAHGPARRVAAEAPEAAAAHAAAAAAPAPAAILAPLAAAG
AAYGVAAAAAPVAAAAPAFAAPVQPPAAAGAAYGVAAAAAPVAAAAPAFAAPVQPPAAAGAAYGVAAAAAPVAAAAP
AFAAPAQPHAAAGAAYGAAAATAPGAAAAPAFAAPAQPPAAAGAAYGAAAAAAPVAAAAPAFAAPAQPPAAAGAAYG
AAAAAAAGPSATAAGLPPRHPGRAQSVSNFDQALRRAFGAPAAPRSVRATPAAGVGLQGAFLALGVPDVLRRRGRTW
ASDATGSSASSYRPSDSASETSSTADTASGRRRAAARELTELSPAGANNGRRVCNNPAYEEEEELQAALARSRRDTG
HPRPPPPAGRAAALLTETAVTVHEMLGGGPTGGLALGGLAPAASPPRLDRARRRHLLNLAASSRPSASAASSSSGRS
GSSRRTTSTSARSGSESMHSARSQQPSASQHIHLHVHPGPSPAVAAAPPAADAGADFFPHLGGLRARYGARLAARLR
RHEAAVAAAAAVTPAAAAPAAAAPAAAAPAAAAPVAATPPAAAAPAAAAPPDDMVQLARVRAAALAHLQRLHAALGP
PAPAAAAAPAAAAAAAAADAPAPPAAAAGPGPDAAAPQPPVSVVNMASSSSSSSAPASAGGCPLWPMPLPAWQPHLL
AQLDHAAAAAAPPAAAAGAPPAALAGGSGGVARMGMAAAHLAAAAPANGAPLSSAGAAWPSAAAMLRPARPIPSWSQ
TGRQQYDGERAGRARFMVDAHGYTEEQAACADINSPYAQDPRLIARALINGASVPESRAWVWNNEELDGGQRTLERH
RGRWVAVGGSLVDWRGQAFIDADGTVRVPHVAHFWLAEFLAAMHTPASFGPSPLFGFTLGATEAATAAAQRFLSLHR
ADTALCHKDMLGKVFSEGVMRTAVTQPWRDLFIMNLTREQKALPEGQVLSVTLVANCLAEAQAAFRGIAAPTPAAAV
HVTAASSAAASPAPAAAPAAPAAPAAPAAPPAPAAAAPAAAAHAHQVNQLPVVSQADLMRRENLVPDTTVNQRKPGD
AQWCLYHQTSHHAADQCRAYSALVSSNRAREAARRIATGEGARAFHKAPAPAPAQAHAHVTAALPAETAQPASAAAL
CPPHRAARRELADDLSVATVRSLYDTGGHSERPPLPSATAAVSHSQVAGASGMELGDCWDAEFGPAHIFHLAARPPS
SGPMQSALPLSFQPLPAAAARRCAPAPADGAATPTASSSTTTAACPSATTGSDAAASTSTRPAAAIPAATAASSDGA
PSGADNRVRAHLNDAKFAALHAVLRDQGLQITRLEGDVRRYARLLRRLDAQAAAGCTHHSAMKLGRGVGPLLLVDGL
SSQGTPYAAALPDPASDVTLTVTEAASSRGLPLQYDTGIYVSSVGGVTSELAQIRGGLPLTVLAGTEQQHTTRVVCF
VVPGPQVFDILLGTNWLIDAYRSVWDAGRNMLGLRLPHQPDASPPAVELPLRQRNRAAGACHAVSSGRAPDTSTSRT
VIAAALACVAAPTARSHTDAVGVPPLTGTGAGTGGCTSGGRNGPESDEESSDYESGEESSDYESGEESSGYESGEES
SGYESGEESSGHESDEESSDDDSGGGGSPSASAGSAPIAPGPPTARPTDVLCERCDSWDHHARSCPWRCDQLPCIRS
FNKAQRRCAEAAVACGPADVAAERARDPARSPCRAFPTASPSPPPPPPGSAASSPPPPPQPPSSGLPPPLQLLAAAA
PTPAYAWAPPSPGSAASSPPPPPQPPGSVWPPPLQLLAAAAPSPAFAWAPPSPGSAAPSPPPPPQHPGSASPPPLQP
PGSVLPPPLQLLAAAAPSPADAWAPPSPGSAASSPPRPPQPPGSVLPPPLQLLAAAAPSPAYAWAPPSPGSAASSPP
PPPQSPGSASPPPLQLLAAAAPSPAHATAPPSPGSAASSPPPPPQSPGSVSPPPLQLVATAAPSPAHATAPPSPGSA
ASSQPRPPQPPGSAPRPPAYTSALPLPGFADLLPPPRRSPGSPPPLLAPSPQRPGSAADPWRSKLPSETWWQFLGRM
RAAGIHLTAMPESEWAGQGRPPPEGAVAAATR

>Cre17.g713150.t2.1|PACid:27571581
MSDASALRTLCIGYGSTPAGVLCVFAQAPCSNLTSARTIEALTKRCPTDLSSFGTILAKIPKYAQWLLDCGTGACDN
AQRTLLMPTDMALDTYLKAKGTTLTKMLAKTGGGRKALQAVLRCHVLQPSIALTSVNITAAAAPLSYKSLQGPSLTF
AESSSGGVTVECGANSVAEVVHADITTGKSVVHLIDTVLQCQAAPSPPPPPSPSPPSLPTPNSSPSPPSPETPPPPS
SPPPSPPPSSPPPPEPSLPDPPSLEPPSLNPPSPDPPSLPPTPPSAPLTPPSPPSPPLPPSPAPSPPDPPSPPAPPS
PPLQPSPPNPPPPPPWYSLGGLDSYPFCAGEVCSRPQLCTAERKCMCGTGPGCGGNTACWATPDMAGWPTPPAASCV
VQERICSTWDGNPRLCLPDFFCYKDIYCMSRGQLRIMLESWTSVGDLDMWVTNPLNQTVWWNSDGWNADETRDPKWL
PYGFEDNGYAVLDTQSDQYGPENVYYIDKNWQDGTPAAPLPSGNFYVCARVYIQDLDPSVWLKFPVIVTTIAESAKY
GINMRTVVTLRDANFEPSTPTLESDCTASSPSLVMTIPIP

>Cre17.g706050.t3.1|PACid:27571609

MPCHATLAPHSLAASVSATATADHVRLAGGSSAREGRLELFNGTVWGTVCNQGFSDADAVVACRELGYVAGAAAGGG
VFGDSASTVGPILLRNANCNPSQHAQLLDCPAEYGVDCCCSHDEDTGIRCYGGVPSSRSPPPAASGPNANSTSGAGP
ASAAAPRLLQVRLAGNGSSLQEGRVELFDGATWGSICDDGMYYGYADLYDADASVLCRELGFSYGLMLNTYAQEFPW
TYDQDVPPFVTGMRSTAYFQQGADTPLQDCFRPSWRRSVYSGCGPTDVVSIFCTSDPLELSAHRASLSAGNDAASLL
PPPPPPSPPPPSFTPGPCEVPFDEYCIHTGALLLNKTVDCDGDGLADVICLNTEPYFSYPINIFWVLRSSDGCAYNS
PPEWSGTYGAVVDPALCPAALGAGDPLKRLEFNLGRDLPQGGIEGQVRLVGAGASSSAGALQLYHAGTWGLVKWEPR
GRDPYSRFIAAAACRQLGFEAGVLKLQAGTVYGSVASPIWALELGCDYYHSRLADCDVFANKGAVALDASLAVDFVG
VECYAAPPPPAHGGVAMETEGALRLVGRPEDVRSGRGTIQLYHAVEWSTIFDWDWNRDAAHGVCVALGYASGNAVLN
SGPTYGILPGPILPEFFRYPLVCNSWRRNFSACLDEYLSLLDAPPFDLYPDNQYSNVAGVECYDDTPGREGDIRLRG
VSSSGSASAGTIEVFHDGTWGTIHQANWDWRGAAVACRQLGFVTGRPVYNSAYFASSLGPVWTSNVRCSDDAVAYGG
RLLDDCSHDDMYDSYYSGRHDQDAGVECQGGPVPEEGALRLVNSPLPNAGQVLIFSGGVWRSVCALAFDILDATVVC
RQLGYNGSAHLVYPSVDTQPPDSMSSTQQTQAVWSKCGSCLGNETRLVDCATECVADSSWWCADAVAAVHCTEGTAP
VNGGLRLVGGGQQGALAAGSGGNSTTTAWGQLQIAVHGVWGVFCDWSWNGYTSWTLAHVACRQLGYDSGRIASSGVY
GVGHRMPQFKSSLQCYGNETRLVDCAQDADAMLGAPWSSIQPYLTNGEYDPTQQWNGNPCGTVGVICSRAGGPKPSQ
PWAVRLVGGPAAGPHMGRLEMWWAAVSTWTTTCEQVGSWRTAAVICRQLGFTGGRFVSAAVFGPGTGGAVTGAWPYN
CRGNETGLAQCDMPSSPNECYGGHDADVGVVCEPAPAVAQQPEGQQQHGSSSPPQHQALGILQQQKEPAAEGTVRL
VDGRGPWEGRLEVYTAGLWGTLSSNLLISYEDTFAKTVCRQLGFSGGVFQRPDYAFNDASSCYFRCGSDFSTPAVWG
APLNCSDDDARLADCAGFESLWDALVPDHGYDVGVRCDPPSTSLHVELELPSAVLQEATQPDSWTANAYCADDSLAS
GAAFRFVDSDRYYTGAYAWGSTGIRFSCYQDGVQLSTLEPPANGRFTSDVTVWNASVECPQQLINGSGGGRMQPYIV
GARLRVSGSFLNGSYGLTAGSDIPAGGVGSPPVPNVLGITGLELRCSNGATASVAGLGVGAWGNWTLCPAGTAVCGA
SQQLGYQDCQQQQEDGTVCLGLVGVRLVCCPVPKGYISETGTRVVRLAGGAGPPGVGRVEVWSEEDKDVEGGGGWRV
LTASGWSAAAAHVVCRQLGWATGTRKALAQALFGAGVLPQEPRIYNCTGSEAALHLCNVTAVDLSQLGSVGVAGVEC
TNEPPPTWGAARLAPDGDAFAAGNTVFGRLQAFWSYDWGTVAGVTYNNLVGATTAVWTNKEATTACRSLGFKSGTAL
YARDLLMGPGAAGLLAGDPIAASGLDCLGTEESLLECGVHDGGLPFEDGMGKVQSQSPWWYYQYYSSNVAVACSSDE
PLPSGSLRLVAGASPSEGRLDSMFNSTWGPVFTYGMFPESFDDMDAQVACAQLGYASGRAVVNGWQRSQQAAWLSRL
VCQGDERDLMSCLPGPNNNMVVAQSSSGDNAQAGVICSNEPRPATGQVRLAGQDPGLPNRGRLEIGAHGQWGTATFK
GGWTWQDSHVACRSLKFKSAEAMAAGTGWDLALLPVWISNISCSGHEAHLVACAGWPLQAAAITPPAALDDNGVIKG
VGQPVADYYRVILTCSDTELPPEGSIRLTGGAVPHIGRLEVFWRGAWSTLSLQSWRGEVFGQSEAAVVCRELGFEPL
HGNTSEISTGNFGPDNMFTPVLSYPINCTGSEPTLWNCARQQQQDEPEVIFNGNPYDNSGARPLYGHDSDVVVRCAP
RAGPDGPDSQLPPRPPSPPPQPPRPPKPSPPPPPPPPSPPPPPDPPSPPSPEPSPPEPPPSPDPPSPGPWPPSPEPS
PPEPPSPEPSPPAPPPPGPEFALFTVVPFTLPLEGGVLTLSGRFDLGGQPVGQQPQNISFGCRFDQFKEGLLSGAGD
QSDSVTSTVQAIRVSRAALRCTAPSATTPSAVLAVTVTRTPARNAGVLLNDASSESLTYRANITYYGNCQNNCSGHG
TCRFGICVCNPGWTGGACADLSPELAVASLDGGPAAGKSVTLVEGSSWQAQTRLLHDVPVNWVLTTSPAGGARGLQI
DDVSGSLVWPVVSSSSPDVLDMNKTAVDVTIAAIAVRDGRTAMYRFSVIVVPLYGVTELSQQGTLNASGGSGASVKA
GGVLALAGRVSYYPRSLAALNQSGNYSTGLPSLKGVPVVLRIMPAAAASSGAANVTTGGAGGSAVELSVLTTTADGR
FVVQWPVPQSARGALDAVAAHPAALRVLNWTDPQLLDWIAQPPATAGGEPAHNASSSNSTASSFMPPQGLGVPLPLA
GQVQRLRTAVSFLYSVVDPRAYSTTGGLDPRFVSPTLNLDPAQSALLDPYVVLVGTVEDLAAVTFSTKVELVSFTGM
PPTLQVSAKLTQEPAPACGGIQSTAGSGSGDNSSTPTAALPPAVSDLIRSVCLTSSSGGAAGGGGGGAAGPARRRLL
QEGSLAPQPGLLAVAHVALQTQTMAGEPGGGTQAAEFEVTVSVRGRGSGDAAETTQRLRVLVEAAEVKLVSDPASGS
LAAVLPPGGSTLLSMAIRNEGNTPSGALSLPAFPPDMSWIEPVSPLPIPSIPPGGAATLQLMLRVPEGATVGQTFTA
QGMLAAASGDVAGAVPLSVELAVATEPTGSLEVSVVDEYTTYSPTQPRVAGARLVLKGPDGSINAQGLTNSSGLYVF
SNITAGYTYSIDAFSSNHSATSRTVAITGGRRQLRIFLARTAVRVTFSVVPTTFEETVEVTVNVEYITFVPMPVIRM
EPALIYFDELNDYSLGQPLTMRVINTGLIAAQNVRVRIPQPGPMYMFDFAGARWLQKENVTANNETASVAFYPPGPA
AELAWSSAVQADENSDGSTLSSSTPRADLYGEGREFVLLIGRMPAMSELALDLSVSRDTELYITSLGAASANRRRHL
LGSETESEGPVAAHDRLPRRRRLQAGCNTYRFGIQFSDACDPSKSTVFGDGPSIIPRHPQCSTGGNLELVEGTGGTG
GGPLNWYVATGSGVGVAEIPTPDICNKCVQGMFSGATCIIKNVAPTEEIKLLGDVLGQIFGVVSKYSVGVVAGRRRR
RLSSHGVHPAAAAGRASATYPYRDMSGPISSWDNDALDISFKSGQELQPRSRGLMGLFKADSIVGAVTGISGPGDLV
VDLIKTVTIKKLKLPGCFVDMALKCAGMWGQISGAVNSATGPIAAGISAAGNAMGFPGSASPGSWLGAATQGAAAGA
SSSGSNRRHLLTMTDDAARGVWGAHHHRYVGDDTLTPQLTSAAVLPRRRQDLGVSAKVPDPDPIFYSPAGKAIVRWS
AAVLMFNTAAMEMWSPEYYEEWISSNYPKDLEAEWRAAWEAATSTASRAGPIVDWDAEAPVLLGEHYDVLVSRAARE
MLIARWNATFDPNGVDPMSYINQTTGLGPMSGILPDGSPRPIDLARVTLAQLTYLNETVAALELGYKSCFDALNDAI
TALVALNVAAQSGGGGGSTCVRVVIQLSQSLVLTRQAFEATLALDNDAEAGGDPITDISVELEVWEQTSGQPASNGT
FAVGEPLLEGAFEGAGPWSLAAGGSGTLRWLMVPRVAAALSNDTWYVIGGTLRYTPAPGLPQEVVPLEPANIRVSPE
GRLDVRYYIEKWVQGDNPFTPEVEPSPPAAFVTLLTNVGGGPARALEMQSLQPKIVENEKGLLVAFNITAVAVNGRP
QPRALQAAVGDLSPNSSALVVWSLRCSLQGTFTGLNASFTTRNPLNDPTLSSVSRVALYDMLRLTYITGAGFDDGLP
DMLVTQLDDDQRKAGLAPAPAPASSNSSNAGSNTTSGAGGNAVGNATDTANNGSSPATSNRHRLRRRAAAESIFNIT
TALPLPTQLHSSKDGAVLPLAVVPAAAVTATSVSAALTGADAAAAAARVDDGRAVVVVEIRVDGSQLQAPKLPPASA

TGGAVLTPATRATNWQYLRVRTPTELMPSSGWSIVRADVSAVAAPSSGGFSGSSSGSRTTQVKLPYNAWSSYRAYSD
ASKADDQLHVLHDGFVLDVTNTVRLVFVRGEVNVSALPPQQPLAPPPQHPSSMPSPVPPAQPSRDIGGDGSGGSGAT
KPPSRPPAAPPPPPTPPLPPRPLAPPAPPSPAPPSPKPPVPPSPAPPSPTPPKPPSPAPPSPRPPSPAPPSPKPPSP
RPPSPPPPPSPPPPSPPPPSPPPPSPPPPNPPPPSPPPPSPPPPSPPPPHPPPPSPPPPNPPPPNPPPPSPAPPSPV
PPSPPPPSPPPPAPPPPSPPPPAPPAPPSPPAPPVRPPSLPPPPPSQRSLLLRSNSGGSSAPSSAAGSRSATANGHR
SQPPPPRYAKAVSGTLRLMTKSKKRRAQQQQEQQERQ

>Cre17.g706050.t1.3|PACid:27571610
MPCHATLAPHSLAASVSATATADHVRLAGGSSAREGRLELFNGTVWGTVCNQGFSDADAVVACRELGYVAGAAAGGG
VFGDSASTVGPILLRNANCNPSQHAQLLDCPAEYGVDCCCSHDEDTGIRCYGGVPSSRSPPPAASGPNANSTSGAGP
ASAAAPRLLQVRLAGNGSSLQEGRVELFDGATWGSICDDGMYYGYADLYDADASVLCRELGFSYGLMLNTYAQEFPW
TYDQDVPPFVTGMRSTAYFQQGADTPLQDCFRPSWRRSVYSGCGPTDVVSIFCTSDPLELSAHRASLSAGNDAASLL
PPPPPPSPPPPSFTPGPCEVPFDEYCIHTGALLLNKTVDCDGDGLADVICLNTEPYFSYPINIFWVLRSSDGCAYNS
PPEWSGTYGAVVDPALCPAALGAGDPLKRLEFNLGRDLPQGGIEGQVRLVGAGASSSAGALQLYHAGTWGLVKWEPR
GRDPYSRFIAAAACRQLGFEAGVLKLQAGTVYGSVASPIWALELGCDYYHSRLADCDVFANKGAVALDASLAVDFVG
VECYAAPPPPAHGGVAMETEGALRLVGRPEDVRSGRGTIQLYHAVEWSTIFDWDWNRDAAHGVCVALGYASGNAVLN
SGPTYGILPGPILPEFFRYPLVCNSWRRNFSACLDEYLSLLDAPPFDLYPDNQYSNVAGVECYDDTPGREGDIRLRG
VSSSGSASAGTIEVFHDGTWGTIHQANWDWRGAAVACRQLGFVTGRPVYNSAYFASSLGPVWTSNVRCSDDAVAYGG
RLLDDCSHDDMYDSYYSGRHDQDAGVECQGGPVPEEGALRLVNSPLPNAGQVLIFSGGVWRSVCALAFDILDATVVC
RQLGYNGSAHLVYPSVDTQPPDSMSSTQQAVWSKCGSCLGNETRLVDCATECVADSSWWCADAVAAVHCTEGTAPVN
GGLRLVGGGQQGALAAGSGGNSTTTAWGQLQIAVHGVWGVFCDWSWNGYTSWTLAHVACRQLGYDSGRIASSGVYGV
GHRMPQFKSSLQCYGNETRLVDCAQDADAMLGAPWSSIQPYLTNGEYDPTQQWNGNPCGTVGVICSRAGGPKPSQPW
AVRLVGGPAAGPHMGRLEMWWAAVSTWTTTCEQVGSWRTAAVICRQLGFTGGRFVSAAVFGPGTGGAVTGAWPYNCR
GNETGLAQCDMPSSPNECYGGHDADVGVVCEPAPAVAQQPEGQQQHGSSSPPQHQALGILQQQQKEPAAEGTVRLVD
GRGPWEGRLEVYTAGLWGTLSSNLLISYEDTFAKTVCRQLGFSGGVFQRPDYAFNDASSCYFRCGSDFSTPAVWGAP
LNCSDDDARLADCAGFESLWDALVPDHGYDVGVRCDPPSTSLHVELELPSAVLQEATQPDSWTANAYCADDSLASGA
AFRFVDSDRYYTGAYAWGSTGIRFSCYQDGVQLSTLEPPANGRFTSDVTVWNASVECPQQLINGSGGGRMQPYIVGA
RLRVSGSFLNGSYGLTAGSDIPAGGVGSPPVPNVLGITGLELRCSNGATASVAGLGVGAWGNWTLCPAGTAVCGASQ
QLGYQDCQQQQEDGTVCLGLVGVRLVCCPVPKGYISETGTRVVRLAGGAGPPGVGRVEVWSEEDKDVEGGGGWRVLT
ASGWSAAAAHVVCRQLGWATGTRKALAQALFGAGVLPQEPRIYNCTGSEAALHLCNVTAVDLSQLGSVGVAGVECTN
EPPPTWGAARLAPDGDAFAAGNTVFGRLQAFWSYDWGTVAGVTYNNLVGATTAVWTNKEATTACRSLGFKSGTALYA
RDLLMGPGAAGLLAGDPIAASGLDCLGTEESLLECGVHDGGLPFEDGMGKVQSQSPWWYYQYYSSNVAVACSSDEPL
PSGSLRLVAGASPSEGRLDSMFNSTWGPVFTYGMFPESFDDMDAQVACAQLGYASGRAVVNGWQRSQQAAWLSRLVC
QGDERDLMSCLPGPNNNMVVAQSSSGDNAQAGVICSNEPRPATGQVRLAGQDPGLPNRGRLEIGAHGQWGTATFKGG
WTWQDSHVACRSLKFKSAEAMAAGTGWDLALLPVWISNISCSGHEAHLVACAGWPLQAAAITPPAALDDNGVIKGVG
QPVADYYRVILTCSDTELPPEGSIRLTGGAVPHIGRLEVFWRGAWSTLSLQSWRGEVFGQSEAAVVCRELGFEPLHG
NTSEISTGNFGPDNMFTPVLSYPINCTGSEPTLWNCARQQQQDEPEVIFNGNPYDNSGARPLYGHDSDVVVRCAPRA
GPDGPDSQLPPRPPSPPPQPPRPPKPSPPPPPPPPSPPPPPDPPSPPSPEPSPPEPPPSPDPPSPGPWPPSPEPSPP
EPPSPEPSPPAPPPPGPEFALFTVVPFTLPLEGGVLTLSGRFDLGGQPVGQQPQNISFGCRFDQFKEGLLSGAGDQS
DSVTSTVQAIRVSRAALRCTAPSATTPSAVLAVTVTRTPARNAGVLLNDASSESLTYRANITYYGNCQNNCSGHGTC
RFGICVCNPGWTGGACADLSPELAVASLDGGPAAGKSVTLVEGSSWQAQTRLLHDVPVNWVLTTSPAGGARGLQIDD
VSGSLVWPVVSSSSPDVLDMNKTAVDVTIAAIAVRDGRTAMYRFSVIVVPLYGVTELSQQGTLNASGGSGASVKAGG
VLALAGRVSYYPRSLAALNQSGNYSTGLPSLKGVPVVLRIMPAAAASSGAANVTTGGAGGSAVELSVLTTTADGRFV
VQWPVPQSARGALDAVAAHPAALRVLNWTDPQLLDWIAQPPATAGGEPAHNASSSNSTASSFMPPQGLGVPLPLAGQ
VQRLRTAVSFLYSVVDPRAYSTTGGLDPRFVSPTLNLDPAQSALLDPYVVLVGTVEDLAAVTFSTKVELVSFTGMPP
TLQVSAKLTQEPAPACGGIQSTAGSGSGDNSSTPTAALPPAVSDLIRSVCLTSSSGGAAGGGGGGAAGPARRRLLQE
GSLAPQPGLLAVAHVALQTQTMAGEPGGGTQAAEFEVTVSVRGRGSGDAAETTQRLRVLVEAAEVKLVSDPASGSLA
AVLPPGGSTLLSMAIRNEGNTPSGALSLPAFPPDMSWIEPVSPLPIPSIPPGGAATLQLMLRVPEGATVGQTFTAQG
MLAAASGDVAGAVPLSVELAVATEPTGSLEVSVVDEYTTYSPTQPRVAGARLVLKGPDGSINAQGLTNSSGLYVFSN
ITAGYTYSIDAFSSNHSATSRTVAITGGRRQLRIFLARTAVRVTFSVVPTTFEETVEVTVNVEYITFVPMPVIRMEP
ALIYFDELNDYSLGQPLTMRVINTGLIAAQNVRVRIPQPGPMYMFDFAGARWLQKENVTANNETASVAFYPPGPAAE
LAWSSAVQADENSDGSTLSSSTPRADLYGEGREFVLLIGRMPAMSELALDLSVSRDTELYITSLGAASANRRRHLLG
SETESEGPVAAHDRLPRRRRLQAGCNTYRFGIQFSDACDPSKSTVFGDGPSIIPRHPQCSTGGNLELVEGTGGTGGG
PLNWYVATGSGVGVAEIPTPDICNKCVQGMFSGATCIIKNVAPTEEIKLLGDVLGQIFGVVSKYSVGVVAGRRRRRL
SSHGVHPAAAAGRASATYPYRDMSGPISSWDNDALDISFKSGQELQPRSRGLMGLFKADSIVGAVTGISGPGDLVVD
LIKTVTIKKLKLPGCFVDMALKCAGMWGQISGAVNSATGPIAAGISAAGNAMGFPGSASPGSWLGAATQGAAAGASS
SGSNRRHLLTMTDDAARGVWGAHHHRYVGDDTLTPQLTSAAVLPRRRQDLGVSAKVPDPDPIFYSPAGKAIVRWSAA

VLMFNTAAMEMWSPEYYEEWISSNYPKDLEAEWRAAWEAATSTASRAGPIVDWDAEAPVLLGEHYDVLVSRAAREML
IARWNATFDPNGVDPMSYINQTTGLGPMSGILPDGSPRPIDLARVTLAQLTYLNETVAALELGYKSCFDALNDAITA
LVALNVAAQSGGGGGSTCVRVVIQLSQSLVLTRQAFEATLALDNDAEAGGDPITDISVELEVWEQTSGQPASNGTFA
VGEPLLEGAFEGAGPWSLAAGGSGTLRWLMVPRVAAALSNDTWYVIGGTLRYTPAPGLPQEVVPLEPANIRVSPEGR
LDVRYYIEKWVQGDNPFTPEVEPSPPAAFVTLLTNVGGGPARALEMQSLQPKIVENEKGLLVAFNITAVAVNGRPQP
RALQAAVGDLSPNSSALVVWSLRCSLQGTFTGLNASFTTRNPLNDPTLSSVSRVALYDMLRLTYITGAGFDDGLPDM
LVTQLDDDQRKAGLAPAPAPASSNSSNAGSNTTSGAGGNAVGNATDTANNGSSPATSNRHRLRRRAAAESIFNITTA
LPLPTQLHSSKDGAVLPLAVVPAAAVTATSVSAALTGADAAAAAARVDDGRAVVVVEIRVDGSQLQAPKLPPASATG
GAVLTPATRATNWQYLRVRTPTELMPSSGWSIVRADVSAVAAPSSGGFSGSSSGSRTTQVKLPYNAWSSYRAYSDAS
KADDQLHVLHDGFVLDVTNTVRLVFVRGEVNVSALPPQQPLAPPPQHPSSMPSPVPPAQPSRDIGGDGSGGSGATKP
PSRPPAAPPPPPTPPLPPRPLAPPAPPSPAPPSPKPPVPPSPAPPSPTPPKPPSPAPPSPRPPSPAPPSPKPPSPRP
PSPPPPPSPPPPSPPPPSPPPPSPPPPNPPPPSPPPPSPPPPSPPPPHPPPPSPPPPNPPPPNPPPPSPAPPSPVPP
SPPPPSPPPPAPPPPSPPPPAPPAPPSPPAPPVRPPSLPPPPPSQRSLLLRSNSGGSSAPSSAAGSRSATANGHRSQ
PPPPRYAKAVSGTLRLMTKSKKRRRAQQQEQQERQ

>Cre17.g706050.t2.1|PACid:27571611
MPCHATLAPHSLAASVSATATADHVRLAGGSSAREGRLELFNGTVWGTVCNQGFSDADAVVACRELGYVAGAAAGGG
VFGDSASTVGPILLRNANCNPSQHAQLLDCPAEYGVDCCCSHDEDTGIRCYGGVPSSRSPPPAASGPNANSTSGGPA
SAAAPRLLQVRLAGNGSSLQEGRVELFDGATWGSICDDGMYYGYADLYDADASVLCRELGFSYGLMLNTYAQEFPWT
YDQDVPPFVTGMRSTAYFQQGADTPLQDCFRPSWRRSVYSGCGPTDVVSIFCTSDPLELSAHRASLSAGNDAASLLP
PPPPPSPPPPSFTPGPCEVPFDEYCIHTGALLLNKTVDCDGDGLADVICLNTEPYFSYPINIFWVLRSSDGCAYNSP
PEWSGTYGAVVDPALCPAALGAGDPLKRLEFNLGRDLPQGGIEGQVRLVGAGASSSAGALQLYHAGTWGLVKWEPRG
RDPYSRFIAAAACRQLGFEAGVLKLQAGTVYGSVASPIWALELGCDYYHSRLADCDVFANKGAVALDASLAVDFVGV
ECYAAPPPPAHGGVAMETEGALRLVGRPEDVRSGRGTIQLYHAVEWSTIFDWDWNRDAAHGVCVALGYASGNAVLNS
GPTYGILPGPILPEFFRYPLVCNSWRRNFSACLDEYLSLLDAPPFDLYPDNQYSNVAGVECYDDTPGREGDIRLRGV
SSSGSASAGTIEVFHDGTWGTIHQANWDWRGAAVACRQLGFVTGRPVYNSAYFASSLGPVWTSNVRCSDDAVAYGGR
LLDDCSHDDMYDSYYSGRHDQDAGVECQGGPVPEEGALRLVNSPLPNAGQVLIFSGGVWRSVCALAFDILDATVVCR
QLGYNGSAHLVYPSVDTQPPDSMSSTQQAVWSKCGSCLGNETRLVDCATECVADSSWWCADAVAAVHCTEGTAPVNG
GLRLVGGGQQGALAAGSGGNSTTTAWGQLQIAVHGVWGVFCDWSWNGYTSWTLAHVACRQLGYDSGRIASSGVYGVG
HRMPQFKSSLQCYGNETRLVDCAQDADAMLGAPWSSIQPYLTNGEYDPTQQWNGNPCGTVGVICSRAGGPKPSQPWA
VRLVGGPAAGPHMGRLEMWWAAVSTWTTTCEQVGSWRTAAVICRQLGFTGGRFVSAAVFGPGTGGAVTGAWPYNCRG
NETGLAQCDMPSSPNECYGGHDADVGVVCEPAPAVAQQPEGQQQHGSSSPPQHQALGILQQQQKEPAAEGTVRLVDG
RGPWEGRLEVYTAGLWGTLSSNLLISYEDTFAKTVCRQLGFSGGVFQRPDYAFNDASSCYFRCGSDFSTPAVWGAPL
NCSDDDARLADCAGFESLWDALVPDHGYDVGVRCDPPSTSLHVELELPSAVLQEATQPDSWTANAYCADDSLASGAA
FRFVDSDRYYTGAYAWGSTGIRFSCYQDGVQLSTLEPPANGRFTSDVTVWNASVECPQQLINGSGGGRMQPYIVGAR
LRVSGSFLNGSYGLTAGSDIPAGGVGSPPVPNVLGITGLELRCSNGATASVAGLGVGAWGNWTLCPAGTAVCGASQQ
LGYQDCQQQQEDGTVCLGLVGVRLVCCPVPKGYISETGTRVVRLAGGAGPPGVGRVEVWSEEDKDVEGGGGWRVLTA
SGWSAAAAHVVCRQLGWATGTRKALAQALFGAGVLPQEPRIYNCTGSEAALHLCNVTAVDLSQLGSVGVAGVECTNE
PPPTWGAARLAPDGDAFAAGNTVFGRLQAFWSYDWGTVAGVTYNNLVGATTAVWTNKEATTACRSLGFKSGTALYAR
DLLMGPGAAGLLAGDPIAASGLDCLGTEESLLECGVHDGGLPFEDGMGKVQSQSPWWYYQYYSSNVAVACSSDEPLP
SGSLRLVAGASPSEGRLDSMFNSTWGPVFTYGMFPESFDDMDAQVACAQLGYASGRAVVNGWQRSQQAAWLSRLVCQ
GDERDLMSCLPGPNNNMVVAQSSSGDNAQAGVICSNEPRPATGQVRLAGQDPGLPNRGRLEIGAHGQWGTATFKGGW
TWQDSHVACRSLKFKSAEAMAAGTGWDLALLPVWISNISCSGHEAHLVACAGWPLQAAAITPPAALDDNGVIKGVGQ
PVADYYRVILTCSDTELPPEGSIRLTGGAVPHIGRLEVFWRGAWSTLSLQSWRGEVFGQSEAAVVCRELGFEPLHGN
TSEISTGNFGPDNMFTPVLSYPINCTGSEPTLWNCARQQQQDEPEVIFNGNPYDNSGARPLYGHDSDVVVRCAPRAG
PDGPDSQLPPRPPSPPPQPPRPPKPSPPPPPPPPSPPPPPDPPSPPSPEPSPPEPPPSPDPPSPGPWPPSPEPSPPE
PPSPEPSPPAPPPPGPEFALFTVVPFTLPLEGGVLTLSGRFDLGGQPVGQQPQNISFGCRFDQFKEGLLSGAGDQSD
SVTSTVQAIRVSRAALRCTAPSATTPSAVLAVTVTRTPARNAGVLLNDASSESLTYRANITYYGNCQNNCSGHGTCR
FGICVCNPGWTGGACADLSPELAVASLDGGPAAGKSVTLVEGSSWQAQTRLLHDVPVNWVLTTSPAGGARGLQIDDV
SGSLVWPVVSSSSPDVLDMNKTAVDVTIAAIAVRDGRTAMYRFSVIVVPLYGVTELSQQGTLNASGGSGASVKAGGV
LALAGRVSYYPRSLAALNQSGNYSTGLPSLKGVPVVLRIMPAAAASSGAANVTTGGAGGSAVELSVLTTTADGRFVV
QWPVPQSARGALDAVAAHPAALRVLNWTDPQLLDWIAQPPATAGGEPAHNASSSNSTASSFMPPQGLGVPLPLAGQV
QRLRTAVSFLYSVVDPRAYSTTGGLDPRFVSPTLNLDPAQSALLDPYVVLVGTVEDLAAVTFSTKVELVSFTGMPPT
LQVSAKLTQEPAPACGGIQSTAGSGSGDNSSTPTAALPPAVSDLIRSVCLTSSSGGAAGGGGGGAAGPARRRLLQEG
SLAPQPGLLAVAHVALQTQTMAGEPGGGTQAAEFEVTVSVRGRGSGDAAETTQRLRVLVEAAEVKLVSDPASGSLAA
VLPPGGSTLLSMAIRNEGNTPSGALSLPAFPPDMSWIEPVSPLPIPSIPPGGAATLQLMLRVPEGATVGQTFTAQGM
LAAASGDVAGAVPLSVELAVATEPTGSLEVSVVDEYTTYSPTQPRVAGARLVLKGPDGSINAQGLTNSSGLYVFSNI

TAGYTYSIDAFSSNHSATSRTVAITGGRRQLRIFLARTAVRVTFSVVPTTFEETVEVTVNVEYITFVPMPVIRMEPA
LIYFDELNDYSLGQPLTMRVINTGLIAAQNVRVRIPQPGPMYMFDFAGARWLQKENVTANNETASVAFYPPGPAAEL
AWSSAVQADENSDGSTLSSSTPRADLYGEGREFVLLIGRMPAMSELALDLSVSRDTELYITSLGAASANRRRHLLGS
ETESEGPVAAHDRLPRRRRLQAGCNTYRFGIQFSDACDPSKSTVFGDGPSIIPRHPQCSTGGNLELVEGTGGTGGGP
LNWYVATGSGVGVAEIPTPDICNKCVQGMFSGATCIIKNVAPTEEIKLLGDVLGQIFGVVSKYSVGVVAGRRRRRLS
SHGVHPAAAAGRASATYPYRDMSGPISSWDNDALDISFKSGQELQPRSRGLMGLFKADSIVGAVTGISGPGDLVVDL
IKTVTIKKLKLPGCFVDMALKCAGMWGQISGAVNSATGPIAAGISAAGNAMGFPGSASPGSWLGAATQGAAAGASSS
GSNRRHLLTMTDDAARGVWGAHHHRYVGDDTLTPQLTSAAVLPRRRQDLGVSAKVPDPDPIFYSPAGKAIVRWSAAV
LMFNTAAMEMWSPEYYEEWISSNYPKDLEAEWRAAWEAATSTASRAGPIVDWDAEAPVLLGEHYDVLVSRAAREMLI
ARWNATFDPNGVDPMSYINQTTGLGPMSGILPDGSPRPIDLARVTLAQLTYLNETVAALELGYKSCFDALNDAITAL
VALNVAAQSGGGGGSTCVRVVIQLSQSLVLTRQAFEATLALDNDAEAGGDPITDISVELEVWEQTSGQPASNGTFAV
GEPLLEGAFEGAGPWSLAAGGSGTLRWLMVPRVAAALSNDTWYVIGGTLRYTPAPGLPQEVVPLEPANIRVSPEGRL
DVRYYIEKWVQGDNPFTPEVEPSPPAAFVTLLTNVGGGPARALEMQSLQPKIVENEKGLLVAFNITAVAVNGRPQPR
ALQAAVGDLSPNSSALVVWSLRCSLQGTFTGLNASFTTRNPLNDPTLSSVSRVALYDMLRLTYITGAGFDDGLPDML
VTQLDDDQRKAGLAPAPAPASSNSSNAGSNTTSGAGGNAVGNATDTANNGSSPATSNRHRLRRRAAAESIFNITTAL
PLPTQLHSSKDGAVLPLAVVPAAAVTATSVSAALTGADAAAAAARVDDGRAVVVVEIRVDGSQLQAPKLPPASATGG
AVLTPATRATNWQYLRVRTPTELMPSSGWSIVRADVSAVAAPSSGGFSGSSSGSRTTQVKLPYNAWSSYRAYSDASK
ADDQLHVLHDGFVLDVTNTVRLVFVRGEVNVSALPPQQPLAPPPQHPSSMPSPVPPAQPSRDIGGDGSGGSGATKPP
SRPPAAPPPPPTPPLPPRPLAPPAPPSPAPPSPKPPVPPSPAPPSPTPPKPPSPAPPSPRPPSPAPPSPKPPSPRPP
SPPPPPSPPPPSPPPPSPPPPSPPPPNPPPPSPPPPSPPPPSPPPPHPPPPSPPPPNPPPPNPPPPSPAPPSPVPPS
PPPPSPPPPAPPPPSPPPPAPPAPPSPPAPPVRPPSLPPPPPSQRSLLLRSNSGGSSAPSSAAGSRSATANGHRSQP
PPPRYAKAVSGTLRLMTKSKKRRRAQQQQEQQERQ

>Cre17.g720150.t1.3|PACid:27571619
MYNLVWLGDARSFGRCAPYVDGWLLQLRSGVTTFYLLQPLLQPGEGDTAQAVATADAVGAAAVGATAGGRGLLRLPL
DVSALEPLGLLPPSGSLVRAVWRPSLKEGTAAPAAAAAAAAAALALPVAGSASEVWEEAGQRDAAAAAAAVADPAAA
VLAAAAAEVQRTPHGALLVLLSLTPLDSGGGRPAGGVAGAGAGWVVLPQPGADAGADAEADAGADASVDAEAGAEAE
GGAAATAGLTGATAAQDSGQSVHRTQRDGQWQQLRRRLLQSFTDFPSPPPPPPRPPRPLRPPAPPSLPAGSPVPQP
SLPQPNHAPPSQPPPGPSPPPPEPSPPPPGPAPPAPKPPIPLSPPSPLPPSPRPPPPRPPLPSPPRLPPRPGPPRPS
PAPPGPPPMPPSPPQPPTPEEGAHAAVVDLYSRSARVAGGGDISTVFIIVDLCGLGGGPAITKEELEELLFEPLPNS
SSSSSSSTGSGNTVGGNRPPPPPSPSPSQSRSPSPSLEDILFEFEAGGATGTTQDPSGSGGMDIDGVSLHDTAPGLT
LESYVSNCSVGAARINRSNSRVVGPYRLPCAYTAPPGGGAAFPSTAATPPPASGTGGRGSGAGESPTAGGVIPSGDA
AAAAGAAAAGGASWSAARCSVADVFGWADAAVAAAAADGVNLSRYAHRLIVLPPNMREWAGPNCSWTGLATLGPVPA
AAVAVAINGGAAAARSAADAPPPQPPPSPPQAADAGVGYAWVSGSYARSLLSYLHELGHNLWLGHAGVGSCDDCDWS
CAMGMCCRARCFNGPHAWQLGWAVPPPSHVMSAATLPLGVRRRLALPAALTSQSSLAVVRADWMAGAAAGAAGTGSR
NATDGAGAAAGGAGPRAAAAPTLFLSYRPRTEPYDADIPQAFGGGVTVHSYNGTSQRDTASTRLLARLLEGNSWWDV
APAAAPPPPPSPASGQVLFSTTAPGAAAGPAAAGAAASVSAPSAAAVVGAGLAAAAGSSAARTATVGGVVGGGSVGS
GLLVTVLYANGTHAELELCRGCVPLPASPPPPAPGSQRPVVIGFSPPPPPPPPLPGGLAPPGAAGPPRPTPSSPALL
SPVPPLEPTAPPASPRRRRTSPPPTSSTSPLPAPQPKSRRAKPPSPPPVAAAPKRARKSPPPRPAPKPPSPPWPSPS
AGSGSGGGGSGGGGSGLSLPVERGSAAGAPRAAPPAPPLPPPRPPQPQLLASPGLQPESTSTPVAAPTAAPPAPL
AGPATSPVAQPPPPPQPRLAPTMPAPPPSPHPPAPGNQGPTRSPQLLPPPAPAPRSPEPLASWPPLLPAARPQPSPT
PTPASLLPPSSPVQAPGSGPAPASAPASGHRRALSARGHATASDSLGDSVAGATGADSGDCHGGGGGGGVDGGDASV
AGGLRVGHAEVTEQRRHAAAPSEDAA

>g17844.t1|PACid:27571621
MCGPAAPSTATTETEVFTLYLQPMFADPSSTCSTTITSSWTGLVCNGTAWYGVGCTGDGSAVTNSTGNVNSIALPNC
FPGGVGVAASLPSEVSVLTALTRLDLTGNKLSPNSGLPADYSALTGLRLVGLASMDLEAGPVPSSGFPQEWSSLVDL
QQLDLSNNRLLGGTIPASWDILASVTALNIANTAACGDIPPTLSNSTTPSTLDSYCELVNCRRYLAETLQPMIDSQV
QPGSACNLTSAWPSSGRIDSWAGITATATTVTTGTPINDVMSFVLNDCYANNSNSLNVVLPSELTCFSSLTELQFMR
NNAAGSLPDVWTQLTQLQALWLADNILSGSLPASYSALTGLTILKVHNNLFTSSLPPEWQAMSLLEEITISDNYDLN
GTVPSAWGNLTSLTSFWAWGVGAGGQLPPSFSALSLLSELTFVSDTLSGSLPSEWSTLTALTLLNIASDRATSTMTG
TIPDVWFGFGGAPAMFALQDITISNTGVSGGLPTALWLVTSLTKVDFSFNRLYGGASPTGFPMDYSMLTGLVSLNLA
DNLFLGGTVPSDWAVTPGSALTYLNLQNTGVCEPFPAGLSSALTPGTSVNSYCDDLSSAYNLFTNFKPIFDSTTPGV
GCSLASWDLGANPLCSNTWSGARCQVSPADSSGVSLGSLTSLELLNCNAGGSPVHFLPRSLTDLGGSLSRLAISNSA
VTGTLPSEMVYLGQLTDLDLSGNSFTGTLPGDWSSFVNLYYLKLNNNPQINGSIPDSWGFGLYSLIQLNLGSMGLLT
GPLPDSLAYLYSLEDLTIQSNSLSGTLPASWSSIYRLSSLVLGAGGNAGPGPSINGAIPQEWFLGAPLPYLWNLRIS
NTKVTGPLPDPGSAFTAGQLGLTSLDLSSNLLSGTLPDSYQGFSILQRIILSDNTQVSGSIPTSYSALASVGLVSLD

VTATSVCGSIPSSINGAVTPNPLPTCPSPPPAPPTPPEPPSPPPSPDPPSPEPPSPAPPSPEPPSPVPPSPPPSPEP
PSPAPPSPEPPSPAPSSPPPSPEPPSPAPPSPEPPSPAPSSPPPSPEPPSPAPPSPEPPSPAPPSPPPSPEPPSPVP
PSPEPPSPAPPSPPPSPEPPSPAPPSPEPPSPAPSSPPPSPEPPSPAPPSPEPPSPAPSSPPPSPEPPSPAPPSPEP
PSPAPPSPPPSPEPPSPAPPSPEPPSPAPPSPPPSPEPPSPAPPSPEPPSPAPSSPPPSPEPPSPAPPSPEPPSPAP
PSPPPSPEPPSPAPPSPEPPSPAPPSPPPSPEPPSPAPPSPEPPSPAPSSPPPSPEPPSPAPPSPEPPSPAPSSPPP
SPEPPSPVPPSPEPPSPAPSSPPPSPEPPSPAPPSPEPPSPAPPSPPPSPEPPSPAPPSPEPPSPAPSSPPPSPEPP
SPAPPSPEPPSPAPPSPPPSPEPPSPAPPSPEPPSPAPPSPPPSPEPPSPVPPSPEPPSPAPPSPRPSPEPPSPVPP
SPEPPSPAPPSPPPSPEPPSPAPPSPEPPSPAPSSPPPSPEPPSPAPPSPEPPSPAPPSPPPSPEPPSPAPPSPEPP
SPAPSSPPPSPEPPSPAPPSPEPPSPAPSSPPPSPEPPSPAPPSPEPPSPAPSSPPPSPEPPSPAPPSPEPPSPAPS
SPPPSPEPPSPVPPSPEPPSPAPSSPPPSPEPPSPAPPSPEPPSPAPSSPPPSPEPPSPAPPSPEPPSPAPPSPPPS
PEPPSPAPPSPEPPSPAPSSPPPSPEPPSPAPPSPEPRSPAPSSPPPSPEPPSPAPPSPEPPSPAPSSPPPSPEPPS
PAPPSPEPPSPAPSSPPPSPEPPSPAPPSPEPPSPAPSSPPPSPEPPSPAPPSPEPPSPAPSSPPPSPEPPSPAPPS
PEPPSPAPPSPPPSPEPPSPAPPSPEPPSPAPPSPPPSPEPPSPAPPSPEPPSPAPSSPPPSPEPPSPAPPSPEPPS
PAPSSPPPSPEPPSPAPPSPEPPSPAPSSPPPSPEPPSPAPPSPEPPSPAPPSPPPSPEPPSPAPPSPEPPSPAPSS
PPPSPEPPSPAPPSPDPPSPAPSSPPPSPEPPSPAPPSPEPPSPAPSSPPPSPEPPSPAPPSPEPPSPAPSSPPPSP
EPPSPAPPSPEPPSPAPSSPPPSPEPPSPAPPSPEPPSPAPSSPPPSPEPPSPAPPSPEPPSPAPPRPPPSPEPPSP
APPSPEPPSPAPSSPPPSPEPPSPAPPSPEPPSPAPPSPPPSPEPPSPAPPSPEPPSPAPSSPPPSPEPPSPAPPSP
EPPSPAPSSPPPSPEPPSPAPPSPEPPSPAPSSPPPSPEPPSPAPPSPEPPSPAPSSPPPSPEPPSPAPPSPEPPSP
APSSPPPSPEPPSPAPPSPEPPSPAPSSPPPSPEPPSPAPPSPEPPSPAPSSPPPSPEPPSPAPPSPEPPSPAPSSP
PPSPEPPSPAPPSPEPPSPAPSSPPPSPEPPSPAPPSPEPPSPAPSSPPPSPEPPSPAPPSPEPPSPAPSSPPPSPE
PPSPAPPSSEPPSPAPSSPPPSPEPPSPAPPSSEPPSPAPSSPPPSPEPPSPAPPSPEPPSPAPSIPPPSPEPPSPV
PPSPEPPSPAPPSHAAPLLNPPSPAPPSPEPPSPVPLLPLPNPPSPEPPAPPSSPPTPNPSLPSPNPAPPDPATPAP
RSPEPPIPVPPSPPSPLPPSPSPFAAAGSDVFIDVNFQGVNLPDVNQTKFAQQSCTELQTSTAAFNCVNNKLVSGSI
IGGYVLTAPPGTPLDVFYTVVQTAVTTDPASLFSGSFLSAYGISSVSGTVLPGAAVPPSPPTPSPPPLPPSPPPPPP
PSPPPPPPPPPPSLPPPDLSPSPPPPSPAPPPLTPPSPPPPSPVPPSPVPLPPPPSPQPPVPPPLVPPTSPSPRPPS
PSPPPPPAPFPPPSPPPPPGQFVSGYEVTYTPTFCAGLASDTGFNVFDPSALDQFGRITAITFVHDAAIPGVVVAVK
LVYGSGSATHTDVLAGSVYDPSSPTLSLSTVRLVSSGGSALQTLSVCCTATGSVAQLAVGFSDGTAASTGLCPAGAR
RRVLLQARVGAGGGSAGVPSGAVVGGVRGGRGAGVNSIGFALAVKKPYPPPRTVPFPPSPAPPSPEPSPPPGRRRPP
PPNAPSPPPPPPPSPPSPPQPVAPGAPARPPLPPRPPSPPPPPPFLPSVPPLLPPPVGTPAAPPSPPPPPAPTTQSV
QVAFQVSAKVDYTRIVANVPPQVPNTVPASTLVLLGPAVPIVRNSLADPLIASTLYGRGRVAAFGGEKMITGCCKPR
GKPTPSDPSLDTIIVNAADWAAWYGKKVGKAIIRVADTRYMPMAKYVVKKRPDSFDTLKSAKIKSYYLSLKAFLKDG
HKACDLYVIGSYDIQYLEPRVSHFLQEFVNYGKGLIVVGPDVMPSIFYPGSLAAPSLAPSTAGRRSMLLQRAAVLLG
DAFEPDVTDAGDNLSDPDAGVDLRHQLHREMAAALVRRRELQQSGSSSTGSSAPGNPLFNSTSIPVNQVSGPMGLVF
SGYVSDPGGNLTVTSPSELQNAELAASQYVEYLQGRMQLTIPELSLAVSTITKARASVPRGTPGASRFWDLSDEATV
LSGKAPALPPLFGAPPPPPQLRPPPPLPPPRPPPPSFLPPLTAYVGCFFEAASRRALNTTLLTGTGVSVDSCLGVAT
NVSNPNMVGTTFLGLQRTACFGGRELSASFVAAQIAEAACPVSCPAASNQKCGGNANATGIPVSVYRILSAAPPPPP
AALSPPPSVTGAIPPVPQPRQQPPSPAPRPPAVSQQPPPSPAGAKKRKRVLMRAY

>Cre17.g738100.t1.3|PACid:27571629
MVVRQHRRRCSGAGTCAFAEFLVALAVLLLVAAVPLLRPTAAAPSTGDRLGNNGRDLLSAAADKTAGTLLAAAPSAN
AGLAMGTPAAAPAAAPAHDMTPRNGREKPPAPGRYGHSDISSGSSSYPGVPPSRPPPTPPTKAPTYPPRRSPGYGDP
DGRNSPPPSYPPTASSPPPPFATLPSYPLPVEPSPPPASPPSSPPPPSSSPPPSSPLPLPEEQPPVPDSPPPSWAPP
DTPASEPPASLQPSSPGAPSPSPSSPPPPSVVDLDGMSIPADLVQQEGAPPAALLTLSRELQLQGRALGQSSEDAQE
LRQLFPAATEVVALSWNVSVVIFAYLTASLHEAGSSGAADNRSTNSSSGGVGTKAMDLVCSSQRLAVLRERLTARWG
VRVGVACLCLGDVGNGSSSGAPSPSPRTAPVPRMPTPSAAAAAAAPSRRRLASTDVHSVRSEGGMGLAQAQARRWDA
ETLAVALPPSLPPPPAYGIYGAECPVYPATAVPVGGPAGSPEALSTPAVGGGSLTNVAAAASQPPPLLVEHSFTLTV
QVQTTADADSGASTGNSTALKNGSGATSNEEGAGGGRRSSARLLQQNSAAGEGNTSSAATPVAGLHNSSSSSSSTMA
GEVYDAVIEWKQEQAQQLAAGGGGLDGGGTVTAVCAAPKQSISVSTQVRALYQVPLSRQGLETFTTACSGGGSLSGG
GSSSSTDTAPLSQALIGSTSGRSSSLACAVQPVALPQASGPSGGILSPAGGPSLVPGDQVPQPQGSPQGAAGGGGGP
AVATVAAAVAVGSAVAIGIAGVAAVVVRRRRRRRVQQLQMDAELAEDTAKALSNPSFVLIHDSVFPVRRARGLSVST
HGGGGGRGGGGAGSPPLSALAVALAASAAARRGGSRTGSGAAFARVSGRVSASGASGGGVSAAASVTGGGRYGCGSP
GLGGITAISSSVESSPAPLALSVAARGIGTGTGAGSVTSRRRPYTRRTALDGGAAGPDGVHSAYGPPGSTTDHACAA
HDDEPPYQRQRRVIRRALSLYGGRVGGSNDGRDAGVASGASSGLPSTGGASSTVGASSGGGMAPATASGAAVAGLAV
AGDLTGRSPASMLRASSLLPHERRLRAPSVAATAATGAGSGSGLAMAAGARGRALALYARALGGGSVTGAGGVGGWA
ASRRPASAGVDSSAASGSPSSSPAGDVTAGSTPTRTPGAGGEISPVLSPGASAARHPLYRDAAGAGAAMGHGYGHAG
GGVGGAGYSHLRPVTAGAAPAQQTRGEREGGSVLAAVAASLAALGPRSQAMPMRNSRAVTGARGTTGGGGGAGGGHG
GAAANSLSRAAAARRALASAVGGATGAARGADASAARLAAGTATSDGAAATYMQQQQEGQWVHGGSPRRAAVLSDAT

AWGTRGLEGANADAGAAASAAVAPCDPVSVFTTTHHLESAAAAAAAAPAGNANGAAAAAAVAEAVELFSALPSWQAP
AQTSTSSEPDVFLPLPAQVPCRGAGDLPGPSGSSSSKAGRSAGSSATPAEGGCGDASSGPSRLLPLPSPYAQEESAM
LPLWASSQPGGSSTILGGSTHVAAGPSGKPGSLHAARPRAVAHACTPSPTAPGRAPATAPFAPAAAAAAAGGATPTS
PVGGGGSGGAGGANRVHPLPLHLEPA

>Cre17.g706700.t1.3|PACid:27571632
MSRVTTLLASIIVALQFLLLQISHAAKPRPWCLDGTALCRGTAQPTDRFAHTLTFVPPNRTSLGDGLFAVLMGGQRS
SAASGIIALTAVDVVDTEDMTYVTYPVPFSVPANVTLRPRSGHVAVPYGSNGLVVFGGVTYNRAVARVPVATNDVLL
LKWGADAGGGGSTSSSTLGSSSSSSSGSSSSSSSSSPLPGAADVLQSWTVLTNSSSGGGEVPVPRQRAAAAVIRDSL
WVFGGLDNFTRILDDTHMFNLTTRTWTRLDTNFSGSTIPRDAAGVAGLHSAKMVASSEGRYLLLWGGRNQGDTPQDG
GLWVLDTRPNPGNPRQARWLGPLNMTVDGVAAGSGSDGVLPASDVAAAWVHLPQPDGSVWRELQVAMLGVYKPTPGA
RPTAWRQFAQVLTTDTGGSLEAAWLPELVAGSRTAPLPFVRKATFINAAANAHVTLLDNTTWTPLPPGSPRRYVWPD
QLPWVKDSAVALSPEGRFLIVNGGQDPGKASTKFLALDLEAGVMYDADSNLRPDYLQSPTSSLVYRTALHVAVPSDL
AAWRAGNDLAVTDFIMYTGLDNVNDDTEPLTTEWRGLDAGRQATGMVCSASYNKAAGNVCRVLPLARGATQITDLPA
PRDGAAWATLYQVFKWRNATARQDVGAVVLLMYGGSSYVGDTANSTQEYDDAGALLTPGEAAVRQALADCDTAGALG
NPQLDAMAQCSLAKFAERPPDGICTGGTGSRTCASSSSSGGGSSSDGTDYADYGASATAYNLYDYPDVYDASQYDSD
IVAAESGAPPATPGLQQPPPPPNAPSAPGSSGTTTGAPLPPPSMTPPDAPPAATGRRRLSDRQQRGLSESDPTGTSG
DAGANPAYDVGAWGNRAAHARGQTEEAAGGGGGGGGGARKSASGRAERLGDAPGAGLMPTVFGTAYAVLVHGREYDD
KRLVDESALYRLKGGSAMSASPVPALLYDILINVNGSMNHSPGPRAAAAIARLPPGADRSDATAADGLLFGGLTAVN
RTAANPTGLPQLTNDVHYLQYIPAKSPYADADTLPPPRLPSCPNTTTASGAPFACRSLSPFAQNGTDRSLPPPPYAA
PAAPNLLTSGATPDMALFTDCPPGVAALPVTATGYRSARFQLRVWRSVNRDYARGMGISFYGLGSGVVVSLEDIGSE
VARGPCCPGTNATAWAWPSASSTTMTPSQVFTLPDDVVEVRLLSAGRGWTAPEDKARPYLCDTLKSPLSCPRMDLYN
VDSGRLVAGFGYNGTWATWDESTNATGLTERYIRLALCTNIGDPPTPPAAPALPPPPPEADPTAQQYSEFPFHVKGS
LNRLRSHEIMLSAAAPASQVPKPRHRHASVYVPTAALSNRSRAALGYGGQGLLLVYGGTTDPLSADPDALLDDLWAL
DLELRTWTRLTSGGDSPGARMRHSMATDGTLVYLTGGYVYDPDKAQWVNTDDVYYMDLVLGYGSTWTRIEGGAVTLA
RNANPTPLSEGAGVSRRRNHLVAPNNKGLVFVPLPTATALSSVYNLSAVPPPPPSPPRPPPAPRDATASPPSQPRPP
RPPPSPPAALPGAFIEKVAAGMYDGDLLLLPEDRDVEVTGNITLTASVVLYGKIMAQYSPPPPPSPSPPPPPPTPAP
PSPPSQPAPSPVLPNATARRRELLLADGDATAAVDGRDEGRPLGNRRQLVLTQEGLDEWAAEARRLGRRLLGDADPG
GDDHDASAAAAGDAAADAAGKLQAVLAEAEAHVEAARRMMQAVTNGTTTTSANASATNTSTNTTNANTAAANTTRGP
TPGSAGSGQGLVWGDGQPPPDFDVTALLALPRTRVSCSNNTTWIIRSTPNVVNIEFVNCNFLITPTGTTAPAVFVNC
VFRNTVGRPAIVVQSGAFLSVKACIFYNNTNSPTTVTTAVNVTTNGTARMVNVTQQVVQYGGNGSAIYVQPRGSIQE
ISDTAFKQNGWGPGSSAGAAVYLAGPGACLNSVSNTVFTLNGPLGAPAAPGASPGTALSPTAGNSTATNSTTASTAS
TTFLAGGAIAMDGADGCDIAFDRVAFLSNTAGGQGQGGAVYLGRTDSRAIRMRNCTFAFNNAKMGGGLYLTNVLGAV
NISSSLITANVAASRGGGVGLDGTLWLNASQVTFTDNSALGGVGGGIAHSRQGLLQLANCTVAHNRALFGGGIGGQQ
DVEVVMTDSMVFNNTAQHAGGGECFMCFRFTSVRSTFSTNAAVSGGGLALTLVELGGALNDTHFLGNTGVPGARPGL
VGSSGSGLKAALSDAGCGREDSGGGGGLCVYLRRSVAVNGGSYVGNIAVTGGALYVHQHCMPGDDDCGPVVMAGGVA
LDSNAAPLGGGGAVFRSLGGLVNVTCAGAAAMLPWANMTAGCSEWRNNTAKYGNITATWAYGIVLQEPPPKYGNGSS
NSSSSSSGTAVTILNGTSTLTGFGSVIPSYRSNDPAPVVARVVDFHGQTITDASREATTSLEARFTGLYLNNSAAAV
AAAAAAAAAAANATANGIAVAATGSSSTNSSSATMALAGNSITTTAGLGNFTQSLRMRAVPGNYTMVIAAPRSILEL
PGVVFNVSVRPCIRGEVVGRSGDECFPCDSGLFSLDPKNLVCDKCPDNANCTVEGYPWMTLPKEGYWRSGAYSPNIL
QCPFEDACKGNSTFDRMEQMARFLVNISTGVQLIDGSTIPPYNRTEFLQMQCTYGYMGSLCGRCIQGYGSKGNGECL
KCADRYINIAYYIFSTWINIFFIWLTVWTQLPNSTGSRKRAERQLVEQHQQEAAQAEQLERQHFEQYQQQQQQQQY
QQQYQQQQQQLHGFQQPGPDQAAYGGPHQPSGNFLPPPPAPPLPPPAAPPPLEAPQAQPSRSGSGRVGSSRADSAAG
SRRASGAWTAAELTPAAGAAATDAAARPPSGRVSGNGAMCSPAGAAVAAAALGNTASGVARAPPSRTNSIRGEAQML
PLISVEPAEAMGMAASSPPPPQVQLPPTPLFPPIHPMEPQPEKKRRGSGSSSSSSSDSSKSGRGRGGGAAAIGGGSG
AAIAGGRRGSDSGSGSGSSDESEGSVVDVDEDMEGPYKGGNHGIVIKILMSYLQVTSLVRQVALAWPSFVMGLLTAA
KSASQTVTTVVSIDCSLDTTLLPISVQKTLITIATPWVVLLLGLVMWCLLFLSLRLRKGGGRFSGLLRHGPNSTRVS
AVTFAGYLQPRIIITIIAIVFYVYPDITGAFMELFSCTTVDPEGADVAYGALMEQRGLYWTSDLDLACYHNPHLWLA
LGVGLPGLILISIGAPVFSWVWLRRHQHKLYTSPSFGVSYGFLYEDYNRNCYYWDSVVMARKLFVVMAIVFLAPYSP
VLQILTTLGIIIVSLVAQISFNPYKNQRMNRLERLSLYATLAMLYCALYFLSDAVPQAAKSAIGAVLLVFNAAVVAY
FALYIAQEFLLMLIWTIDDESNGDGKLTWDDIWAYAHKVHGDKRYSRHLFACLSFAQRCSNCCMVAVTFPARAARAA
AQRIRRLRNKGGEPLDVDDKAAALAAKEGAVSNRTSSRRLGSTTTRKASRTVAGTGGSMTMITAMQPEPLSPPWQQR
MGSVRKSGSGAAGSAAPALDLASTAVGNDAGAAAAALEKSPSGLIGTAVAVAAMPAGQAASKRREGEDSAYLAPPSV
RPGSGASTGTASAAGAGGGGGGGGGGGGGGGGGGGGGEGNTGNRVLRTASGNASAKDASRLGPQPAAAAPLSTIIS
GDLLSARPIPPASQLPPLTSMPRPSLPGGAEPSPAEAAADAAAAAAAAAATAAAARPPLPPSRGASRGPSLSGVPPG
AGGGAAAGAGGNTSRRFAAMSAPLASDALPDASGGAAAAAEAGGSASLLLRAGSNRPSSPLAAAAGIAAATTASPNR
VNSGGRSRGPSGDGDGESGAAAATRGAPSRQGSKQPQRPGGFLSSLIAAATGGGATVVPTNESGGQQPQQAMHSAWG

ADNGDLRPPSDWQQPEEGQQGAQRRVASAGASASSAPGPEQSDSWAAAAEKTERLGSTGTAAAVGRRLYDSPASLER
GLQQEGVRDGSGGGAGEAAAAGPRLRAVQSAMPGARTRGAAGDDAGRSTNQ

>g17454.t1|PACid:27571642
MPFLRIGAALTGGVARAAARLRNNRHDHAKQDDLFFAEDEPAESACISGPPGPKCLFGRERELANPPELAPPSPGHP
EEALGAAQAVQPLVQPELNSFNEAFVQAVASSSCDDTAGHSDFCSVGSFEARRRAVSVKKVRDWDVDCAVEPSTPGP
EPEPELSGLPGSRDSSLGGAEPELTAVAPLVVPTGGAAARLAQPTHLALSTAAPAAGDVSGSSGSSGGDTAASGASV
GQVLSSSSSGSPSSSIKVPDPVVASTAAATGCKGAIEASNAAAGSSKSSFSKPAPGPAPATAAAAMAAFVPASPAPA
SASPVPADGRGAGGAAGCSRLAAVGFGDVVGAGAEPQAAVAAALGGRAPVHVGYSAPHVVKAAAAASSRAVVATAAA
MARPHPAALPAVPPLMILRDANDVPLPAAQQLAARAASMASRGSSARHSLDVTAASAGATVADISAAAVAGADVAVE
AMVVDSPLPPSILETRPPDVSSLLAIRKQVEVLRLMHLQRQAAALLAAPAAGRGSAVDSTAGMRAGVPAAGAPGGTA
SDTRTTRPLDLTSAAAAAVAEVRAAMAAAEAAASAGSGAAADGRSSSGSAPFTASSQQTSASQVPFSASPPSSPVMS
TPSVTVLASSSPPPSLPASPLRPSANGSGHGRGHTRHFSIDASPPHSSPMQLLQQADPGKAATGVGYSVASAQSSGA
RAASPRPSPLQPQYNATVGGGSTAAAAAAMGGLSALSLSAPLPLAPSSVASPAAAPRAASPPPRSPLSVSSTGMSTA
VSPADIQRQQAQLKVQIEALQRQIALKQQAARTPAGSGQPPSPPASYSQQQQQQWQQQWQQQWQQQWQQQMMNPMMQ
MMNPMMPMMTPQQAWPADAAQATISRTCMPMQQQLGSSGHMQAACGQHPGSGAAAAGGSGLGGSPFSTAAMGDGDVA
AEGGGGEGELLSSHPSFRQHLHDPVPVAEAARRTQDWLASTAVGAQDGVPPPPPANGGHDPSILPNGGAYGGIKSAL
KRDPSLRLSPHPTAYSHPPSAAAQPWQQQQQQQQQQQQAPQEQQQPPSRSPSPPPAISRAPSTRYTRFADEPNSPNS
RSPSPSPLARSASQRRNSTGAGGEESSPSGTGASTSQSGMARAPSLNRTPSLSRTPSLMRSSSRRGPVPPELLDASN
GPVPLWRIASFNRQLSVAQPSMVRRQRSITSQNSGGAGGAGDDGGTSSHRSSSRRQRESSKSKARREAAALQAKMAN
GPPPSPLALMSARMLQEAGDKLQPAPRLPTGRAFFVPLRPDLRLDIYEHENTASTASGMSLPDSRDL

>g17202.t1|PACid:27571666
MLLAKGVAYPSYSCRTAELSADPNMGRRATTLLLALLCAGVASTFADRDLLQSPADNRFPPYGCVRDADASRFRLDP
LYTVSGQTICFTAHVVPCNKKGSPCCDAKKVDFYKLELDVVPTCNRAITKVTVNGQRVLAPTFNFYGTDDQSAIYKL
TGLNLTVANAEGAQICMTLDKNGPCPNLVDLCDNVSVPGSCRYAIVESGTCDCCPVGVTGLFPPPPSPPPPPPPSPP
PPSPPPPSPPPPSPRPPRPPPPSPPPPPPPSPPPPSPSPPPSPRPPRPPPPSPPPPPPPSPPPPCVTCLVQTIIPPS
YGLDGRPLFTFNKTICASQGEYIVDALNGQADFVGARMLSPWAVQSCTGKWDPNTNTYPTLRVCGTFFSVEDANLLQ
PWIDDEAPLDDFNAHLVGYEDCPAFIRGYDLVIQFDPFDGPCLTGRSQKACAPSSPPPSPEPPSPRPPSPLPPSPKP
PSPVPPSPKPPSPAPPSPKPPSPAPPSPPPPSPPPPSPPPPSPKPPSPAPPGPKPPSPAPPSPKPPSPPPPSPPPPS
PPPSSPPPPNPRPPSPAPPSPRPPSPSPPSPRPPSPPPPSPEPPSPEPPVQPLTPAVRRPPPPSAQPFPFCKCNRTL
GTVPFVFDPVPVLTRNGANRVICLTLRTKPCVDPSSPCCTQPMAKIEWWTKDACRGSVKQVTLDGTKVDQQWSKGTF
KIPGLNMPASAVPDAGREVCIELDAKGTCPSLNTFCARAATTGSCYYVAFNQAKSCCPVDTFGIPTGARH

>Cre17.g735600.t1.2|PACid:27571700
MPPSPRPALLLAAVPVALLLLAAGPATVSATWVPAAGCQAGVPCTASNTCLAAAATQVAYLPSAPKSCPAGTQQVGA
AYQQVVDYNQCAWTGCSIGGCAAGEQEVTASQCWWGAWNEYCCKLVTNTTLTCRQILNCIFYPNPPSPRPPSPRPPS
PPPSPPPPPPPTNWAAFTNGAWDAAATSNNVRMAYVANTLARSIYSGRLLSSDPGATVFGATYQSRIVARLGGIATT
MRYLDQSTEVALVESTTSYMIVFRGSEQATDWFATNLDIGWSYTTVFGPGVYVWDGFLSALMANEQALRSLVAATYA
AGTTQPRKPLWFAGHSLGAAMAVLFAHYVRTNMGIVPQGIFTFGGPNVGYSDWAAAYQPLLGSRTYIYEDIDDVVVG
QPPWPYNDADTPGVRTRFTACQAAIDYNTALAGRRRAELLADAAAAPAAATPAAANGNGNGNAKEVFRGGPKDNAGG
GGIHGGAKEMFRGGPKDNVGAAAGSSGSGNGGGSGGRRLLAKAPPPRHSGGTAAAATTAAAAAATTLPTETQQPADT
EGLVPLGALGSLDGGSSSNTTGGTATATDAAAGSGTDGDDDGDVQARGNILGLDQHDAQASYMTTIYYCFLSATDKT
KVPSVGEVSSW

>Cre17.g728250.t1.3|PACid:27571704
MFESQVAYYLDRYLGRYVAGLDAAALRISVWSGDVVLTNLALKPEALEELGLPVAVVSGVLGKLTLKVPWSALGRQP
VVVELDRIYVLLRPKARTSATGEACMDVAQYEAAVTAAELEEKRRRVLEAESSWLQELEAKLNAKLQQDKKGSGGTA
GSSSSGGLFNFQAMAHTILGNLQLRLSNIHIRYEDTERLPGGVPLAVGLTLGEVAAHTVDEAGREAFVTEHVLSLMR
KAVVLRQLAVYFDVGLPLLRPPRGGGVWSGAVAAALTPAEWDALFLPAHHLPPLPPPPSSASSSSALPPPGAATAPA
GAATAAAPAGPAGVVPYGRPLYDHHHLVLPISGRMTYIHRSAAHAKDPSVPRTEVRVTIDAVSLALSAPQYGSTLAL
LDALDASAANAPYSRMRPPGRPGAGPAAARLWWRYAFLAVRKQRRRLGEAHVGWGQLRAACLLRRAYVPAYARLLAA
GGALGGDAAIAGLDAQLPEPTILLFRRMAHAKLKSEQPKAAATAAGGSSTAARSGSSASLQAAKAAKAAAGGGSSRT
WSAWLWGSSSSGSSSTAAAGSSAGGAEGSSPAAAVAAPGAVVVGAGADADGSLTAEEWKKLEELLGGQDAEGEGVLD
SAAAATDAANPHALLAAVAVEVATASLELRGGSSSSSSSMAGSAAGAGLAIAPPPPVLLVAAMKGVSAGVQQYPRTH
SLNLQVQETCVDAPGRGELLRAMPMSAQHAAHDGGHVHAAAAAAHTNPPASLLLDVTLAPQDGSADAVVCVHLAPSY
ITADAAAVRPLLDFFTAPTFQQQQQLQPGGGSGGGSGSSGMAGAGGALAAQAAAQAQQLQREAARRLAGLSQERPR

LKLSVVLHAPKVAVPDGKGRTTLLLDLGCLTLSSRSGSSSSKEGQQGLHDEEEQEEEEPGLYQGFALELDHVAASLL
DGSSSGSSSGSTGFRWADLQAMQAAGEGPQGRGGQQRAPHLVPLLSRFGLKAALQVANWAHERLPQARMRLRASPLE
LHVSPFRLHRLLGITAALQQLAAVGGGGGGGGVAAAPGAATAPAATAAGAEAQAAGLLAGSLSAAAEPAWLSGAEY
QTLAQVLTWDALGGLGSHWRRRRLVVWRGRLLVMTATADTSRPAGANTLIDSRTFWRGWRLVAVPPHAVSGAEHVLA
LVPLNGGGGAAARGGGAGAGGGGGGTRAGGGGAASRAVVRPQDAPTHPEALVVRLPDAAGLAALRAAMRQSAAAAAEA
VAEELPVGHGAIDDDSSSGGFGSSSDAGSDDDAAAAEAAASKKAAEAQSKQQQQKQVAGQGTARELFQLRGALPELT
LRLSGRPARSWRTVCAVATAATAMAAGATATATADEATAAEAGGGGDAGVPEPGDAAAAAATAAVAPSAEDAGAAAG
DDANEDGDDDFRSVCSLDSDDDEEQQEEEEDGSGGLRSAIDPVPLAVEVPLLTVRLRHASVALGFRNDRLTADVAMA
SLLCDDELCGGSSSSSSSSGGSSSSGGDAGSSSRVGGLGPAFAVLATSGGGGGGAGVVHAGIGAGVVHAGIGAAGQP
DGAAAAPLARLRFTQHLQSTPSSEQPEADTELGLEVAAMDLRCHRPTVAALMGLGTDMAAAAAALSPTPAAAAHAAP
ASAAGLASSAAGSVATAPDGPQQTGGDLLGDEWVADPLSTKTTTSGLATGAAGSSHRQHGAAATAGGPAGEETSAIV
CYRGLAAGAGSGSGSAAAAAAAPATATAAAAAGGRALVKMHVVMQRLQLTLPYEMEEPPAAVAAAVVAVAGGGGAGG
ARAGEVAARVFAVAAIEAFVMDLAVRPGGLQLDASLGNITAEYGALPAGHPNRTLLTVRQPPGAVAAGSEAGGGGGG
GGSDAALLQVTFRMHSREESLHEPRVPNGVDYSSLSARLRSVNVTLMNRFLLELQTYTTGLLDLQPKARSPPPTPAA
AAAASAGASQKLQPQPQAAAVAVAELQQAPFILLLDVLLESPVVSVPQYSLYRRSSSSTGNSSSAGAAVAAAVAAAA
PVPGLEAWLGRLQLSTTAVAWRDVVAGGEERTATATATAGGFDLLGGGGGATTPAGGRSGRRQVLMDEIQVAITDVS
LMARGGPSGCRDTNMIQDLGPTGMRVALRRPLAARLLLPPSPPAAATAGAPANDAASAASASASASAAAAATAELLP
DASLPPIQVAVSLPRVVACVGEREYTHALAVLAENFSETLGGGGGGGGGVDPVGDLFVRRRLYDKYGASRRRPPPPP
PAAAANKTAGSAAGAASSAFGDGGGSAVSARPPPLDFRLSTRAAMALLSPEADACAVQVTVSIGLAALTLLVPEVEG
GAVLRPLAAVAVRDLWAGYYATSGGSMFLSLALPSATIEDARPGVLPDQRLVLSTSVAGVGASSGAVGMGEAQPATP
SAASASSAEAPWAPAQMTQHPQSAGEGGAPNPSLLVVEFRSIAAAATAATAAAAAASPGSTAAAKPRLPSIGIRIRV
QQPQMVLDLLLLQRLINFAAGGPVLQGAVPRAFDGRDVHVPLGHPGPHSPYTARGGADLWLCPEVRLLADAPAALAT
AGKSSSSIGSGGGNGSGDSSSGGQPRVHVYDGGGGRLVLPPGLAAAEALPLVVVGAGRTLVLRNVRVINAASLPACL
QLGPGAKLVAREEDGVKMIAGGGSSSVVASAASMAGGARPSLYGAAAAGAAAAAAAAAAAGGSGSDGTGSTAAAGAVL
PPYMELAVDVVGASVHVMDSPAGAEGTAYGGAGAGAGAGLATATSLAASARSGSSVSVSVVGRSRAGAARSSRFAPS
HLGSRPAAMAAPLAGRGAATMAPDDDEEEDELDEAATVGRGGGGGWGGRLRRRLVIAADLSASWLAVGDSKQEARAA
LRGLSVRMLTSLRSSGGGGGAGGGGGGVDAFGLLRAQSRNSLSGAALHRAPSIPETASGYGDGTTTVVGGRGGGGGGL
GGDGGALDSVLMEAADITLTYELTVAEMALSVDADSALLLRLTPQAAALLLRLQARLLARLSVPAPNQPLSRCSRYQ
PVCSLATAATAAAGGWSGGGAVRGARGFAGLGTQPTAGGTAANPSTAAGTGLNLLDGSEPSGTSGGSGATADAQPLD
PELDPEAAARGLTIWRPVASSGYVPLGDVICQGNVRPLDSVVTASISSGIISYPTRYEAVGSWSVAGADPDSAAYAV
GDGGGVISIWRPVPPPGYVALGFVGGAAGSSEPPPLTAVGCLAAAAVVEGFAGECFVLPPEFTAAAAAPGHLDVAAA
SAAVSGGGGGGGSGTAVTGGCRVWRLQNQAATFVVTGPGQPLPAPVDVFDLRSPLGVTPAALTPLGKLQQLWRERSER
EAAAAAAAAAAAAATKLDASATAGAAGTTQAAATAAAAAAAAALTVHMRQPPPLSQLCQLSYLRDRRELLRHAARREL
QAPCVEFGRVWSDRALHSRHSGRGRISVWRPRPPPGYAYLGDCVVVGAYTPPPAVSVLRDVVTPPLHDRRGGGRTAL
SPLAKPIGFEKVWDSGAFLVLWRPMPPDGYVALGVVAGVGPYPPVTEAAALRVVRQDLVAPVSVAASRPDWMLPPLG
SNNSGGGGTGTGGSNVYGWIPDYRTGASTIVCQAALPSEGTRHVPRLNLAAGDWLPAAAISGGGGGGGAAPRGKGKA
APSSDAPLQRHELLRLSDEGGEEEEEEEEEEEEEAGDGDEEGRAGGAGADEDPAAAAGPFRDSSLRLRLRHPAVLQLC
APGGRPVLELEARDGRLGWATQGRDASSAYASLIASAWTCSGGGGVLPARGGGGGGGGCWEPLLEPWQLMLRCDDSRS
TGECGSRRPGTWVKLTSAQETAKAVLCHSGLQLLLSAVPAWVDVLVGGPALSPESARRRAEQAANELTAVTAAAGGG
GGGGAWRGAGLGLGGAGPGSLLVTNALGTGVTLLTDDSERQSVTDVPPDAECAAVSLAVGSSSGSSSHGSSSGKGGI
SSGRLQALAAAVALRRQPRMTLAAPAVALYVDVLQLLGAAAAGGATAAGAAAAAATAAAAVPAWLARELGKGGGEGG
AAGGGGYLVGVRVNGPAAEGLPESEWGMVTRAVLQPPPPPPDGSPASSTSAVALPAWRERFLFKLPADLSAALLLPP
YLGGSGSGVGGGSSTGYHAFAGVPVQLDVSLLVSEGAGGGAPRVVAAGTYPLPYDWLLNQVQRLLRGSSTSGGSSTG
GGGGSSNAGGPGGPGPVSHVQLFPTGATSAVSLSFSGGTSGGGAGSSVEPCGLQLQVTLDERQVETSWIPYLASRPT
AAGGNNLLLLPSPSMSSSSLAAAAATAAAGGGEAPTGPAEVFARAAAALRRGLRRGLQLQGSSHWAAVAGEGAAVAA
GAAGALIPVRAGPGLVIEQSPGPGGAVLELLRSRAVVFNSTTVPLEVALAFPAHGGAAAGAALAGVAVEKPEFGADG
QAVPVEELVFENERYLPLRGWSPANLLATERRRYSRAGQSYSIFPNVPLPPGWEWEGPWHMDNSGHVDAHGWFYATT
WVMLKYPPPPEQGRMKRSDLVRRRRWVRRRRWVEGGGPAAATAAAAAGATAAGAGGEAVARKLLGSVAPGERLPLPL
GWEAGGCELQLRPLLLTADPDLDLDLEEQERHAQLLLMGDVAAGGGAGGGAGGGAGSGAGSAPPPLLLDVDGGGVGG
GEEGAAGPRRRRDVEAFHGWSAGPGGSGDPGVLMTRLGDGGARLLCCRVGAASADEQAALPPAHGSAAAATAPGGAS
RSLSSSVTATTLPHAAAAVPAPATQPASAGGMAGQHQPTQQARPPPPPPRVEPDVVELVHQNQRFWPLSGWGRTLKTE
RKAFSLAGNSYADFPEVPLPPGWEWVDTWQVDLTGHVDNEGWFYAADWQLLNYPPTPEQGRRKTLDFIRRRRWIRRR
RWVPPEPEPTPAAPPPRLQPQLPPPSPSPPPPPAPAAAFDLLGDELPVATTTAAASGPLGAGGDLFAGLDLLGGEVE
RVAAPATPSAPAPLQGQQPRTISSAALHGEKPAAAAAAAAAAAAPGGGVAAEQPCWLSVALESEVLAAPPDAEAVAA
AATAGGGGGGGSSSGSAVSDTLVDWRLVVRPPLTLQNLLPLPSHYIVWEAAAAGGQMRLRARGSVAPGEAAAVYSVD
MRKLLYLSYAPEGYDFGEPVLLSEGFARNAAGVSGAAGKARPLPSSFLCSPACFSTATGSTAVGPSLQLEVLRDADT
TAAPVSSLGVGAMPGGAAAATLPDQPVATLVSKAPPTLSEAAAAGYPLTVSLRVPLWLVNATPLLISCALLALWEAP

PASAARLGEAPRPPARVHVVGTQDPRRLTPPSQPHQALQPPPGSHGIPVKPLSTHLLSYPPGVVAPSTEADAGGGGG
AIAAGVPAGSTTSEAALVLSVCGSRWSEMVVLPSLSQSAEGGADSSSAPAGGLGAGSSVLIRALSGDGSRMLEVVVR
LESGLGSSSTNSGGAGLGSSSRLAALRAAAGSLLLRVEPHVTVWNTSGFPLLLLQPSALQPPPSTGAAAAAAAAAVA
TAGGGGGHHADGARSFSAAEPHLQSAHAERSAASVPGGPTPMLGSDASGGAFVTPPPPALTLALAPAGITAAAAGRA
DDAEPAGGAPTPAEGASVPLHWAAAAAAERRHFCLALPAVPGVSDAALRGLHPSVLWSDPITFNPESCVGGTAGASSV
SAGAGGGGGGAGAAAAATAAAAAAAAAAGGWVQEGQVALPVCCVGRPGTMAADEAAGRRARLAVRQLLAAVATLGTGA
GGGGGGISAAGAAGALEALAATAETSVVRLVRQTEVGLVELLAVLLTLRLEQRAPGCLRLSLSGLGGQPRHTLANAS
PTHLHWRLLTGAAGAAAAAAAAGARQWQVAAPYSAQAVVRCGGALGAAWLRPPEVEVTAAAVDPAAAAAGGAPPPGSI
LRLTFGGASDDGREPAPHSQAAAAAAVRLGLVPVRPLASKYLLAQVSEACDALVLTTAGPARLPGSGRHAETLGAGR
PDSTLRMVPLLLPAATTTAPAAAKAATAATAATATAATAGTTARGAAFSAPAATAAPHAAAAGATASTAELLAQAMS
APGVGLLAPTGTAAAATAGGGGDGSSSSGGLHVRLELYQLEVGVVDARPEEVALLTLGGVRVDVGSGASAGGVEYLC
AAVRVDSLQLDDMTPGAAHPVVLQQQGQQGRQQQGRQQGGGVDPVFSMAYTCQPGKLRHALHCPTIVARLLPLRVSV
GEALVWRLWGLASSLTPPELDLPASGAADVPLAVDLLELEDMRLALSFRPDSGSRPHWASGYLQLGLELAQLDNMDM
RLKGVHMEAARMMQSQLVQLVGSLVRQQIVSLGVAVLRSLLRQYGVVGGLSRVLSVSSRGASALVPRRAAGALKVVG
VLASPITMLACAAVGAATSAVGGGGGGGGGGGGGGGGVQGRDVGRRRLQRVCSGDRIVRPFDVHGAMGSALLAALA
ARPNGATLRRERYEEHWVLQSNIILLLTDRRILAVQSPEFAALHHRVVSGGSGSGKGEEPEQLSWVPCADASVSWHL
PWSELLTAEVAAWHDPQTVASWAVAAQPGAAATAGAGAATAAGAGAGARPAGSDTLLLHRRSRGPEGRVVYTAHCWA
MACPAPEVLTRLLEVYHRAWAPLVRRNTPSQQPQQQQQQQQQQQTPSRRREELLAATGDEARGRAALAAAAAAGQT
LPDAVLCADWRLVWAGGEAGGSSGSGGGASGGAPGADGFSIWRAVAPAGYAAVSDVAVAGRDPPGRPARVYKRSAAP
PPVRPAPAPGSFPLAALAPDSAATAPALPLLAAPLGYLLVFRSSGPPALTIWRPVPPPGYAVAGCVAWPAIEEPPPG
LVACVRADCVAPVSFSGARSPIWSGASPEHPSWRCALWPVVTEGGGGGDGSGAFVAIRGGDNQPLTPPPGLMLRLLV

>g17926.t2|PACid:27571738
MSNCCDASRLAAAEVAGRRANKGHLADSGISGHPHAQPRLPTGSLATAVAFAQGPTSASTVAPIAVAAAVAAVAAMW
VGGALALRRLSSVWRPSRRPRRRTAPDSSSRHGMAQPETYGATTCAMAARRRPAPPRRWCGSTCATHTCSTGTLEAN
ARAPAVRCGDSGERAGRGAPAADAAARHGCSSTASYQRGWAAVGKDASASSRVGGCRRDVRGVGEGRSNGANGRSGS
RGHAGSSGVSVGVGVSGGRRLGARRAASASAVLLLQLSLLPLLVTSQVVNIATVSMSPTDKTTSAATLTNDGYYVIA
AGAAPQAVFAVSSSGASSQFNVVIWYRYPATNGNGPLKKSGSSLAICPSGDTSNPIVTGGNTQAIKLTATNETDVYD
STNAGIATGCYVVRVGLVTPGTMNFVGSPAHAGMRVGNPPNIAKCSDVVNIPPNCDSTLFTYNPTGIPGGTAVVSQP
TTGSTCANMTISVPANGCTDPEGSVMTYRWDVYKSGTTTPIQSTVYGQSVSFSTGLPGLDIPAGTWTMVLTVSDSPI
NASLTLAKIVVQQCNINYAPTTPVLTATATCGASSIGVTASSTDANGHALSYQFVLTNSLGATVSTQAWSTSSTATY
SSPTLVAGTYTVQAQARDNATSPLTSSTSTLTFNLGVACTPPPLASPPPRPPSPSPPNPSPPSPQPPSPAPPSPQPP
SPAPPSPPPPSPGPPSPAPPSPQPPSPAPPSPQPPSPPPPSPRPPSPAPPSPLPPSPRPPSPPPPSPVPPSPAPPSP
PPPSPDPPSPAPPSPPPPSPGPPSPAPPSPAPPSPAPPSSPPPSPPPSPAPPSPSPPPSPAPPSPAPPSPVPPSPPP
PSPPPSPQPPFGAPLTPPRPPSPAPSPPPPSPEPPSPAPPSPAPPSPPPSPTLPSPPPPSPEPPAPSPPLPPPSPQP
HSPEPPSPIPPSPPPPSPPPSPHPPSPEPPSPAPPSPPPPSPPPSPLPPSPRPPSPPPSPVPPSPPSPGPPPFGTPS
APFPPPSPEPPSPEPPSPSPPSPPPPSPGPPSPEPPSPEPPSLPPSPAPLNPPSPRPYSPPPRPPRPPPPQQSPPDL
PPPVPPSPEPPSPPPSPTPPSPEPPSPVPPSPPPPSPAPPSPHPPSPPPSPPPPSPEPPSPAPPSSPPPSPPPSPLP
PSPAPPTPPPSPSPEPPSPAPPLRPATWPADPPAPSPPPPGPPSPAPPAPPSPDPPGPPSPAPPAPPSPIPPSPAPP
SPAPPSPHPPSPAPPLPPSPAPPSPLPPSPLPPSPPPPSPPPPSPLPPSPLPPSPRPPPSPPFPAGATVLNISDTST
SGVLIDPPPTLDLIAIDVSDNPITGTLPTSWSQLSALQALDVSFTFVNGTIPVAYSALTALSLLAASNTSVSGTLPA
LLSALSNLAILNVSSSLISGTIPPSLAAITALVVIDTSNTSIAATLPPGLSTLTRLQALDVSSTLVNGTLPSVYSAL
TALTLLAASNTSLTASIPPELLASLTSLQILDLSSTHINGSIPQTASALTALTYLDVANTSTSGSLPAALSSLSALA
VLDVSSTNIGGTIPDQYSALTALQVLNVSFTNVSGSLPISTSALTSLTLLDVSSTHATGTLPGALSTLTNLAILDVS
NTDISSSIPAKYSALTALQTFSADGSQLSGSLPMALSTLSRLQELSIDGTNISSSIPAQYSALTALQTFSADGSQLS
GSLPAALSTLSRLQELSIDGTNISSSIPAQFSALTALQTFSADGSQLSGSLPAALSTLSRLQELSIDGTNISSSIPA
EFSALTALQTFSADGSQLSGSLPAALSTLSRLQELSIDGTNISASIPVQLSALTALQTFSADGSQLSGSLPAALSTL
SRLQELSIDGTKISSSIPAEFSTLTALQTFSADGSQLSGSLPAALSTLSRLQELSIDGTNISSSIPAEFSALTALQT
FSADGSQLSGSLPAALSTLSRLQVFDVSGTAISSSLPEQWSTLAALQIFDGSGTGLTGPIPASWSALSSLSHLDLSS
TGVSGRLPSVLPPALVSLDISDATLTGPLTATNWTGLAALTQLDLSGNAGINGSLPALPPSIRSLDLSGCGVSGTVP
ASWSALAALTSLSASSSGLSGPMPAQLPASLQRLAIANSSLSGPLAASSLTLLASLTFLDLSYNSLTGPLPDLGSMT
NLVAVNLSGNAFAGPLPASWSSLTRLTALSLGGNALSGPLPASWPTSLVALASLNLTGNSPGVCKGVGYAGGFSSGA
SVSGLLDVCLYPAPPSPAPPSPRQPSPAPPIPAPPSPAPPIPLPPSPRPPSPPPPSPWPPSPLPPSPAPPSPPPIPV
PPSPTPPSPPPSPAPPAPRPPSPVPPSPAPPSPPSPRPPSPMPPSPAPLSPTPPSPAPSPQPPSPAPPAPSPPPSPP
SPPPSPSPPPPSPSPPPSPPPLPPSPPPAPSPPNPPAHSPPFPPSPRPSPLPPSPPSPPFPPIVIPNIGIPPEYVAS
TGTATSGFLTVTQAVDVPDSLASDPAALAVLFPNAAEISVDARKVSMPVEVTTAVEATAVMNTGAGSSIEVTVDCTP
PFLEAFRGELVRRSNISTSVLANITCAGLSLGSGGSSSGGHRRSVLATTGSGSIFSGRDDCSGSNSSISLMVLLRVP

ADSNNTNVYKQQLQDALGLWEAESQDPTSNPDGSGSMLRLCGPRPGRITTTSEVRVVTEVPLNNVGTRSLNDVCGAS
GFTSTTAFGGAGSNVACQVAEVQMPSASIPGPAPAPAAASPSSSINWLPIIISAAAVGSMTTIMCAAVLFVLVGRRR
KRRREEEEERKAHRAAAGGGAAAHGSGGGDDDAPWLSGNQLGAGGGGGSPPLTSGLASPTGVRLAAATGGTLSIVPP
PGASSRSAGAPLRRPDLSPNAEDDLKRILASAFHPSQSGAAPVAVAAAAAAAATGSDRRSGSRQLDEMDGSGGGAGT
GTGTAVEFSPSAAVSRQGSRQRNSAATAGGTGTNTNTATRLSGMVGTDSALLSGGRLSPAASHADARFSLTGADAAG
GAIAPVLLGSSGYGAAGLMPPGSGATRLVTDVGLVGGGRPGSGLASPRSGARLQERLWGTGSAGGISAVAVAGGPAS
GGSVMYMNAAFREDPLAEDDMEAAGMEPTAPAATSTTAAAAAFGAAATTTGIGSMPAALASAPPAPPGRATTGGMRA
TLGKFAASFFKRERPSGSGAAGGALQSASTASGRSPMSDTNLDLPEGHVSPVPAHMRDAQSLGGGGGGPASAPHSPQ
RFASPLSPGARLPVPPTAALAAAGAGGAAAVTTSTRGNAWSRAVDSGAAAPADELTPVKGARVKFSHVPENETDALQ
RRPSGLAAAAAAPGGSHSFRHMRTMTQQPDQPSDSLAMHLDDDDEDLPASDTAAARAAIAAAVAGGGAGTRALTPTR
PALGGERNGKWLSMRAAPETRLAPAAGAGPPSASVVGRSSAAGPPPRRATFSGLAAASAAANAERSGLGSAGDAPRG
SSALLLPAKPTPAKPMAWGGDAGSGAGGVGGPERLTQASEVASEPGLRSGLGAGRGGKLRPSPLSPLGR

>Cre17.g715801.t1.2|PACid:27571795
MAHIAHFASASLAASTQITLTGQEEAYTALTSASLPGTHLPTLGYTLGSTQVASDLPSPAVGSEPDPLLALADAVSD
DSDVLPGVAGAEACECAYSAGVDAIAGGAAHDVALRGSTASAPAAADETGQRQHKQRETQDLTLSRYLFGAAADATA
SVTAGTEQDEDLGASPVPRLPHQRVAHAPDHPTQTLVQLPGHARDESAAATSEGEPVQLPVALAAEPQQAGPGAGAD
HRHGLKRQPPPVVEPAMDPEGDPEAKTAVKPEAGREAGAEAKSGAEIGAEPLGDTQPLAGTEPLEVLPPPPPPPPPP
PSPLPHDNTCQTHGGADSAEEQRQQQEQRPRLAALMEGAARAVRGEWLAEVEAIDGLNSRAHGAQTGAACMGVDASP
PPRHHHPHPQNHHRQQQQQQHQGHTGQPEPAMAPAEQPPQLLQPPPPPPHPCPCPVAQLGPTAVQPCQTTAAPALDA
NANSYANTDANVSASANVEVGSESVSFHAWGRTPCVGSEGLTSGCPPPPGSGSPPLADMGAPPSSAHDAAPATAATA
ATAGNAAEGAATPMDVDELAVTARAAAGGGGQDALAAGSAPPPAPAASGQLQGAAANQVVTAASGPPAQHAPHELRR
QVLQQPQQQPTQPQSLLPPRPQAQPPLQPAPQTQPAATQPLPQPASSHLPPPQPSSQARPPPQAPPRPAARPASPPP
PPLQEAWRLCAGSSVAAHYRSLVDIRPDALTSDPVLASLSVLRDHQLVTPAMIAAAGGGGGGGGGGGGSGSAAPILD
RDRERNRAIRLLHAVMVNMATRSDPESEGRRAPGAAGAAGGGGGGGGVGGGGGGGGGASGQDPGQLSRRQLESLVRA
LTQPPPPPPPATPPPLQRVRPQQLETQATAGAAALCAAAAGAVEAAERRERPPLDPAAAPLLVGLDLSGQRLVDNRL
LEDKLLPALTALGTQRCHLRHLGLARCSLTGEALRALALCGPLQRSLRALDLSHNPRLGAPWSGPRWRWALGRCGGL
QLLDVRYTGMDAAGLCELSALLSALQDTRQVLAVLRLGPPTDLAAPAVPATATATATATASASASETAPMVEAAAAA
AAAAAGATAAGVQDAALDGAVPVTAAAAAAAAAAVAGAGATAAGAAGATAATAPTASGDASGNGGERVCGPAAGGVC
WPLDGAALGQLATHLAALPKLQVVELVGMSAQQAEVVVAALAARRSLLGGAGQSWHVKWPAPDTPRLCYKATNGGAG
SGFGGSGGGWSNKWEQVAEDRLAPGVNVPPPLWHDRTLPRPFLAVAAATTGGGGGSGGGGGSAAAAAAVQALRPGAA
QQQTPPVCQQQQQHVDPLQPLMQSNGGGGGGGGGGYGAYGGGYGGEAAGLAGHPSVTGARVPGAGGLGVVGGGGGGG
GGGGGGGGGGGGQQQKPQHRWPGGGGGGSGRGGDGGGGGRMRQQNIGNFLDAGQAPAQHRHSNGGGGSCRARGGGGG
GGAGGGARKGGGGGSKPWRGGGGAAGGPFGGGGGGARDYNPEEEEAGLQSDDDDGGGGGVKRGGRPSAGGGGGGGGK
GGGGGGGGGGGGGGGGGGGGGRRSHGGGGAGGGGWGGGGGAGGGGGGGGGGGGGSRLQKRPSGRAGAVAARPGGAAG
RRQPEWRGRQASLEGLLGLDAAPRAAAGKPARPAAPRPGARPLGAAPDATIYIDDSQGDENDDNDDFVDDGVGAAGR
RARAAPQQAFGGANGGGGGGGEEPEVVEFNFNRPSDEDATGGGGAGGGVYDEEAGDYLRLYARGQAASRWGWRGQHQ
RRRGDRSGSSSSSDDSIVFSDGDMVLTESEDDDDEAVAAAVEVDRAYRQNHFQEDMSDVLNRFLRKGLGQPADYAAA
PGAPSPAPWERQQLERRCRAQREARRRQRSRGPASGPDAGAAAGSRGRRSRSRSIISSRSRSRSRSRSLGREFFDDA
GAAAAAARAKDRDRGRPRSWEGGGEDGGDGDGFIVRDSEEQERRRRRREEKRARREAARLDAAQGEDDGAGDGAAGG
SAALSKEAARLLDQQRLFRAGDSGGAAGAGAGGRVGPDWDSDSDGSWSAGGSSDGGSASDGDGHRSGRRRGQAGGAA
AKVAKKHARPPKPVAPAGAEAAGAGGAAAAGAAARGGRSARLRGAPPEDVAAAAGGGPNLEAALAAAAAVPNSRASR
ELRILQPFGWDKRNKLHPANMDKLKAGGLLLPGGGGGGGGGGAQEEDEELQLAKARSMYDGLADNPLGEDSDDDDQL
LLVARGRTPAVSPPGMAAGGYALPPASAAGGAAAGAATAVAAGAATAVAAGAAAAAAAAGAGAAVLSGAGIDGAASS
GGGSAAVSPAAAAAAPTAATPAAGIAAPGSAGATGSGPSTAPDTAGGTGAELPHAATAGSHEDGDEDEEEGALFWAA
GRRRSSRPVVASAGRDAAEPSGGTGRRLVGSRRRVILDGSEEQDQWLLPPPVPAMELGTEGGKEEEEEEEKEEEEEG
TGKEEAGNAGNAGRRAVASSPSPSAAHSGGPGTSSDGGGGGGDGGGGGKGRASGGRAKRRRPSGPGGKSLKSIPEGY
ELWTERDGGGGGGGGGADAGDPDGSSAGEDEGARPKRRSRGAEGPSSSAGATGSAASHDGGRHRSTGPEGEQGSKPA
REGRGQAERPRVVTGAEKVVGDLQMEVTALEARLQQLQQQQQQQGRRQARQAPAAVEADARAAMESSAGPQAGLAAS
VAVGAAQGTGPSGGAPMEAVAAAEALGAAAEVAAPGTAAAAQELQAGAQEGMQATGEAHAQAQQEGREEREEAEEAA
GLELLLREARKRLRRAEKTWADLKQFSWDSRIGQHPVNEARLLPGAEKRQHRAPKKYSPGREARAEQRLREQARAAA
AAPIDLDGWQVFDPTATSPALAAGRQGKRGAKGGKRSRAERRAAKRRRKAAGASRPAAVDAHRDRMSVDAGSGGSSS
SSSSSSSSSSSDVESSDDEDDMPIGMMVAAGGGNTPRPAAGPARPTALAASGRPSAAAPAAGAATPAVVPKAHVAAA
RAALGAEEAIPGPAFVAALRADGRTPTASATPAAAAGGTGPVTAGSGSKTGQEQEPATGQPTDPRLRAATAADPAAT
TRANTAPVQHAQQQPPAPLQSPAAMASGARPQAQALPPQATPIASGAGSARRDVAALLPPTSFTPAAEAQVPLPPPS
APLPPSGGPAATPSLFRRSPSQGPLSAGAQGSSGMLHHHHRHQLLLLQQSAAQTARTTPSTSQGSTPSQTPSQAPSH
TPASQGSNGTQRRLRLAALLQAPRSAPPLSQQPAVAAPLSQAGPRSGSISPVGMRHGPVRAFSAAQPQLAPPPGPRA

VAPPPLFAAAPLLAHGAAAVSPPTRSGGAGGTAACNAGGAMVAAAAAAAAADAAALAHTCASRAEVTAGAIKPGLLP
VDAASTHSAAAQPAPILARAEAGVGAAGSLDSLALQPGSTSVPGSSPAATGSVHELSSGLRWEVALAAISAGSESVD
DDDVELDSEEGGEDVEERAAREARAYQTQLLSQRATQHATQHATQRMQVTAGQGLADGADTSVAHYRMPGAAL

>g17921.t1|PACid:27571815
MTLKCPSPGPVVAPGTDVAKWLPSPPPPPPPPVDEPPPPSPRPPTPAPPPSPPPSPQQSPTLQTPSPQPPSPSPARP
PPLAPAPPVLEVLWAASASGPVDPYGNSTALGSAGRLLGAPRPGRRQAHHADAAALVPINGFPQQQQQQQPQQPLLV
SVPVSRLQYPALNWLAVEPKAAARPAAKAVAAANGITAATMANTTADGSTAAASYVVLDSWKRGRLCQPGT

>Cre17.g729000.t1.3|PACid:27571821
MASPRSGMASPSEAGSNVASVGSGTALVASPARLDDEPGSSRSEEPSLGLPRSTSDLPEAATRTREAGARLMSEASS
SHAAGATYPPSQAAQELAAAAAHWPPSGGLRPVETDLHFGPHTISVIGPPNRHHQLEVFADGTVLTVSRPVAVAPPT
ASADASPHSPYQQPLYPPPQQPYQQQHEPHQQPAAATEPASLQGAYLRRLGQAELDSTAAAQGAAAQGLHMQPPQVE
STLETQLSFSPFTARPTGGGPAASASGSANPGAASTPAPGPVPSRGPGSREGDSPALFGFNATPPPAYAPRYGAGQG
STAAATPVAMAAAAAAAMASPYGGVVAGTPYGAMATPAPGPVYQSPLGTDPYSPLAGPWPAAQQQQQQQQGLQGLQG
QQGQGRQPSPSRQQPQRQGAFVDPSPQPRGQGLGPGSPGPAQRSSGSFGYGYQEATDAAPRAAGGGMDYITPLRQQQ
PPGASGTVTFTPLPSPALFQQQQQQQQQQQQQRLAQSPQAALFGEAAGREGPLAAVSPRTDATGVSLAPAELRNKYK
RTKKLLQESVREIDTLKSRLTSEGRRAGVLGAQVSALQGQAGMAGDSVALLAATQAEKMVAVQKLREAREQSEALQA
QLATAQAAVRALEQQQAGMLNEFAAERQRREAQWVEERQRLEALRAEDAAQRQALLAAFHADREGWEAAAAEQRRGL
QAHYEQQLAAARAEASAVTQQQRDASAAVQAAATAEADARAVLEEERRRSAALAESVQDLKAKLADSEAACSRAQSD
AATQADTIQGLERRLQERGAQLQQVQEALAAAEREAAGLKQAAAQHDRQIQELQAALHDKQHASAQLAAESSARLSS
LEGELLEARRRERQQPAEVAAFMQQLREQHERELSQVHSVVQQQMLEAQSAASVATEAMTGEMEAQLEVTRREIERV
MGLLESRGSEVSSLRGSLEHASLEREKLVVHARELQEVVVKVQQRMERQDRKHVRALERAAKQREGAVAALKAELEG
LRASVPGRVEKALQTYSKQLAAAQHAAVAAAEARAAATASSVLAAERAKAESALRAAAEAEQQLRAKLAAGEAHAEK
QRAALEVRAAEAEAALAGLRKRCEQLEAAAMEAGEGARTLKAQLAEALEARSVALAQAKKAEEEGSGAVEAARVKAG
AAVEAAEAARAGVEQQLRGVYGVLAQLGLSPQVLEALSAAGSLAGSGAVAAHPHGSAHAYNPFGAAAPFAPAAVAPL
PFPSPLFPQAQQQQQQAPGLLPAMLAGLRVQLTAALGGAAAGTAPTATVAAPAAAAAPPQPAAPAAPAEPTAEVEQL
RRRLQSARVAAAKYQRKMLAVAGAARATRRDAAKALTEAAQGGRRHGHRYSRSHSRHRGPSGDWSRSRARRSSSSRA
APRAAADSWDHGLDYGLDLDPDLGVDLELDMELDSDLGLRAGSDQSPWRRGGGGGGDRRRASSTANRRRRDPDEEHW
PGLLGVRSTSLNDDDGSGRRRRSSSQQRRRQPHHSHRSKSQRRRSYSADDRGGSSPGSSGSNSGGYDSGSGGAHDDG
LRSRSSSRSRRHRSASRPSRRHRSSDSSRSRSRSAGRDHSRGPGRGGGAAAASRAAVAALRLRLRQLQLEVQAVRGR
ESRWLEAVRRADSVVLLLHGTASRAQLLLAGAPRPQDQQLAEELSRALSRASRHRQYVAHLEAQGGAQQPVAAVPQH
QQHLPTALRDPAAAGATGTGRGAAGALGGSVAAGGSAGAGPTVGGGWLQSPIEELERQAVDARAAAEAFSVQATAAR
EEAAEARAAVEDMRRRLLEAAEAHEAELAAARAAAEAAMVQASQLASELEEAGRQQQRLQQEAAGARRQLRSYSQAV
RQLMGDVRQEVDLADLPALAHQVVSSTERAFGLWTLREHDHIQATRRLVSGLRQEAAECRQLLATLAAMQAEAAGQA
AARGVVAGQELARQLEQGRLQAEAAAQQAAAAELQLQRRCDSLLEQVRQLTEQTQRLQDRAEQLQADKEQATTALAE
ARVRLEEATQALAQEREERTSLEGRVKAVVAQAVRVAESQRDAAQAAAVAAAERALKEAQQRDAEADKQRTEWEERL
RKERQAGSAKLRKQRDSHESELARLREAQQAALTAAHEAAAAAAQQAAAEATALQTQLQRHQQQQHNMELQMDGMRA
VLLDWEERLAEAGAQAAAAQQAATEAQATAAEAVREAERAARLEAQASAAAAARSAEAAKASERALRAEARAAAADV
AAAAATAAEQQLQQRAETAVAAADAKKREAEAAVEQASGQMTELQAELQTARAQLAAARAVAVSFETHLASEHAEAD
RLRAALRAAQARVYQLQEAAALGRLSRGAEAAAAAPLPLPASVPATVAVAAGPDVHVVSTGDGAVTVGLRAGSLEAA
ADGVGSAQQKQKQQKSVAFQLQARAPPTLPATGGTGSRHGPPGHAAAAASSLEVAVAEALSPLDVLLPPPSKPLPQP
QLQPPQQPQVHSQHELHQPPESAATVAVAPAAQAALQPAMEVETGPEAAPAPHAELQALLTEVERHCARLPAINSLL
DSLSLPPAVAAAVAAAAAETTDSSYSHTQQRVQGRGAMAADAGCDAVGDSGGDGSGSWSVGLLADPQTAEALTTLAQ
MVAGVLGQVVALEAAAAAQAAAAAAAVSGAGTSSPSAVPRAAPAAAAATAPDGAGVTPEGAGAGAEVTAVAAVARAL
RGHRRQLEDTAARIRAAQERVAAEEAGVLEAAYVRTAPEQTATAEEAGSGPRAEEAPRGRQQQWHQQQQPQEESAGE
ATMRAAGTATGLQPSVAGASGALTPPHRGQGLAQQRYAAAAAADVVAASTAADAIAGGPAAVAAAILTSPDAPQQDS
AVPPQPQLRSQPRTITAQDTAAAASGRAAAAGLSPDRAQQRGASSIRVRADAAAAATATATATAPLSPSFGSVVASP
AGSSSSESPAATRRGSPVATAPVAIAPVATAAAATAAAATAAAAAATATSSGAAAAAATPAAAAAISSSSPHAVMAA
APVASPSAPLLGAVLQDAQVTAEAEVADAQVTAEVEVELAPFRGFTPTATSRGGVWRRAVVSEGGAGTVAVRGAAPI
SSASHSPSTVSRSATATTAPPGGATAAISGGTGATAVQPVQRLAVLTPTHLQAELRSAELRGVNEAADPSMGGRSGS
ISPEPAPRPLPPTLAAVVAAAAAAAPVMPDPGRAVGTALGASSVTVAGPLVFMAAADASPGPAVTEPLLQPVMVAPP
ARLQPVLISLARRSPSPQPRSPAVERLQEQQPQQQPQQQQQATATAAFSSRLEHERERLRERLRALQRRPAAAAAAP
STATAALAPPAAHAAAAMPWATGTGSASASVTAATPGVAAASLDAPSPPPAGGVASPAPRPLAPLSAAELLDAAGST
AAASRATTRQSPSPSPAPSPDLPVSSTAAPGGHSASYGAGQSSLPPGAARLAAMTLPHRPGRYTSPPPPPSPPLPPG
ATALSPMAPTASTAPYHDAPAAAPAAVAAAGTSSTAASAGTAAAAGAAGTQWWATPTQLTATSSAPSWGLALAAGAG
RGAQQQQQQVQQVHPGWLLQPPSASSASSASASLPDAPTASGSWTQAGMAPGGPARAQTTPASAAAVTSSGPAAGQQ

QAAAAPAAAGAGPTSIGDAGAGTVAAGGGGGGGGGGGGGGGGGGGGGGGQGGGGGGARTVPVTGETRRGYATLHLR
GRGAVAKAGEGRGAST

>g17727.t1|PACid:27571822
MTSPPQSDQGRGLRRSLRVQGRATAPAAAGPSAAERVQAHGPARRVAAEAPEAAAAHAAAAAAPAPAAILAPLAAAG
AAYGVAAAAAPVAAAAPAFAAPVQPPAAAGAAYGVAAAAAPVAAAAPAFAAPVQPPAAAGAAYGAAAAAAPVAAAAP
AFAAPAQPHAAAGAAYGAAAATAPGAAAAPAFAAPAQPPATAGAAYGAAAATAPGAAAAPAFAAPAQPPAAAGAAYG
AAAAAAPVAAAAPAFAAPAQPPAAAGAAYGAAAAAAAGPSATAAGLPPRHPGRAQSVSNFDQALRRAFGAPAAPRSV
RATPAAGVGLQGAFLALGVPDVLRRRGRTWASDATGSSASSYRPSDSASETSSTADTASGRRRAAARELTELSPAGA
NNGRRVCNNPAYEEEEELQAALARSRRDTGHPRPPPPAGRAAALLTETAVTVHEMLGGGPTGGLALGGLAPAASPPR
LDRARRRHLLNLAASSGRHESMHSARSQQPSASQHIHLHVHPGPSPAVAAAPPAADAGADFFPHLGGLRARYGARLA
ARLRRHEAAVAAAAAVTPAAAAPAAAAPAAAAPAAAAPVAATPPVAAAPAAAAPPDDMVQLARVRAAALAHLQRLHA
ALGPPAPAAAAPAAAAVPAAAAAADAPAPPAAAAGPGPDAAAPQLPVSVVNMASSSSSSSAPASAGGCPLWPMPLP
AWQPHLLAQLDHAAAAAAPPAAAAGAPPAALAGGSGGVARMGMAAAHLAAAAPANGAPLSSAGAAWPSAAAMLRPAR
PIPSWSQTGRQQYDGERAGRARFMVDAHGYTEEQAACADINSPYAQDPRLIARALINGASVPESRAWVWNNEELDGG
QRTLERHRGRWVAVGGSLVDWRGQAFIDADGTVRVPHVAHFWLAEFLAAMHTPASFGPSPLFGFTLGATEAATAAAQ
RFLSLHRADTALCHKDMLGKVFSEGVMRTAVTQPWRDLFIMNLTREQKALPEGQVLSVTLVANCLAEAQAAFRGIAA
PTPAAAVHVTAASSAAASPAPAAAPAAPAAPAAPAAPPAPAAAAPAAAAHAHQVNQLPVVSQADLMRRENLVPDTTV
NQRKPGDAQWCLYHQTSHHAADQCRAYSALVSSNRAREAARRIATGEGARAFHKAPAPAPAQAHAHVTAALPAETAQ
PASAAALCPPHRAARRELADDLSVATVRSLYDTGGHSERPPLPSATAAVSHSQVAGASGMELGDCWDAEFGPAHIFH
LAARPPSSGPMQSALPLSFQPLPAAAARRCAPAPADGAATPTASSSTTSAACPSATTGSDAAASTSTRPAAAIPAAT
AASSDGAPSGADNRVRAHLNDAKFAALHAVLRDQGLQITRLEGDVRRYARLLRRLDAQAAAGCTHHSAMKLGRGVGP
LLLVDGLSSQGTPYAAALPDPASDVTLTVTEAASSRGLPLQYDTGIYVSSVGGVTSELAQIRGGLPLTVLAGTEQQH
TTRVVCFVVPGPQVFDILLGTNWLIDAYRSVWDAGRNMLGLRLPHQPDASPPAVELPLRQRNRAAGACHAVSSGRAP
DTSTSRTVIAAALACVAAPTARSHTDAVGVPPLTGTGAGTGGCTSGGRNGPESDEESSDYESGEESSDYESGEESSG
YESGEESSGYESGEESSGHESDEESSDDDSGGGGSPSASAGSAPIAPGPPTARPTDVLCERCDSWDHHARSCPWRCD
QLPCIRSFNKAQRRCAEAAVACGPADVAAERARDPARSPCRAFPTASPSPPPPPPGSAASSPPPPPQPPSSGLPPPL
QLLAAAAPTPAYAWAPPSPGSAASSPPPPPQPPGSVWPPPLQLLAAAAPSPAFAWAPPSPGSAAPSPPPPPQPPGSA
SPPPLQLVATAAPSPAYATAPPSPGSAAPSPPPPPQPPGPASPPPLQLLAAAAPSPACAWAPPSPPPPPQHPGSASP
PPLQPPGSVLPPPLQLLAAAAPSPADAWAPPSPGSAASSPPRVWDAGRNMLGLRLPHQPDASPPAVELPLRQRNRAA
GACHAVSSGRAPDTSTSRTVIAAALACVAAPTARSHTDAVGVPPLTGTGAGTGGCTSGGRNGPESDEESSDYESGEE
SSDYESGEESSGYESGEESSGYESGEESSGHESDEESSDDDSGGGGSPSASAGSAPIAPGPPTARPTDVLCERCDSW
DHHARSCPWRCDQLPCIRSFNKAQRRCAEAAVACGPADVAAERARDPARSPCRAFPTASPSPPPPPPGSAASSPPPP
PQPPSSGLPPPLQLLAAAAPTPAYAWAPPSPGSAASSPPPPPQPPGSVWPPPLQLLAAAAPSPAFAWAPPSPGSAAP
SPPPPPQHPGSASPPPLQPPGSVLPPPLQLLAAAAPSPADAWAPPSPGSAASSPPRPPQPPGSVLPPPLQLLAAAAP
SPAYAWAPPSPGSAASSPPPPPQSPGSASPPPLQLLAAAAPSPAHATAPPSPGSAASSPPPPPQSPGSVSPPPLQLV
ATAAPSPAHATAPPSPGSAASSQPRPPQPPGSAPRPPAYTSALPLPGFADLLPPPRRSPGSPPPLLAPSPQRPGSAA
DPWRSKLPSETWWQFLGRMRAAGIHLTAMPESEWAGQGRPPPEADPPPLSASRSASPVHQVALGFAAPPSPAVASPA
AASAAPSPAAGRTPERMTFDALLLRHAAAEAAASSTYPGAPPPGRPAPLSPAAPHTAAAASLLPPNASCAPAAPLVA
PELGLPRLRPIMTRGGSSSAAPLPSGGCDLAPCSPAMPPPVAWPPQSASGPGLPPLPTAALPDQGWSAPPSQPSLGA
PSGLAAGPPPPSPSLLAEAEDLVAAFLHRVPPHALMSAHGAGGEFGGSATYDPEDSEDDEVPPLNPPSDDEEDFDDA
GARGGPSSHMTTGQLPPADSLRVLPTAIPLEAGPALPSCNDPEGRREPLPHDLHTKQPGGWMLYQHPDATDADVNRL
LRLVAQFDTGGSDGLFARSLDDLTGYRGGCDPVEINLSHDRSIYECPRKHTPAEYAIMDAKCEQLRQAGIIVRAPPE
AKYAMNSTMPAKRALDGTMTDTRFCQDARRLNEATIPDSHQLPLPEDLFQLMANAKVWAKADARAAFNQLPIKLEDQ
PKTAFWWRRELWMYTRLLYGLRNATSNFQRVLDTHLRDWGLAHRVVAFVDDLAAWAETVPELIDVLCGLFRMLTSIG
IKLHPEKTVLLADGIEFLGHYVSPRGLQPTAAKIAAFMALQPATCLLECQRLLGSFGYYRGYVDHYSVKMAPITALT
AKAVEWGPDTWTAEQQRALDAIKHEFAEGDLVLRPLDPQRELILHTDWCEVGISGVLGQLDDDALEYMVACVSRTCN
VHERRYGSYKGELLAAVWAVQTLRPYLHGRRFTLVTDHGPLEWLMTQRDLTGQAARWALILQQYVFSVVHRPGKLHQ
NADALSRCPLPTDVDAAGARLDQADEPPPPPPPPVVLYPGRRLPAYAQPPTATTLTTIAEAALPSADGWLPRLAARLA
SSTGAAWPSTTVAGARASAVCLATLHASRAAGPGACVVDGSDLLAGHNGTTSDVREMAPAADTAVRTAVALVNRHGA
SLRQLRLTPRLRLRLCTARGSVVGLDTSLQQHSFFDAAQGDGVAVLEAPGGLAAGLQACLRLGIKVRRYVSMEPQPA
VLGAVEKLAGAPRQTALSGLPLRDGGQWLVFASWGSTNGWSDADALSFVGGLQLALRKQHCAPPAYLLHGPTTTRAE
QEQLGFPFGLPATMDTVQAGVAAHRLVTLYTNLAAAEHLAAVLERLPPEPDRTLAQQLEPGRRPMVALRVEPLPYRP
AHVPGQRLVALPAGSDAPPLRTVPGDEPCHLSVTDWEAVLGHRPGTADTLPPDLAIPALRNPISPRTAATALAACLA
LQRSYLTPHDTHAPPPLRVAPAPSPLGGDEPAGEDDQATVIARGLLQRQPAPDLAFTFSTLAAVAADAQEQATATAE
NQSWTDVWEDAEVLAALRAGGAAAGGAAPRRVTRRMADYRWVNGQLLRNMSDGLTRVCPPPDQRVALTQAVHQRTGH
LGVRRTLALLQTGHWWYGMKNTVRQVLRACRACDMSNARGEALPSRLSPLPVMGLFYRLGVDLAGPLPPSEPDGNRY

VMVVIEHFSKHVELLPLRDKTAARTARALADVFARFGAPAEVLTDNGSEFEGEFAELLERSCVDHRRTSPGHPQADG
AAERVVQVLKQALRKTCYLAADASAWEQALPELLLGYRCSPQASTRYSPYELLFGGVAPVIPPAVRERISVPLDFED
PEAATASLLARMEWVRQAYPAAASNLLIAQHRDTLRYSAIRTGRYLAKPITYAPGDYVYVRRVDAAQRPTLHFGQHD
TILRVECIGPLGVAVLVGRDGARVRRRAEHLRPCHLDIDPAIDPQLFRPQRDLQCEVCGSPHQEAKMVLCDGCNTGW
HTHCLRLPRVPRGQWLCPRCKELDAEEEVLRAGWAAEETGDAAAPAAAAAGLAGTAAGEAGAAAAAPQFGAAGRRER
RGVPAAAAESEELGRLLFPRARTRRLDEAASDLGGRRVRRAEEGPDGVLEYLGALARPRYFRVQWSDGEEQQLTLAE
ARRLLAD

>Cre17.g708950.t1.3|PACid:27571879
MRVAGGRWAGPVALLVTLCAALTAAADESSTGLRSRRLLQSTWTYSNSVDLCGSSYGSPTLQFQSTAAFQQYGAVLG
LEVTSENAQTNSADVKKYEVIYGIAGIHSNYPAPPGVRSTMVLGSPANGRTAFIQTEGNSAASGVQVRTIEVCCTPA
NYVGALNIIFSDNSRKKMGSQQCNKTSVAATAPANGQLASFRIWTGTQPGPGKTNGGSGVAVAKLVAVWAVPGAAQS
PPSPSPPSPRPPSPGPPSPEPPSPAPPSPAPPSPQPPSPAPPSPQPPSPAPPSPQPPSPAPPSPLPPSPQPPSPQPP
SPNPPSPAPPSPQPPSPAPPSPNPPSPAPPSPNPPSPAPPSPQPPSPMPPSPQPPSPAPPSPNPPSPAPPSPPMPPS
ILFAQFNTTEYFFDDASYINPAIQSLDLRNVLGTVLIAKASYSTVSLPSGHNVLYGNRFGNGRVSGTAAQSLVTRCC
KAGSDPKMDSLLLRQVLWTAVLGIKLGGAKVRVSDPKFQPFAEWLVATSATLTGRDKNALKAPPNGNGNNPEWYLPL
SNFTAQYNDPRFNKPGGFVVDLYIISADQVFTDELLINSTLSYVRDTGRGLFVVGSDLTYYDNLFNSTINGTLSNQT
TVGPGGVTRRRHARRTLLSAEEEAALEDLRYGAMLMGRPGLAVDVTADPNGANTLEALTRHYQARRRMQSYTVNTLL
GPMGISYSSTVLPAVSTIVVSSSTLTNCQQASIMYIYYLQGKIALGPADLDLAVKTISIARGSYSLSDPWASQYFWP
YQDDIQFYGGVIPNQPPPSPPPPPPTLLSPPPSLPPPPPPASATLTQLSCYKDNSNARALPYRLGGSAAMTVELCTS
WARSAGYPFIGLQGTNCWAGYDLNLCNTYGTSSSCALRCGGNTAQICGNTGTSPMVSSVWQVL

>Cre17.g736100.t1.3|PACid:27571901
MEDILQRHAFIIRYGGVPVPPVVDWRKLLSAPVNQKPCGTSFSIAITQGIEALLAVEAGAPVKPLAEMQPVLCSEDV
LAGKANATTCPSSGAGGWVGDAYTYAMRVGLASRDWWTEATGREPFVELLKCPVPALRKKLVGTANGTANGTAADIT
RIAGWETVPTSEHALAQALANQPVVALVHAAPDWADYAGGAGSKSASGSGSEIQVYDGACSSDPADANHAVLVVGMY
ADAWVIRNSWGRGWGYGGHMYLRRGVNRCGIANLATYPVLRPAPAARPGKLAQQAACSAVQVVRNTTLRAVAAAADA
PLYDLLRANPHVLGGADAVLGPEPTTVFIPPCTHAVPTPPPAEAACGLSYTVQLAANASGTAAAAVIAGREGQLGNQ
QQGLRRRQLQLLEVPGLLMGGGANEAAAAELQVRMEARRAARREMQRTRSARRQLISGGEQKQDTAASASSSLPRLL
FGEEEEDQLIASLLGGGGGGGSASDAGQQQAQRGRALLQTGGTDAQGCPAIEGYSFLQSVDVRAAGNVTTVNMNDDF
SIYENATQLAARCSKTCECAGFVSIEGMSGFLKTTVAPELRRTFNWSPCSGLYVRDSPCGVADYGNAVPLFQFAAGR
KPAPGTPRAGYVVATSMPLEKAAQICQDMPGPCEMFGATSAGHGVSTTGSPNWVFFLRQQFVTPDQPTYYNGSASFL
DDWSPPPPPPPPPPPPPPPPPPKDAVRSPPFPPRPPPSPVPQGKRVPGYTYYRMWISYDDVVSAGWLNQAPGVSRAD
HRATVAAMCADAYPRCKLFDGSGNMYGWDDAKPITDVLMPLWDDAETTMGVWVSELEPTYNPEAASCPQVSGYDFHP
RTDVVASSDISSVDLFGYGYVFGMPPLQAGLHCWSYCDCNGFVLRGNTSTIIPGSYRSNPAISVQLKMTVLPQLRIK
KDTWGSCEGLYVRRDPCASTTTCPMFDGYTVRIDYEHYGDDITQHASAQEGRADCDGHPRCAGFTWRPSSTGDGSGT
GWTKRAVVPVSPRIGACTYSKIGGAACPTLSGYDIQIDIDAVGGSLALISGGIIAARTNCTADPRCKGFSWYTAGHV
ARTRDSLANTTSSAGNCVYIKRTPFVGAGQPVPGFNYLPDSKPSLSTQYQWSGYIPADSANASEVARLCDRMYHNSC
VMFAAVPNFWAGPVWVFMRTLSLVTVSDPYFNQTVQPPGVYVRENMPPQPPSPPPSPPPRPPHPPRPPRSPPPPRLK
RFPPPPPPPPPPPPPPPPPPPINDGAPRPPPSPPYPLGPYYDVEGYTLVRDVMPGAGATLLGSVWGGSYDNEYTGAR
QAVIWRLVADVCDKLYPRCKMFDHSGAMYGWEEDGVMNYAYAPTGYYNYWQNSLGETFVNGVFLSNKLNTSHISNAK
PPPSPAPRPPGPAPPPPYVSDWGQPLPNYVFVPNAKPSLRAYYSFNAGNLRTDAASLVEVANLCEQIGNTCKMLALV
PASWSGYQFINSLETMTNITDRNYDYGPTPGVYLRQNLPPAPPSPPSPPPAPPSPPPRPFRPPRPPPSSPPPYPPVF
SPPPNPPPPPPPPPPPPPPPPPTMPMPNAPPPPPPRPAPAGYHIDGFRLVYDVYPNSEAAWPSGYIASSFRNSTAFR
NYARVCLIMGPACKMFDFNGNFYTWDEPAPTKLWYGRPQEEYQWGYEVGLYVRLRQPAAGAQLQPPPSPPATAPEDQ
LMQPPPVEEEAADGGARRPPRRPPSPAPWAPQLPPAPPAVSSLSARDLDYLYSRQRKFKTYLEAAAAAYVDSFRQAT
YSLPAGTALKPSQEAPAEPATASAIMVNGPAMPLLVSAADDQVYMAAAEYGMGRVVVLSADVVTRGGAIAARYLPLV
KHAVLWASAGAGKALAPPYRRVALAYDTPGLRALAKVLISDGVAATAKFYDGNVVGPPPPPPAEVLLLNASRSGYGT
DPDSWLRGGGYYNTINNNLDTYSTGVVVLGAAIPPPASYRPLYGRPVLFEFALNQLLLMRVGLALQMDQTSLTGAAP
AAAASSPSPAPSPSELLLAAWPLPPAQPEPTPPAENDNVTVAAPPPPPAAKPFVRPLMWNIHYALVTAALTKESPDY
WEVEAVARSSLAKSLKAYMQIQSGWYWVTWGRLDNYLADAGFSSPPPPSPPPSPPPPRPPPPPRPPRPPLYLLSDLQ
PAFVRAHTQVLNGTRSTKYPVSALVSASATAATAAGAAGATAAGGVEPVPARRLLLLEQSFPLLTDPYGAPLTAAAS
YGGGRVLVAGQAEVWRGEAAMQMAALDWVAGEGWRRYALRADDTPYQLPLNVTILPDFGADKLPKQSRRVVRVTTSL
QPLTSTQLAHQQVVLLSTSQQPLAPATIATLRKWVSRGGGLVVVHTEPRAPAEVPSPTRQPQETWVDGYSFNQLLGP
MGILITPTLLVPRVAPAPLQASQPDAGLLLANSHFAAQRLLAARQDAPLPAAQMRRAMEAVQFAERSLGAQVAAFPT
FATTLSALRAAAGPSSQPPAAKADSAPPPPASSPPPPATPLGGLDPESSVVGPLYDAWIRSPITEPLDPIIIDFAAT
CPPRAFITQLSGKAWSTEDARLTGAMRSGLLGLLVTELRLNCSDGSVVKVGYPTAPKLYAQLTAPLGNSTAAGKASF

GVEWAEAPCPGGYDRARARPHGGDVRNPVKSIQLFFRCRDTGAWTMLTAGIGATAVAPEHVSLMGPGSAAAAAAYAA
VGNHHVGWLTADAGAAAGDGASTAGGGAGRAENALQQFAAAVDLEATPGAVECPGDQVLAGFSGALLEPDLYEWSSA
ATSASSSAFRAFFRPDEGLLLNPAHTSLLAVEGVLCRAAAAAAAAASAAPEAARSPAPAPTSNSTAPGGTAGGSFVD
LACGPGVALVGLQAAMSSESAAAQPKLDYLAVRCSDGSVVSIGAVISGPKTLLVDSSCGGGYRAVKVAGAGATASGN
GTARVGMGGAFALRCGEGGSPRSWTRVGPAVLPLTPPAAPLTASEALANAAEVACAHGSALTRLRVGVAAGGADGTP
ESITDVELHCGPSSTDSASGDQSLTDLAARYGITLAELLAANAGGRPGPQRLNGTSRLSDLKAGADESAAVVVVPQP
CGVSPSPLPATSTVAKCGYWWPSNADERDASAAGNVTCGWLASLFLSGQYRAVATLSRWNGGVCPTADTVLGAGLRL
CVGMAPPPPPPAPAQSFAPPDEEQAAPPPPPPYGGPAYGGGDTPATGPSYGGAAPEASAPPDADGGSSGGGGRRRGL
RWWWNTPPPSPPPRLCTSSVVVAVGDSCDLIAATHGVDVLDLITSNDLDCAELKVGSELCLPAPARDIAAALDLSDL
AAINVVASAADMLEAADPSRHDPPVPPSRPPALWSPPPARRRARPPRPPPTLPAPRTPPSPPSPPPAYPSPDAPAPP
PLPPPPALPSYPPPSSSPPPPVYGTDGGDSCRVPNRCGPAPNRCLDTTTGSYECACAAGFVLRADGATCVPPTENVA
LRKRAFSSSVLRDGSSPDWAHLPHWAVDGDVASAFASAEDGSDPAPFLAVDLGRRYGLGRVEVTGLLRSAAVYVTDM
SVASAVDWAAAQAAPGGVRLCWRQPSKGRAASSYGYSCGGGVEVVGRWVVVTDERAPPQGPAALHVAELRVFGTRA

>Cre17.g737400.t1.3|PACid:27571954
MQAGDTQATAILRSAPVGGLFSSADSLCATDIASTWFNTSSACDAFWYGVQCQSTAAGFVVVSLELPGCFPSGGAFS
TVAPPAALAQLTTLTKLDLSNNKATLDGTGLPLAYSALSNLVQLNLSYSNLQGGPVPSAFHPEWSQLTGIQSGWIDL
SSNRYLGGNVPDSWSALAGVENINVANTGACGTPNAAVAAGLSPASSPLPTRCERVDFRKVLEVDLKPVLNNATSAG
SKCDLNTDWDSTRTIDTWTGVTAVPLTTSGVDIFEITALSIADCWANNGQPFSSALPTNLTPLSSLTSLQYIGNKGE
GSLDGIPALTALRFLDLHDNTFSGSLPANMTDLVALTELRLNINSFTGGAPSSWSAFAIIQKIDLSSNQLLTGPLPA
SWSALNDTLTFLNVSGTALCGDAPGLSSVLATIPPNCPSPPSPPAPPPSPPGPPIPVPVPTNTPPRPPSPAPPSPAP
PSPEPPSPAPPSPEPPSPEPPSPVPPSPVPPSPEPPSPAPPSPEPPSPAPPSPPSPEPPSPVPPNPEPPSPAPPSPV
PPSPASTSPPSPEPPSPVPPSPEPPSPGPPSPQPPSPAPFTLAPPSPAPVPVNAPSSPSPAPPSPTPPSPTPPSPQP
PSPSPPLSPSLAPPSPIPPPPLLVLAPPPSPAVLAFPPPPPPPPPPPSPPAPPPPPSQPPSPAPPVPPSALSPSPPSL
VPPLLPAPSPSTSSPPPSPNPAPLPPLPSPPNPLPPGPSPPDPLPPSPLPPSPVTSTQRPPSPPNPPSPPGIFLNGW
EIVFTPTFCDGHASDKSFTALSSDLLDQYGRVTAITYVYDSKAGGIVVAVKLVFGTGPSTHVETVAGSLYDPGSPTL
SETTASLVSASGSALDSVSVCCSDTGAVAQLTATFTDGSTSSTGPCPSTGGAAAARHRRRTLLQGGAGGGGSAGVPG
GMVVGGLRGGAGAGFGSMGFALAVRRGFPPPITSPPPPVTPPPGLRLPPPPVPPPSPAAPPAPSRPTIPPRPPSPPP
PPPFPPSPPPPDLAIPLPPPIPPSPPSPTRAAVAAAFMFYVKVDYPQVTANVPPTLPNRVPVATLVLTGPALPVVRS
PTDDPIVATSRYGHGRIAVFGGEKLITGCCKPKAKPGRPTSDPGTDQLIVNIATWAAWYGTKVGKALIRVADIKFLP
MAKYVVAQRPDSFQTAKSARMNFVLPLATFLKGGHQKCDVYVIGSYDARYLQTKVSGFLRDFVFKGKGIIVVGPDVM
PTIFYLGTGTASRRRSLFESLELPLGREAASGSQLWNAEDEPLVQEEGSGMDARVVVNSGGRTLLQATAAGAASAAA
ARIGAPQFNSSLIPVNLVSGPMGLLITGYVSDPGGNLTVTSPSELQNAELAASQYVEYLQGQIRLSVPDLKIVLNTI
TRARASVPRSGAASSPRFWDLVDASDKLATSAPALPPLFDDPPPPPVFRAPPPPKPPLPPSPAFLPPLTSYVGCFFD
NAQARALAIQLLVGQPTSVTRCLDAVPPEALTNRTAAITFLAMQRTACFGASQLSAAFVAAQLPDGTCSQPCPLVAQ
QFCGGSASGTRVAVSVYRILSPAPPAGRPPRPSPPLPSPAPANRPQPLAAAPAAKPGTAPPPAARG

>Cre17.g737400.t2.1|PACid:27571955
MEDLSNNKATLDGTGLPLAYSALSNLVQLNLSYSNLQGGPVPSAFHPEWSQLTGIQSGWIDLSSNRYLGGNVPDSWS
ALAGVENINVANTGACGTPNAAVAAGLSPASSPLPTRCERVDFRKVLEVDLKPVLNNATSAGSKCDLNTDWDSTRTI
DTWTGVTAVPLTTSGVDIFEITALSIADCWANNGQPFSSALPTNLTPLSSLTSLQYIGNKGEGSLDGIPALTALRFL
DLHDNTFSGSLPANMTDLVALTELRLNINSFTGGAPSSWSAFAIIQKIDLSSNQLLTGPLPASWSALNDTLTFLNVS
GTALCGDAPGLSSVLATIPPNCPSPPSPPAPPPSPPGPPIPVPVPTNTPPRPPSPAPPSPAPPSPEPPSPAPPSPEP
PSPEPPSPVPPSPVPPSPEPPSPAPPSPEPPSPAPPSPPSPEPPSPVPPNPEPPSPAPPSPVPPSPASTSPPSPEPP
SPVPPSPEPPSPGPPSPQPPSPAPFTLAPPSPAPVPVNAPSSPSPAPPSPTPPSPTPPSPQPPSPSPPLSPSLAPPS
PIPPPPLLVLAPPPSPAVLAFPPPPPPPPPPPSPPAPPPPPSQPPSPAPPVPPSALSPSPPSLVPPLLPAPSPSTSSP
PPSPNPAPLPPLPSPPNPLPPGPSPPDPLPPSPLPPSPVTSTQRPPSPPNPPSPPGIFLNGWEIVFTPTFCDGHASD
KSFTALSSDLLDQYGRVTAITYVYDSKAGGIVVAVKLVFGTGPSTHVETVAGSLYDPGSPTLSETTASLVSASGSAL
DSVSVCCSDTGAVAQLTATFTDGSTSSTGPCPSTGGAAAARHRRRTLLQGGAGGGGSAGVPGGMVVGGLRGGAGAGF
GSMGFALAVRRGFPPPITSPPPPVTPPPGLRLPPPPVPPPSPAAPPAPSRPTIPPRPPSPPPPPPFPPSPPPPDLAI
PLPPPIPPSPPSPTRAAVAAAFMFYVKVDYPQVTANVPPTLPNRVPVATLVLTGPALPVVRSPTDDPIVATSRYGHG
RIAVFGGEKLITGCCKPKAKPGRPTSDPGTDQLIVNIATWAAWYGTKVGKALIRVADIKFLPMAKYVVAQRPDSFQT
AKSARMNFVLPLATFLKGGHQKCDVYVIGSYDARYLQTKVSGFLRDFVFKGKGIIVVGPDVMPTIFYLGTGTASRRR
SLFESLELPLGREAASGSQLWNAEDEPLVQEEGSGMDARVVVNSGGRTLLQATAAGAASAAAARIGAPQFNSSLIPV
NLVSGPMGLLITGYVSDPGGNLTVTSPSELQNAELAASQYVEYLQGQIRLSVPDLKIVLNTITRARASVPRSGAASS
PRFWDLVDASDKLATSAPALPPLFDDPPPPPVFRAPPPPKPPLPPSPAFLPPLTSYVGCFFDNAQARALAIQLLVGQ

PTSVTRCLDAVPPEALTNRTAAITFLAMQRTACFGASQLSAAFVAAQLPDGTCSQPCPLVAQQFCGGSASGTRVAVS
VYRILSPAPPAGRPPRPSPPLPSPAPANRPQPLAAAPAAKPGTAPPPAARG

>Cre17.g740050.t1.3|PACid:27572000
MLSAGARFRRITVPEGSQLVIDDAPLNLTVASLQVYGNLSLGSPTCRLSSAISITFVPATGVATGQMALRGMNTSTV
DIHGELFAPTWTRLAQQTARPSTSWVSLQQPVNWRAGQLVAIPTSLWKDECRNQNEVKQIASVSADGRNITFTTALQ
FMHYGGPEYQTEVVLLSRSILLQGSPETAANLTGGHLRIESKHGRIRGVLGYRMGQQTTMGSYPFHFHMAGEVPPDS
YMTDNSVYGSYWRAFTIHGTHALTVANNAAFHVAGSAFYIEDGAEERNRITGNFAGYIHPLGRADPCDAIGSVFAAP
TIRQSATLLQPADWAAAGFYLSNANNYVDNNAASGGTSGFMFLNLPLPIGLNREVPLEPYKRPPLSFSGNTAHSSGY
HWDNAAAIYCGGDLRYDPADNVTLTYKIERSTFDPRDPNNASIPVKFVLRNTKVWLGGFSVASYGDRFMVDGFESHD
GTMGAAVRGDPNELYNADLNLNSPHGSWLTSNMPNDLQQGYRFGLQFYDTWMRHLVANVTIRNVVHSPTEWLTQAAL
ISMTHSDQFKPYHVASIRGLRLLNVSSAAVLSNAVTRTGSWRFYNFLDADGSFSGTQRPTLVASYSATNSTGPCDTT
SVVPCASPHAWWMADPAACSTTPPNVPAASNIIACDWYPWRTQARLRLEVPTYTLSPEQSSLLSPGINNINIYDAGV
VAQFGFTGANRRASIITRSEGVTGLTGRGGWYVHWNLGAPKTMRVWAETVPRGTSILYASRYPPGTTFNVTRAASAW
LVPDAQLLPASSLDQVLASDSGEYYYFDGSLLFFKVVDIWEQPYQGREPARGEGTGAGLSVPGSRWGLYYQVDAGMP
SCSLSPYPSQNGQDFSAQFCAMNGTVDAYIPPAITAPYNEWAIPYCAEVAPPPGVCAAMGVASVDCTCTALQLQGRC
PALGVGGLWLGATSDADLRTNVEKAQGYCSVACGRCAEGQEQCGDVPLMPLSLSQNMTCEQMVPVGRCYEVAPKGYC
WGTCGVCSGAGLPCVDLSWSDQYSCTDLKSWGLCNETWVTDNNYCRKTCGACMDLTAKPPSPRPPSPLPPSPSSPSP
SPPSPRPPSPLPPSPRPPSPPPPSPRPPSPRPPSPSPPSPKPPSRRPPSPRPPSPAPPSPKPPSPRPPSPRPPSPTP
PSPKPPSPRPSPPSPRPSPPSPPSPPPRPPSPSPPSPLPPSPAPAAPLTSAATATTTAQRTATAREGGSSDPTLST
DGTNAPADAIASSQSSAASAPAAVGSDGLTATINGAQQPSLSSNAAGGDLGGGTTQQGLSGGSASTAGGDGSGSRGG
PAAAVIAGAAAAGVVVALAAAVAATVAVLRARRGGGGSVVPLGAVVLKSEGAASSRDAEAAATSPAVVGAVAAPAAA
AAAQGMLQQPATGPPAGLQRRRSSRKMTGVVVAGAAATATATVGVSSGATAAGITSSRNAVHPAPATAAATALVTGL
RGPSADSGLNNAAAGNSSDAV

>Cre17.g730650.t1.2|PACid:27572041
MGRAATLDSVWPADGDPKTLLCVRVPCLAAELFKTLFVDPELQVQLHKQRNDSPLVETAWVASRDQLPPELYTWQAG
GQRNEPGASLRFRKVRFESPPTALASKPFQNEETHVLTHWAPDQLYVLEAVSNTSAPYGDKFSVFFKYIIRPDASSP
GTASCVLHLLFCVDFLPSMNRMMKPVIAKAVDSGVRGTFKIFRSVLGGLRPGTADVAEAELPPGGLLAGALASYSSS
SGEESESEEDEAEGEGDEDKARKRHHHHHHHAFASAAPIPAPPTGPTPGAHPGMAAAPGLLSVFSQQLVYKDQVVLL
ADLVGAGLRSVTHSELGAQALAALLTVWLISLAVAGLSSWQQLCAGGGGGGGGAGGGEGGGGGGGGLLLPVCWPLRVV
DVPDSTMEVLTSLALVAAINWALIRGSEAAVRYLAATHPALLAAARANAAAAAAAGAGGAVGAGGAGGARGGVGVVG
GLPLGAPPPEQAAAVQAPPAPIAAPKQSSGGGSSGDATASAAKAAGAAAAGAAAAAAAEAPAVTPAPEATAAGSGKE
KGKEKGKGKDKSKDKAEKEAKGKKGSSGGGGAAATQKPPPPPVLAATQSKPLRAAPAPKQFTPEQLRMLEMGAVSSG
GAAGSRGASRAEASPPPSAAAISVGGAAAASAATNPSLISMPDAWATDLPAGMVLPTSPDKAPAALASSSTGALGST
RSGSPPPPAAMTRVGTSIDGRSAAAGAGAGAGAAYAGMSVQQLDAISLARSSASSLSVGGGGITPMPSPAGRSPPPN
AAPPPAVLAAAAAAAAAATGAAAAGAGAGATMSGPSGASNASSFANMFTKEGIDSMAESLRSAFRWDLKPADEHAQE
PTSPSAAAAAAAAKPNGASAAAAAAAAARAAAYRSPPAPRALTVGGGGSLAAMSEPVQSMYSARSDAPGFMTAASNELN
AGGGGAAEPAVVVEEVFENERFQPFRGWGHMWPGHFLPSDRVGHWSDRQGKPGGTASMVWEQVVPQLPAGWRWVEDE
WQIDLDGTEVEAVDGDGWSYALDFYLLKYPPPPQAGKCSLKHFVRRRRLFRTRVRLPDRSLLDSDGSDLLGLQLQPL
SPDASAAAAAAVDAPPADSSAPSALTLDPDAAALPVSTSAAIASAPASTTNGAGAQAAGRPGSGAQAAGGAGQGLEG
LPDQHPYPQQHPQQQQQHLTTSSVLSLAEALPSHSLDSHSAGRLGGGRGLGGLSASAPAAVAGVETLAGSGGGGGGG
GGSGGTVDFRPPASLPSVVGGAAAGGGGGAWPASPGGMGPAAMPRPRSTTTLTSGAGAGASGGGGSAAEQQLHSGSG
TAKRAVAIAEAASGEARAPAEAAALLDLLSGLDAAEAPPPSAAVLPPQPHAPAGRRRSSAGGAGAQGRVGASPSPSP
PKGHAAGGGPARAGQQQQQQQAHPAMGGAGRQADVTAPQGGAAGVARLDSGGATAPAVAVRTAVAGYAASPHAEHDS
EPAARVSTEGYVLSPKTEVKAHDPLGALLQDEAAALAEPSVRPARSAIDAAAAAAATGMVAPGIEEGSLQAAAGGSA
GLPSAARQGGGAGAAAGGGSTGKKAGGKLGAVPLRRGGSDAAGPSSAVGGSESAAGGEGAALKGAAIVGQEERGPAV
GAEGEAGGTPTRADTGASGAGKQQARAARRTAAAHGHGSGAGAEGSGSAGDQAEEDLGGGAVVAADLVD

>Cre17.g700900.t1.3|PACid:27572087
MPNADADSNGIAAPRDLNGMGARPPAPASTQHNTTDSDAGDGVTHVLAAAAGPLAGPVYGGTLFPPPAATAATATAT
GGRQSSRAQDAAEPLSPQGPQQPQARRRRGARTQPLQREQPPSSPPHGEVQSPGGGGGSGVAAELGPLPHPQPPPPR
PPPTGAGSKPWPLGAWSRGGGDNGDGGGGADNGGGGGVLLDAASGQVAAAGVAGSAAAGTQGGAGGVAAGDIGTGAD
TAAAAVLATEPRQEPGVSAGGGGGGGGGADNGAHTRGAEPLGTAQGAVGAAPAAAAAAAAPPPPPARAKRRPRGTWSR
TGAVADEPVTAALAGMSDTSAAAPAPAVAPAEQPVLPLPVSQLRPPEAAAQQQQPPPPPPSPQAQQESGSSLSGAE
ALETHTTQDTDGGSSSAEAVGAQMTQDVGGSAASLLSSTEPPPPPPPPPSPPPPPPPSPPPPGVPKQRKTARGRKTG
TQAVEDVPAMPAAEAALEAAAGGPKQRKSARGRKSGTQAVEDVPATPAAEEALEAAAAVPPPPPLPPPLPVPRTSEL

RTPGAFLSCYDPLVEVVRAEQAAKRKPRRRRSATASAPLQDPAADPETDGAGAAAQAGGGQQPQPGQAPGQAQPREG
SAGVPASGKATRLLRRFTEFEQQQQGPQQQQTGATDAGTGFRRAGDGAGGDGSSSSGSSSGSSSNNHWEEYLATLST
ADDTQQPDGAATAWAAWAGGSSSGSSSGNKERGEEPWLDRTEPLPRELRHAFWLELTQPDHFQHRHFHASGGSSSGV
GRSHSGAASSSSSSTGDSPPLDFTADNAGGAASIGDRGGLPADEQEAALAAATAVAAATTSRFFRTADGGLLRPGCD
ALPVIIGVRQPQPYGPAEVYELPDLGLGPMDEQTWQAWGDLPYSNRPQPLVEIAPAAAAAAPGGAGGGGAAGGPGFE
LCPEEMRGAGGWRTYEVLVGPGYSHDPTGTLHPLILRCNGRVCVNARHVWDLLEFVADPSRDAVRSHLTNIALLQRR
AAGSSSPAGGSSSAAGRSSAGGSSSPAGGCRGAAEGRADGGLSDVEDWAHAEWEAGATATAAASGGSSNVGSGSDGG
HKVHGGRRRGVGSSGGTVTGSGELLSPLFCHWLLLRRWGARGLAHVRWGPGDTLSHVQAVRRNPAYELSRSVSAPAW
MAVARAAAEARLDAAPLPPPPPPPPSSGGAPRAGAEDGGDAGSAGGGGSGLWR

>g17142.t1|PACid:27572116
MGLNELEPEIIGVNATVPVPTFLAALDNGGPDAKQQAGKACVKLIVSSEAADRVLRLSSVEVDMATGRVAGLSALLN
PFHGYEVASWESQPEATAVVLRLREPPTPAAPHERSVGEGARGTQSLQQSQPRSASVLSHQLQQEQPAAAYAYAAAA
FSEGDDPAARARGLAAPASGGGSGGGGRSALLANVDRYTSAAAAEPPPSSRLPMPQHSAGGGGPNVMDTRRSAHSAS
PDICTVPHQRQQQQAHPAPPAGGDYQQQPSASRVMVGGGGAPPPAAGTAAASGPTGSAASSGGGGGMQMPAAEAARH
AQTAQQQAPEGRERQQHVSPPQRQQLAPPLGRRQQQPAHVYGSAAGGGSGSRDHLQQQHHYQAQQQQQPSEEPSDYA
AVQPSSTLQQRPPPPSHLSSAPPLLLASLARHHQQTPPPPSSLGQRKPTAQHLDAAGGYQRRPAAAPLPPPPSHQPP
PLQQASVRSGSAPLLTVSAASAAAVSGGGGGPGRWPTTEPVAAAGGSVNSGGAALPVPAGSAAAALLIPAHHITEAF
KGRRPVDPRGVPVLLSVEDGGGGGGSDRPVSPATTGSPHGSHGSGGGGSRGGGQRTYPGTLVVREDGCGIVHNAPIE
DAVARYQDHTTIGWRLMWLPPPPPPPPSSGHRPMEVAAGAELLVIWRVRNHGGQQQSPPPPPTQAAAPASQHQQLQG
RHPPPLQQVQPAAELPRQQPPQQHQHYHQHPHPHPQQVTAAAFASSAGGHQHGSSTARGPAPGQQHQQHQRDLVSSR
APPAPASTPAAAAAGPPTGADALRPELLLRQQSSMTTNGAGVAGAPTEKHGAPTPQEGDLMGSLGNMLSAAAAANPA
AVVKLLQQLQRELLQGGGGGGGGDTEGRGGDAYAGEERVLAAAVAATTAGSRPTAEDSKSPARQQQLLQPPPPPSSS
VEALQPSLRAALAAAAAAEQPAADCTPAGSLPPAGAATAVAEAGGGLDVIRVEKPDEDMSRPVDCGIGNDGSGGVRA
AAEKDASSAPAVHAVVAAVPKAAAAGGGGSAMDLLGSGAEAGAGSGGDGGRGDGGDSVGEASCGSEAPLPAMPPAAA
LAATAAAAAAEDGAVMSGAGGGLLGSSGAVSPQVKFEVNGARLPKVCVTLIAPAPKAAPTDKSAPDHASMSASAAPS
AQSSSLCGGALPLPWSLLPEGCRIQWRRRTDNVLLVAAAVSESPLDMDDTTTTLLPPGPEHEPCTAGGIACTTGGCK
KRASPAAAVPMPAGAADVAEEAEEAEEAEEADEAEEGGPAANKRRRMCSLAAAAADAMPAGEGGEGGEGTEGSKAGD
MEKACPPSAPQSSAAVAGAAAAAAASAAEGVATPADALQEAAPASGSAASVARSATITAAAPQPPQQTWPVAVKIEP
QAEEPQVQPAAELPRQQPPQQHQHYHQHPHPHPQQVTAAAFASSAGGHQHGSSTARGPAPGQQHQQHQRDLVSSRAP
PAPASTPAAAAAGPPTGADALRPELLLRQQSSMTTNGAGVAGAPTEKHGAPTPQEGDLMGSLGNMLSAAAAANPAAV
VKLLQQLQRELLQGGGGGGGGDTEGRGGDAYAGEERVLAAAVAATTAGSRPTAEDSKSPARQQQLLQPPPPPSSSVE
ALQPSLRAALAAASSGAADVAEEAEEAEEAEEADEAEEGGPAANKRRRMCSLAAAAADAMPAGEGGEGGEGTEGSKA
GDMEKACPPSAPQSSAAVAGAAAAAAASAAEGVATPADALQEAAPASGSAASVARSATITAAAPQPPQQTWPVAVKI
EPQAEEPVAVTLKQERVAAETPGSATDGQPQPQQQQQPQHALQEPPSSPPPASATAVGEEGDAATTATATAASTSPA
AAATGLTADGAAAAVTVNQADMPAPLTGVRPVGFLVLPAAAAAATPAAAAAAVAALTSTELVRQLTAGLHRSDSAAA
AEFAAGGAAPLESAAAGRGAAAAAAAAEAGRAGSYSGSEGSSDDGSGGGSSGGGGTPPHAPPGLSDWLAAASAAAAA
AAVAAGRGSAGGAAGTSTVLAGPAAAPGLTGGLGLPLMHLTGGAGAIGGSGGAVGAAGLSPAGTASGDGSGGAAGGG
GGGGSGGGVRLPNGGSTFSARVVATNLYIGRRQLTALYGPDFVADTRSEMALILNGNLQPDVYSVQWKEGRHKGSFY
PKGAPLGQLRGRFLQDIRWLDEANNRLVAVAWDEPPPGWPVRSRGSGKAVSGGSAGSSGGGGVRGGGVAKPQRRWLW
QRQQQAAEAAAAAALGLSSNGSSGSGAVAGAGAGESGFMSSSGGAAGAATALQTLAMAVPTAAGSAGGDGLFLGGDG
LGGMAAAATRSVRNTHQPHQHQERPPQQGRQQGRQQTPPAAMTDGSAGAVSLLEAARLLQRQLQQPPASAAETAAAH
AADGGGSSSGGLLRVLPLDTPAASGRGDDDSRAGGGSVLGCSPAVEDATGILLSRILVKALSGEAAAAGCGTVGGSA
KYLDIAPDAAGSSPRYAPPPAAAGPAFAPPPLRALATAASRSAAQAVSGSSGVTADDPAGSNGAKLRAAADLLQVLH
IHLQQQAADTQRQQQQQQQQQQQQLQRMSGTTSTRHSTPTAAAAAAATAREGPPSRSNADMQQQLIHACATPHYQQQ
QQQNNPSQTHPLRWLGPPIRVSGVKRYASDQAFGGGPGPAAQAVAVAAIERSGSGAAAGSYAAAVNAATAAAADRTG
SLPPAEDFARLSAEVAALESRSGSGSGSGSLERDLSRAAGTAHGLASISREIEERRRQQQPQPQPHQRQQQPPSLPL
GLRFVPLPPQRQWSPSLLPAAAGAPPPPPPPLQAAAGPRSPAGAFTSAQQPAAESGGGGGGGVADAGWELGADDTAP
SAKRPRGTPPSDLASLLYAGSAQQQQQQSQPSRHQQGRSDYPLEGAVRSSARGYGGNNEEPAAPGSRRFQLLTSGGG
SWTAAEAARRAASGAGGQVAAAQRRQPPQQQREELLHPQLMPVRRTTAPPPPLPSSGGGYAAAMGSSRGGDAFEAGG
GRAARDRSSSRRDAAADDDDGGGCNPSLDEQVEQLAALLETDTAGTFKSLRMVLAQLRKQQQEQQEQQEREGAQQPQ
RLDLSQPLPPPPANPHHLHLQREGSGVSAGLLGNSSPRHSDNFGSSRQLQQQLQPKQPAATHSSGVSLRSHSDSGLQ
QLLAAATATGSLDSWQAEQLLSDCGGGGGRSGGGGGSSTSGGRLGCSGSGDQPSAGYAQANLYRALLDMQQQHLQHQ
HQQHQQQQQQQQQQQQHQQQQQQHQNQQRYRQDASPRAYQDGGAAGAAGSRRMHEVTRQPASAAQGGGAAGGSGCAG
AAAGGALPPSRLGSMNSAGSALAAGSAGGAARSGSVEPAPSGVDLQENTTELVIRTLPWISLHIARRWFPGLWDSPS
AEPFPVTLGIEVDGNLEEERYPVHLKPYPGNNNVFLPGLPVKKVKGRIRTGWRRLADGTLVLQVRTPEDLIMDAD

>Cre17.g742400.t1.3|PACid:27572122
MGACLGRPAAAEHGEVLAATGAQERRGAGSTAAASVIGSAIRQDVFVAEGATGGAAAAEGRGAGLALAAEPAAAAAD
TAPAGRGSGSGRASGDVSTRGRTSGEGSSPDLSRIQSADSPFHILLPRHSPEYAAAAAALAAAAPTTADSLSTEVTL
LDPKRTLRAAAAARRSLSPGRNPRRSAEPPLTGGAAARRPPLRAGSFSSQGSRGVHRGGTSGLNSGGGSGTTGGNST
GRVLRYSLEGSGHGGGRHGSNPASAALLSPSGMSCSSSTSNIIVSIGPTVAVGMPITQLQQQAQLQQLQQQAQLHQL
QLHHQQLLLALPGPSSTGAGPSAGAVMSSEAGGMMTTGAGSGAGVTSGGSGLSGSSAAVGGIDSGQSTNTASSSSRH
RAMLLSAALRQQHQQQQLQQQHLYQQLQQPHQQRQPRQRGGDVPATAAAAAPSLGGARGPGASPEGPTAAFASRAFD
AYLTGNVHGPLGDDGGDGSRHGGGMRDHGRLRELGGSGSPASATTTSSSSDDPSPARGRHRWQGNAAGREGGEAMER
EQQEARIQHSPQSGGDVSSDNEAVVTSSLESTPLALPRLTPAGSETAPDATAATPAPAGGAVGPAAAASTAAASATA
VRGVGALPSTTAAPAPAAGSGAGTGTNNAIGPGTSGPGTGGTSGTAGGSGRLGAKFRERYGTMAAAMGLRTAPDRRS
RVGAQSAPAGLRAQLRQAAAAARAAAAAGAGAGGPGGGGAGGVAAGEGAAQQAAAPPVTGGLAVQLRTSPDPGGGGG
VGGGSGLQGNMARFSWRRGGGIRGSAAGSGLSHSAALPSPFVSVEAGGGGGRAGADDLRRSGAGGAGGGGGADQQEY
EGMVIGDSSGSDGGERESALASRFFGVLGGGVGGAAAMTVVEEGPEGEEMLCETNVMTGAAAEALPAAGAAGGAGTA
PAVVASQQRANSGSGGVGAGRVRGGAGVGLSGQPSGGGGAGAAAVPNFGSHVSSIVSTLSWSSTGTSGDDPVAIAAA
AAAAAADAAANAAAVAAAHDTAAAAAAAAVAHLSGEMPPPLPQLPPHTTTPQPRLSQLPVQAPQPRAVPACASTVAA
PALPLAATFARCSSGRDQPDTPTLAPMFMPCARGPPRTAEAPRVLPTTPPPAQPAAAVSTLTASASLSGGSGYGTSP
FARPPGAVPSHLAGEGSGASAVLSHEGGTAGAVNLNSMWSGALVSHLTLTSSLPSGAASSTGGCGGGGSDGGAHVQH
RRASGGPVPVPIPAPAAGGRGGVLLSPKRDRSASPAATSSHHPFQTDMRAHVNLLQWQQQQRQLARPLPFAPRASTL
QQAGAAATATHLALSPFAPTPPVLAAASGVNVPAAASESAASPALTAAGSGAEAGGGVRRCSDSEAQGPDLVPRGLS
MWSGTAVSTLSPTPSSHDLGAGSVLTNSTTVSNTTAAYPSLQPQQAWLQQPGYELGLPDATAIASATAVLSTPTRPA
IAPAPIATPSPTSRAAPASPVAAASGTRGTAAAGQRAAAATTHSSSRSGRAHQAPVEIDWTLRTAPVDTAALWALAK
AAEEQGGEPQKPEGEGERVGEADDDQQPQQPQQGPHRPSVSGAVRELQKALSAITESTTLPPMTSAARAPLHRAGSS
GVANTGAATPSLSHLLAVTGGRAVQRRRGNDLHPGDLGLPLPSDEAAAAAGAAAALPRTTFTFGEPGVTRDPLLNSR
RRASVLVVVAARSASLALDSRLGRRANGAAARRVGSGGVVPDGASGRAAEFEAAADEAAAAAGMRVSVARQGRGCSM
PELLQHDMDGDTDTGASTAEAQRADDSGGIGAVDIRAAADDARALTRAATTAVFMASGNVASRRYRRRSSLQVGASA
GSGASASTSTAVSPRERDVGAGARRAGAEASAGAGAGADAEQPGPGPSPHDDEARAPAAPAAAAAVAVAPGGGHAQR
SPAPTDAPPPPEVEAAAGGSRRDSFTGGGGFSGTSEYGLHETDDDDDAIDERLHFSSGDMAMQSPFAAAAAAALAST
DSSYSRRLQGRPRHSHVSGRISGLGAGGNMWTPSRSPSPSMLSHSPSPPPPQEQLLPQGEAAAAAARQSAQHHPASL
MLQQSKTEQPRMASRLTGEDSLVGAVCGGRRGGGSTILGGAAPPPFLFTLTSSVPGSEVDNLLALVGNVFQVPVVMV
AAHLPGTIFVRIDQAAVAGAAAAAAAAATTACAASATAAGAISSEPIVPATAAARRAAAVAEAGAAAAAALVADLEW
QMRLGAWAVGSCCLAGGGGAGGGGGGGAAGGTNMAATATAAAGGSSPGDEEGGLGLPDEDEGVVVVEDTVQDTRLAR
EAFVRIRPPVRFVVAAALEDDRPAAAAAAVFAAPVTGMAGGVDSDGDGDSRAGAGACDVALPGGGSFWCDADEGDGG
GRLLGTLCMADFKPRSAATLAPALLANIRQLLLRQLSRDVRLYDERQRSRAVLSELLLSLQRLLDRHNRCLLLVELG
AGPGGRWPVLYANHAWTSLTGMPREEVVGRDIKELITTAAASWSTTTSAPTAETAAAAPGQARRTVSTADGGAGGTV
GRTSPVSALPPRHHSPCTPRRGSSTAAILSNAGADQPIDWVRFSGAFQGLRDFVVENARLPTAASAAGLGPSGDAAA
GAMGAAQQSGLLATLRFRFVAREVAQDSGLLPACWRPLLGAIQERGSGRALLLICPSPPPGAPSPRHTRLVTGTLAA
VEGGAVGGPVAGAPAGGAAVVGGGGTYRATRPGGRQVLLTPVLPPPPPAASTAATAGGGGGGGGAAGGLPPSRTASG
GIPPAAAGGAGAVGGRTSPVLGGGGGGAVAGAAAAGGSGGLGSSGGPVRGLELRHVLGRGGFGAVHYGLWEGLPVAV
KTLDISHSKFSVAGASLEAVMGAALDHPNIVKTLLLVEREREPARPRTVQMAPVAAAAVVPAVRPVAAGPAVAASPP
QAQAAEGTTAALRPAVWAASAEKEASARRARAPSPPRLDLSSATPTSGGGGGGSHQASAVRTPPTLGSGAAFGSTAF
MSQGLMSTLQSMGTAAATEAVPLQSAGGGGGGAGGAPPSHAPALQAEPRPRPPLAPSSSIGPAPQQPHTVIPLYDRF
AGFRTLLRDRDGSIGAAGGVSGPAADGSFGANFFMLGGVGPDASVGAVGDVTAGGLGASVGAGDSAVAGPGGGDASA
GGGPWRLAGTPASGTDGGINGGRPLLLNGGGGSGAGGGNTGSGGFLQHGTGSQLRTNGSAATRYTDVTVFSGAHTGG
GVSTGAHTVHTTDGGVGRSLPPVAEGQSLHMQSSSVGSGALGSAAVRSGPTRTGSSATRTASGPGGSAAAGPARGSG
GFFSCPAANWMPSGGSARRQPGGGDGSGRTRSALTPAQPSPGGSNSVSHLFTGLLNWRGRAPSHAALTAAAAAAAMT
EEHDVELADQATPMTAPPAFPVVDVATVFTTTAAPSAPLSRGPSGAGTGAAGALTGGPAGAAATACPRILEGSSNLT
LSASGGGSSLGGSRGWTSMTSAQIAAASKLTSASAYRRGAGSGVHTGGGGQGGRDAGGGAGDDQHSGVRQAQAAFPL
AAAQVDAATGAAEGAAGASPASPSGLDPRLGHPPHLQLLQQLNKQRVQNGEGCSGPGGAAGWLRRLPSRRPSANVGP
GFGGGVGPTTRGEGGDSSGWVGAGVGGGDGSVGGHDGGDNPVSQALRTLAARRRGSGSSRDLASALAPVPAPAPAPA
GAQAPSSAPQVLAAAGAPGVHTSRTGAGFADTQVSQARGAPSWPAGSTAMLGLPAATAPATASLTAPAAAASAADAA
DAAAAATQVAEAQAAAAEAAAQGSTRSSANFSNYDSDSDLMAPALDSPRTAARKERQAKRAAAAADAAKAVAAAAAA
RDASMGTHHLPLANILSSGGVGGGGGGGGGGFAGGSAGGTSSAAVTGTTGGGGAERSMWPSGGGGVMGGMLPPSGPA
LFGMPLPGGGMAGGNSPDEIWIVMEYCDLGTLQTALEKGLFAQPPDPASPIHPSGGPAAAAAAATAAAVGGRRSSFA
IFSAPVSGTGLAGGPDDGADGAGGGVVGGGGPLGGPLGAPLGGGLGGGLDTRMLSVQMRPRDVDLRALVATALEVAS
ALRYLHALGIMHGDVTGHNVMLVSQDRASLPYNLSYRSRCSSAILTGSPRSGTGGLGTGAFAGGWTAAAGGTGSTAA
GTGGGGVAGALLSGSLRAPASEGGADGGGGTGAITPAAAAAAVPGTRHICHALGLSGGGGNASALHSGHSGHSGIGT
AAGAGCGGGAVRPSSAYAMPAPARMDSNASSGLPSGGVMQGGADAVGGGTAGSGTHLAAAAAAAMPSPQASVAASSN

TSWLPPVAPVSVCRNCGRSFVAKLADFGLSRMLTAQSRIRTRSFGMITFMPPEVLVSGMVSQATDVYSFGVLLWQMY
TGQRPWSGMNTGQIVYHVGMKAAQLPFPPASPPQLVALSRACTHPDPRQRCSFTRVNEELVAWAQQLGLELTEAALP
AAPAVAAAAPASASAVGSGAAGAGRAPQRQ

>Cre17.g699550.t1.3|PACid:27572123
MGQLTCTIAWLFIVAFALSQADECNKEERAYTQRRGLQGPQEAGLQARVLMYADSFTFSVYGTSQTLESLPDVRVTY
DVAPTNTPAQFSREFYDAIVIPKLALFNADPLTDDYYSLIDFIYDGGAVIIGPGTQMLNVTSVFLSSLATKSSSRGD
PGYSTVLVCNSSVGVDVPNAASDAMRQLPGFAPATDATWSSPAAFPDYLAAGGAQASAYLVDCGDLSSVNGTVMYGQ
DSNFIPMPPPWEAAVLSFPRGDRGGRVYYVGVDLDTAPWTATWGQLLRAIVMKAASGALPSNLAYASPPPSAPSAPG
DWQATAPAIDPATSNASQPWPLARGRYSYTSSGATAAADLAEPLSTTAGRSQYTCHIVPRPTVDVDGYADPTSSWLV
LDLGTARRVYSVRIQTLIARMSDEALINTTSVDPVRIQVRVGDTPVQSSLQSAFGQSSNVAANPELCSPAASSTGGS
SDHYLAVGRSPTAYDDPGALVVSCGSAGSLVGRYVSISLLDDVAVPFAMSSVMLCGVDVMGVIPDTWQLVSRSKPVC
ATESKGGVVYPAEAQPSLAVDGLYNNNRNSIMSMCALSDLAASPYIMVDLGGIMVVERVEVTKSVSAAHSHELSGFT
IAVTTENPCVSPESVAASTQPWQFCARGLSLAPGETGVYNCSGMVGRFVSLALPEDFNAPRYLRVCELDVYASLPGS
GLTLAGSWGSESFLMSVNKPTATTPNALVDSLLVGQSNTTAKAMPGLLVDGVTPKDVRAAAAATGSDPVSLPLASMC
VNITSEAAYNTSAWVGVDLGAVLMVRYVKVHYMYDHDRLYTLDFSYYSTVRADVTLRLSNSSVDGVKTFLGLQPACV
QGAGFFTGVEYTTDLYGTGGNHIRRYGCGFMGRFVYLVAERPAGDSSNPVALPPVCEVEVFVASDAARPLGYLSSLN
RLAAVTTGTAVDVIATRFDKTAGSRALQGPNYDPLKSIAESTRCALCLGVAGQAVPRAFWVVDLGSVRLLSMVQMLG
GIQFTNVKIMALDADVPLDPTSPLSTSTALVLPGAAFLTISQTTGIVTWTPAANNITARYVLVMSMQPEQPITLCQV
WVYTSQEGVPGSGARPGVAVALVRGRATQEVGSVSVGITLNVVARRRELLALQQQPPLGGRRAMSAAGAAANAITAF
GSSSSSYDGRSSYDGSSSSGYSGRRRLLQSTFGPALLLLDLALVRSVEVAVLTTPKVSTYAEIANVQIAVSNSSTGA
GGGAFCAFDVTLPLSLTRHVFGCGGAFGRYLVAQRSDGLGLLPGSGLEAFLVDPGNQLNVALNKPTNQSGGVVTGIG
GSNNAVNGYYDSASVAAAPVAGRVVAFGSATAAVAAPYWSVDLGSAVPLVWVEVTAHPTAADGQGLLSFDIVLSNAT
LVTGSEGVTVLTGLSLAAGATGRYPLGGAAARQLVVRQPTATRSMELAQVDVIADKATITANDNQLITPKPLFYPTV
RDTYRTAGAYLDSFGVSTTGELAPLAVDGAAVSDSVQRCSRTEVPLSDLPWLMVDLGAPRKVLRLALLKANDPAAAG
QLDDVDVVLGDSPSGSVVSANPVVLSGLRLPTPGVWAFFELPATAAPGRYLAVRGRSPGATLVVCEIAAYGSSAYEL
ALSPAAPNPPPSPAPPSPAPPAPPSPEPSPPLLPLPPLLPPAPPSPGPSPPATPTPPSPAPSPVPPSLPPAPPLPVG
APMIPAPPSPPPSPPAAPDSPAAASKQSTLTVAPVATDPTAVTVVTASVVAVTVATSLAATLAASAVASAAASGAGG
AIAASVPGGAATGGAGGAAATAFVGHLQFFALTSHASAQVSGSYTDTNSALGWLNLEYDTLLKMGVTWLPDAEQKAY
SQALNVGLVFTGVLLLHVSLVLTVQYIRRNSAACAAFWCWCVRRRPPQPPRVLAAGGPVAEALESRASIAAATSPAS
FRKRKLGIMLPDFLVFPYLEIFIYVFFLVSLANAAGMLVAVGFAASPHRVAPIAVGIGILVLMAALTAAAVVLLTRM
YRRAARLGLEYVPKPRPPPQGEREGRVSRWLRLAERGFWERPEGVEVKLLEPLRTEHYLTAGAFNPAAALAAADPDA
RLTHLSRLATRFSRMASTLTFLNSSRRDRDREMFDTGSPAGKDAAAAAAVTVAVAGAGAGAAAAAAALASDGDGMAG
SGVDGGREGVEGLSAVVVRPSALRIAGMEGALGGSSHSSSVSGIADGCTGGGGGGDSARGTLVGGSSFFNQSRRSNR
VSASNAPLSDGEASSMTGSASAAAVAVRTGPPPFAERRSASGAVSVGAWGSFTPGTGGGSTNGMAPPAPASVDAAAA
GGAIVGRASASGTVVVAAASAAAGSRPGSGMPHRSESGSLAAALLGHFRRGGVGGGSKASSSATSATAGTAAAAAAV
GPTSASRFAGPPAAPSSPPPPVPHSHSAVVDLRDNALFGDSTYSAATATPPLTPLGVAAPDAAKAALVAPHVVQTDS
LLLQSGADYAAAAVLGSPTPTATAGATDVASAAAASAFMSAAAVTAASAAAAGEVDGARRSALSARPQSGRTSSNGA
SPVPAHAAPLLPAVITAAPVAPRVEPLGMSMPSISRRLSRAGGSSLSGAVPAAPTAGMPISSARLPSALIPEDASQD
SSGATAGNAADAADPAIRDVPLPDPASQPNGPDAGPHAVRSALAGSMAAFAAALESHDSFTHASHRSPTRLSGGGGS
GWHGSPRLPGAMVGSPSASHDAARHSQLEQKRRRRKSLIMADGGAALPHDSIGSPRQLSDDEGEALTEAANAAAATL
ISRKSGGGGGGAGHITGGRSRLGAVEMTGGGPGALHMPAPPEAPLPPAPAPPPPAAPVPAGTAGVAAASEPPPQSAA
ARMVAAAKALAEEFAAMEHARAQKLAQQQEQQPQEQQQQQAPPAPPSRRTSLSGADASNGSQGGGILAAGSSALSR
KSPRRTTIDGAAASSDRQMSGRPLSGRPPSGRPPSGRPNASGAAGPGRLPSAAQRNSNSISRRDSGLKGSGGAADDA
VAAAAAGLGAEPSATDTAAGGGAASGFMAAGNAIVASDRLARGRPTSARASDSGLVPAPATVASPGAVVAEPASLQP
AASLRRTGSGSSGRAGRTAMTAAVHAVGATTKLTAASAATAATAAAHAAAAAATAAPATAAAPAVGSTHYAASGGST
SLSSTASPSSPQSTHHYQSPPLPAAIDEKDSRAAAAAWPASVPSAMPPPAIRVPSSSGDKAEMQRAAAAAAAAATAN
GVASGGAAKAPTVVIGSPAGVAALSTRDSVGAAKLGSPAGQEPVLPIELQIRRVEVYERYGQLFEEFRGGKIAAATF
QGALLANAIVPAVLLAVQAGADMAAGSRGAKATNIALLCVKALFAIYMSAVLPFSNAITMTAELVCSWLEAAVCAGL
VALQWYAGAKGISSAMAVCEVLVIALQILRILLTSAIPCIIVVWEWSRIHCCLRCRRRKSGSGTGPAAIASGGHGNA
THEGWNEDDGNADRAAVTGRHSKEHV

>Cre17.g732450.t1.3|PACid:27572158
MPLTVQNRLQAFVVFALLSLSLASAQTACSGGYANCSGGASQTVWASTADVWGVATRSADFSGLLGAPTYTGRCNSP
SSVLPSSLWSLRQDTGLTRSAWLILNFTSALVATEVAVSVAGSSANGQPAWIYLRNGNSGSSLCAADAIPQGCSAAA
SNGTYWYRCDLSAVSPPRAVQALALLVDPDLVIDAVRLMGYPPLVSSARPPPPGNSSTYGGTTPFGLSGSSTSSIPP
GAPMVGNLWPPPPTPSPPLPPSPPPPTPSPARAPPPGMVRPPPPPVAQPPPPPAQATQAVSAPAPSPPSPPRSAAAG

PAIFPPPPLPPSPLPSPDGTATASSPPLSPSGKASPGNKPMKLPPPPQQAGSNSNYSNDTGNGNGGGNNGNGNANGN
GNGNGNGSGGGNNGNGNGNGNGNGNGNGNGNGNGNGNGNGNGNGNGNGNGNGNGNGNGNGNGNGNGNGNGNGNGNGG
GNNGNGNGNGGNNGNGTDTGTGTGTGTGTGTGTGTGTGTGTGTGNGNGNGNGSGITGQVPAPSLPQELLAPSATQAT
AILQLKRDVKVPASSVAAALSSVLRRASTAADGSSSNSTAASGGGGGSNTTGGGTATDLADALKALFPGSTNVEAVA
VDLAVEGLELLLAAGDGGSSINNDTCSQPAQHQALLRMAQDFLRLSGLGASGATCAFACDSGSSNSSNSGSRRRRGV
LAAAGAGDGSTTCLTAAAGGGASSASLDVTVPEGGGSSSGSGGSSSLAGMLDGSLQTQAIAGLQQLAADYASSLCVP
AATPDAVTATAAFKATYQVALNDLGVQTYASAGGSSSLCATLSGSSADGSSSTTDCQLTPPPTTGGVTTASAGSSST
PTAAAAASNSHRGSSKGFAKIIIAGTVSVGVVVVAALAVMGVVTRRRRILLEQDQLQALRMRSYVHVYDGLPARLQR
AAEGAPLDRALARSRKQLRRSASAGDSSSSVRRSVLEQETAAAGGPAGKAVTTRSTGWGASATAAARNYGKAMTEGG
DSNMATSDDSATAFTGGSSSSWTSSLGLAPSPTPRIPANRAAHVVTATAAATATAEDYASTPSLPSPMLSSPRRPAS
IGSGVAAATAAAGAAAALAGAAAAATAASDPGDLVTCASGNARATSGGANPASRQQQQQRRRHQQQQSAARRHSAL
DNALAAAMAAHSTLRASQQHYSPAASQRQLVLPIRRSRRNLMGRGGGIGSDSGSGSDAERGSAGMLLVGCRSRGDWE
DRDRGSRSSGGGSNSSRLQPLPALDSIHQQQQQGPPQVTRVVVRPRLAAQHGDCQKALLMSPPPPSSSAGGAAPPVA
RHGGGSEGTSGITPRHARGSADNDHRLSSDDGEDEHDAFGSWVEGGARAAGGRARGGADGSAASASAVAAAKASPPA
AAAAARPRTAVLSQRCKPIRPRSGARRVRAAAACGGGGGGGAGGGAGAISPPSAARQQAIRPTTALGPPALRAAAVP
SAAAATSAAGGSGATAAAASTLRGGGGSLLARAVATAGWASASYPAASAPTTSCTTSANGLLLPQKRSWPRLAAPPV
AAVAPSAAVTPSAPSAAAAATPSGAAASLQAAPTFNITGAVTKDSNDDIRVWDVTSDDEELLFTPAGMGAEPVSSAV
AAAVRATPPRPVAVAPHFRNAATAQGAFASADAAAAAVATRLMLQAPAQRSSAPSVLSGVRLRLPQRARSSLCRVPQ
PLGQQEGVVVGEVGPSPTATALASPPPAPAPAALLFTVESLTAAAGLGQQQQAPVLDGADSGAAAGSGSVISTGGLR
PALKRPGAPPALQPPAVSAPLASLVPLPLPGAQGPLAAGGSDAAARGQRTAAGGAAASVPGGKVDAVGLRGAGGSSV
TVARRRPRQGSSSSATACRAPHASWRRRRNSSSASAAAMGFTLVVSRVRIGTCASIEGPCLQFRTSRRPLQPPPSGP
APQADMIAAMAPAAGTHSRLPVRSFAASVLSRILPGRARPARIAAPASATATAATEATRMRAAAAAGVMKHGNDEDE
AEADAGAHFDNADDDLPSRSPASAAAAPQWVSLHELMPQPGATAALRAFLRGPVWRAFKRGQSYTAFAAQWRGRLPR
LLHIVTGSSDAAAATRGGGGGGGSGA

>g17814.t1|PACid:27572209
MLEHDLCSLHRAIGDAASGMPIPRRIDRAGQAGSAAAVTSSGSDNGCIRLPGGKLPPLSTTQRVTGTMQHHAACTRG
GAAAQRLRHLMRLLLALALLHLLPGAAAQMCSGTTWVGGGSPYYDCNSGGTTIYAASSTIASSSLKGNLSGPPDFAV
GATCIPSGQPSATVQAQTVYAQNKQSSGGVDITFAGGLFATSVGLLVVSCQQGFIQAGLTLKLQNSSTVTTSCARDG
CTNPATCLGTAAYWYTCNVTASPSLLFVTGMSVTLGDNPLSNSDWKVVDAARLVGYTYYSPPPPLPPSPPPSPPPSP
RPPSPAPPSPMPPSPEPPSPAPPSPRPPSPAPPSPPPPSPAPPSPIPPSPAPPSPAPPSPVPPSPPPPSPAPPSPAP
PSPAPPSPAPPSPAPPSPPPPSPVPPSPGPPSPAPPSPPPPNPAPPSPMPPSPAPPSPEPPSPAPPSPAPPSPPPPS
PAPPSPAPPSPDPPSPEPPSPAPPSPAPPSPSPPSPAPPSPPPPSPAPPSPMPPSPAPPSPAPPSPEPPSPAPPSPA
PPSPAPPFPAPPSPAPPSPSPPSPEPPSPPSPSPPLPPWPAPPSPEPPSPAPPSPAPPSPAPPSPSPPPPAPPSPAP
PSPAPPSPAPPSPAPPSPAPPSPEPPSPAPPSPEPPSPAPPSPPPSPSPPSPTPPSPAPPSPAPPSPAPPSPRPPSP
EPPSPAPPSPVPPSPAPPSPAPPSPQPPSPSPPLPPSPSPPSPPPPSPDPPSPAPPSPAPPSPMPPSPEPPSPVPPS
PAPPSPEPPSPAPPSPAPPSPDPPSPAPPSPLPPSPAPPSPTPPSPTPPIPEPPSPAPPSPEPPSPIPPSPAPPSPL
PPSPAPPSPMPPSPEPPSPAPPSPEPPSPAPPSPLPPSPSPPSPAPPSPDPPSPEPPSPAPPSPPSPAPPSPEPPSP
APPSPDPPSPEPPSPAPPSPAPPSPLPPSPAPPSPAPPSPQPPSPSPPSPPPPSPAPPSPEPPSPAPPSPGPPSPVP
PSPAPPSPTPPSPQPPSPPPSPVPPSPEPPSPPPPSPAPPTPPSPAPPSPLPPSPPPRPPSPPSPPPSPFPPPAAPT
APSPPPPAPPQPSPPPSPPPSPAPPSPRPPYPPPPPYVLPAVTIPPDYMLPEDNTTTATASGFLTVTQTVSVPTSAA
PSLGALTALFPGAQQVNVTGETLSVPVQVTATAAIDATTATATASFVSSRTGQVVNVVVGCNSGFLEAFRSELVARA
NISDEVLANVTCSSNGGSGSGGSASGSGGVDTNVPGRRSLRNTTPERRRRVAAIDATRDQPPQQQQHLASSVATAAA
AAGDYYDYEAGFPARDGASAANVDLSAGAGVDMLPTGRELLQATTTAATTAASATSSTTNGMCPAPGSSNSSSLQLV
AVLKLPAAELNNTDFYKARLRQTLDAWAAEAAVGNGTAGGGLLLCGPASDSDITSTTQVTVSREVALTAAGTTALAS
VCGSSSLTITSALGGAGSSVACQVVTLAAAGGAELPPVQGGTTQASPDSGPAAAPAGANKAGVPMAIIIAAAAVGAM
VSAAACAVAAVVVVRRRKRKREEREPGVSVKHLGQTPTAAAQRPSSAPPASGNEDFWVSWQPMPVPAALAGAAAAAA
AAGAAGGGTPGAEGNQLAAGNNALAAAADAEARREGVVSPRSVVAYFVPHDEAGRSLAAPIEEEDWEEERARLSAPA
YDAAAVAATGAPAAALAAGGGNMARASAAGIDDARASSQQAPRRSFAALMENAFRKALSLSSSRPGSANGGVGGGDL
NGDGGSARTTARGGDGGGIGLMGQDVAPQHFSGSAAVAGSAAAGAAAAAGALSGLSAVATGGGDGLRRRSSYNTAEG
ALALQRPPALRRPSRDGDFEAMARHGTASGDGAGPAPPAAGSPGAADQHSGDANPPRHASGAGGSAGAVAYRFLAGS
RKVRPDRAPEDSGTPGSPSAASSRSSLTGIGPRPVSGSSSAAMAGAGFAGTAVNVIGPSNYKPPSQIRLVGDAGNSN
SLPVPVIGAGRFSDGPRSPAMGSSPLGSPGPGLVMGQRRSMPQTLVEATSVGIVSASALADLRQEEGRPEHSRNNSS
GVGWSSIRREAGGGVAAAVLGGSVSDYGAHSNRSAPLLHVGGVNPYAISRSVLVSPGAGRRSSDCVYLPSSGAASLA
AGAAERGSPRAGPSPTGSSRAQRDAAAEASPGVVVRSGAGALMSPVRRSTLHASSGMGGVGGGRAAASASGMVLSPA
GAHGAVGDWDEEPPLEKLTPLQQVAMKSSADLAAPRERAVVGVSTLLAKSASGAVSGAAARAPSGTSLPLARAGTDV
AAVMAAERAAPLKTATLVGGRRNLLAAGAAPYVRQVTSPTGAASSPGVSMSGQAEPALSSPSGAHQGPRSALLRPAP

AAAFAAVPPAARPPPTALSPRHAEPQTSTPASVAPGGVEATTGGGSWFLPGRGPPKAGASPSPGRPRRAAPAHPASA
PEGAVRAYYGEGISYGPIGGAAPQSPGRSGAGTISPVAGRMPNRRMSEAQAAALPGLGTHRPPLHAASDAGLVYHHS
PLQPPPQQLLHARRSVDAAAPLMMQQQQQQSPPRAQPPGQAPRPR

>g17653.t1|PACid:27572223
MSPGPQPALGSQEVASIGGNPRPGTSSAPSMSVESSIAVVPAAPSLSSGQAFDHGTAPRRGATAAEANVQAGIGAGR
SRRESTPAHGARMQVLVTELVLGSQALRNGVAATGQEEQEEVVYRLLPPLRGEDGEDKRRHEGILKHLARDFQRNKP
EICDRILTGDLNQRRSYPLAGLWVRPRRQAAPAASQQGPGGGGSGSNGTAGAGEREPGSQGEEQLVCAALLRLTLEG
TQDAWGAKSHLQVLYMSTFGPEAGCESRRGQGLGRLLTRCLQRSAHQAELEYVTVVLAGKQVEGFWGKVGFAAPPRQ
AGACLSGAVCACSRRYAAGGSAVWVLHVSELQETAQQAQTAQQAAAVAADVAADVEADEEAAAEAAAEVAAEVAAEA
GASGLQSSGQDTAAAAQQLERVSRAVDGLVRTAMQIAQTKRMQRLLNGPAPDQRKRRREELAAGQEDARSRQPSQPP
QRKLKTTGAAPAPGSSALNLWLPLTEGSMQRKSVYIRHEQAALLRRAGCLGPEAVRWRVGADVAAAGGAAAAPGAAA
GSAGGRDNPEAAAAPAAAAAGAGQATGGQARTRFIGFEPGKGSRNTRLNFVPEWAEQHGAKPGWGFLLSRATGSGGA
GATAGGGADGRAWSPEGAMGDAGVGPAGAGPEPRWVATLLAARPLHCDAPVPGHGADSPARPPQSADAADAEAAGVA
AAAAVPPARAPAAAAAPAKAATAPAAQADIVRKAALAAARGVCTGGRLLSPPPAVRGPRQAVAAVAAAAECSAVGAS
CTVTLTESGVRGCKISIPKTAAAALLKSPTGRGGTDTEAAAADGGGRSPHAETAALTPEVMSLITNSSRAGTNRRRA
MGCGTLSTSKRVWYYSWAENALKPLLSKSRKSPLWHLTCHEIDAATTHPTTGAAAAAAAALVGSADISTGSGVAAGI
GAGACASAGAADIATAAPPGVGAAAHAPSVSTGAAADAVDGAASTGAASEPAVQPAAAEEVPVYEQLQRLLDGRSYL
VASLVWEEKDEAKDPWIPGPVAAIAADAAAEEAAAADTTAADGEAAAGAAADTAAGAAATASNGAAGIYAGAPADAD
APAAAAGAEACATAAPLQASSSAAAGAGALPPVRGMAAAAATTPKAAAAAATSNVTVAAVATEREVTSDRTAAGAVA
ATGAAVQSGSTNAPPRAGNVSGTPAQGCAPPQAGPPGLLLAPLPGGPKLAGLTAARRAAVPPPRPAPLQWRPAAFVT
GHTDFSLDPAPHCSPPPSAERASPAAAPALPRAPAAPAPAPAPITRSDVVAATRAGGTAAFTPAAAASAAAGLAGMA
ADAAEAAAAAAVAVSEQAASPRAREKTPAALQSVADANLFQRMMSVNAANANAAAASASASDALAAARVRSSAVLRF
AALAPVAEPPPPRLRGGLPGPCGLPRPGSLLGPGAVPLNAVTPSSALAAAGVAGAAQRPGRGPPAAVEATGGSDGRG
GGAGSSGKTQWRIPLTEAAAMAGGGYSAAAAGGGGLGSGASAGAGGSVDRRPQELKRPVQAVLAEGTAAGNHREPRT
AAAAGGTEPDRGRPAKLPCHRVEPAAAGLGDPTSTKGVEAPPGPRMRDAALMAGCFGGDAKVAAVAKVNTWQRVQQQ
QQQQQQQSKPAGVEAGSSTHTLAAGDAAAHPAAAMMTAAVVATAATEAAAAPTASQGVLPLPLSHQQRQAEQQVQQV
QLEPLLEIGHVRCQLQAALAPALPSLVRWVLGTTPEGRALLAAGAAGARSARACHEGKAAPGPLQPCSWHLVGVRVG
HAAGLKMLLSFTPLPTGAAAALSEARASAGAATAASAAPAAAAAPAAAASRDATAAGMTGADGDAAGLLLQQWRAAG
ILACVVDIAHTPAAAVATSSTGGGGGGGVRVWARARTGGLEDALQDAGQVALEEVTTWLHGLMRG

>Cre17.g737350.t1.3|PACid:27572233
MTDLVALTELRLKRNSFTGGAPSSWSAFAVIRKIDLSSNTLLTGPLPASWSALNDTLTFLNVSGTALCGDAPGLRSV
LATTPRSCPSPSSRQPPSLSPSLPPSTTSPSPIPSPLFVLGYPPLPAGLAFPPPPPPPPPPPSLPPPPPPATPPSPAP
PVPPSALSPSPSPLVPPPSPAPSPSASSPPPPSPNPAPLPPLPSPPNPLPPGPSPPDPLPPSPLPPSPVTSTQRPPS
PPNPPSPPGIFLNGWEIVFTPIFCDGHASDKSFTALSSDLLDQYGRVTAITYVYDSKAGGIVMAVKLVFGTGPSTHV
ETVAGSLYDPGSPTLSETTASLVSASGSALDSVSVCCSDTGAVAQLTATFTDGSTSSTGPCPSTGGAAAARHRHRRR
TLLQGGAGGGGSAGVPGGMVVGGLRGGAGAGFGSMGFALAVRRGFPPPPGTPPPGLRLPPPPVPPPSPAAPPAPSRP
TIPPRPPSPPPPPPFPPSPPPPDLAIPLPPPIPPSPPSPTRAAVAAAFTFYVKVDYPQVTANVPPTLPNRVPAATLA
LTGPALPVVRSPTDDPVVATSRYGHGRIAVFGGEKLITGCCKPKAKPGRPTSDPGTDQLIVNIATWAAWYGTKVGKA
LIRVADIKFLPMAKYVVAQRPDSFQTAKSAKMNFVLPLATFLKGGHQKCDVYVIGSDDARYLQTKVSGFLRDFVFKG
KGIIVVGPDVMPTTFYPGTGTASRRRSLFESLELPLGREAASGTQLWNAEDEPLVQEERSGMDARVVVNSGGRTLLQ
ATAAGVASAAAARIGAPQFNSSIILANLVSGPMGLLMTGYVSDPGGNLTVTSPSELQNAELAASQYVEYLQGQIRLS
VADLKTMLNTITRARASVPRSGAASSPRFWDLVDASDKLATSAPALPPLFDDPPPPPVFRAPPPPKPPLPPSPAFLP
PLTSYVGCFFDNAQARALAIKLLVGQPTSVTRCLDAIPPEALTNRTAAITFLAMQRTACFGASQLSAAFVAAQLPDG
TCSQPCPLVAQQFCGGSASGTRVAVSVYRILSPAPPAGRPPRPSPPLPSPAPANRPQPLAAAPAAKPGTAPPPAARG

>Cre17.g706000.t1.3|PACid:27572237
MAAVVGERRGGRRGRLGWPAWSASLPGWATRVIILLLLTLPVPVLGARPLVPTAGPAGGADAVPRYLAEVGIGTAGG
SDGSSSGTAPVGLSGYQPPQQWQQQPQQQQQQGQNPIAVVPLGMGELQVATMDRDSTMVLLQERIGRLDAEKRLAE
LQLEVLQLQVAQQQVIRQATATAAAQVTSLQKEVDGLKQQMRLMELAAASAVSKDAAARPPELSPTAPPPFPPRPPS
PYAPDPLQVQQRQPTQQEQQREPPPPQQPEPPGVGGRRLLPPDRPVASLELARDPHASPPSPRPPKPPAPKPPSPVP
PSPVPPSPAPPSPAPPSPVPPSPVPPTPPSPIPPSPAPPSPAPPSPAPPSPVPPSPTPPSPVPPSPAPPSPAPPSPA
PPSPAPPSPAPPSPAPPSPAPPSPVPPAPPSPSPPFAPLMPASPPPPPPPPRPPHPPRPPMFPFQPPPPGLPPPPSR
PPPPSPPPPRPPRPPAPPLDFYQTSFPIYWEDASFNSELADPNSLDAWNIDVCEQLLAFAPEETMCDVYFNRYQVSY
VTLTSLYRGELLLYYMNAMLSTYSHFVFDSEFMGRYGITRVWVYWSMPPARGSPPSAPFPRMPPLPPGPPYPPYANG
LGDTKTVRQRLMLELLSSDTAAADGAVDSEGDYLGQQGTGSGDGGGSGAAVAATRSGGGVRGGAGEKATAPEHPRQR

RQQQQQQGWKEGGEGDGADVEWVDEESEWVDEGSKKAGATTGTAATTTTRGATNNNTTTTADSWLGAGTGHRSYRRD
DWSYGR

>Cre17.g702350.t1.3|PACid:27572284
MQTQWAGVQQPGGGAPAQYDGELHAYAPPQQPAGASAWQQPRAAAVAAAAGPQPSVRPTTEGGFYGPQTPGGIGPPP
PVPPPHAAPSEAAQLHQQQWRHAQHYQWRGAADSNPSRPPAGAMLRGAGDGASSHTAAAATASGCGGVGSAGDRIGG
TVASHDGWTPWGSAPGQGNGPAAAATAAAAAAGVRSQSVPVAAASNYPPVRGGPGATAPPQGPGPGPGAGGSGAGGR
GPPGAAGGDAPQGVLHVAAPGARSVWVAAGPQGGGPGRRPDRPQVAVAVAADPFGPPRHLPPLRLRPPPPQLPLPEA
HPYSPVRRAQPPPPQQHPPSHPHPVYYRSQTPLPPPPPPAGYVYAEVGGEESEQDDEFGAHAAEPELQAHHLQYARH
GQHPPHLLPPYVLPSPQQRQQHQQQHQQQHHHQHHQQHPLQRHDSALGLPQPQEQRQPHQQQLGATWPPQSERWALS
GDVAGQQCRHRPVQLPSQQQAQPPPQPQPPPSQQLQRQPAPQPHAWQPHAHQPQAPVPQPLQPHAHLPQPPVQPARP
QHQWQPSPQERPQHQQHQHQQHQHQRHQQQQMDGEEEPPAAVAWRHAAAAAAAATSPPRRTASASAAAAATPAAISN
AIAGVPAGAGCGAAPAYSRPASSYADAFHGAHAPHYHSHGHDHGRHIAAPLAPVGADGEGGSSWRQSAGAPPALPCA
PGPARDFQHAAYLQAAPFGAAHGGGPGMAADPAGPAPVAAAAVAAVAAGSSGQGRSSGWQEARPYAEPQQQQQQHQ
QQQQQVWARSAQQPPPHAAVATGPAGGPQHSPPQQGLPPVATRSQGHHHQQYPHQHSHQQYPHQHHPLLHGEPRTLS
RHSSLPAAPSGQPPGSAAGFGTGHAVGAAGASTWAEPPPSSQSTKPRLEAAVAGRVAAAAWLERDTSPTAPASVGGS
VAGWREALGGLAAGAAPVAARQASLGSQEGRQLLYLQHQQQQQHQEHQRQQAKQQLQQQQQQMLLARDQRHEVEQLR
QQQQQQQLLQQQQQQQQQQPELPRPHQQREAFPADDLRTWQGHPQRPPQAPPPPAPHHQQQPQPQWQQHQQALPPAH
TQAAEYPAPYVLPPAHAPARQHRQQTVPPPPQPLERHQQLPPHPQRHQQHAMQHHMHQQQQLQAHAAASPYTAQAPT
QPLQQQQPTPPPLQQQTPPPPQQQQQQQQPQQQLSGEGSCATPDADEEEVSATAGGGMQGTVDSPCDGGMNTTHPNA
QVAAPSGTRGYGPPHPKLPQLSLATAPGQAQPHRQEAAAPPPLQHMAAPVAPPPPRGVYGLPPGSGCMQQQHQQNQQ
QQPGSAVVAMAATPRAYAVAAAAQPPPSAAARGGPAPAPVPPHEQYRQALVCASGRPPPRPQEHGAYEEAWELEVVQ
HQQHQQLPPPPHHHQPYSQPSQAAAAAAGAAGDRYYYPEPSESAPPVPNGPVRQAPADYPYQHEQYPPRQHYSAYQG
PPQQQLQQQSPTRQPYYVAYIKQEEYEPHEHAPYQHQHQQHQHQQHQYQRLQQYQGQYAQPPGQHDRPPREPYGMNE
QPPCHTYAQQPQAQQQQQQQQVPQQQQVQNTQQHYQMAEHDMAAHHSQQPQRQQQPQQQLQMLAPQSLAPQLFQPAL
QQQQQQLQQQRQQQLPQEEVQEHAPTQDLQDHYRHQHQHHQQQSQPPLLLPALPGPERPGVSAAHPQPAASLGPAVP
GALAATAGGSHDPGGSAALGSPPPLHTPTVRVKPELVERRQQSLVSQLWSVLRRPGARQQSGQLDQLQEQRQARGVA
IEALLKGAGMGHEGLQAVSPPPRQQQQEQPQFQLERPGQHQQHQQLQGSVEIQQDQQMPPAQGRWPPADDPRHRAAP
LPHATAAAAPAAPTARPPLAVAGSHGSGLGLGYPATASAAVMQAATAPPQLQSQLQPPGAEAYAAHRIYDGYSYSPS
AAAAAATAAGAIGAFAPAVGPAAPGLFPAYQGTTAAEAGLAAAGAKRRRSSSDGCGYADDGVMGAAGAAAAQPGGTA
ASLFEAPAAFAAGPVPQAAAAAHAWQAPQAAAAGAAAEASPRKTRRLVQPAGGGAADAANDAADGAAEGGRGGGERG
GDPGVEAVSTAMLAIERLLQDNGWQRQLQHQQRRHGHQHQQYRQSEPEALDADFDLAAALGLGEPQGEQQVEGVWER
QQPHSSEQPKLVLVPAGGVAASGDDVNGSSPLADHMQQSPAPLQQDPQETARLLAAGWQLAPEGFTPTSCRAPGASG
PRPTQAPRAPVASGVPPHLHEPQLQLQLQPPQRRFVSITELCWLSAGLDAEAVQAARQQLLLLQQQQSPEQRYGTAA
AGADDAAGEDEWVFNVKDLQFEAEALTEGLASDGEDWDVNEEEAGGSGRGRGRSGVVATGAAMWGRGRGRPAGRGRQ
QQQAQQASQQAQPAAQGPGRRRQGLQQGRQALRAQQGPLAAQSQPPVESEAGCARAAAVDRARLGCHRHKPPPVICP
VRPGSGNRPGSVPPLGVQPLGAGMAGAAAAEAAAAARLGVGGAGGSWAARACAAVLPGGGADAAADLHQQRGPVKRK
RCGGRVLVPLEPPVGRALGGVRLKAGDGGGGGPGGGGGPGGGGGGGARGAAGSRGGDVASGGKILNALREAKALLSS
RFVVRRSPIAQLGVFTAAHVPGGALLMEYYGEAVRPTVADVREKRYIAGGRDGCYLFNSGGPREEATVLDATHAGNI
MRFVNHSCGPNCVARTLLVNGQRRIFLFALTAMPPGTELTYDYKLSSVLCLEAFKGLPEEFIERAAEEAGVDLLICR
CGDRRCRKYL

>Cre17.g708400.t1.2|PACid:27572305
MDQAARSNNPMFKDSGGDQHETPSRFASYMEPAVPSLPPLSNAASVRRRAGSPTIDEASPGTPDGLTLGILNPLFKG
SGRAIDLRTLPGFGNESSAPVNSPVPQPAAGRISASGIGSNGSPFTPRQAPRQSNGGSQLAGPPPGLSPITAPALST
VGSSNSTATATGSPYLAAPPPRRSPSGHWLGAAAATATTAAASPLATPMGSIPLADTYSPDTSQQLSRYDSAGSHLS
GSSGSGHAYGRMATGGDGGPISPDRRPSQGGGGGAGLPPAGASLEPTSSITTIATITTKQRSSRLSAVTSPSAAAPS
AAVAVTATAATAMGAAAAAAGASKNVAFSDTGRAVAATAAAVAPSSPEAAIKSVPAPQVVHGAAGGRKPAAAAPTDP
VKAEKVAAAAAQAAGLSTGKKAKKQYRKAAEAEAAKGADPEAARREEEIADAKAVMKEKKRKRYTNLMCCCCCILFA
GIIATAIAVPICVIQGCPPKKPDTNVVTLEEGLYSLRLLARMEDIEQKVSDLIPPLLRDYVNVTRLQESAVEFLDFT
DLAVLREVRDSLLAKYKDQLTFLIPDFPMRFEPDLAEAARAGLSQLGAAATAAASTSLGGISISSATGSATASNTAS
LLASYANGSMSLTALSSLLPDSAKQTLQDLAAQIAAAADLVPGADGTALDGASLLNAALSGSTGSLLSTLRQTLTSA
AGGNTGTGGNDLLAEHASLVSAAAAGAAKDLTGLLGGGGSSSSGSSSAAGTDPNVQWYLKKAEVADAWSLTEGAPDV
IVAVVDTGFDIDHPDLKPNLWVNPGEVPGDGIDNDGNGFIDDINGFDFAGSGDACQGDWRARAAPPPPSPSPPPPPP
PSPPPPSRRPPPPKRAATAKKKVQAAALTSPTVRATYATQLRPMCKPDADVRPESSDTGHGTHVAGIVAAVRNSVAA
VAGAAPRVKVMVLKVFDASGRVYASHVVAAYAYAQRMGAHIVSCSFGPDTPNLQPQPYEVAEMVAQESLYSSAVTPL
ESKGVLLVAAAGNELTDLNGLASVKSNYLPCTLPNSNVMCITGSNPLDRAITGMAGNMPVGVNYGTAVVDMAAPGQD

IYNTFPISLGSYGNKTGSSMATPLVAGVAALVASVVGAVGPLVASPNYYQASSVKSLLLETADKLPGLPVIGGRRLN
AQRAVEAAYAASGGSYLLTPSSDFYTANGAAASLLSAGLAEQYYMAAAPTAANTPISLDSIAVPPAPFDVSVRAPVN
NTPVTRLLSYKYTPATANASAPAGGPSGVLLLRLAGLARFTSSGVWGLQLSGAMAPASVRIMVGQRLLNLTSIGATA
SLMVPTAGLYDFEMLVLAPSDPVELRWATPAAPGVYGSPASDLLVVPSYSPAVHPRFAPNVTFPGAAVAPGWQVMWD
YTTRDDGTSPTAQLSPAFLTTFSTGLPPFSGAPYAPLRNSIMAGADLFRAAGSFGAAATAATQASLAGPSPPTNLAV
PDFASAAAYGVAVAQFTPPSSAQLQFQVTCSSCLLFVQGVLVADASQQQPIGSGSTVRAPEPQTVQSGCMSLTAALN
SLSTGSVYTLELRFAVGDTSRGVLGVTWSPCSPGGSPTGQWGGLAGMLTSSVMWAPPAAGTGIIRSSLRCDLWRSRG
AADASVVPSPRLVPPLASFTLPRVGSRNQNCTLWSSNPNCTAVALATAQDVLPTLVSGSGNYHVRCWAFWNGTFRAG
TFSVRSASITSSPAAVANVFLGNQQIFRSTAPSAQPYVNFNRQSLAPRVNAFAPYLQLLAFEYSDVSPTSVMGLLDG
TEVFAVKETLLIDRRMVVPATLS

>g18266.t1|PACid:27572322
MRCERSQAEWVAGNVPPSWTTLSAGTMTSGRRPLCQQQQQRWVHLSPPAPPQPVAGVTEVAAAGFTAAAGAAAGPVA
AAGGNSEGGRPTPRRASSSTAAALDDHLRANSPNAALLAAHVGAASSDAVAAAAAGAGVSSGFGLQHMQHQQGGSAR
RRRSSDHHVTAAAAEGYGGGDGSGGARSGCSLSGGRGGVAAAAAAAGGDGCDECAECVDSEGEWEAVGGGSDAASSG
GGGSTGCDSSASDEDQSQSEDGEGGDEDESADSEDDDDSGGGGGPPDRNPHHHQQAAAAAAAAAAAGCEGGGAAVSV
AWMASRVSVLHSEVRRLRSRVRRQRRAHAALVAQAAAAEAAKAADAEFAAVGRRRQVAPVPEEEAAAAIAEHRLACN
TGDTTTTTASAADDNSRRQGTPSSAEGGCAGSAVTDGGRGMKRTADAAGLPSPPPLPPPPPPPVTPVRPPPPAPAAL
AKLAAPPPLPVLLQSPLPLPQHRAHPAVAAAAAAVPAAVALSTNCHLPACSSHPKHVQPMTPLATPQLLRTSSSLDA
PPPAARVPSAVPAAAAATVAAAVEAAAGPAAALMAAAPAAPAAAAPAAAAAAALEPHGDAAAAAADVACTSSCCFSF
DGLASSSLMLFDGGGGDEAEDTGACSGGGDGRGSGGGASTCGLSWVGAGGLFLGSGGGGGACPPALQLLQPPSQQLL
QLLPDGGACGYSCGYSDGDGNGVLGVSAACVACGGADCTSCHCFAALTGLIAPSPPPHHHNQLQPQVQQQQQQPHH
QLQPQQQQQQQQPGGMPNEQAAAAACWAPASAAAAATTMAAAAATGAEGTCSSDQQQLLPTPPLVSFPSSQPPAPLT
QQQQKDPAAAVDGGACGAMWIPRCAARRRQRSRGGSSGGAALTAPAAAAAAITANGTLAAAAELLAASLHPQPPAPQ
ALLLQNLLHPSLPAVSISKERLMQAIHAAGQLLTLPLATAISNRLVAAAAVGGGGGGSAAVGGSEEAVAVAAAAVAA
AAALQGDGGSSAALELPQSWTDLVMQAAASMAAAAAAHGDGGAAASAPAAAVRGGGAAATRPRPALGTLPKPIPRPW
KDLSPRQQKMARKRRAQAAAAMRKVKAEIAKAAKEAEEAAAAAADAGDGGDGGADAAGGAAEVCGAPHGIAAAAAGT
TGAGASAAAGAGDVAAGFRARGDTVWRAAGGGGGGGATAVAARTAAVVGRKPAAAAAAHATPKHMVPPPPAGAGAEG
AAGAAVGVKAMATEEEADAVSSSGSLRPGGGAHAAAAAAAAAAAAAAAAAAWCHGCWSPSLGAAAVASWQHPHPHPH
PHPHLPALGHLPPYGSAAAAAAAVPPPSFGAGGGWRSRSHHQHHHYLVTATTAAPPVTHAQHAWPAPPPPPAAGIAA
AAAVLSAPGVAGAAAAVPPPPPAAGTSAAPPPPTPMAGQQQQQQQIAMGPTAPPSCHGPLPHGSNTAAAAGYSCSPH
GAASAAAAAAAASGAAPYPHPYPHPYPYPWPGHPYAPAAGASRPPRPGAYGAQQYPPQPPPAHLAHAHVPFMSPPPP
PMHPHQHQGYYGPTVSAARPAPNFPPPPPHPQPTAPPAGSLYPGGAGGAWRRQSWPASTPAAAAAAAAPPPPGYAYW
PPPPQQQQLQQQEPYGAPMPGPASWPPPSDAPQHPHPHHLQRHPQPHQQHQQQWEAQQLQPQQQQPVTQPRYQQQV
QQHFYGAQIPYPSAAAAAPATEAAPHHAFPPPAPTAAAAAANLPPQQPNAMAPGAGCWQHGEWAAAAAAAAAAAAAV
PECGMPQPPAPSQQQQQQQQAVGMAGNPAAAGHHDGGVWGWSALSVVPPPPPHHPQQQPQPQPHPQEQQPHHQSSWV
NA

>g5290.t1|PACid:27572523
MQATALAVAPDWVVGNVLQQLQRLSATQTLPFSQPFADYAAGLQRARELEVRLLQLDKEAAELRDENAALHRRLSAA
DEAAAASLAAALEGARAGLVRAEGQLAGLYRDKAALLEEVVGSNAELATTRSALSSASTELAQARAEIAALREQAAS
LAASLEAERAARVAAAAEAEAALATRDAALTDSERLRAENGVLVRRLVEIKESEAGRMNELNRMHEELLENAVRMKR
EAEMDRQATDLIRQKAVAAALAGAAAASAMAASTAGGGGGGGPGGGAHPHGGAAGSTASVMISPSAPPPPLAQPLA
SAASLAPAGPGGGAGGGGGGGGGGGGDDASGEWVTPVVPPPAAATGPSTTATPSTATATATAAATAAASSVLGVIPT
SLREVMGLGGRPGSGGAGGAGGGGLGFVPHAHTPYDNDPSLTPPEHRFPSRLPVRVVPNGHKGGCASLAPQVPGHFV
ASCGADRAVALWDISLLVGPGSIPGGGAGASTCPAVLLHGMTGGVNDCAFTCDAAQVLAAGADRSLHLWDATAGRPR
HTLTGHGGPVTAVAPSPMDCRLAVSISESSGAEAARRPYWAAGLDGSGEILGSGDTGVDLDNCYLSDPRYDSGAVAG
QLAAVTTQFLFSQGGGYYARPGDRVWRPSDHRKLVQYYLPADCVYGDNPAQGTAGHGTHTAGSMVGALLALQLAPGY
NGSVLGGAAGDAAAYGPTGLDAGSGAAPRAKLSFVDISLTAAAAGGLQGALGPPEPVDDVYLRLHYDAGARITSDSW
GAVANIYDDFAQAYDAFLWRNPDSIAFIAAGNNGSDALTPGGSIGAPATAKNPVAVGATWRVPAELGLGARLLQVRG
YNPAADLDASTSTELFRLALYPEENPDLPQLLRVIPADSEVPLVVADPPDACTPLTNPAAEVRGAVVLAVRGECWFY
QKVLNVRAAGGGAIIFVNNVPEMMDSPPYPIYPNSNSYAANMATSVISQGLGQWLLGNASAGVLMTLANIVEAAGPD
TVAYFSSYGPMADGRIKPDVVAPGLYITSAGARGGITGGACSPAQANLSGTSMATPHAAGHTALVRQYLRTGFYPTG
SPADAAAAPFTPSGMLLKAAIIAGAKSLMGGLAMALGIPMGPPPDAYQGWGRLSLPDTLPLPGLTPAGFSLQVADRG
QFTASGQQAALTGITATGKGPISIVLTWYDYPADVNSAAQLVNDLDLTVGLGGGRDGSPPQSLMGNNPEGALLPAPD
RLNTVERVYITSPSPGAALTITVTAHSLPSRLLSGPDALLPQRWAVAVVGHFSGTLASELNPAYVRQGQLGTRDTSG

GGGGGMQQASSPPPSPAPPSPRPPAPPQPSPTPPLLPPPPSPPPPSPPPPSPPAPPSPSPTSPRPASQPPSPAPPRR
SPPRPPPKRTRSPPPSRTRPPAPRKKRGSIQVL

>g5228.t1|PACid:27572560
MAASREVGAWRAFLAFGLLLVAIKSNAQTWFDDRDAAVICKTLGLPTSRAKAVFKAWNVYTRGGPWENGKPASAEEI
VAYLNSVDPPIAMTNLRCRGDEASITACERDVTNLGTCTRAQSVGVQCFENDYAVRLVNSGSNPYLGNSLRGGWVQV
WMGSGWSYVSAASFDVRDAYVVCKQLKMPFGGARVFRGVVEGTALFGPANLTSSTRTLLPGLQCTGSEASLGECPID
PAAVDAANDPTAPPQGGASMLCEDAEFDVRLRGGTHVSEGRVEVYSKYGVWQTVCWSTFDASALDVVCRQLGFQTTP
LNPPSYLNFSSSGIPDARPAAGISSIMNCPPGANAISQCGNLTSYAAINSYTSFPLGCTAALDVVIKCYQRNPLYTQ
VEYRSICTDPWSTQAGLSPEAGVTAMLVGEFDDQSQGERSQDVLLLKYGSDGKLIGHAAFGRPWASTFSAEADIDFG
STPKPEVLLLADMTGDGRDDLVVMTSSQVQVAVASGARTFTQLSPWLDLAGSPFYIDTSNTTTRAYIVTDVTDDRAA
DLVFFDQPSLRLGLMPSNRVNKLLDDTDGDGTTWFELSTVSGRCASLGEDCFLLTTDYNRDGLNDVAVVYLDRVTDT
EEFYLTMVATSPPAPLTWHLSAAAIFPRGACTSPMAVALGQFVGADGSMDGTISGTVNPQLVCLSSYDQRIYIGGLG
VWGSLPGPVTSLHVRDVDVDGRDDLLIFTEVGSFYLISTGAAFEPALSTAAFLSPASVSLAVPNTPSLGQTDQTAST
AVASADVVAAAPVEHRCGPPRRLVAYFTNRRPDKSPACVASVADVVATPLQRATAATHLIFAHVRPNAETLGIDLIN
DRDGPVLANLDGNLLAVNPDIKVLVSVGGPGGNDAEFTRLVLNQQSVTGFANATLAFLNTYNLDGLELSWPSLQAEQ
VFGFTALVELLSSSLRPAGKLLSLAVPPREVYLSLAWGRLGSLVDMINFQGFDLEGDEVLGAAPYVETPLFDCLEAE
GLSVNTLIDLILAAGAPPQLVNVVASSMGRSFVLDGDGYVGGPGSPGPCMGLEGLLDQSEIKLLLPPGAAKLDPEAL
ANTGPYASNQFAHWEDGYTIVNKFCFARAHCLGGVGVWDVDGDSYGELLAAVTRTMQGDPAVCEAYSPPECTNAVST
RGSEDLGSPELVATLGDAEYLLYQVRKTWTDARDHCQQIGGDLVSVTSRGEAGVVYSLISSWASSGQLGQDDIYSGR
DVYLWLGGTDAAQEGRFVWAATGAELTYTAWAGGQPDSRYGSEDCLAASVRLGGSGGAGLREVLSPEALWNDLGCSA
VLPFVCQRNRDLARSFLQGAKEVPWLTATYHVMAPVPGSGDPGLMLTQPEANKLCRTMGGELPTLTDPWVREDLTSQ
YHRDLPSHTWLGLRSYGDGQLFWNDGTFTTDGMLNAWEPGEFGDAACGLIIGPNGANVTVSVGALYSFWNATAFQGA
IRVASPPPPPPSPPSPAPPGPTPPRPPPRPANVTPPAPPPSIPSAPFPPDFDTVLIYNLSTYFTDEPELVTVFLPQG
VYSLSCNERMPTTCQTGAPTVSLNPNFYCLSRANGRSYVVPGKELTGMPLYLTTERSCASACMLNIRCVYYTWLPRF
RASFLPSDEMLGRPNQLPGQGSCYLMGRPWASNADRLPKLLSEITDNDRVCFRSGAVFGGDSIPVNDTSLIQPPASI
GRLHGNPTPTTPTPAAPPPSAPFSLLCGGDGSAAPLLSSLTFLVDNATRGIQDVGTTCAGTYTAGVIGYLSAEGYEM
RLRQLQRPIIQSYTARCGPGGVTGLLGSYDNRGMCQVSLMCTGGGVEPVLPPGASRNCSIGASAFDFECPRGHYAVG
LQGVFLNPATYTAADNILATLRMVCAAVPVAQVAPPPPSPPNPPPPPPPAQRRRRAALESSAAWRRSAAAAMASAGR
SSKDGQSAASGSFFEEALSNQQGRRLQQILPPSPSPPSPKPPSPSPPLPPKPPLPPSPPKPPSPSPPSPSPPSPKPP
SPKPPSPSPPSPKPPNPPLPPSPGPKPPSPPLPPSPPAPPPAPPRPPRPPPAPKPPRPPAPRPPSPQPTPPIMSPPP
PPSPPPAPRPPPAPTPTAPGVPNPPTPPPRPPSPPSPPPKPPTPSPPPAPPPRPPSPPPPPPSPPPPPSPSPPPPPP
PSNATSDVIPCRTCSPIFGETAGSNSPFANTSRPGALRCPQGHLLTQLFQPVVINQLLAQQVCCTNFSQPITGLGGV
CGPLAPSAPGADDTMRAGLPATPSQPLSATTPFLTTVVNGSTCSGGIAQVTGLFLSFRPPSPGVGTPSYIMGLTARC
RNVATPLPLPAPASIFQRPFSYTCPPGTVLSEVLWNMQDWPAQPGTARSQPVGVIANIVFKCSNAAFPTPATPMPSP
VPFTQVESSNMALVSINCPAGSYVTSVYGSYDLSPANMGGVAFVRQVGITCSGATTITREITTSLGATASATPFTSA
VCPGGVGALTARALPAPGAPTTSPPALLSLDAHCYDTQAHTRRLSFLPPSLPPSISGSVFELTDRCMGLTLVSGVTM
LRSMFSATPALITVGIPAASFLHGFKLSCTDVPEPSAPALPVSAQLPPNQLPTAPAVAFKYECPAGSKVVSVLSRVD
ADEDLLNLRIECDNAPLGDAEPLAAVAARLTPFFAPPVVFTANCSNCTTAANISSMGLGQRRYISPDVAIAAPRSSY
VIERTCPLGVAGVVNSATTSRLAPELGGLARPVSTADMPNAPTGSQVALFDVLLDWSNAAAFCTAQGGALMTFEDEL
QRVVASSVVAKWAAENTGDFLMGVWIAARRSGNGLYDFSYLDGTSMANYVPPWARGEPNDMSGVEDCVELVIDVTAV
QQASWNDRVCTGLVRRPLCKLLPKAAFDGVATADAAPPNVQIAATNNDELSFYNISVTWSEAATFCQRRGGDLISFR
DQDEIRRFGAAMSDWTRFIYTGRPSPLQVRTWTGLRRQVPASGTPAEALRVATATFQYQNREFLFIPNMAVPEDASA
ACRGFGGELASFLSLVEYNTVFGQFLSTIIAYGITSTASTVTSHIGLIRNNFGTFNWRDGNTWTGFTAVQAPQNLPS
LCGVVLTRCTAGNATAGFSGCSWAISTTSCSFQTNNGFYPSSNSYICSRNTPAAASIVSGSRQYLAFAERRTWPDAA
VACTSIGARLAAFHRPEDYTAFQTAASRSQAFLSATGITNITAGFAHIGMSHNAAGLYTWSDGRPLLYSVWAAGMPD
VLSANTNCSAFTVSCTNTTGLWGSCSSPQLRDVACNTPAPYVCSYDFDDEWAYSDSTPLVYGRDTPAGLGQPAATFA
GNYQWDRVLNAAVCTNANASLDYTGTAIMSFSTPDTYKCCSACSATPGCAWWTHVNNTFVCTLKRGNGAAVTSVNAN
MWSGASGRPAPSLANATALLDDSGLNCVYLTQQMSGGSTYQMPPLQCQQRMGVACRRSSAREAVPEDERRAITNYVQ
PMGRVFTNRGESELSLYGTFQDFPSAQKLCELRGGTLVEPVNMEWQDAVFRLGTDVIYRGASNIIIEYLVGLSERAQ
EGQYVFNSGTRLLTPFWSAGQPANLSNPLYVEDQDCARARHSVNSNPNSTWISWYCRFAPSNAMCQRPAILTPEYAT
IRSMAPAAQTVVGRAHFMFYPRLMDWYQAKTLCEANGGDLAYFDSAQQYEEVMDALLQWLRTRPFVSRSIPANRAAV
GTWLGLNNRLNMNNWRWSARPYVLASATNPSWFARRWNGANITVGGRLGRAYDNPAMGLASPGIDTPFTLCPNITDS
DAFVIGFRAQVDGRSTSSTSAAPNTTHNLLGLMGLRMRCEVIDPDLNSFAMPETAVNSINNVPGVGAWGATEFTCPS
WSPASKIYMVGIQLRAAPRRDQPIMQQVKNCIPSGSNPCATYTISWQATNTTWGDDVGITSMRGICTNTVSSPSSWA
EIVNPAPAADAVWLPAITCATGYAICGISTRVDYAGANYTVRAANTSSNDDSGISGITLKCCPLPNPFNTAVMGFEC
VEARVFGNNGTNTWIQRPCASRNSLISSSNVLCRRELPDYNANAALNVQNTTASVRAADTQTASIPTLPDPLLQQVA

QSVEPLGTERVLYAGSTRANRNIITQKYEVLLYDLVMPWGPAVSFCKANNAELMAIDDQNTANAVTRLVARVMPAAA
FAYFNTTSLQFWLGASNRGGNTAWTWSTGGSVTYYEWSTGAAQPQRPSCAKLTYTINSQSGAAYVKSGWGAEPCGSH
MLVLCRRTTTSGSGRSRAAPTNTKAPAASLTIRDVRTTLFREAVTWDQAKDYCQRRTGVLASINSANEGDVIATMVR
EWARTSTAGEVKVWLGGSWHPDAMRYAWEDEMSFDYVRGGGSLSAPERPTQGCLSWRLRPDGTDVWQADDCAEYALP
LCQSELQPFICNTGPNSTRPIDSNPNDAKAKVFCPPGTVLTGITGSISNASLSGASLTLADGLQFSGICGNPAAPAV
LSTRDNQELPMCRYRWSLPRIEGPDVFIDGTNATTALDSCFRMCSVGDAPLQQVFRTPMLAVLYQVNGVFSCRCGRA
LVGRQLLNDDAIKVAEATGAANFIYGRSYDIYAICTISEELMTAGYFKQISFQLTLSDISAPPKLTSLVRGAKYRVF
TIPQAVDWNAAQRICMLNGGHLATLESTTDIEDLVKLLRENTNVAVDFPRGSGVWLGLYAAQNNMYRWVDGTPLRYP
LGPVLEDWGYAPANCGFLDLWLNSTRDRRIITGDVRAGSCDNINAFVCETSTMVDDSLSDITPGIVRTYDTFGQGTR
GWGKWELKHFAAHRVTWHDASRICRYNNMDLLWFFSPSEEDFVRTQMLAPAGSLTPSTSWLGATPGPDGRISWRTRA
ESASALRSSIWSLPAIWNASAAQYLAENDYGQLCGLLDPRARLSNPARGSFLLAHCSQQAHPFVCKSRVIPWRDLPL
KNQVQPSPPPPPASPPPPPGIGNVLLRVDRPAAQYVLLDQVTSRAEALRMCRAYNGRLPEFTSLSEYDLVSENVRRY
WTGKTSESFVSYWLGGSKKVVGNTTSWLYDSGNVASPTAWGLDEPNNYGGNENCIHVIIYLVDTPTGASRYGAVGDK
RNERTYWNDVKCDAGLPVFCQLSPRTGGPLTPPASDLAAIEYVGTLNGIRYVMYNQLLGWTDAKAYCEANGQVLADF
IAQDELDGVVRGVFHYVYSLPQFQDTLLSLWTGYNSFTSDFRGNSILTANGFAGPS

>Cre05.g240900.t2.1|PACid:27572584
MARCQGRRMGLAVCLIGIAFVLQAPGFADSQRPSRRLQQLGPAEELLPAEDLAVPAEAPAGATKYVEDSVSRPFLKV
ADPGDVDAATALRVYEQGAQVASIAYLLQGAADSLSKSPGSSNALLPYLDPTILRLYSAGPHRCNSHTAELLYDLSA
VLYGSTQLQACHYALYQSLRSVFAWVRDNTPLALVQPPGQPDARVALPDPDLTSHLELVLAVAAALGDPEQLGGETA
ELVQGQGAELVQGSCGLSREAFAGVVPQVAAALAALHQDLPLLQDIAATLHERHWDPEVSEGPGDPSQQRRRGRRML
WLEPGRAVEVFDDALTDPESQPDLEAALGAAAAAEDFDVTIRRRAARRGGGGAAAGARRLLATGASGSITYTMPPST
FTIAAPATLPKLFIPIVFHIMQYKETNGTTGPVNFAQALTYVNRMVRITNYMAKPMNVQFYTSQVRNDAATYPYLTL
PDRNTWLNLPVCTSGTLNCLNTESYVAPLLVDWPRSINVFVASDSLAGTNVPLGYAYVPGSDLKAVNGHVFITWDTL
STDGSNKLSIYNDGPNTLLHELFHHLGLQHPFGPTNDAANSCSDDDYVVDTPTTLGSASSSSFIATASAYCMETFWG
VYGGDWDATYTRWSSSLGIPEGDMNSWADSCPTQAGYDELGNYMTYNTPVCFPALGHFTRGQAEMAHYVSAELNPVM
YSWGQYYAAVSPSPPPAASPPPEAYTNICKATTKNCACKATWSLNGNSYAYCDRTGSSNSLTCEVADPASCADCASF
GTAQCILQCTGTARQCRKSLAPGTFAPPPPPPRPPSPPPMPPPPPPPRAIPALCMKAANGCKCRSSWRFNGQYWSYCA
SPDGDKTLWCQVESTCAGFNVSTAYQYCDTSLTLASCKVQEVFFSQTRNPPWPPAPPASPSPPPDLRPRPPPSPVSP
PPPSPPPTASPPPPVTVASINASVTINANCTVLSVAGNQDALRSNLTAELSRILAVPGGYITIWSFACGSIVANYTV
AFPSGTTAAQVDTVKNAASTVPAAASAAFTSAWGTVTTSSSGNVVVLQPAQLCPAGSPASALCPASPPPPPGTLLVP
TPSPSPAKKKIALPMAAIIGIAAGGGVLLLAILIIIIVCACRSGPKPAHAPPAKGKVVPQPQYAQPQQQQQAYYGGG
GAAPPPGSMPAANFRPAGGYDVAQQQQGNIPYYRQQQQPQPQAYPQYPVYPTGYQ

>Cre05.g240900.t1.2|PACid:27572585
MARCQGRRMGLAVCLIGIAFVLQAPGFADSQRPSRRLQQLGPAEELLPAEDLAVPAEAPAGATKYVEDSVSRPFLKV
ADPGDVDAATALRVYEQGAQVASIAYLLQGAADSLSKSPGSSNALLPYLDPTILRLYSAGPHRCNSHTAELLYDLSA
VLYGSTQLQACHYALYQSLRSVFAWVRDNTPLALVQPPGQPDARVALPDPDLTSHLELVLAVAAALGDPEQLGGETA
ELVQGSCGLSREAFAGVVPQVAAALAALHQDLPLLQDIAATLHERHWDPEVSEGPGDPSQQRRRGRRMLWLEPGRAV
EVFDDALTDPESQPDLEAALGAAAAAEDFDVTIRRRAARRGGGGAAAGARRLLATGASGSITYTMPPSTFTIAAPAT
LPKLFIPIVFHIMQYKETNGTTGPVNFAQALTYVNRMVRITNYMAKPMNVQFYTSQVRNDAATYPYLTLPDRNTWLN
LPVCTSGTLNCLNTESYVAPLLVDWPRSINVFVASDSLAGTNVPLGYAYVPGSDLKAVNGHVFITWDTLSTDGSNKL
SIYNDGPNTLLHELFHHLGLQHPFGPTNDAANSCSDDDYVVDTPTTLGSASSSSFIATASAYCMETFWGVYGGDWDA
TYTRWSSSLGIPEGDMNSWADSCPTQAGYDELGNYMTYNTPVCFPALGHFTRGQAEMAHYVSAELNPVMYSWGQYYA
AVSPSPPPAASPPPEAYTNICKATTKNCACKATWSLNGNSYAYCDRTGSSNSLTCEVADPASCADCASFGTAQCILQ
CTGTARQCRKSLAPGTFAPPPPPPRPPSPPPMPPPPPPPRAIPALCMKAANGCKCRSSWRFNGQYWSYCASPDGDKTL
WCQVESTCAGFNVSTAYQYCDTSLTLASCKVQEVFFSQTRNPPWPPAPPASPSPPPDLRPRPPPSPVSPPPPSPPPT
ASPPPPVTVASINASVTINANCTVLSVAGNQDALRSNLTAELSRILAVPGGYITIWSFACGSIVANYTVAFPSGTTA
AQVDTVKNAASTVPAAASAAFTSAWGTVTTSSSGNVVVLQPAQLCPAGSPASALCPASPPPPPGTLLVPTPSPSPAK
KKIALPMAAIIGIAAGGGVLLLAILIIIIVCACRSGPKPAHAPPAKGKVVPQPQYAQPQQQQQAYYGGGGAAPPPGS
MPAANFRPAGGYDVAQQQQGNIPYYRQQQQPQPQAYPQYPVYPTGYQ

>Cre05.g242150.t1.3|PACid:27572598
MPLLELSNASPWLRAPVVRQLVAVWSLLFHSGLLLYAAALHPLLAPRLGADLVGTAFRRRPQLDRPLPQPPPPPAPA
PQQRPAHRRPCQSPPPPGRGGSPATAGPLSQPPRQQQQLSQPQQQQQQQQQQQQRQLSPPPAAAGRGQGGHVSRQQG
PEPVPAQRPRRSATPTPTPLLGFPIAVYAHRGGSLDTTGLTDPTGPATTTTTTTTTTIGPTAAAAAATAGASTAASA
AVAAGTPRAGARTSASHVQQQHQQHQQQQQQQQLPRRCGCYACRHGLPLIENTLTAFRHSVCAGADLLELDVQLTSD

GQVVVFHDPTLSLLAGEGAGRLTRPSSSSSCSSSSSSCSPSATPSCCVAVSDLALAQLPRLRPRPPGTMYHCGGGGG
GGGGSGGGGGCRRRLQRQGQGREQRRERERQRVWEQQQQGKAWGGSNGDGESAAGAAAAGSGSSSSNSTSNSGSNTY
STSNGSSNIRSCRLLQAAGTATRPQPEAQPGHAQQQQEQAQQEQQGPEKEAPQRGLGLGAGWGSERGSRAAAVAAGA
AQGVLAAGRSAGGAEEQAEEHDGSDTGTGTDVEGAGGGGGGEADAHEYDRIPRLWEVFQAWPHVPVQVDLKSSDPAL
VAAVAELVAAQPGRERLLLWGSFHQRTAAACLAAAPGLPLFASMQRALVMMAAYQLGLLRYLHIYEAAVVVPWRLHL
SPTLTLTLSLGLGLGPLPAWAHRLRAAVNGWLCGGSGGGGGLWRRRSAATATAAAAPSSSAAATAAATATAGGAGGT
GAGVLRAEEPDVELRLTYYFLDAGWFAALNARGVAVVVFGALNDTRRMEACRRSGANAIATDTPSHLAAFLAGGSSS
SSSHSCCSSGSSSSSNGSSSGSAAVDCDGPSAPHLLRVRRRGAISGGGGSRGSSDGGGGGSECASGDTGADGASGRS
SSGGGSRSSFTDVGLGSSSGSASCCSEGGASGPVGPCSSGAAASGVSGACGGGGGGVVVLAGARPLAPLPWLLQPQP
QPQPTHAPPPPPPPTPPLAGAARS

>Cre05.g243450.t1.3|PACid:27572622
MLLPLLLLLLSAPFIASAASAASAAEEASAEAVADHSRGLKALGGGHWHVQRRPQLAAAAAISAAAATSARLTAAEV
AGGAASAAAATDGGSEQLAWSADPQLQQGQTFGHRRSLLKAKKGDSNAGSSGSSISGMTIIAGAAATPPLAPKKHKK
SPPPRSPPPRSPPPPRRHKSPPPVRPLTEPPLPAPPTVLETTDAPPPRSPPPQPAPPPPPPPSPTPSPPPPSPSPPS
PPPPSPRPPSPRPPPPPSPRPPVPPPRPPPPSPPPPSPPPPSPPPTSLPPPPLPSPEESSGPSDGYDGEYGGEDGYM
GGGSGAGNATDADNSMDEPEWPPFPPPHPRDANAPSPPPPPPPAPGSRHGQYEFIGSANIIAVHLMAVPGTDNFLFM
ERPSGRHPDGSHNIAGFLNLPTRAWTHLYSPDGLFCAGHTFLDTGDLLIVGGHQANAGYPDGMKSIRTFNRTCTDLQ
LRKVVEMAWKRWYPSATLLPDGNVLVMGGTQGVGAGTANNPFWEMFYPSNGTTRTFAMKPNYLDSAEQVYYPFNYVL
PSGLLFTFCGRSGFILDYTNNTWMQNLPRLRGWATTQYPFTGTSVMLGLYPETGYTAEVVLFGGQAERATRNLSTIA
AKQAQRLTLSAYDPVTRNYSFGPTGSWVGENLGSPRLMGDSVLLPNGKVVLLNGAQTGLAGDSASGGDTRANYPVLF
AELYDPDRPQGQRVRSLGFSRIPRMYHSTACLTTNGTILVAGCDRCQRFVVDEGWTYDPSPTGKADYRLEIFHPPFV
FFPSDRPIILAPSLDASPPPPPRASPPPPPPPPPTGTKAKQPLPSQHDDLGVAVVMGYGTTHQIRYAWPAGFAGPQP
GSSIGMNATAPPAADPYDVWTPGVITRVVLSAPCSNTHSFDMNQRLVGLEILSNTPDPSTGSSSGTTGSSSGSSSTV
AGGVLTVRSPPDINIAPPGMYHMFLVNGDVYSSAVWVRLARPQ

>g5203.t1|PACid:27572645
MARGSAKPHPARRPRPEDATTSHPRRSARLARIDAAGAESQTPTSKDFGCSPPTSSLACSSRLLALPLDLQLAILLR
LPPNDIAACVRPTCRAFAALLPGPLYTRVLLSQPVNPAEFRWALYRGRLGAHVFNPSPSPAASPSRSPRHLPPPPAS
APNAAAMQDAAAVPDAAAAATVSAPGGSATAEAAAAAPQPRAPAPPTPPRSPPPPSPPPPPRPPPPPPGLDGLTHDE
KTQLSSLLAGAGVLANLVALHSFRPRSAAAMEAAVAARREKGGAAAAGASAGGEAGGGRKRQRAEEQAGEGGGGGGR
ERGRGGAAAAAAGAAAAAGALPAGPGADVQADIAEATAAMVMAGWDLQVGSGGSSGGGGGGGGSAADGDGCPMNCST
PRAAAAASRLHVLRWLDGVGACMCCVRVQTAVGACGSEEVFRWYAAHADAHAPTLFAAACADSHDTRRAAKMQLLLD
RHGHEVATRRALTNAAARGDVATMALLHSTHGVGLDGSALTAAAAAGHAAAVDWLLSRPAAPPVGLLALAAAAETGR
VALLRRLADRLGRPLPPLWRGQLLCSAAARGAAGSVRWLLAAGGWRRQYGGGGGGGGVGVGEGEGDGAAQRHAAAAE
GEGAAAAAEEEGEGEEEEVAGWGLPAMPEGGHGEAGAGGGGGGDGGGGGGGGNGGGWVDVAAELAVASQEALEEAAVG
GHCQVMRLLLQAGLAAPTARVMEYAAEAGSTRCMQLLLVAAMRREAATAAAAATAAATAGAAAAVAASGAEAAAAVA
AAAAGPQASPAAAAAAAAAAAAADAAEAAAYADGRASFVRAVAASPAARALLQPAAYVAAAQSCSSPDPHDGHGARQ
TGYSFSSRDLTAYALGGGVMPTLIGRTHDSGSSSGGNGGSSNGGSGSGGAGGGEGGAGSENGGGAGGGEGGGGVVRP
RSRFICWDDADEAGGGGLGGAPDNSAAAAAAAAEPTLEAAGTAASEAAEAAAAEAGAPPQRKRRRAGAGTGTAAAAAA
ADAAASEAAAYAAAAALGSRSHRCLAPLQLLAAVGCPRGGEEEQQEATVAAALVGSGTRLGWLRRRGWAWGPAALAA
AANSGCVALVEWMAAQGCPLGRRGEAYLAAGANSDFALLAALRRLGCPFAADTLTRAVHGAWGEPSYGGGGGGGGVA
GGAGSYSALAAVTGGAMAVRCRLEVLQWMVARGCPAPAQALRVARDYSRPDVAAWLRRIGVSR

>g5346.t1|PACid:27572689
MRLLCTGAPASPPSPGGGLAVGASAPPSPPPVLLQSAAAVAPAAVSALPSAATSKSAPGPPARCSASASPPSPGGGL
AVGASAPPSPPPVLLQSAAAVAPAAVSALPSAATSKSAPGPPARCSASASPPSPGGGLAVGASAPPSPPPVLLQSAA
AVAPAAVSALPSAATSKSAPGPPARCSASASPPSPGGGLAVGASAPPSPPPVLLQSAAAVAPAAVSALPSAATSKSA
PGPPARCSASASPPSPGGGLAVGASAPPSPPPVLLQSAAAVAPAAVSALPSAATSKSAPGPPARCSASASPPSPGGG
LAVGASAPPSPPPVLLQSAAAVAPAAVSALPSAATSKSAPGPPARCSASASPPSPGGGLPVGASAPPSPPPVLLQSA
AAVAPAAVSALPSAATSKSAPGPPARCSASASPPSPGGGLPVGASAPPSPPPVLLQSAAAVAPAAVSALPSAATSKS
APGPPARCSASAPPPNPPPAQPTGFCAHVLQLQRPIGTAVRGGSWQFT

>g5540.t1|PACid:27572698
MPSGLGAVVSPPTAVWPVLPFTLTPQLMSAITAIPSSPSPRHAATVAAAAVAAQPTCGPGALAALEQLLSDAPGVLL
LGRRQAADDVAQHNAAAAGSSNSSSSSVMTHSRENAAAAADLVTGSRTSLGERRLVLYEAVKAVFAWLGSQQRGAPC
RGGRRGGSSSVAGSSKGSRHDSSSSSSSSGGEAAGVVDGALGVLADGTCVRQQALEALGVGGPLASVADRTRVCCGL

GPEELAAAAQHIAAALAALAAPERAAELAAHAAHVQRAGACTCHRQQQGGSGLPAADGSNGSGSLLLVHPHENDGGG
GGGARSEGSSNGTDSGTDSGTVSGGGRRRRVQQQQLEMEMEVLVTEPLVVRLLGDGAAAKTEPGEREETDADEDAY
DDDDDESGGGGDSAQGHEQFHWQQAARRRLLVMRTEFNLTLPPVTLPEPAAPGPNSPLPDMLVPLIFHVYTYRDAAT
GAIGPPGWDQSPALSARLVSAINVLSRGTGFRFFLREVRWDPNKYSYLSVAGGAGAWQACAVAVVMEDLANDCIMAK
GVLVDWPKAINVHFIGDLLDRSDTTTKLDAAGSSQVFVNNERPIWVGWTVLDGEARGGYNGELLYEYGASVVWHEIG
HYLGLGHTFVSDVKINTECLDADGIDDTPAVLSGASTLISMNFFGRDVNDHCFTVLNRDLKGTIDGAASRLGIPDAD
KNAWADSCSDKPWQWGCELEPGCASLPAAIRDSGSAVVVPCDRAEDMCGSRPPPSPTSQPALYDAICNLPVNLPGCR
CKASWNFGGRTFSGCADLDGGGAPWRCVLDTPCTTTTGHTYGAGVMMGVCDDRAASVCSSGGSSGGSSGGSSGGSSG
GSSGGSSGGSSGAPSSSAGGGGSRPAVCSAPEGLPGCSCLPAWRLQGRAYSGCADVARVLGLDPGFDTKWLCQLQTP
CNSPDGTLLDGTSFSQCSSRVNADLCGSGALADSPPPPPRQKPPPPPSPPPRPPPPSPAPPRTRPPPLPASTPSPSP
SPSPSPSPSPAPRPDASGPTSANPSPSPAAGPKSRPKLKRSPKPRASPKPKASPKPKPSPKPKAPSPPTS

>g5540.t2|PACid:27572699
MPSGLGAVVSPPTAVWPVLPFTLTPQLMSAITAIPSSPSPRHAATVAAAAVAAQPTCGPGALAALEQLLSDAPGVLL
LGRRQAADDVAQHNAAAAGSSNSSSSSVMTHSRENAAAAADLVTGSRTSLGERRLVLYEAVKAVFAWLGSQQRGAPC
RGGRRGGSSSVAGSSKGSRHDSSSSSSSSGGEAAGVVDGALGVLADGTCVRQQALEALGVGGPLASVADRTRVCCGL
GPEELAAAAQHIAAALAALAAPERAAELAAHAAHVQRAGACTCHRQQQGGSGLPAADGSNGSGSLLLVHPHENDGGG
GGGARSEGSSNGTDSGTDSGTVSGGGRRRRVQQQQLEMEMEVLVTEPLVVRLLGDGAAAKTEPGEREETDADEDAY
DDDDDESGGGGDSAQGHEQFHWQQAARRRLLVMRTEFNLTLPPVTLPEPAAPGPNSPLPDMLVPLIFHVYTYRDAAT
GAIGPPGWDQSPALSARLVSAINVLSRGTGFRFFLREVRWDPNKYSYLSVAGGAGAWQACAVAVVMEDLANDCIMAK
GVLVDWPKAINVHFIGDLLDRSDTTTKLDAAGSSQVFVNNERPIWVGWTVLDGEARGGYNGELLYEYGASVVWHEIG
HYLGLGHTFVSDVKINTECLDADGIDDTPAVLSGASTLISMNFFGRDVNDHCFTVLNRDLKGTIDGAASRLGIPDAD
KNAWADSCSDKPWQWGCELEPGCASLPAAIRDSGSAVVVPCDRAEDMCGSRPPPSPTSQPALYDAICNLPVNLPGCR
CKASWNFGGRTFSGCADLDGGGAPWRCVLDTPCTTTTGHTYGAGVMMGVCDDRAASVCSSGGSSGGSSGGSSGGSSG
GSSGGSSGGSSGAPSSSAGGGGSRPAVCSAPEGLPGCSCLPAWRLQGRAYSGCADVARVLGLDPGFDTKWLCQLQTP
CNSPDGTLLDGTSFSQCSSRVNADLCGSGALADSPPPPPRQKPPPPPSPPPRPPPPSPAPPRTRPPPLPASTPSPSP
SPSPSPSPSPAPRPDASGPTSANPSPSPAAGPKSRPKLKRSPKPRASPKPKASPKPKPSPKPKAPSPPTS

>Cre05.g247350.t1.2|PACid:27572701
MAVVVCSPADMQQVTGEAAGGPGMAELGVLPRAMLPRSASLSILNKIQPWGNNKSARLRAALGLTGPQTATAFAIDT
TAVAAAAADPLAATGARALGRSVASAKESWPGLSPAPSPGMPLSDDGAAGCYASPLKAWTASAALLEEADGEGDEQD
EDDAGSDDAGEPGAAGSCPSEGEQAPTGAGGGGGCGGGGAASGVLGLGRPGGRPPMPPGHSSARPGRSPARARDTAA
GPFQQQYQQQQQQQLQHQQQQQQQLQQQQQQQPAAPREPLYPSWAASAASRRSALAAGGGSVRGWVPAHLAAAAGGA
PPPPSSLGAASGGSAGGSRCGSPPPPGSPAEGADGAEGGGGGGADWGGMGGPASGRSSRVGSGHLRPGPGSAAYAAA
AAAAGLTPVNSSANIPSAFAAALAASAAGAAAGPAQHFQLHPRTGAGGGSGGGGHTGGFGRLGGGGGEGVDRDGDGG
SGSDASTPRLTGSGMGGLLRASRMQGYVTAVANPAYTAAATAAAAAAAAAAAAATSTSATPFSPSAAGGGGGGGGSI
WETSRRQRASPAPLVVDVSDAAAAAAAAAGAGSGAMGSGLSGPLLPVPAVSALASPSSGPAGGGGGGALRPSMAQQRD
APRWTSPPPPAVAAAVVAAGLAAPAGPGGAGAGAAGYHTPYHAYLHPSMDPQALAQVQAHAQAVAAAAAVLRLTASG
GPGGPFWPGLPLGGGGAGAAAGPGGMGAGGGGGGGGGAGGSAGPQGQGQGQGQVPTGHRNSQHGAHHHGHRGGHHVG
QGGQHAASNPSNAPAPAQHHQHQQHQQQQLQQQQQQTHHHHGRAGGAGGGGGGRQRRQSLTEFGNVRAQLQHRSADG
EAVAPPGTGSSASQSGAATAVAAVGIVGVSGAAAPSGAGGGSGRVGRGIGASGGAASAPAGEISVHASAPLPGGTLA
ALVLSAAGGQEAAAGAAGGGAAGGTAGGAAAFVSASCDLGALALQAAAAAGAREDLGTSVLVGSGAGGGGGGGAGGG
GGGSDRPTGFSLGASSGPGSTSGRRVLAGGTAAAAALAVAAVGGSGGGGGGGPGRVCHSARGGGASMAGGGGGGAS
STGVLSSPTSWLNGGVSLLQALASGGEGAARRASGVLHGGGLLSGAGSGGGGGSGGGGGGAGGGAAGSFLSMAPRGG
GHSVSRGHVGRGSRVH

>g5241.t1|PACid:27572723
MEGDASSSARSGDHAATAQNATGLERTHARKLAQDLLTSSSSAAQPCTGSFSSPTSQPVSAQPFPSPATCSSSSAAQ
PCTGSFSSPTSQPVSAQPFPSPATCSSSLAAQPCTGSFSSSASQPVSAQPFPSPATCSSSPSAQPCTGSFSSPASQP
VSAQPFPSPATCSSSPAAQPCTGSFSSPTSQPVSAQPFPSPATCSSSPAAQPCTGSFSSPASQPVSAQPFPSPATCS
SSPSAQPCTGSFSSPASQPVSAQPFPSPATCSSSPAAQPCAGSFSSPASQPVSAQPFPSPATCSSSLAPSPAPPVSA
QPFSAPATCSSSPSAQPCTGSFSSPTSQPVSAQPFSSPATCSSSPAAQPCAGSLSSSTSQPVSAQPFPSPATCSSSP
AAQPCTGSFSSPASQPVSAQPFPSPATCSSSSAAQPCTGSFSSPTSQPVSAQPFPSPATCSSSLAAQPCTGSFSSPT
SQPVSAQPFPSPATCSSSPAAQPCTGSFSSPTSQPISAQPFPSPATCSSSSAAQPCTGSFSSPASQPISAHPSPPSP
SPPQPPVPPSPPSPLPRPLVCSPPAGWCGGGNYQVADSRRVDCDGDGTADYVCIDALGNRGVILNNQTAIACEDTWP
SVDRSAARCPVFFLQRCPNVTGYITIPESYPVAQAGQTALYASTALADAARDACSMNDNCTTWVRYPSTPYDQGQYL
PSQPQWIQAGASCTYVKAPGFVTVRLPPSPAPPSPRPPSPSPPRPPSPPPAPPSPPPRPPSPAPAPPSPVPRPPSPP

PAPPTPPSPVPRPIVCSPPAGWCGGGNYQVADSRTVDCDGDGTADYVCIDALGNRGVILNNQTAIACEDTWPSVDRS
AARCPVFFLQRCPNVTGYITIPESYPVAQAGQTALYASTALADAARDACSMNDNCTTWVRYPSTPYDQGQYLPSQPQ
WIQAGASCTYVKAPGFVTVRRPPSPAPPSPRPPSPSPPRPPSPPPAPPSPPPRPPSPAPAPPSPVPRPPSPPPAPPT
PPSPVPRPLVCSPPAGWCGGGNYQVADSRTVDCDGDGTADYVCIDALGNRGVILNNQTAIACEDTWPSVDRSAARCP
VFFLQRCPNVTGYITIPESYPVAQAGQTALYASTALADAARDACSMNDNCTTWPASSSAQPPAPATQPGASSSQPGA
SAAEPTTCSTHPSIAGAPAISPPAGWCGGGNYQVADSRTVDCDGDGTADYVCIDALGNRGVILNNQTAIACEDTWPS
VDRSAARCPVFFLQRCPNVTGYITIPESYPVAQAGQTALYASTALADAARDACSMNDNCTTWVRYPSTPYDQGQYLP
SQPQWIQAGASCTYVKAPGFVTVRLPPSPAPPSPRPPSPSPPRPPSPPRPPSPFPPRPPRPPSPAPSPAPAPPSPAS
PRPPSPPRPPRPPPSPPNPPPLPLACGRPEGWCVGDTYIYRNVDCDGDGVPDHVCYDARGYRGVILGAQETATCVDT
WPWLASSICPPVFNRCTAYPGWVAIPDASWTAASLPGSSSYHDRSAAAVRDYCSASSTCTLWDTDLYPDRWTALNDA
VATLTLLVQRLEQTPVACVYVRPPPQAPPSPPSPPPSPRPPLPPVPKAACPRPDWWCTGSSRAILLEDVDCDGDDLL
DSVCYDLAGNRGIIGSGIDCTDTWPSVAASYCPPVFNVCPEEPGWVALPNAAWSASYLIPTTEMFGWYSAAEAKNYC
FGNSRCTLWYKDTNVWVTYNLDMSRVTMTKRFGAPCLYVRAPPPPPPPPLPPGPPSPPPSPGPRSPKPPNPPLPPRP
PSPPKPPAPPAPPPIACGRPEGWCVSSWNIYRNVDCDGDGVPDHVCYDDAGNRGVILSVQTVTCMDTWPSVSASVCT
PVFNRCPMDPALAGWVALPDVSWVADFSTSLDWSSAEEARTVCTEMDCSFLFYSPPNKWTTVSAFGNRNIVPYAQEP
ASCMYVRGRESPPPLPPLAPPPSPPSPPAPSSPITVCGRPEGWCVGDTSIYRNVDCNGDGVSDHVCYDARGHRGVIL
GAQETATCVDTWPWLAPSICPPVFDRCREEAGWVALPDASWNADTAAAYDPYYTLYLTSADSARDRCNEAANAAFNC
ILWETNVVPNKWRAFFGVLRTMTLLAPTSPTDPAACVYVKGPAQPQWPPSPAPRGLACPDRPEGWCTGAQSMYVQVD
CDLDGRLDHVCYDAAGRRGLILAIQSGPTCGSTWPSVAASVCPPVFDSCPKVAGYLSLPNTAFSSNYGNNQLFIGQT
SSGYAAWQACEANSYIYPCTMFSDKGAYAYGQVDALKYTEGVCSYFKLPMPPPAPEPPLPPPRPPSPKPPAAPAWTI
CTKPANQWCTSGTLRYVDCYGGFSERVLDQVCHNEGGQRGVIRYFTPGTTCTDTWPTATASSCKAAFGNCDRPSGWC
TGVYRYVDCNGDGIPDHTCATEPVGSTRAETFRGVILGNQTIAPGSQCVDTWPSAPISSCPAAFACPPRAGYTVYQD
ATMNSQVYSGVLGLWLAYPPETAASLCAATPGCSVYDTAGYYSIQTELRLTYMVGRCAYISNSVTLDHLTVAPGVTR
PPGTVGTAAAGLAAIDVAASQEQLSGTGVLMDDDGPQPSNEEQLQEVQPLLSGSPPHPAPAATAAAQAGMGASSTTS
SPAPAASSVSREAASSSAPSAADLATTTGATTSSNSEQQQPQQQQQQPQSPLDLPSVLPQGAAVAGAATALDTAHGA
TTADGSGSAQQTNDSSGGHGGSSVPVAGIVGAVVGGVCGAALLAIAVVGVVMWRRRLVHDNMRVAPL

>Cre05.g236501.t1.2|PACid:27572767
MALPECAFCQNNKSSGCLRPSQYKVAQAGAQDLVLFELLPPFGPGALLHQQQQQQQQQQQTGGCGSSCDGDGSSRG
RDGDGSSSSAGLPPCCLHQCYHDVHRLLLEAYPQRASDVWRFVYRGRCPLTAEQLRHSAGGDGDGGGDGDAGGNTAS
FEPYLTTCLWLGGEVVAAAALRLNLPPPPLSASSSAAASTSTTGAGISHVQIVLQATTPRLQRRGLGRLLTRCVMRA
AVQEGHQYITALAPPPPPPSSTSAATAPSAASTTTSTTISTTISTTASTAAAAACFLRSLGFQPAPAARYSGLPLAL
LPLGLHLVLEASSRTHPPFDTQQQGATGAGRGAGGAGGAGGAAEAGEAAGVWYAELPHEPVLQTACLTGDTAGAAEG
AGGAAAAGGAAGRGRAVDMLVNEAAARVAAAFRGAALSVAATSAAAAAPDVAPPRLQGAVVAAQADNLPLPCVGLME
AQARLPPQPMRQEQHQPRQPPLLQPHLQLQQQQQQQQQQQPHVAPPPTPVTVPPLARGRVAATAAAAVAPAPSSVPV
PSPAPAPSPPPVAVLVEQQQQQEQGDHEAVGLVAPTSTASVVVRKRQQDHSEQEHMDAHGGGGGNLGEEVEVTEAAA
AAAAQQQQQQQDQSEQEDMEGENAGAGGGNLEEEGGGNLGQEVEVTEAAAALQQHGWQDAPAAASVPACGDDPARCG
DPPLPLPPPPAAPSPLPPPPPPAAPPLLPPPPNAPSQPPSPHAAPHSADSYRIPTRAVEALFGREVAVSGVLPGWSS
SSSSGGGSGGGGGPLRLPLLVPPNQRHWAPAATLTCEVNVSRAAAGEQQQQQQQEKGPLWRVEGLGEWRRRVVTGPG
WKLKFTPVWRQQELQACVCGVEAGDTAAGGGTAAGDTACGGCTAGGGTTGGGTAGGGSTADGGSCRCALGRVVVAVE
VSAVDTSGARQPNHPATVTAAAAAAEPHPHVTFTPVATTWSSGAAATEAEATGAAAAAAAAAAAAAASSAISPAAASP
APSSVSVAVSYATTAAAVNAAAVSVAVSVSVSGDADEVGQARPPQLAAAAAAADAAAGAAGRSEDGEAQSPEWPNAA
AAAEAAGGRHAEAAEGEEQKASEEEQEASEEEQQASEEDGEQEEKQKQAGTGEEEEEEAEAEAEEAQEEAVEEEQVE
SDKESEGEREVSEEDGERVPEQAAAAARSAVAPVAGPLRHAAEPGCSAGRRHGPAAPSPPVTTTAATAALSPARAAA
AAAAAVSAPNVQVLTRAFAAFNFGHCGVRFSLVQALLGPAAARDPATPPVILYDAATRAPISTGARLQIRAASSSSS
GSSSGLSRANSSSAEAVAADGTGATAAAAAAAAAAAAAAAGAGPRKRSPSSKRLPQCWEVAGLRPWLKAHRAAEGDR
LVFTAAEPPGAIASAAGCCADRHQQQQQQQQPCPSCLTCGGTGRAPRLPRVYLQLVRAPPPPPPQGLRSVAAGPAA
AAAAAAVGTADAGNPTSAGEPTAGLGAGAGAGGTGVASAVSGAGFGCTGGRGGGVPAPGGVFEGARTEAAAACAAAA
GAAAALNVRPLPSPPAAATAAPNAAAATAAAPDGAAAVSASNRLVVAAAQLAVARQLEATAATAAAVARAAAVAAVR
ARAAATASATATATATANAISAAHATVSSAGAAFTASAAAAASTTAGGSTGAAAAAAPAAAAPAATAPAAAAPAAAA
PAIADRDRAPITTATPCVPPRRLGAVPARAAAAAVARPAVAAAAAAQRHSGGSEARLRQVPAGARATGATKRAHEQP
QPPQPLQAPPPPPPPHQQDADADGDDPVMMLLPSSPKRPRLLPARSSIVPTVSLELDDRLHTEPLPVQPAARPQQQQ
QQRQHQSSSSPPQTPQPTQALRRQESPAVMPYDGMGAARSPSQQPAAPPPPTQAPALLQPSAAVLTLGLGGSCGIPR
AAMARGRGGGTARPFGSFGQVTTEVTGQTHVSSQLLEHQPCAVPVTGPSPPLPYSGFTQAAQTQPPAVGPAAGAVAA
TAGGGSEPAPESAAAPASQAPLQHQVVAVAQPQQVVPRQTPEPATAVAARRSPQRVAAHLHEAAAPLDGATAAAAAA
TAPPAGAKAAGGARLVKREGVRTASSCAGASPATSSSSESHGTSGLGAKEMPTAVQPPAKPLLTTTSAGAAARRRPV
CRVGLRGVGAGWEPVSPAAMAAAEVAATAAAVEVVVHQRKAMGTVVNTDLDTALTTSPATAAATASATTAAAAACKG

MGPARSSKGTAGTGRTAPSTAAPAIKHSAAAAARGLATRPPLPAATSRAPAAAAAGVGGFSGSNRNTTGGIKRSASD
FSDDIQPDTTAAADDVPLASRQQRLRQSLPPQPKRPKLQPTTPRISAQRLSPSPPPSLQPQGSQMQPQLRVMRPPPP
PPVGGGQEPVVVAASGPNQRQQQQQLGGGGGSDGDRFAATGEAVAAGGRQALSAAAAPALAEPTPLQLLPEALAEHY
PKVFKLMMAVWKQEHQHQQRRREGEPRRSSPFTATAGSGGGAGGGGGGGAGSGGGGGSGGGAAVQAQPLQVVQRYAD
STGLQLETEFERRRQQRPRPAAAAAAATLAAAAAAAAGGSAGGNADGGGDGEGGGGGGGSQDEVEVYTATLRLTPRSM
PGQATTTATGQESPCKEMARQLAAAACLQLLLLSGVEQSVLWPRPRTTPPQPPSSAAPVAGAAVAAAKPAWRRAPGG
LWHPADSTTATAAVHTAAASPSGPAGCGGGVGGGGGGGAMAGQGYVGVWHDGPPLPPPPPPPPRKQRMSQPPPPPAR
KQQQPPPPPPPPPRKQPPPPPQQWQQPACASEPLCVGGEEDNGGAPGGWAAVDMDSAAAAAAAALVPPPPVPPAYMQP
IMLDAQQQQQQQQQQQQQQQQQQQQQQQQQQQQQQQAAMFHVQQQQAFVMQQHQAVMMQQGQQYAYAAAMMRMGPQM
GGHMGAQLRGCMGGEMGWQMQYEHPFGGAFGAGVGGVGGAGGAGGGAGGGTAFGGFGAFGGAGLAGWVAGMGGGMW

>Cre05.g239450.t1.3|PACid:27572804
MAPNSPDLVKFPRTPHLANLGAATRDDLVLTESDALAILDAGVVLEEKVDGANLGISFPEPNGPARCQKRGHWITPA
SETQFAKLDGWLQSHTEALRAVLGCRYILYGEWAAARHSVPYDCLPDYFLYFDVYDKIERKFLSCERRDDLLRPTGL
HPVPALALPAAAPAANTTTPTNNTALAAAVADATSTTPPVRRRRTGVLTPAQPFSGPGALQQLTQVVLGLRSALCVP
GSQQWAEGVYLRQERDGWLITRAKLVNPAFHAAIEGAASGSEDQQAPTAAAGTQGSSRGKRGGGAAAAATADATAAA
TASGGGTHWSKRTVVWNTLRPDLWADDVNDEGEEAEAVHGRDPAEQQKPVAEAARPAVAFNSPSRTRAATRDTVHTA
VASAAAATATPSTSIPQSPAAASRRTAAAPAAAPAASAAPAAPASRAAPSSSSGTSNNARTSGSGCRLALCCGGAYV
ALVGADVEGAATWALAHADLVPKHFVTSRVRRDGRAHHITLITPPEMCELREAYGMTPREVYDMVLGEMAATAGSDG
DSSSPAAPAAPAAPATKHADTGDHVGSAGSAAGPAAGAWVPLGLGRARCGESGDEAVFLAVAWPAGRAVREALGLNR
AREQHFHITLGFDRSDVHSRSKGLEALLQDGGCPLGLAAAAHGAEAAEAMAQAAAAAGAGRCPVHERAGVLVMHALT
QLAMTAPRGSSSGGAAGSSSTMSPAVHTALSNMLQVALAWLQPALDATATATAAATAAGDASNDEAEAEPYGCHIVA
GAAHVALARLAGRYGEYEQVDEHVRQAEAVAAGAAAAAVADVAAPRGHSDLGADALAAAAACLAGKSLSQQGAFAAA
AAAFERATIARSRMGLPPPSEPAAVSLLPLLRRALEASTPEAPALRSAAAACARRLAREQQHHQHHHHQQQQQQQE
EGQTSQASGGDVSDQDDAAADAAFAGAQELNPRERAVVPGVLLPTSLAATASSATTLSATASSATASGSGAGFVAAD
VTLMRNTSWLVPGCLMGSSTPKRAEQAWALASMGVGLVVTLTEEEPLPPAWFAASVTLRDGAGVGAGGGDGGAAAGE
EAGAEEARQRQHQRGRRPPVTNLFVPVPNYEPPSEQQMDAILDRIERHITRTNTAITRGGNHQQSAGRGVLVHCGGG
KGRAGTVLSCYLVKHGQDPPPAHAAAGANAAAAAATAVPQQPVMSADAAVRLIRQLRPGSIETEQQRHFVGRYADLL
WRRYDQAVAAGGAAAGDDEAGADGGGSSMSGGAGAGAGAGAGAGPSTRGAAASIRAASTLTTTPAAGAGAAAGASAA
GSRQRPAAPQPQAPQPQLASKRASQPPQPPQPPQQPQAPQQPQPPPLLLPGGNYPSTPHLPFSPQVAADDSQMDAAA
CRSALLHVPLVVTEKLDGGNCCIARSPEGAGGQPGAVVVYARTHKHPATHPSFGPIKALAAAGGLLDQRLPPGVMLF
GENMTAVHSITYARVPAHFFLFGVWDGRAQAWCSWPQVKAWAADLELHTVPVVHNGSFPSLEAMERTLSTLAAQPSA
LDGVSPREGFVVRRAAAFAAQGFERSVAKYVRAGHLQTESGWRRTWKAANVRQEPPAAVDPEEEAAEAEAEAEEEAA
EAEADADAAEEAEAEEAAAEAEAEVEEAEEEVAEAEEAEAEAEEEAEEAEAAVGAEVAEAAEVAGAREDVGEAAGAE
QGQAARREGGGLTAPSLTLRNTASGSPPPRRQSPPPQPQLPQAQPPTPASSSSPPPRQQGQKPQQAQHEPPPQRHKG
RSAGGAGSSAAPSSSLPRLVVLVGLPGSGKSTFSQALEASGRWSRISQDDCGSRGAAEGAFGAAMLRGPGGGWKAHV
VLDRCSPDAADRKAWLQLALVGKRDKGVVAVHFDVPAPVCKARVAARVDHPTIPHGRGGRIVDSFAARMEPPTTAEG
FERVYTVRNEEEAEALLAAWGAAGLMRIDTPPPPPPRRLQPWEKGYVEEAEEAEEQLM

>g5138.t1|PACid:27572876
MRRLSSAALGASASGSSAGGEGAGGPGAAQQPPRKKSKTDAPGSQLEAVRMRNLDVLAAAALATADPPGPAVAAEAA
APAAATAAAAAATTSAQAGAGSGAGGATAAAAGSVGAGAFVPAAAAPPDAGPAAPWGVSATGLGTSPRWRSPHSSAA
GWGGGAAGGGGAAGGLPLLPSLATRAAGPSVPLPLHSPPPLQPPQALTENLLQTQQQQLQPWGQALGGQAAGDARAE
LAHRRSSAGGGVTALAIPGSNSGGGGGGFGVGGGNSSNALLTGGSGGAVSASPPGPGLRRGSGGGGSGSMEARGVSA
AATTPPSGSNVAGCVQPGGPATSPPAPEDPGPTGARPAGTGGGSAALLEGLDPPGGQIPGTPMQQQPQQQQQQQQQQ
QQQPAFAISPVTPQTLLQPQQPQPQPQPQPPQPQPQPQPQPQPQPQPQPQPQQHYLQHAAMVHRGHHHLLQQQQ
QQQQHQQQHQPQQHQQHQQQQQHHHHHHHQQQQAPALALRPMQSSHAHAPAPVPASLMLRPAAANGYLAVAATALLH
PSHRSAATAAGGHLSGGSMIPLQPPALPSAASSALQLVPAGLQAPPAAAAAAAVAAPSPAAVADPQPRMQPHRQSQP
LLLSVPPVAVFEDVAAILSTGGDGAVGTGAAVRTARGSSGGGGGGGGAGAANLLPSLLYSPTQATGPAAQQLRGGRA
AAPDAAATTPQALQPQLQPQSHLSYSPLPHADILGLQLPHTGLRFSPLDAMAAAAAVAEAAAAEAAPPPSPPPQPA
LYDYGSTSARGGTGAAAGAVAAAAAASYPAATPGFASGVAASQASAPATSGAAAGASPESESGDVFPTAAAAAAALNN
SLPVEVDALLSIGAGGSRGGGGVGAFGGCSGGGSGGGGGGAPTGSSMLLQPNAQVQQPSGYSGYSYPHHSQQHQQHQ
HHHYPHHHHHHHYSHHHPHHHHPPLDDLLLVASAADLGLVGDTVGSDFAAAGDRGEREREPEAAAAGPPGTAMSAAL
AAAAAAAAEFFGLGSDTDDVDGAEENEEDTARQAAAALVQAAAPPGGLRSKPPPPLPPYSQQHHQHQPPPPQQQQQL
QPQLPQNQGYYQHHQHHQHHQHHHQHHQHHHQHQLPQQYQLQELGDGEDGDYSDGDMAGAASEDGDERLLMLDLAAV
WADNMLGGGGGGGSGGGGGGGAGVTAEAAGAYGGAGFGSSSSGTALGADGQGGGGSSGGSEEGEGDDDRVDTEDLDP
DQGEMDLDSDLAEERAAARARRPGAAARRRKLAARPQRAAPPAAAAAGRPRRRRVPSAIAVAAAEAAEAAAAAEAAE

AAAEAAVRSGGPRAARRRAAAAAGAAAEGSGAAGAAGRKRRGTRGDLDDGQGQGRPGAAAEEEVAALVEVRAPAAPP
SSKPAGKGTSASAKRGPAAAAGGGSSGSGGGGGGGGSGGGGSGGGSRLSDGRIYLPHSFVTTALASLLPPLAAPSVA
AAAPTAAVAAAAATSAATGAAVEGAATPQDIAAAATLAVCDGADTDAGAGAGAGAGAEASAAAAPAAAIAAAATK
AAVQFPLRLSADVVVNDVCVKADTEVRICRSRNASRRSGVGNHWLTGHQRHVRDYKDLRLLAVEVVAPPPAAPLPLA
TPQAGPAPAGPRLRILMSASGRWYGAPPPAATAAAAAAAASPSATAAATASPSVTATASAPFSAAASAVPSAKAAGA
PHVAPNGGPTVSPPPPQPHSQQQMTAVALAPAAAAAPAPAASDAPGFGPPVPPVLPAAPPPQLLAAAAAAASRALL
ARRLLQLQLQLQQQQRQAAVVGGPPVPAPPEQPPHLQSQAQQQQPERQQPERQQQPEQRTPQAPSPAREPDTT
NFIASGPGAAMCGMPIPPAPSDPTGPAAVGASDGATAMEIVADNQQQGQQEQQELAAAPAPNAGAGRVGAAEADTVL
GAGVGVPGLSPLSPMGALVAAAAAAAAVGGEERHVGDKAEAAAAVEPSAVGPAAAAGPVVATPAPVAEPAHQRQALP
LLPLAAAATLVPPCQHQHQHQQQSAAHAAVQPPVAEAPQPTSAPQPLASDPLMQQPAPTGLEASPGPQPAPPPVQPP
VQPPGLSPLRPLAPPAPFAPTPTVVQATLQAAVPAAVPAAVPAAAPAAAPAAAQLQQSLLLLSWMRTLAVAQQQQQQ
QQQLATAATPDRPPQLQPRTSSQQQQQHGARLYDGRVHVPLQVISATFPPDTRYPAEVRVNLWVNSSLVGEGLAAR
VVQHRKHRRTDQFNFFVTGLQKTLARYRDLRQAGVTAEQHPHPHPHPHPHPHPHPHPHPLAAAPPWGADAAAGAGNG
AGAGTGIVDGGGGSCTENGGGDAAGGRGAESGSGCVVLRVNLLAAAAAAE

>Cre05.g242100.t1.3|PACid:27572887
MRPRLLSGLLLLLVGGQPWLKQWAWAQRVAELRTGIIELRTWPDVTAAAAAAAWGPGGGIGSQASTRSAAVSGGGDA
GAAGLDSGAAVEATGAFGLFLVAYTPGPQQLRLRRSLTSRGARLLAHLRPHTWVVAMTAEAAEQVAVESGAAMAPLG
PPHRIDPEWQPLLREVEAVAAEAEAEAGMVAAQRRRRQLLGGGSDAGGEVAGEEEEDTELVAAVAVEVGRRAARALS
QLQPLAGPGGPGALGSECRRRRQAAEQQPTEQGPGQGQGQEEAGSGSAAPPLPMYGVLVTLLPVPQLTAAAVGDLAA
DLPAALAAQLGLTQRQQGAGAGAALPPCWPLVEAPRRPCGSGGGSGPPLLQVHVCAQHLPAAVAWLSGLWQAQWLEP
QWAPQLHNAVANIIAQTGGLLSLSQYTAPLAVRKPYWDAGLDGTGEVVGVTDTGVDMTSCYFYDSQYGPALAALAAN
STPGPFTLPMPAARKVVAYSVVQGAAYGDPGSGAHGTHTSGTVAGAVPAGGPGGPSGLGSGYRLDNATGAVPGARLS
VIDISTAGGGLTVPSPMDQTLLQVHASAGVSITSDSWGVLSRDYTAAANDFDRYLWSHGDHVALVAAGNYGQSPGLR
SGQVTHPAAAKNVIAVGSCLHSPPFTFYYPFYTVRGRRLGSGQAWSQQVWPIECRDVGARDFLTGVGGPSGRNATLE
LVAVSGWSLQPRPPPDPATGTLAQQQGEDGGGELPAPGPSPAPAGPAAFSSSCSLPPGLVASGALAGAVAVAPASPN
CSLVEQVLALQRAGAAAVLLGYTEPSNPPTPVLVSSPSAALNSTGVTIPVGYITQSLCSWLLGNMSASSRPVTFSVS
LSASQGINSVASFSSAGPALDGRVKPDLVAPGFQLTSAWKTGAGAGAGAGTTQGDTCGVSVAARSGTSMSTPTAAGH
TALVRQYLRTGFYPTGSPADAAAAPFTPSGMLLKAMVIAGAKSMEGGEAMATRAPLGPPPDAYQGWGRLSLPDTLPL
PGLTPAGFSLQVADRGQFTASGQQAALTGITATGDGPISIVLTWYDYVSYPNALVKLVNDLDLAVSLTPPAGAASSA
ASVLLGNNPEDAASPSPDRLNTVERVWLSAPPPGAALTITVTAADLPSLLLSADANHTDALLPQRWAVAVVGHFSGT
LASELNPAYVRQGQLGLLQPPPPKASSPRPRKSPPPSASPPALPPTSPSPSRSPTPSPKKPAGGGKSPKAPSPSPSP
SATGGSAGKKKKPPPPERGGKSPPPPKKRGRS

>g5348.t1|PACid:27572889
MARQKSKAAKIRHGLHAANNNLSALFDANAVEDLEGSQLKQLPGALYIVADPTSSSIEVHAYGSMDDPMLVPSITHL
VKCAYAVHQLRQPPPNITIVKAKATGTTAGPAPKPESYLVRETLRFVYQNAVLPGLRELYDTGFTVHMCNGGKWSKD
DGVSAGKIASWIPADGGSCTCDGSTCMDARVKLGFPNVPLVDYMKHELRKEEAKKAISWGVRRSTFVAQKVAELEAE
WKEERADHKASQARSGAAAARVQQSAESDASDSDEEVVNPVVARKVRKQQGATALSQQLLQTHFERFNMDGKGAGEG
SLLQVLKEEGYGLSKAALARALRDASLRWGKLTPTQEEALGHFQEVKEEYGEEEAVGVIAGMLSLTLEHARKRLAAA
KLLRSRVDKQPEGSGRAVQTALSRHTTKKSRGGKEGHSREERRGDDVSRGDDVSHGGEASSSGEASSSDEEGGGEEG
SGGEESSSGEESSSSEESHGCSDVDEDAPCKKCGGADATVFDMVFCDDCNDCYHVNCHEPPLTAVPMGRWSCSTCAQ
KPVGCAGGGLGGGAEALQRAGGPGADLEVAALGRAETAAGATAAADCSSTGGGDGGALAPTGSPPPGLGGLAEALQR
AGGPGADLEVAALGRAETAAGATAAADCSSTGGGDGGALAPTGSPPPGLGGLAEALQRAGGPGADLEVAALGRAETA
AGATAAADCSSTGGGDGGALAPTASPPPGLGGLAEALQRAGGPGADLEVAALGRAETAAGATAAADCSSTGGGDGGA
LAPTASPPPGLGGLAEALQRAGGPGADLEVAALGRAETAAGATAAADCSSTGGGDGGALAPTASPPPGLGGLAEALQ
RAASPPPGLGGLAGAPVHYKRIAVDELPRRNKGAKLQSIPEMLKK

>g15457.t1|PACid:27572950
MDGPTPSTTWPQPSSSPSPSPAPSSSPSPSPTASPKPSPSPSSSSSSSPAPYPPIAAADLVHVSAWAPLALLLRSSA
GGLRGIALSNDRTALVAAADATGSGSGSSSSSVSGSSSVSGSSSGSGSSSGNTTSGAGDWGLSFVGVPHLRLVGCRV
KGLPLSSVAPLLQVIGSNYLTVQGLTLSRLWGEPVVSGSSSGSSSGGSSSGSGVGGSSSGSPRRRVYGPLHAAGLLG
ADLRGVSCADVSDAHGYSCLALLFRAPGSSSGSSSSSGSGSSSNISSSGGVSALLRIADSRFQRGRVVWPGDLTGGG
GAATAGLGGASGSAAVAATAAAALANVTAGAVVVWSPPAAAVAAAAGNASAGSLALGEVWLEGVTADANAGGSGAAL
ALLGGSLGTLTLMKTTLRNNTADPAAANTDGAALVALIFGGVAGSGGGGSGGAVYIETAVQRVVLANRSSLAQNRAA
AHGGALAVGGGPLGSLELYDSSTLEYNTVQYGSGAAVHCGGDLGQMLMYGGSGAYGNAAKGSNGFGGVLYATGNVTR
IQMYNGSSISGNSAVRSGGALYGASSMGEIVLYGASGINGNSVGTGSGGAIHVAQSLALLRLTDGSSIDNNAAATSG

GGVYLESGSLGALELYGGSTMDGNTAANGAGGAACAAAGSLLTVTLDGGSSISRNAAKGTGGGGALFSHDRIAALTL
AGASHMDTNSVLLLRDGSGGAVYCTGGVVSITLSGGSSVSNNSVTGLQRGRGGAIAVDNRLSQLLLTDGSYMVGNVV
NNGNGGAVFAATIKYVDLSSGAYVINNQAVGGDGGAVYGAYLLSDVRLTGGAVVDDNAAGGSGGALYSGGPLGSLVL
SDEGSSMSRNTAAGGPGGAVAVPYGTGVETAGRVSILSLSGGAAMNDNEAHGGPGGALFAGAAIDAVLIEGGSSVTG
NTALDPELGYGGALYAAVGISSITITGSGSSLDGNRSAKEGGAVFCGGPVGLVLLSDGGSMRRNAAAGGNGGAICAA
APYFTNGSALAVPSTAPALSGMGELNGLTVSNGSEVVGNRCWGHGGAVHADSRLVELRVSGANSSLSGNQAGVDVSA
SNVSGGGDSNGGGGSIGGGSGIGSGSGSGGAVFAGSLPGGLYITDGAVVSYNSATGGGSGGAVYVAAGPLPNATIAG
GGTVVSYNAAGGASDSSGGFLAIAVGGSGGGGGGGGAPGLGLLLVTDGAAVYGNSATRGGAVSCGVLGRLRLERGAV
VSGNTARPPGVGGGGIVSYGGAVHVSYRLDALEIRSGGRLEANSADPGHGGGVACFSGSGSSSPNACRVASVLIDGS
SGGRVGGVGGQAAATPSMTTGILNCTARLGNGGGIWVEGNLTAMVMTGRGASLSHNAAVPVSNSGSNGQGGAVFVGG
VLGSVTADGGAAISGNRGKSGGAVYAARSLGAVWVLGGSVVDGNVASSSGGALYTAGTISLLTVAGAGSAFSNNAAR
GGGASRDDGGGAVTAVVGLGNVTLSAGGAMTNNSAAANGGAVLVSRGPLGYLRLLDGAAMTNNAANNSGGAVYGFTA
IYEVHVLGVAAAAADSVGNSSSGSSGSSSSSSSSSSSSSSSMPATAAALSRNYAGYRGGAVATNGTLGLLEVGPSGG
VNDNRARGDGGAVFAAVNLTTLILPPPGPSGAPSTALAWNLADRGCALGRAGFGTAAARTVCRQLGYTGAVAGGEYY
TAGGGVGPIHLLLIESDPARAARLDQCRNTGWGNATGCTHPEDVGVNCEVVYPPPMPPAAPPPLPPSPPPRPPPPPP
PPPPPSPPPPPFPPPPPPLQLPAWSHSCRVELRGTYSAGAAAAAATAPGGGGGGGTGGGSDSVSGGGPRRRLRQKEA
ERESIMGSSDGVGSSKGEEAGAGAAVAAGVEDVLATANEAAAGLALQARHALEEAVAAAEDAPPDDLRLFSPPPPRP
PPPRAPPSPPAPPDIPVFAFEAAYVECWPAGGSSSGGSGSGSSSGSSSGDDVAVRVGSLLMQYLVPGPGVILIDAGA
EAAAAAATAAGVDATAGGGDSSGDSGWGLEFVSGDVSGGVSGAVVSHLRLLDTLVSDLPLSPTGPLLATSGIDFVSL
ANVTLRNLAGRPPPPSSSGSSSGGGSSSSSGSSSREVHGPVHLQEVTGAAVSGLRCEGVTDAWGWSCVLFSYGSDFS
KSSSSGGGSSSSLTGGAGMLSVRGSIVRGNSVVAPHSLLPDYVAFTAAATAATAATAAATASASAAGVAVAAAAAPT
GYGALVVRGTRALGVLQVEDSYFERNIGGNGAAVCATQVAISLAILRGVNATANAARHGAALFSDSRLGDLVIDGAS
TLTANSAAANGGAIAAAAGAASVLLDGGAAFLGNVAGGAGGGIFIGGDVARALVLRGGAAVRGGKAGGGGGGIYVDG
VVGSGVTLAGGAAIDGNMAIGGDGGGLAVAGGASSGGSSSSIGGSGLRLAGGSSISNNEARAGGGGGVWAAGVVAGV
SLAGAGTRVAGNVAAGAGGGLRLGAMPRGGGALTLSGGAALEDNTAGSVGGGGVWIGSSIASIVLTGTGSSSGSSSG
NSSGSSSGSSSGANGTNTRLSGNQALAGGGGGIWVGGALGELLLSAGAAADGNAAATGDGGAVAVGGALGTLSLLGG
GTSMSGNAAARGSGGGVAAAGAVGAVSLQQGAAAAGNVAGADGGFLFAPGVLGSLSLSGRSRLEGNTAAAAGGAVAA
FAVAGGVALSGGSVASGNVAGWRPAPATAGSSTGAGSSSSSGGTGSSSGTGSSAGSSSGRGGFLAVTTDVAGGVTLD
VGSRLFNNSALGRRGGAVGGAIAVQGRLSLLALDGPGSLIAANQAGGHGGAIWVGGVLERLSITGSSSSGSSSAGNG
SSGPGSSSSAATGMVGNVAEDGDGGAIAAGCISEVAVVGPGAVLARNAAARHGGWAAVGGWPAAAPSALAPGVSQW
RFADGASLESNVAAAGDGGFLSARGVGPPDAATSAAAAAAAPFWAVLFESVSLSGNMAGGSGGAWALTTAAAAPLQQ
RAAAAAAAAAAVAEAMTGRVAPLLLVFQNASVESNTAAGGGSVVGGGGGGGGGAIFVDWSMTSLSSSGDGDGASVSV
SDCLVWLEGGTLRNNSVTAAAFVAAAGGKAGDVGGGGSGGGGGGGGSGGGGSGGGGVLRLVGCGAHMTDVAARGNTV
PATGNGGAVSHTYRASAAAVAAALRAAMAAADGGAPAVAGSSDGGSGSSSSSSWSSPPLFPSGYAPWALATASLPKL
SASAQNSCGSSSTRDSDDGNATQSATSAAAGDAAALCPSLPRQPPGAAGVAAPAAQLLAALDAARALALSSGSGSSG
SSSDDSALVDALVAAAAGPGGFTACGTCFAGNVAAAASGRGGAVALSHRLPSAADLLPLAESLIKSGGGNASTGAAA
IAVSTAVASAAARLLRGGGGVAAAAGAMTYALQGCVLTGNAAGLGGGALSFEAEDDTTASPPEPPPQPPAGPPPGLL
LPSASGTSRRLVETMAAAAAAEAATSTAPTIAAAVGLLHVAESDVFGNTVGVGSLLAAWSPRGSFLLPPDGSAVPCG
GGLAISGGGASASVTQSTFTSNAATAPTYNNNSNGMASPSAARGGAVCVRGGAAAAFLATAFHANVAYGDGGSLYGG
GCGFLSLSGGCSIRSSVSGGGGYGGGLAVVGGCSRGLRLQGLLLANNSAASGGGLFVSGGGSSSSVTGDISSGSSSS
SSSGGVMVLAESVTLWNNTAMGWRTAAATATTAAATAATAAMATTGGQQEAAAWRRQHAGRGGGAFLGERAAVVFSG
GSGGGVEAAALLLGGGNTATAAGADIASLQTCALAPAASPAATAQSSQPGASPAGIIDSDVVVGGGGDWAQTWRALA
DTTARGCWPLVLHNTRLRLLATGSSSGSSSSGSSSSSNDTAVKPLWMADPDAAALRVGCSSSSASSGSSSNGTAVAA
GSTDAAAAAALAAREQLDAAVAAVVLRSLTPQSPAALAAQLATCLLLQPQLDGGGGLDPLSAAVALGLFSPGASPSA
VRLRLLLPLATYDRSSRAVQQVLLQPSAAATGGSSSGGGSGSSRRSAAAAASVLLLSTSEPFSLGAELVSVSLGRRV
TGHGPVTVTLSVGPASLARLLQPTAAAGGGSSSATAASAAVASTTASATADMVNGVARWSGLRLAGWPGSGYSLTLR
ATAAAAEPGAAEVLVTVLNVPFAIDTCTPGEVPVPAVLRVVPMPPLAAAGAGTGAALAAPTAPPPPPPVSREAATTR
TTATSAQAPTSPHLVCVPCAAGSYSRWPDPRSNTADAAAAFAADATFASNATASSSTSISSIGGAACLPCPRNAYCG
GGAVLTPRRGFWHTDPGSNIMRPCPNPAACGSAPLVSLGSGFCRLAAALELQAGSGGLGGGGVAGVSEEAVSGVLVA
CQAAWYASRPPGAAVLQLYRAGLDASSGSSSTSGASSGSSSTSTNTTATLAAAAAAAAAAAAGVPVGASSDAQYTPA
DVSAAADAACVAAPPTTALEGLCFLSLPPPAAASSISATTTTTTTTTTTTTTTTTSIPNATSTNTNTNTSTGQLPPS
QQSQPQPQQPRPPSQQQLQRLSYGARQCGEGYAGPLCAACAPGFWAAPGFACLPCNTAAAAAVGLLGFVLTCGLILG
LAAAVLAEAGAEVVEEGEEAEEGEEAEGEGRGRQGGAGGSLTDTLVALVTQLQWLVIVTRLNLTSSSSSNSYSHGSI
SSSSSSGWPASVRGLHTAAAILTGAGGLAAAGFPSCLLPAGGGGGSGGGGPEAQAQARLLNAWVAPLAAAATCLVLW
ASVRLLSGCGDDGDDGDSEDADDGCRRCWPGAGAADRSRRPAADSLRPLVFSDVVVNSRKPVPQETAVGYSAAGSEL
AAAGGGQGGQGASSGGGGDDAGGDILAGAAAAAREAEAVASPPRLSPFQRLAAAISIVGPKAGGGRPAGGRAGSLPG
SGVTMLGPGAVGTVGGGERSSAPAALQGAATATGVAADLFRRARGRHQERRSHVSSRRGGGGGFGAAGSRTGSSGGS

GSEGGGGGRASPRLVYLDDFPRRRQQQLQMQQQQQQQMQQQQEIEEVGPPPHLADTAAAGAAVAAGSAPFAAGGDGW
DGEADGRLGMDSSGGNSFHGLAGGERRTAPARVYGAAAALYANRPPLRRRVTLRSSGGGGSGGGSGSGGGGSRVSNG
GAGGGADAAVVVGGSTIATASVSGRSSGIDTPGSVTPADEEQEEAAAVAADGAAGAVGTGAGAASAAGRIGIRPHPP
PPFYAAAAPSAGGGGRGGQGAGLGGGGGMRPSLGRPMQRRSQQLPPLPTHAQSAVAAAAAATAAAAAKATAAATAAA
AAAAPNSGWAGPGPRVEGGLGPGPLGEATVTEEGGAQEREEEEDAVGGGLWERHTYSGGRGSDGTRAALTAARSARA
SFGGGGVGGGGVGGGGGSRVGGASFLTRGFSLRMFGGGGGGGGGVEGGGGGGDRPSGGRTTATTSSSGRLLSSFGTL
TSSLSGAFGGVSPPPDKPILVRPRSGPLSGATAGATAGAATATAATAAGAKPPLLRSLHGLPPGSRALGSATNAAFP
AGGQHWDRHGGGGGSGGGAAAKRGSVSASGAVPGVGGGWGGGVAGAGGGWAGEGGSVGGWGEEGRSFRVSPNTGTPA
NRQAAYLMQRYGSNSTLRDPVPEVDDGDGDGDAVGLAAAAAARTAKGSRGRDGGGGGGGGWLTRLRLLAAAWRRNRL
TDTDGGDAGGQQQQQPAAVAAVAAVSATPPPPLRLQLLAVGLAAGQVLLPVLAWASLSVFACVDLNLDPDLDPDTEL
AGSVVPSGSSSGGGGGGGGGDPRWQLDLGTTCFSGSPHGSLDTPLGALGLSVFCAAPPALCLAAAAAVLSSRGRREGG
GGGGGGYSSAQALLWSWLCRRFRRPHSALWHAASQLQLLLLLAVDVYGAAWPLQPAATAATAKALSSGSSSSSSSGIS
TAVLQPAVMAVALTSIGVLELVCAPLAGRALALLRAAGLGVLAATAALAALLTSGAGIDEAAREAVGGVIVALNAAM
LTAMCVAACRRGLALLVRLRRRAAAAAGFGVGGGGGQEGGELKGVGPGRSGCGGWLVCWGQQQGRRGPHQQQWRRGS
WRHNAVAPHLHGRGGRGGGEGGGGGGSGEEEP

>Cre15.g635150.t1.3|PACid:27572985
MKLGRASQEAGCSAVAAESATSRNAFDQRPAARARHRVVASACAPETAGRLDGRRGIMSSAWLPPMPPRGGTTLAAE
RASAATSLARTPLCRLNSPASGGWCPAASHQLPLPASAPAAAAAAAGNGHGTAPLGDHVTSPSGNALATTSAARAAH
RSATATAAAAAAAAAAATAAAAAAATAAAAAAAAGVVVVRELEQPIELLPAATATLASGRRRAAPAAALAAAVTGAA
AEPAADTSPGSEPLSTSATALTGRSGAVQFTVSYVVFASPGSGSGWGSGAAPQLVLEGRDGGGGTHRCALTPPPLAT
QPPPPLATPPPPPPPLERKSDPGGSGGGDGGSGDGGGGGAVGAQGDRQQPQPLPGAAPPAVDTDAGAGNGWRQGFAA
AAVAVAAVGAAPGPPTGAGPLKPRPRRRSASATVTATVSPAAPPAQPAPSSQPPQSPAALPPLPVFPLPLRSPRAGA
ASASASASEGGDSPGLQAQSSKAPLQLRRPSAPPAARLLACFTPTTLVRRSGGLRYAPHGARQQPALPPPPLQPLQP
LQPLLTPQQGQRQRLPRWAQLLQGIGHQQPPQPPPPPQPPLPPQPPPPPPATGQPNRSTPAPTQPPPNSNPNPDPAQ
APHRRSGPGSSRGSAPTASASRPAPAAAPAPASGAGPWGRRTTAKAGSPVTRPPSAALPRLPRPVPGQDLGLTRGLH
SLRGGDGGGGGGGGVGGGGDDDLMSRFRPTLAVSRGARVDGGGPVGFGPGVDGDRSEAGAAGARPAAAASGAAALAA
ALAAAASAGQRGPAAAAADARAGGGGRAGAGAGGTWRQQRPAGRPLTAAALDSAPASALRAAAVVRDMEAHWSDGEY
DMGAAAAGGGGGDAAAAAAAGSGGGGQESRQRASPLLLLGFLRAAAPDPLGLLPGAHRAPPLPPPTTTTTTTTTTSA
PAPAAAAAAAAAAAAAPAPARPVAGPAQGSRTGRPGRAQPWPTAPPTAPAEASAEASAEASAEASAEACAEASVEAS
AEACAEASVEASAAAATALDASEPAPRTLEQRLWAASSLEQVWALLAQQQQQPLAATGEATAADGSPAAAAAASGAS
NAASATAAAAAAASATAVAAACDASAAAAAAAVAAAGAAAAAAAAAAAASATAVGDELRELLAGCGAVVALLQRGQHD
AAGGGGVEAGRAGRWAEGSSSSSSGSGSSSSGSRSGSSGSSSSSSGGGARLPWRVSARGVAVHQHPSAMDVRAQQQQ
QQQQQPQGQEKQGPRQGPHQALGPGPRRGGPSAGAVQVAGGEAQEEEEEEEEEEEEQQEERQWARGRRAREEEALGR
RLVGRLARRVLAAVAAAVAARAARSSLSRSLSSSRSRSSGSGSSRVSHGASAQPASHGAGPAADRRGAAVPASDPWA
CGTLGRLPLLAPAEAEAVLAAVWVFSHLADQHDTLELGAGEPGVGKPGEMEGLGVEGLGVGARQLHVTTAAARAATA
AAAAAARAPVPGSVADKAGGDGAAAVAAAQAAAEAAAVAAEAAAVAVAKQAPFRDVMCELWRLLLLQATAPARSPDP
LSSGGLTARAGAGGAHREPHRWEGGAAGGGGGEAVPRQWLLWALWAARRAVPDAVPPLLAVLASEPVSPPPPQQQLR
GREREWERGRGRGRVQEEGVVGSWRPAGGGLAAGSGGAGDDDSSVAGLARLPAWQLASVLWACGRHAEWSAARAAEA
GFSSNAPDRASDDDEEEEDDDDTYGVAAVGRSGGQQRQQQLQQQQQQQQEASELLVLRVAAEAARLIAAGLEPAAQ
PPPPPGTNHPHQTQHHHHKHPHQLLLATPDVLVALRAGLAVAQQATAVAQAAAPLGSPTMHPQHQQQQLQSAYSTAR
LAVQLTNSSSRSSGSSSSSSNSSSSSSSSSSSCRRRLLGSLLECVEGLAAHPRAGLNDITAALHVLAELRLLLPYVA
PAAAVAADAGGGAGGGGEAGPAGPASEPVARTAGGGTGSLRLEEQQQHEQQELEQQPNEQELEAGWQRALRAAERA
VLRLVAGLEADAAAALLAVPGLASQGVAPARGWYRRVLQRRRPRSKGRAATAGATAGATAGVRRWQQHPLPFRLRLL
YGLHRAEEDGAVLLPPPPPSSSSSAAGMGAEAAAAVAAAEGTGAGFTPLELPGDYAGSSSGAGSSSGSTGAAGGWRP
HSLELQLLEQQRLLGGGSSSGAGSSSGASSSSGAPVRQHGTTCLLALLGVLGAVTAASKRAVAASTTAAAAAATSST
SSTRSTTHPASQPQPHLRHRHPATDPVQTLPRPLAPRPATVRAALNSLALRWLRRGAPSAEVAALVGLLYGLRWRPR
SAVAVLVSAWRPWVLLAPHELGDGALAGMAQALAVLTSHQRAVRARARARVRVRAGAAGRQGYREQGSGKGSGKGSG
GLWAANVPWPKLRAFWAALLRLAMVRMAAAAAVGRPPVALSSFPSAPASASTTSTARSAASGAAATAAAATRAGVAA
GAGAVAGAAALAGVAREAVPPLSAACLVRLMWSSARQGARRRAFMSAACAALTPLLLAAAAGQAAPQAAPPGLLHPG
TRQQRHGALPGQWETNGVGRGGTGGGGGGAGGGAGGGGGGAVGGWRLGPREVGWVAWSVGHPRYDDPGVAAALAAL
LRRWLAAGRAQEQQLQQAQEQRQRLQQQYSQQQYSQQHQQQQGQQQQQAGRRPAAQQAAASVGGTAVNWTAAARVAW
ALLRLGRLPSAAASPELWELLLAQLLPALQRAEAAAQAERRRAWQRRAAAQARGAGLEGGEEGEEEERGVTAPLVSP
RALVALLWEVHEQQRQEQRQQQAQQAQGWGQGHKQPQPQQQQQQVRALHQTPSSRSSSGQQQPNAEQLQLQPPPPPP
QPRLLQPRGGAAPATPAWAASAAAAPAPSSLPATLRPSSPPPPPAPRPASSTPPPTPAPPTTPPPTPAPPPPPPLLA
YEFLARGGSDWLARHTAHLPPAALLQLLQIIADGAANGSSSSSTASGSSSSSTSTSTSGAGSSSSTSGPAAVRRCGR
FLSAFGRLVEQRPRWLLRRPEQAAAVLAAYRVLGLWPVGQVEELAARLRQLQAREEEGEEGEGDYIHES

>Cre15.g635950.t1.3|PACid:27573035
MARTFLLGLACVGLLSTLAVAQTPFTITTEYQLLDKLNADQQVMLKDAVKAAYTALRKYIQAKVPTGQLLADPIVHT
YELIPSPPPPPPSPPEPPSAPPEPLAPPFPPSRRLLLSKQQQQQQESERDEMLGLAATSSSSSSSSHSHSHSHSRSL
QQATGSRTATYPDFSPGADPRLSKCGRATINTKHVRMAAGGSLSMADTPGEFTDLYLYVTSDNSGSGICPPMNDIRA
ATCMYDTATKRPVMSAVNICPSALDAYDLNTLVTRILRAMITALGVERVGFFGAAAPSVYSTSIAAETDAGGNDLRF
LATPAVKQAARTFFACADLPGAMMENLRYSYSLLDGADSSVVLNASRAWPWSARASAAAAAAAVVQSWPAAGAPSPP
SGASCFRAVCGGAGGNELSYNVLGTQVRCPSGALVNLTAALPAGSTLLAATLGPCPNNDEVCGVSSPSACTPASCSP
RGGECRDGRCYCKAGYGGADCSSDLMTGLKEDLAGQTSAYSLLEVSVSLKLPLSRFYARQAQFLKVISDNVFSGFTS
SLTAQLSVARIQTASSLPDTLRPLGETNIQVITRFAAANALQGQTVLRTMPPPSPPSPPSPPSPPPMPPPLPPAAPP
TSDGDGGLSGDEKVAIGVGVAFGVLLLLLLFAGWRSFRRWREVQEKIVEGEKQKAIKKLEEDIAKEKARLLSGGVST
KYGHGHSKYGEGASPEEIQLTEGAVSSSAATAAAAAGGGTAGGGRAGPVVMVGQITTGAAAGGAGPEIK

>Cre15.g635950.t2.1|PACid:27573036
MARTFLLGLACVGLLSTLAVAQTPFTITTEYQLLDKLNADQQVMLKDAVKAAYTALRKYIQAKVPTGQLLADPIVHT
YELIPSPPPPPPSPPEPPSAPPEPLAPPFPPSRRLLLSKQQQQQESERDEMLGLAATSSSSSSSSHSHSHSHSRSLQ
QATGSRTATYPDFSPGADPRLSKCGRATINTKHVRMAAGGSLSMADTPGEFTDLYLYVTSDNSGSGICPPMNDIRAA
TCMYDTATKRPVMSAVNICPSALDAYDLNTLVTRILRAMITALGVERVGFFGAAAPSVYSTSIAAETDAGGNDLRFL
ATPAVKQAARTFFACADLPGAMMENLRYSYSLLDGADSSVVLNASRAWPWSARASAAAAAAAVVQSWPAAGAPSPPS
GASCFRAVCGGAGGNELSYNVLGTQVRCPSGALVNLTAALPAGSTLLAATLGPCPNNDEVCGVSSPSACTPASCSPR
GGECRDGRCYCKAGYGGADCSSDLMTGLKEDLAGQTSAYSLLEVSVSLKLPLSRFYARQAQFLKVISDNVFSGFTSS
LTAQLSVARIQTASSLPDTLRPLGETNIQVITRFAAANALQGQTVLRTMPPPSPPSPPSPPSPPPMPPPLPPAAPPT
SDGDGGLSGDEKVAIGVGVAFGVLLLLLLFAGWRSFRRWREVQEKIVEGEKQKAIKKLEEDIAKEKARLLSGGVSTK
YGHGHSKYGEGASPEEIQLTEGAVSSSAATAAAAAGGGTAGGGRAGPVVMVGQITTGAAAGGAGPEIK

>g15313.t1|PACid:27573037
MPCFGCFKSTHAGLSAGAGHNQQRASTQVDEATAKKKFASSSIPSLLSKATTGAQESRAASGVGETANSSSPFSTAQ
QLGDSEHGAAFSGADAQSPSHAATAAGPDGQHATSVALLPRGGTTMATAGAYSASAATTSHSQPYTQGTSQLAAAST
GAGSTATTTMQLRAGHSTAGTTATTAPSRLGSSPFNAAQVVYAFKPPQGAAGSAAPAQPPSPSPGPPPASASMISIS
ASGIPPHLLNSAGTVLGLGTGSQAPEVTARLPLSPAGGGGSNAPPPVPPGVLLASEASTVRLMEYLGGTGGDGLGGG
GGGGGGGSAADATVGLLTTTHASSISVRPSIGVASVVGGGGGGGGGGGGLAAAAPFVPPAAGAGAVAAARSRLGVGG
GGGGFSLFGALKQFTEGLSPDGAGGGAARRPSLGPGGAAVPPALAAAGSSNASEVFGGFGLAAAGSSAGREPPTATL
HRVPQPHHGHLHGRSNLRRQGNSGGSDAAGPAGGGGGGGGAAAARRSGAGGADGGGGGGAVAWPPTLQLQLMTSGGG
EGAYGSETALAAAAGLVIGGQTTNSSGVNAIAVAAAAAAAAAGAGGAGAGHVRGGRAGAAAGGLARLPSAALTDVGV
ESLGSSGSLAQHAGAVAAAAGGGGGGGGGGGGGGGGGGRAASASAGRAAGAAAALRIGAAGGGGGGGGTGAGYGFQS
EAMSFDGMATLFSGASALFTLEGAAGYAGGGGAGSDTDTASNAGGGYGVYGSYGGGGAGLSGTGFLLSGGGLAAGGG
GAGGRQHQGQGHARAGGGGGGGRRRGGAERREFAAAARQLGAVLGDVQIVAVLGAGAHGKVYKGVLGDRFVAVKVIH
HETEVVIGGGRGGGGGSMTNALGLGPLGISNMSHGGGGSGGHTGLLSPSLSLTSGQGAYPGGGGGYGGGYGGGYGGS
GSVYGGGGSVYGGAGGGLRPVVDSSSQLIGNSGGGLAGLLTAAVNISGGAPGSSLTDEPPPPPPPPAAAGAPLLPLP
GHDGGGGGAAAAAGGAALTPAAAAALDGVVSAGHAAAAAAGHHQQLQHHPTAASAMASGATAGGGAHTPMIASGASG
YGTPMLGPTAPATFSGPAGGGGGGGGGGGGVSSPPPPPAGAARAALVAAAAAVAVAAEAVDAVTGDDDSATAAVAAA
ATAAMPEPASVSPAAAPQYPLHYQRAGSSNLHPLFIPGGEGADPAEAGAAPVPAGGGAAGDGAAAGGGGAAAAAGGG
GAGGAGGGAAATTTSGGMWNGLPTLTLPGLPAPVVMPHKHMLEGLISASAAHPNIVETYRIVTQLLSAPGLPAFVVT
RGAPAAGAGGEAAAAAAAAAAAAAVVAGASGGGSEEAADPVPLWETTTISAAALLGLAGGSSSGSAAAAGGGVAAPA
SGQPPPPPQQQHLQHPQHQHPQQPLSALPPGLRVSLTGRRPRLQRASSLGPHRHDWEGGNSPTGGGGGGGGGGGGGG
GGYALVGGGGGGGSGSGMSGGVPAASVVDAVHGGGGLPGGGGGGGGGGGGADAAIGAGPGPGVGRLPALSAGQHPLS
LTALGPLLMQAPAAAAAVAAAAAGPAPVSPLRGPRRMEVYAAGPSAAAAAAAAAAVQLTAAALGSATADRSGGSSGP
MWQSALSGSAYTRRSTDVASVRPAALLSGPAPPRPQVVVAAAPAPLSASAAQAEGAPPAQGEAAGAPRRRPRQAARQ
AYLQAAEPRVLEYLSSLPPPPGATAAAAAADELPAVAAAAAAAAAAADEDELQGAAELAARWNGDAAVAAAAGAGQP
FPGEETVAAATSAAAAASGGDASGIANASADVNAAAHAGANADAVTDSFFVRPEEDLLLTSTSAGGCVAAAAAAAVA
ASASADGGGGGGGGGGLQPPRHGSATATHSTFGLGSSNDGEAGHGSVSLINTAAAALGGGGGSNAGHGGAAAVAPPA
ASSLALSFGEMGRGLSAAVAARVAAAAAAAALEGREALDVGLQDDANAPANAAATLTAATAAAVVSTPVATELRAGT
GGKVSGDSAAPPPLRRPPAKDEASGETDGIQNPDSAELILDLLGAQAAAQRSLSFRARHPQHAQHPQQQHPQQQQG
ASVAASATAVAAAVADSEDAAAGLAAVLAPPTMAAAVCSPGGAASSTSHSHSHSHSTGVGAHSTAGASTEPSLALPA
PLQPLPPMPPMPPMPAASGGAGGGAGITGGGSGSGGGGGHNPAASWQLPTIDELKPGVDGGDLAASGGRGGGGGGGG
PVAGSTSPSPLTRSSTAGSARGSSQLNRTGSGHSANDAAAIAANGMFLGTGTGTALSAIRSDRDRPSNSSGSQNVRV
LLGSAGGILNTGGGGGYVMVGGQLMPSDLQYDKLSWSNATGTSASTNLLLNSGFTALLTSLTTAGGSNQGGGGGGGG

GRRDSAQGQGLGNARRTTNTYASSNADPSLSTIPNHTGGSRQGAATAAPASAASPPPAHQMTRGKSSIGPLAQGGGG
ADVAPYGGAYGGGGVSNLGMDPPDMRAVLAGKMSRSFTDARSSRSRRSLLASATAAAAAASAAAAAASAAGGGGGGG
GAPGSPWSRSTAAAAGRPGGGGGGAANTTIGGGSTSTTFGGGGGGGGGGHQHSHHHHPHHHHHPHQQQLVIREKWQV
LIVQELCIRGCLKDAITGGVFHATNTAAGSATNAYGSVQRGGAGGGGGGGGGGGANGGGGGGGGGRGGSYTAGALST
QDHDDGDGEGEGEGSLLESWDVKSNYRGMGGPLGGGGGGGGGRA

>Cre15.g641950.t1.3|PACid:27573046
MLAPGAAGAEELDQQFAKVAAQVTMLVLSQPKPVKLRAQAWLIKLRDPTNNPVFKRNRNLYGSLLLAQLQQGRLEPP
FTSSPPDGALPNLPQHLAYRGLSPARISTRAFQQHQQRPPRPQHHPQHPPPRSGSASPCDPQSRGRGLVGDAKAPQT
GAPAPQPHAHAHAHAHAHAHAHAPHLGPAQLGCLVAAFHDNMRARGYDLRDQGGGGGGGGGGGGAGAGGGGGGGG
GGGGGGGGGAGGGGAGAGQMKKQAATPRAAGGSGPAAAAPLAASDAGAGGSDIHLSPHPQRSLPQSWQQQPHQQLHP
HSHPHSQQQRPLSPASSRLRRAPSAVSGATGGVSGATGGVSGATGGVSGATGAVSGATGAVSGATGGVSGATGGVSG
ATGGVSGATGAVSGATGGVSGATGAVSGATGGVSEATGAVSGATGGVRPLSPSRSLAAPGRPGTSSARPSSAAASAA
ASGTGRTSARGIGGGGRAPGAEHPPFQQPPFQQPDAGAKGAEEDAEVGNAGEREDEQEEEEETEVVVTAALLAAKEA
ELAARAARRREARLRERSAAGKERLEAVMAAAAGARERWASPPASAASDGRGGSDKAPPLPHQRRRDPQPHRKNAPN
SSDADVGAGRLPASRQGGSRSLVAASSHRHGGASGGGSAGTSGGGASGGGSAGISGGGASGDTAQLLDDWLGQSLLA
AGVEGLPVEPYDFHDGGWGGGSNRFQPLARPLYAAASVDPLSGRSTAGSGSSAADRGSSSRAGGGGGSGAAAADYRA
EADAGQEAGGRAGQEEAGQEEAGQEERELTPAAALFASPPSGGRGGAAAEPPAAGLAFLGAAARRSPARTPGVAPWD
GTGIQAGTRTRASPGRRQQQQQGQQRQPHPWQPHAAPAPCYVTAWAISSGHGPAGVAATGPARSGPAGSGLASSAGV
GVGAGAGRGFALLPRPLPLSRSAVLDARTDGGGGGGYAGSRGTSSRAVTSNAGFLPWGEPTLRLSPPPAATQATPGN
AGATAAGLAAAAAAAAAAAAAASPASLLRRSDPGPVWLASPPPLPPGADGWLRPLLPTHAAASAPRFHSPGGHRPTV
RTSPRGAGVSALHSPDRSPSRSPSRSPSRSPGGRPGGGHWSDTRSPPFRGSRSRSASPSRARRNNINSSHLLHPHAD
PVHAYSLARHLRSSPLALAAAAEEAAAQAAGGGTVAYAADGSGAGAGGGFLIPRLLSAPPVLAADRDGPARFEFSDL
LLRSALQSGPGGDGQEGQEEGGEPEEEGAEAEAGCEAEEGGEAEAGGEAAYEHDDEWHAGEHGPELSEPDLWASPDS
GNSSSRYGGFGGSGGFGGYGGYGGHGNVAGRAAPGALQQASRTIRSRSNPGGGGGGGGGGGGGGGGGGGGGGGTARL
AALSLASPPTSPAQVAAEAAAAARAGLWRPPALRTPILLVPVPPPPPPPQPALILRAPSPVPLGSRAPSISTLAAGA
SQRSRRRSTSVPPSSRRAAAAASTPRSATPPPPGPSRGRGGAGGGAGGGAGGGAGGSRHHTPPRGSSAAAGSVKSAG
RRPPPGSPRHLPPPPLSSPTPVPSSVAVTEMARRKLAAAAAAGAAAAAAAAASGGGGPRGDRASVQLRSGERSIVAAG
GSATANGSDRRSRSLPGSLTPRATAAAIGADGDGGASGASSPTLSSRSAVEVEVGAEAAGQHGSGSAKGGGGKSSGG
GANGGGVNGGGKSSGGGANGGGVNGGGAKGGGGGSATSEDSSVDLSPRHPSRMCGGGAGAGGSDTAAAAAARAMLAQ
ETPKLLRSRVSQSQSPLQEQSPLQPQSPLQEQQRQQHEQRQRQQPLGPEVEAAVARPDADAAAAHATSSGSSGGSST
GGGGSSSGGWEPSAVAPPAGRGGGVGEGEDVEGLIAEFERSLQDHFELTLRPPPSPATTTSAGAATAAASNAAAAAA
ATAAAAASDTARQLSDRLALQSALSDFRRAVGRLKRALAAEGQQQLHQHQQQQHYWMGGGTGTGTGMACPGGGGGGS
SSIAATGGGASGGGGGGGAAEAFRRVAAAALQQYRQDVALLLARVRSVLQPLQPLQQQLLQPGIRSSSSRSSSRMAV
AGAGDFGRLAAEEAEAGGRAAATLREMELRRGLVGRVVSALEESAHQLGIRVLTLAAAPAGLPGPELRQLAAALRQH
LAVAADGGGIGGGTGGGGTGSVGDGWNAGGSAAAPFVAAAAEVAFAAALPPPLHQPQHPYAGVAAGYAASLQLDASG
DRYAGGGAANRGSSWPQSGPGPTGDMPTPAGRLDFSAPESDGRRLLVPGGRPLHSRDGGAGWAGGGVGGGVGVGGYG
EPAAADRDGPGSLLASLFASPPPPPPGLGAWPAPAAAAAAAPVGSVAWYAGGGGGAPRLQPRPLAAGGTPTPPPPPP
HQRASWPPPTPPAGSGAAAAAAAAASAATYADAAAAALLLEDAAAAARQSHLHTPRPHSHSQSHFYPQHQQLPVAYGP
YGYTGAAAGGGGGGGGGGLTTGRPAAYQHHHQQPQRHSPLQHHHHHHPQHPQQAPTSGRDWHAGEAAARLAAEAAAA
REAAAAARAAAESAALAAADAAAAAALARSLSPSRSPAGPAGGAAEAAAAAAAAQQPQQSPSQSPTQSPTRYRAVVY
EVPVDEYPHPHPHPSPHSSPHSSPQPSPQPSPQPSPHPSPHSSPHPHLQPQHADTSQHAALSAAERAAAYAAATTAA
ADAATAAAAATATAAASPRSPRAAAGRTAGAGAGAGGAWELEQRQWQEEEREQREERELTFGGFGTGQAPAWSPPAA
PAAAGGHTTLTFPAALPSGTAAGSGAGASRRFPSPSHLPHADQQQQRQQQHQHQEEEEHHHHHQQQQHHHQQQHHHD
HQHQQQQQHQDEEEQQQQLAAAAALADEGVLLRPQLADPEALALAHFRQLRRQLAASPPPATAAAAAATAAATAAAI
TAATAAATAAASAAASGVTSRFGPYAPQQQQQQHPGPPTSHFSVPAASAAPAVGLARPFGSQVSSPPAGAARVAAAA
ADAAAAEAARAAADRDYAAFQLVKQRLGLSASPPPAAPPESVYGWSAAVRAASPPPPPLPSSEPYPRSGAPAVAAPA
AARSPPQPASYAAPTAAAPTASTAAATAFSATAAASAAAASAAAADRAAAAIAQAVGAGGPGELSSRLQRLAERTSA
LQVRVEQLARPASPAATPSRSLPPGSRLLSPASRLLSPASR

>g15469.t1|PACid:27573080
MRKQPASKQPQAGAAAAAPQEEWPSLAASAQHAAASRFSSAAAAGPLSQHGQSSIRSAATAGTSAATAGTSAAAAAA
AAAAATAGGGAGPAGVGAGTRAPRRAPTADYQHPAQPQIQAQPQPQPHPYQYLYMHYYPGLLAAPPPPLPPMHQPQQ
QPQQPPQQPPQQQQQHHMPSPRDHIGMQMMMHHQQQLLAHHHQQQLQWLHQHHHQQLEQQQQQRQQQQLQQRQEQQ
RQEQQQQQLMPPVQPPGMLLPGMQPQPGMMLQPGMMLQPGMMLQPGMMPAQPPSPAGMSYPYPYPYPYPYGELPFGM
PPYEMPLYGALPYGMPLYGAPPYGMPLYGAPPYRMPLYGASPYGAPPDGPLALVMPPGAASIGGGGGVGGGVGGDGG
GGGGGGVGVGVGGGGGGGGGAPPMHAGGRGGGEVHGWVNRGGMPGPLPPLMPQQAPPEMVHAQPLQYPLQYPLQYPL

PVPYGMVMPPPPGAAPVLRPPYTQQPYELPYGAMPYGPVPNGMPYSPLQQPPPVMQMGSGGSAGGGGPPSEYVGGGS
GPPSLYSGGGGGSGPTPVYGMVPRGPYGPQLPGGALTAAAAAAATAAATAATSTAIASDSAPPPPSSPPSPPPPPPP
PSSEGAPPPLVVPLPAAVARWAAAGGVERLQAALLSSSSGSSSAAAAAAAGPRSTALALASGPGNNRGSSSDIGGGG
GGGGGFGGGGGGFGGGGGGAPPAGLEISGDVDFGVVAIGGPVAAPQAVATSPEDSGGSSRSGKGGVGSGSSSGGAST
QPAATKAVARFRVRNARWAPALLLHVRLAPPFGHCLRLMDDAGLVAATTKPKPAAATAALASAAATAGGRGAGSGGG
GGGGGGGGGCSSGGGVAVLLLPGSEYVLRVELDVAQANFAGRKSERGLLRQLLLLTCLLDLSPLELQQLLLQPLPLP
PHLLQPLPPPQLLQPQLLQPQLLLPPGMQLLPAPAAGAGAGAGAADSATASDNETAIGAAATAAVAAATADANAART
HDRVWCGLALAAAAAAAAAAAAAEEKEEEGGDGNERLLPPPPPPPTGAAVAAAPAAGAGEGKAVAEATKAALAAVAQ
EAAAAGVLGSSSSSSGSNSGSSREGVICSSSTILSRWWRGAGNGNGGLTPPPPPPLPPPSLVAVAVRSVSVAVVDRKR
LMEVNGLLKPTAVPFIPLALRTLFTTPPARLCVCPVPLASALFPPPPTYPILSPPRAAGLPYCPPALPGPALAAAPP
AFAAARALVQALALLQPSEEGWRRLLQPPQAVSRYSRALGAVRGGPLFRMAAEAFATECGLVAAAPAAAAAALAAAA
AVGGGSGSGAGGAAGGMAGVEGVEVLGVATAAAAAPPAVAASASAAAAWAMAAEGGGGGGVGGPAFARLHGLLSFLT
RGDVAGGVGGGGVGGGGVEGLEQRAAAAVGATAAAAGGVGKPGGPGVIATAGGIGGTMGVAACVSDSWAQQLQQTAG
AILTTAAGLGSSGSSSGGGGGSSGGGGGGSSKTARKEAAAAAEAGRLGRVCLDELQGVFVANARALQARLDRLRPPP
PLLTPPLPPPKLPPLAAVGEAGVAAAGVAAAGVAATGVAAGVAAGVATAEGMAAAGVAMSSAAAEAGAGEATPAERH
WAHVCHQARELLRYGALLCEEEGVLERDVRQYNLYNARARTALFDDAMTSYRLLDVWPPPPPPPPQQQQQPPNQPST
ASAAAAAAAAAVGAGGGGGGGTSAPASSQLPSSPPPPEPKPYRGHRLAKLAAQHSCTALPLPPPWRPPPPPPPPPPP
PRQGVVTAADDAATAAGAAAAAVAAAEPPPVVTAAEAAAAAARWLSQEIALVPKMVHVVATALSAIHDVATATAAPD
DTWITSSSGSTATAGGGGGGGRTALRAARDEAVAPMQRLLALQRQLARWVKAAAAPEEVKEEAAAAAEVEATVTFGV
VIPPPARLAVGGIAALVLQSVTAVGAAAVTALDLGADAAAAALAAEGVAAAGKMAAAAAAATVAARLLQGLKELAAA
VAQLTSLTAATELDCAAAAARAAAAAVDECAAAAAGSPAALWVGAADGDGGGSLGLQGEDPAAVLEAVRRQRGSGEA
VMRLRAAAAVRRGMRAAAAAARSGLLAAVFDVPGLVEGRPALVPGDIVYVRLEPQRQHPQQKQPQQKQPQRQQPQQPS
KARRVKEEVEAAALEGEAALGETEFAGQLLAVDGGQVLVLMPPPYWEALYQRHSRCHAAAATAATAAVTAAAPLQPP
PPPQEQQQLLLQPPLAHVCFTYDRTHFVRMHDALLRAALRPHVLLLPPPATATATAAAAAVVAAGPVAVAAATAGAQ
QQQQRQKLSAATAATASGGSGVGSAAAATAGQPNHHPHQTAADTSVAEAVRLAAALVPVGGTDLNPEQRLAVGAMLV
AGANRSNGDGYGGYGFGDGYGYRPPFVLFGPPGTGKTVTLVEAALQILGANGIGPAAIHYGSGHATATSTSTTAATA
ATAAAATAADAADASSGGGSGVDGAGGGGGRGRDGGGGRGRNAAENGNDDTGNGAATAAGATAAGTTAQGASGSSNS
NNVFHIVRRPRPSLAAAAATAAATAPATAAAAATAATTSNPPPPPPPLRLLLTAPANYSADLIAASLAVALGPAAGD
GLLLRLNDPRRRVVEAKADVLPYCLFDDAGGGGGGTAGGGGGGGGGSGTFRLPTLEELQRATVVVSSCSSAALLMEG
EFLTAALRAAAAPPPPPDQHATAAAAAAAAATAAAAAAAGNATAGRSSRSDSRFATAAPAAAAAATAAAAMYGWRVEF
SHVMVDEAGQAQLPEALIPLTLRAEGGVAMLCGDPKQLGPVVRSGTAARAGLGVSLLEQLMSAAAVEGPGGELERAR
AAAAAVEGALRRRQRRQQGRGQQERQQQGRGQQEGRQQQGRQQGQTEMSSGAGDSPEEAEEGEQDESVAAPAAAAPQ
PLSNRQPLPNRQPLRGVMLVRNYRSAAPLLDLPSRLFYDTQLKACVSQSASRPPDWDLLISSGGGGGGNGGGGNGGG
NGGGNGGGGGGGNGSGGGGGRGGGGANGGAAAKRTTAQGDAARLEQEAEAACYGRDDEEEEEEEGGEEAEEEEGEEA
GGPALAAAPAHMLFYGVRGQQAQELDAPSYFNPMEAAAVVDLVDRLIRASWAAAATAAAARQQQHQHQQPQQPPKQP
PPKQQRGSSVGNGGGGGGDGDGGGGDGGGGGSGSSRGAPLVLEGVVVGQQDIGIIATYRKQVFKLRQLLRERGLGGV
RVGTVDDYQGQEERIVFISTVLSRPESLPRYQQPLPPAATATAATATAGAAATAGGGASEPVGGLWSHPKRFNVAIT
RSRALLVVVGHPAVLLADTCWRELVRQCVARGAYRGAGAELIRRQLAPPPPPAAIATTAAGGRNGRDRNGGPQAHLP
MLHKQGAAAAAAVATDSVGAVGAVGSVGGSGFGLLFGTYDDSMLTAPTAVGDDDTTATTAAATATPAAVATDTCRAA
TGDGGAATDPAAGGGAGGGGGWEGVGEGDGEARGGGAGGGAGGGVGGEADCDGESAGAGDGDGAGEGGGAGGGAGGD
EDLAALVAAMAEMAVLGMGCREHVWPSGHPAPHLPPPAAAGALPPAAAAAATRGLGAYGFGGAGAALFSNPAAAAVG
AAAVPAGYGGYGSNNDFYGDGYDDELPFRVQI

>Cre15.g635000.t1.3|PACid:27573198
MRSALEVTLMVALAWFSSAARHDGQNATRLHALNSSAQRLAGGGRTVDVENAKQLFVALKDPSVGVVRLTRDISLAD
PGSPWLDLEPPGVALRRPVIILGASAPLPEGSSSTPSDYGSSGSSSGGSGGSSSSMLISGGACLTVLDTAHLAAKLQ
LQLQPPPPPPPQQRPQLLLPSQGQQQEPGQRLQEQQQEQQGEEQGAEQQPATQQLLLSFVGLRLLNHLPPGDGFVSPL
LTLGVPYPTRPNHPEHLSGQGSGQGQGMGRAAEPAAAATKEEDDAEEDGAEEGEHASAAAGLVSVMEGPFLTTTTTTT
TTSAFDSALVSTLEAAAGNTEPGTDSRGSSPTQVGQQRRQRPNRQSHHQRQLVVGAVSYVGCRLERVVGLPLAATAA
EAAALALAGGAVAAVAGGDAAAGGGGSSSGGSSSGVTVLPRDPACGDYDGYGSRSSSNSSSSSSSSSSGSSSSDACI
PDPSGGVLHYERWAAEGPVAAALLPHPIRSSGSGIGSSESASAGSGAAAVLEAPPLAMPPPAVPVLLVAEAPLLVSF
TDTLYGVRQEVEEACVRAYGAAVCVVQAEARLELAAAAAAAAVAASGSQRVGGLAALTGIDQEVAAAAVARLLAAGD
DTGSSSDDAGSSSSNPLLPLPPPPPAAAAAGSSCPSNGGLSAGAGAGIGVAATLGAVLCVLGLSRWIGSLRAGHRTL
TLAVRPPGATPATTLLITDIMNSTNLWEALPPGVMNEALRLHHRCLRGLLPKCNGYESATEGDSFILAFHTPLDAAR
FALLAQSALMQLEWPEELLAHTDGAAVWAEPAKPKLPVLTPGASSRATGTGTGAHGPHGYLPSGTGMSVGPGALVLS
PSTHSRSGLGAWLSTHGGGGGGTALYDEDDDEDGGGGTAMYGTPEGGGGGGGRLAGLTPPTSPFLRGAINAFTNAAR
KSFQINPRPSLSIDRPTAHLQHFQRAVAPPPPPLSNLRSSAGFSGPAGGSGGSGSAPKAAGGGGGSGDAAAAAAVAA

QEPPARHWSFSARPASPTQPPNQLRAAAAAAAAAASVAAAGPAAADGAGGGGGGAAEGVTAAAAEAAAAAAVQSTIP
GGGAAADGESPGGGAGINTELLLLTALHLSTATLDAPSPHPHPPEMRMAFGSAVPVQPQAGEAPAPVPALAAAAGGG
GGGGLYSGGGLAVMASAAVAAPRVPPTPAQGGGGGGGRSGPGPVAEGLDSPRAAAAANAAAAVAAAFNAGAARGSGS
FRRAGSSLPSPPPPLHRVDEVGRHESESEGGGGEGDDGDDLSSRYSPNGLLPIPHSAAGSNAGGGGGGGGGLNDGNG
DGAGGAASAAAEMAAAFGSPGRGGAGNGGGGSGSGGSLIGGTTHVHLQAAPAVPSYGASVISSDVRRVPSRLGQLAA
LAEAGGSGGGGGRRSAQGYVFTISPAGGGLAAGLGTAATATAAAAAVSGNDRGVRDVLLSALGPGGGGGGGGGGGGG
GGGGGGGDAQGEESTFTRAGTGELLNLLTAVGGGGIGGGGAVNGGSSSSSSEAAPAAMPRPSPPASRASGTHARPPS
PLPPYVAAAAAATSAPLAAALGSPTRQLAAAPAPAYSLTSSPFHSQRGGLVGSGGLGGSYSPLRRHVHGSASGGLVA
VPSDGPLAVSGELQMSHVTAATGGGGGGGAAANSSDSGVGRLRSATHLGFGYTGRAAAAAAARGAEGGGGGGGAADA
SSLLSPPPPAFSHNGMYGRAGPAAAAAVGSGRMQGAVAKVASNHSLARMRTQAMEVVPVAPAAAEVRETWREMLAKM
WPVVMPGAAAAAAATNAGGGGGGGGYWPRPSFAGTGGWLFGGGGGGGGMGSEAVSGVVGYDALVAAGGSGAGGPGLA
THHERSSHSHNQHQQQRPSDVDHENESGGGMAAVGSGGGMLALLQRQASHTFAMSGGLTPNHQPPSPHTGHSHHHHH
HHLGGLLAAVGGGGGASSKNAKSNAVLLFRGFRVRMGLHTGIPLTTDVAFNHTTGHMAYSGGPLKVAKAVGDGAPGG
GILLSNTTFTALHPHLAELPGEAQALYCGDAQIELLIRGLYMLLCRELRPRLGHLPPLRNLKMLQLGNLEAPVGSVA
VTFMMVAGASRAVAEVGQPAVTALAQFKALVTRECCVCGGYVVEATDGLCLAAFISWEALLMHHDWSREVLACSHFR
EVREIRDPAPGSSTAAATGSRSPYGSGGPYGGPHHGGRGKAQGPARRGAAAAAANAGAGGGPVLVARGPRIRTGIAV
GPVTADPSTATARLSYRGRVMNRSARVAAAAADNQVLMSAGAWRRVVATAGVHAVAERWRAAAEAREAEREQELAAA
ATAAAEAATASGSSNQRLYLRQGTGDISMAPGLYGTPGTGENLGIQIQQIQHAHVADASHLDPALASAFGSGILGGP
SGAQVSMGTPFGTAAALSATSVCPSPGGGGSGGATTPVHSAATRPAAAAAAAAAALYDIGSSGGGGSGRRVINGPAS
ASLSVAAAVGSPVAATAAGSFSSTSALARAATHHAGTLAPLGATATGATGVGAAATAGGGGGGGVGGSSSSSTANQL
HAAAEALLTSAAAAGGLDGGEEVEELIAAAAAVASKLPALPGTPQAGGAGGAATGFGGMAGMANRPLAKTAPSLMPV
LVGVSVGNHTLKGVAEPQELFELRVELLPPATATATATATATATAKAAAATASPAAVSAGGKTMAARVTTAVNSNLY
DTSAAPTPGGATDTGASTPRGPTSGPASRSPSFLHPRPQPSTAAAPTATAAAGSSPLGYSHSYSQQQPPQHHYRLNP
PPSPLAYSYSGSFSHAAATGVAAATAAGSFSRAAQLQPLSASASQQQQQQPLALSLSQLHRPSSSALQPLVPPSSRR
QIAPLSPSGFIAAAQDAAAAAPPPLPASPSGSASVAAMAAAALQLSRLQHASTTSYPHQHPHPHPHHPLLAGQELLH
MGSSGFRPGSGVGLGGMMGSGLGLGGPSSGNFGPYGHLYGHGHGLGGPPSAGALVLGSTPSFSAAAATAASLVQWQN
PAASASQLGSPSTRAPPRAGGGGGGGCGGSGAAADAAAAALASSGGGSGTVASGGAFDASLFAIPVASGSIPIAPAA
AAFAAVAAATGAAGGVGAASIAGAVPAAVSRQATGCGDASGSGSGRSRPFRLQLSPLQLQSGQLVPAAGLSGGLESG
LDGSAAAAPAPLPSARSPAALRATSPHEAAVLAASVAAAVAVGDGGDGGDGGDEGEAPPPPPPRALAGDALLEALAA
AASEEDARGPGGDTEPAGAGADTGSVEAEAEAEAEAEAEAEAEAEAEAEAEVEVEVEVEVEAEAEAEAEAEVEVEVE
VEVEAEAEAEARTPAPAQAQAQGCEDEEMVVVA

>g18117.t1|PACid:27573237
MAASSPLVRLRRLALPLALVALCAAAAHGQTLTSAFLTSSTSVTVTLSNSTGPSSDCRNAFSYFDLNNGTKASPFAN
CTANGTFTVLLTLASAGTYAAGDQLNIKVNQTTLNTTAGAPFVAAAVAVAVPPVVGAATLTASRTVVVKLPLSGNTV
PDSFATNMTVCGGAVELRAAGASAAKVAPFEACSLAADVLTLTLKSAGTYAPGDTVNVVNGQSTLRFGTTAYAPLAS
GSVIRPTLTSVSLSSTTTILIGTSGPASLPGNASAAVCNAVFDIALKNGTVLPGALSACSVGPGVTAITAALANGTA
YSDGMTVTLKANQTALKAGDQTAAAPLFVPPTAALAIAPTILSASLTAADTITVVLPVASSLDTSACNSAFELRAAA
SSTAKSSPFSACSLAVNNTQAVLTLASASTYTSGDILDIKSGNDKLLAGSTAYVPQSVTVLPTITTATLVSSTVVRV
ALPVVSSIPAPYSADDCARSIVVAAASSTAAKAISACALSADKKALLVTLAAAYQPGDTVNVRTGQWQLRAGASVTL
GPNYIASAAPAPILPGLSAATLTSATQVVVTLPAASSVPATFSVSDCNAAVDLVSAAGASKDTPFAACALGSGNATL
ILNLTASAYAIGDTVSVKASQTTLKVGNTSYTPYSVPITPVLLPTTTAVLVSETSISVTLPTGFNSYLPSTANASDC
NAAFVLVSATGTAKTAPYTSCTVSGTTLTLGVAAGTYAAGDSVNVKTGNSLFLGASSTGPAYVPAVNATQITPTIVA
ASLVSTTNVFVTLSAAASPSAFNGTLCNDALEVTGGSATVFLNCTLTSDGKGINLLLSTASSVSTSNTINVKASQTM
LKAAGSDAASSPAFARLGSPIAVADARLNAAFLTSASTIVAKLGARAVASDGFSCNSVFALLMTNGSVRASSPFTAC
TLSTDGLTATLTTTTYVSNDRLNVRSSNTIMTVLAATGPNYPNVSADASAVTINPAISSAMATSATTVIVPLPIASN
LAGACSGALEVKSSGGTSKTVDSCTLNTAKTQLTVTLNSGTPFAQGDTIAILSANTGLTATDASGAPYRPVTGASAI
TIAPTVGTAQAISSTTITVVLPATTNAASTLTADACASAIDVSGKSFSSCTISGTTLTIVLAGTYAASDRINVKADQ
KVLRLGTGASAAYYVPLPSSAAVIPTIASAKAISRSVIIATLPVGSTFVSGANASVAVADQTLCAAALSVVSTLLTD
AGACNLTSSTSLRITLAAATYNPGDAITVADAQTALYAGTGLVSGQTYQNRALKISPAYLLSTVVATASDTIVVTLP
VESTVFDSTGTQITADLTGDQCASIVEVKAGTTAKKFASCAVSGVSSGRGTVLTVKLLGNTTDVYASGDTFNFKDTN
ALLLAGTTSAAVAYAALPTAATIAPTIVSAVALTADTIRITLPSASGFMVSGAASTTIAAANCDFLVFNPVRTAKTD
TACNITGSTLTITLNTAITGTTTVNIVTSQTKLVAGTGTSGPAFVPAGSPIAISPGFLTSPAVARSLTQLVVSLPFT
SNVGASASWTGTQAQCNSVVEVLAGGVEGSLRTLHATTNPCVLSGGNVLTLNLAATDSFAPGDTVRIKSGNTRLLMD
TAGTAPYVQGTAVPITLGAVTSAVATKDTEIVVTLPMTVALVKAGAAVTQLGKTDCLLVVTLAGTIADVATACVLTG
GNKLTITTTGSYTPGTTTVQFGNTDASVKLLTGAGLTGPAVVALSSVQSVNPGYISSAATSTASQFVITLPQQIGAT
GTITQVNCDAALEVLQANGTTRVLSGDCTRTTSGGVSTITVPLTTNIAIGDTVRIKASSSGITTATGSLQYAATAAV

PIQPGHVSYAAAIATDKFVVTLPYASTLAAGAVCSDTVNVKSDTNANVETGCTLSLDSTGIYLTVDLADTNTLVATF
KVTFKSAQTALTVGTTKWAPLSAGVYSLDSATWGTSVMKVVAVSSTVIEVTLPMVSYLGELSATACGQVITVAGNTL
ASSGTACKLISTPTGTNNIMQVTLQSAYVAGGLQLTNTGGALKAGSSTGTAYGAVAAVTIKPTFASAVATGLRTVVV
TLPATSTVYKLTPTCTGTGASGACSTGTPTCASGSPACSDGTGTYSCTSSTPSTCADPTTSCAFGTKTCAQTDGSFD
ATQLTCTTAVAGSMSAGCAAGTAVCFDQASTTAAQCASTATTDLSPACTVANVNTPTCVGGSVTSADACLTYAGSPI
SPVCTGGAFACAAAAGPMCVQSATPVCPSTFSSSCSSGSPSCTQTAGLTTPGCTSGGTLTCSKTATCDAEAATPTCA
QVFATFTGGASVTTCTNPSSDSTQPITTTLTLGADWALGDKLTVNAAQATTTATSYLKAGTATGLVYTSKPSGDVVI
YPSITLAVLTSSKTITVTLPVAASVPSTGLSAADCNNIFTLYAGATAKSNVFAGCSLAADKVTFTLTLVAAASATAT
TTTYAAGDFINIKSGQSLLKADSTTAANRLAFAPHPDGLPVVATIVSAVVTAGVGSGTGIVRVQLPIASDLAKADCD
NTDIGVLDNAGTAQALAGTGGCALSTDKTVLTITLAGSVTASWFTTSPYMRVRILSQATLGLKAGSSSGYAYITTDT
SVLATVRAFDVASATPQSIVSATAVGPTRLELALPAVTTINPANGCPSSLTLSRTLSNCSLDDTGKLLTVFTTAAYQ
SADTVQVVASQALKYMAGDTATSYPALGSALTIAPAIVSAYTVDASTIAVTLPVASSFYVTTTRTATLDAVDCDKII
TVLKAGVVNGAGAIASAGNRRTLLTTGACTLGTASAGGTTITVKLSGAIYDAGDAVDIPDTNAGLNGATATSNPLYA
DSNSGPKYVARRSPVVIEPRLVSASAVGAQSVEVVLPAASAIIDSSNAVVATPSSSQCAAIAVVSGKTVTSCSLSTD
GLTLTLGLSATFAPGDTVNIVSGQTMLRVWKASVGAGYEAFGPVYTPSAAALAITIGYFVSAYAPDTQTVVITLPFT
AKLYDYAPATGASTAEITRTATAPTKGNCDSIVRIASRTLASTPAALTDPTCTLSGTTLTVKLASTTLSAGDQAFFG
SGTDAILRGVATGTVGLTSTTYLTTAATTATTGAVTIHPSVTSAIATDSTTIKVTLPFNAWIHASITSLNASDCGNL
LTFTPAKTLAAASSCSFASNVITVKLSGDGTSGTFASGDTVNIAAANAATLTYPLRAGSSSGPLYGPGAAVTIVPKI
AAAQAIAPLTVEITLSAPSYLPDALTQTECAALLTFTPSKTLFATTPCALDSTKTVLTVKLASGSEFANGDQVNIHA
TTNTGTKALRAGSDVTGLLYTASDSPIDINPAIQSAAATSATSIAVTLPVGSTLYVSGATASISAADCAKVLEVKRG
TAALTLESSAACAIATSGSSTTLTVTLASGQAFAAGDTVNIVAANSAGTTTTAVLQATNGGSGKKYVARRSAVVIAP
GYLGGVPATGMAYAVGTSTLMVQLPVVSSIAADNNCANVVTVQKIATPATARTVTGACTVSSSQGAGYYLIVNAPYT
PGDEILIKTGNTALVGGSDPATGAPYVATTALPIRANFDDAILTAPTTVLVTLAAPTAVSLPDKEACDAVFVFSGAG
NTTKTTSPIASCSRASDGLSVTITLTSADAYVPGDALDIKKNQAAATVGSASDTPSTLTTLVPLPVPTTISPRPFGA
AATLVAPTSVAFSLPAVSSLPAATAAADCNAAVRYTDVAGVDAVNPFTSCALTPDTKGVALNTASPIYKAGDVMNIK
WTNTVLRWGPQASGAAYYPADADVPVFATVATATLVDPSTVVVNLPAPSAVPDNFTVPDCLRAIEFKTAAGVTKNGT
VASCMLMPDRLGLQVKLLSAGNFSAGDTVNIKPQQAELRVSALATGPSYVPKLAAQVVNPALFANANLTSATAITVR
LPFASTLATGADCKAVLALLGAGGVAKNVSSCSLGADGVTLAVTIPAASFVGGDVLNIVPGQRALTLADGTTAYVPS
KAGVLVTPNIVSATLTNATIVTVALPTASVLTSTAAADCNAAVVIARNGSAVASPLSACAVSADGLSLTLTAAATYK
PMSGDTVDVAVSQTVLRAGSATGPAYVPRPSPALITVPSPPPSPPPSPAPSPPPSPPLSTANYSARGLATGPLSCNV
LIGDLTVTTTSGNFTGMASYAGKDASYKGSCKDAVTGAVYTDDTVASTLPAGLTGLVLSPVTALASVWDLKSVADLA
NTNKDYLRLLGVPVNASAYGTAVQLLAYDYYVKGFVALETPAVAVLNVEGMVAANLLMYTKFFDGLNASVAGRDITA
AQALAAGQYALAAVLEDSSAPINTTDPASILALLNVTYSVLTANATSAGRRLLQTAPAPFTQLQAQATALAAAAAGS
NALVAAQQARLITAINAGTSISAAELTNIINEASKVIVAQSTVIATAASGLGSGAISPASFTSSYTGSALTTLVAQQ
QLAATPGSDVTAGPAPSPPSDKKKSNTGLIVGLVVGLVGGAIVITLIVVFIVMRRRKQNVAAAGQATA

>Cre13.g586900.t1.3|PACid:27573280
MSYRDRDRDRGDRGYSDRDRDRGRDDRRGGDRGGDRGGGGGGDRGSRAERVSLVVRNLPLDIRMEDLRAKFEKYGEL
KDVYIPRDYYTQRPRGFGFIEFKETRDAEDAMYNLDRSVVNGREISVTFSREGRKTPRDMMRIESKTKGDERRDDRR
RSRSRSPRRSSRRSSRSPRRSRSRSPRRSRSPRADRGRDRSPRDRSPRDRSPRDRSPRDRSPRERSPVRVERERSPE
RERSPERERVREDSRSPPPRERSPPPRDRSPPPRERSPSPRRDSPPRDDYAGDDF

>g14244.t1|PACid:27573352
MAGKRQPPASAVSELEKDAGLVEKDSTRALQNRRPQATKACDQSNSEALETLAPHICTGAQRSENPTAHSAAALPAH
AAASEAADAAGVVVVHPPAAEASSGGAAFGGAGPQGRSSGAGKAPTTTAAAAAAAAAAYAPSASATLNADRSIGDR
ARGRSGSSSGEHVMLRHLPAGGGSGCTSGGGASPPPSAEAAGAAAAASDAAVSSGSDGGSGSSGTGSSTSSSTTGCS
SSSSSIASGGSASGSATTAAAKAGWQEQPRPRPPAAPPAAAVSGTSGGGGSAAACAAATQQPPLRSPPPPLCSRQGP
SVSYLLCSCFKRRSE

>Cre13.g581500.t1.3|PACid:27573369
MFHHACGGVHGSYAFSRVLLREVPSQPPAGTSGTQTRSICVRRANVPASEGQIAARASRRGRSTPQPAADATPSLDV
APSDPAAAPRSAHKACGDAAVEVCGDGAAARPRERLRRGRLQLLRESPAAAVTSQAPAAALTVPSAASAPASPARSP
QTPALAPQPPVPRAPLERVPAPAPAPPPAGPHLSARLVMLAAENAACLSSQQVRQLVAAIRRAAEDPQRRPLQLPPA
AVDRLVAELRRRRQLAELHDLTEAVLGGGGGGGGGQSSGGAGGGGSAAEVAALGMQQPGFLDPVLVSLSAATEAAAV
AAAAGPVQGGAAAGAPEEGLPAAVGGLTGAGAEARAGGRVLSGRARRGSARQQAVLLEPQPQPKDAAGGPAVALMQA
RAGHREAPAPVAAGRVSERLHRGASAVGGGDLAQGSWSGSGSSGEELRRFFDELAAAGGRLLPGEALAALEACVVVR
ALPRRDTFEVLLRTIRRAAATSAAVGAAGAWGPLGAPVEAPVAAAPQQPGFGASAAHAAAPAGTAAETAAGPAERST

GALLTRPRPVTTPPGPPLSASLHSWPLGEVGPPAAAVFNAPPAMSHGRFTAPAASPPLGLPGSLFGDMRGSGAADST
SSSSSSRSSVTAINSISSGGGAGVPLAPARLVAVLHGLTRLGQRLSQPAWRELLSFLQPHLSFSPAPHASTAAATSS
SPHTHHHHHHHAARATPHSQHAPPPPLPAPPPPPPPPPPQPLSHYDLVDLAEAMAAAGCNPSTAFLAAHAAAVAGWA
RQLTGRQAARLLWAYAALDYSPPPAVLGMLAATVQAQVHGVSEPLEVARAAAALVLLAEAAADSAAAAAAAAGAASV
PVNGGVSGTGSETDDNVATTAGDPGQQQQPQGTSGGSSTTTSSSSRLGSGSGNGLLQHLFEAAAACVRHHRRLCRAQ
RTALKAQRRLLWAQQQQQQQQRQSRERLQVQTEGHLQNSDAPGAGASAQAAPSELLPMDDLWSWVPNPNLLLGKPSP
PPSRSPPPTATTTAEATAAADAEIATANAAAGADAVAADAASLAGHAAAPPVPSPAERRKRQAAVEQVRALRQQEAL
LDWLAAAAGLPLGSSGCVTGSSGIGGGEEAEAGTAAGASGAAAASAADTPMLSPSRPHLTLSQGGAGVVLRLDWSSA
GPAGPAAPAAAAALTAHGKAVAESHRPGGSSTGSCSGTGWPPLLPEPLTPDGLVVVSRLLCLRGLGGTGSSTGSLRP
AGASATMAAAGGGALSHGWAAHCWRLLAALPPDQLRPADLAPAAQLHCRLYAAHPRLLHADATPSAPVGPASDVAAA
PAPLRCLVAAAVAGMDRLSYGQLVGLQAAVAAAAAEPGVRAWLQRQQEDWQQQQQQQQGGGGGGGGWLHGDAALLA
AALESAGVGEDSAAKADPGAWLAALLSPPRRHLQPAAAAPTESARAGGRRAARSQPQENEPEPELHSSTPAPAPAAA
STAHAIQTPQSPQHPSAGAPAAAAAAAAAPAMSPAASAAALTCAPAAAPTSAPAAAGTLRILVHLLRSCADARVSLP
PQQLAAMRATWRQQLQLQPQPQAQAAALQPAVQAQRLQRAPAAEVPAAVAAAVDAQEVAELLLAVAAAAPNAGQERD
EAVLELAVVLGDAGGGAAAAAGLRRLPAQQLAEVLHTVTVALGAGVAAWSLATSATAHAGMGTAAEHRSGHVGPALP
LMLQHQHRSPRHRAAAVVRLVDPLLAEIQRRLQGSAAAVGSAPVAGCGSAAAARRAVAEGAAVAAAWPPQAWQLLVR
SLARLAYSGPAWPQRLRQRETAIAAAARNVGLSAAAPTAAVALGAVAPAAAANGVALGALAAQVNSLLLPESRGPPN
AAVARYCLWAIRHLLAPPPPSAGSAAAATAYAGPAPPPPPPQLAAWMRCLAGPEAARHLRAMHPRAAADWLALCAAA
GVAPPPPAMRHAWAQLRRAAAAQLLSTSQLCGVVWCAVRLGIRVPRDVLAAACEAAAAAHTPGESGTAAGASLAVPA
AQQRYQHQHQHQQQVDRQRRALASDAVRLLWAVQYDDRYTPPLPVATALCGLLAGAWHRRPNSGPGAGAGAGAGVGA
GGVDTVSGGGGGGLLPSERSAALWALARLPRPGAERAVAESGLGRAVVAQLMQSAGLHGGRSGGRSRSGSPGRGRSR
VAAASMSSSGGSGAAAAPTREARCLWRRALAVPAAVELTAAGELEQHRQEQQRQEQQLSRGDLVRQCWALAKLRQPL
PLPLLKYLLGRLAAAAAAPQPLDPDDCASAVAQPVSQPGAQPTGSSGQAHLLTPKEAVQALFAAANLRDDNSSSSSS
NSRGSNSRGSKGSSIAGGNKAGAGPSAAHVGAMVALLRPVLLAAPPAALDARCLADGLGVLSRHRHLLQPARAALRR
EQQPLAQQAHAHAHAQWERERQPDEGDSQGEGQQQAHPRTPPAAAAAAAAAPGGGGWRSDPWVVAALERAAELFVAS
VARGLGDRPVPAGCVLEVLCAAGYLRAPVSARLAAAAAALLQLHMPEMAPAELAEVVVLCAAGASAGAGAGAAARVA
GASDGSSVALPTELSWEALRRLVVVEAEARLRGGSSAAAAAEAAAAGPEQDTAEAVATSAGGSSSSGLLEPDELARV
VMAATQAVAADLLPESGEPAVTATAAAATTAAATVAVAAAAAPSSLTQQVALLQRRRLLSGYVMALACRPDEPRMAA
MSADQLSMLVEGVVAAGAAPGAAWLRRATELMAAPPRLAAASGLGLARMAAALLRAEHWPGEGWVEACRRQADALRL
AGRLPKAAAAQLQRALTGLRTLDAATVATLPTLTASSGTTAAFTSSSSSAALDAVHQHTASRPPRSQLQPQQQPQPP
QHVQPEQLQPELQPLLSELLESEVHRIMGLARGVHTAFTAAAAAVLRAPPAHAYLSGPAPDPTRGAAAASAASAQQP
GAAGLDAAGAAAAGADSGAALALRPSAAFSKLLLLYRLAEARVEQVVALTLRVALLQSAYTMHMLQQQHRAALAAQH
ARMLPAPSSPGRNGQRQEGLRAREAQLATSATGAAAAGAAAALYGGGGSGDGAAAWHGGDGPCPLVAAWPRASASDA
RRGTSGRPLPPPPSGSSLRVAPASLEPLAAMLGLPPPGTALGRADATGAAGAAGGGGGEGGGSGDGSSGGSSGGRVR
LHRQRRPHGPEASAASEPSLVPPTAADGQQTTGSSRYPGGCALPPFAFGASMVGGNSSGSSGSGSGSGTGWNVAADA
SRLGGALSGAGPGGALQSAPDEQLLVSLLHLRSLGMVHRRLYD

>Cre13.g569550.t1.3|PACid:27573387
MPALSSVADGNSLGDMAAGCGALPWAGGVDPCDDGAFGNTSTFAYVDCDNFNVVTLDLSGQMLTGCQFPSTWSTSPG
LSFLKTVRVNNATGLALTQDVALPSIETLEFIGAQGIAPSAFVNLAAMTGLKTLRVIDYPFFGPIPSGLGALVQLTE
VRLSGTNLAGPIPSDFASLTNLAILDLSYSPGMCGPVPTGLAACTLLDTNGTYIDQYCPTGGNRNIRKPGGARHGMR
RWRRVIAPSTQPSPPVAASSYPSPPPALPWERQLDTLPERPLQRAGRPLTVLLRTHRAFNVSTNQAWRAALLPPGAS
AAPVPAGELSPWRYYNNEYQWRAVVPSSQLQRAGNYTLYVYATIAGTAATLNVSRNGSLVAIWPGPPAAGGSTLSLT
SLAPGNTTAVGDALAVDVRLVDAFGNPAVAAANVTRGAVRPRLTPHCGELLTAVLHVSGAAAATVTVAVAAVSPAAV
AYGASIGAATVQLYGAGSSTSSNSSSGVSLAPGLLLTGLSEVSNRLALPVVRPSGVAFGGNPGLLAVVSLVGPRPGS
GGAAVVTTTTFNGSWSYGNRSYVAFFRAPSPGLYDVTVSLTHPAMSAATASVAVVFNATQPPLAVTGSLTLSVLNAS
RVGDWAYLTAALNDVAGRPALSTSGVALTARGSASGSLDLALTPAGSAWRARYNFTQQESVAFTLTVAGAVAGTAAV
SVLGVAPSYLSLPASLAGARMINVLSASAAPASSSSTSSSSNTSSSGLLGTDVTTGLTSAAIATGAALVVPLAQAVS
VEVPVLTALGGRWLSDPGLSVVLSLVPSALLEDVSSTYAAALQGSLRRLLRAAGELDAAAWERGDDEEAGASDLRRR
GLQSGGGATYYDVSAWNSSAASFYGSVYSHAYDYPTYTGEFAADNAAGAGVDLGAYAWDPASEPRDPWPVAAAGGAA
PAPPLNADSPEAVSAIQDQLAATPGVLTFKGNFSTFKSAYLLSLWLPASDTYTALLSVRHPAAAGSLRLSRFLLTGM
DMSALASAANASQWDPAVNPLVTLNVEYPGLASAAAYPGGVTALLADVRQLLAGRAGLASRDNLVVWSLGAGAAGTG
VKLVMQIWFDSNWASVSSGFAAMSALQFFYLRLTSLPGPLLTDTAAYPALPATSVAASNVSLSEPFASLLLGSSDAV
NADGSTAGAAAPLTILPISLSDASTVTTASVLAAAAASGDPALAAPPVLTLVGEPYVEVVEGGSFADPGAYMYDPLD
GFSAAAGVRVTVRLCSRPSDVASLMYALNAAAAAASATAGASGNATTAAAVSVAAVAEAAAPPPAVFSCLSSPALSA
AANANSSANATATSGSLTVFASGFTLDASTANAASQMYLLSYSAVNSRGAAAVPRHRALVVRSRCNAAAGEFWCPAL
GACSVGRVCSPSLATLAFALAAVSSSTSASTTSSIDAAAAAAAAGTAASGGSAPLAVLSTPDGGVAVVGADGSTVFL

DPLAYTRTSFASADALVAFATDPSNYGVLGNLLLSATDLSGGAAAAATAATAAAAGFVSADQPTYSRDTLPPVISLL
GSGMPALTADGSAVMVDTVEVGTAWADPGAVARDATDGDLTALLQTYGAGAVDTSRPTPPGAGFSFLVEYAVSDLSG
NAAEPAHRLIVVACPAGQTACTDAGGLPACATPDGICGGDAIAPADGITGATADTTDAAAATAAASSSTTNNMTATT
LLTSASLAPPSLRLIGKAEVTVTQGSSFERCAASLLPATATCDAGAAAWDVRDGSLDLRVTVCGTPLRASRPGQVLL
PLALACSASTATPGTYTLTYAVANSAGLTAYASRRLTVLPACPPGERVCSGGGATGTPVVCSTGGGTCAGDLLEGTS
ALAANFSLSLASASDAASPAAPTLQLLTGAAAPALVTLRRGRPYAPCAAGTDLSMPSTQPCEPGAIAWTTAAAATDG
ASNFTTAANLTDRVVVCPPLACLRALAAASSSNTTFAALSANATGCSADLLRRHTLAAKGLAGCGINTLAPPGAVFY
IDYWVWDDSRPPLNATARRTLVITEPCPEPAAPNFCSDGDGGFLCSPTPCGDATAVFLPPRTDGPAIALLPSGGSGD
ATAAAAGGGGGSIVYVEFGTVPPVYLGPCASGNDTRGCGATASGYSLPLAPGGAVTAADLTPYLSVLATTPCPASAS
ATEPCLACSLEALAVAGACLPGVYTFRYTVSDEQGRVAAADRTVVVYQRARVDAVMALLPSGLTDPTAAGQLLTHLR
NTSHTDYAAALANVTARLTRAGLAVRASDVDITAASVEPASPPPTVAAPASLVVSVAVTLYNPPALHRGAQGVAELA
AAAGSASGTLAGRRRRDLLGLLPDGDAAGDDDSVEWEGGFMDGIEIPVDVSDVMAEVWRGLAVQRLRELRTQSRPLR
ALLALTTSGPTTSSPSAFATQQLQQQDEGRRLLRLELSETGLTAGVAGPELVLLPSGDYEAEQEQGREDEAEQEAAG
RDASERLLARTARMLLQANSTTSTNTATTSNSSAITANLLSAMAAAVTLSLNATSSSASMPASPDMLAGYAVAAASL
ASALAASLTDVSSQLAALPSAINTTFGDAAAAFEEELAARAQAALAALLGDSGAATNASLVGAAEVERLLDLQLAAA
AAASAAIGELGATMTQLAADTRSQTDRALYVAAAVAAQLAMAEEGDAADELALMGAGCYRLQQEGFRTSFTVSRFGG
ASTGSSSSSGRRLLASSGSFGSSSAATAGAGGSSSGVVAWLGYYLTGGSAAAASSALLAGLYGGGNLDFGASLSSAG
ARPRHLGSGADNRVVGGLMLHTTRRVLPVLVLGGDGGSGAGVAAGGGASGRCSGSSRSSEFAELDASCHLYELAYLR
QNGNSSAGNASASDGSSSSSSGSSAATTGLDSGALAALRRLFGSANNSVAPYGVDPVFLRASPLYRADLAAKQDWYY
NTSDPGQTSATGAPYGFHARDLAGRPAGFPVLLESGLGAGRAAALLRYLADGNYLDRRQTQAMTAELLVYNPGLHAF
AFYRGEFDWTPSGAIAGRLSAVGFPAMPYVRPGGSLAQAMPQVFAKELLALWLLTGFFIVVTAVGLVSAALRARRHA
TAAAAAADEALIAEAEALGLPKPAAGDFHGLQQAPGAAKGGRALKPSMTTVRRVLVKGGAGGMTMKMSPHQRMLRDE
ESAEREERLEASGAAGLSLRRAIFREFVRHDGGLLFDVAVCLLLLVAAAYWTAVVDNHLALYDAQAYYNIYDAQGSA
AANWLLPVRARNATLLPAPLAAATASNSSSNGTDASADGSDAAAATLSGNAALLLTQLGLTAPDTAGDPGRWMLPAA
DPVAAGGTDEWDAFNQVINNAHELVDLWTGYGIIQAVVIIFLIAKLVGVLEFQARLGIICRSLMTMANPIAHLVIVL
SVVVVMLAAASNLVMGTRVPAFSTLTGALTDTFALIVGVGMLDLSYLSDPSLQLSAVERSAAGLILAFQVLLLMFVL
VSFFFALMGHIFMKQKHSIDWQAAPGVGADLVNVVFPDMARKLMDMMPGAKERRRRRAAAAALAAAAARAKRAAARP
AADAGAAAASISAESEDAEAAAAAAAAGAGCCAPGCNDLAAPPNRGLLRTLAAGGIDELMCAADLPKTWNGRVRATR
VEGGRRLLDKATMRGLMLQAASASNKYRLPPGKVMSEDGAGGGDGAEHDPAELLSVEQRGQALLAARRVMERVGRTY
GSQTPEFQALERAAAEEAARRRRDERLLDGADALMRGACGADDDEEGGGGTPGEAASPRGPLHHSLSTEIAIHLSIY
DALNAAVDSIVRWQSGVHRWQLRTWKQMASAYLFNQQLMAGLGVAPGPAFVERPAALQDESGGPAPAARTGSASNMG
AEAAAAAAAAAAAEGSFSVLPPWLRVNSPAAAGPPKRASFVEHASGVLSPSQLSMTEAAHRARSTTGPGGSHLGPSPH
PYAHPHNAVAPAFMPAGAGPHAATVAVAPAQQQQQQQQHGAAAQEGGRWASSSPHPYAHPPPLPLGGGYTMSPPGSA
GTGAGTARHMPLLLEENSQSSWGATSPSTVAPPQPEEQLGEAEMAAVMAAWATPLAAPLAAPWRPLSTVAFSQTAAT
AAAAAAVVVAAKDQPPAPQPMTWAAPAPGRSSQSGVAPTSADGAVVGMSPPPRRPSTTGGVRTSGSGSAGGSSRGSG
DADRTTGQPTAGGVRNTPVFTGSVTMPPIRGGAAVAAGAGVGGNASGWLRDRPSPLRDTQSADVQAVLAAAARRMPA
PPPAGPARGARGYSYSHAITPVPEERVGLAAAGSPGGGAGAAGPLGDTAEVAKLRTASEFIPRSGGSIGGGGAGGAF
AGMKERLVGLMRGRSQLASRDGSASGADASSAGNSPTGMARRQGSAGGAAAGGAGRVSAGGSAADRNMVYNMAYTLP
RQNAEGTDASASEAEGGPQ

>Cre13.g607150.t1.3|PACid:27573427
MHLLDTAAAGDDDSGGGGMVGEGTEAAAGYHQQHPGSSYRHLRRRSSVPLLSGGLDVSLLLGAEDGEPWQLGCVASS
PPAGSAGHSAGGGLPRYWIPYAHHPHQHQQHQHHQHQHPHNHPHPDQPQHPQHHHRHPPSRERDMGGGTAVAAASRP
SADASSASPCAPDSSLLLCGSYDEDSGSTAAAQRLLLRSLESPPRAPPLTPLAAGGGGGNTAALGTGSSSTGAGTSA
CWTLPDGHRVLRPQLSMQSSDCGAVECDAEGDDGEDRDSGTRRGRRGGPGGLSTCGSGLMTGSGSGVFPSSGSGSGS
GSGAGSGSSSGDGFVASPYSSPYASAADLMVQSRMKAVAAAARAPFGSAASPASSSSGAAANAAFTAGTAAAAIGLA
RVREQPVAAQSDGSEGRWQAAHTDEIEGGAAAAAAAAPSAGQAPSGGSSVAADISGAHETAAAPQADLAGLMHIRMP
DSISAVVAVSGNETSMTEAAFPVAATHGGAGPLALPSPPSPGAASGASWSGRAAFTQRGRRGGASGSGSPVGSLGAS
STLSGGGGGGGGSGTSGSSGLARLINTMTAGLGSGLRSYRSSSNGGGGGVSSGAHHAWLAGGGGSNGSCGGSASQAWV
PTFTGMEALPAVASPAAPMPASVTEAAAAAGAVAWASPAIAASLGPGAVLGSCEVVRVTLGGLVAGCCSSIASGCVS
GGGGSGVHSGVVTAAVRSSPWEGGGGASGDAAAVMSGVRPRVMSGVSGDLASVDEAQADVEDAEEEAAEDAAAVRSG
SPDTEAEAEEEEGVPPPVSLSGAWRRSVPIQIQIQTAPLLPRRPPPADHRHAWLLDSADGGGEGGCSEALGCRPGEA
DHDGGAAGAASAHDHTDLELVGPLEVEPLLEVAASGGAVAAPVPTAPAGAAAAAAAAAAAAAAAAAAAGGQDGMAPG
YGGPCPAAAAAHGSTQAVHTLSQASVAQPELLPSCGLQLPPLLEAAEEAARSWGPGGDCVQTPRMRRVRTSIELTAV
AAAAAAAAAGPEGAASADGTAVTGRHPMGGGRPSLQQDQQPVATPGARRWGRSVSAVLGDAVRTAAAQHQHQIQPGR
APSFGGAAAGAVSAGGAAGHLLPAAGPAAASKGPRSGPGGSGGGSRLQRMPSQLLRTTGASAAAFLRAAGSALHSWS
SSSALLAAGAPAGGVGGGAVDSDRRGVRSGSSNDAVGVGQLYQYQHQYQYQRQYQQHQSAADVGATAQGLRASRVSC

PQILFGEADGGGGGVEGSVGGGMEFEVERSSAGNARLQHHQHPQQQSPPSPPPLLMSLVPKFARRSLSWRNTQHLSA
HNSTAASNAAVGPAAGELVVAHAGGADSSSGRAALNAVDGVADAGLPPRTQLQLSQQMAAAAAAASAHGASSTRTSE
SESCAAAASAAWLERYGLLAPAPLRPLKPLLMPQSQGAGAGGQLLVGAEADGSGGYASSSAATPAATHSQLQLHAPG
GGGGGVMGWMGSGSGSGSQRPRRLHRASTGVLAMSGLGPGGPGAPGLLVGGSGAAGCGSEVGTPRSPPPPQSAHSRN
GSLSHLGSANGYVGGSGGGAGQPHPAHHHSHHLSLHRMVSCASMHSLSGLGGGGGCSGRDLPYAEWHPCVSVLFADL
VGFTAISNKVPPVAVMEMLNALYCRLDALCNVWGQQHQQSPGSPLRGSAAAASFLSPGAAAAATAAPPCPPGSVYKV
QIIGDAYMVATGLLADDPAHAATACMFALAVLREAEQVLDPVDGQPLRLRIGIHSGPVVSGVVGRLRRQYSVFGDTV
NVASRMESTGTAGVIHVSEDTQRLAAPHMPPGCGSWLRREGVEVKGKGTMDTYWLLPQGHEADRGTPGETTPTRHRR
ASELSHSSPGSQPRTAHFQTKPPPPDQKPSGWAPPESPFHRSLSDSYCDGDGSSCTIAAVSGPGT

>Cre13.g583700.t1.3|PACid:27573439
MSEAANAAGADSRLLPSRHLLRAILLTRVEALELHATANLLCHLGFRPPAPKGALIFLSAIDASGSSGPSSCAVVRR
LVNVVRTEALAAVPYGPPIEELACCVEEGENGSRTYALRCVLDAEPSEQQWRGAVAELCSRLLQGQARHFTMQEVQA
TAHRLAVLRACALKFMQLPYAAPPKQRQHQKQPAGDTQGPGDADADFADGTGVTSGCNGGVGGGRGGGSFGSGGTEA
AVALGGLRQRHREVAALLSAKVAAASQARLADWARSCKGWSEAAAQRVVAAAAAAAAAHDMAAAAAAQAAELAAAAV
AFAAGATRRASLGSGDADSDQRLLMRRSSSGSGGRTAAVGIELAPGGRGLGGMEIDQRDQPPSRPQPQPQPEPEPLP
EPEPEPEPEPEPEPEPEPEPEPEPEPEPEPEPEPELEPEPEPEPERLLQSSPQPQPTPPAHSTLQPAGAGVEMGDAAPS
SAARSDGDRQQHAAATSPAAARGSGPSAEPSGTLTAIARRPQPLLPPPPAQLFPWLARKGLPAPTVLPADVLATAAA
AAAVGGPPSTSAAATSAPNSAGADATGGGAGQQQQQQPACGATEHAGSGARVLPPQEPAAAHAAAAQEGGRSAAGVP
LQRQEDPLPAPLVSEFMAAARELAPAGPAYGAAPVRLWERAWASAGGPADASAELGRSWRQQQQEQEQERAWRDEE
ELLGYRYCRSFSTEAAVGGFAGLPGGAAWGSGLSVQLGWPAAAGGTHGAGGLVSSAPDAAWRPAGWGSVGGEEAVQH
EAEAAAPAPAPQVVSGPVAVAQETSPLAAAAAAATAAAAAAAAAAAATPPAVSPESSPSMDISSGSPACPPPPPPPPP
PPRDSASPSPPPPQDPPVPPPPPSPHGSPCSAPPPPPPPPDALRSPPPPLPPSAAPNDSHQSQQVPVAAPDAAGASR
AASHEAYWPAGAAGSPYTAGYEDAAVVAQLEALAARARAVAQSPPERSDVTFPIRSYSLSSDSAVSYSPTRHVADGN
GEGSCDGARGDRGSDGDSGSRSESDSARHSGGDSDRGKYGDSDGDGGSGSRSDRVGGVNGDGGTGSEVAQSRGLWAG
GERVDLPLPPPPPFPPPPPFPPPAPLHPWGNATTSLDGTGSAGGAAVGGGMGVTDQPPARKRRRLTRWGPEPVAQSD
GEGAADGLGTSGRDADTDAGGGGGGGVHRSGEDEEMHAAKEQSSSPAPRRLGSRSRAECKGPGSHRSRSRSRSRSRS
RRRGREGFRNRSDSRGRDRSRSRNRSNDRNRSRSSSRGWNRDRNRDRDQSRSCSRSHSRSSSRSRSRERGNTNRKSG
RNHRRKAGPGPVNKQEEAYVDKLATFVRHNPGVHLSDLFRRGMDVPREVSSRYKNITGFLKKYANKCWDLTAENPIR
LYAVGKAPPRVLVSGAWRR

>Cre13.g574400.t1.3|PACid:27573460
MPGGLKRLLEKLKAKTKRKQNEEPDPPAGAVAAVAVYTAAAPAVTPVRFPSQHAAAVLPGSHTADQAAAASGLQSVA
AAQGHEETPAATELLIVPAVTSASGTPRLHGPSAVARHSSPATNAIFEQAGSRSAPTGAATSYGLQAPPVATGPWDV
IAADAAVDRVRAQHSDPRHSAVAQPSGERSQPSASGPTPSSTSSSGRTGAPAQGTGLSGTEPGPQAPHHAAGLRLAA
TSRPGGAPLVPATTAQGPTGAASTAGRPAGTGAEARGSPRAGVAKGNPMFDNELRRSMLSQYEAFYAEEEQEEGGSG
QTAEGSTGASVGPAAAGLTRSNHSRPAAGESQHRAPPQARHHGHHGHPGHASSLAALNSAAAQQMNVAATAAAAAAL
AATRSAAAAASVAAARAEAGTGSSPRGRAAVAASGPGALNFARDSAGRDRVNLPLPVAGASVTGAAADGHMSGPRHA
GGVAVVDSIDDIRAIFGVAGDGADSDSNDEEDAGIGSGAAGRSFGAAEGAEDEDVYASGLVRHKSSIVVGHHPGRRS
ISSLTARGSSGATAGSGAHGSDTLAGLQGATGVVGVVHGGHSHSAAASAAGCLDARSPAGSSMTGLISGSGAATTQQ
ATMHRRALRLAVASRWRAALQDAGRQASVPSTPLAASPAGGATPTGARSRRVSSSSGNNASASQPGTPSFAGGSATA
ALLNLADAAAALIGAGRSVSGASQPVVAALRAEAAAYNSSTSGSAYGLGQLASTTTGRLSRASSGRVLSIRYSNVAG
ALTAGAMQQGTGAMVGAGGAAAAPSLLCTADGATASAAIAITSSAYGRSQSGRSQAPPIYACPGLRYRPSSVADQAR
MAVTSGGALVAATAGHDGSAGAAVLAGPGTGTAASNPMLLMAQPASQRRSASSLLAGGGSAAPTAPSTGVTTATGVP
LLSICRMPSSRTLTAAAGSASGYTGTALATGMSQSTAISSSSAAGVLNLATFGAVAAGGLSAGSTPLPPPLYPGSQR
RRSSTSGSAHGGTAPFTGPYGVPVLPPISASAAIISGGRIRRYSAHSVDAAPMPPADTGEQLPEDTEVTASSPGREA
REGAVRDDKHVRFVGAADLSSPAAAVGAVEGLLLLRASPPKPLRPILTAPLRAPPPSESAAAAVAAAAAYVVPADLV
AQEQRDLGLGTSDVAATDVFQSCCPSPSASTSPTLVTGSSVNALSSAAGSPEEPCAVQPGVSLHDTSPFATLGSPPA
EACVEHVPAPPTSAGQARPVVAEDSQLTRVEAALVEQPASGYQPSAIPSAHTRAEGLAPAVKPLLEAACAFAVAAAA
AAACLQQPLTPRRLGSSQAQHVASGGSGAAAAGGASAPVQPAVFVALAAVSLLTTAASWLAPPKGAASPAEGLTGSV
HRVASAALSTAMLMPALFAATPLLGQASLAGVLLLSFLAITALGSAMLVTLRHRMSSATETEAANRRVALPALLAAP
ADVSGKTKGRASFERLARLSQDLGRQQELLHARVGGTAAGTYAAAPTPTAALQWLARQQAVLSQVKLRMLAAPDVAG
VVRMLSCHVAELYDGYGAYLLMALPGETAEVLEHDPSDPAAAAAAAAAAAEPSPPMAVLVPLVHCAALRPWLLQPPA
PPRRSAAGHGLDVTESQEGDAHEGDRQDAGEQDAGGLLDLEELPTMLACLDERAAALFTEIDHCSPVTPQAQWPPAW
ARDLAAEDGDGAGATAAAGVAAGGLGGGGVESGELDVLAAARRQLLPQHEDADTPRAALPIGAAPPNDGMAARASYF
APAPASTSGVLYTTDLGTASAASSSHVTDAVTASLPVLAVRSEVTAGDLITSGIHAAPLEPVPEDPADSGGNQRVGR
ASASDADLDHQEHVNLHEEGSLADSGILKTAVSAEELQVQLGETDARWPLNSGGSVAEAQAAAGLGTAQPEEDELGL

EVAVLPGEDQAEAIAAAAAAASFGRQQLLREAGLPAESDAGGGDVGTGRMATMGEVGDEWAPPYDVMLLAAHLGATS
LMVAPVHCLEHSFGLLVVARPCSGDAGETGGMETWSPEREAQALQRQELDERLLELSCCLADELAAALYLKHVQAEL
AVSEMLMADVMPHDALQLLKRRFQRTLHATASFTAAGAAGSMVGGIAAAAVAGGTPGGGSSASGAPGAAGVLSPVLA
RLGSYGPLVRTASGPPPSHAAAPAGAAASAGHGTESYDGNAQEQATGATSADAAAAPAGIDSASRRRLHHHHQPQAQ
PQQQASPSASQHLRSSPSTPSLPLHTIPSSSSLGGGPSFIASQLSSSYLGTTLPGGTSFSQFSSYAPSITGSAAATM
AGQRTSKPSFTQVAGMGHSHGHNMVGGGGAPAVPNASSRSRYSVPGAMAAAAGQSLWSPPPPGVHSRTVPPSPAGSA
FAPPDGLQGGTTSTNTHGSVSTVLEATILSPQGAPVPASALHAGEGRTGVWSAGTRGPMLRHGGTSARSMRSPCHRP
PPAPSAFLSLDGTAPTVAGDASADAAMRTETSATSTLEVEAALRHQAAAPGTGGGSGRALLHSYSSPLHAGGLGSGL
GAGAAWAGRSSRSGWQLRSREISAVAEETEGREATEAGEEGAAAERPQAPRLEQAAGGQDHSVSQREAQYPHSAAPL
AVPAHVGTAVGPSVFARCSSPPTVDMSGGSLPHSSGEVARLFGARGAKRLLAGPFDSNSGSQQQLSGPAAAAAAAAV
AGAMHNLAGGGSSVDATGSARVSSSGTAGEHMSQVMCSSRGARAEADEAAAIAAAVAAAEAELRAGDFAGDAEWAMD
AAEAVFRGNDDCTDEALVSAEDLCSRRSSLAVQTTPLGAPGVRVLVHAGAAAATRADESAGTGANSPSRPDGQARPS
SATAATAATAAAAATSMAASATAQAAAANIYRLPQLRIVPGGPPSGSRPMVCTPSARAHSTNAGLSAAMAAVHALQG
EVMAPVNHGAAAGGLGLGFALGPMPMVTTSSRRMSAIYLPHASSVGGPGPVTAAVAALAATAGERGFQSGSSREGTP
QPPSARRQARRSIHLPAGIGSHSYSGVGNTPLAAIAASGGGGTLYRENSGLLLQPPTAPISSHSLAGLVSPPPAQLD
RRTSATSRWQQLANAATASAGVGVGGSHHTHQPSMLRRSATEALRSMAQSGGSFGGGSAHGSGGVAGSSGTLLPVGS
VAAAAAGAVDSPRGSGAGHLGHSTAGAAGGGGPGSTTLYGSASGMASSAAAGNAAATFEVPYKQWHSAVSVLFADIV
GYTTLARALDPEQVMAMLHSLFSRYDEMLGPLAVYKVETIGDAYMAATGLLHETPSHASDLVAFGVGMIRAAACVRD
PSTGQPLRIRVGINSGPVCSGIVGSCRARYCLFGDTVNTASRMESTGVPGTIQITEDTYLALPEVARVHWSSRGEVE
VKGKGLMKTYILNVEELEVAAAAEAARPQGEAAAPYHQQHAEDGAPAPPSESGEAGPPA

>Cre13.g567300.t1.3|PACid:27573474
MPPAAPAAGGKAAAANFGAAGADATASPAATAFSAPPFAATAAAAGAPLAATGAAAALRAAAAGGVNVCFANPGTTE
MWIVAALDEVAPAVRPVLCLHENVATGAADGYARVCRRPALTLLHLGPGLANGLANLHNARRARSPVVNLVGDMATW
HRANDPPLNSPLESLAAAVSAEVVVVKRGGGGGGSTPTSTNRGSAGWAVVVASSAADDSEGSSRADSSSSSGNGSGS
SSSSSHRLAAAVAGALGHGSEVLTTGLGGEVVTVIIPHDLSWEPPASPPPPPPPPPPPPMLPAAPASSPPPASAQSE
SSESSASASPAAAPAASPPTGVGEERAAAAAALEEFVRDCAAALCRHAPATAPAASAAAAGGSSSSSAVGSSGSSSS
GGARPGGGAALYLGGGALLAEGGALHAAGQVAAATGAVLLCEGAFARADRGAGLPVLRRLPYFPQEAAAELARYGCV
VLVDAPPPVANFGYRGAPGRLLLPADSDDVAGGSVAGPAVAAVAAAPSRPEVWEFDPQALSAAGWGAACVLTRLAAA
LGPAAAAVRPGVNCRGVFATPSRPPLPPSCTAGGVNAGITSSASASVGGSSGSGSSGGSGSRLTAAELCGVVAALQP
EGCVVVDESLTSGGSYWEASKGCPPFTHLTLTGGAIGCGPPLALGAALALAQQQQQQLGAAAAAREPTAQQAAAGDT
GDGTGEASTVGGGGSAGGVRGRRVINLQADGSAMYSLQALWSQAREALPVTTIICANSSYAILKVEAARQRLPRAGA
ATRALTDLSSPAIDWVALAAGMGVAGERVGSAAALAAALRRGLELQGPFLIHAELTAA

>Cre13.g588959.t1.2|PACid:27573538
MARSNALGLALCASAQHSGALLWSTPAADALPSLEWAAAAQAVAIAATGAGSGVLHLQKQSLAYAASGHGYIIVIQC
DPACPQAAVRLKLAELLNVVDVFLGPAYAAAAAAAPTRDALLQRLSPNSGDVLDALVKESSSSYDARSPPPAPGALQ
ALLLSRLSGLATAAAAAAATPEAATEPPHPAHDTTVASAFSLLHQHTHERYPRISTHHVDLTALSATATSTAAGAAA
TATAAAAVPGAGPEPAVLFSDLVEEAALEEQYGNDVADNMRLVLDMVRPPDSQLWTLILAELQGLVAGGERDAAAAG
RAAPQRQLCSLDLSSLKPGLTVTAIMLRLGAPAPPPAPAVGGRGAGASGTSGSASGGAAGSGVRRHCHVALVLVYLL
EDERLAAELFRGDGGGASAAVRQAAFRGLPYSVAAGPRAAPAAARAALDVAASAYEGAFPDSAAPLLAEARRLPPIA
AAAAAGTGAGAGSSMAGAAAVPPPPKARAGGDVPIVPVGMGAARVDGRAEGGSPAAGPAAATQLGAAAEVPYSLGGR
QGSATGSAAAATRPVPSGGSTTSSVDFGYGPEPSAARQKRPSPLGVFGSSLGPAAVQSQPQAQAQAQARTPHVAAA
AARAAEAAASAPTGAAAIGSPPMSPGDGGGGNGYTPSRISATGTVPTRASVAGAPPARASATGLEPTRSSVTGADPG
AASVAASAAGRGAGHSPLLSGAAPPAWMLEAASPGSSTGSSPGSGQEGGEAVVGVRAGHPGDDSAPRKRPSAEPVPS
RRVADVGETSSGGSSSSSFGPVIMPMGASSQPGAADAPSPALSSTSAATMNAGPMAATVVTPAPAAAHSSPAHATAA
AVAAASASTGVPAARTAPPPRSLLGGPLGGMFSAAGAKAGGGNLYNQMAAEGGLAAPKLLSPTTATGGAAKSLAAPA
ATGAPVAAAATMAPAPAAAPPAAAAAAPQLALVKPIEAGAGAAAAAAAAAAAAGANVAVLRTPQQAAAAASAASLQAS
AAPAVPAPVLLPTPNYSAAADEEAFPQFSMRSQPLAQAQAQAALPPRPKAAPGAPQPMRASVPLLSLMQPLVDPTNR
RSAPALHDLLAASTLTATATATAPAPASVSGPMSRATAAVSEPGAEQTPAWALGATGAAAVSRTTSLGRLAAGGGGQ
LPPPLPQQQQAASRDSGAGMSPPTAASSSGSFGEEPLDALLGLGLGAARRSSASSGRTAPGTAANAAASGARASSSA
GLVGVPVASGQGAIRTSGGAASGPGASGAGEAAAGVVSVSSRAASGGGVLQLAPPAEPPPPRRSTLRRSSSGSSTQS
ASSGSYSGSAALSAAVAGASGRSAPHASASGAPPPSFLKLGDGKATSGTAEVATATTAGEPRLSFAGAVSGRDGVHA
GQDRPGDESSVSGSASPVPSAAAATATSASAAVEQGAAVVVAVAERPQTGGSRALGAASSMPLGGLPATPDTGDGGA
LGAGSDAPDAPLLAPTLTRSSFGCSSSGVSEHAAEADAEEPDPSHAAEQCLAPAPAVAEVREPVSGGPSKLSLVSSE
PSARLGASSGAVAVVSPDAAPGLDTAGAAGASAPAMPEVRVQQRGATAAATSKAAAEVVPDAVEATPLEAARPAATA
SADTVAQAQALAELEAAPSGASAAAVVPPAVFSPAAGILEMAATPSTRGSGPRMALGVPGADAPMAVASDAAHTMKP

AAPATAVAVAAPVPIHSVTPAVVAPDHVSQPDASLLAGTIPASHRGGAVAARSVSRGASGSRAGVAPGATAAPGATD
ASLDTAAATASGGSRGASAAGDASRLPKPALTLDKQQVSPPRRASSPPTSSSPSSSGSSSSPSSSSTSASLSPEPQS
GRDRKRGGPHASSSRSRSRSRGRHKRSRRHHSRRRHSHHHRRHRHHEVVHHYHHYSPSRHPPPPPPERQVSLPPGW
PVRPPPYVPLYPSSSDQDRDWYRQGGTRAGDQGAAAFAAAVATGMPPAVWPWGWPWGAPAPPPGMAAQVGGTVPAEG
RGGWDGAEQPPADYQPYPLQGGARRRSQDQVAAASSSGQEQPEDDERGNASPPPQDLQLDAEAEAEAEAALHTEAAE
VEARTEAQIPDMRHREIVAAHREALAAIVRGNSGAPAVAVAAASSGGRRRGVSSAAAGAEGSLAAGGSRGPGQASAR
RGAAQRLLPDALRAATPLAAFMLPLRDAVKLAADSVTGARSGARAVAATHREAAGGSAEHDRDQAAAGGGRAGAASG
TVAAGSDGQAALAGTDLALIAATAAAAAAAATAAAMVAAPMQQALAALNATRDVTAAGAGGIRQRTQAAQEVGGVAS
LRASSATAPWSLPPDGSPTRPPSHLSSPAASGPPFLGSPIGSPAASAGGPWQAPHHLQHQPLPPSSPTSHPPLPSPY
AATHQTYQVPAVWPAPYPQPAHPIYPQPHHPPHRLHHAYPQPPQQLHYTAQHPAFHPHPGRSHQRLHGHHHLRTAAG
GGGGGAAAGRAAGAAAAAATASGGRQLLLSPRQQHQLYVELLQLQQLQEQQQASVVSHAAAGSPPILQRGTPPVQLV
SATTPRSGPSHRPLHSQGTRVSPASGQHRYPHSSRMQGLAHAHPSSRQQGSGAAPAHGSASAHEAAPAASAVASTGA
SPLRASAKKIAAVTPASWEHNGSGRGTPEAAAAQPRTPQHSTPGSGGKRRDRAQRRQAPGSIRVRLTQHELTPTRLS
KPRPGTTAASSGNATSATESGSSGATVPAAPKSAAAPYGTVVPWLRLMTNTAAHARIERSDALEVEAEVVAALMASA
AAAAAAAAESRARHVREQVAAALATAGHTATGRGPGQHPAGTTRSEGRPQPHQPVALARKGAAIAEAALIHAVGSEH
LTAPLPHVAVLRETRRQHHARHALLVSAQLQSQPQVKAPEVAGVSSAGGVQLPSRPEQQPEREHERLAPGKEHSLLA
EWVQHGEENQTRSLIEERAAEEHQLQLRRQERQQPQRPPQQLQQQGSVALFADFMAAPGSRSSRGSQSSGTPSSAS
ASTAGVRGAAPPALGPTGARVAPTQAASQVVEGEGVQGSGYAIAAPARSSSASVHTIRTTGGIQARSERVQEMLAEK
ERQQDRPQQARVAVSEGARGSAKESLALLSSVAQKLESLASAQ

>g14016.t1|PACid:27573552
MRGNGAGLPPGLSPRPAAPWVAAQTTPPLLPLIGVYGSRPHELAVDARGLLLSHRGSSGSGGSGGAGCAAVAASAAA
ATNATALAAALPGGGCSSNHSNSYSHSSGQDSPFLTPAAGDPLTSTPRGSDAAATAASATAAAAASATAAATAVRSA
ALELSPLSLAPPPLPAPPRLSATDGGCGGGGGNGGGAANHGSAGGGTGASLPSSGSGGLGLGTRGRYGAHGLAARRQ
QQQQQQAQALMPPPAARLPLHSGGGGASPASAPPSAAASAPGGAAGGGSGAGAAGGAAGGGGGELRRSSGSGGLTGA
LVAAAAAAAEEEGLPNDPRLRWKRACRAQQEQQRQQQGQQQRQGQQQQQQQRQGQEQQQEERRKEVLSVTVPLPPP
PPQPQPRVHSPAGAGVPAAPSTGAAFATATARAATAAATAATAAGADAAHAEGGVSPSGAAGVVAAVVQPPHPQLLL
PQQQQQHEEQVLLQPPPGPGAQTPEQAAGNLLTGPPPLWPPDPVPTDPRPQPPPPPPMQPPPPPSQPPQPQQPQQPQ
AQPCLAALVARSPPSQPLRAPPPPPPSPQQSPVSAAEMEDGLDAAVAAVAAAAVAAAAPPATGAPAASHLHHSNAAG
GQTRPAAGRGLPPHLLPLPPPPPQPAPMAATMAVPAAAPLSVPAPHTKPSAATASSTGTAAAAAASSSSGSVGAIAP
GTGQRRRGMLPPPLPPHLLAVVQSGSPPPPPPPRPQPLHRVSLAPRPARGGAHTTHQQQAPAGDGSTNHHCHPQQQ
QQQQQPQHQPHQHPHQHPHPHQQHEGHSSTQKHSSSTPRLHHAQANNHQQAQPHQQPHQLPHRLGSEAAATSAGAGG
SGDGSIGGGGGAGGSADVEAEVEVWALRHAPPQCWADGNAGPGAGAAAAAGVGADAGAGSCLAAPATADGAAAAAAA
ANGHAPPPPPPLPPPPPPLLRDLVRAGHRLQQLLSDLYDAEAVAAAAAAAHVRVGAEEDTEAEVAEEAEEQPAVEAL
DAYGSQSRRRRRPPQLPWPPAGLWDTGADRAMCGSSSGSSGNGLVDGGGGSSSGGRGDAPIGSSTGARVPSGGCSST
TWRRRRVPLTPALAATWLYVPAPRLAELMEVAGWWAAHHCLPPDLLSGLVLLLLLRLQPPATAAKAEAGAEAGVQQR
AGMLQPSRGQAGGLRLEPSALPLTRGLLQSNGGGVGGGGGGGSGGDGDSGGRGGLGAAPEGDGGGFGDAVATAVAAA
AQPVLDARRIAMLQLTEVAVPATSMAAAVAAAMAAGAGNGATMLETRLVLEPAAPLLSAALNPAATTVGTAAGAGCA
RVVCCDLPPEALLQLLLGGSSSSRGAGSSTGGQPTHAQQEQEQKQEQMAPPGGTGGSVCAGPGGGGGNEAWRAVVEH
LGADISAGELLDAASRLVRHTACLPHIRAALRQRRRAQERLLQRLPRSRQRPQPQLPPAAPASTQPPSPPGKDKEHE
EEASAHEAEPTHLQSQQHLPRQLGRLQQEATAAAAAVTAAAKLNPNPDSGIDLDSDPVNAAATAVSEGSSSSSGDEE
QEDKRDSEHGGGGDDGGDGDAIPAAARRHYHAIACALNAPLDSWPELAPLLAHLRAATAPTALLPPVTDPAEAAAAG
SWADVAGRPQQPHWESPEPSMEEVEEAGEANAAAAASRHGAPDPAAAAAATAATDTAFTPAAAAAATATADAVEQVA
ERRRRAPSAAAATAGEPRPRCHSDATLRGDSGTRDLDSRRRLEPGGGGDDANGNDSGGEPPPLPPPPLPGRPYDQLP
YDELLERYERDLYRLLAARPLTLFQIRDRLPIPPLLLGRARPSTFLLRRPHLYCQDMKPGERINSRFKANPAAWEHM
MADTEMATKDSAPVAAAAEGAGGSGTGAAGTRRGAEGSRESRDEGRTRDDSPPPPPPWNSGRGARQTTGADRGGGGG
GGGGGRGEQASRRMAAAAAEEEEEEEEARRPSSSPSASPPLPRNRMPPPPPPPPLPLSERRDLLTAVEATGAVDATGGL
NAAHAVMGELAASHQQSQQQHPLHPPHKAAAAAAAAPPLPPHLAPRPLPPPPAAAASATAAAPPPPPPPPPHQWQEP
QPLQATGPEQPWQRHATDGLPPPPPLPPGLGRHVSGLPARGRSPAARRQSSGGAAPPAAATAAAATAARTRGVGDDG
GSGGGGYGGAAAPHPQPQLRRRRRRRWSTGRGRSVSVSTSPSPPRLRLQPHQHQHQDQHHRQQHQHYYNKDEEEGLP
PPPRHRHSPPRPLDLHAIGRRVFAAAAAAAAGCDGDGDGGNEQRSPGSGSSGTGASSSSGGASSSSSSSSSSSSSSG
DDDADSTECAAGSAQLHDGRRLMRAPPTADDIGGGGGSGGDNGGGIGAAYECPRGGSVHGVGGGGGHEGRQGGGDGG
GPGNLEPWAVEGIRAAVLKQLRGFYARIEGVGGGGGDGGGNAGGGGGGGGSAGGGEGGGGSGGGRHPPGMALAGLAL
PHHLRHALKASRLKLSEFVRRHLAAAGVAVVTAPNSHASLVLLGGGGGGGGGGGGGGGGGGGGGGGGRWRQSGEQQ
PAGGSRQTR

>Cre13.g604250.t1.3|PACid:27573578

MHGLGQTGAGKVHAHAYGSQRLEGRRLTQATTPPQPRPPSPAPRPPLPAPSPSANSSNSTNSSSALPPPRPPAPPAN
SAPPSPAPSLQASLAPPSPQPRPSSPALRPPLPAPSPSANSSNSTNSSSALPPPRPPAPPASSAPPSPAPSLQASLA
PPSPQPRPPSPAPRPPLPPPSPSANSSKSTNSSSALPPPRPPAPPANSAPPSPAPSLQASLAPPSPQPRPPSPAPRP
PLPAPSPSANSSNSTNSSSALPPPRPPAPPANSAPPSPAPSLQASLAPPSPQPRPPSPAPRPPLPAPSPSANSSNST
NSSSALPPPRPPAPPANSAPPSPAPSLQASLAPPSPQPRPPSPAPRPPLPPSPQPRPPPSPTIAGCPQQFEGYTGYA
AYDHTGDDMAGGPYGSPQLAAAACSSDPRCAGFNSAGYTKSTAWPLDPSQVDARIYPGFCIHVKIAPPPSPGPSPLN
PPSPPPQPRPPSPVPPRPPSPVPPRPPAPVPPPSPSPPSPSPSPPRPPPRPPTPPPLPSPPPPPPYHFGCFKDSDAR
RLPVVLAWDQRNMTLEWCDALARAAGVPLYGVQFSWFCFGGNDLAFATSLGPSPNCTRPCGGNSSQICGGEYANNLY
ALNGPPSPPSPSPPSPRPPFPRPPSPPPQPPSPPSPSPPAPGGGLEPPSPPSPSPSPPLPAPTPPPLPSPPPGYVYQ
GCFKDSDARRLPVVLAWDQRNMTLEWCDALARAAGVPLYGVQFSWFCFGGNDLAFATSLGPSPNCTMPCGGNSSQIC
GGQYANGVYAFSGPLSPPSPRPPSPAPPSPPSPRPPSPRPPSPPSPRPPSPRPPAPPPPQPPVPAPPPACTERPQGW
CAGDLSIYLRVDCNGDGMDDQVCYDASGNRGVILAAPPTGRNDTTACADTWPSVAASAAACPPVFSRCPEESAYVAL
PDAYWGWGNYPDSYVALWGETSPAAAKRFCDSYTCYLWDRDGLCATSPILRLMYWEGKPCAYVKAQPSPPSPPQPAS
PRPPGPSPPPSPSPPPLPPRPLPPPSPPSPRPPLPSPPPPPPYHYGCFRDNATRLLPVVLASDQKNLTLEWCAALAR
AAGLKLFGVQFSWFCFGGNDLAFATSLGPSTECTMPCGGNSSQICGGPYSNNVYAQNGPAPPSPPNPPSPKPSPPLP
PSPPRPPPVCVARGAGWCVQPTEVYSHEEDCDGDGYLDHFCNDIFGNRRIIFTAQIGPSCVETRTVAPATPCPQVFN
RSCIDAPGYASLPDTVFGRNPGNLGLMVYTLTPTYNMVYPTLGQCASQYNCIMFSSEGKYAVGTLGQIDTLQSSSYG
GCTYVKLPSPPPSPLPPLPPLPPPLPPLPPPRPPSPRPPSPSPPRPGAPIATSCVPPAGWCVHPGAVLNRSVDCNGD
GWPDAHTCMDATGRRGVILSVQTTTTCVNNWPNATVAECPPVFGTCDRPAGWCATGIFKVMDCDNDGYADFTCADAP
PGQSTQRGVILGGFPKAAGQQCTNSTIAWPNAPVSSCRQAFDPCPPQPGYVLYRNATIDPWSYAYPPDGNAEGAHYR
CNPIKCNPHLYAPCCTAWSTDGASTNDWFIRLKFAPGLCTYVGDYDIDVTMPQGVVRSSSSLTSAADAAGIGNEPSA
AVLLDDSGAGTTAAANGQLVDPQVATMSPPPPAPTSSSSSPKPAAASSAAVSREAAAPSSAAGEGATAATEQQQQQ
PIDMAAADAGGLMEAAAATGGGGATSTSTGTSSSVEDAEQTYPPPPPTSVQSSGSSGSRGSSSSVSAAIVGAAVGGV
AGVAVLAAAVVGLVRWRRGTVHENVRVTPL

>g14592.t2|PACid:27573599
MCGDDAQAPRGRDRTNAGHTAARPALVKTGAGAGMQLCASGLAWQQLQHAPDGAADGYCGSSYCQRNKQQVQALGHN
CHHGVGSSNSSWWRQEVAAPAAAVASGTAAVPAHCGYYCSSGHVVVCGPAVMPTQQRPGHRHTHSASPCAAAAAAAT
AAASTVDGYSSGGVHHSAPPAAAAPAPSLPPPPLWMAAGAAAASPPPPPGGTAPPPAGGTAPPACPFPSSSPPPPSC
PSHSSALLLLHHLFEHYLQQVQLSQALCGRLQGMILQAASIQLKIETGIRQLEDRAAAAEGRPARQTLADTCWGGLH
GSSGGAELIHLLLLVEGGSMGGTKGSSSSSADAGMWEQQLQRLQAHQFQQLRQLQSAVAATAAAAAADDEAATTAGK
QGRQGRREPQQQGAPQGTATADAAAAARFALLAASAAAAASGADTADDIMTDLFDDEDDHEEGGGVDEEQDTDDDDD
IVADGQEGQQAEAAEAAWHLLSHQQQQQQGSFSSFGSAPPAPFGGVACPTINYGAVRSVSPLGLLQPGSASAAPPAV
AAAAGMRSSSTALVGVEAGGGGFAQEMPAPAAGAAAATNAPAPAEANSAAAPDAAWRDGCSRGKSSALAEVSAAQAG
AVVVSDALAATAVQQPAMQRQQQQQLPQQQLEQQQQQQQQLREQERNKLQEQMRQHLLRQQEEQQQLLLQRQMLLQQ
HQMSRQQQELSVQQQQQQQQRFSISTSSSPEAPTAALRSAVAAPAGAAGASAAVSANTAPGLPNRSNSCWLQQPQQL
QQLPQAAVAVAVTARLSGREVPAVVALRGSNSSLHQMEGAVVPAANDAALATYAATTTTAADSPPEPQHAAGGGGGG
APTCTLQQWQQQARGGAVAAAAEGWRAYGGGASTSSSGSKHARRRSGSCHSKQQHLKRRGRGAAESTVEPELSPEEA
RRRALLDAPRPPPVITRIVRSASGATVSTILGASGGSGGDGGGGGGSGNSGSGGSSGDLSAMLAMPLVMLLPSSPAA
AAPSCGPQRPGTGGGTVGTNGFGAEDVSEGGGGGEVGREAVLNVRGGKVRVLPPLHRPTAEHQQQQGISLPRRSPFQ
PADSSRLAPTTAHIQQWQQQ

>g14592.t3|PACid:27573600
MCGDDAQAPRGRDRTNAGHTAARPALVKTGAGAGMQLCASGLAWQQLQHAPDGAADGYCGSSYCQRNKQVQALGHNC
HHGVGSSNSSWWRQEVAAPAAAVASGTAAVPAHCGYYCSSGHVVVCGPAVMPTQQRPGHRHTHSASPCAAAAAAATA
AASTVDGYSSGGVHHSAPPAAAAPAPSLPPPPLWMAAGAAAASPPPPPGGTAPPPAGGTAPPACPFPSSSPPPPSCP
SHSSALLLLHHLFEHYLQQVQLSQALCGRLQGMILQAASIQLKIETGIRQLEDRAAAAEGRPARQTLADTCWGGLHG
SSGGAELIHLLLLVEGGSMGGTKGSSSSSADAGMWEQQLQRLQAHQFQQLRQLQSAVAATAAAAAADDEAATTAGKQ
GRQGRREPQQQGAPQGTATADAAAAARFALLAASAAAAASGADTADDIMTDLFDDEDDHEEGGGVDEEQDTDDDDDI
VADGQEGQQAEAAEAAWHLLSHQQQQQQGSFSSFGSAPPAPFGGVACPTINYGAVRSVSPLGLLQPGSASAAPPAVA
AAAGMRSSSTALVGVEAGGGGFAQEMPAPAAGAAAATNAPAPAEANSAAAPDAAWRDGCSRGKSSALAEVSAAQAGA
VVVSDALAATAVQQPAMQRQQQQQLPQQQLEQQQQQQQQLREQERNKLQEQMRQHLLRQQEEQQQLLLQRQMLLQQH
QMSRQQQELSVQQQQQQQQRFSISTSSSPEAPTAALRSAVAAPAGAAGASAAVSANTAPGLPNRSNSCWLQQPQQLQ
QLPQAAVAVAVTARLSGREVPAVVALRGSNSSLHQMEGAVVPAANDAALATYAATTTTAADSPPEPQHAAGGGGGGA
PTCTLQQWQQQARGGAVAAAAEGWRAYGGGASTSSSGSKHARRRSGSCHSKQQHLKRRGRGAAESTVEPELSPEEAR
RRALLDAPRPPPVITRIVRSASGATVSTILGASGGSGGDGGGGGGSGNSGSGGSSGDLSAMLAMPLVMLLPSSPAAA

APSCGPQRPGTGGGTVGTNGFGAEDVSEGGGGGEVGREAVLNVRGGKVRVLPPLHRPTAEHQQQQGISLPRRSPFQP
ADSSRLAPTTAHIQQWQQQ

>g14592.t1|PACid:27573601
MQLCASGLAWQQLQHAPDGAADGYCGSSYCQRNKQVQALGHNCHHGVGSSNSSWWRQEVAAPAAAVASGTAAVPAHC
GYYCSSGHVVVCGPAVMPTQQRPGHRHTHSASPCAAAAAAATAAASTVDGYSSGGVHHSAPPAAAAPAPSLPPPPLW
MAAGAAAASPPPPPGGTAPPPAGGTAPPACPFPSSSPPPPSCPSHSSALLLLHHLFEHYLQQVQLSQALCGRLQGMI
LQAASIQLKIETGIRQLEDRAAAAEGRPARQTLADTCWGGLHGSSGGAELIHLLLLVEGGSMGGTKGSSSSSADAGM
WEQQLQRLQAHQFQQLRQLQSAVAATAAAAAADDEAATTAGKQGRQGRREPQQQGAPQGTATADAAAAARFALLAAS
AAAAASGADTADDIMTDLFDDEDDHEEGGGVDEEQDTDDDDDIVADGQEGQQAEAAEAAWHLLSHQQQQQQGSFSSF
GSAPPAPFGGVACPTINYGAVRSVSPLGLLQPGSASAAPPAVAAAAGMRSSSTALVGVEAGGGGFAQEMPAPAAGAA
AATNAPAPAEANSAAAPDAAWRDGCSRGKSSALAEVSAAQAGAVVVSDALAATAVQQPAMQRQQQQQLPQQQLEQQQ
QQQQQLREQERNKLQEQMRQHLLRQQEEQQQLLLQRQMLLQQHQMSRQQQELSVQQQQQQQQRFSISTSSSPEAPTA
ALRSAVAAPAGAAGASAAVSANTAPGLPNRSNSCWLQQPQQLQQLPQAAVAVAVTARLSGREVPAVVALRGSNSSLH
QMEGAVVPAANDAALATYAATTTTAADSPPEPQHAAGGGGGGAPTCTLQQWQQQARGGAVAAAAEGWRAYGGGASTS
SSGSKHARRRSGSCHSKQQHLKRRGRGAAESTVEPELSPEEARRRALLDAPRPPPVITRIVRSASGATVSTILGASG
GSGGDGGGGGGSGNSGSGGSSGDLSAMLAMPLVMLLPSSPAAAAPSCGPQRPGTGGGTVGTNGFGAEDVSEGGGGGE
VGREAVLNVRGGKVRVLPPLHRPTAEHQQQQGISLPRRSPFQPADSSRLAPTTAHIQQWQQQ

>Cre13.g566350.t1.3|PACid:27573606
MPRNVLNLICCFRGCVEADLWNGEGATLSRRAPRDLDRLQAIDIVANVSLTGEEKLKALSSYFGAASVSLAVLADDG
IHFVIVATSPSASPAVECFQPGTVIPVAAKSAWAPKAASGCPLDVTAQVHSVPNRDDTANDAAGAQAHSPGVQQPTP
PLAGSSCFGDALGAAFAKLLHEQVAVRSGFGTAHMPPSQQQQQQPQEGLRRQRAGGESGGCSSTSFGEDSGGCSSRD
DGGRLLLVPLWWAQELAGALTIIWGPPAVATAAAAAATPVREGAAAAATAAASGKSKRGGGGKVKSAFLSWRSSAVS
GANRFRNGHAFPSGPGGSTGSTDGGDGCGVAAATGGSTPKPPPPPAHEQEQPAVLLPREVEEQEAAAAARLLTFFLF
RFSLERVPLMRRACGLAAAVYQCARAGSGGGGGASGGSSSGCSSGSSCCVGELLRVLSSGAEALLNDRHQLWHTPQQ
LSVMAATMLFPHTPSAAGVGAADISGCIQLVRPRPRGVQTSGGGNAAAAGAPLQPPPQQQQQRQRQQKSAKPAAAAT
VAFFPLESHALAAAATAAAAVAATPHTDWCYSPLLLPTCSQPGTPLSAVACNHLLLPQRPSFSTHLQLQLQQQLQLQ
QQQQQQQHDSRRLTGCRGYSHIHNRSVQSVDPELTLLGRAAAAAAATAAPATVLLPDCAAELMREEGHVPLDVMMGA
RRLAAPPAVAASASSQAVASGAYHATGAGGGGGGAVGSAAAVCAAPHAAAAAVGCSSSGQAQQAVQPQAQGQPVLGL
VLCSGGAIAEGFLATVAQDASTIVQICDEALRAALSLPPPASVAGTAAAAAAAAGATAPGVTAATCWQDLLLHDGHC
GCHSRTAAAATAAAGATAIQDIVNADVEAAAQQQQERRRMQTEQEEQEEERNARMHAREQQRVCTLLGMMNADAGGG
ASPDTVGLVHDTPLDTARVAAVRQQSYGGAYGRGSGLVAVAAAARAATADGFVPDSVGGGGGNGADCSGGGPGVSTV
SGAIGSFSNRRQPLCMTRPPGGGFGGGFGTGGGGSGGGGRSSDLPAAAGAAAGGRSASAAVGWRSPQPLSAGPIRTA
TNCYVRGGCGTGGGGGHGTVSIGGGDVALIGNHSPMTAYFTSGNLIRTLGGGGSATAMGGVGPATATAHTSLAACMT
PSHPISPMQVMIQTITSSITQTLSAMERADSCTPERQCEELSSLTLGEPLGWGGQGVVFRGTLHALEVAVKVCAYTD
AYSTPTPTPPPPPAPWLPATGSGGVPSSANTTISAANTASATPAYTHHHNVSGLGLAPLGSVGSGGLLAIPAAGGLT
RASGVSAGTVGYGGASPPPPPPPPHLPGAHHSFNSYSSTPIRHNGRTPGGGGGSWATAGGGGSDAGTSGGSSNGGGT
MAATVAAATAAGAAAASATTPTSSSRTGFRAAASASRRGGGATAAAGPGGLAEGAMGYGSSSTTNSSRMRATRFASE
PPAPLAAANMPTGGPATAAAAAATAATAGSGAAGVSPSASPQPYRVRGVSVDSAAATAAATHAATSCGLAAQAAAAA
AFGAASIDGGAAAGSSSNATVFAGSFAGTPGGTTAATAATAAGLQSGPCSNSVNNGCAGGGGGGGGAPAGSCVLQIE
DWSRMVAALGVGAAAKHDMNKVSMLRNAAELAVCSALPCHPHLVQLFGYHTEVQLVMTQPPPPPPHATANAPAASEA
AATASYAASAAAAAEAERPLALVPSSLVPHLGLPVVQLQPQQQHLQQQLQMQLLQPAVYAALQPQQQHVALVMDYCD
GGDLRTAAEAGRLLRAPEPWEEEAAAVAGGGGVWGGRVLDMRAIYTTLLEVVLALRHLHRHGLAHCDVKPSNILLRS
SPRDPRGWTAKLSDFGCVRLLSQPPQQHQQGQYGAGAGALGGGSGRCSRTCSSRSGGTWPGGGSGGLPSNGNMTSPS
AAAAGATAAVAANATADEVPLSPASAAHVGGYSGPLSGSVAAPAPGFRALNQPVGSFTHAAPEVFVRGAWLDASVDV
YALGIVMWELACLAPVYPGLSPPEVGRLVLSGVRPVFGPHVPHEYSALASACWAHDPARRPTSAALVQRLQQLLRAQ
QDTAAAVAAAAATASAAAAAAAAATARPWPHPAQNQQQQQQWPLSMRRTTITSIPSVTSGFGLGSGLTAGLGGSAAS
IAGSDMGQLTPLGPAPITAAALVTAATAHVSPPGVSSPAPARAPGAAAAAGVEPAGMPPGAAGAAAVAAAFAAAEQA
HRASQSQLLQRQPRSQSPSPPPPLPPLHQRPQQQQQLVAAPYQRPYQPPYPATAPNAQPSNANGDDTTSSLLPVPSL
PPALADVISDVAGAGADGGAYGGYGGGYGGYDCTESLMLGGIDVSPRGGGGGGGSVAVVPEDEPLLMMLLGGSGGRGG
GSGGMHAGYAGAVAAAAAASAFGAVGVAAAESDGRRRLTSG

>g14494.t1|PACid:27573620
MWLSSAYTHPGAAWPSSWLRHPNIACLYAIVIPDHLIAAANATAATAITAVPSATASAAQPPAPAPAPAPPLPLSPG
AAGVIAPSSGLPLLLPPVPFAAAVNAEPHARPQQPSHTPQLSHPQPHAASSGATAGYDAATAAHQAAATATAAALQG
GGGAHHQLYHYSGGGGGGGGGHVSPPPSPSSVPSTVLLSEYEVSSGGHDGMSVYGSRTASAAHSVGLSRASTLMAAQ

GSGGGGSGSGGGSSSSGTPPAPALQPSTTPPPLSGLTPAPSQLPPPLAAGSGPVPGMMPADEWQQQQQQQQHQQQQ
QMQQQLPQQQLPQQQQQLPQQQQQQQQQRSARAAAQTRPPPQLAPPPPSAFAGFSATGPQYVSGGGGGGSGGGGGGG
GGGGGGGGSGSGTPTWRSGGGGGGDGGAGGTTGGGGPVDTGGGGRGAPAAAVHAGGSVGGFRSGGGGGGGGGSHGGPPP
GQLYGGAGLQRSGRLPSIRELPSVDASTDDEFAISAAGLIATAGGRGGGGGGSSSGGGGLGSGGGGGGGSFLSSICG
GSSSAGGSASGRRSTSPLFPSGGGGGSGGGGGGGTSGLVASGRANSAEARLVAAVTAAAARLAALEEAAEEAESTEG
SGVSGDNGGGGGGGGGSGGEAKFESLPQGLPSQSPPEQQQQQQQQHGEQQQQQLQLGEQPAPTERPQQQPGQRLQES
LAVAGEEAGKAAVAIAAVPATAGESDEEGDHGYTRYHTAQGSLGGGGSSSASGSRKQGGGDGSSSSPGDTSGGSGND
DVTRRRRRRSGRRQQPETLTYVPSVGGGGGGGGGLGLGVSGTIREEDSAGGGNTESGDLAVSGGGGGQGASGSGGGGQ
GGSGADVHYPVFFISDLFTSGAGGGSDGQRRRRRRHRRRQQRQLDVSGSSAGEGEGGDGGSSIGDSGGGSGGSSSYN
TDEEALMYATAQDGLALLLAGSSSNALASTGGGGNGGGGGGGVGASGSLLFHTARTASTPTPTPTPAVSSAGPLPLP
PPPPPPPAAPSSSADAIAATAATSAVAAAAEGSGGLRGALSPAGPQEAASPPPPPPPPPPPPPAATAHHGAGGSRAA
GAPTTAAAAAPTSAFAPAAAAAQEPEPQQPPQPQLQLQLQLQPPPRPGPPPATAARGLAALSPFAPLSQRQLTMRAA
SLESQLSLLPPSLLQPTPAAGGAGGCSGGAGGGGGDDDGGGGGEGGTAGVTRGASDGAVPAAAAAAAVAATAAAEAE
AAAAAGGGAVEQGAAGGQGAQGEESRQERWRREVQQRRQQQRAAAMAAAPHSVRWAIQQQAAPPPATAAATAGGSAA
EPPPATAVPAGFMQMSPAAAAAAAAAAAVPSRRLDALPPTSQQQLQLQPSPTAATALSSATTMPPPRHSQSPPPPRP
PQPPQPPAPPLRSDAATAAGARGAAAAMTATTGGAVAGSSRSAATAAAAATDLGGISLNLGLGLGLGLGRGRGRGPG
GAAAAGSGGTVTAGTPPLTPAPPAPAATAGPFAARAPPSSLAKPLQPPPPVASAALLRSASGALVRRLGSLGVGLAA
APSSVGSGSGSGSTTGTEAAEAENNRLIANAAIRGPALMSEYLSGHSIRSALRRRSDVVRSGLARIKVALDAAQGMEYL
HSRGVVHLNLKTGNLLVGYAEQQPACKVCDFGLRGPALLAAAIARTAVVAAASGQLPPAAAPAPAPAPPVAVAAATA
QLDSALLARSLPWTAPEVVRCPREVTEKADVYSFGIVMWSLWTRTEPGPAWMTASASPAPAPAPAPAPASAAHPASD
SGIAAAGAAAGAAGTAAGAGAGAAAGVAAGRVQAAGVDRATNAASSSSSNCGRALLDRVLAGEEPRPPVPGSPDWPP
SEPCPSPLAPGWAELMAACWAPQPAARPTFTQVLERLRVMFAEVKSQRRAARAAAATAGRGGGGGGAGGGGSSRASG
GGGRAAVAGR

>Cre13.g606350.t2.1|PACid:27573778
MASGGKDDLFSGLLGPGQGNRTSSAATFLSSAYDAAVAGVSVVADAAAASLGTAPTQPPADEWANLDAPGGPAPHTA
TRTGPSLRLGGGLGSGGGRGGLNPARTTPAPFAPPPWATAAAARPVPAPAPAPIQPPPPAAEDDLFAGLEVDPEVP
QFPGMALGAAAPAAPAPPAPPAPAAVPAVAAVPVAALTLAPVMSRADDLFGEFVAPAAVAVAAAAPAAGPYLASSTL
VTSPSGTGLPPVPASAGSGSAAPVAWVPAQWQPGGMLSPSAGGPTPPAPAAAAGAGGHASGGSSSFSHPTAAVAAGA
HHPGIGSSSVGHGGGAGAAPGLGHGGTRAALTAAASGRSGGLEDLVRAVLTAPPPPSSQPSPAPSPQQPQHAVPNPM
PATAWVASGSGVGGSSSGGGTASRADVSWAFGDSAHSANSGSSGGASGGNRAEPSAAAVAFTASTASTTAVLAPQTS
PAAGHTSGAAGHVSAVGGRSADEDDEWGGSNWVTAGASPAPGPGPAPHSAPEPGAPAPVVAPPAATAAWSGPAPAFA
GAQPSNAAAGAAAAADTPAAAADTPAAAGSGGSGGAGDDDTWAGAAWVAAERQLELAAPMVFTAAPSGNGAVGAPPV
LAASLDEDEWGTPEWATAPAVAPANSGAAAAALAGLAVPAVAPAPHAGADAAASLSHEGGAPAGGDDWGELQWAAAE
PVSAVTSAGGAAASGADAGPGAATLSPAAAGATAAAGAQQAVPAEEEDWGAPQWVAAPQAGAGPAGVHLFASPVATT
ELPSHSAAAVSASVPAAPLDSSGPTLAPMQPASGTADDQPQPASPPSLLSLIAAAAEVAAVEAAAAAAADVKSADAA
SAAAANGLHTSSGAAATAAAAATAAAAAMELQDELSLLPSPPRHVPSDTPTSRPARPLELQSPPHAASDVMSGAEG
LEPHAATGLISRSGGGGGGGGGGGGLATAFGEVADVVGVSPSDPFGSGVSPPVVPVAPSGSFTLADDEAEPEAEVEA
KVEAEGEATGEAPAQAQVEREGEGQPQGDAQVPLEATAPQTVAPDQPPAPAPAPPPPAPPSVPPPRRAGESGFTAHF
EEVITLTTTATADAAAVHDGTSGHVFPMFEPPPQHPHHPHHAPGHAYSEEEDSDAELPDIDADGCLVAVAASAPSSS
SASGYRQQAQQLPPTLAVAEAEQPVLQPFEEQEPAQMQAQAQAQEPAQEQVQVQEPAQEQVQVQEPAQEQVQVQEPA
QEQVQTEADVRGGLLDPEVEAAPQAAAVEEARYAFAEAAAREAAAAEAADAEAAAAEAAAAEAAAAEAAAAEAAALE
AAAAEAAAAAAAAAEAAAAEAAAREAAAREAAAAEAAAAEAAAAEAAAAEAAALEAAAAEAAAVEAAAAEAAAAEAA
ALEAAAREAAAAEAAAAEAAALEAAAAEAAAAAAAAAEAAAAEAAAREAAAREAAAAEAAAAEAAALEAEAAAAEAA
AAEAAAAEAAAVEAAAREAAAAEAAAAEAAALEAAAAEAAAAKAAAAEAAALEAAAAEAAAVQAAAAAAAAAEAAAV
EDAAREAAAAEAAAAEAAAAEAAALEAAAAEVAAAEAAAAEAAAAEAAAVEAAAREAAAAEAAAAEAAALEAAAAEA
AAAEAAAAEAAAAEAAAAEAAAAEAAALEAAAAEAAAREAAAAEAAAAEAAALEVAAAEVAAAEAAAAEAAAAEAAA
VEAAAREAAAAEAAAAEAAALEAAAAKAAAAEAAAAEAAAAEAAAVEAAAREAAAAEAAAAEAAALEAAAAEAAAAE
AAAAEAAAAEAAAVEAAAREAAAAEAAAAEAAALEAAAAEAAAAEAAAAEAAAAEAAALEAAAAEAAAAEAAALEVA
AAEAAAAEAAAVEAAAREAAAAEAAAAEAAAAAAAAAAAEAAARKAVAAEAAAAEAAAAEAAAAEAAAAEAAAAQAAA
REVVDVEATAAAEAVASEAAAAEAAAAEALFSAAAAEASTVRGTAAADASDGDGDDTLEPLDGSRAALAAAEASALA
AAVAGAAVVAGDVANAAALMSDQLDELEPLADVLDVTKMVTMAAGATAHEGVLGLGGAAATPQQLTSLPLQAEAEAA
DALGAAEVMQPQAVPPEPAAGVLDPAELQQVLPGQGEAAAFQRAVPPTALQETEDWGEFFSSGHPQQQQQQQQQQP
APSTPLAGLAAAWPDDMLTSSNISVAADEVTADMTTEAAAAPSAAAAAAPMAAVDLDDWLGLGSAAPAATGSATGGR
LTGGAGSGPVLDDLFGDFVGGSGGAGGSQPAAPPAAAAAATAGAGDPFELLFGALEVPGPAAAVPAPALTDATGAEG
PAVEPAVALTPPSTGASTAADDSGAAMAAADGLEALAAAEDFEPLPLGAEREPQELQQELGGHAVYQHQHQRQAQH
EDAAVGQAEQHTHLELQVPGPRQQGEPPEEPALGPAGGADWRRQSREHWQQQEPPQVAAAAEPRSAVEQGEAAQEER

PQAQAQAQVQAQVVERAAGQPMATLAAAGGGQAEQQEQQQAQEQQEQQQAQAQEQQARGQGGWDLHEVGALAGSGSG
SGGGGAAPGAAAEADEAAVPAEAPAEAVAPTAEAPAEAAAPTAEAAAPTTEAAAPTAEAAAPAAAAPHDGDEDGGGG
DEDDDLFLGLDTAAVPPVAFGTEAELAAAAEQEDQEKVEEEEQGQEQEQGQPGAANMAVDAEARLPAASGDAELAFG
TAGGSASAGATAAPATTDLAAHADLVFGEPEDPTAVAAVAAVAVAAVAAVAVSAGAAGTAAATVPELQDGERGVGQD
QLPVQWGAARAEERELLRAHASQVSMDLAGYAAPAATHAEAPPADAIASAPASAADAAPATYGSAAAGTVEEVRDVD
VDAVLASLVGVDVSAVVPQPQLQPQPLAVATQQPHQHDGAASALDLLLAEYGGYAGEGGAGGNGSAGADALSGNADA
AMAADVGLPVPAEGEGGGSGSRSSDLDALLAQYTAATAGVPPPDPAPQAGAGSVAPPAPPPASEAAVAGSANGAAGD
RNGGDGADEEDGDDEDDLLDLSGVLGDEEQTSPPPPPPPPRPALASASAASPPAALAAGGTAAAAAAVASALGSVPP
PLPNGAFAVVVETRAAVPSPPHTAPDLAPPPASPPPMPPLAASADAAAALGPQTSLQPLPPMGSAAMLGGPGGHEQG
EGGASGPGATAATSLLPAYEVALLRAERAEQALLAGADAAVGGPDELAALMLPPPQPPPSQTQTQPPGAAAATALAG
PGASVAPARRPLVSVTSLGSASSITGASQPLLDPVEGLTALLADATEDWNPAGRPDASGAMVGGSVALRLQELHASL
PDIATHWLTAGRPRVVTLSAGIMAVGTSLGAALVFQLPGSSSSSGSSSGPTAAAAGGSSSSSAGGGGGHGPGVHHGH
HGSGSQGLGGVGADGGGGAAGGGPGAAAGGGGGPIVVVGDARTEAEAVTALALSTVSGPADPLWLLVGHASGTVAVW
DLQRRPPKQVAAITGQHNLPVLHVSFFPGRGAAMALSADRRGRLALHVFSAIMGLKTAVASRPVLDGSLGVIGAVSH
LTPFFATTTTTSSASAASASGAAPASASAGVVASPSGGGGGGGAASGGASGGGAAAPGGDGVSQPPPPSALAPPACR
VGDGLVAVTATSGCHIGRFKPSGELIPLYSIPRPAAVPAPSIPSAAWLPHQRRAARPPPTAADPSPGGATAACAVLA
VGWHDEVMFVDVPLIGDHQLPAPPPPPSPSEAANAASSSAAAAAAAAAAAQQQAQQQQQQQQQAQQGGQAKRTFLR
AATALTGVARGAAAAATAAAAAAATAASAAATATSSALASSTSQLDSLTPTAASALAERLPLVVTRVWNAIATIASS
GSDDGSSVLSTGGVPVAGDDVSVRVVGLHWYDSDALGVVVVLGQGGAAHMMRLVVVDLEGALREQVDCIDTPYVSGL
FPPAPPGGAPAVPDTLSGSGARVVLLGGSSTLYGSRLLTWQERLRTLADIGKWKLGMRFALDFYKLHLARVAAATAS
RSGAAAAADSSYPDALKGWLASLAVGYVKSVLGAALRAAAARGDTHLQLDRLPPDVVAQLRQSAAAAVLGCLAAGRP
ALLADGVFPLCREAGAVGPLVEAVEVAVLADALPRPAPELVQALVEHLAGRGGLGRPDRVERCVLHFDITSIDLDQV
LRLCEAHELYSASAYIYNRLGDYRRPLLDMLAAVAGCGGNEAAAAGNGSASGGGGGSGAGGGGGGGAAAAPHGVATD
AEADKRRRTTGYKLLVYLRCIFQRRSFPPGTGRLPDVIPGAGRYGDPGTADDVKAQLLGLLLYLDTDDLAREWRRRP
QQLPPGLREGPHPALQLLLSIDVAAAVCVLGGAVAGWDAVETDLREAAGRPAAELDSVLVATQVLVNAFIGLLESDR
FRQLTAGTSTTTTTTTTSSTTTSTTNSTGSSLTRVSGAGTGSVTSSALHLAAAQRGAAAAAGSEVVLDFIAGLLAAGR
VVVRPAFALRLLRHVAARAITAAAADAPLAAASGSHHPGSAAAAAGGAADAAVAEAAPPLPRARSRAEVEAEALFER
LVVVVGVNTEGGAAAARHDPDKLSVQETQAAVDLATRAGFHHALATLHHLRGDYAAALAGHLRLGAAAAAAAAAAGGA
AAAAGGPLSPQGSLGPSSGPMGRMAPLASFSGGGRAQSWQSPAPPSPASAFDYVRRFLEGGAASAAQRAAMYSALLA
AAPQLVAADALATAQLLLGHYPGESAAAAFMSALAAAGERRLQYDFLTAAIRTRQALHEAMAAAGEAADALPSAAAS
ARAAAAAAAAAGLPPAAAVAAAALGAWLNQGPVADAYVQLLCEYEPAAVLPFLQDHEADYDVRRCIELARAAGVLDAE
AYLHERLGDTEAALELHLRDVTRRNAELRAAVASGALPAFHPAAYYDQHTAAAAALAADLSHPPHPHPNPNSPSPYL
DHLAWLLALSPRLAQVLQGSLGKAVVEAAERARQQAAQPQRAGSSSSTSGALSAGHGSAAKGTAAVAGAGGGAATAS
VTSPSSYAAAAVTYAASAAGTAIYSAASMATAATSAAASAAAGAAAATVSAASTAASAFASGAAAVASAAAATVGMG
GSAASTPRTAGGAAPSSSSPAMPGHSTGAAATAGGHAGAAGAGADVGAGVGVGAGTARVRSAAGTPPPELVRAWEAL
CSAVAFCGRSCKQQQQQQQQQQQQQHPSGAEAKQRPATWCWQRLLAVYVAEVQDARTPGRQQQQQQQRSGPGGRSPH
HRHHHQQDHTEQQAEEVALEAAAARGDAGAARACQRRVLRHVFGVFLEEVISRMSDQVPASEVVRHIISEHGANDFG
GFRSTLLGLLGACAYEQAIMASAQRLISRGVFASIRQRANLAAGAYPASAVHKRQDKPLAAAGGDGDDDAAAAAAAS
AAGGSDAAAVHAASQPYWRRLRDAHGHGLHRHHGSGGADGADGGGPSSPFLPPGALLGAARVGFGGGVAQPAAGGGG
GSGELRLPLAPAGDLRAAAAAAASSSGLDALGIGGEYGGAGEAEGAAASGGRGVSGFALVAAEGGGHVARVRVSVRVP
PRAQSRRRWVSGAGTSASGGASGGVLASSGVGAGQGGGGLATGGGSLDVDELLNWSRALAS

>Cre13.g606350.t1.3|PACid:27573779
MASGGKDDLFSGLLGPGQGNRTSSAATFLSSAYDAAVAGVSVVADAAAASLGTAPTQPPADEWANLDAPGGPAPHTA
TRTGPSLRLGGGLGSGGGRGGLNPARTTPAPFAPPPWATAAAARPVPAPAPAPIQPPPPAAAEDDLFAGLEVDPEVP
QFPGMALGAAAPAAPAPPAPPAPAAVPAVAAVPVAALTLAPVMSRADDLFGEFVAPAAVAVAAAAPAAGPYLASSTL
VTSPSGTGLPPVPASAGSGSAAPVAWVPAQWQPGGMLSPSAGGPTPPAPAAAAGAGGHASGGSSSFSHPTAAVAAGA
HHPGIGSSSVGHGGGAGAAPGLGHGGTRAALTAAASGRSGGLEDLVRAVLTAPPPPSSQPSPAPSPQQPQHAVPNPM
PATAWVASGSGVGGSSSGGGTASRADVSWAFGDSAHSANSGSSGGASGGNRAEPSAAAVAFTASTASTTAVLAPQTS
PAAGHTSGAAGHVSAVGGRSADEDDEWGGSNWVTAGASPAPGPGPAPHSAPEPGAPAPVVAPPAATAAWSGPAPAFA
GAQPSNAAAGAAAAADTPAAAADTPAAAGSGGSGGAGDDDTWAGAAWVAAERQLELAAPMVFTAAPSGNGAVGAPPV
LAASLDEDEWGTPEWATAPAVAPANSGAAAAALAGLAVPAVAPAPHAGADAAASLSHEGGAPAGGDDWGELQWAAAE
PVSAVTSAGGAAASGADAGPGAATLSPAAAGATAAAGAQQAVPAEEEDWGAPQWVAAPQAGAGPAGVHLFASPVATT
ELPSHSAAAVSASVPAAPLDSSGPTLAPMQPASGTADDQPQPASPPSLLSLIAAAAEVAAVEAAAAAAADVKSADAA
SAAAANGLHTSSGAAATAAAAAATAAAAAMELQDELSLLPSPPRHVPSDTPTSRPARPLELQSPPHAASDVMSGAEG
LEPHAATGLISRSGGGGGGGGGGGGLATAFGEVADVVGVSPSDPFGSGVSPPVVPVAPSGSFTLADDEAEPEAEVEA
KVEAEGEATGEAPAQAQVEREGEGQPQGDAQVPLEATAPQTVAPDQPPAPAPAPPPPAPPSVPPPRRAGESGFTAHF

EEVITLTTTATADAAAVHDGTSGHVFPMFEPPPQHPHHPHHAPGHAYSEEEDSDAELPDIDADGCLVAVAASAPSSS
SASGYRQQAQQLPPTLAVAEAEQPVLQPFEEQEPAQMQAQAQAQEPAQEQVQVQEPAQEQVQVQEPAQEQVQVQEPA
QEQVQTEADVRGGLLDPEVEAAPQAAAVEEARYAFAEAAAREAAAAEAADAEAAAAEAAAAEAAAAEAAAAEAAALE
AAAAEAAAAAAAAAEAAAAEAAAREAAAREAAAAEAAAAEAAAAEAAAAEAAALEAAAAEAAAVEAAAAEAAAAEAA
ALEAAAREAAAAEAAAAEAAALEAAAAEAAAAAAAAAEAAAAEAAAREAAAREAAAAEAAAAEAAALEAEAAAAEAA
AAEAAAAEAAAVEAAAREAAAAEAAAAEAAALEAAAAEAAAAKAAAAEAAALEAAAAEAAAVQAAAAAAAAAEAAAV
EDAAREAAAAEAAAAEAAAAEAAALEAAAAEVAAAEAAAAEAAAAEAAAVEAAAREAAAAEAAAAEAAALEAAAAEA
AAAEAAAAEAAAAEAAAAEAAAAEAAALEAAAAEAAAREAAAAEAAAAEAAALEVAAAEVAAAEAAAAEAAAAEAAA
VEAAAREAAAAEAAAAEAAALEAAAAKAAAAEAAAAEAAAAEAAAVEAAAREAAAAEAAAAEAAALEAAAAEAAAAE
AAAAEAAAAEAAAVEAAAREAAAAEAAAAEAAALEAAAAEAAAAEAAAAEAAAAEAAALEAAAAEAAAAEAAALEVA
AAEAAAAEAAAVEAAAREAAAAEAAAAEAAAAAAAAAAAEAAARKAVAAEAAAAEAAAAEAAAAEAAAAEAAAAQAAA
REVVDVEATAAAEAVASEAAAAEAAAAEALFSAAAAEASTVRGTAAADASDGDGDDTLEPLDGSRAALAAAEASALA
AAVAGAAVVAGDVANAAALMSDQLDELEPLADVLDVTKMVTMAAGATAHEGVLGLGGAAATPQQLTSLPLQAEAEAA
DALGAAEVMQPQAVPPEPAAGVLDPAELQQVLPGQGEAAAFQRAVPPTALQETEDWGEFFSSGHPQQQQQQQQQQP
APSTPLAGLAAAWPDDMLTSSNISVAADEVTADMTTEAAAAPSAAAAAAPMAAVDLDDWLGLGSAAPAATGSATGGR
LTGGAGSGPVLDDLFGDFVGGSGGAGGSQPAAPPAAAAAATAGAGDPFELLFGALEVPGPAAAVPAPALTDATGAEG
PAVEPAVALTPPSTGASTAADDSGAAMAAADGLEALAAAEDFEPLPLGAEREPQELQQELGGHAVYQHQHQQRQAQH
EDAAVGQAEQHTHLELQVPGPRQQGEPPEEPALGPAGGADWRRQSREHWQQQEPPQVAAAAEPRSAVEQGEAAQEER
PQAQAQAQVQAQVVERAAGQPMATLAAAGGGQAEQQEQQQAQEQQEQQQAQAQEQQARGQGGWDLHEVGALAGSGSG
SGGGGAAPGAAAEADEAAVPAEAPAEAVAPTAEAPAEAAAPTAEAAAPTTEAAAPTAEAAAPAAAAPHDGDEDGGGG
DEDDDLFLGLDTAAVPPVAFGTEAELAAAAEQEDQEKVEEEEQGQEQEQGQPGAANMAVDAEARLPAASGDAELAFG
TAGGSASAGATAAPATTDLAAHADLVFGEPEDPTAVAAVAAVAVAAVAAVAVSAGAAGTAAATVPELQDGERGVGQD
QLPVQWGAARAEERELLRAHASQVSMDLAGYAAPAATHAEAPPADAIASAPASAADAAPATYGSAAAGTVEEVRDVD
VDAVLASLVGVDVSAVVPQPQLQPQPLAVATQQPHQHDGAASALDLLLAEYGGYAGEGGAGGNGSAGADALSGNADA
AMAADVGLPVPAEGEGGGSGSRSSDLDALLAQYTAATAGVPPPDPAPQAGAGSVAPPAPPPASEAAVAGSANGAAGD
RNGGDGADEEDGDDEDDLLDLSGVLGDEEQTSPPPPPPPPRPALASASAASPPAALAAGGTAAAAAAVASALGSVPP
PLPNGAFAVVVETRAAVPSPPHTAPDLAPPPASPPPMPPLAASADAAAALGPQTSLQPLPPMGSAAMLGGPGGHEQG
EGGASGPGATAATSLLPAYEVALLRAERAEQALLAGADAAVGGPDELAALMLPPPQPPPSQTQTQPPGAAAATALAG
PGASVAPARRPLVSVTSLGSASSITGASQPLLDPVEGLTALLADATEDWNPAGRPDASGAMVGGSVALRLQELHASL
PDIATHWLTAGRPRVVTLSAGIMAVGTSLGAALVFQLPGSSSSSGSSSGPTAAAAGGSSSSSAGGGGGHGPGVHHGH
HGSGSQGLGGVGADGGGGAAGGGPGAAAGGGGGPIVVVGDARTEAEAVTALALSTVSGPADPLWLLVGHASGTVAVW
DLQRRPPKQVAAITGQHNLPVLHVSFFPGRGAAMALSADRRGRLALHVFSAIMGLKTAVASRPVLDGSLGVIGAVSH
LTPFFATTTTTSSASAASASGAAPASASAGVVASPSGGGGGGGAASGGASGGGAAAPGGDGVSQPPPPSALAPPACR
VGDGLVAVTATSGCHIGRFKPSGELIPLYSIPRPAAVPAPSIPSAAWLPHQRRAARPPPTAADPSPGGATAACAVLA
VGWHDEVMFVDVPLIGDHQLPAPPPPPSPSEAANAASSSAAAAAAAAAAAQQQAQQQQQQQQQAQQGGQAKRTFLR
AATALTGVARGAAAAATAAAAAAATAASAAATATSSALASSTSQLDSLTPTAASALAERLPLVVTRVWNAIATIASS
GSDDGSSVLSTGGVPVAGDDVSVRVVGLHWYDSDALGVVVVLGQGGAAHMMRLVVVDLEGALREQVDCIDTPYVSGL
FPPAPPGGAPAVPDTLSGSGARVVLLGGSSTLYGSRLLTWQERLRTLADIGKWKLGMRFALDFYKLHLARVAAATAS
RSGAAAAADSSYPDALKGWLASLAVGYVKSVLGAALRAAAARGDTHLQLDRLPPDVVAQLRQSAAAAVLGCLAAGRP
ALLADGVFPLCREAGAVGPLVEAVEVAVLADALPRPAPELVQALVEHLAGRGGLGRPDRVERCVLHFDITSIDLDQV
LRLCEAHELYSASAYIYNRLGDYRRPLLDMLAAVAGCGGNEAAAAGNGSASGGGGGSGAGGGGGGGAAAAPHGVATD
AEADKRRRTTGYKLLVYLRCIFQRRSFPPGTGRLPDVIPGAGRYGDPGTADDVKAQLLGLLLYLDTDDLAREWRRRP
QQLPPGLREGPHPALQLLLSIDVAAAVCVLGGAVAGWDAVETDLREAAGRPAAELDSVLVATQVLVNAFIGLLESDR
FRQLTAGTSTTTTTTTSSTTTSTTNSTGSSLTRVSGAGTGSVTSSALHLAAAQRGAAAAAGSEVVLDFIAGLLAAGR
VVVRPAFALRLLRHVAARAITAAAADAPLAAASGSHHPGSAAAAAGGAADAAVAEAAPPLPRARSRAEVEAEALFER
LVVVVGVNTEGGAAAARHDPDKLSVQETQAAVDLATRAGFHHALATLHHLRGDYAAALAGHLRLGAAAAAAAAAGGA
AAAAGGPLSPQGSLGPSSGPMGRMAPLASFSGGGRAQSWQSPAPPSPASAFDYVRRFLEGGAASAAQRAAMYSALLA
AAPQLVAADALATAQLLLGHYPGESAAAAFMSALAAAGERRLQYDFLTAAIRTRQALHEAMAAAGEAADALPSAAAS
ARAAAAAAAAGLPPAAAVAAAALGAWLNQGPVADAYVQLLCEYEPAAVLPFLQDHEADYDVRRCIELARAAGVLDAE
AYLHERLGDTEAALELHLRDVTRRNAELRAAVASGALPAFHPAAYYDQHTAAAAALAADLSHPPHPHPNPNSPSPYL
DHLAWLLALSPRLAQVLQGSLGKAVVEAAERARQQAAQPQRAGSSSSTSGALSAGHGSAAKGTAAVAGAGGGAATAS
VTSPSSYAAAAVTYAASAAGTAIYSAASMATAATSAAASAAAGAAAATVSAASTAASAFASGAAAVASAAAATVGMG
GSAASTPRTAGGAAPSSSSPAMPGHSTGAAATAGGHAGAAGAGADVGAGVGVGAGTARVRSAAGTPPPELVRAWEAL
CSAVAFCGRSCKQQQQQQQQQQQQQHPSGAEAKRPATWCWQRLLAVYVAEVQDARTPGRQQQQQQQRSGPGGRSPHH
RHHHQQDHTEQQAEEVALEAAAARGDAGAARACQRRVLRHVFGVFLEEVISRMSDQVPASEVVRHIISEHGANDFGG
FRSTLLGLLGACAYEQAIMASAQRLISRGVFASIRQRANLAAGAYPASAVHKRQDKPLAAAGGDGDDDAAAAAAASA
AGGSDAAAVHAASQPYWRRLRDAHGHGLHRHHGSGGADGADGGGPSSPFLPPGALLGAARVGFGGGVAQPAAGGGGG

SGELRLPLAPAGDLRAAAAAASSSGLDALGIGGEYGGAGEAEGAAASGGRGVSGFALVAAEGGGHVARVRVSVRVPP
RAQSRRRWVSGAGTSASGGASGGVLASSGVGAGQGGGGLATGGGSLDVDELLNWSRALAS

>g14635.t1|PACid:27573817
MDDSSAGAHPASPDSQAEGHGDDDTPTRQRPAASTYSQPQQPQRHLDLLQPAEPPLLLLLPRQRRRSSDLASVTTAA
APRAFTTAAGAAAGAAVLPLQRGSPPPLRPASGDSTGPRSPRRYAAAAADNHPSAAALAAAALSGHLTVPGAACESD
LAALTAPIASVSTTAAGIARGSGGAAAPSGGTHRALDAATRRPGAPALPPLLSLSGAAAGGLLGLMAAGGGHKRLSS
SIPEAAAAAAAAEPAAGGLGDRRRLLPPPAASAPAGRRLAPPPAKRQRLSDPGLAAGGTAAAALGHLQQVRVAAAAV
GRRRSSSPQGRATTGDRGGEVDWAAAAAAAGRGGSASMALVNDAAAAEEAAAERCYRGLGASAQPPPQLPPSPRRQL
LTAAPSPPPLRGQQPQPPSLPVPADELQAAAELLLAFRRTAAAATAAIAEDDNPQVPSTTAASAGGGSTVSPTAAAP
RTAAWRGEAALSPAAPHSFAAGPAAPAVPPVAVPAAAFPSAAARATSAGTPSPSTCADSAGAGVGARSARHSPAHSP
SPLVHQQHHHQPQAQRAQQQQQAARLLMPPPPPPPLHMARRHSAPQQLLPQQQGPFHAAAGGGGGHQEHMRRASDGA
SIAAPAPSPAAGRHPAVVRHSASTAHQQAPMGPISEYVPLRRAPLMAPPAPPPAASPPLPAAAAAAGGTGSGRHLTA
AAAAAAAAEAAAKERRGASTPDRLLAQTAAAAETAVQRQAASYLDDSSGGVSQQRRRPPRWTLPPGSRLLLARTSPA
ATPPSSSSRPPAPASAPAPAPAPAATASAATVVDEELRALFLAVVDVVTSGGASGRGAAAAAGHEEGAAAAAVRPAG
REALHAALILLTTQHYMDHGLPGLPPATPSTGGPALPPPRLLPAPEPPAPSAPRRLPPQAPSPPQPTAELERQQQPQ
AHRPPHHHQVAPTPRPPHGAITAAAAAAGPDESAATAGGSSAAAAGRRNSSSRNPWAARGAPPGAAAAAGLAVVMEA
AAAPSSRGLGGSSQQHPPGRQTAATAPQTAPTAAGAAGQNSSTAAVTTVTAALNSQQLLRERKRREASGSRSSSSGN
SAVSDQQQQAPKRRRQDL

>Cre13.g591100.t1.3|PACid:27573837
MADDVEDITRRPSIGRDDLGERPRPKARIEERPHIAPRPGAAPSTSANPEISAAHVSAGSDAASAGAEAGGFQLPAG
YSLLLVPTAAVQQAQAQAPQQQALIPDGEPATTASPAPAAPATTAALLSVAPSGASYTSATGTLLQPPQLYAVAGNS
PTTRGGAATPPYAHAHTASQRHLAALAALLAGGGSTAGLAPVPVPPLVPPPVPPPGPGPDPHQLLASQDLHPAAAAA
AAADALPFPVPLSGASLAPFPAPLSGANLAAAAAATAAAPSAAPGWSSAGFSTLTAALQAAPAAAALPPSTATSIIA
PPAPLPGSAAPPAPYGTLASPARTAATGTGAAAAAAAAARQSHHGRHGGRYAASTAAASSVFDDSDSSDEEQELARR
SARMQPRLATTAAAATAAANAAAAAAAAAAAAGVVPGSSPSVGGMGMVAAAGLGGERVLTRAPTSTALAAEAVVANA
LALQQQAAVAAAAQQQQMMALLQLQQQMLQQQAQQMSQQQQINALTRQDGQGQQDAAVLQPQQQVLQPQQQVLQPQQ
QVLQPQQQVLQPQQQVLQPGQQQGQAAGLSGFPSSGLSGTAAAASNSTTATASRAPSGAAAAAAAQGGGAPAAASPP
PPPPPSRPAAACAAGIIGASTPTIAEGDEEEPEEGGGGGGGGRGGGGGALIGGGSLWAGSVSGDVNDGDGGGGGSGGG
GGAGGGGVSGEGDIDPLPSTSTSQRQNAALQLEYLRQWSEVRPYGRSSTGVTAGTGGRGDGGGGGDGDGDGDGDDHG
DATAASGAAAAPGGAGRQQAWPAAAGQQDLGAQREGTLGSSLVAEVEAAAATAAAATAAKAAAAAAAAADGGLRDGG
AAAAATAAAAPAAVQLPPAAASASAAAGATSHLASPHLTSPFENAAPSIIAGTSPPPPPPASAGSAAASPGPTAPAA
PPALLLGGGGGGGGGGTLQQPLLLPPGAMLISAAPPLQPGTAATAASAGTAATATPAATATAAAAPAVPLVAANGSS
LLGALEVPAGALNALLQRQGFSMLPPSHAATSLHQQQNATQQQHQQQHQQHLRVSMDAGSATSLVGLAAHGLLLPPA
PPSQAGLSHQQYTSVGQSQAAADAAAAAAAAAAAAAAAAAVAAQEAAAAGGKPEAPNADQVAENLENIDALALFDSDD
DDDVLEEEQPAWHRRPAAVRLMVSLGLGLVLLLVGVILQNVLSDVSTADNAFRWLYFFAAWPLLFYALGYGTRKAFE
LLEWWFFRESLLYLQSVQLKAAWLIFFLVLLPWFNVVFRWAWCAPTAAQPKRCETAAYLQATHIVWNVLLCLMLFCL
ANLLKAVLAKLLTTHFYRTAHFKKLKKALEKEYVLQVLSRPKRLVMMERAHAAAAEAGAAGGWFGGLSIFHGGGGGA
GGGAVSTRRNNTAAAAAAAAAMASPPEEEQEAGATASAPAVAAAAGATAAAAAAAANGTGGVAVALDPRSGRPHQEEV
EPGIATAAKAADLSAAAAAAATAATAMVAVGGSGVAAPAKLLQPAGSSSASPQRQAGVVAVAAVAGGVEGGAQMAAV
PPPPPPRGLGEGPGQPSLGDGTLPVLQPSQQASLLSAMSAAPQLQPPPPQQQLPQQQQQQPQPPSRNMLPIPMPPPPP
GLLQPPPSQLQPAQQAAAPGPGGGCLNVHTVPAGMLSTTSTLAATPSQPPTATAAAAVAAAAAPAATPLPVSYSAAS
VAGVTPPPPPSPFPPPPVPPSSPQEPTHTPAPLPTPSSTPAPGHGHGPAVKFLSPAPSGATATAAAAGGSSTSAAAG
GGSSVFGGLGPRLAAGLGLRRSGDSHSSSRASHPHKAKGSGGGGGRGGRTLPAVSEVPDGSGPATSAGAAGAAVVSK
EGLTSAMVDVVPPGNTLTANKPLGALNEDELEKVRTAIVIKTQSALIGKYAEKSDEDMAKELGRVRRFAKALFYNVL
PPPAEQQQQGPAGLLGPHAAAAAAAAGGVGGSQGFLPGAQPPPPPPPQGYRDYLVLADFLPFFEDADAATAAAAAAAA
AAGGSAETQAAVAAGAPSQGPGGGGGGGGGGGGGKMPSRRGLAAASMTAAAQGGGKEEGAAAAAAAAAADLAAKVAAK
NAAERARAARLKAAEEAARKAFAVFDADGNGEVTRSEVREAVVNIYKERRNMARSLKDTETIVQSLEFGIGCLIHFV
FAAVYLLIWGVDLLTGFSTFSATVLALTFVFGNSVKNMYESMLFLFVTHPYDVGDCILVGVGADMYRVKKISLLYTD
LVKSNGERVYMPNTALITVGITNWTRSKSSSGSARLVCDLGVAWEVREDLQAALRAYAREHPGEFDGEPSCNFREMA
DPLKVVLVCSWTYSFAPDEFSRLGPARNGMVYVVQQKAHEHGLIYSQAAAR

>g14428.t1|PACid:27573840
MARLWTSGAGGGNATAIATDAVVPVTEVGGGGGGSLVLADSRPGRGPFAPLAVAAAAAAAAAAPPLLPSLPPAPAVL
LPPTAAASTARQPLRQLLPAHLSPCRPGQQLQPQPPALQSSRPGIGTGTGGGKKKAAARPRSALAAARSAATAAAMA
ATATAARDATAIHGGGEGGGAGAPSARQQPGVAAAAALPPPMSSRLSPWPSATPPPPQLAVAPPGLIQPPRQWDWGW

DGAAAGSLGEGGGADAGDAATVVGAQTEGIGQRQAATPEEHEATAVAAAAMEALLPAAPVELLTPRGQWQRQEPPPP
QQQQEHKQQPAVSADQAALQHLLQPATSLPLLPTPPGADVLSILQPHTYALLLLPPSLQPLLSPGGLPPPPPLSSAH
APSSERPPARRRLSPAGGADGRGTTAMQHPEEEQAAPAALPLPPQPMASAPPHWLAAAWANRTSPVGALITNSKAAA
AALPFPADPHSVIRYYGDSKTAATHRGAGGAVATAGWQQPRSWHAPRDPGDWRHRGLERERERERQERGQERGPER
EQQDRYRRDDRIQDVQERGRERERETERHSGRAGAGAAGDQQRGSQRREGPAEHSGGGSRQRSRSRDSYSRRRYDHT
SYSYSYSRSRSRSHERDVRDCSAGRPALPGPISHHRPGGGRRTRSRPPPPSPAVSPCHTRRKLTINPAATSPAATSP
AAPSPAAVSPAAASPAAASSAAAAAAASPAAASLPSPSGKRSRPSRSPSPTGRLQRRLALGRESREGRESREGRESR
QGRESREGSLEGRGGREGREGKVGRSAVGRSNSRSGGRSRSPAARRVVWRRRDSLQEFAQLLETARAAAVVPAVAEP
EAAALQPLPPPPAVMPLPRQWAHQTQSRESDGGAAAAVRVRGSGGGGGAGVARPLMRTVAERVTSRDANRSSSHCYS
PSCSRNCSSSGPLVPALERDDVDAEWAAGAAASWGLGWEAAEEAEAEEAEAEEAAAEEEEEEEEEAEVGKEHAAVEA
AEGRPWRQQQAAAAAINWPQRSAGRVRVSNMSGKTHASPPAAQLSPPAAQLSPPAAQLSPPAQPSPPPPPQPQSPAP
SMLLHLSPGGDLAPPATVTRPSAGPTAPTAIHQPPVLPPGELFTSVSRALRSSARQCTAATTVMYGGGGGAGGGPGG
GGGRDGGPAERLRRRSPAAGVAGGREQQQAAEQWRQPPASRSRSASASRSARDGGGRDGGGDDGGGGGGGGRLFDGG
GDFLAGRRREQTRQGQRYEGDEEWRSCSRSQQPQPQPQQQVPQPQYHNAADWWEPEQQWWQGRDGGRGRGRDWPREE
HEWRRRREEEDRRRHGGVADYSTRRQGSYGREASGGGREAAGGTAAGGGEAAEGWYGGGRRSGGWQQQRHPSDRRGGG
RAGRAGGGGGGGGGGGGGGEGAISPEAVRINLAIRNAGDDFRAVLQVVRERGEEFEPVNAASALHRIGSCGLGGGGG
GGSAALRALLADRHFLRLLRLIADRHLPLFTARNVANSLWALAKMGLGEWLHSPTAVAATAAAATATAATAAAGSSR
LGDQQHVQQQQRQEGQQAPLLVGLCARAAATVGDFTPQHVGNTLWALGQLGHHPGERLLSALCQRLLDPEVLAEALP
QNVSNTLLGLAHMLGGGSSSGSGSSSNTGSSTGSSSTPPPGGEAWLDALARGAVLPRIREFAPQALANTAWSLARFG
IPHRALLEAVFAESSRQLGSFNPQNVVNLVWAAARLELAVGAAPVAAAAVGGDASGSGGEGGRPSGPSSLQPAVMPH
ELVVACCEQAAAAVRTYSPQNLSNLLWATAKLGVRHDPLLRAAARRAAAQLAAQLSNAGSAAAAGGARRRREDDVAA
AAAAAEDREGGGAGGRGGGGGGGRGGGGGGGGGGEWSSQSVANALWAYATLGARPEGELLEQVCRHLSCPRQLRAYS
WQHLANSVWALGSLGHAELTALEAVEREVTDRLLLPPPAAADRLLVQPAGRRGGEERQPSGVNAGGADAGDGGGGGL
AAATGGAAAVTGGVAAGLAAGLDPVQVAALKPQHVGNMLWGYGSLEVAAPGLCGALLRVLAARLPDFSEQEISNSVW
GAARLAHYEPAVMDAVAEHIVSGTAAVAATAVAGSAGVIGGGCGGRLAALLPQHVANVAWAYGRLTHAHRPLFDGLV
ARTVQFCGSHSYSRGGGRGGGDGKAATAATAVAGHGSSTHSAGQGAAGGSGGSGGSSGGSSRAALTMQHLANVCCAV
GVFGWRYPDLIAAAEERLEQCAVAARLLPAAAPAPAREMALLVMEGGSGGDGRGGQQLLAAATAEKAAATAKEAETV
DGDRESGELQEALAVAGEAAGGQLAVAAAAAAAPGSSSSLLDAPGQPPPAQQPPQLQPPQLQPPQLQPHLAITAQQA
CNLAWGLSLVAGCSRRSWQLLLQLLEGAVEGQGGLEEMPAEALTQIYQAYLHVSLDHPDHNPAAATGAATDRLPRSP
SPPPLARGPAGLLQRGAAVWRVNSALNKQVSAFQQDVGRALERLGLPYRMEALTDDGLFSIDLAVEVEVEVEVDVEV
AEKEAGGACVSGASAVAAAGVAGGAPPTTADHDPPPQPRKKAAAAHDERAVAAAPGAEAANGGGSEACGAAIGEAEP
AADASAGAESCRQAAAALALPPALAPPACLPRAPGPDDGTSVSHHGGGHPNPSQYGGSGSGVASKAGGVASNAGGS
SSNVCSCSSSRPLRRLLRVAVEADGPAHFTANSRQPLAMTTYRRRCLEARGWVVVSVPHWRWYRTALGDSAAAAIAA
AAAAQQGRQGQGHVQGQVVVVVEGGRRQSAEQRRQEQGQGQAAMEMDAVGEAGWAGGSRANAGRVTAAGAGAGAGPG
AVGELSPAERDAVLVQLLREAGAGAVLRRRRGGAELPHRGVQG

>Cre13.g565200.t1.3|PACid:27573860
MAPRSALNLLLLFLVIFGGSATGDVGLKDVPVASGLDFVRGLADDNVGTLSFTADVHLREEDWAGIKIPIIIGRNVT
MRGQPDLWRVLDMGWLANKVQNRNGTTVLVQYIILFQFNKASTQAPGVDLYAPLPAGDWSILALETGGMITDYCLPA
SLASAISSAYQRPPFLPGVNQFEATVPQPAGPNCTNDTNAHPLKRCWAHVGRHIDFSGAAAYVDSVGKSVPNGYFSH
LVNVLVMCVHTISEDCVAQYGPLVCMAITLADAEGRNPSPPPPVAAASSPPPLAIGDSGAGGVPASVGGKPLSGSSS
SSGGTSAVAAAVGGAVGGAILLGLVAVALFVVRRRKREAAAAEADGGDWKEKPGELGDMEAGGGCLPCAGKRSGPRP
GSADVFGSERDSSGSVHGVTSIMPAGVNSMRKSEVGTSRKHGGCGATDGGGSHRDSKGDSGVSSRQAGGGLGCNGAD
DAVFTVGTKACEQDTAGNGDGEDSGPTPTASLTGGSAGVSGRALQRQVTANSAAAHTAPPPSGCQQQHPQQHQRQSS
RGEDGSSTPGLGTANGSTQLQAALSTGEAGSGVSGNLGGPNGGSPHSSGTVPTSLGDGGPHSRMSHASTSTDMSDVM
VTRCTPRRPDVQVGPEAADKVVLLPVIRGKGSFGKVYEGMYQGSKVAVKVLSGLPEGDQAETAAARAQAAAGGAAGP
AGPSAERLLFALGQEVEVLGRCRHPNVVRLLAACLQPPRYCLVMELMDTSLEYMVNRAPGRLLPLHTVLQISLDISR
ALEYLHPTVVHRDLKPGNVLINGANSGRPVAKLTDFGLSRLRHTVRSTAHPLAGTPAYIAPECYDVNNRTVTHQADM
YSFGVLLYTLLAGQEPWRNHSVPAIAYKVAALRERPPLDQLGDLRCPQPLRALIRQCFEHDPRRRPAAAEAAKELAV
LEAQATARHLASLTTGAPNSAAAGSGASAGGALDAHSAAAAGGLAGVGVGVPPSGFLGPMQVVADAASLHSNGPHED
TAAAAGASAGAAAASAAAAGAGLDGAPDRDGAGALGAALVVEEHLPLPPSVDASVAAALAAFVGSGFGPAAGMAGIA
PDVRAAGRAVTLEDSTLSSVELMHAKWGDPMCPSGGPTTRNTTASGAAAGHGAAGSALASQASTAANAAVWATSTSS
NGNAAGSFRDGLAFGPSLGLGPDLWMGMGLGLGPEGPVDDGVSQGLLGATEESDMSQSDPMGQLGVVVLGAPPVRGV
HGRRAGGGGGGGSGSGGASPNALAAGLVSFPVLPVGVGVLPGSAAVGGGSGVGGSGGGGGSGGAGGWVSSGGGGGGR
PRGPARSSSSTSLELRKAAVVAAAVAAVAGDGGGGGGRSIGGPSMGTGGGSLEPVSEVDSEYMSL

>g14495.t1|PACid:27573895

MEPAGPQQHEHQLLQGEAVTSSATDGGSESRRQHRQEEHRQQQEEEEQQQQQHVEGGAAAASSSSPSPFSTAASDA
GPSAPPTAAAASEQQLQPQAQDLHNGIEAEAGGGRGGGGRGGGGGGGGGGGGGEALVDPGLEGAVSPPAAADADAAAT
GAAASPQQLPPSPHPGSSSRPALEAHIPPASSAAGAAAGGGGGGGGGGAGSADASSSGGGGGDSGGGGGGGVDTPL
SRSPSLGHSLGHVSSGSSPVGLTSADGGGGGSSGGGGGSSGGGGGGSSGGGGTAVTTPSISLAQMPGSVGGSSTGPS
AATAWAWGASPAAAPQQPHAAAATTTPPTAAAAPPPQLQQQLLLQPQPQLQPQPPAVQHPQQQEQLQPQPPQLQPP
PQLTAVAQGALVGQIHGEMRLTLEGNLMATLMPPSPAPSGPASPPSPSPSAPLPPPPPSPSPSPPPLPQQQQAPMTL
GAAPAAVAPPPPPTAQPPAAGSSTAATAPALGSAAAPLPAATAAAVAPSAGLAETPRLLRPPPPPPPQSDYLGSLQL
PQPAPIPTATQQQQQQQQPQAVPATAASVATAPQPTAAPSATTTAVAPTAAAPAPVAGVVAAPVAAVALAPAAGTAA
PAALLAPAPQLLLVPATAVGAASCGAIIATAATPPPAVASAPPLTAVTASLPVMAPTAAAATAAGFPLLTAAGGGGI
GGGLGGGLGGGIGGGTGTGTGTGTGGLLAANPSLCPLASLQPPQQQQQQQQQQQQQQQQPPQQLPLATQQQPAATTV
ALSTPYGTLLTPALTPAAAAALIGSSSSHSPAGAAACGASAGGAAAGDATAGGATAAGAAGGTGGGLVTLPASALAA
VAHGSSLLQQIQSQLQPQPPLAAGSSIQGGAAAAATPAATAGASSSAAGLTTGLALTPLITPDTGAAATAAAGSAAA
PEPLVLQPMPGLLAPAATAYLQQQQQLLPTIIVPPTAATLALQQAQQQQAAAVAAAVRQQQQQARQQQQAQQAAAAA
AAALAPWKQLLAAQQQQLQQQQEQLQRQQQLLQDQQAQHQQAMQEQVQQAQAQQHQAQVQAQAQQAQAQQALQQAQQ
QQQVPATQQQAQPQQQQQQQQQQQQQQPQQQQQVLRGLQVADLLASKSVTAAAGGAAGGAATAAAGGGGAGLPALLL
PPGMLSTTAGGSPPPASMLGGLLQPVQSDPTAAATAAAPAATAAAANSSTPSAAHTLAPVARRFTGPATSLATVPFR
GGASPPPLAGLATPASATAAAAGGDGASAAQPAATSASAASPTTTPTTATAPAPPPPPAARSSTPTPPPAQRPPPGL
HTHFMPPPQQLPLPPGLLLPKSASSRSSTSSFMSGATAGGGGGSAAALPAGGSAPASPMLLQPTPAVPATAALLAAA
AAPATAPPGGGSGGGSGSSSFRPAVVTGVLRAKPAPSNRAGSAAGVGGGGGGGGGGGGLPATAAGLAATAIPSPPAS
LSSVGMSYGGGGCGAGSGGGGGGAVGGLSTAPPAAGAVITAAGAAPGTATVLPPALLPGGGGGSSSSAGGGGGGSVAV
LGAGGGSSSASTSPSLSALLNAHMQQQQQQQQQQQQHQQQQQQQLQQRAQLQQQQQQQQQQLQQLISNYNQTASAL
SALTAVAEAAKAASAAAASAPAPAPTPAPATAATALALAPATAAAGPSLSTNTSGTAFSPFTAAGPTAASVPLVAIP
AAATAAPAATAATATAAATAAAYAPLAAAAQTIASQLLASGAAASTPAAAATAAGAGSSSTPSGITSAVLQQLQLQQ
AVAALSASSGGLVPASALLQLQQLQQQVLRGAPPPPPPFPATAAAGDAPATAAAPPLPFAGGSGGIAGGGGMAGGGM
SLFTSPGGSGTTAASSATAGGGRHGGSGGGGMHASGGSAAAGTAAAAGGAPAPGHKVKVLVSFGGCFSACRATGHWE
YGGGETRLVAVEEAAGFERFRDSVFAACKGRVRSPDEASLRYELPSCPGTLVDVRSDGDVGMMWEELADFAESSGKP
SYKLHVYVCHHLLLPTSDEGSGVSEQHQQHQHQQHGSGAAVASGGGAGGGGGGGGSSAGGAGGGGGGGASGHGGSGG
GTPRHGSPWISGGGRGSGGRMSGGGGGGGGGGGGSGGAGLAAAAHGPLHHQQLRGSGAAAAAAAGGRGSGSGGAAVLP
QTPAAARAAAAAAAASAAAAGGAAAPMRAAAMGAGYGSSTAGATAATASGGVGSRGGGGGAGGGAATAGRGPSPTGSS
STWLSGSSTITLDLTAPSSAPSLPRSVALEQLSTRVEVVRPSDLTIIRFLGSGGGGDVFQAVRSERK

>Cre13.g575900.t1.2|PACid:27573940
MYGRDHRGPPPPPPRGGGGPGGDWGGDRGYRGEDRRRPRSPPPHMHERDRYNRGRSPPPSGKRMRRDGPYDGRDYDA
PYNGRGSPDYDRRPPSPRHDDREMFERRASVGRDRPEEEKRPMTFKEFMLHKVPDDAAPDVAHRMFQEYLVEYYGSA
IKAEFEQNKDSDMFKYQFDPRNFAKVVASRAEEAQDSAKHVAADLASGTLDPAHEAFAQGMYDLAPKIEKPADKPAA
APKEGEKAAPPPAEDAGPKFAPNTCWSAERVAHDLELSRRLIRKLDAEKSIAENPLLPKLATAPSLGDAGKDGGEDG
DKPEAGAKPEGGADGEGGAAEAGEGAAAAAAPEAAAPEAEPPIVVDDSNYQESVGKLDLQLHWLWKVHGVDYYAGIE
LNEQDWPYRLNCCRLIRGPKPEEGEGEDEATAKADREKLGRQVDETWNRRISLGDPIEAKCMKARIEDELDKWIESQ
VTMVADNKWGSKLSNKLFVGKKYVIKHIKTKHQDKLAVERERILDQLYFDNFRQWKEDEERRRVEEEQHAGEGAPEG
EGAVVESESGYAGRGRGRGGMGMRGRGRMGRGMMGRGPMMGMEGPLMMPMPMAPMFMPPGPMGPFMPILPGPMPLMP
PGPMPLRGRGPMRGRGRGMGPGRGMGGPPKLDPRGMREYFDLDHPANNRAVLDYGDL

>Cre13.g574000.t1.3|PACid:27573957
MDGQLDGGSGAYAAAVASAHGCDAYPGGSYPGAHNGGVAVVAAAAASAAPGGAAALVPAEREVREAQRRLRRAETLL
SKFPRYRANDYAVGESDLRRSLILNTTPRQFAAEAAFSWALTVVIGLLNGVAGFLFNQGIYMLNKWKFETTLKFITS
GGGFGIPYCVYMAFTVLYSATAAILGSYVSPQAAGSGIPEIRCYLNGIHVRGLLTIRTFFAKAFGVLFSIAAGLVVG
KEGPFTHVGAIIGGGVAGLGSTTLTRATGGHWRAVLRTRFGRYFRSAVSHRDHVAAGAAAGVSSAFGAPIGGILFSV
EQGSSFYSASMLSHAFLAAGIAIFVSLLLSAALWHGPDLYRTAFTAPQQFALLNEVPTKGRFWYYAWELPIFALMGV
AGGLAGAAMVAVNNRITALRKRFVPPARPARRVAEVVLLAALTATAWLLPSYLSPCLPVPPRPYLSQLGLVASSGPG
GPNATSGTDAGDGGGLVDEHYVQEQAAAGGPAGGAEALARTPTVLYAGLYPQLWCPAGSYSSYGMLFFLPQMTAISL
LFQFEFPDGTNFLFAPGALAALSSLAWLFMTATFGVGAVTGLFVPSLLVGGAGGRLVGRAVRAIAARIAPLAASVSL
GGAAGAAAATAPAVSICLSSYAVVGAGAVLGGVSRMLLCNTVLVMETAGATALTVPLIVTTFVAKMVADALQPGIFD
VAIARAGYPYLEAEPADPVDIKLQQALAAGDVMSSQLVTLPAVVRIGDLVESLRRWPHYSTFPLTDVTAPAAAPAGA
QQAASPAAGATATPQRPPSATPSANPSRKPTPTSGIASDDDAAAVARSSAATAGRSSASPLQAPGQLQHPQEHPPAH
NFFMPAAAALPRVPLSAAVGSLGAAASPSARGASPPPAAKASRLPDGAHGAEGSAAGAAASAQLERSALLVEHGDSS
GLPQGTDGGGGGPAEGTSAAGVPGPVAEGADNGGAVRGTDAQAQAQHPQQEPQVLLGHLTLLSAFAQPPPSEVSAAR
AGPQPPITAGPNQRPPLPPARPHAQPADSTGTQHPLALQTSAQQPAAPPSSRRHLAGPPEGPADAAATVTSQRPLSP

PPAANGSATALPHAAAAAAAVVGTTPPATPLPRASAAVPQPPPPAAAAPPSIVGVISRSVLLKLLEMRADLAAASST
APRLATASTASSTCSDPAACQPAPTRGWWVWQRLLQPWRGYAPAAVAPAAGGAAAMPGAAAGGKGAVADSNKEGQRR
QEPTEWLGGRRSAMSRQRAVRLLDAVDNYPAKAPADRPGEEKLLSRLSPSDLEGCLDLRHYMQRVPYVVPATASLAR
TYRLFRGLGLHHIFVTAPNIPIIGLITRSDLAPANAYRVAYRLAEREQQQLQSLLAAQQQAQPGRQRRRGYEGEEDV
GDGDELLEGDQEGEDSSEDEEDAAAGMGMGRQGHGAVSTDGKGGGDGPEGDGAGDAAAKPADGTDGGQSARRTSKRL
RAVEGGRPLYPSATAGARDSAPLLGASGAGGRTRVGRSVGNAASSLAGARLASSSLPQFRAANATRPPRDSNELGAA
LGGGGGFDGGISAAASVASTSMGLRRRHAGSIRSRVPSGQRLGGVAGDAPGGLPAPLQSGQQLQQYPSGALQAGSAG
LRGEGAYDEISDGSASLLPAVHTSAAGAGGMMPDGVYDTPHANLGGGGGGAAWRWGGTAVLPYSSVVYGGGGVVGAG
AAGAAGGGSGRGGGASSGTVPRRATGLMATLAAVVNPHAHAQPPLQPPSRPTSPPPTAAASMVGPQFTALSAAASAV
MLTPSAQQSSAAAVSRVVELSGLRSVGLGAASRGVTATVTGDVELQAPAPLQQQPALLLSSASGVASGPGAMAAVGV
ALADQALWPQGTLAPSVQLQGAPYAGSAPDPHAQPQAQLQLLRPMPTRPLPPHRLSAPGTAAPAPRPGPSVAAMHTA
GQGHSPVGALEQQQGSQPHQERRWPRRTASLGTPMSSLTAAAVLSTAATNENTADEPQPTGGPQAVFMQALHNVAAA
FALPSGGQARHRMQPDLEAAAGDGGDDDVGGRRVGSGVLEHEYAQELVDAGAGVGPGHTWERGRGVSDHGGLTAQGL
EEPAAAAAVADRDVLPTTPTAAAAAAAAAAAMMTPRHVALAAPLAEELMFATFAPPLRPLSPPPPPPSATPDTSVLP
ARPERPISPPLPAPATVAHTGETVLPDAPEGTTPEAGVQPL

>Cre13.g577300.t1.2|PACid:27573998
MPFAQGRAAFSLGSADAETTSSAGRSLLQAALPPSPKPPGLPKPPPKPPGLPKPPPKPPSPPPSPPKPPSPFPPSPP
PSPPPPLPPFPPLAPGSPDPAAYFAALPFNAKVISLSMASIPRTRKYVQGWRRINSAWTLMAGTIDLDTGVTTGYVP
GTAIGCSGPIMLEDGSIVYVGGEFGNSGSKPANVDGRFSTTRYDPATGKYTRIGSLSVPRWYPTALRLNDGKVLVVG
GTADADKGPAYSYSELWDSNNPTAPTVPVAHPTGFAANMGLNYYPFMALLPNREILWWGNRGGSITSGDFPFDKIMD
LPPLPSSYGPWHTMYPYTATVALHALRPNADGVYNTFSFTIFGGQNPVKVGPNTPACDKSARLDFTYCGASMTDICI
VPGVNGDWQIEPMPGRRLLADAIVLPNERIFVHGGATTGYAGVTATGLKAANGAPVSLVYDPSKAEGARYQTTAPVL
IMRSYHSTACLDVTGQIFSSGCDECALPVPAGYEGLIDPNPTGDFDYRLTMGTPAEIKDVDRPVITSAPDIIHRGGT
FEVTYTYTGSGIKGVTLTTPCSSTHAIDMNQRVVVLPYTVNTATNTITVTAPPAAQHGVAARGEYVLWLLGDEVGQF
GKTYSQGHWVTLKEA

>Cre13.g566600.t1.3|PACid:27574078
MTMTQVLLLPALLLAGAAAALLLTVALLAGCAAAEDAIDGEATVVKAASAGTDDGDGTRNVTRRRRGATGRSAGNLG
LQGVPGVKPPHHPPPPTAAGVRSAPHQHPQQASSATSLQPVASRQQQRQQQSAAERQEQAATQIEMPPPPPPTRSVRS
LQMPPPAPLPPPPPPASFPSGSESFYSRRQPSSHQSSFEDAAEPYESPFNWSAAAASSSAASLPPTSLPGSVRTSLS
GSGGGGGGGGAGGGTSGSTMGSGGGGGGGRVSHRRAERAAQYAAAATTGRDSGAAIGAAAVTAAAAATAALAAAAAS
GTSSGSSHSADSSSARAQRRERRHAMLATAPPPPDAADPADLTAPPQPAGVRYVTRELLPAATAATASAGRGFRSPP
PPQLRCESAAAAIGANGKAGERHGDGGSGLLASRMDSDLDSGQQGLLHSLSRPPHSPSGWRAPLQPPQPPPLLQPPL
PVVAAVEAALLAPATPEQYHYPAAAAAAAAESRRQRALPPPAVAWSEPGVLQLPSGLPAVQSSAVASAAAGPSTGGG
AGGECYSLFWLDAEGSSSRVMQLAEEPSSSSVLAATYYSHHHGSGLADHEEQFDTTASFAGQVATLGQGAEAPAAA
AAAATAAAATEVAAAVAAGTGDSGIASSAGGEASQEQEHEHEQERQRSPFEGYPPQQPSPRLPSTSTSPQQPLLLPQ
PQPWQRRQAAKAIEAVEAATGWSPATVAAAPPPPQSRVLSKHPSFLAYLRHVGLVAAPDQPPPDQSQQLLPPDRAAP
RQDQTPPPPRLQPPQRQSVRQRRSTGSAAGPSRGGSSLEVIPSSGSSSGGGGVDEAAAMAEQQAKHVRGRGSGRHQS
SSEEGEGVESARSAVLATEADSEVAASAATGGSGDGGLLQPLRAPVPAVTAPSMPLPAQTGNSMLGGGIIRFVHLVT
GAEGVGGVAALLPLSDGSQATGSAGGGGGSSIRGAAGGRSGQTSSCYSHSHVSSGAGGAGTSVVGIGASSSHSCSGT
EAGVEEGSLLLLPGRGSSMSSLGGGGGASGGPMGSGAASHLEPDTSFGSSITEGGGAAAAAVARAAAALLQPSQQQP
PPLQQQQQQQQQHLYGSSCSSMSPFATSHATGRGGEAAVGSSSGGATAGAAFTPSRVAGAAVAAPPLLLPSLQQSS
VRLADLVPPSLLQPPAARPASVAEAAEAAATVAAPGRLQSARPTPLGLGALPSVSALAAAAAAAPCAARPPLPPGGV
PQPPPTSSVLYKSRCSTKTLAVKLEGAQAHRAGTASAAASAAAAAVSGAGGGGCSAAAAGVDHLLSEPAAAAAVAAA
ASRHVASTLLRHVPPGRRLVSVSALAVRGCVQLLLRLQLGPEPPLQPPPQQVQAGLAVAEVGPGEEEQEVLLLPLNG
LGGVQGLEQGMEEMAVAGGSDSSGEDVLAALLRELGAAAATAAAAVAAAAIGADGVAAIGAAAGNAGSAVRAGPATG
DDWTTVPLAAAAAAAAAGPVTPERPTGGDGGSHGWRRRPRPAGAGSAAASMSGPSAAGPPPPSPPQSPPSPSAAGGR
RHRHHAQQQAAPTHTGGMETVQEEGEEEAQAEEIGGGGAGSVPDPAPPPAAAAAVPEGSLLRVHICLQGGGHTRTLA
TQQPLLLPGRSSGGGGVGGGGSGGGGSGGGVGGGRAVAPRAAATAGGGSSPPRHGARSPRAPRTPTQHQEPPSPAAA
AAAAATANAVDATAATAGGSDAGSSVGHSSDSAAAVAAAAAVAAAWVRVEPPALARAASQAPRPSPAAATAAAAGLE
ASAASAAESFAAASAAAAAAAAAAEPEVMEVELLVAQGCEPTTPSAAAAAARYRVVAAVGPEVVADETVQLPPPPPQ
QHPHQHPHLYHWAAAVAEGDAADATAAPSVWLLRQQQPPPSQRHSYQYSEEDESFTADIEGVGDRPPPPPLPPPPQL
QGGMTLLVLPPAAAEEMNRHVRGVLYGGDDGGNCAGGGSGGGGENGGGAAAASSTSPPDKVPAAGSAAGTADKPPAL
AAAAVAAEVADEAEAARCERWRRHLQPLLADFGYVLETAAEVAADVAAAAELAAAHAATAASHGPAAATAAAATLRA
SAATGAATAAAAAWLALAGDVAAHCAAAGMPACVELLRTLTAELLAAEAAGLAAAEAARAEDGAAAAAAAVVAEAEA
EAEAAPTARVIAQQPVTTAMAPGVPEAEVEQGTNAATADAAGPGTAEPDAASHVANAAAVGAAEAQAVGETEAVEAA

APAAPAARESVEDSHRHRRNMQQRHFAAHATAAATAATAAVSGQGGTIRAVPAALTAALLTAPAPAAAEAGVTNFSQ
DAVAWEECCWDVRAAMSEPLQPQLTSLHDCAVAADATADAAGAGAAAVAAAPDVIQPAYVHALLPAFAAEAGGKQAG
GEHGGGGHGGGGCSGSGDSDVMECSPFSTSNVVDDSSGSGGGARDGGSGDGGSGDSAVGATGRQRRRRRRSSSSLA
SPPPAHHHELQAQPLSSSLNSRHGGGVRLGSSSSVLQGLSYSRRTCTLLPPPAAAPQSLSRTSPAAAQLMSPQSYGE
AGRYFVRLPPPPFPAAAAAAAGSRGGGGGGSLVPSSLLPAGYDSASGVFSSDAESESATAYHTAGSAAGVATADAWP
PAVPDTAATATAAIADAEPTALAFHPVCHLAAAGSSSSRRGAGGGGGGGGRRSAASGQGVGGGGSSGGGCQQRLSLV
PPGLEEVECQLLVSKLHPAEMPSRGCSSQGGGGTPANDDVAMLVLQRLQPEAAGPLAGFDVLHCCLLAKELQLLLPP
PQEEEPKAQQQDELQPQPSLLQPQSQTQWGSPRLAAQPADGSAPPQHRAAATGAAPPTGAVPPTAATAATAAAGTGI
AWGGFALLPAGGSSTYTASGAAAEAATAGGGCAASSACGTDEAAGRWPNSYSSGALATAQASAEPGGRYSGGGSGTR
VASEATDGGGGSAAASQDAAVSATAVLQLRQLGYCQVRCDLLLRQLLLLPPAAAVVEAAAACVPADACPSGSQPQHE
HERACLSVQGASGGAVGGAATASGNDAIRTGPVGGSGGSSGAGDAGVYALGGFSEAGDSSSYLSAGSSAKVPEPGGS
GAKVPAEPAGGSSSSGGSCSGGGGADGSDGVGAVAAPAEGGHAATATATALDPGAAAAAVAAAGATAMAAAVAATAA
AGDDGVSAAATALGFVPVSCILLVREEVEVETEVEMTALGGGSGLRLQDPTATAVVPGSSSTSSIGTSSGETAAAAA
TAAAGLWAPRLTPIHQLPTPHCERGSDGGGGGGVSAAAADPLPQCPPLLLPLLPATASTRTQFHSCRLPSRGGGSSS
GSGSVNGSGSGSSSTNCMSTSGSSTGATGAAAAPPPSVAAAATAAAATAAAAAAAADLLLPRYREVRCGLLMRVISR
RQLQPSSSAGSSSRSSGGDGGGGGGGSSGGAATAGGGGGVSRLESP

>Cre02.g117813.t1.2|PACid:27574200
MVRLSLALLVLGLLALAGATRGAPGQGHGRTLLQLDTSASGNSAGSTQPGESDSSTSPTTATIANATTTESLFEALL
SPSVTTIRLLGELVVNPDRARAVVMTSLGAQTGALNLTRDVTIVGVPINTSIATVDFNFTARLVHVAMGTTLNFLRI
RVRGLFERLDTAPVLFTNTSSGSVRFTNTILQRTACLPPTQQLVELINANRVGGTNASGTGGGGDGVGSTSLVASGA
APTIIAGDGTGTSNGSGDGADSGNGYNAFDIAPAYVYDQPFCVSTLTDVGLDPLAVDDCFTGPLVLQDITIIVSSST
GAKHDPSDGTSPDPGLPGAEGGAGGGVGGGNGSLLPSGSGLSLTLAFNNTMILCDVFVGPTCAQQTGEALVACIREL
LDRRNARMPSPPPVSSPPPDSRSPTPSRPSPLPPVGSPRLSSPSGVPLVPVLVPALVGSFALAAVVLGGLVLWRRRQ
RRSGGGGGYVGAKSGLDSQDSDAQQVLALQVNDGGGGGGGGGGGGEGKGGGGAAAAATITAKGAAAAGAGTGKGKPP
ASSAGFGSDTNTLGSGTTSGLTSNLTYGSSTMTMTGLTASSPGASPPDSANTDPTKSIILHNLLGAGSFGRVYYGTW
QGVGVAVKIIPHSEQANAKVQQEVALCIKFNHPNVVRSLHCVTFEASEQHQQLAHMQQDSLRSASTLGGAAAGAGGG
GVGAAGAGVGAGAAHTAHGGNVLSMETWIVLEYCNAGSLSSRLNMKAAVLGTTGLGLLSGGSLALAHDSALSGTGGT
GTGGANALISPGDSVALSTALQREQQKDAALAHMRGVLSVARDIAAGMAYLHSLNVCHGDLKCENVLLCYKAGMDRA
STGGMGASGSVRGLAGAAFTSGPQASAAAAALAGPSSASLLGGGQRDRVPFVAKVADFGLSKTFSETSTHMSTATVG
TVTHMCPVLLLTGQMRPSCDVFSFGIMLWQMATGGVPFAGMRYAEIVYKVAVAGLRPEFPAHTPPRFVALAEACWAA
DPTARPSFEALVGRLGELLEVADELQAEHEAALLAARDSVARPEDGALAPVTTSEADARVASQWAAAAAAALLE

>g1786.t1|PACid:27574237
MARSNLRRPSPAKVASSAFLLALAAMKTATLLVVLAVGLGQVLADNSELFSSGRSLLQAVSGTTVFPYHSCNKKQPA
GSPCYFGSPTTRVINSTVSEFCFTATCPGCRDASNNCCASLSKNIKKIIFNAKLSCRAPLKSIFQPVSWNGKPISSS
LYIDEIPNVATMIRWTSIDIPASQLDGDRLCIRAPAPCNTVDSLFIMESQGFPQVALMDSTDGGNGCCPSRYPDMPP
PSPPPPPPPSPPPPSPPPPSPKPPSPPPPSPPPPSPPPPSPKPPSPKPPSPPPPSPPPPCTTCIDVTIKKTADLAPG
SIFQWDSEMCTDVAPQLVDDLQTRIEDANARVLSAFGIKSCAGEKLTICGTWFSKEDAALVNGPDGEYTRDMLLDWI
NIIAPDCPDYARGYSIRAEAHDQGSTDSTTGCMYGTAQRDCAPPSPPPSPPPPPPPPPPPPPPPAPPPPPPPPPPPP
PPPSPPPPPPPPPPPPPPPPPFPPPPPPPPPPPPSPPPPCTTMCVTWLITNQAVDQAACDLLAQSLQDMVLASYGVL
NGGFTCNGVDTGKREVTACADFATEAGGQAYGSMLDDTGFDMIKVAVGFGNVYVNTPAGPVCDSSSITATLFTKLTP
GAAPCGVWRTADACAPPSPNGFPYCKCDPRLANRTPYSLSFKSKSSRTVNGFPANVYCFSIKVDAGVCGNSRCCNMD
LDKVEWLTDTTNCRQAAVGYTVSIRPTDVKMPVWTRQIDNTVVPATSFDVYKTNQLGLTLGNADGAEICLILKQKSS
CPTLDTFCSQGDIGCQYAVFDKTQNCCAEDWSLNAGDGSFSRRRFRLAL

>g1551.t1|PACid:27574242
MVGYGDAGQRLWEVHSLTPSLTAAVAMVPQLAPSGPSALMMAADVEGLLKLRAFTDGGQRLEQEPGWEADLAAVIGG
GRVLAFGSPLAPALLEGEAPAALVPLLWRQQPAAVDSSTVGVMAGLVRLTLEPQQQHQQSDLLEMRAAGGLRLHLVL
ASSWHVGWIPVDSGSADAAAAAAAPKCALLPQQEDAAGAKGAPAAAACVLAVSGGVRGSQPTAVGGGGGGLMVVADL
AGGSVLLERRYPALPGDVIVYKAGGSEWVFEAPADGDSAAAGIPEVPQPALLPLGVEAIAAAAGGCSCGQAVAAVTE
LVALAPTGPLLRRVRLCRRATGGSGDAEAAGAAGAAEAAVVMAEQVAVAGGEVDVADADLLVAAAASPSAMAAAEAS
SIAGAGAASLEAAWDQDQDRDLQQPELEVVLGVAAADASRVSRSLLQTDPPSPPSPLPPSPAPPSPAPDLPPSPPAP
PPTPPSPPAPPPRPPRPPAPPPRPPAPPSPPPRPPPPPAPPSPPWPSPPSPPPAPPPSPYGCPVMMPGRAPSSRTAA
FYNASKLLNAEADSQQLAFLEAVPKIVPAPITVTLRCNISNSAPINLTAMACEPQGRKRVGGLLPGDLRPYAVNGTE
LLCAAAVPGATVSRVVRTPYRVNVTDANGTVTTVTMFNETVVTTLAPSSAASSSSAPPSSSSSSSSSSSSSSSSSSS
SSSSSSSSSAPPAASTGAAALPEGSPILLSCNATMDSNGTIAMVSQGAEPLVTFDTSSLARCRPVELAPSRPLAATS

FRCAATTTPSPVDVPASPSGFDTHLSCDRFLSDATVGRAGGAYDLQRRLTIALGDPQPLQALRRVLCPTEPCGAPAS
TALDVLSGYSRRAAIDVDLRVYSPPPPAPPNRRVSPPPPPPPAPNSIDWVLLFNLNNTYCLDLTAHAVPRTAFYPPG
GAGVPSPPPPAPMAAPPRSLGELFGSAFGGFSVMPAAVAAVCTPYGYDAYRMSHLSASGAGLRGRLGVDVLQDPLLL
GDGMPVSNLVSLDLSNNSISGFLPNGSVLTPYHVNGTGPGPFTGIDFPCLFWNLSRNQLAGGLPPRFYLHSALVVDL
SYNGLSGPLPDNWTLLETAVVLDLSGNNLNGTLPASWATNLGTPSGLTNRPDYYGLRRRLGAGSSSRGRSSSSSRAD
DNRWEQLQEHAHWLGLTADGLERHEDGSSSSRSQGSSNSRRLQQIGTSDGSASPPPPSPPPPGKPARYRGMHSLLLG
GNQLSGTLPEAWLNLAVAELDLSYNQLTGKVPTDWSGVGSMVRLDLRENAGLYGTIRGISWPDLLRVAKTAGTQVVL
EPEQVVLAGFPRAALWATLMAAVLDGRLSLVNVRRPEVTQEDLPEICDIFRSYRAYQQQGEAAPARDLHGAIDSLSL
GRGLWTRSANTSDPHFPPPGTPSAPVAPLYGLMAEFAAWVAGPAPVPGELEWLPRPEEWCYDRNVAVLLRPVLGLWL
PVMVVVVAMLAPVPLYYYWQRRDKEFEEHGYDMDLDDDDGDHHEGGEGPAASGPSFAARLLERLQSVARKDPSRRGI
LAGRDKDEGGADTGAEQAEKGGEKSVRAGGLGVGKSMKKRGGGVRFAGGGDDAEEGAGGSERPSAQPAAADGGETAA
ASTAAPTAEPSKAGADGWAVGLSRKSMTAGASAGGAAGAGGTLPPLQPLTTLPAPSPEQMLPGRSRASLTADGVLGW
GRGSAFASRAASVAADSGPGTPKAGLSSHGPSAFFSPTPVAGTGAGAGPVAEAAEDDAAAAARASVTGGAPAGGGGG
GGGRGGLRALLARTGGSRSGARPAGAPATPPAEAAAAAVVQPPPPAATPSGSGGRSSLMAPMPAAAPGVRPDVTTPI
ASLKTGGLDHDDVTPPTAATGRTASVIHTGPRLPVPELGSEPSSPTLDQSYGKGSGTASAAMPTSPSKKYGLHALKG
GPSGKSVAAQSGATSRADSLAGGASTSSVPSAAAAAAAAAAVGGGRRALLASSTNGSSNSAAGPAAGGGSSLGRPPS
QSQVLPMPVPAPGGTGPAMLRPMSRDLGLVASLQGSSFSGAGSFTTGGRQMTPPGRRGRRMSQQFQHNELAGEGSAG
SGLSSTAAAAVARSRPSFTGQPAGPLAGLLGGASGAARHMSNMGGLPPELQPTPGHNGIQPHLRATTPSPEPPAVAE
PPAPSAPSFAGSGGSGALPPHRRMSDAEMMEGKDGPKPVPGIHSGRSRPKKGLSNAAAAKEAAAAAVAAEAAEAGDD
VVPSAAGMPAVSAADVAAAAMADRPSGGSLAAGRPGSGGGRGSLAAGRRSDSAASASGEELHLPPPPPPPPHPPPVS
INPALGRVMSVGKDHAPPSPEASGKLAHKPPKAPRGAGAHAAPEAPASTPTAADAAVAAAATITASGAAAAAEPTPT
TPKAPVAPSAPVPAAVAAAAAANAAAAAAAGASARSSESGGFGRGSDAGTPKSDDERFRAASLARRSQSVRSTRSTR
DGGALVPGSALSVSHRGAWDRSRRGSGDSSGDGAATAPPEPQLITVGPPPAPAPKANKEKVQRHPLEHVCFYYWLAW
DFLSPRIGALIYLFSIGSDAYFIGAYVSPNFNAAPPVVVGLVLVIFFSLSWVLRLHVAWGFSTKVSWIAGLLTFPLG
VPYELGWDVATVICAFIIGSPELFNKSIRGFALLCCGGRPGCFSLAPNLRVFRMKLLLEPFANALLQGIVQSTFYMV
SYRSGFVIPPHVYLISAVASATSLIKAAAALHHYAYSGVPLVDVLRQRGEHFNAELLRLDALEDVQDMEALKKQDAV
SLVHRVMGLRAASIATRVRIAPEDGISRRGTSVMPSAATSAAASPAASRPVSRPASRPASAKGAAPAGAEKKAGDEA
AGAGAGGSGEIEVDGRSKSAAVLFSAMFARKASRTNLQASAGGEAGSEAGAAGAAGAAGGEAGASAAASRVGSA
RRIAGNALAAAAAALLGGAQEGAEAVSRAASRANSARSVGRAKSASGLPGGRLRFALSAKEAAEKAAANKELAAAGK
EAAAMQAMDPMAPRREGPKGRYGDMVPKPSAPNAFFGSSDDRPEYHQTVNPLTLLNAAGAKSFANRWKGKAADARSA
ADAAATAATLAATGGAFTTLGGAAATQSSGLEALLRPLSTRPGGAASLAPMSPAGYGAMGAYGTAGMPAAAAAYVAA
PYSPAAAQYTPAQYAQYAQQYAAAQQQQQQYTPEQYAQYAQQYAAMYGTAGAGSFAHGSASMGASAGAVAASPTAAA
APAAQYDQAAYAQQYAAYYAALQQQQLQQYGGVGTAMSPGMTPTGAGAIAGLGSMSPAANAPAAGAGPSGTQ

>g2795.t1|PACid:27574249
MLLAFRGRMRPTPNACFTHKKQSTAAVAPAGVTALPSAATSKSAPGPPARCSASALPPSYGGGLAVGASAPPSPPPV
LLQSTAAVAPAAVTALPSTATSKSAPGPPARCSASAPPPSPGGGLAVGSSAPPSPPPVLLQSAAAVAPAAVTALPSA
ATSKSAPGPPARCSASAPPPSYGVHCRGGAGRCHRPAQCVLRPPL

>Cre02.g145650.t1.3|PACid:27574319
MFLQSVSDGGNSASDGPLEVPLRVDLATVSPGWGDLGLLRAATGICPADGSCAMCDFVDENPCQVADAYGAEAGLCQ
LPVFLGGSTPPEKLCGMGATAEVFLSGDSSSGAGPAAAVATLTAFRTRDSQLVLTLTTRCGWVLVPYASQHDSGSNG
SSSSNTGTRPGASGSSSRRMLGGAAAAAGSQGRQLLLAAADAEQDSAAEQAPAAPAAVVLEGTAQYGGQELYFGAPA
RGYGGSNDSGDGGAADVEERYACCSAALPLRGLPSSCGALELTLSLRLGLKRLVLSGPGASVSGGSGSGEDGGGMGG
EDWVDCVEAASEDGTPEGLRWAEVQLDMRPPQLGRRVQQAVAEATAEQSRAAADARAQVFSNGAAASSSYTATLDPR
TPASAAWLESLSRQFPLVSTSTTHQSLAFLVGALRSNSAAAAAAAASGSGSGSGGGSGSGIVPLLPAGLTLESCGAR
SISLVASAVVAALAGLGVGQQHIADVVCIQPPYGAVASFELPPCARDAAVLFNVSLEMPLAGSSSSGGSSSIAGSST
GLVGTTVPAGAAPADPQALADALTAAAFAAPWTCLSAEGQMRMFSTVTLSVQLPRGPGDGAVQLDPNVLLPGGPALQ
PGISSPAPATAAAPRASVPSGSASDGGSSSLSVTEVTAVAVAVTLAIVVAVMAFFAIRTVRRAKAAARAAQDAAVAA
AAAAGTSSGGGAGNGGGAAAAPPDHKLSWIEVPEHSLPEDVTSMAAAAAMAPGTGAYPLSVSRVGGGSVSAGGAAPQ
QGGPSSLLAPATRPHHSSFSAGGSGAPGRSAHAPAGASASRQQPQDVDLREVHVHSCAGVAPESTGTGGEDDAPLIS
VRSAPPPLLQRPAALAPALAASPAAAAARRLPPPLLGSAFSSPPPDTAKRAMIAPEGVRLWTEEEEEDRGAGYLACG
SGGLPAVDGRAAPLQTPPAPAPAAAAATAAAASWPASGPPCPEASGQFAVAVASAVAACAVLAADAAPVADAAAEAA
AEAAAASAFPAMATATATAEPEAKGGAASRQACLAVSSTFDSDDVAAPEHAVDAAAATPAAAANPLGWIVPVTAAAE
LQSPFLGTSEREDCGGRGSGPPQCGSDQQLRKQKGAEEAPMQLAGVREEALPPAISSVAVAQASVPEAPVALAGMKE
RDSQQAREPPPTDRGSSPFGNSAKLSGPLGVLNAEAADAACAADTDDADLFTLRPPSALTSHNASANVSRNASVGGA
GDGSSYNGTLAGCACVAGSSSRPGSGGGAEDGSDGGGAAAAAAAAPGPAPMSYSTGMPQSLLEAAFEAAATAAAAAA

AAAAAAATGTSTTGSGISSAISLKHKRSMSVSSLPSIAEDEQAPAAETKRHESRRVSTSTGGAAGTASTTAGGNTRS
AAAMEATAAAVAPAVKRAVAVALNGLLGSEPGLASVVASPAGAAATGAVAGPKTTAQEGANVPEAPPSIPATAAAAP
AAAATTAQPTASCASAAAARRSVDAPKAPATAATVATAATAATAGTAANIPAACTTMWTNVLFDTPAASGKKRRANK
DKAATAAAAAGDSQCSSAETSAGGAPPPEAAAEASAAAEAVDASVVTTAEPVAANAAASSLSGSAAGLAPEVVAFLS
ERVRQSRTRDEGAYAADAGAAGAASITTGKAAAAAARAAGSAEGTGVGDGPHQLVPPSPPPAGDATGRNNDSTGAAA
AAASAAISDTNTSTVSAADTASMVAKLLASSSRPASNAGAPILCNARSPVLHQQSLSPTKRGKGAFRRSTSISAASP
TAVEHASSPFKQDSAAAVAVSPVPAAATESPGAKAPASCMSAGTAEWMERSRSLRDSLQNARSSIQGVLAARAARNV
PHPAGPTAALVLANTGPGAGAGAGAGAGLGQGAPAGAVVSAAAEALAAGRRADQGPSVAVEGGGGGGGRGGGGV
SARAAPGARLAAVRLAGNIAPAFVSTSAAGAPAGAGGPTLAAVVTASIAEPPSATSPAAAAAAASGAGGTKPGWRR

>g1529.t1|PACid:27574336
MLPRPAGRAGPRNSRAPAVAFAGSAALLLALLVLFAKPAPAAASPASLTGGPAAVTSANQLDERAIWLTPGDLATAA
ASQRADPTQLQQLRDKLLSRQSDGQASAAPEASTNSSRSRRLLKQQQRQLAAAPQKPSSKAGNSTLVLSGDAAPSPT
SVADLYFLAANALQVKSANPGTAVRFDVPSLPVVSAPTPSFTNSTTPATAGAGTDAAGGTFGAASSGDEDGLGSDVM
TAVAALAASPSASVLHPSGASNDTYVGAVQNDTLDLVRLLLQVLRANPGSNVTVTLRDTGKPTALGPLSIAIEINAA
FSGTGALQTSPSPALPSPEPSPLPSPEPSPQPSPEPSPEPSPQPSPEPSPSPEPSPLPSPEPSPQPSPEPSPQPSPE
PSPQPSPEPSPQPSPEPSPESSPEPSPQPSPEPSPEPSPQPSPEPSPQPSPEPSPSPEPSPLPSPEPSPSPQPSPEP
SPQPSPQPSPEPSPSPEPSPSPEPSPSPQPSPEPSPQPSPEPSPQPSPEPSPEPSPLPSPEPSPQPSPEPSPEPSPS
PEPSPSPSPEPSPLPSPEPSPQPSPEPSPQPSPEPSPEPSPLPSPEPSPQPSPEPSPEPSPSPEPSPSPSPEPSPLP
SPEPSPQPSPEPSPEPSPQPSPEPSPSPEPSPLPSPEPSPQPSPEQSPIDMPASPIPQPSPPSYASPVPNPPSQLPP
PVAYGGSPTGGASPSPVPSPSTPAASPPPSSYPSPAVLSPPVVVASPPPMTPAGPVYGGPASPDPSPSAPTPEPQQP
SASSPAPAPTPTYPATASPSPSPAVADPTTSPPQPAYPSPAPSSPSTTTPTTSPPPPPSPAYPIVPLPPSSPAPAYP
AYPGSSPPPPVGTYPPHGSDGEPPSPSPAVPAYPPSASPSPPAYPLPGTSPPQASPQPQSPAVLQPPQASQPPPHDW
SSTPDTAPSPPPLAPSDTAQPPASQTPPADAASPPINSPPMPPPDGTDRSQPPPSSGPPDHGDNSPQPPPAYSGPHG
SDDNTQPPPDGAAMPPSASEPPAISMPPPEYLMPPGAVAPPDAPQQSPEAWSPPDAATPPEAAPQPPYAEGTPPAYP
ALTPSPPSTASPLPPPPPPPPPPPPPPPPPQPPLTSSPTPPPPADASAPSYPPSYPWVPLPPPSLLPPSPSTEVPSP
SPSSYGPAYGSSPQPPSYPVINPSPPPSAPVPAYPTVPRPPGPYPPTQPAPHPQPSDGPPPATASPPLASPSPTPSS
PSAPPPPQPAAAYGGYGSPSPPSPSPSPYPGVPLPVPPPAPAASPLPPSSPPPSTYPAVPSPSPPPAPHPSYGGGSG
SQPAYPPPVALAPPSPSPAQQSPSVPAYPAAAVSPPPQADPAVPPSPPTPQQQDTPTPPTPAPGYPPVMPSPSTTTS
SPSPPSYQPASSPPPPPQSPPPPPAADSPAPSGAGSPLLPAAAPSPPFPTPAPTPSPLPQQQQPPEAVPASYPPSPA
VPVAPSPPSAPAVEPQPSVPSAQPPPPEPQPVPQPQPATAEPSPPATPSSPSPEEQSPRPSPPLQSPEPSASPVPSP
VGSSPPPSETPSPHQQSPPVPSPSPADASPPSPVPAILPQSPQPPVNDGTPPVDRSPAPPASSPPPAASSPPPAAAG
DEDPELAAIRAQIQAILDRIASRRPNDTAGGAGGNFSPAYTSLNITRVRPAPAATATAAATAAVGSSPPPAAAAAPA
GPTDDELGSLLQSLHQLQRRAAAIQGTTTGGSGLPARGDGEVTDRVAVLPTEQQQSGAAGRRHHLRRLLRGV

>Cre02.g096650.t1.2|PACid:27574371
MTAAASAAAAAAAAATATRGSRCGGSSEESADSGGRRRRGGGGHYGDGGDLATLAAAAAAAASGSAAAAESGDGCWC
VGDSQEQGTAGGRLVGSGGGGCGARGGSLANCGYGGGYGGGGGYGGGSGNGGGGTGSEDGGGGTSAYMTPADVEDNT
ATGELPPLQPWELLQPPPAATAAGLLQPRSRTGAAAAAADAAREGPPAAVLRLPDPVPAAAWAASTPTPTATESGRQ
LGWASLQQQQQQQPQQPRNPSPLAMPLLQLQQQSEAAGAATAGTAASGHPAAAAAGGGAAGRRCATDLLDCAAVLA
AAEAVTEAAAAAGSAAAPPLASRQAARRQRWGSGRGGGGGGGVREITPAAAAATTAATAAMAAARQGQRIALSRRRR
RHWHDESTGSSSSGAGAAGGGRGRAGGCTGGGGGGAAAADAGAWAAAQAAAVAENDEGSGDSEDERLKRLFAHMAHW
GGRGVSVGVGGVGGGGGGGGGGGRGEAVAGRGAVEMRPDCEAAEPAAGVYGEYGSGHEGDDELGGVGGGGGGGGSRR
RGRAASYAAATAAAVAARAARSRPNPAIAAVAPAPAADDMMSGSDGSSRSGRGHHRRHDLAAGATDRRPDDAAATAA
AATAAAAATADGAAAMDTESDRDATAKDMSDDCCGAQGSAVVAATGAAAATATTDATAAGRGLGQGMARAPQRPAAP
PAAGSSSLSPSLSLSFSPVAATYRHYCRSPRGGGGGGAAIWRHHSLALHHRGGGGTPAGGPRDGDAGAATAIAIDTA
TAIAEGFNRPPSRRGVAAGHPQAVAAGGAAAPEGFGSPVAVAAPPPARGLAVAAAAAPDPGYQPLLQPQPALALMQR
HQHQQLEPQQQQQQQQQRQQLPCLFSPPPPRTMRAAAAHLPAAAAGAAAAWRVVFGPPAIAAAAADAARLNAAGLE
APSWGVCGSVWGAAGSGGGGGGSSSGLLTPGGGAVRLGDLGSVSLMAATATAAATAAGSRGGRGSNGGGGSTGGGGR
VRRRRSSATSYSATESGMAAAVAGCSRGRRVVATAAPPLEAVAGGWDGLPARGSSGGGGSGAGGGGDGGGGGGGGGG
GGGGGGGGVHREQQHPRKRSRLGGGGAAAEPAVSTAAAAAVAPAAATAAQDAQGLPRVGPIGWRPAAGGGGSLVRTG
LAGQGGSSAGGGGGGGGGGSGGGAAAGRQRGSGSNSGRAGAAASVAGASMSAAAAAPGALANFIRALKLDSAEREPPA
VAVPAVTAAAAASTAPAAAAAGPAPGSGSGSGALVRTAAAQWRNAVFAAAAAAAAAATAPAGVTAAPQPQPQAQVIT
PMRVQAPPPAACAQAHPASATAEAAAQQQQQQQQQQQQAAAAAAAVASRAAAAARAAAAARMAAAAAKSARDDMVAA
CMYGDPPPLPPEYGSYPGGGGRAGGGGGGGGVGPGAEGEAREVAANAGHDGDVDMQAEEQEPATPTTTAASAAQLAP
DVPTAPAAPSAAAAAAAAAASGQRFHGAAAPAACTTAPAPHLSSSPPPPPPPPPPARRPPPPAPPSPPPPCPGGDAA

LAAALGPLPPALVLEGCLLPSLAERRAASIQAVGCLRARAAMEEKEEEEAEEEGEAEAAAAAEGSGEEDSAGGADAG
VQDMV

>Cre02.g097213.t1.2|PACid:27574424
MLAENVGTVCSAGWDDADADVACRQLGYAAGTAIELKYDFLIPEDAAGSRWRKIWMTDVACPPLAGQQPQQPQPQQP
QQPQQPQQPQRLIDCPHRGAPVTDCPFTEIAAVRCYAVAARAPRAPPPPPPSPPAPSDSCRTCVTLDLSMEPAAGKT
MPYTGRAMCTALAETVTAVVKALDRERAPLPPPPPSLAAAPPPFSNYSLWGFDSPGFEEDLSDGLTPSPVLPIPFEC
VNITSKTMTVCGVVAGSSWAGTWGAPPPPPPPPPSPPPPAKASSSGYSYSYGYTHAGSGPGTSPGSIPGSVPGSSPA
GSIPGSSPAGSGRGSSTGGSTTGSSPGSSSRRSLSVSAGGAGGGGSGGGADGGVYALWEALAAQDNNTAADWLRQLR
NEINLWQPCQGFDLDYDYQIMGLRMTPDLHFEFCPVSGLPPLTYLGMQGVNDLVRTVWLSPPPDAPWAARSGDLEDG
APPEAPRSPGDRTPRPPPIPPTPPPTPPRPPPPRPPAPWPRAANTTNTTNTITYRGCYSLAGIGSSSSSSSSSSSVLA
APGANVSAVALLINVTSSGLNLTLQACASRAAAMSYSRFAVEGYSSTVGGAIRLTATCYGLRWPVPANASTTGNGTL
GGSGLQLIELPASRCTAVPCATATAAAAATSGGQQQRPACGTGKPGGLVLYDVAAP

>Cre02.g110050.t1.3|PACid:27574432
MALQTKGLRAQDDGGAAAVPPGAFEWATVYSFGEREGGQVKCACASQKAVYTSSRAGVRKWDAGTGRSNELISSAAL
PGLVHALCVIGNTVWACTGDGKIALYDTNTGDIKVKTFVAHQGEVLCITPGPGNAYVITGGVDFNARSWQLNGNAIV
ASKYHHGAVECVLVTPLTAPPPSSRPASAKPGAGAGAGDAGGAGTGGKIWTGSADGTVFCWPDPEGKGQINEAEGKS
IKVEGGKVKSMAHSGDCVWVGMDDGRINVLHLSDGSLVKSLKGTHSGPVLTLAKVADQMWSGSGDKLIAAHDCRPGS
SPATLFTLGDQGGYVKGLLTIGWCVWAFNSASTKVYTAESIWHAEAARAEGALKALEDTRADMESQLAKAREEANKA
AMEAAAARAAAEKASQDGDERVKELQAKVDELQSKLDAANAELEDVKRRLEEAEAALVAAKEGATKAGDSAAAELSE
ALAKVAALTAEVAALTATKAALEGDQDAASKAVAKELAATKDALARTEADLEAERAAAAAAAAAATARQAELEAELA
GSAADRAAGEGEVDKLRGEKGELEDRLKKLMDEMAALKAAHAGLESDLVAARASGDAAAADKDVQLAALRDQLAAAQ
ADGGAAAAALRDQLAALQKELDGANGDLAKSAAELDKLRASLAGSDKAGKEAADARNALQKQLDEANAALAAAKAAG
SASADQLKKALEDLAAGQKQLDSKSTDLAALQSQLDAAGRDLEAERRRLADKEAEAADLERQLQEAAAERDAACDAA
REEAERLRSQQRKAEEALRAQMREQEEALRKQLREQEEAARAAAQAAAQAAQAAEAAAREERAKLQAQVEDLRGQLR
DAEARGREAARDRDELRSALEAAKEAGRALEGKLAAAADAAAALERRARAAEEATDSIRRQLAEASDQLSRANSRCD
DLSRRLAEMERRATEAEARAAAAQQELASLRAQVAKLQAEAAAMDAAAKIREKMDEELDRLRNRIRDVEGQLPRLGS
QPPPQYAAPQQQAQPPHIIINTSPGGGQPASPSHASPSMVQTLQLPPHAQPTESLAGTVSAASPVAAAPPPLAASNS
ASYDAERALLQQQLDALRGSSQALEGLVRGLQGTVEELRSAPPPSSPPPAVSSPPPLERSASLGPVYTPAPVAAAPS
PQPPPVVEPAPVVAAAAAPVVAAAPPALDQSASVNALREEVVRLKQQLQDQASELEELRAAKAGLEAALASCQAELN
DVLEDRYQLQVALRAAQKQMADALAAAAAAEQTASQAQLAAAQARAEAASKPPPGPSPEVAARMAQLEGALRQAAND
YQMLLKEVATIRADLGAKDGRIAQLMSELERLGPLAAEAEQLRSQVAILEARVGTLTRQLAECEAGRKLFNDLEATH
SSMQRRLAVARASDEIYGRELLGPGAAGGVEPPQQRPRVDSAGRRERERHLPVTGFM

>Cre02.g110050.t2.1|PACid:27574433
MALQTKGLRAQDDGGAAAVPPGAFEWATVYSFGEREGGQVKCACASQKAVYTSSRAGVRKWDAGTGRSNELISSAAL
PGLVHALCVIGNTVWACTGDGKIALYDTNTGDIKVKTFVAHQGEVLCITPGPGNAYVITGGVDFNARSWQLNGNAIV
ASKYHHGAVECVLVTPLTAPPPSSRPASAKPGAGAGAGDAGGAGTGGKIWTGSADGTVFCWPDPEGKGQINEAEGKS
IKVEGGKVKSMAHSGDCVWVGMDDGRINVLHLSDGSLVKSLKGTHSGPVLTLAKVADQMWSGSGDKLIAAHDCRPGS
SPATLFTLGDQGGYVKGLLTIGWCVWAFNSASTKVYTAESIWHAEAARAEGALKALEDTRADMESQLAKAREEANKA
AMEAAAARAAAEKASQDGDERVKELQAKVDELQSKLDAANAELEDVKRRLEEAEAALVAAKEGATKAGDSAAAELSE
ALAKVAALTAEVAALTATKAALEGDQDAASKAVAKELAATKDALARTEADLEAERAAAAAAAAAATARQAELEAELA
GSAADRAAGEGEVDKLRGEKGELEDRLKKLMDEMAALKAAHAGLESDLVAARASGDAAAADKDVQLAALRDQLAAAQ
ADGGAAAAALRDQLAALQKELDGANGDLAKSAAELDKLRASLAGSDKAGKEAADARNALQKQLDEANAALAAAKAAG
SASADQLKKALEDLAAGQKQLDSKSTDLAALQSQLDAAGRDLEAERRRLADKEAEAADLERQLQAQVEDLRGQLRDA
EARGREAARDRDELRSALEAAKEAGRALEGKLAAAADAAAALERRARAAEEATDSIRRQLAEASDQLSRANSRCDDL
SRRLAEMERRATEAEARAAAAQQELASLRAQVAKLQAEAAAMDAAAKIREKMDEELDRLRNRIRDVEGQLPRLGSQP
PPQYAAPQQQAQPPHIIINTSPGGGQPASPSHASPSMVQTLQLPPHAQPTESLAGTVSAASPVAAAPPPLAASNSAS
YDAERALLQQQLDALRGSSQALEGLVRGLQGTVEELRSAPPPSSPPPAVSSPPPLERSASLGPVYTPAPVAAAPSPQ
PPPVVEPAPVVAAAAAPVVAAAPPALDQSASVNALREEVVRLKQQLQDQASELEELRAAKAGLEAALASCQAELNDV
LEDRYQLQVALRAAQKQMADALAAAAAAEQTASQAQLAAAQARAEAASKPPPGPSPEVAARMAQLEGALRQAANDYQ
MLLKEVATIRADLGAKDGRIAQLMSELERLGPLAAEAEQLRSQVAILEARVGTLTRQLAECEAGRKLFNDLEATHSS
MQRRLAVARASDEIYGRELLGPGAAGGVEPPQQRPRVDSAGRRERERHLPVTGFM

>Cre02.g117450.t1.3|PACid:27574504

MPPKPGRVSWDTTPGSSRSAYSPLDIEAERARGLHLPAPVAHPAAIAPAATLQHATQNPCWNTVDPAAWFGLDPNQV
MHVIHAALVACGAAEQHPTQLTPPPPLQLPPPLLNYDVLALPPTVASLSKWLPSPPPAEVPPPVEVSPPLPPAFDPA
APCWPLPTEEDLQQLSRLYKAYKAPPPGFPPLLAQPLQPLQLLSQLYHHQTCQTYHHLQGQQAGVATSNVQQDVPQH
YPVAAYPPPQHHQHHQHHHPDHTCHQHHQHTPRGEAYHHHHHQQQQQQQQQLERQYHQHQAQQQHEKQQRFEEEQRR
MQQQQHQQQHQQRLEEEQQRRSYLQQQQQQAAHFHAQRQAQHYVQQKAPQAAAMYNQHQHQHQHYNAAAAVATASYQ
QHAPPPYQQPAVMPPPAAAAASPPPPFSYREAAGPHHQRHGAQLYRNRHGQ

>Cre02.g118700.t1.3|PACid:27574524
MPASPPFLQRALQGIWILLLLSEGLGGAPSPAAATASHDCGLLHSLPHGAGPGSVPTHSCRVLLIAASSSKAPDAGA
AAASGGGRAGGADTAVAPADSSFSLNQLVVECWAGDEAPASFSSAAAVGGANASKAVSAGHSPPAPVGADAQPPVLR
VRLGRTLAALVNASLGGGASIHSSSSGVDSTDGAGSSGTGLVVRLSGVAWDVADARPGLSAVLATDGSTLDPDAASS
EAGSGNQGAEDWGLDLLPLDTPAPPPDAGDNGGDTSSSGGGVPHLRLVDSAVAGVPLSSVAPLLQCLGCARRLSVSN
LTLVALEPPLGGGSSSSSSGDGGAAVVDVGSPPGIDSGTPPSGGIGGDGSTSAVVAYYGAARFAGAGGVQGLQSALL
EGVSCSGVRGAQGWACLLLQVAASASNSNNSSSSGAGVLIRGSSFADSRVVPSPSDSSSSSAGSSSGSGHGSAAGIT
DALLSCNVEDPAPRHGFGAVVVAFVALQQQGEGAAQGGAAQEVVVEGSSFIGNNGGCGAGLAVIDAAGVRLLLINTT
AARNVAAGDGGGLLLRGLISAEVEAAAQAAAAAMATASDLQPIVHQDAAAALTAAAANATAAAAAAAHGVGLVLACG
SELAGNAAATSGGGAAVSGMLLSGVELGGGSAARHNVAGDAGGAIHVRAYYAITGSVAISGPDSSSGGAAAAQEAGD
GGDGDGCGGAQLCGNAAGGAGGALRVEAWAGGVQDVVLGGGALAVRNNSAGSAGGALSVLVYGSLQRVAIGGNSNNR
SSNSSGIGSSSSTATSSASETTGAAEAVTLLESNTVLDGDGGAVHVAVLGTGGAAGLLRELVVGGGAALQGNVANNG
GSGGNGGAIAVVLEDGAVGAVSLGEGAVMAGNRAAGGNGGFLYVSAAAGVTSVTVSGGARVVGNRAEAGAGGSDSGI
DAGGDGGGVYVSSSAGDVGLFNVDGGARLEGNTAAGQSSGGEASGSGGAIFAAASGGGVVAGFRLCGNGTAVLGNNA
SLSGGGVHLMQRSNAAAGSGRIIAPLQVLLCDFAVAANNTASPSAASTGAAGPSAANGGFMYAFANDHNVSISITNG
AAVAGNRADTSGGGLHVTGGSIAAVVAYDGSLTNNTAGEDSGGGAYLSARQDLALLALRNGAVVAGNVAGGGGAGGG
VYAFVTGTGGVTKLEVDCGSRLEGNRAGGSGGGFFVQAASGSIKSVRITSGSSLAGNAAVGGDGAGDGGALYLRSDG
GDITQLSFDGAASLHDNAAVAGAGIAAVAAGGAVSVDVSGGSRWMRNTASGGDGGALQVVTRRDADLAAAAVSRVRI
GGGAVVSDNSASGNGGAIAVSGYAELYMGGGAAFTGNTAGGDGGLAAADSLPPLLTVEGGAVLAGNRALRGSGGALS
FTAASATRVGGAAGSRCGACALYQPAAGRRLSDLRLTNCSVANNTARVDGGAIRASPGESCCDAAVTLRLAGVQLAG
NHADGPGGAIAVANPAGAPGAAVSVLVELDRCRLTGNSAGVADGVYTGSGAGAGGAVFMWYQPGTASALVGPAAAGN
TSSGGSGSYEPDRAAACLLRAVDSVFDSNKCEGGHGGALALMACAVELDGCSMSSNAAALSGGAVAALQSGRSSAVA
RLAAAGGGPLTATSIGVDSGLTGASADGIATAVAAAPLGHHHRRRRLSQDDASASSGADEDTLGDEPVEPPLVSPPP
PLFPPPSVPAVISNGSSSGAGGTSPLPSWSAAVDPSCVVSRWRALVRNCTFRGNTAQLEFGGALYAEAATAGQILLY
GSTLEANKASIHGAAAFIATQGRCSGAAAIDLTASANTASFGSAGGVYVLVGQAGARATLRRVNLTGNTAGTGGGGG
AVVDVRVGGVAWVEGLVATGNKASGGLGGGLQLLGAGTSLGSSSSSSGGGAELQLWGSRLEGNTAAEGGGGLAASAG
CGAVLVVRDSALTANRAGGAAGSGAVGDGGGLHLTYRDANGAAGGAAAGDAAAGGGAAAAAAAGTVRRLTAMSCADG
AAASVYGLELRGNSAAGRGAGAYVTPGSAAVVQRLVVSANAAALAGGGLAAVNCSYLAVSDVAVEGNSGGGVYVDGC
ARLLLQSANVSGNAAGVASASNAAGGGLFIGGGQGTGSTSVGISGGAAADVLGFSTAVLHRVLVWNNTARSIASGSS
SDSSSSSSSSSSSGSAAAASLGAGGAVFAAGQVAVALSQCDLARGNAAVLGSSLATTQTCAAASRPPAAAGVRALL
APNATAASAHPGVLQPWAVAWAWLDLAAQQDCWALVLSDTILPRSGSISSGAASSDNTIAGNAVDGSSTGVAQQQPL
WLSDQTAAVLQPRCSSDAGALVGLSGEEAAANARTTPGSAVGGIRTAVQRLLQAANASAFATVASPPPPFPPVPPPP
APGLGPGDPTGGSSNTSSDTSSNNSSSGSSTGSSSGSSSWLGSRYSSLMSSLAACWDGPALSPVERQQAAAALASSG
GYLGLPPTRLGLLPNVGLAAAELERLGFSSGGSSNGVSGSGSSSGAQPQAWGSLTLRLRPGESFDVAVRLYDRFGQP
VTSDASASTVTLELVPQPPGGGTFSAAAAAAVAAAGLNGSSSDNSRPWLNASIAFLDPGVNKSLIAPVAAGVASWRR
VTASGWPGAYLLTFTLTSPGGRGSSVSGQEEQQQVPPLAVATQLLPCAQGQTLDLSLAEKEAAPAWTSCAACGTNQY
GLWEDVRPSITANVYDIPQYLRAMRDAAREARDGPAACLTCPAGGVCPGGAALVPEEGRWHSAPNSTQIHDCPNRAA
CGGTSSGGGSSSSSNFWGSSSVLLSTGSSSSSGSSDSVAVGMPVSGLGARTLHLIRCQQAWYNSWPAGSSVRRAYFL
SSANAASTTGVTGTSAEGSAAPTALPASAPAPSGAAVPAAVSEACLLWGLPASHPDGYMQQLCAPGYTGNLCAACVS
GFYIDTDFNCNPCPPLGQTLGLGLLAFAGSVILVLFTAVANFAEGFAEDAEEEAMNASVKLGAELKISEEEEEEEEE
VEEDVDGKQDRPQGAGAADFQRPPPNFGMRIPSPAGLGAAGAGAPGMRRRLPGSASAAGHNGTGGAWGGSTGPGPVG
EGSEDGEGGAAAQMEVPPPPAEGFPVGPPHSPLLRVHTRKQQQQQQLLPPPHLHLPPLPASPPSPSSAASTPPRRSA
QLRRQQLLRAGTSSVRRRPKRYHADLGDIVKVIVLHLQYFVIVTRLDVDYPHIIPRVQAVFGAITGAEVFLVYSPSC
LRPQQGSAGQARLQLLAGLATPCAVLLAALGLWVTRYLIKHPPTKDDIRAWTNARLAALRRACSGSFPPFQRSGGPG
EQHVYDDADLQRLSPGGGGGGLLSYPSGGSSFYYGLPLQLQQISRPRSGSRAAGAAAAAPRPLAPTDQLLPIPLQPS
HRAALGRGGSLGLRSAFRDVQVVNLDLESAEDFPQPTGTSGTEAASAVGPAGTAATAATAAAADASAVAAAAGRLLS
VRVGSGRGAPPALGSGVAYAHRTSGQLQPRPSATRQQLRQQRSGSIRAAGLSNTAAKGRRSADGRSSDGSRTGAPAA
APAAPAVAPIDELSPFTHGGAQARAALLAESCPDTPHAVRLASGLAANFLDFCSSSSLEHLVTGSRPSARSTAAAMA
DGAPNERAAAALAAPGAGSATGATGGSQRGDAAAAEAVAGGGCALVRGWDVELVPADSPGEEDAAGVSAGVGQGVGT
GGVLAGALRRVSRPLWRRTRQRQLRLQQQQQQQQALQEAGAVDALEWEDAGAAAAAAAGAGGPECIDGALVTCPPSR

AQLSKQPQVTQVPQKRWQPHHRDGQQGLLAGDVVDHGPFAAAAAVTGTYSRTASGISAVEPVGAAATTTDTAQLSDE
ADDGGHLGVGGGGAAASQPAPAALLEVALLGEPGEEDHSGLDEAAVGAQVAPPRLGSQVGSPQWQSLSAAPSSQRPS
LPLSLASRPSVTITTPQPAAQRTSAELIGSSHSGKPLRGILKAGRDSGTLGSCSVAAVPPRHRPGLLPNASSATGPD
NLQLGLACARGRSDTQPPAPTLGLAPSLPSAASLSGVGATAHSAAAPPGLPLRTVVSHTLRTQPSRLSRTPQRLQPA
SSSRLPPQPSHPRPQRASASALLGARGSTGTDGGGTSSGGGGGGALLPLQHSISASRIRGSRSSRASLSEALSTLNK
LRSRAFSAIEPRLTGRATLKRVDQQLTLVAQLGIVFFAAVFILYPAWAHTALSVFACYEIDDGTGPFPEAQQAAGSY
WVRDMKQACYSGTHLSLYVPLGVAAVAVFCAGPPAAAFFFVWRIRHRLEEEHIQRMFGFLYKRYKPRFIWWESMLML
ETLALVAVEVLGRSLIVSYQALLLLAVFIMIAAVNISCSPLRARILVMMEFMSLATLSLTITLSLYFSVEQGLQPSA
QTAVAVLIILLNVGLLAFFTAVVTRKAWGGVEAWAAPRMRAAGGWVAARCWTPAADGGGKGGGGGGGGGGGAGAGGG
GGGCAPCWRAAPLDAGAQQHQQTQLHLYPGGAPPPPPALLPPLGLWARLLPGSSGGLRRGSVTAAGDDAGAAGAAGR
VSNARVSDEGKFGGRASDGRTPGDTGDGKDSCGSCESAAARRGSGNAASDAGAGAARATSAADAVKAMVVFCGGGGS
GGGGVPGSGVDDALVISLYGMAAPGRVAAEAPASVAATAPATAAANNTPAAAMAVPRAAASAAAARSRDAPSPSPAS
ARVTETGDVNLTFMDTTEPAAAAAAAVSVGTSSYPSLGPNPDAAMAMSARSAPAAARPAPPPPPLAGVALGSDEEED
DDDGAGHSPGDNDVDARGRPQLHVESWEEDNAEPALPLLPLYATVQILNGRHTRSGSARSHVVSRSLSGCSLSSHVS
SLSAVSEGALDDAGYGGAGGNGGEKNDVAPAASSAAAAAHAVGGFKGTGTSSGSRARGGASAAGGMGVGKATATTAP
AAHESAPAQSAAAAYAADADAAAHGRTRAAELVWHQNLAAALESGGGVTADDALTTSTVLSEQDVSQAVEEGEGRNG
RQISNAS

>Cre02.g093850.t1.3|PACid:27574570
MLDQGSDAFDRARRLLARIMSRLMQQTFLAWRKHARFETTIKQMEEVIDPVAAVLDKLPENRTPEDIQKLVPLVSQI
EAYKPLHPQVREFLASYLHLALLTDHTELPRQPSDNSCLFSVVFGQLTALVGDPGDPALAAIRDHAVAELMALVPPE
PSEAEVRADAAVGAAASAAAGAPTEQLSVESSLGLASSVAAVAAAEERRLRAQATMSMYGIKGVSGVAAEQVSGSLW
RCIVDTIHNLNKLQKAQAMRALAAQHAATLEDGAGGHGAGGKLGLAGPGAEEAALLELLPVLANLKTQVHIFKAGQS
FSGRELFCHEEAGLVQEVGAMHVELPGRAIAAITPENGLAALVYVSREVYRRAMAMQSEISVRRAVDSCCRLPFLRH
TPLKELYRLSRHMHEATFKPGDMVLQQGRETSFMFFLVSGEVQVVADLPDSSDPLVLGAALAALGGTKPTQAQVKAL
AAHVAASNRRAAAAAASTNGAPGAKGGDTARQDGVMRSRTGTSTGDDGGTVALAAGGGGGGGGSVLLPNFGTTRRVVL
GRRGSGVVLGDDLLREKRLTVGVVATSRCVCLAVSPQRFTMCCDPLTLRLFHKLRLQHSATAVFTDALLAQEERVGL
VTAFRDTVLSASYDHVPDTLKRGELSEPRAWLASSIRPSELDASADDLYQQRAAAKAAAAAAARAAANGDAGAAAAA
AAAAAAAVAAASGRRSVSPHAGAGGRPRSREVSPPPGLAMPARGSTSPPPKQPLHLPPSVAALSLPGLEGAPAAKLY
SGLKVALVQELVGSRAESGSMAVFLQRMKAAETQRIANANAGAGGAPKSIDLAAAPGAAGSGAAAAVTQQQQRSYTD
MLEAACMAVIKVELAPGQLDVKLDTMAQVLDDIVVQWSDLAHRFGLQLVRWRAAEYFLVVPLDLSARRRPLVRIVAD
TLLTMAAEFRKIVAQNEIPHLAMAAGVAIGGLFSSYDSGHFLVGCSGQAHAAAQHLCKMAQEEGGWQGTILVADSVA
QALARTHHVQCASGGGELLGAVGQAPGSPRARRRPSPLLTQEEEEQRRQRLAREQEERRQSSEVGEGGGVKLESKGP
TGSAGAAAAEVLAEDDEDEPVSPGTRPYHRRAFGPGADGSGGGAPNGRGLRSPGSRSMHYLPGGGAACLDGGEPMQD
PALGTPHAGLASNLHAPSPGGDGAASPVSRQPSQGASRRTAGTAAGGGGGPLLPPPLLLGGEPTWATQAGPVTIPGE
DVLSTPNPITSQAAVLRRSQDALRPASLLVPGDPTSAEQSPNSPALFSMRHPNGLAHQQQQQQHQQALRASGGLPPG
AYRSNSSTSQAAPHQLQQLQQQYGGAGGTTGATSPVPGSGAGGAVGGSGRRAPGSVPIGPGWEGGSTGGTTPGWPAA
AGGGVTSTSSSPGGSGLKLAQRRERNSAATSTMMFRQQLRQEVASLRQSSHHQHSYHYDSAVVTQHHMPAVGPSSAP
LPSSPSDRAHSHSHSAPSRGDNLSPSRSRFAPQLPSSAAATHSAAPAPVDAGSGAAAAEVQRAVSPGKRDAWTAASA
NMGAAGLCPSLGPSYRLPTGVPPRRALSPMPQRPVSPYIPFSAVDPASEAAAAARGGASPGAGASRLTPPAGPSAAA
ASSAGGGGGCPAPKLVASNFGYLKVPTNRLRRGSEGMAGIGDSGAVRRASSHTVNFAAGAVDASGRHNIGRSVSHGA
PAAPDSPALHQYPSGGGRAAPNALGQWVPTPSAAAAAGATGWGGFSWAAGAGPAAGIASGESTSGGGVSAATTQYGG
MVGTASSTAYGRSKSSRGRTAQGDNRFESPPPAGQHVGSGGGKGKKNSTWQDLGSQRAAEQYAADLPLGLQHVSFST
AGLSRVPPLQRLLYLESLALSYNHLVDVGSEALAGLSANLKTLTLVCADLSSLPPDFGALLPGLTELNLSNNRLTQL
PPTMGCMTRLRCLSIAHNRLEPPGLPPSLWGMASLEHLLLGHNQLTELPGRSLAELTNLQSLDLRHNRLAALPPSTT
CLSGSLTKLVVSYNNLTAMPPRFMDSMDRLAVLVLDFNPKLVACPQLAMGRPGGDDGSGSLSVHNSTSGGAGLAVKM
SSAVTLTPGGSGSPDKGSTATPTPHANTGSAETAPAGAAAAAATVGTDAAEGTPFAAGSAQAPVTASVEQSPDRGTP
SPLARKDMSPSVSAGGAVPATLTAAPPSAQPSALGLRHSNGAGLWRQVKAVVTEAAAVLHLPSLRVLSLSSVSLLNI
PAWLPAGLQVLDVSRNNLHYLPPWLPRRLAPSLAVLRLHTNRLEALPSDIRTMTALQVLTLEENPIASPERIATPQG
AGWAAEWLYRKKYRSRPEVMASLAAQGSEQPLLSLAATASALMAAQGATANASSTNVSAGGAGGAGGSGGPAKATSQ
AGGGLSANPSTTTVAAAAASQAAALAAEPSVPPSAAAGGAYGIA

>Cre02.g114100.t1.3|PACid:27574593
MQTLGKVIRVYPSTVIAPLLVTACMLGLGLSGMQLASNFYAKSSQVEATEVAVVAAAALQQRVAAALAPLQLLSAVA
QVAPQYEAAAAWFGLLVPVLAAQLPRPQLPEGSLRQLLLAPAGVVRQAHTPAAATPAAATPGAAGADANTSTGGGSS
SDTPTPAGGWPVGTDLLNGSVLGASQRHCGALAAVAARNQTVSSTCDLPVTSAGVGGATALTAYHAVFVPQQRISAS
GNSSSSSSSSMSAAAAVAAAAAAAWGRPDAVNTACGAPCQDDVDTGLQFWGVVGAVLDIPLLLARVPGSPLAAVTDS

GYSYRLEDDANTVIASAGGSGTAFTTASVTVSAPVTLPGGSGPGWTLYVAPAGGGGWGPAWRGGYTAAVVLVAVGLG
LMVFLLLVARRKQELILRALLPKEILHDLSAANSVTSLGRATLGGDTPAALILGLIQGLVTGQRLPDLRHVLLIRTA
LLRGVDVYQPLDLRNRIKDSGLAADVTQALLRQLGAFPGAGGIMDGSSGALVGGAGGPGPGGGGGAAASQPQYYYLP
HVASSNGRVGGGGFMLATGGSTAPDTYVAAAYASGGGAVGDGSFGDVSRFGGWPSGGGGPNRRPPRRGSSKALFAQA
AGTAASAGAAAAAAAPPPDLDTLTGALTFLLTPPPPQPLPPPPPAMLAPAGPAPASANLFSALPAGLGGAPAAVDSP
GTMTAAGGVTAVASANHRRGASTAGVIGCGSGNFSAFRESFGGGPTVASRLGAPAADAGSFGSVSLGGGGPLLCVRG
NHASSIGSTAAMAATFGNNSAFYWSGAATPRASVDATGASSPLPSSLGPMPSKLRASQWLSGTCGAGNSPSAAAATA
AAGALPASSATAQPFQRAATATGMPTAGSNVATDIATTLTTAAASPVPSLLLSAAALPSDPTSNTTTVDGGGGTGGG
YEFGGGRGSCDMPATTAAVAAPAAVAGVGAVADGDVLIDMLALAAGGSCGVAAGAASGGGATSGGILVGSPPGTGSV
NLDNAFISAFSTTAALSGGAGGGGGGGGGGAPAALSPGGAGGSVGVMAGAAAASSGTGGLGGFVGLTIGDRPLVRRP
PTASPSGGLGSFGSRSLTPVLRQFRELRRRASSRFLDGPSGGVGSAAAASALSTATAAATAAALASSSAGGSNTPFG
HLQAMHSHSEALGTVAPASAAQVQLAPFLSHQLSPYQTVGPRQLESLVEDGCMASGDAARLAGSAAAVSAAAAAAAV
AAPCDGGGRRNSLASRVTPLSSPLSMTAPSAVAYTPLDTRCIHRSAATPASSSITSAVLSSGVLPVSASTPGPTHAP
SPSHGPLPHLPPLAVASRSLLYQPPSQQVSSSRLPSSQPQTPLSPTRQLTSPPPAQGALSAAAELLLTSCTGGGSRR
HGGGGGGDCGGGMAAPPSPPPLLDASALAGLVVLPAAASVDMAVEVGAPAAGGGVAWAPASAVPRVRASVDMGVAAA
ALASSPWASGALLPSGALERACLLSRQLQPPPQLRLTQLQQQQQQQQPPQQQQQQPPPPQPPPPPVIEEVERLLAG
ADAWQFDTWRLAEATSGHALSCLGFYLLQREGLIAHFRIRPHRLARLLRALEDGYRADNPYHSATHAADVLQTLHVI
IHGAQLHVHYLDKLGLLAAYVAAVVHDHGHPGLTNDFLIATHDPLAVRYNDRSPLENHHAASAFEQLLQPGHDITTG
MEPSDRAAFRKQVIDMVLATDMKQHFAILSHFNTVHRLQAYKPNSGGEGGGGTGGGPAAATGTGGGAAAPATAERPT
GLRRAFTGELKDKGSSAGTASGGTLSGANGVASGGISGAFSGALSGAVSLAAGFVNNSSKGSVAAATALLQADAAPP
RPLDDSERMLTMQLALKVADIGHLGEDLEVHCKWLSGLEEEFFRQGDKEKELGLPISPLFDRAKQGVSKSQGGFFEF
VALPLTHALTTAFPNASPIMDCFVANYEHWKKVEAATAQQAAGAKEQQVTPKERDATQQQQ

>Cre02.g099050.t1.3|PACid:27574601
MEAMGFVPDDTMRIGSVNFVIRAYAYVPAEDTFRKLDPNSESPAVGYLIATPIPRAPFTMGLPVCLPGGHAVAAAAA
CRQAGYLAGEPVIAGEEGTAVPTVNRTRYSRFLRDLTCSAGAETLAGCSATYVTGAVGQCKSMAAARCFNEVTGPPK
ARRPSPPPPTRRPRQPPPPAAPLPPPPPRPVRRPPVPPPGVPPRAPRVPRPPPSPRPPARPPLLHPAARSPPGLPAL
PPPDGPATDDGSPPPNPLPPRRPRPALPPRPRRAASPPPYTPAAPADDMLSLSDFESVTMRRWSAQFQGWVSPWIGA
VNQVVLCGAEDCLPLCADGSDWGDEAARLLCSYRGYSGGYASRTGPSWAVTPYIGWLSCPGSGSSGGGGAAGPFESI
LQCSAAMVLRGQCRALAGVVCFEREWDVNPPAMPQQPPSSPVHLQPPPPPVPPASPPPPPSPSPLPPSPPPLPLPPP
PPSRPPKRPPPRLLSPPSPASPSPSIPPAAPTEEPDDGHSQLRVVGGSRPGEGRVEIRVGGALGVICNSTRWTATEA
AVVCAMLGFGTEGAEPVSVRAPPGARVVAAGIDCSPRGGPWASLVGFDLGEPGYSAPTVPSSIFECTAFGVVPPTCP
GGGAAGVRCRDPRPLAPAPAPAQQPTAPLPPECDGYTERLRALRAALTSAAAPSSSPAAQAPTPLQAQGALLRSLAA
TKQATAPDWLLEFEEYRTSLPHGSLRLVGGPSPQSGVVLLSWCGYSGTLSMQPTQHLETDLRGLRRDPSSGAATGTS
AAGCSDGSELGSPSLLRERPPQKGLVPADTEGGSDDSGGGIGGGLGQPTPRELVNTLRAAAVCTRLGFMFGRYKKGS
KRFGQGSGLVFDAAPVCFRGTANTTASLIAAGQQVPGGSGAVFPGACRDLDLQLELGGADGGAAAGGAGGGGGGDGH
VHDLNVECFDDPRELQPPAHVLAPGDPPDGALRLMAYDDARQMWAPATGGEGFVQVWSAAAGSWGGICSWGWDDADA
EVACRQLAFGGGGRVHPTLRFPLADGLYRWRAVHWTNVRCGEAGNAGAGSDAATTLSECQPERANADTCLYTDLATV
QCFRAGQPAPPRVAPPPPAVPATDAAAGCAVCLTLDFAASRLLAPNTPFFTGQDCDRAAAAVSRHYAAARVTTAAAA
RLPPLSAPFACDGVVGADAISVCARVPSSDGGGGAKLDAFAAAGADGGSGGGSAAVDHQAVVGVRDAWLAGLYAELA
VELPLRTAGGGDGLYELDYDYAPTYLRSRPRVHIATCTGAANPVMVESSTSAADDDNTDGPITHTLLSPPPHAPAWP
SGGTGGAGPQPLAAQPPPSPPSPRPGPSPSAAAGPLTRASPSPSHLALPPHVSSSTPVASPSKVLPPTASPSHPPSP
QPSPRASATSPVATTSAIASRLSTTAFSTATSPQPAPQPRTSITSAAATTTATTAFLAAAALTSCRDKSGDKAAPPP
NDWAAPPPAQ

>g1725.t1|PACid:27574609
MLHDVTDAESHRIARQRAQLLLHFAQHLRDEEPPDVRGVLSVVEASDIQLPEHLQLLLCAAWIRRRLQYGILETGVA
TLLPGLVPLTEASAQLHVDVRTEACLQLLDSKPVDWGEFDLTVETLFRQPEHQGARGSDHDDGCELAAGGDEQRQEA
TPIPGPGGSEATAAGAGLWPPAIAQLFAERQQQLRLCSRSSRQGLDRKLAQALELLTREGQREGVLHTVDDAVTAAE
ERLGFVNKAFIEQLLAVTAATLPSTSMQRQQQGEEGAQQEQPPPPQQHAAAVQPTAASSLPPGPLSPQPSPQQSRHD
DGVNAATLHADHTTSATEPGADMAGAVAHDTLPGAPAGDAEESRIPHRQNADAPSAASAAATTFVAAHETALVPAAE
DGAVALDPAAPATSPPIATASPAISGTGMGHTPATNVAWPGTGGAPRHDPTSAGQPSAAKATGTGATIAVASGPATQ
APQRRSPLPLPHGSAAGSLAPLQPQTRTSAGPVAGGGAASSASGRATVGQSVQFVHMWSQAGTGRSGTLPGANNSGS
SGALLQQRAGGIARPGPAAALSGVPASGGAVAASAPSRRAVSSSPRDIPLLAGTGVTPPSSPLQGLPSSAPARATSL
PPSTLSSPARLMQGLPGLVGSASHPRAPVTQRAPLAHPAAHVAALLGAPAAPHAQLAAPAAAGAPASSSMGIGGRLG
QIALQQRNAAAAAAAAAAAAAIGAGSAALAGAAAPATANTPLPPTSGPAAPTAASAARAMGSASPAVASTAVVHPAK
PDAQQPQHIEGLLRQVPPAPTGITDPDAQAQAPAAAESVMPRPASSAPPHMEQESRMGQTPPYREQAPAQQATADTS

ADARGVGHSVTAAPLCPPYVASRTGAPALALPDADTSLVAAGTQTAMTAAVLAAVNEVGSGGQTAASVSGEPISGAQ
AAVAADPVGGEEHASRSNSSGADSGASLGGTQEHTGSGGAALAPAPSPAASEAPQANAALPFGPPPNVLAVTSALPA
AQTAPASTGGDMNATDVAAQQPAQPSPPRQPMLPPPPLSQPSQLSPPRSPTTAMPKPDTEQAGQGPEGSQALTAGDG
CITSQSQGSPPPLELLLPLSGVSQARPQDEGVAVVVVPSAGTDEETEQARPTPAAEDDAAPEMHVAAAAAGPSMEV
DAGMGAVASAAEVLGPAAEMPAMLTAAAFAPIFSAATSTQLDAGAVPRTHPTARAALVVSATAAVEVVEPAVDDMSA
AAEPAAGADVLKAAVGEAVEAAEPEQGAPTAVQVSGAVTSGHGTGEGGLPPAVLAQAAATASTAAADQPSSPEFTAA
IEPAAAVSRLYQQAASPASDLDMTPPDAGAAAARVNTLLGLILPPTEPSASGPYQGTGSPHAAGLYAWLGQTLQRVA
GSTAEEPSSPPPQQPQHNEAERGTTPAAGAAVQLLLDVPAGVAATHDAGTAAKAGALAAATIEDALSPLPLLAQAQS
RLAQEQEGRAGQEAEGQEAQGAQPYAHAESGPVVSLEAAVGRGGNSSDRATDDSRQMRDQQLADGGGAPLPGDGAAG
EPKAQPKAQLAASSSGQMDARGSGGGLDQMLEDAPAAEAEVAEVAGSVAAPAAEAAGMAAQDAGSRPAGIAEPVTPH
SARQPVADEEGLARTAKAADNVGAAAGASPVRSGASPWGSDVASPLPQPQHTPVPGAVTDTPEATTAGAPEARAAAV
AMAMQGALLQAPDLGGVAWGLGTLQPLPTGLEEQVLAAVSAAAAAAQQAVGLAAEVVATVVPSPAAEAAIRPGQDEQ
PHQVVEDVSAEAAGGAATPQEHLQGDTDSAFHGQPPYQAQQEQPRGMDKQPEEQEMPQEELDNQAPQVSIAMDRQDD
KQQDGEQQDGAARAVMPDVPSSSSGAQSQPVHGPVPQQEQLPLQCAPGTAQHQREKEQVGYDREALLLRPPSAVPQP
HEDDAQQQKATAPAPRVDAQHTDQSQQTAAMVGTSGGEAVAEVESGAQPALEPPPSQLQVLGDTVAKGAAATPAAST
GSQDGERVPCAAAVALPQAPGWKAYVQLTAANDGRRVATQAGTPGAGGAGGGSALGVQEAAPDLAVHQQRSTVLSRP
HVQPHATTGAVTSRLSLLAASQAQQQRAVPLRQRTVPPGAGDLGCAAAGGAGLLRSGGTAAPLVAGSAAPLASEHRA
GSDGQAPSQSKHPRRLPPSFLASRHGTHGAQQQPTVTTGVLAARAEKHLPPAERQDEGRHGREQESPPQEDRNPEPQ
LAQRSNQLHGGSRPAAPRLQRPIQQQPGAGGERKGSEAAPVQPVSRAGAAGVGAEAAPAAEVPRVRNMPRAKAPEPA
SAAAAPPTAPELAPAAQRPPSVAPRAAPHGKPRIAASGRAGRRLVSKGQPDGMGSGAELPQPARAAAEGAAGMAGAT
QPALKRKAAPVGAAGVKRRCTLFDNGAPPAATTASAAQQVLPQEEAQEQAQEWPSDSYVAPPARQGSSPSRTRSQQR
NRPRGDTAQSGGLSSGAAEADVPYDSTILRQCNDLLLQPPPDGQTHMAVADIPRRVLAFRCSVLLPRTGDPVLVARS
AAAILLLHAMIHESASLPPQLGQQGRRLQGSGSSSGGAGGVAVVGKAATAGALAADCAKVELLQTRWFYVVSPKESE
VKQWQEEWEERQREAPAPAAAAAVPAAPSLAASTAATPRQTDLRAADAEAEAIDMATLPDSPSLVRMQGVAAGRVRT
PTPRGQSPRLRAQPLQQQQQQQQDMNRGVNSGGQLASMGESAGPQQHSPGSTSGSLTRNERHQRWLRRQPGPAELL
AEPMHRPAKRRRSGRSASPASTGAQHPAGEKAGAAGEAQDGPPAGAPAAATVHERPLTPKRARPEASVRAAGAAVAM
KASASTAGTPPAARQAPGVVGPASGSATLPAADTLAPQAPDWSPPRDPRQRPPSTQRLRTPTPVAATATAAATPLP
QVVAWSAPGTGATGAAGAARTHTDTDMQLPRRRRMRPPSTGPERGGDAEAQRLRLREEAAVAAATAGNAVGAAAAPE
SGSPGGSIADATGTDGAARPGASSPRRSTPSKSPLARLARALADALPELVELEGEPQPATAATAAASNGADGGGGGT
AAADVHSAQRSDTAMGVDGSNGAEAVSDAALDALAMGTTPAARREAGVVGACSQAGLAGAAAAASSGDGSPTSEGSS
GSAVSVRIDELLHVRALQHREQVRNLFHSLLGRPPGTPPPATGGQQLPPGAESTSGAAATCGATAGLAGGGAAHDPS
LVAGAASDRTPLATVAPQPQPLQQQPQPRHGQAADVSGTGGMGRHQGPGTAAERHTPETVDLSRHLRRVYAGETPFT
DAVPVQLLERLLRQLDPGRPEPQPPQPPQPPKQQPQQPEAGTHLQDAVAGVARSAAGTEVAGQDGCGRFALLRTLPK
TLFETTAIAAGAARQGAAPSSGLAPETVHRTAAYAQTSLPQPTAGGLEGAAGSHEVLPTTAVAQKPHVPQAPYDTAA
GASGTAGPPTRPPAPPASAPGNAAAHDAIAVSGGRSGVSPEEFFADAPVVPSLLPGLGLDGALKLLRQQQELERTRG
TPMPSPRQAGDGGGAGFEAAAAATTHAAPPMPIHHPKPQSAAPAGPPAAATAHLQQPLVLLRGASAQEQTAPPANTT
SAATFSFGGGHLAAGGHRHAAGDVNSGARSESVGGEPFRARLGGGRPIRGGAAATVPAHTSQQDNHGQGDGAEEQSA
RAQQNRGGAGATASEAGPAFSLAGWQQPSVGQRASCGSASESAPGDSAVAGAGDRGANIGGGAADGAPSAPVQAVAA
ETRSAGSGGAGASQHQRGTAGSGGVGAAATVSAGTGVHGGEPAAEALRLLLQVLLVAQTDPQQAGQPAQQRQGAPSL
DINLLSRGLQQLLQQAIPGGGAGVGTSNGPASGEDGGLWGQAGGGERPGTQQQPGPAAVDAPGSMYQQFQPQQHQPA
HAQAWQATGQQQAQYAYAPQPQQQPAAPMPHGAPPYGAGAMRQPDHVHPAARQQPHGGGPVSQGYATAPPAQHVYD
PMQQAYGAPALPPPYAVNGGGSAYAAGCVQYAGHGGMAGGRQQYPHGGYAAMHGEAEVLEADDIVVSGGYSNHHTQH
AGQQRQHPAYYSQHGHAVAFMAADHGQLPHTAPDDSVRQGRRPHMVAAAAVLREQFGGGSVRAAASEVISNASAAMG
SRAHARQARRIGALAVEDDEAEGSGAYNAIPADARLAAMEGAAAAAAMAAGAPAFSDYPAASTMHPGYNSPTVTVGN
SRWTGRSSLNGAATDGGEQWTGHGHAAAGTGAVGQRAQARGGESRDGTAAAGASPGTWRLPPLRGLSFPRAGGTRAE
APAPVQRAANLFKGATAHAEGSSAAAAGGATQSSGPSQQAQPPISAHQQGVKRPLFAEQQRMPPPTGASKPPAALMA
AAQASRPAKSALKQPSSIFHPIEAEARPTAPAPPPRQNAVPPRQAAPAHPLQPPTRAEASAQMASGTTKRGASAAAP
AASGLAIQPPQRPAAPATSQADDPSQQSSGPAPKRARKVTFDMSGLGHEQHALGSGLEPQPEAGSPKGFVGRHAAPS
PLPPSPPPDAHCAPKSSGRVPQPAAPPGAGAVAADRSTRATAQRREHSAPALLPRHGTAAVGGEGSKPGGVAADAGG
AGGVLRLFDGDPYLMARERR

>Cre02.g142650.t1.3|PACid:27574646
MLYITLSSGQTGDLDSTRVNGCGVSASTSYSSGNIIGSGTDSAGNSYTTTRTVFVAPLPTATCTIVYSDCCRISQLV
TGGDGDFKTSARFTTTTRNSLKTAAAAVMQVGKASSAGAYASVLVPVSSATSSGFSCAINSDGGITPPSELAAAAVA
GGCRIDWYNSARSYTNKIPVPLRVTSTADAHFVDADFILETVNLGGAPVVAMSRSGQSLSSGGAALAFTTWTLAQVTF
VATDPNAGASISVTSSGGLPAGAALSASSGAAPFTTVFSWTPTAASPSSGVVQLIFKDNTNLQASASFSFTMAYTPG
PPSPPAPPSPEPPSPTPPLPPPQPPSPEPPSPEPPTPAPPSPAPPSPAPPSPEPPSPAPPSPEPPSPEPPSPAPPSP

EPPSPQPPSPPPPPSPPPPPPPRPPRPRLPAPPSPEPPTPEPPSPPSREPPSPEPPSPVPPSPQPLALEPPSPAVQP
SPQLPNPEPPSPVPPSPQPQRTVVVISDTCSGADTAHVSGSTVCTKPSVIQEIPPGPVNLTDVYSGAGITLMPLTNG
SSSGNTTTGSYVVTASNPDVVSPDNSTQLLRIVAGLAGIDNCVLVPPGLVGFSGYFATLLANASIQLYTGGMCDSGT
EMGGALAIPAAATAGGATSSPSPSPAAHRRMLLSTANSSAAVVPLQRLQAPADVFSGGHVAFSLRLYAPPSSAGTAG
DTSVRNAIVADQLVFVLRAQSPSPPPRPPAPLPTPVDVVWAAFAVGPTDPAGTHTQPGAASRLLGEPRAGAANSASC
SSDLGMRSWLPTLSAPRWVAVRFAAPSSAASGGNGPAIPTIRLSSVRRLGVYVLNLGSLSPAITRIDLLLLPEAEAA
SAANASLAPARPGSAAGAAGAQQGLLPVAVPVYTGISGGKALQCPALNWFVVKPKAVARAAAKAATALGGTNRRSGG
AQAWVVAGARLSVNNAPVAGRPELPNIHALGLELERAV

>g1367.t1|PACid:27574782
MSFNTELVTFRRGHADSTGGAESTEPALPVSRRYETLGSEPGRGGGGGSSNNEVFSDSDLLLSADTFHNLRRRTCCG
VDVTWLVDRLPPSWLLWWKRLTPGRKRAYTLGFYLSLLLAFILLVVLPINSAVKASAQARAVEMAMRPSPPPPPSPP
SPPAPPPSPPPPPPPPPPPPSPPPPSPPPPNPPPPMPVGLLCGYSGLRLPDLIRPLHYDLSLDLYFDPHPTADPAAE
AGGRRRLVALEQQELQREEDGEVGQGVSGGWRRRRGLLEGGGGGGGGAGAEGADNAPPAAAGGDHTSGTGGGAASPG
ANSTDSGSGTDAGRDTGTGGDAGTGTGTGGTGGADSSTDTGGADTGGGGSELPPPPSDADFVSGLGSEYVLGRVNIS
VLLSFDTTCVVLHGVGMKVDSAQFHYMGGVWRGTVTQPSDPKTAAALQQLIVRFPTPLPRTELPDGLGAANGSAAAL
EAAAGHLSLRFAYKLEDGLDGLYKSTFKDKSGDTHAIVSTQFESIAARKAFPCFDEPQLKATFKLAVTTPASTPALA
VLSNSDVRDTSPVPPDPAAAAGSGGAPARTRTLFYTTPRMSSYLLVLVAGPLSSTSATCGVPAPGKAVKVSVWATPD
KAGQLGVALAVGCSALSTYTAAFATAYPLTKLDLVGLPNFAAGAMENWGCITFRESALLVDPAADDVSTVLWIATVV
SHEISHQWFGNLVTMSDWTELWLNEGFASYLETLAAAAYRPDYDYWSLSASEMTSAALGYDVLRSNHPLSRTARPIA
SLPDIDNMFDPISYEKGGSVLRMLRAYLSGDQLGLGDQLDMRRSRQLLWDTLEQRAQAEADDAAAAAAAVLGDLGAG
AEAGAAVAPQRLSRTARTLLGHLKKKKPPPPPPPAPPLAPAPPTADKRAVSKSGGAGGGGGGDGVHAEPPPHEGDLA
VPPSPPGRANGTARARAKPPHAASTNSSARSPAPKPAAANTTAAPAPAPAPVPAPSSNSSRTAAALPPRALGVYPPS
PPPPLPSPPQRLAAPPSPPPPRPPRPPAYDPNKDLGSDPLFAALRKYLQLYAYGSVTATDLWGSLSASLSLPISSWM
QAWTYTPNYPVLLVSLAAAPPGPFASIVPASPGGLAAASTSPPPPPHPNAPPAAPEPPVKAQTGDNLDQGAGEGEGE
GADASPPPGLPPPGLPGLPPPPAAAPPPGGYVNVRQASVTGTACNRSDPGSPRWWVPMSFRLPTSSSMKWAAFSTCT
AAVPLTTELLTGSSSGSSSSNGSSSNGSSSPLPAPRYVLVNPGRYGYYRVNYSRPLWEALAAAADDPEAVPPVDLAG
ALDDSWQLNKLNMLGPTVFMSLTQALGARLLPEYEPWSVALSALSAWQRLLDTAALLPPGATNATADPARDLAPGAY
YAECGRRLGAYARDFLTGPLLANVSLPPSPAGAPGGGRGLNFSVDSAAQLDGPRLQVRLLRPLALTQAAAHMVSALA
APGAAATTLRSLAFSEPFRSALYLLDAAYEEQQAGGSGGAAAAGSLLHADVRGAVYRVAVASGAPLTYNTVKLMYER
STDATERSRLLSALAQAPDLQRIQRTLAYSLDPHVRLQDIGGLLAAVGFRGGEAHKRTWDFVFNRTADLVRRYGSGP
DAVSSASYSLGRTLKELAKVFVSPGRMDEVLAWSARWPGLMDSGLEAVMRESTARNRAWLAGPARQLCEWLKQRVT

>Cre02.g100450.t1.3|PACid:27574783
MERTRVEPRLALTLTEQGRLLWRSSESEWWCHKLLDPAEAPPATPAAAAAAAAAAAAAAATPAASAAAPIQHPDSTQ
PPGALALAGAGSSGLPAGVAGGLAPGAPVREGGTLRGALESLLPAGVVASLLADVGLQLQAGVEEPEALSLALLHVG
YSYSYSPEPGAAAAGSGSDSVVAPAVATTAAVGPEAQAEAEAVPQAPGPEHGQASGLVAGLGERAGVGMEVASPVAA
SAAAAGQRPHQDEGKEPVARRVSAGGSPASPVPASLATAATPDLGGGTGSGRGSAAPAALAAATSLAAATSPRLSPI
HASSRAPTGSSRGGTPRRSSSAFSGAASTSGGVAALPESPLPALGSLGRLSITSAEQHALLSGMLEPSSIFMSEAVG
LGLFDNLDVMYQHRVQAQAAAAAAGSGGGSAAAAAAAGGGGSGAGAGGAAGGGAGGVAAGVRADATDGSSAARAALQ
LYMIDSVSRNMSEALPAEAPAAAAAAAAAVAAATAAASGAAASRGLPLSAAAAAAAAAVAAHLSALGGASGSGSGAS
GSGGPGAGWMLDPDLRRLLDSAASMYRHGSRTPTADGGGAAGGGAAGGAPGSGGSGRDAAVRRFASPSRNSSSGDGS
GLYGNDVALYGAGGGGGGLLRRGGGPGLAPPGHGGVRPAPLAMQAFLGAPGPAAGFEAYAQGQPPRRSGAEDSAGRA
RGRLSGRSEGGEPVRYLRAPPVRADVDAAAASSSSSGSNASGGIAAGGGGAWPGARAPSTPPIEEDAAGEEAAAAAA
ASGASAAAAGAAAASGTSAASGASAASGASAAATAAVGALHSEAGVGAGAGPQEAGGEAIDVEVSPFTHVLGGMPLT
AAAAAPVSPPPASPSVAAATTAAAGPSGGDGIGAASGAAAVQAAAGGSSLPSGSNDTQANDTNPVPVPAAAPAAAPE
PAPAAAADAGAPGAITAPPPSAPTSPTTAAAASSSLHRSVGAVPARAGGGAHRFPRRRSTRSRMNALVEQYQLQGAA
AAATAAAAPGGAGGGGAGMLPPGILSTGGIATMAPRQLAAAGGGGGGAGVAGGVAGGGSGATASLSSSPAHDQAGLP
PPPIALRAAGRTGSSSSSWLGGVLPPPVAAAAAQPGPVSAAGAAAVDAGAGVGAGAGVAARPPLTALSVESPVAASP
NPSGGTTPETARGPASVPSGAPSGGKIGREESGKGGGGAGASSGASGKSEGDGGSGSKQAAAQAGDESGLLVQAVYR
WHQVSLRLVRAPAPAPGEEPAAAAAAAAVVQLVAQDVDELVHAAAAAAEASLAAMRERQAALEALLASEMKLLEAVFP
RQALEHMIGNMVHQHRQRAAARAAAQAPAAGDMYPVLAAMQRAAASPPPPPPPPPAPPAAMPAVPAVPAVPAVPAPA
VAQQAQMAPQPQLLPALTAPEEDKVAAASSSAAAADAGHDQSMGIPMIPDAAAIAAASGERSPSPTDDAYSGLGGGT
GIGGQHLSASVAWMITAPQYGSAAGPAGAISSAAFVSAAAAAAELGGGSVAGEGSGAARLRGALAAAAGALPAAGAAG
GRGSARASAAAADDGSQFLGSLDSSAVHPRTSNNGSVAPCSIGDLDGGGGGGNTTSCGSSSTRLPGLMLPLGPASWS
GHMATQAAAALAAAPGGATGRASASAMAGGGGQRGASGGSTASGGASTSAVSTGAAAMRGLMKIASGRGGVGSLAGS
LLNTGSGGSAELEAAGAVFTRGSFCLLPDLPRVDRLAAAATAANPATAYGYGAAGDRLPSPLSAPAAGGDAIGAIPF

VAAPLASAHRSVTVLFADIVGFTSMCNELEPIAVMAFLNGLFTRFDCLCDIYGVYKVETIGDCFMAVGGLITVDGEG
FKAVRGDGSEDELHALKVLCFAKAMLREVAHMTMPHNGQPLRLRVGLHSGPVTAGIVGAKMPRFCLFGDTVNTASRM
ESTCEPGAVHVSAATQALLPQEDWAPTGGVQVKGKGEMQTFLWRPPTDPGWARGGLGATSDILDRAPAARAASHSGG
PASGHGHAGAAAGGIAPAGRGSRRGLAGSNLFMEANASGGGAAGGGGGTRVASVSDGEVVRQELDALRLGSPQRAPI
VTATGTSAAATTAKAVAAAGAAGAPTGASSSTPQRSSPSPRGPAPPSGPFAPVAAVATAEAAALAAAAPAPAGLPDR
RVRVSGRGAADLATGPAGGAARP

>Cre02.g107350.t1.3|PACid:27574799
MSTSRTDDKLLNEAPAGRPETSLLYSSGSWRPPGNSLLPTPLNSLGLGGRPGSARPSWSKAGGGSGQPPKVAPLVMA
NAGRAIEEGNLFRPVEVLENAFVNSATVEERAVDCELQRQSLLFSDQPRASRPGLTAMLNYMTNNNAPPGAGTGVGS
SLGGSGGGTRSADYAAVLAGPGMGGGSPYGIYAGFDAIPEEVSGASLDYAADSTLRPGQLSPSTSPMPGAAPSRAGP
TVGGGRTSVITVGAAGGAGGSEAWFGPSSGSIGQGASRPVSSRPGAGRPVSAKPPTGNARPVSAMGNGAGAAGVVTL
SAQERRTIEGKLIASMGLAGTGLVRGESPQGQWAHTRSAKSGAGTAAPGASGSAARPVSAPRAYANTGGSSRPVSAT
MAVAAAAAAASKRRASATQAAGGGAAAVASLRPISALPGSGGMGTGSSRPVSGMPYGSGARGPMTVTVTPGEEVTLQP
HAAPQMLTSPMARPLSPNRPIIARPPSPTRTVPSWMLTRPGSPPPDEYGGTARPMSALSTASASVAAVAAPLLETAG
VPPAPYRQRNEFQQSGKKIPTGCRPLSAFSTLSSATPRNSLSLVPSLAGGLGAGAGPVVPLSESIADQPVYNEEDGP
IKTSYEVVDYFSTHGQNAHKKLFYLNERRPADMMEVVSPFDLVVVPAERVDRAQYWTMSALGVVHVEAGRPEEGQFT
PLGEWIRLSSVYGLLRKMRFFKHFLLTRSFRRWRANVKRSAFERTREVLGQLLFGAVPTFQPVMLRAGQLVQAMRET
AVVSITNRIDRLYALEEFVAEQDNRRSQVAAPALAKLVDELCAELLAVGSRIDMERERMEKLVDIPTLRANEFVTFI
PGPSGGAAAGSAAANKSKSISALKAEKATILARYDAWVAAQERMPALVRLLDYLVGCAAVELGVRGTQELLAYLDNP
AKTRGVLLVTVTFEPRTHRVSEDGPAANPLAPYVVGFGFAPQMRQVREALVHVMEAIVSMLDEMPRPSTHKPVASKY
IKPAGESFVNVAGVIMHGTPLYVTLRSGVLAHVDSSYTKALDYVRATFEDKRPIFDFSLSWDAAAYDAEAASTAAGP
HGHDALPLAFRRDMIQQRRWQRQVDQMKMSNTVGILFVDSKKMRTSLQESIEHTLEDLKLALVAAGRRAVATCLEQM
RAWAKDLEPRPADLDGYAVMRELYGNLVAAREAAFRAVDAANELFELFRDYSGKPDVKDAVALETLAETATHVRSQL
HELGEWVKERSSEMTSDLSTAMQELSSGVMSLLSDITSAALGDPEAEVPDMLDALAGLEMRLLDAEESASRYAQYQT
LFGLPVDEPGNLAHVRREFEHRKAAWGTTAEWAEKSTHWMHAPCAGQDAEAITAAVDEYYRSAYKMAKAARDDRVVA
KLKEDIEGFRKYSQLFMDACNPALQAHHWAQILQGLLGRPADDPAPGDPVSVAHLIEWGAMEQAEALSNISGAASKE
LSLRRALSRMKEDWVGVDFKMVPYKDTGSHVVGHTDEIQMQLDEQLMKIQAMNASPFVKPFRGEAEAWQAALEGLQE
LLDSWLACQSVWMYLEPIFSSPDIVKQMPAEGAKFAQVDMTFRMLVDEVVANPGAIALAKEVDRRTSLAEANRLLDE
VQRGLSRYLEAKRLAFPRFFFLSNDEMLEILSETKDPTRVQPHLRKCFEGIHRLQFEPNTNGVVSAMLSLEGEKVPF
MQHINTAARGKVEQWLLEVEARMFEAVHAVTGRGIEDYAAASAPSGGGKGGAKPKRHEWALRWPGMVVLLAAGVFW
TRGVEDALAAQKLPVYEKQCSAELREIVDLVRGELTGLQRATLGALVVMDVHARDVVAALAADKVEAAADFGWQAQL
RTYWQATDGKPVAGQPDRDFTARCCMMSASLEYGYEYLGNSSRLVITPLTDRCYRTLMGAIHLNLGGAPEGPAGTGK
TETTKDLAKALARQCVVFNCSDTLNYQDMAKFFKGLAAAGAWACFDEFNRINLEVLSVVAQQVLDIQRAIAARLTMF
TFEGSELPLKWSAWCAITMNPGYAGRSELPDNLKALFRSVAMMVPDYAMIAEIILYSYGYLAARPAAKKIVACYKLC
SEQLSSQDHYDYGMRAVMAVLRAAGNLKRRFANAAPSTANQTPEQQETEELGLVLRSIVDVNLCKFLSHDVPLFNGI
VSDLFPGITLPKPDYGPLAAALKTACANSNLQPTEYFITKATQLYEMVLVRHGLMLVGMPFSGKTAAYTTLAAALTA
VAESNHHHPEAAAADSNPHAKQERASFFVLNPKSVTMGQLYGQNDPASHEWTDGVLAVLFRRCAADPSPDRKWLVLD
GPVDAIWIENMNTVLDDNKKLCLNSGEIIAMSAPMNLIFEVGDLAVASPATVSRCGMVYLEPQQLGTGPLLLSWLTS
LPTHLGMAAAAPSTGGAAASHAARASTAGPVLASLAAMSETAAASAAMGGVKARLGACFEGLVSPTIRFVRREVKEA
APSLDANLAVSWMRITQALLMPVIGTPELLRKLGGDVKAMPVVDAVAIMALTWSAGCTAASQSGRALVDQFVRRAVA
GTLSGLEAPSGHHYSLSDAPSVPLMAVSALPPEEGLLYDYYYDLSPLLPSTQAANAAAAASAASNPAAAAIGAAGGA
AAPAATQGQWLPWSAMVADAPPIPADASFNSILVPTVDTAAVTFLLSTAINAKYPFLLVGPTGTGKSVTVVRCLLEL
DPEVFAPANIIGFSAQTSANATQALVDAKVDKRRKGVYGPAGGRRGVVFIDDLNMPQRETYGAQPPIELLRQLLDHN
GWYSRDNAFRAIQDVLMVGAMGPPGGGRAAVTPRFSRHFATLCLTEASTPTLKHIFGTIQNWFLTTRAFPDPVMELR
EPVIAATLDVYSAAMTQLLPTPTKSHYTFNLRDCSRVVQGLQAQDAAALQLSASQMLTFKTAAAAAAQGGAPAAAPS
ASATMRAQQEAHVRLWVHEVLRVFYDRLVDVSEQQWLLDLLRDLAHCHFGMRLDALLSHLTVPDMGPGGAGDSMSGT
ESGAEGSITPGIIRRCLFADYMTESDENGYRPYLEVARPSAAVSRLEDLLADYNGLAGPGKRQLHLAMFLYAVEHVT
RIARVLRQEGGHALCVGVGGSGRQSLARLAAFICGMEAFQVEISRSYGLNEWRDDLRRLARRAGAEAKPTVFIFGDS
QAAQEAFVEDVNSLLNSGEVPNLFPHDERSAICEAVRAAFKRAQPAVADPSPATLWSFFVGRCRANLHVVLAFSPAG
DAFRERLRRFPSIVNCCTIDWFHAWPDDALDAVAAKLLAEVDLQGPGQRGAVKDLCKTFHAQVTSMSGAFRREAGRH
NYVTPTSYLELISCFATLLAQRRAKVLAQQQKYENGLDKLAFTAEQVAVMRAELQALAPVLARTVAETEQLLKEIAR
EKEEVVEPKRAMVDVEVKQADHAAAAARAIKEECEMILSEALPALEAATQALDTIKSADIKLVQTFKSPPAAVKIVM
EAVCVCLDVKPTLVPDPNMAGKKIVDWWDASKRLLMDTTLIQRLKDYDRDNIPQRIIDKIRKEYTGDPDFTPANAAK
ASSAAEGLCKWVHAMDQYDKVAKVVAPKRAALAEAEAEYANVLMGLHGKQAELKELLEKLAGLEAKLAESQARKAEL
EAETELCALKLERAEQLMSGLGGERERWTAEAARLRGSFGAITGDMLLAAGAVAYLGAFTMPYRERALAAWVKQVDA
AGVSTSAGASGHGGGSHSAAFSLTAVLGDPVKIREWVIFGLPNDSFSIDSAVIAATSRRWPLCIDPQGQANRWIKAM

HEAQKIKVLKLSDPDYLRTVEAAVSFGLPVLLENVGEELDPSLEPLLLKQTYRQGAQLVIKLGDNVVEYSPDFRMHL
TTALRNPHYLPETAVKVALLNFSITPEGLADQLLGVAVAAEMPQLEEQRQALVVSSAENAKQLVEIEARILQVLSST
EGNILEDATAVQVLSEAKRLSDDISAKQAVAEKTQAALDAARTRYRAVAEMSAAVFFAIADLASVDYMYQYSLSWFI
GLFVRSIHASAEEAAAAAGAAAPTVAARANGLEGAALAARLRQIESHFLYAAYCNVSRSLFEKDKLLLSFLLASRLA
AHRGNLPHEQLRFLVNGGGGAAPAGSAAGAPPAPLGIPVRAWSELLVLSALPGSSFSSIPAHISRSADEWAELVESP
DPHARPFPAPLDSMLSSFGKLLVLRCLRPDKLVPALEAWVAGELGQAFVSPPPMDLAAAFAEASGPSTPLLFVLSPG
TDPWAALLRFAEERGQAKTLQVISLGQGQGPKAAALIEAARGLGGWVLLQNCHLAPSWMPTLERLWEAIREDNTDAK
FRLWMTCMPTPDFPATLLQASVKMTSEPPQGLRANMRRSMALEPLSEASFWELPPPPAPAAAPSSPSRGGGHRASTT
GGGGRHSSVGGAKAAAEEKPAEGAGADGPPTLKRLLFGLVFLHAAVQERRRYGPIGWNVPYGFDDGDLRISARQMRM
YVQDCLQRSNPVPYEALRYAIGECNYGGRVTDDKDRLLLATMLRRIVAPEVAEEAGAALLADEDGSEGGGHDGGRAA
IRQPADTVLEGYMSYVETLPTAALPDVFGLHLNADISKDLATTNGLLASLTATSGGGGSGAAGGGAAAGGGDAGAAA
LVQGLLDRVPADFNLEAVQAAYPVSYHQSLNQVLVQELARYNRLLSTLRTSLTQLGRAVAGLALLSAELEGVLQAVE
AGQVPAAWKPQSFPSLKPLGGYMTDLVERCAFLSGWAQRGPPAAFWLGAFFFPPAFTTAALQNFARGHQLPIDAVGF
DFEMLRAEPSSLTNPPPSGVYVYGLYLEGAGWDRAGGCLAEPEPKQLYCPAPAIWFKTVRTADSKPTPAYACPVYRT
ADRRGVLATTGHSTNFLMTVRLPLDVGPGAEPRVVQRRSDHWVLRGVAMLTSLPQ

>g2027.t1|PACid:27574915
MLEAPEPAPAALAKQAEPAQQQAQEQQLMTAGASAGASVAAAEETLGGQVASPARDPPGDLAPDHQLPALLPPPRPAC
SRALCAASSEAVDALKAESAAAAAGAAAAIADEDKTSAGAVAKREAAGLAATEAEAVAAAAAAEPPPPPRPTGANAE
ASGGGSSVVAGTSGEHGARPSSPTHRPLGAVSSSARDAAVHRGSGGAVLAAHQQPQKPPQQQQAPAPQPAHVEQQQP
PQPAQALQQASLEKEQRPPLLPPLVPPVVSPPLPQHEQRSEVREEQEGQPQQAKAPQQQASRQQPQEAQGEEPKPPP
VPPPPPRSPVASPAASPLPPQQHEQRPGAKDERELREQHVVHHQQQCNDRDHHHSGGDSCSSGGCGLRGNSGHSRTAA
VVAGAVADGGLQHTHPDGAPQGGSPASDLYRGPGPAGVAGGGGSPHVQPQPQPPQQQQPQQPQQQPQQHQQQQHLLA
GAVGDGGGGGSHVGASPRSFSPRWYVHAAAPNSPCFGAPQQQQQQQQQQQHQQQHMLLQQMAMQQQQVAMQMQAQMQ
AQMQLQRHFSYNHSHGHSFPSPMAASAPYYGGGGASPQQQQHHMQQMQALQQMQAMQPTQAYMGAAGYGPSPWPAAA
AAAQAAAPSVGFPPDGRCGGASGSVVGAAATSTVAAAAAAAAAAASEHFWDGAWPPRGKSLTRATVPYEPATAAVAA
AAPDAAAAPAAPPPSPPPLPLAPLPPPLPPPPSHSPPPLPPVSPPPLPEQTPPPAAPAAAMDGGGPAASAACAVTPL
EQTSSDSSPLQAAAAEAGRGGGTDLSAPVGASPGQEGKSTTDGGKAENGGARDCAEGLLVHSGNAGAASAAADAEVA
AAAVAVAGAAGAAAGADGAAAGAGAASYGLGRDEETAQVSVVSGAAGSSPGDGSPGGGSPGGGGGSPGALSFALQRR
HRVFGVGVAARHQQQYQQHKPVPPSPRQDGPLPLTAAAFQGQPRGSGVEQPAGTGKLQPTPAQQQPPAPLQAPPQPQ
ETARPVCAQQSPPRPSPRAAPQQPAADTAALAVAPGPAHTQCAAAPHASTSLPTSSIMAAAPSPAPSPSPAPAPAYV
TQFNASALAFFTARQRQLQQRQPVLQLEQLRTAGALTPPRVTPRQQLLLQQQLQPVPPDQEVHVRQFLLQLHLAQLQ
AQRQQQTQQQAQTQTQQQAQTHPLLLPPPSPSPVVAAQAMALQPAARQAAVVQGAAAAAAAAPSPHQPQRSQPRFGL
PECAVEAPPQLPPPSPSRLHSPSSPRRPQPASTAATAATAAGIAAMPPRASLARSQSERLLLPTSRSMMLVGLDLVH
SLSLTGSCPAPALPPPSPAAAAAATPAGAAPCPAAAKAAAEAAAAGTAGSPMRLPALPQPEQSSSPPHEPRDTTRAL
PHPPHHPHQQPSPFLSPSPPKPSSPPALALPTTPPAPGPTPPLAALLTASASVPSPAAAAAAMAAVAAGGCASPSPS
PPRPATTSPTRRPFVAPPLEEMQPVGARLVMQGAAALQALPGIRPYFGHGGRGRGRGGIAAASAGAGDGAVAGIDTG
GGSPCGLSAGGVAAAAFGGRGTQQGQQALLGLEQGLGQGLRLEGAGATTSSSPPRSRVAAADEADGAGAGAGAGAG
AGAGAGAGAGAGAGAGAGAGAGAGAGIAVSPGRRRRSGSSSAEGGGEAAVGGQGNKGSMLDIEGPGAGMAAGEGDGT
GPAVVGTEVIERRTVARLRGKAVAEGGGEGDTPAAATASAALPAAAAAESAPAAATGPAGTPAPAPAPALAAPEAAA
APAPAPPVPADTAAAQPPPRTSAVVSAPCWEHLELWADSSSSPAAGRGAASRGLAAAAPVAAAAAVTPGRKPAAG
SGKERQQPHKDEKGGSKQQQGASNQGERERGGKGRAGEDKGRREEEGGGEGRAAAPAAGGGAAAAAGAAAGPKGYVV
ECNGLVGVFYPSLYIIACRCPNCWRPATAVIATAVGATAPTSPGSRSKAAAAGSKQQQPPPPQQQQQQARSRSYRPF
AFEQHAGLGGNKRWKSSVRKVGVPAAGGSSGGAGNDGGGDGGGGSSGSGAGEYMSLGRFIEVNNIFIDQAALNKSGT
APPPPAPPPAPEATDYDGAEAAAPLVAAALAAAERAAAAAVATKRGSSGGNGGGAGETACGVGNRKRTGSTGDGADG
GGGAGDGDNAALTAPAAKRARVAAAEAKSGAAAGTIAGDADHADTTTAPASGEANAGGADKAGGAAAGGGGAAGSKA
HTRTPAKAAGKAQNKPAGGGAEGTSTADAAAAGASAATATRATSREAAATSREASRQDPGTDPGPDPGPGGAPSHKG
TASKHPHPHQQGPPATAPPPPCPDLPSCFAAMSPAQRDTAFPRIKRSEPRAVPPELQGQAMVGHRVRVYWPQDRAFY
SGHVQEYNTWHHRNTPYHVVYDDGDEEYLNLNKQHWQLDKDDVEPLPPALLTRLAAALASGRTLPEMPVVTRRAAAA
AAAAAKAVTSTTGSRGREAGPAGAGEVDGRAATAGGARSTGHAAATSAAETGAAPPAQQGLEKAGHVESVRAAAAAV
EKPPAGKAQGAKKGGRAKAAVEGGEGEARAAALPAGGEGSAECAAPAVPPDAAAVPPGKVRAAAVAAAGEPAAEGTA
AAGVPSAAAAAAPAEGAGRTAGDAGAQELTPPAGDAAAAAAAAAAAAAAGKAALSTPAAGAAAAGAAAEYAAAPPHR
RDVALAAAGPPPPGAVTVIDTGLGRMMELAQQPQSQSPGVAPALRQVEQRSEERSEAAAEAGVEAEAAEAAAHGGDV
KAPGAEEERGVRGEEEAGRQLQEEEEEAELVEEGVKEQEHVQQEEQQEEQQQEQEKEEDQEQDQDQEEELEEEEVQE
APEQERQQGEQERRTQEPEDETDEETTVTEPGAASERDAATATAAVAGGVEGRQEQEQEQAHEQEGTSTKPSSVAAV
VALTGSPRGTTPHVRKRAKPDAATGLIPSAIPPVAAGIEVTAAVATRPYAAAAAPAAASSQAPPPPPTAAPAAAVLK
ARRPRPPPQELDGCLAVTCSGVNGWLLLKHAVVACDCAACTVDAIAGGAGGADGSSSAAAGAAQAAPAGGVWHCWTP

VGWEEHAGMRSSKKWRSSLRVAAPPGGGPAAAATSAEGSGGASGGGGGGGGGGAAGGITVGAWLDRQGVVLRVPSS
GGGTRGRWRELPAAEVRRCIRTPVLVRPRIFPQQEQKRVAGQQPQPQAQPGGSAAAVAGVERAASPSAATTSTVSEG
GGGTGDASASPARRERLHSGGGSSTAGPGAGMAPVAAATAAAAAMPGRVLGGGKADLRRRSEGGSGSAAAAAAAAPA
TAMVSPHTPSALGVTGAAQDAALLLASPTPAQASAQARKRSLPAGAIGAAAPAAEPAPAPAPTSARPPKRLRLSSGA
AEAQEAGGLPAAPALMDTAAAVASAAAAAARAPPQRQAQALAPALVPAPAPEPAALAYGRAPPTAAAATAAAPAATA
AAVTAADANVDVAPGSGGGGGGGAAALDVVARGEGYTVVRTDDPPGYEVYALVSGYETNEVQVRVHDSGRLRIEAVP
ADAARAELWGVRPLRHDIRLPAGGGVRSESAVALMTLHGQLFVRVNV

>g2636.t1|PACid:27574925
MRHWSADIAAFLVLLAVAWTHGVGASHYRGGLIEYTSSGSQTTFTVTQSWRSDSLDYTSVNGCGVSASTSYSSGNII
GSGTDSAGNSYTTTRTTFVVSTPTSTCRVYFSTCCRISALVTGGDGDFKSTAQFTTSTRNSLKTAAAAVMQVGKASS
AGAYASVLVPVSSATSSGFSCAINSDGGITPPSELAAAAVAGGCRIDWYNSARSYTNKIPVPLRVTSTADAHFVDAD
FILETVNLAGAPVVAMSRSGQSLSSGAALTFTTGTMAQVTFVATDPDAGASISVTSSGGLPAGAALSASSGAAPFTT
VFSWTPTAASPSSGVVQLIFKDNTNLQASASFSFTMAYTPAPPSPGPPGPEPPSPEPPSPVPPSPAPPSPEPPSPAP
PSPEPPEPEPPSPEPPSPEPPSPAPPSPEPPSPEPPSPEPPSPEPPSPGPPSPEPPSPEPPSPEPPSPEPPSPEPPL
PEPPSPQPPSPQPPSPEPPSPEPPSPEPPSPEPPSPEPPSPEPPSPEPPSPEPPSPEPPSPEPPSPEPPSPEPPSPG
PPSPEPPSPEPPSPAPPSPEPPSPEPPSPEPPSPAPPSPEPPSPEPPSPEPPSPEPPSPEPPSPVPPSPEPPSPEPP
SPAPPSPEPPSPEPPSPAPPSPEPPSPEPPSPAPPSPEPPSPEPPSPEPPSPAPPSPEPPSPEPPSPVPPSPEPPSP
EPPSPAPPSPEPPSPEPPSPAPPSPEPPSPEPPSPAPPSPEPPSPEPPSPAPPSPEPPSPEPPSPAPPSPEPPSPEP
PSPVPPSPEPPSPEHPSPAPLSPEPPSPEPPSPEPPSPEPPSPERPCPEPPSPEPPSPEPPSPEPPSPAPPSPEPPS
PEPPSPEPPSPEPPSPEPPSPEPPSPEPPSPEPPSPEPPSPAPPSPEPPSPEPPSPAPPSPEPPSPEPPSPEPPSPE
PPSPAPPSPEPPSPEPPSPEPPSPEPPSPGPPSPEPPSPEPPSPEPPSPEPPSPEPPLPEPPSPQPPSPEPPSPEPP
SPDPPSPEPPSPEPPSPEPPSPEPPSPVPPSPEPPSPEPPSPAPPSPEPPSPEPPSPEPPSPAPPSPEPPSPEPPSP
APPSPEPPLPEPPSPVPPSPEPPSPEPPSPAPPSPEPPSPEPPSPAPPSPEPPSPEPPSPAPPSPEPPSPEPPSPAP
RSPEPPSPEPPSPEPPSPEPPSPAPPSPEPPSPEPPSPEPPSPEPPSPAPPSPEPPSPEPPSPEPPSPEPPSPGPPS
PEPPSPEPPSPEPPSPEPPSPEPPLPEPPSPQPPSPEPPSPEPPSPDPPSPEPPSPEPPSPEPPSPEPPSPVPPSPE
PPSPEPPSPAPPSPEPPSPEPPSPEPPSPAPPSPEPPSPEPPSPEPPLPEPPSPVPPSPEPPSPEPPSPAPPSPEPP
SPEPPSPAPPSPEPPSPEPPSPAPPSPEPPSPEPPSPAPRSPEPPSPEPPSPEPPSPEPPSPAPPSPEPPSPEPPSP
EPPSPEPPSPEPPSPGPPSPAPPSPEPPSPQPPSPAPPSPEPPSPEPPSPAPPSPEPPSPEPPSPEPPSPAPPSPEP
PSPQPPSPAPPSPEPPSPEPPSPAPPSPEPPSPEPPSPEPPSPEPPSPEPPILEPPSPEPPSPAPPSPEPPSPEPPS
LEPPSPEPPSPAPPSPEPPSPEPPSLEPPSPEPPSPAPPSPEPPSPEPPSPEPPSSEPPSPAPPSPEPPSPEPPIPE
PPSPEPPSPGPPSPEPPSPEPPSPEPPSPEPPIPAPPSPEPPSPEPPSPEPPSPEPPIPAPPSPEPPSPEPPSPEPP
SPQPPSPAPPSPEPPSPEPPSPGPPSPEPPSPEPPSPDPEPSSPEPPSPQPPSPQPPSPEPPSPEPPSSETPSPEPP
SPEPPGPAPPSPEPPSPGPPSPEPPLPEPPSPEPPSPEPPSPAPPSPDPPSPEPPSPEPPSPEPPSPEPPSPEPPSP
EPPSPEPPSPEPPSPELPSPAPPSPAPPRPEPPAPEPPSPEPPSPAPPSPEPPSPAPPLPEPPSPAPPSPEPPSPDP
PSPEPPSPAPPSPEPPSPEPPSPAPPSPEPPSPEPRSSAPPSLEPPSPEPHSPAPPSPDPPSPEPPSLEPPSPEPPS
PAPPSPEPPSPGPDPPSPEPPSPAPPSAEPSSPEPPSPAPPSPGPPSLQPPIPMPRPGSSLQPSPERPVPQPPGNPI
VAPNTCSSLSSGVNASTVCTDPSLLQGVVPGPVDMTQLYLAAGITFPSSSNSSNTTTGSYVVTASNPDVVSPDNSTQ
LLRIVAGLAGIDNCVLVPPGLVGFSGYFATLLANASIQLYTGGMCDSGTEMGGAMAIPAAATTASATSSPSPSPAAH
RRMLLSASSAMGGRLRRMLEDSNATAGGSGDSAAGASSPEPAPIPLQQLQAPADVFSGGQVAFSLRLYAPLNEAGGA
GDTSVRNAIVADQLIFMLRVLQELVPMPPSPPTPSPRTEVSPDTARTWLPPPPPPPPPVDEAPPSPRPPVPPPPPS
PRPPSPAPSTPSPSTLQTAVSSPPQRPPPPVPSPPLPAAPPPSPSPPPPPSPAPPPPPSPPPGSPPLPPPPPQPPAP
PVLEVLWAASSAGPVDPYGNSTALGSASRLLGAPRPGAAKRTTCGNDWGTLTWVPTLPTPRWVAVRFAAPSTAASDA
NGPATPTINLSSVRRLGVYVLNLGPLSPAITRIDLLLLPEAEAASAETASISGAAQEDWPLVEVPVYLGSAGASDLQ
CPALNWIAVEPKAVARAAAKAATANSTAAAGATARAVSKAPQGWVVVGARLSASNAPVDSKAQLPQIHALGLEWEVR
PPPAGGGTAAGTPAQQAASSRAKRPPPVPPPAAAAAGAAAADRSRIAGSKGRRRHRR

>Cre02.g110700.t1.3|PACid:27575009
MAREEELGLLKAENEQLEQQLLVVSEKISKGLGRDGVLESLQFALRKARGERDAEVREADTLFNQLQDAKAAEQELR
ELRAALTQALLGLEDEWSAVPLLAEEMRRMRARAASGMLDPTQLNQADLNTLLRGLAAIGKQGVQLQAELQALRGEK
AALTTALDAAHGQLQELQAQNEQLQQRPVASTVGSPARSALPVVAGPQGSGASATPAEAPTEALAEAAGLRAQVQEL
SAKLAEYRSYLEDNQQEIEALQGEIQRLQASANNSSQHLHNRGGRSHHASQVGQSQFPTHQAQHQHPQQQQHHGRHS
NGVVDAMKAASSQPLHASQHPTLQPLPPHVPPQAVHGTPTRHHPSSSSPISTVNYASPPPHVAFGGGSASVGAISPS
QLHQQQVAADIAVKVAELQELAVRVQRQAAEREAELAAAHESELAALRSALEAAQEAAQQELEAVRGALVTQHVQDR
RQLEARLRAAAEKQIQEREAALAAAHSQALSELYAQLATLAQQHVAALAEREAALATQHAQALAGVEARLGDLHVEA
VGAQAAAAAQALHEQAARLEAQHAQQLRELEERLVAVAKRQAEELEQRVNQQHAEAATQRDAEVRRRHAERLAEQAA
QLARLHADQAAALGMQHAEALADREAELTRLHSEALALREATLLAEAEKQWREREAELREAFAAEVVEREARLAAAQ

EEQARATVEELGRLQAAVAEAAAREAALASQLREAVREAQAQAEQALRVQLEEVAAAAEAEAAAQAAAAHNAMLAAE
AEKHQSELAAQRSQLLADAESWQAAATEQLENANTALQMRIAELESELSLLQQQLEERPKEAESAAAAQQAQELQAA
VGSVERQYVEMVARLGREHEKSAAESRAAAQLREANLDKVVERHAREAAEARREAAEVKRMAAERVGEMQDQMADLQ
SLLDRERRADAAAAASATEQLDLMEGELRALRGREEALQREVVPLREEVRRCRWLVRDTQTDLSDMEQELARYKAAA
SPPVSPGSAAAPGPAHSPQERRAVPASPTASSSSPAARQQQQQERAPGHDSSPATRNVAGIGAALPATDAASTPGMP
SEASGAEERVGSGDQQARASPTAWADGESVAWPSPQRPTPSRLLAASSAAGLQGTTTADASRPAALTPIPLPLPPPP
PTGTPLFAQAHEQTPVLSQGFQQQQQQQQQHWDPRVSEPDLAAKLVRVLRAGSGGGAEDSPEKDEEDKVSPVDGKR
SSKRRSAAAPRRALLSEGGPAVGGKPASAGGASSFWPWATPQAFSRPAAVTAQAGTDAAGASATVASASGPPRPMRR
MTADDIPSGWDGSAYATASLVDWRSADLVQRPPTISTADAGTSGPSHPPGSELALTAPVEAGGAGQHNTQQLGVLSD
EEWGDLRGASPQRSPVPHPADATPAASPSREAKQAGGSGSVSPVASVSTTSSYNSNTELCFVDEDEEDAEAQQRVLA
AALAESRRVSMAEGQLPEAPPLPPALVEIQQRRMSVSGSTGEAAAAGARVSLSGVAPSSSLRGRAVATASSGGGVAG
GNARSVLPANVRRQGDRAAPRPDEECSGDSIGEAEHSHRIRQAAQSAWTHSGSREGKEGDPDQEQQEEPADFDMEAT
LSSSFAALVIRQARETGSRAGVAAAGALHTVEDEEEDTDAEEAEAQGGEEEAENEEGKDCLDEDGDGDQHYGGGRGR
GGGGEGSTSEHDSYSYSGDGRGHGHQQEPGTQEDGLDMEATLTGPLPALPPGVLLQHGSGGSADGDGAASGQPPSGA
EDNTGTPAYGRAGSFDFVAGSRRNGLPAQAAEASRSAVSSGGSAPGPSPLGLVDTAPMLGECEEQQLAGQEEYDQTG
RRAATSSHFPAERGQDAGPCSGLSSPNYSRGGAADNSAPQSSAGSVQPHAQDGTEEVWRIWGGGVSAGGGDYDTGEG
LGCGLTGEPTGSSLTSTSYPSPNSRNHHHHTQQLQRSPATAGVAGGSHALQAATATSTPALAASTSPQLQQHGGSPP
PRMRSSDGIGSFRLSPGVSPHSQQQQQQPWYRSSVTAVPLMTPNPLFEDRGSARSGVFAGSEPSPPWSAADWSAAGG
SAQHPAADDAPSMPSTPGSVSSPASAGSVNSPGAAPAGPPSGRAAGGRATSGSSDSEEGLAASDGEADGLHAEEMDL
EGNEGLTEAEDDGEAVVALRTLRTSGTPMLGDGTGQPGIDTGAPSRYSGAPAAIPGSPGLLVRQRSGKRSMQGRESG
GLSPTTTAAAEMQRRDRRSSDRLARGGHVPSSWAASDAAAPQGSSGPPVAADEAAETRSLLAAGASLGSSELRKRQR
RRMGDFPSPLALSFALDSPLDAGLLTGNATPAAGVARQPAVSPAPISGPAQVSESQPGPVATSPASPFDPLRQSWEF
SCGVATPLSPGHRSRVQGHVVGHSAPRGYHQHCAASPNAAHSPSQPARAAAQPGFARLSADSAATTGTRGAGVGAGP
APSRLHPGTPGVPQASTPGAQLPPPVPLPASAAEARARGREVLGRVMGRVASLQGLCAHLSPAASGAATPMVASMPG
SANATPRRSASSLSGTPAPVLQGSVISSISAGAPPADTAAAAGGAPAASITTVTPEIIRQLLARHPQQHTLHQHQP
ALPQSPLLAPDDPDLVAGVVGALEELRTDLFELEKLHQLARLPQPGATGAGRQPSAAGAPISAEAAVVAGRAGHGTA
AAPSEPGASAARQQPAASSLLPPGSPMLAPPAVFGASTPAAPEGITAPAAAATATPGGNADPVLMAALAQTVQSQLA
GQLGALQAKIADLQRQNQTCMQRGGDGGCSPASGASTAYATPMLRAATGPAASTGAASAAPGASPIQQLLDLLTPTS
AAPAAAASTAAPAAETAGGMSAAQPPTRTPLHPFSLVAVASAAASGVERGAAAAAAATAAPFGGTSAGATPMAAPAW
GGILAPPVPHSFTSEPRTPLMSSLRSPANAQPAAYGGAASATPMPSAQQAAAGHAPGVSTPPPLSGSVQVGHSGAVL
WANAPPVTPAAAGVVPWASFATPVQHGVPSTGGSRSSGPPSTGTYSSTSPQQQHTSMSLYPFAFGHGGPGSGAGGSA
GAHGGSEPAWSGSGGGTHGAGMLPGHRAMPHQPYSPEELSHLPPGVQLFAPPQLRAAAVAVATNSRQHPWQQAQPQS
LPGLGREV

>Cre02.g111550.t1.3|PACid:27575035
MVVFDGGGGQERGAKRWKRCWSGATLVLLLLLGAAAGQGFLRLNSTAFNVTLVLQPATAEGAARQCLSVPGLDGPGI
LAPATLLLYVIQDVLTRNSYSFQASLRTDPPSYNTGGSSLWVMDPFGSCRALLLPTRIATWSGAVELSVMPCSYTLQ
FICMEYKVAQELSQGLTLPHNLYRTPEPPPLPQAVPYIPSATGPPPGYRLVTSGVKLGHVGAVPEGAVFFSSLQGYL
ENGPIVNLVGRWRPTGLDAVVVVNADDTSNVVETVMTGNSPAAGVHGDPSPAVGIVWVASPLMREPLPPRPEQGSLS
TSYITRVEGCASSNAVERLLLQTAAGAVVAQGVVRCISTFSEEAVPGGFLAAVEGYYVDDIANTSALAAAASASASA
DGTLLPSIVQMWFVWAVPEDASVPDPNVAAGAAGYGLGGVWAIAPRLAPVQSHPNCSDARQLIPATLPASYYVTDAN
GTRVRSECGALSADGYACPSYMCCGAARGRVGQCGVVASACLLSSCDPSYGLCGVVPSDLLEVTAHEGVALVAPAPS
LLGGPPPYSVDLGGPGSDVTTSPVRLASSVYVLDKTGAYQYEDALAQCRSQTLLGLNWTLATLADAMSFEVSLLTRT
PFLQLLDNRVAWVRPRRATVASPSCLTVAWQVDQGSIQLVAVQAADCGTTASVICTATWPALSNAPVGIDNSSGSSI
TTATTTSSASSNGVNESSSVAPVKRAPVLRQWVAGPHSLSYSLVGVGSPNSGPTCTFETIDSLFTADQIVALRKREA
DFINTGGFGSVNGAGSVVPLFMNATLTTPMAGLALRPLLTWSAVSVEYAIAYDAVASMAIQYANVTGGARGILDGNT
DAAASAAAAAGGRAASLSSPPPPSPSMGPAVVTLLNPLILGSLDGTYLSPNDTYGLVPPASPAAAGISAYVSTAGGW
RAFQLDVANGEVVVEVAGCRGGLLEQLVVVTSTGRVWTTAIGTGLACSVPFRLVAPPGGYLIGMQVHAGSFVEELQL
VWGTPMATQPAGGGNSSGGGSPPPHSVVVAANTGNDGERDHQQAVDIGIAVAVVVPTLAVALVVAVWWWRRSNGHGW
CGRAGAGSSDLASEDGTKTISSTGPADSGGADGLAGGRHTGTNGNGGAAAAGTGSSGSDRSMELARVVVDDGTGTNS
TVGTLGILMSGTGDTDVRARTLASSLGTGSGVGSHGIAGGSGLGNSDGAGACPLPRTIGGGGVSGTTMLVDNSGTHD
GMSGTPMLSTSPAGTGGGMVGQPGTGLLGSDTSARENAEAQSGLAAAAAAAGVPLSCESGRVGVAADGVPQVLSFKR
RRSSATSQAIFGTPRSGTGTGAAAPSHRTTASSSSGSVAVAAAAAAAAAATAAAAVGRPQSEPSDGTGGTGSGAAHG
ASAALRFVDGKTRHPAPPSNAPSSYTPRSTQSKVSDAYGSAVAAAAAAAAAAATANGETGGIAGQQRAGGNPLALPA
PQQAIWALPPAVAALQPPPAPVPAASGTPLTPEEWAQQLHAALFHKVLYQWQPNALSGMHSPAAAVPIPQSSASSSH
HPPHPLAGSGSGRGVGASASAASGGVLGASSASPLQGGHPLSAAVATSSTSSANPMLPASPPPSQHTPPLAASLASG
VSAQMRTVGSGAAASTEHSAAPVPSHPGGYDSPAVLQLPQLHQTPPFGPSTTASPTAAVAAAAAAAAAASRRLDPVA

AATLQFLQSSTLTAGSVPSPGVADAAAAAAAAAAAAVVAAAASTTAAHQQQTGSAATSAAASAPAEVWAVHRSPSNA
TALVPSGISPGHSMPAFVPASPSIPTVAVSGGNGPEAVPGASLAAASATAAAVASHLAASTGSGGAAGPASGGHGNA
PAPGAVPLLPAVPHRPQLPAPPFPSQHQHHGEGPAQPSSAQELLLGRDVLVDVDDPTTYLGHGTSGVVRRGMLRRAD
GSWALVAVKLLNRPDDQAIEHYRRHLKTLLQEMTILGSLSHPNIVRLLGGSVSLSSGASFLVEELCGRTLSHAIYDD
STPYSLGLVLRWATDIARGLAYLHPNIMHRDLKPSNVLLDHAGVAKISDFGLARFKVHTTLVTRDAEVGTTCYMSPE
CFVSNDFKVTAACDVYSLGVMINEICTRTRPWSGVRTAVVGFKVAVVGDRPKLPPEDSPLCPPQLRRLIRAMWAQQP
EQRPSSGAVLAKLEAMLEAVEAGLPLPDDGDGAAAAAATYRGQEEAGQEIMGQEEVERVDEEQEQ

>g1842.t1|PACid:27575152
MVSSIGAVREGFFSSVMGTLRAAGVKSPTAAERPGPGASRRGLSAPSAAAATAAGAAMAAEAAGIGDCSLGKRRRRL
QPQQRRVTAGALLSALVVVTALAAAVRGCGAADPAAAAAAAAAGTNGSGDGGGGGPQEAVQRDYRRHLTAAAAASNS
TSPGVAAAASPAATPPTGSTIVGPSSPTTGAVSSSSTSSPATPAAGGGGGAAGSPAAGSNTNNSVGTTTTTTSSSSK
ASISAEASGDSGSSTGGVPSAKGPSPVPVPSPPGSGPGSSPPPRPPPAAATNSSTVTNTSIGSSSCWVATRDFPVRV
CVRWTEDCRLANASQVEGCDMCSKGWGSYPSQRACLDAAGGGFEPSAAGIDPATYPPPPASPYANTCGYNPNSRTRS
SSSACPHLLPDVTLWEAAASWPATGTNPGGVPAPGSAVVLPAGRRIMITGCSLRGTAGVANAFASIRIPASSELIFD
DSDISLVVGAVYVYGRLRAGGEGCRLAAPLSITFAPAAGVPESDLGLQVLPGGQLDLHGADSRPTWTRLTATVRPGS
NWVSLRDPVPAWKPGQLLFVATSIYRDEYENQNEVMSIRSVSADGRTVITNEYFEYSHYGGPEYQSEVGLLSRSITL
RSWPGSAATSRGGHVRIMGQGRLEGVLGYRLGQLNVLGAYPFHWHNAGDASSGVSYARDCAVYAAYYRCFVIHGTSG
LTLSDNTAFHATGSCYYLEDGVEERNVLRRNLGAFVHVIGNPAGGVTQSGELFVQGPNLAHPSDAAAGVFYAANPNN
AFQDNAASGGFAGFNFPSLPAPIGDHRWMASTLNPSKRPLQLFDGNTAHSSGYFWIMAGGIYVGGRLWVNDADGGKL
YYSSGRYEMDTRDDTGTALAFHVFTNNKVWLAQWGISHWGARARVVGWEAHDCVRGANIFGLAQLSNAYINGRSANA
DAKYPAGDHDPIAGFRWYDTRVMTVLTNITFANYPYTPPAAPGGLMGQSVWFSMPHSDEYKPAYISASRNIAYRAVD
GRALVSNPPQSTGASRFFNWIDHDGSATLRGRPTVVGSWPTWWNLGPDCSWQPLGGVYFCDYYPWRTVGRLEVRVPG
YTVPVDSGSAFPPDPAFELGYVAAFGHRGADARSMTVTRNEGITGVSGAGGWYLHLNAGVTPALQVFLTQVPPGTSI
LFATRYPAGTTFSVSRQFLWYPALSSAVSQAGSLQEVLDGAGDKYWFSGKHLYIKMADPGDGSVDPPFSVDGAAVWG
TRYFSAWYWVNITRLPGGQDPYSACPWVSGTPSAPNPSTGTSYPPNPLSPGARFCPLAADPATDVPDSPPAAYNSWM
DPFCTDVAPPGSVLSCSQVAAQGQCGSGAIRAPADPHAAVIGGYCAVSCGVCVQGVRACVDRAPPGGLSCAQRRAAG
NCGADWMRAGSWCGASCGYCTPAQPLPPLPPAPNCFDVPVPPGTYTCKQQLDWGQCSQPWMAANGWCRATCGLCAGM
TPSPSPSPSPSPSPSPSPSPSPPAGCYDVTPPGGYSCAQQAGWGKCSADFIRLNNYCAATCGTCTSGSGGGSSTCSDVA
PPGGYSCAQQASWGKCNDGFMLPYGYCAVTCGRCSSNSAANCYDDPYSACPWVSGTPSAPNPSTGTSYPPNPLSPGA
RFCPLAADPATDVPDSPPAAYNSWMDPFCTDVAPPGSVLSCSQVAAQGQCGSGAIRAPADPHAAVIGGYCAVSCGVC
VQGVRACVDRAPPGGLSCAQRRAAGNCGADWMRAGSWCGASCGYCTPAQPLPPLPPAPNCFDVPVPPGTYTCKQQLD
WGQCSQPWMAANGWCRATCGLCAGMTPSPSPSPSPSPSPSPSPSPSPPAGCYDVTPPGGYSCAQQAGWGKCSADFIRLN
NYCAATCGTCTSGSGGGSSTCSDVAPPGGYSCAQQASWGKCNDGFMLPYGYCAVTCGRCSSSSAANCYDVQPPGGYS
CTQQAGWGKCGAADVMMPYGYCGATCRRCTSGGGSSTTAAGAGSSSPSPSSSPSDPYGSLLGSLNNWLFAAAAAEDD
SAEVGSSAAADSSSSPSSGPTAATGVAAGTPAARQAAAAAPGFVLNPATTTTTTTISAGASPPPPPSSAATAPAPAS
HSSVGSTSTAVAAAPSPSGAVPQRYGANASYAPYQTALWVAAAASLSTDTFAADTDRARAALGPPAAQQPQAPQQQQ
APAPAGSVVTGGVPATKGSSPSASPSPSPTLNAPTPFASSANGGSGGGGGGGAVSGAFNIGSSVSTPAGATAAGSAS
SAGSSSAPQAGGTVTAAEAGAAGGGGGGGTLTPPLNPTFDAALNPFENTTSVASPGSPLAAAKPAFYAAWLRSSGSS
SGSSSTAGSFNSASADDDWVGSVSGSSSSTTGAGPGVVGSDGTFFGLPLLPWASVGVSDGSAGNVSNSSAAGGSHK

>Cre02.g081700.t1.1|PACid:27575177
MPVAAPPTQSAEPPSPPAPPPFLEPFGPCRSSLKDQPSTLSPFTSRFTTHGYSKYPETVFCTTLTLSPPEGASLCSS
MGAYTVSLLIEPKCRAVVRTLLVNGAEKSPSVMDAPTLGALVLKWTQLDIALTAQPQTTELCFVVRRRGVGEDGGDE
GGCGDLSGVCARGLCQMSVLDNPDLPGTNCCPTTYLNLSNVTGSDSPPYVPLPPSPEPPTPSRSPRPGAAPGSRAPP
LPPFYLPDPSPEPATMPTSPPPVYGAPPPSPEPPTPVDTPAVSPQPSRSPRPGAAPGSRAPPLPPFYLPDPSPEPAT
MPTSPPPVYGAPPPSPEPPTPVDTPAVSPQPSRSPRPGAAPGSRAPPLPPFDLPDPSPEPASMPTSPPPVYGAPPPS
PEPPTPVDTPAVSPQPSRSPRPGAAPGSRAPPLPPFYLPDPSPEPATMPTSPPPVYGASPPSPEPPTPVDTPAVSPQ
PSRSPRPGAAPGSRAPPLPPFDLPDPSPEPATMPTAPPPVYGAPPPDTLPGGGYPTLPLGTPPPSPVPEPGNGNGNG
NGNGNGNGNGNGNGNGNGNGNGNGNGNGNGNGNGNGNGNGNGNGN

>g1898.t1|PACid:27575255
MAQPGRSQRGSPALLVAFAMLLALVPGAVAYVKQVTIGTDHQCALLASGCVKCWGRNDRYQLGYPLVPPEGLGADGA
ALAAAPCVDVGANVTVTQVEAGHKFTCVLLSIGEVRCWGWNGLGQLGRPYTNGALEPLPTNSSTAMGDALPFAALGV
GKLAKQISAGYCHVAALTADGLVKTWGCNHAGQLGYEYCYPLSITGTTNYCVNYNGSSSVTASSIARHFLQYNSRGT
LIGVQSRRQLFLEDSRPEVQLGLAAGVAVTQVAAGDSHTCALASDGSVRCWGGGCDLGTALGRLFDADPEAAAVPAQ
HNGSLWASSTTPVLSQPYLQPMCTTLGHTARVTSFGAGSSRDGLNFQMGANLPRVDLGVFGGARKLVSAGTHTCALL

DTGAVKCWGRVESGTLLGNGLAPTGAVSLSAAESSAARLGTGVTVTDIAAGGARTCAILSNGRAKCWGPGSYLGYEQ
PLPAGAAGWGSGDSSLDGGLPYLELWGAGSFAGPVRVVQMALSKRTHNASNAGTICALLSTGRLRCWGADGGHGDTL
PRGSPAAAGRMGAALPDVPLLPDILQPPLPPFPPNPPPPAGLLASPPPPAPLPPLPRLAPGAMKPPAPAPPPPPAGS
DAVLSQAEAARVLGGPGEGVRRYITPTAQYLLTTLGALLIFIAVGGLVLGWLDRRHAKQQAKAQGLAKPKFDKAAFA
RAIEGGVVAAINPRLQVFRWALPMAVLLALLTVLTVALVTSILQEQCVTTCSTVPLQLIRLTDPRRCTPLDVRDTLP
GYGDTGGDSGAAAAALAAAAAAADPAAAAAATLAGAGAAFAGGSLHSYARVGKCSILHSQDCAVAQWSLLARCAAAA
AAWAGNVTTAGGNGSSTLQSWGRLLRDGRGMPDTPLRFSPADRVASPPPPSPAPSPPLAPNFPPPTASASSSSNSSS
GSNGSSSNSSSGLPPGAAPSPPPPPLPPPPPPGMQWPDQPCQLHTSIRYGAACGRPGDPEEGHPGDVCWVLSVETAG
YEPDTRGAEGTGCRNTDTSSAECAKTSVEVVVWEKMGSPQMHGPPACPDVSYVSPSDDPPGLRSPSSGAYLPPPDLS
AGLNVSDPSLCGGDSYTTDHGYRCLYQRSRMCQLVDGGSRTCAEVSSTDESELPALWLGAAGLGFAGLARDPAAHTC
SPWPLQPLDESRLLIKGLLVRMAETATTQVAARLKYFSDATIRVTFDGDFAQVTVGKSRLAAYWSTTQLYCCQPVCP
SVLASLGNALGYAVYIEMVFSLALIVAYMLVFRKEANTLGLRWSHLASIVTEQDIAYDRDTALSLHRAEMRIEAEVA
VAASGMRPGAVGAAPVPRAGGAAVAVGAAAAGGGGVFQRFKGRAKALLQRPRARVAATGTAAGAAAAAAGAVGAGAK
SGKGKGAAAAVAVAAAPAGKVAAEASDGAAGDGGGSGSGRGGVGGFFRRMMPSRLNPRVASDSRPAAATAAATAAAA
AAAAPAASPPASASAAAVAAAAQPPSGNKSMGSKSTKATKAAAAAATAAAAGDAGHASTRAPATLMGAGGKSKVGFA
AGTGGGGGSGSGGGGGEVELAGGGKEWAVLAGQQSKPAGTKSLPAARSKSTSGVAALKEAQSAKSIAIPGGRRMSVE
GAYATPLTIGTGGGGRWVAPPPPPAAAQPVATAAAVITPISTMDGTGTGGGGGGKGAAAAAAPPPPPRDRSAASTAG
AAAQAPGQGGGGQHAGAAAAATATVAAGAMSRHASRASIGGATAAAVAGSRGSTSGASQGTSAGGAGAGAAAATAAT
AATAAGGGGGAASRTNAALSRLGGQQLKLGSPAVPPPVAVPSASPAPAVLGSPAAAGAPGGSGAAAAGQKSTKYAAG
NKSTKYGEKSQRAPPAAAAAAVAAAAAAPARGAADSAKSRRSSVDQLLADLDEV

>Cre02.g089650.t1.3|PACid:27575297
MEYLILTHRRACRAKRPAVSALVLLAVVLGVAHIAGAYRLSTLNNGLERVAVECCGGIEYAVLEGDTCDGVAALFAV
AKRDLLAWNADLSCDPSNVAPGTTPLTPGAAVCVSAPRWNCHGSGEPAPASRAPPPVQLPAIDTAAAAAAAGVAGEG
ADDNLSDHNGVNGADDNDDLYDFMHGSDTASPSATPSTTSASSKAGSALVRNGAPARAIAGMMARRQVQVRRYPPPR
NSPPVRKPRPPAAPPKPPKPPRPPPPYPPPPSPPPPYPPSPPPPPPPPPPSPAPPYPPPPAPPAYPSPPAPEVAAPPP
YEEEFYPPFEWTAPPPSEEGSNLPPRPKRRPPSPKPPRSPHAPKAPVYPPPPPPPPWDSPPAPPSPQPPSPHPSPPPS
PPPAPSPPPAPPPSPSPPPAPPPSPSPPPAPPTEPRSPRSPRRPRSPTPPEPPSTPPSPPSSPSPSPSTAATLSVGP
VRLTTPLGTLVDLDGTTCSVSCDPCFTTAFRDRVLALSNLPPAYLDPLECRPAAASPAPSPVPGSGSGSSSKLASAG
GAAEAAGVALGRRHLLAETTTAVTSAATAAKSCDSPSGELYLPLRLPTDASAAVYASNLILALNGWATAADTAAAAA
AAAAAASPSGSSSKTGDGSTPLQFCVPTIEQVMGLGESPAPGGGDVTAALTAACAGKATGAAVLIGGLTYYCPDSSP
QTDGGGGGGKVSGAAAAAGGSSNGKTLGIAAGAAAIGLAVGVGIAAFVFIRRKRAKKAAEAAAAEPEAQEESSRHSS
AATSRKSSQAGDGEEDDKEEEGADEDENEAGSQVSSRKPSTTTHASRPASASSSKRGAADSAVTSRTGSTTEITPAP
AAAKSSPLDGMLPKLLLAKHAAGATNHGRRKTLNPLPGAMDASSAGTSAAGTPGVSHGGAPPPEAAAAQPPPLMAEV
AMVAGAASGADDDDSAGSEPSSPRRRQPTPPASRPRSPELAANPLPPRPASPPSPGPESEPEPEPEPQPEPEPEPEPEP
AGFILRLKPLRIPTLTRHRGVAGDSLAPAGRGPREAITLNEDAADSRPDSPILIKVPSRPRAAAAPEATLIAVAAAK
KREEEANARKGASGEPAGGSSTSLFALGGAASMAPPPRPSHAASFGVDNNATWVSGMPRLNKATSLNVPAVSAAGAM
PWPSAAAGAAAGSTSTPGQLPPLRRMFDGLKTHAWTSPSEALLAKPSLQAAASAPAPPAALSSAETAARALLDSALE
GLDGRPSLGSSAGSGSGGTAAGERSSNTSSSSGRNSGGASSLWAFSKRSQVVPEALLEEDDDGGEAAASPSRPSPSG
GGLAARPVVTDGAAAARRGAGLPRAMSFMPNRASAAAAAAALASTSGRRSSGGGGGGGGAVTDAGAAGGGVVVPQVG
TAERSSAPTPMNRAAAAAAAAAAQTTTPPPWRPGGGGWLRNARSMPR

>g2385.t1|PACid:27575375
MALKTPLSHASLSSLNTLLRLENYCSFVTDGYLKSSATSVVASGTKDVYVKRKLTDYYLGCYTAFNASGTPTMPYTL
STSSVTVASCAALAQANAWFYYGIINGTTCVVTTDMYRAYSGGDGLNNCTATCAGGGGGNCGGTNYVSLYYLSTDVN
PDPMYNFCFHAGLDIDPSYATTYNLTSAGTTNFTTLAYECLSHSDCQAFTSLGEVRTVANLTYLQARSYSTSGYGVY
LMTDLPAANIARRRSAYASSSLATVDSSWCTSTGGCSANPYATDGILDSAQGVYTHNTSDAQPWVMIDLLQAFTLTE
VWAYTRLDCCHGLINPEIRFGFQPVSSSSVTQFIKCNRLYSSSTLEGVQAMGYKLTPRAANIVGRYLTIQNLGPAAD
SSTALMLTEVQVYGVGVDSYYPTTLVPTAWPETLRMDATVLVESEGPDDSLSRWLGYTNQSSTASLALNITSGSGGA
GGCDWHLSSDGVPLGISFNGTCGMGITGGANISTWNTSATAPFAVVLVLKRPTGSPAGVLFSMGRSPTTNVSQLVWR
TDGVSITNPANTAEELQLFAKTLSADVWSMEVLQRTAAGDFQYLRCSQSICWETTNSSVSVGPLSVLSTDLTLGWDC
ATASAYSNAIFAGVYVYTHALPQSTLNQLADYYRNRLPLASLAPPPSPLPPPFPPRPLPPNPQPPSPVPPDLAPQPP
PSPVSPTPSPAPPSPAPPSQPSPPSPPPAPSPPPSPPPPSPPSPAPPPAPPARPPPPPPMPPVPMDSIVVPDLNASW
LLGSDGGSGAVKDTTAPSITLVGLAELMVERYDPYIEYGAIAYDDVDGVVRVRISIASSGGGVAVLASNNTLNTSVV
TPPGEPVMVTYTATDKAGNSAQQQRQVAVVDSCADVGEFRCPTTRACSVFKVCGIVSGGAATGSGDGSVNDNHQDGS
GSGSGGTGSSRGGTVADVTPPSLLLSVGEDGVAFTTPSGISGVQHTVYVGSSFVDPGVTAVDQVPAPGGTGLPTVLD
LTADVVTSIRDPTGAVVAAVSAAVPTGNATGGGAPFTITYNVADGAGNRAAASVRLVYVVCRQPEVACPDSDGGSSA

FPDLTCSTSGVCGVGNALAQGAADAASGGSSAGSSNTTRTAANTTSEGAPRFDLLGSSAVITVPKGAVVEPCREGSS
GGSACEPGFRASLSAPGDLNSQVVACKWGVPAGATALPFRLAGLRYCGINATRPDTYNITYHLEWPSLPLGEVVLVR
YVVVEPGCDPGELLCAGTQQCSVDGVCPSEPVGNGTAAAATRANQAPSLELTVTPELGLSVSVPRGITYKLCAAGQS
PTTALPCEPLGNATDLEDGNLSSQIALCPPSTSSSASCVSTQCRAHWPDRKQPVDCGVDTMTGAIGTTYRLQLVVYD
SAGASVAAERVVTVVSPCTEGKYYCIVEGSYASDGSQSFECLDKSCEDHAALQASVAALSVNETRAHLYLLPALHSA
NLTQAEADQAIRLAYRQPAGFSLAPCAAYVEGIASAAPTCAAVFDASYANDGDITGDITTLVEMLCNNSSSSNSTG
CSSGCSVVGLSMGYCVPGRYRITYSVRGALGDPIMHRLNVTLEQVSNSVLEFGLYPGGSAAASNDSAAAQAYAAALQ
SNATFREAALLPRLQSLGVSSADMRVLSLVTVSAPEVVSVTAPAPAFVVLVRANLTLGFSTPVDGSTSGSSSSSRRR
GLAALSPAHLAMAWTGSGQRTGPEDYGEGAGAVQLTDTAPPQGLVWVPAAAHGHTAEPQQRLFRARQDLGLAMRLLQ
AAAGEPAHDGDSTGREGADKGEQEEGVDPRVRRLLQSTSAVGCTAPAALSAASSSNASAAAGLASFSSLSTSCSTPS
INAVDVALGVLTGAINDLLNMDAAVSAVQSSMGTAADGIDTAEARLAESVKASALDQFSAASSAAASEESRAATVSQ
LLDRTLALQADNAAVLLGAIDILQTALNEAEINTQRAEVTTARVLDGWGLLTGGSDGSGNGTAAAGLVSNETAAYVE
CVYGRAEAVPFYFMTNATRRSNATAVVQGNSTRRRLSAVLQDQQLEEALQQLRQHAPPAWRARRSLGPSTSRTLVGS
GVCWLIPSERHDNVGPASNGDVSALEPTWAGLLAGWLQRLRARTIGAARRAWCPAPEGGVGNGGNARQQQDSPRVTE
RSILTGSGGASNGRGDSSSSGAVFAGYSLPEDNTFDYSLYDVRNLGRPRFAGLNNRVLAGLLLHQVRYTASDVDASL
LGELATAGSCQPQLYGGRGTSGCAGGNGTQRILASVSELGGIGVDPVFIKQSPLFQPSLDPASYYNTSNGSVELNPA
GVPYGFFHQPLGRRFPDGYPVLLDTRLSALRAEQALLFLRDGRYLSTTLTKSVRAQLTTYNPDARVFGYFRMDLSWS
DSGVIDASVRVLGLPAVSYGESIMQARVEHFLPDFFLVLLAAGYTAMAGFDIMQQLRHQREEREVKEIADRQKQQDR
RRKRRAATRRRRTEGSRDPSTRSTPAANPSMSRHRSGGSRSGDGAGIRPRPFVASYTAKADRAAVSSSGVVLTSRSG
AVAEPAQPAAVTGPAKGIASWLPSANHSDNAALHAQQPQTEDANDSTGHRQQLSAKAANEPSPLRYSAAGGVDRAGS
SISSSSSSSSSDSESDMEKKRYQAQGFVKMPGINKSELRSQHRSYGRLRYQPRLTPGWVLLEVATVAFMWAAIGVYF
TYTVQLSVQNKFQGRYDVYDSDTYSPARYFLLRRGSNTTSTAAVADVAANATLNSTTAAAPAAAAANASELLLDNST
RRLLATAVGTSRLTSVTSPGSGGLRFSTSTSVLGGSGLTGNDGTGSRWTLPADSSQLADAGAMFSRVDAMYDNIVLY
GLLQSFALGMIIIRWVHHISFQARLGVILGSLVAVASDLFYLAIVVVLMCIMFAAAACIGFGYSQLEVSSYGDAIYL
LLKYAMFMEDGGVLKRLVQDRNGQYDTTQGALAALLYALCPLFFGFVMLNFILAILLWPFPGLKFAVRKQPSIPQDL
GVLIKWWWQHLVRAAPKNKRLYERVKASLAKDSHQSLLAKVSSHLSMRELRGGMRRLWTRWAAACGPHQSDSQGKGM
AAALEALDEARGQRRVWQLQPQHLSPLGQRPKHQLPEGKSAAGLAAAHGSSTPDPSDARLSKLSDPSTWGAELQHQQ
HQGPSGKGRLRASLRPSVVMPLPPLSPRPSSSAGMASPSYWKAGGAATLAGMGIGSPDLGQCGSVGSMDASIDSIAI
RNAGGGALTSVVTVAGMHVRLRSGDSAAGSTMLSAEAAGAGAAAAVSDATLAGHEQQQQPQHPEVLAEAVLDNLVRR
VGVWAVSRVPKKLKWGKHRRQAEAAVAGQGAQPARWLEPPTDAAMLRPLAQADSGDTGAGGLSNGKFTPFANTHDTW
DGEQSSAVASYSAAVSPRFKQGVPVLSVAQSTPQCYSPGSLHGPGPAAGGMANSASGSLTGSGSGSDTRGHVMAAGS
VTLPIMPAMAPGMFPDAASSATEQEQAAHALSLPGLGYPASRDVSIRGSTGDGGVPASSVLRDAADVAAAAYATDTG
GAVGLSDNPNLAAVVATSLAVDGAGGALNHTPSWSVRLAARAPSLSSSRPASPRSSYAQHDLSGPLSPGPATAAAAA
TSPLPLGAERPPSAIRKQRTSTLGAGAAAGAPGADRPPSAMRGTLTSTLGAGSMSRAFTAPSIHDAEAEPDVQTHAL
GADESRAHLAAWPSQHIHSAAVFERSQSREKVWPGGAAHAPSRLGRGSGNGGGTPRGSAAGGLFGPGPLFAPAVAPA
AVSAPLPSPSGAFTPVTAVVATGDDADVFLAAVMVVDNGNGSSERVEGRGADALSLDSSIMGRRAAPVPGSARSRAA
VRTLPAILSMGGAPEQEHHEMQAEPAPELSGAVTAVRLAASMEEVEEDDDMEEPRSAAVLDVDAHEPQAAAVAAAQL
PSRRWMRPDAELGGAASSAGPVAEAREHQSRSRLLPRASPALDTSQTGAAAAGTEVARPPPVEQPDVEEMQSWRPAM
AAPKLAPRQGANMAMALSRIESLIAQLRELVGHLQAATADVTAMSAAVARWTEAAALVAAATATATGLQQQVAVEGP
AHRVMFLLQLRAVLAGRSLTSGTAKVATGAQQEAAGKSGGRDVTVDNNSGRTHVDGTSPRGCAALAASGGSSTRNRS
SGDSNRDGPKLEAPTAAPPPCTEAQAAPLGSHASGEASANDPTAFSDAHTDA

>Cre02.g113752.t1.2|PACid:27575387
MCIRVEAQPRVRQTHGIFTIPDTGRCAAGQTLADSSDQAGPSSSSAPGMASAAAPAAPAAQPQPQPPRYSWMPYVRE
LTLVLSPDPPAPEPPQHQPAAAPAHAAADHGPHGPHPQPGPEPAHMQQEEGVGGAAGNGGAAGLPGAGVAVAAAPAL
LLELDLGAPAPHQHHHHHPNWQQVQAQAVAQAQAAGLGGGGQQQQHQQEHQYQHQQHQGAAAAAGGPEPMAVDDGDG
APAPPQPPQPQHQHQQHYDLPQLPLPPQHPLQPHNHQLPHHEVVAAWAQHLADDLQALLAAAAAGEGAAGEGAADGG
GGDAAGGGGGVAGRVEGLVGQVEAVLAHGAAGPPHAADEGGLGVEAEAEAGQGHAGAADVEMAGGVEVEPAEAAAPT
AAAAGPFRAHAEAVVAAVLAAAGLPPHLHQHLGSNQPPQQQQPQQLQQPQQQQPQPQPHALAPAGARVSPAPQAPES
QPSHQPHHPDASGAGPLQPPPQEPLPPPQELLPPPLSEEAAEELLSQQLGALAAGLLPSLPHLHTLSLEAVPRRCMA
AAGAAAAAAEGISELRIERVTSPPPVPPEQPAPPAEDGMNGVAAAAGGAGGADGAGGLGGLGALAGLGNGAGALGGL
ALLQELQALFAGLPAVMPAVLPAVVPAGLHGAGGGGGGGGGGGGGGAGGGGGVGAGGDGDGAGGGGGGGGGGGGGGG
GGGGGGGGGEGGGEAIEGVEAAGGEHVELEMLEDDPFQQLPQQPQQAAPPQQPEQAAVGPHAPGDVHMADAAAGPEA
PGAQQPNEQQQPQPQQQEPLQQEPQEQDLAEEDADAAAAAAGGAEDDAAVAAAAEAAAAAQVLEAVAAHVAMHMQG
VVAEYQDHVAALGHLHGGGGAAGGAVGGGVAGGAGGAGAGAGAGGAGAGAGPALHDHVVAAMAAAGLQQQQQQQQMD
AHHEAHHEAHMAAAAAAAGGNVNGDGAGGGAAGGGGVDVAAAAPPPPAAAAAGAGGGGGHGGGGGGDVDLLASGGSF
TEDGDSGGGEDEDEAEAAAAAEAAAREAAAVAGDAAFWARVAGLRGLRSLVLRGGEMRAADMAGLVALSGLERLAVN

HVINTHPGLACLSRLTALTSLVIEDLQYVGGAIHDEFCGLSALTRLRSLSFLVVGPLPAEVMAWGSLSQLTQLEITN
HYDDNLLPCFGAMLPRLPHLAALHLSNFDLTNALLLALRPCTALTRLQMGRFNVTASPPPPAMTSLRLLHLGFVDDL
QHTEVALVSVFPRLDELALQTRYVLSHESAARIPRELLLPPPHHHVQQQQQQQQQQQGQQPELQPQPMGPHLPQQQQ
QQGGVGVGGGGGGGAAEGRPSPRAHGRALPLTRLHLYDALMSPLLPGALPGLTALRLDWNQTDTAELVGLLGGWGAG
LTQLAWANCDAMQDWGLAMLVGALPRLTELELTRCVSITDGGLAALTALTALTSLTVAAAEQLTHAGFLPVAAGLRP
LRRLVLRGMPALTPRQAQQLEEAAPRRGALQVELLPPGGPGGAGGGHH

>Cre02.g104729.t1.2|PACid:27575400
MDALRSHGVLQAEQAVITAVICRQQRYHVSTDTKAQDGRSPASPVGCFTAHSDTALLMLRPGLPNEGAQRALPAHSA
PLPAVRHHGSAIAGCPRQDRAAGVPEYPMDITVASPPPVAEPGSASLGASDATSNSNAFVRSSRLMLDDDQRALDQL
QHLGPPFSGDCSRRPSSYCSSRSNSGDGAAVDASSSAAPPSSAAVTRYGSLLMGSAGWRCTAVYGIDLHDMLLLDRM
TPRRTSARQQQPMPPPTAQQHHHHQQRTSRPSTPELSAASCAAALRAASSVGSVTGAAPVAAAVAGELAVGEAHVLA
GRRVGSPGTAPPSGVVLGTPPRPGPCTGGGRPPHGPPAAAAAAPTASARDFPPSPFSLDAAAAAGMSPPPPSSIADS
RGPSMLRLVGMGDVYGASAYSTSTKSGAASFHRSPTCELQRPGGRSAAPLPQAVTPRQSHASQPPQPQLSRSATQGA
LRTYSPQIISTRYANIFIPAAASLSEEEEAGQPQSAVSPVSIAGQGLPAGQRCSLPKNSIGVGISAARQLVGITADV
TGRSPRALGSSPNQPTAQQLSSPHTSACRTHACRRLSESQAAKPPGPHNGCAEPMAASQATPADSAAQHDITSLGGA
RCSEGSLRPHLGGPDLLALSRQEGGASGSAGGVSGLRTPSQQRTPGRLSGRGGSLTSWASVSSLQGQLPPPPSAVRF
GPGAGSGYDCGTSPAHDCRAGGSLDGEGGRSLGLTTHPSASSLLGDSGTPHTSRRTSYHSCMVPAGHTPQHPRLQPH
SHSCTGASPAGYLGIRSGSVASWGEVLGVLREQVTPRAASGRAGGSPFCASPAALAAAATVSRGRTLMSSSFEGGRS
NYQSNGGCSPPAPSDGGCGSGCGTGWAAAVLGRMCCTPGRGERGGSSVGVGNARRASGEDFDTGGGGTCWSLVGALP
CVARDGASCDGDACGSAARCRQASNTPDMSWWRKASLGNSISMAKSGTIHFAAAHECDGRDGRTAMPDVRGGKQAAC
AWKGGTGAEEEAAEGKGGASWAHQPGVAPDTVAERDEDAVEVAVVAEESEASKTVALEALSSLDRLYADARRNSFAP
LSAAVFEAQRPLEPSTAAPQGKALQTSLVAAGALVLATCLSPRCGREQATVSPRDAC

>Cre02.g144550.t1.2|PACid:27575549
MGEAGAALLLGLVVGLIMKAAHVGHTLAESVEFRGEIFFYVLLPTIMFDAGYGLDAKVFLRNVGSVCSFAFLGTTIS
TFVVGLIMWGFGRQGWCYQMTLLENLTFGALISATDPVTVLAVFQRLGAQPDLYMNVFGESVLNDAVGLVLFQVITK
FLEGKAVTGPNVMAGIGLFIGIFAASVAIGLAVGFLASLIFRSRFFYSGAIHDPVTGKLVTPAGNSVFEVGVAVTFA
YASYLVADVAHCSGIVAVVTNGMVMNLYVRPNLSHEAEIKIEYLFKVLAALFELFVFCYIGSTLFLGPQHYNVWSYT
ALCLIALAASRAANIYPLSLLCNLLRPIERRITQKEQFMMWWSGLRGAMAFALAVEASESYGEAGQVMKTVTFYLIF
ITVLWNGGSASYLLEVLRLRATDPLPNKYARAQAAKHEADVAASRAVADLQRASAALERGTQELGVRVKQEQQVKAH
AKAGASFEHKSDESSGSGSGSGSTDGVANPGSGAAAPSAATSAAAVVAGGAAAGVSAAAALPEAGAPQVDVADRRIK
RGSTPAAAAAAAAAATAAAVPVPEVEMELAGIQADLEKGRFARVSRSLVDMRQGPDDPFAATAAIAIASPPPASTTA
HGYAIPGRHVGGANGANGGRPLSASGAAGTGSMKRTGSLLQRLQSINDGHRILGGFDRVHSKLTKLLVHPRALHEHE
AASPSALGHALAHAASAAPALSHAPSAPPPLSPAAGSAAAAAGGSPPPASGTGASGSSGSTSGGAALARQVSAPPVQ
LDGGPRSSGSPLGAAPAPAAAQPASLAPVHERASVPEDQ

>Cre02.g100100.t1.3|PACid:27575725
MDLDMHFDAMEVDTAVAAGGGGGEQEMSQAPAPAPPPPQQYHQQQGQQPQHLQQQVEHAQDMQPGPYSGVRLHPQPP
QQLPAGPRQQQQQLQQQHYHYHQQQQQQQQQYPQQQQQPHLQQLVMEAVPLGARRGGGRGSNSSGASASGSSWAVGC
GTAGAAGAGAAGGGAGGGAVPAADNNNRGSAADMTTMLQLNRYGSTAATQLTSAGGGPGGGGPGGGANVGYGYGFD
GAGAGGGGGGWVEASSPPPSGQGGGAAGDNGRGAARPPGSSAFHHHAAAAAVAAATAATAAAAATAVAAAPAAGTTA
PGMAAGGGARFQSYGYLPSQAQAAPQPPAAAAGYPPAPPPPLHRHHSEGALLAPGPQPQPSGPSPAPHRYLQPPPQH
PHYPAPWEQQQPQQPHAAHGGYRPSPSPVPPSLPRQHSFPRAPSAGQLPGGNPTNQHPQQQAQHRPHHHQQHQQQHQ
QQHQQHQPAQQVQQPHQPPYYAYSQPPPGYSQPPPQPPQPPQPPPQQPPQPPQQPYHSNPYYQHHRQQHQQQSHTPP
PPQPPQSAYAPPLPQPPPQPPPQPPPQPPPQPPPQPPQPPQPPTSPFPHFMSPPPPVPAPLPPLPTWSPAASPPAFP
RSGSGFAAGVGLDRTLSGAARGGGGGGGGRQLPPSPLAGMRAPPVPPELQQEMAESFLRALALIMARPATRAPTLGN
VSSSLAIAIDVKSRRVAEPAAAAPAAAAAGTTGSAAGDGGGGGGGAAVKAEGGGGSVDTVGFRTVTTATTGAAGGGG
GGGNIGSAAGGGATAAGGSGNGSSGRLQQLVGDVPSPPAPSPSPGSKPPPAASTPAGGPAAAAAAAPATATTPATPP
GATAAAAPATAAAAPATAGVSATAAASGVSPRYRGVTRHRRTRRWEAHIWEERRQVYLGGFEVEDQAAKAHDVMALR
CRGPDTVLNFLPDTYIELQPLLLPQQGRRPLHRNEVVRLLRSYGKEVTRLAHTAARPPGAAAAGGGGGAPGASAAAA
AARQGSADSGGGAGPVEPPVTIFGDDECDVYCFMPGTLKAQALAAAGSSPPGGGVGGIHRSASVGGVLGGGAAATGG
GGWGAGLSPWSPKAAVVAAAAAAAATAAAGTPASPSTLLSAGLLSPGGGARAFGGAGGGGGGDGGDGNGGGGGALPP
LTPLSPDWAEALASLGVGGGATGLWGLPGGGGGGGSLNGLSGLLPLSDTLDAITSTAAAVEDPDMLDLQYRQTVRDM
ARLQQQQQQQHVLQPEQQQQQPHMLQPSGELQGQQQQQQQQQQQQQQYGGHQGHQGQGHQGQAAPLQRHASGAAAPQ
PPPQPTTQPTTQPPPQPPPPAPRPPQPPQPPPLFSSFSQPQSSLVGLLESPRHFLVDPYGTPWGGHSYSYSPATAAA
GSGTHTNPQYSHPQQQQQLKAEAPQQQQQQQPYQQQHQQQPGPHMPSPYTPHAGSGRPQPQTHPFGIGNGTGSGSG

ASGRGSSPPRQPPEARRLPAPAATPPPPPAWAPPGAQPPPQPPLQPFSSQPYTDPWPPYMPPQYGQRGSGGPAVAAA
PAAVAASGGGGGTTLLGHHSAPAAQLSLFAAVAATAAAAATATAGAEQAPAASSAVRRAAELLLDFGGDGGGADGGG
ADAGAGAAAAGAPAATGGGGGGCRVEFEPISWSAVDARRPGSDGGEQPAEQAPVVGGAAAGEGSGGGAASDADASAG
ATANGDGLDGSMEAFWEGLAAAAAAAIRRQSSRSDGGASGGGAGGAASGNGNGNVGGGGGGGGGSTGSGGASFWDDF
FASQLLMTPGPTDGGRAPSLPPPAVAPAPAAAPAVAVGHSPEVGGGGEQMAGGGDRAAAAPGSPAAAPLPQAPAPVQ
LLPPPPQPWRSISDVGQLTAAAGLHLQPYTCAAAATATATAAAAGPGGATPPPHDGGMAPAGEDAAAAGRERTAFSH
LPPPPPQQQQPQEHPPQPPPWLHAPQPPAHYPQQHNHQQHPHQLQPQGSASGALQVDMEEAEDAGEWGHGPAGPAAV
SLAPPAASAPAAAGGNDTAGAGWGGGGAARWYPQPPPQLQPQQPQQQLQPTPHPQLQPTPGGLLRGGTVWRFGGSTQ
QPGTHTPSHSDGAPAASSHAAATPLPPLPPLPPLPPRPASAAGGAATAATAAPAAAAFILSPRAAQHFSLSLPSGDA
FNSYTSAAAAAAAATPPARSASPAAARSAGDAYTIAAATPTTAAAAVAGGAGTSAPAASAVRRSRRNSSGGGGRNSA
VSGGGSGGTGGAVDPGGTAGDEAASVGGGGGGGARGGSGGATRSRRSGSGNDSLAAAGSAGRMGAEAEVGTSDAGGA
AGTSSGRDGGGAAGTRKRRASFMTARSPPPSLPPPMTTAAAAAPAPPLAPHMPGSGPGPLAPPVPPPATPLASAPPL
PPPSHAFLSHYTPAPPPTHLAAHQWPPPQPPPGPQQQPQQQQQQPGAAAWGSESKEARTPSDGSAHSTHITHTPPLP
YPQQHPQQQPQLYPHLAHMPPQPPQPQQQQQQPQPQPPQPLQPPQPPLSPADMYGRALYGGSYGGGGGVYGGGGGTL
MQPMPAAAGVEAAPTGGGSALPRNGGGGGGRRTVTTPSPTTPGGGVGVSDSGVQQQTPPQQQQQQQQQQQEHWFPQF

>Cre02.g097850.t1.3|PACid:27575789
MALPQWAELWANIAEQSNEARIQPQTGSESHYNGYAEAGLGTLFQAVVLPSAAATELLQRILNTPLHEFEGCGTSIL
LAEPAPSGGVRPVAPDPGLSEQPSTAPAPRRKGATAAAAASEAVIDAGPEPASLHGPLETIAGATGGIRGQGSKAPV
TLAAAAAPGSLPATAPALAPGSRAPTAAAVAGTGAAAAPTAVAAAAARRAGPKDPLQRMRQLHQRERRKREQQEQRG
KKKAQGRAPEPRQQGWSWQTAGEDAISDEEEAAEEEEEEEPVAVWARAGAAAEEDREGGSLHEDEGGEAADTPGAGI
DDRARQPAAAGDAPPAAKRPRRGALGPASAPAASPDDPLAAAEAGPLDGQERDQAPGLQDPGESPASGRGGARGRGR
GRGRGSTATGTGASRGRGRGRGRGARGSGSGSAGVEPAAAADADGLAAPAPGPGAGGRLTQGAAVAAGAAAHPTDDD
VEALFMDSASPPSASPPSSQSGSPPPSPGRGPGGQEPCGPVGGGAGAARVTAGAGLAPAAAGAGAAAAAAAMAADAG
VAAGDEGGERQLVSVISVRPNRSLTTLGSVDVRSLVQLGSGAAAGPAAAAARQRAAAATAAAAAGVAAGAVGSQRAR
QQGQQQQQQQQEEEDAEDAELLSMPLLGGRPLAAAIGGGLEATPAAAGATGLLQQAPSTTAGERKLAALATAKQLLA
SQRSQGALSEPGVQQAAAVSGAAAAASAAATAAAAADWAADIAAGLGDEDAGQGSHLGATETAMAAQAPAPSAPASG
RVAGAPQAQTIAAIRAAAAAAAAKAKAAPAVPEPGGTTALAATPAAAAADVDSEPAGGMAVQWRRWAEPGLPHGESLG
LAKPGELTGGAAGALPSGACPGADGADGAGGDAGGGGGDDRAGGGDAGGGGYDSDGEDGEVVVEEGAGQQHAGGEEA
REGRDDPKPQQQQQQQQQQQVEAAEAAGQQGRQRGGRQGGRGAAEGRGRAAGRATGRSQGRGRGRQQLQEAAPGRRQ
SGRKRGLSDAADDDAAAAGSPPPQQQQQQQQQQQQQPLEVGLYAEVKRFQAQQRKARAIAAAKEGGAGAAAGGVFVD
PDRPMPEVRETPACKYWRLGRCTRGDACTFRHVGQPAPRLLPCRFWRLGRCFRGAAACAFSHDPGTREPCPALLEAG
VCSRPGDCPHEHNYPEDDLHALREYMQFEAGKAAAAAAAAANAAGGASAAAAARAANSAGAATAVAGGGVAAAGLEEV
LLQPPYAAARAPVAAGWVAGWSGAAGAGAGAGAAPGAAQDVGVIGGAGGGGWGAAADALLPQQPALLRAANNNDEDV
F

>Cre02.g094004.t2.1|PACid:27575807
MSEPGDDEFDPPPPGEDETPAPIEVAEAAAAEVQGAAHAGDASAAATATDATAATAATDPAAYAAAYGYPPGYAPNS
YADYYTSAYYYSAYAGYPGYYDYYGTAAAAAGTDTSTAAAKDRPVQVKRAGIDYVVPLNASAKANGSKSAGGQANKS
AGSGSGQGHVAAGGGGGAGGYDDAMALPPPSEPKPTAAGTGVVEAHRPPPAAAHQYPPEEELTEFQRKMLELMEKKK
EEAERKRKGQQQTLHTLRGARMAIAAGAAGAAAAAASNMGGGSAAVAAAARLINSGDPLAAFSEEPPSKPPSKPDPK
AKADAGAAGGTSKKHQIGGLNNLSLIINAPLAALTLPPKFEGPTPAELKYGRPRSRSRSRGRGGRYNSRSRSRSRGR
YSSRSRSPDSSRSRSRSPGRRGRSLSYSPRRGDGYGGFRGRGRGEFRGRGGWRGRGGRFDGGRGRGRGDWAGEGGYN
RGRSRSRSFSRSASRSRSRGRSDSRSPVRRSPVRRSRSPGPPRRDGVPPSAAVGSPRVREGATAPRSRSASPRRRVS
DDGRRPWGDEPLRRGRGDGGDGDSDHEYRGGEGGGRGFGRRGGRGGPWPGSRGGPAGRGGGGGPPHNRDRDYDSGYD
DGPGRGPGRGRGGGYSGRGRGGRGDGAWHSDEERDYHEGAGSRGRGEHGGFRGGRGGYGHDMGRDGGLRGRGRGPFG
PGRDGGYERRYNDHGDDRDDEWRDGYRGGGRGGGGRSGYDDERGYRGYDGYGPGGGRGRGRGGWHGEGRGGGGPLPE
ERWRPDLSPEHEMMLPAERGSERDAGGRSPVRRGSLSPERGLERARDVRRSRDLSNGGQERAAPADASRGAEPPLQR
SAAVAPAAALAPAAGPSVAMAAMEEEQRLQEEERLLAEAAAKAAAAAAAAKAAAEKSAHDFITAFEDLDKLDIIDTS
LALRPLSVGPPAITSGGAADDDGGPAGMWVEVKPSAPAVAPQAQVPPPAPLPSPSLASAPAVNPAAAAPAPEQRPRS
PSPPAVGMEVEAAAEEPKPVPQAQQLQLQQAMPEPQPQYQQPPRRTGAAQECDREQYRPHDRGWDREAPRGQERPRE
RRQGANSERERDWERECELDRSHERGWERERQRDITQEPERAPRTDRDRGRQWNYDAEGDRGREREDEGPSPKPPAS
TPGSASDGEEGGRGGAGAGQAVSGREGDGADVEALGTGSSKKHKKHKKKHKKEKDKEKKEKKDKKKKDKKAGAAAED
EERGRDARGAVDSDASPPVDRYGGGPDGRRGGPDGRHEPAGPGGGYGPGPRGWPPTGGGPPPGPRRDYDSEEDCGSD
RDREYDRGYGGQERGYGRGGRGGRGGDYGLDAPGPAYHDSSYGGGRARGRGPIRGRGGYYGGRGGGDDFYDDHGCGP
GGSYRGGRGGRGAFQGRGGRSGPGGPYGAGGDYHSDEEDEAPRRLAKRGRYEDDGGSPRPPGGADRRMWDDDRGGGP
VSGSGDDDGPPPGRQPQRRYSPGGRSMSPQPAQQRPRLSSHSRSPVGDRDRSPRPGIRGGGSRSPPPRGRAPASASG

PPRHFNRPWHSRSPPPRDGSRSPPPRGPPGSAPAFRGRGGGHAGGPYRGPGPYRGRSQSRSRSRSPPRGATGLSGP
AGRRLPPRGQSRSRSRSPQGRSRSPPARGAGGAAAGVGAATAEAAPVPLEFKRTRGNSPPDTRTKDEIKAELARATE
IARAASQLAAGLMPAAPQQLQLTRHARRVYVGCLPVGMSEVALTQFFNQIMMASGATTQPGPPVMSCFMNNDKRFAF
LEMRCVEETSNAMAFDGIQCQGEVLKVRRPHDYNPAAAKLLGPTDPSPKVNLALLGVINTLVEDGPNKVYIGGLPAC
LSEEQVRQILQAFGTLKAFNLVLDRETGNSKGYGFCEYADPSVTDSAIQGLSALIIQGKPLTARRANTSAETSLTLQ
TLIEQQQAALVSSGAGANNGAPSPQARAALTAAATAVAVAAGAKPAASQPAVPQPAVPQPAASPPAATSPAGGGHTV
VRLSKMVSRDDLLDDGEYADLLDDITEEVGKYGKLVGVEIPRPGAAGAADPPGVGLVFLCYEDTVGAKRAQVALKGR
QFGANVAEATFYDRARFDARDFA

>Cre02.g094004.t1.2|PACid:27575808
MSEPGDDEFDPPPPGEDETPAPIEVAEAAAAEVQGAAHAGDASAAATATDATAATAATDPAAYAAAYGYPPGYAPNS
YADYYTSAYYYSAYAGYPGYYDYYGTAAAAAGTDTSTAAAKDRPVQVVPLNASAKANGSKSAGGQANKSAGSGSGQG
HVAAGGGGGAGGYDDAMALPPPSEPKPTAAGTGVVEAHRPPPAAAHQYPPEEELTEFQRKMLELMEKKKEEAERKRK
GQQQTLHTLRGARMAIAAGAAGAAAAAASNMGGGSAAVAAAARLINSGDPLAAFSEEPPSKPPSKPDPKAKADAGAA
GGTSKKHQIGGLNNLSLIINAPLAALTLPPKFEGPTPAELKYGRPRSRSRSRGRGGRYNSRSRSRSRGRYSSRSRSP
DSSRSRSRSPGRRGRSLSYSPRRGDGYGGFRGRGRGEFRGRGGWRGRGGRFDGGRGRGRGDWAGEGGYNRGRSRSRS
FSRSASRSRSRGRSDSRSPVRRSPVRRSRSPGPPRRDGVPPSAAVGSPRVREGATAPRSRSASPRRRVSDDGRRPWG
DEPLRRGRGDGGDGDSDHEYRGGEGGGRGFGRRGGRGGPWPGSRGGPAGRGGGGGPPHNRDRDYDSGYDDGPGRGPG
RGRGGGYSGRGRGGRGDGAWHSDEERDYHEGAGSRGRGEHGGFRGGRGGYGHDMGRDGGLRGRGRGPFGPGRDGGYE
RRYNDHGDDRDDEWRDGYRGGGRGGGGRSGYDDERGYRGYDGYGPGGGRGRGRGGWHGEGRGGGGPLPEERWRPDLS
PEHEMMLPAERGSERDAGGRSPVRRGSLSPERGLERARDVRRSRDLSNGGQERAAPADASRGAEPPLQRSAAVAPAA
ALAPAAGPSVAMAAMEEEQRLQEEERLLAEAAAKAAAAAAAAKAAAEKSAHDFITAFEDLDKLDIIDTSLALRPLSV
GPPAITSGGAADDDGGPAGMWVEVKPSAPAVAPQAQVPPPAPLPSPSLASAPAVNPAAAAPAPEQRPRSPSPPAVGM
EVEAAAEEPKPVPQAQQLQLQQAMPEPQPQYQQPPRRTGAAQECDREQYRPHDRGWDREAPRGQERPRERRQGANSE
RERDWERECELDRSHERGWERERQRDITQEPERAPRTDRDRGRQWNYDAEGDRGREREDEGPSPKPPASTPGSASDG
EEGGRGGAGAGQAVSGREGDGADVEALGTGSSKKHKKHKKKHKKEKDKEKKEKKDKKKKDKKAGAAAEDEERGRDAR
GAVDSDASPPVDRYGGGPDGRRGGPDGRHEPAGPGGGYGPGPRGWPPTGGGPPPGPRRDYDSEEDCGSDRDREYDRG
YGGQERGYGRGGRGGRGGDYGLDAPGPAYHDSSYGGGRARGRGPIRGRGGYYGGRGGGDDFYDDHGCGPGGSYRGGR
GGRGAFQGRGGRSGPGGPYGAGGDYHSDEEDEAPRRLAKRGRYEDDGGSPRPPGGADRRMWDDDRGGGPVSGSGDDD
GPPPGRQPQRRYSPGGRSMSPQPAQQRPRLSSHSRSPVGDRDRSPRPGIRGGGSRSPPPRGRAPASASGPPRHFNRP
WHSRSPPPRDGSRSPPPRGPPGSAPAFRGRGGGHAGGPYRGPGPYRGRSQSRSRSRSPPRGATGLSGPAGRRLPPR
GQSRSRSRSPQGRSRSPPARGAGGAAAGVGAATAEAAPVPLEFKRTRGNSPPDTRTKDEIKAELARATEIARAASQL
AAGLMPAAPQQLQLTRHARRVYVGCLPVGMSEVALTQFFNQIMMASGATTQPGPPVMSCFMNNDKRFAFLEMRCVEE
TSNAMAFDGIQCQGEVLKVRRPHDYNPAAAKLLGPTDPSPKVNLALLGVINTLVEDGPNKVYIGGLPACLSEEQVRQ
ILQAFGTLKAFNLVLDRETGNSKGYGFCEYADPSVTDSAIQGLSALIIQGKPLTARRANTSAETSLTLQTLIEQQQA
ALVSSGAGANNGAPSPQARAALTAAATAVAVAAGAKPAASQPAVPQPAVPQPAASPPAATSPAGGGHTVVRLSKMVS
RDDLLDDGEYADLLDDITEEVGKYGKLVGVEIPRPGAAGAADPPGVGLVFLCYEDTVGAKRAQVALKGRQFGANVAE
ATFYDRARFDARDFA

>Cre02.g096950.t1.2|PACid:27575881
MEEQTAGEQVVGSESAPPNAMWSVLDFSASQELGSLPPESQSQRPQALSQRPEEADNLAAATSPQVAGATDMEAEPA
SSDEPGADAAPASDELPKEAEARAATPNHGGELTPVSAAAAAAAADGDCGAVAAAAGTTFILPMTLETQAVQEEVSP
EPQHPETAPAPSQEEAGGEEGKEAPEAEQEAEQEAEAAPAAAASPDADAHMTDAAKPPAAIAAAPPQQQQQEQERQA
QPAAGDMELEDELEDADVEPAPTPATAPAAAATTTGADREDGEDAVGGGRRLKRLRRAATPPPEAAAEPAAAPAPAT
GGNGKAATAGPEASPPPKSPPPRQYQYPDLSVDSPSPSPSPDLVARGHKTDGADGAAAAAAARRAAVAAVAAAGGKV
VRKKVVVRRAAADDGLDDLSGGEEGEGKEGGSKKRKGEEGEGEEEGARRRRRSAAEGDGDGEGEDGDKAIGEDGKDG
EDAEEAGAVAGAADKGAAAAGGGGEDAEVLKQRQLRDAALRDRIGGRAAAAEAPVRPLSDVLGKIMARQRGLVERAR
ANAPVSDDAQPPPQQQQPARPSYNVRELLETLAQSKAGARSTGGATAGCASAGATAAAAPAAAAAVAVAAPTQAELE
LRLGGSEEQSAVAGTEQAAAAGRCGSGGEELLLPGGESELVITDSQTEREQQKLPPRSPFAAIRTLKPYTPGGTKTA
GGGAATPGAAAAARAAAALAPPPADTQDMLVYEESATQTDSGSGFGSQQNGGGAGVRTTLVPIVDLARRVPSVLPGS
GFGSASVPDSQDLVPASGTDAAQAGAGGGGGGGGAKSEVDPDDGILMDIDEDDDLIVEPDPEEEARLAALAAAKQQR
AADSKAAAAAAAAGKQDAGAGAAGAPATGGEADVEVEAEAEAVMEGGDDEDDGEAEAESSEEGSEDSEEEAEEAEDE
DEGSEPGEEEEEGQQAAATGNGTAATGAAAAARGEEAEEEEGDDGSEGGEGEDEEEEEGEAEFEVDEEFDSTDEEDA
VEPEALNYEEEEAATADGALTQQAGGGGGGGGGGGADAAERRRQLTEVLIAAAKERRAAQLAAARRQAAAAKGAGAY
FVDMEAELSDEEEGLAGLDDLDDEYDGEDLDEGVLASLLGTAREGAADESRRVELHGAWEAERDAAAVRELLEAVRR
GFRRSRRRGLGLEDDEDITDSDARRRRAQLFASSVGDDDEAPIKVARGHGLDLDLARDDDDDNAGSGSMDPKAAVAA
AAAAAAAGSTEAGADGSQPAAVDGSQQQVSLAAAGSGSLGIRSGSGVGTGAGLLRLRDPVEARRLLRAQSQEAAARQ

AKLAAQHSKLLGGGGGGDSPPDAKTAPAAHTDGAVVELLQRSASAAVGGGAGGALAAHGAGAAAGGAAAPVAAGRPE
AAAAAAAAAAGGHRFTLFGSDITNRTAPDANQGSFLGRSAAPTSLRTTSTLGGGGAGANTRSFIFATDSNSAFPEKS
KDATAVAVAGGATNDGGGEGAGGVGNGGGAGASFAGLRSVIGGSGGGVGQGLLPPRGPAADGAGARKGGGGGGAGLM
GLLGLGSGKGGGSGAGGGGAGGSGAGSALDLKRKLMMGGGGGGGGLLGGEGPAAKRR

>g1431.t1|PACid:27575907
MELTELSRHFVKTSSALRRLPMVAGNMVGAAIIAVVIMASGLGASAASDFSVSSHSGRGLLTTASGFPWCQCIDYSC
ACSPYKVNFESSTQSGSLTTTCFSVAYIGCDTSRACCRGMLAAVDKLTFETTAACGVKSNIANITINGKRWPSWNPY
PHPSGPGTGYELKIYNLKGNNGTFPGSKICITTKAPCSSLKDLCSSSPSGDCTYSFADTSSTTYCPICPLNVYPPPP
PPSPAPPPPSPPPPPPPSPPPPPPPSPPPPPPPSPPPPPPPSPPPPSPPPPSPPPPPPPSPPPPSPPPPSPPPPPPP
PPSPPPPSPAPPTPVPKPSPSPSLVKTHICVQSTNNIPFAISDLVISPTVDQLGLPAVSMCVSASSATCRRGAFCCK
MDFAKMELPITTECKPELRRILINNEATTSYSWGFYDAFTTLKFPSLTKSLPAGPDGATLCWVVRPGACADPANFCL
NGRCQVNIFSSDNKCCPATIVQEAAP

>g2887.t1|PACid:27575966
MHMTACSDVGYAATPGAASAGAAVASGGGSWSLDALPRTGQQSPGLGPGPGSGPGSGPAHGLGASTDQVPRQYWSSP
DPVILTDVAVAATTPAAAVSGCSSGSSGGGLHRSRSWWRLTGLAGRVCCGRGPLPRDHVPHTAHCVDGGLAGGDGTG
SVAASAGRAAVPRPLHPHRLAAAAAEPSAQGSGCGGAASRAPHKPHQQQHQQLYQHSTSCAPSREQRPTSPSQSQLP
SSRRRVSFINEVLAGGGGGGDSDGRSASPGGQPRLPTAAANTAPEAPVAAPEQVRLLAIAASGCRSGYGLVTRSGSG
SGSRHSGAGCGLAAAAAVSGCRGAAEFADDDRCPSSPRGGRGAGGNQLSVEYRPAGGERTSSDCSLHAIRRRSSELA
HGGEGCPAEQQPLLPFGREEEEARTGPQPQAASDADQEWPPQLRDGQQQRLPQTHSSQPQAGRPSGSAWPQQQPEGQ
GQQQRQPAQQQGGDEASRGGELSSYVAGSGAASGGSSACGGTRPQPQSRQDGAAVDVAAALFGDTGGGGRGGGAGAH
KSGSRAWSVSALCAADAIAAGSARSLKSRLSCCSSAAVTLAGAVASAAAAADGDGDHAENGYRTRGSHEAMDAANRE
SQRERRHPAPLDPPAPTTAVGRPPRLQSGGGGDREGGGADCCCEPRRQCGATPGVLVSPQEEVEVGVAPVQATTSAA
AEQFVLEAEPEAADVDTAADRAAAAGVAQRQQQQQQQQQQLAAVGRLPSRTSAPPHSLTAGPDASPPLPQLVSRDS
ANPPQPPPPPRSCQHHRLRRPQPHASPSSCDHNAHSPPFATPIDPVTSDGYRHPCSSTATADSAHSDCRGSGGITRL
TPRRADLRLDAMLLLPSSLPARGSHTAPGAPLPTCAGTAPLLPYAALSSTASPAGGAPSSAGGRAEAGHGLGGGTEL
AAGGDDGNRLELQGHFGPACVAAPQQCRRLHPRSAAQVRPLAACTHRGNAVGGPPAGLLPGGDAAPSLAPAAAAVAV
DASTGYDSVGCLDGVGTPESAVSANVQLLRRDPGSGSGRSPSRGALLVMDGSACRDGGGGVGADVSVDGCGAGAARW
DAAALSSAVATATAVATFADVEPAGSPQSKGPVCGVSDIWNAMVDVPGGGGGSGGGIGGGAGGGAKVLDITSDFDLW
QRPQLQLQLPPPYRAAAGRTQPPTTSLQPLQAHARRQARQAPAAGGREAAGVEAAAGPRHWRRDVEDGNALEEEGAV
AVAETGGASRTQPVLDDGAGVTLLPVLLGTGAAGKVYAGLFQGRRVAVKLLPLGHLCNELAALMAQAAAEAPRSRRT
PPRLSPLPLLRSTQLGAQPGVSATAAAASETPQLSALPSGATAVLTGQLSGCDMDTAAGVPSAYDSSSSSEGDAGGR
DGGDYCGGDGCAGPGGGGGSNVASQVRARMRSARLHQLNQRPSSSSRSRLPTAQPAPQPQLQGMEDKGPASPPPAAA
AAPTHAAAPAGAHAAAGSDAAAPAAAPAADWDAGGAGARDDALGPPPPGPPPVGEDVARAVELYRQEAEVLGRIEHP
NVVRLLAASLSPPAPCFLVMERCDASLESLLLGTAPSPPASAAGTPTPGTAGAPGLRRALMPLPKVLHIAIQIARGL
EYLHPTITHADLKPANVLINDPDSPKPIVKLSDFGLSKLMSGITSTSLKSHAGTPAYMAPECYCSPPPLQPAPETQA
APPDTAQPPLAGAAHHHHHHHQHQHLQDPPPDPPLASRTPRAHKSDMYSLGLVLYAMLTGQQPWKDCGVMAIAYKV
GVLGERPPLEQLSRRRCPGPLRRLVRRCWSQDPARRPDAAVVAEQLQSLLAQLEDEEEEQRLRQDRERGDTAQRDWQ
SGAAAAAALAADQLQLHQSRQAQGPDLGAVGSAADSTTTASTSPRAMSPPARAPALPHQHHPQHHQQHHQQQHQQQQ
WGRARCDDAPLLDTEVVSALRDGGWLAHRRHSCSSGASSGAEGHLQRLAGEWQRSTPPPGAEAGQWQSPPPAGSAAQ
LVSPLVQAHRPAQAGGGPGVGGGAAAGIRWMAFAPGGGGGGHDGSGYLCSPSLSPRHSVAAGRVAGAAHVHSGAVAG
THVGAGGSSALLAPALPTASTVVTAAALARQPLLQAAKPRARQHHLYFPRGHATGPEHGLDGGSSSSSSYMAGPGSM
HQHQPQHQQQQQQEGPMCQSSLFSRSSRRSGSSAASRLSEGALLSQLAGVELEIEL

>g4412.t1|PACid:27575988
MEPVLHKSSHPSLPPAVPQQVVQFLLAKNYHLTALELLVEAQSAGHGDDGAFKDLQAFFSDPDRFPPDELARYQPSN
ALDLQAIGRDRESKLQMAEYELRLAKEDLAEMARRLAKQEQEGLGRAASAASTVGATALSATGSLALAPAGAVAGTA
PAAGPMPTSAAAAAAVGASPAPSDLRALNAAVYGHLVRQGLVTTAMSLEEEYGAQLRPLASAAAAAGAGAGGGGGGG
AGGAGEGAAAADRWDLWAWYQAAAREAVAAAEAAGSGAPAMSAAAAAAVAAQAAAAAASGRSRASTNGGEDGAAPGL
EELLRGGGADVELGLEGESGDEEEADGADGDGDGEASPEQLREQLAATRQRLAAMRARARRAVSAARAAAAAAATAA
ATASSPGPLGAAGAAAAAGDVEGGGAVSAPALVPLPPLEQPVPVVSLPGGDLLMDLLDLGTKPPAAGASPPPPPRGA
SPPPPGALTPTTLALASASSLDSLAAAGGALTITGTDAASEAGGGASTTASAGPPWLQRQAGPAIDVAYHAALEGLL
GAVAELLPRLVPNLTLKSRQDVLPLLVCAAKTSPDWAVRRGLLAAVFNLVPVPDPQQRQAMLDACLDLCRTFGPSWA
AAELLQLCVKQANSTSAERRALVAEALGAAVGVVDAGTQAHVLLTQLSQLAKDRLEQVREAACHSLARLVPHLPLSR
SYYSPLEALLLALATDPAEAVHAAALSRLAPALLTWQGHQPPPDAAAVAAAGAAEAAGAAKARGASTGPLGSLGSLF
GTMATAASRGAALDSSAAAASDPELLVSSCLARVLAEVSQYVRAIADQQQKAAAAAAAAAAADAAAAAPAASAGGG

GGSGFAGAILRYASSASASAAASGQPASSSGASEGGAATGAGHGHGHQHHRTGSGLGGGASSALAASGPVSFGGRGA
PRVFPSAAHLALQQLLALYVALAPALRSAALRSRPAWACPGDHPSSATTSAAPHNHPHHQNHHHTRSAVPGRHQRSG
STADLTAESGADDGVATALGGGGGGPASSALHASATRALSPGHRRTASHPLPGAASPHTAAAVDTAAATPLAKQAGA
AAQGAVEGAGAAAEVEDVEEAMEREREAAEREWEADDASLGLWAGHSQHASWKTLEWVVQHVVPEILTLVCVVGPAA
PESAEVRRAAASAVTATCQALGVPFTRTAMLPIFLAASGTGAAAGNGPMPHAASAATAAPSPPAAPPGTAAVPTADP
FIAPVAVADICAAAQPRSSEALRTARQCVLPVLLACVLPSAGRPAGLLRPHLGELFGTAHELRHRWLLEALPDYTAS
LTFACSLRPDLQPEVLGLFEDLLSGRSSSSGGNGVHGSSSAAALEHGAANGGAAAPEAPGAVSARTSMSGAATGRPP
TSRRSGEGMGPPAPASAGPASTASGVCCVAVASALVPLASLEVAARRLLPGLMALLSLGEPELQRACVGVLAELGLR
YRGEAPGSRVCEQVLAVYDSLLEQGPHAVQLEVLAAGTNLAAAAAHSAAVSAAAAAQVEWLLLAVQSLAARLAAASA
SGGGAAGSTSGAAGLLGGVEQQRALAAALLAAVRTLAPHDLRLPRLQATMGSALDALSRCKPLLPEAAQQATLAALL
KDRQASGHASISSSSAPATAATAPAGSNMAAASGPSATGTSGISSAGAASQSQPSAGSGLATTPLRSSAAAGVPTMT
SPLHGMGSNAVAAGAGAAGGPAAAPGAGGSAAGAHGAEDGSLRSRFDAFKQRLLKRKDEAPAPAAGAPGSSSRAFKE
MNFFSDDEGEM

>Cre03.g156850.t1.3|PACid:27575996
MWSRRHSILVLLICVFSRSIYINALGVAAQVAGVKAPEDAHASVSALGRKLVSQVTTLTGVGVATGVAREADDTPQV
TPASLHSAHRVLAAETTPPAIVPIYFTATVSSTPPLAPADPRLEAVHGLLANATLAQTVYAAVSSRRVRVRVAVGSG
GFIRDCSDASLSAYKARLGSLLALSTVDVLVLCVVQGGGGGGSSNSTQAPRPPACSALSAALVLDTTLRLAPTANVT
AMQYRLAALPGTDTTLRGMCVPSPAYMAPAGASLDVVLSMPDPTRLYAAAATAAAASPPPMPLAASGLTPPPPPAAA
LGNTSNIDNGSGDAAAFLSRVVEALTSDLQLAIALGVGVPLEQVQVVPGTLQMRLPPSPPPSPPLPPPLPPLPSPSA
LPVVVVRDPNTVGTFESSRQWSIPIIISLSVAAGLVALLMIVAYVRSKRKRDSSRMLHRAKSSARAVHFNDDEVMDT
TAPGAGPAGPKDEEVARQMSLAAPEMAAMGLGLAGVSFSGVSKSGVSMSARSLTGGAAAGTTGDAAPAGRRSFKKVL
SKVMGREVPSDEESGIGAAAAAVDEDRRLPPVSRVRSAVQQDMLKRAALRRLLEENKAAAGGGGHGAVDRSAASAAV
VGTRAGAGAGVGAWPSLAAVTTKAPRSQPSGHKGAALALANDNGSAGGGGPSDDASSGSGDDYVGGLTDSDQQGDMG
AAAAAAALLQRRSVTTAGVLSGGSASGSGRPRSAMLRSGAGNGTGSGRNINVSFAQEVAVEDARAARSVSHAGRTAP
VAHHAAMGPAAVVHEGAAGGRVGGLLARMFSRKSKSGAPEDEEGGGHSSRALMRSPSRVGTIVSTRDGDELAAAAAA
VGDDGEDGDDSDDGAALPERGGPDFAMPASAGAAAAAAAAGGSGATSPVASYRSLGGASARSMGGISARRVDSYKSV
VQDETGQGIMVYEQRRPPRGHRSHTDMGLAGTGVAGAAAAAVAAALQGRMSGDPDRARMQAQAREREREQMSPDVLS
RPAQGPTASPPTKPEPQKRNTIAGFLFSPFRSSGTASIPSPGAAATAAILASTRPPAPSPLRRSITAASTEDPNDPN
GVASSTSPFDSKSHGYGGPSRQGTSNSNSQPSDRLSPPPVPPRRAAALSGREPLRSPQRSGGSGSGMAGGGAGYSLR
RQLLSSNSEVMAAEGLLPVMPPAHMVPASLDYDEEDIRVEAAPRGLGNLLASAPSFRAARTNSRTGEAALSSSGTAA
GAAAAAMMASSPQLGGGGNSRPASRAGSITNPGVTGGAPGGVPRAVGRRASQVLGVEEGGIARPASASMGGGRPPSR
PASPLPSDGGWNQVKEQSVSGGSGGGGTGNRHQPPKRRGSRFASGTQSSLMSPSPRSREDEWPAAVASGSGGGGGVS
SGAGSPNVVGVARMMTLTTVGEETGASGRLSGFVGVGAAMPSAGVGSDVDAEMLALGMREVPGDLSLGPGSSTRTTG
QLKGMAAMSLQSDGRLASGGGGAWGTSSASGGHYGVMAAAALASDGAGVARPLGGLRQSVIPSLSAAGGGGSRSFSG
KPPLPRSSAAGQRVGWAPGVAGDDGSRGGSPARSVASPRDQAVASSTGLMGAGGRAGGGGVRNGVRFSGGLNSMDDD
GGSSDGGARWAALKDSVNETLAAAGSELGGGGDAAQPAGRTWRRSVTAFSQRTRASAAGNLQASAGTEAGHDGDGGG
DGEAAASALRAWTTRRALSIRQHAGTDSGFASLATGPRDEPPAAAAGGGGGERLKLDGLLAAGSGGSGSGGGAEPAA
ARKGAWRMHENGLWDTAPSDKSIPPLGKK

>g4424.t1|PACid:27576145
MWSTRFICSLLASWSCAFFILFIANTATYVQGVRLADLLSGSGAAAQTSRNLTLNLQRVVVLVEDVTFHVPASTQLT
VAGQGPESGLDLRAFGSSASPAFSLPGGGSSLELRNLTLLLPPLPEEAATAATAGSAGALPPSLLAHVIEAVGGGGN
RAQVVLRGVTLVAGACSDLNIHRSFFCHDLNRWRARVAAYDEPTILGGTVRYGSRAPGGGLMAVGSADDSGGVAVPP
LAQLLDCAVTCDAGVSGGGGTGSSSSTPYVSSGPSGPWDCMAVTVSDSAGLAALPDLVASQLVLAGATVLVSVEAGA
PVRVDPSTWSTIKVRVQTSIFFVGRGRAASLLDLAGLQSKQFADVDSNQRSQGRVGLMDMTLMGLSYTTRALTQLDL
MAAWVHAVGISGTQDQYLYDVTVGRIVPPEVMAWFKNVRLALPDAEAEWWRNAAPTAAGGTGLPLSSSGWTGPALSD
PDTPLNAWVYGAFPPTASPSSRAADPVRPPEWEAGPVRMRMGWRGLRACDYCWIVPLSRVLEAAAVQQVGAAAGSTS
SGNVLVAQPPPPPPLVLRGTWPLADALGSDVAPKVSLSRVRLMTGVRSAPYVQLAVQCGDDGGGSSGNSSSGRDSPA
VYVMPPADFETPVRLYGGMLYYTLRRTTGLGVNMMPSVGDRDDVSAYGQSNPSVTLGGSGGSAGSGSRCVLLPPPTS
QSAPGPTTWDMTDLSDRIRIRLGPPGSSSDGPSLQIQGFTLYNLAPCAQPQPPEPPQQTPEMPPSPSTAPPPAQPTG
SGTSSLAAAPRISMGRRLQQFIGGVGGSGGICWVCLATPPEPPEQPLQPPQPPHPPALPEPPSHPAQPPSPPPPPPP
PPPPPKLHNPNAPFDGLSLALSLFQLDRFSPLLYAWPQSSPSAALSPPRRLPPLALVNCNLVVPPPELELLRRLLRE
AGRLPAVDGPSGGTEGPQAGTTATTGARRRGRRALLGTGGRGLQQEQAGANSSSGAADEWSWAPPVWPAGVPPASGP
EVPLPAQLLACPRSLLLSYAAVAEVTAVNATAISFRRISFLGWSGRDVVVTSQLPDDAPTGLIRTPAELSQPLFSPI
LEVPCSPPPPEPPSPAPPPERPDPLLPPKQPSVSSGVGTDALPPPPPFLLLADGGGQTESTGDRGAAGAAPAGPDAD
AVSGAPAPLSLHDSGVGTGSGHSESDGSANANADTGQRPSWIVPAAATLATVGGVLLLAIVPLAMVLVWRRRNAMGR

QEQDQHAHGFKDVKGSDEQQPAKGRSSRDDGLAERGTDPSGSTWERLSSATITGTADAGGLGAGSNKRWAFVNYLFS
FGPRSLRSPDSNMAWRRIARARKQGTATGRGSHHSAGTATDTPSAMSAEYSNRNTGAAVSTVTDGALERKTAPVPQT
SVLPELDAAAAAAKASRLWRSLDASRLLKPAESVVARSREMAASSGQLESSSTSRMLVLSRSCRIADTADMARGLGD
HANHAQAAAMAPQHGTDSSAEDSAAALEHDEELWLECIIGRGGFGVVYLGTWRGLPVAVKTLVVHEALLGSEGRRRQ
RAVLEAAVSSALSHANVVQTYAFDVRRLGELPGMGRGRFAPPALEAVAEEEHPEGQEKPLEALASPSAHGTEADSVY
QLLLIQAYCEGGSLWEGIESSQLYMGLAPDSLGGVLLGLCLALDVAAGMAHVHARGIVHGDLSSGNVLLSARPPVVQ
AADLHSASAWGVSLGASGDASVDAGLGGTSQAGTGVAGPLARAELLVDGHGGGVNMPEPESEEAQAAAAEAAARALI
AGVTLPPVTAKIADFGLSARMGEGQTHASNCWQGTPAYTAPEVAVEGRLGKPADVYSFGVVLLELLTGRTVDDGLLS
MAALMAPGGGGGIGGAALAAPEMAVHAAAALSTAAPCLVPSVVSGGNAQLVEMLSACLSPAPQDRPTFTQVMRVLGS
VISDLARA

>Cre03.g157200.t1.3|PACid:27576148
MACCFRPWKGPRDEADATSHQEIVSAPHKPPTSSFASPPPRDDGFASKAPQASVASLGAASNHYDQAPLAWTYAWQD
LRVALLSTTGTPLEKIAACATAISSALHDEASIVSVLGVAPGGCKCVVLGSDGRTAPALPLGLLLPQLLGGGPVIEL
ELPEVAPVADGAASGAQQGLPTEWRRALVDLQLMLACDSERRKNLRVLLLPLIFGSRRAGWLLLALPRTWSPPYPAA
DAGAAGATAVDAALWSLASCVAECCLGPVMARVDQMCDAVEAASSADSLQELVSSVVTAVSATLTYELHVDLGVRLA
LLPCKESPTGVLFYSTAPAAAGCAGEAASGAVPQSPMGKLGGRAPPTASVSMTNLAMRQQLVPPGHQPSLHSHFDPG
PMVVAESAAAAAALLPATSMPTDFVRTSAPGTSGAPALAAPAGAGDVIPSKGHAQVGSGITGDAAYPDMSQSALLAQ
SQLPHGRSQQQDGPSRILQISRSDRRHAATASGMATHSPHGTGAAQSVVLPTDVEHAVALLGTTGAGARMTASGVAA
VTPRGSVAHATMPAAFMTPGHALGAAAAGAVITSATAPSAARLQASCGIAVGRNSRMLYRRAITSGFALSPGGGVVA
TQPLWKAGPFNTNSTLLASLLDGAHGSRRGRPPGGGTGGSAVGSAISPATTGTGMLGHTHSVLNVLSQPMANVNSSS
GFVATALAAAFGLSPPAGAASEHDKSGGIGPVRRGGGMGSMDAAAGFVTPTSAAVAAAAAAAQVDIRSGGSGNGAVA
SHVRLQASVVPDVPSYLQNTSRPTADVFNVVRRTGIAGGLASLIAIVAASTPSISAAAMMGPDAAKGAQAASGAGMA
GTPRRSIRRNASASNVLYAASASIPASVGPVDASQQATVGAQPRGSKLLGQTASASGPALCVYIYSHLPLPASLLHV
AKDRAMSLLRVLAPEVLRALTAGGLLGDEWAFLASRAQTVLGNPGQASGPAVGTPTAPPRDSSTSIPPPPLAPSPPP
TMPPGLAGANYTCPAMPFVAACDSSVGGARAPPVSASSPRARLAPAGFPVFSHVNGGAPGVRSSSPGSATIARTRLR
TGTGPNHDPRQQQQYTSPFVTGSMLAATAVVPHDPLDCPDAPAATSATAGTPDSALAAACSAAGNNTLIELLDMCGD
GSVPGVSLGGLRTQQGSVHSIAHGAASRSITGNGFVSGLGYGGTGGVPGLNSTTLLTDTITDLGGAVTRQTQMATLV
TAFTTTLARARKDNEQEGGMHAEDDIRALKIVRAIGQGGCSVVMLGRLHAMPVAVKVILPVGGDEDEQLQSGPGGRG
GGNPTLQALWPADDRADGNIKGGGGELLLLPNGEDDAPGYQVRFGGGMGAAAADEQSLSEDDLSKRKRRRTTVRRRS
QLAAMMRGARELAVLTTVSHPNIVQVYSYCTRVIVNDTAGGGLPQLEVVPEGEPLTSPLCTALIMEYCDMGSLADAI
DCGAFAKAARAAARASGGSSSSRVSDLHQSASGLLPRRMDVAATAGTPAMRAVYLTLLEVALALRHLHSMNLVHCDV
KPANVLLRSSATDPRGFTAKLTDFGFVDLIRRQGVDGGGCQVLCADDDEEEHAAGENKTPAKPMGTVTHMSPELLTG
APVDSSDDVYAFGILMWEIFTGKAPYAQYADNDFLEVPAKVVKEGLRPRFPSDTPLHFNTLAQECWSAQPGRRPTAP
ALVTRLQSLLDASCS

>g4441.t1|PACid:27576150
MDALLADTNVASGQQAEPPPRRNPRKSGAGQRASRGGDRSQQQQQQQQQQQQQARHPQQHDQGARPAEPGPGAAVRH
GNGSGKSPPPTTQIAATTPPPQPHPQPQPGESSAMARGAAARNQPAGSRDGDGAVLWPAPPQRQRQQQRQQQQRWYQ
AQATPLQAGTGEQLPPPPPPPLPSQQQQQQQQQVQQAQATRRSQRKHVPPSRFVEDTDGGLAHPPLRSHAAQQLQPL
PPQRLLPQAGAAPAGVSGARRPAAPAPTPAPAAAHKRARAVQTATAPEHLTTATTSTTTSTARGQHKHTATAAARAG
THTRPVAAATGGPPAAAAPRDPLITRPGAGNAGAVQGPRQPPTKRPRVAPSDLLPQPQPLPQPLPLPQPWQPLPTGG
PAGTAAGRTAGLQPARFGPAAPYSSAPYSSAPYSSAPWSSAPAPAPALTRPPATGPPLHAATTGPEAAAASASQRPH
TFSPSFAPEVDVELIRQQYGFIPGSPAAGGGGGGGGWASPAGGWPGPAAGPLPALVAPRYALFGPPAAVDPGGGNGG
GGGGGGNGCGGGGGAGSPPRSSVVPWRAVQGQQRRAPAHGALMDVGALLGGGGGGGHGGGGGGGGLGLRPVLSPPRP
FSYAAAMAQLLPPAAPQQQQEEMLQQQLMQMRTRLGLVPFQPQQQLQPPAYAVASAPEYGVTMGLAEEPQEPTPLDS
TGAGGALASGLGGSGVGGGGGGGGAHAAPPLLLRLLPSGRHLTGLPRPVGSPHWEEQQQPQPQQQEQQQQQQQQQQ
QQQQRPALQPLGRDGRDGVAAAAAPTAGPETGSAAVAPAVAPAVVAPSAVAPAADELQRSDTLLDSLIGGGSLFLAH
GESLGQQLDALLSPPPGLISPALPALLMSPPPPPPRMPRMELRPHATEAAASEPAGPVGAAAGSPLGRLRGAGTCRN
ATTQSAFATGAARVSAEGAAQPPEADAHLSGDNGGGGGGPYEADADADADGDADADADDADVYGGLSPMMGSGSSLS
GQLMGSLSMGGLSELLQGLASPRPAVAVAAAVAGRLTPPLQQLPQAPSAATPLPGAVAGAGACAGADAGAGAGAGAP
AAAVAAACTPPPPTCAVAGRTASLAPPIGLQLLRSASAVPAGRASPQQQAGSGAGAFGLSGPGGSRHGQQLQRTPPR
LQQPPQPQQEQQQPSLLRHAQQPPQLQPPQLQPAATTDPLLTSPFTAALAAMAAAPTAAAPTAAGSTHAPSPDGPTT
PGLQQQQMGAAGAGAGAAAVTCTRQQHPHLPEPTALGAEPGAASPPAAAAAGNQGLAAGAAAPSAVAARAAAAAASA
LMPPPMSPPPPKKRRMLQPAAAGGGGGAAAPGQGGGDISPPQLVSTPAGSSNATTSNRPVGVARAAAGGGGGAPDAQ
QPGDGGIRGGRSRSFSRVLSLLPLPASLLLAAAGLRSEGEAPERAGGGDANAGGRGSGGANGGGAAAGAAAAADCGA
AAVPPVLVSGRCVGSSLFYGLEELELGELEGKATAATAAPSSSPRREGGGPQAVNLGARQQQRQGAAAPPVPPVPPG

GRELLETAASLPEAPQKATSAAQVQQRATASTPLSQHQQPHLGSPVPFNTRSPPTGAGAASGVQSIAASPAQTASGN
TLNDGSAVSRSRGHVSPSLQLLPPNPHAPIAAAVRASPGSAVAANAAAAAQRPASAEPAAAAATAPAAAATAPTTNG
TSGGGRGVTRFVLSSLMRLQSPDDGTSLPVHHFQLRPNGMLQPPPSSSPPAAASEMAAPETPRHQVVAAPPWAAWAQ
PPQAVLRPLLQPPAELAVAAVVNAADTTTTSTSTSTRTSSTVTTSSADATATTATTGTAAEASGAAGDTGATAGVVG
AAPAAVHTGPAAANGGRLSAAAAADEAAAARGAGGTPTAAKGGGGGGGGGGGGGGRGGGRDGGLEAGGLPAKQPLAA
ADATAAAARTSAASSSRTSTPSSTVMAPPAAALQAGTGGGGAEGGGADGGGADAALLPVQPQSQGPQAHADYGTGSA
PVAVAAPAVAVAGAAAPATAATADQQEATSQRRRLLQRWEDDDDVLFASETLEELEDGEAAAAAVDAAVAAVGVTGA
GVSPAAAGAMAMSPAVTTALHQQLLQLLMLQQQQQQQQREGSGGAIAGGGAATASGGWGAGLAGAGAGVAGGSSPA
LLEPPGHWRLAAGDGQAPVPALPPPSAPAAPAPGRAPPAVGAGLTMAPQGALAPLVVDRHGLSGAGARAVPRAPGAA
AAAAATSSQRLHPGCLVLSLPLAAEAGVSTLPLPQHQQHQQQAQLQATSAPPPPPGTSSAVQGPQAGAVAAATAAAA
APRPGQTTATSPDEGRGAVGAPPPPPHPQPQRQPAQAMPPHPAPQLQPSPLQTSTQTLQAALSQPLLLAMLQLTQHL
AATPPPPQSGPATNEPARRADTDRPAGPAASRSSPPATAAGRSSTLNLAGRATAAAAATPATAGPPPRIPVVAVLPP
GSGGRYASGGAAGGSSSCAGGGSSTGGGAAGNNRTKSSSYEGLPLVCAIPAPRPPIRPPPTCRAKPGTFYPWRHLRA
FAPFFDDPPRPRRVRVRRADPPPGVFVGYHGSGGGGGGGGGAAARSHGAGSGAGGGGGGGGSNLVGDGDMLAAGTKG
TVKQQPGGGKKGKTATAGGEAARPPRPPPAKKVLAAGSRMAAPGASAAAAVAAAARPASTPSAPLLSADAIAAAKAA
AVAHATAAFAARSNGGAHAAHALPDLAGKPLPQERLPPPPPPPPHQEEEEAQAQAGTQLPPYPLALSQPASGVPLLH
AALPGTRAPPSPAAEAVAACAPVAVARPREAAAPTQLFTSSQGASSSAAVLMGPPMPALQQPPQLPLPPQLQLQPQT
QLLQHVHPNPYRPQPPQPSAQPPPPPPPPHSYFQQYERRQAQGPGLQRMAPPPPPPPPPQLQLRPQMPAAAAPLFPD
LRPPPPPQPPQHPPAWPGVAVGPPPPPQERMGQRPWPPQLPPLPPPPRGSGSPAPGGMVV

>Cre03.g174900.t1.3|PACid:27576184
MEEDSDDFGLGETGELAIEHDDDFGAAPRSQGRAPPVRKRTDVSLFGDGGGDIEIDHAPPARPAAPAMRPGPGAPSR
RRGGDADLFSDDDRDGGLGGLLGDDDLGGDLEIDHDPAPAPPPRPAAPLPPPSRGSSGRRPGRDADLFSDDDRDDGL
LGDSDLGGDLEIDHTPSQAPPPPRPTAPMPAPSGKFTDPEISLDANDAELADLLGEPHRGAPSIPPAAQSLPPRSPA
PPPPDPASSFALGEEDDELAELLGDRPSRVPPPPPPLAPAPQRSLTRRVSFPDEPARPATLPAARGRPPAPDEDFAD
VDDGDGFGVLDDADREVARIASHNSRRGGPGLGGGGLGLGPARSGAPSFRSPAPQDSLADDFDSHHDELSFDARGPP
PGRAPPPPAAQRRADFDPYGEDYGEDSFDAPPGPQRGGRPQPPRGGPPPRGGPPRVAESSELYGEDTYGDSMYDSRE
DAGGFRRVPAPDTRGTPPARQGHGRPQAPGPYDGDDYGDEYDDDYAEDDDDWADDMSWDGEIRHTRGAYRAGGRPGS
AGHPRSQGPHAQGARRRPLSAMAPRDRQAAMIEDMMSRHPSTWDIREVATWVEFIGMGQYRIKFVHHSVDGTLLLSL
GEPELARDMRILPLGHRRALLGAIAELKEAAAAMEKDRADRGLAPGAAQTPRNENERVADLRRRGAQQEISRRPASA
GPNVIPPEPYLGPAVGKITVYEQRAKLLYQLDKARHRAAQHAAIIDQLSNNKQLTEQQMADLRGKLKDLEAKHKDVL
GPGSRRPGMTASVGGAAPGSGSPRHGHGTAALAAAEYGADAAVPWQPVGKGTRKANPNPERIARPGEDPGLDLTFRP
RTVKLSEVPYGAVRDIVSKALGSRVPFWRRMDFEFEKARKDREARDEARSKRRERMGLSRRGGGGGEGEDEEQAKSK
YWNNFWVPGSQKNSSYHKHFKKGSENAELQQYRSFAKDKTRLGRWFRRFIDDGEETEGDVALEKLVNRMIRALVKTK
DQADQEIGETFDKKIRRYFEERGYKFATHTSNTIMVRARGTRRPAPNRPDTTLYDFEEVEQFYPDYLRKRSAAAAED
YGLGGEDEEETRMVPKRTTWKWLTSMYYMDRQKKLRAAIAMVKMSQFVQRHGVRWPPERDKSNDKKAVWVPNSNAGA
LHQVDGRDRTFNTVGQDNIGLHAKLKKALGPPQPVTFQMKMKQMEATEAKADQTYMEMGWPHSSGDPALGPPQEPLL
MPHEDGLGDGGGEGGDDFGLGMEGAEGGGGGGGGRHGGRARAPAMDFGQAIDAFITRALDLRYMHEDWLEAWRRARE
RSYGEEQLSSEPPGVNELDWRGDPLLAMQERAMERQRQEWLLAWEKARRVRDRRAVRLREQGYREEDIEARLGRQQE
AEEVESAVVHHLFVNLTLMARHKPADLVKFRDELTGTKKKMAVWRAICNQKFIDETREKEYKRENTLRAIYDSLRVG
GRKKVLDPDQEDACVGRLVTDVYKRERRMSERIERKLEAVESSLTPWYVLHPPSRPVSRAVSPNRDSVSPGRRNRSP
RSPRPGGGGGARGAAAAVTPPRPRSASRAGRAPTGPDDPEPKAPGDRRKYVFGSSTPKSMWGPQGPVRGGAGRTPGR
GGAAGGGSLLDSPAVQGLTSSVDRRERSPGAGRGGNSTPTRHRPTTPSQPQRPRSAAPGPSPPPRSSAGGAPAPRRP
MSAAGPAPPSPGGRGRPASPGPSRGPPVSPRRSTPSRVGFAPPPSARGRGSGGGAPPPRRGLMDTAGDTTGTDTSPE
RGPPRGGAAPPPPRRGLADESESSPSPPPRRVPSQTTITAAAPSQPAPAAGGALPSRRSSQASGMSGPAARTGSGGA
AAASQPPAGAGSGGASRRASGAATPPGGLSQQPSAASGLGLPTGPPRPGAPPARQPSGTGSQPRASGTGSATGSGGA
GGGGGGSNASGSRRPSNTPPALGAQPSSGDLGAVGGSRRSLHSQGSTSSLGGPGAGGSKPGGSASGGLSGGPSGASG
LGIGTGGGSAGGGSGAGGAGATPQQRSPLSRSPTNASPPSASPPGASPRGQPSSLRLSSEASNPPTAPPPAAAAAAA
PSPSGRPRVEPSTGGGTTDYDDDEFDAGGGELDVEYDLDEADGAELDMGDVGGDEEF

>Cre03.g185350.t1.2|PACid:27576227
MSGTDAGPGSTSSRASQLQELQQRAKSLERRLAAAAGLHDTLYETSSYEYLPPPPGESLLPPKPARTHAADPAAGNG
GAPLGDPYAAYSPLFSNFPIYHSRPLGPWAPAPSPTGSGALSPRGSSGGGASPTSHGTGASNTTIDWVTAAPLQPQS
PSAPRPRPSQKRHAAALTEPEPKVRFRQPPGSAAAVGEGRTSSPLGASTGGDGSVAASGSSTSGVDAFIPSASVGGS
NSPPPVASDTARTQHWLDGVQAASATTLSAALATPAAQMSPPPASSDSPLCRLPPNTSYGRGPGSTSPRSGSVSRSL
HSVGAAASEPIHGPQQQFNQGQHHVHLHLQHQQHQWMQHQQHQQRQQGLLQHTVQRAVWHQGQLRRVAPHGAPASFF
PFSSSPKSAIPYVPALPPPSPRPLPESTAAVARRPLGDRIQNTTWALARAFGFQAFNEKPSAAPHPAHQQQPRQWVP

STAFVAADHLEALLLRNIMPRHLAAMKGGGAGDPAAAAGGDEPQREAAFACAVWELHSHIFLSYEGAAGWCRLLGLR
PRLAEVEAEVLQAPGRTAQDPAVVSVLLLELCLYFLLYSEAANLRHTPELLWFLFWAAAHSDTMQRLCRNSAALTSP
ASGPGGLQLENARQRRLAMRNTLQSALYTAQRYLDHDPAACRPEVCVEAATTLAAQLPGLLPRLNLPGPPASAGPSS
SSSPTKGRTRSAADEQDDAALSCAASRSLFVDLASFGDGGFWTDVVVAPLFTVLAYEVDACSAAGQESAFRLGYDDV
NESLCRRDVLSRLLAELGVQQADAAAGTVHGALDAITRLGYPPSVAVGGAAVATGPDGTVPPADADAHGHSHLRRRH
NRSSGGGASPTFANSATPAISSSSHLDLRAFDVDAAAAFWLDRVFIKTHRERRSWSAIFRAFYRVFSLQLVLLHLTI
AHAFAPFDLAVLSSAVVTHALVQAAERAANWWLSRGTHDPLQRAKARESWVSASPEKLMAAHLAKTKAGKKGKQAQE
AGEDVEASGGSSGTAAEQQQSAAAAGAAGGSRLTDAEAVAARLERRRAVAVEGAPVWGVCGWLEWVIVAAGITGLFA
LQHIGPPALAVMAKSYWPFAVAGYAVAVVGHGLATGRDGYSISLSSALRLPAALRASSARPRAECWLPGHLGVGWRA
ALLTALFWLQVLGAKLAFDYFIIMRPMAGQVRFILHRNWLGCPAAQTHYRLFGAMALPLACADGDWLLVVLRVAPFV
LVCLVDTQIFYQLVLMAWGLVQGLQAMNLGIAGSWEALVAEFHRAPVRWWARCMSAAGNQAARAAAAASVARRQHAI
TGIGSSSAAAAGGARGSEGRTDDEYGSSSGGDDDGDNSEYDSEFEYGSVYGGAGSSKRGGLFGRRSGSASSLHSSSL
HDCAPSPGPFDRRPGTAGVAGPQRGPRPSRPPHRRSPSPAPSAALAAAASSSGTPTSADATSADPSAETIVGRNDRK
RNRDGKGKAKGGKARDAGTAAELVAMLSGQGEEQIAQWMAFAVAWDAVVEDLRRADLISDREQDNLMFTRLPAAATL
LTAALPPTAAGTALAAAGFSPDSPSAAAAAPGRGSDSVSGDSVGAGSSGGSSNSTAAGGGPSSSRPLRPILLPAFFY
AGQVQRAVDSGAASPAQALVLGELRSALVWLGCQLRLLDGRTAAVLLDAPSCSAAIDPAHGRAREAGLAALRRLAAA
LTGLSEPLPAAGSGTVGEALRRRHGLASEAQAALDDVLVVVEVEARAVIKSQLAASRKGSKKGGRKDKTAAAVTGPD
AQPAPAEGAKAASAEEAAAAAADPQVAALEVLQTVESLRTQLKQHPAWLAGALALLHPEGRGCGDVHLPSASSLSSA
AASPGSGDRVLHVSVELPLLRRVLGVVSKMLSLSSAAAQPSGAEARRILSFFVTSLANRQLGRPDTVAATPSWTVLT
PLYAEDVLFPLEARATAEALGLPMPRGDPAAAAASDTAPHPSSHVSSPAGTGALPDLLTETEERVSLMAYLRSMFPK
DWENFKERLGAELGGVQLAAATEADFAAGGPLAEHGLALQLWASYRGQLLARTVRGMAAYGSALRVLAALEAPRPPC
SSRRQHAAEVEDVVGGKFCHVVASQLYGRHRRSPHLRERWLAESTDVLLQANPHMRVSYLDVVHQGPKQGKGQQHQQ
QLQLPQHFAVLIRGRQPGSEGRWESFQSHGGAGSDAGGVTAGGAVRGASRGRTEELYRVRLPTNRFSSRGVILGEGK
PENQNHAVIFCFGEALQTIDMNQDNALAEALKMRNLLKELRPEAVSRPAQKAAAALQEAVALAAADGLPASHRVGSA
TSSSADTGGPSGAAAASGAVALPAASEYRRLLSELRNAERPVAVAGFREWIFSDKAGALGAFAASAEFAFGTIVQRT
MAYPAAVRLHYGHPDLFNKIFVMTRGGLSKATRQLHVSEDIFGGMNHTLRGGQIKYREYISCGKGRDMGFDSINAFE
AKISSGFGEVSLSRDLLRLATRVDLWRCLHLYHSLVGNYINTWLVMGSVYAHIYALVFFALAQAAVYRVFVYYPSPP
PPPPGTPLVPVGPAGAPPPPAGTLKEVLAYDTIRVEHVLQLGLLSLLPYIAEVALEQGLVRALLAALAQLVSGSFSF
FIFKQQTTAASLHSSVMYGGASYIATGRGFSITSSSFLNLFANYGRSHMALGFELAALAIALAATNDCARCSYGGLT
WGTWLAAVSLVFAPCWFNPMAFSPAKVRRDMHAFAAWLRGEVDKELGCTWAQWHRRQLAPARGEAAAGGGQTDRWLN
VLQNLMLRAFPPALLTVAAASRLNLRLDKLPGLPAPLRSASALFLIASAGLWAVLISAMGASRRFAALSDRRRWRLW
SFWVGVSGVSCAVLFLVGLSRWYSGSGLSNLCLLVYANALLATAVHRGLEAVAPRSWTGARRLVDGGHMLLDCCVGW
AVLGMLGALSLLGFVGRVQTMLLFNVTFARSVKRGSLVKTIGTAKTGGGQQQGTTAIVGMVLDQGDAAGAHVGEAEA
EVAGGGAAGAGGRTSAVAVAGRRLRTGGRRGSIGPDRV

>Cre03.g184000.t1.2|PACid:27576244
MAVACLAVAACYEWDFRYNMDFTLVATGTTFPLVFTIQQAFARRERATVLIANMKASIVALYYMHRDWDQTENFPAS
LGNNNLQWARKVKAVLEEFCASCSKYLTAQDMYESVAERRLVAKKNDELYELFVHKLEVEERNTPHFRRRVHRAESR
DPGHVHLTDCYRALSLLSVFNEQLTLKAGYTRGGEGGMSRTNQYLRYIISNLEELRMIREYRTPYMMRTACGILLHI
FAIILAPYFAHFCDSWIGLGNDEDSCPAGYAGAIVFVLIVMLLYHIQVDIEMPFDEKGLDDVFFMSTAELDEAMGMH
ATLSGRNSCDLDSLAGSSPRELSRMGMAGPDGPSLPPQRSYKNLSHHLSAKDLQSNRPLRTIPSDHELNVEENPGRR
LSGGTPHGSDEMLHMLAGRRSPPPPPRVSGGGAGYIVHGGLSSPPPGASPTGTPGHVTWAPPQSHS

>Cre03.g192450.t1.3|PACid:27576287
MGSIRPWHGRCAPLFALLLAAISTPALSDAGHDWYQCMAHAALSKQPACNGTQQGWIDAAIAVWSSDCSTSAAAVNS
SGGAVLGPLAARRALRVLTSSGAPAEWAQRYLLGAAVTVPSTLVSVGSVIDGNSSGGGSFAAASAGSVVQVLLADGS
AFPSVPIYLVYNTAEAYDPGPVVSDAPAGSAFGSGRFSTDNSTSTTRRLLRQAHEAAWTSDQRWRSLQQQSDLGVWA
DAWIVSGVEVGAAMAARHNSGGSLPVPPLDLTALVRRDRAVQWGGGPSGGGGLLPGFEYSLYRMGGSAAALPLQVSV
PLLYFDRRVVTQVPETWDQLLAAAARLHGKDLAGDGKPSYGLCIVSSPPPFLSATTAPDLLPILISVWASLSQAAGP
RQGFWFKQQGLADLQQRSLVSGFDYDLGDDGSALLQPWIDSAAMREALRITEALRRYGPPPGGMTTGSNLTAGSNVS
SAVAAEVLQPPPLPSAWLGGRRHSGLQPPIAATASVNGSRAPPGGYTGHTALALRGACGFIIAPGLSLLKAVAASAT
AEAASWGGSGATSPLYGHLGAAPLPGSPRVWHAGSDTLVACNRTICPFAQPLTMPEGNSSLVNRAPLTSVDVAAMIS
NTGSPSSSPSSSPSPSPSSSPSPSPSSSPSSSPSSSAGAAEGAAEDAAEDAAARQLLAYSLFRARLRGEGAAADVRD
TRSSANPFRYGHLDPGPWAAAGYDAADAAAVLAAYGASMHSPNLALPLSVPGADSYLRELRFMAAGAVRALAAANMS
IGGSSSTAAAMNVSAAANRSDAQAAAAVAALLRPWLKGAQAAVVDAFAPNGASAAVNLVFYARSLRTWSRSREPERL
VAPGDPTDGSPPPVASAAPSTGSQDWIIIVIAIGAGTGLLLLGVLVVDITGRCIRSGWSAKLVFRGAVTAPLPSPSL
TLLLSDIEGSTVLWEELSPMDMDAAMRLHAITVRHLLLVHQGYESATEGDAFITAFANPLAALNFSLQLQAALLELS

WPSQLLAHCHGQEAWVLPRVPALRLPAAWTDTAAAATAVAHAAQQNAQQQAPREGGQQQHSPLNPRRAVRSKASPSF
SVSGAAVAAHRATGGGPLPASASSARVARCDGAAGPGSRLASPLKHAYSNPVYGSLEAPPLAAGSGVAIDVRTLHPP
TTSKRPHSTEVPAQSQHPEHPEQQQHLRMQLAEQARLAHRGADTLNPCMSAPLPLPIARGGSGTITGAGLVPQQQQQ
HEQQDKQQQQEQQDKQQQAERARAQQQRPRSPLGPGLALHNSRHNAEAAAPPAVATSGPHTTEARPQQPPHRPQPSL
SLGSMLSAAAAKAAFSRPQSPPHAVQAAADGRPGVRDPRLLGAGVEAPAFATSSAPLDAACYPNNVSAAMYNRITTG
TYGRTGRSTAPTMVGSCEPGDGMGPPAVWTSRVSTRRASAMQHSQSQPGSSMAASPAPPAEDDDDASLAGASSPVGG
SSSSGLYRPPAWLWSSGQRLGQPPTAQPQLRERSDSGWAAIPAGTSPPQLDDAAFLRMLPQLSINTTNDDPPQNQMD
VSPTRCGQLRSDLSPKHAAGDHGRRFSGRRGRRGSSGSSSSESSSSGSSSSSSGSSNSGGTSATSSRSVGSNEGEHG
HSRPPALGQPGHGQGSPRRAVQMDAGLGTVRAPAQATGVGAAAEGKAIGTAAGNDAPVDFRGHVRTLYEPCKAGTP
GAVRVYCGLRLRLGLHCIGAAALGTGGRLHGATIAACIEMRTNKASSRVMYGGPIMTVARAVSNAGPGGSVVLSEPT
WRALLAGGHLVGGSGGGTALAAADGLPLGLYLGHFGLPRDASYRPPPGARPTASPEEEASAPSSAVAASDSLSEGLR
TATGAVQDQDQQPLMASGGMSGLLGDSGGAARALGSDWARLNRAASGTMPVGTPRLPGLPTPLEQLQHLQQDHRRPQ
HQHPSRHYDHSHLPPPPPNHSYYHRPSSGLSGSRSDLTSRNAAASTAAGFMHGSQAAAAAGISARAGAATHSAGASP
LVHGNPSDQPPPQAPHSRSALLEAVGYGKGSIRSILDVAMARAARARSGPPAVIMPNSTNSYTAVAQLPSPLAAELV
QSQPRRATQDEVVAGHSETSMPRPSPFGLTQQAPAVLAATRSCEGGDITPGDSTTVGFSSLLYTTAAANSGGSGSGS
GGAALSAAALLPAELALYQAVCLAGRHAFLGARVALEPPHISRVGPCLAPGTPNAPVGSNMAIAELRCSGASVLAAW
DISVYGASMALAADSCRRVAARVRGVTLVHESCMQVGSGRAGAGPGGAGTGTGGVGARMARGGSAGAVDPSGTADIV
IRGGAGAAGISHRAGGVVVVGVAADAAVLMAWALECLAAGLQLAWPEALLDHELGEEVLLVPLPPAPAHTLHKAPSV
ASLASEQEQQQALRDGPGGGAGSQQQLAFAGGAAGCDANGVRVPSSGSARAISGRLLAAARRRLRGGGTTGGSGSAN
ALLPTNLRDLAAVAAPTAAAGDLSDSAAAQVLAGSSPSDSVTSGLGRRASLVWHFIRGLQPGARGGGTSPVHARSPF
RAAAATAAVAAGGSTASVASSMSWQRVGSRSISRRYLAAAHRLGTEEVAGGAEADMDTAASASVAKSTAAALALEGL
GSLGGSAAESSAATDAASQSALEMSTGALNVVARGRGGSAAGVQPSQEPANRSPLLPGHLTSLPSLRRTTTLSRMAK
DQWHNQQHDSQRSLDLPPVATDLPVADADGGEVALMSQDSISLAVPDGGPNDREHTHSGAPAGSRRAFGSLVLGAAA
APTTSSVEGAAGSPATALAEAAAMMQAAPLPTGSPAAAADMALQAAVHATAAGSAAGGTEGGAPAFVNSGNRQRRYS
TRTARRSSVVLTAQQRLAMADGRIASSAADGSGGAAADADADGGPKSSAPTHVPGASGSSFTSVASSAAASCGGASS
TRPLKVARRRRRRSVLVMAIEERKQQAQVQLLQQLLLQDANEDGVEPAAAAAAVAGSSHSSLVVATAAGMATSLPDA
DPVSGLDMQFTPVKSAAALAVDDTSGEAAGGGAPPPPVRPHPDGAHTFSLLHHGGKFSPPRVGSASVTAAASVAVRP
VPGCSEDAAAAEDMPQVESWAADTGQLYARQSSKAYPYTTQLAAGASSTACGSGSFCSPPHPDGLRPTSAARSGMAS
HLKLRLHSSGTPPAHMLSRPPCNSSEIAVPAIDAEIMPQDLDEPVVNLRPQQKSPPWAGDVGAAGANELWHRAPDVQ
LHSAPMPAVGGAASALPAQQQQQQQLLRQSQQAYSQAARTVFRGLRLKGAIDVGSLQATLLGSSGRLQYKGWAADRA
AALAAAALSGQLLVTEQAVRAACRLPTFVSLEELHQADVGGGSAHSARTAGGGKGAAGTAAGGAYGPVAQALESPRM
AAGLYGSGLLQAAAAAAGRRFGSDAGGGATAGLPSPSVAAAATYDGCGGAAGARGFATASYSSSCRSGSYNGGSDAN
PRTTKGGPAPPSPLQGGSPVSGRLPLFRRTSLPGDGAPQSEQLPLRADSQKQVPPQQPSVLGRVRRRLQPPVAAAML
RAEGGDSSGAEAASGATTAVWDTPDVGGAVVLAQQLSQRSLGFSAGSPSAQRCFSGKTGAGADRATSPAAAAAAIRS
GDDGHHSRDCPLVPFMESSFSGGGASPTSPALRHLQTTRNELEAIDVAATAAGGRLGSDAVFGTSPDGLGMAAGGAA
AGGAGGGVGRNAAGVARSGVSRLFTRVSAGGSGGVGLGGWPGSSGNGVGGMSPTMQMARERRQQLIAEQWALAGGRP
RGASVTSVLAFSAAGDVMRLTDYLFTQALAAATAAEEQLLSAGLQSLHHHHHHHHHHHHHKHHHSRQHQQALPPPPP
PPPPEVDLDGDGRLVLRVHMPSSGAAAAPAAGTAARATYLGGGHPAASSPSARSPGRQQQAARAASRQLATSHQQQQ
HQLPHDLPHHEQQLLRPGGVLEVVAEVVDSSSCSGAGAGAGAGGVAGGLAAAIADGAIGLPDAAGRAAVGTLLASSA
AGYTQRQQLAAPERAASLAALTAAEPPADGFSAHAPAGSSQQQQRLNRRHNKVHFGKRSPAEGGGAAAAGGIISRPS
CAQCTGVLFEALPHWQQQMVVSRAGKGVGGLLGVSKRQVEILMKGVLVARRQ

>Cre03.g202700.t1.3|PACid:27576355
MPFKVGLPSVDNTEGTGSHCGSRDDDSCYSPRGLKTIEWSDADVVRDALASPSGPGPRVAPSPFPPASDAEPGRRRR
AAGDQSFAAGAMLLDKNHSVTTVALASQRAQAADGGSAALQPAPLATDYASPFSGQQYSLHTAVAAAAAAEGEAAAA
SEADPVSGSLLSSYLAARSSAGAAPTNTCARMASRLHRISINGVGSIISTGDGIVMGSGGGRSRARPSLAGAASMDT
GGAEQAALGLSHALSTAAAADPAADAAPLVGNSPRLRQRRATDGYRPPAADDARPAAPGPGDRRRPRVSGAGSLAAM
SLDAGIVAVSAAASAGWSPPPPLAARGSHLNPQLLLHQQPSSETKLPALPLAGAASYRASADGSGSFGGMRPRAPAH
SLFTDTKEEMTAAAASAAAQLPAPVAAPPAVVSSPLPLLGVEGKGWALRSPSRKHAMQPSPRRSGSGAVGFFQSGAE
VPAPPRTAAAPTSLASPSAAGAELWMAGLVSPPPAPPPAPPLRGGRSGKHLRTMGGAPVSPSASPHYTGFGVNGYRY
SGSSHAFSAHGTTTAAAAAPDGAVSGGGSAVAAAAATGSRSAAALLADARELRQLSRAVSSRVLGSALAAADAAER
RAGLSTYSLPPASLGP

>Cre03.g170850.t1.3|PACid:27576367
MRSFTVLLTVLSALLAGTHAVPHKRGLPPPLLRTRLPPPSAARQRSPPPLPASQRPPRPLRSPSPPPRPSPPPVSRW
KQLSITVQAQVSLSAYDERLSAGLFRRGSVGTILRLVPTMLSSDPTNDITVGSASLRVQLPEEVGRGQTALLTGDIV
EATLVITVPEALAQKLSSLLLINATSHASEAVDIHDLLTALTKANEGKPQPLLPPLIESNQTVPQIPLVTRIARVMA

ATAKGGCKCLIPLPGKGPLNVSSLTFLFNTPACGHDAGFNSSADGPALMRAVANYMGVCSYSHVGAFPDRNLVLGPI
DVANCSDAQPTMGQYDLTHGIDLDAKLYGMWDLAKEWMRKNHPDLYAERLRFDRKTLMIAPGDGVFSLSNMGCPGNP
NVDGVVSDCLNWVSPSEPSSPVADAAALIRSHLINSGYISPGASRLVCDDGPGDGSSSSSNCRTQLLGDPSDPMGAA
EPVNAAGGVVCPAAPQSYTSGWARPLRQLQADDVQLGLGFTEYEVPSMHTAEANFLRVSIDQAGVGSAAGNRLWPER
AVFLSYRVASQTPGGYDSGLPANLSGRVWVHEYNQTANGLPVDPNAPPVLLASLEVEGAALVLPNAFGAGDSLHIRF
VRRSDTAAAVGLCRTTVRVEGSPDNLETCFNGYDDDW

>g2881.t2|PACid:27576379
MSPRQAGLLAVAALLPMWTVNGQVRSTLSSDFFCGLPDNSSAVFSTDEGLVANTSLPLVQFMVWADASVTQGMYITY
SSGDTGHMHGGYRALAQNGAQQNSPGYTFPPAPAPLHRTITAVRACCIDAPQFVSGNGNGVQRLHFRTANGTVIAIG
ATGGDPCAQPQPWVDVPAGYELAGLRSLVPAPYSDTVFLHRIGFVFGGLPSSPPSPPPSPPSQPSPPVAPPGSPTPA
SPVPPMPPKPAVTTSSFQCGSGVADYYVVTRDSDVPYIEAGGALIGLQGLGQRTYMSRLQPLYRGDLSAPAHGLRYQ
ISPIDFALMNMYSDFPDVDLLAAWSLSSPQRVVAVSACCSSGKLNIYTNMVARLLFRRANGDVFSLGAAGSNECQRP
QGWIDVPQGSLLAGFVTDTFSDYASYGALNPTVVIDRIAFVFATPLPGQWTQPDPPPPSTPSPPMKLTRQSRFFCGS
TFDSPATSPGNLNPGAPNNPYVPALGALVESDDDVAALGYPLTAITFSVLQSFGAMNQARSMYGSVPGRWHKAPGFD
VGSDYGADLTTSWGAMSPERVVAVSACCSASGMSGQAMTPIKVVLRTASNRTIAIGYDSSGRLTAYPPSGDPCYSFV
PQPFEPVPPGFLLGGLATESVIGGGGPGFDRLAYVFVQAPPPRPEDYVPPPPAPPPPPSPPPPSPYGAFYRSSSGDG
SGGSSMAIGIGVAVGGVALVGLAALTFLGLRRRARQRYGSDVAFDGATCGFYPAFMPPSPPPPPPRPHQQSTPFATR
SPPLQAPYQQQASAAAAQPLLPNAVSSGRRQLSISQSVALQIQLLMGGGGGGGDGVAGAAVGTRHPAAGEPWHLQDS
GAAAAEGAAAGGSAEAVVESFSASALHAATAGFGDSHLLADLSSDGGGAVYRGSCPQQPSQQDPGCGGGVASATGSS
TCGGGGGRDVAVWRQTAGAVTHDMAAVTRLAALRHPHLLPLLGSCPDDKSLLVYDLAQRATTATTTTTLVDRMLHQQ
RPRAGAGAALSPAAAPAPAPASVLGWRDRVRIGAEVASAVAFLHAQAPPVFCRVPLDAGRVVMDGSGAARLGFVGTA
AVAAGGCGETSVATAAEGEENDTRALGVLLLRLLTGDATGDAAALVARVRGAQQADVAGGGGGRALAALAFDAAAAS
GAAASSSSGDGASDVWPAAEALAFADVALRCCGCGGVSAGGAAASNGTAGVPGLRSAVLPHLLQLSNRTRLYDPHQQ
QQAPTPSAAALDYPPAEVKGAAAADADAEEEQEEEEQDAPPPLFVCPITQDLMEDPVVAADGFTYERAAIAEWMSRG
AVGGRTPRSPLTNLPFEHRTVLPNRAVKSQINSWREEQAAKAKAAQQRRRGTERPQEDRGQ

>g2881.t1|PACid:27576380
MYITYSSGDTGHMHGGYRALAQNGAQQNSPGYTFPPAPAPLHRTITAVRACCIDAPQFVSGNGNGVQRLHFRTANGT
VIAIGATGGDPCAQPQPWVDVPAGYELAGLRSLVPAPYSDTVFLHRIGFVFGGLPSSPPSPPPSPPSQPSPPVAPPG
SPTPASPVPPMPPKPAVTTSSFQCGSGVADYYVVTRDSDVPYIEAGGALIGLQGLGQRTYMSRLQPLYRGDLSAPAH
GLRYQISPIDFALMNMYSDFPDVDLLAAWSLSSPQRVVAVSACCSSGKLNIYTNMVARLLFRRANGDVFSLGAAGSN
ECQRPQGWIDVPQGSLLAGFVTDTFSDYASYGALNPTVVIDRIAFVFATPLPGQWTQPDPPPPSTPSPPMKLTRQSR
FFCGSTFDSPATSPGNLNPGAPNNPYVPALGALVESDDDVAALGYPLTAITFSVLQSFGAMNQARSMYGSVPGRWHK
APGFDVGSDYGADLTTSWGAMSPERVVAVSACCSASGMSGQAMTPIKVVLRTASNRTIAIGYDSSGRLTAYPPSGDP
CYSFVPQPFEPVPPGFLLGGLATESVIGGGGPGFDRLAYVFVQAPPPRPEDYVPPPPAPPPPPSPPPPSPYGAFYRS
SSGDGSGGSSMAIGIGVAVGGVALVGLAALTFLGLRRRARQRYGSDVAFDGATCGFYPAFMPPSPPPPPPRPHQQST
PFATRSPPLQAPYQQQASAAAAQPLLPNAVSSGRRQLSISQSVALQIQLLMGGGGGGGDGVAGAAVGTRHPAAGEPW
HLQDSGAAAAEGAAAGGSAEAVVESFSASALHAATAGFGDSHLLADLSSDGGGAVYRGSCPQQPSQQDPGCGGGVAS
ATGSSTCGGGGGRDVAVWRQTAGAVTHDMAAVTRLAALRHPHLLPLLGSCPDDKSLLVYDLAQRATTATTTTTLVDR
MLHQQRPRAGAGAALSPAAAPAPAPASVLGWRDRVRIGAEVASAVAFLHAQAPPVFCRVPLDAGRVVMDGSGAARLG
FVGTAAVAAGGCGETSVATAAEGEENDTRALGVLLLRLLTGDATGDAAALVARVRGAQQADVAGGGGGRALAALAFD
AAAASGAAASSSSGDGASDVWPAAEALAFADVALRCCGCGGVSAGGAAASNGTAGVPGLRSAVLPHLLQLSNRTRLY
DPHQQQQAPTPSAAALDYPPAEVKGAAAADADAEEEQEEEEQDAPPPLFVCPITQDLMEDPVVAADGFTYERAAIAE
WMSRGAVGGRTPRSPLTNLPFEHRTVLPNRAVKSQINSWREEQAAKAKAAQQRRRGTERPQEDRGQ

>Cre03.g159550.t1.3|PACid:27576471
MDVSVGLRPALLSLNDANLASQGVYAAKLFIDKLLGAQQAGSCVKELLSGLDKCTRAAANSRIVLLSYMLATQSSLN
PVRALPQAIPSLVAYLQNQRHSTNLDELQTLGCRIGQVLAAALDGASAGGGQDWAVSDTRPQLDARGGGSYAAAYAA
SKGPVAADASPCHVDPAWALNHVLLPLLRVACRSPERESMAAAGAVLVPLVQSLGRALQPGRPVMAGTGSVSASQAS
SWLSSPVVAVLERLMGWAFTLMGSAATAPQRSSAVLQMLVSCVQALGEELGADGAKRLALAATAGLDNAEDWLVRRE
AANLVTALAELGASGRGGGAEWMAGPNRREFLTQSLEALRHDKIPQVRSAVQAALSAVAQLPGAELTISARGSLAAA
PATAAPSMAGPGPERGAGPRPRSGSPPQGQAQPARLAEPNSRHSSGTDLMPPQHPARDQPQAPAAQQRQQQQHDQPL
MQHASRPVSRQASIHGKDAGRGGAEGVQRLQQAGRRTSVASASEAWQRSAPTNNDHSRQNPVPSESLRSMGSRQRSS
DGSAAAAPWPLLPRRQDRQGDSTGAGDTADVAVASIGGWMPMADGGPIAVASSSGMGGGEGAWAIAVAQPHAGDADY
RERHVEEAEGAEEGLEEAEGDNSEPLDSTQQGAAGFGSEGGELALEEEGLVRGPTAAYGAAPSRQLQQPYEEEAEGE
EDQQPRMYAGSPQQQGPGSPEGAAEAQHLSDFTGAVATSGTAVAWAIPFSSQGGHASRPASTAGRHGGGASDGSASF

RRSSLSDAGGPQDARLQSYGHDRPAAAAPPPATSGMPRPYGAHLPAPRPPAVDGSWSGAPQPPAGTTQEADHDWYMS
RAGGSYAAGYGAGGGYARAYGGGQYDSLPDDYAAGCSCLARPTDLPTLLPSKSPRFPLDAGRERAVAPPPRQRLPPG
LSYAAMDDAGADPGPGARSARPCSQPRQQALPLVQPPWRCPSRGPSPPLRSRSPGGFYEGADGSGSGGGGGGGGGGA
AAAAGAERQVQRPDPSRMPPAGVKSSAAVRSDAARAAQSGEGALHAGLAATAAVKPPRMSFNGGGGGWGGSGPPTSA
SARQGWTAVGRAGAAAATAEMRAVAGVASSAGDGNRRRNAPAADCCGPVSASSPPRRYQRRDDTRVSLGWPFGYRPP
KASSRFVRPSAPAEHFGVQVFAKEPPPPSPPPPSPPPPPPPPESDPDVQPGSPSHQYVVNVHTESSPGHRTAMVITA
SPSPGKRRADVNNAARHPPITAATPTPRTPRGMVEAAAAAAMAAMAAATTAAGDGSQAGAVHSSVVEGGGEGQQPVR
VTARVRLVGAPGVGDAGQLSSPTWSSSGGLGGDGHSTYGGDDGGGGGRSAAAAAAGALQDQLQSVVEQARALQASLA
QRPGKRPRRRWNTQPHQQGQHPVSPTPAFPSMTAATDPLSAAAAAQQLQMLVQQAASAQAGLQAYLMTAAPALLGSG
PAAGTAAGELLAAAAAMAAGGTSAGAVGGGGGGGAGLVLPQGLAGGATAGPLALQGAVNRLEELVLALQRQAAGGGT
LPSSSASPHPSQPPTRPGSMSAIPGGADAMPGRPAAAGQLSLTGEDSGPAGAGGSTAGILGPEATGALRELIQQVEA
TRESISRTTSGLDARALRHSDSRASVGRDGGGIHPGMRHTNSSIGLGSSMSYSLAQINNQWSVPPIPARRGAQTSGG
GAGPAAAGGIGGGGTGSRGGGEAGTIGSAAVGGSASTPPVGKKTPAASEPSSSGGAPPRPAWGSEAEAGGSPGVAGA
PRVRFAISGGGAAGSGGGSGGAAAAGRGSGAAGDPDSKRLGGIAGGSWRQRAQGSSRARQGRRGTDDEHYDRADEND
TDGMYEEYDEYGRLVHSGGGRGYASGGGDGDRRCGRGTRRDSGRSGTTGALQDLVNRTGSLLLPGEPPFQWRGGGSG
GSGSGLARRRSPQDRDGGTSAHSDAAETAWAEAGYDSTGRPIHVHLHHHNQQQVLRREQHLQQYTQGYAQTAPPGAG
PPARLGAGHQSQSYWPLPLQQQLLSARQRESPERYPALAWAPAAPVAGSGRSLGRAPAGIAHLPPYRAAAGVAGLLS
LPLAPQLEQPVQARTELGMPRVIDFRPVVPIASARPSVLQQGAVSPPSTSASPLLPGSTAAPLPTGAALGLAGAVGL
HPSLHPAAAQPRHPSPTLQASVSRLQSLGAQLRGTVSGAAAAGGWQNESAEAAAAQQQQRVQPLAYELRSEPREPRY
PAPAGPTALPATLTTGGGGGPGGFGGGLAGLAGFTPPRSYTQPTSPSVVTIFAPETSHLTINPPAPTTAATQPGGWG
SLSAAAAAAAARVAAITDVADTAASTQLQPRGGLDLQRAVPAVTGALGTPAAALNPLNISLEEPLFLELYTSPAEAG
GEAPDDVLGGTTDQGGSLRPPEVIGGQAHLPPSPARQATSQAHAAAAATGTSGSAADMVAMASVVDLAAQQDTATSA
LAATRLAAAWGPRPDDGEDEDEAHLYALPMLEDLSSPHASTSPPPAFQACAQPTTALESGPGSGRLAAGAPGDADLE
RLFPATAAAMAARQSTSLTQSPLQPGAHAAPAPGQYPGSPEAAGGGHSGGNAEAQVRSSWAAEAGTLDASFVATGAM
PTPLPIRPATEAHPAGDAQPPPLQLSEEPVPVSRPPKGSVATAAATAGTATAQAMLGVAMPRLLRPENLQALRDSAT
GLARSSIEAVREVEGLVVRLTLLQQQLAEGRRSSAGNAALVPVNPRTVALEAEALEQEAAPLGSVAESAGGVHDAVE
GGEGGDAEEDEVFELPIV

>Cre03.g159550.t2.1|PACid:27576472
MDVSVGLRPALLSLNDANLASQGVYAAKLFIDKLLGAQQAGSCVKELLSGLDKCTRAAANSRIVLLSYMLATQSSLN
PVRALPQAIPSLVAYLQNQRHSTNLDELQTLGCRIGQVLAAALDGASAGGGQDWAVSDTRPQLDARGGGSYAAAYAA
SKGPVAADASPCHVDPAWALNHVLLPLLRVACRSPERESMAAAGAVLVPLVQSLGRALQPGRPVMAGTGSVSASQAS
SWLSSPVVAVLERLMGWAFTLMGSAATAPQRSSAVLQMLVSCVQALGEELGADGAKRLALAATAGLDNAEDWLVRRE
AANLVTALAELGASGRGGGAEWMAGPNRREFLTQSLEALRHDKIPQVRSAVQAALSAVAQLPGAELTISARGSLAAA
PATAAPSMAGPGPERGAGPRPRSGSPPQGQAQPARLAEPNSRHSSGTDLMPPQHPARDQPQAPAAQQRQQQQHDQPL
MQHASRPVSRQASIHGKDAGRGGAEGVQRLQQAGRRTSVASASEAWQRSAPTNNDHSRQNPVPSESLRSMGSRQRSS
DGSAAAAPWPLLPRRQDRQGDSTGAGDTADVAVASIGGWMPMADGGPIAVASSSGMGGGEGAWAIAVAQPHAGDADY
RERHVEEAEGAEEGLEEAEGDNSEPLDSTQQGAAGFGSEGGELALEEEGLVRGPTAAYGAAPSRQLQQPYEEEAEGE
EDQQPRMYAGSPQQQGPGSPEGAAEAQHLSDFTGAVATSGTAVAWAIPFSSQGGHASRPASTAGRHGGGASDGSASF
RRSSLSDAGGPQDARLQSYGHDRPAAAAPPPATSGMPRPYGAHLPAPRPPAVDGSWSGAPQPPAGTTQEADHDWYMS
RAGGSYAAGYGAGGGYARAYGGGQYDSLPDDYAADLPTLLPSKSPRFPLDAGRERAVAPPPRQRLPPGLSYAAMDDA
GADPGPGARSARPCSQPRQQALPLVQPPWRCPSRGPSPPLRSRSPGGFYEGADGSGSGGGGGGGGGGAAAAAGAERQ
VQRPDPSRMPPAGVKSSAAVRSDAARAAQSGEGALHAGLAATAAVKPPRMSFNGGGGGWGGSGPPTSASARQGWTAV
GRAGAAAATAEMRAVAGVASSAGDGNRRRNAPAADCCGPVSASSPPRRYQRRDDTRVSLGWPFGYRPPKASSRFVRP
SAPAEHFGVQVFAKEPPPPSPPPPSPPPPPPPPESDPDVQPGSPSHQYVVNVHTESSPGHRTAMVITASPSPGKRRA
DVNNAARHPPITAATPTPRTPRGMVEAAAAAAMAAMAAATTAAGDGSQAGAVHSSVVEGGGEGQQPVRVTARVRLVG
APGVGDAGQLSSPTWSSSGGLGGDGHSTYGGDDGGGGGRSAAAAAAGALQDQLQSVVEQARALQASLAQRPGKRPRR
RWNTQPHQQGQHPVSPTPAFPSMTAATDPLSAAAAAQQLQMLVQQAASAQAGLQAYLMTAAPALLGSGPAAGTAAGE
LLAAAAAMAAGGTSAGAVGGGGGGGAGLVLPQGLAGGATAGPLALQGAVNRLEELVLALQRQAAGGGTLPSSSASPH
PSQPPTRPGSMSAIPGGADAMPGRPAAAGQLSLTGEDSGPAGAGGSTAGILGPEATGALRELIQQVEATRESISRTT
SGLDARALRHSDSRASVGRDGGGIHPGMRHTNSSIGLGSSMSYSLAQINNQWSVPPIPARRGAQTSGGGAGPAAAGG
IGGGGTGSRGGGEAGTIGSAAVGGSASTPPVGKKTPAASEPSSSGGAPPRPAWGSEAEAGGSPGVAGAPRVRFAISG
GGAAGSGGGSGGAAAAGRGSGAAGDPDSKRLGGIAGGSWRQRAQGSSRARQGRRGTDDEHYDRADENDTDGMYEEYD
EYGRLVHSGGGRGYASGGGDGDRRCGRGTRRDSGRSGTTGALQDLVNRTGSLLLPGEPPFQWRGGGSGGSGSGLARR
RSPQDRDGGTSAHSDAAETAWAEAGYDSTGRPIHVHLHHHNQQQVLRREQHLQQYTQGYAQTAPPGAGPPARLGAGH
QSQSYWPLPLQQQLLSARQRESPERYPALAWAPAAPVAGSGRSLGRAPAGIAHLPPYRAAAGVAGLLSLPLAPQLEQ
PVQARTELGMPRVIDFRPVVPIASARPSVLQQGAVSPPSTSASPLLPGSTAAPLPTGAALGLAGAVGLHPSLHPAAA

QPRHPSPTLQASVSRLQSLGAQLRGTVSGAAAAGGWQNESAEAAAAQQQQRVQPLAYELRSEPREPRYPAPAGPTAL
PATLTTGGGGGPGGFGGGLAGLAGFTPPRSYTQPTSPSVVTIFAPETSHLTINPPAPTTAATQPGGWGSLSAAAAAA
AARVAAITDVADTAASTQLQPRGGLDLQRAVPAVTGALGTPAAALNPLNISLEEPLFLELYTSPAEAGGEAPDDVLG
GTTDQGGSLRPPEVIGGQAHLPPSPARQATSQAHAAAAATGTSGSAADMVAMASVVDLAAQQDTATSALAATRLAAA
WGPRPDDGEDEDEAHLYALPMLEDLSSPHASTSPPPAFQACAQPTTALESGPGSGRLAAGAPGDADLERLFPATAAA
MAARQSTSLTQSPLQPGAHAAPAPGQYPGSPEAAGGGHSGGNAEAQVRSSWAAEAGTLDASFVATGAMPTPLPIRPA
TEAHPAGDAQPPPLQLSEEPVPVSRPPKGSVATAAATAGTATAQAMLGVAMPRLLRPENLQALRDSATGLARSSIEA
VREVEGLVVRLTLLQQQLAEGRRSSAGNAALVPVNPRTVALEAEALEQEAAPLGSVAESAGGVHDAVEGGEGGDAEE
DEVFELPIV

>Cre03.g173100.t1.2|PACid:27576516
MWLSTTGVVGRKSPPSPTPSPLRSPPSPSPPPSANQVLIWSEEFSPCAAGTPNCNNGLNTAIWQYDNGDGSQYTTNS
ITQWGNGQLQCNSDASENVRVESGVAGADGGVLTINAVSKPVTCGITNSYGSSWTSGRLISRGAKPFMYSDPSTPIK
IKARMRVPLRASSQPLIALLPNTARTDCLGCGDFADGWCANGQMDLMNMMGGDGRVQQRIYSGGDNPIYWQGCSTLP
ATPVAASLASAGQAGGTSRWFTVSVVWGPTRISTFVDGVQTSSIDSSQWYTGWASKAALPYAPYDRAFYMVLTLGIC
GYGGCTAPSDTSTMTMQVDWIRAYDMTPASAPAPSPPSPPSPPSPPSPPPPPSPPSPPQPTGGPQLRWIEEFSPCTT
DNCFNGLDTDRWGFESGDGSQYGITQWGTFENTCYFNTSKEVRAENIIDGGVSRSVLTINAWNTKASFACGTSRTTT
RSARMLSRNKAAFRYLDANTPLLIEARMRVPLVASAWPAFWMLPDTPRTNCVGCSAYGDGWCNGGEIDIMETKNGDG
IVSQTLHFGGGSGANYYVGCNYITRQLSPAYSTAGTAAGMSSWFTAQLEWGAGYFKFMIDGVTVSYLSQSPSPTWYT
GAVPTSTNPLAPFDKPFYLIANLAVCGNFVGQPCTPADSTFQIDWIKVWDLFP

>Cre03.g201950.t1.2|PACid:27576565
MPLSLSEAAAASLIPYSGYRIRVRTPAASSPPPAGKPARRLLRGLLALDSTNFTGGLGVNDTPLDIDSFEILGPGDG
SGGAKDTPMGPTNITSVIFLLSFCGYRRPVSAADFRAMWFNNATSTPTSRNMQNYWSTCSNGFAQMPASNQVVVEVD
LPCAGAYLGTKYNSTNNCRTQELYTWLNMADDYARLTLKLNISAIKQRVAVLPTEMDPYCGWAGLASVGCAGTRCYT
WISGSSSKDLSIYFHEMGHNMGLAHANWANTPDDPYGDFTCAMGSGVTCYNAANTWRLGWLDTLPGAALNSSNFATG
RWSTWSLPRQSAGTSPQSIIRIMPDWAPPAWRQNATPGASAVPAYYVSLRARQAPFENWFGTDLTNPGRVLVHTSNL
TQSSNSYQTTTLQAVLAAKGRYTAALPYGITIQVLSINASTGAAVSICRADRSAESPNDESCYDGLDNDCNGLADDA
DPACQPPSPPPSPPPPSPPPSSPPPSPTPSPKPPSPKPPSPTPSPKPPSPTPSPKPPSPKPPSPKPPSPKPPSPKPP
SPKPPSASPKSASPTPPSPTPSPPPPSPTPSPPPPKKKKASPPPPPPSPTPSPPPPKKKKKDASPPPSPPSPTPSPP
PPKKKKKDASPPPPPSAPPSPTPSPPPSKRVRRSPPPATGA

>g2864.t4|PACid:27576568
MEAGGRPAVAVAVPCPSGGEERPLLLRLGGTGEMAAASTSTSGGGNDSDSSLATAAAAAAAASAAAAGAAACSRMGA
AASASSASSASSASSSSAPEGVPLKAAAPDAGGACGAGAVAGSSSGGGTSPVGRRGAAAQATAHLALLLLLLLLGAA
AARAALLVAHSGSSSSSSGRMGLPGSDVQAQGGGRGGGWLAAAAAWPSWGTTRGMPPGTAPAGGGEGAAAAAARGAC
QPHLPQPPVLGRHGGEDLGCGGCSGCGGGTPAAAGSGSSSGAAKSEGSGGGGRGSAAGQDRTWLLLGAGRINGGSGS
GEPPAHERHPREALGPTVGNTRSSGSSGSGSSSSSGGALGLQAAAETRRPNPFAAVAADTSSAAPTTARAGAAAASP
AGAPPDPPFASPGHLAYGYDPGGPDGVGRGGGGGWLYQHYFGRYLNGSSSSNSRGSGSGSSNSRAGGGGDGVWRGLQ
GRPPQLQQQEQKQDEREHLPTRGRRQLLQTDSGSNNDSSGSSGSGSSGSVPPTSCAVMLRYGASVGDPLSSGPHPI
FVASWAAVAAAAQLPPVQSWVLGWSFDAGERLLSAADVFSDTSAASAAGGPGAGGGGAGAGGVGGGSGNGTGSGAQP
PLSLQVVLQEGYTNSSGQVSQQDVAVLGELGNGTLAPGGPQLSFLMFGRKGPTAAAAAAAGVQLAPPLSDTPTPSAA
NAGLPPFNASALPDYAGPPAAPLRVGPPGGVFLNNLHCVPLAQLVALAQEAAQRAAAANATSSSSGGGSGGGSPPPP
PAQQPVAEPLLSQIPRVLQVDYMPITYVDSAGGGTTGAGGAGAGDGSSPGAAGSSPPPPALVVGNATVGPPRPTFFT
QMYLKVSNPGSRRAVSLSEVQVQYYFNGPDESLDPNRQGAAAAGLADGGVDMSGGPGGAPPFRLVCSDTSPGLVGRC
DSVVGRFRVGLPGVRGAQYYATIQFSPGAGWLAANDTAGNSSSTGNTSSSASPSPPPPAPPGSGPLLDYVELIVSIE
PLVPYMRIDERKDYSFIDTPLAPRPDGTTTVAATVVAGEGGSSSGGDGSVQERLVLLRRPEPNPRIPAYLKGFIAWG
SPPSSALPADTTSGGAAAGADGTGGTSPSPSPPPPSGPPGGFVNPFSAAGLPLPPGSSCQRSADGQLSCGVNMVYCC
AAPATGPIATSIPSDWQSTTFRDLQQQWSSAAVAAAAQANGTSGPGPQAPQGQGPAPAAAASPSPGPSPAASPSPGN
TTVGGGGAGGAPAAIATGSAPNTSTDTAAAASPAPGSAPNGSAVVAPQASEGGGKAGGGGGTAAIAGGVAGAAVAAA
LLAAALIALWRRRHRPAGAQEQRRRQKRGRGLAAASSGGEASGPEESDGGGATGAGRHRHRHAQEGAAAGGGGAGRP
RRISQRFQGVDRRVSRYVSLLLGVRRSDTGGSDVSTTSTSSAASPSASASASATAAEQQRRRRAGLPGSAGGAVRPS
GEVAGAAATAGSLGGARGGSRWELQPQQQGQGQRPRFTGSSSEGSDGSEAGGSEAGGGGGLTGGAALAAELQALQQQ
YPYSTEIHVTASEASELREVLDASWWQRGGNVRGVWGWGAAEAKMAAAGLLPLMAHPHAAAAAAAAGPAAAAALAAL
RKYPTLPSEALMAGIAALSRPGSGELRAAAAAVAITAAATRAGGGGGGVRTEGVWDGGGEGEAGGGGPGSEAGVGAP
SSSRLAGSVGSSLLPAGLLLPLTAVDDDGVRLRILCRKAKTWTGRVADTWELLPEFFPHHPPKGGPAGAGAAPLPPG
QLQPAQLLPPPPPGQEPGSPGGEARVGGGLLPRLPPLISKLLPRRRTLAAPLAAPLAAPLAAVPSARGLESQSPLAD

GAGAFEPGDRGEAAAPAAYHLHHHRSRPRDAPAPQLQPLPLPAPPRLGLAGGPAQSSRRLSPPFPAALGGGAAGSLL
RGPAAAAAADAAALGGMGPGGGAGPGAVLPSVVLPSLSLAAVVAPPIQLDVDYAAEVEPFLGRLIGVGGFGRVYEGT
FRGRRVAVKTVVIDSEAQRVALAREAQLCARFRNCERVVQLLAASLGLPQGAGASGTSSGGNTPGLSHPGAADGGTG
GGPPGAAASRWSRGGGGPGGGSSVRSSAGGAGGLFGYGGSVDTSGGGGGHTPTGSTVAGGGGGQATYAGGGAASLFS
EGTFAASGSLGGGGRGQPAHRPPATRLPPGAAAGGGGGSGQQQGDGSGGGVREGQPQTRPQTRPQHPAAAAAHSPPP
QCHAALIMELCEGGNLGGRIHHPGMRRLDYMELLQLSRDVAEGLAYIHRYGVLHRDLKPGNVLLDSTGRAKIADFGI
SRMRDPFRSFISVTQQGGTPNYMAPELFNGTRVDEKSDLYSLGCIMYEAATRKVPFEHLVRGGGGGARGGGGGGGGA
ARGGGREPGGGRGGDEGGGGGGPGGGGGGGGGALPMGGLFAIILAVAIQGQRPPLPDSLPRGLSDLIRACWAEDPRT
RPTAAQVVARLDALMEEERAARAARAAAARRARPSRGTPASSGHSPSGLSADGSSSAIALLAPLGAAGAVRGSGAAGS
AAGSSSANASPLPSIAPAPAPSPYALPPRRPEPSPLAPPAAAAAAGPDATRPPQPQQHLHHHHHRRQAAAAAGMDH
AFSAPPAHPAATPPHQPHLPAGAHRLTPPHPAASAPPHVLLPGEEAGGLGGLGGGPAALDMAVLLGGGGQAGGGGVG
GMQARYEPGVGVAVGGGGSELSQLHSTGAASGSRASSKSGSTAASPIPSSGVAAAQLSGPTAPTPQAGAEVRSHGHA
RHGSRRSGSGSGLTASASAGLLAQLQQQQQQQQLSSSSSSGAAAAAAAARSPYGITAARSGSGDSSGGGSSGMSPL
LVVGLGTPAAGPQPSPSPAWATGQPQQPLVSGGLVAGGGVTAGGGTSEPGSYKGRSSGSSHSAAPSTTQSQRAAPRT
PSVPDPDYLLMPGRGASGGGGSSSSGLDGSAGAGGHPQLPLLQPLQHLYDGAGQVLQPQLQLQPPVPLPYVAEQRPA
HADRSYPPPPPPPPPSAHWGLPPHAQHSPSASSPSTSPTTPDTAPDRPPALESAAAGGPGLPPPAAAFPQLPAFAQRQ
RRKDSWVRPGGVLAPLLPSVAEDRSVAPGELSPQPRHPHHPHLMARRGDTGGGASSGSGEGSSSGSEAGSSSSGSGS
NGEGGNGGTGQAARPVLLPPPASDAGAAASPGVAAAYAPLPLQEALSGGGAPAHEGSAEEGGVGTRRRFGATPSDAT
SAAAAPVGGGSDRFDRFFSARSRYSAGEAEEWPHAVAAGAGAVPSGCVSGRESPASEAGGHGSRASISSSGSYELGG
SNGVEAAPRRHPQGRGQLAGIQESPRAPPDLQGLDQDPQHPHPPGALPSAFAAAAHHTHLRPLRQLRPHTHPHPHPA
DRAAAHRLHPPPPPPPPPEQPLQAGPPAREPAAWPQGPQPQMRPPQQLRQVAYSAQPPPQPPVGAGGAAGAPRRRTT
DTKTEGGGGMGGNSGGSGGSGDSGQTHITGRTTSHTASLVPRSSTTSASAFTPRTEFSSGNGESQPQQLPQQQPQQR
LSYHDPLLLQPGSTAVSFADAAPPGTASPHPYLPYSAGSGPPQPFLQVAGRASPLCGGGAAAARGVELTARPQQQQQ
LQPQQPQPNGQWGASESAQPQGISGARAGSAHHLGAHPPPPPGAMPSPQGEDEAEAAAAAAAAQEEEPEGGGDGGGV
GGGKWWPWWLWRSPPQQHSSHAQQPQQSHPQRFPQSPQQLPQPQPAANQPRPSASATADPPGQQVGRNHHRQNADAP
QAAQRAVPAPPPPLHLAAADHSLGGAVSSSSPRYGAPPSLSSQSHCHWADPSTLDGGTGGMGGSAGPRTAASSALLS
RTVTACSGQHSYAPPSTATGSGTACTDTAAAATAGASPCPVTSSSYGAAATAPGEHVPHQLQYQPRYTCLGRGSERS
DDGSAGGSAVQRQPQPQWGAEAVPAAAPAAGAGAGGAFRATGPGVAVNRPGMGAAPPQPQPQQPSAGLPSPAAAALA
AVWAPAAALAAGGAEPYGGSTPGPAQQ

>g2864.t1|PACid:27576569
MEAGGRPAVAVAVPCPSGGEERPLLLRLGGTGEMAAASTSTSGGGNDSDSSLATAAAAAAAASAAAAGAAACSRMGA
AASASSASSASSASSSSAPEGVPLKAAAPDAGGACGAGAVAGSSSGGGTSPVGRRGAAAQATAHLALLLLLLLLGAA
AARRAALLVAHSGSSSSSSGRMGLPGSDVQAQGGGRGGGWLAAAAAWPSWGTTRGMPPGTAPAGGGEGAAAAAARGAC
QPHLPQPPVLGRHGGEDLGCGGCSGCGGGTPAAAGSGSSSGAAKSEGSGGGGRGSAAGQDRTWLLLGAGRINGGSGS
GEPPAHERHPREALGPTVGNTRSSGGSSGSGSSSSSGGALGLQAAAETRRPNPFAAVAADTSSAAPTTARAGAAAASP
AGAPPDPPFASPGHLAYGYDPGGPDGVGRGGGGGWLYQHYFGRYLNGSSSSNSRGSGSGSSNSRAGGGGDGVWRGLQ
GRPPQLQQQEQKQDEREHLPTRGRRRQLLQTDSGSNNDSSGSSGSGSSSGSVPPTSCAVMLRYGASVGDPLSSGPHPI
FVASWAAVAAAAQLPPVQSWVLGWSFDAGERLLSAADVFSDTSAASAAGGPGAGGGGAGAGGVGGGSGNGTGSGAQP
PLSLQVVLQEGYTNSSGQVSQQDVAVLGNGTLAPGGPQLSFLMFGRKGPTAAAAAAAGVQLAPPLSDTPTPSAANAG
LPPFNASALPDYAGPPAAPLRVGPPGGVFLNNLHCVPLAQLVALAQEAAQRAAAANATSSSSGGGSGGGSPPPPPAQ
QPVAEPLLSQIPRVLQVDYMPITYVDSAGGGTTGAGGAGAGDGSSPGAAGSSPPPPALVVGNATVGPPRPTFFTQMY
LKVSNPGSRRAVSLSEVQVQYYFNGPDESLDPNRQGAAAAGLADGGVDMSGGPGGAPPFRLVCSDTSPGLVGRCDSV
VGRFRVGLPGVRGAQYYATIQFSPGAGWLAANDTAGNSSSTGNTSSSASPSPPPPAPPGSGPLLDYVELIVSIEPLV
PYMRIDERKDYSFIDTPLAPRPDGTTTVAATVVAGEGGSSSGGDGSVQERLVLLRRPEPNPRIPAYLKGFIAWGSPP
SSALPADTTSGGAAAGADGTGGTSPSPSPPPPSGPPGGFVNPFSAAGLPLPPGSSCQRSADGQLSCGVNMVYCCAAP
ATGPIATSIPSDWQSTTFRDLQQQWSSAAVAAAAQANGTSGPGPQAPQGQGPAPAAAASPSPGPSPAASPSPGNTTV
GGGGAGGAPAAIATGSAPNTSTDTAAAASPAPGSAPNGSAVVAPQASEGGGKAGGGGGTAAIAGGVAGAAVAAALLA
AALIALWRRRHRPAGAQEQRRRQKRGRGLAAASSGGEASGPEESDGGGATGAGRHRHRHAQEGAAAGGGGAGRPRRI
SQRFQGVDRRVSRYVSLLLGVRRSDTGGSDVSTTSTSSAASPSASASASATAAEQQRRRRAGLPGSAGGAVRPSGEV
AGAAATAGSLGGARGGSRWELQPQQQGQGQRPRFTGSSSEGSDGSEAGGSEAGGGGGLTGGAALAAELQALQQQYPY
STEIHVTASEASELREVLDASWWQRGGNVRGVWGWGAAEAKMAAAGLLPLMAHPHAAAAAAAAGPAAAAALAALRKY
PTLPSEALMAGIAALSRPGSGELRAAAAAVAITAAATRAGGGGGGVRTEGVWDGGGEGEAGGGGPGSEAGVGAPSSS
RLAGSVGSSLLPAGLLLPLTAVDDDGVRLRILCRKAKTWTGRVADTWELLPEFFPHHPPKGGPAGAGAAPLPPGQLQ
PAQLLPPPPPGQEPGSPGGEARVGGGLLPRLPPLISKLLPRRRTLAAPLAAPLAAPLAAVPSARGLESQSPLADGAG
AFEPGDRGEAAAPAAYHLHHHRSRPRDAPAPQLQPLPLPAPPRLGLAGGPAQSSRRLSPPFPAALGGGAAGSLLRGP
AAAAAADAAALGGMGPGGGAGPGAVLPSVVLPSLSLAAVVAPPIQLDVDYAAEVEPFLGRLIGVGGFGRVYEGTFRG

RRVAVKTVVIDSEAQRVALAREAQLCARFRNCERVVQLLAASLGLPQGAGASGTSSGGNTPGLSHPGAADGGTGGGP
PGAAASRWSRGGGGPGGGSSVRSSAGGAGGLFGYGGSVDTSGGGGGHTPTGSTVAGGGGGQATYAGGGAASLFSEGT
FAASGSLGGGGRGQPAHRPPATRLPPGAAAGGGGGSGQQQGDGSGGGVREGQPQTRPQTRPQHPAAAAAHSPPPQCH
AALIMELCEGGNLGGRIHHPGMRRLDYMELLQLSRDVAEGLAYIHRYGVLHRDLKPGNVLLDSTGRAKIADFGISRM
RDPFRSFISVTQQGGTPNYMAPELFNGTRVDEKSDLYSLGCIMYEAATRKVPFEHLVRGGGGGARGGGGGGGGAARG
GGREPGGGRGGDEGGGGGGPGGGGGGGGGALPMGGLFAIILAVAIQGQRPPLPDSLPRGLSDLIRACWAEDPRTRPT
AAQVVARLDALMEEERAARAARAAARRARPSRGTPASSGHSPSGLSADGSSSAIALLAPLGAAGAVRGSGAAGSAAG
SSSANASPLPSIAPAPAPSPYALPPRRPEPSPLAPPAAAAAAGPDATRPPQPQQHLHHHHHRRQAAAAAGMDHAFS
APPAHPAATPPHQPHLPAGAHRLTPPHPAASAPPHVLLPGEEAGGLGGLGGGPAALDMAVLLGGGGQAGGGGVGGMQ
ARYEPGVGVAVGGGGSELSQLHSTGAASGSRASSKSGSTAASPIPSSGVAAAQLSGPTAPTPQAGAEVRSHGHARHG
SRRSGSGSGLTASASAGLLAQLQQQQQQQQLSSSSSSGAAAAAAAAARSPYGITAARSGSGDSSGGGSSGMSPLLVV
GLGTPAAGPQPSPSPAWATGQPQQPLVSGGLVAGGGVTAGGGTSEPGSYKGRSSGSSHSAAPSTTQSQRAAPRTPSV
PDPDYLLMPGRGASGGGGSSSSGLDGSAGAGGHPQLPLLQPLQHLYDGAGQVLQPQLQLQPPVPLPYVAEQRPAHAD
RSYPPPPPPPPPSAHWGLPPHAQHSPSASSPSTSPTTPDTAPDRPPALESAAAGGPGLPPPAAAFPQLPAFAQRQRRK
DSWVRPGGVLAPLLPSVAEDRSVAPGELSPQPRHPHHPHLMARRGDTGGGASSGSGEGSSSGSEAGSSSSGSGSNGE
GGNGGTGQAARPVLLPPPASDAGAAASPGVAAAYAPLPLQEALSGGGAPAHEGSAEEGGVGTRRRFGATPSDATSAA
AAPVGGGSDRFDRFFSARSRYSAGEAEEWPHAVAAGAGAVPSGCVSGRESPASEAGGHGSRASISSSGSYELGGSNG
VEAAPRRHPQGRGQLAGIQESPRAPPDLQGLDQDPQHPHPPGALPSAFAAAAHHTHLRPLRQLRPHTHPHPHPADRA
AAHRLHPPPPPPPPPPEQPLQAGPPAREPAAWPQGPQPQMRPPQQLRQVAYSAQPPPQPPVGAGGAAGAPRRRTTDTK
TEGGGGMGGNSGGSGGSGDSGQTHITGRTTSHTASLVPRSSTTSASAFTPRTEFSSGNGESQPQQLPQQQPQQRLSY
HDPLLLQPGSTAVSFADAAPPGTASPHPYLPYSAGSGPPQPFLQVAGRASPLCGGGAAAARGVELTARPQQQQQLQP
QQPQPNGQWGASESAQPQGISGARAGSAHHLGAHPPPPPGAMPSPQGEDEAEAAAAAAAAQEEEPEGGGDGGGVGGG
KWWPWWLWRSPPQQHSSHAQQPQQSHPQRFPQSPQQLPQPQPAANQPRPSASATADPPGQQVGRNHHRQNADAPQAA
QRAVPAPPPPLHLAAADHSLGGAVSSSSPRYGAPPSLSSQSHCHWADPSTLDGGTGGMGGSAGPRTAASSALLSRTV
TACSGQHSYAPPSTATGSGTACTDTAAAATAGASPCPVTSSSYGAAATAPGEHVPHQLQYQPRYTCLGRGSERSDDG
SAGGSAVQRQPQPQWGAEAVPAAAPAAGAGAGGAFRATGPGVAVNRPGMGAAPPQPQPQQPSAGLPSPAAAALAAVW
APAAALAAGGAEPYGGSTPGPAQQ

>g2864.t3|PACid:27576570
MEAGGRPAVAVAVPCPSGGEERPLLLRLGGTGEMAAASTSTSGGGNDSDSSLATAAAAAAAASAAAAGAAACSRMGA
AASASSASSASSASSSSAPEGVPLKAAAPDAGGACGAGAVAGSSSGGGTSPVGRRGAAAQATAHLALLLLLLLLGAA
AARAALLVAHSGSSSSSSGRMGLPGSDVQAQGGGRGGGWLAAAAAWPSWGTTRGMPPGTAPAGGGEGAAAAAARGAC
QPHLPQPPVLGRHGGEDLGCGGCSGCGGGTPAAAGSGSSSGAAKSEGSGGGGRGSAAGQDRTWLLLGAGRINGGSGS
GEPPAHERHPREALGPTVGNTRSSGSSGSGSSSSSGGALGLQAAAETRRPNPFAAVAADTSSAAPTTARAGAAAASP
AGAPPDPPFASPGHLAYGYDPGGPDGVGRGGGGGWLYQHYFGRYLNGSSSSNSRGSGSGSSNSRAGGGGDGVWRGLQ
GRPPQLQQQEQKQDEREHLPTRGRRRQLLQTDSGSNNDSSGSSGSGSSGSVPPTSCAVMLRYGASVGDPLSSGPHPI
FVASWAAVAAAAQLPPVQSWVLGWSFDAGERLLSAADVFSDTSAASAAGGPGAGGGGAGAGGVGGGSGNGTGSGAQP
PLSLQVVLQEGYTNSSGQVSQQDVAVLGNGTLAPGGPQLSFLMFGRKGPTAAAAAAAGVQLAPPLSDTPTPSAANAG
LPPFNASALPDYAGPPAAPLRVGPPGGVFLNNLHCVPLAQLVALAQEAAQRAAAANATSSSSGGGSGGGSPPPPPAQ
QPVAEPLLSQIPRVLQVDYMPITYVDSAGGGTTGAGGAGAGDGSSPGAAGSSPPPPALVVGNATVGPPRPTFFTQMY
LKVSNPGSRRAVSLSEVQVQYYFNGPDESLDPNRQGAAAAGLADGGVDMSGGPGGAPPFRLVCSDTSPGLVGRCDSV
VGRFRVGLPGVRGAQYYATIQFSPGAGWLAANDTAGNSSSTGNTSSSASPSPPPPAPPGSGPLLDYVELIVSIEPLV
PYMRIDERKDYSFIDTPLAPRPDGTTTVAATVVAGEGGSSSGGDGSVQERLVLLRRPEPNPRIPAYLKGFIAWGSPP
SSALPADTTSGGAAAGADGTGGTSPSPSPPPPSGPPGGFVNPFSAAGLPLPPGSSCQRSADGQLSCGVNMVYCCAAP
ATGPIATSIPSDWQSTTFRDLQQQWSSAAVAAAAQANGTSGPGPQAPQGQGPAPAAAASPSPGPSPAASPSPGNTTV
GGGGAGGAPAAIATGSAPNTSTDTAAAASPAPGSAPNGSAVVAPQASEGGGKAGGGGGTAAIAGGVAGAAVAAALLA
AALIALWRRRHRPAGAQEQRRRQKRGRGLAAASSGGEASGPEESDGGGATGAGRHRHRHAQEGAAAGGGGAGRPRRI
SQRFQGVDRRVSRYVSLLLGVRRSDTGGSDVSTTSTSSAASPSASASASATAAEQQRRRRAGLPGSAGGAVRPSGEV
AGAAATAGSLGGARGGSRWELQPQQQGQGQRPRFTGSSSEGSDGSEAGGSEAGGGGGLTGGAALAAELQALQQQYPY
STEIHVTASEASELREVLDASWWQRGGNVRGVWGWGAAEAKMAAAGLLPLMAHPHAAAAAAAAGPAAAAALAALRKY
PTLPSEALMAGIAALSRPGSGELRAAAAAVAITAAATRAGGGGGGVRTEGVWDGGGEGEAGGGGPGSEAGVGAPSSS
RLAGSVGSSLLPAGLLLPLTAVDDDGVRLRILCRKAKTWTGRVADTWELLPEFFPHHPPKGGPAGAGAAPLPPGQLQ
PAQLLPPPPPGQEPGSPGGEARVGGGLLPRLPPLISKLLPRRRTLAAPLAAPLAAPLAAVPSARGLESQSPLADGAG
AFEPGDRGEAAAPAAYHLHHHRSRPRDAPAPQLQPLPLPAPPRLGLAGGPAQSSRRLSPPFPAALGGGAAGSLLRGP
AAAAAADAAALGGMGPGGGAGPGAVLPSVVLPSLSLAAVVAPPIQLDVDYAAEVEPFLGRLIGVGGFGRVYEGTFRG
RRVAVKTVVIDSEAQRVALAREAQLCARFRNCERVVQLLAASLGLPQGAGASGTSSGGNTPGLSHPGAADGGTGGGP
PGAAASRWSRGGGGPGGGSSVRSSAGGAGGLFGYGGSVDTSGGGGGHTPTGSTVAGGGGGQATYAGGGAASLFSEGT

FAASGSLGGGGRGQPAHRPPATRLPPGAAAGGGGGSGQQQGDGSGGGVREGQPQTRPQTRPQHPAAAAAHSPPPQCH
AALIMELCEGGNLGGRIHHPGMRRLDYMELLQLSRDVAEGLAYIHRYGVLHRDLKPGNVLLDSTGRAKIADFGISRM
RDPFRSFISVTQQGGTPNYMAPELFNGTRVDEKSDLYSLGCIMYEAATRKVPFEHLVRGGGGGARGGGGGGGGAARG
GGREPGGGRGGDEGGGGGGPGGGGGGGGGALPMGGLFAIILAVAIQGQRPPLPDSLPRGLSDLIRACWAEDPRTRPT
AAQVVARLDALMEEERAARAARAAARRARPSRGTPASSGHSPSGLSADGSSSAIALLAPLGAAGAVRGSGAAGSAAG
SSSANASPLPSIAPAPAPSPYALPPRRPEPSPLAPPAAAAAAGPDATRPPQPQQHLHHHHHRRQAAAAAGMDHAFS
APPAHPAATPPHQPHLPAGAHRLTPPHPAASAPPHVLLPGEEAGGLGGLGGGPAALDMAVLLGGGGQAGGGGVGGMQ
ARYEPGVGVAVGGGGSELSQLHSTGAASGSRASSKSGSTAASPIPSSGVAAAQLSGPTAPTPQAGAEVRSHGHARHG
SRRSGSGSGLTASASAGLLAQLQQQQQQQQLSSSSSSGAAAAAAAARSPYGITAARSGSGDSSGGGSSGMSPLLVV
GLGTPAAGPQPSPSPAWATGQPQQPLVSGGLVAGGGVTAGGGTSEPGSYKGRSSGSSHSAAPSTTQSQRAAPRTPSV
PDPDYLLMPGRGASGGGGSSSSGLDGSAGAGGHPQLPLLQPLQHLYDGAGQVLQPQLQLQPPVPLPYVAEQRPAHAD
RSYPPPPPPPPPSAHWGLPPHAQHSPSASSPSTSPTTPDTAPDRPPALESAAAGGPGLPPPAAAFPQLPAFAQRQRRK
DSWVRPGGVLAPLLPSVAEDRSVAPGELSPQPRHPHHPHLMARRGDTGGGASSGSGEGSSSGSEAGSSSSGSGSNGE
GGNGGTGQAARPVLLPPPASDAGAAASPGVAAAYAPLPLQEALSGGGAPAHEGSAEEGGVGTRRRFGATPSDATSAA
AAPVGGGSDRFDRFFSARSRYSAGEAEEWPHAVAAGAGAVPSGCVSGRESPASEAGGHGSRASISSSGSYELGGSNG
VEAAPRRHPQGRGQLAGIQESPRAPPDLQGLDQDPQHPHPPGALPSAFAAAAHHTHLRPLRQLRPHTHPHPHPADRA
AAHRLHPPPPPPPPPPEQPLQAGPPAREPAAWPQGPQPQMRPPQQLRQVAYSAQPPPQPPVGAGGAAGAPRRRTTDTK
TEGGGGMGGNSGGSGGSGDSGQTHITGRTTSHTASLVPRSSTTSASAFTPRTEFSSGNGESQPQQLPQQQPQQRLSY
HDPLLLQPGSTAVSFADAAPPGTASPHPYLPYSAGSGPPQPFLQVAGRASPLCGGGAAAARGVELTARPQQQQQLQP
QQPQPNGQWGASESAQPQGISGARAGSAHHLGAHPPPPPGAMPSPQGEDEAEAAAAAAAAQEEEPEGGGDGGGVGGG
KWWPWWLWRSPPQQHSSHAQQPQQSHPQRFPQSPQQLPQPQPAANQPRPSASATADPPGQQVGRNHHRQNADAPQAA
QRAVPAPPPPLHLAAADHSLGGAVSSSSPRYGAPPSLSSQSHCHWADPSTLDGGTGGMGGSAGPRTAASSALLSRTV
TACSGQHSYAPPSTATGSGTACTDTAAAATAGASPCPVTSSSYGAAATAPGEHVPHQLQYQPRYTCLGRGSERSDDG
SAGGSAVQRQPQPQWGAEAVPAAAPAAGAGAGGAFRATGPGVAVNRPGMGAAPPQPQPQQPSAGLPSPAAAALAAVW
APAAALAAGGAEPYGGSTPGPAQQ

>g2864.t2|PACid:27576571
MEAGGRPAVAVAVPCPSGGEERPLLLRLGGTGEMAAASTSTSGGGNDSDSSLATAAAAAAAASAAAAGAAACSRMGA
AASASSASSASSASSSSAPEGVPLKAAAPDAGGACGAGAVAGSSSGGGTSPVGRRGAAAQATAHLALLLLLLLGAA
AARAALLVAHSGSSSSSSGRMGLPGSDVQAQGGGRGGGWLAAAAAWPSWGTTRGMPPGTAPAGGGEGAAAAAARGAC
QPHLPQPPVLGRHGGEDLGCGGCSGCGGGTPAAAGSGSSSGAAKSEGSGGGGRGSAAGQDRTWLLLGAGRINGGSGS
GEPPAHERHPREALGPTVGNTRSSGSSGSGSSSSSGGALGLQAAAETRRPNPFAAVAADTSSAAPTTARAGAAAASP
AGAPPDPPFASPGHLAYGYDPGGPDGVGRGGGGGWLYQHYFGRYLNGSSSSNSRGSGSGSSNSRAGGGGDGVWRGLQ
GRPPQLQQQEQKQDEREHLPTRGRRQLLQTDSGSNNDSSGSSGSGSSGSVPPTSCAVMLRYGASVGDPLSSGPHPI
FVASWAAVAAAAQLPPVQSWVLGWSFDAGERLLSAADVFSDTSAASAAGGPGAGGGGAGAGGVGGGSGNGTGSGAQP
PLSLQVVLQEGYTNSSGQVSQQDVAVLGNGTLAPGGPQLSFLMFGRKGPTAAAAAAAGVQLAPPLSDTPTPSAANAG
LPPFNASALPDYAGPPAAPLRVGPPGGVFLNNLHCVPLAQLVALAQEAAQRAAAANATSSSSGGGSGGGSPPPPPAQ
QPVAEPLLSQIPRVLQVDYMPITYVDSAGGGTTGAGGAGAGDGSSPGAAGSSPPPPALVVGNATVGPPRPTFFTQMY
LKVSNPGSRRAVSLSEVQVQYYFNGPDESLDPNRQGAAAAGLADGGVDMSGGPGGAPPFRLVCSDTSPGLVGRCDSV
VGRFRVGLPGVRGAQYYATIQFSPGAGWLAANDTAGNSSSTGNTSSSASPSPPPPAPPGSGPLLDYVELIVSIEPLV
PYMRIDERKDYSFIDTPLAPRPDGTTTVAATVVAGEGGSSSGGDGSVQERLVLLRRPEPNPRIPAYLKGFIAWGSPP
SSALPADTTSGGAAAGADGTGGTSPSPSPPPPSGPPGGFVNPFSAAGLPLPPGSSCQRSADGQLSCGVNMVYCCAAP
ATGPIATSIPSDWQSTTFRDLQQQWSSAAVAAAAQANGTSGPGPQAPQGQGPAPAAAASPSPGPSPAASPSPGNTTV
GGGGAGGAPAAIATGSAPNTSTDTAAAASPAPGSAPNGSAVVAPQASEGGGKAGGGGGTAAIAGGVAGAAVAAALLA
AALIALWRRRHRPAGAQEQRRRQKRGRGLAAASSGGEASGPEESDGGGATGAGRHRHRHAQEGAAAGGGGAGRPRRI
SQRFQGVDRRVSRYVSLLLGVRRSDTGGSDVSTTSTSSAASPSASASASATAAEQQRRRRAGLPGSAGGAVRPSGEV
AGAAATAGSLGGARGGSRWELQPQQQGQGQRPRFTGSSSEGSDGSEAGGSEAGGGGGLTGGAALAAELQALQQQYPY
STEIHVTASEASELREVLDASWWQRGGNVRGVWGWGAAEAKMAAAGLLPLMAHPHAAAAAAAAGPAAAAALAALRKY
PTLPSEALMAGIAALSRPGSGELRAAAAAVAITAAATRAGGGGGGVRTEGVWDGGGEGEAGGGGPGSEAGVGAPSSS
RLAGSVGSSLLPAGLLLPLTAVDDDGVRLRILCRKAKTWTGRVADTWELLPEFFPHHPPKGGPAGAGAAPLPPGQLQ
PAQLLPPPPPGQEPGSPGGEARVGGGLLPRLPPLISKLLPRRRTLAAPLAAPLAAPLAAVPSARGLESQSPLADGAG
AFEPGDRGEAAAPAAYHLHHHRSRPRDAPAPQLQPLPLPAPPRLGLAGGPAQSSRRLSPPFPAALGGGAAGSLLRGP
AAAAAADAAALGGMGPGGGAGPGAVLPSVVLPSLSLAAVVAPPIQLDVDYAAEVEPFLGRLIGVGGFGRVYEGTFRG
RRVAVKTVVIDSEAQRVALAREAQLCARFRNCERVVQLLAASLGLPQGAGASGTSSGGNTPGLSHPGAADGGTGGGP
PGAAASRWSRGGGGPGGGSSVRSSAGGAGGLFGYGGSVDTSGGGGGHTPTGSTVAGGGGGQATYAGGGAASLFSEGT
FAASGSLGGGGRGQPAHRPPATRLPPGAAAGGGGGSGQQQGDGSGGGVREGQPQTRPQTRPQHPAAAAAHSPPPQCH
AALIMELCEGGNLGGRIHHPGMRRLDYMELLQLSRDVAEGLAYIHRYGVLHRDLKPGNVLLDSTGRAKIADFGISRM

RDPFRSFISVTQQGGTAPELFNGTRVDEKSDLYSLGCIMYEAATRKVPFEHLVRGGGGGARGGGGGGGGAARGGGRE
PGGGRGGDEGGGGGGPGGGGGGGGGALPMGGLFAIILAVAIQGQRPPLPDSLPRGLSDLIRACWAEDPRTRPTAAQV
VARLDALMEEERAARAARAAARRARPSRGTPASSGHSPSGLSADGSSSAIALLAPLGAAGAVRGSGAAGSAAGSSSA
NASPLPSIAPAPAPSPYALPPRRPEPSPLAPPAAAAAAGPDATRPPQPQQHLHHHHHRRQAAAAAGMDHAFSAPPA
HPAATPPHQPHLPAGAHRLTPPHPAASAPPHVLLPGEEAGGLGGLGGGPAALDMAVLLGGGGQAGGGGVGGMQARYE
PGVGVAVGGGGSELSQLHSTGAASGSRASSKSGSTAASPIPSSGVAAAQLSGPTAPTPQAGAEVRSHGHARHGSRRS
GSGSGLTASASAGLLAQLQQQQQQQQLSSSSSSGAAAAAAAARSPYGITAARSGSGDSSGGGSSGMSPLLVVGLGT
PAAGPQPSPSPAWATGQPQQPLVSGGLVAGGGVTAGGGTSEPGSYKGRSSGSSHSAAPSTTQSQRAAPRTPSVPDPD
YLLMPGRGASGGGGSSSSGLDGSAGAGGHPQLPLLQPLQHLYDGAGQVLQPQLQLQPPVPLPYVAEQRPAHADRSYP
PPPPPPPPSAHWGLPPHAQHSPSASSPSTSPTTPDTAPDRPPALESAAAGGPGLPPPAAAFPQLPAFAQRQRRKDSWV
RPGGVLAPLLPSVAEDRSVAPGELSPQPRHPHHPHLMARRGDTGGGASSGSGEGSSSGSEAGSSSSGSGSNGEGGNG
GTGQAARPVLLPPPASDAGAAASPGVAAAYAPLPLQEALSGGGAPAHEGSAEEGGVGTRRRFGATPSDATSAAAAPV
GGGSDRFDRFFSARSRYSAGEAEEWPHAVAAGAGAVPSGCVSGRESPASEAGGHGSRASISSSGSYELGGSNGVEAA
PRRHPQGRGQLAGIQESPRAPPDLQGLDQDPQHPHPPGALPSAFAAAAHHTHLRPLRQLRPHTHPHPHPADRAAAHR
LHPPPPPPPPPPEQPLQAGPPAREPAAWPQGPQPQMRPPQQLRQVAYSAQPPPQPPVGAGGAAGAPRRRTTDTKTEGG
GGMGGNSGGSGGSGDSGQTHITGRTTSHTASLVPRSSTTSASAFTPRTEFSSGNGESQPQQLPQQQPQQRLSYHDPL
LLQPGSTAVSFADAAPPGTASPHPYLPYSAGSGPPQPFLQVAGRASPLCGGGAAAARGVELTARPQQQQQLQPQQPQ
PNGQWGASESAQPQGISGARAGSAHHLGAHPPPPPGAMPSPQGEDEAEAAAAAAAAQEEEPEGGGDGGGVGGGKWWP
WWLWRSPPQQHSSHAQQPQQSHPQRFPQSPQQLPQPQPAANQPRPSASATADPPGQQVGRNHHRQNADAPQAAQRAV
PAPPPPLHLAAADHSLGGAVSSSSPRYGAPPSLSSQSHCHWADPSTLDGGTGGMGGSAGPRTAASSALLSRTVTACS
GQHSYAPPSTATGSGTACTDTAAAATAGASPCPVTSSSYGAAATAPGEHVPHQLQYQPRYTCLGRGSERSDDGSAGG
SAVQRQPQPQWGAEAVPAAAPAAGAGAGGAFRATGPGVAVNRPGMGAAPPQPQPQQPSAGLPSPAAAALAAVWAPAA
ALAAGGAEPYGGSTPGPAQQ

>Cre03.g185400.t1.2|PACid:27576589
MRVDPSSFNGSASTAEFTFYVPAGTSPQSAAVQIIRDNSPPLPPKPPTPPSPAPPVPPSPRPPSPAPPSPRPPSPAP
PSPAPPSPVPPSPRPPSPAPPSPHPPSPAPPSPAPPSPKPPSPSPPSPAPPSPSPPSPAPPMSQFCLPASSPLPAIF
PTDSWAVVMMPYNNTSSANLTATVDPAAAGIEGGCAYLLPSTTPMNITVARYTLVFTAPTDPAAQLLAWDPSGAALL
TPGVVATLPVPAAATTATASSGLPVLVSGDGSRQPLRLEAFASFDACDSAVLPNTAPIHLTPSTPGFVNTSRLMSLC
HSIPNVVLPSEQTPEQQAGGVLLDWSFGWDVSAMPPAFATDGFMVELPSYAFAAGRVLPAGWYNVTMQRGAGGAATT
PNIRVTLQPPAAGRHRRSLLQGTSTAAATTVNVSAAGALYLPFAAGIISIQVGVPATVPALSVAVQLRQMPSPPPPP
PSPVPPSPAPPSPAPPSPAPSPAPPSPAPPSPAPPSPPSPPPPSPAPPAPPSPPPAPPSPPSPAPVPPSPPSPPPSP
SPPSPQPPSPTPPNNPPTAPKPPSPRPPSPRPPSPPRPQPSTTTDPNTQSGGGAVPPPPAAGAASPRPLSSAWGALI
AVAITCMLMYVR

>Cre03.g178700.t1.2|PACid:27576633
MPPPPRQPGGAGPISSGKLHKKERILQAINRMNDRDTQKAAADDLLNIVKHMDTEGLPITISCLCSTGPEYKVYARK
ECARALGVIASPLCPLRDTALQQPHLGKVLGQLRKCLQDADSSVRESSTEALTLVAQGLMQLQAVMPPQPPPPAAAG
AAGPAAAAANPVLRLLFECLTEQRKELNAAACATLGVAAPYLGTLEPPLARELLRKLNSPAFLAAPALLAALARTDP
VSGEPTGLIKSGPGAFVPIISALVGQLPQPGGGTPGGSGAAAGTSGSGVVGCLHRQADWQTRQAAADMLRATALLLG
PLMEVEGVWAPGDLRGVTTRAVRGLESCKFDKVREVRDVARAALAVLEDLQAYGSAGGSAAGWPPHIADKLRARGAP
VPSVPAAEPDPVAAAPPPPPPVPGRKSPLRRSSPSPGRRRPVSANVRASPPRLSPERRSSPVFARAGSLSERFQAAH
QDGDLVRDPLRREYSDGWEAAEAAAAAAGGGAGGAEGGAAAAGSRALSRQGSKAGSTSSRAPGPVEPQVSIRSRDGG
RPEDPNATMRGLAGLGLGGDGGQQQHPDPGQQEEEDVLLASGGPPTFARQLSGNSSRAPLPPRRGHDGGQPPLPAWV
TAADTPPQAAQQQAPAYQPSPAVFGGGGGGAGAGAGAAGGGEYVDPVAAMPPPLIPGAVPSAATPPSPVPGWPPHLP
PPGALLPAPANLFPGATAPGSPLPPPSALGPTVTVSSEEWLGQQARLRELEGQTRALLGAFNAMSEQSGRAITGLQA
RVRILEAALEAAQSQLTGGAPASGSYALPPPMALPPPPPSPPPHLPHPLAGGASAASLGGLPPLPPLLTVQDSDVWL
GTGPGGAAAGRGGGGGVLRSSSPMHASSGVGGGSVAAGRLAAYAYENGGVVSPRVSLQLPASVQASQDLALGSLAGA
GSGPGSGLATPTAGGAGAGAGVDLRSAYREVLAGGRPGDMPLMRLMQRSGPVWNELGHELGCQLLVSFISSLQATRP
DGPMLSRILPWLWRLADDDNTRFEAPLPLRAQLLAALRAAHGAASDPALYEKMALLLSTLQTHWSQPDSPTAASRAA
AAAAAQAAPEGYAAAYAAAAAAAGSAAGSGGGGGGGLDDGGHSTMSITPALDVQASPPRPYLQAEPSTARSVAGSVA
GSVAAAGERPLERAGSAAGSVASAAARQVPPLPTAALQSPPPLGSLPPSARSSVAGSGRASVLGSARGPGDAGTTPP
ATPPPLPGGSSSPAPGSARASVAGSVRASAAGSGAGGGDYTPGADGGGDGGGAGGPPRPGSRGGLLGAVPSINTLQH
DFAALQRDFAAMSPAKSRTPPPPSM

>Cre03.g170300.t1.2|PACid:27576641

MISSTQPDGTSSGSRASRGLSAGEAVPEPFFVTYAASAARAALAEFIASQGASIHGYIPQNTWLVYAPGAVVVHAVE
LGLAVSAAPCGPEYKVAPEWFGILASRGVLADAVDVAADGEPEDPATADGAGQTDDFASIDEGGVKKYVITVQLFAP
GGGSRPAAARALADQLAAAWLQPLTAAVMQARQQQSQQPQQEAGAAAGTPGAKRLRRHRRRRGLLQQDPTPNATNAA
DNAGASTAAAAAAASQSADAAGDPCTPALRARGRLLLVIVCAGGDVASALSWLASQPQSHWLEPTPRLTRRDIWGDL
LVQSGARGLGSGVLARPLDVGAHVFWSRGLDGRGQVLGISDTGVDMDSCYFFDPEIPDVWRRLVPDSKFKGAKVFNS
DTHRKIKSYYAYTDFGDADGHGTHVSGIALGACYLSPANASVPLETRSTNSSPPSPRPPSPRPPSPRPSPPPLKLKT
KKAKKPPPPIPRPATPGTDGTGNGGGNVTNRSAAAGELLTNPANYVIDDAVGIAPGARLAFFDIMNKKKEYLLPSYD
DLLTHQLESGVLVQSDSWGNKQTFWYNGLCHQLDEVTWRHPELLVLTAAGNDGNMQPPPANGTVSAPANAKNTLSVG
STLSLTASSTNGGALTLHVWTGGPEPPPEDPALVAASLPAEDAAALMSGDTAALSALYGSSVISALAAQAAAAAQAA
ASGGGSSGDSSIGSSSSDGSAGFDGAVAALVDISFGGRKAPAAATSSPPPAGAAVKAADDETVLITLDNSIAPPPPT
SPPTPPSPVPPPPAALNELIQLPVSQAFRTLRDLEGLTLEAVAASPLNACARLNDSSSASNNNKTAADAASAGAAAP
LPKLLITTRDGCMPYVKAAAAAAAGAAALAIANDAPGPAVAILVNPTGSTLPAAALTQDLGARIFAALAAGQRVFAR
ASWQPQGPYDHVPAFSGWGPTNDGRVKPDIVAPGWRVVSAYSDFVSAGVSDGCAVRMTMEGTSMSAPLVAGAALLAR
QYFESGHYPAGRTDAPEAAPFSPSGVLLKAVLIGGAYDMSRSGGLSFSTLQPLLPAPSAYQGFGRLDLATALPLASD
PPNATVAASSALASVCGGGGEPTLQLVDLAAFATEGEQHVYTLQALGGGPVVATLVWADAPADLLSSVALVNDLDLR
VTHTPAAATATAATATAAAAAASAAAAATAGPGSSSGSGSSSGSGSGGGMGARPTTYWGNAGWAGAAASGSGDRAGA
DERNNVERVVVPAAVGAGSLELRVVAAALNSQFITAYRGEQQRYALAVHGCFSGVLQSRQNPDQRGWPPPSPPASPP
KPPLPPSPQPPAPPPPRPSPSPPPPKPVTQKRRPPPPKATTKNAPTASGCGKRLGKACKPPPPVS

>Cre03.g170300.t2.1|PACid:27576642
MISSTQPDGTSSGSRASRGLSAGEAVPEPFFVTYAASAARAALAEFIASQGASIHGYIPQNTWLVYAPGAVVVHAVE
LGLAVSAAPCGPEYKVAPEWFGILASRGVLADAVDVAADGEPEDPATADGAGQTDDFASIDEGGVKKYVITVQLFAP
GGGSRPAAARALADQLAAAWLQPLTAAVMQARQQQSQQPQQEAGAAAGTPGAKRLRRHRRRRGLLQQDPTPNATNAA
DNAGASTAAAAAAASQSADAAGDPCTPALRARGRLLLVIVCAGGDVASALSWLASQPQSHWLEPTPRLTRRDIWGDL
LVQSGARGLGSGVLARPLDVGAHVFWSRGLDGRGQVLGISDTGVDMDSCYFFDPEIPDVWRRLVPDSKFKGAKVFNS
DTHRKIKSYYAYTDFGDADGHGTHVSGIALGACYLSPANASVPLETRSTNSSPPSPRPPSPRPPSPRPSPPPLKLKT
KKAKKPPPPIPRPATPGTDGTGNGGGNVTNRSAAAGELLTNPANYVIDDAVGIAPGARLAFFDIMNKKKEYLLPSYD
DLLTHQLESGVLVQSDSWGNKQTFWYNGLCHQLDEVTWRHPELLVLTAAGNDGNMQPPPANGTVSAPANAKNTLSVG
STLSLTASSTNGGALTLHVWTGGPEPPPEDPALVAASLPAEDAAALMSGDTAALSALYGSSVISALAAQAAAAAQAA
ASGGGSSGDSSIGSSSSDGSAGFDGAVAALVDISFGGRKAPAAATSSPPPAGAAVKAADDETVLITLDNSIAPPPPT
SPPTPPSPVPPPPAALNELIQLPVSQAFRTLRDLEGLTLEAVAASPLNACARLNDSSSASNNNKTAADAASAGAAAP
LPKLLITTRDGCMPYVKAAAAAAAGAAALAIANDAPGPAVAILVNPTGSTLPAAALTQDLGARIFAALAAGQRVFAR
ASWQPQGPYDHVPAFSGWGPTNDGRVKPDIVAPGWRVVSAYSDFVSAGVSDGCAVRMTMEGTSMSAPLVAGAALLAR
QYFESGHYPAGRTDAPEAAPFSPSGVLLKAVLIGGAYDMSRSGGLSFSTLQPLLPAPSAYQGFGRLDLATALPLASD
PPNATVAASSALASVCGGGGEPTLQLVDLAAFATEGEQHVYTLQALGGGPVVATLVWADAPADLLSSVALVNDLDLR
VTHTPAAATATAATATAAAAAASAAAAATAGPGSSSGSGSSSGSGSGGGMGARPTTYWGNAGWAGAAASGSGDRAGA
DERNNVERVVVPAAVGAGSLELRVVAAALNSQFITAYRGEQQRYALAVHGCFSGVLQSRQNPDQRGWPPPSPPASPP
KPPLPPSPQPPAPPPPRPSPSPPPPKPVTQKRRPPPPKATTKNAPTASGCGKRLGKACKPPPPVS

>Cre03.g204500.t1.3|PACid:27576670
MYELGLDIDGKPAHPAATIVSGDDSSLDGIALSRATTGGLLIVVGSPANLHKTIGAAFDVGSDGRRCDSTSNGGSVT
HGSRPRLHAAAATLFGSEDALDAEWPQTLEAVAVTHSIAACNVGGAVTQVPLYFSPSDGSIVAVAFTSASPRKRTVV
WLGYDWAAGPQTAWGAVLAKLIDTHALLTATSSAAAASLSSASSATAGEPKVVHITTTSSSAAASNTGCGSGTCSHS
HLVMATLAAAASSSAASASAASSSASTLAHTGGSETEFAADAAKDGDDAAAEPVLVDDAAMDAALDSMPDAVSDLSD
GVSEVVRRMMAATTGAGTYPAPPSPEPASPAPPSPAPLTPAPPSPRPPKPRKSPPSPPTTPPPLAPPSPPSPPAPPS
PPPPLPPGLAAEVQGYNSDTSAPPGFNISDTLSVQNFSRDFCENVTRALDPRPDECVVFDIKSHGGATHHHRLLSEA
GALRVWGPEDLAAEHPALAAAGMSRLVHLRTYSVGEAELAAVRAWDLSVSLPVTALPSSVGGRRSLQQSTQTITLAF
VVIYLVEVPLPPSPPPRAPNPPGVMEPPSPPPSPPAAPPHPPRSPPRPPVNLTALASAVGASAVYQVFPPPPSPPSP
PSPMPPSPPPSPHPPPLPLADRITSVRFSRMEAAGALGSNKRVVWWTDGDFESQRKAVWNSPLNLVWRGKAACGKCG
ACGRAWKPRGDKFSVQLFFREPVQLDEIRIRQVWHAGVTEVRLLAWPAVDAVNRVSDVFLGEPVLRQTADNTTCNTW
LTVAIPQERAGTNQTVRPIGSQDAIPPKLRSSTVGGVVITTRTAATVRAGTWIDAVDFSGRVLYPNDPAAYAAYAPG
TGGGATIGRRR

>Cre03.g171850.t1.3|PACid:27576672
MSQEDSVISTAAGIKSKVEQLTGRLQHCVENLEMVSCSGQHFTREPAPVGQHQGQHQRHPSQHAAVQRSAAGERGAD
LLAQARTSATGAQPLPPHREGTRRRSSHASGASHEPENHHRESRGSASGGTDGKFGYGGLRRTAQQEEADEDEEDWA
DMPADPGADDSDENYHDEEQRGRSAMPGFMHQPMPVHGAPSDAPPPPNISVGAALQNLKASLSQQVQSSLSGKHTPT

HGEQLKSQYKQAMSDNVQRRLLGRADNYHNTTSSLDASDSEGAGSGLQSGNLTRTRLMGALGSDNGGTVPGSPLRGA
SPAPTSRAAALLAGGGGSMARSAMRVSGTGSGAGDEDVRDAPQSAASSRRTSMDVQRPPPVPAPAPVPKPAAAPSPP
PPIVPVPATGFKPPAGLTSPAVSSPLPAPVPATGFKMPMAASPAPAVAATSLSPAASFRSDAPALSPSPGSGGIGWK
FGMDSGGSVNLGGPDTDGGGGKGEGGLSPFASGQSLQTSNSFAVAFEQGADVLLQPANNSGVVGFMPPASLTGVGSG
SRPASGPQQATGFVPPASPGVGFMPPASTRSGSVTSESHYPTVGPSVVPPHYPSVGLSSGGLQPPPEPSGRSNSPPR
ADPRMVLNSYQSMQPSRPAATMPLRTVETVRLRTEPAAMQLPPGFALPPGLMGAAAAGGLGGGFGFGGGFGHGMGGG
SSGVGFGTPSNLGQPPREQSRDSGNDSPETFSPMLGVQRPPPGRRRSDGSDNSDVTPIPPAGMSSTPSIPVGSFSFG
INAAVFSSGPSMDSATAGGGGDEHSPAPAASSTSRHSLSQAAHALHGLQTVTEEDAASEDSPPKPVMGWKIGAVSEV
ESCAYAIDVTEEPPPPPPPPSAVGFSPPPAFGFSPPPAQGSSLQPATASGPLSTLGWQPPPVATLPHVPVSPGAAVVP
EHTSFRLSDGASMPGSDGGLRSDRGLQVEGREKLPWEDSTDDDEELQPAPLLRQSSSLRRLPPPIVAVSASGGMAPV
SPGAVQLQPSPGAVTVMSSSLAPVQEADSEGRDSSVRLPKLQDDEVGAFPSYSENDVRPSLVLPAVNAASSSGQSAP
PPTPAAVATLAAVAAPPAAANSSSQLQRQTSTLSSRSSGVVAATTPTGAHPPGDVSITITDADIARSGGVVPALLEI
VRDRFRAKGMSNLAQFAAGSGTGSMAGGNSHGALGDEAHGAGTPQSAALAGETLARRIMARVDTFHSARRGGGPEDL
VAALQSVAEHDEGGPRGVSEDGSSDRAVPVFRRTSTAASSGAAGASSAAGTSTGATAAAAVAAPGLSPTNSGSYAML
MAHSQQAAQQKVAATAIAAAAAAAAAAGGAQAAAITGVAPVTAASTVGMPPNLSRGPSIRIDSVRQALAPPIESPGA
AASAASSHAATAATSAPVRAIRAPPAGGAAASLAHAQSAGTLTSSPPLEPYTSSSLSPRPAAVGTPSSPASTSKVET
ATQTVPPPVADGLPPALGTALDYFASTVVPVSGGTKGRPRNAAAEDERALPSDMPAVRTAADFFKGAANRATINTSA
MAVPPRDTPAGISPAAGAAGVGAVAAASAPPGRVRTARDAMLNSSSATTPARPQRQSVSGRSGVASSGGGGVLTRAG
SAGRRVSAAGRVVIDDEPVDLATYPSRSVSNRHVSAAASPGVAPSALRTSAHGGPGPSGPSDVGVDGLAKQQQLQLQ
HQALMQYQQQGYDRQALTREMLLHLAHMSPSRHPFETTTDEEREEEERRARRHARRTRSPPYPSTRGNGDSASAGQQ
QQPQQQPPTQPSSARRPAPAVGMASVPPVPWPRHQAANAPAGLALNPRISGGVEQLQARFDAEAAAAAGGRGFAAGG
LPPRLADVLAAEAGLGPAAESPAVAAAPGDGIPVPPLSLDAPDMPEPGGGDTASAAGLSGVLLAGGGLSGSLPLPHE
TFFISSPRWRANDSDGLAGTGEVFLASGAGGKKAAGSGRGGAVSMLGRGRISATGGSGPEYTEDGGVSGVLAQRSRR
STTVTNTTSWTSSSDAEEAQLQGRHVDGVSGGGKGRRPDEAAGPRVQQSGGKQAAQLPVRSSAGARSSSSRGVNLER
ADTAGSGTAGGEHRPRLSATGRRRAAYWADEVSPLYGGPDAPYTDPDGLDREPSAASRGGAASPRGSSAAASVLSAR
RYVAVARAHRSVSEANSSKRLGAGAVLEGDDEDEEDEDEEAGGNARAGGADDVEEEDYDGPQDLQLDEEERQEQQPQ
QRRQAGTRAKPALAASRGDGAGAVGSQLRWGVADDEAGMGSTRAMASSSEHDEDEDGHEHSGRAPRDSTDSGGLTPD
GTRVRQSLQRLLDMRAASATSPSRPAASSAKEQSPFAGRGPAPPSAAGTDSPAGLTGGSDGRSNSRSPSVTATAGGT
TDADARDREQLLRRKGRAEGTSRTAAQPTATASAGATTSAVQRALLGNGAGAGAGNAHADGSADGASSDDAPPEDPH
ELVSPKYTQDMAEEEYGWDVATGDADVDNIASPRGRRPAAATTPRGGGSSRGRRGGRAARPPPPPLPYDPAEVPGAG
THDASPSYTRELEQEELRDTMAELIGSGGGGGGTREPPPPVMGTAAAVAAAVAALQADQEPSFSLSAPPAPGSRFGS
APGTAAAAVSAALAAAGSGSPAQHSPRGRHTVGGLPAAAAASTQSLTHTALASDGFNLALTNLLSGLGPVTLSSRAS
ASSSSAAGMAGATSGGAGSASTPGKPIATQTSVGGPAVAVSTQTSLVAADSQQRQLLQGLQALEEQARRAAQATAAL
STSASARGSPSASQPVADVAASEPDPELRRLLLAAWSKWYLRRLSADSGAGHERDSPAGGSAGGASDGQEAATEAAT
AAQARLQALTAALSNGGMDAREEDPSSSPRMQAAASASRLHMAFTRWLGAARSQRSAKAAARAPYQHAASSIPGLAV
GANTNIAPPYPTSPSPAAPTYIGAFQHQHPQPGPGSLTYGSYLPTGTRQLAQPHAHQGSRAPAPAPSPPPVATSWPA
ALRTISSPPPQSAAAAAYQRAGMVAASAAGLRAGASPALPLASAGALSSGAGSVVGEALATNTPTVSRASGVRQVVS
MLQAPGQAQPAHRHQPLATQAYTRVGYQGSAPAPPAAAAAAGAAAPVRRGGYPALGLLGTPAVPSAAAPAPGPASDL
EAAVAQHLLASLVSGSGSGSDSVIVAGMQLATGRALLPATLASGTRGFALGGTLGGTPPAGGSTGGGLDARPRPAST
GRLGPGASRPLAAASPAAAAGGAGGGGILGGRAAAAHSGLASSLGAHRPASPVAARRVPGATSSGADNIRLAPAANL
GGLNLAYYGVGK

>g3001.t1|PACid:27576710
MSLLLLCSSARAGRLAAASDAAASHSAFVNNRYTAIASSGSSGGVEDMHQNQQHRRLLTTRFVGHPLPPRHPPIPAY
GSPSPSYGPGYGASPPPPTYAGQPQAPSPPSPAPPPMLDAPFFAQPPPVNAPPPEIHLSPPEAATPSSTPALNEPPS
PTPAGLEPSPPSPGASPGPSPSPEASPSASPSPEASPSPSPSPEASPSASPLPVASPSSSPEASSSPSPDEACPSPS
PSPEASPSPSPMPSPEASPSPSPSPEASPRPSPSPEASPSASPSLEASPSPSPSPEASPSPSPSPEASPSLSPLPVA
SPSSSPEASPSPSPEASPSPSPSPEASPSPSPRPSPEASPSPSPSLEASPSPSPSPGRSPGASPSPRPSPSPEASPS
PSPSPVASPSSSPEASPSPSPEASPSPSPSPEASPSASPLPVASPSSSPEASSSPSPDEACPSPSPSPEASPSPSPM
PSPEASPSPSPSPEASPRPSPSPEASPSASPSLEASPSPSPSPEASPSPSPSPEASPSLSPLPVASPSSSPEASPSP
SPEASPSPSPSPEASPSPSPRPSPEASPSPSPSLEASPSPSPSPGRSPGASPSPRPSPSPEASPSPSPSPVASPSSS
PEASPSPSPEASPSPSPSPEASPSASPLPVASPSPSPDASPSPSPSPEASPSPSPSPEASPSPSPSPEASPRPSLSP
EASPSPSPSLEASPSPSPSPGRSPGASPSPRPSPSPEASPSPSLEASPSPSPEASPSPSPSPEASPRASPSPGRSPG
ASPSPRPSSSPEASPSPSPSASPGPSPGPAASSPNPRAVPLPSPLYGGTQSSLPPSPPTPESQHSDTPSPQSPSLIP
SPSSPPPSPVDEGVLPALPSPAAPSPSPPSPSPPSPQPPTPPSPGSEEAAPSPSPPSPAPPSPSPPSPQPPTPPSPA
PYASFTWFGGSSTVSLTSLSRSALTVSSLAAAPYASFTWFGGSSTVSLTSLSRSALTVSSLAAAPYASFTWFGGSRT
VSLTSLSRSALTVSSLAAAPYTSFTWFGGSRTVSLTSLSRSALTVSSLAAAPYTSFTWFGGSRTVSLTSLSRSALTV

SSLAAAPYTSFTWFGGSRTVSLTSLSRSALTVSSLAAAPYTSFTWFGGSRTPPTPPSPSPPSPSPPSPAPLSPTPPS
PSPPSPSPPSPAPPPSSAGYFELIETLTAPNVALAMANIPYTRRYWFLERAYGPGEPVPVVFAEPNTGTLNLDTGEK
LKYNAIYNPGCSGTIILPDGQLHTFGGDVLSPVRNLQDGRNKIMAFNAATSSSTVLTNMQWNRWYPSPIVLSSGKVL
IVGGSNACLLPPTWPVAELWDPAAPASPTVNVTMPANFVKYMGLYNWYPFMQLLANGDILWFVEKGGAITDGNFNHI
VDLPPFPASITHCTMFPKTSSISVLAMGPPTYDLSLVIFGGGDCNGSPTTQIISSPAASTSLRLDITKCGSSYCFTK
GWEVEDMLGVPRVMGDSTLLPNGKVLLHGGAQFGGANAGAGWSTKANFQSLMYDPYKPVGQRYSKMDFAPIARVYHS
ANCLDPSGKVLVAGCENCGAYQQLAPGMSLSPDTPLEHRLEWAVPAEIAPGVNRPAIVTAPGSITRGSTITISYSYP
GVDFITGAVLVAPCACTHSLNMNQRVIFLEAQPGGTPGQVLVEAPPPGLFGQALLGPHLLFLLGAGGTYSEGVWVTV
AA

>Cre03.g190050.t1.3|PACid:27576749
MQGELHRRRAVVACPPVIHYEEEGEHESFVKHRLLGYGGFAAVYEVERASDGSRMAAKVLPLSSLADAMKLHRVQNE
IDINQLLQHPHVVTFLGSFQDDQNVYLLQELCCITLSELLRRVGRFSELQAAHILWQVLAALEHLHSCGVLHRDLKL
SNMFLGRDGTVKVGDLGLACHLPSPAARRHSTCGSLNWMAPEVLRPGPAGYGMEVDVWALGVVLYTLLVGRPPFQAE
RRGRDAAGDPVAGSPAALDGGPESTYARILQGFYEFPEDVALSESARGLVHALLVVDPSQRPSLEQVRQHAFFAEHI
GPLALAAMRDGPGSAAAFTTASGDDVQQQQQQHSTGLGTAGVAFSVVGHPHRHAAHTHRAEANAWSPAGTSGAGSRG
PVQLQQLVEGEEDEDAGTGESASNSGHMPRPHSVASGGALTDAHRPPPLQPLPSFNDGHSGPETDLTPPSPFGHLGM
ASSVGAVDLRLLLCDAGLAAVAAAASGMDRGGGGHRRRQRSSGSGYSTSGSYLDLSASEQYSSVATALTGTLTEGGE
ALATVNSQRSSQGQHHSRTGNLASLRQANATHAGHLGRGTGQVELQESEAGHGGGKDRPHKRRHRPEHLRRARSQSH
QAVAATEHVPGSGRQEDEEGEGQHPPGDYMRDHRTGKDAAPASSHAGAGSHGAPILARHSLPARTLSHASTFTADTG
VSNGGRGAGGGSGVVVRAHNAFAVPAEPSRSGEGGSGAPEDGGRLASAATGSSGVLQVGYSSECLATAALLEDVAGA
AASGARASPGPHHARVPMPVPVLVRVQDELAWQTGPGNSTSLSLNPGVSSLGVQPNSHTHQHQFYFHRPPSTGHMQE
LASPGKMSMEGSDAGGVSVLETSTAAGAAVTAAAAAASPGTYTHANTTGEGGLLPAIATRTTGTRNANVTTGPSTGG
DNDVPSNSTEVLALRLGLGPPTGVPSPRLFGGSARGGGSGATADSGPDGLTGFFSPGPVGGGAGLGRRAAAGAMAGI
LKSPQPERGELLRSFTSVSRSVSVAATALGAGEAGPGPAAVRAGKTRSAAEGAAASAGAGAPLTTIYSTGAAALATA
RPSLPSPLHMRSLRRLVGSPMAHEAPTHSSSSALGASTAGGANTNDGAYGGGPGGGAPPPGMSAAFLSAHPHAGSRR
RGQRTRSSRSRTGTGGGVRRMPSGRTTTDAYEHGPYGPADDLDNDISSDTARSYADSLEVMLIGPRRAAAHAGSLAS
ASVYGSATAAAAAAVAPHAAAAAAEVAGSGGLSPPVALCGAAASGPVAVSGAASNGPRVRIMAAPVVASSSPPGRHA
PLTLAASTSHTAASGAGAGADSAQMHPPHVPKGIRERGRSHQHPHVDHVADEYARAAGCGSTSPATTSHLAPFSVVL
RHARGSGGRLRGPRAHSNATTPLTCFAGPIAPAGGAAPGSRSGPGGRSGPGGSTGSGCAGGAGAGGTAVPPSLMALA
AGMLSGCLPSTHSAPCVRHLLSPDASAQNAGTAEATTGDRDNTCDTAAAAAGASGITSGPDAAAHEVTQLGRQDAHA
GLGSTLILGDSASFDIVSTPAVSRSLTTGAGAGVQGALAAFMAATMLERSSRDSGGTGAAQSVAPDLTPGGRGAGVA
TAGTDACTPQLTSPAHSGPLQPGRQQGLVAAARGTAMGVLGGVVAALQGDALGLGCSGEAALEVAATSGSATNNVPL
PLSLDAGLGSGPGGRAPGAAGLLLGFGAGAGVLGSSGRGQETNQPGLGLRSSGRGMMVMESASAMSSGSSGADGCSG
GGGGHDAAAAAAVVVAQLRAAGVDKRGGGSGVSNAGAADLSSRLHAQPQPQQPPQARQPRVSWELDTRPVGEPHRQS
RDQQLEQQGRQQQVSALPAASGITALAGAGTSGVAADTLGTSTNGPQEGTRAEPAVETLASPLPRHSRLHPLPAAQQ
APQAAAAPGASRSPPLVQSHPQGAGDMRIVRKHLYLTQQALAAAVGAGGGAGAPPNAQGAAAPAATVTSSTHPQLRT
SFEQGRSRRSWSAAALLHPWRQMALTRLQQESAQVQRMRRAEQAAEETADEEVVAAAAMAAAQELREWGDTSCSGSG
TGGAGGARLVAMAVAAAAAASAPAPKHPLATPLAEGEPWRASALSSGARGKGGSSGTSGANSQIGGTAGASAGNSTS
AAASVAVAASSATPRMTSGAASTAAAAAGTTSNRSSAEVASTATGASFTTGNATARPPASPPTPAATAALAALGSAG
VPSASTGRNSFSTPVQDASARAPMAGPAPASVAATSSFFASATLAAQQQLTARTGYPASGGKPRCSLEAIAEASPSS
VEEPGLTPAAVAAAAATAFPARAPVAAGLAAVAGPFTVAGRAAAAGPAAAPAGPAAAPAGPAAGHAVADSRPLRSGT
PPGTPPRRVRASVEVEVVLRHLESRDADVLVFSTHSALSTFAASASGGPNSNGTGTSAAGSGPEPVRAAAAGATGAP
VAAAAAGVAGNSSELLGESQRGSGGSQPPQQPQGNGAGTANGISSGSGLLGSSLGTLFQKESPSEAAQALLGHPCEP
GSGILTAPLTPSSGKLQSPGTVGAGSCVEVVPVEADAWAAAVAAAAAAAPDVSAERHLVAPAQPVEAAQGTGGNLTV
WDTVRRSSPGCGAAGLVSIHGQPSATAAAAHGVDNALLPSTAGPQSQAQSVGSPEYSPLLDPQQRREPPPPPLPRSV
PPPGASPASAAAIPTAQQSASAAAAAAAATATAGATGGQQLSAGSSFIRSDWSPAGVPAMERCAPPLLSGPRTAPAR
DAPAGSMHAPAAAAAHRPPGGQGHAYPNGGGGDDDGAFTDDDFGDCDILTTLDCSVQAAATAAAAQAGAAGGALAPA
GLTAALPQAPLRYEPSPIRATHPVYGHLSGSPRANGAGGSTTSALLAAVAAVGSRPHLSEGGADTSAPSQLPQARLD
PTGAAGGAPGSPGAVHEMDPDALAALAERLRSRSFKDGPAGSRNGSFSYRPSRMMPAAAQQQLRAPSYRMSRGPYSA
GVPTATTSGPAGLDGAEATADPAAAVAAAAAAAQSRLSEVLLVKWLDYSSKYGLAFVLSNGTVGVLFRDGSQMLRDRG
AAHAWLLPALRGGGTAAAGEHAPRGGELLQPQPLPAGAQALLLPAGNWQRPGWGTGGTGAGEAAAGVGAGHSQSRAR
AEQGSRRSADSTAAPWVAEAALAPVPGPSYPHDVLKKLKLLCSFESMLVGGGGGGRGGGGDGSNSGGEESDVSCATL
ERRGPLAQKSSERSTGTGAAAVAESGVAAAAAADAAGTAPQAAAAQAQHTAAVPEIHAQHQQPQGAATATAAASPPS
PPPSPPPPPRSPPSVWLTWWARTHKALAVCMSSGAMQVLFMDGSELVLRECEDLVDFLARDGRRQQASLRGVRLAGP
KNSVAKRVQYATTLLHSRGGVGGGGGGGGVGSTGGAAVGPGAGAGPGGGGGGGAGKRWA

>Cre03.g183150.t1.3|PACid:27576808
MRYLFLAGQAEGIPFQAVRRRRSQEVEELLARYYDTGGFMPPPHVLMQQQQPQQQAGQQQSEAQPLGEPVTRDGEVS
APSPSVSTRNGQVLPGDDSFRWRAETAGGALAVWAYDEVSEDAVSQAVLLTDQATANTPPAVLDRVRAHGTRIVIIG
RQQRLTDMPPFRWLLTYQGSGGQGTNGDGRQWVDIRGAGAVAGNPAMAVGEEDLLETYDEVNTGDGWGNESVFMHEL
AHTLIDACQLQRVQQRYAAARDSGAYTPSSYIMTNQHEYYAMAAAVSFDSTLNSPAADNLLRKGELVARDPGTAELI
DWGFGGNPWRYPDDCLTCFYSWPWFFYELYPPAPGRVPYTPLAACPTLRPGISCGDRNTTHCPFWASQGLCTNPDDQ
QYMSVNCMASCGACPYLAAGQPPPPSPPSPPLPPPRGVCRDEYPGDCPSWSSLAGECAKPFMLQYCRVSCRYCSASD
TSISPPPPRRYPPRPPRPPPSPPLPPSPPPPAPRPSGIACRDRNTTYCPEWIAAGYCRSPEYAPSLASQCRLGCGFC
RVGCQETEIRCAGWAAAGECANTPGYMEANCARSCGLCDGPEQGGCGDDNVSCPYWAQIGECNKDAGYMLASCRASC
GVCDKQPGINIPPPWRPPPSPRPLPSLRSPPRPKPSPPRWVRTKPSPKPMRKPKAPKKTG

>g3620.t1|PACid:27576820
MRHHTSTLALASRRPFLPAPFVPTPLLILTFATTPAPGEDPKTCEYVVAIFALRNIFVLKLLNPELACDGSPEISGP
VCVHRFRQWALPPGCLQVHVVTDGETCLSLAAAAGLSLEELLQLNSGLRCDMLLRGMQLCLQAPLSPPDSPPSPAPP
PSPPSYPPAPPSPPPPPPPPPAPSPPPPPAPVSPRPPRPPPRPVGAPPYPAAKCGESYRLIQAQSCENVVSFFGLP
SVAFLVSINPGLDCSGAPRRTITICINSFKPWALPATCIATHTVWDGDTCDGVAAAAGLASPESLYDLNDGLDCYNL
LRGMTLCVATPRPPSPPPSPPVPPLPPPRPPSPPLPPPPPPPAPHPPRRPSPPRPPPTPPYERPAICDKWHIMFKYQ
YCRDVVQLYGLDSEYDLELLNQDVVICPRAPQTRRICVNTFSQWLLPPSCIQFHYVWDGDTCASIAQLFGVPLPDLL
QRNAAGLDCTQLRRGMRLCTRTL

>g4376.t1|PACid:27576886
MEAACGTHLRGLGPLPRVQGGGLSGPSGLFLTFWLPSAARSRSSPSPAAVRTPNDSWDCRAGGVPPAHRPGASGPAY
GTFKPLRGTAGAAPATTAVPPASPLEGASGRHRASICRGSDSGVVWAAAAAPPTASARAVAAAAASSRGATGGGGGS
STSSSTSSRARHLGSPPPSPGADKESTNTAQGGGGAVAQGAPLQRVAAAAPASRAALLRPGWSPALRAATGRDPWRS
QPQPQPPVRPSPAADGPTQEEDWDWAGDGEGADEQTGGQDWGGEDHVDAGAHSSGAATSSSSGGGTAESPWAVADSG
WLAERRPLRPLRALPQRPPTAPGLEQAALQRLQQEWAGGAGGGGSSRALQAGGGAEAEREEQQLLQQVVAAASPEEV
VACLSPALPPLPAASAPPSRLCSARLLLAAAERLAALQQSGQQSGQQAQVARPAGSEPPPALAALCDAILRSAAAGG
FSWPQVEWQQQQQQREWPAREGGSSLGLSAPHAGDGGVGAEAYGRVAALLWRGAELQLQLGPGAGSNGVTAAGTGVG
AAAPPPGVRHTTAAAAAAAVAAGDSGWRQRRRCGGLHAPWRRSGTAAAFWPQLLTCAVRQAGASLQPAELADLLEAT
AAAACREQDEEDGDEREEDVGSRQRDGSGGGGGGLNPRDGTSPLPLPLPPGALVAVEERLLAVGVHGIAPCDAVRLL
AALAALRHPPRAATTRLLTAAAGAGFRGWRWRPPAELQQRLVLTRPQGAAVGATGSDEAAVEAAAAEAGEAAAEPVA
EAMQLGERELRSLLRSVCRLDGAYAAAGAEAWLADWAAAYWAHLHPRPLHWEAHTQHTGARAHTEAEAEAEAGEPTA
GAGAAASAGAAAEVLYLLATLQFVPPWEAWEAAALAAVQPRLMPWLRLLAAARPGRRQQQWRQKRGRQDRAPATPAT
PATPATATATAVEAQVALDCWLSHSPSPVPPQPRSEQHTHQPPPPQSSPPPQSPPPLPQPLDPLALVQLAWAVGAH
AAACPVPHPALPPPAAPAVESSDGGGSGGGGASPVHAAGLAPGDGRSPATGGRLTPRQQLRLSAHRHRLRVFGAALA
ALAAWAEAGSAEGAVAAAAATAAAASGTGGAPGAAGTAARQGAPGMAHRRSGSSGGGHTAGMPPVSSAGGRGPVDAQ
AAAWAACVRDVAWGAAAAGLDAGWVWPAAAGARLQLQAHLGPGGGGDGGSGGGEDRRGAGGLVECMLETAAARGWLL

>Cre03.g201550.t1.2|PACid:27576952
MTGQLAQSVSHSYADTSTSEESAWNIVTNQELVNRLVPVAPQSIDEAAINMRIDLRSEHTMITGDQVDATLALDVPT
SLAEQLGLDIGSGGGPVDADTSNSTASSSGDGGSRRVRRRLTAEQYRARRMILDFHGTRRTLTEFNDLTSVLSQLGV
QNQVPKAVVTGKAKKNGGAKDLMVVGGKPLNISSITFFFTSSACGINPVLTEAQIKARWYDNGDSAPVTATLQRAYR
TCSYNKLSFYPENNLVFGPVDVPCIGTAPTKGPYNLRTGLGNNKNLDAEMYGLYDLAKDWLRRNGRTDVIARLQYLR
RKVLVWPWNNLAQLAKNGTKELVGWPGMGSMGCPGNPDPSGIVPDCLTWMNNDLTTTNLDLQVLFQELGHNIGLAHA
ARRECDAVGCVVNEYGDSSDPMGSAGPVSFEKSFTCFSAPQAYKAGWSSPIANLNKDTVTAGSGWTDYTVPSMHSTD
ANFLRVAIDQTGINSADRLKPERALFVAYRVAQPLGGYDSGLPKELNARVWIHEYNETANGWSADIRKPTVLLTMLD
LPAADPKTKKIGPTVVPGWGPILTKAVFPNAFGRGDSLTVWIKAKTNTSATVGLCRATDVAETPETCYDGLDNDCDG
LPDDLDPDCGGEDPNAQSPPPPLGVLRKAPPPPSPVKRPPPSPPPPTPAKVLSPKKGAGRRLA

>g4423.t1|PACid:27576960
MLQVPVGVFTADSGSLLEVSASEKLALVGAGDGSVLDLRVFGQPAGPGPVVPAFRLLSGDSSLELRNLTLLLPPLPE
EAATAATAGSAGALPPSLLAHVIEAVGGGGNRTQVVLRRVTLVAGACSDLDVHRSFFCHDLNRWRARVAAYDEPTIL
GGTVRYGSRAPGGGLMAVGSAADSGGGDTPPLAQLLDCTVTCDAGVSGGGGTGSSISTPYVASGPSGPWDCMAVTVS
DSAGLAALPDLVASQLVLAGATVLVSVEAGAPVRVNPSTWSTIKVRVQTSIFFVGRGRAASLLDLAGLQSKQFADVD
SHVMSQGRVGLMDMTLMGLSYPTRALTQLELMAAWVHAVGISGVLAPSLYFHTDGRIAPPEVMAWIKNVRLALPDAE
AEWWRNAAPTAAGGTGLPLSSSGWTGPALSDPDTPLNAWVYGAFPPTASPSSRAADPVRPPEREAGPVRIHWLGLRA
CDYCWIAPLSRVLEAAAVQQVGAAAGSTSSGTVLVAQPAPSPPLALRSTWPLADALGSDVAPKVPLSHVILVVGIRG

SPYVEMASACGGSGGSGASSGGDSPALYVMPPADYESVASMYGGSATYTGRLRRSTGLGVSMMPPAGDYYDIAILGE
ANPSVTLGGSGGSAGSGSRCVLLPPPASQSAPGPTTWDMTDLSDRIRIRLGPPGSSSDGPSLQIQGFTLYNLAPYAQ
PQPPEPPQPPPQPTAPRPPGQPAINLGEVIAIEINGQPVDISGNVVTGSGSSGTAASEATSTDGGTGGTAIATEGFA
DGSNGGTATAGGVSGNMDSGSASSGTSTSTSTTVSSGTAVSTSTTSSSSTNDDSSGSTGGSGDATAAALATSDAST
DGVAVGVTLDSGLVTAAPSASTTGPTDVVADGTAVVGGALTQGAELGVVTGGDVVTGGGGSGVGAAVDGAAPGGPLV
GGVAVAGVDGAVDPSGDTGGVAGGVAIGEVTGVVETGGGGSGTSTAGGSSGGRWDTGATGGWTGGGGGGVCWTCLLR
PTQPPQAPSPPEAPPSPPEAPSPPPQAPSEPPSPPPSPPPPPPPDPTDPYHGLSLALPFFKFDRFSPLLYASPQSSP
SAALPPPCRLPPLALVNCNLVVPPPELELLRRLLQEAGRLPAVDGPSGGTEGPQAGTTAAGASRGGGGECYSHSFRR
ISFLGWSGRDVVVTSQLPDDAPTGLMPDLWRAERPFYSPLLEESCSPPPPAPPSQPFVPTSPPLQPHPASRVPPPAS
GAAGAGGGERLPDGTLDLPSDATPAGAGFAAVPPAGGAVPGAVPPGVQASSISGESHEGSGRSTSGSRSHSQPAWVV
PVAATLAAAGGLLLAVLVAALVVGRSRRRRRQELATKSGKCASDDSTHDMHSRGSSSDQQSPSHRNTRGGTPRGGGW
TLVTNLLLSALRSSSVGSGGVRSSRRSCSSSSTPRNAKDAVSLGSTGVGGESGHAVPAGANGVVSMVLESQSEPLPN
VQLSAEAAAAIAAAQGSRLWRSLDLSRLLKSPETRSALSRAAAGGAGGAPSSKVVVLSAACRVSPVAAQVVSIRAVA
EKQQQQQDRQQQMQVPGCRWTVNGIPQFSSEAATLASPSGTDATGTGTGTGSTGSPAVLASPATGAATVTAEVAAMF
AEDEATAGGQKSDEELWLECVIGRGGFGVVYLGTWRGLPVAVKTLVVHEALLGNEGRRRQRAVLEAAVSSALSHANV
VQTYAFDVRRLGELPGMGRGRFAPPALEAVAEEEHPEGQESQEKPLEALASPSAHGTEADSVYQLLLIQAYCEGGSL
WEGIESSQLYMGLAPDSLGGVLLSLCLALDVAAGMAHVHARGIVHGDLSSGNVLLSARPPVVQAADLHSASAWGFSL
GASGDASVDAGLGGTSRAGTGAAGPLARAELLVDGHGGGVNMAGPESEEAQAAAAEAAARALIAGVTLPPVTAKIAD
FGLSARMGEGQTHASNCWQGTPTYTAPELELEGRLSKAADVYSFGVVLLELLTGQTVDGLLSIACIFASDASTSPPA
ANGESQAACGVPPLSLTARGGAAHAAAALRSAVPKVLQVPHTCDKEKLASLVQACLSPSPHARPTFMQVLQDLGVII
ASLDSSMS

>g3450.t1|PACid:27576986
MLNISPFPLVSKGGGLLRVELMMLILFVTVDSLHAQADSAGQARSGGLARSSSRDGTTKEGVISDIKKAQDTLTFDG
LRRRQLDVVAISPAPPPSPRPPSPSPPSPLPPSPPPSPRPPSPAPPSPAPPRPPPSPLPPSPRPPSPSPPGPPIPPQ
PPSPPPSPRPPSPSPPSPLPPSPPPSPRPPSPAPPSPAPPRPPPSPLPPSPRPPSPSPPSPPIPPQPPSPPPSPRPP
SPSPPSPLPPSPPPSPRPPSPAPPSPAPPRPPPSPLPPSPRPPSPSPPSPPVPPQPPSPPPSPPAQPSPFAPSSQAL
PANSTSST

>Cre03.g203300.t1.3|PACid:27576993
MHSALGGLQRQAQRRLLVEAVVIDDAAGPDTKQEVAEGCEDSDGLGTQRASCPSRDRARASLRRLRPTAWRCTQPDA
HAAAINESEGSEPSSQLLQAVATTGKRLPWAHLAAREEAAAAQQTAEGAGTAAESQNGVEGEQATALPTAKTASSAA
DSWAQALGGAEPPAQDWSWLASPPWGVVPLPPELACRVDPHGRLRLSGTIEADVTAATAAAEAAAAGGSPPPSQHRH
ADPDRARMHARLQRTAGAVQQVLAVAAAAAAAAATTAAAAAVGEAAHVRALLKPDWQIVGTSRTPQGGLAGVELPAA
VHTLTAGYAFQVAGSLRQPPPSERPAGPLTPAEEQLWQDLEQLKARIDVQASRRVRVGQVADFYARSPPPPGVAGEE
SDKDVRFAPLRCYGSERQVAQGYMTLLMQDLSAKTRAGSPDPESDTGGGGGWRRLAKLVVPWAEERRKRIWRWLLET
AAGLEEAEAAGGAGKARTAAEGSSSVALQWGAAAEEVLKAAIGTLVLLYLVVQDFLQIQQQAATKFGFCAAEQLNIF
DPLVLASALPAYLLARVEHPDVHVSPADLYNLLLLGQVPAFTHLRWPIPRPATAGVAAALRTELAAVPPPLSYEHVL
AAVEAISLEPDVVHEWGLLLLTTFSSVATVAQLQIQTWLLAWKAHRQKRRQQAEARRQKREMQRRSLSMAWGMGGVD
SVVNALLAIDRAEGGEGQVLDGSSGRRDCGSGSGAGNGGHALAGGSGAGAARSRQNQSAEQGGESAERLEDMLESVG
LAAKSLGLTGVIAAAASLQPVAAALAAAVRGLCLLRIPPTLSAPAPPLLSAKDAERAKAGLAAVIPGPDGGCDVLRL
RLPCDDAAYTTAAVLCLDMSSISSEPAIGSSGASLIFQPIPASKQRRQQQQQQQQQQQQQQQQQQQQQQQQRGDGWH
AGGGRELTEWMELDVCRTFPPMWDAPNARSAWPYPWDYLSYYNAAPEWDDSTSLEAMASHLRRFLVQLRAGLCAAHV
RGVEAIGRLMSALAKVRAEMRDGPRGASRDCAAWVASILDPGDEPQTASELKAQPGGEAGGEARAQGAEPSDTVDNE
DDSEEDADADGAETEGAAKQRRSRAPRGSLVRAWDSACAAGHVVMPLQPEESLRHTLPVVAAAAADAAAVAAAAAAA
AGGSGAKPHSQKGPKHRRRQQLAALQPPAGRAGADPAGDSDGGESSGGAGTPPLWLLEPQPGLLRYGDLLSQYGDAD
AESVAWLELQLAAMVHVLQRREEEPAATQHQEDEGSGSGAHAGHSSRRQQPEQQPPHEQPGKAPARKKQPRLDVKLD
VFFFEVVPGKPWELVPPTSAVVRNLNRAMEKEQLAQLVK

>Cre03.g184950.t1.2|PACid:27577020
MQGRNSSRAGWLAVAIAVLATLTLGARAAVSSFRVDGCSSGTIEVVVGRYGVATVPAGMSCTYNIKPAGSPTELLLK
SQSFNAWVCATNDTNPSTGACAGRVNLTAGAIASLPASSYPDGLFVVIPEPSMLQFSTTMAPCALGPDAPLNSRCSC
IGDGNETIPNALPVRLQPFSASELPLSLCYSIPNLGSLATPSSYLQLEAVFNVAWDFGALPTWFATAGGFTALLSRY
TFPDGALAPPGYYNTSFLHSGDVAMALTVYNVAGGNSTVSSGGVLLVAQPLNAISATVAVTYSAVDAAQGASVIVSL
LPPPPPPSPAPPSPAPPSPPPSPTPPSPFPPSPAPPSPAPPSPTPPSPAPPSPAPPSPAPPSPAPPSPSPRSPPVYG
VYGDTEDWLAPPPVVDGADGQAAPPPAGGEWVEPPTYGDALWGPPPLDVVAPPPGDLAAPPVAEGGNDTWSTPPPME

EPPTGVTAPPRPRRLLSPPSPRPPPIIHSPPPSPVPSLSPSPSPSPLPAPSPDGTATTPSPSSPSPSSGTVVPVTAS
APPPPAPQSGTSQVGEVPSLAAPPPPAPPAGGSASRLGPLGRAEAMLYSTLIGAACLALMML

>g4187.t2|PACid:27577031
MLLIVSVAVQQESFLATLPQLGLALCLDFLGFAACWLTGRYSAPQQVLVHLLSSVVAAVAVLCLQPLFSGLLQLQTS
GTGAGAAGAGARGAARGADTAVVSAGQSVTVATSGLHAAASYPHAAGAAAGDGDEQQSGAALAAEADCRAKPTAAYD
GRTEGDQEQHHRGSVPGTPAAWAHTDNGVAGDPNSGSSSGTTAPTSALSWLHDPAVLDRPLLGPPPGIVQCPERSLL
PVYRRKVFIERTASALVLLHGALWGIAGTGGMAALHLRHRHNQHTQHHSTSPSVSATTGGVGPARVASLGQGAATPL
LHAQAAAAGTLAAATAASLLLLNGLMALLPGRLYDDHATAINAVVTGLHAAAQLAQLLAAPPASRSRAMLVRMMTLL
GANLLGNEEPWVPFCVELAALLGAAAVLDSALQWGNGANAGHGVGAAAIGRGGIVRAGPATEAALAAAALAGRAAAV
VVPSTLVGATAALPHWLQPGAVAAVASGTLNGSGTAAALSVGGALVGVTAAGLAAGGAAATAASTATAAGSAAGSAG
ASFMPFMCYLAVGAAVLVLYGALRTGKYVTMYRLYRRLYLPAPYAAHITLPVPPAEAALLRLVCQSAPAAAILGADF
SGSVASVAVAAPAATAPAGSSAGNANAADSGSTRGYLEGTMFGSSHRRSVSLSNMLTVGRLSTLPAASGSTGPASGS
GHLAWAAALTAAAAAAAAARPAATTGTGAQGHSSVAGGGGAETASMPNLGLKRSMHGSGAAFDAACLAAALLTSPMP
PADGGPGGQGAEAGIASPFTSLADLSSLHAGEQQHHHQQLMSPLRSSMQLPRAQPPQYRASVGGTPGAPAATTAAFA
SVAAGALLLPGRNTRSSGSVTQHDARTGPITVPRGSSLTDPAAPVAQPQATAAEIPASPQGGGAGMSLGGGAVAGSP
LLAGAGAFDGVDEADFISGKVSFTEMVNQMARVHQFTAQVGSAEAMAAAAATATAAAGGGGVTGAPAANSAVILARE
LDRLMSVLGSNGTSSGANTATLPGAGGTPGGGGGGGGGGGSTTVPRLPPSRSPSDISNMYRHSSPAASTQLSYNTRA
TAPATGGVSGEAGQWAAGSTAGTAGGAAADGAGVAGSVGEGGGGCGGEGGGGGGGRSFLASVAASMLTEEQDHREHD
QQQQQQSQQQHQQQGKSEDDGAAAVSPPPRDGAQGPPAPAGPSSPQHVVRLRTAPARATSDLPAIWQQQQRVMPLQQ
QQMAMAAAAAVQQQQQQSHQQNRMGSPGMPVGSHRLPRRSPSDVSYLHRAATAGAGVGRYGWLPGAGTNDLNLQQQY
TLQQQQQQYAAMQYEVMMQQQRQHMAAAALVAAGGRAPVVPPSGGGSRRRGPPSRGASRQSSTMDSTALALLGQNLI
MLGGSSGGRNALHHTSSPSANVSGVNITIPEHSSHVSEEPSKYSQSKSQSYGGMPGLAAAAAAAAAAGSSGAAAPAA
PGGGAGGAADDANVALAFESKLYSLTRSAVEWHASDRAGGGAGNVGAGFSPSVGGALAALEEEEPIGVSTLDVPLFG
RVSPPTSLGSGDGGESRLVAALRKSSATARRTVPAAPPGPGPGPMSSSRDWQQPPLASAVSAAGASVLMLASPEGAP
SSAVSSPPTMERGQAAQQPRLLSNKGSAGGGGLQDGVKVGPGPFSPWQPDGGGGGSIYSPSRRSGMPPFLLSAAAAF
SKGLQAFSRSHHRNQDGLDGTAAIRRSGTDPTLSAQGGAPTAFAPGPSLRYMQTDFTDAAAGRDGLPSPDCLQRGLA
AIAADALASPPLELGERGEPQALAQVQALQGEQRISRGRVHLLWSSGDTVGSRVAASGGAPHGTAAAAAVGGVSPSL
DRLAVSPATTAVTATTVALHLSPGGSSRDVGPPTGNYAAALSGGSGAIHSPAAAVLNGGGAGGVGGAAGAWVARNMV
TGSPPPPQALIPSHLLPGDTQLAHYRLLLAQQQAHALGPHHPHPHLVGSAAAHHHILLSSLPASTDASSAYPHTASD
IWNTGTPAAAAAAAGTAGGNGRSRSASGLPAIRVTSGVSNAAAASAATASALAAAAAEADARLARELAAIWTTTSIS
GATEHATSMSHQGPGSLLSSSLAVGLALGAPSLDDGDGGGGGGGAGGLGGVDGGGGGGGGVGGVGGVGGGGGGGGS
GPPSASTARILPGGREAAGQVLSAVQEVPESDGVGSAAALNSDSTFRLPAGDPARWRGSFQSVPEGVRVGRPSADAG
AEGGRTAESQQHEEEEEEEEEDAEKQCAPPPSPEQQPQDEPQPAGASPTRSAASRESLVAGSPRFGSAGAVAGLSMLG
NAYGGSSAGNRLMAAAAAAARGSSNAGNGSTAMSFDNWAAADDGLVMMLLGGSAAASDLSIEARSIRGPGSRTQPFAA
WASAPAPTATTAITARAGPTPQVLPTASASSGLNGRGAAAAAPGEGISRFASSTSRSGSAAVTRTGQQEPRLPYSRL
GAAVGLPAAAEGAEAAAPAAPLSSPSPLRDQSRTIDSMPLSSGVSVVSAGSFSRSPRGLASAGVAAPAAATALGGGA
FTVGTPHGVATAAARSVSDKGLLQQLAAASSGSCSAPQPYMGAMQQAPGVAPASHQDDVAVAPSWLTAATAAAEATA
AGVPSSPPEHTVLRPPGQQQARSRHPVLRQRSVPHLPVHSYIGPAHQQQLHQQQQLQQQPLYPMPHMAMQQVDGRMA
AYLQPMQPMGGQPFPGMQQGVPPGLLLLPQAPHHHQAGQSALQLQQQPQPPLYLVHSGSGPRYPGHRVPSRSPSVPS
YIGVPAAQQSLLLPGAGGVGGGAAVPSGGLGPSRLMIGSSGIPHVPLPAPGAGRGNSGGGGSSSGVHAVAMHGGPAG
GRLGGAGSPGGLPAAVGQPQRHDVRRTSSMGRSRLSAASLPGSASSGTRSANAAAVAAAALQGDGAFAPAIAAARST
VGTAAGNKAGSGLTAATMTGSSAQKQQQHQQQPDMAALLLTNASSFDGALQQQQLMHVANISTASIGSTEGRLQSWV
APATYQAAPAAAAAVISAAPSSGAATAATGAGAATAAYSMAPSPLPLDGVATAGGAGAELESYSHLLPMRDSQVRLA
ELLPISVLGSNAGPPPAADEPTPPAAAAAAAAPAEAGRRPPPARHASMDQLILRQQQQQLVAVRRLQPSPSSNAHRK
LNRRRSFGQSAEYVPSRFSQDGKISRGSGAASGAQDARSPTERDPSASVHLELSAMTAAGAVMTVGSAGSSSRRRNS
SVPGSTRDPGARQAMGPAHADRASDTGAAVGPGGGCSGSRRGASTPGAPAVQAQGMRTSATGGNYRISAEGSVTNTF
ASTASLQSFADVAGDANYSHFF

>g4187.t1|PACid:27577032
MLLIVSVAVQQESFLATLPQLGLALCLDFLGFAACWLTGRYSAPQQVLVHLLSSVVAAVAVLCLQPLFSGLLQLQTS
GTGAGAAGAGARGAARGADTAVVSAGQSVTVATSGLHAAASYPHAAGAAAGDGDEQQSGAALAAEADCRAKPTAAYD
GRTEGDQEQHHRGSVPGTPAAWAHTDNGVAGDPNSGSSSGTTAPTSALSWLHDPAVLDRPLLGPPPGIVQCPERSLL
PVYRRKVFIERTASALVLLHGALWGIAGTGGMAALHLRHRHNQHTQHHSTSPSVSATTGGVGPARVASLGQGAATPL
LHAQAAAAGTLAAATAASLLLLNGLMALLPGRLYDDHATAINAVVTGLHAAAQLAQLLAAPPASRSRAMLVRMMTLL
GANLLGNEEPWVPFCVELAALLGAAAVLDSALQWGNGANAGHGVGAAAIGRGGIVRAGPATEAALAAAALAGRAAAV
VVPSTLVGATAALPHWLQPGAVAAVASGTLNGSGTAAALSVGGALVGVTAAGLAAGGAAATAASTATAAGSAAGSAG

ASFMPFMCYLAVGAAVLVLYGALRTGKYVTMYRLYRRLYLPAPYAAHITLPVPPAEAALLRLVCQSAPAAAILGADF
SGSVASVAVAAPAATAPAGSSAGNANAADSGSTRGYLEGTMFGSSHRRSVSLSNMLTVGRLSTLPAASGSTGPASGS
GHLAWAAALTAAAAAAAAARPAATTGTGAQGHSSVAGGGGAETASMPNLGLKRSMHGSGAAFDAACLAAALLTSPMP
PADGGPGGQGAEAGIASPFTSLADLSSLHAGEQQHHHQQLMSPLRSSMQLPRAQPPQYRASVGGTPGAPAATTAAFA
SVAAGALLLPGRNTRSSGSVTQHDARTGPITVPRGSSLTDPAAPVAQPQATAAEIPASPQGGGAGMSLGGGAVAGSP
LLAGAGAFDGVDEADFISGKVSFTEMVNQMARVHQFTAQVGSAEAMAAAAATATAAAGGGGVTGAPAANSAVILARE
LDRLMSVLGSNGTSSGANTATLPGAGGTPGGGGGGGGGGGSTTVPRLPPSRSPSDISNMYRHSSPAASTQLSYNTRA
TAPATGGVSGEAGQWAAGSTAGTAGGAAADGAGVAGSVGEGGGGCGGEGGGGGGGRSFLASVAASMLTEEQDHREHD
QQQQQQSQQQHQQQGKSEDDGAAAVSPPPRDGAQGPPAPAGPSSPQHVVRLRTAPARATSDLPAIWQQQQRVMPLQQ
QQMAMAAAAAVQQQQQQSHQQNRMGSPGMPVGSHRLPRRSPSDVSYLHRAATAGAGVGRYGWLPGAGTNDLNLQQQY
TLQQQQQQYAAMQYEVMMQQQRQHMAAAALVAAGGRAPVVPPSGGGSRRRGPPSRGASRQSSTMDSTALALLGQNLI
MLGGSSGGRNALHHTSSPSANVSGVNITIPEHSSHVSEEPSKYSQSKSQSYGGMPGLAAAAAAAAAAGSSGAAAPAA
PGGGAGGAADDANVALAFESKLYSLTRSAVEWHASDRAGGGAGNVGAGFSPSVGGALAALEEEEPIGVSTLDVPLFG
RVSPPTSLGSGDGGESRLVAALRKSSATARRTVPAAPPGPGPGPMSSSRDWQQPPLASAVSAAGASVLMLASPEGAP
SSAVSSPPTMERGQAAQQPRLLSNKGSAGGGGLQDGVKVGPGPFSPWQPDGGGGGSIYSPSRRSGMPPFLLSAAAAF
SKGLQAFSRSHHRNQDGLDGTAAIRRSGTDPTLSAQGGAPTAFAPGPSLRYMQTDFTDAAAGRDGLPSPDCLQRGLA
AIAADALASPPLELGERGEPQALAQVQALQGEQRISRGRVHLLWSSGDTVGSRVAASGGAPHGTAAAAAVGGVSPSL
DRLAVSPATTAVTATTVALHLSPGGSSRDVGPPTGNYAAALSGGSGAIHSPAAAVLNGGGAGGVGGAAGAWVARNMV
TGSPPPPQALIPSHLLPGDTQLAHYRLLLAQQQAHALGPHHPHPHLVGSAAAHHHILLSSLPASTDASSAYPHTASD
IWNTGTPAAAAAAAGTAGGNGRSRSASGLPAIRVTSGVSNAAAASAATASALAAAAAEADARLARELAAIWTTTSIS
GATEHATSMSHQGPGSLLSSSLAVGLALGAPSLDDGDGGGGGGGAGGLGGVDGGGGGGGGVGGVGGVGGGGGGGS
GPPSASTARILPGGREAAGQVLSAVQEVPESDGVGSAAALNSDSTFRLPAGDPARWRGSFQSVPEGVRVGRPSADAG
AEGGRTAESQQHEEEEEEEEDAEKQCAPPPSPEQQPQDEPQPAGASPTRSAASRESLVAGSPRFGSAGAVAGLSMLG
NAYGGSSAGNRLMAAAAAARGSSNAGNGSTAMSFDNWAAADDGLVMMLLGGSAAASDLSIEARSIRGPGSRTQPFAA
WASAPAPTATTAITARAGPTPQVLPTASASSGLNGRGAAAAAPGEGISRFASSTSRSGSAAVTRTGQQEPRLPYSRL
GAAVGLPAAAEGAEAAAPAAPLSSPSPLRDQSRTIDSMPLSSGVSVVSAGSFSRSPRGLASAGVAAPAAATALGGGA
FTVGTPHGVATAAARSVSDKGLLQQLAAASSGSCSAPQPYMGAMQQAPGVAPASHQDDVAVAPSWLTAATAAAEATA
AGVPSSPPEHTVLRPPGQQQARSRHPVLRQRSVPHLPVHSYIGPAHQQQLHQQQQLQQQPLYPMPHMAMQQVDGRMA
AYLQPMQPMGGQPFPGMQQGVPPGLLLLPQAPHHHQAGQSALQLQQQPQPPLYLVHSGSGPRYPGHRVPSRSPSVPS
YIGVPAAQQSLLLPGAGGVGGGAAVPSGGLGPSRLMIGSSGIPHVPLPAPGAGRGNSGGGGSSSGVHAVAMHGGPAG
GRLGGAGSPGGLPAAVGQPQRHDVRRTSSMGRSRLSAASLPGSASSGTRSANAAAVAAAALQGDGAFAPAIAAARST
VGTAAGNKAGSGLTAATMTGSSAQKQQQHQQQPDMAALLLTNASSFDGALQQQQLMHVANISTASIGSTEGRLQSWV
APATYQAAPAAAAAVISAAPSSGAATAATGAGAATAAYSMAPSPLPLDGVATAGGAGAELESYSHLLPMRDSQVRLA
ELLPISVLGSNAGPPPAADEPTPPAAAAAAAAPAEAGRRPPPARHASMDQLILRQQQQLVAVRRLQPSPSSNAHRKL
NRRRSFGQSAEYVPSRFSQDGKISRGSGAASGAQDARSPTERDPSASVHLELSAMTAAGAVMTVGSAGSSSRRRNSS
VPGSTRDPGARQAMGPAHADRASDTGAAVGPGGGCSGSRRGASTPGAPAVQAQGMRTSATGGNYRISAEGSVTNTFA
STASLQSFADVAGDANYSHFF

>Cre03.g206300.t1.2|PACid:27577090
MVAATHHDSLQRAVLSAAACLTLLLVLPLTSPFGVGATAASSVAQQQPQCQQQERFDFEEHTLQAFAAPAVSTRAGP
VSPAGSDPPAPSDSKQPQPHSHSHLHAEQQQHHHQLRLRAAVADLVAAFSAAAHATPAVAAAAAPTTHSNNEQALWV
LAAALASIAVLAVLMLAAVTVLALVLLLRERRGTSSAGGGATPVATTCFTAAAERRKLLLRRRVAARQQQRLVQRPL
EAQERQPSPPPSMTPASGVPGAPASPTLPTAHALAPATAVAAADAAPSAAAAAALDGIATTASPAPTAAASTLQPVE
ATAPQQLPLPPPAAAPAAALAPAASSPTSRAPGMPVMAPGPATHIASSSAASLAATNSSATVGWHPPPMTTGRDPAA
TPATCATPAASATPAAFAAAHRPPRRRRSLTSAVISTLSTINSLSNATSISTTAGSSSSAGAPPPHLLLPPRMVSEG
GAAVFRTLRLLDPPSPDRAPQPAAPASPSGSSGVAGGREGGVAAAVVAAAVAAAAAAAVGAEAQAQTHTHTPLQQEP
QRLVAFGSGADDSPAPGTAGFLSAATGAGDNAGVATGNDSSGDDSRSAPSPQRSPLPDPWWTESPTRAQPSPLQLQQ
PSIGPERPSAEQQLQPQPHQQHPAPSANGRFVFRAASEDGRRAFSFLPPVGASTAVLGLASSRCYGSPTPGGGGAAS
HPPASTPRGMSARIPQHVPQSQPPPARLHPLPHLTLSRSEYQVAQLRQQQLQRVRQARLLQRLGSSGPINFGGAGSS
TGGGGALALPSLTSTPAGAGAAAFAMPRALSSAGGGAALPLGLPMPGSGSARCVAAAPMSRPAGPTPRRALFQAAVV
PAPEAAGGTGTALPLLAPSPTQPLQPLEGVRLLPARSGSGGALAAGGGHVSGSGSSGNHGGGGTHGGGGGGGLLCGL
RGCGVALLPGRQRSAIPATIPEEVEDSASAVTGAELSGAELSPSPSAITAPYRSSPWCPGAPASPPHPLLPASTSSG
APAALDTAELLQPAEPVQEPQPEQQYDGPSHQAPDQAQELLSSHDDLSSVFSTQGAWAGCSGAYATDASTVMPSAAS
PAAASGGERGRGHGHGHGHAAHPGSMTLQDDPHRHFQQQEQQQEQELPCRGWSAFGDVFTTAGAMAAGHGGGSGARG
GSSGRNNGGASSAAGGAACNSAAAVGAAAAAAAAAAAAAAAAAADDDDDDDGGAQAIEDVHGGSPPPPPTGTAAGSC
SDDFVSPWSSHAAPTLPPGFYYSGSAVAGGSPYAGARGAGGHPAGTVDTLPHNATAAAAAAAAAATLAADPYCAPFY
SVASACGLDPYAVADAIAADADYDGNTFGAGGDGNADDDGGGGGGPLDLPVPPPAPVLRSCTTNADAFSQQLADVF

LSAADVGGLGSLAGGSMILDPEVDHVLPPLPDPMVAAAPPPLPLGLCYAGGMGVSGYAEAAGMYGGTAADGCESPPP
YLPLPTPPTLPRRAPSLAEFPGLPPRRGSSVGRYHRRTPASAAGSRRHSGDRGSSSAGSGQRHSNDGTYYTGGSSVV
VSGGGGGGGGGGGGGGGLSRSGFGGSSGSPSRVARQLFPPNEVQQALHAPHAAATATAAAAGPHGGHMAFSGILGGG
AAAAAPAAAAVAEPGGGSSTPRRRNYSPAAESDSDVELLQLAPSRTASPTEAVARSVLRSPSPLRPPPPSQQRPSAA
PHPSPLPFRRASSPAAASVVSVSATGDGAANTCFTSPPPAALPPRSPSRRSSEVLPPLPPVAPSGHGGGRTPRGSRP
LPALAAAAAAAAARLQASSNNGSARRLVSGGAAVGGGGSISDGSGSGSDGGHRGGCGGGGGGSGSAYLRRSGSFLGGQ
ASQRQRLSTSAPAGTSPAAAATAAPAAIGAPATGAAGTSIIAHIGYCLYAPPAESEQQHHQRHSRTIRRLPWDLPLL
PPAPPQSPTSPQRQQHQHQHQHHHQCQPQLTDNGTSAGTGSPGSSPRSPHGPHSPHEGDHQLCLSDLQATAALPAAL
LAAPDHNTAARSDLTRFGLSPSAAAASSTAAGAAVGVPLLPSSRSVIAGAAVPPPPRSASLSLPLSPSLISLPGHLG
LQRTTSSSSSSSVTGVAASTSNASATQGRMPRRNSTASGTSRGSLLGKGHGSSKLRRKLGSPSMSGSGGVKERVPSS
SGGKGSPRKAAAAPATSTNTTTTAGSAATTGGGASVAAAAAVTWPHRRHSRLIPHLTAVLRPQPHVQHYPSPPAAAA
QPPHADGSEVAAAPGSGAAAVQSSVAVATAQPAPPPSVLYRSHTRTRYFSVKVHDHSAGARAAAPRLAAAALRALVQ
PHLHRHPPPTALREAPAPAATAAADGPTSPATPPPAVTAAGRSSSPQHSARLVLNRRMTSSRAMAAAAVAAAAAADP
VMPPHLQSGTRTAPPISSRQPVAVAALRGMQQPRHNADSGEGGSISSSGGRGVVLASAHLATGPVAASVLVLPGCLQ
VLAWRRHVMPSPPTARTAPAAPAAPAAAAGGGRTVPVSQNDSGDDRPQQQQDADEEELASGLLASLKAALSTAAISS
SSSDTPAFAAFAAGAGAGVAGVAGAADGTSDVDAAEAAAAYSTLTKLVVHDCSLGRVSVHRLQPPHQLQPLQPPLVA
QPSTASSSPSLLSWCSEAAAVSTAAAGAVGGGHETTAAVGPAVVLPEALPHIPHALYEQQQWQTDAPDGCGFAWVEP
ACWAAGEPADLVLQLGMEALAPLVEAVVPDRAGSDGSGGGYGAGGAGSTVRVRVVATANAPDVLATPSAAAIANLQQ
REAAAVVVVADEEVEVAMSDGCARLHVPASATASCAAGGAMQVFALTASGATAPGHSAAGSDGFSAAAAATACEAGL
LLGRATLWLAPAAAAANAADAISSSSGQLAAELAGLQQRSTQCLLAADAAAACGAAADAAAAGSDADGAAASSRSAA
GSAAARCRAWQGHVQPLLSDLDFVSAVMSHWQQQQPQRVREPEWEPLQRHEQVEVEVGKEEEEVQVFREAVEVAADL
VAFADAQGCDALAALVRGMVSAAVAAAGQAAAAAVVPASRRNSHGHDQLTQLPGGPVITTEAAAAASAAGFAQAPPL
PQLQQQPPAAMPPPLAALLLAPQHPKSLSVAAVTGSLLAEVVAATAAAAALDVRAAHACATAAVEWAPSLPAPRATA
SLNDVTPPVPCFFLQPDGVGSGGGNSSGDSGRRSGGEAAGGGGGGGVLSGGCCRHVFLEDC

>Cre03.g181750.t1.2|PACid:27577120
MHEPPRKKSALKYVPWVAIIAFLFIFVGVIVWATGANTALSNTNDALTTLSVEADGYVQTIHSVAVGASVVCCLMAA
LMVLMSGGRTCLERAVDQRGKAVCGSWLWLATEAVLCAVWWLILFWLVFVIFGSCLWYGATYTVRGVLQVSINTAVA
YGNSTVLPPSPFPSLPPSPPLNITNSTVLKLSPPPRSPPPPFVCASSCLNLALFNFLVQKDICVCSVALMSQALYKT
NKAYDALGSVVAGAFLMWCGASFLLMSSVADFAKTKRERELLLRAQRAAVDAAAGQLALGTAPGYAPGYGGDTLPLI
KTQQMQQAALQQQQMQQMQMAQMMAQAQAAHMRPPGQVSTGASQQNMGHQV

>Cre03.g181750.t2.1|PACid:27577121
MHEPPRKKSALKYVPWVAIIAFLFIFVGVIVWATGANTALSNTNDALTTLSVEADGYVQTIHSVAVGASVVCCLMAA
LMVLMSGGRTCLERAVDQRGKAVCGSWLWLATEAVLCAVWWLILFWLVFVIFGSCLWYGATYTVRGVLQVSINTAVA
YGNSTVLPPSPFPSLPPSPPLNITNSTVLKLSPPPRSPPPPFVCASSCLNLALFNFLVQKDICVCSVALMSQALYKT
NKAYDALGSVVAGAFLMWCGASFLLMSSVADFAKTKRERELLLRAQRAAVDAAAGQLALGTAPGYAPGYGGDTLPLI
KTQQMQQAALQQQQMQQMQMAQMMAQAQAAHMRPPGQVSTGASQQNMGHQV

>g4420.t1|PACid:27577129
MSTCVATPPTRNHMMPLRVASLLHLRNGPGAVLHGNCSDTLRRSAVRGLLWTTPAVANPHAAADALPLASSPHALGP
SYAKPSAQHSNDAGTQITESSASGASNKSAPGASVFLELNGKPAASGSGTTSSAAATTTTGPRQAGALVGGGSERPP
QATQANSNRDRVNAERQGAGAGRKQGRSPGTPGTRPSGRGAGGGGSGAGGGRGDRGAARGGGPKAAPSPPPPPSPPP
PPPPPPPDHSNGGYTLVSTPEELELMLDQLAVAAAGGQAAGGGSSSSGGNGSTGGSSNSSSSSSSGGGGHGLGALAI
DCEGLELGRPGGKLCLLQLSARVGGRSGRCLSIWLVDVAALGARAFAHRSRQPGPGLAGAPFSLRGLLEDEHVPKLL
YDVRSDAAALWSEYGVRLRGVTDLQLADVAARQAEEGARRAAWVEGLVRCLSRRLAADGRGGAGVPPRLADDLAAAT
AIAKRYHDGGNTQVWTQRPLTPELSEYAATDVRYLHALEEALRRRLSRELLDKVAVETRKRITTPAPNSKDARAAAP
LMVSSRPGSGSGGGASLVGTADRGSGSGSGATSSSGAGPAGASGGAGGGGGGGHMGLMEGLVAALLLHYDTEQRRRE
QQAQAQAQTQRPVTQYGTQAASEGTSSTAAETAAAETTPPTPATAANPSSFFPQPHSQSSAASGTPSRGATAVAAAS
APGPAPGSGGSSATRSGGLVAARPPPPPRPPLLVPAEQTLDTAARELLELGLRPEAPRGAWAAPPPRPRLPPATAAT
AQPSPVAGQPAAGGSLSGGNSTGGGGEGCNEATQAAPEGELGALEAPEAAAARRARLAAVWWLLRAERALSQPGSRL
LQDLAVARDPGLRWLVGTAEPGAGRVGRRQA

>g3533.t1|PACid:27577130
MVYSMQYSAWYCGRIVEIHGDMLRITYPGWTSEHDDEVHRHSDRLDCSVGGRRKWKPIKGASGAFKRVNPTWIPTGA
KLDAAARQLLTDPTQSLTGGRDSELAATDTEVAGGAAAGAEADSPAARGRGRGRGLGRGRGRGRLQGRGQGAGRSGS
AERPAKRQRRQEGPGGEAGAAGGEAGVATGAASAAQDVEILDLVLTLGEEVGVGEEQEDEGKESEEHESEDEDEHEE

EDEGGEEEAEEEEEEEEEEEEEEEEEEEEAEAEEEEEGEDEDEEEGDDEEEQGEEDEDEDADSDAGEEGGAEAEETAE
AVQARACAAAMAGAAAKEQRLPPVEQQQERDQSSLQPQSQPVSALLQPLVAEGEDGEAGVDEAEQQGVAGRHVASNG
WLGGADAGSDSLGSTQEEEPQQEKQQDEEQAGASGSVAAVATAVDAAAPPQSHVQAEERDTDPLQAQGAQHQAAKAQ
QQAPAQAGLAHEGLQAGPHAVPLQRAEANAGSTASPSVAGPSAAQPVESPVGAPLGELARGLQSHGQELQPQEQQRV
QPGRPLSFAKRFSQMLSDPEFVAGLSSAGVKPWVAAATAEPPAVADAGLAAGAVRPQLGPQVGAGTDALPTESAARA
APATEREGCGAAVEGRVKATAAAAAAKDATAATAAAAAVAPGAAGAGARMAAGMQAGADTAARAGTERAQGDEAVPG
GAGADPQPQAGGSGELEDGEAAAGAEDPGSGGAGTHGQAAGGQEPRQAEAAAEGPGKEAEAEGAGEDVAAEGGGSGA
QVEPEDAGQAQPLQPLQTAEGANGADTSAGRTDVQMTDAEAHEGAAAAAAAADMDTGTGMGIGVGTGLAAACDACAA
AAGIVPCAAATATAGIDAAGAAGPHGTAAESGEAQEVLQPAVDPGDSGARRAASDEARAGAAAASPTVPTPEPPPPP
LPQSQHKHLHHPCMGAEAEVAPAQGALDGGRPDPQPNPGAGDQQPQEQPQHTSPGQAIGGSAAAGGGPSQSPAQLQP
RSGRRKRARPQRQGLPPGVCDPGAVGSASVLLHIDVGTVLVRKGAAQAVAAVIAGMRGEADGGGGAAAAATGAAADG
RNVLDAGEGAGPLGRLASGAARSGRGAPAGGAGGAPEAGGGAAVAAAAVAAAAADAAAEMRARRLLEKELERERRAR
EAAEAQVADLQSRLQQQQQQQGAQLLGMSAAASAAAAAAAGPAQRPPPHALQEAEPAPQPQQAPGSRSPGAGAGAGA
GARAGDDVEANTRLATDMLLSYAMKLQGDCLAASRSAALLRAQLEGISPAGSAAAAAAAAAAVAPDESAGGAEGAAAA
AATACALRSENLQQACNVLGSQLGSLHRGLLQLRKSQPEMWEVLRPHLPPQIQHTSMSLPPSSRVRALAEHLAELKS
RDVGVAALHTHLQAARELAAERAALLSAPAEAAFLAVLPHAPRERSAVLRLTQRLGVELPGGSILVLPAAEVPHELL
AAAEEIRVPAAAAAGVNEPRQPHAHTGQPSPRPPRQQPHPHQQQQQQPQQPQQPHPGSAPELRRLSPPPAAHDGAPF
AARMAAGPGPGPAEDGGTGPAPPFAAAGGGGIRPPRSAMSAAAAAAAAGLLNGVADDADLAAAVAAAAAAAETGLLP
EGLLLHSGHSPSPGSGLGPGLGPPGMHRPSDELGHLAGRVAAAAGGRAGGSGAQRASPQQHQQQQQQHHHQQQQQQ
QERLHRASGAGGNWADLPPGFYRAGAGAGAGTGAWGPGEGEGELSGRGLQEPHAKRPRVAPSPPPPPAMAAMHGLHW
DDTDGGAVMRATQGLFDQMAQRRQAVEEQQVRQRQQQQQQQQQQQQQLRQQLQRGKEPQQQQRQRVPSLDLNARGP
SLLHVPPEPQQQQQQRLQLSRPPGGAAAAAAAAAAAWPPPRPAPRGPPGHDHAADFDADLDIQLRSYGGGGSGRDDLF
VPPPFMCQPQLQPQPPQPSQRHPQDPLPSRGLGAGASPAGGAAGQWLQNQPLGAGHAPRAPQAPLLPQQPQHQHHNQ
PALRPLGGVTDLGGAAGAGAHAGPGGSALGGEPAPRGLGGRLSASVAGGGGGGAAGSGGVYGSPSPMARAAAAAARSP
LRPAVDELSDTDTQALLRQLARGGAADGLAARLPRDAAGLLLRPLDGPHQHLEEQLLQPPVARSAATATGARAGGGA
LPPLELRYELHGGRGGAGSSGGGVDGGGGGIGGVQARQQQQQLHTHMHGAPPPAQYWPLKQQPAAGGPLTKEYHDDV
L

>Cre03.g204450.t1.2|PACid:27577252
MSKRVLGLHCNSWIVALVVAGCVAAASAQSSLPVISRPVVACYEGPSVSRSWDPHGFSSIADQLAELERERVLAVGP
SGRPADAATGSAFVVPPLSGDESLLESHEDIQALADYVDAGGLVILHPSLRDSSETPELISQILSYNGEWKLCKPLT
DNRRSQLGRAHALPHAESFLQGPGWPKELEDAAIVQAFTRCHHSDPEAFSIPLYAAGDDNSKIVAQAFGRAHTHGAV
VLLGYSWRDGPQPGWGDLLAKLVTDFADGAYTAHAAGRFTTVAAATASSHDAADAADMGSDGTDNNSPYDLDSSYSQ
DLDLDSVLEGAGGLSDEAAEVVRRFLTATMGYPGYPGYPGYPGYPGYPGYPGVPAYPPPIYISPPPAVLDSPPPATA
PSPSPAESSPSPSPSPSPSPSPVYPSPSPSPSPVYPSPSPSPSPVYPSPSPSPSPVYPTPSPSPSPVYPSPSPSPSP
VYPSPSPSPSPVDPSPSPSPSPIDPSPSPSPSPVDPAPSPSPSPVDPSPSPSPVDPSPSPSPSPSPSPVDPSPSPSP
PVYAPPPSPAPPAPARVLFMDFANLPATGVQAPTRPVVWYDDPDFDSLLAASPNNSKLWMVKPDREPCVGCGQCPRA
WYPTNTSDTTNGFNYTTIVVYFREPMLIKSVRLNEVYQPGGVQQIFLLQWPAVPVPLVFPNATALGPESVKKQDIVF
NGPDTTKTCPGVLNVDVGVQRSAMKAPVRKGFTQKRLQRGLKKRAVGGVGITVALPLPSGPGGSNGNGNGNGNGHDN
GNNGNGWGARRQLRAAGGNGNGNSGGNGNGNGGGNGNGGGNGNGNGGGNGNGGGNGNGNGGDDWEQLLTTFIGDIRF
DGRVLYPQDPSIFVPYAPPKPYNKKLKNTA

>Cre03.g174100.t3.1|PACid:27577285
MPLAAATATKPPPTKPASSEPAASQPASSQPAASQPGTSQPCPAESRTAFACTPVACPPKPTPTHPAPPNPAPPSPT
PPSPAPPSPAPPSPVPHSPPPPSPAPPSPPPPSPEPPSPAPPSLPPPIPEPPSPAPPSPPPPSPEPPSPAPPSPEPP
SPEPPSPAPPSPEPPSPAPPSPEPPSPPPPSPEPPSPPPPSPGPPSPAPPSPAPPSPEPPSPPPPSPGLSSPLPPSP
EPPSPEPRSPPPPSLKPPSPPPPSPGLPSPLPPSPDPPSSAPPSPLPPIPDPPIPEPPSPAPPSPAPPSPDPPSSAP
PSPLPPSPEPPSPPPPSLEPPSPAPPSSIPAPPSPPQPSAAQMNPLPPKLAGPPSQPPQPPPAALGGGTLQSLRSPP
PSPPPAAVTASPLWLPPSPPAPASPNLLQALGSPAAPPPPPPSVRADALPMLEVTDLQDAAFSASNRLVGGVSDDYT
DPDEDMLLADASFVISEGATGDQAGTDGWSAQQGGLAAGSPPAAPSSLGSAVAQAPPLLIALRGSAHVRVAVYEPYT
ELGAAAQQLVMASSPGVSGSSSSAGSSSSGSSSSGSSSSRTVPVAVEVQGTVDTQAVTPSGRPLTLTYVARDAAGNT
ARVERTVTVYDACAAVHEHRCSDSMQCSVLGACDARLLATADLVAAARAAAIATTNSSSNSSSNSSWSFSAGASALV
AGSLLTSAAEYITAAATAPDAAAALPPSVVVSELELAEQEADLAAAGGASQQPQNARRLLADTTPPAITLLGEGQLY
DTGTGTGSVYGMIHTVFVGETFLDPGAVALDLVDTYPDEPKQESLLDPSLIAVTITAPSGALVSAVRTDVPTGNAAA
GGSLPYVLAYDVADAAGNRARTVYRRVYVFCRTPEVACFGSGGAARSCSVAGVCGAGAAADLLLAPAALAVSVPAAA
TVAAVAAGAQAASSSTAAAPTLTLLGSSSVRVRAGVPYDRCQGLDTESCDPGANATVAAAGDLAGRVVACADRAAAA
GVAAPRPFVLVGLRYCGLDTRRPGRHIISFHLPLLSDGGAAASAAAAGDELVVRRVVVVEELCEEGEQPCGDGRCSA

GGTISTTTGAASSAGLLAALKSQLAYAATATSGGSSSSDAATAAAAYVSPLGAANQPPQLRLRSYPPAVLEGQAVAV
RQGTAYRRCAAGQVPTPQLPCEPNATAWDAEDGNITQLVALCPPADCAPSQCRAHALAAKQPADCGVDTTTAPAGAT
LRLSLAVFDTAGAVAVAERVIRVVSPCGEANGQVAAAQYVCDAPSGAAAGAATVGRYVCSEVPCEALAALNQIAAAA
GAATSSAPRLFLLPASHQLLPLPANGSSVSGGGVLAASGPPVALDRGAAANQTLTLTYGTPSPIDLAPCASFSALTS
SAVLSGSPTSAGPAQQQQQQSRPCAAVANSTAEGDLTPFIEVHVEPVCDDSSSGSSDGSSGSADMPATTPACGNSCS
VAQLSSGACLPGTYRVVYRVQAAAAAGAAAAGAATAAATALQLLVAVEEVRVTRLTELVFVPWVEQLLPAQQQQAAG
AAAVAAAAAAAATNRTAVEDLAAAMRLLASAGGGTSGATASGLRTAAAAAMSAALTALGMAPGTVRGMELGAGSSSSS
SSSSSSSSSSSSSTVTILELSPAEQQQLNLTAGNAAAATTIYALRVSSMSIITAVAAFRPTPAQLLPSAATASGGRR
HSLRRLAQLGAVAADVTSLTACGVPDPSLPAAPYLTSAAANTSNTAAAAASGGALVSLGGLSSACTTPQISWVDYLA
AQLAGAVEDVDRAAAEAASAAEAAAALAAAQAAAFEGRDAQYEALVGQWVREGAAAEAAAEARAAEALRVLVATLAA
QANVTAAAAAVQSLFQAALTEATQSARLALLTAETVWGGVESLTGDGGSTDTAINGTSDNTTELNAFLACARLRGAG
NGAVFAFTVAANTRTAESKTNNPQTSFGTATEPNTTNDASNSQLHHHRRRLMTSTGRDGRFDGYVVASGRDFDYSLW
DVRAAERARHVGSSKNRVLAGLLLHQVRRTAQEVRDRSSADGALCRRTTHFSLVPSCTAAPEAVAAAASPTAAASAA
GRAVSYISSGGDLGGIGIDPHFELRSGMFQPDLNASDYYNTSQGSPELNPAGVPYGFFHLPLPGVPRRLAAGYPLLL
DVRLSAQRAAQALDYLSRGSYLSAALTKSLEATLVFYSADAAVFGVWSASFTWQDSGVIHATARLLGLPAISYGEAL
SRLDVKAFQRRLLPDLALLLLIACYCLLAGWDAYHTLAEQAALRRERRRLKEKWEQIHEGREKGLGLAAGWQDSDSA
RGESAAGMGTGTGGASAFVVAAAQMLARTADGKAEGASSGLLPPPPDRTPPPRSTQQLVLAAGGPQAVAEAVEAAAE
GAQAWGRDPTASTPAASAPPAGRQHQQGARGGILHGGLPAAAANHDMDTDGRRHRRHSRWGSRSLAAAAGPNAKANG
SGGGSRRGTSGAADRGVHYRPAMSVLWLCYEAVVCGLMAAALGVFFTYAIRLSVKDDFTAGGSVYDADTFSPARFFL
IRRQEPQSAPSVTVNGSATTAAAAVPAPGGAGRWRLPADTSAWEAVGAEFGRVDDMYSAVVLYSFLQALVLALLLVR
LLHALSFQPRLSVISGTLARALPGLTHLFAVATVVVVMLAAAVMATQGASRAAQLSSYSAAVAWMYKLVLLGDDEGV
FQALLLSASSLAPGERVVTGLVYGLSCFAFQILLGSFVLVLLLGPFFELKQATAGQPGVPQDIMRILRWWLEHNLRR
APKNKALACRIDDWLLQPPPAGGRLYRAWARLLLPLAMLRLGKACDRGGGSSYWGGGGGGAGTGAAPGKAKIDSSGG
GGGGGSGGGGGYFRPDTPGGTRFTNLADIKVSTSHVAAAAAAGFASDISNRGLRLGAPAPLLLQLPTPLFPGGAASA
ASAAAAAAAAGSVDATASPGPLPIKRSRTATATTLRFMDSSGGGKGGGRDGGGGGGGGGGAALDISDVRFALRTMSL
KQIAIASASGSGGGSSATSPFLTTTGGAAAAAATGSFTGVLSRRGSRLSRGRQHDLVGNSGGQMLIASRRSFRSGRS
QRSLVAGAGVLGSPGGGMTVTGAGAAAAMTRSFSSRFAVGFAALGGAGRRGTAATEAGQHDSVDGGVGGGGSAKNLI
ARMFSVTRQGSSPKAPAVASGPSGAAAAAGSSSGGGRMHQMLLLPQLPPTLLEDPRSEEDEVAAASHMDAPAPVGSS
PAAPFPSHRSTQSFSTAVSDAEAHGEADDIFSVSKWRSEPDSPPAFGAGEAELPEPATGLLRPQAAWGLQSPSGNRL
APGPAPLLTSPTEGSPLIRSPAGSFSTNRSLMPRPLMTVGIAAAVSAAAAAAAASQAAAHRLPRRTTSSPQQHQFGS
QSQLQLQVPSSSSRHRAALSRMARDSDGTPLPAAAAHVLAGADPSRWSTDFGVQAAAAEGSGGGGRRRFSAIQPAVA
GVSASGDAVTWAGANQDSRASGYAALQALEPPDLPSVLGNTRSPQRQTSPELLHSPDQVAAAAADAAAAAEAAALAN
TVLHNLMLRFGGGGGSKEAAAAVAPSPARHQQQLHIQPPLSPLPGRDSGEEATQGSSGGATPHYSQGPASSGAIGSR
AFQPWTDTGFLRVSIASTSGHGGSTSGMVMPTGAAAAAVAAAAAAVAASRSVTLSPRLPLRSRLASFRRSSAVTAQE
LLTATGQPLGLGFAAAAAGIGRGASRGVIHSAAGAAVDASSAVPAAVGTDGGVTLAPQPQLPRTLVLRSSAAGLGGS
GSGGGVRMSAGSEQTQEQAAKAVPGIDIEAPAVLGSASGAASPVPVGGQVGSVQPGQQQLRARSLGRLAARVESGPI
TLQQSYKALRNLLAVASPTAAPPTTPVAPAAAHVAPAAMPVGEFDGDGSGAESALGARGGGALLRRVLPRATVDGDG
AATAESPRAPIIRVSSQSSFGALGRTQTVDGAVAVPGPVVAAYASTAAGSMDAVSLAFRHNPPPKRGDSVPGLLSRV
PGAPVPPPGAGVQAEHQRPAPQQVWLQNPSNSAAGAAADGSSGRQPAGGGSAVGWSAHVAAAATAAASTALAAPAES
TATANATAAVVFGRLPVTRSGGSIAMGPAEPQIRPVTAGASTFTVGMPNPAAAPVVPLARFASTTGKRSHHASQTSA
APVAAAAADTPSSPPTTSSAHIAALVAQHEQRQRHQEQPRLRPAGTSTFDSLAFEEGEEEDVGGDGVQVAGVALQKH
WEQPAGAGGGLPRPLPTRVSSVPDWRLFVNAAAAAEHAGQSGHGAEDGVEEHHLHAAAVGASASGATRAAAPGPASF
GGGQSTSRLTRFAYGMWMAAGAATTTHNASLGGGGGGTGASREHAIGSITAHSGLQSPMLRPLLVTGAGMAGAAAAA
ASRTAVLSTSPAPSTAAATTLTSPRSAAGLGGSAAGGTATTSRLAAVVAGAWAADEPAASSPVPAAAPGPGPDTRGV
LAAGPGPVAEALEVTSEEEVDGSTGLRVATHRNLTEAERREQCKQRRGGVAAAAGEASRYASHTRTVLVLVAAMEVM
VSELRAAEQQLQAWMAVLDRLHMWLLRDGGGDGSSGGYGLAAVRRTAAAAAAGVAAVAMPGLRTDTACASVRSSSSV
ELSGTDVTADALAATAASSDLRPNSSGGPFAQTCSAAGPAPMLSSPAAHSRLPLPPAPVSPQMQPPPAQAFPPVQRN
RGISSGFHRLSHSSFALPSLPAGTTAVAPAATTPGLAPSAAASGGVRGSGTSGSAVDGIGHSSSTDDDGAEGLLRCR
QPSPIGARFRGQDGPAVRMSLDRGASSVSRMGSSGTRMPARGGGANGGGGDSSPLRTASSPSTGVAQLALKASRLRQ
RVYELTSPTPQQQRQPQHPQWHPQQSWPDQ

>Cre03.g174100.t2.1|PACid:27577286
MPLAAATATKPPPTKPASSEPAASQPASSQPAASQPGTSQPCPAESRTAFACTPVACPPKPTPTHPAPPNPAPPSPT
PPSPAPPSPAPPSPVPHSPPPPSPAPPSPPPPSPEPPSPAPPSLPPPIPEPPSPAPPSPPPPSPEPPSPAPPSPEPP
SPEPPSPAPPSPEPPSPAPPSPEPPSPPPPSPEPPSPPPPSPGPPSPAPPSPAPPSPEPPSPPPPSPGLSSPLPPSP
EPPSPEPRSPPPPSLKPPSPPPPSPGLPSPLPPSPDPPSSAPPSPLPPIPDPPIPEPPSPAPPSPAPPSPDPPSSAP
PSPLPPSPEPPSPPPPSLEPPSPAPPSSIPAPPSPPQPSAAQMNPLPPKLAGPPSQPPQPPPAALGGTLQSLRSPP

PSPPPAAVTASPLWLPPSPPAPASPNLLQALGSPAAPPPPPPSVRADALPMLEVTDLQDAAFSASNRLVGGVSDDYT
DPDEDMLLADASFVISEGATGDQAGTDGWSAQQGGLAAGSPPAAPSSLGSAVAQAPPLLIALRGSAHVRVAVYEPYT
ELGAAAQQLVMASSPGVSGSSSSAGSSSSGSSSSGSSSSRTVPVAVEVQGTVDTQAVTPSGRPLTLTYVARDAAGNT
ARVERTVTVYDACAAVHEHRCSDSMQCSVLGACDARLLATADLVAAARAAAIATTNSSSNSSSNSSWSFSAGASALV
AGSLLTSAAEYITAAATAPDAAAALPPSVVVSELELAEQEADLAAAGGASQQPQNARRLLADTTPPAITLLGEGQLY
DTGTGTGSVYGMIHTVFVGETFLDPGAVALDLVDTYPDEPKQESLLDPSLIAVTITAPSGALVSAVRTDVPTGNAAA
GGSLPYVLAYDVADAAGNRARTVYRRVYVFCRTPEVACFGSGGAARSCSVAGVCGAGAAADLLLAPAALAVSVPAAA
TVAAVAAGAQAASSSTAAAPTLTLLGSSSVRVRAGVPYDRCQGLDTESCDPGANATVAAAGDLAGRVVACADRAAAA
GVAAPRPFVLVGLRYCGLDTRRPGRHIISFHLPLLSDGGAAASAAAAGDELVVRRVVVVEELCEEGEQPCGDGRCSA
GGTISTTTGAASSAGLLAALKSQLAYAATATSGGSSSSDAATAAAAYVSPLGAANQPPQLRLRSYPPAVLEGQAVAV
RQGTAYRRCAAGQVPTPQLPCEPNATAWDAEDGNITQLVALCPPADCAPSQCRAHALAAKQPADCGVDTTTAPAGAT
LRLSLAVFDTAGAVAVAERVIRVVSPCGEANGQVAAAQYVCDAPSGAAAGAATVGRYVCSEVPCEALAALNQIAAAA
GAATSSAPRLFLLPASHQLLPLPANGSSVSGGGVLAASGPPVALDRGAAANQTLTLTYGTPSPIDLAPCASFSALTS
SAVLSGSPTSAGPAQQQQQQSRPCAAVANSTAEGDLTPFIEVHVEPVCDDSSSGSSDGSSGSADMPATTPACGNSCS
VAQLSSGACLPGTYRVVYRVQAAAAAGAAAAGAATAAATALQLLVAVEEVRVTRLTELVFVPWVEQLLPAQQQQAAG
AAAVAAAAAAAATNRTAVEDLAAAMRLLASAGGGTSGATASGLRTAAAAAMSAALTALGMAPGTVRGMELGAGSSSSS
SSSSSSSSSSSSSTVTILELSPAEQQQLNLTAGNAAAATTIYALRVSSMSIITAVAAFRPTPAQLLPSAATASGGRR
HSLRRLAQLGAVAADVTSLTACGVPDPSLPAAPYLTSAAANTSNTAAAAASGGALVSLGGLSSACTTPQISWVDYLA
AQLAGAVEDVDRAAAEAASAAEAAAALAAAQAAAFEGRDAQYEALVGQWVREGAAAEAAAEARAAEALRVLVATLAA
QANVTAAAAAVQSLFQAALTEATQSARLALLTAETVWGGVESLTGDGGSTDTAINGTSDNTTELNAFLACARLRGAG
NGAVFAFTVAANTRTAESKTNNPQTSFGTATEPNTTNDASNSQLHHHRRRLMTSTGRDGRFDGYVVASGRDFDYSLW
DVRAAERARHVGSSKNRVLAGLLLHQVRRTAQEVRDRSSADGALCRRTTHFSLVPSCTAAPEAVAAAASPTAAASAA
GRAVSYISSGGDLGGIGIDPHFELRSGMFQPDLNASDYYNTSQGSPELNPAGVPYGFFHLPLPGVPRRLAAGYPLLL
DVRLSAQRAAQALDYLSRGSYLSAALTKSLEATLVFYSADAAVFGVWSASFTWQDSGVIHATARLLGLPAISYGEAL
SRLDVKAFQRRLLPDLALLLLIACYCLLAGWDAYHTLAEQAALRRERRRLKEKWEQIHEGREKGLGLAAGWQDSDSA
RGESAAGMGTGTGGASAFVVAAAQMLARTADGKAEGASSGLLPPPPDRTPPPRSTQQLVLAAGGPQAVAEAVEAAAE
GAQAWGRDPTASTPAASAPPAGRQHQQGARGGILHGGLPAAAANHDMDTDGRRHRRHSRWGSRSLAAAAGPNAKANG
SGGGSRRGTSGAADRGVHYRPAMSVLWLCYEAVVCGLMAAALGVFFTYAIRLSVKDDFTAGGSVYDADTFSPARFFL
IRRQEPQSAPSVTVNGSATTAAAAVPAPGGAGRWRLPADTSAWEAVGAEFGRVDDMYSAVVLYSFLQALVLALLLVR
LLHALSFQPRLSVISGTLARALPGLTHLFAVATVVVVMLAAAVMATQGASRAAQLSSYSAAVAWMYKLVLLGDDEGV
FQALLLSASSLAPGERVVTGLVYGLSCFAFQILLGSFVLVLLLGPFFELKQATAGQPGVPQDIMRILRWWLEHNLRR
APKNKALACRIDDWLLQPPPAGGRLYRAWARLLLPLAMLRLGKACDRGGGSSYWGGGGGGAGTGAAPGKAKIDSSGG
GGGGGSGGGGGYFRPDTPGGTRFTNLADIKVSTSHVAAAAAAGFASDISNRGLRLGAPAPLLLQLPTPLFPGGAASA
ASAAAAAAAAAGSVDATASPGPLPIKRSRTATATTLRFMDSSGGGKGGGRDGGGGGGGGGGAALDISDVRFALRTMSL
KQIAIASASGSGGGSSATSPFLTTTGGAAAAAATGSFTGVLSRRGSRLSRGRQHDLVGNSGGQMLIASRRSFRSGRS
QRSLVAGAGVLGSPGGGMTVTGAGAAAAMTRSFSSRFAVGFAALGGAGRRGTAATEAGQHDSVDGGVGGGGSAKNLI
ARMFSVTRQGSSPKAPAVASGPSGAAAAAGSSSGGGRMHQMLLLPQLPPTLLEDPRSEEDEVAAASHMDAPAPVGSS
PAAPFPSHRSTQSFSTAVSDAEAHGEADDIFSVSKWRSEPDSPPAFGAGEAELPEPATGLLRPQAAWGLQSPSGNRL
APGPAPLLTSPTEGSPLIRSPAGSFSTNRSLMPRPLMTVGIAAAVSAAAAAAAASQAAAHRLPRRTTSSPQQHQFGS
QSQLQLQVPSSSSRHRAALSRMARDSDGTPLPAAAAHVLAGADPSRWSTDFGVQAAAAEGSGGGGRRRFSAIQPAVA
GVSASGDAVTWAGANQDSRASGYAALQALEPPDLPSVLGNTRSPQRQTSPELLHSPDQVAAAAADAAAAAEAAALAN
TVLHNLMLRFGGGGGSKEAAAAVAPSPARHQQQLHIQPPLSPLPGRDSGEEATQGSSGGATPHYSQGPASSGAIGSR
AFQPWTDTGFLRVSIASTSGHGGSTSGMVMPTGAAAAAVAAAAAAVAASRSVTLSPRLPLRSRLASFRRSSAVTAQE
LLTATGQPLGLGFAAAAAGIGRGASRGVIHSAAGAAVDASSAVPAAVGTDGGVTLAPQPQLPRTLVLRSSAAGLGGS
GSGGGVRMSAGSEQTQEQAAKAVPGIDIEAPAVLGSASGAASPVPVGGQVGSVQPGQQQLRARSLGRLAARVESGPI
TLQQSYKALRNLLAVASPTAAPPTTPVAPAAAHVAPAAMPVGEFDGDGSGAESALGARGGGALLRRVLPRATVDGDG
AATAESPRAPIIRVSSQSSFGALGRTQTVDGAVAVPGPVVAAYASTAAGSMDAVSLAFRHNPPPKRGDSVPGLLSRV
PGAPVPPPGAGVQAEHQRPAPQQVWLQNPSNSAAGAAADGSSGRQPAGGGSAVGWSAHVAAAATAAASTALAAPAES
TATANATAAVVFGRLPVTRSGGSIAMGPAEPQIRPVTAGASTFTVGMPNPAAAPVVPLARFASTTGKRSHHASQTSA
APVAAAAADTPSSPPTTSSAHIAALVAQHEQRQRHQEQPRLRPAGTSTFDSLAFEEGEEEDVGGDGVQVAGVALQKH
WEQPAGAGGGLPRPLPTRVSSVPDWRLFVNAAAAAEHAGQSGHGAEDGVEEHHLHAAAVGASASGATRAAAPGPASF
GGGQSTSRLTRFAYGMWMAAGAATTTHNASLGGGGGGTGASREHAIGSITAHSGLQSPMLRPLLVTGAGMAGAAAAA
ASRTAVLSTSPAPSTAAATTLTSPRSAAGLGGSAAGGTATTSRLAAVVAGAWAADEPAASSPVPAAAPGPGPDTRGV
LAAGPGPVAEALEVTSEEEVDGSTGLRVATHRNLTEAERREQCKQRRGGVAAAAGEASRYASHTRTVLVLVAAMEVM
VSELRAAEQQLQAWMAVLDRLHMWLLRDGGGDGSSGGYGLAAVRRTAAAAAAGVAAVAMPGLRTDTACASVRSSSSV
ELSGTDVTADALAATAASSDLRPNSSGGPFAQTCSAAGPAPMLSSPAAHSRLPLPPAPVSPQMQPPPAQAFPPVQRN
RGISSGFHRLSHSSFALPSLPAGTTAVAPAATTPGLAPSAAASGGVRGSGTSGSAVDGIGHSSSTDDDGAEGLLRCR

QPSPIGARFRGQDGPAVRMSLDRGASSVSRMGSSGTRMPARGGGANGGGGDSSPLRTASSPSTGVAQLALKASRLRQ
RVYELTSPTPQQQRQPQHPQWHPQQSWPDQ

>Cre03.g174100.t1.3|PACid:27577287
MPLAAATATKPPPTKPASSEPAASQPASSQPAASQPGTSQPCPAESRTAFACTPVACPPKPTPTHPAPPNPAPPSPT
PPSPAPPSPAPPSPVPHSPPPPSPAPPSPPPPSPEPPSPAPPSLPPPIPEPPSPAPPSPPPPSPEPPSPAPPSPEPP
SPEPPSPAPPSPEPPSPAPPSPEPPSPPPPSPEPPSPPPPSPGPPSPAPPSPAPPSPEPPSPPPPSPGLSSPLPPSP
EPPSPEPRSPPPPSLKPPSPPPPSPGLPSPLPPSPDPPSSAPPSPLPPIPDPPIPEPPSPAPPSPAPPSPDPPSSAP
PSPLPPSPEPPSPPPPSLEPPSPAPPSSIPAPPSPPQPSAAQMNPLPPKLAGPPSQPPQPPPAALGGGTLQSLRSPP
PSPPPAAVTASPLWLPPSPPAPASPNLLQALGSPAAPPPPPPSVRADALPMLEVTDLQDAAFSASNRLVGGVSDDYT
DPDEDMLLADASFVISEGATGDQAGTDGWSAQQGGLAAGSPPAAPSSLGSAVAQAPPLLIALRGSAHVRVAVYEPYT
ELGAAAQQLVMASSPGVSGSSSSAGSSSSGSSSSGSSSSRTVPVAVEVQGTVDTQAVTPSGRPLTLTYVARDAAGNT
ARVERTVTVYDACAAVHEHRHVRHEDLVAAARAAAIATTNSSSNSSSNSSWSFSAGASALVAGSLLTSAAEYITAAA
TAPDAAAALPPSVVVSELELAEQEADLAAAGGASQQPQNARRLLADTTPPAITLLGEGQLYDTGTGTGSVYGMIHTV
FVGETFLDPGAVALDLVDTYPDEPKQESLLDPSLIAVTITAPSGALVSAVRTDVPTGNAAAGGSLPYVLAYDVADAA
GNRARTVYRRVYVFCRTPEVACFGSGGAARSCSVAGVCGAGAAADLLLAPAALAVSVPAAATVAAVAAGAQAASSST
AAAPTLTLLGSSSVRVRAGVPYDRCQGLDTESCDPGANATVAAAGDLAGRVVACADRAAAAGVAAPRPFVLVGLRYC
GLDTRRPGRHIISFHLPLLSDGGAAASAAAAGDELVVRRVVVVEELCEEGEQPCGDGRCSAGGTISTTTGAASSAGL
LAALKSQLAYAATATSGGSSSSDAATAAAAYVSPLGAANQPPQLRLRSYPPAVLEGQAVAVRQGTAYRRCAAGQVPT
PQLPCEPNATAWDAEDGNITQLVALCPPADCAPSQCRAHALAAKQPADCGVDTTTAPAGATLRLSLAVFDTAGAVAV
AERVIRVVSPCGEANGQVAAAQYVCDAPSGAAAGAATVGRYVCSEVPCEALAALNQIAAAAGAATSSAPRLFLLPAS
HQLLPLPANGSSVSGGGVLAASGPPVALDRGAAANQTLTLTYGTPSPIDLAPCASFSALTSSAVLSGSPTSAGPAQQ
QQQQSRPCAAVANSTAEGDLTPFIEVHVEPVCDDSSSGSSDGSSGSADMPATTPACGNSCSVAQLSSGACLPGTYRV
VYRVQAAAAAGAAAAGAATAAATALQLLVAVEEVRVTRLTELVFVPWVEQLLPAQQQQAAGAAAVAAAAAAATNRTA
VEDLAAAMRLLASAGGGTSGATASGLRTAAAAAMSAALTALGMAPGTVRGMELGAGSSSSSSSSSSSSSSSSSSSTVT
ILELSPAEQQQLNLTAGNAAAATTIYALRVSSMSIITAVAAFRPTPAQLLPSAATASGGRRHSLRRLAQLGAVAADV
TSLTACGVPDPSLPAAPYLTSAAANTSNTAAAAASGGALVSLGGLSSACTTPQISWVDYLAAQLAGAVEDVDRAAAE
AASAAEAAAALAAAQAAAFEGRDAQYEALVGQWVREGAAAEAAAEARAAEALRVLVATLAAQANVTAAAAAVQSLFQ
AALTEATQSARLALLTAETVWGGVESLTGDGGSTDTAINGTSDNTTELNAFLACARLRGAGNGAVFAFTVAANTRTA
ESKTNNPQTSFGTATEPNTTNDASNSQLHHHRRRLMTSTGRDGRFDGYVVASGRDFDYSLWDVRAAERARHVGSSKN
RVLAGLLLHQVRRTAQEVRDRSSADGALCRRTTHFSLVPSCTAAPEAVAAAASPTAAASAAGRAVSYISSGGDLGGI
GIDPHFELRSGMFQPDLNASDYYNTSQGSPELNPAGVPYGFFHLPLPGVPRRLAAGYPLLLDVRLSAQRAAQALDYL
SRGSYLSAALTKSLEATLVFYSADAAVFGVWSASFTWQDSGVIHATARLLGLPAISYGEALSRLDVKAFQRRLLPDL
ALLLLIACYCLLAGWDAYHTLAEQAALRRERRRLKEKWEQIHEGREKGLGLAAGWQDSDSARGESAAGMGTGTGGAS
AFVVAAAQMLARTADGKAEGASSGLLPPPPDRTPPPRSTQQLVLAAGGPQAVAEAVEAAAEGAQAWGRDPTASTPAA
SAPPAGRQHQQGARGGILHGGLPAAAANHDMDTDGRRHRRHSRWGSRSLAAAAGPNAKANGSGGGSRRGTSGAADRG
VHYRPAMSVLWLCYEAVVCGLMAAALGVFFTYAIRLSVKDDFTAGGSVYDADTFSPARFFLIRRQEPQSAPSVTVNG
SATTAAAAVPAPGGAGRWRLPADTSAWEAVGAEFGRVDDMYSAVVLYSFLQALVLALLLVRLLHALSFQPRLSVISG
TLARALPGLTHLFAVATVVVVMLAAAVMATQGASRAAQLSSYSAAVAWMYKLVLLGDDEGVFQALLLSASSLAPGER
VVTGLVYGLSCFAFQILLGSFVLVLLLGPFFELKQATAGQPGVPQDIMRILRWWLEHNLRRAPKNKALACRIDDWLL
QPPPAGGRLYRAWARLLLPLAMLRLGKACDRGGGSSYWGGGGGGAGTGAAPGKAKIDSSGGGGGGGSGGGGGYFRPD
TPGGTRFTNLADIKVSTSHVAAAAAAGFASDISNRGLRLGAPAPLLLQLPTPLFPGGAASAASAAAAAAAAGSVDAT
ASPGPLPIKRSRTATATTLRFMDSSGGGKGGGRDGGGGGGGGGGAALDISDVRFALRTMSLKQIAIASASGSGGGSS
ATSPFLTTTGGAAAAAATGSFTGVLSRRGSRLSRGRQHDLVGNSGGQMLIASRRSFRSGRSQRSLVAGAGVLGSPGG
GMTVTGAGAAAAMTRSFSSRFAVGFAALGGAGRRGTAATEAGQHDSVDGGVGGGGSAKNLIARMFSVTRQGSSPKAP
AVASGPSGAAAAAGSSSGGGRMHQMLLLPQLPPTLLEDPRSEEDEVAAASHMDAPAPVGSSPAAPFPSHRSTQSFST
AVSDAEAHGEADDIFSVSKWRSEPDSPPAFGAGEAELPEPATGLLRPQAAWGLQSPSGNRLAPGPAPLLTSPTEGSP
LIRSPAGSFSTNRSLMPRPLMTVGIAAAVSAAAAAAAASQAAAHRLPRRTTSSPQQHQFGSQSQLQLQVPSSSSRHR
AALSRMARDSDGTPLPAAAAHVLAGADPSRWSTDFGVQAAAAEGSGGGGRRRFSAIQPAVAGVSASGDAVTWAGANQ
DSRASGYAALQALEPPDLPSVLGNTRSPQRQTSPELLHSPDQVAAAAADAAAAAEAAALANTVLHNLMLRFGGGGGS
KEAAAAVAPSPARHQQQLHIQPPLSPLPGRDSGEEATQGSSGGATPHYSQGPASSGAIGSRAFQPWTDTGFLRVSIA
STSGHGGSTSGMVMPTGAAAAAVAAAAAAVAASRSVTLSPRLPLRSRLASFRRSSAVTAQELLTATGQPLGLGFAAA
AAGIGRGASRGVIHSAAGAAVDASSAVPAAVGTDGGVTLAPQPQLPRTLVLRSSAAGLGGSGSGGGVRMSAGSEQTQ
EQAAKAVPGIDIEAPAVLGSASGAASPVPVGGQVGSVQPGQQQLRARSLGRLAARVESGPITLQQSYKALRNLLAVA
SPTAAPPTTPVAPAAAHVAPAAMPVGEFDGDGSGAESALGARGGGALLRRVLPRATVDGDGAATAESPRAPIIRVSS
QSSFGALGRTQTVDGAVAVPGPVVAAYASTAAGSMDAVSLAFRHNPPPKRGDSVPGLLSRVPGAPVPPPGAGVQAEH
QRPAPQQVWLQNPSNSAAGAAADGSSGRQPAGGGSAVGWSAHVAAAATAAASTALAAPAESTATANATAAVVFGRLP

VTRSGGSIAMGPAEPQIRPVTAGASTFTVGMPNPAAAPVVPLARFASTTGKRSHHASQTSAAPVAAAAADTPSSPPT
TSSAHIAALVAQHEQRQRHQEQPRLRPAGTSTFDSLAFEEGEEEDVGGDGVQVAGVALQKHWEQPAGAGGGLPRPLP
TRVSSVPDWRLFVNAAAAAEHAGQSGHGAEDGVEEHHLHAAAVGASASGATRAAAPGPASFGGGQSTSRLTRFAYGM
WMAAGAATTTHNASLGGGGGGTGASREHAIGSITAHSGLQSPMLRPLLVTGAGMAGAAAAAASRTAVLSTSPAPSTA
AATTLTSPRSAAGLGGSAAGGTATTSRLAAVVAGAWAADEPAASSPVPAAAPGPGPDTRGVLAAGPGPVAEALEVTS
EEEVDGSTGLRVATHRNLTEAERREQCKQRRGGVAAAAGEASRYASHTRTVLVLVAAMEVMVSELRAAEQQLQAWMA
VLDRLHMWLLRDGGGDGSSGGYGLAAVRRTAAAAAAGVAAVAMPGLRTDTACASVRSSSSVELSGTDVTADALAATA
ASSDLRPNSSGGPFAQTCSAAGPAPMLSSPAAHSRLPLPPAPVSPQMQPPPAQAFPPVQRNRGISSGFHRLSHSSFA
LPSLPAGTTAVAPAATTPGLAPSAAASGGVRGSGTSGSAVDGIGHSSSTDDDGAEGLLRCRQPSPIGARFRGQDGPA
VRMSLDRGASSVSRMGSSGTRMPARGGGANGGGGDSSPLRTASSPSTGVAQLALKASRLRQRVYELTSPTPQQQRQP
QHPQWHPQQSWPDQ

>Cre03.g151400.t1.3|PACid:27577346
MSCERDWRRQFRAVLWKNFVLQTRSRKSILGVGGWAGLLLQLFLPVSFFLVMYLPKYYIKPIRHGETLGFAPVAFES
RSWSWDPYDGPATTKAHGGRARLALAPDTPATRGLAVVLAKALACPRNPALRTCPVPNLPASFHCMFMPPPPPAPPP
TPRPAPPPRPAAAGQMKQGQEGGGETEEHNEGWEGVAGRPSGRLVRWLRRRRLGAMRSGGVDSAGGGAAIGGEAEGR
AGAAGWAGAGVGVGVGVDACADFRTCVSTAACYEHVLRRQITFMPSIEAARANTSVSPGVFDAVLYFPHAVTPPAAP
DPDTPDIAADAAADPNLQSQPLGPLLQPPQQLQPQPQLQPQLQPSSTPPPASAAAAPLSPLLRSLGRAVLGMNHTAV
PPTRLLLDLFEVVPTRGNGLGLYRRYWFFSNLQLAVDRSLIGLHVAENTPVPPPDPDPAPSPPPPAATDDSPPPPPP
PSQQQQPAQPPADPAQRRRRRGLAADTTASSGGSSSSRTSTSIGGSSRTSSGTATGGTGSSSTAEGPNSSASASAS
SASAPSSVYDRAVPAAITVSYKPFPWPAVTEDLGAASAAVFFNLLLVYAFLPPTRAVVADIVREKELRLREGMRVLG
LSEASYWLSWGITHWVLLALSGALCAAAGTYPFANTSALIMLLFFWLYAAALVSYSYCLSCLFSSSRVAGTASQLLY
AASMMPGFVVPVVYQYGGWSWYAACLCPPSAASLFAAALVNWEKVAEGITARTLWTPIAAANSFSAGSVLLLLAADV
GLYAGLTWYLDKVLPKSYGQRLPWWFPLDRRYWPRSLRRRAPAPTRAQRLGAAAKAAAAADEEGGLLEPLTSAAGG
SAAAASGADAAAADRDADDLGGERDADGGSGSGSGSDGDVAVRVRGLVKTFLCASGGTTTAVDRLSMQIERGRITA
LLGHNGAGKTTTIHILTGMLQPSAGSATVNGFDVATQMNQVRSSLGVCPQFDILWPDLTVEEHLRLYGAIKGQPAKA
LGRIAREAAAGVGLEPKLSAAAGELSGGQRRKLSVAIAFMGSPAVVFLDEPTSGMDPYSRRFTWEVIRRHRAAAAIV
LTTHSMEEADLLGDRVVILARGRVAAEGTGMELKAAYGVGYNLTVVLQEEALRRQQHQQQGAAAAQAAAAEASGAAE
AGTGAYGGGGGGSAAVREAAAVEQQSRALGEALLQMARRHVGPAAQLLGAAGAEVVLRLPREAAAAFPPLLRELDGE
GGRAAGVASYGLSLTTLEEVFLAITAAAEQSQPQPPQQPTQPPTPPPPRQPPPGGVAELAQASTLPPAPTPPQATSP
PTAAANGPATAPASSPTTTTASSASSSASPTALALYLQQLRALLTKRALCAARDRLAVLTQLAVPLALVGVALWISS
LQVREPSEPALELSRPTALFGRPMALSAAPAARNASDGCVGGGGGGGGGGGGGGGGGGPAAPGSCLWAFLAGHPAV
AGGAGAGGAVDSGATALFAGDVKLRNTTLEDYLLSHWYSGGSSSGSSSGSGGSSRVVAPPLYDALHVTQLPPPAAVA
AASGPYGPGGGGGGGGGGDAAAALSFVLMTNQSAVSALPAALSGAMEAALAYISTGLTAAAADADTTGQAAAQAGAR
VAAQAAAAAAGGGGGGGGGVFQAVNWPLPTLPTEPVVQVQRDAASLMLVLCLVLASAVLSASFVVFLVRQPRLRPAG
GGGGGGVFQAVNWPLPTLPTEPVVQVQRDAASLMLVLCLVLASAVLSASFVVFLVREQDNNSKHLQLVSGAPASAYW
AANYMWDLTSYSASAAGLVALIAAYRLPQYSGQRLAAVAGLLWGFGPAGISLTYLLHFAFRDEMRALQRLNTAYFLT
GYLGFVATWIMDLIQLLVPHPGGVAAWNGWLKAGLRTASPHYCFARGVYDVQATFDSGFPFGKRGKPFDWDVYGSPM
AHMAAQAFVYGSLAVLHDTGLAAAAAARVHDAAAAALAAARRLLLRRTATCAAAGRRPRSSLLDRSSSGHDPTMGPM
GHLFERLLLAAPHSGGGGGGGGADVEGQRGREGEGEEEAHEDEDEDEDVAAEREAVQAGRRASSQVLLQGVRKSYGM
GLFRPPVAAVRGLWAGVPAGECFGLLGVNGAGKTTTFRMLTGEVAPDAGDARVGGASVRTQLAAARRLLGYCPQFEA
LPGAMTGREVATMYARLRGVPEPQVAALVKSLLGRLGLAAAADKACGEYSGGMKRKLGVAVALVGEPPVSALDEPST
GMDPGARRGLWSCLQAEVMGAGRTVILTSHSMEECEALCGRLTIMAAGRLRCLGTPQHLKGRFGGGYILDVKLIPRS
ASSAVLLGSPRTGATTTTPGVAGASSPPPLAPAAAAAAAAAAASSAVTAATAAAAGATAGALSPHPSGVAAAESADA
ARLQEWMSGVCPPAHVTMIDHNRVVFGIPRGGADLAGLFEAVEGARRSLGITDYALNQTTLEQVFVTMAAAAAAAAA
AAGEEEEMQGREGHSRQQQG

>Cre03.g205350.t1.3|PACid:27577398
MRRASGLIAHAGGRRGSTPVVPAAFEPTSGPGLGTSGPPSAGGPPGAPALPAQVSQGRSPANGIAAEAATAAAAATA
AAATGTGAGTGAAPQKLRRDAPSGCSGLVTDRTGSWQQQLGLAAPSAPGLVARPRGGSGSGVGGGVGGGTPHGTLWD
TALRQAAAAVAAAAAAATVAAAVPYASRDLAHRALAATGRAAGEASGQPGLGERGCSSCGGGGSSTAGGMGGGGSSA
PLIWVHRSAPSNAGLQTVAATAAATSVRHASSTAAAGDTAAAASDAADAGRSVLQPPPHQDLSPPLAKLLPAIEDRY
PAEMGVAAGWVKARVAAALVAAAGREDGRLSEEGCAARRLAGLYDWVVAEWRRDKGPATTLPPLLRDARALVAALAA
DPLFRLSPPPPTTPTTPLSPSWLSAQVGAGSAASDVSSSSSVGGSSSSGSSCGNGAGEEQAATPPEAVYVDIYVDPL
TPGRGGGGGGGGGGGGGGGLTAGGLGLSLWQARTQRRTYTEWRRQLQAAEREAAALRAALDKAAAAILPATTAVAAPG
GAAVAPAAAAVAAALGRLRARYRPGGYVCGTADEARHRVAEELLRQPGAEAPLEIAVAAVRLEALLEAARRLPAGLA
VAEPPSAAAVERVLRDDPAVFELVSSSGSSGSGSSSSGSGSSGGGGGGAGMSVRLRQWLLEADAQQQQQPAAAAGAA

ASAAGASAPPPAATADAALAATAGAAPVDSAVAAPPAVAEGAAAAAAAAQTLAAAAGGPPQEPAVGDAAAAATALQA
PAAEAVCVDAGAIAGSAIANAGSATATSAAATAAADAATAPTWLDKAAAVNKTLQQRMRDRGTIVSLPEKLAPLLPH
IDARYPLQRSAADAARNLAAKLLLCAPPCAALRLSQQDVANYLYAQQASAWPAAEQLPTGGVFPTLFAGDPAFDLRA
RAPAHALSTYRGRLTHVQLRVRGLGLPPDVEAAAAAHVTAAAATAAKAAAAEAAAAAAAEAAAEVTAEVTAAAAAAT
APVGSEGPAAAASSPPPPPPTGSSSSSSSSRSRSSSSSSSRSSSSSDSPPPRRTSPDAAAVAAAAAALVAGLDKDAAK
RLPVRRTPLPAATAAAAAATSQQQQQLAGAVPPADAAALAALSERLQRRARLRGHLVSLPPALEVLLPYIDTRYPP
DGTHLAWARNLAARMLLCSPPSAAPFRQDKSLVASYLGAMLRARTGQATGPLPDKFIAGVFHDDPAFVTVSGAGPAP
AAPPTAAPSAAAAAPPVDAALPSATALAPASSGRAYLELNLSALQLPPQVVAAVAGSFHTGTQLQPGTQLQPKAQLA
APQEGPVVEPAAPGVAAPSAAVAEGCPLPELAVAAEPVQLEPPPLQPPLQPAAAVAPDDEARTRDFLAAFLRRQGRA
ATADASEAAAAAAATAAGGAEGGAVPVPAAAADGLGHAAGGAAVADAETLGLVHSAPGPAPPPSATAAATAAATAAA
DGSAVWWKHGLNAGRAGAIAGAGAGPATANWSGRAGAAGDAAVAGQEQQQPQQQAATPAGGGGGWTLTGAWQHVRAA
WAAVSAGGVGGGGGPLTPPDAAATAYDAAQQFGAAAATQPQTSAATAATTAAAVHVPYGSSSNPRWSELHGRLQRLQ
AELRSLAPVIAGSNDATQQQLSGQWAAAASPPPPPPQPPRHPHRHHTAASTLLGTPLSLNDPDPDPDPADLGTSQLT
SATATTTSAAATAAAAATRHAAAAAAVGHADVVYPQTGHYYSWCKHTLVEVLARHGRYVAAAPPLAAAEGAAGVEVV
AAAALAEAARGPGASEAGAGVAAGPPRGPTLRMSLMAAGALMDQHYAARPHLSPTDPRTAARRKSLRHQLAAVVEDD
PRFAVLLPPPDADSAATAAAGSAATAGSGSRSGSGSSSGASGIYAELLSSRTAGGGGGGAGGAGAGAEVALVLDELE
AAAAAGQALDAVPPRIRRSIEKVKAAMSPMAVAEVLLAAPASIIRRLAAERLAATEAAAAMAAAPPPPWLTAAAAAA
SDVADAGVAVVDVGVEKARREHAGLLQLLREVAAPRPGEAAGPPAAPGGAARHLEVQPPRYYTRYWAAKSSAAAAMA
SGGGGGGGADLGPAGQETAVADVGEEPAVVAAAADVLAAAAEAAPATGVAAVAAEVAAAEVETEADPQSEHEATAQT
ASQAAAPSELAKEGDEQPSNRLGGSSGAATETGSSSSRGDGTSGSGGRSRVRSRGNGGGGGQQMHIDVGGVRVRVVV
LASAEQLTQHGGGGRWDSGSGSSSGSSFGSSNSSSNGSSNGSSNGSSSGGDGSSGGVLDHLSRVSHMAVDTEAAHRQ
LALLQIYAPAVEGHYPATCYLIDPLMAGSPAARRRLLAAVAAPLEDERITKVGFDPRADKELLYMYEIRMRRVVDVQ
EVYGVARAAWTQGSGSGAAAAAAAAAAAGSQEAAVAAGAGPRAGMGAGASESEAAVAPPAPPTAPAGATATLTREGLA
ASRVGLGEVLRAAGYEHTHKDLFRGAYNRGEAPWSQRPLTREMIKYAVQDVHPLLDTYRALLRHLLDDPAAQPAFLM
QQPQQQPAQQQAQAQQQQQQQQQEH

>g3634.t1|PACid:27577405
MCQAGLFIAGLCILLLLPQPSLASVQKASFNGSLADLVGVSANQGYDSPTYQQQVTGLGRRRLTQTSNSTSNTTSRY
LQSNCLCNKFAQVGNFTADYHYLCFTADACGGPQYSVPRNYLQSTECNATNTTGCEVLPTQYDSCRSWISATGNTSV
PCPSYPCCQTVQQPANVTYCASLGQYCFNVGPSPDGSTYDRFCTTGPQQTTGLQQTCTVKLVSPPPSPSPPNPPRPP
RPPSPNPSPPLPPRPPRPPPPQPPAPPPSPPSPPAPPSPPPVQEPSPKPPSPEPPSPQPPAPSPPHLPPPSPAPTSP
APPSPDPPTPAPPSPAPPSPAPPSPAPPSPAPPSPAPPSPAPPSPEPPSPAPPSPAPPLPPPPSPTPPSPQPPSPFP
PSPVPPSPAPPSPAPPSPAPPSPEPPSPELPSPFPPSPAPPSPAPPSPAPPLPAPPSPAPPSPAPPSPAPPLPAPPS
PAPPSPEPPSPVPPSPTPPLPAPPSPAPPSPEPPSPVPPSPAPPSPAPPVPPAPPSPAPPVPPAPPSPAPPVPPAPA
PPPPSPLPPAPTVPADPPLSPEPPSPEPPSPLPPSPNPPSPAPPSPEPPSPEPPSPMPPSPHPPSPLPPSPAPPSPE
PPSPEPPSPMPPSPHPPSPLPPSPAPPSPEPPSPEPPSPMPPSPHPPSPLPPSPAPPSPEPPSPEPPSPMPPSPHPP
SPLPPSPAPPSPEPPSPEPPSPMPPSPHLSSPLPPSPAPPSPEPPSPEPPSPMPPSPHLSSPLPPSPAPPSPEPPSP
EPPSPMPPSPHPPSPLPPSPAPPSPEPPSPEPPSPMPPSPHPPSPLPPSPAPPSPEPPSPEPPSPMPPSPHPPSPAP
PYPGPPNPAPLSPAPPSPAPPSPAPGLPPPSPAPPSPAPPYPGPPAPAPPSPALSSTAFGVPPPSQFTAPPSIWVGW
SPPAIAPPLPYLQSLNSTQPPPQLPPALMTPAAAPTLNVLVPPLYAPGSWAGHPVVPPPAPPFTWSQPPSTLLQPLP
GYPPSPWPPTAAPPPPPDAPASLPAVPTPRLPPAPQQPPQPEPPASPQPPVARPPPVSPPLPAPLLPPPSPLATPTP
SPTSSASPAAPPSAPAATPAAASLPAPPPMPSPAAPLPPPLALALPPEPPVAPPAKPQVPPSTPSPKAPSPAEPPPP
SRQPPPASPQQQNSTGRSNSSGSGSSSSRNTTGGVMVSPPLAPEAPAAPDSPTPSTGSTGDLQSAHLPPPPPPPPQP
GSGTAGGKPSNRRRQAVAVAVGSIFGACVLVCCCAVLYKMYDRGASPRRGWLGMGGRGRRDRSAAYREEPQGSYAEE
PTKGYHWSQRDKGVQAGPGGGSPYPPPPPVPLGGGASAAAAFGDRRKGGGATPRELLYGDERYGGNGNGNGNGGGAM
PWHGGGGGEKYGDGRNDNVRAYISNGPPPSPGRRPPPPPSPRGHSTAEVYPVTWEGSRGDGYGHAGGYPNGYGDGPH
GNDGGTAAASRGGRSEAGLAAAVGGALTSCLKVCSRGGGGGGGRGPVRYDDDEYYDDGGNREEEYDDVRYAGRTGGG
GAVASGGGGGGCFGRGAANAPPQGRHGYGPSKMPYPPPAGQVYGPPPYTDGYDDDAYDDGYYGNEGLDARWPPPPPS
PRQGDSRRPKMVMNKKVQADIDDGAGGGGMTAPSVDVAAAAAAGSRQQRQGSREHQQRQRQAAATGRRDAAVGGGLG
LDGGGGGGPCDDDVSVGVTDGGSDISSLAHRQPQRRQPPAAAAAPPPPPAQHPQQRVHWAGGPDGDTEGDTEGDGAS
EASFATAPSARVQPQTPRHGLVMALPPEGRQSMAVAAAATAGVGQQSPPLRPKGSRSRRRGSAVAPEGGPPAASPRN
RSRAVEHATDETNSEEEEEEEVEEEGDNGAANTPRRLRAAAHVPSGGASRRPSTGARPASGGRLRHNTAVAASASAT
ATGTGGDETPVVDVSDEGYVQELLKQVNGLLADMNAGVVLGGLGSGGGGAAAATDESPPAAVASRRPSVPPRGSVSL
SRGLSEAALAAAAAAAAGGADLAPLPPLAAAEAPPPRSSYSGMSGISDIGRHVEEATRAALGRLASHSPSPTSAPPPP
YYGARSVPSSPLASVSYASPTRAGGGATGSVGGGAMGSVSYSSFPYGSGGGVGGLQQQYDMTLQQQQPSRSPSLLQQ
QQQQWQQQAQGSSVVSAVAEESDLDREYRAYYDSSASATAGAAAAATASFRHQQHQQHQRRYQPSLLSQQDPQQPPA
SPTTMLLAGGHRYTAFDNPLAELAAAQPAPAPEPHMGGGGGARRDLGGSLGGARGGGEGGPTARSTPLLSDVLFSSL

APELAWEVASALGGVGLMGMDGEQQQQQQPQHQHEQHDGDRHHQQHPHQYAGVDGYYDHDDAGEGGVAARRVALPSM
PQSPHTATAAATRPSKTIINIPPGAEADELIYRHHQHQQHQHQHQQQQRQQPQHFAAAAGPHAGRPASPPASVSSVL
ASMTSSYTRPREHSAVCLSDKLRTLASFATDAAMQLQTFAPRLTASTSAGAEGAEPDGQQVRLQPFAAGNDTPQGRA
STALSAASAAAAAAAAAWPAGLHGDQQQQHHQHGDSGPYARAQSPSLSSIADDYTPAAAAAAAPLAQQQLLQLQHAR
EVVSYSQPALAMGATASAQQQLHLQPPSARMPFLSSPGPISGVGGNSNGGAVKPPPQPPTPRHGSTAWPAGGVGGSE
GASPLPDVPASPPLPPRKTGSASSSGGNGSRGVANGSGNGEGCASPQHQQAQLRQYQQPQLTPGQLRARMLSEEVER
RQAAAATMSANANLTRHQHQAGSGDAYNAAGAYLQSPGVGNGATGGGTSSGHQGRGGVVEGDEDASFLAMAADVLQR
QRSSLRLGPGAAAAPDPNCPR

>g3447.t1|PACid:27577415
MCASQICGGPYASNVYRLDVLSPPPSPNPPSPPLPPTPPPGGQTGLYYGCFHDSDKRTMPVVLAWDKKDLTLEDCAA
LARAAGLRLYGVQFSWFCFGGNDLSLATSLGHSEECTRPCGGNSSQVCGGPYTNGVYIIHELYKVQVSDYMCGSVTT
TYVNTVSDGDLAAYTKLAALAARTDTNSGPMWVEISAYAAAGTPLGALHGDSSSSVGAEYLVSNVDGLDIMAVRACC
AGSSGWGNAAQTLQLQLSNGTILYVGSSTLCTSGQSWVPVPAGHWFAGIQTQSQPSPDNYVHRVAFLFAAPLLLPLP
PAPPSPSPLPPLPPPSPPPLPPPLPPQADAGATFKLIRNSASELCLVAQPWPGAGLLTAPCNPSSLDQQFWLDADSD
GSSYTLKLRANAGLTLAYFGGLDSPPPPSDRRVTVAQYVCESYQAAHLMAPYVPKQGLDQTFEFLDGNSAKHAAPPV
SASTCFVPVLASHVAR

>Cre03.g191600.t1.3|PACid:27577436
MNDLDARAFVALDGTASSLLRPQPEALCLEACNGGLAGALARRQRSALVALACPDLPPSALSDSGLHLQAQQRARCA
LGRHERACVSGYRPHLRAAATARAVMAVDAGLAPDEAAEARERQELSIHNVAEDVWRSVYQAARSYEGHHRAFKEAD
DYSMEESAALMGMRDATLGLREAVRQVDERDVAAQLAALQPTSAAEAEERQRTTALGPVLSQPLEGVSTPAWLLRAE
AARMLPARAALLLHCHAVLGCPPVDMEEACKKCNVSISGDPSTPACGRSAASAALLAAALLRYWQLQTCGAGHGSGG
GSGSRSGSSGVEARAWLSSDFTDFAIVMSALQALALWLDHGRGVGATGALAALTRPAVLLRVAAVLSHDVVAGTPPE
HLDTVVRQFVMQALQNPVREAVYTVLGLDAKGNESEDRKAAAAVGGGSGKAAARAPPAPGTAASGDAGLPDEEAQGL
LLERVRSNFITCQQRARKNAKAQIEQLAAAGRKNKAKAGCSEGAATVDALGEELANQRVLEMRVAAGERLVAALFDP
LPPLLASTPPGVSGAAPRGGQDVAALAKSPPPPPPQPAQVVRMLWRQGSALLPSRTQGKAPPDIVKIQWDTEVAEVA
GQAALARAMVGALAPCWEANMGCTLFGAAAPCANSLTACNPTYSPGPHSSLVLQGSPAICYSLARTAAVNYVLDRVA
RGMAAAAAIEAAAMAMAREGLASECVGQASQPLPTAAAWCKQADRPAVTAAPLPTVAAAATAARVASQLPRLPYKEH
SEAVGLLVAAIALPDTLGFFHRAVGWPLVMTLSGSEEERAQHARQVPAELLKPLDPTGVETQKLGRVCLTANWLAGR
EPGRGLVAAAEATPATDGTAAGGGPAGGDTTRGNARDWTASTGGASSLPEAVTKRAWQSKTVSIYGGSPGAITRAVC
DVDVEVCHSALRACCLKPSNAHQTAEAAAKLAVLSRELRSDAAAPRELVVVPLLEPGALVCDTDALRRLRGIVCLVL
RDLTQPAPAAAGAAAPGIGAGAREQRPPAHCEDEDEATEAAAVAAAGFKAPRVLRPDWRVRMPLILNRALCRVLGVP
LGAIRWQLSELSPPPPDGGAGVQQGVKQRPLFDNSVGGGDGGGGAGSSNGAGAGAAAADLAARLQRAAQYLQSRAGR
GVLFQPKIDSAGEGLGAAASGVHVGSNGTAAAKALPAAQVQAGLLALQEALADWKGLDEERIAGDGDGGLPFMLASV
SRSDARRLLHSKAYRDLLGNKLDNLVPCEEGDSGYQGMYYGRQCAQSLQLLIVEGCVGRPTELLAERPAGEVAAAAV
GSRWDELD

>g2885.t1|PACid:27577510
MLDERGRGAPGQAFAPAPAESRFASVPSSLHRGAFAPGPAQHMQHVGYASQQLQQQPQYMDTGASEPPPMSAVPQA
PPQPQQLHGCQAWLDGSDPRQQQQRQWAQQQWAWEQQEAAVAHPDEQQQQPQQQPLFREWLPRAASDSRLEATAGPT
GAHHDQGPRGQGDSQSLGRAAGTYPGPAVQGASGRPLGPPAGQPPPPAPPAAHLHTYHQHRYHRQSHQHSYQQPQEQ
EHDQQWYRSTGGGYQAAAAAPWGPGDRGPHPPGGTGGASGGTGGTVAAMSGGSGDGSNGRHGSSSSSASGTTATGAA
PDTASWGARGPPVHSAWAAAASASPYPQPQPQPPQCRAPQQPRIPPLPLSECSGHEAMEWQQQQHPPQHPLQHQQAA
GPWLPYGHPPHAPVPGSSALAATGRSHSSGPYGCRQLTAAPSAFASAFAAHAACAAVGVWESPPARSSGARQQLPGD
SGSGGGSGGGSGGGSGGGSGGGSGGGNPPQPYDVPAQPQPHQVPPGAYYPSPQHTPSPYSSSALSSPPPPPQQQQPH
MPTPPHLPQMQRGKYPSQELPRFCSPPQPPPPQQGYGPPPHHQHHQQHQPQTPQQPHQPQQPPQPLLYGVQYGAPPP
SPPHGQQAGPYQQQQQQQTQQQTQMAAAAVGRPLLPPAPSSPAGTYPYPYPYAYPYPHSHPHQPPQHPYPHHQAWPQ
PHQPHQPPTSMAGPPGGPPEPPPPQEQQQQWPSGDWQQQQQQQQAQQASPQPPQPEPLNAGLAPSPPQSLSCRGKPR
AKARARPTKAAAGGGGSGSGEGALAVIPGQPPQPQQQPQEQQTFGATGDSAAAMGVVGGDGGGAGAGADGAGGGGGG
GSGGGGGGGGGGPEGKRRNRSGPKSRSSPYIGVSQYKRTGRWEAHIWDSGGGGVGGDASGGAGGGGGGGKGRQLHLG
SFPTAEAAARAYDRAALCMRGPGAEINCPLESYTHDPVLAQLRQMPKRAMILAVRATANTTDITGTATGPGGASAGA
GDGYGPLGGGAAQAGAATAALAAAAAYAAAVMDAAAAPGACAGAPSAAGCAPVGWGGQQQQQQQQQQQQQQQQQEWL
DQAASAACCMGGPGPSGHLGGPGHITAVVVKAEAEPEMECVGVGAWQPPEGEGDRRMSASPPPPPPPPPPLPPGLGG
RVPSLLTSPAAQAAAGDAGEAAAAAAAAAGLLQTPLQPQQQRQQQADDVDMLLATPPQREPQLGPQQPMQAAGGGCPP
QLNQVSTSAFDAGTAAAGTAELRTGAAATHDAARHDAYRCQQQYWQHPHQFPAGHVRAVTATRAASGPPQFWSQQAQ
PPTHVGPPPGGAAAVQLQPQAQQLQPNQLQPQLRRSHHHRAAAAATPGSTGARHRRCSAGPAAAAAATSLSGSSPVS

AAAAAAAAPAALVAAVAAASHGSSHGSPRSPPATHHGGVRKHRAGGGAASGVTSSSPNSVSGNAAAAGASPLAAGAA
TAHGNARRGAAAATGVSCALATGLADERAAAGAGGGGGGFEGDDAARGMVTVAGLPVPPTAATAAASMGAGAWGAAY
TPPSSPAGVPPLAVVPGRAVGQLPPGRAYSGAAGAAAAVEARPAPAAAAPGQAAEEEAAQQPAAGPQPHWPPPEQQQ
QRAHPSQWCWPHTPVVATGVEAAAPAAAAATAATAAAWGAQPCAVGPYGGGGVGAAAGSPQHTAAAATADADASMVM
TRAESFSGPMQPPPYMQQQQQEQHQHQQQQQYAAACPWLYTSSVPPYGQAAAEAPPPQLGAVSASPHAPAAAAAAAA
AQAGAAAAAAAAAAVSPLRRLNLNPAASAPLPLPPFDVAAAAAAAASGPASTSRAATGAPAAGSPYLLVYGQAAVTAAA
AAPAQAAGASVQPGQYQQQWGPVAGGFWPHSSAADATPPPAFALGPGPGEAMAMEWGTTPAVDRAAAYLAQTPAAAA
GTTAAGNSAAPHHLQQQAAAGAAAAAAAAAAAATASSCLSAGLAAHTTLDWPPPQQLLQPPPQQLLQPPPPLAAVADS
AAMVTGGSPCGALAGRAGLHQHIGTGAMTGGPPQQQEQQIRVAGRAESAPHPLTMLPGAEVALVMPVPSAAAGAVVG
QQEWAVAATATAATSFPRTALPAGSPPPPPVPRHWSHATAGAAAAPPAATCVSGMGAAWGGLGAGAAAGTLSGGVGG
GVGGSPASQLPNMPSPDGAPREPLPPPPSAGAWQQAFTVAAGASPAGAQPPPATPSPTQPCLQPYQLQSPYPQHQQR
PPQPTAQQHPPPQLPLPYLSAVSGPTPSVDMAAAAAAALDATHAANANAAIAAGSAFPAAAASFPAAAAAASFPAAT
AAVAAAVAAAAAPAGEKAPMPSRASVSLGALMEAWLHSEDSGGGAGAGAGGAGGAGGAGGGAGGTAGCAQQGVD
APAAAAAAMELQLQQQQQAASVEAAGGEVPGSGGSDGCGGGGGDAGSLLGTRQSSSLSSLGGDEDMEALERSCGLVL
DLGLGLGLEEGAPGGCGGGGEFTDDAGGGVGCGVISAMAVGGSEMMMGVDTAGGAGAGGAAEAMAGVEALPATGLAA
AAAGAAAAGTQSQLALPPPQCVAASGERPPGPQQEEAMDVDALAAVPAVQQQQYQQVPEGFPAGRPPVLPAPPASPF
GTVRGLMGAAAAAVAAGSAYYGGLPCGGSTGGQEQCWPAVVATAAAAGGVDASPMAAALAAHRQRQAAAAQPQQSPP
QPPPMQQQQHPYSQPPSLGLIALQQLPSLPDFGSLDSSS

>Cre03.g157751.t1.3|PACid:27577561
MTLDLLLADIADGSILTSGGDSLVCFEKTGQRRWLFRFNRKTEVVADNQTFTYSARQDGASPFIAVSSTGLVYLAAA
DAVLYALDVRTGDLVWHWRDSPSTFESITYGDGRVYVSTTDFKTYALDVVTGEVLWGTQFRRVSSWTTGWFKGVFVR
PDVDGRVWGVDAETGDLIWMFNMGAEIQYRWDPELHFHPSGIGFMMARPSQIWAYNVTSGDVLWKMNAREGYMSPDN
DGDNRQTFEVWGDLVIAQDEGAVETNPNKYGILQALDVFTGVRVWIRDDVYPGKYGMVVTEGLLVYTTYIPDMSTLD
VSSSLLISAMGPDHQDVWNWQRDGAIPAGQIQALDGLVFALQGEIANTTFNGLGSSTVLVALPATPSVICPPDVKSP
PITVNNNQQGTNAVIAPPPPPFVSGKPFCWTHSFSYKPLGSPTSDGVRAYGQDNAGLVIAVYLDTGAIAWKATCGPD
SNSLYYPSNVVVVDGVVYLGCSNLDVQARDAATGHLLWRRPFQLTPDRAAYRGGIIGNPAVANGKIVISKPDGFLQA
LNITNGQLVWSYDTGILAADSVGIRDGVAYAIARHNAEVRYSTIVAVDLDTGVEVFAKPLEPKWAMEPGRLNFRHDN
TLVYATWWPDAQLQATTIGRDGGDLAWNYPISDSTGVYSVLVVSGEDLGLPHDALFYNDNEGLVTALALGSDGPTEV
MWTVDLTDARLWPGVENLLTPGQSDWAQITYSNQTLYVPTTQGMFVVGATNGEALWSDLRSRLNANIHVIERPGGPQ
VIYGRYLSNFESNTPQCRPPKPSGLVTKISVRGSVPADGGEEPSTPTDAPAPDSRPVPVTASPPPSVTPAPAPSPSP
SPPPSPSPAPVRSPSRSSSPPPPPVCPPCPAINCSAPAKRPSSPAAVAVALGLSGLPYNIVKADSKLRDRVAGAVEK
LVSGKVTGFVRVRVLGVESTAGGTAAVLRLSLQRSGGASATAVASAIEDAALNGKLQEVVLAAKVPAKVRSVVLKV

>g3002.t1|PACid:27577621
MARQQHQPSQLRATAGCAGLLVALLLVLPQLCSFAAAARISSSSSSSLHSSKHQAVDRQQAISGSDGDGFDGVERGD
HGAVGSGSSSRSSANAIPGGAWGRRQLLTAGGKQAGKPDKASGKSGWPGKQAKPGKQQQQQQQASYPAAADDWSPQP
QPQLQLQNDGDLDDQASGGLHSGVGGGSYNYYQPRAASPGIPNTSFALPPTYYTNTPSPSYPPPPLPPSPRPPPPSP
EPPSPRPPSPEPPSPSPPSPSPPSPEPPSPIPPSPRPPSPRPPRPPSPKPPRPPRPPRPPSPPPRPPSPPRPPSPPP
SPPRPPSPPPSPKPPSPSPPLPPSPSPPSPSPPSPEPPSPSPPSPSPPSPSPPSPTPPPTYPGTYGGGSYPSYPPTP
AAGITPSASPPSPPSPPSPPSPPSPPSPPSPPSPPSPPSPPSPPPSSAGYFELIQNLTAPNVALAMANIPYTRRYWF
LERAYGPGEGLGTNPSTGTLDLDSREKLQFNAIFNPGCSGTIILPDGHLHTFGGDVLSPVRNLQDGRNKIMAFNPSS
TAMVQLGTMQKNRWYPSPIVLSSGKVLIVGGSNACLLPPTWPFAELWDPATPASPTVNVTMPANFVKYMGLYNWYPF
MQLLSNGDILWFVEKGGAITDGNFNHIVDLPPFPASITHCTMFPKTSSISVLAMGPPNYDLSFVIFGGGDCSGNLTA
PAASTSLRLDISKCGSSYCFTKGWEVEDMLGVPRVMGDSTLLPNGKVLLHGGAQFGGANAGEGWSTKANFQSLMYDP
YKPVGQRYSKMDFAPIARVYHSANCLDPSGKVLVAGCENCGAYQQLAPNMSLSPDAPLEHRLEWAVPAEIAPGVNRP
AITYAAPAVDRGVTITISYSYPGVDFITGAVLAAPCACTHSLNMNQRVIFLEVENILQGKVAITAPPPGLFGQALLG
PHLLFLLGAGGTYSEGVWINITDPAVINGGSR

>Cre03.g192250.t1.3|PACid:27577677
MTSSWLSKRVAALLLLVPLLAAGNHVRGSDVLGRRARHLAADGPSNATSANSSSGLTPSPAAANTTASAQPSPAALP
ATYGSQPPTAAAPPYYGGGGGGGWAAAPPYGGLYGGGGGGGNVTNKPPSPVPPSPAPPAGPLCHQPACFPALSAAAT
NATNATNATNATSATNATALAVCPYLLLTAAEWAAAAADAADPKLAAAGRVLAANWTQLLESGYFRLGSGTPADLTA
NTFLFTEPPALTCFLRRQLAAPPPATAPALLPLRAMVRVARPVPPPPANSTTEAGSGCTLAEPVAYYGPWVAEMLAL
KLNLLLQPSLVTPQPPPGGAPALGAAVFAQLAPTANASAANATVQPCSCMTVGSVLAAAEAAVSGVTVNGRNATIWS
YDSCARAIALTFNNQTSCAAAAAAGDRALCTPLSAPPSPPRSPTPPPLPPIGCHGASCYATQTPSPSSCPFLAYAPE
DWAALNRSTPAARLLTLNWTSVFATPGANGSLTLGRPSAGPQLHLTSPASVQCFLWLQLRAPPATQVPAPLPLGGVN

VTFTARVLDNGTAACELLPPDLLRLGVGTWAAELLAMELDARLGEALASSNVTAAATLTAAGARYQYASELVRPSLA
GSIFHASYSRRQCACQSVRGVLNSAWALYISNDTAAAPAAGGAVSDCAADLLRTYVDGTCTSNRSLPLLCPGLGGGG
GGGGGGGNGTASPSLPPPAPAAVPSNATSPPPASRLPPAPGNSSAVGAPPPSPAALPGNGTSPSPPSPAPPPSPAAL
PGNGTSPSPPSPAPPPRPPSPPMPWPPAPALACTGPCIAPGTPCPYRLYVGGAWSPGNLTSVNAATRLLNNTWELFA
DTGLPDDPTFILGSRNGSRLEFLDPASLQCFMRLQYSRSSVLMPDGVTPANVTNATLPLPLTRVLGFGATINATTGR
CVLAAAEDATRFVGAWLADLVAFKLNLRLSAALRAGAVPGGENASVALGDTVFANVWPPSPPGVLSVRGCACAGVTA
TLAKAEAGLAGLPGSKVNDAPAAAGCGNAVAALYTDNTCARVPAVAPPLVCAPLPPPPVPPSPPVTGNAPPVPPVPP
SPVPPSPQPPSPSRDIPTPSAYPPPPSPQPRPPSPKPPVPPAPVLNKPPSPRAPSPSPPSPAPSPRPPSPRPPVPPS
PFPPAPPACTDTCFPPGQPCPFLVYGAPAWFAPYDASPASTLLNQYWANLADFNDDGIFILGSSMGPHFEFLDPDSL
SCFMRLQYNASSVQPPATPGAQLPLTTVIGFGASLDPDTGACTINTADGTGTDFFYGRWMADVVALKLNMKLSELVA
AGEIAGVPPPPLGAPLLGAALFGAAWPEAEPGSPAAATAPCGCHSVGATLGTAEGYIGGADALLTEADAATAGGCAV
GLTEVYGAAVCGAVPPPGPPLLCTAFAPPPVPSTPSSPIPPSPEPPTPKPPSPAPLSPPPSPSPPVPPSPAPPYPPS
PKPPVPPSPAPPYPPSPKPPSPPPPYPPSPEPPVPPSPAPPYPPSPKPPVPPSPAPPLPPPSPQPPVPPSPAPPYPP
SPEPPVPPSPAPPYPPSPTPPVPPSPAPPYPPSPMPPVPPSPAPPYPPSPTPPAPPNPAPPQPPSPEPPSPSPPYPP
SPQPPVPPSPAPPYPPSPEPPVPPSPAPPYPPSPEPPSPAPPYPPSPEPPVPPSPAPPYPPSPEPPSPAPLYPPSPE
PPSPAPPSPEPPSPEPPFPPAPPACSEDCVAPGTPCPFLVYSASEWASTLERTDAAELLNTLWPLFADFEDLIWIFG
SRTGPHFEFLDPASLQCFMQLQLNVSQVLLPNGTSVPPPSEVLPLPLSTVLAPSGAINAAGQCALTNAAEAGWYMGP
VAANAMALKLNVQLSRFIADGGSVPQLNLSSSGSPVSLHQAVFAHVWPPLPTDAPGAGTGACGCAGVAMTVARAESF
VGAETDMGASMEDAINAGVCGAALVEVYGAGVCGAEPAVGPPLLCPTLSPPPAPPSPEPPSPEPPSPAPPYPPSPAP
PYPPSPAPPYPPSPEPPLPPSPAPPYPPSPEPPLPPSPAPPYPPSPEPPLPPSPAPPYPPPPSPFPPAPPACDTGCV
APGLPCPFTIYSGAEWSITSDFSLAAALLRAYWAQFATYADDQIWIYGIPDGPHFEFLDPDSLTCFMQMQYNASSVL
VPASDAPPAAVPLPLDTVVGFGAVLSADDGTTCVLASAEDAGKLYGPWIADAIALKLNKQLAELVAAGEIAGQVPGP
LPLAAAQFAKYYPPSTNASAAEARAGMCGCSSVESVVAASEAFVGGAEGATQEAGEAAGVCGAALTEVYGVETCGAE
PAAGPPLLCMPLSPPPVPPSPEPPSPEPPLPPSPEPPSPSPPYPPSPEPPMPPTPPPPYPPSPEPPVPPSPAPPYPP
SPAPPYPPSPAPPYPPSPAPPYPPSPEPPLPPLPPAPPTCTGQCFGPSNPCAYNIYGAHDWSDTSYSAWPAQALKQS
WPLFATYQGDDIYIFGEPDGPHLEFLDPVSLQCFIIMQFNRTAIAPPVRWPDSGVDGDGNNATARLPLTTVIGFGAS
LDPVTGRCVAHGPADLDLYYGAWMGDAIGLKLNVRLSELIVQDLLTPQRPHAPPPPSPAPPSPVPPSPAPPSPAPPS
PVPPSPSPPSPPPSPPHPPLPPSPAPSPPSPAPSPPHPPLPPSPAPSPPSPAPSPPHPPSPPSPAPSPPSPAPSPPH
PPSPPSPPPSPPHPPLPPSPAPSPPSPAPSPPHPPSPPSPAPSPPHPPSPPSPAPSPPSPAPSPPHPPSPPSPAPSP
PSPAPSPPHPPSPPSPAPSPPSPAPSPPSPAPLPPSPAPSPPSPVRQSPPSPAPSPSPVRQSPPLPQPTMSRVLRRR
LLELESMPPSAEAPSALPLFGGDNDTIVISPRPPQPPPLYSAFFGQVWPALPPGAPGAGMAACGCEGVAYVVARAES
FVGAGNEGATLAEAKSAGMCGAALVEVFGADVCWRTPPPGPPLLCAAPPAPPPSPEPPSPEPPSPSPPYPPSPEPPP
YPPSPEPPVLEPPPPPRSPRLRPPPPSPPSPKPPRRPRPPPPDAPPPPDAPPPPLPPSPRPPRSPRPPPPSPAPPPD
VPPPSPKPPRRPRPPPPDAPPPPEVSPPPPPSPKPPRSPRPPPPSSPLLFSPPPPPSGSGQTNEFPFYKCYPKQGM
PYQLALSQQYVDNAGLNRYCFRFNQITCNQNQNLCCNMYFEKTEFLIDPVCINTFQNTTVNGNTVFASFNTYDRGTA
FYSIAKIRLGFDQATANNSEFCFSLNNVCSTLQSLCAGDSCMYTISNTADPACCPVTTQVWW

>g3056.t1|PACid:27577708
MDSGCGPRPAVVCSAAATLIQASAAGGPIVWRCAGCGKPGRAPQASCSALAAAQASNCCCGASTSGGHRAAAWARGG
VTSPCSQTAAGASNSSCCIWGYADVPRRTCSQAAPLSEASCHGSSSTSSSTSEDDATQRPSSCEACCSCGSRIREAA
HDAAYSTRLGKQNIGGCRDQRSQWRHSTAQPTAPAVAGASAAKAPGKHESVGTAAAWSAAVRRPCRAALLVLLAWLT
FTASPAAAAATEAAAGIGTGGAGMVAVAEGPRWVPHRLDGGATYTTLDATAKYERQEAVVSSRQMLQAAPVYPPPVL
PTAAPPMAPPPGPPSPMPVLPGIPPPESPEPVVPLPSPQPSPVQPAPLPSPDGLGTADAPPPPPASPVVSPSPPDAP
DVASLPAPRNCRRNCPPPEAPEPRRPRRPKQPPPAPPPDLPTATRPPALPAMPPSPPSDTNGEGNPGLPNFPDPSPP
VPELAQPLSPELVAPPPLSLPPAPQPPQSPSSPVPTAPTPPSPEPPLPPSPFLLAPQQPTPPASPAPPQPPSSPWPP
PPFLPPSPLLLPSPAPTSPEHPSPAPLSPPLLPPSPEILNPSPAPLTPPASPPPSLPVPLPLFNPAPSSPAAPPLEP
SASPPPPRRPRRPKPSSPAPPSPEPSVPPPSLPPPPPPSPAPGTPSQSPMPLSPAPAAPVPPSPAPPSPEPPSPVPP
SPEPGTPLPGAYIPSSEPGAAQPSATRPPAQPRSAQPDPWDPSSNPNATKPGPAQPGPPAPQPVPPAPAPPPPPCRL
CVSLRVFPGPTVSLPLSITTDDMCNAGIDFAAMVNDNVLSQAVTEPGGGDFEAFVPPDGEDETACSAVWNVDTNTNP
FTTMCGDFTPGGDAIRMLNEMSVDDWSQFFYFLRGDDPCARKLAGMTSVVYFEPLPASCFAHEPIVSPACALDAPPS
SPEPLSAEPPSPEPPSPEPPSPEPPLPAPPSPHPPSPAPPSLEPPSPAPPHPSPSLPPPSLPPPSPPPPHPPPPPYP
EPPSPPPPSPEPPSPHPPNPPPSPAPPSPAPPSPSPPPPSPPPPSPEPPSPPQLPPPPPLLPEQFIVTVNTGGGTAA
SCARIQKFVNDTSIAAIGSLPDLFRCRNVTSSRSSPTPAYSATSIFATQGKAMSVMNAVQANMSAFVLAVAVGCGGN
VLTASTDTISFFLCGGGATGGSNVPELCCPPPPPLPPSPQPPQPPSPEPPQPPQPPSPEPPSPEPPSPAPPSPAPPH
PAPPHPPPSPAPPLPAPPGPPRPPPPSPPRPPSPQPPPSPPPPAPTASIEVRMSGLNLADIPDLDAFKTQSAAEVKN
ATSAATATTIYVLPGSIVAGFQLTFLATPAAPPPRPPPPPGATGAAPPEPPAPPPADPLAALMELAAEPDRLFSPAF

RAIYNVSTVTAFVVAMPPLAPQQQARLPPPGDAQGETLISPPPPAVIPPPVASASSANVAPIVVAAVVPAVALIGLA
GGLYYWNKRRHLDRAMAELMAVEVEGEVHVDSGGPDGSGGSADSMGLRGIRPIATQEVLPAERR

>Cre01.g052650.t1.3|PACid:27577718
MPRARPTEAAAAAAEAAAAVGAAGAAAAGARQPMQSNGSGSSSGGGGGGGGGRVGAECADGRGPTGDFRAAAGSAAA
GDAAGGEATASPTPGATASGSAAAGGTAAAAGSGGGGGGRGSFMRCSSAPVWQLQPSLQPPAAAASGPAAPATPGNV
AGASATSTGGATPIRDISGRPSSSGCSPSSTSGSSSGGAGSSCTRTSTCDSGGGSSITSSSGSGSAFSNSATPGGFA
GRLRLGVCAMARKADSKPMREILRRLNTFNEFELVMFPEEVILGQPASEWPVVDCLMAWHSDGFPLSRAQEYVALRR
PFCVNDLGAQELLLDRRRVYRLLQDSCIPVPRHVVVSREGLAPGQSPPGFVEGEDFVEMDGVRIAKPFVEKPASGED
HNIHIYYPAAMGGGVKRLFRKVANRSGAYDPAHSGAVRREGSFIYEEFLATGGTDVKVYTVGPRYAHAEARKSPVVD
GRVARSADGKEVRFPVVLSPQEKEVARMVCLAFGQKICGFDLLRSERGGSYVCDVNGLSLVKNSTKFYADAADILRS
ILLQALAPHRLHLMPALPATPTFGLMLESQRTMAAAEAAADAMAAATAAAVAGGGAAAAAAAVAAAGDGGSSIPDGG
VELIRAPTWLRPGGGGEGSSSSGGGGGVSGGGSSGGGLPGEELRCVLAVVRHGDRTPKQKVKLEVSHPAFLALYQRH
VDPSKPPGPHRQAKLKSASQLQELLDVTRQLLQQHPQLQPQPHPQPQPHNRPSSGGGGGGSGAASDGGAGATPAPKA
GDLPAGQQKAAAAAAAAAAAAAAPAAAGQQQQPQQPPLVDADTLAVLAVVRGVLEAGGRFSGVNRKAQLKPRGWVTA
AAAAAGEAAPPPSPPPATAVATGVGVGHVDERLLQGRQRGDSPAALGWASGSSSSAAVTPAASRVGSMTPPPPAPPA
SDSATATISASDAATASATASATASGPAAATSPAAATAATAVAGGGRPGGGPSSLLLIVKWGGVLTHAGRAQAEMLG
VRFRCGMYPRAGPAGGGLLRLHSTYRHDFKVYSSDEGRVQTSAAAFAQGLLSLEGASLTPITASLVNKDALLLEAYG
KGASADMAAAKQELYARLTWDPEAGVSRCKPPGGSSSGSGSSSPAPPPAATALASMPSLATSTTSGSSSSQQTTPLP
SPLRPTPPAASPTATAAAPATPLPPSQSPPPSRPAAAVAAADAAAVVQNTAAATAADTLSTVRPLPDRPLELLQLLR
AQLQDLVEAMEGPAVAAMNEAVAAAEGGGGGGRGGADDDGGAAATAGEEGAIAATAAAAVARAGVTAEVAPAAAAPA
AAAATAKPTAQEQGGGRGGGGGAAGSSNPGGSSRSSSGSGNGSSSGRGISSGCYYSSLSMSPDEWRAVPGVPCGGEH
WMLVYDRWHKLLDSFYSAKKKQYDISKIPDIYDAAKHALIHNAFIPLTPPPPPPTPPPPSASAATQPPPPPPAQQPP
PPPPPQQSHHQQRQAPQQPQPQPQPQPQPQPQPQPQPQQPQQPQQQPRSPPDGVVPLLLGEVVAPLYLTARALADAV
VPHEYGLTPQAQLHVGSAICRELLGKLLCDMGAMREESLATSYSHTGGGGCSSSGYNSYSSYSSYSAGGSSGAAAPA
AAQRALAAAAAPAAPPRRSHPGFIAQPPAAHTPQPPQPPQPPQQPQQLPHLRSPFSSAASIGPAPPALAAATATPLP
GRAGAATAIPAPGTAAAAGAALVLLPPPPPPHSMVSEFGNASLEDEMLLPTGMAAARQAAVAAAPSPPASPARSAAA
GVGSSSRRGGATAGGPAVAPAAAGARAGGAGAGSSTVSPPLYTVMEECAAHEQQADDREEEGAGGEQAAAAAVEGDG
SPPLLHPLGVAGFVGAGEEREQEGEARAAGAAGAAGGSCGGGGPPEQQQQEGEEQVEGEGDDDGLVGGPPPPPPLEA
LHRLCPQYATDINSPLRHVRTRVYFTSESHIHALLNVLRYCHLRQQQQQQQQQPPAPTAATAATAAAQPSSPAASAT
SLGPSGGSSTSAAGVAGSTAAPAAAAATSVPADDLSPAQPQPQPQPPEQPATLLGAAGEALLAGVPEYDYMTQIVFR
MFENRHVSAAAGGQEEAGGGGGSGCRFRVEVLFSPGASRAALEARAQSMLARGRGRGRRRGRRRRQGAGMARGEEAA
GGEEGERQRQQRLGGGCSSSDSSSGEGEEEEEEEGGREGGVGVGGGGGGDALEADRHCLPLEPLVHLNAGPGAARAL
EALEALLAPLAAPRKAQPNTYALQVALKAPGAATADSATATATFGGGQLPAGSSTSGSSTSGSSTSYMRRELAAAVA
SRAPSTALRGPAPLAPDMLLPALGAMATAVADEAAAATAAARAAVLGGSTAAAAVQPRPPAAAAAEGAPGAGSGGDA
ATANTGARGGNGGAAGTGGLPPGAATARLHPYMQDGLQSAASLPVLPSHRLRAAAALMAAGGGGAAAAAAAAAADVA
AAAKEAGDGGRAGDPTPAEP

>Cre01.g011700.t1.2|PACid:27577808
MLAAAAPPSPKPPRRPPRPPRPPRPPPRPPRPRPPPRPPRPPRPPRPPALPRPPTPPRPPPPCSPPPPPRPPPLPPP
RPPPRLVTLGMPVLLGTRALPPRPPPPPSPPPPDEVTLPTVTGTMLPGSNCPDAVGVVEASNTYRAWHQAAALTWDA
TLAASAQAYADVLAPQQCALVHGGVAGENLYRMTSYPKPDGSCRRAIDMFYTEVSRYVWTSTPFSSNKDNGIGHFAQ
MVWRASKTLGCGVGLGEVPWPSMPGGVVGCKIVVCRYSSGALASDTLWLTNVLPRV

>Cre01.g011700.t2.1|PACid:27577809
MLAAAAPPSPKPPRRPPRPPRPPRPPPRPPRPRPPPRPPRPPRPPRPPALPRPPTPPRPPPPCSPPPPPRPPPLPPP
RPPPRLVTLGMPVLLGTRALPPRPPPPPSPPPPDEVTLPTVTGTMLPGSNCPDAVGVVEASNTYRAWHQAAALTWDA
TLAASAQAYADVLAPQQCALVHGGVAGENLYRMTSYPKPDGSCRRAIDMFYTEVSRYVWTSTPFSSNKDNGIGHFAQ
MVWRASKTLGCGVGLGEVPWPSMPGGVVGCKIVVCRYSSGALASDTLWLTNVLPRV

>g765.t1|PACid:27577821
MDEDYDTANMHNYAAGSNVARWGRLEELDPGDEAFLKSLERKQQLRKPLRCQVGGCTADLTDVKKYFQRYRICERHL
KAPSLKMDGRAVRFCDQCSKFHDIGAFDGARRTCTEKLEKTRQARVVRRMRRGGGGGGGGGAGGGGGGGGDGEGSGR
RGRRQQRGEGGSDGGGGGEGSSSDGGDGDGDGDGDGDGGGSGSGRYGGASGSGSEHAVKRRRWEAAEDEDRGGGDDY
DMPADGMHGSGGLSGGGLGGGLGGGGLGGGLGGGLGGGLGGGGGSVYGSDRAAAATAAADRELLPGGADNRSLRDFD
GLAAAGAAGTAPPEPLDLRLPPMGLAGGGLLQPPLLPPADLLGRGGGGGSGSGGLEPMGGLLQALNGGGGGGSMSLD
LEQAPLLGMHASSPASLGGGGGGGGGGAGGGDLGRSLLLSRTSLLLPGGGAGAGAGGINGGGGGHGRLLGGDDGVMG

AVEAGLQRPLSATRLLLRGLSGVPLGRSGGSSNLSAEAVAAAAAGLAAAVAGTGGGGGGGAGGRGRGSGGGGGGVRS
GGGATNVVPGGGKPDDVHMAGSDDDDVVIVSDAAAIAAARQARATRPGSGGSNAAAAAAAASAGAAAGGNGGDALDG
AAAGARASQTHRLADLPQPGGGAAGGGGGAAGGGGAPQVPSPAPPLTSPVAAAAASAAQRPPPPAQPSPGLGEALQL
QLQQLQLPGRATAHLLGLVQALDEAGAAPEDVAAVLARVPLMLVRHRTAAAAAAVATDGVNGNGNGAGKGNGNGAVD
GGLSGGGGGGSGAGAGDVVVKVERDVEEGGGGGVGPELLLRLRHQQQQQQQQQQRQQQLRDELLGRNDGGRASEYSP
RRPAAVAALTGGGFGGGGGGGAAAPDDVYTACSQQLMARQLGVRINGYPSRPSPPPSPPPLAQQQQQQKQQQVQQHK
QQQQQQLQQQLQQLQQLQKLQQLHNHQQQQPQQQQRPLQPPARQLSGASNLHQELRAQMERLAPAGGASGAGERGGG
GGGGGGGGQMYRSLPALSGRQQLLQPDQAPGLLLQLGGGGGGGGGGGGGAAGPAAEFGRSRNEVVSLGDGCGGDGGG
GGGGAGGGGGGGDGLEVQYRYLSVMQERLLRQLGLPKQAWQQQ

>g765.t2|PACid:27577822
MDEDYDTANMHNYAAGSNVARWGRLEELDPGDEAFLKSLERKQQLRKPLRCQVGGCTADLTDVKKYFQRYRICERHL
KAPSLKMDGRAVRFCDQCSKFHDIGAFDGARRTCTEKLEKTRQARVVRRMRRGGGGGGGGGAGGGGGGGGDGEGSGR
RGRRQQRGEGGSDGGGGGEGSSSDGGDGDGDGDGDGDGGGSGSGRYGGASGSGSEHAVKRRRWEAAEDEDRGGGDDY
DMPADGMHGSGGLSGGGLGGGLGGGGLGGGLGGGLGGGLGGGGGSVYGSDRAAAATAAADRELLPGGADNRSLRDFD
GLAAAGAAGTAPPEPLDLRLPPMGLAGGGLLQPPLLPPADLLGRGGGGGSGSGGLEPMGGLLQALNGGGGGGSMSLD
LEAPLLGMHASSPASLGGGGGGGGGGGAGGGDLGRSLLLSRTSLLLPGGGAGAGAGGINGGGGGHGRLLGGDDGVMGA
VEAGLQRPLSATRLLLRGLSGVPLGRSGGSSNLSAEAVAAAAAGLAAAVAGTGGGGGGGAGGRGRGSGGGGGGVRSG
GGATNVVPGGGKPDDVHMAGSDDDDVVIVSDAAAIAAARQARATRPGSGGSNAAAAAAAASAGAAAGGNGGDALDGA
AAGARASQTHRLADLPQPGGGAAGGGGGAAGGGGAPQVPSPAPPLTSPVAAAAASAAQRPPPPAQPSPGLGEALQLQ
LQQLQLPGRATAHLLGLVQALDEAGAAPEDVAAVLARVPLMLVRHRTAAAAAAVATDGVNGNGNGAGKGNGNGAVDG
GLSGGGGGGSGAGAGDVVVKVERDVEEGGGGGVGPELLLRLRHQQQQQQQQQQRQQQLRDELLGRNDGGRASEYSPR
RPAAVAALTGGGFGGGGGGGAAAPDDVYTACSQQLMARQLGVRINGYPSRPSPPPSPPPLAQQQQQQKQQQVQQHKQ
QQQQQLQQQLQQLQQLQKLQQLHNHQQQQPQQQQRPLQPPARQLSGASNLHQELRAQMERLAPAGGASGAGERGGGG
GGGGGGGQMYRSLPALSGRQQLLQPDQAPGLLLQLGGGGGGGGGGGGGAAGPAAEFGRSRNEVVSLGDGCGGDGGGG
GGGAGGGGGGGDGLEVQYRYLSVMQERLLRQLGLPKQAWQQQ

>Cre01.g017600.t1.3|PACid:27577844
MTAYCALVAKSQTPQVCWNRDLGDYGGQQLSTTDIADGSILTSGGDSLVCFEKTGQRRWLFRFNRKTEVVADNQTFT
YSARQDGASPFIAVSSTGLVYLAAANAILYALDVRTGDIVWHWRDSPSTFESITYGDGRVYVSTSGFRRCYLAACAP
DFKAYALDVVTGEVLWGTQFRRVSSWTTGWFKGVFVRPDVDGRVWGVDAETGDLIWMFNMGAEIQYRWDPELHFHPS
GIGFMMARPSQIWAYNVTSGDVLWKMNAREGYMSPDNDGDNRQTFEVWGDLVIAQDEAAIETNPNKYGILQGLDVFT
GVRVWIRDDVYPGKYGMVVTEGLLVYTTYIPDMSTLDVSSSLLISAMGPDHQDVWNWQRDGATPAGQIQALDGLVFE
LQGDISNITFNNLDPSMVLVALPATPSVICPPDVKSPPITDNNNQQSTNAVKAPPPPPPFVSGKPFCWTHSFSYKPL
GSPTSDGVRAYGQDNAGLVIAVYLDTGAIAWKATCGPDSNSLYYPSNVVVVDGVVYLGCSNLDVQARDAATGHLLWR
RPFTLTPDRAVYRGGIIGIPAVANGKIVISKADGFLQALNITNGQLVWSYDTGILVADSVGIRDGVAYAIARHSVEI
RYSTIVAVDLDTGVEVFAKPLEPKWAMEPGRLNFRHDNTLVYSTGWPNAQLQATTIGRDGGDLAWNYPISDSTGVYS
VLVVSGEDLGLPHDALFYNDNEGLVTALALGSDGPTEVMWTVDLTDPRLWPGVENLLTPGQPDWAQITYSNHTLYIP
TTQGVFVVGATNGEALWSDLRSRLNANIHVIERPGGPQVIYGRYLSNFESNTPQCRPPKPTGLVTKIPVRGSMPADG
GEEPSTPTDAPAPDSRPVPVTASPPPSVAPAPAPSPSSSPSPSPSPAPVRSPSRSSSPPPPPVCPPCPAINCSAPAK
RPSSPAAVAVALGLSGLPYNIVKADSKLRDRVAGAVEKLVSGQVIGFVRVRVLDVESAAGGTAAVLRLSLQRSGGAS
ATAVASAVEDAALSGKLQEVVLAAKVPAKVRSVVLKV

>Cre01.g017600.t2.1|PACid:27577845
MTAYCALVAKSQTPQVCWNRDLGDYGGQQLSTTDIADGSILTSGGDSLVCFEKTGQRRWLFRFNRKTEVVADNQTFT
YSARQDGASPFIAVSSTGLVYLAAANAILYALDVRTGDIVWHWRDSPSTFESITYGDGRVYVSTTDFKAYALDVVTG
EVLWGTQFRRVSSWTTGWFKGVFVRPDVDGRVWGVDAETGDLIWMFNMGAEIQYRWDPELHFHPSGIGFMMARPSQI
WAYNVTSGDVLWKMNAREGYMSPDNDGDNRQTFEVWGDLVIAQDEAAIETNPNKYGILQGLDVFTGVRVWIRDDVYP
GKYGMVVTEGLLVYTTYIPDMSTLDVSSSLLISAMGPDHQDVWNWQRDGATPAGQIQALDGLVFELQGDISNITFNN
LDPSMVLVALPATPSVICPPDVKSPPITDNNNQQSTNAVKAPPPPPPFVSGKPFCWTHSFSYKPLGSPTSDGVRAYG
QDNAGLVIAVYLDTGAIAWKATCGPDSNSLYYPSNVVVVDGVVYLGCSNLDVQARDAATGHLLWRRPFTLTPDRAVY
RGGIIGIPAVANGKIVISKADGFLQALNITNGQLVWSYDTGILVADSVGIRDGVAYAIARHSVEIRYSTIVAVDLDT
GVEVFAKPLEPKWAMEPGRLNFRHDNTLVYSTGWPNAQLQATTIGRDGGDLAWNYPISDSTGVYSVLVVSGEDLGLP
HDALFYNDNEGLVTALALGSDGPTEVMWTVDLTDPRLWPGVENLLTPGQPDWAQITYSNHTLYIPTTQGVFVVGATN
GEALWSDLRSRLNANIHVIERPGGPQVIYGRYLSNFESNTPQCRPPKPTGLVTKIPVRGSMPADGGEEPSTPTDAPA
PDSRPVPVTASPPPSVAPAPAPSPSSSPSPSPSPAPVRSPSRSSSPPPPPVCPPCPAINCSAPAKRPSSPAAVAVAL

GLSGLPYNIVKADSKLRDRVAGAVEKLVSGQVIGFVRVRVLDVESAAGGTAAVLRLSLQRSGGASATAVASAVEDAA
LSGKLQEVVLAAKVPAKVRSVVLKV

>g545.t1|PACid:27577886
MTATTTLVQRRAVLSVTVSPSSDATPDGPTANTAGAPLPPMPCPAGGAPPPPPAAARRGSTNGSAPAGAPPAAGGAG
SIGGSGAGGGGSRQWQQSSLLLAQPAGVARAPAPRRRRANTDTVKLLMLGYRQELAREFTLFNCFGSSFNALSSLTA
LAGTFSIGLTYGGPVVMMWGWLVVMFFTTSVALSMAELASAYPTSGALYYWSFKLAPRRLRNLACWMTAWILTMGQA
AFTASNLYTFVNLVNAVIEVVYAVGLASFHRFLVLLGTVAVVGLLNLGSARLTAFMTTAGALWHIVALAAFCVVVPL
LAPERQSAKYVFTSWQPSYDVTGLTSPVYTVLVGLLMSQWTFTGYDGSAHVAEETYHAEVNVPRAIILTILGVGVTG
YAFMMSLLFVKVDVGALFDPDNETGGVNVVLQILIEVSKSAFNGSTRAGVALFSIPIVGTFFCCYQSIANNSRMVYA
FARDDGVPWSAWAKRVHPKTQAPLYGVLYMLVLATLLSTPMCFNTYVFPAVTSFAVVGCYLAYCIPVLCKLATRRRY
WLPGPFSLGPRLSFANNLVTALWVLTVTALFTLPQYYPITPLNMNWSAPILALALAFSLGWYYLPGPLGAKKWFIGP
RANLGQFHDVLPSDIAKARRQQLEAEEAERAADKAMAAAAAAFAKRGGGTGSGTGSGSRGGSGTGISRRSGAGGGGG
GDSGRGGGSGASGTLMSVMAAAAAAAAFSSPSTPVSRRRMHSPPPPPSAATTARPEGAATAAAAAAAAAAAKTAVHD
EPPPYNGRSPPDPPAVRGGTRDGNALFERISNGNYVGGGGGGDSGCGGGAAAAALAAAGGGITSGRRASYGSSLLL
GGGGGGGGGGGSGPLHPHRHPHLQLPPRRGSGTGFLGRTQQTHQPSHQQQPQHHLSRHAIQQHGQHHAAAVAAAAAA
GAGTRGTADGILMPQAGGGGSGSRHFMPRAAIVSGTHAVGPTAGGGGAAAAAAHQLLSSAASFTLASTAASAAVSVI
VADSSSSIPAAAGAAGAAGAASIQSGGGGAAAAAAAAAPPPGAATPVATHAHRQAAAAGLLAGRGRRGSVLALLSGT
DGGRIMAGPRNPSESQIAYLLTLQEQQLRGGGGGGGLRDGESGPNTAAATAAAGLAASAPTPVATPCAAAAACDSTD
EGSVAIVLSSSGANAITQQASSAATAAAAGAAAATAAGASHGAANAIVRERPRPGKAQRRMSLYSGTTAAARRASGA
LMAVAGGFRRGSGSGAGAGGSGSGPFQPAAAAFNPGGTRARALTADRNTGGGGGGGGGGGRWLPSSLNLSTLLRGVG
VSHSGHLPPQPSPPPPPQQLAYSSPPQTTTALLALGHQAAAAAGTYYQYLQALAPPLQPPSPPPPLEGIDEAAADSQ
MPSSAMATAAAAGEAAAAAPPPPIGLGIAPAAAAAVAPTLRRSVDVPPHLVHLVYGGGGGGGGGGGGGGNGVGDGG
GARSVTSATARTSSRSGLGPAAAGAAVALPGDSLYSPTHTAAVTSDSDLHPGGGGGGGRGGGGGGGGGDGGGGGGDR
WVSSNPTGQDGNGGTGAGRRRSTFEMLHVAAAAHAPPYHQQHHHAQGLPQVRAPTRLSGPGVAPAAGATAGAAAGVS
SSSGLLLLSSPSSLHSAAAVAAVAAPLPLPLPLPAAGGAVNPAPSSLLPVSLLDPPLLHPPHPLYPQPHLPSPRASS
RNLAPHTVTAAAGLVINQPPSAAAAAAAAAAAHPADRLSTRARMRRASVSVMLVPSPPPDADDGSDGGGGGGGGGGGV
GGVGGLMGMAFAPDSFAAVAVAAAMAAADAAEEAHLTAAQAEVVVGAGGARYTGGSGGVTDGSSASLRHSAGGRSTP
PEMQVLAAAAQRPHLSPLSGGRRGSTSSYQQPHWWQQHEQQQQHEQQHEQQHEQQQQQQHEQQQQQQQHEQQQQQQ
WQWQARSATGYFYGCMPPAGTSAAALAAAFHGGASGGGGSGGGRRGSAACYYTPPAPPPPRLSHDMASAGGGVGGGG
DAPERRRRSRRVSVYSRLLLLPPPPPPHDAQYQYQQQQQQPHGPEVLQPAKSASAGCSRDGGGGGGGGCSSTGLSSL
LGVGGGGGGGGGSSLLGSSIHVGTTGGAGEGGGANSSSWGVAGATSQQKQRQRQSTGGPHRPADEAGGGGGGGGPGAA
SSASLAGLQQAPGRV

>Cre01.g001400.t1.1|PACid:27578001
MHRRRGAAAAAAVPHAGAAAGAGPALLLLALLSAAALPRCVSPCGHHVVSGEVRDLVALHLAHQAAHPLSGAWGAAL
RVGGRQQQQQQQQRVHEGHEEQEEREESEQGLQQQEVVVDVLGRLRHQHEHDHGSHGHDHGHDHHDHKHEHEHEHEE
HEDEHDHDHEHEEEHDHDHDDDDDDEEEDEDEEQDAEEEEGSLAIERRRLQQTVPPPAPLRLAVSYQHLEALDAGQQ
QMLTRVVEAVRRILQKFISVQRPPSGAMLADPYCLSYSSTRGCLSYYPDFVSRRPAPDNLLCGLATVRPEHVVNPAN
CTRRASLADVLALGSGTSGPASGAGGGASGYGSGCSSYAGTPGEAADMYMYITAVQNSDCDAGAAAWARPCLLDLGN
NRPLMGAANICPPALSMLDESSLTAVLAHEMIHALGFTESMFNLTRRPDGSPRPQSEVVASAVIDGRTVSRLITPRV
RDAARAHFGCDSLAGAQLENEGSEGSAGSHWEYNLFQGEVMVASTIFAADGSPPALSNLTLSYLEDTGWYVANRSSA
GLLAWGAGAGCALPEKSCQQYMAAVPDQRLFCDASNPPTSGDGSPAFRCSDDFKSTGVCRSLNFTGGCGLLLSRNAA
QTCTSPDATYDMPDVFGWGTGSNSGRCFPVVYRFKAAAANSIYTFPGTGVDGDVDAACFDTACSADGSRVYVKMFGQ
SFECPAGQYLNLASLLPSRYRAGRIGPCPPAASLCATQSCPAASCTPGGGDCLNGQCYCRLAFTGPSCSVNLITGQA
ITNDATGTSPGSGSGTNGTSSGGSGTNGTSSGSSSPPPAAVWTQVVQLSLSLYNPVGDVTARSSALLATIAFWAELP
TAAVRLALVAASGNSTSTSSGGGGSSGNSTAGPQLSAGEGNGGATTITQAGQAYDATAASSPPPAHRHSSRHLLDNS
TSPSPSPSPAPSPPAPPQSMVVTHLTMPSSRATTILVGRLRDGTQVSQLFSALAAAGFATQPSSLTVATLLTKATTS
PPPPPPGGSSGRPGATAAPASASEDKSNSRMVIIIAIAAGAVAAIGIIAALVTIAVVRHRRARRHQQQASQQAAMQQ
YLAPAAPPAVPPPSKRAARYTYQQEPAPGGPVPPAPPPPYMAHPPQPPPPAGGFSALPTSAPASAGGGAAYYPAVPA
PAAWNQTPPRGSAPQPAAAAAGSPSNVPSGYAHYGARAGSPPQQPQPSPPAYGGQYASYR

>Cre01.g037750.t1.3|PACid:27578011
MTGNSLRAWLQYLRIDKYHDQLARAGHDARSVAQLDDSALAALGLPLGPRKKIRLNAGLILQSCESSAATSGPSAAR
TPSQQQPADPAAAAGCLHTGQPPQQQQSGLGTAGSAGGQAAPLACELWMGALMAPRPAPPAPAVGGPRPQSGPIYGH
GHPPPTHPPNQHQHLQQHLHQHGQQPQQCGHQRQGHVPIPAAAAGAQTAAPAAHARPQGHAQAQTSSWSQPHPHEQQ
PHQPQQQQQQQQQQQHHQQQQPTASCLPLTSSSMPGVVRPHSGGSSRSRPGPGSASQPGVQAGPAQPSDSWTSRPPS

PNPTAPQAAAVAAAVAAPTWEVGASVRPGPRGDPLSRVHAPPPPSRSASPSTSAGRSAPASPPPQPAAAASANGFGR
YGVTEAQRPASSGTAAATAASAGAEGAPSPPPPPSALVLAEQRALTELYSYPYRSSAGAHGRRQPGLHGRPGQGHVR
GAIPPREAPRPARPPALLPAGRALPVLKRSRSWDGSCLVRTGAPAQSAVSGGAARPEPGASGQLPEGQDLYAAAASA
GPVEVDFGPLKMEAAEAAVAARQRRQQVQEQRQQQEQAERLLHVVLEGVAGRAVAGGALQAPARQALASAGAGAGCS
AASGAGRPRQAQATGAGTGATAAATPGAATAVAAAAAASSLLDDPRNFETPELLALLLQEDRAAAEAADAAAAAALA
AAEEAEAASEQAGEAGGAAGAQPTREQRQARLRALREEVAATERLLDTLRAMVAEEERALGAETGGPGGQGGGRAGG
AGSGVGAGAGRSAGVAAGGELLRVDAGRGWSGSTPAFDAGTEAQLWQEDAPTQVWGVGEDMLLTERLGLLAD

>g949.t1|PACid:27578066
MLLLAFLIGAQAIAAPPPGVPAPPDAADCSSCGATLSPVCTSGGLTIANACLAKCQGLLDFIDGACNADPLFQSLAA
PPPPAAPVPPEPAAQAADAVVEQGSIVDTEAVTRYWTEGFVYLGLAADLGVAGVPETTDPAAERRRRAMQELGSSEL
LFRSFRLHADGSLYAEARPWALDVSSLGSERQGSVTDAELLRRRERHRLLRQQQAQQPERSRGNGHEGENLEQQQWE
SASDEGDDGQAEERQQAPPASLHRAVAGQGVRRRRRGLLQKEDARYPVRDTGYWPNNAVVQMYFVTTGTNTATSCSG
TWVSGYDVLTATHCVYNWAAGATYTNFVIRPAMSGDVWNGTYKAVFTTWYRSEWNRTNYLNEVNYYDIAMIRAEASD
TATIMPYPTYLGYKYDCAKQHSQLRAQSLYSASTCGYPNDRRLGDFAASASYTQMCCDFSFPGTTCQADFQPLTSCY
TYPGASGSSVFDLGDYKAIGVVSGRPSDNTSYSRWTPLTAQHFGALERWRYQGGVVAPLPPAPPSPPPPPPLNYTRY
ACASNGTVRLVSSSVDGRGRVEICAPDIGGKLWWSSVCSRGWGYPEAQVVCRQLGYTTTTAQPTQKGYFGAASSDQY
FFFISNVTCVGTEASLSDCRQTAWGNLDSCSSLDVAGVVCDGSTGSGSTQDSVTSTPYTNDTCTQWVTRVNNATTYA
NGTVVGRIEVCINGVWGTVCRRLWDDVDATVACKGLSYTYGVAKRLNESWSSTEPWQVAQPSVPIWLADLNCTGAEV
DLSACNQLLAYGSTQCTHGMDAGIVCSNTTLGPQYSDTAQCSTLGDIRLVPTDDASVGGAEVAGRVEVCYNRRWGTI
CADSYWGVEEAEVLCRHLGYSYFFVNMTASMGSLPVWASYTQCTGNEDRFFKCQMWGLREVTDCTSGTRAVVACSNS
PTIAAPPPPASPYACTTPGALRLAPNDTSTKTYMYSDTTAGTAGGRLEFCYNGQWGTLCAEYWSTPEARVACRQLGG
GLEYGGVVANGTYGFAPSGQPVWLSIANCTGAETSFAECARYSNYNYTITNTTFYPGQLGVLSLESTLYGGKTVCQS
HLADLNIWCSKTWLSPPPPPSPPPPAPPPVGDTCSAGEDGKLRLVTGNGTSYVYDGTSGQTQTGFLQVCYSGTWGRV
CAARFGTPEAHVACWQLGFAGGSKMTYSGLLPLYVWGPAKLGPAWIDGLICNGTEAMLTYCYFKGWGSLVDSCPPVQ
GVDAGADLVVQCLTSVNSPPPPSPPVPPVANYGCDTQGALRLVDKSGAVVDTSSAATVEGAVHVCMNSSWSYVCDYL
FSSNFAATVVCKQLGFGGGTPYSWTQLAARGYNNSLVQPAGMPRGPSQLQVCYGDEPTLLDCEWTANDVFNCPTSNS
VAGVFCTRQAPPPSPPAPPPSPPPYWSQTSCSRNYDIRFVDGPNPTAGRVEVCYNGTWGLICARYWDDALSNIVCRQ
AMGSSSAIGEAMIDWDGVATAAANGTYPRFAYLLASVTVTYHLDGGFIKPFCTGNENKLSECVPLEAFGKAASTCSR
FSEVGINCWASPSAKVSVPATTAPYSCAGFQNGTVRLVNGATPAMGRVEVCWNGNWSVVCGSGWANAAAATVCRQMG
YTSGYVNNLGGISLTSLAPNSRYAIWGTPASDQPVALHRVTCTSTATHILNCTLNSGAASNQFYSSGVAPIGSCIAP
ARVVCTGLGFYMPPPSPPSPPSPPPVPAPPSPQPPSPAPLPPSPRPPSPAPPSPPPKPPSPEPPSPAPSPVPPSPAP
PSPEPPSPVPPSPEPASAPPSPSPPSPVPPSPEPPSPAPSPVPPSPAPPSPEPPSPVPPSPEPASAPPSPSPPSPAP
PSPEPPSPAPSPVPPSPAPPSPEPPSPVPPSPEPASAPPSPSPPSPAPPSPEPPSPAPSPVPPSPAPPWPEPPSPVP
PSPEPASAPPSPSPPSPAPPSPEPPPEPAFAPPSPSPPSPAPPSPEPPSPAPSPVPPSPAPPSPEPPSPVPPSPEPA
SAPPSPSPPSPAPPSPEPPSPAPSPVPPSPAPPSPEPPSPVPPSPEPASAPPSPSPPSPAPPSPEPPSPAPSPVPPS
PAPPSPEPPSPVPPSPEPASAPPSPSPPSPAPPSPEPPSPAPSPVPPSPALPSPEPPSPVPPSPEPASAPPSPSPPS
PAPPSPEPPSPAPSPVPPSPAPPWPEPPSPVPPSPEPASAPPSPSPPSPAPPSPEPPSPAPSPVPPSPAPPSPEPPS
PVPPSPEPASAPPSPSPPSPAPPSPEPPSPAPSPVPPSPAPPSPEPPSPVPPSPEPASAPPSPSPPSPVPPSPEPPS
PAPSPVPPSPAPPSPEPPSPVPPSPEPAFAPPSPSPPSPAPPSPEPPSPAPSPVPPSPAPPAPEPPSPVPPSPEPAS
APPSPSPPSPAPPSPEPPSPAPSPVPPSPAPPSPEPPSPVPPSPEPASAPPSPSPPSPAPPSPEPPSPAPSPVPPSP
APPSPEPPSPVPPSPEPASAPPSPSPPSPAPPSPEPPSPAPSPVPPSPAPPSPEPPSPVPPSPEPASAPPSPSPPSP
APPSPEPPSPAPSPVPPSPAPPSPEPPSPVPPSPEPASAPPSPSPPSPAPPSPEPPSPAPSPVPPSPAPPSPEPPSP
VPPSPEPASAPPSPSPPSPAPPSPEPPSPAPSPVPPSPAPPSPEPPSPVPPSPEPASAPPSPSPPSPAPPSPEPPSP
APSPVPPSPAPPSPEPPSPVPPSPEPASAPPSPSPPSPAPPSPEPPSPAPSPVPPSPAPPWPEPPSPVPPSPEPASA
PPSPSPPSPAPPSPEPPSPAPSPVPPSPSPPSPEPPSPVPPSPEPASAPPSPSPPSPAPPSPEPPSPAPSPVPPSPA
PPSPEPPSPVPPSPEPASAPPSPSPPSPAPPSPEPPSPAPSPVPPSPAPPWPEPPSPVPPSPEPASAPPSPSPPSPA
PPSPEPPSPAPSPVPPSPAPPAPEPPSPVPPSPEPASAPPSPSPPSPAPPSPEPPSPAPSPVPPSPAPPWPEPPSPV
PPSPEPASAPPSPSPPSPAPPSPEPPSPAPSPVPPSPAPPAPEPPSPVPPSPEPASAPPSPSPPSPAPPSPEPPSPA
PSPVPPSPAPPWPEPPLPVPPSPEPASAPPSPSPPSPAPPSPEPPSPAPSPVPPSPAPPSPEPPSPVPPSPEPASAP
PSPSPSPAPPSPEPPSPAPSPVPPSPAPPAPEPPSPVPPSPEPASAPPSPSPPSPAPPSPEPPSPAPSPVPPSPAPP
WPEPPSPVPPSPEPASAPPSPSPPSPAPPSPEPPSPAPSPVPPSPAPPWPEPPSPVPPSPEPASAPPSPSPPSPAPP
SPEPPSPAPSPVPPSPAPPWPEPPSPVPPSPEPASAPPSPSPPSPAPPSPEPPSPAPSPVPPSPAPPAPEPPSPVPP
SPEPASAPPSPSPPSPAPPSPEPPSPAPSPVPPSPAPPWPEPPSPVPPSPEPASAPPSPSPPSPAPPSPEPPSPAPS
PVPPSPAPPSPEPPSPVPPSPEPASAPPSPSPPSPAPPSPEPPSPAPSPVPPSPAPPSPEPPSPVPPSPEPASAPPS
PSPPSPAPPSPEPPSPAPSPVPPSPAPPAPEPPSPVPPSPEPASAPPSPSPPSPAPPSPEPPSPAPSPVPPSPAPPA
PEPPSPVPPSPEPASAPPSPSPPSPAPPSPEPPSPAPSPVPPSPAPPWPEPPLPVPPSPEPASAPPSPSPPSPAPPS

PEPPSPAPSPVSPSPAPPWPEPPSPVPPSPEPASAPPSPSPPSPAPPSPEPPSPAPSPVPPSPAPPWPEPPSPVPPS
PEPASAPPSPSPPSPVPPSPEPPSPAPSPVPPSPAPPSPEPPFPAPPSPEPPSPVPPSPEPASAPPSPSPPSPAPPS
PEPPSPAPSPVPPSPAPPAPEPPSPVPPSPEPASAPPSPSPPSPAPPSPEPPSPAPSPVPPSPAPPWPEPPSPVPPS
PEPASAPPSPSPPSPAPPSPEPPSPAPSPVPPSPAPPAPEPPSPVPPSPEPASAPPSPSPPSPAPPSPEPPSPAPSP
VPPSPAPPAPEPPSPVPPSPEPASAPPSPSPPSPAPPSPEPPSPAPSPVPPSPAPPSPEPPSPVPPSPEPASAPPSP
SPPSPAPPSPEPPSPAPSPVPPSPAPPSPEPPSPVPPSPEPASAPPSPSPPSPAPPSPEPPSPAPSPVPPSPAPPSP
EPPSPVPPSPEPASAPPSPSPPSPAPPSPEPPSPAPSPVPPSPAPPSPEPPSPVPPSPEPASAPPSPSPPSPAPPSP
EPPSPAPSPVPPSPAPPSPEPPSPVPPSPEPASAPPSPSPPSPAPPSPEPPSPAPSPVPPSPAPPSPEPPSPVPPSP
EPASAPPSPSPPSPAPPSPEPPSPAPSPVPPSPRPPGPWPPPPLPPGLALTLNDFSATFVNATIIADLDVTNTSSVM
SFLQEFKQMTAAALGVSVDQIIVRAVTVDGQQIGRRRAQEEEPLGDRQSLEPSAEFQEFGRVTLLQHRSYVGDHETI
AALQALEERLLAPQLPRGAASRGRRHRQLAEAASDVIVDFTVVQYEEVELPPLPPPMPPLSPGLVAPPGTPLRPPSR
PPRPPPRPPPLNLNRLAQELGAQEVIVVYSPPSPQPPSPAPPAPQPPPSPQPPSPAPSPPAGSGDALPAALEATIVGA
EYAAVVGSPTFEVEFCADLISSSGSKLCLVIYILPGSGGVVVGAMLVPQDATSQADFDTMVQSMLDYPNGVFTIVFK
SAYGVSSVILKRAILSPSPPPPFSPSPPPPPFFKSMSPPPPPPPPPPETPPSPSPSSPPPPFPLPPGWPTPPPLAPP
PRLPPPPSPASSVPESLLAPPSPAPQEPSQPPASPVPSPVPQDLPPPPHARSPKPPRRKKQPPFPQKPPKSSKPPKP
RRSPPSPPSPPPPAPYPPPGKPKRPPRVRAPPRPLLQAAKEDPPSAQPESPTPPALPPPPMQRRRDKSPPRLPPSAP
PPDAPPAGAPPPSRRNRSGLAG

>Cre01.g008850.t1.3|PACid:27578068
MDQTTSGILDGWGDVDLDDDEDDPFSQPVRPVTAADTAGSDPFASDGGDSPEWPSSPTASGPAATTASAAAPPAPSR
LAPVAAATAATAALPPPPQPSTLAPLQASSPPAVAAVPPRPPSLPGAAAAAAAGPPRTSASGAPPLAPRPAAPTVTA
PAVPHQPPNLAAASVPPGANAAAPSAAAAAPAQAQAHAGSAAPSSHAPTAVPVAAPAPVPQAPAAPVPPPAPPPAAT
AAATPPAPGLASPPAPQLPSPPAPVAVPAASVAAAEHAPVAAATASHFTPPAHAPVTAATAPHGVPVAAHPVAPAHA
AAPTPPAAPVPVPAPPTAYAPAAPPAPPPTAAPPAAAPLAAVAPAAAATKDESASFFDDMGDDQDGAGDWGAGDWSL
PPAAPAAAVPAAAAPAPPAASQPPLVAVSNGGPAAHAPPPLPLVASAAHPPPAAKLPSPQQPAAAAQPPVAPAAAPA
PAPVTAPVAVPPPAAQAVPAAATPAAAAAAAGDGGDEALMALKPRRVSVGFQLPGDEGEDHQSHTAFPPTPFGPPRT
ADEAARARISPEELAAAMAPTPADSATSTPRAPPPAAAPPAAAPPLHAAANGAGGAAATAAPPPPGLTTTASMRRHR
LSVSSSMREHGAGAAPGEGLSESGQAPDLLPPPTAPTAPPPPPTAEPAPRALADGGWSQLAPGGAGAEQAATVAAMV
AATEAPLEAVAAGGPDGSSAPGVAPRSHPGVSRVAAETGRAGSGSGSLASGAASVTGSELAAFDDAIEQLMAAPDTV
STAATALAGPTAAVATPAAATAPSPLPPVAALDGTAAGAGAVLAAVDAGGEQQAAAAAPSPGAGALSGFYVAAQNPF
GPTDSGVVDTGDDDDVFAAIAAGGGGGGGGGAAAAPAAPAASGRPPVPGGAPAPTSYVVAESSRKGLSHLFGAASGS
VSSQLSQAGDSAAAAAGGDGGVAFRQASRPLFADSSAPSAASQNDLPAVSVLLSGPGPDQDQGAGQTLAAPPPAAEE
PPQPPPPPPPAAMAAPATTFQQAATGVAALFGGGAEDDDADDPFAAVGGSDAGGGMFGGAAAAPPPAAAVPAAAAYG
SAGQQPQQPPQQPQAQQGWAQAPAATTVVPAAAGGPFGGPDAGESFFESLGGAGTDAAAAPSPQAAPAPQQYAQQPC
AALPPAAAAYPAVAPVAPVAVAAAAPAVGFTALGTAADGGASAFFDSIPSPEPSPQAPHAQTVPPPMAAPEPVPPPP
VQQHPHPPPVQPPPQAAAPAAPSPAAAVPSAAGAFGAPIYGAGPFGGPAMAAADDAASFFDAPPSPPAPAPEPAAPA
AAAAPPPAAHPPAAVQPPPQLAAPAPVVPGPTAAPHPEQPQQHPQQQPGYPQPSTSQGGWPDQAPAAAGVSAGGFAA
APFAAAVVPPAVPGVDDGRSFFAESPGAASPAAAPPGAVHLPPQQQLQSQQPQLQAGFPAVAASTPAPFAAAPAAAA
GGLFGGAADAATGDSFFDNLGAASPDGGAAAAAAATAAPAQVHPPHAATGAPHQQHLHQQQHPHHAHPHQQPHPPVE
PQPQSQPQPQPPLGQGPGHQDGHPPGPALQPVATAFAHGGIQGVVQHHIAASPGLPTPPYNAAAPVPAAPPATPPPP
AVEEEEPEEPEEPEEPEEVVEEEPPVQVMSEEQVTAAAAAAAAPAWEAAGGYSLPNATETYQQYYTYYYQYYQQQQQ
QQQQQERERAERMQAKHAAAAERKRAREEGRRQRQAAREEARRQRQAAREEARRKAAEAEALRKRQEEEAAAAATAA
AAATAAAAAPAVAAAAPAVAAASQHGQQQQQYPPPAAQQQYGQQYGQQYGDYGAASNNHYGGQYGQPQQSQPQPQPQ
QPQQQATAPPTDPYAAWHASQHQQQQPQPQAATQQQPQPQGVTQQQQQQQQPPSQGQQWPGQGAQQPQTEYGQAATA
AQQQQQPSQQQQGWHGQPQPQPQTLPQAQQAYPGYNQQQQPQQDAYAQASQQQQQEPQQPQQHAYGAYGEQQWNQQQ
QPQQPQQPQQGQQPQPQTGYGGYGQQQQPQPQQPQQAPQQGYGYGYGYDAYGGQQQQQPHAYGAQSQYGQQSYSQSG
AYGQQNQQYGQQYGQYDAAAGYGQQPYAAYGQQQQQQQTQHNVNWLPTTPELARSCPHGRPAHTVFTFGFGGRAVIM
RPPAAGSDTSISAVFGAPGPLQLLPVASLTAAHLAGGPGAGGPAAPTSASGDKKKPPRGGTQPAGHPLFEPVLKQLA
AFPGPLGAATPKDKLLKWLADQAAAAAAAAAGELQARQEQQQAGGVGGEPPPGAAAQRLLLWEALRLLVKHADAAAA
AGKAGGKQAAKGGKEEPSMESELARLLLHPSSLSTGGAAAGSNGAAKGAASRASGDGAAGAAEADVSALVSGAFAVG
GPPVLAAPPPEAAQQAAAQRMQRLLVLGRKAEALRVASDAGLWGPALLLATAVGGGDGRAFQEAATAMARALLLPGT
PARTLALVLAGRPDLVHAADGVAAAAAGGPDGAAAPPSASAAMPFGVPAPPGAGAHAPPAGFFNPTMPSAAAQPAPA
GGAAASAGGSCTLLGSWHEHLAVLLGTRGPADASEAVLRLGDRLRGDPVQVLAAHLCYCLSSCPPALPDAPGCRYSL
LGEAAATAATAATAATGHWTPLGDGGAALQRTEVLEWVLVQRAVVVATAGASGAAAAAAAAAAAADTVHLQTYSLLPY
KLALAAELVEYGRIQEALSYCAAVEAGLRAGGVIVAAPPVAAAGQPGGQPPAAPPQQQAAAAVRLPGGAWLAATEAA
LRRLQDRLVNYAQANAIALSKGSGESAITKVGRFLDGTINKLLWGGDTGSTAASEAAAAAAAAAAAAQAGAAAGPQI
VMGGASVPPAALGPIAPWAVDVGGLAAAAAAAAAPAAPVGAPSFPPPSAPPPAAGGYGYGFGGAAQTAPISGGAFGA

VGGGGGGGGMTQSVSTLALSAAADGGAGNSSSTWYAGPAAPPGGASRSVPGGSTLPPYGQPQAPYGQAPSAAAGATA
PSDPSAAAAYGWGGAPAGGGGGGYGQPSQFQAPTSFDSQALPSQQQQDQQQQQQPGHRRIASAMELPAAGAGAPHGP
SPPSQTQSTSGAAPAADAKSADDKAAAAGGKGGSSKGAGGRGGGKTEKPPPSPDPKSTKSGRWLVNPLRLLAGGGRS
QSNSRGAAAGAAADGPKQGSYGEENQFYYDETRKRWVMKGQEDAGGGADDMPAAPPKSLGGGAWAGGGAAGAAGPSS
SAYATPPPGGAATAGSTPGTGGAPGAPPSAAGSTVTPGGTPPPAPGNFFQRGKGINSRYVNTFNPGGGSGSGGANGG
AGGGMGGAPPGAPGTAALPATGFFVPPPPAPMRPGEADSYAEADPARAWADQQQQQGLQQQGLQQGDPQQPQQHAQA
QANPQQPYAPPGQQQPQQQQQLQQYASYGAPATDAWGQQQQQYQQQQPQQQQQQAPASGAMPPSSGGGAMPPPPHQQ
QPPQPQQQPQQPQQPAYGAGPSQPPPPPQQPSPPQGPPPGQQQQWGQATHPQPQPQQHPQPQQQPQPQQQPQQQWAQ
QAPPQPQPQQPQWGAAPAPPPEQQQPQWQQPPQQQQPQWQQPPQQWQQAQAGPFSPSTSTSGAAPPGSSPQQSLQQE
QPPQPQPPYGQPPAAPQQQPQQWQGGYGQGAEQAAPPPQQPQQPQQQWQQPPQQQPQQPQWGQHPQGPPSTSSQASP
PPPPPHQHHQQQHHHQHQQHQPPAPATAGHTGAVGGPAQGYPQPGQPQQQHQQPQPLQQGPPSSSQQQQQQPHSYGQ
GPPAPYGAPPAAPVHGQQQQQHPVQQQHPQQHYAPPPQQQPQAPQPPAPQPQYGQPPPHPQYQQQQPPQQPQQQYQQ
PPPPPSPPPAAAAAPATQMVSSASAATAVVDDLLGGASSTGAAPYGYGSGAPPPMAAPPPSNGYGAPVAVPYGSSSG
MPPMGYGSSSGYGAPGYGGGAAAAYGSGGWGSGAVGYGSGTYAGGGGGPPVPAGGHGEGEDMREVEL

>Cre01.g011900.t1.3|PACid:27578118
MSATPPKAIPGLSPSPWPQSPAPPPHPSPAAQPPAAASTPPVPLLQPQPPPVSGSMPPTAAGSSPPPVGLPKPPPQP
AAVPGDVGAVPTPLLLLLPPSRPAPTPSLVAGTSSNGTTAPAATNSSSPAGLAPGSSSSNTSGSTTSSVIPPVARAP
PPLPLLRPPPPVASPPDTESEPEPPPPPPRHHRGKARQRRKRPPPRPPKPPKPAPGQLSRPSTEQSQDVELPLATIE
ARPPRPSPPPFRAPRAPPFPPSPKPPRPPPRPPAPPRTDGRPNFVFILTDDLDDVYNSSSARFMPVLNRLIGDVGTR
LSNFIVSTGVCCPSRTSILTGKLAHCHNVTSNYFPEGSFTKFYDENLEDNWLPGWLQSAGYFTGLSGKFLNQYFETS
ARKGYVPKGWDAFDALTINAYNLTNSCFSLNGAASTCFPGEYQTDLIATKARGQIDMALASGKPFMLYVAPTAPHRS
STDGATWYPPTPPKRYANLYQGENLQAPRTPNFGVRNPTLPRKGTPRVDAAFGAYMDELFVARLRALRAVDDLVGNL
VARLNESGVLNNTYVIFTSDNGYHLGAFALMDGKNLPIEEDIRLPLFIRGPGIPAGKVVPYQMNMVDVAPTLLALAG
LALPGHLDGLPMPLNPQLAAAHTQVLLASNGLTDAPSRPPTPPVPAPAPAPSPAATTPLPPPHTGSATHLPPPPAPT
PGAAPPRPGPGPSPSPMPTPPHAPASGGSSPAQSANATSNHITTPAQPPPSPPQPTPSPAALTPPPMPVQQSPAAVP
TRPATSPSPPQPAVAQPPLPLASLSPPLAQLPPTSALPPVPRPPSPQPQPTPAPPQPTVTAPRPPQPPPLAPINGTG
IGGGGGDGDGGGPVMSPPSAGPAVPAPRSAAPPPLPQTSTMPPVQQPSPPPLLTPAPGPDGNTSSTDSRAPSPPVPR
PASPPPLPPLPLPSGTSQRQPAHQFHLHQLQHQHQHPHQHPHQHPHQHPHQHPHQQWTQPPALLAPSPAAMPSSPTP
VPVWPAPVAAPPLPPPSNQQPPQQAPPSPPSPPRPPRPPPPPPMPQSSPPLAQPPSPTRPVLPSPAVGTAPPPPSPA
VSTSTPSSSAAGPSAAVGNGAASPPSSPGPLNISTSSNGGGDMDSGRQPSQPGDGYIPVVSSANGVYGMYGAVYGGG
YGGYGGYAPTAVYGGYGATAANESYTGQQQTGVARAPGAAADGSSRPPPAAPAPAVAPDVAAAAGNNMTYGNASSGG
GNGTLAAASSNSTSAAAVNATSSSTNSTSAAGGSGGGGGGGNSGGSPPPAPPAAVGLLRRDAMILEGWNGDGTEKTF
TLAAHYKSLRVCTAARLLPLPPTNAPRAINSTNATSSSTSTSTSTGTANLFSARYVLDPGVTCYKYTVWCQGNRELY
DLAADPYELSNRIQSAPVRAVNRLDAVLSALVHCSGASCRNPYSLLHPGGGVWDFAGAMDAAYDRMYDRLAKLSIKK
CVNAYLPTNEVTWTQGVGPQPFQFAAGP

>Cre01.g008600.t1.3|PACid:27578135
MRVQSRYAARRDDGALHSLFAGVQPAQRGPRARRRQNLFGMRNMLIAAVCVTSLLFWLAVQYYVRPTQHWKARVFGE
DPYDSFYGDARALSDEELQSLATDLQENEEGYYYGDVGRSDDAGNAGSSSSSSSSSGGADGGNGNAAAGSSSSSSSS
SGKDGGDGGGGVAEDLQESSAKMTKVWAALWERGVGRNRFKGTGGGDDGGQQLQRGSAGRPPPPPPLDSCRRCAAFS
ATDFLPGAADAALAAQDPFWAVLQPGKAGAATAEAVGSQAGSSSNSSSSGNSTVGRGSGSGSGSSSGGKGAGLDVRR
RQCRQHAPYIGGEVRGPFLERYHHRIIRHIQSPEYRARAARIAAHGGRGLLIPAGGPRYTSNLLVTLHVLRRHLRCT
LPVEVAWQGDDEMDAVTWAAIERHFAPIRGFNVKRQPHPVPGLHGKTFDATSYSGKVFALALSEFKEVLMVDADSLP
LLDPNTLFEDPRFQAHGSLFWPDAWTGQASRGIMEVYGIDAEKAERVLAMGRGTGRRDAESGQLLLDRSRHLDVIEA
LLFLNRHPSSNAGLLWGDKDTFWLAAALVGKAHCYNQVAVPPSGMFHWAPDMLLNKITNTKGPGWQLGGFVQNLEDE
PFVYGVNSMAGAPAPPPPSPPPEPPLPPADPSPPQAPPSPPSLPSPPLRWHRKKRHRPGHGLDVSSSTETTTRSESL
HVHRKVGRTVGDIVQGMLEKDRARQQEQEAAEAAVAAKEARDAAKAAAAAAAAAAGKQEGRRRLLAAAADADADGDD
GDADAGDDATEANAQAASDDYMAAAARAAAATPSPPLPAPSHPAPPKLPPLPPLPPPRAPDATRNESVLAAARAAAA
ADTADEDAVESDAAAAADEQAAAEAGAAAEVDGALAAAVAAAGLNTTEAHKHEGRWRQLVAAVARATSGLEAGLLAG
RAGEALDGSSSSGSSSSNSSSSSSSSGSSGSDTTSAPPPSPTAAEGRVSTAAVTPQPPPPPPPPSPPPAAIPAAAAP
SPPPVPSPLPQPQPRLQPPPTPQLPPQPPSLPPPPAQPAPPSRPPYQPPAWPPVVLRAPQYGSRGHGRAPRPGESLL
APLGPGLTRPVFLHRTINKWGQGAAPWLPEVISAPVTQRWAGYYLAHESEGPTRGIPWDFVVPAHALTILEATAPDP
AAVAAAAAAAAVARAVAPHGKGTAEEADGGGGGSSGGGGGGSSGGGGGGSSRGSERQQPELSGLRWLPAGVTGSGSQ
SRGVAAWLAEWRSIGGRRGGSSRSSGGGGGPDDAKLAEWRTWGLDGAGSAADEEDDEGDGGGGGGALWGWWLSGKS
KAGAGGRRRSDSAVAEAPAAAPALAANGSSSTGSSMSSSSSSSSIAPKLCPLIGGGSSGGSSSAEKAAAAAAAAAAA
AAAGVGGDVGGGGHSCPWGRADVEAYLKAIDAGLPLDRHPALEAACRTQLLAAAAAAAANATAAAAARSEAILAAVDE

QRRLRRRDRMRNAAGHDDEGEDDDFDWLPWFFRGAVDSYRDARYDGILDVLPLPRTTVAPFAYLETETGEGGGLAPL
SPENLAARRPEAAVDSIGGGGTAAAGTTGTTAKSFLYYTWDGDGLDAPPPMPALRQDWSSPWPCSDAPTAAALRAAY
AAYAWVRRHRRRFPLLQK

>Cre01.g023800.t1.3|PACid:27578188
MESEVHSRLAAAQNAPPGQLNATSAPGGSSMEHSGLPADAGGRAQVSGQVWQHTAPQAHSPSAYPHPSPPAALLQGR
LMALVQSDMRCQMRGLLESRAKLDLAARLTATLAAQPPMAPPGFFPGSPQAAGGGGPGGASGTHLAAAMLMALERAP
PPPLAPLHGAAGYPAAAPVSTGWWAAGLAAQPASGAELAAVAPLSSLAPQPRQQQQRQHAYQQHPYQQHPYQQHQPP
LLLSSLSLEAEQLRRRRQELEARKQAAEERLAAAGAAATAEEAAAAEAEGAAAAAAQEAEAAQAAVAARQATDARAA
AEARAAAAAATGGAAATAAAAVAAAQQQLPARQPQVPLAALLLPSAPSPSSRAVSPPAPPSPRQQARLGELLQLASRS
AAQLLAVTATLGYAERPGPLGPLLGRLREAGRTAEVEVEAAAAAAEALAAAAPPQGAEAAAARAGGTGGTTARAGGV
ASDSAEARPLGSSADRAAGEERPRAQGQEPQAVQQRKRGPEQQVQPERPASVRRGAPASPARDAALESAPPAAANPA
ARASGAATAGAAGARGALAGAAQGSGPGSGSVRLLPPGAADRSSPAPQEQAPQPQPALAHQLPPPLTGTRRKWGPPM
IPQQPAAGTTTTATTTATTATTTATTTATTTANTTATVAATATASSSQPRREGEQPSHTTAAAVATERRSVSVGLAA
DAAPPAPAPVTQPPAPASLTPAPALAPALALAPAPAPAPAPAPAPAPAPAPAPAPAQAPASAPAPASAPAQAPAPAPVQ
APALVPAPAPAPTPAADATAAAAPAATSATQPSPEQLAAPAPTPSQMRLLAAAFPGDRGRPTVHEANRVYRALGAFL
APIPKKGDWRAMTAVLSVAAAPVRKALPRAEQAQAKEALRARLLQQQEGGGGGGNGSGGGGGGGPSASDGGAKGVFV
LETVPAAAEAGTSEPLEVVRLDWEALTAAVSAVRVKAKAKGAADRAKAKAAAAAAPAADRAKAKAVAAAAVTAVLQS
AVSGGLETAPAPAQAGAAAGASAAVGEEAPAPATAAVAEAGAAAAPAVASALQPDAAVSQAPAAAAATVDTALPAAA
AEPEPEAAGTEAAVTTAEAGAEAGTVPVRQLEADAEASAAALQAPAAAVAATAAAPAPELSPRTQPHESAGAVASRD
AAAASKDAASLEGAAGAGAQAAAAPAAVNRQCGSSADGAPEGARASSIAGAEGKATAAARETAEGKDAGQERERQRE
WDRERGRDSRDRERSRDRDRERDRERDLERERDRERLRVRERERERDARGLGSRERERERDLHSRDHGYDRDRDRDR
LRDRERERDRDREQDRRRASMDARSRSHSQGRTRSRSRSRGAGVRDREGDRGRSLVGASRRSMDGVRERERDRALRL
SSDREGAGTRSATPAGNAAGATAAAAAGADSDRESGEVVSEAAAAAGLDGQPGAEGQSAPQADTGAAGAEPAGRAAQ
RQREEARKEEEAGGRAGGATGSRRVEARDRDRDRDATWDRERQREYDRERDDRQRQRERDRDRERERERQRERERER
EREKEREKEREKEREKEREKEREKEREKERERDREKERERERERKERAAGHKRRRSRDGVDEAAGTAAAAPTAAAPAG
TALTTAADATAGSAHHATAAPPPLPAEAEAVVAAEAAPQASEVADTDNMDVEDDSDVVEVEAPPPLPPASPPPQVLT
FSLAGRRGAAATVKPATFAGAPPAAGGAAAGAGDTPGDPAHEDTSPSASPPPPPPPPSPPPPAAAPAQQQPQPQPQP
QHPVLDQQGTGYQAYPPQAYGYPPYDPAQQQQQPYVQPLHLQQEQYPPGAAYPHPGSSYLQHGLPPADDGLQPQQPY
AGMGFGDGGGGGGAPPPLHGQTQHYDPVSGPPPPHWRHYGGPGGPGEPLPYGPMRPLSPPPHGHPHPHPHGPPFSPV
GRHPPDWELSRERERERWQLRGIGRERERERWEWERERAQERADWDWHRDGLPGLRPPPGRGPRPMFAADRMPPPGAGP
YGPPPLPWELDRERGQGPRPGSRGRDAPPGSPRDGADGGAGGRRQQRQKERSTQRAQAHQQEDQRRRRGSTGQGTEA
SPPKQQQQQPSQQQQQQQQQQRGRPSSRSQAQALAEQQAQEAQEATPAAEREQGERQREPDASGASQRNDGGAAAPA
AAAAASTSPRQPAPGTGAEAGAEAEASTGSLQADSATAVSTAGAAPGDSSRPPAAAPAARTRRQGRERPAHEHGEAV
RLGSRSQQPLRDAPPLPRPQSAGKPSGKLTESARIGVELQKSRKEIKNAKAARKLDADMLLHKKSTASAPATASATA
SVNAAATDTATAAAAAVGSGSAAGGGGGEAATDRQPRVHEQIQWTDPTLSKQPQPQPPAESGAPAGRRASLRDAPPL
PQPQHQPQQLQQPQQPALTKKQRKQQQKHQQQQQAQERAAVVWQERGQGKGSATDAGPPEAGGDGGEAPPGSQNQQL
EQAAAAMAAAAFAMDPLVQQGLFGPMLGASPFGHLPPELLFPWGMPPPLPMLPPFGPGGGGGGGGPGGLGYPPGGGG
GDGEGGAGGGGGGVQQQQQRLPGARGGKRRRRQM

>Cre01.g031450.t1.2|PACid:27578204
MGARRLELQDQSAGPDARSSSLEDDIFIPFWQPPPPRPPRPPPQPASPPSPPASPPQPSSPPSPPPSPVPRELQYSP
WYCGMPFHEDDGYYATTSDAAAASPAPQLHWLTGMSSSGGVVLESLRSSYDSGALAGPQHGSNATAAAAGSSRGAVI
DRTLQLPQTGPGRVEYVAACCSGEGLYGNSVQRLMLYTAGRELLALGGPPRWCRTPLPYTRLPEGFLLAGVETQRPT
APGGGTWVHRLRFVFARAPEPAAMPDTPPSPPSPDPPEPPMEPLGAEPPGTDSGARPPGAGGDLPPGVEPPEASPPE
GARPPDQATDILMSKWYCGRVPDSPYTYNARTTDLVQESLRDMVMVGIRGGVAWPPYDGSGYEDTNDLTGSGANATG
GGAPPPPANGRTAGLLSWLQSVYEPGGAMGPQHGTYFDPAGGQDAPVNGTCRGGPGSTCAVRFDATGPKRVVAYSTC
CAGRGQAHGVQRLLLWTADGGLLSVGDPLGCPAFQTVGAVPSGYRIAGIETGSTTYGKYSGLIHRVRFIIMADYYQG
GGGGSSSASPPPPAPLPYGGYGGYGGYGGGCAPPPPGCGGYGGYGGYSDYGGGGSYARPQRFVPL

>Cre01.g012000.t1.3|PACid:27578232
MNDTAADEAHNAAFAAAGRSVAGVFSRSELVEAISPSLGPSRVASSSLLRLGPGGTAVMPAAHAAGGRRVRAAAARV
QVAVGQAPTLATTPYGQLGHQYHTMGLLAPNPQPSVVAATASDAAAGAAHARDMGMACAAAEARNLAVVSTGANPIH
LFQLAECIDILSEVKDKLNLQPHPGGGAAATAVNLVSTAGHTLAHAPPALQPTSAPYRGSLDNLQDVQQRLDGGAAA
VAGVASGAAPSDGAGGHLHAWAAAGVAHGGHTQHQPLYKYQHQPYSMYYHQQPLQPLHSLLGVDVGAGVAAGGSGQE
DTAAVAQGGEELDSFVDGLFAEPAFPTDPVGEAGGAGAAATGSAAAEAEAEAETIPGTEAVGGTAAVAEVAAEPQPQ
PQPQPQPQPQPQPQPQPQPQPEPQPEPQPELEPEPQPQLQPQPQPEPEPQPEPEPEPEPKAATRAATGAGAEAAAVE

AEAAAEPGLTVGACMPQGCPGDDADSVGSGLDDGDDQEVNSSPVVREVVVPGNAAAAAAQPEPLHCAPQLPAGAAAG
AEAVGVEVAAAETPGAAAAGVEAESGVVAGAEAHEAAAEPAAEEAVQPATQAAGASPTGAATGQAHKDATPSVDAAD
PAPAAELLPTAGDVARGAAVAMQAGPVAAGQGPGLGPAMAVLVAPLAAATAPGGSTELPGFKDRASFAAAIKDAHPA
TGGGDGAEDAGPGAAESGAVGVGGKDVDSEGAVPATSLTAAAVIPPGGAATQTRTAAAEAGADGEVGTAAAAAGALD
GHGQWDPAGSEQLLQPQAPALQALAGGGSMDLQQLLPPQVRIKQEQVDEVQYQDEAVDLVMDMEDGEEDQQEEQAEG
EQAAAEQQAQQQAQQQAQQQGQQQAEEGSEAEGEDGQRSQGRGLSPQLHSQQLGAAADDASADPSSHSAVPDGMDAS
SPSAAGAATAAAVAAARPQPPLAVEAPAAGLDLRTLEHALLAAATAAAAAQAVAATLEPPAAGAAAGADPVALPTAV
QTAEAAGGAQASGREAPARAPGACAAGCAGVATATVSEAEALRQLARCSKAQVKAAVGAVAEAEVAQAEAAAVPLAQ
PQPPRLAVTGGGCVCAPADLAQRAQGVLTLIENLMADLRATTAAAAATAAATAAVDAVSAPTAAAAVLPGLTGRVYA
GAAPPAAASAGAGSSQVSPRVLEQPADDGMASDAVGMVSPFTGRAYPAHLVRCPGDVPLRRQLEVPGLLVSDPHRAV
QAIVDEMHSAAAGCTTYLVKWRGYELDPGHPGGAETAFVAGARAEGGGVVGTESTGGEARAERQGGSGAGACSASGS
GAGTCTARGRGGHWMRESVLRSWAAVQPAATAAMAAWEAGARPRLTSAGEVPVHPPVDLPGAASDTPEAAAAAAAAV
VVAAVAAPAEAVPAGASGGVEAATVKAAAAPAQLPELPPVTPAPRPGGPGVGTDTHEPQQAPRPAQEHEGEGTREAA
PAAPPQLQPRRRGRSAQGEAGAVKPAKTPGGRELARLFESTMFRTGNDSGEVVGGGRRSRAAARASAAAAAGGAPSA
GAAAPRSARQLRESTAGRVRAGNLPQPLPLLLVEQGPAQERPLGEPATAQLDRATQAAATTVAKRGRGNRASKSRAA
AAHDPQPREAHCVADTGGNAAAASSESGGVQATAQPQPQGPQAPPEQTLQQQGPQAPPEQTQQQGQLQPCPKRAS
VQEDLAERQPRALAEPTSGVTAQAAQGPLELPQAAPHQAQAPHQHLHQPQPDWLLLAGLPQRLAPDVLAQLVVGADA
AAAQPNQAQPVPAPETGAAGMAGPVNAESEQWALVLAAQPPPMPQRQPNDQLQLQLQIQLPNQLQLSPPQPSTVVPA
ATALVPGQPHRQLAHPQLLQLLPAVPMLAPAAVPAGELEMSPQGQQRLALEAQESALGAQAAAAPEVLVPVPAPIAL
QAEPEQAVREQLGQNQAQAQHMQDQTRTTLAEQADGQQQRITQVRTEQEAPEALQQEAPEALLQEAPEALQQEAPNE
AQQQGLMQQDARQRLQPAAPLPAAQDQEAGGWEPQQQDQEQQQQQQSAAAAQAVGTQAPAARSTGARKRPRHDLESE
PGPAVAQLEGGSEGAGGPEALEAAEPCRKRGRAPQKPALPLPPPPPQGLRTQPVRQPASRRSVRLASRAASPCDLGP
TASCPATAASSPAASQLERQHVGESPPPPAASALASPPPVQVLLDQSRFGSAAQQLQGRVRPQEQEQPHPLPQPQPQ
PQPLPLPLPQPHPQARTQPLRVGKYPSQQVPTAPGAIEPDAAILMGAAAEAEAAVGGGAGIAVAQMTAQRQGSQTPV
PEQVSHQQPQQPQQPQQPQQQLDGMLPFPSPSSRAAPPSAAPQPPPLPTSLRRRHLAPVHPNQRAASVTPHPDDGGA
LQPRVASVGGDAAAAVGVPLGSGALAIGVTGSNPTGAEGALKRKRELRRGQREREALVAAAGASLHLVSGPRLRATA
RRRQTAGQQVGSAAALGSPAAGPPANRRGGARAARARVGRRAQADTSADEEASEVIVVDSVDAGEEAEAHGKGVESK
DAVLEREAETDEEPEQADAATQPAVRGRKQNGTQTKAPRAATVTAAAAAATAAAGRAASAVRSRATGHGPSAWRTHL
EAAPLQQAAPAAVAAPAAVAQPRHPPRHPPQQQQQQQQQQQQQQQQQQQQQQQQPMAGQRRRGGRRGEEPGEGQVGP
EAADSDHADAGAAGGDRTAAAHLPRPTRVAQLESPLYQQPGADGGRSKRRAKRPRRDEDFVTVSAIISGNTSSSDGD
GDDDGEASLNTAEDDSALRARRGRGRGGGGMRGRGWSRGRGRRRSSRTRAVARRIAKDWAEGSGRETDSETRKLACG
QFAQSLRRQQERRRGGEAAAAAAPPVRRVQQRQVRQRRVPQPCTRIAGRRGAEDDAAAEETEVEDEAESKAEAQEQA
EGNDGGEAVAAAPARAARRGSVSACVQPAAAAKRASAGPVRRRRTAKRCAADAEAETEEASEEASAEEESAEEESAE
EPRKESEADLEAGSEAEADGECMSGAGSGAAAAARRVSRCRRGSRRRVDGLHASGNGAGGGHDGGGQGKTRKPRNAA
RGGGGGYGTRALQGAGAAPCGDHAPAVGEGEEGAARPQAKKRGRPSQEALWRKEVVAGDYVESPITGRRYPASACYR
PGDIRVADYVPEEDDTEGVVERIMDQVEDAAGHTYFLIKWKGFELQPGFGWGYPAHPGSVVTLWAGEGKGHWEPAEH
VLPSDPSRVAWELAKPFWYTEPAAQQTMQVAVAAATGAAGGATAVAKPATAAGEAAGSSLRTGKPPPAKRGR

>Cre01.g002950.t1.3|PACid:27578259
MLADNCPDCQVPLMKARDSQERLCVNCSTTYVPDGQGLRPIGSAGGEDAAGDSGAGGSASTSGRVGQSAGVAAGSAQ
SNGGAHGGRRGEPALHGAEGAQVAAPNGVGHSGDEDKEEEEEGADYDPRADPRLGPFSSAANASAPDSKAAGAAVAT
AGATAASAGSGRKQQQQQAEVDVADLLSSKMLQGWALLDKYCPRCCTVLVRSKATRQMFCVACDAWVVPEGEQPPQQ
QQSPPPPAQQQEPLEQLSPPQNQPHTTPAAAAEGGRPHAAASPAAAAAGVPDAAQPNSAAGPNSAAGTSTSSPRHQV
QAAPAATAAAVPAPLASAAVHTGGGAAGFLADVLGGVGPAAGGAAAGVAYSGYESSSRGSSPPPVFPVDAPFLVGGA
PSRLLQPSQHPHQQPPQQHPAANASAHANAHTHAGSHQPSQHLRGNFAAGLGAGSGSGGKSPAVGGNAMLLSVSRTV
ALKMTEAQQQLERISVLELDRARGLVGFIAECLDLLAKLHRLQAQ

>g189.t1|PACid:27578351
MSLWSCCVRATRHQSTADLSPATPRRASSDGSHPSTICCSLQRSSSSGAGAGAGAGAGAAGAAAGGASTRAAAAGGG
VLDAPEALRSRLLQELERRASATPPGSLDGWLGSGAHLLLRHGGQQAPPSAAAHSAAAHGLRLQQIKGVEAAQEARV
ARGPAAAAHDAERISPEAPLASGRPLEQTDAGGQSVAGSQPPVLPPAAGLAGLAHVRTNAAAGGAATCSRRHSPLPS
PRSPAHPCTFRNLPPSSTPAPAPSHAGSDACGDASAHQLRARSTSISTTAASTAGGTTTASSYLSISSFSASASTSS
SVLRPIGVLSAHLRQGGRGASDSAPTAANGLHHGVNRDSGSDSLGRRRLLADGVLADGDEAVAPWMGMSDLKTSPSA
AVEQQQQQQRKQPWLPQEPEQGPRADGAESAADEGVASGSDAGISAALLPASRRHRSMSCTGSHNGGIGSNSCGSLS
AMGSSGGVRASRSRASVVGGGGSSSKSMLAVAEALPPPKQQELEQQHQQQQRRISQTVVASGPVPPLLALRQVLSGK
NLSATRGTGAEPLICQRNSSQNGSGVAGGGISASGLADSYAGTGSGACQNTSRRTSSHRSRRGDKGSEASGLAPLSP
GRLSPAGLPLSPNFKHTALSPPVHPAFALTSPAPSPPLSPPPPEHADTPPAPPPAAPPALSPAPSPPPQQEAPATLL

ARPRGLGSCQQHAGDLAATGETMLDAVVITAFISPTATASGGEAFAGALGGRAGGVATRFGGDKKAPSPVQDPSVEH
ADVFASSSLAPPSGVEAAAPPQQHSLQERPPQKQQQQQLQPAGGGSVGGSAGGGSPAQQLPLLDTQQFGRISSPISA
SVGGRVSLDDDTYLQHGADSCSLGGGGPGCGGGDGTGSDASAQLLTAPLAPHPIFPAPLQPPIRHGSYCTASAAAAA
AAACAYGSLMAGGGGCGSSSDGGAGAAAMGFHGGGAGGRQPHVAPQRCASSGVSAFASAVAQRQQLLPRQPVFDQEG
HCGGAAGSTAGGNAGGADITGSATSSTGGGGATAGCPESPFACLCCSPLVLEEGEDCAVQAAAAAAEGAAAVCDACA
RSGKDTSSRSSGGSSHVSGSGKRGGSGAAATNASARPRGAKGHLLASKLGVAAVVAVTEETAAAAATGDAAAAAVAT
APADAPEPIDADLPAPTSMSSTPSISVGDGAEGADGLTASIADACRGSGVSVSGSSSLTAAARSASEAGSAAVSSAV
ALMMRDVMWPPAVRYGGGMDPEDSRSSSIGLAPGLSSLSMGVGRSNARDAATADYLDATTAATMGGGTGANSAFSTT
AAAAAAAALAGVTGDAASPIYHLHRSPRYLWGVSPDDTTLMRALGDGGLGPCAHLTIWSSSRLGSRMGSLLPASSLA
GGMGGGSGSLYGSLAGASGMPSFATGCMSRGSISGSPVAASGVGGVSGAVDIDFPLRQPSREGSGGSAGTGAGAGGA
AAVSEARPRSARVRQRRSQQQPQVMTPTATASAFPAQPLATAMPPRPPKQASPSPAPAALASLRRSCSKAGVTSGGL
GAPPRQIRWQQRLACVVGGHSASEAEAEEDEGEAPTAASASASNGAGCIGAGDLQEALATASVSQLMAALVSRLVGA
VLAAPARMAAALLALLLSWVALAAAGSRAATGVVLDRTASVLHGLYVTAPARLLEMAAKAMADGLGAGTGAAGVVNA
GAPGNDEGDGSADVGYAGVRR

>Cre01.g011400.t2.1|PACid:27578360
MPKSSDGSGEGSSDEGSHERRLSSLVLRPAITNLPSIGGDGASDDDDDSETEVVFSHINWAAQESMIGDEELVSPGR
ARLPAAESPRVAVRPQPVAPSAAVPGALPSSKQTAGAEFATLPGHSHDRSSLSTPARTEQAARRSAAGPAVPAARLQ
ARASQKAGSDAEDDEDEDGSSAGGGAADTEYEGRGWLSGAAGRLANVEERKRAAAAASDADHVAAAARDAAALQLQL
QQLAIAASLAADALGATVAGGPAAAGSSAAAGSVVGDDGSGSLAGAESSTSPDRAAVLRKLREVDREREQRIAADFH
AAIQEAEAEPLLVAATGLSLDAGPVAGLGLDDLDALIARLSSGATDMGAGGSGTGPAKSSAAAAVAPAASGSGKSAW
ATELPAADSLWSDEEEEKKPTPPAPRPAAAPPVKPSPSVHAPAPPTPATATTKPPPAAGPPAPPSPSSGAASAPLL
PPMPAKFKFFAAPPQAGAAAPAAAGAPAAQSGADSSSSGPVKLDLRQSIAETVERNRREADEAAVKALEAEAAARAS
EAKAAAARRRVVDSDDDSEDDEAAEAARRAAKVRPQPPPKQSPPPAPAPPPAPAAAPAAAPAPAPAATAVTAPQKKV
TATASGGASTDLAVSGAPGSTVTTGAGSATAVSRRPALSDSSTSSSSVAVASGTAAGSTTSRSVTLSGAAGGGSATA
VASSSTGTDSSDLRASASKSVFVGTSENKTAEAEEEERQQDIRAYLKARQQKQEQEREAQRQARAAEQEGSEDEDDA
DAGPTGAAAAAAAAKSASERRWPSPPPAVAQREAAIGAHPLSNGVGPTAKKPVAATSAPAVATSTAVGPDGDDEEEE
GAPARAFAQCSTEGDEQWVAEPTPSTVVHASRERGARMAAAGTVLAPLGPAAAAVTTASASPRSSTTNDIGITAVSP
VATRAAPAPAAPAAPAAPAAPAPGGADAARPASLQLASAAAGRASISGVSDGVGAASLATPSSAPHTDLGRLPAWLP
PSSAFTLLLASPSNLPTAEVGQLLEWLLNAAAASGGAAAASGAVKGAPAFSVVGARLLGVSPSSPLLPAAAAACFAR
PAAAPASASAAGGGGGEVRVLAVACVARAGVDPDSYCHDALPAEAAAAAAPQLLTALGAVQWADVLPKGIRSSGGGE
QAAATTPQALQSVGAWATARAGAEAALQLFDVLRVPTQALHQHGGAFSTPRSSLTGAATTPSGSAAAAAGVAADPPG
VSTFVSLVSEGVLREPELLRALLRRACRLGLSLRGLQVLYATEEATASLVATTPHRPAPFTGGALLALSGRHEPLRR
WQAQLGPPDARLAALAEPGSLHALYGAAVRHITASYDAAAGAREVAALFCGGAGGVDAASPRPATPGAAAASPLAAG
AATTGAAVWGLTSCCPTQSQWLALSGAGAPRQAAALWCVQQLVWCGYSLRGLLLSALPPAALAGAYASASASAAGPL
AVVVEVSKEEAAQQLPVLLSELPPLPGGGGAAAAAGAAAVVSQSPLCSSDALRQQLTPAELHSLPPPYTADQLLSAA
AAVAPDLAALADGAGRASWDGSGAGFASGAGASDAASAAAGHAHAEDGVAVVALQARPLHAAAAVLAALVAYVTAPA
APAAIGVAAGGAAAQQMELVGVRYLRQVPSDVAGALTVLQPQPAGATATSSSPSAAAGAAAAAATGRHRLVISHPAL
LAAFNGPAAAARVAALLQVAVSRLATAAAATQDGGAATAAARPGDTSADLTAWSAVVDKLLRDYPTLALASPLASGA
TAAALVPPPPSGTGAAAQPPLLAAVSRTQAAAKAALARAFSLAQVYLDPGMHDGVHRPPLPEAAAVAAAAAAATTLP
SPSALCRLLAGPQRLPAVVALDFGAVGRNLLPRLIKAMAREGYALHGAATVTVGLRSALAAAMPALAELAPAVVLHV
SRSNAVAHLRHLAAALAPPHAASAGSGGQGAGAHVSGAIGGGVYTCDTWRDAAEVVAEVWPAAGGTAIVSAAAAAAP
PVAPGAPARRFVRPEGSRLLQLTAMVVTPGTTHAAGTDAAYPSLGGLGGPAAAAAPTLDWARLADALEALHAEGFRL
AGLRALPTTGRDLAALASRGAFRISPAQQQAVHASAAAAQAAAAAAAAAAAELVGVAAAVMQPLHGQSQQQQQPEQQ
QQQQLEAEPAASNVVLVALTRDNAVTTLQILLDEASAAAAAAAAQRGGPGRRQAAAALAALGPGCVSVSHSPAAGEQQ
VLTAFEGGAFGAGGVALLKWE

>Cre01.g011400.t1.3|PACid:27578361
MPKSSDGSGEGSSDEGSHERRLSSLVLRPAITNLPSIGGDGASDDDDDSETEVVFSHINWAAQESMIGDEELVSPGR
ARLPAAESPRVAVRPQPVAPSAAVPGALPSSKQTAGAEFATLPGHSHDRSSLSTPARTEQAARRSAGPAVPAARLQA
RASQKAGSDAEDDEDEDGSSAGGGAADTEYEGRGWLSGAAGRLANVEERKRAAAAASDADHVAAAARDAAALQLQLQ
QLAIAASLAADALGATVAGGPAAAGSSAAAGSVVGDDGSGSLAGAESSTSPDRAAVLRKLREVDREREQRIAADFHA
AIQEAEAEPLLVAATGLSLDAGPVAGLGLDDLDALIARLSSGATDMGAGGSGTGPAKSSAAAAVAPAASGSGKSAWA
TELPAADSLWSDEEEEKKPTPPAPRPAAAPPVKPSPSVHAPAPPTPATATTKPPPAAGPPAPPSPSSGAASAPLLP
PMPAKFKFFAAPPQAGAAAPAAAGAPAAQSGADSSSSGPVKLDLRQSIAETVERNRREADEAAVKALEAEAAARASE
AKAAAARRRVVDSDDDSEDDEAAEAARRAAKVRPQPPPKQSPPPAPAPPPAPAAAPAAAPAPAPAATAVTAPQKKVT
ATASGGASTDLAVSGAPGSTVTTGAGSATAVSRRPALSDSSTSSSSVAVASGTAAGSTTSRSVTLSGAAGGGSATAV

ASSSTGTDSSDLRASASKSVFVGTSENKTAEAEEEERQQDIRAYLKARQQKQEQEREAQRQARAAEQEGSEDEDDAD
AGPTGAAAAAAAAKSASERRWPSPPPAVAQREAAIGAHPLSNGVGPTAKKPVAATSAPAVATSTAVGPDGDDEEEEG
APARAFAQCSTEGDEQWVAEPTPSTVVHASRERGARMAAAGTVLAPLGPAAAAVTTASASPRSSTTNDIGITAVSPV
ATRAAPAPAAPAAPAAPAAPAPGGADAARPASLQLASAAAGRASISGVSDGVGAASLATPSSAPHTDLGRLPAWLPP
SSAFTLLLASPSNLPTAEVGQLLEWLLNAAAASGGAAAASGAVKGAPAFSVVGARLLGVSPSSPLLPAAAAACFARP
AAAPASASAAGGGGGEVRVLAVACVARAGVDPDSYCHDALPAEAAAAAAPQLLTALGAVQWADVLPKGIRSSGGGEQ
AAATTPQALQSVGAWATARAGAEAALQLFDVLRVPTQALHQHGGAFSTPRSSLTGAATTPSGSAAAAAGVAADPPGV
STFVSLVSEGVLREPELLRALLRRACRLGLSLRGLQVLYATEEATASLVATTPHRPAPFTGGALLALSGRHEPLRRW
QAQLGPPDARLAALAEPGSLHALYGAAVRHITASYDAAAGAREVAALFCGGAGGVDAASPRPATPGAAAASPLAAGA
ATTGAAVWGLTSCCPTQSQWLALSGAGAPRQAAALWCVQQLVWCGYSLRGLLLSALPPAALAGAYASASASAAGPLA
VVVEVSKEEAAQQLPVLLSELPPLPGGGGAAAAAGAAAVVSQSPLCSSDALRQQLTPAELHSLPPPYTADQLLSAAA
AVAPDLAALADGAGRASWDGSGAGFASGAGASDAASAAAGHAHAEDGVAVVALQARPLHAAAAVLAALVAYVTAPAA
PAAIGVAAGGAAAQQMELVGVRYLRQVPSDVAGALTVLQPQPAGATATSSSPSAAAGAAAAAATGRHRLVISHPALL
AAFNGPAAAARVAALLQVAVSRLATAAAATQDGGAATAAARPGDTSADLTAWSAVVDKLLRDYPTLALASPLASGAT
AAALVPPPPSGTGAAAQPPLLAAVSRTQAAAKAALARAFSLAQVYLDPGMHDGVHRPPLPEAAAVAAAAAAATTLPS
PSALCRLLAGPQRLPAVVALDFGAVGRNLLPRLIKAMAREGYALHGAATVTVGLRSALAAAMPALAELAPAVVLHVS
RSNAVAHLRHLAAALAPPHAASAGSGGQGAGAHVSGAIGGGVYTCDTWRDAAEVVAEVWPAAGGTAIVSAAAAAAPP
VAPGAPARRFVRPEGSRLLQLTAMVVTPGTTHAAGTDAAYPSLGGLGGPAAAAAPTLDWARLADALEALHAEGFRLA
GLRALPTTGRDLAALASRGAFRISPAQQQAVHASAAAAQAAAAAAAAAAAELVGVAAAVMQPLHGQSQQQQQPEQQQ
QQQLEAEPAASNVVLVALTRDNAVTTLQILLDEASAAAAAAAQRGGPGRRQAAAALAALGPGCVSVSHSPAAGEQQV
LTAFEGGAFGAGGVALLKWE

>Cre01.g017150.t1.3|PACid:27578389
MARAAAAGSASQTGHLHNNAVPTGRAGHERSSAVRSRTAAPEGLPVRASRVRASAPRQPSPVAARGRRTPASSEGSE
GSDVETWVRAASGRPRLRTGVTSPAAAAAAACHSAKPSAAAAPGPGPSRRRRLGDEDPALAWLLRSSSSSGSSGGGT
GRAHGVGLAGDSSADDSDGYGRADSRGSAAVASLDGWSRHQQYQQQYQQQYQQYQHQQYQHQQYQHQQYQQQYHART
AAGYGQDGPGGAGGSPLRGISHLGSPPRFASAAGRSGGGGGGSAGSPLRGSSFQQHQHYHQHQHPGSPQPARDYARL
ACGGSPSSRHAAPPPRASPPQPPYTRDMASQPRQQTPPLPQPDPHHHLRGHIPLPPQLLHPAEPMQAPDRQPHHEQQ
QRDPQHQQDPQRQQHQQQRDPQGYLSHTQLHPSHRPGSLHGPPHGAASRIHVPSRQPVHSDASSGQPPHPPPQPPLA
SSARSHVGPVAAAGRLPAPHPQPGESLHATAQAVAQAAATVAGGWRALQEACLASAGASPATSPRLGTASEMDLGLG
QRHHHQHQQHQQHQQHQQQQPQYDQAALRQLYRREGLEPGADTAAAARMAGQLYALQAQPLAHSQTHLLHLAAGGPW
VDLPLDDSPGPAPGPTAVPQGGPGSGLGARRASRMPGSSGVRHSALGVDVNGLGLEVADGLELDEAFSPALRRAVAA
AEAAAAAVSAAMAALGPEEGGGGAEEEAAAAQEQGGRLHKPRAWGKVGWAPPQLQAGAGIPAAAVSAPSATSSVRAT
ATDGSLHAAYAAAREHDGGAAGHASHPYSSVRAASSTSAAAAAAAAAAAAAPASSSPPSLAHSAAKGAHARRASRSW
DGSVVSHGVSGRDRRLLQDERDGAESVQDNFSTSPTDVVGQLVWGPWAGAAAMGGGPAQVAQGRGPEPWSGDDVGSD
ARSGLQPRMTAAGGGSGGGGGGSVGGSEGGGSVEHVAESGFRGAPSFSELLTAAALEAAALAAHRRGGAGGGGSDAA
AGFGHGYGAAEDVAATTAAAATTAAAAIGPDAALTAAAWFADPVSALTAAALLRAREALAGPGAAGSHPASLLGSRR
STATEAPIPPVTAWPLAELSRHAMALALPPEELAALAAALAAVAGAAAAASDRTDTGGAGGGIDGGGGGGQGGESGE
GGGLRRLRAVLGAGGLAGAQAAEAVEAAPDWPPRVQVPLAASGVIQRVGGGYAVRPGGEGSGSGGGGDGGGGLGGMG
AEPPTASPSASPAGGRKSYSRLPSPPPSPGATAATAGADAGRGGTAAATGAGASRGRVSPSPTSPSPTSRSPASRSR
SPRWSTAGASEAGGSSRAASPRRRSSVSGGGGFGGGRGGGGGGGGGGRGSATSPSRIPAPPPQQHQRQQQQRGRSLD
GGLSAWVGAASPRGIATSPGDAAARVAAASPGRHRAQSAAGTAVTSPASGGSGLAESLAWGRQGTSWAQDGGGTDGR
GGVGDGDGVGGVGMQPATNLTGSVQLPPQPEAVTTDIMAAKGAGTSTATDTAAIWGPAASAVLSAVAQAPLLPDTQP
FAPTSVPDAAVNPSRNPSPPTTCNTATSTTSTTALRASANGGGAGSGGAGGNTAIAAAGNTAIAAAAPCGGLPPHHP
LVYLPSTATLRSSGSGAAAGGGVPGLPLPSSAEASGSLGLSWSVSSVGWARLREEADRALEALMTQGQRQAVQVPAP
VPVPAEQAAARGTMPMPMPMPVVASAGAVTSQAGAPGAGTDAEVAGAAAGPVPDVAAECEAAAAAVRRLTAALAAAE
AAAAEAEERDYAAYVEARESGGGAGAAGAGPGAEAGSSSSSGRGGSSSSGGGGGGGGGGGATGVRSLSLAGDGCGSI
GSLGSGSGSGFLLGAGDSAGPTSAGELSGEGVRARERECEGGAAGHGQPCAGSWQSVAGVAAAAAQGEEEAGVEAED
GSGSCWAGHGGDVRWLPEAADAVGSGAAAELSSPTHGVAAVPVSPPPPRLPLSPGALDTPLCLTASPAAGAAGAIAA
ATRSPGAGSDPPSPQLCLSPYLPPAAAGSSTVEFGSGFGFLGTAPTQPPPDQTPPVAGGSHEGGGGGEAEAPGTTFY
LALSRAPGWASESGRGAREEAQQAAGPAWTHAVAAAQQKLQTQKGLRAEAHADDDDDAVGFGFAAAVAAGGIRVGDP
QPKARSLLGEQPDGEEDGGAGGAAGSDGGGGPGSGFGFGGFAPGAGEGGGGGAGGGPLTVFGAAFGVPASTCGEALG
PGDEAPAAATSAGALVGQEQEQVGSEAAGEESFEEVCTPVSHAHRAGNPFAAAAGADTGRGADSAGGAAAAPAPPHG
PSAAWVQQQVEGGGSSHSSHPHPQHNHQQQQQQGSYSLGGAMSRSLGFSAFGSPDPPRASPTASTTATAYEASSGAA
AVLSFSPGPGTGLSPGPGAGAGAGGAGFGTLVTPGTGSGGGGMLSTAAARLPRSPEADWRSAADSLGSTPYRSIYEF
RGASGGGDGGGGALDAAAGSSTVSTPGRSPGAAGVLARHHPRSSPSRRRDSPRRASTAGAPQGVGAALLAGVGRGWR
RFRDVAASVARSASPSPSPAPSPLASPSPGPSLGGGGGFGGGGRDDGGGGVFGGFASVRYNRGVGGVGGSGVGGSPL

SPLSPLSASSAHGSAAWAASPSPSPSPLGSPSPFPSNPLHEQHPRQHRHHSRGHDRRRGHDRRRGHDRPAAEQAASD
AERHSRRAADLGELAAGGLLGLASPGVSPSVAAEASAGGWDSGGDLGGGGGEGGRRGWGGGGGGGGGGGGGAVVRGG
GSEQSEWGGVRGLGGAGGGGGMGSGAGDGAGSGGGASGSPRKRVRSFTGLLLGAAEEGRAKSGRRGGGGGGG

>Cre01.g051300.t1.3|PACid:27578421
MPDNSAASPLYEGPDATSSGNAVKKEETRPPGRPSRTVCWIITAVVICIAVGVGIGVGVGVGLSSGNGDDSTSSPSP
APLVTPGNLTVIKNKVLQYIPVDIQNADTDEVIANYVAQAARIQPVTDLLASVLPALGANSSIASGLAGTVVQPQAI
QMYVSKWLEDNKVDSSSLAATLDNLAQLAQGVTDWQARLLTITRNLGNVTALVNSKVESLLNSTTSTLTGGRRRMQE
KDVFAILNTTVRSVLSSEAAKPYVAALQAAGYDPATLPALAGSLLTMARRLLGASTELRDLMERLDTHFDLNLFPSL
PIFNPRTGINLTYLVDSVPFDKRTTLVLSPAKRRRLQTTDPLRAALSLPRTALPANFSFAKPEALPRVYIPLVFHVM
TYSTTAGGYGPPNWDKTPDYVTRIVNIANAMAWRTNFQFFVNEVRYKPDSNPYLVKADRASWMGCASKPNYFYLACA
DVLNGALDHPRSINIFVVGDKADASYAGYGWVPSSATDISRGHIALLWSVLDPSQWNGQAGWEYGGKVLWHELMHHL
GLYHTFGKGNTGTGCTLASDDGVADTPIVAGPVYNQASFSSQARDYCLSYFNKDLGSSWTRVMQEWRNRLGIPTADS
SHTFDSCPSLPGVDELGNYISYTHDVCIPALGHQTPGQVLVMHNTTSRSMKVLYQWGQYLATLAPAAFETPAPPPSP
SPPPVSPPPSPIVVASPPPPPPSSPPPPPPPSPCLATRSGCRCRVNWSWNKGTYSGCARPDGDPVGLWCLVEQDGNC
PSAINGFWDYCPAGATDERCGAEFPRPPSPSPARPPPPLQPCGAGHVTASGSICSREPWAYTGVDGGEFNATYLGCA
NPDNDPQGSWCRLQAGFTGLLGRTWDYCGPVCDPAGGSSTGSSTTGPNSAANCESSQGLPAGCVCRETWGGVFLTPG
ATTGRRYTTARSCTYVPELAGQVAAGGGVCEVASCNDATLNGRVMTCASSCTVLRASDGETVDV

>Cre01.g052750.t1.3|PACid:27578565
MGRALIYLVFALALAAKLGDVDAESRPWCLDGVALCRGKTNPTETFSHTFTLVPGIGGKRTKAYSLGGQRSSAVSSY
VSLARVEALDLTDMTLLATDKIGSSIGAAARPSRSGHTALPYNESVLVFGGSVINKTESKLPVASNEVMWLAQGDAF
TPPQWLVLANRTRGEPGSGTPPPRSKAAAVVLSPGGGGGGDVLWVYGGLDNSSKPLEDAWAFNMTSGTWRQESLSSP
PTDAYGGRGLIRPAALASSDGRYMVLYGGRGSSNQTAQTKLYLMDPVAKAWKQIRINLVPSADGGLPGQLLPGNETA
IAIVQLASPNATAASNCTNTTSGGSSSSAAAPCSNATAAAAPAPTELLLVTTGPWMAKATSKPTSYKLWVSVLQSDT
GGRFEQLLPAVANGSVTSLNFVRRGLFVAMSGSGNDTRLLPDNTTIDTNVTRRFGVSDRIPYLRETAMAVGADGNRV
YISGGQDAASRASSKLLIVDLLEGAVIDTSITLKPDSNTLNSRSAFRYGAHVVAPKTLEPNTTSNQTWSPTQAGGYG
SSDLLFYTGWDDLEDDNEPALTEWRVLDAELQQVLPDCSAQNQLNNSCKVLPVPRGTNAADELPAARDGAAWAPLHQ
VFTWSDGTTTKPVGGRLVLLYGGSSMVGDQLNATAIDTSSDEDLGPDEIVMRDVISACDSAGSLDATQLDNLAQCGA
ASLGLICASTIASYFSSYYDYSYGDYSSGWYYDYSYDYSYDASPPWEYHHAAPPGASGGGSGHGGPPSNTDAPPPAQ
PATPPPPPKPPPPPPPRPPSPPHPPSPPPVPPLPPRPPSPAPPSPRPPAPPVPPLPDIAPPPEGVTEPPSPAPTSPKP
PSPTPPSPNPPSPKPPSPEPASPMPPSPGPAPPMPPSPPSPSPPSPQPPSPQPPSPEPPSPEPPSPAPPDQPVVASP
PGVTGRRLLSAAADVYGDEEGALQADPWDDSYNVATEDAASAAAAAADGEGAAARELAQGTLGRQGKQQQQRRGAGK
AQGGRRGGPRAQRQDRGGAGGGAPGGIRTRAASPGKAGGGDDDEWRRRLHSGGATDTELAAAYSGDTTAAGGVRGGR
ERGGSGGGGGGRRRSAAQSGGGEFGFSDDSVIDGGDEDAGNDESGESETEKEARSAQLRQRVKDYLLQLSQRQRLPP
HQEAHLQLLVSKAEAEAEAAAEREAEREQQGREGRRGRGRKGRKGHQGAGAGEEGGAGEAAQGRDREPGRRRRAAEG
RSGRGRQQQQGQRRRRQMAMGRGAASSSSSGCGGGGSRQKLQQLQQQGPGQQRRRSLIGDVDRSGDTPLDLSAWGNR
ATLNAAAQKASFYKSSKAGNSSSSSSNGWVRMYDAAGNLLNSDGSVKLPTLFHAAYAVLWQSKEELAATPLVNTRKP
YAKRGTSPTLVNAGPAFYFDPLLNVNGSDMHSPGQRAGSTLASLPYVTATNADVLLFGGLTAVNRSAANAWIGVPAV
TNDVHYLQYIPAAPGGRAATLAAAPPSRFASCPFAPGPCKSLAPHPQNGTHTVPPPPPAAPADDTASGAQSYSDCPA
QLSSLGPADKGIYQLRIYRGTDKAKVRSMAVGFSSVSTYAWTSPWFSTKTVLRTPVYAGNSSAWVWPPNVTEANSAP
NATIFDDYYFDGAEDYWFELAYDVYDVYLFSAGYGWTSADDLAMGCTAAAPTKCPRFELYNADSGRLIKGMGGGGQY
GSIWIPDPALGNATEWVRSFRLSLCSNASTAAAAAAAAPPAPPLPYTPNPYPFAVDDSPNLLRSHELQLAGDHDPAA
IPKPRMWHASAFVESSGLDSRLGFGKMGLLAVHGGVTNPLLDTLDVDTDLLGDLWVFDLQFRRWIQLTGGGDSPGRR
LRHNMRSDGSVIYLTGGYTYDEGTQQWPYTTDVYYLDLQLGVDAVWTQVDGDSVELGRRSSPGAIAQGMGVSKKRSQ
LVTLNNNGIVFVPLPTSATVDAPGSGGNGSAPAAVSAAQLELLVDKMYDGDLLLLPQDKDLAINGTLTVGASVVFHG
KIILRGLGGATTPRPPPGPAAPPSPPAPAPPLSPAAGRHRRAAAEAGEGAKAGVDDGATTGVLEPACERSPEDCVLE
EAEAALAGAAARRLSAGDSDDNVDLQGEQQEQPQQQRPARARQMLQQSELEAALAGHLGFRGQGGEDGSNGSSGGSG
SGGNGSGLQLRRDEAARTASRWGVLEALAAVRRVMQAAPAGPASSFSGAPTHVDVAAWMSIPRTLVKCEPGSVWMLQ
SSPNFVNMEFVNCHIIVNNPIGTLPVTFVNCVFRDNVNRPALAVMSGGTLSLKGCLFMNGTYNYDLISAAATAAVNA
AIAAANVTSSSNATATNSTSGGANATISAITNTTFVAFPPPPPAKSTSGAKLSALQLEVPYSGLGAAVYVMARGTLV
EISTSAFLGNGYLNGTQGGGAIYLAAGSCLGAVSGSVFEGNGRNATGYSGGAISAEGMGSEACVTSFTNTNFTGNVA
DTGGAVSVSTSTRPFNFTNVRFWNNTATREGGGLYMASILGRVGFSNAQFVANTAAERGGGLALEAVNAVAAEALRL
EGNAATTGMGGGLAHSRQGTLTMTRSVVTGNRAVYGGGLGLYQDLDVRLYDSLIERNTALLHAGGLECLQCYEVYMS
RCSFAYNVAESAGAIALTQVERGGAILSSNISHNVGRPTTPTDNTDQAGCARDAPGGGGGLCVFLRRNVTLSGVRFV
NNSALAGGGMYVQQRCLPGEKGCGPAVLINSTFTDNEATRGGGGALFRATHDLANVTCPLTNVTPNWTDLHTACSES
WTGNRALYGNTTATIAYNIWTATPQFVRQYRSNDPLEVVVEMRDYHNQTIIDGTREAVTTVEVVPAASAARNVSLSG

NSISTLRGVGNFSSTLRLQARAGVYGLWIATPRTIQDVPRVNLTVEVRPCILGEVTDVDGTSCFPCTSGFFSLDPQK
LECDDCPDNANCSVDGHPWITLPEDGYWHSGPRSTNILACVVEEACKSTNKWPREDNIGNFLKSLDVYTESSSSTNA
SSAATSGAAAATSLAAYDLAAYRDLQCASGYYGNLCGRCRHGYGRRSSGACAKCASRALNTLFYILSSAVNIFFIWL
TVHAQLPGSLTSRRRMERLDRERERMQSQQQRTALSWLKRSQNAPGVPRQASNMGGNTADSQLLAAGPPPPPPAPFQ
TWEPQASPYGGGARAVGEATEAPAADPAAAAAAAPGADPSADTGSAKGPKKAKRDKKQNGGDSSSSDGSSSSGSDSG
NGRRRPGARYTSGEPQQQQQQQNQKDKNGEASSDEDNDDEGKVVVDEGTGPYKGGNHGIVIKILMSYLQVSSMVRS
IAAPWPAFVMGLLSAAKSASNSITTVVSIDCSLKDGSVPVSVQRVIIQIATPFVILGLALLVWCALFSRLRLRRKGT
TETWASYLRPRIIITTIAVIFYVYPDITNTLLSMFSCPPLDPMSGPYTQYMEARGKYWASDYDLLCYAQPHLWLVAA
VGIPGVVLFAAGAPVFSWLWLKRHRDLLYTSRDFGVSYGFMYEDYNRASYFWDSVIMFRKLAVVTVIVFLQPQSANL
QVLSALGVIITAMLLQITIKPYKNEKMNNLERLSLYATMAMLYCALFFLSDLPKVAKEVIGSLLLIFNCLVVAFFVW
HIWHEFMVGFIWTIDDHSNGDGKLEWSDVWAYVDKEHSGRAYTRVLIKVLKGLQKVTGFLDTAVVRPAAAVTRAASA
AARRINTTLGRRRRSVDTAALAATAEAGAHPLSGPSVIMDGAPSSSIGKPHLAQSQKGDGSGAAAAAAAAPPPPLEE
EPSSRVHPFTSVTGSGSRRRTSSLKQLAVGSVTEPATAATAATAATAGAGAEAPDAGVGVGGGGGGGSSWGAAV
FGLGGGKNKVAPEPPGSGGSMVARRSGEAPVAAVGPAAAAGRTGVAAGSSPGPGATRQSPVLRPGPSADYLLGSGAA
EVDPGTISELSSPAAAGTGAGHHSTAAAAAAFASPSLLRRAADDAAAAAAAGAAGPAPAAAGASSKPTTPPLQPVII
MGPGAVSEGVLHSRGGTATWPQPPLALRASPPPRAGSGAVAAAAPAGGASSNNVAGTSSSRGELRGVETSEITELSG
DAFDALWAPSDAAAAAAARDGSSSSSRLKLRSAAGPVGGGSSGGGAGPH

>Cre01.g002800.t1.3|PACid:27578583
MPVSAQYQLRLTDDYTPANAVKYEGRVEVVSPLGSICSFGEFDDKDAAVICRQLGLPTARARALYQAYKFMSGPWND
PQASPPEVLSLVSAGAPPIVLTNLRCKGDETNIGSCLSDTSTLALSACSRGQAAGVQCFEEDYTVRLMTNSVNPANA
TAVAALTEGRVEVWLGEGWGLVGATNFGPDTARVVCAQLGLPVSGATVVPNGAAAYGAPASNTAVVLDGLACSGKEE
NIGFCSPPSNTPPRGTAFAAVACRQADFSVRLSGGGHPSEGRLEVYNRGQWGTVCLGGFDRSALFTVCVQAGWRTSD
ARRPSFTAFNASRLPADPRPGTGANALTSLRWCPDDAESITQCGEYKYDAAATCPAGLDVVVRCYESPPLSLNVRPT
SLCTDPWTTSATISPRPGRTTMLVGEFDGASGGARGQDVLLASYANDGTLVGHVAYSRPWGSSFIAEADINLGPTPE
PRALVLADLNGDGLDDVVAITSELVSVATATSSRSFTPLTVWLDLSTTSDSIDTSNTTTRAYILTDVTGDAAADLVF
FDRPTLRLAFFPSNRISQLMDDTDGEGITWFELSTISARCKSLGEDCFLLAADVNGDSLNDAVLVYLDRVTADQEVY
MTFVAASPPAPLTWHMAAPAFFPRGACTSPWAVVMGQFVGPDGSIDNVNAGSSVPQVACISSFDARIYVAGLGSWGV
VPGPPTRVHARDVDLDGRDDLVLFTEAGSYYLLSTGSAFEPPVSTARFGDGYTASVPTSGDRSLGETDQTASTIIAS
SQPVGDAPVEQRCGAPDRVIAYFSNRRPDRSAACTAAASADPSVSPSALTRAASSATHVIFSRLLPDADALDVALAN
DRDGPVLANLAGNLHSASPGTKVLVSVGGPGAGSDALSRLVLNAQSVGRFANASADYLQRQGLDGMELSWPELRAEQ
VPAFLSLVWALSHEVRARRGLLLGLAVPPRAVYLDLPWAGLGAGLDLINFHAFDLEGDEVLGAAPYVETPLFSCLEA
TGLSVNTMVDTMLASGAPPQKVTLVASALGRSFVLDADGWVGGPGSAGPCLGSEGLLDQAELRLLVPPGGAQLDTEA
FANTAPFAGNQWTHFDDPFTIANKVCFARYHCFGGIGLWDADADSFGALLGAAASTVNGDPALCSEYETPECTNTVS
SYGTTDLGQPELVGSVGDAEYSLFQVRKSWSDARSHCKALGGDLAAVTSRSEAGVLYSLLSNWAGSGQLGEADVYSG
RDVFVWLGGSDAVQEGRFRWVNTSADLTYTAWASGQPDGRYGGEDCMAAAIRLGGSSGGGLRIVVSQEAQWYDMGCT
AALPFVCQRSRAGGMGVAGVKLVPYLTTSLLVFPPLNRGEPGLQLTMAQGQKLCRTYGAELPSLTDYWTRSDMTSPT
GWSLDLPPYMWLGLRSYGEGQLFWSDGSFSTDGVLNAWEPGEFGDAACAIIVGPQGVELELEVDSLYSRWNATATGG
AVRISPPPPPPPNPSPPPPPPQPPPPRPSNSTPSPPSPPNPAPNSSPPPSPPGTPPDPRQRLQLSQGVYSLSCQERMP
VVCQKSSPPVSLSPTFHCLTRPNGLAFMVPGETLAPGGNALTPVASELECAVGCMIAPRCVYYIYLPGWRPAGWVPP
NNQPPEDFAPNTCYLMGRPWVTSWAPKKLSEVVTAADRVCFRSNSIFSGDTAPMSDPLVLDPPTAAGPLFGYPAPSP
TATALPFSLLCSPTAGAPVLASVSLTVANTSRNTQDVGVGCTGADGGGAEQRLWLYRPAAGGVYEAEADCGLSGVQG
VSGAFDAAGLCWLQLQCGSGAVLPVLAPADARPGCPMPQAAGSGGGGNFQYTCPPGMVGTGLQGSALSAPSPATNSI
ATLRLMCGARPPALTPPPPSQPPPAPAPSPLAVGRRRLLHSVAAPVSVVKDPFLAIAESGARRHLNQFSVCTTSALA
GRRYTTGGFSFNPSQPGGTSVAATTASACCSACAAQATRMYGYTLISTYINPMSPTLYEVACYCYSNVPSGGTVDAA
TTATADVVSGSLTYRCSGGVVQWPAPPPPRPPPPPLPPKPPLPPPRPPPSPAPLPPRPPLPPSPPSPRPSPPSPPPP
SPPRPPSPPPSPPPPSPAPRPPPAPKPPKPPGPRPPSPPSPSPPSPSPPSPQPPPSPPAPSPGVPPPSPKPPGPLPP
SPRPRPPSPPSPPPRPAPPPSPPPAPPPPPAPPPPSPPPPQPPSPPPPPPPPGPITSDAPACSDCSPWFGDSTSDSP
FYSSGTVQQQQQRGAATCPPGFGVSSLTAWFPPASQYGTQPNASQPYSSLSAACTPLLPTPAGQDHTVRVGGGWLP
PSNVTFSQTALNATACDTLGGIASVVGAFTHFTPQRPGFITAMSTRCRLTATPLAQPALGSPFVGWNFTCPAGLVVV
EVLWAQQTLTRPGLGPLPLITRVAFRCGLSAGLLPSPPVPYVSGFTATESSGLEPLSLLCPPGEYVTQVFGRYDVPT
ALSGVALFIRDLGLVCSGGAAVTKDLDTGIATVNSSSLAFTTASCPAGIAVLTGRALRHPSPPAPSATGTPGLMLSL
DALCSDTADHTSRLTYSPLLSKVGEPYSERCRELTLLSGLSLLRSRWVVGGVTTGYLHGPRLVCMDVPEPEARLPLA
AQLPTVGGVVSSPATPFLYECPEGSKAVSALALMDASGDVLSLRIECDNAPLASGAQTSLQRIAASGVPNTPIVSNT
VINVTATGDLTPMNAINTTATNTTGTGSSSGSTPPPDSGVLRYISPELIPSPPQASYLLQRSCPTGISGLSTRASQT
DVAQLGTAVVPVARMDLPDAAPAGSTAALYDVLLDWGQAALFCRNQGGSLITFTSNVQVASVALMIQEWAFGSVVDD
VLGVWVGAERTGPGLYDWSWVNGTLFSASFVPAWGANQPDDAGGVEDCAELGVNVTDAAVDWRDRGCTGLVRRPLCM

LVPKSAQAGVSSSDAAVPVVALSSNDGADIISLYNVQLPWDAASTFCQRRGGALASFDAQLEIRRLSLAVSAYMQPI
ATQPGPVQAWTGLRARLPSAGTISTALRAAVAAVQVGSREFLYVQQQRMSFDQASAWCRNEGGELASISGDEYDATV
SVAFEPAVNTLLLAQQPVPPATFTANAWIGLSRVGAGAWTWLDKSPYVASRTIIDAANTSLSCVALVTTCLNTTGST
TSAPINCSSSYVAVSCANDSYGFVCARPTDAAASAIVSGRHFRLYPQRLAWQDAAQACTDRGGLLGTFRTQDEYAQF
TAAVSRSQPLLNATGLAFINATLGTKAPGFMHVGLSRRSDGSFLWVDDKPSPAINWAANQPNGTAQQREDCAAFSVM
CNNFSGGLWSNCSLPELHDSECSLAAPYVCSFDFMSAWGYSDGTALMSQQPGGMGPVTPINYAWQSDTCGDMLKNTH
YGNLLPDLAVLTNVANATACCQSCSAVDGCAWYTHDSAARTCALKRDEGPGRLVNNTLAQYTTGPSGRPRDLIPGLF
DPAVALTDCGALSSSLTQNVSAPDYRFMPVNCATPLPVVCKRPAGLLDLTQQPTASGGGPELPEEERLALSTYLTPL
ATGFTSAGTVELSLYDTLMDAFAAQQLCRVLGGTLAELIDLDVQAAAFRVAQEALLQQPRLFAPLSSITYHIGLTDI
TDENSWRWISVGGKLQLDSALWAAGEPSNVASPVFSSDAIPLSDEDCAVATQSGNTSQWVARPCRFQFTRPMCQRPT
SASAYGAQLRSLAPAAQTLQGTAHFQLFTRLMDWPAAEMLCQAQGGNLAWFDSPLQFEAVTAMTTSWMRRNEQASNF
GTWFGLNDRVTEDDFRYSANPRVRVNASAWHPWAAGSRGRDFVDLPVSGATNFGSWSATVTCPNGTVVRGFSARVDN
RTAAVSTATDLMGVMNMEFQCYNTSTDSMAAPLRPVADTTATTIGTWGPVAKCPGNSSNYAVGLQLRAVAKQDYSIF
KLVRTSANRTTLTTATVYRDDVAVTSARLLCRYGQTVEVTNPSSLGNWMAETRCDAGYSVCGIRLQTQSFNTFATVS
MANSSASDNTMVNGAYIRCCSDGPSVNDCAEIRTEIRDGSATTGVARWADQPCTTTRSQVSVNHVLCRRNVVPSASQ
LQRVALITSNTSVLSRAEGATGDGGVGGGGASWRPTAATPLMPAIAQSVAPQASERFTYYVLETDDDGGSGGGGGDD
DGDSAATSLFSTLQCRARRKESCPRGRPAIPRRVLRTDEFLLFDVPMTWSLAKSFCAAHSAELAPVMDSMEQQSASD
LVASWARGRVGRSVSFWFGASNTGNGAPWRYTTGERLRWSAWGRDSVAPIRPACGEMRFGVELVPVSLSSLLSGSRV
SQSLLREVLTSNTTSGIAQSGTFQLPRVGRRKPG

>Cre01.g049200.t1.3|PACid:27578621
MGQPSPAPPSPSPPSPRPPSPAPSPRPPSPRPPSPRPPSPSPPSPAPPVPPAPPSPAPSPAPPSPSPPSPRPPSPAP
SPLPPSPRPPSPRPPSPSPPSPAPPVPPAPPSPAPSPAPPSPSPPSPAPSPAPPSPSPPSPRPPSPAPSPLPPSPRP
PSPRPPSPRPPSPAPPSPAPPTPFVQGAVPFKISADWAPIRGTDGLPPAGTFTNVSGCPDPREALQYHNQVRAKHQA
PPLTWSQDIASQAQAWAEMLAKNNSCGLYHSSIGENLAASWGSASNVANCTRASVWWYGEEPQYDYSNPGNPKNASD
VIGHFTAMVWRNLTRVGCGAAVATHPQYGYCWVVACQYRASMNIYGYYGRNVLPPIASSGPANCTAPGTGWCNTTAG
AVQSVVDCDGDGGLDVACREPGTTRRGVVLSTRSCRNDTGSYMNPDLGTMAWPSASPVFCPAVFGYDLDLVTAWAYG
GGAYEVWRGNASLTSRGVSWGGNNVDDTAVNNEAITWSSGSSPDPTQYHVCVRWYAAVRYFLLNVTLTIMRNNQTAA
TFTSAVDTSTFHSATCKPGANGFLGTYDIAYSVFYTPNGTMIGYLPPSPAPPSPAPPRPPAPPSPPPAPPSPPPSPA
PPSPSPPSPRPPSPPPSPRPPSPRPPSPSPPIPAPPSPPAPPSPPVPPPVPPSPPPSPRPPLPQPPAPSPPIPTFTP
GFNLRFRLMWSQPDTGINTTVLDNDIYVLWYAGSVLNVASYFSNSTKGTYFGDNVGLKVNEETVYWPPGTMPDVAEY
SVCLRPYVTRYLELDVRLEVFVNSSTVPTAVISTRLDTGRYGFIGEVESCSSTTPGFMGRFNYTGANIVQTAAPAAP
PNPPTTSLRIRSRWDVKNPGTSNVGSSSTDLDMVVQWTNGTTTYFVSNNKANSGGASYVYDGGYSFRVEDMQWADGT
PPAPSTVINVCLAFYNPNGTAPFSVNAAVAVALNGEALTTRFWKELFSTGLRSVAGSWTSCTSTSPGFIGSYTYTPP
VTVSSMSADGDGALLMSDGLLDGTDATLYDTSAGDPFWSGLGPSGLGGSQVSKQLDTGAAPSSGYSATKLQTISQLE
GKAGAAARQVDSPAASQPEQDLPLLPSPDGGAGGAPGAAPAAGALTTAATTTTTGSSGSGSEAGGAADTAGVLPSA
VAVSTSSNGNPGVPVATVVAAAVGGVVGAAVLTVSVMALVRWRRGAVREHTRVVPLGVA

>g1103.t1|PACid:27578651
MPGSAISGPALVHSTRVRRRKNRRALPLMTLIVMGIACLGSIMAATAYAAGDVEEESGLLFVEQPPSTDSSTSVNFF
SSSSSQLPAGGTTSSQSTTCTQGSCCDVVMYPSNSTGQLIGGKGPVVTCGRQAACLGVYLAGSTCKVQAEAGTSKVY
TYCEVCIFWSNASGACSKSLSDTISHVCSADRQYGVVQAPGQAPQTSDTNKLEGWKSGLDDGYCQWIRWNTTSSAWA
ETLWSVKDGSGSCLANGAAPVTIDLPRGANASCTGPRLTSGNNRVGCANGNQANECLWRIWLPRPGGDVIPCTASPP
SPQPPDPPNPPSPPSPRPPAPPSPAPPTPSSPKPPLPPSPKSPVPPSPRPPSPSPPQPPSPAPPLPPSPKPPVPPSP
RPPSPSPPQPPSPAPPLPPSPKPPVPPSPGPPSPSPPQPPSPAPPSLAPPSPNPPAPPSPAPPSPQPSSPSPPSPHP
PSPAPPSPTPPSPAPPAPPSPKPPSPTPPIPPSPKPPAPPSPKPPVPPSPRPPSPPRPPLPPLPSNDCVYRGSFDTP
VDVKRGPAVTCADQAACLDVIFDGSQCEQDYADVDEVEDLQQGDGDSSSTTSTDGQQGSSSSRRRRLQTTSSSDDGP
YQQDVYTYCKVCLAWSNARGGCGKDTRDTLSHVCSADRFMAVVPGAGQAPVVGSTNKLNNWESGDQNRYCQWVRWNS
SSTWTEVYWSIKDGNGECLPAGQTAPLSVTLQGLRAQCGGRRRINSTTVAGCDKGSQSNECLWRIDLPLPGSPDFQC
RAPSPPSPRPPVPPSPSPPAPPSPRPPAPPSPKPPSPQPPSPAPPSPAPPSPRPPAPPSPAPPAPPSPAPPSPQPPS
PAPPSPAPPSPAPPAPPSPAPPSPAPPSPRPPAPPSPAPPAPPSPAPPSPQPPSPAPPSPAPPSPAPPAPPSPAPPS
PAPPSPAPPSPAPAPGVPPPSPVPPSPAPGVPPPSPAPPSPAPGVPPPSPAPPSPSPGVPPPSPAPPSPSPGVPPPS
PEPPSPAPGVPPPSPEPPSPAPGVPPPSPEPPSPAPGVPPPSPEPPSPAPGVPPPSPAPPSPSPGVPPPSPEPPSPA
PGVPPPSPEPPSPAPGVPPPSPEPPSPAPGVPPPSPEPPSPAPGVPPPSPEPPSPAPGVPPPSPAPPSPAPGVPPPS
PAPPSPAPGVPPPSPEPPSPAPGVPPTSPAPPSPAPGVPPPSPAPPSPAPGVPPPSPEPPSPAPGVPPPSPAPPSPA
PGVPPPSPAPPSPAPGVPPPSPAPPSPAPGVPPPSPEPPSPAPGVPPPSPEPPSPAPGVPPPSPAPPSPSPGVPPPS
PEPPSPAPGVPPPSPAPPSPSPGVPPPSPEPPSPAPGVPPPSPEPPSPAPGVPPPSPEPPSPAPGVPPPSPAPPSPA

PGVPPPSPAPPSPAPGVPPPSPAPPSPAPGVPPPSPEPPSPAPGVPPTSPAPPSPAPGVPPPSPAPPSPAPGVPPPS
PEPPSPAPGVPPPSPAPPSPAPGVPPPSPAPPSPAPGVPPPSPAPPSPAPGVPPPSPEPPSPAPGATPPSPEPPSPS
PVPPSPAPPSPEPPSPTPTGPPNPAPYSPSPPTPAPPSPVPPSPAPLEPPNPTPASPEPPSPSPLSPTSPSPAPVEP
PSPAPLSPEPPILAPPSPAPPSPAPLTPPSPSPLPPPVTICASCVEFQLSNSPFASTSFTFDSSTCASLEALVADDL
DLLLTGSGLVVETPFAISICDSTAVRICGAYSPSSVIDISLDLELWLEAASASNVCNGVSIRAGAILDPNFTTGSAS
PVCLTGAAQRDCALELPPSPDPPSPEPPSSPAPPPPATIASFLPPPPPPPPPESPPPPPPPPPDTPPQPPPSPPPPP
PDAPPPPPPPDAPPLPPSPPPPPSPPPPRPPPTGQGLFGQPPLRNIPPFAPDAPPPSAPPQDCAYKGPSTATDGAPPG
SRSCGMQASCLDVIMDSSECMYRTTTGRKYLYCPVHLTYPREGSMCEITSAARASSPPPSGRNRGGSTTAAGGDTRA
STGAIVAAGSSSSSSAGTSAVEIVEIDIAYSDVLVCAGDRLGTVIEGPGGVPVPGGVNQVMWLPSSNYTQWARWEAD
DLSPALITFSIRNGNKACAANSNPTQILVNGMTATCSNPRYDPDSGAVAGCQSNDGTVFNECLWTITVPRPGGIGWT
SAVCVRDSPSPPSPAPRPPPRPGASPNIRAPAAPPDPCVLQGPSSDGGGLSNAIACGTQGSCLDIYYNSADCKYQTL
SGDPFASQQYLMCPICLTFPRPGSPCALNALTPIDYVCAGDENSLVIDGVTGQPVVGGGNIKRGWEEGTRYCQWARW
SSDDMAPQTIAFSIKNGANRCIRRNSIDITVRGLTASCGAPRVNPFNGFAGCAGNDDVDNECLWNITVPRPGGVGWT
GNVCVTPPPPMPPSPKPPRPGAKPSKGNPPVPPSPTPPRPPKPVAKPGGRSPANLVPPPPPPPPSSHRIPFPFCACKK
RSPRNAPYRLVYRNTTQLATLSDGLARKRHCFSVEVTPCDETTSCCGMGIKKIELFARNECRKSVRLVLVDGMSVSW
SFTQDVFQSATYTTFKLPTLTMSRVDVGSGFDVCMQVIEPCASLEDFCWGDSGSCRSTFFSLDEQCCPTGDTVFGAA
PSRSPDPEGAPPPVMEVEVGRRLRRAT

>Cre01.g003450.t2.1|PACid:27578666
MGHSRSRSRSLGRSPKRQRRDRGGSGSPRGRSRSPNGGSRRHYSRSPSRSRSPGGGFGGRPAGYSPSRAGGLLGRGP
PGSALGAGGGRGGPGGGPAGGPGGGGPGGGGGGGSSGGVSPPAWVTNARLAPQVKHCLENLYGRGKLGASDLEYACV
EFLELLGPQGGMAVLDEFASLDFFRIRNLTAFFIGICKRVQNRAPPPPGRLGRGGPEGGSPPPSLERGAGGGGAGGA
GAGGAGLGSGGGSLRGRTMARGGGGGAGGAGGRSSRSPGSAAGMRRASPPLHFMLDTAGDSPPPRRWVSKSPPPRSR
WISKSPPPRRYGSKSPPLKRWVSRSPPPRRWISKSPPRGRVGSKSPPMKRWVSRSPPRRQGSKSPRRGRSRSRSPPD
AGQQPGTSHGQPHHASHLHNHRHRSSSPSAQQQQHPHMQAGGAATGQADGGAASGAFSQAAAAAAAAVGQQAHPYTH
THTHRHHLPHSASANNISTSSRTSASAGGVGAAGGAAAAGGAQFDTATAAAAGAGAGAALGAVNGGEGLAAGSGPSR
GGSLMLELPDGHGLAAAAAPGVDGAAAAALPPLPPPPEPEPEPAEDWARVEAELLGVPAALLDGRILEAAAEALEAA
AAAAATCSVRADAAGGPGGASAGGGTGAEAGAAAGGNGPTAAGGDASPAARGDAPGGSAAGPASPMPQPARGCCGES
GLLSASAARITAAACSGTGAERSSAAAAAAAAASCYCPEPPLPAAACPLGGWLSLELGVGLDAAAGRGGWAASGTGT
GPPLQGGGEEEEDEGEEGEVAPAGGDPLQFARQVLTRAAKTDADAAAAFAAAAEEEQQASAALAAAAAGSDEDGPDS
LPPWLLRGLLATALDTASAAAAAAAAAAARDGGGGTGAAPASVPAAAGVAASLAGASGAACNGGALAGSGPSGAVAAG
GGGGGSGVGLGTPAGGAGPSGAGFVGEGGLLRQAVTEHVKEALKPAWRANRLTRETFKAVAKKAVDKVLDMLPDGGA
GGAYDTPGGVAAFFGDPGRRDSIARVVESLLS

>Cre01.g003450.t1.3|PACid:27578667
MGHSRSRSRSLGRSPKRQRRDRGGSGSPRGRSRSPNGGSRRHYSRSPSRSRSPGGGFGGRPAGYSPSRAGGLLGRGP
PGSALGAGGGRGGPGGGPAGGPGGGGPGGGGGGGSSGGVSPPAWVTNARLAPQVKHCLENLYGRGKLGASDLEYACV
EFLELLGPQGGMAVLDEFASLDFFRIRNLTAFFIGICKRVQNRAPPPPGRLGRGGPEGGSPPPSLERGAGGGGAGGA
GAGGAGLGSGGGSLRGRTMARGGGGGAGGAGGRSSRSPGSAAGMRRASPPLHFMLDTAGDSPPPRRWVSKSPPPRRW
ISKSPPPRRYGSKSPPLKRWVSRSPPPRRWISKSPPRGRVGSKSPPMKRWVSRSPPRRQGSKSPRRGRSRSRSPPDA
GQQPGTSHGQPHHASHLHNHRHRSSSPSAQQQQHPHMQAGGAATGQADGGAASGAFSQAAAAAAAAVGQQAHPYTHT
HTHRHHLPHSASANNISTSSRTSASAGGVGAAGGAAAAGGAQFDTATAAAAGAGAGAALGAVNGGEGLAAGSGPSRG
GSLMLELPDGHGLAAAAAPGVDGAAAAALPPLPPPPEPEPEPAEDWARVEAELLGVPAALLDGRILEAAAEALEAAA
AAAATCSVRADAAGGPGGASAGGGTGAEAGAAAGGNGPTAAGGDASPAARGDAPGGSAAGPASPMPQPARGCCGESG
LLSASAARITAAACSGTGAERSSAAAAAAAAASCYCPEPPLPAAACPLGGWLSLELGVGLDAAAGRGGWAASGTGTG
PPLQGGGEEEEDEGEEGEVAPAGGDPLQFARQVLTRAAKTDADAAAAFAAAAEEEQQASAALAAAAAGSDEDGPDSL
PPWLLRGLLATALDTASAAAAAAAAAAARDGGGGTGAAPASVPAAAGVAASLAGASGAACNGGALAGSGPSGAVAAGG
GGGGSGVGLGTPAGGAGPSGAGFVGEGGLLRQAVTEHVKEALKPAWRANRLTRETFKAVAKKAVDKVLDMLPDGGAG
GAYDTPGGVAAFFGDPGRRDSIARVVESLLS

>Cre01.g004600.t1.3|PACid:27578766
MATVGEPPLEQLRELFHLSARDACITLNISQSRLKKICRQHGISRWPHRKLASLDSLRQQLKGDSGLTTQDRAVLLA
RLEAQVAAVIAEPDRPLEKLFEDMRQTSYKVRYHARNKLRRGTGAAGEGADDGDAAGTASGGDASSPAGRALSTERG
GGRRGRRGAASPRRGPQRRAASSGPASGSRLRRAGRRRGSDWGGSSGEEEAASDSTEAASDDEEQAAGGAAAGGYSE
RDDDEDDEGDDGEDSDEDDEDDEEDEDWVAGGRAGKAGGRQARADMAAAATAGGSAGRADRLRRRATGLQGLRVPAA
ASPATPRSRRRRRSAAPLDSGDTEPSPGGRSAAAAAPLATTACGGGDTNSSDSAGRSLEQRQPAAREGAGDAAGRPP
TAPQPQLSGRSRTVASRSASARAGAPAGLPGFSPVRDAATPSSHTGPAAASRGRLHSGGHGHGHAHGRAAKPERGGA

AATLSAFAAAERAVAGASAAGAAGGGGGGGGGGGGSAVKQEREQSAGQLVGPGGGGGVAGAAGRGADGAAARDDARAA
AGDVAVEAGAGTGGSSQLDGSSQWGMKRARSAPQPQPLGAAGVAGTTSSHQTTSSDRSQHQQQQAPLPPLQARTSRE
WPPMDMMPSGWQQPYRQPMPLDATRTTPATATTATTAAGGGADVGSGRHQDRRLDHPSHLQQAPQHPHPQEPLHQRA
QRPASSPTASMGGPATKQQAHGSYGAAAPLHHTAAQSHSHSYSHAPPTATTAPPAEPHPFFSPAAAAAAAAAAGPGI
ESSLGLGSSVPRSGALEHGVEDWEWRWREEGQQRHQRALLQQQQQQQEYMPPAPHHHMPHEQQQRHQQLLQSQSQSQ
SQSQSQPQSQQQRHDLHREQLPPLPPQPEALSRQQQEQRQQEHFELQQLQLLQQQQQSQQLQQPQQHLMPSSSSFQR
DWIPPPQPDPQPLPSWRQSEPLHAEPQPRASRSQQQPQQLQSPGHVQQGQPLQHGGASGSAPTYRGGGFGSELDGGW
HWQQQQQQQQQQQQLLQQQQAASLSHQLQEREQQELQRQQAQARHHMQLQQQQQGAPSSSWHGQYPPLHQRPDQAH
AQYPHLQQPQEHQHPQQERYPPLHQQTQQQLQQQQQQPQLQQQQQAQWPGADPRLLAPGRQPLPPPPSWSARQGPGS
APPSTSAGHPTLQQQLYPPMYQQQTQQQPQPQQQNAGQQPAYHHHPLLSPHSPQYQQRMQASAPVGSGGAHRQSQSI
IPGPPSALLSLPSPASNEPSPRDFAYPSPLPPHHQPPHQPHPHHHSLSGSGFDSEGGADAAAAARRPSNQSQSFDQQ
HQAAAIASGTLRNWSSSSSASSLLVWGSAWPPGSGLGGMGPGGMGPGGVGSGGMGPGPLGPGGSNPHGAAGSGGGSA
YGAAGFRSGLHSGGGVGGAGGAGFGGSAGGGYGWSSGGSSYSGGGFAHAMGLPSSGGGAAAASGTGGDGGGGDENRM
LHEGLRGRSAYVSDAGDRRDEAVAEAAAAAAATVAAGPGGGRMGGRDAAGTRGGGGGTGSGAATTAAVAAAAAAAAA
VAAVAATSGAADHETRHRSPALQPPTHHGAPTDLLSSRHHARHSHVLPPAGMNYAAYLPPPLSLPPALDSPHQPPYS
ASHGPAHGRLNPAAYSRVVSTGFPGIHPGSLPGSSSPSPSGGGGGGGGANAGGGRVGGGSGTAARRRGGDRAASPLGS
YGSGAAGGGGGIAAGVKAEDRGPSGGSGGDGGFGGDDTRDVEPHLGHVAFHGGNAGQQQTAAYRRTSAPAAAAAATA
DPRAQSASGERDSGGGVAVDLRRVPYTGRLSEPVMRAPLRALLGLGSDLADEGEGEAELAASMAAAARDEERFRMLS
TGSSTVAIFAPEAQPQPHQQTQHQQPHQPSRPVEGRPHHGYGLPRLQQGAPEGRVASASAADRGGRRTSGDMERGGN
GGRRPHTVPDARGWAGPADLLLACQETDAIAQAEAADGVRGAVGHLHQATYQAAASLHVAQQPRGGAAYEEPPPRRQ
PHQAAGAGGPSPVCSADLTTPTAAHQRPQQLRPHAAAPPSATSSRHQVSRRAAAAEAAVVTATPVAATGSSGRPGPA
AGPSPAAAPVTAAAASGAAFLSPVASVAAGSGVAGGPATSSASSGRRSSGGGSSSSGGRTLLVWTGSQPRWGQVLPP
LQHQSQPQQQQPQQQQQPQQQPELQSPSMRPPQLQPLPLMPSSAPLRTGPQRRISGAGAAGAEVTTGTATASGPGQE
STSAAAAGAAGALPPMPPLPASLLAALAAGGGAGGDLSPDAYPVTALQPPAVGAADRGTDAGQREQDRPQGRRASPP
PAATDPANATTAGGVPLSILEYLRHGSGSEPPLDLEALEDLDMDLDLDFQTPQAGDAAAALALMRPWLTSTPGLGPT
PMEISPRAALPATRRISAAGTEAPAPQRQQAPPATGQPTCEDAAGSTAAGGGTAAAPAARMTSAGQAAASTALSLSA
SAAGPSRPPHATCAAADATTFASPVAAAAADAARQHEFASPAPHAAAATAAAPAAVSPCTPAAAVIPGSGAGGAAGA
GAPASPPPAPVKRPPPARLFLHTVRRHSGTGEGPAAEPTAARAHAPAAHAEGGGMDAPPLPHHQSQQQHQSQQHPQH
HHQQASPYGGAAAHMYAAWCSSPAATPTRHNASGGGAAAAAAGSTGGAAFAAAPAPPQAPRAAAAADADTRSVSVTG
GLGGSGSGGSAHGGGTGGLGGVSPRSRAALLVDVAEPLGPYGPQQPTTATSTAAAAVAAGEAGGASGRQLQPAPAPP
TAASRYPWNMGWGAGASGAAGRENTPLRQLPPFPPPQPELAALPLHRAQATGTVATGTAGPGEQGPQGYQQRSGGGD
TTEAHVPPPLLQPPPRATSPTGYAVASYRRDSEGGGVIPVVSPPPQLMPQSVRPPPRLAPLVAPTAAAGPPQPPPLT
SPPAPRKPAKQPALMAPGPPAGQRRSSGGSVDTGGSAGGAAGRNSPASLAGAGTGAAGPLPKLPPHVLERPLPPRMQ
VHMPAPAPSPAAVQGPVGATAHSGAAAPGSRLLAAAMSPVGGTNAGGAAAGASPPPLLKVTIGGRPGSLQSAIGSFS
VQPTQLPQPPQLPALPQSQHQSQRQHQVAEQGRFGMPGAAGWAVPGAAVGAAGSHLPQLHQHQHAAAGILDGDTQSA
GGTGGGGVSSKRKREGDSE

>g952.t1|PACid:27578814
MPPEELLPLVAQADAGRGVSLGPPPAPPNSPPPPEPPAQPAAQAADAVVEQGSIVDTEAVTRYWTEGFVYLGLAADL
GVAGVPETTDPATERRRRATQELGSSELLFRSFRLHADGSLYAEARPWALDVSSLGSERQGSVTDAELLMRRRERHR
LLRQQRQQQAQQPERSRGNGHEGEDPEQQQWESDSNEGDDGQAEERQQAQPASLHRAAAGQGVRRRRRGLLQKEDAR
YPVRDTGYWPNNAVVQMYFVTTGTNTATSCSGTWVSGYDVLTATHCVYNWAAGATYTNFVIRPAMSGDVWNGTYKAV
FTTWYRSEWNRTNYLNEVNYYDIAMIRAEASDTSTIMPYPTYLGYKYDCAKSLYSASTCGYPNDRRLGDFAASASYT
QMCCDFSFPGTTCQADFQPLTSCYTYPGASGSSVFDLGDYKAIGVVSGRPSDNTSYSRWTPLTAQHFGALERWRYQG
GVVAPLPPAPPSPPPPPPLNYTRYACASNGTVRLVSSSFDGRGRVEICAPDIGGKLWWSSVCSRGWGYPEAQVVCRQ
LGYTTTTAQPTQKGYFGAASSDQYFFFISNVTCVGTEASLSDCRQTAWGNLDSCSSLDVAGVVCDGSTGSGSTQDSV
TAIPYTNDTCTQWATRVNNATTYANGTVVGRIEVCINGVWGTVCRRLWDDVDATVACKGLSYTYGVAKRLNESWSST
EPWQVAQPSVPIWLADLNCTGAEADLSACNQLLAYGSTQCTHGMDAGIVCSNTTLGPQYSDTAQCSTLGDIRLVPTD
DASVGGAEVAGRVEVCYNRRWGTICADSYWGVEEAEVLCRHLGYSYFFVNMTASMGSLPVWASYTQCTGNEDRFFKC
QMWGLREVTDCTSGTRAVVACSNSPTIAAPPPPASPYACTTPGALRLAPNDTSTKTYMYSDTTAGTAGGRLEFCYNG
QWGTLCAEYWSTPEARVACRQLGGGLEYGGVVANGTYGFAPSGQPVWLSIANCTGAETSFAECARYSNYNYTITNTT
FYPGQLGVLSLESTLYGGKTVCQSHLADLNIWCSKTWLSPPPPPSPPPPAPPPVGDTCSAGEDGKLRLVTGNGTSYV
YDGTSGQTQTGFLQVCYSGTWGRVCAARFGTPEAHVACRQLGFAGGSKMTYSGLLPLYVWGPAKLGPAWIDGLICNG
TEAMLTYCYFKGWGSLVDSCPPVQGVDAGADLVVQCLTSVNSPPPPSPPVPPVANYGCDTQGALRLVDKSGAVVDTS
SAATVEGAVHVCMNSSWSYVCDYLFSSNFAATVVCKQLGFGGGTPYSWTQLAARGYNNSLVQPAGMPRGPSQLQVCY
GDEPTLLDCEWTANDVFNCPTSNSVAGVFCTRQAPPPSPPAPPPSPPPYWSQTSCSRNYDIRFVDGPNPTAGRVEVC
YNGTWGLICARYWDDSLSNIICRQAMGSSSAIGEAMIDWDGVATAAANGTYPRFAYLLASVTVTYHLDGGFIKPLCT

GNENKLSECVPLEAFGKAASTCSRFSEVGINCWASPSAKVSVPATTAPYSCAGFQNGTVRLVNGATPAMGRVEVCWN
GNWSVVCGSGWANAAAATVCRQMGYTSGYVNNLGGISLTSLAPNSRYAIWGTPASDQPVALHRVTCTSTATHILNCT
LNSGTASNQFYSSGVAPIGSCIAPARVVCTGLGFYMPPPSPPSPPSPPPSPAPPSPQPPSPPPLPPSPRPPSPAPPS
PPPKPPSPEPPSPAPSPMPPSPAPPSPEPPSPDPPSPEPASAPPSPSPPSPAPPGPEPPSPVPPSPAPPSPEPPSPD
PPSPEPASAPPSPSPPSPAPPSPEPPSPAPLPVPPSPAPPSPEPPSPDPPSPEPASAPPSPSPPSPAPPSPEPPSPA
PLPVPPSPAPPSPEPPSPDPPSPEPASAPPSPCPPSPAPPSPEPPSPAPSPVPLSPAPRSPAPPSPDPPSPEPASTP
PSPSPPSPAPPSPEPPSPAPLPVPPSPAPPSPAPPSPDPPSPEPASAPPSPSPPSPAPPSPEPPSPAPLPVPPSPAP
PSPAPPSPDPPSPEPASAPPSPSPPSPAPPSPEPPSPAPLPVPPSPAPPSPEPPSPDPPSPEPASAPPSPSPPSPAP
PSPEPPSPAPPVPSPVPPSPAPPSPSPPSPAPPSPAPSPDPPSPAPPSPAPSPAPPSPEPPSPAPLPVPPSPAPPSP
EPPSPDPPSPEPASAPPSPSPPSPAPPSPEPPSPAPPAPSPVPPSPAPPSPTPPSPDPPSPAPSPDPPSPAPPSPAP
SPAPPSPEPPSPAPPAPSPVPPSPAPPSPTPPSPDPPSPAPSPDPPSPAPPSPAPSPAPPSPEPPSPAPPAPSPVPP
SPAPPSPTPPSPDPPSPAPSPDPPSPAPPSPAPSPAPPSPEPPSPAPPAPSPVPPSPAPPSPTPPSPDPPSPAPSPD
PPSPAPPSPAPSPAPPSPEPPSPAPPAPSPVPPSPAPPSPTPPSPDPPSPAPSPDPPSPAPPSPAPSPAPPSPEPPS
PAPPAPSPVPPSPAPPSPTPPSPDPPSPAPSPDPPSPAPPSPAPSPAPPSPEPPSPAPPAPSPVPPSPAPPSPTPPS
PDRPSPAPSPDPPSPAPPSPAPSPAPPSPDPAPPSPSPPVPPSPQPPSPAPVDAPPSPAPPVPPSPEPPSPAPPSPA
PPVPPSPQPPSPAPVDAPPTPAPPVPPSPEPPSPAPPSPSPPVPPSPQPPSPAPVDAPPSPAPPVPPSPEPPSPAPP
SPAPPVPPSPQPPSPAPVDAPPSPAPPVPPSPEPPSPAPPSPAPPVPPSPDPPSPAPVDAPPSPAPPVPPSPKPPRP
APPSPAPPVPPSPQPPSPAPVDAPPSPAPPVPPSPEPPSPAPPSPAPPVPPSPQPPSPAPVDAPPSPAPPVPPSPEP
PSPAPPSPAPPVPPSPQPPSPAPVDAPPSPAPPVPPSPEPPSPAPPSPAPPVPPSPQPPSPAPVDAPPTPAPPVPPS
PEPPSPAPPSPAPPVPPSPQPPSPAPVDAPPTPVLPVPPSPEPPSPAPPSPAPPVPPSPQPPSPAPVDAPPSPAPPV
PPSPEPPSPAPPSPAPPVPPSPQPPSPAPVDAPPSPAPPVPPSPEPPSPAPPSPAPPVPPSPQPPSPAPVDAPPSPA
PTVPPSPEPPSPAPPSPAPPVPPSPQPPSPAPVDAPPSPAPPPPSPAPVDAPPSPAPPVPPSPEPPSPAPPSPAPPV
PPSPQPPSPAPVDAPPSPGPPVPPSPERPSPAPPSPSPPVPPSRQPPSPAPVDAPPTPAPPVPPSPEPPSPAPPSPA
PPVPPSFAPSPVPPSQRPPGPSPPPPLPPGLALMLNDFSATFVNSTIIADLDVTNTSSVMSFLQEFKQMTAAALGLS
VDQIIVRAVTVDGQQLARRRAQEDEPIGDWQSLEPSAEFQKFGRVTLLQHRAYVGDYETIAALKALEERRLLAPQLP
RGAASKGRRHRQLAEAASDVIVDFTVVQYEEVELPPSPPPLPPLSPGLVAPPGTPLRPPSRPPRPPRPPPLNLNKLA
QELGAQEVIVVYSPPSPQPPGPAPPPPLSPQPPSPAPSPPAGSGAALPAALEATIVGAEYAAVVGSPTFEVEFCADL
ISSSGSKLCLVIYILPGSGGVVVGAMLVPQDATSQADFDTVVQSMLDYPNGVFTIVFTSAYRVSSVILKRSPSPRPP
FSPSPTPPPFFMSMSPPPPPPPPPPETPPSPSPSSPPPPFPLPPGWPTPPPLAPPPRLPPPLSPAPSVPESLLAPPS
PAPQEPSQPPASPVPSPVPHDLPPPPHARSPKPPRRKRQPPFPQKPPKSPKPPKPRRSPPSPPSPQPPAPYPPPGKP
KRPPRGRAPPRPLLQAAKEDPPPAQPESPTPPTLPPPPMQRRRDKSPPLLPPPAPPPDAPPASAPPPSRRNRSGRPA

>Cre01.g051950.t1.2|PACid:27578838
MVPLKQRQQEPSWRRHLLRSGYMPVQGSVREIGYFYTTLKLGTPERTFSVIIDTGSTITYIPCKDCSHCGKHTAEWF
DPDKSTTAKKLACGDPLCNCGTPSCTCNNDRCYYSRTYAERSSSEGWMIEDTFGFPDSDSPVRLVFGCENGETGEIY
RQMADGIMGMGNNHNAFQSQLVQRKVIEDVFSLCFGYPKDGILLLGDVTLPEGANTVYTPLLTHLHLHYYNVKMDGI
TVNGQTLAFDASVFDRGYGTVLDSGTTFTYLPTDAFKAMAKAVGDYVEKKGLQSTPGADPQYNDICWKGAPDQFKDL
DKYFPPAEFVFGGGAKLTLPPLRYLFLSKPAEYCLGIFDNGNSGALVGGVSVRDVVVTYDRRNSKVGFTTMACADVA
RKLAERSTAAPNATVIKVSPPPPNPAPPAPKPPSPAPTPKPAAPKPVPSPAKDAETKNGDSSSGSTDGKADSGKPKV
SNTTGTSKPAATPSPAATPQPAATPRPSASTSGAGKGTGVVMEDYDYEAVQGGQGSGEGENFPLDHEEDYDETLKEI
EEELAKVAPVMPLPMASPPPPPPNGKQGQGAARPMQYKKPKPTHSGSGIGGGGHGSGSGGAGATTSATTSSPSPSPS
PPAGGSGASSTGEAGSGSAAPYHAQVVKESSGMGGYGLIIVAVVVVGGVIVAAELFVFRRHKLRAWLASLSRARSAQ
DETELAPLAGAKGILGGSRV

>g51.t1|PACid:27578878
MSPRRHALPLLLAAVAGGMLLGLQAPPVIAAVTDGQGAGGRSGAVPPQQQPQSPFPEPPRPPRSAPSAGRSDSAGSS
ISSSSSSSSLAGADSTSKYSSPAASRSSYSSIGSSSSSNYSSSSTDASSRSSGRLALVLHTPEQLWAALRQLLLLRH
AAAEADAETEPPSDGGGDGGGSNDAVLVVGGDMYFSGIGSGSTGAGEGAGAAGAAAAPHSSAAAGGTASRPRQLRVT
LQGASGGTAAAGGAGGPRPRFDLPGLRALLSQLTLPPPSAPPPPRTPAPDPTRSGPAPSPPPPLPPPLPAAGVDLRL
ALVGLRVMDVAGPELGGDSDLLFLPPQPPGRQPSQPTAGPYLLPPATEARAAPAAPSEPWQQQWQQQWQQQWQQVQA
GVPGRHHNHQMQQREPQPQQQQPAGSPGTAAAAVQPAQSPGLSPTGALRAAAPAGSAAGTANTTTTRANTNTGSTGG
TSGAAAAAAHQHQHQHQHQHQHQHQHQHQHQHQHQHQHQHQHQHQHQHQHQHQHQHQHQHQHQHQHQHQHQHQHQHQ
HQHQHQHQCQQPQQAPAAAAGAAAAAVPAPSTAQQQSEAPGAGAGATAAPQPPSPTAAGLRKPPPPPQLPPPVPPAG
RQPSPSPPPPPPPPSPPPPPPPSAALQRAVTGMQRTAPPVALSPSPPSPIALPSPQQLQAGLSQQNQSRSSQTRPQP
QPHSSRASTEAPAQAPMAAVAAAAAGNLTGQRRPMGGVGAPPAGARAINGTALTLANAGKPYVNPSPSSSPSPSPSP
EAAGMARPLQQTQPQQPATLQHPSPSLATAEQLWRRWQEQPQPPLAPAAPHNRAVPTTAATATGYLLPGRNTTAGTV
GARFLVAASKASATAAESRNRSAADGGGGRITAATAAPMPLPSVAANALIAPAAPAPTPPSAPPAPVARAQRQAAPS

SRNLAIPAAPIAASIGGATLLLLAAGLGLAAFDRRRRLWRLAEAAPAAAAAAAAAAAAAQPPPLAPPAQAPLRQQSV
AVAGGAPGAGGGAGAGGAGGGGGGGGGGAAAPTVPAAGGGSGGGVASPFSPLQQLRRL

>g303.t1|PACid:27578883
MALPPDPEAGGAQAFVYRDASARGMYAGLAPHFNDRRLSDIVILRPDGRSIYAHQVVLAACSRKFSNVFGHGEATGK
ELPIQGVDSEALELVLSSFYRGECKLTPTTVVPIYDVCLKLEVPGLTSACEQFLQRSMGPDTCCMYLEAALAVLLEH
TVKMCLSYAKDRFEEIAAAPSFLRLSTKSVRLLLTSTRNSGSGINDISLARALARWAVSKPDHLSECESIFKELNVT
TTALLQLLQSADFVHALQGQQKPGTGQGSTAAAGGGGGGSGGAGGGGSTTGRQPPPPLPRQQQQQAGLAAPSAAPQQ
QQLQQQQIGAGHGREGDRGTFGYAPDANSRDAREGAAGGGGMGNGRLGGASGSPAHVAGAHMQQGQAAGTGGPPPPH
AGGHSAQQQLRNSPMLASGGSGSRAAERGGPTDYGEGPPGGMRPPAAAAGTTAERGDRYDRTGWYSGGGSGAGRGLA
GQAGPPGSGLTAGRPGGGRSSPPPPFGRQSHLVEHAAGAGAGSERVGGIDGGPFQRRSLPSELLAGGPGIGGGGGDD
DRQAYARRYGGGSDRAAPDSELAGAAATTRGGGGGGGGSGGGGGGERDNLVAVPLPVLEQLGLTVRLTSSGALAATG
GPLGSKQVPLSVSGSGRSGPLMAMLPAGLVKYLQQVGSGGPMPYEAGGGSGRSGAGGAAAGRLDDGSPPLPPGLLPP
QQHQQQPRAYGSADRALDRNGGSGLSPHAAGPSGRGGAGLGPHQRSSLDMLSTVAASAEEEAGFVDIPGLPRLGQTG
GGRLMAPRSASLGLGGLATEVGASGGRSGRFDLLGLGGLAPHLTADGGVAGRWSVPGAIAGTSGSLLPLGGSLGGPG
DLLGGGLNGGSRAPLGGMSKRQRPSPEDLDPDHEEPGLGLGGAGGGRGGGLTPGGGARARGGGGYGSKGPLLCHVDD
CNVDLSSLKEYHQRFRICDFHLKAEVVLREGIPQRFCQQCGRFHLLSEFDGTKRSCRARLQRHNARRRKRPDQRGDS
DDVPRRQRPVYQTMRQPPYLSAGVAAGNMALAAAAAAAAGVGAPLSGGDAGGLHIPDLTSLPPAGSSGDGPHAQLPL
DLQQLLAQQHTLPTGGVAGWPDGWPGGGGTADDADGDGDYDAEDNGGPSGDDAAMEGTPEDGDNRFTPQQLQQQQQA
GAMRGGGSAAAGSGIRSQIIRDILMQAEERQQSEQQQRQQAAGQGQGQQNAAPPAAASGARASLLDLAMAVRAQEGE
ARPPDAAGGTAGGGRTPKQGVDMDQQQSAGAARGAGLDGRSGAAGEAADTDGVPPATATAGGGGASNVGDGNSPRRP
QQPSTAAMPQPPPQAPSPPPAVKIKLEPTTSADVADGARMPADAAATATSAAGDDTRAAAAAAGGGK

>g303.t2|PACid:27578884
MALPPDPEAGGAQAFVYRDASARGMYAGLAPHFNDRRLSDIVILRPDGRSIYAHQVVLAACSRKFSNVFGHGEATGK
ELPIQGVDSEALELVLSSFYRGECKLTPTTVVPIYDVCLKLEVPGLTSACEQFLQRSMGPDTCCMYLEAALAVLLEH
TVKMCLSYAKDRFEEIAAAPSFLRLSTKSVRLLLTSTRNSGSGINDISLARALARWAVSKPDHLSECESIFKELNVT
TTALLQLLQSADFVHALQGQQKPGTGQGSTAAAGGGGGGSGGAGGGGSTTGRQPPPPLPRQQQQQAGLAAPSAAPQQ
QQLQQQQIGAGHGREGDRGTFGYAPDANSRDAREGAAGGGGMGNGRLGGASGSPAHVAGAHMQQGQAAGTGGPPPPH
AGGHSAQQQLRNSPMLASGGSGSRAAERGGPTDYGEGPPGGMRPPAAAAGTTAERGDRYDRTGWYSGGGSGAGRGLA
GQAGPPGSGLTAGRPGGGRSSPPPPFGRQSHLVEHAAGAGAGSERVGGIDGGPFQRRSLPSELLAGGPGIGGGGGDD
DRQAYARRYGGGSDRAAPDSELAGAAATTRGGGGGGGGSGGGGGGERDNLVAVPLPVLEQLGLTVRLTSSGALAATG
GPLGSKQVPLSVSGSGRSGPLMAMLPAGLVKYLQQVGSGGPMPYEAGGGSGRSGAGGAAAGRLDDGSPPLPPGLLPP
QQHQQQPRAYGSADRALDRNGGSGLSPHAAGPSGRGGAGLGPHQRSSLDMLSTVAASAEEEAGFVDIPGLPRLGQTG
GGRLMAPRSASLGLGGLATEVGASGGRSGRFDLLGLGGLAPHLTADGGVAGRWSVPGAIAGTSGSLLPLGGSLGGPG
DLLGGGLNGGSRAPLGGMSKRQRPSPEDLDPDHEEPGLGLGGAGGGRGGGLTPGGGARARGGGGYGSKGPLLCHVDD
CNVDLSSLKEYHQRFRICDFHLKAEVVLREGIPQRFCQQCGRFHLLSEFDGTKRSCRARLQRHNARRRKRPDQRGDS
DDVPRRRPVYQTMRQPPYLSAGVAAGNMALAAAAAAAAGVGAPLSGGDAGGLHIPDLTSLPPAGSSGDGPHAQLPLD
LQQLLAQQHTLPTGGVAGWPDGWPGGGGTADDADGDGDYDAEDNGGPSGDDAAMEGTPEDGDNRFTPQQLQQQQQAG
AMRGGGSAAAGSGIRSQIIRDILMQAEERQQSEQQQRQQAAGQGQGQQNAAPPAAASGARASLLDLAMAVRAQEGEA
RPPDAAGGTAGGGRTPKQGVDMDQQQSAGAARGAGLDGRSGAAGEAADTDGVPPATATAGGGGASNVGDGNSPRRPQ
QPSTAAMPQPPPQAPSPPPAVKIKLEPTTSADVADGARMPADAAATATSAAGDDTRAAAAAAGGGK

>Cre01.g027650.t1.2|PACid:27578950
MSTYGSILAATASSRPLNDSGRYGVQLQPRADSQAGTSGMSNSHGVRAWYHSQHPLGATWSPSRSPPPASTANNHWP
RTTSVNPHPSSALAASLPRPASLTSTTTTLSPNGKVHVAASPGSVTGQSASPSPAPRLSAAQAPYSNALMNQVLLDS
LRNIKALRQHSQARMQQQRQQEQQQPQSPRSPRSARQSPRHSQRTSPNQPRTSPLRSDHPRQPQQQQQPSPPAHQ
PHQQHQQHQHQQLPQGQELQDRQQQHQYQQQQRQENQDRFYRESQGQPHQSPPPPQRQQPHAHLDLHRFQSLQPAA
RGVAPVLGGAASRLPDGPGSPYGRASAPLSSYGARATQRAASASSATAGAAGPDAADRTFHLPRRAPAHPSPSAPLV
LQPDPNASIAGLPVSPHQRLVSDQHRPTAFQARPLSPRARPGSGAAERAAAAAAAALPPPPPPLPPHLRNEIGTVPA
LADMWPPAVESLRAVAAEPGGQPRAGYDPVLAASLPPLRSLPTRTLLAAAVTGLQQQLNNTMEALGCLSSLSALSAL
SCLQPALQAPMHVVRERAAAAATAVAAAAADAARGLQAEQERAREAWLAEQQRLQQKQELYQQQQQAVQSQQQQRTA
APPPSRQVVPAVAAVAAVPVTAAVAAPSIATTAPAAQAIADEAAAAPAPPEATAPVAAAAPAAVELQAPADPPAHDA
PVPAVARADSMKQPQSQPEVALAAEGSAANAAAAAPPSPAPAGSAPAAVAAATAPAAEGGGGGGGGGAGGWGGGDDG
HLALSPRSSIASELAPPSKAIWPFVELEHPPPPQQQQQQQQPPPPLQTPGTAEDRTSPAAGAGAGAGASSVKGATGRAG
SSIRSSAEIVAPSNAAAASAAAALAAAAAAASPAAPAGGVASFAAAADVNVTLSPAALQQLLAETARASAASALDLY
HHRHVMQLQQQLYDERIQQRSVQLDRERELMRQLREAAEAAAATAAGHAQSSAAAARRDGAAVAASQAEAAEARAQA

EQAQLAAAKARSDAAVARWIAAASADSWNASDPALRTMITLVPAASPMLGTATASATALTATAAVTVSLPEPERERV
RERAAPVAGSAQLLKSAAAAAAAAAIEAAAVDAGAAAGAEAEDAVEAEMRRLGLRDGLQGMGPGSGAEAGNTEVGTA
AGKEQGPERRQEDEEAQEVEEEPEQEEEEEPEGEATLSIPPPVASLRAYVAAAKAAGGSREGAEAGAGEAAGPPAAR
AGRSGSAPQDGPAASAAAYVFRRDGESWPSAGLEAGQGRAAARDEEAMGSPADEKDGGSAHKATVKRTDSGGSSSS
SSKASSSSSNSISSSESGRGKDGGGSRQHAAGAGGAGAHHAGDLVPGDGGAQEEEADVAAVVAGPAPARPVLGQGAG
AFGAGAAGPRPQARARLPVAEGPPATSAAAAVAVVGEAAAPGAVTGPAGAGGGAGAGRGGSRAASRSGSMGHGRAAE
PKAGVGPAAANAGPVPPASAAAAAAAAVGDVDESEDGGVWGRGVRRGGSGGSDSGDSGGSGGSWRYVELFPSPQSQA
LSEGVASGRYGSSTATAAATPAAGAGAGPVAVAEEGRGPVGTGRRQREWRHGSDSGTEEPEEDPQEATAAGRTREQP
PQPSPAQPLPRPRPDDDEDDELMDVEDVPDLEEVEEVGEVKEVPGQNGASAEAATAKDGVEVASGAPQPQPQSQRPV
AVDGDATDAVTAAPARAPSARSVLLPAADADAGAGAEGRSVAVEGPATEVQATPAESSVGSGGRSDGRYSQAQQSHG
VHITSTAAAAEDAPRRGYATGASDGAHKDDAADAATSPFSRARPPPPTAEEAHAPAPHASPPAAQPAAPLAAAAAAA
AADADIPEEEALTRQGSASAHRGSASGAAAAGDRRQPLPLPPPAALAALLQHSHSLGGDEVADVSFGDSLAFVDPYD
PFERSLPLSISFGALLAAEAAGLSTAAAGAAAGGAGGGRRGRVSGSGSGGLPAGLPRPASGSQLPALGGRFGALPPV

>Cre01.g006900.t1.3|PACid:27578951
MGLYLQVTVLLGWVRTQPQPTQHAGHGDGPNVQPEAAPSPPYGAYAPAATPAPPYPVGYGNHGDGGYGGYSGGGGGY
YGMHYPPLAYPGGYATPPPLIPGGYPPPAAATGQLFPPAEGILASGAPGTSPPQTPSPPPLLPGSVDELPSPPLTPA
PPSFPDPPAPPSPPPRPPPPPLSFLPIDGIVEYISSNEGSTYLLRLDNSTGSGSNAGAAAQLLAAGIRLLRLEPPAD
ASEAQGGGLTAQMRRVGVRLGDRVSLVCGLLIQAPTSALTGQPQASCVSVLGLKRLSTQYGSGEEAGSALGGRQRAL
VALVEMDCSAGWPAGMGPLELAVLGQPQKVTSLTVDDGTEWLRRLDHELSGCSRGALRITVNESEVLPVAVPCVLDW
LRRVQTCATWRPAYDRADVRIVVLPAGAVANCPWRGVSLPSDRQALLPAGNDVKGTSLPWVGVHETLHLFGLQHAGT
ADYELSSRPRSTEGDPTSPMGCERCGSDVSICPNAPQMYYLGWADPLAELDADLLPVGRPLQLTIPLQSKSRQSLVR
LLPNWLPAMSQTSNVYLSYRLADDDESGSDGIEIGLDAAYSGKLLLHQVAVDADVRRVPGLQHGDTQLVRAVSPGDS
ATLGSLRLLLRTGFPVYLDEAGYNVELPIILCRFTSPTTATAECPMRISPSPPPRPPAAPRPPAPATFQRNRVAVGI
SHVCLLMDGGVVKCFGDNAVGQLCVGDDQVRGDQPREMGDALPAAQLGAGVVRSVVSGSSHSCAVLERPTGDVVVKC
WGDGQLGQLGLGSTASRGDSRSSVGEALPAVDLTGLVPAALSAGGWHTCALFEAGFIRWLGLDGRLGVNDRANRGDK
PGDMGSSLRAVALPPGTIATAITSSSWHNCALVMAAREDDPSATRVQQVMCWGDSSLGQMGRGDTRDRGDSATSPIR
PVDFGTGLTPVLVAAGGQHTCAILVAASTGAAGGGGTPQRLVKCWGSNSEGQLGLGDTNNRGDNPGEMGDALPAVDL
GRGYTPVDLGLGWRHSCAVLQPDAAPWLRVLKRNGGYQLGYGDLNNRGDQPGEMGDALPVVSLGAGLSVSEVRASVT
GSATCVMLNPGGIFKCWGYADFGLGQGSLGAVGTRPEQMGDALPPIQLLGPGSTPPSPPRLPSGPPLPSAPSPPRRP
PLPPSVPSPPSPRPPAPPLPSGLGILQVVTGLHHACVGLEGGFVKCWGDNGSGQLGLGLPSSVSLGDGPGEMGAALP
LVDLGWGMRAVQLAAGNSHMCALLQSVEGGGAVVKCWGINNSGNLGYGDTRDRGTSPSDMGDSLPAVDLGPGLNPIS
IVAGHWHTCAILQPTSGGIYQVKCWGYGASGQLGHDCTCNEGNTPNKMGANLKPIDLGLAAPDGSAMEPVALASGAG
HVCALMRPAANGGSNSGGGSGNITYASNRAVCWGYNAYGQLGRGDKNDRGNEPNEMGPNLVWLDLGPGLAPLSLTAG
RYHTCALLGGVSPASAAVRAVKCFGDNAAGELGLGSTSVRGDQPGEMGFTLQAVQLGSGLVPLQVVAGAEHTCVLCA
PANTSAVPPAQRRLVKCFGSADNGQLGLGDTNNRGDQFGEMGNALPAVDFGPGLVPVQVATSCSASFTCVLLEPTAA
APGGGGDAAAVGTQPRLRSLKCFGSNDKGQLGQGDTYNRGDSPGEMGPYLPPLDLQVPPPPPPSPPPSPPLPPFAGL
ALITVVTGSSHACVLLEASVVKCWGSNAYGQLGIAGVASWGRNGGETIAALTPLPLAGTTPGAPGGGDFTVTAITAG
SRHTCVLMSADRPAPAASPAAAQAAPSEIRCWGYNDNGQLGMGHMLALGGGPGEMAALLPVQLGRGLRPVAVQAGCW
HTCALLEPMVTGVAGAPTRVMKCWGWNGYGQLGLGDTSARGYRPGGMAEALPPLQFGDGVTPLQMAPGYMHTCALLE
EDDGLGPTRVIKCWGSNGVGQIGTENGEVFGDEGGEVPAALPPLELGEPAGYVPVALEAGDDHNCAILHPPSSPESP
PLDSGGGAGASVAAGVGGGTVVPAVLGAIVKCWGRNDEGQLGRGQPGNVGASPGDMAALQPVDLGAPSGDPAAVLV
PVQLALGWQHTCVVAEEWAPQAGGEQAGQLVRKGSLLKCWGRNAHGQLGLGDSRARGLQPGDIGSGLPAVDLGTRQP
VQVAAQRRCSRCRWRRRRAAASRAR

>Cre01.g006900.t2.1|PACid:27578952
MGLYLQVTVLLGWVRTQPQPTQHAGHGDGPNVQPEAAPSPPYGAYAPAATPAPPYPVGYGNHGDGGYGGYSGGGGGY
YGMHYPPLAYPGGYATPPPLIPGGYPPPAAATGQLFPPAEGILASGAPGTSPPQTPSPPPLLPGSVDELPSPPLTPA
PPSFPDPPAPPSPPPRPPPPPLSFLPIDGIVEYISSNEGSTYLLRLDNSTGSGSNAGAAAQLLAAGIRLLRLEPPAD
ASEAQGGGLTAQMRRVGVRLGDRVSLVCGLLIQAPTSALTGQPQASCVSVLGLKRLSTQYGSGEEAGSALGGRQRAL
VALVEMDCSAGWPAGMGPLELAVLGQPQKVTSLTVDDGTEWLRRLDHELSGCSRGALRITVNESEVLPVAVPCVLDW
LRRVQTCATWRPAYDRADVRIVVLPAGAVANCPWRGVSLPSDRQALLPAGNDVKGTSLPWVGVHETLHLFGLQHAGT
ADYELSSRPRSTEGDPTSPMGCERCGSDVSICPNAPQMYYLGWADPLAELDADLLPVGRPLQLTIPLQSKSRQSLVR
LLPNWLPAMSQTSNVYLSYRLADDDESGSDGIEIGLDAAYSGKLLLHQVAVDADVRRVPGLQHGDTQLVRAVSPGDS
ATLGSLRLLLRTGFPVYLDEAGYNVELPIILCRFTSPTTATAECPMRISPSPPPRPPAAPRPPAPATFQRNRVAVGI
SHVCLLMDGGVVKCFGDNAVGQLCVGDDQVRGDQPREMGDALPAAQLGAGVVRSVVSGSSHSCAVLERPTGDVVVKC
WGDGQLGQLGLGSTASRGDSRSSVGEALPAVDLTGLVPAALSAGGWHTCALFEAGFIRWLGLDGRLGVNDRANRGDK

PGDMGSSLRAVALPPGTIATAITSSSWHNCALVMAAREDDPSATRVQQVMCWGDSSLGQMGRDTRDRGDSATSPIR
PVDFGTGLTPVLVAAGGQHTCAILVAASTGAAGGGGTPQRLVKCWGSNSEGQLGLGDTNNRGDNPGEMGDALPAVDL
GRGYTPVDLGLGWRHSCAVLQPDAAPWLRVLKRNGGYQLGYGDLNNRGDQPGEMGDALPVVSLGAGLSVSEVRASVT
GSATCVMLNPGGIFKCWGYADFGLGQGSLGAVGTRPEQMGDALPPIQLLGPGSTPPSPPRLPSGPPLPSAPSPPRRP
PLPPSVPSPPSPRPPAPPLPSGLGILQVVTGLHHACVGLEGGFVKCWGDNGSGQLGLGLPSSVSLGDGPGEMGAALP
LVDLGHMCALLQSVEGGGAVVKCINNSGNLGYGDTRDRGTSPSDMGDSLPAVDLGPGLNPISIVAGHWHTCAILQPT
SGGIYQVKCWGYGASGQLGHDCTCNEGNTPNKMGANLKPIDLGLAAPDGSAMEPVALASGAGHVCALMRPAANGGSN
SGGGSGNITYASNRAVCWGYNAYGQLGRGDKNDRGNEPNEMGPNLVWLDLGPGLAPLSLTAGRYHTCALLGGVSPAS
AAVRAVKCFGDNAAGELGLGSTSVRGDQPGEMGFTLQAVQLGSGLVPLQVVAGAEHTCVLCAPANTSAVPPAQRRLV
KCFGSADNGQLGLGDTNNRGDQFGEMGNALPAVDFGPGLVPVQVATSCSASFTCVLLEPTAAAPGGGGDAAAVGTQP
RLRSLKCFGSNDKGQLGQGDTYNRGDSPGEMGPYLPPLDLQVPPPPPPSPPPSPPLPPFAGLALITVVTGSSHACVL
LEASVVKCWGSNAYGQLGIAGVASWGRNGGETIAALTPLPLAGTTPGAPGGGDFTVTAITAGSRHTCVLMSADRPAP
AASPAAAQAAPSEIRCWGYNDNGQLGMGHMLALGGGPGEMAALLPVQLGRGLRPVAVQAGCWHTCALLEPMVTGVAG
APTRVMKCWGWNGYGQLGLGDTSARGYRPGGMAEALPPLQFGDGVTPLQMAPGYMHTCALLEEDDGLGPTRVIKCWG
SNGVGQIGTENGEVFGDEGGEVPAALPPLELGEPAGYVPVALEAGDDHNCAILHPPSSPESPPLDSGGGAGASVAAG
VGGGTVVPAVLGAIVKCWGRNDEGQLGRGQPGNVGASPGDMAALQPVDLGAPPSGDPAAVLVPVQLALGWQHTCVVA
EEWAPQAGGEQAGQLVRKGSLLKCWGRNAHGQLGLGDSRARGLQPGDIGSGLPAVDLGTRQPVQVAAQRRCSRCRWR
RRRAAASRAR

>g29.t1|PACid:27578978
MVLGAPRSGRGSANSLRGPLALLCLILLVELNDCHAAVGADARPADKSTRRGWGSRTLQRRRRVQQASASGDPPTPT
SSAPTTPTPSVATSAAPDLYSHLAASFPGRALWERILAPGLAPLAPQLADPTFRGTLLWPKYSVFNTDRTWRALLTA
DGSQPVNDTALAVVRRLLSNHLLPGVLLLPPDSSSGGGGGQEAGEVASGSVVATAADGGSGSGEAAAAVVRVTWRDA
GNSSAAAGAGAGAGAGTGAGVAPLEVTVVAASAGAGAGAAGIADAADTGAPTSRGVGAQWPVLVGQAVVWVVDGLLL
PLGVAELPTTNTSSSATNLYDYFSGALDPALWGIMRGAMTAADVAALQDPTAATTAFVPSAQAVMSNPALLPLVLQP
AANGPAIRRLWLRHLVLGSGLSAYAQLADGLVLHSWAGEALTVRHNATVPLPLVNGFGVHTPVRVGKSGVWTLDGVI
MPPPPPPSPPAPPRPPPSLPGPPRPPRAPPSPPRPPPAPRPPPPAPPLPPLVPAPPPSPSPAPATPLPPAAPTRPPS
PPAAAAGGPQEQPTGAAGVPSPPPPSPPPPSPPPPPPLPPPPPPPPPSPPSPPSPPAPPVPPPAPPPAAPASSTPR
RNLTFGSTGKHFRDMYQLVAGVPELSGVALMEAAIAAASGASLIGVIPPQIQFTLFAPQASAVAARATRLALAPLDV
ALDMVALVLVDRALPSSDFAEGLRLRTGTNSPELALTFDCVMHVDAATGAVTVQGPYNAIEVLGADVFVADPSDAEE
PGGWGPPFGVVHLVSGFLLREPPPPPPPSRSPSPPPPPLLVSPPPPAQQPYDPDCAGEGGLASDRDPPVVGMAADCG
VVGDVSAMTQNGVCSDGGGKCPWYAAEDRVGDKPPLDVTFRLVLQLPDGSRSSGRNALALPCRRRPAASDPAGLASR
YYQPQLYLQLEPPLQLRPGQALVLQARDAACADPGRWGDALVLAPAGWRSFLWLEGGVPRPACPERAPPVPPAPAPP
VAPPLAPLPPPPPPPEPPAPPVVRSLHLPLPPVTHHCRIVVTRHAAGGATAAAAATATATAASIDCWTAGGSSSSSS
SSSSSSSSSSSSSSSSSSSSSSAAPLQLQLQLGRSLAAALQSSPSAPDSGSVRGAQVVAWSRHQQQQPPAVAATTAA
VAAAAAAVGGGDASAAASDDGGQSAEDVQAQAGQEEDEDWGLTMTMTGSAGSGAGDGAGVPYPYPYLHLRLVDCVWR
GLPLSRTGPLMHLRMTGGAAAGAAGAAAASGPTPAAFLTLENVTLSDLTGSSSSGSGSSSSSSGGSSSSGTSTGSAS
SSSSSSSSPAAAAAAAAAAAGSGAGSPTSTSTSTSTPAEAEAEAEAEAEAEAEAEAGSAAAAAASSLLVPPVPTLL
LVGSVVEGNEVVAASAGGGGSGGGGGGLQCGGSGGGGGLMDTAAAADGLLGFGAVVVTAGGGSSSAGDDGGGGGVAV
WVEDSVLRGNVGGCGAALAVLLLVQAQAAAAAAGGASAAAAVVLSGVTAEANSAAHAGGVLYMQQIIERPVAVADAS
AAAGGSGATAADDADAAAALAAPLQLQLQVVGRSRLLRNAAGARGGAVCADGGGGAAVNISLAGGSVLEDNTAGEAG
GGLWAARLAGLSLAGGSFLRRNAAVAGSGGGLFVEGGAEWVSVVDGSGVVGNTAAAAGGGLRVGGDLIALTLARGGR
MAGNVAGGAGGGLACGGELKNVAVRDGAAAEGNRARLGGWLHVAAKVGGLLVSGGGAVVSGNSAATSGGAVWSGSAA
STVWVTGGGEVVGNTAGDTGGALHLTGGVGTLTLSNRSVVARNSAGGSGGFLYADFSVLALVVSGNASLSNNTAATG
DGGAVHALQVRRLALQEGAVVSGNVAGRYGGAFYVFELLETLAAAGGATLSGNRAMYGGAVYAGLLVRNISLTEGAT
ASGNTAAYDGGAFNVQGDLQSLALGGGASSLRGNTAAAGDGGGVYVQRRLEALAVGEGAAMEGNSAGVNGGAVAVAG
DVRELALTGGARLARNTARAGDGGAIRIGGDLGAALVSGGGTAVEHNTAGGGSRGSGGGGGSGGAIWVGGGVGWVRV
AAGAALLSNSAAAEGGALYVGGAELGSLELVGGAAAAANAAGSAGGVVFAAGAVANLTVAGNCSLSRNSAGARGGVL
AVAGGGGVRWGRLADSSFVGNRGGEGGVLHLAAAVGFSAWGSLDLTGCVLTGNRATSGSGGVLAVPGGGASGGGSAA
TATTATTAATAATATAAAGGAVPAQQPSTGSGGGSPTPQPPQLPPSTSTSSPSSSSSSAPSSRLVFRGCVLSGNSAR
VHGGVAALARLPDLLTLEGVEAAGNTALSGSGGVLYVSPESNVLLLSSATTPGDEEQDPQDAGAAEAAAAAAGGSGS
SSSSSSSSSSSSSSSSSSSSSSSTAAPAACGGAAASVSLLLLRDARLYGNRANMDGGAIYIRTAVATTAADTTAAA
CSRSEVDVQATAFTGNSAGGGGGVLFIINAQSAPASSGSSSGSSSGSSTLAPGSLSVRVRGGSLFESNTAGLRQQQQ
QQQQPGGGGAAGRYATATAYAAFEAAAQSAVSGFRGYGGALFVWQQRPDRLLAGSSSTSSSSSSSGDGSSSSAGQQQ
QQQQPQECVLVVEDATFRGNAGGGGSGGDVAAFLCAVRAARCRFEGGGAAGGEGGALAVWGSLRAEAASAAGSTANA
SSDTADGAVEAARRRHLQGSAPACVPLGQQQVPAAAALPAAAATAAPPLPPPLLLDLSDCAFVNTSAGGGGGAVMAR
AAGAVVRLTRTNFTGSRSSGGDGGALLLELLGSGSSSSGSSSSSSSSSNGGGWAALTNCSFASCAAAAGSGGGAWVA

LAAGARADVAGCAFTGNSAAQDGGGLAYLQLPPPPPPTDGGEAGLRGGGLSVGPLGAAAVDAASFTGNTARAGAGVS
AQGCAALRLQATALVAGRAATLGGGAAVLGCGAAVVEGCEVRGNEALVGAGLYLAAAGAGDGSSSGSGSSSGSSNNG
SRRLQQAGGPATAAAAAPAVALLLRTALDANSAVPSRTQLAQLDYGALAAAESGGSSSSSGSSGSSGSSSTGTAGAA
GGMLGSAAANTVGRGGALFVGDGVALAVVGCGFGGANRGLSGAALASAQSCPARQSWRGGGAAGGAAAGGGEAAGGE
AAVSTAVGAADWLVQSMAASSALLLLLLAPAVGGSSGGAGGGGGAPGAWGDKATATAALLAALGDGLAGSSSSSAPA
PAPAPGSGSSCAALLVRDSALPLSAEDGQASAAPLWLLDPAMSAVLAACDLPAHTAATGDTTTSSSTAGTSSASASV
PASSQVVSPQAAQAQAQAQAQAQAQVQARALGSQGAADVVAALLQCARRTPQQAAADDPAPSAGGDGSGGAAGAFLE
LPPMRLRLRAPCELVVGGGAAAAAAAAAAAAAAAAAGQQAAGQCDAAAGLSVPVEAAFSLAVQLVDVNGRDVTSAAYP
YTASLAVRALPPGGSAHNTSLADLDTLCSSSTGAAAAAATAAGQQPPPPPAWCDPQFVAVDSAGGGGGGGGGSGAGG
ALTLTLARGGGVWSRLVLRGWPGEYGLFVNVSGREWRVRELALPLSLQACRLGQTLDARAAVVGGVATWTSCRACDS
GQVGLLRDLRPPYAAYYRGSGGGSGGSGSSGGGGGGGSGSGGGSSGGSGVLSGEVADGGGEGGGASGGNLTAAAVRA
VAAAGSSSFCMNCPVNAFCPGGAVVVPAPGFWHSAANSSRIHRCPNPAACSGNSASPSSSSSSSASASSSADLAYTD
SRTRALVSCQRDWYASAVPGAALLARWRATGGSSSSAGGSSSGSGGSSNSSGNATGQDDTGPCLLWMDGDVAAITGS
SITGSSITSNSITGSSSSSSSSSSSSSSSSSSYSLYGDMLCAPGYTGNLCAACRPGYVINADFDCSSCPRLDRTIGLG
VLAFAVNLLLILYTVFANMDIGEDPDEASIGDILKVIIIHAQHFVILTRLNIAYPAAIGVIKGTLSTLTGAEAYLAF
NYGCVVAGRSSAARAQAQLLGNLVSPVAVAAVSLVLWALRYKFLHRALLRKAPAMPRSHLHSSSRRRMLDGLVAAAA
ALEEQEGEEDLGEGEGAEREGEEGQEEWEEERGASRQSGDVETATAAAALEYDHCNAGAGGGGGGGGGGGGGGGGG
AAGVALTAAAAAGGSGAGPDSAVLPVDDVGEQEEPWQEQVAAGEGAPVPLPLPLPPAQAGEQRCPRTHPHPQSEAEA
ATQQQPAAQPHPHPQPEPEPEPEPEPEPEPQPEPALGLPVGGYPDIYPHTHHPYRPAAAQPPAATAAATAGAAAAATAG
VGGSQQPPGLLQGLLGSLRRSLTLPRQQQQQQHTQPSTDTAEAAPLPPPPPAEHEPPRPPPPLQLHPQLPPLPLRER
LQLQQQQYYPPPLQQHPQEAAAAHSTAAPAPPAAAATATAAATAATAANAAPAATATAAGGDGGGGSVWDRPSPFAM
LGAGDVEAVEGRSSSRSSVRVHVRLPAAAAAAGSAAPPPPPPDSSSAAAAAVVAADSAPAAAAAAAAAAAAAAATVSS
FIALPASQQHPHALPSKAPQRGPSRLATATTTTTTTTTSRTPRPSPTTTNIKPRLSLRTSGSGAADAGERPSRPSRP
SRRSSTSGGVAGPGTPLAELRSRPGAALRYAFSTVVLGHAPTSSSSAVFMNKNSLAGRSSGGRSSSSAGGGGRSSAG
AGLGGGGGGGLALVHVDTALGLVPQLGIVMMVAVFVLYPALAQASLSVFSCIIIDDGAGPLAANQLATWPWGYWVRD
MGMRCYTGLHAALYVPLGAVCVLAFCLAPPAVAAATMYRLRAQLPAFHTQAVYGFLYRRYRPNWYFWESVLLLQTLT
LVAVDVFSRSVPTLYKALLLLAALIIISSVNMVCNPIRSRLVVVLEFISQMVLCMTITLSLFFIDTDSESGYLSSVS
AGVVGVIIICLNLGLLALFTALMVRRSWAGLAARLRSAADFAAVMLGGGGGGGGGGGGGGGGGEDEGGGEDGSSSSV
TSSCHGRGGGGGGGGGGGGRGHVGASQQPEAAGAPRKPNGGGGGGGGVEQQQHCYHSAAAPPAPHRLSGSGVDPSSGG
AAAASGGGGGGGPAAGSSSRSGRTTAAGEGGGGSRRVLRPCVQRSGAAEVGLELEVVEAGAAVGGQGRAGAQDAAAA
GDDARQQLQAAGSGAAAAASGALPQPWRSPAQRAVYSDSGAGGSGSVWRGA

>Cre01.g042300.t1.3|PACid:27578985
MLSIGRGLALVLLFLGAFSSGVAAKRSSRVGSAAGAWDCDECPEIAAPVCVAGVTFRHACLAACQGLPSGANGPCAG
DKLIATATGVLDSDPEAAAADVASTSLQGSADATAVSSSGRGAAAVSSRSSAAGEDSGKHHKRKHARGQDMHRFRQH
GFVLVGSLPLASAAEASPQAPPSQPPSQSKGGSGDGGGGNGDGGGGSVDSDDTILGESSWDPERANMRTPKSRFNPG
SSSSRDGSSSSSSSSSSSGSDGMQVRALRYSVADGLLYAELAARPALEPAVRRALSAATSLDGAAWLAPAAGGDQDQD
QEQEQELGRFESFEAMATTNGSVEDGGASAAVAAAALAGAGYSKRGSGRAAGPLELLEPESESAAGGLLRRQWQVPR
QQQAQPQQRQRHRRSLQQGLQWSGDARWRVRDAAAWPFSAVAYVVYKQPSTGSRYQCSATFISPVDVLTAAHCVWDF
DNQTAYRDWRVWPGLASASSAPSNAAAFMAEYVTFYRTEAAAAAVGDTTVFNDLDRANRRGPGGVGNVTDEQQYGEQ
RYDVNYFDIAIIRVNRSHNSWLGLKYDCSVQSYPKTMACGYPATWPASYWQHCSQCFLATSSCRPLWQMYNFCYSER
GQSGMAITDLHDSRVLGVLSGGPANGWDYSFWTPIDAFHFHNIVRWLAPNDGTLATPPPPPPPVPPSGGEKQSPLRQ
ERLPDAPPPPNVPEAPQPPPRPPRPPPPPPPSPPPPPPLPPDAPADPPRPPRPPPKHSKPPPANKPPLILKPKPPGR
AVRKKFPPPRFAPHPSPAPSGIRSAYVALEHSVVVDQEALSSATAAAAKATAASTRAAATTTAAAARSAALPLDGGG
AADGEPPLEPLLPLAAALSDPPPPPPAPPGPEAPPVVEIRGAGIAPSSGGVSGSAAAVGVATRATAPASSSPSTSTS
TTETVRNTNTTSTDTASTGTRECTEGDVRLSDGPNAWSGRVELCSGGRWGTLCDAGWGWDDARVVCRQLGLGEAGGE
AMLGGWFPPGAAAMPIHAGGIACVGSEARLSACPQAGLSSSLCTGHEFDAGVICNNPSVASPPPSAGATNGSGWGRG
GYPCTGYADGALRLVPATGLPPSPQSPPPASASVPVDVTTGRLEVCLGGEWGSVCNDGWDDSDASVACRQLGFASGV
AVTSPTPAADGAVTNSPFAGPNNMTIWLTGMDCGGGEDGLTACPHTVGLGFGAAAPPACSHQEDAGVACSRRPAPPS
PPRPPPPQCSEDGALRLQPLPAAVAAAAGLNATAYATGRLEVCYSGRYGLVCDDNFGAAEARVACRQLGYLYGKVLG
PEDMAAAGNPLAAGAPAGAATTFWMDEVDCTAFDTAFTGWLGRLTQCMFSGWGNSDCDAKIEAAGVVCSNDPSVLVQ
PPPPSPPPPPPPAPYACNTPFSLRMGAGNGTAGRLEMCSRGGTWGTICEVGWDNVDAGIACRQLGFDGGVALPSATT
DPKRGFPSAGSSQPLLFYDITCSGSEATLTQCLGRANAANNSGCVNPRRDAGIACFNTRSPPPAVADTFSCSNTYAL
RLMMGNATITTAAGSGRVEVCYGGRWGSVCRDGWDDADAGVVCRQLGLSGGTAVTDDRFGFASKDLPVWQDEVACRG
EEAAFAACSLREWGNSDCWRWPRLDAGVMCGPPLSSLPSPVPAPSPLHVAPSPGYYPGSPSPPPASLSPGYYPGSAA
PPLLGPMAYPPTQSPAPTQLRPPLVVSSPPGGRLYACSSEGALRLVGFSTSSSGFNVASGRVDVCFGGQWGAVCGRE

WTYYPSSSSSSSSSSSSSSGGGNGTDGARSDGTGDVMWDDDTATVVCRQYVTALLAAKGSSTSADDVLFSATGLLPGD
ASFPGLAPGQGFAVSSVQCDGTEATLDDCSRSGWGTVPAACGSADAVGVQCVMRPF

>Cre01.g009700.t1.3|PACid:27579014
MLQHRNFPGTWIVGAKQRQILGLSDASIHDYARRRTARSRLAVVAGKFTVNVAGLNGASRQAIQTIPAPQPVLTPTP
FLGGMLPYLAGGMLALTGAAALVKGVLDRRRASEAGAGKPAESADAALATASAAAAELKQMSSKVQEAAATAQSIQE
RVTEEAAAAAQRQQQLEQEQARAAQAAEELQAEIRAKERELQQLRVASPAAAAAVEAGARQELKESQRKSELLEAMV
GALRERLKREEDRNTVLAVEVQEGRVELARAEVERRELLARVDDLRAELADTQEQLAAALSHATRLEARVAALVVEN
ADLEARVGGLEAALAGRADEAGAVSAAADERIRTLQAAADEDRLALARLQGKLAEREAQLQSASETEAELQQAVEAA
KAAAQEMAAVLRHRDADVQLLREQLQTAGTSEELAALRKQLSELQKQRDGLQAARHAELTALRDRVRAAEEDNSRDL
KQLKEKLAEAEAERDAQVANLRAALEETARQAAALSDASREEGRAEAEEELEELRGQLQEAKLKAEQASARRVAEVS
ELRSSLEVSLREARGEALAAREALAAVKQQLDGLLSEEWVEALEEELRQSVQPGYTPVAKPLPLSPELTEAMKSLEA
RESRLQEQLESLQRELAELQSAAVARLGPALVARAQELEGAAAKCRQVMRAREAVKDEPGMTRVVQQLAALTSELGS
LYRTVMTPEQRSRERAVRAELQDRETEMRGVVEQRIKLEQQRELEEADAEEKRKKAKAAAHGPGQDALRQLVQGRVR
QLLREREAQMQQQARAREAQLQAQLDAVMVNSSDAQRRELKVSQEAVAAQVRHLETSWKAQLEASNRALQEAAADKN
RLAAEATLSTTQVRQQLSELDSLRSELSAAQSAVAALRRAAESSKQAAAQQSAGAASEVSQLKAQVARLEQALQERE
RQYAATQPRQSPPTTSFVANPAAAAAGPLRPPANAPLTPLRTAPPVPPAPRPTQQAQPQAPRAVSQAPPMPPGPPRP
VQQQAPPQRPPPPPVQRPPPARPTPTAATQLPTPPSLPRRPMPPTSTPMPQQQAGSSGPRPAPSTQPAAATPKPRPP
QPPPPQLQSQQQAASGGDFAPGSANSNGASHKPITPLRSDWPSQYAATAPGSGGSSSGMGSGSDAAAAVGALGSAIA
AAAAQVAAGASATPGAAGGLVGGAGSSPGLVGAVTQTLAEVASAVAGEVAVGLVGAIAAAAGPPAQAPSGGASSSFG
ASSPPAPAGTNGAPDSARPRQQPQQQQPTSRGVTSAAAWGQKPPAPPEATAPTGATPPIASTSATSASVAGGAGFSG
QADGDGGDVRTIWSKILTSPKDVPPPQPSSPSWPPPQPPQPPQPGPDLPQIQLRPEFSKPGAAAAPAAPVAPSAPSS
GDVGASGSSNGAAEADPWARSSAPKSSTSSSSSSTAGRPAPGPSVSPPPSTSTFAQAPGGSSDAAILNTIADLKSAT
ARLDQQASAAAKQPPPQLPPPQAAPQQEQQWQQPLTPLAAAPPQSAYLSDFRRPLPPQHPASDAASPASAGDDDDDD
GYIDNGAPTPSSSSSTSSSSASSGGGAGARRAPPAVPVVETWKQRTTGMPLMQQPSSAAAPPSAKPPQPSPPPVVPP
KGSRQEPLLPQAQAPPGKVPEPPPLTPSRPAAVPSSSSAAADAAQQPVRSPSPQRPAPAKPAAPKRPSAAEEAAMAAA
ANAPAVAKRAAAAMPPPAKAPAGFSTPPISEWGRPKPAPEGQDTAAVPAGAAIAAAAAPSGSTAAATSAATSSSGSG
AAAAGSAPVSPVDSAFLSQDELGAEIAAITQSIVQRAGARGVDITRPGTAAAKVPLPAAAGPTTTTPGPLAVVPSAA
AAASRRLQLSVSGYVLPQLGKENGSEDAWFSVTPLGGTATNGVVSAGAQPAGTVSALGVADGVGGWAQANVDPGQYS
REMMAAVARAVEGKTSVSDPRDLLAAAQSAVRTVGSSTACFAVLDGSRALLSIANLGDSGCRVVRRGALVLATSPQE
HTFNMPYQLAHPDNLPDTDTAEDAQVYQLALEPGDVIIMGTDGLYDNMWDEQIVALATGAVTALGPALGVTAGMAAA
ATAQPAAQQLAATLANTAFRHAQDPSFRSPWAVELANQPDATWLQRLFPRGGKMDDITVVVAVVTLATVYQE

>Cre01.g015050.t1.3|PACid:27579029
MPGTWPVDWGEDGKYRGIISKYDATHPRAPWTIYYPKDGDEENGIFKRNFTEWHVEDEENPDDPFTVRRILKWRDGK
LPPSMVPPTPPDLDPQGFPTLLGKHEALPQPPSGSEDEGGGGSGSSSSSSDNDGEDDGELAVRARRRRNSRVQSSSS
SSEDEDEDGRKKARGGKGRGGAAAGARGLKRSSSGLTSTGVAGAASPPAKRGPGRPRKHPRPDGEEPPAKRPRGRPR
KDAGASAGSGEGTAPKRKPGRPPKNAAAATVAGGSPPTETGDGGDGGGSRGPSAEPAAGREGSPKVERGAAAEEGTG
NGPAVSSEPPAGSGAGKAAGRASRDPRLAKEPNAEEDGGADAKQLGPGASRPPPLLTAEQLAKRKMFNPGPPPRTGG
LGAVPTAKSQREAGYSGTVKLDPGKPGGASGPGLGAGSAKPARPVAGMPVPAKLATAAGVSGAVRGSGSGATAVAAA
AATVKSESGAAGTGQEPAGDVQAKPSTKVSDEALAQIPQPPQLPQPIAVSLPQPARPLPSLPASLAAGPAVAVKASL
PSRVTSVVSLAAAVGTAAEQGQGAAGGGGGGGGGMTGSTARLLPAVPALAAIMPAAASAGAGAAPGAGAAGTAAAAA
ASAVVAAEALSAAAEAALRNGSTPEAAPADVQQLLGQQLGRALDALTNSQNAIKATANAAAAMVRAVGANRVARGFF
DKLKELGEEAAVLAPEGCGGDATGDERVAGVLSRRLHVLYVMDSLLYRSTKGQEAAMVGWPHMLSRGLQSALRLKAV
TADHVRAVRKVVEVWKRRDKLDGGVLAMAEAILAEQEGLLRPESQQHLQPPQHNAQQQQGPQAANGGPGQDGGDPDG
GRGHLRRRKHYIVALPRPVRLHRILYGSSSSGYGGCTEMEEYYVNGFGPLANRCMLDNPYGSLSGAHAAGGAAAGEE
GAAVGSGKGEPWGRLVEVDFALDDDEPDFSGTVTWRDVVAVQLPQFNPASPARGGPPPAVVTPPHPDLEHLLRSGRR
GAQHTPSVTRYPGAASGGQAFGTPLMSSGAGGGGDAALAEPPLRTTSGPPIIGLTPPAVDDELDTWAYGGELGTGGA
SRPAAAAAGPSAPGTAGRGGSAAPPSVGPGGRGTGAGGEEEGGANLFDMPAAPSHEVASTGRRSDWGDDVDMDVSDE
EGEAPDWQPPLPDAGGGAAGPPPGAPPALPGMPSAATLSLLQAVGPMAGMHMDPIAAMLGGAAALTSLGFVPPPAPP
PGMLLGMPLPPPGWLPGLVPPPPHSGGLAMPPPPGAPPPPHHHHHHHHNQPPPLPPSSSPHPPLPAPPVPPPPPSP
PPPPPLPMAPGGSPPPLPHHSPPPLPPPMPHHSPPGSDHRPPLPADAPPMPQGGGPDAAKANSTSPSAAGPGGRRQL
PPHLQPTLHASASKPTTAAAATPAHAAPHVTAASNGGGGGGGSKVQEPATGTTAAAATTSPGPEQGSTAVGASRPDV
RAPEPSARPGPGSRPAAIAEARQAYRPLADRLQRSPSDSGSDSGSSSDFEDRMPRQRSISDAGTGGGGAAGGRGSSR
EREKKKKHKKKKHKDKSKKDKEREKEKERGEREKEREREREREREREREGERSKEPRGKEREWERQRETAAAVTQPQ
PQPRMDRDRSDRRDRERAGYDRAERMEGDTRDRGAPPPPLPPLPAHTRVGRDREQERDRDRDRHYDRERDARERERQ
EQEWQRERDRDRDRDRRSAPLPQQQPPPGRMDARDEPRNVGRGGRGDYRPPFLPPPQPGPPHRGHHDPHDRDRDWER

QQDRDLRDHRDSREQREQRVQREQEREQERERGEQRAGRGRGEPAPAPQPPRSNVHREQERTLVGEDSADGGAKGGR
DKPQHDASAAVSGHPTANGHSAVQHASRAEPSGGRGATGHDKAPLEPGPPRREDGVRPEQAAAAAEAGTAGAAVRGT
PAGAAAVAGRGSAGAGPAGAARPADAGAARGKVSEDGEVADGDVSPGVASHLGRAAGAATLDGAALDGARAARAAGR
PPLHRPAEQQRPPQQQAEVEEGEVELPGPPPLGKQDSSHGRRQPIVWGTGGKAPASAATRSPDASLQPQPGHLSQQQ
QQQQQRKRDPISFTLAPRSASPPAESRPRAPTTLAGGGRQPPAQTQEHGRQPPAGGGTGGAGGGGGKARLRPGLALR
LSHLEGDDADEEHLHAPPSAGHGGGGKRRRRGSGDGEGRGGRQKRSQSPMD

>g38.t1|PACid:27579030
MFNTDGNRYSNYYKFEIENGPSPKRPSFIDRARFWIKDNPKWFWGGLFGVVAVVLIAVIVSSACGDGNCVGEGKQET
AALSLPPSPPLPSPPSENQGSGIDGAAGPPSPAPSTPPSPPPPVPPSSPGAVDSPPPPSPAPMTPPPSPAPSPGGVY
GGSTQPSPAVYGGASPSPPSPDPPSPDPPSPDPPSPDPPSPDPPSPDPPSPDPPSPDPPSPDPPEPPTAPEPTGAGR
RLRLR

>Cre01.g045601.t1.2|PACid:27579106
MSDAAEGSSSDGSSGSSSRGAGVDVVTRFATEGFVYAGHAALSDEGPTGFDQGGFVSKLTFDDSTGTGGDGDSSAAG
GYKEAADSSTTTIGADGSSSSSVRSSSSSSSSSASVTQSIESSGDSGTGSGRGWAAALTEQGVPTQLRVRAVRVSPD
GAVYIESSGVDLDLTQLPASAQTESKSSFEELDGLDPNGLDQSSPENATKSAALVGLASRRRRRLLRRGGQEGAQPL
AGDWEADEASELPDGGDEDADGWFAARRRRLDQRRRRRLVDDSYRSDTGVWPYRIIGQLMYKVNTLAYICSGTWVSP
YDVLTAAHCVFNITSGQAHRDFAFTAAKAGTTGPDGTVRYSSVTFYRAEYPRAKGDGVNYFDMALLRMSAPGPGAYM
GIKYDCGQIDYPKTETCGYPKEKSTPGSNYWQYCDDCYYTSNACNPMIQPVNWCYTAAGQSGSPIYDLADYRVMGVL
SGGPVTWEFASDLSFWTPIDAFHFASLTRWMWKEGDGTPAEAPLYVSAPPFPPPPPVGPYACSGKGSVRLAGGPDGA
SGRVQVCRPDGVWGSVCDLNQYSAGNADVVCRQLGYGSGVFVAGDFFPANDASVPVTLEPQCAGSEKSLSECFPLSE
GDWSTRASCSRSTDMAVLCGRAARNLTTGAAATAGATLPPRAFPCSTEGAVRLQDGADDASGRVEICARGQWGSVCQ
DGWDDLDARVVCRQLGYVSGAATWGPLGSGTAPPGPAGMPVWMAQVACTGGEARLAACATEVPVGAAVCSHRADAGV
VCGTAPLPVPGVAPIVSGSCAEPGALRLAVGTSGAGTAAPVAGGSVAGRLEVCYAGRWGAVCEAKFNDYNAQVACRQ
LGYTQGRAVPADVLPGQLIRMSEIRCQRLTGGAYLAWPWRLTSCPFWGMEDVSCATGSVAGVECTSDPNGFPLTTLP
CSSQGGLRLRGGVAPNMGRLEVCNAGQWGTVCENNFGPADADVACRQLGYATGSVLSRTATPDGHPDSSIWMDELDC
KGRENRLDECPFNGWGQSLCGVHSFDIGVVCTPKAAPLSPPPSRRPPPPVLYGCTTPSQLRLVDATGKPGFTAGRLE
LCLKGQWGTICDEGFGVQSANVACRQLGFAAGVPVRTDTPDGGFGYGAYNAPVHLTNVSCTGAESRLTQCAANNRTT
CAHITDIGVRCFWPDEVPSVAAPPWPRPPAPVSPYACSDLYAVRLTGGVPGLSGRLDICYGNTWGTVCGTGFSAASA
AVVCRELGLGGGRAVVPGGASGAAIAPADMPMQLDGVACDGGEARLVDCPRDPWGMVACRREDAVVVVCEPLSPTLS
PLPLSPPLRLPPSPTRPSPPPPSRPPPSPPSPHPPSAPVSPPPAFPREPCPAIGAVRLYGGVLPDSGLLQVCNRDIS
RSSAAANNPPVWGAVCGTGFKGPEARAVCRQLGYTDGVVVNKPYLPSFRTTGLYYTFAPYPPGTPVWLDNVHCRANA
TRITDCSSNAGTTCTRNQEIALVCTPAKRPTKRYACTGSVGAVRLVGGRNGSAWEGNVQVCQSGTWGTVCDVGWDDT
DARVLCRQLGYSHGLAVGGGGVVSLNGTAAGPFPAAPSNMATHWDTGVECGGSEARLTACRRGGAGGAQGCADHSRD
AGAVCWGKAG

>Cre01.g013650.t1.3|PACid:27579117
MPRGGRATRSKAGKTLDSSPPEPEKQSEAELEEAPAKEETQAPEASGDSSDGDSDSSGSDGSEASGDSVADAKRRQQ
EEKLKQEQDQDSETQEEEEEVEPQTEEEARDALRENWKFAAVLQFARIFTEALQLKGFSADILESAIVNPSEHQAFL
GELVFKLIREDPAAHYVERDAEGYWEADLANKIHRLWRQYFPRNPLHKRGYFELSTEQRVDVLYALCDWRIHDCPVL
RETMRTMVDDPEVTADVLRAEPVGEDGRGARYYFLSGDKHEDCYLFKEVLPKPSRGGKRGSAAAELPPDAEAEWSTE
CTTLEELQEFAKRMSASRNKVDKELGELIDDEILPELVSTADARRRAAEKAAAYEALPSKRSGRLHVLQKKKEEEDA
ARKAREAEERRKKEEETREKERKRREKEREQRLKEMEEARRKFLGITAQSGPTREERMRARMVGRRWSEEPRSTTEA
GSSSDSDDEGEGEDDEGESRSSSNSSGGEEEEDANAELPQKRTRRPPRRNLSPSPPPAQQAKKQRRASAPAGDSRAE
RSTRSAARGAKAEAKEPKEPKEVKKEQKAKRTPEPERPPPEPAPPHVRLYPGTWSPLEGVSVVRREGAARAADIFRP
RVKYEVLIQASRGATGDMPYWVAPAARRMAEEDRKAAARLRVRDSAAFDEPGSGSQSPPPPPTSRVGRPRRSSAAAR
AAEEADDEEDFDMEVEEAARPPPRSRRKPHAIRRDTVDEDGDVEMKPSTSGPAGEAGPVDEEMEEAGAGPRAGTRGR
RQTMEEGDDEPTLSPTGRPQRHRRKPKRNQSSDEGDVPEEPDEGGELPPPLPAPAPKARPQQVEEDEEEYRAPRKKQ
TARAQRQAQRQGSGRPQPPAANGGGVAAGAAAGMGALQLPLPMANVNQAQYLQAFMAKALEVMPEANRQQILALPHQ
ARQAVFTTLIQQQQMLATMIQQQQVVAAAAAAAAAAGAASVGHPAGTAGSAAATAATVLPAGTLPPAAAAGTKAVAA
APAAHVQGAASAAGQPVPPLVAPAAAAAAAAAPAGPAASPQEAAAESCAAAPSAVDGTAQVQAAPLATEAHAQAPSA
AVDAEPLTETPATAVMPAAEASTSIVTAAPAGPSSAAGAVEDVVAAPVQPSLAAPEAQALAPPLDAQALAAPGAAAV
PDLSTATEAQGPALAQPLEAAQIDMASLAAQQQALQAHMEAMQAQLQTQPAQGHSEEHQQVLAAAHAQAVAAFAQAQ
AAQAAFAAAGFDPMAVAAMQAVQAGMVQADPLAGAAASYDPAAAAQAQVQAQPVVAGTPPLPVGMEMPGLTHEQIQA
QAMAQAQATMQAQMQMQLQEAQAVAADLQAQAHAAAAALAAAPPEAQEAAAVHAATTAAAAAQAQAALQAQIQAIEQ
VTHAMQAQVAAATAALPGAAAGGGVDMTALYAQQQLQVYQQPHLMLQQPQQVVATGAVDAGQDSAAAAAAAAAAAAA

AAAASGTWPGAVPGVVDPAQAAAAAMAWDPAQIAAMQAMYEQMGMTGMVRDTGAAAADVAGAAGAVVPGHAPDGSAA
AAPATG

>Cre10.g438800.t1.3|PACid:27579138
MQQPQPAVAGAPLPERQSAQPYYLDQSPPGYRLYGANLEPHQPAPTPSGHAATPTGVSGGCLPPSPSQSLCAHAQLP
CASVAATPSSAGGAPAPTTVITAAAAGGGDSGAHGDVVGSATPAAAAVMAPALQHAISHVAAPSGALPYFPHHSHLQ
PQPHAHYQHQYQHQYSHQYPHQYPHHYQHQHHYQHQHRPQPQPQPKHPQHFHSHQEQPWGTGQHVSPLAPGGMSSLT
PSPFSPATAAAATAAAAHGAPSGGAATSPLVPSFSSPLPWQQPQQEPRQTSCAPAPCEQQQRQQLQQPGQGQEPHAS
AADTAVPLPQTMSAPAALNHLQLAPPPHYHYSYPGPQPPAQQHSQQPHMPVQPVPPPAPQPPGPPPQPPPQLQPPMV
WAAPPYWPHSGPPGVSPRPCAPQPATAPQQQPPPQPQHPWQQPRAPPFHQPPSSPPAPPPAALPQPRPPPLPIPPRP
PPSLPAPLVWLPKPNWCAYHILRWQAAANHILFYLVDHDYKAVLAVVGTDLRFSGHFVYSTLPDGPVAGSPLRCTNR
NRVMDWLAQQGAVDPADASRLRLPPLAPHELALLESSDPLFSRPAESVLDRRYSSWREEVGVLPDGRHIKQFWVSAA
PPAGCGEETGGGDGACEGRPGQRSAAAALPERLVVVAEDSHRRDRRYTYRALPEFGGFVFENGHCAKEWLSFVVGRR
LQPTALPQRPPRRKGAAATAGAAATAFTRVPPQANGGVRRNMSRTNGASSPSSDASDGGGPQEAGGTSSTPGSASGQ
QQRQTPSAAAEATASATAAVAAAAAQPSPGADGSRRGVAAAVAAAVAGVAGMDWSQLRLGGSGSVPPAAGVGAGVAP
PSPFSPPPQPPQLPPPPPPPPPPVWAAQGHGVGSAPSHVPPNNQSPLLHPGTHPPHHHLQHPHPHLHPPADPHPLLH
PGVPQLHPGVDPGSCGPASSAWTAAGQHPLLATPPPPHHHHHNRNAHGRADVSGGGHAGAVPHTAAPPAQQQQFAAA
TARPVTPPLPSPPPHPDSQLLADLCMTVDEDGDDFADWLLRTCSGGEGLLTDWWKGPAASGDGGAVGAAGGVASVTG
GHGGGGGVDAMDVQHVKSETEVPAGGDSPHAEQKPSYGGTGGAAGFAAVVAVAAGSPLPASPPPSRSRSGSPPAPRA
PPRAQHSGPGSDHPQPMDMHGFGGGGGVTVVASDATDGEASTAPAAAAAAIHQEQPLQPEPRPPLPPRQHAASAGLV
TGMGMEPGVQALGGGGFGEGLGAGLGGLLAWDGGPDADFDVFTAPDVRAAFQHSPRLLDWVAGVPDAGSLSAYRSWA
ALLRGFASQAAAGCNTGAAGDARQPAAGALTGPTEAHVRELLDELSRQCVSLRVCVELSGAVVALRDACRGRQPGVA
AAAERLLEEWHGVAAQALGTAAQALSVSRANSGSGSGASAGGAGAGLGLSDDPMDWLW

>Cre10.g438800.t2.1|PACid:27579139
MQQPQPAVAGAPLPERQSAQPYYLDQSPPGYRLYGANLEPHQPAPTPSGHAATPTGVSGGCLPPSPSQSLCAHAQLP
CASVAATPSSAGGAPAPTTVITAAAAGGGDSGAHGDVVGSATPAAAAVMAPALQHAISHVAAPSGALPYFPHHSHLQ
PQPHAHYQHQYQHQYSHQYPHQYPHHYQHQHHYQHQHRPQPQPQPKHPQHFHSHQEQPWGTGQHVSPLAPGGMSSLT
PSPFSPATAAAATAAAAHGAPSGGAATSPLVPSFSSPLPWQQPQQEPRQTSCAPAPCEQQQRQQLQQPGQGQEPHAS
AADTAVPLPQTMSAPAALNHLQLAPPPHYHYSYPGPQPPAQQHSQQPHMPVQPVPPPAPQPPGPPPQPPPQLQPPMV
WAAPPYWPHSGPPGVSPRPCAPQPATAPQQQPPPQPQHPWQQPRAPPFHQPPSSPPAPPPAALPQPRPPPLPIPPRP
PPSLPAPLVWLPKPNWCAYHILRWQAAANHILFYLVDHDYKAVLAVVGTDLRFSGHFVYSTLPDGPVAGSPLRCTNR
NRVMDWLAQQGAVDPADASRLRLPPLAPHELALLESSDPLFSRPAESVLDRRYSSWREEVGVLPDGRHIKQFWVSAA
PPAGCGEETGGGDGACEGRPGQRSAAAALPERLVVVAEDSHRRDRRYTYRALPEFGGFVFENGHCAKEWLSFVVGRR
LQPTALPQRPPRRKGAAATAGAAATANGGVRRNMSRTNGASSPSSDASDGGGPQEAGGTSSTPGSASGQQQRQTPSA
AAEATASATAAVAAAAAQPSPGADGSRRGVAAAVAAAVAGVAGMDWSQLRLGGSGSVPPAAGVGAGVAPPSPFSPPP
QPPQLPPPPPPPPPPVWAAQGHGVGSAPSHVPPNNQSPLLHPGTHPPHHHLQHPHPHLHPPADPHPLLHPGVPQLHP
GVDPGSCGPASSAWTAAGQHPLLATPPPPHHHHHNRNAHGRADVSGGGHAGAVPHTAAPPAQQQQFAAATARPVTPP
LPSPPPHPDSQLLADLCMTVDEDGDDFADWLLRTCSGGEGLLTDWWKGPAASGDGGAVGAAGGVASVTGGHGGGGGV
DAMDVQHVKSETEVPAGGDSPHAEQKPSYGGTGGAAGFAAVVAVAAGSPLPASPPPSRSRSGSPPAPRAPPRAQHSG
PGSDHPQPMDMHGFGGGGGVTVVASDATDGEASTAPAAAAAAIHQEQPLQPEPRPPLPPRQHAASAGLVTGMGMEPG
VQALGGGGFGEGLGAGLGGLLAWDGGPDADFDVFTAPDVRAAFQHSPRLLDWVAGVPDAGSLSAYRSWAALLRGFAS
QAAAGCNTGAAGDARQPAAGALTGPTEAHVRELLDELSRQCVSLRVCVELSGAVVALRDACRGRQPGVAAAAERLLE
EWHGVAAQALGTAAQALSVSRANSGSGSGASAGGAGAGLGLSDDPMDWLW

>Cre10.g450100.t1.3|PACid:27579245
MESAPRSWAEAREQLLQELHAQAQGRTSPPAGPSPAPATSGGGTAKPTADPGGTPPRRSQAGAAGTGEPPDGPNASP
GRSRRRSVKNAAVGLPYLQPLPSAGTAAAAAPPPTTASIPAAAAAQLNQYLQDLPPTTDAAAATTAAAIGSGSAPAA
ASDDAAARPHRLSVGHLVHGPPPYAQHSSQPHSQLRHSRSGVGAGAGAGASAADGGYGLGLGGEGPLERLAEGAELG
EPGQGPAPRARRVSRADGRKVTGDVAIVASRPRGPRLSYGAGYGVGGDGQAPPSSGPASSAAGLAAGRLGPGGEPRS
GAAGRGQPRVSFGGAAAAEMAAAAVAAAEGSPQPPHPRRPSVVGIGMSGVTAVPAGVAGTAGVPAFQGSPAKAPPGD
ADRSVRRPRPSNTSGGIPEADGGPGPGLGPGSGQGRPPGKGRGGRKSSTGLDDAAGVSTVAAAAAAAASPPPAVGAS
ASGPGAGGKGAESSPGRARRESRRAAAAGGGAAQAAPSEPEAAAAAAALGPAGALGTAAGAAAYSPAKWVGAESDGRQ
RAAAVPPPRRSSSPKLGPSSQPPQEPPSRQPPRRPYAEDGVVAAGGAAAAAVAAAVVELPGAASSGPAPPSAAASPI
KLTAAALALHTAGLHGTAAWEETAAAVVAAADAEAAMEAAAVKEAAEQQQQQQQERGFETQLSLAPRASQQQQQQQQ
QQQLQAQASRGASAQGATAVRQLDAFLSFGLHPQPLMQELDVRAALGGGAGAGAGMAARPTAAAAAAPAPASASAAG
IGAWAAVRTARVSDVSGVDNAIDGLAGFGGPGGLAVCGSPVILPDAAGGIMLTSHSRRNSTGGGGPTSPALAAVLAA

AGGGSFMGSPASPPPPVGSVRTSEAGDGVRIVGSRRTSDAGLSTRLRRMSNLSDTTAGGGGGGGGSFVLAALNLAVA
QSLTSGAAAGSGGGFGHGGGGGSPSASRHHSTSGVPGATSPLLMQVQLQQLRQQYAHMYGGPASPSPPPAASPPSAV
PAAAAAEAAPVRQGYRPTAGSLTGSMMGCGAGGGGREGSAHGGRPSDSSAAEDSPHRQPHPHSHPHQPHRRRTSTQG
VERSRLSMELGPSKLALEAGITTTAVATGASASASVAASDGPPGAAGTSSTPPHEVPSAAGALSPAGTPAAHTPAAQ
VSGGEAAAQSSHPSLVVPPPPQQDPARKASAGGAGGGSAGAADEESGALGMLSPCLPPPPRSAILESSPNAGDEASV
GDGDGRARPPPAGPPAPALQAAPAAEAARPSALQHSSSRGERRRATFAVAQSEDDDGGGGGGGRGGAGTAGDAAPGE
DDGRKVYVPARERAAAGAAAAAGAPRGKSARGKSARRTNRPLAGSGGEGGLDSVDVVTLDDIRQHRRSHSRPHHHYD
DGDEDARPYSAASRRSRHDAGAGGRRRASHRRHAAGNGNDSDGGGGGGGGEPDSALRSLRSGRGRSESMMSRASRSS
RRGGERGDRGDGDGGGEGTVAALSRGGGAVVARAERTGRSVVMDVMVVQRPARRNFHGDRRVSEFDAPDWDEDGHGG
RGGSDDSDVEQLGRTVGRHALPGAAARSRPTTARAQRPTTATPQRPTTARQRPTTAAPQRPTTARQRPTTATPQRPT
TARQRPTTASRRADDAGRVPHGPAPASGGGSRRPAGGRRASESGSDAATDLSDLSDLSGISIFRKDGNHRKAAGRGT
SAAAAAAVATAGVAAASAAAAVAAGAAAGASRSGASFLETAKALLNSVAGKPKAGVSGSWVAPRRRSLRPDGTATIS
GGAGSGRLGADADAAAGAVRGAKWGMGGAGAEAGEGYGAEEEGGSEEDGLENLRSASRVSHLSRSFSSKHPSHAAAS
HRTHVRRSSSARAAAAADYTNDKDGKDVGAGGHTATTTTTTTTKKRHGDDGGEGSVGRAGAGSGRSLLRVDSRMSAS
NKPFTALADRRRPPRTVGFAGRGGGGEEELIEEEEAEHTDQDGEEDGYGQEEEEKEEGRAASGLASKAKAFLSDTNT
TAASAAAAGSAAAASGAGPRAGPTMAMVREAHAQLQRQKTATGGEGEASGEEEPALRRQDAGADSYMLRIFGTAYTK
VAGTDGDEGGGVLKVWLHPYSSVRMRLDMVWVAVGLLMSVVVPLQVAFVTPATALPYLVLLLVCSAAWAVNIAANFL
TGYDEDGVVVMEAAAVRARYLRTWLLLDLLGALPYVAVPLAVCGSVHAGLPEEAGGGGGGGGGGGGGGDCTVRSGQ
MLRLWSLLGLATLPRLFHYLNKWQDTWDIDIHKMRLVKLLGLAMVFTHLFACMSYFVQLLEGFPSDGWVVVNGLDGL
GPWITYNFALLRSLQLVMGSNVDNVSTSSDVEVVFTIVCYIVGVFFYAMLIGVMSSIVLSLNRSGQMYVEKLQIWRD
YCHYRRLPGELRRRVMAYINTAHSNKRVLDDTSMLLELSPGLRTDVNLQLCAELVATVPVFLECSPIVVRAMVSKLQ
RETHVAGDFVFRVGDIADAVYFILKGTVAISDETGQLLTRLAQGSHFGEFGILAYLDGQLGVRTAAARADDCVELYK
LHCEDFAALADYYPELVDFFRDVAVVRSAYNATLRANRAMTEYDDDEDEEEYEDEEEYRDDDDDNKYDDEYDDEEDD
YDADDDDDGQSRGGGARAKVAAAHRLLSSSEEDGEGDDDDGYEDLLRADVALGSDLTVPGGGGGSGGIGGGRSRSIT
AGGPGGGSSRRRHQPHLSPSGSMGRSTGSGEGGVEGGEAAAAGSAARGLRKNGGGSMLRRALGAPAEGEGDDGVSAS
GGSAAARRSVKFSISIPPTPTTPSGVAAAAAAGSAADGSGGGLSEGGGIGDAHGPTPVPAPISGSASGSAGSSPARS
PGAHSTGGAAALGAFSSLPLPLPSMRTSIAGVYDGAPPRSPALLAAEAAGGAPPSVLSATSMLLAALQGGGGAAASS
GGTGLSARSLLNRIAPQESMRSHVTVSDAGEEADGPADHSEPNSPSPAAALAARPAAPWSSNTASRLSMPRRSASAS
LAAAAAVRAIPLPPIPAVGAAATGTFGGLSPPVPAAPGAAAPGGGSGGALPQVGAPPLGRGASRLRQMASMKNSVAP
LPAVAAAAAAPGTSTGGGAADQGPVQFADLLMDGTIGGPGAAGSSPGGGAGHGLDGEAAPPTAGPEAAAAAAAAELAS
SVPMLARMRKVTDVVSFLNAVAGGGASGGTSPAGGTSGGLSPGGFPAGAKSQTGSALGPPPPAAASSGRGFGGGMSP
GGGGGGGGGGSGGFMRAATTLVDGPIEAPERPTPRTRHHANASHGNSSSNTAAGGGGAGGSGHTSTHVSTDGRPSGS
GPAPTSAAASGTGNGGGGGMTGLWQRLMKHKPAGKEAETFEMEGAPSFGSTAGGTGGGGHARGSFSAAAAAVMDLRR
KSQAPGPPPASRSGGSFFSAGMMKDNRAQAQLAADLEVGAAPPGVAAAAAVTALRVASITGVAASRLGSISAAAAGL
RLPGATASITGPSALGPGGAASGHGHGGGGGGGGGGERPSGGAHHGAGLRGSTTRDLLAGGGGAYHGQHPRGPGVVNL
GFG

>Cre10.g449750.t1.3|PACid:27579274
MGNSGSGRAESGQELGDEEAPPPGPAPEPFPEPQLPQPANPPAVPSTAAAAPPAEAAALAPAPAPAAPLTAAGAAVG
GPEAAAPGPTTVPVAGPQPASTAVDEDNVAADEAAAAAPSLDAWACLGYAACRFGSRLALVDLDPSPLAAPPAPAPA
PATAATASRAGSTAAPPPSPAPAQTATPAPTATPAPVPCGPATGAPSGAATGARLYTYRQLHDRSLGAAAALLAAGC
GGGGRVGVLLRNCGEVVEAHFAAAALRAVVVNVNVNLAPPELSYILHDSETQVLITHAGDFGALLAAALALPPPPAA
ASPPASPSASPEPGAEPAAAATAAAAPPPAPAALRLHTVFLVDGGGYGGSQDGGGAAVEAAAATAAAAAAAAAGLN
LKFIPYHSAVAAAIAAASADDGAAAGTRRRLTLRAVRAAALAASATSTGPTTAPTAATATAPQPQPPPPPHPLLEPD
APYHMYYTSGTTGRPKGVLLSHRIVMLHALGTIAEMRLHGGDVWAHLAPLFHLVDVFAIYAVTLASGRHVLLRAFSP
QEALAALERERITVTNIASTMLAMLSSNPALATANTASLRLLSCGGSPQSPAVLRRAVAALGCEVFLSYGMTECCGK
ISMSILPYGWWARCRLAAATAAGRSSRAGGGALLQPGDVGPHTTAAQLMELVVSSGRPFVLMEVRVVSEGDAEAAEA
EGQQGAASDATAAAAATTTAAAAGAAACGGVGELWCRGPTVFSAGYWRRPAATREAFAPGGWFRTGDLALMPPPQPD
TTVEAEGAGAGAGGRAVRGYLHVVDRKKDMMLVGGENVYGSEVEAALAEHPAVAAAAAFGQPHPLLGEVVAAAVVLR
PPAPAPPAAAAATAAPATAATAAGGAAGAGAATAGVTESELVAWCRQRLAHYKVPAKVHFLDRMPVTGSGKILKTEL
RARLIPTPTTSTATATSASASASAAAGGRPATAAATTAAATAVATFTGTAPGLVHSDVPAAAAILAAARTALAPAAV
PVSGSSSSRYSRPLLPLLPLHGATDPSQTYLLVLQPLQPDGTAAAMTAAAATASSSLARMLYGGARSVLVLQPLLAP
VPDDMQVQLQQLVAALSDVARRHTAAAATAAAATRAAGGVRTECDVRVVVVHAAAAAADPHVLRFAVLSAAAAASAV
AAAGSTAGARAPAMPPLAAVAAAAVDVGPPHDAAAQQPSSLNPLLRTLHRALPQVRLLPLLEATAPAAPAAVAAAAG
VVTRRRQLSGQQCTYVLALGELGDGLAAAAAPGAEPVSPEAVALVAEAVVAGARRLLLVARAPAVAAGAAATRAAAA
AAVALLLPQVTGAGAADVQARLVLLPHDGGDDKARQQQAAAAYDEVVRYALLSAAAAEGWPPIAHICHPAPPSTSAA
AATASAAASTATHPHAAAPVAGTAEVAPAAVVGAAAATATAAAAAAAGPSPREGPATSAASIAAAVSSAVAEVLGVA

VAGPAAAAATAAATAGTAAATAGRITGSSTLMAAGLNSTAAVALVAALEERLGVPLPPTLAFDHPSIDDMAAALADM
LLLQPPQPQQPPQQPPQPQPALQQQQQQRPSAGRSSSVPFTTAAPATATTIATAAAAAAAPAAPAAEQLVPRVLAA
VRQVLGGGGEAATATSTVQLQPDTPLMAAGVNSTAAVALTSTLEDVFGVSLPPTLVFDHPTAAEIAEYLATSTTASP
GATAALTPSPAGATAAPHSAPPLQAGYGGSQQRVQQQHAAALAHVRQAVAEVLGAAATAGLSDDAPLMSAGLNSTA
AVQLTSSLEAALGATASGGGAAAAAAAVELPPTLVFDYPTIAAIAEFVTTSGLIPSPPPPPPGALVAAGALVPATAA
AATAAAAAAAPPLALRMAPRPLVPHPLGLGVGGGARRDLVAVVATADRVPGGGVAGPWGGGASADRTTRVPLTRWDL
DRLAAAAAGTAEPPPACFGAFLSEVDMWDAAAFGISRAEAALMDPQQRLLMDAFGQLMMTRPPPPLPSRLADRAAVA
GIVPGVGGGGGGATGVFVGVSQLEYARMTLEQRTDLNAYYATGAHLSVTSGRLSYTFGLRGPAVTVDTACSSSLVAA
HLAAASLRAGECGEAAALGVNLTLVSSWTQACQRAGMLADDGRCKTLDASADGYVRAEGVGAMLLRRLQQGPPQAGA
AEAVAEDGGGAPLALLLGSAVNQDGRSSSLTAPNGPAQQAAIRSALALGSVDAAAVWALEMHGTGTPLGDPIETYAA
VSVYMQQQTAAAQEPGTRRGRGGHQPLQMTALKSHMGHAEPAAATLEMAGGGAAAGSPSSQQQPLVLLPRSAAGSSS
PWAGLAADDRCPELAVVGVSAFAFQGTNAHAVLGPAATGGSLQAFTVHDNPHQHPYKQVLGMQQAQQRQRYWVHPPL
HPMLGGPRLAASGTALVMECVLALPGSGGGGGSSSSSSGGAVLLPRLAYLRDHVVAGRVLLPAAAMAELCLAAADVA
LEDAARAAATAVEGGGTAAAAAGAGAGSGGCWGIGGLRILAPVVLPALPAGAGAGTGGGSGSGSGEGGGAVVLSCRV
DLQSGQVTLHNHTGATGRGVGVGGVGDGGGRLVARGSVARARACAPAPAPTSPTAAADKVDDSSNNALLLLLASILF
SSPEGSSIRASATPMPGPIGQIATPSDAAAGAAAGAFSGSTATASAAAARQDGYLSHPASVDAAFHLGVLQPPAPGA
TAAAKIPVGIGFFAAQQQHLGGGAGWGHGGDVGAAANSAAALNGAGEALGWACMTPPPAPAGAALSEAAGGRSSSSG
GESARRRSVSSFALLPEGTDSACGAASVVISGLETVELQLPAAGAVAAATAAATATATASPGRPSTQAEAAAAAAEA
AALYGTQLLVTSPAAASAVADAAGDGLPIPLLPRRSHPVAQLQVQGLQRAAAAGGATSGGVVAIAAAISGGAGAGGL
SGSLGEAAAAAAVDGVLRTAASEMAAVPMALTAAALQPTSGSRGAGGGVAEAAALSRPQPSQAMSGGVLAAAALPSG
AHGPLALRQLLPASGSSLPTATTTTAAISSPGVDAIPGLAIGGGVIQPESYHMEARPRGAFSNLCIVPTAPAPDGVA
AAGCGRGQVAISVKAVGLNFRDVLNVLGMYPGDPGAPGSDVAGVVQSVGWPAAAGGSGAGPRLKVGDAVFGLAHGCL
GSHVVGPANLLAPLPPHVSFAEAATLPTVFITVVAALRAAAALQPGERVLLHAGAGGVGLAAAQVATALGAEVVATA
GSPAKRALLRRQGIRHVANSRDLSFAEEVVTATGGRGVCVVLNSLTSPGMVGASLSAVSRGGRWVEIGKRDIWSAAR
VSGERPDVRYGLLAVDFMPCKVLHAALLEVAALLATRAIRPLRTLAYPLASVATALRALAQASHVGKVVAYAPLAAA
PSAASPGAGAGLARGARVLITGGTGGLGLLVARWLAGGGGGVRVAELCLVSRSGRVAAGGGGGGGGGGGKGEGEAVE
DAFAWAVQCGVSVRVAAADVACAEDAAALWSSSDGVTSPAAATGLPTAASRRGWDAVFHASGALADRMLPRQSLAAA
RTVAAPKLTAAHKLFGGGDGGGGSGGLHAHGAPLGRVVLFSSVAALIGAAGQANYVAANAGLDAAAEAWSSAGHDVT
AVQWGAWTSAGMASAAVLGRLQRTGQGALPPAVGLAALATVLRGLSLQQPTAGPAPPLALAALPPRVVTVNPFVWEK
YLQTVGGSSSAAVADGSSTWPHPLYAFFADAVAERRRRQEQHEREQQAVTAAAATTTATGTPVDPTGRLQPSSPQVL
QPPPLRYSREAVRREVQGALAEVLGAPLPDSEPLMSAGGLDSLGAVEYVNLLGRRLGGAVPLPATLIFDYPTPAAIT
NYLSDKLAAAAAAAAAIVQPRPAATAKAASAAAAAAAAAAATGIGGDADFLQQLLEPPTDQPPTLAAGLAPPAAARL
ALPPPPWALPQAHLRLQVPVVGVLSLEMAPLMPTATTASVFASSFARASSSHMAAPSAVPLADTLRSIPLQRWDRTA
AADPLLADALGSGAADAATQQLQPQQLLPAQFGGFLAGVEDFDAAVFGLSAGEAAAMDPQHRLLLQLAVTASASASS
ASASAAATAAGAEAVAAAAATAAARRGVFLGISWTEYHALSRAHGSAAAAGPYAAQGAVLSVAAGRIAYHLGLGGPA
MAVDTACSSSLVATALARGYLTAPTTTTATVSFTNSQQLQQYESAGIGAGVAEALSGATALVGGINMMLLPSTTAMF
QTAGMLSPDGRCKALDAAADGYVRSEAAVMMMLAAMGSQVGGAGSAAGPKALLLGAAVNQDGRSSSLTAPNGPAQQS
LLRQVLREAGLTPAAVAALQLHGTGTPLGDPIEVGAALEVFAAAANCTSAQQGTGDAATAAATATARRQQQLQLLAA
KSVLGHAEPAAGLSGAAFTLWQLQQRELVPALHLRTPNPHVAAALEAAAATAGAAAAVAGMPRQPQPLGIAAAEREE
EGQRALEALFGVSAFAFQGTNAHALLAAAPQPTGPPADDYEAQRLRSAASASASAPLWRPSRTWVHPPPDPWCGRFL
FASTPKSLVTSATATLRSRGVGGSSSSSSVAVLETVISSARHARMWDHRVAGAALLPAAAFLEVGRAAAASLLLPTA
TASTAAAMLALVGVSIPAPLLLPEGNLHQARGPTTSSSSKEAGGKGHGRVVVSCEVDLASGRVCVFTAPRRRDGQAG
SLQHLQQQKTTHLTAGIAILDTTTTSGSTGPAGAGSNNADPLAALCRTLFGAAVTAGGADASSAAVAAAVAAPSTTS
VDGEDVAPGGVDPARLDCVLQLGALSALLAGCGTDLRVPAALAAYCPTTARQTPALTPAPPGSAPPPDAGGPSGTTE
MHAVGRVRPGASGALAVVDFASAGACAIHGLEARRVQPRALIATAAATAAAAAAAAPAPGAALARQGSTAASIPPAA
RSAHTPLAQPQRPPVAGGEAEEELAEVLYRAVPSAFEPHVDRADRVAVPGAAPSVMQLRVEGAGGAAGVAFAALGLA
QQAVAAAVPGIRIEVQSRDSGVSATAAVTFEGGVPALLRTMEAETGLPCSACVVASSGGGQDSSSGHDTDGKEVTSR
PSQADACRVVVHATALAPGEDAYGAARDAGLLLRDVLVPAPLAPGGEAFLQPGGDFQLLPRPRGALSNLVPQPLTAP
DIDSVALASAVPAGKVAMRVRAVGLNFRDVLNVLGMYPGDPGAPGGDCAGVVVAAGPSNATDAQPSRLAPGTAVFGL
AVGSLGTLVHASAQTLVAMPGCLSFEEAASMPTVFITAQQAFAQAAALQPGQRVLLHGAAGGVGLASLQVVGAMGGE
VVATAGSPSKRSLLRRLGVRHVSSSRDLGFVEELTAAAGGSSGGGCVDVALNTLTSPGLVSATLALLRRGGRFVEIS
KRDVWSVRRLQSERPDVCGSFVAVDFLPDAAVQSAMRRVAAGVAGGLLRPLPVAAHSLSSASSALRQMSQARHVGKV
VVFNPAPAALATAIAAAPSAPVLITGGLGSLGLLTATWLLGQGVRRLHLAGRSGRPAPGSAAEAALCALLQQGQRHG
ALITISAGDAAATGDQPHLLLQPGIGGSGLVGMGGVAAVFHAGGVLADSTLGRQSAAALRAVAGAKGAAAAMLRRLG
GAAPVTQHVFFSSVASLLGSAGQASYSLANGALDALATASQSTGVCATAVQWGAWAGGGMAAENAAVARKVARLGMA
MLQPVAGLAALERLLRACVPPSVAAVVPFRPARLLASVRSRRRPAATTPAAGGSDAAAAVPVPVMFQYFEERVQSSG
GLGAHPSGEPLLQRQQREAEAARALEEALLGLVLSAVSDIAGAAVAPDAPLMGSGGLDSLGAVELRNALEARLGLQL

PGTLVFDYPTPTAIAAYVAERVAPVASQAPAAFPPPPTSQPDLWYEPDDDIRDPASPTLSSTVDVTAAVLDALSEVL
GSSAAAAVAADAPLMAAGLDSLGAVELRNTLQATFRVALPPTLAVDYPTPATIAKLIASRLPQQLQQHPAAATSTAA
SRRQQAAAGAGTPGSFQHPPPGPVARMPSGASRRPPSFAASILSLAVSLPASKTNHNINGSHSILTGAATAAGAAAA
VDAITAVPYSRYDTDGGDASRGAAAAAGGAVAALFEGSPPARFGGWLGGVSAFDAAAFGLSDAEAAAMDPHQRLLMA
CAAEALRAADTSPASSSSSPGGVVLQRDRCGVFIGTSALDYGKLAARIQWRSGRGRRAGGPAPPSVYDATGSLSLSV
ASGRLSYTFGLRGPAVTVDTACSSSLVAAHAAMAALALDFRQQPYISAGEYEDRTGGGGGGGAAGCGVALAGGANLM
LIPDTPAMFHKAGMLSPDGRCKTLDASADGYVRAEAVGVMCLAPLQPQQHAGRSGGGGAPLALLLGAAVNQDGRSSS
LTAPNGPAQQAVLRAALHAAALSPQAVAALSMHGTGTPLGDPIEVGAALAVLNTHNNSSKLAAGASSLAKPLRGARA
LLSSKSSYGHAEPGAGMVGIATALRAAAGAAAPALTHLRDLNEHVAVSLMEAGAAAGVAAGAAWSLPRQPQPLLWDA
SQEAGGSSAGASTAAAAGGVPKSLGGEEELLCVGVSAFAFQGTNAHVLVASSGGSNAINQQAAAVKQQHSNNGSSAL
MWRKQHQWLHPPPLVLVEAVAIPAPAPAAAVAASAATATAVFECAVSRTSRLAAVWDHVVRGRALFPAAGFVEMAMG
CMASLVQQQQQQQQQMTAAAISTGATAVAFTGATIPAPLVLPPLASSSAGSRAGSGGDSSSTVVLLRCGVSLATGR
LAIRSEQRQLLQPTGDGITAAAGAAVSRVVVHFRAQAVLVPAAAEPSGIYAQAGASQATEAAFTSRQRDVLLCALLN
ALGLRVTDAAMATGSGSSNGGGGAAVAQLDGSGSSDGFLLHPALLDSGLQVAGAISTTPSTASALAGAGSSAAGPEP
ARAALRVPAAIGAVRMPLDPEGAAGIGASGATGSGAGSGAAAAGAWVVAREQSSSAWSGSTATATAAAGGNGGEGAA
TGDACWDYSFRAGDGSSGGGGGEGGAVLCSIQGLIAKEVRSSGSGSAAVGATATAAAAGPAPSDAKGTSTSTSSAGG
QGMLYTSHYLVSEPEEVTGRGILASALPNETSLHLATPAASYAAASAIPVAAAAALSALQGAPDRSPLTLSAHVAPS
RLGLSPDGLVSSALGGIARSLALEQSQRGVAVAATNADALSARMEPSVAISLGAPAGDVFGWRSRSGLVAMQQLQSA
AQAVSGGDFQLLPRPRGALSNLVPQPLTAPDVGSTAAASAVPAGKVAMRVRAVGLNFRDVLNVLGMYPGDPGAPGGD
CAGVVVAAGPSISGTDAQPSRLAPGTAVFGLAVGSLGTLVHASAQTLVAMPGCLSFEEAASMPTVFITAQQAFAQAA
ALQPGQRVLLHGAAGGVGLASLQVVGAMGGEVVATAGSPSKRSLLRRLGVRHVSSSRDLGFVEELTAAASGSSGRDG
GCVDVALNTLTSPGLVSATLGLLRRGGRFVEISKRDVWSVRRLQSERPDVCGSFVAVDFLPDAAVQSAMRRVAAAVA
GGLLRPLPLAAHSLSSASSALRQMSQARHVGKVVVFNPAPAPQLSSPAGRVVITGGLGSLGLLLAGWLSRQHARCRI
QLLGRSGRADGGAGTAGPLESLLGGAQGMVEVCKADVSLAADCAAVFGAHASDCGSPVLAVLHAGGVLADSVLRNQT
ASAARRVFAPKLAQLGRWAGAVGLQPCGGVGSSGCAHVLFSSVASLLGSPGQANYAAANAGLDAAATALSSAGLGFV
SVQWGAWAGGGMAAADSQTAARVKRMGMSLIQPQQGLAALCAALGGLSGAGSPAASFSGVGVGVVAAVPFNWPVFLQ
RRKTPSAFFAAVGKGGNTTVSPSAAEKQHVSVPGGATTPKAAPATAAASQASAQQLLVEVQDAVSGVVGGALAPDAP
LMGSGGLDSLGAVELRNALEARLGLQLPGTLVFDYPTPAAIAAYVAERIATRQGPSSLAVEGGRAAAAGMSDILTDA
EDPDADLLFGGSSSLALAKRQPLAAMAPAAARAAPLPGGAEARLIGSSAIRRSTSAAVVLGLSQRFPGEGGPAAVSQ
AVDAVGCIPLNRWDVEALRLGGAAGSEPPVVVPFSALMGGVEVFDAAAFGISGPEAALMDPQQRLLLECAAEVLAGS
AGAAAAAAGHQRAAAAAGGVITAGPDVRAWLAACGAFVGVSSQDYRALTQRVAPNTTSFNATASALSVASGRLSYTF
GLRGPAVTVDTACSSSLVAAHAAMAALALGQCGGALAGGANLILSPETPLHFQRAGMLSPDGRCKTLDASADGYVRA
EAVGVMCLAPLQPQQHEGSSGGGGVPLALMLGAAVNQDGRSSSLTAPNGPAQQDVIRAALSAARLSPAALAALSTHG
TGTPLGDPIELGAAAAVVAGARNRAAAGSAATAAAITVLSSKSWYGHAEPGAGMVGMVFGLSALNTSAALPLAHLRA
LNPHVASALEESGAAASAAEVTGGSSSPAWRLPLQPQPLLSELPLAAAGYKAASSATAEEAALAAVVGVSAFAFQGT
NAHVLLAAAPQQMATSAGVAIEAPGGFGASDARASVNTAVWSGRQRLWVLPPLSALIGSLTAVSKQRSTLLFEVTLP
AGGASSGGGGSSRLAFLHDHVVGGRALVPAAALLEMATAAAAATAMYSAAAAGGSVSAAAQHDTASAAGSSDAASIR
SQLALTGVRLLSPAVLSKDSQLTLSCSLDTFSGRLRITSGGGTAAPAAAAAQPRRSSRATAPRATAAHGAAAATTHV
VADVAVVLEQQTAVTSSASPSGCVAAALAALARVLLPGVAATAAPASVTAGAAGTAASAPAPAAHGQLYLPAAAPDF
LLCHPAALDSAFHLAALHDDTAARSSGSSSGGAPPRPQLKVPVGVDCLLLPLAGLAARGSQPMCARPPAAASAGATD
SGGVLASCVAGPASATQMDVAVLGDGGSAHGFKVCGLLAKPLAPFQRPAEMAAAASPPAIAGAGTTPRPAAQQASSG
SISYRTVMVAAAPAADDASAAGSTAALPPSGSLTLSTSGATSSSSQCVALLPVLQAFAGGSAMVLRVEQQQHAPVAP
GLPWGQSTAVSGLLRTVAQELPSGLQLQSAAHSGRGSSGVMRTAAAVTMTATATGIMTANSSAGTHPEVELDAAIAF
EPRLLPDIAPVASGGDFQLLPRPRGALSNLVPQPLTAPDVDSAAAASAVPAGKVAMRVRAVGLNFRDVLNVLGMYPG
DPGAPGGDCAGVVVAAGPSISGTDAQPSLLAPGTSVFGLAVGSLGTLVHASAQTLVAMPGCLSFEEAASMPTVFITA
QQAFAQAAALQPGQRVLLHGAAGGVGLASLQVVGAMGGEVVATAGSPSKRSLLRRLGVRHVSSSRDLGFVEELTAAA
GGSSGGGCVDVALNTLTSPGLVSATLGLLRLGGRFVEISKRDVWSVRRLQSERPDVCGSFVAVDFLPDAAVQSAMRR
VAAGVAGGLLRPLPVAAHSLSSASSALRQMSQARHVGKVVVFNPAPAPQLSSPAGRVVITGGLGSLGLLLAGWLSRQ
HARCRIQLLGRSGRADGGAGTAGPLESLLGGTQGMVEVCKADVSSAADCAAVFGAHASDCGSPVLAVLHAGGVLADS
VLRNQTASAARRVFAPKLTQLGRWAGAVGLQPCGGVGSSGCAHVLFSSVASLLGSPGQANYAAANAGLDAAATALSS
AGLGFVSVQWGAWAGGGMAAADSQTAARVERMGMSLIQPQQGLAALCAALGGLSGAGSPAASVSGGVGVVAAVPFNW
PVFLQRRKVADFFEAVTPASASSAVEREAQVQSTAAARTPRRRQPQLLATSVAGVPPPRQLDDIVAGVREAAAAILG
AAAAALPAVDGPLMEAGLDSLGATELRSALEARFGLPLPGTLVFDYPTPAAIAAYVAERLAPAAHAAPTVAPLPSRR
RGPQRQGPRSRRLLHATAAPAALSEHQQDSSASGGVPSLAEVRQRVVEAAADIIGLTPQLPQQLSPGAPLMEAGLDS
LGATELRSSLEAAFGLTLPGTLVFDHPSAAAVAELLHARLREQRQPHPARTIVEEDWEEGETEEDWDEEEGSWEEEK
EADEDEEEEFWSEAELEVAVSPGGRFVASAPRRGFQRSRPGQIARSLSLRGSSRRTSAGVLVGVMSSAFRLPADAAA
AAATAAGHTSDLVAATHVCDAVSAVPLDRWDVDGAAVAALSAAAAGSGSSSSTSAAAASAPAGGLPPHFGSFLSGRV

DLFDAAAFGISPAEAALMDPQQRLLMEGLHEALALLPPPPQSHAAARQQPQHPQQQQPHVGAVRTLWDRQRFGVFVG
VSSPDYMKLAVASKQGITAFSATGATLSVASGRLSYTFGLRGPAVTVDTACSSSLVAADAAVTALRTGQAAAAAVAG
VNLTLSPDTSAMFQRAGMLSPDGRCKTLDASADGYVRAEAVGVMCLAPLQPQQHEGRSGGGGAPLALLLGAAVNQDG
RSSSLTAPNGPAQQDVIRAALSAARLSPAALAALSTHGTGTPLGDPIELGAAAAVVAVTAPLGRRTPAPPAPLVITA
AKSWMGHAEPASGVVGLLHAAKLATAHVALPITHLSAVNPNVAAVLEGGGGAGALAARWAAAPRQAGPLPAAVGNAH
ASRPTSAAAEEAAIAAVVGVSAFAFQGTNAHVLLAAAPQTADVVGGAAEAPGGYGARTRASAAAGAAATAGSHWLSP
RPQLVLATGFQLTYPTCPAGPASLTPGRMSGGGGALSRTESGAPRTADAIAAAVSTMPQQPGAGAAAGQQQLLPPAS
PQRQPCVLSRTGSISRVPSATTGLSRSGSLSALPPPPAPASIPWLLPPATSLGYNGPPLAVFTASPSCARLAMLWDH
KVNGRPVFPGAAYLEMVAAAVALLRPEAGSSSSGGGSSSLEVPLLTGVEIPAPLLLGFPGVWPEVELRCEVQLLPPS
AVDAATAAGAALGRVTIASLAGAAAGMLPSPRTARARRRTVHMTGYIEAAVAVPEVAAAAGSSSSGGHGGTSAYSAA
DLAALARLLFSDSGATAGAMASSTSCGAPAVASVLYGDGAKGGAAGGPGGSSGFVLHPATLDCVLQLAAAADADAAA
AADDGTAQPARAPAAKVPSRIAAVSLSPFQRPGTCFSSGVGGSSPEDAFLFASAASRNADASSSPDASSASDSAAGG
PRAEASAGPATFTSYSVQTPATAGGMLSSIVDLEARSIGHATRTSGVQDPQQMQAATSASTATEAAAATGAAPALPA
DITYHTQWLVDEPLAAVVEQEDAPAQQPLFLLSPGGMSGTSTIAAHVLAAVQQAISGNLPMGATVVDAGGHAPYVRL
TTGSAAPPVTTADLAAAVLRAAAAESLPVAHSLLAPHVRQQPRSSAPPLVLHGPPDGTDGPLLLKDVYGGRDSLDAG
ARLAARLVPAPRLEMAPGGDFQLLPRPRGALSNLVPQALTAPDVDSAAAVSAVPAGKVAMRVRAVGLNFRDVLNVLG
MYPGDPGAPGGDCAGVVVAAGPSTSDTDAQPSLLAPGTAVFGLAVGSLGTLVHASAQTLVAMPGCLSFEEAASMPTV
FITAQQAFAQAAELQPGQRVLLHGAAGGVGLASLQVVGAMGGEVVATAGSPSKRSLLRRLGVRHVSSSRDLGFVEEL
TAAAGGSSGRDGGGCVDVALNTLTSPGLVSATLGLLRRGGRFVEISKRDVWSVRRLQSERPDVCGSFVAVDFLPDTA
VQSAMRRVAAGVACGLLRPLPVAAHSLSSASSALRQMSQARHVGKVVVFNPAPASQLSSPAGRVVITGGLGSLGLLL
AGWLSRQHARCRIQLLGRSGRADGIAGIAGPLESLLGGAQGMVEVCKADVSLAADCAAVFGAHASDGGSPVLAVLHA
GGVLADSVLRNQTASAARRVFAPKLAQLGRWAGAVGLQPCGGVGSSGCAHVLFSSVASLLGSPGQANYAAANAGLDA
AATALSSAGLGFVSVQWGAWAGGGMAAADSQTAARVERMGMSLIQPQQGLAALCAALGGLSGAGSPAVSVSGGGGVV
AAVPFNWPVFLKSRMHQDYFAVLRERVVHQQQQSAAASWPAQQPQRSGSSRRVRPGASRGRRGAPVTAAAVSVESHL
QSMLAEVQDAVSGVAGGALAPDAPLMGSGGLDSLGAVELRNALEARLGLQLPGTLVFDYPTPAAIAAYVAGRTMPQP
PPVSEEEGDVSAGETDDGGLTSAPVGLVAPTTTARSGRVLASIDQLLPSRSADVSRLLGTSAAAAAAAPRVLIGVLA
SVCRSVGDALAPGRLDATDAPSRVPTSRWDVEAQASRTLAGALPVPFGAWLPAVDLFDAGAFGISVPEAALMDPQQR
LLLLCAAEALLAPSTSASLGSFAASATTAPAAWRSRTGTFVGVSSMDYNKITLRHEPTASAFAATGASLSVAPGRVA
FTFGLGGPALSVDTACSSSLVAAHAALGALRLGQVSAALVAGVNLTLSPDTPAAFQKAGMLSPDGRCKTLDAAADGY
VRGEACGVFLLGALPPAGAEGSAASAPLAWIAAAAVNQDGRSSSLTAPNGPAQQAVMCEALAAASAAASAMQWLHMH
GTGTPLGDPIEVGAITAVFGGSSDSGALGKPTITGTVAASSISAAGPSPPALTLAAAKSWLGHAEPGAGVQGLAHAA
TALTHGSEPELLHLRALNPHVTSALEAAGPVALARMRAPRQLGPKPLPPRGGAGRNGGRSAVAGMVEDMSEAEAEAL
CGVSAFAFQGTNAHVLLATRSRVEGSAAAAESAGQLAARGCAWRRERHWLHPALTPLLTWVAGGDVGGSIGSSRVIL
AFETQLSLPQVASVAASLALTQPRAPGSRTAVAAAAAELQPPLLVLPASLLMAVAAAVHTACATADNAPATTSCTT
PEGPIRVLLTSGRLQPVAAAHQQQLLTVVDAASGCVEVWVLPAAGAAARASSQRQSQPLLAFSATAGFTLGGTSEPA
VVTQGPGASQEATALTGLARLLGLVPVEAPSIGNAASAPACAMAAQPSTGAAGSSGSSDEAEAPGWGLIAGLAATLQ
LASAAGAAEAAAGHITGFDATSCDVRPPSATAGNLQHYCAVAPHFGGGGGGAAGSALVDACLSGAGGGLLTGVRIAP
LDVAAAAAIAGHMVSGTAAQQHALAVVADEPADDSERCRYVVEWLVEEVVPEQLDGTGDPPAAADAAGSAYFEIATS
AAGLGLSDIAGSVAALQGFMIKGSQQQQAQVQQLQLRSCGAHLPAAAALAPPAAGGGSANNAAAAASWALARVLANE
RSGSVAVTTSDVSAAAAGSGRKRGIAWSCWPQEVADTPAAAAKRASSMYGQTTAGRVAYTARLTRALLPSPQPTPTS
PAATEVATATASRAPAAPPLLLPRQGCYLVTGGSGMAAAHVTRWLAEVCGVAHVHLASRTGRLPDASLAALGAAGTP
GAAAVSAGGATLITASKADWSSAEDLAWLTGAACSISGTSSVYSSLQQQSSSRPLLGVFHAAGVLQDALLAAVTLPA
LRCVLAAKLGGMHGGGSSGSRSCHRDLVGGSCSSRLQVQPLATHVMFSSVAALLGSPGQAAYAAANAGLDAAAAAGA
LAGAPMVSLQFGAWAGAGMVAQSAGTAARLERLGLGLLSPEAALSALSAAVAGAAAARPAQLPAVKSLLLPQLQSCG
VIVVNPFRWPAFLRGLQEQQQQHQATSRGAGVPSFFSNFVAQAPPHTTKQRRTAAGAGAAEARPPPSSQRRLLADVG
TEVRAAVSAILGAGASEGQGLSEHQPLMEAGLDSLGAVELARSLEARLRLALPQTLVFDHPTVAALTDHLQRLQEQQ
LAVAEEDLLEEVEGEETAIVQRRRSAAGAHLRLPASVAASAPLGAPAGLASDVVVVQALVQRSPAAAAAATSAVAAD
PVGLAPFSRWDAEAAAGRVLEARFGAFLPGVDLFDAAAFGISQAEAALMDPQQRLLMEGLHEALVGSASSPGGSSSS
NGARGRGMGVYVGVAASDYGSLVKAHGTAAGQPGGGAFHATANALSVTAGRLAYTFGLTGPALAVDTACSASLVAAA
LAYDALTRGGSASSGTAGGGGRLRAAAVAGVHVQATPTSSAYVWNAGMLSPAGRCQVLDSAADGYVRGEALQVMILG
AKSSASAAAGGGAPLALLLGAAVNQDGRSSSLTAPNGPAQQAVIQAALSQAGVSAGGLCLLSMHGTGTPLGDPIEVS
AAAGAFAPAMPGTRSAARAAPPQPLVLAASKSVQGHAEPGAGLVALSHAAAALAQALRPQVLHLRGLNPGVAAAMET
AAASQVTMAAGRSSAPLAAHAARGSTAAAAATADADGEALLLAGVSSFAFMGTNAHVVITSPAPPAVAATAASGSTA
TASAAAAGEREQQPQQQQAAVLWQRQSHWAGPAEPHLLAVRALQQATRATAAAAPAVSRSHVVTLAGDLSHPRLAHL
WDHVVGGRHVLPGAAYMEMAVAAARAALLLPQQPAAAASGSGVGSGSGSAGDGMAAAALLAITDATIPAPLILPPLQ
ASQLTSKLAPALHCAIDCVTGRLLVYTRSKGSGGGSTVVHMRATVSAALAMAVATAAPAAAQAPAQASANPDLLASL
LAALQLRPYVAGIVAPTASNAGGGGAAEPAAAVATATLLQPHNADDAAAAARTALQSEGQAAGVGLLDCFLQLGQVF

IHQREDDEQAPRSSPRAGPRRTYIPSGFGSLLLGAPAAAALCGTAAGGGAGGGYLGASAACVVAARRSMPAAAAASQ
QDAEAVESDYFLTPAAAGGVAVSTGQLPAAAGPHAAVCIRGMKARPLAGSGATAATAAASTAAVASGARQDTDADEV
ATGEPRLLYGVSWLVAEPAAADDGNEDQRAADPPQLQLLPGAAGGAAVAAGVSVLQQLAAAAARAGPSAAGGVTLVH
AADASSGRRGTAMSAPAAVVAGAMRVAALEAPNLRLAVQLSAASALAESTLTLRASPGNSSSRAGASAAAAGAVFGE
AASGGAKAVPRLLPSPQQQAAGPYQLLPQPKGSLNSLRPAAVVLLAELAALVPTSPAFGGRPPSPSAGAGADSCIVA
VRAVGLNFRDVLNVLGMYPGDPGAPGSDFAGIVEAGPGAGGAVFGLTTGCLGSHVVCRTDTCAPLPPHVPFEDGAAS
PTIFVTVEAALVEAAGLVAGERVLLHAGAGGVGLAAAQVAAALGAEVVATAGSPAKRALLRRQGIRHVANSRDLSFA
EEVVTATGGRGVCVVLNSLTSPGMVGASLSAVSRGGRWVEIGKRDIWSAARVSCERPDVRYGLLAVDFMPPSVLQRH
LHTLSRNLATGAVRPIPRITHSLASVAAALRQMSQARHVGKIVLSNSACAQRPQHATAGAIGSSSSGAGGGDGGGAV
LVTGGTGALGALVAAWLYSSLCVGGRSAPHIILAGRTGRLSRGSSNSKGDGDTSSYSPVDALSSGDGRSPLHAAYVS
IVRCDAASSADAAWLMRGAAAGRSIAAVMHAGGVLADATLANQSLRGARAVAAPKLGALAALEPLLTLAPAAVPHVL
FSSVASLLGSPGQANYAAANAGLDAAATAMTLRGVPVVSVQWGPWAGGGMAVATAARNEAAGVGALHPHLGLRALAA
ALAAVDMTPPQPVLAAAPFNWRRLAAAQSGPLPPLLSEVASPAAAPAAGEAAAGPAPKQQPLVAPAAPQPRRQQAQQ
QTAAVHREHVLAEVQDAVRGVAGVPLSPDAPLMGSGGLDSLGAVELRNALEARLGLQLPGTLVFDYPTIDAIATFIT
TTLSTSSSGSSSGRGDRDSGRRVGQSAAAAPAWDDHAGDGDQALLGAALTSFDDNMEFVDEEDEPSTGDAALGRPRV
RLAAGSRAAAAVPRGAVAPAGALPQPGMLVAVVAAAHAVPGDCPARLPGLGGSGAALHDVVTPVPLDRWDAERALTD
DLPPRFGAFLPPGRITTFDAAAFGISGPEAAAMDPQQRLLLELAAEVLTSGVAAAATAVAMSATGGSSHQALGRLSD
EASASAVGVFVGVSTPDYADLQTAHAPIGVYSATGAALSVASGRLSYTFGLRGPAVTVDTACSSSLVAAHAAVRCLA
GGDCGRALLAGVRTLLTPNLSAMFNTAGMLSPEGRCKTLDASADGYVRGEAAAAMVLQSWRQHEGHSGSGGGGGDSE
RAQPTGVLALLAASAVNQDGRSSSLTAPNGPAQQVVLRAALAAAGASASAVAFLQMHGTGTPLGDPIEVGAAAAVML
QPQAKPAAAASLPRSAPLPSSPLVFASDKSGLGHTEPAAGLMGLLHSTGGLAAAATAPLLHLTAPNPHLHELLQASQ
SAAAESSGGEGARGIALPRSGAGRPAPRAAAHHGAVAAGQADADEEALLLCGVSSFAFQGTNAHVLLCPPAPDTAGA
APRTSDGGRQKFGAWQRQYAFVLPPAHALCRSAAVTSATAKITWCADLTHPTVQFLRDHVVAGRTVFPGAGYLEMAV
AAAHIASHGSTSSASGGGGVVALAHVSIQAPLILPATTTAAEPAKAAAGAAVVAASVAAAKAAGVQLQVVLDTTSGA
ITVQSISGSSSSASSSRRSRRDTAAPVVHLTAQVAFVAADGSSSAAAASTAKGGPLPATRPPTTAPAAASTAPSTDS
SPTVPAFAGRRQQLLSADALRAAAPEPLPAAAMYGSLAAAGLQYGPAFRRLRNLHQQGSSGGGAGGLATACLAPVSV
TAGAVSEASDAGAVVGPRDGAAAAAAAGGYLVHPALLDSAFQLGGAVQDGGGDGDSSSGRHSGATYVPVAAQLVCVA
VSAQTGAGAAATVADGSRPLFAVALAGSSTGNSNSTSSSSHVQGAAAAASAAAAATAPLIRHVHMYDWHGQPLVQVV
GLESRAVSAGAVGAASSSSSSAAAAGGQRSAGAAEVAASAAAATGAGAAEPDAGLYCVQWVAEQPLAAELQAEEDVR
AEEQASAQHRPPGAAWLDMAVHGGRSQQLLSQALQALQGGTRAGVAAVALCAPAGGASVFPVTQPAVSAAAASDCAG
LQGLLRTAAQESGGSTGSGLHVQSLQRDPWAAALPCRGPGLTAAFGMGTKDALTQGGSAGTGPGFDGYGVSTAAAVT
YVPRLLPSAAAASAPPPPFKLLPLPKGSLNSLKPVPLVPVTAADGITAGGLPACGPGTVLLRVHAVGINFRDVLNVL
GMYPGDPGAPGGDCAGVVVAAGADVAAAPTAPLPGRAVFGLATGSLGSHVLASAQTLVPMPPTLSFEQAATTPTVFV
TVAAALRQAAALCRGESLLLPAAAGGVGLAAAQVAAALGAEVVATAGSPAKRALLRRQGIRHVANSRDLTFAEEVVT
ATGGRGVCVVLNSLTSPGMVGASLSAVSRGGRWVEIGKRDIWSAARVSCERPDVRYGLLAVDFMPAAVLHAGLSAVS
AGLASGSLRPLPAAVHSLSSVAAALRQMSQARHVGKVVVSAAATTVPQALPAGAPSASTISGMVRGGRVVITGGTGT
LGLLLAKWFTQQHAASEVVLLSRSGALPTAAAAELLPSSPDAVITLVSCDAAAAGELLSAASTAAAASDQHSACRPP
SLLVHAGGVLADGILPRQTLAALRRVLAPKAAALQHLNTIASRYPMAGQVLFSSIASLLGAPGQANYAAANAGLDGH
ATATGAAGLPVVSVQWGAWAGGGMAAADSQTAARVERMGMSLIQPQQGLAALQGVLSALTAARVPSTSGPDAVVAAN
PFRWPTFLRRLPAGPPALLSHFATTAAAAGAGGSQPQALSSGATTSRERGAAAVARGSTSSRRRARIQSPTGATSGP
TSVVPAAAAAASPSAAAILATVTDTAASVMGVASLDPAAGLMEAGLDSLGAVELRNALSRAFGLELPPTLMFDYPSA
AAVAAYVEGELAAGGGAVAAAVAAAAGSGEEDAEGVDEEDEYMSEGWDQEEEEEASELGTEVAVAARNRSSRMGRQI
RGGGARDVSGSKLVVVSGISTRFPGGVDSLQRLHQASRDCAELHSPAPFERWDPELAYLAPGDGVATRLGAYVPGVH
DFDAAAFGLSTGEAAALDPQTRLLLEQCAAACWEAGRPLAGLVGAPVGVFVGCIWLEFGELLAAVGAAGGGGGGAHV
VTGNGLAFMAGRVSYTFGLTGPCVPTNTACSSSLVAAHLAAASLRAGESAPAALAAGVNAMLLPRGASAAMTRVAAL
SPDGRCKAFGAEADGYGRGEGFTALLLEALDATAGSAAGGAPVAATADVGRARAMGPLAVLAGSAVNQDGRSSGLTA
PHGPSQAALVAAALAAAGADALSFVATHGTGTPLGDPIESNALRKALQLAASARGGSSSGSSSATAPTAAVQRQLVT
LGAVKSLTGHLEGTAGLAGLLQAVAALTQRAAPPLRYRNINPHVASTLEGAGGGAGGGAAIRLPIGPAPSPDLDLAD
ESELFAGTSSFGMSGVNAHAVVRLLGGSSSAGQAVATVPGAEAEAPGLMQQPQQWRPLDFARLVPLPLGHPLVHRVL
QPAWLPPPAAAATAAAGSPVVELVLPLAPCCTAAVSYLCDHVVAGQPLFPGAGYLEMAVAAAVAAAPSAFAGATAAA
AGAVEGTTAAVASLVLTGVRFLAPCVLPAPDSSDGEPVRSQPRQPRELVAQLNTDTGEVRILSRGGGSSAGSGSSGS
GGNSAMTVHMTAAVALLPTMGAATTGKGAASWQPIATAASAAGEGGQQTQPQPLSQPSVARRSPLSSDALRARCPEP
LAARGLYSALHAAGLQYGPAFRALQSVHTTATATATSASGAGGAANGATAAAAAGRLSPRPAATAAAAGGGDAHVTG
GYFLHVAALDGLLQLGAALPTTAGSSGSGGSGGGHTFVPYALDAFTVSLKQRADNAPLWGSAEAAAAPSASAGAAAA
AAAPAELHRNHTLLDEDGAVVCSLHNLTTRPITLGGGASGSAGAATRGRAGAGRAGGIDGTQPAGQILTDVAWLVSD
PCEDEVLHEQAQAGDAAESYVLTSSAAAPAAAAAAANALAAMQQLAGAGVSGAAQLQLPVAATHNASGLDAVAALTG
LGGMLRTFNAEHPAARAISLTAHAEGGEPIFANRMAVGGAAATFRVSAHLQQAAATEAPDGYGPSAAAGAVSRAVLV

PSAAAASAPPPPFQLLPLPKGSLNSLKPVPLVPVTAADGNTAGGLPACGPGTVLLRVHAVGINFRDVLNVLGMYPGD
PGAPGSDCAGVVVSAGADVAAAPTAPLPGRAVFGLATGSLGSHVLASAQTLVPMPPTLSFEQAATTPTVFVTVEAAL
RQAAALCRGESLLLPAAAGGVGLAAAQVAAALGAEVVATAGSPAKRALLRRQDIRHVANSRDLSFAEEVVTATGGRG
VCVVLNSLTSPGMVGASLSAVSRGGRWVEIGKRDIWSAARVSVERPDVRYGLLAVDFMPAAVLHAGLSAVSAGLASG
SLRPLPAAVHSLSSVAAALRQMSQARHVGKVVVSAAAGPLAHGAAASAAGDLSRSALSTAGGTVMVLGGTGTLGVLV
TKWLAAEAGVKRLVLLGRTGRTAAPAAAAADASALHAAATRDAAVTVFAADASTRSDLAAAVAAAASGGDGISAVIH
AGGVLADATFASQTLAALRHVVSAKSSAAARLASLVASLQPAAAHVLFSSVASLLGSPGQANYAAANAGLDAAAAAM
TSRGVPYASVQWGAWAGGGMAAADSQTAARVERMGMSLIQPQQGLAALEAVIGALSGGPAAVPPLLPPPAPPGVVAA
VPFNWPALLGGFGRKGQSIPHLFSHMADVTGVSSSSSSSSMEGGAAAGAQALAASASPSARAGRGSGGMGSRQQIAA
AVDECIRGVVGGGNGAGDLSPDAPLMGSGGLDSLGAVELRNALEARLGLQLPGTLVFDYPTPAAITEFIAAQLVKED
AAQDDAALTAAATAGGGGALLPLTLAPAASPALHRAAASSAVVGITGISSRSPGGAATSLTVVDAITPVPLSRWDID
RLWAAGSSSSSGVPPARFGAWLPAIDLFDAAAFGISAPEAALMDPQQRLLMHVAHEAVCQAVASAAGPGNGAGGGGG
SGRALPAAGLEVAVAIGIASAEYTNWVCRRAGVGASAYGATGGALSVASGRLSYLYGFTGPSVSVDTACSSSLVATH
WALQEALLQAAGGGAGAGGGRGAARRRAPLAALASGVGVLLSPEPTAMFQKAGMLSPDGRCKTLDATADGYVRGEAV
GALALSVLTSGDSQQAPQQAQPCLAVMRTSAVNQDGRSSSLTAPNGPAQQAVLRAALAAAGLQASHVSTSQLHGTGT
PLGDPIELGALAAVLRPPPAASMATKGTTTTITAHAPSQAAPQTQTLPLSLAAVKSKLGHTEAASGVMGIAHGLAGL
GAAAADSLLHLLCVSPYVAAALGYGDGGGGDGDISSDAGGGSGRWLLPRQSAPAAAAASGGGADTEPEAAVTGVSAF
AFQGTNAHVLLQRDTSNGGVRSATSPSGPLHWRRRRVWVVPPASLLLSAAAAFSFSSRVPGTPAAGAAAVFEVDLRQ
PALASVLSGGLTLTTPRGRASALLPTGFLLEVCAAALRQLHHSTAVGGVEEEAAVAAFAAAGGSGSGRGSVDVAAGG
CGLLLALSHVTFHSPVDVFGLLGGGEGGTAAVEQRQPQQLVCVVRPASGTLEVVTVRRYQEEHEDRVDEEEEEESSE
GAVAAESAQRHVSASFTRLLHCGPGSTLAASAPPPADSTDVLAALARALHLPLPQQLGLAAPAAAATCGIAPEVLSS
SSSSLSAAAAARGSGASAAGGGGGVGGVFGSPCGLEASLQLQAVLVVSQGRSGDAEGPSGGRIQVVSHIDCVHLPAA
ATAADGAGRLHASAVASAASAAAAPSTDLRLSGCLAAATADNTGHGGPAVSPAAAISQTLWKLNGVRLRSAAIAAHP
LTANTAGISGSHGGATDAATAAVLAPTGAAAASVDTAHIRRVVREAVTAALGGGDVADDAPLMSSGLDSLAAAELHS
GLQSSLAISLPPTLVFDFPTIAAVADHIAERLRQQQQQDAGAGYAAMGGGLRRQSRLRGGAVSATASTVVSIVGMS
ALPWSLLQPAGAGSSSSATCSGDAVSPVPLSRWDAADPRVLQLPAGAAGGALPPQFGAFLPGVDLFDAAAFGISPAE
AALMDPQQRMLLAAVLEARPQQQAAAAAPSGAAGAGGADVGVYVGIGSGDYEALAQRHGVQIGPYAFTAASAAVAS
GRVSYTFGLRGPSASVDTACSASLVAAHLAAVALRSGECGEAIAAGVQLNLVPQSTLMVAAAGLLAADGRSKVMDAA
ADGYGRGEACRALWLRAERRRAPGRAAGAAATFSSATAAPAPLAVLVGSAVNTNGKASGLTAPHGPSQQALLAAALA
AAGLAASAVAGLQLHANGTPLGDPIELGAAAAVFGKSGTSTGAGISAPGGAAGAGGAGQGAGRLQPFAVMTVKGYGG
HQEAAAGAVELAETVAALRARAVPPAFNLRSLNPLVEEPLAAFGGAAGRGAVVARGGVMGLPAAAASAAAAAAGAAA
GAEPELLIVGVSSFGAQGTNAHALIGTPAHATSAAAAGSSTTGLAIGGVGGPAAAGALTWSSVRRHWVHPEPQLLLC
AARLQALRPSAARRGGGGLLGSAGLLRRTTLLGRLDHPSLAYLWDGTAADVGDSAVEPAAVAVAAAPPLLTNAAVLA
AAASAVRLLLQQDGGGGGGGGFLLQDVMMAPPLPLPAAAHGLGGAAGSGTASRADPQEIRVTLSPPAAAAPAQASAL
AAGQPMTPRGGVHAGAGSGGGGSGGGMQLRVAVGGSQQLTARVAMLPPSQPGSAAAALAAAPAASGAEHEVWAPAEA
AQLPALLRALLPADAWPLHLSSSASAIDDENHRHRAPHDHVFAAAALDGDSPQQQQRPQRHSTEALDPRAAEAAMQA
AALAASDADLELWLGPCYLASVAAVAVGGAGASQQAGASGGGGGGGCGGTWLAAECEASAADVAAASAALRPIHMPT
AAVQQQQQQHQHTAAAAGGATAALRLLLHGAVVAPLDAAAADVARAAAATLHPGEAEHAAAAEHAAAGADGGGGGIG
GDGGNWALLAMSPEERQLYLAGRIMTEVRALVGRPVHPSEPLIAAGVDSRAAMELRRSLGEALAAGAPAAAATAAGD
AGAGATAGGGGGLSLPVTLLYDYQSVDDIVGYIEGQLQRLGQQQQQQQRGGGGGGGATAAEVRRRLPGHDADDGGYY
GTDDDEDDDGEGVEEVRDARRAAAREAAATADAAEAAGSSSAAAAGPSALLKVLRPPSAPRPLFLAAPGVANAQSAY
FSFSAFLQWADQPIYVLDKDNDLDIDSLARRNALDMLAVQPPGSGPYLLGGHSYGGVVAVAIALLEEWGHDVGLVV
IMDTPRPEQVRPAQPHATHCTEADALELMEMILGALGRDAIGLGSSIVHPRESDEWRGMSLAQKFEFFAPIWRVMRD
DPMSVEEVRRQVEFVALAVKQGSQISDMRRHRFSGRLLQSRVLYFRADRRGACDYVNDSRFWAYAHGVCWEGLCTDL
QVVDVPGDHFSLLRQSPRDMGYLVAALQAALGDFGWAETVGRRAGAGAGPEEGAAGGAGAGGGWEGGQQQLAPAQAE
ELAEYLTRMGVDSSRAQQLAGAAGGGGGAGAGTGAAAAGQAGAAASAGGGATAAGAGVVSAQPPRVGSSLGASAAPL
NEAARRAVSVATAVAAAAPATAAGRGSGSLARPSGSIVTPVAILPPAAARAPPPALLPSPFAQQQQQQPQQQQQQQP
QQQQQQPQQAQQSPPPLLPVQGLDLVLVLADAGQRLPPMLESALAGLGLPAFTLQLPPNSVLPDLRSASAVAAELVA
AAREAAAAVAVQLQQQEAEAAAAAAAAATPAVGGRLPSLRRRPIGGAGRGAAGGSAAAAAAAAAVMPAADWTARPPL
AAAVIGVGFAAAVAFEVAVQLQPPAPHTAPTGAAAAPQAAAAAHGARAAPARSPQPPPTTPQPQPQPPPPPPALHAA
VLVLVEDPLLRRCCEVCAQPWFQLRDWAAGWRPDLDLSEFAAVGRLLEEQGHAAQLEYVAGLVPAGLSRGEWEAMAE
EAARAANGRGGYSWEEMQYSWAVLYGMIQNLHERYGVMEAAAAAMTAAAPSGSGGAAAAAQLLAVGGSSGGGSGGSG
TGTGGGTESGARLSGAGSTVGSGAGCVPLPGWESFLLDMYALADRPELQLDYLARYRPPAALGEVQWDTLVRDSLWT
ASYLKSLSATHKPTAPFLGCSLFLHAAPSNAVVITGAVRDVVAGWAAGATRSIAALVQPLRLVRLPPTAEQQPPPAA
DASLVGLAEEGEGGSSVAGAAQRSGGAGGGAERDEVLLGTAATAATVAAEERAEQQQQQQQQRPAEDGAPEPYASW
SANPVLPLSPQQLQQLLTEAAGAQARTADVRRRLADSVAGAPPSPLAGAAAAPSLPIVAYDRQVAANAAGMSLAPPA
DASASDAPSQSQPQAAVAQRSSTAGSTMSFVLVEGQRSSGGGSGGGAAAAVAGSPSTAGGGGTPPGAASPAPATAAL

ALLQPLPPPPPNTPAVRIVALNRLCPLGLPAYAATVAAAAGSAASTPLQQGRRPAAATAPQPSQPQPQPSAGGGGGA
EAGTAALATSLPVWLIHDETGSTGSGAARTLAELLPFPVYGIDMPDFPTAAAPPPARSGAVLQPSSLEELAERYADA
MVAAQPQGPYCVAGSSVFSCATAFAVAVALEHRRGADVMLVLLDGPPYVSLSGSGAGGSGGGAAGTGYGAAPSPSSS
LSMDPVYYSLHQTLVQHQAAAAAAVSRGEHGASGARPGAGAAGTGAVLLSTAGGGDGGEAAGARGSDAPAGGTNGAP
AVPDLAAFAATLQSRGGGPDDVAALLATAASFRPPSMAAEAWASAVRGAVRRGAVLRRLMLAAASSPRLTATPLPTL
GGPVAVVLPEDRQGAAFLAAARACCDNSLTALTLECRHGAVLATAELRRAAAAAVTEAVGEMLQML

>Cre10.g461850.t1.2|PACid:27579344
MNSRHTAGLESIIKPLMMTAGQAMGPDAPSTASAAAAVPTFKSDGSDAANEQRDGDDIGTPRPVAHLLSGTPPEVPS
ASSELEAAGSAPRRPVIAPNSSSNSNVPAVRIAPAPTAIQTSTEYRNTEITAWAPEPPEAAASVTPPPAGTAGYGAA
ACPSIAVTSLPPLSSSATAPTSLATDDACSPPPPPPSPPPPHHARHADLSDVPLDVLSSLILPRLPPGHRLDVRAVS
RRWAAWVDGGLAPATHINLVMDRKELAAAAAAAAPVAPAVSAAHSAAVAVPSAAAADAAAAVAPGQRLPHPSPQEGL
LDRRFPSAVAVRLWVGCRPPASVAGTNGGGLAAQAAAETAAAAAPHAAGSQRRGVASNGRGLGSTGPLLGLRSLSSA
SFAAAMEDCCSRAVGAPGRIAAGIADLLLASSSCVFPMTSAAMRRRLGGGGGYGYGYYGGGGAGGGGGAAGGFQPSY
RWHVHTQKLEAGGGSRGRPAAVRNADGTFQLLRRYSQDALEPQALPPLPQCLPLLGGSTALRRLSVVSAYDMSTETL
ALLAAAFPNLTALSLHAHQAPGVDAIPVYLRHSPGATMIATSLTEARANTSASRNLASASTGTTGAAGTACACSAPR
ASAAADASAGASDDASGGGAAGASLGRLSRRHSDNMPAESVAAAAAAAATAAVAAGEEVAEQPRWVLRSPLVAGLPQ
LQTLALCGLWLGDAEAQVLGALGGLRALVLHRVTCRVHRLLPHLEAGLTRLTRLELRRLDYFHSLPPDPPDTAALEA
RERRRAAADAGRGGVNRHAARRQRQLDTATRRAARLTARRRPHLLTSLLGRGTGSGTAEGGGGGGAAAAAANHTAAA
AIAAAEVTEAAAFADLLSGPDSDAEEEERREEAALDGDLEEEALPGAPWMTRQSRGLFPDWAGALWSLGRALRRLVG
LQVLVADVECPARGQQDAWAALLLGVKRLQRLRELRLPHLAFGSAWQVRSLAAALPQLTCLEFGSLPPVPRTPEVCA
WLAAGGRPAGFRAHMSPSGGGPTAHGAAAAAAAGGGGGSRLRGGGGGGGGGGGASGYWAAAAAAPPPPAIQPAFGSR
ETLGWCYLGGGGGGGGDAGEGLTAPTATVGPRRGGAGVVGMQQPLPRRHGGGLEGAGGGRSGLSSGAGSGSGAGGAG
ADTEDRRCRGSDSGQLLMRGRAARPDCTANDCLAAGSGSGSSSGSDSRSSSTSRRRVSGSGHGLLLPSRFSGSGAAA
AAAVAAAAAAAHGHPSPLGLSGGGEAAAGTAAGVGGLCCFRALRHLVLRRVGLEELALHGLPLPPRLTKVELPLQAA
VGPGAESQPIQLSDLLLLHSSVLPALAALPSLEVWASLGPARGADGPVLVAMLAAWLGPGGVKGLDVRGLTHPSNAS
GCEVTETLQRIAAGLPRLERLSCYLWGGPGGLLQPQQQQPLAPGRSSNNGNGAAAASGCSGSSNGSSRSWTQRALQP
ITQLGGMRRTPSGSSGPAGPVAAPAVAPVAQPRRWRAPAGRALGSSAGEGGGGRYSEAWLAAADAAGVALGGAALEA
VNRLTLLDELRVKVLSLPAAACNSSGDGDLLAAALRRPTAASRFGSCAVPVRVPLGLLRGLRRAVRVVDCSPLEAWC
SSPVAVGRGGGGGGGGRGGGRGGGGGGSVGAIGAGAEADPRAALAYARGLLAAEGVCSSLVAEAEEWAAGAIGVGPD
LEERWRGEALRPAWSEPGCDEEPPGETATVTGWILGNI

>Cre10.g427800.t1.2|PACid:27579379
MECTGQPRAWRWRQSELVDYSCPSLLTRTRLSGWSKLVLIALLVLSASSPRQAEAALKLRGARRSLQQPNFEVLDNA
VNLKYGDLLCSNFDEDPTGQPTFVHAGEADTALVTGIDADYTDPYVDTTSGKFYPRGIYNMGIFDSGTFVPGGGSVG
TRLELVRRVSGSAKAGASPQVPLTDAVLTRRQAGPFATSKLGSLLKAIVCCSRDNFVESIRLVRELVGDTIIGNPTC
YGYAIEVPRPADAPLWAGLSIGMGKPVGSTEYVVSSIRFVWASYDPILSSPPPPSPQPSPPPPKSPPNPPPNPPPSP
PPSPRRNATGAVRAPSTPPPPPSPKPPSPKPPSPKPPVRKGKSPPPPSPSLMDKLPPGTNKDWYVITKGVASIELLD
YTGTVQMVGPTFPVVTTPSLDTVIAAGRYSKGTLFGLGSENMAMTCCKAPVGAKLPGLDQIILQGAAWAGYYGIKCG
GAKVRVSHAKYVPFATWLITQRPDLFLKDVEIYTPVADFVRAVKGTVPQGKRPPMDLYIVGPGDGDQAGGNSTYTPT
FLAALRDYLNSGKGVIFLGQNPLESIANGGTTTTTGTTTGTTGKNKKGKAGKNVVVTVDVGRRRQRSLLESSAAYAL
NAVSGPTGMVFAGQVLPPVPPGPVGVGPLPPSDLLNAEQAAGQYLLYLQGQAGKIYDLTVVAGVVSKARAAMPRGSP
GSENLYTLIDSITQLGGTIPVYNPAAQPSSPPPPSARPSPVRSPAPSPSPSPSPRASNATAGNSTTAGNSTSNGNST
AASFKYLGCYVDNSATRALPFRLSVSDRNLTSARCASLAANASLKYFGMQGANCFGGSNGTLATGYGTSTNSAECSV
QCSGNKAEMCGFASANKLLLSLYQWT

>g11256.t1|PACid:27579408
MGQTYGLVGGLVMVIIALASIPSDVEARDFKLNVQKGQQTLWQKSPPPKHRTSSPPPKRRAVAPPPPSPEPPSAPLP
DPANSREKNGVRLVLGDGPRGRLELSSTEGWLTDYVDGAVAWLPVCQMIGFDDSKAQIMCELLGYTYGRLYYDDEVA
WRPPNDTASYTDLPIENLDCSPNYPDAPPPSSEGGDQTAGGHRRRLLAPLRADINIPKNAPYTCSFYSYATKRCDYS
GPLVGVECGDDVFPPAPPPPPRPPSPPNAPPDRNVDLKLYGVGVGTVADLESNLCDSEEQDGCRSYERAEVLIADPA
NPGSQIWAPICSVDNNDDLAILVGDTLCKQVVNYPDPGYVSYSGEVQLPLQIPEEPDAAAEGNLFRPSKYTQWVTFT
GGEPDARYPALQQLEYTVASACESAQLFAVFCSYADD

>Cre10.g448900.t1.3|PACid:27579468
MAASSYFKGKRTTRSGAELPVSSCVARLLVLALALALPYAGAQLVTPSLGNGTSPPGRTVQMLCNINGRSSFVINIY
GSYDSRIVNSLGVTCSDGNFFDAGEPFGDFTFSVTDTRGFYSARSEKGFAEPVRLGFRNSTGVWYNGPTGKPIGAPS

GAAARTYDTPACKNPGQVIAGFTVVSGQPVSVPQFLNAIIRIDVVCINTAGLSPTVPLSPPPPPDLGPPDLPPPPPP
PPPPPPRPEVPSPPVEGPSPPPPPPPPPPPPEPPPPSPPPRPPPPNPRPPPNPRPSPPPKPPGPSPPPPRPPPRNPRE
PRAPPPDKPPPKAPREPRMPPPDKPPPLLPNEPSLPPGTRRSPPLRPPPPLRPPPPMQPPSPPLAPVSPPPQSPSPP
PPSPPPALEPASAPPLASSSPPAASPQPSPEPSPEPSREPSPDPSPANSPSPTASPPLGSSSPSAARSPEPQPSPQL
EPAPAVTGPPPVKSSKKLPPPTRAHPPPRKRFNRPPNMINIGKGRRRSE

>Cre10.g434850.t1.3|PACid:27579481
MRAISLSLPLVCLLLVACHVALPIAQPLVKREVLDVTVDNSAPPPLGLAEWAPPPDSSDIANGQPPPPTEDLEAASP
PPRSWRAPSPRRSPSSGSGSGGSSRGGPARRPPVQRWHWRHPPSVLVLRRHPPPPHGRVASRGHPGNSPPPRSGTGT
AARPPSPPPVRLQSAECSLTLIGSQAARANRSGAAGGAAGASSTSTSTSGGGGGSGILRATALLAAELHCTSDDGRP
VAVAAGAQLLSLLTAANAAGSSAAPAPAASGVALSHRPGDDEWGLSFAGPLEALVLTRSRLADLPLSRRGPLLHCRS
CRRLELRDTTLSNLGGVGTSATQAGVLPYGVYGAVHVEGAAAVRLSNSSCSGVRDAHGWACLLVDYDAAEPAPLGPA
GAAAVAADAVSYDALPPAGVAAVAAGLGGPGAVTGGGGFSNRSVSGSGRGNSSSGSGNGSSSASDRSGGAADAAAAV
VGWSLVLEAGSRVGNNSVIVGGKHGTAWGGFADPDAQTMSYGYGAILLRVGRSSSSSSNVTDDVNAGNSSSTGALAW
ARMPSSPPPPLPTVEAAAVTTVALAPLVVVCDSGSVLDQNVGHRGAALAALGAEWPVVRLLRGSVVSNNKVYVGDGG
GLWLSAAPPPAASARRRVPSRPVPPGLRPGFAVLGGSSLRGNSAEQGSGGGLWADVPLDLLVGGAGSSLDDNAAAGG
GGAVSMTAGDSAAAGGAWAALLRTYGAAGACSSNGTRGGGSSSNTNSSISSNSSSSGAGGGGSGSGSRGWVYWPGTL
RVVVAGGGGLSGNQAQGSGGAVALDVRRPVLVQVVGNSSVQANTASGEGGALWVAAACEGVGPLDGRCPAGLRLSGG
GAVSDNLAAGGSGGAVCVEGVMGLLELDASSAMTGNAATDMGGAVYGGKGLSEGRINGTFDGNVALIGHGGALMFGA
ANAATGASSGSGSGSGSADPASAGSPSGRVAVGPGAVFRANSAGGRGGGACFLGGVNSLLVTGAVFDSNEANTDGGG
LAVVGPLPAANITNTTFTANKAAGSGGAIFHQVVGGALLPTRMGISSCSLQGNQADGGSGGGLAAVVFPAPVGRLRF
AWATPNPEIPESAAGPDPTSGAGVEVDGGSPPPAEEGGEGGSGGRRRELGAAAGGDSSELPAARRAALSVVARLSYS
LVLDSSVLQGNSAAGDGGAVFVAADGRAVRLRVWGRGSAVSQNTAGGGVGAGAGFSYLGLGGSACGGGGIFVGTVRY
DNPNTTKAAGSSSSTGGGAAEFNGCRTSLTDMRGDFNTAGGGGCGGGAVLVTNCQVNANGCAYVSNRASGGGAALAV
LNGGGGTTAAAAAGGAGSGVATRSGQRGLFEGAEGLGRAELQVGVVAAGNAEELRLAARRQTSTAAIRDARLALLHD
SGRGGSSDGSSSNKSGSSSSSSSSSSHRRKEAGVLPLELQQLHRLLLHTATMAPTPLLRSSGEWQGQLQGGHGAGGA
AAPDDVMQDFARVMAEVRRVLAHTAGAAAAAAAASANSASSGPATEPTGIPGLEAEQPDAAQESSGLGGGVWRHRQR
HARRLSQQNDTTDGAAPPPSDPDAPPAPPSPPFRPPSPPSTPSSPYPPYFPDPPPPPPPAPPVRQPPPDSASLNNCI
FFNNAADSGAGGALWVRGIGGVLMTACAFSYNSAPGGGAVAAEVVAAPSTLALYREDDRAPADAPVAAPSAAVPPPP
PPPPSPPLASQAPTAWLRLSGCMLTFNSAQAVTDSADEQDGGGGAASAAAAPDGGGLLIWVRSHGGAAGNWTAAGGS
GAAAPSAGAGGTGGAAGAGGGGGGLLLVASSQISDNIAGGCGGAAAVRQRLLPAAAAAGVAAAAGGGGAAAVTTSPL
LRVRFQDTAMSRNKAQQGSGGALFADTAAAHITLRKVAATSNQAYAARASGSAAPAAATASVGLGAGGGAVAMSGGA
GEGVAAVLEVIDSTVAGNLAVAGDGGALLVSAAAARVLLSGGLISNNVAAAGHGGALAVLPPPPAAAPAAAIGGFLL
SGAGVAPQAAASEVVLRSMSLRTNSAAGSGGGVMVAAASGSLTVAACVIDSNTAGSTSGASGGGGGAPIGSPGGGGG
GGLACWLYGGGGAANASRSSTADPSATAAKEPYVFLINSTTIVSNKALTGDGAGVLVAAAGAPVGGSVASQRPLQAA
PPLSAAVLESLINNNYAPLGSGGGMSINVFVVLINSTTADSVGASAAPALSDPAAAPPGADLPSPPPPTSIQTGRGR
TGLDVRLQGCVMSDNAAAAAGGGLHVAMAGVAAPGGAAAADEEEEDDVEALRLWRDAGAVARVHLKGCSVGNNLAAV
SGGGVAILYGAVAGAAAATAAATLAAASAPSVRLESCDISYNSISYSQVMATSAAMEAAAAVAARARLPGVGATGGG
VAALGRLRVTARSCTIMGNNVAAADTASTTSIAGQQQQVPWVNGGGGLCAGGAVSLIVAACSLATNAVDLGAAGNAS
GGPAGAAVAAGGGGGVLAWGVGHVEVLASSVKAGQCGGGGCLGGGLHLAAAGQVALVQCDITGNTAGGGGGVYLSSQ
ADGTGAAAVAAATPAGGAPQPATTAVLFGNWVGDNIATGYGVYAASDGSSGGDSGGGGAAPYSGYGGGLLLQGDRLA
AAVVSGNLSHHNSLEAAAGGQGAAVAAALGCGGGGAGGGSADAASGDAATPQVWLSPVAASLGGTWAALRAHLAGSG
GNSSSTGAGGRCSSLLLLSNVLLPTGRRVPSGPYLAPGPPSPSPAPAQAADGGGGAEAATVDAVWVADAAALVVGCS
AAGNATAAAATGINVGGNTDGVGGVSYLPVLGGELGVAAALTAEEAELVAALQAAALSSLAGRGAGGSGAAGGAGGG
GADALELQMATCRLPGSPPPLGA

>Cre10.g456950.t1.3|PACid:27579494
MLASELPATGLQYPRANDELVRSAAFVQRYGSVVAPPQMDWRSVPGVISPPADQNPCGACFAFAAAHAIEARVAISK
GDAASSVRLAEMQAVVCAQDGNGCKGGWVGQAYRYAATSGLVPKAAWAEMTGNAYVESQYCPSRRLADYIRKVDVAD
PAAVASGGSSSGSGTTTAKIAGWETAPRTAMALMKVLANQPAVALVHAAPDWSDYRGGMYRGDCSAAADDANHAVVV
VGYTADAWIVKNSWGAGWGAGGFMLLPRTGNSTNKCGVLNSVTYPVLDKVSPERRGELLRQGFCRAAGKVSLADVAA
GNVSLAALAVRFGVPLNEMIRVNSHVQVGVDTPLELMTNYYIPPCTKEVPPQPVPKASCGTTYRIDAVADSDSDAVV
RMLNAFEGAGEAGRAAQAQHAEQLRMRQRAVERQRLVAFEEAQRLRRRQLSQAGRMDAMAQTGEAAGVVGRRSLKSR
RSLMQGGDTTSGSDPNSEPLPPTAWQHWREDYDVLAANINHHRFPRDMNTSDYSSVATTRLLLLREAFPVLLSPDSQ
PIAAATRYGAGRVLVLGQREMWREFYQPTDPTETGHVAQQLLFNGFGWLAGYGRPAAAQDGINATCYATGFAESVEE
VFDALDVLAAGGPDGSFDGPPSYRETQYDGFPVDVLTAGVQETGRPYLQVLVLTTSQVLAGYLQDAIRNYVAAGGGL
LVVHSSRNVEELPNAQFAPDETFAEFYSCNDLLGEMGILISPTPVEEAPLWRPPLPVPHPAPLSYLLLNAEAAVDTL

RRYYAGLLRPGELDVDDFQAAVAIMEYAKSALKTEADLFPRLFNAIATVRGPPPPPQAPPSPPPPSPPPQGDDLTGV
TRLGPVYTSWLNSYALEAISDYLDPYDVVCPRGTHVTGFKGRAWSTFDPAPWGATSGGMLVTEFALVCSNGKMLSVG
YPTAPSKYDRYTNDPPYGNEGGLIPTGFEDSNDDGLNDSGYDSGDDVAPSFGVDWSEQSCPGGYDSIRVRPHSDDTV
NFAKPLQMFFRCRDTAAFTTYTAGVGILQLSPEYIYDYDPATWYITSLRNLARGTQQLTELQYLAQLEDVPGVAACP
PGQALAGLQGSLYVPHVTDLFDPEPYDQFLAPTGPLINPGYTVILSIQDAYCRPIGDLPTPRPAPAVASPRPPLSIP
APNGTAYLTSVTATGLNFDAGAAGGAAAFVDTVLTCGSGSVITGIVAAQEGATDINYLGVRCSDGSSQGVGSYHPSA
ESSGYLVDNPCLLGFDAVKAIGGRGVSYFDGSLGAGLGGFAVRCGSPLNEDGGGEAGSAWTRLGSSVLTGVNLLQQP
SGGSSNSSGGGSLLYALTEASINAPGAVCSPGQRIAALRVQAVPLEVAGGAAGAGDAAGEGSLSIAAVHFYCGEVPG
PPKDRSFLDIATRYGITLSDLYGANPTLDRTQPVAAYNGTIINVPQLCGAPPTQPPVTTIASGCPLWWPLPNITVTG
AETCGDVARGFLRGNLPYLNTVNNGLCPNASTTIRRGIRVCIAPPAGVLSVGHHRHRHHHRSLRADNTTTQNPAAGP
AAAQQACRASYLVSVGDSCLGVAAIHGIEVESLLNWNRGLNCSLLDAGRELCVAKRTANARVPLPPPPLLASSLPAP
PPPPPSPPSPPPRPPAPPTDQWNLQPPGVPHAPPPPGFTTSPPPLPAVASPPPPTTTDTDSAPSGGQSSFSSPPPRP
PPPSPRPSPPQAPPLSPDSPTAPPRFPQFPPSAPRAAAADGLALTPCAASPSPCGPPPNTCTNLPRSAGSALGYACA
CGPLFLASADNATCVPLRNRAVFRPSFSSSHLRGVSDTDSDYRFSSLWAIDGSASAQDLQVTSGYFSSGRGDTQPWL
SVDLGSPFYITAVVIRLRPGGVGASRLGNAEVRVGLTPIRSEPDDRPGLILNRLCGGRIATAAAPLAQGQDVVSVEC
GRPGSPPAGQWVTLQNFHPAGELLQVAELEVYVRGPVLSLGCGTEGVDCGGDGELVDDGAGGVQAETSPDAPPASAS
DPSGPRVTRLTNVTKPYRLGVSSLPVFASSSAGPDNAPEYAFDGIGVAPIVYASGSSSGSGSADPEYASSLDSSAGS
GFFQSGPGDMSPFLSVDLGSSYVVTYVRLFNRADAYGNRLRNVSVWLSDAPATDAASVAALATAGGGARLCAVLDWV
ADEGEVVQLNCTSSGSSGRWVVVRKAAATAGTPPPPAGDEVLALAEVQVFGHRGEPPGPAKAVPPPGTGASSPPPPT
GRPSLRRPPVSKRPRLSARPPRRPPPGPKRQRVRIQYRDD

>Cre10.g451600.t3.1|PACid:27579499
MLFRDEVRQKQRAGRLVAVLGARARDLLRVCIVAIASGSRGFSPLTTWLDLTGSPFSIDVTNTTTRSYVFTDVTGDR
ASDMVFFDQPTLRLAYMPSNRNNKLVDDTDGDGTTWFELSTVSGRCASLGEDCFLITSDVNNDKLNDIALVYLDRVT
ETEEFYLTLVTTSPPAPLNWYMTSAAVFPRGACTSPQAVALGQFVGLDGSLDGTIAGTNQPQLLCLSSYDQRIYVGG
LGVWGSLPGPVTSLHVRDVDVDGRDDLLIFTEVGSYYLVSTGASFGPALPTAAWLSPSSVSLAVPLTLDLGQTDQTA
SSVVASADVIGAAPVEHRCGPPRRVVAYYTNRRPDKSPACLASVADVETTALQRAAAATHVIFAHVKPNAETISVDL
ANERDGPVLANLDGNLFNVNPDIRVLVSVGGPGGLDSDYSRLVLNEQSVVGFANATVTFLNAYNLDGAELSWPNLQS
EQVEGYTALLEILSATLRPAGKLLSLAVPPREVYVSMDWGRLGLLVDMINFQGFDLEGDEVLGASPYVETPLFDCQE
AEGLSVNSLVDLILAAGAPPQLLTVVASSMGRSFVLDGDGFVGGPGSPGPCMGVEGLLDQSEIKLLLPPGAARHDPE
AFANVGPFAANQYAHWEDAYSIVNKVCFARSHCLGGVGLWDVDGDSYGELLGAVGRAVLGDPAICTEYSPPECTNTV
STRGSEDLGSPELVATLGDSEFLLYQVRKNWPDARDHCQQIGGDLVSVTSRGEAGVVYSLITAWASSGQLGPDDIYS
GRDVYVWLGGSDAAQEGRFVWAESGADFTFTSWAGGQPDGRFGGEDCVATTVRLAGSSGAGLRQVISSEALWNDVGC
TAALPFVCQRDTALARSLLRAAKEVPWLSATYHVLAPAEEGDPGLMLTQPEANKLCRSLGGELPTLTDPWVREDLTS
QYHRDLPTHTWLGLRSYGDGQLFWNDGTFTTDGMLNAWEPGEFGDAACGLIVGPSGANVTVSAGTLYSFWNATTFQG
AIRVASPPPPPPRPPSPGPPGPKPPSPPPRPANLTPPAPPPGLPNAPPSPVYEDLTIYNLTDYFAPQVQMYSVFLPQ
GVYSLSCNERMPTTCQTGMPAVSLTPNFYCLTRPNGRAYVVPGKELPSMPLYLSTERACAAACMLNFRCVYYTWLPR
FRASFLPTDEVLGRPNQLQDQGSCYLMGRPWMPNSDRLPKLLSEITDSDRVCFRSGSIFSGDTLPISDDTLIQPPAA
VGPLFGNPAAVAANPAGTPPATPFSLLCGSDGAAAPLLSSLTFLVDNGTRGIQDVGAACAGTVSAGVLGSVSAEGYE
LRVRQLQRPVIQSYTARCGPGGVTGMLGSYDNRGMCQVSLTCASGAVEPVLPPTASRNCTAAASFFDYECPPGQLAI
GLQGLFLNAATYTASDNILAAIRMLCAAVPVAMVAPSPPSPPLPPPSPPPAGRRRAALETASAAWRRAAAAATDAGS
SSSSSSSSNTQAAATGAFFQELMQSRRVDLGINGGGSARRRLQQTSPPSPSPPSPKPPSPSPPSPTPPSPKPPSPKP
PSPSPPSPLPPAPPKPPLPPSPKPPSPSPPLPPRPPSPSPPSPKPPSPSPPSPKPPSPSPPSPKPPTLPVPPSPRSP
PSPPAPPPAPPRPPRPPPAPKPPRPPAPKPPSPVPFPPRASPPFPPSPPPVPSPPNPPPSPAPQIPTPPSPPPRPPA
PPSPPPIPPTPSPPPAPPPRPPSPPPPPPSPPPPPSPSPPPPPPPANVTYADVILCRDCSPLFGEGPGAASPLVTTS
RQGVLRCPQRHIITQLQTPIVSAGALPLQSGAVANQPLTGLGGVCAPLASAPGAVDTVSAGLPAVPTLPPISGQPFN
VSIVGGAACPTGIVQVTGLHLSFRIGPPANPSYVNNLAVRCRTVATPLPLPTAPVGYPSHRPFSYTCPPSTVLSEIF
WNTQEWPASSPGTVRNQPVPVITSLVFKCTPWTVPTPAAPIPPPSPFSQIEANSMTSLTLACPLGQFLTSVYGRFDS
APANMGGVAFVRSLSITCSGPTTVTREVSTIMAPTAATTPFTSAVCPGGIGALTARTLAVPGAPTTTPPALLSLDAH
CYDTQLHTRKLSFLPPALSNAAPQVADRISELTDRCLGLTLVSGITVLRSMFSGGVLITSGVPTSSFLHGIKVSCFD
VPEPNPPALAVSAQLPPNQLPAAPAVTFKYECPAGSKVVSVLSRVDADDDLLNVRIECDNAPLGDAESLASIAARLT
PFFAPPQIFVANCTNCTTAANVSSMGYGQRRYISPDIAISAPRTSYVIERTCPLGIAGIINTATSSRIVPELGGAAT
PVSSAALPSSVVTGATIALFDVLLDWGNAVAFCMAQDGALMTIEDELQRLAVGSVVQEWAAENTGDAVLGVWIGARR
TGNGLYDFTYQDNTPMGGYVAPWAPGEPNDLSGVEDCVELVVNVTSGLVSWNDRVCTGLVRRPLCKLLPKATVEGAN
SADVAPPNVQISATNNDELSFYNVSLTWYEGLNFCQRRGGNLASFRDQDEIKRFSAAITDWIGYAEPGRFLYQYTTL
PIAWTGLRRQMPAAGTPAEALRSASAVFQYGGREFLFIPTLATPDEADSACQGFGGNLATLYTLDEYNAVLTEFLPA
IIRYGINSTASVVATHIGLIRTNFSSYGWRDGLGWNGYTPVGVPVNMASLCGVINTRCAAGNTSVSFSSCSWNVSTT

SCSFQANSIYPARNPYICSRLTPATSSIVSGYRQYLAFAERRTWAGAAAGCAAIGARLASFQLPEDYSAFLAVANRS
QVFLAATGMTTSLAGSAHIGLSHDSTGAYSWSDGRPLLYGVWAAGQPDALAANTTCGSFALSCTNSSGVWMNCATPQ
LRDVACGTLAPYVCSYDFDDEWAFSDGTPLLYTRATPAALGQPAAIMAGNYQWDQFNRTGCTGATASRDYTGTPFLT
FSTNDTEKCCSTCSATPGCGWFTIVTNTSTCTLKRANGVLVASANPNMRSGASGRPLASLADPGALLNPLGSPDCVT
LTSGLDQRLLIGSGEPSTVGSVYRMPNQQCLQKFPVACRRSNAKEAVPETERRSLASYIQPVGRVYTSNGQLELSLY
KTWQDSPSAQKFCELRGGTLVEPTTYQIQEAAFRLGLDILYRGQSTVFVDYHIGASDKVQQGQYVYNSGARVLDTYW
GWSTSTPVPIFLDNRDCVKARHNSNMSPNQTWAPTFCRFAGSYPMCQRPATLSPEWVVARSIAPAAQTVQGRSHFMF
FPRLMDWYQAKAFCEANSGDLAYFDSAQQYEEVASSLRQWAQTRPFASGSISRSQSYVSVWLGLNNRFGSWRWSARP
YVDVNPVWAPWTGYNATGFRVRNRAYDAPAMGLMSPGIDTPFTMCPNETDAESFVVGFRAQVDTRSLSGTPTSASNL
LGLMGLRMRCRSKSFVGTFENLMPEQDISSITSVSGVGAWGTSETLCNNWNNSGFGNWVVGLQLQAVPRRDRPIMSQ
VRNCIPSGGNPCASYTVSWQATRTTWGDDVGITSIRGICANQRADNASWAQIVNPAPATDAQWLPPITCESGFAVCG
ISTRVDFSGANFTIANASNSASDDSGVSGVTLRCCPLRDQFALSTECAEAYVFGNNGTSAWQQRQCATRRSLVSISN
VLCRRELPDSNANAAMIVTNTSATTRAADKELASIPTLSSPLLPAVAQSVEPLGAERVLTTITSRRGTTDQVQLYEV
LLFDIPMPWQPAVSFCQAHGAELLVIDDDSTAAAANRLVARVMPATAFQSSTTFSLQFWLGASNRGSGSAWAWSSGG
GVTYENWVTGAERPQRPSCGKMTYSVFNQSTTYVNAGWGAESCGAHLLVLCRRRSGDGGGVARATPSMSTAPAASLT
IRDVRTTLYRETMTWSQAVDYCQRRSGILASINSANEADVIAAMVREWARTTTAGEVKVWLGGSWSQSEKMYKWEDE
MPFDYVRGGGLLAPEGPTQLCLSWRLLPDGTDVWQTDECAEFALALCQSELQPFICNTGANSTRPVDANPYDAKAK
VYCPPGTVLTGLSGSIANSSLSGASLALADGISFSGICGNPAAPAILAKRDNEELPQCRYRWSLPRIEGPEVTINTA
NSTTALDSCFKMCAVGDAPLAYIYRTPLLAVLYQINGVFSCRCGRALVGRQLINDDAIKAAEATGAATFLYGRSYEV
YAICTVSEEFMTAGYFQQITFALTLQDITAQPPKLTSLVRGSKYRVFSGQRTDWNAAQRICTLHGGHLATLESIGDI
DDLNQLLRANLNITVEFPQGTGIWLGLYAAQTGSYRWIDGTPLRYPLGPVLEDWGYAPPNCGYFDPWLNSNRGGRLI
NADVRAGSCDNANAFVCETSTMVDDSLTDITPGIRASYTPSTATRGAAWDLVQFKAHRVSWHDASRICRYNNMDLMW
FTDYLEESIVLSTGTAWLAATPDVTGRISWRTRAESAAALRDSRWTLPLNWNGTAAQLLARTDYTQLCGIFDATIFV
SPNPSTRQSPFSLAHCTNEAYPFVCKSKVERWRGLPATNLVTPSPPPPPTPPSPPPGIGNVLLRIERPAATFVVLDQ
VTSRAEASRICRANGGKLPEFTDLSEYDIVTESVRRYSTGKTSEAFWEFWFGASRDYLTVPNTTVWRYESGEIASPT
VWGPDEPNNVGGNENCVHILVWLQDTPTAQRSSSPIRNERTYWNDVPCDRRSNVVCQLPPRAPGPLPPPNSDINAIE
YVGTLRGIRYVMYSQQLSWAQSKAYCEANGQTLADFVTSDELDGMVRGIFHYVYTLSDFNDAVLKTWTGYNSRSADY
RGNSILTANSFAAKSTTGYYAPTQLASGSCYGLTLDPKTQVLQRLFEPVECTELRRPICRYDQALAAAAYETTISGR
RFAYFKEERTWGRARDVCRGLFQGDIATFRTDAEWASVKTWLNGIFWSGLVDTLQAATLDGSIMTEQAWSGFTNLYS
AEWAMVDNLTRPLTGTLRNAAWLNASEPTVTSECGYVDIRGTVRPSVDSFWRASRCNITRPFLCAYSPTGAPPQNPA
TYVPARSQFELATSIAMRINRYGYTYTIITDPKLRGSYAEAQSMCSMLPGFPGGMLATLFSAEDLEYFSRVGAVAEG
DAPNLWVGFGDLLSPGFPFWMSSNVAPGGDLSEQWRRDFSPVATLEPVTNSMACGYLDTSSGQLRRDFCLLRKYGFI
CMSPGSYGETQHVAVTEMTGDGMPDVVVATNCPWDNGASIITFQQPAPASFSTAADQLIRPTLTARHCGDGIPLMAR
EECDEGFDIEEVITWDFGYNATVAKPAATANRNGPGACATSCKLQPRFVVQAGAREVFIALGSGNDTIKHLGVNYNL
YGQARGFRELADPPDSPLVLDGFTNITADNQFPANEPIAIHKGRALMPGTPFQFRAFMQTDTSKPVLPNWANVETAS
VSDPSFITATSTCGCTPTNPSGQPVDLSIRQQGGAVEFSWVVGSACESSVSITRSLVDEVNNELLNTTTVAQLSIGL
ACGTAYRPSKAEVDEDIARDKLQVGRTYRYCIVVTSAATSTYFLDATKPLEPIRFVSEPICRDVRIQWTSTITGEVR
TRYDTPAPGVRLSARLIGTPYVVSAVTDDSGRYELVMQTDVYNCDPVLAPEECLKQRVRAVASARTKLRSGRILLHD
FSINGRPGAVQMLALQHLQVQQSVRILDESAMPITGFVRWPGSEANRGFDQTRGCPITNARVCAVTARENSSVSCAM
SDPGTGAFLLAAGVGSFLRLDVTYENHTFAINMLGTVDVDDTGAFELMAPIYDVDIRDLTRRTVRVGLVGGQCEIKL
GAAVLQFSAYDCDLQSGQRALDRRVRLDPSVPYTDIEVPALPWDISYVGIEDAVPDVSPEAIERFLTITNQITGFVN
LTIGTDTELFDPTAADLDIGEEPVLPEPPVVKFQFTAPNEMEVQVCKDFKGSLINCGEFSLNRRCDGNTSINAYQAV
YSAFGPPDTSLVKKDVLLLKRAARYKMRIKLFQMYGRLRCDNLKTTVLVQDSIGGEVEENQCSVKRRGCVKNVGFDE
LTNTTEFTYDLVPGAISFAVGTAYALPLSAVAASPGWPSRSLLLYSIVEGTQSRTGTGYIEIPIPVPLLILRDPPGD
RSYATLEGSISTAVKLSQVTHDKQTCGGVGDDEVFSKAMPVNLLNAVPFSKFGKTSKINEGVMKATKSAAKVATAQD
KIRRGVPKTSLWGQVKRFGSKGAAAPGKAVKGVVKKVKSWTGSGTKKSDIVIGGQRKGAVPVKQQEWVRATANRGAT
TATQTSSAAQKGTQLPSSLTKAVSTAKKVVDKVDDSLEQMALFYQKYFQENAIKKFYDDYHISKYIKVLKRLYKSDA
LTPEDTTGEQLQDPDGLEMGRGAGLSYRKTENGGQLAFELFEGLGVPTDPDTLAAVIAEMKTLGEDESAELLAGLVE
QRDYARDAVEEDPMLPSVNLIDMNVAFNRDPYGLRPGYKHLGKETSRIPDIYRPQDKAEFRLRSGGFSGLNPICAKR
HIGVGYDLDILSCAGLGYMVCLPSIRDRGQAGMLTDMAWYNEEEEESYYKLTIKREEGFSTPEESTPELTDGGGDMI
LAPTFAIMFILQDELKFDTRTCTPTARMGIPGWDLQENMHGTAWHSVHHIRNVVIPELSSKYAAEMSKPAAQQSPDI
LANIYQGMRGWEDVMTIYDEMNTMAKQQEESFPNYVVGDQEINGMVQGTVEGVWQATLDDHPDRHMDAFEARSIDPE
RLEQSPVWKLGYYEGSEKLAQLMLRTQAYMDNSTSAGNIVAMPRAKAAMGGVATGKHVDILQEKYGVQFNTMSFSGG
GGSYSYRFKTMSTISTKIQFQISFKDMFGWKGGGGGGVGFWIETTDENLYGFELEFDSSLLEETDRERTVSFTLKDK
DVGDSFLVKVKPDMAYGTPMFELLAGRSKCPYEHGTLQREGAAVSVLGGQNVQFNVKQGTEALYELLIENQSGTDEQ
VELQLGPDLATNNQSMYVQLMGAPWIEPVGFSLRGPFAAQTRALVSAKCGPRYPSASIDIVAKSTCDSSELSRTPLT
LQCFTPCPAVMWPQEWPQQQPLIYNLSDITASKTIRLKVFNPAYSIQKWNTHPRLNGTNGTTSIIIEYTNVVSGGGI

WLPVRYLNGSRVDFSKIEDPAYGYATFDWSPASVVDGEYLLRYVAYCDILVGGGTDSRYEGPSISLLVDKAPPVPSR
FSAWPNMSYVPGDQLFVDFTEPLDCRKPFAFGVVATQRDSAGSTFIIPSTDLYATCSGNRINFHWDSINRPTALMPN
RTVTIQLLNVKDLAGNAVPRAINLTFATGTLTANAPVTLNYVLGPLPTPARRLLFADRSGVLPVQLVNRLALRDESA
AESLEALVEQRMEARRQLLARVDGVLQSCTASGEALAAVAAMTSARGRLQATDLLEDEDLLSMVIEFQATGGSAEET
AAAAVQLVAAARDNSSCLSTQLAANFAQLRRVFAEKGIDDQLLEGAGEEELRAVRAAATARAARMGLAQAWEASVAE
AQREQAKLLAASEQATTEAQELLAHMTAVSEADGVSANQQAAAAVVVSGAEEGGRGGMDTNRRHLVNPAAVSAAASA
TVTPQQQQQQQPAAAGGLVHWLRSHPYISLAVCVLVSNAVALLLVGGIWRYRKRNSGLSELNSKKRGTGLLPTRRRR
GLAAILRPGAASSGSTGDGGSSAGDQHQA

>Cre10.g451600.t2.1|PACid:27579500
MLFRDEVRQKQRAGRLVAVLGARARDLLRVCIVAIASGSRGFSPLTTWLDLTGSPFSIDVTNTTTRSYVFTDVTGDR
ASDMVFFDQPTLRLAYMPSNRNNKLVDDTDGDGTTWFELSTVSGRCASLGEDCFLITSDVNNDKLNDIALVYLDRVT
ETEEFYLTLVTTSPPAPLNWYMTSAAVFPRGACTSPQAVALGQFVGLDGSLDGTIAGTNQPQLLCLSSYDQRIYVGG
LGVWGSLPGPVTSLHVRDVDVDGRDDLLIFTEVGSYYLVSTGASFGPALPTAAWLSPSSVSLAVPLTLDLGQTDQTA
SSVVASADVIGAAPVEHRCGPPRRVVAYYTNRRPDKSPACLASVADVETTALQRAAAATHVIFAHVKPNAETISVDL
ANERDGPVLANLDGNLFNVNPDIRVLVSVGGPGGLDSDYSRLVLNEQSVVGFANATVTFLNAYNLDGAELSWPNLQS
EQVEGYTALLEILSATLRPAGKLLSLAVPREVYVSMDWGRLGLLVDMINFQGFDLEGDEVLGASPYVETPLFDCQE
AEGLSVNSLVDLILAAGAPPQLLTVVASSMGRSFVLDGDGFVGGPGSPGPCMGVEGLLDQSEIKLLLPPGAARHDPE
AFANVGPFAANQYAHWEDAYSIVNKVCFARSHCLGGVGLWDVDGDSYGELLGAVGRAVLGDPAICTEYSPPECTNTV
STRGSEDLGSPELVATLGDSEFLLYQVRKNWPDARDHCQQIGGDLVSVTSRGEAGVVYSLITAWASSGQLGPDDIYS
GRDVYVWLGGSDAAQEGRFVWAESGADFTFTSWAGGQPDGRFGGEDCVATTVRLAGSSGAGLRQVISSEALWNDVGC
TAALPFVCQRDTALARSLLRAAKEVPWLSATYHVLAPAEEGDPGLMLTQPEANKLCRSLGGELPTLTDPWVREDLTS
QYHRDLPTHTWLGLRSYGDGQLFWNDGTFTTDGMLNAWEPGEFGDAACGLIVGPSGANVTVSAGTLYSFWNATTFQG
AIRVASPPPPPPRPPSPGPPGPKPPSPPPRPANLTPPAPPPGLPNAPPSPVYEDLTIYNLTDYFAPQVQMYSVFLPQ
GVYSLSCNERMPTTCQTGMPAVSLTPNFYCLTRPNGRAYVVPGKELPSMPLYLSTERACAAACMLNFRCVYYTWLPR
FRASFLPTDEVLGRPNQLQDQGSCYLMGRPWMPNSDRLPKLLSEITDSDRVCFRSGSIFSGDTLPISDDTLIQPPAA
VGPLFGNPAAVAANPAGTPPATPFSLLCGSDGAAAPLLSSLTFLVDNGTRGIQDVGAACAGTVSAGVLGSVSAEGYE
LRVRQLQRPVIQSYTARCGPGGVTGMLGSYDNRGMCQVSLTCASGAVEPVLPPTASRNCTAAASFFDYECPPGQLAI
GLQGLFLNAATYTASDNILAAIRMLCAAVPVAMVAPSPPSPPLPPPSPPPAGRRRAALETASAAWRRAAAAATDAGS
SSSSSSSSNTQAAATGAFFQELMQSRRVDLGINGGGSARRRLQQTSPPSPSPPSPKPPSPSPPSPTPPSPKPPSPKP
PSPSPPSPLPPAPPKPPLPPSPKPPSPSPPLPPRPPSPSPPSPKPPSPSPPSPKPPSPSPPSPKPPTLPVPPSPRSP
PSPPAPPPAPPRPPRPPPAPKPPRPPAPKPPSPVPFPPRASPPFPPSPPPVPSPPNPPPSPAPQIPTPPSPPPRPPA
PPSPPPIPPTPSPPPAPPPRPPSPPPPPPSPPPPPSPSPPPPPPPANVTYADVILCRDCSPLFGEGPGAASPLVTTS
RQGVLRCPQRHIITQLQTPIVSAGALPLQSGAVANQPLTGLGGVCAPLASAPGAVDTVSAGLPAVPTLPPISGQPFN
VSIVGGAACPTGIVQVTGLHLSFRIGPPANPSYVNNLAVRCRTVATPLPLPTAPVGYPSHRPFSYTCPPSTVLSEIF
WNTQEWPASSPGTVRNQPVPVITSLVFKCTPWTVPTPAAPIPPPSPFSQIEANSMTSLTLACPLGQFLTSVYGRFDS
APANMGGVAFVRSLSITCSGPTTVTREVSTIMAPTAATTPFTSAVCPGGIGALTARTLAVPGAPTTTPPALLSLDAH
CYDTQLHTRKLSFLPPALSNAAPQVADRISELTDRCLGLTLVSGITVLRSMFSGGVLITSGVPTSSFLHGIKVSCFD
VPEPNPPALAVSAQLPPNQLPAAPAVTFKYECPAGSKVVSVLSRVDADDDLLNVRIECDNAPLGDAESLASIAARLT
PFFAPPQIFVANCTNCTTAANVSSMGYGQRRYISPDIAISAPRTSYVIERTCPLGIAGIINTATSSRIVPELGGAAT
PVSSAALPSSVVTGATIALFDVLLDWGNAVAFCMAQDGALMTIEDELQRLAVGSVVQEWAAENTGDAVLGVWIGARR
TGNGLYDFTYQDNTPMGGYVAPWAPGEPNDLSGVEDCVELVVNVTSGLVSWNDRVCTGLVRRPLCKLLPKATVEGAN
SADVAPPNVQISATNNDELSFYNVSLTWYEGLNFCQRRGGNLASFRDQDEIKRFSAAITDWIGYAEPGRFLYQYTTL
PIAWTGLRRQMPAAGTPAEALRSASAVFQYGGREFLFIPTLATPDEADSACQGFGGNLATLYTLDEYNAVLTEFLPA
IIRYGINSTASVVATHIGLIRTNFSSYGWRDGLGWNGYTPVGVPVNMASLCGVINTRCAAGNTSVSFSSCSWNVSTT
SCSFQANSIYPARNPYICSRLTPATSSIVSGYRQYLAFAERRTWAGAAAGCAAIGARLASFQLPEDYSAFLAVANRS
QVFLAATGMTTSLAGSAHIGLSHDSTGAYSWSDGRPLLYGVWAAGQPDALAANTTCGSFALSCTNSSGVWMNCATPQ
LRDVACGTLAPYVCSYDFDDEWAFSDGTPLLYTRATPAALGQPAAIMAGNYQWDQFNRTGCTGATASRDYTGTPFLT
FSTNDTEKCCSTCSATPGCGWFTIVTNTSTCTLKRANGVLVASANPNMRSGASGRPLASLADPGALLNPLGSPDCVT
LTSGLDQRLLIGSGEPSTVGSVYRMPNQQCLQKFPVACRRSNAKEAVPETERRSLASYIQPVGRVYTSNGQLELSLY
KTWQDSPSAQKFCELRGGTLVEPTTYQIQEAAFRLGLDILYRGQSTVFVDYHIGASDKVQQGQYVYNSGARVLDTYW
GWSTSTPVPIFLDNRDCVKARHNSNMSPNQTWAPTFCRFAGSYPMCQRPATLSPEWVVARSIAPAAQTVQGRSHFMF
FPRLMDWYQAKAFCEANSGDLAYFDSAQQYEEVASSLRQWAQTRPFASGSISRSQSYVSVWLGLNNRFGSWRWSARP
YVDVNPVWAPWTGYNATGFRVRNRAYDAPAMGLMSPGIDTPFTMCPNETDAESFVVGFRAQVDTRSLSGTPTSASNL
LGLMGLRMRCRSKSFVGTFENLMPEQDISSITSVSGVGAWGTSETLCNNWNNSGFGNWVVGLQLQAVPRRDRPIMSQ
VRNCIPSGGNPCASYTVSWQATRTTWGDDVGITSIRGICANQRADNASWAQIVNPAPATDAQWLPPITCESGFAVCG
ISTRVDFSGANFTIANASNSASDDSGVSGVTLRCCPLRDQFALSTECAEAYVFGNNGTSAWQQRQCATRRSLVSISN

VLCRRELPDSNANAAMIVTNTSATTRAADKELASIPTLSSPLLPAVAQSVEPLGAERVLTTITSRRGTTDQVQLYEV
LLFDIPMPWQPAVSFCQAHGAELLVIDDDSTAAAANRLVARVMPATAFQSSTTFSLQFWLGASNRGSGSAWAWSSGG
GVTYENWVTGAERPQRPSCGKMTYSVFNQSTTYVNAGWGAESCGAHLLVLCRRRSGDGGGVARATPSMSTAPAASLT
IRDVRTTLYRETMTWSQAVDYCQRRSGILASINSANEADVIAAMVREWARTTTAGEVKVWLGGSWSQSEKMYKWEDE
MPFDYVRGGGLLAPEGPTQLCLSWRLLPDGTDVWQTDECAEFALALCQSELQPFICNTGANSTRPVDANPYDAKAK
VYCPPGTVLTGLSGSIANSSLSGASLALADGISFSGICGNPAAPAILAKRDNEELPQCRYRWSLPRIEGPEVTINTA
NSTTALDSCFKMCAVGDAPLAYIYRTPLLAVLYQINGVFSCRCGRALVGRQLINDDAIKAAEATGAATFLYGRSYEV
YAICTVSEEFMTAGYFQQITFALTLQDITAQPPKLTSLVRGSKYRVFSGQRTDWNAAQRICTLHGGHLATLESIGDI
DDLNQLLRANLNITVEFPQGTGIWLGLYAAQTGSYRWIDGTPLRYPLGPVLEDWGYAPPNCGYFDPWLNSNRGGRLI
NADVRAGSCDNANAFVCETSTMVDDSLTGIYTGRDGTS

>Cre10.g451600.t1.3|PACid:27579501
MDLMWFTDYLEESIVLSTGTAWLAATPDVTGRISWRTRAESAAALRDSRWTLPLNWNGTAAQLLARTDYTQLCGIFD
ATIFVSPNPSTRQSPFSLAHCTNEAYPFVCKSKVERWRGLPATNLVTPSPPPPTPPSPPPGIGNVLLRIERPAATF
VVLDQVTSRAEASRICRANGGKLPEFTDLSEYDIVTESVRRYSTGKTSEAFWEFWFGASRDYLTVPNTTVWRYESGE
IASPTVWGPDEPNNVGGNENCVHILVWLQDTPTAQRSSSPIRNERTYWNDVPCDRRSNVVCQLPPRAPGPLPPPNSD
INAIEYVGTLRGIRYVMYSQQLSWAQSKAYCEANGQTLADFVTSDELDGMVRGIFHYVYTLSDFNDAVLKTWTGYNS
RSADYRGNSILTANSFAAKSTTGYYAPTQLASGSCYGLTLDPKTQVLQRLFEPVECTELRRPICRYDQALAAAAYET
TISGRRFAYFKEERTWGRARDVCRGLFQGDIATFRTDAEWASVKTWLNGIFWSGLVDTLQAATLDGSIMTEQAWSGF
TNLYSAEWAMVDNLTRPLTGTLRNAAWLNASEPTVTSECGYVDIRGTVRPSVDSFWRASRCNITRPFLCAYSPTGAP
PQNPATYVPARSQFELATSIAMRINRYGYTYTIITDPKLRGSYAEAQSMCSMLPGFPGGMLATLFSAEDLEYFSRVG
AVAEGDAPNLWVGFGDLLSPGFPFWMSSNVAPGGDLSEQWRRDFSPVATLEPVTNSMACGYLDTSSGQLRRDFCLLR
KYGFICMSPGSYGETQHVAVTEMTGDGMPDVVVATNCPWDNGASIITFQQPAPASFSTAADQLIRPTLTARHCGDGI
PLMAREECDEGFDIEEVITWDFGYNATVAKPAATANRNGPGACATSCKLQPRFVVQAGAREVFIALGSGNDTIKHLG
VNYNLYGQARGFRELADPPDSPLVLDGFTNITADNQFPANEPIAIHKGRALMPGTPFQFRAFMQTDTSKPVLPNWAN
VETASVSDPSFITATSTCGCTPTNPSGQPVDLSIRQQGGAVEFSWVVGSACESSVSITRSLVDEVNNELLNTTTVAQ
LSIGLACGTAYRPSKAEVDEDIARDKLQVGRTYRYCIVVTSAATSTYFLDATKPLEPIRFVSEPICRDVRIQWTSTI
TGEVRTRYDTPAPGVRLSARLIGTPYVVSAVTDDSGRYELVMQTDVYNCDPVLAPEECLKQRVRAVASARTKLRSGR
ILLHDFSINGRPGAVQMLALQHLQVQQSVRILDESAMPITGFVRWPGSEANRGFDQTRGCPITNARVCAVTARENSS
VSCAMSDPGTGAFLLAAGVGSFLRLDVTYENHTFAINMLGTVDVDDTGAFELMAPIYDVDIRDLTRRTVRVGLVGGQ
CEIKLGAAVLQFSAYDCDLQSGQRALDRRVRLDPSVPYTDIEVPALPWDISYVGIEDAVPDVSPEAIERFLTITNQI
TGFVNLTIGTDTELFDPTAADLDIGEEPVLPEPPVVKFQFTAPNEMEVQVCKDFKGSLINCGEFSLNRRCDGNTSIN
AYQAVYSAFGPPDTSLVKKDVLLLKRAARYKMRIKLFQMYGRLRCDNLKTTVLVQDSIGGEVEENQCSVKRRGCVKN
VGFDELTNTTEFTYDLVPGAISFAVGTAYALPLSAVAASPGWPSRSLLLYSIVEGTQSRTGTGYIEIPIPVPLLILR
DPPGDRSYATLEGSISTAVKLSQVTHDKQTCGGVGDDEVFSKAMPVNLLNAVPFSKFGKTSKINEGVMKATKSAAKV
ATAQDKIRRGVPKTSLWGQVKRFGSKGAAAPGKAVKGVVKKVKSWTGSGTKKSDIVIGGQRKGAVPVKQQEWVRATA
NRGATTATQTSSAAQKGTQLPSSLTKAVSTAKKVVDKVDDSLEQMALFYQKYFQENAIKKFYDDYHISKYIKVLKRL
YKSDALTPEDTTGEQLQDPDGLEMGRGAGLSYRKTENGGQLAFELFEGLGVPTDPDTLAAVIAEMKTLGEDESAELL
AGLVEQRDYARDAVEEDPMLPSVNLIDMNVAFNRDPYGLRPGYKHLGKETSRIPDIYRPQDKAEFRLRSGGFSGLNP
ICAKRHIGVGYDLDILSCAGLGYMVCLPSIRDRGQAGMLTDMAWYNEEEEESYYKLTIKREEGFSTPEESTPELTDG
GGDMILAPTFAIMFILQDELKFDTRTCTPTARMGIPGWDLQENMHGTAWHSVHHIRNVVIPELSSKYAAEMSKPAAQ
QSPDILANIYQGMRGWEDVMTIYDEMNTMAKQQEESFPNYVVGDQEINGMVQGTVEGVWQATLDDHPDRHMDAFEAR
SIDPERLEQSPVWKLGYYEGSEKLAQLMLRTQAYMDNSTSAGNIVAMPRAKAAMGGVATGKHVDILQEKYGVQFNTM
SFSGGGGSYSYRFKTMSTISTKIQFQISFKDMFGWKGGGGGGVGFWIETTDENLYGFELEFDSSLLEETDRERTVSF
TLKDKDVGDSFLVKVKPDMAYGTPMFELLAGRSKCPYEHGTLQREGAAVSVLGGQNVQFNVKQGTEALYELLIENQS
GTDEQVELQLGPDLATNNQSMYVQLMGAPWIEPVGFSLRGPFAAQTRALVSAKCGPRYPSASIDIVAKSTCDSSELS
RTPLTLQCFTPCPAVMWPQEWPQQQPLIYNLSDITASKTIRLKVFNPAYSIQKWNTHPRLNGTNGTTSIIIEYTNVV
SGGGIWLPVRYLNGSRVDFSKIEDPAYGYATFDWSPASVVDGEYLLRYVAYCDILVGGGTDSRYEGPSISLLVDKAP
PVPSRFSAWPNMSYVPGDQLFVDFTEPLDCRKPFAFGVVATQRDSAGSTFIIPSTDLYATCSGNRINFHWDSINRPT
ALMPNRTVTIQLLNVKDLAGNAVPRAINLTFATGTLTANAPVTLNYVLGPLPTPARRLLFADRSGVLPVQLVNRLAL
RDESAAESLEALVEQRMEARRQLLARVDGVLQSCTASGEALAAVAAMTSARGRLQATDLLEDEDLLSMVIEFQATGG
SAEETAAAAVQLVAAARDNSSCLSTQLAANFAQLRRVFAEKGIDDQLLEGAGEEELRAVRAAATARAARMGLAQAWE
ASVAEAQREQAKLLAASEQATTEAQELLAHMTAVSEADGVSANQQAAAAVVVSGAEEGGRGGMDTNRRHLVNPAAVS
AAASATVTPQQQQQQQPAAAGGLVHWLRSHPYISLAVCVLVSNAVALLLVGGIWRYRKRNSGLSELNSKKRGTGLLP
TRRRRGLAAILRPGAASSGSTGDGGSSAGDQHQA

>Cre10.g451700.t1.3|PACid:27579544

MAGDEKCLVDVVVAFPEFYRRLDAASAARLATTCRAARTVFFSSCTRVRLRSGQAPLLSEGSIKKEREAFRKVLEKS
KSKAIDLVLGGLATLLDRGCHPTEVELQCDYNLPASAILLPVAQHFGGCWLTRLAVHLAHLPRLAPWTPNQWGKGGS
PTVWAQASRPDFHFSPTLGLPAPAAPAAEGAAAAEAAGVPGGGRGGGRGRGVRGRAGGGRGAAGEALVAAGGEGTAA
AVDAGRDGGWAVQLAAALPHLTSLSLHLDASDEERAADPLLRAVGPGLTELELACGWNRRLPPCFAGADVATTFARL
RRLRLSALLNGLTPDEDDATWGPWQDLFPVSAETTVVALARLPGLTHLDLQSAPNDMYHFGVRRPEEELERLHDLTS
LATLRLDIICPSNEDVRPQWSFRPTHHYFLSWVMAPMLVCLTRLVHLDLPSAHVRASDLPHLASSLVCLTRLSALGL
ATPGVAHCSRWWERRGLRGGAGGFAQRPRLVGGHGALGGGSRGGGGSAAGGTAEPKAAEVAKGKGGKAGRGGKGGRG
GKGGRGGRGRGRGKGKGKGKGKADAAAEAEAEKMAAAAVAEAAAAEAAAAAEEAAETAETHAVAATDAEEATQEARE
LAALAAEMIAVPLPPALQLLEVSCRLPTLHLAASLAAHPRGPVRLMLAPTAPPPAPHCATAASQAFWDPPHLHAYPS
FDEARWAAESVEAEEDFAAISDLDEKASDAGHGQKPCNGGAAGPGAAGRRSGGGAAGGSCSGSGGGVGAHTSRVWAS
PLSLLGGVAAQYDNMLPLRIVEQASAAAAAATGAAAAAAGAAAAEAAAVAVPGETFPGVVETLPWLAAAARVGARVI
RAAAEEWRQAGERQNWLRAGGGSTEQPAGAAAAVKAAAPAAEAPVSGQAAEEAAVCAEKASGGGWRLRITSVSVYEG
LHRSCDDALVRPGEVNAASVPGGGAAGCAGGGGSGGGGARRAGQRWRRRRQAPLRGAIVYGAGSDDLDAFEDGFGDH
LDEFDHELDAFDDGLDDGYGGGGGFGGGGFGGGGFGGGGGGMGLIGEAIRIERGDSDLDDLGGGDGGGGDGGGAAAI
PDILGQESLYDDGWYNEFEAYGGWDGYYDIQDCYGRSRQRRRRDIYRTADGSGKPERWEYEQRAQRSADLEPLPAVQ
GRHGSWLAAVADVLRPYFCTERSGSAASDIDEVYRSYAGTFTPPKPAANNGGGNGGRGRRGGGGSHHGRGWGGGGGG
CGVRWDLGESPLAHSLLQGVVLRAMDVAALRGVWPEADLRVASKIRTAAQHAARSTPKTVVQLEGHTLRDEAELEDP
EAQARAEAIERELTKAANVAAAARQQAKAQAKKQQKVHLKALMAGAKAQAGSASGAGGAGGAGGAGGASGSGGGRPE
GKAAGVAHTGQGGKGGAGGSVPPPKQNGGAAATAARGAAGKAPLTKAGAKAAKAGQAARAAQAAAPLAAPGAARGPP
STPAAATAPAPAQKPAAAAITAPTANQAPAAAKSAPAAAGAAGVAGAATAAAAPAAKPAPSAQTSKAAPNAGSAPAA
RKGPAPAAASAAAPGRAQPAPARAGSERGHGGGQGGGGERGGGERGGGERGGFGRGVERRGERGDERGGGSANRGGL
SRPSPPRPSASARHPARPLTRSRSRSPGRGPRGPLPPRTGSAGSGRGRRSPSPPRRARPASPVPRPGRRSRSPERRY
PRRSRSPEYRRTRRSPSPEHWRTRRSPSPGRLPRGPLHTHPHTEAHPPCKRPRGRSRSPPPSVRGGSGTGRGGTPPR
RMSPPPSPHRRNHPGPKPLRRSASPRGGTGSGGRRGSPRPYGGSRRSPVRPVARPRSRSRSRSPPRPHPKGPPQPER
MQRSHSGGSLHRTGGGPAPGQRERRSPSPPPRSRQQQLPLQQPPLQQPLLLGQAGAGVGAVIGGGVPVTGNGGSGGS
GGFVTGGYGGRLLLPVLPQQPPHSHQHQHQTPLHYQQMPPALHEQPPPYWGVPHQQPHHQPPPHHPPPALLHHQPPY
HQQHQPYQQYQHLGGPPPPQRQGPGGWAPPPAPGGGWAAPPPGIGYPPPSHAPPPSGWW

>g10543.t2|PACid:27579554
MAPAGRASAAPLSLLALVAYCAALLAAPASAGSQGFLPRRALLQTCLTCSGLTTCIKATCLYTDGTVNYYVSVNLTSC
KGASISWFCCQQNSCTTTVCDSTQVNGATCNNVLTSVVTTAVGATSVMLQVHDGRLIGDYDCGAPGNSCCGGDGGAC
GSGSNVCDDVVVPIAGVDTCSSRPPSPAPPSPKPPSPAPPSPKPPSPPPSPAPPSPKPPSPPPSPAPPSPKPPSPPP
SPPPSPPPSPAPPSPEPPSPKPPSPAPPSPKPPSPAPPSPAPPTCPPGCRCTDTAWGFPLPSQFRNADLSSTAELPL
TVDPGFGGAVFWNSRQVGGAWGGWFRITAPGSGSLTLGICGGCGQNVVGKGYYFGNGFSITFNSFTT

>g10543.t1|PACid:27579555
MYTTRTTQYRTTCLTCSGLTTCIKATCLYTDGTVNYYVSVNLTSCKGASISWFCCQQNSCTTTVCDSTQVNGATCNNV
LTSVVTTAVGATSVMLQVHDGRLIGDYDCGAPGNSCCGGDGGACGSGSNVCDDVVVPIAGVDTCSSRPPSPAPPSPK
PPSPAPPSPKPPSPPPSPAPPSPKPPSPPPSPAPPSPKPPSPPPSPPPSPPPSPAPPSPEPPSPKPPSPAPPSPKPP
SPAPPSPAPPTCPPGCRCTDTAWGFPLPSQFRNADLSSTAELPLTVDPGFGGAVFWNSRQVGGAWGGWFRITAPGSG
SLTLGICGGCGQNVVGKGYYFGNGFSITFNSFTT

>Cre10.g429600.t1.2|PACid:27579561
MRRHGLQSPLDPHQLASWLIFFALVSGVYAFYMPFVEDAGARWFLVALYSLLVIAIVALDLYTSYLDPSDPGLMGAT
DGEFFCGLCQASVARSSKHCRACDRCVEGFDHHCKWLNNCVGAKNYWHFFALISSTVSLLTLQLAWGLWLFIISFTQ
KQEMKGRVAEKYGSSVVYGGWQAALALYMAMLVAAVVMLGELFFFHVVLISKGMTTYDYIIAQRDAKLAAPPPSSGD
GGGHAHGLKACRSSRVADQTTAKRKVRVGINPCAAIKTSKPIGNPAHYGKAAGAGGAAASAAGGGAAAGLHAAGAGA
GSGKGLEGVVGNGSLKGSLGISDKAGAGSMQDMVARAVAEQHHHAAKAGLGADGNFDSALISALHGGAGAGAGAGAG
GLREPALLGAAAPSGTVSAQGCVPECSGEHRGGAGAGGMDSSRQNFTSLASQSPPPAPSAHHQHHYHQSPGKAGPGP
GSHAAAAAAAVHGSPARSPGQLTLGVSDTPPGSSGPTPAPAPSSNAAAGSFDAALPAVAGRHLGGQAAAAAATGSPA
AATLVAAPHPPPAAAGSPALGMPPASARVGSLPPIRLPSGSGAVPASPPPASAAQRSLMFPAKTPSGNARGAAEARA
FGGGRGQVAVMPE

>Cre10.g458350.t3.1|PACid:27579585
MIRYAGAIMAARSNAVLVLVILLTLAPFAACIDVKLVNDFAPADAAVVYEGRVEVTSPTSGQICSSLFDDRDASVICRQ
LGLPTASATAVFEAWRVYGKEGPWAGGAAASDADILAYLNTNYPPITLARLRCRGRETSLDSCTLDTAEGGDGAAA
VALCNRAQAVGVRCYPADYTLRLVNNGNSGFGVTEGRVEVWLYHGWGPVGSKSFDTSDAAVVCRQLGLPSGGATVLT

GAAAAARYGSPTASTATVLIDGLQCTGDEASVAACDRSVSAADLAAAASAAAAAAAAGAAPRPAFVGVQCREPEYAA
RLVDGEHPSEGRLELYKGGMWGSVCASGIDTSALLTACRQLGFFTSPDLGPTVQTLSASGLDVPAATPPLVAGGLAS
CPAGATSLSQCRASLPNGAAGDPTATCANGSAPSDSAAVLRCYAVPPLLASDVEPRALCADPWSTAAGLAPSAAGAT
ALLVGEFDDLSAGAATRAQDALLLSYGADGRLLGHAAFGRPWASSFGSEADIDFGVLPAAPGALLLADVTGDGRDDL
VALTSDAVLANAQLHVAVASGARSFAPLATWLLPNGAGGAASGAGSISSSSTATTAPAATGSGLGGADTIDTSNTTS
RSYLFIDVTGDNAADMVVFDADTLRVSYYPSNMVNELQDDTDGGGITWFELSTVPARCVSLGEDCFFVAGDFNGDGL
NDWALVYRDRFTAGPASSASSTAAASGPQEVFLTLVGTSPPSPLTWHLAAPASFPRGACSSPWAVLLGRFSGGGADS
EPQVACASSADAHVYVAGYERAWGVLPGPPTLIAVRDVDLDGRDDLLVLTADGSYYLTSTGSGFRPPVSTAAYGSAA
TGSVPLAAALALGQTDQTSAKVLAAPAPIAAAPVEARCGAPSRLVVYYSNRRLDPTPGCSSAGSSTTATAGSSSSSS
SSAAAATADQQQQVLQRLAGSGASHVIFSRLRPNADGLNVTLAAPRDRPVLANLARSLRRLSPGVKVLAAIGGEDS
GSGSSGPEDAAAFARLVRNPQSIERFAAAAAAFVQSYGLDGLELNWPGLTEEQVPGYASLVQALSFSLRRLSTNTNT
SAASGAPLLLALALSPASSPQLALPWYLLAPYVDLFNFQAFDMGGDEVLGATPYIETPLYDCTEAYGLSVNSLVDQL
LAAGVPPQRLNLIASALARTYVLDGDGAVGGPGSPGPCLGAEGLLDQAELRLLLPAGGSAGSGSSSSGSAGGARLAP
AAAAVYSPYSYGGGSQWAHWEDAYTLGSKACFARIHCLGGLGLWDADGDSFGQLLGALGSAAAADPASCVDFTPPSC
PNTASASGTTDLGAPDLVATLGDTEYSLFQVRKTFDDARAHCAAFGDGGDLAAVTSAAEAGAVYGLLAGWAASGQLG
AEDLWSGRDVAMWLGGSDAAQEGRFVWLSRAGADLTYTAWAAGQPDGRFGGEDCLGAVVRLAGGSGAGLRQVVSPAA
QWYDLGCTTALPFVCARPRSVTEGSGLTAAATAAAGGYRRVSAGPTASLLVFPPSAAAGSDGGQQPMMMTMAEGQKL
CRSLGAELPSLTDPLVRQQLLGSFSSGGSYGTRAGLPPYIWLGLRSYGDGELYWSDGSQTSDGLLNAWEQGEFGEAA
CAVLVPGPQSANVTLQIDGLYSFWNESVALGAQRVSPPPPPPPNTPPPPTPPPLAPFGPDRPAGLPPQPEPAGLRPA
LPPPGLPVAPPDAPEADQIAAGFAPPPQAPPLAGVPPVVDLLLSPGVWSLSCLELAPVVCLDGPPEVSLTPDFYCLS
RPSGPGGAPLPMPVPGLLLPPGDSAGGSSNPLSVRSELECAAACMVAQRCVFFSYLPGYPSSTGSSSSSSSSSSSNL
INACFLLQEPWSTAAGATVPQRPGSSSSGSGSSSSGSNAYSYTTADRVCFRSGAAFMGDVITVADIAAVAASASNAS
STSNSSTDAAASGTGSSSTAAGASLAPVTAISPMFGFPAPTPVPGLDPPAPRAAPWRLSPGVPPQESGYGGYGGGGV
YGSHGGYGGYGGTTVTEYGEQHLAFSLRCALDSALPVLSSVSLVLDAAGYIADVGTSCGGGTASGSGGGSSSVSSNS
TTGDDLGPAASTDMLKSAEQRAVLPPLPAVTASIYRESCGPSGAVVGISGTFDAAGICTAVLSCASGATIPLLPSSS
AGDGSSADAAAGDGRNLPRPCAAVGGAFAFSCPSGMVAYGLQGTFASAYGASSAISLATLQLLCATAPALPTAQQAA
PPAAPLPPRPPVPLLALPLPAPTGGNGSATSGRRLHEEGDPVIIGSTGLVGSIQGSGSGNGGSGSGHNRAIGVQLPK
PRRTLLQQADDNTSMPSPPSPPPPPPPSPPPRPPPPPRPPRPSPPVPPPQGSPPVPPPSPKPPTPPSPPPVPPAPPS
PKPPSPLPPQPSPTPPTPPPAPYRPDAPPSPPNPPPRPPRPPAPRPPTSSPPPVPAPPSPPAPVASGVPACNGCSPV
FGSLGNDMGAAPAVATVCPPGYLISRLVFSSARSGSSSSGDAPYTGLGAVCMDAATAAATNSSSSMRGSSSSLTAGV
PATVGPFDQFHALACGNANSDFITSVSGLYYYPTADSTGYITSVTLGCRGDTGSHQRRRMIEDAAVGVSWSYDCPSG
TVLTEIRWAVAPAAASTDSGTSDTGLLSGVAFRCGLTLAGLPPALPPATPSNPIATFTASDSSGSGQQQQEVVALQC
PPGQFLTSLYGRYMAAVEPAEDGSGGSSAQINSLGVVCSEAALALREASARVHNATANNSSSTYVAFTAAACPAGVS
AVVGRRTGGNTSAAGDATAVAPLLLASLDVLCYDTVDYSLNNLYGSTTAVDGTVFGERCPQLTFASGVTLVRSAAGG
DGFTSLRLLCTPVPDTTLEATAYAGQLPAVGQLPALPAASPAGSTTQQQPYHYECPAGSKVVSVLARIDSTAIATGS
AGADTSALLNLRFECDNAAPDPSRPSLAAIAAAAELAASSPYSAAVLLAATGTSSGAGAVTSGAGSGGSSTGAAGGG
GLGQLGQERYISPDLTSSAPPTSFLIETSCPLGVAGLDADRLSALDDGAAALLPVARLDLPYYTTDDAAMASGGAAQ
TSVRRGEVYYAALYDIVLDANQATAFCRLQSGDLMSFRNDIERDAVASMLQEWAYGSGVTDAVFSVWIGARRTAGAS
GAAGGLYNFSYIDGSALYGQYVPPWTAGEPNNIGGVEDCVELAVNALTGVASWNDRACLGLVRRPLCKIVPPAYRPG
VSPSDAATPDVVVGLGGPSSGGGQQLSYDVPLTWDRARTFCQRRGGALASFSSTDDLRRLGLAVGQSYFSRTSLSGD
ALKDRQDLWVGLRRTLPGPDSPAAALRPAVAAEQYGSSLFLYVPQPNRTFEQARSYCQSLGADLAVFSSARQYDTVM
TGIQGSVVRDLQADSSIAWIGIRRGSGSSNAFVTAASGRPATFTRWAVGSPGGSGSGSCVAVRTRCSTGGGGNCTSE
YVTLGCDGAAGSAPADAFICERATDASASALLPGARQVAVYAADGSSSGWQAASGSCSGRGGSLASLVAQDEYDTLL
AALARSVAAAVAAGNSSSGAGSSAVAGLLPGGLPADGRRARVELYTGLRKAAGDAAYSWSDGTLAAFLPWASGAAPS
NGSSGSSVTRCGRLAVACFKSGGTSNSTTDPTPISSVTVSLGGGDGDAVTCELEGLYERDCVVAAPYACTYAYEGSW
AFLDGSVLKAVFTPVPANTFFLSDGGGGTTLRDGVRVDTIGHVWGPTFVLFASEYAGGTLITTITDTVGSESCAAAC
AATPGCDFWSYGPLNGILGPATCYLKRDPGGRGKFTGYGQRPFFKAGNTTKPYGPGPYWKLLATAPPSTPPLAPAPT
ESANACAVLSQQLLDPSATSLQLTSMKTVSVSIAGPADETELLRYFQFMRPADCAAERPVVCTQPPAPGGSSSAAIA
SASPEEADALSNYLTPLASAFAAGGAVQLDLYDMLVDSTYARQLCRQLGGALVEPSSPELVAAALGLALDAIDLVRS
AMVAAAKDGTTTDDAGGVAVALRYHIGLTDTVVEGSWLWDSGAKLLDAAAAWAPNEPNNAINAAFGNASTGADEDCV
SAMVISGGSGATAAAQWFDVPCRGYVARPICQRPVSAGGSSSRVGSGLAALPPVAQSLQGNAHFTLFAQLMDWPSAE
LLCQAAGGNLAYFDSPRQFDAVTDMVVTWMRSNGRQPPGTDTTSSDAAAQQLGVWQGLNDRLVKGTWRYSVGNDVAD
PSWQPWAPGTRQSDTIELPVSSADNYGTLQPTVACPVGTYVRGVSGRIDVRNPDTLADSDPDALGLVALRMTCGGPG
PLLAVTDPANADPPLPVGEDPAAISAGVADWTPSVYCSASDTDFAVGVQLQALETQLTYQGGYATLVFYTANDKVAA
TNLRLVCDHDINPPADENGNPGVVSDTPGTELPLPGGATAAGSWRTAAMCEPGQYVCGIGVQSLPYNASVTHGDTKA
WYRFYSSYIGGPPTTLYSAPPDNVMVNSLVIKCCSARASTKHCAEVRYTLEATSTTGSAQVYDQPCRTTRTQTSVNH
VLCRRDVQDAEAASATIQIARNNSVTPRGGDATASALTPTLSTPLVASVAQSIFPVASAPFASTSVQTSTSAAVTGV

SDSSGGCGYLTSSGRTCVNLSPPTPTVEVTQEEFVLYDVPMQWASASSFCSLQGGALAAIDDDTQMAVVSRLVGSWA
AGRFYPPTVSVWFGASNAGSGAAWLYTSLSAVTYNAWGGGATVPQQPSCGELVFSLNPSSIDPATSANTPSWGAADC
SSHRHVLCRRPLARRPSPLRGLAPAASLSLPPQTRTSLHRQAMNWTQALQHCMRQDGGYMVSVTTASAADAFAAAIF
EASHSSRSAAATTRVWLGARFAAPPDSSSSGARVWTWEVDNSPVTYTRRGLVLPTAPSAVDLCLVWEIQSGSGSSGA
VGDSWRVESCDALALPLCQTDLQPFVCRTGASGGAAGPSTSMPLLCPPGTALSGFTGAIEQTTTLPSAAPTNITGGL
PIPGIPGPGSPGQQLAPVVTNPSLTFSAVCVNPQATGELVQQATAAEPSCRPGWSVPRLEGPEVPVLLPDAVADIAD
LEVAYAQLVTRCVAACGVGASRPASPYTSTSLAVVYVDGTCRCARTLQGVELSPAAIAMGDSDGSGPGRHVYGFGTQ
EDVVTLCAASEDVLTTGLVQPVQYELSLSDTRWTPALTLLLRDSKYRVFTDPADSWDSAQRVCTLHGGHLAILRSAD
ELTELAARLAASLPAAATAMAGPFYWVGLLGTQRPAYRWLDGTALQQPIAVEDAFAWDYDSNCGFMMTSELGSSASG
LMAGSCNANKFPFICETSTMIDTSLSDLTPGESWTVPGLADRGLGEWKLSLHAAHAVTWREAQRVCRYQGAELLWFF
DTDEQQAVSDWLSGMMAAVAADGVWTAMAEDAGGRYVWRADSAADSVALSTWQIPRDLAGAGSSRDGACGQLQLLGS
SGNGSSNAVSVRSCSDALPFVCKSGTPWAGPPVVTPAAPTAAVDTAPPPAPRELMYTPDAVVDTGGKRFALFGVDSP
TKVWADAQITCAQAGGSLAEWASVDEFDTVGSAFRTALSVRTTLQVFEAFIGLIRDPNDNSQFRYYGSQLAPDPTVA
ATLWDRDQPDNSQNAESCVEMRIWRVIPLKRRFTWNDINCLLKLPYICMLPDPSLSSNTGDAIGSVVAPSTPQQLLP
YTPDAVIDYRTKRFVLFGLTSPAKIWIDAQVTCAQAGGTLAEWTSVDEFNNVANAIRNAVQGKTSVAFFEVFFGLTR
DPNNNAVFRYYTSQLPPNADVASTLWDAGQPDNYQNAEACVEMRVWRPYPFPPKALNTWNDVTCTARLPYLCMLAAP
AAPAAAPNVDLRKLDVEVAVRGARYVMYGQPVGWQEAKAVCEANDQALADFDSPAAYDTVVRFITSHWVYTQPFFND
PTIAVWTGYNSRFSDTTGSKYFTANGLPAGAATGLYVPPATPSGLSGVDATCYGLALNPKSQSRSFGLQDCGSARRP
LCRAVDRALARPLFQSDNINGRRFALFSTLVNYGTARDVCRTLPGGDLSVFKSDSEWAQVSGFLKDQFWPLVVDQNK
ALKNPTDGGLYAPLWAGFNKLATPTWTMLDDPLRDVTSNAAWYPGQPVQTSSCGSVYIYQQGDRPGVTANWLATDCN
LLQYFLCSYTSPIDTMQSPAPPAPVTPLPAASLYALRSGVSASIARGAYIYTLVTDPSLRASWQQAQLMCQLMPGAP
GGGLATLFTREDAAFFAAVAAEAEPTSAGVTNVWVGFADFMSPDSPYWTATNVAPPGDQQGLVGGWTQDDANPITRS
TPSGDMVMREAATCGYLDPSGGGRIRRGVCSWAFSFLCMAPGYTSAPQHVAVAEISGDGLPDLVVATNCPFDNGASL
VALQQPSPDPAALELPIAGPDSLPVSATEPALSQRTCGDGIPIWQREECDVGPDGSELCSTTCSVTPKFTVRASSKE
AYIVLGNTPPASTAVAVSSPASAGALVPSTSSYFLPHLGVNFNLYGQARGFKQLVEAPGDPLLFRGFSNITADNVAP
VNQPVAVHRVRKLSAGTPFQYRALWTSDLAKPALPDWSDVETVAEDDRSAFTTATTTCGCTADNPGGGPRGLTVSQQ
FSSIVFSWTVGSACEASASITRTPLDPLTRQPVPSANATTASGSADAGAAGASGNGTTVVAQLSIGLPCGSVYRPSK
TEVDEDIVRDKLQVGRTYRYCVVVASAASSGYFLDPANPTAADRFVSAPVCADVTIAWAARVEGEVRTKYDTPAPGV
RLTARLLGSPYVVSAVTDDSGRYVIELQAGGSSSSSGSAAGTLLPLPGGCDPVLAPEACLNQLVRLTASSRTRLRSG
RIILHSFSLNGRPGGAQTLALAHLQVATSVRITDESSLPISGYVRWPGTAANAGFDQTRGCPITDAKVCVVSAADNS
SIACVSSDPGTGAFLVAAGIGAYVRLTAAYMNHSMTIDLPAAAEADAGGAFELTQPLYDVDVRDVQTRQLRLGLIGG
LCQYGLGAPVLQFTAYDCDDQAGQPPLQRQVTLDPAEPFSVFTLPALPWVIGFAGLTQPIPGLLPESVSTYLTLTNQ
NSRFVNLTTAPDDTTDGTDGGGGTVPSVGLQTIAFTYTAPNDVELEVCKESNGLYVFCGDYSLANKCDGSSSTGAYA
AVYSAFAPPDSALVGKDVVLLRRAANYKMRVRLYQMYGRLRCDNVQSTVLIQDSIAGDVDTNLCSVSRKGCSQQVAP
VPATNTSEFVYDLVPGAPNFAAATAYARPLSVVASSAGWPSRSFNLYAIVEGTLSRTGSGYIEIPVPVPLLILRDPP
GDRSFARLDSSISTAVKLSMITNDKSTCGGLGEREILSKALPWSLSNAIPFSKLFKLKNIKSTYSTLKLFGGRAFNF
LENGASKAFGKLKTLGTKVFKGAEAAEKATAIAKAKPRLNAGVVPVNPTAWARTKPTSAAGGAKTFLRRGEAVAKVA
KPRTTAVGAVDTALDYLPFGPTIKKGAKAVAGAQKALVGAVEQTIAFAKKFDPEAIYKAAEDAIPAVVKETKEVYDE
QYKKQVDMVTKYFKRLKRLKDTPPNQQQPDPNGLQMGRGAGLSWRRIDSTEDLVTQLFADAGIENVSPDTAAAVLGE
MLVLEPDQTEDLLANLIARYDNHQLAAAENNGDGSSSDDGDYDEGAGDGSGPKAVKPVVQLGAISIAFNKDPYAMGP
GNNGFGDQKSVVPDSYRPNGAGKSGGDFELNPRFPSTNLIYPQCSRQTLGVGFDLDVSQCTGLGVSICTPFLRDRGV
AGVTTDKAWYNEGDEESYYQLKITRTEGFSTPEESSPEMSGGGSDMILAPTFAIMFILQDSLSFDTATCTPTAQLGV
PGWDLQENSHGTAWHSVWHIKNVMIPELASKLEAENAKPPREQRPDIASNLQQGLRGWSDILTIYNQLNDLASANDE
ELPNYQVGQQDVNGLTQGTVEPIWHTTMDEHPDRAADAYEGRGVDPERLEESPVWKLGFFEGSEKLQQLMLRTQSYM
DNSSEAGNIVIAPYSRAAVGGVTAGKQLDTLQRAYGVQFNTFSFSGGGASYSYSLTTTSTMSTRIGFSISFKNMFGL
QGGGGGGTGLWVETQDDYNYGFELEFNSQLVQETERQRVVSFTLSDKDVGDQFLVKIKPDLAFGTPYFELKAGRSRC
PHEPGTQQREGADLSVLGGQNVQTNLKQGDEALFELVLENQSGTDEAVQLELGPDLGTNGNGMYLQLMGAPWIQPVP
MDLRGPFAASTRALVAARCGPRYSNASVDIVARATCDASELSRTPLTLQCYSPCPQVQWPVAWPPIQPLIYNFSDAA
AKNTIRLTLFNPAYAIQKWNTHPRLQGGNGTAVILVEYLNRVSGGGIWSPVVDPAAGGPVNYATREDPAFGFAPFDW
DASVLPDGEYLIRYVTICDNKVGGGTDSRVEGTPISLLVDKRPPLPTYNSLLPGLAYVPGDEVYVEFSEPLDCRQPV
AFSWTGDHVDAADPSGSDPRPVQPDDFSPVCDGRRVGLSWSPLSRPSALAPGRLVRLQLRGVRDVAGNTFAPPLNLS
FVTASGLPSNNPVTLTLALGPVQAPTRRLQFATSPQPSATSASSSTASSDDVDEEWAQYGGEVARQEAIGSTRRLLH
EVDAALDDCLNRHIIRTAATATGAVATAAATGTADGVADEPQAQQQAQQEQPQNVGSGGRRQRRSLLAEVAEAVSAV
SSRLFGGEASGGAATAGDEEPPVRAVSILRDDDLMSVVLELEVTDPQASRTAFKPHSVPAPQTSTAAAVSAATSSPA
DGSSSDGVPDGKGEGDNDGEGDAVGLASRVLRDARNPASCLAGRLSAAVGWSRSLPPQTEAGSTTSSGTGSSGTSAV
RAAYVTQSIVQQGVSGVSEEALVAARRGAADRAAALGLLPQWASGLESLRAELVAAAEKAGLPVDVVIGSGNAVAAV

STDDNARAAGAASVSAAVGAADAAELAAGRAGSGALNEQQQQNTWALPSFTASRRMSGGAGGPAAAGGAGGGGGYV
GMVAVVLICNIAAVALVGGLWRRWRGQQRGASGQQGKRSPRV

>Cre10.g458350.t2.1|PACid:27579586
MIRYAGAIMAARSNAVLVILLTLAPFAACIDVKLVNDFAPADAAVVYEGRVEVTSPTSGQICSSLFDDRDASVICRQ
LGLPTASATAVFEAWRVYGKEGPWAGGAAASDADILAYLNTNYPPITLARLRCRGRETSLDSCTLDTAESGGDGAAA
VALCNRAQAVGVRCYPADYTLRLVNNGNSGFGVTEGRVEVWLYHGWGPVGSKSFDTSDAAVVCRQLGLPSGGATVLT
GAAAAARYGSPTASTATVLIDGLQCTGDEASVAACDRSVSAADLAAAASAAAAAAAAGAAPRPAFVGVQCREPEYAA
RLVDGEHPSEGRLELYKGGMWGSVCASGIDTSALLTACRQLGFFTSPDLGPTVQTLSASGLDVPAATPPLVAGGLAS
CPAGATSLSQCRASLPNGAAGDPTATCANGSAPSDSAAVLRCYAVPPLLASDVEPRALCADPWSTAAGLAPSAAGAT
ALLVGEFDDLSAGAATRAQDALLLSYGADGRLLGHAAFGRPWASSFGSEADIDFGVLPAAPGALLLADVTGDGRDDL
VALTSDAVLANAQLHVAVASGARSFAPLATWLLPNGAGGAASGAGSISSSSTATTAPAATGSGLGGADTIDTSNTTS
RSYLFIDVTGDNAADMVVFDADTLRVSYYPSNMVNELQDDTDGGGITWFELSTVPARCVSLGEDCFFVAGDFNGDGL
NDWALVYRDRFTAGPASSASSTAAASGPQEVFLTLVGTSPPSPLTWHLAAPASFPRGACSSPWAVLLGRFSGGGADS
EPQVACASSADAHVYVAGYERAWGVLPGPPTLIAVRDVDLDGRDDLLVLTADGSYYLTSTGSGFRPPVSTAAYGSAA
TGSVPLAAALALGQTDQTSAKVLAAPAPIAAAPVEARCGAPSRLVVYYSNRRLDPTPGCSSAGSSTTATAGSSSSSS
SSAAAATADQQQQQVLQRLAGSGASHVIFSRLRPNADGLNVTLAAPRDRPVLANLARSLRRLSPGVKVLAAIGGEDS
GSGSSGPEDAAAFARLVRNPQSIERFAAAAAAFVQSYGLDGLELNWPGLTEEQVPGYASLVQALSFSLRRLSTNTNT
SAASGAPLLLALALSPASSPQLALPWYLLAPYVDLFNFQAFDMGGDEVLGATPYIETPLYDCTEAYGLSVNSLVDQL
LAAGVPPQRLNLIASALARTYVLDGDGAVGGPGSPGPCLGAEGLLDQAELRLLLPAGGSAGSGSSSSGSAGGARLAP
AAAAVYSPYSYGGGSQWAHWEDAYTLGSKACFARIHCLGGLGLWDADGDSFGQLLGALGSAAAADPASCVDFTPPSC
PNTASASGTTDLGAPDLVATLGDTEYSLFQVRKTFDDARAHCAAFGDGGDLAAVTSAAEAGAVYGLLAGWAASGQLG
AEDLWSGRDVAMWLGGSDAAQEGRFVWLSRAGADLTYTAWAAGQPDGRFGGEDCLGAVVRLAGGSGAGLRQVVSPAA
QWYDLGCTTALPFVCARPRSVTEGSGLTAAATAAAGGYRRVSAGPTASLLVFPPSAAAGSDGGQQPMMMTMAEGQKL
CRSLGAELPSLTDPLVRQQLLGSFSSGGSYGTRAGLPPYIWLGLRSYGDGELYWSDGSQTSDGLLNAWEQGEFGEAA
CAVLVPGPQSANVTLQIDGLYSFWNESVALGAQRVSPPPPPPPNTPPPPTPPPLAPFGPDRPAGLPPQPEPAGLRPA
LPPPGLPVAPPDAPEADQIAAGFAPPPQAPPLAGVPPVVDLLLSPGVWSLSCLELAPVVCLDGPPEVSLTPDFYCLS
RPSGPGGAPLPMPVPGLLLPPGDSAGGSSNPLSVRSELECAAACMVAQRCVFFSYLPGYPSSTGSSSSSSSSSSSNL
INACFLLQEPWSTAAGATVPQRPGSSSSGSGSSSSGSNAYSYTTADRVCFRSGAAFMGDVITVADIAAVAASASNAS
STSNSSTDAAASGTGSSSTAAGASLAPVTAISPMFGFPAPTPVPGLDPPAPRAAPWRLSPGVPPQESGYGGYGGGGV
YGSHGGYGGYGGTTVTEYGEQHLAFSLRCALDSALPVLSSVSLVLDAAGYIADVGTSCGGGTASGSGGGSSSVSSNS
TTGDDLGPAASTDMLKSAEQRAVLPPLPAVTASIYRESCGPSGAVVGISGTFDAAGICTAVLSCASGATIPLLPSSS
AGDGSSADAAAGDGRNLPRPCAAVGGAFAFSCPSGMVAYGLQGTFASAYGASSAISLATLQLLCATAPALPTAQQAA
PPAAPLPPRPPVPLLALPLPAPTGGNGSATSGRRLHEEGDPVIIGSTGLVGSIQGSGSGNGGSGSGHNRAIGVQLPK
PRRTLLQQADDNTSMPSPPSPPPPPPPSPPPRPPPPPRPPRPSPPVPPPQGSPPVPPPSPKPPTPPSPPPVPPAPPS
PKPPSPLPPQPSPTPPTPPPAPYRPDAPPSPPNPPPRPPRPPAPRPPTSSPPPVPAPPSPPAPVASGVPACNGCSPV
FGSLGNDMGAAPAVATVCPPGYLISRLVFSSARSGSSSSGDAPYTGLGAVCMDAATAAATNSSSSMRGSSSSLTAGV
PATVGPFDQFHALACGNANSDFITSVSGLYYYPTADSTGYITSVTLGCRGDTGSHQRRRMIEDAAVGVSWSYDCPSG
TVLTEIRWAVAPAAASTDSGTSDTGLLSGVAFRCGLTLAGLPPALPPATPSNPIATFTASDSSGSGQQQQEVVALQC
PPGQFLTSLYGRYMAAVEPAEDGSGGSSAQINSLGVVCSEAALALREASARVHNATANNSSSTYVAFTAAACPAGVS
AVVGRRTGGNTSAAGDATAVAPLLLASLDVLCYDTVDYSLNNLYGSTTAVDGTVFGERCPQLTFASGVTLVRSAAGG
DGFTSLRLLCTPVPDTTLEATAYAGQLPAVGQLPALPAASPAGSTTQQQPYHYECPAGSKVVSVLARIDSTAIATGS
AGADTSALLNLRFECDNAAPDPSRPSLAAIAAAAELAASSPYSAAVLLAATGTSSGAGAVTSGAGSGGSSTGAAGGG
GLGQLGQERYISPDLTSSAPPTSFLIETSCPLGVAGLDADRLSALDDGAAALLPVARLDLPYYTTDDAAMASGGAAQ
TSVRRGEVYYAALYDIVLDANQATAFCRLQSGDLMSFRNDIERDAVASMLQEWAYGSGVTDAVFSVWIGARRTAGAS
GAAGGLYNFSYIDGSALYGQYVPPWTAGEPNNIGGVEDCVELAVNALTGVASWNDRACLGLVRRPLCKIVPPAYRPG
VSPSDAATPDVVVGLGGPSSGGGQQLSYDVPLTWDRARTFCQRRGGALASFSSTDDLRRLGLAVGQSYFSRTSLSGD
ALKDRQDLWVGLRRTLPDSPAAALRPAVAAEQYGSSLFLYVPQPNRTFEQARSYCQSLGADLAVFSSARQYDTVMTG
IQGSVVRDLQADSSIAWIGIRRGSGSSNAFVTAASGRPATFTRWAVGSPGGSGSGSCVAVRTRCSTGGGGNCTSEYV
TLGCDGAAGSAPADAFICERATDASASALLPGARQVAVYAADGSSSGWQAASGSCSGRGGSLASLVAQDEYDTLLAA
LARSVAAAVAAGNSSSGAGSSAVAGLLPGGLPADGRRARVELYTGLRKAAGDAAYSWSDGTLAAFLPWASGAAPSNG
SSGSSVTRCGRLAVACFKSGGTSNSTTDPTPISSVTVSLGGGDGDAVTCELEGLYERDCVVAAPYACTYAYEGSWAF
LDGSVLKAVFTPVPANTFFLSDGGGGTTLRDGVRVDTIGHVWGPTFVLFASEYAGGTLITTITDTVGSESCAAACAA
TPGCDFWSYGPLNGILGPATCYLKRDPGGRGKFTGYGQRPFFKAGNTTKPYGPGPYWKLLATAPPSTPPLAPAPTES
ANACAVLSQQLLDPSATSLQLTSMKTVSVSIAGPADETELLRYFQFMRPADCAAERPVVCTQPPAPGGSSSAAIASA
SPEEADALSNYLTPLASAFAAGGAVQLDLYDMLVDSTYARQLCRQLGGALVEPSSPELVAAALGLALDAIDLVRSAM
VAAAKDGTTTDDAGGVAVALRYHIGLTDTVVEGSWLWDSGAKLLDAAAAWAPNEPNNAINAAFGNASTGADEDCVSA

MVISGGSGATAAAQWFDVPCRGYVARPICQRPVSAGGSSSRVGSGLAALPPVAQSLQGNAHFTLFAQLMDWPSAELL
CQAAGGNLAYFDSPRQFDAVTDMVVTWMRSNGRQPPGTDTTSSDAAAQQLGVWQGLNDRLVKGTWRYSVGNDVADPS
WQPWAPGTRQSDTIELPVSSADNYGTLQPTVACPVGTYVRGVSGRIDVRNPDTLADSDPDALGLVALRMTCGGPGPL
LAVTDPANADPPLPVGEDPAAISAGVADWTPSVYCSASDTDFAVGVQLQALETQLTYQGGYATLVFYTANDKVAATN
LRLVCDHDINPPADENGNPGVVSDTPGTELPLPGGATAAGSWRTAAMCEPGQYVCGIGVQSLPYNASVTHGDTKAWY
RFYSSYIGGPPTTLYSAPPDNVMVNSLVIKCCSARASTKHCAEVRYTLEATSTTGSAQVYDQPCRTTRTQTSVNHVL
CRRDVQDAEAASATIQIARNNSVTPRGGDATASALTPTLSTPLVASVAQSIFPVASAPFASTSVQTSTSAAVTGVSD
SSGGCGYLTSSGRTCVNLSPPTPTVEVTQEEFVLYDVPMQWASASSFCSLQGGALAAIDDDTQMAVVSRLVGSWAAG
RFYPPTVSVWFGASNAGSGAAWLYTSLSAVTYNAWGGGATVPQQPSCGELVFSLNPSSIDPATSANTPSWGAADCSS
HRHVLCRRPLARRPSPLRGLAPAASLSLPPQTRTSLHRQAMNWTQALQHCMRQDGGYMVSVTTASAADAFAAAIFEA
SHSSRSAAATTRVWLGARFAAPPDSSSSGARVWTWEVDNSPVTYTRRGLVLPTAPSAVDLCLVWEIQSGSGSSGAVG
DSWRVESCDALALPLCQTDLQPFVCRTGASGGAAGPSTSMPLLCPPGTALSGFTGAIEQTTTLPSAAPTNITGGLPI
PGIPGPGSPGQQLAPVVTNPSLTFSAVCVNPQATGELVQQATAAEPSCRPGWSVPRLEGPEVPVLLPDAVADIADLE
VAYAQLVTRCVAACGVGASRPASPYTSTSLAVVYVDGTCRCARTLQGVELSPAAIAMGDSDGSGPGRHVYGFGTQED
VVTLCAASEDVLTTGLVQPVQYELSLSDTRWTPALTLLLRDSKYRVFTDPADSWDSAQRVCTLHGGHLAILRSADEL
TELAARLAASLPAAATAMAGPFYWVGLLGTQRPAYRWLDGTALQQPIAVEDAFAWDYDSNCGFMMTSELGSSASGLM
AGSCNANKFPFICETSTMIDTSLSDLTPGESWTVPGLADRGLGEWKLSLHAAHAVTWREAQRVCRYQGAELLWFFDT
DEQQAVSDWLSGMMAAVAADGVWTAMAEDAGGRYVWRADSAADSVALSTWQIPRDLAGAGSSRDGACGQLQLLGSSG
NGSSNAVSVRSCSDALPFVCKSGTPWAGPPVVTPAAPTAAVDTAPPPAPRELMYTPDAVVDTGGKRFALFGVDSPTK
VWADAQITCAQAGGSLAEWASVDEFDTVGSAFRTALSVRTTLQVFEAFIGLIRDPNDNSQFRYYGSQLAPDPTVAAT
LWDRDQPDNSQNAESCVEMRIWRVIPLKRRFTWNDINCLLKLPYICMLPDPSLSSNTGDAIGSVVAPSTPQQLLPYT
PDAVIDYRTKRFVLFGLTSPAKIWIDAQVTCAQAGGTLAEWTSVDEFNNVANAIRNAVQGKTSVAFFEVFFGLTRDP
NNNAVFRYYTSQLPPNADVASTLWDAGQPDNYQNAEACVEMRVWRPYPFPPKALNTWNDVTCTARLPYLCMLAAPAA
PAAAPNVDLRKLDVEVAVRGARYVMYGQPVGWQEAKAVCEANDQALADFDSPAAYDTVVRFITSHWVYTQPFFNDPT
IAVWTGYNSRFSDTTGSKYFTANGLPAGAATGLYVPPATPSGLSGVDATCYGLALNPKSQSRSFGLQDCGSARRPLC
RAVDRALARPLFQSDNINGRRFALFSTLVNYGTARDVCRTLPGGDLSVFKSDSEWAQVSGFLKDQFWPLVVDQNKAL
KNPTDGGLYAPLWAGFNKLATPTWTMLDDPLRDVTSNAAWYPGQPVQTSSCGSVYIYQQGDRPGVTANWLATDCNLL
QYFLCSYTSPIDTMQSPAPPAPVTPLPAASLYALRSGVSASIARGAYIYTLVTDPSLRASWQQAQLMCQLMPGAPGG
GLATLFTREDAAFFAAVAAEAEPTSAGVTNVWVGFADFMSPDSPYWTATNVAPPGDQQGLVGGWTQDDANPITRSTP
SGDMVMREAATCGYLDPSGGGRIRRGVCSWAFSFLCMAPGYTSAPQHVAVAEISGDGLPDLVVATNCPFDNGASLVA
LQQPSPDPAALELPIAGPDSLPVSATEPALSQRTCGDGIPIWQREECDVGPDGSELCSTTCSVTPKFTVRASSKEAY
IVLGNTPPASTAVAVSSPASAGALVPSTSSYFLPHLGVNFNLYGQARGFKQLVEAPGDPLLFRGFSNITADNVAPVN
QPVAVHRVRKLSAGTPFQYRALWTSDLAKPALPDWSDVETVAEDDRSAFTTATTTCGCTADNPGGGPRGLTVSQQFS
SIVFSWTVGSACEASASITRTPLDPLTRQPVPSANATTASGSADAGAAGASGNGTTVVAQLSIGLPCGSVYRPSKTE
VDEDIVRDKLQVGRTYRYCVVVASAASSGYFLDPANPTAADRFVSAPVCADVTIAWAARVEGEVRTKYDTPAPGVRL
TARLLGSPYVVSAVTDDSGRYVIELQAGGSSSSSGSAAGTLLPLPGGCDPVLAPEACLNQLVRLTASSRTRLRSGRI
ILHSFSLNGRPGGAQTLALAHLQVATSVRITDESSLPISGYVRWPGTAANAGFDQTRGCPITDAKVCVVSAADNSSI
ACVSSDPGTGAFLVAAGIGAYVRLTAAYMNHSMTIDLPAAAEADAGGAFELTQPLYDVDVRDVQTRQLRLGLIGGLC
QYGLGAPVLQFTAYDCDDQAGQPPLQRQVTLDPAEPFSVFTLPALPWVIGFAGLTQPIPGLLPESVSTYLTLTNQNS
RFVNLTTAPDDTTDGTDGGGGTVPSVGLQTIAFTYTAPNDVELEVCKESNGLYVFCGDYSLANKCDGSSSTGAYAAV
YSAFAPPDSALVGKDVVLLRRAANYKMRVRLYQMYGRLRCDNVQSTVLIQDSIAGDVDTNLCSVSRKGCSQQVAPVP
ATNTSEFVYDLVPGAPNFAAATAYARPLSVVASSAGWPSRSFNLYAIVEGTLSRTGSGYIEIPVPVPLLILRDPPGD
RSFARLDSSISTAVKLSMITNDKSTCGGLGEREILSKALPWSLSNAIPFSKLFKLKNIKSTYSTLKLFGGRAFNFLE
NGASKAFGKLKTLGTKVFKGAEAAEKATAIAKAKPRLNAGVVPVNPTAWARTKPTSAAGGAKTFLRRGEAVAKVAKP
RTTAVGAVDTALDYLPFGPTIKKGAKAVAGAQKALVGAVEQTIAFAKKFDPEAIYKAAEDAIPAVVKETKEVYDEQY
KKQVDMVTKYFKRLKRLKDTPPNQQQPDPNGLQMGRGAGLSWRRIDSTEDLVTQLFADAGIENVSPDTAAAVLGEML
VLEPDQTEDLLANLIARYDNHQLAAAENNGDGSSSDDGDYDEGAGDGSGPKAVKPVVQLGAISIAFNKDPYAMGPGN
NGFGDQKSVVPDSYRPNGAGKSGGDFELNPRFPSTNLIYPQCSRQTLGVGFDLDVSQCTGLGVSICTPFLRDRGVAG
VTTDKAWYNEGDEESYYQLKITRTEGFSTPEESSPEMSGGGSDMILAPTFAIMFILQDSLSFDTATCTPTAQLGVPG
WDLQENSHGTAWHSVWHIKNVMIPELASKLEAENAKPPREQRPDIASNLQQGLRGWSDILTIYNQLNDLASANDEEL
PNYQVGQQDVNGLTQGTVEPIWHTTMDEHPDRAADAYEGRGVDPERLEESPVWKLGFFEGSEKLQQLMLRTQSYMDN
SSEAGNIVIAPYSRAAVGGVTAGKQLDTLQRAYGVQFNTFSFSGGGASYSYSLTTTSTMSTRIGFSISFKNMFGLQG
GGGGGTGLWVETQDDYNYGFELEFNSQLVQETERQRVVSFTLSDKDVGDQFLVKIKPDLAFGTPYFELKAGRSRCPH
EPGTQQREGADLSVLGGQNVQTNLKQGDEALFELVLENQSGTDEAVQLELGPDLGTNGNGMYLQLMGAPWIQPVPMD
LRGPFAASTRALVAARCGPRYSNASVDIVARATCDASELSRTPLTLQCYSPCPQVQWPVAWPPIQPLIYNFSDAAAK
NTIRLTLFNPAYAIQKWNTHPRLQGGNGTAVILVEYLNRVSGGGIWSPVVDPAAGGPVNYATREDPAFGFAPFDWDA
SVLPDGEYLIRYVTICDNKVGGGTDSRVEGTPISLLVDKRPPLPTYNSLLPGLAYVPGDEVYVEFSEPLDCRQPVAF

SWTGDHVDAADPSGSDPRPVQPDDFSPVCDGRRVGLSWSPLSRPSALAPGRLVRLQLRGVRDVAGNTFAPPLNLSFV
TASGLPSNNPVTLTLALGPVQAPTRRLQFATSPQPSATSASSSTASSDDVDEEWAQYGGEVARQEAIGSTRRLLHEV
DAALDDCLNRHIIRTAATATGAVATAAATGTADGVADEPQAQQQAQQEQPQNVGSGGRRQRRSLLAEVAEAVSAVSS
RLFGGEASGGAATAGDEEPPVRAVSILRDDDLMSVVLELEVTDPQASRTAFKPHSVPAPQTSTAAAVSAATSSPADG
SSSDGVPDGKGEGDNDGEGDAVGLASRVLRDARNPASCLAGRLSAAVGWSRSLPPQTEAGSTTSSGTGSSGTSAVRA
AYVTQSIVQQGVSGVSEEALVAARRGAADRAAALGLLPQWASGLESLRAELVAAAEKAGLPVDVVIGSGNAVAAVST
DDNARAAGAASVSAAVGAADAAELAAGRAGSGALNEQQQQNTWALPSFTASRRMSGGAGGPAAAGGAGGGGGYVGM
VAVVLICNIAAVALVGGLWRRWRGQQRGASGQQGKRSPRV

>Cre10.g458350.t1.3|PACid:27579587
MIRYAGAIMAARSNAVLVILLTLAPFAACIDVKLVNDFAPADAAVVYEGRVEVTSPTSGQICSSLFDDRDASVICRQ
LGLPTASATAVFEAWRVYGKEGPWAGGAAASDADILAYLNTNYPPITLARLRCRGRETSLDSCTLDTAESGGDGAAA
VALCNRAQAVGVRCYPADYTLRLVNNGNSGFGVTEGRVEVWLYHGWGPVGSKSFDTSDAAVVCRQLGLPSGGATVLT
GAAAAARYGSPTASTATVLIDGLQCTGDEASVAACDRSVSAADLAAAASAAAAAAAAGAAPRPAFVGVQCREPEYAA
RLVDGEHPSEGRLELYKGGMWGSVCASGIDTSALLTACRQLGFFTSPDLGPTVQTLSASGLDVPAATPPLVAGGLAS
CPAGATSLSQCRASLPNGAAGDPTATCANGSAPSDSAAVLRCYAVPPLLASDVEPRALCADPWSTAAGLAPSAAGAT
ALLVGEFDDLSAGAATRAQDALLLSYGADGRLLGHAAFGRPWASSFGSEADIDFGVLPAAPGALLLADVTGDGRDDL
VALTSDAVLVAVASGARSFAPLATWLLPNGAGGAASGAGSISSSSTATTAPAATGSGLGGADTIDTSNTTSRSYLFI
DVTGDNAADMVVFDADTLRVSYYPSNMVNELQDDTDGGGITWFELSTVPARCVSLGEDCFFVAGDFNGDGLNDWALV
YRDRFTAGPASSASSTAAASGPQEVFLTLVGTSPPSPLTWHLAAPASFPRGACSSPWAVLLGRFSGGGADSEPQVAC
ASSADAHVYYVAGYERAWGVLPGPPTLIAVRDVDLDGRDDLLVLTADGSYYLTSTGSGFRPPVSTAAYGSAATGSVPL
AAALALGQTDQTSAKVLAAPAPIAAAPVEARCGAPSRLVVYYSNRRLDPTPGCSSAGSSTTATAGSSSSSSSSAAAA
TADQQQQQVLQRLAGSGASHVIFSRLRPNADGLNVTLAAPRDRPVLANLARSLRRLSPGVKVLAAIGGEDSGSGSSG
PEDAAAFARLVRNPQSIERFAAAAAAFVQSYGLDGLELNWPGLTEEQVPGYASLVQALSFSLRRLSTNTNTSAASGA
PLLLALALSPASSPQLALPWYLLAPYVDLFNFQAFDMGGDEVLGATPYIETPLYDCTEAYGLSVNSLVDQLLAAGVP
PQRLNLIASALARTYVLDGDGAVGGPGSPGPCLGAEGLLDQAELRLLLPAGGSAGSGSSSSGSAGGARLAPAAAAVY
SPYSYGGGSQWAHWEDAYTLGSKACFARIHCLGGLGLWDADGDSFGQLLGALGSAAAADPASCVDFTPPSCPNTASA
SGTTDLGAPDLVATLGDTEYSLFQVRKTFDDARAHCAAFGDGGDLAAVTSAAEAGAVYGLLAGWAASGQLGAEDLWS
GRDVAMWLGGSDAAQEGRFVWLSRAGADLTYTAWAAGQPDGRFGGEDCLGAVVRLAGGSGAGLRQVVSPAAQWYDLG
CTTALPFVCARPRSVTEGSGLTAAATAAAGGYRRVSAGPTASLLVFPPSAAAGSDGGQQPMMMTMAEGQKLCRSLGA
ELPSLTDPLVRQQLLGSFSSGGSYGTRAGLPPYIWLGLRSYGDGELYWSDGSQTSDGLLNAWEQGEFGEAACAVLVP
GPQSANVTLQIDGLYSFWNESVALGAQRVSPPPPPPPNTPPPPTPPPLAPFGPDRPAGLPPQPEPAGLRPALPPPGL
PVAPPDAPEADQIAAGFAPPPQAPPLAGVPPVVDLLLSPGVWSLSCLELAPVVCLDGPPEVSLTPDFYCLSRPSGPG
GAPLPMPVPGLLLPPGDSAGGSSNPLSVRSELECAAACMVAQRCVFFSYLPGYPSSTGSSSSSSSSSSNLINACFL
LQEPWSTAAGATVPQRPGSSSSGSGSSSSGSNAYSYTTADRVCFRSGAAFMGDVITVADIAAVAASASNASSTSNSS
TDAAASGTGSSSTAAGASLAPVTAISPMFGFPAPTPVPGLDPPAPRAAPWRLSPGVPPQESGYGGYGGGGVYGSHGG
YGGYGGTTVTEYGEQHLAFSLRCALDSALPVLSSVSLVLDAAGYIADVGTSCGGGTASGSGGGSSSVSSNSTTGDDL
GPAASTDMLKSAEQRAVLPPLPAVTASIYRESCGPSGAVVGISGTFDAAGICTAVLSCASGATIPLLPSSSAGDGSS
ADAAAGDGRNLPRPCAAVGGAFAFSCPSGMVAYGLQGTFASAYGASSAISLATLQLLCATAPALPTAQQAAPPAAPL
PPRPPVPLLALPLPAPTGGNGSATSGRRLHEEGDPVIIGSTGLVGSIQGSGSGNGGSGSGHNRAIGVQLPKPRRTLL
QQADDNTSMPSPPSPPPPPPPSPPPRPPPPPRPPRPSPPVPPPQGSPPVPPPSPKPPTPPSPPPVPPAPPSPKPPSP
LPPQPSPTPPTPPPAPYRPDAPPSPPNPPPRPPRPPAPRPPTSSPPPVPAPPSPPAPVASGVPACNGCSPVFGSLGN
DMGAAPAVATVCPPGYLISRLVFSSARSGSSSSGDAPYTGLGAVCMDAATAAATNSSSSMRGSSSSLTAGVPATVGP
FDQFHALACGNANSDFITSVSGLYYYPTADSTGYITSVTLGCRGDTGSHQRRRMIEDAAVGVSWSYDCPSGTVLTEI
RWAVAPAAASTDSGTSDTGLLSGVAFRCGLTLAGLPPALPPATPSNPIATFTASDSSGSGQQQQEVVALQCPPGQFL
TSLYGRYMAAVEPAEDGSGGSSAQINSLGVVCSEAALALREASARVHNATANNSSSTYVAFTAAACPAGVSAVVGRR
TGGNTSAAGDATAVAPLLLASLDVLCYDTVDYSLNNLYGSTTAVDGTVFGERCPQLTFASGVTLVRSAAGGDGFTSL
RLLCTPVPDTTLEATAYAGQLPAVGQLPALPAASPAGSTTQQQPYHYECPAGSKVVSVLARIDSTAIATGSAGADTS
ALLNLRFECDNAAPDPSRPSLAAIAAAAELAASSPYSAAVLLAATGTSSGAGAVTSGAGSGGSSTGAAGGGGLGQLG
QERYISPDLTSSAPPTSFLIETSCPLGVAGLDADRLSALDDGAAALLPVARLDLPYYTTDDAAMASGGAAQTSVRRG
EVYYAALYDIVLDANQATAFCRLQSGDLMSFRNDIERDAVASMLQEWAYGSGVTDAVFSVWIGARRTAGASGAAGGL
YNFSYIDGSALYGQYVPPWTAGEPNNIGGVEDCVELAVNALTGVASWNDRACLGLVRRPLCKIVPPAYRPGVSPSDA
ATPDVVVGLGGPSSGGGQQLSYDVPLTWDRARTFCQRRGGALASFSSTDDLRRLGLAVGQSYFSRTSLSGDALKDRQ
DLWVGLRRTLPGPDSPAAALRPAVAAEQYGSSLFLYVPQPNRTFEQARSYCQSLGADLAVFSSARQYDTVMTGIQGS
VVRDLQADSSIAWIGIRRGSGSSNAFVTAASGRPATFTRWAVGSPGGSGSGSCVAVRTRCSTGGGGNCTSEYVTLGC
DGAAGSAPADAFICERATDASASALLPGARQVAVYAADGSSSGWQAASGSCSGRGGSLASLVAQDEYDTLLAALARS
VAAAVAAGNSSSGAGSSAVAGLLPGGLPADGRRARVELYTGLRKAAGDAAYSWSDGTLAAFLPWASGAAPSNGSSGS

SVTRCGRLAVACFKSGGTSNSTTDPTPISSVTVSLGGGDGDAVTCELEGLYERDCVVAAPYACTYAYEGSWAFLDGS
VLKAVFTPVPANTFFLSDGGGGTTLRDGVRVDTIGHVWGPTFVLFASEYAGGTLITTITDTVGSESCAAACAATPGC
DFWSYGPLNGILGPATCYLKRDPGGRGKFTGYGQRPFFKAGNTTKPYGPGPYWKLLATAPPSTPPLAPAPTESANAC
AVLSQQLLDPSATSLQLTSMKTVSVSIAGPADETELLRYFQFMRPADCAAERPVVCTQPPAPGGSSSAAIASASPEE
ADALSNYLTPLASAFAAGGAVQLDLYDMLVDSTYARQLCRQLGGALVEPSSPELVAAALGLALDAIDLVRSAMVAAA
KDGTTTDDAGGVAVALRYHIGLTDTVVEGSWLWDSGAKLLDAAAAWAPNEPNNAINAAFGNASTGADEDCVSAMVIS
GGSGATAAAQWFDVPCRGYVARPICQRPVSAGGSSSRVGSGLAALPPVAQSLQGNAHFTLFAQLMDWPSAELLCQAA
GGNLAYFDSPRQFDAVTDMVVTWMRSNGRQPPGTDTTSSDAAAQQLGVWQGLNDRLVKGTWRYSVGNDVADPSWQPW
APGTRQSDTIELPVSSADNYGTLQPTVACPVGTYVRGVSGRIDVRNPDTLADSDPDALGLVALRMTCGGPGPLLAVT
DPANADPPLPVGEDPAAISAGVADWTPSVYCSASDTDFAVGVQLQALETQLTYQGGYATLVFYTANDKVAATNLRLV
CDHDINPPADENGNPGVVSDTPGTELPLPGGATAAGSWRTAAMCEPGQYVCGIGVQSLPYNASVTHGDTKAWYRFYS
SYIGGPPTTLYSAPPDNVMVNSLVIKCCSARASTKHCAEVRYTLEATSTTGSAQVYDQPCRTTRTQTSVNHVLCRRD
VQDAEAASATIQIARNNSVTPRGGDATASALTPTLSTPLVASVAQSIFPVASAPFASTSVQTSTSAAVTGVSDSSGG
CGYLTSSGRTCVNLSPPTPTVEVTQEEFVLYDVPMQWASASSFCSLQGGALAAIDDDTQMAVVSRLVGSWAAGRFYP
PTVSVWFGASNAGSGAAWLYTSLSAVTYNAWGGGATVPQQPSCGELVFSLNPSSIDPATSANTPSWGAADCSSHRHV
LCRRPLARRPSPLRGLAPAASLSLPPQTRTSLHRQAMNWTQALQHCMRQDGGYMVSVTTASAADAFAAAIFEASHSS
RSAAATTRVWLGARFAAPPDSSSSGARVWTWEVDNSPVTYTRRGLVLPTAPSAVDLCLVWEIQSGSGSSGAVGDSWR
VESCDALALPLCQTDLQPFVCRTGASGGAAGPSTSMPLLCPPGTALSGFTGAIEQTTTLPSAAPTNITGGLPIPGIP
GPGSPGQQLAPVVTNPSLTFSAVCVNPQATGELVQQATAAEPSCRPGWSVPRLEGPEVPVLLPDAVADIADLEVAYA
QLVTRCVAACGVGASRPASPYTSTSLAVVYVDGTCRCARTLQGVELSPAAIAMGDSDGSGPGRHVYGFGTQEDVVTL
CAASEDVLTTGLVQPVQYELSLSDTRWTPALTLLLRDSKYRVFTDPADSWDSAQRVCTLHGGHLAILRSADELTELA
ARLAASLPAAATAMAGPFYWVGLLGTQRPAYRWLDGTALQQPIAVEDAFAWDYDSNCGFMMTSELGSSASGLMAGSC
NANKFPFICETSTMIDTSLSDLTPGESWTVPGLADRGLGEWKLSLHAAHAVTWREAQRVCRYQGAELLWFFDTDEQQ
AVSDWLSGMMAAVAADGVWTAMAEDAGGRYVWRADSAADSVALSTWQIPRDLAGAGSSRDGACGQLQLLGSSGNGSS
NAVSVRSCSDALPFVCKSGTPWAGPPVVTPAAPTAAVDTAPPPAPRELMYTPDAVVDTGGKRFALFGVDSPTKVWAD
AQITCAQAGGSLAEWASVDEFDTVGSAFRTALSVRTTLQVFEAFIGLIRDPNDNSQFRYYGSQLAPDPTVAATLWDR
DQPDNSQNAESCVEMRIWRVIPLKRRFTWNDINCLLKLPYICMLPDPSLSSNTGDAIGSVVAPSTPQQLLPYTPDAV
IDYRTKRFVLFGLTSPAKIWIDAQVTCAQAGGTLAEWTSVDEFNNVANAIRNAVQGKTSVAFFEVFFGLTRDPNNNA
VFRYYTSQLPPNADVASTLWDAGQPDNYQNAEACVEMRVWRPYPFPPKALNTWNDVTCTARLPYLCMLAAPAAPAAA
PNVDLRKLDVEVAVRGARYVMYGQPVGWQEAKAVCEANDQALADFDSPAAYDTVVRFITSHWVYTQPFFNDPTIAVW
TGYNSRFSDTTGSKYFTANGLPAGAATGLYVPPATPSGLSGVDATCYGLALNPKSQSRSFGLQDCGSARRPLCRAVD
RALARPLFQSDNINGRRFALFSTLVNYGTARDVCRTLPGGDLSVFKSDSEWAQVSGFLKDQFWPLVVDQNKALKNPT
DGGLYAPLWAGFNKLATPTWTMLDDPLRDVTSNAAWYPGQPVQTSSCGSVYIYQQGDRPGVTANWLATDCNLLQYFL
CSYTSPIDTMQSPAPPAPVTPLPAASLYALRSGVSASIARGAYIYTLVTDPSLRASWQQAQLMCQLMPGAPGGGLAT
LFTREDAAFFAAVAAEAEPTSAGVTNVWVGFADFMSPDSPYWTATNVAPPGDQQGLVGGWTQDDANPITRSTPSGDM
VMREAATCGYLDPSGGGRIRRGVCSWAFSFLCMAPGYTSAPQHVAVAEISGDGLPDLVVATNCPFDNGASLVALQQP
SPDPAALELPIAGPDSLPVSATEPALSQRTCGDGIPIWQREECDVGPDGSELCSTTCSVTPKFTVRASSKEAYIVLG
NTPPASTAVAVSSPASAGALVPSTSSYFLPHLGVNFNLYGQARGFKQLVEAPGDPLLFRGFSNITADNVAPVNQPVA
VHRVRKLSAGTPFQYRALWTSDLAKPALPDWSDVETVAEDDRSAFTTATTTCGCTADNPGGGPRGLTVSQQFSSIVF
SWTVGSACEASASITRTPLDPLTRQPVPSANATTASGSADAGAAGASGNGTTVVAQLSIGLPCGSVYRPSKTEVDED
IVRDKLQVGRTYRYCVVVASAASSGYFLDPANPTAADRFVSAPVCADVTIAWAARVEGEVRTKYDTPAPGVRLTARL
LGSPYVVSAVTDDSGRYVIELQAGGSSSSSGSAAGTLLPLPGGCDPVLAPEACLNQLVRLTASSRTRLRSGRIILHS
FSLNGRPGGAQTLALAHLQVATSVRITDESSLPISGYVRWPGTAANAGFDQTRGCPITDAKVCVVSAADNSSIACVS
SDPGTGAFLVAAGIGAYVRLTAAYMNHSMTIDLPAAAEADAGGAFELTQPLYDVDVRDVQTRQLRLGLIGGLCQYGL
GAPVLQFTAYDCDDQAGQPPLQRQVTLDPAEPFSVFTLPALPWVIGFAGLTQPIPGLLPESVSTYLTLTNQNSRFVN
LTTAPDDTTDGTDGGGGTVPSVGLQTIAFTYTAPNDVELEVCKESNGLYVFCGDYSLANKCDGSSSTGAYAAVYSAF
APPDSALVGKDVVLLRRAANYKMRVRLYQMYGRLRCDNVQSTVLIQDSIAGDVDTNLCSVSRKGCSQQVAPVPATNT
SEFVYDLVPGAPNFAAATAYARPLSVVASSAGWPSRSFNLYAIVEGTLSRTGSGYIEIPVPVPLLILRDPPGDRSFA
RLDSSISTAVKLSMITNDKSTCGGLGEREILSKALPWSLSNAIPFSKLFKLKNIKSTYSTLKLFGGRAFNFLENGAS
KAFGKLKTLGTKVFKGAEAAEKATAIAKAKPRLNAGVVPVNPTAWARTKPTSAAGGAKTFLRRGEAVAKVAKPRTTA
VGAVDTALDYLPFGPTIKKGAKAVAGAQKALVGAVEQTIAFAKKFDPEAIYKAAEDAIPAVVKETKEVYDEQYKKQV
DMVTKYFKRLKRLKDTPPNQQQPDPNGLQMGRGAGLSWRRIDSTEDLVTQLFADAGIENVSPDTAAAVLGEMLVLEP
DQTEDLLANLIARYDNHQLAAAENNGDGSSSDDGDYDEGAGDGSGPKAVKPVVQLGAISIAFNKDPYAMGPGNNGFG
DQKSVVPDSYRPNGAGKSGGDFELNPRFPSTNLIYPQCSRQTLGVGFDLDVSQCTGLGVSICTPFLRDRGVAGVTTD
KAWYNEGDEESYYQLKITRTEGFSTPEESSPEMSGGGSDMILAPTFAIMFILQDSLSFDTATCTPTAQLGVPGWDLQ
ENSHGTAWHSVWHIKNVMIPELASKLEAENAKPPREQRPDIASNLQQGLRGWSDILTIYNQLNDLASANDEELPNYQ
VGQQDVNGLTQGTVEPIWHTTMDEHPDRAADAYEGRGVDPERLEESPVWKLGFFEGSEKLQQLMLRTQSYMDNSSEA

GNIVIAPYSRAAVGGVTAGKQLDTLQRAYGVQFNTFSFSGGGASYSYSLTTTSTMSTRIGFSISFKNMFGLQGGGGG
GTGLWVETQDDYNYGFELEFNSQLVQETERQRVVSFTLSDKDVGDQFLVKIKPDLAFGTPYFELKAGRSRCPHEPGT
QQREGADLSVLGGQNVQTNLKQGDEALFELVLENQSGTDEAVQLELGPDLGTNGNGMYLQLMGAPWIQPVPMDLRGP
FAASTRALVAARCGPRYSNASVDIVARATCDASELSRTPLTLQCYSPCPQVQWPVAWPPIQPLIYNFSDAAAKNTIR
LTLFNPAYAIQKWNTHPRLQGGNGTAVILVEYLNRVSGGGIWSPVVDPAAGGPVNYATREDPAFGFAPFDWDASVLP
DGEYLIRYVTICDNKVGGGTDSRVEGTPISLLVDKRPPLPTYNSLLPGLAYVPGDEVYVEFSEPLDCRQPVAFSWTG
DHVDAADPSGSDPRPVQPDDFSPVCDGRRVGLSWSPLSRPSALAPGRLVRLQLRGVRDVAGNTFAPPLNLSFVTASG
LPSNNPVTLTLALGPVQAPTRRLQFATSPQPSATSASSSTASSDDVDEEWAQYGGEVARQEAIGSTRRLLHEVDAAL
DDCLNRHIIRTAATATGAVATAAATGTADGVADEPQAQQQAQQEQPQNVGSGGRRQRRSLLAEVAEAVSAVSSRLFG
GEASGGAATAGDEEPPVRAVSILRDDDLMSVVLELEVTDPQASRTAFKPHSVPAPQTSTAAAVSAATSSPADGSSSD
GVPDGKGEGDNDGEGDAVGLASRVLRDARNPASCLAGRLSAAVGWSRSLPPQTEAGSTTSSGTGSSGTSAVRAAYVT
QSIVQQGVSGVSEEALVAARRGAADRAAALGLLPQWASGLESLRAELVAAAEKAGLPVDVVIGSGNAVAAVSTDDNA
RAAGAASVSAAVGAADAAELAAGRAGSGALNEQQQQNTWALPSFTASRRMSGGAGGPAAAGGAGGGGGYVGMVAVV
LICNIAAVALVGGLWRRWRGQQRGASGQQGKRSPRV

>Cre10.g454000.t1.2|PACid:27579604
MDRARIRSLVKAFQADPESDEVDRLELVENLHQVISRATALHNFSAVVAEETGLLDLLLFLLKSGPGMDMPAVPLPG
APPPSGAHERPERKYQRLLLEMFAGDEKEEWGLIYWPSSRGALVPLALRPETDAFGVVAQLLRDIADAPPEQQLDSG
GVLSAASRVLVRLILSTDGLYAQYGASKESVRKGVESGALPALVAGLRSYIAAYLLEAGTSSGAATAAAATGGGNAA
GGAPAATSNAAGILKSMTMVRAARASSSGIGISGGAGGGGGGGGGIMRLPRSTASGKQAAFGHGAKSQSQRRREVA
HRQQVAAAAVTHAVMLTIGYVCQALTPAELRETGVAELVLEPVVSLMAGLAEQLAAAEKLAAELAAAAAAAAQAAA
AGATTPTANAPANNRPRTGTIEHQTVNIRLSYVIPPGPDSDADPDASAAAAQPPAPAPRREASPPPVAAVSSVLVRA
LDGLLNCIEGRADGHVESLRQQRLIAFLAARPAAATTSSTPSPLRALMSALSRLLRRQEEDEEARRRQLGRGGSAAS
RHAPLPPGAAAALAAASHSAAQHEESGRARGKLHAARDALAPYKVLFDLQGLTQQAAATAAAALDDGAGSAAADADV
DDGQGGAALSALASELGDLSTAAVRATLLPTLLARGELHQAVDTAGALEDLFRHLPQPGQAALAGVLADAAAQPAAP
LGLVVAVAAAAGALTSAAAGAAIAAAEAQHAGAKSGTSGPRQKKQPQPTAAQVAAAVAGAGLGAGAASSPVPSVAGA
PQLSPRGGAASSAAAQHLHAAVAAVSACGTLSHVSSYASGLGGLGGGAGAGDDTATLMGTLPAAAMVGRMRELTTHH
QAHHQAAEAAAAAARDTADASAAAAEAAGGGAAAAGAASGRGTHAPVGLVEAVLVGLAENLQSFLTAAVDLLPPSEA
VAAAEEDGGDSGGAHSGHVDELVGLVCRCTQRFQERLPVVLQHLQAHLHVSPADDRHGGHDAAAAAAAAETAGMSPF
AASSSAAASAEGGGGGGGGAPEGGATTASPQGQDLLVSTACVAATRALESCASSLTAPAASAASPSGGAAAGAGGAA
AAAAAAAADAGSPGEQAAAALRILATIIKSPRAPLQARVQASAAAAQALKRLLLSTWTADGGGGAAAGGDAAAAATA
APAAAATAGSSSGSSSQPHPLLAHEATEAAQACLDALFGVLSDPVVHAADPDLFEDDVCAGLAVPLAELVVDRPPPN
PLAAALDCKHSLVVAARSLKALVKGVPLGQLTKNQETLVRCFVNAHDDGLRHSAFNLDLLFAEGAVGALLRRMHDTP
PDQPLEEEALQFFASLTDWVPSCRARLRDVGLLKRVSRRMEQPGCGGFELVLLVSICKGMSESEALHTFMKELGVAR
ALAAKLVGDEEAAAVAAGGDLSLLPVGSPGGLDALLTLANLNGGTTSPDDQLCQDLIVKHDISAQIAQFAAEAAASD
RGLATYTFPDGTWSIYDLPSLLTSCLSLTRTAVNCERLVQHGLLPTLLCVLRAANAAAAAAKAAAAPAGAAASAAAE
PDAGGVGDGGEKGSSSGLGGCSRNVDPALGVVAARTLFNLAQVSALHPALKEAGVQELLKACLARDDLRVVDAARGT
LLQLGELTDTAATRALKASQAAEEAAGVKAKAAKQIADAAAAAADAAAADAAATGAPHIAPGAFSNEPPLYDVFLSH
KRTDARDFARALWNLLVSNGYTVFLDFEFKQELGSLEEMVGRCTHFLFIMTDNVFKSEWCIKELVAAVKHNVNIVLM
IKDGARWADVDGNPTCDFPPPHLLRTLPAEVQHVFSKKPVVHNDEYYRAFVEALFDKLYRPPVLESSSRMASLDEVM
QMQMDAIANGMTVTPDGTPVDLNLLDPAAAAAAAAAGLVPGGPGGGAGGAPPPPHSAHPSSADGSAHGSSAHPHHGP
HPHSHHLHSAQHHAGHHGPHSAHLHSALPGTAHHGGHGHGHGGHHGPHSAHSAHSGFPHHSALPHHPGLPSAHHTAH
HAHAAHHLHSAHGGHHGSHHGGPGSAHSAGHHGSHYGPHHSSAHSAGGSHRSAHGHGHGHSHSGNHHGNHHSPHSSA
HLLHDPAHAAAAAAYAATYAAHHQGTHNGAHTAAPSHPSHFPALVHHPASAYTMIGNPWPAAAHAAQAAIMLSHAAQ
HHGRHSAHGGGGGDGAASGSESAGPHGHGHGHGHGHHHDGRGHSAGSMAAAHGGGGSSATAAEMAAHAAYQAELHRQ
MQQMRNEAEQQQQQQAAGSGGEGAAAEGEAGEGGAKSGGAGGEGLAAAVSDTEAAVAAQQKAMAAAAAAAAAAAAAA
GGVSPPPHILFQHAISDPGGYGGAAGMRLDSLSGAAAMAGEHGHGRTHGVLSELQAELAAVRRDLAAEVAGLGRSLI
SGQQELRSGLGGEVAAAARLVSGEVRALVLETQAQVHGLKVELGDVRQELGASRQAAAEMRGLVAQVLEGMSALIQR
NVQLESLLHGGSGGGGGGAVSSHGYGHGHGHGHGSTSGGAGHHHSHATGTGSGISPPSLSGMSAAAAAAVINASAAG
MHRDSSILAQLLSGGNSNSGGVAGGGAGAGAGAGAVNSGGGGGAGGGGGGGGGSGGGLHRVNSGGGVLTPRRARLSA
SCAGYGPYGAGGGMELVAADDMIIIGGGGGGGHGGHGGHSGGPNSGSTPMLVMPNGAGVNRSAQSATGLVPLSVYEA
AAASCPGGAGPSPPAAPYSPRLSPIMLSVAGAAATTTVQGGAAGNGSRRGSETGLPPLQRDQLVAGGPPSMDSPRGR
RIYVSQPLVIKDGAAVEPASHAPPLGGGGGAGSGNGSGSGRGAGLAAAGAAAAANGGAAGSLADGITSSGLVLPPLA
AAAVLHHHHQQLAGSAAPSSGLAAAMVAADRVGPGGGGGGGGEGGRGVVAGGGFQMRPPPGMPQSQLRGKAAKA

>Cre10.g461500.t1.2|PACid:27579643

MATKPGLQEWTPAPAPAPASYPLPTTVVAAGASAASRPQPEPPEQPQPQQPLLQPLSSFPLTAGELHRAGSSGLLDE
DACCRPPEDLQPHALREAQLRALLPSPPPPPQQHQQLQPQQQQLQPISRQHFLGPLARGCDPATALAAEAHLGSAST
PGTAFSTSGDAAASALAAAGAAAGDASDGPVMTLLRAALAHRKAQRHQHQHQHQPQPQRQWQGAALAASVKAPDTAE
AAAAASLAGAAARASHAYGDNVASAGGAFVGAFAEELCRVGGHLTHELRGLRSLLDSAASRLRAAALVSGLVGRPPP
PAAGRPTPQPPPPAAEGASSCAAACDGGANAVVLAAAAVAEENEEEERAAAAAGAAAAAGASRAEALRAVCRQLRRE
CDDLGARLVGLEAFERFNSARFAQLGFEFDSRLLHRASNASAAAAGALSSVAKLQVLERRREYQRCVQELLLDGLVY
DSLLISLSDVYEGVRHAAQRAAAALDGGGGGTGSGASSGTTPWVPPDQFKRSTTKYWVAPRHVMRVQLAVLRHLPLL
IFGRKSNPLFHHWRHEDELEELPADGNGLQQQQLQRLPDLKQHPQHCSAAAVAAGAVNGSVGSGNGSGNGNGRTAGS
GGARVSSSNNGSCGSTSAFAPLARGASMLLPPLPETPHDMQPDGNGLGAGSQRLPTADESTAPADQWPPQHLWPGGK
GPTAGGGGYGSYSLGGGGAAALVTATAEPASEPASCADAAVSSATADAAAAGTLNANSSRSSAPAAVGSSSTASTTK
ATGGGGSSSPITSVYFDNADLQVYHQRLVRGDGASLVRIRWYGSRAPGPDQTVFVERKRHRDAWTGEWSLKERASIP
HREVPDFLSGRLLPGAVPAPPLATAASVPPPPSRSAPPPPPPPPPPQPPLSVSTGRTASPDVFASSASTSDEGGGGG
GAGGSSSPSSSTNSGSSALQHRPGSADGGSSSGRDGGWAPAAAVHVPEAIKVSASPLQQQQVHATAPAAPPEVAAAA
AAASAKAVQLLREVQQDILQRKQEPYLRTRYRRTAFQLSSTNAVRISLDTQLSMLREKLPPDHQELRRLRLLQPLAP
SAVAGPAAAAAIGGQQANWCRQLPADGDDDDDAASAAATATTALLPLEDVVRFPYAVLEIKLQDEDSCPAWVLDLRS
SGLLVEAPKFSKFLHGMALLYPARLQHTPHWFLPELPEQEDENENGVETETAGSAVASGGRVWGREIEAAGGMGLQA
VGIGVGGSNGNGNGSGGRGHDNGGGGGGGAWPWTVGMRLLGRLHFGGGGPATAVANGARGRSRGGGCQPPGNQLQLL
QPPSRAPAAAAAALAAAAGGGCRRVLRSLLLPAPPAPPPPPPRHVHPRMSPATLEELADPGDIYAKEVPRWLFPANI
RPDQALDIIPPPPGCSEADFEMATTEVAAAAPTAATVPAAGGAGGGSAGGGGGGGPQLLPHPSLSPPPHLLGHNEGG
DEGDQRRHSSGGSHRSGSSSSSSSSSTDDYSRPSSVRSSMVPTITEPLVPSSSMGLLASPTTAAAATAATGCTPGPA
ATAASLAAGGFAVYGNGGCGPRSGGAAASSAFADPRAAGAALPSAASCGVLRTAVGGGGGGLHMCGSSTPLLLPASY
SSGGALHAHASSPVGVGGFCAGAGAVVGPVGGGMGGGMGGAVLAPACSALGGASLQAQHIGGPTARAASNDSAAAAE
LVSIAGTAPTGSSSSAAPRPGSSCSGGSAVNGGGSGSSTGTTGTGAGGAKKLAAAAMAVCRAGVCGGGGGGGDGMVR
VNEEEQQQEHRMEITSYEQSSSAMVHVSKHHVQPGPTTVTCCFTGLPAAAAAAAAAATAGGGAGGAAGSSRGAGGGG
GATAAAAGAASGMAAAAALHGAYWLRRARAAVTGGGGGSGGGGGAGATQQQHFRTLVAALRRASGSFTTHGGGSGGG
GGRFNGRSGGGGGGGRWSAFMRRFGRGRGRGGGGGGGGSGGSSDDLELAGGGGGGANGYGGRYGSGGASGDGGGGGG
GGANGYGGRYGSGGASGDGGGGGGGGGGGGGGGGGGVAGLLQLPRVSSMVRTRVEPKTFFAAERTFLSWVNIAVLLM
FTSLTLMTSRITTFSGHQGIAAPNIPTPGGAAGADIGVLGASGGGGVAAAGQALGAAAGGSLMQPNATLLFAGNDTA
AAAGVAAAAAGVANSTLAPMQAALLQAAAGIGAGAGAGGGGGSGRVDSCAPGSMCHNSQVAGMVMAPLALVFMLYAL
YVYRKRSAQILRREAVRYDDQRGPVLLVAMLLILLLVAYVLSARAATGF

>Cre10.g436800.t1.3|PACid:27579649
MKGSAWPLSGAALGLSLCVALLLAAGASAQLYITDVDPTSGSLGGGTRMVIQGSGFSEDRYTGSNVVFIGTYPCTVI
NHLTSDSVITCLTSPAPLEGKYDITVVVDGLDSVTYSTPYSYDMDYTPTLNFVFPSAGPPGVNPFVYGTPTWGINNE
CSSNADTGYMYSSTPACVESVLAGDYSATLPSGADGYTYLGYLSYYTSLYRINFTMPLDNTGMGLEGLGTAGNMNLT
ISFGGNAYGSQPYVARSAYRTGLSDNLPYMYQQYAEVASVSPQTGSRAGGTVLTITGRGFPTLTPDSTETVTVTAAG
APCRVLSSNFTTVLCETGPASVLVPGYRAPVPAGGLKGLYPAMRGIEYELYNASINANCSPYTEGGDALNRLWRFNN
TIKLSDCNNSYSRILTGAWEAPPEDTQLELSTYNTKAFFIAPRDGNYTFHVSCDDQCRLNGTYQLANGTVVTGVPLA
YMPQWSSFNEWFKYDEQMSDPILLKAGQAVLLEATGANNRGPGGIQVGATVPSTVARYNSLPEIQTINVNGAALNRM
VEIKYMWGGGFKHVYNITVTAPTVALLVNSEVGVRVNITNSTNAAKRWDPRFMLDDKASDIARSFAYYLGISGDSVG
VSMSIVGSTMRLQVGISAPVFASTNFNFSIGFFMVAQPPFPPSPPPPALGCAYFNPRSLREMEADALAADGSSWLDR
RSLIPNITSWTVTIAASAPVAAVTPGGQWRLISGGSERVLDWNADAATVNAAFAALAGTGVDIASSYSKVEGAYLTN
VWTVVYPIWILNNVPAVTVANASGVPAGARVVMTVRNGTTALGGSFRVAFGSYCESVVINLTDSADDVQVKLSTLPG
VKAPLSVVKTGASYGGSIKWRVTFDPQGNPGNMPQLRVIDAKNVTGTDVRVFVEETLQGSTRQFFAPIPTEMTALAS
TVPVDLAVRVNNIPSACASPRGAAGCTFTYSAALTPSITAVSPAAYIFPSTAGFTTLTMNISGAGFNSSIAGNAVSL
DDDTQCAVTFASAGLITCQLPNTGAGGARLVRVRVAGKGFAGSANTAVVTVSVRVLAVTGVSPAVVSATAGATVLNV
TGRGFDDVVCDRNRVSVGAAACGVLTCAYDQLQVLCATPVVAGAAPASPTPVPVGVRVFNAAGALLDFSDNTTTTLA
DAAGPSIAVITPLSMGSSGGVIQLATTGLAAPFANGTDIGPFPLVWLVSGNVAAVNTSSATAMAALASALDNEDLRY
PCANATYDNSTGLITCTVPYVPSGTYSVALEPAPYNGGLNVTTWLLSRQAIVFTMTIAGVAPQAGSVGGGVVLVING
TGFAPGDRMRENAVFVTVPVSTTFLNGIVPCDVINASTTEIRCRTRAHIAADADATDPFALNVQPRATTANSVVVVA
CGQSFNSSATVDALRFYCWAQPTSARAVCTGTVGACTFAYTWAETPSLTSVATPNMDGVVFPGDNITVNGIQLADVV
QLDLRSPNGTTSGSCAGADLTRGATAVSCNIPAGTPAGVYQVVLLTSREGRSVDPRKVGRIVVTARVVAVAGSVGSL
GGGGSLTVSLAGAIFNTSFPDRNTITVDTLSCNITSFNATSANCTLPPIIGSVKAEFWNLPAESWSVDVKNYTKPDF
VTYYQSISNVWWSTPAYAMWPSGLSPDLDTILPDYFGGRFTFYMRVNRTARYSFVFNSIDDVARLYVDDVYIGYHGE
QPLRVNLTAGRPYKFEVMYYENLGGTSISMVWDAGDGGAIDRALPWGTVSPQPPAAPIDVTVTINGVPAVSACPAQD
LNLTMPGTSPSPIPGEPAVLSLPNNTCAYVFTGYRTPTILAASGLPAVLPGVVSFTGNFLTTAVADFSITVSGQNCT
GIVVRAAGAGFPTGSSIVSCTLPSLPGRVSKPILVSVAGRGVARAGRDLDASALWPSFPLAITSIVPDYTSHFGGFD

IYINGFGFVPLTGPMANMGRQMRVVLGGGTNPGNLTTVPMPIVSASTTQIRIKFPRILPAPGYIADFNIWKSITVVL
VDQNTDSSNWQSQWEAIQFDRTSTPVFNATTQAVVPAVTSLGAVNATTVNLSYSIYYGWSIAVNATPIGNFSRMSVS
LTPTPTALPAALNYTGVFPVCVNATVTNSSWVRPVYRESLSCTLPAGTPANVYTAWICQPGGTLGTGCSPAPGTVTV
PLVLTGISPRKGSYAGGMSVFIQGRGFVSNPALIQVFFGDVPCNVTAANNLGVTCLLGSALPAIPTGSNPIALMPSK
PLLAGLRISPADGAPLVNYTSANVTFTFDPVQTPVVISVSMTRGSTEGGTQLEYGLINMAGVFAQNVTVLLGDNITC
TNLTLTPGYLSSGDLGAVVRCLTPKPPTRQIGPKPVRIMITGRGMAACSATYEYVDLWSRRTTWGGGAPPVEGSLVS
IPPGINVMLDVSPPLLGALVVEGRLTFDESLTDEIELRANYIIVRNGGVLSVGTEDVPYGGKKAKITLVARPHEILE
LPLYGGKALAVRDGLVVFHGQHKTPTFTRLTQTADVNSTVLNLAGVVNWVAGDEIAVASSSFFTDESETAIIVSVEV
DDYYNVSTIRIDRKLNFTHLGVVEEIAGGAAGASIDMRAEVAVLTRNVVFQGDEDSERYMFGAQIMVNTPSTRPRAN
IRFEQAEFRQTGQAFRLGRYSIHFHMHGDLAYQSWVKGCAIHHTYNRAVTIHGSHRVILRDNVAYQCMGHTFFLEDG
IETGNIIEGNLGFSTKASPALLNTDTTPATFWITNPNNTYRNNVAAGSDAYGYWVRLLDHPEGPSYTTSVCPKFTPL
GRFENNVAHSNMFYGLRVHPEYYPKKEPCADNKFQQVPAVFNGLTTYKNGVKGAIATQVGLVVFSNITAADNGAGPK
MHIVNGKDNGAGIEFSWIVDDRNRANVPLDLMAGLRDSIIVSRTVGGFVGTAGQWPARDRKITGIITQSPPLGDPKH
SALLSITNVTFVDYTGGQFFALEGCGKCKTFQGGATSFMKQLRFVQSNAGSYPALSSWSYGHQAVYLDTDGSLINSQ
NLPPELMSALPWSLATPGGTWHSAVESELFDPAECVYVRGKGTSNDGAICSPDLVFRRIMLNEHLPTSIEFRDLRVT
SLATNRTSLVHFTKYNEKGYQFTAATHRDYWLQWESPFRVDPTTARFHKMDLMNDTDGWIHMSFKFLARNDRVEVNG
AVTAGAYPGPPPEGAPHGSSYYSKLLQPNSWWWGNFTYNDTKFTMYMAGDQDDILRVTPQACPVTGCTFDFNVTADL
RGTLFWSDDNTWLNRAGGKPKAGENVTVPFGWNLILDEDTPPLADVVVLGNLTFETASRNVLTANNIFVAAGGRLSA
GAPGTPLPPAARATIRLSGYRGDASRAMASDLVFGSKFISVFRGGTLDLHGSSVGSRWTRLGATAAAGSNYLVLDPP
QPGWAVGSSILVTSSSYNVWQAEERTITAVQNNGGTIFLDSPLVHPHYAFTASFSGAPTVVDMRAEVALLSSNVVLE
SLEGTGVEAAVGGERYGCRVIVNGASTARISNTALRYCGQAGLDRATILFDRLTALQGGAPNPSFVADSVLYKSQEA
AVFVDGDNFTSPITLSGNVIFGSYDKDTVEVMTSGNLIRGNLALGLVKIMDGVSGFDTRLPAVFNFHSARNNVTGNV
VAGSERLGYRLAGPPCSAVNTTSLGVGTPYVFLNNSAHSTLVGMVLEASNESLAEGCTALANFTSYMAWDFDLLTLK
GIETHVLLRRINSLNPKHAGVLVLKVGEMTQKAEVEMFDSIIAGRTHPEVCALCEQQGQPGCHPKLSVQSYNSWNPF
TPSVGLVSATFATEFTPGPEQKPWDGLKGYQTIHGIMRVYGTTFANWAGASACPGSAQGAYALANHPKAPDAFHPHY
LYRSNVVNVRSTTTESGLFLFTGPNPDWRNEADCGFQTYTQPDGSILELNCAGPKHAHFRDLDGTLLSGLTGAGWNA
TAGDTAVGYFGSPRSFPYDQGTPVIPGPCTFLPAIDGYACAANSSAYALPNADMKPVVPAAGGIGGNQQLFVLESR
DADTEDRNFGPVFFNVSGSIDMTVVAMDQGWCFAYTCQKRLSTFWTYLPIGHVVGINFTGTPATVFRTWLPYAAPEE
EIVVEIYYMMIPNRRFVWLPESGRVPASNFKPRPYDGATAHGDYYWDQNTTTLTVKITGGRTLEVRGENAVVVGWGL
ALSINQFYDTQELFLTNLANFLGIPRSRIYVAKIVPGSSRRRAALEGGSGGSSGSQVDIMIYDDPATSNNDPLPEPQ
IDTSGNVDSESAAPLLGASQSEREAARAEVLGQLSPPPMPPPMPVLLNMVVFDPPAPPSPPSPPLPPSPAPSPPPSP
APPSPAPPSPPSPAPPSPTPPGPPSPRLPSPAPPPSPGIAVSPPVPPSPAPPRPSPPVPPPSPAPPSPPQPPAPPSP
NPSGVSFTAALSNLKALIVDTNTASAVLGVTVAEVADVQVLAGVSSSDGGSGGADGGSGTNDDFNNGAGDLLKALGR
APPSPAPPSPDPPSPPPLPPPSPPTPPSPRPPSPRPPSPRPPSPVPPSPVPPSPAPPSPRPPLPSPPAPRPPRPLPP
SPRPPRPPSPPSPSPPSPRPPAPPSPSPPSPRPPSPAPPSPAPPSPVPPSPAPPSPAPPGPLPPSPRPPAPPSPSPP
SPRPPSPNPPSPRPPAPPSPPPKPPVPPSPPPKPPVPPSPPPAPPMPPRPPSPSPPSPRPPSPPPRPPSPTPPSPKP
PGPSPPPAPPSPSPPSPAPPSPPSPAPPVPPSPVPPSPSPPSPAPPSPSPPSPLPPVPPSPLPPRPPSPTPPSPKPP
RPPSPPPAPPVPPSPPPSPPVPPGPPPKPPSPPRPPSPSPPSPRPPSPPPRPPSPTPPSPKPPGPSPPPAPPGPPTP
PSPPPTPPSPEPPSPAPPSPPAPPSPQPPSPQPPSPAPSPLPPSPSPPSPRPPVPPSPSPPSPRPPSPAPSPRPPSP
SPPSPRPPSPAPSPVPPSPAPPSPRPPVPPSPSPPSPRPPSPAPSPRPPSPSPPSPRPPSPAPSPVPPSPAPPSPRP
PSPAPSPVPPSPAPPSPRPPVPPSPSPPSPRPPSPAPSPVPPSPAPPSPRPPSPAPSPRPPSPSPPSPRPPLPPSPQ
PPSRSPPTPPPWPKPPSPAP

>Cre10.g436800.t2.1|PACid:27579650
MKGSAWPLSGAALGLSLCVALLLAAGASAQLYITDVDPTSGSLGGGTRMVIQGSGFSEDRYTGSNVVFIGTYPCTVI
NHLTSDSVITCLTSPAPLEGKYDITVVVDGLDSVTYSTPYSYDMDYTPTLNFVFPSAGPPGVNPFVYGTPTWGINNE
CSSNADTGYMYSSTPACVESVLAGDYSATLPSGADGYTYLGYLSYYTSLYRINFTMPLDNTGMGLEGLGTAGNMNLT
ISFGGNAYGSQPYVARSAYRTGLSDNLPYMYQQYAEVASVSPQTGSRAGGTVLTITGRGFPTLTPDSTETVTVTAAG
APCRVLSSNFTTVLCETGPASVLVPGYRAPVPAGGLKGLYPAMRGIEYELYNASINANCSPYTEGGDALNRLWRFNN
TIKLSDCNNSYSRILTGAWEAPPEDTQLELSTYNTKAFFIAPRDGNYTFHVSCDDQCRLNGTYQLANGTVVTGVPLA
YMPQWSSFNEWFKYDEQMSDPILLKAGQAVLLEATGANNRGPGGIQVGATVPSTVARYNSLPEIQTINVNGAALNRM
VEIKYMWGGGFKHVYNITVTAPTVALLVNSEVGVRVNITNSTNAAKRWDPRFMLDDKASDIARSFAYYLGISGDSVG
VSMSIVGSTMRLQVGISAPVFASTNFNFSIGFFMVAQPPFPPSPPPPALGCAYFNPRSLREMEADALAADGSSWLDR
RSLIPNITSWTVTIAASAPVAAVTPGGQWRLISGGSERVLDWNADAATVNAAFAALAGTGVDIASSYSKVEGAYLTN
VWTVVYPIWILNNVPAVTVANASGVPAGARVVMTVRNGTTALGGSFRVAFGSYCESVVINLTDSADDVQVKLSTLPG
VKAPLSVVKTGASYGGSIKWRVTFDPQGNPGNMPQLRVIDAKNVTGTDVRVFVEETLQGSTRQFFAPIPTEMTALAS
TVPVDLAVRVNNIPSACASPRGAAGCTFTYSAALTPSITAVSPAAYIFPSTAGFTTLTMNISGAGFNSSIAGNAVSL

DDDTQCAVTFASAGLITCQLPNTGAGGARLVRVRVAGKGFAGSANTAVVTVSVRVLAVTGVSPAVVSATAGATVLNV
TGRGFDDVVCDRNRVSVGAAACGVLTCAYDQLQVLCATPVVAGAAPASPTPVPVGVRVFNAAGALLDFSDNTTTTLA
DAAGPSIAVITPLSMGSSGGVIQLATTGLAAPFANGTDIGPFPLVWLVSGNVAAVNTSSATAMAALASALDNEDLRY
PCANATYDNSTGLITCTVPYVPSGTYSVALEPAPYNGGLNVTTWLLSRQAIVFTMTIAGVAPQAGSVGGGVVLVING
TGFAPGDRMRENAVFVTVPVSTTFLNGIVPCDVINASTTEIRCRTRAHIAADADATDPFALNVQPRATTANSVVVVA
CGQSFNSSATVDALRFYCWAQPTSARAVCTGTVGACTFAYTWAETPSLTSVATPNMDGVVFPGDNITVNGIQLADVV
QLDLRSPNGTTSGSCAGADLTRGATAVSCNIPAGTPAGVYQVVLLTSREGRSVDPRKVGRIVVTARVVAVAGSVGSL
GGGGSLTVSLAGAIFNTSFPDRNTITVDTLSCNITSFNATSANCTLPPIIGSVKAEFWNLPAESWSVDVKNYTKPDF
VTYYQSISNVWWSTPAYAMWPSGLSPDLDTILPDYFGGRFTFYMRVNRTARYSFVFNSIDDVARLYVDDVYIGYHGE
QPLRVNLTAGRPYKFEVMYYENLGGTSISMVWDAGDGGAIDRALPWGTVSPQPPAAPIDVTVTINGVPAVSACPAQD
LNLTMPGTSPSPIPGEPAVLSLPNNTCAYVFTGYRTPTILAASGLPAVLPGVVSFTGNFLTTAVADFSITVSGQNCT
GIVVRAAGAGFPTGSSIVSCTLPSLPGRVSKPILVSVAGRGVARAGRDLDASALWPSFPLAITSIVPDYTSHFGGFD
IYINGFGFVPLTGPMANMGRQMRVVLGGGTNPGNLTTVPMPIVSASTTQIRIKFPRILPAPGYIADFNIWKSITVVL
VDQNTDSSNWQSQWEAIQFDRTSTPVFNATTQAVVPAVTSLGAVNATTVNLSYSIYYGWSIAVNATPIGNFSRMSVS
LTPTPTALPAALNYTGVFPVCVNATVTNSSWVRPVYRESLSCTLPAGTPANVYTAWICQPGGTLGTGCSPAPGTVTV
PLVLTGISPRKGSYAGGMSVFIQGRGFVSNPALIQVFFGDVPCNVTAANNLGVTCLLGSALPAIPTGSNPIALMPSK
PLLAGLRISPADGAPLVNYTSANVTFTFDPVQTPVVISVSMTRGSTEGGTQLEYGLINMAGVFAQNVTVLLGDNITC
TNLTLTPGYLSSGDLGAVVRCLTPKPPTRQIGPKPVRIMITGRGMAACSATYEYVDLWSRRTTWGGGAPPVEGSLVS
IPPGINVMLDVSPPLLGALVVEGRLTFDESLTDEIELRANYIIVRNGGVLSVGTEDVPYGGKKAKITLVARPHEILE
LPLYGGKALAVRDGLVVFHGQHKTPTFTRLTQTADVNSTVLNLAGVVNWVAGDEIAVASSSFFTDESETAIIVSVEV
DDYYNVSTIRIDRKLNFTHLGVVEEIAGGAAGASIDMRAEVAVLTRNVVFQGDEDSERYMFGAQIMVNTPSTRPRAN
IRFEQAEFRQTGQAFRLGRYSIHFHMHGDLAYQSWVKGCAIHHTYNRAVTIHGSHRVILRDNVAYQCMGHTFFLEDG
IETGNIIEGNLGFSTKASPALLNTDTTPATFWITNPNNTYRNNVAAGSDAYGYWVRLLDHPEGPSYTTSVCPKFTPL
GRFENNVAHSNMFYGLRVHPEYYPKKEPCADNKFQQVPAVFNGLTTYKNGVKGAIATQVGLVVFSNITAADNGAGPK
MHIVNGKDNGAGIEFSWIVDDRNRANVPLDLMAGLRDSIIVSRTVGGFVGTAGQWPARDRKITGIITQSPPLGDPKH
SALLSITNVTFVDYTGGQFFALEGCGKCKTFQGGATSFMKQLRFVQSNAGSYPALSSWSYGHQAVYLDTDGSLINSQ
NLPPELMSALPWSLATPGGTWHSAVESELFDPAECVYVRGKGTSNDGAICSPDLVFRRIMLNEHLPTSIEFRDLRVT
SLATNRTSLVHFTKYNEKGYQFTAATHRDYWLQWESPFRVDPTTARFHKMDLMNDTDGWIHMSFKFLARNDRVEVNG
AVTAGAYPGPPPEGAPHGSSYYSKLLQPNSWWWGNFTYNDTKFTMYMAGDQDDILRVTPQACPVTGCTFDFNVTADL
RGTLFWSDDNTWLNRAGGKPKAGENVTVPFGWNLILDEDTPPLADVVVLGNLTFETASRNVLTANNIFVAAGGRLSA
GAPGTPLPPAARATIRLSGYRGDASRAMASDLVFGSKFISVFRGGTLDLHGSSVGSRWTRLGATAAAGSNYLVLDPP
QPGWAVGSSILVTSSSYNVWQAEERTITAVQNNGGTIFLDSPLVHPHYAFTASFSGAPTVVDMRAEVALLSSNVVLE
SLEGTGVEAAVGGERYGCRVIVNGASTARISNTALRYCGQAGLDRATILFDRLTALQGGAPNPSFVADSVLYKSQEA
AVFVDGDNFTSPITLSGNVIFGSYDKDTVEVMTSGNLIRGNLALGLVKIMDGVSGFDTRLPAVFNFHSARNNVTGNV
VAGSERLGYRLAGPPCSAVNTTSLGVGTPYVFLNNSAHSTLVGMVLEASNESLAEGCTALANFTSYMAWDFDLLTLK
GIETHVLLRRINSLNPKHAGVLVLKVGEMTQKAEVEMFDSIIAGRTHPEVCALCEQQGQPGCHPKLSVQSYNSWNPF
TPSVGLVSATFATEFTPGPEQKPWDGLKGYQTIHGIMRVYGTTFANWAGASACPGSAQGAYALANHPKAPDAFHPHY
LYRSNVVNVRSTTTESGLFLFTGPNPDWRNEADCGFQTYTQPDGSILELNCAGPKHAHFRDLDGTLLSGLTGAGWNA
TAGDTAVGYFGSPRSFPYDQGTPVIPGPCTFLPAIDGYACAANSSAYALPNADMKPVVPAAGGIGGNQQLFVLESR
DADTEDRNFGPVFFNVSGSIDMTVVAMDQGWCFAYTCQKRLSTFWTYLPIGHVVGINFTGTPATVFRTWLPYAAPEE
EIVVEIYYMMIPNRRFVWLPESGRVPASNFKPRPYDGATAHGDYYWDQNTTTLTVKITGGRTLEVRGENAVVVGWGL
ALSINQFYDTQELFLTNLANFLGIPRSRIYVAKIVPGSSRRRAALEGGSGGSSGSQVDIMIYDDPATSNNDPLPEPQ
IDTSGNVDSESAAPLLGASQSEREAARAEVLGQLSPPPMPPPMPVLLNMVVFDPPAPPSPPSPPLPPSPAPSPPPSP
APPSPAPPSPPSPAPPSPTPPGPPSPRLPSPAPPPSPGIAVSPPVPPSPAPPRPSPPVPPPSPAPPSPPQPPAPPSP
NPSGVSFTAALSNLKALIVDTNTASAVLGVTVAEVADVQVLAGVSSSDGGSGGADGGSGTNDDFNNGAGDLLKALGR
APPSPAPPSPDPPSPPPLPPPSPPTPPSPRPPSPRPPSPRPPSPVPPSPVPPSPAPPSPRPPLPSPPAPRPPRPLPP
SPRPPRPPSPPSPSPPSPRPPAPPSPSPPSPRPPSPAPPSPAPPSPVPPSPAPPSPAPPGPLPPSPRPPAPPSPSPP
SPRPPSPNPPSPRPPAPPSPPPKPPVPPSPPPKPPVPPSPPPAPPMPPRPPSPSPPSPRPPSPPPRPPSPTPPSPKP
PGPSPPPAPPSPSPPSPAPPSPPSPAPPVPPSPVPPSPSPPSPAPPSPSPPSPLPPVPPSPLPPRPPSPTPPSPKPP
RPPSPPPAPPVPPSPPPSPPVPPGPPPKPPSPPRPPSPSPPSPRPPSPPPRPPSPTPPSPKPPGPSPPPAPPGPPTP
PSPPPTPPSPEPPSPAPPSPPAPPSPQPPSPQPPSPAPSPLPPSPSPPSPRPPVPPSPSPPSPRPPSPAPSPRPPSP
SPPSPRPPSPAPSPVPPSPAPPSPRPPVPPSPSPPSPRPPSPAPSPRPPSPSPPSPRPPSPAPSPVPPSPAPPSPRP
PSPAPSPVPPSPAPPSPRPPVPPSPSPPSPRPPSPAPSPVPPSPAPPSPRPPSPAPSPRPPSPSPPSPRPPLPPSPQ
PPSRSPPTPPPWPKPPSPAP

>Cre10.g432600.t1.3|PACid:27579678

MALQGMRSNMTRGAIALGLLMIALGAAKGETRPNIIVIVTDDQDYMLNSTNREFMPQLNRHLADSGLQLRNFLISTA
ACCPSRSILMTGRYTHNNNVTSNIEPHGSFWKFMSQDLDADYLPVWLQRAGYRTMHVGKFLNAMDPTDSRFRCPRGW
STWDALVEPYVYMYYTPAFSLNCGPTQVLENQYSTDVISDKVDAHIRQAVAASAAAASPAADGSSGSSTGGSSSGSG
QPFYLQITPIAPHTQCDYINARGTCVFPIPAERHRTLFSHAVLPMNPNFNVAPPAELGLVNEMTSDTGVQKHFLARI
RALAAVDEMIGRMVNTLTELGVLDNTYILYTSDNGFQLGNHAQKQGKQFHWEEITRVPFYMRGPGIPPGLVTDWQGN
MVDIPATVMALTGAGVPSLADGSPIPLPDILPNGFPRPYNTAPPAAAPPPSPPAATAAGAAGRRLLRTAGGGAGLGA
GTLTYVESGFEGPDLLVGEVVEVAGEAAEAALGGGGGGGSGGLRRLAQAAPTGFNRGMRDSMPIEMWGHAWDRRITL
KDYRAIRICTSHLAFGAGQDYIRALPNATLGWGDTFFGTAARAYAGPGLNGGISSYAFVPPPDDGTPSDEILPARRR
SAQQQDGTQGPWEEVDGKVEQSEEEEAARLAREHEIELHVKGIIESVRQRLRADASSGSGTSGSSTGIAGAAALAPQ
AHRLHLAFSYSQPVDAQAWQEHLEATCVYVSAATGAAACTPQHGGSSSSSSGGGQQAAVGGGGGGSSSYLASDGLSS
VLELDLLQPAVGLPAADFASAVADVAARPLEFLPLPYRTHNRVVSISSSSSSSSSSSGSSSVESGAVSAAAATMGQ
HRRVLLEADAPSPPSPAAASAPIGDAARPPPQPTPSPSPSPSPSPSPAQSPSPSPSPAQSPSPFPSPAQSPSPLPSP
SPVPVPAPAPVPSPAVVLQPPEPPPTPVSEPPPTPVSEPPPTPVSEPPPTPVSEPSPLPPPPPQSVDLQPPLPVYAP
PVASPRPPSPRPPPRPSPPPRPPPRPSPPPQPPRPPSPAPPQPPAPPPSPPLVRQLLLTLTYKSPMSPTRYPTQQDS
TCLFLKTALFAASCTPASSAGPGGAWLADDGLAALFSVVLVQGTGTTGPEFESLVLELQTRTETFLGPSYRQLYDID
TLVISWPSPPPPPPPPPPTPPSPPLPVALRTEVLPVGLLFYQSVLEQVYVDPAAYLRLISPIACAAVRKVASQAVPA
PVQVVECGGVSMELGGDPKFAVFGVVAPDLPDLPALRAVLEVIRSDPLPYFANSLPVLGLVAVLPAVMPPPSPPLPP
SPPPSPPSPRPPSPPPSPPSPPSPAPPLPPSPPPLPPSPQPGTPPSPAPPGLPQPPSPSPPPSPSPEQPSPPPPSPS
PPPYAAVARPPPRPPRPPFWWRPSPPYPHSPPLPPRPAGEFVDSPAPPPYASSPALQPPPGYSPTQPPPYFSPSPSV
PYGSYSPPSPSPAAPYGSYSPPSPGPAVPYGGWSPPPWPSPEPASYPPPSPPYPPSPQPSPPSPPQPPSPRPSPPHP
PSPRPSPPPRPSPPPAPSPPPPVPMPPAPPAIIEVVLTFTYNLPMDSTRWYTQQDSTCLFLQIAVAAGSCAPRPVAL
AADGLASSFGVALVQGPFIERQLFQLLGQQLVNEPGSFLGFSYRQLYEIDTLGISWPSPPPPSPPEPWAPKPPSPPP
SPPSPPKPPGPPPSPPVPPPVPPSPPSPPPSPPPPPPPRPPRLPPSPSPPTPPSPPPPGPPPPSPPPPGPPPPSPPP
PAPPPPSPPPPNPPPPSPPPPSPPSPPPPRPPERRGTLGTCYKYVVWCMSNFRELYDLVLDPYELNNRISTAPVALI
DRLDALMTAAGYCRGGGGCSNPYAVLHPDGSVTSFEQAMDPKYDDLYSKLRKFQFQRCSLSYTPNNEVSWYTPLLKS
PPPAKKKQ

>g10449.t1|PACid:27579684
MTDCAWICSNECPSGSAYTGQYSGNNCYLPGKSQCQCCNKEDTIECAAYKQCIWAPDVPSPPPPGDRGGCSYDNYEA
YVGRDQLDYTCEVSTPMTVTASVSSEAGDSLAIEVRSGSSCSGGTLQTTDRSSNPSLSYGPLSYGTFCVSVVCKNWI
EACGNAVISISYSEALSPSPSQPSEPSAPPPPGDRGGCSYDNYEAYVGRDQLDYTCEVSTPMTVTVSVSSEAGDSLA
IEVRSGSSCSGGTLQTTDGSSNPSLSYGPLSYGTFCVSVVCKNWIEACGNAVISISYSEALSPSPSQPSEPSAPPPP
GDRGGCSYDNYEAYVGRDQLDYTCEVSTPMTVTASVSSEAGDSLAIEVRSGSSCSGGTLQTTDGSRNPSLSYGPLSY
GTFCVSVVCKNWIEACGNAVISISYSEALSPSPSQPSEPSAPPPPGDRGGCSYDNYEAYVGRDQLDYTCEVSTPMTV
TVSVSSEAGDSLAIEVRSGSSCSGGTLQTTDGSRNPSLSYGPLSYGTFCVSVVCKNWIEACGNAVISISYSEALSPS
PSQPSEPSAPPPPGDRGGCSYDNYEAYVGRDQLDYTCEVSTPMTVTASVSSEAGDSLAIEVRSGSSCSGGTLQTTDG
SSNPSLSYGPLSYGTFCVSVVCKNWIEACGNAVISISYSELPSPPPPRATHSPPPPRLLHSPPPPPPRSPSPPPHSP
PPPPHSPPPPPPHSPSPPPPHSPPPPPPHSPPPPSPHSPPPPPPHSPPPPSPHSPPPPSPHSPPPPSPHSPPPPSPH
SPPPPSPHSPPPPSQHSPPPPSPRSPSPPSMHSPPPPSTAGVTVIGSPLPTSSPPRASLPPPPLPLPPPSPSPAGFG
AIGSPPLPAPTSPPRGNCTYDHYYVKSIGHYDKVNFICSNYPVGDAPLIEVVVTAESSVKDVLLIEFRSGSSCSGTS
IAESSRSSHPSFSYSFVPRSTNNCLSLACKNTASGCKGAWISIDYRRPARSPPPKLSSSG

>Cre10.g431050.t1.3|PACid:27579820
MQRLRVQMVSIISRDTQWESRLCKVDVRTRGGQSYSFGEHCPGGLAQTLDLFPGERILSSTLVFRQRARVARGNVRL
SGNDTFVDGALCLKTSYGDRLASGYATCDRTTDITSDVASGLLAGIGGSVALDDGGSEYITSLYLMMHPWPDRLTYN
INSFRLLDPPTRQNVGLNRCEGRPGYCAMRDDQVLASCDLTTTLTRTFTLHTSDSFTLSFGVSNSAAVSLSNTTGNE
FGTNRQVTEDRSNSTGAVTGVTVSRGNSQGNEVANGISFSKQRSDSQSTSEGSADTSDWSDAATSGTTNSFSRQESS
EDTTSTEQARMQEQSRGRTQTSSESIGTSTTDESGGSEMSGTTSNNAFSNSVMAGTENTAGTTDTSTQSSTDGSQHS
ESTSVSGTLNAPFGLGSATAGHTWSDETSHSTTTGSEHATSSSSANTRQNTAGTENSFGSTAERTSTWSQGRTSSRE
RTTGEELSDTSTTSNTLTQGHSQTLGQVLGEDKTFSTEQTSTRGGSSEVNRQNTYGVDVTQGSTQDQSRTQSLISTS
DVSNNAELNQQLTESASKSVGDSQSSFQNVMTDRTNQISKDVNNATESTQGADYSYEISTTVGSSVQFDPTKLVISL
YLTVEEFVDARYTALVDITTRGWGGVITLPTFGVMNGVFSNAQTTYDTTDIIDCGGPKPWDIVAQGMDIIYENSQSK
LLLSRSPTSYRNAADACKYYGGLMVSVATAAKEALVVKALDSAPQSEVSTDLLWVGAYKGLPSVGSDSVWLWIDGSP
FTYTDWAPNPKVQGTSTCMSFKRQVDPNGPQQRWGTVSDCKELQTFICEIKTPQTQCPTGMAGTPPSCVPCTGDQFT
DAPGLLDCLTCPDIADVKDLDRNGYNDACLCRVGSAGSYETGCTPCDNTTYAPNQGLPACLTCVNGTAADLDTLPGN
EACVPLTPSAGGSGNSSARRRGLQSSQQGQRQASASGGGAGAGVASTGAGSNGVSGPLPAGLNLRSEWERPARGVRE
AVPFDPVLIRELKHRRLLTQSNRRSPPPPQKQQSRPPPPPLPPSPPPGPPFPPHPPPGHPPSPRPPSPSPPSHADLC

GRLWQQLEAVKDGNYTAFNASGGAAALPGRTARGSCAAVVAANALLPTVNVSAFIDEEGGFIPGTNYLDKICISNVT
VKGCIQELAPVVDAMVQFQCCDQLAKVVEAFRPPRALCISRHFMPLRSAFGSYQDVAQCEGHVRLVNGWPDGSSGRV
EVYHNGRMGTVCDDGWDDDDATVVCRQLGFDGGYAEWGGAFGPGFEWQPIYMTRVACNGTESGLAQCTRWGNRGSLK
WGNHSCSHWEDAGVTCFNCPSDRCPRQNTLRLAANRTLQAASGAIWTEGRLEIWHNLNWGTVCSDWFHRVNADVACR
LLGYEAGMLLPPDLAAPYLLSVNGSGAGAVAGAAPVNHTASSVPIQLDNLDCLGDESSLAACRRNAWGFHDCDHSQ
DVALRCYRYPEGTVRIVDGWEGLQQPGVAVAGRLEILRGGTFGGVCADGFDAAPDGLVVCAELGFSAATVGGNVTQW
GPPLGLPAQMTSVACQPDSRTLFDCGAVLPPSAALGGNGTAANGTAEPLCSPSQYVNITCYNPSPPPPSPRPPHPPK
PPSPHPPKKPPSPHPPKKPPSPHPPKKPPSPHPPKKPPSPPPPQPPPPSPPPPSPPPSPPPPSPPPPPPPPSPPPPSPPPP
PPPPPPPSPPPPPPPDAPRGVCWPGQDVLGTDVDIRRDTVYNEGSCRDACIANSACTYFILLTDGGCVLKNNLLAGP
NGSDQSGASWVAETCLQRTNYAGKFLCVAGYDVNGRHVNGVGPFQVDSLDACRRACEQQSQCLFFLYFTDSNCALKY
SAFNGDWGNTGPNSYLEWACFRVFA

>Cre10.g431050.t2.1|PACid:27579821
MQRLRVQMVSIISRDTQWESRLCKVDVRTRGGQSYSFGEHCPGGLAQTLDLFPGERILSSTLVFRQRARVARGNVRL
SGNDTFVDGALCLKTSYGDRLASGYATCDRTTDITSDVASGLLAGIGGSVALDDGGSEYITSLYLMMHPWPDRLTYN
INSFRLLDPPTRQNVGLNRCEGRPGYCAMRDDQVLASCDLTTTLTRTFTLHTSDSFTLSFGVSNSAAVSLSNTTGNE
FGTNRQVTEDRSNSTGAVTGVTVSRGNSQGNEVANGISFSKQRSDSQSTSEGSADTSDWSDAATSGTTNSFSRQESS
EDTTSTEQARMQEQSRGRTQTSSESIGTSTTDESGGSEMSGTTSNNAFSNSVMAGTENTAGTTDTSTQSSTDGSQHS
ESTSVSGTLNAPFGLGSATAGHTWSDETSHSTTTGSEHATSSSSANTRQNTAGTENSFGSTAERTSTWSQGRTSSRE
RTTGEELSDTSTTSNTLTQGHSQTLGQVLGEDKTFSTEQTSTRGGSSEVNRQNTYGVDVTQGSTQDQSRTQSLISTS
DVSNNAELNQQLTESASKSVGDSQSSFQNVMTDRTNQISKDVNNATESTQGADYSYEISTTVGSSVQFDPTKLVISL
YLTVEEFVDARYTALVDITTRGWGGVITLPTFGVMNGVFSNAQTTYDTTDIIDCGGPKPWDIVAQGMDIIYENSQSK
LLLSRSPTSYRNAADACKYYGGLMVSVATAAKEALVVKALDSAPQSEVSTDLLWVGAYKGLPSVGSDSVWLWIDGSP
FTYTDWAPNPKVQGTSTCMSFKRQVDPNGPQQRWGTVSDCKELQTFICEIKTPQTQCPTGMAGTPPSCVPCTGDQFT
DAPGLLDCLTCPDIADVKDLDRNGYNDACLCRVGSAGSYETGCTPCDNTTYAPNQGLPACLTCVNGTAADLDTLPGN
EACVPLTPSAGGSGNSSARRRGLQSSQQGQRQASASGGGAGAGVASTGAGSNGVSGPLPAGLNLRSEWERPARGVRE
AVPFDPVLIRELKHRRLLTQSNRRSPPPPQKQQSRPPPPPLPPSPPPGPPFPPHPPPGHPPSPRPPSPSPPSHADLC
GRLWQQLEAVKDGNYTAFNASGGAAALPGRTARGSCAAVVAANALLPTVNVSAFIDEEGGFIPGTNYLDKICISNVT
VKGCIQELAPVVDAMVQFQCCDQLAKVVEAFRPPRALCISRHFMPLRSAFGSYQDVAQCEGHVRLVNGWPDGSSGRV
EVYHNGRMGTVCDDGWDDDDATVVCRQLGFDGGYAEWGGAFGPGFEWQPIYMTRVACNGTESGLAQCTRWGNRGSLK
WGNHSCSHWEDAGVTCFNCPSDRCPRQNTLRLAANRTLQAASGAIWTEGRLEIWHNLNWGTVCSDWFHRVNADVACR
LLGYEAGMLLPPDLAAPYLLSVNGSGAGAVAGAAPVNHTASSVPIQLDNLDCLGDESSLAACRRNAWGFHDCDHSQ
DVALRCYRYPEGTVRIVDGWEGLQQPGVAVAGRLEILRGGTFGGVCADGFDAAPDGLVVCAELGFSAATVGGNVTQW
GPPLGLPAQMTSVACQPDSRTLFDCGAVLPPSAALGGNGTAANGTAEPLCSPSQYVNITCYNPSPPPPSPRPPHPPK
PPSPHPPKKPPSPHPPKKPPSPHPPKKPPSPHPPKKPPSPPPPQPPPPSPPPPSPPPSPPPPSPPPPPPPPSPPPPSPPPP
PPPPPPPSPPPPPPPDAPRGVCWPGQDVLGTDVDIRRDTVYNEGSCRDACIANSACTYFILLTDGGCVLKNNLLAGP
NGSDQSGASWVAETCLQRTNYGKFLCVAGYDVNGRHVNGVGPFQVDSLDACRRACEQQSQCLFFLYFTDSNCALKYS
AFNGDWGNTGPNSYLEWACFRVFA

>Cre10.g434350.t1.3|PACid:27579855
MFALCGNSSLTPPIFSALCSTWRIASSLCSDDTDVVASASELCSGIRAAYADDAQYRSCLQPYTGIRSTSKVLADTL
GACADMNGEPMGGCDTCTSATACANPLVSYTDGCIDMDMGQCTNWRSFCNSDAAPTGAAAAVLCRRPGPARNSVVAP
PRPPSPPPRSPPPLVVSASPPPPEPSSSSPVPSPSPALVDPTAASPPPAAVVVPASPAPSLAPSPAPAPSPSPSGTL
QSDGSGSGDSSTPAASPPPSTSLAVVCVADGTNPMCVSYVYPNTSVAADLLNLCAAMPYMPGCSIRDACRVGTVTGD
FCRPMTLLASICLDMPTMGGCKSYRGLCGNGTVVQQCRDFPAIPGLPTTKEAQASVDAMCASMYMERCDSCNQVSCD
NYIDAMSSMCTDMPYMEGCPTWADWCKAAAGWEGAAPGNSMAGFCAGASAYGGSSGSGIPSMLMFFHQRTKELLLFR
EWMPTNEGQAVASFIAISAMGVAAVGLRTANSILQTAAAAGRLGPRLAPGSAAGGPWWLPSGSQALLNLISSGLSLL
VTTLDLFTMLIAMTFNGAYFAAVVLGYMLGALFLGHLRDAYARHIGRPPSAADAGAAAELGQGCCAGAPGATGLLAG
LDSSDESLSGRPAAQAAAPGKAGRAANQLQSSQQHPGGEVALSGAGVAYGRPVVESTTPAAEGCDGSVRATGLAAAS
SEKPGAVGGPLPIAPPQQLGSGYGHVPQHQQPFHKQQDALHHHKLHADHLAQSQGYAVQRHDNGLSMGSSADSA

>Cre10.g428966.t1.2|PACid:27579862
MPPQLLPVAFELLPPYSPSVPDGERHKYDSVRVLLEEYFVPQRKIVRNAVYCGRSGVDPTRKLDASTKYEACAVAGL
WLGPELVCGAALRLNFPPTSCAGLGRAPPAEVAAALVAGRWRPQLQVLFMRTAPGHGRRGLGRLLSCLIGRQAARAQ
HELVSVAMSGDDAAPFWTNAGFAAPPLQPPQLPPTASLLHRAEAARRAAAARRAVTDGEPHAMRCEVWVRSLLPPLP
SAEPSHREEQEEGELVGEGEEGSVMEDADEETEESVPGKAAHTRRLAQHLLQALSHAKEQVRAAAAQVVAKYQQAAA
AAAAAAAAGETLLAAAGTQAVPPPDVSLLQLDEAVAATDIATADADAAAVEATRPAQLYACAGAAVSAHAGDAARRG

AKRKAAGRRQQQEGESAAVAGTGSSGAVAAELGRAVGAASGSRGAADARTVTTSSCGNSAGALDPSEAAAAAESAAP
ESLTPVQQQRKRQRRQQPSRTSTPEAAPAPAPAPAPAPASGLASPVMAPQVAAPAPARTAVPWTSSRRVAAAAAPPSQD
DRAAGGARTARTAALGTAAAAPMPLPSPAVAISGAVDTSSVSLIRWWEEPPGGQNAEELRQPPTPAATAAAATRPPT
PHASPVLLLQQPARNSPRLQRHPHLTQPPQLPPQPPQPMPPLLKLSLQQEEHQRVEAPGRLSSPHGVRPWQPSAQLQ
AQTPPTRPGGGSGRGVGPGGSLTSALLAAAGFGQARSVGPGASDPEDQAACQPPLEPEPEPEPDLGLELEEVAGFGR
ALVDLIGRFEGIVKRQHRQQQEHQQQRWRDQGQMQQPKAGCDAEQAGAPAAAVADACTQHDTAVAWGRKAASQAGAP
VVAMADACTQHDTAVAWGRKAASQAGAPVVAMADACTQSDKAVAWGREAASQAGAPVVAMADACTQSDKAAAADDRF
KEAAVQAGPSVTESGTCTAEGGPAGAVAVAAVRACDEQLAASRTPADADGEKAAAVRSSDSDGSVAVAAGDAVMTEG
REAPPPPPPAPLLPPPPLKPPQPSQAPQPPRPLQHCGQVVEAPRAVELRDTKGSATATPKQDGQQQAAVQRMQAAAG
LALLAPVSPPSAAAAGARGPQGSSPVTPSTAAGCRTAVASYGSCGGGGASRGGGSGSESGGSAIREVDNSAGEGLAA
FYDATEEAEQEQAQRAERLLRWARLRANAEKPADPTAAAAAAEPAQAAVAALGRPLQRPMPLPPQVPASPPPQLCGI
GSPVAAAGAAFGTEAPPAPAPAACREAGAAAVVPRGAGDRAAADGEQPGVEMAEEGPPDSTLSAGECASDGTPVAAP
TAKGSASLRKVLARKLGVLGNTSTSSTRCRQSRWGAPVSAAKAPDAPPKTPQPQPQQPQHVWLAQNLLALPQLPVLP
PAPPATASSATAAPAAPRLQLQASFEAPSPSRASAALVASTAHGTAAAGPDASATAGQGEAAPRCSEPVVTGVLHPR
RGLGLARGTQASSSTSSRRGSRWGSPPPPAATAGSAAAAPLAPPPHSPARAPLGHTAGDGGVPVLTPTRVSDGPRAA
EQRAPQPLQPQAGGPPLPPSHQRPELTAEVACLARAATVTTCHPTSVTWAAAGAAGPNVDASAGTAAAAPHAGNGTE
RGGAAAPADVGEVSGGGGGGGGVIPVAVDRPVVTVGAGSSHNNRPGPSLAGGGQLRSPAAPKVLAPAPAAATVAAEC
REGPPIERSSPARVQQAAAATPRPLSGAAPGAAAVPAAAAAAAAAAAAAAAAAAAEVVGVRGTGKAVSDRGRVVRES
GGPRSRGSCAQPCSGPAHEGHQSPRHSLKRSRSHSHSHSRSRSRSRSRSRSRSRSRSRSRSRSRSRSQGRSRGRSRD
RGKGQDWTVGQALVWKRRKSLSGSRGHSRGRSPPGVRAESRAQEGLDRRGRSAGGSQGRPRSASRGRPRDRSRSRNV
EWQCRRSPGGRREESRRRGPDADQAQQQGGSRGAGGGRSSSRDGQRGSGSSRSPSRGQQQQTQPPAVLRPDSAPTTG
VEATSQHGEREQSYQVLSYLSPELVSRSARGDFSWLGVKKGLQGGITYQRSWQEGLYAPGIACVLVKLVGDVTHAAL
FRNARMLRQTRGVKIAPFLAPEVRALRDQRMNQYIALQCAGLKPKWDGVTANLSILNGRGARVLYQF

>Cre10.g423850.t1.3|PACid:27579885
MAPTSFRLLVLALAIDVTVGGRLQKLERRSLQDGTLRLVPDAYQGRVEVLYNGVWGTICDSGFSDRTANVICRQLGY
AGGRAHWGAWWGEGSGPIQVTNLQCKGTESDIQECSSSPWGAESGCSHADDAGVVCFDEQPVRLADGTAPGTGRLEV
LSMDGIWGSVCELGWRPENTYKACTQLGFPDNLDVAVLVGGSAFGSGDLRVAMADVACSGLERYFAACSFAGWGVSG
GCDPETQTVGLDCLGTLGHSPGPDQNQPPEAAEASPPDLPPPSPSAPPMEGDAPPPRRPPFNPAGDGAAPPPGNEPI
GADMPPPPLAPPPNRPPLSPDTPAAAPPPDNAPDEWSPPSDSPPDSPPNHEPDPGTDWSPPPPPYVIPAPVPDPSPP
PYYGSSYPPPSPPPPPYYGSSYPPPSPPPPPYYGNGVPPPYYTTSPPAPVYGPPYGSAPPPYYNHTHKPPAGYPPPG
GQSGHQAPPLPPFPADDNQGSYLEAGTIGFPPPIRPGSSGALFLEVLCPPGFYATAIQVLNQETASGLGLECISIPA
CADPPPPPPSRSIKIHYRAPGCGPRRSRRSLRGLGSELAAEDPDSRLSSGSQQESNGQLRSSTAAGGSARVRSSSRML
GSATDVGAIVGNAFVLSTARLTDGAGNTVDQLAVYFGDPSSPDLQTKVVIRLNSEWVDGFSVPDYRFDCPSGFSAVR
AQTGGMSQPPTRVGFRCGSDAAAAGGWTSPELGVGDYGPAVPLSEVQCPEGSRLAGLLASTRRIWDGGPLLVEAFTA
YCTKACAA

>Cre10.g417600.t1.3|PACid:27579905
MGKSGRGFKWHLSLLAVVVMSSALKGSAQPGRPAKIATMPTAPPPPAPVAPATYPSYPLRPPPGPYGPPGPTYPPTY
SPPIYSPPMYSPPIYSPPIYSPPIYTPPIYSPPVYPPVMPSPEPSPEESPAPSPEPESSPSPAVASPLPLPSPSPSP
SPSPSPSPSPSPSPSPSPSPSPSPSPSPSPSPSPSPSPSPSPSPSPSPSPSPSPSPSPSPSPSPSPSPSPSPSPSPS
PSPSPSPSPSPPPSPSPSPSPSPSPSPSPSPSPSPSPSPSPSPSPSPSPSPSPVPGPDDSPREYKCKPDVGLWNGKVIADIKL
RPPHDDFEAVTLCEKECNTRDGECTGYHYEKDGYCTLWSGDVRFTRGVPSNIIACIFIGYKMPDPPSPEPPLVPLPP
PPSPEPSPSPSPSPVPAPEASPNPSPSPSPITIPSPSPSPLPAPLPMPSPSPAPVPSPRPDPSPSDDSPRNYKCKDD
VGLWNGDLIDRLKLRPPLNDVDAAARCRAECNKRDGVCAGYDYASDGYCKLWGGDVRYNRGSPGDIIACIFTGYKMP
DPPSPEPPSPPPPPPPPSPLPPAPPPPSPPPPSPPPPSPPPPSPSPPSPSSSPSPSPSPSPSPKSSPSPSPSPKPSA
SPSSPGSGRSYSCKNGLSLWNGNQLEVITIKLPTNDEAVAKCQENCNNKPGCTGFHYKTTTRCYLWGGANLLFTKPY
PSDVMACIWTQQFSRSSSNR

>Cre10.g451250.t1.3|PACid:27580046
MRLPLLLLLVVVVVTATPSRRQLNEVDPRHELLVELYNATGGPRWSHEFVSPAPWGVAPDPCDWAGVICCTSSGLSG
QANLTYTYTRRRRTDTPAEVVVTRPCGAAGAVVGLALPAANLSGSLPASLAALQLEVLLLPDNPGLRGPLPSALWAV
ERLLEVDIRNTSMLCKDAVATGGSSTDTSGGGAAAALLSACTVPVPLLASRATLVFTSTGAEGSLPLVPPGTSYACP
LLHYRNSSGAAASVNSSISISANTTTAFTVAALVPSSNSATASTVATAGTSSNIAGGLAAAGPRELDGDGVPAGAAV
VHADPRYWLYQSCACLQLPGVVATAARPPSLLLLVAGANATAAGDLAAGSAGSGEVVAASCAAAAAALSADDTDASG
NGSSGGSGGQVSLSVGALVGMVVGIAVGAALLTACCGVGALLCYASLRSRRQRGDRGRTSSAKSLRSRAARGGAGAH
SGAPVWWSALAGWLWDSDGHAPSADWEGLVGAPGGSMSQSSRLHGAAAAGQQLLLTGVSAGVGTGLLGDMPGPSRKP

MPGSLFEAKRARPPGCHPELFGHEVTLVATDVEGSTELWEWQPDVMNAALALHDRTVRLTLAACCGYEVTTEGDAFL
VAFHEPVDAVRWALLLQSALLKLDWPPLLLEHSLCRPRPLVPVAGLGKDGAGRDAPLLVLFKGLAVRMGIATGVPSA
VREHPVTRRMQYTGAVLRLATGIAELGCGGQVLLEPMTFKGIHNKFEELDVEEIEVQELTDLISGQPWGEEEQVGRN
SRSLQHQHQLQPLQPPIGGRESLVLSEHNAGSYSPAGAPGHNSTGGGTGLSSYGVYGSGPAATGGPGGPGSAGRGAA
AGGGGGGAGGGGGPGSTMGPSAARSAPGSAVEGDYLSSATSSPALHQPHPHAPPQPMPQQQHHHHLQNPPHHYPVH
HQHPHPHQALNQHPYMYPHPASQHAQHPQHLQHVHAQLHTHSYPHLHPHQQPRPSRDAAATAPVAGAAAAAPSGGPP
VHRGRSAIALGQDDVVFLPPGACRTQQSIPRQHSNRMLTLAGRPSVTGARASTGGHTDGSGLLSPPHGPTLASTATG
STTVSPGLALIERSAWEAVAAAVPSTMAAPGVGRTSGNRVSGGRRSSGNRASAERDAASRGFAGPSISNGGGPPLFS
APLPSSVVDGLHSAPSQTTGSAGKLPLLLQSVAASQALHAPADGAGGAGAPSREPTARASGFVPGGGGGGGSTSSAG
FSLALGSSVGVFAGLGPGSSLVPPSPTAPYLQQVMNQVQLQNQTQALVQQQQQQQQYQQYQQYQQLLQQYQQPQQQP
QQYQQQYLQEGPGPNLEPALGQGHGQGRMPTQVQVPGQSSAQWEGHALEPGASSGAGVAVSAAFQRQLLSTQDLVSS
VGRSASGLSIEMSSSAAPSRAGTAFGAALAEPGAAGPGMGGAAPAASPSPAVVLDQGHAIGGVTIEGYVAPAPPLPQ
PPPPPRPPPPAPPPPPPPSGRGPVLEPGQLASSTAAAASASTAAGPMCEGRELDPRLSNSDKGQNGDRAVVAAVAVA
GAAAAAAEAAQAGAGAAAAGAAPHGAATPPGGATPPSQPMLPVQPPSSSSPGFVGITLASTSPGPHQLLQPALALLP
APSAAGSDSTAAARSSPFSAVQMAAERSFRRSPAGSGGGPPQLHRLASMSNAGGGAAALLPRGSATATSSVVPSAAA
SGLASASAPHGSGSGSLGEGAPVGLPSGGNAGASSSPASTALSSADPPFAAANPHCAPGAGRASAAAAAAAASAEGG
KNGSSRMAPPHDRSTGQLSLVASGWEGALGPTDNRTARAASKLPLAADGVRLFEGMAAPATSSSSMLAAARVSTGSD
NLSGAPLPAVSAEPITPPHQDLQQQAQPHSAQEGGGRAGGANASTASGPSASSPLRTSLSLAVSGREDATWSPGHMG
DTGVLGVSLARPGALSPLHPDSATAAAGATEGSGDACYYPHAALVGALTGGGSVCIGASAGGPSSDTAAASWQQHGR
YTAPQILEGAAAHSLAAGPGPGSGPGPMHAAPYALLPHMPGYHACHPQEQAAVYYQHLHLLQAQLGQQQPQQLQPHH
QQHGSIHGPHAPHPLPVDPRGGSSTNASGLELPDLVSPRRMHLYAFLAGTALPSTAAAGADAAGGGVAGAGPDRGGV
SGLAGLPGVSGLAGTLLARHGGDAGGADGFQSAEGGRGGAGAASVAWRRSSDPDNGAAADAAVTDAAAASRTAMLAT
VAAAAAGSSGGGVAAQLLRNMGDHSMRNLMDLMTLAASYGGGSGYPGGHGSSAAAAAGGLYDSIGSGGGTGGTGQGP
RLLALRSAMALQASGSNANRAAKEALDLSMGMDTDFMDPISPRVGSASAAPVPSQRSGGATQPAQSQAGRASTTSSS
RFRPSRKTSTRSQLLDLLRAPSFGGRPSGPQQQQQPQVGALQEPSSQQKQQQQHRASPAGHMEAAAGGNAGCAQQMW
GPGMAAISPTSQAQSPSLMPPTSRTSQAQLRRFGAGASDPESHRTQQLSKSHTAVLVSGGTDDGGSVRGGTMGEAAG
PLTTSEAVGQLDMDPLACVAEEGQSGAIGSGSRGHGASGIGGAGGGDGGQQGLPSGIFEVTLRGGDTTQESGQLLQA
AAPSGAERAGQAVLTPMGAGAKADAAAAPTAAVEAVASRPDLGTVYGSNSGSGAAESPRTDVQKLKERYTKRSFLKS
LSTRRRNAATAAAATCVPASDREPRSPSSRLRLRGVAAGLLGGSSSKDGGGGASASGAVTTGPGSATVSRRQLQAQT
TTASSLRSPLAAADRHSVVVVDMGTHQLKMPALGAAYNDIYDGVQVIQILPEHLKFRAAYNPPLKSLAQLSPSFFDA
PGARQALTPLAPGQPPSFPRCSLLFIQPAGYSAVANSRPEVAERSGAVFSGAVREVLSIFGAYECQEYENTFMVACH
GPRVAAELALALHCWLLDADWPTELLVDHAEGRVQLAEDGQPLLRGFRAKVGIFTGVPLSVVPHATTGRADYFGALV
NRAARLMAGAKGGQTLLDKSAGLEVLKEWRHMAAAHLQQQQHQLRLQQQQQQLAAAMQTHMHSQAHSQVLMLPML
GHSTTGPLSPPPLQLHASPTPGSPWSAIMVAAGRSSVGMDEGAPSPLVFGGQAGAAASQPGLPPLPPAAMLSMPSLA
HPYHPPLQPRHSDPQPHPQQPGAAATAAAATAAVHAAATAGRPHLTRFGSSQSRAPSSAASEAAVAPAVMPQLHSQP
SSFRAGSGRSSAPALSSPPPSSGVPPGTRIPPPRVEPQTGQRNIMDVTQDSASTTAIAAADVAAAEAAAAPAAGAVG
AAPGVLGLSVAGAPQAVPPSPPWQPASQPIAVAIASPQVVLSGGHSGGGDLTPHLAAAAAVLSFGDGDADAHGNQLN
SRQVLQPPLPTAPAPALPCVPHAAGDELSERTSPAASSHVHVDQQAAPAPAAAHPQQRHSHSDRVLLHHRTLGQGDM
WQAAPSPGFSPAPSPGPSPGAFFPPSLLPTMASGLLPYNLPSLGPLPSAAGVAHGLMSGGITGVGSAGVGGAAGGGS
AGSGAPSAAPQLESMGVGAAAHDRLPDWWAALQASRNPQLQGRVHTADLVVQVCDLGLFKLKGLAEGQPVIAVQLKR
LLAGGTEPSVAAAATAARSAGGSGPGTAPGAEQHGDGGAGGAGAGTGGAGGGAGSAGGGAGGGSKAEQLRGTALGLM
GEVVVTLPTRAHAHGRTTALGLQL

>Cre10.g440350.t1.3|PACid:27580061
MFSRIRVPPELKHTPPNVLEDYVRVRLTQYISLLSGRLNHYAPGLSKDALDRAAAEALAYLPPGEHGQLSLEDKQLQ
LVFRHDCLALGCLDPQCALCTHNPSRRCTVNFDRKYLVNDVLKARCGAGIRVELIDPNTGQPVDDKLPEITLQISVL
DGNLYDQKFLDSGKQYEGGTEPAEELDACTLLQNKKKSTALLVCGSGGSNDAAGYVLLNMLEGKVPLVDLHVTDSSE
AILSGRKPPFRLLVRAMLPPGGPRLNIRHAVSEGFVVATRRTRTAGKADIPSVDDPVSKLEHMGKETVKKLGDLATA
AEQAGVEIDIPENCVQKVGEFKMLALRADQDGHLRQKLQHVLKLSKEKWDEARDHAMRAVVADNRMRAWYADRRSCE
LGVLFTARMGNVDLDRPVGLLQTRMEAGERKTEAILVAQLNPNQREMLRHMQPQAVAAWWAPGHTGWAIFPMDSDTF
LQTGALTTAAGLASALAAPTSTAGGIADAPPSPTHSTAVSLSTLPQRRHTTGSGGGGGAGGSAGMNGGASQSGDVGL
GADPFKAPPLQPALPSAPAALPSGLPLPGMPAGPNSGPTGMSGLHSGGSLPSAFGGGLPSAFGAGMGSGRSSRANSM
TDSPPPSMRMAAAMPMQQPPQQPPQQTGLSLTHHHSLPQPPHGQPQLQGHPSMSPHPSLHQNASLQGHPSLQGHQSL
QQHPSFHSHPSMQQHPSLQQHPSFHSHPSLHHNPSLQGHPSLQGHPSLQGHPSMQGHPGLQQHSGLRQGGPPGMAPS
SGGSFTGSQHSQHSHDGSVPPGAPPQPLQQGSAPPPSATQPSAPSPGQPLPQPQPGLHTLMPPPAAPPMPMGPPGSR
SMMLPPQGPPPSFRMPPPQQPMGPPTSHPHAAHYRPMGPPDPFSQVQMPPQQHVHPHAHMQPPQSPHHLAAHQQQQQ
QQQQHQQQHSLPPPMQPPSGGEPPLLQPTTALGRPQMSAPPNPFSNAGALGPSSGPNSSSSLGGNDGTNTSMPPPP

PPGMRMTASAGFGSHQHQQQQQLMRPPLGPSLPPATAGALQDADDLQSRGSANPRPHKMPSLSRTLDGVFGPGGDNK
PDFLESTSVAAAAAAAAATAPALGAPGGAASGTAAANGVKQDPAMGVAQAGALGPSDSKPQASGNASSPSGLPPGPR
AAPPVPGQAPPGASPPPPGPPPNPFARVQSGRVGGPTMGPVDDSDDSDSDDPLAALQSLPFTNSMLQAALGAGPSVP
RGNGHLAGGGAAGGGGAGVQGGAAGGAAGGAGGGGATANGGLGAPLPSLQMIDSQQLDMVLGDNMILETSSLNTGLS
YGLFLPAGMGLPDGLAGTQSKELLKTTTSGMLKPTSSGMLKPTTSGLLKPTHSGLDTMASMELALPGLRKNGENHAA
PPPALQPAQPLQDSRAGPSTGGANGTASAADRADAAGAAGAAAANGGAGSSRAEPYAR

>Cre10.g456500.t1.3|PACid:27580063
MSAAEAVVEDWCVAAPRANSDSQAQAQKQVVPALPATLVSPPGAPADCISCFTSDAAFSRLSLSSGLASCSSISGGV
HAIHSLLLGSVAATAPSFGGLAAVAEAAPAHGAAADCQADVIAPPASAAHGGRGRGGRRAFGSDGHLMPPTRRAASA
TSSHPAYTQQHPLRAAAGAAAGAAGLPLEARAAAASFTVGSMAAASVRAPAADAGLAVVASEGCISPAAAASGARSH
SFTRPGNRRARQSMIQGQGSRASAHDDVSSPDSARFHGGSPSEAHSNGDGAADAAPLPAADMAGEGDLLRASAAVAM
ALLPPATLPVRIITPRSTKDLAATGGGGGAASVSTTAAATPSADPISSAAAGVPVEDCAIVAACDVGVPEGLTRVPS
RSHRGGGRGGGAGEQPHGLQHGHPHEGTSADSAAAPGGSAAGAGSSEPAPPVADVTSDELTAVAAAAAAAAAAAAVA
KASTITVVRSRSINTGPTLHPGASRAAAAAACDSAGHNTGSFTRAAPSSALGTPPADQMGSAGGSGSAAIGSSSSRR
RLLREAGAMQVAVEERADPGSAGASRPPALHSSGSRSGAAAAAFGSGSTGPVSAQQQQQQQQQTHPQQRGSLRRQA
VVPAEEDGTGDTMHDAELDSDEEELMAAIRFQQSPRGPQQGWAADARAGAGAGTGTGGVPPPPARESPRPGGATAAG
SPLPAPPPPPPPPPGSRTPPPQRSPLPAEPNGGLRRTHCGVVGAGGGASAAPDAADLDAARVAAAVSSRARNSYGGV
LSSVEWAAGGGGAAEADQRGRSRVGGGRGLGGGAGGGRSPTLSTGGMRSLTGGLAAAEGGGGSGLQDDMPTRTWLDL
QPPPPPPPPPPPRRPGAGFAEGGGDAPASLHGDHHSRNSPLPSPPPPPARQQPPNVSGPPPPPPPPPPQRQHQSPQPR
SQVPSPPPPPPPRVSSPQPPPPPPPPPRPLSQGSSPMHRQPPPPPPPPPPPPRLASPQSNFTQPVIGQPPSANSSVSQP
PPPPPPPPPPPPSAEHLPSRQQSAIAGAAAAGGSRGAASGGIQSHNADFRQARHGSGAAGISNHDGITFSGGFAEDGG
IGGGRLGLETLGMAAVPAATSDADAVETGAVDGDLPPRGGSQMDVKWEQSVADAAVAAAQGASRSIWADAATAAVAE
AQAAGGGAGGVASLQSQPSGPEHLDLGAPIRLQPSQHSAASTSADLDTTPWPMLLGSVAAAANSPSAAGSAAAAGGR
DARQSTGHGDEEAALAAVSAAARARLIAAVGCLGPDSLNSSDEGVRSGGAGPAHVSLSGGIASGGIAGSPLAPGGAA
MAAASGRRTQQPLPPEWAGGADAAASAAASEAAAAAAVDAHLAALGLGQLDAHTALAAAAASAAAAGGYSNEHRAAM
AQEQVQRQHQQLQQHHHQQQLQQQQHQAAPQRRSPPPPPPIQLPATLGGWALDATASTGLISSPPSTSHSQHLHNLH
SDSSTSAGGGTTPGGAGPSHLPMPSPPLSPQLLQPHILAEYDLGPRDGSVTSAVSGSGSSFLSQRHSQTPLHTGGGG
GPGVSAAGGSAGAVAAGGRSGEFTLDAISAVDWSLGAFGGSGGGGGGGTGGGAGAAGGVPTSLRSPPAPTSPGSHPH
LVHLGPGCSSASSASGISAMSGASGAGSNAGSTVASSRLAALHFAALRVPSSSGGSHSAAFSPLGAASSLSSASLHQ
AVYSPVVGGPSRSGGSASSAGVAAGFSPLVSARSPLGFSPLGAGAAGAGSSAAGALAQAGSTSSAAAAAAAAAHAAR
YRGAAAAAGAAESELVMRTSSFPTLQQHLQQHGVGDGGMADGLGPDLQRAGMAAGGSGHSYTSYSHQYQHRQQQQQH
QQSRFGAAAAAAAAAGGGGAYGNVAAEYGGGGEYGAAADPFASLACRSSISMPHFHFTPGAGAGGHGGDEYEHVAADE
VVAALAAARQLMPQGMGSLGGALRAASYTPGSGSADAAVAAAVAAAAATAAGMGHPHPHQHLQRQQHLESPDYGPAS
GQLRYSSSYMLQQQQQQQQHPQLQHLQHPQLPGQGR

>Cre10.g440000.t1.3|PACid:27580095
MLRVAGAGKFPGDANAEAATAAAARGFGGSRPGRGVRGSSGTGSRQGDGFGSVRGGRSGGRSWGGRSGSSGGRGPGS
RGPPPQVGTRVPPASPDDVAAWVGTADGGDSGGGIMGRITAATLWQQLTAILESEAGAVAAAAAAAAATATATATAA
AFPAAAATAAVGSMAASSTSTSIPPPGGDRAEEAAMPGEAGGGASTRTTPPPPPPSLGEPELLLLLWRAVCGCLQTF
TDGSLLLLPPPPLPVGSQSGTGAALFPHMPALPGSTATTHAATPSPFGATMGGGGAIDDLFSFSASGVDLTNGLGTG
AARAPLAAAGAAVTTVPMWTVAPLAGSGVARQRCLRALAAALARLAPRLSPAGLAAGVQLAARLGQVAAGGNTGGGG
GGGAGGYVQEGHRQAAGAAAWELAAPLLQATAATAAAATAAAAVTAGAGDGGPALVAVAPETATALVDVLVALPLLQ
ALEPDHGPGSHQQAGLQAQQQRRRPPQQQHDGQGDRPSEACPLSRRVLETALGTLCVRAGAVAPSTRELAVCLAAAA
RCGLAPPPAWLALLQCEVRSQLLLLTPAQGAVLLAAVQGLERHAGGSGGRGAAGMPAPLVAPAWADMVWREVRGRAG
AAPAAGGGGGPSCGLGLGFGFSGGGVGGGVFGPQALVDPDLQELGWFADAGLLLMPPPPLPPSPSDTPQELGGQGGA
GAGDGHGGREDPVGWLVQHLAVRTARQWLLPTEAAAPQAAGPGPQELVAVLAALARVAPFAGAAATAGKDAGGGATA
TDSAGAGADGGMVADAAATQLGGGAGWRQELLAAAVAAVLQCLGGTPALAAPAPAAQPATAELASRAGCSMEWLQLA
QCTEALAALAQAAAEDEAQGNRAAAAVPTGPATAATAATAASAGSATAAAVGSAKLPDLDALVWRLQQALLEAAAAA
GSRAAVATMAAAVGPGELVWLLRAEAALLRMQELWPRQAHHLPPARGLARRLLAALIAPLLRQAPLAQAAHNPASHA
AAATAAAAMPPAQLLQLLAAAQHPWTAGMFDGELLAAGSSSNSSSSSSSSGRSTSRSGEAAAAGRLTVPAQAASARG
VVVTALLDSLPLTTASAGARGDAAAAAAAAAALDLAVALLPAAVHALRTPADAVAALRAAADRQQRHQRQQQALQQQ
GQRTGRLRQADALAAAALPAALSRLLALAIPPPPHQEPGFMDVGAAAPAPAALVAPEVWCEALAALLDGVRALDPAN
AKAASSTSSPAAAVGAPAGPRRAATSSAAAAGYFAWPPQAFSPPPPPPPLVQLRRLWGALDRCWPLMAPGQLVRVLG
LLADLHAPLSAMSSRGAAAAGGEPTAADLQALQRAVASRMADMTPTELAEAVTATARLPAAAPPCDEWLAALTTVLA
APPPPPAPLAGGLQGLRAAEALALVLACLDMKAAAPVTARDAVMVPVLPLLEQLGLALVADHGPPGPVRDAAKALRA
APDEAAAATAVAAAVAGAAAAPATPAAPSPPQPTRPLPPPPSCGELCRLLPRLAALATHPATAQAMGSAAAPMLSYL

AAVGFAGAGPADLAACVSGLGRLLVLPGGHWVAALCEATRPLLLSRGTSLAELAALSHAMATALLLPPPQWQRDLEA
ATAQRMARAAAEAAQLAAAGVAEAPGSGRGGSRSAGGGGGGGGGLRSAADLKALDQLLTAQALWWRLSSGSSTSSSS
SSSSSSSSSSSSSSSSSSSSSRKDSGAEAAAVTELRPSPGWLDALVECAAALMAALGPAASPQSALAGLPYTLHVLG
HDPGPDFISALLAACSRYLPLLPLARDPGLAHEHTAPGAPTPTLSPTPNANPGGGGGGGTQQQQAQEAAGPHVVVQV
PASAPAFTGPQLSRLALGLAGLGYQPEDAWVRCLQMESALRLSGCDPHELADLLEGLLLLAEGCEAGGGSSSSSASG
SSSTSGSSGEAQWVDSRLVPAFLGCWWAAAEIAMHNDTAVAAAALERRARRVDLRLATAMRAELRARLSDRSGAGAA
WGYWSEQLPGLDPDRLFEMSDQDIEAELAALQFPVFDAGMALAAAQEEQQADQQEQEEQEQEQGRTGRWRRPQPDGG
SGGGGGPIKDGNKVSFDVGMPVLQRYTLWAILRGGGSSSTAAGEASEPAGRIPGPLPEGAAAWVGSLAVRSVALLSG
PRAAPAADGRLTAAQLQQLAAAAAAPGSAVVVAPPAFWLESMLACQAAAMMLGAYGGAGSLSLAEYESAVGHDADGA
EAGEPWRRQLLQRRAAAAQRAALLQAAAAQRAALLQAAAAARAWALPPVPEWGEAFLRASRLAFLSPPPPPLPQPSR
QSSPPAAGVGPGASAADAADTAAAVTGPGGGDGSGGDGGSGGSASCLSELLQLLLAAVALDMQPGEGWLRAAEATSV
DLMRGGGGSGVGPEEAVALVLALQRLGHTPSRLWAEAVLERMAQGLPTPTQLSEPGAPGAYATAAPSVTGNPAPMLG
AAAAAAEAAGAAALLPPASVAGVVVALAAMRVRPSDGWVEAALAALHERHGDLPAPLRARLLAALPQLDVTPSAVWL
GPAVEAFWEGVPLLLEPLRQRLRQLSRGGTETGAAAGAAAGAAGASSDTQPGLEAAGEGAGAPGDAAAAALVAPLVS
GLTAVSELGVPVKQQQLTALLGRLSDLMAPPPTAAAAAAAAAAAAAARSPGAATTSSAAATTTAAAATLAAAEVGRVL
VCLAAMRRATPSRANQRVLDSLFAAAAERLPLSGPLTAELLDAWDKLVPP

>Cre10.g435950.t1.3|PACid:27580100
MLVLLNPTLRAAAPGLLLAKVAASRSVGHGAVPCGGALPRPLLPLQLEGAAPCREAAAQNAAAGGAVPLAATCRVAD
ARRAASASSRGSRDAGRTAAALGAAAAANAAAAAVAGSVAELFLRINGGGLSATAASGAGGHSLAAQHPWPSLSSWR
TNGGFPQPPLLDTRSSCSLLDACGDIQDSPRQPGSASSTARRAYRHATAAGHLTSSHSRGYGGSASTGSGSSTADTS
PDGRQQQHGRLTHDDQHYHQRQQELLPGGGSSNGGSSSGGSSSGIGGGGLHAGGTGLLRSHGPSPGLPVQPAVGSPP
PPLQRPDRLAHETVTSAAVAGPGGRHEADAYEGDPREVVLRVLRSATAGACCSPGAGADLWCSDEDEAEAAELLGCS
TTTVVAAAASGAAGAGAGLDVEADGIATERAVDSCLAASAATQRQLMRLPGRELAAAALAVLPRLPPWRVAALAVAL
ARMQLLGAEGFKSGLVEHVVGRLYQFSPQQLADVAWALATARQYDVSFLEALAGRLTATAAQWDARSLAQVLWAFGR
FSFVLGGSTGAGGGGGGAGGAQSDWAQSPPPPVGAEAVAAMVEKLQGELDGGSVAEVVYAAGVMCGSAGDWQAGRLQ
RLVGDFAADNMTAFGPEALGKLAAGLTGLGLRDLGGGQRRRLCAAVAGRAAELAARLEPEDLCRVVGLLAANDYCDA
EDGGGGASGGSSCSSNSSSSLRALAARAEELVGTTAAAAAAAVAGGRHPHMLLPACELLEPGQAEALAAGFQALGVP
LRGLPLVSSPLGPYAGGGGGGGGGGVSCGPAAFR

>Cre10.g429050.t1.3|PACid:27580102
MGVKGLWNLLEPVGRRINIEAIANKRLAIDASIWLFQFMKAMRDDKGDMMRNAHLLGFFRRICRLLFHRVRPVFVFD
GATPALKRHTNIARRRRREAQQGVLRKTAEKLLIAQMKKQALQAAKAQQQGARGAGAGAAAAAGAGAAAGAGAAAGP
AVGSAAPTESASGAAAGGRGGRGTARAQPPVPPRPPTASLQAASAAGAGPSRPGPGPSGSGGAGPSGSGGAGPSGSG
GAGPSGSGFRAGPSGSGSGSQAPAAAAEVIDVDDEGAMRRLATGKRPMTREEEAAWLARKAAQEMPPPLPRPPRGPA
AEALAAADEALARELDLQLNSRTAAAAVPAAGAAAAGPSGAGGAGAASGTRAVGSAAGAAGGAAGGAGGAAGAAGRG
AAAGVRLAAGRAAGRPAVAAAAGVAAAAAAVAGTGAYGSAEDWEDVAGGALGEGGDGMSEADAAAIAAALAEDERAG
DDEMNVEDIDPTAAMRFDPYFNAYVLDADASRRPTAAATTAAATSSILEAQRRSARPDGDGDDDLGDDDEEDDDDAE
LLASMIALPDDPSAVDPAVLSTLPTSLQLELLDKMRDAAAAANREKFQVAARVRPESFCEVQMSTYLKASAFRQQLE
AYKQNLGGAGGSGGANGDNVQVRRIMGEEGREYFLARDNEEDGGAGAGGRGRGSGSGSGGARGRGRGRGAGAGGKHD
QLAIAAAARAAARNVGMPPTLTASIPLAAFKGLAAVTNTKLPHAASAPAAVAAGGRGAGGAAGGAAGAGRAGATAGG
GGGAGGGEGAGELLAGAGVVAKGATLELRLSDGEGGGSSDSAKGMFGSSSSGSDAERDAGGAAGARAPWGLASSVLA
ADGLSSGAGATVTAATAAAAGPPASSGATAAATASATAATASSAPAALLDDVEWEDVDVAKEAAAPSVAAAAVAGTV
ARPRDWRERMQSRQKLWSSSHGFRMGRKLADWGAAAAAAGQQQGLTARGRGRRRKQAQQQRKRKEAAAGGTGSGSGG
RRTSSSGAEGEGEGAEEEEDGEELDAEVVELGDEEEEDEDALLQEAIRQSLLDSAPAGGGGGGGSGGAATLPAGAQP
RAQGQQPHLQQRSQPSQRQPSSTQENGSEQEDDGFEWEDVSEAVQVVELGTSKQAAVTAGKRPAESTAATAATASAA
TAATAARQSPGTQIPTPATAAALVSHTGAVGGGAAATAALLAPRRGPLALAPAGAAAAVADALGRPRTSATRTAPAV
GAAAAAIPSATTATATATTTATATATVTAAATAAVGDGDGDSVSDGDVIWEAADADLDALEAAMTAPRPAATASATA
ATAATAAAAATARVGAAASATAAGRTSAAAAAPAAAQAAAQVPAGPSLAGDDDDFEWEDAPVVWEAAAAVGAVRLGS
PAPAAAAAGGAGGGSSRLGLLGLSRLGAQAHTAAAAAVSGAAAAGVASGGGVGAGAAPASPEPRVKASVAASRAELL
AAMAAAQRRAGGGGGGSGGGGGSGSGDGGDGADDVDDVDDAVGAGVSAAAARSLIAAINSSSKELLDAPAGGSAGGV
AASGSVLLAAPSRAALQAGSSGVLGPAAAAAAAAAAAPAPVVVSAAAPVVNSRPELAQTNSGADKQQFAAYELADED
AEDEMDFAFDFVEEPIVDQPPASPPPAQLQRAPQLTAQSPATPPELHTAPSAADAAATAAAATSSPPSSPQHQQQPQ
GAAAFPTGVRALPPPPLAAPQQPTAAAQPQPRLNPSLPASAAVTAATAVTAAVAHPATNAATAAAAAAAAAAAADDD
PSLLDDEDAGEQAAADGGGGSVDGWDDEDADWFDEDIEFVPERDKRPPPSPPPQQRQLQQPAVAAAAVAGGGAPAVA
APQPRQQQHQPQQQPLMRSADPLQPLVDRPPEAPQGTFFFTSPPVVATAPATAAPAPPQAAAGGVAGRQGGTGAYGY
ESEAEAAAAEAGAGAGGAAGVAGAGPDIDELLEAVEVEEAQLRGAQARASRNLEAPTTEMYQECQELLQMFGLPFIV

APTEAEAQCAYLEQAGLVDGVVTDDNDVFLFGGRHVYRHIFENKKYVEEYNMADVERELGLGRERLVELALLLGSDY
TEGCAGIGIVNALEVVQAFPGREGLARFRTWVEAPDAGLVAAARHLAGEEAGDGDLLPDDTPAQQRFKRQHRGVRKN
WQVPASFPSAPVLEAYLAPRLDPNKNKFGFGRPDLQLLRQFCSDKFGWGGDKVDELLLPVLREFDNRQAQLRMEQFL
SFSTRFAKIKSKRLQHAVAGMTGHAPAEELVLGDGIKVTPKKHKSGAAAVAAAAAEVAAASASPNSGANVGSGQGTP
AASPGDTDRSGGRGRGRGRGRGGGGGGRGRGRGRGRRGGTAAASGGGTRRRRRSSGDAEEEDGAEGEDAGGDGGGEA
AGEDTGAAAAAGGAASSPGQSPPTQSEAAGQNAAAAVAVGKPPPAKRRRTGTKVAKQATTAAAASAADAAGGDRND

>Cre10.g446600.t1.2|PACid:27580316
MTSPPQSDQGRGLRRSLRVQGRATAAAAGPSAAERVQAHGPARRVAAEAPEAAAAHAAAAAAPAPAAILAPLAAAGA
AYGVAAAAAPVAAAAPAFAAPAQPPAAAGAAYGVAAAAAPVAAAAPAFAAPAQPPAAAGAAYGAAAATAPGAAAAPA
FAAPAQPPAAAGAAYGAAAATAPGAAAAPAFAAPAQPPAAAGAAYGAAAATAPGAAAAPAFAAPAQPPAAAGAAYGA
AAAAAPVAAAAPAFAAPVRPPAAAVAAYGAAAAAAPVAAAAPAFAAPAQPPAAAGAAYGAAAAAAAGPSATAAGLPP
RHPGRAQSVSNFDQALRRAFGAPAAPRSVRATPAAGVGLQGAFLALGVPDALRRRGRTWASDATGSSASSYRPSDSA
SETSSTADTASGRRRAAARELTELSPAGANHGRRVCNNPAYEEEEELQAAIARSRRDTGHPRPPPPAGRAAALLTET
AVTVHEMLGGGPTGGLALGGLAPAASPPRLDRARRRHLLNLAASSRPSASAASSSSGRSGSSRRTTSTSARSGSESM
HSARSQQPSASQHIHLHVHPGPSPAVAAAPPAADAGADFFPHLGGLRARYGRLHAALGPPAPAAAAAPAAAALPAAA
AAADAPAPPAAAAGPGPDAAAPQPPVSVVNMASSSSSSSAPASAGGCPLWPMPLPAWQPHLLAQLDHAAAAAAPPAA
AAGAPPAALAGGSGGVARTGMAAAHLAAAGPANGAPLSSAGAAWPSAAAMLRPARPIPSWSQTGRQQYDGERAGRAR
FMVDAHGYTEEQAACADINSPYAQDPRLIARALINGASVPESRAWVWNNEELDGGQRTLERHRGRWVAVGGSLVDWR
GQAFIDADGTVRVPHVAHFWLAEFLAAMHTPASFGPSPLFGFTLGATEAATAAAQRFLSLHRADTALCHKDMLGKVF
SEGVMRTAVTQPWRDLFIMNLTREQKALPEGQVLSVTLVANCLAEAQAAFRGIAAPTPAAAVHVTAASSAAASPAPA
AAPAAPAAPAAPAAPPAPAAAAPAAAAHAHQVNQLPVVSQADLMRRENLVPDTTVNQRKPGDAQWCLYHQTSHHAAD
QCRAYSALVSSNRAREAARRIATGEGARAFHKAPAPAPAQAHAHVTAALPAETAQPASAAALCPPHRAARRELADDL
SVATVRSLYDTGGHSERPPLPSATAAVSHSQVAGASGMELGDCWDAEFGPAHIFHLAARPPSSGPMQSALPLSFQPL
PAAAARRCAPAPADGAATPTASSSTTSAACPSATTGSDAAASTSTRPAAAIPAATAASSDGAPSGADNRVRAHLNDA
KFAALHAVLRDQGLQITRLEGDVRRYARLLRRLDAQAAAGCTHHSAMKLGRGVGPLLLVDGLSSQGTPYAAALPDPA
SDVTLTVTEAASSRGLPLQYDTGIYVSSVGGVTSELAQIRGGLPLTVLAGTEQQHTTRVVCFVVPGPQVFDILLGTN
WLIDAYRSVWDAGRNMLGLRLPHQPDASPPAVELPLRQRNRAAGACHAVSSGRAPDTSTSRTVIAAALACVAAPTAR
SHTDAVGVPPLTGTGAGTGGCTSGGRNGPESDEESSDYESGEESSDYESGEESSGYESGEESSGYESGEESSGHESD
EESSDDDSGGGGSPSASAGSAPIAPGPPTARPTDVLCERCDSWDHHARSCPWRCDQLPCIRSFNEAQRRCAEAAVAC
GPADVAAERARDPARSPCRAFPTASPSPPPPPPGSAASSPPPPPQPPSSGLPPPLQPPGSVLPPPLQLLAAAAPSPA
DAWAPPSPGSAASSPPRPPQPPGSVLPPPLQLLAATAPSPAYAWAPPSPGSAASSPPPPPQSPGSASPPPLQLLAAA
APSPAHATAPPSPGSAASSQPRPPQPPGSAPRPPAYTSALPLPGFADLLPPPRRSPGSPPPLLAPSPQRPGSAADPW
RSKLPSETWWQFLGRMRAAGIHLTAMPESEWAGQGRPPPEADPPPLSASRSASPVHQVALGFAAPPSPAVASSAAAS
AAPSPAAGRTPERMTFDALLLRHAAAEAAASSTYPGAPPPGRPAPLSPAAPHTAAAASLLPPNASCAPAAPLVAPEL
GLPRLRPIMTRGGSSSAAPLPSGGCDLAPCSPAMPPPVAWPPQSASGPGLPPLPTAALPDQAWSAPPSQPSLGAPSG
LAAGPPPPSPSLLAEAEDLVAAFLHRVPPHALMSAHGAGGEFGGSATYDPEDSEDDEVPPLNPPSDDEEDFDDAGAR
GGPSSHMTTGQLPPADSLRVLPTAIPLEAGPALPSCNDPEGRREPLPHDLHTKQPGGWMLYQNPDATDADVNRLLRL
VAQFDTGGSDGLFARSLDDLTGYRGGCDPVEINLSHDRSIYECPRKHTPAEYAIMDAKCEQLRQAGIIVRAPPEAKY
AMNSTMPAKRALDGTMTDTRFCQDARRLNEATIPDSHQLPLPEDLFQLMANAKVWAKADARAAFNQLPIKLEDQPKT
AFWWRRELWMYTRLLYGLRNATSNFQRVLDTHLRDWGLAHRVVAFVDDLAAWAETVPELIDVLCGLFRMLTSIGIKL
HPEKTVLLADGIEFLGHYVSPRGLQPTAAKIAAFMALQPATCLLECQRLLGSFGYYRGYVDHYSVKMAPITALTAKA
VEWGPDTWTAEQQRALDAIKHEFAEGDLVLRPLDPQRELILHTDWCEVGISGVLGQLDDDALEYMVACVSRTCNVHE
RRYGSYKGELLAAVWAVQTLRPYLHGRRFTLVSDHGPLEWLMTQRDLTGQAARWALILQQYVFSVVHRPGKLHQNAD
ALSRCPLPTDVDAAGARLDQADEPPPPPPPVVLYPGRRLPAYAQPPTATALTTIAEAALPSADGWLPRLAAHLASST
GAAWPSTTVAGARASAVCLATLHASRAAGPGACVVDGSDLLAGHNGTTSDVREMAPAADTAVRTAVALVNRHGASLR
QLRLTPRLRLRLCTARGSVVGLDTSLQQHSFFDAAQGDGVAVLEAPGGLAAGLQACLRLGIKVRRYVSMEPQPAVLG
AVEKLVRALAAANSLCLPLAAWRQAGAPRQTALSGLPLRDGGQWLVFASWGSTNGWSDADALSFVGGLQLALRKQHC
APPAYLLHGPTTTRAEQEQLGFPFGLPATMDTVQAGVAAHRLVTLYTNLAAAEHLAAVLERLPPEPDRTLAQQLEPG
RRPMVALRVEPLPYRPAHVPGQRLVALPAGSDAPPLRTVPGDEPCHLSVTDWEAVLGHRPGTADTLPPDLAIPALRN
PISPRTAATALAACLALQRSYLTPHDTHAPPPLRVAPAPSPLGGGEPAGEDDQATVIARGLLQRQPAPDLAFTFSTL
AAVAADAQEQATATAENQSWTDVWEDAEVLAALRAGGAAAGGAAPRRVTRRMADYRWVNGQLLRNMSDGLTRVCPPP
DQRVALTQAVHQRTGHLGVRRTLALLQTGHWWYGMKNTVRQVLRACRACDMSNARGEALPSRLSPLPVMGLFYRLGV
DLAGPLPPSEPDGNRYVMVVIEHFSKHVELLPLRDKTAARTARALADVFARFGAPAEVLTDNGSEFEGEFAELLERS
YVDHRRTSPGHPQADGAAERVVQVLKQALRKTCYLTADASAWEQALPELLLGYRCSPQASTRYSPYELLFGGVAPVI
PPAVRERISVPLDFEDPEAATASLLARMEWVRQAYPAAASNLLIAQHRDTLRYSAIRTGRYLAKPITYAPGDYVYVR
RVDAAQRPTLHFGQHDTILRVECIGPLGVAVLVGRDGARVRRRAEHLRPCHLDIDPAIDPQLFRPQRDLQCEVCGSP

HQEAKMVLCDGCNTGWHTHCLRLPRVPRGQWLCPRCKELDAEEEVLRAGWAAEETGDAAAPAAAAAGLAGTAAGEAG
AAAAAPQCGAAGRRERRGVPAAAAESEELGRLLFPRARTRRLDEAASDLGGRRVRRAEEGPDGVLEYLGALARPRYF
RVQWSDGEEQQLTLAEARRLLAD

>Cre10.g421050.t1.3|PACid:27580344
MPILPDNLFPSPVCTGFGVLGMPTICNLRDGATFAPALPLLTHNLTANTTLTGLPELPWPHAGGYLGGAPGWPRLQL
WSPPASHAVWPFIRVPAGLRLMLQGLTLTSYPPYANRTAFSSLLIPDDLTRAALLPAVLALVRPESDVLPGGVAARE
APTPGGELALVNVTAQLPDCEVWEELRLQLCRGQPSDNVQVFSNLIHIKSFSYRGLIAADVFLTCINTSSAPWAGRQ
LAWPPPPPSPPPPMYRSWLPIRSGCAATVRSASDLATVIDMADQEQLPTYDVEQDDPVFAIRIQGVVRLSDLVAPQQ
SRGGQTGLLENGALPVRRAYQLWGPDTGGATLMDSSSSSTRAHLDMGGLPRPFALQLPNPPPNRVDGLRRLELRLTG
LTLSGLAAGDPSTIPHGLLTAVGWALGINTAIVERGQSQAAPARLILQDCVVVVSADELAYWRAAAGLPPRLAGSQT
GGSGGAGVRGGEGAVLLSGASARQWGGWLNSSIDLEVDAVSLNGSSSLDSLTLRYLDYPALAFPTIMFRNITLVTAA
ADPSEAPPTTTSQPAGSPSGSNSTSNSSSSTGEAGSGSSSAEQGATAWEPLWPGAADGWLLRLAAPPEAVLAEAAGA
ATADVEGAARAATAAGWSTLPLSHVPSMQGFIPAAAWTTAFAAFVKDPSYLDDGSLPSPPPSPLPASAPPTLPGAVS
LTLTGAGTATPVAAAGSSQPVFIQDSALIRMLGGKDGAVGEKSLPPAEPPLPPDAPRGNGGIVPGGGGGFTGGGINA
GAGLRRRQLLQRLRQRLRQLQWQQQPMYPGWRQRRTLLGGGVGSGGADGGGGDQAGGFQSLSVPGNVSLFGHPWFPT
ILDLSDMVAGLQLINGSSVALRQLVLVGLAGGRGTQLSSAVPDSGVASGGDGKTGSPGQQQQAPAPPSDPASGGVGT
SGGKEPSAGARRSGTRRRRLQQAGAGACAAGSYINPATEMCMACGPGTFQPVSGTNATACRPCPRGSFTPLAGATDC
SACPPGLSSNEPGAADCESLWLPAASLLVFGFDRTYGTAPRVFLRDVLLVLPDLELQVLTALAIELSAALSNATNSS
SSSPGTGGATVVPASPAALVTSAAVAAAVEAVAGSYAEQLSSSGGGSSSGGGSSSGGGSSSGDAAVRALAELLLSSE
FELLESGDATNTSTGSNDTISSSSSSSSGGGGLADAAGDPLGAELVLRRVAYAGVRGVNVRLASRSTYYAKELQAEL
GVPALPDMPPGAPMPPTAPALPPDPPSPPLTPLRPPVPPRRLPPSVFQDGGGKPLPPSATTQPPPPPGTLGTGGSGG
AGASPAAASPSAPGSSLVGEGVGPGSGGAAAGAAAPAPGGGSSSGGGGTVYLQPWQAGVMVAGIVVGVAVAATLGGI
ILWNRCGPPPKAGAPPPPPPVPKPLGGGAAVGDGGHAAVAGTAVAGGAGRGAAAAGSSIELVDMAGGRDGTRA

>Cre04.g220400.t1.2|PACid:27580374
MLQSMLRAALSSMTWWLLAAALSGAIAWWRHAGRPRAKPTSAAFGWRAGSAIFGCAQEFDRGAPHRTLLDFATQDQD
ASSLLSIGFGVLRGPLVLVSEPGVAVGLLRLDGASARSSKGPGYDAIRSVTPGHLLVSEGQQWRLMHALATPAFAAA
HRRHTPTTLARAARDFAEGLPLRAATPPPPVPPRQQQMQTLLSGGGSPTSRATQGEATSAAGHGGGDSSSCGADGSG
GSSSTTISGSSSTTSGSSSRRGSSTDGAFVVEDLAAEVRSWSLGVVMEVVFGSSSSGSGSSSCGSGSSGSGSGRSSS
GSVSGSSSSSVSSSSDRSSGSSKAELLERGAFAAALRGVMEEWHRRAVALLPLWRLQAAVAAAAHATAAAAATAAAT
ATAASSVAAVKGGDAYAPRLLLRRLLCACTVISIGGGLLRRGRAWALAVAARVLGWAADALMVADCGGVAAARRLRR
SQEVVAAFVRREVRAALAEAAAAVASCVEPAEAEAAAVVGDRGGGESGDSGSGGGGGGGGGGGKVTVANVAAAAPP
PPPPPKPLVRVIAAEAVRQSAARAPAPAPAPAPAPALASDGGAGGGHTGRTAAAAASAASAGGAGSEHGERLAVAAE
GVALTLVGMGHENVSATAAWALLLLAAHPEQQQALYRELRQWAAAAAPAPTATPAAAVAPAAITAAAESLPGGGGGG
FATPTAAVAAATAVATPAAGGAGLDTADPGGSSRTAALLRLPYLDAVLRETLRLYPPVPMLSRQLMQDTTIGGVMLP
KDVELVVSPYVLHRLPRLWGPHAACFQPERFMPPPPRPPPPPLPSALLPLSPTPPPPAPTQQPASASPRPAPEPGQS
ELSAGTPSPTPPPTPPPTPPPMSPQKHPQKLSAAWSAPLALLQPQQPLHSLPQPHHTQRALSADAGFVSHGGDHAAA
APYAGAGGAGSGLSGVGNSLSSSISISSRSSAAGVTAGCGDDGWQSQHHQRHTACVRGLSSPGVLSLPPPPPPPPPS
PPSPPPSALAASTVATTPIAAVAVATAAAPASPQQQPSPPPPAAGGGCTEPAAAGPYLPFGAGPRACPGASFGSAEV
KLLVAHVVMRYSLELLQPPPPSPRLQPAAAAATAAAIAAAAAGRDGDGGGPVVSRRATSNVKGGGSSGSGSGSGSGS
GSGGGGGSISTSDAAAPVPSDVDLLLPQQLFVSLRPGPGVRVCFVPRHQQQVE

>g4706.t1|PACid:27580376
MQARVQLPPVLLRLYDTVESVFDYLVRLSAATHPNLASILLQPGDGPDYRRVLLGSYVCWVGPAPAASCFRAGQPLQ
NSCSQQEVLYRAIDTLVRAGADNVLRSGYRKRRPNSNWAGYARGMRHAEPWCATSAGVALLTPAWRLLLARVGDDVM
LHLLTTGSLFVPLPVPAPAAPLKAAARATAPASKAAVPATGGVGVARGAAGCVGGGARSSVAVPAGSRGVGGARGGL
EGQRGSGGADGNGNLLQVSGRAITELARERYQAHGRSGCPEPPRTGTTAAATAAAAAAAAGPSAAGGAAGCSGVRGG
AVCSAADGVARPPPLRYSWFAASLAEALAAAAARRRARGAAGAAAAAAAAAGGAAAGAGRSAGAAAAEPSESAAAAP
ARLAGRGADVKNVGVESSSARLAMRDAGADAAAAKGPEAGPGSPPEQRQEQRRRRPRAWQRRKEAKAKAATAAAAAT
ATAAAGAAVAVAVGAQGRGSGDAEVAFAAGQEVGEDQEAWEVLSCGSLDEPATQVLCTSPWPLWEFGCDSPPPLATP
PHRQRYGHRLLERYGEGEGEGEGDENGRVPANAAAATARTMAPGAQGHGLATGVDGAGTGGGGGRGGAGGDGITGGA
SGTGAGCGGLGLGARAVVAEAAAAVGDGAAGARDDVEPPAAGVYMEAAAAAAAAAAAAAVAGQQLQGLQAAVGAARG
GHGTAPQAVQGPAEAGATRWRRRSSRRRRQPAASKPTAGDANAGAGAGAEGAAASEAQPGLGPSTVAGGRAARAFPH
PAGWLPPNQQVIPRAHVFYCATFHPRPGLPAKHMLNVLVRAAAAATPAAARTHHRLFAHIFTPQLLPPPAAAQAQQP
AAAQPAAMDAATAAAAAAAAAASAGFAEAAATAAGGSGAAVAAGSATAVAAAPAAVATAGAGPPAAPVAATSTSTAA
AAHAAAAAHAAAAAAQAAAALMPGQLRPGLPDTLKPAVRRRHRVLLRLLRSLLVRARRCRFALLLERHCPLPPALQR

RGRQPQPPQLPEAAGAAAAAPAGPSAGPSAAAHAATARAAQTASVAASMEPEAGGTSQPPAQPSQPPQPPPQPHPQP
PSQPRAQEARAGAPAASASAAGAASSHGAAHLTQGGDAHQPHSRHWAPPDFSLGAAVGFETQALPPSPVARRFGAGT
AAWQAPPWGAGAGVVADAGTGTGVGDMVSAAGAHAPTQPLPEAAATGAGAAPAPAPTASRRSALERAIAELAFQAPL
LPPVSLSQRRAQAAAGAAGGTGGAGGSQVLPTQPLLQPTQLQPTQQPAQSASLRLGRGGACRRKRGWAAGASSCGGG
TGAGAGVGAGAGAGAGAPQGGGASQPAGGVGDAGPTQQPAGTAAAAAAAAAAAAAAAAAAAAAAAAAPGRALPVPA
GAAANVAVQGAGATAHMAMQEAGAAAAAAGVGDGAGGGSGSLLPCCVPLARVTSFLWSVLRHLVPPPLLGNARNRQC
LRRTLARLVSMRRHEHLTLHQALQGLSPGLGRMSWLPKLKGGPAAGRPASRAAADRRLLSCWVLWLLRDVAVPLLRA
NFYATESEPYRQQVFYYRKPVWARLQGAALADLQQQRLQAISSEEARQLLSSRTLGPARLRLVPKMSGMRPIVNLGK
PTIVRFEVGKARRQRGRTAGLAAGVRPKRRQRREEAAHPNPAAASVGTSAAAAQCGAAAAAPEANASRKRPASGGSA
AAVFGPFGGAAAAGGVGVGGDGCGVGGDTGAAAAAAAATLAFKSVNSVLQNAQLALKASLEGRPGCLGSSVFGLDGI
YKAYAPFVRRWRAGRAAAVGAGGGGGGGGGGGGGIGGGGGRGGGGDVGGGGGGGVVGGDGGGGGGGRPAPAGGSQPCG
RQRQRQQGGGGGGGGGGGGGGGGGGGGGGGGGGGGGGRQDAAARDLQWLLGARRRALRFRLGRGGSSGDRGGCGGGGGG
AALEGGARGRLDVGGTAAIPQPAAAAAVAGAAVAEGAAAPKATRPLPRAVVAAAAAGAAAAATAAAGTAPGVACRGA
EGSDASKAGARAVQAGPVAVAQEPSAAVAQGHSAATAAAAAVAAAALPPKQLSGRKRALAEFGGSGGGGGAEVCAVA
GAAPQSFGGPQHTCGTGAHAPAAAKRSRLSLAIAAAGQAAAANAAVAAKGPPAVAPAVAVAAGPLPAPASAAGGSGG
GGDSPASIYIVSADISKAFDSLLQTADLHLYLADTHSTRLSQVTSLFTTASLHLPHCNCLAAPASLHLPRCTCLAAP
ASLHLPRCTCLAAPASLHQRRPLLPPASAEVTPAVGLHGVRSRPAHAAHPAAASYPGLPAWLAAHRSDAFNTVFMDK
LPLLRPLPRAEVVGLLRQAVCGNVMRIGRRWYRQTTGIAQGAVTSSLLCSLYMAALEAEHLLPLLPGGAAGAAGGTG
AAGAAGAAGGTGAAGEPPAAWLRSPTTPAAAAAAATTAGASPSTGGLTALAAAMSHAAAAAAAASAAAEAAMPMTAA
AAAAAEPGGIGPGGQSTAPHAIPAAVTATRHSATPVPASRAGAAVANPPVAAAPAIAIGSAPATAGTLAAVAHRGDA
PVGGGGGGGAPGGGVGVSPGGSGGTTTTTTGGGGSGPGLTALAAVFSAAAAAATTGGTTTGALQTPGSGSKTVGQNT
NTATPDPGSGSKSTAAGAAVAGAGAAAAGAAAARAVGVGAVETVGVETPVALGGGAGNGPGGQPLWPQQLLQPPPPG
LFVTPLQQPVGRPGAAAAAMAAAAAAQGAAATAEAAATVAASAAATGLPTSAPATAVFGGLQLGPAGAARRPGATAL
LARAATNAVAVAAAGGAAAAVAVRHQHQPGSVVASAPHGGRRLLAGGPTATARGEGEEGDGTPLRHLRLTQTAVAAS
QLQPPEEPAALSQAAAAVAAAPPPPPGDGSAGSNGAGGGPREVSGDEVVAASEEDPQLYGTAPQMYGTASQHLFGSC
HAGPAASGGPGAGVMSSGVGVILETPMHLLPATLLPAALQPGSSGSAGLLQPPPNAALLQPPGAGAAGSEPPLTSSE
PQTQTELPPAPVLQPSPQALLQPPLQPPPGSTSSGSGPGSEPADKAAGAAVAVAVAVAEDAQAAVAADAAACAGDPA
GARHGSGPAPVLQPPPFGTHAANRQSAGGGAGGGGGCAGGMGTAAASPASSGHHGSQPSAGTGAVGAHHYIQHRSGG
VLLSGSQTSGGSGSSGGAAAVASSGVAATAAAAVAAPTAVTGALVPPPALLLPQPQPQQRQCYSPGTHDHSPRPSPR
KGAPQGPTGTTAAALAAGGASEAAMRTNTNTNTNTNISTAAAAAEATTRPAAGHGTDATVAATIQAATQWRLQQPGG
KAGGGQVAQEADGGDGSGDGGGEGGGKGGGIAEVEAAQPSAAREADAADNAATPDGPAGAQLGTGDGEEEDEDEDMP
DLELRLFLTAPPMPLPMRTPRPRLSTQAVGADVAAARGPAAAAVGVAGGTATAAAAGGPRPGLEGLESIAVEAEAAA
DVGPAPEADLKAEAGAEAEAEAEVEVEVVDLLDTPSQERHSRDASGSQSQGGQGGDGDVAARDAKRQRRCSDALGAG
AAAAAAAAAGAGAVMVGGSAIGTGAAGALTGSGTATGMGAADATMGGHGGTGMAAADAVPVMQPSQPSPMAPTQLLL
FAMPPPPGTQRPPRHPQQAPDVGPLPPLALLQPTQPLHDGAVAVAASGLAGTTGLQPGHAPPQRGWQETACGVRGTH
GQPLQPELLLPLAQQVLLPSPAAGTAGSAPQLLLQPSPSPAPVGALLQPAPFQSGLLPPTQDLFPPVPGAGAAVAAV
GAVAAVGAVAAIGAVAANGATAAGAQGQEAARPAAASAQVLGDEPDAEEAKAEEEEEAEEEVEAEEAPPLSLLPPTL
AATQLLPPGPPASQLPLPGSEPQTDVLVDLAPPPPPPTHQLLHSHPGNHQERYYSSAADRTPSAQRSHAPGCDAVRP
PRPPSAAAAAALSGFLGNAHAAVPEEGEPGLHGFLPLTLPLTVPLAAVRAAGGSAGGAAGPDPEAAAAQGRPGAVAA
AAAAAAAAGLSTRATASASAPAVTSSQCLASDEDVWRLGRAGGGTAAAAVAAAAAAAVAATAAGAAINTTATATATA
ASAGAAVAAAAAARAAPGWHPALPSVPPLRAVAPIADNPPAAGAEPIPATATVGAAAAAAAAAAATAAAATPWTPGL
RLRHRCPQEVLGCSQPSPLADGAAAPDDELDLDFGPPDPDPGLWSGPGTQELLPAPGTQDLGLCVDTQAQQQGQQHQ
PQQPQAAEAEAGAEAEAEAQALKVEVEAMEPGPRAPEGCAGHALGRGSSAAAAAAPAAGGAAAAAVAVADEHQRQR
QQVLASLLRSLMESDDSPSKSGSKSTARAAADGPGPSGSAPPSGSSGDPEQLPRPGSRSAGPARAHELCPQLGGEAM
DVDGGAEVTAMAAAEAAAVAAGAPSADAATAAVALAAGGNGALATGSMQAGLDLQQQPAYEGQAGPQAAGTGATAAR
LMQAGPDLQTAAQQHLADDEGQAGPQAAAAEVQRRRGCRGGRKRRRNQLIRQWQGQARQRVQEGAAVAAGPRPAAP
EAVAGPAATVVALAAPRAAPAAAAAAAATAATAAPSAAALASPPEAQPSAAAAEAAATTAVPTAAVAAGGPGLGGSS
NGGGGGGGGCSTTIGGGGRQVPASLLLRLIDDFLFITTSRAAAEAVARRLREGFVSDYGCRINPDKTQANFPLPPHP
HHHHHHHQQQQQTQQADAAQGLPHAAAAAAAAAAQLAAGAAGPVGQQPGPAPAATAATAATAAGSGEVRVTLQQLPLR
PQGQGWAAQGQGEGSGGGGAGGDGDGGGGNLWTSKDGKRFIRWCGLLINADNLEVQADYTRYAGVHVRSTMSVPAET
RPGKQLVVKVCQYLRPKVHALLLDSGLNSGATVRLNIFQAFLLAAAKLHWYVRCLPRGPARDGRLVLAAVQSAISYM
CRLVRSRASAGGLAARRLGPGCRCDVSPCHVKWLGLVAFRRVLGRKQAAYGGLVLGCLDRQLAAPGFRHLPRQLAAV
VGRAANASFEELRY

>Cre04.g228208.t2.1|PACid:27580419
MTTASLASTRHSLDARANAELSSYRNPPRLAIDLSGLAGSTDEPLAPSPSLVTAPSPSGTTNAPDCGSSDSASPSSK
KVGLLKELKQMFSSNRRHRGQDQQGHGRPRDFTSDGGADPIAATAASSSPVATAAASRAAAADDDPTRAATASKPSA

GRSSSSFRAVRMNFSRKIAAADAAQLDKSTSHHHHHHAHHHHRQLASPPPLEPPHGAHGSGSLSSIFARIKSPMRLL
STLSVGGLKSPVESLASAPCSPPPLALSCVGDVTPPLPSPSCQQQQHNPNGAAGSVKRSSSVAASRTLTRGVGSNSV
AASSGGTGDVRNYRNAAASATAALAAVSVASNTVAAGFGSGSGCRAVAPVEASGDDDTAAVAKSSGSLNGGHTAAAQ
RRLLQAVVAAACQTDKDGAAAAATTTTVTVAMSRFNSCPAPRVPCVLSAGPARPHHAAPDPAAGGGAAGTSGSSAAG
DASLQGSSMLHNGSAEDGRNSYWSAMHTTGSDGVSGSGGGGDNSAQADYFATDTPQIPQIPLLPSAVPPSSTPVPAH
YQHYGSNSYKVIAVAAAHEAAAASISTRAATASDGAPHQQQHPSNRNGGGGGGSGCYGMHGKPARIYDSLWRGVTRS
AAAAAAAAAAEAGDAAGIAASSGLGPGRNGSSSGRAGGAAAAASIGAGAVGDTGDYTSLLLSACVLTPLDEEEDDED
SGYAAARSAAAAGRVAAGTMSQLAAAATAVPGAATAAASEPCTAPASAAPPPSEVHLAGATAATAAKAAAATVATAA
VAVTSSTTGTPGATAADAGQHQEEGEGGAALLAMNDAVPPTMPRRTWDLSDYDITKRIYKGALSCVYRATCRKSGLP
VALKVYFMARVPANTLHMLRREIELHIGLAHKNIIMLYAAFHDSKHLVLVQEWAERGDLFGVHRRMKCRLTETQTTE
LLLAPFLDALASLHRRGIVHRDIKPENILYTQGWTLKIADFGVSICLRDERAVTRAGTVEYMAPEVERCPLKRTSHE
NKYNPSLAYTASADIWSVGVLAYEMLVGFLPFVSEANEMPPKAGGDGVAVAAFMAAEANTRALSFPSSLSEPARDFI
RAALSENPCDRPTVEQLLQHPWIVPALEKHERLRARMT

>Cre04.g228208.t1.2|PACid:27580420
MTTASLASTRHSLDARANAELSSYRNPPRLAIDLSGLAGSTDEPLAPSPSLVTAPSPSGTTNAPDCGSSDSASPSSK
KELKQMFSSNRRHRGQDQQGHGRPRDFTSDGGADPIAATAASSSPVATAAASRAAAADDDPTRAATASKPSAGRSSS
SFRAVRMNFSRKIAAADAAQLDKSTSHHHHHHAHHHHRQLASPPPLEPPHGAHGSGSLSSIFARIKSPMRLLSTLSV
GGLKSPVESLASAPCSPPPLALSCVGDVTPPLPSPSCQQQQHNPNGAAGSVKRSSSVAASRTLTRGVGSNSVAASSG
GTGDVRNYRNAAASATAALAAVSVASNTVAAGFGSGSGCRAVAPVEASGDDDTAAVAKSSGSLNGGHTAAAQRRLLQ
AVVAAACQTDKDGAAAAATTTTVTVAMSRFNSCPAPRVPCVLSAGPARPHHAAPDPAAGGGAAGTSGSSAAGDASLQ
GSSMLHNGSAEDGRNSYWSAMHTTGSDGVSGSGGGGDNSAQADYFATDTPQIPQIPLLPSAVPPSSTPVPAHYQHYG
SNSYKVIAVAAAHEAAAASISTRAATASDGAPHQQQHPSNRNGGGGGGSGCYGMHGKPARIYDSLWRGVTRSAAAAA
AAAAAEAGDAAGIAASSGLGPGRNGSSSGRAGGAAAAASIGAGAVGDTGDYTSLLLSACVLTPLDEEEDDEDSGYAA
ARSAAAAGRVAAGTMSQLAAAATAVPGAATAAASEPCTAPASAAPPPSEVHLAGATAATAAKAAAATVATAAVAVTS
STTGTPGATAADAGQHQEEGEGGAALLAMNDAVPPTMPRRTWDLSDYDITKRIYKGALSCVYRATCRKSGLPVALKV
YFMARVPANTLHMLRREIELHIGLAHKNIIMLYAAFHDSKHLVLVQEWAERGDLFGVHRRMKCRLTETQTTELLLAP
FLDALASLHRRGIVHRDIKPENILYTQGWTLKIADFGVSICLRDERAVTRAGTVEYMAPEVERCPLKRTSHENKYNP
SLAYTASADIWSVGVLAYEMLVGFLPFVSEANEMPPKAGGDGVAVAAFMAAEANTRALSFPSSLSEPARDFIRAALS
ENPCDRPTVEQLLQHPWIVPALEKHERLRARMT

>Cre04.g214300.t1.3|PACid:27580425
MQLGPPAAQQRYTVHVVSSSSGSRCSSRGRHSACSSVASDTGEAANSAPSSALPAWLRSQLCFELLAPAPAAEPAQA
SSQASAALVDQQQPQQPRPEPQPRSQELVAVWHAPSADVAAAADEAAASDQPARLPAMWVRRHGCLLQALGCCAAGD
VVLLLGGVHDITHQHQRHRLKQERQQQQELLPKEQVQQEMQEQQERPSGDMGQGAWATDAAAGPAWIAPMALGDVVI
PAGVTLLGENPADPSHAMIRHSGLCVGASASLLPGPAPASRLVTCAIGVTLVHVCQGGAQPAQTPSQPQAPPGEAAP
AAALSVACGRLSMRGCNVQLGAGGGGGGGGGVGIWVGPGCGLELRHSHMCGTAQVGILVCVGASVCLVGTACRVTGR
AVVAMQAAAAQPAPQPAPQPAPQPAPQPAPQPAPQPEPHPEPEPAQSQLQDAEAATQSSAAVGAARGRAMSNSGQVP
PPASVAPVAPTLLVGQECILSAAGLDGGSSSASAGDGAGGSGGRGRAEAYTVLLLRPAATRGADAGAARQAAASPLR
GCRVCIHPDVVFAGQQPTARPAAAPQATAVEQQQPPQPPQAERHAAADVAAAAASTQRPRVLRLRGSSAAPFSLGVA
TRVPCEAAGSASGAASSWPITGVGLVSTKRFPLALGDRGGAVPSTWLVFGVAGGSQAVARLPRELGGWVPAAGLIPL
INSVAASYAAAHPAAPAVVASAPALASAASAGAGAGAGHGAGSIHAGATPAHAPSPPLLQQAVSGAAVDCSGTHGCS
GVRQSASVVVAPAPPNYTTPLRPLRRRGCFSPQGAGQSALPCTSAAAAPADSNADGATQSSSGGVSTMRRSVLGSEG
AAQSSSGGVTEMLRSVLDSAARTRAPAASPGVGGSGNASDRVELASSVTRPSPIPFRNPGRALRSAELQAAGEDSAG
RAGIGAAGTAAAGPGRNGVSGCVAVCPTAQLPTPAIPTLQSERCCKGSGTDAVGTAERLVAVGGSGGGCSRARVAAT
AAEVSRAVARAHGAEGPLPSPPPGPERSCGHASPALAAAPGAASPTAAPQPQQQWMQQWADQDLDPQMQLLLQQLPA
RAGNPFGGDANLMQAYLAFRDESPPPPQQQQQQQQQQQLQLQLQLQLQQQQQQQQPLQLQVQQQQVQACSVAAGGG
QGGANGGNSLSYECSNGNDRITTGATGSETSLAHSTFQHSPAAQHPPAAQHPPAVQLRAGPQLASAKAADVQRYLDQ
AAAAHAAAESAARHAHAHLSAAATVAAAALAGHTSRHGGFRAAAGEVGEATAADAATGAGAPAAGTLAAGGAAGECD
FSRREPGVEGSISHRVKRRRGGDAANKPAGLGYGSGASASDGDEEVGADDDPDYGPDGHHCGGDDDAGAAAAVPSWV
IPTCVSTCYPALPPQVLWPAPPHGQEEQQAQQAQQPLWDAQVPMRCAWPAREVEAAAAAVAVAEAAAAAPASAPVAA
GGSAMRQPPPLAAMQRAHAAALLMQQRPQAAAAGSAAALLLGRTAQGAPAGSHAGSLPLPPPPPQQLQLQPVPARPR
RVNGAASSSVASHKRSRHSGMPTGARAGSSRVSGARLGAAGDAGCGGGGGSGGGGGGGGGGGGGGGGGGGGGGGGGG
GGGGGGGGGGGGGGGGGGGGGGGGCGGDVLAAAHPHHLAAAAAEAAAAAAVAHYGGIERSSSSSSRRASARPAPLAS
QATTTRAAARSAAAAAAQAAAAQAAASLLCSHGCRAGAAELLPAVGLLAASAPWQHQQQQPQGPQYVCLQEQASHLQ
QQRQRQQQQQQGFPPHAAQGGLHGGVVGVIMGAAGGAHTTVVSPSGARRPLMPCQPPLAAVVPAHPSQALGCVAKPG
AAAPKPAMAAASAGLHWSAAAGNGRGEFLGSAPAVPMQGLQMQQPWHLQHVASLYPAPPQQQLQQQQQQQQRGGDGG

WGAAAGGAAAGVPQEHARRPQLHFAAGHALQQQQQQQQQQLQQQQQQLQQVVGQQQAHQAGLQQLPFWVLAQLPPAV
LVRLQHISASDPAGWQELLQRLLCPPQHVQQGQEQH

>Cre04.g227800.t1.3|PACid:27580455
MARLLTQSLVLLLALLAASAAVDAATEDLCNSTQKRCNGTGASAPRCVQTSVNTTRCECGAGAIYKPLVGCVQPFTG
YESLVIGAAQVCPQSAAAAGGSCPPPPGVAPKSCGLRTRVGGNASASGFAASYACDRLWSMSRSHYLQLREDGTLVV
THNATGTAVWTAETAGKPGVNSGSSSSGGGSQQQAATAAALLLRSNGTWQVVARLGDGGGVGAAAAVVLYDSSSPWS
FKAWPAPGQIFPDNGGIGNAPYTMRVSDAGAVTLSGRDGRQVWTSLPAVSILPTPTTAAGNTSSAATTAEGAGYYCS
ALHLVAAGETCAAVAYGVGLPLPALEQRNPPPACARLEPGDVLCVDGYGHGGNTNPEAIAPCRLNYTIARHDTCVGI
ARAANISVPGLKTLNPGLNCSRLFVGRVLCTARAAAAPPPPGPTPLSPGGSGGSTVAGCGPVARVSNPGTTCSTLAA
ESKIALSELRRLNPGLDCGGSGAGGREPALPLGNPVCLGDEATADAGGDIASASGAAAGCDPVRRARANDTCASVAA
AAGGGLSLFQLQLLNPGLTCGGGDGAAGHVPLVAGQLVCMGPAGGGPATGDASSGDGGGGGDGSSGDGGGGGDGSSG
GLQHSGFYDALTCRQAYAVSPIDTCHSFARAHNLTFAELAVLNPGIPCTSSNTSSNTSSNVGGLQPLLEQLLMLGYG
GSGAQPRTPVLCVAFVGCPCPNGGIGKIACCASPPPPPAPPAPPSPRPPRPKPPTQAPPPVLPPRPPFPPAAPPPRE
PSPPLPRAALPPPPYYHDLPRYQPPSPPPYGSGYGGYHGGYGGHGQQPPPYNYGGLQPPPPMYASYPPPPSYSPAPT
YTYASPPAATYAVSYLQPDMTAATYGGGSGSSIEGGGGPAVFAAGNENDSDGTMTGVEGRRHLGEVDASLPAADNGG
GGGGGDGGGGGGGGGGGGGGGGGSGGGGGGGNGNAEPPSGGGDGGGGGGGGNGNAEPPSGGGGGGGGGGGGGGGGGG
GGGGGGNGNAEPPSGGGGGGGGGGGGNGNAEPPSGGGGGGGGGGGGGGGGDGPAEPPTGGGGDGPAEPPTGGGGDGP
AEPPTGGGGDGPAEPLTGGGGDGPAEPPTGGGGDGPAEPPTGGGGDGPAEPPTGGGGDGPAEPPTGGGGDGPAEPPT
GGGGNGTAEPPTGGGGDGTAEPPTGGGDGTAEPPAGDGASLAEPPVGGNGLAEPPGGGGGNAGTPPGEDGGGGGGGG
GSDGDPSQGCGVLSCLPRFSLDQLQQDPSSGGSAPGLVIGGQSLFGAFRVGDMVSFAASLSDSEGGNCPFLVLTGAE
IKAYSKDVQRVKGLGISALGSAAKACVKIAGLLEQDESFILGALGWDAKKKPKGEAQDPQKITWGATSMAVCAAISP
CKGVYAFGFAGAQVTIPIWEDPPITVTLALETVSFSTGGADLTLHIPHLWDFGDGTGMGSIQTAYPFAANAIFQGSA
TLSLGIKSSEGKDVATIDMTGTLSLAINFDVNGDGRGGGDYGFATTLFGAPAVTIGTKFRLDLSKMISAELNLVWMH
RSANDWKLQITMRRSFDLVGLVEGISKEVADIIRGLIDIKGTVTFSISVDNNRIGILLDVAGDIKILPSLGLPEIQG
ELGILLSVSYNGQGDPFEVGLIINGHLYCISAYGRGAGRGIDVCPPGTVKDPGGLLCYPPCRDGFYMSGPVCWQNCP
DQFSAFLGSCIKPQPYGRGGGYPWKFGDWFNLDGARRRCEADNSQGCEQWGAVIYPKCAAGFHNVACCVCSPDCPNG
MSDTGIGCAQSSYPNGWGQAFQCGDNEDQGVAFCYPKCRENYVGVGPMCWRVC

>g4830.t1|PACid:27580491
MYIAPSAPTSAAQVDNTGFTEHQASDHAAGAGQGPDYARKLGACCGVAPSAPGLGVAFRRTSDSRSTPQPAPPDSLE
SPDHPFRGTLRASCESPFALKAPSMQAPDVVMSEASSGAAGGAADCVADARCDKAGGDMWSRASSASKPTVAGSASA
AGASAVASPEGNAAAVAAAAPRTSSPPPTAVAMAAAAAAAATATAATMAAPLHCHASSASPSPDGLGFSSGPLSVSA
LADAAMEPAAAHGSGGSRTGAAGPTAAAAAAAAADAVFAIAGNEAPRAHSDAAAAAAALYQASDTAQAFRNDLAKQGQ
LQRHLRDRTTPGWPADGGAGGGPNPQTPRGAAASAATATAAAPAAAATAATPPGPTRGPTPSGAHSGAVGPSYSHSL
QPPPKTLVPNHWLSPSPTHSSGDSHRSSPDGSSPPPAAARTQSTCCPAASPATAPAIATATTATATTAATAADDATP
PGPAGPPALAAAASPPRSGAVGDAPPTGAGHQHQHLLQPPPLPLSPHVRRVLVEWMGEMCAQLGLGSAVLFSAVAYL
DCFSAVQVVRTPDGVLQLLAITCLALACRQEGSISRLSTAKWAGVVVDPTDGQPLYTAEDVGRMEWLLLESLEWRTQ
LPHPLSSLTRLLHALGEAPGLEAWARRVAGGGQQPQLQPPQMGGGLAAAAEHQQQGQQLPPAGALPAPASPLPAAPS
ARPATGGGHGGGHAGMAAAAAAAAAGVVPMQTDDAATDGDELYADEQLSPTEVGAWHQPYHQQQQQPAGYGGGAAPP
AAAQPAAPLLLLLQHQRHQPDLRVLALQILELSLMGCSYTCFAPSTMALTCLATAVAAAATAAGTCAPLPRQRQQQG
GREQQQEVGEGEEGPPATPIGVTVAAARRQSRPLLEGAVYPPLLQAIRDEATALAAALAVAFGIVQSELAPNLHSCI
TAVHALITGSIPWVQ

>g5060.t1|PACid:27580504
MPRARFRAASTAVALLALLLVVQCPPSAQAARGEPRRPRVSRSPSESESGDNAFGDDYVSPLTSGLAPSSSSSSSNS
NAGGVDGAAAAAAAGSGGSFPLDALDLDPDTARRAALREQLLARAAAAAAELRPPGDPERLAAATADGAALLLLEQV
VPPPPPPHSYEGLVAPSPVGSHGGGGAGPGVDLAAPWLDRVPVSGAFAEGVQPHAAPAPAPASTASPTSSGELGATS
SSSSSSSSSSSSSSSPSPRRIGPASMPLASPPLPPAHATATSWGGGGGGGHGEEASGAAAGTVHTATPSLDAAAAAE
EHGIQVLTSRSRGGDSHRIQKPGQDAEATAVAGTAAAAAAAAARKPHLLLPRIAPHMGRGLAHDRWAVAAAEAAAGV
AAAGGTSSAARRRRLGTNADYSYCLTSPGADCFPCMSGYDDDPCATDDLYGRDDTKCSMPYYFYGNVDDESICGDLV
RPTNIGEMPLEAWLGAAGGPQQLVTVGQMLVFMQMSGMLTVTLRLDCPWLALADPARPGAAVRLGLRHPNGTEWVRW
LNTSATGSRFVSCASFSVQAKSLNASRPGDNGCDPLTYTASVSFLAETYQADPGPDADTPGTCTVNDPTTANSRFSF
SLDYTPIQFQPPSPPPLPPPLPPRPPSPPRPFVPDMPEEPPAMPPWPPSPPPLPPQPPQPPSPPPPAPPQAGCLRVT
ARTGKLYTDSTLTDSGNITVAAVNARTIRFDISLAAGRFLTAAPTAIGLDMVPYFSGMALPSFCPQPSSVGTPQSEI
NYVGGYGYRTSAPDCANRSNQLFVDVSRVIDCFYVNGPVSATYHVWLALDTAASSCSSPSPTGALWAGTTVSRLALA
GGCAYLPVTLVCDNSCYQPPPPPPPSPPSPTPPSPAPPSPAPPSPGLAPGDPLLTSGLNGLNGTLLQLDYNYLTATA

QFQGDSPYASCEPAVTGALVDTLSGLLSFPAASMTTACAAAPTSGNTARRRRQQEAEEEAGEEEAAAEQRRRLQQEA
SCFPKVKTDITFKVPPSVPLEGFKAAVYGALQSGGLAGVCPLGLLAGSWAVTGAVVRALAAPGGAFPNCQAFSSAVQ
SALAAAATPGASVQGCQVVTYYGALPTETSGGATGGGGAAAAGGGGGSKPPFAIIGAAIGGVVALAAAAAAYMWWKK
KQAGFNVSASAEPGMAVGAAAAAAAGAPPGAAGAPPGHAGGPGGPAAKAGAPPFGAGPEDAAAAAGGGGPEGAAHWA
SQPPGTPFAQDGAAAGAAAAAAAAAAGAGAAAAKAGGGGLFSRMLRAVGLGGGGGEAAGGAPPAAGVAASAQLTSGA
WSDDVPPMPGGGGGGSAVDAAAIAVAGAGAAMGAGALFRKTKADLPAIKAKAGKGGAGASGDGGGGGGDAESPGGSS
EGLSAASAAAAAAAGGGGGAGGPGGDGIPTLGDSASPGGGGGGGPDAFGAFGVAAGAAGGALAAKRALPAIRAAKLP
SLADTSLSPSAKSFSAGGAAAGAAAAGGGAAFFKKAVLPDIAPGSLPKARGAALPPIAAAARGARGGGTLSPLAAHP
PLAGEGASAGGAASPDGGATSPTFDPFAKSKLPDLPAHRSAGGAADVSMEAGAGGGGGGGGGGGALAAAGLAAAAGG
AAGASMVPPGMMSTASLDAATARELVRQLDAASRTAGAGAGLEPLVEEAHGSGPSLSVAQRGGHLSASGLFVRSPAA
VGPGGGGGGGGGGEGVSAGQVPTLLEDNMSAELPAGTAAALMAAAAAAGGGAAMHRAGAATPAAGADGTGAAAAAGA
AAAAPPKGFFSRLFGGLFGGGGGAAAAKSGGGAVSGAAGSVPGEGGAQPPSPRELPSLAQLDQEAALATAAAGAAA
GGALAAGRVSASGVAARKGSGSSFMSAASGGSGGASPYASAAAAAGAGVAGGGAAGAVGLDLSSPATAGAAAARAGG
GYSQDGGASPGGGAGGGSAFATSAGSAAGAGAAAGAAAGAAAAGGRVWKQGTGLGPEDELGAGGAASAADPAVRTGA
RLWQQGSGVPGSDSPIAEPPPAAAGKASSLPPIFMKTNAAYVPPRGDAGYSGGDFEPDPQFPAASGPSASRAGRAFK
RPGGGHEGEFDPFGAGAAAGAAGAAAAGHRTVRFNRPSGGHDGDFDPDAAAAAGAAAGAATAAGHRTARFNRPSGGH
DGDFDPDAMDAGAAAAVAGAAAAAGGRMGNFKRPGGATDGEFDADGAAAAAGAAAAAAAGGNRTVRFNRPGDADNGG
MDDDEAAAAAAAALGGGAAAKGARTVRFNRPGDGLSGGFDADPTAAAAGPAAAAAAPSGNRTVRFNRPGDAGGGGLD
DEDEAAAVAAAAALGGGAGGGGRTARQFQRPDRGGSGGDLDAEVAPPADVPEEVVSPGGGLPPNLRDFNRPGMGEHD
GDFDPEAAAAAAAAGGAAAAGTAAALGKAARKIKFAPELAGLPEDAPDGGGGGGSEHKPPSRAGSQTGAGHRSVRQF
RFADTRAGRSIRPGDVDFDDEDELLDGGQEEAGAGGRQEALPENATEEERRAHAMRHARTGAFGDVGRIGPVDNIQL
QREMRRRALLEQMKNNAWAGSFAPQREPGPGPAGGDA

>Cre04.g222650.t1.3|PACid:27580553
MQTAPLAPPPSPFPPPPPPPPPPPPPLPSPRSPPSWPPESFPPPIEVQPQPSPSPPPADDGSSDSTSPPEAPEVTQT
ANVQTASIFCDGLDGSAANVTVYVGEALYNYLWLRKWASTRGVNLVKGRAFAGTAASGLAASHEQSSNHNSPFMEDW
GVTFVNLAHLNLDGSSITDLPLSPVAPLLQCLNCSRVSAAGLTLARLTGSSGRPPFPAANGGACPAAATSPADAVAA
AGLAGQRLPAYGAMYVTGASAVHLVSFTCTDVTEAHGFACVSFELVAPAAAAPDAVGGGVDVGGGGGAGSGAGAGPA
GEEASTGAAAAAAVFRCDGCVVQRNRVSWGEPEPAAATGIGASGVSGSSAVGRGAVVLRSRAAAALQTGAEVEVAAV
LNDCSWDGNHGGAGAALSADAGVRLVSLAIARSRLAHNFAELGHGGALLLEGGLGSLTLTGGSIFANNSASGFGGAV
AVGAAFVPGFSLPAASGTAASGAFGAADALGELVVEGGSLVQENRALLGGGGVFVNGSLGVLVVSGDGSGVSGNVAD
WGSGGAVLVAGRLGALTLSGGGGGGGRGGAGGGGSNGGAGGDAAVAATVAAGFCTLCGNSAYDSGGAIYVAGSTGSS
TGSSSTGSGSTGSSSTGSSTGSSSTGNSAGGSSSVAAGVVIEVGYGARVEDNTAVNGAGGALCLTGAVARLRVAGPG
TRLLRNAAALTGGAVMVRQGGVSGSITLADGAVVANCSARKGGAFKVDGDVGSLLITGGARLDANNATRGPGGGAQV
EGRLTLLLLSGGGSSASGNAAFLRGGAFYVGGGLGSAVVEAGAAAEDNAAQFAGGALGVDVGLGSLTVRGAGSSISR
NTATLDAGGGVWVRGDLGELKVSAAARLAENFALNAGGGVYVSGGLAGGGVWVLDGGAIDANRVSRSRGGGLAVRGN
MSSLTLARGGSISGNTAGDVAPPLSAGARSHMPMAAGVWVGGAVGAIRIWDGSRISNNIARKGGAAIYVGNPDDAAG
SGGSGHVGSLELTNGSCICDNLSGVIPDDGSRHAVVFIAGRAGSIRIQNSSVSRNRVPYGDSVSLPYGTTHGGALLA
WQGVGSFVATAGSDLSDNAGGDGAVLCVPDQPASPTSTTALTSAAASASDVTSGSIVSAAAANQTAAAAEAAAAAAA
RFYAIGEFVLEGSQMERNVARGAGGALWSMGRVGRVLISGASSVSGNRADGTPGGGAVYLGLHLLSNVTIVDSVVAA
NAATQGSGGFLMHQVNATGVEEVAARLPPLLAPGTISTNHTGSASSASSTAFPASVLTLLRSNVTDNVAVRDGGAFM
FDMNVSVPLLGAATKPGATAAAAAAAAAAAAALAARPGPPLLQLRVEGSELSGNWARIGAGGAVAMLNALPSTVATA
GNSSNTNSSDYSAQQQQQQQQQQQQQRRRLADVWLGFELLIVGGSQLRRNRAGDDSVRFEQSVEDVSPLAGQGGALF
LWAQPTTTTTTFATTTTTLHAAAAPEDGGSRGSGNASAADSAACGGSAAQLAQQQMAAGMGSVEGAVAWPAGSRACR
LYVEGATLADNQATGGYGGGAAVVNCAVAVRRSRFAGNAATLRGGGLAFADYGLPAEAVAAAAVGAAAKGEPGDCGG
GGGVVADANSNGSAVDAVTARHLLQQPQPQPQPQPQPQPQPQPQPQPQPQPQPQPQPVHSLWLDISRSDFESNSAVG
GGGGLFVEVNTTAAARLADSSLTGNSVPREGGGALIVVAATAASAASATATTNAADLNTTATNGSLPSSNGSGSGSS
GSSSNSTSVANAATAAVTVLRVSCVGNQAGEAGGCLAVRAAAPGASVAVQELWATANAAGSGAGVSVSGVRGSAVTA
DGCIFESNTAQTTGGGWATGASFPAAGGASNDATAGADIAAAAVAAVAPAVMSVRLSNCSFRGNRALLGGGAAFVPD
PAVASLVQSCNFTLNSASGSGGGVAVDGSVRPGGSVIVSGCAFDFNEALVSGGAAHVLAAADGGSSSSGSGSSGSSC
GGLLQLLDGCSLNSNTAGSTAGAVMVEVGFGSSSSSSSNNSSGGGSSSALALPAAGNASTDASTTTTVNTCLLQPAL
RLQDASVSACSAPLGGAVFVAAGAVALLNNSRLINNSATISGGAVAGVGCGALRMTGCELVGGDAKLSGGGVFVDSC
GLVVVDRSVLRNNSASAGGAVHVSGLRSNASSSSSSSSSSAGSSSSAGSSSSTGSSSSTGSSSSTGSSSSSTLPVVL
LHRVDMSGNRASGVFGDASLLDVNYPSYAGFLPYGSHGGAVLVSNVIGVAISGSDVASSNTAEVGSAIASTQRCDGS
SSSSSSSSGVEAGGLVALAALSAAAAAQQAAAEGGLVSEAAWRAALGELRRALAARCALLTIANTHLPMSDDNGTKA
EGGVALPPLPAVWMEDLRATSLEARCSLTMDGSSSSSSSGSSSGSSSDDGGISSIADMVQQSLAEMPAPNTALAVAT
VAAAGVPPPAAAAFAAANGSSGVAVSTISMRTRITLGTLLQMLHNCVRGGGAIASSSSSSSSSSNNTSANNTNRSSVT

LEASGVNSVGSGSSSSSSSTAQVAIAVSATHLRVAYVTDALLLANISGRVRPAFQTSAQAPSQQQEQQQASAVADAL
AAGAAAGEPSDGSSSSSSSTALAAGALAGLLVAPGGRFGLRLQLHDGLGQPVAVDIPKYDIRLDLIRMDDNASTVAG
SAAGGTATAVLRGADFPVTTSYGVATWDDLEAYGWPGRHVLRARAEVSLDAVGPRAYPISTLRIPLVLVPCELGQEL
QQAPGSGSNGGGSDSAVVLTSCRQCRRDQLGLWNDTRPPLPSSSPPPAGASSTSTSNSSDAGAGAGAAAGVNATAAI
LEALAGAAAVTRDLLGLSTEAGADDACDACCVPCPDGATCAGGALLIPQPGYWHSAANSTAVHACINPAACGDGWDE
SEPWSKSVEATLAAADPDTLVWSQAQPALALAYAAVDIITSDPRSRLLGRCQQWWYHNLPPQQSRAWAAAGYSPALT
AAGAVLSLEGSSSNSSSGNGPPPPWCQLYGGGADPSSYLQLQCVEGYTGNLCATCQPGYTLGPDFDCSPCPSMARTI
TLGVLAFFGTVLLIVYTTFTNMAQDFEEISMKLEAEKVKRKAKKARKRAEKEQLKSVEKAAEPAEKHEEKSAMIGHD
YDDDEDDFSASDVLKAIITHAQFYIIVTKLGISYPHVVTKYQAAVSAFTGAENYVAYSPTCLTPDSGPEGQASTAVA
LGLLTPCVATAVAALLWAARRLTLNQSHHHCPPPVPGSCVGGAAGSSSPDISRSATRGASAATNACNGDNAAAPAAA
GPAAPTGGEDRREAQAAASAADTAAALLQQEAVTSGVSAALELQEIHAYVPLPVPIDSAAGLEILRDSHALASFHTS
RSATPRTHTAANTPRGPLSAFATPIRSQPHHHSAHPAAGEASSVARSSVGHGNLNGSWRGSGLTGGPQSAERDPRVP
PPPPPPPAGAAAAAAAAAAAAAATGAAGPVAESLQEEGAGRGAVPEDVATTAAGVVGIALWSSNNHAGGGSSTWLDV
LMAAPQPQPPPIAAAASANGAPASTSAAASYAAAVAGAATPPAPMPVGDDDGYALQRAEKATAAQPPGGSAAAGASE
RAGAAAAGIRKGRGCWNWEAHKAKAHAWVIHTKDQLRHPLRGVAYADSKLPVLKQLCVLLIVASFILYPSLAQVSLS
IFACYELDTGAGTFPENQLTLSSDGWWLRNMNQRCYTGLHRSLYLPIGIVAVIVFCAAPPLSYFLITFVNKDRLQKP
GILVQFGFLYQQYRRGWHWWGSVRQLQTLALVVVEVFGSAVSTLSQSLMLLAVLILFSMVNMACPAERSKTLRMIEF
LSTAVLCLTITLSLYFIQIPDDQQLSDAAMAAVGIVIVVINVALVAGFVMVMLKSGPVVPKRVQKAWKRFRKQAKHK
LKVFGCCCKGGVCALWKGGKVDGNQESDRKPAVV

>Cre04.g214200.t1.3|PACid:27580578
MPTNIWPFLANTAASQAASASASPSAPTAAAEPAGNLAGPGGPQGPTADDASAPSSPTAAATPAHPALSQDDPQTTA
TAPSGVTAPSSPDGATATPTSSSAGDDPVDACIVAIAPAGLGSGATEASSSPNVVKELPADPKPRGAVNATPSSSAG
DGTGGRAAGSVGDVDITHAADGAVANVTRPLRARGDDAVKPQPEAQSSQQLKPQPSVLQRVRSRGAGTIPGSGAGGW
DSEGCVFVENALLDSYSWHGIKKLYAYGIFFIITFLAVWFQTETGATAFKSSVIASFASYDTGNAWESSNLNGVGST
SEAFEYMIQYLQNALRSSGEVAPEAGSFLYLGDFAHVTYAQLEIKFASTCPSADAKSRVRETMQKLRGSLTPLLDDT
NLRRYTLGGAAAGGGGVSDLPTTVPLLGVKQQLPTSQLTSGILDLADNCVDLSPRTLVVDPADGPEDPLTNNIRNVL
FNSRYSQELSDFEVDYYSFLDLVGRLTRKEGPGSGGAQPYDYYEDGSGVLDLPPDGKTCAAGYIRRCAPIAGGGGGG
GGGGGGGGSEQQAGAAGSACFCAHPETGLPRTLRAYMQEDAVKGYSAPGSGGAAAKAPRIFRRDAKNRAVVELVRLW
PDFAGKVSLEEMKESLAYLLGSCRGVVGKYATQVQLGLHAHDRRNNDSNVLFYMEATRDSNRGRLETRTLSLTYNLN
WRPAGNFFMAMVIFASIADIVATVHAVLLARQQGRGLWAYIVSGSRHPFTWHGMDILHQLTMVAWICIWLHLHAVTR
AAMASYPQSAKELASLSSSAQLFTAWLYMGLAMFLIAGLRSLRYLKHHDGLRIFYTLFRLAYYDILEFATFVIGIAV
LLATAIFGVFIISGGNSVFTAFGSGLSALTRLSFGFLDYDTFVNSGNGMGSYGAAVVVLFWVSVLLLVLVVQNTLLA
IFTRAYETAKQGAAASGAQDSIFLLHAFYDFCFLLTYRARNYLLTLVWALVTDPLGVTCYDAHLRKQQARAKAASPG
EKTQGSVGTTGRVAGAGEAQGRLPGPGGSSGAGMQQWVYWSRAYALQNGVSDDAVSDADGLVVGGSSGGGAPAAGGA
GATAGLGVGTAASGTSPGQPELQRAQTFRMAGEQEQQQRLGSIRRYLRPQVTQNGRPGPGSAGGAQQAPVALTAASH
SGDLGAGAGTGAGPTPSPQPSLPLSSPAGAADPAQQPPQPQQPQLQPQQPLPQLQPHPQQPQPLPQPASRAVPAWRL
ALWRAGWYLYGLPYRLRLYWAEAHLRDFELLAAGDARKAAAPSRFVQQQRQKLLILSKGSTLRDPTRTFSFTLARWR
LASSPQMAALVAFFDDEAMSRLNVLPVHAVEEARSEAAAAAAAAELATRAANKPIALSYSVAATHLTPGTAAGGRDGA
DPAPGGSSPAGGGSSTGGGVGLLSEGSNKALRPAPVLAVLAEEPEDLDDSTAGDGGGNGSGHRAGSAAAGNNEAAPP
PPPLTAAAARHQASPFATTEPPAAALLAIEAAGKVPRLSSGGGAKEGGGAARGGGGAAEEGQPPEEEEADLADLEDA
EAVYSMWRLRFRGSGGGGGGGGGGGGGGGGGGGGRLAPMGSSNVAAAAAGGGSGAAAQWGPVRLLPQLQTFSSRPKK
ASARTSAPVKVAPATPPPPSSPPPPPGKGAAPAAAPLHAAHVHPMEDEVADPDRASVVLLDTVELKFYVEAYEAEVM
RLLVEGPCRAGLALQAQVERAWQRCWDYKQQHFGRPRASAAAAAAAAYGGAVAPAPPLAPADPAPVADDGSGAGGKGS
SPPPSPSPGPGPQPSPGPEQQQQQQQTQTQTQTQQQTQQTQQTQQPLQTQQQASPVVAGWRQRLSSWWRRSWCGRA
ATLSGLWCRAVFMTTRPNPFWPFLASSLDGHDENLKKLVRALVAVYGPMAPRISERQQRELAELRGSRAQVRSIRKL
LQSKQPGQQQLLALGQTGRSGVRGGVASATDPRAFLVPRVSFKPEVASPAAAHAAAMPSPGGYHQGPGREEGATVVD
VERLLRDAMEKALAAALAGGAAAGSSAAAAGAARQQV

>Cre04.g219150.t1.3|PACid:27580591
MQTQLRLLAQWRLHAQWRLLALATLIFVALTTAQGFVAGSPVNAVPALRGRLLLGSGATPPPTYGGSGDGGQSPGTY
GGSGPGPGYGGGDASGSSSSPAPAPNEPVCPSGPPSPPPVTHTCTLRLVGDVVRGPSPPGYGGGAAASPPVYGGTGG
SAAAGFGGSYGGSDGAPTITVSGSLQCATGSSAAAGGSAGGNVMVELGPLLFSMWESGSFTVSGVEVEPRARRPLIG
GSASPPPGVPEADSGAGDTPPPPAAPAGEAPAAPPGGEFPTGTAGDGSSGGATAAPTLTAGLDSSSSGSSDDWGLTF
LGELHLQLVDSEVRGVPLSSANPLLLCPSCGFLSLTNVTLTQLASQASLLQAAAAGAGSSSSSSSSSSSSSSSSSR
SGSSSGRAAEADAAAPALIFGALAAPGVRGASLSNVTCSDVQAHIWACALLYAQPAFGDAGVQVRDSVFERNVVLAT
PGDAVFGPTGEDLWGAMASIAAALGIGNFGCAGSLVSTQMQFGAVLVLTPYAVDDANGAGGATTAIGDCVPVEVVRS

RFTDNRGGAGGGMALAFGTGHATLCIEGCTVSRNQATLAGSGTGGGMSVCLSYEGSGAVRSMAVFNSTFISNRAAAV
GGGALYVRVEDGGGMPMAAEVRGCTFRGNWAAATVAGEAGEADGGAVLLFPTPGFTATTLADSVFEGNSAADTGGAV
ALRVEGTSSYGALHVTNCSFRGNVAHGSGGGALSVVKPTNNDVPLTLLRIAGGSRFLNNSCGGIDSAGGGAVYTPGV
ERVDIAGGIEFSSNAAAFGSGGALFLGSPTSVSVSDAVFSSNRAPTGDGGVICVRSELLAVDSVVLGPNVTARSNSA
NNGGVLFVQFVGNRDPNEKYPNDPNTTCALLALRGVQLVGNSARQVSDTSSVGVGSGGAVFAAVSELGTLELSGCYM
EGNEADNRGGAVDLELTGSVANVTVAGTVIRGSWAGQEGAGLALKARNGVGMVQITNLTVGPGVAVAPALSAPTGSV
RTIIADNAAGAYGGAGGGGIYMELGTGSVGLLNISAAAFVNNSAPCLACSGGALHVDASGGGAVWAAHVTDGARLVN
NSAGVDGGALYLIANQDPEGVSIGFIALSASGSSVISGNSAGLGRGGGVYVRSGNRDAGRIAEYATKPSLQQSGGGA
GSSNSNSSTTSSSNSTGNSTGSGDVELVVAGVLRVTGGSAWSGNTAPGGGGAVFADGFFEVELTAGATFSGNRVTGS
GGGGGAIALNRFPPRLVMAAATFDSNSVSDGSGGAVSIEVSPEQFIDGTVRDVCLRAIMAPREAFAANGTALRSNVW
RSLIAISNTSLLDNYASEDGGALYLKLHARSCAGDVGLSIGNGTRFVGNRAAGNGGALTFHNPRNPDRATTLSASIA
DSAFELNAAGQSPSWRGFSNTASQGGAIFIWNARDFNSAPAGASVAADLTQDCNVTVTTSAFTSNVCQGGYGGAVMM
AGCASQLLGCSFAGNRARLGGGAVASVQYGFREEAELPDPRIFSVLASAAGFGSSSSSGAGGSSTSTGASERRRQAM
DATAGGGFNTDARHIVLPYDDGVGNDIGMGSQMFVADAGAFSTAIQQASMAAAKQAAALRFTRRRQQQQQSQQQPTA
NGTAAAAAGGDAAAPAAAAGSAAAAVAAALAPYGVPRCQPRLWFTWVAGCNFTENAADLEYGGGLYADAGGSGLIVV
NASRFETNVAASQHGGGAFLVASEGCSAVNVTGSIFERNKASDAAGGGGAAYLLLGPEDGCTANVSGSTFVGNSAAY
GGGMVWYAPIASSVGLQRNTFAANGATTFGGGLASVSDCGASVVAAGDTHVANYAGWAGGAMAVVQLASSSSGLGFC
RNGSAPVWEGATATNNTAGYFGGSLFVAPGAAANVRGLVASGEMAGSSGSGSAGSGGSIAALNCTFLTVTDSSIRGS
TAVRSGGGVLASGCGWVVLERTEVVKCAAGAAGGGVHLAAPGLVAAATMPVAVSVSASSGSSSSSSNASEAGDTSAY
GRVVLSGVTLAGNRAGGSSSGTGTGNGSGSGSSSSSGGYIPRGGGLFVEGQLLAVAVSASDLSSLSNSAALGSALAS
TQRCNVSVAAAALAASFLAGAINTSNAPVPLLMAVLYPPNADLQRWAPAWRTLGVASASGCGALVLSNSLLPMVTAA
GNSSSSSNGSSSNGSSSSSSSSGGSSSGGSSSSSPAVPLWMQDTSVSALAAECSDDTDALVRALQLSLASGLQLRAAV
QAAPGTASLGTFGAAAAAPDPASASAAPQSPQQSSPPAAGEDAETAAIRVLDSLSYLLSSPSSTNTTNANGTTNGTA
SASVAALLAARYRGLTSALARCVPRQPSADGSAAGGLSRYAVSLPPTQMTLTVEEGGVQAGDGTWLLQAGGSLMASV
ALRDRFNQPVTSDMASIRITLQMLPRDPSGAPFNATAAALSTSTSASSTSASDALARPWLNATVANLDPGPLANSSL
AQPVAAGSATWRGVVPRGWPGDYHLLFTAEVLSEDTGVPPVAPLRVRARLLRCLVGDTLELDTAQQLDAPSWTACAS
CRLGQYGLWQDDRPSLTDIQTANYLKLMNATANDDYNGPAACTSCPDNAACPGGALLVPALGYWHSAPNSTAVHACP
NPAACGNSADADWAAPNAVVGPDGFVVVPYDAVLQDARSQLLGRCQREWYGSIPQGADATATYLARYNVSSLAEAER
LLGTAAGVDAFEGGGDFDQLPCLLWGLSASTPDSYMQRQCASGYTGNLCAACLPGYYVNSDFECQGCPSMGRTVFLG
LLAFLGNVILILFTTYGNFRDAADEDEGRQGIEPGEVLKAMVTHVQYFIIITRLNINYPPSITKCQAVLNSVTGAEN
YVAYSPSCLLPREDSAGQARIQLLAGLLTPVAVALLCLAVWALRYRFGSTAMVVRTRGGRLRYAPDPGAPNSVQRAT
WFNRIFGSSLRRHDHHDQQLDDWHSVQCTAASGGGGGGGADAPLVASDALGAGSPYASGSVPGAWPAAAAAAVAGAG
VSGYSTWVLSSNGVTPSHSGYPAAVAGGGGGGGAGGGVPAVAGMGSRPVSARYPTITPSSSGRLEPVLAMPPVAVAA
LPAVAVNAAAAAAAAAPDRPPAVPNSIAAPAPLSRLGPRGVNSNGGAAARNAGGAAVVGILVDRPDGRAAASGGVGP
RPVAEGAEAPAGTSPRRRAAPDVAGAVEPQAQAPQPPPLMQQPAALVGPTAASGEQRSLGSAASARSAHPHPAGAVT
AAAVAAAAVADPAAVAVAVAGSVDEARSLEAAAGMYSGYPASYPGIAGGLSPSGRVYNGQDSGYPGSYAPDGAFPEA
RNGGGAAGSAADTWADEGEEGGEQGDASAAGGGRPGGGRSSVLRQVRARARSLLEPHIKRRSALVHVDQTLTLWEQL
AIVLMVAVFVLYPGLAQAALSVFACYRIDTGDGNFPDAQLATWSRGYWVRNMQQECYSGTHLALYVPIGVVAVLAYC
LAPPLVSFLLMARHRHKLDDHHVGQVYGFLYKRYRPEWWYWESILQLETLALVAVEVFGRALVTAHQALLLLAVFLV
IGAVNMTCSAMRSHFLVVLEFLSLSVLSLTVTLGLYFAVDNNTVTGGAGAAVGVIILVVNAALLLGFLALAAVAVWP
SLVESTSPRLKQLGGTLARKLRLKQQDAAAGGGGAAGGGAAGGGKGGDDGGAAGDSGDVGEGVSGQEEKGKGPSEGG
GCGCGAARCRVAACDWCGLRR

>Cre04.g214000.t1.3|PACid:27580655
MWSARWLQQHYWRCSHRRRWSGDRPAKSFIAAAVKFLYLAAMMIALPVAAQVPSTSRFPPPPFSRSPAPPSPQPPRP
PSPAANSSVQSEALAPSPSALDSTYSDYSDLLEELVSSLPVPTATAGDATAYEQAGLASPAARYCASLSASLDLLYY
GLLPSLNRTLNASVGGTPPSSRRFGLMINAVPHCRTLQSLFPWLLQALGDGGIGSGCYSVAFSSSVELTLTPVTSAD
LLLETGRAVASNLINLSLDFCNGDMPGDWGQPYGSEIFFVTKFATATILRGGDAAGSSPPARGFDWWTASSPPPPTL
YDNGYGYSSSGGWYYDPNYGLTYGRRRLLSLLEEDAASGRDRSLLAADSYGNGGGGSDSEEGFMSVFADAMRESGAA
GRAIADGAHKPGGDGHDITISRLRCGSAGGSGVGSGLSGIWRRLLSTLRLRGGGEEAVAAGERAPAAVARSHRRRRH
RGRYLMQSGTNSTNSSNLYVVLANRSTDSTGRVLRGRPEAGVAPNGDLGSICDDNFSEGSAAVICRQLGYPGGGLAY
SSAVFGDATRPFILDELNCTGLESNIGRCRSNPLWTHNCFPNEAAGVVCFPSFNVSSYCADVVANVPRVYPTPTNTT
PDPSTWSRDQRLQRCGELMGLAAAASTLVGYKFPGQYISYREVQSYACGRENVVPAMTAWVAAWRREVAASGCTELI
VPYLWMQRLGGTVPTAPSPPPVSFNTSAFRRPPPPPLPPAPPSRNNDSCPTAMAVWPTPADRAASDAAAPYGTPAVP
APTTYMILDYVSAPSSTSGSSGSTNSSVSSPLDMTCTPPSAWLAAGGAPSRYVRSSNGGSSSVARGPAPSTFNSSFR
SPPPSRAPPPPFGYSFGFPPPPSRFGNNISSPSSSTFNSSISGAGRRLFRRALVDATLGAAVTATTGAPAPPPPPPP
PPPLSAAAASLLRYGASGAMWASASAAFASVWSPRDLVNVSTLTRTLARGGPSWLPSRLSDRRHLLSTLELFSRPGG

IDLVSSGILDTVRWALASLPSPPPPPDANRPPRPPSPPPFRWTDIGDIKDVAVTDAAPPRPPLRNIVSGGLSDGGSN
SPSPPAPPSPPSPPPPGPRAPLSPLAAAMPLKPTCVAYDSSWTQADGFRSDFAMLDFLASNAASSDGGRDGWGQQVS
QTETELLYCNARQELKTRHTVSGIAITGLGLAGSAVSTVRALLGGARPFNGTAAIPAANLAFLDLSGNALTGGVPAI
PVPVLHVNLSSNRLSGDLQNQLKNNLTAAAFTATLVLDLSNNGFTGQLPTRIDVAAPATNSSAWGALLTSAVRIDFS
GNGFTGSLPADWAPLLAQMDLVVSRNPGLTGGVPAEWIAAARGLPPCGDGGSGPFSLGSLITATAPPPSSPPSPAPG
GGEAALGDSASSNPPRQGALLIDLSGCTGLTGRFTHTADFSTLRVRGTQLQLDWNKLPAAERKWTYVVDRVARDHMD
FEQVAYRKSGISNVLTRASLLDPDEVMLVCDAIDEQRNAERRTAISAVGSSTGSSTGSSSTSGGSSIGGDATVVVAV
TEPWPRVRNYLEVHDSYTEWQTDPAVWCYGGAEARGIVALKVWLPVIIISVLACGVALPAHYIRERVRNRAAARRAA
WKQQQQQQQQQNQLAESKDGKKRPVNTLAAPKGRFTRAWHKFKGVMVPVIDVVEPRLVLALYWVDLISDIVFIAGYK
GWNRQPAPAIAVIVILLANLTAGWVLHLIHLRKQLGDGPAGGEGWSVLRCIVVGILTAPFGIALEWLWNGFLTVVTC
VTTWHAVAKGDLKAHVTAVQRITRGWPLLLSYEANIQIFETTVLLEPVSESLPQAIVQTTCYVVGRNAGMVILTPIF
LFSAILQAISICKCCLVLAWRAYKSGSWTAVLTLQDEGAGKSFSMEDKEEDSDDDKNNKKKGEGKGEGDGEGSDGAG
SGAVEGGAGGGGGGAKGGKQSPHCSRATTSSVSGTLTAMSSSGLQASGPPPPPPVQPPPPPSNAQVANFAGVSAFLN
QQQQMAAQMQMPMQMQAPPPAMVVAASAPPFAPPPPLQPMGPMLMFPSAVAPVPQPPQSALLQPMPFSAPQPSASPV
TKGATAWGLRDWASLAAAASSRFTDGFAEAQEGEEEAAEVEAEAAGEAEQAEAQAADTEQGVVAAHDIAIQFAPAAY
STPAPPSQQPMQALPMQAPPMQQQQQQQQQPVELPLGYSPLTAHLHGYRAPGPAPDSAAEDGTSSLRGAWSAMRGMW
RGGRN

>g5069.t1|PACid:27580669
MGCCQSLPEVVEPVVQRRKGGTPVVESCFPEKHGPGGYKVLVTTSSAELDFCPTSGTARGRAYARTVVGPTWLFACV
FECLPVPGDSGDVRSRGVYSDAAAQYCADTAYEVFAQQQQARRSTGHAQGAGDMAGLLGAVLAALDRRFLNDPAVMP
QDRLGSGCTGVLLHMDLAAPPEGAEAGGRVAAVAAVGPCAAVLGQRQGGFASATAAPQLLAGGNAASHAAVCELLER
LGAERSAELVRVRQQLQQAQAGAKAKPGKGVQQPPQPVLSAVVSAAPQEPSYAPQPEAPAVLGFGACKGMGQLVALP
PGGVGPVPAGAAPGAAPGEVPPTPPPCVGLAVQTARHVLQPADESLMLLGAGAVAALSPAEAVLLMHRFGQFAFISS
EDKPAPLDSPASSSSSSLSQAPAQPQAVQADSHGKAGPRPPTTARGPGHQTSAATAAAGGGAGVAPFNAARLVSQQA
AMAVGRRRAQRTAAAAAAGGSAPPLAGFPSCCAVLALSLDWSPAGAGRDPASPAGVVAGSPAALAAAAAAALREGRR
GLYRWQQLRAYYKLASRRRAALIARWHDLFDRVVGAAAQEAKAKEVTAWKRMGAALQVSTAGKVKVIEGSSGQQPQQ
PAAAVAAPAAAALAAAAAARGSPMRPAAPSASTTAATSGQAASRSPSPPPPPPPATAAAAVSQQQQPVAARVAQAPA
KAEAHAARAPSPPAAAAAAQEHSPPPLHHSAIRLEERPLVGVAQRYAVAVEGASEQDTAVSATAVATPPAQPAASAA
GTVAGPHTAVTPATAFPSSRGAASPAAGVAALPAASPHMTPRGSTGGSGIPTPPATTPSRSSGGAPAASPVPLLGSM
TRSGSSRLPAAPGAGSSGGASSGGASAVPQQRNSPPVARTVAPSAARPGAPPALSPPLLEAEGEAGADLMSPDADQL
ALWAAEAAEALAAGPQQPQQQSGGGALQGAARGSMGRATAAAAGSRIPLSPSRADNISSGGPAGAKATSGAALAGGV
AASR

>g4508.t1|PACid:27580749
MEGGHRCNARSGRGGGDDISCAGQQTPGGAAGDCRTVCRGRSSARSGRVWRPDPGPDIGTQGVASCLTVSRHVGGSS
RRCRRQTHGPGSWAAAGPAFSLALLLLLVMGPVATLAADAVTVAGGGGGSGGGSGSTGMGDTGADVCDILPAWSPAN
PPIAGERVARFQVVARIAAPSPPLLATLQYGDEDGRQRTYTTRAYGQPDAYGRTPYILDRWAGASVYDSTPEASTAR
LYGKSGWYNVTCRVQDSAGRLATGTRRVYVYAATPRAALGGWPTLLTGPGTGTDAPTPATESLPVTWADPGLPPATF
RVLDLQIQDSGLGSGFPYGIVHGYLSARALSPDPAADPGDPDSWSLATTRRVDFVRRQGGDGTTWALAPEDATDPSH
PADPNPQPGSAISTFKLRVPGRYRLTWTVPDPQNPDSDTPYASDPEFVELLVRAQDPGYVAPPLAPPPFTPSPPAEP
PGLASPPNLYDDSPPPPPPPPASLPSPSPQPPSRPQPPQQPPEGADEPLSQARARPPAPPDLPPPEVSARGGQQGDP
SPPPAPPPVRRQPPPSRRPPPRWRRPLPPHRRAGGKVEVSVHVVVEVEVRVRGGRRLAKQEEAQVEEQRQER

>g4982.t1|PACid:27580755
MSGWYRRACRHFSDPEALLAAAVAGQDYGGGGGGGATAASLGGAATGMAAAAAATAGGGAAATAAAATAGCGGGGSS
SWLLAPGHLQLNGSDWARAYARTKYLYEATYGELFDPPHTQYVSLCVPHPLAAPASDLAPLLELLELRRPRRQGPWP
VRMLLPDWVDAAAATAAAAAAAATEGGDATASDDDEAEAEALAVAGGAGGSDEHEDSDPDSDDGEGRRAGIRLQRGR
NSGIRGRPHHHHHPHHQHQNHHDHGHHHHQQQQQQHQQHPGSSGGGGGGGGSRAGSHSSPRHPHHPHLRIQPSPSL
PSLPNGGGGGGGGGSGAAASGASASYSPRSGQQQHPHQQPHQHSKRRDGPAAAAAATISNALALGSTSMTAAAAAAAA
TAAATAAATASAAATAAATASPLLQPPGCVMLLMASTTTKADALESPYDPRVVRQPRAGAHALFLAWRLARGVHDRA
VRTGVFEEQGCCGCAPNTFTLAWPDRRPVNCLINFVKLRQGWLQLEGLPHDSSHPYLLDVTSLWDDDTLEFRAVHTV
PWAAAAAAAPGLAAGGAATLSLPASPAGTGGTAAGPAATPEDGGVTRAVGDGGGGGGGGGWGWGRGGTQDDVEARIS
HGGGGGGGARISGGGGRISGAGGGGGLVVAGAARPVLRGRTQSSYGQSGSGQLEPSDPFDTAGGGGGGGGGGGGGGG
GGGGGGGLWRRTTVSGVASAGGGAAATAVGSRLGGHSLRRRQLADDELDDVEWRRQATTSGGGGAAAAGTATARHSN
AGHGGGGGGGASAADHDALDSPPLRPTPAAAISQAAWQQASRAAAAAALAKERSSHQSRGSVPSRKVLLSMIAFASN
GGGGGGGGGGGGGTGAMTVSYSGVPALPMPPPGSKPGGGAAAAVDTASFKTKLSGLRILTGGASSGAEVTAAAAAAA

EEPAGFVLPAGGGAAFGGTARGVAEGTEVGSGSPVSPVGSRAVELTRATSAAAAAAVAAAAAAAAAAGDGGFSGGAG
GGGASPGPSAQWAPPLTSPIGSMRRSSLMLNASSAAGGGGGGGGGGGGGGSSGGGSLALSPRGTGGGGGAAAVALGAI
AAAAATPEGGRGSHVNGNGPWSGAMPASALLDTDAAAFAASPRALQRLGSLHAAVRLARTQSAARFASSSIQQPSPP
PPYATATAAAAGGADGPGGGGSNVMRVSGGGGGGGAGPSSPQRFQHMAAEQRLRVASSPLRSSRTGGLAAPGSAAAV
GTAAMAAAAAAAAAQLPSPAGLAASPPPPPPAVRRSTHSGLSSPAASNLSSPARYPGSTSGFHGGGGGPGFSEPGDLF
AAAAAALTMHPGIEQLQLLSPGSGSGSGGSPRAAPPVPRSGGRGPSVDGVVAGGGGVGGGGGGGGGGGGGAPGGGGG
RSRLATSSMPHPAYAAVTAAAPAAGAETGEGAAVVAVTAAAAAAAAAAAAHAASRTSVSVGVVECEGEGEEAGVDAGT
ALVAAIVRHPYDALPGFTPGRGVTTPEQAEAFLPEASGWQEALQELLCLQDTYGGVYGTVYGRGLRNARRRQQLRAL
ADGTMESAMAAAAEAAGAAAAMAAADGGGGGGKGKGPAAARSPAAVAAAAADAAEEDAASVPCTVLTWAQVRAWGRA
VFWYPCGKPLASCWSALEPHSRRTLAVCTFIIPVVPILVILMGAAAAAALAVLAVPLLIAGAVLVTADVIDNGCWSC
VRRRLCCLVSYIGPVPQSDSVEEEAARDACEAELAAAAAAAAWHAQLTPPLGVAGQAEYLLRKQQLRQQWPRVAASQ
GAAEAGCRAATASPPAVRPQALEAAAAAAAAAAAAASFGSVGGALAALNVGGGGGSVLPPSAHLAATLPPGLQTMLRS
GGGSPAIASGGGADVDGWTLPPSATNSLVIGTGGGSMYGAIGGGAAAVMSGGGPGSQLPQQCSRSYSHQQQQQQYAY
AAYSHPHNRFRQGASPAAAAHHPSPGLRLQHPQPQAHQHMASPPTSATAAAAAAAAAGGGYYSYSQGYSPVRHHSQGH
ALPPAAVAAANSNNWSGSMASAAAAASGGGPGSFGCLATAESPYFRPQPQHHHHHHPFSQHRPQPQHPQQPPPYRER
LSPQPPPSPRHYQQHGSPGALVPAAIGGVGVEEAVPLAAGPAASGSPSRGAGGGYELPAGRMAAAAAAAAAAAPTYL
RRSPLSRSPSAAGTPDPAFGPGGGGGGPGGGGAAAGGSAAGLGLRHSYSGRTYPLAPHPSAATATSGTSTTTSLLTS
ATAGAQGSLQGPLQGALLSDGDSGYALPAPPTLAQVQGQPAQQQPGQGQQPGQGQQPGQGQQRGQGQQQRLRPSGSG
VAKALFGRTSQSALAQWPVGGSASGTAVEPSPAEGARCPAPPPPPLATSPLGRAPAAPPPPQRFGSRPSLGTAFGSA
TVMEAVLADAGDAGGDSSYGDGGAAAAEVAAASAATAGPSGGGGGSSRYGGGGGGYSHGVVGNDPRGYGLSGAIADR
SAPTSRSALGSEMYSLAAAGAAAAAAAAAAAAVAGATPGGPRSPLPSPPVAPGQGNGRSLPLGPSPGSGGGGGGGSGG
GGGTPGDYTRTEPYSHSGGGAAGGGGGVPANYARYSPGGAFALAASGLPAVAESLVGSPATTEAGGPPSVGSRAPTA
YSITSAGGVVSLGGGYGYGYTAVASAANSPMPRGLPPTAAGGLGSAGLTALSASRRREGGSAHRSGYGSGGGGEAGG
GYTSGGGGGGGRRSGYMSGGGEDGARRPASGGSGGGGGGGSGGGGGGGAARGGLAAVRTRTRRRSSGAGYESAGTGSS
SRTPPAARYASVSATSPTAAAYGFGQVAPGHGAAGTLAYRLSGGRASGPGGGAAAAAAGFGGMVLVGGQLYALGGGA
GVGGGGGIGIGINLQAAADAVLTASRARRSSTGGGAGAGGSPGPNSYSLGSFAGGATAGAAGAAGGGVLGTPPRPSG
GSAQGPTLAEAAAAAATYASLGIGQPPLLMAVPRSPGLSPPPHDLIRRASGGGGGGVGGGSSASPFRSPYFPLGGGP
ADVASPARLPSPGFASPALAQPPLPPLLPPRQSSAGSGSGPGSGLGHPLVVLPSHSMPHYASGSMAVQAAPYSKPQQ
QTQPQQQFQPQQHRPQPQPPSPLSKSPSAGAVAFAAHAPAATAAAATAAAASTASAGSSMSSGMSGAATAQASGAG
AATSVKPGSRGGGGGGGGGGGGGGAATAAAALARPAWGQRVPPTKQPSQRQAQQAQHALQQVSQQAQQQQQQPHQ
PQQQFYQPTLWDRRRAGSVAASDSGDSSVSSSPAPAPPQSVHFAAPALRIRTRSGGDDVVGAPAAVATATAAAIATA
VGVSDSAAAAVAGRPGLSVPAPPVVSLPPDVIMPRGDGYSLASAAAAGGGGGRGGRGGDPLSPPAAGASSWASPSGG
GGGGGAAETPTGASASVTPRAGALGVGPHARSSNALPATPTSSGKPPLPPKAFGGSPRLRQAAPGEDGGTAAGGGGG
GGGSWGGGAV

>Cre04.g213950.t1.3|PACid:27580792
MITLPVAAQVPAGNTPPDFLDFFFQPPPPRGRSNASTALPPPPIPGVATAYERAGLASPAARYCASLSASLDLLYYG
LLPSLNRTLNASVGGTPPSSRRFGLMINAVPHCRTLQSLFPWLLQALGDGGIGSGCYTVSFDASSSSSVELTLTPVT
SAGLLLQTGRAVASNLINLSLDYCNGDVPGDWGQPYGSELFFVTKFATATILRAGDATGSSPPADGFSGFDWWTASS
PPPPPSLYDNGYGYSNSFGSGGWYYDPNYGLTYGRRRLLSLLEEDAASGRDRSLLAADSYGNGGGGSDSEEGFMSVF
ADAMRESVASGRAIADGAHKPGGDRGRDITSRLRGGAGGSGAGGGLSGMWRRLLSTWGMGGGGDEAAAAGERAPAAF
ARSHRRRGRYLMQSGTNSSNLYVVLANRSTDSTGRVLRGRPEAGVAPNGDLGSICDDGFSEGSAAVICRQLGYPGGG
LAYSSATFGDATRPFILDELNCTGLESNIGRCRSNPLWTHNCSPSEAAGVVCFPYFNVSSYCADVVANVSRVYPTPT
PTTPDPSTWSRDQRLQRCGELMGLASAASTLVGYKFPGQYISYYEVQSYACGRENVVPAMTAWVAAWRREVAASGCT
ELIVPYLWMQRLGGTVPTAPSPPPVAFNTSAFRRPPPPPLPPAPPSRNNDSCPTAMAVWPTPADRTASDAAAPYGTP
AVPAPTTYMILDYVSAPSPSSTSGSSGSTNNTVSSPLDMTCTPPSAWLAAGGAPNRYVRRSNSSSVARGPAPGPSNS
SFRSPPPSRAPPLPFGYSFGFPPPPFRSNISSPSSSTFNSSAFNFNFSFISGAGRRLFRRALVDATLGAAVTATTGA
PAPPPPPPPPLSAAAASLLRYGASGAMWASASAAFASVWSPRDLVNVSTLTRTLARGGPSWLPSRLSDRRHLLSTLE
SFSRPGGIDLMSSGILDTVRWALASLPSPPPPPDANRPPRPPSPPPFRWTDIGDIKDVAVTDAAPPRPPLRNIVSGG
LSDGGSNSPSPPAPPSPPSPPPPGPRAPLSPLAAAMPLKPTCVAYDSSWSQADGFRSNFAMLDFLASNAASSDGGWD
GWGQQVSQTETELLYCNARQELKTRHTVSGIAITGLGLAGSAVSTVRSLLRGARPFNGSVTSDEGTAAIPAANLAFL
DLSGNALTGGVPAIPVPVLHVNLSSNRLSGDLQSQLKNNLTAAAFTATLVLDLSNNGFTGQLPTRTDVAAPATNSSA
WGALLTSAVRIDFSGNSFSGPLPADWAPLLAQMDLVVSRNPGLTGGVPAEWIAAARGLPPYGDGGSGPFSLGSMITA
TAPPPSPPSPPSPPRPPAPAGGGGAASGDGASSSTPPRQGALLIDLSGCTGLTGRFTHTADFSTLRVRGTQLQLDWN
KLPAAERKWTYVVDRVARDHMDFEQVAYRKSGISNVLTRASLLDPDEVMYVCDALDEQRNAERRTAISAVGSSTGSS
TGSSSTSGGSSIGGDATVVVAVTEPWPRVRNYLEVHDSYTEWQTDPAVWCYGGAEARGIVALKVWLPVIIISVLACG
VALPAHYIRERVHKRAAARRAAWKQQQQQQQQDQLAESKDGKKRPVNTLAAPEGRFARAWHKFKGVMVPVIDVVEP

RLVLALYWVDLISDIVFIAGYKGWNRQPAPAIAVIIIILIANLTAGWVLHLIHLRKQLGDGPAGGEGWSVRRCIVVGI
LTAPFGIALEWLWNGFLTIVTFVTTWHAVAKGDLKAHVTAVQRITRGWPLLLSYEANIQIFETTVLLEPVNESLPQA
IVQTTCYVVGRNAGMVILTPIFLFSAILQAISICKSCLVLAWRAYKSGSWIAVLTLQDEGAGKSFSMEDSDDDKNNK
KKGEGKGEGDGEGSDGAGSGAAEGGAGGGGGGAKGGKQSPHCSRATTSSVSGTLTAMSSSGLQASGPPPPPPVQPPP
PPSNAQVANFAGVSAFLNQQQQMAAQMQMPMQMQAPPPAMVVAASAPPFAPPPPPQPMGPMLMFPSAVAPVPQPPQS
AFLQPMPFSAPQPSAPPVPHAVTAAPPAAKGATAWGLRDWASLAAAASSRFTDGFAEAEEGEEEAAEVEAEAAAGDA
EQAEVQAADADQGVVAAQDIAIQFAPAEYYTPAPPLQQPVQQQPMQAPPMQAAPMQAPPMQAPPMQAPPMQQQQQQQ
QQQQQQQQQPMEPPLGYSPLTAHLYGYRAPAPASEATAEGTSALKGAWSAMRGMWRGGRN

>Cre04.g220150.t1.2|PACid:27580814
MASLATSLGGVLGRSGGVSGASGGGSSRRRLFVPGPASTGGAAAADDLGGSPHPSAPPLPPDLLLPAAAIMQATGAG
GGGPRDASSGPAAAAAAAAQQQRLQQQQQQQQQQQAYPEVPGRSRGGGEGGSSIGSPAAVAGEGLPSWPQPAAAAPA
ATPVATAYAAAEVAAAADAGSSNLLLPAAAEQSEDVVGVIGRLAAAARRPLARFLRGGSSSGGGPGSGGGGSSSGSS
STGEDGRRALNQAMTAAAARSEHATAAAGGSSGGGGAFGGSSSDNATAAGAAAAGRELMWRHRDVLMADVSLFLVTP
TQQPQAPAAVRALVEGDALRLRLLPATAAATATAEGGGGGAGGGSSGSGGVVRMQVGDVFEVVLNPPPPLLPPHHHQ
HRNQHHSPPPQQQQQLLPLMRAGPGIYTFRLPAAEAAAAATAAIPTATTTSSGSSSGSSSSSSSSSSSYYCLTVSPT
TADSCSLDELDAVFAAYTCYSRTPPAAATATASATAATADYGINGGAAGGSQGGWRAAAAAAPAGPWVAHAEPAAAA
AAAPGGGGGGSPYVGATAPSWPPPRDTSGAAGGLRNRRGGKEQRQQQQQQLQQQQRQQAGFGYSGRSYPAIAPPAA
ASATVVYPQQQQQQLQLAVATSGGDGEWLLQYGGGGSSSTAAAAATAAPAAVAAMAWAAVAGASSPAEAANRVAAVS
VWAARAAGRGLVAAAHVSAYGIRRKADDIIRGLPASAVAGEDDEGTTTTTHLQQHPAVAGGGGSSRSGHKREVLRRF
FYALIHGSSSDATATAADAGTRRRRRRGARRRSFPSSNSVATAMGTAALLVPVRVWVVLVWVAARAAAAAVGLVCEL
LARLGAAAADAVIWASGRAGHLAPHLPSSPPPSPPPYWQQQRYPAAAPVVGWPAPAGGGGGGWQQQEQEQQEHGEGG
HAAERGPAAAAAAWRLLAAALVALSLPADVAKAAAGAVLLIYGELHGAAEEAARLVLGASAAAAAAVAEAAGGAAAG
GLTRGALEAVGYALDAATGIRKVGLRAVALEYQKRHAAAAVLRHLPTGTSSTGARGGGGGVGYAAAPPPPPLLPPPP
PVTAPPVVPPRGRYTFNQQEW

>g4507.t1|PACid:27580857
MWGSRIDPTKRHMRRLGIWAPVFMLSIYAWSIYSRPLATNADSAISASHQDPYPQRARRAQLSEPNAENSCSISLAS
RAPIANSRPAFFQVVVKMSDPQPPLSASLSYGDGFERKYWAAPAGGPDSGGLRWFALGRAMGVRLADGSPEASTGRL
YVAAGWYDLDCLVLDSAGNSAAGTRRVYVYESEPRQAAGGRLDLLAPPAVPPPAAGAAGGGGGGGGGGGGGGGGGGG
GGGGGAVGVEVEVEVDPGAPALRFQVTDLTVSDSAPHQLPHTTALPAALHAWRLEADGVEGDPGSWRLMDTRRVDFV
RRQGGDGTTWALAPPDAGGGGGGGDPGFTTDAAAATSTFTLKLRAPGRYRLTWLVPDVEHPDSDMPYAADPAIPAGS
GPDFGSGSGVAALGGVEVVAVSTTPGYVVPAAPPPPGLPPPFPRVRSPGDYWLAPPQQGDGWTDTEPQQAKPEYNPP
PPPPPPPPPQLPSLSPPTPPPAPPPPAPPPPSPPPPPPPSPPSPSPPRPSPPRPPSPAPAPSAQAAQKAPPLNPLPP
SAPEPLAPSKFRPWGSRLSLPSPPPPPPPPPPPPPPPLSGNSGGDDGGGGIDGECESLLIAAYELLAIATATAASTP
ELQSSTVGARRRAAATAGAAATADVISAATAVPSQPFWDMRAHGVAWTWGAHAQDWALITQQPQLQQPGPAPRAVVW
VSDPDYPALRASRTPGYGPLALLGSSSSGSSSGSSSSSSSSSSSSSVGGTGSSEGGGSQQLERDVWRPARYSASELQ
PRVSAPPLKQPPPPPPLPLLVLYFYEPVQLSAVRLQLLLGGAGGGGGGGRGGAAGAVSVKAWLLPWAVAQRRTQQQQ
GQQQGQQQGQQQGQQQGQQQGQEADPREGAWLLLSGSLNASTSTSTAGSSSSQNGDDAGCGDGGSGGGGSGGGGSGN
GGGGGGAGCGVWLELRVPPAGSPGEELAAAAAVAAAAATGRGDDGNAVGGGGAWEQPAAPVPWLRAVGGVVLQVAQQ
GPGREETTAEGRERRESGVQGKEEEAVDEASVGGGGQQEGLGPGVAAVLFDGRVLLPRNPAVYAYWASPRRPAAEGG
GSGESGDGH

>g4500.t1|PACid:27580889
MPAQPASAAAAAKQPEQQQQPTQTHRAGQQQQKQHHPHMISPHPGPGGPQGGHGGGHGAALGWGAFAGLAWASVGVI
FGDIATSPLYVFTSVFTELRSTAGGGPGMPDRADVLGAACLVLWVLTAQAAKYCGVVLAADDRGQGGTFALYSLLCR
HMRIKPHGARGPPPPPPAAAAAVAANGSAAAAAEAAEQKQQRQDQWRRRRHMRWWQWGCADAEAVRGFFRRSRGAQ
LALWGVSVVATSMLIGDGVLTPSISVLSAVSGLRVAVPALSQEVVVGVTVALLVGLFTAQQAGTGQLGVVFAPVVAL
WLAVNAGLALYGMATLPGGWGVVAALNPAHGVSLFARHGAAAWRCLGGVMLAVTGTEALFADLGHFSAGSVRAAFLG
LAYPCLLLTYLGQAAHLLHRPSDWTDVFWKAAPQQLLVPLLVLATAATLVASQALISGVFSIVRQAMLLGAFPTVRV
VHTGGRRVEAATQVYVPLANAALLLLCVGCVSGFRDTVALGKAYGLAVMSDMLTTTALVTLVMLAVWETSLALVALW
AVLLLVLEGALWSANLVKIPEGGWFTLAVAAAVASVMLVWWAGSRRVAERMAAAAAGARLRAVPRRRRRQGPPGLGP
PQEGAGGGGGGGDGKLGLKGGLGGGGGDHEGGPAARPLLLPPPPPLLPPYGHILRVSSDRQRPEPSGGGGARGVVAG
EESPRLGQQRQQGPDWDSNDTAASSDSHPLMHAAAAAAATAAFGDTGGGGGSPVGQLTPPRPLPRQQPLQPLLLQPA
AVVDSQLQSTPTYLPAPLPPPATATAVPAATAPRAATAAGAGATASGRLLGCSSYSPPAMSQPPRGRQEHRLLAARS
AAPAAAVAAAAAAAAAAAGGGSGGWAAVRRRFMAPTRLSPPPPPPPPQQQQQPPFQSQAQAHAAAARAAAAFLLSPP
ATAAIKAQLQQQSQQQRERAGEDAAEGAQPPVPLLQPPLLGRSVSLAVAAARSLAASTGGLGGGGGIRRRSVNLQQ

QQQQQAGGGRGGGGGGGGGGGGGGGGGGGAGDGDSDGGGGLGGSSSRALGGPPSGGGCVALAAPQPQPQSPDRRLLL
RLGSRAEQQAAAAAAAAAAAAAVGDLGLGLGRTPDMPPSLPPLTERQQQQQLGYPATAAPDPAPASNGAATASGSDY
DYGYDLVLELPSAHPHTQHQQQRLQQQQQQSAAAAVPAAYAAAAAAAAGKPPPPPPPPAEGGVSAALSPPPPPPPPL
SVRLTVLEGVGIYYMDELTPQQQPEGAAALPPVLVHFLRNVQAIQAVGVFLSVRQLPVPSVPRSRRLEVLLPHSGAA
GSGADMGVGSCLPPNFYRVISRYGYLDQVDHGPAFLSALCGALLQQLLARGLQAAEEEEEGEAEEEEEAGEEEAGEE
EEEGEAAAPAAVRLGGSGRGRDGDSDVGSGDGSGGEDGEDEDDDEGEREAAAADAAEGGAGRWLFCPQAHPHPHPRY
HTPHYPLHGGPQRQEVGQEEEELEVDAADEAAAMMAAGDGGGGGGSSSGEHGEGNEAAAAAAAARPTRSRGPLQRRG
HGRIHGGGSSSSSMWPMRIASATAAAAAAPTSPATAAVASAREGQQQAPQPQPLQQPQPPPPAHDRCRRRRGHVRPP
QSGQQWGVAEVEAAVSEFLRARLHGVVYFTSRPQLRTGGAAAIRAPPRPLLLHPTPPPRPTPPPRPPPPPPPGSCAA
TAAPSCPSPPAPMGPRRLLQRLRAGLVWWLAAVAYDDSERWRLPGEALVEVGMAIEVD

>g4853.t1|PACid:27580900
MSAVTSGNVAVFELLIDSFTMDMGMTPSPSPMPPSPAPSPPRTIPWPPSPEPPSPEPPSPVFANTRTVSVDTLPLGD
ITNTSLQALGIRLGQSIYTRSPAWGEVVDCAAGWSYRGSTMTATGCTKTLRFPYIGVSGTFLTLPSPSSKFYSLTLT
YRTTLLYVKLNLYLGGRSCGLNLSPTEPIAFPKPDAFTYTRSNTRTVTPADCGWPAGSTFDTFQINPRDTPDSLEEE
YVAADVFELLIDSFTMETGMALPPTPPSPEPPSPYEPPSPEPPSPEPPSPQPPSPQPPSPEPASPPPLPPSPAPPEP
MGFGSTRSVDFGTIPLKYLTATSLDALGIVIGNTPSSGTDINQPIHDLGKVEDCAIGWPYRDSITQYMTSTGCSRTL
AYNIEGTSSWFQALPLSSPFWRLTLTYRTSSPHIGVGFHETGDVEFSRGCELELDRTEPIIFQPPPDAFTYTAPPKT
VTLTPADCGWAGSTFNNFFLFSHQMNAVTSGNVAVFELLIDSFTMDMGMTPSPSPMPPSPAPSPPRTIPWPPSPEPP
SPEPPSPVFANTRTVSVDTLPLGDITNTSLQALGIRLGQSIYTRSPAWGEVVDCAAGWSYRGSTMTATGCTKTLRFP
YIGVSGTFLTLPSPSSKFYSLTLTYRTTLLYVKLNLYLGGRSCGLNLSPTEPIAFPKPDAFTYTRSNTRTVTPADCG
WPAGSTFDTFQINPRDTPDSLEEEYVAADVFELLIDSFTMETGTTLPPAPPSPEPPSPQQPPSPQPPSPEPPSPEPA
SPPPLPPSPSPPSPEPMGFGSTRSVNFGTVPLGSLTATSLDALGIGLVNAPNGVDIFEPNSNWGTVKDCAAGWSNPN
RRTTYMTTTGGCSRTLVYTSESLSSWFQALPPSSPFWRLTLTYRTSSPHVGVGFYATGDVEFNHKCELELDRTEPII
FEPPPDAFTYTAPSKTVTLTPADCGWPGSTFDNFFLFSYQMNAVAYGNVAVFELLIDSFTMDMGMTPSPSPMPPSPA
PSPPRTIPWPPSPEPPSPEPPSPVFANTRTVSVDTLPLGDLTGTSLQALGIRLGQSIYTRSPAWGEVVDCAAGWSYR
GSTMTATGCTKTLRFPYIGVSGTFLTLPSPSSKFYSLTLTYRTTLLYVKLNLYLGGRSCGLNLSPTEPIAFPKPDAF
TYTRSNTRTVTPADCGWPAGSTFDTFQINPRDSLEEDKEEYVAADVFELLIDSFTMETGMALPPNPPSPEPPSPQPP
SPQPPSPQPPSPEPPSPEPASPPPSPAPPSPPPPSPPPPPPPPPPSRPLTDLPSSLPPSPSPTAPPSPLTPSPDAPS
SLAPPSPLPPAPPTLSPNPPPSQSSPAPPSPAVVAAAPPSLPAPRQEPSPPPSPAIVATFRSYFLGCFAFAFDTTAY
AAASRALPITLATNDEAMTVAKCAGLAQAAGLDHYGVTNGQTCVGGDVSRQQATQHGSLPASACDRPCPGDASQTCG
GGPTNASTQLSAMSLYSFNPDSPPPQPPQRAPRKPSRPGSAAKPPKPPSPAPPLPSPPSPSRQVETQTLIQTAAADL
PALQKDVSQAVAVPSARSLILLQPTAVPIFTAGVITKPSATSAAALNAPVIASARVGQQGRLVAFGSAVMLTGCCGQ
QGSSAAAVAGFDSAELDKIIANAASWAAAAGTVGVSISISISTVCTYF

>Cre04.g219742.t1.2|PACid:27580926
MAQRGPSTAGHRTTHRPLIELDAMRSTDLEQGPSASSGDEAPLGPVLGARARSAWLHALLPGQILTPCGGDRSDSAK
QQLPEVCDPLISSSPEQHQGPRGLAASVRGPEPERRGSRGQPATSHASPPSGDASPAPPTTVAMQTGAQTPPPAPAS
AVVGLSTSPPPRAGLSGSGATPAHGSALELAAGIPASTLVASPPPDSLGLRPRPASAAPAPGGSVVAASNAASAAAV
SGPAGLRLRQRRRDSPLARWYSGNRNRSRPESGTDEDLEASYTLLDYPPPPPGSAEFGGGAGGTALDADGCEGGDSD
GDEDGNAKYRRRGAGAGGMGAAAQQYLRAAWLGLPLPLPTALRSGSAAAALSLSAAGAAGAGAGAGAGAGAAALGGA
SGSGWWYLPSTLAATAAAAAGAAGASVGFYGSGAGDGDMARAASGRWLASETERCRAAAAAAGRQLDRQDSEARDRA
TGCDVAAYLAADRARCLAISAAAGRRLEQERREARVKAAAQMVRRAPGG

>Cre04.g221200.t1.3|PACid:27580984
MVKPGAGATRGYRVWDAAAEDALKAGVRKHGLGAWEHIRKDPQFAILSDRTGVQLKDKWRNLVKFRHIDPDEQRTYK
PKTQGPWSKKYKRDRSLGASGTASPASGDGGGVSEDEDGMQLALPGLTHQASGALPDGAALVLTAAAGVLEEQQEQQ
QQQQQQQQQQQRQSARTNAQKRKAITDLALLLEGPDGADNGEGGEAGGAGSGDDGDGGDSRGRPRMLLGGGGSRVLS
ARQKKLQEDRRFGIYDRRRYGFAEDGTRLEGSAGDDLSDLEDGGLDGAPAGAAGVPGFGGRGGGVSSRPRRRAAGAT
SRRARRGSDDEEEEVDPEDDEELEGEGSEEGGGGGAGEQYTIACPCGVTHDDGEMMIECESCAAWAHVSCLKRQMET
EPERYTYDFDTYVCINCTSSQPLQGPGQGRGAATTARGRTRRGGAGAEARNGTAAAAAVAAGEASGEQDTASEDSQR
KASELSNILLGLANGRMGSSSGGGGGAGPGGSRRAGAPSKLGPFGRNSMGSSGGGGGGGGGGSNAAAAAAGQMPSPS
GNVAGRGGGGGIAHGNGLGRAASLGRTSSTGLRDGAGGGRGGGGAGGGGGLGVMDAGGKRPLVTVSVSAVPGPASSS
GSRGRLDGAASPPPLVDGIPLFQAPGSSLGGAQQLGGLGGGSGGGVPGVGPGGTKIFKPIPKLATAVSTSPGPGQQA
PHMLVAGGGGAGSGLGAGPGGQPAQLSAPKARPHPALHHSTSAQNMQRMLEAANPLGGAGGGGMGGGIGGGLVGGIG
GLGGLGGLGLGAGGVPQRGVPLASVLDAAGPLSVAAANLCAASGRSKAAAAAAAAAAAAGGAASAGGGGPAASDGVA
STGDAAGGGGGLPPRPPSRPPSSMAREVANRIFGESDDDDPGAAALLRPLSALRGTVGGCDPWFLMPPSLSRAATPS

AFIDTLLRATPDIMPLSAAAAAAAAAGSSPPPHLAAALAAARSGSPGAAYLPALLAQQQHHLAMAQFLQQTAAPSP
APGAGGTPGAGGLAGVLLPPPPLVRPSPLGPPPPPPPFSAAPALAMPGGIAGVPLGWPPGAPPPPPPPPPLHASNSG
TNMPEAVQLHPVLRSLLLSTAQQQAAAVQQQQQAAAAAATHPQGQGPPGQPPAGPGQPSAPVPTPPAQPGLAAGMP
DGAAAAAAAASAPVGAAVPPAAGTRDAPGSGEAAAMAAAATAAVAAGGKLGGMEVAPQAGPGPAVKQDPDAAVAAAQS
QLPPPLATDPAAAATAGLLAMPPPLRQPASGTPPLPQAGSVTSAPADAAAAAAAGQSSVAAAPPAIPGLTSITAAQL
PIGPTANSLAHLAAAVGLPPGAVGLPGLFSGGGAAADPLAAAAAGNGAAGAGAGTNPQAAAAAAQAAAAAQAANNQV
AALLGLGAGVHLQPVLDHLRSSSPDPWGIFRTSTPGPEALLQSFLHPPPGPLPTDINSLPWLAKSATPPLSVAELMA
HTQAAAPPES

>Cre12.g544450.t1.2|PACid:27581115
MYRQSALLGLLAVLIGCAASSATTVDVYLNAASGVIDLSWDNGPQNLIAQLSSAFTAQVASGAPMGAAEPAAFVLPA
QTGQASFSSVEDMDAISQYIAAGRVVIALDSSSHSGADFAAKALNYDGNWISCKAAGDNAQAAAVPSVTEQASEFLA
GSWPKTLENVRITSASTVCSHEDSSAVVIPLYTYEDGEDVRVVAQAFGKVGVAGAVVVLGYNWKAGPQEQWGALLNK
IIEDFAAGTYTAPEEGVQSTVFDSVLDGAADAAADATEVVRRFLQTAAAGVYPGPPSPPSPTPASPSPPSPLPPVPP
SPPPNPPGIARVVENYVMTIEGAKLNFQTPEDVKAYVESLKLAFANAWKVLASQVLIRQVTINGVSLNALVGVQYWR
RRAAEGKAADAVQAEPSVALATVLEAMGPELARGVVRLVLEERVPATADMVMSLMDTDAASLSDTAVIRRALADDNG
NGNGNGNGNGNGNGNGNGNGNGNGNGGTNNGNDLKGTLSIAFSIVLFVEVPLPVSPPLPPGGTNPPSPPPRPPRPPPVD
LKALEKQLGAKKIAAGFPPPPRGSGLEGAPFLNFRNLTGQGSVGKTRALVWNTDADFKNELTPYGPLQLVDLNKTDC
TAGKALCAGCAQAWKATSSVVSVPIFFKDPMNINRIYIKQLKNSGVVKVQALKWSYPAAGVVSDATLGRVLYTKPTD
DSTACQTWLTVRVGKVRSGIMAPVPANGTQATLPAKLLAKTIGGVVITVARPAKAGPNYGPYIEYVRFGGRAIYPTN
PAEYLAPGVAKKKL

>g12111.t1|PACid:27581126
MGIANTRCRPGGRTQGTANGGPRAGEPGPTGSPTTPPPSPSPSPSPPAPTASAPPSPPAPLQQQSSPPPASPMIVAT
FRSFSLGCFAFDVVSASPSLRALPVTLATNDAAMTVAKCAGLTQAAGLDHYGLTNGHTCVGGVNRQQATQYGSLPES
ACGRPCPGDSRQTCGGGPIDSADSAGTQLSALSLYSFNPASPPPRPPPRAPRTRPARPGSAAKPPSPAPPQPTPPSP
SRRAEAQALTRTAAADLPALQKDVSPTINGIPSARSLLLLSPTAVPVFTATLLGASAAELRAPVLASARVGMGRLVA
FGSEAMLTSCCNQQGAAAAADGSESAAEINKIIANAANWACAAAGAAGRKANIRVADPRLDAVARFIVSMLPDIFAK
SRQDYYMSLTTFAKGGHARCDVYLVLGADPQQRYDTKVRDALRSFLENGKGVLLAGPVVADAGVSGAAAPVPSDEWT
LPDIMFTKQNGVTAVASATAVAPSGVAAEHFRELLSRLLQIKARSSSPEFISLKARVLRARSDIAATDMSQFDEVLK
AKIAEFDRDIAEP

>g12033.t2|PACid:27581229
MTGWAYTFAAQIRQRIDADGHRYTFPWPASIFTRADREALARCQEHFVFTLTDKSASTLQVVCKQFYIRACLHNLGC
PPPPPPPPPAPPAAAPSPPPPDHPPLHHHHPAPPPLAPPPPPPPPPPPPPPPPAATAAARRLAAILRATGQLQRRR
RAPLPGILPAQLALPPHLRPPYSALALHANPVAVATAEAAAEPAPTPLSTRAADAPTAAPATATLTAAPAPAAPAPP
PGPTARFPATMPPVAAAATPATPAPAARADAPGPATATPSAPPRFTAAAASRPAARAPHAHAPAQGAAAHITSMPPS
RAAAAPAAAPAPAAAAASLAPSRTALAAPSPPPARAARAHATARGAAADTAATPSPSATAAPNDAPAPAAHTPPPGP
AVNVSATGPPFSANATPAAAAPATLANAARPIPPSAATDRPTTATGPSTAGAECRRAPATPTDAAAVGPATHTRLAR
PLACLAALSGRRRTPAATCGVDPGDPRRMAHPTTACPAAAAHPDTAAAGPAAPPPHPAPGPHPAHAHSPAAPILPAP
IPAAAPAAPIPAAAPAAPIPAAAAAAPAPAAAPIPAAAPAAPIPAAAAAAPIPAAAAAAPAAPIPAAAPAAPVAPAA
GPGPPPAPAQHQYYATATVDQMHAALTQQSATLAQAGISHLQYDVTKDAAYYAITMKMHKTPPGLRFLACSHACPVT
AISDVVTASLRTLADAFRDMCRDRIGTDPWFCLHSGAVMDSEMRNSYRALFLVLFRSLVCSTFIRFGTFALVQQTCG
IPMGISAAPFIANLFLAWFEYRFLTQPAATAQRQRVLHAFDLTKRYLDDLLALNNPFITRLLSVDQLYAGLHGLYPA
SQVEAQSHPHLQAQLAAGTAAMPFLDILLILRTTPAGHARITTRLYDKRVQPVFDGVRLSRFIGTDSNVNEASKRNI
FTGQFHRLRRVVTEVENFAFETANLITALTHMGYGRPRAGRLHGLHERGLLVSQPAGVEDRRQLSTAGGLALHTVQL
NHRVQPLVKVLRVKS

>g12033.t1|PACid:27581230
MTGWAYTFAAQIRQRIDADGHRYTFPWPASIFTRADREALARCQEHFVFTLTDKSASTLQVVCKQFYIRACLHNLGC
PPPPPPPPPAPPAAAPSPPPPDHPPLHHHHPAPPPLAPPPPPPPPPPPPPPPPAATAAARRLAAILRATGQLQRRR
RAPLPGILPAQLALPPHLRPPYSALALHANPVAVATAEAAAEPAPTPLSTRAADAPTAAPATATLTAAPAPAAPAPP
PGPTARFPATMPPVAAAATPATPAPAARADAPGPATATPSAPPRFTAAAASRPAARAPHAHAPAQGAAAHITSMPPS
RAAAAPAAAPAPAAAAASLAPSRTALAAPSPPPARAARAHATARGAAADTAATPSPSATAAPNDAPAPAAHTPPPGP
AVNVSATGPPFSANATPAAAAPATLANAARPIPPSAATDRPTTATGPSTAGAECRRAPATPTDAAAVGPATHTRLAR
PLACLAALSGRRRTPAATCGVDPGDPRRMAHPTTACPAAAAHPDTAAAGPAAPPPHPAPGPHPAHAHSPAAPILPAP
IPAAAPAAPIPAAAPAAPIPAAAAAAPAPAAAPIPAAAPAAPIPAAAAAAPIPAAAAAAPAAPIPAAAPAAPVAPAA

GPGPPPAPAQHQYYATATVDQMHAALTQQSATLAQAGISHLQYDVTKDAAYYAITMKMHKTPPGLRFLACSHACPVT
AISDVVTASLRTLADAFRDMCRDRIGTDPWFCLHSGAVMDSEMRNSYRALRVAIAVTVTPPKTPDGRRKYEATLLTS
DAAHNAPAYRRDPMTDVAVHTFTRD

>Cre12.g560000.t1.3|PACid:27581256
MTDPALPGAGPSTASPAASNAPAADTGSSGSSGSSSRLRSGRGLGTDPVEADRAGDSNPPPLLLEARLSAAAAPPPP
VVIMSEGPVAGWERLASGVLDAAAAAAAVAAQGSGVGRAGDGGSVGVGVGVRPVVPAAEAEAAEAMKAEAVALAKDA
DDGRGGDGHDEKRGQDAPPPVLHAVPQPAAAAPTPATAAAASMPAATPAVAPPVPSAVAAVAPVSVTAPSAAVGALP
ALSPAALDAAAPPPQPLSPPPPPPTNAPHVATAVSPHLAAIMLDPATATAATAGDGGADSRAVAAATAATVAAVAT
AGLEVRVVAYERDLLRHQLKASAAEKEQLRIQLRALQQQLDETTEALNMAAAAAAAAGAASSAVGSVNGVDVGGLTS
GLSGSVGGGGGDDSVSGGGGGGVAVRGVSGPGTGILIPRLMMARGSASPPPPPPPAAAISPAASASDAAAAAAGAAA
AAAGGSPWAGVVTPPPSLPPLRTGLPLLPPAVATASTAASPAQPPLLQPPPSSSGPLLPPAASGGSVASGGSGSRLH
SVFDTATAAALAAAGGGGADGGMRSVFANPSAAAVAADLLGGGGGGGGGVFAVGGLNSGLPQGSVSVADHRSYLPFG
ATSTSTFSTTTAAAGVATPAGPDTSSSPLLPSPLLPPRGSSYRAASPNSSCQLLLLPGGASTTLAVAPPPQATLQPQ
ASTGGGGGAGGGLQAFAQPTLPSPFSSPPPSRRRSVFGGGGATAATSALTSALQSRNASVATSPRYGSVWGGSVAHS
PMRRHTAAVAGGGAAIAAAAAAAAAVAGAPDAAAGGGLLLPSPLLPPALSTPASISTVGTGYPYHAQHYPPGGPPHP
TAAAAGWLQLLPSRGTATAGTATAGTGTAGGTRAGTTAAGSRLPSRPVSPRRHLPHGPGGDLMAAIMKLEALTGSGG
ELRPAVEAELEGLGLAGGAAELEAGAAAAVAVVVEGGGGGGGGLGGGGGGGGGGGLAVVAGLREEVERLRSQMEQQ
VRLREHLEARLRLALQSQQQQQQPAAAQAPGSPQAAAGGAETAAPPPLPAPAPAAAPARERLSSAPASAAAATAAEA
PAPAAAPALPSTTTTTTTAEAGAPVQAEGHVGPDDAASSAAATNTAAAATTGHARHLPAAAVHSGDSFRFPPPPRGA
APGATAEAAAAAEVPVARAATAATGEGGGGGGGGGGGGGGGGGGTEAPPAEAGAFPLMSGAPPPPPAGVSPGRTATA
AAEGAEEDAGQQQVLQPQQLGGESGATAGSGAATAAAAAVEDPERQQCRDLLDDILGPAAPTAPTSAGASPPAATAA
APAAGYSQHPAAGPPATATPPSSPSRSDLPTITIAAPVSPDSTRFPFPTASPFPASSSAPNLSATSSPQSAVGSPPA
AHTRRRRPPIGGRPPLPPPAAATAGGAAAAAGAGASPPRHGAAAIFTTPAPVPGSRYSRAGGGAAGGGSSTMIAAAG
GGGGYSRRGGRADLAPYLDEVAATAASATASQKPVATAAVAAAAEVADAAAAALVLPEDEEIGEAAAAAAAGEGGGG
TGLFAAAGPRQQRQQSAAAFLSGYSAGVTAGASAATAMDLPRVASLANMLFTAQELGEGEEGGEGEEAEQGEEGEES
GEDRIAEEEERMEVEAEAEGGQEGPRQRPQQLQLTRRNSSGAAGGGGSGYSLRHRRQNTAEEGAFEEPVGVEEALEA
ERGRLRQHQEALAAAVSLAAASGVLRAAGGSVLPPGTAAAVAESLRERAQAAAAAAAAAASGGDGDGSGGAGGAAAG
GGGGSMAAAAVSSWRTPLMSRLSRAGSAPPPPPPPPRVSTPAEAEAGDKTGGTGDDGTATAEGLNGSLQQQQRQQQ
QQQSSGPATPAAHRPLLFAASPPTPSTSTPTTAPAAATTSAFGGFVPSPVGGAGGAGGAGGATNGARAAGGGAFGGE
AAAGTATAATERGLIGSSSTGGGVSATAAGADAGREGGRMLFGAFRNGGVGAGAGSGSSNGEYSYGGGGGGGSDAAV
RAATAAAGAGAAAGPEAGALQLHRWAIAELVAQLTEAESELAAARAAAAAREAALRAARTSLRQVHVEAAGRAAALQ
ASLADALGQVNTLTQQVSEHLSRERAALRAAAAAEARAAAAAEERARGVRSPEEIEADVRVVVPSGETDELELLTSRL
VAAEQQLAAARAAAAEAARREAAAAGLAAELAFKEAQVQALTEALGRAALLLEQARALGDGGGGGGGGDGEAGGEAG
GGGSGVGGALPPRARRSSTGEAGYGAGSSSATHGGSSGNLAARSRPPPPLPPSLSTTSAPAAPSGSAVSAGSGGGGG
GGDGGGGGGFSRRRRSGSGLPATPPGSPAEELSRAASAAAAAAAATAAAAAAGPAPQGGTAMLASLAGPVAPSAAPS
GLGVSSSGGRRRLYDDLSLVAAGAAATAAATAAATAAASSGYMPGMNVVPEATEALAAAGSSGSEYDESRNGGGGI
GGEGREPTAVMAAVAADPQQQQQQQQHLQVLSEAEAERAAVAVAAGGLLPPPAEAEMVRLRLLLESSAREVFALRQ
RLAAADALEQAERAAEMEGLRGALDGSGRLLGRLRADVRGLQLRGRGGALAAAAAAAEQSLARLQARVRGRGRTGEE
EEEDEDEEEVEAEEEAVAASLLQDADAATQSAKPSSDSADGATNADAAAPGTDLGSEPGAAAAGGAAAGRGPLVSAA
AVSTARRLLSAMGSQMGALQQQLEAEAGLRAAAERELAELRRLFDGYVASAVARVQQAQQAAAGGAS

>Cre12.g515350.t3.1|PACid:27581271
MGDWEQRARRQPWATCSLLRAFCWLSCLLWKSFADGQSSCPGFNGYQFYQGGQAVGNNLGSALTSSLPAIAASCTAD
CSCQGFTWNGVTGQLKSQAAGALLMPDTTNLNQTCLGTYVRLEPCQTTSGGACKAFTGYAFQQGKDVTNNLLGTAVT
GSLETAAAVCSSNCACQSFTSTQLIKSDAVVVTGGGTGTCDGTFVRTINCTAATLTCPTSTYFSKGYNFYAQQDGTG
ADLYSGQPLLRGGIADFMDHCDLTCACTAVSTWGAIKATNTRGASGNFSTQCQGVYTRTGSMVASVAGSKPVSASSE
DTGGGYQKSYLVDNNIGNVFSTASGGTTTPWVAIDLQADYTVTNVQLYNDGTYGNRLGWAEVRVGMTRIDDSGDGAL
VSQNALCYKFTGRGTNGAVYSYFCTGGAPDNTAPRISLFGRYVTIQNFNTDPGNDADYYRLMLKEVMVDIACVMPQL
GSPSASYNNYDVYVDWSVDGAALTAPTYPVFSWKACADYCRTTTGCLGFKYRATSRQCDLFSTRGKLERVWDPADTA
TFGATGYGDVAGVKDHSGCGTLSQSAGGSADLTLPFPQTISFGKTFSTPPLVLASFAGQNADSASHEVVITEVNTTS
FNVEVLSSGLSTVRISWVALDGLPSQGLAFRRNGFMCTERECYNADLLASQGYIERKWTHSMKVLTGHFPKIFLSTR
KISGSTGTSTVNTVFATNAYALLWMTPVGAMPTSYSGFTMDVLLANHPLVDAGTDSSYNCAAGSRTNYAACPLAMAG
AGNNVCASRTITTSISFARTFTQAPLVSVSLTAVEWATSFDFFVSAGSITATGFTLTMTANCMSQVYSASVSWIAVD
ASTTAVPPVCPATPTSGQLKYASPAIGGAGSSLCARTSAGKLKCWGNNGQSKLGNNVTTLVGDGPGEMGSALPDVDL
GTGTSAVSTCMGHEHSCALLTDGRVKCWSSNAAYGILGIVDGSSNAILSVFNTTGYGMGDLLPAVNLGTGRTAIQLS
CGHYASCALLDNYSVKWCVRGLRPAFPYQHAGMSCGVTAGLPTSGALATCSWGGGWAGQLGQGGTGNIKDPSTISSI

NLGTGRTARSITVGDQFACAILDNGQVKCWGANSFGQLGQGHATDWGKTAGQLGDGLPATDLGTNLKATYINAGPDS
TCVVVSSGDPLLDGRVKCWGRNNVGQLGLGDYANRGDNATEMGDYLPFVDLGTGVKAVQVACGFMHCCAVTTKGGLK
CWGENGSGRLGLGDTVARDTIALLGDALPYSSVGTGRTVLFVFNRGYTTCALRDDYTVVCFGANGSGQNGAGDTVAR
CSTTAYCGDNLTAIDYGAGVFIVPYAGVAALQTLTPYPSFPPIPPAPPSPPPSPTPPSPKPPSPLPPSPAPPPLPPS
PDPPSPAPPSPAPPSPVHSTVAVAASKPRAPKPCPPIPAASATPSTLASTFTTPKPNATKSSATPATGPAGPSFSSA
STPPFSSASTASFSSASTPSVSQPTASISSSAATAEPQAPDLSIESDPPPPPAIVPDTQPPLLSLLGDATLKLQIYD
PYVEYGATAMDARDGPVPVVTSGVVNTNVTSVDGKHPVVTYTARDRAGNVASIRRVVMVSDACTASGERRCPNTLKC
STLSLCDSSFTAVLNFFSNSFGDVAASKSGSSDGGGSDGGTDSSGGGSSGSGSGGSGSSGSSSGSSGTTSISAQRSR
KLLPDTMPPRLSLRGSGTLFVTPGGASGMIHSLYVDEPWVDEGVDAEDDVDESFDGKTNKVVALPATAIVTSVIAPS
GAKADAIKTDTPTGDQESGGLPWVITYDVSDGAGNRAPTMRRRVYVLCRPPERECPRTDPYEPRTCSVEKICGLQVQ
PSDGQGGKSSRVNSGMIFSAGGDGGGDSGWSGEDGSGSDSGYSGSGYGGNSGSGGSGSSSSSNSGGSSSSGGKNAN
STGSGSSTRTGSGATNDVRVVQLPGTAYTAAAAAAAAGVTLTTSGSSSTGSSRAALVAATGKSGSSSGSSNSRGSSG
SSSAAEVDIPDTQRIPKLTLVSDWFNRFKAGKPYDKCKRLEVADCDPGVTATLRVPGDLNGKVIACADRARAAGIAS
PRPFDLMGLKYCDLDTNTPGVVTVSFHLTWPSVGELVVTRTIIIEDNCPDGERLCDNGRCSTGPTTCLSEIGLGDGG
GGTTVVPTAENAWAEGGSGSGGTTAVDLASAMTAGGSAAAAGSGASGSGSGGSGGSGSAGSGGSGSGSSSSGGGSSG
SGGSSGSGGGSSGGGSASGSDGSTGTGNSGSGSSSSGGGGTSGGSSGAGGSGAPAAVSAALATVVGSGTVASGVTLY
TVPGSSSASSATASVVAATLPPRLLLAVTDVLGRLVHVKRGTPYLRCAADQVPTPELPCEPLGLASDDLDGDLTDWV
TLCPPADCARSQCREHVSKQPAACGVDTVNGDVGSTFTLRMVVFNSRGLNATVERVVMVDSPCPDGQYLCERAPNSA
NAGGQGASAAYMCSDLPCDRRKALLEVVAIAVKATMSNATSGGGSNMTDDPTVVDVEEVVFSPVPRIYLLPSLHLAT
NLSDALANQTLSLVYRTPAPFSLAPCNVFSQGVPLSTFSPRPPSPAPSAWRPPSPAPPTPPGPASGNSSSDSGAGRR
RGLRQAAPLPPPAFQPPPVPAASSGSSSSAAAGAMAGACAAVANDNNGNDLTPYIKTSVAQLCPAGAPAGSCGGGCS
VAQLTTGSCLPGMYRVLYRVEADGGTEAVAQLDVAVEEIRATQLNLTLQPSLPTIIQLLPPPPPGELPPTPEDVAGN
RTQVEAMAVALAANASALTAALRPLLPGLGLDPEDIRSLNLSAAVMVVELPPPPPPPSPPLSPPPPGAPAFPPMPPN
ASYPPPSPSPPPPSPPPPSPPAGPPRYGLQLAVQLTTGVSDCLYTATGAAGGGAASASFADLSTSRHRSQRRRLLRV
RQEADAVWRTDEDAALLETATAAQRRKLLDTQSEPSAEASVVDQQLQWRRRAGEEAATPLERSLRRLQSVSATLEAA
LEALATGRYTEPAWMAEWSEEDELEAEQDAENEAGESEDVFDFSPRQQRRLLQQVLAGGGGCGCNPAPSRTVFASST
AANASAAASAVVRGAVTGTACVTPAIDGASAAVGLLLGAVSTLDAFGQDMDVTQVALIAAVSSINGRMDEGDVRSEQ
GSTNWAFTARDSVKNITTTATKLTSVVQAAAKTRGNQELSSVVGTGVADVLQVMVSEVAESAKTALASAEGILSGYG
INSSTPADENERKFCNCIFNRSEPTGNSLSFVADHAAKATDGNATESMPPMPPAPPPGVPTRPPLSPDAGGGSSAST
AAARRRGLRAAPHDTGVVPEEDQAAMDPDKASCPDTPSGPRAIRRPANSQQQRRQLQQSSGSTTTSGGSTSSSSGSS
ASDERAPSSTRGGSVKVGSNLEFASGHGVLINDTFDYSLYQVRSGMRTRKLTQVGRNNRVLLGLLLHQVRLSDRDVR
KRSGRYMCTSASKTRQLITACAGENAGARVVGGDLGGIGVDPVFLPRSPLFRAGVNPDDYYNTSAGSSDINPNGVPF
GFFHEEIAGRPDGYPLLLDIGMSEQRAREAFIFLRDGSYLSAQLTQSMSAELVTYNPSASVFGYFRLMFSWTANGLI
RCDATLVGLPAVSYADAFAEFSKYYRQVLPDIFMVMLCAGYVCLVAYDISQQLKEQRRSRLMAARAAALPQYEAPVI
EWPTAPELSRANSRSTHHQGLSRPVTAENSRTGTRSRPASHRQGVGHLVRSRAGSAAPSEAGGVSSYGNSDSESPVR
AGIADGPQPVAEGYEWLQQPGHGHYDAEPMSMPGGSPTLSMRLPAGKPGTSSLKAAAGNSRAAKMKCVSMAMGDVPE
LPSTVALEPVDDGDGDEDAQRIGAAYEGYAAGGGAAGANLSMVLRSRQNHEKPLRRDPYSKRYRPSLLNRDMRPDVS
QMLLMHQQEPMRHGGLRPIGKAMTQVVYRPRMKPFWMAFEAVVCCLMVAAMTICFVYCLKLSDRNQFTARYQVYEAD
TYAPAHYWLLKRQEGAVVVQGADTAAAVSSLSGNSTLSGNSTSNSNSLSDAVNGTVAASIQVLSGNGSSTGGSSGMI
SSSSPNYNAAGRWRAPEDPAPLESSGTMFQRVRHMYTMYVTYMLLQSLVLAMVIIRLIHYVSFQPRLSLIAGTLSLA
FSDLMHLALVIMVAAIMYAGAVMCTFSEQAEQLSGYEYTILYILKYLLFGDDEGVFYNILWAPLIKSTGQTFLARLL
YVLGPILFGLILIICIMVILFHPYFIMKQVVRNAPGVQRDIYLIVRWRWQHYRHQAPKNKQLMCALDGFMAGGDAEA
TSRRQQLINALATKLAGPSHMLRKLNERLGSRPVSAAPQQQQQQQPVSGGKGAKSSVDLGKPGGMAGIGSGSDSSS
GLESAALAAGLMSHGATTSDGQSGPLPRVLASPVSRGASVHTATPSRVSLAISSMAQRDWLQQQGQDGGGSTRSGG
LAKVKASNSGGLLSRSTSASNKVAPLPAAAAAAAAAADMEWVGGGDRKAPSTEPSPLPGGTAPGGSAHGRMRSRFAT
ASTPDVRPSLETDDGAWRGPFGGAAGTESGNGADPLMPRGSLGYSRRSRLDSQESSAVPGVGPGSDRPSPKAASAAS
SAAAAAQLADAVMANLTSRFGNITVPEDERAKEQQAAASAFVALARADSLRSHSSNSRPSSAQRLRLADGGASPRPP
SALAGRSASGAVVGNSPTSSGSGAQPGLATENSWAQRGPLAHAASAPKAMLSPPLTAPDRFSRASSGNEALQAEASR
AEGGTDGAGSAAAAGASGEQRHLMQLMSFAPDKAAMELHMARDSGGGAGDDAAAPGAGGYAVQARLPQPVRMQRPSS
GVPTSRPGARPSSAIRANAALGAFAAARRSSQAGPGPVAEAEEDDQEQAMGGTRDAAALDRLPDLDSDSGESDSGRV
AELAKQKRQALRKRAAPGGVSFGHVLVYELQAEQQQQHGGAGAGGWTYAAALAQVQQLVAGLCSMVAQMRECIAQV
QAMSAALEAVHKELALAGPGSGAAAALRSPEVWRAAKQALDKAQGLLMPKASGLLTTADGPAGAGAVVPAGLFGPTS
SAGLPGGVLEDRSSAVGQLQLALAQRLGPHEVQEAMMKVKAGPQMVNAAEERHGGHDDGHRRQMMHHAVPGI

>Cre12.g515350.t2.1|PACid:27581272
MPDTTNLNQTCLGTYVRLEPCQTTSGGACKAFTGYAFQQGKDVTNNLLGTAVTGSLETAAAVCSSNCACQSFTSTQL
IKSDAVVVTGGGTGTCDGTFVRTINCTAATLTCPTSTYFSKGYNFYAQQDGTGADLYSGQPLLRGGIADFMDHCDLT

CACTAVSTWGAIKATNTRGASGNFSTQCQGVYTRTGSMVASVAGSKPVSASSEDTGGGYQKSYLVDNNIGNVFSTAS
GGTTTPWVAIDLQADYTVTNVQLYNDGTYGNRLGWAEVRVGMTRIDDSGDGALVSQNALCYKFTGRGTNGAVYSYFC
TGGAPDNTAPRISLFGRYVTIQNFNTDPGNDADYYRLMLKEVMVDIACVMPQLGSPSASYNNYDVYVDWSVDGAALT
APTYPVFSWKACADYCRTTTGCLGFKYRATSRQCDLFSTRGKLERVWDPADTATFGATGYGDVAGVKDHSGCGTLSQ
SAGGSADLTLPFPQTISFGKTFSTPPLVLASFAGQNADSASHEVVITEVNTTSFNVEVLSSGLSTVRISWVALDGLP
SQGLAFRRNGFMCTERECYNADLLASQGYIERKWTHSMKVLTGHFPKIFLSTRKISGSTGTSTVNTVFATNAYALLW
MTPVGAMPTSYSGFTMDVLLANHPLVDAGTDSSYNCAAGSRTNYAACPLAMAGAGNNVCASRTITTSISFARTFTQA
PLVSVSLTAVEWATSFDFFVSAGSITATGFTLTMTANCMSQVYSASVSWIAVDASTTAVPPVCPATPTSGQLKYASP
AIGGAGSSLCARTSAGKLKCWGNNGQSKLGNNVTTLVGDGPGEMGSALPDVDLGTGTSAVSTCMGHEHSCALLTDGR
VKCWSSNAAYGILGIVDGSSNAILSVFNTTGYGMGDLLPAVNLGTGRTAIQLSCGHYASCALLDNYSVKWCVRGLRP
AFPYQHAGMSCGVTAGLPTSGALATCSWGGGWAGQLGQGGTGNIKDPSTISSINLGTGRTARSITVGDQFACAILDN
GQVKCWGANSFGQLGQGHATDWGKTAGQLGDGLPATDLGTNLKATYINAGPDSTCVVVSSGDPLLDGRVKCWGRNNV
GQLGLGDYANRGDNATEMGDYLPFVDLGTGVKAVQVACGFMHCCAVTTKGGLKCWGENGSGRLGLGDTVARDTIALL
GDALPYSSVGTGRTVLFVFNRGYTTCALRDDYTVVCFGANGSGQNGAGDTVARCSTTAYCGDNLTAIDYGAGVFIVP
YAGVAALQTLTPYPSFPPIPPAPPSPPPSPTPPSPKPPSPLPPSPAPPPLPPSPDPPSPAPPSPAPPSPVHSTVAVA
ASKPRAPKPCPPIPAASATPSTLASTFTTPKPNATKSSATPATGPAGPSFSSASTPPFSSASTASFSSASTPSVSQP
TASISSSAATAEPQAPDLSIESDPPPPPAIVPDTQPPLLSLLGDATLKLQIYDPYVEYGATAMDARDGPVPVVTSGV
VNTNVTSVDGKHPVVTYTARDRAGNVASIRRVVMVSDACTASGERRCPNTLKCSTLSLCDSSFTAVLNFFSNSFGDV
AASKSGSSDGGGSDGGTDSSGGGSSGSGSGGSGSSGSSSGSSGTTSISAQRSRKLLPDTMPPRLSLRGSGTLFVTPG
GASGMIHSLYVDEPWVDEGVDAEDDVDESFDGKTNKVVALPATAIVTSVIAPSGAKADAIKTDTPTGDQESGGLPWV
ITYDVSDGAGNRAPTMRRRVYVLCRPPERECPRTDPYEPRTCSVEKICGLQVQPSDGQGGKSSRVNSGMIFSAGGDG
GGGDSGWSGEDGSGSDSGYSGSGYGGNSGSGGSGSSSSSNSGGSSSSGGKNANSTGSGSSTRTGSGATNDVRVVQLP
GTAYTAAAAAAAAGVTLTTSGSSSTGSSRAALVAATGKSGSSSGSSNSRGSSGSSSAAEVDIPDTQRIPKLTLVSDW
FNRFKAGKPYDKCKRLEVADCDPGVTATLRVPGDLNGKVIACADRARAAGIASPRPFDLMGLKYCDLDTNTPGVVTV
SFHLTWPSVGELVVTRTIIIEDNCPDGERLCDNGRCSTGPTTCLSEIGLGDGGGGTTVVPTAENAWAEGGSGSGGTT
AVDLASAMTAGGSAAAAGSGASGSGSGGSGGSGSAGSGGSGSGSSSSGGGSSGSGGSSGSGGGSSGGGSASGSDGST
GTGNSGSGSSSSGGGGTSGGSSGAGGSGAPAAVSAALATVVGSGTVASGVTLYTVPGSSSASSATASVVAATLPPRL
LLAVTDVLGRLVHVKRGTPYLRCAADQVPTPELPCEPLGLASDDLDGDLTDWVTLCPPADCARSQCREHVSKQPAAC
GVDTVNGDVGSTFTLRMVVFNSRGLNATVERVVMVDSPCPDGQYLCERAPNSANAGGQGASAAYMCSDLPCDRRKAL
LEVVAIAVKATMSNATSGGGSNMTDDPTVVDVEEVVFSPVPRIYLLPSLHLATNLSDALANQTLSLVYRTPAPFSLA
PCNVFSQGVPLSTFSPRPPSPAPSAWRPPSPAPPTPPGPASGNSSSDSGAGRRRGLRQAAPLPPPAFQPPPVPAASS
GSSSSAAAGAMAGACAAVANDNNGNDLTPYIKTSVAQLCPAGAPAGSCGGGCSVAQLTTGSCLPGMYRVLYRVEADG
GTEAVAQLDVAVEEIRATQLNLTLQPSLPTIIQLLPPPPPGELPPTPEDVAGNRTQVEAMAVALAANASALTAALRP
LLPGLGLDPEDIRSLNLSAAVMVVELPPPPPPPSPPLSPPPPGAPAFPPMPPNASYPPPSPSPPPPSPPPPSPPAGP
PRYGLQLAVQLTTGVSDCLYTATGAAGGGAASASFADLSTSRHRSQRRRLLRVRQEADAVWRTDEDAALLETATAAQ
RRKLLDTQSEPSAEASVVDQQLQWRRRAGEEAATPLERSLRRLQSVSATLEAALEALATGRYTEPAWMAEWSEEDEL
EAEQDAENEAGESEDVFDFSPRQQRRLLQQVLAGGGGCGCNPAPSRTVFASSTAANASAAASAVVRGAVTGTACVTP
AIDGASAAVGLLLGAVSTLDAFGQDMDVTQVALIAAVSSINGRMDEGDVRSEQGSTNWAFTARDSVKNITTTATKLT
SVVQAAAKTRGNQELSSVVGTGVADVLQVMVSEVAESAKTALASAEGILSGYGINSSTPADENERKFCNCIFNRSEP
TGNSLSFVADHAAKATDGNATESMPPMPPAPPPGVPTRPPLSPDAGGGSSASTAAARRRGLRAAPHDTGVVPEEDQA
AMDPDKASCPDTPSGPRAIRRPANSQQQRRQLQQSSGSTTTSGGSTSSSSGSSASDERAPSSTRGGSVKVGSNLEFA
SGHGVLINDTFDYSLYQVRSGMRTRKLTQVGRNNRVLLGLLLHQVRLSDRDVRKRSGRYMCTSASKTRQLITACAGE
NAGARVVGGDLGGIGVDPVFLPRSPLFRAGVNPDDYYNTSAGSSDINPNGVPFGFFHEEIAGRPDGYPLLLDIGMSE
QRAREAFIFLRDGSYLSAQLTQSMSAELVTYNPSASVFGYFRLMFSWTANGLIRCDATLVGLPAVSYADAFAEFSKY
YRQVLPDIFMVMLCAGYVCLVAYDISQQLKEQRRSRLMAARAAALPQYEAPVIEWPTAPELSRANSRSTHHQGLSRP
VTAENSRTGTRSRPASHRQGVGHLVRSRAGSAAPSEAGGVSSYGNSDSESPVRAGIADGPQPVAEGYEWLQQPGHGH
YDAEPMSMPGGSPTLSMRLPAGKPGTSSLKAAAGNSRAAKMKCVSMAMGDVPELPSTVALEPVDDGDGDEDAQRIGA
AYEGYAAGGGAAGANLSMVLRSRQNHEKPLRRDPYSKRYRPSLLNRDMRPDVSQMLLMHQQEPMRHGGLRPIGKAMT
QVVYRPRMKPFWMAFEAVVCCLMVAAMTICFVYCLKLSDRNQFTARYQVYEADTYAPAHYWLLKRQEGAVVVQGADT
AAAVSSLSGNSTLSGNSTSNSNSLSDAVNGTVAASIQVLSGNGSSTGGSSGMISSSSPNYNAAGRWRAPEDPAPLES
SGTMFQRVRHMYTMYVTYMLLQSLVLAMVIIRLIHYVSFQPRLSLIAGTLSLAFSDLMHLALVIMVAAIMYAGAVMC
TFSEQAEQLSGYEYTILYILKYLLFGDDEGVFYNILWAPLIKSTGQTFLARLLYVLGPILFGLILIICIMVILFHPY
FIMKQVVRNAPGVQRDIYLIVRWRWQHYRHQAPKNKQLMCALDGFMAGGDAEATSRRQQLINALATKLAGPSHMLRK
LNERLGSRPVSAAPQQQQQQQPVSGGKGAKSSVDLGKPGGMAGIGSGSDSSSGLESAALAAGLMSHGATTSDGQSG
PLPRVLASPVSRGASVHTATPSRVSLAISSMAQRDWLQQQQGQDGGGSTRSGGLAKVKASNSGGLLSRSTSASNKVA
PLPAAAAAAAAAADMEWVGGGDRKAPSTEPSPLPGGTAPGGSAHGRMRSRFATASTPDVRPSLETDDGAWRGPFGGA
AGTESGNGADPLMPRGSLGYSRRSRLDSQESSAVPGVGPGSDRPSPKAASAASSAAAAAQLADAVMANLTSRFGNIT

VPEDERAKEQQAAASAFVALARADSLRSHSSNSRPSSAQRLRLADGGASPRPPSALAGRSASGAVVGNSPTSSGSGA
QPGLATENSWAQRGPLAHAASAPKAMLSPPLTAPDRFSRASSGNEALQAEASRAEGGTDGAGSAAAAGASGEQRHLM
QLMSFAPDKAAMELHMARDSGGGAGDDAAAPGAGGYAVQARLPQPVRMQRPSSGVPTSRPGARPSSAIRANAALGAF
AAARRSSQAGPGPVAEAEEDDQEQAMGGTRDAAALDRLPDLDSDSGESDSGRVAELAKQKRQALRKRAAPGGVSFGH
VLVYELQAEQQQQQHGGAGAGGWTYAAALAQVQQLVAGLCSMVAQMRECIAQVQAMSAALEAVHKELALAGPGSGAA
AALRSPEVWRAAKQALDKAQGLLMPKASGLLTTADGPAGAGAVVPAGLFGPTSSAGLPGGVLEDRSSAVGQLQLALA
QRLGPHEVQEAMMKVKAGPQMVNAAEERHGGHDDGHRRQMMHHAVPGI

>g12149.t1|PACid:27581335
MTATSCNRSLRYKNQRVSSAFRPLSRVSEPPPFWRLTLTYRTTSPYVGVGFYETGDVEYSYPCPVVLEPTEAIISVP
VPAAFTYTASPNTVTLTPADCGWAAGSTFDSFFLITYVEGDDAPVSELLIDSFTIETGIAPSPPPSPAPPPSPEPPS
PAPLAFGNTRAVAFSSLPVGALTSSSLSAQGIELSAGSNPVSPAWGEVVDCAAGWSYGGGTIMTAAGCTQALRFPYD
DFSESFRPASPPSMFWSLSLTYRTTLPYVTLYLYPTSGQGNANACKVRLNRTEPIASPRPPAFVYSASNSVTVTASE
CGWSAGSTFFSFSFYVGVEGSSAGIDVFELLLDSFTMVTGMAPSPSPSPPSPAPPSPEPQSPAPPSPEPPSPMPPSP
EPPSPAPPSPEPPSPAPPSPAPRAFGSSSLTVSFDALPLGSLTLTSLEAQDIRLAHQNESNALPGWGQVVDCAAGWT
TDDSWAMTATGCNKTLRLTNALSDFFLPLSPPSTFWKLSITYRTTFRYTVLLLYGTGDVSHVCDRVLQRSDPTPLPN
PTKFTYTAAPITFTVTPADCGWAAGSTFDRFAISISTYLAFGDDRVDLLLDSFTMDTGSAPSPPPPPPSPPMPPPLP
PSPPPLPPSPPSPEPMVFGSTRTVSFDTFELGSLTGTSLEAKGLALSNYPDGTDHLRGEVVDCAAGWYNNVPMNAAG
CNRSLLYKDEGSSNAFRPLSRVSGPPPFWRLTLTYRTTSPNVGVGFYGTGDVEYSYSCAVRLKPTETIAEPAVAEFT
YTASPNTVTLTPADCGWAAGSTFDSFFLYMSMTAGVAPVFELLIDSFTIETGIAPSPPPSPAPPPPRTIPWPPSPEP
PSPAPPIPLFASTRVVSVEALPLGNLASTSLLGLGIGLDADMYTSSPAWGEVVDCAGGWSYEGTTMTAAGCNQTLRF
PYQGFTGRFEPLWAPHGGFYSLSLTYRTTLPWVQLELFNFQYCTLRLNGTESIVRPRPAAFTYAVSKTVTVTPEACG
WAAGSTFLNFNFDVKYIPGGIDVYELLIDSLTLEMEPPPQPNPPSPPPLPPPSPAPDQRIASFTSLPVGALTSSSLS
AQGIELSAGSNPVSPAWGEVVDCAAGWSYGGGTTMTAAGCTQALRFPYDDFSESFRPASPPSMFWSLSLTYRTTLPY
VTLYLGQGNASACKVRLNRTEPIASPRPPAFVYSASNSVTVTASECGWGAGSTFFSFSFYVGVEGSSAGIDVSELLL
DSFTMETGMAPLPSPSPPSPAPPSPEPQSPAPPSPQPPSPMPPSPEPPSLAPPSPEPPSPAPPSPAPQAFGSTRTVS
FDALPLGSLTLTSLEAQDIRLAHQNESNALPGWGQVVDCAAGWTTDDSWAMTATGCNKTLRLTNELSDFFLPLSPPS
TFWKLSITYRTTFAYTGLVLYDTGDVNNFCEQTLERSDPTPLPNPTTFTYTAAPITFTVTPADCGWDAGSTFDRFAI
FIYTFMAVGRDRVELLLDSFTMKTGSAPSPPPPPPSPPMPPPRPPSPPPLPPSPPSPEHMVFGSTRTVSFDTLEIGL
LTETSLEAQGLALSDYPFGTGHFKMTWGQVVDCAAGWYLAAAAVSMNAAGCKRSLQYKDEGFSSAFRPLSRVSEPPP
FWRLTLTYRTTSPNVRVGFYKTGDVEYSNFCQVELKPTETIAGPAPAAFTYTAGPNTVTMTPADCDWDAGSTFDSFF
FYIAMADDDAPVSELLTGKQFVGGAPGSSLVYGAAWYVL

>g12249.t1|PACid:27581361
MAQHRRLLPLIYALTVLHAILYDGVLGAAASARIAAVGASSSGATAITRVSRKLTQDGEIKGTIDAQLDPNGAFSLT
SAEQARLLAAGNCTEIYTERNFPLVAKGWSGLGMVAVSRGPVIDGVTHMRIVVDVSLTSGLYIAQPSNPDGHNIRFA
LAGKLPLTCESSEYYHAGVFVDPATAGQQATQAMQNMTQDASSPPNSTSSSSLSSSSGAILSISAHVPLPAAVFNCG
GASTLTAFLRVLFLRVDIDLSDQPQGPTTLIVTAGDPRDFVVPEPSCPYNVIRAICNPEACAQEQAAAAAAAPAAAG
AADNGTVAHSHGGAADPMTPAASPAPSSSGNDRLTGMPTESTGTATGATGAADLGTAAANSQGSDPTLAAAITTTTT
TDDGAAVGGGGAGTGAGAVAAAGAAASGGSGSGGGGNSSTPTIAVAVGAAVGGAALLALVVGGTVYLRKRKRNAGSG
VSAASSATDSGAAALRHRRHGDASPSGSSSGRSSSSNISPLSISPSADALPLATRAAPAADHSPPPAVSVLVMDAPA
TACGSPPPAPATSSAPVKKQRTFFGSFWTSPRNAAASSAISRTAPAPASVDMPASLTRASGSGQQTRAGSPNQLLAS
LPSLPRALPSGAATAAGGGAAAASSAASLGAGAAASPMFQYVNPAYAGAAAAMPPPAALSTRADDMLATGPAAGVEP
DGGDSAGVAGGIAAATPPGAKLAGGAAMGRGRATAGTAAAKKATAYAVMAAGEDEGAAAAEAAAAAAGGGKAGRVAG
GGRFAAMAGSCFGGRPLNELDDGDEYLDDAAALAAGRAVAAAAAGLDDDGTSLPMPPRCGKPRSPAPAALPLRNAPD
PDAGMDDAPAAVGAAAKGGCFDDDAASVVSFADAPKMLGCIPTSLPTTSTAARPLLSSSAVARQPRSDAAALPGPGR
RTDASAVAAVPAAEAEGASAAEGSSSPSVPRPGSLAGMAPEVAMILADEEEEEGQGKHGGRRPWKWWRPAPSPAAGV
TAGRRA

>Cre12.g506550.t1.3|PACid:27581432
MSAYGSSHCQTVDELGAKSAVCVLPAFLGGTIDPGRLLSGASAPLLLDSDLSNIGGPVQLGSVNAFGLGASQLVVTA
TAECGWLLLAPPAVTFGTADGDAEQVSQDLTSTDGAAAPDSSSADPDPAFRQLYSCGSTQLDLPQELSQQLTSAGEG
DAAAPSLPLTVRLRMARLGAAASGTGGGSSCSSLGPEVLITRRLELRPPSLPSEAVTQRWAESSKSTSAGESAQLLS
AAVAPEGFDLVTTASLQVRVGEEEQLVVRQLRGTGDVLDASLAAYNVSYALAGLRLPGTTQSDDGSSSSGTGSSSHA
SRCQAAAAAQAELLAAALGLPPANVTVTCSAAEAAAKQGLMVVAPSEAEADAASGGAAASMAALASAADASAAAFAA
ASAFASATCPGGLAAQQTLSEQTAAAGVALVSLSFLLSPDVDPRAFAATVRRLLTPLASQAQAQALQGQAQQGQAQP
LAVYLCLSSAASFSEAVGAGAGVGAGSTATAATASAASVGTATSGGDDLQGADILSVLALRMRVDVAGGSWSVAAGG

GLTLRNGGPAANAIGPAGSGGGSSSGSSSSSGGGSSSEGVTLELGVESLTARAGLVGPGAGGSGGAGPGGAAIAPVL
NPEGVIPGGSPPTVGGPPPSGSRASPPPGVGPVVGGVGTGVGAAGGSSTSDTGVSTTILGVVAGASATATLLLVALI
ALILRKYKAHRTQLYLSSIGKPLGKDGRSRRASSAGDDLSPSASTISLHRTPRAATLPRATVAFTLDPADATDGCRG
YPGAIVSAAAVAVGNAGGGSDGWSRVPPPHASAAGAAALGAAASPPPPPLPHGQSPLATQGSFMQSALRRLISVSSI
PSVTSVGDLSALEMRALEAGEQRMSGGGAAPLSPPLPPPPRPLAVSPAGGRGAGMAVGAGAAGGGGSYCSSPYASSA
PAPAAAEVSLFTAPAAAVRAVAGGGRRGSAGGVSGGAVRQPAASITLGPKAATTSVVLGPAMAAAPGPGPLPRAAPP
AAAAAALRPAPQPASAAAAPPPVQQAQSPRIAPPVLPLQWLSPPRSAPASPALQPPPAAPPPAPIRHVPAVLPEPQA
MVASAVLPSTQPRAPEVMMLITNPLAESHQ

>Cre12.g551350.t1.2|PACid:27581518
MKRVNSVAALVALLALSLGSSSDAAKCQLAFLDYHAHTLSRYSDFGVSIVKSLRKDCDGLGRALKTCMADMRCKTGK
CSQRCMHAAAKISDSCWKELQCAFEFDPILVDYVPFITSMTSRCLKNTLPKCQAPPPPLPPPSPRPPPPPSPPPSP
PSPSPPRPTPVAPTELSRFGAATSSILSGVTVPATYAVYWSSGTVPAVKNASAPAGSRERFGNTTEQAISILERMST
LLAEAGLTLADATYLRVFLVADPHLNNTVDYQGWFNAYALYFSKPGSVKTARSTMAVTSLVNADWLIEIELWAAYPT
KGAVAPDVVSAGGPDSLLRLGSATSSILSGVSVPTGHAYYHSSGTVPAAKNASAPRGSRERYGNTTEQAISVLTRIQ
ALLREQGLGMQDAVYVRCYLVADPFLNGTVDFAGWNVAYGQFFNIPGSTRVARSTLAVAGLVDAEWLVEIELVAVYP
ERQPLVYVPTGTPGVPSDLVRYGANGTGNILSGVTVPADRAVYWSSGTVPPTANASAPVGSRERLGNTTVQALGILR
RLAELSNETGLSLADAVFMRVYLTPDPFLNNTVDYQGWFNAYAQVMSLPGVTPKVARSTMGVPTLVNRDWLIEIELV
SVFPKEGSPPRSPPPPPSPSPLPPSPMPPPSPSPPRPTPVAPTELSRFGAATSSILSGVTVPATYAVYWSSGTVPAV
KNASAPAGSRERFGNTTEQAISILERMSTLLAEAGLTLADATYLRVFLVADPHLNYTVDYQGWFNAYALYFNKPGSV
KTARSTMAVTSLVNADWLIEIELWAAYPTKGAVAPDVVSTSGGPDSLLRLGSATSSILSGVSVPTGHAYYHSSGTVP
AAKNASAPRGSRERYGNTTEQAISVLTRIQALLREQGLGMQDAVYVRCYLVADPFLNGTVDFAGWNVAYAQFFNIPG
STRVARSTLAVAGLVDAEWLVEIELVAVYPERQPLVYVPTGTPGVPSDLVRYGANGTGNILSGVTVPADRAVYWSSG
TVPPTANASAPVGSRERLGNTTVQALGILRRLAELSNETGLSLADAVFMRVYLTPDPFLNNTVDYQGWFNAYAQVMS
LPGLTPKVARSTMGVPTLVNRDWLIEIELVSLYPKEAGPAAGHRRHQLL

>g12152.t1|PACid:27581526
METEAPVLASPPSPAPPSPAPPSPEPPSPAPPSPEPPSPAQPSPAPLVFGTSRAVAFTSLPVGALTSSSLSAQGIEL
SAGSNPVSPAWGEVVDCAAGWRYGGGTTMTAAGCTQALRFPYDDFSESFRPASPPIMFWSLSLTYRTTLPYVTLYLY
PTSGQGNANACKVRLNRTEPIASPRPSAFVYSASNSVTVTASECGWSAGSTFFSFSFYVGVEGSSAGIDVFELLLDS
LTMETGMAPLSSPSPPSPAPPSPEPQSPAPPSPQPPSPMPPSPEPPSLAPPSPEPPSPAPPSPAPLAFGSTRTASFD
ALPLGSLTLTSLEAQDIRLAHQNESNALPGWGQVVDCAAGWTNNGSWAMTATGCNKTLRLTNQLSDFFLPLSPPSTF
WKLSITYRTFAFTGLVLYGTGDVNKFCEWTLQRTGDVKYNYRCEVVLKPTETIAGPEPAEFTYTASPNTVTMTPADC
GWAAGSTFNSFFLFMYVEGDSFTIETGIAPSPPPSPAPPPPRTIPWPPSPEPPSPAPPIPLLASTRVVSIEVLPLGN
LASTSLLGLGIGLDDADMYTSSPAWGEVVDCAGGWSYEGTTMTAAGCNQTLRFPYQGSTGKFSPLWAPNDGFYSLSLT
YRTTLPSVQLELFNWQDCTLRLNGTESIVRPRPAAFTYAVSKTVTVTPEACGWAAGSTFINFYFYVPPNEYIPGGID
VYELLIDSFTMETRMAPLPSPSPPSPAPLSPEPQNPAPPSPEPPSPMPPSPEPPSPAPPSLEPPSPAPPSPAPLAFG
STLTVSFDALPLGSLTLTSLEAQDIRLAHQNESNALPGWGQVVDCAAGWTTDDSWAMTATGCNKTLRLTNELSDFFL
PLSPPSTFWKLSITYRTTFAYTGLVLYDTGDVNNFCERTLERSDPTPLPNPTTFTYIAAPITFTVTPADCGWDAGST
FDRFAIFIYTFMAVGRDRVELLLDSFTMKTGSAPSPPPPPSPPMPPPRPPSPPPLPPSPPSPEHMVFGSTRTVSFD
TLEIGLLTETSLEAQGLALSDYPFGTGHFKMTWGQVVDCAAGWYLAAAAVSMNAAGCKRSLQYKDEGFSSAFRPLSR
VSEPPPFWRLTLTYRTTSPNVRVGFYKTGDVEYSNFCQVELKPTETIAGPAPAAFTYTAGPNTVTMTPADCDWDAGS
TFDSFFFYIAMADDDAPVSELLTDSFTMETGMAPSPSPSPPSPAPPSPEPQSPAPPSPEPPTPMPPSPEPPSPVPPS
PEPPSPAPPSPAPPNPPSPPPLPPPSPPPVSPPPPPPPPPSPPPPDPLPPISSPLPPSPSPASSPPSPSPASPSPA
PPSPAPVVAPSTLPSPAPPTPSPTGSPATPPPSPSPSPSPPSPIASALPSPPAPLQQSSSPPASPMIVATFRSFSLG
CFAFDVVSASPSLRALPVTLATNDAAMTVAKCAGLTQAAGLDHYGLTNGHTCVGGVNRQQATQYGSLPESACDRPCP
GDSRQTCGGGPIDSADSAGTQLSALSLYSFNPASPPPRPPPRAPRTRPARPGSAAKPPSPAPPQPTPPSPSRRAEAQ
ALTRTAATDLPALQKDVSPTINGIPSARSLLLLSPTAVPVFTATLLGASAAELRAPVLASARVGMGRLVAFGSEAML
TSCCNQPGAAAAANGTESAAEINKIIANAANWARAAAGAAGRKANIRVADPRLDAVARFIVSMLPDIFAKSRQDYYM
SLTTFAKGGHERCDVYLVLGADPQQRYDTKVRDALRSFLENGKGVLLAGPVVADAGISGAAAPVPSDEWTLPDIMFT
KQNGVTAVASATAVAPSGVAAEHFRELLSRLLQIKARSSSPEFISLKARVMRARSDIAVTDMSQFDEVLKAKIAEFD
RDIAEL

>Cre12.g509150.t1.3|PACid:27581535
MDARAEWTGGSQSPLGESLLPCTSAFLVPGQEDVYLRDSQYFDGRTSVQGLNAGYGSPGQLMGATWPTRHASAFAAP
TAFPGQSQYPAADPPLQPSASPAQEAAQYHQAGQLSPENPTLLAALDLAPAAASASPAYQHSGGAPARSPGGGPMNLS
AASSPFHAQGAGGYGMPPQPAEAWGAYQQQVRASQQALDARFAVAAAAGAGGGGGGGASPSPPFRMAAAPIASPVRA

SQPLPSQYAQHQARSSLQASLAEPYMVTTVEQLRTSPLPSGVSESPALGGPRSGGGAGQGGSSGGDKGASSQRYQAQ
ILSLERQNAELQKRLADVQAALEAAQQGRHALEAKNDMLLDEVAQAKSDAKARESALETARRANDAMSTELRGVQEE
LSSARDALAEAKDTAVQWQDRAMEAAKELDASRRKIETLGEEERRLSAENMVLRKEVQQLNDAIIKGRIETSELRDK
LRQAQQEGARATSEVHMLASRAESEQESAAVQLVLMRNRVAELEARNSQMVFDLAAAREESVRARQAAEATKAALAA
KPAGIISELGPASRHYGTLSQAWLGTEGMGGAPTSAAAAAGAGPRHAAADRGPAVGSGSSAQSAEVYAPASTTAAVL
RELPRDVAFSRLDPETFRSMVRAEAEALARASGGRTAASHDAGGHGTSAYDAGPHSPPPPRYGGYGSAGASADALAA
EASSGSVASGLPAPSSHPAWAAPQSYADNNRQSYDSKPYDSRSYEAAKPYEVRDSLAAMLPDPRSSWVASLAEARNA
PGYGDGPGAYGARTGAAGAGQRPPLPPSAGTNQQQSPGPTSPPPPYAIHTADSSYAYRRPPNAVGAGAAAGVGGGSA
DSNTPFGTEDTSKALLARSKALEDQLMVLCAEKGGLEAEYARMPLGAGRSLRERNRKAVVEQRLELLNKEISAVRMQ
LKRIGLK

>Cre12.g501900.t1.2|PACid:27581598
METPCTQPQQHQQPPAGITSSANGSGGNTSHGSGSGGDAAVVTAAAALLPFRQSIPGACLPPLPQCMGPVHEAPATC
STYPEHNIHAHAAPDAPAMQQLPLLAAATGGSSGGGASPAPLLAPPLEPPTAAATATASPFSAVCTSSSSGSGGAAP
AATAGLPAAAIAAATAATCYGQQPACGPTSPDTEGWQEACCAALLQRHASEGAPALPLNAPGSPAASDLEDFTRQIR
VQHQRQHQHHDSFWRLADEGSGVASEAETAPTAGRHHMGAAAAVAPSTDGGHAQPPPHFMTPADFLHQKHVEHHHLQ
QQQQQQEQVKQRQRQRQRARRLSTQQRDAAQVAAAPAGPEEEVEAEKPAAVTAAPGDATTAAALAAAGAQDSATAAA
ADATAPARAGPAAAAAAASAAATAAADSCKPLFHVMPLSGWGSDPNGPIFYKGRYHLFYQSRPGTCQWYWGMRWDHV
VSSDLAHWSRLPPALCPTPGGIDADGCFSGSIQVDPHSGIPVCFYTAARLRTNRDVTLPHPPAEHDMGLKHIETQCC
AICDPDDDLLVRWRKVPMPLMQLPPSGQLTAWRDPWFVEHGDGRGRQWTMLIGSGLKGGGGTALVYRSADITRGWYF
AGHLCSWPDPGTGICWECPFLVQLQPLPLCAHVLPTTDLAAVAGAAAEATAVAEALTTAMDYMAAMASAAGNGGGGG
GSGSAADGEGGGGGGDAAAAGTHAGSWRATAQAGKVSTAAVAAATAAAEAAGDASPDAAVSVAMAAAAAAAVATVNA
ATAAADAASSATQPHHAPAAGSGTAADADVDGRGACTSVRDGGSGGGDGGCDGGLVDTLLHVVTSVSAVTPPTADGF
AGGSEAGGGGGLPTLPSSSGIVPGGGGGGGSSAVHAVLPTADGAGIGISVTAAAAPAGPDGAPGGADGSTITVTVTR
AGAAAVTGPADGGGATDDVSTAAAVGGSITTAVATVTGGIEGGAPATATIDVVVTPLASPFAAPAGGAAAAAAAAGA
AGSSADGAANGVCAPPSAPPPACPPPHPSSQRGALAPASGRLGCAADGAPSDAAAPAADGPLLPRQGSLTSTSVTAS
AKMPPASASCSGSGSGSGGVLQDLHAMIAQVMEAHRARTAAAAAVSSCSAAAATTAGCLPAAGAGADAKPVAAAAPA
AAAPTPSAAPGAVSSKTDVNGRTGGLRGSLTPRLHAVGRNGAVAPLGTFIAGVAQPATKSNTSNTSNSGGSPPPASS
STCSRSSSASSTAASSPVAGDFDGVYVLGSADDQPAANAAAADASATASATADADAADGGGELLQPHQLWYRPGMVG
HCASPERAIPVEPLAADLLLQPQPQPELQPPQLPPHPTDGAATAATPATTLTMSYHKAQPLLPPGASYSACVAAASA
LPLHGDVDSATSHAHAVALPPGRRWLFCCAPDACTYSILYWISEYDSAAARYDMGGAEAGGRPRKLDLGNVLYAPTC
LKDPQGRNVLWGYMKELRNVPAPPCLCNKYSYAGCLSLPRALYLRGDKLFQVPLPELTSLRSDVAVHVSALGLAPGG
HWRLSGVRGLHLDMELAISPGRSRRVVVLFRSWRPRGRGAAALVYEWRTRRLFVVFEALSPAGRRKWSSGGGEGEEG
HRHHHHRHAPERDQEQQEPDGLQRELRRVQREQQVGGAGGGGAGGGGAGGGEVAVGAPSVLATALPAPLVREPRGGT
AAAEEGSPPPAPVPPQQGPQEPQGELRKDGAADGGARESGSSSSSTCSEQDHEVDGLGGAEDGDEDEEEDEEENDEY
DVIRDPDFIPDPDMNPLVEDWIRMKRDEAGGELDLPPGCPLRLRLFLDASCLEVFTGTGQALTTRIYRGHPPQHCHH
VPPWGSPAATSAPACAPGGPAPDPGIEIWAIGGDGDGGGAAAATANGAGSSNGSGSSTTSGSSRHVGCTLDDVHVYE
MSSGWVREQDVPREEPPPATAAAAPPAPPAPPATMAPLKQQRGVRQSLSQPASPLPPSVLRGQRERAS

>Cre12.g552700.t1.3|PACid:27581606
MANSERWRRKRGSVSGLALLLAAYIAAAPLARVIDTGVDAPPPFDGTPSESPDFNTTITTNNAPPPPDDQALPPPPP
GAGGNQPPSPAAAGTAAQPPPPTQLGGGGSDDEMPPDRAHTDVLPPPPPAKAIPPLRLVGGSTSYSGRLEIFLDGAW
GTICSDGWVNYPDNAKVACKQLGFSGWSYGSRWYTSSTSVPWDYFQTAWNARIVMFSVRCSFMDTYLAGCWWARSPV
GFASCTHAQDVGVSCGGSRPPMPPPDSPPQPPRSPPPPSPPPTPRAPRLPPAAPKPPPPPPQQGDAAVPLPDWSHSC
QVSLRDPGSLNPNAPAPGGGSSSGTPYIRCEAAAATAAAAAVAAAGVVAAAAGGGAGGPAEQGATAAVGMVAVMLGP
SLAPLHAAGLLQLSGVQVLQWIRTGSSSSSSSSTAATGSSNSTSSGPDFGITFVGVPHLLLRDSELTGMPLSGAAPL
VVCVNCTFLTVERLTVEDLTGEPLVPSANGIPYVRRVTYGAVEANGLVAARLAGVSCSRVVNSYGFACVRLSYLPLD
VRRSALLAAAKVTGAAAAASSSSSSSSGEAGVAAATTTADVEVGSAPTAPAPVASLDATGTAAAAEVTLADVRPPPV
LHLLDSSFANTSVVWSASKDVVPAKMFVPTASCGASANNKTMLGYGAVLIDFDARYPMPTNDTTTAISTITGSSISN
DTTSSSSGEPINVTMGPVVMSGNAPLAASVNGSSSNGSNSSTAGGGGDEDASAWSAVVVQGARFVGNRGGCGAGLAL
DLKKLPVKLSLSRVNATENDVMTGTFESGDGGAFYVLGGGRSRVTISGGSVLSRNGATHGGAVYAESSTSLVFEVSG
GSRLVLNRAGFAAGAISVGGKQEVAIRITGSSAVSRNSVSGFGGAILAFAKTGSLVVSDHSSVSGNVANLYSPSDAK
TNGGNGGGAWLEFQNTTLVEVSGGSAVSNNAAYNNGGGLYVWKDLGTFTLNGSIMSGNSARGGPFCEGGALFIDRSA
ANIILDGGAVMNDNRAGLWGGGIGIAGNEISSSTNLTVLGRLVVKGASTMSGNSAGQSGGAVRVSGTDGDPVPSVEL
TEGSRFINNTAKLGGGALYFGGDVLSLFTSGALLSANGVPPTFREGDYDNGGAIYVSGSVRNMTLVNTTLTRNVARG
GGAIAVYRNATVTIINCTITQNQALLYDGGALWFGTVDTLRLAGGTVIANNSAARCDIAETLPCKSGANRARGGAIM
VQSNAGEVVLEGGVVAADNRAGVSGGLLHVGGALTSLRVDTGSVVRGNAALWAGGAVFGTMAVGAVEVAGGSSVSGN

VAQEFGGFMAAAGRLATFTVSGGSSLTQNSVVNGSGAVLAVDRNISSFTITGGSDVSYNSAPYGSGAVLYVGGSVNG
FSVGGGSTCARNTVGRAGGVVAAEGAVRNVLISEGSRVEDCAAAAYGGVIHAKGSLQNVTVKNSTIRNCTAGIGGGA
FSTAGDVRNLSLAGGAALWDCTATDAAATRTGGAVFAGRDLEGLTLTGGSSIEGGGADQGGCVFVYGGARRIVLAGG
SSIRRCTARLGGALFVGSEGLTGLSLTGGSSIGSSNAAARGGCVYVGAANGGGGAAGGASDTDSGSSSDGGSGSGSS
SGGGMRRVTLTEGSEIFGCTAQEGGAVFVALGAAADWRLSGGAAIRANRALLDGGAIWAAAAGSWTLEGNSSISDNA
CGRDGAAMYVSDGAVSTSSSDSSDGSSSGTGASGSGSGSSSSSSGSAVQSAGAAGAGRTKSSSTASPPRPPPLLAPL
PPPPVPLLLQLTLASGGRVSSNRAERFGAIFLKGTGGLQLLNVTGAGSAVSSNSALSGGAFYVTGPVALVSVAAGGA
VTGNRATGGRGGSSGGDGGFLATLQDLGRLEVVGAGSALSGNLALDGNGGAASVTGGAASVVVADGAEVSGNRARFG
GAVAVKGRVAAFELTGGAAATNQYAQYSGAVLHAGGGAAAVRLAGGARAAGNRAGLHGGLLWAAGVGEVVMTGGVMV
ERNRAVLGAGGVLAVEGSGLGRLDVTDGCVVRNNAAATTGGFVYSEDLVAAAAAPVAADATASASIATADAAAPATA
TDVTAAAASAEPAAAGTDAAVATQVRLASSSFTGNSAGTAGGVLAFGSTAVAVAGCVVANNTAAASGGFLFLGRQQA
PDAQDAVLEPTPAGADSSSSSSSSCTARPAAAFFSSEDSTYAGNQAGAYGGVVALYGSSGSSSSSSSSSSGGSGSSS
SSACGVPRQQLVASVARSQFVGNGAAAGGGAIAVLMMTADASGANSSSSGSSSSAVAGSSNGQQQQQLPLGGQPAEV
RVSISGTRLVRNVAGRIPGEAGSSTTGSSSGSSSTPQVVVSGGGAVLLWVEQPPLAVAAANAVGTAAAVGVNSSLVI
DGCEFASNAAWGGGSGGDVAVWSATVSQRQRLLAALAGGGIATAATTARSNNSSSALSALAAAAAAGGDGIQTVRIA
NSTFQNSSSSADGGSVLAVCGSSSFGSSSSTGASSGVEGGSSSVVPQLTGANACTLAISNSSFRGATAGQDGGALAV
AAVAGGGGATADTAASNGGSSIGRMAAAAGAAAVELEGCRFEINTASGRGGAISYVELAWVQAAESPPCGASSSSNG
NTSSNNAGCFAFNTSRARVGLLVRASRFTSNRGAGGGGALALAGRSAAHILDSTFSGNAAAAGGGAVLARGCGLVLI
NGSSLSGNSAISGGGLFVSPAEAVAEAPAAAGAAVAVAAVPGGSSSAPQRRRTQQQQAAVTAVSTRNFAALIVLDTV
FANNMAVLPPATAGVAAAGAGAGAGLGKGGGVFVQAGVSALFRNASFATSNTAASGSALASTQRCSTGGSSSSSNSG
SSSSNSSSSGLLVEDLVKQFEANGAAGVSDWLAALYTAHQHQQGTADYCWPLLLLDTAVPPPSVPDAATQRRPRPVW
MEDANMTAMAAWCQGASTAAASSDSAAGGGGRVAALLQTLAQCSLPQLSSVSELSAAAAAAVEFPPTQLRLQLPCSM
LQSGSSSDSSRSSSSSSSADTGSSSGGMCVVGGRAASAYGAGAAAPQQQPRPPATTTSTATATATATVVSVLPEEPF
AVQVQLVDAHGSATAPVASIYKVTLSVRALPPPSAAVWSAAGSINSSSNNTPSATAAQATPGTAAWLDSICAPQLGK
LQPAVADTGGAWWWCDPDFANLDVGRSQSLTAVMEAGAAEWPRVLLRGWPGDYLLVLRAAGPLQISELSVPLEVRRC
AAGQTPDNRFAATSQVPAWTGCRTCERGQVGLLPDRRATASELATAAATAATAAAGGGAGAGGAVAVAVATRQQLLL
PQLTGRAMAGEVVRAYDGAEGVCRECPDNAECPGGRAVVPLPGYWHSAPNSAAVHACYVPDACAGPSDLAYLLPTTD
ARSSTGGSSRSSLNTYPPPPALGSSSTGTGGSSSDGSGSSSSSSSSGVGGVGVNERTQALLACQVALYSSGSPPYGD
ASAGWASNATSSGGAGCSLGAYMQLQCGDGYTGNLCAACLPGYAVSQDLECGACPSMARTLGLSLLAFFGSVALILY
TAIASLGAATASSEDASGVTKGDIMKILIVHCQYTVIITRLNIDYPAVITTLKAALGSITGAASQGISFNYGCLVAA
APTAQRAMAQLLGSLLVPCVVVAFSLALWALRYRFLHQAVLKRTRATHGRRRTTPDALPSFNFYNDLMPPLDEASSS
QQEGRSEEPEEEQDGEGEGIGQGSGSGSGSSRGSGRRVSSGEAAAGHTPRHDRCSEPAAIDAAAAGGLDDATLAASHGL
CKPAGAAGAAAAVPAAEVEAASALTPAGGGVAWGAGALVSAAAAADAATACCSAHEVLLQPQKQRRSHTSHHSRTTN
SADDDDAAHTAEAPTSLPQHEEQDEHRQQQQPQSATCQMLPLPPAAPLPPPAAPLPPALPSRAPSYDPGVSQGPAA
AAAATAAAASAAHRTHSHTSSWQKPATSTHSTSSQSSPHTYQQHPPTGPGSSAPQSRQHSASGAPPPPAPHTQPSQP
GSRRASHVAILQGPSPSAASATQQRTHAPPLGPSQPPPALPPSAGSGTSLQQQPSTQRPSSVHQPHQHQHHHQPRTS
GPRGPPTRDSAGSHNPWSSNLASRYARDRSSAGGAGGGYERYSHLSHVSSAMRKLGSRALMLVRPRDAAPTSALALA
DTSLGFWQQLGLVLLSGVFVLYAGWAQAALSVFSCYRIDEGGSLVPENERANWPRGYWFLDMQQACYSGTHLALYLP
IGIVSVCLFCLAPPLSSFLLMWRARHRLAEYRTQQMWGFMYKRYRQRFFFWESVLQLQTLALVAVDVFGRGMPVLYQ
ALLLLAVLLSIASVNMFCAPLRTRRLVVLEFSSHMVLCLTITLSLYFIPTDALPLSDGEATAVGAIMFALNITLLIA
YVAYILALSWPAFKPHVDRVRGSAAHAARRVLRRRGAVSSEGPSQHAEHGARNVEGKGGAAVGEGGGHDGSYEVEEG
RPAGGGGGELRQPGLEGRWAEAGSCGREDAALGTGATGVRRELGGAPELPTGSSGAAKEDGKAKAC

>g13542.t1|PACid:27581708
MFKSRSAAALHELRAAAAARTYPTDPAQYELLEECGAGVTSTVHRARCVPLGGEVVAIKRCDLTDLADADLQVVIEE
VAQMRRYQHPAVLPLLCSFVTAAGELWLVMPYMEGGSVAHVMRYAHPDGLEEAVVATIAREVLRALDYLHKQGAIHR
DVKAGNVLLGGDGAARLGDLGVAATMERSGTWGRGRVGRRTLAGTPCWMAPEVLQDCVYDDKADMWSFGITLLEMAH
GSAPFANQPPLKVLLNTLQNPPPQLEDRIGARTFSKSMREVVALCLQKEPAARPPARQLLEHRFFRHHARDRAYLVK
HLLAGLPPLTERVRQLRAGHGGRVPTRQAHSRELKSRASYMTGVLGWDFNLPCAQPQQQLPQLQPVTELQPSGSPAP
PLHQAASGLSCVSSSTTGASLATVTGGGGGSSATSSYSPGANTAAGTGGSSPTAATAPTTPAAAPPSPAGSASTTPP
GGGSSIPGGGSSTPGGGSPTSSAGPVLGSRAQQSPSHFHPQPRHWPFLSPGPPSVSPTRQPRPGGGGAASPFGAAVS
QAAAASAAAAAGGGSGTDTDHSSMPGTGGARGAAPDEGEEDEEEWAAAEGVPRRPSLRSSVSSPDLHRRRTQQPPP
HALPPPPDLPSVALPQPYQSPKQQQPLLQPQPLLPSLSPAAQPLTPAPAAGAAPAVASPTAVSRSPFSGAGGGGGGP
GGGGGCHHSGHHHRVSFAPDVAGLGSSSSGPPPPTPLLGSGGSSGALSVQRKGRFQVIETTQPAAKPQHPPPPWLTS
PGAAAASAHARGGGASTASTSGTGAAACGGAGRSSSGGHVFTSPTSTSPLTSPRSHSGQHGQQHQHQHQHQQLTQLP
QPQQPLQLARAHSGGKPPLPPRPPPPAATAAANHPTAAAGASNHPHQLLPMAVGSHGHGHGHGHSSHLLARTPSLR
TSTSTSGSSTRSPPPSPPGSVAAAVAASGGGSAAAPPSPPPLSFPSVTSPLLPLPQPPAAPTLPPSSMLPSAPASPF

ASAVGGMAAAAPPPAVSVGAARSDWLKGLYKRWSDAAVHSLLAAVHRPDESHQLSLALTSAAAPRHVPTIRVTAAPP
ELAAALVAAAALKPPQSSLTAPRAAPSSAAQSLTSAGTSTEASAAAAAGAPPAKAPSPRLPIITLAHLVPSQPHHWF
TPLLDRLMPHHPAAGTPATATSAPATLATDSTGGTGGATVTAAAMERAQQRRLQQAELRGGGGGRHRRLLVPSIYPP
LEMDSGWCGCGSEADASASSDSSSGEGGAVDTAGGAMGGAGGEGLWGGHTRGHTDGSTQHTLTGSSTAPSTSTAPST
ATGATATGTSGTGSQASGSDSAGHRGNREARAAAAAAAAPSTDVVPAADASASVTTSRSEALQTKHRRPHHHHHHHH
HDLHDRGAAGGSMYGSMYGGGGGGGLVVVEVDPSHPDYNALAECYAGAGGTCWAPTARLLQPLLRTLPVELERAQHT
CGDGNSGGGSGAAGGGGEAQGAATAAAAALDAENASTTTPVVTPGLATSPTPPLSLPPPPEPLLLPHRPLQPPAASL
MPGAPRRTPHAQPSLPTLQEKPAAAPGGTFPTAHDGGAAATIVPSRVPFHGVVPEAAAAAAEAQRRRRQAELQRHLE
AALMTFGVAPEEIEELERVQSGGGGDGGAGLWSPKLVPAAAAAAEGKREVAGKAAMERVEEGEEGEEGDSSESTVSD
ADEEAAEDEPPDGVSPAAPATLASQGRGQGQGDGLDRGGGARSSTTAAAVSRRTTANTAGAPQQAWVPAGPAQHGPQ
AQAAGPAPAANAAAAVAAPPQTPAQVTLGGSSTRTAGGLGGSSSNSSSSNSTSGSAAASALAQGTGAGAAVAACALQ
AAATGALSPAAAAQLLPIVSATSLVPSLPSPPHAQAQAQALGAPGEAPPPLQPFQSYWFAVSPPRSAVLPQPPRTGP
GGGSSTCWRATAAAPAATAGASPATAAQAASAFEAWRQQGDLAGIPPLAPLAPSSQLQPLQPPPPLPMGLGGAAPLL
PRHSSSSANSGSRRSSLNLPPGSHTPHGAGLEGSSPMLEGTGSGGCSNAAVAAAVAAAAAATAATVLGSSGGSGSSG
GGGGAHSPGLDSAGVPRPDAAVSAGEDSGGAGPVQLRGGAGVAVTGAADHPGGSGPRGIADGPMTRWHAGGMAPTTP
ASPNSAMGAGSGVSAPGRLVLPAGPRAPGAAGRLQPPQRHRQPPQTAQERAVAAAAAAATAAAMAAAAAGRGVARGR
FVVVNETMQQPQAGRGRG

>g13542.t2|PACid:27581709
MFKSRSAAALHELRAAAAARTYPTDPAQYELLEECGAGVTSTVHRARCVPLGGEVVAIKRCDLTDLADADLQVVIEE
VAQMRRYQHPAVLPLLCSFVTAAGELWLVMPYMEGGSVAHVMRYAHPDGLEEAVVATIAREVLRALDYLHKQGAIHR
DVKAGNVLLGGDGAARLGDLGVAATMERSGTWGRGRVGRRTLAGTPCWMAPEVLQDCVYDDKADMWSFGITLLEMAH
GSAPFANQPPLKVLLNTLQNPPPQLEDRIGARTFSKSMREVVALCLQKEPAARPPARQLLEHRFFRHHARDRAYLVK
HLLAGLPPLTERVRQLRAGHGGRVPTRQAHSRELKSRASYMTGVLGWDFNLPCAQPQQQLPQLQPVTELQPSGSPAP
PLHQAASGLSCVSSSTTGASLATVTGGGGGSSATSSYSPGANTAAGTGGSSPTAATAPTTPAAAPPSPAGSASTTPP
GGGSSIPGGGSSTPGGGSPTSSAGPVLGSRAQQSPSHFHPQPRHWPFLSPGPPSVSPTRQPRPGGGGAASPFGAAVS
QAAAASAAAAAGGGSGTDTDHSSMPGTGGARGAAPDEGEEDEEEEWAAAEGVPRRPSLRSSVSSPDLHRRRTQQPPP
HALPPPPPDLPSVALPQPYQSPKQQQPLLQPQPLLPSLSPAAQPLTPAPAAGAAPAVASPTAVSRSPFSGAGGGGGGP
GGGGGCHHSGHHHRVSFAPDVAGLGSSSSGPPPPTPLLGSGGSSGALSVQRKGRFQVIETTQPAAKPQHPPPPWLTS
PGAAAASAHARGGGASTASTSGTGAAACGGAGRSSSGGHVFTSPTSTSPLTSPRSHSGQHGQQHQHQHQHQQLTQLP
QPQQPLQLARAHSGGKPPLPPRPPPPPAATAAANHPTAAAGASNHPHQLLPMAVGSHGHGHGHGHSSHLLARTPSLR
TSTSTSGSSTRSPPPSPPGSVAAAVAASGGGSAAAPPSPPPLSFPSVTSPLLPLPQPPAAPTLPPSSMLPSAPASPF
ASAVGGMAAAAPPPAVSVGAARTSAG

>Cre12.g553850.t1.3|PACid:27581790
MWARLSSALDEKLKRKSKSKTRAASSNNSRSGSSSCSFEAPSDGSTHKQQHQQPQQQQQQQQQQQPQQPQVLQQRIR
QQLARADGPALATQQQQQQPQAFAIASPGPESGRVLALRDACLGNSSTRGNSSSNSSPCHHGSSSARNMLGAAPVAP
SDALLVTMPCGASPHGKAAPPLCTTGGAGSSISTFLDGVDDPHDKNDCVGAAGAVDPADDRPPSPSSTLAAFRTGRL
RRRGRAVTAAADVAAAAAGATGLLSEEHGPPPPPALGAVAAVAAAAGRTSTEKQQRQEQEQEQRSVATAAALGSAAA
DAQGRCRHGGSANGTSSGSSSGCCGPAGSSSCSTTAGMLCSSSAIGSAGGLEGFSSGGSAAAAAAAAFGLIGGAAAA
SRDFRRAAMSLVSDTAYDTSVDSSTASMGLLMGNHRCYPEAAGLLGTVGSAGAAAAAAAAAAATATAAAAAAAPAA
TAAVAAATGSPAGGRGLVLGSAAGAAAAGSPTGRSKPTVIPAAFAGLAASSPPPTTTIVAALPSTGTTKVAVDNAAG
GASAAATDPPLILQPRVPYVPSPPSEPQAVASPPLTPAATHPAAVAVVPVAVAPVAVAPVAVAPMPVAPVAGPAPQV
VVSGGVGRGGGSAPGGGGGGADGGGAGGGGGGLQVLAAQQVLYARPVPPPVRPPPASTQQPGRVLLQPQQQQQQQQL
LQPQPLQSQQQLLQLQPQPPQPHRRTPSLLRYRGLTSVQAVSVKVHAPTGAPDQFALRLSAAAAPLLFPEAAATAAA
ATPAGLPAVLSPAVAVGDAATTVRATSDGSDTAVVAAPPLGRHSPAALAPGGPVAAVGSSDDPSSACCTSRSSSASL
STSSTFPKSGASEDRAAPSPPAVAAAAAPPLQPPLPLPQPAAGAAAAAVVVPQDLLLPPAGRWSTGGIVVVAGCVQL
IAWLRGLSEAAVAASLAGGGAAAAGGGLVGGGGGAVLLPLSEAELAAVAAAMGLESTSVVEMLQLQLLQPPRPGHHI
PLQEHQHQHQFQGITGAATSADVVPAVAARQAVAAASLPPTFPAAAPLADALAAALPDAALTGPWQLQGAGGELIDL
GGGGGNGRRGGGGDDDAAAAALGSVAAVAPPKAAAEPADTMSAAAAGCSRAGSSARWSLDLEWTPCVAVAPDADAPP
AYVELDFAVLPAGGDGGGGSGGSGGGGPEPLAAAPPAEMRLMLVQHGRLLADFKCVLAAPAGGGGGGGDGSGVLQLV
IAPVATTGTAHGGGGGGGQGRAAESRATAATAAVAAAPPPLVYDIATLLALPADVAAELSQLSQVMRMDYCGSSSCS
GDTGSSTGGAGGAAATAVTAIAGGGWRDHYAPLVSDLAFLMERAELAEAAAAAATQQAAAGAAAGGTAAAVAAALPP
DGAAAVLQVASQLLEYLKGLSQDLQEQSAVAAGAAEAAAEAEDASDRNATAMGAGGVAATAAAPVTGGMPQTAAFVA
AVARTALRPYLASGALQVVAAAAGAGGGGTGAGAGAVVGAGGEAAPAADGGPSLRAAGEKATGGGGGRPAAAGDSGG
CSGGGGGGGGGGGGGGSGRSGAALWSPFIKDQHEVNNNETTTMVGCSDLAHMPPAAASVPASASGSAFGAAAGTAPD
DDAAGSLQPSQVRSPQARPMGALSRSDVASKSDALHALHRDRLMGAAMAWMGTGAGGSSAGGAGRGGGAASRGGSGP

SGIGMAVAASGAGEMVGWAGGSSSNRALRRSLDWALGGRGGGAAAGALLPDGAAGGAGGNGAQQDFHAAATAGNFF
RASAGTSPRDASALSTATAATAVSAFLGLDPGGFVDDGDDAGAPHQHLPVFRSQPHEHLQDFSPPPSPHHLQHHHHH
HQQQQPFTATGATAATAAAAATAATAAGACSSSSPLLPGCWPATPLPSGDLHATRADSSGLLLPPTLLAALLPPLLP
SAAPPAGQPPAAVLLAAGAAMPNEAAAAAVAAVEEAAAVAASKEAGDDIAGEAMAASAGRLGGSISSASAVGAGSST
MAGGISSGIGRIINSHLMGARALLAACFRGFPDAQLEAEYGRYHARAAAASDGWTLLNHTALVVLMGCRLLAAAVAA
AARGGAATTATAAAVAAAAAAREGGTGVSGDAGGDAAGAMLPSAAAAAATAVASRPPPFLLPNRTLWLVQGVVRLTG
AAAARLAGPVRREAALLAAWALETTTLAVVLLGEGCVMDEGGGATSAGAAGVLLPALASADEAASGGSGASCLAGNG
MAAAAAAAEASCWWCCDDTQLSPNVSCSGWAGTAASTAAATAAPVGAYAAAAACWLPSACCFLYCCLRRALLLLLPG
TRPGNSTWLSWLWWWPWFQNGSSSSSSLRMAAVAACCYCRRLLSWDGSINGALGALPIEVLMHGLVRPLCDQIRLRR
RLLLAAVHAGLLLVCELRFARAELAPAAACLRAAALLVLACGVCLALELPRRRAFLRSRAAPRPQ

>Cre12.g547250.t1.2|PACid:27581796
MHHAHFQWQTHRQGTRRAVVVASGSTGAPSEAALRQSAEALLEAQLRLLEAEKEESLQIAQACTQRSKRLLARLDNL
EQYKQGSGMRAGPGPGSAQGQRRSSGGSSSSSSSGSGSSNSTGSRYTAPGSMAGSRGGGGSSRATQGGPAPDPLESQ
TRRLVSQALNRNIARGKNNVALAEKLQIYIHLLQDELYRVRRGDSQLAARVVRQHSACSTATSGAGASAASASGSAS
GLGSGSGSASASAAAATAAPAGRVGGRQGSSSGPSSTARTASTSASGGDPPSGTAAVKSAATSAAAAGDAHGTATAA
ATATPAVSPVSHSSSNTSTDSSSSNAVGSASRAATATAASSPPPPAQPAATIAAASDNLTARDGATNATVAPPHSPP
PPPPPTDPVLAVSLASAGSLGSGDDGAGAAGGPEDAAGLRRIFSRTRTAQLPGDEAGETEAEHLPEAAVAAAAAAF
APVEAAAAAGSAEAGGAAEASEAVSGAASEAAGLEEGGGSASPVAGLSLEDATATEGVAPTTTQVPAPATAPEPEPA
SEPEPSGAEPPDRVCRGMNLEVARSLADDSESARGALMGLLSPAGGLLPGPLQALQAGGGAAASLLPPTPSPAADGA
MEGADLWGEAAGASDRAQPPAGAASLASAGSEPKFGLESGSGVEEPGSVSASEGASSSGNGRDAGGSSAASVSTSFG
ASSSAAAAAAGSAFRYALAGEQVPQRTVRVAQTAAAAAAAAAAATARARAAVAAVAAAEAAAAAAVPFRAAGSGDNK
ASSSAGSASNSSSQSTARSPSASSSAGAASRDVAAVVGLVTALTSELRRRGYVGCEDSVEEAAARLEKVVAAARAAR
N

>g12818.t1|PACid:27581870
MATPATPPAARSQVEAAQAPSIVLSVTDLANFIHLRRCGRFLALGGFKAPAAVASGPSSAPGPAHARCDRPLDPELA
RSGHDLEHVVAAELLSRASRAPRSHPPQGSSTPWSEFWAQVTTLPPPPAPCSLAGSSNSSNSRNGENGENRRGQAPP
AVQVGYWASEVALQGRPTPDAAAHLSGRADFLMLLWRRRGAGTSGGGSGGASGGSSSGNSGGTIWVPVLRLVECKAS
REPQTKYLAQLVAYRMLLLRLLEQEAGRAPARGAGGASGGGSANGSRSRVLIGGRWWALPHSVGSSSSCVDGSSSSS
GCSSGGADELEIECVLRINGGAEDSGAGGGDEDDGTDAREGGEEEGVAAGAAAHTARQDVVDAEPLPRREVESITRQ
LASLLAPLSGRVAVALETPLQEVPYELGSHCVSCPRSPACWSDAAARGALQLSGCGADTAQALRLAGVADIEDLASL
RPPPLQPQQQSPQPQPHQATTRLQQVAEQVGLSRPELYRLSLTAQVRAARWLPQAASAVGAAVTAATADLEALSTAI
PQQSRRSSLAPPFLLLPGSGPSCLPARAPWPFTDRHEKQRLQPPPPLVRLYLTVATDPILCRVVGVAAHVAAADGTE
TSEAWWRPPPQLEQRGWDVVELITRAPSQSAEGPQGAAEGLVKGKEGHAADARGAEADSDVWGSWDEAEAALLERFS
TQVCAALEEAAASLAPPSAGSATHQPPQPLLHVYVHSQSELQSLVRRCSALDHMTAGSGGGGASGAATPSNRHSSSG
RGSGCGGVGGDGGSSGGGRLAWLPHLLGLRAGLPGEQLVSSALEEEVRRYATPWQGGGLLAACAVDWGAQEQHQPQQ
PPQPQPQAQQPPPHNRSRADNGGLYQWRAPAFLVDAGAASGGACAGDVDLQAVFSPGGCLSAIVPIHPELHGALQQQ
EQQTAAAGATGAAIGAAGPPQQRQPQGRPRQIPSSASAGIAGSHVPLLVQVQPDLPGGSIPPAFIRALWHSAAATAN
PAAAQQDDASRAAHVHDNGKDKSAGCAGGGGVFAAGARHLRALLAARAHAVRWLEERLVRLPGGGRIAITNSKLDNP
LPLPLSPQLRHFRLHAGAAPAALMEGPTGLRESVASAVTERSAAAVAVALAATDAVRLNRGAEVRQFWRQLAAAPPA
ARAEGGRGCAVLGDLRAVPQGKYSTKVLRGRVLYPPLNAADAADGSRDAARVLADAFSELTGLKDKDGVVVSKVAAV
PAAAGPGGGSAGAAAALVPDPWLCQSEEQVMNHFALHALKLLPERPEGGGGDGGGDGDGDGDGGGLWVELHYNYVSK
ELDPEDFFVAGPNTAARFAEWAGGMVALDAAPNIAALVATSCVEGLQEAAADSAYVARRLRLPSPPPRLRAGRRPCR
PPPLQQAQQQAAAAGGAGAQPGGAPPHMAPVRGQEEEDVEEEPTTPRQQQQGKPIAGAVTPGSVLGSAAQALFSPPP
GAAAGAASPASPSASSPGITRAVTAASGPARGLATCASPSPAAAGGNRAGGRDAAAAAPSLPPSLSPPRQAILEALS
CWRPGRPLHPDQAACVAAFLSHDTGGLPNTGGRGSRSSRGSSNSGGGSSSSGSGSSSRGSAGDGMGSQVLLQGPPGT
GKTETLALCVIGWLAARLVEGGPTGPPGQGGVVLLSGPTHTAIDTVMERIAQRIQEGALKEAVAAAATAATAATGAG
APAGSCQRRVHLVRLNNSRTSLAPHLTALGMNSKIPKTFEPGVWYLVAGTLRHLQTAAEQLRQCEAQRACRQGAAAM
AAGGRGALASALVLDEASMVFAPQLLALAGLLVEAAALLVAGDHQQLSTILAADLAADLRPNLRRHRPDRSAFDYMR
LLASAGSGSGSSGGNSSGAGGSGGNSSGPGSSGDQQQQDPPQWATGFTATPRVRVVALSYTHRLPPAVTALLAPLYA
RDGVQLVGRRHPHREAVEVVEAEAEGVGAAANRLVVRRHGATGAVVGGGGGGGAGVGPSRAPGSGDRIGPLGTATAI
GGGQLLAPPLPQPGRALHTSTTSTSGSSGGSGTSGSSGSSGSGGGVQQLPAQLLKQLWAPGGGEGDDSSGGGGVYLL
LHNEDRSQRVNRLEVELVCQLMEASPFARQEQQGHAAAAATAAAAPAAAPGRNAGDAVPAAPRPAVAIITPHRAQRR
ALRAAAQGRPWGGVCRVETVERMQGDEADTVIFSATVSSCAALEAAAAFYTDLCRANVAFSRTRTRLLVLASRAFLT
HMPTSYSHYRGLELWRQLQHMCEVEGRLLGELVGLPGGGGGGGGGGGDSAVSYRCDVFAARSPGQ

>Cre12.g514400.t2.1|PACid:27581933
MAHQFNGPGGPGSSVSPPPQLKQEAVPAAQNAEPPPSKTRKQYTVSKRRERWTDDEHQRFVEALKLYGRAWRKIEE
YVGTKTAVQIRSHAQKYFNKLEKAGDAAEIQVPPPRPKRKSVTAAQRAAAAAAAAEQQLNPRSGSEERGYGDLQDDN
DAGSEGGAEDGEPGYGQHDQMGSPDDVPLAADLQGLLLQPPRNGAAAAAAAAAAAAAAGDLGPLAAAAGLPQLPVLQ
QQQQEQSAVMQQQLQELQQLQLQPFTAPGLMPFRGAAGAAAVQLPVLAAAAAAGQLLAAAAAGALALPPAPSPPPVL
LGGALPPVGVGALGQQPQQQPQQATPSGTTLSAQAPMQMQASPQQQQSQQQQQQQAPAGSAGLSAGAGAPGASNGGF
SLADSIARNIAINMAQWAQAQVQVPDAAPAPAGAPDRQTVEAVAAAAAAAAAAAATAVISAAGEAIQQRIQEKAAAG
YLPFLVHPLAELIPPSLAARSGSAATTSDQSAPPWALPVPQQSLMHVLAGSVSTGGGAAGTHPHSAPPVSSRQQQQQ
QQQQQQQQQQQQQQQQQQQQQQTAPFSLGAASGGAAGNAEQQLTATRDDTHTGQHTGGTGGTVTATPGSSAQQQGGA
VSGPGNSPQHMSASFAAVAAGGGSGGVASVSMAGGLGRLPSSSLVASLGGPSVSLGGAAAMSLGVDLQPPQLLAPVA
SPPRMEWEVAVTVARASQQQLQLQLQQQQQQQQQRQTAPGFGLLAPQPMAREGSAAAGLLRTLSLPAAWDSGGAAAQ
AAAALQSREVLSRLAAAYTSDVPQLQAVVAATAAQQQGLPQGLLQPQPLLQLVEQLPSRPSVELAQSPVTLPGGGG
SSSAFQVLRPATSQQQLEVSLDPRVRAVVDPRLQPQQQPRHVRSQSAAPGGAQPLPQARAEAQGQQEAPAQAQAQAQ
AQQEAQAQAQAQAPYIKAEVLTEDEERGRERERDPHRHHHHHHHAHHHHHHHHSHHGSNHGGTYGGAHSRGIGSSAE
PERGSRNPVDRRSRSVASIRSGGGCEERQMHSGSGGGAGGNGLTRIGAAAAAAAAAVAAAAEEAAAQEQVDKSSGAS
GCDNGSPAVSPDPRQLPLAVRGHGISPSAGAEGQLAHAGGGGTTGARVRFEAHVRSSTSEGRAHTGQQPQATAAAAV
AAAAVAGAGHRATAHRHGVHSSHRHRSSGGRGSAAAAAAATAAASGAMDTDASAEPGGGARHRAGSRAAWLMDDYGA
AASGDGAYQLGQLQYLPQQFMPQQSGPDNGGLSGTTGSAFNSLQEVLQGPDPAAMVPLLAPTSGVGACGVQDAATTA
QLARLQLGAAHRSQAAERRVAAAERAAAAERAADGEGQEGGSGGNGVTDVNTYIRGDYSLLSPSGGNGGSGGYAAQL
AGAVATAAGSGGGGGSGGGSGGGGVPLSSSGPSSVPSTQQQGANGGAATAMAAHRHSAQQTKQRAAAAAAAAAGAGG
SDGNGAGASNEMQQGSNQNAGSGAGSGQAGEAAGGVAQATGVALALPPPLPRSLLHVAARQGAPEAPSAERADAVGQ
GACGGAAGLNAVSPTAGQPGGGSNTGSGSGDGSGAGGPPDVYQSSGPTGLARAAAHAAGLRQETPPTGVSAGTRRGA
GGSGSGNADGGGGATGSGSGAGQGSNPSGNGAGEGSGSGAAPAAGTAAGAANTGGVGSTQAAAGPHALQHPHAHGNA
HQQQLQQVQLQQQQQQQQQGSGSGGERGGSGSGAGSGSRVGSGVPAGTYAAREAMPGASGIGRHVPPYVVAAQRAQH
ERVEAAAAKAAAATVGRDGEAGQADGSKPISSPEADAEGALLGSGGGSGGGAVVDLAVALRHKQRGSPGGSGGMMGS
QARGDSPAAALTDPRLLQRGGSALALAAGAAGRVASPPPNGQSTLPVPGDTLALLHQMLSQQQQQLQQAQAQVVQQQ
QAQAAAAAAAGQDGGAAAVGSLAPMAPPPHGLMTTLAGLSAGTQLAATLLALHEQQQQQQQQEQLLATNRGGGSAAA
GAGRSNGFLSLAAAAADPTSSGGGSSATAPPPLMALGPGALTALLPYLTGERPVESEHVARLEEVARALQYASSLAG
PLAAVLHPPGTSGGGAVDAPPTDPRRPDLAALFSSGGGGGGSSTTSRLTADVLDVMRGAGPSRLLGPPAAQLASARD
GLPSSDLAAAALMAQWQNAATMYGLQPLLRTSSAAAAAAAAPGSATSGGSAAAPPPPSSAAAAAALLGFPPHPLLIQ
QHHLQQQLAASGQAQAAASYLQSLGLASGLGLGFNMAANMGSRSALGALSLGPMGLSLPTFASGGGGGNGTASGGAL
GAAAAAGGGGGAEQSPRPSAGTIGSGGGVHGLMPAPTRSHHQQHSHQQHQQAAGAPSDPRLSGGNPRRGSGDGAVGF
KPPRPLASLGGVDGSGGVARGLGSGPSMSGEKLAAAAAAAAAAGGNGGTSKRSLNELYGALVAAGAGAGGAAGGGGG
GSGQGVQRSGRQRNGRRRHHRGHSPTRSVGSGASSSGFHCGDKSGGEEANYGPRPSREYKEGSPQGGGAVEQQDTAT
SSGREEQGSAGAARGCGAAGEVGTAGQSGGGAGGGAAAANAAAAANAAADGGGGMALPPAKHARHDLMTGPNGKLAA
AAGHGGGAGGASGQGTGGAAAKAAAQRPPKDATPSSGGGSGGSGGGSTGGNKSGTSNDAYDGSMNASDEGSNEPGSA
PADGPMGQALAPSDGMGMLGAGDPGLNVQGMQMPGAGMMGAGGARSFSVAQPEPGPGHASIMAAQPGGCGGGGSTGV
AGAGAGAGGGAAGGGGGTAGHKRVRYDAGPAGGGTAAAAGAPAAGAPLTPAAAGAAGAMQQRAGALQADGAGTGTSS
PSETGGGDDSRGAAQPPHKVQRTNAGGAAAVAEAGAGGAAGHAADACDGNNRSGGSAGAGSGQAQSGRGRFAAPPSN
QQHPHGAAAAAAAAGGAGGGAAQRTSGSLPSHLGYVLEQASSKQGGNDGSGNNNSGTGPRSGVGSNEGGAAGGAGGS
RSGEGGNGNGSQGNGGSHGHGGSHGNGQGSNANGSHGHGSNGNNGHGSNGNGASTNLPGGAPYAYFRTPLSLQQQPR
GGGGGGGGGGGGGAGSGGSGGSGGNRGGGAQGSNGGNGDGAGSGGHGSGAHDGASGGAAGPSPYDAGAPTSGAAADH
MHRHLHHHHFTHAHHHYGGGGGGGPQEGTSMRTPLPLQPQQPQPQPQQQQQQQQQQQQQAGTGAANAGQGMALAAVA
AAAAAPAVAGTSGGAAVVAAAAAAAAAVSQQQPVHVEGQGQDGNEPMLQDG

>Cre12.g514400.t1.2|PACid:27581934
MAHQFNGPGGPGSSVSPPPQLKQEAVPAAQNAEPPPSKTRKQYTVSKRRERWTDDEHQRFVEALKLYGRAWRKIEE
YVGTKTAVQIRSHAQKYFNKLEKAGDAAEIQVPPPRPKRKSVTAAQRAAAAAAAAEQQLNPRSGSEERGYGDLQDDN
DAGSEGGAEDGEPGYGQHDQMGSPDDVPLAADLQGLLLQPPRNGAAAAAAAAAAAAAAGDLGPLAAAAGLPQLPVLQ
QQQQEQSAVMQQQLQELQQLQLQPFTAPGLMPFRGAAGAAAVQLPVLAAAAAAGQLLAAAAAGALALPPAPSPPPVL
LGGALPPVGVGALGQQPQQQPQQATPSGTTLSAQAPMQMQASPQQQQSQQQQQQQAPAGSAGLSAGAGAPGASNGGF
SLADSIARNIAINMAQWAQAQVQVPDAAPAPAGAPDRQTVEAVAAAAAAAAAAAATAVISAAGEAIQQRIQEKAAAG
YLPFLVHPLAELIPPSLAARSGSAATTSDQSAPPWALPVPQQSLMHVLAGSVSTGGGAAGTHPHSAPPVSSRQQQQQ
QQQQQQQQQQQQQQQQQQQQQQTAPFSLGAASGGAAGNAEQQLTATRDDTHTGQHTGGTGGTVTATPGSSAQQQGGA
VSGPGNSPQHMSASFAAVAAGGGSGGVASVSMAGGLGRLPSSSLVASLGGPSVSLGGAAAMSLGVDLQPPQLLAPVA
SPPRMEWEVAVTVARASQQQLQLQLQQQQQQQQQRQTAPGFGLLAPQPMAREGSAAAGLLRTLSLPAAWDSGGAAAQ
AAAALQSREVLSRLAAAYTSDVPQLQAVVAATAAQQQGLPQGLLQPQPLLQLVEQLPSRPSVELAQSPVTLPGGGG

SSSAFQVLRPATSQQQLEVSLDPRVRAVVDPRLQPQQQPRHVRSQSAAPGGAQPLPQARAEAQGQQEAPAQAQAQAQ
AQQEAQAQAQAQAPYIKAEVLTEDEERGREREREDPHRHHHHHHHHAHHHHHHHSHHGSNHGGTYGGAHSRGIGSSAE
PERGSRNPVDRRSRSVASIRSGGGCEERQMHSGSGGGAGGNGLTRIGAAAAAAAAAVAAAAEEAAAQEQVDKSSGAS
GCDNGSPAVSPDPRQLPLAVRGHGISPSAGAEGQLAHAGGGGTTGARVRFEAHVRSSTSEGRAHTGQQPQATAAAAV
AAAAVAGAGHRATAHRHGVHSSHRHRSSGGRGSAAAAAAATAAASGAMDTDASAEPGGGARHRAGSRAAWLMDDYGA
AASGDGAYQLGQLQYLPQQFMPQQSGPDNGGLSGTTGSAFNSLQEVLQGPDPAAMVPLLAPTSGVGACGVQDAATTA
QLARLQLGAAHRSQAAERRVAAAERAAAAERAADGEGQEGGSGGNGVTDVNTYIRGDYSLLSPSGGNGGSGGYAAQL
AGAVATAAGSGGGGGSGGGSGGGGVPLSSSGPSSVPSTQQQGANGGAATAMAAHRHSAQQTKQRAAAAAAAAAGAGG
SDGNGAGASNEMQQGSNQNAGSGAGSGQAGEAAGGVAQATGVALALPPPLPRSLLHVAARQGAPEAPSAERADAVGQ
GACGGAAGLNAVSPTAGQPGGGSNTGSGSGDGSGAGGPPDVYQSSGPTGLARAAAHAAGLRQETPPTGVSAGTRRGA
GGSGSGNADGGGGATGSGSGAGQGSNPSGNGAGEGSGSGAAPAAGTAAGAANTGGVGSTQAAAGPHALQHPHAHGNA
HQQQLQQVQLQQQQQQQQQGSGSGGERGGSGSGAGSGSRVGSGVPAGTYAAREAMPGASGIGRHVPPYVVAAQRAQH
ERVEAAAAKAAAATVGRDGEAGQADGSKPISSPEADAEGALLGSGGGSGGGAVVDLAVALRHKQRGSPGGSGGMMGS
QARGDSPAAALTDPRLLQRGGSALALAAGAAGRVASPPPNGQSTLPVPGDTLALLHQMLSQQQQQLQQAQAQVVQQQ
QAQAAAAAAAAGQDGGAAAVGSLAPMAPPPHGLMTTLAGLSAGTQLAATLLALHEQQQQQQQQEQLLATNRGGGSAAA
GAGRSNGFLSLAAAAADPTSSGGGSSATAPPPLMALGPGALTALLPYLTGERPVESEHVARLEEVARALQYASSLAG
PLAAVLHPPGTSGPPAAQLASARDGLPSSDLAAAALMAQWQNAATMYGLQPLLRTSSAAAAAAAAPGSATSGGSAAA
PPPPSSAAAAAALLGFPPHPLLIQQHHLQQQLAASGQAQAAASYLQSLGLASGLGLGFNMAANMGSRSALGALSLGP
MGLSLPTFASGGGGGNGTASGGALGAAAAAGGGGGAEQSPRPSAGTIGSGGGVHGLMPAPTRSHHQQHSHQQHQQAA
GAPSDPRLSGGNPRRGSGDGAVGFKPPRPLASLGGVDGSGGVARGLGSGPSMSGEKLAAAAAAAAAAAGGNGGTSKRS
LNELYGALVAAGAGAGGAAGGGGGGSGQGVQRSGRQRNGRRRHHRGHSPTRSVGSGASSSGFHCGDKSGGEEANYGP
RPSREYKEGSPQGGGAVEQQDTATSSGREEQGSAGAARGCGAAGEVGTAGQSGGGAGGGAAAANAAAAANAAADGGG
GMALPPAKHARHDLMTGPNGKLAAAAGHGGGAGGASGQGTGGAAAKAAAQRPPKDATPSSGGGSGGSGGGSTGGNKS
GTSNDAYDGSMNASDEGSNEPGSAPADGPMGQALAPSDGMGMLGAGDPGLNVQGMQMPGAGMMGAGGARSFSVAQPE
PGPGHASIMAAQPGGCGGGGSTGVAGAGAGAGGGAAGGGGGTAGHKRVRYDAGPAGGGTAAAAGAPAAGAPLTPAAA
GAAGAMQQRAGALQADGAGTGTSSPSETGGGDDSRGAAQPPHKVQRTNAGGAAAVAEAGAGGAAGHAADACDGNNRS
GGSAGAGSGQAQSGRGRFAAPPSNQQHPHGAAAAAAAAAGGAGGGAAQRTSGSLPSHLGYVLEQASSKQGGNDGSGNN
NSGTGPRSGVGSNEGGAAGGAGGSRSGEGGNGNGSQGNGGSHGHGGSHGNGQGSNANGSHGHGSNGNNGHGSNGNGA
STNLPGGAPYAYFRTPLSLQQQPRGGGGGGGGGGGGGAGSGGSGGSGGNRGGGAQGSNGGNGDGAGSGGHGSGAHDG
ASGGAAGPSPYDAGAPTSGAAADHMHRHLHHHHFTHAHHHYGGGGGGGPQEGTSMRTPLPLQPQQPQPQPQQQQQQQ
QQQQQQAGTGAANAGQGMALAAVAAAAAAAPAVAGTSGGAAVVAAAAAAAAAAVSQQQPVHVEGQGQDGNEPMLQDG

>Cre12.g510350.t1.3|PACid:27581979
MDLTMSCPRSVPMTVLVSSSAGCLNNDCGLHGLPSVTDNNNETYLATAATTAATEVVIGFGSWQRDIVQAVLMAPPG
TGQEAWLQGVSITLQSGTGVDLASCGAGVSAAAPRDRITLHCQRITGSVDRVVVRRPASAAPVPLAFSGLDLYADDC
PGVLSGQLSASPAAITWLPAASRFSNGYRSGSASGYGLAYAADSTCGANSTSSELGAPGISGPTLTVKLSQAFSGVL
VVTSQHTAAHQNDDVWVSRTTTYMDGMRCARGPAVAAGATRVLYCPAMRGIAYVTIVRAPQQVVGSGYLDMCELALT
GHRALDTGIPQVPEVAHFDAAYGLVKDSYTGTLIAWRDVDSASRPRNILNFTGGCTEWASGPGSTTYGVNFDGSCMG
RNYYDLPYTWNRNMTAQFTVVTVARFGVDPSTVPQYLLSLSRSPASSDTSMFWASNEFGITDITTPGSGADTYTPIG
HMPLGSWTMEAVTRHWNGINTVYFHKSEVTDGFSTDISAGTNAIGPDNLVVGADYATGSKYLTNGQIAVVLIYNRML
NPTELNQLIDYYRPRFGLSGPPDTPRLNGMAAQGGAWGSVNLPRPGLYFSWFGEAYVTYRVRWLYADGTRGTTIGNM
AITDGTYACPTMDFEFTMPMSWQITTDRIMLVQRYGYQADQAGNLPPGYPAVASYWMDLWRIPWTHTSFWILGWANA
DRAIGFTDCLVNGPVPPTVAGWRWSGGLVGSPNLYDATSMPRLTATGTTAFISFFHLVEEAGLVSRYFPSVLRLIGV
TNSQPAIYVQLTAPWPTDRTLVLQRSDDGTDVYDVDYIPAESAAGRTWFVLRGWNAPGRNVTQLTPMPPQSCESVAP
LDPGSGYSDSSGCYQANCVIWGATSFASKLSDNNIATGVISSSVGTTVTLRFNLASGQLNVTRVIITPMAPPASLTD
ATWFQGIQVTLLDGAAAVVSTCSNAASIMYMPQDQISLTCDPTTATVWAVQMERIPGANGIPATGGIGVAELSVIVD
ACPEIAHAQLSTSTSAATWTADAVVMYPGCGSVTGADCFMYGPKQAQDSDLSNTYALTSEDSVSGPYIQFALDEVVS
GTVQVSLAYSAVGTSPLHLSIWVSQDPAFERSGKKCAYGVSGPSVTAAAFLKFSCPVMKGVAYVTVHRELRSYSAGS
PGSLQVSEISFGVRKWTVPSPPPPPPPPTSGIGGCTATVPLSSAYLSPVGCYANNCTAWGFANLVDGKLSTTFRTEA
VQDSALSIVFPFTAATTNIVEVQYQMPLDNIQDAQNITIEVAFADSGRSLCASHAAGYRAGEFVTVSCQPRAGTATG
IQITRNNATGSLAFAELIVVRDSCPLIAPSSLSPAPVVCPWNSTLAVMYPGYWDNNIGYSAASANDGDYINTFAHTL
PGSETGPGSYIQVPFTSPAGVSPVSGSFRVLLYRRECCYNQQAHYTVWLSGGTASNFNVTGRKCASGAWLMAPVGLP
SIFACPAMTNVNLITIQRVAPSPAAPNMGNFVVELTEIIIQGYLTPVVPAPPPPSPPPVAAPPTGYLSPPPPLTSSC
ITPASIASIDGPYGCYLSDCATYGLAAAQDGNPLTALRTGGSAGAEPSVDFVLTTPTKRITEIKLKGPAYGSSELQA
IRVELVAANGTRVMCASHVSAYQPSDDIMVVCPFTPLSITRVRLGRRITDVPNYKVGYFLGVAEIVVLVDACPDVSE
FRFTDTSFYPVRWSSAGGVLWPATLNGDTARWGPQNANNDVVSVLSAPWDFYLAHTISGPSQGPGAYFQVPLETPAT
GAVRVTLWHRTQSPLHATYLEVWVSSTSSHRTSGRLCASGVHLAERPTNFTGPYTFNCPPFNSAAYVTIQRNNPPVF

```
NTEYVVELTEVDLEMMYADGSPATAPQPMVTLPVSYSASAVSAGFSPLVAACRSNGVVQALWVAAAGSGGSWITYLG
IILCRDGTGTGPANPLAFGVAPTGGAGLNVTCPGGFDAIRGEESAMGQTLLPITALSFRCSTTNAWSSSVGSGDAGV
ARNATVSACPPGYVIYGFNLMGNASTLGGIQPLCVLKPGSKNWDPVKRCPQAAGYTLVPGWYPAGPTTLYTMAALPP
WVDVDIAATACRADPSCTSIGYSVDSFGNTTFYTYNSTDPAEFGVAMGKTLYTGVDGCSGTLAPTTWAGGPSQWFCA
TGVRTDASALSTTSVVGPNDCATACDSDPNCESWTMDASGTTCTTREYGFAATQWVDDSSTGVTCVIANDPWLCPVY
GWTFRGALLNSSIHVNPETCKAACAFAAPDCTHYTYTSATLVCELYGSSLMSGGAGINGPVVASIPDRVCVATPQHP
KLGQPVASTRAAHSSMCWPGVQLVGTGAATLGSAPPPPPPASPAPTPAPTPAPTTTTSAGCARRCVAGSTCVHWSLR
MDGSCYLYGADAVVAGQAAGGYGPDPSVAQSCLANVQGTFHCVPARQGIKYELDSTFSNATSRTHCAQLCKASRYCL
GFVYYIPSSTGTTTCRLMARVFAGAADATSVHQLYAPLNFQSCLRTHNLRQMYSNVADEPQACTATPGYTFYPAPSG
VSAYSVLSVASASNAVGWQGLCDVDPSCVGVQVAAGVVTLMGGSTQFQSLSVQPGLPSVNGSCDGFYARSPRDIKPF
MCIPGLYMPAALDTISTVVATARECELLCRGYPSNFCHVFYFEETAPGNVACEIKTNLFAAGTLSPYLAGIGATNTT
TCVPATDPWYCLPYGIGITGEALPGYPTSSGTAEACAYACQTTPTCSAYLHSPDSATASCTLFRKVFNGPDGTAALL
ADGRARACLKTPQFSHPTGSVPFWSHMCYTGVDLGGTVVSTLSNVDGHECAKWCAAYGYGCSHWQLSTAGVCEARTG
PLWTNAGGMVVYGPQSGIATACLRSTGTFMCLRNGAEVAYTLLSSSNGVASSSACANLCLGDAACEVMAYDVNTTCV
LGRGPFLGSSGINGFDWSTSTLTTTYCLRTPYFGGPLFGSSLTPSPPPSPVPPSPAPPSPVPPSPAPPSPEPPSVVV
SGGSAATAGIIVNTILSGEQSPPPPNAGVVPDTTAPVITLFGLEEVVVEVYGTYFESGALAVDDRDGLLAVDIQGSV
NTNVISLGLPTQILYLSADRAGNIGSITRGVYVVDTCASKDEFRCPVTKECSVFRQCLFLPDPQAINKTVAVNSQAA
SAKLTDTTPPNITFVGTDLELYDVQTAAGNLTVAVEVVYAGYTYTDRGVLAFDYLSDGSAEGAGGLIALNLTDRAVA
TVSAPATADPDAAGVSTTIFTDIPTGNSTSEGSPYTIQYDVADDAGNKATALKVVFILCEPPEFVCPDNTTFEGTCS
QDGLCQYKTSKITTNNTGLPRSNETAVALITGPPDPDNLIDALMVPVFVINGASSVSISQYLPYVPCRAGVISNNCD
PGAKASLRSLGDLNPRIIACADKAQKPQPYSVVGLQYCGIDTTTPGTYMITYHLTWPSVGTLVVKRQLLVVERCVGE
QLCPDGFCSVDLSCARNVSSTISSYIVVTGDANGLLPNSTSLSTNNPPTLSLRNGEVVNTTVVVPRGTPYMVCAADQ
VPSQSRPCEPLGVALDTEDGNITATITLCPPDDCAATQCRDHRADRKQPSECGIDTVNATVGSSFRMHLVVFDSKGA
NATAERLVTVVKPCELLESYCRITRNGMPVYVCSNVTCETLEPLNDEITRSGGGQPAVPPRLFLLPGYLTSNLTLAE
RDQSLFLTYGTAAPVSLAPCVSFTGTGCAAIANDTTDGDITRDITVTVAARCPTGRTCVACSVAALATGDCLPGRYL
VRYSVADTDGNSASVQLSVAVEQLTVTLLDFSLYPGGSALASTSLTAATNYANTLATNTAARSRVLSPIFTAFGIQS
GSIRAIVVDTQPAIEARYFDNTTQIYYIVRMVLRVTTGSSDFTSATSTLDTSSSDQSAAGGTSAASVRRRALPAVAS
RALRKVLEPMATGPAAGGILLGTAAQSPLRSLMATNPEASTRTPAQQLWEERFAGLHSALDRLGALLGLEEKPEEEG
DGEDPLGSPRRSMFALEARAASQSPGMAPWHLPAGARLAGARAAAAAPRRALSADAGAQAASGGDGRISAHALLASR
RQLQVVANGGCGGPALAAPPNSSFSALGLADVRTISSSCSTPQAGDMDVALTVLAGMMADLERMSASMLEEEASMDT
TLMALDPKFSVVDNAYQTSYTDFAAAAGVAYASMLERLQALLVAVDRTAAAQAVTTTAVTTLLSLMQALLNEMQQIT
ARAELTTKVVLDGLAQDGNLTDEELANYTSCLFLRGKAAAFSFRTGRAGGADAGANVSTAAAASGGARRRALLGPDL
LYSAEGAPAGAAAGPAAMRAAGGVDTRPRLAALASSWVPKWVWGALSPAAWFRRAGSPQGEGDALDAQQAQQRRSLL
ALANGEAVQSFYGYDLPDGGDYDYSLWDVRNIDRARWIGLNNRVLVGLLLHQVRRSPAELERAKGTDGYICRDSSYT
NLIVGCNTSNTSTTSAALGNSTTATARAKDLGGIGNDPVFSRHSALYQADLDPAEFYNTSKGSPELNPGGLPYGFFY
QPAGRLPAGYPLVFDTHLSAKRAEQAITYIRDGGYLSETVTKTLRVQLVSYNADAQVFGYFRLDLDWLDSGLIWGAT
RLLALPAISYGAAVENAQVQKFLPDFFLIVLCCGYLLVTAYDIFVQLHRQAQMRKLRTLLREGKRKEGLETIHEADD
DNDDDDHVLGIGDAERKVLYKHDGTKVTVKLYKPRMSVGWVVFETAVCVLMFTSLGFLFAYTAKLADKDAFPSRFEL
YDADGYAPARYFLPRRQENVTVTQQANGTSNAATASPTPGSANRWQLPEDTQLGSASDFYNRVDNMYDVLIIYTFLQ
GFVLAMIVIRWLHYLSFQPRLSAISGTLALAAPDVIHFFIVIVICAVMFGAAGCVVFGPDEARLSGYGDALQLMLSY
VLLREDGGVFKALLDKVKQRDEGTTQVMAGLLYAIGPLFFVFIMSNFIMAFLAWPYGLLKFATRDAPGVPQDIRRML
GMLWQRMRHRAPSNRHMEKWFKRQVVPEVRDRGLMYRIRETMANAYQSIAIRGKAANGTVAPASGQAAQAASTAPAL
SAPPVAAAATRRKSLVKLAPGMPAMNADALAAALMSASRINIDIGDSPQHAASKLDPLVDSDEEHSGSTPSGFAAAA
AAAAGGSRLDSAPTLASPQTPHGPESADGANALPASQDASGRSTPVPKLSGSGVSSRLRGGKSSRMSAPGQAAEASA
LVHTVMANLLARYGHEKVIAADAPPPAPRSGDSTIGRGEGADSTEVSEFGEARRRITVDGSLGSMTEAGTLRGPGLG
GPSEWAQLAPGAHVRRSIAGQVVGGEGAGSSGYASPALAGNDGSGSPLSAATPGSQVASPPPGANDDGAGASASASP
RVGSLAARPPRLQTMGVSSRLGRRTSQSSFKGLAGAEGGVSPSGSAQSPAMTSGGGLAALTSPHGELSPAPTSASGA
KAGRMSMPGPSPLAAGAAMMAGAVRHSVDAQNTQDASGTGGFVAGETKLPKLPPIGVKPQFGGLPAITAAGERGRRR
AGELDSPGSSARQSGPGTPGVAQDAHLDQLNEHRGEDWTGPAPDPARNLGQAKAWLTFKQAVNRREGTPGGDASAGG
NGGGADAQEGLGSLAPRIGSVMVEGQVKVLDPAAAAALEAEERAAAEGPVVQGLRAPMRRASAMLLPEPDMMALAPI
LSVDPAAQLPALPSTQVEQPMVTLLDHDAQLPVGTPLGGPSPGGASPSGAAEARRAGMKQARAWGSVKRSAAREAAQ
AMLGMLDNLRGSGAGQTAPQQPEARAPTAAQPEPQEPQEPQPWFSRQTGLNALAPSTQRRLSVAAGGAEQELLVLDS
DGADTDGSGRGSGARNLRARGPGGAGPVAEAVEAGSVPGGADGEQDGDPAALSAADAPAASRRTQGVKFAIEPLPTS
DSDGDNTAGPFAAHGRSAALVAVAGGGSRDAMAGGLASMVLSMLQHLEALLAQLQAAIAELKLMSEVLAAMLAQFSA
AGLGPDSAPEARQVAWQAARKTARDLLLAYKRDALLTAPLPVLETKRLPPLPLALPAVGINALGGSKAPLPPLRTNP
MFRQRSTRRTGSTRRGTLPPMPMPGVLYSLQSLARSDGAVLAAAQSSGGAALEILHHVEEQLETRLGMPLAQRKSLK
RVADEVAAELVRAERAESRAHMLQQVASLRHGSVGHSGYSTSGYGVSGYGASGYGGAAALAAAAAAADSAYGAAANM
```

SHPHQHRADAGRSVQAAGSHVSGTHRRGWGARRRAMAAAEEKWLEELPADEQMAALTGRTGSYAPGKRKSRLLLGGA
DSRAASRVMERDFSPAALIGGGREVSVGGAGADAMEAGTPLARSDAGKAAFKSGGTGAGGIGGLGGLLGSPAAGPPA
RMRLPPLNARGTQPQSDLPL

>Cre12.g513350.t1.3|PACid:27582125
MRVSACWSLLASLAALALLGSALAQRSVPASSAELLAAWGNATVVTSLYPQTISNDGAASLLAELQGPGCPRGGSRT
LYLVFPRASANQQDVSILGTLSDTCNSVTFRAATSIAFQSIQPGVYAIQVTSGRPQAPLTFIGAASKSGASGGDTAA
AKSGAALVTVVDAAVGQLAGDVNVAATSSSLAVTLTAQLAAPALLPATMSLQLSDPSAAAVSPPALTWRAGEMGPKH
FTLQLRRPVAAEGAAQGILAEAVAVGNMVFGNGQSRSLPPRVSVYVPPPTFYVPVNTAIYRDGPPPVRRPRQGAAGA
AGNGTAAAGNSSATGSNSGASAGNAAVSDAATGSGGSSQRAGSSADNTTVVMVPVRRASASPFPSTLAFKVQLLESV
GCASRVRCAAAVRGGGQLRFTGDQDVVGAPVWVDYSVVPQEAVLRVAVILSAVDNARVLSYSDMLLLQWQQQQQQD
QSQQMPGWVGTRDDPVFVDADNVTSLFVFGTTYGMCPAGTAGPWQEPQLAPQESAGPAAGPTSGPGAGTDITNSDSS
QQVIPPPRDRVCDDSAVAFALKGYASSLAWLPALSACAAGSAMLVLPVGAAPLAPPDTATNTSTTPSLAPSPAPSPS
PLPRPSNSTSPAPSAGAGWAAVQDAGSRSAPVELRVPYGMDLAAVFLPLASAEETVVVSNTSCSVLPVWQSVVLAAP
STDSVAGAGAAAQSRPWARWVLPAAVEDCTIELAVFATVAVNPAPTMSSPRPSSPAQTSSPAPAAASSSPASRSPPP
GKRRMLAASATSGPSPAPSPVSITAGVAGNSAANSSSNATASNATAKGTASNSTSSGLQLPVYGLIQVRAYTLTLRP
QSDPDLFPAMALALVHELAQEPQAAGSNSTGNSTSRHISTSSSISSMLVPLCGLPQPLLHAAARQGYGAAFNASSPA
PEQLAWAAMTLSRAIRANPDAISSLRQAAGKQAAIGNSSQAGAAGSVPPKRLLEEMAQVARLSGAWLAEALSAPGKD
SDENGVKRPGSRLLRGGSGDRVDGTDSWRQRRLQQAGSGSPSPLPAPSPFPTASPTTSPAPGTSPGPSSASPSSSTS
TKPSPAATGSPSPSASPSPILAVAARAPEGFDLIVAGGSDDGQDFAARVLFNPQFVERCDPQDSLVLDLGLLPETSA
ALPLTLQASSGALPLRHSVQPPANSGDGGDSGLELGGSELVVTPATRAAVRVRVDAREAAVALAAAAAAAAAAAAS
GSPGSNVLLPSGGGGVITTSSKVAMDTSRGTGGLSPSLPSPAPAVRLATGADGGSEDTNSSGTVISFMSLEGSSPGP
VKALATPSPPAPAFSTYNTSTSGNTTTTNSTSNSTSDATGCDADALRPVADQALLVALLAPDGYSTASITLELFLDY
GAAAADAVAAAADCEARGLLLRIDGGVGSGSNSSSNGTDSSMTGAAAAGVAGGNGTDAGGVNATLPQPGTLQVEWDL
LPVSPLDACRRCGAGSYSSSSNASTCLLCGPGTFAGDVVNSVCSPCAAGTYTFTWNSPACRLCPAGTFSGRAGSTAC
SVCAAGTTTMYDGASSCAAAPPPPSGGSGSSQSALLVSFQMLINMSSSAQAMTSLGANNTGIDASPDSIVKLLIAAD
TADALRIPPSQVHVSSLFELSPSAFLVNVSATVPLGGGPSGSGSGGSGSDGSPGKDMDADTLISRLVDNPGTSFPRT
TEATGGSVQVQYVDMKVVTWGGSSSGLNLHVVLWPTLAGTFVLAVVVGLACNRYCSRRRGGRAASGVSLPPITDTFG
RLANVWKTPSSPPPASGGAYLMYGQLTSLELQDVAPGSLARHRGRHSRKPSLQQLHGLGPSPQEALAGGSAGAAADW
PSSNSNANGDLDMFAMYTRDVMAAPRI

>Cre12.g515400.t1.3|PACid:27582126
MSLDGSDTHEEWALKFFGSPSAAHVFGTCAVSNVFLSAALKCGTSTSSKDACTAQPYCAYVRVDPTYHICELRRLEP
LVSSDPAIFTSDPWGAALASLTAKCMPLLDANTGRRYGSSDCGEYRQSTEGRQGLTAQFDAAALLRAPSAVLNPNNL
RPSFYTDQAAPAPPSPKPPSPIPPSPKPPSPPSPPSPAPPPPRPSPPSPPPPSPPPPPLRSLKSLLFSPSPPPSPPP
AIIAPDLSIESEPPPPPAIVPDTQPPLISLLGDATLKLKIYDPYVEYGATAMDARDGPVPVVTSGVVNTNVTSVDGK
HPVVTYTARDRAGNVASIRRVVMVSDACTASGERRCPNTLKCSTLSLCDSALTAVLNFFSNSFGDVAASNSGSSDGG
GSDDGTDSSGGGSGGSGSSGSSGTRSTSAQRSRKLLPDTMPPRLSLRGTGTLFVTPGGASGMIHSLYVDEPWVDEGV
DAEDDVDESFDGKTNKVVALPATAIVTSVIAPSGAKADAIKTDTPTGDQESGGLPWVITYDVSDGAGNRAPTMRRRV
YVLCRPPERECPRTDPYEPRTCSVEKICGLQVQPSDGQGGMSSRVNSGMIFSAGDDGGGDGHSSGVSGSSTGSGSST
RTGSGATNDVRVVQLPGTAYTAAAAAAAAGVTFTTSGSSSTGSSRAALVAATGKSGSSSGGSSNSRSSSGGSSAAEV
DIPDTQRIPKLTLVSDWFNRFKAGKPYDKCKRLEVAYCDPGVTATLRVPGDLNGKVIACADRARAAGIASPRPFDLM
GLKYCDLDTNTPGVVTVSFHLTWPSVGELVVTRTIIIEDTCPDGEKLCDNGHCSTGPTTCLSEIGLGGGGGSTTVVP
TAENAWAEGGSGSGGTTAADLASVVPAAGSAAAAGSGASGSGSGGGGGSGSAGPGGSGSGSSSSGGGSGGSGGSSGS
GGGSSGGGSASGSDGSTGTGNSGSGSSSSGGGGTSGGSSAAGGSGAQAAASAALVTVVGSGTVASGVTLYTVPGSSS
ASSATASVVAAALPPRLLLAVTDVLGRLVHVKRGTPYLRCAGEQVPTLELPCEPLGLASDDLDGDLTDWVTLCPPAD
CARSQCREHVSKQPAACGVDTVNGDVGSTFTLRMVVFNSRGLNATVERVVMVDSPCPDGQYLCERAPNSANAGGQGA
SAAYMCSDLPCDRRKALLEVVAIAAKATSGGGSNMTVDPTVVDVEEVVFSPVPRIYLLPSLHLATNLSDALANQTLS
LVYRTPAPFSLAPCNVFSQGVPLSTFSPRPPSPAPSACRPPSPAPPRPPGPASGNSSSASGAGRRRGLLQAAPLPPP
AFQPPPVPAASSGSSSSAAAGAMAGACAAVANDNNGNDLTPYIKSSVAQLCPAGAPAGSCGGGCSVAQLTTGSCLPG
MYRVLYRVEADGGTEAVAQLDVAVEEIRATQLNLTLQPSLPTIIQLLQPPSPGELPPTPEDVAGNRTQVEAMAVALA
ANASALTAALQPLLPGLGLDPDDIRNLNLSAAVMVVELPPPPPPPSPPLSPPPPGAPAFPPMPPNASYPPPSPSPSP
PSPPPPSPPAGPPRYGLQLAVQLTTGVSDCLYTATGAAGGGAASVSFADLSTSRHRSQRRRLLRVRQEADAVWRTDE
DAALLETATAAERRKLLDTQSEPSAEASVVDQQLQWRRRAGEEAATPLERSLRRLQSVSATLEAALEALATGRYTEP
AWMAEWSEEDELEAEQDAENEAGESEDVFDFSPRQQRRLLQQVLAGGGGCGCNPAPSRTVFASSTAANASAAASAVV
RGAVTGTACVTPAIDGASAAVGLLLGAVSTLDAFGQDMDVTQVALIAAVSSINGRMDEGDVRSEQGSTNWAFTARDS
VKNITTTATKLTSVVQAAAKTRGNQELSSVVGTGVADVLQVMVSEVAESAKTALASAEGILSGYGINSSTPAEENER

KFCSCIFNRSEPTGTSLNFVANRTAKATDGNATESLPPMPPAPPPGVPTRPPLSPDAGGGSSASTAAARRRGLRAAP
RDTGVVPEDDQAAMDPDKASCPDTPSGPRAIRRPANSQQQRRQLQQSSGSTTTSSGSTSSSSSASSSGTESSSSRGS
MTAAGNQLEFASGHGVLINDTFDYSLHQVRSGMRTRKLTQVGRNNRVLLGLLLHQVRLSDRDVRKRSGRYMCTSASK
TRQLITACGGENAGARVVGGDLGGIGVDPVFLPRSPLFRAGINPDDYYNTSAGSSDINPNGVPFGFFHEEIAGRPDG
YPLLLDIGMSEQRAREAFIFLRDGSYLSAQLTQSMSAELVTYNPSASVFGYFRLMFSWTANGLIRCDATLVGLPAVS
YADAFAEFSKYYRQVLPDIFMVMLCAGYVCLVAYDISQQLKEQRRSRLMAARAAALPQYEAPVIEWPTAPELSRANS
RSTHHQGLSRPMTAENSRTGTRSRPASHRQGVGHLVRSRAGSAAPSEAGGVSSHGNSDSESPVRAGIADGPQPVAEG
YEWLQQPGHDHYDAEPMSMPGGSPTLSMRLPARKPGTSSLKAAAGKTRAAKMKCVSMAMGDVPELPSTVALEPVNDG
DGDDDAQRMRAAYEGYAAGGGAAGANVSMVLRSRQNHEKPLRRDPYSKRYRPSLLNRDMRPDVSQMLLMHQQEPMRH
GGLRPIGKAMTQVVYRPRMKPFWMAFEAVVCCLMVAAMTIYFVYCLKLSDRNQFTARYQVYEADTYAPAHYWLLKRQ
EGAVVVQGADTAAAVSSSLSGNSSLSGNGTSTSSGLTDAVNGTVAASIQVLSGNGSSSTGGSSGMISSSSPNYNAAG
RWRAPEDPAPLKSSGTMFQRVRHMYTMYVTYMLLQSLVLAMVIIRLIHYVSFQPRLSLIAGTLSLAFSDLMHLALVI
MVAAIMYAGAVMCTFSEQAEQLSGYEYTILYILKYLLLGDDEKVFFNILWAPLIKSTGQTFLARLLYVLGPILFGLI
LIICVMVILFHPYFMMKQVVRNAPGVQRDIYLIVRWRWQHYRHQAPKNKQLMRALDGFMAGGDAEATSRRQQLINAL
ATTLAGPSHIWRRLNEGLGSRPVSAAPQQQQQQQPVSGSKGAKSSVDLGKPGGVAGIGSGSDSSSGLESAALAAALG
KLLPQGAIADSNAEGGPPARSLANPVTRRASSTAVTPSSVSLLSKSVARREQLQRSIEGGGLAKVKASNSGGLLSRS
TSASNKVAPLPADAAAAAAAADMEWVGGGGGKEGSAVPGVGPGSDRPSPKAASAASSAAAAAQLADAVMANLTSRFG
NITAPEDERAKEQQAAASAFVALARADPLRSHSSNSRPSSAQRLRLANGGASPRPPSALAGRSASGAVVGNSPTSSG
SGAQPGLATENSWAQRGPLAHAASAPKAMLSPPLTAPDRFSRASGNEALQAEASRAEGGTDGAGSAAAAGASGEQRH
LMQLMSFAPERPATYAHLASGAVGGANGTGQGNAISTLAAQPPLPQPVCMQRPTSTFPTRRGLRSSSAIRTTAELAT
LATGTGSLSQAGPGPVAEAEEDAQEQAVGGTRDAAALDRLPDLDSDSGESDSGRVAELAKQKRQALRKRAAPGGVSF
GHALVYELQAEQQQQQHGGAGAGGWTYAAALAQVQQLVAGLCSMVAQMRECIAQVQAMSAALEAVHKELALAGPGSG
AAAALRSPEVWRAAKQALDKAQGLLMPKASGLLTTADGPAGAGAVVPAGLFGPTSSAGLPGGVLEDRSSAVGQLQLA
LVQRLGPHEVQEAMMKVKAGPQMVNAAEERHGGHDDGHRRQMMHHAVPGI

>Cre12.g492850.t1.3|PACid:27582382
MYGRNNPVLRHFGADDLAAQATLQAVDQRRAAGEYVPPSEEVVPRPTPDYDDYFEFDRPVPSGVEPDAHDFLVEEAM
GAQRYGATQVPVHYQGASAGPSSASPVPSSPPPGSYDSPNEQAYQGYRSGGQASSRSGGGSPSPRVHDPRSEASRRP
YGAYNSQSGGGSAGASPNRSGDSRGGSPTRHSYQQAPSYVQPPFRAGVPATGPVRPLVSTPTGDRRRQRRTTGSTAS
PPDSRTASPSQPRSSNTDATRGAGASTSGDAVSGSEGHSAAAAVAAAGAIDAATRQARGIGAKISGRPTSARPGSPS
ASPAARTGATRRPVSASPASAGAGAAGARRSQARAPAWSPAAGIANVRLDEHDEATRVAMMLNDPSTSREVRSAASA
AQQQQQAAAAHGTGAVRGSHQQPYASPRAPPSPGAAVSPPRHEASSPGQGQYMTDGQRNRSSEISEQYYNTYRLRKH
REAQGVPDHRYSQPRSLGAHAPIQQKYFSFDEAHPAIATEHCLRSPGQNHMLQAERQKMQQAKMRAARMAARDRMLS
PTPTTQQLAADVDRLARVREAGRARLRQLEREGQALRQETSQIQQRAVDAIARAEAITVASGLPHSRSPSASPSPPP
HYARRSRSPSPAAYYSGGGGGGYGRGGFGPPPRAYYNRSPSRSPGRQRPVSASPYGYYGAEVDEGYYSGGSPGGGAA
GHRPVSAPPQPPVSPPPGPQHGGAGHPVVAPAMPTSPPPGPAEAQYILDMINQVERLPQHAQVPVASSTSGAASGSE
PSQREADHMARASAIFNSRAPLGVMDDSETRIAMGAYDKARYEEYQRQVDLEMQRRAVDGGYDSDVDRDEAAALLVE
QPQEEEDDESLARRMQLEASWKQQNEWMDAYRERFGPEDGYLPERATSEGGGSPLQGELSVMSNAEGGMVGSPYVPI
VSKDFPLSAGYASEGPDAYNPHRRTRRKLFFKSTVPKPFEFEERERSRPKSIAKVKFEQDVAIRLAEEAAARNRKFR
ANPLPSAVVEPRYERMLLEAEVKRQLSHQRRLEELASLMEQPFSFFYRDQERELAREELAKAAKDPNRFQVAFRARD
APTSTKQERFRMMQLELEAKRESTRRRVEEARRAAKERVEEEAKRRQQAANRAYQERLRSVDPAVHYSPNAPHPTKP
MGAVPDFNGLHRQWEVQMARARANIRKRITVPQEFRLNGADLSEQAARDRKAEERRQRIILDMQVDAELLPEMRWPY
KSPRGKVRPTPMPAYLVERMGRSPGNHAATARKSATKAAAQGGAYTLREEKEAAARKASEKLRAEALRRADRLLKDA
AARKQAEAEAAGGGGDFGLASDVGATAAGGVDQSSSGRGAANRRRVEVAGRQDNPQVYVEARHMQIERKVKEVVEDA
LLDQGIEAYRYVEGVGQGRGKDGSAADAADA

>Cre12.g529250.t1.3|PACid:27582394
MTRQGKKEGEVGILSREESAGERAEETLRRGPSTIGLVCICIIVLLCGLGGGFGIGWAARGDNNGSPAAPAPVVMVD
NIDRLQPLRPLSILGEASNDAVLASEEQWRVMTPALRVFAALVSAMANDTAVVEASTLRAYLASLPEGPSSSSLLSG
VYGAAQRLLVNPEVQRRLYDVASSLTPVFKWARAAQDRYYGSSSSSSSRRRSDRRLRVLLESEQAAVAAAAEAEAES
GLVLEAGLQVGGAMEEVMQQRRRRRLQQGSITGGRGGVNAPLYNMIDDALNSPALVGARAVLQELYEWESLQDMLVS
VWLALRGLAASMPALKALAAAQHPDPLRVALAVTQEVLVAAGAGGAWGNGTSITLLPTLPPVVVLEDEISGAASAAA
HHHHHRHRSLAADAAAVLPTLPAGFSPRPPSDVPNLLVPLVFHILLYQSGDGSLGPPGYNQAAAAVTRLVGVANSLA
APSGIQLFVREVRSDAAKYPYLLAYGANRTTWLNCAAESAFAYEQCGDIIRTASSDFPRSINVFVVSEGPPAKYYGY
AFSGGSSEDPLYGHIGLTYPTISTSQLNGQLSYETGAVTLWHEVMHHLGLKHTFSADGSCTEDAADDLVETPVSSGP
VWGQPWAMAAYLACMQAVQYDLANDWGRIYAAWRTTLGIPAGDQRSFAKSCPGRPGQDELGNYVTYSYDVCYAAFGH
VTQQQVRSMHAFTAASNPSMYAWAQYYAANPPPEAKQTAAAIVTASPPPPPPPSPSPPAAAVAEALQQFLRPCPPQY

TRTGCRCKDTWAVMGTTYSGCTTVPGMAQGGPWCAVVLEDGCASARNGWWDMCTPVQTLGCRATSPPPSPPLDPSPP
PPLAPSPPPPSATFNATAMCGQGAPTRQGFTCATGRWTTTATGTNRYSGCANPDNNPQGLWCALPRGVTTASGASWD
LCLQACNTAAAVGSSGSGSAGTPSPSPLPSPSPTSGSTVATGCTTTALSLPSGCKCASSWKASVGASTGTHIGCTLF
PGVDPRPVCAVEGCDAKRNNGKVVTCSCP

>Cre12.g554950.t1.3|PACid:27582543
MRGDAAGELLAAAGGGVADTGAGGAGGRATGGDRAAAINGGGGGDGGSGHMSSGGHSTRGGGGGGGGGGGGLSSLDG
SWAAVDDGSGSGGGGAGGGGGDQASAGSASVAPSALSAPSTLWLGEPAIALPASASASAQRQHSPPPQQQQQQQQQ
QQQQHGMEVEAEAEVEHQGQGQQGRGQEAVAAEMEAEAEARGSRRRRRVSAGDGGRAGGGDGAVAGAPASAQAQLLQ
QHYAAEAGGRLRAQAGEPQQAQHVPGLLELMTGMDTDTAAAAGAVPAAGRTRPSGGGPAISGTGETTGGGGAGGGGG
SGGGGGGGGGVTRRISGASTSREPVVRTESEIMEEEMVASGARPGAAAARAAAVQAAEAAEAAVTGGRGRERERERR
YPGTSHSSSSRRGSGGGGGGTVTGTGTGASSGSSDNPAAELLQRFLLDEAQIASPVLRPQVAATAPPPPLSLLQPAA
AVAAPRGQGRGAATAVAPAAADPVPGPGSSADAVAGGSSVGGGGGVTEPGATTQLQPLLAQLQPQRQPQQRQTHGSD
TPRLHPLPSPPLPPPPPLPPPLTHAALLPGAATTAAVAAPPPAVPLDMEPALSLASGISAAAPASDVVHAAAEAAAT
AAAGATTPDDAATASPAATAYSALVHRPDEVLQDQDAEAAEEALAALRRSRWRQEYSRVSFEVSGIITQPPPPPPPP
PPPPPPQTPAGAPMLDHYSGGGGSNRRVGGGGGMPAVSESPVEGDEAGRGASGAGAGAGAGAGAGGGGGGGGIAA
GRFGSAGSTWTGGSGGDTDALAPAGVHEASEAALDDRDNEFEFAVPPLQATPTMRRVPPSVSAPSALPALATAAALG
GGRSAAAGGGRAGKGSGGPGGVRGAAAGAAAAPLHRRGGGGGGGRMVPHWYFLDAGGGAATGTDVEDPAAAAAARHA
ALWRDADLHGGTPTTVDSGDEAARSICTENEEILPGQDAASFAHHSALQLAAARAAAGADDGGGGVPMHALHPHSRG
HGGGGLASAAYGLSGRGVGGVRGVSRNEREEEWSFGVARAGAGAGAAQGGPARGGPQQHQPPPQAALPQAQQVVHPY
AQYAQPQPTPQPTTTQQQQVPQQPQQQGHHHQHHHHNLPHLPPPGARAGGSASLSRQQHGRPLNHPPAATTGGGAR
PTSPALAAVAPPPPPAPLAVAAVAPGAHHGERTYRGATHPAMGPEISARTGTPDSGVRNSSGSGGSGGTAGTGAQGR
HATAVCRHPPAQQLPLQPQQPAVAEQPAARRAVGGVPLRPTALPSPPPQPQPPHHPPRHRVSFGAYGGRAGPRPPSP
SASLPPGRDAGGGGGGGGGRAGSRVSWSGAIGSAGLSGQDNDSGSSTQQLAPPAAPVAPQPPRRRTGGILRTGSVAS
ATSYLSHGSAIPPLDDTNPPHPHPHPPSPYLPAQQQPHQPPYTRKTVSFFDDEQYEPPQQIWGRRQYGGGGSGGRRR
SSSTYDDSGASDYGSDSAASEAAGDAGGRGFRDRDRDGDRDSGPDVGLLYDVAFGGGTGTAGGGGGALPYDVGSERG
STSEVTDVTELTTSTTAGARYSRLRSYLASASAAATAGGGSGEAGATAAPPAPPRVGGGSWDDPGSSSYRSEATLTW
GSPPRHPNLPATATAAAAPVPAQGVPRQRQKQAGQAPRQAPVPVPASPSAAAASAGFHPILGNIKLQLQPQPQPELA
PTLYAGPVVPAAVPATAATAGPPLPPTGSSSGVGGQARDWTFTRTTQFGRASFDVPMAAAVPGGSGGRLCALAPYGA
TAAASQPGSGAAGQPSAAFGSTYTAGGAASIGGTGSQASPLASVTSHQQQQQQQQQQQQQQQQQQGATAVQLLPPY
NSWPVTAVAARPAAAAPPAAAAANPLSSPTPAPAVAGDPNPMTTTAVAPMSPGGGAPAATAAAGYGAFQQPADLLWS
PFATPSEGRSAPPPPAGMPPAAAAPPPPDATGPPVHPILSAGALAAAAGGNGGGGGASVSGASGPGGVAASGAAGGV
PPAPQVGRRLPTALPPPPPPAQWPPPPDQAQEAEEEGGTGYMGGMRVLRWRRSGGGGGRRLSPQPEGQEEEEDAGQR
WQRHAVDEEAELEAERQAVMAAMFHQDQAGRTAPAAAVESYAAQQPVRTGSRPGPVAAAEPTAARQVAVTADAPVLG
IPVQQQPVQLQAMQLREQQQQQRQQQQEPAVGVPVLDAASSLVFVRSPGLLQPLQEQLLQPSQQQQLLPPQPQPPSA
PPPPQPQVAARHPALVPPPAPPPAAAQSAAPQPPLAPPPAFVAVPGNQPISAALPAAAEPAPVLQPAAATAAPLDAA
ASSHQRHLSQGSSRDLELGLGTVSHAAGRRRRADAADASSAASNGVGEAGDALEDEASELRDGDGRWRQAAAATATV
SRATSATPPPGCAGREARGEDEEGDAATATAAAATATGVAPPPLPAPRVVLAAAALPSVAAGALPAPGGGGGAAVAA
PLLLPPAPPAAAALLQPLLEVDRDVALTAPMYLLQLGPSGAAVAAGVAPPAAGVTAAAAGLPGFGGGVATVGGVGSG
GAVTDGSGSATGSGGLGSGVDLDVRPSVTLRRLSISQARPHLSTSASVPPPPPPSQLYGESHSEPLPSTARYAALAG
AATGAAAPPPPPLPAGAAAVTSGGSEDPPPAPTSPRAAALPPHPPPSPPQPSPQGMNLAPGSSPQLPAASHSSISAI
GKTVDSAAKAPKALAGDEELVAAEAKAAAAAPAAEQQEDSSEPATPRYGVLAGGSGLNSPFIDTPSTSLADAGDGHI
ASPEWAGVGRTSLSLGTQTSMPAALPLPLLPLLPLPGPILAAHSVPPLPAATPAADTAATATPIQPAAGGTGSSSNI
QAAAPDTATIAPAPATAPPPPPPQRLAAAAAAGGGAPSAAAAGGSSTPLRLVLLPSGASTSSGPGSSLVAYRGSESG
CSDSVSPPPPRPAAVPVAHFFRHTSSSGASTGGDAGATAGAGAGATAVSPHLAAQQLQPPQLLPPQLQPQPPGLEMR
PLVPPPPPRPHGPSGPGIAATDTVSSSPITSPPGAQPVHTHPSPPHTAASTTTSCSRHAAAAAANSALAQQQERQEQ
QQRSRTGAAPPPPLPAAAPQPPPPASAFAGFAASPLPPDSAAVRTTASTTAAAGPPPGLPLQPLPPPSPVPRAEAAE
PWRASSPASSGQRSLSLGLSAPLASSGSLPVPTAAATAAASPAAPPSRSASNVSDARRGAADLTGGGGGRAGSGGGG
GGLDGGGGGFHDLYDERLVRMHRSASAGLGSSPAGAAGAGGGIAASRSRQGSASRLAPAAPVAAAAAPGTLLTAAAA
RATSGGAAGRAAGGGRKAGGAQQAVGAAASGGRQESSAFSAGGLLTPLPAAAASQFASLLRGGSAAAGSAAHHGPRS
DAASASSEAAAPAPAPAGPVPEGGASAPPSPPPAGARSGPGSGPGSSSGPGSGGSGRSRDRHPWQLLRAAANAVAAV
AGSCSGSSVTGSRAGISSGGGRGGGKGRESAGAAGSGMGSGGASSGPSSVRTSGSSTTTATTGASGDAGGRSSGGTA
AAGSSGQAPSSSSSSSRGGALPLQPATAVRDEPAAAPPAAAPPVAVTPAPHSAAPTQGTQHAARLFHAVTAGGLVAG
IRSISRIRWQSQPQVQPQPRAATPADRSSGTTSGQASRGVPGIWGRAGRALLLILMALLLPSLSLWMQQTAQPLPLS
LSPPPPPPPSLFGLGTNLPVFPGAGGGSAAAGLSHADLDQLMMLTAPGSMLAEVEAPSRLQQQQQQPLAAAWGQWP
AAAAGGLDGGGVAAYGALVWRLRQHAAGAAAWFCASAAANVPAGGAPVSPAAASAGAGAGGYRGGVLAAAAAAQLAA
ADLAEAMDAYGDALDGGEAGGGGGGGARAAPLNELLRKVLLRPLPHDEYRQELLLQAEAARRQLAAAAAPGLAAAAE

RLAAAALEDAAAEGGLAAHMRALAGDVWHVFVAAASPAALKPRAEDGGAEYAEAQAAAAAAAVQHLQQQHLQRLASH
LRHAAGAAEAGLAPAAAVLARAADAAAGLLLRHQTALRLALEAASATFWTSPPADYDTGTGVVATDTFAGGGAVGSS
SGGGSGSSGALQVFPAAWSEHLTRLQRLQTALRAVAAECGGGGGGGDAGGGVEGRVEGLAAAAESLRELLLAVGAAG
TADGGGARGSSRGGGGGGVGGLCIAAELHGQAAAGEEHEAVAPAPARASASPGGRTAWRVVWRLHAAPRCGDGDADC
VARSAAGLGGSRSGDGGMGYVDDLLDDAAAARRLAAANRQAQQVAALERAAAPGWVWAVAEPGSPPREPSAAAAAAA
AHGEEAGVGSGPSEGAGGKVGSGGGGTGGPEGAVQRVVVSLPLAVQARLWRQAWEDVAGVLDTCAELCAASAASGAY
GRR

>Cre12.g553750.t1.3|PACid:27582625
MTAAEEKPAAEESAAARRERRKRSRWGEETEAGRKVLEETARQLEGTPGPDQPQQDAEREGSVGPPTGDAPRKRRSR
WEPEETKSVTLPGLSAAITLPPSLAHLVDINPETLELQMQLNNISQRISLLQAGKLMDDTPPEQRSPSPEPIYGESG
MRINTREQRLKDKLTERRTEIIMELLKKNPNYKPPADFRPPKKTRKLFIPQRDHPSYNFIGLIIGPRGNTQKRMEKE
TNTKIAIRGKGSVKEGRTRRDLSGRPEPGEDEELHVLISGDTDDDVDRAAALIEKLLQPNDDTLNEHKRLQLRELAA
LNGTLRDYELMELQRQAEEGLGELFKLPEHLKEKVEEQYRRDVIAVHGEGAGAMEDEYKSFLRDLGGDVPHVPRELE
GLLGGPGGRGRPGLGRAADRDADDPRTVYVGFIPHFMAEAELRQLFGTCGEVVDCNIIMDRHTGTSRGFGFVRFATE
EGAQAAVMQLNGYVLANKRLVVRPKGNPNNSNIKPGFPPPPGAGGGGYGEGGFGLPPPPLQGREQQQPEDPIPAYRP
PPQWLFTDEPPPGLELAAAAVAGAVAGSGAVASHVPLHAGAVRPPPHLAECGAMHRYMVAQAEWQQQVAAQWAEAAE
AEAEADAAAAAEAAAGGLPPPAPRERPAEWVAAAAAAAAEVLPPGMHYYLRERDREREAELAEQAQQQGAPEQHQSG
HPQWQGGVGPSAAAAAAAAGEPPAPGSEDAPPGTSAEAGAGSGAAGAFGSATGTGPGAAGPSQPRPHGPHSRAPTLP
LTPAPVWLSPPVEVEPGPPGEEPNTATRPMLPGETIESYNAALYAEMYGAYGAAAYGGYDMAAAYGADPAAAAAAWA
AMYGAMPGGVDMSGGAVGGGATGGGGTMGAAAQHGADDGAGGGGGGAGGRRGRGGRGKPAPAVVQVVSLEPEEMERA
RKRAAEEEERARRIKEAAERMKAEQERAKAGVGGQDGDAGAGAGPGPGSAPADGGPGGPGGGRPPPYSAGRAPVSNG
PGGPGPGGRRPTRSPSPDILRGRFASRSPPPRPGPGAGGGGGPPPPPLGMRGRGGGPPQGGRYSSRSPPPRGGPPPQ
GGRFGSPGSRSPPPRGGPPPQGGRYYGSRSPSPDRGPPPQRGGRPPGARQGPPGQPYGNGPSPDRGRRFSRSPSPMG
RGPGGPPGPRGGGGGGGGGLVGGGGGGPYGSRSPPPRGGPPPGGRFSSRSRSPPARVRGGPPGPGPGPEYRGGPPP
YGGGAYGDEEGPRLRRPPPPRGKDSESGELSDGEVDDGEVAGPGHSGRRHGGGSGGEEWRGAGPRGGGGGGGGPRGG
DWHDGDGAGPRGGRGGGGGRDWDRDRDYHRGPQGDWDRDYDRPGPRDGGGVGGGYGRGGGGGGGRGRGSGGEGRGSR
GRGGGGGRGGRGGRGGPPGHGQGMQPFGMFPAGFPEEAYGWGYAWEGPWEGGPEMWYGFEGAEYGDFYAAGPYGGYD
EEYEDDYGDGGGGGGYTGGSNPRPSGGGGGRGGGGTGAGARGGGRGGPPGGRGSGGGGYQGGSRPAGGSGGRGQA

>g12162.t1|PACid:27582697
MVLQAHGSPVSSARERSPLGNAPPTSDQHHHSSSQQHGFQQHQQQHQQRNYQQLPAVRDLRGRVALVTGATSGLGLE
AAAALAARGATVLFGARNPDKAARVAVDIRARFAPDADLDLILPSKHWPQPHTSTHSANASDTKSSDSPQHASGGGR
HGGLCVPALDLADPPSISRFAAALLGPTTGSGGRGGLVQENEKEQHQAEGAAPGNGGGNGGGGAGGGGGEGVWRLPL
HILINNAGSSLLPPGPRSGITAWGVNGLAQVNYLGAYQLTRLLAPKLIEGVRAEAAAAAAAAATAPAATTGPEATSS
NNDNIHSGTTGSSSSSSSSSSGSAGGGISRVAGRVVAVSSVTHRCYELPADPRLFLHATADITYAWTKSANVLFAYE
LQRRLGEYGIQSCAVDPGGVRTAIWDEVPALAVPPAKWVIEALYAPPSDGAAVLVEAATRPWDQGPWRVWWRVVKPS
LYGTSVILHSLADYPVRHWSGGRLFNAVVPVRSAGQTYSGALAAALWEYSAHLIDTITAATAATATGTPAAPGSAPA
HGWAAAAAAKAARTHDEGFTFAQPTPAPHGPGAAPGPGTGTGGGGGGGGEYDSPQATPTRKRAVMSPLQPQFGFSPG
FLGAAAGRAAEAGAGGAMSPPPPPAVVGGAATGQAAPPSGSGSGTGGGGGGDVQRQGSASRRRLVVAAHGRGGDVAG
GGADAAAAAAEGEEVVHGGLAAAASPPPPGGGGRVWGSNVPSPVAMPPPPRSLQHVAEEHGIVLPGPTTTTTTTTTT
SSDTSAKPAAAKAASGAGAVTGAGAGGGAANGAAAAATVAAPAEVSSTGAAALAAAPGPLQTGPEFDKGAAAGAMAA
SERADSMPAPPPLKRPHTTPAAAADVEAAAAAGGGSDTATTTASYRPAPHGAPAAAAETPASITPPLPTGGAAAVMH
GAAAIPGKRGQDAGPQQPPPLPPSKSPPPATAEQAPTPHALAPSEAAAPAAVRDTAVAAAAPHTGGGGGGDGGKKKQ
GGSGGGTSSGGGAAAPTPAASTHSAPAPGPAPALVVGKQAAGGGGYSASAAGGTMSAGAGAVEGVGGGSKSVGADTA
GTASAGGGSAGGGSKHKKKNRRH

>Cre12.g496400.t1.3|PACid:27582790
MDACSETATVEAPGAAAGYIVHQGVGAANEAPHGGMDPSHDSPNPNTCSPAHTVDGRVDKVACTAVAAGQHAASGGS
APCNTAAVFGATCGISCDDETLSGHANASALPPSPPHCSSVAQLLPRATPGRAVTVTASPSALGARRQNSSLAAAAA
SVLCATAGAGAGTLLGSSRAAGAPPAVLSVQTELQQDDFESRLLEAAAAADAAAAAAAVKATSNSSSLKFWGLGAAP
YGSRGAAAADPLDTGALSSSSLRRQGTATPPVPPTSAFAAVPSTKGANAGVVGSRASRSTGAGLAAPLPLPLSHAPH
QMQFTGALAQPVVQRPLSNQHQHLHQHQYQQQQQQQQQVPPGRRQPQKCQEPRAMAAAAGRSGAASPPPRAAAVA
SPSTAAASALQAPGAGAVRSKGVLSPKVPIIMPRQFAERVMDPATMLQHGIAVVVRRLGPLPPPPPPPHPGAPGADA
AGGAGVGGGVVQDPAALAAAAATPAGVDAVLDVVLAAQVEQFFHAQGYSQYRVLGLKEISCGYHNWRIEMWEALGDR
VVRLTISAPAQAAKSASPSASLHLVPALPAPPPASAPAMAHVSAKAAAAPPVTAPSASGGLGYRTVSGSDLGGQGDG
GGLLGAGAMRLVTAGSAPLLLSSAARRPGMPVAPRPGGRFSGAAGRGAGRRPGLSLPASLDAAIAEHTAKQSLGLEC

MEEDVGAGEELGPAHALASGAGAVSSGAQLLAAAAAAAAAATGAPAAAGRRGSLDRTAARAARPVSSSAVAELKAAE
YWHQPQHVQKQQQPTRGGRKRQHMEFDRAGGDRAVVDDSGDEEEESVRGSWQRTSGSGMAGGGVKQRRHSCNGTGRT
SLDAGRPRELPPRAPRRGGRTWGGMGGGAMAGGGDGGAGTAFGGYSSLPAEDMPPELAGALASSPHPCRKGGATVLS
PEHFIHLPRLFFQDNFDTAAVLAGGLRVQVQAGGVLDGTVSSASVASYQNHTGYRYYRLLGFKDVPRRYAGWRVAAW
DKVGPDAVRVRLAPPLGVPLGTALARAAALGAAVGGAAMTAGGSAYAELGLGAAGVVGGEESDAEEAEVQPRLRRRR
TAQGMVSGSGGGDGGWPTGNDEDAAAAAGLAMIMSAAAAAGAHRQQRPQRGAVALDVPPSAWETAEVDEALPLQRPS
GWRHATAAAAADHTEDEDDGAGEEQQYHQHHAYTTSEQDEDGSEDDGYHYGVGAAGRRTSGLLRTRGGGAAAARRDP
EYQPPRAGAARGAAGIGAAAAAAPATRQPPSPTAITAAAAATGTPCPTSAAGAAAAVSASTPNAAEVRRIMEAGVAA
AVARLSVPASRVRSVVSQNQSVLRPDKGRVYLQKPFVESSLAVTATPLAVQVLINAGGRVDAATYPTKVTLSSSGAH
GKAEYLLRLTAPMKHAHTGQVIVGWSALGRQQLLMHLATGTPQPHPPQQQDQAQDLPQAQDQGTPPPPGRVSAISG
VQAAAGDALAVRGAAPVRQFRGTGSASNAEDGEGGLDGDASACQAVAEEAEGRNAARASPRAQAASQEPQPRASPGW
SAHPPTADAAPLPRLPPHLQYRQRYRTAGGPAAALHSGAAAGSDAEADTALPPAMAPVAAGDVAAMCSSGDDEPSPL
LGISMQATRRARSRTGQRPRAAGSWSHPTCGPQLLALAPASAAAAAAASTDGAAESDGSGSGFTPGGQRPMQLGRYH
RSRLLAAAAARSRPATAAPAGAADSADASEGAANVDRTPHHSRNPSLPESLLHAAEQLLHLAGAGDAGDARGTSDGG
SGDEGGEAEAEVQQQARGEILCEPAVKSEQALVPPASGARERAACQVKCEEAVGEAWLASEQAAEGVTAGAAAAGCA
KPEPAHAGQADGWDFGPRRAAALAAMRAAANAAPAVASTIMANAGAGGSQAAGAPSRAEEAAGLCVRLSASMEEERL
ACMAAAAAGNKRCSDADLDSPRRPSLDEHRTSRDDGGMRSGSRGATSSPRTSGPAMNSATTSGAAGDAAQGRGEDGL
AVAPGIDASYPSPPPAPLKTQPGVEKPHAPSGGPSGHDNLNAVAADTADIAEAPAVGKAAATELPAPHGSRKRPSSD
AYGRTADEAGAPEQEHQQRPRCAPRRDADKATAVPAGMSLNGESWPAAGSHAAHSHSYRHASRSPKPCTSETQQQAA
AGAAGAAGTAGGPVSQVAAALSPSSAASIAALAVDPTASFAAAAAAAAASMWPFGLPLPMAAAMNPMALPAAAMSAMG
MGAMGAMSAVSAMQLQMAMSAMGAMGAMGLNLAAAAAAGGGVPLGVSPLAAGAMVARTQEQQQVEQQEQQEQQRQQ
QEQEQQRREARRLEKVRLRDSASATPSAAATASKEVRPVPEPGGIAHAPTTTGSQERLAPGPRPSPSPQPSPSPAPQ
RNSPVATGTTATSGSAATATPVGEMAAPRVSKGTTLAPSPAPVAAPAFTAAAMPAVSPFSGPAQLPLQMWPGGPAAG
DALSFGGLSLMLLAAQQHHHQQQQAAAAAVVATAAAAMASPTASAATLSAGASGRTSSGLLLPPVPPLLPSASGPAA
LPGLAHQSSAGMLLPQRSGSTSALVGHHVCAAATTTDAAGAAPPAAHAASGDASAGGAGGPAVALTSAASGGLAAAA
EPHPLAAVGPALQHLVVQSRLRHQAAELSAAAALAATLAGVAPRPNGPTAGGASGGAGLR

>g13804.t1|PACid:27582854
MDEVLRRKSLVEQILARAAKIKTHMQEVNQSIANMDQIAEEKKARAAREAEEAAKPVLRLMDLPDDLLRLVAAAVAR
GQSANPSPILGSVLTRGSLAAGGGGGGGGGMGAAGLAALALTCRRWRRLASQEPGLWADMCGARWGVFQSDPAAVLV
LDPRQLHLAAGWPRAAVAAAVALPVSGGTSGGLGGAGSGAGAGTLLVAGRQSGGVVAGPRNGGGGAAARAQQAAAAV
VSTAGPMRGGISGPAAGGAGRLSASGGAQSPSQPQSQSQSQLPALLAPGHPPRLRSARSQGASTLGPAVAASSAASV
TSASGAAAPAGTSGPAARGVLAPLPPAAAAAGAGGTGAAGEAPGAGPGGDTSPPPPASPPVSSGRRPLLPALSLPHS
PSAPMTATAATAPQPSPSPSPSPSPSASRQRFSGCGARGSSPPPPQPLQECGDTVVRPGAWGGPGQADAGAKGVVGG
GGGGAVPQAAGQWAPDASQRSDVDPDPGPDPDPDPELGLGLGLRMGMGSDGRRAAVSASALPGPSGAAPEAAPHGVS
TGPPPSPRLPPASSPASARPSMVQPLPGAPSPAGAGAVAAAVAAAGPAQPAPGGNSKQQQQPAGVGLPLLPGGDALE
PVPLGLVLLRAGGGGSSFKSTCSPNSGWAGSGGVTGAEASAATAGQDVAGTSCAEAAPPAQNPWRLVYRDRLVGWRG
VLAVFRWLQSAGCPPGVSGALHRQQLLLALRYCHMLLQVNAAPPEPAAVAAAAAAVPYPSAAASSAAGAAGGAGGGA
VWGQERHPEGAGSGAPVGRQSRPWASAGRARADWVRQRAAVALAAELLAAAPDTVPCLHALLSNESGLVVELAAAGL
ADLLALDRAVGEEGDRRGQGEEGRQRGAATAGGGGSGSQDTGLLAQARQAHAAAAAAAAAAAAAASAPSTAVTTHGSG
KTAVAAAAAAAAVAAAAARRAAEQPSVLAHVLLSSNSGRAIAAQLRDGLRSPGIVAELTRCLVNLWAPEHLVPPVLPL
PPPPPPLGLPRGTAHSHSHLPHGRAAAAARLPPLEDGVWQLQEFSARGDPLRLIHTLVSFRPGVTTAAAAAAHMGGT
GATGRTSGSGAATGHAAHMMAASSARHSDSGVRGGSGTGGAGGGKEPPPLPSVSESGAAAAYRPGHEDGEEGLGGGG
RAPASLQYSPGPAPPHPSGNASSTGSGRRTTYSGGASASSSSSSPGGPGGRSAATRQSCAGGRPSPCDTGAASQGAG
LGLLAGGVLTGLGPMSSGAAAAAAAAASLGLLAGRTAWPLVGPDGRPLGGTAAALVALSGGTRAGERVVVELLGGGYD
TSLSRGGGGGGGGEEGVGGAEEFRLSGFVDAASGEWQLQRRCINVPFGLTYRGYSDAHGCWGTYTVQQQDSGAGARAA
AAAAGSGAGPGPAAAAGPGPGLAAAHGGPQASAPGGLGGATAASSGSTSSRGAPRVFRLFRDPRLPPPPELLRLLGA
MRGEQQQQQAVEEEEEGDSGGSEEEGDAAERSGGGDCRGQNGLSSNTGGDGVSGVVGGGGGGGEPGGAGDWGDLPHL
EPLPVASSSSSGGSGGR

>Cre12.g548050.t1.3|PACid:27582991
MVCGCLCFGKKRVDGSQNDERSAKSKQPGGAPPLYNALPIAAPSDAAPGVSQAKEADDGIRSQREEYREELERRLSQ
KSLAKHLLERQPSSRNQLSRQHNLHSKSFGGASNVPSAQRWQPDSSAHAGTASGLAPPAHTSQPAAAPLLLLQEVKP
LRDPVAAPSLTRVGASSHSGAVFGSAGGGGGAGATGISGSMASSCSMSFARSVSRLNAFATSGIKSRRASMAVMDMD
GSARGTSAAAAALAANLQAMLGLHGVNLGPTAQFARGSASSITTTSQLGMVTPGTGVGGGWGEPWLLHTLLRPGAGH
GTGPGAYTIPGTISHMNTSAGGGGSSLVVGSMATGALGKMPSHTRLMLSGMPDRASAGAGSTPLGPGPPSLGTLPAA
GAAAGGGGGAPSSGAPSVAAAVAGGSGNLGRPYGRPSMSLDRDPSRSSLSQYLGPGHAAIPSGNTAGGHGLGGGAAA

QPLALLTRAVCDRAAALPTPLMVLVLRPAAAGPGGTSGGVSASEGAAAMVDADVDADTAAGRGTSKDAAGHAGVPVV
SMTNDGFAGLTISGSELRTQSLSSLSRVVASMYLDSLRPQAKPGRRRPGAAAAAAGSSAANSGAAQQRAGAPQSLDG
TPEAEARAALAAAVAALAGGDVDLELVWQNVAAGALAGAAAAVSGHDVRVVSATTTTTAAAGGMSVSEVGVGQLSMS
LFAAANAPAASVVPAPPASPPGVGPTWAALVSGAGGSAARAAPISAGPATAGAISSRLLGGQATPSSAPTTARSPSS
ASAPNLPAADAPGAAAASQPSNSSPSNAITSSPFSATAAPEAAVGAGATSHATAAATASAAAATGDAATAASVWALR
ELLEADPCLLLLARDLVRQAALRPEHVPPATHAVSPGGYCQLLGGREPLPAAVAALMAALAAASAGGGGGPTPAVSA
SNAALGAAAGRSAASAAVGGAAALLAAVQSGAVRVHAVAFQATAAAAAPSAATTAAQAASPDAASAAAAAAVAAAAS
AWLPAIVLEWPQPTAAALAAAGVPLGLYGPAGMSGANIGAVVAAAASAAASGVLPSLPSGTAVTGSLPPLPRPPPRP
HSFFAGRTSYTDHSHNPMLSADTSPPPAVAMVQPAVSHPSDAAAGLGSFLLSMPLGLASANTSYQGPAGVGGAANGA
AAAHITTGSTNQATHPFMISSGTVPNAQPPGSVGGSEPRHSTAALPSLSLGMGVVMDGSGFAGLGQQEPSFGNIIAG
GGSSQRVDATAAAAAAMTAAAAAAPGSAWPMPNSQAAPVVPSSGGAGMGVGAGTGIGTDAGAGAGTGEMAGVVGLLE
AAASDAGSLAPLAGEELLYVVQTWQRHHLLLASVPYAITLFDCGGRVLQQNQGSVDFMGELTFAEEAQQQQQNSDA
QQQQQQPQQAKPGPPLGLLSARNSLPSDPRESVAYMAKAQAEAAGKVAGGSASSAAAGAAASSASPHRRSPTGGGSG
GFSTGFGGGAGQAATLLDVSELDVLSVLFCLCPEQLDEMLEEVICGNMYRSLIPVPRVMRPCIRAAQRWGARVLAEQ
QANAAAAPASVDPAAAGAATTASAVRATAAALLGEGSGATASGGGGGVDSPVDATTGLAPAQLSALTHSGTFVASHG
GTAAAAAAAVAAVAAGGRLSGSQARIAAGPSGRSLLSSADVEMPSGRGQADGRGNSVGGAAAANDLTTTQPRSSSRD
YSTTAHSSAAVAAEALGVSDRVAAATALTRPPMRHGTGSFTSYSSGSAPRKGAYHPSPRRGGAAAAASAATSAAASP
LPFHRLISGAAAAITATSGTAGTDPGEVQATLPSFGFGHKPSHQRRAQLLGAVGTSASFTAASSPAFSARVTADDNQ
DDGVGPYAVEAADCFTGEPDLAPSHDSGNGSFSRSPSTGVAAAGGAPRRRPPQRSYSLRGLLSRSFTQGASQPHAPL
HPQLQPQPRPHHHPPLPGSPLLRGAAADGQHRVIGSGSGSGSQASTPTAAAAAAAASSQAPPAHASRLGLLNSVTSAV
SAPDPRLPATMPTTASASAVSGDDGQKLTPPSLEPEPVAEAPSPVPTARAGVVAPAPDPEAAASGGSAARRRDGSLL
QTLQRADTSGGAPAMAVGSSGGSFSSGAGAHAGKPRASVLAFAERLRSMGSSRTPAVIDGPLTNGADADATARPAAP
VVDGLMPDANGSAAAQVDVQSQADSAPNVEEQHAAALEAPASSTAAAVAPKQHPRTGALIDAAVSALPGRVLSAVVE
GGEGEHSMRLGTLGSGPEVQRRLQLQAMADPADPAPLGPGDGPAISEDMAHVTGGGAAAATAAAAARAGVVLGSGFG
SEGTGGGGGGDLNLIPAAGDSGAAGGPTSPSSTSYSHGQSHSQSYSLHGAGRSGHLSASGSGAPGPGGAAADDAGSA
VTATGLGTYASTSSMSPGAAGTGANGGAAASTSTAAGGAASGGGAVHSRGSVRRQGHALKRPLSRNSTRSYLAQALP
TSTLTLIGRVHSQRDLEALDSAADGAAASAAAAAFGGGAAAVLSPGNGGGSRARGLGGRRGGGLGEVGRSMSFNVAP
SEPRLTVPRIFSFMRSASKRVLEIQQKAASGNGLKQAGGGAGGSGAFGGGGSFAGGGGSNEGALATMSDCMAFAATA
AGGQVPSPTQPQPQSPRVGPSINAIVSAPDTGAGALAFGRESDAPTGEHAFGQLGTFAQMAGGAAAVTATRESASTA
ANTVSGSHSHSHPADGAALAAPRGSHEPGALPEPAHFGGAAAGAAAAAQLPSPPPSLPVLQPPAMLRLRAEERVLRD
AASGGAGAAAAVQSPRDMSPAEMARRVGSAWHEVAATSFTDPVTGARVVAIVQNDVSEKVETEMQLTELMEVEHRLL
EQIFPRHVLEYIAVQNMTMYASGAGGGAAAGGGGPGGGAGNTGGGRSRRASQFELLQSQDCSQLAHHHEAVTVLFCD
VKGFTAMCSVVEPAVVMAFLNSLYTQFDSLLGVHDVYKVETIGDCYMVAGGLMRRGPDGVGALLEHGEQDPDHARKV
VRFAKALLEVVGHMRMPTGQPLQVRVGIHTGPVTSGVVGTRMPRFCLFGDTINTASRCESTCPPCAIHVTTAVRDLV
PEEPWVPTGGVAAKGKGVMRTWLLGNTAAPPGAPDAASPETIASPTATAAAAAAAAAAAAAATAAAAAAAASAAAAA
ASPESTGFGSASSGNASPVSAAAAAAAAARRRAVGVNRRASVRTLPYAGSALLPSGAAAPAHSSASGMLASPPADSA
AFAAALAAQGGATGLGMAELPPQSEARSLPGGGSVPGASMASGGAAFRQQSGEPEAASGPTTMLRQLQPPQLSAAST
SSDAATAAAQAGFSFASGSSSTAGGAAAATAPGDSASPFLAPPSAVGMSSTGQASFTRGGGGAVGGFMTAAGGELPV
LSSAAHSNLLLTGEARGTAESSLMGPAAPGTNIAGGAFTTAGGLAAAAALHRTGSRLGRTGMGLPPATLQAVLPGAA
AGAGGAAAFDVTSAGSGAGRGGRVGEPVVPAGAATAPLLGSQELATPLTTPMTQTLTYEQKQEAEWRGEQGRQQEAA
ARMQMQLLAMRQFEELGDEGQPQEGAGDRGDRAVTAADDDARLVTVDGMICGIVGGGGAASRLPSRLGSDASITLAA
AVSPSVGIAGPSFPLGRSIGLSGGSSGGGGSGIGSNADSRVGSRHYSTLVEIMGPDFSRVSAVSGGAGVSVSMHRNI
YSSGGPSFMAVAATAGGPTAAEAAAAVAQYGGYRDAQPRDLDDGPMQ

>Cre12.g560050.t1.3|PACid:27583043
MPLDLKALGVTLGVDVAIAAVALGLLTVLRAVRPTRRFYAPRRWLAGGGGYASAAVAYGGGGGADGGPGTPLRPRPP
RLRWWAWLWQLWRLPPERVRQLAGSDGAQYLNALKLGLHISLYLSVWCLAVILPVNMAGSEVDRLIASQQQQRQQPQ
RPPPPPAPPPPLHPASAAAVAPPSPPPPPPPPAATAAADPPPSGSASAHSPSPPTSEPPPSPPEDILTPAEAAASAA
VAAARATADAAVATAARFHSNTTSNNNTADAAINASGRARNASEVTPPPPPPPPAHTANEARRGHISTPAEAAADA
AREALNVAAEEAVAVAAEAGHGGEGPAGGGGGGEAAGAATFAAAAAAEPPAAAAGGAGGGGGRNASSGGGGGGGTSY
VFSDLDRLSLANVAPGSALLWAHLVSVYMVTAITLRVLWQQSKEAVALRVLHVTHTPPGGSSHTVLIRDIPGLSYGT
TAHQARHLLARATLFRFLPRPLRSRLEGALVGLLGGTRQRLEAAARTAGRLNCVSPHSSSDWVAGMHAAQRRFIADH
LAPAATLPGLKVWAGDYNFVCNAAIDTLHRGGRLLDPGTTSLFASTCASPVDSFRHLHPTRRSFTFINHMGASRIDR
IMLSPELLSSLRACAHVPGRPSDHRLVVATLAPVDGAVSPGPGLRRLSDAYLAFPDLRRSLLDWNRGACAAAPEAPV
PLLRWFVAYKRDLVRTVGRLNRIARGRILEETAEEAAARAAEAAALAVVEAGGPDAAQAAAAAVAARSAAAAAAQRA
VARHARAARLQWLRTRETASPAITRLVRPPGGAARIAAIRRPDGSVCDSLPEIPTHIVDYWRRISAAVATDPDACDR
VLAALAAFGRRCTDEDAVRLGADTVSSAEVEAALAAAPAGSTPGRDGIPAELYRELGGPTARVLARVFTASLAAGAP

PRGFLDGVITCFSKPGDPLQPSSYRPITLLGTDYRTLARVLARRLIPVFSNVIHPAQTAFLPGRRIADNVLLLQLLP
ELMRVAEGGGPGGGGGGGGAAGSTADADRGVVAFLDFYKAYDTLDRNFLYRCLAVMGVGSGFLAWVKLLLTGTRSAA
LANGYLSAFVLIIAGVRQGCPLAPPLYLAPAQALFAYLDRAGFGVSWADIRLVATAYADDAAPFLRRMANVPGFLAA
METFRAASGQRLNLDKVELLPIGARRRATPAPARAVAAGLAAGGAAAGNMAAHGVAATSVAAAGGMAAGGAAAGVAV
AGGGPGASHSGGGSLAAGGAVPSRAGAVWAAAGVVVTGGVTAGGSAAGAGGAVAAPAPPNTVAMAHGLRVVSHSRTL
GVVFYDLPLGTAAVQGAPSLSPAIGTLGTLCGLPLTAFGRGFCASSYAVSRVLYHMEFAGLPPQTVLADFLKTLVRF
VDRRIIVATPQGAGRARLPGLPSDCVALPPRDGGFGLLPVVEHVRARHAVLAVRWLQHMHGSAAGTYVPPWTAAATA
LVQQLHTAAHPLCVLTMQRAAGGVGPAADGCAIFGQPVAATCPALVRLLGALSYLPPVTILRPAVLVPGPWCFALPL
WGNPVLPCAAGLQAGGLERDFPALMALPGLLTLGTAVRCWEALAAVRQLVASKPAGALGPRLARRCTMQYKRSVLRP
ILPIADMARVPPELQSGPDAAAQFSALLARVPAAWRVAASATLHAPGGAASAPPAPQVWLMLAQSLGWRHGAADVPL
LTLTVKQATQLQLAPAYDALRVRHLAFIQEAYSGAAPPAEAIHALRAALARLWALVWEPRHKEPLWRLAVNGFTGFG
MLAAWAADGRVEKCPCGTQMTAGARVHHFWDCVVAEALRDVMREHANVDITRNQLWYLDRVDAGLGLEAPSTSSVSP
AHHAHGPAAAAAAVAAATAAQPQVQVLQPAALLQPGGHVVNTLYAGRVRSVLPVVVSTGQMPAAAAAAAADAEVGR
GSSDGGYGTTSDGGQGQGQQPTSRTAPAPAPVPEQYLLAAAAAASAAVAAVVTPGAQGTAAPSPASSVRGALPQQTA
PAAAPCHYPALASPYAPAPLPPAASPPPPPSPSSSSTASSSTMLLRRRRRQPQAEAGEAGSGGSGRAGGGAIEAGGA
GGGIGTGSGSAATAIAATATAAELLPPALPPSAWSRAEAALRVMEPPEYVVWELGHVFPPCRPVALHLVRDERRLQP
HVTAYGAVRRSLEDLLDNYERQLAAARAAAAAVGSAVGPAAGGGGWWRSCCWRRRPPTVQRRKVRVLGPLLGSWAVD
LYGVRPVRVDELLHLRTHLEVLYEQVRQGLDESRHDLPYVPTAFVTFSRRWEAAVVSSALWDRDEGVWRAAPAPEPG
EVLWGNLRLRLWQLNYREGVLRLAFGALLLTYAVPISALQGLLQIRRLEKLPVFRALVRVPIVRSLLAGLLPGVVLR
LLLLLLPAALGRLVRWAGAPSRSQVDFRTTTLVFGFQVVAVFVASFLAGALLNQIGRFVSEPGAVLTVLGTGAPQTA
SFFLTYILFTGLVLGPLAALRLWGLLQFAVRYGLAPTRRARARLLAAPPLTFAATVPSHSIALLLGLVFSCVNPLVL
PAAAAHFLLSGLLERHSHCYVWAREYESGGRMWSQVFGQVMTGVYLFQAVMIALLAIKRFPWAPLALGAPLAAAAFQ
RSVVRMYSRPWNTTSLRDAADLDAADRKGHQQQQQEQQEQHEQQQQGQQQQQQQQQQQQQQQQQQQQQQQQQQQQ
QQQQQQQQQQQQQQQQQLLSHRSHDLRSPPPQPPATTASPPPPPAPHPHHPHHPHQPHHRHGSRRNQPHQTHDPHHP
RRHPSGTRPPATDTRATAVLSPLAPPPPLHPAASAAAAAAAAAGGDGAAAPPPPLPPHATAAAALPLPQSEVVPATV
APMAGADDSVGGGGGGGGSGGAGGGGGGGAGGGGGGGGTGGDSIGPQGGSSGARARQSAPAGPQQAGAAGAGGAGGD
GEGRGGDKARDDGAAGGGTVDGGSGPPSAPCKTSSPPPPPAPAPAPAPAPAPAPAAPGLLTSLYLSPARQLVCGW
GAAAHRRLVEEANVMDRRLQVAAALAPQASVGVSPLGLGAPGYSSGSSSGSDSDVEQEEEEEERGSDENDAEQGLVR
RG

Volvox carteri

>Vocar20001532m|PACid:23123887
MAPRIWQKWLPQDIAQGIQDEEPGRFAALSRCVCAEVDAILRDDVDPNRWHRLVKWLSHVNTFSNRHQDFPAEHVLP
LAEALWRSVSESWRDLEAQVRITECLTKLLRTHKRTLRRAHLQLPWRPLFAVLKRLHGLPLPKLEGVGLIMARGTAL
VKLVSKARRFFEPNALGEVWCELRPSILAVDRRPSEGYEALGYLQLFAPTHGMCRCNWQLSTAGCWSVCRVFRFRVL
NWQPHLPHIFASLTGFFDVPIGTSRAVGDIITRSTPGRASVLFGDQMGYGSFHAARLVVHLLRCTADPQRGPPTAAA
AAAAASHLEHLTSLLEQYYHPSNTGEGEGKWCRDLGTLLKHLVKAAVKQLERQQPPPPQPPLAAATAADGRMACNDD
EDVHGGNDDDDELADHEFELLQLEGGGDGGQGSPAVAAVAATGVASSRPWVSELREAELQRLASCCVTLAARAQFSK
DSGISTVSVKALSQLALVAPGRVLPLVLERFRTALATATATHQLVAATTTLALCVRPLLLAGWAAGGESEGEERETA
AQLVSEALTAVLPGFDANDEPKTNAVLQLYVSAISSLPRLRGAGEGDETDEAAAAEEAEEHSFGRAVQPAGGTGSTA
AAVAPMGVDAAAAAAAANSGAAAGGSSGGVGGELTLSLYVEEWAEEVLERCLALLQNLDSGPGQGGTDMASRGGGSA
ASTSSFLTRLTPTRATSAAADIGDASLALGQLWLAVTVRVAVAAAAVAAVADITAVIIAPWLGPSSEHLALANQLIR
LRLLRPAQRLRALMRGAQTTTATTTTASAASAATTTAGTGGGVDGGGVVAKLHVRGELLKVSVRVGRHGKHQIRGML
LNALSMLRDFHFGEGANGDATISSSSGSGSGSGSGSGSGSGSGSGSGSGSGGAVDDDGGNGVPLAVFGGLGVTVGDP
RVREQLAEALVDICSALGSSGDPELLAVASRCAGVLLTAGSIEHAAQVDERGQKATDDTQSDVPAATRLMAESWTSS
APLVWRRRMPPWLVQARLQNQLVSRSADAAFRSAAWCTTRHPQMTRLEQFPVAYLSLLRTQCMMAQHSHTSLARNAA
TVVDVATKRFPCLAPVYLPYFLATLADVPYDWAPTTPLSPPPLTDFCASVRAAAFGGGSTAVAASGAAAGALACIFS
PPPLSEIDRDGKVVGACHTMNKALNFWRLISRSPTWLGATLAALLAARTHNASVPQKALGDLLLTFAGRFLHPPALS
YADSGGVNGGLYGDLVRSLVEVARPGAMARRGAPFRYTIIANVMLTLMLPYRYGASADVSDYGGAGGADPEIAGLLL
RHMLGLLVGSDAPQLRQLGLSGTFLPLCVVVEYGAPLPQLKDVLRATIASASASASASTSTVAGAEPPPGASGAPSS
GCGGAAGGGDGGDSGSGSVWGFRLIHALALNHTELDSAEALKDKRGAGLLALHQRVLRMTFEELAGGLASVPLERLS
KWPNDGLPTPAAVMEGLFAVRHARLVQLLAAAAPEEMNVKNNPCHVPRPPPRTAILTLPNLTYAFLHSGGFLHGNAM
LSALRGHIELLTAAGHVTATGGEGGDKAEMATAAEVLAGLIASGAPWVAADGGTAGGAGSSGAGAGGGVGGGWAVAA
LARAISGTNLDMSETWALSYRYVVQQCSTPFATSYIFSSCIRILKGIPYTGIPTHPALVRLYAVRGILDGLVPEGP
SAAADRRPLLPRPPLAAGLTDGTLVTALGDLLRLTVPSPLAPPPVTAAASTLAAEADVNAAAGGSTAVTASGGGGGG
GMPARIKRLRCVGAVVRELTRLDPCNELPLPCELNMINRGTASCVFYLAMYNSLPPRKQLHSFVVRLSRLSRLKPRN
PPDQLRGPITDTLAYFGATTDGVLLRALARHQQQQQQQGGSGRGDPSGRNGGGGDGGGNGGSTTNGSNGEGELVHVS
AEPSGVSTPAEVEVEVELDGAGSSGGVAGAVMLLVRPGGSQLPSYHASLQDLRTRARNLALTVAARFNDAAVQAQDA
RAAEANTMAAAVAPPPASPRASPGVATAAAASDSPVSRAATDDLVLVDAVDVVSVPASASASETTSPGGGAAAGLAT
AAEPMDVDTVAAAAGGGAGASSASAAVAHLGLGLEVQLDAAARHRSPGLRELVVGMLPGLLRLQDVAGRWSWALGEG
RAAGAV

>Vocar20001547m|PACid:23124013
RQPPSPPPVPLAPPRAPPSKGAPPSPSPPPPPRSIIPGAQYVCRWANLAAGRPTYASSYLGGSAAVGYCENDQCLPS
YATDGNTSSPLRTFRTDQGDINPWLSIDLGSPMMLVQRIVLFYRLDCCGDAMGGVELRVGNTSISRPSDVGLIRSNP
LVWSEKDAGATGGVLDVSLRAPVAGRWLTLQNSRNTAYYMTRVLEVVELQAYGIDTSEMHRATERFSALVLVAVHCD
DTCALWNRVARTRTRAGVRGSMHTHEKRDTHGDTDAVGFRAWMEVQSNTFGQLGVGDLDNRGSQPGQMGSALPTVNL
GPRRVVAVTAGCFHTCALLQPGGFVKCWGLNDVGQLGLGDTRNRGGLAWDMGSALPPVDLGPGLSATALAAGCNHTC
AILQPGNIVKCWGLNTNGQLGLGDTRNRGGDSADMGAALPAVDLAGSGSNLTAAALSLGASHSCALMNPGGLVKCWG
SNSQGQLGLGDTENRGDGVDSTGNITKEISRDLPEVSLGRNFSASVLAVGEFHTCAILQPGGIEPAATAAVSCSVLS
PARSACGSKLTGNKSSQLGLGDKKARGDSAGEMGNSLPVVDLGPGLEATALAAGARHTCALLQPGNIVKCWGSNDYG
ALGLGDRSDRGQLPEDMGVNLPPHGSKQVRQGCRCALVKVCGTRSVREISHWPLMGDARVNPYMAVRLSAQLPYFDL
RGGVARSCPFLVAYHKAAAAAAAGGQQDIPSMGCP

>Vocar20001605m|PACid:23124017
MYRFLNPQVFTHTERKEEAPLAVSASQLRSRTHTATPFDDPPSIAVPKLQTVRFSYHRYPSSDGTEAPPAHRHNVDG
EDTEDDEGDSDPGIIGTLTSILVGGVADLLTDLSPHPSRATTTAASASTATAARRYASTHSSNDGTFSKPVAADSSW
ATAQAPPAAHRRSTRACSLPEPIAGDHSGGGGGGGSDHFTPRTAEAQWLKSSRSLGRGDVGGGDDGAGGGGGNWVFP
PQHRTRPVTAPTGAPPSGRSRSGVSGDAAVSNSLVGSPTGGGDGGGSTGLLGAMLTGFSSLGQKATDLLTQLDHDFE
TIFNGVLEGDPEAEGGGSGEYDRESSAGVNGGPRKGSEGRRHSCSSGGGAGGGSDAADGRPSGKSNPAAARGGRISS
TGTAQMPPPYSAAAVAGGRTAVVTGAMPSAAKASSAAATRTTPMPPPVSRSSGGGAATAAASGGGGAAGRRTGSVVK
GRSHKLDLARSSGGAAAAWAKMEDDDDSWGWGTCDKKVAGAGGVQGEPSGSGRGPAAPPPPASAAANTKTKSMTITK
TTSMANATAKVTTKVTAKPQPQAGAATAKAAAGGPPPALQPLTGPGMSTAALAPSPARTRSCRSVSVSVSAAAALTS
PPPSASASASESRTNRSGGGKLIIIKPAAGTATSRTQPGTGTSAKCTTAAAAAVAVAAARSSSVVPSAPTKSITRAA
AAASPPGSPARQAIVSPAGSPSSPGCRTAAGVTVSDKAVAVVMTSKVLPSREPSSPDRRHPPNDHNKGPFTHPTHDP

TQQHQRIHASAAAAASEDSSATAAAAAVAAAVTSGGSGSNHDMAGFTSEAKVAAAAAAAVTKQQAELREELEEERRR
ADAAEARCAELQQQLDAAAAEAARLSELADALGEQLASSEKRAADLAKEMFVSVVVLVLVLEMSVMVMVVVMVVVMV
VVVVMVVVMVVVGGAIATAATAAAHTVDSPAVAASLATAALAAPPLVATATAAAAELLSYTAQHATYAHGDGGIADD
EGYGYDGLDGAAYDTTYWQYDDLSTTPLTRPPDAAAAAAAAPPPPSPPPGVAVGSPSGSGGRPSSSYGDLLYDFRDG
CQRHVGASGPISWGTPEPPVPIYKTRQRRSNAVAGFETHSALSDRSPASGGRIGVLEFPPPDGSPLLGRRTGDGDAC
GDGQSTDAVRTCRAAAMCAGGGAVGGGEEWRPAADLPANDGQEGASEQMETCKEAEAGRHTWQERRPPAVCSSVGRP
FSDFFQGLERHRAHLGLWPGRTLGRRSAGGILAVM

>Vocar20013421m|PACid:23124125
MQRARGGFGDLNFLRYRSRKESMGTKSWGAVPRGFNGGIGRTLKAMIRVLNTHTHIRNSLPAAGEQRDSRQNCSADL
EASTEAASGSKPSASAAEGTRARSHSRHPQLLLSNSAPRLLSFTLNIVNTLHWGKKSIVRLSRTSHVSVLNSPPSPP
PSPPPSPPPSPPPHSLHSYHRCGPHHEQEQEQTRGATPMPRHSSPPNDTSLLDIRGSAVHFNSTTHSATAHPWQGPI
TAFTAQPFPEMQKA

>Vocar20011598m|PACid:23124190
MVADSEYGNGNGSTAATGGGTVSGGGLGSGSGGMSKSNNKRSRCDLEPSGVEAVAVALLKGQGPQQSAPTSLFATNP
PGAGGASGGGGGGGGSSSGLTNYDAAGNLLPAGITRPLPQHLGGTTAALLSEMAGQLGRISALAREVEGLKEENTALK
RSMEALTEAYGNVRLLGGDGGGCGGGSGPVGATPSGPAGDVTGDGLAGGDGVGFLPYDGDGVADGGAAASGASEGAA
GGSTGSGPLEGTSAAGAAGTGAAAAGAAPTAGPVANVMPLSQAVEVQQKRIWDQKLRHDDLEKQLQAQREVSAVQAA
KTAALEAALAANTAKAVHDAAMFGRVLEERVTAQSEVVRDRLEAQERANKVAQDVNQRLEARVAELEAQLSGGIQRR
VTPVPQLGHQRATSNLFGMCMVVISTVDPPVSVVGPPHFWMVRPLLTSPPAEEIPGSNRAISASSCMVTRWARIRVE
ARLWSPPPVPSNLPVNNCNNNNNNNNLNPCNRQPPLDWPDAAALGVRAIHHQGAGAQVPAHGSVAIADGSGDLAAAP
GVPDGELGAPGLVGSSPPPPAPQQQRLMLAFSPEAELYAKSIEDRLWGTVEQIVNSADSPADGASVGRATRAGPEVA
AAKAGMFHDPYGTGANDGGGHYNGTQAFSEHNAVRWEAAEGANSREGSGSCPPPPVPPPVPLPMPPSVGCGGSGVVV
NQHASEAAAAAVVVSRAAVATTTGTATVAAAAAAGEVVDGEHSGVAAPQWNEHADSDHMDMGV

>Vocar20011305m|PACid:23124215
MALPPLAPPRSASPDRAASPPTISPRGSRISSIAPLASFRATITSETNLGSPTTGNTRGSAVQGARSTSGGLPPATT
ASITAQTTSPASSPSLSPARRGSAPSTAAVGPRVASGPRKLTHSQLAAQLHGQLTDVLRMEWRRIKTSNLGVLSGTA
VVEELLLRRIRSWCSELHMLAISSPTASYKLGKLCQSMEGTELQQLLVMFDQTGHPDLSDFVRVLVFNTADPAAKEA
VGDLRALRGIKSAGRRARSTPSTSGSSRRRAPPVLLPTPRTVSHEPDDPASLRFAGLLPEGHRLVAGAHAAAERRRQ
REEDERRAAEAREARARARFEAAGGRRRLGLHCDRRLSGPPTTPPLPPSPPPLVRLSPPPPPPTARSSGHVGPPTVK
THPPGRPYCEELLPYYLADVEEQWRRTVATRVPAEGVYDSVIMSHEALPVYESMPDAALVRREAEEAAAAPPSRHGL
AAGSVNARAAQPFSGLFNTFGSGGGAAGGGGGGRPNGGSGTAAAASGPRRITSGRPGSGRGPQLLPPGGDHSSTSTA

>Vocar20011392m|PACid:23124309
MPLPQLPHCILGPLPSTGTAAHLTWTPPPAPTPPAPSSKAGSSPQQECSPPPPPPPPPPPPSPSPPSPPPIRGPPQH
PDIATAGPPAAPQPRLWRAVTSWRPCAVHTETPPPRRTAAPVGRPCPPTRPPLSVGSPVSRANSVSLGLNTVRHDSS
SGAIGPSTPPASSTTGTPAALASCATSMLTDSGISRCSSTAPAQESSCFTPSERLQAAWLAPPTMMMEFSPPACSRI
NAVPQGPATSWACERSTPQPSNALRSSAALGSSLPNWPMKKVG

>Vocar20011770m|PACid:23124508
MMRRTPFTGGCLLRRAASRPPQFAAFPWISFRHHGCCANKLKARASTLEDRTGSRQSVALEHVLEELRKAQGIDKLG
PDVEAVVSGRGKHLGIAVSWSLRWTASGSFTEEIRGPQLTFKWGYDGRKDGGCWEDLIVLDPDLAFAVVVDSAGLVR
VMECDDHEAVLMTTYIRTATHISSGGSGSTTTARGRKTAAVALTHGAPGSPAADEVVAIGLRVRGRKLRVRVFVRRR
DWRLLGFNHRMCADVEMWELGEWKEWAEGVSYPSSALHKASNGGQHAYLTNSGAAERVAPLPPPPPLLLLQPPEPLR
TSPPPLPRVASAEAGSLPSPPPPQQLLQLPPPTDFGLPLLPPIPGDTSYSPPGGGGGQGVPLWRAASGHYLVRPTIN
GQEGGGYFVVDTGASGFVLTPQAAARLGGQSFGETHAASISGKVAARFVRLGSWSLGGLSIRQPVLMVMALEGLVRG
APGEVVGIVGHDLFSRDGVATATATAHISSRYAGSGAAGPVLGPPPPAARSTAAFEDPVATAAAGAAGSSGRAAAAA
DGGGGGKGHPIANLPHVHLRFADPGGGAPHAALLMVDSGACGADLMLHARAQKELGLMQMPPAAAPGAGAGDGDVVV
GGGGSSNGSRSEGHYLRGVGQDPKDYVQLQVRLVRK

>Vocar20011633m|PACid:23124569
MHLSSSEVVSIEGPFKSAYPYAGPPGTAVNLYGDTTWRLQYSDCPAKTWGDIDCVGSITFGDYLCRQDPSTNDTGIT
FKYGTQRYGSNLYILGCTLPTPDANNNQPAIGTANNLNISIHFEASMRGGMPRPLRYSYHYTTDGTPYQFQLYPEVT
GLSPAVGSNAGGTLLRISGRGFPDLGLGLGDTANVSISGVPCSVVNSTYDTIFCVTGPKPAYTSLASIPANGLYPGM
RGVEYEFYNMTQTISYGDLWRLNKTILLNNTSGSGSYRTILKGAMESIHYETSNHCTRLKAFFVAPRTSTYTFYLSA

DDYGQLNGTFVNQTGVVVTKTLIELTSYTYLDNYAMRSTQKALPLALMADQPILLETAHCNVWGSGALQVAVRMEVP
KPQANSMVERQTITVSSVLVPRSLAIKYLYGAGAGTMAFTVNVISTDNSKLDSASLGLELTFNCTVGARTVVLPLLA
TGLDMTALIEVAFGGKYSLGSNGTSFGVYKARTPGLLTVQIGIDAGRASTLGFTVTLARLVVMPAPAPYTSVSCGGA
ANNQTAPTRHLLAATTDVNISTGGMVVILEQSRPVTAVIPGGFFQLRLANHADAVVLSYDTNTTIMWSAIRNLTSLD
NIVAINSVRREGAFLARVWNVTFAISAFNNVPDLVVETNDRTPDGVEMGMEVHIANPPVTGTFWVAFGENCGSVEID
VTDVSEAIRLKLASLPGMAAPQSVVVSGGPAYGYTYTVYFDPIGNPGDQPMLKVTDISGLSGIGTNASVVTDENGST
DAFYAPIPTEFLRLAVSQPGTINLQVNGVPSACADASGICGFLYSDAATPRITGVSPTRLAFSSQASLPLTITGSGF
SSGPSVEVRVGTALCNVTSVTATQITCNVPDTAPAGVRLVSVNVAGLGFADGAPNVTLETLYVTGSAPSPAVLSSAG
ISVLNFTGKGFDYVDCANNKFSIANVSCGVVACGAHFLTVAYHGNGNADVASASVTAEVYEDSSMIDWDIPPSVQVQ
ISQSASSISGLSPAHMPGCGGVITLSLTGTTASQVTSVFMVPFISSLLNYTEASAAYKARVACTDVTAGSSGTLTCT
SPALRSGRYHTLAVLSSGLQLLSTDTILFELFITSVSPNLGSIGGGTLVTITGSGFSPLVAANAVFMAVPVSSTFLN
GIIECVTESVTTTTLTCRTLPSMAANADADDPRATKVMPVATQPRLVSVVICDPGYNTTILRGYCWSLPETPRANCA
ASDASLCSFGYVGGALGYVHCLSPGNFLLSQAISPSRGYYGHSVNLGGTYMDDVINVQFLQDNVVKGLGSRVSAGYS
GVTCLVPDLLPGVYKVLLLKANGEMSVDPYKIGIFTYHAVIAGLENDVGSLTGGLQLGITVGGAGLAESAGDNQVTI
AGLTCPILSKENRTHLTCQAPGINGYVYAEYWNLGYNTNSMPDIISFTDPLVARLEKGLDLAWNGSSPVPGTIQSDY
FAARFTFYYQIAQTENVTFRLDSDDESRLYVDEQLIGSCWNNLNVSLAAGVHKIVTTFVEYSGWATVKLRVMLTRAD
GTMGPLSTVEWQKVTPVPPGEALPVELTVSGVSSDKWCPTSTKILQLPGQPALSWEPPQVTVPYGTCGYIYSTYRTP
SLSILSGITYSGIMQPTFNSTNTTLSIFGNFLTDPSLPAASQISVTLANRLCSIVGVAPVTVNGTNTTNINCTAPAL
PAGQWPVSIMVEGLGLTRPTATAATDLPIVTYTVRVLNYTFNNPNSKCYFSLFGGGTFNITGYGFVKDLVDTTLQVN
MTAAWETVFCGSGFSNSPPCQAVNSSRLNLVPLASDGFTASFGLTRFKVPNPEAFAGNNSISGVNLRYKPRVYYTSN
GTYAATAGAELLYFCGGRTPALSGVTPLSAPPDAGATLSLTWYLSGVGTLNNIITAPAAKNGASNATVEFEVGPTVL
PCRNPVVTDFNITSTTYREAISCTMPAYMPAATYTLWLCIQPFGCGLLPAYTVPLTITAMSATTGGSAGGITVVITG
KGFDTNTTLVSVRFGNSTCKVISSATTSLTCVTGPLDTKPVSPVTWPLSITPTAGTNETTLSSFTFTFDPALESTVS
GIVPARGSTEGGTPVTITGADFRTGVSTTVSIGDVACQSVVVVNSTAITCTTGKPPNSILRVPLPVTVLQQGRGYAR
SSAQYQYIDVWSRNSTWGGGPLPGYEDSVVIPAGVTVLLDISPPKLYVIVLEGNLVFDDTKDYINLQAHYIIVKGGN
FTIGSAEKPYPGRANITMHGMPNSLDLPQYGAKALGVRTGIVSWYGQPKIPHYTKLSQTANPGDTSIVVNGAINWQV
GDRIVIASSSFLPYEVDESTITALDNTTVPSCTVISLDVPLKYTHIGEIHSQEDVARPLDMRAEVAVLTRNIVLQGD
YTSAKYQYGVQVMVNTPSYLPKGVVRFDNIEITQSGQAFRLGRYSMHWHLMGDMAWQTWVRGCAIHHTYNRAITVHG
THRAIIQNVTAYHTMGHTFFLEDGIESSNLIEGTLAIYVRVSDALLNTDTTPGAFWITNPNNTVRNNVAVGSHGYGF
WYRMLDYPDGPSATTTICPKFTPLLEFTNNTAHSNQFYGLRIHPEFYPRNVPCTGFSGIFQQVPAVFNGLVAYKNGV
KGVVAGQVGLVQFVNMVLGDNGGGPKQHSISGKDNGASMEISWVVDDRNRYDVKLAEMAGFQNCTIYAQTSTGMQGT
AGQWPSSRRITAIISQSPVQGDSKHSALMSMINMTYVDFTADKPGFSVLEACGKCKTYQGGATTFTANTTFIQSDGT
KPVLSLWSWGHQGIFLDTDGTMLNAKSLPDSLLPDPFGNWTLGPGATWHSAVENDLFDPKECVYVRDMFTSNNGAYC
SPALTFRRVMLHNHGPEALKYKNLNVVSLATNRTSVVHFSGYNEDGYQFTTATNRNYWLTWDMVLYRLDPEYYQLHK
MDLMNGSDFVYYSSKYIQVKDHFTVNNALSNRTAVPLALPNTTHGDSFYNKTFNQNSWWWSNRTYNDTKFTIFLNGR
IDGGLYVQSYTCPDTGCTDLPEVVVDVRDGTLYWSNTSTWNTSAVNNFRKPVAGDNVTIPYGWDLVIDESPPPLLAL
TVQGNLRFDTTRDINLTATYIIVMGQGVLSAGSATEPHPAKATIRLKGARDTPDYGIDNNLNLGSKVLAALSGGTIN
LYGRQVAKRWLRLGSRASPGDITITMSDPNHGWSVGDKILISSTSFSWKQSEIRTIASVLKNGSQLLLDSPLAHPHG
ARVKAYPGGPTVDMRAEVGLVSSNVLITADDGESTHSYNGEMFGARVVVSGNSTGRFDNIAMEYCGQGGLSNRSCIF
YDRMASVKLFNKDNITNTTSQVTVPNPSFLKNSALVWGMQSNVRIGGEPEQADPVSITNNVMYEALDIHSVEVYTTQ
NIIRGNLVIGTIKDMSGKSVFDIAMPSSFFISNASNWVTDNVAAGAERYGYTYFGIPCGANFTTGVFRNNTAHGCLA
GLWLQASSDSSREGCTVLRNFTTYMNWDFGIISTRGITTNVVLEDVNVLDNKHAGIHLMYKGGLTDNGAMWWYGGLI
AGQSSDDVCSVCTTLSDPGCHPKMSIQSFNQYEPFSPAVGLQASQFAINFYDGPEKKPWDKAKSYAIVHGMFNITGV
TLADFLGPGGCGGAQTGTYALANQPKAPETFYPHFFSNMNVVNVTKGPSQGMFYHTGPDPGWRGELCGEANYTREDG
SVIMLTCAGPNHVHWRDLDGSLLGDVGSVSGYFQDGPRVFPMEQGTPVVPGPCTYDPILRSYGCVQGSSSFLLDAPM
KPKPIPADGIWGDPQYFVLESRDKDSEDRNFGPVFFNVSGSIDLVTVQMDHGCCFTYKCQKRLSTFATYVPSGQTVY
INFTGTPSQIFRLWLPYADPDKEIVVVINYLYTMNRRFVVLPGGVSNVTGRIPPEASAPTIGDGKGHGAYYWDQFNT
LMHVKMTGGKHLEIRTENAVQISQSFALTVDQFYDIKELFLKNLAYAMNISMDRIYIVKVVPGTVTATIAIAPAVQD
VSELTIADALDAGDPLPTVLPPAEPPVVLTDEDKINLASMGDNAPQNLTWSTLLVLRQLWALFDTYTSVVNSVAQGT
PGLEGVVLGAPTAYIPSLETLEQMFPNTTIPSSTPSSPPPSSPSQGEVIPVENSGNAGSTGSNTGSNTGSTGGSGAG
AGSNNGDDKAGMIAGAVLGGVFGACLIAGAVMFAIIAAQRRKAKRLRPVSPARSTGAQPESAALPELPPRRNSSQGG
GSQSGVREAFTAGHSTGPGAVSERERRPTVSAELDAERPMSARAVAHISALNAPLPSAGSATCSLAAGYVAPSEPEV
IRPLVSANKQPHTSSPVVLPTFPQLGSPKTANTAGDGKPDRRSSMGESVTKTPAELQRLDSLTRNSAPHKWESDMAP
PVVMGRLSQSITAVRPASGAHPGSGLPETSPLDSSRTNAARPGSARPGFTLLVSDVEPSGVTIAAEQAMPFEADTQV
VAGMETPVVRALETQESWRPKSRAATGSRPPSGGVPHAAAPEDQAPVQGVGGSTADLSPGFPISPPQEVYRRTSSPR
QPTMMLPGGMMEFSASPSLLRSEVAAMIVAAGSNQGLDSVAAAVMQEDQQQGSPHAARTPAGLRSDSMRWQGGEQS

>Vocar20011249m|PACid:23124633
MGVKGLWNLLEPVGRRVNIEAIANKKLAVDASIWLFQFMKAMRDEKGDMMRNAHLLGFFRRICRLLFHRVRPVFVFD
GATPALKRHTNIARRRRREAQQGVLRKTAEKLLIAQLKKQALHAAQAQQLQRAGRQGQGRAEKGPPGGKPPGGPAVA
PAAPGGAAAAAAAAGGGPAAAAAGPASSAPGKAAAAGPGPAAVTNSVPEIPRKRSRVPSGSGAGAGPSGSGAGPPASA
GAGPGSGGVDVIDVEDEAVMLRLQLGKVPMTREEEAAWLGEKRGVQRNHPAAREGRPRPPAAAAADGADDESDSDRD
NGDEDEDYEERMAADEALARELHRQWNGAEERQGAQHQRQRRTHPATVADRGVGSGGGGAYGSAEDWAGSGDASAAA
AVVSEADAAAIAAALSEDEGVEELDVELLDPTAGMTYDPNANGYRQPHSHQPTRGNRAAAAITTTTATTTRGPAIPD
PAAADGGNDDGDDDDDDDAAAADLLDCMMALPEDLSTLDPAVLSTLPTSLQLDILEKMRDAQMAANREKFQVAARVR
PESFCEVQMCTYLRASAFRQQLEAYKQNLGGGGGQGTEGVQVRKLMNEEGREFILAREPDQPADGRHQQKRGAGGGG
LFGGSRRGGGAGGGGGKVDQLAMAAAARAAQRNVGMPLKLNVAIPLAAFKGVPTVSEPAAVEELPPPPPPAVSAGST
APAPPGAAQVPAAAAAVAMEGVLVRGAVLELQLSDAEAASSGSAQGMFGSSSTDDDDVGVSATASGAHVAANPGAAS
LAATGVASATAAAVAAAAPGGAAATGLDDVEWEDVEAEAAAPSVAGASARGLITASATVALTGARPRDWRERMAARQ
KLWSTSHGFRMGRKLADWGQGDQGGTDGQRRHRTSGGGRGGGTTRGSAERRRQLRRQELVAAAAGGGRGSGVDAAAA
AAAGEEEEEQYDGDGDVEAEEEDAAVQEAIRQSLQHQRQQLQPQQLQPQQLQSQSAVAPLNGGDEDEFEWEDVVEAS
PLQAAVPSAAASGPAQEAAAAAVAVGVSAPPSRATQGSFLGALGLSRLARSQPAGGDGDGGDPRPLAPRRGLFAAAG
AAAAVVEQPNARGSDGGDTRPAVAAAAARQPPAAVAASPDSVSDGDIIWEAADADLDELEAAMRVASAASNAPAAAT
AAAAAVAAGRAVSPPPHGSQVLADGADGISTTGGKKTSRVAAARRLLDLPPARPSQPPPAATAATGIAAATAGTAAT
AATAAGSGPAVAALGSGERSLKPQAVFPGGGDLGQLGGEASGKVCSEAVAAAATAAVGVGSSTRSLISQINSSSARR
LQELSSGAGLSGGSAGGPSPAAAGGAAEAAAAAASASGGSGGGGVEGLRHAGDAAEDIDDFDFGFVGLVSLSAPAAA
TAIPTAAAAVAVPPQLTCTVTAVTATAGARTAAAAAAAAAAGVAGAGAGAAAGALDVEPPGPAATTASADMEMDFDF
GFVEAEEMLLPTTAQPPPSAGPAPKPPSPPPPSRQEPLLPPAQPPPPPPPPPPPPPQQQQQQEQRQEATQPSQERD
AVAAASTAAPAADPGVLESRVPDLDPVRAVLGRDLDLHLDPDVEDADADAGEEAADDADWFDEDPDFDPERDLRHPA
VQQVRQARETAQPPSSPLRNQPVQMAVAPGSTASDALAPNPGPAAAAFSATVDSVPYHAFLPPPLPPGTWVARMPSA
VPMPQLPAAPPLPRAGPYGETVAAPLRAAAVPPARGTFGGGGGGGGSGGGGGSVAGNAAAAVEGPTLDEMLEEVEAE
EQELRSAQAEAQCAFLEVSGLVDGVVTDDNDVFLFGARHVYRHIFENKKKWKGRVRWGGVSATHDRMFPEPHGEVVP
LHRHTLMHNPARDGQPDDTPAQREFKRQHRAVRKNWQLPASFPSPPVLEAYLSPRLDSNTAKFTFGRPDLQLLRQFC
SDKFGWTPDKVDELLLPVLKEFDNRQAQLRMEQFLSFSTRFAKIRSKRLQSAVAKITGQAPGEELVLGDAVQLTPSR
RKHDVAAAVTAATEPALGPEGGLAAEMTAGDAAVPTAAAAAAAAAVAASRDGDEAGGGGSGGRQAGGHSSRGRGRGR
GGGRGRRRARDDGEAAAAATATVLPGDAGAVPWGEEKQAEGRQPASKRRRKKAVAGAPGVPGSVRVDAVEVVDLANG
DAAAAAAVATGVPASRQSAGPAGLERVRRPLGGQEDSRGACTAVAADAIAGSRARRRAAAAAAARIAGVDIRGAGVQ
LDSDGGDNDGDNGDNGDEGAPGQDSEWEVGGLEDGNSD

>Vocar20011722m|PACid:23124666
MRMTNPASVLRLLAAGVLIMTVLATGAASSKAHPAPSHSKPSHRGLMTTDSNAGFDLLEMEADPAVTRQLLAAKKAP
KKRSPPPKKQPPPSVTRGLLESSESVSGSVFESEAEATIERQLLAAKKAHKKHSPPPKKQPPPSVTRGLLEDDSVFQ
IEAEASVRRQALAAKKAPKKRSPPPKKQPPPSVTRGLLESSESVSDSVFDFEVEAAAKRHLLAVKPPVGKVQPPLTG
KTHPPPVGKVQPPLTGKTHPPPVGKVQPPPLKA

>Vocar20011677m|PACid:23124682
MDFHLPALTRYLAETKVVSPRTAQGPTEEGGQRDALQPRTSEVTRVLSDHLATTIPPTRLPKAVEAYYLGPGARGYH
PIDLDAQYLVPFNKALPQSQSHSPSRSPPRSPPRSSPLLASPELDPDADHAVRPLGRTSMPLALGPPRHSSLTSGQV
ACIVTAAVNTAPLDWSFLFGSPPPPSPQHPGARTSSLSAAIATKLHPVKLGSSTSIGDVAAAATAATSTAATAGTGT
SPTGRKSSLLVRKQPRTSDGLFGRRVNPSVAAMPPTPEAKFCSAVMNVMERLAATQSIQTGRYRSLRLQAAEHREAI
ISTVTMPPPPKTGAFSSKAAANRLATVLQRSGASLDDMQAIVRQIQQLKGLSDDLRNAVAQQFQLFSYPPGSYLYEQ
NCACDEAFVLISGTVMLHAEGRPPHRLQAGTVLGMELMLRRQTVPLTVTTDPHFEAQLAVLSWHSYEATVRRWAADK
VVALMPELQASQVARLKAGLMRDISYAQRQPGEALYVGGQPTEHLVLLVQGQVLMAAPLEANTLVTQAQMVSQLRVM
IGPAASSMVLNEMPRAALQTRGGLRGGLKGAQRPSFGPSGSSVRLSGDSGGDSAPAAAAGGSIATVISVESGVGGSG
RRRRGSDSGLELASKKGVTVSPWSQPGEEGEGSDGGAGGSLKDGGRSGGGRVQQQRHVSLVRALRNGNSRNARHDAG
EAAGARAEDAAGGSGKAASAVDAATAAADGGDSASEAATPPPGPASEDSEPSVGSTGREDAAVDGGAAADATGDADI
AAAAAAVDGGDMDLDPEAERESAKESTAAAASRLSRVSLRFADAADAASTVQTEAGGDVGVEAFQTEASPADAEASG
PTTAGVPAAGPHPKSLHPQPEDLVPEPSMLPAVNSFDLAADSLHGSLHMHEALSPVKALLSGSTSVDVIAAEASFSN
GAAAAAIATAKTADGFAFSEDTANGVSAEYGNEPAVLWTGTPAAADAQGQADAADAGQGGSDVAAQAAAAAAPRGEG
SGDSVGSAAAAPGPENSKGDADAVTAAAATDTGAAPGSADTQRSLPPKSGLRSKIGGVSSHLQHAVSRNGSRGASPF
ASGASSPRPVTLSRGDSLNKIATVGSSRGANGSRGDESTSSSPLRNSQRGLSSLASAEGKIRPVSQGALSESSVRQR
RPSSPTSEAPSIDERRPESTTLLVHPILPPPAPPPPSSKPRLTDSDDDGEAAAAATAAGGGGKRTPITVRGSGGGRR
RSSEAADADMARASTEGQPPTPPPQSPPPAAAPPLHQPRQQPSRMSTTAGPRATAAADSDSNVDYSVACVSGTALP
PTGAANRKAGARSGRNAAPERANLHPWPRRGGGGVGSRKQRESELLVSPGASVSPGGGGPGGAITGASQPLANALAS

QCKLHQHRVVAVYGAPVVFCEEALPETKEPDALKPSKPLTAHSAVALQRCEYILIPAALLRSLLPAEALPQLAEAAV
AAQRRLVTASGHVEDTGPVTWPAAADKIRAILSTSTPGHRQPWQVELLAQAFSHLPAFSRLTWQVRLRVFQDLEYRK
LEPGEHLDLAKLEVTELPPRASEISYRKSSATAEGAPDARSRSSGDSAVADGSTPPDDAATAAPPEARSLDRAEAEA
GVEAADAAVALNADTGVAAVVRRPTVGMPRPSEAPSSLPSRSPSYTGDMPEPPPYDPAATSTAGWNSVMGSAAGKPG
SRGSNVTGAATDDGAKVPDLQQLPPTPPTPASAPSLTLLPPSTTGGKKSWAEKYGSRRAIGAAGASGDATQAGRDDA
EAEARAEVEQAEADGVAEAAAAGESEERAHAVSMTQLPEVPIAASADKFPAVDGGLDLDATGGLAETSSEPSAGTLP
REASAAVYTASPSCRSPTFAGTSSRSASEPGDVGLAGPAAGNNTEVLEPYTSRRLSNAPGVPHPVEALFNPCEEEPL
IPSLALRRRESAADDSKSGDGDDKGSPFATARAAAMATTSGQPTADAVAAAKGLHGSHDSSADGGGGGGIEFPVLPD
GPINSPKLLPRAASSNASEQQRGDTAETVGTMDSEPPSTSPYSTNRRQRGGTAETVGTMDSEPLSYSASSILPSTPQ
GDSFVAMSVLAMPTEGTARGGNTVAPPSKERLTAIAPRRSGTQPRREAVVEKLDDRTYWCLSGTVVLEAMLLPEESD
SAERSSSSLRSDNDRRHSQSRGHRHSLSHGHDPSHGHGHGHGHGHGHGHGHGHGQGQAQDQGHNKNLDKTHEKDH
DEREDHPAQPRQLQVANPGDAFSGVAMAEAAAAAFSSSPAAGGQQQQQSAVEPPPQELQQLLQVLPLGSSVLRARV
VAAAEAGPPAAAGAAAGTAAGAEVLCMSRTMFELLSNGIGRSVPPGVLPVLARHVDWLMGVDLQALATDLTGHYRLE
EHKQGTELLRAGEAAPVVFLVASGEYTLVVSSSSAKVISLDMPALRTWLLTSPLGRAAAADVVAAVAEVDIALYGRA
VKQIAVQVLSEKLELSLFNAPMKKILRRVMDDDGPELEAILERHHERERQQQQQQDAKEGGKGGKEGGKGGSSASG
AAASTAKRPRDVVLEGWNQVEAIAASLARPYDPQAQSWELIAQARYNASRVNHSRYLDSLLAASQLTTQEDRSGHPT
GDQRIAMPAHGLEAEAMADPGPVRQARQFDVRTVPKDLRGSEVETSRLSWDCSYVPLHELTTMMSAQVHVKQAVALQ
LLRPVNHPSQQLVQPQPPHHRGEQHDQKHESASSPPQQQLRPPGTSAVIGASNCLLANKAAATTSIVRQHGSGAVTS
ISTSLSTAISGGSPSLASPLRASVTLIPTAPWDAKGRLLRARGSVTADDWRLTTSPSRLARERAAAQSPSAWSMAPL
GALAGPSPSNLSLGASCNSLVLLDPEIRAQPDTAPLPEEMPSMTTAAASPRNPHGYRDSAAVVGAPAAAAAAEAAPL
QERVHRLAATASMESPFNNRTPHLDLIPEDRSYMPLMQSLDLGSSPRAPPTARALPPLPHPHDSGALFRRAEAVDDP
ARSFWVRDKLHDPQGLAESTMTDAAARAVGQAAAAAAAANSAHGTGSPRPDEVADAARRSVGANVLMAAEARTGLTS
GNRSLRLQPLRRKASRQGPGGSAAVAAAAAAAVAAAAAAASGTARGGSAGGLMDRSESRGTAAEVAAAEVRSTAPVN
PEMLYVTLELPEAPKILPEFPSPAAQREAAEAAEAAERTAREARMAAAIAHPSTRPLLIRPDMKYVPQYLRQRQRAP
VIPLPSRPLPEGASENWVVPSYNSEVGFQDGFYDENSQVIYFD

>Vocar20011717m|PACid:23124692
MLALRLTLLAFLAGLLYRAVGQPVINRVDPSVGSLAGGTRLVIYGNNFGDMYTTSSSVYVGPYPCNLISHYSSSEVL
TCETSPGSAALKAVLPRAGPPGADVFIYGDRTWYMAYRDCIESNWGDTSCVGSILFGDYLCRQDPADQDSPIIMTTN
QRLGGYLYRIGCTMPAQDSNNTQPALGTAAKLNATIHFEASMRGGAPTALPSSFLYDVDSVPYQFQLYPEVTGVSPA
TGSNAGGTLVKVTGRGFPDLMLDLNDTIAISIAGVPCDIVNSTYDTLFCVTGPKPAATASPIGGLYPAMRGIEYEFY
NGSRLSYGNLWQLNNTIKVQALNGSYRAVLTGSWEGNEYSTPYYCSRSKAFFTAPRAGNYSFYMSADDYAQLNGTWK
QSNGALVSTTLINLQSWTYIDNFFQYASQRSPEVTLSAGQSILLEMNHCNGPSVGAPQLAVRMPVPEPRLNSLSEIQ
KISISATFFPRSVAIKYLYGAGAAMTAFNITISALNDTALDDPQIGLYVQINGGWDIVLPLGATTGAMDDLVEASLG
GNFTKNDTLFGIRKTRAYGTLTLQMAALDGSMGSFDVTAARLVLLPPPPPPMANTCPSNSSTAINVTSIYPPPAPEA
LQSVGTGFAVVVSRAQAVPAVVPTGDFLLGLYGSVNTYNVSATSNSTAMVAAIQNVTGYNSYYYSVNVADTREGAYF
ARIWNITFTYSWLDNVPALVVGPSSTTPPGALLAMESRSASPRLQGSFKVAFGSSCASVKINLGDSSETIKAALGSL
PGLAPPKQVDVSGDGTSGYVYTITFDPINNPGDQPELRIADASDLQGVNPTGSITTVWNGTTDAFYAPIPTEFLRLA
VSQPGTINLQVNGVPSACADASGICGFLYSDAATPRITGVSPTRLAFSSQASLPLTITGSGFSSGPSVEVRVGTALC
NVTSVTATQIICNVPDTAPAGVRLVSVNVAGLGFADGAPNVTLETLYVTGSAPSPAVLSSAGISVLNFTGKGFDYVD
CANNKLTIGGVRCSIIKCQPNMLTVIYPGNNGANAAAASVTAEVYEKGQLIDSDTPSSLTVSVSSSAPSISTVSPAR
LPGAGGVITWSLTGATASQVISASMVPFISSFTNYTEASVAFQEPIPCTDVTAGSSGTLTCISPALRNGRYHILAVL
SSGLQLLSSDTILFELFITSVSPNLGSIGGGTTLTITGSGFSQVVSENVVFITVPVSTTFLNGVVQCIATSVTSTSL
TCKTLPNLAADASADDPFAKQVMPVDTTPGRAVSVVICDSSYNSSFLQEYCWSLPETARARCDATGSATCNFGYSTE
LTPAVDYLYPSSGSLGTRITVVGSYLDTVTEVQFLQGGVLKGTGSNVDTTSMYVFCDAPDLPPGVYTILLLKANGEK
SVDPYGKGTFTFTASIDSVTSNVGSLAGGLPLVLSVGGAGLAVGPDNLTANAITIADLPCPILSVKDRTEVTCRAPG
INGYVSAEYWNLGVSTFSMPDIFSFTKPRISRIEKGTSFSWGTKSPEPGVINLDYWAARFTFYYQISVTENVTFYMY
ADDDARLYIDDVLLGGRYTTLTRIRPSAGIHTRHALNLGSYPTRIPLVSRRQPDGSRGPFANLEWQKVTPVPPGTPL
PVKLTVNGIDSVTQCPVTNLTVVLPGQPKAPFEPSWVQTPNTTCSYVYSICRTPSLSILSGITLSGIMQPTFNSTNT
TLSIFGDFLTDPSLPAASQISVTLANQLCSIVGVAPVTVNGTNTTNINCTAPALPAGQWPVSIMVEGLGLTRPTATA
ATDLPIVTYTVRVLNYTFNNPNSKCYFSLFGGGTFNITGYGFVKDLVDTTLQVNMTAAWETVFCGSGFSNSPCQAV
NSSRLNLVPLASDGFTASFGLTRFKVPNPEAFAGNNSISGVNLRYKPRVYYTSNGTYAATAGAELLYFCGGRTPALS
GVTPLSAPPDAGATLSLTWYLSGVGTLNNTITAPAAKNGASNATVEFEVGPTVLPCRNPVVTDFNITSTTYREAISC
TMPAYMPAATYTLWLCIQPFGCGLLPAYTVPLTITAMSATTGGSAGGITVVITGKGFDTNTTLVSVRFGNSTCKVIS
STATSLTCVTGPLDTKPVSPVTWPLSITPTAGTNETTLSSFTFTFDPALESTVSGIVPARGSTEGGTPVTITGADFR
TGVSTTVSIGDVACQSVVVVNSTAITCTTGKPPNSILRVPLPVTVLQQGRGYARSSAQYQYIDVWSRNSTWGGGPLP
GYEDSVVIPAGVTVLLDISPPKLFIIVLEGNLVFDDTKDYINLQAHYIIVKGGNFTIGSAEKPYPGRANITMHGAPN

SRDLPMYGAKALAVRQGVVTFFGQPKIPHYTKLNRTADPGDTSIVVNGAINWQVGDRIVIASSSFYGSEVDEATITA
LDNRTVSGCTVITLDTPLQYVHLGELHSVQGTKPLDMRAEVAVLTRNILLQGDYTSAKNMYGVQVMVNSPKYLPRAL
VRFDNIEITQSGQAFRLGRYSMHWHMHGDVAYQSWLRGCAIHHTYNRATTIHGTHRAILQNNVAYNTMGHTFFLEDG
IESGNLIEGNLAIHVKVSDALLNTDTTPAAFWITNPNNTVRNNVAAGSAAYGYWYRMLDNPEGPSYTTTICPKFTPL
LEFTNNTAHSSMFYGLRIHPEFYPRNIPCNGFSGTFEQVPAVFNGLVAYKNGMKGAVGTQVGLVQLVNVITGDNGGG
PKQHVVNGKDHGGDLELSWVVDDRSRVGLQITEMAGIVNATIYARTSVGKRGNAGQWPSCRRVAGIITQSPVLGDSK
HSALMSLINVTFVDFKGWDDCTLFSMLEACGKCKTFQGGSTTFTANISIITSDNSSSMMSYWSWGHQGVYLDTDGTL
LNNKSLPASLLPNVGTNVTLGPGATWHSSVESELFDPNECKYVRNTRTSNNGAYCSPGLSFRRVMLHGHTPESIMFK
DLILTSLATNRTSLVHFTKYNENGYQFTVATTRDYWVQWDMPFRFDPDTYQLHKMDLMNTSDYVYITTKHIQVKDHF
TINNNDQWNNLTAMPPPTNSTHGMFYYNKTKAPNSWWWSNRTYNDTKFTVFLDGRLDTTLDMKAFTCPNTGCSNVPD
AVVDIRDGTLYWSNTSTWNTSAVNNFRKPVAGDNVTIPYGWDLVIDESPPPLLALTVQGNLRFDTTRDINLTATYII
VMGQGVLSAGSATEPHPAKATIRLNGARDTPDYGIDNNLNLGSKVLAALSGGTINLYGKPARKRWIKLGSAAQIGNN
AINVSDPNHGWSVGDKILITSTSFNWNQSEFRIITGIRNNGSQLLLDSPLAHPHGARVKAYPGGPTVDMRAEVGLVS
SNVLITADDGESTHSYDGEMFGARVVVSGNSTGRFDSMGMAYCGQAGFDDRACIYFDRMAPVTVLRTDNVTNMTTLS
AIPNPSFVKGSVLLYGMASNVLINGKPSVANPVTLADNIMYEAYDTHSVDVYTTGNIIRGNLVIGTIKDMTGKSHFD
IMMPSSFSILYASNWVTDNVAAGSERYGFAYYGLPCGNFTNGSFRNNTAHSSLAGLWFQASPEALIQGCAQLTNFTT
YMNWDFGIISTRGVSTDIVLKDVNILDNKHAGIHLMRYGEMTDKGWMRWDGGLLVGQSSNDVCSACTKRSDPGCHPK
LSTQSFNQYDPFTPAVGLQSSQFALGFAVGPEKKPYDKPMGYSLIHGMFNISGITLADFLGPAGCGGSQSGTYALAN
QPKAPEAFYPHYFSKINVQNVGIGVSQGMFYHTPPDPDWRNEADCGEAAYTRPDGSQIMLNCAGPAHAYWRDLDGSL
TGTVSTTAGIFTSKRVFPMDQGSPVLPGACTYSAYQDSYQCVVNSTSYIAETGLDPRYKPNPVPADGIWGDPQMLVL
ESRDKDTEDRNFGPVFFNVSGSIDLVTAAMDHGWCFAYTCQKRLSTFWTYVPTGQTVYINFTGTPSQNFRIWFPYAD
PDKEIVLVINMLYTLNRRFTWLPPGNGNTTGRVMPWDKPIRIGDGTPHGSYFWDQDNTLLYVKIKGGMTVETRTESA
VMVSQSFAVTIDNFYAQQAVFLKNLAAVMGIDMNRLYVAKIVAGSVSATIGVEPDKSIPVDALPENSVSAAVDPTDP
VVTNPAAAAVTSTTSSSTSPPPPPPLTSSSAVLADLTAAYNAFVAAVTDPNASSNLGFQPIGVPTADWSNVGVAASP
PPPLMSSGANSPSGGVTLSPPPPLILPNTEAAPAGGGSGGSSTGSGGSSTGSGGSSIGSGGSSSNTGSGSNNRGWAL
SSAAVVGIAVGMVGGCIFVAAVVAVIVVRRHAKRPRPVLPRGSLSTGEVSSSMSPAPAAGRPGSANTRQAFVLTNPA
YETPGERAAALRVSRTAVPVQESEENPTSPRASVLAAGPTPLSPAQFIGPLTSTARMPHSSLPLVPPGAQSGAVDAV
EGEAMLAPDASVSVAAAASGAIATTASGSVGGSRSPAQLTPGQPTTRRPSDTTMKYAPNMATGSTVWDADSAEIEQL
PDHDQTKAAAAAAGSGPVSPKVAVEAAATSVGAGAPASGTPVETLNRSMGVSESMSPRVKSVGGSNDIRAGAEQMSG
NGDEGRKRAAAMEPLDPQQQRPGSGRQTEQAPVLMLPGGMMEYVATASLLRSEVASLMVAGGEAGPAVTGTGESDSA
NTPKARTPAGLQSDSMRRREHS

>Vocar20008597m|PACid:23124783
MGIKYPSGDNNFPIPYPGLSIPYSMKARTSTRKQDAAKHPGFRSAIWTLQRLLSYSAPLSQTSVTTSDGVDAAAAPS
SSTSARSSTCRRLSAAFAEGRSSWVSALTSALFRSGGGGAAAAGSVNVAPLVYANPVGGINAASPAASRAAAARLLS
MLGSAPVQEILARDPTSGVTPFRRLDRMTTRTNESTAPLESTSPSSLQLLQQQVSAVMVAPATQQDLDLADLTERL
WDHPNAAPALRVPGAETLWPYFDAAIAELSPYNSPQLSLTPSTNLPYADDEPPETPAAAVTAALTLLSRKLPAALEA
ALPSPAQRRTEYRASLSYRQPVLTQRVVAQPSSSRLRVKLIVGLTLGLGGGAVLLAARVILQSRRTLRRLRGTSSGG
DDSSNRRNTATYVVLDIQGLSEPSLPETVREQALRLCHDVVKQLARRYDGELVPLPHDEATPQLSASAAVRHGVGGD
RLPESPAAALQAALAAAMRGSTADQAAAAVAAAAAAAGRNAGWRCATVPDAASDMYCPDGDGDDDGGSNGLIRTDVA
SANPALFLEQFKPATGKEHLSAGHLSDMLLPLPSSATITTKAAAGAAVAANAAAGGGPWATMSLNSEPHLPQSPFTV
QYCTSSGEIPRPLQDSDGASLSGLPGQGLTLGGQVLGHQYLSGAWPSPSVLPTLPEGVTDSGMTTVDRAVVAAAAAV
SGPRSGGGGAAATSGGVSDDLLAAMHAGRILPHAQSTPTTPLLGQLHALSPLRLRPMPYTTLAGASSQLSPAVPPAV
AAAAAPSSQPLSSRGGGALAVAMAAARAAAGGASLASQRQLSTRLALIFASVHDAVCFCVHMQAMLTNCRWPSEVLE
LSSCQPIAVRPRAVSADVQQQACGAGGSPRADARKGGVDGVDGAAAAAGAMSTGVSGMAGCTDPGGDGVVMASTASS
FITSATPSPFTPTGAVDRMKSLISGGAKGRSGRALRQGHLSPRLAVHGLGGGAAAAAAVPVATAAGLAAAPERSALS
VSRFATASLGPGTSAGGCGTVARPFTSRSLMPYTSFGGSAHLGSDPWVVGGQISSAGCSRSGSTTVWRPPLLNGSFN
SRGGSGGGRYTAAVPGGTAMMTSGLQYGGGGDFCPGSHIQPPRSALGRVSRSGGGGGGGGGGGVSPAYGSSLARVPH
MGAVSQPLPKLNTVQFSPSFPEQASLCTATSTTAMRFPMPHGCGRTAAIQRSGCVVPDVATTAPPMATGGGARASSV
LTSPPGIGGVSGIGGGTITAAAAAVAAGGGGGGAITGAAAALESGDSGLPGRIGGCSDRDTAMGIILGSLDYDTDGE
AAGLQHVPSPPPYFTIASTAEGKDVDGGAAAMMTAYGGTGTTGDVSDRVVAAQVGSGGGGGDGGANCYPVQAASPAQ
LPGVTYDNVYDKAAGTSPTGGFQARRHSSYGDGSATSPAAPMPPMPQLFIIPAVRNMQQAMPTPEQVDAHAAMPPSW
KSPPDTSSLRSAATPVTCEIGRTYPLYGAPATLKLNTDIDAPPLLSVPPFAAAATVVSDVNAGGGCDVGELSLSLAA
GFRKVPTAEVLPLPPPPPAPPPAPSLAEMAEEMVVGSHGAWIHPPQPWYGRPVAECKEEISSSTNNLNPASTIGSAL
TATSLSGDCRTSRDVTRRAATAVLEAGDGSATGLGPSRMVGAVQDTMYDTAPVVSGGGGGHGLRNPVYDGTNANSLG
SGGGQPSHVVVAAVAPPPLPPAAPPPSRRSVRSFGEQVRLTFEELMGVERSNLEDGVTVIFRGPRVVCGISTWQYGD
EECTDMYGRRRTAIDSGDQHLYATQSNPLFYKTANGVLPALTPSYGGGTDITSDHVDDDVPNPEGAPATSVGGLGSG

ADDGGGGAPDDARVGVVRRRTTIMSPEARKYLRRRRMPAPLRRALAICESGQGGFVLLDGATYSASSQEALQEQCMI
LHMGEYVLAGVRRPLDLYLALDFAHLARVAYLRPLACAEQLSLGLPSAPVNTASICFSLVVGMQTLLAWNTEVAKAA
LAVLRDAVLPALLSHGGYLVEDADGLLLTAFSSSRSGLAWALECQEEAKHLDWPKELLGHDLGKELYMDDLDMEGKE
EEEEDGDRESGNDILPVGLPPAPPVPHVNGGDGGDGSTAAAAAAAPYPRRHLVFRGLRIKSGLDCGDVLARVHATTG
RMTYRGRVMNRAARIAGMATGGQVLCSRGLWDSSGLAAAPDLCQQALAVGASLGDVQLKGLSTKMEIIIIPVAHEDV
VLENSIGPIGTAPFQFHQKPGMQEQQHQQQEQDEDAEDNGADVDVEARDGADAIPPKPGMAPNCAVRTVPSGGGNYP
GTATATAAPCPLDGGGSTPTGAYPGSLASAVPALSEYDSDGSGKECAGGSGGGSIRIGPGAAAGESSAAVVAAAAAV
ESATAGSVAPAAAVVTAAAESVPYVSKALAVARMRHASQLRLNRCVHGSGGGIRGGSGAVQAAVLAAQDEDDDMADR
LVAGYSSDGSGVDSGDGGPGSGSDGGSAAEPDRVRVVVNGTSGGVAALHGMLRRQRPAGSTTTAGLSSAASGGAGQG
NAAGAAVLSAAATAAAAGGGSGGTARGGHLAMAAAANFKKAFRVVKRSILEEPDGGGLASASTAGGYGSDRSSDGDG
GTGAGVSGGVEATKLRRPPPRRASELVKSSAAAVTAAAGAGGARLFNRLRGLAVVRGGGGINLRGRGGGEALSRFDE
LEEDDESRFRPTSSATSAFFPSLSRPRLRDGSGGGLSYTTTERSSSCNPSPLHLVSVAGQASRTVAGGGGGGTGSGD
ATAAGGSGVFARASSRLGGNSLVAEMSLASMTMRRQMPVSRFLKSSASSGGGGGGGGGSGVGGMGTALPPTSPVTLL
STLPMTSSSASTPGEAAAAAQSMSPKQRGPPQLYGRPRSNSGEGARIFRVGGVGTGGEATTYGIQEADGEEEESELE
GAEAAGTGDDTVKISTAGSFTAEAETEATAPGWASTLRRGSETRSFHVSPLRGGGGSASAIGTVPSLPFRLASFTSS
FLQRAVSRKLSPTAAAASAAAVIAPGEDVVRPYSGSTVAGTVSPAGVALDGGAQRSVSFLRARGGGGSSIGDGKQGS
GDGSVSPPPPPPLQPASAQLAVMDDSRNGGTAVHMPTAAAAAAAMSPPTQTTATHLSRVDSLRRNSSKESSATVHTA
IHEVLQIRPSDGLYGHVFPSPTATTSAVSPSGTASLSGPPPPPSFQPVRLGRSATGLGFGAATEAAGKSVNGQEHRS
TSAGQEGQGGVASPPVSAASRYGNPLTSALEATPPSPRCDGSTPPLEPHMSIYTGDGDGGGDSGISNYGVYGGSSAR
MHQQTVVNAGVVANPADTHAVGRRRAVSHYLMDESVDGGGADGGGLGSPANAAAAAPPTGQRSQRRRSVIEVSGNVL
FLNARSSYADSGWQLPAESETYGCGGSSTMLADQSCDGGAGDAVEPDAMYDVYEFADLLHHDVYSGQLAAAAKASPP
SPPPPLSLAAAVPSAPRRRRSRRSTNLLAGVGSGSGGGNGGGGGETNLRTDVGSRPYSQFRQLSSSFKGGSHDVGGS
NSQQRRSQPVMHSPRPLAHFAAVTAAAATAGDDSGIGNGNGSGSGAAFGQVQRSNTSAAHDLEPGGAVSGLGRLVRG
LNGLIGRANSTTCNGGRRHLSGGSRQVFGSQRRGSVNAASGVMRSSGSATVDLYCDRGSSGDDRLERGTVVVAATAA
AAAATDCGGGSASGEPTTATASGRRLVPELARSARELVSRVWSMRSTHSIGNESDGGGGGGGGGSVSAGATSSSGGVR
RDVAVSPELTAAATAAMPQLACGSLAAEAALAAVMRTIKQER

>Vocar20009914m|PACid:23124821
MPVCPTCHSEPPYQVVLRDRYVRKNIECVGPYLLGQPNQSELLQVTTTSLRSAQARRIRDIDGVLQKYVQLGVSLWA
YLGFLLRLLLMGVNCTAIFFAFITYKSNKSSGWKYEIPTRVVVWAEFIIICCYLLWAIWRLLVLLWAVLRELNSKPD
NLYKNNLEVSTAARNWYNSLMLLDFNLLLLLRYVSPPSVAAALGKARRRVYGSYGTRVHRDQYCLDLCCCCCFYGRL
VLCTLLQWSLVLLLAAAALFGVLIKVLQLRFLGTADIQNWNITELLLFGQFVVNMAWLDTRQKDRRSAKLELLFHGS
DAEQSSEEAEAVKLVEDLALLVLRYHYSLLTAYIYIDDSVEEEKPRNRVKHEHKKKSGAGGGGGESASYGGRGADWG
RHGAINGPYADLPPSALQAGQVVVEMSPRQARRSTTPAATAATPAVRGGGFGGGSSSSNSSGTDSGPSAPPILAPLE
RFPYDFYTYGHAVGAPLRRNMQIPELKERTYERGQGQRPPPSAVLFCCEFTGAFADDAWTEARGKTLPGPASGVGQG
PARRVSGKVRFRSQRCNNRRDRRSKELSPKGKQEISAVPSGSGHTLPVPVAQPQLTLVPGAPERCDYYDCECSPYQL
EYLGTAILNATFSRTCFRMNYIGCDISKPCCTSLLNSVDKVVVETTPQCGNKSNLVSVTVNNATHLSWNPYFHNNTG
DVPGYELKIYNLKRNVTTFPGTRICITTRNTCYSIGDICRDSKTLGGCRYSFAESSSGVFCPVCEPAPPPPFPPSPR
PSPPLSPAPSPPSPVPPAPSPPSPVPPAPSPPSPMPPAPLPPSPAPVIPCTMCAELTILPPDSLPGPLYTFGSGGAP
NCTEMFDLVSGDIISSAALYGAIISSPFTLEVCSTTFDAQLFEYPFIKLCGSFASADSAVKLGDWLEGALARWVDEM
SGGVCVPGYSFNSDVYGIGDVSCLSGSLQKTCDPDSPPPSPSPLHPPSLPPPHSPPPPPPPPPPPPLPPSPIPPSPS
PPSPLPPLCAYVFIEPPKVLGSRPLYTFGSGARSCDDVASYIVQDISYNAAALNVSISKPFSLSTCVTEYDNTVEPP
IVPYVEVCGTFASYDEGARMAVWLDVDSSGILPWVDFTTNGVCLDTNGPFKNYTLYAAIFDPLGEGTCLAGQIEQSC
QSNPDPSPPMPPPSPPVVQPSPAVPPPPQFVRTHDCEQAYNVPYSVSPLYNFSSMEAGVPATTMCVKVSVRSCRAGY
CCPMEFAKIEVPVAQNCRGDLRRIYINNNTVSVSWGELNNIPQLLWQFSLNRCC

>Vocar20009738m|PACid:23124828
PVLTGRGGTSGVMGADGSMPGWRSDSPYIQSGTVGEVWVLRAAPCPLPRPGEDPRATHGRHEGPGRGAAEPTGAAAA
GAATAAAAATAKLAMRPAAPRNGTATNTATNTSGGACRGYGLESSRTWNVAYGSEVDDDDDDEEEEGARVNVEEEAA
LGDMEGFADRKVWDMASLDPPSSSAAAVVAAAAVGTELPNGWCSEVPQEKGRQPRLHTYLDNHHHLTHHHHHHQQQQ
HLSYKSQSYEARRRRQPEGFLGVAEDEGTGGGGGGEGSRRRRRRQNQLDRGRGRRRRHVRQLSQGQMHARQVYAEEY
NGQMYGHGHENLQALLYQADCGTHQVVRISHGHAMEAPDFAPQLILPPLPPPPPQQQQLLHVEQRSSQGRGRPGTGS
EAAAGVCACGINTTSAAAEAAAAATSTLDIHAGGLGNGGAEPADFTRSMSRPANPAHHCQHQHHQQQQQQEMLSLDN
TVLAAPLLGPGLTSVWTASASAAGGGGGGSAGPVVLLMEPDLPRVDVEETFELQHQEHAAVYEQQEQQQQHLEKQQQ
QQHLEKQQQQQQQQQHLQQHPEFHGCYQPPHHQHDPEWQLELARQHPQGEVRQQGCVRGAGIGVATGGCRSPVVRP
HSASANYDHPHWRVTTTTWASTANSPPAHRPSAQTLVMGPTTAGGDWDDDAGGCYTPPEWLRAAAAGGIGGLRCGGS
WGGGDGGGGGGSFVTVRPVASLGSPPHCNSARPPPSMTGVAGAGGGGAVAGVRTGATSAPSTPPPATVVEPFSDAAG

QAFYRSLSGSRRYDLSERRRGISDNYKAALAAAAAAANEGRSRPTTPSGAPGTARPPDSPPLPPMSPAAAAQQQAAA
ATEDLGPHSRSSPPVLHSSPPPPSPPPMSPTLATNTTCGVLPGSISLEAPAEVALLSSENSMPLSADPVTSAADGVF
ANGGGGNSPGGAATADRLFSTPGQSDAPSAVAFITTDVAAVPTAAAAVVEAEEGEQQKSCHSRRVAEALDVRGAA

>Vocar20009379m|PACid:23124835
MSSLFAGLKEKIADKLAEYKGSTAPQDPNEKHVVELVEAATSGELIAPDWNLNLSVVDFINGDVHQNSGRALKAIKR
CIAKPNTKVQLLSLTLLEACVKNCAVEFHAQLATSELWSDVVKIAEDTSITRVAVEVRDKVLGMVEDFSRYLQPKQF
QATYEGLLDRGVDFPIRAPDDSVPFITPPASAAAAVAGPPAVNAATAAAGELPARNGPVGNAEAAAAAADPLAGLDE
QDRAAIQAALADLEAEAEAERIAAEADAAEWRQQRQQHHQAMQLTQQQQAAAEAGAALGLLAAVQSVEAVAAAQAAA
ATTTTTTLRWVAGIERSGNGEGCEPVGVDMRQDAHVIGNAALRERERERERERECVYTANTTTMLLSEMLASVPDSE
PLAVREPYVAEVADTCVRLRSRLERLLETLADEVLLASALNQHEELSRVLQRYDELLEAAEAVAPTVPRNAAAVALS
HAAASRGLLAAAAGHAPGGQRQQQLSQSSDPPPPQQHGWGSATAAAEAGAGSAGVPAGQKVAAVAAALKERFAGAID
KLMAAAAATRASGAQLQGAAGPADAAGFGAASGLTVNDASIPLLGVGGAFTIVEDEEDEAEDGISLVTKRAPQPQML
AAGQAAPPLQQLLNTATAPVPAPPPPPPAPPVEAGPDLINFDDDVPSPPPPPQPAAASPTSLLSTQQQQQESVSGR
GVGDLILFFSSPERTARLKRLPFAEVSNLYRPPDVDLFMINSLPRPLPYKSKLTITWYNYLIHLNAVTGGLDELLAG
LTIHTQSAAVPDVAAASAQSPPPPPAASVSTTTTTTTAGGGGSDRRVSQGGPTGDQLVDALDLLVSQASAAASPEVG
TPLARGP

>Vocar20008314m|PACid:23124874
MASKMLRKDPRTLGAFIGSGTTHPALSFRAGPTWTRIVQPPAASQRDRVPGGLGTYTLSPLAKTIRRDRQDLGEAAA
VDKEGWKPQHGSLSRRYVQSSQSPQLAPRVATRTTAPPAAKQTRRDSLNMYPRDPDRSWSKRPQQVSDKAGRCPPPS
QRPRSAGVSEEKAPPQSPPPSPPPGAVAVADLRSELGCARFGSCSGCSLQDGLAAPPLYGEAVEFFRGLGLTEVPAV
MKAARGWRCRAKLAVRGPVGRPLIGLFREGSHEVVDIVGPISPLPPACRAHHPRINEAAALIKEVRPGTEAAAVAAG
GLLRYLQLTAVPSQPGGRAEDDPLAAVQVVLVLNAPPECEPDDPRKEPKSRARAKAQKPPSVAPLVHSIWLNFQPDA
ARNSVLGEGWRHMAGPAAAWQEFGGVAACVGPGSFVQANYGAMQLVLGEIANMVPYGSRVTELHAGIGVIGLSLATR
RRLAALRCVEVNPAAAEPFRTSLERAMRTTWASTQEQQQQVAGSQPERFLEGCDVLVVDPPRKGLGQSLLAALTRQ
PQSPSPSQSPTRGSVDAISNAHIPSAGAVDPFKSESALSSSSSSSSSSSSSKAAAAVAATVAATHGEALIGATGRG
GPSAAPRVTAAAAGAVPEAAGCSPALPSRLVYLSCGFRALQGDLAVLLSGGWRLRSATAYLFFPGTDSLETLVLLSR
GEGDEADQELEEGQEGLRKVQGKVQGNRTRGGCVWGKECYPA

>Vocar20009331m|PACid:23124955
MLIGTSQWTGLCSLRPPHVAWRDALASSKANNISVSSHHNHNQQAAPRRSTRLSGPSLVPPYHGGGGSSGQGREGGH
SEHDASAEEERSQDDVEDDDERKVGFVDNSPQLQQQPMTSAKPATAAGGAGGGSSGPSRDAGGLSVRRTSARLLNHQ
SQHPAGNATSDSGRSGGEDDDSDGIGDDDHDAEDDRRPRRTSGGVGEAKDGRSGRTGQHRRANGHRDGQEAEEDEDD
EEGIAEEDEEESEGEEEDEEDEDEGVYKGRYHLRGHQVNALRKHPAKRPRYSEEQPGEGPSRRSGGGRGDEDDDDDE
DGDEEEEGEEEDGEEDGEAKRRRDRVRSRSRGGDRSRSVGRRRGKGDGGKGPARRYTLRDRSRMVPMQVQEQQRQRE
ELRQRELMRARGTADELLAGGGSPHKHCCTRPDLARKRFRDRSRGQGASGGKHGGGGGGGGGGGSNRRSGGGWGWR
SGISDDDVPDVDCTSGGNPWRNVHQGIGQCGLWRGGPRSHAPPATPLPGQPITAHGGAQVVPSSAAHMGPGAGAQGA
AAAAAAAVAAATPGPLGGGHGGVGGAAPLAAWEQALLADAAALAAGAGGKEKAGNAEINPVAVDPSVGFDQVGGLDS
YIDALKEMVFLPLVYPELFTRFSVQPPRGVLFYGPPGTGKTLVARALAAHASRYGGRKVSFYMRKGADVLSKWVGEA
ERQLRLLFEEAQRNAPAIIFFDEIDGLAPVRSSRQDQIHNSIVSTLLALMDGLDSRGQVVVIGATNRPDALDGALRR
PGRFDRELLFPLPGLQYTGAAHSMLHVASDGCHPSVLIPACCIGSPPPEACNQPCTATFSPRATRGSGTRKFMHLRI
SCWWSPPPCAWSGRTSWRPLAPSHLPPTAPLLRTRGRCPAAPRSAWRRAWPLSWAASKVLSHQRRSAYRPALPGRAE
LVEPAPPRPSSSRRADGAFGALLARLPSFLVSRPRLLLCGPAACGQAQLAAALLYALEGLPAHAIGLPALLANPGAR
SPEEALVWAFVEARRSAPAILFLPHLQLWWQTATPQLRATLVMLLRDLPPELPLLLLATADVPFGELDRGVRAGVFS
GTEDVVVLQRPMEQQRREMLQEPIMAAAEPVDPALLAAPADVSGSAATAAAEPLPKDPSVAEARAAEARSAEELAAR
RAYEEDQAALRSLRMALRGVTLTLLNERRWKQFSAPVDPAEDPDYWRLVSNPMDLATVLARVDARHYVTIGQYLSDV
ALILQGAREYYVDSVRDLSRATALADEAEHLVKARVPLELAKRCEDILARGGPAPPPRGMELVEELAVARAKVVAAA
RTAAVGTAAAGGGGTGSAGAGGVAATGGGSGPGAGGYATRQRAGGHGGHSDGPCMPMFDDPEALLRKTRLLQQRERE
QQVSGAGAAQACRLSQAHVHAQLAGPRPDDGSAGHDAAVDDGKEATSPPGKAHTAEPKQQQPQTGARRVSDGVQAGP
SGLGGTSAAVAAVSTVAAAGNVDIPARANGGTLDATGTRLQAGEPGETQPAEGQDGAMEVDAGTQAEAAGSQGQLRE
GTPAPARGRVDDDNHNDHDDDDILKDVAGGHASDELRAQPEYPGHQAQGMGSEVALVVRDPQDPVALERAKALLERT
VSTTADWSFEALELLYARAAGVVLEHARTRDRQLVTAALDESLAAAVAAGNGAAVGLPNKLRARN

>Vocar20009581m|PACid:23125060
MSVEAWQDFGQKVDLTARIREILLNYPEGTSILKELVQNADDARATCIKFCLDCRQHGTRSLLSPAMAPFQGPALLA
FNDGVFSDRDLESISRIGDSKKKDEEGKTGRFGVGFNSCYHLTDVPSFVSGRHLVIFDPHCRHLPNISSTNPGKRID

FVQYGDVAAQYADQVAPYAGAFGCTLGAGGGGAVSGGGSGSLGPWSGTLFRFPLRSAELAEASTISKQIYTIESIRT
LLEQLATESFQILLFLKHISRVEIHEWAPDAAAPHILFSCYVSNLSREVAWERGLFARVSRDRPSQQNVDQAASGGG
GIRVPLPEVLASYRLELLREWHDGGGGIGAVVRRPERRSYIISQMRGGGEAAEMAEQLSKLFGAVVAWGAVAADVTT
VAGAAGGGGDAAAAAAAGQLEDGRAFCFLPLPIRTGLPVHVNGYFELSSNRRDIWYGEDMTGSGARRAQWNIQLLLR
VIAPAYVAALTAAAAVLGHGEAYDRLWPAADVAQPWQSLLEEFFRRVADMALCWSTIRGGCWVAPRSGVLRDQVVAA
SPPLAAALVEVAELPLLELPPGVGQLMLRHMWQHLGHYGLRHLRRRLWGRHQQELLLLQLLQLVRKLPKRVAAQLVG
LRLLPLLDGTTATLCAAVPGGKQHPQTQQQSSPQHRGGHPAAPYPSCFLPTAEECTLLARAGGLLVDANELSPTGRS
KLEALAAARVLNFAKLDSLAMAAVVLPQLLPPAWLPARAVGGAAAGGRVVDWEIDPAGQAAGVDREWLQLLWRWLAT
HGSLPCFVGLPLLPILGGRLAPFADAASSISVLPPAEVVQRAAAGAGGRVVSTADGGVGAAADRAAQLAELLTQLGL
QIVDLETFPDLPVSELLRGGHVHRCDGPGILAALQRLSAATHHPQAGRTTQAPVPQPAGGGPPIDGVGEQPASDGNG
RGRPVLSAGLAARVTALAAADKHLLRSILLTEDCLTALTASGPSRSATSAGKPALPQAEARGLLQLLAALPIYESAK
GAGAAATAAAAAGSSGGSGLQAAEPVFMALHGSGGTCFLAPHGVDVRVLGSLHGSIFVVAASEAEGRLMVTYLGVRA
ITAAEAFRHHVLPGLVLLPGELRNATVLDMLRGLPQLSAQDRGFGAWLAQVPFVPNNKVWCIAARRPVWTESRATSH
DVCCNALVTQGELHTPGELYDPRNPDLAALLDPEADFPAASLLLQAAGSASSAATNGGGSTGNGGEASGGAPAAAAA
SSTDEDGAGDASLVLYMLQQLGLRTAATLDTLLQAARFVERIADAAAAAAVAATTATAAAADAPERPSAPQAPPVAA
STTSAAAEDSDMAVARGKALLAYLEAEAGRIMGPVGASPAPAMSASVGAAGTTGAAGAGSGGGSGLGTGGGARRFAA
GLFSKARELFGQGTQEQPAVAEVHNFWQELARIRWCPVLQDPPAPGLPWPARHAVPRLAAPRTIRPPSDMWLCSSCM
FLVDGECRSSALAAGLGWSGRLGGSVLAQQLLQLGEMHTQVTDPALSQVLAGVVPLLYRSLAELGLHEAPVARGLLY
GSRCVWVGNGFAPAGKVAFKGSLDLSPWLYVMPAELAPFKDLLLSYGAADGFSAVQYCSVLQDMAEATGAVGSASVS
AATAAAAASSSPALPPPPPPPQPLMEVQLGQVVAVAQALADLTLPAGAIIYLPDERGVLARAGDLAFNDAPWLAGQP
SAASVRLVHPRISAHVAARLGTPSLRRLLLAASADCMALGTVGGAEAFGQSEALTTRLRHIIADYPEGPGVLMELLQ
NADDAGATSLELLLDDTTYPASSILSPAMAVWQGPALLVANDAVFSPADFANISRIGQDSKASRPTSIGRFGLGFNA
VYHFTDLPCFVSGDYLVMFDPHAKYLPGVSAAQPGLKIAFARAQLLQQFPDAFTPFTHLGCNMQERFMGTLFRFPLR
GPAAAAASDIKTTPCSPEDVAALLESFRRQLPAALLFLKNIRLVTAYYRSAPSPPAADSGGEACLQLLFRASREMVP
PAGAAETASLAAGGDGGAEAAALQLSVSELSGAGEQNGLLQSAITRFIAGSASDPSDLASFYKRLANTSATGLPSEM
GLMQLQMETNLTLPPGGIAAVASGGGGAASATSASSAAEASSKPGHRAVPGMVTERWLVCNALGGGGARDMALKSFR
NHSTKMVPWVGVAARVPSAVGGAVGGEVLATAKGDAGGDDSGGGLDGRAFCFLPLPIRTGLPVHVNGYFELSSNRRD
IWYGGDMAGAGAARSSWNVALMADALGPCYGRVLVAVARRVGPGQQLYRLLPSLETPQPWSHLVSALYQHQLGQLPI
VWTRTRAGDGRWITPTAALFSDAACAAEPLLRNLLVALGMPLACDMPPEAEARLLRGVPSATAVEPQHVRRHLANML
RAAGGDSAAVGQQITAAAAAAADADIVGGGAPQHKAASATMGGGQLPQPLAEDAGGGGKAPIGASGSAGGQQAAQSQ
QQNQQQQQQQLQKLQQELDGLPLLPLADGTLGRLQVTHQGAPASGKSARGRNSAPAPAHVYVLTHGSLELEMLAGL
RNRCLAPGLPGPLVERLQRVVDLRLFNVQRLSPALLDSTLLSLLLPPSWQGAVEPQQPQQPQQQVPVQDPQSPPVLL
PPPSPGPTAEWIRALWAWLANREDVLQLASWPVLPIQGGRLGTLQEKSLVLREGDWTEGVTSALLRLGCRLLDTALL
EKAAGAGAGAAASPALQACVQQPSLSGVMAAIAAAKARGARPTSGPMAEPWSVRLSGLTAVERRQLRSYLLQSRWFT
GSTPPLAQEGLDLLRSLPIFEVFPDPVPAASAAKAPPPPPAEPARAAAGSSASASAQQQQQQQQQQPSQQQQQQAA
AQLQHFTALDPKRHRLAPSDTDPRVLSSAFLRVDSPAEEALLERHLGVPRLSHPQLLQQHVGPRASELPRDALGTYV
AGLLCRLAEVAGMWGSAANVEAQALRQALSEHPLIPTAAPSGDLRRPSELHDPRLPVLTSLLPPDSAFFPAASLTVP
AAAGAVTAAGAPPSRLLDALGFLGMARAVDLRVLLTAARALQTDHAAWASASMASVTQAAAAAASASSSALSAVTDA
SLLSRARALLQQLELWAGQYGSSGGGAGASPGREEDPKRAWEQLSGLSWCPVLREAPEPGLPWPSMPTPLLAPPRLV
RPPADAWLAALGWNAMPRVSVLAAQLIQLGRLHAYKPKRLTQTEAEPQLPPPPQAQTEAVADGGADAGGGANSDTES
SGGSADAQLSSVPEPAKSSEGKPTTDGAADAAASTLNKALESAVHRLYDSLSAAIDGPEGDLVAMSFERPDPDSPCV
WVGAGFVLPGVAVLRSEGDFRPYLWVVPEPLHQYGRLLSLMGVMDRFTAQHYASGLASLAAAAAGSPLDDDSLALAL
QLADCAADALAAYGRPTGHFFVPDAAAVMTPGPELFFNDAEWLDARHVQMAHGALPQTTAEALGVRSLRYAHEVEAQ
LTAALPCPSPGELRERLGLAAAQASEGRGVADPSGASAAGASAGEAAAATFLFELLEVADALGLRGVRLVLDVRQHP
AQSLLQPALAAFQGPALCVVLPEVVLSTEEVAGLLCSRAQPPSIRGRVAAYSAGLQSAFLVSEILQVVSGDTTYMFD
PSGIYLGSGTGSTAGSGGGGGGAGRGRGGALSPRAKQYKHASSDLLSTFADQFAVWSFADEYDIRSPINATLLRLPL
RRASPPPLGSVAAGAAGAAAIARARGESVAAADTRGLRNGGLAAATLESVEAALRQFVVHAPRSLLFLHCLHGIMVS
LLRPQPAASEATASTASAGAATGSTAAGTGAATGSRAVREVLLQARVVTLDTDRLRPDFAALGAAARQRSSGPAALK
ALAQAFGRSLGRGGNQDNPKHYLCPLELHVMAHNALLLPTEPAAVSESPPSASASTPPPPVKPRGGGSGALGLNSVE
GAVQGATVYRERWLVSSCHDLDGVPLSPAAAAASGGGGRPELVAAAAVCIGRDDQRNLFITSGLYAPVYLPATPSGS
GSGGSGGGAALGRMPFLVAGHFYLLRRNGKHVVPPFARAEGAAGGGSGATPNTDAGVPGPGGPVTPPLLPQLQHRCE
HNRRVLDLAATAWQNLALFFCSQDQYNGPRERLYDIFPDMAAATAANAGGIADETALYCVRQMYAAAARLPLWRLRT
GRFVHLPEGCFLQPTTEGLGTAAMGFMARQLPLFDVPWITKQHLESADVHGLRTVSPAVVRPLLKNLGRRQVAGAGS
AKIAWPGLTVLEATELLLFCSADLVAEVTAASTAPAADAASGASAPAGVGSARAGGRGQAAAPAAPPMGGGGSSPG
QQQAGGVGGLLDGLTSQVRNVVGELVGPALYDQLRQQVRSVESLGSTLLLVAPPLPALPTPAVLLPPHCGPEFVHPD
CVAKMVEHFKDPQYRSSLELRLYTLDNLARHLRTALPPGWDHPMASGAALQPSPSPAQQQQRQQNQHSDPRVGPTGL
GRTWDGGSGGQQGDGPSGLWLWQLWALVNGLLEAAREWQAIGGGSGSEGRLEPLHNWPLLPVLGSRAGTAVLLPEPA

LPAPWNWLAPALHAIGCPLLDPRFRAAAGRHCAPHADGLPARGLAEAAAVAGSAATRGSAGAAAGAAVAALVEKMRL
CNAACGGALPLRCGAEWDDATRAAVLGLLAEATPTNIDPADIIFLRRLPIYPTHGGVYTALEEGSPGPSTTSSSAAD
AAAAAAVETGAETTAAAAPPVVCSAELLHLVPGLDAALPASAPRRLLLPQPGASRLYTLLGVASLGAAGFLAAVALP
HLERLPEEVRVMLLTHVQANWSRLRSDDALLAVLRDTPFVLVADGALKRPGELYDPDHPLFAAAFLGCPVFPRDQFA
TPAWLTVLRDAGMQHKVTAAAFMSAAEAVAARGAALHMTLPVEGLPEHGTSLEDPFLADGATEVPSPVAGARAKQGP
ALGGGREWWASLAKVAFVPASLGLPGSRKARQLLTRYSDAAAASDWPLVWSVLPTVAAERQVPAALGQGQLRIKSPP
PLAAVVAHLRRVGADGGEEALGGWPASAGGVEDAFRTVLSYLDREGVTGQKAAQLRDVAFVPVARGTLLAPPRLYV
RLKEDFAPFAFEVPPSLASFTPLLKSLGAQDEPRAQDLVESLRALAAAVGPQPLSPNQRAAVIRLMSHVAALGGAGA
ANGPGGGAGGGGGTVGGSAADLTYLATARRERRLLVLSSDGRLVPAHSAVSVGEGGGVGAGAGRLLGRLDPGALTLA
HPALSESVTRWLGCPRLADVAEERLDPGHPLEPVEEIQGLTLRDARALLASPAFVSACHALLRTHAPLVRGITTHGS
LHEVAAVLRTAAPHLTFVRSLRTTAVLRSTGAALSPAAEASRVAFDFVEVAAVAPPPSAAAPSPPFGVISSLAPKSS
PGFGAGSGLGRIFIAEPPQHLPVSWLLASVVSRVLGSPVVLPLQPLFTTPSSELQQLQPVLLPGGFDAGLETAAQAG
TPGAPLLPSDAALLQLKPLRRYCAGEVVAYQRNAAAVPAAAAAAAAAARRAVATPDVPLAITSNMCYGRVAAHCAPT
DSAAATGGAAGAGAASGGVHRVLVEVEPGVVQHLLSTQVFCFSEAALTLDCASRHGGHPYSQLVPTLYIPRSPSSDT
ASAATTTADPALAATATTSQPADHPTIADRSGGTSSGSGAPLPSSVLSRGGGGGPEAAAPRPSSGGGVMAPVSSQEM
LAAVRDMMAAAGLPLDPAAGQLMGRVAALQEQLSEVQGQLAQAKREATSSASEAEAARGAWQCKICFSRDVDSAYTG
CGHTICALCANAASTNRCPVCRKPSQSLLRLYRA

>Vocar20009585m|PACid:23125137
MITDVLKFPDDQPPVNLVFGCVNGERGELYRQMADGLMGMGNNHNAFQSQLVANGIIDDVFSLCFGFPRNGVLLLGD
VPLPEALLASTATSTVYTPLISSMHLHFYNVRIEGIEVKGERLPLDPVMFDRGYGTVLDSGTTFTYLPSLAFEAMSR
AVGQYAEERGLQRTPGADPQYNDICWKGASDNVDALLEFFPYAEFVLGGDVRLKLPPVRYLFLSRPGEYCLSVFDNG
GSGTLIGTGSVQNVLVTVTPLEEDNVQLQLKVTPLEDNVQLQLKYDRRNSRVGFTDIDCEELASALVATANATAASS
PSPPPPFPAPPSPPPPPPSPSPPPRASRGSRSSRGSPSPAASRPHPHMMLHHHKHHHPSGGGSQLRKEVAQRSHWLRL
R

>Vocar20008642m|PACid:23125204
MLVMHSNVDECQVMLNTGRSESRGSSAPSPSPSPLPPSPLPPSAPPPASPPPVVLKPPPAPTPPSPPPPAPPPPSPA
PPPQQQRQQEGSTKQASSASRAAWHEYDVSKLISTEVSKTATDVHSRVSDWSDFDLPRTQADIDRLMKLPHVDECYY
CKVCIPNSALKLGQQQQQQQQQKPGAGGRRRRLQSTERASATYLENPAEAEGLIYGLKPVDQALPVDQSGGAGCDSC
SGGSSSSSSAAGSAGNYLNRKLREQVRASQGGGSDSVGAAEQLDNGVGLVAEEALLGTDSGGLGAGSRRRLTEAYQA
LDTTESGPDYHIGGRIYNISDIAPGRRIFQSKQHLPNCSICRGCDHTLSGWAVVKVWCVPVLKPKQRGLGMCDPKKV
YAQVKLLLAQQKLTAECGLNEIVPKVWVEPLNGVMPGSGFHVKWLGLWADIADGVSVQNIQEAGKPPLKPEVMLDLF
SKRINHHELVQGAIFDLLFSQCDRHQQNIFLTETGKFWLIDNDQVYATAWRKCGIDSLLVPTTQKFMINHLGFFYVL
KYPQKNPPRGPAKFLSPLLLLDYRCHVPGGAIAKNFTPELNECLSHISSLSPEVIQEQYGYPVLRMADAVRNRSIDL
LTHGYEWTLLYGQPTNQPMHRYKIAPKCCEMHFNASKSNYECDTPGYKQMTEIPFGNAWHGGPWHGTAGLDTGTYEG
GTSFLPN

>Vocar20009884m|PACid:23125208
VIQAAICPERKGGGDMLSAVASITNTYVSNDLQVVKVFVSVYSDDYGKRVAMQNLARLEPYVRVLIGQRVRLRRTPE
VRFVYDDSEEEADLVVRVLGRAEVERYRSEIESELAPNVEPASGVSEEDGRKAAEEEEGFDEEEDEFGEDGDEDDED
FFEDPDELPQEYPDPFGDLFGDVSGDTLLPSRRSTELKPVPEGDGGSTAGDGTAASTPSPFTNSLGPGDAVRKGRGG
SGAGRAAAATAGAPPAGARVASSSSAAPGEGPSAVQSRSDMRPTLSSRSQNHPLGRTSLVMRLQQGGRQLIDLGRLQ
ADWLSNTEVKGPEAQGYVNEADQGFVALLARHVEVPPASWQKLQNALGGQDGHYWQSSHAPGDFTRTSNLPQAREGP
QGGAETATAEEGRGLEAAGHKPRNSIFGLPVIQPYNLFAVLWTMVILFLDLTYTAFWVPMNVAFCTAEYGRLSSGCT
TSDIVGGALYLANVLLCFQIGVVATNGIRKRTVVDGRLVSWLYIWHGRFVLDAVASVPMLYLIVVLAGQSHFRLNKG
WVNCLSLLRLLRMVVYMDSLSGRFQDSWLARVLSVTVLYSIFLAYQLAVAINLFACIMVLCAYFEGYENSWMTAVDW
AHVPDASPLYQWYCAIYWMIVTATTTGFGDFQPRSIAEQVVANVAMVGGMIMFGVLVASIGQALSRATREAHQAYSG
RQKVLHVMEWAKIRKLPEHLQNRVQNFFADKYGGKEEEARDHVMLDALPSRLRAKVALSICGPLLREVHILSNMPRS
IRDLLAEHMRPLRLPAGEDLCQQGDVASCLWILYEGVIEAVRYKEAPVPVSSKGQPRLLGESILLGEAVEASRIRPW
TIRTKSPCRLWGIALRELYPLMRMYPCIGQMAAEYVKHKAVTMFREAMIEEGRGREWAESIRPNELCRDEEWCEVAD
VINRYLLCAPYDEATEIVGALAQASALDGSLPALLEQLLALCAIRRVADPTSTSRRDTNPRLHSVASPPPPPPLSSS
AGGLGGGLDASPAAALGGGSSGPVAENPPSQTSPPPPGPQQQQQQQQRPNLGASAAGARIIAGPQAAAAANASPLSA
VVPASGGSGGNGSVVHCPGCQRCICPSCGQGMDAAPPGPLAAAAAAAQPLAAATTGGGAAIFTPAAGRPAGLAPGML
AWVNRQATLRRGLGNVKRLCYVGGEGMLEGGGGLGQSGSTCGTRVWRCQSARLLYLCTTVTTDDDTAGHMSLTGRSE
GPQTGEPPHSGTLDALSSEATSGGEPSEEGLKRANLSSSCGCSNSQVCQCCCCCSCGGDATHMIDGGAGGGEGCIGS
SGGGAGSPCCTCGRRHRARRNGILSIRRRRNGFLSIRRRRNGVLAVEEDGGDGSWDGAAKRPASGLACRTQGPFDVP

DGTRCCYCAGCCRGGVLSDGGEAEDGGAAADGGSPCSATRGLLLSGGGGGIRTDRLVGAVPTLPSPQQKPTARESRQ
SDEAAAARVPRRGTVPIMLPGPPPSPALQQQQQQQQQQQQQQQQQQDQHQQRQQLQTVGSHSKPYPNPDLNSNPNLI
LVSNPNACIVQQRLEEGPPGRKGALGPTSPPQQLQQRQQQQEEHELLQEGLSSCPVADATAGEVGEYGGAATSAVVE
IGSRGDIGVYDIYSYPRSCLSSPVVRPHPPVFGSSVSGRVLAQPQLGKSTFPGAAQQLSVPGSTQPGGAVSTPSLYR
YDMGSRCSSQGGSDAALGVVVTIAGGGGGGGGGAETAPPPSPPPPRPPQHHSHLESGPVVRHAPGAAATAVICNPRG
SGGGSSSSSSSCSTATATMMIGTPNSSSSHVGGVLGAHPAGGNQVGSSGKPHRPRSPQLATASAGTATAGTAASTAT
AAAPVFKAISPGAANGAGLLRLCTNPEQAAPNRAAPAAGAPAGITAAASSPYISSQSSYTPGYHRKLMPSASQCEHL
RQPESITAYPAAAAAASNGSGGATVGASSVATPSIASTTAGAGATAAAAAAAATATVLVAAPCKVSHRPLPRRARSL
ATLAAPPHVHPARQPLPGPAHVGLAAHVSLLPLLHSPCQSTTADWAGGAMRARGPGSSPKDRPRRSVSTLTAVGYQI
SLEPPPLGAAAAAAAAAASSAGRPQGALQLVGVFGVAVGAPPGGSGWMGASSSTTSGPPPPPRPRRPSLWSSATSSA
VPEVEVEDGVGGGDDVNHDASVGKADVAEELGSRLHPQLYGFADRGARSAASPSNLGGNGGGGGGGVGGDSGGSEIP
VPDWSGTACGGGGAIEAMHLSPGADRQQQQPRQQPSPSPPTQQQLQQQQYLAAVTATTLAPPLSYRSSGVPRALSGP
EPVNGYGIRGPAFGSLMAAGPAAAAAAASSVGGGAVGGGMHSTLPLATTPSTPPSSQQHQHQQHPRMSVAAPSPLSP
RGNSTVTQGARLSRWVQPLRQLMRSSAGSGQSAKAPSPPSSPSTSDISRQLDGRADSGSAGSGGGVASSPPPPPPPP
LSPSGPVNERTLKHRRPWTSATPPPLASASASAAAAASGGAAASPRRELQQPTSISLPPIGTAVASRGFDTGCSCGG
CRTGACDGRAPAAVGAGVAAAAGAARGAPFPLPVVCGSSFPPPLPPSPLPPGERGGLCCGDA

>Vocar20009206m|PACid:23125273
MRSPASPHARQPPLLLFPPTHTHSSLFPASHSPLLPSPPPPRQVVAVQGRARSSRMTLPHFTAHTPMFMSVGTQGSV
KGLTTQQLEELDCHVILGNTYHLQNRPGSELVAEMGGLHDFINWPRGMLTDSGGFQMVSLLHLAEITEQGVTFQSPV
DGSTLLLTPEESIAIQNRLGADIIMALDDVVPTTAKEVKGGRGREKTNHERMKLLPRKECCGRGRVGGKQLVARDLP
GYTVGGLAGGESKDDFWRVVSQCTAGLPAGKPRYVMGIGYPLDIVVCSALGADMFDSVYPTRTARFGVTLVPEGTLK
LKNSIYARDFRPLDPGCSCMVCQRYSRAYLHNVVTRGAISSAAILVTYHNVAYTQSLTRGMRQAIEEQRFPEWVRNY
LKTMFPKGDVPGWVRDAMAAADVSLEGVVAEDLPSPPPSTALAAAAAAATAAAAPSPAPPAEQ

>Vocar20009084m|PACid:23125359
MSDGHFMTQGDFLHFEVVGFPVNGGVQALQLTNEPFSVFTADTLVPQLHHGRRLLANSYSYCTSTASSCNACDTSFF
SIDKCYTADALGSNSAVCQLPYPDGEVSSSSMCWSPYGSTTVPAPDGNSTAAGELLAWRSSDGQVLTATLKLSCNWM
LVAAAVTSLCRPYTCLPTNQPVTRFRCGCMSMCPVKQGLQMRRREMPCCIHLEGSQHVRPPESEPDAKHSSVGIRPT
YWIALTLLCKRKSPPGKAGRSFVLFPPPYSNPSPPPPPPSPSPPPNPNPPPTPPSPPSPTAPNTTTLVTFNEEIDAE
SFRAAVMSQPDLLTCFVIAEVLSYELYVPYRLLLAPDTDTASRPGCSTLQASYSASITSIPGWNDSQVSVSCNYDSQ
AGSSTFSPTTFPLLSQPVVAPPSDQWGSSSGGINANALLSCSSASGWVNITVQLTAASWNSSGGLSGSAGVLSLLEA
ALQSTAGSPVGGDCAAALRPRCRALRTPGGGHIAADTRERCSTVRVQAVLSARLPSPATPALLLDPRPPPLPPATAL
PDPPGGRGQLATDLVPPPPQAPGGAAAAAAAGAVSRSSKIAPWVWVVVAIGVILLFTGCFVLGTAGRRFTSRARYRE
DHAVTAAQARAAVLQGNTVVALPALDPADREAALAAAAADAAAAATMAVSPPVEQTDPGRSPELSDIRDVSIRISRR
LSANLGLRSDSRQGSRLIKGGADSAGAGATVVAASVAAIPGSPLRQHTTSPPSVFPSVARGSVSGAISGPAGALSPL
GGGGGGSDASARLSVAAAANGSEVPRAPMDIEPPGLSSGGGNSSATYGIDSGGVVVADILRSRGSIEVPSGGGGGDT
ASARPHGRRSSILGNAPRSLAATAAAAATAPGASARNRIRRRSSVPLLLGSDILAMHSAGGGGGGGGGGSGEDRLGA
FAARLFSSSGKAPAVPAGGAAAGAEAAMIAPGQRPATTSAVGPLRNAGPASGPAAFVFRRGSVDTGDVAIATAAAPG
GSGAAATATTAASGPLILSSSRSAFLNLAGLRSEGSRQSLLGRGGSRPSNRVPATPPEGSSTGSSAPLPTPQQKRPP
ALQIPNQLTVAGNTTRVAWVGQLTPPPVAGKGGGVAAAAGSADEPGAAPRPMPNWTAPKESSPLPSPRSRDVMSASM
SRDVHSSPLTRTNMRSHGTGTAVGGWSGASAATGCVPPGATQTAVTLTKTGADADADEQQQERSELARRNGWRLSGE
TLRWSAGLPRSSTLGIAPASDGTVRTLFHSSSFMKMSSAVRNEPGGALQDSARVPAGSPIQPARSFTSAAAAAAATA
AAAAAALEAVFTVEDSNPVDTAHATVQAGRTLCRGSGSRPASPAGPTPGERGSGNSESHNKAADGSQVQQLRSHPAR
SQPMLRAAVSRLRQLSFGPGNSPDISISPDPRVTGQVDRDGPSPRAYPASLPTPDDDGDDSAPSVPRGHNVMVAGAP
FAKERVAQVRITTRQPLEESASAASTAGSGPSDSSYSPASPLIPELRSGALPVTPRSPNSTRLASLVPLTMESQHRT
EDEPPPPPRNPTAAAATAAAAVPDGRAADGSKSTPRVVRVGPLVATAATATSNATAVTGEDETADPGLLDTFLPPPP
PSTVLQTLNTISDTNDVTRQSLSVGTLFERTERVAAADASLETHMSPNASVTEDARIAAAAAAVLAAAAAIGGNDGS
GPYRVQVVRRAQPQPPLPPLLLPASGTQPRPGSAWPNGMKGKEPPYEVPARLHAPPNDQDLRDHSKTAPSAAAVFAD
RPNAGTAADGAPSRPSSGRVRPGALPLRFEIPAALLGSVADAQEDPAAQSTGGADGADGASDKGRTRPGWRQLVLQA
AAGELRAGSAAKGTEDST

>Vocar20008360m|PACid:23125433
MEGFYPFAQANLWSAVPSNNGTQHLQGGAAHGASQGLTLSGVTGGPADGETPGVPGPLADPTSSSNIAGYPATLPSG
LFAALDELAAAIGFTSGANEPPAPLAQPPGFATGAPAWSGHDLAPAAAAAAGSRPLEVAPPTPATAANTDPAAAQQP
PPAVLQPHTSQQAAGELPQSQLSAPQQVATIPAASPQQQCPQALPLQQEPASVQQQAQPPPAPVAQTFKAPTIAMLR
AMRNNAWAAAAAAPAPATSAPSSAPAAAYGGKGQLEPAAAKGVVRAAPPEQPGSVAAGPGPLAGAADAATAAAASAT

ATAASASAPAAAAAGDAAGYDPAVQLHLQQQPQQPQQLQVQLVPAAGGGGVLQVVAFSSGSGGGGPMPLVPEALPPA
AAALLGTGLAQGPAVALQGQTHAPLQQLQYGEVVQMAWPAPGGQAPPVIAYNAPESFPYPQAQPQPQPQPQLHQQNE
PQPHGAFQPHGQDHAYGYRQQDERHYQHGRDDRQHLYAPYNEYGNMNQPPRHQPPHPQYASSASGAYDRPDAPRHPT
PYIYRPPAAEPPPADSHSAWPENEPRSLGVGYVPLRRRPPSTHPVEGWRTTGYRTRQREHPQHLLWSPPPSVLYTPT
REVEERSESPPPEMGPGGSGCDGEGEGPREAELEASAAVSAAAREGEGAAGVAARAADKVRGRSSSPTSQEGSPPPP
VLEQPRGPSTATVAAIQLIEARRKQAQAKEQAAKELRARELKAKEEEQQQQQLAASEQGPASTDLVAKRAAATAVE
PPPKDAKAQQAKGQLQQSRLLASQDQPQATAATTAMATAAQHQQHQQQGQGQEQGLQQEQEQGLQQEDQEQVQEDHP
TAAAAAAPSTMQRPSRSAPLRGAHAAAAAAAAAPDALEPKPLGRNKDQARAEMDAATQAMLEKARRLRQQQLQERIQ
QERQRKEQEEARRQQKEQEEERLRERLRERLSQQQRAKAGGGTAGLVLAKPQGSAAAALAARLAAGGKGGTAAASAA
AAAAAAAASTPAGATDAAATAEPDGNAVASGSGDCGALNEAELEELEADLEALEQQIASAPTYGMNARALVLRAAEL
VAKREAAARPAGTAVAAGAAAGAAAGAGASKPGAVPAADPAAGLITVVKPLADAVPVPERAFVTYVPRSSIPRLVRQ
EAAHSLTGGYLALALQRARVPEARAVQAIANEPELRKVAVAAAGQHEMAIYRQVTSKMSYELLFRKTKAEPERLQMS
EELVQLLRKEYGSGGAKGKSPSPSGGHAATAAAPKATPGSAASRVAAAAPAGAGGSGGGSGGGGGSVMAGTKRRLAS
ADGTATSAAPVAGPAAKRVAVTAATAAGGPAMAKPASRATAAAAGVAKVGGHTAGRRGGGAAGDGGSKAADAAVDEL
FANSDSGDEDRPNHRNSGSALGDAAAATTTAGPEGTDTALSAGAAKAAMTATTTASPTVDSTVAAATAAAAPLLAAA
TRGAGDAADRLGGGISDGGEDAPDASYEDLFGSDTESGEDGTRTTAKQTTEDAPKPVPQAVAPVAPQARALAEPTTA
TAGAVATVPAGGATPVRHAAAVQSRAAAGLASTAVATVQVAEGNAGSASGTATAGGGDARLPKRRRIDEGGAGSDGG
RVGGGDNTGSRGRGKSSERDSNRHDHRREKHRSSRSRSRSRPRDGGGDAAARELEPRGSGGAGRQGTGPEGQQVQQQ
QQQPAVVVTGPMSRGAMSQEEWEQTDVRQKLRQQVVALGGRVLAFIHATLEPLYRAGLLGKESYRRVAAKTSEKLLS
AHPDDTTADFLVREHAHVSKFIMKVVEREQQRDAERQQQESQQQAK

>Vocar20009961m|PACid:23125482
MSGSFLKSTGPSRWHNGASRTRKTSFDYVALVDQFQQEELSQEELYESSVRPFINRCLQGCSGSIITYGARGSGKSY
TLGTLDPQQLSLAARGNGTPGVLSRCTQQLYEWLGEDAAVEVTCVEVQGDDIVDLLEQRPGASAFNKAAATTIDGLN
GGPSSTSVAPGCASPVSALSSPQAGFSRSPIAFAAASASSRRKPLTATVVRNLAEALLTLQRAVSNRQVFSTATRHN
SSRTHAVFQLRWRNPAAPAATGGCGCVASAVTGAAGYGSCGGAALRPFSAPPRSALTGAPLGSPPPGTRVPDGGGSG
GPVCTLTLVDTCSFCPLLPPKEASAVKRSLLALGRVLSALQRRHSMTLRESKLTRMLEGAVDQILLLACMCGVGDES
TVQLVKTLQQGSHMRDNALRRSRRASSSDSRQASGAWESAASLCAVSAVSLAGVAGLAQHGVVTGSTADAIGTPPRV
LVGKAAGLGPSRALFRSPKSPHGQGYGIQRAAAELTELPLALSTVPSGDGKSSSGGGGTDTDAVAMPPMSESSPGES
SSSPAASSRSGGGDGDGASVATSAAVLPLAAAAKAGRPEPLTIDVMSTDTLTLCSPGATGVAAPERRTPLSSGCRRA
GSAIPSPPGMSPVGPSASRLSGTLSSRERPASVTARAAPGPEVRRSQGFSVLQGKWSPPPPPVSHANDKTFPIVPCC
IASGASATGQSLSLMRTNAAISTDSGVPLAAPGKPAAARGGIGTSGTRGRTSPGRGSLAKSGASSGRGSSGSASALT
ALSLTAAAKRPSVRASPSVNSATLISLAEVPPTRLTSTRYDNTNASHDSTHEGMATAAASAASALLTEPLADGPSAS
QIRLSLTLRNQTEGAALGGGAGLAAGARARSASGVSSALSVKRSISSGTRWPSPLLRARRTEWLEDTRRSRSALPRY
KGAPGTDCHVGCNEWDPPVTEIPEPQPDSALGPRKPPDGFRCAPMSRPTLSAQYKDLLLDFEASTPQDSARPVSGSC
ARPPPVFSRAHSPTADEGLLSLTAAAAAAATAGGSPRTAVSVGTPPSGRPRPGVSRGSGAAMSPRLLRSPERPATPV
SPTTVLDMSRLFAAKSSPTAAAGTQPPSSQQPGSGRGPRLSAGSARQLASGGSDRYKRMVEDDVDSVRTSEAEGDSE
GERAHSDAVSYPSGDSDSDRDNLLGPGGLENLGLMLEAGGSVAALHDSISRQGESSVTSPAGSMATPASQRRTSARS
AATATPTSVRICALQAAGARRGYDRRPDEELPAVSFFIPRFTPRGDSSNASPAGVLRSVATAPVATALAAAGTSIAG
VSATGAAAQVTSTSAAGSRPRAQTGGTELLTVPVEAAQEAACSGGRQLNAGSGARRTLAPALLCADNQSQPEAQPEY
EEAAAELQPRRERVSGAVAAGSAPGAVAAAAAAAVAQLAQLQLLYIARDRVSGSSDVLQGSCTTLPADLDGATACKY
SDGGQALPPPSQPEEEEEEENEEEVEEAALQGKPLEGRSGSARASPYRKRQAKPVLSTAVMGKAALGPPSHSSISLR
QLLQQQLMQQRPGRRTAGRVAAAIAAAAGPRKGSPKPNSPAQGYTAPTPQAAAGLLAQRAVVRPNHMDLDEHSTVLL
VDGDCASESDATVLALAAARAAAAAVPRRASAWGNQVTQAGDAGSAGTAGGVSAAGGDDDVSKTHLVVSGCPPLSAP
SVPLPESPFAIRITKRSPRQPPQLPTPSSLSQTSVRERVAALEGVQSPGTAANVPPHHTPSQTMKSWAQEVSGDGGS
HSSSCALVSAAVPGGRLGVAQTRSAPSPSATTLTNAVADALQRQAVIAAASRTAAVVIPQPSVRSGSGSGSGTGSGS
RSGADHQPYPSLYSPTGTLDSGGGRYGSSPTLPGGAGARCPAMAVAAGLASRPSAATPAVQVARSIDGDPGTHRSTP
RQHALQLLPQQQHQQQRPQHHNQSKHQSQQQQQSQFMATLRASGNVAAAWRAAAAKSTSSYSSVRSCFRSNSGQHQH
SMLGSDATAVATVVAVSRAAPLGVGEEKHALGRTSGHVAAAAATAAAVKWSGGLEHLPGNGAFTTVLGGGVMPVTGG
TTPMAGSSPASPPRLPPHPRGHQSGTTFANRLPSSETYVSKTLPPSNANEAAAVTPAAAAIANPATPAVVTASAAAG
LTTPGILAKAKSARGERMTPLTARLWAFKSYRTPRPAVATPVARLGEEEAEPLPPPPAYPPGFVGWATKMFSKSKKN
KLASLK

>Vocar20009619m|PACid:23125553
MQDKLLRAVAESENIRNRKEARNYSVIALGKAIIITVPDASCLDPQESLYGWQLAWSFSSYPIYSTRDLGEAVLVTS
GSELSPVRVVNTAGAASISPAGGTANFTFSVEPTTSSEGWSEAPLEWTSVQLNGQRCAQLPSRSLGLPACGMPYSQL
VTKASTAAAPPPPTTTPKSPPGGAPTGNPADLACASVFCCGQLDKVSGQGSGPSPAYNNARDGGGGGGGGGGITDH

SPIAVRSAAATMPAPPVDPMAQSRTTTGSGASGASEVSGLKPFLFAGNTSSSTSGGAGGGSGGGGTDGVILSPEAVV
RLQNLLTRAGDGTDVSAELVATLQNIVRQGGSRDGGVTATAGGGGDLDALLDKAAQAAKEANKAQPRFLPLAPTPAP
QAMPPDAPVFVPPFPTIAIKDAYEGSREFTDPRVMQQSVDPGLPGPSTPAAVGSASEPPPLTSKSPPPPSPPPPYTA
RFFQPPRLDRPFGFAPTAPAAPYGSVPSAPRQTAPDTFVVQIPAVRTPGGRSDSTSVTVAAADTGSADSGGGSGRPS
SPSTLAVVGASIGGVVAVLLISFLGCVLAAHYYRRWERRQQAGSNGEVAADAPGTAAWIHRATTAAKIAVCGLTGSE
CPSAADEAVPRGGGSAVGPAAGAAASAAAAISRTVAAAAASDTAAHPDGPFGLSIPPPFAIEDDAMAAAPVPPNGAA
AVAMFNARRAGNGRSAASSGGSGTSTTAGTAHAVRRATFLAHPTSAPSSLPLQPPQPQQQCPMPPSPPPPPPSPPP
SPQGQTWSSYDEDVNAANPLAAGSGAVHGDNNAQAILTSSLSLPKGQQLTEGGDRAVAAALGAAAAPGAAAAPPTPA
VLHSGLGSWPPRPKPPVPPSEWGAAATAAAAAARDGPENDVTTLLAQGAGGGGGSAFQAAIRPRACRGMSLGAFMAA
MAQELLMFGPQPDIYNIARLPTLLGGGGVGAREERHVFKSGSTGVAVTDGEAIVVAGEQATAELSAASAAAAPASLV
FGSPPRLQDAEPNDPMLPPLLPPSPFLSVPPGGTTSSALGWGSAGGPRSLTLPTPSGIAMAAAAAAATTTGITAITN
ARLPSQGEKQGQEKGAALPAALSSPAQLLDRMVLPATGAKPGRTSGVGGSGNVTPRLPGPIGGAASAAAATADAADA
AEPNPILADRATAVIGGGAGGDGDGGRVRPPCSRLLTPPLAASCQCSPLPGTRAFPGCMGAPRLAAGGHGAWSGATA
AGPGVPIGDPVGTEAEAEAVPAAAAAVPDRSPVPATACGTAASPFARSNTVAARVLLDLARPSGGRAAGGAAAAPAA
AGPGPDAHFTFLRQSQQQPQQLQQQQPGVHPPYPVMLPGAIVNGPVAAAVAPVLPPPAPALGSGVVVGLGGYGGDGS
GSGAAAHGPAGGAVANSGGSLVARANTVAAVAVRSTAVPADNCNNCSGNGNGNGNNVQRAPVMNRPVAWASIDFARD
VVLQGQLGSGAFGTVYRGTWLGRPVACKMVQLMDETMVLETRVVKTIRQEAEVIRVGLEVASAMAYLHGRRIVHRGG
EQMGPHCGYCAFAMTMYECLTGVLPWSWLDGDVQVCRGMCELSSLRPRPTTVWSLMMLLLHTIARRHHIRPYCPHRP
LNRLTPPAPRRSSFVAVCCAVSRPGKSVRCKILSRLRWPGIDFPQVSAPVIELYINQEPWRRKMPYTHIGALRCRAI
WVYHFARNSFPPF

>Vocar20008794m|PACid:23125571
MALRKAKEDNPTDMMLPAFRRCNPQPMSVGTRCLLLVVAALLLMPTSGPALLAKAASDTSSSSTLTGGHEGSARLRG
SLGRAGAAGERRAVAEAAADDIIFPLFRPPPPPPPQCRRLVGGIPIWVPCPKPPPPRSPPPPCYVIKVIQGTPVKLP
CMSPPPPSPPLPPPLPPSPPRSPPPPPRPPLPPPPCFVTITVGGQIIKIPCDLVKRPPPPPSASSAPRPPCVLYAVD
KDGSLIPISCDLVPQLATPQFNLLVVLADNKRLSQ

>Vocar20009755m|PACid:23125646
MEGSRTASGVPLLPRVALPLVDIQAVRELLSTISSGGYNAQLAAAVQLAVLLETEEGRFRDVVGDPWGGPAGQRSAH
SIGRLLACIKERESVYDTVCESWLLSRSAPLEQQVAGSRLVLACVSCWGFQYPLTEDRCMDLITEWAGDGGDKTPTP
ADPPPADDESLLREARSVYATGLLSIAMTNEDYAGPAASTPQVAWVAKYMRTALIENIHVPVALAPYCAALLAAVGE
FVDALGPVLAERGAEALLELMRRAAAAADGRLLYDTLAACVALTAHRRFSEVFVESGGVELALTLPRRVCRCRRRRR
GRLGRYVPALGDLPSANLCGLEPHTLQSYPLYLGVGAAGGIAMNPHTFSGLAMLLYSLAAAPLAFERLITPGPPPPS
SPPPPLPSPPSPPSPPPPQAPAQQQQQQGTQAPAAAPPPPPAPQYSGAAATTAGGGSAAVPRAGPGAVIAAALSLLG
CGHDLVRKTADGVRRMLNLLRALLTSLRSDSPADLRMERQVGYYAALTLRQFVATHLVLHVGAIKRTLAAAAAAAAS
NGADAGRSSSTIAAEAQAPVLPYRPVDTSREALEALTASLESDRRLAEAFARCRWPALEHLLEMQAPALLLELVSAL
PPERWFHEAAQAALEAVRLLTLAPLARRAVLAAVQQQQQHRNGVGVLLSAATLTTSAFFSSDPEVIGGAGFNMYGSG
DGHRGELRRGGQRECLWSRGCRTSAALSLTGTRLSLPCPWTSSPSPPRARVRACVDGWMCWVACIGVGVGVLVGVGV
GGGGGYRFPPVSPPDDKRRARARPPFITRAGPVGPRHGDKVTAAAANDATAPALLKIERAAIAAASHVSYSPQELLL
LVYEHLMASGLHASAATLASEANLARASAALHKLLQLSSNMAATTGGGGGGGGGGGGGGGATAAHGDAGHALEAGH
GGHGGNGGGGGGCSAAVQHPHQQQHQVYGAATGLPSAVCTPYTGGGSEAAGAAAAAATATAAAAPMSSDADYLVRAF
GTQRQQQRSSTSTATPQVLIRARAMASPFADGTATADRPARPVGVGFGGFGLAAAAAGTPFGAAAAAAATATATAAA
AAHTAAPNPLLANLPSRRPGPPATTAAAGLRRHSSNTTATAAAAAAAAAAAASSAAAPPAGGGSGAAAVATTVPVSE
LPIHVEAAARELHLLSPPPSCHAVGSAHRHAAAHHPLPAIRHASGTATHALEAPFNVTRRICRRELVEARGGRGGAR
ADAQLIYSRFRPLRTLRDDGSFSLWASFVHPLLLSSARADVKLWALRPEGGESEGEGLLPRGPRATWDGVARGRFSP
DGAQVVAVSCTSARQAMIFDVRTSARLTVLDPPPLAAATAGGPSPGGSGGRTPRPGGGLGGLGGLGGGLRLGLGAVA
GGDVRRSGRGSAAAAASACYSPTGTMLLWGNCLYDLRTRTAVHQFDMFTDSGSGTFHPAGLEVILNSEVWDLRTFKL
LRSVCSLDGASVAFNGGGDVMYAWRRHSEESFSQLFHPRRARHPLHTAFRTLDAATYTDIATVAVERVVLDLAVEPT
DSLVAVVAVDLTEEEVTAAARVYEVGRRRPADDESDAEEEEDDDSDGGSEVGREFVSVTLTGSLFRLLFMQDEEEEE
MDLDGSDPDTPGAGRVQRNNRQRGVGGGAAAALRRLAQGGFGEDEGGERGEEEEEEGGGGGLGALRGALGRGGGGGG
ADGGDGDDDDGDDDDDAGAGDGVVSDDEELGLAMLEDAYAALEDAYGDDDEEGGEHDMDEPDDEDGEFDEEDLEAFL
EGQEDDDDDEETEGESEDDDDHDDDMNEGG

>Vocar20008337m|PACid:23125688
MEYYRCLYTKHLVKKRKTWEDGFLVVKAGVAVLHDCDGRQMQEGVRLPASALKLSSDDGPLKYFSGLLVMVEDKCDL
QELPGSLRDPGKQSSTNDDILALIGLVPEQPPSSPGDRHGGLRCVGAGRLGLTSGSCSGVYGPPPRQRLGTTSVGNC
GAGHAPGSNPWSSSCPQQPQQQPLPHHYTQQQQAQKGPARPQLSDSWADLDLELTDPDDPWDGITVRVDGSTTAQHA

QSHGSFKPAYVGFKPNWPLTHMGNTGGAGPSMAADQTDSGVSTAPPPKHHAPGATCGWSADPQTALPRAPAAAAVAF
GSAGPGTASDDGGHGAPAASRDVALARRRWGLVASSAEHASGSPSGLLLSSAPAPCPDSDEGRGGRVLSANMIPPNL
SNSSTGGEFGEGRARGDGAHAAWGTACSGTIPLAGMGTVPGVDRPMVHSSKPCADTWTPAATAAPGAATTANTRIAW
DAFEDGDAVLHDVDDEEGDGNDRRALALIVADMPAAAAAAAAATATTTAIAAVPWRRSGGAPCRDVAGTGLVIRTDV
GCERVPPATGMHSAPQAAENTVLGAVGSGTASAGGGCSGGTGDLGPTALAGKVAAARARWSAPQPAAGNSSSGPLA
TAGAAGSRLERVNHLRPVVAALPLPSPAGTSGQGGPASKPSPSVLQPAAYSGPAAAADGTGGGGSMCGTGFGALALP
PLDQPAVPQRLVALPCQFSGPEHYRTAWVSALVEEINLRLAESVQQLYGIVRRLLAAGPAAMPPVPSGGGNSGTCGG
HQHQQLPWQRQQQQQGFQLAARAAAHNAHHQGQKQGFGAATSAAASGGFARLPPPMQASELERACLPVHVPYFASCT
LTCWVHREGGGFGGGGSGGAKQKSKKKRGRGGTAAEDDGDDEEVEEARSAGPKVSYYLTLPSVRNRLKNTRRSDLWI
VSSHPLMQGSGLGQVGGPGTRRGPGAPAAGGSTGVSVSGWRAVCRSLWHGPDRDGKFEVEMLTAPPPGNVRSQAVYA
LRGPDVQSEVQLVQLLLGGIGPIQTSGGGNSLPLLQHLLSLRPPDAPLDLSTARGSGGSVAVAVGSGSGSGGFKRPR
IEGKAGRAAAAADETLVAQLEDSATIAAGYAGGNGKDVAAIGAVGKSGSSASASFPPPPGVAGGCGFDPVAVAREYI
ARFKLNADQAAVLMHCTSWFKAAPRQGTQATAAAAAADADAKWGCTTSRRTAAADRRVTAAAEADDDDDDDGDDDDG
NTAEEGKEENGAEEDGVFNGDEDEDDHDFVVQKPGRRQTVDISGGAAPTSVVAAVGRRGRRLHGIVVDSDGEEKEAE
EARVTKKTTQQQQKQEEDDAEDVFPGSAGGGPGGLRWGAGGGGGGGAPEDGGAAPLECQAPVCLVHGPFGSGKSSLL
VALILMLVQLGKQQQSAATAAAGGGGGGGAAWKGRGGRRPAAPALAGPGRNGGGLSGGGAPPVRVLLASHTNVAVDR
VLMGLQDAGFTDFLRLGSVDRIAKPVLHRSLRAGDDSSGNRDTATELRRALKDATSPTDVAYIEAELAALAAGAEKQ
RRKALRTCPVVAATVCALLQPSAEDSCGGTFTVVVLDECSQMTEPLCLVPLLRARARFLVAAGDPLQLPPVIASPAN
LTAGATPALHPPSSSLGTSSTHGSLSVVPAAPAAGTSTGAGPLDSLLRPMFVRLSQLGHKPHLLSYQYRCHPLLAGI
ANGAFYGGRLRDGCSSSNRAPLLPGLPTLVFVEAQGQVQVDVATRSSFNTAEAHVVARAVTALLDRGVAPEALGVIC
FYRAQVAAIRRALLAAQQGTAAAAADTVPPHPHSHSHPHPQPSRRDYGDGGAAVVKGDAAPRDCGVGGGLSDVQVAT
VDSFQGAEKEVIFLATTVNRAADFAADAKRLNVALTRARRHLLLVGSPAALSQTSAVFKNIIHICQDAMRASHQQQH
QQYQQQQQQGHAVAEVAAAAAAAAAAAAALPLPSAPGTPGPGALYVSAAGFYRLLASLHPGPSATSDQPPHPAPERHS
GTGTGTGTGDGDGDGNRCNATRHGVDAATPPQRQPLQQQQPSLQWQGQQAVHGQASPAACVVTSGIQHQQLHHRYQH
QHQHFGGCQQHHPTMRQQHQHHDYDQRQHHHHDHNHNHDQQQQQQQQQLQPARPPPLEGSEAPSKGGFRGAPAWRRL
AAESHQAVPSSSSPPPPPPLSASCSGPAWPRTAEVEGEEEELDDECPHPPSDDTGILNQSPSPVSPGPASCVHSSAM
TSPNCRNKCTAGSSICRGGYVPISPPPPLPLLLKGFIMSGTSQTSATRGVALATAPPAAAAATARFRTGSSSLPFVP
AVTPPRSSAFSSSSSTTPKGDGVEPTAGPSLSVYPS

>Vocar20008400m|PACid:23125751
MEIIVIDDREGYLQAHAQSPAGSFTRNATVFLSSNYDATTAQAIRDGNAAAVHAVSILLSHTPCLKPSYWETLTIIQ
LSDYEYFEAAGKAVCTASTGSSNLYGATDGSLSTSGASIALPTGTRSAWLRVTLPSATNITRVGIRGVFPSGVANTL
YLEFASGAQVPVLYNLTSYSFVPALGQWTGVVSLLINSTSAFSVTELAAQYGACFENATVDLGAVQTIGSIRIRYWP
GQNGLATWLLASTDGASYDDVVSGLLGLSPLDPTRLGALTLTLPTPVLLKSLVAFRCRTLQARYLTVRHKVAETDWA
KVYVWGIDAYSPAPPPSPSPPPPPSPTPPPSPNPPDPPLPPSPPPSSWNNYAGIVRAYSYQGTVTVSSNPDPDALPH
IADGNDNTQWQSAACFPTGYTGRPSMNPLMQLCSGTGPHGAPASYCTASAGSSNLPATTDLSVYTGASIAVDPARVA
AGVTDGAAFFAAAVPGGPRAVTRVSYKAFSSSNVTVWLVMQPSGSSAAQRVQVATMVPAVDNYAWVSVTGQWQDVVA
LRLESNGSFTLTEVAVQSGPCMEWAVVDMGSVREVGVLRFRHWSVDAEQTNMSVSADGVNWTVMKAFLKPDQLEAVE
MYLPSVIYVGDVCDLPCCWVFRMVVGIIIWMFGTWMMIRYLKITHKVKEDADWRKVYVWGIDAYDVYGRWGAPPAPS
PHPLTLRGMLGVNGIWGWGFNQFSNNLHKVGKGPDMYTSVASWGRNYHGMNWDVRAPGNNPKYDIMSKNGTDTFWWL
DWDKEYVGWKAAGLKVDASFQFVSDVFGPPTWGPNATDTAYKLGFAFGSHFGPGRAPPNVSLDAVEFGNEPWVGYNA
SFYSSHTAPRTPRYCYYYCDSHAAAPRVRYRAREADNPYGEDNNSGNSVGAVVDDGGDGVDDGVVVVDDGGDDDGAR
IPPDVAPLIDVVNLHVYSWFRPQELNGTIIGVHPEHPGSTMNAVNNMLAWRSYNMPGKPVWVTEWGWDAHLPGEVCE
TTRCVSQHAQALYGIRGLLVLARKGVEVADWFFYANADEGSGVFARSGLRGSNNTNFPRLPVFDAFAAFMQLVGNQS
FLGVVAERTDLYGYLLGPVNASTDAATHLVVWRPVAAGDGPEEPTAAAALPLNRTPFAAWTVSGAGSAPVSLSSVVQ
VAAGGLWTVQVSAIPLVLKLT

>Vocar20009316m|PACid:23126033
MDVSCGFTFSVKRVSDPHTRNILWSLAGTIAATVIIFASLGSIQNQCRFEDPSLLSGISGFSGAVLGCKKLYRYYWF
ITVLSFLTAVYGAWTAAHGFLAGRRGAVLGLLVVANLLTINMTDAFLSIVDVPKWQSGPARDRVRTMIAGCIITAFF
QAVSIILVGYDPSAVAPVAKSAPKEASKDETPAVELATAVPTAAQPAEAAAEPAVTADVEAPAAPAAEEPQSAVAVE
GGLRMDVGNGSNAATVAAIATSGAVRSMGQAPTVAPAGAPAADVAANLLPAERDIREAKRRLQEAETLLSKFPLYRA
NDYAVEESDLRRSLILNTTDSQFRWDKAFSWLLSLVIGVLNGIVGFLFNQGINWLNRAKFETTLRVIQSRETTGIAS
YLVYLSFSLLYALVGALLGSYVSPQAAGSGIPEIRCYLNGIHVPGLLTVRTFLAKSFGVVLSVASGLIVGKEGPFTH
VGAIIGGGVGHLGSTSLTRATKGHLRAVLHTRFGRYFKSAVSHRDYVAAGAASGVASAFAAPIGGILFSVEQGASYY
NFNMLSHAFLSAGIAVYVNALLQAAFSQGSSLYKTAMTAKQAFVLLSQVPENTRLWYYSWEIPIFALVGLLGGLLGA
AMVVLNNAVTRLRRRFIPPTKPARRITEVLLLAALTASAWFLASYLSPCAQYPPKHLLLNNQGNVTGLGDPVVPFGG

YYPQAGSCWWALPNGASFIYNIGALVCLFALGWLFMSVTFGIGAVTGLFVPSLLVGGLGAYAIVGAGSVLGGVSRML
LCNTVLVMETAAATSLIMPLIVATFIAKAVADLLTPVGIFDLAIQRAGYPYLESELTNLTDTKLQHALTAGDVMSRQ
IAALPAVLPPPSRMTFLSAFAGGVVAAAAAMPGALSSEVLRVMGGGGNKSPGLGWNSGGRSGGGLKTGGAGAPCRRT
ASAPSPLALPPLAASRAEHTGNTAGMASACSGEGDVATSNVNSGGHAAVTAASVVELQPSASPADQMPAELGGGGGG
GVMIGIISRSVLLKLLEMQLELNTRTWRALSSTSANGGSKGAGSCSGPAGGPSWLTGRRAAMSRERAERLMDALDNY
PAKAPSDRAGEEALFARLGPGDMATYLDLRHYMQRVPYIVPATASLARTYQLFRGLGLHHIFVTAPNIPIIGLVTRT
DVTTANAYKAAYDLAKREQAQLQATLARTTEPGEGRPGGGGGGGGAGDHADGGDERGCGGRITPSTAVDQQPGAPST
ANQIPYWASLEDGLGAGAVERRPPAGGLPLTQQLQQSKEPQRSQWRRRSLGGSREDGITSLAPYIDERNIAGAMAAG
GRAGHLYGSSGDVPLPSGPRQESQLGPVRALSPETGGSIGVATLSRDLRLSSDGRDSAVAAAPALTAPLDQQRQQRR
YSERSCLHQGVSLGSTGSGRAAAEAPSPGSADGQQHASEGETSQPAASLAADGHGGDGPKGSRRDPAAHGRGPDGGA
GMASGAAALVAVGESQGGRRPLFPRVLTEADAGEVLPGTGGRSSSPAGQQRLRRRFRSGQRESSGDMDAGAATVGTP
CDTTLAPVYGQPWPVAAPFTALVYGASGVGGVSRRATTPAACGATGAAAAAAAAAAAAPEAAGLTRRPMSGTISRGL
AGRIVNRLVGNGSVRTDMASGDVESPRQPTVSVQQAAFVPHGGAPPGQLTRTSVSMALGRSSGVVSTARHSERGLME
QSPPPLQDVGAAAPANIDEEQLQSQLLLNQPLLLQMSGEDPAGDDPSEWPAVAAPAPPVEALLAATFLPSPPPPQLQ
QHLPPSTQPEQQPLPSPQLPPPSLPS

>Vocar20009264m|PACid:23126040
MSTGIYRLMDRGQGQPSPAGPEVELPVSTKRHHASTYSSEGGQGGGGPFPPQLDDMPSESDHLIPDDIIDKELLRKI
QRIWCCGVDVSWLARRLPTRWRLWWRQQPRPRKRVYIAGCCCFNLLLVVLMIVLLVRMALTQHQGYLLAHTPSPPLP
PPSPPQPPQPPLPPPSPPQPPQPPLPPSPPPQPPQPPLPPSPQPQPPLPPPLQPSPWPPSPGPTPPSPPSPGPPSPP
PTPPPPPPPPAPPPPPMPPSPPAPPPPLPVGFFCTFNGSQLSDDVRPEHYDLKLGIYFDPHPGQSAVQEGTGGAG
SGGGGGGDGGAGRRMLRDANPDGGAAINAASILSRLGPDHVLGSVNISITLLEDTQCLVLNAVGMEVYNISYRWNGQ
EWRGTRTCRREVGVCRRALAGGPLPANLASGVGAAAAAAANSRALLHGRSRCALEGWVLQTHCRCFPIILSTPTPAP
SPNPPTRAAPLPPGNVTSQRDQTATLMFESPLPATLSSGSSSGSSSSSAPLNGSVQSLGHLSIDFAYNLSYGLDGLY
RTVYQGADGKQHALVATQFESASARKAFPCFDEPRVKASFNLTLETPPGLAVLSNMPPSPDQPSQVTASGRALSVFS
RTPPMSTYLLAFTVGALEGKQGPCNDSKSTPVSVWATEGKINQLDVALEAACVAVQTYEAAFNMSYPLPKLDLVALP
DFQAGAMENFGCIFFRWVGLVVHGCEEEKLLLKADSDSDLDVGSEIAIAATVSHEISHQWFGDLVTMAQWEELWLNE
GFASYLEIMGVDAFRPQYGFYQLSYTSMTATALSYDVLPSVHALSAAAPLTSVADIEAMFDDISYEKGGAVLRMVRA
FLDGNLLTNGAVQNLRRRHLLQQEQPPPLPPPPSPSPSPSPINQSSPAMAPPPPPSSPAAPYQQTRTNHHHPRARHS
ALFPPALDARTLPAPPPSPMNLRNHAGLPFSSWMRSWTHSPNYPVVQVSLEAQNPGTPVVLPSAGGLDAATASSTNS
TDTPAASYLAVSQSSVTGAPCNDTAGPEGSAQWWIPLSFLPEVKNFLPGFSSKGATTMSWAPFNSCSAAVPLPTNVS
YVVINPGRYGYYRANYSEEVWRKLAAAATDPRPPVHTHTASSDLAADWLKGSWNNMAINLRNQTALRAPPPTPFLPL
LL

>Vocar20008410m|PACid:23126064
MSENDTLDVAFAAMLELCLELDDDNATAHIAVRTQACLDLLSRAPVEWDASTQMVADLFTLPDPDPDPDPDPDPNLQ
SKPNVNSEADFDPQSQLQPQSRPHGGAQGPHGSSHISAAVRHMFAGQREQLLSCLPASIAGADGCADGGGSSGGGSS
SMSSGGGPSAAALLQRVGFVLEANPRELAVQLVDQALRQMERRLGLVNQLFIRRLETELRRQQQQQQQRQEHYRQQQ
QQQQFRDAAPEPESASQDTTGWRRERLTATASTSGRPLGPMRHPPTPRSSQRPSLESVGALQSAAPTAPTAPLPPPA
PAARSTAGVSAEPAATSDRPLPAGRIAAASPRGAASAPAPSQGDEGSAGADRPLESCSTAGAAALLVRAAVLPLPAS
APTPVLSRAAGTQSKATTALATVAASPEPVPAVVPAVVPSPLPPAVARLHTAASPASAAAATTGAVAAHSREGSVLA
AATAANPLRDQVLIPETAPELEVEPEIALQSASGAPPGTASASSGGVSGPAGAPATTAGASSAGQKGTAAEAVAGRP
SQLLGAVPQAKRVGQPVHFVHTYIRSDGTGGGAGGKTAYPLGNLGLSPLQPRKPLSGTSTAAAGATGRALPPPSPPP
PPAPRQQSAAAAAAAPPAPFPAAAARLASLAAPAARLLTSRLTPPRLTSSAPQVPPPPSPSTTAATATTAAAVTGGS
VPNAAYCVTTQEGVAVLLSVEPPPQRTTLSVPPQPLMPRALVPSQSVASASAAPSQCATFPLPVHGSPTQHASQHLT
ASTPLAQRTAVAVPSPPPQMVAQLPAPSFATAPPFEGPMGQTATATTQLVDGCVAASTATPAASATRVSYLPALQQH
MTCTMTAPSPGLASAPSCSETGGPSGALSCELAPGNTGADASEDLSAGAAAAVAVAIVTAGDPPRTAATILPPQGL
LLPESSAWPAIAPSRDEAAGRLLQAAAGACAPEPPDQFLEAGPAGGGGAVPEHMHRIKLSDQPTGDSHSLRSAYHPT
RQAGSCGQLEAPGQRGASGEADLAVQQDEHVRFGTGDRQPVLSGMWRGEIDDHIDGVALRDAAHGSLDEAASGLQIS
GDDGDDGHAGDAGDGSGSGDPAAVSSVPRVAGDEEGGSGEGDVAVSGRCMGTAAGRAAELPPPTLPPSGAMTGSAGC
PVMRGAEPPTVHLDQPPIATVEEEELDSNSHTRSGRRGGGGGGGGGGGGGGGGGGGGGGGRGGGATPDAVDAGGVAT
AITADSHLPRGITASAQPALPALEVTGDPTAVAPCASAVATEAMAPAGADAVPAMAVVGPPPATIEHPMVTNGPVPA
TTEYSTATTEPPMAIVLTEVRTAVADATPGAVAARAPNPTGPGPLAAVAVATAPAGDRSGGEEGLNSDAPLNAASEA
QGAAMAAASPCVGGGAPATVCAYGASATAATAATAACATTADQAAGTASPSPSSKVSGLPPLLLQPVPSSPESPPQD
DLRNVRRHVGDQQLTEEPHGSFHRGENGDGAEEDAAGRPSSSSPENMEATAVAKAVAAAVAEAAAVAAAVAAAEVAA
EVAAEAVAEAEAVAEAEAVAEAETVAEAAAVAEGAAEGAAAAEAEEVADAAAEADAEAGSSGGAVARKTVPSPGGVL
SNDQEGLDRAGQPHAVSSERLSPESVWAAVARVAEPGSSTGDYSDGEGGGVEVCGGGIAAEQPNRSGTRLASEARDD

VSSASDGLRARTVDSINSGDGGGGGDATAVAAQAVAGLLALVLPAGGSSAVYGGSSGSGVEDEEDSYGQRDQQQRRQ
PPLLLRVLHPPCQQTLEVAAAKATQADSAGLGDKRAAAAAAAGLAAPVMTAKHTGGSDGQQEPGGSVVLPAMKLAPL
AAGGAGAVRSGLAAGGRDPVPTARAAGDSRRSASVEVGTGAAAADVNEDGAAAAAAAGAGANRLRSSPAEDAAPAVV
GATAAPSIAAADIGATTPITPQRQLSATASAAAQAAVGQQEQQQQQQLTAVQNAFQFQPGSVVQHQSPRGATPDVLL
PQSRTAPEPERDQVRDLWASWNPRLGGAGGASQTRDADDAATSFKGSPSGSPSGRGGGCSGGSPGVGGDSPWGTDIE
ASPERRRSPTAAAVGGVAVMRGGSLSIRSDPCGSGTTGGTRSGTAVPGQVVDERDVAMTAVVSERHEELSAVLREDR
EDREVELSKAVGGCDDGTTAVDSSVHSVARSPPNGLLAVDDSTAAAADTTNEATADAAVVPWGGGGGAAASGDVGGG
MAAASLHEDLESLALAPPGELCTAKYEVEHEGNSPAVTLEGTAASGDTPRIASGAVQVREDEVTEEVVVVVRGSPTA
GELRNGPMGPRQPPPEAHGGAGPDNTMQVFPQQAAADVVVEVAASEGEYFRRAVGSGAERGHVPAGPLQSKVAAAAA
AASMGSGGAPADVVTVPGPSADAAADTSLQPAVIPAMHFAKTACGAGSTARMEALIAKRPPPADVVPLAEPLAAEAD
VKHQGLHHQNLPPPLPPPPPPSAAPVESPCNDDPPPFATAAVSPDGNRSSDYGEADPAGSQGLATPPTPPTPQTPHV
QLALPQPGVPAAGPTRSAAAAAAAAAAAGLLSRAGCCDSVSLPKGPDTEPCMVPSCDQRVQQLAQQKEQEPGRAEEG
EGGGGCEGEGGGGGGEGAGEAGGEGEGGGQRQVEEEPELQQYTLERQQQPQPSLAPQQPQPSLAPQQPLGEQLGEQQ
QQQQQLERSAQEQERDDGGGCLPTAPACTAPVSCHDAGGDVALTPLELGQSMSSGSRSNSHRRRRASAAAQLQPAAL
LRIPDVPHVRSCASPGSSDQQKTQLACAEDSVSRARPGDLPGAAQQRCVSELGGASPAAVEVAVEQEACAAGAPTGA
AVAAAPSPAVLDPAEAATAAAAAMGAVARPGKASVDSRAGSSGAPASRRALAGPRIDVPAGGGLPGAGGRPAAVYWD
HMEGTWGTSMTTATAPVCVRRLCGASPPSGADGGGGGGGGSWEGVREPHSPSGADAFDSADRVLGVGGERTKEKGAH
TVLRVAIARSPGGAGAAATAATGPPTPLSSHAAAPVIPRPASAVPSPQQLHEMQQPPSPSPSPSPSLADAGGPAAAA
AQPPLAGAAATDKHEEYTFLAKAAKEVTQPFAPAAAMNNEPESPGTAAGPQGQLQKRDSVVPGGGADGQPPAASEYQ
SLRDHVPIAPPQDKGATVQWRLETSGTEPLLQNVVDVAQRGGTAQRPGCKSPCTGAQLQNHPAVASDPQSAVDSYAS
GDADRRVKRTCRLHAAATVTHGHSVACTLPPPPQLPPPQTSTTNNVGLRSSPVRSPASSVGAAAATAATAVARAVER
STEPVTAAAAAAASRLGDPVAAMERPGAPAAVAQRPASPAAAAEQPSAAAATDPRTGAAATLRQPGLQALVRAADAG
RTPRGRGDLGAQAHELLLQKVPDGPYMSLQRLLELVLGLRCSVVLPRWATPEVVAQAAAVMLTLHEAVCCGKVRLRL
QQTPRFYVVSPAEDLLAEWAAADAAADTAAAAQRQQADDGGDGSGGRRSIVRERDDMHVAHGMMRGRGGLGRQHEEE
EKVPRCSGGLTEPEVAAAAAPPPPRPWAVRSGIVSDKQPVREKEKEKGTAVEGGAQRGRQRQRRRRQQPMQQRRSAG
VAVAAVGVDGNGEGIDGRPLKRPRPAGSSPLAPTAARPLTGVGASSLPPSPHDATDERPTVPRTGSLPAPPPPQLPP
ARQVTTSTSEAPPSPLVLQRNTVEKQHQLHQLQQVQAQEQQQPPRPTLPKQSPAAVLPEAQHPSTCPHRLHGGPPAR
QHPEGLKPHQPQQQPQQQPQQQPQQQPQQQPQQQPQQQPQQQPQQQPHPSLFVSPARRPSGPPPAEQGGGDLDHQHG
SSPAGGRRTDTDERLPRVRRMRPPSVRKPAAPAGPAQAGAGGVEVPSWTAAPTAPTAPAPTAPTAPASPPAPAPAPA
PAPAPPGQGPPAPPAPAPVLRAGQGMLCAGGGPVTEEAEPTATGVVTMATAAVAPTPAMGAAVVTEAVMTEAAAVHA
PARKSASVAAAQVMEGTSIAMAAAAAAAAVSPRMVLEVTANAPGALRNAVTPSRPADMVVEELAGEVEARDEAVVGT
AAVHSPWRSGHVLERVPERFGRVVAAAPGDTIRAAAAAAAAAAVGGGGGGGGGGGAVTGALWETAACAPLARLVSVL
AAAEVVPELQVPGPEVPDLAGPGGPERQQEQVMEDEGRGEEEPACVDMDADGEASPRMDFSSQRSGAAASKEDGEEE
EEEEEEEEEEGEEGDDGFGGFSLHLDEARRARAILERDKVRALFTRLLGGWAHRGASGARVSLSTGCGGGDNGIAHH
APEPLTAAAAADAAGAAADAAGAAADAAGAAADAAAGTAAAALSTGERRRTASDAPAAAAVAAASAARQTGGDGDAG
LRLSRGRSSRHDTAATVEDRAGTGLLYIYRRHLTTQQTAPARLTEAAGSATVSVGGADGGSVTEAASGTEPGVGRRQ
GPGAGSGSGSGSGREEVFGGGFLEPMKHGNSNRPWRARRGNGAADATAAKRGNDDTRDISRAAAAFAPSALKPPGRE
VAAAAEREEEEPNPGGSLQDSPTALTPPCPEGMEEGEDLTTGPERDDVDGDDGDDGDDGDGDDDGAPSHRRAPAADL
LLRRAVTAEGTLGHEGAAPVAAGGSSAATSSAPARHLLPPSGLSPEEFFADSPRLESLGDADLVLQYAARDLAFRQR
LGSGPKQTRQQAPPPPPPKPAGPTHGPEVLRVSPGLPAGLSSGLVVQLLKSLDPWGGASACQQQAGGGRRGTEEGTQ
QQQQQQQEQEPVSVEAIGPVPPLGTVPPLRHVPPPPPQHQQQQQLQQSETGHAGIQKGHQQPQPQIHQAAAAQATAA
PPPPPPPHPHTRQGGGGGAAAGSAEREVAGGAAAGRARQLVWLGGGGEGGQRRETGTTGTLAAAAAVAAAAAAGHRT
AEVAAVAPDGVGVATADVSTKLPPLRQHAEHRQQVRAPEAASGDGDARVPSPPPPPPPPEVALSQKHQALSQLQPQQ
DVVALLLRALSSSTGADLGALGVGTVRQGLIEILGLTTTTATATATPAGQPGSARWDGLDAGCGNGGEGSGNGAGGA
VEEGAQEAPQPGAKIPRCHPGAPGPGLGAEVRGVRGEVGAGQGRAPAVPAATTAVREVEKDIHAVPYNQPDDAVAGA
QKAPSAQPGEIPATAAAMPYRGSDGMTGTIPPHPHTRAHPHAPPPGYAPMHGMTYPAMPHATPYAPYGQHLHMPYME
YAPDGVQDSAAVSSGGYSNHITRPPEPHPLPHPQHVHQHQHGQLPPQRLQWRQRPGPPHSRERGAVPDVGAQPAASE
VISNATAAAAASRMHNLPPRPPPPPPQTHFRADAEAAKAADDGAATAAAAAMRGGGAREVHREVYVDVEDGAAATAD
ASSAWSPAVRRHSRSSWAAEYMSPTVSTVTRGFSGIGTAAASGGGGAEQSDAEPPPAGYQLEGSPSSQPQSRAGWRQ
EARGGLPFRGTEDGGGGERRPPADAPKRDAEGPRVSHQLDAPGGGGPQENTARRGQCAVGTAANTKQPPLGGRTAAA
DVTTTAGTAAAGGGITAAASAAAAAEASRGRSTTARKRPLFAGAEMVQGALKSSLKAATAAPGEEQQVALEQPLKRP
ATQHQHQQQHQHQQQHQHQQQHQHQQQHQHQQQHQHQQQHQQQHQHQQQHQHQQQQPAAAAVARQPELLLPAAKRAR
RVTFDDAALVVHQRRREHDVGEAAAGGTTRRGNGSGSGSVTATTAAAAPAAATAVPRAQRHPQPLYSPEPAAKLPYT
VRPLVAAVAAGGEQDESPEGDAGRSTPLGASPFRLFAPLLQNQLQPHRQQHQQHNDHHHHQQQQQQQQQQHPLLGQQ
QQGGQHRLSNVGSQRAVVVSDVGVDVGGENVEGSRAAAAATQGGGGGGGGGGATAGGGGGGGGGALAAASGTGRGMG
ALLCRHSGESNSLLRRLRDGRTSVAAAAEAAAVGVRKRGSGGGGGAAAALPVQHRMADGYTGSRSRSLLGDADSHDG
GGIGDACGGSGGGGGLRGLLNPWAPPQAPPAQHPPAAAPTHPLSTDGTLGRGGGGGQVSHADGRAGATPATTTTTAT

ATKAMPPPPGISGLVPPPPPVAAASVAGSLARMGTAATLCRISRLSPPPPGTPKQRPRGAPIDTNGLRVARVSCAWR
CIPL

>Vocar20010019m|PACid:23126131
MAAAHLLGHFGAVLAGRREPSLEGRLSPSALIDWALAMLSPSSLLPAAQRMTADAKESALMALHACIAAMPPADVAP
FAQPLLRLTTALLEATTTPPRVLGPLLRLLLAVLPVVPLAVLGQGFGDLADLLCGWALEPLVASDDRQLITLILHAL
RPQWHSHPDLTASLTDNMLNDVKAAAASTAAATAEGGADAAVQMASCMQLVQLLCDIFSAASLRQLPLAGLSPSEDL
AQVPQNLIPQPQTSTQPYLQANTGVLLQRPSRVVLAGGVLKSTTAGQYSIQTNNQMPQKQVGLVGKHAQPGALAASP
HPTTNTLGQAADAFMPKQHAPPIPPLALTAILRTRPGAATVRHLIGLYRRLLATLTEVRSSVGRLDEDADARASSNT
RPNGAGTWTSRLCGCIETLVDSVASCVTGIDPRDVDDVLEGFDDDITRHLAALTLRDQGQGSTASTSASQASTVAAP
SLSWSLDEAAGIFGPVGAVVNADGAARGGSEDGRSSAPRTLAVEPCNALLTSGIESRGTALRLWIFRTALEWALSSL
GAAVDGEEGARPGADAEALMGCALRLLDRASAPGEARCMLLELVLEGQAGSIGVSSPCASSVAAAAKLLHALLSRRE
LATTTLEWLLGDLCLHLAALQPGAAMLAALQTHSHLYRSPLAGATGLEGGVGDTGAAAAAAFDCAVLQAACQRGWLS
DKSLFRIEALIWAALCASRTKPASQATPTTESRESSTAQAVLLSSKGVRARGGLAVLPAKDRGGTSKRTATAPTRLE
QRPSLPSSVALALCDAWAAAAVGLIAVVYNKGRTSCAAATIGTRRSHEHAQPESAAAAQDARWQAIANGLAAAPPTV
ARACCAAYLHTIHSLQAGSLIAGNDTSVDTGLKLRTLLAVQRLIKHPVVAGSGALRCEQLFRLAMEAISAGAVSWDA
RIRVASAECAADLVANVLRPLQQPAPLAMSASGIGAPAIGVLEWTLAGPVTPPQPPRPESFGPLVALALDAMADLSP
DVATAALRLATELSEAALLAMLRGPPSCCTWVPDWQDSLGVAPAMRAPRVQQVARLLELIFQSAATTQGNVLLRKRI
PAASSAGSGAGEDGDGGGRLEALQRLALGMLPAEVSVQAAADSAGGGSTVAASNSSLPVAVTGGKWNHASGAVDRSG
AALVWLALQEGSKHIVAARLRTHLGGPTQTLGVLERMLQMTYARLSQERRDAGSGGVGASGHESAWLALELVGALER
AVAHAAEGHAGRDLLPQPVLAFLTANKKVCEEWFTRGRELLARIAALASAHHHVAHHGLMRLSDLRLTMRQLLNSLA
KERSKAAAVSGGAPQADAKGDGAGGGGAARPLGVAGAAAASAPGSNAAVDAPSQAAHTASRHASKQLQRRGGTVGDA
APAQQPPHPSAVAPAPAAAGGAVPGVAMADEAAAAPSPMQQLESSLSRLALSVVEILKLVGSALLAMGEADSLQGLH
TWAVREFEPLLQGAHLPGASALAAATAAAAADDAFAWLLGLSQQAAGSYEQALLSYNSFLAPAQAPATSLSATGSPG
SGNGAAAAPGPSPSTALELAAAAQSFVVERVAECYAALTDWSGLQGLVGAFSRTAATESAVAGWWPAMAASIEHRFR
ALAAFDTAVPGDSSNTVLLAALRALEGTAAAAAPRSAHPSGSLAPLSPPPRPRSMDLAAVREATSLELDRILDQLRA
SALSDPAGQRQLLLQISYLRSALRSLDGELAAHGSGAAGAHGPWCGLLLPQVLHSGQVAGETTTGAVTSAGFSFGLG
RGLLSPDGGLQTAVVRDVAVTAGLLRAVRASDPRAGLPGTQALLIENIRAGAATGNWSLATRLVQAAAARVSGLEVD
AAAGAREVLSITHSLVCAASGRLMPGQVVELQLRALLPHLGSVDGPALRRLPPGAPLHRVVDADADDLACALCTLAR
WMLKAGAQQQQEQRSLQHAQPGVTGTDTNVSRPSPATSSVPQWPSALDWPALSSSLLRELVSEHGISSCAMAPGPGG
GGSAPAAQLAFLLPEALLQSYYAPASCCLRALQLSPDLARAWKAWGDLVFKATKEHRARAARPPPQAPSPAGGLASS
TLGSSSGAAVSPTGASVDDASGAWAAVGYGAAAVAYCRYLALSFSHDTAARPEDMLPVLLQLLHIVVRHATPIEHLL
EAQLSVVPPLAWSTITPQLLAQLPSASGAARRLLGLLLHAVGRAAPSLVLYPAVVEVRAADMAAATAAAAAATAASS
GEAPAKAAMESSVTGASMVPELRALLADLGRSRAGLLAGVEVLVAEMERLTVLWDERWAALLAEVEVEIARRAVSLQ
AEASRVADDASINADQRQALLSSRYSTLMAPAVMLLERQLRVMAAMPAETPHERRFAEAVLPRLRAAAASLRDGVGV
RDWARPSQAWAPLRAAAASLARQQRQPLPPIAELSPRLAALQGSEVPMPGCHADDGAGGGLVPFATLASLGITESAA
AATAAMAATVTVAGVCSEVVALNTKTRPKRVALVGSDGQTYTYLLKGREDLRMDERLMQVLRAINSMLQADPSAAAR
GLATVRCYSVTPLGPRAGLIQWVPSTTSLFAVFRQWQAATLERHGAMVAARQEGVAKALAEGSPPPPEVEPPPPAAA
SRPMDLFYSTLVSALQERGLSSATPRRNWPADLLRAVFASLAAVAPRQLLARVLWAGGGSAALSWRRLQRYSRSLAV
MSCVGHLLGLGDRHPDNILLEGREAGVVHIDYNVCWDKGAKLRVPEVVPFRLTQMLSTALGVGGLEGVFRAACETTF
ACLRRRREALVGLADAVLSDPGVDWAVEREDMAARQDMELAVALNLFVSRSGRVSCHPFLTCGAEEAQRQLQQAEED
LPAVLDGPVGVLLAYVEAQAFAEAMRSAATEAQKRIQQAKSALEIASRAEAEASAIVAAAAQEAASLAAEGSSLSNA
VPQLLQQCGAWAQQHAGTLGVLREGSFLEGTLASGASWRAVESGCALGLLTPVPVPGHAPLNAATPHALLPAVMGSD
DLGIGQLPEELLLSCHECDRQAADVLGRREAALCEAVATLTQYGTIVRKLLPPAYPATSYHHRWAQALSALVAGGLS
VPAVAQAQALAPREPRPLDVAAAWQALRGSQRLATAAAVVLAPGSPDAAAALSGHVAGVRVPGRELAGAVVAAGRTL
GDAMVGIAADAQAQVHERQAAASATGAAGRAGGLVGLSTNQQVRYVAEVLQRHSPPHQRPGLGATDGGNGSNGGDPS
AAAIVSAASDALLRLVRAASNRMEARMLTGQQLCPSDPQRALSHGGEPVADSATGLQDFLRLSGDVAGLAAATATLA
NARLSAPVLGIADPGSGLSCTRGSSTQSLTQHYLQGNEPQPGGSPHAIIRAATPGVQGGIEAAAACPLPWLAAAADI
CSRWPELMHRLAADVAPELAGQLHIGCGFGGAGGTGGGAGSAPAAQPDTPFAQVVADLYALIRPVEESLHAGRDAHT
RFEALAELTATYQDRRADLLFRLAGGTTGDAATAGGGVSPLLTPPERESLEADLQALDAAWESRDVVAAGLSTAIAT
ATEALAEALRRFYATVLGPGRLPVELQTYDTAAAAYAAAGMEGWAEAGGCGHAGLAALAAWWDAVTESLEGIQSCYG
NGEAETVTSDSLVGVPPTMRPLWRCVEVAHMVEQSLRLCGQAVRLTAPVGTDGALAEAYSAVIQALAPRAAGYITGR
LFPHLRHYLSALESQLVTSLELSRQKMGSNNNSGGGGGIGTDATSVEICEPRSGGGDVALGAGTPELVPFTDFDPRI
AGEGELLGGLDDDEDEAGGGPGDSDDGAGWLGYVDGPEGVNNGDGSGGDAEGFDIDLGLDLDLDDGFGGGSEGLDEG
RVGFDSPSQMEGSYGEGGSDAGSRAGGRAATAAAATLPCASPEEVYTLLAYVAACASVAAVGSVTAAIDPSAAAGQP
TATSAWAAGPEARASRAARLQRLAAYEWMHEHHLLQALPAGDPRIGQALAQFFAAQAADPLQPSARVTATSRLALLT
QLQSALDALPLLEQTVSSWEAASSAAVSQVMAMLRGLPERFPVDTAVAVQRAAVVMSRRQQWLAESRAVVMRLCQVS

EALLQFEYSRQGITWSSGSTAMADGFAAHFQLLGRAEVLATLAAAGSEAGRAAAVAEASQRAAEAAGQAADARYALE
VAQYEEVAASSQLQAHLPLLVSRSLELGSAVAEAEPAVRAVVQLLSGKLGPALKELSSVAAQHSAASDVASVAAAVT
SHFERSRQAAAALAAALPATLAALVAADAAASLPADMATEVGFLSAQLLGGPAAAAATRERAMALLQHLAAPAAALQ
PVLHTVLELPRALGQLSNEISTLAAAAGSIAAAVESKHLLPAMDEQQQGQPEALTPPQMSSTGASSTPQDTVGRTTR
QDRMPGDVAATGASTLEGSQAAAATSGSGSLLSAVKAKSATSTSALAGPSGLNRLMLQRLAHADHVQHHRSRRTAAD
EARRRAFAAGALRRFVAKLEGREVEGGLSAGGVNSASNQHLSVAEQVDLLVRQATSTDRLSQMYEGWTAWL

>Vocar20009176m|PACid:23126167
VSAPHPESVATARAELSTLLASVQGSLADLRVEVAAAKRISTNASAVVAGYSSPVTRGATSRSGSAQSRPRDHRSAS
EITPPMHLRTSAAMGPYGQPFGPLFPSPPGPDGLAVSPPALLAASISTPPASISSSTAPQNEELVKTIDQRLLELGH
KIERSLEEQSLRAQLQAVQAAAAMQQQQQMLMRSSAPPQLFTGFGNLAAAAAAAAAGAHPALEAPAAATAPVTVASLP
AAAGGAAANGSGGVGSSAGPSPLVLLAMNKADAAEKMSADISKELHVLQKSAAKEAEDVEKRLASLLDASRQHWEKM
CHVSSQVESLLTQRSGAAELQAEVARVATKLVQLETNQDRTVTSIDEHQRLMRQMTTALQKLAAKLEAAAARAGELE
ARMARVGDVETRMARLTDAEVKLARIAELEARTARLPDVEARIARLADLEVKVARMGDLEARFSRLGDLEGKLSRLA
DLEARVGRLGDVEQRLARLADAEFKLARIGDVEAKMARLGDVEGKLARLADMELRWARMGDADARSKQIADLEARVL
ALQTSVAAATASRSTPMSASPSSGSDLDTQEVMRRLATLEQHMARLAAAEQSSQVSGSLCPGLRSSSGADRRRSEQY
GAGQPKDILERVKVLEETVLQGSFGQGMSSKSMQAILARMERLEQIVRAENSIEAMAQEARLAVAEEFVLELRKDFQ
DVVGPTLEKLAAHVSELRKGQVQNQGSPDAGKMVTELRILSDRVDALDAQVAEVAAQELHTRDLSNSGVATMFQERV
GMLELQVKELSGAMEDHNSIFGRLQRHQAHLDRLQHELEALRNGLAAEMSELKARMTSVDGSIGQMHVDADDVRHQL
AALLRLDEQLASVSEAMVGLTERLGGQERQVAALEQQALEQRVNTDAAMRELSERLEGQEDKLEVLEGRQTEQQGQR
TAEEETIQELGERLDDHDGKLEQLEQQLRQQFSSRATAKLEERLEEQENKLEQLEEKQTAQQKVVEGLRERLGGQEA
AVAELRKQQQEQNSTANAAVQGLSRRVAAQEDKMVELAEQQEEQRKAFEAASAAAAAVAQTAIGAAATAGAGAGTVS
RALPGSGVGTAMDKEGAEELGNELRTKLEGLRASIGDVRKELGSKVQVLDAQTRELLAFREEILSSYVELQDGVERA
SNTAEDALRKAADHDGKLQALSEASKTQGLASKMSELQQRVSDNAATQQGHLEELEAKVHALSELLTKASQDSQAAA
ETRIRAEELAYELSQAQASLESLALDSGRLVEQLAKVASCGEKTAGQVDRITADLQGHSAAIAKLQDGLQRMESRPN
ATPPPKGARSPLPLPPRPVPDPAAAVAENTPRVGQSPSREASDTGARLSMTLPATRSGASQAILNPLYSASSEDNVF
GAIVPSSTVGDVHVSGTDVHTSSNEPSSQQHTPGEAARPPIVLEEATVRDKAQFYENLASKTEKAGVIPRRARGALG
TSDGAPSQDSSMMGTQPLRVTGCEDTGDAGGPFASSAAILGDLKDAEPMSPGAAATTAAGAVGLAGLRHAALASGNS
RSLPQTPTGAVAQAASTVAAAGALAAVRAARSGGLSGDLSSPRATADNDDEKATGASPLSTGGTPRVFGREQSYRRG
VEDEEVAISSDDDEGGARVGGPGLLNRERTFRRGDADEEVSISSDSDDAGEVRLGATDDDGGEDALDAPAPHINLAT
TQGFDDDDDLDLAGLAPPAAAAAALSTNPGTNEPRDKGAIEGQDAPGSSSSSSSSSSSSSDSGDIDEDIADEFGVNQ
PAFDDDEDISSAWPGPAASTAATSPPPALQPGSVAADAEDQPAGAAASASPSTNAPKTLSQASSVAPPADTAAAAEG
PANQLSTSGALGGPQLAPLQLSSSAAAMFSLSGDNLSRRKLPPLGSLPPVGPTGSGLNPALSSSRGLDLGPPLLRPS
LASLQNSGNISGHISGDLQRAGGLPPVAGPTGEAVNRSGPAKQALPGDSPFVGTMVEPGSPLRSLLSQPVQQPHPSE
SVDVDLPAPAMPEAHTDTSGSMPYVDNMGDLSGLSQEPSTSTHPFAAVSSQAPLAPLPTADPANNALGAGAAPLPTV
KSGPSDRGLAALLDSDSESFHGEHSSGAAVSDTVVADSGNSFAAAATNAGGGEHDASAADGITASPLPFPPLSPVGA
KVLPPLPRLGALPPLARAGALAPLGSSPPGSPLTGNASQLGNSPGHAVAALPPGSMDGGHGSQNTGATGASSGQEAS
PSAEMPLRGSTATVHESAPAGGNKDDDDVEAEEVAEEYDDDFDDGANSSAPRGVSPAVTNAPSAASLKVALPRDDSD
DGASNADLEVPRSVANRGNLSASDSLSSLAPPLPSRGSAAAPASSLPPLPPGLRRTADPVTAPPPATMSSRMSLLTS
EDSLALIAPPEGVGGGLLEDGSDEDDIFGDSTDAEATGGMVEAPVGGTAEQWSSSRNPIQSQGSKDQSLPQGQPRGR
PGESVSAQRKSDAGGLAIEPSSTSMDLNGELSVEAERIAAMLSSAQPSIGASSADGATSMSGAIAGFGASTSKGPAT
MEPSVIVPMRPLPDSDELIVSPPPMPMTGLRSIYAPRPGITDGTGAGAGASAGGNTAGGVRSIYASTAAAAAAVQAG
ARVHGSLDFYDEEEEVSSSGDDGDIGDAGRSGGDGGRQEGMSVAYSRGPAGGGADASRAGNSGLSRTYSNDSMELEV
FRPPPPASAAQPQQPAPAGRNRARRLSNESNTSASSLRMHLQEQERRAVPAIPFADSLGDLSTSSGAYRVQVVTGSR
SGAGCSQGTVRLTLYGSEGTHVAQLRPAARSAKHGPSGGQNADEDECLREGSTDDFMIDTAEDLGDLSAIHIWHQGE
GFSASWYVETVTVEQLMTGLEWAFTVQEWVRGGQRGGRVVRLPGANLSLSRENSLARLASAGLAGSQPPSPLPGTAV
GGGGAPKRRGLAAIGDESMDVSSDMPSPVDMAGATGSRSVRGSLSESLSRAAQATLQSHDVAASAAPSSRIRVERSI
GNTTNTSGSVHFSDGDDGDDLLPGMGSGRTSRAATAPASTPQSVFAGAAAAATAAVGATARPRMAANVPQGIGVVLE
EDIEEVDGMLSTTEDFEEGSADLGSGAAARRQPLPPTPPLNRRNSGSSSSFSAGRRLSAPALETECSLESLAGVGGG
YNTRRRVRLSGNGEDGGDLASELSVEGSELSIASAASLSLYAPEGVRPPPLSGCVIGVDYVGTSRKRVSWAEYAEEF
EFLRDDASSSGSVASLTEDDILKAQGETVRPDSSSVGYDDLMRGSSAVGVDSMVAGASQDLTAGQHQSANSDGQDGG
GFTEVPLKPRRGTHANSYGYKIKIFTADKLNAGLGNSMVHLELLGREATVAQALPKERGTFQRGCVDRFTLYSNTGD
MGQLLALKIWHEGKTLGPGNWGFEQVVIDDRLKGNRYVFRNPDSECVLRKGRRNCLVLKPSVSRIDESEDLAEVARE
YETQLSANRDLSEAERAGLRGKLDKINQRLSKLGTKLAQSLSGNFGFP

>Vocar20009276m|PACid:23126275

MAVTRVAPLKSPRTSREGDPPKASSEQSRDRCIVANPRIALALCSVSILSVGLDQFTVLHVTGKNPECGSICPIGSQ
LPALKAVLAQPGGGSSSRLRYNSIQVLQLLQSSLLYSFSDPLQGSQEIKEPPYDWQALYGTRGLTDFCALPLIHAKS
VVGAVTLAFDPPPPPGQQAPGLGRRGGLPSVSSGPCFRAALLMRCDESSLRALGLLLSVLLAGQDLKLAQQVGDILA
GVHDARTLQQVVAGVAAGVERVLQLKTHVTVAVTVALLPGSAAAAAAAVGAAGGNVGGGGGGVTAPSSNGGRAIFFE
ERGGSGAAAAGGGNSGGGGGSGCAASGGGGGGGGQSANPNEQYGARGIMARSTSAATREAQGGSSHDGCTGQNGSYG
ALLWQASTVQARALGLRHTLLANLPSGGTVVHDCASYMQQVGCPKRDLYLLSSSKGIPPFSLAIAPAPLPLLRDVPA
TGGGGGGAAAAAQLVLYVSVGMRLPLELLNEVLAQAQHLSLRFLAPLIGHKLARGDLAEEWILLVGKCFSGRGGGGG
GRGASVGGGTLAATESHPDLRPTTSAVCNGTSGGETAAAAAAATAGNGGSGCGEGSRGQCAVSPDDGCSRGSSNAAP
AGTAGCGGVSAAAGVPPAPHPGHPREKATPTPTPTSPPPTCMDASTSRPAGGEAAAAAAATVDAGPPVAECPVVTPG
PDRMLSAIGTPPEPRGPVLSTALAGAFAGDTRAHMGLLVSSFKETINALQALRADEYEELSVLKLGKVLGRGGSGLV
FQGYLHMGLEVAVKLFENPDDTDREAVSSSEHASTITSQPHPGGGGAAADGLAATEAAAAAAGGIDGGIDGGTDTAA
AKGAVKGQPPPALPSTTAAAALATKRQRDLLHNALELGVASSLSHPNIVQGYCHWVNVVLMQDASLNRCWLMGQAEY
LALAPPGSPPPPLCSALVMEYCDMGSLVHALKRGTFTAADGKTNMEFVYMCLLEIALALRHLHTAKLAHCDLKPGNV
LLRSSPRDPRGFVCKLGDFGYAAILKDGLLPGRPAVLPDEACGTLQYMAPELFVSGRPVDASIDVYAFGMLMWELIT
GRTPYSECDYPSRSLMKAVYHGKRPSFPGNTLSAYRSLAVCCWSPDLNSRPSASALVKLLRQQLESLKHQSSTGSSS
APPAVGPRAHV

>Vocar20009967m|PACid:23126317
MPLEEISQAAWSLDAATYAPTIAVLTVVLGLIVLLSLAFPRAGPKCSNEALESAPIDVASSSDPRVVTELLSGAAEP
SSDAAATQEGLLALLAHQEQFSSRGPSSHALSLREDEEEQEQDDEFHHDSTAFPRRGDVVDDDSGGSAARGDDITSP
FDSAAATATSPPAGDEVSNCDNSNSDNNVSGDGGGALEGLERVIPRLRPRPPSATSAAAAAAAFTDPSVSPSSSAAS
SEIWHQAYQARPGSQGTALSQRDLDSAMHAPATFDGGGRSGDGGDGGGGGSSATATPWPVELATSPSATTSVAWGGG
YALPQGWSSASRGGVGEAPFASCGDGGLSSYLVQSFPTDECPLSPASVADAAAAAVAAASHGGRMIRIGRGGFGGGA
TAAFAHRTSAEPSPPPPTPGEGSGGGSEASGRWKPPGLSYVPAVVAEGSALPLPLPPFFSRSGASCWAAEGAAAGGG
RGANTTADDVAAGAEAGSSGMEAVAAEEQSRALCSLRHGGNLPQPLDTPLQWYRRRGVSGGGNGEKECTDTAAARGG
VQMLSAELTGVVPPPEPHVTAAQQPVQVLLAATDDDRLRGCLPAPRPTQESVLLPNAPLMPPALPPPPPPPPSPPRA
SPPSAASMPYQHSTFLSHYSQLGVSGGGGSIISSPDDVQPMQSVPNTAAVGSAPAQAPPPLNVAARTSRTGAESVGG
SGAATIPSLIPSGAMTLTPFMGPPETMSDPVCGHGGGGGGITAGVFVSSAPSEVSVHVTGTSLPLSDGSRADALSHA
IFTSRSEARSSLTGGGAAAAACGTSSTRSSMSGGGGGGGGTSSARSSVSGGGSGGGGGAVNPRSSAVYSVVGFHEPR
LSGGAMGAVHPVVEEQEMEGAAEASGSTVSLAGQCLEFSSTSTTGGDGGSGAIVLQLRASPPDADAALATAVTPSPF
ATRSAAQRSFDAPPQPAVHPSPQVPLPPPAAAASAARRPQLCINLGELLHPPQRPRDPASMPASTVPYAAGPGTCAA
APPAGPHAGGAGARGTASVLYTSRCSSQLLSIKLEGAAAAAAAPGSQPAAAAAAGAISRHVASTLMRHIPPGRLVRV
SALAVRGCIQLLIRVQLGPPLQPAAAAGMRVRPSVGAAAAAQLTAAPNHVASGQAPAGPTEMPSPARGGSGGGGGGG
FATLLPHGWREVTEATALAEPSAVITAAIPEPAVLGTPAPDAADDGGEDGDMVHVRLELLYNTAAAGGGDGGGGGEE
GPAALRIVAAAEERVLLDEVYGMRGRRPYIDLTIPAWSLAGLPPGPLHLWLLRSPLDDPRVTEVTSPSLLAGSTELL
LLPQQAAAELENHIRQILSEAAQHDPATTAVQPAGGGIVDTEGDAPAAAASRGVLTRGQVWSQHLQPLVADFGYVLA
AAGELRAGSVAVAAATAATMAGGEMAAEAEAWWLDLATDLLGHCEAAGLAACGEVLQQAIADGGGDFGGGGGGDAAD
DDGAVGHTGVNAGFPWTGSPPSASSLVVASAVRPINGPGFLPTASADPDKGAAAATTHADTSVAAAEKAVTPPPALT
PPSRRPSQATNRVTLPYTGGRLSPHGGSVDGDEDPLREGGEEDAAVNPYGSPFSTNNVYGSGDEEKWPEGYSRRVCT
VLIRPLPPCRAWKTPASALLPRPYGSAAAGKAAAAGGCTDPQRACTKKAITAADMSPAVSPPPPLSLAPPPPPCPPR
QPSEPATGSSSTAAAAAAAGAAMYGILPHSATSAAAVVSHCGSGSGSGRTQDRPCSDAMSAAFAGDTDVPSFTGCYL
EPQQSGVHTGNTITDPLCATAGQMLSGVASTAAESDTAGFGFGGGGGGGGGGRRSLDGRVSGGGGGGVSSVPRRARA
TKSVQLPAGGGSYSILPTPIPLHMDGDKDREAAAEAAEAATSAGVTADAIAAAVGGGWLQRESLSTAGGHGAAATAA
AAPAEAEAATAELPAGYEEVEVELLVASVNSLGRPQVARGASQGRTASPAAAGMDNLNNGTSAPSARRHHRSSSTAT
NGGGATATAPSPGHATAGSSTTAAPSANLSLLQLRELPPGPLEGLKTPTQPPGAAEADMDCLECDANREEGNPGAAV
TTTREADGARVGGGGAGGGAGSNGGGRGAAERHPLQPRAAAVPTRRSSREGYGHGGNGGGARDGGGGNGNSDDDSDG
LCCLRCLGYQEVECGLLLRGIDPTCGSVFAATAAATAAAAGGEDGGATTAAGREMELTRGQSAVTAASAAPASAAAA
TIAKREARVDVGCAGGNSARSPRDAAGAAGRLAPGADASSSQEPPPVLLAGQPGPGAQPPPSVLPPAPTAMAAGKRP
LGMEAAVAAALALGYRDVTCTLLVKDDSATHGSSSSHGSSLGPGWLDMSSGEGTSESLDTQSGDDGRRRQQPYLEVR
CGLLLRVLQRRRVAATEILPAAAAPSKNRFDASVGTEALKLRRGS

>Vocar20009338m|PACid:23126339
MNHANGTVAALDTLAKLNTAGDSCFLSRGTDAIVAPGPYHLAETPMAGRKRCHSESFLVGQTDVAECVESLRGSGAG
SGGDSCLPPHAHGRDVTCCMWSVGELQLDGAAAGPGRPWSAPGPVQVSTGSFPVDGLAVGTAVLDDTDPPSTPTGAA
VVVAAAAAVPLGLRFSGSSPSEQPTAAAVTGCGCGGCINSGGFSAERSVCAGGGCSITPCIVRGSGCSGAVEGHMAV
SEVAITSRDNPSALAPPPPPPPLPPPPIASGLLIPLPPVTTGLPEPCCQPAFTYHCNLQAVQLRSLSFQHQKPRPSQ
SQQAGAATSAVAIEPPANAGIAGSSTAQQSDSGVKRPASCRSPTAANPAAAQAPPPPLQPPPPLQPATAAATVLGGA

TTEPGAASGCLHANQDGAVDAMDHDATPRWTADGAWLLSTSLRGVPPATAPPPLPPPPAPALPASGAGLAVGNAPGI
LLHNSTPTSSGKHQKQHQHQPLLGNVITTGNSIECGLPPLIQCGSGGKQQQQQVGIAGNASGAAVVAAAVADPTAKS
SPPPPQQPHPPPQQQQQQQHESNEPKDMGLLPRPPPRPAVAPNAVTTGAGPFAVEATGITFAAAGLAGLNFGGGAC
QDGIGGCCTAGQPQKQHQQQHRQWGMQGPFSARRELAKPLGRFNVHAAREGEAETPALAAAATAAAAAAAAPWEGF
ADDATSSCSSKDMRARPTTAAASAAAAVKPSPGRQQRDKEGMLHEQDNDDDDVVHEDEDDEEEDEEDDKEEREQAEV
KDQLGGGEEQDEQQDEDEDGEGEYGQEYEQNQRRRRRGQALLSIAFPPPGPEIQQGDRSCLEGAPTSKPQSPDNVVL
PLPHHSSLHCAYRQLNPHHVKQHAPHQPQHQHQHQHPDSGGAQGAVQDLRTVAQLPPPGCSSGRGTAPCEPPRLPGS
GAAEVHGQEGGWTMLSEGFEPYNTAEPAQPPWALGWANFSSGAGGMSAGAAAAAAAVPRATDGVSSVQLPRTATRVV
QPSDGGSGRVRSSYWDEMEPMYGSACVSDELPPPPPSSVLMPPEYERDAAGAALSTSGTVLQHVEPPAAAPAPVPQP
PQPQRSAEPVSASAATSSYTAEPCQRSLHALLPPPPPSAQLASPLQQQQQQQPEHQNEFHHAADRAVHPISGSSAA
AATAAATASFVHAASGFAPALTYPHAPPQPVALYTPFANQSDVWRCDGRVKTPTPPSQQQQQPLPPQLQPPQPAPPN
DYCPSHRSSINRPEWSAPLLPTGSLPKAREQWQRPGPREPASGGEHGATAPPPSPPPPAAVPDCCYCSYCHAGAGCK
DGGSSACAVHASYDICTACCHAVSQLGENPHHGHPQPYHHQQQGATHHHQHGKVSAYCTKKGAGEGSTAAVDGGDGR
KHRLDVSGGSCGGDGGDGDADKELHNSKIQRSAYGNADADVSVLHGVSQQEQAQCHVSGDTACASGIRERRVFGYGA
AAPYGTLSPRRSLYGIDPLSGAVTSAPQWQQPVSPPGPGRAPTPVGGGGAAARPAVSPLYGTCSCPFCTAQLRQQPS
SAIVLAGHTATPGPGVGPAAGFAPTTAASASSITAAAAAAVTAGSGPAPRPPCPCEVCRRAGVSMLPSGTAPPPPSL
RPPPPPPASTVHQPCHSHCQKARQSPNRASALTQPPALPRTTLHALGATNLQPAPHSPNLTSTHPNSTGSFAHAATS
SHIASPFSSVSYQVPYSATHRPEAGRPPTTTAASCSCPACVGAPPGTAAGAAALNAVPSADAAAASIGTDPPQSQPL
PPPQLYDHTNTQRRPASLVAASYTTSAAAPPPPFPPVSCISLTRGVSASGGGSTREPDGSNRPSAGVAPPPWSGSWY
DNAAYNSSNMPSAQGPASLPHQPPPYAPHHVQTSVRAPPLPSPGSSRLPSSYSSSGPGAVATAITAAAPSQAIPQYG
NHDESPPEPQQRQQLLQQPLPQPLAPHVCAPAPATQRDSQDCLPTCPPTCRPTLRASLSVPDPYAYQIQPCQQLQLH
ACHSSLPTTKSAATASAAAGATVTGNCTIPVRPGIDMLQYGTASYCAAAALTPPPSVLAHKVSMQLDGSATRYASCT
TPSQPTAVAAVVAAAAAGIEGPEVTQHSQLMSQSDLGQRESHALAPPPPPLPHSSGAQAGSVQQTGFAPGAAASGGA
GLIVLAGMYGESVTATDGTCTGTAPSGFWDHGHLPAVCSAPAAHTEYCGGGGGGESQPWSRWAPSLPAAQQQHHNHL
NHQPQNQQQPQLQQRGPPMPTAAGGPAAAAAAAAGGPDAAIGGYSSSDADVYRSLPQHPQIPHISTATTQMTLGSVA
ADEEAAMWAEDPISPDLASPGAPHRALAVRRQTKRQLSTTDLNQEWSAH

>Vocar20009711m|PACid:23126388
MLLSLSNTDWTVSAARAPKPTASPSPPPSSSPPPSPSPAPSPAPSPSPSAPPPSPAAPYSPEVCTSPTLLWSDEFDA
ATLDTSSWNYFTGTAYNNELEYYTSRPENVRLENGSLVIEARAEAYGGMNYTSARIDTRLKRAFYPGVSVNGTVVPK
IRYEARMKLPKGQGMWPAFWLAPNSQVCDACGPYGSWPYSGEIYILEAINNMTYAFGTVHYGGFRSDGQTFNNQGHY
RPTSFNLGLEWHTYAFEWSYNQMWWFIDDVLYYTTHSYFLSDEGWWTSSQTTTPTGPNSPFDAPFYIILNLAVGGDW
PRAPDASTVFPSQLLVDYVRVLGYW

>Vocar20009936m|PACid:23126447
MYGRERGPPPPPPPGMMRGPPPDYMDRGPRDYPPRDIDRRRPRSPPPPMHERDRYGRGRSPPPIPKRSRRDGPYDRD
FHEPPFGRGSPEYDRRPPSPKGAYDEREAYERRGSVGRERPEEEKRPMTFKEFTLRKVPDDAAPDVAHRMFQEYLVE
YYGSAIKAEFEQNKDSDVFKYQFDPRYFAKVVASRAEEAQDSAKHIAADVASGALDPSHENFCQGMYDLAPKVEKDK
QPGNAEEGPKFAPNLCWRMPRVAHDLDLSRKLIRKLDAEKGIEENPLIPKLSAGASGGVASAADGAPEGDGAGGDAA
APANGGESATDGPPNNQGGGAVTTTAVPAGGTGDEEPAVVVSEANYTDSIGKLDLQLHWLWKVHGVDYYAGIELNEQ
DWPYRLNCCRLIRGPKPEEGESEEGAEKAEKEKLTRTVDETWKARIAHGDPIEAKCLKARIEEELDKWIESQVTMVA
DNKWGSKLSNKLFVGKKYVIKHIKTKHQDKLAAERERILDMLYFENFRTWKEDEERRRSEEEHHAGEPMEGEGAGME
GEGGYSGRGRGRGGVLGARGRGRGPGRGMMGRGPMMGMEGPMMVPMPMAPMFIPPGPMGPFMPIIPGPVPLLPPGPI
PIRGRGPRPLRGRGIPGRGMGPPKIDPRGMREYFDLDHPANNRAVLDYGDL

>Vocar20008225m|PACid:23126448
MELEDKARATLQELLDVVRGYREAAERSEQEASRARVECEAWRSRAQEEHRRNVRLRRMLTIASCSQSKCFPPSLRR
VTRYGPNSDAPAYHFSSMLAVAIALSAPAAQGLQGGYPRSQVDSDLNQVCSSTTAVATAAAAASAAAISAFAAVSPR
ASHGIQEGDLSLALQRTSASPGPGPGPGPGPGSSISAQVSPAEQQQSGGLRWSPGHVGHGPASAGHQGLLLVAGPGS
RRAGGGGGSELLQGASGAGGGGPATVDSGTRNTSCSSLSSMGTGGGGGGGGGGSTRGHSRAGFKLVLRGGNGECGWG
WDVSVVGGGRGIGGWGWGVGFARRKGQGRCGWMGGWTHTHTQYTQMSLMFVNLKPHLAAGASVPVSPTTFPTLLLIR
GSRPGSRLLYNPYHHPTSPRTLIPRCFPLPPQPASLVCGDSASLRQLSNYGAMFPPPAGSGSRTSRADGPGAHTGPS
KVHESVSACELTRAGTGTDTTGGGKRGRGSSPPGAKSYANTSSAIAMMTSGGGGGGGGGGGGSRGSGGGEDEAVVPA
GRAHSVSSAGLDARLAGMQVSVAGPLPAGPPVSPPPPPPPPRPAATAGAAGSSADLTPSCSSTPTSRGSVTTSFSSD
RDRDGGAGGVQQGQGQLARREGGGGAGCCCGGGGTGGLGGRAVLLPGEDRVSGGGRSVGGRAAVQVQGEEEQEEEGH
HLADVTVVFSEPGPGPGPGQEELSVARQHHHQQQQHEHHLRAPQGPRGATTTTTTTTLHEASSPLQPPSHHPLPPP
APPPPQQQQQQTASAPPPPPPPPGSATFGSVTQPPSTFNSVPGSGSGSGSSHVGRTQSVGFARSPLSAALMGMSAGS

GVPRRETGPAAAPGGPGAAAGGGGGGMVPGGGGSFHEELSWHAGSLHKQRTKLQDDLASIQQSSNLIRCNRATCNLA
PGSPRHTGAAAVASPPRAGGGMMSAAAAAAAAAAAPLARWEVPLLPPHEPAAAAAGALAPFGVVTSEAAGSPAAHEG
AAAPGDGSGHPGPDHHLPPRIGSCSMPMPATSGGDVENVSASPSRGVPPPVRSCPSTPSSTPLAAAAAPPSQPATAP
PSPPLVAVAGQPLPGTAPEEAAAAAVPLEAATAAAPLGGGPSEPSCRTCQAMSPEPAFVALYWVTPPSAVLVVAKPS
PGVRPTFERVLAWLHQRGVATYVEPSLLRGWPGLACKPPSPPPAVLQRRGSSYMRRRAPLPATGAESSSSSSGQERG
DGSSSSGRSGGVVGGEGWVMEGEEEEGEEGWVEAGEGELFEEGGGGGGEEAEAEEAEEGVLMPGCYTGVPMLLSWPA
DEGDDCHSQVVPGDVAAAVDFARSLASLPPPLHSRDAAARRLEIHRPLLPSPLSSSPPSLPPCTPALHSLPPLPPFT
PSSPSLTAGPSRCRVPLTRCKLLHPPFPLLASGFPIMLRHRLHCHIVRAAEVGQCGGLGPAAAAAACARGPPGQGQC
QEGAGGPSVGALLPPPPPAAAAAAGSVAPREWVVLNEVVIDRGISSFLTNLECYCDGTFVTHVQGDGLIVATPTGST
AYNLAAGGSMVHPQVPGILFTPICPHSLSFRPLIFPDHVSLCVQVPANSRAQMWCSFDGKDRQALNAGDAVVIRMSA
WPVPTVCSKDASRDWFSGVREGLHWNMRRLQAGAGQ

>Vocar20008276m|PACid:23126463
MQLYGVATAGDPALLFSRLVIARGHILAAVFEGHISPSAAEYCRSHCWEAFVQATASRQQTPAAATSRPVPSSCVLA
AFSFSCGFTLHPPHGALRALDRGFLTCSKHSLESRLSSGCSATLLHIDLATRTVTTASVGAHGAVGLGSSTSTSSGG
VPGVGRSTDGGGGGAAVTVAADAPAATASAGRRGALGFGCAKDLPVLVAAYENAANAAAAAAAATAAAAAAHVHAQP
AGRFGAASRHLHCGCEVRVSSRPLAAGDECLVLLAGCGGAGGRTASGGLGARAGGRGSSGAGGVVAAAAAASMNSGA
GGESNGGGNASAARSGGAVASGATGSGAISSGGASGGLIGSSALLPLPLPCAWAPAGALSAADAVSLAHELAAAAAA
WPPAVKEGGRGASNVGGATAANSAASVAAVRLWGPAMDDRFGVLYDSYDAEATAVATATPTPTAVNDSSVGDRSAMA
VASYAVTAALRNRGGLGGLVRGMGASAPPATPASLSAACITIWLDWGIAAPEGAAAAGGAVARPATSRIEMRPPPPP
QQLPRAAARWDLLRAYCRFVVARRRRIIAMWHALAAAVAAAAPQRPSPSAAAPSPLAQQIASVLSLGAGGAGQASFE
SCTGGGTATVMNRTASFTSRPPVMMEFVEELPTPASSGAVVGRHDPYGGSVMAAASAVTATAAASASAAIGSSMATA
AANAAAATLDESHTHSASAAAPYGISGLVMAPMGSTEDMDVADGDGDAGGFSVEASTAQPPPPPPPLLSQRSFVMPYP
LGNRSVMAALSLRPSQSMSDLNNLEAKARAQSINPREPSVSRHGSDGGAAAAVAAATTNESTSCTDLAAPHEAEAHL
PYHRYYSNQMSTSSAASTAGFGAGAGGGGGGGASGSHFDWSLVMTSVQQSSDSRYENSAGHTPIPSTAANRFASSLG
YGHDGVAAAAASHQMTFGGGGAVAGGRSPAGESHAVVGSGGYLAIPGGGGGGGANTRLRQKSSAPPPGSVSFSSYVS
RDALSAASVAAVAPPPSVRTSSRSDSVGSGGPSVGRVGVGSLLLPSSSGCSLDVQPTPGIVIGRRSAGDSDGDDSTS
PAAAASAGDAETETELISEDLPQLRIESTAELAALMAEIIITDDGEVVRGAGGAAMPPPYNSRSHGLHVGGLHSHKG
SRSLSRQGSRGWQPRLAIVREDAREANTSTSCSQLLLAEGVRFTAISEERGSSTSQRGRQRRPLALQELHAPLLEQQ
RGRQQQRPCDADWLRTTPSATTVIDAGVTAFTTVAATDVGVGVDGEGASSRNPLPLRQEQERVAVTAAAAAAAASSA
DVATRTTTKLPLSAAAAPPPPLPSELPSTGLADVLDDLRPSLESHGLTEKDAAAAPSRTDVAAGAVASLLRITTSPS
VARYRDVQYTREPAVPVAAAAVAAAAVERISTPTSVLGPHWSINELATGDGFGEDVDCAVRDTGAAAVAGGGWQRQS
PYQLRSYRTAAAATRSDATAAAAAAAAAAALSALESFHEAASAGGYPDDADAAAAFQSATFQMVGRARSASVSRSAGL
SNHRNHHQSSFPFQSLHHVQHRQRQHSACQYTSVDAASADGDGDDDGGDDVPVSCQTTAPQLPVLGPPSGDEHHSIP
SHPAAARRTRRLYGLSEAVPPLSAGSAARSCCSAAAATASATTAAAAAYDTSGAAANTVTTIDRSWSMGSGSGPLYS
LLQSTLSKGSITAATTSTAIPTPACDSAQCSGTRLPIVRPLSPPPLSPLQASSAAAPLSSPQSPASAGAAASSTAGP
SRVGVPCTQPESGGGGGGGHSRRPSLLLAADTDADAVVVSSSAAAVAGLLPSLPADCASSSRLEGSASFSVTLHAPR
FSDWEPQGAGAAAIASGVSVGIGVNSSGSVATVTASPVPGFGFRIDIPPATAAAAGGGGTQSAALMRTFGGVMYGTY
GAIAGPSNNAIGEAASAYDDGAVTNRSSNISRSDASVWNSTSAWTTSTGRTTDTMMSNRRVLASNGGVGGGSSCLML
PPGIAVGGGGYGLGAVGGGGGDTSGLLAYNAAYSSISGLVMTADAGAPGSNTQLLVQMAAPGTFCFGNGGGAANGQN
SSQLFPSSRQLGMFPAPNGGGGAGGGGESFAYTAGQTTAAMCAQVTHTQLITNSTIINTSTSRALLETRGAQWNDPW
ASPSSSSSPPQPSAAAAAAAAVAAQNVSGGATAAAAAVLQPRQPPLPRLSPPPPPLALPLQVASASEPGEGGRYDRPA
VGVYDSSSQAPLIHETGIYDSASGCFFLAGGSTTNNATNNSSL

>Vocar20008616m|PACid:23126490
MATLVEVAKHGSALHVDAAVQKLAGQLNKPSNSEVYPLHVAVWRNHLTIIERLLEAGAWTHCTDGESGWRVLGAVRA
GGGESCSGVGGLCLGAGASVEATDLQGRTPVDLVSSELRRMPSEGAGRCSAIYSWGSGANYQLGTGEKGGVAEVLTV
VGESAGGSTDFHLNPLRVEALQGLGVVAVVAAKFHSAALTADGRLLTWGWGRGGRLGHPDYNIHSGESALIHPWQVA
GLGRRVVVAMAAGKHHMLVATSAGDVWSWGGNRDGKLGYPNVDTQPTPRKIDFKGRAVLVAASNRHSACLTSAGEVW
TWGANNDGQLGYGTNNSANNPTPRVVEAMKGKVLVGLSLSKRHTVVVSAEGDVFTWGHRVVTPRRVQLAGCRDTARL
AAVQLQQLQQAGGGGPGPPVAASAAAPAAPLPLPALPAAAAAAAAAAPCGGGGLEVRFHRNHNEVVRPEAALAAAGY
SHTTVVTRSGEVLCWRSADPALRPQEVAGPLAGKCVVAAAAGKTRTVVVTDTGQVYMWECKEPQHQQQRATPGGGGA
AGAGAGGGFGSAGAGAKGTEAAAAPGTGGSGTGAASGAAAAAVAAAGGGGGGSGAGAVTGQSGAGMGTGEEAQIVPV
RVTGLTRVTQVAVGEKHTLALQAWSVPPIPDEHGVMPGMQRHRRQRRSGASGSGGDGSSSSGSDDEVCGGGGGGRWW
RSGGNSFRAGGAAAAAAVPVGSLTRGGSGLGSSVGTGFTPPPGSSPMARSFGTSLDLVPSPLAADRAAAAAVAAAGG
NLTGFFAVGPRRRGDAAAALAAIDEPGPPGWELFSHRTGGEPGAGAPSSPLAGARRRSAGGGGGFPYDGDDMFGFND
GDSVPGARRTRGGGGGGGGGGGSVPSLQVLCQRLVATQLVEPRTALPILEYADVAGAALLRCYCLAVAVANLDCTLL

EAPGGLISLPSHLLAELEHLYRAVLLAPAPTSHPQGSLPALGSGGGGGSAEDILGTSPPIINTALLQMGRRPTAAPA
WLEGASATDRMTPQASSRLGAAALAALAVVGHGAAPHGCAERSAPPRSGNGGANLAAGAASDSGGGGGEAVSEVQRL
ARNLQRKLQQISSLEELQRGGRQLDAQQHAKLSQRGALQEALMALAAGAAVESVKQAMAHSAEVASKLAARPDAVQA
ATASASGSGGDGRAGGGWARSSGGNASGAGTAPLSSQPGQQQPGGSSGGPGRKRSTKEAGGGGGGDGSSEPLTVTPT
AVAAATATTASVTDSTVNSTTFSPATTAAGASRVTTTTTISSSSSLGNAPTTPATAVMTAVVSGTGPARKQQQRRGA
LSVFLSGALDQPLSPPPAASPPPPVAAAAAPKAPAWGGAKLPATPPGGSSLRDLLSQQPTSSAATVGPLQQQPQQQQ
TAAAASSQAAPSRSSAGLGLAATPGASSLPARTPSGPLPLPVAATTGASASSFPIATAAGAAPGGRKPTSSPFTTAV
PKAAASSPAIASPGPAAAAVKASPIAASAGGGGGGGGGGGVKLTLAEFMSGRPITAPLQVASLRGRDQEGEKEPAGP
AWGGVQGGSPVGAKSLLDIQAEQALVQKRAAASWARQGVPGGPGGGLGPGGASATPNAVPGGPAAIGAVAAVPAGTS
PPVGRSLLVGLTQAGAPPLQQQQPSKWYVPEQCQPAVAAKPLTAIQTEERAIKEITARYGGRVVARVVPYNAVEAPT
PSASSLAASAGTGAGCGVAGETASGRLSMPAASGHAGSALAAAARSKQKAAKGNTS

>Vocar20009885m|PACid:23126539
MDHGHAQGGKAAIDPHDVTDVLQQALWELKRLKNQLAKPYATRSTVAANIARLEDDIRRQALAIAHATGVEASLALP
APSPPPVGLSLHPKAPAHLPPPSTIRADQFLATRSGPLLDNPITETARQYLQERFAVPYQPKQPPPPGNLAKGRTPA
AAPGVILPKHIREDPHGLPPPEVTDLELDKGLLHVLNRGLLPARSDLTPALCGATGPIRTAPVPVHPYQKQFERGPV
TSALEDALTVKQDFKLDLLTPVIRPQQESKQISHHTLSIASVAGGGIMAQPRPPHAPVYFPGVDSSSEPQSGSQYRQ
SVSGLDAQQQQQAAGTAPSRAFESLMDAFSLHEVMIRKGAVVRDTPEFESYQRTFTSVWPVVEGLLQHLAALCAQYA
VPLAVVNGKSLADLAEQVGAAGYQPAMEDLLVCLTNIQEVAALLKQPGRRFLGPGGRDAAAQLIQAQYRGHLARRKD
SVRGQASLKIQHAWRTSRLRKQLRERMRMARLERDMRFTELRDQLEQQWPLMQSNAHVVVHVPNLLPTPVVAPGDSA
PQRRPEPHLIQAMLLREAAQLARLCDLAEPLVDLILVLPSPPDPEVMHYWNKLLEVGGVRDPASRYRIVVPENYVRL
PGHMSVTAKLLSSPRALKRIAAAVHGRLAYIVPGNVHDEEVELSVHLGVPLLGPSPSVCRALSRKSAARELFKTIDA
NIAPGCAIRPQHASTASHQLNPQLQALTRSGRDSGDGATGDILTTPLRAGTAFAFAPDGELIMREPMSGDAVHGSAA
AAAAGSGSPPPRGSSSFEHDEQRILMTLAEAMVRHPLVPKWLFKIDDEIMGMGHAFFDTASIKGCDGVLERVIGEVT
GSGPRSPGDALRLELGLSGSGDGGGAATGGSVPPLTEVQRVAMFRLYELLFRQLPRRLHLTCRESYPTYRDYVTTLA
RRGGVLEGCPHMAVGSPCANLFIDPNGAVTVLSTHEKIFCYPYRAVGTTFPQSSVPHRALYDAALAVGRSAYTAGLL
GHVMVDFVTLLEPPGGGADVPPPGSANNPGGLRLWLVDLRPGITPSLTAFQLFDFLAAGTFNPMTGAYLVELDPIED
TDQGGDEDGRGQGKNVMPPGADAMALGPAPQSLRYYFVLDQLQHPAIRSMPCSKFFYYCWQHGFHFDVDHRQGVIFN
LSDRYLTSGALGCMTVGLTPASTYSVMQRVLSFISATELDAAQRSDWSLTVEATTFQDVQALVKFMAEQSASQEREA
RVGHGLHVPGAGLAGFGRGAA

>Vocar20003560m|PACid:23126735
MAAGQSAFHPPFLPIPLLRPPASHPSPPPRTRAPVDPFHVPSHLPRPTPPFPGPTLPPSPFLYTLSLAAASLVRGWS
FPPPSPPPLPALPKPIHPDPQQHRLHSPLLFWQYTPRVFVYQLNEDGPEDDDEGEEGVPSYRELVLPAADLQGQWES
LHYDSAIKQRLLSYATSALLFADRGVNNNLVAWNRVVLLYGPPGTGKTSLCKALAHKLSIRLGDRYLRHGCLVEVNA
HSLFSKYFSESGKLVSRLFSKITELVEDLDSLVFVLIDEVESLTSARKAAVAGSEPSDAIRAVNALLTQLDALRRYP
NVMVLTTSNITQAIDVAFVDRADIKAYIGPPTLAARYEMLRGSVQELGRAGIIAGERVIDAAVGVANGCGSSGAAGT
AAAAAASPSASLSALLLAAAAACEGLSGRTLRKLPFLAHAGSPQLAQMVPCGGRQFLRALREAAAREQSDRSQLLSS

>Vocar20003669m|PACid:23126751
MTRRNNLDLAPRNVNQKYVLQPNLNVSRPALATYAPGLSGLLLPGMAPIATQYPKQQQQELVAPRHQAPARQRFSTP
SSPSLRGRQSQANPNFAPPASNISTRLPTPVRINASPGPSPSPTSVAPMLPKPPSPSRSLSQGGAPVTNPFACASAY
SSPPATFAATNAFMLPTPPSILSNGPPTRVTPIQQAAARSTPSPMPWASSAAQGQNNLTPTSVRPSPPPPERSILAS
VQLLNQSQQQRSTGYGSAVVGTSSLSAAIPGHSEWLAPVAAPAPSSVITGVAPVLTAPAIQAALQQALQGAMERKPE
AFRVDDFERFFDSMMHPGDTGKLDGPLPHELADDIRYSDNWYKHCRDWNAHNKLTTPYSAPAPSGTAPEMSTFCRLD
DTPFAGEQYTCTAPAATQQEPEAHGQPQWRLGESSMSEACCKALDIAHHGLCKVVTPTARSRSATDMSGMAGCASTG
QGTSAPASLLDQHQQSLTRRRATAAVAPGELWDTGPSHAQQGAYTAAALTSGLETLARDQIATVATSAAAADGPVNS
QRHLAPSLSLVASTAENPLTAAVHGEDSGAAASGVSVDVSATPIRTSINLPGGRKFLVPALRLGSLDVLPRAAGAAL
SPPPDSARPPSTAATTPGPRTPGEGGGGAPNSTVTTAANTPVGRDATGGAVTPTTTTSRVNVEASPFFEAASQDLEV
AVAPPPSKLSRDVAATDYVRDVAVVGSVELQGALQIQTLSTAEAPAPSASAANATADVTTLTETWLPAFPTAVSQQA
VTKRAAAAAAGPQAFFNTGGASVPSAGPSSLAAAVTAVAVPGKAASAALKSTAGPSTSSASATADPLKEIVVGIEKL
STLKQNILAEDKEVDVASITQMIQLKLAVEQLLARTRSASKAQAAAVTATAAPAASSAKCILGASGANAAGQAIVTP
ASPSTTVVGVKASRPMSASALPRQAASPSLADKSRYAELHQRAAARLAAAAAEPASGTRQAQGASGQVQRGGIWQRY
EEKIAMHRHQLDTTKAQLDEAVRKLNDMQAEFDELLLCLGMESAKNKALCDAMRAAGLDPEPILAAIEEQWMSGALA

>Vocar20003739m|PACid:23126865

MTGSFTSAMWQRFCPVGGITTELLGINATSVPPRLQVVVFPGNPGSAAYFKPFMLAVHRLLRGRADVLAVTHAGHDP
DTDHGGRLWDLSEQVAHKVSYLREHVLVPGRAPVLLVGHSIGATMMFRAVSQIEGLDPPPHDPQHQHLSVKQQQQRP
PILKELSRPWDWSLLSALGPRVHVMGCTADTWLSRGQYEDLLARVPGVQATWHPDLRHAFCVSKRQSLAVAEIISAV
ARSIPDLNLNLDLDLDQAASTATGTCVGMMSGDPATAALPPATPAPGCCDVDTIRADIGGAADQAAVDDTTGGIATT
ALYGKIASDAAGGFREGAAAGFEWGVMRGAVKTLAALDSQAVGNRGLRDQIQALKAAADVDPRAAMLATFRHILSSP
PTPPSHEQQDVPQQLPAISPEPAPSSRGPPPRRGTGHCSSHTLAGNSDGLAREPLSASAKDDPGVAPSNSSNSRGIR
GASTDLEVEQLNGVRAAVAGMSLSSSSSAAVAPLSGQRGTIAAAAAAAGREAATAEVSTGGRLKAEYEAGEAFGCSR
DAATLGISATPDAPPSPGRAGTDGAVKNAPDTAATASLSAIPAAPGPRGAGRPAVPIALPPDAAALHLPDMVALMGE
LRGRLADLGFRVESAAPSPPPPPPPPPRSPPPGTPQGAARPSPS

>Vocar20003630m|PACid:23126878
MNHGACMKVFGAEKSPASAMEVLEKPPCTVLYPQGCVQALCGFDGSSCGVKYASFAAFAPAVLDTDAGMQAMFSDLL
SQRGLRSTAPLPGPLPMPGARPARPSFLQQLAERRRRVIGGMSCPGLRDECYACLYGCSQAGVLGPRDIEAAIMDTG
GDAGRALAWYPGLLMAWRFDKADTFFRDNQLPAYYGEIDSIRSGRLQQDYSRNGTFLNGIPLQPGRQTTLANGDRIS
LVLSINPLAEVSYVYEEASLMALMSVDRPEECELFNDSCGGSSPLKSSLPRPRPPLSYIDGDACQGQVKVLLRPCTS
SLPCNVQRAVSAEAGLAAVAAAGPSSPHPLHPQQPSVPLAHLRSPDRRRLSRTASSTAGFGGHSGGGGSVAVGTVRR
RPRHVPPTEPMGCRTVDGGGGDGNDFIGPMKHGIGGSSDDAVVSPTSTPHRRCCTAPGRGYPISHVRSGGVAAVTAS
ASTSAAARGAADFGRRDGTVPGAVSAGLAHRTDRTTANRATAWDAFAFMEAEAFAARDVALIRWPSSVPSPPGIEPG
SINTAMPPPGAASVFAPDRRLRCAHSPIQSCPLPQTSSPASPPACPRQPIGRALPPPVPQPSLSPSPADAAATAGGS
CGGSMRSGAGSGAGRPRWRPSRLLHSSSPGSLLSDMKGTAAEHDATPPATGQKLQRPSPPPQSSARDAVAANVQEDD
APGMAAAKLVAALAVEPRRGRRRNTRTGADGEDGCEAAGGGGESSQTTSEDDAHSFSFSIAWRPRVSPPPTPPPSSC
GDAAAAAAGGSPHWADVRESICSAAGEGPGPGLLRGRFTQAAGASGCDRGDEKGISASAAAAAAAATAAPGKPQSQL
QRKAARAAAASTDTGSICPYLPYTPITLRQRLLSSCYRDAHTTGVEGSGVRSVCDSNDNYGSGGSDRDGSGNPHSAQ
GSSRTSAGNWSNQCCGDSWMDISVDGCGRGTASGNDGGGDDAPVSSSYSCVSGAHPTGNAAVVVGAANAASAASAAA
RRRYSSPDSEESVYDNLRSGIYCGRDTVEGVLGDHHRDRPSVAAVMVDRTPDVIRRRRAVALGSYESMTLGNDPQSV
PRRARCHILSPSAANPGAFLPSLALRTNLTPAAVASTHATPGTDDDDDVDDDNGYDGAVTAAATCVASRLVQQRHQQ
QLREALDSPQPPSPPSPPSLSFVMPPPTSSGVAVRPSPALAMQHLMTCSGSDALDPVVPGTCRYSPRCGPEALLCGL
CGDYLQRAIAVHPCGHTYCGSCLSEHLSSLMAAGCRLGCPEGCTVFTHLAANQPARRLEEILVGPRHNDGGCEQERI
GTGSSGGVAGATAAAAMALPLGGGGLAATALTPGAAGNLAALNPGPRDSGGSGTADAVAAGASNAGAETPARFQTTT
ADIGGVQGPSSAVPRARPERMQHEDSPAPHHRNLTGRHNAVPSPSQSPLQRCLLYTPAGRFPQTESTTTATAASDAS
STAGSYGGEDGGGDGDGARIGERRTRHRRDGDAAGGILHDMGALGRTVDGGVSELCPLPDGILPLPAVRLHLRQAEH
LLLRLRGVMGLRPEAAPNAGAVPDADDVAAARCLELLCPLTRLATTQPEAREALSAMGAIQGCLLVMEQQQRIQLQR
DRQHLLEQLNRHRQRQHLPQQQRQQHHHQQPMPSSHGGRCNCHPNCNSCYCSTDRLDVDPEPLRNPCNGDQSNDGNA
DVGAGAGGGMSFHCDELDISDRNLDPAGRHNVVTTAAVASTTRNNGQGGSSSGGPRMTASSPFPRASASSPSPSYDD
GASTWHSSFTAAADHVVVPCSGTAATPAAAVSAHRESWGPAPAQTLLAKGSTAAAPTDDLAVCRQSLMAQRAACGLL
VALLTAVGPDDGCQQSNQWYMARLGAVDVLLRLLRRAADTVAAMETAAAAAAAEAETKTEAVNLTLVRERRTYGEGQ
GVLDHLRQVQRRQCWQQLQLEDEADFLAIAVLTVLRLMVAENTMTQAHLACDGTAAVLGLLRDMPRSVGVQTAGLQL
VAQMAQGDDVTHTAIRHCLVEKGAVQLAVAALGGCGDAVCGGSGRRGVGAGNAAAYHSQCQSQGSSVGDERVTGRAG
GGSSGGGARGPLSAAALAALKALLTAPIPDLLRKALASELRRVGALARIRSSLREFEAEVEDRWLLTTARRLEKQIV
EVMAANHWWRWRRSSVACGSGGGGGGSWELSWGLLAAAAGVGFALGASASAVVVGAFLV

>Vocar20002267m|PACid:23127061
MFGPYSHTATRCGGNPTGRAAPDTLHALMLADLGRQPVLSSITVSETGQVLPQSGGDIIVYVGVPVTVVFTVTDPDA
GDPPPQRPPKSFRFQPSNLTSSSSPPPHPLPPPPLTPLPSLPLPPPPSPPSPPSPPPLPPPSPIPSPFAPKRLRSPP
SPSPAPSPAPSPTPATAFWDATPRRPRTATYCLLISSNASLVSPNAECAGMDTVRRVDMVHNKHMSTDRV

>Vocar20002176m|PACid:23127091
MDKAYAFCRLRRASVVLVILACAFAHVHGARKLHQDLHKVEDIMQGGEFFDAQSESHMKAIESALAALTAEDSDTLH
RVLLEGAAAYPPSQPPSPPTPSPSSSPPAPSPPPPLPPGLAIEIGEYSAKFVNSSLSRGLDPTDKESLEAFVGGFKN
ITADFFGISPNNIIVRGVYKGGQQLTRRMMLARSERTWTHRQLATALPANSNMRLLIHVRSYVADGATVGALRQAER
DLMQSVPSIPAAAATVYTAPLAPLNRAMAETSNDNSVVIVEFTVVQYVEVVLPPSPPPSPPLSPGEIAPPNVPPSPP
QRPTHPPPRPPFSFERLADSLGALKPPSLPLTPPPQLPSSPQPPSPKPPSPPSPPSPSPPPPAPSPPPPLPPGLAIE
VAEYNAKFVNSSLSGDLDLTDKKSLEAFVGDFKNITADFFGISPDNIIVRGVYKGGQQLTRRMMLARSERTWTHRQL
ATALPANSNMRLLIHVRSYVADGATVGALRQAERDLMQSVPNAGTVRTRPVYEVSSTAAGRMAQTNSDGGVVIVEFT
IVQYIEVSLPPSPPPSPPLSPDEIAPPNAPQSPPQRPTHPPPRPPFSFKGLADSLGADIVSPSKRTDDGDLEPGTVV
WSDDPIFRTYRKPRSALNLVFASKHMTCAVMRGVCAPCPRAWEATKTFRTVPIYFRQPVQIDTIKILQLQNPGVVSV
ELLPWPATAIPELPSLQSRNGTLGAPVWKVEEDSSECGMDLVIQVSPEVSGMNIAVPPRGSQESLPPRFRSSAAGGL

LITVKEQRRTERPTVIESGGSNPTAVTNDRYENVSNPIGLHGSWDDNLLAAGVRGSGARKGPFKERTAGSTQTAAAD
CSSARLQAHLKPECSGSAGTGSASRAVSARPNSYWPGSVEEALTLVVTVSFNGESGRLERCRTGLAWCER

>Vocar20002174m|PACid:23127201
MIPQGIMWIRGMTEIRTTALLSLSVLRGKIMLFSGLQRRDDFLRPSFINDGAAAIVNGAISAACRPVDYLDNNSGDD
VTEDGSGAPGGDGESSPRGRGRLGELAAAPTSTRQHHQADDHGGRKLIQGQRLLHSGADDTTVVISSRNIGSSAPAS
SPGPQRVGSDEDLTRGPDRVPAAGTTGATTGAAAGGGTVATHTVSLVTNLDSDLGRRTHSHTLAVGGGGGGGGGGAA
ATGPGNVVSGTVLPCSQVALTMQCGTATLAPGATLLGNLETPAVIATIKGHGLDPPSPPSPKQQEMQQKPQELLGTR
TRSTVNESGNAYATSHEGVAAAPGGGEQGPAAPADTVFRSYLRAADAGPDTGVGSRGFVGLRVGRISRPREQLGGGR
CLQVPQSTQRQDWFEGKGGQRCQSHGALAGGNSEGRGGREYVSSRSQVDREQHNRDAEERCVYDDRRDDGGLAKDAR
TSGDIQSYGRESDKVRGRHEYGGAPRTDVGRDNGRRDYGRYDYGGHLLKCERPCDSVGGRRGYVIGRVYGGYYDNGS
HREVCRSDADRGRGDGNCTDAGRREGVRPGDGPSRDGAGPIGNADPHRQETGPRGSVAAVVKSDTGRGEPQEDQQER
QRIKGEDAAAAAAGLDSSRHGAVARAGDAAVAAVIRVLRPGLGPGLEIHTGSGPVLPANAEAGAVGDRGCGTTGEGM
TERAAASAAAAGDPAAAPPLSQRQQSCGQHGALLHAQLWAVKGELEGEEKGEQGEGNRRRQRTGRSASSSDKGFGGS
GSNGGVRDAHELSLPLQTASPLRRQPGTSPPPPPPPLPSSRSLSRAPRHGCMPYSGKACQTHDDVYGVAEREQEGRG
VSQRFNPREQQPEQLNGGGDRGDQLERLLATPKASQEYGLFSERHGGNGDGNVGSVAERQGDIADAAVGAAAATATT
TDYPEPRDLAARPSNERDCECGHDYEWDPESRRATDRDRDRGLDRDPERDPGREAYLQREMELLRKRHLEWQLQQKR
KRDQLEWERDRERERERERERDRDRERPPHRNRDRHRRATSHGDVPLVPEQVARPERCHPEHHPGSARRERRGLDRD
DRSRERRCDQQQVWELNRDRDRDRGPPPDRGVDSCSFYVGSPDRGRHHQGQEWNPEHDRGKEREGERERERERGREG
EGVLGQELDRCRRGQDSAPAIGFRAKWPARIFSRQSELVQEGERRRMPAGSLEHAGAAGQRRGQAAPRGIAAARPQQ
QRGECAQQSSHSHLAQPTERDVMEPPPGPPPPSPSPLHPLMGGGMHLYAHVQLERHDEGLSCERREHEPGPELVADR
EQGRKRQRREDAPMPTPAASFALEPAATPGEQITSVGSPLQSSEGVLLALPSQQPLQLPGQQQIGVLQLPGQQQDRL
GTRNQGQVRAAATRQVTTGYMGAEGEVTGQEQGKYGQGLRCREDEEGEGEGEEGENKGAEEEEEEEEEEEEEEEGQG
PSGGDTGARRLGDGSRSGGDIIPDVSAAAAAAIVMGDVGRTGIGGGGGGDGCVPSRSDMLKEAQAEEEAAAAAAAAE
EEEVEMAAPARKSELVLGDIDRGRRLGLHCGEGADGNGRREEKEVVGSRLDRQVADIWKPQELPQHLEPSPPSEHRL
GRHREDEQAPRYVSGISAASGAASAEHGAGSPRFLGFAVLPHVRHSPTLLQRQQERKSAARQGQRQPPYSPLYGARH
TPRHYPRAVPPKDEERRFQSPPPSRTDPPEPRPSSSYRGQWLRSQTMPQRMSRESSPPPPPPPPPPLPSPAEATWGL
WQEPPGSRSGGNGGHGDVGGHRRWHHDREGRGGKIREDDLGLRDQAIRDRKIPENGIRMDPTVQNQELDGRDTRKTR
NYRDQETREVRQVRHWRSEARQEHQDAMMAGRNQLGGSGPGRPGLGREATADDHDLDRDGGYHGESRRDTDRGREAA
GTVRGLGGSLDWAPYERRYGGWREREREQLPVGSPPGEWVMAGRAASPARGDGDGGGGGGGGRGDGDGRGDGDGDGR
GAAGVSYQLDRVVGRQEGLTAVEGQGGEEARGLPGHGYGSGSGGGEVEGYGAPSSCKRRRSGDGDGGGRVALPPPAQ
DRRQSREEASTPPARYRLLEEPGHGAGSSGAATIAVTGSGGRSGSGGGGSSAEHIVEGHTGRKRSRSNSSDVIGGRR
QIDVQHQPPPLASVVEELQSPQSRWFRVSARMSTAAGLQHTTTSRHRDDVSAYDREEPSYRNAAAEHGSRSAAAALP
TRGAASAAAEERSGDGGPGRHRGEERSLGCDKGRQQRTEYGGHRRGPAGGGGGDEGPFQTDAVGPVGIRINQAILGA
GTNYKNILTLVRERGPQFEPVNVAAALHRLGSCGLAPGSTAVRQLMADPQFKELERMASVTLGQFTPQHVGNALWAF
GTLGYHPGEPLLQGLTTRLLEVLPEALPQNISNGLLGLAKLGWSPGPHVLDQLARGSVGKVPEFNAQALVNTLWAMA
HLNYVHEGLQTAMFEQALKRILEFNPQNVANFVWAAATLGLGSSAGGGGGGGGGGCQQGLMPQRLLRKCCEQALATL
QVCRHLSTRALLASYTWQHVANTVWALATLGHQDATALAEVEREVADRLAAAAAAARDGAAAAPAAASGQSAESAT
VSPTGPQESSPSPQTPDNRVWALRPQHIANLLWAYGTLEQPAPVLFSALLPTLLRRVAEFSEQELSNSVWAAARLAL
YDKRLMDVIGDQICSSRLPSLLPQHVANVAWAYGRLTHAHRPLFDGLVARAVSYMAASGGSAAATISLTWQNLTNIC
CAAGVFGWRYPELLEATERRLAAVYRAATTAPDGRGFHFPQQPHQHFQLSAQQACNLAWGLSLVGGCGALLWSELM
VVLEAVVEAQGGIEELPAEALTQVYQAYLYIQLDHPGVFLSPGPPGLLHRGEAVWRSNSAQRSQVSGFQREVSRALC
RMGLPHRVEMLTDDGLFSIDLAVEVVMMVEVENEEDEDNRLDVGEGLREERQEPGRGHPEQKEDDQEEGGKEERRQQ
KRQRLEREQQEQPEGEGGGIDGGITGGAAGRRDLTSCARRSGATVAATDAPSSADATATANTSRPPSLSREPETATA
ASAPIVSDIAEVSATRNRTTTVAATEAPWLSQSISSGPHPKQQHADALAPSRPPSTTPSSPPPQRRCGLTTKLLRVA
VEVDGPAHFTANTKQPLSMTTYRRRCLEARGWVVVSVPHWRWFEFRSGQPERDVLLAEIMHEAGLGDMLKKAAAGEA
GGPGPRSISKARDSEAYQQHEYIFVRSTNSQGYTFHNDNCAIINGPVLLPRCKNAHTEFSHTQFLPLSAP

>Vocar20011081m|PACid:23127282
MRNRPTIAAAISRASTLIPATYNTSTTSAIFRRSLLLAGCLHHAAAQAPVPVDNLADPALALVATGPQLIAALSNPA
IRVALLTTDVFVSAADWRPYIPLLPLVRDADFAISGVPGPQTSWPQLDLNNAHDKVRLGTNIAFTLQYVVVRNFRYT
PFLISPGIDLVAPTAVDWSSRVTLLDSVMVLRVCWPDFSNANSVAATIAAAPGIRVLFGAASRVPQTGCTNSTSPS
VRAVSRCWADRGVYEAIVSPGWNIDDAGVTTLTNYMLYLIRAGFLSPPAPLPDPNAPDVSVIRTGRDLVVALANPGK
RYGIIVNSIQVSFADWTGFARPTVNRDSDFTLLGTPAPLTAWPTVDLGNSRAKDVITPAVNMDDQNTTAAGYIVAM
QRVPYLCEQQMPYLCLLALGPVGCYISTLQTNRPPSPPPPPPLPTRPPNPPPSPPPVPPAMPPGPRLPPMPYTYAPG
VSVIRTARDLALALYDNSISYAIIRNDISMDASAWSGIPSPAVRFQPITIAGSYSGGPPESWPILDLGFVKNKVKLS
GNVTVNFQNVMLQAYRDAFDAFDTFSSPGLDLITKSNFFAGALVRIQDCALLLPVCLPRSVVTLSFTASYRPLLIAG

ERQVINAGVSQAGCVNSAAARPVSRCWADRGVYYVNVATYASMVDAFGQQVLGDYMFYLVNTVYLCEQQMTMECVAAY
GELGCYSFIRSQTAG

>Vocar20011092m|PACid:23127283
MQTLLCTTSQGCASSVAAWATKLSGQQPTPAPAALPPATTTASAGGGAATPAAATSPALSSAEAVAAATRAGSTMSC
LRHGSAAATSVSVLLTEELASGRSQLERGYRQLPLGVPLKEWGDLHVRMWLLTLPAVLRPYTDMLCSPGGLLLEWSD
ELLAEAGVSNAFHRKQLLEYVRQLQADVATHLTEQQDRQRQLQRHQGGAGPVAPDRRPKSLAPPGLIPGSIDSPQS
DGGDDDSAYCERTGASVTAAEGHGGGLRTTAARSPSAKQHPSDGPMGRDASPDLGPGLRTPAKSVSPRRRRSLGNSG
SVNSSSGGGVRASGRELLAEYEQQLHSFVVRLSPHPHSFVVRLSPPPHSFVVRLSPHPHSFVVRLSPHPHSFVVRLS
PHPHSFVVRLSPPPHSFVVRLSPHPHSFVVRLSPHPHSFVVRLSPHPHSFVVRLSPHPHSFVGLLEKALPTLKLLVR
EAGSMCPWPGDAAAQLLASLLDMGERALVNKRNWQQLNTRAVDLLQLIARNKQLQSDTHSYRNIMHRLINTLKGIRE
YYTDFTNRNWLSRIVGLGRDQYMFKQVGCQYCYSCS

>Vocar20004810m|PACid:23127399
MVRLTYVVEPSAPAPSEVQPVNLAFAAAAAARASWASKEREDILWDFEQLDDGAPAGAALGDGGFDGSIFVRGTDTG
AGQPAAIMRISGTELSINGGRLTGGSASNQDAPGIHDIPRNSVLFRTHAAEQTMTSSLPPAEPNAPTAVEVAVVMEE
RKSRGLLQLPEDLSLPDGTQLPQRSASFSPGIDQHTNNGVCSIANTASGSSSAATNVLPNSRKASYNHPYVVGAGTA
GSAAAALGALSSSGGMPGSLAGPAGEGEGEGEGDGEGGERDGCLVLPVLRTLLAMQQDESPRPPSLRLANQSHSLLQ
GLSTHGGYGSGGHSLGGGHVHGGGSGGSSSNNYSRQASVHVLRASVTSAFMGGGGGGGVARQGSSLPSGPPVPARRR
VVVGGGGAAAPMDSMMAMGKASAAPPRRQASSLRTAQTQHSGHHSAVSPPPPLPPPPDDDDSPLGIGTFESAARRGS
YNTSAVLGSPGHSFTQPPQQTANRHQAAARGLANGGPSTTAVAGGSGDTNNHGGRGASPPPVTRHGGGGIAAAATSA
AARGSPGKAAAAQQQQQQTQPQPQQPAAPATSKSSRAGGKALFAWTHKLGKGRWSWFPGGGGTAAASRASAAATTA
ASNAVSAPGPVGAGGVVGGQELPPSVHGSATTDGVGVGGVRPAAALSLSSSTSGNGRTSATGRSSGNGQQAHSGPPP
ASQASPTPPPVQQHHQQQQGTAGLYIVPRDLVQLPPIESSYPRRKSTAASEPLSPTGLRAGGGAVGGPLSPRRGQRG
PL

>Vocar20011994m|PACid:23127410
MEATGQSEGQGQDPQRLLANLAAEYTRSRRFLEELENSFVRETSPGPPSRPTYPPAEDHDEPNDQLATAPNPREVGH
KKPPESAPSNLRSKLRSFFSRRALLRNEAIPEPSVPQALIRPSSESQSTIPGRRYLSPSTTMVSERQIPASSPVRRT
SQVCYTTGPAETVPRTAPRAPASAAADAGGRPATARRSSIVYVSGDAMDSTSQGWIARVACPDTGDRPATGRRTSIV
FYADSSPTTGATPGRSAAGSGCAGPAVGVTGNIAGGGSGGGGSSGGTTAGEHPIDVIAPAAAAAAASGRVRDDSNSD
QFRHQHNGENGTGGAGAAAAGNSRRNLSVHSAVGVGSLQDTADPYEFMSLMAGPRRTTSFQHRSGTKLQPTAQRLRS
AVSTRRTSIEAYCNRELAQPPPPPPPPQQQHPPLLQQQPRLQGSRASFEALCQPVMSTTVDQASTTVRRTGSLEIPRP
YSAVPRNHSAAAAAVVPTAVNISNSTLFAPASISGYTAPVAAVPATATAAAAAAAVPNPPQAAAAAAAAVPGPRMRV
LHHQSGVAWAAPAALSPTGSTGSSTGPMSPSDAAAVLTVGRIRTVGGGGGGGGPAIRTSMSSLNSKRLLDLADIAQQ
LMMTASASASAAYCMGGVERPRAGSGSGMIDVAAAAAAAAPPSPPAAAVRAARGGPALHIHVTADGADEAAAAAVAA
AAATAATAAAAPSPNTSPTGRSNSLSPKLAAPYTAALHDRNSPPPFTSPPPPTQPHAPPQVPPQPHNAAAGLPFLPS
GPWLSGYLDQAYTVCSGFASNALKRVGGDLPGRLTFRTGGRLFFFFFFWSFSVILGLPITTKCVEEVDMTYKHMINR
VHSKLALTGWRDQ

>Vocar20012031m|PACid:23127431
MQHTYEVRLENLPYFWANVCCGMRNGNITDFTTLFFRVLASVAFTVYGSQYTLTSEICVRQDIPPVLPKRGRAVQFA
AAKADSEARKQKCDAGRGGPPLERRNVFSLVSRWLHSEGGVGRLRQLVREVDSHADAGGGDGVLDMEEVKKLLALAV
PSISDLETQLFVAMVDMNGDGALSEGELVQSVVDCAGIDEAVSAGGGGEGAGGLAGSGSVAAGAGAGPGSPGSLLGC
LEAAVQHLRSNVTAVEAKLVQMNPPGGYLTFTQLSELFQAVVPESLGGRAAVRALMAHVGRTAILSPLDPGVHLGEL
RRCFGLQAGTATDGPAAAAAGWVPGRPRDVFKLLRYHVKHNRAQLDELFPRGSDLTVNQVRHLVRQMLESRVGEPPL
TGREARHLVAVLDVNGSGTLSREEFDSGLKNCRDISAALTQIQIQGRGLDPAALQGQLDTPMYPLGPPGGAAGGGGG
GGGADARAPTWADLLGALRQLHELLGGQNEDVEAVWRRHDSRGKGRLDACELRKVLLELSPPGLLPLPLLRAVLMCL
DLWDPSEHALMSRKHLLTALRGDRPPDSLQPPRVVPLASVGQGVTMATRPLPSISQGLTQALYGGNVDTGLPGLLPD
PAQWAEWHGNVKTANRPSGAVTLGGSQLPPSPGFWRAQWKWHTAWLAANTAHRQTAVAGPPATAATTSASTSTLGQG
QSTAAAPAAVAATSPAATTPSPNCLECRMWPHVSVAAVPYINAVYGSELAAWTKAGAAAAGGASPPPQGPGGRPGPE
PPPVGLEGGLGSVVPVTYPAAPPAGRPAGQGAGPVVEAVEVEGGPGAPDAAALGGGPHVAEQPEGPLATQLPQQPQP
QQQQAVETPRDGGDSSLAPGKDQPASHRPPVPAAGPLADPQAAVLGATSSPVAPAAVQQPAPVADTPQQPGTTQAGL
PQQPGTTQAGLPQQPGTTQAGLPQQPGTTQAGLPQQPGTTQAGLPQQPGTTQAGLPQQPGTTQAGLPQQPGTTQAGL
PQQPGTTQAGLPQQPGTTQAGLPQQPGTTQAGLPQQPGTTQAGLPQQPGTTQAGLPQQPGTTQAGLPQQPGTTQAGL
PQQPGTTQAGLPQQPPAAAEVAEAGAPGPEPLPSPPPQKDVTPRSPLPGGVLEPSDVKGSVAGSDLPAAAAPAAAAA
TAPVDVASADAADAAPPVVGESSSAALADVPAAVVAQAPEKPPADAAAAVAAVAAPSVAPPVAGVAAIRAAGPVELP

AAVTGVIAATAPTAAAATATAALEVPPPEAPQPGEGPEVREVTRGKPVSEPRQPPAGQQQLVQVPPLLVPARVLSYK
VVLAPGAQLGDVDSVMLKTSSGTRTGSSAAAPCYLDKVEVMCSSSDPPLLWEVTVRRWLRPGEPLGPLRPDPVAWEE
WDMGQAGDSSGAPGCVVYLNRNLGEVSWRTPPRSPLPALPAAPACAFVHILGCSAWGVFCRKAYTGIHPPGLQLASG
EWAQLSGVPPLSVPRVRVRFLDCLKSPTVFRAVRVRSTYELGFGGQLGAVPGQEVRVRGECCGHSIAVMARGGFKAG
DPEDTNSKRLQPAMYGKGTSGAAPGPSAASTAATAASTAPFEDPLGGGPFPGARECVVLSYSLEAAAEQLGDGKYLW
LDGYVALDDSSGTPPAPVRFSVVRDGGLVWAKWVRAAGEVHRCCVPLLGCRQVGQLQLVVETDFSGGCRAVWLDPCL
LCGPLVDKALTAALERAASRENNEVADCSGGDVKVSFVTEAGGAVMYMAPAHSVPAEVSRASSTASYRISVFTGPEK
NGGTRGKVFMRLQGRQGTGANSTAVKSSLVCINPDGTVLKEGDKYSVLEPLSLPWMDEVEGVVLQHCPAPAGLLGAG
GAVGGASDWSIQHVEVSCEETGRTWYFVAGGWFLELARKQAAGAAGQRTPGAVNRLPPAQLELVLPRVTSPTQLKVV
FHIISSGKGVGMGIDGDLQMVLVGMDGGGYQYAPLTLPGRSIDGPHESRLLESPLPLGELQSVELSCKQQGLLGTRL
QFVEVVRLVDAKHFLFLPPALDPSEAADTAGPSRLVLRAATQPYFRISTFTSAIPEVSGRVRRSMDALYCAEPARLQ
GLLPCRPYPHGPASLKASTDARVYIDLFGARGKLLDLYLRDATGDSFNEGCEDVFFFPDPGLGDLGSVCISHDDSGD
SPNWHLDRVEITHTGTGRTSVFVCKQWIGLGANRRDGGQPEGRFGPDRVLERVLYPTHVLAAVKNGTPLLRYHVIFH
TESPRGELSHTAEALQLVGQQQQVSGDGGGFQFSEVVILDSGRADMDLELDMQYVRHIQTISRGEAQRGLEDGDAAP
PPPTGAAARRTYQVVLKKLAVFQPRANIVLQGGLGARQLAVVLHGSLDSCRPILLGADNCTTPWINPFTSQADDVFQ
VWRVGGCKAPGIRRVRGGRCGGRGGGGDRGSAAARGVWVTSDTPLGSRLLRVDVRLPGTASPDWGILLESVTIRDPS
TAMDDPWEFFAGSRGQWVGVSPDTPEGAAGGTQAVHLLPAGCCAVSFVSRPLQDLVALHVRHDSPSLRPGWGLRCID
VLVSPAPGAPVRGDMSTTAAAAEVASPAATAGQQQQQQPLQGVPAVPPPAATTQPQLEEETGGGGLMAPVDTAATE
ALMQTNQEPDLERLQALTEEYRAEVAAYLQSLSSPGAAGTDLFTLMRARMRAEPNLLPREFRRLDRDKDGKLTSQQI
YTLVPGLLPVEVKCSEQQSIYLGAMITLGSEEPLDQAQVMSAIKACRGAYKEAASMVRMVESGGVDWYQLAQEDGLG
VGLALCRLAEQLVQPDNIQGAVEAFQQYDTDGSGYLEIGELCKALQSLPDLTLGRREMRLLLAYLFHFGDKDKDLRM
SVLELQNSLVPFVPRPPAEELEWAYQTYKASHNLAALLRAVNKLQPALLPSACMKVKAAAVSAVAAQPSEAAVTAAL
LGEGVDSVPLALLVDVVDAVVPGLEVHRQDVAHLQLLIALDSGNNETLFLEKELQSAERVLQRLAGVLVQDEGGQLA
AAFQVLDMDHSGYLDGQELCTAIRKVEENITDEELRILLVGFWAFCIHTSRQDCPAGEVNHPVIETHGGLQGSGAV

>Vocar20014507m|PACid:23127553
MNRDFAAISLLEDKLGRELEHEELRSAIADFRLVHGQLPSIRQLALELASRADVTLGTEDVSESPAGGTPYGSQLPT
PLSVIRHDLGASPCPAPRGDASFFSQLRALQLSGTHLMSLSHDPVGRRHSPGILPRQSPSRFSTSASPESCTPVGRH
PGFRVQAAGRTSPAPEELHLPLGGNTCLAASGSVGVAAADPFDLQQEQASRRPVPGLNLRNLQKKKTTTTQTPQGNG
NSSPVASTTVSPTPQIPAPCPGGSGQRTPDTNSPTEILRMLLPSACKPRASISLGPGRGWSALRAPPRPLLEEQGSE
QRVEVSMDCSVVESLNMGQMGFPRVSLDDSDQIQSDLYRDQPTLTPHGSAFEKQQRATPTMRSRRHHLQDQHDQDAS
PHRTAQSKRQLVLQQEQPAGNDSVVVDALVGSGCCSAIDSDLRKDTPSKAATAARLAAGDVVATVHCTRTGRSPLHE
PSMATPASAVRSRGLSQPSPSQLPWVTPVPPLRLPGPCQTVPPPSGASASANGARSSARRRAWSTNSLQHRSMATVA
RALRLSVCLPSALVYGEGPLTTRLIAAGANKGITSHALCTVFWAESEDDLEDDDDSDVYDSDELDDEKERTQRGELD
DISGLGEHELTGGRYASAAQRPRGRNRRGNGDADDPDRSSRGLMLPEQLWDAWERLSDGPKGRQYSLLESLTHVERI
TCTSYSTVSKACHQGQPVVVKIYDVAEKDKLANAFAEIGVLLHLASWAGAVRLLDYGRHEDKVMLMLESCDHSLKDW
CDGQRFETATGSDYIREVLRLWVSLAELLTELHERWHVAHCDLKPGNVLLSGGRLRLADFSESMLFHETPLLQDQAR
GTVPYQPPEMVHGHCVDARKADVWAMGCMLYEMVTGELLFRGNNDCMRAVAAGAAAARLRQQSTAAHQHGRRRGARN
GRDGSGGTPSQTRVQTRTPQLPRQQQEPPSLQHRSAPPAATTVAPSPPTTTTTAPPPLDVYPAQLAAQTPSRPVSEQ
GSGIEPWRVAETLEPASDAVAATAVVQPPSPPPPFVPRLPLALTRGPGMSSSPWQYPLTPGAGGSLPPLPPLTLPMT
FLMGTGGLSSARVTGRSLELIAEGCGTEDASSLTTDPWVVPSIVNTAPTTARSTARSQMPYGYDTARSVASSSTAAA
GAVGYWPPAPPPSTGTARSVADSEWTLAVNSTTNPVGGVNTAVITAGAGGSYGGATRSCPGGEPSSYRSVGGHYSTG
GADSQRDDVAPLLPPQPSPPPSLLPPHRRLRHSIATQCADPAGPDWLTTEEADRLRIMCDEPPSFLLVHYHGGGSSS
RATGAGRGGGDDGVAAVAAELRNVYEPRPLSGADGPSLCDGVLGLLRAVLVEDHARPSMRQPPQPQPRSPQPLPLSK
RSCACAAASGEGNTLGAAAGDTPSNTGRTGVRDDLMDCRGTMSGHGLPNTAADRSLGGGGHNGRVGKGALVQELVPT
TTVLPSPPLCLSVLKERSPSQCDVNIGNGTGGAGGISSAGAGPQTAGADGGSCIDSSRKENNSSATATATTTVAADS
NGSAGAAGPDAVVSAPGDGPRSRSVSLFDICEPLVTSRGPGGSAPAPRPHSRSWRRVFKKPY

>Vocar20014569m|PACid:23127581
MSYAYPLARLHEPYVSSYFEDIYFRSEYEGLAVTPDRRGPKGSTFDLWNYTTWLCDRKNGIPYDLTEFGIPGFVWSA
QNMASQSYFAIMLGKPLEVVYGPTSRELSGRRAVGRSIGYVATWAVGPSCRATCGWRTHQSALFSVGISYGISANSK
YTYAYMLNIPFVNVFTSYLQHPCGLVRAMVERRLSSLNPNLALRHVAMEKCSFSPHTEFNAMRWSIRASLESSAELR
KALEYIKYGNQTLPKESYPLAARPFLPDTLKRLTIKRTNDGSHPAKAWKGRPVIKGFLPAEWALLRNLEYLDLSDDM
GQGLIEGPIPSTWLMMTRLRTMIVAYFTDQYGIRLRNRFAVDGVYYGREKSYYGRPFYLYLNGRWRLYYHTNVTLFN
LDGVGWQWYDPRTGEGGIPLPDNYEVGWAGYTNVIAPDGRCCWDLYSDQFAARNYTLQGPSGTSGPWDPWGVGYEAA
DFCDRNNTGFWPRATGSRPPIPPAAPKPPDIPAAPPLPSAADNNAASSPGRPPGFPQIPSTSAMLIPNAPPFPPFPP
GIPGMPAWYRAQWAGAWPLRDLIPPRPPPPRRPAPLPPPPPPPPPPPPPPPPKPPPPKPPPPKPPPPKPPPPAPPPP

PPPSPPPPPPTPPPPTPPSPTTPPPTPPPPTPSPPTPSPPTPPPPTPASPKPPPPTPPPPTPPSPPPPSHPPPPPTP
PPPKPPPPKPPPPTPPPPTPPPPMPPTPVARPPTLPPTPTPTPSAQRPPTQLQGAPPIALPPAASRGPGPPPPLSRP
LPAPRQPPLNTDPPRQPTEIQIPPSPEPTSQELRPPPTKPTSKQGQPNSASPTPGIPLPPSPAIPAPTSINCSLAIK
EVSFAIPSISRGPFYIAISVLHGGSAYCKLCGCRVSYIILERGTASIYDPQQLARTGSSPATLPKLPSLDVLFTLAA
NQTAQGSGVLKVNACFLAAIVATLTIR

>Vocar20014543m|PACid:23127589
MLDGRSAGQELRGATSSAYSLWPRFHLRQNSRRAATSHLQQSIDEARIGSGQLACSSISCCCSKARSTHVVTCPAEQ
LQIETGRADLGRAQGLAAWRSGSQSRARKPPSSPPPSAGQTQQQQPQQHLQAQRQRKEPLQDGPLPPGVSSAAAPVP
APGPAAGESRSSFPQPTGGLQAGFRSTTGVRQSATATSDAAATGGEGADAGLVSEGSGPEGLGLVASLANIGQLPAQ
QRSLQPPPQQQLQQQQEGAIGARSSDSSSSSSASFTSTSASAARNAAAWPLDAPLLTHRIGACSSWRELSSLVSQHA
PRMNAVHVTAVATRLARFSPPLPAVRDDGGGGGGVDADVSGDGNSVESQQQRRRFRQMRRWQRGQEDEELHEWMSFV
RSILALVERHAAILDARGIANISWALARALPAPLLQPPSPVVAAVAVAAGAEVGSSETGGQCDMGTAAAAGVAAGMG
PRRGEDRAVGLRRRRHHGGDGVAGDEGDGSGEWRQPGGSCTAGGGGEAPAAVRFSALRAAARHTVGRLLALPAADPG
EWSKTRERESCRSGGGIMTPRHIANAMWAAASLGVLSAPGPEGREAAAEGLVRQGSTHGGGSGDVEGPRRWVMRALA
AARGALRTGGFTEEGISQLVWSVSELGLTPDAAWMEDFYGAARRQASELSAHSAANVLLSLANMATAAAGGHRDGDD
DVDDDATDTPAGLAESHLTGRRNRIDSGRANSSGAVAATAPAAVAFLLPSDSWLLSFLVDTAPSLESADPVSLANSV
WALSVLMRVRRSQLAAASPLPRSLSPPPPSCWLRHLYGALSQPATLRRFTDAQLGQLVWSLADLSDVRPGGLPLVPS
AGMFGLVAAELRRRLVAMADKHFAMASWGMQRLQLRLEQRQHLLQLRQLEEELLQLRPEVEELPRRKRGLIAAAAAA
AADRDPDDQQQEQKQLASHVRHSYGRELASSAYSDGQNADWSSAWLAAAEKRMPYMADRWLLLTLQVAAASGECFAT
AASASSGDGDAVLAWFAVATDAVLLPPRRRMGHLPTPAGTDMGRQSQQHCGDSRMAAGSSATAVSAAAATAIASLGQ
LLDDQPPSGDGVAAAAATQGGGSGGTNPSGTTPALDGVGPDSRTTASSSSSSRRRRRLLPRLLQALLLAAEACLPPG
PALSAVMAAAAAEVLAATLAVHPSGSEGGGGGGAEEATSWTVTELLRCGAALARAGYEPPPAWRQAFFGRVLQLAGV
STAAAAPPPQELRPSHPGFAEATVPSESDRYAAGLPDQASSATAGSADGPDGEDQGPEQQQRRRRQQPLRRHRHGHH
LSASDVGLLLSCSQRWAATASDATLAALLYGVPYELWTPRAAAWVCRLLASQVTTQQPQLQQQGLMQQQKQLSPFER
NTQGLEQGSAHPLPDQLQPSLPPPSPPPQQQQPCDEGVAHNNNNNNRITSGSGSGGSAGLKAADPFRCEGPATASEA
LQDPSLTAASPASPLLDQSSAELDPACTQPAAIQGPPPAAPPPPLPEGSVLASSAAAAAATSSAAAAKLDWDVDSDV
EDLDCNTGVNGGTRKVAVREYDVVLVAPRPRPLLPGGLMAAGTDGSGSSSSNARGPFPKAGAAGSLSSRAGAAVPDL
NGDAAAAASDGPLGEGKGNGGGDGDLPAPASEAGAAVLPYTPGADGAGARRVEVLQRGPGRPAAAAALVTASAAAAQ
PLWLATGFQPVGAHPHTRGSAMASATAASSSSSAASLPSRPICWSTFSLSQLLWAAARLRLRPSPRQRAVLRITLSA
ALVRWAAQPPYALIPLLWSCRRLGVPLGAGPTRLLRRALLLHPGLQPAALADGPLLMLLEALLGWRLDPPMEWSRRV
PYRLASWLARASPAVAIATAAASPTFAGKLGFRGARWAARTDAGAVGSAAKVGRDSVGPTDSGGRSNSSSNNSGGGG
VVEARQLLLVLRALRRLQNAARSEAAGSGGDDDDTRHIGFSRRDGGPQAAPGLRIAAGGSIDADVSSAAPGARPYFF
RLQLPPRWSPSWAPVPPSKLATRGSRHGRCRSTPAATLTPLDDALWPELWPMLRIAVRQRLTEVFGLTPGVAAPPAT
SPRERLAALMCGAACGLVDWEAWGVAAMRRLAGELPPRDYHAWLALAVGLQFRVTRGTLEDLLLAWCIASGPQLDAM
SPRQLHTTLRLAGRVAETLLQPLSFDWVSVALRRYSRCITALPPAQLAQVPAVLRRLRRLPPPQWQASYAQLVVRHA
LPYMSGRSAQAMLRAAVGLSYASERHRFTQMQMQD

>Vocar20014535m|PACid:23127622
MYIAWLETPTAHVYAHGTSCPFLGVLAVPTDENSPTPAADAPAASPEADKPAATPTTAPAAAPVVVPAAVPGTVPDV
APAAKPAATPADATAAKPAAASDAAPKSASPAAAPAAAHTVAPEAAPAPPAAAVTVPAAARPAGASSPDDDSGVGGS
SRNNNNNNNNNTSRNAATVSGSGRGSGGGDAGIGSEPTSEVTDHRMARGSPQKAPKDPQQVAKVKAMLKDPKSTKPD
DVLDELDKNGDGKLDKSEVADLLDLIVGLRAQSAFFKTLVLVQAIALLVLLAALAGMAAAIVYFAKDTKLNGGTLVD
KKTGNPVLVYSADFLVTPSGVLVSRSSTADSSSSSSSGSGVAVTASNTSAPPPPVTSQAPPPPAATSPAPATGRRSR
TLFEELMYPDEREQPGYQEPLPQHKGVGEPHWPQQQQQHRAQEGTGGGSSSAALSSETKASTSPSKMSRSSGGRAFS
RILQQQQPPLPPQPPGGQQSPQPQPPQQSPPLPAPPSTPSAPSTPPPAPSPQQSQSPPPASPPSPLPSPPPPTPSPT
AAQPMNSTSNSTSSNDTAASAAAEAALQSALGTVPSGAVPLSTSASTVAMGLHSALPMSVLKELRYIQACALLSVCI
HVLVFLRNLVMFTCVRFIEGINSSSTGADLNLFVLAVAKLPAQLEEGAPYSSCEVVRIVTYIGTVTVHGSELEFGQE
DSVGDVFRDAGFMVRGSGRRRMLSDSYGITGHFNFIKDIEIVNVPPGEPKPNIAPIYQMKMRIYDECPKLPDSSGTA
AEADGCLDPDTGLDRPGTVVLDGVRYMTTIQTVTRQNDVVLTTTEYPNYGPNLVRKELMNETSGEIFVWQELFQATR
VTHCEYLSGSAGDGDSLRSLISESIRSFKFLGYELKRGVPARHFRLTTRQPVLRERDAGSSNALENGSIEYDMKLVD
VDFWDTSDIGSVCSIARNNYPVAFEMVHPVAGNSKIHVMSFRAGILQPEDELAVPASFNDTDSPRGCPGPRFTSVPT
MTSPFGPVWEAPPEDQLDSVLFTSYTKVIADPNDPDIFPGLVEASNKDYPPSSPCTATTAKSTKPYATFTITPITPT
ITSIATPATAVTATAATPTPRAAVSSAATTEATTSTFSDCITTSRRSRHQQRAARSEAHGLGEVLRGVLRWADGRIA
LTSVTKCRSRRTGNSDTLSALGITAADLTSMLAAGTVSKSDSEAKVEEDFAPGKGRNRALLRHTRNYTLSNVCGVSF
SSDDQDARFSYNIDCGGRLSWPPNADVTGQMAVQLNLTRKTSTVEIRACQIAGCINVTVDIRQMLQNITGTGLANLL

ASYGISPSLNLEGICASYDSMDKMGVLSSTMKVPNNNNVAWLLWQLRFETDFEVRKKLLRRMYAPICFERVRWITAV
LFLHPVLCFVPLRCIRVHESHWFTSQGSASTFWQPDDQL

>Vocar20014505m|PACid:23127661
MLISKAYCDSNCDTLDIFRDCTPTALDPGKSTSTATSTAAAASQQLAVVAAGAPHRTTGGATHESGGTASGTHRRRR
RGGEVMGAEGGGGGKGVKAGVDGDVGDPTRGDRVAGPSTAVHGGDGGGSTKTTTNPTVPPGTAAANKRHPEKPLETP
PWMVLELLQEGGLLGPLAPLAARLNVSLPPLPRPSLDAIIASTGLNVSVLLGITDILTDTLNDLTDTISTGRLSVTV
RGLLSQLPAFSLPAGTGGNGSTATTAAGLRPGLVMARRGYRAKHPVVIVPGFVTSGLELWQGLPCGRRYFRQRMWGT
LAMVQDPAGIKIRAAVGLEAVDYFIQGYWVWGKLVEALADVGYDSNSLVSMPYDWRLAMPLLEERDGYFTRLRLTIE
GLAEVSGERVVITAHSYGENVVRSFMSWVEAARSGWVTRYVAAIANIAGTTLGVPKSVSALLSAAPTQNIPSSYNFL
SLRHDLIRTGETRDTAQLGALAGFLTSNLVPRGTRTRVWRTWGASYAMLPITRLDVSGFLALLREVGGPLVHQHMRE
WGAVDLSAGPDAASSTKGAGEGEGEGEGEAANVGEPPPPSPSPSSSSSSSPPPPSPGAAAAAAGAATAAAVGGNCTT
AAQLGEPEVGTAAAAVCSGSGSDRSTSSSSPPPRPPLPHNPAASGEEANPESGPAAGWTILKDVSDPGGSLDVGVQM
SDGDGTVPLLSLGLMCRRGWRPGGSLNPGGMRVITREFKHRSVSLLQDARGGPATAAHIEILGNEAVLQDVIRIAAG
RLDELSDVIHSDIDRIAASVEF

>Vocar20014525m|PACid:23127671
MTSGHLPFRSRSHTSAPASAPSLPRSVALEQLSGRVEIVRPSELTIVRFLGSGGSGDVFQARWHGTDVAVKCLLTSP
GEDEMAVLDELLVQSRLLCRLRHANIARLYAIVIPDHLLDQATGGKATAAAPLPLPSPVPVHLVAEAEAAAAGPLA
VPARAHGPSPPPSPSSVPSSILLSEYEPSSGAHEGLSLYGSRTVSAVTPSAILSRASTHPGGMGSTGSAGGPGGGGG
GGGGGIVSPAPLPSRGGGGGGGGASEEADVEKAAAEADTVVSGPWTPRVVPPVSVFLGFAAPFTGGGGGGSAATAAS
GCGGDDDESGDDEQGGAARGGGGGSGGGGAVLSRSVTPTIVLSGLQRSRRLPSIRECSSLGTRRSTEDEPQSTSLTA
VVAAVVAAPLQEQQQQQQQTHSTGSSNDDSSGTFASLTPPAADTAAAAAGRKTAQGDAAVAVRRPVGGSGAGAAVS
TGSAADNTANAGGSSSGHKRGGRVVTLHGAEEEKEGSRARVGGGGRHHGDGDDPAGAGSDGGDGGDGSDGGDGGDGC
DGGDGGDGGDGGDGGDGGDGGDDRSGNLRRHDHVARSHHLPDARQRHHHYQHHQHHHQHYDHLPQPHSVRFSLPGPL
ERHDGPALEPPLLGPYESRPVAVPGEMTGFARHTYHHHQQRRRRRREEEEEEEKAGGGGGGGDGGGASTTHRRCFSN
GSGYVSSSSDEYERYDSALGSFGGIVVGDGGAWGVSQRQGKRSGGGGVDGGGDSAALSSGTSGVGVSGGDSSSGGDI
GGGSSGSDADSDMLYSTAHGDMMDAMYDTAGHQLWTRSAAAQEPASAGSDASTTAFTPAASAADFDPGFAADDTGAG
AASAHRLPHPSPPPPAAAAAAAAPTHGSFLSPFAPLSQRQLTIRAASLESQLQALLTGAGGTPAAPSLAPGGTANAA
AAAATAAAVEPPPILPLPQRTRGGSDGGLCGNGGASWTAVRLRDEPGVQQQEQQQQQEGGENRGGEYGKRQQQQQPL
SLQLPGGATATSQIGSPQGPFQNHHHPFTGLASQSAVAAAAAPPPRRYGDPAAVSPSPLSRRSSSSTLLRSASGAPV
RRLGSKVLLAAAPSVVAAGSTGGGTSAGTEAAELENSRLVACHAISGPALLSEYLTGNSIRSALRRHSDVVGGGWRS
GDRLVVLLGLVRREGMEYLHSRGVVHLNLKTGNLLVGFSEKQPSCKVSSYYIVRYGRYGMRLRFAAPHRVGGGGDGA
DGLDTAASAVAVTVTVAIAGCSSIVTSTSSSSRRRHWRRCRYRGAEYECKYEYECKCEYEYEYQYEYGDEAWRRRRR
GASRRQDQRNQNQLIPASGGGGGGGQPPHHGGHQAPQALLARSLPWTAPEVLRTPHDVTEKADVYSYGMIMWSLWTR
AEPWAGADMRALLTAVVSGGAPRPPVPGDPDWPPSEPRPPELAPGWSALMRRCWSTDPRDRPTFNEVLERMRVRGSQ
PHENVFLL

>Vocar20015099m|PACid:23127715
MGPACETPWWSMPEICTHCGGGDVEPEGRRVLVLCSACFAAGTHTGCHEDVTGEPLSSEITHGDGLYFCGKECQRSY
EALEAATGRRSRIRDEPEQYTFELVHYKQDDRTVRSAVETAMRMFRTSFAPLIMENGRDLLEMVCTAYETPDEEVEE
EGGGHNFSAFRLAILRMGGTIITAATLRVFGNKFAEMPFVSTREGHRRSGHCKRLMKAVEDLLLAGGVHCLVIPSIN
ELLPMWTNKFGFAKIATDEVEGIEDWIVDTDRDTCTLVKKHILPPGQPQPQPQPQRKLPPVTTASQPQQTLPAPPTG
AAGALSIRPGPAARGGARASVKNSRKRGRAPKIVLSKSLRRSYSDYSDEDESEEESLSGSDGDGDGGGNGKRRGGGH
HRPGGRRSSGGGSGRHPSRRSRRDSGSESDFVPDKEEEEEEEKEGGSGADEDLVPEESLAGGSAGGKCARGGKRAGG
GGGRRSKGKPAPDSDAAGASTAAADPKPKRTRRSGPRAPRPAGPRLWPPSESLPLHPVYGTEVSVIRIPENLRPAPP
PMPLEVDMARPGLPNEPFELSIHLQAFIAKKAAREAARAAALAAAAAGGSGDAAAAAATAAQSGGGAIAAGVSATGT
AAAAAPPSTAAIAGAAAAAAPPINLTAVTQPTPSPPLQQQQQQALPQQPPPPVQQQLQTDKQGADKKRRRKRARRNE
QDGQQPPRRHRGRRPANSPAQEAARQRRLKIRLAARQRRRAHRELMMGMIVADMVRMELCPLEPRKLSDPLSILRAP
RQEQQQNDPRDIELTRLREEHAALQRSHQALELRLAAAESALQQQQQQRQQQQQTLLLPPPPPPWPRLSPLSLPPGQ
HPPAEVSPPPPAAATTAARDSPPSPCSPLAAAPDNLKLDKGSEASAMATAAAGAAADIAGGTAATPLGHPLFSGFWS
TPAALEHRRPQVLSPLPLSPASLHPYAAAASTPSLERPDITGGRRSGGAAGALEAAAAAAGAAKAAHNGCAMNPGAM
MMRTPLIVRIARSGDGSAGCGDGSGDGSGDGAVAVDVDDVTSSSANGNAMNKGGGHGPGRPLECEDGGGGNEAQEAE
DGGGGRSVGGTAEKRRPAVECTESLVTMQVEGFRTFEAPADGPSSVVPLDAAVGTSAAAAMAAAPLAAPAMAVTGIV
RSGISPPSEAPGCKSAAAAADGAGGADPAHAARLVDEAPTAGLDPGAAAASSVRQTSSVTLNKAFRTQLDLVILAAI
GADDTPPDGEYAQRPPRPLPSPPPPPPPPAGETVVQSSVEFRPAEVAPGGLQEDRFGMKTGTNAAEADDNVLAAAAK
CNTDDTVAAATAAVPTGSVRIHTFSIAANAAGGGGTAAACAPGPGDPCARSPVSGGGCRHGDKSPPTTPPFSLADLS

QHVRQSVLASGRQLYGSPPSPTPDTAAIAIPNRGEVEGCGGLADSAADGCVNGGVVHGGCGGGGGSSAGGGCIGNMQ
TQVLPITTLSQTALRLTTDTPHPWGVRRQLVGLMTDRTAAAAVATSPPRRHGGASAIDDQMEPPPPFDGPPVSAPLS
HNSLASSSCGSSSSGADDGSSIVSRSRPRKRRLVVTASNAAAGAAPVSKKRIGSSSSSSLSSGAGGGGERPSSCYP
PGAAAQRETSATTPNVRSACEAEGTYGTAAASRGCGLSCQNGSVFVGSTADCALDAVEAAGVGQLTAMAAAGTTGCF
PTAAVSSPNADGSRPPRPPAQQQPQSQHQQQGPTEGELSTSSSVVAPLAGATDLEHGRAPAAAAAAAAAVGPADAM
TAAADHNATDCIASPASLRRFELSCLYPLLHHVVAVLLADTPSPSQRRKSPHFQDGLSTARRRRQGAAGSPLGEGG
DLGPEALMVAAVAAGSTADAEADGLGGCGGGGGGDGLATTRPALMAAFEACATAAEVVCGAAEEAEAMAAGASTGTS
AAAAAQAFTPGMADGSLNAVPLGSAAAAATLLAPVNAHTPLLLQLELAGTAAAAAAGAVAEDVDCFPVPEPVIGTPD
AGGFEGSTVAAAAAVAGGGVAGAPAPPMPSSSGRLAIVSSPATGPSSGSRATRSPGLRPTMAVAHGGGGSTGCATVD
VLYGSPALPKHGGRLVMLAALSGAGGGSMREASAVRHVVDPYDAVIADEGAVTVADDGGGVDKPRTSTGAAVAATSA
AVAAAAAIDIVGGRTSPTVSANGSAGAAGRRTYSGSPSDATTTAGLLFRKKRLTRRKKTAVDLFGEDIVLSASAPSD
SPDSTPGSSSVPPPTPNSGGREEQQADGGGTKACCRTIARSAAAGEGATTAAMPALKAAAARGLEFGPAAVAGDEKA
EGAATVIAVTGVAGGAPAAGQGLDSGGSGGGAASSSTAPDKDPTGSTLVIATILPSGKPAVRTVSTVAAGCSSNAVG
IVVQTPAPGAHPCTAGATPVATGGTAAATRPDDLKTTTSAAEGPASASAGAVAARSPPHPSGVPTAGQYNATGCRVP
DGGGGGSNGGGSCNGGIGAENAASEEALLDWLVTGTPPEEDILFGVTEGREGGGGRVAGGRGRGRGRARGGARDRSG
GVLDSPVSSSAYLDEPLRSLFHTIDADMAAERYEEIATSAFGGSAAATTAAGGPAADGGGSELASQGSLYGQDQQLQ
VRRRRCRRQIPVQEHALPSQPMSQVAQQQQQQQLSPLRSAPQAVSRAINGPDPQGAGVQAADAAATGAALGALDSLA
EFCGRVGSEGPTSEDVGLARQGVSALVDMLVQD

>Vocar20014934m|PACid:23127719
MFIIKICTNLQTGTPCQECPGEGLHALAPPYQTPPPQQNMQLYQTPYAGSLPHPPPIPPHPPMQPPQLQQAQQQQQQ
QQFIPHPSQLVPRLPYQPPLHQPQHHQHPAQYQYHRQPMQHHNHQLQQNPQDLATRAAHPGVRGPAGQSLRALKAGD
DESLQHTAVLHSNTRQRALHTMFRSNLEHSWSIPDVFIRGGCFRSPVGHATALVCVTWLGALVEMEDKDGIRPLESG
NGASLPSPPPPTGHWDLAANGGGHDGGGEGSRTQLPPSPSPMRLRLPLRVAYEELCVADPLLRARLLGDLAVRWLEK
NAWRSPLEALLGRGVRQRSMDRIGPFECLDEQQQANWDRNRRTLYSHVVVVARAAMETTAAVAAAAAPMVPKVEHND
HKYTRGSGGSDGAGAGDGDGSSGTAAATSIATSFGRASGDGNPAVPAPEPRPSLSSIRRFLQVTDYLAEGLAAHLLA
PPSPPPMTLTASPTPTLATSPLLPVAVRPPPGAGPAADPLAPRGAEAVLAANCADGEGGHGSGAPSAHSSAQSQAP
CSSFINLTAMRANRLAVAVDWVPSQAGLAQVPAGRTATAGATNGDGNQLNLAMPGATAGHAAGCQARSPPAAAANIN
AADGPAAVPLKRPPPPSYDGDVIRQDHDVPYAGELSRGVSGRGGKRRGRRRISTADVATAAAAAAAAVPQGPIPTPQ
LSPPPQPPPHGSMAPREVPGCKREGGEEEVEYFVPWAKKHVLCDVLPALATMSGAT

>Vocar20015071m|PACid:23127749
MGWSRRPTLPTCSFVIISQVLLALWGGSARAGLPFPVDPTVVCYITVAGEPALWRIAAFGSDNVPPWRDVWTPSLIS
IECINNLNPGAPVNVTLGKQLMQYIQPSVPLPPSMLLPPVYSSPPVYGNVAQPSPPPPSNPPRSLTATVSTSGVEVF
ESESDSGWSIVFNDQIQHLQLENSVLRGTPLSYDPLISCPSCKYVTVANVTMEELYGAAVEPIGDDYCWGRTVGALS
FGGVLEAYVDRLTCTNVQGATDFACIWLGVVARPDVDHGVPAPGETPQGDPAPAVDPAGNTQVAKLDRIIRISNSVF
LNNSVTTGECFDFSDTEQLGFGAVVVYGPNSVGARSLSSVELLNTVMLGNKGGYGSGLSIYRDAYGFLSLEDIMHVG
VLNISRSSLEMNTATKHGGAVFMNLYSHLEVLHITQGSKVDDNSAGGSGGAVYHANKTDTITVEGSSSVSDNTAAES
GGAFFVLSYIQQVSILSGSTVSGNKATSGGGGAVLVQNTDKTVTPLSITVHGSSHVDGNMAAKCGGAFGILGNVAEL
LVAQGSSLNNNTVGVSDGAQEELRGGAVYIGSCFAEGLQASSGNISRLLVSDSSSISYNVALYQGGAVFVEQDVIDG
ITIEGGSQANNNSVTGPLAEGGGAIAVYGSCSSLTIQDSSSISGNMAAGDITYEKGSQGGGVWIGGLLQKLVLTGGS
RVSWNKAWNGGSAIVSGMSAEEELADVKHVMELLEVSNSSCICNNTAGQYRSSLYGAMYFRNTRVVNFTVTDRSCMC
SNKVQSGSGFGGAVYAGKGLESFRICNGSSVSDNQGGNGGAIYIPNVQLVGDDANGTKLLPLQRFELCGGSRMDGNL
AGISGGAIFIEGPAGEIIISNSSISHNRALVTGGAIHLTAMLKNLSISDSNLFNNSAVFGEGGFMHLMMVPSNQGQV
SGTPTDISNSDTSQKLPDDGPYYLNIARSNISENSALKDGGAISINLRHWIRTVPDDMKRLQLQKDRVLYLKIADST
WDSNWAHYGAGGALAFLSLTPVLSTQQRMASVLMGARINITNTTFHTNTAGDDSTRLLYIQDVSPLRGNGGALFIWA
EPFLLEDSLSEKKLLTNQTGYVMYDKSSGCDTTEADLVPGIQAFSNMTCWPEGSQACGVRLEGVSIRDNMAKGGYGG
GFLLAHCAAKIINSNFRNNSADLDGGGFAFMDYAIPAYVAQPSYVRTTKDSSSSNSNSNIGVNPYPHAPILPISPPD
TSPQTPRPSASTFGQSPLSINHYVDPQTPGAADVLLSRLQPSLEALQNVDTTRALQQPWLVIQHTTFTQNSGTNGGG
VFLEVNRTAAIILDCTLADNTAQVQGGGAMLIGASTFAQVTAVLRNTTFEDNEAGTVGGGLAVQLTRSRAAVVLFND
TFKGNSATNGGGLFNVGVRGTSLLAWGCILHDNSANYGGGAALSYIMSASSSKFSTSPSPPPSPRPPSPAPAPPRPP
RPPPPPPPPINNIPAFIPVTAIVHFQQCRFTENQVLLSGGGIYLAAAGQNVLTLLEQSEVSLSSAALGGAIYLNATDG
CIMRVSSCNITNNTANLQGGGAYSATTCGGQLMVGNGSYWSGNTAGVYGGAIMVNSADDVLISSSAGIAGVASGTGS
SSSSQQPSACMYASLDLSNSSVVSNMAQQGGGIYASEDTVVVLCAALCSNS

>Vocar20015127m|PACid:23127757

CPRGKAGEPPYCEPCTGEYFSGQAGQAKCLKCPFEVADLDHNGYNDDCLCPPGSAGNYTAGCTPCLDITYAPTAGMR
ACSICVNGTAVRRLGATPSEGNTDCQPPTTNITRRLQLGVTGNMKDALDVPTPRHLSSITMFQQDILSSPQPPSSSQ
ISQPNNCSALWERLELLKANPNISACGNIIATNRRISNIANATITNCASNTTVKACLQELGPIVDAMIASRCCDLLA
RVVEVFRPPRALCIMRNFAPIRHAFGFYQDAAQCDGHIRLVNGWPDGSSGRVEIFHDGQLGTICDDAWDDDDASVVC
RQLGFDGGYAEWGGAFGPGLDWQPIWMNRVGCKGDESSITACGRWGGPDSWGNHTCSHWEDAGVTCFNCPGCPQQHS
LRLAGNKTRRAASGQSWIEGRLEIWHNLNWGTVCSDWFHRINAEVACRLMGYHTGELLPPEQAAAYLVNTTLIQVGI
HNVLSNAGTRYSTIPPIQLDNLDCLGNETSLADCSHNSWGFHDCNHKQDVALRCWRYQQGKVRLVDGWPDQQQPGIR
IAGRLEVLLGEQFGGVCATGFDAWDATVACRQLGYAEAILTAGNETQWAPPLGQPAAMSEVYCLPDETQLYNCGSDY
SSKGLTCNQQQFVNITCYNPSPPPPPSPFPPRPLPPSPLPPSKPPPSPLPPSPQPPSPLPGAPLLPSPPPPSPPPPS
PSPPSPQPLSPSPPSPQPPSPKPPSPQPPLLPQPPSPKPPSPPPASPPPPIPPQPPNPFPPKPPNPNPPSPLPPPPP
PPPPPAMSTTYAKLSIVCSFSEQALLRVLRVLGRRSRCLTPNPVTPGEV

>Vocar20015004m|PACid:23127772
MSATLTARPLDAPAASAQNDDSHQNPLPNLQDISASSSFLGPALGGVWFDCSGWLLIHAAVSTMAPLEGVNGGAQGS
SAHSRRLSVSFLSRFNCLCPPADDAEAPKARALQPPPHKQASSGLSGEAVALSDAAERQVVLSSCTILSSRGEPVGR
EDDVTLSCMDGQALDTKADHQPGLFATLTFGGQPTSVASQRLSPANLEVDTTAAGTVVSLYQYCHSSYAINAPTLTP
PIANGQASPQPETYIDLPKISGSPTPVSPIPLGPPVTRALRKAPPAAPVIPNVTTVAVAVNPSDRTADEVPVAYASP
YSTGGVERIGKSVLENVGVRTQVAQAQVAAAVAAAKAGAAASAPGGVAAAVVATPSQPPPATTIGGHPWLGSFQTLS
ELASAARPPPPWPDPPRAAGLRLAEQPSAPLPSVQLWLQVSTMQGPGEFQQQPRRTVVSERQERPRRGPIMPEASGS
FTSLTPRGGVTGLSPSLDAATTAAAAAAAAACSAADASPFVSAIAGSHVASGAQQSISGPLSCGYGNVYGNAGLGY
GFLDPHGLLTDGRSALMSGQISGTLSPRQTSFQCGTTTNATVAALYAQPPQSPQRSSVGLAAPSRVSVSGAGSVLSL
PFETSSLFDVTAPRTGLLQEQTALEMSMQAPALDLWAAAMHVSPTSRIAQGAYGVVYRGLWHGCTCAVKMMLTASTE
NLSSQLTEVFLCKALSCHPNIVQTFGCNVWRITTDTLQAVRSLSASGGAASPFTALVGGGGGFTGDSPMAPAAMLLQ
SAASGWLGQSPSMLLSASPSPAPSVSSYTGPYGITALATAPQVSLDVPCSVYSPGQGSTTGVATEAAAALAGAVGTS
TATASAATQRSLPPASMEPWITPQGAVPMSTQADSPPLPPAAGSAAAAAAGAAVVPAWASSVYPALTAGTTGPRNDG
ADAQLDERESAAASTGEKMLARPPCTDSMASSPPLLPSPPPHGAHPQPPTPQPQGPKDPHHHHHHPASTQASSLRG
PGNHAGCGTTHITPQATHGSFADDLPQLPTLGLPVGAVLEQGFLPGAQISQGLAPPLNPPASSSLGVSQPGTGTVDV
APSSCPADVVSSTPPLGRGLAGTGNESEEVACEEPCTVGPCEQPPTPPRYARPLSPPPQRGVPAVRQPLLQIPQAPQ
LPQQSGFQHHRQEEVQQEVQQEEVHQRDSKGKEEEEGAQQQQQFSQQQQQQQQQQQQEVQQQQQQQQQQQQQQQGG
AALDAGGRAGSATAAAAATSSTSAHKSQQVATAATAIPSHYVAPQTGAMRLLTAHSASMQAVSQLGAAACGCGGGGN
GDGGGGGRCTVGGSTPGTPRGTAAGAIVGALEAHLAPSGVEVPACTSPFRQAPYSCFNTTSMVATATAAGAVAGAAI
NGDTGGGGAGGGIAARLVASNNNTGQIFLEGRWPSPDTSNRSPSPAPAFNSSYSQVGGNSSAARRTAVRHAASGSTG
PMPVSASQVATVFNLAPGFNGAGAGLGLCSSPLSSGMLHASPQPGSASGTGRLFSDRGLNLPMATGGGPGCGGSVGG
VGLGGLGSLLARSAVATGDEGDAAAAMAGARNLAQVLNLLGARDGQLLVMVIMEECERGSLQHLLSKAAASPAAAAA
DATTAREVALGMAFLHHHNIIHGALPLPDVVPGNVLLKSSRQDQRGFVVKISDFGLSRVLHSSNGPPHHLLATVTST
GGSGGGSNDDADKNGDSTAAAGGALDGTVPYLAPELINNGQRSKASDVWAYGVLLWQLVTGLRPYDGLTQWQVRATG
VGCGGGNREKLTGVAVESFGHRIFG

>Vocar20015010m|PACid:23127773
MNPPNAFRLVNSRTLLFAGLLAFGVFISRAEATPRRIRLLLSKDDPPSAPPQAPSPPPPPPPSPPPPPPPPPPPPPP
PPPPKPAARRSPPPPPPPECEICVTLTGSFAGVLSKGKDVYLSPGACQHLSNFITGAVISSSAACPSSGPYFLRVCT
SAPLPFDFYAPWWSDMWELAVRSWSQAALDAVGGCTPGKVGIKYTFKGTIEDRNLCLLDRNIASYENSFTCPW

>Vocar20014977m|PACid:23127820
MNNGSTGFRLVANFPCTCPTALYRRWAGDIRTSEQLAVGEAGRTRNLLGKKFRAVARLTGHTGTGITVCLTCKHSAS
NTRMFCAIDDIHAVPYSAQFKESSSTRKQMIPRLKQARCSDYSRLLAVSWRAEEDVEDVCDVISTQQQLDSISRPCQ
AVEGLRSALTEKIFEGEYESEIVLKYTFKQLCKVLLLTAAGIVAYLNKAVTENRAFDHIHMCDRPPRPPSPRPPSPR
SPPPPPPSPPPSPPPSPRPLPPSPRKPPSPPRPPPSPPKPPAKPRLPPRKPRAPQKPPALPPSPPRRPPSPPLPPTQ
PRADEVAKTLSARSPYASRVIEVIEDCPTYIPTYLFSSARAGTRNGVRLTRGAGRIGRLEVSFKQEYYPAQWGYTEN
GGWAPLCDDGSVGPDEAIFFCQQLGFKTGRQYYAAGISDFGADEVITPSPVGGLLCDGFPPEIPVGMIAFQNDDDPT
CFLFRSDCSEGVLVALQCSNGADMYDGRKQKQNSLEVMYSWVERSTARSCLRTPDGLIALQVHNMVLCMHCDCAYAA
NFTTPSRPPPPRAAGPPPRAPPPPDMPDAIKVLDLEPNLVDAGGMRAGNGRVEVLVNTSAGEPVWAPLCATAEDLAL
FNPPEWGSDYIPDPVANTSCNQPVALSMVVPQEDPRTNTWNTEVIGFIRQPVPIPNDTLPVNSEANLFNPAKYTHWV
TVVGGKNESKYALQAMDLQVSTTPCATGYMYSLICQYLTMYG

>Vocar20012184m|PACid:23127906

MEAARRSALSSRPYPGEDEEPVSPPPASPPPPGYFDGLGDFQDDELHAYGMNDDAGGGSDAGGGYKVSEADADAETY
GDTGEPLKRRRRDSGLGEEQVPEQEDEDRPRGRLTSWGFDVDEPCPGCPMYEGYGFD

>Vocar20012157m|PACid:23127912
MRGPLLHRGCSGSLRGPAFSVTDVLSTPARAAAGGADGVTTPELLQSSNEPIVTSYQNCTDVSSTKPCPAAFHRGAP
NTLNTDVMPAAVQHITSRKGSTAAQTHWPTSPIAPTQPSPIHIDSLASGLVHHYHCGHGGPVAAAGEETAFHFLPSP
RHANTPAASCSVGRLGCLDPLSKGRRTTASPSGPFPCRANPAGHVASMASAAYPEPHRASRGPPQQSAGFLYVGGSR
DTGGGSDGGGGTGGGLAGGSDAAGGSESSCYTDPAQSRQGLQAAEFGPAEEADRLAALLGEDPLPAPVPTAAWLPAS
SGRTAAPAAAAEADLINALASSMAHDGAHEAQLPRLRNVYDTGDVSGGDGAPRSVLVAQTGLSDIAAATAVGGSGSA
ADAADGEAAAAHSPPPQGRAALRIDSSRAHTLSRIAHKSLAVHHAYRLRLQQAAVTAAAAAAATAREDGSAAAAASA
APRSSAAAPAGARLTSPNPTALRFAPAVAHAVRHGLDANTSAARQRIDWQAAEVEESVAAAAAAAAAAMSSKGNGL
AALAASRRGGDVGALKGLAMSRLARPPQQPPPQQQQLPQQDRQQQYGKQSELLLRALGRGSPLRERLVSPPKTMAEE
TPTPTSTAATSTSTAAAVHLAGTREIGLRLAQLRQQQQQLPEQLHHPQQQQQQQLPYAMIGQDEAVATTAAAAAAA
AAAPEAVVASSPEAPLWIQARQHAAAAETAATAEAVGIEVPERLPHAATGPSAYAVRSDAAATAAHATSWRGRLGAL
RLAMSAWSLPALQQGIRDAVRHSSPSARQPSQQLKLQQEEQQQEHIWIGEVAPPPTSPPAAATLPTAQADSFLGQSL
TAATAELGAADVVAGTAASPPPPQAAAAVAEAAIASSRGPERGFWMEALWSMWTVRPQREGTTVRHAERSDVAEMQK
AVEAEEGGGVVAELRNRLPVLDAAPAAATEVAVTEAEAAVRTTAEMVAGPLSSPPPHLPSTLPAAAAMPSMVDVEAL
AVDAGGRQQHPEAVEAAAAAAAAENGMGQQLEAAVAAAAAEVTGVAAPAAADSSRSLLDLIRSPLAAEAAARFADVRA
SLERRRQQKEEERAAAAAAAAEAEALEATAAEADTGPLIPPGVFTAVAEAGPVAPSPSSTEEAIRQLLSQIQIQTPAG
GGVPGVSDPVLPLQPSASPFWTSGGGGGGRREPSYQWPAAADAGAGGGMGVRNRRLQVLEPAAGEEDWARSMASLYG
TAANAGQAAAAAAAESYTDETSAALDPRGSAAAAAAVSPSPLMPPTTSGRRILFGEPQQVLIPEGVTVRGLSALLGI
TTTRLETFLRDQLGEVVRSDREEVAPESAELAALEFGKLGIVKRDGSPTSSGAMEPRQPVVTILGHVDHGKTSLLDA
LRSTAVAAGEAGGITQHIGAFEVRLATSTAAESSPSSPAAITFVDTPGHEAFSAMRARGAAVTDIAVLVVAADEGVK
PQTREGLAHARSAGCPVVVAITKCDKPTARPDMVKAQLAAEGLDLEDFGGTVQVVHTSATSGEGLRELEEALLLQAE
LMDLRAPREGPASGSVIESRLERGLGVVATVIVKSGTLRVRGGEVGGLEGGDGCVGGSGCGGVLYG

>Vocar20012192m|PACid:23127925
MVSSIRGMLVVCGPSGVGKGTLIGRLMAEHGDKFGFSVSHTTRGPRPGEQDGVHYHFTNRESMQREVESGMFLEYAD
VHGNMYGTSLGAVAAVGQSGRIAVLDIDVQGATKIKSSRAASKARYVFVDPPSLEVLEARLRGRGTETEDKVRLRLA
NATAEIDRSREPGFFDARLVNDDLEAAYHRFKTTIEKLLPGTFSAEEIKPPPPPAAPPAPSPPPPPAAPPAPSPPPA
RVRTPTPPSQPQPTAAAARGGPASTAAVGATAAAAGLPVRQYMDATVVPVLREGLRALNEARPDDPLKFLADFLLDA
RSANGGRHSRTVGGGGNGKTVY

>Vocar20010190m|PACid:23128063
MHLMCQCPRHQRFAALLVLLLVPNAVAQDNGDSATSLTEELKFLLTPNRCTAFPSTPGALRLLAYVGVAQSASNDTA
DLGFTTCTPGSLTRVVRDAIAALFSSPSPSSSSSSSAAAAAAGPGLLPSNAWPLSIEVHAQVTSCSGAVASSGAPPA
AGEGLLAATQTTPTSSNWTQPAPSSLPAAAAPANDAATIITAPAIKDAAAAAAVAGAAAGAQGIITDAAHAFSDLVP
AGELVSLRDLAAIDGSFSFGDILPPLRRKLALGEVGGAAVVPLDAPVLLLVYRRDVLAKMGREIPRTWDELADLVEL
YGISRPAGAPSAALCATTGEPCSSLSLFSAIWHSLAQSRGLSQGVYFDRETAAPLVRTAAFRAAAQLFLRLWRPALP
QPDGMCGFVHPGLRLGTCAFSVGTFEQVKDVARIAPSQQQPPSDTAGQNQSASGVRSSPDVATAGAAAATNANALFG
VAQLPGSRFVLSPEDSSRLVLCSTGNCPLGDISATGAGQQQQQQEQERRRRGRGRRRRHVLQTEAPSDAVLVNRAP
YIPSSLLVAGISTRSTTAQRLLAWYSLSHVAGPEGSLQLLREPGANTAPFRTSHFSAAARAAWAAAGYDGDLMGRAL
DEQQAALAHPNLAWGLHMAGGAFYRSALSDFLAELLNTASSSPSAATDQLDLLIEQLHAKLCSHYNSNSNSTSNSSS
TSAATWGASITAARYAAFLNIGSWPRLEAQAGGPATEPAGAVSAPASGSGRGRLVPLAVALIVAGLAVLAVALYVPY
RRGCGPLRRRQRPSWSPDAPAVSPNTTLLITDIESSTKLWEELPEVVMGCALRLHHACLRKVLVRHRGYESATEGDS
FILAFHKPETALAFAVETQQELLEVNWPPELLANEACAPVYTSAPVGFPFRRPATLETVLSRMSMRPHSHTSIGGGG
GAASGPCAFAASPSTLQLLASMLPASFARATASRGSMAPQSPTLLQGRLHSPFCSRQRMSIDMGLLHHLSREEVRTP
TSRGDILETDATFNPLFASTQVPLAASPSPSPSVLRRGAERADAAIRALTSMPLASTAAAAAAATAVAAAASATHSV
TPTATTAEVAAAALSAHVPASRGSTSSRLIMARLSGRSEMSDLAAAGGSSSSPPPPPSPPSPPPPQRQQPQGLVPPR
KPGALSRKLQELQRGAQQQPLQPPQQVEVLHHRYHHFLTQSHHQQSQQQQQQSQQQPSPRQHQLQLMEEQFAPASSM
NSSGAWAIIHGGETAAALEDPTSTNTNVAAVTAATAAIGCVKNVIEVMPEYVSAPLNLLPTTSTSRTPTGSGMAAPF
TSGGNGIIEPGVDATSVSEGLFEISHNGSGPALNIGAGNRARHASPSQSPGLSMLGGRGERRGTSRRAQTQPVAVAP
GRSPLPTAPGGSPELVCVTGGTLINLEACTLEESLKAAIRHLDEAGGGDCKRRLLWGRLRIRMGLHSGIASESELTH
NRASARTVYSGECASIVRLVSDSAQGGLILLSGACWGALMAAAAAAGGLHGAAAEALLINIGRYRVMPPSSAAAAGG
PVDHEPAGVMPLYIATSPELAARAAVLPPPRKISAVGLSLYAAPVGRVAVASLNVADVGALREWDEQALRQGLALLR
RTVLEQLACHRGYLVSEDWEQGTLSVAFAFPLDAVRWALRCREVLGSAAVDWPAKLLAHELCREEQLQRLQQAVAGR
ASTGGGGGGGTLTLYGSRSGGIALAGSCTGPPPSPVSGRGRIRTLELATSYDSNTALWTSPSLAAAAAAVAAAAAAA

SEPAGSAAVAVSSRAPRSAASGGPAGDGARTLSREALSATVERGLQQSYTMALGPRAGSTPADLDLVPTTGLQPPAP
VVRQAPAGVAGGSGGSAAATAGVRGRSPTVHGTASGVMACHGSYRRRSGKQLIALGSQGALGSGAAIAASIPAAGTT
LTGSGLVATDSGSVSCTTATTAGITPKPSGYLSTKFAALSSRLRISAGGGSHADGGGGGGGGGTAAALSHSHVPAA
TAAAEAGMLSARASGAADATARSSGAGSPGGPRDSAAKTSLAWALRDEMEDAEDGGDIFSLHLGGMDGCGLLGRAPG
VSLSGGCAIYGSSTGREAAAVVAGGASVGGHQTAPAVADRRCVTTTLRGPRIRVGVDSGPVEWGISSSHHCLTYSGA
PTAMAEKLAAVAAPGQVLATYSVMLELYRMQANLGPPSEAEEATAAAERGSRAGFGSGGCGGGVGGVGGSGAFSAVV
GTALPPPSGRSGLTSGRQRRQQQIFACRFTNDWEEALREYDTLMAQQAGAYQAAATSGWSSPRSGRMSPFSSPWNWS
ATGRRSKRPSGTAAGAAAVMAVAPPPPPLPSPRIGESRHTQPNRGSFDLFGRGSTSLTQRGAAAQPPLPQRPTAQCA
AADALYGSGGGAGVPSPLPSSMSHPLPSAHAPAAAASPREYLDGRVVAVAAGCSREFTSAGLMCCPPGGPSRSGDGR
SSGNAAGRVVVGNSFASYVSMSAPSNALPPTPPPPPQQQRQQQQQKKTQEQLSLLAAAGGALAPAPLTYIFMPPPV
AVELNRPGRTSSASWRTLSAARMEPPPSPPPQQQQQQQPLSTSSSQGTLAESCPWFCQNGGAGGAGGGGAAAAIAFP
VGSMDDSISLSLRHQLPADVKRTPSRATIASGTVAGGPAGAALLSSTPEHEEVTEVGDVEEEFELTPRTLPRPLPPT
PPHQTYGMDPPTSEFASGLVGAVMPAVRTAASATVAAAAAASNSPSASIGSTLPSYPRSGPGAPYGSTGEEGSSSAG
GGGGGGGFGSGGSSSASGMLQQALRQHQSYARRRRQQRESSLQHDHVIAAAEALAVQDLSVSSHSPLHQETSPRLQQ
AALATPTPPPQQQPPTPLPAQPPPQASAIPVRSRSGRQAAAGRRGGGDPATGTVVWRASSTITGHGSSSSSSWPLEV
TAAAQQGYRPASLGQAGTGTETSDEGAASAAAAAAAMAAAARLVVSKSGVTAGGREQAGKAGAAAAVPAAAVVLVPA
SAAPATVAGFDLAGATGDGSRRPYSEGCSEGPPCADQLSSPPGLHAPADQQVLPIRTAVPVQQSAAQPAPYGGVQAG
LRWGRPDVGARGGCLAATSQVGEAEVADVKVVAAGEECAVVAAAAAAAAAAATLLDGEVNPAVAASTAPLKDVRSGG
SALAQFAAAISQKGHHTPPPPPPPPAASYSGLQPRRTGSGAPHQQRNLAAYGDDSAEVDVASELCGTFECRGRWRRP
THDGAIAGGFGSTIGSSGISGGGGIGSSGISGGGGGGRSVVEVGSYVGRAARSIRALSLQGSHGGAAAAGAAGFGSL
AGSSNRWHDTAVRASPTCRTSMADRTTSNLWYMQPQPMQLQRCGPLPPGAAVGGGGGGGGDVSLSPFLSPARFEVYE
PHEYGGVSDDCDDRSVSVRAPAGWRPGIHPPPSAGGGSLPPPRRSSFARVGSLAPQSESVSMHGGLQSAVTGLTESS
AGVCAGNRAGGGYGISTRGGGGGGGGGAIMTLPCGSACTLSLVRGASACYALDEPELWQDEAYTDPLEM

>Vocar20006420m|PACid:23128229
METVIQFRTKVQQILDKECKVDWGKEGKFWGIISKYDATHLEAPWTIFYPNDGDEENGVFTPDFSEWHVEDEEHPKD
PFTVRKILAWRDGKPLGLPRPPDELDTAEFEPVLGHDPKLPHNQSSDSDGSAQEREDGDDDGSASSSSGDDEKDSNG
DALRARRLSSGGSLSRALSGTPRLPPKQSARRNTPDTDAKPRRSVPFGEAQPGSSHSQPDEQQQSAKRKPGRPRKHP
LPEGDQPPAKRPRGRPPKNANTAAGGGSGGAAKAKPGRPRSAALEGSLVKPASQPSAAMASDAAAGGSGGRGGSAEP
VPAPIKQQRLVRLDSDEFDEKPDEKPPVSKSHGKVHIKAAAVAAEADAPGPSCASPISDDGGLGTQAAAAIKDGDAS
PGDPAEASIAVGANKGAKAGRSTQAAAATDSQLGQQKAAGAPDAKPVVGTGRPKPLLNEEQLAKRKMSFNTAAPQRP
AGLLGVPTARAQRDSGHTGTVKLEGPLAVKPTGKATAASSKPAGAGTATEHGQSQSAAAGSGTGMAKDTQGAAGAAA
TAGAEGAKPSTAVEFSAGPAAAAADAPAQAKEQQEDDGPKRGDIPSQLSPPPQQQQQPKAAAPGPASTTALPAAAVL
PQPSLPVSLLAPAAVPPVVSKATLPPLPVVSLPPRTASTVSLAAAVPQPALVASPASAGAAVTGATVTAALPASQSM
PHLPQCDSQQSQDPLLSFSQAYHPGPSQGSQRDLAAAGEPVESPPSKGAISAATFGRPSALLPAAAATALHGAAVVP
AAVGAASLAARPSLPPTAALAALIPSGIIATASLPPATTTPVPPGAPAVVTTAALAPPGSTAATSTAAPAVPPLASG
STAAEALAAAASAAIASGATPETANPGVQQLLLQLLGRALDALSNSQSAIKSTANSAILMAKAVGANRVARGFFSKL
GLLGEEVALLAPESCTGDATGCDRVAAVLAKRLNVFYVMDSMLHRCTKEEAIASWPKWINSGLQASFRLKVFTEHHL
RAVRKVTDVWKKRNLLDASVLDMADRILTEQESMLKSRSAAGAAAAAAAAATASMTGAAAAAAAVAAAAASGLGPIP
GRGDLEGPFMAPGGAGPRGGPAGLHVEMGGEGAAGPQRMSHAGAPIVAGVRRRRPYCMVMPRPSNLARFNYHMGEYE
MYYINRFGPMTNRCFIDNPYGSLEDSKDELTGEPLAKLIEVDLSEDDNEPDFSGTVTWREVIAVQLPQFNPESPLRG
GPPAPNVTPPHPHLEEELKLAALGLSRTPAAAPRPSLAPKFITSMPLSSAAAGAGAGAGTSANGATAAAAAASTIAV
PAPPVPASPPPDAARVGIPAISGLTPPPGNDMDDLDTWAYGGEPAPADSATAGAAAAIATAAASQSTGPAAAGSRSV
TEEDTQPNIFDMPAAPSHEVSSTGRRSEWDDVDMDMSEDEDGEWQPPLPSAPPPPPPPSGGGGGAAPQPPSTAIPPP
VPPPATVLPPLPGLLQPQPHGMGMGAPDGLLALMGPGPMGFPLGLQPPPFMMPMPPVPPLWPGQPPLPHPLAAPLPP
PPLPPAPPLPFHGPPPPTEPPPPPPQEPAPAPPPHGPPSPPPPPPSHPPLPGAGSSGGGSPPPLPPPMPALPPLPPPM
PPPMPPMPTSGAGPSPPHLADLHLPPLPPEAPPQPPSVAPKQAAPPSPARLPPPPLPLDGPMLPNPTSPPVKTPTSL
PPHLQPTRPGSATKTAAVLPSAAATITLTTAAAAGPQALSPSCVTSPKATATPAAAAAAAAAAATQTSTTRSGRDRK
QERQRERDGEEEMGLSPEAGREDRDKERERQRGRDRDKDREEPEDKERGKGKSREEKEQPRILKLGLKEDRGRDRAQ
EKDREKERDREKDRSRKRSRSSRAASEERQDEEQQLQTVPRDRQQQQQQSSQGYQASPPASQQQHGMEADRLEKESA
RGEREGDRGRGRDREGDRGREKDRGLDRERERERARDGRGPLVQQQQQQQREPTTLPLRDADRDRDRDRDRDHDVR
DWEPSLRQSQPPPLPPLPPQSSAGPQRDMSRRRYDDRDPRDWPPPLHPGPPYERGWDPRDVDPRDRDPRDWDGGGSA
WDRDRIRDRDRDRDRGGDPWDRGGGPPGNRGRRESDPVRGGPPGGPLPPHPHMHPREYREAEWEASGAPLPPYMRGP
PPGGPGRAGPGGPGAARPQSPPPPPHGRYGYRDSPGREQPPPLPPQHCNDYPPSANGRGGRAAPRHSGGEPMPDLPY
RDSGAGGGYGRGPSHAVVSGDAGVSGGGIGAPDERRKSLGSNLKRVVPSDSLPDELSPRHEFEDAGKQRHHDRDRER
DRDRDHRDRERDRELRREQKRERKKARQRRREEADRERGERGGERDTDPPAAGSSPRREDGEDGGEAEGGGEEGEEG
ELPEAVDAAGPASPGPGPGPGGSGATLVRNHHRKPHVPGGPAASGGGASGRRLISKDTDQGIEGEAEEGEVELPPPP

KLSSPLLSPHGPQQLRPSSPRGTSSSHQPPCPQNPKAPHLPGPNPPNATQTASSLGPELRAPGQAAAEHPPKQHSPF
AQAGDQGAESQQGSAQQHQQHQQPRKRNPIVYNLNPRDSRDAFTFAAPLLGLKPSSPRVVSPPAAATAATGGSRPQP
LASARSASPRRAGGGGGGGGGGGSGKEKATAQTAGRGSVSSSQHQQHESGGGGGGSSGAANRPPRAPLSLGPSQGS
GAGSGGGEGGGGSGAAGTSNHGASNRNAHSHQQQRQRALSPPSQQPHPQQQHRHCRGADGGLGAAVTAGIAASAT
SALHEAAASTAGDARQREGHSGGGGGGGSKRRRELDWEGEMMGAGGCRPVCLPDA

>Vocar20006313m|PACid:23128536
MHRLCIALCSRSRQRRKSYIVSILSIVFIFLSWGWCDDSVDDGKSQQRGAASQPYLPAYPWKPQQPETPPSLPDNPA
KPPSPPPRAPSPPPIFCPRIPPYPGISSIQILDCDIEYPEPDTCGLSLLNGVRMQLQPGQALAIQFRNLNSSSAQLS
RLVISAEHMSATLTFQAGSSVGGGDAVIIGPQRLFLISGMCPNIAALTSCLHDAFHGVPQYRACQSCFGINVVAELW
DLVSSTGYEHLPMVGTAATVGDTSYFRSQLPADGSVLYFLASASSGTVLLDYARAARLLYLPSAQVSYSPPGKKSFG
GIRGTENGPPGVRRGPSVVVGGGSSSSDSKGADGSSTGNDTATDGSSKGSNTAGDRQPPPGSIPSCDDRGTSSSSCR
ANISDNNSGINSPPSPSFYSTTGGAKCQQRIPLLYLMVLAAVAQRLGDGVLLPLAP

>Vocar20006629m|PACid:23128602
MVSREPVRRSSGSRDAADLRFLPRSCWANLGRWNFLLIFLAVFAITSCLAGPLAGTSFPFESCEKTVGTSRISVSLK
NTSWSATRGETRVCFQFSVSAPELCDAGPRRCCNTGFHKFKFYPDPMCKQAVNSVTYQVKGGPVVGPISSVYYERSG
DTFIGKITMLPFTINTTDGAVLCWTLRSPCSTLRDLAHPKTRDLNLLEVALYDYKVDGYEVNTANPPPPPKLSPPSP
PPSSPPPSPRLPPPSPPPPSPPPPSPPPPSQNTPGQDLTKPAVRFYQDSRVFGIFLLPGISFDDARARCGRGGGFLV
SLVSSSEWNEVRAALAELSSYSGFGNSLSVWVGVDPAGNSTWLDGGAIRFWTNNQPPRVGGLCYTAVCSGTDGQQAV
AASCTLEPIPCTSRTVQGFICKTYTGVITYINAAASYGKTYVYSTYGLNSNAASTYCGRLGGQMVSYNTPDEFNMVL
QPLIYGTIRVQRSAIDSNGTVTVWLGFSDPAASRWMDGTPVTFSKLNTTSATGCYIIRCPYAQRQSAGAGAAGAGAA
AAAGMVSPLDACTWELQPSCMRLSLFICELPGVFEGSSGAALLVSP

>Vocar20006473m|PACid:23128621
MTEDAKPTVEETAAARRERRKRSRWGEETEAGKKVLEETAQQPEAAAGTTEGDQQQQAAGEREGSVGPGTGDAAPRK
RRTRWEPEEQKVIPGLSTAITLPPSLAHLVDLNPETLELQMQLNNVNQRIALLQAGKLLDDTPPEQRSPSPEPIYNE
FGIRLNTREQRTREKLQERRTELIMELIKKNPNYKPPADFRPPRKTRKIYIPQNEYPSYNFIGLIIGPRGNTQKRME
KETNTKIAIRGKGSIKEGRTRRDPMGRPEPGEDDELHVLITGDTDEDVDKAAALIEKLLQPQDETLNEHKRLQLREL
AALNGTLRDHEIIELQRQAEEGGEVFRLPEHLKEKVEEQYRRDIIAVHGEGAASLEDEYKSFLRDLGGDVPHIPREL
EGMLGPGGRGRPGLGLGAGGRHDEADPRTVYVGFIPHHTTEHELRQLFSTCGEVADCSIITDRHTGASRGFGFVKYA
TDEQALVAIERLHNYVLGGRRLVVRAKGAQRPGPGIPGGPQPPGGGGSGGIGPGGLPLPPPPPGRPGLDNYLDFSGG
SLRPPQWVFTDDPPPGFDLVAAGAVHHPVDPAAAPPPEHLADCSGMHRYMVAQSEWQAVQATYGSAPHLRLDAVAIG
AVAAAASGGYNYYLQERERMRQQDLQQHLGGGDGGATGEQGAGGGGGGGGGYHDDRFGDGGAEARVGRTLLEEVATV
WLSPPMTLEPGPPGADSWPAGEAPLVALPGETAESYNAYCAAYHAAATYGYGYDMYGDGVVYDPALWGDSAAYGAQG
VGGGMDAGGAVAKAAAESAAAVAAAAAADAPPGDDAAAAAAVAAAAASGAAGGSGGLGGGTGGGGGGGHRRGGRNR
NKAGPAVVQVVSLDPAEMEKAKKKKQEEEERARRIREAEERMKGPSAAAAAGSYNSRLLPAVPTTHHPGIAALPYLN
RFGSRQLVNTFDVLVYRILTFRARSILCRHSAQADQERRAKEEAERQQRAAGTSGAGFAAGDAAGGEAGLGPGLGSG
PGMGQGASAGAIRTGAPPPPPHPGGPSDGLGPRRGTRSPSPPLRRFNSRSPPMRGRFVASRSPPPRGGRYVASRSPP
PRGRLGAGRYSSRSPPPRGGGGGSRYGSRSPSPPGRGRHYGGRYGNGPAAASPLADHRGRRFSRSPSPLGRRGSLRG
PGSRYGSRSPGPYRGPDRYDSGYPEEDELPGGRRGGPRDRGREDRVLGGAAAGTGAGPAGPAEQQQQPAASPEPTES
GEISEGEVPGSDAEGPAGPSGRTRGEPGQRAGRGGGGGGGGSGHNADGAEARRGYAAGSSYSGGYEKGSRKGEGPGG
YRGGGPGGGSGRGRGGGGGGGGRGGRGPGGPALGPGMGYAFGSHATYEGYDEEAYGWYDDGYGYGYDEEGEGWEGDF
GYAYADPAYFGAFYAAAPAMMAGGRGGGRGGGGEYEMYGYSGTGGIASSGSGGGGGGGGGYSGGSRPAGGGGGTGKG
KGRGAG

>Vocar20007438m|PACid:23128780
MEASARSYAWEGKRMPHQRSSADGQPSSSGRPEARRSGSGDLQLRSLARLPRASQNGSEPLALQQPAEDALLQSGEE
QAARRTIDLKARPPNTAQRSNSTSTGRRVRRGSIADFLGFKSGSPSQESSAAPSRDVTLHGMAMPSAPGARSRRSSF
LGTFGNNTAQQTEVDNNGESGAGAAPLSDAMSGPVDLAKSLPASSQGRWRRASMLDLFRSMHSVETSGECGEPGQQG
EPLPRTKSTPKQRRASVIDFFRSSNTGMANVDDPGTPGNAQMGRRLPRGDSRGQRRRSVIDILGHSSKKVFAKLMHH
EFDHPLDEDDLGVEGKLWFTSLNDFKPLTFQYLSGRYSSAFFACSVKTGQHYILKKFDKGKMNLSDERGVRRALHFA
EVLNHEHIVQCCGLWEDEQALYIVEEYAVKGDLLQDSLSHPEKYSEAFMATKVVKPLLDVLAYLHAANVVHRAIFPE
YVMFGREDKLKLGHFTSAIDQRMDPPTERIPFLDYMAPEMLSVRSEDCGGGGLPYVVAAAAGPAAAGTGRGKVGDVN
GTDRDDGCCGDGGDGGCCDAPGTPVFDFGNGHDSFLTSPKQQQQLLQQQQQQQQQLQQYNHNQENHPYQQYQPPTV
QLPPPPQQQQYQQQQNQQQQLANMASLTPSRRQTTSSGAQLLEFFGLSPHSPVNKTGADAAGGAALDKCPLSPVSRS
STRSNLNLFRSARRLTPAGQLQQQQQVLQPMISSDDDRSGMSYGSGMPSAAPPPAMMMPQRRRKSALEEMNAPWLRP

IGSDSQSTCTAETRDGADALWEPADGGGGGGGSDTTDHGPVTTPVVETTSAGPTADGSGGDAVANAVSGDGSSSSVG
VGVGSSMPAAGQGGNDGGGVGNPRSQSQLARADSVQQNVVITAAAAAAAAQLDEGQGDHSPVERLRPSPPGSSTQLD
QAPSSPSPPPASQPPSHHHLHHHHHQHPHLHQQQQYQQQQQQYRQQGVLTDGTATAAADDNSPPLRPAPSWPQPPRP
PSACRSPGSSSVHRSEPSPAFRSGSGALCSGGGSGGGKSSSGTPLSPPLPRPGPGLTPGPLAAANSRLPPFALGSGG
SGENGRLADRDECLPGYAAEPLAHRCSTSLQGRPPPPPPPLTGLHRLQAADSAAAPSSPPPSPPFPPSPGPVVESLI
ATEHTPMMPYDGGVGSPPDATEVKPSRAVVSFTTAAGDCSSPTGVDAYGDDARPSGRSSRLVSQEPSSSFSQGAQMM
AGLRTIAKNITTRLGMGDTSGWGDVADAGAGGGGGGGGGPREMSPRPSCPGAGEATALIMPNNPWEWQDHYNEKVD
LWQVGCLVHEILCFSLPFETEDKLLASALILWADIVSFPDHLSPECHDFMRACLTKNPVERPSAAELLQHPWIVRHA
AGEVLKSVREHRENETLAAFSSAQQLTLLQRAAAAIGLGWVVGAGGGAASSHPSSSSSSSSPRAASPWGRWFWLLR
RWNWNWSSKVAPSGEDEGGEKDEGEAKLAMAMAMVQEQHQQQHQQQLGSSCRTRTTTDSEKTSAADSTRNLRR

>Vocar20007654m|PACid:23128862
MSCRRMSCLGVTHRPGFRVPSVGSAKAGLMDSIRSINPNARLNYAKGLTIEVPDGSAVGSGGPESLAAGLPPGHNFL
SSLHLHHQQAAPALGAGLMYGGGGSGGIPLTPHSTASLAALTAAASGGLAVPPYVPVPVPVPYMMPPTPHGGGVNAA
GGSMMGYLLPYQQDMYQALMDRLTTPRTSFAGSRSGRRTSISSVHTPRMSEAGTAYSGAGSDGTPLSSDHEDEAAAA
AAAPSPPPPPPQSLRSSYPSPPPLDTAAIPPPPPPYVEPPPPPYPNEAQPLPYGTNYSRRTSYPGSYYGSAHAPYHP
QHPPHMYDNPSGPYGAQQYGSVSPATGNGNGFGAVSELERIVTNDPAAFHNNFSASSRRVPISSVAAVRRPYTVRQP
SGYTYVVPRAALGQVECIRWVRQLTTSIQPNLADQRRLNLVPVYVCVYGGGPEREGDMMAVAPVPGEEDESCEVCTS
LINRVYPGLRDRERAYLVGVLAACLPYKMASYTEDDVLHAGAAIERAVQERRLPGDMVAVAARLADDVMGNAATYGT
ARLTLSQVTTVHQLGAQCLPPSEGLLTLTGIDPGISRGQWSRLTSGAKPYVCMTAFQEADRFRRGYLPLTEAARMLT
RLNAVPPTAVAWLMAGLAHYAATALQGAADLSYKELGAGLLYMSDSAATAAAAPSAQLPYYSSYDQKDSYAVPPDST
TPYPSPFTGPHYPVQQPPYGAGGPSYGAAAPSYVERYPSLSSVPSMGTAPPQSPGSYDSRYPLSPRVSYSGPPPPYG
SPHPYNHPPNPYGGPPSPHGVSPNPYSAYPMGPYGSPPLSPHVGSLSYPGMPPPPPPHSPYGSYTAASHIPVATALD
YPTAPLAAGGGPAAAAGLSLGTAYMMWTHDNRQAVVQKHLSEVLKGRVAELEVQKGALEKEAGLQTKILDLSNALLA
NETAQRDVLGLIQSYTGAGTPPVSVAEMVGRIRTTLEEALQLAKSASAPSPLGGPGLGQGASGPSDFDSLLDALTNA
RWVGGQPVSTETLRTALSLRRDAFALRRAHTAAMLELEARRQGMQVASQEAQQAMSIQLQLLQVPHAGTAAMVPGLG
TAAASSLAAAAAAAASVNPPPLVLSPRTWQLEPAAVVPAGTATSPSALPLPPQLLPVNAANVSAPYSFMTGSRAPST
GGLPPAIYPAPLHD

>Vocar20007526m|PACid:23128897
MSSDLEEQKKAILPFMQRAQEIQQADPKVAYYCRMYAVDQALKLPQRAKEVTALLSATLNQLERDKAQIKLDPVADR
IHCLGFALRIFDNADRVDRAGRATERTAVAYYAASVFVEILNQFEGGVDADLLEKQRYCAWRAAEIRKALREGRQPT
PPPSANPLPAATSEGGNAATSADLPPASGSSAWAYGGQSQPHALPPPPLPPVPPARGGGEDAMGGEGFGSSDSSFRS
TGGDSFVYPRPPPPSVSSSFGRPESPMGQAAAASGGPARFWPGAKLLVHTEEGLDVGGEGGLSSGGGGGGGGVHPP
GTQVGTVGQVVRRPDGGFSYKVALTDRLVEVPEEAAVAALREGDVACMALGHQQTQPFTVKVLRINYASWPPTYLVQ
TPDGKEQVVTAQQLTPPPRDGLSAATSTTAVPQLAPSAPAASAPPPPMHPPQSPPPPAPSSPPRAPSAPAYPAYGGG
VATPPGAGPYPPGGAVHPPSLPSHAAGAPSAYPYPAPTPTHSTSTPVQGYGSIAVPPSTAALPPGYKPPVQMIMDAQ
KQAKYAVSALSFEDIPTAVKHLSEALRLLTSPPPPSAAANLTMPR

>Vocar20007631m|PACid:23128899
MDCDANLYPRGDAPVSSCPPVFGRTVQRGSQSQPLPAPPPPSPSSPSLPEPDWDYGSDLPESTRNADGVADSSPPPS
LPPSPPPPLPSPLPSYPWLPASVTASPPQAPPLLPPPPPLQPSAPLQPSAPLQPSPPPPPSPSPKPPPPPPPLPPPP
PPEQPPATVQPLPASSPQVQGSPQNSFNASPSVQRLPPRRPRSPPRRPQPPSRPPSPPPKPPQMPRPPPSPRPPPPP
RRPPSPPRPRNPPWIPRAPALSSKASGEVLFSGDYYSSDEGADVTLGTEMIVLPAVLPDNDPQSNGQWVLKAVGNVV
AVHMSLIPGTDKFLFMERPSGRHPDRGSNIVGYYDYLTNRFTNVNYTDSVFCAGHTVTQDGHVMVVGGHIAKSGYAD
GLKAVRIFSRRTLTMHRITNMSYPRWYPTATLLPSGKVTIMGGTVLPGSGTGNNPIYEIWEPETPSQLLLRKQSPGL
IANSNDIYYPFTYVLPTGDLFIFCNAYGEIIEPMTGDVRALPLFCGIPALTDCVLPPLLLKRFVFSCGVWARSAVLP
VRPSVPSPPSALHSSPPGPQSPEAFEPSTPSRAPAPCFRSHPRMDTRLRWSSLAVSMTGAGSTPQPAGWRSGSRCFM
IRSRAPTPSVSASDLRGLVGGAHSQLVGGAYSQLVGGAYSQLVGGAHSQLVGGAYSQLVGGAYSQLVGSAHSQLVGG
AYSQLVGGAHSQLVGGAHSQLVGCAYSQLVGGAHSQFVGGAYSQLVGGAYSQLVGGAHSQFVGGAYSQLVGGAHSQL
VGGAYSQLVGGAYSQLVGGAHSQLVGGAHSQLVGGAYSQLVGGAYSQLVGGAHSQLVGGAYSQLVGGAHSQLVGGAY
SQLVGGAHSQLVGGAYSQLVGGAYSQLVGGAHSQLVGGAHCTGLCYSLCRSSSSQSFGSPSGGIHPAEESATECFSK
QQCSSSTHKPLLFLRGSVASIAGDGWTAEKMPLPRVMGDAVVLPNGKVVVLNGAVKGLAGDSASGGVAKANEPNLWP
VLYDPDAPSGSRMRLMARSMIPRLYHSTAALTTDGSVLVAGCDRCDRYWWTTPGGISKSPTMFAEYRIEVFRPPCWF
NVTAKPQIISMDAATWDEYDSVNVMQYGEPFVLQYSMFYATDSVTSAVLVSPGSTTHSTNMNQRVVGLEILAQDVDA
RRLVLNGPPDINIAPPGWYMLFLLNGDVYGQSAWVRLPGTAPRLDRFLTPASLAT

>Vocar20010321m|PACid:23128970
MGNSCTHAADAVAHLVTETEIHQAVRSIEEWQKVQSATVKNGGPGQNMYGPLLQRVGGPDVVKKVVELFYRKLYGDQ
RLILFLHDQDVMHLRAKQSAFISWLFGPPNVPYLGRSVRIAHLRIVKQRGFNGDDFELGMKYFEQAMAELGASETII
GEVMRRLRPFKDAFFTPSAKDAEEEARWVLEEREKEEAARLQELQAQEEREFHDRERRLQEQITLAHRAAEVAEAEA
EAEAEAAEDGKARERTAEVPAAVAMASSVGGCQVATTTSGTMTIRPASHGPSCPFTGGGQSRGSISPQGCRTPSRQE
AGATLRQVDGDDVTQEDSNGHIERQRPSNGNGISTAAGAAVAGSMAPPSPTAQTTLSPLSPPPLSPPPAAAAADRPG
SIPSTATIPNTTVTVPGTMEAANGNNGNNGNNGNENGSSSSTRTAPTPPPAGGPAAPSATSTAVLAPGTAAVAVAVA
KSQPSLAAAATPAAAAPAISLDFGAELNDLEKGSSHDKGRMQQEEEPLLPLTLKPSSSS

>Vocar20010315m|PACid:23129065
MGGISSAIPSVMRRNDSFPGFPSRQLRTCAAALAVAISHQTYGEQLDCIHVSAALVRLAKIYRNNAAELTVPYAAAL
AEAEAAPPPLTAPVGSGGGAIRTKGSSSSGGGGGGRGARFARAWKRARGTGEALAAEGAETPMWLVTAEERRLRRSV
AERHPGGMRRCTVLTALLPPPSALLPPTTGTVAAATALLPPTTGTVAAATALLPPTTGTVAAAVVDHVARLLDSLLV
RIPGLMPRACCRTVSNMLWALGVLQPLAAPRPQWRRRGSGAAAVGVPRVAAAASTLAAKMRHFWREAGPHSLSLALW
GLARLGLRPKPVWVAELEFASGRLLPRFSARELSCCLWALATLRVRPDALWLAAAQRSVTAALAAPDANLRCVATLL
WSLAVLQVRLEGPTAAAITSALAVFLQAECGGGGSGDDAVPGLLPSICMSLWGLARLGLRPRSELLAEWRRAASGPL
LAAAPARAVSCMLWSLSRLRCAPPAAWVDEAAAALLARALDGRLGCQSLCLAVAALAQLDSRISPEWLRAFSAAAAP
LVRHLDPSELGVVLHGMGRLAVRCGGCNEALAAEFLAASDAAAAAALSTASADDVAGIGGGLANLGAARSVRLHDAF
VRHTAGLVQLRALAPRHAAACLQYFVASSPRHGSVASAHAAAAASAGGGGGGAASAGRPDGPQTGPDSGRELWYDTV
RARVPDDTKQPLPPPQPPPPPRKLVVALLMAVLTAPLDQYDVFDVGQALCAAAELGLKARTPAARSAVTAAAARVCG
HAPGANGVLRLLQGMYGLRVRPRTTWLTRVASQIGCRLRSLTAVEELELLLRLLAALQWPSPAAAAAGAAHLAQQPP
PPQQQQQPAAPGEELKGEDDSTLSSSSALLALLRAAQPALSSAPIQHLLQLAAALVAASTTRSTAAGAAAAAPAAAM
APSRTSPPPPAVARLEFPHGWMPAFEGATAGKLSQPCVTAASLVELLACFACFGHAPGPTWTAAWYDSTADRFRSLR
PAHLCMALHLQRRLGLRPPLPWLRNHAAALMAAVVPSATEAPLPPLEVLAAVAELQRLGLDDASAPLSGMVATAATQ
LRAELVAAEPAAQLATLAALQAILDPGVAAPAPHAEGWAVGVLAMAHSVITQRPLHLFLAGLRRTAVAPPSLSGELD
DAAGGGHGAAASPRSSEAIVTLLASMVRCGHVPDQRWMSGIVRHICRQAESIAAADGGDGDGASGSGGGWPAADVAR
AVCCLAALGYVPDAAVAERLQQLLQPVLAERDGLSPGLAAELCCAWASVRRRPPPSWWCAVEAGALAPAALAEVPQP
ALVRLPYSMKQLGHLPSYEWLACYLPHLSIALAAAAPPPPLAHSAAASTTRAADLANLAWALGSAVHDGGYPPDVLS
SSAGLVGGGPGQGPPPSAPGLGDCFCGTSDKTASCMRRTAQLLPPLLRSQEAYRVDDLAWAVVKLDPRGAVAVPFLE
LLQRQRAAAVAAAAESSAWRRRAVACSGAADGASDAADVETHRSGGGGGGAAAATLRRTYVRRRRRGRLSKSLDNGT
HGDIAAAAAAAAADIPADGGATPAGSLSPAEAEAVNDSGPAAAVRRVQRANVAPGTFDMYVCVLYFTMRQIYMSSGK
VGRFDLFCAPATSISRAATFTSPLRAAGALAQLTRVALAAASSPVAHSALHTAAPGSPQSLTKQSDVSSSGGGGGAG
GTDSGPSGHGSGAPYHTQHKYGKRSSDSGGVAGGGGVGLGFLVSLPPPKGASVSGAPAVAAASSTFHHHLPSHQQSA
AQQQQQGTTGSQGSAGRRLQQLGQRLNFGLALGRKKHATFAPGLSGGGGGGGQQLSYPEDASGAGGNGGGGGGYATT
ALRLKRDGMPPASYMWLWLGNFKRWHRRYFVASEAPGKLLIYKRANMKGKVWSTSLRDAAVVQDDSHPRQIRLEAPS
GAIFLRVLRPEERQPWIACLRESQEQYRKHKEVVDTLAAAAGGEGGEGGGADTQSLPPIHRHTISTPNLPNVSPPSP
GAVEQDAEQRRRIRARLNDRLAELAPLVSEVERHLVLVGHHLAGAAAGIAHLTAPLTQFNSVTQLSGRGLRSPGPTA
ASASAVAAAGPEFYGAGHFPPAVITTTTTATAAVTSTAPPPPPPPHRAASPLVAGGGGGGGGDGAAAAAAAACHSAP
AAAAAMGRPSAGGGGAGRRPETAAAAAAAAAAGGLLLPLTVSEADAMAGLPDRGAPSLAGAIAALVDAFRATLHNDA
VRIATLEAENAALNKTLNILRANTHRNATRTRGVSTPGFNPNGPGGGGAAAPGGGAAAAAGSQGPARSSGSVPAVPM
AGVTSASGTSAASGGPGGAGAGAGGAMTAAAVMNDVDAASEGGSACTADEDAFDVVEAFDDGVDYSILDDDAPHLHH
RLGVDDYEPPYVQHGNYPGAADEEESDEDAEEDAAELAQQAEVLNALEVVRQVEYITAANDNNAQLKTLLTADSPPP
PDAEEEEEEEEGGKAKHKSCGKGEVRVGPGEKTRRQGEGRGGEGKQGEEKQGEGRGDRTRMAAEEKVLRFHFYFSSC
RAIPLALHPLVHSIPLPPP

>Vocar20010558m|PACid:23129191
MRGSLQTLGGEGALSLLRNSVTSASSDFKKASKPAGKAFQTAHDSSGWDRKARWQRAFNLAKSKMDVQGMDESVDKW
GARDAEGGDRRSASAQPARRGSTLAGGGVGPGGVLLGAPGTIAELRTPVALKEHLKRGRGARESGIQLVALEAGSAS
DMPLSEQDAAAVVAHFDQRRQMLGAPPSPSRPALTPADRALRAWTNNTNNTNNTNNTNNTNNTNNNSNKVPGVIVRG
VGGNGSGGSSHSGRGSFSGNGGSGGGGALFQRRFSESGDMCGGSFSGLDGAGLPTARRNAAGTAAPDSIIAQLQQQQ
LQPPPPPPPGESASLHRPVQETSASAAISLERQALAAGGASGAAAALLPTRAGSLSQQSACGARAAGAAVPTAAAAAT
GEHTEPPAGWVSVPLVSGWVDAMGGSGGGIDGGDGALRPVLQPSSADHLQVAQVQPGGSSSGEQGNVDDGNNDDGAA
DSATVPGGGPSPPAPSAAAAPPQAATSAPPVLRSLLVPVLSAGELSSPPPDRPGSGRAGLAPDKQPSAAAGGGVGSS
GNGSEGEAGIRSRFLSTPSAAVSIPQSPGLHLAVNSPVGTAAGGSCSGLVPVPPSASPDCPGLLIAGLRRPRPRVSV
ANDGTPSGGQLPQLTQAGWVPADGWSGVDGGGVTPNAAATTTTATPSLRLVMHISPPSPPPHSPSRAGAGPTASAVA
YRRNRTSSPGFANLATPSGALSPTHSPSSSSPPLMLRPSAQPPTPNTPTPPSPSSQRRASLSTPVVVATNPGGSGAG
RATAAATTATTAAFAAVSSLCRSPPQGHLSHLQQQHQKASSAVPVRQYVFKPGNTGVTGVRGRLTDDGGGVLVMGP

VGVATASGSGVHASGGGAATAATKPRTTPVSDAVLAGLRVVATEVADELSREAEVAAMTRQAVASQRSRLRTSDSGK
PTRYISLPGDPWRKWG

>Vocar20010867m|PACid:23129193
MGPMVKSKRTLRSSVLIADFHYEIVCLSSNLARHGPRPDPSFCPSPHSSSPPPPSPPSPSPSSSPPPPPPPPSPPAP
ATVQLVHVAVNLGHAIRPWALCRALSDAAAAVEEGEGEGEAPATKVGHSRCFPFPNCEEQKQKQQQQQQQEEEEGRR
PLKGTAAAGGDVGFAVRRQALRELVLIRGSTRPALRLLVCALGGSRERMDAGWEDGDKRLEGLAVKGQMSRQLLGAG
VGPLLGPGALRALFDNLDQNELMGYDLFTTTYQPSLRRTDACLVFHHGLADHSERHGEVLAHLCASLGMPVYTYDAH
GHGRSGPHDPVGRALIRSYRHIIDDLLDFTRIFVAEAEDADTDLGVEAEGEAEGEGEASPAGPAPPARQGNGDDGNV
RQFGQQQQQQRRRRRRKLRAFVLGYSMGGLATTLAVAETCPTHAATASTTTAIAVAAAATGSSLFEGLMLTSCLTDP
LYGNHPVLRMVKVAYVTALSWLAPAVPMFKRNPVESGIRDPAAVAAMAEDTLWYRGRFKVATIASLLAGCARLRRTA
RRLTAVPLYVQHATVDMSCSLPSMHAFLARTDRQAHTHRGMVKGMRMCVYVCVCVSTRGKGVVAAVVAAEVPESAGG
VRGKGGPCWRGR

>Vocar20010555m|PACid:23129220
MTALLSANTASDSFLYSFTPAAVGRGGQLTGSRRLRAYSVPGEPILPIPEGGEGHSDLQGIALSTSLSVPAMPVGRA
AALGMSRCGSADRSQQQHHHGTSSSLSSAAALSVNNLAGGGAAAAMPVIGGGGGTSSERILPPMRHSISGNPGIGL
PKLDLPFLAFGGGGGTGGGGGGVPLERPVLGLSSSGRRSLSGTLCEVQSVSAPASFLDLPSPPPPTAAGPFEGSAPP
SPPRSGAPAVLAPLRVSGVFAASGAGGCAAAAAPSFSSGPAAVGESSGGGWCGGGGGGGLLSSSSSRRHTSSAGLSE
GGSFPPQPPPQGSLLPCTSEGAVASPLTPGLPYGSPYGPPRRSHSLTGSGGGSRLLADSDGGSAGGKRISDAREGPS
EGANRACGSSDGGGGGGGSGDATVNSSAAKALNDITADLQRSTGALLALRAPAFRSRSSPPPSAGAAKAAASAEASVA
AGPPAAATVPPLSRTTLTALKESAPELQWALRVSVRRFFHMLRQELSSAVPAGRQQLGSTIHEAWATLDAYYESTAE
LVEALVENALDVTAAQAPPPPPPRTSANPDRQQRQVDAAGNGVIIETERAAEKLRAANEELRKQLEECRARLAESET
RLTAAAARGAQPGGGGGAAAAPRPPPATTVTPPLESCYGTSPRRGKHAVVQPSRDGVAPTASDGTDGATGRTPGGGR
GAAAVASPAPASPAAAATASRPPPATNACERPLRRFERSAVLARGATCDVMVIEEDDSDGDDDADTAEGSVDARRHM
LTYHSASFAEWVRGARRRLPEVPERFLLEMAKIKKTPTCPTSWFGIVCSPDSVKLV

>Vocar20010414m|PACid:23129244
MNNKGAVWTSGPTLGAPESPAAESGSSGSGSGFGFKAEGGSVTTGSGGDLAVLLPQGVTSTAANSLTVQLDASVAGC
YDAFTGLKLPYDISSSLSLTSPLVVTPVTTLLAAAVAAVTSGGDDSLSSGTVVERAYGMVGVKVTASAVAGGLVALR
QADIDERRTGVRMVFADAALSSLVITGAAALAAFPAGTLPSCTTEAALVNAVYGSLAAHINTSASLAISLDGTSLLS
DVVTAAAKDKCGASTATMSNKDVQALLTQGSLLYRMVQSSLLSNGSSSSSNGGSIIDDELAVLPLEALSTASRVSYV
VQTQAPTVLKALSSNTMAVQDLTKVWAAHLADAPVDLAGMASEVGLGSLAPSAGTLRIRVRALGGLKQCQVQLQSPY
LANSTNVTTDSRGFANASGVCAPMVTVPSGCMDSALSANNASVPMPPFKIYVHVCVRVWGVQLVFKPSPTSLGHLLA
PAFPFIGNSPAGPVKALVPFNTTAVINPAAAIATAAFMHLRTNEAAAAGGNDERASHITAADYALPYTYFGVGLVGR
KDALPANTSFVSQEWKAGSLLDQRAFLVNLRLLLVLNTSSEVVLGLQRPAGTPADEVQKDRVVSALVSQIAADMLSN
KLMITERSYLEGLIMSVYKETRGSTTGGSRRALQQSEQVKKVLAITASVLSNCTKRLEGLDANCTAAAADGGGDGSD
TLDAMSKQAANVTAMMLYAVVPDLRNLTTARSDAAIAAMEQNIVNSYMVDNPEIPAGIFIDTTEFSNKDGDALASID
NGNPTPALEAPSRSLIPPMAAAAGSPLSGQIAIAPDIAGGGSTREAGRGTLLPMSIALSATIGSVLVLAAAVFAVRH
VKARNAFRFKLDENAFRLALDDGSPPPLARLSSPCMSGCSKDGRRGSLASSELKAHSNFSNTNDPEAASSSPPEAAA
PPPPLPLAVTASASAPGLLYDSEMSPRRHHEHHHGHQEFLVQTQEPQQEWQRLVQTREPHGSGSPVGLLLPPPEPQL
LNRQQQQQQQQVEVQSIRDLKSASTARGPGPDADDSKETPVELNSNDKYPSYHSGSSSNNSTMPYGSPPRASSSGTG
SADQPQAYAESARGGPKCTAVTMGCTEITPAAAGPEEEAAADNHFDGRAGTGGNPMQRVNGGGGDGAYPAARSADSI
PIAAAGQSAELPSEALAAVLAALRAVRTLGSSMDGNHEGAAGLDPRSGSGSGPSSKALHSSRSMVKSRSTTADPSPW
PTPPGQVTSPAEDAPLQRPSMDAVPSPPPPPPLPGSGFP

>Vocar20010913m|PACid:23129245
MSGTGPLHPAFRSRCAAVSPVPPRIIGSHKRQAALVDLIKQQALYPTDVNLRVSSPALGTVADVPGSALLRAISEEA
QVNITAAQASTSFQFIQLLAELCGRVAAVGSGCQQGGLNTYALRMAPVGWAGQFAGGAHGSLGQAQGWRRLPVPEAA
TASPPLRAAPAPAPAMAMAAAASGRGPLPQLEATAMGAPAALVGFEPASDTAGLADRCVHVAQYGPPFLPAQLPHGD
SVNFCPRPLGDWLVAQPAVLSCWDKVSLEPCGPPKAAQYYVVCGEQHSSAARMFIRASSGLQYHVKSPSFALFTPLA
LRHTGRIVTILAVQDVSATFLAMRLGTHTPGRTPAHLHSIDGVIPVPLDPGPGPPQAAATADGATAADATAAAATAP
FSWHPLARRPDPSVVRSAILGFRSNHALASAASEPAWTPDEPSYRPGMAACTAAPAAKGGAAACGAGPWGTAAAAAA
RAPVPLCHYWRNLRHQQQQHQDLGPAAAAVAGCKGVGLGSAAAGGGSSASIGPGDLPDVGPPALVVYVVPPSDCEE
DVLRALMEVCACLAPVTPAARVPAAPTPLAAPPAPGLRDSVTAAAASRETLSHHHQQYMRLQQQHHKQKQDHAAQPS
SNAAPGCHWVGEGCSGGSGGTAAPAASGSEAIAAAAAAASGGSAASTPPEPPAGYPVTASASSCSSVLPYGGVAPVS
NDSADPALLARLGGAARHLYRTCPSVIGNHGSVSPQLDLSQLAAIDLTVQVVLPSSLHDVTGASVRATACAAYSKLC

RRRAGYGAAAAGASAWSASSSSSSSSLTLSAGNTAAAAPVGCRDSGIGGGSNQSWADRCCVASSCSGRTVLYEPLVA
LAPLSLPASGDDDPAALPLPPPPSPPPPPPSLPPHQHGLPPPLHATRAGGASVSPLSAAEPAAIHQYGAGPAAGTVQ
AVTPPAPPPTLHGCYAWWRFPEASARAASCGPYTPAGAGTAQTSGGHFPQPPQQQERCWLAMAFCDAGGKLMDARAF
ALCFDAHAHKDRAAAVADVAATATSGSGGGTAPDGLSMQAAAQSSSSGPCNCKVDGGGGDGSRTGTGGSGDARQDCA
DPAGRFRSSGGDAIGDVGMVGPQLQSADREQQQPVQVMAQPVSLLPAPTAAATAAKATATAAAADSHASGEANSLDA
LVCRTVLGHAKLLSRQVAEAYSGDVSAAAAAGACTDGAVSPNGAVAASGTRGRSRAAMAPLALAKLGPPTAAEAAEW
RLLLPYVPVVAGGGGSLRRRWDQAAAPVPSAASPAAAPVTLAWLEPHPSIAVQPGCRLPQGGFAVACSGCSSELPAS
AAAPQHSIPLPPPPPPPANPYAQPAAGEFRLPITLVAFPAQRPPSTTCAAAEALNHHLRLLSVHPWPRIPSPPPPPP
IGAPLEAAAAGPAGGSEEGQRQGGPTQTTAKTAAVMNILKRPSGYSPALATSAGYDNGGGGGTASVVGPGNQGLGCQ
QHQQMQKRRRWGERSDGLAADGGPVGPAGCSADGGVSGAVGGWSDPGGSMLRELYGLCLLRDWLLTAQSGARGGPTP
GRRQVAVTGSGSGGVGWGAAGVADADGDSGDAGWDAPLQLLPHHVAVCQQLLRMLWAWHASRFAVSREPEVTGAVSA
W

>Vocar20010491m|PACid:23129265
MASSLPLAAASNPKSLPTKPSSTLPPSPSSLPIPPPSPQLSPEPPPSPAQRSPPPPRPQPSPRPQPKKHPSPAPSPK
PPSPRLPAPRRSPPIKRSPPRVSSPPPSPLRVAKTPSPASPFKIASPQRPQSPLVWAALKPPPPPSPHVPDAPLAPP
ATPSIQPYNSQPENALLRTTIRASFLSSYLLYCRARAPPTRIGDSYSAAAWWGAQQYDALETGDAVRLIGQQRVTAA
TAAAAVAQAAAGKGSGLTAAAADQDVDSDYIQADTLSVISSSFYRYLRHISYEVPRPRPFTSITYILNICGQVNPLK
RQDLLPYWDGSAMDGATLPQFISDCSHGVESFSQENNVLLEPIDVPCYATPSNNRSYDSQRCTDREVFGWMQYAMDV
VQQSKTSLGLQPWKYERRIFLIPDLPACGEWWSLASVGCSADCPLVQKVMPGSSVRTVVSQLIHDLGHSYSLSHGGA
REGALPSGLGRAGGARADPTCPMGSASEAAGLVCPNAPNSYKNGWLTLLDGKTRSSTGNITGPFIGTIKLPPTNTRR
DAVVAVTVPAAAPTGATGLRGGSPPNGRFFISYRLSGSSSTGSAYDKALAAELSGQVYYVHQYDGMTARVGTPYKPLL
VGASTDVDVYKRLRLGATASPYFAVRVVSTDANYATIQVCSAGTGLLPQQEPCKLCADGLDNDCNGLIDEQDLACQA
C

>Vocar20010633m|PACid:23129271
MGTNKDDPIKALADSLREKKGVQWGTAKLEAPNGEGHYVEYFRGKDFARYFRSNPEKLNAHVPPKPGKTVDDQIAEL
IMVFMKRKLVRKTDRKYKKPKPGKKRLVKFPRTLVPHPDGSGWTENAFYYWTYDRPTSMWYYVATVALPFLVIAACL
FPLAPWWVRMTLVYFLMAVLTLLLGLIALRYSLFGLVWIVTGHSFWLFPNMMSDEVGVLDAFSPVLSYDRPKKGSRT
QIVARLITLALVSGIVYMLYQHTPDAEKLAASAKEAHDSILGYLDVYGKGKMFITGNTNVTGNGTGTGAGTGASADA
GQTAGGTGASAGAGKGPGPGSGPGGRASGRRGSGPDLASTPPSGVLVAWESTIGSVEAFSSDGQPLWSSLGRWSSLA
GALHAGSSVLLLGTPASTSGNLLSLASIHTTTSEPKWELPLPRQFSMHHAPLSLTLLDLGRTALLAVGPDAGVKQQL
GLHDRDPDAATLPLLTLAFVDVLTGHTRVTVIAGVSGVGQVLSIGQPLEYDRDSGAARTPQLLLLPFTYLPSANAAE
SDGELGAGPRGGLLAFRRTAATRVAAVAEREGDQELQPGGAAAAARADAWDPAAEVIAVGDTAAELLSSWELAQAAG
PLSPATQHAQQQEHQAIGWKQPHNHLASWPYSHIGDWATGGPLDAAPGTPRCSLLRQHEAVICLLSSAPTSQTVASG
AAAAVTASGTTAAGSSIGGSSRLVLTDLRNGETLLWRDYPTPGDLTHHGDAGGRASNGEQRGRRSRDSSGDGAGEAS
WSLRLHGGDESSSAAAALEALAVFPADGASETSAEAIAPAAEASGCGQLAAVVAELTAEVQKAGGSRVRMALLCRNA
REGAAAKLPQQQQDGAHVAWDDLATDGAAEMGEKETLELDVLERIPKLLPGRITVTLVCDVANTAAFKVETLNCGYN
ETKRVGGGAILPTVANGTRLSCTRDSFPTRPLNITLIYCDAVSTPSAVDVPPSRNGLNTHLGCERFFGGRAGSGGAS
GMVDGAPEGLQRRLSALLAAPEPLQAFTELLCPVKPCGHPASAALDLLAGYSRRANIDVDLKVFSPPPPQPPSRRVS
PPPPPPPAPNHVNWVITFDSSDTFCLDLSLHTLLPGAVAPYPPPPEGVVAPPPSPPPTQSSLPGLFSAAFGGWNILP
TPEAAVCTPFGYRPYRMAHFSAAGAGLGGFLGTDILRDSLLLADGMPVSNLVSIDLSSNAIRGTLPDGTGGFQGYDF
PCLFWNLSRNAISGSIPASFYLHSALLVDLSYNDLRGPLPENWTLLETVVVLDLTGNNLEGPLPRSWGTTLGPSSNR
TTRRDYGGLRRRLDQSSDPWRGMHSLRLGGNRLSGTIPPEWFHLAVAELDLSNNQLTPTCPPARGPGAVPTDWSSIA
SMRLLDLSGNPGLEGTISGISLPDALRIVKVAGTQVRLDPGVLADGYPRSALWVVLVKAVLGGHIDLHNVQRPELRS
DDLKEICSIYQAYNAYRQQGGSAPQRDLHGVTDSLTLGSSLLAASLNPDGSAPTSRHMYGIMAAFAVWTAGGRVPNN
FSSNDTLHWVPLPDDTAWVARPHEWCYERNVAVLLRPVLGLWLPVLAFILAMLLPIPCYYYWQWRDEKLAQQAVKKE
DDGVLHVVSRKQLQAQEQGFIKQLMYRLHVVAASQANLSRYSAEAANGGGGDGGAAAGGEAAASKRHSPFGKLFSKF
GRGKKQPAAPSAADSRQEGDGGAPPSAPVRRPGQNGGSGTAAGTLGEPQISAAEGGGDPDGTGAAAAAAPGVGGNRA
TARASAVLVTTEDGWTRGVPRLSGSGGPALLAVDALPGADFPPPTHGSLTAASVLGWGASAPSRANLTLQSTGASRA
ALNRGGGRGGSSAGDGDGASRGGTGNGDGAGPVAEAKEDDEVPAATVAGKSDQGAPARGGLRALLGRNTGTGSGTGS
FFGGSAAAAAAAAATASTTGPAKGSSRIASLAPAPITVTENRQDLTTPISMLTNGDIVSPVPVPRRKSTIKRGPHLP
IPDYASEPESPSRERYDEDVAAGRRASPMRSVRGTAKDGPAGAAAAARGGGGGTAASGTVSDHGTAAASLSRTVSHS
QLQTLPLPDAAAQNQGPRPMSRGNLGLVASLQSGPSLNPSISQRFRGRRISMTPTETTPAATQDGAQAVVLRPPRPS
GTGTPPPGSVVDGPLAGLLAGRSRKSGIVDLPPELMPEPGQGGRRLSGLHASGAPVKGRGREDGDADDEGDGSGVGM
SGRQRRPSNAAAEIVPSAAGIPAGAGMDHPSASGTLPHIGRPGSGGARGSLAARSSTAVSVGSVEELTLPGPPVDGS
PRAKPERRRSKKSREPAAAAAVLTASEATAAEGSADAAKLERRKAREAGAETTEVPRGEDGKLKKRKSSKSREAATT

GGETTAAEGEAEPKLEKRKSRKSRDGKATDEATPEKEGDGSSKLRRKKSSKSLESAGVDEASVSAGADAAGKLERRR
SRKSREGRTADEATEATTEGEGGKPRRKKSSKSLDPVEADGVTAAAAATAVDDAAKLERRKSRKSRDTGTAAKTGDE
AAEVEADGKVRLKKSSKLPVEDASAAGEDAAKLKRRKSRKSREEAPTAGETTAAEGEGKSKEDKQQLRKKSSKVWDS
KPTPDGTAGEAAATATEAEGEGGKILKRRKSRKAWDEGATTTAGETTAVEGEDDKEKSKDKKSLKKKSSKRHEDSKT
PETAAEITSEGKLERRKSRKAWDGAAGESTAAEGAAEEKEKDKKLLKKKSSKLRDAKPAEATTEGEDAAKLERRKSR
KSREAGAESLEKERGEDGKLKKRKSSKSREAATTGGETTAAEGEAEPKLEKRKSRKSRDAAVEAESGAASDGKLKKH
KSRKSRDADEATEGEEGKLKKKKKSKKSKKEGEGSEDGKHKKRKSRKSREAATIPADGADGGAVDSTAAPEGSGGAE
LPPRTPPPPAVAAALAANAAADAYSDYTSDDDYDDDDGTSSYYTDSAAPSRASSMSRRSSMSRRSLSIRSNRSMRVS
YTATGAGSRHAWDRSRRNSLDLDGTAMAPPEPHLITVGPSLAPPPPSKPLIRRHLLEHVCVYYWGAWVQLGPRLAAI
LYFFSIATDIAFIAAYVDPALSPAPPVVVGAILLVFFTTSWGIRLNVAWGLSPRSATITALFTFPFGLLYELAWDVV
TVLLAILLGKEELLNRAVRGCGVWCWGGRPGNFSLAPNLRVFRIKLVMEPLLNALLQGVVQSTFYMVAYRSGFVIWP
AVYLASGVASALSLLRWAAALHHYAYSGVPLADVFRQRGRHFNEELLSLDALEDIKDMEVVNKDDPVNLVHRVIGKS
ASITSRVRVAPEDGVSVRGSALPSAVGSPAGSRTASRPDSGSKRDDKEGTERGGKDSYNKKAAVKPVVAAAGAKSTS
AGAAAAPADPSVLPAGTGKEAVPEIAAESEAPSPSQKSALLGPLPPIGRGGGSTAGASRVSSTAPSRLGSARKDSSG
GEPGAGGAGLSSSLAAAAQALLATVGAGGSGAGGSRISSGRVGSGRAGSGRSRSASGLPGGRLRFAASVKSAGEESE
GSEADSRGGSSAAVAAAPGAAGATGRYGNYIPKPSAPNAFFGSATDRPEYHFQQSPIGMVNSVVSAKGLAAKMRSRS
ATARTAAETAATAATLAANAGTQGNLAQYGSVPVGMYGAYGGATAATATAAGMPYAQFAQPGTNAALYGTYGSGTMY
GTGTLAASGSMPAGGTAGMPAYDPGVYQQYAAYYAAMQQQYAAAAGGQSMAYDPYASLSRSYGAGMGSTAIPGAVSV
SPKRRVAV

>Vocar20010725m|PACid:23129287
MVVRLVALRHEAQRYYSAIVGGRFCFDVKPACDVFGVNVRATVNGVYTKVAPSFDYAADGPIGSTILRVTQLGLGPA
DDGAEICIILQASKTGEGCTTLEDMCVAPAGDAPGVCAVAAFDSSNECCPISKVNVPSPPPPSPPPPSPPPPSPPPP
CRTCIYISVKAVSPSAGRFTFSQNDCSAYGSDIGADLNDGANSLGIALISPFELAECAATYFKVCGTFFSEAEGASL
QPYIDDTLAFFLTYVVGEGPSCPAGLYGYEVTAAIGGDGSIAAVPRGCLNGVQHEARRNTLRRQSACGSTTTLLKAE
FWADDAQRRAVAGIGIQPAGGRMRYLSPTWGAVGEQTLKATPLMWDLNQANGGKICLELDKATDLGSFCNNVPGSPP
TCWVNFFDESKNCCPLFKSALL

>Vocar20010528m|PACid:23129322
MAADGPRLRKGFITFGVAVLIAAASISGVSSNPEEHSLAHRRRRGLQDAFSDIQAIVADDNTIYSDSAYVQRIAGAE
ATRFPFCKCKTYACECSPYTVSLSSVTKVTNPAATKYCFKAKYNGCPTPVQPCCQALSLNVEKIAFTTTSKCAKRNV
SRVDVRGRMWSSWETKSWPQVTTVTPFTEPCQWCATIRGTDVSSPVAFSKSVCEYVTSRMRANITAGFAAVGATVTF
NGTTCDNTQTSFSVCGSSSPKEAVMSQVLADSFIYEWLLNIAQQPSGVCPPGTLQTSVTATVTASFGTCFAGSNNYG
VNCAFGQPPPPPSPPPPPPPPPPSTPTKRTPPPPPPPPPPSPPPPPTPPTPPPPPPPPPPPPPPPPPLPPSPPPPPP
PPPPP

>Vocar20010863m|PACid:23129362
MKATCSTTIQVRALRALPLAVAPGGKLPVQGTICEGIAAKRACQTALSVPTPVIAVYRNDKQFAYTFCVRKHHGPCN
TFNMQAPKRFCRLMLTSVNSTMRLAALLFIISCVVRPAKSAVDGHNVLTLRGEFVWWCQLAGADRETHEYRLGKGKL
RIAIWGRRRSIFLLMVYFSMLGPFAREVLRLFPARFRFIAGRLVQVDLDFSQSSEISPDRSANKDTHCILIDANDTV
WGLNFGRGQEPPNFHGLLVEVDVPAADLEATAAGIAPGIDQRRSLAGWDAATGRAIKASGIKPSTASTTASRRSVAG
DEPALVNGGQLTVVNWRNVASKAERADAPGAVTREATTTQGPDPTTFTYNSGSRIVMDDVSTVVYILDMCEQGPAAS
KEELESVLLGDRQDTLQSYLSYCSSGLARFSHENVLILGPVKVPCSGSYTFTYPGTEIEREAFWYTSSCRPQDYKGW
LRYAQQWARGNGYDVDGYRHHVALVSPNQEDWMTGGDGFGSSCSWTGLGFVGPYGNSDNTEFTEEYGMMTYRRGVFG
FAWINGRYWRNISGWAHELGHNYYLKHSMTLNCEYCDTTSAMGFCCDTRCFHAPHMWQLGWRNPQEQLDWYSLPAGM
WVERTLSAATNSTPLGSALRLEARMWVDNDIRSTVRFYQSYTFYTAYVWVAYRKASKPFDDLEESVANVVSIYVTYV
DIVGATAGTVRLATLRPGDKPWHHDELMLHVEVTQLTSERGMDVAKLKICRMDAVRENTWRSCTFDNLDNDCDGLVN
IQDPDCAAFADYSSEEQPSGEKTRTETWVDTSPSFVITPGRRPPSPVSLQLPPPPPPPPPPPPPPPPPPPPHRFHEMP
FNPAPGIYTPPTPMLPAYPGVPDPTLSPFADPPQSIPYPTMPSTHPGVYASDHFSPPWPSSPPPSDAVDSWDPSLKD
RNELLGPEFSTNSPPPHVGIEFPELYGPSVSEVETGRSYRKLELSRNNFAPTDSQSPTEEPDGTAAPARHRTLAGVL
QLGSCPWAHHSVIQTGTELQVPT

>Vocar20010418m|PACid:23129433
MGTQALCKIQSPIWFFLIVGLIVLRPGPSAGIPLINSDCNPDNGTVNPATIFTRAAVKANADHLGDAAVTIQYGGER
RKWVFTTMADKLVAEKVLPITLKALGRLPLEQGLREMGASLDPSWSDPTGQQLAGEGSGGEGRTRRRLRGPKEGGDD
ARGGDSGDSHSGGGGGDGLSGRALSSLLVVVTWDLGGYVACERLRRKELFGHHCVRQELHELHSEGSSNMSAFHSPE
FNALGYAKIKYLYDALSLGYDVMAVDADVLVLKSPLPFILQQRAQVAALTERCEVVDPFMQLQLGKARFPNQNIGVV

VARSDGPVVRCVERWFASMVVHMDNPLLWDQEEFNRVIAHCASHLGMRWHSLDNRRFVSLCYSQCGCTYPDSDMELL
TDPAMRRKHFEWSFRRGVPYEDPVARTCGPSAWADWVLAHFPCQGSIRDKVRYMGQLLEGYLVGEPRVIKNLAKRKS
FPVAEPSPPPSSRVDCPPGPPPVSLSPPRPAPLHPPWSDVPAAGCGLEGGNAEPRPPARVAAAVTTTVGMTGGHFPA
LPSPPPDWGGRACYFLLGAGLLAAWNSLITATDYFGAVYPGWHTDRLFTVSYLPVCLLMLVVGVRYPDLLPAAVRIR
LGYAGFTLAMAAVPLLDALLLMEPAGSGGGDGGDGTAAAATAVPAAVGGLLAVLVCVALVGACDGLCQGAVYGEAAQ
LPPPYMQALVSGTASSGLLVGLMRITSKAVFENVPGAPARDEGGDSVAGEEEEVFKRKREGLRDGTRLYFALAGLLS
FACLIVYDAVLPRLRTLPSRGTAVAAAAAASDVSDGDHGGNHVYEHHPLLLPLPLELSTECKSGAGSGEGSWRSDGG
GGGAATAVNVDVDGSWSATVPSLYPQTVDGDVGGGGGGAASIAYPPGGGGDGSHGGGSGGDYPFGPHAGAVASAAAV
GDRGGGDGGGLCSAAQYSRGSDGHAGGRLSGSDGAVRVQILHCLFLSWALCGSLFLTYTVTLSIFPGFLAEDVHSAQ
LGDWYPILLITAFNLADLVGKSLPVMEPLRPPLQSPQPLQPPQPLPQARLSRTPPIRQEEQPPPQLPLPTETSPYLA
HHNHNHNQHGREQERGYEGAHDGYHDHQDDAVGNEPGHPLLLPLPKRAAPGGRGSSKAVRLRTCGCLAAAATVLQPL
RRPGGLLTAAVVRGCLALPAFLGAAHWRAPAWVMAALTAILGVTNGYLTTQVLTMGPSAVLDSPHDNQNVRNNHNHR
NHHAPQPQLPQAARAVTMTMTTTAAMTESALASSAAGDCQTASRVVPTAVVDGCGGGGGRATEEGEVGGGGLLGGAL
QRCSLQGLGAAAAAAAAAGAPAVISRHSDAEVVEILLVISLVAGLNAGAYLGWLWLL

>Vocar20010654m|PACid:23129456
MSGVKSRHRDNGNAGFFSRIWTSDILDCVVAFLPPNVVACTIRLVNKLAAEQFKRYSTTRLSQLVPCHAFRRRWADP
SSLRTMTYEERMRFLCLVASSGSIYNLKVAMQSTGLQLHSRILCAAAKAGRIDLCAWLVDEGCPSDAMGLGDAVGYS
QSKDLFHFIFSDGIFFDIHVIADDDESYGNNADGFVAAIWAALCAAARNGHDDFFRWLLQQLPQIPRLSAHSDSFGI
FEPADLFEAVAEGCKLSTLKWLSRRKRLPPLWMGPNGTGCLTAVRMLLELGPTSVVAAALEAANAGHLHVLQALHAF
RHLAQDAAEDGEPEKWLLHEALVGGHLQVVAWLVEVLGVDVRNDADLDLMLAAAKSGSLDMVVWLHERGCPWHVDGF
MNAVKVGNTAVLEYMAENGCPVPQCGTPYVHAVRVNDLSVMRCLRRLGVQWHPRGRTFTACVKVGRGLWDPAVGRVK
DAAPYIQTKRLFYCIVLGASYLQAKVTGVRRSWTPQLHSMADASTVPRYQATAAVNTRHTFNEAHEHGNGAWDDLKH
DITPSPPPRSLEPLTRGSAGVRSSLLSMGQTSPPPPNTTFVSYEAEITLPAALAAGTSLPAVPGAVLHLSYNFLTVT
LRFQLSSPYGTCTAQGAHSTLSAVVAAAFGLPPSNVTTTSCKLDQAISLESFGTAVFRTLYGSGSGGATGVLTGLCP
GETDTSLAVPGALVRGVSTADSSFTGCQAFVAVLLAAMEPLTVSAASSTGPSLSVALTQASCRATLYGRTASSLPPP
PPSPLPPALLAARPTADGGGAAADMMPDGDGSGGASSSGRASVSNGYSNKSVAAIAAPAAVGGVVVLVVLVVLLLCC
YRRRRRELVTEVEAAGTAVDGGKDTVAEVRTPPAAAAAAELTPPTPKISGRSWLWRSLFGRRSSGSSGGSFSGRGGS
WRGTRVAPNELKGRPLEDTGEGDDGVVPTRRVRSVDSDALLSASAASLPSSPPRSSAEDTARRTGTGIGVPSSQLPP
LPPPPPPGQVPQEPMLLVLPPTTTSADSCDGARRCDGGHIRANNYGGGGIASGGGSGDDAGNGGLFSTVMESGGSGP
GLPSVRKPEPAALETEMERVSEASAATTAAAAVKETAAVAETEPSTPLPAAAAIKAPAAIEAELQRASAATAAAPLA
DASSLTAPAEPMMLVAAAPAASASPSMLGADVGAGGPAAAAAGLDSPSGPPLAPASPAVTSSHMTLVSGAGPRLPLL
PPIGGNLEHLPQLGPALAPRSLPVRNVLPIVIPHHPYSSHNHDSSAASVSSPTAVAAAAAASEAGGDGGSGGKDILS
GRLPSPAVRRSLLPSLATTVPLAAGSLDVAKAGSPFAGPLSLGQQRTGCADARGREGGGGVAAPLTEPPPPTANGTA
DSAGDTAVAMRNTAVITLASSSCSSFSPLAAVPFSPSGGAFGESPRPVEERRREKGVSSPGSGGSGKGSTAAEAAAA
VEAAAAAVAAAAVAAASAASSSAAAAAAAVGGDGWSDPIFARPDGPDAGGSLRGGDGAAASGGGDAPADGNNSAVRQ
LLRPGDGGASGDIGDGGDGGAAASGGGDAPADGNNSAVRQLLRPGDGGASGDIGDGGDRGAAASGGGDAPADGNNSA
VRQLLRPGGGGASGDIGDGGDRGAAASGGGDTPAGGSSSAVRQFRRPGDGGASGDIGDGGAAASGGGDAPACGSNSA
VRQLLRPGDGGASGDGGDDGATASGGGDAPACGGNSAIRQFHRPGVGGLSSSDLQGDDDVAAAATGAGARTADWNPL
LAARNFKRPDGDGDGGEELGGDSDSRAKPPPAKDLAAPSPPPGAAARAMKYGAGIAPEAALAESSVSVAESELPSSS
PLPAASPAATPPAAAAAAAGRQFKFGGMADGKDPDDVDGGLDSGSNDEEELNGMADGAGGVEGQKEQRTRLRALRPA
AARHGGGSLAPDEALGSVDLMQLQLERRRKAQLEQMRNKAAWGVAAGGGGEEQLDHKEQERGDGLSSVQPVAEPL

>Vocar20010705m|PACid:23129497
MRFVTEGGEGPKGRGAQVVCTRGKERRRERLNEKQRATLLLILLAAFFFLFTLVRPPPLPLDLWLLTYGSAIFVSDR
HHLRALELLDNLSHAATGLSSSGVAAFRGLPPQLAESRLVVESLLQAEMLGAAMYTEAHLVLERAVAESAQELGLAA
AAAPLALSPSDSNGITGGPLTSSSVSGSEATVMSREASLRSAGSSSLLAAELAAAREQLHDRLLPVVLGLHRCARLE
AALRQFAVNRAAEVKAIVREVVERLLPAYLAHTLLVGTLVEDALRGGRAPAAALVTPKREVHSALQAVVDVATGSRL
KVYEFRALLSACEAFAALPDMHGLRPGAALRPTLQALCRAHLENTHSRCAMQMQHLLEAEQWVPAEAHASFQAIVDR
LEDRCPGARPQSPQQQLQEGDEMAQQQRREGQEQAKAASGAADGKLEGQRPTQGLLRLGGRHYWPVNALLMLLKLLD
EHYMGLLPASPPPTAATAAALTAAAEAVSGAGTATLRRWGAVAGQAPSQAASWALSTVGPDVAQRCMELLRSFNTVT
AQQVLGAGAMRTAGLKSISAKHLALAAQSLAALLAVLPLLRWQLAAAIAEAPRRALLMPEFDRLAQDLNLHVEEIHG
KLVDIMQDRVLAACRQISADSDAWGRAAASLQAQQAAQPVPSEALRLLARQLGTLRSVLQPILQPEEVSYIFGRVAA
ACSESLAGLLDSLTPPTVVPSPSAPLPSGASGLLRGLGSSGAAATAAATAAVEAQALATAAWEASRRANALFMLQAL
LSLPLDSSRSSSYVTRLTTFYTKHYGLLPTDGQARPVPAGSAGLGGAPAAAANPTTAPPGPAAAGELPAAVTVPVSV
LQSPASVSIVATGASPDARLAPPPVAPARHPDTAACVGPPPLAVPMVPAGTAVVAAATPATTSGYGGRDVSDDMQRL
STESGEPFSAAIRNASDSDAASSSVTLLSSKVPAILMSPPPQQQQEQPPPVERRRSLQDGGSSVPARNDAINGPKPG

DGDDRSATGEQVQSQSVEGDADCQHRAAAAAADSGAAAAAAAPATERAASLAVDAVAQVEGFADGTMVGLTTESEAA
AASLVIGSGEALVEPRASTSNVQGAAVAAREEAALTGTGAQDALSLPSTSTPVAGAALPMTMTGAEALDAEASSAGE
EQEPQPPSPLQPQEQQEYGTTSAEPPARTTGTQAPAAVRVPDVTTAPMVLLDPLGGLEVGRR

>Vocar20012979m|PACid:23129651
MAPVKASTMGTPPPPTSSPPPPVRSPPPTRSPPLRRSPPPRPPPPASGMVLANTLVSDKSVIRLGEAPIGDFICDAM
MWYGEMVTGMKPDLCMVNSGSIRANLPAGPVTSGTHSVYDIVHPRIRVSLSVRAGNHSTARCGFLTKKPSRHSIFRL
RSLTNISQIAAALPYGNTLVLKAISGKGLLAALENSVAGDIAEGEALGQFAQVR

>Vocar20012874m|PACid:23129684
MRTDLGQLSRPGASPRLLTTSIGYTARPGWMPTTAFTSAKRQRTFSALLPTHSSASAGLPAAKPCSPPPTRQQHFLA
ASAAGSSSSSSSSKQALKPRASIACASAANSDATAVAAAASSGTTARLPFWKTYISRKPLLLQALFDCLTHIRTQGG
PLPDVIDVAMVYVSSEYLEVAVATDAFRSVLRFIHSCPGSSFNRVWFVFSPSLPLSPPPSRPPSSTFCNFLGHPDFG
SLVQALRAELPGLKNVFGCTGFGVIGVDEEGALEVEAQPALSLSLVSIPQAEVIVRHLDETNVPDGDAPPDRWSAVL
GVPAFPESPLSLVVLSDPSFAAVQDLLGGLDFAFPTAAKIASTALPATSAVATAAATTIDTASEPGSSSSSSRAGGG
GGGGGGGGGDGGGDGIGVFTRGVVVMSIFGEVQMDLMIAQGCRPLTSTTWTVDHVDPANPSRVTALSAPSVARGRSL
RALEAFQIELQSVLSGMSEAQLRKTVANLTVGVAPEGLKASLEPHDFLIRALRGFDSEQSLVVGEYLRVGQRIRFMV
RDKQGAQEDLTSHGLSYKRKQLQGGAAEQPPPPFGMFMFTCNGRGSGLYGEDSYDARTMSSYVPVPCAGFQCNGGNG
HPGRRPVVIIIIAITIIATDTDSFTAFIA

>Vocar20012901m|PACid:23129746
MSSLTNGQDVTQFYLHADGGQMYQLSFCDNVTVEGLTPNSQVIISYSSAENGILHSCTPPQQDQHRRVLFGNTITAP
TTPTFLFYIPRFCGYENASIASYDVSCSRLLNRTLDGYYRQCSYGQVALGNVVVVDNLYIPCYGSLDLPFTFPSGNS
FDTKSCNNDNMLKWHYYLDSLMPADTPPSDFNHKVMMLPKGLTSKFPGCNGIAGSASIGPWIRQFSDANTWGTGLIW
WSADAFGDVELLFHEAGHNMGMAHANVPGGCDLGDQCDFACPMGGAGGQGVRCLNAPHNWQVGWGRPFLELTDSSLR
YGAPMTVAVPQQMSNRDSSIAVALTGMAANQRFYISARINTPPYDLPFFKWQNGRPFVLIHSYAGTAAASYARTVVL
GKTEVGETFRDSASGLVVTFNRWDGAVYATVTLCRRMGFTEASCGDGLDDDCDGLPDSLDPCAGRMAATSSETTTR
DQYNTVPMQGASGGTVTVPPESVRSPTRGPPPRRSQPLPRSYPPPPPPPPPPPPPPSPPSPRRRRPPPPSPPPVRTS
PPPSPPPVRRPPFRSPPPPPPPPPLPSPPPRLPPSPPPLVPPSPRPPRSRPPPPSLRPPPSPPPLLSPKPTFPPPPT
APPPGPPSWPPDRHSLHLCHHQLAAPQPLTS

>Vocar20012843m|PACid:23129759
MADPRTLGIGLKQAVDVLSLPPSQRSQAQVSKLASFLRNQDQGDFGESMYIVIGGACEFVTPFDSLSGQKGKQPLDP
QKTLNYAKKASLFLKSNILLKKNTGYRHTQRIAYVSSFADTSHALEAVLAACCLGVLRHPPGSRSPEEVDMLTEFMK
SMEFTPPHLTSPHLTSPHLTSPHLTSPHLTSPHLTSPHLTPHLTSFHTTISPGPCYLLLLQAFQKLPPDMVWRLASD
CSHVRLPPGRVLFEEDAPGNCMYVVVSGQCQVRARPLALRQKTASGGGRGLMRPGSLGIGLTMTPRRLHVTGVTGGG
GGGGSRMLETTPRSLVSFTSTSNAGAATEAAAGPAAAAPPPPPAGGSVVRYRADGKVATVMTLPGDARRRRQTNGSS
GATSTAGGGGGGGAGGSGGGEDDLTDEEQQQQRRARVSAPPAAAAAGSTGGGATGNKDSPFWIARYMEDTLKRLHLS
DSTAGLGQQMTPLAAVAAAKWRQATSGRRTGDSTAGGGGGSGSGSGSGTGSGSDPATTAPAGVAAADGDGAEEEGME
ALLGRAAARALRQAVSEQRARKGQNYGLDADVAHDEDAGGGGGSSSGSAGSSNGSSSGSGAASRPTTATAAAASGAA
TATAAAAAPTPAPTAPAPAQEHTLPSGGGSGDGAAVGPAAILPATAISPVTASSPSPPPPPLTAPAVALEDPAKRLL
SKIGSRDPANVSRTLLAELVQRADVGVVLYEVAKKLVKRGGGPRRINHTTSQRINRVLHHQIRAGPDQLLQGTLQGQ
KVLGRHSNLAPSRTSSLWTKGLAAAVLVSAHPPHHQNTSQHHHHHPLRPASRQGVALVTVPPPPQLAVSHTWGEFPS
VQGPPSPPPPRCIHELTQKQANPRSSHNLAPGTRPGSALTSAVSGYADVDVEVGDDVSEVDWNGSELYSLASYKPGT
VGPYGRRMPAADVDHSALINAGRVRGFYGRIDAAAAAAAVAKLTDARSFHEESSGRRVMFVSGATNSNRGAAASGGS
GSSSGGAAASATGGGGGGGAADGPPLRYISPAALNSLGGILTHMGPGGIFGEGLLMTAGVPAAAPPPPPPPPQQQQP
LLPPAPAAPPPLPAPSLPSPPLLPHPQQQQQQAVASQVTAGEDDDAPRTRKARGRGPAPVVPPPYPCTVIARRATR
LLTISASDLSRFLDVVAAPLAELAAARSEFLASRVASVSAAKDAVSERVGTMRRTLRMMRAAEDAADSNPGLAVDLR
KAAAAAAAEAAAAAAPAAVPAAPAPASRGASISVNSSASTASQGASQGARSAAAAAAAPPSPPLLPPAAPPAPGRTA
SLDGGVLRLTGATRTVRDLAVAWVGGTGGAAGTGSGRYSFSGGSSGTVLSVSGRKSMQLLPGALLRRLQMGPTNPVA
TAVATAAAVADALRTSGISTRPEQPHAAGSRLAALLYDNTPRLPEPRPPMDEPSGYAPEEPYGWEEPQYELGGLGVS
VSGGVGPLRTIESFGGVGAAAGGMRCLSTITEESSGGLLRTIGSSQVMIRSRGSVEGGGSSAASAMVAAQSSSSSSS
SSTGLPAREEFPLALTQEKPCDPRVTAGLPTDISNPEGTAATVPVDAATAPWITAADPRVTGADPGAVDDEHSAMAG
DPWFGSDPWVGAPGASRISTQRTSIAASSHGGGSGVTSDEEEEVDGYEGGNEEHQRQRHLFEVVSAVDPLYDSLGTV
EEGDEEAALAQRSDEEEDQAAAAEEEDWEPGVTAAPPAAAEDRVSYAVRSSRPQLPQLRLPIQMILSPVCSSRPSTS
GSPGRASLPQLPSLRPSSATPLASTSRRRAHSSCGLPSLESGPSSRVAGDFFPPLLLPRPPPRRPSLTPVVRHPRGS
GGTPNASSSTVQSYLTDISLAASTGISAAAAVAAAAAAAAAAGVSARRRPHSASDAVGSGRVVQVGDGSAGSDGSRS

ASDSFLIYTDDGSVSPDLTHVGFEFPGGGKRGGGWAPSRVSAGRVDDGGLRTPYISPQSRSPRPPSRSPTGASHTTR
ISDGLTPLSGFASFATSSSLHLVPATTTTTTTAAAVVPPLYAVHGSVSRSGSVSRSGSVSRSSSDDGSGTGSGEVVM
EAHGASLDRLVAGRPPRPLARTTTTTAVAASGGNAHFVGGGGSGRSSSSNSSSNSVVRMPVSHPRLPLAGHTVDSDA
SALSLASYVSSSAAAAAASTAGAGAPPQASALLSDMADLAAAAAAAAAGPALPWSYVDGQMRRPPSASASSTDLSNQ
RSPPLLSPPPPIVPPLALAAALRRHLVESEGGGGGRGSGSDGGASARSCSPCAALTDSPLRPRRHTVGLIDSPIHSP
RLLSQVY

>Vocar20012594m|PACid:23129799
MGMWVEGEVKEQGGYCVCAVASARWVSEAIKQPAISGGGLFPCLHASMHARGIACNKLSRCYSEESRFRPLRLSCRV
VSQLIDSHLLLHLNLTLTVPWILTHYRSGDLAVLARRFKNLQALFLRPDSATDFQKRVDAETCLCVLTPWPSSLRAF
HLSGWPLPYQLRFCCSLPYWAPALTTLVLEGPAPGAHEGAALAELPSLRRLAVRFGPLYGLKLPYTCPYLGALTQLT
ALQLAGAVPNQATVSSLSPLTGLRHLHLELLHDGYDKGMQVLDLRGHSCLSSLVLPIRRVLQCLPASLESLAVDLPW
WGPGAVAPPPPPPPSAALAVAAAAAAAAGSFQPAIRFLACPPNLVSCLLGLGIDMASLVGLRLCAVPSVTASNGCCC
GGGGVQPLAPLRRPLLQWVVPAPAPPPVRLKAAPAVLMALTALFPQRDLAPAALAASLASAAGDAVRHLALFDLDLS
YSSHLKRDPCSYQSYEAESLAAATAAARLVQGPVHLPSVETAPLPREFSCAAPTDGESSKEEEEEGEEGGEEEGEFP
GATIAASAAAAAGAGGRTMAESSQADPRAAAISSLRGDPRSELELFPMAPVTSMAAAATSLRFVAACCRLRQLRSLT
LQPVRNLLCALRLLAGHPTLRVLCLLCVEEDGAHATDRPLLPRLRALVLALGGNGGGDPWVSRGRIGGGGGVEATAL
GEGDGGRDNDPRISGGSVDSGVMEKGPLLSDPWVGDRARSKPEVAIAAAPAAASRAPSGRQGGEGPWLGSRGVVVVV
PQLAAWEVEEVTFTELAFADTSSLTLGYHARMTAQLQTFAASVLGLELDYVTPDIQGLSGWVAGARPSTTHPVIAHF
RAPRDSENAAPILAALQPRSLPPEHNEYVSLAQYLHEIPAELLADDTAPRSAHSRAPSHRHTTSAAAATPPPAPSAC
TTCHALPAYNPSHPNLAVLYDVIQDRAALVTTRAAPETLQETADNESATATASPLPPPAPEPPPLRLAPTQPLPLGV
ATVGASWYPGCSLADCLRFPAAAASCGLLPGGGGRVGGGGGGSSGGPPGGDQHLRFLLLQLVAAVCHLHSHGYDLGG
GGGITTATVLLQPGGWLQLLPPPMPPQPLSQQQQPQQQSIGKQQQQVAAPPPIPLLPACLRRPLPTLPALTRAWQHH
ALSTLDYLMLVNLLAGRRLGDRTFHPFVPWVTDFSVPPQEMLAAAAAAIGGSGWSCGGSSRGGSGSGGASGGGASGG
GGGGWHDLTRSRYRQAKGDLQLDVTFSSSEVPHHIPQEPLSELSFCIYMARVTPRAVLQRAVRSHFEPREYPPSLET
MLARTPDECLPQLYTDPGIFSSIHPELPDLQLPEWASSPQEFIRWHRALLESDHVSRHLHHWLDLFFGYKLVGAAAV
AAKNVYLTNTQPQTAAAAGPAAAAASGPAAAAMAWSTAVAEGSAASSLLPYAPVGMHPPLFLSPHPPRLPSPHPPAP
AAAAGPAAARTGTCGGGGGGSVSTHRHHRRGRLPSGDLVHSTGNAGGGCRVGFGSGAGAAEGGGGGGGSIVSGIGAV
LCQLDSLELLGAVAAREGGLGLHEDYFAPLMKPLPLPRRRPEPASPAAAAAHDLGACMELGFEEEPEEAEEGEEEGL
TKGLVGSAAAVGGNTTASAAGGRTWGQQGGMAEESVLARKRQDCWAIGQVAVQLYLGRCCYGPQQHAGYWLRLADGL
PAAAKRFVRLCFDPLTTSERLLSDPFFSPALHAAHELLCTTLYDGKAAAAFNSTPASLPPPPLLRVPERLSALAAVA
TVGTRLRPVLDLCLPHIIHLLWQAAAVCNSLGHGPAAVGGGGGSGCSTNANGYGAVAACFPQPPAATATAAATSTVT
GEPTLATEVSSCVYDILMALLGLLPPDKVAAHVLPYVCACLASFIEQPLDGGGGGVGGPRQRRRQLALQHRLLSASL
QTQLLGGSDLELYMQHVLPFLLAALLCGEEGSSSGTTAAAADSAAAASGPPSGSSGGDAAVGGVARALAVSTISDAA
AAAVVALAARLPLPLVLERIVSPLLSYMTFGTSVPRLLAQLCAEMGGQLTARHVAPALLQLAVVPAPPPPPPQSVAP
SRGVGRGSRSQQSYSSLRASGGTKSEFTSASASSAAAAGTSESAATVPPSPPPTEPWLQHQIYTSLSVLTAVMDLLP
REYIPRVFLHGLAPSWVLGPASPSTHGSPAASPGVADAAPLPPPPLNPMSAAASPRTSITAAADGVPSLVNCFLYPL
SYRVAPGSLTMLAGLALRAFARLARPENVAWVVLPHLTPLLSYASAARRASYRANGTTRYWGGSDVAHIHDQGLPAV
AMAQYGGSAGAAAAAAAAAAIASQGNGSNASWTLHRSRSSPSGEGVGGVAGGGIGSGGSTAAAAQGACDDEDGYWEL
VHCVYGHLVDSSGLLTVRNLLPGWQIVEAGLSRRLGWSPAAAGLWGVGGLDSGLGGSSTAAGSAGSAESAAGSCGET
TAGTAEGGGGGGGGGGAAAAGSGGGGGGSSRRSALSALEMQVRQEIASKQQRLAAIHGTHLFIKEVGWSAPTSLATA
VPPRSSPKDVPYAVNGGIGGGQGALSAAGGAGGGSTAAAAVGGGGYHFALGGGYGFGPHGVGRDVCGPEEGFVWPQL
LAIGSPTVVEGLAVQGGGAAAGMGVTGGSGTGGALLGRQASLGGALVSITPNGARRTGSGGSGGGAAGSGGGRNTTV
PLGTVAAAGARAARGPGIGKEAAAPRREVDSRTHGVGPSSSSSPAVSSCGGGGRGRSARVDDLGTAAEQEQAKAAGP
AGQHVGEAAAAAVDSLSIAKSDSSNTTASNFLRRRSDRDSTPIQDAFSEGDDLAAASVVAPVGAPATPTSTATTSSV
NAPGKEMKQSCDSGSAVTSPVTALTRHAGSVVASGHSSAAVEAMAEGSKPQLLPPPPAVAVVADTEVVSGREGEGPR
ESPSTGAVPARTGRVSNVKRPPVQTELMTASSDSPLYPPPATERSPEELSPPPQPQPPPPLQLGRLAGVDGSGSAVE
VLGATATAAAAALPPSPFPSSAAPDVLTAPPPSPPVEPLPPPPPPAQHHLGSGTSTASPQQPPLASEAGCSAAGRSA
GRRGNGAAAGHSLSPLLIPPPVVSDSQVPDTGSQRWHWLPPLTSAANAVHTAAAAAACGASSGSSCRAGSRARLLAA
AAAPPAAGDVWSLQAAVVHSWPAHRDRIRALAAHPGECAVLTAGRGMVAGREGPVVRVWSLADCQARVQYGGHRAAV
HALLLIRAARAGGAVVASLDAAAQLHVWAYDTGQQLACFAPVGGTGAGGGLASGGGGGVGNAAGSGSAAAAAVGETP
VPSDSSPEVSLSSSAWGELIDRSGHYYMSSQGLEPRGWQSLGGLKTSRQLSASAVAAAAATEASSGPGAGAGSGGGS
GGGIQGLSSGAATQLGAVPAATASPPTPGLAATAVTAGQAGAGALRLGFTTMCCAPEEHRVLYAGTADARVCALDLE
CRRVVAEWLAAPAIRHDSEDAVTALCTPGGGADTAAATFGAGWPAAHPEVVAAGSRAGRVVVLDRRCGTAAAAFRAH
GGEVTSLASSGDFTLITAGADKQLRLWDLRRCAALGSGGGGGSAGASTWTTCTTGSGYDSRTGPWSGWQGPRGCGGA
GGAAAEPFRGILDAMSPVAVGAAVSGGDGLYGGGMAPGGALDVTFPTQALAPGAAAAVASSMLTGVAGPPQLQLQQH

PQQHVAWLGVAAPALLRSYPLPRECVQGLRVLRDCVLMTSGSSIGVMALPGEAAAAGGGGGGPSFVRLRNGRGGRES
AAVTGLAVLPCSRLLVVAGDDGAVRICR

>Vocar20012710m|PACid:23129818
MDAVRVEPAGGADDSASASLALTPSALLQQSISPTALTLPINCHPCHPCYRPVRMQRGPALFPASLKPTPNLSTAHS
LQFGATGVGSFGAYTSAEHITKSVAQHSFTWLLIHTVNYIATGAPRHVVAAITTAGTYYLRAGLRSATAAVSVAARR
PVGILPGHDSSLSKRSTSPSSWRFCMLLPSRFLARQRTQPQQRKLVLQPPPPPPPPQQQQRRMAVLGANILPGAPRR
LLSAKLSPSPLPLLPSSMSTASSPAPAGSSSTPCSVCIHVEVIPPPPPSPSPPPLGQLLLRGRSALRPDPPSLSRQS
CDDLASRIATFLNGIPSRTDYYNYNYYDDDGYSHGSLDNGNANASAPGITLRQQFRLESCDASSLRVCGSYNNPEGV
DSRAVAVWSFRLGDFLQELLMGLPARVLSTSEASGSSACESRGGQLAGYTLRAYGQPVSGGSDGSDSSCIGFKEMGN
PSGSSCPQEARVSFQLQLMYKIKDAANSNNLKLDWSQQVCTSTDIIFCTSVNTTSNFADLSEMLGIVLPSIVKKLCN
DSGAPLASRKNILWYGSYPLISSQPVTALTFSSCLIPPAPPPSRNSQPPPSLMSPPPRPLVPLPCVYGSNASSVGPL
RLRREVQIESNISIQGVALGRSIPCFALEVASDSSTTPPNACNATRLTGLIFRASDTAKSALKSIIFRTALGDIQAN
SSSIWKLWNTLPGSSSTNNSSSSSNVSTTVPDTLAVLEQLMDPKDFPSRWTVDQVRSQSAVGRARVCLDIEPKLLRD
FCMGDTCIVHLYYSASCCPADSATFPVTS

>Vocar20012657m|PACid:23129876
MYIETDVYVSHRNGSNPHELLLSIVHIFLIPALWPWADIKVVPEADSRPFLALKDPINAGSEDVQRLYEQASQVSAI
AYLLFAAARPLSEAPGSSNALLPYLTPTLLRLYTSQPGRCSSTLTRQLYDLSLKLYGTTELQSCHYDLYTSLGAAFS
WVRDNIPLRLQEVPGQPLASAAWPDMSRTTPLKLAARVAEALLPNNSLGDTASQLNASCGLPAADFASAVPNISTAL
QRLHADLPLIQGLAGEMHSARWAPDHPDVDERRRALLEQRTGGYINPQLHIASELDTADADGSSSRGIMISDNDDIL
NNNDDDIVAHGDESTHRFHRRSAATLPSNFFYSLPPANFVLLPPGKMSQLMVPLVFHIMLYQDGRSIGPARYDQSLQ
YVQRMVQVTNIMAKPSSVQFFVKEVRNDPATYPYLLLSDRATWLQVPLQNCTGPSCFSNATFMSTSVWDFPRSVNVF
IASDSTVGVPLGYAYIGGPKRLSATCLASQISKRYLGNARGGASVIMVRILRPLKTRSPLYSPVCSSTRLGSGNSYG
EDAGVTQGIRALPPCIKPLGKYLKDALNWQFKGGVMRYEVITARISGGSDIQPYTGHVFVSWDSLSFDGANSITSYD
DGPNTLLHELFHHLGLQHPFQNSYGALGQCVDGDDVIDTPASLRPISSSNFFATAQAYCMELFWGQYGGDWDAAYVR
WSSTLGIPESDMNAWADSCPALPGYDELGNYMTYNTPVCFAALGHFTPAQVQRAMLMTSEVNPLLYAWGQYYAQNAA
PPPPQRSPPPVVVNNTCRSTAKSACPCKSEWQLGGNTYFYCDRTGTNNTLTCEVADPITCADCTSQWQCILPCAARL
PPPPPPRPPSPPPMPPPPPPRSVPSECMKSRTGCACRSTWNYQGAFYSYCASPDGSENLWCQVSSSCPSFSNNQSYQ
NCLPGLNESYCGGRVYFSSTRVPPRPPRPPLPPPDLRPRPPPGPPTPTDGPIATITGRITINASCALLSTNGSALQS
NLLAELSQTLQVPLSYVKITGMACGSILAYYSVAFPTGATIEQINSVAQAASALSFTVSIGFASRWGSVTQSSSDVN
VLQPSASSTSSYKWIAIGVIVGVAILAIVAVLVALVIYSRIKNGRVAPQAQYLITAAFTTALSCPQAQAALVDATPA
TLQHP

>Vocar20012909m|PACid:23130003
MSWISCGWKGRGGSGKIVCMICCERRTVRCWVKRDKGRPVPEESADNPIAVDAKSSPSRGRSGTTAGEYYSSRWASK
GCIPSISEDQGCSPCIDAHQRPMQQSGSPNRHSSQDHQGRHYNDSYVMCGQSNEGGEAAAAAYSNDRHVNEDGSSAV
ADDMYDSAYGADDYDYADADAAEYGADCSDEPPSPNPGDQPSTLPYDGAVADADTDADAGGGRSSSGRNGRCSPATS
PQNPSLAEGDAKQERLSFDAHPAVPEPLNLNLTQDMDRDREKRCTTTADGAASSRQQLSRGRSRSPAPRSHPQRQPP
RGSRRRASHVSLIGRTSRSVGIRCRRLQGEGGPGWWRRLREVEEEGGLEEGLRGIGRRARRRRSRRRQHIVDHWAAA
CRHHTTTGAAAAAMGHIPMVSEDLVLGQRRRLYDFRERASGEAAAAGPMAVPYSHHDVAPAHLPDVPLVLTCRVPTT
TTTTTTTTTTIRITTRSVRRHSTDHHRPPAAAGAAAAGDGAVEAAEETDSTTRSTVTLRTMSSISISSHGGDCRRRC
CLTRSVLGPMLLLRRRHGPGPVSDMMGRVHQVPEHPGLGEGGRQHRATEMTGARQRRGGAGGDTMAAAAVNPAARGL
VLTVRRTMAHRRGLLVAAAAAWKAGLGPQMPSVDRGQYGHSGVPDPRPPHPGRAPGEIPLYRALHDRRGSPPPPRDP
GLRIHPMSSQDPAPHSGSGGGGAVFDRYGGGGGGGSWAAGRSPPLAPPPPQQQWGASRSGPRNNSPPPLQRLQAVRR
SPPRYLRGELGPPPRPNEAAAAAAAAAAVVGSPLHMRGGFQAAPRKDYERRPQPLPAFDGPGRGPGRPHSYGPYDGE
NGGPPSYNNRRALSPRSLDGPLPPPKRRPPPSSCASGRGPCSPGRGRGPGFESGMGPRSDVSLSRSGVPHGRPGTEY
PSGSCGGAGDTAPGRQHGSNSGVAAAAPTSSEHVSGGGGRGRHDDGGGGGGGGGGRYHDVRDEAAVVIAGTSREADR
IENNGPMDYQSSSPQQQQRRPRSPSSSLQQQLQLQQQVMPPALNGTPACQHAGGSSTGTPSKGTDGGGDGFKLRVVG
DCGGGSSIGVAWPSHATANGTTGGCAAASAAAAAAAAAIPPTSSFSTAATAATAAGVSPDEAAEEWFYTDPSGQVQG
PCSIGQFRSWIKYLAQDEDFKEEYDKFCSCVVWRKQGSQPPPPPHLASGFGESRATLLDLLRSLGG

>Vocar20012556m|PACid:23130101
MRRGDIGLIIVRQVLLLACVLHSTCGHQQRTKLVELNLGPKDSSSPDYIKLVDPDAVRPFLALKDPTAASGNDIQQL
YEQGASISAIAYLLFAAAEPLSKAQGSTAALLPYLSPTVLRLYATQPERCSSDLVRRLYGLSVALCGNDVLKSCHYD
LYTSLGAAFSWVRDNIPLRLQEVPGQPLASATWPDMSRTPPLKLAARVAEALLSSQPDDQTLGVNTAILLSNGCELA
AADFAALVPAISAALTDLHADLLLLQQLAKQLFVSHWTMHDPSSSSDGSTDSITGTALSAASADSSPDMAASTRLTS

ATASSSVSSSSTYFYSMPPDDFVPAPPAAQAPLLLPLVFHIMLYWDRNGAVAPSQYTQAPSFLERLVRVGNTMARPA
NIQFFIKDVRNDPAAYPYLMLSSRVAWLLCPSGGSYEDECIRNRTFMRSIITDFPRSVNVFVVSDSTVMKELPVGRA
WIPGSDLTPGYGHVFMTWDGVSPSGSNSLTLYNDGPNTLVHEIFHHLGLRHTFTEASSTTTLATTGGCVDADYGTMG
SSTFAATATSYCMELFWGQYGGDWDATYVRWSSTLGIPDTDMNAWADSCPGNPGYDELGNYMTYSTPVCFAALGHFT
PAQVQRAHYVTAELNPVLYAWGQYYAQMAAPPPPTASPPPEPYNNTCRMSRGGCACKSSWTLSNGSTTYSYCDRIFN
NNTLVCEVLNPATCSACNTTNPVVKCLATCSGTPRSCKRPPAPGTRDPPPPPPRPPSPPPMPPPPPPRDVPSECKTA
ASGCACRSTWQYGAYFYGYCASPSGSPQLQCQVEPSCPSFDPENPLQPCAPSLTASLCGGRIRIATSLQAAPMLPPS
SSRPPAPPRPPRPPRPPRLPAVPILPSPLSKRPPFPLEPDLPAPISACGNPGDESTCFGAPPPRQPPPSSPASGPAA
TASNRSDLTSMIVGIAIGTSAGLIILTLLVLAVVWYRRMSQVRNRVNPVAQRASSAEGDRRGHVPGGAGSGSSWAVR
GRGTPGPAPTVLGSPAGSGYEEVMQIPGPAVTGPTPATASSTTVTPPLTAGGRSWRQTAMVPAAAVAAAVIPSNNTT
STAVTGAVGHLGRFGSVALPAALAGPPLQASPTSPPPPLPSATAVAATASATGEEPPREVLATQPAESTSLPSSWAP
TAAMTVVPPPRRLAPLRTGPALASPRMVLPPFRRTTAASATAALPHTLHTAAMEGVAAPSGLRGNVVAVDDEEDDRH
RRDHDEQQVAVIPGAL

>Vocar20012717m|PACid:23130182
MVGSCVLQAIQVSYAILRCSSQAASSFVPSGACYYRAGVYVSGAVVSGWIKWLGCCDLQSWSSSVRLATALFLTAVL
VVHAQDIPTVELRSGILELKLPSEIDLTGGLPPGGDKGVPIGFDLNARNQYLVQSRTDDTLWPLRNELVKQSVSVLE
VVATNTLLVFGLPGAIVKAASSYNALVGEYRGDYKISPETLVVISSAAPAAPAAGGGRRRLQEGDENLLSDLLRGNG
VPGTPSALSGLMTWDMYDIGRNGPGAKTGDNSRTAPPPPRPQLKANATLYGLVARLVSGLVRDPLTVQVSNWRYRML
DLFGLSANDPCAPRLVDSGQANSLSTSILVFVCFQDFNRTIDWLKREPLVTWVEPLLRAESTNAIAGWILQTGNLTA
TQYTNPTAALRPYWNATLRGEGVIVGVGDTGVDMSHCSFIDNKYRPGSLRSMFVGNPLRLYLPNHRKVVQYVLPEGS
SVAWFGDENDGHGTHVSGSVAGSVLDSNGQLVNDASTGSAPFARISMFDIAYANMSGLYIPIPLDDKVLPYHYAVGA
RLSSESWSQPFGLATAYMEYARQFDAFAWRNPDFLSVVAAGNFGFNGLMSTVTSPGTAKMVVPYVFPQNVLTIGASL
PTIKGSTDPTVNRAFLFRYNNTATSRQGFALWPRRGSSMANWTAVANNREVPVRMANPEHACTSLTINATGSIVLID
LGAPGATCTLAQRATNAVARGAVGVIFMLESDNTFVEEFAIVNPPNIPLPFVYGFTNRATGIAVRTYARGNVTLTNP
TMICLTYPNINLGIDGIASFSSAGPTRDGRFKPDLVAPGGAPGILSADSSNFPIDGLNADVTDGTCSKSTIRYQGTS
MATPLTAGHLALMRQYFRDGFYPSGVKNDVLSASFEPSGMLIKALAIAGADSLAGGYALSGGRVLGNAPDGFQGWGR
LDLSGSLPLPGLTNPNVKVQVADYGVITNGQMVFLRGLKATGTGPVFATLVWYDYPASGSASKDLYNDLDFGFQINQ
RNTSITTYSVTRNDSVNTVERIELTTLKANDLITFVVHGRNIRHSLLTTPDAQLPQRWAVAVVGHFNGTLRTQYNPS
YVAPNRLSTDGLPAPMLVQSSATTSFPCAGSLRNGTLVLSTALCNDTSSSTLVFTEEQDVRTGYYYYVVRDSVGLCM
AAPSSLSGTRVIFQNCSGSSSQKWALFRNRLTNDGLFMVTPQNALGVLPEERRCLRTGLSGTATILLLQACSDEDVA
QGFRIARNTIVRSPPPISAPPPSPSPPAPVNQVVDKPWYPYPLTFRLDFYPIEGHPNDYDYDYSNDPERRDYFGVPN
LSDMDLVVAWNSGGQDYQVTPDNFAVAPTVDGVGGAVHGGDNKMYTSMYEVVHFRTGLTPPLTTYHICAAWSETETP
SMKAVATVYQGYQVASSSKVFDVTAQVDYTMPCNPSAPGYIFSFTLTNTTAPPAPPSPPPPLLSYPYPLAFRADWTV
VGGMITRENNRPVDMDLVVSWRYFDVYKELGYYLRVNMDGRYNGDNMEERSNYELIYWPEDRIPPSAKYDVCVRWYG
PRKPLAQITLSVFYQGQLVRMNTTIVDTTFPRNTACNASARGYLGSFDLSWVPLAPPRPPPLAPPLMPNIPAAPPLP
PSPPPSPTPPSPPSPPVPDACSAPASTASLFGLTFRTRWFPSSGATNVIYDWDMVVAWTFGGSSFELAQYNRFVAGG
VHGGDNINIANRTNGEVAFWPTGVTGQQPQPADYHVCVRWYQTAILNVTLTVSLGANVVKTTSTVWSSSDIISKICA
PGVAGYVGTYTYTGINSSSLTGGVSSIWASGQAASMSTLQKGSVDVASTERVEPVKALPSITQAGASMSSAGSFAED
DVSVAGGEAASSAEGPASSLPAERSVNGGSGSGSAKNSVAVAAGAAVGGLVGVAIVAAVAMQLRRSRQRMTRVAAL

>Vocar20013255m|PACid:23130289
MFEAQVAYYLNKYLGQYLQGLDADSLRISVWRGDVELRNLSLRPEALQDLDLPVTVKAGLLGRLTLKVPWTALGREP
VVVEFDRLYILACPKDQTPGQATQVNVSQYEADVAAGELEAKRRRVMEAETQWLQFMWGGGRGGASEPPFSLASTST
SGTLLPWQQLHSLVVRLFPPELQAKLILKAQQQQQQQQQQQVSSDGKAGGDTKGKSKSRGGGGLFHLNLTALIHTI
LGNLQLRLTNVHIRYEDSSSLPGGQRLAGGILLGGVSAHTVDETGREAFITNDVLNLLRKAVVLQELGVYFDMGSPF
LEAPRDWSALMAREWDLLFSPARHAPGSGDGGSGGDGGNGGPHYRHHYLLLPISGQMNYVRRSAKVLLADTSSAGGG
GGGGSGGGGGGGRPPRQEGRIALRAVSLALSRDQYVGARALLETLDSYTANAPYRVMRPQCRPAPGAAARVWWRYAF
LAVRKQLRLQRGGSCSWSQLQTACRLRKKYVPAYARCLMAASAASTSTAASGRSGGGGGGGSSRSPERQPPASAMAS
AGGLGGDATIAEMDAQLPEGTILLFRRMAHAMAQSQRPATTGGGVQGQPAVAAGAAAPAASTSTSSGGWVGWLMGSR
RTAAAASAATAVGGGGKGDAAAAAAAAAAAAAPAATVTVSGSTAVSASAVGTATAASLTPEEWDRLQQLLHQQEDA
DQDEEEEEEVEPGATGGGGGDHPYCLTALLEVAVERCSLELGMSAADGVQPLISASMQAVGLRVLRYPQTLGVSLRV
GWAGLDSPQGMLLCTGQLQNNSPQQELAYGTTLPAISGGGSGSGGGGGDSGRPRMSVKELKGEGKGVSGGKGGSESR
PGTPSMPAAVITGSSVGGGDAGGDGSGDDSYQPTCAFMLDFVRRPQDGSADATLSVRLAPSYVTYNAAAVAAVGAFF
SSSQQIGQLGNLQAQAAARVQRLQRLAQVRLRALSRERPKMVLRVVMHAPKVALPDDKGRGTLLLDLGCFSLSSSTP
KAQGLVPSSDEASLYECFNFGIQRVEALVGGRFAWPATSPQLPAALEEVAGGAVAASAAAASAAAVAAATFALLGP
LPAGPDAREDRRGGRGAGGGGGGGSTAPLGLAFVAALAKEGPVAADELPPEGTHDSGEPPAAASSRSPAAATPPAPE

LCYESHDDDDSDGGSSLVELVRHIAGKDETSSSPPVVVTLLPRFGAQAELQVAAAQDPRLPAVRLRVVASPLRVEFS
PWRYHRLMAVIGSLTASGSHGDPTGEAGGCWSSNRTRWVGGADNAVALVPEHVAVRREAVAESPEALVMRLGDSGEV
AALRRSLARSAALVESVAGLLGAEAEDAESDSVAGQDLAKAGGGAGSGAGGGGLGINRDLLDELLAHRDVFELTGSL
PSLEVRLSGRPPRAWRAVRRLAAAEPPPPPGAVDINAAAGGTGEVLGSMYQPSESHAGTDLEGTPRRPGGSVSSLSD
FAGGASASAASPSLAAATKQPGRPGSSSSRFGGGGGGGGEVETASAAPPPQPPPQPQPVNTVGPTSSLHGSRVGPGS
ETESDVEFVDAAEYDNDDVDGGGSTYGVGGGDEHDGGDHEDHDHHQRLQDLTLEQEVPLLGVALTSAAVRVQYSADR
LAVSLRMHGLRVENELMGSALGKQFAVLAVSSSLCTSTSATETTGAVDAAAASAGGEEKAATEGTDVESSSPLVQLS
FTSNSSKSDRYKGLDTEICLQLASLFLTCHRPTVAALMGLGADMAYANSRLAEDKNADAAAAAAEPLPPAPSAAAAP
AAAAAVVRSGGEGRTLMRLRISMARLQLVMPYEVLLPVQLPTAPSPPTIAHAVQQPPRQQEQQEGSVREQAAAGARS
GPGPRLLAVAEMDAFSFCLDVRPSSLELTTSLGNFTAQYGALPASSPYRTICTMRSGTSGSLIEVAFRMHSAAESYS
QPRVPPGQPYSSLSASLSAVQVVYMGRFLNELLSYLTAASSSAAVPATAAAAVAATSGSAGATADTAGTPMVLLLDV
SLASPDISLPQNSSSGLEHRVGIDLGTLRLSNSVAWRRGVAAAAAASRAGGGVTTAGVDIAAPGAEGWEVLMDEMKI
HLTDMSLVVGHADEGDGGQAYAGGSGKGQNIIRDVGSGVTVSLHRPLAIKPVGPAAATTDIEGTAAAAAAVPPPMPP
LHAAVTLPQLSAVVSETEYRRLLAVLSGNFSEVAQNDPRVTELHQILQRLGGASSPPPRPPPPPPAPLSPHPPLTVP
SPVKSTSTAAGGGGAGGGGGGNGVLRSPVLDFTRSIRSVLAALGDNCTVQATVAVGTARLTLLMSAAADGGGALAPL
TAVDLRDFWLGYYATARGGMFLSATLPTFTIEDLRPGVSLEQRLVLSTAAAAVAAADGGSRGPSAPSQPQTLSHSAP
SPSLLALEYRSSPGSTEVHGGNGAGTATAFPAVQALKLRLQRPTLVLDFGLLLRLVNFVTPSPVLQYPSIQGVPVHP
RYQLPTSVVRLSLPSQPCDMWQALTSIFRMFGRFPFPCQGATPRPYETRDITVPLHMPYSAYGNDLWLSPEVRLLAD
APAAGAAPPATGFLHVYDGGGGRLVLPPGLSPVESLPLIVVGANRALKLKNVRIVNCASLPSCLSLGPGAQLQARDE
DGVKFILADPEVDRQYGAVMAAAAEVAAAAAPAGAAGGSIAVTAPPADLVPATVPPPPPVFEMSVDALGVSLHLIE
APSRGIGAVLDENGGDGQPYGSSGYGSALDLTLGLTSATATPPRPPRLGADADATSALGGAMAQRLRRRLVLQLDAS
AKVTMQGQRQEAAATLRSLTMRTVTIFPTAAAATATAPVAASPPPSKVGSVSNLAAAAGCGIRPAAAPPEAAPTAPA
MTIATSLLEPTDIEMRYSLSSSNQDLSLDVDALTLRLSPAALHLLLQLQELVLQPLAAPPPDKPLTRCDRFERLWSS
HWGAPRTATAGATAPGGGGGGGGGGGGGGGRGPRGLELGVGGVDSLAEERGVTVWRPQPAHGYVALGDLLTAGTRMP
SCQVATLAINSGLVAFPVAFEPVWLGPEAEDLALWRPVGPPGYVALGCVAGPRAAPPPLAAVGCVAARAVVEARLGE
CLLLTDRGNLWAVQNAAASFEVSPTEKHLPVVHLYDLRIPLGVPPAALTPLPQLLPTAAAVVPLGLVREASLPLGLG
AASEPPPLSLLCQQRYLRSRSALIRRALSQASQLPCVEFTRVWWDKSLPAPSANRLSIWRPNPPPGYVFLGDCAATG
IYAPPPSVVVLRDSDPGAALRDGRPPPLARPINFIRVWVDEHRERQGDPGALVLWRPVPPAGYVPLGLLAGLGSARV
PSVQIPLRCVRADLVTSETLSRSAPDWHLPAFRHRTPLSGWLTDRRTGTFTVMAGPGAAPGDSSVGGGGGGGGFSEG
FGGGTGSPAGKSFGQGQQGQGGGATFDAYRMKLDADDPWVASPGAGGGNGGGAGGGGGGAGGGDGTGTAGAGVTWGP
IPGAVMAGKVTGAPRAYVFPDLSRSYAYLTVSIWTHSPSLGVWEPLLEPWAVLAHLDDNRTARPVAGVSPGTTIRIT
STQSSVQVALAATALKSLLEALPEWTGLTSPEGLAAVRRLAYGEAAAGGSLYGSLAAQQGLMTNALGCPVSLLTDL
GDRQAVVELQTDAEDVPVLLQPPPTAAAAAAADSRAAGLAPPPVPAVRMFVDAVSAGPLPAELLREGGLMVRIKVKR
QAPKGAAGRDVEGGSSEGRRVGTPITDSVVRWDERFLLKLPVELSEQLLCPAPGDNQFAGVPLQVDDSWEGLGASDW
LANEVDTLRNTAAAAAAAAAAAGTLLPVLGAAAEGGGGAGAGGAAGGGSDVSETVDVPLFPTRVVVAVHNGDGGGVG
GRAKSGGRPVGRARGDEAPVMLQLRLSLDVHQVDSSWQPFLSRTRRRLPYASPDGGPGGGSEGRTGGAVAAAAAIRG
GRRAVRLYGSDVWVPFGQTGGIIISGPGGDMLQASSCIPIRAGPGLVLERSPGPGGQPLELLRSLAVLVNGSGVPLE
VSLVAGDPEVASWTMLAGEAKPPPALPSAAAAAAAAGGHAEAATAAAPPPSPVEEDVYENERYIPLFGWSPNNLLPT
ERRRYSRHGQSYTSFPLVPLPPGWEWEGPWQVENAGHVDANGWFYALDWPLLRYPPTPEQARRKVTDMVRRRRWLRR
RRWVGVGSGAVKGAAAVDGGPPRRLLGVVSPSDKLPLPLGWEVEGVELQVRPVAATSTSTSTAAAGGLGDTEAHESH
EWSAGLVMSGLDEGGARLLCCRPMSALPNPGSEITSSTSATTGTASTSAFAGLALMPVAEAEEAATAATAPSAPPPL
VDPLHPAWLSVSLECEALVATSGFLSGPGGGLDTLVDWRLVVRPPLTVHNTLPVAGRYIVWERDGRVGAREPPRVRQ
SGRVAAGGAAPVFSADMRMQASPHVYLSFAPDGCEYGESSPVIISDGYMRNAAGVAAAPRLPDSFRCTYSHSASFSA
LRIQLIRDVDTSAASAGFLAASAGGAGASRRGGGGGGLLFSPGEAVARGYPLTVSLHVPIWLVNATQLPVSCGILEV
EAPPAPPSSMAAGVGGEGGGGGGGGGGGGIRPELSAVSSGVGGFGATNQPPPHVLRIIDTEAFKNSPRPQSYVSVLPR
SLELLSYPAGVTGPLESGDGDGGLQVAVVLRLHDSRWTQPIYLPRPGEQMQPRPGAGGVPLPSDRLLVRALCTDGCI
HEVAVRLESAPLGLGLEFGLGLGTGLGAGLGAGLTTAGGLGGVAAATRASVAVVVRLEHHVVLTNTTGYPLQLLQPE
LAASADLGGGAGGRGFRGGAGMTFTAGGGAGRGSASGTTAAAGAPSLAAALTMSLPPGSLGVPLRWAAAAASRRHLC
LALPLAGETATSATAYGLSPSIMWSEPLKIDMPGGQDAQVAVPVYCLGPVGRYHSAHASGPAAGALAAVAALANMTP
QQQRLFELQRAHTVARAMFASLCGGGPGGGGGLLRLTRHASSGEDGGGGGGGGGGGGGGGGGEEQQQHQQQQGGEAA
ATWMARGVPVGASPPGRLHPPSLEPNLVRIVRPTELGNVEFLAVLLNVRLVMRAPGCLVLRLASLGGQPQQLLHNTS
PHDLMWRQASPRAAWARLPGYSAAAVARGVLGESWGSPVEVELRDTEPQSESSLLLTTDLMAVGTGVSAGVGGFPGR
GGIGGVGGGVGGGLLAAGGGGGGGGGPLGAAAGDLGRPVLQGIFSVGQKHREVMAQVAGGWGGKGEGEGEGKLTAG
RLTRVLRVGRGKGVDFMHLSLRLRSAQLDDQVPGSAYPVVLQPLEQPGGGGTSDPLFVLQYCCQFQFQFQFQFQFPV
SGSDLPMAIDLLVVEDLVLRCSFRADAASRPRWAAGGFLKLGLDLAQLEDLSLVLPGIEVERTRLLRSELLKKVYDR
LQERVFSLALAVLRSAGLMGGLSKVRSLQGGVLEGGKLIAKGFLQGATGIITKPVEGAERGGLSGFIGGLAKGIVGA
AVNPVSGALAAVSKVTEGADATRRNIAGVLTGAAGVRRAGVGRKRLQRAFTGDNVLRPFKLHSALGQALLYSVAAWT

GPSNPASGNDPRVAGGGAGGSGLLGLGAAGLGLVGAALPGSVRGGAGARPDLRQDRYEQHWILPGAVVLLITDRRVL
AMRAAEFCALQERALAGDIAHVTDVPVSDAVVLWHLPWPEMLSAELAWFERPDRAAAESAGRFQQQQQQQGMYGGMY
GGGGGSGGGGYVRRSAADGLVLHRKHKSEGDRLYYEVHCTPGSAMAAEIRNRLQEERGWRDSQPQQMQLAAAAAMAA
AAAAAAATTTAATAGSRSAAALPTSSAAAASSGSVNHYRSGTGGPDVVLPDTTLSQDFKLVWAATGSRASGGRSMSI
WRPVGPPGYVALGDVAVTGREPPPRPVRLYKDSPALAAAAVNASGSGDAPASEAPRLAPPAGYSLLFRDSARPPLTL
WRPEPPQGYVEMGCVAWPEIEEPPLGLVRCLRKDLVGPGRVFDSPVWAGGSGDNPYWKVSMWPVDCPAGTFVAVPFE
VSKKPTRVFEAVF

>Vocar20013223m|PACid:23130306
MESTGQAALQPGGLASRRDLEGCVALVTGATSGLGLEAAAQLAERGAKVIFGVRNPAKAERVVAAIRKRYPADAAAA
LDFVLPPAVPPLDVSDPASITRFARALLSDTATPINVLVNNAGTSMLAGPSVDERGINRLVQVNYLGAYHLTRLLEA
KLVASGSRVVCVSSVTHRCYEVPTDPRVFLHSTSRMTYAWTKVANVLFAYELQRRLGPLGVQSCAVDPGGVRTSIWD
EVPALAHPPAKWVIEALYAPPADGAEVLVAAATLPWERDRVPHVLSPAQDLRYYARGAFTSPLMTLIDGAWRDWSHV
VKPALYGSSVVLHSLLDYPIRHFSGGRMFNQTMAVRSARQTYDMYVAAELWSYSEKVTGLTDSWRSPPPPLAEAAAH
AVLSGSGANVDSTAATEHDKAKTADRPPAVPPLQGRSNTTSATATAAAGGSPPPAALTSNRKRPVMSPNKTSAADVL
QPLDNRPLAIDAAAVGSPKCDAVGGMFPATAAGVENAGGVGGGGSSTTRRRLVATRRDSDAEEGGGGGGGGGTAVSV
SAAGDAAMPLVALPPPPETLQHVAEEHGVVLPGQSAVQNAGAGASASVIGGDVLIAVPSFEGDGPTAGTPAPPPHKR
LHTQSPVVSLQDETDVAAGDTAVVPVGSVKVGGTDGVGERRGAGADAGGIAGSGEVPAPATVVGLGEDAAVAAAAVA
AAVTPTAGVRAGGKQAISNAVSGPRSSEVKGLAAATMVSSNGGAHGNIATPGRTSASGSDGGGSSSAGKGKNKGGKQ
KGKRH

>Vocar20013298m|PACid:23130315
MECTVIRDGGGGGSGGGAATDTTSLQPPVLPLSPQVRPSVYGSVFRTPPSFLQPLLNAVVAVSTGRLSSLPAAAVAW
LLRALLSLGHMPPTAWIQEAYDSLAAGARELHPSQAANAYLAIAGLAIVSRTARTGGGNSRADDAKDAAELAVSASP
PPPPPPSALLTGLYEALARPGPFGVMDPRVALACLEAAAAVESAVTALAEALIRRAAGIQPSAAAAPLLDETTGRMG
AVPEADATAAAAAAAVAPTGGAHLAAASAAAATPTATTVVSPALVVAAARALVAAARLENPPRPTAVLDLASSAPLP
PPIVAALISGVEGHLPYLDAVQISYCVTVVQRLAAMQSSAGSAAADLVLRFARGPAAAELARKLPTLPPARLAAAVA
ALPPPLRQPPPAEVAAAAHEALRRTLAAADSDDLAEVLRQLSAADALPYYGWQGALAAATSQRLRDAAALISSQELC
NTLFVLAALNYRPGFSWTAAAAAALQPRLLSLTAPQLTNALWTLAKFGYRPEPSWSTPAVQALQLQLPALRPHQLSY
GLGSLQRMGCTVSAAVLDEMLTVAAADQLRQYPPGDLVLLLMTVARLRHVPSYSFTEAVTERLRPHLTASPAMTAAA
SAGLVEPLAAAAGNSAVVAAAPPPPPLTCQELASVGYALAKLHCRPEADWMSAFMDCCLAVMYDFTTDLLASLMWAL
GELGQVPSPAWMGAFLAASRPRLGEFAAQDLVSVLSSLAALRSPPPADWLSEALTVLGARVGELDGEGVTALLKALV
DLDARVSNPDWYDALCDQVASFLPLLSAQGLVDLVASLGTLGHTPRPEWLERCSSAAVRAHAQAFFAATAANLTVMS
PDRVTALASALAGAKVAPEPAWLDSLTTAVRNDVMSYTLVQLDALTRALAVFQGLVPEHGAGGGVKPRNTAAASRGE
EWEHQLPRHRS

>Vocar20001212m|PACid:23130480
MIQTTRAPGKALHFKSVVPGRVGSGARARGAMRRLLAALLANALSAPRLRRTIQVTTVAAAERQSDWTRNGPTLLEE
SASTHGGDDNLQQLRQLESALSHHNDLYYNQQRTEISDAAYDTLKLQYRQLAAQLRGELPLEELELPVGAPPPASGG
LRKVPHRVAMLSLAAVNSREEAETWLDKTLAKLQADAGGGGGNDAGGGGGAGMVRGGGGGGGGGGQKGVQKKKATKK
SKTGADVPASSVGRSYRWVMEPKVDGLAVRVLYGRTAHGTYILKEAATRGDGWVGEDVTHNALYGGINGLPTCLAVG
PPASSWSDRMSSSSSSSSSSSSSSSSSSSSSSSSSSSSSSSSSSSGQQPPLPPPSQLGRGSHIAASISPSPAGAANH
DGDGNEWENGSAASAPAADCGAPEWVEARGEVFCRTADLELVNREQEAVGQPPFANTRNAASGAVRLLDPAECRRRR
LSFVAYAALAPSPPPSPSQQRRPEPATTTPPPSSSSPPPYPSSPSNRSSGNAAAVQPLHPSQWDTLQWLKSAGFSVS
PDNRLCDSREEVLGAVEEWMEKRATLGYDADGAVIKLDDTTLYDILGTAGSDPRWAVAWKFPGEEAVTRVTGVELTV
GRQGQITPVALLSPVVVGGVTIRRASLHNLGLARALGLHLPGDAVVVRRSGDVIPQVVRVLPELRPPGAELWQPPSA
CPACGGPLKAITAPAATTATAMAEGRGGDGEGGSSARGPEPEGVGSGGGVGRGGGWKGRAGRGPREAPTATTITTTT
TTTANMGGSGGGGGGGEQLACDNPNCAAKSERRMLHFAQVCLSGSGVSRGVVGQLLEVGLLDGDISDWYKLDEASVG
ECSGLGVGRLGGRASLSALPGFGPARTAALMSSLESSRHMSPGTLLRGLNIPRVGEVAAEALGRAFPSLELLAAEVP
YADRIAAAAGVAPSVARNVANWFQQPANQRLLARLREAGVRCITGNTITTAAAAAAVSTASTAAPAPSPPSRTEIAG
TEATEEQEEGPERAPGDGGRGVAEDVAVGRGGGGGGGGGGGGGSGGRSAGPRLVGLEGMRVCVTGTLMGPYGKGLVR
NEMRRMLEAAGAEFHLTVKKDTQVLLAGTGAGERKTRKAAASGLLVMGEREFLQQFWSEGGEPPGAA

>Vocar20001132m|PACid:23130493
MGSCSTKLRNIFQMAQKPEGPLGKNTYRHQSSHSQSPKAPKSTVEARDNGLCGARLDSPGPQNPSTAAAAAAPLHPP
SDFYCSSGIEQLSATRSRSVERHKSPTPTMYDDPAAQQRRQDCVPKHHSDLSADAISLHHPLISRNAGLQQPHSATQ
AAAAAATATAAAAASAVLGATSSLQGDERERVCTAAIGEQFAAAVGAGVSGEPSMAQRHRGSTAVISRGSIGVFEGT

AGGSAGGGTADVYAPYIPSWQLPVATAGAAVPGQREAVAGDTQLAALQARGSLALLASGGSVAGVGRCTDMGVSASP
PEGQRPQSPPPPPLVKLEDELELRSQPCIADESSGAGHLAASGTVPQAHGSQYATTGTQPRPIQTSPPPSVLAARDF
RGLVPSPAALSIPGTLVTDLYEQDRVNGGAVQSLPSVPAPPPPAIMQQPEFLSGGRKAGNASDSPHDSAHYHTPSQQ
AGSGPHRVVEMGGPEGDLAAAADRLTAGLTGDGIVHVVHAAAVAALSSLSVPSAAAPAWQYAAGAVGAASPSGPFTS
QPPGGMPVSAGHPGLESPRAQVRKLQDIEKRLRQMEADLSAANRQTQTAQRDKLRVIENCDQLEDHVRQLKDLVRNQ
EQQITQLRNQSTRVTASERRLQGEVQLLQRQNQEIRSQHTSVLRKLHAVQQEVTALLDERDELQERCSELTGNVDDL
HVQLSLLEHQLKQQEEEERGRMGSAVQKGGGGAIGGGGGGGGSFQLNRPDLGYRAPTAPPPPVLWLDASRLRVGADQ
EIGFGVSELKKHLYDLALNLEDMVTMQVQIPRGDVRGRLIGHQLLLWLLRELWSDTTGLICRAGLGQGALRVRHGTA
TATAAVPSQRGDPRVIAESAPGSWHLGPLPLLPEERNLQSLLRSHAARRWYDVVRDVAAQEQASTASGGSGPVQLRL
NGGGNGNTAVATEAEAAVRHVVDFVASSLQDVVTEAAKGLCGVRQPLQAPSTAAAVRILRVLSYNAVLVGLFVPAAH
PLLRLAVSPAADPAAAAAAAAATVSAAAAASSVGLRACPKIANDQSSTGIENGARFDPSFQINAKMLSLSLPPPPPP
PPQPQPPHQQQLLSSHGRPSEAQDHAARQPHVLCCVKPGAVYDDVMAGRAVCAVQEEVLTWTSAPPTAVTAAVPATA
AAASNAELLSTQQAAAEGKVAVVANMAVTSVCAGGSEATGEASVASVTEVKMAARKTVIKTGVGKHPAAAVDSSQTG
VDETGAAAAAAAAAASSTVDAKPPSPSSSLMAVSAGGGGRQPAAATAPTAAPTRGAAGGAADEKAAHALSHVNDLST
AGTATAALVSAQRAPTAASLSQMGPTTTRPAASAAPATAAATPTTTTTTTTITTTTTIAYNAPANQQFVTLAQVPQP
NVPDSQTAKPGGESPGSPGAPPPATATQPPAPPHTPPRTSKQAENRSGPSGVPPPSAPPPPPLPPPSPPAVYVSPGT
TAATANIPAGSHSGPAMRSYATVAAAAPAKPTAGRMPSPSSSSSSSPSGTACPAAPPPGNTSTGAATAAVHAQQGRC
GLASFITNTTNTTNTTTTTTTTNNNNKSDSYASIAKRNLDDGQAADRQIGYSDEGSQRRIPVASPGGKLPCNVQQDG
CGSASFSDLLAFGVTSSDLNIPCSSTTSSWDSKHDFVGLSLASLPDLVVRPSHYLTILNCDGYYLQQETARSPDECR
HHGSDAAATALELEAARQASQSKEPADSLNTTSQPTSQERAINERVLAVRALCGGFSSQQQDLAIPRTARHFPTVVA
TDSVHGNEAVSEPTALASTSAVRQQIRPMSDTGFPEAPSCTTVTERLCLRANSPHQPVPTPPKVPPHCREADSLGAT
SRRGLSSGQLTSFGVCSGPSSSLPSQGVSSSYRTSSVPYTGSAAVGTATAATAIDERSRFVRCKCDEALALPDQLPD
DSVHGGRPRHPSRDATMQPSDGPPLLALAEAESAAHNRPILDPSASVLPTAPVTVCFGAVTSSAGSVVLPGMLAGDR
GKRRGAEAAAPGHAAQQLLGDLHMQLRPLCMAAIWLLLLALALPRLVWGYLAVPLAHPLVRIVAARLQKGSLAQFVW
LGSEEPRGYLQSDSGNVTCSVS

>Vocar20001252m|PACid:23130501
WGDLDFDDDDDPFHPVAAGAPTGSRAGFANSESDDSPEWPSSPMASAAQPPPPVPPAALPPSQDPTPVPPLQGTSIP
RAAAVAPLADSRTQPLTAQPPSKLSNPTPQLPNPALTTSGPLAHAVAASAHQAPGVLSHPSTSESIPVTPLPPSPPA
PLGALQLAPPAAVAVPGPHTGAAVATLACPTATGPYAVLPGNLLATGATLPPPSSLPMQSPQPQAAQSAQLGTPPSA
QHAMAERASASPPFASPSWATTSARLQIAGNQQQADDSLSFFDGFGEESEGAGDAKLLGAFPATSAGRHSEAQSEGK
GAELHRNAGQAAGSTVAVPSYSFPSTQPPTAADQESDAADNELMATREVSFSVTPFFTSASTGATSAWGVAAAGAAG
SEPADAAAGPDPLAPLPPPPLPSLLPPTRKLSVGFILPEDPAVASPGEEGDKQHKAFPDMSYAAPGSVADAACASAA
GGGGGGGGGGSHVEAEPLGGDGAGEPHSLTFTSAAGLGLLPTSQVLLMPPPPQGMVAPSAEESLPLDFPPSVGPLPP
PPGLSGSGNSVLSRTPSQRHRLSVSSSFRDRDPDPERFSDADFATALVAGDMAAPVAGALSNPEMAGGQAGQPHAAA
APSEASVDAGLAGGDDDSGGYGAAAVEGMGYCDALNPGILAAANTAAAVQSPPPSSRGEQQQQQQEQASGCDVGMPS
SSAMSVQERGDLGSAATATPCPSSTPGPVSAAVPVSGGSPVVAFHSGQESAEGDLVTDMSTPGSFDAATELLSEADF
AQMPMGQTEGGSGGVAGAALRARPGMSRLYRTSIASSETATPSELAAFDDAFDELMADSFDTEPEPGALATGDADDI
FASIAAAEPAVAAEAEGNLAGAAAAAVGLQGQQLQTPGQYTIVRGSRSNLSHLFEPPQVAAVAAAGTAAVISPPAAA
VARNPAPVSTYQEVVGDVSALFGSSGGQDEDDVFGVAAADETFSGTGVGSGEAVATATAAATAGGAQDRYPFQVPAA
GEIAEWTQPRGQWQQPAIPAVQQPQWLPQQQHQPQWQYGSKTHHAGATDVDVGASFFDSLGAAELPAAAAAAVAAPM
AATATAAASAAGAPRTHGEPGPGATATAPSGEAFAPQPLQSVPADDAASFFDSLDDGDEEREPPQRPQPADTGPHVK
HYLGQGSLPQMQPQQQGLAERHAGQHAIAQPIAFSLDLGATRAAGFGGVLSATQDDGTSFFDTPRPQGVNGAPEGAL
VVGGGADSMTAAIAGLPAPFFSPLEQLPPSQQQQQQRLEPLQQLASRPPQLPQQQESHHHQQQQPETQQAAQFNSV
SHSTALRPLQQQQQQLPPPPLPVAQQSPQGASTGDLDGSDFFDNLPDTFGGDGGISDSAQHQQHQQPPQELAAPAGA
ATTPGGVTAVTAAPTDQVNDVAAPHGHSAAQELASAGPAGGQQQPPYFQQPGYRAHEHQEHTAYNQSASAFGLPAAP
RQQQPAYEQLPGSSNGRAYGQPHASALQTYGVESLAQDQARETQAQAAGEASRTFGCPTPGDTASVAAGPGPGAAAA
AVPGGSAPHEAPSVLTDSSRQAHVLHDLAASPNPQPSSSHAIATAAVAVAGDTAYFAAGDPIRHAFGYGQAQQTQRL
QEGQDSEQRLWSQEQGRQHGQAQGQDGQAAVHQRQATGAEWPAVADEQGALTDGTVATQLNRGRHVEPQPQGQLPDQ
GQEPQKRMTEVAGGATAAEGPGDVGAAAGAIVVMSAEEPKAQHQPSSSQEPNTQQQQQQQDHPHYAQQYQYHQQQYP
QQPAVAWSNFTSPASYSAQQSPPPQHPAAQQQQPMYGQQVTLPYTSGGPYGGSASASASSFGHQQLDYDVTQFAQPY
GQPHGGGSQSAAAYGGYGQQNAAQMFAPGTYAGGMYGQQQYGMQQANVNYLPTTPELARTCPHGRPTHVVFAFGFGG
RAVLMRPPASGTDTGTMAVFGGPGPLQLLPLAALTQTHLAGGNPRQPPSQQQPSSTSGSSGGPTKADKRIPRGGQAA
ASTPVLEPLLRQLAAFPGPLGPATSKDKVLKWLSDHAAACGADDAPWEQQTNAGDAGGPTAAAAAATAAQRQLLWEV
LQLMVKHSEAGKTKQARGWLMGQSKEDQSMESDLVKLLLKRRHNPPRHHHSQHHNQQQQQNTSTASGLAAAAGGFFQ
SAAAAAAAAAAAATGAASSSTAGGNATTAVSGDQGHNEHQADGDDAVDDDDALDGNHAMGGPPVLAAGPSEALQQAA
AKRMQRLLVLGRKADALKVATEAGLWGPALLLATAVSPDGRAFQEAVVAMSQSLLLPGTPARTLALVLAGRPDLVHA

TPDDAADAAVPAAAAVMTATGPSPTRSVSSGGVSLTSPSFGAAAPSPAPQMPPAGFYNPMAAVASTSGDGAGKAHSG
GGSAAAVAAAADGGGGMLAAWREHLVVLLTTRGPVDCSEAVVRLGDRLRTEASQVLPAHVCYCLSNCPPSLPDTVD
FRYSLLGATTAAVLQHPLADGALALQRTEVLEWLLLRQAAAAAGGAGGSVSTAAAASATAASIAAAADALLLQMWAL
LPFKLIHAWELVEYGKISEALQYCAAVESVLRAGGVAMSPGQGQPAQPAAPTAAGRLPAGAWLNTTETQLRRLQDRL
MHFAQANSINLQSRTESTITKVGRFLDGTINKLLWGGEQIDAGAQASATAAVAGASTPASGAAGGTDGAAATAARPT
GPQVVLGGPSVRPTDMGPVSPWLLDLHPHPHVPGSSGAPGMQPIEPGYQNTTGGGSVGANGGRMQHSASTAALAAAA
DGVVGTSWLPGNTSAGPVTAPVASASVVQQQPPRTSFSGTVVPPAPRSVPGGSLLPGYGFDAGVGGGFGANGAGSGS
TASGIGGSVSTPGVEDPRSQPLQQQQQPQPLGHRRIVSAMDLPPQGLIQSPSMSVPARGVPAEGSTAVPGGGGGGG
DQGKGGGKGGGGKQGSPPTRAVSSSGSSRWLINPLKLLGGRKGGGGGGGGTGPKQGSLGEENQFYYDETRKRWVMRG
QEDAVVEEDKPPEPPKMLGGGWAAAAAAAAVATGVESTAFPGAQGGMQSSSQSPLTTPPSSAKGGPPGDLGSSAGPG
SAGGPPSASSSLASTATPPMTSQYFQVGWGRVSGGSGGGRGPGAIIACAG

>Vocar20001353m|PACid:23130511
MAYDLCKTCAGRVLIKSAAAVLPVEALLHQHVHQPGSNPTAFPRWRPAHARSSPRDRCPHPAKVPVSPGPSAGPITP
HIAPGTLTGSGSPRGRSRSPPGAKQRRFSSRSASRSRSPPGTAYTGGRPNGYSPTRGIAYGGVGGGVGGGRFASGGS
APGSGRAMVAGLAGGSNGAASAGAGMTPPPWVSNARLAPQVRMVLEGLYGRGKLGPTDLEYACIEFLELLGPVGGMA
VLEEFASLDFFRIRNLTAFFIGICKRVQARAPPPPGRPLRGPDGGSPPPGAGGLRGRTMSRGGGPAAAAAGGAGGGG
GGSSTGGLSARVVSRSPGAGPGMRRASPPLHFMMEDGSPPPRRWVSKSPPPRRWVSKSPPPRRFESKSPPLRRFNSK
SPPRRRWVSRSPPLRRFESKSPPPRRWVSRSPPPRRTGAGSRSPLQRRWVSRSPPPRRATSRSPQRRGRRASEQGNA
GSRSPSLSRQQATPLPPPEQSQQQLRSPSPPPPQQQQPAHSAGGNQPPAVLPGASAVTMSTSSRTSASANESGGVTA
AAALPWDRVGDSTVAAAPPPPPPPAAPLGSSWGAERPAGSDRPDVAAAAATGTATSEATAAGYGGGGGGSAGFGGGT
DGPAGLTGAVAASSCRWDEGSCLLGGLAVASERCSETAAPPGHVAALAKQQQQQHLRRQQIWQQQQQQQQQQQQQQ
QQQQQQQQQQQQQQQQQQQQQQQQQQQAHLQGLRHSSAEHPLDSAPGGSISVPRPLTPAAASSAVLQGRPAPIPGPS
HAAPGVLAAAAAPPSRLISPPAPAYGVGHGQGGYGGLRSQHNSQQHHSELQNGDRPGRPGGGGATAMPSISPAVAGP
APASSGTSGSGIRDTDYGGASTVPAAELWGGGKDGPAVGASNGAVHLPGFGSSTASAADGARAFPFGGEFNDDGGGG
GDGGDAETLLEPAALLDTRLTTAVATALQGAAATVVTGNGIGAAAAATAGGGSCSAAAGTALTAGIAYVPEPPVPVG
AYPLGGWLAADVAAATGGPRPRRCSAASGKPVPSGPPLCGALVPYTETRHKQEEKGEDEGVQAVTASTMEVEVEMSE
GGDGWRLGARGGSGEGGEDGSGAAAGLGGWVLGSMLAQALSAAAAAAASGGSSGCEGGILRQAVTEHVKEALKPAWR
ANKLTRETFKAVAKKAVDRVLVTLPNNGVGSLYDCPATVATFFSDARREGISRLVDALLLRLVSGHVTATAAVPATS
AAAPLPAAGGGASSADCAVECHGAS

>Vocar20001376m|PACid:23130540
MAGIGFIWVICGNTWYRWPQSSRSKTNAAGVSVVVVEAAPDLFPGSASARFSVDLVEKYPDLAALLSLPEFRPQTNV
RPDDLHQYLLAYVEHFKLRQHTIFSCKLEHAQPRPGGGVGEDGRWCIVCSDLLDPQRLHRLTSDYLIISAGACLEPS
IPQLPQGSADFAGVQYHARELKGRELREVAPLVSGQHVVVAGDDRTAVECARGAADAGAASVTLVFRQPHWPLPAKL
LGRALPHLAYDRCLPSLLFPPYYTAGGPMRALNSALAPLRRLFWWRLQRRLVRKYGVDELTRPPVNVMRDMFYSLQA
CPGEDNWVGVLQGTSVRLVRGDISRLGTDCVVLKNNSSLRATVVVHCTPYTRNYVVFDPELQATLGVRKDGLHLYRS
LVPPAVPSLAFLGCQTTSPYSWLTTSLQAAWVAQLFSGRMRLPPLEVMKRDVYRQRRWRRRAFPPQHNRGSLVHWYA
QSYHDQLLQDMGLPVEVKDRGGCAPLFSSCAPYTPADYRPLFQADGGGASLSSSPYKRRKTRESVSDDVEAVELTLR
GDGASMMSNAAAAAAAAAAAANRTAPSTVTGVSPSPSRRALLDRRLSMDRRRSIEAPRRVSMEAASRAALAQVSVTSN
ASRLQLLLNSRTSNAPGVEVSEVEAEPQLEIFTSAHLLMGSAAAGVSAAAAAAAAGVGGGGGGGGGSSAPVSLFGAS
ALVPPAGTSSRRGSSLGCSRLGSQRGSTMGMAVAAAAAASAATGTVTGPYAVLQYGGSSTGPEAHLNSHPEIEVDVL
GPEPRQCGDDAIEAAIAASKSPGGSSGAANVLAPEAGTAAAGARHTVPFVMPSPQSLPSSPPPPAAASTVSLGIRVG
GEGPDPDYVPIPEPWVTPAAPSTAIAAASSVPDGGGDMFISPGSLNLGLPYSRSALPSGGSGSAATVILNSAVQRLT
HGSPARSGSAGQWSSGPNAGRAAAFASNGGSAGGGGGGSNAQQPGVLDSAYGGQVSSSGGVRTTQRDIGSLLPPAML
SGYEVLPVGDSYVLRPALAAGMKQPSGETAGKGLAATVAAAAETAARAHARPYIDMYESFPAAGGVMPPHRSSFGDQ
FSAGDGGGGGGGGAMAGAAAGSSTATAAGIVSATIPGVAGGGGGGGLLVTSQPRGNLSALLAASRGGAIARSSVNGV
RGVAAAGPPRSSQLRSAAGFGGDGGGGILLSSMQDIDVLESPRLRSRALHPNAQMYGSNSLMFYINERGSPPGGGGL
GGGGGGGGGAALRMGITGAGGGSGPIQLTSPPPPLSAAAAAGVASGSLRSGAQRAKRRGSTVAFSNVLGNDDFVQLL
LEAGNNDGRPEGVIDVRQGSGLGGRSGDFPPAAMPAGEEGTGRGGGGGSSGSGIRTATGMAAGPVAALTRRHASVQS
LLLVERGGGSPGATSSSGVSGAAQLASVVLPVSAKGPRAGRGAPLGLAAVSGSAAGRAPHPTATASAAGGGGGGGSG
SGLIFPAVAAAARLARMGSRGPGGFATASGFISSGQMRELDGAVYWGGRDGRGGGAASGSWRRADFVRQLTHQHASS
SGMLPETVRPHQARSATLDAIVDQLRLHQHSPKQHSRREPLSPSPAAKSVASPVTRSAAAYTAGLKPDEAPQPQQHL
TKAAPAASATATAAAVAAQPRRLTSAPSQEYGRSLSKMALVRGWNFLKTALGLKRSRASGSGGARRPMSGSAAATEI
TGGAAATAAAAATGQDVLDLKSRDARAALMYDTPPSPYRAASISRLSRQSSRGAAALNPSVSSSASAAAATPAAAFH
SYDMPVAMALGRSRSYCLPHDVQRGVGGVGGVGGLAAIAGGGGGEVLYGSGLPAEAARRGGGSFTAGSRSPVSSQVH
HHQGSAALRGSSRDSRGTASPWAGVGAGWGAAMASSSIRRKSTLGLGALTRLNSTGAGTGGGGGGGGGFGLSSMIGT

GSVSRGDGADTSAMEYDSFRTGLGSPRLTSQDVVTPVALPLPPPPASVGGDGVGGGGGGSGSTGTNALAVSGAFGS
SKNPPHGGWDTASRPSLRRLPFRQSSSVALAVDAAAAVLAAGVVSPSPSIPLLPGRGTGLGIVPPSPPSHAAAGGSA
AVLMPPSPSGRATPTPQPLVHCLDERTMYGGFSCQALIRQESVRGASAAVAGMSGGGPGGGGGGGVITAGASIVLPS
DGEGAFMLSDGLDHSGTGAAAAPPLPVLLPPPQRVASELPASMQYAGVVVPSLEPSGSAAAIAGVGIVHRRQYAGGS
SMGARRSGTGMGAAGPSHVASSATGYFSSESFGAPPTAVSASPTHSVAGRAPGGGGGGQTAPVGRRGRVSRRDSIIG
NLLAEMAGVPGGDGLVSELPAAACGSSGGGTAAGASQIRQSTSLIGPAPAAGYDPHAPEGVIAWERPRGWEGAGGLL
DGGGNAAVGGGGGSGGESEEQQRSLRGAVSELRENSSSRASRQLAAAAAAVLPSVMLGHVTVTAGTQPPAAPSPVMY
SGGNALSNVPPHAVVGPSWRAVGGSRAGG

>Vocar20001344m|PACid:23130666
MRLQSEVQRAQHLFCVSAQQRHLLLGELSRKKPRARGDGELPEGGTKGIFRNYAADLTGCCVAQTKRSAFAKACEYP
FFIIRQSKCNAIRCLPFFVQQQRRSSGCCNALQNIQDLPDASLICISKACSLLVYLYKDMVILLAFLLQVISVFHRA
QANAVIGQQEQQQHDRRKSSAASALLRRAVEVLPQRQVALVPDGPLQGITSLPYSIASRIEPLPQLPRAPAGDHSNH
EAVLEALLCQHFDIVFRIATCKESGNHHSKGGRKSNVSSSNSSSHKAAPATAEATHGSSDSSHPYRSTAGGKGHSRP
RRRVAVPPASVMQPPQPPPQPPPPPSVSVRGSSGRRGAASMSHHFGSTALCLWSYRLVTSAANTTHHDEGTEGDDRS
GGGGGAGSRRRILGQLLGVLGPDGLVSGTGGLAATHQVVQRQQQQQQREREEMQPELEAKQTQLQGALDGWNGWRR
RKLQHLLRTNRSEAAEAAEMEKGSNRGSGDGNAGTVVLTEEDLSPYNSVWPMEALAYRAHLTALRLCSELLLPPPPP
VQQQQHAHDLAVAREMAAAETEPRLLLPPPAARGGSAGACAVITDHVGWVAAADIGALERALRREGVSSAAAVVAAA
AATSASPPPMKSGPSRPQRPIFAGRDMVEINATLSALVDSFDGRSGTLTATSIVSASVDVTAAAAETETAVPMRRES
LLGVVPWGNKYLNAVKSSVDGGGGGGDAMACTGVYVSREEWLSDLADGLTELRRRRVPTAAKTAGDVDSEAKDTTDA
VVLSVRDDDASDGDGGGSGGPGGGVLGIGWDVLALPSACAGWTVSGHVDANANADAGADASSCADTVAAVDGNLHNG
GGDGGGTHHNDNQNGMSNIGTSSRSSSVSTGRSEIPRRRRRGRLVPPPRPSSGVAGCILAFSAEAAAALGLLAARTY
PYNATDAVVDITSHGANLGVAAPSLPIVPGDLSGTPASGASNCTAVASAAASGPVTAASAVAASQPGNASCISPQPP
PRHSYTLLPVINTPLFAKLSLLARQGVLSLAISSPPPPRSPATPQRPEADGSDTASATAAAAPPPPPRQRRRFLLYT
CAGDQGIWRMWANPNRNYDLLVAYYGVLDLDDPRVRGGPTGGVGVTDTRVGTRCTDSTGSKVCGGIPGASSRLATDP
WVAGASGGRPDYVYRAHGSKHQNLYKLHSLHPGLLASYDAVAVWDDDLEATAAHVSGMFERFSELSGPPGHTDGIWL
AQPSLRYGSKVDHALLVNQPMLRLSYTSFVENNACVFRSDKLREVLESPLYSGELMGWGVDYAYLAVLGPRLRNRFA
VLHDFEVLNPPSEAKAEGREILKLASQEDRIRQWESYKAKFGIEVEPEVVYECIPALPDLPPWQYPTYCPEYQPQPP
LPVRPGVNRQASKQVSITVQRSGVRIGDGRSGR

>Vocar20001343m|PACid:23130709
MQGAGPPGGHNPFGPASQGAYPAYNVNTTAQFADEVMPAAPQGPPPSASFAPVQQGAWGPPAPPGAGYGPQHVALQF
TDTTAPQYVAGNIQRQEERQILDGGPGGSASASTPKEDYTKYPFYNVRRYREYFDVDTKDVLWRVGNSMIGAFRPNF
MEVTMKNPDLYGPFWTATTLIFVTAVVGNFVDYIEWRRGTGTSSPPPPSPLMSSSPPPTALSNGAGRLLLSSSDTDF
TKNQWFTDYTKLATSAAIFYGYIFIVGLVLFFIVKWFKGELRLANVFCIYGYCMTIYIPVSIACIVPYDWARWVMVM
TATALSAGFLFMNFRATIYSAAPARAVLVLLLIVLAHIALGLGLKLYFFQY

>Vocar20003023m|PACid:23130762
MDGTKVGSNVGWLVGRLRRLRRWRTFDLRGGQGGAQPSRQGPRFNSITEDVDRTAWANRLCVHFATCVWASKKQSRR
PRIQAMGTRQTLLPCLYLSSRTSRLTSRNSRARYRHVVMVPAVAADSPCPDKYGAGPGQPATAARGHGQQQPRKCSF
CNKPITLIGPMRPGDNLTCQQCRQVAQTGQAASMHMQAPTLKHQPLRGLSTAGPSFNRLANPRPMQQSSFYGSGDGG
NTGGRGLDLRAPIVSAAVVPAAAAAAQEEQQAASRTRRGQQLLLLQQQQQQQQQQQQQQQQPVFRRSVSDAADEEP
SQLGRRTAGAAKARGRRSAPVEPEDKMQVGVLLAQGRPLKASYGSCVPIGLTWWTAAMTTVVLTRMLPSASRPVARQ
APKNKFDKLSAQVAGLKCCFPKEGGKHSVQVFAEDLARLDSGEFLNDTCIDFYLKYIEAHLQTEIRRRYHFFNSFFL
KKLQEKPAKGVKLSKAERLKLDHERVKKWTKHVDLFSKDFIFVPIHGTLHWSLVLICHPGNVVQQADHLRPPEGGPE
GSRDEGGGAGTPLLLHLDSLDGNHAPKAIFEALRSYLEHEWRRNMEDETQDSVPRRWKARFLAAGRDVPEVRFTLQT
LPGLSMAARLPKQDNHTDCGLFLLSYVDFFVAANPRCIVSEGSNAQNVHALDPRSDAANAATLLQKNWFHKCNAARL
RDHLRALVCKLMLECVPAEDQRFKTLECVVNSYAEKPHEMGQRYLQPEEYLKYVTMRPNEPWPILDDGVLSNESDHE
GHMEDPVVVLQNPTRHMTRLAAKQAGVAPAAALPTSGNRRRKGSAASEAAPAPPSQPPGPATNPESVVDLSTPDKVG
DVPRATAAVMAGPTEQAPAGGSTAAAAGPQAGEAPPIARAKAESMEIEDEEHEDDEGVELVAVSHGPASGGEARSDD
QPAAKRPRLLAEAVPETLAQVPATTGRGAAAKVEVEVEDLGASSSDEDKDSPPRRPRSRWSTGRGCKQGDLHGAPPS
GSVDDVGADGQKVAGQDVPPSQEDPEALPKHGAAAAAIAAQPVAAAPPGGVRGELPCPDSGAPDEGGGASGGSRTKK
FCRKVKRGPEAPGGTAPPPGGYRRGLQTEDTGVASHAQVEQPPINGLVLGDSEDIGEDDINQDPAWLLEHQARFNLS
AGGGGGGGGGSGGHESSSGSEDERGALQSVADRGREPGGRVKPHPPDPQQKPQRAGCGPVADAAGAAGPPARGQGAT
LGGPCRKHTRFPDDGAEPITKMVPIRPEEAPPPSPPPSPPPPRPHTGAAQQPQPQPQLEAYSIEDSDSDNNPDRAKR
RRCGEEDAAMSAYPPAMESSAHWQVSVVGVGDSQAAIDAAVDGALQGTPQGRCTLPVTHQQQQEQPMSNGQLSEQVV
ASNRDVPTPGIATQAHQPIAEHIAPGPAVATPFTACRKPALMVSRFAQKHLSRPRAAHAAAAAAAVTIAGAAAITGA

GAAATATRAAASADVKGAAAATATTHTHTHAHTARQPLEVVAASASAARPLDVSGAREHVDAKAISRRDVDDDDDEE
EEVEEVEDGPYADDLSANLDYPPTRGAHASTSMRWSAQESPPLSSKRLTAHGTQVPDLSRPANGSQAHGAQGSHQQQ
PPPPPPPPQQQPHDHVYQRQHQHHTGDPHVPQTHQAGPVGSLARMIHALPALMGEEGVGSGSQGFSHGGDVGTTSHG
AASSALPQPSGSAVSHQRESFVDTQLCGWNPQSLPPFLPPQLQQQWQQQRVLPMGLLKPVYPNTRTARTAVADLPAA
ASHRHKDLNGGVRNQASEAPSWAGDESQGRTTDGHAVSVHRPASFGGGAEQHLQGWADTPRPPQAAKAGVNRIRQEA
DKVKKRWRTSELGRGTQPLPGGVPWHLPGPELETVAVTKGPLPTATQPAPAALYAGTSCTCQPQEAGPSRGDPHTCV
AAGPSPTGGDRKRRRRSELDSLDSQQQLPPERGDAGAGPGQPHAEGCPHNQPKWQSQTELQQQGGGERASVASYATA
TAATTSPKGSQPQPQPQPEQLGEESPTLSPQLVLVLDCGEGSQEQQQLQQDQAFRLVEYGGSLTADDAAGASQSLGG
EALSPGGIPGTPSDILTERDGTGTASPAQQTQMQTQTQTQAAASTRGALHATGPLVAEDAFRSLRWREVANACTMLA
VSEAAAIGRVLFPRPDSAVQTPGAPTGPTAGPTAGAAAAAAQGQGPTTASAVGPGGPSSSCRPAAGAMQCVSRLDLT
EKGAFERGDPPAAAGRQGAQGAGAAQGLIRAEVGAAEAAWQAAAREVAGRPFADVAAAAAATCGSIGRGRAVEAMPS
DSLLFSPGSGGEASGLTISIGDDMEENQAAREEPCKDEQVSGGGDGGGQPHLRAQAQGEGGSSGQGVADGGRPAGRG
TENESAGLEGSPEEEDSVDIVEEWRPGDATEEDSSDAFVDGRGTGSGRVGSVSAMKHKGPGRGKQAAGRQPTEGSRR
RRSESATGRSKSRKAGQAGGSSKPSSSTKGVTLSRGGAAAASRGGGKPQNQRGSLAIDALTGPMDAYYGRAEHARRN
PKSTDAGPPGTGQSRDEPCDLTSE

>Vocar20003050m|PACid:23130927
MIQDILSGMHVYKIRRLHLDYLMHHPRQQNKLSDQPNELRHMSRTWLLLICGILLCPISSCSMLSNVATVAHDDRPQ
NLPPNDQPLHGEIPALSSLAQNPDGLRWLLAAAEAALRDSSSCRGDGDTLCKQGEAVVTQLRQMADTLLPQKQQQGQ
DSSAAQPVSGQDDGDPLLVGFLRGPDHGEGQDLRRVLNGNGGGSAEHAGNASSGRRPRRSQSRGRPRSSSGGAGNGS
AAVDVDLDALLSDEADIERDAAFAAQQWQLWLDAQDDWVADDPTSPPQSPPQPQQQQQQAGRSPQALNPDRGADGEG
QDRGGAVVDSSGKRQLVETTAGADELWDTRGDGGLGVTSGTLPGGAGASAPLPSEEIKEGTSHGGDGSLLAKGSRRP
LKHSPLHRQHRQQSPPPSPSPPSPRPPPSPSPPLPSPPSPRPSSPSPLPPPQRAPSPSSPSPRPPPPSPSPPPQRQP
PFPRPSPPLPLQRPPPSSKRSPPKAITAPPPPPPPPPPPPDFVGYLPDFPNSPTAPLAPPPFTGRRSPSPLPPPPSP
PLPPSPPVQFTPSEEGPLDQVGVYYLVGISFAVAVHLVHIPGTTRYLFMERPSGKHPDKKTFIAGYYDIATNKYTNV
PNTDSLFCNGATIMANGNIAVVGGHIAKSGYLDGLRSLRIYDRQEGTLLTVASMRYPRWYPSANLLPDGRILIMGGT
QSPGSGTKNNPVCEVWDPQNAPLAPTVQWKLPDTFVAKAGDIFYPNNYILPTGDMFVYCDTAGLVLNPYDGTVITSI
PSHTAVAKTVRLEYPFSSCSVMLPLTPQNNYTAEFVFFGGQFGYGWTNTPAVDLALRLQVNYDSTVRNYTFGAWQLE
KMNARRVMGDAVLLPNGCVVVLNGAQNGVAGDSATGGSSKAHFPQFNPVLYDPYAPNGTRFTRLAHSQIPRMYHSTA
ALTPDGTIIVAGCDRCDYFNVTVPYSPSPWGLPEYRVEIFYPPFYFWPTRPVLLSAPAAITYKQAFNAVYDTTAAKV
DIDGAVLMAPSSTTHSTNFNQRAVGLAIVGDNGRGTLRLRGPPSKYVAPPGHYMLFLLSGQAYSSAVWLQVTSSLVV
A

>Vocar20003024m|PACid:23131045
MSYAAPVYDPSDGLLQQGDILSYDCCPPAPLACRSTSKEGSYNTSLTRSQEGRRQRRARRKASFESPRWSPSEPAAS
RSEWLQGAKEQRTEVLLAGLLDPDSIPLARLPVLLTLVEGRSDLEDCVIAAGLPAVAAAVLRSDMLLRLLPGGTFLE
LLMDLGLVALQWWLEWRGDGVELTGGADFGGLDGFGLAGAAKAAALGAAASSAQGTKEKELEGVRAAVAALLHSVDI
GAAAEPSTLLIVLDAAVQYGVDVPSYFVQAAARMAAASAVLSPPSSSSHSSSSSVHSAASDPGQLAARLVAHAFSST
TTTTTTTTITTAASTGGADAGAGASGGSSTYLTAAEVRGLMTPYLAPHRGEAHHVAFLSALMTALRQLLKQRRGIAT
AAAAAGVTPREAAIEAADGLVEALAAAAGHLGPAPLALAMECAADAAALLAEAPLADAAALGSGAAGGDAVRRLLAA
AAALADQRQPSAAAAAGGGCPPDGLCRLAAAAVRLQRRGVFGSAAAADVSKLLAAVQATSLQEVSIDAAAALVAVAA
RAAFTSTVAEGTAAASGEEGVLSEAASEGRDGEVTIALGLPEPLLEALLRRLASEAEAEAAPATATTSRTVVRRLGD
RISPADSPPPQLTPPPPAPSLSYDQAMGVLAAVVAAAEAAAEATPSTSSSSAAAPAVPCIPAERAAMLRAAGIVACR
ALTPAVRELDSARAIRLLKLIGRAANFGVRPDFPPLLWALHERLRALAGASALDPPDVLDVLSGCVSLRWRPALLLR
ELLLPLLRWLQHAEDAAAAAAAAAGGAAAAAPRVEESAAAPGARVGVGMEETAEGEGEGGRGAGPLGARSWTPRELK
VCLALLGKLRYSGPIAAAVVKLGVGQLLRAAEAEAAAAGGGSGSGGGGSLAAQGAELSASDLAMLLWVCVAMRYRGA
TVLRPLLQLLLLAPPSTVPVKAAAQAVWAAGEDKLEAAAPQSLALLCWGLAKLGVRPPRAFVNAAMAASRRQLPHFK
AQELATLLFVLATWGGKLKGDDDAVSVVQHVVATRARYDGPALCTTVWALQQLTQPASALQTGAGGTPVTDDDDHDH
EHDHATAAAAAAAALSATALRALENRLLAVDIAAGPRIGFPDHHLLRFCSAAAAAGYQPKRLLNRYTTRLRLRLRTM
RGLSPDVAPTEAERRSRMLWSAARIMATFDLRDSELLTLLELCAERCQSLLRPGHLAGLVNAMAALDHYPAHWARHG
LLRRMGLALRRSTLTEAGLIMEAIASWRPYMTAAVVAGPSPPQGGGESAGEGQAAEAGNGPATTAAAAMRGPGRRGP
TPQRVDLLRRVALARLTQLCTSSPPPPSQQPPTAAVSSAGTTATTATAPPYSPAAAAATVAAAAPAPASAASPQATP
RSGGVAAAAGISLDDAMRLLTALARLRWQCPPVEALLVRVASRLPLERRSRVPQLTTLMWALASLRQDTPELLEDLQ
AALLGLPPQPSLASEMSLLESGRAASVRVAHSSSGSGRDVAANAAAATDLQLLSRPAKPVLPAAAAEPAAAAASQGA
AGFDPAAAMVAAAAEANQRLEQQLDGPAGRFQLPSEPWAPADIFKTLWACAKLNRHPGPLILAAAERSWLQYTAGGG
PTAAAAAAAPSSSSSPPPLHTVTGLLWSLSVFRHHNGSFAQRLSQQLGAWLREGGTLSTASAGASAAHAATGAATRA

VAACLLAAQADRVDSPLNAALPPEVRARLVAAWRYQAELVSVLRKMGLTAAANVATPDGCALVDVAVALRQPAPPAA
AAGGSPASPLPPLPPPRLLALELIGRHNTAANSPRILGEAVIKYRLLQDGGKSGREAISRVVFGSGPEWAL

>Vocar20002956m|PACid:23131213
MEAPYPAQLVGWVVWGKVKSLPWWPGQVMHPSKGPPEVRKMAAMKSNAAKLLVMFLGDNKYAFLPWESVVDFSEHKE
RHSSTNYASKYWKLGVEEAEELLARRAGLQPSTDHQPVDFATKKTNFSNLAKAWVGGSGSTGRQQCSSGAAGRTTER
NKASTAQLEASKIGAPSATTPPDAKAAAHATKSQHRAGGTATDDGRPKSIGGVMLEWARYMPQVAATRKSAELSSSD
ATIRFARRLARGEELSEVNPDDQLALQRLHSFALQQRLRGVDALVRPATGLTAANPDDGSKPGAVITRQQQQAGDVA
CFGSSAGAGGDLKDADSASSGPLSPRGPPRHAASGLVATTQPVVVQETFTISDAAAVPVPARVVEMLLKSLTGPAAV
TETAAPGGTATCPSPRARRAAAGSGAGGGGGSPSPDEARTTGTDTGAAGVEVKPTGTADGDDGDGDNGGKAADAQDA
AAAAERAGSPAAAGGADGGLQPAAVVSLQGGGAAVADSAEEETILEVDVALRVNGKLLPYLSVGEQLLLRTRRSSSS
SSSPRPGTQQELGGGSGDGGSTPQRPVALSSIAKRPEQQRHGQQRQSGQSAALMSMGDAAEIPTPAPAAEITVAAAA
AAAAAAFVKEVDGMPAAAAAAATREMSPSDGPHPGARPHEGGRQAPTQPLGQQQQEQQQEKQGVAETVPAACAPAGA
DADNAAPMTEAPSAAAAAAVAANPAAVQGTPGAPARAEDQAALCPSPVPVSPPPTAAAPNSPPAAAAAAAISNAAAT
IDLSRSLAPWLRGEVRVTCWTVCAPRLLLVAVSADRKRDGGLGGIKREARLLLAEAKERRAASPGGPDDDLAEGAGG
EQQLKRRLRSATSPPAPAAVQALGQSPASGSAGGTGRKSSPPPPPLLHTGTAAAAVVHQGKAQQQQHQAEAAKAAGG
LPQISATGPAAAALMRSRLLQQKRQASAAEAAAVAATAGAGSGNGGAGTGGTQQPGSDASKSASSAGGGGGTDTRPS
KKVKLVRFPGPPVGGNVAAPCSASRVSSGGLPAAAAAAAAAATGAAAAPAGGASAQTDGSGAPAARRRVSYSQLMLN
SGGDALDDSQRYGGGRGTGASATGNAGYSCSSYDADWELSYPPWLPQPAQRGAFITVSHLLLNKNRPTQNHHSHHSH
QFQTHKADHVPGNYHRNHQHNAPQQVVLQGARGNAATTAAAAAGGQLSHSTFRQPNYNHSYNQHHHHHSHHHHHHHH
HHDGQHTIHPIASPRNNGGGGGGRAGSRRSSGHDAWQRDETEELPYPERRMDGWSSDAGTALDIEYGTGAASAAAWL
STSAYGVAQAPTSGTVPPPPPPPPPPPLGGGGSVTDASVSTAPAGFGACEQILLDIRVGDETDAEGPTDPQRRLSGG
GAPGDAAIAVTIHLPPRDTAATAVPPPGAPPPPLMVGASYGSVTAGYLPLPHITSYVDLAVAAAILADRHMYVCESA
ALKPNGAAETEAQQPQSGATAAGEGATVGAAAEGVSGGGAVAAAAARPTLCEGEVVRLRQECDTLREQVKLRLAPGS
LDPLPQQQLPYAELVAQVSERILALALQLPSLSGPTKQTFIQYSRTLMQEAAACSQETTVMATMATSAPPQAAAAAA
TAVFPADDGVTPAVPAAASSASSVSSGRSLAVARVAADLVRSAAAPGLPLHPEDRWAFINLAAELLRP

>Vocar20002824m|PACid:23131224
MWVRPPSRKTSSMKPPRRVWLWASPGRPSAAPPCTWRRPGWRGARPRAPSPPPGSWGTSFGSPPPSPTPSRATSYPR
ETPPPPTPSSAARPCTCTCPPAPPPRTAPRRAARW

>Vocar20002796m|PACid:23131240
MQHDGHAHEANAPHLHGEGDAHKMLKGYDDLPSSTSALLQPLLNSGRHADDISSQKRALASASGSSATNNSVEESSS
YEPTLEIRRPSSGGGLHSDGDDGGSSGDGGPLAEGTATTATSNNSSSSSSSSALSPPSPPPRLYVPQFDPHTHQQLV
CSYDRVTAPEPAIGCGPNRVEVEFQALIVGGRGEAGGGLSAPPPPAASDNDNDGGGGGGGGSGGGGGGSTPPAAADV
PLRMADPQPASTTCKCAAATAAAAAGDGDGGGGGGCAAGLVPLIATIVPLQNAVSSTTASDNRNDGPATFSRANVT
LWPSGSYGSSDGGGGEPAPFVRCVKPPLYDKFSRRFVFSACWRYACLPAALTRRPYELMLGPVSYTAAVTPLPAGRY
RMTTSLPYGVGMSYEYTTAVGGPWTTVTANRSDGGGGLVVNLPYLGAGQMRIRIQIPFDGNVDDGGAPPPPPMPRWW
WFRSFGCPAAGVSTVSELGEIIRRGIEDADAAATAAAATASPYSQGNVSASVALVGFRLTMPIQFKDVPGVPDCSDE
SLAELRAAAAAAAAAAAATAMTPSGNDSSTTAVTLLSVGCSFVASRRRHHHRRRLQQLQQQETCTAHVNTPVSVDTP
VLDLDAFISSSTASLSYMYGGASCLLDPTSADIDVTSKLTVSVSMPAARVAMASSATATADGGGADASASASACTAG
AQVLASSLGLSDGEVMVVGCQLSAEAPPSAVPQSGRQGASPPPGSNSSGGGGGSSGSSANISRHGSKANGAAIVAVA
VSVAAVAAVAAAVLLGVALVRKRRRQNRSARFISSSEATTGRGERAFDNISSTPFFTNDAMKAKTEDIAQFSRVAAA
PLPPPPPPPAMRAALGEAIRGPEGRCGTSGAMMPSSSKSLVSYRIRACSSTYGSGAALGRAVSGSGAAGGMGAEGSG
GDRWTASPPAASAASASAAAASPRCNTYSSGDGGGAGAGAEVGGGTPWSPEQIVRRRGAFHWGHVNQPGCTNGSDDG
LGGAAAAAPAVGDMVCCTLGDAAVQGTSDRGSVARSSSSRRASGGADGGGGGSAAGGWYDLFGLDEAMAVGGSDFSI
SNAGNLPLEFFPPPPSSYDSCGDRAVGGAPRLVGQQQTSAKDLYPGQRQRLTAPAGAVATAVAVASTPPPPPPFSPR
SSTPALPQLHLRFALPGMDWYGVRDNTPCDAEQIGGRGSGGGGDGGDGPGDLPATNDGGEEGDLLRVQSRRPAFLDG
TGGTLAAAVAATVRSSCSGSGGGGGSSGGTTSRSTGCPRELSTRVEPRRGGFIHRNSVAPYTGHQPSFTSYIRNRNC
ACSATEAIGGGGGGGGACFEAWSPSSMSPLNSPLRGAVAGEICTTLAGVIGDRRRSSGYEGFGGGSGSSSAAANAAA
ASDRMRSAAGAKTDASDDEQDASTVRTTAELPLAPPPSPSAAEMAPQWMGGLSRRARPSPVRHSADGECAARSACVP
PPAAVAAVPAGDGGAAAPDVAASATVAAGASAVPPNLDPNSDSVKYNVRQPPYVQPSGTLDSNLKISGPAAPRSGSG
DDDAVQDSADAGGGNDDAVQHGYDSGGGGRRPAATQPASPRPLTGLSISRGVGLAYCTAPAAPLHDSSSGSNSPYSH
NSTNGSGPNGYVGSGGGGGGGRRRVEELSPAFGPSPAMRARQLVRRSSSCGSSDGAVPYGMGGLVDPYVEANAGSGA
ARLAMMKRLAVGPPRPAWGLVHAGVAPMKRSIASVQYVKSLNQAEASGAAAALAAPPSAAVMAAAAAAAGGGCCGGR
HSSVPGRLFLSGSSSPGGDGEGSSRASSETSVPFLRLVV

>Vocar20002483m|PACid:23131285
MEVDMHSDPAMWRAKIGKLQSLIRRLRCVVIHVNTAISDEMLFQNIALVQQLKGVSELKLHMREGCDLPPAAAAAIA
AMPYLTSLSIVGGGLKSDAAATLLSGLAATAAAAAACGGGGGGLRSLTLLPAAGIDIYVVFAVFVGKLWVFQTGQYV
CRDVIYGHSRLTSSGLATLSLGLPRLTRLAITSLDSDSGYELACLERLAQLAELDLALDNAPTGAASRRALCALRPG
RSLSLSFRWTDKAQGLLAEYGNRLANLTHLDVGACRVGSESLFEAVAQLTGLRELRMAVYSNADHCIPCHLSRLSAL
GALQRFHLEKRRAYFPEARRFGESSIAVPVTAEGLRSLAGRWSRLRSLRLALSRKDYSPEALSVLSSFTGLRELGIV
VERYAGCSGSSSSSSNSQHHQQEQQQHQEQQQQPQQYPILGSSPGGSSGLGFGRLYRRAVELPPLLDLSCLPSGLQS
LELSNLLLDFTEPAAATTSAALPPSPPPLPPPPPLSQLSSLDVDGCVVRPPVLAQVARRCGSSLRSLRLVGLVGLCN
GVLAECLPVLTMLRSLTVSAPGNRAVSQASLAAMSSPPPPAGDAPPPFPSPPPPPPAVMAVGSPTTAASWRLPQQQQ
PPPPLPSPLSPPPPPPAPPRRSLLPLLRHLTWESDDLTARGPDLDALAPLTSLRHLYLSCTDKTAERALGSLRALQD
ALPYCHVDMVWTAARPTGDVP

>Vocar20002730m|PACid:23131321
MFTGEGSSMDDYQCKLQLQDASDGEPRVIALVATRDRFDLLWSRCMESILAQTLLPQLIVLVDDSEEISGELDSGKL
EEPYDCSIDGEYMHILGVLQQALLARSHGKYVPNPEVRGKNESMANLFNSRLGGRDGGGGEGSSSSSRPAAPPIVYL
HNGETPGASGAWNTGIMFIAEAVACGAYFGPAAGRDPRGGTECECDSGDGGIEPDQETPLSRRGWPMVDATTAAALA
MYGDSCRTSLPNNRYNLGGTAVVPPSGGRMLYDKLHAVWVAICDDDDAWAENHLQLCFEAAAQAPSDMVVSGLIRVE
GDDTDEHRPPPPPPLTTTGPAPAPAADAAGAAAAAPRQEHHQPQQQHSTSGRGGILERPLPLPPAGPLEPSWFLRGN
PGIQGSNLFLRLGVLLRAGLFDEWLPSTTDRWLRCRAGDVLLGGLAVVILENGAGDGGGGLAAAATAYVARGLQVSY
FSREAQQAVCRQVLQEVAGLTKEKSCTSRDGNGRSSSGAAAVAAAGDCSSSSSSIMPPAPPPSLSLVLPLDLAEVVA
AAAPGTPLPIGVTRTLLQHLVFYFVQMEVPEGVRDTAVAWILDDDKRLPRLRRPSEPRRPSPWGHGPLSLGPLAAGG
PSNPVTGRARARQEQPQRQPQQGQEGERISTSVCEDRPGRRGGGAGSGGSSDAVMVGATDWAKAGEGDDIDVGFLEV
LQRLRGAADPGATLPRPLPAPPDVVLGVDIGSPPLPAASCARLALLDLVTALRRQLHQQQEDQKQRQGRDQQEDGT
RCQAAAAAAPATGATAAPGTAVAVGTGTGTGDVSKMMTAVGGGGTEEALAALQTAARPPSPSSPWANEPMGPFDVLR
AACAILERLYSPENLDVLMRQLVLRDQRRDADTVRRILMQTAAPPLATVSDQLDRLQVLGHGQEGVVLSGCKPGDVI
FKYIFHVASRVSRGARRWGDGGGSGEERRAFLRTLPNRRPRPTACLYDIYDWVDDGPSALAVYGPYEDGGVLLSRQV
RLEGTAHVRKGKISREANDCFIMGICQPPAFATCPFLAAVASITSDAGGDGAGHMCPGGLLIMLARECIAAGVVLGN
VHPSNMVLLLLPSPPPPPPVPAATPPRRRRHQHAAKETEGKEEDAASADDDGSAAKRDTPPNDGCGRTAGDDREGGC
GGGGGGGDGKGGKLLAVVGMIRKLYLCWRWWRREDLRELLRASLTLPSERTDSAADPPCKALAHLHHYDDDVNSVPE
LRGFRRFRDAVYGAYDTAEAVLLPELLRARHASEGGLAVLDYGAGSGRLLAALHGELPGEVALAGCDPGVERPRHWQ
PQVAVAEGRRAAVWTTDCAQAVAMQEYDVVVCSLVLCVLEDEDEYRTALKNLASALRPPPPSPPAPLRRPGRVGMPT
VTAPAEAKSGGDNNVNDDGDDDGADEGGGVLILAVCNPFFTSHGSTVMQHRFPATKSGNDDISSSTGASRRGSGRGS
GDGGDAGEGGGTGDGGDAGERGGTADGAGDRDGGRAGDSGGAGDGGGSGSAYRYLQRYAWTKQLTMWPRAASRKLTE
DGGGGGICTATVTNAGDQLRHGGGGDFNVETSSRSRKARKVHSPGADGADGAGGSTLAASGAEGKVALAGEARPAVR
RRTDVHRPLEVLLLDLRRLGLELLSVRQTASLDPDLLDEPASDFLLISLRRRVAKPTSGPATAPPSSLGHGSAPEAP
LPTVMPPVPPAPPLPSQGPQAASGRWLRVGPTSPGPGGVAGRPRVALLIKACAMDHERIEQQVLHLTSHLADPELPF
TSVVLLVDSFPGPYLRQYARPDPAALAARLETLRRLGVLDDVIWAQPAGSPENCSLTHSAEGASVTPVLQAVQQLAS
SEHHTTCEYALQVDCDLVVVRGAAVMCPSDAAAAANSSGSYGAGSYLRRMVEVMESDPAAVTAALDPVPTSPQPLPR
PWRPSQPSGSGWSGSDGLGDDTSGGGGGSGAPAITFCGPTGPWRAEVRGCLLHLPRLLGLLPLPSLQEGMGLSPGPP
APTASGTRQDHVGAPLKDAAFATAATPNAAMRSAGQPCQGSSPPAGKAAGACGRAAAAKAVQATDADVGDGADVLPE
AGALAAVSPPKWHRALDACIVASEGRIRSYRGACGGRIAVLHPPNIPIKLPSPPPWCPAWYGKYPPRQRGNMDVVGG
VDDWAAALGRLEEVVVVVCGRNVAPGKFGRCLESLADQDIDPRRVGVVVVDDSSDAGACGPYMEACCRHLMPSRWRS
QITFIRMHRRRRMLANIDFVVRHIVRHPDAVIVTLDADDCLLRRDALSAVYRMHFDKGHDLVIGGQLRTDRTASPAA
SAFGTAHVVFGGPPRQRALRGGGDVWRHLRSFRARLYNRIRTEDLLDPVTGWFREPANDWAFMVPIAEMATRPAVLP
LPVYLYEPSQQECPSLERRAKREAAIGAIMALPPYCPRHGPFCSAAAEGTRGERRLGGDGGEATACPLVLGLRPLVA
VVGDANLEVERVGDREEKKHLAFEVGRLLVDSGCRVLTGGEGGVMEWASKGARASARNRFVDVPMATGLGEARNATI
GTAADAVIAIGGGPGTVSELCFAWMAQALAVVVLSSVQGLSAALAGRALDGRVPARRVVKDACSAAEAVELVVQTLH
RAGFHVEVQNHYNNA

>Vocar20002646m|PACid:23131337
MLIRKARAAGQRLRKTSWLLAAVCIGAFSAVSVATTPGLDKWCAFSPGHQTSSACLRTTACQTVDEWWSSPDHCIIP
GVPGVFPYSISVNDVCGGLEGITSLPLADAAGRQLGTAVLFRSNDVPLLHLTVVLDALDDAVVLYRPGEMPGDTASL
LLSPSLHTHTGSLRGQYSLLVRQPAPAAAVSMAAAAAGTADPAAATSYSCFTWSLDLRSVCDPRTSHQVGLEDLIGG
GGAHPCACRDGMEGFSCPPLDLSRLAMLYFSLRLNVTLTQPHGAGCGLEEGRSVTITTRFPSTAVDGPAAAAAVVAA
APAAPSGGPIRSGCWEPSAPPALEAVAVTTLARRQRRLTHDGVPTYPPPPAAAATPAVTVTEAVIIVRSPLTALVQA
VQCSQAVLQLYPLYVTTAESFSCSLQNRPQGAGTELAFRLTFPSSTGLTGFNGSMANSRSWEGLLGAMAVGCGSLGT
YSDTAGESYSVCGDAAQAANCRLNDVILLPGASAVVPFSCDMGKDRNSVSVTGVLASSADVAAFLANANITPNVETL

ASFFNLSCGDSLVVDASTCVGPTGRIVYTGQGFPANVCRRRPPSPPSPPPQPSPNPPSPSPPPPRTWIYPPYPPYSP
DPPDPLYPIDSPPPVSTAPLAPSALSPPLLKEPSSPSASPPPSQPVQLLQATVSMSAATTTTTMATFNDKSSSHPPT
YLAVGCQMCSAFQMFAAAADAEKRVGTAMGLKKEGPKKDLLLHQLHQGVREAADLHPGDECEDTVVMAEEVHTTHMA
SHSRDVFRAVGKLDANT

>Vocar20002617m|PACid:23131359
MAMVSLRAPLSWKPGIQLDHRESGDVEASVGRAGLLLARRRCNVARCAVHRRTRRIRANSRPHGGVSRAVSAFGAYG
ASTDAGDLTEVMDFSNNPLSQACRRLRLESADELQDLIASLILSECVYKKLELDEAGVVAKVTEFVSSFPPGWVELQ
AVQLSLSGIPQHYLIATSPTSMYVAFMGTKQFQDILADANLLHTPVWAESARLAADRQSIPAAHRGFLERARAIHVE
QLYELAVSRGLRLVLCGHSLGGAVAKLCTLRLLRELPDWPRPRVRCIAFATPAVGNAALAELVESAGWAGHFATYYL
PEDQLVRLISFSQARTSPGSSSGGTSSSSGGTGSSSGAAAGSAAAAGVCRRGDTAAGNGSGGRKGSRARAGRHRKGK
EGSGAGAGVSGTGAGVVGGEGMGLLPPPPTRAAAAASAAEPARLDKAALGRLNTAAATAAAATDVQQMQLQPQAQLP
KHLARAPSASSIASSSSSSCASGDTTTSSTDVSSTGPSIRRHRRRDDSLSFGGAVMSLESQNDAYCSSASSSCSPIS
RSVSIGDRTRSSCSSGGGGGGSDAGSLTGYIPLQTRAAAALAAQEHGRSGGGGGGGGVGRMSLVESLDEDQVGVALA
MEEILSPGPDELEAAAAAGVALSGQTPMDLAAAQREALYGNAGGSWGLRLALGRKRVMRRLRRLAARARIPLPKALL
PVSRYHTFGAQWFLTEHGALSPEELENQLRAQREPDQQLQQLQQPVHQQQQQQAMSSVAGPAMATPAAAGATAAAG
GSAPPEAARGFFSFHRMVAYRQRYTDLISRLMRDDAEGVVGVAELKRPQLPAVTAAAAAATATAADDLVQLQQGGVP
QGGNAATANMSTAAVAVADAMSCAPPHSLAAPYTCTVQLCDSLLPRISVQRAVLQGVLPESRVVSAAAAAASETAPA
EAFQTRAHAKSHHQHQGDSLTRLSPGEEGQQSSALAAKRRAGSYPAGGDISSSNSSGGGGGGGAPAGRSGDAAARPP
APVAPVVGHLLRWVWGSGGGGAGVYRSPGSGVGRARGSPDELVRLDLELEGHNLQYCRKVSVMLVRDGVVGTAPVAC
AGNVNADASASTNAASQAGPGAGAAAAAAVGGQVQARWVPFPAALGGGSVVAGDGSSIPGATAAGRVVAPRQNQQPG
ALSSAPQPTAAAASQLTAMTAKATADMSPPPLAASSPAVRLPGHTNPGPMAAVPSSSSSATAVPCEVVQVLYAQPYS
DEANPPPLHPVSLFRNTLHHFAGNFMRRCRPQGQGQDQGQVGWGSFASLAATSATPASAPSSAGAGGGSGPLMAPAA
AGMNGHVGPWRRFRFQQQNNRPASNNTAAITEGVAGGGVGGGGTVNAPPGRHTSSSSCVKARSDECASFSMSGRSPG
GFIGGMAATRTDGAAAAAAAVATDAVVAMPSRLLLRVTLPLSVAQGLLDGTLPARLVVSARSDFHALSDVPVAVHRP
RVAILGTSPYAASLVQAALSAPAALAASRLDARHPAGGTAGGGALGASAGLAAWVPPPVTWALSAVFSAVATANVRR
AATAAAGAAAVARAGTIAASQAGTAGSVPSASLPPPPPPAPAPSASAAGAAAPLPSARPGSSLSARTHNTLGTKPGP
AAAGVKAAGQRGKGGFAPWWIRLAAQGRVLRVRTRWGIRQSLAAAAVGAAAASAAGATGAAAAVGAAAVVASTVAAF
HSDGGVSVFANAAVTMAAVVAAAAAAATAFTGSEVAVLVAAAASAAAVAMAAATSSGVGISSEQPSPPPPPPPMTTP
QQQRPAPSKETATAPGKSPPAALLLRAIPHGLPISIPAPRLGPASSRTAAGTASVSAGTSAAASAPGLASVSESRTG
STSQWGPPRWVLRFGAAAQAASGRAQQNRSTGSTKTTSSATQLSAAAAESSRRSGPWFPPPLGGTAAQLRRMATAQP
GTGATASVAVAADVQPTRGRGGGGGGGGGGVAAAAAVSLAQLRSQLLRRLTAGSGRAALPLPPRVLSDGLELCNVMV
EPASRTWGSGRSGSGVAIAANAAGQRHLLPLRRPRGGSGGAPAPPPPAQLELVPAAQLWRGRCYGSSPAGGEAPQSS
TAAAAPAPAPAAPATNSVSAAVAPGVPPASLLLGPGAAAAAAARLAVGGAATAAVAATAALGDMPFFHGGPRGADRS
ELVGVAPFVAVQRMEEGLRRQVLQRGLWWRALLGGVVGALLRPVALVGMLGRLPGWGLQVLRRGHSGAGGPVMSHGS
SATCHHRHHHPLETQPGGERPPSSPAQAGSVAAAPGGGSGASTTATASASPPPRTSSSDWGVLVVVASCRDPLRVLR
SRDMEALRNRATEAACPLVPVLLTCAESPPGRRAAALRTLAAACGVSVSEVRVVRLDAALLHQPAAAIPRADLAAVW
QGRGDVAAFQRPPKAPSNRVLSVLSVGVQGLRAVVVVRPSHNPTADPGQQPHMAP

>Vocar20002925m|PACid:23131402
MSRRPLNFTGADRTGSSGGGGLALIASAYVPPPAADPAATAAAVTTSLGTHASSFKPSALYAQSDTSSDATPGQLGA
TTASTLPTAATAKPPTDVVMPPTVHPSPVGADDPQVAQLGLGRQLQSSNSLQGRPRGSNIASFTNREGGSNSTPLTA
TSAVAGDAVAAATAAGHPGTPFSTSVAAAAAAAMGSETAGSDKARPVPIASSISTPAASASAASCRTNAPRPASSPL
TSPLTSFQIRKAENGGGSGSMFLGARSGSSTIVPAAAAGAATGSCTTLLRTVSERRIQRLLHRIDSMCNGGSASHSA
TSPGFSALHDNELDRDSGGGERSYKWKGLSLPPSPPLPPRLDEQGLQELLKMLPASPEAAGVEPSAGTTVYDGSSVA
AAAAIRFRDGADAADVVVGVKADGGLVDGTAARGGPTALAASWVEREAAIAADPVEAAAALAAAEAALKPGSAAAAE
PRERLLELTLRPLVGNPNGVVSERKGAPSGVTDRLCALVGAGGPDGGDGPALGNAGGGTHAVVYAWAADNGCAAAGS
STDGRSGGGDGGGKAVRQSRRSSSGDDSDSLSDSAERELSVRQLVDRRLAAREGVSTRRPGASMSSLTLAPRALADT
AAAAADGPPTPSASALLTSPPPPLPLVVAMVEAVPAAGSPRVGLRKQGNRCSRKAWSSSSSSGGGGEGSGSDVGGGV
AYRVVAERTQIPHAAAEGRNAGGDGGSDSDGEGAEERDDSVGDRSVPAAAAAEAAKWPNLQPKPPADTGPRRASTQS
HLGHVGGPQPLLAATATATATADGSHGNGTSMAAAEGRRSGAVLMSSPLQARLSPAAGRSTRSRSESSALLREPVVA
GATATATATSGGQIKSAKAPTYRGGGAEDDSTSSDEREFEMRLGGLTPPPAGSSSAAVLQAAASLVAAIDKDLNRST
ASVAAISGTQVRHGNDVMACTTVTAEHGASSAPAVSSPPPTMYPRPPPALPAAAAAAAAAAAAEATLALQRELSGRS
QRRLASLQRSLKHAALSPPPALQQQPDPSWGRRPSTRLLGTASATDDGSGGPSFVSSTGGRSFVARHAAGCHGAAAA
AALGTAAEPPPPPLPAPSSVSRACAGVSVGPSSASSPAVDLYGNGNGSGGSTNGLLAVDSIIDHLDTDDSTAPMCVD
RIKRDLERLLTVKVLAEEGIPVGPRVIVIVVVVLVLVVVPVMAV

>Vocar20000527m|PACid:23131478
MIISFLLYCSRTSELLAVEDFPVQALALDPDVGPDAAAHYSSPFSSSSRFLFSSSPSPPVVRLNTPPYPPIPAAPCI
LRSPPRPPPRPKRHDVGPTGGVGLWAATSASHINMWVVPRGPAHGSVGGADSGRAFSVARRSMGVHANMRNRMSLEQ
NRQPLARIPEATIPGIPALVAHEVLSDKRRVLTRDMGGHVGLWDVLQGTQVASYGKVDFGAKSQELWEPRSVPSWFT
ADTRLGCLCISLEPPSAFMAEEYGVALELGYVADDFKSPPPALSMVPHPRVNYGKLVLDCVFAKWRYKLQAAQHQIP
NPAANPTSPNAPITPTSPASSPGAAAAAVALAAPSGGAAALATAPGGSSWRGRGRGGGARGCWWSAVLLGLVSKLLG
MQGAAGGEACAEIASRYNQGDSKGDIIAICIISCRRAWHALQQHCSFFHPFDGSEAEPAEIPTWVADVVLRNANVTP
KEAKCAFVLLPAEGSDLPPLQQSKLNAPRILQVYKVANYCSGKLQDMDLPLVVRPFYLTGGPHPPDPHHLPLESPPP
KSTSTGKEGEPHVLELTCNGMAVPYEMSLASVKKFIWKKSDDLLFHYRTSALIQPPTDTNHPVLLAALVSSHFPTHP
RKKSERELEIVAVAAVAIRCGSSGLWWGLMVWQVAIGGFASVLGGNGIGDPRFKGGGDDGGGRSAALYTAGCQPFEE
NMVLGYACPPNFPFFRSSPNPSRNVPANPPTSQHQPAKENSLGNRAGVLATLYHYDHQCISEQCPGISKPADVLSYS
LYSFSSLRFSNSRASPSPLGH

>Vocar20000492m|PACid:23131548
MLSPKDSGAEIGEPNPLFDKEQPGSVRAVIGNLSGDVAAAPSPPVEDPNAGQKMGHFKYGTSFSVPNTKETAKDEGN
NSSTRTSGFKAWVSKHPFMFAGLAVLGVLGLLAVIIVPSVCGTIGCKIKKDGAHEDPPGSLRVMVRFTQADATVKQL
LPLLSKYFNSSKFNSSGLEVLQFDSPEMLKQVKKLLQDRYSAILDLLVDDFVMRISPTINGGTRQNSTSPPSTGDQS
SGTTPGGNSTGRRLQVTDKYPYDDTVSDPLETQMWHLEAVSAAQAWAVTRGVKEVIVAVMDTGIDIDHTDLNESIWR
NVKEIPNNGIDDDGNGYIDDYMGYDFAGPCSVDWRSSIPPPAPPLPSPPPVPRPPSPRPPPRRLRSLLQSRSQPLRP
PPTRPLPSPPSPAPSPPVPVDRINQQGRCGADANPRPDASDTGHGTHVAGLVAAVRGNGQGGSGLAPRIKVMALKVA
DPTGAFYASNVMAAYDYALRMGAHVVSNSFGPLRPNFNPNDYDKADDAKKYRLYERAVQPLAQKGVLLVAAAGNENG
NLDDLRKVNMNYLPCTLDLPNVLCVAASSPDGRLWSETQWNQQVGTNYGKMSVDIAAPGSRILSTLPGNRYDLKTGS
SMATPLVSAAAALVLSVLGSSDGNYFQASRVKTILLETGDTRPGLEVGSQRTLNAARAVQSAAALSISGAFLLAPMI
VNVSAVSGALVRAYSEEYYRVSATARDTLQAAPTTSLTPSPALSIAGLTPMAIAARSASSQFRGFKIVGNDVVVAVR
AKVLLTTRGSYTLNLYTTAPLPRVSLTLGQRQVSWNATSTRNMYQAVVSVNTVPGWYEFELLYGNGGNEGIDLNWLR
PGQTAYGPLPEDWIFTASMAPATPLPYVPRDLPSATNSPASEVAVSPGGWQVLWSTMSPPPPDSDIPINSVADPAGA
RVFNPTFLASLSNPLQYFPYSVFVDELNFLNNVTMVTALAGGGVDPRQADARVVSAAASGPVYGIATTYIQSPVDAP
LTVAFRVTCVQCSLYIDGVLFHETIDTSITPGSANPPPVSTRITPCITLDPVRTNNTVRPATRPRHALQVRFAARTR
VPAAGLLVQQAACVPGQALPTTFANVRDIAAPGPWYQAAGASAPSSPGSIQCDMWDYEVIGQAGYMGLGSIPRPEDF
PPAARFRLPNSRASITPCSPFGAFCPARLNFTTRPAVFQSLGYGDTNPYSSQSRAPNTTSLAGNYRQILAVEWHGVG
AWTRLQLVDGSAALPPDGSAVVIDGSFFAPAP

>Vocar20000447m|PACid:23131577
MSAAFLSLVAWSRHLAYSSHDAAFSLLQWFRRVHGFSAALPEVSAKAATAAARRSMPLLQLPTPFESELEEVLAERG
LKPRDARRLGQELAERLKSLSHTPQRGGFGPPSSTEPLPPRGGRLRGPELLAAREALEAGQILLMDERAVNAALGLP
PPLRGDERHGNVLRGVFEGAASEGRAPAPQMQKIAPKYKTEPEILAYVVARLAPEYAVCRRVLHEAVRLLEAAGALP
GAAAVEWRPREVLVHGAGVGAAVVATLEAFATSRSLLRLVALEPSHLRQVLGARVERAYRAKQQQQQHVLSDRDEV
SLTHQQQEQQQAGGGGDGAPRSRAVAGAADSGSEDRAARGRGMSGRQQLPAGPVVSWVQTLPPLTKAAGAQRRRYDL
VIAPYQLTVLPTVEERERLVRELWDRCGDVLVLVEPGTPRGFAALAEARELLLGREGRKRAQLEAAAGATSGGSKSG
SGQHRLPSHGGGGTDPRVAIKLRSAGAHVVAPCPHDGICPMQVVVGSKRHWCHFSQSLQLPAFMQRAMAPPGGKALG
RNRALNVQDERFSYVILRRGPRPNFRVAISRDFDDGGAAGTSNLASSPPPPSRLPPRVVLNQIMLDRAAAAAVARVG
APSAAALAADGGVGPLPGTGIVDVSKRPAVSRRTPLFSASPPPQSSPADDSPQPTAAVPPQPPPPAPTVADSDMHPV
GTLDVTVQPAMPAAAEAVSPPRPRARGGSSFGGAIGAGRVDGGGTGGGGADVGGEAGGGVEWASLGGKGDESSYADA
AEEEAALEGADAGTIESEDDDGGDDSGDDSGDGSSSRRAPGLDAAVAELFMSHLAGPTALAANGGDGGGGGGGGDAV
PAIGQRCRQGGVASADSTRVAPAAAAADRGFGAGAGALRQDLDLDMSLSSPCLHHSGAAASAAFPSYGSQQQQRLTR
ADVSSEEYDRVELQLVDRIYGSAAARVWPTAANRRDRADSTGPGSPGPTGASATRVGSVSADNGLEEEEEEEAAEVE
EEFRQHVTARQADGHHLQQHQQQQEEEVEEEVEEEGSAPSPGPGTTTPATTSGALQAAEASSYVWGRLVRSPRLRGG
HAILDMCVGPQHHYPDEGGDMGGEGANTTGNSSATSTVAAASAAGAVRTTGCVDGGVAVEADLCQPLSSGRPEGRLV
QQVVAKSSRRGWMGAPAYRLARGLTWGDCWPDWYIRGHNTRETTAPPRITASAGRAVGLGWPVLKRLQLAACPSLFS
LCFPFSFFAAHLVVVVVVVVGMGMGREFILG

>Vocar20000342m|PACid:23131621
VPSFVDVCPVTIMTFPSKHSEKFAPRSAAAPTPLLVDRQITTTGIFAVSEADCTCMCVCRPLLKAVSELIAGGGFDG
VTSSQVWANTLYAMGLMDHYDPRVFQALSQALFKDNSNSSGGKSDSSRSPDVNSLTSPPPQSSPPPPQPPSLPSPPS
PQLPPSSSSSSSPSQLLRFATAQTCGNVLFAMGALRHVDEQIMDALLTRMLQIVRGGGRGGGGSGGVSVVDLALAV
RACAHLNYRSSLLDELLEAAMPALKAQAARLMAYNVLWSMLILGILGEVRHQPLVQYLVSCVNRYGTSTWKSAADPS
SPSSPSISSSPPPPSSETAAYDDLTQLVQYHLESRYLGLTGTAYDLTPPQGMDALIRQIRERRQQLAAQSSTVVHQK

VVATLQEMAAAPNCLTAGVRHPRILAVESEVLLEPELVVVDVLLTLEADPDANLNSNSNSSPFEASSPVAAAAAPAV
AATTAAFAGNNNNNKNNNNNNNSSPTDYVPSSPPSPSTSSATSTSSAPIQYKLAVEVDGPHHFMRNRLDHQDGTSAY
RDRVLQRRLGQQAGGGGVAVVRTRTWVEQLPDDEARRRYLAGLLAASVPPRS

>Vocar20000396m|PACid:23131645
TPLMYAAQSGHVAAVRWLLSRGADPWARDRCGKRTALHYSAMRVCGGSGRAGAAWNASARFWMSGKTHRSCTDINVR
IIPLDLDRYIEVGSISGLTPLHYAVAMRQADAVRLLLERGADLRAVNTIGYAYDLVNVPLRSTPLHVAGMLNELPCA
LVMLQYYQHHLAGPNFLDPRRRVDALGRTPHRVAASRGHTGLIAELLHPAANLEAIFSREGGPGGGGAGTAAAAGVP
RLAALAAAVVRQQLVEDVERAEKVILNFRRRQNPPGCPSPEQLVKAQLATPLPDPNPNLNPYLNSGLVITAAADDGV
SARPYTAPAAAAAVAAGLEVEGELAEAEVALWDRQGTGPSLRLKGLACSPPPLQPRPPPPPPPPSEQPTAGEGCCGSE
SSPPPPPPPPPPPPPPQQQLQSSEARVDPQPRPGSRRGSRGDIPQLLLPPLRMTLPAASAATSVVNVSPCLVAEGVRQ
SLRRLSCGPQAPQTPQGRPSPGAVVAGACRTTSCLTTRCPSSTNGRPSLSSSAAPPAPAPDPDGQLLAVLTTTSSTE
PATAAVAASATASATATATGGSSCCALRHPGLFVRRSAGYVESAFSRAPLLPLAQCAVCLEECYSLVVEPCHHRLCG
NCARDVTAAAAEMPLTCPVCRLVVCQFTSPVC

>Vocar20005315m|PACid:23131683
LFHSWRNNMEYEDYSTAMGHGNACPSAAEISRMGWATPATAGDQLDKDELSPAGSPRTFYLPATSLTGNYSFLRVLP
NWLNDTYNSPNGKNLYIAFRVAKNGDEALEAEFASKINVHEVNATMDNGYPKDMYKFTDRKIQFIGATESMSQMVLD
AYKLIVYGGATINTDTMRVHLCRYVTSPVECPALRMLEDIGVKISPPFITPPPSQRPLSPSPPSPLPPSPIPTSPPP
PPSPPPPPSFRPPRSSPPSPRPPRPQPGELTSTPNHVTHTHTYIHVHTYIHTSRQAAGRG

>Vocar20007034m|PACid:23131811
MDRQPPPGSVFRLAPGERQILSSNPRLCILRGHQSLSWRASVPYHRITSVAWALGPTTPCYSLTLAIQPQHRPSSPA
STQHLAGSPRRTAPSQAGEGGMYLSLLPGGPVVTSCLCSTWPPPHIKSTGSSSLCPSVCPSDGDDDDDDDDDGDADA
DAGRTRGTFLRGFVWSLLLAGGPDGGLGASRWRRGGGGGDGSAAAGGAAGDDGGSGAAEPSGGSTESSSSSGHGGSS
GGGSTDGVSGSSTAPASGGSGGGGGSDRGGAVVASPPPRGAEGALPGEGGDVEELPATPPRRPPQLSPPEVPLYGSS
SASAAAAAEGLARPADGGNPVASAGAGGGGGGGGSGGGGGGGGGADRFSGFRERLGDLRELFIGAVEGLWRSLDTWY
TRLAFVVGLMCVIVTAVINAVLVPVVNAHVMPTWEQRAAVILQRDVSLGRIRWVGLGGVTGVVPLIRLGPVTVGPQW
GGAERSHAVLPAVSLYLEPLTSTVYRQAVLRMHLEAPDVTVVQGPNFSWFGYPEDTPDNARNFVPGLGSEEELALRD
GRTTTLSYGPPPPPPPSAELMPHQSRQPHQSPPSQPAREGTATPPPGSGTVRQSTADTVGLRGSGLSNGELANQGPG
RGPTVPPLAALVHSPFPTAPLQPPPSPSLTREAASTGNHAAATISAAGGAGTSSVRPVQQLPQQQQDLMQVMLEAQR
RLQRHVCEQLLQLPVVDWVLQPFTQTIATATAPAEPVSSGGEGVREPVVPSTQQYGITPLSELHAGAAAGRRRAVGG
GSSGQPPPPLASVSGAAGAVRTLLRGGGGGALTRASLHFWDAPDDISSADMDLVLERGRMYLLGAVGRFGAVPITLT
GDMDLNPDTGTYRLQANVPGGGVEVNALRATLGVRPVPIPVAGAVRGVLHCTGPLERPIFSGSAIAVRPSREQLSAG
LEDTPALAALVRAYGRTSSGDSRSSSSTTGSSGTGGSSGSSGVAVAAYDKVPLLGAQAVFTLDMATQMLNLHSVQDV
ELPDGVRLGSASVRGVMSGSHLAPAVDLTTEAPAARIAGSASFGQKAISLSARSPAFSVDGTVHTSLPYLDELRASD
TQAEATYFARPRFLGADLELHCAAPGADLLPLATGPTTPPPFDPLAANQPLHLRLAGHAKLALRPAAAAPAAAPAAA
SLHLATRGGQQPPPPPPQQQRQVVAVVAPAAPPPRVPDGPRVGGPKRFEGTISLSGIRVNSLELVRSLSGSAAIDGE
AGTVAVRARGPGAAGAAAIGTHTGALSSGGAPASTAAAPPAAAGGGSSAGPLAADVLDFELAIPDLMAALNLEAAGL
PPPAAIYTPPATSPLATTTTTATTTTTTSPSINHGSSSRTASARTQRSSTSSSSSAAAAAHHAGSPPPPPPPPPQTG
PPLEGTDSRFLLRRGPLLLEAEVVSGGAEVSLQIANLQLDDLEGGSLRGMLRSARLAADVAGRRGRGSCTAEGLRLS
SLAVGSFGGAVRWEGDIVKLEETVLEQQGSRYEVSGEVFLPGFGAPTTPPPSPPTTTTTTTTTNNNNNNNNNNNNNN
NNNGTAASAAADSPPTTLTKTTGNAPTPPQPSPSQLPALTSTLSTAGRRRGGDGNGTADEVDAVTAAVAVAAAATEE
EEEVAPPDCRWRLQVNVPSAQISDLLPAVQLATSASRPGTDYGAAKARFLDAIQSGAASAISPPDVRLQVDLYSRLP
SILRAAPAATAPKIWRTAATDTSSAGSASSPAAAATATATATGAPPSTALGTAPVVVGGNGAVPALSELSGEWSGGI
QLYGGLSGPSALEFSLAGREWRWGPRYRLDRLVAVGSADAAEGVALEELAVDAGPARLRAAGSLLCPRQEARLALYD
FPLDAFAPLTSTAWPALERAAAAARARGAAQASPLSADGPSPLSPAITHSWLWLWRWLRL

>Vocar20006777m|PACid:23131844
MSEEHALGRVAPTTSHHPEPGTAGDAAREHYEVLTSAYPERSCFIFRKASRLRKTSVLISHNRLFENFVFFVIICNC
VTLGLGSNRQGFDETSLGKIVARIDYVFIAVFTLEMLLKVISMGFVLEKGTYLRDGWNVLDFLVVVVSYVGLVAPQN
LTGLRTIRALRPLRTVKRIKGMRVLVTTIIGSLPMLFDVFVLCTFTFFLFGIIAVQLFAGVLRNRCGSPDLAGAYNV
TTAGIADGGGHGGGGYGGVVVANVSYLVPDDQTEDMCSGPLSSEVEWYSSNDGPKAVPGKPYAGRACDSGMYCTWYG
NPYNGFLSYDNILWSWLTIFQHITLSGWSDVMYLVMDAVNYWVWIFYVGLVIFGAFFMINLALAVLSVNFSTDNLRE
QLKGEEGGQEEGQPGRADTSAPAAAAEPDTAGRDGIGAAAGEKNRNGKDARLDGKEEEEEEEEGDGQLPPLSLYNVM
AKLRMRKMGATDDEDPTIATGSPPPPDLGRLRRLTWRVAVSRGMELTTAALILVNTAVMCVNWYGMPYKVERATDYI
NYGLTGYFAVELALRITAFGWVRFFRQGMNIFDAIVVAVSLIEMIIDLVPSLSGAGPLSVLRALRLLRIFRLARNWR

EFNAIILGMYNSVQASIMLVGLMLLFLFISSLLGRELFGYNVLYDSGRFMFCDYVSGSRPVCPPGHRVWGQCPNHFH
CYLPCAEDQLGAWVEAPGSFYNGLAYCERFCDNAEAAAAEAADASNGNNGSNTTSGSPVSGGAVLGGCEYLAMVGKS
QVPRANFDNVFWAMYSVFQVLTGENWNDMMYDGMRTVSQWASLYFVAVIVIGNYLVFNLFIAILLDNLSVKVEETPP
EDSEARADGTTSTNSTTSSSGQVSIAAPSELPPGSGGGGDGGRGGVTDAAEAAGGRQLDSGRSAAAEGRGRQRTSLS
SRGDGTERDEWAGSPAAAAEPETGTDQQQQQQQGPSPNSSPSLGKALGHQPGAAVRSSSAPQGPPGDGGGRSPPGAA
ATAAANPVGGGGGGGATAVPVAEGRLVTGGKEEDDETEAAEKKETAESAMRVTIDMHAWAFCQPTRQMESPAAALAE
ASEVAPPPGGGAGGGGAASSPRALHGGKGGLKGGKEEAGGSVHTAAKVRWDDSAAALPPPPTSTSDKPKDKEQQQEP
SGLEDGWARVQEDGADNGPMMSGMSHQREISLAAFAGLLEAAGDVSVASWPHPEHPRPRREVSQSRIPRQSSKSVLV
HLKSFSSKLGAKGRAAVGPVWQGAVQGRSLLVFRHNSWLRHGAATLVHNGWFEAVILLLILASCVCLVLDAPSLNPE
SRLALALRILDYIFTGAFTLEAVLKIVTFGFAFTGRHAYIRNGWNVLDLLIVLVGWVFIIVEVIGVGAENVKMLRVL
RTLRALRPLRAVQRFPGLRVVVNALFAVLPAMLNVALVCVFFYLIFAILAVNLFKGKLYNCVDAASGERIDPYYVLP
YGQQLTRSWCEAGNQTISQSAYYSALNVSMPEYSILTQWVNAANNFDNVGVALLTLFQAATLSLWVDITFSAVDAVG
ADKQPLWNHSPLIILLFILFIVVCSFFVLNLFIGVTLEKFAELHDAEKAAMSVDLTPAQQAWVDTQMLLLRTRMQYK
PPRPSNRVRLLLYIAVSHRLFDAFMLVVIVCNVVLMAMVHVGMSSTWQDLMSYSNVVFTCIFVVEALLKLSAFGPLG
YFRDRWNCFDCFVVLVSVASVALDYSNTRNVSFMPLLRVLRVARVATGMQKLLLTLYNSLPALANVGGVMLLFFFIF
AVIGVNLFAGIKQGDNLNRHANFDNFPNAMLLLFRMLTGEGWNGLMLDCMRTGDCVTVLHDINITTTTTTTTTTSA
TTTTSASLSPLSSPVGPFLSPPPSSSSSSSSSYLTPNTATSSSFPITSPANSTPTSLAATTVFLSAGTYLDPSDPLL
AALPSDATDNQCPLSPFAAVVYFPVFVVLCTFILLQLVIAVLLENLTQAESEAGLPIPRTCFDSFVDAWSNVDCSAS
GFIHASQLVSLVAATEEPLGRAGRVNCRLDAQELLLQLDIPLYAGNKVCFIEVLYALSGAVCGAVLPPEVEDDMVEV
LSRRLPPEEPCRDHAARHYHAAVAVAAAIRGFLLRHTFRSQLLLVDPANPDGSPAAATAAAIGAQVGCETLINGHRL
GDIRRELGAGAGAGAEGSSYTLLPSSRLRRPILAKLPPIAPLRGRQPPGGGGPTAAGVPGGGGNSGGGDHKPPLGLL
QPAPMAAAGVKGEEQEERAERRVGDDGARGPGRWPPPSVSLSSLSSSSRRGRWREVSLPAVRPQSVRLQERSRLGVM
AGTSDPTAPPSLPRALGLPSEQQQQSQSRLSVDGRLPGRRNVVLLPSLSGYSPRSPSASASASASGGPGAGAMSVSA
GASGVGGAGRL

>Vocar20001811m|PACid:23131865
MQKLIDEVVGRLPPPTPTARRRLLQQQLPPRAASPPPTQSPEWGPFAHAQLWLLTLGPTLHWQEGGQHHLANGGKNG
YRHRIGAEAGEDTAAVAVAAAAGLPPPQHFGPAQARLLSMLHSAGCAVVSFLPPAAWLLAVADDSDLAPLLDAFPDV
RLAPYGPPSLRVAPELGAALDLLRRAGSAVAERIAGGERLDQLMEEAQAEQSDFGSPELLLVAVCPVALEATVEWLA
AAPQYFSSTQHRKLRYYLALVQDMVDSFGHGTHVSGTLVGSRYDRASPTDEPLAMGTGQAPSAKIAFLDLSRGNSDG
VWTPGDLYRSYFNLTYRMGARVHSDSWGSELTAYDSMSASLDRFTFTNQDFVSVFAAGNYGDKSASSARGTVTSPAV
AKNCIAVGATLSTVPVSGAIRPNWFSSVTNMTANLDLAGGQRFTRSVRVLKATFGVDLEAAGLSNSQMAVASPETAC
TTLANAGAASGKVLLVQRGGCYFTDKARFAAAAGAKAIIVYNNVKDSGYFSMSPPSDYVPGSISVAAGSVPLSTGLW
LKTSVAAGTVSLFFSKVPQTDYPSYEDVAPYSSVGPTTDGRIKPDIVAPGTLWSANSNRTSPVFGRSPSCDSSVALT
SMSGTSMATPVVAGSALLVRQYFMEGFYPDGVRNPANSFTPSGPLIKAVLLGGADHLTGVVADTGMPLEEGPSYRQG
FGRLSLLHSLPLASNPISPGWRLQVVDGEGASLAQSQQHQFCVSATGGPLRITLAWYDYPGDLSAARALVNNLDLQV
RAAGRGLMLDYGNGVQDNVNTVEQVWYDDLPAGDVAITVTAVSIFQKAVKQPYALVVQGKFSGTLRAPRNSSDSSTA
LNTCAAQMVVIWDELSSAPITNSRNVTFFFGTQDNNTPQAGFQCRLVSEAVASNQTDPYASWASCVTSSSYLNLKDG
KYTFQVRVYGEQVGATRSVLVDTTPPVVNLTSTLPARVGNRITTASDNATFIFVAADAATAVNISCRVEFENGPKGG
TTPPFLRFGPRTAVAAASGISGRPAGQLVPVWQNFLCASPLELIGMLYGDYRLLVSAVDAAGNRFDADPVSWSIRYS
SNTTYARVVSGPLGLVPARDLQYKVLGFNGAAAATTPPELLGLLGAYEYTQALQPPATAASTRSISGSSSGSSGWTL
RSISEGATWQPLSGGNVTVSNLTDGSYLVLVRPSRSSDPITWGVAAVQVDTTPPSVVITRKPNAYQSDSQVTIKFAG
DGTDDDTRAFFCRWMGTRLTESEPDPNTPETVVPYSPCRDSVTFNVTEGHWLFQVKGQDAAGNIGAPASASFVTDLQ
PPSILTTLPYFVNNPYVEWNYTLSDTGTGVANYSCSFRYLKLNNGAAPLDSNATFPTTTDKMDWRSPCPPPPIRYAL
REGQYLWSLEATDGAGLKSSLDINLFTMTVISRDLPMTINSGVVKQLCRINATSAPPAPPPPPPPIPPPSPPPPPP
PLSSVSPSGGRTYASAAASASSTTTTTSTTTSTTTSTTTSTTTSTTTSSSTTTSSSTTSSSTPDSSGGVGPRLLNGS
SIRLDEWHECDTGGDAQNISYEGFSSGFYTFSIRALDAAGNLGDPATPYFFQVDATLPDDGVETVQVNRSEGGGSSD
NTKKIVIGVAVLVAGVLLILSAGAIWVGRRRYLRKRFRATGTPPATVSTHDIPMQNLPPPPPAGTVPPYTNGHGNS
NGYSNGSHAPPLVISPPPAPPMVGAVTPAVAYYHTVPVLGGGMVAGVPLATADPALAAALAASSAEEAQRARAEEEE
RLRRAIAVSIEEENVRRAKEASLAAAVAQSSEQADLDAAIAASIATAERDRQRQQLQAQWQQSPPSAPPARSPRPPD
YDLI

>Vocar20001759m|PACid:23131925
MPVRTRYRRAAASEICGGCRAVDFEPSLQNQAMQVVHAAGTLQIRRQMQLPTCFFGYIGKIGCGVQQQPPDMAVQSA
ASPLPRPPPKPAASAVQAAATACPKSLVRAACGAVRHQNFGELRPALEQLKTCGQPLTRSQAAELVECAAVENIVRL
LESPGQCLGDWTLLRWQPAATDNVVYNSLLHEVVLRTYSCTEESEDSDEAQEMLERGLAVVRLICERRPAAVVDFQS
RLAGVTPVEALLDKGNTEMDEWLEELLKAVAPLNWVLTKPGWAGQRRALAICDQLVAQPAVAEALGIDPEGWVIRGS

LLQAMLDEHLNDPVSLVMMENPERMPRPAASRFIVLPRADVLHDKAPVPVTAAAAKAASTVSPKGNSHIKPAPVAPG
AAAAATAPPPPPGRTGAPHAVTSGSRARAAPEPLQTTGPVTPQLAQLPPSSGMMIFKGIFSNEAAQGKARAPPAAVP
AAPAMRAAAAAAPTHVSRTPHLQQLQNSIMTVRNALGHLDAAVMLRGDIAAIERAVQHLRTATAKEAMTPKALMDHL
AKEWRQGDAKSKGSDKAAALVMLLKSAAGSQCDAEVQLLGAVVSTLRKCAWHAATELHNLDMLAMLATVELSPVILA
LKGNAPRQLVIQLAASCVKFRKDRTEADDEATALGWAMRQGRMDLLRELLPLIDPRLPAEGDMPPLHHAAEIGDDTA
AAVLLDPCGKWWMAHGLTVQELLAQPHEGQTPLYRAVAGRHVGLVRQLVAAGAAPLEPVSADGYKDTPVHLAVYMKQ
VDILRLLLENCKVATRPDCFGSAAVDESQAQLNRAVDGERQTALMLAQRERHIECVGLLLEAGANPTLLRDTASGGG
GGKGGKKGGMGGGGAGGSRLMMQNARLRGLLAAKGGVVDGTEGASGKVGAAGGADGTVMPAPASSSSSSSTFPESVL
SRALEDGVEDVLRVILECWRPCFNGSALKPELGPAACNRVRAWLKEHARVTLWPTVKALTDLADASPSGVASPSWLS
APLLELLLELCNEASGRDWRACDGASWQGPDGTGPLPRLVVWGVMSGAVVGTAPPPGPVPTFVTPALLKELLRHGVA
SLTERVPAGATRPRQLQEGSSLLHLVCFTGDVAMLEVLLAAGADIGCMDDLGNTPIHVAAACPHREEVAEQLLRALS
AGTAQTKEGQGPRHEQQQQGKEHALAMDLATAEAAVAAGEARLQALTGRNKNGLRPQDMATSRKVKKLLQQLQETAG
GALQSALKALQERQGPRDATSDAAKEVVSKAATKKGNADDNKRRKGDGGGSKGVSKSGIAATGVGDGPRVIRSAADL
ADDEALRSAALAQLVAVPEPLVLPLASKLVKLLPANLARGGPHAGAGSDGGAAAAADTDAANAATNRELQRIVAEMR
VNCGEEDGDEGVEGDDAAAYSDDDAPPPGEALVDDEVLLQLPARGASGLSVATATATASTEASTTSAPATAASAVDG
ESGEHPLRGLAWRFVITRQAYEAWGRLPDVWRRLVMSRLRVIGSGYWARCRGAKKITADDPTLAQQELWRLKVTKGG
RILFEVAVEYDEESRTWAEMIRLWFITLSHKAYEDMIGLVQASFRRTLAMRERLRLQPIPQHEAPMPSPLPGGRTAT
ASAAAAAGRTAQRLPRHYREAGKTTDGEAAAANSGGGKTRGSRKTQQQMPPAGDLREHFPPASWAVDTYTLMKFYNL
DSFLVAAVLQGLETAKVDFPFKLSPEEYDLITLAPNPPSSIILLGRSGTGKTTCAVYRLWANWLRHYTNPGLDPVHA
VFVTASATLRERVALAFRRLQSAVMAPAEFARVVAVANATYHTFHDVPSDAFPLFLSSRAFLRILDGSTGRPFFPRQ
PNGAILQLPDDGEELDPDGAALVVALDEDVSDDEDEVETETAAGNQGAGDGGAAGPDSASGNAEDTAAGGVVALEEE
MEAAAAAAAAVAARAAALADLEAGGGDGHAASDSYGLGVAGRARLNREVTYQYFVNSMWKKITTPDQRDSVAPGLVY
QEILSYLKGSAEAIASPAGHLSLPAYLALGRKRAPNFSEEIRKDLVWPIFEKYERLKRHEWRYDLLDLVGHVYREMS
VSGYTGTPIHALYRDEVQDFTQGELLLDLAVAADPNTLFYCGDTAQTIARGIGFRFTDICTLFYEENQRRQQQLQQ
QMAAAAAAAAAAEDASGEGRRKGALMARRGHGVTVATPPIHQLTMNYRTHQGVLDVAALVVDVLRRYFPRQLDRLER
ERALFPGPHPLLLGGISSDDLAILLSGSDRNTSQVEFGAHQVILVRTMASVAQLPQDIQDSNAIIMTVPQAKGLEFD
DVFIVDFFNDSDANSEWRVLSSYLAELAGTGGVGAGDGYQYDMQQANAGAVRPLAFDERSHVLLAEELKHLYTALTR
AKNNVVIFDRNPTKRAPFYHLLQSLGIARTVHRSLLEDGRDAVRYGLTQQATSSRAEWAKRARNLLDNRNYLMAKKA
FVQAADMVRTEVAEAYLKRRRAADEESPTEQRRLLAGAALQLLAAASRREQSPDPVTAGELRRWLGLAGRCLQACGL
AGPAAELAFKAGNHTAALRVLLAGKEHAAAAECCLDAAAEALRQHAVYQRQHLAEGGSAAAVVALLESLVVPPAGTA
VAAAAAARKRALAWLAKAVEQVYLANDLETLTALLAPAGANASGANDSEDDELGIKDDGRGAAAASTGAPTDVFAML
VPELHDMLRRRWVSSYGRALQRVALRCHARGEHGRARRMVRLMPVEAERDALLARMGDWREQARLKRLAANQRAVRR
SSDPLGAAQLLLDNGDFERAARLVSDYIASKAPAPQQPQAKLNSVPGGAGGRIVAARGVAAVGVDAVATAWAPVEER
AWDLVYRCIMAQTDAAGARRLRRELDRWAVAAAGTPEAALGSAVSSSPSAGQAGRRVLLYRGHAVLLEARLLLQPWV
SAGGVSGAGGVGVGTRNHPQQSVPASWEQLNDEAVAAWEAAAPLLREAADCFSRCEFWPGHLEALTLMLMFGPESDA
ADAAVATAAAAAAITSATSVPGSASDSPRLRQADGAAAFVRTVQAVVEAFHASGPWGLTKLQGQHLVALEQLYGLPG
LSRWDERLPAKQATHVWWAVLGPDSAAAARAAATAASAGSASLAPRQAVEAVASAAAQRAIKGLSYAAAAAKGVQTT
APPPQVAAAAAAVPPLATTRQLLTHQLGIAARQAVPPDAAGTMQRSAVVAELVRDLAVKAACVSFQALRSAIDTYLA
IPSETTLPPATSKMVLAALRPVVRSIRVSRAALALLNAAEADFGLYPVTTYQEDLLQPPLFYGGPSPPPHGELLKLL
LSESLQKMDLHEWIKEAIVFSDALNGRLALAPTVSYTAWRTTMLVLMHDELAGRVFKLRRARLPPDVPASRMIRDRE
NTALPEYVLHQAFLHFSTNLPHKAIGDLLYYLAWCCRRDGLLVLSWAASSTAAAAGAVAATGGAAVPASAGLLTINA
AQGSSGAPSAPVSSAAGGSARGRCLALEAYVELTELAVGQLLLAACDNAFLSGNIAGKLAGLPKHRDHRLSELALRW
LWALGFRPPTRPSVAPGLQRGLGNTTQEYFRYAKVAQEALGKQLLLCARLVMVLAGHLGRLLVVAEQGEGQGTGGGG
FGAGGTANAVVMEELLPLLEAPSIAAALELMGIKGNHGGEHCASVAIPSPVGATPSARGPRLIALARRILLAAGSAW
LSLEVQKRMPQQQLPQQQHSAAAVAAELQDAAATRGRWPTDVVPLFREAVGLLAEPLVGAAAIRVLCSDLTLSGLAD
QLRQLAAAWNGHYYGSMLLVDARADREFEGFGPQHQAKQFKGLQGPLAALYTVVQARVQGPSAMQQERAVLTGAKQA
PAQAADVPVIAGLLRSAAEAVGVVASKDYRSTRDELTKQSAAAAIIQRSWRQWRRRREEAAAAAQQRQREEAALRQL
REGMSFGLRIALRVYLSRLRRSIEKRKQQEAQGNGQDGQWDEFGFAAVEASVQEGEKRRFIDESSCPVCQPKSIPQE
QQQQQLWKEEQEQQSQQQSLAVEDRAACGSSGSKLNLSLTAAVAEFLPHKSRTEHQTALHFFRLCHAKYRETVSGLV
RHAQSLLKYMEQVQGQLKADPEGSNFLVIVMESSMQLDTALKELCDQLHKIVAERLWMTSPQLLGPKWYACMNACDI
AASQLNLYTTAYRREVVQQFAQGDAQQQEVARQETHGTFAASQGPAHQQAKEADVAPHVGAWQEAGPRQGQQAGCTS
NVGMEAEHQYTRSHHGHQLQYQQPAPPVPHELLNRLKFQEGSNVWQGDETQQQLAAPHRYPHKEPSVAPSYQSQLHG
QVITAHMRQQQEYPMMQPQPLQPQLHHALQQQLQQQLQSYQVVQQQQQRQQLQQGQPYGSSLNAWAAGPPAPAAPQF
SQRMPPQQQRMAAGAGAWGVRQMPGVSVWGGIAGARPQLPNVQAHGAAQQAYAARGPAAAAPAPTWASMSSTPMLQL
MAQQNQQQHHMLQAPYAFVAASTAPCRNADLSPGLIPGGGGSAAAATGAMPYMHIMGGGGAAAAATAAGSGLRTDDE
LLFPDESATSFGSLPVPKLARYAWMPTNDKVNNENFHKMLEEYRATKKRERELELKIKQLGAQLARTEEAAKRALVQ
TDASAKGGAAQKLLDAERAIAKLRAENAELASKLAREKKKSAEFKTLADTYKQRLDRFLRDQRNLVKRVGELERSQA

VSRKRPEDDYWGEEAGRRFVAQQEELVALKEENAALKRLMESDGPLAQCKAYEGQINELQNLIRFYERKLTSLAEAG
LIGPEALGGPDNQPGPDDWVLEEFWHNDEMHLLDRKNGKLFTVPGDNNWPKPIGIRINKDIKMGVHNGMERFLGTLD
AFLTNNAARLQEVFTQFDLDNSGQLDRRELARLLQTLMPDLSMSQVAELRTMLDVDGDGLITLEEMLECIKEAYAAR
TAAKLGKSVEVNDALDRIRDTLRENKQAIKESFDELDTDKDGCLTHMEVVRLVRRFLKDMYQKEVRYLLARLQQWDV
VGDSRVTFDELYQAMELVRIFRVGPGLGGVIRTSSPLRSPPRTVSPTKPRGGTIASAHGLRPSLRSTGGGAGMKETW
MRERMTQLETELRDANRRNAELEDGNKKLDELQRDVALYKARIEELERDFMRIDVLGNLEGAAGDEQLQKAWEIAST
FKKRFMEHKGELDNIRLMYARMQAQLDETHKLLHEEHRKRFKLEDEITRLNVELMRLGDLEARLAHEKGERVKLERE
YLALQQKALNAPGEALAEVRALREELFAVKREKATAQQKEAEVRLELSHVRALLDGMNLESYRTMQGESDTLKKRIA
SLTLELQAARDKLAVYMRTALPNSANADLLLEDDDLLFGSDKRPDSDKTPEELRRELMQLRDVLETESAITMEAKAA
LEEAHRELERVKRELQADLKKLEREIERRDEKIRKLELQLRGAYSGINRALRSSRRGGVGTGELRDSLRSDTDDFSE
IGEDQNIFELHITEAQVNEEALGKDPSVFFTFDFFMHETQATPIMASNSPSFNTIIQYVVDNDPFLLEYMDTHVLVL
DLCRARGYDYDVLGMARLPLRQVLEDLEIGAALGYNRAYHYADVFGADGKRLGRVRYGYCFRRPLDSLLKEYRVQAR
TKRPSDPDERDPATQAVQLALSQPGNNSCIKVIIERCEQLVPAGGTALTIKPYVHYRFPGTRDYHDTRILAGTSPVF
NDEAVWPITRTPELEADLLRRHLSVVVFDDAQGDDPLRAIVGVASIPMASLASGIPVEGAFKLTNPVNRKPAGRMVL
GLGWHNPLQLPGATPRVPPPRVPISSEVQDGESAHDFGLPHDAKELLPVSGGLPPGGFGGRMGVRVANPGSGLLPGH
QPPSVLPRQYENRSLAAELGSTGSRAVSSQGYGLAAEQHLQPQQRSGYGNSAQSPLRPQPQRGADFSGFGLAVETAS
QHQQQLNQQPPTPYLGAPHDQEFGLDADTRSTGYSQHQQPRGLQPSPYRQQSPYRQGSGGPPSSGFTSRTPSEYGL
GAEMTAAPSAAPQPAGHSGLPRTGSWGRPPLPVGSPPPPAGRPGSLTGGRRGQSEETFGLDAEAGSQREVPIPGAWS
QLSRQQPVDEGFGLGAELEDAGTSGSPPHGMILSGRGSGSGNNGQRRYADTQVSLESSAAPYTQLQQPNAGPLSQDA
LLGPVLRRELPDPAAWSELDHRLVLCLHALELTPQALRNPRLRNVVLMHSLLSEQEGISELDTCTQPVAKGPGQLQI
GYCVSYNVLAGAAPSELLDALAGGDDFMLEVKLMSTAASRLDMNSIAQPGTLQTLGSCMLPLADIWSGRRGDLMQER
VEILDDSDDTTQVAVLTVSLVAEAALHTLRQRQH

>Vocar20001854m|PACid:23131964
MDWVLAGATAARRPQGLAAMLNLSQAGASGRTAALQAAATSEPLAPQAESEPIFLPSQPDRSSGTRQRLVQLAQPPP
RRTQRGRQDAVADSNSRLFFMGRRRWFGGQLKWRRSPAGRVPPSNTTSASTCNTSGVTETPLEGDRVHPSTAGTRQK
QRRRPPTQIASGPYNLVGCHDPRVALRSWMRRREQTGGPDLDTVRVHDFPEGGQQHMHHRHHHHQEVEEAAAQRHGL
RPPRDPGGMASTTTRVFPPVGISPEEGSPATAVTSAGGGTSSLGLPVPWATGDGPSLGMQAGAGAGSGYSGGPGHLR
ARVGSSSSSGGGNLYGLISRCSSWRELQQLQLTHSGHLLPQHIPALMAAVARLQQHHRRGSSPRQQQYSHHHNHHHH
RQQQQQQQHTAEEQRQVLRYMWELAGSALRLARSRELPPRSLATALWAVAKMPPPPPGELELEASPPPDVSQTLWAV
ATLECTPLPRGFLPGLLEAAKPALHSFGPQAFSNTLWALSRLRCQPPPGWLDAACAAAAPQLPTFNPQALATTAAAL
AHLEHSPPPDWWEIFFVACARSMWLPYPHHQSPPPPLPPSSPSPSSPTPPALEPAVATAAAAIAAESGGDCATGGGR
TAAAVAPQSLAQTVWAVARLRLTPPPGWLEGLAAAAAVAAPRMNGQDVANVLWALGALRFRYNHHRQNNDEQQQQQQ
QQQQQQHYPALRSSSSAAAVASSPPSPTSAAASRPTAAPSGPPGLAPLLAAWRRCLDSMQPQQLSACLVASVHLGLS
LDPSRNPNRDQAAAPPAAAAAVPDDLVARTLVVVGCRLASCSAQVVSNSLWAAARLGCRPSSSWVGAAVERFLVCLE
DDDAGPQEVANVWWALGRLRWAPPADTASELLSRTLDLAAGGDLRSGELAAVMWSLGRLGLRLPKPLMDRLVCILRA
SLHEEAQQQQQAGEGQPPPVPQPPRHLTPAPPATLVTLLRALAARQATSQRSTAMALLERSLRHMATTIRASGFGDG
GDGGGAAAARQVALALHSAASLGVPLSAGSTAAAEGRGEGEARPGGGSSRGLAGAIIAALRAVLPYANTRDLAMALS
AAAAMEAVVPLGLLAAALQRAGELYARDSATAAAVSGSAVDDGKDSTPVLTIHGNDVDGGRAVAVVPEGADGAGEGG
EGEVESGADRVLGWVRGRPDGSEGVMGVRGGGGDVREVVDGAGGDLATLLCSLGRLLELHARRRAEMTAQQQQLQD
AGRRGWRRGRQQQELQLQRQQWKSGPKHSGRRLERGGGGGKGRSGSGDDSAGDKSTGSSSNHHHHRRVDMDVYRDAA
GDVAVKAAAAAAMQEARARWDELLRKRPRLVAARTALLQFVRVGLERSSSSAIVDADGGSSSSSPPPPTPCLSAATS
LQQLVLGLSKARLAPPPRLLRMLARALVGAVGDLPASYLAACLSHLAAMGAELDPQDAMALVGRLRAVHVATAARRS
AGGRSRSRGWMVAACGGVSCMVALWVLRRKAKRKTRVSRGGAARALRTASPRNRTGRSRLRAAAAAADPEVMQRVER
ALTRLREAGECRRLA

>Vocar20011064m|PACid:23132161
MKMLLRCLECFASNAYSCFSKAALTSMAVVKVLVTISIIVIVAVKLEDVRVVMQVPDLTTVEGLKNLTDSLMAISGG
GEIAVMQLPPGAAASDASSPPSPPFAPFGSETTDFNGAAGPFHVNASCLLQKRGVPPQIHHYTRTMCSYVYAVSGLS
LLATFAMACLLWCTCHLCGVGPILELSFALLGTAWWLAAALVLQENTTVSVDARDAVKQFIFGDAMSPPPPLGPESL
PLPSPPPSQPPPPLGHDTASQLQALMQQYMPRSLQQWRHALVGLCWAMMGMFAASSVLMLIEACGCLLDCCTCVCRC
CCCPSRPESRWRTNFRKRMRAGGEEYMEMMGCCSDLCRKLFVGLLSLIKVGVSTAIIVIVAVHLYKADITWKKGDLP
EVKPSCLMTGSVRGESVCEYTFAVCGLSMLLSMVTSLLLCVTCDLCGLGDWFQFAVQGLQTAWWVIAAIIISKNVRD
SNEYEGVGLPKEGWRDAVAVMSWVNAGIAFLCAVIFLIQVSVSVGGLGACNCLRNCCSCCCGGDGDGDDYAKRANVV

>Vocar20004069m|PACid:23132240

MQTTKRHVGATHFKGSWRARTNGHDLRLGDALEPTLNWATASPVPPLLVSRMQDNLCIWVFYFSSLYNAKLWYSQGL
DPPFIPTERQGAPGTFRSPNGSRIFRNHPNHPKPPFPPYHTAGHAPNPIHTPGTDMPALATPGAPASGTPAGRFAAV
SLGDGHVAAITVRGELLTWGRNTRGQCGHKTPTSERVTTCHHNRPQSPGTATRNASPQLVRFIPPLHSPATATGRLI
TQLQVQVRAVACGGNHTLAITSSGVFAWGCNAVGQCGLGYSTAPPGGFVVQPVAVPALINVPVSQVAAGSSHSLALT
SACEVLSWGEGTWGQLGLGDHKMRHEPTPVEALWALPVVQIAAGHTHSAVVTSSGHAFVWGCNSYGQLGLLPEPEAA
QEATTGTTSPPPPPPSQDKQHQEQQERCPCGMAQGEMRPGDAEGPGECERGPVGGRRRVVPVDPGPEPWTDGPGPPG
RCSVRGAAGGPAGQPGVAAQQLLQLPDSPDVGQRGSGSQVRHIGGAPQAAHQQELPQGHQHHHHRQQERHRVGLRVP
RIPQPRMSQSPSPSGSPSRTGSTVRASLRRNAGAGAINPRWLLAMTEMGIPQRHAQQLHSFVVRLPPSPGQIALEET
GNSGVEVATEWLFAADRSAVLEEPPDCEDGHNEATAAAAAAASTLNPGAAAPRQCGGTAVHAAASRLECQRRRLGGG
EGSVEGGSRVASVGIISSPELQGVVRFLKGEELEGTEKAVKEEEEEGGGGCGDSRRGGVDDKLEGLVVLEPRRVPLK
DVRFLAAGARHTVAVTDSGVFAWGDGSWGALGLGHQEPRDLPCRLEAFSSPPPPAAAAATAGSPPCSAAADSVTAAT
AAADLPTARHYLRSRLGDGSSSSGGGGGANAGPAVVTAAKAAAPAPPPPVIQRVACGTQHTLFLAQDGTVYWCGTME
FGGGGAAGDDVSDGSRVVRSLVPRRLKLVSGRGGGGGGRCGKLGEPPAAAAGAPAAVFDVVARGASSAMLMCSPREL
PQVLHPPGGPRLLDKLRGAVRRAALARTRAGLSDGGGGGGGGGGRDLARLSASLKDVATGGGGGGGHRKPLPPGVRT
VLSSPAAVSAAFGRRSGMGLDGPLLEEVLESVRQLFSDPPRSASGSRDIAGGERADGHGSGGSSSSSSACSTGNRAG
GIGGNSRGTGSSGRSSSSSSRGGDISCTVCELHRYSVVSELQAAAGALVGDLAAHVRMLGTPERAQVILAALQHPLL
SEPRIARQLLPRLVATVTSAPAVVRTWLVHWWSDYPGPLLEARVVGPLQDFLTRELGATKKLTVVLMNVIKVLALVE
EAASLSQSLPPEAFYNALISEKLDVQDHYSAWRQAQELISSGCPDPPLREAPFSFCSYPFLLDARAKSHLLHLEARF
RMEQSVAHARMVVQLYGGRCAAAQRRAAEGLVLQKQAANSCATCSRSSSSSSSSSSSPRRSGGGGVAAAAASSLSSS
STGTGASHGGSSSSSSNSCIGTGSNIFSGSLRSLFSLLRRRLVSSTSNSGRSGGCSSGGTDDARDRDQHDKRAGLEC
AMPGVHSDMCIVRVRRTHLVEDALEELGRQVKSDLLKPLRVHFIGEEGIDAGGVKKEFFALLMERLLRPDYGMMLYD
ETSRTYWFNPASLEPPDSYFLLGLVLGLAVYNRVLLAFPAPLLLYQKLRGAREPGLRDLETWQPELARGLKQLLEYN
GPEPVSEVFGLTFSVDVDRFGHKETVPLKPGGEVIAVTETNRAEYVALLASWYLGGAVASQFEAFAAGFRTVCAGPT
LVLFNAQELERLVCGSPRLDFAALRDSARYEGGYGRDSRVVGWLWEVVLQELSEEDQRAFLKFFTGSDRSPQGGLGS
LRPVIQRDGPDSHKLPTAHTCFNTLLLPEYGSPTKLAALLRLAINNSEGFGLQ

>Vocar20004064m|PACid:23132285
MATFAYFGSGARPPGANEPPNDTSKPAGGTVPQVLPFNGASTAGGKAKAGTVSGVKAATIASAKVAGTGATAAVKPA
AAAAGSTATEKLDDDEDMELPPPSEPKPLSVEGVAAAAASHEEELSEFQRKMLELMEKKKEEAEKKTKKAAAPTLHT
LRGARLAIPAAAAAAAAAPTTGGTNATVAVPRAAGPGTDAPAGAAGPRGVPVPAAFTEDPPKPKGEGATTGTTKKMT
IGGLSGLSLVMSAPLSTPATFDGPTPAELKYGRPRTPPSRGRSHSPRGRSRSRSRSYSRSWSRGRGRSYSRSRSPFR
GRSRSYSRSPRRHDNYGYRGRGRGDFRGRGGWRGRGGFYEGRGRGRGGWQGRGRSQSRSPSHSRSPVRSGSRSPGRR
SPPRDGPAPQRMPQGGPFGGRAPGGSPSPSGSGSPPRRDDRAWKEIPCGEGRAYGCSGCKGTREDRPRRRSDASDDE
YGGGGYRGRGRGAPWHGRGRPQYDRDPYDGYDEGPGGRGPGRGRGYYGSGGRSGPARPYDDDGYYEGGGRGERGYRA
RGRGPPPGFQRGRGPRHGWDYDDGGYDRRYDDRYDGYDDSRGGYREGGRGGGGGGWYPDRGGAASEERWRPEMPPEL
DEEEPAANPRSGGGAQGDRSRSERRAAGGATAAPAAPNRPSAAAAAAAEEEETAEEERRRTEAARKAAAAKAAAEKS
TLDFMSEFEELDRLDRIDTTAILPGAISVPAASEPPSQQQQPKSGAEAADGATATQPLFSVAAGPAVATSASWAEQN
LRSHEQGGDLAQGYERLGGSRTARPGAGGAEPGIPVRNSGAGGAGELAEVVLGPGGEARGISTEGVGGSDGEGGGKK
KKHKKKHKKEKEKEKEKDKKEKKEKKKKEKGRPPAEGEGDEAERGFGGKEAGAEGDGGAEGAGRRYSGPDQSPYYDD
VDERGGAGPRAEYDRTNDFDSGRRGRYGPDGGGKRGGYYDDGSGPGPAFDDGYGRGRGGYRGRGAYGGRGNYYDNDY
GDYGYARGRGGRGDFPPGGFRGRGRGGRFGSGGGAAEDYYDDYDRPIKRQRYDGEEGLQGGPSRRPMGRWPDDGSDD
RRFGGCGGESPGRGPRRPSPGGHSLSPPPRRRPTSRSRSPGPGAGRSPPPRGRPRGAGGMGSGPTRPRIDWRSRSRS
RTPRSRSHSRSPSRDSRSPPPRAGGRPRGGGPPYRRGPSWSRSPRPYRGTSRSRSPGRSGSPPRRAGSRGPADGGSG
GEGRTKEEIAEELARAAELARAASQLSGLALPLQAALATAAPNQQQVSRHARRVYVGGLPAGMSEVSLTQLFNNVMM
AAGATTQPGGPVISCYMNNEKRFAFLEFRSVEETSNAMAFDGLQCQGETLKVRRPHDYNPAAAKLLGPTEPSAKINL
ALLGVVNTLVEDGPNKVFVGGLPGYLSEEQVRQILQAFGPLRAFNLVTDRDTGASKGYGFCEYADPNITDVAIQGLS
ALIVGGKPLTVRRANTAGEASATLQTLIQQQQAALVGAPPVAAATAAAAAVAALNAANAAAAAAHAKAAAAAGGGTA
AAGGAAGATPSAHTVVRLSNMVSREDLVDDGEYMDLMEDVTQEVGKYGKLVGVEIPRPPPDGGPDPPGVGFVYLCYE
DPRGAERAQVALNGRKFGDNLAEATFYDRSRFDAKDLS

>Vocar20004074m|PACid:23132343
MEPDIPCVPHMFVQRVDALPEDISERLLTALAPQAEDRRDLGWLPNFGQANDTKSSVGLEPPSKAELRNLLHMRRRD
ELAADADTADYSSEPYDSIPRQMQLQWRVYARFHPDEVAENEKLASMLTQPRQDRQLSGTDRERQSSSHNSRSSGEG
ASTLFGALPSVTSLQQSLRNLMPLPVQPASPAVPPSPPSREAGRSIATPATATALLPLGGGELWRRQDPVFLKLSRS
LDRWHELQREHSRPLRRGRRARLLVVRGVDLPAEWKDVMRPVAAAADSAGARRAWLGGIFHAFTVAAGAAAGVIGLQ
RRHRVWEVAGVAARTQQQSLQHREQQQGPPGALPVSSMRREANEEDAAAGLICFVVFMDLLYGWQIGGSHRAYTFPR
PRQREQALESSPAFGAADDLPTTDFNAVDTMAAPSGGGSMHTHKPPLPPLPSPRSAGHGGRGAKPLAPPPGTAVATT

AAAPRRAAVQAMEPVPRLAKEAKTAAVNRTGATGSSSSSSGEGIPYIGLQGGDVHAHGNVNMSQAEGGGLEALAWDM
QQQQQPQQQQPQQQQPQQPQPLPAWEGVPLEASRLEAALAVMQRSQELRSLAAISPQHHTAMAAALTAALALLPPAP
PPLELVAAMRAQGPSVACTHDPTLPVAGEVPTAAAVPKGDGGNRTNSTCHGTTALSPPPPRLRNVSSGGGVIVAGKS
SSASGPVPYTGPVAVAMYESNSYDATPVWLDSLASLLDVLGVPNAIMTRGSLRRIVGQHRIELLASLRNKVLDTALL
HGLKQMTHGSQPQYRTVSFRPDRLLFINDVYACAPDLLRLLQLGYGVGADLACGLDFDRPGDRRRGRRRRRRGLLAS
QDDSEPDLNPDREGKDPGRRGPIMATEAAAEATELGAGMAGTAAVSIQRDNEEIKEEEEEEEEDEDEDLDEEALQAA
NLHRLLRRLSARPATTSGEAAAAAADDDDDDDDDWGSAVPPRPPPQAHASGLGDQTDKDLTRSLGSSVTAAAVGPSP
VVSDPRVGAGRKAHEILVAVLEAQRMAAAGHRWDPARQYEFYDTWVARDVGGRRMDKRLPLAVHHGRTALRLALGLP
AAAYCCWNGAAVLNTAPFLPPPPPPPPPSPSPSKQQQQHLAADECDATGSDATAAVPSGNGSRGATGGTPYPLHEKR
GVVRLPGGAELRFRRSLPGECDASECSLLCDDLHRLGYGKVVIDPSIRLSYMPYHDAGLHKDWFLGTPLLQWGELAS
LAAAASPPPPLVGRGKAPAASSNRRRRSQVLALKVILGRLWWDGAWGEAQPPDIDAALKSGPNAQVGCPPVQPLQPP
PVQPTALRQVPAVEAVEAVEAVEAEVEVLRGGHDPVWRRRESPPLADVPWVSPLPRLHSCLNMEWQMLAQRVRALPP
TPPTPPTLPRSRTAAASAPSSTAASPLAAASGGGGGAGGRSESGVGGDVSPSDSSKVKLVPLAAQRPQRPAAGGPGG
PRGGDSWLGLQGPWSWWSSSPPPPPPSSSPSSSPLSSSGDQLLAVPSLEVECIDLFPGSDMANFAGFMLVHPAQPNF
TDTYLRQQRSLMRLRPLMTHPCPNLSLRGGPVGA

>Vocar20011894m|PACid:23132347
MSPAQLPPPHYSPPRTPSTDCRTSALGYQCMVAKGKVTVHWSVNTTSAPANNGCTPRTPTEVNATSLGRYGTLHMAV
QANITGYVGIGFLGRSLLHMFPSDIVFGYSKSSMPYINTFYANAEGLSATNQYPPAGSPSWAYDMGVAQSSSNGVVT
TTICFSRRLSDSRPRVSQNITTDGSKNTTFIWAISTVDGFKEHSSVNRGGFYLDLGLAGKAKPPPPSPLPRPSTSPS
PSRSPPPPSLPPPRRSPPPARSTDCRTSTLGYQCMTTKV

>Vocar20015157m|PACid:23132449
MAAGTPDDKFQKLREENERRRKKKEEEEKRRKQEDEQKAKEALERRRQERLEQDERMRKPWQQPPAPKKPILRKGAK
QKPVQPGGSPSVDPVRQSQAEEKRKEWRQGLQDFIKQQRQQAAAANKAPAAAAPAGVTPPGADGAGPVVPLTWEELR
MAAEAGFDELLRQQELQAEQRAAEEPTSRAARHRSPFKSPFRGAPAPPQETVQVALVPTQQYNSNGVAAPAAVAAAT
HGSPRPSPRQMVPDSGSGSAAAAQRGSYKYNDDQGPNGSSATPVRPSVPLPPSPNQHQQHQQHQQHQQAQRGANASN
GSDSRPQQHSPDAATGGDSAFEGTPEAVLYPPFPADRNPQGSLGSLAGGGEGVVTGDAQLAAAAGGGSLGGSGFGGA
GSVTSSGGLRRGVTSTARHRAAPVDPTSLDMAERSAAPAEAASDPWVAGAPPLPPDEDGLDDSAAASPRVSPRSPTG
RRAPLPGPYDAGSQQQWPPQAAVTEPYAPAGAAAVAAGSRAQLGIPYDPLEASLQAEETLAAQQHAEGFRLSSPRSS
GSLPPPAVDQPPALQPPQSQQPAARQDQRHHARRGWGMPAQQPPPQPSAAASAGNGAHLPADPSPPPWVLNGAAAVG
ASAAAVPATAQWGSNSVEASEVPSKSGFGAEFGMAAAYGSGGGAASAAELPLRSDAGPALHQAPSPAAIRHSVAVGD
SGGPDMWQRESVVLLNGSPQYGSPPRPLSAVSPSPPPDPPDVPLPPQLPVPSSSSPSSSPRPDDEGSDGRLADRTAA
TRGSAASPQQGPTTSPGAVGVAPRAATSRVDVSGDLVRFSSPPGPSSRAPSDGSAHTGSPQQLQPVSRSQELQRADG
AQQHTPPQPLPQQQDQQQQQQQQQQQAFEPTFPSASSSASNVPPEPPQGFSAVRQSQSQPQGPLLLPPIAPGRPLS
GSAAVRQSSSGVQVASAPPISLPPLASQGGGPAGHSEGDRDAAAATAAVMQLQPPPPFRAPSPPASPPRSSGASQRY
LRQHSSASLLAAGGPGSSGGGAAAVTAAARRASSDGQSVTAAVAAPEPALSGDAGHLGTRRDAVAVAPAAAAAALQL
PEAQQLQPQPPALPNACRPLSPPGPPGAASAPGQSRHDLQAPPPPPPPQQQPPPPPQPQQQQPCVAAQAQEPAGNPE
RHSAPARQASAAATDAFAAGIPRKAGLVSRAHGTTPTTPAAGAALTAANRPAMPAMTVRHTRQQTPQLQPQPPAPPP
QQQRMLASTEHPQAQPQPLPASPSQLQPMAMQQQQEQQQQQQQQQQQQQLPYSTAAPVSSPDVSSSGIPMQLERLAA
EGSPTGTSRRFDGAPIGPRSSQMGSHGTSVDCRTSDRAAPPVVAEESSEFGTSCGAAGLASSAAVAAAPAAAAVDNG
LVYGRAEDAQAAADVELEGDGDLEVEVEVEAEDSAAAIMDGGGGGGGISGVRMDAAMDSSLERPPGTAEGSVLTGSH
ISARPWSGPSSGGLGGGGRIAGSGYQTALLDDSVSSFGFRLHSAPTSRPTSSSASASASCVPLAPSSPSSLVYPAT
LPPSPSRRAAASTGGAAKATVPNGGPLSSAVGMPSRSGTWKEPNGAEGPPLNAVPHSACESSVTRDTVRRSKGPTGP
GLGATRGSAVTTNLAPRNGVAATGAAGAPPAPPGPLAVAPNSFQVLQRPAGGRVSGGGYMYGNPDDADGGGEGQPFV
EEPVDEPDGLYDGGSGDAATAVGGSDAADGVQAPSATAAADPDLDALMREIQELSALRKALKAEFEIMGHGDAVAGL
RTLSANFMELSPPAAAAAAATDAGGGGGGVDAGPSPLPPPPPHLGPEPLLQWNLPGQPAVQGPIVAKAAAAPITAAA
TAAVPVPSAVLQHMHPLAQQQTSPPLPNAQYSHSHSQQQTPLLPPPPQQHHQNHDLVAAAAIQRHSAPHPPAPGAGG
PPPPSLQPTMAVNPPSAFPAHSVTPDGFPMAFPPGAELGRPAVQVQGHGNHPDGWTAAVPAVVPATATATAVLAAAP
AVAPSGGAEDGQGGSFAFASPAAPALDSVGLIAGGGVEMPVQHVQPAALVRAPAAASAPGHQPLQPLQWTIPVSGGD
MAAVPPSTSGSAGATPRKAAAASSPPPPQQQQQQQPKRSPSGGGYSVPAVAAAAVDSPGSASGAGAARRAGGIATAQ
MAGGTAPVGRQAANANAAVSGGGGGGGGPVAAAPAERPLINEGSYAQAAVQARQRAERESAAREALQAESAAAKAAI
ARRSKSERTAVVAAASPTAGGGGGGGDGVPAPAAAVTPPRPSREDSLGSDFDFLERASDISDTETDSPEAYGVFSKA
PPPRREAFASPPPAGASPAASTAYRERLAEARPHPLRIPSSAAATPMGGASTASTVASRAVPTGGRGGAAAAAGPGA
VRHGGRGPSTPFTAAIRELMGLPGSAPLEQSRAAAGKQQQQQRGQASVPQGRGQRGEGAPQQQQPQPPQGGGGGGGG
GGGGPIRLLSEEEDKLLASLKRLDADILRRGLMAAGIINDPLLQQQQQQNSVAGGPPPQAAAAAAVGAAAKGSRVAP
PRAAASKAGGGAAAAAAAASPPLTNSLQQDQLRESLERLDVRLGALRRKMEERACSLDSPAAGGGGGGGGGAPRQDA

APLSREERRGPPAALAKSRTPTPNKAKAIRDQRQQQMSAGQGPTPPPARAAALSSAAPPPKPASAPTMGTMGTAGAV
AAAAAEPSLLQAQQQFLQPAAAAAAAGGGAGSGAALAGAQPYGMPPRAPAAKAPSSAPSGPAVVNAAQQQQQQQQQ
QQQHQQQQHFQQQQMMAAAAAAFGGMQGYPPGGGGGGMMPMFAPPPPPSQQQQQQQQQQQGSGPAAGAQQQVAYVPAM
LPNGQMAFVPQYIQPGGGGGGGASAAAGVFPGFLQGAAAPAVGFGQAGPAQQPMMLMMPTGQMMPTGTTAFSGMQPM
QQQQQQYGGAGPMLAWGPGPVQVAPPPPQQQQQLQLQNVPYGSGGGAGPSLYSFDRSGPSGHGGGGYRRSSQDGSTA
GGYGQPQPPQQQQQQPAAPTLPYATGPPPSGPMPHYQSTVMRSRSIGSRDGEAVAAAAAAVGLISPPQQHPTSQHQH
HQHHQHHQYDAQQHQQLQNMQIPSHAFPAQQQHHMPYGDQYGAVPQQSRDSYSQQQHQHNPHPHSHPGQAPSSISPS
PSSSGHGTSSQQHTVQVQSFGPNGSSRGLGVAGGWGPGGAGGGEDAGVIKAVNLGLLLS

>Vocar20015133m|PACid:23132454
MNPTACRCGETKDPGYYDLPIWGALIKNTAECMPYETEVANQTAYGTFWEINAKREFESYLEEFNNATGLNIGFQSR
PPGDYFDENNATEWPTGEAPGPFDFWMVGAEVFGEGIAHEAFADLTFLLDREDHYKFTDIPPALRSAVSSAFTGSYT
ALPFMVMPWLLLYRRDVFNAYGIAVPQTWDEAIAVAEEHGNGKLGPGQPDLGFCFDSNLASGNARRALAYILASVVQ
LRGPSHGSWFDPEDLKPLYATEALREALRIFTTLKRYSVDDVTACQEVYDMMAGGRCLMLWGFSFAFKRGSSVSGPL
RGRQGVAAVPGSTLVLDRPTGRLVQCTPELCPYATPITTAANKIVANATATNATNATVAANGSGTAVTLVNRVLPLD
SVSFTINAFAPLPRQAASFALLHTFTSPSQRQKVMLSPTSDLGPLRTSELAEGIWVAAGYEPEDVKSFLSESRRYFV
QEATVYQELRIPGVFDFYALIQTVLQRSVWGNYTYDELMSYAATTEDIVRRVGGKEYLLELYRKSIDYSPPPTGKL
YDMRQQQQQQKIVLVSVLVPTVTMVLLVVAVMWGYLRYSRLRAAARAALSAAPGAGPTTTLLVTDIQDSTVLWEGLP
AEVMDAAVKVHHRVVRELLLPYRGYESATEGDSFILAFGKPDRALSFSLAAQEALLKADWPTQLITNESAATVVVRC
DAAACAALLPYICRASRLRDALASSLAQVVGAATAGGCVGGDAGDGCDGASPPPPPPHPPPSSLLMSSFRKSLDRRP
AKDDFNSSVCRTPRARTATMATALINAMRAASSSERSCTISGHGGNNGGSAGGGGGGGSGSGQEPVLTERFSPQSAE
SLRRNTSERTRRPVSVFARRPVSGGAGLAGGRSGVAGSPAAAAAAACGGRASTDQGYSTRTTPGFLFSTDTPSTSAT
EMRPSTAATAAAELLSETSYTPSAAAAVLESDGDGVSGAASGGVSSMYGEDLGLLRPSAASGWQRISRAGNGWTASG
GAGGGSGGTAITSANLGEALAIMFPSVPGLSGCAAAAAAATTTIGFGGGDILLFRGLRVRMGMHCGVSPKEVQYNRA
SSRTVYPGRTLQLAKAVSDAGRGGQILMSSAVQTALKAGGGGASGGSDVPYIVLSAGRHLLKVGTQVAELYDVFPRD
LLARAGHIPGLRSLQDQVPGVLAAPLGRVAMALAHVQDSEEFAGWGLEASLEGHRAMSRIAATHVRQQGGYLLRTPP
GSFHAVFTRPAAAISWVLELQEELSPAPGNPSTSPPPQPPGPQSQLPQPPPWPLQLTAPSVNSSSDSDAAGTLLRRA
VAGFQLSTFHFKGGVDVGPLTAALGADGEMSYSGGAAKRTACLAANATWHEVLVSIEVAGEVLGEDHPAAAALRRAA
AAAAAPSSAPPAAPSSSSSRGITSGRGKRACRLRALRPASNNNNNNNNTFHTCPSQASAAGLFKHISGGADPAADLG
PAGLSSCQEAVTETVGPARFFSSADTAGGSADRPMGRGRESAAAAAAAAAAAAMPAACTGDAAGMPMESPFVFGPAV
RMFKFKGGVIEACAVALARDGGGGGGTATTATTATGTASSSSSWGPSVV

>Vocar20015244m|PACid:23132515
MQPYPLPPPLEMQPYQTPPPQQNMQPYQTPYAGSLPHPPPIPPHPPMQSPQLQQAQQQQQQQFIPHPSQLVPRLPY
QPPLHQPQHHQHPAQYQYHRQPMQHHNHQLQQNPQDLASNLATGRDGTPLALAALPPRPPPPPPPPGLLAAGPGAAA
AAAAAVAGPGGSSYPLAPSCCPLSPPRNPLSPPVALVEMEDKDGIRLLESGNGASPPPPLPPPPTGHWDFAANGGGH
DGGDEGSRTQLPPSPPPMRLRSPLRVAYEELCVADPLLRARLLGDLAVRWLEVSSVVTSFLFARFLLPDLLHIPYRT
SRGSVLLGHHDPADLVYLPRPVYGDLLGRLSPGWGACQWPRGSRACM

>Vocar20015297m|PACid:23132540
KGYLPLDLFFGVESGYWWVCMMCVDVDMKAHRSFHLFGSMHLQHACLATSSLLAAWPMSFINIVILCFNQFLKKNRR
AFPVRIFGMVSERIFDMKKGWMALESWDNCEGRLGWVSGCRDAMRCDAEWWRGSAPHKSSPSPSHSTSTAPSFLPYS
NNSNNKNNDATSSSPAAAAGAGAVLPASSPHQKHRHQRHRILNLPFPPTDTRNIDMPCPVPAGHVSPFSNDWETLVS
GECRTTEWPQIQHGGIPTSAACGGGSGGQTNTCTSTKQQPLLLRLPHFQQQSRVCKASYQGSDKAAETLTHRNAAAA
MAAQQPRSQPSLSRVAAAAVLPALLVLLALGGPAAPRLVYGSVYTGTDFPFENCVQEQRNSPYYATLYSYRENRLYN
TSTICIQVRVLSTCTPGKYRCCTTGINKLKVFPALKCRGSVASALVNGNLIMSYYWEEHSGYDILKVTPLSQWLSTP
EKADGAILCLNLQAPCWQASTFSYNSQLLEYALYDKKANNYECCPTGTVLFSASEPGFPSRPPPSRMPPMPPDVPSE
PVAASPPPKVRKPKPPPALPPSPPPSPSPPPPKRVSPPPPLQRSPPPPTPLPLSPLLPPPSKTKRPPPPPPLPSASP
PPPPRGCAVTVIVHRSAASTQGAFTNDVCTLYGTLVTLPLYDGTASPQATYTCAISDPSNMALNITLATAEDARALL
SIYGDAVQRRNIAALLGLTDCSNDFATLSTSCMQDLAPVTGATLNTWIGLTIRTDQGAAIPGYNTDGSRLSFIPDDF
QYDRARSAVRYYMLSCNAATSSCGFRYSPGGATDVQPSYICEFL

>Vocar20015165m|PACid:23132563
MCTAQIRLVQEYCDLGSLRDKLKTGAFLKLAPSAVVADGVLTPPSPPPSPSPPPPPAAAAATATAAATPPHSSPPSC
VLASPPPHLRVVDLAAVLDTAIDVARALAHLHREGIIHADLKPGNVLLKGSTSDPRGFVAKVADFGLSMRLENNETH
VSNAYHGTLLHGHVSRASDVYSFGILLYELYTGDVAFRSVPKALIGHAITKENLRPVFPPTGPLGAPFEYQLLACRC
WESNPEIRPEFDFILEDLKRMRVRLCATVPDGGGGSIGNMGRLTPEAAGGGGGGGPTGFGFPSEAAPAVGRLWGLSA

VVHSRQGLNYLVQTSQGIGAKPFFRECGGVGGVGEGSLESDRSLSYSNSTSMCSTDAVLTRPSHNVTGGAAATGAKD
GGGGSVGPGVVQGFAGGSGLHGDVRTAPYPGCRDAPAQDEAAVDEEEAAAVVRSL

>Vocar20015245m|PACid:23132590
MLRLGGSRPESSGGERFPTSSLAAAAAAALAADGGATCQGNSPSGTSDDGDADVYSLPIPSPASLRSLRLTQGVSNS
DTHGSTNSSRQASPNQAVRELTSDHFLGDVNTSNSGSRARSASLVCTPTRCIPSAGDMRVLDLTCLQQERRRRTIHL
PAHHSPAEAAALGTPSEAQATVEDALGANLLSKPSEAGSVDSAVSEVAPLRELPTLSPCVADGLAECAWGALLDGPD
RERAAVELPLQPSSTDGYTSATSIASWAAAAVADNIHSGDIRELPVAALPALNVPAMRRSGLVEESSSTLEPHIHAP
IILAAATEVQLQGADMMAIARRLERRGDDMIAAALEVQRQSEDMMAAAHEARRQSMTLLAEGVSVKMQSAEMVAAAR
KVQEQGADLMVAAAREQEVEENIVVEVEQAVSSEVKHADGPEDSADGRPPCGDTRSCDAPLNQTAISPAAISCSTLP
EGAALTGSARAATPQDCCKGADSSSSAADHAVTSTASTAPDVPASGVAFAVLEVEAVVPVVCVTSEAAVMAAPNASL
SQPELAAEASIYSPEEMAELLELALEVSAEAMGIRAAETMRANAGEEDALEGLALILAESARCPTIAADRSTAEGGG
VSLPVLQVGQRLDSAADTNNEGTETLTLSPRSAGVVAPALAAIKGEKESAAALPSHGRLYAEVAAASVAVAAAGAAT
VPALPCVITADVDGFGLLTARGAPPPQLNDGGTGSGNGGMHCQQTPNGAAAIMTPNSTSSWCSPKPKSKAAALAAAA
AVAKAKAGSVGLPAAPTPASPSASAPSSPAWSTVASHKGQRSRSAAMMQLDPVAFTNAAVSSCATSSIKSNNGARQS
IASTNSSCSFVDNTKGAGTRHKPTASRAHQPDIQLPGRSCARGSLIRTQSERHSKDGSNEAARACAGATSGNSVSSP
MAATVVPGDPASQRLLWAKVKSFLFRSSAYSTEEEDIEDEAEDEPGRDGSWLTFGTGLLAGAVLVFASALLLSEVTK
NQQPPIQNTYRQCRLGPLGQMSNGTPLPAAVPWSAEGASNDVTRNSRLEWGGPLQQSASGWRASQCSDLDADDRACQ
EEASSTTYATCHGLGHSLSESESPTEDESKTDRSVVVGSRAENVGSRAVDGGAQSRMGPEGQRAAEALVSHPQYVGS
GVPAWLTIHPQAIFDRLEGWLAKDVAHLDGAGSDSQSSERLESVASSLQPLDSPQGLEQMISAQWPPYGEQLLLGDK
DDGGMTYKVQVTQVLETAHGVTTEANTADLPDDEVDPGAEASNTAGAADPQATTLEEAAGAADPQASTLEEATGAAD
PQADTLEEAAGAADPQATTLEEAAGAADPQVSPSEDGAYAADPYAATPEEPADAPEPHTGPSEDAASGPEPSWEGAS
SALDRQAGPAEDATFAPDSQLVPAAADAICSRPAEILPAAFEGHGMHRVTAAQPMVADGDIPAMNAPAQHVAGATAR
AADASTAPTAISVIAPLRGAGLGLSAAVAATVSTLLWAGRRRRPRRHRNWGGTWKSVWDVGPVPSARRNIPPATAQV
DGAGPIGQPHDTANGLYSAYGSGVAYDAYGGTDPYGYSAAGAAGFDAGAVLGDAGPHGAVGNGHGQPTEYDTEYHNQ
PMSPRRFLRDVPADEGLLDCYYLRSNRLVPRGSPRSPRSPGGSGVTFGSGAAAGNAEADVAAGQAAVARQRDVWHAA
SREVAWGPLLEALLDASGPPATLNVVLPAAAAIVRFMPEVVPTSCCGALVTLLDPPAGVCGGGPNGQLIAGAHAVSC
LTALVPIWRSAALQPHWRATRGSTRLLAHVRRCVTAAAASAATAAQLMRNPGSHPRLVRASLPDMSSSAPAAAAAGG
TTGCGSGGGGGCLAQLTAALRFTLTMVHLGGEVRADMLEQVRPWVLLLLDAHQAASAIAAAAAAAAMDPQEPSGGGP
GKAVTGARIRAHGLGIGVVSDSTVSYFDGPLRAQALVLELLQLLSTSRRFWAAVLRPSQPQPQSQANIPDVAAMRKG
TPGATSGVGVATRSSAGLGSAPSAVAAVTVAAAGSGRPVLSCRLLAVLAEHLGPPRRAAPPGAGSPSSPSPPQLSSP
VSAHLRASAALLCHPGLWRPSATYHDGTVAGAGGAGAGGGGPDPLEAARCAVLEAVLGYLEPHTRADTGSSSSSSSS
CRAAWPPSTAILSVLAAALMPAPIPYGSKAMERELPYSSVWSKALAPILALAVWAVLQGTAGGGLNAVASVANPDVP
RGPQQDRSGSETGDTGVGMAAVPVAAGGFGFVGGPTGVLKEVEGAAAEATGWLLLRLLAPRATRETLLAACGIASCW
AAGCRDTLRPGPGNTAPAPGRVSTTSPAAGVPSSPAGASAAADADADADSPTWDSRPGTLAALITRVVMEQYAARGG
SGFGSGGGGYAALVVSALNYTSLGALSETRAQPGPQRRLTLLKVLLAAAASISADAGAGTLTSRPGQGCSAPPVPSV
DLQRLLVNTLSCCCGELLASPLPPPTGPVGHALVLHACLGSMLVAAGHLPPGERAAAVVSALPVLMAGAAGSAGVVR
KMAAAAEVPTASHVVHTAGSALRLCRQLTALATGPNLDDAGIDGMSSSHQSYQSYDRTHGQEQQGKGVGPASMAAAA
SAAAAGGLAAISHSQDNRSRMDPSWGCWWWAEAEVAAAEAEAEAEAAVTGAVTGQRNGVAAVGGGLRYGGGGAGAPG
GSDSGSDLDGDADGDVSGDPEDDGGIYECSADMSSGAALAATAPARLCGAVRHLVWAEIRCAATALLSSIFGVLLSG
GAPPPPPPPPPLPLQPTQECLCAAESLAAEPEAGAPLFGSAGCGGDGSGSGDGCTVAPFAPAELLGEFCDATFSFGA
ACNAEGQAPAAAAAVAAAAAVAAPAALIAAGCPALAAKLRQALLQPPAAASRDVGVGGSRGSSEGGNVSGRTPRSSS
SSSGRGGGGGGGSGGGGAAAAVAAAAAAVAVVAWPRVIEVQLGARVPHAAFRTVLDYLVQGSARLTPYKAAAATATA
VREGPAGDDALAAAAAAAAKREVSSLALALGLPELRATARSSFPHPGSSPPHATPCLPPLLPRHLLLLPPQPQPSPP
LPSPPPSPPQRRAAHDTSSPSPPPPPPSPPPPTPAAAAAAAVPAVETGEPVTICMGKYGHGGGMDGGPGVDLGDAAG
GDDGDGCGGGTNGNGVYAGGSGSLNNGDDRRCGDGSDGISSGVQLISSSHFCKRRSVHWRLEEQLAAVARWEQEQDE
RSRWAGPAAASSPASVATSASARAAAALPRIPAVEVGCLAGGAGDGGGRGRGGGIHDGGSEATGTSSTRWAHPCPWP
ADCVLAVPLLRQYGSAVHLAAANAAFAGAGPLGPDTLAARGGAMSVLDGSAGAGGSGWGPVGAESLVKASGGGGGST
AAGDNCSISTTGCRTVLIPTHRVILAAGSEYFAAALSADRWGAGGGEGDGGGGDVYLRPAAAIPAAARNSTHGGGGG
SGASAAAVALRVRLLRVPGCDADTVVAVLHYLHHRRELRCCGPHQRRYKGDGEVDQALSGTAQGGRTPTPPGSCAAT
RPTAKKKHPKVPPCPARTAAAAEAEVEAPGPSPHQAADGHGGAGGSGGGLSVGCLLTAVRDGAVLQLPWLISAAVEA
LTQRYSSSEAQQAEEWGDLPEGLQRAALDSWARKARAAAAGAGSRSSGAGMGAAATNSGAVNYVFDELLFSKDTWAD
WKLENPLADMNPSDLLPKAKERFSGYDEADFNSLDGPKSRLPPGHKTAITGDGPDVDPLDDENLVEPAAKAAQCSTC
HVLVAVVWHRLIARLLSGETLTRNTVEELLQDACEKDAVDVMLRKHTILKMESGGGAGSLTFFVVRERKDGGRGPAA
TEAEEGAVELSCKGLVEEHGSDLTDVIITQLANYRREALRLLVKHDRDGGNTQDEPVTATDTEATAPAPSAATSGSG
SRGQLPRLGGGGRGAAGADDDDDYVDPDDLDEKGEVLPGARGSKGGVQQQQQPAKTAAALPLRVRGLVIKVNPDEPR
CEDQHHLCSSWAAAGECSKNPNYMIGTDKHGGSCRKSCGVCHPDDLLSSSPKLSAELDSVSSALLAVKNLGCGAGG

PCLSGVSGMAMGSGLLKQHTAAYKMATTQVVTSEGLEGGRKELPPGMKEFPGGFILGKPCHGEVDRVLAPALAHRVM
SIPSDFFIYEYLYKNHTRHYHETPNGDVTEDWLLGRFTRESVSLMSSNTGEVAGLEELDLLPGSSHAELLKSNTWPY
VKQVYAGGDECVLAGGRRVVRRVEARIACSPDKHIYMLLCELDYYKPRPKATAGGYSKSASVAGAGGGSGTEEGASD
GGRKRRRMAGGGRT

>Vocar20000614m|PACid:23132651
VPLESALLHAKLDLTLIAILSKGNDYLPGLKGVALEGPGRSGLWARYEAAAAKLGGDGGGGGGGGGAGGRNPQQQP
QQAPSSPSPRSPRSPPPAVAVAVAALPAGAISATGSVTSVDPVTGAPRLDAAALAKMLKLAGVAAATAVAAMAVEVV
VEEEEEMYLTGECPDYRFVYDGVAPSAAAICAVCESLVASGQTYITLNTFGTADPRAALPLLPVACALALLPANCRH
LAAPCVRHLMHSPPDDSSDSDDGVPDMAVAGTSELPRFGRDLFYELYGDCAKCKKYGAEMAKIQKNYLTARQVHAIA
TKRHHAVQKGTAPVGEPPEEELMAELEAAHRKMQECKAQLHQVEQQRKAHISSTHPYRRFPVEQLEAVAEAALSGGS
IGAEERAATEFGRPLVLRAFNSKPEAAEFIRMHGTANQLQRPQSSYGQPPPYQQWQQQRGMSSWQRPQQLQQPRQED
YEAIYVPPPPPFNVERPAFFAAIVRQEVLYDQYGSTAPVQLRIASKAPNEVVRYLMTQDSRPLVSVAAAATATAAAA
AEAATTGIIGVGVAAVAEAAAAVESHKRRRATTLQQPELVEAVGGGVEAESGMEEEEEEALATAAAAAAAVPVLTGR
GSAPAEAQKRRVRKAKAAATATGDSVASPSEAAAAAAAAAVAVAAVSTAGGGAGGSAGRGRGSRKGSAGGGRDKSRA
NASGAAAAAAAAAVAVTSATTGAGDVRARVVAGPAVASGCVNLGPAVRVEAAAAAATSWTAPPAAAAPGGPLPMPSA
RLLRTAAGGCGCGGGGGPLELLSEAPRVGGTPACVQPYRPPQLHTQTPDYGEGLGQRPVSPEYSTFYGRGNAPYDVT
WAPPPAPVPPPGGYLQHSQYYPAPSAQQYGTRYSYNVQRGLAPNAPYPAPVAMPYVGSGYASGPYSAARPPYRPMSP
PPLAAAAAAAAAAAAAAANRGRMASGPAGPRMN

>Vocar20000582m|PACid:23132660
MTTYTVNLTGAAGSQVEREVRRLAGLTSAFTRPSAAAAAASANDTANTAQGASFDTVEEMGAASPGSGAGAGGEVDA
ICSGGGGGSLAQPSPRPSFFGGSVRGGGGGGGTAAGRGPIRPAFGAAAASSTGSGDACRPSVNGDVASVRHSVELAD
VTSIPSSSNMNIPGLEGRQGTGRQGRRPSLPQAFLGADGAVAAATAAATSAPPHGSSGSLSLSSVIPSSSSQLLSGQ
LGLMRDRKASRHNMLLDGCSPPPTPPPPTTTTMTGGLTCPATPGGGGGGIGAATAGASASSPVIKLPTLPSATPGRA
QSGSQIADPSTSPPPRRIHATIVSASTVTPAAIAVVALSPFTSAAPSATIGGGGGGGYPLALTDSRPGSSRLVLARG
YTTNILTVPDRRSSVENIDLGAAVTVGSSGGGGGGRWFQQDADDMEAMLSGNDELLERLMVAGMSYGSGSGAPLVAG
GVGGGAGSRVFSGCGAGDSGGSGGGEPAGGRLLPRLADVSSSSVGGPQDRATAQLQELWRVQASVEDQLNRERLAMA
AGRGARKASVLEDVAGQHQPRWQVSYNVDAPVTHAKLGTVDASRRSELDRAHLTKLVKEQKAAAGGQQHPGPVVPAA
AVSPVVARARRLSTVLVA

>Vocar20000674m|PACid:23132671
MPASITSGVLVNCSSGYSAGLPRFTSTVALESGLLANATWHIVTEHIGEIVVASGMGCGGTVIVTWWSSDAEVFACP
KQPGWVHTAELCCPPPPPSPGPPELPPSPEPPSPPSPSPPPPPKPPSPAPPSPRPPRPLPPRPPPPLPSPNPPSPPP
FPLPPRFPPPSPRPASPLLPEPMSSSAGPPSPAPSPSSPQSPIPRPSHPPPSPEPSPQAPSPKPFPWPSSPAPSPEP
PLPDTPSPLAEAKKPPQLLAMPPAPMEPYTPPPRGVDANDTQPPLPPQATPNNQLAALPGGSSGTSPGPIVAAAVVP
TLALVAVAAGLYYYNKRGPTGRQQLLSGDVEPATALPRNFILQEQASTAYGYLVDARVCSRLLGAGRGVLSHPLRTN
PCGRHVNQALDNLMGGGEA

>Vocar20000578m|PACid:23132724
MANPSFPWLPGATPVYLSSFKHNIVAFLKEYGNLVPLKLQLTTAWIVPLKHKNGVVKLHVYEEHLDEKQGTPPICDQ
CRNMGWQHHPVSNCRYHFILPSPAVLSDPDQLPSLVEAAAAGRTAQLGGLGTRHVECTPEPWFMKVESCKPRGHSLG
SRKRKAPRRGFARLPPLPRPPLLLLQVVWIRPRQRAYNAPASIFDSTAHCLHGVIHGNGFGHLLRVNGRDGAAATAA
AMAAAAQDDGAAAAAAAAAAAAAAASVGTPLVGPDLMNLWDTLCSTLRAREVSVEDVSNKASMLLRLLYSAAHMRTWY
GKWGYGFGRAAFNYGAEEYMAAVRAVHEAPVSELLRDMEGVDLTAQKILRTYCALPPSLPPGATFGEVLQRMLFFIS
RPDLLKEARVQHIAAVKAAMTAKASTGQDPPPPPPGPSGLGQDGGAESGGGGGSGAGRRTSKGGGKVPLSGGTLPPA
KRTRRSAGPEEAPGAAVEVSAAGGGGALGGSVATVTAAAAPPPPSAVSTTSLPPLPPLAGAAGAPSGTTASATTPGF
PGAPPPAAVAPPGASAAGGTCAAGAVPAVGGLGSFVPLQPGTSLPGPPAAPNSLVPQAGLNNATAPALFQMRPPPSL
AAASPSAAAAWSSVMAASAAAAAAAQRHSLAAAAAAAAGPAAWAAGQPHQQPQSAALSTAAIAAAAAAAAMAAMAAQ
AAGNVSGAAGYRPLLRPGLQLPLLQSPLFRGPQKPQLQPQLQGSGGIPLPQIPQPGLLQLPAVRPLLGVQQHQQQSQ
SPAPGQQQQQLQQLLQQQHQQQQLQPLPSLFLQQQQGPSHAGPQPLMPSAAAAAAAQTAAGLLPTHGSSAPSSVPAP
VVYPISAQAGMPSQVLPAHADGTPPGTATPNPHQLPTTKFMPMQPPSAFLTLPNQPLSATTNQNQNQYQQQQQQQQG
AAGAQNIPGPAALPAGFVPVGSPLPPPTAVCPSAASSEANPPAAAPSATGQPVPPLSAAQPQSQSQQQPQPPLPSSQ
LSPQPSPPATAAVPQPPPQQQQQQGASQPSESSRLEAVSMSTDGAVLASLQQLSTLAKDDAPGALLRPGTSMPPPPP
QQQQQQQQLGLQSGSGVLPHSSWLASPALSLMPQAPAAETTSLAPPPPPPPPHQQQQQQQQMGVNAVQGTSTTGSTL
PLVPLAGATLPPPQPRQQPPSPSQPQQLQQQLQSLQPLQQQIQAQQLQLQQQLQQLQQLQQLQQLVMQTQALLAPLI
RQANNNNNNSNVAAGGPQATQPIISGPVAGAGGGTGGSGGAAAVTDGSIVQQQQQQQQQGVAGMALQPPPYAAGVQQ

QGGGGGVAGVSGSVPVSLPGGLLRQSLAMPGELPAAAAAAPSGVQPGDDDAGGGGGGARQPSSQLPPLQEAVGAAA
GPESLLHGAAGPAETTLTAAPPSPKLSGEAPGDVAATAVAVLPLPPALSLLPSSVDPEDVAPQQLQQQLQEQQLQQQ
QPVARPVPDACQDAAVVAVPTAMAETAAAAAVMVAGTGPTLPATSCTTACQSSHAASMPGSWSHDNDLSPLHHHQQQ
QPLAHVWFPGFVPMLPQPSGVSSYADGGCLGALVPSQGAAASARPSEPHAILPGLETSVPAQEAAAKAAAGEGKAAS
ASAAAAAAADAASASCQEPLAGTPTALPDGASSMPYHVVHGHALPYCADYACPVAPRNDPVLAPAIAAAATATAAAV
QSMDVPGATPLETLKGSDGGDGGGCGDDAVCGMEYDPCSALQSAAGSAGLTATGGGEVGMRRTTTADLAAGVGADGA
AAQLGAQGSPAAAAEEEKAAWQTANGVMAGPADGTCGYGGGGGGVEERSRSNGAEAAPQPQLFFQLLKEKQQVQMGA
TATLAGGPRTNSGDGGFGADGGSGTSSRGTAALGVVGTDTEPRSPQPAGVGEPPAALDLSTASAAAVDVDVCGAGAG
AVTAAAEEEENSTVRRVTAAGGNSDETERERGTTAVAAAPLLPTPVSTSPAEAEAEVETDAPDSAIENAAAAAAAQT
QQQQQQQLCGSDSGEALPPPTSPTICPLLISSWDIAAAAGPSRQQDWPPPPPPLTVRGGHISEASAASAMELDFADP
GLQGEGGAAAAAASPRQEARTTAALGTGALFDSVMGSDGVWPSDGVSDPLPEPGGGGGGDVMGPGLSSPSPSEAMVS
EGTAAADQAAIVESLVTSPERATAAAGGAAAAGCGVLLAAMPLELAAAAVAAAPTVTLLPLEPERSGATAVEAVAND
TTAETPDTAAEAGANEGSGLAVPRDLSLAALRPMGLSSVEQEAAPETAEAPQALLQPLAGQGLEAVAVMVTGAHGHR
AEAGLAVLGSEVAPAAASATDNGPPGDPAAAAAAAAAAAAAAAGAGGGAGGGVDDAFPAALLACAADAAAAGTTVSD
AAATSRTASASAAAVMMEAAATTAHPSVPFLGNAVGNRSSAMADGNAGGSPVTCIAADTLAPAADGLLVLAPTAAAN
SHIATAADAAPSPPPETLAAAAAAAGPGPGASGAAIDQDDSPEAAGRATAARSGAAATTSHPGAAAALDAAMPSSAA
ADFDTADGLWTDLPPLLTTTSSFTDPLAAVTVRRVVATAAAAARGSAQSPLRGDGTEPCAAVPMLSPPPAVATAAVS
SPECPGDDAWGLYDGLALPDHQDLYDGLGAADSGMGVSPREVSPGGWSGFVLEEDLVPGALAMPVLAAAVAAPPPPP
LGPVPGAEAGARPPRSAGAGADALLSNPVGTAEAVSIGGGDGAPAVAFPPAAAAERAAPLGLDATDADTAAASTVST
DLPCAIVASDGGDGDGDGVLLDGDADTDGDASGGGGSGGGGLENGALAAAGATGSGGGGGSGGRSRSSVKASGAHIA
PAPDDLAREPVASLGELHAEPEADMHLRDCQRAMDAMLQALQSHCFQQWLARLQLRDLMMMAYRDYKVLDWVLKQVA
NVRIGGYVVYRSKYTVTRSYYYRLERLPDTPPDTPLSYTVPMPAHGPYARKGNRLIKPRGSGSQAHAGGGGAAALLV
PPPPPPPPPAPPSGAADDPAEVPPLAAATEGSGAGAVHEETAPVAAAAVDTSGHVDGPAGSGGSCSGEAAVPQPSAD
VSACHDGLAVPSASPGAEAAAAGGLDSCIDVRDSVHEVVPAAVQPAAPPDPETTDSDGADSAPSAPPCAQPPPADLK
AEVDAEADACRTTRCMDAGEGEIEEMGNTDVATVPLAAVVCEGNEESSLVSEHEVAAVAVAAAAAAAETEEAEEAQQ
AQAEDERRRSCAAAGPSHGGSPAQVLGPKKATAGATKSKPQTAKAPRPKGHHKPPGLGPVRSPVKLRSKAGLLASSA
AASASAPAAAVAAVAAAAAAADHRATVSLPPSTPTVTSTAAAAAAAAPSSCGATRFPPVTSSWRDLVKEHQSEGQAL
LDSLLESRIDQQVRQQQQQQQQRSGGLEPGAGGGGGGGGATRSAAARVPRELRYLMSQSTLSKLKKQQQQQQQQQQQ
QWQEKQEKQDEHRERQRQLEVQQKQHGAAEGAAEGAADGKGDGVATLAEGSPPPPPPPQQQQEQEQQEQEQRPEAEG
GERDVGEQGVAGRSGAATGAAALLPVSALCVSAPADDEEEGGGGGVVDRGPRIKQDQDRELPPASCGSETRATGFVV
GCKTPCGGGGGGGGGNDCLHVVAPVPVGRTRAESASAGAGAGTGDAAIEPVEGAAAAAAAAAGGKGEGTGKPGLAEGR
AGAAGLPGRLQNPAELHIVIEPPPPSTALEAPIDVAPEPAARGSGGSGVDGGEVIDVVQRAPSLTSVQPLSPPPPPP
VVKEELSEEEQGSKKGFAGMKVGAATGAMAAANDGTLIKPPPPPQQQQQQSASRLRFGQRPQRARLAAQAAQAAAAE
VEAEAQRRRTAAMKLKTEEEEEEEEEKAPPPTTLAPGGGDDKAPSAAVFLESLTVPRKPRSVKVRRRPSAAALAAATA
IIAAVLDTQPQSRKSPSVTTALKGHATTSGGGASGAIGSCAANPVSRRRGGCASTTGDDGASGAGAAVVPRSAAANS
NVNANAAGAVAAAKTSGSGGQVKAGASTSKVKSAPGKGGGGTAAAICGAKEPSAAGGAAAAASSGCVLTKAAPPVRS
KPKSQPAAAAAAAAAAAATPGPNAARAAGSGAARPMRAAAIAGKALREGGKETAAASPPRLDKSPAAASCTSPPPPPP
PPSGSLRIRLGRVTRPVVLTSPLRAGNGRAPALPPTTATAAAAAGATAAAAATTTAGISEPGVANTAGALKVRQRGD
AAVTGPPASKNTATAAGQASARDAGTGHKRAKTSHSETQSLVHSLVQSQSPSQLQSQTVSHRKSRERPSARSPQKNG
KRRKVTTSGTFTTVTTVTTAATPCRTAGAATAVPSGGGGASTAVGGTATAGGGGGGCGPQDTACSPVTAAACASGPL
TSLPRSTRQTMGAPAEAAKDGRDATGAGGSGGSCDGSRSQKGFQGGGGGGRTRGRKGPAGGPVYDSTAPPSEHALVD
SVATRPSPGPPPPTSASVPDAATSAPSPDSAAGATPSPLPPPPAAADGGPLVSPSPAAAASTAVVASLGPSALQEPY
EGLLRTLPLTYIHKAMVAAASAGRAPDSQGKGSGAGSLGGGSSSKRGRPGGCLATLVMSVGAGGGGGGGNGTSFDPE
PPAPTNPYVVPGKAHHNTSLTYINDQTQCDIQYLYCSVLRLYKPALGAAVALVSKRFTAVKPLALPYMAAAVQMLKD
TKYFVKDYGGVSAAAAASATAADGKVRVLVALQLDDDTALAGVPPPPPPQQQQQGPSRRSGATRASAAAAAAAAAV
SVAATAAHAPPPRVPRQLTLTPPEIVVLEPSGSTAGSTADGTTAPVVTVGDLKRAAAAAFRGTYRMCCPRSGGGMVV
TSVVRGLDEQVLAEGDTAVLSAPSTTANPSTTAVSETVMETEPQRPPQLPLVVVRCSGLDLDRRWRHAGGPEDWRVA
CLCGTQDDDGERMIACDMCGVWSHTRCNDIPDEVDEPPAFVCRECAAASTAAAGATKRGSAARSG

>Vocar20000685m|PACid:23132727
MFEGWASEFLAASLGRFVDVQRDKLRISLWSGSGVLENVRLRPEAFDYLNLPFAIHEGVVGRLRIKTCDLTRMSGMS
ATQIPWGNWLTGSLVAELSDVVLCATERSDSEWEEAAVLRREQAAKQADLAAAELAKLSRRMVVQQQQQNQQQQQQQ
QQPPHSQSQGGLTSAVGVRGAGSASQRRSQGGGSCFGAAPEGTAALTSGAPWLPTAPPLHNGVSFSAGLALDSLSTV
PEPKAPKSVLARLYGAALGRHSHAKLARHVAVRGLGVYWHPPLPDEAQTAAGAVAGPPPLPRPPPHSDDYLITPMDV
VLRLTATAVATSGQGAHMQHAHHQHTQGPSGSQAGGVAAGSGAGDGAMAAAAAAGAAAAAAAAEFRQGSSGSGVRG
AAGSGGLQPPTHRLSISIHQHHYHNLSQQQQQQVLSNAAAAPIPPGAVAVAAAAAAGAGTHVEIVAGTDAVQIQIRP
EQLQSMAQLADDLAVWTKRNRYGRFRPQGWRTAAAQAKRVCVKEAAAPAAAAATRTGAGGAAGSGAAASHAHQIQRS

SSDPGRIQTLSRIVLQSVQEEDEVQAAARCVLHLQSDGAPTATGSDGADGGAHLTSPLTYAASTLLCESDADAMTAP
SAGEARALANETESGGGGGGGDAAAVEGAHGSKGRRSPGAQNGVGRPGGGNALLPLSPRSPGTLTPAVSPGASRCGA
GPQPSGVGRERVSEASASGSLNAPASPTGTGTGTSTSTPLVRWSQLTEWTLEEEQERERAAAAAAAGAAAVASGSAG
GTSEAVAAASGQQHPDAAPAQHRSWLAWGLSGVSGFFTYGSSGGAGAGAKASTAVPQASFTDAELAELYRLLQGTSL
PTAEASQTQASAGRHHHHHHHHHHHHHHQHAQQHFQAQLQHQGAGVPGGGTTGSSSSAAAASSMPFSVQFRVLQLKL
DIRGPGPIPAPHHNDRVGAGAGAGAEPCSAEGSPPTPGFGISSPTCVPSPARTRRGSSGTRPPASSPTLLSLTLGST
VLALESDGSGTAVQASIGTVHAHDLCSDPGVAECILAQADDMDTGGGAAAAAASGGGGGGGAAAGGPSAAREAGQRR
RRRGAAHRSWASFGQLLSGTGSRSSGVHASLSTDSLDSDAGHWAMGYQSQAAAPAPPPQGDVWYGGPPVVSVHQGFG
GGGAGQVDIHVRPLRLQYRAQCLASVAAALAVDSCSFLEHHAMTAIAGFESRDARVLAMLQHLGCTASSQKLRLHVE
GFEVVMPLEQPSPALSTRGSGVTYDTSRNAHTPAGGPTTAAPGHLTVGLYDIELASINGMPPPPSQPQAADRAASIQ
TGRITECTEGPAVDGSAAGEHPSGLAAASDEALPATNSGLVRGPLEREELAEGAYGSGTVQYLQDLMQRRLSPCSTT
ADLLRLAAEAAGREPVHDFSTSDAGPAEATTAAGGAADVVTSAAAAAAEGAYSPVGTAGSPRRHRRSSGTLPPRPDP
CSVEDLARLFLYEHVRFAVHARHAAEPLTRASSELNEQRQTKRSVARSETRPLLLLLPPALQLAGYLSRSRLEQGVGS
PASLLQLSLSGASTTVKASEVLDLKRLAHCLDHSHAENAAPPQPQWQPQRQPAAEAVACSPATVAAEQLQVPEPPVA
TKNQAQGCSPALDAASGPAAAVSPPPPGPLGARAALPGASAQGGIGEEAAGEAGTLSSTASVDFPPGVITATVNNQQ
SCAPPAAAAAAGAGGSQGRNRDGSGPTGSESVQVSYTRSAPTSPLPGAARPLLLPRSATTAAAMCLGGDVEDSVVVQ
YGRRRGSMGSGLSNLETAPRQWDSIRTRPCTRALAGTTDPAAAGLEAPGMELQGREQPYSQRETVDRQEGGIRGCAS
PRLPLLPRTTCLLLSVEPFEVNYVQDGCVGSNDGDDGSTAAAAALVVRVDSMMDLCASYSSGGGGGGGGGSTRPAIE
STVSLQLRDLEVHVVPATAAAAAGAGGRGNNDADRTGGLIWSLPRTLWMASLTASAVKTSGGALSACGAIRGVRLLG
SADQPGDFLLSNGPGPDDATVAVRYWTAGAEPPAEPADAAAAAAARVAAGWWQPAEHVSVAKGLGNSPRGRVKSWNA
HESGVGTTDGDDGGGGGGGGDDGGGGGGGGRGDGDGDGDGDGDGDGDGDGDDSGGRDGGADVQRQTGGSEPPGLG
GEVRTMELGGRGDDGAIGGVEVVVSDAAVGMGLLMSLASASGPSGSAAAAAADGVSEGHAIGEAVAGGGCVAAVAEA
EAPSAASPPLAMAVHIRNSAVLAVSPCRTPFAAASGGAGAGNLACNALLSVDKVSYSLQPLHVSLPAPQKPSLSSCS
SAQSSGGAAEAAAEQIRESDNAGRPAASIAATHRVAVTRLLVYLADNPDSAYLSPVLLLPELCACYGPTTAAGADGA
ASLASMANSAAGRTRGDYYAMGGIADHGEVARVVGLGVSGVAAAGVDGRPRVTEAGVAAWLSVDLGRVEANFQPQQS
GVLCALMGELAPKEAQQGQEHGFSEVPTGQSSRSQQQQPRAAASPELGTAAVRRESRRSGAAPSPQRRPVHEPAASV
AAAVAAVEPSRVLAASLPYMLVSVKLGFLTALLGSDTLSEEATVLYWRDVRLSYDRTDVDVESSLAWRELTLSSVGS
RLHPAPRMVMQGVVRGGGGGDDDAAAVASSKIHAGSLAIMLELSSYAESTLRRTAAAAATAAAAAGPAAFDAAKADA
QFFRSAAPPLVSPAWPLPSRGSGFAPAAAVPPRSLTVPRSPAAAAAAAALVPGSALGSFPRGGGGGLQQHTDSGGGG
GADADAEFYSIEGGSSVLDDLEVEAEVEMEVEADTEEELHLDGLDGDVHQREEELWPCPCGTGTGSAAASDPGSGRR
GTALRAHLSSCKSIVQLSTLPSAHVPSDPVAWCEPAQPQPQPPVGEDGAGRAADACFGGGGAGASAAAAADTTIASP
SGPAVVLLSAGATCRTNPGPLASASAAAVPAATATVDDPAGAAWSHVPDAAASPISTVLVRIQLSNFVRHRAALQPP
AVLAEGLGSPRRRGSPQHHGTPPGGAVEAGVGPDGRQDAFPSPATTGYGDGNADADDDNDDNAASYGANRGHAGGGD
APAAGSGRLHFDLDVSGVHALVSLTQWHRLQDSLAACGQHWSTSAANQLRARARQQQLVALEQGRRGSGRLGRPGSS
VGVRSAAPARDLPGSRPVNATSQELTSAAGGGARSVAGHAAAAAAAAATTTAAAAAVSSRGLAAVVTRVPVLGASGG
SREAAAECDGSGGGGGGAAASADGDGMLRLLLDLPVTASVSVQDMSMVAYSARELAFGGRAGRVACRGPDGQDSDGP
QSRAPQLYPATATAAMQQQRCVRLELMMGASLEAAAGGCMDRLTLVLPEVSVSTGALVLEHDPRVGTWHEVVGAWRL
ALLEGLSCAAVPHLRRASSGSAPGAVDEVQGAGSLHIQTAVGKAGGKALAATISAVSAAATATATAVPSNKLWVGDS
TDGDPHAVAGGPLMNPGGVSQPVKHLSVGAAPPAVPPRLSAAAAAAAAAPGMSSALGEGLSTAGAGYDSGSDAAAAGS
ARQQRQTTDGDVLDGNGGAATGVDDIVVHALLEASLQHASLYAARHAVLLLLGLVYDTLDSWEHAAAVTNRRHVLEG
SDSEANGNGDGDGDVFAPGILDEQTLRLVGSPPDIAATAAAAAAAARLGVPPWSPAGAHRASGAASAAAGDLGAATA
SGTAPHELEYGMWPHDADEEDRDGAEGWSGGPDLLYHYHYDVPHHYSRRHTSKQRAGRAVAGRSESKGSGDATGAGG
AGGAALGDWLGVPLWPAWLPLCCLTLRVGKAAAVVYTDTQGYDVPLFEAAVAPLEIQLKLSTLGDAAAAAAGGHAAS
DGDADVNADTAAAAAAVAPDNGSLPTPFMRSFIPKKAENDNANVRFATTTQPAYVRHRAAAGAAAASGTTGGGGGGG
GSGGPTFGLLHRRNSNRRHHGRGGLSLPDGSAGGSTVATARPPPPLWVSSLHVVLDARLQLDVYNLDKLGWEPVLEP
WSLQVLYMARQPAAAAPRYPPRPAAGAPPSSAALSSPAPPPPRHSISITTDTLLEATLSPGLIPAALAALHLAEELQ
GAMTDPSTVSTAGAVPTAATHPGWRSASPAASPKNVVPEVGQEPPLGRHGVAEPPLTIAGRVGDGGPHVPSDPWVRD
PAATALRHLGQLPRPDALPRLWLQNHLGQPVSFVTLEQRRPRASFLAAVGALGAVDGVVTHGSLVVVDSLRHAGRRF
AGRHVAGLPHHLHHHHDHQGFHHHHHHHHPSSSSAKINGKFQQCSPQDGKMEMLAADGSGHAEDTDYNMALSSSHLR
TTRCSEGAPAASVSFSGELSRLARAGDGDGDGGGGGGNDTSGGGGGGRILYFRLYGGSTWLGPISCNASPSSRYYFS
LPPAARLAGTVASPPPGLRLVCDIRPHHLCSRRLELRSNVRLRNESGLWLSVSDWAVVAEASAGGSGGGGGGGQVL
QHTLPPGGSTWLSTGLLQQSPAVLAVRTAPPPAFASGNIPVVEEGLVVSTAGGVAFGDEPATRFRSCGGGPAAETA
ERHAPLKRLGATFRGAKVQNASPWSDWSNPIDLTSMLFDAVAQGLDGAAASGAAAAGRVGVARRLYCTLPTTKTYRL
PTSDAASAMPMLEKVHKSSVPASAAAISVEEGRMCHLIVHLAPAASGAGRGRGGGGDGGAASRRLEWELVLRAPVVL
RNDLPVPARFALHAYSGLGGGAVAAAAPTACALPAPELRESLHALSSLHLHTFPAHRLVSVACRPAGYAWSSAQPVY
GRPGENEHDHDHDGGDGGGGFEPLHIGLEPLPGAGDGGSAGGLMPYCERALMVRPTQAGLQDLLLVLRTWRHRPTGQ
VQLELSCPVWVYNCTGLPVQLRPTARLVIPPGQHAQVVRGSVAEPNAPPGTGCAAPGAPSPGLAPECSDTGETAWVP

ACVSLCGAPTLAAAAGPCTSQLAALQAAAGLFIAAAAGAGRGMQPYGVHGDIAAIARDAMGGSAAAAAAVAFTARSS
SSARSNCSIGGHTAASSARSAGAGVPGCVVRGGGSGFGSTGLLAALADGEDQGGDDAGDSGDVILDGGGAHPYGAHL
AGLLAQPSPRHSYYGGSGGATGTTAPPGYGVHVADFAAAGRASPSAAYPHHHPHPRQPPAAMVRHPSLDRLNPARTA
VRYDRTRSAAAPSSAPFARIEYSNQHSMSLHCSSSRASRSQRNTQSSYRAASGGGAAAAAVAAAHLQLSLNRPLSRS
LSSTCAGHSDSPSTSAAGASGGGGFGTVDAAYRPQGLQPCMYGSMPVGIGQVELRVWPGGSSSNDWSPPIWPAIDGS
PVHITLPCPLPDSDGGISPAGAAAAAAATAAAAAVGAGGKGTSGGGGSGPGKPAFHVVARLMHATAGTGAGGSGGGS
GGGVRSVALHLRPRYVVHNGLSAAVQVRQQGTSHASSLGPGERRPVHWAHIGLPLKLQIRIQNPGWTWSGGVALDAV
TPGDFLLKIRHRNRAETQLVRLDVSLSAAGTTLLSLSHHHDDFAPYRIDNCSGEVLHVQQVGCLDQEDVIRPYACLP
YTWDEHTAPQRVLVSLPGRRRLGEYELEKVGTSRVVSVVASQTAVAGEEWEVEVTAVVAGIAVSVVSEQAEVLYALA
QGLHGTLLVGPAKISASAALLHARWDNCMRDAAFPIVVYSPVGRSMFNTAVLLPVPPPPSATAPGSAAAGGGGGGGG
GSVAAWRLGSQFRQSAYASLTAAPLTVSLEETHLREILRLTSKFSAAAASAAAARADGGGDQCTRLTAPAAPPWHGE
PHVPYVPDPSLHRPTSPSFHGGAYHGAVSTSASSYAAYEVLLNGFPYQGASGGGSGGGFAVSAQRRVHSPTGAVGGL
SHPVVGAAPRSPGSQRGGGDGGDGGGGNSFSGHPVSCPPGGAREHLFAAPSRPGGGGGGWAGARASAAGFHVLQDAH
DMLVPAPRSHRIYIEQLHIGEIRISASFSPGPYGGGSAAAAAAAAVLTADGGGGFLADDDVDATAAGGSGAVGPADI
GSGDGEDGGGGGGGLVDAAVRLAVSLAHLENAWVLLRPLDMRYALMRREALVQNVVRHYMSCALLELIKVVGSLDIF
GDVVRLLQGLGLGVWHLISMPVAGALRGDVRKFAAGLVSGLGSMAPRPESVPVTFIAHRHPPGATGGEVEVRSVTYA
ASNSVVKASRRARTTLANLAPEPLPYPAMAAAAGGGSSGSGSVGLRAASGGLGGGVGAASRSGRATATGRTGAGSSG
GGSGHVMPYGRRVSGSGARVHGSTPGQRDLLFSTWEGYAAILQRVAAEASRLSPTGTARELILGGLGAVALPSMAVL
QLVEVAAASLRAAVSPHHGSGWGWGWGPGGPLGAGRGGVRYCWLESLCRLLLREVRGGVFAEEVYVGGVQISDSSFA
VVTLQHLLVVSPRQPGPPPAPEWRLATHPADLALTLALPLEGLQHQHQQLQSTAEVAAQPTAAATRKVSQSAGGGGG
GGNDGGVGGGGGSGWAGSLFRSSSESGSASLLSLRYRQTAPAATAVAAAAAGPVHSREHEGASGAAGGSATSLGPAG
PGGGGGLSSGSGSGVALSPPEPVRGGVQAIRGQRYVGGVGGMSASLTGGVAKEDEDEEDEAAGCGLPPQSPWLCVTT
LEVTAEADAVRLVSLLEEVLCGDLGSEVGCRL

>Vocar20000643m|PACid:23132746
MESRLTAHMDSEDRRVKELTEGILYDFLPSALNVMLPRCEDHLQELDTDRCRAVASAALAVSALMNAPAGFLEFLLC
VPWRKLSQLYPKTPNLAARSDSIDKLVSNLQVTDEERRKLANTRIDPALMGTVLDFLYKDKHADSVPPMLQLTPVNP
LQPDDKSAPLVVRETVAAVLDGATPLHCAALRGNPAQVDHLLYCGADPTLKTAAGELPIELVPVCGDRATGSGCSGA
AAAAVAANAVVEGSTSPPLSSSSSSQRNCRCMGPHDQEVWECRSRLARSLIARRCFFSFGVGLFSWLRLVVFCLLCL
LGQAGCYTSINRPVVERHIDTRRQQKVAAARARAHALVTRMRAEAEAGLHHLEQAKRECMESAAFHRGPPDISQMCF
TDPNKSSPPPPPPISQIHDINLHLQESGTSSTGEELASEKAFKCFVRSVHALQSLDLHGDVVPQVFAELVAAGSAES
RETHVLEDEQAVRMAHIQATRLFGKVDRVKQSHTERWRAVGQALARVVYVHICLLLETEAQASPTRSLVWRAEQCLR
EWDRLEKAELTQGAGVLDPRHVAALQLWAKTADSDLYLAEALHGQSMPPTKTLQEVVQGAVIYGADNTPILARPVSA
DVVASLEQALTLSSLPSPMLANIVRGVVANAVAELAAAARLREFVGTRGAAALGGAGSGTSETVAALTEALRAAEPF
RSRLGPEMDAAQELHGRWTQRAQALEKLESAVEEVRAYITAHPLSFSLAGAAAAAAALAASTSAAAAAEAGAGGPAT
RAAPWPGPGLPTVTATASSSGPESAAGKPGGSGHGATSREVGSGGSGGGESSSTGASSSGSGGATGTAAAACGEGTL
AADLAEWDRRMRQLEAAMNDAKDANISVTKAKRLVKEMTTVAAAAEASRQLEAVMARRPSGPMQLKLALNKAEAAAS
TLGSLGGAPLPGLADVLGPLVAAARRRLDTERAAEALSKAAASYRTLADLARLEAAIYNAKKIGAEELDPESYRTSL
ELRAHLQEAARMRTTLETALRNLQNQLRPEDAEVLERALQDAARWEDLLAPDLARARKALEHWRAMTVSDAKLARLL
REGASTGVLARAIQEAAASGVKVQQAKRVLKLMQALETVLAGGGIDGGAAERYAAVKAKLEAAELGGVTAGPLPDAA
RRMLARLAAQIAREALEAALKPHSDWSMAQRISVLRDALEKAEGVLAAAANEQQQQQEEGSDGKASSDATAAAATA
IATAVAVGVTGTQQEQHQTAPKLSNASATGKTSRGKRCSGSSVTVRELVRKARRVLEEDQAEMARIERERQEADRAK
KEAQERQRQERQRQEQERQKREKAEAAERERLQAEKRERVRAERARQLHQNQQHQSQHQHKAKAPAATAAAAAASDP
GSLGGPEKGGKASAPGRAASGKGASTHHHQGSGPNGGGGNARSGSASITVSAVAMAAGKAGSALREPQVLPQLAASA
VMAAARHQPSAQCQQQHQQHQQQQHQHQQQQQVSAVTTAAQSSAAASAVEAVPAGDQVATSAASAAVSSESFDPPPT
TVFVDPPQSAAIADPGTALGIFAVQLLSANDAALQSLTHQAAATNNVVDGAIVPACLPPNAVAAVHTRGDSMGSLSE
VVRNLNLDVIEALDMDHDVSLSCAYSPLDAGAVEATVAVACGGCGDSGTPADGGGSGGGSVTRTMSGVTAAAESVLD
RTPSQRTSSVMSSVASADDDMLHSAAGAARACSVAEVGGGGGGGCALGSLSSISSGQPGASNASLEVSSLTSLSATA
NSATAGPDGTAACRVAAAGAAGPVAGGPLGGGAGASNSSNGNGGGAAAKPRFGLVDLGGASWGLDPNPVLTGVSVRT
ASLFGSLQPSMGSSMGEFGSMLCAPKPDAGGGGWAAAPLGAAVGAMLYNGQAAVQGLPVGGPDDAGNMLGSAATVRH
VLGGSGGGNGMHVRMTDASFSSLVPVHASPRTTVATASTSVDGILQVRPLGAGGMPGNAPPLLQLAPTVAAAAGSGG
SSGSGRSSLHLAMPLQQPQVSNGAGLAPRSGYWTPPSCQAPIDIPSDGAAAAATAAAAASACNGGAPSSGTISSLQV
GGGVGLHHLSLGTSPVGSSFQMLDWSLSQQHQKLQPQLPSPPPPHQHLDGGLQLVHLGGGGGGGGPPTAGGSGGGG
SGVQVLRSAGGTGATGSRICHFFLSGYCQKGERCRFLHHIPAPAAGSPLPSAHVAISGGSSTVLQLGQGSPPPPQLS
PHHLRGQVGCFGVSGGMGPPGGSGVGAGAGGGGGGNGSGAVGSFAFGVPGAEAFAPLGPGGGGRVSRASTGMDSGPS
GQPAAVAVAEQQQQQQVLLLSSKRASVNHLRHNGVAATGPPGGAGVMSGTPLGLILMDRNQPGPVGGGLSGPGSSS

SSGGGGGNGEVVLGSHQQPVQLRDSATGATFAAVLTVPGVPIGGGSFHAKQLQQQQLQPTSARDHAAPQQQQQQQQ
PMLLTLPDDLSYSPRSSSSRSGPSSR

>Vocar20000780m|PACid:23132750
MRGLGRATCKEQTRDELLEAVRAERAARSGVKQRARSAGVIQRFWRGYRARRHLREMLISEWAMSYGPMVAVQEARL
SGRDVADKLLPPLLYLLRPQPAAAAAAAAAAAQRDSRGSTAATANDDAVLLPPQGSPLSNMIRGSLALLLRTCSASD
PRDSYLAIALEGDEARRVAWRDQAWRLLCTCCRLVAAPRGVTTPPQPPQPPQPPSSLSATGSPRVQHPQQQHPQGQS
HGGSNAAAAGGPSAAPCGVGSGVGDPVVDAAAIRVAVLLTDSGTWKCLGQGQGGGGGPAATTGGSSSSAGGGCGGSS
GISERVAAAEAATAHFRSRLVQQPHSLFAALARLLAARRQAQQQQDGGGSSTAATASPAVAGQQQQQLSRLLGLALA
VLRSALVPDLNPPGKADPDPSPKKSAGHSPPPPASELNREGSPPGCLAVCWWCGRVICFEKHRDVDVGALQVRFRSS
GAFACLCRSVRQQLDERTVGDATAAAWCLANLLQLLLGPHMHSVGGTATAAAAGGGGGGRGFVGVLSPPPDSFWGAG
GEVASELAATAAALMEHIMARGRLYGSPELAAAVSGLWPLAQRGLLVPMLAALESRPGGVESYTDMCCAAMELMPRD
SLSAAFSEKGIRAADVPGLLNTLAFSPGLLPRLWRWLCFAVHLPLSCPAGATRCLDVAALAGGVYAQLLLVLDDGEF
YELQQPLELSAARAVGTCLNSLVFHTYLPKLPSDGARAAPGPGPALGPGDPDFALLLEWAPRLLRALYERDVRRPYC
QSLLWLAPYREMFGSSDEQPGPPPPPRPNHDVDDPFAAAAAAAAAAAPDPADARIGPFTAGTVLQALMYSAGAGGAG
RDDEDEDGGAASGSGGGGTAPALQPQVCMCMLCSESVPFPRRVDLLRAVLAEDKRRGSEAGLDFGGLQKELLERVVS
AGLDANYGLFTSTPDGLIYPNPAAERLDGGLALLEFMGLMFGKALYEGILLPVPLAHFFISRLQLLTAVRPPGRQPL
FDDLASLDPELHKNLLMVKRYEGDVADLGLTFSADTDYLGCPAHHELLTGRGDMPVTNESRLLYCHLLADWHLNGKL
GAPTAAFTRGLLSLIPQGCTAQYTAAVSQRRSQLGYPSASLRLFNPREFNQLLSGAAADGPGGGGGAAPLDVADMRR
WTRYSGGYSPDSTTVKLFWRVVAELTPNQQSALLRFVTSCSRPPLGGFRYLQPPLTVHKVECEAGLFAALGGRDVDR
LPSASTCYNMLKLPNYRRASTLKAKLLYSITSGAGFELS

>Vocar20000782m|PACid:23132874
MDIDMTVTSRDMAHRLETENGRAFCEPVPVVLVTYLYVRMRNIAQRPNIYTFGDLLIMVHEADRHVAVCCRVWPNGD
VHEKPPIPHGAEGSPTASNCSTPSCSSRARALSCKPADESTETKCGGLPRTQPYHPCPGHSPVAATASEQQRVPSLA
LLQQRQQPMTSQDVASDRQRSSHCQHPRARAPSRSRTAGSWVQGPLYLFTMLLAAWVSAAAAAGAGPASLSTAVSGA
STQSVAAGEAREELLGLLHGVPLHAPEVSTGGPRNLIQSMAPDTVHRPLRRLASYYPSYYSSWTLPPPPPLPATLPP
PPLQATLPPPPMSQDASPPFLPPDCPEIPEPPCEDEPEEPLLPPSPMSLPPPPPPPSPSPPPPQPPPPPPSPSPPPP
QPPPPPPSPSPPPPQPPPPPPSPSPPPSQPPPPPPSPSPPPPPQPPLAPSPPTKQPVARCAPWRASDEDATGTNRAA
GMHRCCLPYVQSATCFILFAGSRETPIFASERALCSGSHRRRGAQARAKLGRVRLLFLQPRAARLAPPIDSASQPRV
PSCGSSGTSHGPIVAAAVVPTLALVAVAAGLYYYNRRRHVNHALDNLMGGEEA

>Vocar20013601m|PACid:23132907
MRRYTGRALLAAAALAAEAYSSLSPAGALFLALFLAWGALATRSSLAPARAAARTNLVLWSCAAAAACAVLLLQLLL
PLLPYNGIGPGSTLWAVLGLRNLSQCSAWELLAKNFFCWHISREVRHHGYFNYKYAYGYLGLHDTYQQPTTAIKTLI
EIPDSRARQSSLVRLPFKLSHVVLSPPLLCALTALAEAQDAHTACCGGGSGRGAGGGTVGGGGGAGGAGGGTVGGGG
GGGAGGGGGGTVGGGGGGSTSEVCKAILEHRTTATRSACPPNSAWSAGSGGAAASAAGLLVVLAALLWPAVFNLPWL
LLASYGIAAWALRLSLPLATHAPLLRLLQLYCGVALAALYGWQLQPPDAWHPMVASGGRALGLYRLYGAAATQDVDD
IVLMALHAGTLTALYAALGFAAGAAGEAWRRRRLRHATRVSLASSGWPPSSSSRNLTVGTTVRHGRVDTALREPLLP
PSPPAPEALPLAASASPAGAASSPLRSPLAATAASGSFRPLSPAAAEGPSTVAPGDGAIPPSHPPVLASGSSLPPSH
PAELHHRGISDRGGLQLQLQLQLQPQIQASRHQGQDPSLRDHHQPPPPQQQQQHQTRAAWQQPTHHQQQHQHQHHGG
GCGGGPAVLARAAAWSIEAVEGLSRHPGMAAVALAGLSMVEVSVLGWVLLAVGMWALLVPGRGLGLSHGGRGAKGRE
GRGTECQPKDCPSGGGGGGGGGGNMEPSVALKSAADVVEHADRVYGKYGTSCGRKVLRRASPVLTLLLLVWNTAVYV
VTCLSASYPHLLPPVLHSLGLFMYVSPPPVVLPLLGHAFAIVAMAGLSRSSTAGRTSNSESGGGGGASANASAGRGR
DAGNIVVSSSSCSVGGGAGGRHDSVANTSLGLTFVLALYHILHVLVPVSWILIGSYKLDLLHGVYLLAVLLYCGSTA
VRLIPDPRVGAIMPEVRQREGVVHGRVPHRAASSSDDDVAGDVAVGGGGNGGSGTSGGVSYALSYPFPATPPAHSML
RLYASLHLLALYAAMCGQLPGLGFLQPDPWVTTLRLVGLWAPCRTADCLPLLATLVLATVHAVAGKFLKVVGAANGK
PPAAAESSQQLQPSPPPPPPQQQQNPAGRAAVVQHWLLALGSTLADLTSSAGYLLLAVLLYVLVLYDVRIGLLGLGY
MVLGVPLLLGPPRRGHYVGLLLLRQRRRYIA

>Vocar20004224m|PACid:23132989
MDRSQDLDLIQLHTGTTRMWGMERPNVFEDPPPTAVLEAIAELSAAEASPRPPAPSPPPTPPPWTHSCRITLTDEEA
AVHCVANPDLPNLPPTLTAWTGRELLLSLMRPASPPPSSTDSHVGSGTSDTANHAAFSGVDLRLRVPPEEALRAAEK
ELQQSSTWTAKPFPDMGITFRGVPHLRIVDSVIRSQRGRVHMQPLAPLLQCEGCPYLTVENVTVKGIEGPEVYIFED
SPWVNDPDFPTFGPIHASGLIGVEVSDFSCTDIAFSQGWSCLHLQYNDLAVDEGDDGDGVDAVAPGLQQLYDTAAGE
GFLNIHDSAFISTETSWGGAYGASALYSEKLYGDYDFNTGVAPTPVHRYGGYGSVLVSRLAAGAAARADAYKAPLVR
EATGSLQIRVYDSLFKDNYGNFGGVLAVLGSSRDYVEFSRCQISGSIARDGAVLYVNGSLSGLALRNMQIVQSIAND

RGGVAFVSGSVDRFIVTASNAFNNTASAGGMLYAPYIDKVSISDSVVTENVATLYGGVIAAEGLGDVSVARSVISGN
RALNGSGAVLYATNGFDSMNLGNDTAISGNTARDAGGV

>Vocar20004139m|PACid:23132990
MRSNVNSRRTSSFIASSSRPEKEKPTNAAPAVTGPTTSINRNRVTANVAPVQAQGRDLNTATYQHAAPALAAPTAAT
AVKLSTAVATVVPSDIARNDSPAAVAVVASGSRAAVTAGGTMRRSSGPQPSTQWDPQHHHQQCYVQQQEHQQQPPPQ
QQSMPSHEGTLTHGYKQDMRLGTSPTGQRQGAASTAHAPLGAETHGSAAYTTADISGAANLRRQKLEALKARRARAS
PAAAASPAAVVPGPMIAASAIAGDLVSMVSYAEEHQRAVRDPAWSRSTSLSPAKSPPPHHQHQHYHGQVPRGRRSPS
PPPPPGYGSPPRDARRGGGNGGVGSAATAAASAGGMRPAAVAKMRSPDNGTQTDPSDLDMSSLQQRQRAHYQQHHQQ
QQQQQHQQVLVDELRPAGRLSAGKSPPGADGAGAGVWAAAASADDTRDLADQSQQQRQQQSPHVTRPASSRRQHAAS
RDSSATATVLVSKSAVGAAEAGPESSARRHSGDNSSQQRRDQNHSGRPADDKLLPRQQPEQPQQQFPQRDSEGNNRR
IRMAEMVRRFREEPVTVLAEDEEEEEEDEEEEGEGAEMPGAGDRVMKGEGGSSDGAVDGDADQDQDQDHPVDDLAAA
VLQRSRAMLRERDAIQQRIEAALSKFGVASGAGGPSGAGLLQPQPQPQPQQQPQQDLRSGLSAAAPVRDHPHKEGEG
PRGSSPRPDLDLDLGLGKDRKSTAGRSFGKGFGENEGVGRPGYNGGSRRHHHRHHPDDGDNGEDRYMGESQMEGGGA
AVDGCVQGFRSDAALWHQRVGSGSGGLGGAGGGHGSGSSSSNSCTGSESSGEVPRALLAQAGVDPAPAAAPASPSIT
MAGGVGGSGPSLASLSPTTSSPLPPPGSSPRPGLLLKRGEAVPNDPLQAWRQLRQQQQRSPVGFLPDLSAMAMSSV
ANGPATGSPSASAAAAIGAHGGGGAQQELVVRFDGTLGTHLPGGFSAARTALADWLGQVSGAAAAAVVAGRCVGSGG
RDSGLPGGSDEPPRSGAVASGLRAAVTAAPGALAGAASGFLGLSGAGGGASGHSWGAGMAAVGPSFTASASAAATDP
LVPSVDDLLARLAQRFGLVPGTASLPSSSKTAFGSVAGTSPASASAAQSLGAVLAPAAADAVHALAAAATIASPAPP
MTSPDAAVILHSCVVSTAAATSGAAAPSATSAAAFRAAGGDMDKDEASCSSSTTGVRRAAAHQTNGGTASSRLYGPC
EPLQVPPPLPPRDADADAGPCGEPAPQHAGGRLVEVAAVAGAAAATASNGVMSGAAGGGSRRLADAFEEAAVEDAAV
RRAAVGQYVTNPKAPVVAEMEETEVAVEDVAVEDVDMERSGSLKSDDEAAAPAVASAAWPGWLAALGIPRPVDAALL
PAAFRDDGSGGGGSGESERGHDAAGARAREADVAPEYVEPHDMALRVVKGNPDSAEASLRKSPGAGGGSGDVSASGS
FSPASSPGSRAVVTAAAAVAAPTATATSSATSGTRDHGASSSRVASPQAASGDAAPGSSASAAADAEVGFSTPVSLR
LLSTDTRGAAAAAAAGGDFGPRPTAFTPVLGSLLDLAVGGSLFADTPPTSARRTHAQPRSHSHLQVDLGLDLDPGGH
WPGGPALPSGITQNGEGEEGGGAVGAGGGDDVGDGTGRVVDSADGDVMQAFMPIASPEPVAAGLSPSRRRRLVGRGG
GGAGRWGGDETGSSGGGASPGGLMVRVPGDVSSSTPFSSIASSGGSRFAAILHHTVTAALFGDSPVTSLEVGGDSSR
FSTTAAGGGGAGGGSNSNDGGSGRFAAAAAAAAAAAMTGREDVGAAVRRRSGSVERELSPLGRGRYAAVAAGEVPGR
MAEAGCDDPADAPPGASAAGISRAVAAAAAARGPALQHGAETAFVMPWLPGAERTSSSWDRDRDHHLRPAVPPAVPL
AAVAGTLREASMASIDPHEGVAAVATNNRETQAAAADAGGGGVPEFRGDGRWYTGVHLEAGSSSSSSSHSRSHSED
GALRQRSASTVVSEVYEEDFVEPSPQSSGSDISLGAIAAAAAAAAPAASAYEASSSPPAAPAASAAGPLSPLTFDD
RRKGSTEGTGAPSTVQQRLGWERSRDGGATAAAAAAATAAVAVAGQLHSTHHVSAPRGEPLSAASRSAARPYQEAAA
AFAAELSGDVLLGVSGLTCGDAGSGSGSGSGSRSGSGDGSVCGVSVAAAAAAPAGVDIYAATARIAMGAAAVPADGG
SCGGTASASAVDHGPAAALSSTAAPPGQHAGTEGSGASTDGTTDPSSRGQEADRSNSSSTESVYDFGASAHPVLYSL
DIEELLMLGASHQDATIGGDSVRSSGAAAAAAAATAALAAAAAARGMSIPEAAAAAAAAAASGSFEIEIDPVTGESG
LVFMVDDPLEAMFLSGGGGGSSMPMGRRVPPVIITEIGSSDESSSSSSSAAVVAVAAEDGTAAEAATSSPSSRAGDD
EGNGRNLGGATAAATVAPSAAAATSGLIGPSRGANDPRVATHGSFPGPASPGHLAALLPYQRNGVQVKEGQQHGVQR
QCDNVSPQIRQPSSPPSTSRNTSFRPSDHTAMAQAPPAPAAAAAVPKLGSAIQLAKGPNPGGGRLPPIRGSPSSATA
TATATGAATATATDIATGAATAIDSVATATAVNAAAADDDDDDFARREVLGMRPLGTPQGTSMHSPLHLQPQDATRA
PPLRQEEDAWSGKQQEQQQQQQEQQQQQQAAHQSTGVRAHTRRSLFLDAREEDATLDQDLGHFQSFQSPTALVLQQQ
QQQQCQPDSVDSLSPPAGSHQIQGSAALTATARAAATAAVAAGANVGPLASDASGFMQQQPEAAWGNGPTCTAQQHA
ARRTFDAVPFPVGAPSQQPQRPEDGVHTDLTAGQPTVHHSAFNAAMQLGQQQQQQQQQQQQQQQQRQRSAVVAGEAS
AVTAATVVAAATTMATVAAAASSFQRPSSASHTLLSGSSTLHSLAQPALPLGSMHQPQPQPSQPSQPSPPSPPPIQP
LPPPQRPDGSGKVLDGREAAAATAPGRRTGHPDEYMPALPAAAAAPHGSLLDFNTSVSLPPYSQSCLQPLGRQAPS
MSVAYPGPLGQDPHQQRLRPAAAVATAEADKAWPGGDPEAAAEDVIVAMLKRKMDACMDKLRCVVCALFLSLYVFGK
GVCGGITQWYFFLYYSSSRAQRPLPTSGLYLPIGRASLRRLGDTAGPTLRSRLSCALSPAATAPPTCRHRHCAYPPL
SLHLFLPPRRLSASFRSLERDLRRSPPPPASR

>Vocar20004158m|PACid:23133054
MMHSAAETYLLKKFEQFRQRKEEAAAARRQAAAQQVRQSLASKLPPSLALALGTGNSTISLPARGSHVAPGSRASSV
ERADSTAAAAAPADATPAGETAKDRALRALRLQKQRTEAAEAGEQVGSGAAAAAPATTSITGRKLPSAARRNAGAGG
PPPTPLHTPAASEPEPAPVGYVPISRQRGGSASGGSGGGGSVPAAAAAAGGGGAVPTAPSAKASSAPPGYKPPAPAG
FKPPVPGGFKPPVPAGFKPPGPPGFKPPVPTSLPRPLGHAGFKPPVPAGFKMPMSGGGSGATGGGAGASLAGNKRPV
PEPPPPSSSSYGSDDEDDYDPSALHGNCMDTIPQYDGADDAMHDGTGGDGPDAGLASAATAAGAGLTVPLILSDPVV
ATAAALGALSGLPLGTDGLGVLGGGDGVAAGVPGCDALLVPAVPAVSPEEAQSALAEATAALVQATAALAEATAALA
EATSTSPVPLPMPCLTDANGLPVLSDTNGLPPCLGDPNAGSFMAGVDGSAVWGVIPFAAPFAPIALDDGTAAFAALS
ASAAAGGDGAVAGVAATAAAGAASVGVGALVPDGFDPMLPSLQQLQPLPSLDNSAATSDFGLQTFLSGVPFALPDAA

NTAAAAAADTAALTSSTGNTAVGGGAAAAAATAGSAAAAGANAAPGSATAMDFSVIAALTMADGSELEAAAALTFDD
DGDDLDASFHALIQSDDELCEDNELAVRAPGPGLWLPAGIGPGTASPGGLLPPPQQQAHQHPDQQQQQGQILEVLHQ
RQPSMRPGPMEWAPGQPHSPPPPAPQQQQQQQPQGSGRHGASGSAMQLSGACYGLDDDDNDDVDDDDQPHAMAICGI
NEGHARAQLHQERRQKQQQQQQQEGQQQQGQQHERGGQRKPQQLGQQHGQQHGQQHGPRVQESRAQLRAVKLEPAAE
DAAAGRRPPPPPPPPLPRQPQHHHQRHMAQREQQQQRRQQQPQQRQEAAATPSHQKQHQLQQQMQAQPKQQQMQLGK
VQESTSASVHGCGGGGGNGGVQAGRVQPPHAAPPPPPSPNTPGLGEPSGQRARQVTLPAAQADENEDDGEEGCISKT
CTVKARGNAGGSGTVIRVKLGPYRATATAVPPPPLPLPPPPGSNLTPRVNSAVSPPQTIPPPPPPPRPRALSSLPLMP
ARSTGFGVPESSPFAGGNGAGGSGGGGGGGGGSPVRGRPARGYGLQIQVQVQITWCKYGPYVGTAWRCPAAAAAVPL
AVAISISSVPPEAAQLTVPAAAGRRGGARSPPPPVSYYRGGGGGGGGNGADPGKRGGRRRRRRQRGGRRRGQQGYAH
AQAAAGPPGNTAMAAPAAAAAAAAAAPPLLQLAAPGAGGNGSGAGNTSPEGSATRPLPPPPPPSDNRGSGGGSSGAG
SGIGGGGGGGGAAATQPAGGFATAAQHHRDMDRRQDRNRDRDRERGDPRDRDRDRDRSAVREWDAQGQRGGGGPRAC
DRSDGRDRDRERERSFGGSGGGGGGGGSDTRSLELACTLFVGDLPHGISVAELRNVFATYSKEVVNARLVSGQLYGF
VRFSSPEAATAVLDLSRSMGVEAGGHRLTVARAQGSLPDWKKTGPSQQRRDYTVSEGYGVPFDFPPIAIAPGPMRMP
MRQPPMFGMFGMGSPMASGMTKALELSGAADFDDVPTPASSSCGCVELQLLVPGPAVGLVNLGNTCFFNSAIQLLLA
CPQLNKAATPIWGQAGTKSGLPGGTGGGSSPGKPGVLGRGPLGFALQQAFINANGLHGRGTLGLSSQPNNAHHNHGH
HHAQHGNKGAYNPQALLAAVCRVAPQFKGRQQHDSHELLRLLLDGLQEEEKRAMEMTRRRPGKDPASPGAATAAVAT
DSSAPSPPAAGGGGGAAGAGSDVYGAAADDSSSVVASLFGGELASCIRCNTCGHTSVSSESFYDLSLPIPDDLPALD
ERELKALGAAAATAAAAAAAAGQHQAGGSAAVVAAAATATAGKGRKLSKREKRELERKEKKAKEAAAKAAVGAQKGG
REAAGKVKGKGRGAGKAQTEEGPDPEEVAEAEAEDSKKQQEERLLEKQDVGGGKTLAGKGKGPAGRVPAAEKAATAA
VATAAAAEGGDKKKLSQKMRTFHILINPQILTSAYAVDISTNSCFSSYLSYTHIPTWALFIMSLSRRHPKQLRKLEK
EQRKLKIVAKRQAKRADKVKAAAGGLEPESGDEGSEAAAAEPSSPTSSHTAGGSQVEPAAAEDAASVSAVAANGLNQ
TLVADDDDGDLRVAQIMSEPLEPARARVATETLESTEDAAAAAAAKTPHRLKTVASDKGAAGPPPVDGTEPAGTDAL
SQSVSPAGGIALPAESPRHGNLPADIQTGRQTELQAGSQAELHAEIGAQLQAEMEAGIQNESQVDPVESLPESTRQE
LEERAPFGAEDSVREQTLPSSPLPLQPLRPPRVLDVGACLAAFFAVESVSWECPRERAEALDAAASASAGHSVAGSV
AATPLTPTLRKQVSWSPGAPEVFLVPGSAEQRGTDLGEALRGNAPFFRPAKAHGASFIVQAQLDPFETETMAGDEAK
VARDADGTSSTSRASSAAMPITVSKKGNGPTVRRRAATGGHETWEYPTALRRPAEHPAFAHVGARALSGALTPPNRS
SSSSSSSSSSSSSLESSASTSPDMSPADSDTESERREEAVGLHGQRPPSPPPPPPVAQRGAVSLAAAAAAAAAVSTV
TPPSPPPPPLPSPSALANRAAIEAEVGTLPPSPSPSPSPGQLAMSRAGTPTGAAWKTRRQAPKLMLLSCTCMELTWT
LPCILPTTTIINIINIINIIIIIITTIIIITMNTSNPAALGRSQGRFLARVAAAAAPPPRMQMGFLATPAPAPVDADTM
FDMDDLAPPSSSATVHLIEESLSGQQGQPPTTYELVSPSAAILSLLSPPPLDRSPVPPVDGPLPAAMTSPTRSMPTP
AATSSAQDFDIGGAAAAAAVAASSMISAAILPAPPACRVLDAVSFNPAAAADTDVDAVKRSAPNELGACGGASVDDL
HNLGDAPGPPPPPLPLQPQPHVVSPHDTSVASEFKSGAGSGSGCRSDNVVPAVRHETAAEPPGIAIDSSSPRAPAGP
TGPTSSSSSSQPIRIPSSTLSSSPSPFCTPLLPAAAILEGDNGGDGRRLLSVPSAVAPTAPGGGSAAATAAATIPNG
RLRCRSSSSGGGGGGGLRRSSGSSFQRQSTKQYCLRRPPEVLVLHLKRFSVDSRGRLTKIDKHVAFDTTLDLGPFVA
AAAAAAAAAAASPAVVAALSQPQPPLSPASRSPACLSFLRDNGYAGAAVPMVPAPATATAPTPASASSKENGAVAAA
SAQTLGVYTASTSDVETAISGTSVRQPAEAEVVAAISTRVSGAAGSTVACHPLAGRSAVCNGGEGELLAAVLNFRSS
WAPVITPTSARIPTAAVVMYVAYVKRSNPAAGGGPQWYYVSDTQVRPVAEDQVLRAQAYILMYVRRREPSSLSRSHS
LSAAEAAAATAVMAPAASPSHPSGQVLDEQRQQQRQPNEEPQSSHS

>Vocar20005028m|PACid:23133244
MLPEMINSSNGRVLIDPCLRREPPVSSTSVKSPCEPSLAANIHAGMIMKIILLLGLLSVAIVSEAATKRVDPEEFTG
LPMVPLPPSKYDTKNQLSSDSVSYTASMPLAPPPAWSDMADTAPAAVRVSFQEVGANPSVPTYPPSSPSVNSGSLPP
SYNYLPSDSRPPSPPVPALVNMTGVAFFIGPAASCTGSAGVGPLHAPVSTNTRGEISGNGLVVAGREGHKVTIEAQN
CTDAFTGQQLPFSISAPWLIPPPENSTARVIVTPATRLLVHVLRSQNGSNFSADTFGSGLGISSVAYSLLGVYASDV
EGGAFQALRNNFLERLKGVYMLYADTALAALVVIAAPAVSFAAPNSALCPTPDAYVDAIFDSLVALRPEQSFALDLT
NTTLCTNVIRVATEQCGGGAAAAATAAATVCRLYGNAVATLLSPDNSPEDLALVPLAAVALAARVSYVVQVKAVAAA
AAALAGDGTAINALIEELIFSKAVATPAVPHIRKSPPPPPAYGPRDPTGALGLDAVPTVGAISVHVRASGSLTGCTV
NYFQVTDVLNSQNASTNDKGVATFSNVTFGLATAYPGCRDAALSVGGKTVRSQMTMRAFVPPSVIGSSVFINPAAYL
ASLVYMYNLSEQAPRTISAADYAAVYNYFGVGVAGAADALPSDADFVRQNWRDSSPLVARVYLLNQRLLNAIGPASS
FMSGLLNYTISTDDVAQSYFANMSYHLIQKTLQPDNKTYLKGLMRDSYIEWTGQLSNGSDGNRSPARNRRLQQILDL
TTLDQLLTAVSSAVAEVSSMLVQLDRQVTTAAAAGQTVDVTAVLLSAAKVASVAQSSLTTVLTDLAVAASTGNTAAL
TALTTALAANFSTAGLQAQVQNAQVDESGIGAVSDATLQGSGNAASPPPPAPPASKKKKTVSMPVLAGIIIGGAFAA
GIAVAAVAAVIVHKRRSKQRVGDGKAMAAAPSGPSGYNNASVVNVGQQAPPPGMTTVVVVGPSAGISQPQQLATTSR
GYAGAMPVAERVEMYGTAEGETAAAAAAAAVPWASPRGEAMCNSNTPRQQPRGFNVTIVSAPAANAACGVGGDAATP
RDSVPRQNMVCPEPAPTAAAAAAAAAGAAAANSAGIPFSGVSKAPDSSRLDENGTWWAAAY

>Vocar20005062m|PACid:23133257

MYFTCIAHSLNRPQSVRDACGEGGCGACTVAIEEERDGHPHLRTLNSCLAPVYSVLGRSVITAEGLPAAEPYGTSLV
AGRLSEFHASQCGYCTPGFTVACHAALHNAAAAAADGGADATATPATPDDGSLLRALDGNLCRCTGYRPIVAACRSL
AAEVTDIEDLCRRRTTGATAAGSISPVAATACGSGTADVTSSGSSGSSGSCGDSSCTAISLGSRQVLKVPRSLEQLV
SEAGRLAAAAAGGSTPSWRLVAGHTGHGVFKDWPLEEEVLVAVAEVPELQRLEVVAANDGLANCEEATSAREVSYHL
HIGACTTLERLAAHLAHRAQGVAAEEEVAVAAVAPGGTGADNNSDVVGTAFGVRQWAAETSAHLYRIAGRHVRNSAT
VGGNLVLARERSLPSDVATLLAAAGSEVELVVVSSDPETHGSVRRADLLEFVTGAAPSLPPGASYILTAVRVPLPPA
DPRVRFWSHRIAERYSNAAAVANAAVLVRLSAAEEAGGPRPVVEEVRIVVGVRTPGGGASAPGAPHDSVSEHNAAKL
KDATVVLLDGWQCLRAKAAEAALRNQAATAATVVAALEALAAMDLAEVTAAASATAAAGGDEEKAVAAVAIAQGLVA
QGLMACLGLSPTPPAAESQAAPGVDVIKAATGVENGDGDGGADCVHGAAKVAVHGGVNGDADGAAEAAVIISANGHG
ITAHSRPDQQPPQQQPQCSSKSSPPPRRMPSPPLVEGRQFLTDAVPELLPVTQAVPKLGSKLQASGTALYTEDVPVH
RDALYGAFVLSARACGRLAGIDASPAVSLPGVVRFLGASDLAGVGPGGALNACEKPPVGGGAAEKTFLEVGEKCEFA
GQLYIFAVERTLSHLHPCGPYRGLSPGDLDSHVAAQRGAKAVHLMYDTSTDGDAPLLSIADAVRARSFYQLPDVLPS
LLSGNTYGDKNSVLSKGGSDVGEVLSTAAAPAAAALTAAALGGGERDGSDAIAGVPGSASPPPRPTTMSVVSGRYVT
PSQLHFYMETQSAVAWPDEDGCVVVHSSCQGPDFVQGGVSAALQLPLNKVLVRCRRVGGGFGGKLTFARRVAVVAAV
AAVATGRQVRISVPRNTDAVMWGGRCETDVSYVAVLDDCPAEDGDPDASSGNVNGQQNQTSSPQQRRAPPRFRALDI
HAVMMGGAQRDISFIDAMGMIAAVDSVYDIPAFRLEVALARCNLPPRTAVRGPGDINATMVIEQIMEHVAAELKVDP
EALREANFLKAPPPPPPSPEPDCANDVATAPCQGVAAAAPMEPVVTTALGKTIPLRQYTLPYMWSRLQRVADWERI
KAAVESFNATSPWRKRGVAMIPTRRVGGAAQGYTMYRGKKPAYVSLLQDGTVQVACHGVEMGQGLWTKVAQVAALTL
SEVLPHSRRPLDISFIRICDNSTELLPHSGVTGASTTSEIACAAVRLACLQLLKNLREFALPKMAGREDFGLRDLVA
AVSTPMGVMVRQEVYGIGRERGFTREALGSMAYCYGRWRAPLSAFAWGGQSDLPESSRGVAVGPVAPQYHVMGVALG
VVEVDILTGDRRVLRSDIMFDLGRPVNPAVDLGQVEGAFVQGLGMMLQEEVTYDSTTGALVQNSTWNYKPPSVSCVP
QQLNVHMLNDAPLASEGPVSSKACGEPPLLLSAVVLAALQRATAAARTATSTSVGSTSNDGDGSGAGQVMKGSGGGS
ARSGEFSAKVKVALFAKGLCFTPLAVPCGSTRSQEFLAVNPLGKIPVLCVNFEDGRREVVLESEVILEYLEDQFPNP
PLLPADPLERSKARLISRFHDLYLEPALRRLYPQVAPSARSEQVVQEAAAAFLERLAELETLLPPPSSPLESAATAT
AASSTSRSSSSSTLALGGAQLSLADCAYPALLLYAELILPALGLPPLRGTVGASGGGEGVAGAAACSRGVAAKEAEK
LRKRERRRVDEGAGCPKRGCSLISQAHIAGGLLLARFMRMARVSGAHHALWLACTFMCVGYACTYVRSLGKRLHVC
VTGAPPVLYRRQMRSEVVASVSLTDPVLSFRYAVQQNRYRTTQTALMFLSHREYLEPRHGLTPPDQPPYRRFVTPPP
PTPLPRPRTPKPAPRPPPPPPAPPLRPPSVFPGPSPPPTTVPGATTPPNNNSRDAANEPPTTPAGAPAVALAAAAA
LLRRPDRPPPPPTPPPRAAPFPLSPLAFPLACRIMPSKGSSTPAAASAPPAAGPGGHRNYRRCIQSTPEEAPAVVLS
AEAAALQQQAEVALAARIRRPVWLWDPGS

>Vocar20005010m|PACid:23133287
MPSTNNGTTSAITINTFTTLITTRTNLDLSRTQSSVIAALTLMLASPGGMQSQRQRRQQLTLGVPRQVSGGRIEAGL
RRTRTPRLTHPRHEALLQRLLERFEAFRSAGRAATAEVAPLPGRASDCTALDLSNMALGMAKSGVTSPVLWEGLTEA
AAARRLRDFGTQETANLAWCLAQAHRDFCRREAAAVCGSDVAGGCRTSAVANTAAADHHYGIVGVVEGVDAQCRVFV
PWPGAPSSPPPPSAARALHPAWLPWAGAFVEAVLAPRIRCMLRFYTNQQAANAAWALAALGHADSVPLTAQGVSMFV
WAMATMVATMRSNGIDPHHAAVSTSSSTSSDIISSSSTDMSGSSSSLGNSSSDTAQQGSFTLVDGTGGNSSSSRKHP
ARSAPQPPTSPASRSSTAIHPKALVALTRVACAQTDRFSAQGLSNLMWGLGVLGFRSPRELRHLAAREALLRLPELQ
PLGLATLLEDWARARRYHPALFATAAQLAVTQLQNRDNMDGGGGGFFQPRTLARLLRACGAVGHANRRLFLAAAEVL
APAQANAGVRQRRVGGSAAAPLDELSPGELYQLAVAYGRVGVHAPRLMDELLARLAPVLQAAEEDPHGNSPPLLPRS
AGRLLCALAVLEHRPAALTAWRLLNGETNSSADNNGGATAAAAAAAAAVTFRKLGRSVLTGLSLMTSRQALTAVWA
LVRLRAAGAPAVMRLVYLLVRRAAPSADGLAAGLVAVARTPSVSGNPQTMARFVWVLGRGATAAAADAQRHRRTSVS
VDRVDGCRDAADAGAPAMAAAASVDTEDAEDAEARGVTAVFVALSQGLLAVAEVLLAAGQQGDGEGAEEGPPAGGE
ALVALAEALAGVLVVAQAPTTVGGGANGSSTQPAMPQWLYDELRLCFVAVADTALRSCSGAAGPRPSRLAARDLAAL
VHALYDSGAAAGRPDMRVAAARQVLYRLQAAVPSANGAASAAGSEATSPTPHGGGDRYTQRQPRRFSQPPRNHRRLE
QQRPRQRGPALKLPPPPPRLSPDDLARLLAAAARLELFEHPLAQPLLAEAVHTLMRSTPAAAAGADDGGAASYEHVT
AMAAAAAGPLGYPAGLAALQELVRHCRCQYDDVEADGEGRCGDQSAATAPVQDVRMAGAAAVSSSKGRALRHTLYGW
AARQALPLLPWLCEGTRALISEEVEHVQQGAPSRTIMDEQGTYGRRGGRGAYPDREIGGLTVRRRYVEFVERFAGVR
MVDGVDLGGGFGEVPIVAVGTINDGFGPAGFGSDDEEPIFLSPFQRRLRERGGLFGYGGGTLGMLNSMAFSSLSSGL
TLRHMRAAAGGSATATAGNSNATNSDTRGDSNSIGSGAAGGADGGSSAGLLQPRQIGLLDLHNRVSDLTRNRIQMLE
HNGDGSDGSGEGSGDNEDEDNCRDEEDGGDAGNVDGLDDDEEEEEEEDLWVYSEDDWEGDEAEEVYTEYFDDEAEEF
EEEEEDGYDTDSYSWGSLAAMAPGVGLHGSSSSGGRGDAELPQRTATAANGSAIESAARGLLVADASPDAVLGPRPA
SDGSGSSCSSRASGATDGRGGCAGSLDLFADEGEPLLAAYRAAEADEAQRPRSPPNGGPGAANTASTPPARRASSVR
LDATSIAGGLGNSGREEEGAPLEAQRQPLAADAVTLSASVYVTEEGEGHGQRHGRPPSGVEGRRRSRSLSLAWAAAR
FALSAVLVPLRLVMFVAGLSSDAFAVIARRPPPLRAMRVRQQLVASAWCSTGTVSAAEAPVASPTGTLATPSLPFSM
WLQRRRQQRELGLGQQPGGSSPPDNNAVAPAAAITTVELSLAQMAVGTATDLTTIDIAEQHPSSPVAAQGQPARRSA
AGLATTAATAGGTRVAGGRSRSTLPRQQAGQGILDIEDAGAVHLGSSAAAATGDALQGSPIHRCRDLDAEEADGEST

SSGLGSDADLDSDSSVFRFWRQLADADVEELVSAAGIGCSGFSGEASEQGPCELGDSKRQAASRCRGSGGSGHAAAA
AAASPTAIAASAARSPHTAGTQETAGGLRSDEVTTARMRALVHQVPAARRPRSAAGCPRPFGSARALLRPIAPPGVP
EGAARPLQQPLPPDHPSTGLEPRNRVSAPLAAIGGGSGADDMQPTTATATGAAAGAPLLLRGRQYGVPEASDRLQRL
LLRRVASMENMWNPVQATQLPPPRPAAAGAAGDELGGLAVPSCAAAAAMLCTAAVPCFPPRLLRLPNTELGVEALNQ
LSETGPWQSAGAGPVRVVETNTSTGGHSPRLRSLCPSALAAAAGSTFGGSSGGAGETDGAHITAWRNLDNGGDDERG
REARSGRRQVASFGGHRRPQQRQRHQQQRAGRQSWDSLDDGADGGDGNTAFDGRSPGRQWQWCYYYSDGNTDDYRGL
PDRTCCTLGGASETHVNPRLPSHLGLRPLSRGPAAVAGGGQRVGGGSGAARDLRRLSHCHPAHPVPPSVERAGGRAV
APVYTAQGQQGEQQQQLQQYQQPRVATCEEERQLRRQRRRQGLVEPPLSEPNGGRLDVLNALGAVDTAADDTPPHLA
MIRNTSATLRFARQTGNARHAVSVRSANGAETSSASFTCPRPEFRFTTREDDRGVAGSRSEILSTGERDLSALARMI
ATGAFCSPVAAPSPPEATAAGAAYTAPGTSPGAAGAAAASVGQSARRRHAQQRHERRALPVLPHRRRLASTEASGSL
SLGHATLPEGDLELEEIAACLSPRPTMPVAAAAAPVPRDRRRVTVPWRLGAAASISVDVGESVSGGDDGDDAGDALS
TPPPFEPLYAAEDGSVFGDAGDVDAWAQGLQVLVSDSDESETARPARCRLPHPRRWLHERHEGPVALPGAVSVAGRG
EGVRGVRGAGPEGGCPAAAGGGGAEDGQVDGSSGARDPSYRSDSSAGWWRLGQQRRRPGCEDVGGAAGLLLGQRRAS
GTGVSVGGDGSSDDENEDEDGSWREWWALPRGRRRASGGPEHGLGGVSHLGARQPAAVDNEQRGKCGGGSGCGGHDG
SSVGTPQSNSTDVGEGHVDREVGKAACGDSSIRDGASAPQLLNNEDGGAGAGVPCADGDGGDDSDGDGDLVQQPLLG
EEGSDDVASRNSGYGHDVDVDANGLTGTGSFGAEFDDHLQGLDDILADELYEHGYGGWDVAAGETTVDSDEGSDDVL
PPLNRLHYSSTGANGNGSTEGAAVAGGGSVTAPAGGQRGFGSTTTTTITTTTITNNNGSWSLGGGATNTSSGLSIYR
RMARERPRDRNGPNAAAGACGDEDDTYGGESDSSGGRSSPRSLPPLLSNSDTPRGGGSSSSMSTGSNHSDSTPSLVH
SETSSDGEPSGQRRRRFAWVESQGNQSSQRWPQRPPAAAAAATVTATTGTRATPRPASVSDLQRTGRRGPSSSSPSS
SSSRQTAAAISSTVQAREGISNTQSGAETSWAAAMEALLRALLDFRTVASGAGGGASGNSAESSAWSLGLEPVSGLR
GQPTAATATATASFSGLLRPRTPAAFSGGGGSGGGGGATSQIVASLVRIPADMLINVPHGSVVSITIPAGGFRGAGA
GGGGGGGGTASTSGSDRPGGDSSPSTTATTTAATTITSAGTTTTTAARILPIQLGGMSSFPLSWRRYLPPEDSDGSH
PNDRPGLPGSYEEQDIPSWAVRFAPPRVTAAGAGRSELSDAPLRIADGGAGPGSAAAANLASGRGLQGPVSEHDGDA
GGTGPANLAATSPGFPTLWEDFSFSFVNDDDGDGDGDTDNSHDDDDGDDDHHDDEEEEEEEEEEEDYNGEAEHGQDS
DTESFPDSLPDLIPLDVLLGASGLAQQLPQPPPEPPAAGAASRGLAAVGELEAAGDQGSGGDREPAEGADGAFGGVA
AGIERDGGAGPAGPAAGDGSSVAEQIHSATAHEPHEHGNSPGEGREGGPARQTVDAGAVPPNAAGSAAGPRGLNTVS
RYAGPLMDGDDTDEGNVAAAPGGDPGEGGGGGELRTTGGTGVMAPAPAAAAAGPRPPGTVPARRNAVAYPRSVLLSM
LEALRLAETAVGNAMQDNGARGVAGSGSGAGDAAAAATAATRAPGLVALGSPPPPPPPRPARHVAAAAALLRDGAFV
AQLLNELPEIHAMGVFGLITQLVNAGAKPRVVSLQNGEADGRPDNLLLVDAFNVLYYLVSKIPDAIDWKSTEFHGQL
YQETCRYFGGLQRSGLRCVLIADSATAPRHERLYTESTRHALTIGQVIPPTAELVMFQACRDLGLEIIRSVHSADDL
LLAWAHRHRDAVFAVLSKDSDFLVLGVPKIVHPQDVIIDARCVCLHVWSTEDAWRAWHMAAAGPAGAYMPPLLRRAQ
VAAVLGNDMGMDLRHRLMRHRTAAFSGAGGGLRPGVVPPISPGAAARAVVHLPSNVKHLDVNFFRKRPISLREFSED
DLTQINDIVQAYLDQDRAARDGSILRDLTLTEAALPWLDPAVANRFMAAGPLPSFLAEISCRGLATMTLVDPFWQQL
RVLRRAVAEALLDERAPGAAFEYEPSTNTLHPLTGGRAATVERSVVDWFQPKVHLPELPVVDAAAAAAKPSPQRQAT
LQQTVTYLREMEPELPASLAVVYSLMLHAVDPDPAAWDEQAVREVLVPGVPPRSGADAPPAMVAAAVEADEADAAAA
AVGLRTTLIDVGSALNERDWNDIDDKEVVGQVRSGGGYKLLKEIQPWDQLAAVRGRSGAAAMGGNTLFALARRALEA
GPRSSEWAAAQDLLDMVDEHLKGAERQPQRLRARPLHVILSTPTGSGKTFTAVMLHLHVLRDMERPVAGATGRGGAG
KGGAAGAVDAVAAEGRTGHPQTILVYSVPTKQVLKRVGQECEAHGVVYWTAARDGDFFQVRRPYSIRTKRGGGSTGA
GTMRQQLEENAKRGATNEDLGGGKPDVIIADIYATAALVQVASEAPHDAFYRSSNLVLYLDEPNMGIHLDKEVRQVV
RQIMSFAPPTTILASATLPGWDDLPAWWKGSGATAATHAVITQEPYELPTCRLTVLDDVSGQLVPVSPLELFDDVGQ
LATTLERSPRARVLLLRYFTSEQANQLLGLEAGAPAAAANRSEAKASPDGKKAGAAAVANGSGGTAGAEEEGKEKKK
KEKKEKKISKKGKEEEPDERGQEVQGEERKEQPQQEEEEEQEQDEGWKMLDLDVRGLRSELLEPALRALSTQPDRF
QELREAWRQPAVPAPGDMRDVLSKQGVTLVATMEPRKLALKLAGKSPADQERWAAEAHALRAKVREAAADKRAADKA
RERAAKRGDEEGGRAGVRDDGLSGAGRVALRPGLTVWADEADDVTDVDTLVMLSKGVAYTASKDVEPLVKRLYQQAL
LYVPERAGTRPPLHTLVVDYSAVYGTDCPAVDTIILCSDLAEHLSWEDHQQFMGRLRRDGTVIYPSMGLLRRAVLGG
GRQQWAEREGGRQEQQRRQVEEVLDRHVAPTTEGAAADATKAATRELLRLVRPGSLSRADVASLVLMYALRDVVRVS
AGSGATQLCAAALRRLKQWGPLKPLQLLEFLKLNRAAEQRRLVSALEELCMAPAGPDSTAALLPCAGRLLQLLAEEE
LVEEEGMAMWCEAAAAAALQGPAGAPGAGQAAAAATAAFQKAATAVRNWLQEGSEEEED

>Vocar20004970m|PACid:23133295
MDDRSGARPSYLRSSIAFGCMLAGSAGAKPPKRQPRWPAAAACRRQPEFSTRQLAAGVVFSVRSPEEVQLGEPSVTT
ATHPVVAPTIGGNITPGATGGNGDDDDDDDDDGNEDEESFADANPCTAVPGAGVPPPPHPEGLGENEAGVAAAAHHR
DEGQQRWPGAGSGGGGGGGGAAMDACGGERVDGPDVLPPFVDPDQEEGRLSPARIEALHYIRQLVVGRITASADAKL
LVDSLDWCLPLMDMRAVREAFRMIRIASTVYGTHIMDWPYMLCVVAGMSAGRVRDLELMQVLHDRGLQMLRQSEQAR
ARWKVASVQQGGGGGGEGGGEGGGEGGGGGDEGSHSRRRPYRHRGRGAAPTKAAHVPAIGMRDLVSRFLLPLHHLGQ
PIDPEYMQLLEDMLTRALPPRPTPPPKLWLLRDMLELYSLTGTAPSTTLLRGVREVCMAAAGELRAAAPQATAAVTA
NGDHCNSGLGLPAQASSGRQGWKGDDGAAADRDDQRLGRTTSSSGGGGSEGQWQRRTGLHGRRMSSRMWARVGPNRS

PVMQHGSSDAADATDVGGGSRSGGAAVQREYEVLAGTLASVLYLLSRMVRRKDGENFFDDSDRDSDRDSDRGSGGGG
GGGSRASEVAVREADGAAVRCLVRNTDGGGAPSGLSPLPQLPGAALFTVVQEQQQLPSQRRLGSGGGGGSQAADVG
QHPAAAVGATAAETAAATALLSVPPLPSSASPLLTHLPPSLPDTKPRINAADTLLMPYPDTAIAPSAAAAAATSSIP
SSSTGSGDHSSGDHSSGDHMSPSALTWAHDALESLVPWMPTLTPLQLSGLLRTTVWLGAVDRLLLPPPPPSPPPEDS
ASCPAGGGGGGGGGAPPRQSEPPRRTTAFLHAYFTSLARAISQHPTADTVTIGNVVEAAVGVAASGVAAYGVPPEAG
NLYGKLLFRANAIMAYQATQRNGFLPPYALNLVPALAAAVRDAGSTANVPAKLVAENLRLGERLLPGWRADGLAQVA
AALETLKWRPVATGPSSAAAGGGVGGGSPHTEPTYGVMPEPLAECLAARLAYMLAEGEGLYDEDLADLVLLCRRGTS
PSPPPPSPGEPSKASVSSGCGGGKEEAAAEGLLRPVQQLPAAVPVGGSSVEGAKRAMAQGSSLTLRLLDVLADDADA
VPPGQQTRRGNPGAPAAAAAAAASTRNLRRSSSVYNQGPGEARCALAETRTRHIILSGLEQALVLQLRGGHPAVASR
GVDALSRSLERALRRSPGPVWLAAVADALGARSDAALAEAWRRNPAVGAATAVPTPLPTHAVAFKVVQTANDLLDFR
ATAAATAGHQNASWFGSAARDHPDWWCDVSHSAGICVDFLRLAPGAKASGAAATAAGSKRVRATAAWAGSPRTTEAA
GPKVQLNTHRSENEGTDQSRLDSDLELSQDRPSDQNLDLSLDPGLQPRLAVDSLIRYAQHLGRWLDASRAAAIIDPE
PSQAAATVAVAEAEGAAVAAIQVPPPPREPGDPFFRPPTSTPLPPEQPAQSPASYRPQDQQRQQQQHRQPVPELK
RHTYFLVGLFKLLQPMLLQEHRPPPAAAAHHGGSLRSPGSRPLPLPPPPPPVPEGSSWDVAAPSDSPGFSGGGGGSY
GSIGVSATQLVRLAELLAFSRFQPRRFPPLEGWPEAYLSQAWRPSPEFMDGLVDRVMANGGITHVCTTPPWPWVKRV
LAAAGTAAAAASAADATVNIRAHTKFPDTAAGGGGVDDDNSRTATRQASSASASAVQTSDAHLPSQLAALLWRWSAV
AAAATPSDGEDGSGGGGAAAAAVEAFAGEMAAATLPLLAHMEPIDLVRCAEGMAALQQLMTRRNTSGGDGGGGGGGK
GGGDQADGTLAACAVADTASTSAVLQRTLESCMDGVGNGRGYDGRDGDDGTTLTTVTAVAADGTAEAQKNDLLTCAV
SMSVSGLLHALEPYRHRDGCSDGGGDVVGRTAVASAGFGVDASIGCCGAVAAAAAASVADDRLGLRRWWQELQRATA
NVLLTRHAASAKPLLYTSKRLKQPRERLQPPPQLKPYNLSMVLRCMAAAGHVPSCAWLQRYCQHTLPLLPSYNARDL
AVSMWAFAHMCFSFAGQSTRAVQSVQTAAGGPLLRAEHGIRSSSCSGGGGGGGSRSAVANGRREELTSPSAPPWHPPA
SLEKSLSKASSARASTSSSSTSSLTGNARRKGYQGRLQQPQQLPAAAAATSAAHVEAPPALGRWMEAAVALLPPLLP
YCTSADLQNVAWALVRLDFPPPAGWLHQYELATQPRLPYLSVTALLYILWMYGKFGHAPSESYLEAALQALRPALSA
ARSQPAAVSPSLVATADSANTTTISTNPTIASRTVTSLAPLAPSLPANNGAAAAAGDLIGNINVNNEDESGAGGGDG
GGSGGGACATSGNVRELLDAQSLPLLLHSLSQLDYLPAEGWLVEYMQAVLHALPRMTPLALAVMLRAVATLQQLESF
LSALADLDRPATLPPSAAPLQRFLWACLAAAPDSRKVAAYYRLLVPYTLPVAAVSEQGAAVAVVLPARQHSRSSPST
MCLSRGLLHEGASRASAHHLWLLLAVLVALFLHLCWRGHHLQECKPRMWKVRGPWAPNHQAVQC

>Vocar20005032m|PACid:23133412
MVVQSTYAPNDSHPHLRTLNSCLAPVYSVLGRSVITAEGLPAAEPYGTSLVAGRLSEFHASQCGYCTPGFTVACHAA
LHNAAAAAADGGAAATAMPATPDDGSLLRALDGNLCRCTGYRPIVAACRSLAAEVTDIEDLCRRRTAGATAAGSISP
VAATACGSGTADVTSSGSSGSSGSCGDSSCTEISLGSRQVLKVPRSLEQLVSEAGRLAAAAAGRSTPSWRLVAGHTG
HGVFKDWPLEEEVLVAVAEVPELRRLEIMAANDGLVNCEEATSAREVSYHLHIGACTTLERLAAHLAHRAQGVAAEE
EVAVAAVAPGGTGADNNSDVAGTAFGVRQWAAETSAHLYRIAGRHVRNSATVGGNLVLARERSLPSDVATLLAAAGS
EVELVVVSSDPETHGSVRRADLLEFVTGAAPSLPPGASYILTAVRVPLPPADPRVRFWSHRIAERYSNAAAVANAAV
LVRLSAAEEAEGPRPVVEEVRIVVGVRTPGGGASAPGAPHDSLSEHNAAKLKDATVVLLDGWQCLRAKAAEAALRNQ
AATAATVVAALEALAATDLAEVTAAASATAAAAGDEEKAVAAVTIAQGLVAQGLMACLGLSPTPPAAEIQAAPGVDV
IKAAAGVENGDGDGGADCVHGAAKVAVHEGVNGDADGAAEAAVIISANGHGITAHSRPDQQPQQPQQPQCSSMSSPP
PRRMPPPPLVEGRQFLTDAVPELLPVTQPVPKLGSKLQASGTALYTEDVPVHRGALYGAFVLSARACGRLAGIDASP
AVSLPGVVRFLGASDLAGVGPGGALNACEKPPVGAGAAEKTFLEVGGKCEFAGQLVGVVLADSHVAAQRGAKAVHLM
YDTSTDGDAPLLSIADAVRARSFYQLPGLLPSFPSGNTYGDKNSVLSKGGSDVDKVLSTAAAPAAAALTAAALGGGE
RDESTAIAGVPGSASPPPRPSTLRVVCGRYFTPSQLHFYMETQSAVAWPDEDGCVVVHSSCQGPDFVQGGVSAALQL
PLNKVLVRCRRVGGGFGGKLTFARRVAVVAAVAAVATGRQVRISVPRNTDAVMWGGRCETDVSYVAVLDDCPAEDGD
PDASSSNVHGQQDQTSSPHQRRAPPRFRALDIHAVMMGGAQKDISFIDAMGMIAAVDSVYDIPAFRLEVALARCNLP
PRTAVRGPGEINATMVIEQIMEHVAAELKVDPEALREANFLKAPPPPPPPSPEPDCANDVATAPGQGVAAAAPMEPV
VTTALGKTIPLRQYTLPYMWSRLQRVADWERIKAAVESFNATSPWRKRGVAMIPTRRVGGAAQGYTMYRGKKPAYVS
LLQDGTVQVACHGVEMGQGLWTKVAQVAALTLSEVLPHSRRPLDISFIRICDNSTELLPHSGVTGASTTSEIACAAV
RLACLQLLKNLREFALPKMAGREDFGLRAPLSAFAWGGQSDLPESSRGVAVGPLAPQYHVMGVALGVVELSAFHPTH
IKRFLKTFLHLRQVDILTGDRRVLRSDIMFDLGRPVNPAVDLGQVEGAFVQGLGMMLQEEVTYDSTTGALVQNSTWN
YKPPSVSCVPQQLNVHMLNDAPLASEGPVSSKACGEPPLLLSAVVLAALQRATAAARTATSTSVGSTSNGGDGSGAG
QVMKGSGGGSARSGFVPLLAPATVERVRVMCGREGAAEIIKRNLSNW

>Vocar20004820m|PACid:23133421
MVLLGMDGMASAVEYSIQQQHHVQQHHVHHQHHRNHATTSYPHPGQTPALPQLRPPVPLVSEAAAEMEEAAQPASPL
DPATAATSRWPAAAAAAPADAHPPPDYQHFVPLPHQRERRKKLLDRFAFRGRLDAPGEEPIFAKTVEVEDVREGRAG
EGLRAWLVNRLLLDPCLHPPSCRTRNGYLLLAFEVLFMGGESVQVEVPYKHSAEDTTGTHGNFVAEVLLQRLHSKLR
LRDELVLYPKTDKNVFQMVVLPNVCAECLEPIADREDEALICDNCYDFVDLGCCGMAELPEADDGWRCRWCREGESY

GDVGPEATMTETGTLTCTAGTAGGPAGRSVLQGAGAEPEYDPDTATTLAAPSPSARGWRRRSSGSGGNGGGDGDGDR
RGGEAAATVTVGAGGAATAEAPELVADADSGAPRNGGPAAVAAAAAAPPAAGKAGIASDVAAAAGDVSLVAAMAMTA
ACPSPARVPEPACTAVGEGLAAAAAAAVHVVLPPPPPPLPQRTSPEVAAAAMATVPPQPLPPQRQPGQLPPPLRAQP
PPPPPPPPPPPPAEPPASRPEAAGSSVVPLPAPKLPLQQPLHQAVAWQAAAAAVGPGLGSVALQAVPSQPSQPARQP
EAVTEPPLPTPPPPPLPPPPPLPPHPSPLLPPLPPVLQQPRAEPQLEARQPPLPPLPPPPPPPLPSSPPPPLPPEPE
PPVPPHLEPQPQPPPPPPQQQQQQQLLLQLLLGSAAPAPAPQDGLGPALVGLPEPMHVAAAREALRPPPPQEQQQL
QQEHPPFRGRGGDGGGGGGGGGGNDEMLTGSRIEAAGTAMAAVAASGRTAELPVQGSRGVGFLAAAAAAQDGGLLQ
PLGPSFEQVIAAAEAQPLQPSAAVPLPPPPPPPQPQEQQQLQPYSWPPAPPQPPDLCSPLSFSSVSAGLSSFGGHH
HHHHGAAPLASGGVAVEGPAAASTGPICRTESHSLQALMMTVEVTAGDDDIASSGLPDTLRTAEAAVRETAAVAQVT
SLAQAQAAHAYASAAAAASVALASAAVASPPQPRPPPPPPADAVRPPPPPPPPPPPPPQGPPPATAGFVPTTYGPTWD
PALRSGPQGTGIGYAPGIGQMIPMDQLLLHPQAYSRLPAHPYPQSPLLPPPLPLQLPQHQHQQPQQPPPPPAPPPPP
RPPQHEQQQQQQQQQQQQPFHQQMHQAHHQFFAQAQQYLMQQASVVGAVGPPYIHQAPLGPSPPAAAPPPPPHSPLP
PQLRKAGQRGPLRQFSAAPPPPPPPPPPPPAAGRFSRSASAGVTRAVAAAAAAAAMYESDDGIWRPDATDDDSISVA
DPSSDDSGSDYDARGRSARGRGPRGGRGRGSRGRGRSHSQGHTAGRVPRGRGRTSAASVYNYNVDACAEDSLTPLRS
RAAAAKATGRGGTHASMAGDTAAAAATAAAAAITTPSPSALTAQSPLEGAGAVGPYTSLRDGCEEAVGGGGGGGPGG
PLLAELGAIASAGGLAAVGPSATAAEAEAVANGGGPDSLVYLQMLSAKVAGHPAPQIALESPVPAVSEALPARIPEQ
DGAAGLALGAGGEAAAAVAAAAAVAAVADNAAITTLPPVGVTVAAELTAVATDAVPAAAVAAGPEVPAAASARPERM
ALETEPAVTSSLRDDPLVATSSTGVLSALVAEEVAPTPPPPLVAPPPPPPLVVPSGLTSEAVGEPSATGGAAETSAS
SALGEPHRDLGQPISCSSTHPQQQQQQQQQQKKDAGGSRPAAEQPRRPGTTLRIRFRGGLGVPPSAGLPSAAAHAAP
AKHARPPPQPQRQRQPVNAAEAAGTLPPACNRPVAEAPGVPEAAAVAVVTEAANADGAAAGPNKRGQASGQWLSTVG
VAVDAGVGGSGGGAATGPGVSAGTDPGAADAVASAPPPAPPPPLPQPLPPPPMVLGEQAPLGAAAAAAALLEAAPTA
ADLSGVQQQQQQPVPPATAEATLAEQPQLRSQPRPALPLQPPPSPSPSPAHQLSQHQQQRLLTQPSSPSPLPPPPPP
PPPPVQDESGGLASLSLLQPLGSGQAIGPDMDGIEHVTADLQLRLQEPELTLQEGGGRGGEPSAPASEDGLGGQVAA
DSALVSSVVTVTAVSALADVQPSRAPAAPPPQASPAQLAAVQQPVSLPHEQGEDRDGGAVLDTELAVVMDGGSAGVV
AGFQGTAGAAPAEAEAAGSGRISDGTTHSATYLIAAAADSVFMEAAVGAAAAAAASSAWTPLSDMMLEQVVERPGDGN
GTQLPPPPPPPQPPPLLPPTQPLTLETPDIPPLPPPLLELLLQASQRQFPTSPLPEQQAELQLQLQLHLEQELQQEL
LQHQQARLQEEEEVLGGRQGETGGFPSGKADAANTGSPVNPAEALESAVRGEDAGESEETQAPSGMAPAAPAAAAAA
AATQVLDGTLSREPSRPNGKLVETECSPTGREPSPQLPLPPPPPLPPSPPPPQPPEIPAATNAQVPQPVPDSPPEDT
PAAVPAQAMEFASAAAAEPAGGAEDDHVPSAPLTTWPSRPSPQHLERPSLPWGRGRSPTGGPQTQSQTRTPLPGTED
GTEDQAAALTATGVVGITNDGGGTATAIAAATIAASAPAASAAGEEEGGGVPSIAPAVPAPAGPAPPPDGVSVNASW
IAHETPPATAVAVQQQPTQRVGQKTPQQPQRVQPLRQSRLPAAPMPRFPSAARLPESVAALLNTSDVMANGGSGSGS
GVGIGAMSPVKAAAAAGAAATAAHPASTAASVASAVTVAPAAARGATSCIASPPTLPSQLSGCFQPGAVAFRPVGTT
VGSLRSKRIAGCAVSLDAGTAAAPAGGAARAGGNVVTGTCIGASKAAPMPTRSRANPSVVVPDTDGDSVAAAPAPEP
AAAPMAAAPLEPPAPAEPPPPAPAPAAAPAPGKTLPPAPAAAPAPETQKDTEATTRAASGTTEQAPPRAHREEEDGG
RDAASGRGRGRGRGRGRGRGRSSSGRLEPPPPVLPPPSEQEPDGAVPAGHGGPFGTGKATLRPAVPPPPPPVDPAPT
VPQQLSGERDPRGLSGTDRPSATTAATADHLSAGNAINAAGVSTGGSGRRSSRTTLTARTAGSEKSGREPVHAGAMA
IVPENCIDGLPSAAVAGQGCGGGGGGVAGGHYSGEDNGLVKDPVVMVQPPRKRKRLMQNSSPADAPAAAPVSREPVD
VDLEGSEGIAADKVVYAQPSHPLQPRARSNGNAATAAAQPRRRGRKRLSRHVVLSDGESGGEGGEEAAARPGPRTHA
VVVDHGLAGEGSDDVTGLGGDVDGGSKGGATRRAVAKRAPPEPEEEPSGTPARKARRARGGGGGGGGGGGGGGGGG
IDQARLPATVEAATAPGGVPAEAETELAAGTASAAAVVAESVAAGAASVDLAAAAAAAISGFGRVEEHAAGRRRRRG
EGQEGVAEGSRRVEDGIAGGAEGASPRHVGEEQESEEREGPPRKRRVGRPPAAAKRAAALAALRPTSEPGTRATGDE
QDAAPPPPQELPCLPQPLTEDAQTEKDSSSRLPLRPSPQQDLHQQRPLRLQRQRARRSLHAGRGGADQPLPQPQLHD
LCDPHHPQDSQEAARSPAAARQQQQPQLQRRQQPGEAMPSVPAVGEQQDDQAEQQRRQTTQASHPLVIRHPSPPADE
LSLSAAQQTQPQEQSQPPQPPQPQPPPPQQQQQQHQELELEQQQLAGQVALLWPQPPQQELWDQVPLRQLREGFGAG
RPDARLARTDADSRGRALTGISDDGTDHLVPLQHHHDNRQNQQQQQQQQQGLQLTHLHAFERMVSELQAGMLELQAR
ADMAVDRALAAEGRASEAESRIAEMERRVTEMESSTADLRARTAAAEDRAAMAEGRAAAAGDRAVMAEDRAAAADDR
VAAAEGRTMDVEQQLAAMKERALGLEDRVTELETGLAAAHKKEEDYVAFVTAITLALANARERISLIHLGLEPGSQQ
QQQQAHSEVVLEAPEEAAADADADAAAAAAAAAGPGPSNPTLALQLCSVGSGRVLAGREPGSGFAAGGWV

>Vocar20004997m|PACid:23133451
MDLIAAALRANLPYAVTLEDGLRELLRRTLGSVEVGAAAAATAGPPTDLRSVDAGDAEMHKEDSESESDLDSDLDLG
LGRDTATATGLSMEGSGGDSGGGRAGRRGRRGGGGSSGGARRPRQRQRNGGGGGGGGGVAEIALLAGDPVQTSVFVQ
GLSRLLAMQWLSRRRGPETRGQLLVEGGGGGGVGAGGGLGGGSGAGPSLELLSEPPPSAGSFCRELLGLIMPLRNER
EEEEEAGTAASVKEGGEDEAAAGTTAGTTFYDNFKAVVNRAVLFDPEVSVCHILRALAARERTAAAAGSAAAAAAAV
STTAEAVLAGVCESLLRSRAAVAAVARARPAAAALGGPPPPPLPPLPPPSALDLHLSAFMASCRRHLHLLQPLARKG
AGSSGSSGPSLESQLLLLRVLRLLLQLDAAGLLQPGPSLDFILDCYCEALAPSAAAASAGALTQALQLLPALAAGLP
DGGPLERLFGGGAAASGGAPGVVISLVPYVCPTNSRRDHPDPTAPAAVDMSMRLHALMETLVQLSYAAAAAAEVATA

AAGGGGGGSGGSRGGAARAAAAASLLKALLIVVQDMGGPTGHLLQTRLVRMFVQMAAAAWHGAPSVTEDAAKDVTNP
YALSAAAAAAARAWPGDALLSYSFSWLLLDPGFRGMREMRLALLHHVVMPLLAAAPAPYRVEWYGTYVRRMVHHVQD
TAGGGGGAGGGAGPGTSQLLNSWVCYTLLQAMYGMCTAQEIDRIWDKSNRDIRAPTPPMSEAGPYAAAAAAATSTTA
AAGVGVGVGVGGGEVLKNSTLMGLCKRELCRPLPAPRGALNAHELQYQDIDRAVRCAAWAASAALLLRTQNRATFFA
KLLRMPDDRNGDPAFVWKRLLPDKAHFRFAADPEDQQQQQQEEEGAAGAEGEGVGLGVGAPPSRLRSSEPNRRQRA
RWDQEVVRTAFREMRRQREEELAVRRGGGGGGGGPSASMLMGGTLLGVGGGSGGEDDVASLTAKVSASQYDFGLGLG
VSSGTASLDSGGGGGGMAASLLSSGGGGGSGTVAAAALAYTWTASGGGGGGGPLVGDVFARPAAENSAPGAAGEAA
AGSATAGGGVGDEGVDGDLIDDNPMNDELPYVDDEMDEHVCMLPLVQLIEKFSDPATWDSVAEAGAIATAKEAAAAG
AAAAGVAAAASSALPGPDAPMPRWLSTLLEALSRTDTPRYIRLFLLKAVLHVEERVRQRKIRIQVQSEQLNQQTEQT
ERPMSPLGMQYSGIATTISSSSRSSRVEEGSQQTTVRKESCTGDGGNGDGDGGHGGAYGFSKGGRQGGGPGVVGTGA
GEEGEQEEEGGGASAVAAETRVSELPASDTVFAIWAVRFFPALADAEAAEAEAEAEAVPLSSPPQQQPLAGAAEAAA
IGDDDDGDGGEEPLLGQRPPVEAAAAAGGTGGVASSTAAGGSGSGAVFSYVLRSMCQTFMSWRGLGDLGDTEGETQS
QTTATATATTTKALLRGAAEQLLRRVVRLCLSPRTPLELCRLNAHHVMALMYFWRRSGVKLRGPELLTLLSPRLETK
QRVEGLRVVRAALGYGQEHAVLYGEARDQLWRALRALLSGSPPPASSAAAASKRNHRAAANPSSTHTPPPPPPPPPP
ALRIVAGPLGELLGLYLAKLAEKEEEGKEGNAVTAGVTGAGDGGGGGGGQEGGGGEGGDVWEVGELAGSGGLALGRQ
RRTSFGSLLEQRQYDIQDGLRRRVGGGGGGGGSTRHWDALVVVLRGMTAPSPLGYPPSVRHLVGEVVRGLGLGLVGT
AREVACEAAFEVLGRYVPLANPTQRQELFIELVTALDRLPLRTMRPETQRGALKLMQALAESARLDDLNCLLRNLTS
SRGLASGPSPTRLAMLQLSPALSAAAAAAARNPAAPAAIATSPSSLVSEAASRLLIRWPNVACALLLEPSLALTDND
QPLYTSQPYECTFRDYTPSLGLLYGGGVGGTTALGMYGGGTAAAAALRTPAFLLTQDMYGSYTGAAAAAAARRRPLG
LLRATATAHFVPTVTLSVTGDVFAASGGVALDSTYGTGTASQSTAVAAGAPTQSVAAALPPSYGGNRTVRLAARRQQ
SGSRYARTAAIRGLQQRELSLRAARHEGRASRVRLFRSYRTGELPDTLSVRVASFLRPLAGVVEVDGPAATAALAAI
VEAVAAAAAAAAAPVGPQQRRELLDALAVAFAARPAAPSYVTALQAMAARLIDAGGAAVAAPLLLLPADLTAAAAAS
GGYQAAAVLLEAQQAVQERELDTAKAAANASSSGSGRYSTAIVKRASAGLSATLEAVAHVYGELDEQDIVLGTRRRL
CLCPGSFAMLAAEAAGRPRLAVRRAGQLTAALDLLTATVTEAAAAETAETKQGPGVASKLVPERPQDRGVALGSSAV
EPLLPPPQEGADGAGGSSGGGCGGGGDGAAATSTVAALLGEAVMARAGGGEAGGGGDGGGTAAAAAVCSRLVAPAEA
ALWRETVAVCADALGDWSLLEGELQRSLVKLGVTLPGGGEGGGGGGGSSGGLYSSSSAVTAVAEALPRSLLLSGAGP
SGGSSGGGGAGLVLRRVLRSALFQLSEDDFQLAAAAAAAAEGRGDGAAGAGTAGVAATAGSAPSTDGPALLVLRLLE
QLDHGGSGGGGGGGREAASCAAAAVPLELVAAEVFRACRRGSWERCLTVLQASLAGLRCRWTGLHPLSTAARTATLT
VLQPLAEIHEMIRQLVHAGGGGVSGGVGRGRDGVVVTPASSAVRHLETLVEVWRRRGLGGATVGVARVATATATGTR
PGGGGNGGPSGTGSALGTLLQVRRLSLEAAANCILPRAVLAGAAGVGGANSASCRGQLQLAARAAQASLRFAAAEAA
VQAGCADTAAEALPPPLLALAQSPPQQPQQPQQEPQQQLRLFRAHIDTAAAQAAAEPADACVYGAVSELLSTMRPWH
REAVRTGILGGRLAAESELAQSQALLVLSGRHLPFLVLGVAVVRGDPDPTVRQQYLAVRLATALCHAATATAYQPPN
HNVLQPPRPQIHDGHHDGQSAVAAGLSVMTEAAEEAAAAAAAVSTAARASAHFALLCGRLLRATHESATERALDPTM
SSLEELYGTAKRKQVAAAVRTGGGDLSACVVRHLLRAVALAGSGTAGCYGGGEDNGVGGKGSEERLQTLAAHLRLQV
PVVLSVLRDSAAAAEAFRQGWTAVPLGVFLPWVSQMTSMSVDSWCYIAFTTLRESPPWQVFSGRITQALARGDVDAA
VEVWSSEAWPNLFSRYHRPHHQHQHYQTGLPAAAAATPRPPVSRRQVAGAAPPAEPYNVAFARRYRAAFEEAFGGAD
GSGLRQLAERAREAFLLQQQQQQQQQQQQQQQQQQQQQQQQQQQQQQQQQQQQQQQQQQQQQQQQQQQQQQQQQQQQQQQ
QQQQQQQQQQQQQQQQQQQQQQQEQQHGKALGRALVRAFLSAAAAAGSGNARAGGLGQWEGLQAAGLRSLDLLSPWF
RSYEQVAADANASMLSSSHPFSSSTGLARQGRRKQQMLLLLPSMAFATATSPPPAEPGAYGSRCSGGGLPPLGLQDD
AGAVAVVGFKRVVTVFSSKQRPKLLTLYCSDLSTREFVVKGGEDVRLDERIQQLYDAMNGVTAHDAACARRSLRVPV
YDVIPLSPTVGLLQFVPGTRPLQSCLVDNPERAEQEAQAIELYANYIRSAAASTVQPRQQHQAHAAPGLAAPGQLPG
PRDYWQMYIQADSASTVRHFKQGGLVSGLAAGSVFGYLAGVGDRHTSNLLLQPATGRLVHIDFGYSLGAATQ

>Vocar20013661m|PACid:23133564
MSSPVFSRPFLGSLGGARPAARSNTRDGTGRGHDGCSKSRSCCSRGSSSSSRGSSRDSSSGAKGSDASGVFSSSTPS
HTCLLLRLSSYRARTPPSSCPGSAAAAAAAAAAAAAATSSVDPATERAAVMESDGVLTAADNAAAVAVPGAGAVVVT
MSGTKRALLPPAAPVDATAGSGSSCGSDVGNRNGSTGELNRCTANGTEYGISCSQSAGRWHDPAPSEPMGRSSIDTT
CISSLSSSAAAADAADGAGGNGNGAEVDTCGVTVTWGADGSRITAVATRTAAAAAGLEGWDGDPPPAAEGCRTPGEG
AVTAAAAPPPPPPSPGETRPQRLPSPPPPEQGLGIQTKPKQQPADNSQQQQQPGGHSRASLTSPEPAQLANGPDDL
HLAPGPFSPKPIADDDRQRNVGAGMVAPVAAAAAGDASAAAASVAGGGGGEEHAAADVRIAAPAPIAQVEATATAEG
LASASELSRDVTYRDVGAAAVALPFSAATAMTAAAVEIPRPVGASASMTAPATSAEANAAAPSPPPAAATAASPSAR
QEAATTAAVATGPPASAMAVAVTKEADVAVGNTGLAASSILAAATAAAAVAAGQRNIPEASQPHATDAAAATPAVAS
PTRGSLADPSAPSSAVTSREGSCVPDEEAGKETLPGVPPPGGSSSSGGGGGGAQEHTTAGAGAAATTDAWKWAKIAE
KQERRRKEEVVSGLDDAFVHDPQDLPDYKTLVKRPMWLKEIQKRQKKKHYTPDDEGFRRLEEDFTLIGRNAVHYNTE
YLRERQRQQGVNALISPVAAAAAAGAAAAGAPLPPGAELDEELREHDNVRREGEDLIRRTAEVFANCRRELQDEVAR
TGNAYRSGR

>Vocar20012042m|PACid:23133579
MTMDNITAGLPTFNVSQCTSIALRIGSSFAAAAPTYGSVITSGFTLASCSPLNVVLRGSFLSSFDGSRMSDWLYGPT
GMQGWMLSIFGAPLCNGLSTYSVFMNVAGANGASCLTGSLLQDCAPGFGQPSPPPPKLKKRTPPPAQPPTLIPQLLS
SPSPPPPASPPLPPPPSPMPSPVPSPPPSPPPSPAPPSPDFPFIVQTHICRTATTNVPYSVGPLYTASTKDQNGDAA
VAMCTNVFRETCSTGTCCGMDFAKIEVPVSNACKADLRRLTINGTPFAYTWGLYPDNLTTIKFTSLVTNLPNPVGGT
MCWVVRSGNCSTPANFCLNGHCQVAIFSSDNNCCPTEYIS

>Vocar20012070m|PACid:23133605
MTARCLRDFISIVVVSALAITLSVGGANGALDTIRSRQLLQATSEFPPNCVCERNASSSPYRLTPSNATSTTDFSVY
CFKIEKTGCNSTSTCCNGNQGVMKVELDVVPTCKPSLKRVTVNGVFYTAWEFNTKLGTLRVTSLNKTATTASGTQVC
LYLTKSNTSCTNLAQLCALGSGACKYAIFNADRNCCPVSVLSYFPPPPPPQFGPVVPAFSKPPTPPNPPEPSPPPLP
PSPSPPPSPPPPSPPPPPPPPPPPPPPPPPPPPRPPPPPPPSPPPLPPPPPPPPVFPNCSCLRSPSASRLALSPEIK
VSPADNGLTSICFTVTSKDSCDRGSRCCTFTLHKLEFAVKPSCNNTLIYATANGEHASRVFQTRPYPAIKVTNLALA
FGDTEGAEICLFMRPPCNSISALSSNGDGTITVGLFNPPIAATNCCPLYTLG

>Vocar20012311m|PACid:23133722
MLGDLPWPYSTITPTSSGAVRGRPRSGALYRYLVLPGNESVLLREALERRPWWEPASSSTAQQGPNQQQHPQHQQHQ
QRASDNTSNWNLWAGLNGQRFTQWDVMRQCGSGSGGNTEPTSRRLVNRLQEHKVICTKSGLASVLAAIQAAAVATTA
TAASAASAIESSEIRSKTGSGSGLDTSWIPETYVVPAGPKAVATGGSSLALFKAAFSRHAAAGRRVWIAKPTCLNRG
NGIEVFDSLDRILEHIKGRPAGSSLILQKYIERPMLLGGRKFDIRAYVLVGPDGSVWFHREAYVRTSSTPYDSSDLS
NRSAHLTNDAVQKHLDTYHAFEDHCKLSLEDLGPALRGGAPAPAPAPAPASASQTPAAPETTMTTTTSATAITSPLT
SEPAAGPTATATATASSVALDTRPGCESGLWGAMRRCVAALFSVAGPRLNPRCLVHCYELLGLDFMLDDEGKLYLIE
VNTSPAPFRAGAYLSDLLPRLIEEVVQKVVDPVFPSPPPPPSPPPSQPTGEAAAGEPEVKRSAAEAAPRSPLLDGFV
RVELDEQLAAAAAAAASSLTSSGKAAGSRRVAGSPPAVGLSFMGGATLLPPRGESKPPRAAAAAAAATAAAAAGGRG
RNGRPGTK

>Vocar20012380m|PACid:23133732
MQPPLYSWEEVLAHRAKPATDNTESATAPDFSEIAFPWAASSKPSSMAKTRIIHDAELWGLKHADRFHVVASPCWWW
RCYCCAPVAAEPLLHRWDSHRLPASGTAPSTERAPPPPPKKKRPPPVSSSPNRLGISLVSRPNYLQTAKSSLAQLRI
SEATSYPETPKSSLATSFAFRATVPRLQQYRKDPERLETDALRQKPTFSRLWSGAKEAFLAVTDVYSQCRPYLTILT
EADGHGLLSLAALVHASFPSSANGTDCGCGSSPYSLVWGDDVQIGTSSSSHTAVVPLCSHARGLLSWLTIQMDPTRA
AACYRVDSTAGTKPGSLGLTFRGGAYFTPFSPFAGNYPVGLPVPSLADAQPISPPPPRPPPSPPPPKSTRSPPFSIR
NATAQAAVALPLSVCYSLREGDLVPLEPPESPYPPSPRPPRPRSPPPAVQTSQPPPQQPPPVRQRPPPLSPFAAISL
PLPLNPTSINSLFPPPLAPLTQIHRQLPRNHCHRHQRHQPYGTLLTDLAATVSETAQTQAAAYSTGTAISPSADAPT
AVAAAKTPPPEAALSASEAPLTQTTTPIHAAAAQATAAAAAATSGIQEKFPFGLFHSNVCSCDAAPYVLRFGSEIIY
RSVSYYTFRLYPKPCNESIVIDGEPVCNTIRSLLNKIAFRLEATSAPCFDNVTTSIERAANWRGVRFWNDTMSEPSA
WIQARAGFTTSRRHIELYIYGLRGDWTQGTFMMAKCNQLGSFRNVCKRSDYGMCLYSFVDTPGHRFVTNCRTQGLTD
SY

>Vocar20012354m|PACid:23133820
MLYEPSSPTAACHILPAGLISKPLPLTPYRGALPALGDGGAAAAAAAGTNPVAAAAIAAAGGGSAAVGGAGRHHHLP
APPIWDQALELEKRLRAELRKKSFWDHDVRRLRGALQGTYEALLFGHYEFSCAHEVEPNLWKAVFYKPIEEFRSRTR
ALEALAKGTAAAATAAAPAATTTISPNGAGVSPPAPAAAAVQLATPEEARAQLARTTGAYLRFLDDALAFYRKTVWK
LQWVHGSVGAVVDLDAALQNEIQECVPRATGPRAPEVRQSVHRCLIYLGDLCRYQSMALKERPTSARPLWERAMSYY
RQAARVLPGSGNPYNQMAVMSYYTNDELRAVYYYFRSLAVAMPFATARENLMLLFEKNRNRYGSIAASHAAAALQGG
HEGGDSRAAAGHVADVSVRFVRLHGMLFDKINLHQFPEVLAAAMHDLEALLGHPGCRTILQKAPEADQLLFHLAIMN
IFSVHNVTTGGGVTGGGDTSASYAAAAKRAELLCYALAVAMRFAAAVTAVTASMAGAAAAAGGVGDVDTGFGAVSTA
CHVMLAWMVSNPEVLSPKPPGMAGAAAAAAVAAGGGGSEAPPEVVAEVTARSSFLVAAARLASHLSDLTRRRAHDQD
WQDPAVRHRSLPEDSELVGFGPLMPVVTVAATWPAASEASGGAAVLAVRVARMLQALRPPPHTRMGHRLLPRTAATK
ASITATRPDIRDTGMDMDTRQQHQHQQQQQQLSGSPHPSPPPPPLPRMLNGSGAAATAVAATTMASANNVARKAQQS
GPGPGQPAATIQRHRTSNGDSPTAAAAAGATGGGSTGHGGGRAAAGRGGDDDVAEDEIVYQPPRPGSALPRPLSVGN
GLHRRSSSGGGGGSSTAIGDGGGTALHSQDGRCLLSKQPSGSSAAPPSLIPSRDASRDGSSSLFLRSPSVLSTGDLA
NAGGAPAAAAPGAVAAAAAAAAATPWPPQGLLLNVGNLSGSGGGDGGRRSGSGLASPASGYGGGGGTLPPSPQDQQL
QPTPSFLNPHPHAHPPVQQQLLLPHPTFQRSSQSAYHQEQPGWGDVFTSGGSGGGGGGGSGTGMAPAPATNTAAAAA
GPPPAGSLARPTSPAVGELPGAGTIGLLQNLLHGGGGGGLASHSAAQMQSGRRISAGGSVTPGVPSVPSAGFAADTS
NIAGGAGGGRPASSGSNAVRRLPSAASLGGPAGAALQRPASAAAAAPAVAAGPAAATTATPGLLPHPTQVLQLDHSP
PPPLQQQQQLLLQQGHEPSLPSTLTTSTTGIPTVSAPFAGLPASLQQRAQQQQQQHQYGFLGHGQGGVSGMLLLTA

EGQPPPRPSAAAAAVGLGLGRGPGPGQGITGPLELPYDAFTQPQGAMHFGGVPGGNRSAGGGTTTSAAAAAATFSGL
PVPLGAQHSPSGAVTTTAAQGQLCWPAGGGSGIGGGLDVSSYFNVAAGNYQAAALAEATAEATAVAIADELLQAMDE
DELGAAGRNGGGGGNGGGFASPACRSSGSGSGAGFAGGSGAAAAAIAAAATGRARISCDGDGPATLGAWTGATTGLC
EIQAPPLKNLEGFGSWGVVPAALPERLGEYHQRLRCSSGVEARSPPLVGQAPGPPWEQHATAVGAAAAAAGGVGARQ
VSLPGAVQFLQHVSPHQQATTAAIPTPRPIPPITKRPTSYTSIAPPIRISLPSTSTSAVALSTTRQHHYPTRPPTPL
LIRAT

>Vocar20012353m|PACid:23133835
MATRPSPPSSPPPRRPPPSPRPPKLPPLSPPPSSPSLPSRPRPSPSPPTTPRPPPKTSTRSPPPPRAVLRPLPPPPM
KQLRPLAPAELEVGFSGGESTRMWPTVFEDIPPSPPRRIVRSPPRSAGSGSVEDIECSVDGGGTITTDNEQFPFGDF
KSSVCGCSASPYGIRFGPEITYRGMTYYSFRLYRKSCDTSLFAEDAPVCSTINTTINKIAFRYEAAAAQCFDMALPT
VDFGLNWRGVRFWPDSEPILPTSSWLSARIGYSYDLNHIELFIYGLKRAYTDGTFLITKCAALGSFSNVCKDTSEGN
CVFAFVDTPGHRFVTICKAQGLPAI

>Vocar20013065m|PACid:23133894
MDATSDVLDFNWNDGPNNLLEQLEQLGFHVQASHAHQPNLLGTDKPSAYVVPVQNGHLFYSSVEDMGAVAAYVHSGG
LVIILDANHGEGEALRNFVGSSLGYEGTWSHCKQLQGNSRGLAVLNGYVSSSFLSDAADSWPQTLEDARAITYTTWC
RHEDEFAVTLPLYTSQLDSTRVAVQAFSKVNNPGAVVWLGYDWTDGPQDQWGALLKKLVTDFAQGAYYAPLFGNADA
HPLFAVDAVLETASDVVADAAEIVRRFLAVPGMYPSTPANPPPSSPPELTGFPPLPLGKAMEVAKYTAVYQEPAGEE
FDFMGLINRLKISVAAANDVPTGNVIVNFIIFPNGTGLRGHWRRRAIETSERIFTDTEIAEMYAKTDVRPLELYLVN
KAPITDEMMLQLAQEETEFIRRAMLDGTADNAAQGGLLQIGYLVIKIISVAYPPSPPPSPPLEPGQIAPPMMPPSPP
ERPSFPPPSPPVNFTRIELETGAFGVKDGWSPPSPSPPQQPSPPPMPPSSPLSPSHPSPSALPSQPPPVFPVQSIPS
PPSPSRPSLSPPSPSPPSQSPPAPPSQVSLSPPLPSPSLPSPLLPSPKPPSPKPPAPSPLPKPRSPKAPRKPPSPKP
PSPAAPPPSLFIKVTNVSAHGARGSSRSLVWNDDGDFKSQLTKQSPLQLVYPAKPSCPTTCTACQYAWKATADQLSV
AVFFKEPMQISRIYLKQIRNSGVITVQFIRWVYPPRGVVEGNLGRTVWNVTDDTSMCQSVLAINVRPAKSGMNLAVP
AGGSPANLPTKLQATAIGGVLVTMERPRNAGLNYGPFLEWIRFSGRALYPSGLVPYN

>Vocar20013175m|PACid:23133922
MQATPSHIGVQKAMRQQAMRDQDRERAMLKDAAYRANLESALQRQIALSDVEQRQQAEAELQHLSDVYDRRLEGVGE
AHLEAAVYMQQQSEARAVQERRFEELNQRREQRFHSALSAVKAQKGVAKAQAAAVQQRRAQILRAERDKAQAFTAEQ
AGRTHTQKRQQADLARQELERRKRSPNGLAMDFRQTRLHEGQGSPAPLAVPAVVAPPLPSVPDPLESAEELQHRAAM
DKRRREAAAVEAEIRARERFLAAQAKLRADAQKQAVEAELAELAAQRLRAKKQALAAHRLHLRDQRKQQDLQRVFED
NFLSGAAGAMPRWSKCRTAPLGHPPPLPPLPFAWMPSEGLTAPPPLPPPPPPPPPAPKEEAAVQTDPELIAPFNNQQ
QTHKDMHQDGPCIIPGEDAVSAVPVSPTPAVAERSMSHLSWSDPEPGGTPSDAATASVVSHPRPSESEATASAARVD
SSSRTVPPDAARVDDLQGVLGDEDDVDAWGEEEVDRELARMKKELGIEDSDLRSSVSSMDSGVSVPAFLKDVAAVAA
ARQASSHAAAEIAAARAGAAGPTNAPAPAPPGPAAQGADNLIATAMAGTGTGTDKPRQSTAANATANITTSPVSRSR
GAATAAERQHQTAGGVADAVAALDALGAMLRPTTAAAGQHSTPVSSSPADTLDSSILRILDSTSSASTPTANRGATP
TLSARAMGGGAVRRSGGGGVGGPSAAYTPGSSSLPSDLDDVLLLTELDWEELENLAGLRGTGVKKGRIAASPTPPAT
GTFGTAAAGTKGTPPAPLPLPLPLRIIGIGDGVDGAGGGFRGESPSSTFSGVSSSVSDDSLRRLVSQGILPPEIATL
LASTPPSTSRADPPPKPAASAPAVAAAATSTAVPAPTIAPGGRAAAPPPAAAPVAKPSEASAASPASSPSLSDINSL
LGRFRLPTEAVSAPSAAASVNSLQGNLSLSSTSASGQGARTVPAGASGSGGAAATAFPPKPAMRGLFTIPEGGSISL
GDLSAGAAPSGPAGPATRAAPPHLPQPQQQNQHQQRGLQGLLQLQPPRAEVQGPVPANDAMRPELSRLPEPIRLNLA
DLNRQLLASIGGAASSRGRASPGAASPSGQLRPAKPGPLVITSGAEPSPARGPPLQPQPRPQPAELGGPRADAAVPT
ISLSSSSSASSLSLSTMSSLSLSPKAQVRGHQAATPQLRIGAGLAAVDAGAPSAAAAAAVAGGAAATAATALDISDT
SSTLSSLGLGRFAGIVANAGSTSRSDYSHAMDPTARTEGQAAAAAAAVASPPAAKAMPAASARQTSSPYDLYDKEAA
RLQELMATINASLRQSAEAVSRAGGGSGGGTSSSYTAGVAASPSTGIATSSTTTPSPDLHRRPVHNRRGPAAGGSAA
AADPRLSASESSVSPPSTCGSSWTVSSMGPLPSPPPLRLAALQPGSQSPVAAMLHSARAVAAAAAATAASAKAVPSV
SGRSNRNAGAAASIGGTSSSVNSRGAAAAAAPPTALDRNATRTDGYRKQVLAGPTSPSSASVSSVTSAEPGYGRTLA
ATAAAGTATAPVAADAAGIKGGVREHSHSIPHARNPPITPTRPATSASTEGASTDTISTPSSALSGLSSVFEMLRMG
LAPESRPPEPDGASPAPPDDVTLPHMPQLGSPPPPLPLPAASPVPSILPTAPPTPFSPILSESSDSSLSLPSLSPAA
GVPVLPLAALGTAAAAAAASRPAQDAAPVGPGGISLSLSMSSSDGVSSVAEAIAGFLAQRFRGVGAGQSGPAAPPPP
PSSSSPSSPLPTPLPEQQPGQQPPVNKQQQQQQPAASIVDANNNGTGDDDVRAHVSLRKPAGEGQLIPGAAAFEDA
AAPMGVGGGDRRRSGGDGRHGIEVWASLTGQTLAEALSGLGLSGSGEPYEDLLRCSLALSTSSLTSGGGSGGGGAPD
GGDVDGDYDLTSSVQGTLAPSEAQQMAPGTATAGIEAFGEGDGEREEDGRQPRMALPLSALSVLPPASSIPGLSGQV
QETKGDVQVAPVAAPLPGSARQLLADVDAVLARMASRTSGNTSELSPTSVHSPKANPAAAKPSSPQPVPPEPLPGQL
LQQQQLQQQQQQQPAFAADADLGVTATAAPAAAPAAAAAAATASAVPLPRPLPAPAAVVTAFSEDLLSSSDSDDF
STIMAGLMQDIQRGLATLKAPLSPQQQRPEQLQLQQEGTKRQEEAAPANPAGELAGSPPGGGTTAAAAALAGAAAAL

ESAISSVPSVPWDDDSEDEKDGDAEEEAANRERGDGVRVGWWPGMSPKSPRATAMGSTRGVEWDTAEGQHSAAAAAT
AHPGPPDSNPCASAALSSSSSSSEAPAATTATAKAGRKFLPMPTLSRGIADAPARGSAGDDADSLDLLRLGEAAAAT
AGAVDDAVDAAELTFRAESIRRISRASAASSGSASSVDLAPVELLLTVAAKSSAGHSSTAAVISAADSIAAYSFLQP
PRASTARNRTPPSVQSGAESPSHAPSGSGSSGSPSTVSSLDSFSQALGQYPEFGGARHSLVGSQSPPSSDGGADGTS
SGPDAAGPAVSCGVGARQGDDYGVGDIANFGRPWLPIQYQPPDPLPLPLPVAPTPYSSLDQEPWRGAWDPPQPPQRP
AEARESAAAGAALLGYRLEAVAAEAQTRHGPLEHEPGPPAPPVELDISFGCGSTSADVDGEFYGGDDAGEDEQLRVP
GFRVQPVGGLAGEWRDDDDDDEEEEEEEGEQRWNSSQLSAGTQRQVQFPVSRLGVEDGPKPSTPLQARTLDTLADVA
FPLSDAREQQEQQQQQQAVTPSLRRQILGLQPLALEIPTVGGADHPASGGITAGVPSSEGGLASRIAAGVITPATPV
AAPAGQLASGDADDALPPPRTPLLGRRAAVEAVTSQGGEGPPQPPQQQHVVASTRGENAAARRCGTAGAAGQESGAG
GAGVGAGAGSGGGYAGALSEELLSDLLMWTVSDFFSSPGSGSANDGE

>Vocar20013155m|PACid:23133938
MDAGVANPAFGGSSARDSSLAGTNTVVPQFVFKGILRAPTLALQLPSVAASVTGTAAPVASGASTASLKTSAVILRQ
QPQRQASPQPQLRAPRCSSPPMVSSGIASRRRSSPADTGASGCGGGGGVGPDGGSASPRGGRATYGCDNVAGSPRAT
GVSRCRSSPRDRHVSVVSNGVRGSGGGDGGSGLLTAGQGGPSMATGGDVAGGEGAADSGAAQPARGRPRNTITRTAA
LRALKQQRSTTPDIIAELRAVASIKAAQQADFVEAASLPTAARVVGGVELEPTSAGDTSATIRQQQEQQYKPNGPHE
AGGASAKAGSASGTQPCNATLGDSVPASHTSYGASTTAGRAGGLIAGSAADTSPHPPAHPPAQQHLQPPQKRCAPHE
SQGHVQKQLRQQQSQPQPQLQLPQQLKQLQVQQPKSQEPSTASPTAAIGRRGTNLPLEAVAAGSHCSGPALVATVAA
VLPQPASASSVSACRDAGAVWEIGEEPSFRSPSPQRAKGAASREFGQAVPQVPAAIAAPVSAALGPVAAAAAAAPTA
AAIMAVAQRQQRAMRQHVQRQSSPSPGRRNGQAPGPLTDKQTAARTSLSPQPQHRLRGLSMEGGSWQSDVVMAAQAR
IRRLEEQVVSLSAQVAELQASHADLEFQLEEEQQHTRAAAARADAAAPACTPQTADAGQAHAMASSRWESDQHLQEQ
VREVEERLKAREAELDERLKAREAELEGRLKAREAELDERLKAREAELEEQVMRERAANDSLAKQNRALYSTLEETL
SAAEHEVHDTSQKEVSQLKEDLELRERALSAVQAEFSALRRRHEEMSTAAEAARRDNALRDGRLRREVEVATQDAAI
LAAQLTKLQREHQQLHSRKAEKDRQLATLAKRADAQQEQLTAAERLAQEQAALVRELESRATLLRNRNAALDRAVAA
EKEARQAAQEQVRQMEEEIALLVSIVRQSDPNLETLTRYKRDPAAAAAMAAAAAAAAAAAALLQASPSSAHGIRGEEG
TEVEGGQAADGDYGVVAPIVPPLILSGRGNGGLRRQHDPYGHSVVTITAGSGPAAATRTTAPQPLPFAIPGSAPLSV
RAPPPGSQPLSHRQQRADAEAQADISFADAASVDCDAILQPSPQAATGDTPRQDPDSGSAHAPNRRYAAASREDSPL
TMPYPAKHNMTGGVFLDIGGNRGPFSTSPRGVLSPIAAGRAHTSGGAGLHGTGFTSASPRLRQETRLAATTGGGGGN
GCRNARTPLSRRGSSAGLRGRRRSDVSTAGFCSAASNGGHLAATWSGADVSGGSAVAKAAAPVWRGAGIARRSATPP
GTSAVRRGRDSQSLLGPDGAGAFVTVPSGAVSFGGVAAKARTAVTAPSAATVPKAAGIGTDVPQPSAAAAVPVEAVL
DGSLSDRALWAKPRRSSRSDGSCANGRSGGGGGQPAGPRVGSQVQQLSYAAIHTAQRALATSALTVPAAAAARCHSR
SRSCSAMEWIRPRVTTRSRASRRAMTPEPRTVAAWLKLSEEAAAAAAAAAAAGDALKHSHSEITIGRGSYKLGPLSS
SDGGAFAGAGAGAVRCSDAASELAALLEQHPLGLRAQPQGGRAGASRAVCILTPHAEGQECEREEESTGAAKHSGDS
CGHGSPAGAANESRGGDGGESDGNGGCSWGVGLDDSKMEMASPVKLSRASGGQAGARHCLADSRDSHEDVVGDASTG
RRRTHSPDGVNGGKDNSSGSPSRENTRGVCSATGRPSAIIRASYAFDSPLRRQKQQPQQAAGAAKWDHVSSPTAARS
SLVAADGSDEVDPLGSVLRSLQKRQAALLIELATGGGGNGGSPSAVVKPLLLIPGSSPRNSLALNRSKPAEDPSFVA
SFTFANPKPQTSLLSASSEKPNGGRGTADEGTDSDKNWTADAARGSRPQRRRSMSFTGEEAKGVPKGGAGGASGDPR
PLAQPLRNVSSSREGGCWAAPQVIRQPTPPPPPPPPRSPHRPSVTAAAAAAAAAGAGVGGPLTALLGQLEHSGGTWR
GNAAHGEQGGPAGPHRLSDGSTDVVPSSPLPSPPPQQQQQQPSQQQQWQKHEQPEQGHIVTHFSVTAALQMPTGKFE
GEKSGEDQQRQRSHQNGKADACEPRGSNQESNQESLRVAKPSAARCHDGEGSNGEGIEGEEVFAADDDEVFGEDEDN
QGIKDGDDKEAFGADVGMEAATGDLKELRLLLPTVDTLTDARDASCAGGADCESNNTAAPPSPQPRDERSMGGSELF
PDDDYPPACDADAGSLVSRLGVSLYDNVAYDISRATSARAEYYNSSDGGAGHPRVPWRSSSGFPLVPVICVPTSLAK
AAGDGGRGGGGSSDGTALDPRAVARSALANLEQHFSPPPPSHPSDSGPLPGANAASSNLLDLELCESGISATSVATV
SGSVSAITSSVSHPMSSHDTSLRASGQPSWAFDQAPWDRQDVCEEAGCCITGDAAPVAAVEVEVEGVEAYEGCESRA
VGEEAASGWGTLGSVEQASRHPQPAGMDCDGISSTAAAATAPDGGADASGCVAGGKEQHAVAAAMAAAVCESPAMEE
EGGDAAAAAPTAAAEAVAGALPAPAADADVGLKAPAEESATVPPVTPLQATATAHCAGGLPSSSLQLSSSSSQPNTG
AAAAGGGAAGSFPRSAGAYGPMLGSWLLSRTEPTLLDRQFQQISGALPIARKSIGHATAATGGSVSSGGGGSSSSSS
VTAATATSSTSSHDWLQQRRPPSPSVKPLSLAEVLRRPPSAR

>Vocar20013151m|PACid:23133940
MDATSDVLDFDWNDGPNNLLVQFQQLGFNVQVSHAHQPNLLGSDKPSAYVVPVQNGHLFYSSVEDMSAVAAYVQSGG
LVIIILDANHGEGEALRNFVSSCLGYEGTWSHCKQLKANSRGLAVLNGYASKSFLSDAADPWPQALENASTITYLTWC
RHEDISAVTLPLYELQDDSTRVAVQAFSKVNNPGAVVWLGYDWTDGPQDQWGALLKKLVTDFAQGAYYAPLFGNADA
HPLFAVDTVLETASDVVADVAEIVRRFLAVSGTYPSPPANPPPSSPPELTGFPPLPLGKAMEVAKYTAVYEEPAGEE
FDFTGLINRLKISVAAANDVPTGNVIVNFMIFPNGTGLRGHWRRRAIETSERILTDTEIAEMYAKTDMRPLELYLVS
KAPITDEMMLQLAQEETEFIRRAMLDGTADNAAQGGLLQIGYLVIKIISVAYPPSPPPSPPLEPGQIAPPMMPPSPP
ERPSFPPPSPPVNFTRIELETGAFGVKDGWSPPSPSPPQQPSPPPMPPSSPLSPSHPSPSALPSQPPPVFPSPPAPP

SQVSLSPPLPSPSLPSPKPSSPKPSSPKPPSPKPPAPSPLPKPRSPKAPRKPPSPKPPSPAAPPPSLFIKVTNVSAH
GARGSSRSLVWNDDGDFKSQLTKQSPLQLVYPAKPSCPTTCTACQYAWKATADQLSVAVFFKEPIQISRIYLKQIRN
SGVITVQFIRWVYPPRGVVEGNLGRTVWNVTDDTSMCQSVLAINVGPAKSGMNLAVPAGGSPANLPTKLQATAIGGV
LVTMERPRNAGLNYGPFLEWIRFSGRALYPSGLVPYN

>Vocar20013159m|PACid:23133966
MKWWWTAAQGSSPFPKEFALKAADADSIPNAYDLTIVERKGQAFCWGTAQYSSLSELKKKLLDPWHEVIYPILDDEG
PAAAPSPRLSYSTDEAALRNAVWQRAAAGRATASGAETTSDPLPPPLQRRANLLVDMFGGVIAHSAAPESLCWWEPS
PFFPLSRGGLRQLPNLLPLHAAAHRDRQDLLLAAVVRDVTEANGMVAAAAAAGEVAHTVDIALAAGQLQNQPRFFLV
VAGEATATAAASSHIAGIADKAGRLLSGSGISAAAMDESTTAAATEEVVQLRPLRTGTSPAAVAALWEFVLIAVVAA
GAGDADDDMGFGMPPAATATAVEAATPGAANGGEEHEPTAGAAAAAAAELLLDLLCTNWAGLQEGTLVCSLPFRQLQ
LCSTADRRSEHPPPPPPSSSQHSRTAAGKAVVPPGGYPAATATATGAVTDLLRLYLDCTADAMAYGVDSAPVSGDGDD
GKDTAFGHTVEEKGGAVAAIPVGSSAAAAAAAAKVGTRESNSLLSRARAAVAAALSWLPPRRRPGLAELLLPPLPLT
TATAAADNCNRLRPCNDAANNGSNEKLPGAGSVRRGDRQQRPSGLWIPAVRQPAPLTSRRSQQQLRRPDDYLLWPP
QRHPPAARWGLLDARAAAATATVAVAEVESDARSLAAVPEMQSPYARAAEQAARSLGGAAAGSRAAAADDAPAVAAP
AAAPTAVVPLSLPGLQHLPAEAAAALERAFMDAAAAAAADTARWKSHLGSRPVPAGTTTAAAPGVYLSRGAAAAAAA
AAPAVTGVAMARPQDAPSARSTAAGAVAITATSGSQEGSAALASSPPPPPPPLARPGGTRLGQLSPETAGPVLATFR
QAASSGYRTYHLGSAPAPVLRPTAVTPPPPPPLQPAPAGRGSFFGGCCDGVGGGSSCRGAAAAAAVAFPSGDGGGSG
DTACLNNSNNLTRPPVRSSPLHRHSGYPFSDEEHRAHALAPPPPPPVPSPWFGASSHAANRLEGKGDVRMEQYQDFV
RHSQRESGVVGGAGGIYAHLYLGKAGNPRADGCVRIMGSASSGRRCHHPLCRVTSPPPTHTHTHTHAVMGVVLGEEV
AMEPYERSVERAEGPPPQPFRGPSSF

>Vocar20013780m|PACid:23134072
AQRSQANRLPKQEEPWRAAAAAAGRVADVRQGLTPLEDLIGPKAQERLLHLQQLADGNEDDKEVYGMYDDKAAEAAT
ADWEAWRASRAAAAAAGWGLDTETVDAGTALNPNPDLDPDPTARVPNLADLGVDLEQLREQVLRQLADLDPDTLDEL
LVDSPYGDAAGDAVAQWQQAQEQEVQGPPRTASSAAASTAAGPGLTVDDVGRLAERQRRLWQEVQGSLESAAAAEGQ
LVVQAESSLLPEAAAAAPPPPLLQGHRRDPPPSPDSSSEASSPFSELDNLLGQLAPPSGGGSGAASARGTAGGGFGA
AVPASAASAPLSSSSIGHSRQVTNFGGPAGSGGAAASAAATAASGGGVSGGGGGGGGLLQQVREAARSGTAPQEIQR
MLDERYGVASPPPPPPKPDARATLPPASSPSTAAGAAEEPGGAVSKRRRGSLFAGLKSAKAPAAAGAPPPPPLPPQA
PPAAARAQRRRPYGPSPPPDPDPVMKPLLELRPDIARAVQGERELGCI

>Vocar20014012m|PACid:23134098
MSITLPSWHVVPSVFVGLLLGSIFGSSSDTWILLRGAFVKFSGRSPSQRRQDAALTAAQSRWLVQNLVARLCALTLA
YSQLCVLVNNVTRDRAGSHSHTPSSPSSSSHHHRHYNDIPHQQPSPYHHHHYHQPHNATPAGPATLDFSRVLALCGA
LMLTGVHATSLFWLALDGRGYVAKAPRWHPRLSLLQGLAVLACHCSGRMPVQFQVQAISHGLLLILQQDSKDLFLLQ
LALAGLTYQVAHAAQPYVHKLGTAVPGSGGGGGGVCDAAAAAACPAVFDSSLSYSPPSVNAAMQSASFLGIMLALIF
LYNVYTKSFSLPQPSSSHVERNALAASYMSACSSSLPVTGGGGSSLAGTAPGASESGGGGGSGGGGGGGLEPSAAGD
SRGSDSPRRRISGLAPSLLPWVTRRRAQGRSSSTAGAGGTVTPSPPPRNSISGTSAAAAGVGAGGCGTCPSTSASVI
SCGIPSATQTFFASNFLTLRPMTRAYLLKVGLSRLSAVVAGTVFCWALVMDLVAGAPPLSRMVSLLSTVALVGANAA
HAVLIWRHPYVYLRAVRKYNFATWAVTVAGLVVKHVEPWVFRQNPELMAVAVSFASLHGFVHDDSGLVFAGLELVIL
AAAVLQTLLWGPGLTASPGGQLEILRDCTIYGAICGLACMAHAVNQQFRLTGRQTSFRAAVPQTLRVSSSSSAVVDP
RGITRASFDSFLADSQQTPSPPPQPSLLPPDFLLQPQGQPNLQLHPIGPHVYPPHGQPLLPSPSRWAALRHPPKRHM
SLLTPLAEGHAGAPAPTLSPRTTGGEASRLAISSIATPFCSAAAAPADDSFSATLGAEVLGDRPQVRMERGGSVGDL
SPLPYSTAAIRVSGSAAATRILNVHSSTSMDQVLDLSASAAIGDIARSELGSGDGTAISGSSVGGRRGSRAPPCRHV
SIRMAALREPVRRRRSTATTPTLFEAPRLILGSPACTAGGSGELDGNIWDAALPSPESRPSSSGPGSGKLPTLSTQH
LSRGGSSKDLASLPLLQALGGGGGGTGRASGSMTTAGGEGLGSSLRRLALSTQIGCMRRSSGGGGGMGPVTGWDSAT
TSPSNSKSLTAAVAAVAAARHRSTQPEPEEVDALLLAACRSLEGSPRSSPRKGAFGPPAAGRGPAVPADFRLPPPPP
PPHHPSAIPSVLTNDDDMSSWSAALAAAGGSPPFQVYGTRNKSLTSNTRSDSAAQVSVGGVEELTAVAAEGRALEGT
LCAVRPAGSTEATTEATAVAADLSRPGETVMPSSTTATETHVRQLVDIPHLIAVAGGLFRRSRQVIPTPSPLGLSTT
SGGGGGGVAFVPDSMDGTDDAAAAAAPEAAWAPLAAEQQSGSEDRGPLSPELSPGRPLKVSTSLPTLALRTVVDAAT
TPVPTQSGAATAAAAAAAGPRAAERAADATATAATAAAAAAGGALRALGHQLRRLTSAPASRGSLSGNQGLQGAASL
GGASAMDAVVGGLEGYVSPIRMGLGSLLFSWRGAAAGYGDTSVAAAGSGSGDPSRIEDSTPRDVVFGIIYTAQHALD
LGHLLRAFKPSEVTGTWVAAALLYGITLVAGLLYLAALRRGDRWATSQRFRRVFWALRLTVLHALRSTIYTVAGMQV
PSLLGRSLVSSVAFGLLGLPADIFLRAMPVKIVVVYCLSCVKDATRLEPLRLLVSLATYFVASTLAFTAAAAAVNRA
ALRLHLHAAVHCTIDAAAATAALESKLAERATAAAADGAAGAVDDDNDFDIGDSASDLIAADERDMGDLAEDVLTNH
RRMGCGCCSLTTADFGVPTMAERDGSGGRAGPVSAALSLLACALRNGLPAAFGAAASAHAAESSAESSQQQQQQQP
RVVPNPLLCYWNTFCSDGNTLASTVFALLTLLFAFVTRLLLRYGEHKSGYEPDPYFLLEQWILHGVEVGVRAVVVRL

NAAEGVSGLPQTALALSLFLLAVSCVVISQVVALAFSASVDPRVALYQQAMAELSAAVSSMQLQEASRHAITASVDK
YMGEALDSCSALLVLVEAQTTRPATFDGMVSPLLFKSVGGPQNSRRNLGAAATGSGGHGGGGGGGSAGATSHGMEYD
THATVYVLHDRLLGPDLPPYKLPLEELPSVTAALQGCVPLFTNDTTKASEDLMNRFGDWRSARDVEGARSLCVVPMC
FSGQPQGALVLHSPLADVMDSLFVSMLQALAAQLGQVMYLKRALEDVRADEHLLSDLMPSHVAHTLKRRFMSSHEDA
LTLPMFASKAASQGPPGLGPPMDAASLGSFGNASFDGGSLLWKQSGGGGLGGGGGSGGFATVSAAAAAAVAAGMPGG
GGEAAAAAPAQIASALASGGSGGGGGGGFLQGYASGGPSLGSGQGGMSTRRCGLAAAGWSAAVLAQQQQQNSGGGGS
AAGGGTAQDLTGLTEALPAGAAAATATAAVAAVAAASRIGPAVTSLETAVGLVYQQWHSDVTVLFADIVQFTPMSQE
MEPQQVMMMLHELFSRYDALLDRHNVYKVETIGDCYMAATGLLADDPDHAAHMLDFAVGMLAAAATVAVPLPGRGNV
RIRVGMHTGRVMSGVVGAVRARYCLFGKCASETTYLSLPSERQLHWQFRGAIEVKGKGSMNTYLFVPQNNNNNNNNI
IINNIINNNINADNTVTA

>Vocar20014335m|PACid:23134125
MVSGTLEALRSILYCRISALAFWKAPGTFRKKNCGITIGIKTGNPVESGLVRYRPVAAAETNLVEDALSWQGDNCSI
LGIITGFSIHRSVVTLRSRVNPRQQGCSLQAFAYSIIIITILTNNANQAFSLAHGPPSTFQKFQSSATHYAYSPFWS
FTRIAFGWEHFRSSHRPAVAAHAARRTPQVDNPADSSVAAASGTTPLLATAPLTPSSRRRVSRRKNTPNSGLTSDSC
PKNDKAEISQQQQTACGTDRVRSGSQPTSPQATSNTQPKPSRSSPRDLDGRARRQTASAPFPPPPRRESTATPNATP
LPADAVEPGGGVRRCRESISGSQYGSGGGGRDGSDGGNKSSARLPGGKNRARSYGDGGAARPWPSSLSPLSPPPLPS
ATQAPSALWHPGRGPGPSPPSPPPPAAPPGDIRPPPGSVMSGWGLAGAQPQMNSVYRLIRKKELQHHYNHHYDDHNH
LYERHPDKGASASAAAVAAAAASGDGIGNRSGVDLHGTAGPSGRVGQQQLQPSSSSSSSITTPRVTLSGRDVSPRLG
DVHRHSPPMPPPPPPGPPLQLQEHHLPYQRQCQHHHDHHHHQEQDCDDPHQKRQRKGAEDSPWEGNPGLMRAPPPPP
LPLAPFASGHTAVDRVVRIRQIRIRQRQQQKWSTGGEVGDGASGREYGNCQTSPRDARPPTAGYGVRSDSAQVAAA
PVAAREGVWAADRVPTALLGGCAGAVWGSAWDERTAGGGGAGLGVDPNTSRIEVTLLGPPSSSSSPSSPGDTVAGGG
GAGGSDGYGSGVSNLLRETLISDLSLLNPVYRLHVEAISVLCQPAKGELAAASERDKDGFSWSRMIRKHELLLHHRP
QQPSNKTQAAGASSASSFSAAAAAAPGARAGGVSGAASSSVSSTLTSAASSAEPWAADDFADVMLGGLILPHLHPHH
PPGASELQSGSGFDAIKESESRLRSEVAAGAARGHNSCSSSYYTSRHVVETAEAEAEMEMPGGVAQPQGRLNPVMRL
IRKKQLQRRLRLRGRLGPEPAEAVPPVVRQQAAASVVLPAAAAGDAASAGAPACRTGEERDPGGFGGSGGGGGSSSS
SGGDEAAWEIGPADAVGGRAVMNPVYRLIRKKQLQSKRASQGFAAGSGFAGADSARPPQGGYAALAAANAGGDGGGN
DSVWQRRSAPFFDLGFGGFGTQLLKHPASTLAAALDPMAGSTGSRSKSESWLESGSRPPDWLPDRQGRAVSDRLPHH
DLIVQISAKDGESEGGGGKDGEGGAAAALAAAAAAAAAPLSSLVSDQLASSMVGERPHMNHVYRLIRKKELQLRQRG
VLLRAAPPEAAEPSAGEAAAATATSAATEIGIDGQPAPQLPPGPPPPAAAVGPPALLRPFDLVDELDEDRLQPMVYG
SMSAGGAGVSSRRGLTRYSSRLLMGRTYGEEYDDVDEQYGNVGDDDGAFWSADEGSVVELDEQQAAAVCGDVSSGKA
AAAAAVAEIEVLAKPMAAAPQRRRRKKDTNVAAAAGGAAQVQKTELQPAAEAAPAAGTAAKDSMGVRHHSSGAAASA
VLVTSAAATPAAEQECATAGRQVKDLRRGRPSRRRDGQAAALPGAIATATATTEDTAADGATAGGSTRGSSYSYSYS
GGGSGSGSGRGIRRVLTVITPRQHRMEELKASLRAKLLQSAAAAAEAGAGGGGRSDPAAAVTAAAAAAAAADDGNSG
SCESPCTAISQSSPQRYDTFSGRTLAPRPSGMRTTPLPSRDGGGAPPLLAVSVQCGGADIADAASTTNATTALTAVA
VAAATSLHDDAVAAPPPPFPPTPKDSGSVAAANQAETGLAATTRHDAVPVAVQARENELSRHGAAALPMVTARPLPW
QQARRGVAAAAAATATATAVGSGDASRSAGIPLPCRTGAPHRPSGLLRFLQETSPGPDCAETMYDTQAAAATYDGIG
DRRQLRAAGCSDTAAATVGGVVRPPRRGLFSGIAQLWDAVGLRVGPMPPPARRLAADPDTAAAGAAVVRNLTQLEDL
LLNQGVDANWLVQSRGWRPEDVVSRLRGLQRVLGGGGTPPTSKTKSLRQPPAAAAATAAAVAVASPREQLPAPQTSP
PPPPQRLPESPTAHGGPGTPVVHSDILPTKVGPAEDEQEEEYDDDDGGGGGSMAGLAEAAAAAVKQPNLLTRDPAML
EINLRALASTLRMDRSWAVQLKAMYVCGSHMQPSLLSYRAVPCCTLNVLKLKDFLRRFLSGDAGMRRMRARVGCRTE
LRPTSLTERLTALAAELTHPTYQKSDKILSWGCQGGCDGEGHVGSMSGRCCEGGEDVLGGRAVPEGRFAGRGVDDWV
GAGLVAASAAPVVAGEEVSMEEATEAGDGSGGVAAAAAAAAAAAAAAEFVRRYPPALALTPAAVGDRIRALSEAIAAT
LLTERQQQQQQQREEEQQEDKLEEEEEEEEKSKEHGFRYARQQQQMHQAVVTAEPPPPSPLLQPSPPPSLLLQPEPSE
DHNHARGSSTAVSGITPHELQPCCENTSSRRSRSCSSSSNVQASHGGATPSSVGSAKTAMTAAVAATETNAMSAATA
RALDLFSGHPGLLGYSPDALAARWRALDAAIARAEVELMLTSTLPPPVARAPPGEQSLDATLRTHVLDEDPDNLGVN
QDRRRVPERLLDLSLGGGNSGDHNNGGGDAGRSDRVRIHGSLSLTLPWRQQLLEGPPAAVARCLAASEARLQRLVFV
AAAAATGGTAAATPAPALLSMPATADMGTATCKAPAAAATASAGQGAVGSVATGSADSFTHSELGEEVAVALPGASA
VAAIAPTAAVGAAAMAAVAASVAARRPRGRPRKAPWGLHLQVPASTSAAVAVAAAENPKVDVPEGAAAAAAALVQAT
VLEAGSNKLPSSLSLSTILAMSAVRFDERFPGFKIWLQNRQQEQEVQLQEEQQQQGWRRRQLLL

>Vocar20013969m|PACid:23134208
MSSSVVVLPPSPNWFCGQVVSVLPSRQLVAYGGHNCVVLCNAATRTVQRLLSCGLCRVTSVSLVPELAGRPLVAAGC
EDGSLRCWNWQSGEPVRSHRKTSPEVTALVALVSTPASASVAKSLVVAGVADGSIRLVDVAAGQVLLAVQQAHRCAV
RSLQLAALPYLSGLPAAAAAGASGAGSPSLEASAGRVSPPGQQRDGPGTGSPPPGNANVTATAPADAAANSATAAAA
AAGDAVLSNVEDDGSLVQTNGTDGASPSPAGKDPPKARAAASDACTAADAHGGGGDNAADPVRWPEHSWGPGQEQAE
PSAAAAASPSAAVAAAAAAAAPTDLWLLSSGEDGLAKVWSFPAMKAAAALMAAAAALPAMVPPPPPQPLAVQSGPGG

GGAASRHPWVPAALLPGCDVGGGGSELVVALAAPWGHVLMYHVSLATGKVTLTARLKHHNRPVFTLHAMRLPSQDRS
LVVTDLTMQRGDTPGHATAVAAGSGSGSGREGPPSEATGATEGGLRQAGSARLPGCRTAAGQGATEGAGKWRQGQGQ
GQQEEGAGGCVWWRLVGLGGYPHALQVADRSPSALLAVGCGDRTLRTVGLQLGAGEGEREGEGEGKGGDTGTIAVKH
QPQPPNQQSQPTSPAAAAAAPVSVSVSAAPPEPGPQPPGVAAVAVAATSGLTHGLKEQQQQQEEVEEEGQQEQEQG
DRLDIATPSSSPPPTAFRQVPELGVRGEDTTGVGGSEGEGEGEVVRVAWHPADPRYLAFGCADGSVGVMDVRKQNSR
VFAVRHSGPVTHLAWMASPAAPPPQPPLQQQHAATVTSSSRLSPAAPAPAAAPAPAAAAPAAAAAPAAAAAAARS
GGGGGSEGGSVSGGIGGGRRQQPRPQSRHSPHAQGQAQAEDRPAGHKEHREHQRLGQDPQQSQPPDEGHSVAQRLTA
APTDPLSPPPPTSPILPPQQTPPPPPPPPPSPPPPPPPPPPPPPPPPSASDNPEADVRHGEEQILQLPPQQSPPPEAA
MQPRAPELCPSASEVVSEPGPLAHHPPSQSPWSQTRGAVQRPSQGGHISARGRARGGMSANAVCGGGAGGGNGASRE
GGIGGNSGTSTWHQPSFSVSRGPGGGSAGFRGRGRSGAPSAAPGWGLMGPRGGGGGGVGEEDPPARVSERPELLVRS
CVGDLGIEPGCPEGGDGGDAEAALGARPAAGPEEGKEDVEDLGPRGGGGGGDGAVRRVECGEAVAAERRRTRGRPHE
PGTGHRGEADAEGSCGGEGRLLRWPGPSLDEHSAEPGLGPTMAAAKPVDVGAQLEALLAAERALGPPAATATAATAA
ATTIAEAMEPSIGPAAATTAATPHPNPHTHLHAQSSTHRRHNPPSHQQPQQQQHSGSRAAAASGGGFGLPVWAMAVH
PRRGWLAVALKAGEAAAAGGGHGGGGGGGGLVVVVEQQPQLGAGRGGGSRGPAAAWRLLAMTSNAGSQEARLLLWGD
DCEYVNGGDRDGFYSDAAAAEGNCGDGNSDNYGAGSAFLAALYGKNSVSVYGMVAQPYGRLSPCRQLRHVSCFSTEP
RQQQLVAACWLGRHCLALGADDGNVQLWTVRQPADGKAAVHGSPRPMSATDGAGGGDHEVAQPGTASAASAASGGGA
GPAGPAGMTAVQVETEVKAAGVQALRVRTLAAGGHHGHHDSITSLTAVRWWAEGGPLPPQAPAAAAGEQVGGQHRGR
SAGPVRRGPGGPGWLLFSGGRDQSVRCWRLEAGQLRTWELEAARRRAGGRAADADEESDVGGTANKAAVKTLPVTSA
LSSQPQQQQQQQQRQQDAIAAAGPYAVPVSGPFGDDAIGNGTAAGGGCGAGGAGDVDEAAAAPLAEVTSSGTCAGRR
SRLPLSEGNPAEAPPAADVAALAGAAAAGAAAAAAPGPLPDTHPSPPISPSSATAAPLPPRLIEPTAASRGRRGRRP
PAAASRPLLPDLPDLQQANTAAAAHSLLLKLATVMYPAPTPTPAATHTPARAALFPDAARMHEGAGLPAGSGDVGPS
PVAVTGVRQQGTAANPRPDGETGTKGAGTGGEARAGDEAAGLLGLSEAETQLAGHLLHDLGEVWQMERPMGQEGRQQ
SGEEATVPELRPLPAELRFVAAMWEGDVGGALALAAVTEGMMSADVVAMAAAGGRAAWEAAARLYASRMEAMGCATE
AALALAAIGDKAAAVGVYQRAGMVWEAQKMLEGTVESGAGEGDLG

>Vocar20014070m|PACid:23134438
MTVGMCALYACASQTMAAVPSSRNRSLGAQVTSGRSAVLVLTLAAVLLAVAGLGAVEGAPLPPKPPKPSPRPPSPRP
PSPRPPSPRPPSPRPPSPRPPPPRSPPPRSPPPRLSPPPPRPNKAPRPPPPTPPPEGGLRLVRGTAGNVGRLEVQLT
YPDEWVALCDDGTFTAYEAGLFCKMLGYSYGRQFYAQGISTYGPDESDPPELRGNLVCTGDNDSRPPYAFFLQMINF
SSQQEVSCEVFHVACPRNMLVAMECSETPFKYNGPPPPPSLPSPPPPPPPPPSMQDFIRLIRLTGSNPWMPDGLVVL
LVNSSADGRTGEPVWAPLCADGIAGVDKIANMLCKQNDGFRDSPIRIILGSPREAIEITQQASTISDFNPSEYSHWV
TVVGGISSSAYTVYDTQLQVTTQPCSSGYLFHVICYTIMA

>Vocar20014071m|PACid:23134439
MTVGMCALYACASQTMAAVPSSRNRSLGAQVTSGRSAVLVLTLAAVLLAVAGLGAVEGAPLPPKPPKPSPRPPSPRP
PSPRPPSPRPPSPRPPSPRPPPPRSPPPRSPPPRLSPPPPRPNKAPRPPPPTPPPGGLRLVRGTAGNVGRLEVQLTY
PDEWVALCDDGTFTAYEAGLFCKMLGYSYGRQFYAQGISTYGPDESDPPELRGNLVCTGDNDSRPPYAFFLQMINFS
SQQEVSCEVFHVACPRNMLVAMECSETPFKYNGPPPPPSLPSPPPPPPPPPSMQDFIRLIRLTGSNPWMPDGLVVLL
VNSSADGRTGEPVWAPLCADGIAGVDKIANMLCKQNDGFRDSPIRIILGSPREAIEITQQASTISDFNPSEYSHWVT
VVGGISSSAYTVYDTQLQVTTQPCSSGYLFHVICYTIMA

>Vocar20014201m|PACid:23134445
MEDDAMADHLEPNDDSDSETHLERVVSGQLHGEAGKRRGDITLAELQEQFARLQHEFEIFQEGRTRPPTRSREHVSA
APVPPPRQRSPRAVGRADGEAGRVPAADGPRSQSVPPGGRMLRNPSPADGRLDQMRVRQPSAQPPPRASRPPPGIAA
NPAAGAAASAPSRPRPVSERRLPSPPPPHHPRSASKAPFDGPGASPAAGHGKPPAGRAEKKLVKKKVERPPWRDDFA
ADSSPWSRELAQEEAAAAEAAATAAADSGAAVGCNAGGGSGMAIPRAPDSGRGRGDKERAQRWRTGGSSDGGGRDRS
SSPVPAVTRAESPGAPDKGVPAQARRRPQPAAAANARAAAVAAAMEAGRQQQMLEQHQQGTRQQQSKGGMLLVIPSG
PGAVAAQQGSPQPGSEVRRISPCRASQIPTSPSPGLDVRPRTQQSRIPTFPSPQRQQQQQHQQLDAQGDGGRWQSGP
GTSAALNGIAVAAIVDRLVVDSSVSSSFSYDDPLSTSAESSAQTPPSNACGNPHQQRRRRQWEVPGGAAAGSRGTAA
AAVAGGDANRRVSSPEAVADAVTAQAAARPPPAGAVSPAAVGRVYNPTPPSSRPPSRPGSRPGSRPTSSTGEATAAG
AIPPSIGHHPPLTPMAYTEQDARQHSSPERPTGHRVSSAGARHPLHARGGGESISEVTMPDVTFAAGFSGGGCTEDQ
TSVRQAPPLQAAAAAAVDAAPGAAAGLTHAPAAIALVAPGMRADLNLPEVVHGGGAPGVSKTGTARGPGPGGPRDKE
DYFRKLSALKRSVPNDPLRRVTEDGGNQAAAGAAAAGSGGGAGCGSAAQSRKWMDPGTGDASLSAAPMDAVGSAPAA
VRAARSRNLAADVLGGIARSSGGCGDAADGGEDVSGDAAAGWKPRPGKGRSQLQLQRLGSGSMGDVTAPASAGPALG
GAAAAGGRTSESGYSARAASQAPQYMAFAVQNPEVAMAAAQAAINANGSRARARWNVPPPLPEAVVATAVHPPAPPG
FVAAAAAAVSSASPPPWVHSHQPPAPPPPPPQQHHQLAAAMVSSTSTGSTAVPLVCSPGQAPQPNLGAMHSLGDNHH
DLRRANSEGPRLSHGRDAGSGMLEMAPPSGALPTSAPSGPGPGGLGGLPPPPGGPYYVGAGHLPPPQGPIMQGLFPV

GSNVGGPAGWAGAPGPGAMNEGALDGSGRLPGFGPAGGPYTSPLQHIRPAMVPPGEMWAGGPGAGPGPPPHMHMAGF
PPPPHPPPHWNGYGYGQGGGRPPGYLGPGAALPGTLEGHGPLAHTGPIADPYKPQLFKRNSNGHYDGFGGAGLGYAS
APPGASGAGPSYSSGGGAAASPDLPDYLRVSMSRMVAGWSAPNAKHGALSGFNDVWQHRNNSRPPAGPGMAVHGSGG
VPGPGPSGVPDGGAPVRGPSSGGGGGGGGRETGFEGLSLEAVLASKPKKEMPNFTKKT

>Vocar20014106m|PACid:23134472
DEDEEGLLPEVAEAAVAAAAHEQLGKGVSDGHDNEGQVQQGHGGGWVAAVGPPCPHGEVQGLGRESGAVTVSRQEPL
QQGRPGCQDAPATAATAAGASPTGVPTGSTPAATAAVATARSGPYTPSADAADVAQHHQHHHLQRLRAYWHRPLSRW
DPHERLLVAGAVVAAQLRSALLRELGYSCSAGVAHYKLMAKLGSGLHKPNQQTVVLARAVPTLLRNLPLAKLRSLGP
KFGEQVQEGLGIQTLGELWSVPASRLESLYGPEAAAGLMRLAAGMDEGDMVSPRLAPKTLSCGKTFRGSSALQDIRQ
VSPRLMQLAAEMSERIEADRRDHGRLPTQLTLTLQTGAPGLAGAGAGEAAGGPGAGGGGAGGGGNGSTHSRSCRLAR
TSTECIAEVATGLVRKWAAERSGWRITGLSITTSRFDSAPSGPSTLARFLQQQQRQAPQQQQQQQQSKQSHSSVT
DSRQPSPLPSPTLTSPSPSPSPALASPPPPSSTESPSPTPTPSPSRNPTPGMATPAAAASAASLATVAPSVEPAPTS
PSTGPSASASPSSSHHHHHEQQQQRRRQHKVVGSGARRVKASSTRGSPGAGVHSADVRALLLVSPRRPTAPGNLLRE
TTATTATTTAAATATPAAGSTVTAAAAAVAGRFLPGATAPGASADTGPVQAAAAEGGGGAGAGAGGLVDSTTDSTAA
TLPPPPAQCPSAQPPRPPSPPPRHPSPAHQHQEPGHQLAGPQPPPSANPRAGMSPPSPPPPARPKWSRGALAALLLH
RQPLAAHTHQPPEPVTNTTNTQRPLILIAQQEQQWQHEQEAADHQVLQPPSRLPSPSSSPSPAPEVWMGTAAAQQYD
DHDHHYHQHQQQQGHLLLHTPPSSHTWGSPAGSAGGVLQVHPPGPSGGHPGGGLLAAGPETRRTPERESGSGPVYGR
HGHSGELGQGQYDSGGGGGGGSGGGGDRAGGTAWAAGVGAAALGGFGELYGAGPRSEVGVPSSPPGSASQPLVYSMG
DWLADWLEPPPPPPAPDNPEAAEGGPAAAAAAKGVAVPAATQPPSPATHVIQEHLDRQAQQQLSAVQHQPQLSGPLA
VAEDTEAALEQQEQEEDPPQLRQRRPSSHDHAPHLHGPTSLNSKSSSALFAFVSPPLNLTVPRTSTGIPLGVAAGER
GGGGSCGPGPGAGPAVSAAATDWSQLEVDERVLAELPWEIQEVGSACLWGQGRGRGGSYWPEPLPATGACAMPQLQG
TALLEQWTAVVQPQLPCIW

>Vocar20013965m|PACid:23134482
MVFAAATPASSPRTSRRALAAAQLQALSTVVSRSSINPKHHIQATWPCATGWQLTRPCRPTGCYIPASASTAATTSN
PHLAKPPSWPTSSAFSASAAPHLPVSRRSFAIATCGHRGGVSRRALLVLCRAGKFDDERPLSQLLKHSCPYCGYSIS
SKNYGGHHTKSCKALAEDPRERERRVQESEAKFKRLRDADGGGGGRRVSGGLEGRGSPPPPPLAAATSAPARAAPGP
RDSVARARSAATLQAPVRGVFSPSPLRGAAPEARESPPPLRPPATPNPAWGGANGSPPLPAAASRAPPSPPPGPPQP
PAGGPSSQSTRGSGPVSEPEGGGLPGRYGTVSAPAPGTFGSIPLGAALPGQDGVRAGASYVAAQQPLQQQSPWQQSL
TPQPQPHESGNNGNNGTGHRVGLTSNGNGTGFGAGAVIRQAASSSSSSRKQYRKPKMEPLRWPGFDEQGEMDKLALN
AAGFWNVVSVPNGAPAATSASPSSPPEQLFVRPGERDQLRARVKLHYSYLDSFLMALPADVGRCRFTLATDNDPAGG
ALRRELLRRLGRERCWEVVSWDNEEEVALEEAEAAAGADGAGGFHLAAAAAAVPPGAAGLLAAGGEGAAAGLHPPAL
PRRGRGGFRKDANEVLMLDGPEQLVAVLESAQPAKVSGLATFREFEMEIAAYFQRQDPLQLGVSTGWRCLDPFYRVA
PGELTIVTGETRGGRGGCARVCMCVCEFGIRGLLIDPYNELDSRRPRDTSETDFIRELLTKKVLTGQAPGLYDISGS
AHWFNKTDNGIVVHRRFEERTHPDTGKRYRVALPEVDIKLQKVRNKDIGTQGEAYLLYDKATGRYEDPDVLLREAAV
SYRGGGAGGGGGGGGGSGGGGGGLDRIQLRNLEVGYVFNSGSYTPHHQQQQQQLHPQPLGPGPDNNNPNGKQHSQI
IDVTLAPVPPAPSQPPSPAPNQLATAAPAAAAAALAPPHRSQAPAQLASAQLPPPHPSQPQQLQLQPGSQPFYPPPL
PSSPPQQPPLGSPDIGRGGAAAPPPASPPPQRLGITSSVESGSFSPPAAAAAAATPAAAAPKAPGPAASKEAQGRQQ
RAAAKAAGTRGGSLGGFGGSSSNNSRASRSSKGNGVRKVEGFLSPELLRLAMEEEAGRGAVRLRDNGSACGWDADES
LSGAEEEEEDEEREEEDEEGAARGGWRMGRRRRRSGGISRGLGARDGGGVMMFRDGVWAPKEPESPGSLASTLAENE
CGI

>Vocar20014156m|PACid:23134497
MEPYTSSRLLWRGGLQASWEEVESEFWRLVEEGEEQVEVLMAVDLDSAVYGTGFPRCDGSAAPPSPYAVHKWNLNNL
PRLEGPHPSLLRHVSAPLPGLTTPWLQVGMMFSSTTWHLEEHLMYDVSYNHLGDPRRCYAVPNSHRAAFEAAVRDAM
PAGASGAGDGSQQLMLAQLSHGGGSGSAGGGAGSAAAVMLSPRALRAAGVLVYSVTQAAGEFVVTWPGAYHAAVGLG
VHVEEHISMAPPDWLRFAEEAERRQRLSRRKPAFNQQEMLLHAARGECSPSLATFLVPELCRVIEQEHRLRLALWEQ
GVIRSRRWVSETAAALLPAGPAAAAAAAAADVPVASGAPAGRGPGGSKASGRGGGGRGRNGGGGKVRRAVRPGAGSG
RGGSSSEGTAVAGGTGLASAASSSTCCEGDESSDEECAVCRSMLHLSGVECCRCPAGRIVCLHHAGALCGCPPDRRR
LAFRHSIKELHQLLEDVSRRARAEERKGALEGPQQAAGAVAAVAPFLATSAAMDAVDPPELSPALQQIQGPGAPPPA
PAPPPCSPKMEPDTRLGVGSEGIGAGPGSSSSHDGGGRGGSCATSTMAIHQQQQQQSVLGVKPQPSAVFGPVHGAVL
VAGPGQQGILAELAARHGSAAHAWCRATRALLASSAARPSAVDEALVGMEQFTWGDRGVDEAREVELQCRRAQDWVA
QVASCSRGRPILEQVEELLDWEPPPVALPALPRLREMRAVGKEWRERCVAALAPQGGAGGGAAAGGSGSRAASPSAA
GSGGGVGGGRGDGGQEEREEHRLVTLAALEELAAQGSRLPVEVRELHELRHRIEAAHDLAAAAVELLGPGGVPMATA
TTATTTATTTTKGTRRTGGPDVPRSVSPEDLPTLAQLRQLQQQYGALRVVVPELAGLAGLLERAEAAAAVAKECVLT
RATLEQLDGFMELATGLPVYLPEVRQVRDLLDRATEWRRRVAEASSARQQPPLKELRLLLHAGERLGVQMPEAEALR

QAIRRREWEDAARRLLSPPPAAASKPSLNALAEAVEDAARMGAQGCELYEQLRSKLEAATAWDRRVQRLLAMGASLP
EEREGEEEREREREEQQQQQEGGENTGTVEHGAEAQAHGPPTVQPVIRPGSRRRKMRKGGGGDGIDSASGGAGGGNI
MAAGSDGAGAMGAPDGTGVAAAGPGGAGGASGVNAADAISPPRSVEELDALVSEGSQLGVRLERLPVMQKLLDGAKK
WVEMAARMVKNAEAPTVARLEAAMAEAAALGFAAAALPSALVIRLRVLRNGAALWQDRVHTLLGLPPSRVEAIMGGD
VAEAEAEELLDVFRSGVAGLDAVDAGRLQGWLEALRWNKMVRTTFASVIKRSQEGSRAAAETDVPGGGAAAKPRSRR
SSPPQVPLSVARDALDRAAALGLPVDPKLRQALSDAVVEATAFEMCVRSRLAAVEGAAAAGSEVVSQEEAGPLREEQ
VVAWREAAATLPLAPDVAGSLEVLLSEHQSLKKVLELEAAYTRSLESPLELDPDMAAAVAKALEEAEAFRERLRKAL
PRRNSSLKVRTGGGMEVEALDKILSVLASSVALALDGLNRQRARLQASGVGLSASAEAAAEGGAEPARSSAGNGNGN
GEQADGGDGDAGEEGNRRKRHQRSEGRKAGAAAHVDADADSDDIKLRKVKGDEEADGLASYGRQRPRSAVPDPNPVS
EGSKNRRRTAAAADAATTAGGSGRGGPVLVGSDAPRGSVFWLVQPQQLSPDTQQGAVPGRRPGEPALMCLCQSTAGT
EDMQLQCGECGDVYHHRCLGMSVMATRAARAARWSCPICTALVGKDDMDVIEPLCGRFRKTKRPQLEEVEVMLSAAG
ALLVELPEEEMLRRARDTYKCWEPLARELLSRYEALLERLKARVSTARTATANGGSNTHASQLTATTTLDEPPLDAA
LAVTSRAVREVIKQACVCEMDAGEVAVEALRLLKLESWWSAGGGTVQRPGNSKVPLDTVARLLRDAGTFGVAPDEPL
LGQLETQMAAGRHWLDKARVLTDALHQAAGRQANGPGAAELRELLQDAQEHVKAGQSLPIKVEGELDKLAEMSTVYC
LCQRVYDEDEPMVACDHCDQWYHHRCVGLPRTVEEGLGEPGARRRPLGASSEYRCPCCCIKECKPYLYESSMPFQMR
DVMVWMRANKTAIDDMVENPLLTSGAAAAPATEASPSPPPAEEPPPPPPPRARTSSRAQSSSGREPRSFATMAKQRD
HMLHRQQQAAAASAAAAASAAAAQAQAQAQLHAVAAAASGLSGLGGLGSLLPGAPSSTSFGGRGGRPGLLSGGMGVA
GPTAGASPWGPGMFLAGGGGGGASAAVVAPTGVSAGPDVDPLMLERWARLEEMERQAREEKSRLMSNNWHRTVSLAA
AAAAAGTDLLPGMFGGAGDSAVGGPSNGLWGGMSAHQHQQLAAMMLSMQMGIGAGGGAGGYGAAAQAALLATRLQHP
NPTAAQLQMLGALGPQQMSWQLAGELAALERAELLQRQLWQHVDGEQQRWRGQDQGQQEYDEGQEQEQEGAGKEQEQ
RHKQKQKMRSRRRPPTEAADTYEAYGSNE

>Vocar20014304m|PACid:23134575
MRGALPSDQVREIMGAANLSSTAELKAARKAAKASYAAELQAQMRANEEAKVKDRMERLGIARPPPLQAPPPGGLPG
GGGAVSPGAMGGGMMGFGQASPPGAPLGPYPQAAPPAFPPAGPGPVAGMPLSGSPGRGAAPPPPLWNQPLQYQLSPP
PYQPSMQQPYQPGGPLGNLYGMPPPNPSPPPPFGGANGYGPQPPGYSSSPQPGFGPVGVGGGFGPAPGGGYALPAPP
PPPPPPLLPGPAPPPGPGRDTMHRLEQQAKKDQYRLELEAQIREREERKASEKARQREEDMRKEAEMAAAARDAMGR
GGGGAPLRDAAGNVVANLRGAMGGMGPPQGLPSPGGMGMMPMGMGPMMQQQPPPPLMGSPPGMGMGIGIGMGGPPPP
PPQQPPMMGGPALLGSPSGMGPPLGMMGALGAGGGGPMDAGPPGVLPNYRFRSDNAYLTPKELDNKQRQMRELQEAL
EQQIQEKRRAKELEKHREIAEIEREEARFRAGLEQQRGGGQEGGAGAGGEARSKEEVLADAWEKARQEAEELRRNKF
ASKRGAGGVSHGPGGGGGGGRASPGAGGRGGGSWVDGADEGSEAPPPMATHHSAPAPQAGGGFGDVFSPPMAAPVAG
RPSTPMVVEKVRQVAAAELAAVKQELAAEAAQLREVVAAQGQQVAALKTHAERMEAEAERAREQVTAMRLGLIQQRS
GPEFDPDHALAAVPPGPLPPALVDIDLDLPPTGAMLRRNAAPTPVYDMGPAIPAVNSSVLMVDNAAASAINASAAAA
APGAGGPVASGRPPSRLRAVNPLEASMAGESVFIYPNGREMARQPNGVPTRPGGAPLPQALSPGGPLPFQSIHLAGG
VGAGGADLPTLVTVQELEPGPVPPTPQGGALASSTVMLGSTGPASSGYGAAAASVNASVAAAPMTVSVASSAAACEA
TGGAAAGPRQQSISPEGRRAAASATPLDVDMVMSRNKEKLEMLKGITDLAVGAASVEALDEFLARYAAQRPGLPERT
ATPVLRICTPAVESAGAPTPAAAELPALRNGSLPSSTPLYSSRTKSGTRMSRSRSLSAQGEIRPGKDSAPVSTSQRE
HEEEPRRPEPLPLPPPPEDTLDVEMRKGLGAAAAAPPTPPSAGPHSSLPGTPVGKGSNQQLGTGDSGIPPFQIPRSP
MLAGARRTGSAASQRGSGGP

>Vocar20006099m|PACid:23134651
MAMGIIELAVAPPPDDPMDTTETTDEQEPPPQPPSRIDAVAARARGQRRCGGGGGGGSGTCGKEAMNGLYSQSHPPM
PLPPLILPLLPHLPAKQPLLQPPPSAGAAAAASAARSAILSEGRRRRRQLPTNDGQTEGSAAAAAAGLVAGGGSSES
NPSASKRCRHCIGDGRATAAGLALPSAAATAAASGALAQYIRALSLDPPPPPPSSPPPQLPPPSMALQDPPPPSPRI
LYDFRIREAVPSWDCDPDAAAIPATPPAGVAAAAAATASAACSALTAATMHGDPPSPPPEYGGTPQLQRLEAAWQQQ
AQLQPIELLQGRWQPLLQLLQDDKPFQRQLQRQMMKWTVCEGGEGMEAGSPPDAVPEGAGVVPVVVVGEVPLRRAQQ
GQEEEGKEEGKEGEGEETGEEKVGEVREGEEEGEKKVGEEVWEIGRRGRNTQEEELEEEEKENMGEVVEKEDLPPCP
GDLPAAQLGPIPATSVLDSELLPSLVGRRTESIRPVGAWRKGGGCGGVGGLGQMEAWR

>Vocar20006075m|PACid:23134661
MEIGPLFGNTISEGETFSVLDGFLDSDAGWDGALIGTEKLHGYGVSGLYQPAVQLSALGPTASNDMDLNAVDWDAIY
SAPVPESSPSSPGSSLQFVSSPSAANGAAPQAPWETQPLSQPQPQPPQPAPPPEPTTTSLQPPLPPPQPSAWQDCAG
APACSPPAHWHSNTPLLCSGPPHAPPGSAAVPALTTNGVAASQMGCHTSVLPYSGGGTSSAPAFQSAFPTPGACSSA
TASVAMAATPPGTDDGVGGCYSSCAETGTIAPAAEALSMLPSRGSGAGSVVMTMAASVPTLLVVRPAAATAAYDTHP
RELVTALSQSSNCGGGSGGGGGAAADLSDGGSRVAPVVVDQSSLRRPPPPSLRGPCCDTPAAPMVPTAFAPPPSPPS
SPPPPSVHARQLARAAAAIAAAAMLGQPPPDLEAMGLLPPGYPQCRLPLLQLPAATATVSHFGSSPSHQQNPLHQI
HQCQHHHRCQHTAAALNAVDMGGGAVADCGDGGGGSGAAAATASACGDYFGCTGGCSCGGGSYAEYEGLDRSRWRAA

QRYHPYGRGACGNRTCASGGGGGSGSGGVQASATSIAGQQNFQKQQQRPCLTCHACWCCRTAWISPPPPSPAPPRC
CALMERRHSITSTIPRKCCGCGCGQDSSSPLAAAAVAMSPSGAASCGGVSCGGDNSCGGGVCGKHNNRGMRRGSGEL
HFGLRRVEKGFSCMEVDGAKQPGETGGGSCGGGGSGASIAAARASSSGGQQPCCVGLAQVAARPPVRPPERTAGRPP
PPLPPPPSAPKNEFPAWTPISQNRPLGCTCPACCRTLSFGPYSDPSQAPPASASPSVSSPPPPPPPSSHPSQPYTSA
PSPHTSLGKPYGTGAPMVPRCTLPCCNPSLTATAAPPPPPTPLSPSPPAWTSSHCSLPCCDPAAAARRGWEGPRQGG
SAPSSCTAVRECAYDPNCSTSLRSPVLGPQPGLMLPSPPQPPRGQGHQRCRQYGGGWDDAIVIVNAVVRVIWETGPG
TPDVLSGRCTMRRGVAAPVSAGALDFVRTGPPPRFRRRVQRRRHKPITASGGGPGGGAGRGLTVRHFSAAAAANVVV
CNGHLYCLPGNVAVSFPYVLAAAAGAAAAPPATLSTTTSTATACAMATALPALAPLP

>Vocar20006209m|PACid:23134721
MQRSESNLDLQFQQTAAQLTILALSQPRVAKLRVQAWLAKLREPTNNAVWKRNRNMYCALLLAQLQQGQLELPFTTM
PPDGALPNLPNHLLYRGLPPSHTAAQPRSNASSPLDSAQHSHASLAHGQLQGLVQAFHSNMQARGFDLTRSKPGCAG
PRYVFNCGAYRASSTSWHKMSTGRSGCGRPSANRAEDAVEPNLYPGDTAMMQTHRDQQRAPLNAAADWSACGHGGIE
TYADQDADAAALLAAKEAEIAARVAAKRQRQQEAARKQRQIREAEEARCRQQQQQQQRNAAAKAHLDDVISRYSDA
RRQWASPQAADAAGSPRPGAPPGAARAAWTRLYLAMIAMSVCLAHRNLLSALLFRSCEPHTRPFFMLTTLRQACIHF
ACAAASQLACRIVLRRGFSSAVCNFCGTAKSFGPHLTYMMRLSPRPLGAPHAATTARSPNTAAAITEGASQGFIAVT
ATQLQRTNSMSTRLSPASGTIGPEDGPPKPELYGPASSEKLGQDKARVTAPDRQPDGAAGSPSPAPSGQAVTWVERS
TATGWHGADAGGGSTTTDVPARLSATSHEGTPTNGAQSISCMFASPRNAVLPHYYPATSCSQHSASPRRHSRSSSPA
TSPSCRKAWPSPDPAIVYSVTRKMRTSPLALEVLVKEALAPVPNIAGAQPSGVTSCSVLVPQPLVSEGSGGVRRRAA
PAKFEFSGLITSMLPQPPASVLAATAAPVSPFQQSGGSTSPHNTKQLLPDAPLVGPLFSGISSGIAAHGGESGGEDL
IDAAPYVPDSRTHNDFESPGSSLHSGGSYIRTGPIREERPANLGTSGNASGVVMSNTVGSTAGVCGSQPALALLLAS
PPASPRRLAARAAAAIAARPSPRRSSPRTARAPPPDTGNVKLLHRQPSGDQSTERMRRGEAKTGPRSASAGGSRPAS
VPSSRRLPSTTLSRQASLATTRPSSVPPASRACTERRPVAPARSRRSSAGVLTAAPSLHGEQGPSTAAAAPMERTRS
FISRAPSMQLSSLRGFTSASTMASMPSSTQLTEMARRNLAASLPQNLSFASSYGGSRAAARQPGGNGSSVSAAVGSS
LGDGSGGAPGGSTDAGGSGAGSRLGDQHGGSGLLQTTWSRMESAARDGRTSQTSEFAGGSSSVGISALAGQYPWRGN
RYGHSRLARSHDGLIKSGSSFSNTDPNAAYASGSDGLIGHSGSFGRGLDLAAHVGSSDSFATSAQTRQPLRSSTAAA
TAGFNGSPSVETLGSDWMVNSSNEKRCDGFSNPSDDRDGWSTQAGIAGSSGGGSEPLEFGSSNGTVDLQQSLGNLAQ
RVQETPTLLRNIAASAPAARIQPDLTLTGSAAATSRRNALSAAPTMPSSEPQWHQMGRTQAHLDRDGFTTWSWTGTH
KAAGELALLDVEGVIQEFERSLQNHLELSGGGAQPGLQSVHHSGGGIGSSGGSGALFPSAASRLADRFALHSALADF
RQAFDRLRRRLALHVSNPAVADALYGTSGSGGATAASGAGAHRYSSNPSHTRGSGRSERLATSTSQHVQRRSGGVAR
AGSVDSSLRTAATRRTLGSGSNSADAGFDLGSRHLNPTERFSRVATAAVAHYRDDVALLLSRVRAVLRPSLDGSATV
GLRRRSSGAGAGADRDGGGMALGSSISRTLENLSEEEEALRELALRRRLVSEMLQTLEESAHQLGLQLVTLAAGLVD
MPPPALSRLRAALHGHLDAGLAATSSILAGNCTGDSRPTGLGESRPRNIPGTSNADLGLHGSHDSGEAGDLSPSRGG
PFARPAAQSIRDVLEAMYPNGGSSRDGRDCAGRYSAPAARHHRYDAGSSGVSGNGELAEAVEAATAAGTSSRGRLST
VGATAASGSLESEVLGMQTQLARWQREGKSRDWLGDMPTPGAGPADLSSQRGHQGAMDKSWSQLHQDLAGAHMDADF
RNVYSDGRHAGGSYLGGASTEAGATGQLPSFLSPLHNPAGGAASGVRSAVLGTGGGGGRHTAASADLFATAFASPAV
DIIPPSGSALEVLMMAGVSPHASYSTPSSRNEQHHLHVAGTQGTNQPAVSVQLPVLASPSPPPSASLPRPSGCSSTG
PTPQTGRPSANASLQGTTTAATPGSASHEPPPLSPAGRQLTLAGPPAAQGQSLELGLRQDGDAPEEPWHAGAAARQL
SREARAIWATAAARGLARANTFSLELAGMRDGAVVAVEGAATAVSGAAATVAMAAATEWAREAKESARDAIGSQRGA
KESALEALESARETKQSVHEAREMTQETKDAAQEAKESAREARELARDANQSAWEAKESAREARELAREARETAREA
SEREAATIAQRQAALAAERQSVQKAALEAALEAARDVARKTALEVATEAAREVAIAGLQEAVAVGRQEAQEEMEELR
QELAAAQENAAVSSVEIEGLHNLLEAARKDAAAKSEEVDSLRDQLAAAEAAEAASALQLQTLCRELVAVKDAAAMNS
KEVDRLRSQLEAAKEDAAIRSQEVDKLQKLLEAATMAAAASSRQVDNLQTQLESAKAEVAISSRQVEELHVQLAVAK
EEASTAAAAGSKDAEQLQQQLREAERAVTARSQEVDEARRKLAAQRGALEAALQEAEALRQQLAAAEMAAVARGRDA
DELQRRLTEVQAAAGASNAEAALLRGRLVEYQQLAEARGHDAEELRRQVVAATAALEQQVRETEAMRGEKARLQAAV
ETSDQRLSELHHKQRVMMEKQAVEAQSCDLAQMRPHLGPGQAGAVRPKEESGSIGRQGEAAAAAAAAAAAAAERQT
VQAGAFEQALSVAREAAEVAAHGAAAAAAERETATAAAVLAVEAASSLALARKALLEASQVAAPFMQPPDGPLPPPS
EYNAVGTASQHQHHARHHKYDLTAASSHTVVAPEPPRGSGAEEVAGAKIFQSSSHAPTGIAAGQAETSGTLLPPPHP
ASYHAVVWELHSAHANSIHHPVPSPGGMDNAGPPRPPQPPMLPADFHLHDHSTPHGEDPAGSPGRPRVIVEAHAPDV
LLDEASSPAVAAAVAAVAAMHREAIFTGPGPAAPPPPPPVVNAPIARSPRRPEYDEPDWSPRMARQRGNAMPPPPAS
PSPPPPRDWGTCSLAPGPPYDDPDGSRCHIRGLRVDSYPKAATDPNADRRVEAVARSVSSSSTEMTLQDFRIRREQL
ATSPPSRLNTGPGTAARRHGSMPHERSGVRLRSGAILTPDISSHDSFGSSLPQAKQLQPGLRSLSDRFTANGSSSSS
SSGFKLPQEAADALARVVRRGEEEVEEEEEAGNDRGRDLRNEEQALNPRELAARLRQLTSRTTQLQHRVAKLTRPRG
ELPQ

>Vocar20006165m|PACid:23134819

MEHQELEPLATSLLPLDVDGDDDDDEYQVDSDSETDDTESDEGYEQEALPQEEGMAANFGLLSAKSHERIRTVASSA
GQSAQEPDTLHLLDIADDLLGSLEEEVLSDSECVESDAEDEDEEAGADAAPANLQKCQQPAAEGMGEHAVDSTPKLG
RLARGAAGRRAEQTVVHDAGGARDAADDGGCSGATLLHGTAQKPRRGAQVSSTQQGLQQHVQHGVSSVGDVPAWEAL
GAAVASTPQQEDLPSHQQLGPLNGQASLEGNTHGPAGTSVHVNHHQQDHQDRMPPGPGHGQAAAQERSQPVELCPPS
GGICRRTRAHMSLRDVDLAELEQQLPQHVDLEEIELAEDDEEYGKFLQALFNDTASLPEDSSDEDPDFAADLDLDPD
GASKDEDDDRAWLKLLKSPDREDGGSAGGPGGPTTLDSPARRTRAKSRRGNAKGGEAAAATSDIGEPMSLPEMTAEQ
LSILHAQVHCHTQMLVQTYALFFGDADSEAQDLAAKAEAMLEQLMFCRDTQITTRKALGLASYTPEDLLRPPPQPPP
PDGPAPVASTGLVFSGGAFADRTTVSVTAEGGSSSGPAAAAAAAAATDSSGGGGGGRSCGQTSQVLLLQQQQKLPVG
VVPSAAAGAPVTTAAAVAATSLLGHSEISQDEVSAAAASYEWDNDCGAGSAGAGVMNAQKAAAAAKTAALSTAILTF
RPAWCPGPDGGVWSVLDVVPLRRFPQVQDILHVLPAMQYSALQQQRELQVIAKSKRKSRRRRMDDPLSVVLEWARPA
LNCLQGHLVTALMYNPSPPVPLSYWTTAEDELMMYGMTRYGTNWRLIARNLLITRSASQIGNRYKNLNARRHKPEEM
LEEGRADALQARAVGLVCAPVPAAAAAAAAAAAARKSKTTAPTAHPKVPNAPALGSDAVAAVVAGVGGGQMAGTAVP
AAPKPTLQPAAATLPATGLSGAGANTAAAAAAAVAAALGAPPITTAPPMAPELAAMVQAARIVGLPPAVAAAHIMQA
HQRLRPQNPAPARKPLPQPAPPQVPLAVALQLMSQLQKPGGVTGPAAAAARLLAAQLQQPSTAPAAIAALQLAAQQA
ASPSGLAVGAPATGVTASAAGIAAAATNGDLGGLGGLLSGQSGTVGAGGDGNGGWDTGMPSLLAMAAQLLQASRAAN
QATALGALVPQQQVPLTAPQLVPLAAASGGVTLPVPLAAQAVVAPQNLSLLLTLGQASTTQLVTAATSVLGTSTATA
NPAATGPPSVPQPVAADGRPSGMATAADGVVVVGGGSDAGMYGVRHQEATVMFAGVPPAGGSEVRPAPMNVPTCWPE
HQHDGEPDEAMNWTPPSPQSPPGPTVQGNSGDCTAALLPAGQTGMARPPPPAAAAPRLGGAADLGVRVPEGVPPGAA
MPAPWPQAVYSGLAGDPLSRYTMTVAGGAPAASVGAAGSAAADDAAHGCGPTDPRLPAVPRDLGIHPPLDFTTPQIP
PPSRHTEEHQLPNLQRNPQHPGSQQDQDQHQRSEASPRTHALNPASLHRDHHHRHHDAVPHTHQQHQHQHQQHQQHH
ELPSLSENFTIGGISELLSYGGPKSGAATPATCVSPPGGFQSACFSRGHTVSLLLGSGLGVSALLQGDAEGDSTPTR
SAVAGPQRPVTSDGGNGGNLRSHDQYRHLNNHNHDYSKHKYHSHMQQGSGGGGQVARGMPEEPRTKTTGDVDADGVV
NAGAGAGEHDVEDRGLGTPREDFLPSRCRQQDGTAGAVPPQPQPQLRLPPPPPPKQQQQQVPFSPSSPVSPFVAGGP
PVPIRVWGHGGPAAAMPPPVQRLAALRGGVDVTPSSDPKAKRRSRICAAFFAAEDAARRDAVATLDTAPSPFEGTAQ
NDHREGRGARAGAATAGYRPRPPLFADEGGGSGGDAGQRRGGEGQGLHEVSTATGEDDDEADAAAEGLARARKRLRL
DLEEGGRCGGKTPGRAQRTRRLGLEEGGVAAASGEMRGAWGSAANSVAASPVKPTSSPGTRAAAATAAAAAAALVAR
GFAAQPPDCGSRQPEQQQQQQHSIGTGSSGGSKRRSGGTATTSGASTKRVRPGQTSGCTGSSGAATDADAATLIEDT
VVEAPTTVAPVAAAAVDDVGSSRDGGQRQGQGPGLGPGPVQPASRLSGLKRSRSGAGSKPPPADKDTRDSKDRARQA
GPYITHGEPLAVVDSNANASTNTNSPIVTASEDGVLNVLSTAPWDGSCAAAGVSSGPTKPPQLQCQLSAAVPPSGPG
GHVAGKAAMAGTATEAAAAGPRSSMVPVAAAKPPKAPARRGTAGAQRLTRASAAAAAAAAAAAAAGSCPAPAPSVRKP
HSAALNGTIAHATAAATSSPPPVRALPDAHAATAAATDGAVHQLAAVAPLTSSPQPPVPMLTTPSSAHAVSEVSCGG
LSLHSLLFGGDSLDLATLLPCISSRSLAAAANVCSPTSRPTALFQPAAGAGMVSSQHPSPAAVAAAVAANALAASGS
AAVAAAPPPAGFTSPPHSQLLQLTPFAPSVCTLGSLGFLPLFSASTDPWQALQVASACGTTSRGPAMRVVAGSTAAG
DGDGRGEVPNGPLAASASALAADGTPMDPWSRFMAACSPAAANSNAATAAAPLPLPSYLSADYSLTAFHLHMFPPGS
AGLRDKDADATPAAPPPVTTAARTTAEVEPERSREPGRELTSCRGDTAAAGAADAAAEAHIRDGHDMDDGPPSVETG
AAVAMEEVHISGGGGKDSPPVQLDATGSPPPPPSSEALAAGAAAAVGSIAAKTAAVLAGTRHAEPAVLELEATEPLA
PPPPPPLPPSPPVDVGTAARPLASGRVFCPATAEVEVRQLPLDSEAAAGVLMSPPPASCGHLPQEHDQRQQQQQQDA
ACARYSTAGKHAGGEVATACGGLLPPPLPPPFPRHSNVPSPLGQSPLPDSRALPSETVRLFGHTVAKPLPIASPTMP
AAPKLLRSAALATAAAAPPGIAAAIAALQPLQLPAYPDRKARPPPVPHGDDVTAATAAVATAAAYTVDDSSNSRSQ
SQPSEIAAVQQLAAVPPLPLALQTPAHGQARAGGSLFRNVLSPADTPRCATPGTGFDMPRLADATAAAAATPMPTPM
QPSPGADVAAAAAAAAQYTPAIVAPAGAFAIPDMHTAVAVAAPLSLTPLPPALVATAAKTVGRGDMWQARRHVVGAP
TGTLGPSISHWASNDHDPGHPDFSAPGVEVDRQGSSCLRGGAAVPYWRGVQTAGGAAAGGGGSGAAADTPGHVTCFS
KVARRVPVAAVDPLMLPSPSVLQPTPQGRLRPVDDGEEVVPLRAAEAEQSPFREDEDDDGDDERNRREGDGDGRGND
GGRKYGSAAAEDDDHEVDRAGDAEEADDEGASEGEDCGLPESPSPRPESPGGHVSVIAAVKPSGAAATAAAAAAVAT
GGVVKNGEGKSAAQPKEQVQARQPPAPPKPAAAAAPAAAGPSWTKEQDQLVMREIFRLGEGASTWRCISALLGGPPW
TMEAVEARGKVLMERMMQMM

>Vocar20006210m|PACid:23134845
MLWRRIRSRFVLLSTQCNSRPAALLRSNTVYSCVLYFVNEAIIGDYSVRVHKDYLYLSSNISAPSESAWKLLAEGES
LSEYSVLTGDVVRVNYKNSSNNSSASGSRRFLLNPSPEIESIDVVTEAQGHEIYTGTQIRVKSMIYIISTCGWAPSA
TVEQLKGTLFNNPLNISGYYNTCSYGKVVFDPSENVVFGPVEIPCKGDVVNGIPKYDASVACGDAEQFAWRQYSEAA
ARAAGYGSFLDTTAHRRIVHILPKEVLCPWSGLASVGCSGLACTVFIKGGYAQDLTVNMHELGHTQGLSHAGRGLNK
VGEQYGDYSDVMGTAPSEGYLCMNVGNQYRIGWNKPLATLLPDNTTNTPGNTGVAGRWVLPATAATDTNALYLRYKS
NATAFPNIFVSFRSRTSRYDNALPSSMNNKVFVHNFNGTATVRDYNRTLLVAVMAAGDVYTSPFLDQIGFDNSGLGV
KICVLNIIAGVSATVEVCRLTEKVETSCFDGDDNDCNGLTDELDPACSRVPPPPAPPELPMTPPPTPPPPSPPPPSP
PPPSPPPPAPPPPSPPPPSPPPPSPPPPAPSLPVSPRPRKFFPPPPSPRPPRPPPPPRPPPKPPSPPRSPPSPRVPP
PS

>Vocar20006220m|PACid:23134986
MTFLPLMESPCRPDVVKLPLPPGVKAGAVVGRGGHNVRWLHFHSGGAHITVNDTAITIRAKEDHVRERAVLLVQLQL
GAARKLLGATHDDDLPPLPRPGFILLEAAFGTDPRASFSQRQDEVAAALGLRERLFELVPVTIPCSRDQDGQRDQDK
ASIGRDGNNAAGVHAAATAAAAAAVARALAGLGPKRRGNPPLLAAVGLALAAAAHKAACLEPAFDVLKLRFKLGKNL
FRNLGPLADKTELSASDLAASSGRGAYQSVFSNYVPASTVPRVTSWLTDVLGFSLTDTKTTATLHVINQELNVHYSI
ALTMEGGDDSTASLRKLKVGGSKNPSVTLMSGPQQLDLRLKLITQFREHPGLMYDVDAHSTARRVAAAVRTEGFDGF
IRSKRGLPRNRHLEYGRRKTKFVYEGFMPTSEGEGGCGQRLRVSVTQVQDDKGTHYEISGCLPDVDAKLRRLLAQHR
SPPPSPPPPAASVDDSGCFVPEWRRRGGSSGGSSVGPGSSLGLAGDRVLYKRFRSSGGGPVGPSAGPCWARSISGGG
GGVLHDSASSSLTASFSTSNPKPTCDAEGHVTAEWAEAALVACMVQELQDGLQRGVWR

>Vocar20005932m|PACid:23135000
MQPYPLPPPLQMQPYQTPPPQQNMQPYQTPYAGSLPHPPPIPPHPPMQSPQLQQAQQQQQQQFIPHPSQLVPRLPY
QPPLHQPQHHQHPAQYQYHRQLMQHHNHQLQQNPQDLASNLATGRDGTPLALAALPPRPPPPPPPPGLLAAGPGAAA
AAAAAVAGPGGSSYPLAPSCCPLSPPRNPLSPPVALVEMEDKDGIRLLESGNGASLPSPPPPTGHWDLAANGGGHDG
GGEGSRTQLPPSPSPMRLRSPLRVAYEELCVADPFLRARLLGDLAVRWLEKNAWRSPLEALLGRGVRQRSMDRIGPF
ECLDEQQQGLAAHLLGQPPPPPLPAPNQSIQALTTPSLQPPPAPPPALAQQHQELPPPPPPLPPPSPRPSSLPAPPS
PPPMPLTASPTPSLAASPLPPVAVRPSPGAGPAADPLAPRGAEAVLAAANCADGEGGHGSGAVGASPSGRLALRVPT
PGDPFVLEPLKPPPPQPQPLLLQPSAHSSAQSQVPCSSFINLTAKRANRLAVAVDWVPSRAGLAQVPAGRTAAAGAT
NGDGNQLNVDMPGATAGHAAGCQARSPPAAAANINAADGPAAVPLKRPPPPSYDGDVIRQDHDVPYAGELQPRRVRQ
RRQEEERPSQDQHSEDRAQLASSVDAAMTGMTYQADVATAAAAAAAVPQGPIPTPQPSPPPPPPPLPLAGSAAPPPP
SHPTPDVNTLRIQET

>Vocar20005422m|PACid:23135017
DKHPCCGCKHGHGAGPSLLDATLQPDGTVRIIELDDRALKRRRRLVTRTWCKAARHAAGASPLSGDHYLCDEHLNLL
FVQSQYLRLPLKVRDVRVGAAAAAAGTSAGSGTRPGWLGLLPRLTDTQVEELRTSLAPMMEMLLYPPGLTEQELADV
RFGNEVPVMRAGEEEEEGEEEEEEEEGDGEDEDMDGRGHGGKATEIEEQAGRDWSRGGNGGSCSSSSGRSQRKLVCL
PLRGRNWCFECGCNPAVYIFKFRNDLASDRVLCPTCWVALRDSRARTAAQQQHHHRQHAGVDPASLALFCCLREEML
LRLQVMFPRCSATAAAAAAAAAAAAASEQQAVDTGVGDAGERKHIIADSQPASQSTTPEQQQLRQEQQQQQCSPPPLQ
HRRPEEPHQRQQRQRLVDLPAPAAPAQLPVPMSAQQPQCQLLQLVPPGLEIPSPELQQQQQQHELGPPRHAQLHLSP
PPPPPPSLQQPEQRLQQQPPALQQQQRPVVRPELGATVRLGGAASDQETAQMRFPMSPQGYQPLQQQQQQQQPPWVQ
VLASHQPPQAPLSAPRGPSQLRRPEQPQPDPLPGGRRDLGSISAAAVELYQRPRGVFQMAQHLATHASPKQQQQQPL
QLQQLRPQQPPSSSLLSMPPRPPPQQQQQPLQHQVAHQHQQQHPSQSQPQPQPWQQHVPSHQQHQQQPPPQEQVSQH
QHRPWQQQLSSYQLQHQHHQRQQQQQQSSVPRTLIQAGDSPPSSFGWFGDLSRSFDPTERSDGRGAAATSLTWSAAS
FQRRSDSRNMDCTNGGGGGGSNGLDGGWSADGTVQDVRMYGRPSAISQCGDEAVLSVPEPTELPTGVRTYGIRGTRS
WSAADLPSRPMATAMGTASELQRSAPWPARDDGRHTNVREENWDLCTAGAAGLRPQVDGVQRICGGLVCTELHRVTG
GSGGGGGGTAAAPRQMLAPLAAVGRSAAAAVGPAANGSTEVARAGGCEVGVHGSDGGGDGGGGGGHAQRHSSVTAVP
GPVSATKTVAAATATATATTAVGTAPVTGASPAPVASVPAPPPTPPRNELFPSCSRPELSTSRYNTHIHLPGQHMAA
KLGLPPPPRTPDSAGRREWYDASAVAQDVPYFGGALARCAPHLGPVALRAGGGGAATSAAAAAGRGGAGAATRIREE
DCFYLIRKEAMLRLPEQKRRQLLELARDHNARGGPVLFCGPLGLVHDPTDMLSDILEATQECERAMRDAKPRRGGSG
GDGGDGDGDAHLEGPFPWSAGGYEWKRLSPTGRGMDDASAADAAAAPPAAPPTGTASCSAAGAAAVAGVSSRGSGAQ
GHSGGVGGGVPPPPPPPLRDHFYGPVHLCAMDMAHRYEHKLGSTPVSAAFRPFLGKHSPEEEVDHAHPVNPAKLLSY
MRHMLDAYGVLSDPRVIQVREQCVAMQSWAAMRGQDAAMAASASASDDDDASAAATTDPAERLAKLRLRNRDPHEAC
IAALKAAVAAAPAKRSAAAAELPLPPRGSRAAAKRAAPCPQASRRTLSRAGRGGGGNAANSSDYDLEDDVMDGAAAA
AAAAAAAAAAAAAASAEDGGVLVVGGAQVPVGLKVPVYPLTADDTLFMTSGDLMRLAHRTLVLHAPLWLRDILHERH
NDALDFGTCRPTDGGLGVFAHFANMLRRPLLALELATFAHVDRSQPATLMSFATPFAVPADLDAALSRLQKGLGERK
HALWMQVRNLTDQWRWQHPAAAAKRPPVAAAAAPSGGDTQPLLNASPDRTPRRGSVWVVVPGCRLHQLAQALNKSAK
RGWPRNHSGFFSVKYGRDLDALVTMCWRYGVPFHATYHCEGDLLLPGMGAAHAVMPLHCASMKISKDFLTPSGPALR
LLWELDALRQEHHLMWAARRAKEQKIDPPPRSDSAVATAAAAAAVVPPPPPRAKERGRVRLREEPQVDVIEGIAEEG
PAASLLPPSPPLPPPQQQEHPEDEEEVKVEEQGAAPQLPGQAEGQAAAANAIGFFCALGIRDAAAAAVTDVVVTEAT
GAADVAVASPVAAMVDVTEEAVVMGALHAPTAPCAPDGLAGGCPVEVAVRGEGGMSGLWPGDVACGFLSPPTPATAT
VVLAQEETKKQALLRAAARSHARAFTAVAAADAGTTAVAAQAPRPPPSAAAIDHGDVRQQQQQRTLQAIPEALLKEV
QQQQQQQQHCARPTSSTFKPILAELGLITYMAEDEENQDEKGVKEGVESDGKGIRGRELGREGVREDSRGASGQCCE
GRRQLQRAHCQAAVGATVYTSRQADCSSVEPGTGSDAQLPPLPLRSLPPAEVPDSGVVEGDGAATTLYGNGSCGGGG
GTTTLGVATTEPAVCGKASAAAAATACAGVARDDGAGAGTVAAPTVAIAAAAAGATKTCRCAASMAGLSAAEGVHGT
VQCTSCREEDNGGNVRPSSSKPTEASGPWLSDGTANIVTSAFAVATLLTVATARMAPGLSSSPEPGGTMSAASDGYG
DGGGGGGGGGGGNGAVAAAAAADGGELSGVCSADGGRGGCGEAAGGKAGCADGFAAAAAAVGVEGSVAAADVDADVA

GEVAPAVVVPLASPATHITVSGTGPGDMTEYGSEEGLRNQGQQQQRQQHQRALGVDSEIGTAVVADGEAPRVQPCT
AAEVPPPHLKQLLIKETVDAIAALLPPQPCIRVQAAALLSNLALVHRARAAAKEALARAMVNRHQHMMYPHLEERGT
MSRLALLLQLTPPLPPLAAAVNRTPATRSLSDALSPQMPAAEVLQPDSSVCACPVREEPEHAAQLMISLPNQPQPPP
PPPQQQQQQRQPAVLRQQPKLPTVEDQAARTARRNAPAAGGAFTAGLPGPHRDADADLGLGLGVDQAPPRRTTRGSK
QRLVKEQHAAAAQERQATGQDQPRKQQQQQQPFGGTVARAASAAAAQAGGGGGGVVATTDHGGGDVLEDLPLTMPF
ALGSGGAGRRAKGASAAAVKRGKKGPGSAAGGAAAGFRMQGAQRGAALGPVLPPEAEQRKRGPLEPAGGAAKKACGG
GGGGCGTAVGPLKVAAAAAAKPRAEKADRGPHVGAAAAATMGMGALGVPTSENGVALGNARDAFLTQSILQPVFHAG
PRPNFIPQAGIAHLAPPILVPPAWHSSSPSPGAQA

>Vocar20005484m|PACid:23135163
MAATWLSKTLVPSGVVEHVVCGNFVATCQPGHAQVVLLHAGSNLELLAVGASGAAASVCSQPLQAGDAVVDMRVLPR
AARLDTTAVAVRPCPDPDSLLLLTGSGRVTVLRFDEQCNRFVLVQEVLLTGPGTQPRFQPRLMATSPSGRHGVAIAA
FQDRIFYLPPASLVASTPPSACAHTHPSASTATAAAGREAGGSPRTTPAAAAGAEAAGGGTTTTTTAGVPAASGGG
AAVAGRYPAVLSNTPVPYIHRRPRGAAAPPPVLTAGPVAGTAPRDLRSATVDALAASDSRFIAGSSFLPLLPEAGFG
AIWDLAILEGPGLITAGRTGAGASGGGGNGGGGGNLVELRRRQRREPGDEGAAVVDGGSSGARGVDYFRVAALVHRS
STDSGEVLLLRWTRGSPDLLLCDLVVCLGGCSPPAALNAPPDDAGTRRASPRPAATSAPAGTTHGSLAPAPASATRP
RPDSVIGTRFGPASGAGAPSSSSLSPPPPPPPPPGAAPTAPPPPADLLGVGHLLRAAPPGPAAAGGQAEDAGAAALV
RSNARSVARLNERGRGLLVVGTHGTVFLMAALLARHALPPAGSGGGGGTVSAAAATPATAGCSAAYSSAAAAAAAAA
ALPPPPVLYLEDLMATAATVTLAALGTAYPAAVAARELPRCGGDSSSSSGGDGGGGGIGGSTDDGDDDVFDFCFPDL
HFRRAVLSPLGQQPVRGQGAPERAPAAASGHGWAYLRFAETYFGPAAAAPAATAPPGAAAAAAVGAGGVHAADVGMV
RVSAGDGDGGGGGTAAAMDLDDDGENYARRGGTSSAAGFGGAYGGGGGGRVDRTAAGEEEDDEGTTDSRAASTRILP
RRRPRRLGALHDAFLRYRATESQGAAAATTAAAAGGEGSRHGDSLRDRLAPYSRGGRGGEEAAQEEEAPPPPPPPRF
PPHAAPLLQLLNRDRGTRPLNARSLRTMMDPEHLRLVRKRKEGADTVSARAAVDAATAAMAAIAAAALDEQDIHGAR
PLPPLKQAPPVLGVEQLPFAEPQCKGHICTVAEWLSAPIPPPTLPPPPPPAPPHFSGIARAASPAAAVAAGHAPPPP
PPSLPPPASAFTSRVNGSGPDIATAATSRAAAAACAPLPPPQRQLTHGSVAQMARRLLLCSAAGPALCLTVTFPTFS
TPPPVPPPVPPASPPPEKHRHGSPVAAAVAAEYGAADDVELLDAAAMGMQPNISVDARDLEHEEEGGDPGGAAEGDV
GLPQLPEMRSPPLAQSRWLSSGFTTDTAAAAAAAGTCAAPTAAARATATLIGFARSSSSSLPTSGGAVGAPASSTTI
GSSPPEVAKGRRHVAPPPGVSGRAAGISGGRQAAAASWATRGSGGTGNVPGGRRTKTSALPGRGGGLGRWSCGEGGG
GGDGPGPRRPGGVKEAAAAAAGVLALRLLGRVPQPRPMVDFVVTEAAGVSGRGRMLQVTGAVLGLEAPGSAHSPPHP
AGGTAAPEHKLQVLRGCLAAPVVASVPNQGAIPNGLWSIPLRTAVALCTSGGALGGGSGAPQPPRAAAAAAAAAANS
LPAGGSLVVLSYVGGSRALAPVLDTDFAAESNPGGSGASLPYGDVGPADVAPSMCDVTEALQLRHWEPTLAAGLVAE
SVLVQVGWYDRTELTENCLGYVLCFSLQITPSGVLLVSLDTLANPALAAAAAGAAAASAAAAAATAPSGSSVLPDDV
AATGGGGGGGGSSWDAPLARSTTDGGALTERPFQVGFFGQAAEVAEVAAAATAGGGGCSESRCPSISGYSSVAEGMG
AMVLASDMLLDDEVQEGVRSTGDNLALYSPDILPPPPPLLPSSAPLPGFGNAAGDTAAVEGLAGLRRMLSMPPPPPP
QLLTTAAGSQMSGGPDTGGGGGASAPAEAEVSPGGVGWWSAAAPDITLGSVAPGCVALVRKLDRRITVLGVVQRGSL
GGGGGGGDGSAPSRLSRKRPRSTVTVRELGCSPPLDCEPSCCALRALPPPPAQPPPPRAHALHTFRRTPSASSSAFV
AAAATAAMTAAAAAAADAVDPGGAAGAAAPAVHWGVGLPPLPPRTGPISSIQLLLLTHGSSGLRCSLLARISTSVPP
GLGPSAGPAGPLTASVPESVLFIAPAPIPMAAAAVAAGRTAGGGGGGRLSIANRWGSPGTSEAVFCLVGLRSGGVAA
FSYGAPAATSEAALAAAPSHYRASSDGAEDAAACNAGDGSDFDGVEAMENDGPEAAKAQVVHSVAPTAAAEVLRLVW
HSPLEQVPLRLTSLAPFRPWQALAVGTAVHLLELTSHSGRLLCSTVLPASGKWAPDCGTSKAANTAPPHPLEAILAV
PMHLPAASAATAVATTAAAAVGGEAAGAGFDAFLQRPVRLPPPPPPPGHPGPQELALVVLNDGSWRVLSLQRLSRLG
RQPPSVMPLGPPDGVGDGATRGSVGNGVSERRPASLPVPGTAPLGRPRQLLFVPQLLPALSSSSLSPRAVVHDDDRL
PAGVSAAPGGGAGRACDGYLILLADDPQGSQLQPHGQSAGSRFIHVLEAASGRLVTSFDVDAAASGFLATCASVWQV
RPEAGVSIDVDGGGPGGGVGPPPWERLMGGGGGGSGLLNDDDGGEVDLGEEGVHGGDGGGGGGGEVLMGDAREALPP
GHLWQRRLWDQGGGWEAAEHEALQVAELAAAAAAPPAAGGAEEHREAARGAAAADAAAADPFMVEDWELEPLMAGGE
AAAAAAALAAAAADANQPQEPPPEQPHMHRWRWQGERAGAPAVAAAFDVEEFLRELDEQRGAAAAAAPAAAGIGADG
EGRRGMDMEEPWRRMRHFVGFLGQVPAAARPGGGGSSAATAAAGDNTRRARPAGTPAVLLAVGCSGQGGGEVMVLQM
VPALLQLLPRLRGGDGASSAATAATAAAAAAADSPNGSPSLDKLGDDWFTWPVARRRSPLQHWLGRMAGNAAAAAAA
TAVPPPPPPPLLPAREAVAVANASDGAEEQAPPPPSSAAAAAVRTAAAADDSGGLPTTRVVHLQVVHRLLLARPVTA
VCPYDAETLVVAAGRRLLIYALRCGRLHRMGWTPTRNSVTAMSACPARSLLACTDGTTGVMLYTAVHAGYGTGASAM
AVGPPIALRLIAADAVHRPVTSLLLLPPPPPQLQPSRSASDASVSNAGGGGVAAAASPSGEPMDTAPGAADGQPDAT
TATATDAAAAAAAEMDCVVIAATDSSGQLLMLAPGPPSSGLPCRTLVVVAAAQCPDTGLRLRAHLPRPVPRYNQARP
FQPGAPALLLAGLSGAVHVVRPCPEAAVPLLVLLERALAGYWAVRHVTGGRHRCIGEAAVEDVDDVAGRWWWWCSHQ
HHPGVLDGDLLEQLLDLPRPMGLQVLKGVPPGALTAALAAARTGLDALYHRAVAEADSRLQAMTLREDREGGPQQPQ
QQPQQQVPAPNGLLLAGRDSELGFGASPAGGEEESLDLVLQVLQEVLLLPP

>Vocar20005388m|PACid:23135190

MATCTSVLCPSFLWHPQARSSSPGAGADLHGGHNETSALTYPFHPIYPLPRVQTIERGDVTAVLLDETEALQFAGQL
VRLSVEAGGEGDGATTDQDSAATTRSSTTVGDEQSSKAHHRRRRRLQTSPVRAKIIESFGFASKDAPKDAPSGARNI
TSVVFLLSFCGWNNLFTAASFRDFWLNGPDTPPTARTVQNYWSFCSQGATRMTNATQLIFEVSLPCEGTYGGRSYDF
AKKCDSSLELYAWMDLADDYVQNTLGQNISSVKQRIAVLPYEVNQNCFWAGMGSVGCSGSRCYTWINGRYSQDPSTY
VHELGHNLGLQHSGRVGVAYEYADSSCTMGQGRTCYNTPNMWRLGWLSPVPGGDLNGTTFEVGRPRRFILPGQNKSP
QSFIRIDPTWVLKGTESKSLTSTPAPVFYVSLRTQEPPFENLSPATNRATNSYMRSVQQAMLNTDNEFRTSQTYGLL
IQVNSLSASNATVTVCRYSGESEYQASTNSCSDGRDNDCDGLMDDADPDCSGVPASLSAPTPLTRRVPPSPSPPPPP
RPPPPSPPPSPPPPPPPPSPPPPRITPLKSPPRLPKRASPPPPPPPQGVPPPPQDRAPAAKRPKKRPPRAPKTTKGG
GT

>Vocar20005537m|PACid:23135245
MWFPSRILAYVHTNHLCLSRHTPGNWWDKSVGPRDVMYRYDPAVWPHAYCKHTLDHGQHQQQMRGLVFLHFCWRFC
SQVEQLVRKVLRNRKQVYTADIAISTSDLPTNGCAPYAGLSCSWQYASQTGDSSTIMGSGIACPNAPELAYMGWATP
AAGGDRIDSEALPVGTALSFELPATYLSPDGNYLRVVPDWLPSYSNKSAAKNLYIAVRVNKGGDSGMTPLNANKVHI
HELNATMDNDPAAFIYGDRMISMLATTPPLTQTNLTAYRLVVYGGLWVDSDILRVYLCRYEASPKECPSLASLFVQR
PPPPSPPPPAKRRNPVTAGPAPASTVNNVAASGPVTVSGELVFVATHSEQTAQWAIRQDDGSLIPIELGYGAPKTDD
VGQPISPGALVTISCNIDALGNCKPVPSSKTVVLSAQPAFLPDNQVIYQRMLVIVLDYSTCGVSAAINGTTLRSIFL
GPNGDGSGGIAEKYRQCSYGKLKLNTTAFKVINVKADCTDGVVQSCNWLNMGRTGDTGAKALLGSTAFAEFTHFAYI
PPPQVPCGWVDFGYAVLPGNRIWLSSKKDGVYNWATVMEKSLHNYGLWHSWKDGIEYNDETTVMGRGLTCPNAAELA
YLGWATPAPGGDRIDSTRLRVGATLTFSLPATYLSPDGNYLRVVPDWLPSYSNKTLAKNLYIAVRVNKGGDALLDKT
LYANRVHIHELNAAKDNAFPELDLYLDRKISYLTAITPLSQVTLTTYKLVVYGGSWVGTDVLRVHLCHYKSSPQDCP
SVQFLELSPPPPSPQPSPPPPKPSSKQVGPVKPRKRPPRRDTATGASAATIVDVSGELVLLNSHLAPQWTIRKSDGS
LVPIALGYEPPTMDNLGQAISPGALVTIVSCVVGAYLVIGLYNLLWSVYTMRHHIDKVVSDGLLTLRSDQLPLASPA
GTPCLVLHSMPVFEMPVSVIQVRGYLGMIRAIGLYD

>Vocar20005504m|PACid:23135252
MAHTAQYHTYIRCEGNTLLRKLPEDARLSLRYYETVGSLRPFHPLPPLASSRKVIPGKQYYYVAVTDWSLYLLTKDN
KVEGSVLLEVPWLGIRELEKTEADSDEFLNHDPRKPGSVTRSMAISIYPRASLTEITPAELRKAGLDTGPTRGSSSR
RSNGQKPPTARHTTSTDSAGKGDGVAGAGMQLQPSPELAFVVRQQQQLYDNATQVLQQQQQQHHHQQQQQLTPQGK
QAPDQASMRPLHVDVMAQRIPPGYYYPEYFSPNSYTVPYSGHYPDLRHVSHTPIPAAMGLQPPVFCPKLREMWGSWH
RQQQQREARPSVQSYVAAPRLPTCSDPRTGELYDEGYWWAGPRAGGTADAAAAVVQPAALHTFGQPYMTYSLYRMTP
GPLGDAIERPGFDPAALHLSSSPAGTTVQGPAAAAPAPPPPPQLPLPSGRRENLFSTLRRRGQDGSTTQASSAIAFT
GTLPSARPSDTAGGGAASAASAAPPSPSLVSRKDDELHRAVEAGMRQLENSLRQQPRPQLVVQSAAPSGHASASRAG
RSATADGAVYGGAPVAAATPATPPGAAGINRFSISLRPYLAAAKAAAPLATAAAATPQDSRLNSTYPRLGSRSPEEA
PGGLSNTAALHPQLQPQPAQVLLHPTAVAGPAAAVRQGPAPSSPRRPAPASRAAVAVAAAPPPPAPGIATAVASEDD
GDAAGFPHLTRRGRRSRNLPPEPGSFQLPFYPSCQPVMFSGPSYGGTASGGGGGSVSLPSAVTEPLIRLGEQVLRRQ
LRTAPRTLWEEYERQEAARLAEGQQGSGGGGSTKEYDTVEEEYEDFWISDPDDDDFSAEDDILSQYRLILVTIERKS
NLFFHIQRAWLGAHMRLALTEAAVRGMPLWYAPPFVPRRIVVDLPANGAYDDAVRQISPAELPEVRAAPDATQQALQ
YFSDPALLAEGLRSAARAVGTTEALGLLPGDSPVLYGHPPSGEGSATPALPTTPAAPKSYHPWPNQAQRNQQQQMAR
VITSQQLSLSPSTSRRSLVRLGSLRGTLGGAGSGGGGGGSGGGGLVAAAAGAAAAAEQPGRLGRKMSDGDASGGGGG
ELDLSVSVSSALLSLYDAQRSRRRSRQINALGTPSGSAPAPLQQSYDTAAASHRAMAERSDGGDAFNAAFRKNLSAF
NNSWQSLQLQHQDSYGGDGGGGDGRRTSAPTVGANISRTITWHPDVAGDGADERSSYATRAPTAAATTGWQRPTPTP
TSNHLRAAASIAAAANLAAEWYGADVDAREARAFYAEIEVAMGYGCSLFDDGDANLIQEIILERSFSQAPGVRQLSK
CFFTSRRVFSFILSRLYAWGAVCAMFGSSASRKTTSPTRTRAAATTTTTTTTAATDHGTDAQSVHNDNLTSRVSQRR
SVRRSMTALKRDGRGGGGGATAAAATAVGSSYISPPSSPKPSSSSSAFQQQRRWLQQQHLAGAEQPLSPPPPSPPA
ASSGGVTGGWREGGAQVSGQRGGDSYGKPFSGPVAESLGAEAATSGNRARLRASNDPRVIDLDVPGPQGQSRSQSPQ
FSLPTALRGGGVGSGDGSRSPPRHSSFAFPSLPATAPSPPSPPPYTYSDDLVPPLAPPRKPRLANGGGAAAAAATPK
SPLPPSTSSSSPLSAEPPFTLRPKKSVLDQVADLDEELSFFQRLHNSRTWSMFNRGARRQKHLLRRLGLSASEPTSI
RATVQALAEILELQQQWAAKGPHQQLYGNGDGSGGDGGSSGISSLMDMLTGRTARSEEMVRSGMWLYGDGDGGGDGG
GGSGRRRSSSAAAGVRSAYLRAILQSRTSPTEEELLGGPAVELWERLAGRLAARLAYKRLRRMLYWVHQELAFSEGC
ENRIHWLSEALVETEPGAPPELDWSFSRCSRTLVALVTQMADPNPPIREGGRYPPEVEPAKPLSGSDMMYWIRVGGR
SHFRIPMLRCTPRGTVLLRTDEQYEIKSYTLDWSVGILYEMTGLSEEAQALGQYENEDIVLQVLRQLPPELLLQFVR
RMFLRLACFVMGYAELYGIARQPSLAALVTAFRYGSVLLALVRSIKSCAEMLADEFFLQLYYLFRTQQVQDRLRVVR
GGKDAVLAESTQHLFEAVLQIIGEVATRQGQAAGGVGSSGTAGALSVGQITSRAAFSKMPGRLT

>Vocar20014893m|PACid:23135449

MGIPVTVAYDMIVNGVMHSCSSPIFLSETPEQHEEEEDEGREEEGAEGGVGKQPGWRRAAQRRLQSVGLGDAITTPT
APRILVYLPTFCGFAGPAATTPEAVTDLLVFGKNMWSNSSLAGYLSTCSYGQVKLLPENVKVLDGVQVPCSGNLQRG
HPFSTGSSFTTRTCNEADNMAKWHYWLDEWAAATHGVRAADYHHRVMVLPKSFVDLVTGCGGFSGAATPGRWSFERT
TVNDWGTSLVWWGGDSYGDLEMLLHEIGHTYGMGHATIPNGCDLSDQCDNTCTMGAVGGQGIRCLSAPHNWQIGWGG
PFLQLNDASLPYGKTTAVRIPQQLTATNSSVMITGAGLSPTQRLFIATRINTYPYDLPWSYKRNGMPYIVLHKYNGT
IWHPGIQTVLVGQVEVSGIWRDDVSGLAVRFDSWNSTTGAAVRICRRRYATELNCLDGIDDDCDFLTDFDDPDCFAS
IYKPRPPLPPPAPPPLPPLRPMPPPLPSPPPRPPPKPPGPPKPPAKPPVPRPSPPPPRQPPSAPPPTLPLLPPPSPR
PPRPPPSPSPSPPVPPLPPPSPSPPRPSSPNPRPPLKPPGLPRSPPVKPPIQRTPPTLSPK

>Vocar20014826m|PACid:23135506
MSVIEAKLIDVKTKLESDKVSIRKDGVKLLFTIMDSQQALQMLDSETARMWPDEQCKKITTWPRLLRVFIDFVEREL
LMGGGPGGGGGGAGSGSRTSRSKGTSAAGSRGVRSSSSSSLCAATKVSEAGHQLRRLVALADDVQRSGRRGNLQLWV
WRLWHHVAKMIEELGLLQSRLAPDYISILTRHLLPNTEYTRAPLEPFRAHSLLSCFCTALEGLVTSQGQSESASAAV
TGQAQAALEKSARWLAKIRDTGRLALSWFNCAVQLLLAGGLDGLATIRVCWGVWRKPVLEEIMTRYGLLQLQLGGLV
SESEGASNGGDVEALVELYDMTLYDVSYEVAKWDLNPCDGDIGPLALLQLQLLAGLFFAMQQVYACLRVLLPFAAGG
HGTDGGGGAAAAAAAAASLLRPHDLAVKQLGAVWCLRCLEVLAQQNVYATSMVTHGSRFFGCGFTVPAAPAAGGSSG
GWSASASSSAASPSASPPPGTAEPTAAKSRDAVAAAAAAAAAWQQVVGFLTARVAGRNLLVPQTPGLPNALQAEALW
ALAAALQLQKTSAVSYVVQAPPGLLRTVVDVMQGSLQADDAAVDATTASDGGCTGTVAAAAADADSPAARSYIWQAA
AATIVAALNAAPAADAAAAAACQAAAASCMTAVLRLAITPPNELLHALLAATSGASYMAAPQKRSSGAPPPPPLPPL
PLDLAPDQYTHPYTRLPRWCPTDSETIAFCCNVLAVQRRATEVMAWQEARERAFRHYQTHGSPYGSTASAPTKTTAA
VSTEAVAAGRRLLSRIAAAALSRTRNVVLDMTESGGGEEVEVARLLGCAQLLLSAIMHGLALQPCDEGGSSAAAAPG
APQPPPSPPPPSSIPPSTQQQQHQQLMAAQTVAPAQAWDVLCKAVAAAVRPVLGESSGGGSSSGAGFGGAAAVAAT
QRTAGNGGGASRAGRGGAGRGGIAYCASSRSPVFRLGNGNFPSHLRFIPYPSPVVGIVWRQALYDVLVRHASPGALT
AAAVPQPAHPIDSQRASQQTQPSTAMLAAAVAAVDAVLQRQVGVLRRVADPAATSAAVAIGGGGGSGGGYGWQTGGG
HGAEDDDDDDFGGVTRRPVLRSRLGGGAVAAAGTAGTAGGGGGGAGAGGGAAQVVGVAAATQMLTGPGTAAAVMAAS
GTTATPYEQALMAALRAVELLEAFAPLYGSGVSQVVARTVGVLMERRQQPPPPPQQQQYQTPQGRRQQRIGRRTGGT
DDDDGDDEYMEVDDDEIEEPDDVSDGGDDAVASGGSAYGAPSTRRAVATQRRPPPPPPPPPEELMAALARVAVSAAIA
LATDAAAAAAVAASDAGDGDSGGRTGIASSDSAIRSAANSTAGGGDGGSAAATAAARTALAVALTALGPEQGLLSLR
FIKYDEPRATAAGTLSYKVIGKQVTEYQMVVAAVELRRLARCLGTTAAALMAGKSGNARSADGAANAAGLDELLAVG
GVQVLRDAVQAMDEAFNTNDFVPYSWQLRAAVAALTVELLPLNTPRFSPGMAQYDMPDLVGSLRLLPSWQEPSYRAR
RAGAVLTRILFRRFTAHDRLLEELDLPPLAGDAAERTAAAATAAAAAVPPPSSRSLTETAMLAYGEIIQLSSKLEVV
VVRRLLAHGSLCRRDQALVAGCLRLAAARLGYGTAADYLATIMRGSVLLPLVIGDLQQPAQLVASLLGVKLPALLSE
HVPYIFGTLLLLQAAGQGRWLDEVVRNSLVSGNLDVEADVDRQLSNRTYLFLANMLLDVVPDEEVAAAAAAAGHEGE
LGGGGVPGLPVLPTFSAETCCKAVLDIPGLPTLESNEEFLWTSLLPPYSVAWAAAALHESMSGGGSHGGSGGGECRT
TSTAAAPRHRAWRIAGLGALLRLLGPRAAEPATGRYLAHILMQQLSEPTLQLHSCDLLHRLLGTMGLRELTAPTSPA
THGSLAPAPATATTVNEADRQAVLSELLPPLVSALAEALEAAEAFADATEYSPMTGLDERTAVVRAGRPALVGLLLR
VTLGLPPEVAAGLALCRLLPELPELRDAVELQQRLRARVALPEQLAHLASVLRRGGRGSFGGGGGGGGLGASARARL
VTAVRKDVEARWSELLPVSDAAGPTTGSGDGDLTAAAAASAWELAAAAAELDDTALSELAGRLLAAAGLRDPRAPPP
PRGLTAEGSEIRSGGGNSRGHFASSGPPGTGPMSPAAGTAAVTGGTAAGRASRRTAGAAAAPASAATAATAGANGGG
RRALRRSSAAAAGDAGGAATATATSSTEPGGGGGDGGGDGGNGDSWLVSVLRLMAERYLVSDDPMLIVECQSTALQL
LALPAGRDALNGMGTDSHTHRHLRIYLSSSSSSASALQRQQQQQEKGVGTAAGAGGNADDKSGAAGGGGGGGGSGGN
NGGDSVNLDELLSFKSGVVSERLWRVPAAAAGGADAWLRRLTPSLLELVTDPALKLMRGLARLQSEIVLRSLEHLRR
LHRRVVSERKPPGQVHSTRGGSAAAVGGGGGRGELDSAVDGWEFVYGLEIDYLVVARPATALLYVTHWSEKAAAAVR
LAHRVHADREQAEERAAAGQGKGGGGGRQGGSKHRGGGGGGDALSVVLDAYGAIDDPDALYALPSTYMLIGNGGGGG
DVGGGGGGGGGGGGNGGGHPQGHLLSMHLFELEGMWPQALAGRDLQLRPSTMAVAGPLYGTAGSGCGAASSVTAAGA
AAGIVSALRNLGGDVTAQYFLAGLAATGVGGGGLPDGSSAGGGGCAAAAAATPLPELQYELSWRLAVWAPQTQQEAR
PPSLPPPPTAAAAVGLTAMRSAGGGGGGTAIPSGSGGGFHEAVYRTLAALVAEDRVQADALLSAASHAAVCRLAAKM
GPAGNGGGGGETYGSYGGGTVSCLVELRMLSALRVASQLVGAAAPPVAAVAAAAGTILLPGGGTGGGGGGDLEQAAA
LEPLLALRCAALAAAGLRREQAAALAAAVAAARRRGATAAGLAAVQPATDPWVGDMRYGVAASWRLDEARLVRHRGQ
TTLAAHMALALVSELEQQQSAAIAAAGKYGSSGGGSGGMLQLATTLSLAGAWCAEAQVECGAYGGVMGLMGRAADIA
VQALEGRQDGGSGGSDMVGTAAAAAAAAASWESQAACKVLYRLASYTDQRYRELVAVLESPEHVKRMGVVAAKRREA
EGIRAKKDAAAGQMKAYLEGQLRRVLAPVLADEEADAALRSALLALRSTAIANYGRCLCAGDRHDLAAVYRLCQMWL
QPADGEDVSTAFAQATAAVPSYKWLPLVYQMASRLSAAPPAETFQRASGSSCTSSSCTSSSCTSSSCTSSSCTSSSC
TSSSCTSSSCTSSGMVFTFHISAVVCKRVLQSLLLRLACEHPFHVIYKLFALRNGNLDKMGRLQAAADAAGFVQVVD
VSKIKAAESLLQQLVARGGRVAVIVKQAEKLIRMYIDLAAQPAPQEDNPPPQTFPANLKRQVDGGAMDALPVVSSLV
PVDPTAKYDGLPHLVSFDSTVSYAGGINKPKIIVANDSNGAKHKQLVKSGNDDLRQDAVMQQTFALINGLLARDSAA
SRRGLRIATYRVVPFSPTAGLLQWVEDTQPLNLYLLGKNYASGAHARYQPPGEWTWIQCYNHMKSTTEAVRKGSTDA

GAELLRGPFRRGCEETLRATALLGNADAARAVLRVKQKLEGRMDAGGLSRTAATTNTGGTAAGGGAIGGGGGRAAAA
AAAAATAAAAAAAGAATTGSAALAAAAAAAAADAGGAPLNVEGQVRALLAEAQDPHRLSRMYVGWSAWL

>Vocar20014716m|PACid:23135521
MLIPNDISMAGRVSMKGTSLTLPTQEQLPVLRAKTWTTMFARSPPSPPPPRPPPPSPPLAKAQKSPRLPGSPPPSPP
PPLRKAPKAPRVPKSPENPSLPSPPKPPKAPRFPRPPKMPGSPPPLAPPSPRPPNPSRFPPSPPPPAPALRSPPVKR
PNPPPPPPPPEEQAQSDDAYVPVRDPKTLTSVVMAVDVCGEPSRANLTALQIAWATVLPNFFRQSSFGALLMPTDPKY
NRVLRDHVNIGCPPGDRDLRVWGARADADARQRLQQDVSAYYSHVYIMPPSFGGVSYGVIMCPNYGTSDVPGVWTTV
SAIPWTMVHEMGHNLGLFHSKGYDSNGVEQEYGDPSCPMGTNVVAHYNAPHSVWLGWTTPQRVLMDTDLIVGTWSTY
TIKGLANTPVSSLQIYAGSWMFGRTESPLVWDRNDLLYVSFRHYNTSSVDVGLPEAQRNKVHIHRHARDVEDCGAPV
LLASLDDGRIQSTWPKRGSKLMQDALSPLLTVTVTRIDTKAGIATVKLCRASQLTAETGAQCHDNLDNNCDGLVDNC

>Vocar20014771m|PACid:23135546
MLICTCSTGRRRWQPACMNICFLLAPLVTCLFHTGSVFQQAFYKKAVYYCIDLFWGRFGGDWEAVYARWSTALGIPD
LDKNAWADSCPRDPGYDELGNHMAYNTPACLAALGHLTPEQVQFAKYRMSEYNPVMYAWAQYYAYRASPPPPQASPP
PLPANTSCMFSDEFGCPCNSSWVFDGESYSYCDRVPGYETLMCMVADPSSCISCSKSPCLLSCAGTSELCRKPSAPR
ELLPPPGPPPPPFPPSPPPKPPPPPPESIPSSCKISSHNCTCRSSWTTIISRNSVYGHYCSPVPNKPTLGCVVTPSC
PTFLESPFQLCDRTLNATYCAGTRATRPPPPPRLRSPKTPRLATTRNN

>Vocar20014728m|PACid:23135562
MSQLQFAGRRSILSGLCGTGVDAAGSGEAAVDGGDAAARHSPRPDASPCTASKYVLVATAASPQPPIQARAGATLQA
TAATAKSGTGARVLHMLATSVPLSCCRTFAESPRAFGTSAYNAVVASDGAGSTSLSPSLPPPSQPPPKPLASPLEDA
VALPSASRNPPPLAVPLPPGGSLQISSEDGGAGGGAWSAATASATASAAAALPAATAPPPLDATDGFFAEDTATRAG
YNRRRCLFRPVTSRGAAAPGSPYLMGPLQTIRAANRPAQPPPSQLNHGSVGPKNIRLSPLDHGMLLTCLNPKALWLP
YDLDGRQLAIALSYTLDEFPLLAGRLRPSINGGRSRLQRLAARRSLAFVVQLCNSGATLREAVSCTAPLEYFLPLAA
LNRRAFTQMGSELPPYFEPMDTDAVLYGGEPLLKLPAGPRSDRRGSLYASGTSCSIGGGGGGGSSSSCHLPAPVLNV
AAVPVREPATAPTERIPTREWEREAPQGPGLGDGGSRKKGVWYCADGRSACRFLERLAANYREVTAAATTIPPMPAP
DSPPPPQPPQSQLPSGSSSRQLLVLPYESPADPKSESSAAAEPAPLEAVVAADVAAVAAAAAVAAAAAAAATTDPVA
TTAVGTAAATQPRLDEAGSPDQEKGGMRGLSAVAVADVGATAKDLEPRQTVSTRRMGYSGADVRSQQQRSNSNGSSS
GSGSSGSSTAGSSSNILFRAAPGGSCGHASGNTDYGSSNGDGGGSDNGNGSGGGGGGCGGSSGDVDVDGGDPWRNTK
SPPTCQHPSEKPGAGAGICTIIRKRTAIQPTQPPDLPRFSISVAASTSATYPSPSHPPPPPPPPPLPPQQLSPAPARP
SPQMPIHPEQLPLPPSLLPPPQQQPSQHQQQQQLMLSSPPPTQPQQVTFDRSLLFPARDVSSWEPEPGSSRAAVDVD
ASYGTPAGGGGGGTRSGGGPDRGDEGGIGAGGSQGLLSSLTIQQVAAAGWARSLRSMGKMLRNVVSEERRWRGASGR
ALVCEVLHLPPDQVARLKALAAPARPVPPSPTTATISTNDALAALVWVLMCDLRGRPLPGTLPAGADAARNSVSSGS
GGYMGLAIDLRRNGRLHPRPSQQLFSTDSSGDGGGVAPADTVPPGFFGNGVWCLHIPSPLLPPVSAAAAPASDPDGN
DAVKGLFHAAHSDAGGLRCTACGIGRICGAGSTVDSRFRGGSYCVVCCLRSGAAAIRGCLRELRERPDGAEQVLQTA
QSQLTAPMSSQMGMMAEVCCRQDAMLTSWQMPYWDLEFSSGATDGDIRLQNRPVQFQSLLTPSPPWSAAVMAAVPPD
RVRVLSPTAATATTTVAAHMAPYSSGYSSGRQTASSTGPSKGDAASSIAPPQGGVNLFIVVPEGAAAALRCSQVIRE
LVPGGVFH

>Vocar20014786m|PACid:23135592
MLICTCSTGYRRWQPACMSICFLLAPLVACLFHTDSVFRQAFAKKAVYYCIDLFWGRFGGDWKAVYARWSTALGIPD
LDKNAWADSCPNNPGYDELGNLMAYNTPACLAALGHLTPEQVQFAKYRMSEYNPVMYAWAQYYAYRASPPPPQASPP
PLPANTSCMFTVEFGCPCNSNWVFEGETYSYCDRVPGYETLMCMVADPSSCISCSTSPCLLSCAGTSKLCRKPSAPR
ELLPPPGPPPPPFPPSPPPKPPPPPPESIPSSCKLDDCRWLQCSLRPLLLTSFNYGTNAWLRRDTVLPNLLGEPFPA
LRQNTECHLLCRYTGNEATTTTPSEIP

>Vocar20005252m|PACid:23135726
MIRQNWRPPGEGEVELADIQLSDRKAGDLSSVHGGYEPIGWGTSSTGGGATGAPSSQGGGGAHDQMSSGGAPGDWYD
TQYDTYTDKFADGDGTRLLASSGISAGGKGAGGGGGGRMERLSDYLAVRWKVVVLAWLAVAVVGIVIALPVSLLTIK
PRSISQPPPPLPPQLPPPLLLSLPPSPPPPSSPPPSPLPPPPPVPLPPPPPASLIELCSHNSSSLPADPRAVPQSYQ
LHLSLPAYGAFTTPTPASGTAPSPPPPTFSAVASLQLLVTGAPTSCLVLHAANLNISRLDVEQLGSSGSSGRENEGT
AAGGRRRLQQQPPPPPPPAALLSRCLCGCNDSSPACSVATIQSVHRPQPEWTGAGHRRPVAPHTHLHRKCTALLGRL
RTVHVRPVGRGHTRWSRTIEQRVADDPVRGQLCQANFSVAVELPDNLTVLSNTPPLSSVPVTTTATTTTTTGGRKLV
TFATTPVMPVYALAIAAGNMTSLNSNITTFPMATSSPPPEHLSPQPPNPRMYSGGSSSSSSMQIRVWGPASMNATRR
LAGSLGIAEAAYSFYLNYTGMALPLSKLDLVAVPGKSYAMENWGLLMFDTERFGILLFPRLKTYSSVRTGKFTGKIE
KKNTGGKVYIVACREGLDGPLHSAVLGREPVGDVLGTMSQGVMMTCPSCGTCAPNYLVMPQDNLALNEGVASYIEYD

CIAAVLPSVLPLSSYGLSLAEPLRRLVVPPLGMAAGVHEGVLWRSRWADEDPLVGAPLDANASSVLVYSKAAAILDA
AAGLAGQEGLRRALQGLLASNMYGTATVSDLVDRIAAEAAGQWNGTLLGDQEAVAAGVMGWFTKPGVALVVAADATP
APPPPPPSPAPPPPAPPAPLPPPLPEPLPPSPAPPVPPPAPLLEPVQVLSPPSPVLPGLLPPSPLLPPSPVPPAPPA
AAAAAAAGRCRGVQPATAVIPFTVIPFTVTPLTVTPFTVTPFTVSTAAAPSDSDERPSALPAAAVQLGSVARGELPA
AVRAALGQVDAARSGFHGDVRYCVRQSCRGERNGYVLRVHCVFVYGMSSINTSGWRLLVGAMCVMVVLDAQMVLRDT
LALAFSLPATTSTTNPTAAGAPLGDPSALLLLPSGSVPGVAAAGNTAPLPAVLAAIDTALTSPLARTGAGMYSLGQP
ALMALDYLQGLFASSSNSSASCNADLQRWVVRRVGPLARAATNNTAKPDNLVDQFLLRLAQSQIVTEAALWGDSGAR
DFACSLAAQIYAATPSSAAEGGTGAVDPDWLPAALAVPLGRWPHGPYQHPRRKRSPHQFAPGYSLPLQISYNPPVVG
VPSGTRERLLHDRCGGSDDVMSPQEAGRLSLPNGRFRTCCDQQSLNLIGVGGLWGDGWPVHTATSIRSRPCVTALKQ
LKKYDLGFDR

>Vocar20006912m|PACid:23135739
MTLDLCRSLALEKDLPYFGLEAGQECFGGIDILRVLVRQQSTSCDMPCAGNARQVCGGVLALSVYATQPQSPPPPPP
PPLYPHAMISHDLVGCYSDDETYRVLPYLLDVSYTMMNMEYCRQKALAEGLTLYGMESGRECWAGLDAARARSLGPS
SDCNTPCTGSSSSSSDAMCGGDGAILIFALAPYVWPPSMLPLPIYNVPIYNSPPPVYGPYSPNPAYPPNPAYPPNPA
YPPNPSHPSYPPSPPLVEVDNLLPERLRPGAAYFDASFGVSVYSDPYYEDDIAMAWEEVEIPQRASQGNTFLLQGDC
SLTSPPATSSGESAAIRFGGSSCFGVFVNGLLPNWDTSPTSAFSIVWIGRFDNSKRGTEEQTYVTFSRTGVDYDREL
YWTDEQAFIYSYAKGFSFDLVTPRPAAEQWTMQVLSRRPDGKGDVVMSYSSYNMSGVMSTWTLPGGACEVASDHLVI
GKDFRDGNKFFGGDLAIFLVYNRFLDEGELQGLVEFYAPRFAWPTNPK

>Vocar20003155m|PACid:23135805
VTSPPFPCAWVTVPGTDFRTRRFLFPPPLDPQLLAATDPTAAPLSFGYTVEVTDGAAGATTTRPVVICSSPEPCTRP
LEAESAAEDEGEENEGEENEEEVARAGAAVLNMVALNDRAAFASTNSRVQLPSTNYPEGVFVLASSDGSGQRYTVEV
CTVASPPAAVACAESFGVLSVLRPADAWPSEADNLKNVTYSATHWSSKAAQRAAAASDSPAAVLTSTCAPPPGIDFR
RKVVFSWGWDFDGLPPSLAPTGFVAVYRRYPFDHMAPGSYGLQFAKHDNVRLAVIVRFTDANPDVAGTPAAVREVVA
AAENASLSIPGGRVSDITFVYFVPPLKLLSNITKMSASVTMTWRPSPPSPPPSPPSPPSPPPAPPPPSPTPPSPSPP
PPPSPPPRPPQPPSPPPSPPSPPSPPPSRLPSLPQSHCVRVNQQSAPQQLLGSSATLRGVSRSLVALLAAAFGTLML
LA

>Vocar20003210m|PACid:23135823
MRCVGLVQLASRIEHSGPLAVASILCFVVSLLAAVPPLVGTLLSERTAAALQTAALCGTYLLSGLPQAVSSVALAAG
GHLDTHVLMSLAGALLLLLFQVSHFLEAKFTSRAQGSLERLLAAIPERATLVAMTQPPPHVRQPSQPPQPPPSHGTE
EEVDGAKDMNNGAEAVSMASSSRHGRSSSSGSGSSSSSSSGIGPDLSSCREVLVESVAVGELVLVRPGEQVPLDGEV
VWGAASVSAAHISGESAPSRVAAGGWVPAGALSTDGALVVRVTAAAADSTPARIARMASEAQASKPQLQRLLDRVGA
VWSRGVVAATLLAAAVLLAAGVPLMAAPGGAVYRALGVLVAGSPCAVVLVPLAYVCAMAAVTRKGVLLKGASALDAL
AACTTVALDKTGTLTTGELRLTEATLLDYSWMAEPLRPLSSPPPSPPPPQQPSPQPAALSAPASAALMRSVEEEVPG
SGVRAVCRLRGRAFHVAFGSRDFAEQALADAVRAAAATEAEAAAQTAVSRLRAVLARSDVGGDRVGGAAGEAAAAAA
VKAISLLVVTPLAAPTSMADGSVAAGVGLTPGGGGGGGGGTAVGDPRVALFCFEDVVRPGVREAVAALQNGSWRRRR
GERVGADEAAPPPPSGATAALRVVMLTGDNPAVAASVAGGLAISEYRAAMLPQDKLQFVQQEQRHLRELQQRREEAE
AEKGRSPLTALLFGRAEGPGSISSSSSPAGAGAGGGAVLMMGDGINDAPALAAAHVGVAVASSPRDLVAAASDVVVL
NGQGAAALPWLLRLAHRTRAVGGGDHDHEHGSGNDRKRSEVVAMEAPPGGPQGHQAKPHQAQQRSSDQVEEEEGEEA
GKAADGNGGAARGPVHVRGPVGRDVSTAASAAAAAEPAPAIQAPHARGHARHCGGGGGGGNTRSCCTVWGCISGPGA
AANAVNRRRYGVSSRLGPLLALGDGVGVGFLPSGGCAAAVGSLDAGGALRRRRRVSGLVGPGVRVGVGVVAAAAPTA
AAATAAAAAALRSDTGDRVAL

>Vocar20003270m|PACid:23135827
MTVTKEGRERGGRGEGEGRERGGRGEGEGRERGGRGEGEGRERGGRGEGEGRERGGRGEGEKGGRGGRRPGEEVGLS
ATKAQTRYKTKNAFVVWSLLHEHNSSVCFAPSLCAAGRGAKADPWVAGLGFRLRLSEEVWVACASSVGLQLHNANTD
RAAAPAPPPGGAPPPAARDGDRGRGESGGEPHGGLAVPELRKLQAQIELQPVEMRLVTPLVCDDVLPTALMRRLAMA
FDPAGATPYWRQHRYGRRKTPFFSYLYRLDQPPTSVVEEAIQELHRRLLLPLLQTAKSEGHQQLQQQPESASFPAPL
GAPTSPPPPPRPHPEADSVLEFRTTGSAAERAAGFTDLAVPSATGRRQHAVAVVEWWAHHRDPAAAHQLHFDVNEDV
LRRGLDAYHLVHPMISSVLFLPTAPLPLPVAVTAAAAAAGANVAEAANVAEVAVAAALRGEEAATARSLKSVSDLPL
PSSSPPPPPKPFSYSGRALPGSGPGFGFGFGSRGEGNDGQEAQGHVRSDPIAAADHPAPSTSPSPSPVALRAWAIGP
RQNRLLLFPGNLLHGVVPQPFPPKPKLPPLRPSSSPPRVQCPLAPPPPLPQRDDAASRSPLPPFPDPTRQRQQPPPP
PPSRPPVVTRSQLGTAAAAAVPLPPRRITLMMAWCFQTAPITSTTKAENNICDNVGVDNSGNWRTVDTQTRDDEKQD
VTVKPAVVPAASAVATARAIGGSSGDGAAGRALPPPPPRPPRDDSLTAPAGRRTSQPQLSWRHWFAALQDRNPVSEP
SYNADGSSSGGGGGGGGGGVTRLRWAVPECVSPAWVTVGATQTRKEQEAEEEEEARWELARQALRGMAIALGLLPEP

LIAEVVQWRQQQQQQQQQQEQKDGAEGLQEGEHKDEEGKEGEEGGEGEGGGSLQEPSAALRRVMEALQLPDLRLFLR
HPRELEHLYLPPAAGV

>Vocar20003132m|PACid:23135846
MADMRLGDSQFPASGSQQADLTFGLDANSVLARIETTVEAMVTEIAEGGVPRLQLGSRPRARSRQHHQQQLQERGVE
RCLLSNQGAAALPYARVLAVLNAVHDLLRQGRQATQRDLYYTRPGGDVCGAWGGGGAAAAARRTRFTLGGLQYRRWE
WVGPSGRALPGDMATIARFGLQLMSPAAAAAAAAGGSAAAAAAAAGGSGGGSGGSCYLLVVEKDAVFQRLAEDRIWDH
LPCVLLTARGMPDLATRAFAARLAASFPAPRLQPVGLVDYNPAGVVILATYKYGSDRLGPEGRAHLLPGLRWLGLRS
RQLADVEHHHLQPLSERDRVMVRGLRERLGDMEPGWAGELDVMEASGYKGGGKRDAQAKIKHQHTHSAAPHRHRAPR
RKGEQKMPWNVQSPDEPKAQRRHRGGVPGGCRNLGKIRTLVKSGLDPNGLVSNTSCFDNTTWVLKDRKPLVPLLVFS
CFNGVPAAVLDLLIRLGALPNAVTDCGLPAPLAVLCAKITAEEHRALFQVLRQHRCSTFNSSVCSARNDHPIHITCK
VLDLSDWTSPRKVEGIELLIKFGTFQLSDGGLDERAMYDFVATAVRHSCGHKLLDCLWRHFPQQLLPSLPASDCLLL
RALADSWLEPFWRRSAERQHTVSMGLVLRALLEHPAMQPPQREAVAYWLRTNMAVLLYRPELVALLLDRCRVCPAQS
TSSRAASGPGSPVSPRSAAATAAAVPLHFHPDDRDLVLPVGLLSGPEAAQGDVPLAPGMSLMLAYVVDPDEQLARQG
GIHAVPPQPCPRERLLDLLDSNVLDYVDNRNLDLVEVTLAELLPQLASLSPSAAAAADLDDDQGAAGGSGVTLSDGG
IGDRGGGDGSRSAACGGGRSDGKGAAANLAAQLSVVSERLERAHACLDELYDSERSDAELANDPEVWRAEAAVEALA
AVQRCLQRAATAAGGDGDASARFRCELSTGVAFVDVVAARIVGQPEWGFSRGLLLQLGLCQMRRALRSLQAAAGSG
GAADRGADGGVQGVEAQMEVLRLYFEELLADTSVSERRLLEDLVSALEAAERERDLEEAQVAEEVAAAAAAAAAAAV
TTAAMETNTEASHALQPPRATEYGSMAEIPYDANPSEALLRGMTKPLSPPPPQRQQQMHEVVGRCEQEEAQQETEGQ
NIDVALVPRAGVEGGDGGSDGCGGSAGQQLSACSSRHPSSSSCRCDSSSSSGVSGSVGNIGGPEAAAAATTLPVVAD
STAATAAAMAAMRQAFLTPQARAAMHRAMLPGESMGEWTSDDEDGSDGGDDDDDDDDIGVGGGRGGSSSSAESWRRQ
AKDVAVSARGVGDAVADSGASQGSNAGCSRSIFAGRGGSGVKGKGSCSTRRCALENGSDTLGAGPGRQCSQLSPNLA
KPPPPPPQQQQQAQQMRQVQQRRRRLKLVGVVDGGAATGRRRRGRRLYGSESGAERPEGFSTPTVDGWRDPDLLLGG
LNVMSLPPNIRHGSGGCGGSGSSSDSSGRELAPYEYLGAAVMFQDYEDSGSGGSSDANAGFGVGVGVRCNGSAASSA
YDTPSEGSGGDAASLLEAQARGWSGGGAADAVVAAAAAPRRLSHSSDQSSELSYGSAQGGYGSVHGYGPEYGLESVG
GDMGALDSGVANRPTCGGSSNSGGGGGGASRRDMHTIAAAAALAMASATGGSGPAPLPAPQGGGNITQQRRHRNGSG
QQSKHAAMRSLSCRAAYAAGPTVAVSYYERANGCDSVTSMEQSGDLRGLRTSVPDGSGTPSTAGVNAMVLMAERPLA
CHVSSSTSCPSASAASSSSPSGASCCSSLDSTRACIVHKPFVATAGATEARRWSRVPTKPHVQIRALEPPAAVAAAA
AAAAAVLQPPSERHTASIHTYSRPPEGGGGGDSSGDEYVHTYDASNEYAAEGVCIMKPTGSSAAAKEDSAVNGRQRY
DANAPAYGDLDDSGVGLGRRGDVRSGGGGAAAAAIGATARAETAGGRRRAVVRRTRAKQTPLVVAEAASPLRQQAAS
SPPPLPALLLPQSFGLRSCSDASAGNETESSDSGGRRRRSTSKVSYGSDGSVVATVAPRRPAASSSGGGGAGEDVDA
SDDASSTSTQEWPPRRPPAIAKSITLAAGSFPDVSTATAVAPIATAVAPIATDASSENDSDAPGSVGFSGRRPEHGS
SEDFGSGSGSETEPGGSTEGRRTTATRSSTVPQPAPPSLPLQSGSNSEFRGNDKCRCRSASATSSDSSVTSRPTPVG
GNAVATRPESLVAAAGRRRCAAAADGAIDAVAASEEISISRLCLAAPFTAAAATAATATAAPAVAVLAEEKEDGSDG
DSRGYGSVTEGVGADHNGVDGDDEDGSPDMSGRRQQQHCPVASAQPPPEVAAPAPATGPVSLSAHTAAAAADKTLA
ALLSNLATSANVLPRPAGAIPNRGSDPIGAASGGGGVKRPRSGACTWFGDGTAAAATAAAAAGGSMPSSCCYVLRLS
GGGGAAAGECAGVPPPDAVMRALSNGPCGPEALYSWAGELRTALVTSSGCVHQRLLVVADSTTRAPGPTWLTALRAA
VAGAVLGPVLDGAGAGSGGGDARSGGGRRRDHPMGGGGRRRYCPDWLRQTVLLLPLSHLIVPYGGSYLYQAASRAYQ
GKTTFGRRFAHEM

>Vocar20003078m|PACid:23135866
MGIGFRKEDSDIAPVAETQQSPQPEQQEQKDQDMPPQPQLQPQALQLAAQQHLQQSAQQPRPLEQMALDQNTELASA
ATPAGGSNGGYDAVKPLSHIRVQSPGRGSTPLALRPILYSMTVRNADGTPQTARQLPSISAPCSALSAYGPVTSGEL
PYDESIEPPLTSVAAYPRSGRVGGSSGGGAAGPNGEDDGLSQGSPSEPELRPATTATLQSAIGSLMVTEGPATSTAG
GELLGADLPSNEGVQQQQQIKQQEEQQEEQQEEQQEEQQEKQQEKQQEEQEQQDQQRLAGPHSASPPEAVAVSAVSD
SEEEVGRGSVSSGTARPQPSSPTAAEVTGEAIEVAAVPPVQPQNIQEYQSSPPPPALPQQSPPPPGQPKRASAGQSP
EASDDAVQPNENAGQGAGGSSADGGDVAGANTTDTCAEAPGQPNQDLAQNLEELVAALPPGPIQVTVAVRVGAGGRR
PRGGGSRVPEEDTRSGYMRGHACGVFDVPRYLAGRDCIHNGSKWMSRSQFEKVGGSKMAKWYRSIRVLPDLEPLGEW
LERHGMPVTKGPARRSRKRAAESGDGEEQQGQGAEQEGVAASPDDVQGQGVADPTAALPAVPPGPAPPPAAPASAAA
AMPPMAPLSSGTPLTPRTPMGQLSPQAAAAAVAAAAAAAAAAAAAASGSAGVQAVGSAGPSPQLKAEQMAEIPLEALA
ERLLQQHQRSGRPIDARFLRVLLNTLPMQPEMPLSRGAAAEAAAAVAAAAAAAAAAAATNNEEFDRDAYGAYAAYGD
YGAHGSVSLTHGPAGRVSGAASVSPPPRGAAGVPGADMQGLAKVQAALKSEAAGAAIGVTPSAAAVAAAAAAAASVR
YNRAADVSSVAGDTARLRMRQQQQQQQGGGLERSQSFHEALATPPMLHLDLQDPHISRDILAGLAAAGVVDGVGRG
VSGRMPAAHFRTEQQLQLLRRLLAQQQHLMAPPPPPPPSQVPQARRALSGMALPNGARVRRVEPSESAALDPASAGF
GAVASAATRSAAAAAAATAGGPPPRQERRLPPPLPSAPLQQASALVLDAATLARGLAALQSLDVAGGGLSGHSRDHL
TRTPASVLNQYLGQSYTGQRRYRPHAPEPEHISASGDLGYGRSDTYHGEGSRRAAASNAATAAAAAAASLRRRRRPW
EDEEEEDEAEEEEDVWRQRESVCEAPSRFLPRLPAELKPNLLQQQQQQQQQQQLPHKRQRLGEGECQPPFPQLNGH

DPIDVDPYTVRQHMARLQDPPPSMLLQQQHRRRAHAVFAAAADELGEDDEHQEGEEEEEEEEEEGGGVAEYWADDTR
RHAYRSGFNAVGDAKGEHPQVPPLAMSLPAVAARRPAYYTPQRPVMAANREGAASSWRRPRGGPVLQGESQGRRTDW
PQQARRSGDDSLSD

>Vocar20003183m|PACid:23135893
MANEGVLAALEKEFRPLLAPKLLAEAKAAQVVWARVKGYPAWPAQVLTEAAALRRKDLGQERHSKGNSVPVMFFGTV
EVAWVKDADVVDFKEGLYQGFFDRKTKHFRLALEQVCEYLALDRKRKAPPKWWCKPPSNGLDEQSDGPSPKLVSASG
NGAAKGAGQRQDASKSSRQRAIAPESGSGTSSGEDEAHAAHASGRKGSAKPAGGKEAAGGKPVKEAAATGLPAKLRD
TSAGRVPRENGSTAASTNTAAQAQAKKPPTAPQAAASRQQVLVKESNPAGDAAREVKDTRQGASAANSEGRAPPPPP
QQRLRSSAGASSASVKAGDAGLPPRKRVKVEAGAGAVVGPTAASASSLPPPPVVAAKRTASVGDDACELGAVCTNEV
DVLKLPAEIRRLAGIRLPALPKKPGARVEESSREQPLPKTEHAAALNVANGPATASKRASAVGENAGMELRSTRPKR
TSSAAGANQPAGGRSTEAGQKKRSRGRSSSGESSQSAASSDGQEEEEDKEGQSSGEEDDTDAAACSSSSGSSSDEES
SASEEVSDDDTAIAAARRSGRGGVAQSHRKALRSKSLSQPPPARDRRGEKQRRLARLAAPEPANRRTSSRLARKPPE
AAPSKKAINEDSDTADSGDPSDEASDSDSDEGGGGEDERSSSEAANSSSSSSGESDSDDEVTAKESGGSGDVGIKQR
LRTRAVQAAHVRPQRAPALQKASAGPSSQPPIREASQPADQLAKASTGRADRHVDTPDDDKGAQRRQGSGSVLAPPP
RAIPLELRRLQTSPSSGIQAVDTNEPRTLRDRSSPAQRTLASTGVTPTQTATAGASAGFKRGEERVSEDGSGTSMSR
VVERSMSPEHRRDGQKTSSEAEAKMPPRPARSQVVDGRSSKAGSVAGSASPPPAPMPGVVPVRKKPASLGVPSTARS
AGQVGARQLPAITPAKAVLRSLTSMGDGLPSSTMHASGCGGTSAAGALIMPIAVCVPKKPSTGPPTRRLVEPSLEAS
AFSTGMPDVVSVDCDADAKHASSPDIAAAGAVDAGDGYLPRSPDVSAKVSGMRMLEEGLAGDRQGVAMDEQQQVELL
VHLQLGPAEHCAEHDRGQAMPLADERASSADAEHGFEHGTVPGAEATEATKDLAKQMQRQAPEQCEAPEAAVRCIQP
RLELDVNLAAKLEQQDQVDHQPSSCCQQTLTNTLTDPVEQAAALTSRSSVVHDQENEPVNRSPARALSAPAQLQQVA
AVQAASALGPQVRSSPGSPIHDEVLLSDEQLPDQGEAMAEHDGYALPPEERLTLGSSGGIRGSACLRQVAVADSEAA
REAPTMASPSLAAPFPPLEGHGAVSAPPACAGSRASSEAGCGDGAPAGVSGASCSATLWGGDDDAAARARAMELEEL
GEVAAALADLQQAEVPRPAAELRPASHPAASQLPAALQADSLLIDRSRSRGSRGLPHSSGSQGNLVASESLVSEVFR
WPQPEPLLPAQDQSFALPRDWLVTRPPRYEPIRRNVWLSRNKPKRLPKDEINICACRPPPFAAPGELQRMGCAQNCL
NRLSSVLCDAKLCPCGELCSNRSLHLLRQPKTEVFLTENRGWGVRTMEPLSKGAFIIEYAGEVIDDRELGRRMEHAR
MNGEPHFYIMELAAGLYIDARRKGNIARLINSSCDPNCETQKWHDASTGEIRVGIFASRDIPPGEELVYDYFFSTYG
AIKQSAASFVCMCGSKNCRGTMDLHPEKRRDLGRRVEVFWDADGQYYRAVIVAYHPTSKKHTVMYEDGGTEKLSLDE
VPHRWMDDDQPRTSAQQPELPHGGPSGDAQTVVADPPQSQPQPQQRLGMQTPEVELPQQRLQEVSAVDDELADVLPG
GRSVPIAAVPSQVPPRPLIPIDQRCLLPPLGSTSVEVVGIQSHGQVGKHRQPGSELHLPLKKRPRMVQHLTLSAHQY
AGLQPSPLSNTLGTSIRAGETVQEATQPQTSGGSAGAVVLPVSGHNRYSIGGADSADGEPNGLSARTSARRESSVTA
SGRISSVAADEDDLNEDLDSLLEAAEAVAALRTAPISNPAALHAQHEAMLPLQPRRVRSGSLAAAGLGTVAMVLTAK
AASPRHLAAVQRSGPPNGLPPHGGSRPSATTAPHPSSSSSGFIPHQAGGCRQHPAGNGSGHHTSDGGVGGLYESYQH
QSHGRNRRLSGEHDGGTGPASWNRGKNGNGSHCSGDVNREGLLWKLVGIEKPKDLLPLFSGECEKSRNGGVKGRAAS
SAWIPPTHPYDKLLRALEASELLPHADQLRLFAHQHSQQQQLQQHQQPQHQQLQQHQQPPQQIQSPPPQQQQQRVHQ
QQQPQISYGQQGIAGIGAWLLLLTIQKLRSRRQLA

>Vocar20003200m|PACid:23135959
MSIWRSFGSSIPFLSMSPRTASPPPPPPDGFCTVRILSYGLQALVAKYGTDWVAVAEGFGGRFDRHQVRDRWYGVMT
ASGPRRVGKWSADEDAQLVKAVDELGQKWTAVARHVAGRTARQCRERYVNLLQPGLKFGPFLGGAPARSAQGGPDLT
GLHMLSNGSVCGLPYCPCGMHPDVDCYQPVRMQRGPALFPASLKPTPNLSKEEQKVLAEACRELQAAYGRITWARVA
ERLPGRTDDQCSRAYEGLVKHDKPLLPPCTPPSPPPHTHTAYTPRAILRPTSVISAEAAATSCCKTRGAAAAGRGTQ
ESGDGAAGWRRAGRKRRPRREGRRITGRGLPDQEGQGALGASGPGGATAASPQVTEGEQGVEGDGGGSGGGARAAGG
GVVEGRDGEEQQMVLAVLLLNGRARTSFPNLRRLRLLGGGGGGGTATAARMTMNPDYGTKQWQIPGAAAFAVYILAF
PAL

>Vocar20003105m|PACid:23135995
MYQQASGKVTSPPFPCAWVTVPGTDFRTRRFLFPPPLDPQLLAATDPTAAAAAAPLSFGYTVEVGTARVQLASTNYP
EGVFVLASSDGSYNSYSVEVCTVVPSAAAACSDSYGVMSVLRPADAWPSEADPYGEYGAVFPGSEIWSSNAAQRAAA
ASDSPAAVLTSTCAPPPGMNSGGKAVVFSWGWDFDGLPPSLAPTGFVAVYRRYPFDHMAPGSYGLQFAKHDNVRLAV
IVRFTDANPDVAGTPAAVREVVAAAENASLSIPGGRVSDITFVFRVPPLSQLSHVAQMSAAVKLSWRLAPPSPPPAP
PPPSPSPPRPPTPPSPSPPPRPPRPPRPPSRPPAPPEPPAPPPAPPPMPPEPPCLDTNEQFSFQPAAGSADSVGSLS
NGLTVLIAAFLGYAFLLIAV

>Vocar20007271m|PACid:23136147
MACCRRLQPEIPALCGWRLAVIAREAFGSPAYCTPPRYVALWQSKTASPYVHYYYYGPVTPNQPPSPPPPSPPPPLP
SQAFIDGQYIRTGTARGNCLSLQPNVSLWQNELVQQPCNGKDKLQRFAFMHIAGGTFRISPSWKRDVCLAAGDIEWG

GARLVLQPCSGGVSISQEFFFHPLTGRWWMIGGRYRPGDCLNVVDGSPDAGKLVRLYSCSGNSAQRFILPKFKPGGA
RPFSGFEEADTRLVEFFDKRYFILFKGLGIQY

>Vocar20007294m|PACid:23136163
MVPTSTWHGCFCGKGQSLSKACRLGLALIAISLFYLSRTATAQGCPQVLGYQFYVQREVANAYKNWTAPSPFTVTSL
SATCNATARCTAFNTRGMLMTAPMRPAFSYLDNTKAGPCDGVYISNRTITGLLLPPNTVEDLRQEAKIIDMVAASTA
AMRVAAQLKTAKVPITAATIKTPTSQVTAAFAAVQTPLAQVSNVTGNYTYAAVATAILYPTWDSRSANGTNNNLISP
VKDQGSCGSCVSFAATAGAEAAVATALKSASNSNDFSEQWLFFCNGLYNPTCDTGWTADEAADVLAKFSIPQETNYP
YKGTKTCVLNAKPESRPAGRFQYISITNLDTAKDHIRLYGAVLTYFAVYNDFYNWRKGSAPYVWDGKSALSGYHQVL
CIGYNDTGHYWIAKNSWGTSWGDNGFFLMSYTNRAGFMSASGDNVAGLTFTPTQPPALSPSPPPPTLSPPPAKSSPP
PLRPPPPAKSPPPGVARPRPPSPRPPSPFPPSPRPPPPLPPPRPPPPPPPSPPAPVCGDRLCNGDETCLSCPGDCGR
CARCGDGICSGECSGLKGSTSG

>Vocar20007299m|PACid:23136167
MAPLAPPGADLHGALAAAMSAAAMDAVSRHALHHGPPYPKQLQQPFSYTIRNRGSSGPQPGAPQADCPSSSGDPTMS
PGSPWTPAAASSGPLDLLPLLPLPLPPSLQPPEWGDPDGGSDGGSTAMRFGAEGDVMNGMAADCNGSVGSCGGRGAA
ADGDTNDETEGEVADDGASWSSRYCSSRDPTVDGIAAGGTDAGGTDAGGTNAGGTDAGGTDAGGTDAGGTDADGTDA
GGTDAGGTDAGGTDPEGSGSGQVAGASSVGLRDGEDVYGDSTAVAAAVDAVVDAGQATPAAAAAAAADGSVDSNTVA
AAAAAAAAAAAAADNAAVEMDRAATGDPFTRGLMNADIAAKLALAVSNHDDAVIIDAAASAPPVGVFRSFSDGVTGV
AILPYSSLPGGFVPPRGPSPGPAAAEPGAARITRPSWAGSHDSSPPSTSGSMHSAPAATSVGLVAGYPSARGGGVGG
GRRTLAPPAAAEWSSHGTVSVSGGVGSSGSGLELGLVGSGVVASASGQAPPLAVQSSTFPSADRAMAAKINIEVSAG
AVCMDLNIQGRGRASGAASPVAVQKRSRSTASVTTAASAAAGAAAGVSPSTGKVNSGPSSSASLSSLANGTPARTQP
PPPSTSSALWSSSRRLRISLHRMHRQYQNYHQAMQGPPLDAAPLAVLQQQRSYGTGAGGSGSSPRLKPEVSVGSNAA
VAAAAAAAAPARRRIPNQGSEAAAADGTAAAAAAAVVGASGNHGTPRAASRPAGLRSQSAPSARPSPPPPPRVPGLR
QKLDAESSWGTARAVHSTASLSELDKLLESRWLQLLQPTDGGSGGGSSARSLLRHARSLPASPRAAKSRPHTNTRRA
RSAGGGAAAAADEPKSSSSNPSAMASPRASKSTPTLSSPRLGRRTAAVHPVLIVPSMVAPTASEDPDLAKVVEKARR
RAALEKEKRRAAREAAAKAVAGGAAVTATVSDGEDTSRRRAGVSGPRTAAERSKATELLFQRQAEGWARTHRQMPPR
RRLAPSVTKMVEKWFSLADGDGSGYLETKELAAALQAAGVGVSEDDVLNLVSAIDVNRDGAVSLSEFLAFLLKASKP
MIARLKRLKILDDVMKGGKARNRYVDMLYNRGTLAAEIGLSEQAAAELNAIAMTLDPKHQGDPNDPAAGGAGGTGRR
PGRVRLQQLFQQYRETMRRATEDGEVLTGRDPAATAAAASAADPRAMSSVGGGSVSGGEGGRVGGGDDRPGQAPDSG
AFNVGGDVGGGEDEHDVDVAAGFGEKVSGVSYFPRHSPEVAMLQGACCSHRASVGDAVLLPPPPGADPSLVFKLTEL
PVISPLPPVKRPNTRAFQRALLDVRHSAPEYLMQRMGLPGPMEAAAAAAATAGQMFPHMERSSSSPTRGHRRTGGEG
GVTASAATASAATGHATESFRPPASTAPAGLSAVVAEPSGSSALISTTASDPVTAAATAANAAAAAKAAAVAAGSYY
TTYGAGFTRYKRWSMYDIDPAAENPDYWQAKAAALLMRRTNRAVKDVLGAGVAALSAAAAAAAAAIAAADAAKDIEL
CSSVAPAAAAATAAADSKGQSNAASGRQLLPSPPSQRRLLQPSQPSKKQQHSDGLAVGYHLPCLQAAGTSGGGASAT
AAAATETATSHAGAIANAPDGAPAAVAASAVRSPLRLPRRRVEEPTSSRSPTQNMGRGGGGAPHGRGVGACALGRGG
AAVVLLSPQNGPSASGTVGSSGGSPVQYVIRPQHGANPRMAAEALRRRQPVPPPFLDGDPLLQPPSPPSPPPPHHQQ
QQQSQPQSQRQQASGGGGRLLPQRRPHAASTLPPVTPSAAHQAH

>Vocar20007145m|PACid:23136196
MSRLQYVVESGAQDGPPAWTFYESTTSPTCTLEDDWCISSSNDGLECSEDMPYEDAETLVEAPDTTCCQRLGSAQPS
SLPGADTYVPVGALIRADLGTQQEVYASDTQFLLRSVFAAPRQSRSCPWAADTTFHSSASPHGRVGPTDRGDPRAAA
WTYQHTEAERIYGGWPESTLGDRRLLVAWMMELKNAAQLQRETLFLAISLLDRFVAASSGYSVDRPERMLQLLAMAC
VSVAMKFEEVAPLHSTELVRLAVDQTTGMPLYQCSDLGRMEWRLLWMLDWRTRVPTSLTFLQHFLLCYCYCSCSNTK
CSSRNDWLQAASVITEGSNNQSAGSSSSSNTSGSTSGAGGSSGTGGKLRPGTHVPGTGIGSVTANLGPGDLHLPEGN
AAAGGGAPSAAAVACGLPQSETVDHLVDHYADLEVWANRILEVALLHHCDLLHCQSTVAMACLALAEQATVAAMAAE
SPVLTATLAHTADPHTSLPSAPSPPSSPPPPSSPPPPAPASSNGGCPGATAVAAREHGFCCLIAVQAVTGLPIEELA
PNLSSCIALLKHIWVPLRFPSAASAIPATALAVGGVQARRRAGTGAGAQAQSQAQAQQRLRQQCNGVQLLLVWHLEA
NIGYVHIRPCPIPTETSPSEYEGGVG

>Vocar20007182m|PACid:23136214
MHQAHEFSHTIIHGRPKPGQLVVGAGLSALAGSPGAAGGAAAGQAPLAAALPGPSRLGAAAAAAAPLQPPPAAGGSP
AAPPRPPAAAAATPGAAARVGSAAAQAPVRQSAASPNMMPRNGAALGAAGGGLAAGRAGAVAPGRGAVAPPALGGAG
AAALKQAQEAAKPREDAVMALARKGQPQQLQPQSPYQQPPQQRQWSRPLSPSRRTSMARQPPSSPPPAPDRLPPLSP
SLAKRAADAEANAKRELERAKAREMAALGAQREIERLRLEEQRKKLEGEKARLEAERKKREMAKVEEERRAKAAEEA
RARLKEEEARRQRETEAKRRKAEEEARKKDAERAAREKEREDMRAKIAAQRKEFQDRQAEAARNRAAVEQQMGRAAQ
PEEGGRGNGVADEPGPSGAGPGAGAGAGGRRNNEDYLSAEQRRAVWEEQRAAAERNRRAVQQAERHGGIAEFLGVPR

DSDDGSRGVRPGGGRPSPPPAARGGGGGGGGGAAGSDISPDVRRAAYLEMRAAAERNRASAENSERGRGTPPGQRAA
GAPGRAPSPAGQGAPAAASPVSRPTDPWAAKRARLEEERRKREQEILEFQRQHWQEVREARERNKRQIMAEVRGGAG
GGSPAGDAAQASPPDGQAPADAATPSAAAAGGPAGRGGAAAAAAAVEDDDDDMDFAAMVKDMQDVLMQDGANAVEVR
GGSGEGDDEEDDEIYNDEMHNAALSGQFMLNGQVIQLPGVREGDSLALKVEALRVFLEQNLGTDAFLKVYRMLESLT
PQDDEAQVSALFLEVLGQEKLPYLQLVHQLIVCEENMHMEGA

>Vocar20007298m|PACid:23136222
MKSITQNFYVFVGPCDFASRLGSSRAPRDIPRIRSSFFLHRRSSPRPPPRNVSPLIDILESQRPTADPDAALLYSYS
RPDDAKFSALSLDLYTPLQALRFFLEAPAVSLYICDTLIRMRLISIICSQMSQQTARFFPISHNQSLVQCTASWPNG
STLPTTPSSVDTFNIVCICSNLLNNVALITVIITIMTKTSRPFSFRHVVLTSLNFAVRCFAVLCRELAAVHPLSAAC
ATSRDKNPARAPALAIETFKKNGACKSVRKTQFSFSLTVNLLLYIDNLLSNRITCQGTRQEREYARRNGFFNDSDSG
DGGGSGSCETRFGLRKRAVSAATAAACGPPLGPCETVVQLPPADPRVSRAMLPRDYDRRAWLLLARMALSVVLFAVL
TSAAVTIVVSLVGVRQLLVLLAAAEAAFAVYFRQRYSALNSQPIKHAPESHDPLRAFNRLLELASNSSYNIDVHDYL
SSWFCGADPGTIRQGNLADLIAYGFWYKRRGEMESEGQGPLLWDCVSKMQSSLGFSLPAGYTHGLQVMTHLWEPLRV
WYRCLVAADLPVVAVEYKHVSMRLCSIIPSADDIAAAVMGLMDRLEMEQICPSNNFPFLSTSPPPPPPDLISLPPVA
DRMFLPSLLANFIYRPPRSSSLQHWIQDVPCRTLVTMAGGDELIHVGAVMEFVRHYAAKVLFHPEHCHAQLLLDPTW
QQQLVADVRSMASSGLGASGAREVQRRLTEQQPLQPLAATTPAAAAAAAKLPQRRMTVSELQRLLRQQALQQWKGGT
GSGGGDAVPSHVGGSNGVNTASAVRRRWTHGALGDKLVGVTTEYDSGRGFFGGISEERSMSPDDIAAAVGNGSLTQL
ASSSVEPPASYDILARRTADDEADDSAEMTEAGMAALAEDLRRCIGNSRAAAAAAAAKGTLLPPPPHGLPPQQPPPQ
SLQTNRNGGGAAAELDSLAGKPAAVPPPLPSSSPPPPAPLKGGGGGGSCGIDDDVSLGPLRWLSASATAPPPDLAAR
RAWPDLWDRRLLLEFELAEPTAAPAAAMQEPPAAAEPTGTQGGGER

>Vocar20007392m|PACid:23136279
MAELGFCNSANSLCDEHAAVEYFTTQLLQPFAISSLNSYIFEKITTALCAALVGYGIGVLASRRRHRHMPQPSQQAL
HHTTTLHSRLHAALELLRDLLPGHVVDELLNDTQVFQEEQQERDESQPTSSQSQQQAFADPLSIDETFEGEVPCDRI
DSGFRVPLSPLARDSPFTTTGAASASCSSCGSSWHRSPGKSGVGVGVGSNGAASSAVRSYLSYMSSSHPTTEMSDRS
AGPGFHNHPYVQCTASWPAGSTLRESEAHCNNHPYHQTLSPTAYERRVSYCAEIIPYTFSDGTAASDGTDVAALAMP
LSRRRSVSASGLAGCLPTSTAAGFTTNVPYRHRCSLADAQRSVAAGGDDAFVTGPSVGIDDVQEAQAWPSHTAGSAC
CRTSSLRRLAPEMIRWRGEMQEAGGDGSSVTMRIPASPAAVVVTPPELSYSNPVALAVILTDGQSCCCRVGGVGVGG
RAGDVGSDRVGNAGGSSSSSSGGGGGGGGGDHSHSDTSLNAMAGSNANFVPPPPTAAVAVTVSGPAIAGAAGTVQVD
EMSPLPSWSAEENVEEGTDGQHHQHHQHHQHHQHHQHRGHQQKERHHQAQQHPQHLRLVDQEQQPCGRQSMRREQQP
HGRGSYQGDDRSGEELKGGDCCRGGLGAGPSSYSVAREADVVASAAAAATAASRSSGSSDGGGRQTPDELLPPLVHN
CSEAQLRPKPTFLAAAAAAAAAAAAAAAAAAATTAVVTAAAEENEKEEAKMVSERGNVAIVVVSAHMRGRGSWDDTA
KTETMVWERRNDSYGGTAPPSLSAAAPPLPPPAPYGERSRAPSPAESPTGVTVPTSSPSVAVTSAASINLSAASPPL
PHVTKATTTVVTADAAPTPAGAPGPLVAVKWDASVRTTSGRVLELSGGEEGMAAAAAAAAAAAMGAAGTPDRDGDGD
MLGVAREWNCLQDCGCGGCGCGCGGGGCGSGGSNGSSSGSPTDGTTDRTTATGAAPVGSGLATDSQTKPSDEAAFKW
AERGLQRPGYSGQCTTQLAARVDVQQSNEGMTDPFKSRPPAEFGEPYGQSRGVPSIQKWLGVAGTDPRHCLLVSEEG
SSAKKLRPVNFMHKVVRQRSSGQGLPAPAQPPAATTTTTSAAAVTAESTAAGAATAGVTAESTAAGAATAAVTAEST
AAGAATAGVTAESTAAGAAAAAGAAESTAAGAAAAGVTAESTAAGAAMAAVTAESTAAGAAAAGVTAESMASGAATA
GVTAESTAAGAAAAAVAAESTAAGAAAAAVTAVEPYYYEPMAFPPASGWEKVMVRSCTTALYDMQTGTVAAPPSAVG
REMITISSEHGTIPAPAEAIRGALPGVSCCCNGGSSSGGGGGGGGGRAVMPLHAGMLSVSPRPLAEEAAAAAAAADA
GGGMYDKAGVTGGSTTQSCYSSYSFGCVAAEGSRGGNAVYVWASAAEAIPGAVQWLAVQPPQTLPPAPRTTSTQSHV
GPQLASAPTCPAAGGSGPNSPQRWGLLSRSLKNGSQESCSTPPPPPEQQQQQQGCSALTTAAAPPTIAATESELVRL
QDASRSASVQPHEAKLAEAAAEAATADASVDVYGQTTSAPTVADRTGATDPAAVTLFFNPANDTATAASLLWAKGAA
LGGNSDAKACTVSRRLDELPEHVLSVGAVRPAVANNGNGPAGAATDTAAASAAAPAPSCELLFGARMSGCGDSYSSS
GRTSNRSMASESTVRRSTATACVDGLWTAPPPCGVCASSTGSVLPSAHMYGGGRGTVTGAATELYDEITVADFSDGG
ALSVAARDPPDGCELPDSPHEHPNVKLRLRRVKTSIAELFSIGSGGDGRSGGRCSGGGGGGGGAADGWSRGGRTNVR
IGETTQRAWGGDPAANRGDAYDTTKHDKGKDYRQGRRGGLGVLRTTSGAAAFFRAVGSAASGVVGSILYGTSAPSHE
PLDKAATAPPPSPPPSSNGSPLRASRRDTGCSDSCVYEGARAAAPASPGAATKTATEGGGGEVGSGGGGCGGGGVGS
GGGGGGGVGSGGGGGGGEHPSMPTARARRIHRVSTGLPFYSGRGSSCGSVEAGGGGGGGRGGGRRSISSAALGTTAV
CSNTVSSAGGSAGIAASNIAGSYNNGGAGGGGGGGGGGYNLGRDLPYAEWHPCVSVLFADICGFTQISNRVSAVAVM
EMLNALYSRLDALCASWHQSHGPTTHGSQPVYKVQIIGDAYMVATGLLADDPVHAATACLFALAVLREAAQVVDPCD
GRPLRLRVGIHSGPVVSGLVGRMRRQYSVFGDTVNVASRMESTGAPGAIHVSEETQRLAAPYMPPYTVTWRRREGVE
QQQQQEQEQQLQKLWSFGDLSKLPYMSSPSPPPPSPPRQPLPPASRRQQSATAPSAPPPFGQEVREYEQERRVASQP
LPRMLSVPSRTDTAESYIGGLAPVLPPVVMSSSEPQPEAAAAVAPGV

>Vocar20007288m|PACid:23136315

MAAVPCCLLAPCIGELLSGADRTAVICRENLEKKTLLPKQGCAMLRRSPLPSPPPVQSPSRPLIPKPPLPQQQPPPS
LPSSPPPVTNIYVFMRLKPAAPEIPKPDRQFGRDINCSNLADIISANLMKVAAQAQLIFSSPLQVTACNDTTFRMTG
AVVYDPAACVATESLLEASWTLSDWLKLAMTENRNCTASPDIRAAVSLHSYGPMGESQCIDLCRSRIFCADASMRIR
ISEY

>Vocar20007141m|PACid:23136321
MPRAKNGFVASSGDRNIDGNGCSAVLEVLLLPKLRLGHFVVQDGSCRLRWKPAPLENPSSRSLLVRLIKEDCTKTCW
RMTDAQILAAECESSNRTLGVRGALLIFNKTSPVDLQLRSLAYLDPAAAATQTALRNPQNAPVVRTRIVRHGSLDGT
AFLAAATSAATLPPPPPLPPPLQTNTHRQHRHRRHSFHANSQNVPPQTASMASPALIQTAAEFDKEQQPMAPIAPKA
PPPMGATVTRAPVVSSQGGRVTKATGLVLREWGALYPQAWAPAGGAAPPSLRTLTGSLLPPASALESVAVVERQDQA
DLRRAAASTVHQASEDDTPGPGQYEVATVGAVGRTGPAFTIGGRTAPPPSAALVNPEAAKVPGPGAYNVSRPVADGP
AFTIKGRPREPHQSTQHEDVPGPGEYYDPTKTSIREGPAFTLAGKAKRKFDTDAPGPGAYLVVGSSRGGPAVTIAGR
PRARAPEPTPGPQDYQQGRATSPGRDGPAYTIAGRLDAKCDTNVPAPGEYDLPVAWRTGPAVSFAGKPVPKPDPETS
PGPGEYHMPHCPGEGLPAFTFGGRPPETDPEDLPGPGAYYRPVAKEGPSFTIAGRPASPGPANRDAEHLPGPGAYDP
QPGAAAGPASPPGPAFTFGARPRERSPPPSPGPGTYHPEEGRAAGEAGGGGGPAYTLLGRPVARDPPDSPGPGAYSP
TDPALSPGPAFTMAGRVVEPDPRVDLPAPGEYDSGVVTQPGGPSYTMRGRPVEAKRPDSPGPGEYSSPAPRRGPAFT
MASRPASPDAQDPDTGHQVPGPGAYDTMTPQSPSGPAYTMRGKPGERSPPPSPGPGDYPRHPGPRGPAFSIRGRAAD
PEPQLTFVGPGAYNPDNPGLPGAPAFTLRARPRERETPSPSPGPGEYDASAGGGDVDRGPAWTMGARPVAPQVPDVP
PPGAYELPASPRGPAYTMRARAAAEPLSREPSGPGPGMYSLPSSPRGPAYTFRTRPPAPSAAAVDGPGPADYQQLSG
GPSGPAFTIQGRHAKGGSPADPSIPGPGGESPGPGAYSPSGNPDGPAFTIAPQVRQPEQEPTPAPGDYGAADGGPSG
PAWSISQRLAEPSGLEGPGPGEYYSSPAAGGPAYSMGAKPRSRRRRKRSQLPPEPGQYWSPLSPAGPAWSFGEKPQG
PMPYKPDAGPGDYNDPRDMVTGPAWSLRPRTPSPPRDHVPGPGSYNDSKPFPNPLGTVVSQPRAHTELDPMYLTDMG
GPYDDTLRARKSPSRPGRRVTFHQDTMFRDSLDPTHGGRVRLEESAVSGRGGAAVPPPRVKTHAVPPPVPTLAPSPP
RESGVSSGTTFPGYSPRAAQAQRPGPPGIPSPRHQLLQQRPVAAAAAAARLVAPAAAGSGGGNSLDAEVERAMRLYR
RVALEQRYLGSGGGGGGDGDGGGDGGGGEQSRSGARGPSRQSSQQQKPPAAPPAPAPQQRRQAGRDSAIPAAAVVAA
AQQSYMADLATRQAAASEQQRRGASSRSSSAVPPQLQQQPAPGRGEASVSGAATTAAAAAARAQQAAGRQALWAAAA
EGRTAPGLGGPPTMTQGPLGVPVPRARRRLEMPGAPSGLSPREAALARAAAYMAGRGASGLTGGAEWEGPAGSSAGG
AAAAGAARASSVGVQAAPEYAPHAPRVMSAPAGGVGGAAGRTASRGRSISSARFRGK

>Vocar20007123m|PACid:23136407
MPPPTLSAADRLLSFYKDSELLARSGTLASVKSFGSQASAAPSMAVGGATDNRFGSRNGRPDVTDVVIDPYELNSSD
MESSSDSGFESDGLQQPLSEPAEDADWTASGYGDSEVYDYDGDGNLESPAGGGGSVAATPSRQLRGGAAAAVNARSR
TGSVAGGSRAVSVRSVAISSVAGVDAFSTGEYRTTRSRGGYAANSIRSRFGMPSGGGGGTVSPSLKSILGGGGGGSS
TGGGRDLLSFKRRAGAVAAAAAGGVRGLAGSVKSRASTRHSGGAGGGAVSRGVVQSSFKSLASSGPGGMPSVAGSTI
SRITVGRTGDTPKKNRLSGKIVRRRLLRRRGWDFAVTPGEVDFQLGRQLPHVLTRAQPTVNSEDEGEAEGNGNSDSG
DDDGSLSAGGDEPPPGPVLEAIKTYAKERAPEVMWSERLRAAKMQARTTGQTVTVRGRLDGKRRRDLARKIQKWDNK
EWFSVRKRTPPAPPLHPAVARVIEQWFALVDDDGSGTLDHAELLAALRASQVPVSPDNINEMINLMDMDGDGVINWK
EFETFFMYEFAAGKNLLSGEYVLPSGVALPFGVMIAKLKRAKLLSDLTQGGAARAKWMQVADNPVEMEDELGMMAAV
ELAMEEVHNPALSQRRLRAAKALNRLPPHLRTKSMGRHLERHPEIADKLLRASSITPLLLGSMKRCGSSTSQGGRNV
DLNVAAALAAGLEWPVSGANFYNRRARTILRAICQRPAETLLKNQTAAAAKLAAGLLSSMRMSRGRSRRRGSDDDDN
SDGGGSGGGGRARRRRPGLNRSTNSTARNGGKAVLRKRLRRRRRHWFQLRVTAATSSWPLGSVDTPSRAGGGGGGES
LGTPLSGAPWALGTPGTAPAVEGGKPRKPRRRMRFEDEQDAAETPTDAGADADKSTPTPAGAAAAAAAGPGMLAAEP
SNLSINSSLAGATAGGSVGGNATAAATPPLLRSALSHPTVMPAMYGTSGGDTGSVREVSTEDGNGDDRSSPAVVVTV
AGRTFPLRSSLASVELSGPDSSRTSVSELPSLPLQPPAAALAAVGTAAVGANEHHGARNHYSARVLELPGAASPAGG
VRYWPAVPGASSGASPLPLPRSSVTSVTYDEEMRTPFRQIGGGGGGGGGHVPYRDSEVDVLDADEARELMAALTLDF
RKDEALYDLHGGHVAVRPSTSHFRAALAKIRTEASSLLQSYSLPSYPAYSGGAASAAAAPSSSSTAAAAAAVAPPVV
AVALHPRPASPPPTGPSFLTATLSGTTAAAAAAAGPMPRGHARTSIGGGGSGGAGASMDSGGGTSTTNTRARDGGRS
VKQLATRHRSLLISGVHLRSLASAVLTAPGAPGPTAAKTGAAGPTAARRRRPSAPGCRTKPFPQQVVALRGPSYTSL
QLSVTGAPFAGDMPYAPGICVSSGGSAAAVTTRGPHRPGRISPVASVAPMPSTQLPESWDAHVAAMLQRRTETELAA
VQRAHRSVHTPRDGAASPTGRERSPDVGNHRRTGGGGGGGGDAVSGSSTGIGDTDVPYTSVLMGPSGTALPDFVPVP
GVRPPLQSRYVPKRLPEEAGEQGTMAAAAAAAAELPQPSGALGGGDGGAGGGGGGTAAGTSHPNINRSSAVDKEVAKL
RRCRHVLSCGLTDPALPLLSVDEALMMSGFGKPQTPALARIERAAGRVPLSNGTATTRRNIEHGALAVDPAAGPASP
RGGGGGGGGSGSSFTLPAPSRLKLNGPHLQLQIHQHQNVPPPPPPPPPAPGSPRSLAPVTTRGSASGGASQAAEATG
TLARGSGSGGAAAAATSAPAGHTHVGDGGGGGGTAAGAKFTASKSARVRQAMTESLPALPMRMHSQGNLALQHHNAK
GGAGGGGGGGTAATATGDGGSPRPPPHGTSSRALSNGSGIAVQGGGSGGDRDRIMLPHPPLPREHSSSQTAAAAAQR
RAALVAEPLAAPSLSSRLLAPSLSAPLSSPPPPPALPPAATLFAPAPPAAPLVAPVTCLTDLPDIVTLLSSEPRVPA
RSGPTRIRRMDY

>Vocar20007095m|PACid:23136647
MAVGMCALYACASQTMAAVRSSRNRSLGAVTSGRSALLVLTLAAVLLAATGLNAVEGAPRPPKSPKPSPRPPSPRPP
SPRPPSPRSSSPPPLRQSPPPPRPNKAPRPPPPTPRPEGGLRLVRGTAGNVGRLEVQLTYPDEWVALCDDGTFTAYE
AGLFCKMLGYSYGRQFYAQGISTYGPDESDPPEPRGNLVCTGDNDSRPPYAFFSQMINFTSQQEVSCQAFHVACPRN
MLVAMECSKTPFKYNGPPPPPSIPSPPPPPPPPPSMQDLIRLIRLKISSPWAPEGIVVLLVNSADGGEPVWAPLCAD
GIAGEDTIANMLCKQNDGFRDTPTRMVEGSAWKAIEITQASTISDFNPSEYSHWVTVVGGISSSAYTVYDTQLQVTT
QPCSSGYLFHVVCYTKMMEF

>Vocar20007093m|PACid:23136651
RIASSNISEEPVASLMKRVWATRRAIEKEISISMYSSALQSNADTPGHWPSLPAAVLIVANPLNVLLVGLNAVEGAP
RPPKPPKPSPRPPSPRPPSPRPPSPRPPSPRSSSPPPLRQSPPPPRPNKAPRPPPPLPRHPEDHACLPHTPSSYLAH
KHIATKSEAVCWYGSRTYMCCMLFNDVLIWPLLRLFLIRHLLVEGGLRLVRGKAGNVGRLEVQLTYPDEWVALCDDG
TFTAYEAGLFCKMLGYSYGRQFYAQGISTYGPDESDPPELRGNLVCTGDNDSWPPYAFVVQEINFTSQQEVSCQAFH
VACPRNMLVAMECSKTPFKYNGPPPPPSIPSPPPPPPPPPNMQDLIRLIRLTDSAPWAPDGLVVLLVNSADGGEPVW
APLCADGIAEVDTIANMLCKQNDGFRDSPIRTIIGSAWEAIEITQASTISDFNPSEYSHWVTVVGGISSSAYTVYDT
QLQ

>Vocar20003405m|PACid:23136737
MSCIRRPLPRGSPSIAKKRRGSGRSQLLPELLSGKISAQPLFPLSPFCHLVPLSAGVWCSSSSSSNSTSSSSSSSST
TNFSRTPSNSSGSSRAAAPAAATSSIAGEFTTFPRPWLENGNSPPPYDTCAAVDWCYLVDDRTRCPFETYGPEEFAG
LVASSAAPSYIRNLWRIFTAASGGQFVVGRRDDAATRILFNDADSLICFLRLQNDSAPLTGAATLPLSARAAANGGG
GGGGGGPLVVSATLDPVMNVCVFADADAATRGDYNFGSWMAAALTLRLNAELSAARKALGSQEMYGDLQATLVTRT
GPLTDLPCGCRTVAEYLNAADAVVSGPSDLPSPGEGFLAEHCLQRVSLAVAAEYHAGVCGQRGSRTASFLCKPAAPK
YDDDISSPPTTAALPPPPPAVPPPPLATCHQACYPDQAAATGCIFKFYTAAQWGSLSASSPASAVLDNSWPWLTRNG
PFYLGAGGEDRTGPSFEFADRRALQCFIRTQLTATPPDAAYGLPLGHSIPVASPGTWSSSIAGALPSSAATGSGMTA
GCTFAFNVSSYGPWVAHILALKLNVLLQPVLSPGSSTAAAPKLGDFVFSQYGGTACACLSVNEVLRRAEAAITGWRL
PGMPKLGLNGPIWLYGDCAKAAASQYANGACNWEDPCPVRQLYDTASMADVTPTSEAAAVLSAFWLYILEELFPADL
VFVLGSPTGSGTERHIEFPSATSLQCFVRMQLNRSDVLLPYGGPAEPSPAVPIVRPLRTVLPFDAEVDASVGRCVLA
ATWMQELYYGPYMADVLALKVNVALSRVRAGGGGGSGSESGSNYGRKPAGRFKLNVARFGNGWQPGDGGVDTAPCWC
RTVSEVLAWADNFVGGEPNSTLMDALTAGACAAALMKVYGTRTCSTTPGPGLLCADDWELAAPLYPFDSPPPPPPPP
PPPPPPPPPPPRHATMMYPPPPPYAAVGAPLYPPLYHPPPLPYPPHPCPTRQLPLYFLPTQDPSLYPPSCHSSPPSY
APQAASPPPSNPHIGHPNCAVHDSGSGSGSGLKLCGNGCIAHAELHSRGAPAGRSGRCRMLLRYICEAAHAALLPRA
VLLGGTAVCTWARLDPDSLDLAYRPARTAMWRIGTIARVEESG

>Vocar20004379m|PACid:23136978
TAGTLQSIARGPPPPPVPLPPYQQPQEQSPPVSAPRLHPTPCGLYSSSITQELSQDGYLLDLGELVEDEQYGLDWFG
VGQSYRLHVVPYAGRKFAIPLDGTLLHLFYREDVLEALGLAQVPQTWTQLVEFTLSYTALLASSSSPSGGGATSAGS
YSHQSLPPYPICIAQDFGCRHESYLQAIWSSLAQTHGSSQPIHFEPATMRPLLDGPASAAAFRILAQLLATSAPPEA
GEGCSQGSLSFARGKCVLALGGFVQQMQMLAESRANGTGLPPGAVVRVSELPGSELVSSVPPQVAAEEEGLTPCTAD
SCPYATRVTINVAAGAEVEAEAGGVAGLGAALPAPPPGSSLDIWVAAGYDTELMRTAMPVLYGSMTHANIAWGLRMP
GQHVYSNFLGDVIGSILTGNISVEPWISASLSAGAGAPPPKSDPSSPTLPRVLSAVRAELADYYKGENASRLYEVSR
QPWVFAALTGSNCNARVQQATAVPPPRSDTSRSHSRHVPLLPVLLSALVPGALLLLALGLGLLLLVGMQSDGGGWRL
VPFAAGGSSGGSWWSSGELSAGGPCGGGGVAVWEDSPPHQRHHRHPPQHLRGLWGCLVVARRGARKVHLPGAGSEVV
LCVTDIQDSTALWESLPADTLARAVDIHHRCVRRLALRHGGYESSTEGDSFILAFSSVRAAVGFALELQSQLLLAPG
WPKLLLEQEVCKPVYTRLPAGFPFSLHASSQASTTTLSPRVRKVRKSSWGGEALANAAAAAAAAAAGPAAAAAAPSS
LAEEQAQANGRGSRGGAAADAVLATSPRACGNSDGGAAARRVSAAVPLVPYTVRPLSSTGTMAQQPPRWELRQLPSP
TSSGATTPTWGPNGASTPRGTFPRGRSLTDMTYHAVQQQDSITITGGGGAGGSSYRSELHPLPEAADASAAPREVTS
DLPGGGGGGGPAVTMSIWCGGGKTCSSGLLKKQLSWLGEAAGGGDIITARPPGFVGAAGSGSQRGPAARSRVLCSAV
GGEEEEQEEEQEEEQRHGVVVGVTTLSDDTDMQQPPRQPQLQPQLQPQQEQQEQQEQEQQEEQAQGDGDSDQLPVWC
DSGPLAAAPDFSMYSNATYVPTAHGAGGAGGSVSLTSGVRGGLWDEGDGEFISSASNAVISRRSFQNRTQNVPLSWG
VGGAPSGPAAAVTPAMATIRGGGGLGGGRGGGGSGGGGGCRTLLEVLRTAAAAGMAGSSSSGLSGYARTSQSQQLHA
QHNQQNNRHLNHHNHHHEHLMFRGLRVRIGISVAVPTAAELQYNAAAARMVYGGPCAAACKALADLAHGGQVLLSSG
AHRALAAECQGLGGKPPGTMVLQLALRQCASPPPPPPLLPLTTTSPTALRPTAAAAAAATAATETDAAASHGDGLRS
GFPLNPFKRHGPEVPELHSPLPRVPEHGPGAALKQQTKSQQPPPPPPHLQSLLQRKLLNEQSGQHNQTQTQTQTKLR
VDLEGVYLVTSPRLSQRLALVPPLRFPADQPPVQDVYRAPLGHVSYVVVRVPAAASLVRWDSRVAAASFQLFVRTAI
SELRRTPGSYLVSASPAEVRATFSSPALAATWALKLRAALLRAPWPDELLLHEMGEPVEAYDNADGGGGGEISGPHS

EGGTPRMRWTSFAAFTRVNGSREGSVHRCGGGGSGSRGGSVSGTTVVAESPPVQLRLRGSAHRRMSGRPSDTRAASR
AELFSTGSEMEEPAGPDSECGAAPLLAAAATAAGGAAKATGHIMGVEAQAVTGNSRSSRSSGSSRSVDRQTRALALL
AEHLLRTTGTAADVAEVGECESADSGNEGGGGGGGGGCSAPGCHLPNGPGGSPGELSALGGRGQSPMGLCAVRRATV
DGAVSLSPAPPSLPSSAPAQRQPSQQPPRVAALSSEFGSRGRGGHPDAGGLGAAASATATGAAAGGAVSPPPSRQQA
AGEGRGTGRLIFRGLRAVSRAVRLSEVAPCGEVLCDRGTQRGILQLQQLPHPLDRVQQQQEEEEGEGEGEAEGALPH
SIVVPMASGGAQLPSRSSPYRTRTPTTPSLSSPLAQLLSPSPPPPSPQQGQPPPDATPGDGGGGGGGDDDVVVAERV
AARELQLVRLVARSRLSVRAMDEVFVCQFRSPGVNDPPAPAATLCRTRVLTRPQSPPPQHPLTPRPAPAAGAPGAAV
PHVSAPTNLVAARGSCSAASPAITPPPPPPPWPICPPPRLQLPSRTSLPLTLGSSPRGPAYSPTAQQSPPYPRLALM
PEPLDLDLTASSGNGISNGNGNGRGGVASVNFGGGARHAAGGRYGRRSTLQPYHAVLEMNNNSDEHQHARLVVGCGG
RRGGGASCPGLLPSHHQPSSSPGGPMGSGAGFGTKSRAGCGPGATGPNLQLCLQRNMGPAVSAAAAATATGFATGGS
SACGDVLIMPPAAVAAASGATEASAPPPLTRHASGWSSLSGASDCGLGPSVMLYTPGGGGGGGGSTTAPSSGLLSTA
SALQHSSIVRMSPGQRFAGRSVTTDGHMPYIICERVDEEVEGFSEGASITVAEGGRGAGGGSRESGCGLIRNQKADT
ESNTRSRPAAWTPGPGSNYGGCSFQGGAAAAAAVAAAGPHPGGAAAAAELAQCGPRDSPPRTPGGGSSCSDQDNANN
NANDDDDMTGWLPGNSGLGLAAAVAAAAAYSSSGSDRQQELPPHVRHGAFASHRVSASASFGAGFGAAAPGAAPLAA
GLTAGAAQGTATAVVAPGLPTERWLPSQQSARTWRRSPSSERGEPLRGGGSGSGSGGGGGGGGGGLRGYRFVEGFGG
EVIDREVVVEGEFEFMPPSCGRGWSGPGSRRGGGGRQGDCGLRAEATVRSLAQGRLMAQMAAAAAAGPFTVVKPEAV
GTIPPQSLPPMSPAILLRIGSSGRRGSVTFSGSSHLYNSGSGSGGVLCNLPTFAGLAQLGPGPPSEAGSCSAGSSFR
LAAPPGAAAGQYHLPEWKQMERPTGHGDGHIREEG

>Vocar20004510m|PACid:23137041
MANGSSAASLDGTVSGAAAVRKSNSGGDDVAAAVAAEGETAAVAARGRLDGHRRRLQQAKTCGSYTVCPAADAAARF
LAGPGVVTSNAVFSTGTCKTTVDSVVSSVVPPQQQWALIPNNATWTPGHAIQSFFPQGSLVLSTGSVQTVNCNFNSR
TSTSTVIDGGLDGDADLDAIAPGQLLYDTVVLEFDVTFNASGAFTMTYIFASDEYNDWVGSSFADVLGVFVSPANRA
SDNASSGTTNVALVPDANVPILVNTVNAAANNFTWISNPADGFTRRLLTKPYNVVANQSYHIKLAIADMGDQMVDSW
VYFGGASLRLVPKLIDSFDVAVPSENDCSTNKFVNLTAKSAVTGAQPVFTWTLTAAVPCMPAISKTGPSLRVPIGDF
IEGFAYTVKLTAFWNGFWDVQYKTVTIPSGCGAANQPNCVVSGIGGNNTVEATPARPTVTVPTMATPPNGGTAVVTW
GGTAIVPCQGFLAIEPRGINVTALLKGLNVPAASFTWGLYDVLDPELSSPIAFAATSYILGNDLSSSVVRFSAANEL
RQGTAVQRYMVRLHVGTQPRWPEELGGWGGGVGSFAPGQTWVQVNACPVSATTREPATFAFNNVSPLDPITLRCAAA
VKLDGSVPRARAEQLNNPKRPGRFTSLIWLVQLTGPQSMEWRQEVSTSDTLYVFDPLKLLVNAGLPYDTYSTFIVEA
QARAVTGDLLTDSKWRIKVLVMAFYSDSKIPPGFQAPAGGALLPDEQTINEFVSQFKASASRALGFSPSQIFILSMG
VQDTAVAIMSRRLAERQQPHAEEDRNSRTDAVQGGNESLDGGFWSSRELEELAPGSGMEMMFERSQATVSEVYGNGK
SEEDAVLEATNQEREALSAFRSLLSPPSPTRARTHTHTHACRRTCKVLISNVYEYRKAIVIGAHLSVIFNVMEIRPF
ASPPPPPRPPPLPPGTSAPPSPPPRPPGWPTLRLGALADAIGSSSLFRLAGSESILVSRVVGAGTVGATRPVVWFDD
PDFLSLRETETILNLVYPNALTCPYTNECGECGTWTIERDPYKLYIYFKDPIQITNIEIRELQNPAVQLIALLPWP
AVDENLFARTDILGEPIYMDDGTTRPCPSTLSVEIPPERSGINLAVRKNGTQENLPKALRSSAAGGVFINFWIATQF
EAVTIVESVRFSGRVLYPKDPQMYSKMTDLSKK

>Vocar20004487m|PACid:23137058
MDVTPSRLACFKQLRDELTCAVCLDICVRPCTTPCGHNYCRSCLRRNTELNRPCPKCRANLPAGHTTGPTGNVAAGS
NVLAFNAGMPTLSQQGAGSGRSLSNNPFLRLPRPAAAMASATASRGPGEVTAVVDTRSAGLLQVVAGRQDTGIVAAA
APHFNASQRALLSACISTQAQAGARDALNRTGSGALATFRSDGMDAAAGPAPREASITSQDSGSCTTAGGESSSPST
LGSNWSTKAFDDASELELLPENDDLCGLGSCTTVNESSAASRGVAGGAGCSTLASNGRCNNPRRPPPEDLRQHPSLR
LDDESDISRDLELSVTTDSGIGNGLTALSTDVPGAAVARAQGNVQDNRCIAETQGTPQPCLGTFADAPSPSWSELDA
QVARAMEPLHISPPNLPRLRVSLRRDPMPMPAPASHQTVSLDAGSKPPEGLVIRTRVCSHSQHNTNPDLASNYDGEE
PSGADTFFSSPFFDRALLMESLQEAAEGSEPGSEVGFSPRWLAGGACDGDWLIDDSGRPFVGDRLLTADAGAAGELA
SGDRLATAETALIALAESGRLQEFVARGTGTSRPVRDGLPSSMPVGHGSTAQAQADSAANRTGLLPGDRGCPIELLS
DSDEERGNETFLNLELRVASPECEAATCHMQYLQNQKNPLVPQEEAFALTVGTNLVRVSSLHAPGGKDSPGNHPVTI
VLHSPTTQQVSGRPATRGKPPAIQAPVGRLQQHKSTSSIEISGSDLSGSIDIMHEDNPRPTAARGPGAAPGQGMRPS
TAGRPPPLSGRSANLPPAGMQPRSVGGGRGIGGGRRPATAGSGGSDSLSISALSSDDLSADLDITGGGGGGAGGGQD
TLVEVLSLEGPKKTKTHITRAGLNQEVVRESTHTVAMSEPESLMSLSGGALGSVSGSLVIDNSEDMGLGVGIGGGVN
EAGRRGMQQGAGRAGQGTHQPQPRMRPPAAASSGTIEYDEDFLGNSDIGVSGGGGLGSASIEYDVSGSNEGGGGMLA
RATASAVYASGAVGRQVAGRQVASSAPRYGDSKTPSISGSTSLSSIGVDSAASGSGAWRGGRDSSQSLQPRPGYSAL
HPDPLNRTTGSVRLSRPQSIGGLTAEPAFASGSSRSGVTPSRSTGDGSAKRSDGRSRQSSSGGGALGVNLMDASALE
PVSGGMDESYGVYSSTEIVRHATDVNRSLDRSDRSRGGRRAVDSGGARSSHSSGGSSSSGSSSYGVGGVRLHGIEDL
GTSGDAVSGGLTQPVASATPEVSYSDLGIEEEAGVSYGDDAGVSVIHGKLKPQSVAAAPPSPPPPKQLSEAPRAGGD
SGSESERKSAGRDLNRVSHRAGSSDGSLVVREGSSSSGSSSSSGGENIMTVDQLMPSHSSEGGSSHRGRTASGGGDD
DAYESDFVAESVATESVGRSLSRAASSRRRGETAATPSLGLPIRQREFSFGRQLAAAADSAFTPRQLAKSGSLSEEE

SYGRGTGGGGGKGGSAGAGPATVPRPPPPPSKAQQQQQQRPVPDPRVAVVADSGSYGSVEDEDEALMAAAVRSPED
DGASRWRRRQQRIQLHQSADTLSPENSSPSLSRERHGGRRRGPAAEDGSGDPSGTFDASASHLSQSGGLGPGPVAPA
WTTTPAAATGRDGGGTTAAAGAAIRGPPARLDPNQFSRDSDLSEEFIPSQAMQRGLTEQPIFRPLMPNTSAPRVSA
GAQGAVPAFTARGGGVISPPSPPLGPSPSPADLRRFPDPHFHPTEPHLPPHPPRPPPEPLSPAVAAAAAPPPPRHAF
AATQTDVYTNVVDLDPVSRPGTVKLRTVAMQWGTLNEASTQVAFDPVAERLMEASTGLYDTEALPPHLRRQLQAHLL
QQQQLYGPFTKRGGGGGLLQAGADALLLGESPGAAFSGIGPGFGILSLAFHSVKTAALLEQLERSLSSATIAAGVAP
PPAYPPHVSHGAGAMPYYPYEWWPGQPGAAAGQPPMTPGQAAAAAYTASTAAASAAAAAQSPPPSPPAASGSPPLSP
PRPAGPPGDARDMDGASGGAAPGPASWPTLRTVTSPGATAGASRPTSPLPLTSSPSRLRTMSVAGGSDISRVRSSGG
FLRESSGTFGVDRGVTYRRSRLSSSEMGDFEDAVEEESGLVGGSGDVETVEEENEVEEENEDVARGAARREKETAEI
EEEVEEEVLEDSSIPEEDVVRSSGSGSGSESSGHYSEDFETESVPAQSRSVSRTTSLLHRSSLVGSTSRGKLNPHLS
ATSFSGRSMLRRGSVVVSTLAPIAGSEAVSREDEEEGSPPQGSRMLSRVGSVIKSSSGFKRTASISITMADDPMLPN
QMARILPKTFSTNASGSSAGGGLAAQASGRRLSLDGTGRAHHHHNHPHHGAGAEAEPPPGGVSAATTAAASSAAGGD
QHAQQQQQQQPGAYPPYYSYPPYPYGTPFGMMPYTPYPPYPPYTPAPAANVASVLQYLPPPIPYVYPPYPTPNATAA
PAPAPAPAHGLIPGHRLPATGATVGGPAGIAIGGSAAGAPPRPQSAMSPGGSHHQQHHFHVKFADGMAPGGSDGAAG
VSTAAAPAVTQQPGARVPGSMQTAGAAVAAFDAGTVQAPGMAPHTSQQQQPGYSYTHTQPPSHHHSQQQQAATAATS
YYGDSPRFMGGVPGGGSFLGYGGFGPGSSSLYSAQSTLQRFGAVLSAGAFPGLDPATVRTIRADLVKTAAAVSGQVG
YEDIVAEAGAADEYRAQLARVRARLAAARASMPSVIPAQEKVVLLRTTTRAAAAAGPTAQSQQQQTHPQAALAGTRI
LEVSTTAPGSYRYTTLEDTRRLIEVTRPYTSVGVS

>Vocar20004581m|PACid:23137101
MILMSSLPVGDLSQYYLTTDAGSMYRLDFCDGLKLETIAPNSRVTISYSRITDGVMYSCQQPQQVGVRRALMGDTLS
TPDKPTILVYVVTLCGYDVGPAATPEQVTNLLTGDGNGRTLAKYYDACSYKQVSLNSAQVKVLGPEEIPCSGSLNLP
FTFPSGNIFDTKSCNNDNMIKWHYYLDTVAARRGITATDYNHKVILLPQGYMALFPGCNGVSGSASIGPWLREYSDV
NKFGTGLVWWAGDSFGSLEILFHEIGHTLGMAHASVPDGCDLIDQCDHTCPMGAIGGQGIRCLNAPHLDQLGWGQPF
RDLSDKDFPTGAKQLLTIPPQMTTSRSFVRLDFSPRPGSRKLYAAARINTPPYDLPYGINQNGQPFIVVHSWNGTST
NKYVRTILLADVELGDMFVDDVSGLVITFVDWDDKKGASATFCRRKGPKEKTCGDGIDDDCDNLVDENDPDCAGRMP
GGAQSDRPVQAPATRSPSPPPRGAWGGAVRLPPPSPPPPSPPPPPPPSTPPPRRPPPPSPLSPLRRPPFSPPPIKPP
PPVFLPTPSVSRGFPKPPPPLQSSSIERPFIIRRHPSPLPPSLSPPRLHSRSPPPPSSSPPPPWIFNPRPPRPPPKE
GFSWDFTLTWSRSPPRGAAVREKLLLFCQRVVLSGVIWNKANSATYCVDAEAGGGSMKRVSFPNTKSSTYIEYIDNP
LAKFFKKKEVTVAPRRKRARKEINLKKTGGHGSGAGEPNLDAAPVPAADVHEDNTGADFSNIDEEPQAELSDDEHGP
PNGPPESNYEAVAKVYLQQVAAARSLLMLMSSIHGGQDASKYYLRQDDGQMYSLSFCSELGPLGLVSNLRVQVNYNR
IEDGVMYSCQIPVPLGDDSPSSTPVSASKRRELFGPTISTPKRPTFLIYIVTLCGFSQPAVATASRMRDFFFRNNSI
SLSGYYDTCSYGQISVLPEQVEIVTGLEIPCNGTLNLPFSFPTGNKFDTRNCENDNLLKWQYYLDDVVSDMGITPTD
FHHKVIYLPPTFVGYRCDNFTGTASLGPWIKTDSKINEYGTGLIWMAGDVFPNIEYFFHEVGHTLSMGHATIPNGCD
SRDQCDHTCPMGPTDGQGIRCLNAPHMWQLGWSQPMRRLTESDLPYGASQEILIPRQMSTKDSYVVVSGVRGLVPTD
AMYFAVRMNEYPYELPFEVNEPYIVVHSYNGTSTVPYMQTVLLGEISVNNRFVHNTSKVVVKFIGWDAVRGATARFC
RRSSTREQTCGDGLDDDCDFLPDELDPDCAGRPVTGNRERPPRPPPQRSYRPPTPRPSPPRPGGLQTG

>Vocar20003848m|PACid:23137412
MSYPKSGRKAGPSSPTPSQAASEGERKVYEDTYEHSTAAVLKGGRSGVTPGVTAAAMPHAAGTFDRPILCEDNDQGS
ATAAADNTGGASTLHRGLLSLPSPSVRASANENASPGGGDASKVEASDRRTNTITDTVVFLLSFCGWKNPHTPESFR
KFWLNDPSTRPDEWTMQNYFNYCSQGAVQLTTDTLRVFVVPLPYCMGEFFDKSTARTKTYDFNTTCGVAEEDAWHSL
SMQYVTDVLGQRVSGATHQIAVLPAEGPCEYAGKATVGCRGSNCRINVNGKHASEHWVYMHEMGHNLKLAHSGAARR
GGGTDEYADYSCTMGRTTPTHASLSAYCENSENPNPPAHLPTPVVPLCDLLLFGHARVKQKFEGTATATLQQAVLGP
SGEYRDVLPFGLVVRVVSINRTSAVVTVCRSGGLREAQGGDSCTDGSDNDCDGLVDDADPDCKDVVTSPSPPPSTLP
LRPPWPRTPPSPRPPSLPSKPSPPPKPSPPPKPSPPPRPSRPPPKPPLSSWRGRP

>Vocar20013483m|PACid:23137582
MHQPDGRAGPSPGSTLPDGSARPSTALPQQLTLSSTPYLSTSARSPLQCFRSSSRPMVVAARAASSLGGLRFGRAAF
LPALPRLAALAFSTQPSSIPKPGFAPGAPGATPPPPGTAPNSAAQAATASPPPPSGISATAASGSTRPPPRGAASSA
PPGGGGEGSGSGGAAAAPTAIKTRSSRLGWVIPIIGLPLIAAYYNYQKEYDKEWDTKVEKLMQEFMAERRPPPRTLT
TAPPPPPPPPPPPPPPQAPEPVPTPSPSLPAAPPPSASPKATETAPLQGVASEPKAKPEAMSATPPPVQELAEAAAP
AAAAPPPPPSPPPPPPPSDTEAAGVILTATEAAQKAADVAPSSTSTAGFAATPAATPVVATEAKSPEEGSTPAPAP
APVQSPEAAVATATAAALALLQTGAVPAAPLPADLPPGKLGLEGMDLSPVGLIQHAVSSAPVVDWSQWPERHAQAVA
DSQLMFDALKTLTRWHEQQMAEVRGQLAAAVQRSEQLQSAGEAAVQRFKALLRQVVESSKEMLELEVRQAEKRTRLE
AENVRLAEAVKRGEQLEQLRLAVGALGLAHEARVRQAAAAQAGHALAAGAAAVAAAADSGAPLGPAAAALRSGAAAV

DSLVESVVAALPDGPVATRRVFRVSELLQQFRRLRRDLSALSYLPGEKAGMVAVAVARLAAALKADESEAVKVLGSQ
YGSGGVDAALARTEALVTAGQLAAAAELLAAALHGTAAAALAADWLDAVHNRTAAEAAVAALNAHAAATWASMA

>Vocar20003971m|PACid:23137636
MRMAIAAFMNYLLACAGLLLFLTPAWKSNVLAFTYPPLIASPSSFTSPPLPSTPSPPPPLLPALASPPPPPPNEDVD
RPPLVKDNTPTSPASSQPAIPPPSPPPSTPPTPPVSYSSIWDFLVKNNSFPTISLALSTANEVATFNDSSQEVTFFL
PTETAFDKLSDALGVARSNRAGLLPYLPVIKRALSYHVLPTRISLQSVANQSVGGTEYYNTTLTMGQSSSIGVRVSP
PSSPPATSPEIFILGVSSTAKVLQADVAAGASCINVVDTVLQYWYNSVDEVFASISGASTMYQALKTAQLLKPANVT
SPYTIFVPTDEAFVSAFGASAATTILANLRSYESLLRHHVAYGWVVTDTTSEEYVRTSYITLNSNNVTVVVPSNDKA
DAGVKPTVASAAVPGSPVFSILNTFQVGIEPQVIVQVINGVLNPASSRQTAGGAAVMVAPSPQLLLLLLLLLAGLML
I

>Vocar20000912m|PACid:23137779
MAPPTVRASQTSVPSTQQRQQQQIDAAEYYGLYKTARTLARSYQAVAHQKDDQVKALTREMEELKLKLQQMEAQQQ
RLTASYPGCFRYFHTAPASAATTCDTAAAAFTGSKSFCHHWRDLQLCDRLQHVSDVSLPAPAPVAAPTQLVYPEMPM
PPPVLAPPPAGGQNLAGMFVRSARSARVAGDAWSARYSEPQPPGLEDAEDADTADASKGKKSKEAKGSITKAFKKLM
SKSWRGGRDENPHRQDAGEGGGAAAVAGTDILDPHQNYHQYSQQQQHLSYPHTVAHNWGPMEQAPPLSLDSHDLG
TGHGVHNPSLLPFAAEPLSMAPPPLALPAAAVTDRSLAAQQQALRPGVKGPLQSQSQVQSPGRVLQFQGSLGRTNPL
NSSTSEIIVARNAHSQSQISSAGIGVAAAAARGLHPQASTSTGASSILLPPSAAAPLPLDSRASAFSHSVVGDGGNW
GVRVSRSAMGQFAQRLKNSVPAPAMRRSYDGSMDMAAAAVAGVSGLVSSTASVHQSWSSDGAAAAAWAVAGSHPTEL
PMPPSANSLRPNSVGRSGGVTTSLSSLNFRAAAAQLNHLPPRPAGRATAVSPAAAPAGPNVLPGGHGGVVAAGVGAT
AIAGSGLTHMIEQGAAPWSGPQAAAPEAAPASIASAEIVIAVRSPVANRTAPAHMTQLSTPVKNFMADAADAAKSTL
KPKPPASPIAAAVVAVPSSEAYLEGILGRLKTPSVLLTASTAAQLAAAVQADLDQVQQQPAPPPPPPSPPPPPTTTE
MADAVVGAAAVASVSVLHLTADEAVQTESPVKACASQPEQGTAPASTATSSLTPHGGEDDTVLRRDLRRSVGVGWRS
KALGYKNAFPSPSFHEKTLPRASMSAGMLQSLASPPPSPPPPLLARCTAMVEESLLAGTPISPQQKSSQAQLEPTAA
SVDLVPMPASTRVSRTAPASPAVPPVMQVLGRTPTRASSSSAAAAAAELFALDSPKAPAPPSSPRGPGGEDVDELEA
VEQRAEAVSLLQSFSPRVRVGTRAAADKTEQSFTNKSEQRAAGGGGGGGSQGGSEDAKAAGAAAFATATKGSRVGDI
ITSIGTEAAENVTGGAKGGIVEPAEPQQPQMMRRMDMIGEGGRRIAVHNDKKVVEAVGATKTKALPTTKTTATATVN
EIVTTTLTATIITTGHVPSTDLDCQPDGEGGTVGALWLAAPVSARTVAASVSVKPARGYGGRYAATVAECDSSSNPS
TSRSGACSTEPSPRRQHPTHDTQFRTHDPSDTKVAGCPGADGSATLALASAKLSDVRKTVSSSGVTHETVSAAPISS
RTGTLLHVADVALSDYPSARSNPSASARLREREGVSNAHSGGATPSSCRQISLAVAPSPSPPVSASDSSAGSWDPVP
SSQRGSSGCAAPRRGIVPSSLAASAATATGPTPAEISQAAGVASGIRHQQLPASSSRPDGGSAMGAQPPPQHLLLLD
SSTGGVTPAALRLQQNLTEAANSVLEPIAARKRGAVRRRSLAAGLDAAGDAMKEPASAVGGASAATAAAGATHELSL
LAPRNRHRRWWSQGDSENLREDVPSAVADELPARTTVSTSIDAAPVAGTSAAAGNIVPTVVSSHCSDGQAVSRRVTT
AMDIKPATSASLEFVLTECQRHHAASVIQSSWRDARAVRSVGAGAEGLLAAASKADKARSVQRQHLEQLQQRQQQLQ
KRQHARLQHSKSAMTIQRAWRAYMAAKAAAKREEHRRQEPVHQGPGLSPSAATPVLVLLPLSQQQPVCVRLPSARMC
SSGGLKRRLFQPVTYSLDGMGRGPLLDASERLRLLRGSMSQSEGGWSGSVNESTAMDAQQPECRRRSASAGGASPAL
DAPFPQPAFMLPDNADDDIGDVAGPVPVKSAPMAAVRAAILPPAGPAVGGLLATAAAAITAGSSAMPVAAAAPAAGT
STPVTRSPAPPAPPPQPPPPPPPQPAHQQQQVRASLDLLSMETAAAALPLPEANSDYSSAATQSWPAGLKISCPPRPH
SESTAGVVALASAASPSTQPTTANSPMPPNPLASPTFLFCGDVVTRAGAVHSDLIHGRTRSRVHSSCGSDIRTVDDH
DGDDGVRDRPMGRGGVSLISASWDGGRMLLERSGPSLDPKSAVRRSYGDGSNNNAAGGGFMDSLRGIRTDVTLLAQL
AGAAMNSCLSSSPARAQQHTPKHQPAPLPPRQPPAPSPPSQGLAAATTMTASITATVTSTAAAIPKYAGADTLPTGL
VVSRQPPLNTAISPLGVPLDSFCRIGTEEELAEFEAIEAAVAAAGSRSGSDRSDALPAGPPAVGLSAARALATEFTG
DDDDEAPITSSRCLTTTPPTSRLLSCSSSNDEGDDDDDRLMLRIEQCELSSNPAPATAPLEAQEVVQERDFVGREEN
NDGSLSPLRQTQASDRTPQAYSTSCLAAGIGSPPSISTATTTTGGDADKLLGVGNAPSPVRWHTSELWEIAHLHPH
TPTPLSASHRFDDVLTCAPPPPTPSTPPPLQTAAAAAASTTQPLATASMRLHVLSGDTVNRLKLRAAEPAPLHSSPT
LSAASGQCQVAAMPATTKGIAAVATTVTGATTAVAKTTAAAAALTTPAAAAKSIPAAAASATAATAKPGGTTSSLRS
SLSKIPSLNTSALSGSAACQSESRAQSQSARSSTTRDASTPLQPQPQQSQPSSASGAMPISAAQSTATTTTTSATTL
KGIARSDSQSRAVLTEATNSIAIAATQAATMAAATAAAAAVLSPSLKTQRLISSSSSSGSSNCSSRSGETTATSVNP
TSYDSATAVAYIFPKSSINKGDTPAAPVAAGAATASTADGPAALDRRSSAVVGMLCPAPSGTAAAAAAAAMLPVAPA
AIGSSGVCSHQERRRYASSPSMISARMTSPMLDGDSDASPRLGLPPHNTLYPSAAPRRLSTGSATASSEGSSPRYAG
ISSKLPHALSYYQNPYFDIGNGSGGNSMAAQARGLCHRRSSSSPGSLEDMSQGATESGVAAAAVLSGGLAAAAAGSA
GVDSAAALCHSSFAGSKRQQADAEAFSLYGGLALSYESSDSEL

>Vocar20001048m|PACid:23137838
MAGGTQFSVISGLMIWRPWDRCRRRKPLRSPEESPYSYGNASRGAYSPPPGPSSPPPPLDFYDSSPSVAYQATGADS
GGYGDGGSGSGGSSPEPYTPDAESAQHYSYDGYGYRRDRSGGSGGGGSSGGPDTGISPLRRPAPPHVLLPRCPSQQP

SNDPATSEQQPSASEVTSPVGGGGGGRGSAGSTSTTPRRGLGAKISGRPSGASGSGSGSGGTPRVGATPRRPLGAPS
PRGAPPAVPSARKDAGGGGGTGRGSATGNRPAVPRLPAWAPVSGIANMRLEDVDEATRVALMLNDPGIDREGVPVRS
PGATHREEGAAAAAAAAEQARSPQQLPQLQLQPQQRHQASRSPGPGESPQPSPGAQHSVRSQSPNLGNRARASDVSE
QFYGQYRLRKHREAHGVPDYRFSQPRSFGAHAPLQQRCFSFDEAHPAIAEQHYARSPAQERELEAERRKLWQEKARA
AIAEARRQYYASPRGSPSTQQLAADVDRLASAREAGRARLRELEEEGETLRQATRQVQRRALDAIARSEQMTAAVAT
GSRSPSPGASPGPTRSGSRSPSRVGYYSTSASAYSRGAFGPPPPHPSLYSHSPGRLQRPASASPGGVYDSAGPSRGD
GGGGLYSARAYYGSPAAAVLASLPEQQEYVGAMYRRSGQGQQQQQQSQSRPLSAPPTSQGGFGAAAPATTTATEEEA
EEVLQLLQGVERLPRHEQQPAAAAGPAVDYLGRARSVATSRQDLGLLGSDEQRIAMGAYDRSRYEEYQRQVDMEMQR
RAVDGDYDSDLERDEATEMLVQDSEKIAAEEEARRRAEEAEREQRDAWMDEYRQRFGPEDGYLPQRRVQSASSAGGS
TAAAVRVVRCGGGAAEGALRASGVTGDAAVKLEQDLALRRAEEEEAHKRRFRAKPVPSAVIEPRYERMLLEAEAKRQ
LNQEQRLDELASMLERPFSFYYRDKQQLEEREALARAAKDPNRFQVSFRARDVPQSTKELEARRESTKKRVEEARQA
ARERAAWEAQQSREPRVFTGYSCPEKPPSQITAWFHLCCFRFAYRRKAADRAYRERLRAVDPALNPHPPKPLGAVPD
FNSLHRQFEVHMAQTRANVRKRVTVPQEFALNGSTPDEQSARARKAQERRQRIILDMQLDAELLPETRWPFKSARGK
ASFLVPDPACKHVIFSLSAVKLQGHCVLRASDRAPVVWCIQVRRTPPPPYLVEWMINKPPDNRAASARKTSTSAAAA
GGAYRTRAEMYGVAAGEVAERKVSEKLRSEALKRADRLLKAAQARRQQEAAAAGGGDGGEEFGLASDVAATAAGGVE
PSDAAGSAGRGGAQRRRAAVAGRQDNPEVYVEARHAQVERRVREVVEEALLDQGIEAYRYVEGIPGDKKHKGARGG

>Vocar20001894m|PACid:23138022
MDTGYTMCFGSGFGFSFSRSFGSSGFDSRGHPDSTHQPGRASGEPLSAVLLSARARRSRARRQDDPPVLHPLQELKG
PLRHRYNPPFGRHESPPSRPPIGPSWHPHMGPGVTGEAPNGAGPGPAPAAAATAAVGHSRCWLRVLLAGVAIRWRRR
RGRGGGRWPLDSTIYIRKAICCQRWRCFLRPADFCFSFLCWEWCPCGCCACCDLRRSVCCAATWQFVVGVRVGGVLG
SGYAAVTALSFGFAPHVVNPDTQFFALMIHLLVFWWLYFLCGIVGCCTSLDRQGILTYDPRQVDSNSNNNNMASGKL
GVFPGPRVPGSQPALCHVPHHAGGAQQGRVEESAQQTGTIPPCGFWEVGGRLRVPALPVPIPRLTSPLPPSSSSSSS
FCSPAGGGDGDGGVGVGGVGGAVNIFLSFLPLLWDWYIIYLAWSLIRKLQEQRKGIFRGRPAMDKQRKPSQLLELHI
GHNLALSPPPDQVIVMLAATAGGGAAQSTAAFDANGPGHVTDTCSVQQAGGVDGLPAGVMTTPQQYGMERTSPPASA
SAGGDGGGGGGGGGWGSPWSEHSNVSSAVWNVADSSFSGACSVGGVGGVRHSSRRRRRQVIDSDGADGGDDADGDGD
VNRPYHGWDRHQQQVQGDGEEQQQQRGPPPPPPPLLLAMPPAMATAVATEEDAHAHMYTRHTLPCQDGQQPQQPQQP
PSPSQSPSPSPSPSLQQQQQQPYGAARQSPTQASPPPPATHRSRSSHSRRRIWPQDDNGDSSQGGVGDPPPSPGPPP
AGGSVASPSGHNGPIERSISSLRSRRAASASTPAASTTATRVRVGSAPTPAAAAAGAVAGASGAAMEPSAATSSAAD
VAAVFEIRRRSTRPRGAPPDGVMTHGAAAGTTAAAALENEGEAAAAPWSRWRGVLGAGVLRFPGGSRPPRKTSGSEA
SYASAQPRNPPLPTLEPVMLSVPATATALFVQQLYSLVVRLIPPPPSDPAELSLQRTQDHLSRGSTPAREHQAHAQA
ATAPDPARSRTPTTAAAAQHPPAAATRAVPLDLAVGVPDPYVGLGHEEDTMSHAPPLSPVPGTAPRRRAAYAAPAPW
SLGSPSADPRATPWGSPRLATRSTSLSAAAAAAAAGTRAAAAEVQVTEEVALGATALTVAVVTGGEEPLAGMFGESR
GPALPYTRPAAAAAALAHSNKHWEENDTGVGPSTASGSPPVVGLDQAGIRSPESAGGGAVALSSSDDEERRQTAAAA
AAEVKTTRGSSELLPPRNRPRMGSPASLLLPSGLTAMAVAARDGARIMYDAYVPYVGRAIHRSSVGAILWGSGKAGT
TAEMGRDTAAPKAAVAVDIQVAQEAQEAKQWIH

>Vocar20001901m|PACid:23138052
MEAWALAAGEQNIQVTALAVDDRRHSLGGSTTTTTASRAMLIRQSPMEISRGPSVGSASRHSLGDVRDQFQSDDDEA
EPGADSLDDALSLTELQSVYHLPSKEAARSLNVSLSRLKRSCRAHNVLRWPHRKLSSLSNLQETIRNDKNMRLLDKQ
RILQQIVNEISAVRANPDHTIDPRLDEIRQAKYKLKYYHKTKHTLGSSSASSRKRRQSAPYPDQALNLNLSAAAMAP
ISAVAAATANTTAYNGEGVSPPPPRQQQQQQLLLSPSPWASPGGRPGPEGGGPFATVSAPAAPAASLYTATVHAASA
SAAVFRGGGSGGSGGGGDSVDGQLAASFPSGSALARFTSDRASFGGSGFCLPGVGGSVIDEMHLSRDGCSAHAVGGG
IGSGCNSSSGGVGQVHRDMTMMGPMAPVAAPSQRPTQDSRLSEPGVGLPYGFPLSMQPGPAPLAGGTTAVSLNVQHS
QHQLQMLQLQHGHRENHHHYYAFQQQQQWEQQQQQQLVGGLRRPSVGMMDETTGVVYPLPGMGPWGRKSGRLSDASM
DGVSSAPPAEGAAVATSVAPNHSAPSSSLWPSPPPPLPPQSATSCQMQLSNPSPHLAQALGSPPLSTGGVGGSATAA
AATVTAAAALSAGPNSAGSVSGGACAAESFPLGACAAIAPHSGANNLSLSSSSSLAANGFAAFAGTFGGGNVDGGRA
SGGGARLGAPYSHGVVNTCCPPPRCSSTGDPHIMAAAVAPPPGLAAMALPRQVSAPPPMYGSVATGGGGGASAGGST
CSGGGGFYGGSTTPMCGMPDGAARARFLSTDGGYQEALGMPQPARSSHTGGGAVLSYSTTAATAAAGGVGSHHTTLH
FEAHVGISTVGSAALASSSPPWFGIEARVRSGIVPPVAAAQASAAMAPAPEDSSRTATTAAALETHVYQEQQPLMQQ
QQMHMQQQQMHMQHTQGLRQAEQRHHHQQDCVSMQHQHEQYQQQQRQHARQHPQYQHKDLQREQHMHQQQQYQQQNQ
QARHLRQAYLHQQEQAQQKEQVRQQEQQQGQARQQQIGHGHVHAGAQAQQLVPSSASCMAPLQMTAPKHQLQPLLQQ
AEWQQQQRPFPYVQQFGGSLDGGNSGSSPPATETGGFQNVPYKPYGMDGTAARHSVVVKTGDGAAATIAAAVAKPPL
LNAAGTDVRMSSSTGGAAAALGWVGREESSPSAAARGSGASDGCCGELPISAGGNSASGVAPNGHSVACGTSSTTFL
PGRSVTTGVPMRAPSPLQGRPGQYPQHTIADAMAYVGASWRSPVRGSAGADGTCADAGGRAWSKPSASVEAPEGLLS
VQESSPPPQQEQQQQQQQALQQPQQLQQPQQVQLQQCYAAKLEPWGSSSDLFCPFPHDGASPTVAAVAAAGAPAPVP
APAAGPSAAGNTGGTGLGTACGRAETAATGGDGCDADGSAGVMLASFDWLLE

>Vocar20001912m|PACid:23138067
MMDGTASVARMRVCISNKTKHSINVSLMMTVSGRMDTVHLDDGEGVHNSRPAVQVQAPFYLGASPALAASDAFGQEP
SLGTSIRHRLQQARQALSSPHDKEIAAVAFPALVGLALEPVVNAFNAGACVSRVAAQSMWVALLCGVATAAAVGLGA
SNIVAALNPPEPAVAAQATQYIMVRSLAIPAVLLGFVATAVFRGFKDTRTPLFGTGTSVAVSFGLHVLLLNVLQLGV
EGAAAAAALASVSSCCVMMGILIRGGKLRLRHLASPPPLSAILPILQRGFVLGARNMVSFGMILFASTLCARMGSAF
QASFEVIRQMWMLTMPFFECLNVATQSLCSVALGQQVLVALPLVCAFFPIDAAGSIMDGSLLAAKQSNYMSAVQIAG
SIVQYVMLMGIAGTGVVDTLSIWVPIKVYLVCSCYGVAPTGWAHPHPHPHPHHCNHQPPQPPSPPPAAITQLTSPAA

>Vocar20001953m|PACid:23138093
MNGVNHASRSTPSTVGLGSTDGELRGAGENLIVNADSTSIKPTQPSSLLPPPPPPPPPPPPPPRKFSSFGPLVWCYFN
FPQWPDMPEMPPSPAQPSSPAGFAGRDTAAFESPSQPPSPRKTATKAVQQKPPSTKPPPSPKMRPPPRLFSPPPPPS
PPPPSPKPPSPSRSPPRPRSSQPTSRPTTGSPAPKTPRSCGYPFRNPAGICSSLLLATCSYLCTKMPCQPCTVIRCS
FAAYPPHLPSSPPLEMIPALAGLPPLAPT

>Vocar20001992m|PACid:23138107
MDPPGPPHTTRLAVHFRTLVAIQSLQHAAHADGTANFSTTAAAASAAAAVQTQMPAGVHGNSFTLSAAAAVAAASPK
PGQFAPVLLIDRVVFEHGSDSFERLCWGGGPEALLAAATKQHVYIFSLRPFFQASGGPAAMLPRHITPSLPLPSTAF
AVSGVYRTAMPATALNFTQNSRGLLLTDVGNNVMMLKLVPKPGLGLYQSSSQQQWQQPQQQQLLKKKKTQYGIMTAG
TAAATAAAAAAPVRFSVHEAWRVRADAAHTLASASFDCYSPCATTTAVAAASGGPHKVTIWWPSQPSSSGSGHLGAS
AVGAEIIRHPVRVLSLDWSPPLVGSRPAEKVFAFDSEPPGLSLTPFSRPGMRVCWARPMADIGVAAAVAAVPSPPPT
GGGGGGGAGDDREQEGNDSSGGAAAAVESGSRLQWIVASVGILGSDEGEHHDSGSGHGGGGGGSLLEEAVCVWAVDG
LSAVVLSGLMPRGAVSSSSASSSSAKTAASPKAVLWGLEHRTVSWLQPGKLLPSPDSALSLDHYRISAYVTYPGGMP
LLRAADSCIGMQGAYAEVRTYQIAPTATSTPGGGGHTATFLRVGAVARQAMTGSPETISNMLVHPAFDVALIRDRTG
GVALWSLTPLCELPLCVAPAAASARRVLLAAAGQLAAVAWLSCCSRAPLTPQYGSGGSGGGNDCVFAYAACIHGTKL
LIYAVSLPCTAAAAATTAGSAVRSSTAATDWGATFVSEVLRVDLPPDVGAVRALYEISYDTDGDAPYATATLIALAS
RSPAAPDSATAAGTRSVGSGGGAAAARPGSFGGAVDGSSFSFSHVLHQHQQDLPPPAPQDVWVTWRLLVQPPCASTG
LRTIELVSQHVDGMAAALATHGSSSTHGSPGEVVAEDAAAAAAGPQAEGRIVSTGRRDPPRATAIVSCCCTSLPSPI
DGGILADASGRSGGGGSCSDAGTSTVEAVGSSSSNSSSSSSRTRRVPCLMSGTSDGHVQFWRPAAAAGGSTASSSGG
GGGGGLELVQSLPCGFRRRSSASGEGDGTAAAAAAAGLVVAVALDEMSGYAAAATTCGDGEQEEAVHIWRLREDPDG
DGSGGGSAATTFGQYELEAVVPVHDTVSALSWLAAAGLSACLAVGFASGIVSLMVRDRVGGWREVSSYSGTVAVAAF
GSPRGAAVVMAAGNHLLCFSNEMDSGSPDGRGPEPTLAAVAADAAGPLPPYHPGVVRALVAKGKLVTACHVMRRLLD
WLRDAADAAVTSREASDGGVVAGGGAAVASSAAAADGAKLGVTDGTTVARGSKRPMGRPPLVIGLPELLGDSLTASR
GLAAFQQALTAPVLVAAAASPATPSPPALLPVHAAAAGPSGDATSRTDAVYGNLARGASGGRADDPSAPLRPFLPAA
VAGGGGGNGSTVLAAARKQPLAAAYGGSALDSGVLDMSAFGEFGEGGGSGFATAASPPPPPPQPPKSVPAHPQETGL
LDMSAFGGFGSLSTPGVATEPPRQESPPRRSALETGTLDMSAFGGFNAAAVDTTPPPPLLSTAPPSHAEATFETDPN
MSAFVDSVSMPSKSTTASPVPPPQPPPAEPTLSQPQPLLSLPPAPLQTGLLDASVFGDFLGGGGGGGLGAVLPFQPP
QTSSQSSPPSQRPAVQQFPSRSASASPKSSTHTAPLGLAAAVGGVGGGHVSEAVLPLPPPLSSEQLSSPLLPNTTSA
TQSAPQAGPPSVQSSALQTGTLDMFAFGDFIGGGMPPPPPAAPPPPPPPPPPAPAPSPLPQQRSDPMLSGMLDMSAF
GDFQFAGPAQSSVEETDPWVSGTAAGATETSSAAASAATDAAASMQGTSAAVAPAAELAATVAFSAVHSPAVAAAGG
RSVATAFAYPPAPVRSALETGAAGGHPAAAWDTPSLPPSVTAAAATQLPAAGPSGLSSDRTGSSSLLFTPAATLTQQ
DFGPQEHASPAPYGTAASGAVAAAASDAADEAENAMVVFPRVPEPATPADPNLPDLLLTPSEVAELHRLLGQNPPSK
SPYTGGTAPAASGGGAPSSPPGAEAAAAAATVPPGLSVDGSAADSAVVDAVTALTALGLTPDDGADLCRLAAALSRR
SVEQLGRDGGASSSTTSSVAADVGTTDVTVGLDHPARVFLIQARVAAEGAAAALAAEAGKDDGALAAAVNRLFRPVS
VTRNTPPPAAAAAAAVASGSDGAAAGSGVDLTGFGLRGKSSGRDGLASSIPTPSATAGGAAGNPPGSFTFPSMEGSS
RERSSTTKPTSSYSYLPYARGTAPGGGGSGLHTHASSDTVSRSRSVMSVMSVLSFSSVPEHAYGARTSATGQGDDQG
GAGGVQSSNLKIADFLLRDFNQAEPRKSAAKNAFALLGQHRYELAAAFFILAGQLYDAVSVLVREQRDPQLALLVAR
LLDPPGSGPGGPVARRLIEQELMPLARVCGDPCAVATLEVLAGEPVRAVLHLVDSRSAAAGGTASPPPASPPAAAAA
LDVAALDYCIRIGVQYMSGKTVPLAACRALRQLALRTSRALEAAGLAAAALEALLLAAALRPAATAGAPQQPLPPGA
RRRYNRLVASCLSFGLSEVQKRVQEQQQPQVAEEKVSAAAAATPSRPCAAGAVPAASGLTWQREALTALTAASAVL
GPAVDEPAVLCLLGRHITALERIRQQPLVPPDLIMSRPRPPMTHGSHPAPPAPPRQAAAAAAGVAPDGSGSSRREAM
SSASATAAAGLQHPLGSAAGGGGGGALRTMHPQPRLSTQQQLRPSTSSQQQPYPYMHQQPPRPSPHISTAPTAAAG
GPPLLLQPPPRAGSSGGAATMPPDTGLTMARGGSTHGGGSGVSSVERLSIDSSASSLPGSTASPPLHAGPPVDGSSY
GGAMSHRDRTAAASSTSGTSDVLRRGEGGGGGGGMLPPQFPAGSSWGAFEAAWDVMAVEGDRCRSVTVQRGRGPDEE
GQLPFAVATAKSGLLGGMLGPPALLRPSRPAGPSSVFLGLMQCVGRHGRAITFVGDSSSQLLVAGQGDRGGLVGWWD
TLVAPAAACVAEIRGRKAVPGVLALLRPDAGGVLVFGDESGELVATDLRMMSAREFIWTLPRVHSGPITALAQWGPA
APGILNLPQQGSPLAVPGFGSGSGPGSRSSVLPNPQHAQHHFSHGGPTQARMSNLLVTGGRDGSLALVDISSGRVRG

VTSGASGWHTFIIPLSLPVKVVSFLEKAHYTTRTGLPGLLAAAAASAGGGGGGSGARLVDRSTVRRTRPAGTSGAAV
GGLCCMPDCSGVLSCGADGVVRYHPLSQQLLDLRRG

>Vocar20001990m|PACid:23138112
MRSLAALQAPPDALRTCPCARGTRRIIPHRFRLSKSKANSPASDMARVQQNFNSAGVGLFFSLFAGSQSLALPHVAV
PDIRHVDWAKLRSAGFRGLVFDKDNTLSRPFALEVEPSLRGALDRCLTAFEGRAVLYSNSAGLKQYDPDGAEAQQLE
AALGIPVLRHTEKKPGGGCAELESHFGCPAADLIMVGDRYLTDVAFGNRHGMLTIHVQPLTSRGEPLGVLMARRVEE
FWVARWTAAGVHPPAHTRAPYKSLQGFITEPSPPPPSPPPLRPESGPPPPSAAAVTGSSAAVAAAAAVLPQATPAAQ
K

>Vocar20002058m|PACid:23138131
GPFLIGVPGLARPVSVLAPITILRPLSLRDRPLIRNQTLSMPFTCAHRLDSATSRYGVTDKTDFAGKPATGLQLKRG
SKSLVRECLLRICNDAMVKQTRGYRVWDSASEGALKAGVRKHGLGAWEHIRKDPEFAILSDRTGVQLKDKWRNLVKF
RHIDPDEQRTYKPKTQGPWSKKYKRERSQGLTGTVSPVSGDGGGGVSEDEGTQHVADPQALDDACMHVGSSSSSTID
DWKLALRKVAVLQKVHVKQGMGFVGLWRLPGASCSEQAATFYMGPADVMARRSAVAAAAAAAESVVAGSGVAAASSS
SSAAASAAVTRTPNNTSTSVVPAQRYQRRQSRNSSDENCGANGVIGKEFSWPRPCMHMACIRQGKPPLGQQFYILLP
SAAAAAIDTDTLKLLFLLLEQHISIEHIAQCGVASSVTLNNRFGVWLRTNVCMYECMYITYAHTYVCIDVRLPSWGN
DWSPAAAVRKGANMIGGRICRSHGSSNSAIAAGCATVLAAAAPVVAATAAAVAAAAAFWRQSGRSNAQKRKAISDLA
LMLEGDGADFYTGSEEDDPAGAAAGWSGSGAGAVSTGAGAADGGGGAGGVVDGEGGLKRGRPRSAARSSVGRGSASR
HRKAMEDRRFGIYDRRRYGFAEDGTALRGRSYDGEGSGNASESDGEGAVGAGAGAGAAPGTALPPKGRRRGGARRGR
DGSDDEDEEDDDVGDPDSDSDGEAAMDEAGGAATVAATGGGGGSGRSRGRGGGSGAIEEDVISCPCGVTYDDGELMI
ECESCAAWAHIDCLRQQVATAPDRYQYNFDTYHCSGCQAALRSLQHGSRQPHGYGSRQGGYSAAAGAGTGGLEAGGS
VGAGLGSAAATAAAAAGRSRRPPMAQGMVAPSAVRNGVHRGPPAAPEQERQRDGEGEGDTASEDSQRKASELSNILL
GLANGRIVTGSSSGAAGARRGGGAAAAAGAGGSFSKLGPFGRASAQPMPGSASAHRGGSAGGAADANGFGGGAGGS
PIARTGSVGHTSSSGLGGAAAAAATAGGDQTASFARDVSGSGRLVKPGPQGAAPDGLRKVTPIAVGGGGGVAATAAG
STGAGPANSSGSRGGLDGAGSPPPLLDGGLPLFQGSAPGAGAGGGIIGGGGCLGVSASGPKIFKPVPKHATMGGLS
GGQLQPQQPQQAPQHHLLSAPKARPHPALQRGASQHSLQRMLEAVNPSAAAAAAGGCGGAVSGGSAAAASARGLPLT
SVLEAAGSLQVAAANLCAASGRKAAAAASASTGGAAAASDGVGSCDAAAGGGGGALPPRPPSRPPSSMAREVANRIF
GESDDDDAGGVLRPLSALRGGDPWFLMPASLSRAATPSAFIDSILRATPDIVPLPGTSPPPYLAAALAAAASPRCTD
LNALLVQQQQQHAALLQLMSQSAATAPSPASAGLPPPAAAASASAILPPPPLARLPPPLAGAGLLPPFVPSPLTIGG
LPSAWPASTPPPPPLPPPPPVSLHPPSNNGEPVALHPVLRNLLITTAQQQQQQQQQQQQQPQPGDGVPQQQPHQIG
PGSGSAGTANGGGGATVKEEEGAMGPPQQQQQHSTQASSGQKAAAAAAGLSSGGGPAACLVVSPLGRPSTTPPLPSA
GSSGGTAAAAAAAAGPLRDVAMAAAAAAAAAAAAAGGSNGSVPPPPGLDSSGSSVAALFGLGVGGLNLSVLDHLRSSS
PDPYVFFRTSTPGPEAILQSFFPPPPGDLPWLKSTTPPLTGA

>Vocar20001932m|PACid:23138137
MPVASQGKVAGTAAKTTAGNNAASKPKSDATAAKSLGPVSPKQAAAKAAPASPAKKAAGNGAAPRPAADPQPANAAP
PSSAAAKAGAVAKTAAANEAAKAQPGRTAQAGGGNGGAVAAPATATSAAKQASVILSTAQPVAPEAPALTADIAVPV
AAAPTAAVEPAATGGAATATVVAVSPVAKADGTGDGDSGGGVVAAVTGSQTPEGSGGPQPPTGAAPPPADTEAVCAG
AGATSPPPLPPAVGPAVATADTVAAPPPRSDGAGNGHPEPRTSLSGQSPVVLAAAAAGEVNGAGGVSAPAAASSTNP
EVSVRTLTTASVATNAPSLAATASVPGAAAALTEVTPVTQIAPAPDVDCIAVKPAPPAPTIKGPPSLKVISAPPLRS
ASGGGSGGDSGSVPAAAAAATDAANDSDGKVLRAPLSTTAEAAPKPPPRTRTGSAAWVEEPSAEAPPRPAEGAASAP
VLRFDSSASRKSIRATEVEAEAEVPQPTAPPTPPPPAQPALSLSTHSPPPPSLPRALSDPPAGSVVVTLEGNPGGPC
PVELAVSALVPGGVGEVMVADLMTAAMKALRAKYGTVQAGAAVSAFFVESLHDPGSRPGDQRRLLLPMFDAVPRELL
AAAARGGGAASVVLQGVTCGEAAADKAASGRPPVAPAAVPPVFPLPGFTEPSESGSGALPHTRVTFLSENGSGRPSP
NQPGGTANAANAALLPMRSLLRGASRGGGGASGGGGAGAGGRILGAGHRSVRIRLEENTVETLCVESAAPSVTSGHS
QFRGAKLPPLRTDSDASRGSDGDGDGDAAATPTEERSTGPSAFRGRLGTTRSSRRLLVPAYDIAAESIGSSDFDQVP
PPPLPPPPPPAAVVKQDPVQILMATASVGADLEARDRVSNTGMDGVAGATGSVVSAVGKKSSALFVRPDAVSPSSGE
DLEAAFFLPPAADWTQLMGRGEVEDAPEPSAGGGGGCLPCFSSGSKVAQQRQWRQRLRAALDARDLEEVAAWVGFHG
GRNGTREQEELDAAMWLAVEARAPDIVRVLLRGGARPDAPRPTVPSAGGASAGRGSAGNSVSTARPASASRVKDSSV
AAAASMATAPPPSIAWWPGTMATATGALEGLTPVMRAAELASHAVVEVLLAGGANPMAVHPASGWTAVHRAVLATPP
SQSAWANRRLTPTGTMAATAAGAGSSNSSGSAAAAAAAAAGGAAAEPGSGDGGGSAAAGLHCLRVLVQHIGLQRAAA
LTTDGRYCDDLLRFSVRYGRSAALKWLLSAGLSAEAAAAGVDDDNNGDDEASCAFLAGLLGLTLDVWDMPPPLRPPS
SAGPLRDPARPLSWLYGPEERLFADRRRQLLMGAAAGGRVEVLAAPNLPIPFFSSPLFSSPNGNSTAMRTHDQILDK
PPPFDRLSRDELEAASGACRLAQPRNQRQVEELLTRKAHTYMSWEREVVAALRRFDEAVVGDWLLRDRPSPLEVDRA
LTTLLCSERLGSPIWQDSATASVQALLDAGADVEHEWVVDPAAAPLVAAVAAKAKASTRDLATLRPLQVALLAPAPA
TVRQLLKAGADPRRRDADGENALFFAVRVAAAAAAAARENDDPDVVRAVQSQPSGVSRANSIGHGDAAAAEAAAAEC

IDVLLATEHVAELLAVRNDKGLTPAVVAVLAGLPAVLSKLCAAGADPWASPVPAAPGAPDVPRELLPSAAVTGSGKR
PAAAAGLWYVGHEAARMGALSALAVLLGGPSSAVGRKAPLAAVSAEQAAARRRDALIGAIRGGRVGAAKLALDADGL
GGSLYEGELAAALALCKASAAAPPLPPPPGAASIVAAATAASGADPPRYDTLAAFLSSRAHVYMSWEGEVLPAVRAS
NVGVVRRWITYDKISAADATRALCALVEAAQQCNISGSTVDAAAAAAAAAAAAATAPGVDAADGVLSRLSSVAAATR
LTSPGKAAADVAAELVKAGADVNFQREPSAGGESLLMQVSLKGHLPLLELLLSLGARVDAEDAAGLTAVDHAVLARA
RLADNGCLSALLAAYGARRLNAHRTHAGDSALMLAVRAGDTAAAQLLLSAGATPAAADASAPPRTGSTTAAAAAGGI
STAVGAASRTRSSQYYCDGGEAAAAAAAAAGGGGGDAASTSSAPISSAPSGSQLVPSGPRTLVTLASGVKASGLGRR
GSAVYLAARMDDKAMLQQLLRMASADESAMESCRLEVQLGALHGARLALLEASLGLPELLRTVPGPAEGGGAGALGG
AAAVCARLSEQAAMRLAEVQGLLRELEAAEAEAADAPPAAAAAVAPGPSGPSAATASGGPSPLLASGHVARLRRYLT
AKCGVLEGVRGAQSRMGEFYGLARMHLESLEGAMEAKKELVSATIRDGSDAQVKLMAATTRLTASRESVLRSVLARR
DAQLGRVEALLDKKLAGLTAAQKRAEAKATSEVEGIIMDMRKLVGRRLKEIEAKKAEGLGRLGALERKLDTVWGRRQ
RLLAGRPPAARVALNQRMGALMQQLGSRREALNEAVAAAVARMEGGQRRGTALVNLEGMREALGRRPPNDPDGGGLG
PGVALGGQPSAKTAVAERAEDLLRNDKHDQLLRIVGHMTAEVTRGSASTAASSAKTLAALQQAFAVAVADLERRVAL
ARSRMQEHLSSLVGTHSDLVKALVAADEPAAGGAVQPPGAPSQEVKAAWEDTSSSPPPTPPPDVATMLLGFEGASAL
EGVEGVPRPGSGTARGQEAGGGTDGSAADGDGLSVLRRVGTVEDMIEDMIADMAAHVLRAFPHDGICDTLAPSDLTA
LLEPDLDISTAGLGLERLRQDIAAIDFGLPNLDLEIGDLDLSALDDDVGGAGGMRAFGVGGGVGGMGMGGYDMYDEE
DMDNGGGGGGAGAAVVPPPGRYGAGGGDGGGGPGGYNRHGRGRNSEDDGDTDPGGRGRYHAADYGDRHEDDFALSYS

>Vocar20001893m|PACid:23138151
MAQAAGVAPLISGEHGATSTNSSVDVIAILRNLKLRRESCTSITAAGLASPNYEKLLPRSRSNLHAEAGKSFASGLQ
SYGTGTITFDHETNNGDTNSAAAVAAAAAEVARDIADSVGGRQSPSARLRSPTATKPAAAAAAASVPAALGSVLTTA
VNSPSRTASLHGVAANNGSGDGGGGGGSPPLYDNGLSRLHKSSLEYQIARLRGALESHVSAAAPTVPTASTANGHFS
NGHANGHVPAAAGAAMMMEFQRWSAASPAPSAAGPSHDSGNAGRLSPPPTQQKAARPTAPPADADGKSSGGGSGGSA
SSSGPLSPTSYVGSGRGGVGSGAAPPAASAVRTPPGAPSSSTGTTLAAAAASQRAQLLDEIHLAHKRELALAEQLGR
ARAEVLELTAQLVRQVEAATAAAALADAEAAAAAELRHQLRKERAEAGEEVRRWRSRAAAAEEQLQTALTQLACLRS
AAAATAAVGTTTAGVAWGPSRHHSAGGRSSSGGDGGGGNRGTASKDAPSPIQPITAAAIADAGPSPPPSQSPVHSPG
SPSSSRRRRSLRSSPSELRESAATAVTTPTPLRRSLSYQSLGVELGREAAAAAAVSKTPRAVSTGGAKEMGDEYASD
STDATAVVSAAAVTAGVSGDGVPPGSPQIIASSVATAAAAASFSTGPQSGHRTAASAAVAGKAQTARPPRPPATAAA
THSPSHSVAVLAVPDDGGASTAASLTSSYGASYGSPPSQGCSPQGSSAARDGGSSSAGGAVRELRGDSEYGNAGVGP
KAAADDAATCTGSGSDDSGDGGGAAGVEPAGGWMTWAAAATAAAAAADGGGGSAAATAEVPVSYSVPIPSGQEQSVQ
LDSPAAARPSRLEPSASACRAVPGVHVTGARASAAVVGGAAAAAARGLPVGMAALTAVPEGADGTAVLQSEVAPAVP
LAEAASFRGSVVSKGDAAATALLVSEVERLKQAGNRAYRSDQFPLARHCYTCALELLLPPANTGGSAGSSSGGGAAP
ARGAAAVAAGAVSSNGGSAEHPTPAAVAAARDRAAAAAAAAAAAAGRGVGFPGERRMIAALYCSRAAAHVAMRLYTDA
VADCCAANAHDESYAKPWKCRAEALASVNAFGTAAVALEQCIQLVTEAAAAAAAAAAAAAASAPQPKGDGGAADSDIA
ACATAGGGGGRDHLVENSDVDPRELPLLRHILQRLQELARAAAAPPPAAAVALLAAVKGAHSAAAPPPVDYYAVLGV
PYHASAVDVRAAYRRLALRYHPDKAAAVAAAPGAGAAMPAAGAATALSSPMRAAVVVAANTVFPMVAEAYAVLSDSA
SRRAYDLRRARVALSTRRQGAN

>Vocar20002105m|PACid:23138155
MRAGREPKESQPWAAQKRGSSQALPVILDDDEETGSTSCGAPDSSGPANELLTSSGDDEQWSIGNEIGVDSGFLEMS
SVSPVGLYSTNGLRGRIAALPFAQGQSDAASIAGYHSGQQQGEEAGDATVGPSRIEAQAQQRLVSVLPAPSSNSSP
NAPLLSKRASSSQRLAALVTDAAIAASSAATAMGGALYGRDEITVPRRTTSPDASPAPAPGRQSIVVPTRSSNLPLH
DQTPQYGLPPPQQQQQQQQRPQLGLQQQRDRPKSFGRSRSVSFAGASLPVPTGAATRGVGLAPSPPPPPPPRSEAGG
LPAAAAASSSGGGMAAEISFTNNIIPTTRNSASSILEHRDAVQHSPSYGELPNLQPQPLEPQPPSLTTQPQPPPAAR
LPFAALRLQAGSSQGRLRISPRGTSPPPPPITSPDGTNVRQQNSQQQQQQAWWGLPSPGAAAAAAAAAAAAASRLGT
TAQAVAAAHTAAVSPIAAARTISPTPMALSRQVQLSRGPVGTAATADGNMDNEGVWLQPLSSPSATSTSLLPLPPPQ
QQLQLQPEPIQPSYKAQHDTQDSSRQPQQLLLLNAFSAPVLRLTEATIGLGGAVDMAAAAGAPSRPGPPGRTTPPSS
LAPFVHTRAARQSAPALGRGLQLPDLRTAGGTAGGAAAAGAAAGSAAAAAGPPGEAHISGVGVLAAAGGAAGPAAGE
VPAAPEITLRPPKWLKPSAFQVTEHLRSGRLQELFDKLTTVNCPTFFDLGGCEFSGPFTPAAAAAAAGADGSVGGGA
AAAADLGAALATAAAAAMSNTTPRHQTLRVGFNQTVATGSAGHLSARSRYLRNLPDTRLQVMSIPPGQHITLYNATL
LLTADQYIHVGPGASLTLKGVEILGCSREAARQSAAVRRRALSMAAAAAAAASASPGAQAAAAAAVAAVTSTAATGYS
KLVRRTSSPGRTTRDAGVYGVGGPGRRASVSDGTSDGNGGGGGSNGGPGLPPVRRRAVSTMGGTPFVLSIPVPGSGG
VAAATAHELVDPNEYVDNHGLVEVVGGYARVSNCRIWPGRCQMALCVSDGGGCSLLYSTAGACCAAGSKSCLVSEQT
TYEGGAAGCVSALHGARLGLTDCVLRYADNTKPAAAAAVAAGAAEPAAAATAANGNAGGCDLSIEARGSGTLAVLTN
CDVTNGGVAAALAARLYLQSTRVADARGHGLLSCDPGTALYVRDCRIDDSGRSAVVACGGAAVRVADATLAGSAGSG
VLVVPTCECRENRESGLCVTGAAAVELSYSRFAANQHDGLSVGGTGSTATAHGCILNGNVAKGAVVCMGGAAEMSEC
AVHCNGSKSVQVSDEDSRLVLGRTCALDRQAVAIGGGSLVYVDAFPVR

>Vocar20002016m|PACid:23138203
MQRSRSYAPAAKTANRCWGVGTANLPSKISYDIGGFAEDDIPKVPIANANNADGGDNPAQPSSLLPPPPPPPPPPPP
PPLSSPFSLAYQFIWCFLNRAQWPDVPDMPPSPAQPSSPAGFAGRDAAVFESPSQPPSPRKTATKAVQQKAPSTKPP
PSPKMRPPPRLPSPPPPPSPPPPSPKPPSPSPSPGRPRMTKPPPSPRLQTSPRLATGSPALKVRVLLELCSRTGFGV
AVHMYGSLSGSLFGGSSHGVPKGMFHCCVRLDKLLLPAYVPCPARPRVLVLGNRLSKLLVL

>Vocar20000261m|PACid:23138345
MAPPTHRPTHRPGAILSVTVGGRTGWEQINNNGNDNNNGNDNNNCNDNNNGNDNNGPGRSGSGGEGFRCGVFAVSRA
GLHPRARNPQEETDVRTSFASFAREGAQRTTAPQPQRLPHHQTTHPKWRQTIFAVVPNPSFIFPLAYIVQCLFSHAP
SLCSTPLPLVIPVPSDTYRLTLMLLAILVLWTFGSGFFIRTANAYDFGVVLSLSYSFPNGAPSSASAVAVNVIMYVV
MMLVVILVSVHINRYDPFLQVLGITCVALFGIVLSRRTHAPALEDLGDHTASSGSFFLTQDLVMQRHQLFILSAYPA
GVLRVQIFVWMALLHMAVLSTMRRIMPLMVRHNWLLVRCKITERRDLLDPAAVASLIDFPPGYSPTPDRRVAACYIR
YISPWWAPFLGHYESITYVGELDAGGHPSGYGEWRDTHWHGEHLTGFWHGGLPTGPFRSRETGSASGFVNLRIAYFR
NRGEALRKAACLPRLKPLQFGIVACEVSTSGAWFKSLPRVRELLPPVDIAEGLIAEEDAAIEEGRVAPAAVHRPVRS
LTVPTGMPGMRHAATGAAGGGSGSGGGDGRGMTAETERVRSSGSGAAAAPACVGILKRHDGEAAALAAAATAAAAAA
AAATRTSTGLTGTGAGGSPAPLAAAARIGRLMRTLTPLSQLMTVVPRRVSGGDTRERGGGDFGTGVASRERAHGSRN
LNGLIEAERALRAPSLRHPGGCGCNGGGGACSVPTSSMASSPPPPASAPTASLVTGLGHVPRLVSMLMPLQPSQKTS
TKHGHNRDRAREGAAKAARPAAGTAGPGGRVDGGQGGSWDMAGIRPWGPRRQWGPGWGSGTRSRELRNAPGVTEAMA
AEVAPAAASPRSSSVRMRLRVRSAAARMGSGLATPSGGVRDGRLSLVGRLRQMQTVNPAAPSSTAAAAPAFADGPGG
PVHHRALLSFIRSAQPVVATGQSGDVAAGRTRPDGATAAAVSSRGAYSCMEGNQSTATRGSRLRLRLESVRLGPGRA
AGTPGMYREPVRRLAATAAAAAAGTSGVAAALGEMDKIQRQASSQPPLMFSFDQPPEALLFVHGLGVDMHKALRHYA
QMMALIGFPPYITPVVFSWPSSVPVAYFCARYRGAEPPETGLALAATVRALAADGFAGLHLLVHSMGTRVLNNALPH
LEAILGGDGGDGGDRGEGGGGAPPRPRPRMKLRTVTICNPDFGLRRFVEDMGPRLRALCPHVTLYGDTADGALALSE
IVNALARPFTSAGAAIAEAGAVVPSLAASAAAAVHARTMSAAAMVYDGAVELVRWERIPAPHNRNNNNNNNNNNNNN
SVSKCSNPLLLVPESLSAQPRPQLDKPPQLPPYNPEAATATLGQQRYGFQHPSCPHTAVCSTPQQQQQQLQQQQKLR
RQILSKASKCGVPAVLDGEHGTHGGEPPLPSQQQQQRQHQTQERHQQCGAKASTALAASATASRTEWSTARPRDRLP
GGAAAAPFPAAAAGSEVGTSPSEPQPPPPQPQYDVQLPFPPQAPPPPAPSPPPPPPPPSPQHPATEPRHVQRPRRNM
SRLQAPQPQPQPAQLEMTAESASGSGDASAGGMAPLADVLTSAVKASSTPHEVSVPATTEVVITVADNGVARHPDGS
LTGAAVAAVAAAQTSANEQSTPDRPVGHAAAASAAAARGTSGGGHDPGGGLSWWNERVVNYRRRLHTPPLYGWLEHS
LGRRIYQVQDPVSGLPLDIA

>Vocar20000005m|PACid:23138487
MHVLISLTFHVINPPPSPPTHTHTRTHTHTSWTQRAATCSAAVGLQTIQSNESSCRNAGVGVGGSIFASHDAVRWPS
TASPPPSPPPPLDAVAGPNSPAPLPPPAGGDVDAETLAVRHGFNSQPSSMTSLAHGVAGGKSGDAAGAAAAASSAVA
ATARSLVSGRGLPPVQEGSGGALEGLTARHLSSPLLYQVPSATSVGAAGAAASVTGFGAGRVVDCMDVWHEVVVSGL
RHPRTDEQLILVTQHDVSARVWAEQQLARVMEAEHSLLEAIFPAHVLEHIAILAAATSTEGHGVDDADGAAGCGGGG
GGSPSVLAARPTLKGLEAAAFAEAEAAGLVTAPSAGGGGGTYTRRWEAASPPALPITGDTFLHLATSHSALTVLFCD
IQVAGGDGGGFTTMCNSVKPATVMSFLNDLYTRLDAMLDAFGVYKVETIGDCYVAAGGLMKVDAETGAVTVRSGDVD
PQHAHRTVQFAKAILRAASAVLLPTSGEPVRLRVGIHSGPAMSGVVGTRMPRFCLFGDTINTASRMESTGVPGAIHV
SKATRDLTPDEPWEPTGAVEAKGKGLMETYLLKPQNSIAPPPQQQHNHHNHQPV

>Vocar20007906m|PACid:23138671
MQDLSGRFGTIYLRSGDSCIHHIMLSGNMSSLELRSQGLTNGSHARAIYCPAPDCVASKGTGLRDDTPALLEPADYP
NGIWINVSVPTIGAGGSGIAAAVEFNSALASCSELGTILLPLFLTPPDDARHSSRPSYSLCVPAPNNHPDFGLSFSW
SWSMADLPRVFEDGFYAVLKPFPLTLPLGVYDINLLKAVAPRGAMEITLDLLAGNGTVLASFDDTTTGFYIPAANDI
TQASLQFYLPAGIGSQSTAVYTVRHMPSAPPLPPAPMEQNCTPASSPYIVQRRGRSTFVTLRMPYTKYVDALLRTNV
GDVDNPSVLTGGCAFSFPATTTAGDSILRHILSFTSAPSADAAALAWLSSSPASSKVRMRPGTTVSLPAPSPADSLT
VLLSGPGSISQPLVLDYMAELASCAAPNTVPLHLTALGPQTAGNKMTLSVCNAAPNVISNDTRTTTTMLDVSIGWNT
SRLPAVFSDQASVLAAEMRNSSFGQGKRLPSGWYRVALLHHPSIVLRLTIGNTTTSANSTIFLPDVVTNLSLYATVP
LSPTTRAMSAAVRLAPLPFPPPQPNSPPSPRPPSPAPSPSQPPSSPAPPNPVPPSPATPSPAPPKPLPPIPPLPSPL
PPSPPSPSPFPPSAPSPPAPSPSPPNPLAPSLPPPTYPLAPLLPTSPLGPDLSPPPEPAPSTQPYGMPPPPSSSSSP
PTSYGYPSDRSSPTSSPAGPPPLGQGATAPPSPRSTPPSGFSSSQLHSPPPSSGTPRSPNRTAPLDVSTLAGSGSRA
GGRACMAVSAALGIAVALLVRVTLC

>Vocar20008056m|PACid:23138808

MAGCWVLAAGCWRCFPCWRRAASTYPISTSPQQVKDLFLTGKWTWRNRYIAEYLRSCSLGGVNLLPDNLKVLGPIVV
PCSGALKEPQPFRTGSSFTTSHCTQNDNMKKWHYWLDAWAADQGVNASDYHHRILILPSGFTAKIAGCGSFSGMATP
GRWSLDRTAVNDWGSGLVWWSSDNFADLEVIFHEISHNYGMAHANIPGGCNVGWGDQCDYTCAMGSASTGQGIRCFN
APHNWQVGWGSKPVLQLDGKGLVHGNATTVRIPLQTSNVQSSLIVTGNGLLLGQRLFISARVNTYRYDLPWDFWYDN
VPYVLLHTYSGTDAVSYVTTVLVGEIRVGQAWRDRNSSISVRFDSYDRYTGAAVRICSRVADAEQNCGDGLDDDCDF
LSDLDDPDCKGRNGPRPLSPPRPSNPPPTPTPAPSPPPRLYPPPSPLRQAFPRIRPSSPSPRSPPQSPMPSPRPRPP
SPPPSSPKPPPSPRKPPAPSRKTNPTALKRNQGPQ

>Vocar20007930m|PACid:23138922
MPPLPIQVLQLLRSWSARAWNEGLPTDLSKLNAVNAQRASNENNATTAATHIPADSRSFVTPSHADSPAADKRTTDL
PATNTYGATEPYGHQPNLAEPSTAQPNATQPIAAQSITAELGVTQPSTAEPSSELTNSNTADPHAPDPRAAQDLSAF
EPSSAASRLKLPATAGYTAYTTCALELLHKCPVHATFAVATPASSSAEELSMCKAGSPVGDLFVVVDTADSAVVVVP
THDNVAPEPIGFVIVMAAVVLVFFLISMPDSVLLLALDCRAVLRAYISRFTGTPRAKTNSVTTSLDSSSVGDQSISE
CASNVTPVAAMQSTAPATAPRAFPKREAAFDSRLHNGNVPTAANCQHTSEEGGIRDASSSVGGGDDGMASTTSQSLA
RHTAHLAETQDRGNAAGVNHEPDDGGGGSPDNNPVADPDNSATSKPAAKTTAAAAVPTQRLATDTTTPKADDATASG
RDNAACICTAPLGNWGLAAPAAARNPRDSARYDTCSTDSLTASPDASGVMVGGSPSEQNEIGCSAINGQDELFTGAE
GGPATPLLDRDEVNEDFLVCMASPGPIEGSVELATQAATSATPSASIASLQSWATGGVVPSAVGLPSQAASALPVLA
PGGATPANGYESDPGDAPQLPVAPVALYEPAAVVESSPPLSASQSPLRSPSQSPSQSSSQKMPHSGDPLASPPRSPW
RPPSQSTPPSLLRYGTPMTSPRRSPSKSSSESQSPSQSTSPAGPRYRTPLMSPRRFQQQSSPSTSASPSPSASPSAS
PSPSHPISGSAPLSSPPPPSSVLAGSGFVVPPGDLTTTGKSRIPRSIGGSGFAAPPRAGHSKLPAGVPPLAFGHVNA
MALTALTARTPAGTATPGTGRRTGVTAIPLFIAKRSGIPAPPKALEVPATEDAAMGVANADERPVMQAMAAMTVEEA
SAFAQPQLPPLRIAAVTLQPKEAMGAAMAAGSEVIADTEAAPCSPTRNAPVPANLAPAVAATLRPGRSRSWRGLIRV
ERVVYRWRKRLAESNKTLARRWLCALVRTRGEANIVKYVTQVIGSGNMSPRALTPPSPLAGDPAGNGAVVNTDGGEG
GNSSGTGAASGAPDAQATNGTFSESAAACIQRMVLRFIEQRISSTSAEVSTSDSLQLVSDGGCASEIKLVLASNDGS
LAQSSPSCTTRDATGNSGGGSTLHASAPQLTRMNSSSRDCEGDGTGGGHGRMVAMSPTAAHGGVALSADDEAWVCVV
ARPSPPPQFQIHGVKAGDEVTQVERCTAELASEAWSSDDELPLAKGFAGAFVEAALAAAAVKGPTNVLCPASRRNLL
QGSLPCSE

>Vocar20008187m|PACid:23139016
MQLHNLRSYINRPGGIGRRRVASRMRMVFTAAQAKEDGFLGSFKAVAKKVQGSLPIVGLVSRLAAPEGGFDELAYPE
FCRSIIDKATVSYRVAQAELEKAYGKPANSRWVLLVLWMSKMGVGLVPAKDIIAASRRLRVTQDIEIEVDRFENAKG
AVLKKYAMMQRPEGKLEDKVNVAVDALCTLCIGLKEGEAVPDTAVPLLRDIVKGAFPEANVNQAVEFSGSANSGQLM
PEPNAPHSEQAAGPAQPARQAQPVGPIREAAMAQMTEMAIAVAKRLRLEQLDSSENAPPAASSETPRPPSASKSHKH
HSGSINRSNSRTTSGYSALPHAVPSLSQTNTPIKRSESTASTSGHGASPSPNRSRSRPGSASRGPAPDPTGSSRDAH
ISRNSSLTERAAAGANRSRRRPDPLEKDFQDNCELVPSPNRSSSRSHLAARPPSGRIRPESARSRGSGGGAAPDGSP
VAASSRSSRAVGNGGVSSSRNGRSSLEPLGYPSWPSGVDGGYEEATRSLSRSGSRGHHASGSGGGGSGRLDNQSRPS
GSPTRSTRSGRDASAGGAGNGGGVSGYNGSAGKSSRALSRDDQRGFDNPDVYGPSPRDASRLLPPRPPSAQSGRHQQ
REPSPSLSPAPPLHMELQQGLHQRQLTPSPPHRRREYNLEPAQQQQQQQHPEQVQQKDDRHHRGRSEMEMWHKRPEN
QQKKHHREQDSRSPSRSRSSSRRDHTLHRPASGGPGGSGSSGCSGSGHISAVGGRLERSASFSSRSDAHTSIRSDIE
VAIRAELAAMVAAAVANSSPSSAISAGGAHGPTLVSPSGVRTTSPPPRQLQPHPLSPGALKIAPDTAAAAAATVAEQ
TTSHLHAIQAAGIGPTTSMDLEQRLPSAAPAAASSQSPSQPVAEAALPQNRSQPWVGALDPQLSEPVLGAVAPPAPP
PFATPVSSQAQQRRTALDYEEEIVPAFLPESELRPPSSSAAADAVAPAAALTPAALVARLQMQSPRTLRSSISSLR
SAASGTLAAAAAASSPLVLRPSARSPLGISRDDEGDGNPAASVTSLPDSTSQVQRVESSLQPDGSRDSPGNAPAGGR
SGADGSSNGQSGGAYGDGGGSSGVCDGRGDWDYGFGCPQDPGKVGSHSEMQAAAGGALPLLYGSEWEELTHEVVWRV
ARAYGFQVFNLNPETKSNPAPQWTPATAFLVSEHLCRILVRNMLPRMKPPASAMHPAASSLVGENASASASAAGVSN
SMYSQARPASTAAAFPGESVTSAAAYSLFPNIAPGGAAAAAETVATAAGGPRFAMAVYELHNTVFCMYEQRWCPHLD
LRPRPRDLQRTLETSRWADGAALNGLLSELALYFLLYSEAANLRHTPELMWFLFWAAIHSPPMERLWREGFPSVQGE
NRGAWGRRIRLRNQFQREIAALQAEVGAPPGANPRPPPPPDRLPGPVPPDLAAVALPAFSAAVTEALLAAAAAAAAA
AAASAPPPPPSRTSCLGLLSPTDADLLADLAAYGDGGFVTDRIVTPLFYFLSHEMDALFDAGVEVAHRLGYDDVNES
MCRRDVVDNLLYTLGVTEEALVFGEVLDLWRALSDLGCPCPEQPPMPSHIPRAPGPFDAQLAANFWSAEVFVKTFRE
RRSWAAVFRAFFRVYCLQLVLLTLIMATAFAPGDAGALSSAVISHVWLAAAERVANWWLSRNPPDPLMSQLPLPLLQ
PVARMAGGAAAVAQAWVRVVVAEVEVPVEVDFGVSAYSEFAVAERTGLTVGVMLCPSPHVEVSKPQPNRTVRSGRRT
SRQPRQQNPEHIPLRRSPSSTLLLTYFLRTIGAEASGLLAGAPGSSAVAGSEEPREEDTAVPPPPVVVEAESPHAAR
ARRRLVRMEGQPLLGGLVGCFDWALGAMILTALYVLYLKAPGGGGVLHRLHVGLQRWWLPFSAAYGGVVVLLGVLTT
RMGYRVSALSSLPTLRAVLSCHSAMPPAACWLEGHLGVDWRAWAVNTALWALILTMKVAFEYYIIAKPLVRPMRVLL
SHNLPGCSSWPCNNVSWVLAALRVLPLLLVCLADTSIIYNVVMAMFGILRGLLRLDLGTVASWEELRAAFHRGPVRW
WRRCMSAEGVSSHRRALRRQLMEGGQDRAAVEAFVPETPPPAPVSNSSDDEDGSAAVGGDDDDSGAGAEDDVDAEGA

GNNVDAEGYDSKDEEVRRGGGAATATTRASGMSALIWPLRPAGREARWARERRMLGRIRENGGASAMGLMEVRDADS
LQTEMLQPAGRWATEPALPPMMVTSTTGGGRRRMGTATGGSASSSLRAGGGGDGGDAGELGQRRRGPMRRLPTPSDG
GSMDRSRRRFSEGGGARAVAEGIELAINGNVALNYPRSGGGAAACGRVRGRWFVSSGGAARATSSASVSAGPADVTV
GHLRRTRSSGSDMEVEPQAGGLVSPVVRTPAKAPDVTGLWQRISRRPTHVEGARGGGGRRSHGGGGGGGSATTAVGS
AASGNTPSRGWNIFTRNNSVVPLPLSPPSAVPPAAPPVHQLSPPRISCRASSLGGGTAGTSGVGCHDITRRRLAFVD
VCESPGPVAEATTSTLVGHTRLTRREAASPALNGGQPPPSPPSSVYGSRRGVTTPSPDGWRQRDQDYWQRRQRQLQL
VAAESTAPPSTPPPPPPPPPPPLQATPSRSQPPKLLTRPSLDSISGLSTDRDEPELAGDGEREVRQAASRSRQATPA
AAAEERPSGAGLGFAAIHRGRLRRRSAGSGAETTERDRVVGLQRTEDLSLGVLLSRQNGDLAMWDAFAQVWDGIVDD
LRAADLISDREAGNLRFMRLGHFQGRHALRPILLPAFFYAGVAQAYVDTGLLGPGGGGSGGGGATTTILTELRSLLV
WLACELRLLSEVQAEVVMDAAFLHATADVEHANCRGKMLQAGQALVKQLAELCAAATKHQQQQLKQHRELLRQQHHY
QQRQQQHQQQAATASPVSRVHFGEKAMSQQRSSPQSGDSGGGVGAGGGAGGDGELGSRAAVAGRATLSQRQAAAAAA
HALVSSHVGAIGGSLREVLEALAGEARAMQRAVSSGRLASESLAAAVRLEEVAKQVLADLSTHPEFLSSCLGNLVSR
NFPVAPTTPPSGATAAAFAAGAAAPEPEDGAAESPTASSSGSVVGGAPMACEPVSALSATHMQSPQSVLPSNFAGAT
PGPAEAHLGGRAGNPRPGVGSTHPHRIADMEVSPWICTPPAAAEATTMATAPYAATATTASLPPALVPPVRVRTPEG
LFLAAGGGGEPTRPGGRFGSSWGSGVSNSAAAAPPLEPEVALRIRVVEVLVKMLTTPASACRPAGAEALRILGFFIN
SLSNPGLKKPPPLSDMLSWSVLTPCYEEDVLYPLSADVAARQLGLAPPPPSGPGRPPDLLSETEDNVSLMAYLRSVF
PADWKNFMERLSDMLGGADLSRVTENDFAPMGPLHALAPELQLWATYRGQLLGRTVRGMMCYRRAVRMLVELEYPRP
AGVSLAAYNSWAEALVDCKFQYVCTCQVYGKNRKAADIRRRWLAEGVDSLCLEFPALRVAYLDTAVTSYGPTDYSVL
LRGNPNHPDALAGAARGVASAEAAATAGAAGDGRLGVAPVQPPPPPPLQPQPLTPSPPPPPKLQPQLPTPVPRALFS
QHSLSVADDACCQIGRADSKGGMKCKDSGSSNKDCEVAAACCTTPAHDPADAAGGGAAAASSGTAATATTAAGNDSP
TLELYRVRLPYNRYGKRGVILGEGKPENQNHAAIFCFGEALQTIDMNQDNALAEALKMRNLLGELAPDPAPRRLQAV
ASHPRGSTSSESHRRAIAARTAREVPVALVGFREWIFSDVSGALGTFAAACELAFGTIVQRTMSYPGRVRLHYGHPD
VFNKMHIMTRGGVSKATRQLHISEDVFGGFNQLLRGGQIKYKEYISCGKGRDMGFDSINAFEIKISGGGGECVVSRD
VARLGPRMDLARLLHFYHSGPGYYINSLFIMTAVWLNIWVVAVFALARASTVQRVGADGELHLEDTLRVEHALSLGP
LMLLPYAAQLLLEWGVLRTFATLALQIVSGSVAFAVFRQQTTAYYFKDDITYGGARYISTGRGFSITSSAFTTLFTN
YARSHLYPGMELLHLLILYASVRDCKTCSFAAVTWGTWLVAIALLFSPFWFNPMAFTREKVSRDWSSWLGWMRGEVD
QATGNNWHSWNRKQLEKVRNERGTVTDPGLNVVSRLLEEVVPRLVLVVAAVSRLDLRIDVGHRQLTSPLIFFTGATA
IIWLVTLLSWALQRHFQERGRGRAWRLYRVMLSALVAAALVSYSVFAVRFFRGPAMSNLALLLYANSQLVLAVHRAL
EQLAPTSAAARALVDQGYWLIDWGLQTRLLFNSNFAASIRRGKLVRSTGLMKLDEEQRWRNEGQARAALARSVAMSL
RKHAAPGMHYAATPIHPASAAAQQQQQQQQQW

>Vocar20008121m|PACid:23139060
MGGCLSNESKEARPASQPAPASTGLVGPLPQQSQEPTKPAASHQQLTSNSGHRDETTTAGAPAVAVRDCDGRGSPFR
VAELPPDAKPTEEPSGPVSLPASGPVPGTPGGACGDPPPDTPGQPPQQSQSHQAADGAADNDVSLRTLAAGGTLLAP
PVAASRDSTIPAATTDPSRPVPTAALVHAVNSSETGSFQNPDGSSLTPSNVQPSGFQCMPASILAKEVAQLSWVGHG
GGCNCMPGAGVLGLLGRTVPFSCCPLSPPPSPLYPAFPPAPPHPAPGELPHVRPKPYPSGGVVLLPKVFPPNVLPRT
VSHPRRPSVSPPPPGAPPHTNAHAHANAKYCDRGSLHQAIAKGLFKSSSKWNNKIALRALLRTAREIAQGMSHLHTC
RVVHGDLKPGNVLLMSSRADRRGFTAKVSDFGLSTYCMASHTSVTRWSTVSYMAPEVGGWVGWVGVNESVCVCACLN
DLSRVCMCECVCVGGVYVGE

>Vocar20007931m|PACid:23139061
MSTSVTAVTHRTMKMAALVTASTRRTWRKGIRCCRRNRTAGGRNPRCCTGLPPPWRQRRQLVSLGLPEAATELQRRR
RWQGPPQRRRVVLQLQLEKGAGSMTQPPSTWLAELVVAVAVVREEEMGGAMGPTSGGSWGRFCGGETWCCSWLPLRS
WATASERLTPFCECRLLGGRAGEITCFSVHMQCVFVEGEEILGQEDLGRYRKGVCVCMCVSVRERRSWGPQGGVKRV
EEEEEGGAGERGGEGVCLACTADAGYGRRRWEVVGAGFRVLGRMGWEAGGEVGLYMGATGTLMGLTLTITCAAEVPA
FQLQDRLLDSWGVPAVLDLVQAVYVVRLALYAALPYAGNVMWVLPVEVLHGITFACGWGAGTVNCKLLAPPEMAATL
QGAFQGLYFGLGYGLGSLVGGWQGTKLGWQDDDDDNGADDDDDDDDDDDDASEEAPPRRRKAAAPARTRARRSTRSS
KSYKDESDEDVDMNDDDDEEEEEEEDDDDDDAGDDDEDDDEDEEDGGDEDDDDEEDYAPRKRRSRAMSASAAARPRR
QAAQQAKARLRQDSVEDDDEEIARAIDRQINGLRARPRRVRVCVLTRVAKVVSYAESDDDDDDDEGGGGGGGGRGGG
RGGGGGKAKEALSAFEDPDEDEVEDEIDKVLAHRESETLSPVAGDPWASREFYIKWKRYSYIHCSWDSRAVLEHLRG
YKRVLIYIKRVDDVEEGRRGFSREEQEMQDVEREMEAQLNQQHMQVERVLAEKQACVYCSPPDEKEDEAEGARGSVG
PPGQRRRELEAADVEMVNVTKYLVKWEGLPYAECTWETVNDVIKAGGSTQIDLYLQREQRLLEPGRSVDVARKQFKI
KGTRALEKQPEYLRGGTLRDYQMESLNWMIYSWSENRNIILADEMGLGKTVQCVSFVGYLAEALQIRGPFLVVVPLS
TVPNWIREFRRWVPFVNAVVYVGDSRSREVLRAYECDPASHHKSSRPHKFEVLLTTYELILKDAQILSRIKWAYLLV
DEAHRLKNAESALYQELMEWHFKNKLLVTGTPLQNSLKELWALLHFLDPDKFPTCERFEAEYSLETADSVSGLHGVL
RPHLLRRVIKDVEKSLPPKNERILRVDMTPLQKQYYKWILTRNFKELNKGSLGSHGGGHVSLLNIIGELKKCCNHPF
LFESAEENYRGSEDDKSAMVRVLDIISDYMRHRGFTHQRLDGSTPAAARHAAMEHFNRPESPDFAFLLSTRAGGLGI

NLATADTVIIFDSDWNPQNDLQAMSRAHRIGQTETVNIYRFVTSGSVEEDILERAKRKMVLDHLAGASAKQMFGKDE
LAAILRFGAEDLFKQAPEDDSNAAERVRSALYEDDIDAILERAEVVDTRALAAAGAEENAAAAAELLSSFNVATFKN
EEDDAAFWSKLIPPSERPKEEEEDLLPRSARRALAADSDDDNGGGGGAGGGGAGGPGGLGGRRAGGGAGGGGRGGRG
GGGARGGGGGRSKPGAASGGLEPGPPVDGAALRVDEWIVEVDKEGIPLMKLLQHLNPPSPTNKVTNQPANEPQEDGS
EPESRSLSRRDAAAFVRAVRRYGRRERLADVASEVGRSLEESSAGQRLSLLTFSRRPNIVMHLPLSAIAAADRLLGC
SAAGHPLMMLVVVCWREGVQDAILDFFGVSVKAAELLAVCSCLRLLAKKVAAAREPAVQHFRLDATSQLTVPKWGKT
INWTSREDALLLLGVHLYGMGHWDRVAADEALRPHLADKLAGAVGGAAAARERERDDAAAGKDGMGIGLPKASHLET
RALGLLRKMHAVHKAATAPPVRRPAAGAAARKKGIAAAAVAAAAGGSGGGGAGGAQAPVHPPPPHDRDGGRADGATA
AGVVPGPVRERERELPPALAPPAAAAPAAGRDPCAVGVVNGAAAASAPGGAAAGGGAGGGAGGHAAGGKRKAGGGSV
AAADAAAPGGAAAAAGPSEKRPRMDARVGVSAEPKAVTTAANTAAAAPAADGPVGREELKTLLEPAMSDIRKLRKLQ
DGSITDKSVIVQNTKLYLTAIGGHIERVMSVRGPNVSKKLWDLAARNIGNGKTGAELRQLHTRIQQSQQQQQSAQPQ
PQKHQQQQKPPPPHSGQVELRSPRHEREAAAAATQGGPGLRSPKPKPKASPPAVPSAAPPLSQRPSASRSVAVSTTP
LPPLPPLSGTAAAAHPSPPPPPHPPQGYQASRRHPSEPLATSHHAVVAAAAAAVAPAGPTMHHRQHSASPPGPASPL
SPPPPALPPPMPPPAAPPPPAPPPLPPMPPPAPPPPLPLPIPPPAPPPGGPPPLHPSYHPHLHRHPHHHHQHLAHH
HNFNHLHPPTSRVPPPQLLLPGDMAIQFPGLGSGLSSQAGEPSSGGDLCHVSPPGPCFGAGGGAVSPPVTRWPPELS
PTAIAVAAPSASTAVGTTAVAGVDMPQELGFHTLPPSLPPQQPAPQPPPPPQQQQPQIASPPGLRDAVGGSGGECGG
MELSPRDCRGGGVSAGGQPPPPPQRNSAEAPDTRWDRDPRDPRDRDRGRDRDRDRDPRDGRGSDPGRSAHHEDRDRD
RGGGSSGGGGGNGGGAREWRERERDRRTSRSRSRERERERDFRSREYDRDRDGRDPPRDRRDRDVRDRDRDQRRDPR
DRDWDRDRDRDRDTARGRDNRDHREFDRDRDRGRPLRGAHYEDRGRDYGGDRARDVRDRSRERERERRDRSRSRSRD
RRCRSRSRSRGRERGRGERERDRIRRSRSTSRDRFDRERERERDEPAGRSGGGGGNGLSRGASFGPTGGGGGHGGLA
GGGPGLRGGGGSSGGALAAGALASGPTPLGDNASVPPGSHLGGGGVAAAPLPPPQTLPPPPPPSSAAAPPGLS

>Vocar20012236m|PACid:23139113
IPSPPPSPPPRPPPSPPPPPPAELPYGSDGKFFVLTIVILKDGGLSFNLRLVNGTRAGEGRVEIFFNGTWGTVCDDN
FDGNAATAVCRQLGFLRGEPFGQAFFGQGTGPILLDGFNCNSTVSRLDQCTIPAMASHDCGHVEDAGVRCTTDIRLV
NGSTAGEGRVEVFFNGAWGTVCDDNFDANAANAVCRQLGFPLGTPFGNAAFGQGTGPIQLDGFTCSSTASRLDSCTI
PAWATHDCSHSEDAGVRCGSQSFDVRLVNGSRAGEGRVEILYGGAWGTVCDDNFDGNAATAVCRQLGFLRGEPFGQA
FFGQGTGPILLDGFNCTSSVSRLDQCTIPAMASHDCSHVEDAGVRCTTDIRLVNGSRAGEGRVEVFFNGAWGTVCDD
NFDANAANAVCRQLGFPPGIPFGNAAFGQGTGPIQLDGFICSSTASRLDSCTIPAWATHDCSHSEDAGVRCGGSQSF
DVRLVSGSRAGEGRVEILYGGAWGTVCDDNFDGNAATAVCRQLGFLRGEPFGQAFFGQGTGPILLDGFNCTSSVSRL
DQCTIPAMASHDCSHVEDAGVRCTILCHTCNHYPPADIRLVNGSTAGEGRVEVFYNGTWGTVCDDNFDANAANAVCR
QLGFLSGTPFGNAAFGQASRLDSCTILAWATHDCSHSEDAGVRCGGKMVLHTNLVTLIGFSDNS

>Vocar20009294m|PACid:23125207
NSRKPNWSLELWFFGLWLSNSMCNDTTRRRGVRAPGAVDDLRSTSVQYGCHAWVHTYNRMAVQLTQFAIAQAEELSR
LDVSTIVCLDESVQLNTSCGGELQELAAQAFAHVRGVPALRFTPYSGIPESIRNAVRAALGAGLFPDLPGALEPINP
KATADESRSCFSVLDQSSASDMEEVPLIMRCKKARRCNSLRGPCTRELVQPASVTPRNERALSEMPIDDLLRLVSGL
DPELTSSAFRSGSLDVVAPATQNPSIPRPAHAIWPGYDKCEQGLGISEARNDTRRATVPTGTGVEPAPQAYGRHLAH
SDEPAPLSDDVVAPHASTGTAQRQRDERERTGQAGQQKSKHRPFSPGAKQPPPQHQQQQQQQQEQHQEQQQQQQQQ
QRQPDVVHGENRFQQARKRAPLWLNQMLPSRSRLRLEGSLQEAERRSGGSRQRRQDTAGPATAPPSVAHPAAVPVAC
DQIAERSSSGEVVGRTMAARQLRDEQHDDLPHQPPASVAPLSQSPPPQPPQPSPELQGPRGDAVPPTAAGEPALETT
GPRGPELTATLLDRQRSGRTASVAADVKTSEEEQRHDMQLYCVLSLFGYPEAHVLVGAPFIGIGITAGQGDAVPRCN
MSEMEGEEGSAAAAPAAAAAGVTVGGTRDAAAPGGDGISRDIPAGSTDPREGQLLVASAESALRGTVPRRQPEEQQQ
QQQQQQQAEKQTKEQLQQSQLDRDGLSVSQQDQQQQESGAPQQQHPRRRTRAAAAPASGVPQLASAHICGTRKCTAL
KASGGGMGHHTAPPAAPARDSLSRSPDSEQKHAGQKGAKGPGKRRKPNPPEEAPAADAAAPISPAIAVPAAVTGSPG
GKRGAGRGGRRKTSAAESEPPQRPLSPAQRGPPTEAPGRGGEDGDAEKPAKDLGRELRDGLKGDLSAAVKASRSPGL
VSASPAKDQAAPCSHSSAEMERRSAGRAKFLRGASPPPVKPGELQPPSATAAAGADGARGGVEACVSPGLPGAERAP
VAAQGTAAAAEAEAKTSAAAAGTRSCTGGAAAKRSRAQAVEEDLTLDPRVGDRLMDVPLAKRGVGSRKQLAVRAEGK
AALETEERHVAVLKVIEATQAAGKVAPATTTVRAGSVPTAEPAPPAVGPDGQTAGAHAASGHESTSKQVKGCVSGLS
LPGTAGNVGPVIRTCMANAAALDASAAEAEVSIPAPSTRPQQPPSNSQHGVPSTTVVAGVPSALGPAASLTEAEMGS
KIRPSQPGVSEAELVSTAAAAAAAGASVAAPEPCDKAGATTTTGAPAGADATSADPAAEEHAATAPVDPSPSTAASD
QAPAEAPVGLATAAAAATAATAAAAAAAQHRPHSSGPSVCPTRRVPGSLSRANESLMTAVRTLAPGRAVPTTELQGS
VAGAAAAATAPAAASDPAKPERRYRPVAPARRPAHTRAPAGDLPRTHVELAAVAAAWAAAQHSSSGGVSGGGEGPAG
DVTAQGAAVTDGIAVVAAGAGAATETATATAAAVGAAAAAMPPAAVQEGAPVTAAPAGGHGVCRGAAQQPPLSSADV
PCSTAQAGANAAPPPPVVPPSSSPACTQSPEEKLHPAWEGVLRPTVEGPEGAHPDVDGGCGPAAASGASNHLSAAAA
AAVAAAAAAAVAASDPHKPASGNNPGENTPSRPPPSDTATSPVRPSVPEADINFLAEPSGRDAPAAVAVAGAGAAGV
APGSGGGEDGREGGQSPAEADRLSGDGVGDGELSRRNSSGSGSGGAGGGREGSGCEGRPEGPSSAEGWRAGHATADV

ATTAAATTTTAAADPHAVADAAGGSAGGAEGGGRHPDDDDAGMSAVEREEWRRRRVYNMSLPLVSMMTPQAEIERQR
AEAAAEREAARRARLAAQAASGELERRMKQRLADHHQLASAAAAEAAAKEGIRTRIRDSLQERLRKALDEHSLSRLL
RQLEVLPKGFRPHFHAFLTSGFAALLIITCGGVALLSAHTGATLRTAEQRNKALKTAKIRFHPDKVTGSLEERVYAE
EVSKLLNSWNWNK

>Vocar20015001m|PACid:23127828
MIMGTPRSALQARRFPSASRALLLAALLACAGLLVHGHVISNPPPPRKSPPSPPKTKPPPPPNNKSPPPFPRAKPPP
PSPLAKKSPPPLKRQPPRFPPKSPPLTPGFPFFCNFRVRADRKVNDQSIEFPANVGVCQAANERIVADLTSIFTAVG
AFSVQINPDPCTVDEDPAFSVTGTYAHRNATAVAAVDGYDLANEWLLRLWSSLRSCTGGGNLFVTISTESPSSTQCI
VGSAALALVC

>Vocar20010577m|PACid:23129462
MGGRPLRLAFPATSLFLVLCSSWAQVGLPLTTEAEDRSSSTSHIKQRYGRHLRGAASSSSPLFAADSQQRVAHTPPG
APSSSQRKLAAISSKSQPAKPVTTTKLVDLYSLVETALNLKSQNPNATVSFSLPPGVLHAGIGSGSSYSPGSFSAAA
EDEEIADMTSPLSGAASTVLVNASTGTYYKAGIENDAVELMRMLIQVMRQNSGSSVTITLRDTGAVAPGESPLQVSV
SLDGDFSGQYGTPVVSRDDGDGAISGPDATLPSPAAAGPQPSSPSKRDTAGPSQLPAQPGALPPPASPLPDDVMSAP
SPLLPLPSSPPLQLPQSPLPKLSQLPTPSPPPIALAVPSQLLASPVPVPPLQSPPPLPLASPSPVPYPPPSAVLLPS
PSPSSLPSLPLQSPLPLASPSPVPYPPPSAVLLPSPSPSSPPPLPLQSPSPVPLASPSPVPYPPPSAVLLPSPSPSS
PPSLPLQSPSPVPLASPSPVPYPPPSAVLLPSPTPSSPAPLPLQSPSPLPLASPSPVPYPPPSAVLLPSPSPVPLAP
PSPRHPPSPLSPVFPLTLASPLQLPSIPMSPMPQPSPPLLQLPSPLPLLSTSTSDTELPLPPAGIPTTSPSSWSSSP
SPSQLGSPPSYADASAPPSPSLYGSLPPPQTASPPAYPEVPLRPPSQLGSPPLYADASTPPSLALYGSLPPPQTASP
PAYPEVPLRPPSQLGSPPLYADASTPPSPSLYGSLPPPQTASPPAYPEVPLRPPSQLGSPPSYADASTPPSPALYGS
LPPPQTASPPAYPEVPLPPPSQLGSPSYTNASTPPSPALYGSLPPPQTASPPAYPEVPLRPPSQLGSPPSYADASTP
PSPALYGSLPPPQTASPPAYPEVPLSPQSQLGSPPSYADASAPPSPALYGSLPPPQTASPPAYPEVPLPPPSQLGSP
PSYAYGSAPPSPALYESNHPMQPSTPASVPQSSAFPPSFCPQSPLRPAASPPSPPALSPPPLEPAAPAPAPQPSPSL
AHSPLYYSSPAPAGQLDSVPSPPPSAAAPSYPSELGIRPPAYAPAMPTSGRSASPGQHPPMYGSPAPIQRSSSSAGR
PAVASSTPPPSYVPAPSLALTWQLPNPAPLASPPSVLEPAEPPAPPSGGVPMGWQPPSPASLASPPLVLEPTESPAP
PSDSVPMGWQPPNPAPLAPQSLPPVSRGDGPAAYQPYSPLPAPTTSLPPPVPDLASPWPALLKSSPSSPGAVSPYRG
EEEHSASSPPPPTTPSSSPLPSSPFLVPTPWWQSPLPSPSLATPSPASRPTLPSPPSVPPSSPPRKLQHAVLSLKEE
SVARSSARDSQLESIRTQVQFLLDRIVASNPGSSSQLTVTRSRSAAGLTLPSAAAAAAGSEPSIPQQRQPSTTVATL
AANTQQSAGGTVASGPRRMMLRGSPEP

Klebsormidium flaccidum

>kfl00030_0300
MAKSPAPSRSGSSSYTTSASGSSYSSSVSRPAPATSKAEKKRIETDKKSAAPPVAKGSPPPVAARQGRPASPAPRKA
PASPPPKAKTPPPPQPSILHVGSLTRNVNEAHIKEIFGNYGEVVNVELAIDRIVNLSKGYGYVEFAKREDAEQAVLH
MHGGQIDGNVITAQFVLQPRKKASPPPRPVPAPNREGAVRGPGGPPPPPRHRSPPPPRRRPAEPARRRRPAPARRRP
SPARRRGASPPPSRRPLPAPRPGRSRYPPAPLPFRGGRPAPRGARRAPPAFRKRPAPLPARAGRRSPPPRRPRSRPA
PQGSRPLVHPPAVAAAGATAEARRIPRMTTAQFDGLLHHLRSRARRRRARLGGGVPVLEAAQAAGVGPGRAASNEEP
I

>kfl00080_0260
MQLLAFIALVGLALLNGSAAVYYGDGPKSTHYGDLNGNGATDGGACLWGNVRPVFGVNTFAGGNAIFNNREGCGLCF
DVQCDSSRDDWRAQLQPGPDCIAGASVRVYLTNQCPECAATHFDLSYPAWKQIGNDRAGILPIIATPCGKVDRNGNS
VIGGGDSFRATVSTSAPDTPAPTQSAPTPGPTQPAPTQPAPTPPATQPAPTQPAPTPPATQPAPTQPAPTPSATPAP
PPATPPATNDPPAIPTQTLSVSSAKITSADVIAPSSNPTSTPTPSTPNPATPAPTVAAPTAAPTPQPTSPPPTTPAP
TAPAPTSAPTPPATESTPPPTTPTDAPTDAPTNPPQTPPATSAPVTDVFAPEPTSTSAPPIVYCDAPPRPRTLLPRR
ESLPPRGVFPAPGVFPAPDRAPPANPLPLPNNPPPASPPPALPAPGTPLAPPPPLLNPPPPANPPPRVPCIPRPLRL
R

>kfl00196_0130
MAQTSSVLMLLGIVALFGAAHAQVLTYPDDVSALMSIKQAIGNNDNGAFATWGIGDPCNNQWQYLVCAATLSLPVAK
DSQLMSNYKVVTSLNLNGRGISATLPAAVASLTNLVTLGLSNNKFGGDLTSYDFSGLVKLQSLALDINAFSGTVPKS
LASLSATLTELNVANNQFSGVAQELKALKKLKVLTLNNNPLASTIPSWLDGTFTALTDLLLDNNKLGDSDLNGNGLT
TCATPTSADLIAYPGHGGETWCINPKLAALKSSLVQLDLSTNDWSGNIPPQLGSLTNLQTLKIWDNELQGNIPVELA
NLKNLNTLNLNNNALSGSIPVEITTMPKLSIFWVQTNRLTGAIPPEIGNMAALTDIELQLNQLSGPIPQSIANLGPK
MNKLQMQNNLLYGAIPDFSKFTNIVRLDLNGNSLGATDEFGNLNTTNPIGIPATLGNLTTLQYLDLSNNKLGGVLPA
QLGQLSNLEFFDISGNRFTGALPDSLAKLKKLEQMFVQVNDFSGPIPEWIKNMTSLEDLYLSENGFTGSLPLAIFQA
PALKSVTVHTNKLEGPLPTNCNGSTTLAQLDFSDNAFTGPFPLCLTTLELTFLNLENNELTGTLPDALGNWTSIQLL
GLGYNKFTGAVPAALCNLLDTLIALDISNNQLAGNISSCLIDHAVEHADDFFFWYDGNSFSCENLDSCPDSDTPDDN
PFSGNSTFGKSPPPMPSTPRSPPPPPSVLTPPPPPKKAGASHTFPAVVTMVVLAAAGTFASVF

>kfl00361_0120
MALGAGQRAAGLLTLLWLASWGTGSLGAGTPATFRVAYNAEVDIPAASCKAHFVEGWTFPYTTGGQTWRDMGLDGDQ
RVSNVYVTAPAGYNTKYQYGWNNGAWRISWYTSTSSLSYTVSYDKDNAIRQCGGNATTGVPGTYTARFATGKWSQQM
NALIVTFKLGFVPSTRPTLAVEVNGLTRPLNPQLSGDGSTVTATLNGPLFSNQNAVFTLTYDYGSPARPTCSCSSAS
STSSGKGGVLGGIIAGAVFLIILVSCGIGCLGLCMSTKTTCPCSRCNGRGYLGAGGVLMPPAPNGMYRDVAPSSPPP
PPGYYDQAPVTGVPYPGIPQTDAKDFAPGTSSGYQYPPPPPAGYPQANPGYNPGYSQAPPGYNPAYSQAPPGYPQVG
PPGYPPQPMYASSPPPGYPGAGYGQYPPPQQFAPGMTIPMQPPGTTVCPQCQGKGTEVRRTVPTWAKVFGAMGIACF
LCFCCCSGGGYHQQYGYNSWWNGGGGVGGNGWSSGGGGNSGGGGGGGGDGGGGGG

>kfl00697_0070p
MFDLEKQVMAAFLARHLIAVFVIACLLSVDASLGSTEDAFSGAQPDVGPRRATLQLGGTDTPYPNADTNNNSPPPNF
HSINPYRYPNADTNSNSPPPNRHSINPYRYPHANSNSHTPSANFYSINPCRYPHPDTYSNTPAP

Physcomitrella patens

>Pp1s136_208V6.1|PACid:18036826
MASARRPRTSSKPVAKIAGGVDRNEDGTEKSKSRKRKLTDMLGSPWSKEDLEMFYQAFRKYGKDWKKVSASLHKRTA
EMVEALYTTNKAYLSLPEGDVSAAGLKAMMTDHYNLLDVSQSAGAESSDDGVGSEERSNYIPLTKKPVTSTGMDSSS
IRFDGLLGASGFGGPSPVKRPRTSSIGPVGKRTPRFQTGKASEKRIKIIKVPDSKPQNVVEEDSDSDVAAARTLVSK
PTTSPSASNNLSRRSFRQSSVHQNGDKESLWRREPDGSVKPTASTSQADNQLDGSPESKVLQNEGATKASSSTHTSD
ADRKTPKVGDIKSKLKKPFFKKIQLQNLDDKHEAAQIREKRKENVKELLEAAERQRIGADEPGEGSSPPPFVQRRRR
PGSPPPKQKKRSRQLFSGGTYTSPAVVSNSGLDALATLAASSLELEGLEFATFEAYTEYLKPKARKQSSLDGTEPKH
VSKSLRAEEMRSLGNDNFEGEGDLKTEEEEAASEDRRPCTSPADSKKRKRKFSGDKVLKVVAPVPDGEAFVSLKPRT
KTKRPTGTKLLSSKSWHKLLKQQSEIDAVDGAGASSLGKQTPKNAEDHSMDIDESSLSPKLGSKKNGVSEKLLPERI
GTPSKSVNGVNSPRQESKRREKNFCPHSTPELGLTSAKAKIIHCLCPKVRRWCMCEWFYSAIDLPWFARNEFVKYLN
HAGLGHVPRLTRIEWGVIRGSLGKPRRLSKRFLQEEREKLETYRESVRTHYHELRTGLREGLPTDLARPLTVGQKVI
ARHPRTREIHDGSILTVDRSRCRVQFDT

>Pp1s195_51V6.1|PACid:18040277
MLAQIQPSPAAKCEFIRRPWERDENRKPRHPFSHHLLRLRAVRPSDVFRPRVQLCFDGECGCWAPPLATVVHPTVIH
PTVTNSVAFVTRNMCQSSPRTKPAKWESESSATLIGRAGCWTTHDSKTPFLITYENRTTLNVQTESCCCPRWALGTM
SPFVLGFPISPPPSSPPARLAFNQAQVAEAPSGHFFNDHEPHHNLHPRSPHTSLRRLRHLLILWRVAANEQHSSNNN
TKRGGRGRQRHTREVSFGACAAQKQAMNNGKEGDAKEAGLLREQPAPEHNTTEQNSAGGDEERCAGSKAASPPPRIL
LPTVTNSRNISATPHAWLSSARPVDVWLGLRHEAMKRCCASKPVSEEGQEGKGKTKNGEAMRGGSK

>Pp1s191_108V6.1|PACid:18044911
MAYYNEHERLRPYAPRDEYVSQGHDGPEFTLGRAPPSNDPYWQPPSFRTAPDYQPFPVREEYPRGVPHEVYPRTLTS
TTFTPDDILGGPLFDFGPSQDPDYGRVPSHRSFDSGHDSEYDLRRPLHDFPQSDYDRRPSHDFDHERRPQSQYAPRP
PEFDSRSRPYYDQGSPPKFDRGPPSEFDLGHQPQHERSRPVDFDRRPPPPDFDRRHPSADFDRRYPPSEFDRRPPPP
DFDRRHPPADFDSRYPPSEYDRRPFPPEFDRRPPPPEFDRRPPPEFDRRPPPPEFDRRPPPEFDRRSPPPEFDRRPP
PEFDRRYPPSDFDNRSLHGHDHRPHDIDRRPEPHYGQRPPEVEHRPPPPPEVEHRPPPEVEHRPLPEVVLTPPPEVE
QRHVSAEVPSDAGFQPPLPYEPEQHLNLPESPKRPASPPPPVVIYPPAPVVACPPQEFATERAISPPHPPADDVGPQ
FPPMPYEPDAAPQPQVETSRDIPHEHRPPDVVPQFVPPMPYEPEGRSPMHHHHDEYPDGYPPHMDLESRGVEGAVPY
PMEGGPPPLHQDAYYGQVTDHGHVKKRHFQACKDYWAAFLFFLHFWAVVGVCIYLGVRGLIKSNQNEELLRAYALAP
SPTPLNSESLYNIKHWAPQLAAATGAGWWFAFIWQSLIRAHPLPMIKICLCLGAVSTGLLGIILLSTGTAKGLIGLL
FVAFALFQGLYVHLVRHRMEFAGIMLRRALLTVHEYKSLYVISMWTVFLAMFWLAIWIFGVSGALSFQHGGYYVALL
VISLAWSMEVLRNTVNVTVASVIGTYYYEMGNMPHLPVLRSYQRAWTLSFGSVCLGSIFVTPVTTLHAIAKRLANEQ
GANEFLFSCVNCFLGVLEFFIKHFNKWAFVGVGLHGKSFVRSAKETWILFKEQETMLLVNDDLSGAVLLTGCIIGGV
VTALVGGCWTFATHRNLTVGVSIVSFLLGFFVTYLTMVVSESAVAAYFVCFSEDPRVLAKHDPALAQYMTRRNDQLK
QELED

>Pp1s53_68V6.1|PACid:18046262
MGCANSKVDNEEGVARCNQRKRLMKQTVASRHNLAASHAQFVVSLKGVGSAFRQFAEGEVKDVETHMICPPETPRTP
RTPSLSLGPPPPPLLSPRSFSSGLSSMFLPRPLPHSPPQVNHVELRSPNSRAASSPDSSSMDFLPPPPPIIAKEYRM
EHTLSVVPTKIYRFEESPNSYMPELYKLEESPPPPLIQPVQLDDDWRYSTQNLPVSRIARKSSPPPTPTISRGSWQ
DLFMDPFRPSPPTFNYMQSRRNQESEEKQSQKMEERRREQESEQRRIQEAEQRRKNEVEQRRFDEKQRKAKELEDSR
IWERKQMKVEEMEKSRAEAIPPKQPLEIIPHRSSSVEVECLEAVLDSNQKFLDQVSTSYPELEEADVPDLEDVDEVI
WQPAKDTNDEKVDKFVEGPEPEPETPRRGSPQVQEQSLKPSKVAKAVKEAKSPTLGKSTSKPPKPEQREYATAKFNT
NLAVIRAEKDDRDLLDVLKEVDDYFLKAAESGENVARMLETKKAHYHSSFSDGLKVVGESARINFLKLSGKSGPTGA
QHSRTSSNTSVLTDDSASVTSMRSVSNMSVARWSEECGLTGSHAGTLDKLNAWEKKLYLEVKEAEAIRIELERKYHL
FRNQDAKNEDQTNIDKTRASIKSLQTRMVVAIQAVDSAAQQVQKLKDDELYPQLLELLEGLGTMWRDMSLCHQAQLK
AVVAMKKLDYSAACAPTTSSHRHSTAQLELALNKWSEGLGRFVQSQRDYLKNLTAWLRLSLMQFGNEELERSGSRSP
LRSPGSPLQNTGASPVYSLCQRWQDALDQLADRVVLEAIGSFAAVVREMLSLQWEELRIKKRAEAHHKELEKREAAL
MSAAMRDPGSMPPPVPQTPSTTSGSDMDDGRSDSRTDSRYDIVPREVYSERAEVAEKRLKMEATRRKLEDELESERK
AHMDTRAYTLNSLQAGLPQLFQAVIAFSNLEAEVHDKLNTGFKQPRH

>Pp1s297_39V6.1|PACid:18046494
MAGEDAKKVSKKADDKDDREKEAAPSPPPPAPSPPPPKPGYFSVWAEPAHYDSTLHLIMTQLMEQHEGPAFLPHVTI
MSHHWSSPEEEAEASLKALCDCTLPFTLKVLRVESGTTYHQCIYLLMDKTQEVVKAHVEALECFKQLKEDSNLEEITE

RICSEYKPHVSLLYCHMRDEELDLAKAKVEAEHGKILLGKEYNITHFSLWYTEVNDESCISWNKYMDWPLKEPERPI
WAQGQGTPRSSLKPEPKLEA

>Pp1s11_22V6.1|PACid:18048660
MAKKKGGAGRAAKAGGAAAGGSAEVPPREFNEGDLVLAKVKGWPAWPAQIGKLETQGKHAGKYYVTYFGPGQQIGWC
LPVELSEFDADQREASIQKSLKKTADKKFISAVKEICALQDERVGDTPKDSAEENGELVEDDSTRDSGLDGREEGTV
EKEVKQEVFSMPVVTLNQQDESVYFNEGNPEEDADDGDGEKNWSERPEYNGSGLQMGKDSDGGGQEQEASHDGAVGE
GLKSFAEVEKPDSEANGGRRRAFGNALMRINQTSDKDVEEKVEIPENDTQVLTYQYGRKKNRNKTNMDESPGVVNGA
ASNPGQEIVEDSAAVHAVTSEFSKKIKPKVGRPSKASQQSKMGQDSKSTTQAVRKLSAAEKRSELGKGSGGGGRDGG
GKPGKDDDSALKRVSSDNGHNRKESDSKKKDVYSSSVKGEVLGGEKRERKLEGKKDVKGLASEVDRLKKGPKKSQIL
TKSSEESKKHSGTPKQRGEHVKGSVDDLEKKVPTSSPGSFGGSKVKSEVVGEASLKRRRLSDGEVEGEEGSAGGEKG
DDGVSPKVDAVEKSLPLLKRPKMLASEGKSWEGKQKHVSVGRSGVDGNNAENDSPRDGKSRERGTSLPPKADKDRMR
KAALDGEAALPPSKRRHRAYAAMSACEAEAATASAAESREQIGNGVSSNQDGNDVTAAASLETLSKEGIPVCGPPIG
SVASPFKNATVTPDIFESTDGGHKQQKVGSGHDNAGAQDRIESKKHMLLKDAQATDSPRKHSSSEDPSKVQTAKSEG
RAKSSKSKSPGPTRSSLEVATEKRKGHLSGSESGKLSTSMLGGAEAFGSLAKPSFEDSSIRNAVALANEKRNALKRE
GDASASMKVLIAVAQAKQRARQNSTGGSGSFAFAELDKFTNSMVCSPSPSQRRGSSGLTSPVQHGQGGVLTPHDGHK
RQDLDYIRHGETAVDTEATIARDTFVGMLETVSRTKDSIGRTTRQAMDCAKHGIADQIVEVIVRKLENEPSFHRRVD
YWFLLDSVTQVAHTQKVAAYLPIVQSFLPRILNAAAPPGGAARENRKQCLKVLNLWLERKVMPESVLRHFMTEIESH
SEDKGLPGNGPRRLSRSERAVDDPVRDMDGMLVDEYGSNASLNLPDGFSIIPQLYEEEEEDSEDGDKKLETEFLSAI
NTTRSTEGEALVERHPTVLEDVDGELEMEDVSPTADSEVPAMKSREQLLADRFAGDARTNSGQPPLPLDPPPSVPSP
PPLPADPPPSPPPPPRSPPPPLISSQAAYPSNGQSSAHSQDLSTHQASDSPSTPTTHSGLAGHQGQGNSGFSQVGSR
TNSYPSGPGSQSGASLDSPIPPLPPQHCSSGFALGTRLPPPPPLPPSCRPQRVWPESFASPAGGSDVSPRLGRPGEE
QVRQGNQEVSVSERAGDLSGGQQDAGPSQSGGCTSSGPSEGVQAGDQASGGSMSGYSSNAPLYLSQDNLYRSNAGGR
PGPGAQFPYGPPGIQSAMRGRSVGGHFSPYAQYQTPPMNPPGGQGPMHGFRPGGPSVSSWRSN

>Pp1s15_49V6.1|PACid:18050638
MLSWCSVCYSWVSYVDFGRGSFAALIQRLQKMGCNNSKIDNEEGVARCKQRKRVMRDVVASRHNFAASHVMFIKSLG
DVGVAFRVFAEGETTKEHIMYSEVSTPRTPTLSLGPPPATLHPPPPFSPGFSPSPPPHSPPSPHVKLSALRFQNGSP
ITRAVSSPESFAQEFFPPPPPPVALKEHRMEHTPSASITNIYRFNEGSSNSYKSYRLEGSPPPPPFQPIQMDDDWKY
SGRRAPPSNAARENNPPPPPPVTRSSWQDLFMDPFRPSPPVFNYMEERRNPDVGSKQSQEIEEQKRRQESERKRNHE
AEHRRMKEEELGRKHDIEQRIHEERRKAHELEERRNRELEQRRREESSARPPPVRIVEVKPKNFVDDIPDLEEDDIP
ELEDVDEDIDWPPPEDLANGKNIHAVKKPQSAPEPSPKRAPEPSPKPAPEQSPKPSPKPIPEPPPKPAAELSPEPTP
EKIPEPVPKPAAPKPAAPEPAASEPALPKSVPEPVPDPKTPPKTPSQTPPKFQEPPPKAGKAAKTPGKPPKPEFSIA
KTNKDLAVSKAEKGGRDLLEVLKEVDECFVKAAESGEIVSTLLDTKKAHYHSNFSDSLRGVGESARMNLLRLSGKSG
SSSFRTTSNLSSLSNTSVLNDESPSIMSIRRSISNLSSARFAEECGPPTHASTLDKLFAWEKKLYLEVKEAEALRVE
LERKFYLYRSQDAKNEDPAIIDKTRSSIKTLQTRMAVAVHAVESAAQQVQRLRDVELYPQLVDLLDGLGTMWKNMSQ
AHQAQLRAVETMRRLDNSAACEPTTSFHRQATLNLEQALNKWSEGVSRFVNSHKEYLKNLTLWLKLTLQQSSNDEGD
SRSGSRSPSRSPRSSSSEGMIGSSIYDLCQRWEEALALLPHQVVLEGIGSFSSVVREMLRLQWEELRIKKRVENYQK
DLERRELALHTVASKDPGPGFHDGSRSQTLTLRSPTTTSGSDADDGRFDMVVSSGFSERSEVAEKRLKFEAARRKYE
AELEAERKAYTDTRAFTLSSLQSGLPQLFQAVTTFANSEADIYDQLSTAHSLLNK

>Pp1s87_107V6.1|PACid:18054365
MEEFAEAYSVRLLAHTGRYLWADPDLKSVSQKKGKDTKGVIWRVELVPERGAIRLKSVHDLYLHASDYAFLLGATGK
KVTQSYASKADSSLEWVPEKYTPYVKLKARSNKYLRANRGVPPYRNSVTHDLPVLQGNQHVVLWTVEVVRTKDAPSL
QPSRLLTPRHSDATSSKSSSQHNQKEDSSDDEAPHFTPARGSTSRHKLKPTEIPPSAEPSSSSESLSPVSIRRTRAV
RVDADSSPPPRWNPPVREDGSPLPPRRRTPPARQDDSPPLPHIRTPLAREDGDSPRPRSPNPRFLEANNSPPPHMKI
PSTDDSDSPSPRIRPPTTLESSRNPPRESSESSVRRSRFKPPDNDEENESSSRGRASFKTAEPKSANARRINYEVA
EDDGEVLALEDGTFGTFMFEGSTVDELIEELRRKTKIDDIILCMRNNQKGTLFKMRLALPSNKAPLPVVVVRQNSSF
GRKLSTR

>Pp1s134_173V6.1|PACid:18054780
MWICGQEVCFGIQRGRGSGEGVKMERKLSVGILALLLVPFLCVLDLVAGQNCNSTVPCADPNNCCSQYGYCGTDDAY
CVIGCQNGPCRDSPSPPPPPAPPSPPSPPPPPRPSVSPTPSSGAGRLITRKLFEKLYPNYNKTFYSYDAFIVAANAF
PKFLNEGCRESRLRELAAWSAHVQQETAGLVYVEEISKSSVYCDATSTRYPCEPYQKYFGRGPLQLSWNFNYGPAGE
ALGIDILKRPFLVSFDPVLAFKASIWFWNTAREGGIPSIHDVIIGKYRPSAADKAANRTVGFGYTININGGIECGK
GTATPQAANRVKYFLEFSEKLEVSPGKNLDCTNQKSFA

>Pp1s134_145V6.1|PACid:18054792
MSAPAPTGGITPPVNSTTPPVAVVPPVAATPPVVSPPPAIVPPPVIAPPPVELPPPPPAPVISPPPAIPPAVVPPPP
DPTPPAATPPVPVTPDVPPPTAVAPPPPTPTNSPPAPTPPTDSPPAPPPPTGTNPSPPGLSPPSAVRGPPPAVLSPP
STPSAGGTPPSSSGSSSLSTGAVVGIAAGGGILALFFLFALIAFCRKRKYKKDSLPYTAAGGGGGGAATAAAYEVG
GMDDPKGYAAGGYAAGGNGRLPPGSHGGSVPLPPDGTSSVGNSRSWFTYDELHAATNGFAIENILGEGGFGRVYKGE
LPNGKVVAVKQLTLGGGQGDKEFRAEVEIISRVHHRHLVSLVGYCIADKQRLLVYDFVPNGTLDVNLYGNGRPIMNW
EMRMRVAVGAARGLAYLHEDCHPRIIHRDIKSSNILLDDKYEAQVADFGLAKLASDTHTHVSTRVMGTFGYLAPEYA
QSGKLTEKSDVYSFGVVLLELITGRKPIDTRNPAGQESLVEWTRPLLGEALAGNMEELVDPRLDGRYNYKEMFRMIE
VAASCVRHTASKRPKMGQVVRVLESEEENAGLYHDLRPGHSSEHEPSFDRYGGGSDYDTQEYNSDVLRRKRRDTNKS
HGSEYTSEYSSSLYPTGTVDSSNEFESGNSRPGDTRSNIVEQPPRRPPVSVRTGRASLATIPVPPNVTHTSLSGLPP
TRAAPPRPGQLGNSSVASSDTSGSVSSSTFAPPPPPPNFSDEYDPIDINPKGIRKSYDDLRDGR

>Pp1s35_281V6.1|PACid:18055126
MAKTVGKPRAASPSVTTGSSSYTGSSSASGSRSRSPSSSSSGSEVSGSRSPPAKTSGVSGAKKIRSSSPPPKRISPA
KKLRTPTPESTVLHVGCLTRNVNETHLREIFGNYGEIVHVELAMDRTVNLPKGYGYVEFKTRVDAEKALAHMDGGQI
DGNIVSAQFILVPRKKASPPLKRGVSPPPKREIMGRDGPPRDGLKDGSRRRDSPPPRRGRSPSPPPRRRSVSPRRRM
ADSPPRRRPALASPRRRSPSPFRRRRRSPIRSPPRRIRGSPVGRQRSPPPPYRRRSPPPGRRLRSPPPRRSPPRRRT
SRSPPPRRPLRSRSRSRSPPRRARSPVGKRRKSTSYPSSPSPAKKRTSPPRGAKDFSKSRSPKKTIANSSPSGRSSS
SPSPSKSSQALAKSIPANN

>Pp1s16_181V6.1|PACid:18055578
MASEQPFKKRRRYEQLMAEGVAVASSSAVLSSPLRESSQVVAGPDAAENLSASVGVEQPSDRDGGKNNAVVSVGKHQ
SEKDGLVSDKRIAIARERQSAKVKEEAVSEKKDTTLLEGQGVNLKEEAVIERKDADLREIRGVTVVEEVVRGIIDEN
VGEHQSVDEGVMSEGKVEEPAVVKVESAGLERPQDVVQGSGEDEKLRKKRNREEIGNFYKAYRRVRICLTQRDDSAT
FGVKHDLEAAYHSMIELSQGCVSVRRIAAELVPRYVIFCPTALEAAARMTIQLSDWSSSILLKGGKEGEGLAGETAE
ACFDGLVNIVTAAVNSAFELSSLSAMCSEVCRNIYLYLLRQLDGRDLLNVFNSSGKDEVKSIKHKIPPNGEDIDAIV
DSINPEKLGALIISSTVQVFTCDPQGVLTVCFELLRATDADSRKAGQHFLSQIVKFRSNTKVSNASPVEKVQSSAVA
ITSEVDGSKQSEVEETVVNIDFSSSGPDREEATIADSSLVATVIHSKPELERWLVSAVRHFRRTASNSVIDEALPVL
LPLLKSLHTFTPLDLELATLEELDVGPSAEDEEKSRGRPTYRFPRPGEQPAVSWNGTQEFSKEHWDDLDNPSRASES
TESNRWGFGPSSQSGYMRGPFSSGPTECSAIPATSPVPGKEEIWRRGKDHTIVSASMELLAQTEKPSPMWDIEGSQP
VHPWEGPSGSWGSGRPASRGYEHVPPKEFGSHKSPPGADPWRLRERDSVTNKDGSLREPNDVSPFRNLEVDVPTTWP
SMSWHAGGRGPQDKREQSSGSDMNGWNHVREFPNDGSRVEANTVGSSLLPSPQQPLTPRAQKSSPNQCGWFSDGDAV
AMDVYAASTHLLVGPINPPISESGIKFHVEKCVTIDSFLRNQDYAVLGFRTVRDAAKAREVLQASIWSKALRIKFIE
NSTKTSDSAANIVAVGPSCFVWVGGISSQNAKEDLLKDVLGAGLKQPRSVTALVNASAISLEFESSEDAAAVLGHIR
QRRREGGGPLLANSKAISDRSIMGSMVQPADIPSPVPNRHLWIGRVDPLICEEELLSAFNHFGDITGWKFLRQSGCC
FIDFRSPECAAMAKATLNGTRFGNQCINVEYKNAPAHRNASGPLLNSPPHPPALLHTPPFNGSSSGRSPANPALNQA
LVNISNKLVNFTGGSMGNFHAGRSRGGRGGGGREAAERVPTNTLWVGLPDMVGPNFMNEAELKSVFNLAATGVGVVT
KVRSARTTRGPCRFVEFDRVDAAAVALRAVSGRLDPAIQIEYSNSAISLQHTEHPHPHGHNHGHGGPGHPQSPFMDI
RRRGWDYQHEKERPLGTRQREWDKEGEERDSPNRMDFDDGNMASGQGHPGVPPGWGNIREKDLTEEEYHLGSRDQHQ
RLGRFESSVEYRDDSSSRIDMTTPRHDSSANFQNSNSATVDVGPVVVTESSSMVSQSSSHNVLGRKPIERQSSSSAS
LIRPPLPPQGATLAPLPMLKHPSRAHLERVPSNNSWSNPMGGVSSSPLGPVSPVTSTPVPKSVLASPLGTPLQYRGP
TKLDKSGGRSGPPLLQSPVTPALSALSVSQPLEHLNQPTEQVSSLPEQLSSPLEPVPNALDRVVSTFERANNHVEVV
HPPLPPLPPISPPPPPPSETPPPPPSSPPPPPPMPPPPSSPPPPLPSTPHGFSAHASVAGHERRESAAVHVRGAPAV
EEHQWRGTLCKSGMQYCQVTAHRQNSTYSSYDRAPFEPTGWPEKLDVTKRADFKSVKSTFQNTPPTQREVCRLIAVP
EHRDGFEQFVTYLKQRDRAGVVKLPASDRLWQRMLYILPWSAEICSMLEIPQLPSLCLIGIILPAPPSMAGS

>Pp1s5_354V6.1|PACid:18056352
MSEMTREGEGEQPQAETDPSSIFDQLIIDEDVQISNSPRRKDGKKRAAKPARSPSSLTVSPPPRDNSAFFQPQQQQQ
PSYLSSPPPFDMSIFMPSPTAPWIKFPLVLHSPQSVDSHCLNLPNSTGGNLTPNHNVQLSAAPFDQLSPPDSDLLDS
SPEYPWFPAPQNASPSASNFGPLLQLLNLSPMAVKVGERSLPDMSSYNGDMNSVAASSGMSSDAVSNQESSSRRGRS
PSPTPSRLSDVSPMNSPLTSTPNSPFDSPFGSEGGYFSDGGTPIDIIGVLERSVSSSKRKTPDRALDESEETQSLDE
PDTKKQATKKEKKGTVKLNKRVREPRYAIQTRSEVDVLEDGYKWRKYGQKAVKNSTHPRSYYRCTSHTCPVRKRIER
KADDPGLVITTYEGTHNHLSPVTEAANAVAASLKNSDTSSNTAPSEENRAAAGTSFPFPPIQERNEFDLSVQIRSGE
IDLDAANRDVTPAANAMSQASQSLPAGPHMPHIPTSTDSYRVHNPPQTLNQAQLEAERLLKELQGPMPASMVIPDPQ
LGRSSQEMFDLSNPLPHAQSYQGLLDDMLRQSGQHHL

>Pp1s157_87V6.1|PACid:18056644

MAKSTGKPRAASPSVTTESSSYTGSSSASGSRSRSQSSSSSGSETSRSRSPPAKTRKLRSSIPESTVLHVGFLTRNV
SEAHVKEIFGNYEEIVMWSWRWIALFLFEVFSQQFWTRVTFKVNLPKGYGYVEFKTRVDAEKALAHMDGGQIDGNIV
SAQVLLVPRKKASALPLKRGVSPPPKRELSGRDVPPRDGLKDGPRRLDSPPPRGGRQPSPPSQRHSVSPRRRMAASP
SRRRPPVVSPRRRSPSPFRRRRRSPVRSPPRRIRGNPVGRQRSPPPCRRSPPSGRRVRSPPPRRSPPRHRTSRSPPP
RRPFRSRSRSHSAPSRAIANSNPSGRAYSPPSPSKSSQ

>Pp1s65_286V6.1|PACid:18057740
MSRYDDRYRGSHRRDDRGGGGGGGYRGGGGRGGDAVPDAGATRLYVGRLSTRTRGRDLEDLFAKYGRVRDVDVKHD
FAFVEFADARDADDARHYVNGKDFDGNRLIVEFARRGPRGAPGGAREYLGRGPPPGTGRCYNCGNDGHWARDCKAGD
WRDKCYRCGQRGHIERNCRNSPRPTSSPSLSRSRSPRGQKRSSRKSVSRSRSRSHSRSRSRSYSRSPPRRGRSPVKG
DRSPAKDDRSPMKDDRSPAKSDRKKSARSKSRSLTPLPKDEKRSLSPSPVPNGRSLSRSPGPRSPSPEAHRGEQSPP
PREKRRSISPSPDQDMDKDSPAKDLSPPPRRSLTPEEE

>Pp1s82_27V6.1|PACid:18057833
MLEKREVLPEASVWRNQQNNMLSDVAMQFAPGAELMVGRSKMQPWSEHNESSCMTELASPPANPTAFWDQTMHSQIE
SSNLFGMTTSNMDIDFQPSTPTFYSYARETSQGSASTGSSTYSDQEGERMKRRRMLQFTGVNTGGFAHDSPPPYESV
IGSPTKTFSSYSCSSSYANSLSSVYSGDDYMLQPSESPGLPSSSWVSGSDVSTSCMLPNIQEPLDKWSSLTSFDNIQ
SYSASMQIGDLQIPTDSYIPNSFGEIHSPQQWQRQSSPPPQSSFCRQRQSPPQMQFQQPTQCEQTQQRWLQAKAEPG
QLHSPLTAFAKPPTPGELPFQHSFRIFIRKVRYDMCEMNPEKLCIDMYTSYCVNDVDNGLTWAYAGRRHLGPSRFKT
KSMTPIALPFAMLKPSPAQGDVTLSDINKFLMNPPTTLTDGPSPVEEKKPPTPPGAGLSGKSVFACTKIRTEGAGTI
TILRTKG

>Pp1s38_278V6.1|PACid:18057960
MRELSSNDVFRGQFPMKQDCGGAGSDITGRCPARPSSCSKTSELQGHNIFGAFLNPSQPSPPPPMPPYLPEEIRLPP
DKIRRNPQKLASHFTLGGPEYLDPPGPKSESSWARQRMLYGSVIDEMQGDGLPVSLVSDLKARELAGHTNLFGIPPR
FSPAFSPPPERRIIPHCVAGNDPDDGRQNHRKKIQDLVGYKADLISDDRSNTHRPCRHPVSDAKAHELRVQEDVS

>Pp1s140_87V6.1|PACid:18059532
MTREDDGEQPHVETNSSPEIDYLMRSLGEDVKLSNSPRRPGLKKRAAKPAKSPSSLTVSPPPRDNSTFFQPQQQQQQ
SHPSPPPQFDVAIFMPSPTAPWIKFPPLLLSPQPVDTHGLNLPNTTIGNPTPHQSVQLTASPFDHLSTSNSDLLEPS
PEYPWFPASQDAQPATPNFTPLVQLLNLSPVATKVGERTLPTLSTYSDGMVSMPASSRISSEAVGNQDGFTGQGRLP
SPTPSLVGDVSPLNSPLTSTPNSPFDSPFDSPFGSEGGYFSDGGTLIDIVGVLESNTSSAKRKTPDRALDESEDIQP
LDEPDPKKQTTKKEKKGAVKLNKRVRVPRYAIHTRSEIDVMEDGYKWRKYGQKAVKDSPFPRSYYRCTNQTCPVRKR
VERKAGDAGLVVTTYEGTHSHLSPVTEAATVAAASAKGSDGFNATLSEDSRPAAATCFPFQPVQERNMFDLTMQIQA
GEIDLEAANRDLASVAKTSNEESQPPPVGLHTHNSLYSDSHLPNDSHILNQAQLDPDRLLELQGEIPASVVIPDPQL
GRSQLEMFDLSGQLSNTESYVSLLGDILQNSGPLHHL

>Pp1s115_10V6.1|PACid:18060186
MAKDTQSAGSGINFSFLLAWFAYKIDKAALVLCPSKRCIPPLLCPLDKKHKPASGKLSQAFFAFPITTELEFTCLGE
SSAVLRVILILNTFLWRRCKAFIQSWKAREVKTARSITCFVYYSELATFVEGYHRGYSMSEMARKNASDQRPVADSS
SGIEQLMRSLGEDVNLSNSRNDTTKTTTKLANASSLTVSPPPRDSTIFFASCLGLVNSPPPFDMSLFMPSPTAPWIH
ASPVGDTTVQSAQQTSHGLNVNPNTVSNLGLTNNVQSTPFEALSPSDSTDLLDASPEYPWFPRDSNALMSNYNPLLQ
HLNLSPVATIPEERGLPSSSYNTGICIAGYQTLSDPFDLSSLAVSNEDKSPKRGSSPSSTRLNDVSPLTSTPNSPFS
SEDGYVSDCIADDSAVGVSHNGSIIKRKTPDRALNESEENQFPSEPSPKKQTTNNLKKEKGAKSKRERKPRYAIQTR
SDVDIMEDGYKWRKYGQKAVKNSPYPRSYYRCTNPDCPVRKRVERKADDHGLVVTTYEGTHNHLSHTTAGCLSKSSD
TSATAPFSDQDPGTGVANFSLQPLSRERSEFDLSIQLIGQQGNQACHDFATTGTSASSSLSATQGMTMLYSLSRNPN
MLSEALKENPLLGNDQFNNDRLSELQGTMLGSMAFPDPQLGRAHEIQMILGHDLLPNSYQGLLDDILRQSNGLHRHL

>Pp1s133_139V6.1|PACid:18061689
MGLTPFSCVTVQGYVRVVYPDGHVENLSKSCSVHDLLLGNPDYYVCGSTPYTITNRMAAEEVLEYGVTYFVCATPNA
QPFLERQPKVVHRGSKILPRFSKHGVHVRELRSPTHGSQQSRKVFDYHSVTMQQLESIRNEGPEPHLAGDRPSKHLK
LVFIRHCLRALRLPRISIDLMESPLPNLSGEALSPTATAEDEITQMILKSAARSELGMYVSKRQEFYLRRARRRRKF
AWKPVLQSISEMKPVMEFHTPMAYRDSGSPPKNASTPSLPGPKNISPPRQVSVPQRSSPPPKNVSPPPQPAFVARTA
SKYSAASQQVQRNRGNAKSLYMA

>Pp1s97_6V6.1|PACid:18062368

MEHGNALSRRKWEQWRLVMTVLAIGLSSLQAVDAGLGQWRLVTQNAGISAMHAAVAPVSGVVVLLHHTNSGPSNITF
PDGRCRNTTYDRLLKKDCFAHSVILDPWTGVVRPVMVMTDTQCSSGQFLGDGTLSQTGGDAEGTKRTRKLSPKCYTT
VAQKRYCDWVEGGNGTVLLSQRWYSTNTLLPDARQIIVGGRNTFTLEYFPRYSPPSPKAGLIYLQFLNDTADAKGPN
NYYPFVHLLPTNQLYIFANRDSILYDYKQNVVLRKFDKIPGNPRNYPSGGSSVMLPLLYNDNFKKVEILVCGGAAVG
SVANVKAQMNCSTSCGRLDVLRKNSTWAMETMPMPRCMGDMVLLPDLNVMIINGAKRGFQGWKCGSEPTLNPVLYEP
RKIAGNRFTVLNPTQTPRVYHSTANLLTDGSIMLAGSNTNRYYAFKPKTSTVDFPTELSVETFMPPYAENQPNSGGR
PVIISVNATTVRYRSTLELLFWDYPSNGASAAPSPPPSPSSSSSSPPPPPPPVSNPDRFDFTMNSSPWSTHSFSHGQ
RVVTLYPISLTTQPESRFENGRWVNVRTVQLRVPSYSTVLPQTYYMLWVVKNGNPSTSCVWIRVRG

>Pp1s196_28V6.1|PACid:18062419
MNMKKPGSTALGQADLYETTSSEPSVVVITCASIVEPFLAPKSHGISSREDFPKLIRGAEVDILVEVPDLSEKGSGY
SGEHSVCWLPGQLLAVVDVPAAGAALQDLLDVHGGSVKGVWEVGWALAPVEDNAQQLHSLLTSEILTETQLQEDVAS
QHDLKGDKRPVAHAVAGVSRLGMAAAAATKIAVLSVGSHGDSSALNTCLKKRHQLSKVSNGLSKMPTGHVIAEQKRR
GDSLIAVGSPFGALSPLHFQNSVSVGIVSNLWPPTRGPPSLLMADVRCLPGMEGGPVFDERGNLVGMLTRPLRQRGG
AAEVQDVTHFVANMHMQLVMTTDVLLPVLQRVGINVGVLCNTKSPDVQLQPSASAMLVENSSFETQPCRPGSIESHS
TYQAMQYVPSAVERAVTSVVLITIGDGAWASGVILNKTGLILTNAHLLEPWRFGKPRMVPSPVNGSIPKDSGFPLSC
DESQEDLALNQLQEEEISKGVSTSRTSSWPTDVSQKNYQRIRVRLDHRQPRSWHAARPVYVSQGPLDIALLQLESPP
PGLHPITPDKECPTPGSTAVVLGHGLFGPRSELRPSVSAGVVARVVKAGSSPFLTGIGGRKEGKASKAAMLQTTAAV
HPGGSGGAVVSGEGHMIGLVTSNARHSGGTVIPFLNFSVPYAALVPVFEFASSADSDWSRLEELDKPNDQLAAVWAL
VPPTPPRPSPPPFFRRHPRVPVSQEIDPDKGVPKGSRFAEFIKEKGGLDWKSPKSLPPPLPEESPHRGSPPCHYEEP
SYAARASFPIHSRL

>Pp1s78_56V6.1|PACid:18063419
MASRRAAILARRGASCISSSRRGGAKPVCSTGELLECSFQSGEFSAERRFSTVPSFNGRVERAADLSSRFTLQSKSP
TVPKNYSRGQKSSLHASSVNSVMEIGMPSLSPTMTQGNIAVWRKKEGDEVVAGDVLCEIETDKATLEMESMEDGFLG
KILVKDGAKDIPVGQAICLMVDTKEELESIGDYKPSGGGGDSSPPPKKEESTPSPPPPPSKKQDKSEPTPSKPGHAT
PSPPSGGNRIFATPAARKFAEEKKLSLTSIEGTGPDGGIVKADVEAYLASKPFPSEKSVSRTDQHVSGGAPPKGVAP
IDDLSYTDIPNTQIRRITAKRLLQSKQTIPHYYLSLDIRVDKLLQLRGDLNASLDASKKKDAPTKKLSLNDFVIKAA
ALALQKVPEVNSTWTDEYIRQYHNVNISVAVQTEHGLMVPVVKDADKKGLATITEDVKTLAGKARSNTMKPSDYEGG
TFTISNLGGPFGIKQFCAIINPPQAAILAVGTTEKRLVPGLTPDQYDVGTFMTVTMSCDHRVIDGAVGAQWLGAFKS
YIEDPVTLML

>Pp1s198_51V6.2|PACid:18067874
MGRGRGPNTADSYIGSLISLTSKSEIRYEGILYTVDTENSNIALQNVRSFGTEGRKKDGPQIPASDKVYDYIIFRGS
DIKDLQVKSSPPPPLQPPPLPQQTDPAIISLQSQPQYPPPSSLGSYNSGLSAAGSSSEPSASPPPPPPPYMGMPSQY
PRAVPPLYPPGGMQSWGPPPPPGANGSGLAMPMYWQGYYRPPPAGHMQHQPMPPQPPPSLATPPQGQPQQQQQQHG
QQQIQQGQQAPSLAQQLPAVSRTPGPVTGASPSGGVTPASQTQASSSNVAAAPMSSPATSVAPLAAEASPPASTVMS
STAPTVTTAVTPLASSAGTAASASPSAVSVSAPMQGVSVVITPVAKNPRRPVANVTQVPQAPSQTVGSSSNGSQSTS
SATVVSSGSMVRPQSEAMQSSGIVATAAKSGAVVAQSQAPPKVRQKPADQENAARAPIVQTSSLAAPAQEQVSQPLL
PLPPADQKQQQRGSHGVPTGQRNHWQGNGYMGNGNYNRRGRGRGNGSGRGMALANPTQQFTEDFDFTAMNEKFNKDE
VWGTLGGKDEEEDYEYENGNAEDEAADTTHLDVSKKALYNKDDFFDSLSCDASEGRGERTKFSEQRKIDTETFGAFP
VRSRGGRGGRGGRRGGYRGGYYGGGGGSYGIGRGGGYGRGRGTGRVSTGNL

>Pp1s75_115V6.2|PACid:18068234
MKALIAAAQVKQRAKQNFTGGSGPFVFAELDKFPNSMVCSPSPSQRRGSSGLTPPVQHGQGGVLSHEGHKRQDLEYT
RYGEAAVDTEATIARDTFVGMLETVSRTKDSIGRTTRQAMDCAKHGIAEQIVEVIVRKLENEPSFHRRVDYWFLLDS
VTQVAHTQKVVAYLPIVQSFLPRILNAAAPPGGGARENRKQCLKVLNLWLERKVMSESVLRHFMTEIESHNDDKGLP
GNGGRRLSRSERAVDDPIRDMDGMLVDEYGSNASLSLPDGFSIIPQLYEDEEEGSEDGSRQLETESVSAINTTRSTE
GEEPIDRHRTVLEDVDGELEMEDVSPTAESEVSGMKLREQLLGDGYSEDARTNCGQPPLPLGPPPSVPSPPPLPADP
PPSPPPPPRSPPPPVTGSQAAYPSIGRNPAHSQDSSTHQGSDSPSTPSTYSGLAGYQGQGSGGFSQAGSRSNAYSSG
AGSQSGASLGSPIPPLPSQHSSPGFALGTRLPPPPPLPASYHPQRVWSESRTSPGGDSDVSPRSGRGGEEPVRQGNQ
EVSGSERVGQLGGGRHDVGPFPSGGCTSSGPSEGVQAGDQGSGVGMSGYSSNGPLYEKEDKSYRVNAGVRPGPAGAQ
FPYGPPGVQPAMRGGPVGGHFGSHAQYQGPPMNPSGGQGPMPTLGFRPGGPSHGSWRPS

>Pp1s236_45V6.2|PACid:18068933

MAMPWRELSKGWRRRGASGSRVGMLEWTGLRAQDSGVLFGPVNSGSVLDLRSHHCHHYHHHHHHRKSPPPPSPPPPP
PPPRAILSSLELSNAFILDPPIAPSIHKYTTTVGEYVSKVHIIVSVPPDIQAYRLTVNSSNLLSGEESPPIVIGNGE
WVDVVVTISAPSHSSNRYLISIYRLLHVGSLRPAQLSKPLLLRHRLHKQACHHPRLLQQV

>Pp1s118_176V6.1|PACid:18069093
MAKLGGKSKAASPSVTSGSSSYSGSSSASGSRSRSRSSSSSGSETSRSRSPPAKSSDVAKKTRASPPPPTKSSAPKK
LRTPTPESTVLHVGCLTRNVNEAHLKEIFGLYGEIVHVELAMDRTVNLPKGYGYVEFKRRGDAEKALAHMDGGQIDG
NVVSAQFILVPRRKPTPPPVKRQVSPAPKREIVGREVPPRDGVKDGLRRRDSPPIRRGRSPSPPPRRRSGSPRRRMP
DSPPRRRAPAVSPRRRRSPSPFRRRRSPIRSPPRRIRGSPVRRPRSPPPYRRRSPPPGRRARSPPPRRSPPRRRASR
SPPPRRPLRSRSRSRSAPRRARSPINKRKRSTSYSTSPSPVKRVSPPGAKKFLKSRSPKKAFANSSPSGRSSSPSPS
KSSQALVNPITVSN

>Pp1s27_365V6.1|PACid:18070714
MERSVAMVMVECAASLPSSSLALRAHVQTRIASVASTWAYFPVQETEGSRVRGLQCDDAMRGVGGWRRGGGRRREVV
RMAVGGEPGAAAGGSGGSASWKERAVIFEESKKLLAMQKQLLDQIEERQKLYSSPTVDTDQPSPSIVTESSSEPQAG
TPEPSVETSETFTSTPTIPSSSARPLTAAWPSPPPSKRSVGSGRAVAQANSPAPSDRVETSPKTSPPPSPLAQSKRD
FHSDHSRESTGGNNSEVLQQTRTDAAAPSAAEKPPPLAGPNVMNVIVVAAECAPWSKTGGLGDVVGALPKALARRGH
RVMVVAPRYSNYAEAWETGVRRIYNVGGQDMEVGYFHSFIDGVDFVFIDNPIFHAFANNIYGGSREDVLTRMILLSK
AAVETPWHVPCGGVCYGDGNLVFIANDWHTALVPVYLQAYYRDNGYMDFTRSVLVIHNMAHQGRGPLADYHRLGLPG
HYIDKFRLFDPLGGEHNNIFMAGLITAHRIVTVSHGYAWEVQTPEGGWGLDGVLRDHNWKLRGVVNGIDDKEWNPEL
DVHLKSDGYTNYNLHSLDTGKAACKAALQRELGLPVRADVPLLGFIGRLDHQKGIDIIGQAMPWMMDQDIQLVMLGT
GRKDYEDMLRHFEGSHRDKVRGWVGFSVTTSHRITAGVDILLMPSRFEPCGLNQLYAMRYGTVPVVHAVGGLKDTVQ
SFNPFNESGLGWTFETLHVDAFIHALGNAIWTYRDFKSSWKGIQQRGMSQDLSWDHAAQQYEEVLLAAKYTW

>Pp1s34_212V6.1|PACid:18072425
MADWESRAKRGMKSASQQSNAELLRAIAARRQAQLLRNNKAPSPPPDEVPTEKAEHVHAEVRLAASKILPESSEASG
ASSDDDGSFDSTSLPGEGGRCKESTPKDKSEGSSMDDLRTVLDQMSIKDKNGAQSSDRPGILEKQSERKPRDLILNI
RKRLVKKSTIVDSDNESESESFDGVDPVSSPSIDYNSADDGDVDSPVVSKVKSKIKPAGVAPRRYPSVDTRPSSSPN
ADVEHVRAPGFRSVAKGFTETTLNTVDSPPPSAAVGNLINESDVPEKQQELVLSDGKHRFCLRPKTANMLYPHQRSG
IEWLWSLHMKGMGGILGDDMGLGKTMQVAAFLAGLFCTQNIKCALIVAPKTLIAHWVKELKVVGLSRKTYDYSGTSV
KAREYALQNVLQMGGVLLTTYDMVRCNFKALRGDFDGRDGYGDTSDDIITWDYMILDEGHLVKNPNTQRAKSLREIP
AAHRIVISGTPIQNHLQEMWALFDFCCPDLLGDRKEFKDKYERQILAGTDKNASDRQKRIGIQVAEELRQKFGPFFL
RRLKSEVFPDSEDKKERKLSRKNDLIVWLPLSEGQEKLYRAFLNSNTAEETLSTGTKVLSALTVMKKICDHPMLLTK
RAANDIAEGMEGYLDAEDIQAAEAMTHSLAGMVQDDEDMSATSCKIDFLMALLDNLVAEGHRTLIFAQTRKMLNIIQ
DEILERGWIFRRIDGTIKAADRELCVQEFQSDDEIPLFLLTSQVGGLGLTLTGADRVVIVDPAWNPSTDNQSVDRAY
RIGQKNDVVIYRLMTCGTIEEKIYRKQVFKGHLMKVATEKKNQMRYFSQGELGEMFKVPEVGFRVSETQLQLHKEHS
SQHKIDEGLQKHIEFLDGLGIAGVSHHDLLFTKEAEPLPPPGSEDQDLNATWEPRNPSYVGHRYKAASKPEKYSWEA
SQAVGRGILATATAGLQSQGVDNSNALKVEFLRGKLERLSRTLDDKAMIARLPDHGVGLEKKLKETQKELREAELIA
GSKENIGAHLPSRSAAQVPGADLSADETYAVTNIAEGQSAQRPKPLGFNNNQLTVPTLVSKKNSCFQETVPATQTAS
VPGVQRGVHLPSWSLLSDSPTSSRGSPAPTSREDVDDMAEALSSLRLRLH

>Pp1s57_170V6.1|PACid:18063894
MGLHRSILELVVVLACSALLLPVAMAVEYVVGGPGGWTSVPTASHYTDWATEKHFVTGDKLNFRYDPTEYNLQQVSS
NDYSTCNTLHPIRQYQSGNDVVKLRTAGTYYYISGFAGDCNEGGMLMKVVVAQSLGPASAGESPPPLNHSPPPQNHP
PPAASNSPPPANIPPPPHHSPPPPPPPPHSSPPPTPASSPPPPPPTDISPPSPPNQLTNPPPPPPPAPANKTSHAHP
AKTAGAPSASTLLPPTPSTDTAPSPSESTPDAANPRRVGTAMVLAVSVLVTAALL

Selaginella moellendorffii

>417164|PACid:15401545
MMSSAWGYTDLSSGGVPWPQTGIAGNRCDCDSFDSLERERKIGGWDDVPYGTGGGSFPAAKEVVVDQECPSWPLRLC
STTTQGFQGGIETSREFEALVSVPESLTARSSSSSSNVASTSAARFDSFLDENGDASVASSSCHFPLSEECLGQAPS
YKEVADGWDAIGSLADMDDFLGHNPPFGQANSLEVQSQMMPPVPRISPESCKKKLTFEQPILIQHEATSAKRLKTLS
PWISGKPSQRHTFHQEFNSLALPVVEPQGTATASSIEHLLLLQRAMNYNGAIQASNSVLGGAYNYAAPPAFGDGSLE
ASVLYQLGCTMSKLDVSTRLCIRNALYRLATSACHRSGDEGASSIESSAKRPGGIPCLETCTNPIDRTVASLLFYKP
EADTTQDGSVSPQSTVVSSEGSWIQQPVAMAGVTPQATLPRCAGYFARGAAGLDRGLEPALVGDDFGFFSGECEVAA
AFHNPFVAAAVAHRDAVAAAAAPGATFTTVLAAAKRIRLGERCRGSGLEEEDSAPEKTRSQSCRRNAVKEKLRDRWK
EGMLPSFWDKSGGYVLPKYASEPFSPARVPEPVPSNDDDWDKELAPEDLEEFVLSALSPEALDNGKPDIENGIGAKG
DGGVVSAKPGLDRGQQSHSHSQTGVSSSERIKGKAAAVEPELSADALVLQELETVVSKLESRSRLCLRDAFYRLADN
ATRRTGVQADAAELQRLSWRTASLSLRQELSTNTIDRSVASMLFCKESPPPPLPLPAPSSPPPLPPLPPRPRSSPGH
PPRPKSSASSSSSIPKKQHQHHQAPLGNNTNKKLSLAPAHKHRCAGSTSVLRTATTLRATPAAIPGAARNTKSLILL
PPSAATKRRSPNATTTFTSTSTTTTTNKRGKL

>408701|PACid:15401715
MMMRQKRGPPPLPLSPPPPPPPLSPPPPSPPPPTLYFNMLLDALTSFQQDLHATTTTEECTGKRRNSLWDRFTICGA
AYEQGHGQGPWTCKFCNELKSGLLKRLIAHVGRVPGEEIEFFSDYHHCEPPQKDGLAICTANAQYVNIKGEIFKTPF
RRSASATCPPIVTHVTSSFPLGSNAPSQQSLAATMDSAASNDNNAADAQTGQALSGGLSSRQQRSNMSGPMNSSGGM
QLGLGMAAGRQQSLHTTWQNRSYIDADVVVRRFIY

>409370|PACid:15401746
MEALALAFSWLRRAPAQASTQASTQATGLKVLLLETPAATADKKAEGFPALPSQLEAVIASSGPASSVPEYDAPVLE
EDDEHDLEEPVAPIELEDSSSPAPPSQLEAVIASSHAAASSVSKYDPPPLPVLEDENHDSQELEADMVGTVDSVRYT
SLGGFAIDDDDDNLSWEDIQSYFRTWTFPSVQLPSRAEMHLEVEELRQLVGEWTWKLPTTLQSIKEEMWSRLLNAGL
EEDEVTSQELWEELKNFLGWHRFLVISSRRMLLEAAPGPPIFVSNENGPSLFRWVDGFIAPLRAQQNGEEQASVFWQ
RLRGDGTSSVRKHRRPPDKNSEGQELAAAMAEESSYKVLKSHAAAGSRKLLRQGDVSSTPASVFWRSLRGDGVRTHR
RLPYKNSEELAAAMADETSYKVLKSHAATGFPSQKLLAGGTGDSKEASSNASPPPASPPPFMWRWFPRSLGEDGAKA
KRINTETFQEAEGTSISSSQSLVYDVGVSSSSASASDFIELFEGEGISSSDAFLNSLLEGEGGGSSSNAYLQLASSN
DLNLQATAVAATPLLLPFLAGLLVALLFLFFLYFLSVFIGWRDGGSNVIKAEEISYKTEVAGGSGALSNFLHRLMVL
VGKQKKLPGLEVTITGLCS

>415984|PACid:15401904
MEALALAFSWLRRAPAQASTQATGLKVLLLETPAATADKKAEDFPALSSQLEAVIASSEPASTVPEYDAPVLEEDDE
HDLQEPVAPIELEDSSSPAPPSHLEAVIAGAASSVPEYDPPPLPVLEDDNHDLQELEAVMVGAVDSVRYTSLGGKTL
AIDDDDDNLSWEDIQSYFRTWTFPSVQLPSRAEMHLEVEELKKLVGEWTWKLPTTLQSIKEEMWSRLLNAGLEEDEV
TSQELWEELKNFLGWHRFPVISSRRMLLEAAPGPPIFVSNENGPSLFRFIAPLRTQQDGEEQASVFWQRLRGDGTSS
VRKHRRPPDKNSEGQELVAAMAEESSYKVLKSHAAAGSRKLLRQADVSSPPASVFWRSLRGDGVGMHRRLPYKNIEE
LAAAMADESSYKVLKSHAAKGFPSQKLLAGGTGDTKEASSNASPPPASPPPFMWRWFPRSLGEDGGKAKRINTETFQ
EAEGTSISSSQSLVYDIGVSSGSGSASDFIELFEGEGISSSDAFLNSLLEVEGGGSSGNVDLQQASSSDLNLQATAV
AATPLLLPILAGLLVALLFLFFLYFLSIFIGWRDSGSNVIKARSEWRYLTKQRLLVVPEKNSFRGLLCLTLDRAAAM
NIASIKPPHKVFATVLQPRAFQKLCDQHKF

>402258|PACid:15402752
MSPHLVGYSVHSENLRAQNLLLRLREQGRNPVRDVSHEEAMILDTADDSYSLQAVVDGASSSYNHSPPPLPPSGFIH
LSPVVCVLEPCAACSCACCCQCPWCWTEKAAPFQVQWVLEQQQCEELDCSELFLLSQCAAAAAAAMENFTDSSPPPI
DAAAEDERAAFFYSFTDGCLASMDDAIVADECWNSLLDPRVLLEDEKQQWGKVAASTPPVNVRAQETKFQCGDGNCQ
EHARQENARIREFRAAFKRALRDNLVIDVDAATDVKCEVVDDDDDFTLPGLREYHPTMDPLQRSPEVAGGVESRNKR
VRKTQSDDSFLVDALRRKKARTKLDFHIPEADVYAMHPALLLRNSPAGTRYFFSRTKYYFCRPARSRTTLEGWRWKQ
QGPSLPIKMNGEVVAYKTYLSFVPLKNNASWLEMLLEAAEGRRSRRWARYTMEEIVLKEDRAIELQMLKACLIPRPF
RVLCRISSRQDP

>418095|PACid:15403721
MEEPSPPPAYYAGGDYGGDETLGMRLVDTMAGFARRLWTSSYTVLTVDLRRASITYDGPPLLRRRRRQVTRSRSSSP
PPSHRRLVSSGHGYLVNERDAESSGGWADGVEATRAQARLKVMQVWELSTRKETPDPWRLNLGIGGEFEVDKGEVKP

KLRLRAKHASLHLLPLPAIELRRKWPLPPSSYTHLGIDVRCRIPLASPDKMLDPASGPRLMVNVYNIGGTGIHFTPG
GLLFEDNVIELGRFTKMRVAAALEFPRELPWSEEERPFKLEVRRVGIKARTL

>409661|PACid:15403921
MADERVVMFRYGKTLFPMVGLQQMGESLKEVVRIVDVPDSRHEKLAFYFGGRHIDIDCRSEETPGDIDFYVTTDRIA
KIIQCVHAAGREGGTTTFLKSIFAPAHYISPNTLEKGSKSSEELSPARKIASPPPPSPHRRGGAPPSKPSPKRARSP
PPPSPPHSHNTRSSGPPSKALPTGASSPSPPPQQHSHNRPKPSPRRAPPPSPSRYRPAKSPPPRHQAATPPAASSNK
HDTITWRPRRFGPPPPSSITWHPRLPSPPPLTMPGLATSAPPPCLASSASPSSSAAAAAAVFVTSPGLLAAGIVIFL
LAMVAIIIGIWIHCTRRRSKVRDEVLVHYAGSGHHPGAGGRSQREYLIPLSKVENGLEHRPSQLNADPRIREFLRPP
RRLPPSALLGGACHDSGSGNKVAVNPLFRANPVPDHQKCWGNVVESCLIVSEQQRPFSASLLQVDPLLRATDPDQQG
SRENVVENPLIFSEPFSASLLQVDTLLPATDPYQQGNQGNMGEDPLIAITPEEVHEQPRPFSASLLRLRHPEDPFSA
AHFEPAGKNMAANELPVCPRKGMEQSHGREQGGKVADPFFRSGIVFDPGDGAAAVQKQILKVMEMEMEKKIHMMEPR
IKRFSHRMTDAKTSDSESSLLASPGHGSQSTT

>403152|PACid:15404879
MEALALAFSWLRRAPAQASTQASRGLKILLLETPAATADKEVEDFPALPSQLEAVIASSDPASSVPEYDAPVLEEDD
EHDLQEPVAPIELEDSSSPASPSQLEAVSHAAASSVPEYDPPPLPVLEDDNHDSQELEAVMAGVEDSVRYTSLGGKT
LAIDDNDDNLSWEDIQSFFQTWTLPSIELPSRADLHLEVEELKKLVGEWTWKLPTSLQSIKEEMWSRLLNAGLEEDE
VTSQELWEELTNFLGWHRVPVTSSRRMLLEAAPGPPIFVSNENGPSLFRWVDRFIAPLSAQQDGEEQGGVSSPPASV
FWQRLRGDGTSSMRKHRRPPDKNSEGQELMAAMDEESSYKVLKSHAAAGSRKLLRQADVSSTPASVFWRSLRGDGVG
MHRRLPYKNSEELAAAMAEESSYKVLKSHAATGFPSQKLLAGGTVDTKEARSNASPPPASPPPFMWRWFPRSLGKDG
GKAKRINTETFQEAEETSISSSHSLVDDVGVGSSSGSASDFIELFEGEGISSSDAFLNSLLESKGGGSNSNADLQLA
SSNDLNLQATAVAATPLLLPILAGLLVALLFLFVLYFLSVIRRRWGRRKRGLLPH

>410398|PACid:15404937
MQIMRLYENLDQVSRKRIRCAQERASASPGHRRATPPKTRLPPPGSIPPPKPSPRRARGAPPPHSSAHSQAPPPAPA
EQHHGAAKQHSHRGAPPPKQDSHGAPPPPPASHGAAKQHQHSPPPTNNGAAKQHQHSPPPPPASHGAAKQHQYSPPP
PSTSHGSKQHSHGGGAKQHPPPPAKQHTGPPPSSGTTTWRPRPAHDTITWQPGSSVFHTFSLPPPPGPLSIAPPLWS
SSSTAAAAAAAFSTPGLLAAGIVLFLLAMVAIVNGIWILVKGRRKKANNILVRYVGSSHLPGGSSGSQQEYLVPLSK
IEDAELRDKLDSFVQERSKQQLRPSQLNASTKVASPPLTRPPRRLPPSAFLKMNPIYGAATNSVDDRLTPSLGRDPP
AAIHGVDEHKKEQALDPLSCRGTVFDPGDAFIVRLPAAAEKIREVENLEKPVVEANHSANSGGLHLAARVHDSDAKA
QNEDS

>103093|PACid:15406058
MVSPPLPANASPPPPSSAPPPPIASPPLPSAPPPTFLVLPPILSAPPPASTVPSPILSAPPPTILPSPPPPLPSAPP
LLPPPLSTPPPSFVPPPPSASIRPSLPPLSSAAPEVPHNISSPPASPLSSPLPSPPSPS

>404053|PACid:15407027
MTLKGKKRFSKDFRAEVLFGETDGFSSPVAPVPSLIEDNGGLPMEAFAKVQGLFSSGDWLDSGGDAALKFLQQLTTR
NRAMESEQKQAVEHHHYSKNNDKEKLAYEETETTYPLGTWSLPRCSSTGSRAQFNKVQAVRNTSRHTRTGSWQRRGA
IDNLSRGLDVGSPFFYPDSTLGLAVENAIEAVSCSDSVGSQEKPGTPEWWIMAIILTVDRQEAIPLMEDRHGGTGTA
LTTLVARPYGAPENRPIHPQKLRTLRTFHRRGKAIRGQRAMIAQQQQQQQGEKMIKKRRKRYGAKFAIPTGFVYRK
VWDVDLDPILAEPSARRILAALGLDEFAAQPLPPLDRSLCDEFLTNFDPWNSSAMVRGERVVIDAASVSAATRLPIG
DDGGEISTSAVLTKEERSALGAIVLDLKEYSSRGYSVDALVLGENVEAWKAICMMLMVKLVARRECFHCVPVEVLLA
LDRSYKLGIKMRWAEYLGKMIARGAKTSSRRKSTFAHGEWLTGIIKNKLGQQDQGRVEEDHKLGQQDRAEKDHAGVL
TDKMVIAEDVHSSIQRKQQCDASVQTMIQEENRDRVIIARQREDIRLLRGEVERGRAMVASLRDKYLKIKQEGITPP
RKKIADHDQSMVSAPVAAAGSKSFREEWIAPFDEDVEMEDAFPPPLPSSPPPPLPSSPPPDR

>404055|PACid:15407032
MAPSKKRGRSSSIAAAAAAEWSVGDLVLAKVKGFPAWPAQVSKPEAFGQHHNPARVFVVFFGTKQIRFCHHSEISKF
TPEAKASLVAKAHSKCTPADLRLAVSEICDVAEKVEVGSGDGDMNGRSGIEFRELEDGEASCVMDLGGKLDVEGQAG
NGGTSEMEDYDLGWGSGSSLDGNSDDDTTPKGIVLHEKVQTNFASHEELDTFDSFDFFNEEVVNVPSAVRADKKKAG
KAEKPAKQRTPAKSRKSAPVNSTTPVFPPNFCRKKRKQMTVKASTPRGVVRKDDNSDEDHATPPTADEFQEYTFRRK
VTLKKRLRPAFESRSAETDSDHNKDKPDTSNETSPITQDPDSNLPIVKRARARAAKEPALDKHSEEEEKRGAPHSPP
SSTTDTPTCNGSESTKATEERASDGKPRFEEKYTGLSLPPKAEKYRMRNSLADSEAALPPSKRLHRALEAMTACVAE
AAKEETARDAKVEETPDNVSQKSDSTEQTRLSEGVVVKNCVADDADKLQEVTVTSKEFVDKEMDPASVGLGSEDLQQ
SEVLSVTSIQVSVKAEATQDTPVSPAVQEVVEVAEELVVKAESAISSQAKVQASPSGNTLGEDRRDVETGPPSDAGG

TQNVTVVDNPEDRTPNPEDRTPLSSALSPVSMDLSEGKSDVKAGVLEFESKSDELQSTAENSEAKTPSMADMKYSPV
SPRADDGQIARFHAESLVRRDEPVSKGRLSIDGQGAPKERRQPEKNSPSAWWGRSSHLSKFHRGEDKRKLENHFEVS
LYRKDGALAKTALERLQSMSPAKISPGSMRYSPVSPALREEGNKVLFDHAVTVKSSREGGTKVQLEKLDTSGRGSRH
SSKPGISETSPGTTKARGLSTDKPKTSNSKMRRHSPVRHGTETSSLVSERGTTESHSGESLASENEHTSARTSSLRT
KAAGHQDSFKLNDISASKPHSDNNVRKAYEAAKELKQKLASKDGDTSTSMKYIMAAVQSKVPGPVTPMDVGTGPEIV
MEQASGASKDLPSLPTANDEAETARNVFEGLLDNLSRAKESIHCATRQAIDCAKLNLADQVIDIIAKRLEAEQSLSR
RIDLFFLVDSITVSSHNLRGTPAGVYRHIVHNALPRLLSSAAPPGNFARENRRQCLKVFKLWHERNVFPPTVIRHFM
REIELHNCDRVLMNMSRRPARNERPVDDPIREMEGMQVDEYGSNASFTLPGVSIPRMFEDEEEANAGGTEANAGEDY
AVATVTPHDPVERHRHVLEDVDGELEMEDVSPETDTWQRQYVSPASILTGPPLPICPPPSPPPLPSSPPPSTPPPPP
PSSPPPPPQDVADPGAYPDSFSPGYHHARNAVQQPSNGPESYEHYNPGVYGDEGYGMPVDHGYNSYGGFSDMPPGPI
SPQYDGHHGHHYGGQHYPADGHSYQGDYPYQAYGEAGGHWSEPSPRSRYMHSAPDDHYYHSHHDNRYGDHGADYYHQ
NGPHYMQSHYDGGYSSHGRYSSGYMTPPPDHQMHHGSSGRSSRRHGHYSHDAGGGYSHDYGGHDGGGYSRSHSHSHS
HDGGGGGYQHSHSHSHDGGGGGYSHSHPHHDGYSHYPNW

>410699|PACid:15407218
MGMGGDGDGDGERLTDRAAPAPPAPLPRMAPAPPAPRPGMESAAPALPEMKCEERFRDLAALALPAPLPEMNPATPA
LPETECEDKERFKDPAALALPAPLDPAAPVPPVMECDDEEECLNDLVALVLPAPPPAAPVPPVMECDDEEERLNDLV
ALVLPAPPPAAPVPPVMECDDEEERLNDLVALVLPAPPPAAPVPPPEIEPAAPVPPELECDEQKRKRPRFLKGEAVP
LDDADPAPHTPEAAPHAPETDADPAALQPPLPADLAPVLPAEPVLVPPPAAALLPAEPVLSPPPAAALLPAEPVLSP
PPAAAHAHGASSSTPMDVFKLRAWRRLQLRGLIPPTAPAVAREMPEVGFCVEDDDWEWKVDIKELFGSCFQ

>417964|PACid:15407583
MAHKLSTPPPPPPHREHVRRTPPPKPSPKRVPTTSSPPPKRSPPAPAKRHTKPPLSSPEHHGAGHPLLSSPEYHGAP
PRSPEHHGAGHPPPSSPPTKEHPAAEPKRHHGDGAATGPPPSSSPPNKAGTVTWRPRRAGPPSPPPSSITWQPRPLG
LGTAPPTAPSAPSSAAAAFVASPGVLGRHRYAPARTGSHRHWSLDTLEAEKEQGARRHHPGGHQPGGGNSGSSSSQE
HLITKVEPRPSSQLNAGRKKAATGGPLVLATSLHPETIHSFVLLIQCMASREAGETSWRILSSSFLRMKLTSSQGCP
QPLRRMIHSSRLVATLITQQAREWLTTRQGSNEGKV

>417968|PACid:15407588
MAHKISTPPPPPPHREHVRIMELDIHRRALSTTELSAHQGTQQQSQRGTMDIDELPNKADTVTWRPRRAGPSSPPPS
SITPPPSSITWNPRPPSPPPSTITWKPRPTVPSAPSSAAAAAAFVASPGVLAAGIVMLLLALVAIVTGVWIHLKRRR
NKVHGDIILVEYGHQPSGGNSGSSRNQREHLITKVEPRPSSRRENGCKRAPNAPIISTYNYNIR

>417975|PACid:15407607
MARKISTPPPPPPHREHARTPKPSPKRAPTTSSPPPKRSPPAPTKRHSHSPTDGHHSPAKPPPSSPEHHGAGHPPPS
SPPTKEHPAAEPKRHHGHRRAGDGAATGPSPSSSPPNKADTVTWRPRRAGPSSPPPSSIMWNPRSPSPPPSTITWNP
RPPSPPPSTITWQPRPLGLDTSPPTASSAPSSAAAAAAAAFVASPGVLAAGIVMLLLALVAIVTGVWILLKRRRNKV
HGDIHHDDILVEDGHQPSGGNSGSSSSQREHLITKVEPRPSSQLNAGRKKAAADPLIRELLRPPRRLPPSALPAGAR
HKSASGNDPLFRAADPVHGQQGSRGNVMMEDPLVLFPAYEVDEQPRLFSATPEDDPLFSSGGYSHHPAGEKMAANEL
PVRLSSRRDRVPEQFHGREQGPKVADRSGTVFDPGDVATLAKKRAEIEKQMQKFIMMEPRIRPFSHRKRDVENSPEI
TSDENLVRKLTANDPKRVKSDLEVEPSAPGHASLTGENADQALQETKRKNK

>417977|PACid:15407611
MARKISTPPPPPPHREHVRRTPPPKPSPKRAPTTSSPPPKRSPPAPTKRHSPPKPTDGHHSPAKPPPSSPEHHGAGH
PPPSSPEHHGAGHPPPSSPPTKEHPAAEPKRHHGHRRAGDGAATGPSPSSSPPNKADTVTWRPRRAGPSSPPPSSIT
WNPRPPSPPPSSITWNPRPPSPPPSTITWQPRPLGLDTSPPMASSAPSSAAAAAAAFVASPGVLAAGIVMLLLALVA
IVTGVWILLKRRRNKVHGDIHHDDILVEDGHEPSDGNSGSSSNQREHLITKVEPRPSSQLNAGRNKAAADPLIRELL
RPPRRLPPSALPAGARHKSASGNDPLFRAADPVHGQQGSRGNVMMEDPLVVFPAYEVDEQRRLFSATPEDDPLFSSG
GYSHHPAGEKMAANELPVRLSSRRDRVPEQFHGREQGRKVADRSGTVFDPGDVAALAKKRAEIEKQMQKFIMMEPRI
RPFSHRKRDVENSPEITSDENLVRKLTANDPKRVKSDLEVEPSAPGHASLTGENADQTKHKNK

>102692|PACid:15407659
MAAASINVMLAIHAKKTTPVDLHKPLRLYISQHYSEREAREADDDLQSVQEMRSEIEKATDSLDSRRDLLQRYFRAL
CVMESRFPISSEREHVNSLSFTWYDAFKQGRKASQQNIHFEKAAIAFNLGAVYSQIALSADRSLPEGLKQACNSFQA
AAGAFAFLRDNVSMKASGNSTTTDISVECAGMLERLMLAQAQECFFEKVVSDNRPSALCAKVARQVGLFYDEAHAAL
VLPPLNQHFDRSWVSHVQLKAAQFQAEACYRVSLELHEKENIAEEIARLKAASSMLTEAKKSGRGVIGPLLDAVTKL
DGSVSRNLERAVKENDRVYLMRVPNPDSLPPLPSAVLVKSAAVAELLDASKEKMFSNLVPDSSAKALSKYTEMLDEI

IRVQAEKLQQESEITRVKLKEMNLPDSLLALEGNASLPEHLRDDVEAVQIDGGPAGLESEMAQLRDLRRVNEELLVQ
TEELLEKESREDSQIRAQFGTRWTRPQSSTLTKNLHDRANGFAANLKQSGESDRRIERAIKDNTAFLSILDARPIES
ALPSLARPILSLNGNEDAVVGALRQKLSELDRLGLQRASLEDMLKDMKRQDNILPKLMTTSGSYEDLFKRELAKYEP
VSIEVARNVQAQEQLLHEIEAHNNAFAATFNLQDYVVARERAYKQLSAAIAKYREIRENINEGLKFYVTLQDAITNL
KQQCSDYVMTRNIQSREMMEDLQRQITGLSFNDTGPSRTYPPVTPHQSQAPHHSPSPPQHSPPPQHSPQQNSPYYPP
NMPPPPYYSQSQHQQAQTTQAPPSQAPPHRQQQSPPPPQHVYGQPAYPGWQGTYYNNAAPPPPPPQPSSTPPPSSSH
SHFAGPYAPGPGYYSSYYKQ

>410730|PACid:15407855
MASASVEAQALQPEEAPTAPPTSPPPPLVPPPLPDEQADDSRTFPLDVSSAGADLLRGTVQTKLSEFMGSYTDEVLA
EYVVVLVGHGKQQKQATADLEAFLGVQSEAFVSWLWEHLTENLHVYTKAGEKKAANDALPKDTPEAKDKGENEKKEN
PTQENAKEENHVTKREPQALRQELKRSRSPEGWARRRRSRAEDKPSAIERLSPPPPAPPAPRLKASRRLLEFAVRDA
VAPVGGSRRVADANTKRLRSIVSGSADHENAGAPLDSNGKLKARPAITKPAKNSPAMMIALKAAAAAAEDVNNRKVS
KPVSRFAASIWDRLGHKSDEDDQPDQDLDDADEITEKMEYVHVDSAPRNSKSDRHMRKSRTADRLGLVERRTADTAM
PPPPAASHDAYDHGEQFERRIEEVPESLNKKEPSKRHTLRKERRQKKVEEPLQYKRVFHTDDDTKHKDILPLERRTS
VEQEKLVDIPNGKGQDLQDVTEMKRRMRQVELEVTKLRTKQDEAAKVSEKSSAPHPPAATTNIPSVPAATTSMQTAP
LTSFKTPQSSSDDTDSKVILVTNVHFATTKEAISTHFGSCGEVVKVTMLTDGVSGKPKGSAYVEFSSKDAADRALSL
NESSLLSRPIRVARKDANVAETSSPAAAVMRPRPPVRMPMRAAFPRAAFMRGSPYMMRPPPRLVRPPYPVGAPHLQW
KRDGGASQMGPSSAAKNGFRSSVGPIRLKRSLSYVRPVVPVTPTPPEATAAPAAAAATTAAAGTAAALSTSTETQKG
S

>404287|PACid:15408701
MAAAATCPSPTRLLCSLASSPGKFASPSSPLSPRSSAAAAVKEELLIASIGGGGDGDPAPPASLKRKRPPRLTIPAS
ERESSTAAECVDDEYSDLTHAGHGYGVACKKGRRQIMEDSFTALPKQGFFGVFDGHGGREAARFAAHNLLDNIVKAA
CPTDEAGAMQIGAQEIRMGYHTTDYEFLRQGSSSGASCVSALIARNELLVANAGDCRALLVKSGGAAVQLTQDHRFS
SESERRRVESLGGIVDRYTGTWRVQGVLAVSRGIGDIDLKQFISCDPHVVSLPLTSDCEFLILASDGLWDLVSNQEA
AECALLALKVGAKRESVQLDSRGAASTPGLSSLGAACRRLLDLTLKRGCLDDTSVLFDKMQATEMVEFFPLVLSPAL
HCDPHSIYCFSQESKCLFQSKCFGPTQSPSQAPAPSPSPSPSPPPPSPPPPPPPLPPPPSPSPPPPPPPLPPPPSPS
PPPPPPPPPSPLCPPCGYGQKCCCMFPSCLFLFKCCPVTTVCGYGPHC

>426517|PACid:15408752
MGSDALSNHHQAQFVSLAADQSGEIICAGTRIPGVLGFLVTGQNRAPVDVFEGKGRVENFTHTHDVLTVVYCSDGKQ
LGCSTLDGQIHFWDPIDGVLMGTIEGRRARANALHDGSLLLAGGTSNYICMYDAADQALLQRLQISHSYSLDGVLDF
LNSKRMTAAVRGKLAQDAAGPIMCTKCVRISPTGSSGNNGKPTQWTTLFLIPVDVEPEAINDALESKRYSQALMLAL
RLNKPLLIQKCMEAVDISVISAVVCNVSLSYLGDALAQYLEKTPHLEFLLVGALHWQNNPVEEHGAHARFQVPILQQ
QLPPKMTTFSHYLCSAPTKSQSVEQSLDLYGEPNNFTKLSALSGPGVVGPTIPLLLHVRNHCLTCVPVTTLAGSTSW
VRLAAPLSCRGPWGSLAWPSASPPPTGIPLRQARHIRAHPSSVLATLIQALPHLKLYTPATASGTVNGSPPPLTRSA
S

>404364|PACid:15409460
MIAQQHQQQGEKMIKKRRKRYGAKFAMPTGFIYRKVWDVDLDPILAEPSARRILATLGLDEFAAQPLPPLDRSLCDE
FLKNFDPWNSSAMVRGERVVIDAASVSAATRLPIGDDGGEISTSAVLTKEERSALGAIVLDLKEYSSRGYSVDALVL
GENVEAWKAICMMLMVKLVARRECFHCVPVEVLLALDRSYKLGIKMRWAEYLGKMIARGAKTSSRRKSTFAHGEWLT
GIIKNKLGQQDQGRVEENHKLGQQDRAEKEKEHAGVLTDKMVIAEDVHSSIQRKQQCDASVQTMIQEESRDRVIIAR
QREDIRLLRSEVERGRAMVASLRDKYLKIKQEGITPPRKKIADHDQSMDSAPVAAAGSNSFREELIAPFDEDVEMED
AFPPPLPSSPPPPLPSSPPPDR

>412471|PACid:15411631
MSQRCASRRFRPGLFLRHCLPGTGAQLDCYNLETGARYFPSVHVFDGIRVHGIVAAAAESSALPPAIVAVHGEGKAK
LFRLSTEALEPWQCVPRFSKWILDVQIFTKDDGHNVVLAIGLSDNTVCLWDTSSKKIVSNFHCPERCLLYSMRLCGD
SIQSLQVAAGTIYNELNLADPLSSARIWNLQTKAVIVLFGHTARVWDLHIDAKMFITASEDCTCGVWSLSGELLRTL
KGHTGRGIWRCIFDRQLSMLVTAGADSSIKLYSLSERALIPTTAKEMVMYRIDTGKNDGFLDSRSEYVRCLCFEGPS
SLFIATNQGLLQRASLLPSGDVKQVSILQTPERSPLVCLDIFKEQLVTPEGCPSKEVLVACGDGQGRATVLGITSEG
QIAWQSTWIAEAERQLLGAFWCKSLGSRFVFTTDPKGGVRCWAMNGETSEGPRHLAYFKSSLSSRIVLVCGDQKGSL
LFFHFDSILFEMEDPAVVEELDSFRGAHGISAVANVSISEFSNEGTKTISTGRDGCVCEVFYQRGQLSSVLSCHRVE
KIAAITVLESVLCLSGSKRRIGLGFSAADFVLWDITNEVEEQTICICRNWDDSDDSSPLQALQKQSHGREVHSILFF
PDTDWLFSGSEDGRVQISRYAQEDLTVFASLEEHVGGSAVRALALSTRVYNSKTEASGIDDMQHAVLFSAGAKEVLT

CWELFDKPEQISARWLSTHSTFSKKSGVNKDDLRYMALTVFAVESDGVPVYFVVAAMSNAILTVKAFDSSSKTWSRI
AVLQHHKCPVLTLHHAVLPCFSQGTLANRFFVFSGCTDGSIAVWDLTNSVASFTVGEREKAQGLRPLSGRGSQGGKA
RRRLNHQRHPSQQQILKDPQSSPDLSQDAAVLSPVLVLSCCHQSGVNSLYVCATSSSTLSIVSGGDDQSLHFSVLSM
QEGAGSTIRVLSRQNVANAHSSAVKGKDSFLLLTILGVWSDSEWIFSTGLDQRVRCWQIAQDNQLTRSCTCIIDVPE
TESIDAHRSSESSYRIAVAGRGVQHCSTQEQYERMWELRDDAELRRLATSCTDLLASLGIHDPVWIPPLTLTPATIA
AEVSPLEISSPAMAAEVQPEVSSSCAVEQEIPPCEMASSSSLGHGDAELSPATDLGEATATALSCASSPSITMAIVK
SEPMEPDPMRIKFLQQKARFHEAVAAHDALQEQPTLLLGSGDGGDGSDPSDAHGVRRRKKSRTDLAVAAQFHKREDF
LHHRKRQIGSIPGVEVGDTFSCRAEMQVMGIHSLPVAGIDTMDWEPGVPVAIAVVLKRSSVYGDNCDAGERVIYSGQ
GGFKKGNTEDQKLEGGNVALENSMKNKLAVRLIRGYLGAEFGRFKTLYSYDGLYLVKRMLYGPGRRGNLVYQFEMER
IKNQPPLRFYDRFDPPLPALPAAALPSGMIVALPARGFGANVRQSSCSSALEVFHSPPPPTPPPPQTPPTPPPPPPP
QTPPPPPSPPPPPSAIAKKRKFPRLCLVYRYERSMEAAVRRRAGIALRQSVSCSFSRTIDSSTALRQRIDDGNAAQS
SSENPMAGIDHNPVPGRGSGDRGAGKIKLEELSFKPGIADKPIASPAVARFRRLAISAEAQELLEILETEMEEVREH
KLREYCGKLSTDIVVDVLNHARDVEPTIQFFEWCHGRHGYTHTAFAFNRLLEFLVNKRQYKRAHQMLIAESKPSSFQ
ANAFTYSTIVRGYCEDGETRQALALLEHMKKAEVPANAKLYNIILTHLCKHGKEKEALDLLHSMATTSCAPDIYTYN
ILINALCKVGRLEDAQGLVEMMQARECPTDIVTYNTLIHGLCRKGNGRFRVERAFRLFEEMVDKGHTPTSVTYTVVI
DALLRVKMDEQAQKVLDLMHENGCAFDLVTYNMLINRFAKAGMMDKAMDLFVEMPTRGCIPDVVTYNSIVDGFCKAG
KVEEANKLFAEMKARGIFPNGRTYTSITDACLKAGRMQDAHAYLVEMRKEGLVDAISYVALISTICKLGKGKLGFQL
LREMLEKGFKPNLVTFNVVFEALSKESNTDDAKIMIQELRQQHSSPPLNK

>441945|PACid:15411749
MKKVSSCKKCREKVLEILKHTAGVKEVEYAPKSGKIVVTGEVTLEDILAVVERFKKKKKLVFLSIEDLPPPPEKEKE
KEKEKPKPKDPPPAAPTPPSPKCCDHRHGHHCKCPCHHHHHHKSPPPTPPPPPPAPKEPKPHHSCCDNEHHHHHKS
PPPTPSPPPPAPKEPKCCDNEQDHEHHHHHDHDHDHDHDHHHHHHDCPPEIPFCPPTIIVTGCDASDPPCYSQYDP
DPCCGGGGRNDSMDLAVLIGLVIVVMVLLASLSSSSSSSTSEAAAEAMKGFFTSIIGTLGSLFLIILALFAIMGFL
CLVDPRPSQSCKC

>404698|PACid:15411877
MQQQQMGGFRPPTLPGSFGQSFGPPRPPPPPPPPPPPPPSFSYAAPPPTPSSTNFGAPPRARSPPPPPPYHSPPRGQM
RREFDPPPPPPPPRPPAFSDLAAAQLMLSEVDSLDSFSLASLVLLRQLIDQLLMFYQASGPSRSEMEETCWPDEESST
SWPRSPPPPPLYNDGAYSDMTSERSGSPGFYDRYYDYDDEDFAMPPRRPPPPSRPRPRSRGDYYYPDDDQWGGGGSY
GPRQPPAPRFVERSRRRGDGYGVNPSFYNEGDDSDSMSSRDASPSSHRRRRRQHKDRSLKFKVKDRSRWVLDPDESG
SIEFNSGVRTQVSQSDDDSRPASVSFSYRPFAFQRFSSPRPREDSA

>405447|PACid:15413174
MKFHRPTIIQEKFKKLKLLWSLCTIASRKQMKMKRITGDTATRKFEEVKEGASSWIIELVENLKVTDSVLGSISLSG
LGCFSIHEQSRKRRGDDLGDDFLTSWKPGKLTAAPGLDLDDSPPRPMAKTGKTFDLSKFDMDLDLDGGFNKLASFNI
DLDLPGIDATMEKNADTGSKTKERLKFFRIQSMDISPVSRPPARDTDRSDNNVETGSKAKEVSSGLEASSKFLRIQS
MEISPVSPPPARDKLRVDLSGLDSMNEKNADTGSKTKERPSFKFLRIQSMEISPVSRSPARDKLRISGLDSDKNPDT
GSKTKDHLAPLLDTSYVLISPVWILGMTRTLTLDPKLKKPSSKFLRIQSSPVSRTPARDKLRVDLSGLDSRNDKNAD
TGSKTKDSSPVSRTAAGDKLRVDLSGPDSRNDNNTDAGSTSKDVPADKLDSFEVDHSDAAKEKSNGSKGKDVVRSVD
EYSSSPPPARENNPAIYAINVQEMEWRPEPAPPLAIDAQEMGWRPEAPPPLAIDAQEMEWRPEPAPPLLEKPENEVV
DKVDCYTEVLEEMCKMLKQKKEEATDLLVRAIVANNKLLLLNSPLHDEKISLHFSVSKSLLPFLTTFLFTSSFSGAL

>421190|PACid:15413687
MWSVSVVELVGEDNFTVIPRAFVKPIHAVAEEKENKNDPLVFLSAPPPAPPSPSPPPPPPSPPPPPPSPPPPPVESP
PPPLPPGLKRRRIRDGLRGAAEDTRASRNTLQEIKFILSQNEKPIQLHLDHFARLLHLSSFHDELRAVGRDTECHGV
LADIESMLDLRASPSPSLS

>443419|PACid:15413738
MQHAVTQLGGLPPLGTHSSGLFGDGRIGSLAFSAPKEAFFLSRTMSATTRAAYSGSFGQNPPVPPRNNAPPPPLPFD
NAATPPPPMPGNPQMQTPPAIPPPGPFATETNALPSERPSAIAPNAPTDAKFTSARPPSSSPGLNFPGSQIPGLPGS
PRPKPGTPRPYDPTKQKSKVMEWLTPPPPPTELLQREHFRLSPTCTCTIYFGDLSKWFVDGKSDAVIAPANKRLNTG
PAVNAVLFKAAGSRLLDATQRLPDVAPQGIKAEVGDAISTRLSTAFNLPVSRVIHTVGPVYKKNDQTNVRESDESLI
KAYKSALDIARKENLVNLAFPPLSCRIYGYPYIEGAEVGLRTLKENCEGFTQIDIVIRNIEGYEAFIEEATKQLEVI
KEKKTIKAPSMAPRTGAEAVAAPPSPPVTASEVGAPAAPASGLEKTEAAGPETPSAAAPDTPSAAAPETPSAAAPTP
PSTTSGYSSNFQAASNPFQSTSGPATESPVAPAASSSSAPTSQSPAAPSPFQSAPVTPSPFQSAPATQSSFSQTPPP
PSAPQTPSPFQSPPATPGPFQTPPATSSPFQTPPATPGPFSSPPASRSPFQSSPAMSSSFPGTPAAPPSPFQSTPAP

PNTTTPPAFQSAPFQSPSASPPSGSQFQSPPPPSEFQKAPPPFGTPTPSPSAGSPFQRAPPSPFQSAPPPPSSGSPA
PPSPGSPFQNAPPSPGSPPPPGSPFQSASPPPSPPHFQRSPYSQGGYQQSSPPPPYQNAPPPPYQQQQQQYNQPPP
YQSQAPPQYPPYQQNATPPPPSPPPPPPPPSFESSTPTYSPYSPFAPAPEPTPPPKPLIPPHQQKYVPPPPPPPPES
KPVPPRERLSPFPPPNRPPRPPSSPTSTYEQPLF

>420676|PACid:15414594
MASIIRRSVPPSSPPRRAAHRSVHPPRPGHRSTTPPKTRVPPPGSMPPPKPSPRRARGAPPPHSSAHSQAPPPAPAE
QHHGAAKQHSHGRAPPPKQDSHGAPPPPPTSHGAAKKHQHSPPPTSHGAAKQHQHSPPPPPASHGAAKQHSHGGGAK
QHPPPPAKQHTGPPPSSGTTTWRPRRFGPPPPPPAKEHTGPPPSSGTTTWWPRPAHDTITWQPGSPVFHTFSSPPPP
GPLSIAPPLGSSSSTAAAAAAAFATPGLLVAGIVLFLLAMVAIITGIWILVKGRRKKANDILVRYVGSSHLPGGGSS
SSGSQREYLVPLSKIEDAELRDKLNSFVQERSKQHLRPSQLNPSTKVASPPLARPPRRLPPSAFLKMNPIYGAATDS
VDDRLTPPLGRDPPAAIHGVDEHKKVPRASLPEDPFLSSAARRSGNDRRAQEQALDPVSHRGTVFDPGDAFVVRLAA
AAEKRAEMEKHMQRITGFGMRRPGNKHSHRIREVENLEKPVVEANHSANSGGSHLAACVHDSDAKAQNEDS

>430005|PACid:15416953
MLHQDYKRAFFYLTLPFILKNENFCAATTSIKVEGYIYCTNCGSLSQSGIFNAKVNLVCPRDSDSSFTNNRGYFNIF
KLVKTGTDIESCRVSVDTSRAPRGCSFLTDINGGSSGQSLRTCINNGNNKFTCGPYYFTPNACSPPPPPSPPPPPPP
RRPPPPPRPRMPLPPRPRTPPRTQPPPLSPTPTPSLSPTPSLS

>445144|PACid:15417274
MVSVRPGRIEQHKQVSPDRMMFKVPANSPPPPPSSSSQAPADMAGKRKWQEAFKGESCPSVPAPPHRSPQLTIFYAG
AVNVYDNVPENMVKALMTLAAAKSSRPAALMNQIELQRSCSSNKTGFGFQANQSSCNGGGSARAGVKSSSNVASSST
SGSFSFPVLETTSTSLSLNSSYKAANEERSSVPAPAPAAAAAPAPEQSSGTAVQASSSPPSPAASPDLRGMSSRARM
QMLPAGLPQARRASIQRFLQKRKQKGTVIVNRQVNNNGEDDDEDAATAEMKPTSPPPVGPPTSASTAHIAAQVPHFL
KKR

>421625|PACid:15417283
MSVEQQHRQASLALVCSLAAMAIIILPWASLCGAQNVTVVLENSAPVNTNFSTFSRLLSMTNVTAEINSRSSLTILV
PDNSILDSYVGANLEGMHVWAVADFCRYHVLLQYLDTQEIMQMTNQSGLLITTLYQTTGRAEGIDGFVNMTFGNDIQ
FGLPPPHASGPVATVLNNFVRYPYNMSFMMISKPLMPVRLAQMNRPDHFPSISNALFKGNIYKTFQKLMQDTGTLAA
AEDIEHQPFTSGVTVFAPTDSAFQNLPSGSLAALTQSQRQLLVRYHLLPSFFTFGSLRTLKAPLTTLATSNRNFEVN
ASGEGPSGGLAIATGVSTANVIATLLEDDPVGVYALDAVLLPPEIFPLHSTPPASIAPSPAPPATVPPPKAVQAPAP
APKPAPPPSPSSSPSPPPSATSPPPVQSSSNSSSSSSSSSPAAENGTSAEAPVAAEGPLAERSDARKFGLGFTTRY
WDLEHSLTPAHLSTNARVVLDHSKTVETSSEMPAVRGREEAGGARSAATGRIASGRLEGQLKVTREDEAGRGEDEAN
GDLRRARPWWDEASAAMPCYDFNRI

>421629|PACid:15417289
MLATSNRNFEVNASGEGPSGGLAIVTGVSTANVIATLLEDDPVGVYALDAVLLPPEIFPLHSTLPASIAPSPTPPAT
VPPPKAVQAPAPAPKPAPPPSPSSSPSPPPPAASPSPPPVATSPPPVQSSSNSSSSSSSSSPAAENGTSAEAPVAA
EGPLAERSDTRKFAALATTALLF

>165961|PACid:15417303
MAAASSARHASLSSPPSSSSSSISQSRATAAAASPRSPSPRSSPRRSSSVLEHTDRAGKVDVGKEIEQQRRQRQDLE
SKAKSGLVGFDKFVRSNPLTDRFKVLCFHHVELWCGDATNTWRRFSWGLGMPLVAKSDQTTGNHVYCSYALQSEELV
LVFSAPYSDKHRYQPASPPPHPAMTKPAMLDFFDAHGLAVRAVGVRVEDAATAFRTSVEHGAIAVLEPTTMHDVHSG
QELVLSEVALYGDVVLRFVSGGFDGPFLPGYERVSPPPFSSSPSGLGVGLCRLDHAVGNVHNLLDAARYVAGFTGFH
EFAEFTAEDIGTCESGLNSMVLASNNEMVLLPMNEPVHGTRRKSQIQTYLDHNEGPGLQHLAISCRDIFHTLREMRM
RSHIGGFEFMPRPNKKYYRDLPERIGSSLTPEQVEMCEELGILVDKDDQGILLQIFTKPVGDRPTLFLEIIQRVGCM
EENVQRGGCGGFGKGNFAALFKSIEDYEKTLER

>415080|PACid:15417334
MAELKAEASARPHADRDRNCEFLADSREDPSSRVEQEGVGLDSRRDDVRKDEQLDLERASVAPGASDAEEDKNVEVV
RRVDDPGIDQKEGERRFHGEKVEDDLNEGEKNIQDDGREEKKLEDEREKKIQEEKKLEDEGEKKIQEEEGKVEDDRS
EREKKIQDEQEKKIEDDRNEGEKKIQDEQEKKIEDDRNEGEKKLQDDLEVAGSQEEMPMEAAGDPTKPKILRTFERR
KKPMKVFERRKKKVDGEASGGNEDTEKKEAIGAEKKKLKRPRPDDGEDTKLAQEGKVAKKPKEGKQRNLKEKLREEK
QGTSKEQSKEGKLKNVKQTKKLLKCQSDDSKKESGDANGLSSERKRTQDSEKKKVEKKALKRSSDADIKDTKQAKKN
KASKETTEKQDILKEQLKEGKKETLKEGKKEKLKEGLKDKEKEKWNDVKIKDRPNLFRSQSNDSKKKEAKVSTSKES

SDGKRPEVEDAKKPLKTLHVEPEALNNTLEAISTPVSRLLDGGGSNKVREAYEAAKELKQRRLALKEETASASMKFL
KAAAESKQQTQSDSPVFKEPTPDNTSPAEKEKHGSDAKKLKEREKGIALETFQGMLETLTRAKESIQKTTLFAMDCA
NKGIAKQVMDAIMRKLKREREHKKRVDLLFLVDSMAQYHHHKGGPDYASIVEGCLARFLTAVAPPNSPSENRQQCVK
VVNLWVKRNIMKETLLKDTIARLGMEAGLNTTQRQYQRHDRAFHDPMREMGSDMLVDEYGSNTSFQLPGFLVRRDDE
EDEEMEMEMEMEMGMDVSPVKDHKLKVAMEEAPEPQNGRTLVVKRGVERLAPLPTSPPPSPPPLPAMPPPSPPRPPS
SPPPPYPSTIIQQTWQQHRRQ

>68846|PACid:15417424
MARVYVGNLDPRSTERELEDEFRSFGVLRSVWVARKPPGFAFIEFDDYRDAQDAIQNMNGKNGWRVELSRSSNGGRG
GGRDPGAREGRGGGGDELKCYECGEAGHFARECVLRVGAAGMGKGGGRRRSRSPPPRYRRSPSYGRGRSISPTARRR
YSYSRSPPPPARRYNSLSRSPPPRRER

>443953|PACid:15417591
MTTSPRTMSSDPEDSSCYSSPYETNRYQSPSERRNVAVDQGGVSYVFEPFMETSLAMEKPDVVGIGGGAWLPPTAPV
EPPRPQTCPPLEFQLPKRLIIAARDKESLLEEVITLKTELQHQNRKIAAQRIQASTVENENRKLEKDLEDLELQRTD
ARRTGGPERLRSKVYERSAGLKTRLEDLHMVCEEQRDELFTLRRDVRLTRSRELEVERNVFADEIIRLLKYMKRLKA
GQNKLSCENRELALVKDKAVLLEIANKRLESCLTAIHKNLEKTKQETAAFKQKALETSNSNKADEKRRRELEDKAAK
LTEQLDTETAEKEKWVRKASKLENEIRKLSKELREAEAAKAKFELLNTSKSPPTPPPPAAKKAATAPAAPPSPPPPA
AGKKAAASPAAPPSPPPPAAGKKAAASPAAPPSPPQAGKKVAAAPAAPASPPVAAKKAPVTPDKSAKTKPGGDETRG
KSAANEASLREAEEDTKRKAAEALRRKQEEEMQRKQEEEQRRKEEARKKAAAEAQAKSRAAAGNDAKKKGGETQKND
EDKKKKSDLAGEGGGGREDKDAGGEAEEEAKRKALAAAEKEARRKRLLAEEDEEARRKKLLAEEEEARRKRLLAEEE
EEAKRKRLLTEEEEARRKRLLAEEEEEEAKRKRLLTEEEEARRKRLLAEEEEEEAKRKRLLTEEEEARRKRLLTEEEEA
RRKRLLAEEEEEEEARRKRLLAEEGEARKKGGITGAGDEEEARRKAAAEAEAKRLEEELAEQKRRLKQREQELAEQKQ
SVGSNDEGKKLNEDEERRKAVEGLRIKEEEERRLREEEEAKKKRAQEEEMKRRMEEEERKRKEGEEELRRKREEEER
KHREEEARRRKHQEEERERKRREAEEEEARKRRIQEEEERKRREAEKKRLDDEERARKEEDAVARKAEEDRKNAAGG
GRPRIRVFKARGTTFAEDTKQVVDRGAPTFEGRSRKNFGTAYMAPAPASPASSSTTESSSSARRTTLRVDPKSGGDP
LE

>422324|PACid:15417904
MVGTVDSVRYTSLCGKTLAIDDDNDDNDDNLSWEDIQSFSRTWTFPSVQPPSRAELHLEVEELKKLVGEWTWKLPTT
LQSIKEEMWSRLLNAGLEDVPGIMGRTQKLPGVASIPSYQFPTDASGGSARSSYLRLQREWIFSVPLGGLQTRIVKD
KNLLAAMAEESSYKVLKRHAAAGSRKLLRQADVSSTPASVFWRSLQGDGVGMHRRLPYKNSEELAAAMADKSSYKVL
KSHAATGFPSQKLLAGGTGDTKEASSNASPPPASPPPFMWWWFPRSLGEDGGKAKRINTETFQEAEETSLSISQSLV
YDVGVSSGSASDFIELFEGEGISSSDAFLNSLLEGEGGSSSSNAYLQLASSNGLNLQATAVAATPLLLPILAGLLVA
LLLLFFLYFLLGWGRRKRGLLPH

>422329|PACid:15417909
MEALALAFSWLRRAPAQASTQATGLKVLLLETPAATADKKAEDFPVQLEAVIISSDPASSVPEYDAPVLEEDDEHDL
QGTPDVANLLLQPVTPIELEDSSSPAPPSQLEAVLASSHAAASSVPEYDPPPLPVHNHEDDDNNDDDNLSWEDIQSF
FRTWTFPSVQLPSRAESHLEVEELKKLVGKWTWKLLTTLQSIKEEMWNRLLNAGLEEDEVTSQEEQASVFWQRLRGD
GTSSVRKHRRSPDKNSERQELVAAMAEESSHKVLKSHAAAGSRKLLRQADVSSTQLQSFGGACEVLKSHAATGFPSQ
KLLAGGTGDTKEASSNVSPPPASPPPFIEAWARMVGRPRGSTLKRFKKLKRLQYQTLRV

>414593|PACid:15418273
MAELEPALKKRRHEEAAKNPSPPPEISPRYDPSEDMEMSPEPPPPPPPPQQQDDELEEGEASANEGDEELDRGTIST
AAKMEEEVVKRRHRGEIRSVYRAYRAFKSCLARHPSIGLRVCVRKRASLSGCSSVRRIAAEIIPRYAICDWTSSILV
HDKGHDDVIAETAQACFSGLVQLSKAANTVAGSFLGLGDMCAGVCRNIASYLLCQLNGNELLCEEDEESSSSSAGGR
KIGQVISESMSKLFRNDPENVIASCFELLRADAPEQRKHALHFLAQIAVEEIDEKNTDTEKKPSPVKSGLVSKVIHP
RPALEKWIVSTVRMFRKIEKADIVGECLPKLLALVKSLRTFTPSDLELDEEPVEERLLSRSRSRLVVTNLSDEGDSC
SRSVRAVDRTGRGYSPDADGWYREQESRPRRSVRSVTIERPSDRMEDTTWSRRDSKYSDSEASQLRSSPGAMDVFTA
SESLWVSLPSSGVTESMLQAQFSIFGDLDSVTTVRDQGYGIVAYKNIRDAVDAREEMQGSTVWGKALRVRFADSDTR
STSSCHVWVGRISSQAVKEELLNELTSAGMKPPASVVTLISSSALLLQYDSPEDANAVMVYVRQRRRESYRSSYGTE
AGGRHLCIVRIDPLVSEDDILSAVSQYGEITGLKFSRQSGYCVIDFRSSETAATARARLNAARFGNQAMVVDYRNKI
PAESVLSPTYSLYSPPARTASGTSVAATLENLCSKFSLGSPAPAGKTRRSKFDIIENVPTNTLWIGLPESVPQEYMS
ENELRVIFNIACDGTGAVTKVRSARNSRGPCRFVEFDGIDAASTALQNITGHFDPSIHIEFSNSVLSHANDHHYRDH
PRRQDKAASRAWDECYRSPSAEFEECRTRPRPHAWEGSAYDETFQYNRKQASPGVASAIGSPVPGKLHSQPPPLAPL
PVKNLPGYGQLDRAATWQGSSPLHPISPAALTPRRRSCSVTIGGIPTSLSSLQSPVTPAMRNASVSGNRDQAMLPAS

PVPPLPPEAPPPPLPPSSPPPPPPPPSPPPPLPSSPPPRAPSDRQAKSFWRGPLCKSGLQYCEVMLCREDFSSGPLR
GWPDKLDVTKRADWRLHASPASGENYNGFLHFKEYLKQRDRAGVVKLPGAKGLWPRMLCKRLGIEPRSFCKVYSSGG
GGGGGEDGGDKNAQDALVEIVRLQKGQIEVDEFVKERAELLKNIAEGANDEYQRIADDVNKGLEEIGSKVIESMEAD
VGELEQEMAQSRAQIEADEREFAEFEKKLVQDRSEGLFFKSLYQPLKKSRKPPAASHQLMEEQTKKVEKVTKRAAKS
SSRITVYAALAILLTLTIVEAIATGEELYKVGIYSALLLCLALQFGYERLFANQSEVDKPKD

>406775|PACid:15418404
MEALALAFSWLRRAPAQASTQATGLKVLLLETPADKKAEGFPALPSQLEAVIASSDPASTVPEYELEEDDEHDLQEP
VAPIELEDSSSPAPPSHLEAVIAGAASSVPEYDPPPLPVLEDENHDSQELEAVMVGAVDSVQYTSLGGKTLAIDDDD
DDNLSWEDIQSYFRTWTFPSVQLPSRAEMHLEVEEFKKLVGEWSWKLPTTFQSIKEEMWSRLLNAGLEEDEVTSQEL
WEELKNFLGWHRFPVISSRRMLLEAAPGPPIFVSNENGPSLFRWVDGFIAPLRAQQDGEEQASVFWQRLRGDGTSSV
RKHRRPPDNNSEGQELVAAMAEESSYKVLKSHAAAGSRKLLRQADVSSTPASVFWRSLRGDGVRMHRRLPYKNSEEL
AAAMADESSYKVLKSHAATGFPSQKLLASGTGDTKEASSNASPPPASPPPFMWRWFPRSLGQDGGKAKRINTETFQE
AEGTSISSSQSLVYDVGVSSSSASASDFIELFEGEGISSSDAFLNSLLEGEGGGSSSSNAYLQLASSNDLNLQATAV
AATPLLLPILAGLLVALLFLFFLYFLSVIRRRFSSGGEKVALTSSRQEVSGDILQNRERMHAKSSCVPIFALVMAWE
PRTKGRTVRTELDDQESTATISSPKNQDEGSMS

>444786|PACid:15419097
MQLAAVSQARVSPPLGIHSSGLFGDVRPGNFAYLPAATRNSSLSLSVRACTGAPGSPPPSGTNGTADSLPKELPTRP
TASTATSDRKFSRPPPFSGFSGSLGLNFPGSQIPGLPGSPRPKPGTPRPYDPTKQKSKVMEWLTPPKPPTELLQREH
FKLSPTCLCTIYFGDLSKWFVDGKQDAVIAPANKKLNTGPAVNAVLFKAAGSKLLECTQRLPDVAPQGIKAEMGDAI
STRAFDLPVHRVIHTVGAVHKKNEKTGVRESDPTLEKAYRTALLVARKEKLKYLAFPPLSCNIYGYPYADGAEVALR
TLKDSCEGFERIDIVIRNIEGYEAFIDEANKVLEVIKEKKTIKVPAMAPRTTDYLPPAAAAAAAATQPASGVPTTPA
ELYPMPAAQSAGAPPAEAAQPAAPAPEATSDASGGRLSSQAATPAAASAPAPTPAPAQASQAPAGYSQPQTGYGTQQ
QAQAPGGYGATQSPYSQPPASGGGYGQSPYSQAPSQGGYAAAGTGQSPYAQGGQPPYSQQGGYGAAQSPYSQAPAPG
GYSVAGAASGQSPYSQQAPAQGGYSAAGGGGQAPYSQQPPRQGGYSAAGAPGGYSAAGTQAPPAGGGGYSQGAGSPY
SQSGGYGAGYSQAPPASGAPQYQNGPSSPPPYQNAPPYQQQQYNQNAPPPQYNQYQNSPPPQQQYPPQQHKQYGSKE
VSHFQHFQNRAKVLHLRRRGSLYAILSAATLESPPRSCQSFHVSWRPPVDLTQLWTGCVVVLMVARKHK

>422567|PACid:15419535
MEALALAFSWLRRAPAQASGLKILLLETPAATADKEAEDFPALPSQLEAVIASSDPASSVPGYDAPVLEEDDEHDLQ
EPVAPIELEDSSSPAPPSQLEAVSHAAASSVPEYDPPPLPVLEDDNHDSQELEAVMAGSEDSVRYTSLSGKTLAIDD
DDDNLSWEDIQSFFRTWTFPSVQLPSRADLHMEVEELKKLAGEWTWKLPTSLQSIKEEMWSRLLNAGLEEDEVTSQE
LWEELKNFLGWHRVPVTSSRRMLLEVPPGPPIFVSNENGPSLFRWVDRFVAPLSAQQDGEEQGGVSSPPASVVWQRL
RGDGTSSMRKHRRPPDKNSEGQELMAAMDEESSYKVLKSHAAAGSRKLLRQADVSSTPASVFWRSLRGNGVGMHRRL
PYKNSEELAAAMAEESSDKVLKSHAATGFPSQKLLAGGTGDTKEASSNASPPPASPPPFMWRWFPRSLGEDGGKAKR
INTETFQEAKETSISSSQSLVDDVGVGSSSGSASDFIELFEGEGISSSDAFLNSLLEGEGGGSSSNAYLQLASSNDL
NLQATAVAATPLLLPILAGLLVALLFLVVLYFLSGVIELWWQIFIGWREGGSNVIKTRSERRYLTKQRPLVVLELFL
VVHNFLYRLVVKNSFLFSSEAWWETKEVAIMTRSNTISTCSRNELLPCVRSESMEGRNSSFVLQFELSYNQDHDKPV
YLIRKLASRQIPVISSGSVHRRNCQTVWSWTKARSLTNYKRNGILQVLKEPATI

>421975|PACid:15419720
MATIIRRSVPPSSPPRRAPPPAAHRSVHPPRPGHRSATPPKTRVPPPGSMPPPKPSPRRAGGAPPPHSSAHSQAPPP
APAEQHHGAAKQHSHGGAPPPKQDSHGAPPPPPTSHGAAKKHQHSPPPPPASHGAAKQHQHSPPPTSHGAAKQHQHS
PPPPPASHGAAKQHQHSPPPTNHGAPKQHQHSPPPPPASHGAAKQHSHGGGAKQHPPPPAKQHTGPPPSSGTTTWRP
RRFGPPPPPPAKEHTGPPPSSGTTTWWPRPAHDTITWQPGSPVFHTFSSPPLPGPLSIAPPLGSSSSTAAAAAAAFA
TPGLLVAGIVLFLLAMVVIITGIWILVKGRRKKANDILVHYVGSSHLPGGGSSSSGSQREYLVPLSKIEDAELRDKL
NSFVQERSKQHLRPSQLNPSTKVANPPLARPPRRLPPSAFLKMNPIYGAATDSVDDCLTPPLGRDPPAAIHGIDEHK
KVPRGSLPEDPFSSSAACRSGNDRRAQEQALDPVSHRGTVFDPGDAFVVRLAVAAEKRAEMEKHRVWDEEARQQALP
QVENLEKPVVEANHSANSGGSHLAARVHDSDAKAKAGPWYSIEIITDGEVWRHLNQEIMICKFQTRQQDNNFSLR

>406925|PACid:15419813
MEALALAFSWLRRAPAQASTQANTQASTQATGLKVLLLETPAATADKKAEGFPAQLEAVIAGGDPASSVPEYDAPVL
EEDDEHDLQGTPDVANLLLQPVTPIELEDSSSPAPPLQLEAVLTSSHAAASSVPEYDLPPLPVQTHDSQELEAVMVG
TVDSVRYTSLCGKTLAIDDDNDDNLSWEDIQSFSRTWTFPLVQLPSRAEMHPEVEELKKLVGEWTWKLPTTLQSIKE
EMWSRLLNAGLEDVPGIMGRTQKLPGVASIPSYQFPTDASGGSARLRGDGTSSVRKHRRPPDKNSEGQEPSGSNGRG
VILKASVFWRSLQGDGVGMHRRLPYKNSEELAAAMADKSSYKVLKSHAATGFPSQKLLAGGTGDTKEASSNASPPPA

SPPPFMWWWFPRSLGEDGGKAKRINTETFQEAEETSLSISQSLVYDVGVSSGSASDFIELFEGEGISSSDAFLNSLL
EGEGGSSSSNAYLQLASSNDLNLQATAVAATPLLLPILGGLLVALLLLFFLYFLSGWGRRKRGLLPH

>406928|PACid:15419817
MEALALAFSWLRRPPAQASTQATGLKVLLLETPAATADKKAEDFPAQLEAVIISSSDPASSVPEYDAPVLEEDDEHD
LQETPDVANLLLQPVTPIELEDSSSPAPPSQLEAVLASSHAAASSVPEYDPPPLPVHNHDSQELEVVMVGIVDSVWY
TSLGGKTLADDTDDNDDNLSWEDIQSFFQTWTFPSVQLPSRAELHLEVEELKKLVGKWTWKLLTTLQSIKEEMWNRL
LNAAWRTSQELWKELKNFLGWHRFPVISSRWMLLEAAPGPPIFVSNENGPSLFRWVDRCTAPLCTQQDGEEQASVFW
QRLRGDGTSSVRKHRRPPDKNSEGQELVAAMAEESSYKVLKSHAATGFPSQKLLAGGTGDTKKVSSNASPPPASPPP
FMWRWFPRSLGEDGGKAKRINTETFQEAEETSISSSQSLVYDVGVSSGSASDFIELFEGEGISSSDAFLKSLLEGEG
GSSSSNAYLQLASSNDLNLQATAVAATLLLLPILAGLLVALLFLFFLYFL

>407767|PACid:15421204
MARKISTPPPPPPHREHARRTPPPKPSPKRTPTTSSPPPKRSPPAPAKRHTEGHHSPAKPPPSSPEHHGAGHPPPSS
PEHHGAGHPPPSSPPTKEHPAAEPNRHHGHRRAGDGAATGPPPSSSPPNKADTVTWRPRRAGPSSPPPSSIMWNPRS
PSPPPSSITWNPRPPSPPPSTITWQPRPLGLDTSPPTASSAPSSAAAAAAAAFVASPGVLAAGIVMLLLALVAIVTG
VWILLKRRRNKVHSDIHHDNILVEDGHEPSGGNSGSSSGSISLPKKKAAADPLIRELLRPPRRLPPSALPAGARHKS
ASGNDPLFRAADPVHGQQGSRGNVIMEDPLVVFPAYEVDEQPRLFSATPEDDPLFSTGGYSHHPAGEKMAANELPVC
ISSRCDRVPEQFHGREQGRKVADRSGTVFDPGDVAALAKKRAEMEKQMQKFIMMEPRIRPFFHRKRDVENSPENLVR
KLTANDPKRVKSDLEVEPSAPGHASLTGENADQILDNTRIRTVVFLMQLKSNFTVVLVAGPQNGHFETVFVIDNVLP
RVTPRLWEEPKDVHQRRFHQHQHHNRFCISLQSLMKIAIIAGTGAYRFASGYLQIRSYMLIPLFSAILRYLVLTDHV
KIERAKNVSISPKSLL

>407772|PACid:15421223
MARPPHQEHTRRTPPPKPSPKRAPTTSSPPPKRSPPAPAKRHSHSPANPTEGYHSPAKPPPSSPEHHGAGHPPPSSP
PTKEHRAAEPKRHHGHRRADDGAPPSSSPPNKADTVTWRPRRAGPSSPPPSSITWNPRAPSPPPSTITWNPRPPSPP
PSTITWQPRPLGLDTSPPTASSAPSSAAAAAAAAFVASPGVLAAGIVMLLLALVAIVTGVWILLKRRRNKVHDHDDI
LVEYGHQPSGGNSGSSSNQREHLITKVEPRPSSQLNTGRKKAAADPLIRELLRPPRRLPPSALPAGARHKSASGNDP
LFRAADPVHGQQGSRGNVMMEDPLVVFPANEVDQQPRLFSATPEDDPLFSTGGYSHHPAGKKMAANELPVRISSRRD
RVLEQFHGREQGRKMADRSGTVFDPGDVVALAKKRAEMEKQMQKFIMMEPRIRPFPHRKRDVENSPEMTSDKNLVQK
LTGNDTERVKSDLEVEPSLFAAPGHASLTGENADQALWEA

>407777|PACid:15421229
MARKISTPPPPPPHREHTRRTPPPKPSPKRAPTTSSPPPKRSPPAPAKRHSHSPANPTEGHHSPAKPPPSSPEHHGA
GHPPPSSPPTKEHPAAEPKRHPGHRRAGDGAATGPPPSSSPPNKADTVTWRPRRAGPSSPPPSSITWNPRPSPPPST
ITWNPRPPSPPPSTITWQPRPLGLGTSPPTASSAPSSAAAFVASPGVLAAGIVLLLLALVAIGTGVWILLKRRRNKV
HGDIHHDDILVEYGHQPSGGNSGSSSNQREHLITKVEPRPSSQLKAGRKKPAADHRIRELLRPPRRLPPSALPAGAR
HKSASGNDPLFRAADPVHGQQGSRENVTMEDPLVVFPAHEVDDKPRLFSATPEDDPLFSTGGYSHHPAGEKMAANEL
PVRLSSRRDGVPEQFHGREQGRKVADRSGTVFDPGDVAALAKKRAEMEKQMQKFIMMEPRIRPFSHRLVVSALELTI
WTDEAESIRKRDVENSPQIMSDENLVWKLTANDPKRVKSDLEVEPSAPGHPPLTGTAREKQIAGRAKARHLSVSPFY
GLDVNDHVKIEQAENVSISMKSLF

>407779|PACid:15421231
MPPASSPEHHGDGHPPASSPEHHGAGHPPPSPPPTKRHHGHRRAGDGDVTGPPPFSSAPNKADTVTWRPRRAGPPSP
PPSSITWNPRPPSPPFDHNKAPWLGHLSAYCVVSTIFSSSSIRGITWCARGRHPARAGSHRHWSLNTLEAEKEQVEC
RKKAAADPRIHKLLRPPRRLRPSALPAGARHNAGNVMMEDPLVVFPAHEVDDKPRLFSATPEDDPLFSTGGYSHHPA
GEKMAANELPVRLSSRRDGVSEQFHGREQGRKVADRSGTVFDPGDVAPLAKKQAEMEKQMQKFIMMDPRIRPFSHRL
VVSALELTIWTDEAETTRKGDVENSPQIMSDENLVWKLTANDPKRVKSDLEVEPSAPGHPPLTCENADQALQETKHK
NK

>437863|PACid:15421238
MEERPCSSSEELLPRPLTRLRTRFPSPPPSPLLLPLAPSPPPESPTRLAAPLAMWEREELGAFSDGSLPQPGSVESS
LPRLGQLLGVQRIRACEEAGAILPAAIRRVSWGDHRHELFACEDSPGKVCSRFLPICCPSSSPSPSSSPRNAAAGGE
ECDGSKEPGWIWKAANDPDEVALANLFSREEEEHESQGGGDGGNDSAVSVCSSGGGWKADFSSHFKSNSSSSPAVPP
LARITSATSSPSSTSSPLLPRAATSSSPKKSPKVVPAAATSPKKIESPSAMPGPPNIRSATPTPRSATQAMPRDQCS
SKHMKNAAAASASPDTATDKSSDGACSEHRIMDCSCCGRKDKSHLSIQAGVERELHVEGPVRKRLPTTTLLTGPPGR
KPPMRFGLGPAKSEFLNRAAAMNNAAAKARFTVLPVVTLSKASKQQAPQPRRERPLRSSRLNPEFKGGWAPKCSSVL

PRPHSCQSHRSHDRSHDHSHSHGRSVPSPPELDLATPSLNSTARLMERLEEAKREKFDPRAVLEQQQQQLHQHQHH
HDHDHGHCSDAELAERYKDLCKISDGSSPSHPRVPRKHRRERVSSLNFKKPKMSDLWVPSEQCPQVCSTAPIRIEPP
YWLNADKAARDASAEEAEREVFQSSRNAMSLYGFPCLF

>407782|PACid:15421249
MARTPPPPPPHREHARRMPPPKPSPKRAPTTSSPPPKRSPPAPAKRHSHSPPKPTEGHHSPTKLPPEHHGAVHPPPS
SSEHHGAPPRFPEHHGAGPPPPSSPPTKEHPAAEPKRHPGHRRAGDGAATGPPPSSSPPNKADTVTWRPRRAGPSSP
PPSSITWNPGPSPPPSTIAWNPRPPSPPPSTITWQPRPLGLGTSPPTASSAPSSAAAAAAFVASPGVLAAGIVLLLL
ALVAIGTGVWILLKRRRNKVHGDIHHATSWWSMAINLAVATAAAAATSGSTSLPKKKPAADHRIRELLRPPRRLPPS
ALPAGARHKSASGNDPLFRAADPVHGQQGSRENVTMEDPLVVFPAHEVGDKPRLFSATPEDDPLFSTGGYSHHPAGE
KMAANELPVRLSSRRDGVPEQFHGREQGRKVADRSGTVFDPGDVAALAKKRAEMEKQMQKFIMMDPRIRPFSHGLVV
SALELTIWTDEAESIRKRDVENSPEITSDENLVRKLTANDPDRVKSDLEVEPSAPGHTSLTGENADQALQEKNK

>439181|PACid:15421773
MQHAVTQLGGLPPLGTHSSCLFGDGRIGSLAFSAPKEAFFLSRTMSATTRAAYSGSFGQNPPVPPRNNAPPPPLPFD
NAATPPPPMPGNPQMQTPPAIPPPGPFATETNAFPSERPSAIAPNAPTDAKFTSARPPSSSPGLNFPGSQIPGLPGS
PRPKPGTPRPYDPTKQKSKVMEWLTPPPPPTELLQREHFRLSPTCTCTIYFGDLSKWFVDGKSDAVIAPANKRLNTG
PAVNAVLFKAAGSRLLDATQRLPDVAPQGIKAEVGDAISTRAFNLPVSRVIHTVGPVYKKNDQTNVRESDESLIKAY
KSALDIARKENLVNLAFPPLSCRIYGYPYIEGAEVGLRTLKENCEGFTQIDIVIRNIEGYEAFIEEATKQLEVIKEK
KTIKAPSMAPRTGAEAVAAPPSPPVTASESTSGPATESPVAPAASSSSAPTSQSPAAPSPFQSAPVTSAPETQSSFS
QTPPPPSAPQTPSPFQSPPATPGPFQTPPATSSPFQTPPATPGPFSSPPASRSPFQSSPAMSSSFPGTPAAPPSPFQ
STPAPPNTTTPPAFQSAPFQSPPASPPSGSQFQSPPPPSEFQKAPPPFGTPTPSPSAGSPFQRAPPSPFQSAPPPS
SGSPAPPSPGSPFQNAPPSPGSLPPPGSPFQSASPPPSPPHFQRSPYSQGGYQQSSPPPPYQNAPPPPYQQQQYNQP
PPYQSQAPPQYPPYQQNATPPPPSPPPPPPSFESSTPTYSPYSPFAPAPEPTPPPKPLIPPHQQKYVPPPPPPPPES
KPIPPRERVSPFPPPNRPPRPPSSPTSTYEQPLF

>445983|PACid:15423384
MTTSPRTMSSDPEDSSCCSSPYETNRYQSPSERRNVAVDQGGVSYVFEPFMETSLAMEKPDVVGIGGGAWLPATAPV
EPPRPQTCPPLEFQLPKRLIIAARDKESLLEEVITLKTELQHQNRKIAAQRIQASTVANENRKLEKDLEDLELQRTD
ARRTGGPERLRSKVYERNAGLKTRLEDLHMVCEEQRDELFTLRRDVRLTRSRELEVERNVFADEIIRLLKYMKRLKA
GQNKLSCENRELALVKDKAVLLEIANKRLESCLTAIHKNLEKTKQETAAFKQKALETSNSNKADEKRRRELEDKAAK
LTEQLDTETAEKEKWVRKASKLENEIRKLSKELREAEAAKAKFELLNTSKSPPTPPPPAARKAATAPAAPPSPPPPA
AGKKTAASPAAPPSPPPPAAGKKAAASPAAPPSPPQAGKKVAAAPAAPASPPPAAKKAPVTPDKSAKKKWLPHGSYV
DSRDPQAGTRWSKDKAVKAPVTHSSVLNAQTKPGGDETRGKSAANEASPREAEEDTERKAAEALRRKQEEEMQRKQE
EEQRRKEEARKKAAAEAQAKSRAAAGNDAKKKGGETQKNYEDRKKKSDLAGEGGGVREDKDAGGETEEEAKRKALAA
AEKEARRKRFLADEDEEARRKRLLAEEEARRKRLLAEEEEAKRKKLLTEEEEARKRLLAEEEAKRKRLLTEEEEARR
KRLLAEEEEEEARRKRLLAEEGEARKKGGITGAGDDEEARRRKAAAEAEAKRLEEALAEQKRRLKQREQELAEQKQSV
ASNDEGKKLNEDEARRKAAEGLRIKEEEERRLREEEEVKKKRAQEEEMKRRMEEEEEEIKRRMEEEEGKRKEAEEEL
RRKQEEEERKHREEEARRRKLQEEERERKRREAEEEEARKRRIQEEEEMKRREGEKKRLDDEERARKEKEDAVARKA
EEDRKNAAGGGRPRIRVFKARGTTFAEDTKQVVDRGAPTFEGRSRKNFSTAYMAPAPASPASSSTTESSSSARRTTL
RVDPKSGGDPLE

>438797|PACid:15423465
MGGVSNTITTTATITTTTNNKCFSSDSTLVNPPPPPPPPPPPPSSAAALDLNSPPPSRLSSRASSSSSSSSSPPRSIG
GGGGVVAEAEPSTANGGGAASGGTCQAAKKPASPVAAEAEAIKQQLAATTFIQADVLTFREVVQKHTGAAEDAQDKM
PVTVPARQSGGSKNHHQHQQHQQQQQQIASQPADPPGNAVATSAAGPGAVPHGTAGGMPLRRHMSRLHERKQNNLR
NLDINRGSPFKRTGCSPRGSDVSPKLVYSDALHTAAAAAGGLLPSPVTPLSSEASFETSNPQRPHHHLQPPLTPTEL
LRQSFDRSLHCSDDLKSPPPPPPPPPPVHHQPNGIVASPAANGGGVFERFPFHHHGMILHTQQQQQPQAVVATHLQL
GNPHHLHHHPAAAAHHLHHHHVAATNQHFVYEHHHQQFQEIQHHQQFQHHHLQREPELLDLFPVHATSFKDRS

>438863|PACid:15401938
MENKTFLYLILLLAIILKNENFCAATTSIKVDGYVYCTNCGSLSQSGIFNAKVNLVCPGDSHSSFTNSRGYFDIFEL
VKTGTDIGSCRVSVDPSRAPRGCSFLTDINGGSSGQSLSTCTNNGNNKFTCGPYYFTPNACSPPPPPSSPPPPPPRK
PPPPPPPRRPPPPPPTTTPPPPPPRTPPPPPPKTPPPPPPRTPPPPPPRTPPPPPPRTPPPPPPRTPPPPPPRTPPPPL
PRTPPPPPPRTPPPPPPRKPPPPPPRTPPPPPPRKPPPPPPRTPPPPPPRTPPSPTPAPSLSPTPPPLSPPLSSPP
APPPESPPLEPGPTSTPS

Pinus taeda

>PITA_000053745.RA protein AED:0.31 eAED:0.33 QI:0|0|0|1|1|1|13|0|941
MALKIMQLIRSTLLTFTVLTGLHLSIAQQTDLLTEVSNGDSCGSIQYTNISDFYFSLTTVLDSLNSNLNMTGFETSY
SYTGQNISSSVYGLLQCRGDLSFYDCQLCASTMTQMLLQKCQNTAGFIQLIGCFQTYAGACNENISVQPETFASDVL
KLLSTITAKAVRNPNLFAADGFQADSSPSRLIYGLAQCWRDLSKASCHSCLSFALSNITAQTINSLGCATGSLGAHL
YAMGCHLRYEIYLFYNSPTSSPPPQSPPPKRNVLAITLGAVAASVGLIAAICLFRFRPKRWEQISRTRGKEGEDSVS
PPIANLQLIFKYDTLRQATSDFKAENKLGEGGFGSVFKTWRLYNAERALEVMDPTLESSYSWEEGLRVIKIGLLCTQ
AGATLRPSMSQVLLMLTGEQQHIPSPTRPAFVDEESVLPAPDQAERATVTPIQNVHSAGGNISLSNMDPSCGNSQHA
NGSLFKLHLNQVLESMVVNVSSSGFNNSSVEGKNKNSTIYGLLQCRGDLSSPNCQKYAFTIKAVLIETCQNSASGFI
QLDGCFLRYDNHYFYNYSEGIQPYGSRPTRDVLVCCLNLLSNITERAMGSPNLFVADVFEEYSSASLLIYSLAQCWR
VLFQASYQSCLSALSKGKKLNVLAITLGAVAASVGLIATIGLFRFCPKRSKPISRTRGEVSVSPPMANLELIFKYDT
LRQATSDFKVENKLGEGGFGSVFKESSEIAWLLRLNIRKADSLRIPPQHSYEIIVGNTRGLAYLHEESDILIIHRNI
KEGNILLDNKYRPKIADFGLAKLFGEDESHVSTRVTGTRFGVLLLEVISRRKNHSLLQHIEFLIETRYEIIVGTTRG
LAYLHEESEIRIMHRDIKASNILLDNKYRPKIENFGLAKLFGEDESHLTEKADVFNFGVLLLEIISVRKNYSFPQHV
EFLIEVVSRYLFEVEIF

>PITA_000086908.RA protein AED:0.10 eAED:0.10
QI:0|0.5|0.42|1|0.83|0.71|7|0|694
MALKIMQLFRSTLLTFTVLTSLHLSICQQTNILTDVSNGDSCGGSRYTDGSVFHVSLMKVLDSLAVNVSTTGFNTSS
VEEQSSSGTVYGLLQCRGDLSSQDCQKCAFTIKAELLETCQNSTSGFIQLEGCFLRYDTHYFYNYSEGIQTYAGACN
NDVSDQPETFAPAVLKVLSNITARAVRNPNHFAADEFLEDSSPSRLIYGLAQCWRDLSKASCHSCLSLALSNMTTTT
TNSITSLGCKIGSRGAHLYAMGCHLRYETYLFYYSPTSSPPPQLPQSPPPKRNVIAITLGAVAASVGLTAAICLFCF
RPKSWERISRTRGKEGEDSVSPPIANLQLIFKYDTLRQATSDFKAANKLGEGGFGSVFKGVLPDGREVAIKRLNIGS
RQGDNEFLNEANLITRVQHRNLVRLLGCSVERSERLLVYEYLHNSSLDKIIFDTTKRHLLNWTERYEIIVGTARGLA
YLHEHSEIRIVHRDIKASNILLDNKYRPKISDFGLAKLFAEDESHVSTRVAGTLGYIAPEALRGQLTEKADVFSYGV
LLLEIISGRKNHSLLQHMEFLIEATWRLYNAERAIEVMDPTLESSYSWEEGLRVIKIGLLCTQAGATLRPSMSQVVL
MLTGEQQHIPSPTRPAFVDDESVLPAPDQAERATVTPIQHLNNTSTNTHSLQLALYCGTTSHVHSAGGNLTLSTMEP
R

>PITA_000043793.RA protein AED:0.15 eAED:0.15 QI:320|1|1|1|0.33|0.5|4|133|488
MDCRRLHSSKTLLVVLYISVLLIPTGANEPEASPQPNPGIGVNYGTEGNNLPSPKQVAKMLGNTIVNRVKIYNSEPS
ILQAFANTGIAVVVGLENEKVQRISESPTAGQAWVAQRIAPYYPSTLIILITVGNEVLTNSDGSLDSALVPAIQNIH
SGLVALGLDDKIKVSTPHSMAILSSSFPPSASTFATSLLPIMKPLLKFLDDTGAPFMVNAYPYFAYRDNPKEVSLDY
VLFGQSPGVSDPKGFTYNNMLDAQIDSIYSAMAALGHNDTIPVTVSETGWPSKGDPDDIGVNPDNARNYNTRVIKHV
TSNAGTPMRPNRVIETFIFALFNENHKPGPTTERNFGLLNPDGSKVYDVDLSCGWCASPPPPYSPPASPPPSPISLF
SGPPKGSRGGLGFAVYCIAKPSSDPSVLQESLDFSCGLGGADCASIRQGGPCYNPNTVHAHASYAMNSYYQIHGRNY
WNCDFKSTGLVTFTDPSYGSCHYPQE

>PITA_000064608.RA protein AED:0.09 eAED:0.09 QI:0|1|0|1|0|1|2|0|525
MRVVMESSVRTLCLVMVAFAVHVVLWNDGVCCGEPKTSLVTGMVFCDQCLLGTLSTLSQPLAGAQVSLEYSRGSTNA
AFWASTDENGVFSIIVEEDDFQIHGDGRCTVKLVRSTDMDCYIPTDIANGSSGATISSAGGVYRERAVYYVGPFAYR
MSRPATACNALQLGSRHGIILESSELPVQSLGALSRGLKAVGRSFAEAMPTVPSSASTIPSTATSTAKPPSYSIPVT
RPTPAHGDHSRVKHSGVPTSNNHSPTKCIPLPPVPLDPELITKLHYPPCKDEPPTGGGATPPPSTPRASSPIGPGAP
FLPPTASPPKVGSAPPKANSPSYGGSPKPKLPLFPLLSPPPPTSPSYGGSPIPKLPPSSQLSSPPPASGFKPAPLSP
TPPSPASPPHKHPIHRPQPHHRPSPRAPLPPTKPETPISHPSLPPYKNPLAPPPPTNAKTPISHPSPSPYKNPPPPA
GSQVRYPLPPPSYVKPSPPITPPPQPPSTPKMPPHHHQTPHHIPPPQPPSTIPLPFPHPPPPL

>PITA_000077687.RA protein AED:0.00 eAED:0.00 QI:82|1|1|1|1|0.5|2|303|151
MAMAITGLKLTAVFAFIMLIDHTQAVQYHVEGTTGWTLSTLNYSQWAAQNTYNVGDTLLFVYTAGDHTVLQVSKADY
DTCIAAAAIKSYRTGKDVVPLPTAGSFWFICGVPGRCPIMAFGITVTAAAAASPPPKATPVPPPRKASSPPPKS

>PITA_000016346.RA protein AED:0.05 eAED:0.05 QI:0|0.66|0.42|1|1|0.85|7|0|707
MPFCQLKPRQGTKKLGLAMAMKHLTGSILIIILCLCTSIRFAAADPETSTVWSNCGESGNYTYKNGSAFQVNLNKVLD
SLVNNVYASGFNTSSIQGSNSNSTVYGLVQCRGDLDSSDCRQCASTAKANLVQVCQNTSGLIQLDGCFLRYDDHNFY
NDIESRKETPISILCNTGNSSQPQQFTTAVRALLSNSTAIAQQSPKLFAADSVAAPRNISQTIYSLAQCWRDLSRTS

CGSCLTFAVENIFQCQSGALGAQVGSQNCYLRYEVYEFFNATVLSPPPSPPPSSPPGESPPASSAGKKPSVLGISLG
VVAATIALIAVIILWKWNICSLLKRPEEIYRTGGGEENSILGPSIANPDLIFKYDILREATSNFNLANKLGDGGFGS
VYKGVLPDGRVVAVKRLYMGTRHADAEFLNEANLISRVQHRNLVKLLGCAVEGSERLLVYEYLENSSLDKFLFDATK
RHLLDWKERYEIIVGTARGLAYLHEESQIRVIHRDIKASNILLDDKHKPKIADFGLARLFAEDQSHVSTRVAGTLGY
MAPEYALRGQLTEKADVFSFGVLLLEIISGRKNRSSSEDMEFLIERTWRLYTTDRALEIMDLTLEGSYSSEEGIRVI
KIGLLCTQAAAALRPSMFRVVAMLTSETEHLPSPTQPAFVDLDNAGGPHKLKSGKVNDEDKTSSSTATSSSTPSVIH
SVAADPTSGSLEPR

>PITA_000007355.RA protein AED:0.29 eAED:0.37 QI:0|0|0|1|0|0|2|0|376
MANRHRILLLLSSFLLLVAVRTQAQDSQPNITGYACSSAPSPCKTYAFYKAQAPDFLNLRNISDLFGAVQSSHIANA
SGLPADTSSIADGQPLLVPITCGCMKNYSQANISYTIVKDDTFSGVSTRKFENLTTYQAVEVANPTLVPTDLQIGTI
ATFPIRCQCPSKAQLGSGTKMLITYVVEKGDSLSSISKKFGAEQQKLKSQNGKNSTLDPYSTILIPLSKLPKLAQVH
SPSPSPSPTAPTASPPSPPVSPSPSASPPPSPSPSPSSTPPPPTTASPPSSSSPTSSPPPSKSESGLHRGVLIGASL
GMARSLLCIGSLAYCNGQQNPNMVKPEEVCISRQGQSCLVYKYVECVFLNSWVGFRMQSLFRIRVLPA

Picea abies

>MA_148308g0010 high_confidence
MVLIVSCIYAANPSPASYYKSPPPMPYPYKSPPPPPSHVHYNPPPLPTPVPKPTAVEKLPPVYSKPPTKAVPVSPPY
NYKSSAPPPTVDVSPPKPTPTPYAKPPPVYKLLPRPSPPKSWPPLPPYHKSFPVTKPPKATPSPPEKPYGYKSPPPA
TYVASPPPEVPTFPHPHPNHKPPLYSSPPTHHYAYKSPPLPPN

>MA_6426538g0010 high_confidence
NSSVATYNKSPPPKPYPYKSPPTPSNVPSPVYYKPRDVLTPIPKPPIVEKPSPIHSKPPPKAVPISPPYKYKSPPPP
KVEISPPKPTPTPYAKRTPIHKPLPPPSPLKSWPTPLPSHHKPSLLTKLPNPTPSPPEKPYGYKYPPPPTHVVSPTP
PKGVPTFPSPHPKPPLYP

>MA_8038g0010 high_confidence
MSDGKKVRLHSSKTLLIALYISILLIPTGANEPEASPQPNPGIGVNYGTEGNNLPSPKQVAKMLGNTIVNRIKIYNS
EPSILQAFANTGIAVVVGLENEKVQSISESPNAGQAWVAQRIAPYYPSTLIILITVGNEVLTDSDGTLDSALVPAIQ
NIHSGLVALGLDDKIKVSTPHSMAILSSSFPPSASTFATSLLPIMKPLLKFLDDTRAPFMVNAYPYFAYRDNPKEVS
LDYVLFGQSPGVSDPKGFTYNNMLDAQIDSIYSAMAALGHNDTIPVTVSETGWPSKGDPDDIGVNPQNARNYNTRVI
KHVTSNAGTPMRPNRVIETFIFALFNENHKPGPTTERNFGLLNPDGSKAYDVDLSCGWCASPPPPYSPPASPPPSPI
SLFSGPPRGSRGGLGSAVYCIAKPNSEPSVLQESLDFSCGLGGADCASIHQGGPCYNPNTAHAHASYAMNSYYQIHG
RNYWNCDFKGTGLVTFTDPSYGTCHYPQE

>MA_8201g0010 high_confidence
MYPHAGVNNGQIVYASPPSPPSVICTTCDTCENPCTPVVPASPPPPSPPPPAVAQNECPPPPDTYVYPYSPPPPVKA
GTQYPYYPYYTISRSAPLVNHGSALFFLVSPLILILSL

>MA_8395g0010 high_confidence
MAYKAVIRLDMQCSKCKKVALHSVTNIEGIDSLTINMKESTLTVIGDADPACMTMLLRKKFRCAELVSFGPNPPPKQ
PPPPKPPSPKPISPPPPPPPKPKCEKCGKEIDPPKYCHACSPPPPKPKLKCEKCGQEYDELKCCPACMAKCIPPCPP
CSCSQGNCRQCARPPPPTTPPEEKWVNCPPVCESPKCCPPSSACSCRLVKCCPPSHGSSSKSEILWPQYHNTFAPEE
VWYVWNENEHGSCTIV

Brachypodium distachyon

>Bradi2g54840.1|PACid:21803118
MKALGLQGTDLLCGPRILRAAVLAVACAAAFLVGYSWSGASPSLLFFSPASSTSRFSSRSPSVALSPNSNVSFDPAL
IPTPAAPAAAPPASSTPDASPPPSMPPPTPMPTPSPPPPIPPPPMPPSASLGIVGDDGAMRDDFDIGAAANDTDLAT
DEDAGQEASDAGAGGGNRAQVGKFPVCPETMREYIPCLDNDDEIRRLPSTNRGERFERHCPAKDKALSCLVPAPKGY
KAPIPWPRSRDEVWFSNVPHTRLVDDKGGQNWITKAKDKFKFPGGGTQFIHGANQYLDQISQMVPDIAFGSRTRVVL
DVGCGVASFGAYLLSRDVLTLSVAPKDVHENQIQFALERGVPAMVAAFATHRLLYPSQAFEIIHCSRCRINWTRDDG
ILLLEVNRMLRAGGYFAWAAQPVYKHEEAQQEAWKEMEDLTNRLCWELVKKEGYVAIWRKPLNNSCYMNRDPAVRPP
LCDADDNPDDIWYVNLKVCISRLPENGDGSTPFTWPARLMEPPKRLQGVEMDAYSSKSELFKAETKFWDDILEGYIR
VFKWRKFKLRNVMDMRAGFGGFAAALINRKLDYWVMNVVPVTEPNTLPVIYDRGLLGVVHDWCEPFDTYPRTYDLLH
AFGLFSKEQKRCNTSSILLEMDRILRPGGRAYIRDKKEIIQDIKEITNAMGWRGIIRDTSEGPYASRKILMCDKPMV
R

>Bradi2g45680.1|PACid:21803120
MASPIRSPPPEQAAEAQAPPPPPPPPSQLPAVDPPHLSPDNPTPTGPTPPLAAAPATAAPPPPGGTGDSSPPSLPTA
PTPPPETAVEAQAPPPPPPTSELPAVDPPRLPPDNPIPAPPLHATPATTAPPPSPPSPPKVVPTPPPLSTTNPSPPL
SPGNQTSAPRPMPPPAPALAPTSPPPPAPVSPEAKSNQETDGAAGESENMTLAQTEEAIRPTPQKAAAVADSSTESP
QKESALTIAKLLTGEDPAATEAKPAADKVAPVVVTGSVAAAGGGGGGGGVGSKRWLLGRVPEKVRRTELKRAELGFR
VSAALFCLVSLSVMSAGTTPGWAGDTFRRYNEYRYTLAASVIAFTYSVFQLVAEVHYHVTGRRIIRTPWINYFNLAM
DQVLAYLLLSASSAALSRNDVWVSRFGVDQFAKLINASASMAFLAFIALGLSSIISAYHVFSLIS

>Bradi2g38080.1|PACid:21804731
MPPPAAMSTTTIFPILLLLSLTNNGATAVRFDYATLTLSTLRLLGDAHLNNNTIRLTKDLPVPTSAAGRALYASPVP
LLAGFSTSFAFTVTTLNRGSVGGGLAFVLAPDALSVGEAGAYLGLSPTADLAAVEFDTLMDAQFGDVNGNHVGVDLA
GDSLMVSSAAAADLGDVGVELTSGRTVYAWVEYGKKVMEVFVSYGSKRPAAPVLSAPVDLGLYVKDQAFVGFSASTQ
GSTEIHAIEWWSFSTPPSSSSSPAPATAPANSTQRPPVAAAAPPPAPVGTGGVNPSSPLLPGVSPPPAAPGNVSSSP
PEDGSVIAASAPANSTDGVKRNAAASASASPRAAVAGGRDGGAFVASRSRGSALSLASAAAMARSPREFTYKELSAA
TRGFDASRVIGHGAFGVVYKGIVPDTGAMVAVKRCTATATTASANNAEARSEFLSELSIIAGLRHRNLLRLQGWCHH
DERGEILLVYDYMRNGSLDRALFDDASPVLPWRHRREILSGVASALAYLHHECDRRVIHRDVKSSNVMLDEHYRARL
GDFGLARQAEHGASPDATAAAGTMGYLAPEYLLTGRANEGTDVFSFGALVLETACGRRPIGTTEGRCNNLVEWVWSL
HGEGRVLDAVDARLGGEYEEGEMRRAMLVGLACSSPDPALRPGMRAVVQVLAGEADPPFVPAARPSMSFSGANRQLL
ISLQDSVSDYDALGGLNLSDDDDTDSDSLSSSSLTSTLRKGGHDIGFSSTAGDAR

>Bradi2g20087.1|PACid:21805494
MSHHHHHHDPPPPAACCGCGCGCSAPAPPCGYYPAPLPQPASGSDHLLHAIAAHLLLNSGAPPPSPQQHLQPQPQAP
PPAPHHANLYSYAAYQSQYQQAPAPPLHHHHQQQQQTNPPGPDHGQLLLHSLLRRVAALETTLPGCFPSPPPPLHPN
HHHPRPQHRASAAHQEEAPPSPPPPPRRRRGPSEAELAARTIQEHFRRFLARRSRTLRQLKELAMLRSKAASLRGSL
SGRRRCADPAVVTESVMRLLQHLEGIQGGDPMIREGKHAVSRELCRILEFVNKVLVKDHHENKDEYPEGCNGPPGLN
RPTVNKKVSFHCNGAEKEANESSDSSSSAEADERKGKDSKKTDNGKPGLAAPVPVHMEDF

>Bradi2g12720.1|PACid:21805504
MGALTDSRKRVSADHRLHPSFPPSPPPPSKRTKLAPLLPVSSPPPLHYASPSSAAPGPSSSAAAAAATASTSSHSSL
PHPRRRLPPAPPISRPIHGPQRVRRSFRGGNSRPNSNPPWYSPSPPPKPLGLDQYADLVYSVTHPPRPTPAVHVPRG
TEAIPEVVMVDDNEDIRQDKEDEQDVEEEAKAKVVGRKVPLYKELYEKSSRQRDARLRTLEFEVQLAEKGRLGLERL
AEVLPRITPNKEEVPEPFVPLTDEDEDNVRHALGGRKRSETLSVHEASNIVITREILQCLNDKEWLNDEVINLYLEL
LKERELREPNKFLKCHFFNTFFYKKLINGGYDYKSVRRWTTKRKLGYNLIDCDKIFVPIHKDVHWCLAVINIKEKKF
QYLDSLGYMDMKALRILAKYLVDEVKDKSGKQIDVHAWKQEGVQNLPLQENGWDCGMFMLKYIDFYSRDMELVFGQK
HMSYFRRRTAKEILDLKAG

>Bradi2g12720.3|PACid:21805505
MGALTDSRKRVSADHRLHPSFPPSPPPPSKRTKLAPLLPVSSPPPLHYASPSSAAPGPSSSAAAAAATASTSSHSSL
PHPRRRLPPAPPISRPIHGPQRVRRSFRGGNSRPNSNPPWYSPSPPPKPLGLDQYADLVYSVTHPPRPTPAVHVPRG
TEAIPEVVMVDDNEDIRQDKEDEQDVEEEAKAKVVGRKVPLYKELYEKSSRQRDARLRTLEFEVQLAEKGRLGLERL
AEVLPRITPNKEEVPEPFVPLTDEDEDNVRHALGGRKRSETLSVHEASNIVITREILQCLNDKEWLNDEVINLYLEL

LKERELREPNKFLKCHFFNTFFYKKLINGGYDYKSVRRWTTKRKLGYNLIDCDKIFVPIHKDVHWCLAVINIKEKKF
QYLDSLGYMDMKALRILAKYLVDEVKDKSGKQIDVHAWKQEGVQNLPLQENGYFSYLCSSPITDTIIFLLDLVP

>Bradi2g42310.1|PACid:21805712
MHRKLPPLPHLALRSSSTSSSASPPPPRRLPPPVPLRDLLAHRLPPPQTPPSRSPPPVHPHAGLLLPRRGEEAAPER
LHLELVKRGLTHDLFLSNHLVNLYAKGSRLAAARQVFDGMLERNAVSWTCLVSGYVLSGITDEAFRVFKAMLWEGSE
FSRPTPFTFGSVLRACQDAGPDLLAFAVQVHGLVSKTIYASNTTVCNALISMYGNCSVGLPLQAQQVFDTTPVRDLI
TWNALMSVYAKKGYVVSTFTLFMAMLHDDSAIELRPNEHTFGSLITATSLSSCSSGVLDQVFARVLKSGSSSDLYVG
SALVSAFARHGMLDEAKDIFINLKERNAVTLNGLIVGLVKQHCSEEAVGIFMGTRDSFVVNTDTFVVLLSAVAEFSI
PEDGLMRGREVHGHILRTGLIDLKIALSNGLVNMYAKCGAIDKASRVFRLLCARDRVSWNTIISVLDQNGFCEGAMM
NYCMMRQGCISPSNFAAISGLSSCASLRLLTAGQQVHCDAVKWGLDLDTSVSNALVKMYGDCGARSESWEIFNSMAE
HDIVSWNSIMGVMVSSHAPTAESVEVFSNMMRSGLTPNKVTFVNLLSALSPLSVLELGKQVHAVVLKHGAIEDNAVD
NALMSCYAKSGDMDSCEQLFSSMSGRRDAVSWNSMISGYIYNGHLQETMDCVWLMMHSNQMLDCCTFSIVLNACASV
AALERGMEMHAFGIRSQLESDVVVESALLDMYSKCGRIDYASKVFNSMSQKNEFSWNSMISGYARHGLGEKALEIFE
EMQRNGACPDHVTFVSVLSACSHAGLVDRGLDYFEMMEDHGILPHIEHYSCVIDLLGRAGKLLKIQEYINRMPMKPN
TLIWRTVLVACRQSKDGDRIDLGKEASRMLLELEPQNPVNYVLASNFYAATGRWEDTAKARAAMGGAAMKKEAGQSW
VTLGDGVHTFIAGDRSHPNTKEIYEKLNFLIQKIKNAGYVPMTEFALYDLEEENKEELLSYHSEKLAVAFVLTRSSS
DVPIRIMKNLRVCGDCHTAFRYISQIVCRQIILRDSIRFHHFEDGKCSCGDYW

>Bradi2g38200.1|PACid:21805837
MTPAPTVHLPALLIPFLLVLLLLAGGASASTLAVSAGSAVCAVASENGTLYCAVPALPNATANASSSSAQPVAPSLV
FSRVSGGAGFLCALGTARGSGVGDSVDRALLCWPPTDPGSLRRVYRGAATLADLAVGDTHVAAYDAQARGIRWWRRG
SDQSQSQFPELVLGAFRSIVSGDGFTCALETTNSSGDSKVRCWGPQGSAVEAAFANATSVSSLAAGGSRACGVEEGS
GGQVICSGSGAGALAQTGIYPHGLAVGDSHACGLLRPNHTAVCWSLAGPTPTLYYPAYGTPFELLVAGGNLTCGLVS
TNFTVECWSSSSSSVAVELPLPPVLPGACVPDGSSCACGVLERSAELCAGSGGGVICKRLCELSPPPASPPPQSPPP
AEAPSKGVSKGWIAFAAVGAVGAFIGLCSLVYCFVFGFCSHKRIHNSVQPNLPGAPAIAAAAADTNVGVAASPYGSP
NGSRARGVGGGGGLFLRRQLSRAMTRQRSGPSSFKESTEEYTFAQLSAATGGFAPEAKIGEGSFGAVYRGKLPDGRE
VAIKRAGDSPARRFQEKESAFRSELAFLSRLHHKHLFMKNGALYDHLHPKLDSPSSSPSPVVASWRLRIKILLDASR
GIDYLHSYAVPPIIHRDIKSSNILLDGNWVARVSDFGLSLMGPPETEETTAAAAPQHYMKAAGTVGYMDPEYYGLH
HLTVKSDVYGFGVVMLEALTGKRAIFKDADEGAPVSVVDYAQPSIVAGELGKVLDGRAPEPTPHEAEAVELVAYTAV
HCVRLEGKDRPAMADIVANLETAFALCEGSAGGSRGRGSGNGAGGGGFGNSSSSASISLTSMELSGRRED

>Bradi2g61230.1|PACid:21806631
MAASKLPEEKSLIGGAADNLQDGKKVGRLRWPGFVHFFFLLTVVTCAFVYAPRFFLAPSTSGVVASVDFFAPAKAAM
SSSSPPPSSSSSTPQAALLPPPPSSSSNSSSTPQAALSPPPQPSSSSTNSTVRAGGGQDHADGGGGGGGDNASSSSS
GDDEDLVLDNQVGSPCESMGKHGRICCDRSDFNTDVCFMSGDVRTHAASLSFLLFNANATVEEQRIRPYTRKWERHL
MASIHEVRLRAPTASESETSKCDVVHEAPALVMTAGGYTGNLFHAFNDGFLPAWLTSSHLRHGVVLAVLAYNPWWAG
TFRELISELSGRRGVVDLVHDTRTHCFPAGAIVGSRFHGVLSVDPARTRDHKSLLDFHTFLARAYEADNAALKQEEQ
QGRRPRLGILARKGNRVIENQGAVARLAESIGFEVSILETANGAPLSASYAAVSACDVLLGVHGADLTKLLFLRPSN
NTNSNSTAAVLQIAPLGVGPIARGCYAEATVSMGLRYEQYDVVAGESSLRLKYAADDVIVADPETAKKGAGWELVAK
VYLGSQNVTLDLDRFGDTLRDLHSAILAAAAPGPAARP

>Bradi2g61380.1|PACid:21806817
MAKPRRGRSGSRSSSGSSSRSASSGSGSSRSRSRSRSLSSSSSASRSRSPPAAKRSSPGARKGRSPSPPPKKGSPSR
KGRSASPPPKKASPPRKASPAPESVVLHVDHLSRNVNEAHLKEIFGSFGEVVNVELSMDRIVNLPRGYGYIEFKKRA
DAEKALLYMDGGQIDGNVVKLRFTLTPRQKASSPIKPPPPPPKRDVPQNDKGAASAEKDAQPRPRESSPRRKPPSPP
RKRSPPNRRVESPRRPSDPSPRRRADSPIRRRPDPSPVRRGDRRPGSPIRRRSPSPPRRHRSPMRRGRGSPSPRRRS
PGPLRRSPGPPRRRSPPPRRLRSPPRRPPPPPPRRHSRSPPPRRPLHSRSRSISPRRGRGPPLRRGRSDSSYSGSPS
PPRKGPRRVSRSRSPRRPPRGRSISSNSGSSSSSPRRR

>Bradi2g39730.1|PACid:21807430
MANRAAAGRSPSPLSFSSSSSRSPSPFSSSSSSSRARSPPRRSSSAARRPRSSPPPPQKRKGGRRSPSPDKPSPPPP
SPPPAPPLRKLSPVRDQPSLVLHVDRLSRNVNEAHLKEIFENYGEVVNVELSMDRVVNLPRGYGYVEFKRRADTEKA
LLYMDGAQIDGNVVKVRFAPAPQQTAAAPSSPKALPHPPKKDVPANDRVGQSTGKATQQRPRESSPQRKLPSSPRRR
TPPSRRVDSPRRRPDSPTIRRRTDPSPIRRGETPPSRRPGYPVRRRSPSPLPQRLTSPRRISPRRGRGSPIWRRSPL
PPRRMTPPRRMRSPPRRLPPLHRRSRSPIRRPFRSPSRSVSPRRGRGAPVRRGRSSSSSGSLTPPRRGAGRVPRGRS
PRRPFRGRSASSYSRSSGSPSPVRN

>Bradi2g50880.1|PACid:21807431
MASSADPPPKKRKLVEAQTPSPSSTLLTRPPPPLCPGLLPTPPPPQTLAAAAHSSPPPPPQSPPPSSEEIVRKRRNQ
EELRKLFECYRRIRLCVEGKDARLMPELEQVYLALITASRGT

>Bradi2g02280.1|PACid:21808369
MNSASKAMLLQPVSRSGSSSHRSRVNAVLSILLVFSIGYALGLISNSTFQNSYIPPFFLAPLLHPSSMPSSAPEKSP
PPCKGLPTATDLFLSPSSGGGGSAMHNMTDEELLWRASMAPPKATHGRTPKRRVPKVAFLFLAKGELPLRPLWDKFF
SGHDGLYSIYVHANPGHTAISPPPADSVFHGRTIPSKNTSWGHPSLADAERRLLANALLDISNERFALLSESCIPIF
DFPRIHAHLLSFSPSSGAGNGGMSFVDSIDDGISRARYNPAHAAHGVPITVWRRGSQWFEMERSMALEVVSDEFLYP
VVREQCYDPKYGGVPDEHYVPSLVSLLELSARIANRSLTYLEWHAGTAHPWTHGPEKVTEEIFRKMRAGGEGGNCSF
SGGDHGGLSGICFLFARKFEGSALGKLLELAPKAMGFGSVI

>Bradi2g51620.1|PACid:21809387
MPAAAASGGGVADAEDLFRTKRIAEIRAAESATRREISAKEEELRQLVGRSYRDLLDSADSILLIKQSSDSISDNLS
RVSESLSSLSPPPEAPSASANAASPSPSGGRARLYAAAARAKYLVDTPEHIWGRLDEGMLLEAAGRYMRAQVVHRLL
SRDAAAAARFPLLAHQAQLVEAFRPQIAQRARERLADRRLSSALASDPSSYTSVLCDVARIVRVTLGHVGQLFVPAL
TDLPLFYKTVLESPPPAQLFGGIPDPDEETRLWREHWDRLEATMVLLETEAVARTCTDWLKECCDEIFGVIAGAQRL
VDAIESGEGLGSVQKLMREALDERKGLEGSLEQWLKSVFGSEIESPWDQIRGLILKEGKDIFEDWVEEAFVRRMKDI
VHSEFDSLGGSVNVMESMEAIGANADPKDAGDFLLYMRKASTGGSVWFSESKIKKGGILAHLKPIADENDFHSCLTS
YFGPEVSRIKNAIDNKCKSILEDLLSFVESHNSVPRLKELVPYLQEKCYRTISEILNKLEAELGKLSASLGTQRKDN
SIPAAPIIVERSLFIGRLLFALRYHSSHVPLILSSPRQWLKDSGGAAFARLSSPTPRHSRTSFDSSMPFAPRRHTLD
SPSSPGRQFSDSPRRPIASAAASLFGADDSSNPRLDELNKTLKALCITAHTLWITWVSTELSDLLSYALNSDDSLSS
STALRGWEVTVIKQEQPTDGPLEMQIALPSMPSLYIISFLYQACLEIHKIGGHVLDKIILHNFAWDLLQKVIKIYKN
FLVSIELGNSQVSEKGVLQILLDLRFIGDVLSGGKNSSSNPSETQIKQDSSPSTMAKTSFRRKQSQFQADSATIEQT
NKLIDQFSQRLDPIDWATYESYLWENEKQSYKRCVVLFGFLVQLNHMYTGTVQKLPTKSNTDSNIMRCSQIPRFKYL
PISAPALSSRTPKSSLQSPSDDSTSRSPWKSYSNGERSTSSEYDNDASLGSAAPLLKSFVTQVGSKFGENTSRWGSM
LSDGQVGKLSDILPGPAAGFFSSFTSGVRYDS

>Bradi4g11210.1|PACid:21810514
MASASSLPLALKAAAIAAIVSMLVLPSLGRCPNSPARAPPPPPSPPPPSPPPPSPPPPAPPASCEECRSECYSTCTA
FALKTICNECLSIHIRCDSCKTPLIKKCEAETGCTPAGGCHDCEGAGNANCTDACSAMSCSFCMRGQQKQCISTCET
QCSPNCARATIPEKCSDPCDSVAGSCNSCQSQVFKNCKDQCTGSSCDGSCNAAAENTCRGSACTRRYCDACMFGNDT
GCRDTCTKDCNAANCIRD

>Bradi4g32050.1|PACid:21810963
MERCGSWSDCDAAAAQAAAQKAVPAPFLTKTYQLVDDPATDHIVSWGDDRVSTFVVWRPPEFARDILPNYFKHNNFS
SFVRQLNTYGFRKVVPERWEFANEFFRKGEKQLLCEIHRRKTSGSTTSPSPPPFFAPPHFPLFHHPGVSVAQHHHQQ
FVGDDGVIAAAAHGMGVLPFMQPHWQQREQSQSAAPVATRLLALGPSSEGGSRAANNAAAAGALMAENERLRRSNAA
LLQELSHMRKLYNDIIYFVQNHVRPVAPSPAAATFLQGLGLQAPARKKQPATAAAGNGLNTSGGSTTSSSSLTIADE
LSPPPPHHLAAEKSGGEGAGSSSAARSSAAAPTKLFGVHLSATPFGAGSKRPPSPEAELPSTPPATKPRLLLECDDL
SLSVAQPCAATSSPARASS

>Bradi4g39190.1|PACid:21811556
MGAETDLSPPPPPLEPTASLEPEILTRDDRSWKADMMSALGESVSFGRFLAEPLEWGKWSAFEHKRYIEEAAGQSRP
GSVAQKKAFFEDHYAKKRKSEADDADHAGCDEEDVEVDPHDPADGGVAASSADSSCMTDGEEETRGVDPDAPDRGGG
VDAAGPVEALQELKAETDGVGSSCSLVDTDDQSHEKDDVPQVAEAREGLQLHGHYGMVAAVDSVEKQPLQESSIVNQ
GITDSVKKRRLPMPSFFHKPTECSSPPSSGKKTSMRKRSMLRSAKENSSPPPSTDSTKQPETSVPHKRSTLDSMHMS
MNFRRCETGNATSRSRNLGSTIASRISQLEYASRPVKDTHPKVSQFGPTRKGLCRGMPEIVSRTSQLDEQRSSDVSV
MGVKEKLFGSTSPPVHRKTNIITEKMGKANNEPELKELRQGICFKAPHFCRKNKEPKDSSQQSAQEITHLPNNDHLL
NDASNPHRMGKGSAKDKHTCCFPLRRLC

>Bradi4g39190.2|PACid:21811557
MGAETDLSPPPPPLEPTASLEPEILTRDDRSWKADMMSALGESVSFGRFLAEPLEWGKWSAFEHKRYIEEAAGQSRP
GSVAQKKAFFEDHYAKKRKSEADDADHAGCDEEDVEVDPHDPADGGVAASSADSSCMTDGEEETRGVDPDAPDRGGG
VDAAGPVEALQELKAETDGVGSSCSLVDTDDQSHEKDDVPQVAEAREGLQLHGHYGMVAAVDSVEKQPLQESSIVNQ
GITDSVKKRRLPMPSFFHKPTECSSPPSSGKKTSMRKRSMLRSAKENSSPPPSTDSTKQPETSVPHKRSTLDSMHMS

MNFRRCETGNATSRSRNLGSTIASRISQLEYASRPVKDTHPKVSQFGPTRKGLCRGMPEIVSRTSQLDEQRSSDVSV
MGVKEKLFGSTSPPVHRKTNIITEKMGKANNEPELKELRQGICFKAPHFCRKNKEPKDSSQQCSCGCRV

>Bradi4g01990.1|PACid:21813009
MASPPPAPAVDVDLGKLSYEIFSFLESKFLFPGGGGPCSLPGTPSRPGNGPRVRVLAIDGCGPGPADALLAAAAMAR
LEAALRAKVGDPDARVADFFDAAAGAGAGGVLAAMLFLKGDDGRPRYSAAEVLAFVAASLGKGGWANGGGGGGWFRG
RFAALFRRGDRDRSSSSCTAMGRSSSSSTAASLRRVFGESTLRDTVAPLLVPCYDLSTGAPFLFSRADAMETDSFDF
RLRDVCAATCAPAAAVRSIDGRTAIAAASGGVAAMGNPAAAAITHVLHNKARVPDGPHPSTTCSSSPSGSCSGAGSG
VGFVDARVGARRWCPGSPFPGRRWVRAHRLGAVADMVDQAVAMAFSGCSAHNYVRIQAATAAAAMAGKEAKKAVAMA
DAMLTQRNVEAELFRGRRLSEKSNREKLDAFAAELVREHRRRRTMSSPPPPPGIFLPNVDIKQAATPPRLSLSSAAT
TASSSSGATAGTMPSPASQEDFSRH

>Bradi4g22160.1|PACid:21813043
MALALRLRRALAAASTSTSAPLLLRPAVSVAASSSRSLLLPAPFVPPLPRPFLPGAAAAAGFRSTAAAAARGDYGRG
ADENNIGPDEILFEGCDYNHWLITMEFPDPKPSREEMIETFLQTLAQVVGSYEEAKKRMYALSTTTYVGFQAEITEE
MSEKFRGMPGVVFILPDSYLYPETKEYGGDKYDNGVITPRPPPVQYSKPQRTDRNRSYGGNYQNSPPRGNFQNSPPP
RGNYQNSPPPPGNYQNSPPPQGNFQTYSAHQDRRGYAPQQNYAQTGQDSRGYGRNDSANQSGYNGPPGGYQGQANQA
GQGYQNPQEHRNFSQGQAGDFRSTGTSAPGNYGQPSAPGTYGQPSGSATFGQPSGPGAYGQPAAPTNPGANQGGPGY
GGDNRQGAGPAYGGDNLQRGSSQYPSPNEGQGNWQGRQ

>Bradi4g03620.1|PACid:21813317
MGQEGSFEVGDKVFSRSILWIGVGYFDDFASTPLDWGFPIDPLSHHPSPPPPPSPSHHMNIDLPQSSVAAEFQPPPA
DGDLVSTPSDAGFPIDQLWSSPPLPSYQMNVDPPKSPVAVELQPPPGGDLVPTPVRHVPPPSPSRHMNIDLPQSSLA
ELSSDAGFPIDQLSSPPPLPSYHMNIDLPRSSLAAELQPPSSGDSVSSTVRPVPPPLPGQTDRPRSAIEEELQAWRL
PDLPDVTDVELLLKHLDPACERLPRKYISQPRRRAVVNADLPVKNPQELPGVDRGEGRIGIHYLTDNLKLCFAQNTQ
LLENGQRKKFTLETPDGYWTLRDSAGERNDIWCIVGFKKTFEFRTKSGTKTKWIMNVYSRVNGSYFVEDDLALCHVF
EQDSPGYDPRPKCPTFHEGRCDGLSKVTVHHYQDQRSCPRFIPFTPNPGFLVGDPDPDTSTQRGVPPTADHAESKKR
KVGSDVWEYFTKIFARDIKGNVLTFAVCNHCSKVLTGGSSGGTTHLKNHTCACKLKPVLAGRNQKASVSVSSS

>Bradi4g03620.4|PACid:21813318
MGQEGSFEVGDKVFSRSILWIGVGYFDDFASTPLDWGFPIDPLSHHPSPPPPPSPSHHMNIDLPQSSVAAEFQPPPA
DGDLVSTPSDAGFPIDQLWSSPPLPSYQMNVDPPKSPVAVELQPPPGGDLVPTPVRHVPPPSPSRHMNIDLPQSSLA
ELSSDAGFPIDQLSSPPPLPSYHMNIDLPRSSLAAELQPPSSGDSVSSTVRPVPPPLPGQTDRPRSAIEEELQAWRL
PDLPDVTDVELLLKHLDPACERLPRKYISQPRRRAVVNADLPVKNPQELPGVDRGEGRIGIHYLTDNLKLCFAQNTQ
LLENGQRKKFTLETPDGYWTLRDSAGERNDIWCIVGFKKTFEFRTKSGTKTKWIMNVYSRVNGSYFVEDDLALCHVF
EQDSPGYDPRPKCPTFHEGRCDAICCYKELRMSHASC

>Bradi4g03620.2|PACid:21813319
MGQEGSFEVGDKVFSRSILWIGVGYFDDFASTPLDWGFPIDPLSHHPSPPPPPSPSHHMNIDLPQSSVAAEFQPPPA
DGDLVSTPSDAGFPIDQLWSSPPLPSYQMNVDPPKSPVAVELQPPPGGDLVPTPVRHVPPPSPSRHMNIDLPQSSLA
ELSSDAGFPIDQLSSPPPLPSYHMNIDLPRSSLAAELQPPSSGDSVSSTVRPVPPPLPGQTDRPRSAIEEELQAWRL
PDLPDVTDVELLLKHLDPACERLPRKYISQPRRRAVVNADLPVKNPQELPGVDRGEGRIGIHYLTDNLKLCFAQNTQ
LLENGQRKKFTLETPDGYWTLRDSAGERNDIWCIVGFKKTFEFRTKSGTKTKWIMNVYSRVNGSYFVEVCKIRVHAM
QAGKHHA

>Bradi4g03620.3|PACid:21813320
MGQEGSFEVGDKVFSRSILWIGVGYFDDFASTPLDWGFPIDPLSHHPSPPPPPSPSHHMNIDLPQSSVAAEFQPPPA
DGDLVSTPSDAGFPIDQLWSSPPLPSYQMNVDPPKSPVAVELQPPPGGDLVPTPVRHVPPPSPSRHMNIDLPQSSLA
ELSSDAGFPIDQLSSPPPLPSYHMNIDLPRSSLAAELQPPSSGDSVSSTVRPVPPPLPGQTDRPRSAIEEELQAWRL
PDLPDVTDVELLLKHLDPACERLPRKYISQPRRRAVVNADLPVKNPQELPGVDRGEGRIGIHYLTDNLKLCFAQNTQ
LLENGQRKKFTLETPDGYWTLRDSAGERNDIWCIVGFKKTFEFRTKSGTKTKWIMNVYSRVNGSYFVEVCKIRVHAM
QAG

>Bradi4g30240.1|PACid:21813595
MDVKYKPVAFPNGSAKKPVKPAAAAVAMAPPPPSSQVVTYQDCLRNHAANLGAHAVDGCREFLPTPENNPADPWSLK
CAACGCHRNFHRRVLVEDSPPPLPQQRRGEEETPVERLPGVDSDSDSDGSGSGYDDERSMSPPPPTQQHQPPVAHNP
APVAQQPPAPPGAYFSPALAPAPHMLLSLNSSSAPGAAQQRLPVSPVAAAQMVPAGPVPAQHLGAMPMMPAQRKRFR

TKFTLEQKKRMQELSERLGWRLQKRDEAIVDDRCRDIGVSKGVFKVWMHNNKHNYLGGHSARRSASASSAVATTTTA
SGADAAAAPPPPFNPSEAPAATGFNMNGTGGHPVNGGASSSQSA

>Bradi1g70200.1|PACid:21814823
MSHLAAPASPPPAPNPSPDAGEDPSCSSPPPTERARRSAVVVVMGATGAGKSRLAVDLAAHFPGVEVVSADSMQVYR
GLDVLTNKVPLHEQNGVPHHLLSIIDPSVEFTCRDFRDHAVPIIDDILGRGGLPVIVGGTNFYIQALVSPFLFDDMA
EETKGCTSSDHLDDIGLADDNQGNGCYERLKDIDPIAAQRIHPNNHRKIKRYLELHATTGALPSDLFQGETAEEKWG
RPSNSKYECCFLWVDADLHVLDNYVNERVDCMVNAGLLDEVCNIYDPDAIYTQGLRQAIGVREFDEFFRLYLTKIVT
DKIKLGCSSITKLDVGDDKLESLLDEAVCQLKANTRRLVRRQRRRLHRLNKDFGWNLHHIDATEAFQCKPVCTTGDS
WHNKVVKPCEDIVKRFLSGDTTLASEDSSSNIGGTRAASRELWTQYVCEACDNRVLRGAHEWEQHKQGRGHRKRMQR
LKQKTKIIIA

>Bradi1g70200.2|PACid:21814824
MSHLAAPASPPPAPNPSPDAGEDPSCSSPPPTERARRSAVVVVMGATGAGKSRLAVDLAAHFPGVEVVSADSMQVYR
GLDVLTNKVPLHEQNGVPHHLLSIIDPSVEFTCRDFRDHAVPIIDDILGRGGLPVIVGGTNFYIQALVSPFLFDDMA
EETKGCTSSDHLDDIGLADDNQGNGCYERLKDIDPIAAQRIHPNNHRKIKRYLELHATTGALPSDLFQGETAEEKWG
RPSNSKYECCFLWVDADLHVLDNYVNERVDCMVNAGLLDEVCNIYDPDAIYTQGLRQAIGVREFDEFFRLYLTKIVT
DKIKLGCSSITKLDVGDDKLESLLDEAVCQLKANTRRLVRRQRRRLHRLNKDFGWNLHHIDATEAFQCTTGDSWHNK
VVKPCEDIVKRFLSGDTTLASEDSSSNIGGTRAASRELWTQYVCEACDNRVLRGAHEWEQHKQGRGHRKRMQRLKQK
TKIIIA

>Bradi1g74470.2|PACid:21814987
METLVISQQRSHHHHSGRRRKPSSHFSSPQSMRGYNCRAFHSTVSIGILRSPPPPPPTAPARTRSSPEPKTPKQQQL
GKKRSRVIPITPSGSPPSRPELWAGPAYSNSPPPSSLPIPKFSLHQKRSVSLELPPTGPSVHVEVLVHAKSAPSTPT
AGSGSDFFGNDTAIATENLRRILNLEIADH

>Bradi1g04527.1|PACid:21815244
MDTEPFDEADLLAFPTSPAAAASPPRRLKRLRKSSETTTTTTTTPPAVSPPLSPPSPPPPPQEEAGAVSPHLESPPPR
HNPSPPPQQPASDADVLAPAPSSPSPDPISSSPLPPADTAAEDDEEEDDGLDPLFSESGSGGAGWDPLGMPGGEGD
DGEEELLGGGGLIEELRREKSAKKRLDMDEAEDGAEMAVEAEVTRKRSKRRKKDAAPKESAQSKKRAEKERRAQLDS
IHVESQRLLRETRKASFKPTAEPVYKPISSVLEKIRLRKLEILKKSSTPIEEEDDDDDDDSSESVSEAAGHPCMPGV
KEVGSDDKDLKNDDANDEVGANGSDLNDHDSLPEDEDALPSKKDGSQAPDKDLGISQDNHEDSAQPSDDPINAVEEA
QSHASSSPTKSTYDSLSEDEEYNDKENIAPGTQQNDVNTHEELRETHADDSCPDVAVLKNFLDVEAEEEDDSDDDMM
RFKDNEEDDGDDENEVLNALIAAGFEEEEVDHDKRNELHQKWLQQQDAAETNNVIQRLKFGHQEQKELMDEDEDDIE
DCEDESETEMPYELTPTNVVRQNSEKAKQMIAKMFTDENDTYEHSDDEEIEEHLARQRISKRDVHNDSFISPLEDDS
SREVFSLIKKLNIAPQPKRRGKQATSNLEMLMVGSNSSSSSKSSFLGRTTSGPVSSSHRSVYKTYVFGRDDSNSSSK
SCLATTESNSDVDQTNPSQPKKAKFSTQAKAAATSTNSKGDANSGASLFEILRRSAGTSNKQEPSRQESFGIITESQ
AVHQFSAFKLSRKFSKIGAIN

>Bradi1g45280.1|PACid:21815431
MGCKGSKHSLLYGGGAPESGHHVARHSVALRSSTLGTLSLDRAVSFAAAEGMTTTAMAAAAAKSVEEESAGRPGRAW
ATTWSVSNGWLFPASSSPQMEPGTKQRQIRQPPRTPTKTPARDPEEINVWELMDGLHDDDTEEEEECDGVQEEPSPE
FDPDVLSAFRDALDELSPPPSPPPREAAADAVKKNVFQEQSVDGGGNPEEKKPSPSQPPPPPESARRVVVYTTSLRG
IRQTYEDCWAAATILSSYGVRVDERDLSMHAGFKDELRDALALAAHGCRLPQVFVDGRHLGGAEEVRRMHESGELAD
ALEACDAAPCAGAGKEGGFAAAESCGGCGGARFVPCDVCSGSCKVFVVEDEDGAGAFRRCPECNENGLLRCHVCY

>Bradi1g07010.1|PACid:21815588
MSSNSSAPPPSAAPPPSPDLSPPPSPPTANSSSPAPTNSDGPSPPPAPSNSSSHGAPSLPAGNPSPPSESSRGSSRP
SSSQASTRRVGSNTTAEIIFAVAGAAALLALLVTACVCCSRRTAPRRRRRKPQNPMHLYADSSAYKGNSSYYTSEPQ
PQWQSDANAGAPSMSAPGLGSPGCSSWHVPPPDTSSGTHGQPMPGLGKGKGTFTYEELALATGDFSAANLLGQGGFG
HVHKGVLPCGRAVAVKQLKSGSGQGEREFRAEVDIIGRVHHRHLVSLVGHCVAGDRRMLVYEFVPNKTLEFHLHGKG
LPAMAWATRLRIALGAAKGLAYLHEDCHPRIIHRDIKSANILLDDNFEAMVADFGLAKLTSGDSDSETHVSTRVMGT
FGYLAPEYASSGKLTEKSDVFSYGVTLLELVTGRRPVDARRPLLEEDDGLVDWARPAMARALASGDYGGVSDPRLEG
GYEPAEMARVVACAACVRQSAKKRPKMGQVVRALEGDMSLEDLNEGVRPGRSGVFDAAAESSGYRKEAPAATGS

>Bradi1g54650.1|PACid:21815613
MPTASRQRLCRAALLVAGAGYLAFLLLFEVPSLPFSSSSSSSAAASQQLPTHSSRRRELEASASTSPFSPARPSKPA

FPAAGSPAPSPPPLPIFSSLLVLPRSNATATPFDGTAADAFAAAKPHLTHLRTAAASASVAASPPPSSPTCPASISL
HADKLPANGVRTVELPCGLAVGSHVTVVARPRAARPEYDPQIAERKDGKTPLMVSQFMVELVGTKVVDGEAPPRILH
FNPRIRGDYSGKPVIEMNSCYRMQWGRSQRCEGFASRPAEETVDDQLKCEKWIRDDDNKSEESKMKWWVKRLIGRPK
DVHISWPYPFAEGKLFVLTLTAGLEGYHVNVDGRHVASFPYRTGYTLEDATGLSLNGDIDIESIFASSLPNSHPSFS
PERYLEMSEQWRAPPLPTEPVELFIGILSAANHFAERMAVRKSWMMYTRKSSNIVARFFVALNGKKEVNAELKREAE
FFHDIVIVPFMDSYDLVVLKTIAIAEYGVRVIPAKYVMKCDDDTFVRIDSVLDQVKKVQSDKSVYVGSMNYFHRPLR
SGKWAVTYEEWPEEVYPNYANGPGYVISADIASYIVSEFDNQTLRLFKMEDVSMGMWVEKFNNTRRPVEYRHDVRFY
QAGCYDGYFTAHYQSPQHMICLWRKLQSGSTHCCNVR

>Bradi1g77920.1|PACid:21816583
MGVHAAPALPPPPPPPRGGAREADDSDHGLLRVGAVAVAESPPPMSSCGRYILHRVCRFDTLAGVAIKYGVEVADVK
RVNGLTADLQMFAHKTLRIPLPGRHPPAAPHSPPPPPSSSPNARDWTMRRPPKNAALDPFLKPPRSMVSPSMSLLQG
YYGLAPTPNMDPTDEGVEMAMAIKGQHRKARSISTGFNLENGDASRETDDAEKPIRRRQKADLELSAREDSTGGLLP
RAGQGLALRPKSGNRQDTNSSQQDLVATGLVPSYGDGLLAVRKSSSTPEFHDSDNSIASVWLRSKWNLKPDAFSIPL
PILLLDSIQKPFFDSIPKPIAAWRSKAARD

>Bradi1g51450.1|PACid:21817847
MASPSLTPAAHPPLPDADSPPPLPDADSPPPPPDDDAPPPPAQDPQASLPADLLEDIFLRLPTTSDLARASAACAAF
RQIVTALPFRRRFRSVHRPPVLGFLRDDFYPCKPPHASAPAARALAQAADFAFSFLPNAAGWSPRDARDGRVLFSAV
PASHGRGDFADATSTTFVDLVVCDPLSRRYVQIPPVPDDVAASIEGCGMLDFEPFLAPASSEDLQEVEPPFRVIGKV
LCETKVAVFAFSSRTGKWSCHGSSALSNDVVLDALYERRHYVHGCFCWLLEWMEKLLMLDAHTMEFSIIDLPPGNDE
RRLAILEAGEGRLGLLNIGRNTLDLYYKVWRNRGDGKQEWKHETMEHPLPDYHWRIIGSDEEYLLLRGISLDWPWFG
NSSSQGPDIEYFALELKALRLERMYVSKHKMLHAHLYRGFSPAFSSPSI

>Bradi1g65760.1|PACid:21818204
MEYSNEATNTAAPRRPSMSPPSSRPVSSSCSPPPPAVFPLASSSPTVVLSPCAACKLLRRRCADGCMLAPYFPPTDP
AKFTTAHRVFGASNIIKLLQELPESARADAVSSMVYEAEARLRDPVYGCAGAVCRLQEEANDLKVRLARAQADLLSV
QAQHANLLALVCVELARYQQPHPSPPPLVDDGLDHGAMYQSLYDSDLDLAAWEETRLWT

>Bradi1g68920.1|PACid:21818346
MASSSSSSSDGSNSSGDEWVPKSQRPLRQKRQGAKRTTSEQISSLDLKFRFGSEYVKFRFGSEYVTKGIVEQIRINP
VVSTIEVEHGVIAPSHSPTPPEHLVVDCAMPKSASKLSTGPQIVETKFPYHSTDCASTSRSLDQMALSSSSSSNESS
SSGDEWVPKSQRPRRRKRRGVKRSTSVQVTEGIVEQIGINPVVSTIQVEHGVNTHSHSPTPPKHLVIDCALVKNASK
LSTNASKLSTNTEIMETKFPDRSADCARAKNTPKLQTNPNVLETKSPINNEDFVGPNIIKRLPASTEILKPKSPDQL
PASANTEILRRTSPDNKPGSISNHYFRRESPALSSVSPRGTTVMASPPQHPPATAPLPTATPAHPATASQPLPSAFL
ATSAPQRAAAASPPPAPQFVFTATSATQRAAAASPPPFVFTATSAPQRAAAASPAPSPQFVFTATSAPQRAATASPP
SFIFTATSAPQRAVAASPPPFVFTATSAPQRAAAATPPPAVPFVFTATSAPQRVAAASPPAAAPFVFTGRALHTNPR
PASTSHGIIYPVATSSTAAANQRRAPHVAVGYPRANAVALPIAPSQQPQVPTQSRLPAAAPRSVVAGVTPSPRPELP
PRGVPIAPQPQPEVNPVPAVALTPFPQPREQSNAQERGSTNEDSRTVVIHGRNVNLSDSESGSLYALCRSWVRNGVQ
HEIQPSFVGNVAPVLPRPLPASVVDSRMSGKDKEAENEEPKEKKNDTGEYTTAELLKELVDRAKKIRAEYVFFFSQP
FCTFLLEVQSITR

>Bradi1g24940.1|PACid:21818567
MPPRRKGRGRGRGKPKPKSTAPEPVDETVETEVDAAEDAKPEEQSDEDSKGDSSESGAEDMDAEDAEEEAADSKAEE
MDAEEAADAKANGAEVETKGEEMDAKAEAEAEGAESDSEGEADADADLEAEAEETDGENEEKAAGTGGESEEEAAET
GGENEEEADGTDGEDGEEGAETGGENEDDGADSDPEGQGTDAEPDAVEESPPPSPLARSRRKRATTPDPAPEAEDH
EAEETPVPPRRRRRRKSGDRGNSPSPPPDHLRCRRSDGKKWRCQALALPTVSFCEYHYARASKGKKPPADGEVLAVA
LQRQKKNRRKGRRSLNLTPASPPKATKDLPNGLMTISPGSSGAAGSPITMKVGVDIPVPLRRCYRSKNAEPLPVGPV
KVVPRAMGMAKAAAQNACHRCGLKKVARVVKCKNCNNQYFCNSCINKWYSGMSKKDIKMQCPVCRGSCDCEECTLGQ
SRGAMSKGSASDHSKLVRIKICNHQLYKLLPLNLNQEQLDELEIESKIQGTKISNIRVQVAEDDHSGSLYCNNCKLS
MHQALRSCPRCPFKLCLSCCQKIREGNMSDSTPEDKFTQRLLQQESVQEDGSISCPSIELGGCGDSLLNLIYAPPSG
QSEEFSSGDEQDAPGNHSGDKDAQPHSSPLAESNGSLARLSGGQQESMST

>Bradi1g23380.1|PACid:21819612
MAPQPPHAPPTVAERHHAAPRRCHVAALHHARSLHASRSATPRCSPPPCTRHYTHGAMTATTAGKLHRKPRHAGSSI
SNHGSGATTDGASCSGQRSGIDTAGARKSGTVGPTPSRAAAGEDPHRIRPPPLWIRRQWPQNRRRRPPPSSEGPGCE
GERRGDKDASNEASPPPGKAAWGGGRGWVVGRVRGVAPESWGDGVASRVAPKTFNCTQKELKREWTLWDFPIARRIG

SEIKRPTQRRSSYSSIYRRDANDFLPVFSAKPVSNNVSLNVCYFSKVVPAHRWQKRSSVGTKELRGNGPFGGFFMKQ
ENGGEDEFFAQETSSST
>Bradi1g29180.1|PACid:21820207
MPQSNEVRYTARSITPPADRDSSSKSPPPKRRAASRSPPLPPPPPLPKGTHIIARSSPCKRRSVSRSPLPRRRSVSR
SPPPKRRGRSRSRSRSRNRSRSRSADEVRNPGNNLYVTGLSTRTSGDDLEKFFSKEGKVKDCHVVMDPRTKESRGFA
FVTMDNIEDARRCIKYLHRTVLEGRLISVAKAKRTRKRTPTPGEYCGPRGGRSQGRARSPSPPRSRRRSRDRSRSPG
GRRDRNGKHN

>Bradi1g29180.2|PACid:21820208
MPQSNEVRYTARSITPPADRDSSSKSPPPKRRAASSVSRSPLPRRRSVSRSPPPKRRGRSRSRSRSRNRSRSRSADE
VRNPGNNLYVTGLSTRTSGDDLEKFFSKEGKVKDCHVVMDPRTKESRGFAFVTMDNIEDARRCIKYLHRTVLEGRLI
SVAKAKRTRKRTPTPGEYCGPRGGRSQGRARSPSPPRSRRRSRDRSRSPGGRRDRNGKHN

>Bradi1g29180.3|PACid:21820209
MPQSNEVRYTARSITPPADRDSSSKSPPPKRRAASRSPPLPPPPPLPKGTHIIARSSPCKRRSVSRSPLPRRRSVSR
SPPPKRRGRSRSRSRSRNRSRSRSADEVRNPGNNLYVTGLSTRTSGDDLEKFFSKEGKVKDCHVVMDPRTKESRGFA
FVTMDNIEDARRCIKYLHRTVLEGRLISVAKKRWFILQSKFIYWGGHGFCTAHCVGALV

>Bradi1g42707.1|PACid:21821318
MEMVECSLAAARGPRGPRALLGRVPRPSPAVLGAGRTKLRVRSERRGQPPPVVRASPPPEHDGRALGKAAVGLAAAA
VVSLNGLAGDFSPPPALAESLTVAFPVSKAREVNRVQRTLVEAWGLIRETFVDPTFNHQDWDQKLQQTMVEMFPLKS
ADAAYGKISGMVSTLGDPFTKIISPKEYQSFRIGSDGNLQGVGIFINKEPGSGRLLVMNCIEGGPADRAGIREGDEL
VDIDGNSVFGLDGETVAQRLRGRAGTTVEVKLLDGTGNDRSGRTRQKEVQLRREVINLSPVSTAIISHSSGDGHEEK
TGYVRLAAFSQTAAAEMESAIKKMEDEGVQSYILDLRNNPGGLVKAGLDVAQIWLDGDETLVNTVDREGNVRPINMV
QGQSLTHDPLVVLVNEESASASEILAGALHDNGRAILVGHKTFGKGRIQSVTELDDGSALFITVAKYLSPALHEIDQ
VGIQPDIQCTPDMLSLPRAPLLSENGEAPSLEMDSCIMVAEQALEIQQSKGSASYMVSQIVALMPC

>Bradi1g46290.1|PACid:21821473
MATPGGPPSSGCRDALVPRGPSPPPPTTAMPPSTAATDVNDLGISLTSLSLQVTPSPPPPLLAQTHLPEPAPSDLAQ
VHVPNALETSGPMSGPPPSPSLPSSPRVEDFIAAICTDVPAPVLATPATAVQIVSPTPAVLPPTTVHRRARLAARPL
SGISSTLAAQTLVARRLGSLPPAAPFDDEAQEAYVKLFKEPLSEQAIAAIESLVAEARRCKKTPRAAGIGWGMGRGA
DRGRGIAVN

>Bradi1g01210.1|PACid:21821641
MVQLKDMVLKLSGTHRQPGGGPRRRGGSPPPTRTTSLYRSGYYRPGVVQDDMAVPPATYLGHGHGGGASSTASSTPA
WERPPGNGDAAARGEWVAQVEPGVQITFVSLSGTGGGAGGGNDLKRIRFSREMYDKWQAQRWWAENNERIMELYNVR
RFSPRHDHVLPPSSDAGDPEQRESFYSQMGSTRASSPAATPSPAPETSATWAAAFARAAPPPPPSAARQHSFRGPLS
PPPPSSSNPSERAWQQQKQSQQNDGGVEPARTTTSSCRDDDASVSNASELEVTEWVIQDQPGVYITVRELPDGAREL
RRVRFSREKFAELNAKLWWEENKERIHAQYL

>Bradi1g34867.1|PACid:21821989
MAYSKGVGGGGGGMSAVDAILAEAADLVALEQIARLNTAHLDGSPLPSSLESRFRKLKSLPTAPAPPAKTLGRSATA
PLPPHQLRDDDPLPQAPARASQELRPDDAPGKLNSPPPVTTVPAVRKEDEDDEEEDLERLFGPARRGRPTLRERNRG
MDSDDSLSPPPPRQACCFGFSSSPKKPPSTRRKKSHPEGAGNDVLGLDADEWGDENRRMVTELKAQQRKMKEALEEQ
VKVSRETAKMARWVKQASARMTHTAAIDDLLSDIDDEEELK

>Bradi1g70790.1|PACid:21822345
MPPSGWNPLDPVLRTVSAFSRRLLIAPDTAPDDLRLRPLLSLSLSPPSPPPPSPSPPPEVLKAKDAKVAPLTKEEVG
RATWMLLHTIAAQFPDEPTRQQKRDAKELMALISRLYPCKECADHFKEVLKANPVQAGSQAEFSQWLCYVHNVVNRS
LGKTIFPCQRVNARWGKLDCPERLCDLEGSNDIMPNR

>Bradi1g31440.1|PACid:21822403
MAPPPLPWAWRLALPLVAFVSVPFLLPLTLPFILFLRPGSSSSPHPLSFHRLTWLPSPQIPSPPPPATNAPPYPPPQ
TQITSPSPPPPPPPPLPSPPAIEKKARCDIYDGTWVRSTDASRPLYAAGTCPFVDEAYACAANGRPDSAYTRWRWAP
RRCALPPFNATDFLSRLRGRRLVLVGDSMNRNQFESMLCVLRQALPDKSRLVETHGWRISKGRGFFVFKFLDYGCTV
AFVRSHFLVREGVRVNRQGSTNPTLQIDRIDKTASRWKKADVLVFNTGHWWTHGKTARGKNYYKEGDTLYPQFDSTE

AYRRALTTWARWVDKNMDPDKTVVFYRGYSTAHFRGGEWDAGGSCSGETEPAFRGAVVESYPEKTRIAAEVIARMRF
PVRLLNVTRLTSFRKDAHPSVYGKAAGAAERRRKKKQDCSHWCLPGVPDVWNELIYASFVMEPSPSSWNRR

>Bradi1g71450.2|PACid:21822715
MGSSSPSPSPPPPPPPMIGRAGNLTVFITPPSPASTPRSSRPSESPRSDLSTPASRTAPAPPVSPSPQKRASPPPSP
VKFSPPALPVKVFPSPVQVPPAQYEKASAGDKHDGSAFAFFWDAVARVQEAHASLDEYVANWFGLDQSKYQWALNDY
YENTGKEVECGKAGKPKELLTTKVQKV

>Bradi1g29990.1|PACid:21822831
MADSPRKRYSSRPSRSPSPYKGRTKSRSRSPAAQSQSRSPPPDPRSQARSRSRSPEREPDAGNHGNTLYVTGLSSKV
TDRELKDYFNKEGKVVSCHVVLEPHTRVSRGFAFITMDTVEDAERCIKYLNQSELQGRHITVEKSRRGRPRTPTPGS
YLGHRYERREQQRGGRFRRGYGRDEYYGNSYRRSPPPMYSYRDTRDYPPYRDSRDYSPHRDARDYYDGRGGRGYSPQ
RSPPPYGGRSRRERSRSLPYSPYRMPERGYGRRTGGGGYDR

>Bradi1g65637.1|PACid:21822935
MAAPKPKSIATARGKMANKSPTGNVVSAGTAPQPRPRRAFGTVRSSNSLSDKPPPPPLKPSKLSPPLPLKAPKVSPP
PPQKSSKVSPPPLQKSLKLSPPNPIRAPRTSRPAAKPLKKACPGPDLKAQAKKKSQRVSFQDDAAVVAVPRSREKVE
ASAEDAVGHTPMVSVKALEKKPVKVVAAETPFFSAQNCSSCTLDQLESAEYWLAQIRLAESVGKHSVSAVFFRLAFE
CQAQPFHRIQSELRNYTVRHQSASTLTPLFHELLLAHGMLVNQPKFDTDGSEKVDTLITTNTVGNKLDATTLEHECS
ECDCGGDRVDVREVNVVKQGEKGMDQPSFERKLEGSFEFDDCEAVIVDQLLQEHSDVAKNEGAEVPHGSEIMLSARC
SIDKLSLKVSPVARVSSERRLSSDKFSPPARSLSAKRLSSGSPFDNKSLFGSCSVKRLTSSCPSYKKSSTKGLSSKR
MSSGHSGVCSDKENNSPAGAVDSSKVTQKGEPMCHASVDPLKLKEHGDYADIDEIH

>Bradi1g42790.1|PACid:21823046
MGSDTEPAKGLLPYLQRADELQKHEPLVAYYCRLYAMEKGLVIPQKERTKTTNSILVSLMNQLEKDKKSLTLGPDDH
LHLEGFASSVFAKADKQDRAGRADINTAKTFYAASIFFEILNQFGELQPDLEQKQKYAIWKAAEIRKALKEGRKPEA
GPPGGDKDEAPANTTEHANDMGRSQSFGNRQNGNEASSQPIDKDFSRRDSFSAVQPGNSASQQSAEKFNDHVSAQPP
YSSPPPPSEFSSPQSQFSSPHPSYSSPSYHGTDYPPSDVHTPQPNYSSPSYTSTDYATNEVHRPPSNYSPPPYTRTD
HTMDDGYNPQSNDKLDVSAYPHTYHQPPYTIEPQHTSQNYYSTETPPAPYSYPNFQSYPSLQDSTLPSVTTHQSAFR
PVSDGVTAQTYSPSTSNNSVPTTQYRSSADAAPPASQYKYDSSYQPAVEKIAEAHKAARFAVGALAFDDVSIAVDHL
KRALDLLTNPSAETH

>Bradi1g52350.1|PACid:21823318
MAVRVLSTRTVRPPPPETPCSPRLRIIPLASWDVAMLSCNYIQKGLLFSPPPPSMSSTAALVQHLAAALSETLADYY
PVAGRFVTDKHHDSSVGCSVSIDCARQGVEVIHAVADGVSMADVIPPDADVPPVVESFFPLNGAVCHDGHELPLFAA
QITELAHDGTVFLGFACNHALADGAAFWDFLNAWAQISRSGSPPPSSRRRPLLDRWSPSAAKPIVLPCGDVADLIDR
SPPPGPPLRSRMLRFSADTLAALKNRARTELLASGDAAGAEALTRFQALSSLMWRCITRARNNHTLPPETETKSTTT
CRAAINNRGRLHPPLPPEYFGNCIDAVSTDSVSSSDLLLLGHGWAAAAVGRSVAAHTDEDIRARVAAWVESPVVYTA
RWYDPRGTFMGSSPRFDMYGCDFGWGKPAAVRSGKANKVDGRVSLYPGREGGMDAEVSLTPEHMAALELDHEFWAAV
LPEPGGKAKA

>Bradi5g23750.1|PACid:21824459
MVAAAAARSKRSHHRPPANKRPSPPPPPPQLTTLLAHLASSVSLALRFASDRDLLLRPSQALALDPLLLAAARAVSR
LLALLPLHLQTLTLTCLSLSPPTPSPPPSSSSSWFLRLSSSPSLPDSAWRDAFGMSRPAFFHLLHSLALSDPTTTTF
SPSSSLPLPADHKLAAALYRLAHGAPLRAVARRFGLATPAMAARAFYEVIRAIADRLATLLDLAAPDRISRAVPGFC
ALSLPNCCGALGYARFGADGAVAAQALVDAECRFLDVSAGWDPSMPAPDILRRSKLYASQSRVLANAPHGELIGGSV
PRYFLGPACVPLLPWLVTPYKDAADGMSKESIFNGVHAHGARLVERAFGHVRARWKLLGESWKGECQEALPYVVVAG
CLLHNFLIKCGEPMPDADGVPAEADVFVDFEGERDREGERIRDVLAAHLSLVSRNQWQ

>Bradi5g07090.1|PACid:21824787
MAASPPPPPAAAPAAAAPGSTPPAQVVGNAFVQQYYNILHQSPDLVYRFYHEASRIGRPASTGAEMDTVTTMEAINE
KIMSMDIARAEIRGVDAQESLCGGVTVLVTGHLTGKDDVCREFAQSFFLAPQEKGYFVLNDILRYVGQGQAETSPPP
PQQQQPAPEVDAVVAPAAVLANGTVSGPTESVPREPETLPEPEQDLSELATHPHEEEDPKEEVYNPPNDAEVPVVEE
TPVPEVIDEVPNNVATSVPVSAPTILHEEAPKKSYASIVKVMKAVLPPNSVVPYRPAPPKKEKQAPPTPAPAPVVDA
PAFSPNPESSNIQDPEVDALAVYIKNLPLHATPSQLEEEFKRFGTIKHDGIQVRSHKIQGFCYGFIEFEDASSVQSA
LAASPVTIDDRPCHVEEKRTPGSRGSSRGRFPTGRGGNFRGEGMRGRGSYTGGRGYGRGDFNYRSEYGGRGGGRGVS
SRGGDVGYQRVDHSGAGGRGARAAAK

>Bradi5g07090.2|PACid:21824788
MAASPPPPPAAAPAAAAPGSTPPAQVVGNAFVQQYYNILHQSPDLVYRFYHEASRIGRPASTGAEMDTVTTMEAINE
KIMSMDIARAEIRGVDAQESLCGGVTVLVTGHLTGKDDVCREFAQSFFLAPQEKGYFVLNDILRYVGQGQAETSPPP
PQQQQPAPEVDAVVAPAAVLANGTVSGPTESVPREPETLPEPEQDLSELATHPHEEEDPKEEVYNPPNDAEVPVVEE
TPVPEVIDEVPNNVATSVPVSAPTILHEEAPKKSYASIVKVMKAVLPPNSVVPYRPAPPKKEKQAPPTPAPAPVVDA
PAFSPNPESSNIQDPEVDALAVYIKNLPLHATPSQLEEEFKRFGTIKHDGIQVRSHKIQGFCYGFIEFEDASSVQSA
LAASPVTIDDRPCHVEEKRTPGSRGSSRGRFPTGRGGNFRGEEVVAEGSHLVEVMLATSVLITLALVVVVPGQLQSE
FRW

>Bradi5g26567.1|PACid:21825335
MGELASVSPPPPAATAAAAAVLQVVFADGDRSVDLGTVTVQPSLGVRKLQAVVADRVGGGVAPHQISACLARPRRTR
RVPLDEAATPDLAAAVAREGAGCYVLASLRRSRRDRRGGGGGGGRSRRERKAAGGGGAESLMMMAAAPERTILKRLP
PTDLASLASSIGPAPAMPSPSSFGAAWDYEAQLRELQRQREWYLMSTAAADPYLQGALLLPDREDLLWQQQQQPQRS
SSCPECDSAAAAMRSPAFHWCVRDAVTVGFRSPVGPIERPSKKSPSPPPHQANAPARLVPSFAAGVMPVCY

>Bradi5g26670.1|PACid:21826284
MHFLSLPTASSPTHHHLPPKPLFKPISSSSASFRRPTQPPPPPPKPSPPPPPPPPPRNPNPLASKLWLSSRLSPPPP
PPPPPKTAHQPPPPPPPPPEEPEQEPEPEDADARKEEKQEEDFRQKGKVFVGNLPLRARKPEVAEFFRQFGPLDKVEL
VRAHDDPERNAGFCFLYYAGDAAEAEAAAGRAAEVDGVDFRGRSLTVRLDDGRKGRARAEERARWVDHGRRPEARSP
WHQGREEACREFRRVVESRPEDWQAVVSAFERIPKPSRREFGLMIVYYAKRGDKHHARATFENMRARGIEPNAFVFT
SLVHAYAVARDMRGVLSCVEEMKAEGIELTIVTYSIIIAGFAKINDAQSADNLFKEAKAKLGDLNGIIYSNIIHAHC
QSGNMDRAEELVREMEEDGIDAPIDAYHSMMHGYTIIQDEKKCLIVFERLKECCFTPSIISYGCLINLYIKIGKVTK
AIAISKEMESYGIKHNNKTYSMLISGFIHLHDFANAFSIFEEMLKSGLQPDRAIYNLLIEAFCKMGNMDRAIRILEK
MQKERMQPSNRAFRPIIEGFAVAGDMKRALNILDLMRRSGCAPTVMTYNALIHGLIKKNQVERAVSVLNKMSIAGIT
PNEHTYTIIMRGYAANGDIGKAFEYFTKIKEGGLKLDVYIYETLLRACCKSGRMQSALAVTREMSTQKIARNTFVYN
ILIDGWARRGDVWEAADLMKQMKEDGVPPNIHTYTSYINACCKAGDMQRAQKVIEEMADVGLKPNLKTYTTLIKGWA
KASLPDRALKSFEEMKLAGLKPDEAAYHCLVTSLLSRATVMEGSTYTGILSICREMFENDLTVDMRTAVHWSRWLHK
IERTGGALTEALQRIFPPDWNSLEVLGEVSDSPSTGDSDYSCDSDLSDVDENQDADE

>Bradi5g22320.1|PACid:21826675
MAATRKPRSQAAAAGDHLRFLRPGALARLRDARLRRRKPASRPSPSPPPSPLPPSAPATAEGVGESGGPFVPYFGSA
SRLLSPRCPQRKKLTAAKGVKLFSPPPPSSDLPIEVLVDFLNASDMVVAAH

>Bradi3g30370.1|PACid:21828133
MGGHGGLNILPQKRWNVYRFDNQEKVRVDEAEAARQDQLEREATRRRESHSRLLALRRNRGLQADSPSPPRASSPPP
HAYPVAPSPPPATPSADPSPIVSDGGHINLFSGGGGAADFAALASANGGRGAAREREPAADTKPNPKKRKKEEETRT
AGPDDEKYRLGYGLAGKGVAAPWYMSKPLASSSKPRKDCEEGREGKRNGGKKSIEELREERRKREAKEKERERALLA
ATARKGRQPERERSSRYVRR

>Bradi3g06720.1|PACid:21828285
MDRPPHLDIDLNEEPSPPPSPPPPPVPLLPPPREVPPPIFALPPTPPPPPPPPLPLWPVPAAIAQEQLRFMHEREAR
ELAVMYHQAELARAAAGRAIAVPPFPHPGPGEAGWGNPPGPCAVCGKPETPWETIICDACDRAFHQSCVRVWGPPVG
TQPLPPPPPATPGVRRPAVTMNEDWMCPQCERNGKRSERWKLGPVRLDINAAPPPWTEDAAPTHWTEGPVLTRHLSQ
IPLYIQLWFATNLIRDLPLL

>Bradi3g16570.1|PACid:21829328
MVSHRKATPGRGLKFKVKLRGSRSAGSGGDDDREPSPPPPFLYPDLTTLSTSPLPPQPPRGSSGDDDDDREPSPPPP
FFYPDPSAVSTSAVPPQPRRVSSGDDDGEPAPPPPVYFDPSAVSIKYTDELLDDEDWKKEKPTGSSDEKFYFALFMS
IQKSLPRDIFRQDSVPLNKDVYFSLWKNWDRLLDGKRTYLRPPKDHCATSGIFRCPDARTRTLGGPEPLLDLVKEYP
NKYRIREMIKILSQHYVLFRRTRRDGNCFYRAFLFSYLENLGQMQDSQAEATRLMERVAMYRENFCRLKWDMAYFLN
PEEYFSSVVSEFNDLVNSVANGGSSDKLYKRSLQEIISLRILSLLRLLTETEIRTREEDYRAFCPAGVSVFQYCWTN
VRPMDVEVLTLPMRALTYALGIPLRVEAVGGGKTDGIIQVKRLDFFPRSESGKGLLHLVESYWSSMTAPEPLEMGSG
NLFSSDGTPLLTLLCRYNQCGILYRK

>Bradi3g16570.2|PACid:21829329
MVSHRKATPGRGLKFKVKLRGSRSAGSGGDDDREPSPPPPFLYPDLTTLSTSPLPPQPPRGSSGDDDDDREPSPPPP

FFYPDPSAVSTSAVPPQPRRVSSGDDDGEPAPPPPVYFDPSAVSIKYTDELLDDEDWKKEKPTGSSDEKFYFALFMS
IQKSLPRDIFRQDSVPLNKDVYFSLWKNWDRLLDGKRTYLRPPKDHCATSGIFRCPDARTRTLGGPEPLLDLVKEYP
NKYRIREMIKILSQHYVLFRRTRRDGNCFYRAFLFSYLENLGQMQDSQAEATRLMERVAMYRENFCRLKWDMAYFLN
PEEYFSSVVSEFNDLVNSVANGGSSDKLYKRSLQEIISLRSEDM

>Bradi3g10010.1|PACid:21830049
MSTCGMCAGSSYCPHHQRASWPAHDHRSPPPVEYHDFFHHSVQGQAAAWLRLDNPPLPQPAEHDQAAAGMIHGGHRH
GTFELNRPLMDDQHLLQMPPPPTIMPFGGGTFGDTMGREAIMAVDGEMMMVAAHHPTMHEREAKVMRYREKKKRRRY
EKQIRYESRKAYAEMRPRVKGRFAKVPGSAAPPSPPPSGSYDPSKLDLGWLR

>Bradi3g44040.1|PACid:21830415
MKTLCSKSSKGGKIHPSPISGDVDPTAAALRLLPAAILVLTAALCPEDQQVLAYLVTRSLQGAATPTPEPQVARGRR
RRAHPPAIGCGCFDCYTSFWSRWDCSPNRELIHVVIDAFEDHLAAAESAPSTPSSSSKRRDKGKRKPPPTPPTPPLR
SLMSPESSESQSEKVQEPPSAPASPPPPPSPPPAVPAATTLESSDEEKAPEDSSEVKLEMDAEAVVENADAGEEEEE
RKRGWADVMGGMLNLRLWGIWSPAVESAT

>Bradi3g22960.1|PACid:21830660
MLLRRSHHSPRRAPLLPVLHRLTMCSSAPASTSPPPASLAAVDYHCRTKHSLTAGYARGPGRLDWANQPNPFLRFSP
SPVIPLPNPPPPVASRVHYPALFHSPPPPPQPLSLDSLSDLLFHSLALSAWKSAGASTWSLRVNPSSGNLHPTEAHV
LFRDPEDPGRLVVSHYAPRDHLLEIRATVPATSDCSALLPPPATAVLALSSIFWREAWKYGERALRYCNHDAGHALA
AVAIAAATLGWDARVLDGLSDQDLGRLVGVDKGCPAPPEGIPDKMVRGKTPWVERHHPDCAVLLFPAGSEPEVDYAR
MSQALRGFHGLEWVGNSNGLSKDHVVWDVIYRTAEAVKKHGPKPGEVSLGEQQGPQSALPFRVLPWDAEVHAALFIH
RISGLPKGLYFLVRNEEHLDKLRRAMRQDFEWARPEGCPDGLPLYRLMEGDCQRLAMQRSCLQDIASHGCFSLGMIA
RFEPVLHEKGEWMYPRLFWETGVLGQVLYLQAHAVGISATGIGCYFDDAVHEVLGLKDLEFQSLYHFTVGAPVVDRR
ILSLPAYPGPGIDA

>Bradi3g36437.1|PACid:21830663
MGSCLLDRLLDMGLGRFNEVGPHQRPRPLEALDGLGDLVAFLRCAGAAQVDQTGWRRDEILIRILRTRKNPKIYYGL
GLLLPPSAGGEVEQEKGPIENPEEWRRRRGGFAAQADTSAAGIHRRLLGRVPGRTGVKRGGKSRGEFPFLRNTAHAR
NPARKGRERSEWGGRVQIPAEAQGTHSHTHGTEKSQESRKATVLGLMRKAGAKEDIEGSIGFERAVDVNPSLVSAET
EGRLSDLMIRLCSCSGRSSALRTLSKTYISMTCKGCGGESIVDRGGPSCSIKLNTMGLELPRPIDPEVNWNTVNRRQ
RAPRRARTTFSGEDRMTDEIKSFYGCVDAPSREMAREDAPVSDSEKLGVSILGRRFSDSMENVPIKKRKFHMDCSPS
PPPTPLLVDPYEKIPSRSCGGTSSYGKHRKVKMLGGKQMEEKKGPFDTADFSGISILAAVACESELDGAILNGECSK
SAHSLEERKPENIIGSSRLGLLHEIKEDKLNIADASHCKVGRPLISSETAPDMKPLFPSTLNSSESAPDMKPTLFPT
TLNSLENDAESAAALKANTTPNSSLKNADKTDDASDKSADVIMSNNSSNPDKFVGCFGDTVMHTKHSDGTHDSRLHW
DLNVPMEVWDTHCGGDDHLSMVGSAPVTSDVRECNDAEKDMNNPQDLCDPIVAGDILHLSGDKIHVVEVPKDVKTKD
QSGFPADGSFHPLCSQSPQHLQLLDSESLNGNGSSVETNDLPDQQKSSYVSKMELHIGSNPDLIKEPLSLTADVEKL
DVNLDVSHATSLDRKGLPHLVSADGHGGGSLIQTGDLGSRVKPPASRIVSEESTNIATVTVSNKSFTDAGWSDDKLG
QVSLQSIAEYKNEELLDVDSGTSKTDLSVNDKAEHSADLLCVANRAPDADNDLNLPDHHPENNLGTSDCNMANVHEE
DGVDPPINCKNHLVTCANSSSAEPNYITSDAPQALDLSSECTKLAVTDVDSTVDSRSAAQSYPDGYKNGLQKVSSSN
RLEQCYQTDTSHVSKNLSVTGKVDVEEDDSQYEDGELRESGDRYWADDGSEEVKCADYQVSDYKDEEATPDIHPVPA
GSVLKNLGIPTADYDGTLSRKEDRDVSPVSSKRSRSSNCLDGGSGIICAASIGEEARDVHLTMKSEAQMYESNPGHV
IAESAATVSQSERCSDGLGDDPLNIRTKPTGWDMLPEDQEHSHHDSRDGVDSSNQCVLGTLDPAEGGESFRRMGLSK
RDVQSQLDRPRSFDRSHRNEHCRSDDGYGSGSKAERTIDAHRSHGRGGASRHGQTSSQVEQWVENSNNSRSTQRGSP
DYYNYGPPGPRNAAEAAVAKMESNGFVVAPDGTLVRAVDAANAGTMARRMKSSSSSYLPLSGRGSPIDRDGGCVLSR
GSAHAREAPSDRRFGASGNRSGRFGPEVGKDDTADGNISSVRCSLSNRQQRRFPPHRASLKLSRAHSQSPSGSRSRS
PHAWTSPRNRRDIMENGGSSIRRHSRSPNYMTEVRIGRVTSPRRQPGFGDRVMRDSSSSRNRTYTQHDSTWVDGRNY
QTADISDNKKRYSRRSPPPRITSRNDRFDVMDSQGRSRSGEFHCPTHERLPYSFERGNKHDGNGDNKREYDNRYETV
KPYDRNGTVKHFRNHTGDKLHPRISAPRSPGPQRRGSPRRF

>Bradi3g05720.1|PACid:21831019
MSSRQQLSPGTGTMPPISGDADFGSCYTSTHHPYAAFDGSPSAAVDHRPPLLHHHHQQLYDTTGLDYAALFPFAPQQ
DNNPPAHLFPNQLPPFTANSTTMLLQPPMLTPLPGLPTSSPPPAPGDAYQLHHPFGGFQLKRENEGGLFPFSDAMAA
SGVSGVGGGSGGRIGLNLGRRTYFSPADVLAVDRLLMRTRGGLGGGGMGVLGLGLGGGIMQQPPRCQAEGCKADLSA
AKHYHRRHKVCEYHAKAAAVAANGKQQRFCQQCSRFHVLAEFDEAKRSCRKRLTEHNRRRRKPVGVQGKDSPPPPPS
KKLEAGITTSSYAGDHTTTNKSSSTAAAAVLSPSASAFSCLQQQEEELDDNNEGSGGRPTTLSLAAPPQRDYGGALD

TMLLMHHHHHQAAAAVVQDDDQEQDFMMTSLVQSHHHHQQQQQQGEVSGNILSCSPTASDQRRQNHQQMQINDGGDD
SGCCNNSGMQQQQHFFEVDFM

>Bradi3g03280.1|PACid:21831344
MPPPRALPLPHFTLPPLAGEDLVFVTALRSHLSSSSPPPAPSSLSPFLPQLTPLRLSHLLLHPPRVPHHLLASLLPS
PPPPLPFALLLHSLPPRRSSELLSSLLPRHAAAFHDLLHHLLLTARLAAGGGGAAAPAIDVLFSACARANKLSQATL
AFRAMRAHGLLPRVESCNTFISAALRLKRPEIAVAFFRQMRRCCISPDTYTANMMLAAFCALGRVADAAKMLDEMPE
WGVYRTVVSFNTLIAAYCRGHGGLENALELKRRMEKEGCAPDAVTYNTIIHGLCKERQMRQANQLLIEMRVMRVAPN
TVTYNMLIHGHVMLGDNKTASRVREEMVKNGLQLDIVTYNTLILGLCREGKVKESEHLLQELDKRGLEPNASTFAAL
ITGHWKTQNSERALQLLNVMKKSGFHPNYDTFKIVVSTFCKNKDFEGAVDVMKDILGRCMAPDKALLHEFFDGLSKA
KKLHLAEDLRSADRAGRLIPAVYYTGDYRIKGEEETAY

>Bradi3g29340.1|PACid:21831639
MATWEVSWVDRLPPVRNQGNRPSCVLESSMYAAQVVRNTNIRNGLLVPPLTTFHHESFIVDYELSTQTFLDEWDNLY
YPEQERRVQNALELFRTQGVYATSEDWFGFERVNISNYQAHDYLSFEQIHEYVCMGHPVLGTFGVRSDYTIFPFELI
YELSPPANERDYTYMVMICGCGELMGRAYVLYLNTFGWWWGYWGLGRVYWDQILQFPGTQHCLWTIEMDGIQVPVPP
VDPRSPPPYPPPSKRRRTRYAHQPRPFPPGYAPQSPPPPGSTANFSGLKSPPALLLCTEAARNILLPPDGTASKAAD
CIMNASASDSAWQYKICDSSENQIALEASNFTSQTRGKLKGSKIILERVMEDMVKAKGEHLKSYDRYIKEFHKRNNQ
IHQEKLDMTQREKLSLLKFDEAEGYLCIVQDAKCDHVMQLRRETEKCLSRLQVDNSLDGSGPYVFLKKIEDEIYEPQ
ICSVLNSVMELTNTCNQIHYSQLHIEEIEGYLHSHYLPFVVRLAEKIERLDKFISKLKATGHRVEDCAPGLYLPSVM
RLGAKLEMLETLVSKLKAASHKSDYRHFQEALRKCISLYNVATTSSRAVLILKQCRLTEDKHAELLPIEWTDNI

>Bradi3g27050.2|PACid:21832781
MEALVVMSEQRNHRSSGQRKSTGPNFSSPPSSRGFRGMNCRSSHSAVCAGLLPSPPPPPARTYSSPEPKTPKQQHVR
HSGKRSRPISISPSMSPPSRPELWAGPAFSNSPPPSSLPIPKFSLWEKRSVSLELPPVERCDDVEVRPHAKSAPSSP
VGGSGFGFFNDKDTAIATENLRRILQLDITDH

>Bradi3g46380.1|PACid:21832837
MDPDIPALKPQWLMQGQVTATGAASLWAAASPSLRKDSQGKGGPSRNRSSGHNRDQSSRQSSSRKSSVSSGPRRLDR
DRDEMGKTRGYANFGRNKDKEREKDFDSRDRESRSVAADRDGFQSFSSCKPERDRMNRARSKTDTWSKGVGNNGSTS
RSNAVGVSFERDFPQLSSEDNGKQEISRIPSPGITTPIQSPPPYTVLAEAPVSSETKKNLVASSVPQAAPSKKPEVV
LNSGAVLSMAETVMQAPQKIYLGPQLSIDAQKIEERTLRQNTLRPLTSTASKSFVTSSSKTKGTRGDPAGPSKAIQQ
ALLPPANGSVRAPVKTELSKLSLSGSFKILTREQNGTAQAPKDSPGNPVSPPPAPVASVGPQKKPYQNQKLKIATHD
LPLTQGAYGDTKLKSKIRSNFFRTLRSKSSSGSSSVIESGCEPSPSSIVDVEHDSCLKPGKAFSCMGNGKCSCEEAN
SSEGSQRHLSDNEENNSSLEPADMADGGSRQLLLENRESDSSELADTGDEGFQVSLSDNIDGSSSSAPADSDDGCKN
SQSGNEEASSSSEATEPEDEEYPAEAIFTAEDLDFMTSLGWSKDEEVQPLGLEEIADYVRHHKGLEQRLLSMESNAH
IKIVLLYLCSQS

>Bradi3g37800.1|PACid:21833071
MSASSPPPPDAAAEPATSAGPEASAEPAVFYCYECDSTVSLPAPAATQPSRLLLCPRCRSDFLEEDPNQSNSSPPPP
PPPPPGFLSDSSSEEPEDLDLGVDQTAARAYLSRLVRRLYDDPTSVATAAAAAVSVLQQQGHRGAGQGLPPATAASI
AAVPTVEVSETAEVCAICKDDLPLAAAARRLPCGHLYHSDCIVQWLEMRNSCPVCRSCLPSTDLEEVEPSELDPPPT
RITIRFTTNRRSRVRGANGAVAPVATSPTQLAQAMTGEGGAGPANSQETVSSEWPPPPESDAAMSEARQGEGFFD

>Bradi3g08580.1|PACid:21833091
MEPHMERALREALTEAERRSLAPVVAAHHTFPGSGQSSPSPSPGKKKTCTSLVTQRVDAPLAAVWAIVRGFATPQRY
KHFIKSCALAAGDGATVGSVREVTVVSGLPASTSTERLEILDDDRHVLSFRVVGGEHRLRNYRSVTSVTEFSSPPPP
EPEEKEKEETQDAAASSSYCVVVESYVVDVPEGNTEEDTRMFTDTVVKLNQKLAAIATTSTSSPSASPSSPPPPQP
SDGAQADH

>Bradi3g29350.1|PACid:21833179
MHQNYLETEESKSLLAVDPFTAEAQASSGEEAMAALGAAATGDARPGLLVPPQASPPPPTLVAAPDLISQAQRQPQE
ESETNAASTTTTLLPLPNQQTSVATKSPPPPIQGSVAREVSSAGEQGLQQLSRVLTSLGYNEMASAAPLLTNSPPLA
RWPGAITVFAAPDVFLQASCPTCSRRHLLLQHIAMGYYPYSELAAAPTMKIPSASVGFCLKIVSERGPFGIHYARIY
ADGVEVSHPELYNDGRYVVHGLHGFLRPLTHSCFDGSHHLTARSAATSTATAASVVRIMIREAIARLRDGGYGFMAL
AMRVKFAELERFANLTVFALDDQAIFVGGGHDYVSAVRFHIVPEHRLTRADLLRLRPGTILPTLAGEGQNLVVTHVA

GSASDDVRINYIPIKESDVVINSRIAVHGVYVPFPRLHLANLAASVAVASAIQMNGTCGVGGPFSDCASSAMTSPKI
PAAHGYGEGQ

>Bradi3g55930.1|PACid:21833305
MAAGALLLPLAATLLLLFLLHDCWLSPVLKRRRLRLAGFLGPAPSFPLGDLPEIAATMAAMPPSSASVSIGSADIHG
GVFPYFARWRGSFGKVFVYWLGTEPFVYVSDPAFLRSAAGGALGKLWGKPDVFRRDRMPMFGRGLVMAEGDLWAATA
TSSPPPSPPPTSTYVNANASL

>Bradi2g22250.1|PACid:21803275
MGSRRFTGLVVLLLAIALLPDPARARFLQGEKPSSSEGSPAPTIATGGSEKSATSKESGHAAQNPDKQPKQAPSQET
TQTTKDSPPPPLGVPEEDGSKPQVSVPPVPSTTVRKESPPPGGPEPSGQSGQEGGIPEKPTDESKQVVKCHDPVYTC
LVPGELYACLQVSQTASAGQFVIVQNRGQNTVTVNVKATPDISIEPKLPPLRKGESKRINISYMNPNGGEISLNVGT
KHCVLRTRQAVSNWQQQFQQLEAYATSMKPIYGAYFFVFTVVLVGAICACCKFARRRSNDGITYQQLEMGSQAPDTS
GANNTTSTVNGWEDGWDDDWDDDEAPAKSPEKVPAGSVSANGLSLRSQTNSKDGWDVDWDD

>Bradi2g01050.1|PACid:21804418
MARRVQQQLVVFFFFFFAVAAMGDDGGEKCTGGCGNPCGIPCTYSSPPPPVPEPVYYPPPPVYSPPPPAPVYSPPP
APIEYPPPTPTANCPPPPYGGGYEPTPTTPSGGYNPSPSGGGGYNPTPSGGWFSPPNMPSYLTPPGTLYPQDPGFRP
NDAPGSRGRLSWRAAALAAMAGVLLLAS

>Bradi1g13030.1|PACid:21816261
MERSHHLLLVLGLLAALLPAAAATFGTTQPEPGAPCEPTLLATQVSLFCAPDMPTAQCCEPVVASVDLGGGVPCLCR
VAAEPQLVMAGLNATHLLTLYTSCGGLRPGGAHLAAACEDEAPPPPPSTEKPSPPPQQDNVTAHGKAIPTHAATSPL
APAASMIHMSPPPACNPCSGSAASSAEGPLLIAALLLVITAIIVGTLDDK

>Bradi1g23200.1|PACid:21815454
MALIFILFFLATAAADSELPPLWNFPPLPPLPPLPSLPPLPALPPLPPGTPPSNGPQPSPSPPPETSSCMLPLLRAA
PCLDFLTNTSVPAPSSRCCDRFRSLVGNASESICICHLVLSDPNVTSIVGNVDLGRLVALQLRCNVLVPPMLIFSCV
SRPIPPLTMIPSSEPAAASMSTESSYEHFDLGTMSASHEKVQQPELVSTIEQPPPSPPPPKPAGTGENKGV

>Bradi3g37245.1|PACid:21827101
MAKKQKLWQRLFSALLSFLLHGKRPPISSRSSSTSSPPPREQHPASLHFHPPPGMAAEKLATDGRTLVLDIDGGLLV
SSSSSSSSSLFPYFMLVALEAGGILRGALLLLLYPIILCLSACVGKGGGEDAAVRLMAMAAFCGLRENRFHAGRAVL
PRWLMEDVADGGIDIEAMTSGGSRVVCVTRRMPTVMVEGFLREYLGAEAVVGREMKVLWGFYTGLMEEDGRGDEALK
KKITVDDAVGFSGSVEFLQHPISRYCKEIYLVTRDQEAPAALPKPRPRQLHHRKLKPVVFHDGRLAFLPTAGATLAM
LTWLPFGMALGAARLAVSLTVPYRYSTPILAATGMSWRLKGSPPPPAGVGGRGRHGQLFVCNHRTLIDPVYVSVALD
RPVRAVSYSLSRLSELISPIGRTVRLTRDRERDGHAMARLLDRGDLVVVCPEGTTCREPYLLRFSPLFAELADNVVP
VGIAVETAMFYATTAGGLKCLDPLYYIVNPRMCYTVQFMDRVRTAPAKEGKVPSTDVANLVQRKMGEELGYGCTMLT
RKDKYLMLAGNDGKCSSTPAPAATKI

>Bradi3g41430.1|PACid:21827313
MAMAARAVLLSRLSPLPAAASRFVLLRPLAAAATLLPAAAASTIPAAAARGAAVRCFATQPATSSLRDSSPNWSNRP
PKETILLDGCDFEHWLVVMEPPPGDASNPEITRDEIIDSYIKTLAQIVGSEEEAKQKIYSVSTRHYFAFGALVSEEL
SYKLKELPKVRWVLPDSYLDVRNKDYGGEPFINGEAVPYDPKYHEEWVRNNARANERSRRTDRPRNFDRSRNFERRR
ENQQNFQNRDAPPGGFNSPPGPPPPPGSFNSPPPPPGQGFNSPPPPPGQGFNSPPPPPGQGFNSPPPPPGQNRGMPP
PPPPLHTAGGPPHYQSHMQNPQAAYTPGGSPHMPNPQAGYTQGSQPGYAPGGAQNYQQSGAPGYQGGPSGYQGNQGG
PPGYQGNQGGPPGYQGSGNQGGPPGYQGGPGPAHPGSNPGYQGGNTPPHESHGGRFYGNAPDRYNQQ

>Bradi3g01100.1|PACid:21829243
MKRLGVLAVALLLLIAWQPIKMAAAQSPAPLLPPPPDDQDDDPPARSLPPPPADDQDDDPPSWPGLPLLPSPPTPVE
SASPPPEPEARTPTSPPPPPPPHPRHAQLPPRQDDPPETEPEPTEPPPRQRQAPREPAPPRTVVPPQEPGWAAVPLP
LPPMPPPMVPFNYSATGYTTMLVFGDSTVDPGNNNRLQTVMRANFLPYGASFLGGRRPTGRFSNGRLITDLLAEKLG
IARSIPGFHEPRLRLRQLRRGVSFASAGSGYDDATARISSALSFSNQVEDLWRYKRNLQRLVGPRRAEQLFRRATFI
ISAGTTDVFFHYLATNHSGAANSWPQYENLLISRVSNYTQVMRALGGRRFVFVGVPPVGCLPLVRTLLGTGTEKCHE
NINLLATSFNRGLAEVVRLLKNERDTRATFIDIYTIVAMATVDPRTFGLTETSRGCCGTGVIEVGQTCRGRLTCTDP
SRYMYWDAVHQTERMNQIITDHAIMNSIGEIYV

>Bradi3g48460.2|PACid:21829362
MARVYVGNLDPRATAREIEDEFRVFGILRSVWVARKPPGFAFIDFDESRDAKDAIRELDGKNGWRVELSTKSGSGRG
RERERPGGSDMKCYECGESGHFARECRLRIGSGGLGSGRRRSRSPRRSRSRSPRYRRSPSYGRRSYSPRDRSPRRRS
YSRSPPPPRARSISRSPPPPRARSISRSPPPPRARSISRSPPPPRARTISKSPPPPRERSYSRSPAQPPQREESPYA
NNA

Zea mays

>GRMZM2G414826_T01|PACid:20819907
MPSTPWLPWRLAVPIAAALASVPFILPLVLPLLLRSNASPRALSVNRLTWLPSPAIRTATTVSPPLAPLLSPSQPSA
PSPPVIHMSPSPSPPPPSPPASLAPPSPPMIQAPRSPPPPPPIQAPLSPPSQSIQAPHPPPPPHSSSPPPPESMATD
EKAGETETRRCDVYDGEWVQDEEARPLYPPGTCPYVDEAYACAANGRPDSRYTRWRWAPRHCSLPRFNATDFLHRLR
GKRLLLVGDSMNRNQFESMLCVLREALPDKARMFETHGYRISKGRGYFVFKFPDYGCTVEFVRSHFLVREGVRFNRR
GNSNPILQIDRIDKTANRWKKADVLVFNTGHWWTHGKTARGQNYYKEGGTLYARFDSTEAYRRALKTWARWVDKNMD
PARSVVFYRGYSTAHFRGGDWDSGGSCNGETDPAFRGAIVDSYPLKMRIVQEAIARMRFPVRLLNVTKLTNFRKDGH
PSVYGRAGDKKLSRRKQDCSHWCLPGVPDAWNELIYASLVMEPNPVAWGNR

>GRMZM2G138585_T01|PACid:20820443
MRALSRVGVGLAVVSALLLLALTAELYYIFVHKRRLRRRAAAISDASSSPSSSSRELLQLFCFKKPPALASTYAVPE
AGDDADADDETVEAQLMRLGGVVGPTRLLFTIKEETREDLESEDGRGRSRSLGELMHCSETPPFLTPRASPSPAMDN
SYNPLFESPAASPGPRPGPTVSPPPMFQFLKDAEEKLYRRALAEEAMRARRSPPPQQATSPAAGEEDGGYITIMVGK
NSRVIIPVPSAPATGGGRHE

>GRMZM2G386095_T01|PACid:20821011
MPPVAQEGAPTWSLSPGSGAASSASASSNPSSSLSLPPPVPTSSSAPSSSSPPPSPPPSPTSPCAACKHQRRRCLPD
CLLKPYFPADEPDMFKNALRMFGVRKLQHMVRSVRPERRDACARAIALESKSRAEDPVRGCSGVIQDLEEQLMDTAV
ELEVLLRRLDSYRHARDSLPQRPPNPHDAGAAADYSPATLQ

>GRMZM5G869788_T01|PACid:20821834
MQLPQHHQKPSAHSCCSSWIRRSPPPSPPPHKRAGGVGRSRYACRLVPLLVLTVYSVVTVLRIPSSSLVVSTADSER
VERREDLEALKTHLPSNQNGLEAREETRSVASLPCSAFISGEAGHGAAADAEGVLCCDRSHYRSDVCYLRGDVRTDP
STTSVLLYNAPRGSAPEKVRPYTRKFEGSVMSSIDEVTIVPVPPGGGGGGAHSNMSVGVGGGGRRCDVRHPRGVAAV
VFSTGGYTGNVYHEFSDGLVPLFVTAQRFAGEVVFVVLEYHYWWLGRYGAVLEQLTNYRVVDFRHDRRVHCFDEMVV
GLRIHGELVVDPKLMPDGKSIKDFQALLHQGYSRTTTTASASSSSPTPLLPLAPPTRPCPRSANKPKMLIFIRKQNR
VLLNLPQVVTACRRAGFAPHVMNLRRQTPLAAIHAALASADAMVAVHGAAVTHFLFMRPGSVLLQIVPVGLDWAADA
FYGKPAQQLGLDYLEYRVAPEESSLAAEYGLNSTVVRTPWVISSRGWWEMKKVYMDRQNVTVNVKRFADLLRAARAR
LRNTTACAAKPAAPLR

>GRMZM2G436404_T01|PACid:20822473
MAPPSPQPPLVRVLDRIRVSPPPADSEQEPALPLTFFDVAWLFTGPVERLFFFRHPEPASTLPLLRSSLSLALRRFY
PLAGTIRPHAPFLCSYTHGTDALTMVVAESNSPDDFDSLVARSRRDLDRIRPLVPQLPPPGEDGAFALAAVQATVFR
GRGLCLGVSVHHAACDDASTMHFVRTWAATCRLGLENDDGSEDAVLPPRPVLDRSVVADPDDLRGKTLAGMARLAPP
PSPPPPQQEEKTPMVMASFLLGREQIDRIKEGAAAKASSFVAASALAWVCLLKCGSVGVAGARRSHMLFSAECRSRL
TPPLPAEYFGNCLRPCFVEAATADLLSGETEDGVAAAASAIGSAIQEMEQGVLEGAEGWLGRVLSVLPERPMSVGGS
PRHGVYETTDFGWGRPARVEMVSVEKTPGTVALADSPEGDGGIELGVVLPPDAMDAFASCFAHAVGANV

>GRMZM2G156099_T02|PACid:20823244
MADVLEPASDAPRVRRPPPPPPDSPEDRSPRLPPPPPGGPPAPSRKRSRSPPPPPPLGSSRPQRYRDHRGGGGRGGS
SPSPPPYRGSRRHSPPRRSPSPPFKRSRRDDGYDRRGGRGSPPRYGYDDRRRGYDHDRGGGRGGYDDDRNHGRYLNR
GPDWPDSGYGAANDGPGVTQREGLMTYKQFMLVLEDDISPSEAERRYQEYRTEYITTQKRAYFDLHKNEDWLKDKYH
PTNLLSVIERRNERCKVIAKDFFLDLQNGTLDLGPGITGGAASKPGGSDGNSDDDTDNDKRRKHGKDSSKGTDSISG
ALKAHPVSSESRRVQADIEQTLALVRKLDSEKGIEENVLSSGDHDKSDVDKSHIGSMGPIIIIRGLTTVKGLEGVEL
LDTILTYLWRIHGVDYYGTSESHEAKGLRHVRVDSKTSSTSDVSAADWEKKLDTFWQERLNGQDPLVILTAKDKIDA
AAAEVLEPLVRKIRDEKYGWKYGCGAKGCTKLFHAPEFVHKHLRLKHPDLVLESTSKVREDLYFQNYMKDPNAPGGT
PVMQLSAPDRGRRRPSMDSRLKFDRGSNKEHDKAERDGGRYGRDDHSPSRDGPDDQMFDGFRGRPSNAPFVAEFPPP
PILMPVPGAGPLGPFVPAPPEIAMHMLREQGPPPPFEPNGAPHGSTGMLGPMLGGSAPIIAMPPNFRHDPRRLRSSY
VAFVAVITTSMPLMRKLLSLTTEACRVQHPLEIWAVSSLYEGEKRKVMDK

>GRMZM2G156099_T01|PACid:20823245
MADVLEPASDAPRVRRPPPPPPDSPEDRSPRLPPPPPGGPPAPSRKRSRSPPPPPPLGSSRPQRYRDHRGGGGRGGS
SPSPPPYRGSRRHSPPRRSPSPPFKRSRRDDGYDRRGGRGSPPRYGYDDRRRGYDHDRGGGRGGYDDDRNHGRYLNR
GPDWPDSGYGAANDGPGVTQREGLMTYKQFMLVLEDDISPSEAERRYQEYRTEYITTQKRAYFDLHKNEDWLKDKYH

PTNLLSVIERRNERCKVIAKDFFLDLQNGTLDLGPGITGGAASKPGGSDGNSDDDTDNDKRRKHGKDSSKGTDSISG
ALKAHPVSSESRRVQADIEQTLALVRKLDSEKGIEENVLSSGDHDKSDVDKSHIGSMGPIIIIRGLTTVKGLEGVEL
LDTILTYLWRIHGVDYYGTSESHEAKGLRHVRVDSKTSSTSDVSAADWEKKLDTFWQERLNGQDPLVILTAKDKIDA
AAAEVLEPLVRKIRDEKYGWKYGCGAKGCTKLFHAPEFVHKHLRLKHPDLVLESTSKVREDLYFQNYMKDPNAPGGT
PVMQLSAPDRGRRRPSMDSRLKFDRGSNKEHDKAERDGGRYGRDDHSPSRDGPDDQMFDGFRGRPSNAPFVAEFPPP
PILMPVPGAGPLGPFVPAPPEIAMHMLREQGPPPPFEPNGAPHGSTGMLGPMLGGSAPIIAMPPNFRHDPRRLRSYN
DLDAPDEEVTVLDYRSL

>GRMZM2G389881_T01|PACid:20823449
MDSSCGRQPSPRSAPSRERRAPADGLSTIAAVARTPSPPPADATSGRRESRGNGPARRSSSPAAKRTQEQRRAGAAS
ATATQRKPPVPTRPCGRASPRRGQQAPPEPAQPREPEDSASPPPVQPEENAGSTTTAVAGDGEGKESLDNPLVAMEC
FIFL

>AC204573.3_FGT015|PACid:20823752
MAGSEESLAGRVEVGGEDGGGRMKKRTRRGQRKRRSENQRDSDDQEAAVAVPSQRRADQSHDRVMSNYGCAADVSDA
LAVRFNLDAQTLDLHRVAPNSYVAFLPNEEVACQGLETAVQLLDDHCLIRDVHPDSAEGVDLSVFKLSAWCDEPELV
PAELDLLAEEPQLSASDIGLCPRTLVFPISVRVFRSDVFLGRSPSPPPPLPYSGDDREQDHDKRRRNPRSTPTAPVR
KPVHARLGPSVCAGSVPGETVETVALPVPLGAETAASVSPPLARLGPCAHAGSAPGGAFVTVELPDPLGAEDAASGS
PNSALTPSVSPPPALIPSVEAPQATTALITTHSTKVPGPASLNCVISFDLKDSGKPAVGDSLELALSYPPHDISSEM
SVIEEIGGYPRIDQSLESHCADVSGRASVPIMQDPVDPVVNAAQVRDPWEIITYRLQAKPPCFKVYLRRRDDIPVHA
QAVSSAASTPLQEFSQHISKAINGLLSPPPIPKRRKKTLLRNFRPRRSRRVAKFPPELGSEAAATICKKLGFYDDHG
NISLGDASKYVVLYNSPLSREHIAGLATLFGWDTSELA

>GRMZM2G101702_T01|PACid:20824068
MEGDSSPPPPRPWPPRRATGSPSPFARSSPGRSPPPPPPGLRQAPAPQRCRTSAAASPSPADGHATCDDRQAGGNDR
LNAFLTARGVPKETSHVAKYNSNATAAYHDRITALAEGKPWTDPPVVKETSGFGAPAPARKPPLHASGGGGGWDDWD
DDFWLDMRRNQSVGSLAARGRSQGGSRRGPNQHRTSTLGSSWRRRRLIRMISSPGGWRRTSPSLRVSRHPRAASTLG
SDPAQRHRLIGM

>GRMZM2G025518_T01|PACid:20824572
MVIMAGASDRRRHHHDHHTATAKPAPPAAASPSPTPAPANRTRLHDFAFPTQSWGAHRLLRCSKDVGGPASPPPHP
QTPSPDKEKHQQASSPGAAAASQPPRPWNLRTRRSPTAAPLASRSDAAAPAGGAQPPQLQSPPPPAAPTKRAFSAAL
SRDEIAEDFATIRGTRPPRRPKKRPRAVQRQLDMLYPGLSLADVNLDSYKIEEQR

>GRMZM2G159169_T01|PACid:20824632
MATSRASSRRSSPSSPTSPCSTSTPTASATYSPARFAASASSTSSTSATTASSARSRTSSWTSLRCASWVSASTTSR
GRGHRPASPLRPPARRHLPQPQPQPPPLPAPGQLRQLTSVCRCPRAQRLRRLPPGKRRNHVRHTQGDPSHQQRPLLL
LPAGDRSAAGAHRARRQLQPARRPAAAGARPDEEAGAAGRRAQPAHRRGTVGDMRPAAPQELHVGLQLLHVRAAVLC
AHRGAGRRPEQLPAQPLVAADAAAVRRLLRPPTCQLRRIPVQIVRPAHVAAAAASFTAPPSPPPPSPPPPSPPPPLP
SPPPPQLLSSPPLPHSPPPPSLPPPAPVYHPPCLVLPPPLPCTPTHPWPSPPPCYPGPLPPTNPVRYASSPPPQQHH
PWPSVHPVQYGSPLPPLLHP

>AC186406.4_FGT006|PACid:20824633
MWQYTGDGDSDRHSAENLTKEELRARILAITAGVTSDFLDESGPLPFNQSRLSDLRKAEKKRARLARAEPRRSITPS
TDTEDLTSTDAEASSNVQPQRVGDAAEAASPAALEDRRGRGPEAAGSSSSPTTTTVRDGLGEMGSPPPVAAATVERR
EEAEPTAVPPPAPSAPLVAMPRVRRAVVARTRSSRAYVQGKTVKSVALAAKGGTSTTQPSLVVAGVTALASSAPKGT
LGASVVATGAPELLAPVPDAPAVDTTVVVPSPLSLAPVAAEATAGEAPAADTTMVVLPPPPPVLLPSGPAAVAAGSS
AEPSLPVVVAGPQGRPPLAVAVSSFPGGVLSPGGEGLEDPPPFVLEGTVGEEMEIEEWHLVEVAAGRADRSLEAIER
ILGNFGEAVSIARSSVTQGLIPHGQRFFSQSVEWREALLAAQREVETLRAQAARAEDRKRELSTLRAEVDRLRNEAS
CLRKGRDEVRRDANRLHEERSRLRGIWTSFLHPAPCMVPCSSSPLSLIIFCGAELQKDFSTAGDAMRAAEEAKEAVE
TEVRRILLACDGAIVARSQMEDSLKERSAELQELRRVAGEVCDRLSPPPSVGVLLSERLLALPGHVERVVLKGAYLG
SSMVLGQMVSHFDEIDAAVIAEGYATGRSEEELDVIEEQVRPHARSLASRIDVKLLLTSSLSPEASAGGPSAPA

>GRMZM2G075628_T01|PACid:20824637
MPPGGRRGAKQRPNWTREPQLGDLVLAKIKGYPRWPAKISRPEEFNHEPTPKKFFVYFYGTDEIAFVPLADLEEFTE
KTKNDLLDRAPNMKVKRKYVQVFNDAVEQICKAYNELPKTSEAANGALPDQSEKPSEHLVRSQDVGEIPGLGQMEGD
SPTDYSHTSAKRPGTEEDVKDDGHEICDHSLAVYQKKTSLLEDPKHPMTKKSAASKSSLDIYMEQEHSPTSVRAERE

AEEVKIEKESCPLDGFILEPNLEVVCALEVPKKSKSNKPLRNAERKENKRAGIGSPIERIVAEAASDVVLNMSADKE
SREFKKSKIMTKQSLTTDSEKMDYNKIVHGGPDEQLTGKSSAGFSSNKKSLPDSGQHKMDSSADTRPGKKPRLMDRA
GETDKTVDKSEMKSNIDYGKHYAVKHERPTSMETCKNTILKTSTSDDRALRSGGVVSPISRLHSEVMEPACGSVTLS
TVADSVKKGSSMKEDASRVDRQLVKPKRRACRFDEDEDEGQRTPLHRTSAKSLSTHIVPTEKAGIRGKFSSHVGNVF
GKKLGPAREEKSRSVRMSPVKHEPIFSSPSQDKVHARHQVVEKRLITGLVETPAVIGNKMSLVDGKSSAQVKMPASS
EVKKLRSSSSKPSHLTSGNSHSRNYPAPEKIPLLPQPEETKAKSKLATSVEHKVGTTLSTEQSGKRDHLKEERSSYV
DKAVSSEPNPGSAKSIKHLIAAAQARRNLMASVHAKFDGSSTDNADITSTPYGLPGLSPSPVFRIPSPPRIAFPGSP
GQRILKSPIEFDNGHGKSPKSRQASGSPSGGTDAAIARDALEGMIETLSRTKDSIGRATRHAIECSKYGIAGEIVEL
LIQKLQSEPNFHRRIDLLFLVDSITQCSHSQRGVARASYVPTVQAALPRLLGAAAPPGAGARENRRQCLKVLRLWLE
RKIMPEDILRKYMGDIEVPNDDTSTTLMLKRPSRAERSVDDPIREMDDMLVDEYGSNATFELSGILSSKVFEDDEDF
PKNNGSSPFVSQARPVENDVIRKTEDTIAPVYVEEHIIIVENVTTDAAMEDALELPGNKQQTDGAVLIEHDSIQDPG
SEQAIDQNDFPSLPDGPPPLPSDSPPPPPPLPPSPPPVTPPPPPPPPLSPASPPPPPPPPLPSGPPPQPAPPPLATQ
VTPLPSIPPPVPSSPPSLSYQPPAPEYFVTANGDQLTQMTGNILIQAIGNATTFIPSGPVNGQAAVNFVTSMQAEYG
NNNIFMAPQASNGNYQFRPTGVPFQQGNFSAFPSAQTHPVQPHSHMAHMNPMGQQAVPPTLNPYAVQSFPNSQSHYP
SEEHWRMASGNFSPDGQHNWLAGGGALPCSEGPYVQDGYSRSNIDRSSINPMNHQHTVLNHLPYGAPLPGHVPQMLP
ARSDVHTLNCWRPS

>GRMZM2G105598_T03|PACid:20824802
MGSESEPAKGLLPYLQRADELQKHEPLVAYYCRLYAMEKGLRIPQKERTKTTNSILISLMNQLEKDKKSLTLGPDDN
LHLEGFALNVFVKADKQDRAGRADINTAKTFYAASIFFEILNQFGELQPDIEQKQKYAIWKAAEIRKALKEGRKPEA
GPPGGDKDEAPVSTTTISQDMGQSPSFSSVHQGSEASPRPVNMDFSRRDSFSAVQPGNNVPHQSTEFNDHPSTQLPY
SPPPPSQSQHPSPSQSYSSPSYQATTDYPSSDFHKPSPNYSTPGYTSTDFPTNEVHKPPSNYSPPPYTRTDRPSNDG
YNPHSNGKPDVSAYPQAYQPSPYTFDPQHNSQNYYSTEAPAVPYNYPNFQSYPSFQDNTSASVLTHQSSFYPESDDP
AATSHTPASNSSVPTHYHPTADCSPQVTPPAAPPASQYRYDSSYQPEVEKIAEAHKAARFAVGALAFDDVSVAVDHL
KRALDLLTNPSAEPH

>GRMZM2G105598_T02|PACid:20824803
MGSESEPAKGLLPYLQRADELQKHEPLVAYYCRLYAMEKGLRIPQKERTKTTNSILISLMNQLEKDKKSLTLGPDDN
LHLEGFALNVFVKADKQDRAGRADINTAKTFYAASIFFEILNQFGELQPDIEQKQKYAIWKAAEIRKALKEGRKPEA
GPPGGDKDEAPDMGQSPSFSSVHQGSEASPRPVNMDFSRRDSFSAVQPGNNVPHQSTEFNDHPSTQLPYSPPPPSQS
QHPSPSQSYSSPSYQATTDYPSSDFHKPSPNYSTPGYTSTDFPTNEVHKPPSNYSPPPYTRTDRPSNDGYNPHSNGK
PDVSAYPQAYQPSPYTFDPQHNSQNYYSTEAPAVPYNYPNFQSYPSFQDNTSASVLTHQSSFYPESDDPAATSHTPA
SNSSVPTHYHPTADCSPQVTPPAAPPASQYRYDSSYQPEVEKIAEAHKAARFAVGALAFDDVSVAVDHLKRALDLLT
NPSAEPH

>GRMZM2G105598_T04|PACid:20824804
MEKGLRIPQKERTKTTNSILISLMNQLEKDKKSLTLGPDDNLHLEGFALNVFVKADKQDRAGRADINTAKTFYAASI
FFEILNQFGELQPDIEQKQKYAIWKAAEIRKALKEGRKPEAGPPGGDKDEAPVSTTTISQDMGQSPSFSSVHQGSEA
SPRPVNMDFSRRDSFSAVQPGNNVPHQSTEFNDHPSTQLPYSPPPPSQSQHPSPSQSYSSPSYQATTDYPSSDFHKP
SPNYSTPGYTSTDFPTNEVHKPPSNYSPPPYTRTDRPSNDGYNPHSNGKPDVSAYPQAYQPSPYTFDPQHNSQNYYS
TEAPAVPYNYPNFQSYPSFQDNTSASVLTHQSSFYPESDDPAATSHTPASNSSVPTHYHPTADCSPQVTPPAAPPAS
QYRYDSSYQPEVEKIAEAHKAARFAVGALAFDDVSVAVDHLKRALDLLTNPSAEPH

>GRMZM2G159498_T01|PACid:20824998
MASPTRTPSPPPAVEPIAPSLSSEHPPVNPSCDGSFSPENPAPAPPPPPATPHPHTPSDSSPSSTPHPSAPQPLPSP
PPHTPDDSSPSSTPHPSAPQPPPSPPPPSADASPPLSRQGETSPPRPSPPPSPAPVSPPTPAPASSEAKSEQEEAES
ASESGSMTLAIVLAKTEDATPPKASSAEASPTRSPQKESAVTIAKLLSAEDLAAMEAKATPDKVTPPSDAGSLAAAA
AVTTVGGVSGGVGSKRWLLGGVPEKVRRAELRRAELGFRVSAAVFCLVALSVMAADTTTGWSGDSFRRYNEYRYVLA
ASVVTFTYCGFQLVAEVHYVVTGRHMIRAPFRSYFNLAMDQMLAYLLLSASSAALSRNGVWMSRFGGDQFTKLINVS
ASMAFLAFIALGLNSVISAYCFFSLVS

>GRMZM2G080503_T01|PACid:20826488
MVSTTLCSSPPPLLCFVLIIIIFFASGDGGAAAAALELDTQAAYLANMKEQFAGPGMSRWWDFMSPAPDYCSFHGIA
CDRSGNVTGIDVTSWRLVGRLPPGVCAALPALRELRMAYNDVRGGFPLGVLNCTSLEVLNLSYSGVSGAVPPNLSRL
RALRVLDLSNNLFTGAFPTSIANVTSLEVVNLNENPGFDVWRPPESLFVPLRRIRVLILSTTSMRGGIPAWFGNMTS
LTDLELSGNFLTGRIPESLARLPRLQFLELYYNELEGGVPAELGNLTQLTDMDLSENRLTGGIPDSLCALRNLRVLQ
IYTNRLTGPIPAVLGNSTQLRILSVYRNQLTGEIPADLGRYSDLNVIEVSENQLTGPLPPYACANGQLQYILVLSNL

LTGPILPAYAECTPLLRFRVSNNHLEGDVPPGIFGLPHASIVDLSYNHFTGPVAATVAGATNLTSLFASNNRMSGQL
PPEIAAASGLVKIDLSDNLIAGPIPESVGLLSKLNQLSLQGNRLNGSIPETLAGLKALNVLNLSDNALSGEIPESLC
KLLPNSLDFSNNNLSGPVPLQLIKEGLLESVAGNPGLCVAFRLNLTDPALPLCPRPSLRRGLARNVWVVGVCALVCA
VAMLALARRWVLRARRCAEQEGALALSPASSASYDVRSFHKLSFEQHEILEALIDKNIVGHGGSGTVYKIELSSGEL
VAVKKLWLSSSKRLLRGPSSKQVDWAAAAAMTNTTNTRDSTTSDGGGGGWLGDRELRTEVETLGSIRHKNIVKLYCC
YSGADCNLLVYEYMPNGNLWEALHGCYLLLDWPTRHRVALGVAQGLAYLHHDLLFPIVHRDIKSSNILLDADFEPKV
ADFGIAKVLQARGGGGVDRDRDASTTTIAGTYGYLAPEYAYSSKATTKCDVYSFGVVLMELATGRKPIEPEFGDTRD
IVHWVSGKVAAGAGAEADALDKRLAWSPYKEEMVQALRVAVRCTCSMPGLRPTMADVVQMLAEAGPPAGRTTKDDSS
GEPKVLVADHHHASPPPPPP

>GRMZM2G141632_T01|PACid:20826699
MGIGSNINSHQHFEAKTIHILSFSRSAKHGLAMKMSTRGQPAEGHLWLVFTMLFVTSAVRSVGASSFAPPPSVSVSS
PAYDPVIKVVGKVYCYRCFDEAHPDESHGKKHLQGAMVKVTCQADDQALVAFGYTRSNGKYSVVLKGLPISSSSSSN
YGAADSCKVELHGAPGGSDCNVPIELNLSGLSVYSRSSEEVVFKANQIMAFASKNAFGCSSKPQTQSSSPVHPYYSS
PPLIPHQYPSPPVPAATHRSPPPLPLPLPLPYQYSAPPPAAPAANQLPSPAYQYISPPPYQQQQSMLPNSYQTPPPP
QGTESPVLQPRKYLPPPYYYYNSPPAPQHHQHSYVPPPPLAYQYPPPPLIHKSPLLPSSSPAAPYHYNSPPTYQYPP
PPYSYQPSPPPAGLYSPPLLPPNAPEHLHPNVPHAKSPQPPLHTPPPPNGAVPSPSPTTPPLRYYTSPPPTDQLS

>GRMZM2G064111_T01|PACid:20827098
MRLQGSSNQSEFMQGVQEQHNILRNSIEQLKLSESLRATLISHLKEALNEQELKVEQVRSLLQAAQPRFKRAAELCV
GLGSNMEMHEPSNQGHKRSSLSDPLSTVAPGAEKSLQKGQSSAVLYSQEGNGFEHIMTETKLAAGDASDNIRGGDLP
SQVNGGNTKLKIEEHTSGNKRQKLEDDTDISQPQSQSPPPPPPFPHPDAFEPPPPPPPEVPPSPEPSPPPPPTSPPP
NMIPPPPPAMPPQMISHVPPPAGTFVPFPAGPPGPMYSSYPPFTPVVNFPITSPPAGFPSPSPPPPFQGLVGTFYGP
QFPTAPPPADRK

>GRMZM2G019874_T01|PACid:20827914
MHRKVRVPPLPHLTLRCASSSSAAASVSPPPPRRLPPPVPLCDLLAHRLGPPRSPLSPPPPRPHADLLPLLRRGGDA
NSPENLHVELIKRGLNHDLFLCNHLVNSYAKGARLAAASQVFDEMPERNAVSWTCLVSGYVLHGIAEEAFRVFRAML
REVQAGCRPTSFTFGTLLRACQDGGPDRLGFAVQVHGLVSKTEYASNTTVCNALISMYGSCTVGPILAQRVFDGTP
IRDLITWNALMSVYAKKGDVASTFTLFKDMQRGDSRIQLRPTEHTFGSLITAASLSSGSSAVLDQVLVWVLKSGCSS
DLYVGSALVSAFARHGLTDEAKDIFLSLKQKNAVTLNGLIVGLVRQDFSEEAVKIFVGTRNTVDVNADTYVVLLSAL
AEYSISEEGLRIGRVVHGHMLRTGLTDLKIAVSNGLVNMYAKCGAIESASKIFQLMEATDRISWNTIISALDQNGNC
EEAVMHYSLMRQSCISPSNFALISSLSSCAGLKLLTAGQQVHCDAVKWGLDLDTSVSNVLVKMYGECGAMSDYWKVF
NSMAEHDEVSWNTMMGVMASSQTPISEIVKVFNNMMRGGLIPNKVTFINLLAALSPLSVLELGKQVHAAVMKHGVME
DNVVDNALISCYAKSGDMGSCEHLFTNMSDRRDAISWNSMISGYIYNGNLQEAMDCVWLMIHSGQIMDCCTFSIILN
ACASVAALERGMELHAFGIRSHLESDVVVESALVDMYSKCGRVDYASKLFNSMTQRNEFSWNSMISGYARHGLGRKA
IEIFEEMLRSRESPDHVTFVSVLSACSHAGLVERGLEYFEMMPDHGILPQIEHYSCVIDLLGRAGKIDKIKEYIQRM
PIEPNALIWRTVLVACRQSKDGSNIDLGREASRVLLEIEPQNPVNYVLASNFHAATGMWEDTAKARTAMRQATEKKE
AGRSWVTLNDGVHTFIAGDRSHPNTKEIYEKLNFLIQNIRNAGYVPLTEYALYDLEEENKEELLSYHSEKLAIAFVL
TRSSSGPIRIMKNLRVCGDCHIAFRYISQMISRQIILRDSIRFHHFKDGKCSCGDYW

>GRMZM2G151444_T01|PACid:20828197
MFPSPGRAVMALGHGGQHMTASSTAGGMAASSSSTPTITFAFQQPSPPPPPTATSSLALAHPHHHGVLGYGSACLLL
DHHHPTTATSSASPSHAAPSSVVPAPTLHHHFHGHAAGAPHVSLSPPTRASPPPHHPWSTAACEEEPEPHHDHRQQ
AGQGGRPPRGKGAAVSEGSAAALGVGAVRMKKAGGGGGGKARRKVREPRFCFKTMSDVDVLDDGYKWRKYGQKVVKN
TQHPRSYYRCTQDNCRVKKRVERLAEDPRMVITTYEGRHVHSPSRDEDDDAARANAEMSFIW

>GRMZM2G047961_T01|PACid:20828230
MASTPRTPAPDRSPPPPLVTHTMPSTPRTPVSDRSPPPVTHTMPSTPRTPAPDRSPPPVPTPPPPLETPPPPSPSSQ
PGDEYHTPPPSLADGSPREEASFPSDGAPKSPQLSPMRLAAPRLLPPPSPPTPTAQNGQGEEGGAKAAAREPLRLAT
GLARSPSSQRSLATATNSLSPSPSPTPPSPLTPAPVVDTSSNNNNNNRSGQSTPKRAETKVPLPLPSPAATAAVAVRF
DPVEEAVTSPLHLGKARLDQQQEQHAAAAENAGSSVPPDVAAVAAVGERRELSVTLRLATAVLSLAAFSVIASARTS
GWAGDYYAHHLQYRYAVAVNVIVCAYSIAQSFGEIRRLISPRFIFRSMSSYYCSLFLDQALAYLLMSASSAAASRND
LWVSRFGTDAFNRKITSALWLSFIAFLMLALNALISTANLFSMV

>GRMZM2G060540_T01|PACid:20828317

MAKPRRGRSASRSSSGSSSRSLSHSVSSGSVSSRSRSRSRSFSSSSSQSRSRSPPQAKRSSPGARKGRSPSPPPKKG
SPSKKVRSPSPPLKKASPPRKVSPPAESAVLCIDHLSRNVNEAHLKEIFGNFGEVVNVELSMDRMVNLPRGYGYIEF
KKRADAEKALLHMDGGQIDGNIVKLRFTLQPRQRAASPVKAPPPPPKRDALQIEKGVSSAEKDAQQRPKESSPRRKP
ASPLRKRSPNRRAESPRRPPDPSPRRRPDSPPIRRRPDPSPVRRGDTPRRRPPSPVRRRSPSPPPRRHRSPMRLSPR
RGRGSPSPRRRSPGPLRRRSPPPRRLRSPPRRPPPPRRFSRSPPRRPLRSRSRSISPRRGRGPPLRRGRSDSSYSRS
PTPPPRKGPRRVSRSRSPRKPPRAGRSVSSDSRSSSSPSPRRGR

>GRMZM2G060540_T02|PACid:20828318
MAKPRRGRSASRSSSGSSSRSLSHSVSSGSVSSRSRSRSRSFSSSSSQSRSRSPPQAKRSSPGARKGRSPSPPPKKG
SPSKKVRSPSPPLKKASPPRKVSPPAESAVLCIDHLSRNVNEAHLKEIFGNFGEVVNVELSMDRMVNLPRGYGYIEF
KKRADAEKALLHMDGGQIDGNIVKLRFTLQPRQRAASPVKAPPPPPKRDALQIEKGVSSAEKDAQQRPKESSPRRKP
ASPLRKRSPNRRAESPRRPPDPSPRRRPDSPPIRRRPDPSPVRRGDTPRRRPPSPVRRRSPSPPPRRHRSPMRLSPR
RGRGSPSPRRRSPGPLRRRSPPPRRLRSPPRRPPPPRRFSRSPPRRPLRSRSRSISPRRGRGPPLRRGRSDSSYSRS
PTPPPRKGPRRVSRSRSPRK

>GRMZM2G390221_T01|PACid:20829313
MASPPADPHPKKRRVADAQDPSSSSNPAPTRRPSPAPPPPETLGAVASSTSSPPPTETASLPPEGVRLQKLRNQEEL
RNVFQRYSRIRKYIQEHKDGGLTPELEQDYLYLISASRGCESVQCFLSLPIPRFASRCPTALEAATKVTINMYNCNM
ATVKRGEDLKSVPYKTAKACIIGLTYICSAASSEELKSSVMKGICSVVYRTVLSFFISTLEGKDIYRMDYTKRAMLQ
DPVTLLDTLKLELDNAKQPTIDNLSELGAICLLCTFLLFPENILEACFKLLDSAECDDVKGEGLYLLNQLTCHLTCN
AANDAMGDKIDEQCPVMEGNLSDTNKFVDSNPVVSENAMEPNECYITMAISRHPFLRRWILSRYKKLCDSCKPAVVS
EVSSCLKVLGSLSEPSDDKSHTGNESSVLEKLDNNVRENMRPDELISSSEQGALAKTESVDNYGNKFSQNKNVDMVR
SDNQKSDELTDAKLDDFKGGTVVSDAQHQGSRSDLLMPKSVYDSAGGSTSLTSPGQHFGRAKHLFSEPFDIYGTYVA
RDVISVSKELWVGSLGNRATEALVRSKFEEFGPLVNFLFYPSRKFSLVEYRNILHAVHACGYMQGSSIWGGFLQVKY
LDRLIGSKGVIRGIAIGESRHVYVAKVKNKKDKDEVFDDLKMAGLKWPSGITDIPGENALLLEFETAVDAATAKFYI
RHQARPNVCSRDMNLPGHQLLVQNIDHSVPDIDLINAFSQFGEVIRWQINRADSNCFIVYRSQDAAARAKSHLHGAR
FGLKSISIESRTYSAGSVHDKTVSPVAPLLGQSVPDNSVRHEIRNPIVPGYHAGYAAPGDRPIYGPPPPNTSRAPQG
NFPCPPVSTHHGSVMPPPPIQTSFVRPMYPGPGSPWENTTPNPPPFSHVSPRMMPGSNFRVNPASLPFVPSCVTPVS
QLPGGSAQHSEKMPPSLPLPTVAPPPFTPLDMPPTHPPPLPISQPPSVPPPPNSPPPQPFADSSDSQKPSSHPQWQG
PLLKSGLHYCRIYASRIEFGCLQI

>GRMZM2G168959_T03|PACid:20830140
MVDLPLFRNACLPKVVSTQSSPPPSVALARDLPSRLASRHCWSYSYATFYPSMAPPPPPPLHPHHHCVLTRFRCPAT
GTARFLIRLGIKPFCCSSVPSRTPPPLHGPSLRRGRRPLDYPDPFARTFDLAALRVPAAACAPLERRLRGHLLNWPR
VHNVIRLRNDQGLLSLSPPPPGYPAEEPGGPAPRATAVLRREKLVRELNARGFLRFPNLARLSRPSPLARKRRERKG
DGGDEETCEPDKD

>GRMZM2G411786_T01|PACid:20830377
MALQLFREMQTLSFDRHTLPFASLLSVAGALPDIKIGRQIHAQLILLGLTLEDFMGNALIDMYSKCGMLDAAKTIFA
NKSDKTAISWTALITGCVQNGQHEEALQLFCNMRRAGLSPDRATCSSIIKSSSSLGVIGIGRQLHAYLTTSGHMSSV
FSGSALLDMYAKCGCLDEAIRTFNEMPEKNSITWNAVISAYAQYGQAKNAIRMFDSMLHCGFCPDPVTFLSVLAACS
HNGLAEECMKYFDLMRYYNSMSPWKEHYSCVIDALGRAGCFDKIQKVLDEMPFEADPVIWSSILHSCRIYGNQDLAT
VAAENLFTMVPTDATAYVILSNIYAKAGNWEGAARVKKIMRDRGVRKESGNSWVEIKQKIYMFSSNDHTNPMIDEIK
EELERLYEEMDKQGYEPDTSCALHMVDNKQKLESLKYHSERLAIAFALINTPPGTPITVMKNLSACLDCHAAIKMIS
KIVNRDIILGASDFGSFPHTLVFPITVRVCRSDAILDRRPSPPPPPPFSGDDREQNHGKRRRDQHSIPTVPGRRPVH
ARLGPCVRADSAPGGTCETIALPVPLGAESTASASPPPVRLGPCGGAGSAPGWTVGTISAPGPLSAEVAVGAEEAAS
GSTIPTPAPSVSPPTALVTSVIAPRAANALTTGSLSEDPGPASIIGAISSGLLDSERPILHDTSFNVAAFEEAGGYQ
RLGQGPESLCADASGRASVPFSHVPHVPDVPADNAAQVRNPWDIIKYSLQAKPPCFKVYSRRRDTVPVQSQEVSSPS
PTPLQEFSQHISKAINGLLSPPPIPKRRKKTLLRNFRPRRSRRVAKFPPELGSEAAASVCKKLGFCDDHGIITLEDA
SKYVVVLFNSPLSREHIAALAALFGWDTSELA

>GRMZM2G111907_T01|PACid:20831410
MRPRGRGDDVEDDEDEYEDITPPRRTPEPEADPVRSPSPSPAPAPAPPHPARAPLSSLVVRPPPQQQENGGGSSPPS
SSARSLSPSTGGHRGRGASPPRRRRDFSPPPPRGWVRRRSPLPPPPERRRPGSPPPQRRRFSPPPRSHLPRHPRFHD
EQEGYGMHAGPSPPRRAEASKFDDDVSPRYTHGYQGGGRGGARFREGPPPYGRGRSYGRGYGAPHGKDLINIDGEYV
HRNDPNLSPREGDWICQNPNCGNLNFARRTHCNNCNKFRYSAREAYEPRRSPPFRSYPSPPRGPPRMVGPPGDRAPA
REMARYRSPPHGWGASDPRAYAGRSPPERAGRFTDPSTKGRMGFRGERDPRDRVKYEWSATDDYVQRERPYDGYLDR

SRRRSGSPRGNWGNDLRDRSRSPARNRPMKSSFTGRGRPDDYAADPYGSRGRPNGMEAGRGRGHAYRAGSGPYPGET
RGDQRAAPRARNEDGY

>GRMZM2G104231_T02|PACid:20832642
MMMMMNRPQPQQAGPLQLLRSLGLGVGGGGGARRGAMKSATSLLLGAALATAFFLLYTSLCRDLGSAAAAAPRSGSP
PPTTPRREQETGDTAAARQEAATGEGGEERVAASSDGDHRGGDATAEENGQSKEEEQRRIVMPATSTTHEATSHHRP
QPKQQKKSPPPASQDLADLLRRAATADKTVLMTAINEAWAAPGSFLDLFLESFRQGEDTAGLPRHLLVVAMDGRALE
RCNAVHPFCYLFRVDGMDFAAEQKYMEGDYLEMMWRRNRFQQSVLELGYSFLFTDVDILWFRSPFPRLPRAAQVVMS
ADFFVGDADSPNNYPNGGLLYVRSSPATVGFYRHWQASRARFPGHHEQYVFDRIVKEGYAGARVQFLDTAVFGGFCQ
HGDDLGRVATMHANCCVGLDNKLFDLKNVLQDWKTYRARAAAGNAQGFSWRVPGKCIH

>GRMZM2G032071_T01|PACid:20832783
MPPRRGRRKGKGRAKPRAKATEPEPELEEAADSEIEAGDAKAEESGEAKEDESASDAESMDADANEEAVENGADEMD
AEPEAKAEPEAVATEAEAKPDVSDAEDEEAGETCSDREADAKDVDSKAKAEESDGEDEEGATAETDGEDEEGATAET
DGESEEEAAETGGENEEDGGDSDTEGDAEESPPPPPSRSRRRKRAATPDDEAEETPTPSRRRRRRKSRQRGDSPPPL
PDHLRCRRSDGKKWRCSGRALPTVSFCEYHYARANKGKKLPADGELLAVALQRQKNKRKGRRRINPSTSPQAATSDR
QTRDLPNGLMTILPGSSGPAALSTPVTTKVGVEISAPMQRCYRSKNAEPLPVGPVKVVPRAMSMTKAAPRMCHCCGV
KKAARVANCKNCDTNFCNSCINKWYSKLSRKDIKARCPACRGLCNCKLCSLGHTKGATHKEPPSGERKILSIKISNH
QFYKLLPVKLDREQLDELELEAKIQGTKTSNVRVQVAENGQSESLYCNNCKLSVSQFLRCCPTCPFKLCLSCCQKIR
QGNMSNSNPEDKFKNRLLQQESVHEDGSITCPSIELGGCGDAMLNLIYASPSSQSEELSSECELDAAGNHSGVKDAE
VHGSPVPESNGRLAAASQAETMST

>GRMZM2G032071_T02|PACid:20832784
MPPRRGRRKGKGRAKPRAKATEPEPELEEAADSEIEAGDAKAEESGEAKEDESASDAESMDADANEEAVENGADEMD
AEPEAKAEPEAVATEAEAKPDVSDAEDEEAGETCSDREADAKDVDSKAKAEESDGEDEEGATAETDGEDEEGATAET
DGESEEEAAETGGENEEDGGDSDTEGDAEESPPPPPSRSRRRKRAATPDDEAEETPTPSRRRRRRKSRQRGDSPPPL
PDHLRCRRSDGKKWRCSGRALPTVSFCEYHYARANKGKKLPADGELLAVALQRQKNKRKGRRRINPSTSPQAATSDR
QTRDLPNGLMTILPGSSGPAALSTPVTTKVGVEISAPMQRCYRSKNAEPLPVGPVKVVPRAMSMTKAAPRMCHCCGV
KKAARVANCKNCDTNFCNSCINKWYSKLSRKDIKARCPACRGLCNCKLCSLGHTKGATHKEPPSGERKILSIKISNH
QFYKLLPVKLDREQLDELELEAKIQGTKTSNVRVQVAENGQSESLYW

>GRMZM2G161315_T01|PACid:20833540
MEAMDVKYKPVMFPNGAGLKKPKPAAAVALAGEPLYRECLKNHAASLGGHAVDGCGEFMPSPGANPADPTSLKCAAC
GCHRNFHRRTLEGSPPPPAPAPLALPPPPSVMHGQPHRAREETPEDRHPGVVDADDSDSDSEGSEYDEERSVSPPPP
PHHLLPAPVAQQPPPPSYFPTAPHMVLSLGSGVPGPAVAVSPAQRLAPTQLTPSSAPPHAGGAMPMPRKRFRTKFTA
EQKQRMQELSERLGWRLQKRDEAIVDEWCRDIGVGKGVFKVWMHNNKHNFLGGHSARRSASSPAPAPAAASLQTPAA
GAGAAAAPSFHPSRITPPPPVLTSSPTAATGFNINGAASSAPTVATDYTTDNANGASSPHSA

>GRMZM2G370473_T01|PACid:20835475
MFHYVNSFCCRFVSTKEGVHSTGGSASVQSALPTAAAINAHADPPRSSPPPPQSMPTRILDPPHRHGRSAITVAGVT
WYFGQKSWHRRPRPSTGHHADPLCSPWKGTIRADLDRALHGRSAWFHGRSDPAPFNRPKSTRIHSVLVVPWKVSGLG
AAARDFSSRALWKGAFVDAFLARIKKNRENMNGKKIWSRRSSILPEFVGSTVLIYNGKNHVRCKITEGKTKAPSNNN
RKGKSRKGQKVKVEMCMKGVHSTGGSASVQSALPTAAEINAHADPPRSSPPPPQSTPTRILDPPHRHGRSAITVAGV
TWYFGQASWHRRPRPGTGHHADPLCSPWKGTIRADLDRALHGRSAWFHGRFSGLGAAARDFSSRALWKGAFVDAFLA
RIKKNRENMNGVHSTGGSASVQSALPTAAAINAHVDPPRSSPPPPQSTPTRILEPPHRHGRSAITVAGVTWYFRQAS
WHRRPRPGTGHHADPLCSPWKGTICADLDRALHGRSTWFHGRFSGLGVAARDFSSRALWKGAFIDAFLARIKKNREN
MNGKKIWSRRSSILSEFVGSTVLIYNWKSHVRCKITEGKVGHKFGEFAFTQRRRPHRTITGKGNQGKGRKFSSLGAA
ARDFSSRALWKGAFVDALLARIKKNRENMNGKKIWSRRSSILPEFVGSTVLIYNGKNHVRCKITEGKTKAPSNNNRK
GKSRKGQKVKVEMCMKGVHSTGGSASVQSALPTAAEINAHADPPRSSPPPPQSTPTRILNPPHRHGRSAITVAGVTW
YFGQASWHRRPRPGTGHHADPLCSPWKGTIRADLDRALHGRSAWFHGRFSGLGAAARDFSSRALWKGAFVDALLARI
KKNRENMNGKKIWSRRSSILPEFVGSTVLIYNWKSHVRCKITEGKVGHKFGEFAFTQRRRPHRTITGKGNQGKGRK

>GRMZM2G058197_T01|PACid:20836223
MPPPHPTDAEAGPEQVAPTEAVPLAAAAPAPVKKKRNLPGTPDPDAEVIALSPGTLLATNRFVCEVCGKGFQRDQNL
QLHRRGHNLPWRLRQRGPGAAPPRRRVYVCPEPGCVHHSPTRALGDLTGIKKHFCRKHGEKRWACPRCGKRYAVQAD
LKAHAKTCGTREYRCDCGTLFTRRDSFVTHRAFCGALGEETGRALAPPAPPSPRPPDLEAEENVDNDKEEEENEDSA
LAEVEVPQRVEVAVPEEPEPEPQRIPSPPPLPQEPPCRPSPPPLPQEQPRRVSPPPLPKEPPCHHPTPPPLPKEPQR

LPFPPEQQAVVALVPNVDEPEVAAVGTVAAKLEDETDQDETTCFQEADQYKDAELEVSNLLDKDTPMLPCFLPSPSE
AIGTDGSSTTCGAGGSVSNSIVPSTTTNTFAGLFASATTSTTSQSRSLRDLIGVDPTFLCLAIGAPSLFPQTSASNS
GTFAPPPAPHMSATALLQKATEAGATQSSSSFLKEFGLASSSSSPRPKQQQPHGRVAETSTDPWQYRNSNQQMEMER
HQSHQRREMESSSQPWQHPRITQQMEMERHHQRSHQQREMESSSQPWQHPRSTQQMEMERHHQRSHQQREMESSSQP
WQHPRSTQQMEMERHHQRNHRQRETESSSQQWHHHHHHPNDQMMGLESYRSNQQMEMDPSSQQRWAQHQRSNQQQQM
EMMERHHLSNQQIRSTQQMERRAMVSGGGLGLGLAYESGNPGLPLPDLMTGPSPLLGPKPATLDFLGLGIGGTMGGS
TASRGLPALMVGRELDMGPSAQVPPAAAAAPWEDAKRKTNGRTIL

>GRMZM2G047178_T01|PACid:20836867
MERWEDSVEKRVTTWGASQQNYHHDRKLLRHLLFPSRRSSSCNDSSPKSALPLSPPSSPTSGDLSRQIWAVLALGHL
RPTMGSESEPAKGLLPYLQRADELQKHEPLVAYYCRLYAMEKGLRIPQKERTKTTNSILISLMNQLEKDKKSLTLGP
DDNLHLEGFALNVFAKADKQDRAGRADINTAKTFYAASIFFEILNQFGELQPDIEQKQKYAIWKAAEIRKALKEGRQ
PEAGPPGGGKDEAPVNTTTISQDMARSHSFSSANQGSEASPRPVNMDFSRRGSFSAVQPGNNLPPQSTEFNDHPSTQ
SPYSSPPPSQSQHPSPSQSYSSPSYQATDYPSSDIHKPSPNYSSSPYTSTDYPTNELHKPPSNYSPPPYTRTDHPSN
DGYNPHSNDKPDVSAYPQAYQPPPYTIDPQHISQNYYSTKAPAAPYNYPNFQSYPSFQDSTSPSVPTEPTYQSSFYP
ATDGPAATSYTPASNSSAPTHYHSTADYSPQVTPPAAPPASQYKYDSSYQPEVEKIAEAHKAARFAVGALAFDDVSV
AVDHLKRALDLLTNPSAEPR

>GRMZM2G087350_T01|PACid:20839032
MAGYLPPGFPFLSPPPPPRSAWSPPPPQTTPRPQFSPKSRDNDAASHGGVIAGVAISVGAFFLVLSFLCSLCQGYRN
RRANAAVDAAATAAAAALAARPRAPPRPEPWDNEEQWLRRHRSDREDDGRTRRASPTAGLPSFTYNRAVRHNVKGSG
EEAATCSVCLGAFQPGETVRLLPVCMHLYHVECIDPWLQAHSTCPLCRSGTDDPTMHGGLLLPV

>GRMZM2G021483_T01|PACid:20839431
MAPAKRKRAAAAAAAAAAAKWKVGDLVLAKMKGFPAWPAMISEPEQWKMPSTKKKPLVYFYGTKQIAFCNYADLEAF
TEEKKRSLLAKRHGKGADFLRAVDEIIEVYDSLKDIGSNKVDLPAEEVKPGVEKFAENNSRMDTEDLVSSSYMHNDR
KIEDHYVTTRSHDMVNSDRPSVDERCVVNSAPDEPIENVSILDEMRDISLRTNSFSNKQRDAQPQNCYTRTRVPSLR
KSRSSLSLESRKAQGSGNFFDHTNLASINLVPGEHKEPSHHRHEDDKANSSSVSTSDNVWLHSGEGTSNQPVTLGAS
NGNRMLNTPAEVDSTSNREASENGTTETELKSNGTSDLPMNTAVNFKRKRKSDRKPGPHYRDCTTPNKDDQLCAEYS
EILPDSPNSKNEVSKSDGDEHLPLVKRARVRMGRSQLEDSPVDEIDVSNKKPELATALDQCDRNGKPASPANDYSAD
QVSAVVSSAPNLSCKLDTTILSKEGHLPWKNKEYHPKILALDVEAALPPSKRLHRALEAMSANVAENTKNIPEVTGP
NEMTLNGSLLTASSLSNKSADAVVTVSNRPVIVQSPEPSLDTQFVHNPSGKCTSESILQNNSIPDSASVPSKENDHI
VLKGDICEETLMDTKTANGSWDCSELNNDSCGKTSALCMKLNRPALNVTQATSVPDRLSSSLEKASENVAASGVKET
KTFGSAVCDVDRSAEPIDRSNNNVTSNTMRHSETIVADSVNNMSAFEIAYAKLGLCDDLRPRRIHATSFCWHGFRLP
LEQRFYKTQEYPSMKKFYEVLIASLCFAIFAHGFRHTFSSLALKELNHRKIKDRSTSPDSMPMKELIAVAQARRFSR
STSFPDNFLNAKYIPETSINIPSKEGPHRQLSPSNRIIRSTSGNDNVNSRSPFDNIQQKKLAAHGANAARRSFKDFL
STLTRTKESISRATRLAMECAKFGIAGEAIDIIVEHLEKESNLYKRVDLFFLVDSITQCSRNQKGGAGDVYPSLIQA
VLPRILYAAAPGNSAWENRKQCLKVLKLWLERKTLSEYIIRHHIREIETINEASFGSSRRPSRTERALNDPLRDNE
GMLVDEYGSNAGFQLPNLICTKVLEEEEENSSEGRSLSFEAVTPEQDAPYNDDNEESQMPVEKHHHILEEVDGELEM
EDVAPPSDIGATTKCQPEQSDTNRAPSDQRPSDAGPPLPVDRPPSPPPLPSSPPPVPVPQSAQMQPKLQMASDPMAP
HPQRATYSVQSQQQHSIAEHPVQDMEGNKIRYHLPHQWHHSILLVPMAVSLLPQHLTMGTTIIGLQPHPCLTRDIIS
NHHPLHRLQINSLLCHQNTNIGHITGVITVHRIPRDIGTMDMIEVITDMIEGIMDMTDNTIMMIEDTTTMIEDITMM
IEAITLMIEDIILTIEDITLMRELLGGQCTMMPLTREDILFLQVLLGFLTILKLHQPQCTMGDHQILRQGLVQAGLG
RLGYLITILPRDILWSLQFHIQLEVMVDGDLDNLFRYRSGALMLSLVCGGLTCGRVCLVSLLQVEGTHGGGALQESG
SWYLPPCTDVSHGFYGGRAA

>GRMZM2G021483_T02|PACid:20839432
MAPAKRKRAAAAAAAAAAAKWKVGDLVLAKMKGFPAWPAMISEPEQWKMPSTKKKPLVYFYGTKQIAFCNYADLEAF
TEEKKRSLLAKRHGKGADFLRAVDEIIEVYDSLKDIGSNKVDLPAEEVKPGVEKFAENNSRMDTEDLVSSSYMHNDR
KIEDHYVTTRSHDMVNSDRPSVDERCVVNSAPDEPIENVSILDEMRDISLRTNSFSNKQRDAQPQNCYTRTRVPSLR
KSRSSLSLESRKAQGSGNFFDHTNLASINLVPGEHKEPSHHRHEDDKANSSSVSTSDNVWLHSGEGTSNQPVTLGAS
NGNRMLNTPAEVDSTSNREASENGTTETELKSNGTSDLPMNTAVNFKRKRKSDRKPGPHYRDCTTPNKDDQLCAEYS
EILPDSPNSKNEVSKSDGDEHLPLVKRARVRMGRSQLEDSPVDEIDVSNKKPELATALDQCDRNGKPASPANDYSAD
QVSAVVSSAPNLSCKLDTTILSKEGHLPWKNKEYHPKILALDVEAALPPSKRLHRALEAMSANVAENTKNIPEVTGP
NEMTLNGSLLTASSLSNKSADAVVTVSNRPVIVQSPEPSLDTQFVHNPSGKCTSESILQNNSIPDSASVPSKENDHI
VLKGDICEETLMDTKTANGSWDCSELNNDSCGKTSALCMKLNRPALNVTQATSVPDRLSSSLEKASENVAASGVKET
KTFGSAVCDVDRSAEPIDRSNNNVTSNTMRHSETIVADSVNNVGDTASNSSLATKSSSIQSDADTRTSELHTFSSLA

LKELNHRKIKDRSTSPDSMPMKELIAVAQARRFSRSTSFPDNFLNAKYIPETSINIPSKEGPHRQLSPSNRIIRSTS
GNDNVNSRSPFDNIQQKKLAAHGANAARRSFKDFLSTLTRTKESISRATRLAMECAKFGIAGEAIDIIVEHLEKESN
LYKRVDLFFLVDSITQCSRNQKGGAGDVYPSLIQAVLPRILYAAAPPGNSAWENRKQCLKVLKLWLERKTLSEYIIR
HHIREIETINEASFGSSRRPSRTERALNDPLRDNEGMLVDEYGSNAGFQLPNLICTKVLEEEEENSSEGRSLSFEAV
TPEQDAPYNDDNEESQMPVEKHHHILEEVDGELEMEDVAPPSDIGATTKCQPEQSDTNRAPSDQRPSDAGPPLPVDR
PPSPPPLPSSPPPVPVPQSAQMQPKLQMASDPMAPHPQRATYSVQSQQQHSIAEHPGNMNSSVAPLPPPPFNSSGYG
GQQNQIPPPPPMAPLNPPGPHGSFPAPPAPYHGNNYHRPPTTSMPNEGYHQQPPPPPPPPNQFPSVPPEHQHRPHHW
GNNCPPYPERYRYNGHDRGNHRHDRRHHGHDRQHHYDDRGYHYDDRGYHYDDRGHYFDDRRHHFDDRGHHFDERAIR
GPMHNDAADQGRYSFPPGPPRIPDHFEAPPAPMHYGRPSDPPPRPCAGWSRPPRISDNYSPSRHSVEPPVSHTAGGH
GGWRPR

>GRMZM2G108981_T01|PACid:20841657
MSLLLKPKPHLSATGLIIFLRHLCEGSPSQPGQTGTDTSPPPLTRDDTKLVDALLAALLDHRRANPAAELPVPSLSN
TLPSLSSAISDLLPSPPPPHLPLHLLLRLLALRRGVPLPEAVAFFHHVSPSLPADSLPDLYASMIVLLAKHRHFGLA
RHLLDEMRERAVPISRQLILALTRRYVRAEMPSEAADLFRRMEEYGAGAPDPATLASLLAALSKKRLASEAQALFDS
CRTVFTPDVVLYTAVVHAWCRAGRLDEAERVFTEMQQSGIMPNVYTYTAVIDAMYRAGQVPRAQELLCQMIDSGCPP
NTTTFNAIMRSHVKAGRSEQVLQVHNQMQQLRCEPDVITYNFLIETHCGKGQGNLDAALKVLDKMTAKKCVPDCHTF
NPMFRLLLVLGNIGAAHKLYEKMRELQCKPNVVTYNCLLRLFNKEKSMDMVLRMKKDMDAEGIEPNMHTYAALIEAF
CGRGNWKRAHTTLKEMIEEKSFKPSKQVRDMVLVLLRKAGQLKKHEELVELMVDRGFISQPPNDALWSALSAC

>GRMZM2G504910_T02|PACid:20841762
MTCATRSPGFLREAELRLLRCTLPSLPSSPPPPASPPPAHPLGPAAASAIAAVEAGDYEAALATAAPHFFPAFAPSR
PPGSAAQFYADLGAAARAFLLVDGDGAAAGEGFDCRCAVVLSAAVAALLAFTQQNVTGQMDVVEGTGNCVDLASIIT
TDMTIKSDLPFHVNLNLLDFLGRCYSNLGDTWDAWASDNLASFGSHVHGKFSLLQFIVFARLLFTSVQSLDLSDCWS
VSWWLFRISMLQQNILDELSSSLFDQVQVYKNKMLDHFGELEKVSTYWDCLLSDGEGSYFVSAAFLEAGIVEYKYGR
VDASREHIRCNHSYWCNRATKTQGLRLHLNSAQEACGLHLSLTGILGFRTIYQVDAKSQMVLVAKTNKSGPDEGQVT
EPTVAQNDNAALKNARSFVPVEDDEFCDILRMPRLVQDGSNSASEDATNPSAKISLSAIQQAAVLAQCLYVSRRSRV
DEMSGWEMAPYIESIDSQDESYFVVRSLCDILRVRWESTRSRTKQRALLMMENLFHYIFSFPITGVFAVPGFSLEL

>GRMZM2G504910_T01|PACid:20841763
MTCATRSPGFLREAELRLLRCTLPSLPSSPPPPASPPPAHPLGPAAASAIAAVEAGDYEAALATAAPHFFPAFAPSR
PPGSAAQFYADLGAAARAFLLVDGDGAAAGEGFDCRCAVVLSAAVAALLAFTQQNVTGQMDVVEGTGNCVDLASIIT
TDMTIKSDLPFHVNLNLLDFLGRCYSNLGDTWDAWASDNLASFGSHVHGKFSLLQFIVFARLLFTSVQSLDLSDCWS
VSWWLFRISMLQQNILDELSSSLFDQVQVYKNKMLDHFGELEKVSTYWDCLLSDGEGSYFVSAAFLEAGIVEYKYGR
VDASRLHLNSAQEACGLHLSLTGILGFRTIYQVDAKSQMVLVAKTNKSGPDEGQVTEPTVAQNDNAALKNARSFVPV
EDDEFCDILRMPRLVQDGSNSASEDATNPSAKISLSAIQQAAVLAQCLYVSRRSRVDEMSGWEMAPYIESIDSQDES
YFVVRSLCDILRVRWESTRSRTKQRALLMMENLFHYIFSFPITGVFAVPGFSLEL

>GRMZM2G088242_T01|PACid:20842025
MERCGSWDCDAAAAQKAVPAPFLTKTYQLVDDPATDHIVSWGDDRVSTFVVWRPPEFARDILPNYFKHNNFSSFVRQ
LNTYGFRKVVPERWEFGNEFFRKGEKQLLCEIHRRKTSTSAAPPSPPPFFAAPHFPLFHPDAPHHHHHHQFVGGGDD
GAVAAAHVGGVGVALAFPHPHCWREPPHSAAPVATRLLALGAAPPAPSSPLPAADAGGGGRAAVLVEENERLRRSNA
ALLQELAHMRKLYNDIIYFVQNHVRPAAPSPAAAAFLQGLGLQARKKPAAGPLNHSGGSTTSSSSLTIAEEEPSPPP
PPQQADKSAGSSGTARSAADRAPPTMLFGVHLSAARGSKRPASPEEEDDQSPTSPAGKPRLALECAGLSLAVAVPSA
GASSPASTS

>GRMZM2G022213_T01|PACid:20842808
MPHPTDPEAGSEQKAEAPAVPLAAAAPAPVKKKRNLPGTPDPDAEVIALSPGTLLATNRFVCEVCGKGFQRDQNLQL
HRRGHNLPWRLRQRGPGAPPPRRRVYVCPEPACVHHSPARALGDLTGIKKHFCRKHGEKRWACPRCAKRYAVQADLK
AHAKTCGTREYRCDCGTLFTRRDSFVTHRAFCGALGEETGRVLAPPAPPSPRPPDLEADENVADLEADENVDKDNDK
EGEEENEDSAVAEVEEPRRVEVVPEEPQRIPSPPPLPKEPPCRPSPPPLPQEQLSPPPSPKEPPCCHPSPPPLPKEP
QRLPFPPEQHAVVVALVPNVDEPEVVAVPSVTAELEDETDQDEDACFQEADQYKDSELEVSNLLDKDTPMLPCFLAS
PSEAIGTDGSGTTTCGAGGSVSNSIAPSTTTNTFAGLFASATTSTTSHSTSLRDLIGVDPTFLCLAIGAPSLFPQTS
ASNSGTFALLPPAPHMSATALLQKATEAGATQSSSSFLKEFGLASSSSSSSSQPSSKQVPQGRVGAADTSAEPPWPY
RSNQQVDMERRQSHQRREMESCSQQWHPSSTQQTEMERRHRNHHQEREMESSSQQWHRHHRNDRQMGSESYRSNQQM
QMESSSHQWLQHRSSEQMVGRRHHLERVESRGMLLSGGGLGLGLAYESGKSGLPDLMTGPSPSPSPLLGPKPATLDF
LGLGIGGTMVGSTGLPALVAGGELDMGSSAQVPAPWDDAQRKANGRTIL

>GRMZM2G305311_T01|PACid:20844056
MDDLTFPATSTAPPEPPAAVAGPLPAAAAACRQLPSPRFAVVARSPPPLSPPPPPAAAPPPVTSSGAPTLTAVAAPA
GAGGDATEEDRMDLLWEDFNEELARGACCCCRAAAAGPWSPSDSESEPAAARGCAPVLRPSSRAGAVRHCRRRAGTW
ALLMRIFRRLFVVEKTVVSRRQRHRAAAKARAR

>GRMZM5G876379_T01|PACid:20844142
MGELAAASSPPPPAVLQVVFVDGEQSVDLGTVAVQPSLSGVKRLQAVVADRVGVAPYQISASLARPRRARRVPLDDA
TDLAAAVAREGSGCYVVAGLRRSRRERRGGRSRRDRKGAKDAGAPAEKTILKRLPPTDLASLVGTPPPPLPFPHAVA
AAPPLALDGWGYDDYEARLRELQLQRDWYMLSTVGVGAGPDPAYLHLAAGGHPEHEDPAAPWSPGRRPSPCPECEAA
AAAMRQPAFHWCVHDAVVSAVFRSHVGPIERPPKKTPSPPPLLPLPPSPGRLLGMPVYEFIDQHLLFRRRRLPHSSS
NCT

>GRMZM2G043501_T01|PACid:20845148
MTRHNISKSLAVVLRARMQPNPISSPPPPTPPPPPTDPDPAAAPLVASVSHWLHVSASAASPPPAALDSFSDGYRSLD
LVGRREVLRSLAVDYDVPRARVRDLMKQYMSVTSAEPSGVDDVEAEEGKEGAASALYRMERGLRDALRPRYAGFLEA
MNAQPGGLKLLAVLRADLLALLGEENVSALRALDSYLKEKLVTWLSPAALTLHQITWDDPASLLEKIVAYEAVHPIR
NLIDLKRRLGIGRRCFGYFHPAIPGEPLIFIEVALLKDMEASIQEVLWDDPPTPESEASCALFYSISSTQPGLSGIN
LGKFLLKRVIDMLRRDMPSVQNFATLSPIPGLMQWLLAKLASQIKLSEAESREGNSLGACSTFKESILLPDEERMIH
DAIEQADGKQGIELLQDILKSRQWVKSDKLSAALKSPLMRLCARYLAREKKRGKALDAVANFHLQNGAMIERINWMA
DQSEKGIQQSGGIMVNYLYRLENIEEYALSYSSTGLIHSSPSLSEYLEVSNI

>GRMZM2G128322_T01|PACid:20846264
MASWQPPAALHLRTHLRDLFSLATATKSVLAGCCAKSAGLGRLLRQIGRSWQVALATHRLAGEMRAGGCTVQQSLTA
EAAAVVKQAVSLARRRGNAQVTPLHVASAMLAAAPAGQLLRAACLRSHSHPLQCKALELCFNVALNRLPASASSPLL
GGNHHCYHPPSLSNALVAAFKRAQAHQRRGGSVESQHQQPVVAAVKIELDQLVVSILDDPSVSRVMRDAGFSSTQVK
ANVEQAVCSSSTMATAAAAPSKNPNPSSSATTTSPPPKEAKAKPPLALHHQARDEDVAAVLDCLASRSKRRVVVIAE
SAAAAEAMAHAAVDKIKRGEVKHEHDALRGAQVVSLRVSSFREMPREEAERRLGELRCLVKQGRRQRVVLVVEDLKW
AAEFWAGHDGQSGRRGGYYYCAVEHVVNELRALASGGSLCWLLGFGTYQAYTKCRVGQPSLESLWGLQTLTVPAGSL
ALSLTCAFDDSALGTVNQSLKAGSDADGNGPASCWPLLGGSQLSLSSRCCGDGDCSAARIDTKAALPRPSVSSSNLP
SWLQHCRDHQEPTTSHLTDLSKSKTWSSICSRPSQRMTLHFSAPVSPASSISSYEHGGDQSSQTPPQHSWLLAGLDA
AHHRWEPKRETSGNKAASRSHDSGASNGGSVEVECRARFKELSAENLKLLCGALEKEVPWQKEIIPEVASAVLQCRS
GIAKRRDKSRSADAKEETWMLFLGGDADGKERVARELASLVFGSRDSFLAIRPGASSSSPPRSGSSEGHRSNKRPRM
SPPPPGEESAPACLERLHDAVSENPHRVIFMEGVEQAGRDCQLGIKEAIESGVVRNRAGVEVGVGDAIVVLSCEGFG
STGRSRACSPPSKKVKVEMEEAKEERTGDIEHNGDGGSSSSPSCIDLNVDMESDQADEGSLDDDVCLLTAVDRTLFF
RRQG

>GRMZM2G315734_T01|PACid:20846887
MDEAGREIMEDVDLEEQPLRQHSPPPPSPPPPPLRKPSRKKPAGSRLLLATPVRALSLVVLVVGLLFSVHLLVHDAR
TLVLLAAEWLCIFFVMSCVAAWERDHDDGAGGGCNGLGTVAEVALWSFAMALTVTMTFWVAAGLPLPAVAVLYLLSL
LAVSACFAVLLN

>GRMZM2G341253_T01|PACid:20847445
MAPPSPQPPLVRVLDRIRVSPPPADSEQEPALPLTFFDVAWLFTGPVERLFFFRHPEPASTLPLLRSSLSLALRRFY
PLAGTIRPHAPFLCSYTHGTDALTMVVAESNSPDDFDSLVARSRRDLDRIRPLVPQLPPPGEDGAFALAAVQATVFR
GRGLCLGVSVHHAACDDASTMHFVRTWAATCRLGLENDDGSEDAVLPPRPVLDRSVVADPDDLRGKTLAGMARLAPP
PSPPPPQQEEKTPMVMASFLLGREQIDRIKEGAAAKASSFVAASALAWVCLLKCGSVGVAGARRSHMLFSAECRSRL
TPPLPAEYFGNCLRPCFVEAATADLLSGETEDGVAAAASAIGSAIQEMEQGVLEGAEGWLGRVLSVLPERPMSVGGS
PRHGVYETTDFGWGRPARVEMVSVEKTPGTVALADSPEGDGGIELGVVLPPDAMDAFASCFAHAVGANV

>GRMZM2G159246_T01|PACid:20848040
MSAIGLVQGSRAGKEAKQEPESEQEADSEPHQRKWVALVSVPVLLGNADDDRAQAMALGTDVQLDLKDPPLPSYLVL
HPRVSPNPRSNEEPLSAYILAADRSGYILLQVVEGNRPDFFLCDTHSRTVTIIPPVCSYLRTGLDIDIRPNLSIGLI
ADPLQGHYVVAQLHPATLHRYRLLCYSTAKREWNVRYLTRQEDHLMINTSIDNAVLAHNGCLWWIALGYGIFFCDPV
TPVLELAELRFLPFPAGCQMNAYASLCPKHLKQRRCVRPSEGKLRFVEIRGFYCNVDWSIRYDDVLFEPTVRMWTLE
DPERPDPWTLEYEVPLADIWNNETYANARLPPGEVPQVALVDPNNHCVVYFFQGSKLFGFDMQNKQVIACKKCLIDR

NKVIMFQSSRPIVDAWELSSPPPFMLSEDDGDLSSEDIVFPRGKKRRVCKFVHSWTSSRPVVGRTWKIASPPPDASS
HTSGDQADDAPSSPKGAE

>GRMZM2G164428_T01|PACid:20848329
MSPGAGTAKKRLGVGAVAGGGFALGCGCRDAKAVAVAVASSSVSPYSAATSATETTTSTATTATWRRARTTTHPSSA
SGSTGTLTVPSASSSSFPWEDMDAEGDGEEVDCKRESSAATTSFSGLLRQLNELEQSVVSWGRKSASKDYLSPPPPP
PLPARSSAKQRAVHSGGGDSKEGHGNFSPPPQALRPFQFQTTQQHRKSKSMDKDKAARQVEVVHSKQPPPPPPPPPP
SLSVPPEQLKVKSTDDKGCKKEAANVSPAPQAPNHRKARSCDAGAGTGPGPARLDGSVAVAVVKQSDDPLSDFRRSM
VNMIVENRIATCDELRDLLRHFLALNAPHHHDAILRAFTEIWDEAFSAKTARGHREPVVATRTTTPPRPRPRAPTPP
RRRHAPPPPAWR

>GRMZM2G081790_T01|PACid:20848668
MAIGHYNNSRATARTAAMRPSVESPMYAAMEGSTGYLMTPSRLLFVFFLALAFLAQCGGARVLRPEKMLLGHYSYSS
TPAMPPYAELFAPPNPPSHPPGSPNCVLTPDTPPPPPAANTVQTPAFVYSSPPPPEVEYYYPPPYISPNPPDISPSP
PPTVAPLPPIVYPSPPEVTPSPPEVAPYPSPSPPEISPSPPEISPSPPEVAPSPPPTVTPLPPIIYPSPPGATPSPP
EVAPYPSPNPPEISPSPPEISPSPPEISPSPPEIAPSPPEISPSPPSYEPTPPSIVPSPPEITPSPPEISPSPPSYE
PTPPSIVPSPPEITPSPPSYEPTPPSIVPSPPEITPNPPEISPSPPSYEPTPPTIVPSPPEYAPEPPAYVPSPPEYA
PEPPTYVPSPPVYAPYPPGIIPSPPEYAPEPPGIAPSPPEYAPEPPGFVPSPPAVVPGPPEFAPEPPGPPAVPSPGG
GFLPPVVFPPPFATPLPGGTTGSEWCVAKPSVPGAIVQQAMDYACGSGADCDSILPSHPCFRPDTMLAHASYAFNSY
WQRTKANGATCDFGGTAMLITKDPSYDGCHYILM

>GRMZM2G106819_T01|PACid:20849560
MSYRLGGAADREEGDEELFETAPSVSGGEGSCDQERRAFVPPLRRMNSDSIYDMASMVSQQLPAKKGLSRYYEGKSQ
SFACMSEVRCLEDLRKKGSPYKHRTNKSYGALGLGLGGMMWPRSPPPAPAPTSASPPPTGSGRPTAPIQNGYHQ

>GRMZM2G106819_T03|PACid:20849561
MNRGPGWLACRKGLSRYYEGKSQSFACMSEVRCLEDLRKKGSPYKHRTNKSYGALGLGLGGMMWPRSPPPAPAPTSA
SPPPTGSGRPTAPIQNGYHQ

>GRMZM2G106819_T02|PACid:20849562
MSEVRCLEDLRKKGSPYKHRTNKSYGALGLGLGGMMWPRSPPPAPAPTSASPPPTGSGRPTAPIQNGYHQ

>GRMZM5G872442_T01|PACid:20850062
MASSPSPSPITIRATSPPSTATPSPATTTPKPDSPAPVTQPNATPADPPSSPAAPPPLPPPASTPPPQLPSPPPSLP
PPPPDAVPPPPVVVATPPPAPAAVVPPPSSPAAVPPPPTPAAPPKASPIRPPAAASPPPSNLPAPNPPADPTPPTVA
QPPPPRRRPPRSPQTEPPPLAPPPSGIPVEPSPTSPSPTSADPSIPTPSSPSSPGTTPSTPGSDGTPSVPAPATPGD
SVSPVTTGGQGSNKSSSPASQSSSSSVDSGGLSSGAKAGIGVVVAIIVLSLVGAAFWYKKKRRRVHGYHAGFVMPSP
ASTPTQVLGN

>GRMZM2G487776_T01|PACid:20850064
MGSWHDGTSSPWWGRGEAAAAPDAGFVASSLRPCSPPPHPPHPTPPPPSGALDATYRRIKSDSPARATHRCLDNGLE
PIYAAAPDSPPSHPPHPSPPSPSGAPDVSCHCLNSDSPPRATHRRLNNGSKHIYAAAPASPPPHPPRSSPPPPPQPV
SPGATCSRLNNDSATIRDSVAPTSTPPVAPRADAGDHVMMLFPGATERPAQHGTLQSLLSRGVTSVEGQAQCKRCGA
RKAIAYDLESKFRELHDYIVMNRHAMYNRAPKAWRLPVLPNCDACGQKGSMWPEIASDKREINWLFLFLGEMLGCCT
LEQLKYFCMNNGQHHRTGAKDRVLYYAYIEMSNQLFSFD

>GRMZM2G342647_T01|PACid:20850637
MNPNPKLPSPPPCPLIDRLMDPMNVPGVPQAANRFTDHGASPSRAGSLENRIIGSEEVPRRFGYEALELRNGGSDYF
GKGVLKSSSPISHRRTPIPLPSRPETLSSGNRRPRPPISSPETLSLPSTPAVLEFSVLIRSDSDAAGTDEDEDATPS
PPPRHRHQQQLVTRELFLAGAGPPAPAPWHWAELGFFRTDRQQQQAPTDSSNRRRVEEETGIKLNIEDMVDLMTLLD
PATGGRMLPSLQDEWYDPAALAASSAWDVAKLRSRFCVPSTPVEDTQCRDLHAGARLCVSCSLDDVNLKFYDAVLDS
KLPSLPESKRLTTDQKTLQAQLSVSYRAPAQTSTFDIFGDWQDISQPNFEANPPDGVLAVPLLRHQSWPGLPRGVEF
NPSDSDLLWHLAAEVGNALAERHPFINEFIKFVDDVREFICTHPQHIPGVRQDGRASYFFHRSFEPYINENDVNNCL
KKIGSHRSIILDGTLMGCKEVFALYADMPSDKRSQETDWRLHQYHLQNTVKAESEIVASKIFLASRNSLCELAEETH
IESEWVLLSYQCPFVYIRLEIPIIFVL

>GRMZM5G865151_T01|PACid:20851308

MGCCFSKNPRTRPVAGASDFPRRCGPEDRDPPSPEEETVKEVLSETPSAKPRANPKLVGNAVVPAAADERKAEKAKK
QDSVDAAASDVGSCVSLSLATDERSEAASESSVATSSVAGPERSPRTKPARRRPASADLGPARRERDRAVAASYGVR
SRSARASASPPPRHVPRDRSVRRSPSPAAKRRASSEPRRAASPTPSVQRKPPVPARPAGRVSPRRRAQEATPRPASP
PPPSQLEDDAVTAASEPSIPDGSDGGDVQSGRGGDDGKESLENPLVSLECFIFL

>GRMZM2G113652_T02|PACid:20851629
MEPDPVAAGASSSPPPSPAPATSPPWQTRDDDEPGAVDCADPGGDLNPAPPPSLQQGTVENAAVGSEVEQEEPKKEA
MAGVGETLRSFMEEFGDQGEDSIILSPRLKDISTPDRPAALGFLGEKYNNLMERYKKYKKQVVKCSEECEPRYNGLK
KKYTDECAERRRLYNELIELRGNIRVFCRCRPLSSDEVNRGCLSVVEIDPSQESELQFVPSEKERKPFKFDHVFGPE
DDQEAVFSETVHVVRSVMDGFNVCIFAYGQTGTGKTFTMEGVPENRGVNYRALEELFRTSEKRSASVAYTFSVSILE
VYNEKIRDLLDESNDQSKRLDIKQNADGTQEVHGLVEAPVYNIDDVWEKLKFGAQNRSVGSTNSNELSSRSHSLVRV
TVRSEHLVTYQRSRSHMWLVDLAGSERIAKTGVEGDRLKESQFINKSLSALGDVISALASKNSHIPYRNSKLTHLLQ
SSLGGDCKTLMFVQISPSSMDSGETLSSLNFASRVRTVEHGPARKQVDPAESLKFKQMTEKLRHEEKENAQLNQSLQ
LMQLKYASRENVFRTLNEKVKDAEQACRNYQQRIRELESELGNEKRVSRDSARSSRPPLVPTRQKQPHGRNAPPSGP
SRWRLKAPTIHNKENIPVMMNKSNPGDDPNKAVGRARRVSLTPVIRQIPIQPKRRSSIAILPSVREHLSVLNEKRAA
SRLSQVNMPRRSVATFGLNLGPGTPLAGHGGPVDATPDGAKFRRFDLGSSSSKFTSPPPNVTPQRKMGMAPAGGPPN
AAKLCFSIQKRVTPAHVRAKPGMGIFDPALRENMVVGRTGNALRVLNGKRRQSVV

>GRMZM2G133926_T01|PACid:20851836
MAKPRRGRSASRSTSGSSSRSPSHSVSSGSVSSRSRSFSSSSSQSRSRSPPPAKRSSPGARKGRSPSPPPKKGSPSK
KVRSPSPPPKKASPPRKASPPAESVVLCIDHLSRNVNEAHLKEIFGNFGEVVNVELSMDRMVNLPRGYGYIEFKKRA
DAEKALLYMDGGQIDGNVVKLRFTLQPRQRAVSPMKAPPPPPKRDVAQIEKGLSSAEKDAQQRPRESSPRRKPASPP
RKRSPNRRAESPRRPPDPSPRRRPDSPPIRRRPDPSPVRRGGTPPRRRPLSPVRRRSPSPPRRHRSPMRPSPRRGRG
SPSPRRRSPGPLRRRSPPPRRLRSPPRRPPPPRRYSRSPPRRPLRSRSISPRRGRGPLLRRGRSGSSYSRSPTPPPR
KGPRRVSRSRSPRRPPRGRSISSDSRSSSSPSPRRGR

>GRMZM2G151205_T01|PACid:20853576
MGRSPCCDKTRVKRGPWSQEEDAVLRSFVQRFGNAGNWIALPQRAGLKRCGKSCRLRWLNYLRPELRHGGFTDEEDR
LILSLYGEIGSKWSVMASRLPGRTDNDVKNYWNTKLKKRYLASSSTSTTQGRSPPPPSPPPAPAPPSGDNSSNAAAA
ADIHHTHQDEKPCLPPTPPALTEFDTTFADDTLLFKSEQLYAELVGLIGKQSSSTGDRDEASTPSSSSSAARPRRLA
AQVAALCGPWTVDVRDTAALLSEPTTALLDAYGAADDAFGPALSACSFQDLLASSYVDL

>GRMZM2G079034_T01|PACid:20853702
MADDQQPPTQRIWFPYWTAPAPAHGPRPAARPQLSRRESRPAPQASPPPTTASTGAGGGVPAPPAQPTPLSSRPSPP
PARAPPLSPIGEPNARAPEPSPVPVAKQHRPPSPLRPNYIARDVPKQKDIVVPQEKTIQEPALVASKTPAKAPEKEK
EDKEKREEEKTKEKDKGHKMKKEKDHEEKQEKDKEKEGGQPHKELKAGVADMVRKLGTPAPSSGGGHERAGSAAAEG
TTIITLAGENKGASMKVDGSAVADGRADAAGSKERRGRTLDGSAVVSGTERAWGGRGFTAFVNSNVQVINNSLLLQS
SCNGGGPGVHLKLSTKAKKKDGEGAGGKSGSAAAPKK

>GRMZM5G886055_T01|PACid:20853932
MAKVAPAAALTTLPNTGSSSGSQSSQPRPPSPNQQQQTGGRGGGGSFQQRGKRGMRGGRTFGGNRGAPPVGSSQVPT
PPGGHSYYNPWMRSIYMWPGSRSPAPPRPPTSGPPAPAQPPQAFLAGPSCQWAGSWGVPPQLTLLLDLLGVLRRPAP
PGFSPIGSTSPSAAGKSTPVGAPPGFSPIGSISTPAAGTRTPVDPAPTGGTSSTGPTTLPGPPSPFPGHFLGKVYSR
WQTNTVAPTSPPPSPCIPASGTSPPPSVPAGAVPVPPVTNNMA

>GRMZM2G171223_T02|PACid:20854258
MAATADPRAKPTAAAAHHLAVPWAHQASPAAHRMPPPVLAAAAGGGGCGARDRHRSSSAHRQVQAVGEDNPYDGGIE
ALRAKLMGHLRDAADRLRVPQPLWSLAASRPPYPRPPPPKAATASSPPPPGTDSEPELRAPPPLRPPTPAPPQAEAA
VATATGRPWNLRERTRRRPASTRSWAASPPPPSSSSRRRRKRAPFSVSLKAEEIEEDIYALTGSRPRRRPRKRPRAV
QRHVDLQRAAFTCQPVPLPSSRSTHFFELVVRCHSPQPAPGKPAQGRVWWCRVVEESETGAESREPRVGDRCVPRPR
CRGQGLNTMDLDPAAEDEEAERQLEDHNEAMDARQALLGVGVTHLDPVDLEDFVASRTGTGTATDSTSPPASTTASA
GGGSRKRSKVWNNFDELTNLVNGKRVSI

>GRMZM2G171223_T01|PACid:20854259
MAATADPRAKPTAAAAHHLAVPWAHQASPAAHRMPPPVLAAAAGGGGCGARDRHRSSSAHRQVQAVGEDNPYDGGIE
ALRAKLMGHLRDAADRLRVPQPLWSLAASRPPYPRPPPPKAATASSPPPPGTDSEPELRAPPPLRPPTPAPPQAEAA

VATATGRPWNLRERTRRRPASTRSWAASPPPPSSSSRRRRKRAPFSVSLKAEEIEEDIYALTGSRPRRRPRKRPRAV
QRHVDSLFPGLWLTEITADAYRVPDE

>GRMZM2G117007_T01|PACid:20854587
MYNFKVKPCSRAYSHDWTECPFVHPGENARRRDPRRYSYSCVPCPEFRKGGACRKGDGCEYAHGVFECWLHPAQYRT
RLCKDEVGCARRICFFAHKREELRAVNPSAVSVGMQMQPTVSPPPPSGLGDMLSPAAWPSSPASRLNKAALGGGRDL
DLDQYQHMLFDTVSSPRANWGSAGGIGSPLPPARAVPDYADLLGSVDAASMLSQLHALSLKQAGGDMPAYGSMVADT
QPHMATTSPMVGLGSSTAFGLDHSMAKAILSSRASAFAKRSQSFVDRGGRAPAARSLMSQQAAAAGAPPVLSDWASP
DGRLDWGVQGDELHKFRKSASFAFRARSPPPPAEPDVSWVSSLVKDGHAGDIFAQWPELEQMVA

>GRMZM2G447406_T01|PACid:20854826
MAAAGHCYYHDDYGDDDHNPWSPFMQSEAQISPLRQQVQSLALIDIWEDRKIFDTQAQSLKDDFFRRLKDIRNQLKN
TGSEQLEKVVSSYKHVLNAPMDEDTLMRKCQAALINFDELNKTYGNNSILGGCSYCANGLHHTFVATEISLSLDGLY
KEMHCSSNRSGFMQELQEQHSILRNSIEQLKVSESLRATLIFHLKEALNEQELKVEHLRSQLQAAQPRFKRASELCV
GLGSNMERRQPSNQGLKRSSLSDPLSTVAPEAVKSLQKGQSSAVLYSQEGNGVEHSVTETKLAAGAASDDIRGGALS
SRVNGGNTMLKIEEHTSGNKRQKLEDDAHISQPRSESPPPPPPPFPHPDTFQPPPPPPEYPRSPDPSPPPPRTSLPP
HIIPPPPPPPPAMPLHMFSHLPPPAGTFVPFPAGPPGPMYCTYPSFTPVVNFPMTSPPGFRNLTPPPSFRGLAGTFY
GPRFPTAPPPADRK

>GRMZM2G363038_T01|PACid:20855639
MPPGGRRGAKQRSNWTREPQLGDLVLAKIKGYPSWPAKISKPEDWKRKPTPKKFFVCFYGTGEIAFVPLADLEEFTE
KKKNDLLDRALKVQRKYVQQFNVAVEQICKAYNELPKTSEAANVALPDQSEKTTEHLVRSPDIGGTPGLGQMEDDPP
TDCSSTSGQGSGTEEDVKDGGHEIGDNSLAVPQKKASLQQDPKHPKTKKSAASKSGFDMYTEQEHSPTSLRAERQIE
EVKIEEESCHLEGFVLEPNLEVVCALEVPKNFKSNKLLRNAESKENEHVGVGSPTGRIAVEAASDVVLNMSAEEESR
EFKKSKIMTKQSLTTGSEKRDNKIVHGKPDKQLTGKSSAGFSLNQKSLTGSGQHKMDSSADTRPAKKARLMDRAGET
DKTVAKSETKLNINYENHNAVKHEQSTAVETCKNTILKTGIGDDRARKSGGAVSPISKLHSEIMEPASGSSVTQSTV
ADSLKKGSSRKEDVSRVDRKLVKPKRRACRFDDDEDEGQQTPLHRTSAKSLSTHIVPTEKPGSRGNFASHVGNASGK
KPGPAREEKSRSVRMSPVKHEPVFSSPSQDKVHARHQLMGRNLITGSVETPVIGNKMSLVDGKSSAQVKMPACSEVK
KLHSTSKPLQVTSGKSHSRNYPASEKIALVPKSEETKAKSKPATSVEHKVATTLSTERTGKRDHLKEERSSSVDKAA
YSEPNPDSAKSIKHLIAAAQARRNLMASALAKFDGSSTDNSAITSTSYGLPRLSPSPVFRIPSPPRIAFPESPGQRI
LKSPMELDNGHGKSPKSRQASGSPSGGSDAAIARDALEGMIETLSRTKDSIGRATRHAIECSKYGIAGEIVELLIQK
LENEHNLHRRIDLLFLVDSITQCSHSHRGVAGASYVPTVQAALPRLFGAAAPPGAGARENRRQCLKVLRLWLERKIM
PEDILRKYMGDIEVPNDDTSTSLMLKRPSRSERSVDDPIREMDDMLVDEYGSNATFELSGILSSKVFEDDEDFPQNN
GSSSFVSRPVEGGVIHETEDNIAPASVEEHIIRVEHVATDAAMEDALELLGNNQQTHGAVIIEHDSSQGPGSEQALI
AQNELPPLPDGPPPLPSDSPPPPPPLPPSPPPATPPPPPPLSPASPLPPPPPPLPSGPPPQPAPPPLPTQDPPLPSI
PLPVSSSPLSLGYQPPAPEYFMTPNGNQLAQMTVNTSIQAIGNTTTFIPGGPVNGQAAVNFVTSMPAEFGNTSVFMA
QASNGNYQFRPTGVPFQQGNFSAFPSAQTPPVHPHSRMAHMNPMGQQAVAPPHNPYAAQSFPNSQSHYPSEEHWRMA
SGNFSPDDQHNWIASGRSLPCSEGPFVHDGYSRSNIDRSSMNPMNHQHTVLNHLPSGAPLPGHISQMLPARSDMHTL
NCWRPS

>GRMZM2G123996_T01|PACid:20855843
MAEKMSTLIIVVDLDCRKCYHKIRKILCQLQDHERIRTISFDDKSKTVTMVGPFDPQRLACKLRCKGGKVVRDVYIV
DANGGGGGGGKPPPPQNVPDDGPPAPAPAPVRSGGKSKNKNKNKQQQQQQPPPTERPPSPPPPPPPETAPGPPPPEA
MMPPSPVHVHHHPPPPPDRGMAAVVPQPQYVEERPPRAELEPQPPMSPPPPKEMRPPMDLPPSPQHQHHPPPPMPMP
MPGNERPPPPPMPMLMPAECVIPTVEIPSWPAAPPVGPCGCPCCAPCYQGYYEGCRCYCCGRLYGTTVRPLPAPCGA
YRGCRTFSDDDPSAACSVM

>GRMZM2G031846_T01|PACid:20856667
MSQAKEVRYTARSITPPADRNGTSRSPPPKRRSPSRSPPPKSASRSPRPRSPKRRSTSRSPPRRRGRSRSRDRSRSR
SPDDRNPGNNLYVTGLSTRVTEDDLEKFFSKEGKVKNCHVVLDPRTKESRGFAFVTMDTLEGARRSIKYLHRTVLEG
RLVTVEKAKRTRERTPTPGKYCGRRGGSQRSPSPYRPRRRERSRSRERRRERSRSRDRRERSRSRDRRERSRSRERR
RERSRSRDRRERSRSRDRRRERSRSRDRRRERSRSRDRRGSSPHGRRHRDRSRSPTSNGNHKAD

>GRMZM2G031846_T04|PACid:20856670
MSQAKEVRYTARSITPPADRNGTSRSPPPKRRSPSRSPPPKSASRSPRPRSPKRRSTSRSPPRRRGRSRSRDRSRSR
SPDDRNPGNNLYVTGLSTRVTEDDLEKFFSKEGKVKNCHVVLDPRTKESRGFAFVTMDTLEGARRSIKYLHRTVLEG
RLVTVEKFNMVLGCLYVNLVGPGCAVSNHGCLQRMCS

>GRMZM2G031846_T05|PACid:20856671
MSQAKEVRYTARSITPPADRNGTSRSPPPKRRSPSRSPPPKSASRSPRPRSPKRRSTSRSPPRRRGRSRSRDRSRSR
NPGNNLYVTGLSTRVTEDDLEKFFSKEGKVKNCHVVLDPRTKESRGFAFVTMDTLEGARRSIKYLHRTVLEGRLVTV
EKVRC

>GRMZM2G161248_T01|PACid:20857224
MAVGRACWVPMIATPNAMGPGLLTYTAYPERHELVSRPTSRTLATPDLTGGRHSGLHKFYRCPGGGRGQHTVGGDNR
FEGAPMASAYPTLLYHPPDSKGRQPSKARMTMNCGFFYQGLSPMNVRYLYAALFLVANVVAWFSRENHSSYFLNQRV
GGCQGKIAHLGAGIFLLVQLVWLKLHLATMGPPPLLAPIHHLSMNCLPAWANWWVYFVPVPVSDYKCFVFGLLGTVY
STFSTGSDYKCMQLWNIVESEDDVPYGYGFFHFVFAVGSMYVGMVFVGWDTHHTMKQWNVDIGWMSTWVHIVNEALV
VVFYIAILLARIFGIGWLRHLLAKVFGTGDQLHSGESQTTTTSDEMYTTSSSLSPFYQTTEETMTTSDGMYTASSSL
SSLYLTPEETPDNNAISISNYNTDEPPSPPPISLVSDEIDISEASPPPSPPPHTVDEMDNSEASPPPLSHTIELKMF
EVPTTSGNNEEDEEDDDSIKEWQSD

>GRMZM2G370815_T02|PACid:20857392
MAESGESSPNSAAATDDTHHEMPEAGAAAAAVHAQPRPPPPPSKVRLMVSYGGRIQPRPNDNQLAYVNGETKILSLE
RPLCFPDFAARLAALVGNAADVRVKYQLPGEDLDALVSVTNDEDLEHLVHEYDRLHVHRHATASSAGSSRGGSAPRL
RVFLFPVQSPPPPPQPAGILEPKVEQRQWFVDALNTVLLPSSKQDPPPVPPQQQKQEQESVFAQQSPPPQAKHEAVF
VQQSPPQPQTVVQMQMPPPQHVVLAAASPDYLFGLDNGFVHPPAVKVKDPAGDPPAVRESVPVELPAKNEDRHPIPN
PAGDNVAVSPAEYQRQIPGLEKLQVADNAAHQPPPPAPAPAPAPSLAPTPVPAALPRNGSDDSLTRGYPLATPTPNS
SAEYYLPKFPEKPPVPPPPQSSAPPPPTYLQVQGRYTSVAPGSGADRGPVFFIPAPHGYFAATASPGATSYPAVYAV
APPSTAANGSAASPAASNATAYSPAPTQVAYDSNGRAIYYTSMLPQYPSAVNGMSSAGAVLGTEPVNPVAVKPTVS

>GRMZM2G370815_T01|PACid:20857393
MAESGESSPNSAAATDDTHHEMPEAGAAAAAVHAQPRPPPPPSKVRLMVSYGGRIQPRPNDNQLAYVNGETKILSLE
RPLCFPDFAARLAALVGNAADVRVKYQLPGEDLDALVSVTNDEDLEHLVHEYDRLHVHRHATASSAGSSRGGSAPRL
RVFLFPVQSPPPPPQPAGILEPKVEQRQWFVDALNTVLLPSSKQDPPPVPPQQQKQEQESVFAQQSPPPQAKHEAVF
VQQSPPQPQTVVQMQMPPPQHVVLAAASPDYLFGLDNGFVHPPAVKVKDPAGDPPAVRESVPVELPAKNEDRHPIPN
PAGDNVAVSPAEYQRQIPGLEKLQVADNAAHQPPPPAPAPAPAPSLAPTPVPAALPRNGSDDSLTRGYPLATPTPNS
SAEYYLPKFPEKPPVPPPPQSSAPPPPTYLQVQGRYTSVAPGSGADRGPVFFIPAPHGYFAATASPGKTTASSVVLR

>GRMZM2G444222_T01|PACid:20857649
MPLAAGDPSPPPSTAHAITTPDGWHPRTAERHLLHLLHHCPLARGRPLELLAFAVRRALPYSPPSPHHHALAALLLL
SSPPPPALPILRLLPPEPTPPLPLLNAAVKALSASSPPDDAFRLLSTLRQFHAPDRLTFLPLIGCASSLPLLYALHS
LLLRLGFLSHHVISLALLKPYPLPQARVLFDDMPQKNKCTVAYNTLITACLRVRDIRAARHLFDEMQWHRRSRRSVV
SWNLMIAGCARCGRDDMAVSCFERMIREGEVAPDDGTLVAVLPACGRTGNVRAGRWAHEYARKSGLLDGTVHVANAL
MDMYCKSGDVNSAREVFMTTRPRSVVSWNTLIAGFSLNGRGVEGIDLFQEMRRQGMEPNAVTFLAVLGCCAHAGAVD
IGREIFRSMLSEHGTEAAIEHYGCMVDLFGRSGLLEEAHVLIQGMPMKPNAAIWGALLSACRAHAGLGLAEVALKEL
INLEPWNSGNYVLLANLYVETERWDEAGEVRRLMRRMSVNKAPGQSFMEEAEFS

>GRMZM2G109159_T01|PACid:20858377
MEAMDTEPSSPAPAAAVNLPPPPYHHCPAPADEVAATPQPKPKQSTAGPGLRTPSPPPSVQVSGYSLHDLLLLSPPP
PSSRRHRSSTRGAAGTSVDESLEIVAVAGTPTPPRRRRRGGAEQCVTPVLASPRNARRARRRLEKDVEAVEDAGRRA
RRRKSTRAAPKAAAAADKAEAALNEEDITMAVVPACPAATCGTDFLEQSDLEGLWERIVELVMWRNMAKSALWFGFG
SMFFSCSLSRDVTFSPISALCHLGVMILGLAFFKDSVPQRPQVENERRFRLTEEDMLRASQAVLPFANSMISTAQVI
FSGEPSMTLKVLPLLLFGAKYGGLVTVWRLLAAGFFISFTVPKLYSCYSSRIHKRVEILRDQALEAWKSCPRKKLVA
AIIVTMFWNMFSVKTRVMAAFVSLVILRYNKKHHKAAEVGSYRDEQGQTMEMDS

>GRMZM2G107896_T01|PACid:20858752
MARVFVGNLDPRVTAREIEDEFRTFGVLKSVWVARKPPGFAFIDFDDRRDAQDAIRDLDGKNGWRVELSTKDGGGRG
RDRNGSDMKCYECGEVGHFARECRLRIGSGGLGSGRRRSRSPRYRSRSRSRSRSPRHRRSPSYGRRSYSPRDRSPRR
RSYSRSPPLPPPPPRNNSMSPRGAREMSRSSPPPPKNNSSSPREAREMSRSPPPPAKNNRSSPREAREMSRSPPPPP
PRNNSRSPPGARDVSRSPPPPPDARRSYSRSPEQQPQHESPYANDA

>GRMZM2G417859_T01|PACid:20860143

MEDTLTISRLSVLILVRLAAKRRNRVKFPFFDPPIAVTLDSPPPPRCSARRAIRPASSHRRPHPRPPPLARASPPPS
NAPSSKRRRSEVLDEDTYVAAIERIIERDYFPDLPRLRDRLDWLQAVRSHDPLILRDAQLKILDRRRRAQRHAAGPV
PTPTPATSTALRSPSFLTTPAGSVAGGVRGPDADHDDDVAAALSLDGFFHRFTSEDNDSFSRILDKVNQRRRERYAH
LLEPPPPVEAGTKPSEDAKRNRITDGYGTSGQPPSTLEGAKFTAKNLLMYYPADQGEAPLTEEERAERLRGMTKEID
KSNTRLHGRATADDARPKEEEGAAILYALVAGSTPGGMAYHDPDKLKKYDLEDLRKTPNPFYLESDKKAGNGYSFVR
TPSPAPGVDESPFMTWGEIDGTPLRLDPDETPGGSERAHFKMPPPPARDVKAHLLSRDAARKIKERSKPFHRPPLPS
PARGVSASPRTLSPAAQKFVRNAISKSSKSSSAIDESLRASYRGSTPSASTPKSRFSRDPGLASRSPSTRQGSTPPW

>GRMZM2G397540_T01|PACid:20861037
MASPPPSPSPAALIAHLAAVLSSPDWRFHPSLPQLPALLAPSLPRTLLVPLPLRLAAAVARAAAPSRHLLDISLPVV
LRLHALSPPPLRPFFDRPFRSLLTHFSRYALTPLMLRLFAHMYRHAPPAPTGATYNALIRALCRRADLRHAQRYLSL
MVRSGWRPDAFTFNSLILGYCRTQQLEVAHDLFCKMPFRGFSQDAVSYAALIEGFCEAGRIDEALELFREMTQPDMY
THAALVKGLCDAGRGEEGLCMLQKMKELGWRPTTRAYAALVDLWCREQKAEEAEKILNEMFDSGLMPCVVTCTIVVN
AYCREGRMSGAVRVFESMRFKGCEPNVWTYNAIVQGFCNAGKVYKAMALLDQMRECGVEPDVVTYNLLIRGQCIDGH
IGSAFRLLRLMEGNGLAADQYTYNVLIDALCKTGKVDEACSLFDGLEYRGIRPNSVTFNTVINGLCKAGKFDVACTF
LENMISAGYAPDTYTYSPFIENLCKTKGSQEGLFFIDEMLQKDVKPSTVNYTIVINRLFNERNYGLATRIWGQMVSQ
GCSPDVVTYTTSVRAYCNEGRLDEAENVVTEMKKCRTIVDAMAYNTLIDGHTSIGQTDRAVTILKHMTGVASMPNHF
TFFILLRHLLQRRLAEHVPLKATSVWKTIELADVFELFELMKKNSVPSSARTYLSILEGFSEERRLDEVTSLVSLMK
EENLPLNEDIYNALVNCFCKLRMYSDAWALLCSMIGHGFLPNLIFYQYLLSGLTAEGQADRAKEIFRSSRWKEYNTD
EIVWKVIIDGFIRKGHADMCHDMISMLEQMKCKPSDETYAMLTEELPTRE

>GRMZM2G065298_T02|PACid:20864084
TAHHPSRIAPSLGPRSYGASVALHAFLFLVWHREPPHPTHPPTGHSPPPSPGLSPAAAGAVLRPSPCAPGKTMRRYS
PPYRSPPRRGYGGRGRSPPPPRRGYGGRKEGSGSLLVRNIPLSVRAEDLRVPFERFGPVRDVYIPKDYYSGIPLRTG
ASRGSLLDMTKPSQP

>GRMZM2G065298_T01|PACid:20864085
TAHHPSRIAPSLGPRSYGASVALHAFLFLVWHREPPHPTHPPTGHSPPPSPGLSPAAAGAVLRPSPCAPGKTMRRYS
PPYRSPPRRGYGGRGRSPPPPRRGYGGRKEGSGSLLVRNIPLSVRAEDLRVPFERFGPVRDVYIPKDYYSG

>GRMZM2G449274_T01|PACid:20865256
MDRRQRAAARVFEDFDPEVEWKLAGEEQDVVEIALPGFRKDQVRVQVDNHGVLRATGERPARGGRWARFKKDLRLPD
NCDSDGVRARFEGEKLIITLPIVAALSHSPTPSPSPPPPPPQQQPRRRPSPPEPSVPAPEPPPLTRQNPSPTQAPPL
PPQPRRHPSRPEPSIPAPEPPPPQPPPPAAEELTEAGTEERKQSPPPHQTPGAAPGPKTPPPWSWQPPVPVSPPSPA
PAPPHGAEEQPKKRQKQPQPQETNATKRAEELGGGARGTKVVDKAARDEKRPKKDSQAGASPAVPSHAQTTETVRPE
PARQLLVNAAAAVAVLAGVIAAVWRTLQ

>GRMZM2G064503_T01|PACid:20865405
MDTEPFDEAELLALPPSPVASPPRRLKRLKKSSSQATATTATTTSPHVGSPPPPLSPPPPPLLAEATPGEETLAFPS
RDTSPHPPTPPDADAPTPLPHPSPAPVSPPLQPTVTPDNDSDEEDDGLDPLFSGAAGVAQWDPLGAPAEGDGEGDDD
DDGLGGGGLIEELRREQRSAKKRLDMDEADGEVASGAGARAEAKGKRSKRKRKEEAPTMESAREKKRSEKERRAQLD
SIHAESQRLLRETRKASFKPTVQPVHKPISSVLEKIRLRKMEVLKKYRFFSLRSNTSIEDSDDNDAVPEPVVDLAEQ
LDVPQSKEVALDKDLRIDGAEKELGANGHSLDRCDSVQEDEDGLSCKEKYLYNCGTKASDEEFSDRLQENHENTQPS
NNHNDSVDQSQLPPSSSPTESTDEISSEDEENNDKENMDPSAQNNDVHTLEHLQQAIGGNSRPDDAILKDFLDDEAE
EEDDSDNDMTRFKDNEEDDGSDENEVFNDLIAAGYEEKEVDHEKRNELHQKWLQQQDAAETNNVDNNTLISPLEDDN
SREVFSLIKKLNIAPKPKMRGKQSTSNHEMLMAGRNTIASKSSFLGRTASGSLASSHRSVYRSYVFGRDDSNSSSRS
CISTSESNLDLDQTNSSQPKKAKFSSQPKPEGTRATPECNTSSGVSLFDVLRRTSEQSSQESCSTITESQAVHQFSA
FKLSRRFSKVGSKN

>GRMZM2G100020_T02|PACid:20866701
MLSCIACVNKEEDGGRDREEHGGDTPSCRDPVKSLTSQLKDMVLKLSGTHHRQHGAQHRRGGSPPPPRGRATSLYRS
GYYRPGVVQDDMAVPPATYLGGGGAGASSASSTPAWDLPAAARADGEACREWVAQVEPGVQITFVSLPGGAGNDLKR
IRFSREMYDKWQAQKWWGDNNERIMELYNVRRFSRQVLPTPPRSDDAERESFYSQSQVGSPSATPSPAPLTPERISW
GAFARQVAAPPASSGATGAARQHSFRPMSPPPPSSSNPSERAWQQQQRHIGGGGGGGAAGGKSPAASEAAAAATEA
ARTTTSSRDDVSVSNASELEVSEWIIQDQPGVYITVRELADGSRELRRVRFSRERFAELNAKLWWEENKERIQAYL

>GRMZM2G029514_T01|PACid:20867398

MPVPPAQQRTLPLPHFTLPPLTGQDHLFVAALRSHLSASPPPAVASLSRFLPGITPLRFSHLVVLLAGRVRPPHNLL
TALLPSPPPSLPLAILLHSLPPRRCAELLTSVLPSVSPRAFPDLLHHVILTARLAAGTQRAAVVPAIDVLFSSCARD
KKLSWATLTYRAMRAHGMFPTVESCNVFISGALRLRRPEIAISFFREMRRCRVSPNVYTVNMVMRAKCALGRVVDAA
QLLDEMPCWGFSRTAASFNTLIAAYCHDSGGMETVLRLKERMEREGLVPNEVTYNTIVHGLCKEGRMHQARRIVNEM
RVKGVTPNTITFNTLIHSYVTLGDNEVAMKMHEEMMKAGLSADLVTYNALILGLCNEGKTWKASQLVQELCAAKLEP
NASTFSALIIGQCKKQNPERALDLLNAMKKAGFQPNYYTYSILISTFSKNKDFEGAIDVLKDMLMRCIAPDKALLHE
FFEGLSKAKKLHLAADLRSVANGTRFISDIYYTDDYRNIDEE

>GRMZM2G371271_T01|PACid:20867827
MEGDSSPPPPRSWPPRRTAGPPPPSARSSPGRSPPPAGLRRAPAPPRCRTSAAALPNLRRCKSFSCGRSCDVRGRST
LWALFHQDDRQAGGNDRLNAFLTARGVPKETSHVAKYNSNATAAYRDRIATLAEGKPWTDPPVVKETPRSGAPAPAR
KPPLHASGGGGGWDDWDDDFWPDMRRNQSVGSSPAPSANRNGGAAQGDVLQVVSQGFRRLSLVAASAAQSTASVVQV
GTKEIQSKRRRLKGLSPVRTEQHHGAGAGDRAMAAPAPSPIPGGGAASSPGDGGARCLTLQRGLLYHDLALSRVHDN
QGEVKIGDIVLILTAILHKSHTVHCVDEPGKQVRSVVRIKNINKSHVAFKTQEPLRQQIHFNGKEVEHREAQCRWG
PTSERKAKDVIKSVKKCIGSRSKTTQLFTVMLLEMLLNNCGEPVHRQVIDNGLLPILVKIVKKKTDLPVREKIFLLL
DATQTSLGGAKARFPQYYEAYYELVIIVHVLNFFVLHSAENFRALCTGEKGVGSNGKPLHYKGTPFHRIIPGFMIQG
GDIVRGDGKGSESIYGGIFPDENFTVKHTHPGVVAMANSGLDSNGSQFYITTIKTSWLDGEHVVFGRVIQGMDTVYA
IEGGAGTYNGKPRKKAVITDAGEIPKEKWGDQEA

>GRMZM2G371271_T03|PACid:20867828
MEGDSSPPPPRSWPPRRTAGPPPPSARSSPGRSPPPAGLRRAPAPPRCRTSAAALPNLRRCKSFSCGRSCDVRGRST
LWALFHQDDRQAGGNDRLNAFLTARGVPKETSHVAKYNSNATAAYRDRIATLAEGKPWTDPPVVKETPRSGAPAPAR
KPPLHASGGGGGWDDWDDDFWPDMRRNQSVGSSPAPSANRNGGAAQGDVLQVVSQGFRRLSLVAASAAQSTASVVQV
GTKEIQSKRRRLKGLSPVRTEQHHGAGAGDRAMAAPAPSPIPGGGAASSPGDGGARCLTLQRGLLYHDLALSRVHDN
QGEVKIGDIVLILTAILHKSHTVHCVDEPGKQVRSVVRIKNINKSHVAFKTQEPLRQQIHFNGKEVEHREAQCRWG
PTSERKAKDVIKSVKKCIGSRSKTTQLFTVMLLEMLLNNCGEPVHRQVIDNGLLPILVKIVKKKTDLPVREKIFLLL
DATQTSLGGAKARFPQYYEAYYELVVKKVSGPMANLFTIREHHSIVLSLAS

>GRMZM2G371271_T02|PACid:20867829
MEGDSSPPPPRSWPPRRTAGPPPPSARSSPGRSPPPAGLRRAPAPPRCRTSAAALPNLRRCKSFSCGRSCDVRGRST
LWALFHQDDRQAGGNDRLNAFLTARGVPKETSHVAKYNSNATAAYRDRIATLAEGKPWTDPPVVKETPRSGAPAPAR
KPPLHASGGGGGWDDWDDDFWPDMRRNQSVGSSPAPSANRNGGAAQGDVLQVVSQGFRRLSLVAASAAQSTASVVQV
GTKEIQSKRRRLKGLSPVRTEQHHGAGAGDRAMAAPAPSPIPGGGAASSPGDGGARCLTLQRGLLYHDLALSRVHDN
QGEVKIGDIVLILTAILHKSHTVHCVVGQVYRSLWHQRCMRSTTSWWTSTCLECVCSRW

>GRMZM2G086123_T02|PACid:20868190
MAPPAGTGAGTEGRPARRRGTGTSPGRTKVWVEPPGKSHGHHHHQAPPPPSPPKRVAVVYYLCRNRHLEHPHFIEVP
LAVPEEGLYLRDVMNRLNVLRGKGMASMYSWSCKRSYKNGFVWHDLSDDDLVLPAQGSEYILKGSELLDRSPPPPPD
RQHDGGASNPNVEENLKHRKEEESPQSRGSQEGCSSSSSPSASAAGKEISPPPATPRPQQQAQSAALPPPPSPSPAS
TSREDDPCRTAHSGSSGNQSPEPAARNAPPSEASSPGPSEYRVCRPVGAQDAATQTDDSERDAAENHSTRVAGASTM
DTTSDAEIQECHRRSSPLSPKVPETVRESPPAVCSSDAASPGGRVETLESLIRAEASRRSSFRTLEEGHMLGPVGVK
LKPANLLMQLITCGFVSVKEHRGFGFIPTYRPRFTQVEFPSPVFSTPVALRHLDRIPCNTKATGSTRAPEPECFSGS
LVVETKQQDEPGRGIASLKRSSSYDEDRVYRAAHPKSNTESSCEAGSFRCLPQTIRIISCKQSRSGTVLSPPASDVR
NGSIQQEHSTMSSPLGSSKSASNRMADPSPPGKLSCARAESLFREEKDMIKIEERLPSGARVIIQSAPLCEEESDDS
SE

>GRMZM2G086123_T01|PACid:20868192
MAPPAGTGAGTEGRPARRRGTGTSPGRTKVWVEPPGKSHGHHHHQAPPPPSPPKRVAVVYYLCRNRHLEHPHFIEVP
LAVPEEGLYLRDVMNRLNVLRGKGMASMYSWSCKRSYKNGFVWHDLSDDDLVLPAQGSEYILKGSELLDRSPPPPPD
RQHDGGASNPNVEENLKHRKEEESPQSRGSQEGCSSSSSPSASAAGKEISPPPATPRPQQQAQSAALPPPPSPSPAS
TSREDDPCRTAHSGSSGNQSPEPAARNAPPSEASSPGPSEYRVCRPVGAQDAATQTDDSERDAAENHSTRVAGASTM
DTTSDAEIQECHRRSSPLSPKVPETVRESPPAVCSSDAASPGGRVETLESLIRAEASRRSSFRTLEEGHMLGPVGVK
LKPANLLMQLITCGFVSVKEHRGFGFIPTYRPRFTQVEFPSPVFSTPVALRHLDRIPCNTKATGSTRAPEPECFSGS
LVVETKQQDEPGRGIASLKRSSSYDEDRVYRAAHPKSNTESSCEAGSFRCLPQTIRIISCKQSRSGTVLSPPASDVR
NGSIQQEHSTMSSPLGSSKSASNRMADPSPPGKLSCARAESLFREEKDMIKIEES

>GRMZM2G006673_T02|PACid:20869094

MAEPSPPPVSNAGLRILLSKDRPAPSPPPTAAVSSHADRDRIIGVFRSALSRNEPPETFSLQTVQEAIKPQKETVLV
LEENQSLENALRTLLQELVSSAVQSDKKIMQYGNSLDSGESNCLITRLLDIVLYLCERGHVEGGMVFQLLEDLTEMS
TIKDCKDIFGYIESQQDVLGKQELFGRGKLVMLRTCNQLLRRLSKANDVVFCGRIIMFLAHFFPLSERSALNIKGVF
NTSNVTKYEKDAMDGISVDFNFYKTLWSLQEHFSNPALTSTNPAKWQKFSSNLAVVLNTFEAQPLSDDDGKLNNLNE
EEDAAFNIKYLTSSKLMGLELKDPSFRRHILVQCLIFFDYLKAPGKNDKEGPTGSMIEEIKSCEEHVKKLLEIIPPK
GKEFLKSIEHILEREKNWVWWKRDGCLAFEKPPFEKKPGQAGARKRKPRWRLGSKELSQLWKWAEQNPNVLTDPDRV
RMPSITEYWKPLAEDMDPSAGIEEEYHHKSNRVYCWKGLRFSARQDLDGFARFSDYGIEGVVPSELLPPEVNAKFSS
KPAEKVKRTRKEDSKSVSAHQPREHQVAATPETDGGVDPEEGEGAAPMDSDNGAVGDSQKRSPGEVSGPESGQCEPE
ADADDNVRTETTSRDARAGEMK

>GRMZM2G121957_T01|PACid:20869359
MPRRALTLETYLRFFLDPWNTRITVDQLNQILFMHGFVKLHRTKKAKIMERLVGQVDLLPPRRSTLHRASLPSAAAI
TAAQARDDVEAIGWAECPIGRVAAFAAPSADAPEPVERVPQPADFVLTGRRPRSKRTRGSAYGPSDVAAASKKLKAG
APQKHANVIVKEEENDHELVSPPPPPPDVALPPPRKLQPTLARIPAPPAWGTPMVLPYPSHQLFWGVPLPSRPTAQP
CPATPPPGWSVPTPPPSYPAPFCGAPVMLPPLPRHVAWRSPPPLLLKRPPPHLQGKPLPALLPVQHHHLLIHRSCSC
SSAAAAVALTGPVGSSHLLRFFRLRNLSK

>GRMZM2G306348_T01|PACid:20872112
MGNLCWSTNRERWRRTAPSPSVPACSDLPAAVSSFADAFVDFAVSDIFFPTFPSSSSLPPRPKTPTTFLPSPSRLVA
IGDLHGDLPKSLSVLRLAGLVPPSCTPTPPPPPASSWPAGPTLAVQLGDILNRSDDELRLLYLLHRLSLSAETRGGA
FLPILGNHEVMNVSGDFRFATPQGFHDRAVCGGVLACAGFAAWAAEAGVAGAAGCWRTGVAGAGAVYTASLIVSRDC
LKLNSTLGLWTCFCPIRSKPNPLPSYIDIGSWAGLVNPRRRKKYMLPSANWQEMKKLSSGKKSGKGDGLGRPGESLD
SQARQIAVRSPLPWREAYQSSPQIPRYSPLRLRSRLRRRAPPSPPAPHRATPMYSQLVDGDSDSEGSLISDISASPP
RGRSPSPPPPPPPPPEHRPNPKHTATVKPKPPKSKAKPKLAASACTAAVSPPPLPSAALSDPHGLVSRIAAAPALVT
ATSTVSSSSFRRLVQSRNPSFDPAAAFSAPTPAPTLAPTEIATAATAQCPAPDAPPQTRPKRVHPNSVSEVVPPAAE
AEQPKRPRGGAEGNFVRLNINGYGRRRTFRNAQARRPTKYRSWRRQRHGGAKPQGRGDEEGDFVAEAMIEREKKRAG
GDGGVLEAVEAAREDPSEQNLENLLRIIYGYNSFREGQLEAIQKVVAGESTMLVLPTGAGKSLCYQVPAMILPGLTL
VVSPLLSLMVDQLMKLPAFLPGGFLASNQTSDEFHETLQRLRGGEIKVLFVSPERFLNEEFLLIFRDGLQISLVAID
EAHCISEWSHNFRPSYLRLRASLLRRKLNVQSILAMTATATTQTLEEIVTVLEIPSDNLIKTSQIRDNLQLFISMSN
NRLKDLLLLLKASPFVDMKSIIVYCKFQGETDYVSKYLCDNNITAKSYHSGLLMKNRNRVQELFCSNKIRVVVATVA
FGMGLDKSDVEGVIHYSLPESLEEYIQETGRAGRDGRLSYCHLFLDSTTFYKSRSLLHSDGVDEYAMSKFLFQVFSC
ENSVGCICSLVKESTSRKFDIKEEVLFTVLTQLEIGGQQYLRLLPQFSVTCTLYFHKTSPQLLADKDVLVRSVLDKS
ETKGGHFVFDMTKVANDLSITVNEVFDHLQQLKFSGEISFELKDPACIYVILKKPDDLNALSADITRWLSEVENSKI
SKLDAMFDLANFAVKGCQRTDGCSGSQHTPCIQKKIIQYFSKNYSTSDSGQCTQPQRGSPFLQADIKAGILVGRGRY
LEVDFPVVMEAAKAELVKLVGKGE

>GRMZM2G036534_T01|PACid:20872300
MAGLLHLQSTLLPSASALRRRAGAPVPCSSRRRCRVEAKIREIFMPALSSTMTEGKIVSWTAAEGDRLSKGDPVVVV
ESDKADMDVETFHDGFLAAVLVPAGESAPVGSAIALLAESEEEIPVAQSQAASFSSSSPSPPPPQETAQEASPSPPP
PPPSAPVAVSAPALPSPATQGGGRVVASPYAKKLAKDLGVDLFSVTGSGPGGRIVAKDVEAALAAPKKAAPVTAARP
DVPLGSTVPFTTMQGAVSKNMVESLAVPTFRVGYTITTDALDQLYKKIKSKGVTMTALLAKATAMALVQHPVVNCSC
RDGKSFTYNKSINIAVAVAIDGGLITPVLQDADKLDIYSLSRKWKELVDKARAKQLQPHEYNSGTFTLSNLGMFGVD
RFDAILPPGTGAIMAVGASEPTVVGTKDGRIGIKSQMQVNVTADHRVIYGADLAAFLQTLAKIIEDPKDLTF

>AC218932.3_FGT009|PACid:20872392
MAGGNASCSVRPDHGGNGDVVATLERIGRKLARKQADNQAVVDLIRELQAVPMTYEALEATMIGRTVNGLRKTAPSA
LARLLADTLYRQWKALAHEWHASSKTPTPMLPEADNGIKEPSYSSPPPPKVDDDKDTKKRSSSPPPPQPLDAQQSAG
PEADREMGALPTTASVRKPNILRFRMVTVTEPEKPDQRRVRLIPLATPHPAPPSNDAMQNDSGNKQQACCSATAASH
HQGTNADNACVGSTDVAPLTTVVKPSTALSNHIASSGACKRKEEVSTDFDEERLAKARARLHEGYEEASAAREKRKT
KLIDVIDVPEKAKRRPAGHRTRKKQPPIRSLNLSQDA

>GRMZM2G044394_T01|PACid:20872427
MAGADTSGSDVLATVARIDRRLAREKDNDVVVELIRELQAVDMTYEVLEATKIGHTVNALRKSAPTALARLLADTLY
RQWKVLANEWITISTTQQQPSPPPNTDTDSNKRSSSSPPPKAVDDKATNKPSSSPRPASPVDAEQSARPEPDHEKS
APTTISAAPVRKNILRFRMVTVKEPGKLDRCRLMIIPSEAPDPAPASNYATQNGGDNRQQAYCTATTSHQATNADNK
GVYPSDNVPLTRTVKSPTALPNPVASTGTCNKRKGEPSTNFDEEKLAKARVRHHEGYKAASVAKEKRKTKVVNVKEL
EKPDQHRLTIIPLEASDLAPASNDATQNGGDSRQQAYCAATASHQTTNADNKDVYPTDDSPLTMVVKPPTALVNHVA

SSGACKKHKGKAPADFDEERPAKTRVRLHEGYKEASVANEKRKTKHVNIQESGKPDQRRLTIISSEAPDPAPASNDA
TQNGGDSTQQAYCAATASHQTTNAGNKGVCPTDDSPLTMAVKPPTASANPVASPGACKKHKSEAPTDFDEERLAKAM
ARPALHNVARGFVYTQWDSRTNEPGRQSVGLVNEPGRQCRISQRAEQITDHPLLCAFVTLLVLRGTVGVNRRLVYIA
GSPDNRADPATATGSARPVEDVERILREIRADSDDDPDAGPNNDEAPTSTSSSAATGDHYDRGAAWTTTTYRLQPRV
TRWNAKRRRWLHQNPGFPSRDARGGPRVLLVTASPQGPCGSPDGDRFLLRATKNRLDYCRLHGVEMVHTTARLEDPE
LRSPGDGWAKLALLRRLMLSHPEVEWLWWLDAGALVTDMGFELPLARYEGAHLVVRGDSYQLFQRRAWDAASTASFL
LRNCQWALDLLDAWAVMAPRGRARHDAGALLTATLAGRPAGEADDQSALVHLLITEKERWMDRVYLENQYYLHGVWT
ALVGKYEKAMEKHHPGYGDDRWPFVTNFAGCNPCDDGKNRSDEYPLDRCASGMERAFNFADNQVLRLYGFRHESLAS
TEVRRVANRSTDPLEAKEEALAFLKKPNEPDLWSNDVRKNRKRKGKGDLDSVLARILRRLGWRSQC

>GRMZM2G003090_T01|PACid:20873095
MWISDSAFVEPSSSSPSASASPPPSPPPARLLRQRNRRRRRSSRKQGSASAVDGVADPEAEDVWRGAQWEAAWPARR
DSKPVVLAGGDDAVGRSRSLTDDDLEELKGCADLGFGFNYDEIPELRSTLPALELCYSMSQRLQLDDEKPQQHEAAA
PDAATPSPPVTNWKISSPGDSPDEVKARLKYWAQAVACTVRLCS

>GRMZM2G003090_T03|PACid:20873096
MWISDSAFVEPSSSSPSASASPPPSPPPARLLRQRNRRRRRSSRKQGSASAVDGVADPEAEDVWRGAQWEAAWPARR
DSKPVVLAGGDDAVGRSRSLTDDDLEELKGCADLGFGFNYDEIPELRSTLPALELCYSMSQRLQLDDEKPQQHEAAA
PDAATPSPPVTNWKISSPGKQHIADRRLSSSMT

>GRMZM2G003090_T02|PACid:20873097
MWISDSAFVEPSSSSPSASASPPPSPPPARLLRQRNRRRRRSSRKQGSASAVDGVADPEAEDVWRGAQWEAAWPARR
DSKPVVLAGGDDAVGRSRSLTDDDLEELKGCADLGFGFNYDEIPELRSTLPALELCYSMSQRLQLDDEKPQQHEAAA
PDAATPSPPVTNWKISSPAQVTALTR

>GRMZM2G040673_T01|PACid:20873645
MSAAAAGAKKRLSVGAIAGGGFALGCGCRDAKAVAVAVASSAAASTSPYSAAGTETTTTTATTATWRRARTPPHRPS
SSASGSTGTLTVPSASSSSSFPWERADAEGDGDAGEEVDCKGEATTTTSFSGLLRQLNELEQSVVSWGRKSASKDHL
SPPPRPLPALPVKQRLAHSCDGDRKEGHGNFSPPPPPPHFQFQITQQRQKAKSMDKGDRQVEVAHSKSMQPPPPPPP
PPPPLPLQPERQFKAQSTDDDKGGKKEDASMFPTPPQAPYHLKSKSSVLDGSVGVAVVKQSDDPLGDFRRSMVNMIV
ENRIVTGEELRELLRHFLALNAPRHHEAILAAFTEIWDEAFSARTTTTMATPPRPRPSAPTPPRRRHAPPTRSWR

>GRMZM2G010323_T01|PACid:20874112
MASAEIPNSSPDATNPAPAAAAATGPDLSSSPPPLPPRKRRLSPSPPPTRSASRSRSPRSRSPHGRRSRSRSRSPQQ
YPTDGKRQRHNGLTVDACRDFLRDRCTRSDLECRYAHPHNSVSIDLDNKVTACADSLRNNCFRGMTCRYYHPPPHIQ
EQLLRSVGVEDPKVKTQICRDFTRGKCSRSANECRFLHHSSVEDVAIVCQDFLRGQCNRIACRYSHVVAHPMPPMNH
VPMPYPEMLYMPPPPPPPLGVPMMGPLPSPPIPFADNTNRVEVCRDFLKNMCNRESCRFVHPETHTTAVNANVEVCR
DFKRGECNRSACRFFHPHTS

>GRMZM2G010323_T02|PACid:20874113
MASAEIPNSSPDATNPAPAAAAATGPDLSSSPPPLPPRKRRLSPSPPPTRSASRSRSPRSRSPHGRRSRSRSRSPQQ
YPTDGKRQRHNGLTVDACRDFLRDRCTRSDLECRYAHPHNSVSIDLDNKVTACADSLRNNCFRGMTCRYYHPPPHIQ
EQLLRSVGVEDPKVKTICRDFTRGKCSRSANECRFLHHSSVEDVAIVCQDFLRGQCNRIACRYSHVVAHPMPPMNHV
PMPYPEMLYMPPPPPPPLGVPMMGPLPSPPIPFADNTNRVEVCRDFLKNMCNRESCRFVHPETHTTAVNANVEVCRD
FKRGECNRSACRFFHPHTS

>AC194104.3_FGT007|PACid:20874367
MAGGNASCSVRPDHGGNGDVVATVERISRRLARKQADNQAVVDLIRELEALPMTYEALEATMIGRTVNGLRKTAPSA
LARLLADTLYRQWKALAHEWHASSKTQPQTAPPEADNGIKKRPSLSSPPPKVDDDKDTRKRSSSPPPPQPVDAQQSA
APEADREMGAPPSTTRAASVIRKPNILRFRMVTVTEPEKPHQRRVRLIPLATPDPAPSSNDAMQNDADNKQQATCCP
ATAASHQSTSADNEHVGTTDATPLTTVVKPSTALPNSVSSSEACKRKEEAPTDFEEERLAKARARLLEGYEEASAAR
EKRKTKLIDVIDVPEKARQRPATHRTWKKQPPVP

>AC196290.3_FGT007|PACid:20875206
MDHGWQAAAEDGPGQSMIHSRKGRHRTRGRRPRSRSAANLPPVDDDSSVRLPSHQSAVAKSAGLAARPVCVLDRARD
LTLVENNLRRCALFAVVIGSRPPVSAELLAAEIAAEYELDPASFSVHRSAPEDFVIILQNEQAALRIFNNGAVLNSP
SGSFKFIKWSRLAHAEAVSLPSFVSVSFEGIPLHAWSVETARSLLRAHCTALELHPDTAARLNLSSFRVLGWCRHPE

LIPVAVDLLIPEPVQSFEVEATVKNLLAYQVTASVSLISPPAGEVSPPPSPPASDDDRDTRSRRRRLQQAPPAGQGG
PAEALAVRRPVHERLGPLPICPRHVAISQEVLEQEVSKETSLSGSSNFEELVPAVKELNIEALIEASEDNVDGFGRS
HEQDCMVEAGASGLSGGCPVHSPLPVPSSPSSPLRAPADCCSLGGCEKEQAIQGSPLGSPSRATVNMGGQMSALVDA
RTSSRLSRRGPIITYFRQRRKKDSQPSQLLGASPSPPPPPSGANEFIDKLTRPAGGILRAPVRRPRQGKASVTEVIP
RRSRRVAGAGVERLPTPPPATQGKKRILKELGIVKADEAVTIRPEDLDEYARVFSKGLSEVQVTAHGSVIRLV

>GRMZM2G079913_T01|PACid:20875777
MALPSATVLWTASITFRLAQNMVVLFFLAALLAAPARSSSPSSNNGAGLQQPTLTLCSGHSTVAKSGEFASFLAAVC
AGAYGGGVAHMSHSMSSYVAAENRTMYGVAQCRPDVSASDCTACLAAASSKLITGTACATSAVWHDACFLRYSDRDT
GRFREDEHTETVFNASRALSSLPPHVGANKAVVVRRLLAAVADSTRSGHMCSDIGVGTGAAGGGRIYGLTRCAAEIS
CADCRRCLGGALAVVEREYNGSAGMQVLRLSCMARYESYPFYNTKFLTRRLLASATSGSPRPTHGGIVFNSSISFGP
FGLVHSGGPNPPPSPPVSPPPPAFTTISVPASVGTAQPTPPPSRPAQAQPSPPPPAASMIVSGPATATPRAAQAQPT
PAPPGGHNPPPASPPPASIMNISGPATATPTPRAAQPTPAPPGGPTPQPVAAATNENKGKGTLNTPKWQIVSIATGL
VAAFIILALTVWCRRKQQLSQREHVIEPHVLNHQADGVPGETMHHLEKKHLGSEEDDDGGRTSCQLYSYLVLEAATC
RFSSRNKLGSGGFGTVYKGTLENGKDVAVKRLRDSKRAIQELEREISIVASLRHKNIVRFLGYCFQEEGRFFIYEYV
LNNSLDKFWYKASSQCQKLEWATWFNIILGVARGLLFLHDMGIIHRDLKPHNILLDRNFNPKIADFDLMRMYDQQKT
HESTEKVAGTL

>GRMZM2G152111_T06|PACid:20876482
MSSQADSPRRRYSRSPSYSRGHPKGRSRSQSPARSQSRSPVPDPRSQARSRSRSQEREEDAVNRGNTLYVTGLSSRV
TERDVKDYFSKHGRVVGCHVVLEPHTRVSRGFAFVSMDTVEEAERCIKYLNGSVMEGRNITVEKSRRGRPRTPTPGS
YLGHRYERRERGRFHRGYGGGRDEYYGNGGGGYGYRRSPPPMYSSYRESRDYYPSYKDSRDYPPYRDGRDYSPPHRD
PRDYYESRGGRGYSPPPYGGGRSRRDRSVSPYRMPERGYGGGRRVGGGGYDR

>GRMZM2G152111_T03|PACid:20876483
MADSPRRRYSRSPSYSRGHPKGRSRSQSPARSQSRSPVPDPRSQARSRSRSQEREEDAVNRGNTLYVTGLSSRVTER
DVKDYFSKHGRVVGCHVVLEPHTRVSRGFAFVSMDTVEEAERCIKYLNGSVMEGRNITVEKSRRGRPRTPTPGSYLG
HRYERRERGRFHRGYGGGRDEYYGNGGGGYGYRRSPPPMYSSYRESRDYYPSYKDSRDYPPYRDGRDYSPPHRDPRD
YYESRGGRGYSPPPYGGGRSRRDRSVSPYRMPERGYGGGRRVGGGGYDR

>GRMZM2G430392_T01|PACid:20878874
MPEKPPPLRRRPTAAGSWVRSLHCKSMAADDVAARVAAAPKKLLPRAGCGGSSGDTLNHVVVSSKPSRPKPKPKPKP
SPEPKTTNKPKPVPVPPSPPPGPLGPVPALTELPAGHSSRQVVEIIFLSSWSSPPPGAPTSSSSTFPGAVVEMLFRV
HNPARAVARFEDYRAAVRARAGGASRSAADGNEMMRFTPAPPYGSSAAAAAAAGEDGPRVRTFDGSGGAHASARGPA
TGRRAMFLCRVIAGRVAEGAGAHAAAAAGKEHFDSVRVGRGGELVVFDRRAVLPCFLIIYKL

>AC234522.1_FGT005|PACid:20878960
MAPPPTSPSFLRPPPLPHHPHPRLLLRPPSASFRVAEILGGRGLCNGEVGVRKELASGSTTSSPAPSPPPSTDSPPP
AVDPDAFEKEMMGLTGGFPGGEVGLKDFVAKNPPPPKKSESQPQATLSAPPRPPELPLFLPGMVVLVKNPNNAYHMY
CGIVQRVSDGKVAVLFEGGVWDRLITFNLDELEGREKGPPMANPKSVVLEDLVAELEDDDDDKEDEAAKKKEPEGAA
AAAT

>AC231183.1_FGT002|PACid:20878985
MLSYLFVGCLAKRFKGEAYLSATTSCTWYFNPDNPEAQMYYNKLLNTKFQMLRPQATAEELQASQTLNIEDKTIEEL
LQLDPDMFPEQGFRCTVTISRLIQNGRWWFPSCIKCNKSSSQTSTGYQCTSCNGTETEYRHDANLRILKLYTTQMLM
NRFQLKSIKTASRHLLRSVHMWPCSASQDVGRDIPATLHPPPPSSTSTPSTFHGSSSAPVGVHKCTVPPLPSSPPPL
HEMPVADGPLPRMPRQAASSPPPPRPPSILACALGTSIRTSLHSIRSNRSLPAFLPVRVPHDGGFQNLRATALHKEK
AWVVVQRKVEVMDHAELTKAYYHRDKGKIVPPPSSRDAQRWYAFLWYGALIITSHRTPTPNPTRMMPRESLHPPRLA
HDQPSWPPFSRALSSVPSRALLPALHASKPCAAPSKGFTEGFLTTCLYPALEYSSKKNAKGNKPRKERNIIFEGFRT
PREAIEVQYFFSCPTN

>GRMZM2G027105_T01|PACid:20879366
MARVYVGNLDPRVTAREIEDEFRMFGVLRSVWVARKPPGFAFIDFDDRRDAQDAIRELDGKNGWRVELSTKVGGGRG
RDRNGSDMKCYECGEAGHFARECRLRIGSGGLGSGRRRSRSPRYRSRSRSRSPRYRRSPSYSRSPRDRSPRRRSYSR
SPLGSPARSLSRSPPLPPPPPRNNSRSPPGAREVSRSPPPPPPRNNSRSPPAARDVSRSPPAARDVSRSPPPPPDAR
RSYSGSPGQQPQRDESPYANDA

>GRMZM2G114634_T02|PACid:20879497
PPPPSSSFARRSLRRGREPARRPTLPQSSDPPWPPPPWPSLQSLSSSRCCSSPPPRRPPAPRFPSSPPPPTAPPPPP
PTRSWRTWPRSPPSSRRSSTPSRRSTTGIRTPRSGSGRSTPAPSASAPCSGTSSGRGRAGRRRWRGSPTRPWSGDSP
PRRPSRRWMGRTGSTSSPPKARSGSTPASGTWSWLGRWSSSLLATRTVASSSSSYLR

>GRMZM2G408897_T01|PACid:20879545
MPSRRALRRSPPPRQSPQAAADAKEAASPWLPSSSSTSSSSSSASALVRRSAAFSAAGPRGGSGTRVYPLRDFPGGD
AAALGGAFRDNVRWLLKQWSCVPGSVSAWRALLSDERTGALVPVIAVEELAASSPLPLCDLCRCAGWSHHWVSKRKY
HFIIPAVVDWDQPFRADGLLGHSEHLLHGLIHSNGFGHLLTLRGRVGGSTFLSGCQIMDIWDQLCTALRVRAVSVVD
LTQKYSVDLRLLLGVAQSKTWFTRWGYCLAKGCFSVSRSTYTAALEALAALPVDCLRSRHVRRVVTIYRRLSSKPLV
TVREFLLCLLDWKHREPPLSPPPVKASSRLMFLLPKSCMMKRPRQPCQRFEDVVDLLECRWSKKRLLSAANVVVAKL
REHADGTKITRQAVRDAARGGIGDTGLLDFVIKSLNNTVVGNHIVRRVPDPENRVLHFSLEEYAEPEPQPQADHELE
PVELDAENTPPAVRWPNAAEAERDLRAVYRAMVGARSEAAQAVLDCKHWVKWWGLRDESDDQLRFLVEWRPQPWEAT
ELTRPMPPGDIVVVPLHASIGELLVEAEHALRDTYCFFENFQAESLDGITGDKWDPVMLGGAESGDTIGVHGHGADM
ETGLRCQGGADAWEVQCVCGAQDDDGERMVACDACNVWHHTRCVGIADGAPVPPLFLCISCSGALMAAGPISG

>GRMZM2G066449_T01|PACid:20881801
MPSPPKDRSSGRLGRFLAALRPSRAGPLPVQTGFPTSLADLVVKNHGRLKKQPSPSSKRGRRAAAAAAAASPSPSPC
SPTTSPSPSPSPPPASPPPPAAAAAVSPSDRPRPDLPPAQPPRGFGLGFLAVSGVVSLALLVIWSKKVVAAVTVAAF
SLYLLESVRSSSLPRRRPRPRPGPGAAERRLCLDGRGRVSPIREVDAADTEPSRPSCSDSDRASEAEGRSGVLDESS
NTKAKARNKSWKKLLAVAGAKKLQRGRRSKEADSSGSFRSDGDREDAATACGGGGNARAADSSGSRRGSASQSDVLA
EDGAAREADSSMGSRRSQGVEVEIDADVIEVEEEEEQAGTGFPALVLVAVVLVGLVAGKAVALALTVLCSAFLSSDR
RSPCGGGGGGCSQGRRLEPSMP

>GRMZM5G817439_T01|PACid:20882871
MMLPSSTISCISPRRLPSPITRSNPSPTARMADVLDPASDAPRARRPPPPPPDSPEDRSPQLPPPPPGGPPAASRKR
SRSPPPPPPPPLGSSRPQRYRDHRSGGGRGGSSPSPPPYRGSRRHSPSRRSPSPPFKRSRRDDGYDRRGGRGSPPRY
GYDDRRRGYDHERGGGRGGYEDDRNQGRYLNRAPDWPDSGYGAANDGPGVTQREGLMTYKQFMQVLEDDISPSEAER
RYQEYRTEYITTQKRAYFDLHKNEDWLKDKYHPTNLLSVIERRNERCKVIAKDFFLDLQNGTLDLGPGITAGAASKL
GGSDGNSEDDMDNDKRRKHGKDSSKATDSLSGAPKAHPVSSESRRVQADIEQTLALVRKLDAEKGIDGNVLSSGDHD
KSDVDKSHIGSMGPIVIIRGLTTVKGLEGVELLDTILTYLWRIHGVDYYGTSESHEAKGLRHVRVDSKTSSTSDVSS
ADWEKKLDTFWQERLNGQDQLVILTAKDKIDAAAAEVLEPLVRKIRDEKYGWKYGCGAKGCTKLFHAPEFVHKHLKL
KHPDLVLESTSKVREDLYFQNYMNDPNAPGGTPVMQQSAPDRGRRRPSMDSRLKFDRGSNRDHDKADRDGVRYGRGD
HSPSRDGPDDQMFDGFRGRPSNAPFVAEFPAPPILMPVPGAGPLGPFVPAPPEIAMHMLREQGPPPPFEPNGAPHGN
AGMLGPMMGGPAPIIAMPPNFRHDPRRLRSYNDLDAPDEEVTVLDYRSL

>GRMZM2G055204_T01|PACid:20883056
MAAAIDMYKYYNTSAHQIPSSSPSDQELAKALEPFITSASSSSSSPYHGYSSSPSMSQDSYMPTPSYTSYATSPLP
TPAAASSSQLPPLYSSPYAAPCMAGQMGLNQLGPAQIQQIQAQFMFQQQQQQQRGLHAAFLGPRAQPMKQSGSPSPP
PPLAPAQSKLYRGVRQRHWGKWVAEIRLPKNRTRLWLGTFDTAEDAALAYDKAAFRLRGDTARLNFPALRRGAHLA
GPLHASVDAKLTAICQSLSESKSKSGSSGDESAASPPDSPKCSASTTEGEGEEESGSAGSPPPPPPPPTLAPPVPEM
AKLDFTEAPWDETEAFHLRKYPSWEIDWDSILS

>GRMZM2G329710_T01|PACid:20824124
MSRRCSRAAFCLARSRRCSRAAMAPPPFYLPLLLAFLLLSTAAAHSQHLHLQHHGHGPHHHHHVSPSMTATARFYTA
PSMHQNRMESEDARQSLRVFSPFLTPVAAQAPSAEEAMSAMVAAADAGDPARARLDPPSPPSSLLAAGDLMPSSLPL
LQPQAEVVGSFESEPAAPPSVVDEQYRDEASPSPPPPAVYEPYRDAASAPPSVVDEPYSDAASPPPPPFVVYEPYRD
TASPTPPVVYEPYLDAASPPPPPPVVYELYRGVASPPPPSPVVYEPYRAASPQSPVHDYAAGVVYSGSGDDLGLQQI
AKVLASLGYNEMASSATLLAGSASVATWPGPITVFAAPDVFLQADSCPESECSRRHLLLDHMALGYFPYAELAAAPT
TKLPSASVGFCLDVAAQPQRGPFSVHHASLYVDGVMVSEPELYDDGRYVVHGLHGFIPPLSRASCAEDAHEHPHHQV
HLHQHRRHHHLSARSSAASAAIAASAVRVMIREAISRLRDGGFGFVALAMRVKFAELEKLSNLTVFALNDQAIFTGE
GHGYVSAVRFHIVPEHRLTRADLLRLRPGTILPTLAGEDQKLVVTLGSGSATDEVRINYIPVKEPDVVINSRVAVHG
VYVPFPRLHLANLATLVAVASDLQTNDSCGGAGGPLGDCASTPMTSTTISVDQGYGKGQ

>GRMZM2G470914_T01|PACid:20825099
MRHLALLALLLAPVLGSTGTTLSPSPAATDSPANQGQASSPPQSAFALGPVAVPTAPTTPSASPSPEKAAAVSPAAP
TEPRIAPAPVAPPKEYSAPPPVEVAPTDPTDEVPPPVAPPQAAVGNPTPILPGTPALLPYVQAPAPSVVLKPNPPVV

VSPPPANNQPSRPVGSVPPYPPPASPPPANGIPPYPPSGRFPAIPPATSGNAVPPANTYPPTNLKKHHVPHASPPKG
STGQTVPGHKSPVTGSAPATSPLPQNTNMPSIPKNASSVPHAHPSPPSIAPELAPTGRSHAQGWKSSNPNNKTNRLH
APSYPPPRAQGPEVSRTPRQTGTKNYTHHAPPPFPQELLDSTGNHISPTLPSIPPLPEPKAPSAHPIWTLPPPPPNS
DCNSLSCPEPLTDPPAGAPCACVLPIRVGIRLSVDLYSFFPLVSDFAEEVASGVNMAQRQVRVMGANVAGDQPDKTM
VLVDLVPVQVKFDNATAFSAFESLWSKKVSLKPSVFGNYEILYVVYPGLPPSPPSGQGVGDGAFGNNRNARAVKPLG
VDLFDS

>GRMZM2G552644_T01|PACid:20863064
MSVFVEASIPLPPQTGCREAPPPPPPSPGGHVEFPPPPPGECALALPHPKASVGAPPPPPPPGGHVESPPPPPPPGE
CALALPHPKASVGAPPPPPPPGGHVESPPPPPPPGECALALPHPRASVGAPPPPPPPGGHVESPPPPPPPGECALAL
PHPKASVGAPPPPPPPGGHVESPPPPPPPGECALALPHPKASVGAPPPPPPPGGYVGAPPPPPPPGECALALPHPKA
SVGAPPPPPPPGGYVGAPSPPPPPGGYIGAPPPLPYGGIGGVPPPPPPFGGLGGTPPPPPPAGFRGGAPAPPPPPGG
HGGPPPPPPRGHGGVGGPPPPPGAPSPPMPPGMPGGPP

>GRMZM2G097884_T01|PACid:20878768
MASLLRPLVLVLLIASCAAQQSPPAQPPPTPNAPPANSPPQAPPAGNPPPAPQAPPAGNPPPAPTATPPPAPTTPPP
APTTPPPAPTTPPPAPTTPPPAPTTPPPSPPASPPPAPTTPPPSPPASPPPAPATPPPSPPMAPPPATPPPPATPPP
PAAPTPAPSVAPTPPPVATPAASPKSPKTPSPAAATSPAPSLSPAGTPTNEDSGASARAVGFATVVALAGAGLAVLL

Oryza sativa

```
>LOC_Os09g24720.1|PACid:21927556
MGPHRHLLSLSPSPLSLSSLLPPTTGLLRPPPKVAASFALRPMVAAAVSPPPRHRLLLPPPPGRCLLHPPPTVAASF
THRPRSPPCLQNSKSGSPPPSHLHHARSGSYRAKDEDNAGARTIGEWGTTPLPNAIDKPLRPSPLISGRRQASTSPT
LPLTVPATMNSGAPDLDTMNSEVADPTIPSLGAPDPAVLASATPDPFVRHWGLYLC

>LOC_Os08g03980.1|PACid:21886802
MDQSEHHRPAASPAADWCFRLHGWPLCRRCWASTSSPTGLPSPPPNGVSACMAGLYAAVVGRLRLHRPACLCRRRLV
FPPARLAFMPPLLGVYVFTDRLAFAAADWCFRLHGWPIMPPLLGVYVFTDRPPRPPPTGASACTAGLLCCRCWASTS
SPTGLPSPLSTGVSACTAGLYVAVVGRLRLHRPAASPAADWCLRLHGWPIMPPLLGVYVFTDRPPRPPPTGVSACTA
GLLCRRCWASTSSPTGRLARRRLVSPPARLAYYAAVVARLRLHRPAASPAADWCLRLHGWPIMLPLLGVYVFTDRLA
FAAADWCFRLHGWPIMPPLLGVYVFTERPPRPPPTGASACTAGLLCRRCWASTSSPTGCLARRRLVSPPARLAYYAA
VVGRLRLHRPAASPAADWCLRLHGWPIMPPLLRVYVFTDRPPRPPPTGVSACTAGLLCCRCWASTSSPTGLPSPPPT
GASACTAGLLCRRCWASTSSPNGRLVRRRLVLPPARLAYYAAVVGRLRLHRPAASPAADWCLRLHGWPIMPPLLGVY
VFTDRPPRPPPTGVSACTAGLLCRRCWASTSSPTGRLARRRLVSPPARLAYYAAIVGRLRLHRPAASPAADWCFRLH
GWPIMPPLLGVYVFTDRPPRPPPTGVSACTASLLCCRCWASTSSPTGRLARRRLVFPPARLAYYAAVVGRLRLHRPA
AASAADWYHRLHGWPVLTPTDAIFFTAGYIAVTTNRHQYLQHKLPTFAMAADWFLHFHGCMILPVLIGLTIFIAGRL
VCC

>LOC_Os12g28770.1|PACid:21936243
MEEHYTMKKPRLLLLSLEVILIALLLPLRGAHCESSTQGEGGGGGAGAGAANLTVTGTVFCDACSSSSFSNHSYFLP
GVKVRIDCMISVKSASKEEIKITAEKVTNTFGAYQLDIPAIDGFECATSAAAAADSFCRAAVIDNPSPLCNVPAVTT
TVGHISSSSSGHDHDQPNACLYNLNSLYYRPGGAKNGGQCGGTGGGGDVPPAALNTSLFYCPPWPWPPIPFCTPRPW
FPPIPFLTPPPPPFLPFPLPPIPFLTPPSPPPPAFPFPLPPWPWAPPPAFPFPHLPPIFSPPSPPPPPPPAFPFPFP
QLPPLPHFPPLPSFYPSPPPPPPPPPPPPPPSFPWPFPPLAPLFPPYPSPPPSMYSRKDPSTWSSSSKQQP

>LOC_Os02g06670.1|PACid:21918001
MVKGTSGYSYGLGLACFALVVAMAGATQLKVGGGNGWSVPAANAESYNDWAEKMRFQIGDTLVFVYPKDKDSVLVVE
PADYNACNTSSFDQKFADGNTVFTLDRAGAFFFISGVDANCRAGEKLIVMVLASRNGTATATAPSPPPASSTAPPPT
SPAPASPPPSSPSPPPASPPSPSSSGAAAPTTPPPASSPPSTPTPASPAPSASSPPAPPSANAPSAQGARNPSATSS
PPPAANGAAHAAVAASGLAAGIIGYAMLAL

>LOC_Os02g28970.1|PACid:21921589
MALLFRISLLLLVPLIPTAAASHHHSPAGGGGAAVPLHPRRHHRSVANTATALFYPAPSMHQNHIEAEEGQLLHVL
ADPFAAAPAAAEAPSGETAIAAVGAAAEEATPTLIDDSPQQAAAASPPPPPPPPPPPPPLFAKPDLDSTAPPQPKEE
GVDGYGSTTATATVTAAPPLDEPAAATATTTTTTTTTLPLPRYSHVASPPPPPVHAGVAGLGDEQRLEQLARVLSSL
GYNEMASAALLLANSALLAAWPGSITVFAAPDVFLRASCPMCSRRHVLLEHIALGYFPYTELAAASTAKLPSASPGL
CLNLASDHGPFAIHHVRLYVDGVEVSHPELYNDGRYVVHGLHGFLPPLSHGSCSHGSNHRHHYHYQYHHHHHHIIAS
SAASSAATAASVVRIMIREAIARLRDSGYGFVALAMRVKFAELERLANMTVFALDDQAIFVGGGHDYVSAVRFHVVP
GHRLTHADLQRLHPGTMLPTLAGEGQNLVVTQGASGSGSGPRDVRINYIPIKDPDVVINSRIALHGVYVTFPRLHLA
NLAAAVALASSNQINATCGVFGDCASAAATSTTVPAAHRYGEGQ

>LOC_Os01g10480.1|PACid:21906559
MTRRVAGVAAAAAAVAAAVVVLAAASCCEARDFYVGGRDGWTTNPAEPYNRWAERNRFQVNDRLVFRYNKEDSVVVV
SQGHYDGCNATDPLLRDAGGDSTFVFDSSGPFFFISGDPARCQAGERLIVVVLAVRGNATATPTTPSPPPPTVPAA
PTPRPSPPPPAAGTNGTARAPSPPVPAPAPAGSPPPPPPPAGGNFTAPSPAGGMNFTAPAPGTNGTAAPPPRPSSA
PSVRGGALLMLLVVATAGAMALV

>LOC_Os10g37960.1|PACid:21883031
MARQAVATFGRSTRPACHADTSRLDERTNDHGETGRRHASADALEHRDAAATSPPPPPLALPPRSPLHPLASSAAA
SPCRHSLASLAAVLASPPPRLLRRRFASPPPPRLPCRCARLSTPSPPPLPPPPPRLASPRSCLCRRRLKEKRERESN
PYLQDCKLTSIGLFGPPDDSGLQLDENKIDDIPQVSQLENEALTQEFTEDEIKKAIFQMEHNKAPGPDGFPAEFYQV
FWNVIKSDLLDLFIEFHNGTLPLFSLNFGPIILLPKCMEAMKIQQYRPICLLNVSFKIFTKVATNRIIGVAQKVISP
TQTAFIPGRNIMEGVVILHETIYELHRKNKSGVIFKIDFKKAFDKVKWSFVQQTLRMKGFSPKRCQWIASFIQGGHV
GIKVNDQVGNNFQTYKGLRQGDPLSPILFNIVADMLALLIKRAKDDGMLSEVIPHLMDDGLSILQYADDTIIFLEHD
```

LQEAKNLKLILTVFEKLSGLKINFHKKSLAIKIGRLSNKELRRNLVVGKVVFRATHWLRSWAQLQKCEEDSELLKAA
CRILETTVMQLFVNFGWRFTNRIQ

>LOC_Os10g29240.1|PACid:21883931
MSPPHQSPPPPPLLASSLLSSHPRESPPPHSLHPIPRCRQTSRPLLLSRARNPRRLAPPDSPMPLQSTAGPATPSSL
LLPPPLSRVNARRSPSLDQRRRYRASTAGPPQSLRASS

>LOC_Os10g28710.1|PACid:21885902
MEGLVVISQQRNHHNQHSGGRGKMTGPHFSSPPSSHGFRGMNCRSFHSGVCAGLLPSPPPPPARTYSSPEPKTPKQQ
QQLQRRGGKRSRPISISPSTSPPSRPELWAGPAFSNSPPPSSLPIPKFSLRQNRSISLELPLFERSDEVEVKPHAKS
APSSPVGGSGFDFFNDNETAVATENLRRILHLDISDH

>LOC_Os10g42560.1|PACid:21886565
MPPLAVGSPPPPLPLQPRVVLSPRSRRAHLGPKPPARHYGVRRVGHGVPAPAPPSPPPHPMVLRPPLPLVHRDHRAR
LCRMGCLFESPETEAAAGTTDAGSSSAAASTSHGRFLSSTSGQGERRVELEEAT

>LOC_Os08g02580.1|PACid:21888066
MAIAARLLSTAPCRSPSSWPLPPSKPYLRFPSAPLLLLLRSPAAASVIHLSPPPVTPLAVACDDRHALPDSDEPRNR
NPPDHPAPLLAAAAAAAAAAVASPHAALALSGGSMGGCSDTSSSYSSSFSSSSSSDSFSSWSSSSDSWRSSSSSSSSP
PKKKKVVVVESADLETHESVGTAASPPPPPPVALTPWEKFWISVAVVLGVGGLVFGLIFLIKRSIPPPRTISVVKLQ
IALGGVAAAKSFQKDLNRIAERVQGSSRRWYKFILSDLFKKYYYQVIAFFAEINLFVHRTQFSIKVMVDIKDSDSWN
GHFKKISLEERGKFDEETLSNLEGVKRNKEYSTKMDGSKNEYIVLTILVATDGTMDFPKLITNAADLKVALTKLYST
PETGLEGIHVLWAPQDKDDILSKERMQKDYPYLKPLSV

>LOC_Os04g28640.1|PACid:21892460
MGGEAASSRGQRLPEAVAPAAAAPSPPPDPAGGEAASSRGGRLPEAAAAAVAPSPPPNPAGGEAASSRGWRLPEAAA
AAAAPSPPPDLAEGEAASSRGRRRQLQRTTAATAAVPQRRISRGRRPEATQGRRTKDELRYWKGWKGAKRFCEWWCH
K

>LOC_Os04g14090.1|PACid:21895309
MPPLLGVYVFTDRLAFVAADWCFRLHGWPIMPPLLGVYVFTDRLAFAAADWCFRLHGWPIMPPLLGVYIFTDLLAFA
AADWCFRLHGWPIMPPLLGVYIFTDLLAFAAADWCFRLHGWPLCRRCWASTSSPTGLPSPPPTGVSACTAGLLCRRC
WASTSSPTGLPSPPPTGVSACTAGLYAAVVGRLRLH

>LOC_Os07g46340.3|PACid:21899530
MPMPSSSSSLLSAIRRLLASLARSPPPAAAAAMPPSSPEPEEEGMGRSDAGVGGGGGGEGGGGGGGRALSYGKAEYW
DARYVEEGGAPYDWYQRYAALRPFVRRFAPPESRVLMIGCGSALMSEDMVDDGYTEIMNIDISSVVIEIMRKKHFNI
PQLQCILNCFSILFLSPPPLKFTIHWSPYDT

>LOC_Os02g06960.1|PACid:21923247
MASAVASTMPSPPPPAVSFGWLAPTRVSPPPALPAVAVAAEQAVSKEFIDFEFSLGGSATMLPADELFADGKLLPLR
PHPVAAEKAVAMEAEKAVPELVKAVRPAVAVAAEVFDPYVFSPKAPTCSSRWRELLRLKKVQTPTKPSASTSASPST
ATAATPSRSSNSSAARSLKLLLLQRNGGRASGAAASDLSVAPLLRDSSDSEASLSLASSRFSLSSSSSSSCHEHDDF
PRHSLDSVDPTPRPRLRLVRSQPQPHPPAAAAAAAAAVAKPRAAIQSPARRRPSPPPPPPPQVASVDSPRMNSSGKI
VFQGLERSSSSPAGSVHSSLRSRSRVMDRSYSAGVRAATPVVLNVPVCSRPVFGFFKDRKDAAAKDSSASRPRSALG
RKTAPHAGNGGGASRDLGTSN

>LOC_Os09g36920.1|PACid:21924942
MSPAAGGAAPTQLPPPPVAASPSPPPLAAAAAAAAAIAGPEGDRAPPPQGNRRDPAGSAEGADPGNAAARKTAWNVPA
PPLAAAAAAGAGVGVPGGGIMGGGPGSWPALAESAAARGSWPKSASSDSLKSLSDGSAPSASEDLIVPSVQPHPVAN
PISGGSNPTSSPPPPNATVVVTSEQNGNTDQSNPVRHSSGGHCAGSNSSRDGNTSDGGDGSWNDGGLGSGSGSNSSY
GHGNSTNGAGGANMNNIIHSSGTSSSANDSSRRISGNNNWNNNGRSAGSNHNAAGSGDGSNRNLWNNNGRNGGGSSN
GFVGRGGHRNRRDHERGGSFSPRNYPRHTPMPPQQQQPGIYQPGPFPRPPPPPPPPGHFMVPQPFVPYVPHFAYPADV
QGYPFYLPPMEQFQNMHLVRPQMQPLWVPQDQQNLQEDIRTQIEFYFSTNNLCHDTFLRRQMDDQGWVHIDVITKFN
RMRRFTNLVDTNYILDAVRGSELVEVQGNTVRRRNNWAEWLLL

>LOC_Os09g19750.1|PACid:21927900
MVFGRSKSSSSTASPPPAAAASSAAAAACSELRAAYHECFNRWYAEKFAKGQWHKDDCVGEWHKYRACLEVTPFSPP
PNSITASLIYYRSPQFVAKTTALGIALVFSQFRMGFHLSRFLVYMARNRVVF

>LOC_Os10g35750.1|PACid:21882985
MLLHRRGLLLRGGGGLLRRLSVAVEESVSPSPPPPPPRQPQEDSVDDEETASPSPGKPQEGSVAAEERASPSPPPPR
RKKDSLFRRVAAAADPRLPLSPVLEQWCLAEERPIAKPEIQSIIKYLCRRRRFSQALQLSMWMTERLHLHLSPGDVA
YRLELITKVHGLDRAVEYFDSMPDQLKQQQCYGSLLKCYAEAKCVEKAEELFEKMRGMGMASSYAYNVMMRLYLQDG
QVERVHSMHRTMEESGIVADVFTTDTLVAAYVVAEDIEAIEKVLEKADTCNDLMTWHSYATIGKVLMQSGMEERALQ
AFQESEKKIAKKSNRVAYGFLLTMYADLGMNSEVDRIWDVYKSKVPASACNSMYMCRISVLLKMNDIVGAEKAYEEW
ESKHVYHDSRLINLLLTAYCKEGLMEKAEALVDQFVKKGRTPFGNTWYKLAGGYFKVGQASKAADLTKKALASGSNE
WTPDLTNVLMSLNYFAEQKNVEAAEEMASLLQRLITPTRDIYHGLLKTYVNAGKPVSDLLDRMKKDGMEADEETEKI
LAGEVH

>LOC_Os10g17110.1|PACid:21884413
MPPLLGVYVFTDRLAFAAADWCFRLHGWPLCRRCWASTSSPTGLPSPPPTGVSACTAGLYAAVVGRLRLHRPAASSA
ADWCFRLHGWPIMLPLLGVYVFTDRLAFAATDWCLRLHGWPIMPPLLGVYVFTDRLAFAAADWCFRLHGWPLCRRCW
ASTSSPTGLPSPPPTGVSACTAGLYAAVVGRLRLHRPAASSAADWCFRLHGWPIMLPLLGVYVFTDRLAFAATDWCL
RLHGWPIMPSLLGVYDSTTGLPPPPPIGVSACTAGHYAVVVGRLRLHHRPASTVADWCFRLHAGLLCHR

>LOC_Os10g11954.1|PACid:21884892
MAAPPLPASSFSFLGAGHPPAACAASPVRRLTSPPPSSASQVSLYAGLPSVSHGDHRRHVLAGVQWRCRHCTTSSRL
CRFAGASAFVVAASPGTGTPSSSLARPRRRPYFAGKLSCRTGLSILLAGISPVDRVVVSSGLVAARAGGEKGLPLCG
GIKTRPIPPQFKFPPVPRYSGETDPKEFLSIYESAIEAAHGDKNTKAKVIHLALDGIARSWYFNLPANSIYSWEQLH
DVFVLNFRGTNEEPKTQQHLLGIRQRQGESIREYMRRFLQARCQVQDITEASVINATSAGLLEDELTRKITNKEPQT
LEHLLRIIDGFARGEENSKRQQAIQAEYDKASIAAAQAQAQVQVVEPPPLFVRQSQPAIQGQPPRQSQAPITWRKFR
TDHASKAVMAVEEVQALRKEFDAQQASNHQQPAHKKVRKDLYYAFHGCSSHTTEQCRNIRQCGNAQDLRPQQGTIVE
APREAVQEQTPPTEQRQDVQRRVIQVITRADPPSQLSKRQKMMQIRMVHSITSAGEGAPQYLNQLISFGPEDAEGVI
FPHQDPLVISAEIAGFEVRRILVDGGSSADVIFAEAYAKMGLPTQALTPAPASLRGFSREAVQVLGQTLLLIAFGSG
ENRREEQILFDVVDIPYNYNAIFGRATLNKFEAISHHNYLKLKMPGPTGIDDADPTKLVSLGGDMGEEEVESILEVL
KKNIDIFAWSPNEVGGVPVDLIMHHLAVKPDIKPRKQKLRKMSADRQEAAKAEVQKLLRAGVIQEIDHPEWLVNPVL
LVDSTAGCELMSFLDAYSGYHQIHMNPLDIPKTSFITPFGTFCHLRMPFGLTNAGATFARLVYKVLGKQLGQNVEAY
VDDIIVKSRKAFDHAIDLQETFDSLRTAGTRLNPEKCVFGVRAGKLLGFLVSERGIEANPEKIDAIQQMKPPSSVHE
VQKLAGRIVALSRFLSKAAERGLPFFKTLRGAGKFNWIPECQAAFDVLKQYLQSPPVLISPPPGSELLLYLAASPVA
VSAALVQETEFGQKPVYFVSEALQGAKTRYIEMEKLTDALVMASRKLKHYFQAHKVIVPSQYPLGEILRGKEVTGRL
SKWATELSPFDLNFVARSAIKGAGIAAVLFLPNGVPIRYVARLQFDTTNNAAEYEAVLLGLRKAKALGVRRLLIRTD
SKLVAGHVDKSFEVKKEGMKRYLEAVRSMEKCFTGITVEHLPRDQNEEADALAKSAACGGPHSPGIFFKVLHAPSVP
MDSLEVMAIDQEKLGEDPYDWRTSFVKHLETGWLPVDEAEAKHLQLRATKYKMVSGQLYRFGAARTMASKVFRQGVY
WPTVLKVCVEQIKKCESCQRHGRSQAAPQYDLQPIAPVWPFARWRLDIIGPFPVARNGYKFAIVAVEYFSRWIEAEP
LGAITSAAVQKFVWKNIVYRFGVPKEFITDNGKQFDYDKFREMCEGLNLEIRFSSVAHPQSNGAAERTNGKILEALK
KRLEGAAKGKWPEELLSVLWALRTTPTRPTKFSPFMLFYGDEAMTPVKLGANSPRVMFSGGEEGCEVSLELLEGVRV
EALEHMHKYTTSTSATYNKKFDPRS

>LOC_Os10g09880.1|PACid:21886243
MPPLSGAYVFTDRLAFAAADWCFRLHGWPLCRRCWASTSSPTGLPSPPPTGVSACTAGLYAAVVGRLRLHRLACLRR
CRLVFPPARLAFMPPLLGVYVFTDRLAFAAADWCFRLHGWPLCRRCWASTSSPTGLPSPPPTGVSACTAGLYAAVVG
RLRLHRPACLRRRRLVFPPARLAFMPPLLGVYVFTDRLAFAAADWCFRLHGWPLCRRCWASTSSPTGRLVRRRLVFP
PARLAFMPPLLGVYVFTDRLAFAAADWCFRLHGWPLCRRCWASTSSPTGLPSPLPTGVSACTAGLYAAVVGRLRLHR
PAASFAADWCFRLHGWPLCRRCRAPTSSPTGLPSPPPTGVSACTAGLYAAVVGRLRLHRPACLRRCQLVFPPARLAF
MPPLLGVYVFTDRPPRSPPTGVSACTAGLYAAVVGRLRLHRPACLRRCRLVFPPARLAFMPPLLGVYVFTDRPPRSP
PTGVSACTAGLYAAVVGRLRLRPRQTRPAGRLVFPPARLAFMPPLLGVYVFTDRLAFAAADWCFRLHGWPIMTPTDA
IFFTAGYITVTANRHHHLQRKLSTSTAD

>LOC_Os08g14370.1|PACid:21886677
MPPLLGIRRPAFVAADWCLRLHGWPIMPLLLGIYDFTADWYLRLHGWPIMPPLLGIHIFTDRPAFAAANWCLRLHGW
PIMPPLLGIHVFTDRLAFVAADWCLRLHGWPIMPPLLGIYDFTAGLPSPPPTGVSAYTAGLLCRRCSASTSSPTGLP

SSPPTGVSACTAGLLYAAVVGRSSTGVLAYTVGWSHHRTWASTSSPTGWPSPPPTSVSACTAGLLCRRCWASTTSPP
VCLCRH

>LOC_Os08g03160.1|PACid:21887042
MAIFSLITCAADLVIFFSVCYMQPPAPVAALSFNYPTFASSHNQYIEIEGNASVSVGYIDISANSVGNNVGRVFYKP
PVQLWDAATGEVASFTTRFSFNIIAPSDRSKKGDGMAFFLTSYPSRLPVGHEGGENLGLTNQTVGNVSTGQNRFVAV
EFDTFVNPFDPNTTNDHIGIDVNSVVSVTNESLPNFSLIGNMTATVDYNNNSRILSVKLWINGSTTPYTLSSMVDLK
RALPENITVGFSASIGSAYEQHQLTSWYFKSSSSFEQKLAAKVASPPPPSSPSPPPPSLTPITSHSRRGGVVAGATV
GAVMFVILLFAMVAVLVRRRQSKKRREAEDGGWHGSDDDDDGEPIVEIEMGMGPRRFPYHELVDATKSFATEEKLGQ
GGFGAVYRGYLRELGLAVAIKRFAKDSSKQGRKEYKSEIKVISRLRHRNLVQLIGWCHGRTELLLVYELVPNRSLDV
HLHGNGTFLTWPMRINIVHGLGSALLYLHEEWDQCVVHRDIKPSNVMLDESFNAKLGDFGLARLIDHAVGVQTMTHP
SGTPGYLDPECVITGKASAESDVYSFGVVLLEVACGRRPMSLLDNQNNSLFRLVEWVWDLYGQGVVLKAADERLNND
YDATSMECVMAVGLWCVHPDRYARPSIRAAMTVLQSNGPLPVLPSKMPVPTYAPPMASSEGQLSSSTGMSSSSLTLT
SITPR

>LOC_Os08g36170.1|PACid:21887346
MSASSPPPPPAPADAEAVPLFYCYECESTVSLPPPPPPPSRPLFCPRCRGEFLEEENPNPPPEPEEEEEVSSPPPPP
PPPPGFLSDSSSDDEEGGDLDLGMGGMDAAAARAYLSRLVHHHLYDDEPIDVAAAAVSLLQRSGLHQGGGGGESAPA
AAASIAALPTVEVSEPATACAICKDDLPLAAPARRLPCGHLYHSECIVQWLEMRNSCPVCRSRLPSDEPQDAAAPSD
PGPAPMRITIRLSTNRRRVRTSSDAAPPVAASPTQLAQAVTGEGGGGPANSTETVSSEWPPPSESDAVMSDAREGQR
FFD

>LOC_Os08g21710.1|PACid:21887512
MPPLLGVYVFTDRLAFAAADWCFRLHGWPLCRRCWASTSSPTGLPSPPPTGVSACTAGLYAAVVGRLRLHRPAASSA
ADWCFRLHGWPIMPPLLGVYVFTDRPPRPPPTGASACTAGLLCRRCWASTSSPTGLPSPPPTGVFACTAGLYAAVVG
RLRLHRPACLRRRRLVFPPARLAFMSPLLGVYVFTDRPPRSPPTGASACTAGLYAAVVGRLRLHRPACLRRRRLVFP
PARLAFMSPLLGIYVFTDRPPRSPPTGASVCTAGLYAAVVGRLRLHRPACLRRGRLVFPPARLTFMPPLLGVYVFTD
RLAFAAADWCFRLHGWPIMPPLLGVYVFTDRPPRPPPTGASACTAGLLCRRCWASMSSPTGLPSPPPTGVFACTAGL
YAAVVGRLRLHRPACLRRRRLVLPPARLAYYAAVVGRLRLHRPAASSAADWCFRLHGWPIMLPLLGVHVFTDRPPRP
PPTGASACTAGLYAAVVGRLRLHRPAASTAADWCFRLHGWPLCRRCWASTSSSTGRLVRRRLVSPPTRLAYYAAVVG
HLHLHRRSSRLLLTSALTSTSGYTATTTDGHHRLPSPTGYAAADWCFYLHNCASSLPVCFCCRRLVFTSSRQYNFVI
MVERLHLYYLRYQQTLLLLLHKFYYFTNHDVFVNLDISACNAMLFYYNKWLSNIQDFYLDIFNTYLQSSNDYSTTRS
YNSRLYVQLFPN

>LOC_Os08g12300.1|PACid:21887893
MAQGRCPAARGSVLPTCPGGSAVGGIGSVLLAMARMGWKLCHVFRLYTVVMAGSISRRGLDLTGFVLELVDYQGRPL
PEQEGDDAEPHAAWEVTAVILTSSPERTSLAVTAGGDSFSAACQNAALLAIGTLHQRYPDELQHSPYRYHPRHGGAR
DFATFRDASSEDDATIMHLARMVEAYDATCIDFHQMVRRGMVENNMKILEPRQENLQLKKDLDAVEAQLHQLKIAQG
EVCRPKRRRVCRSQKITARKSTSRPELVRQSLAWTCFVQTPRAEPAPVVPQEGEASGVGSTEDALLLTFRPGPSQRR
SANTGDGNQPEGSNHNNHGSPPPPPPPPPPPPPDTNAILTQILAQQAYMMTAFLHHLQNPPQQNAPPPPPQHSKLAE
FLRIRPPTFSSSNNPVDALDWLHAVGKKLDTWYTSGQSKTIRIAVPAVMGWSNNVFPPGLEWYIPGLEIDLYSRMVT
TRRSAATGEGESHPEGSNHNNHDSPPPPPPPPPPPPPDTNSILTQILAQQANMMAAFIHHIQIPPQHNAPPPPPPQH
SKLAEFLRIRPPTFSSSNNPVDALDWLHAVGKKLDTVQCNDEEKVIFAAHQLQGPASLWWDHFQATQLEGQPITWAR
FTAAFRRTHVPAGVMALKKRKFRELKQGNRSVMEYLHEFNNLARYAPEDVHEDEEKQEKFLAGMDPELSVRLVSGDY
PDFQRLVDKSIRLEAKHKELESHKRRLANFRNQQGANQRVRYTNPYPGGSSSQQQQQQPRSAPRPQFVVRVSQPQQQ
QSQQGTRAPRPPAPAVQPGQGRRDAQGQQRLCFNCFEPGHFADKCPKPRRQQGQAPPRPNNGGKDVIPGRVNHVTAE
DVLTTPDVIVSTFLIHSIPATILFDSGASHSFISVPFVGRNQLGVERLRNPLLITTPGGVMTAKYYSPAVPIEIQGI
PFPSDLILLLDTKNLDVILGMNWLAQFQGVVDCVRRTVTLYRGPEQPVVFFAPSTSVGSSELHQIGLSETPIVREFGD
VFPEELPGMPPKREIEFRIDLAPGTTPLYKRPYRMATNELAEVKKQLEELKEKGSLNEVTIKNKYPLPRIDDLFDQL
RGATVFSKIDLRSGYHQLRIREEDIPKTAFTTRYGLYEFTVMSFGLTNAPAFFMNLMNKVFMEYLDKFVVVFIDDIL
VYSQSEEDHQQHLCLVLGKLREHQLYAKLIKCEFWLSEVKFLGHMISAKGVVVDPEIVTAFTDWKQPKTVTQVRNFL
GLAGYYRRFIENFSKIARPMTQLLKKEEKFVWSLQCEKAFQTLKEKLVSSPVLILPDTRKDFLVYCDASRQGLGCVL
MQEGHVVAYASRQLRPYEGNYPTHDLELATVVHALKIWRHYLIGNHYEIYTDHKSLKYIFTQSDLNLRQRRWLELIK
HYDVAIHYHPGKANVVTDALSRKSRCNTLGVRGIPPELNQQMEALNLSIVSHGFLAILEAKPTLLDQIRDAQKNDPD
MHGILKNMKQGKAAGFTEDEHGTLWNGNREAHESPYSIHPGSTKMYLDLKEKYWWVSMKREIAEFVALCDVCQRVKA
EHQRPAGLLQPLQVPEWKWDEIRMDFITGLPKTQGGYDSIWVVMDRLTKVARFIPVKTTYEGNKLAELYFARIVSLH

GVPKKIVSDRGSQFTSHFWTKLQEELGTRLNFSTAYHPQTDGQTERLNQILEDMLRACVLDLGKTWDKSLPIAEFSY
NNSYQASIQMAPYEALYGRKCRTPLLWDQVRESQVFGTDILREAEAKVRTIRDNLKVA

>LOC_Os08g07900.1|PACid:21888309
MARPGDLANRLDSIQRCIPWSTAVREEEGLLSRSAAVVRITGILDEGCLVQDLEEPKTETKTACLCAWIWSEDPDAI
PKAVHFQLEEPLLQNSPDSFWLEDLGESGLERRRTVSSKLLTYQVLIHLDQVLDSSPPPMEPVWGYSPSSQGSGMIS
SASVTEEVPKRHDIIWSLGVKDGQRAPREHVAARLGPGRNRSHSCSRSPPAHEHRRPSGWDVPPARTTAPTRRSSSR
PPDHRGGSYGGYQRAATSPVRRERTRSPRNRQSPPPAPRPLVPQTASPPPPPSEGLTEFRPQESQIVPFPLTTPFAT
VLQVDQLANSIAGFDAPMMAIPLSVDPMLEEVGLGNIAPPPEQSLEQPQAQPPEQVMIGRLFAPLYPASPILSIMML
LMKKLKVSSTASFVLIDPALHYMFSCPGSAVQETKLDFIDQANFNRLLGPTFSENFCFPSTSGTRGGVLLAANDNKY
ELKDAHTGDFNLIVHDRDKNNANLNRGMMQAFRAALNHLEVLEIKLNGQRYTWSSETESPTLTKIDRVFCSQGWEER
FPNCLLAAISTLVSDHCPLLLTANPSRPKSNIFRFEKYWLRLPDFLDTVQQAWNKTVHTTDAIKRLHTKLSRTAKAL
SVWNKSRVRGIKLRTDIASELIFRLDLAQEERSESRSRSTAINYSFLQEQSQDLTDLEDPFTEDEIKQVIFQARSDN
APGPDGFTGAFFNKCWEIIQEDVVLAINQVANHDTQSLWLLNSAYIALLPKRTEALQTQIPGVWRQVDWMDHFYPIY
ILIKDPPKRESRSSYKKKQMTRLHGGGLQMEPTRQTPHIGYNSSEASRITGRSSLPTSSISVVGTTVTSALFEVWNR
RQPLTSSWTAPLQSKSGDSSGRRREMAKEQRRNFDGLFIYTTWGIWLQRNARIFNGAYSTVMQVVESIIAMCKAYDG
AHNIDE

>LOC_Os08g15630.1|PACid:21888528
MDPLSPALARPTWSSSSGKPPLQEDNCLERSSSQASTPPSWRYRRRRLDHVARVPAMGHLVASPSPPSSHHDSLAAA
AHHPCRACTTAPSAPTFHGPCQSSTATSLHRRPPEPPGTPLITTVPAYYHHPLLPVGSSPSPPPSPLASHRRRRGCA
PTVDVVVIAPFYLQTSPPPPPRCRVSLPPARGLAPRATLGEAALTPRTSAASTPPRPDTVVSRGSGRKAAGFGPSHH
LHVHTATEAHVATSTQPRHRHHRIWTGGCRIWPAAPPRRRGVSRTVGVVGVNYAT

>LOC_Os08g06150.1|PACid:21888899
MGVHVFTDRLAFAAADWCFRLHGWPIMPPLLGVYDFTTGLPPSPPTGVIAYTVGRSHHRSWASMSSPTGWPSPPPTG
VSACTAGLLCRRCWASTTSPPACLRRRRLVSPPIRSVGHTTVDGHRHLHCRPASVAADWCHRLYGWSVTPPFMGVHV
FTDRLAFATADWCFRLHGWPIMPPLLGVYDFTTGLPPSPPTGVIAYTVGRSHHRSWASMSSPTGWPSPPPTGVSACT
AGLLCRRCWASTTSPPACLRRRRLVSSPIRLVGHTTVDGHRHLHRRPASVAADWCPRLYGRLVTPPFMGVHVFTDRL
AFADWCFRLHGWPIMPPLLDVYIFTVGSPSLPSTGVSAYTAGLLCRRWWASSLSPSTAFVAADWCLHCY

>LOC_Os08g43334.1|PACid:21889175
MKGSRLAVKESCLPMTMPMPETFAQYSNPLRSTRAYGQLCRVPGRRRHACVDGWGQDRARTRGDDGQRRYAQCRGGW
GDIWRWGGYITCGSPRETGMVNGAPSPPPPSPMVMSFGPLDSPWVKQPDTTVYPGQICAAAGGGGMADQTAAAVVV
GGGAAATMGEPSPPPPAPAAEAAGVGVGQQQRTVPTPFLTKTYQLVDDPAVDDVISWNDDGSTFVVWRPAEFARDLL
PKYFKHNNFSSFVRQLNTYGFRKIVPDRWEFANDCFRRGERRLLCEIHRRKVTPPAPAATTAAVAAAIPMALPVTTT
RDGSPVLSGEEQVISSSSSPEPPLVLPQAPSGSGSGGVASGDVGDENERLRRENAQLARELSQMRKLCNNILLLMSK
YASTQQLDAANASSAAGNNNNNNCSGESAEAATPLPLPAVLDLMPSCPGAASAAAPVSDNEEGMMSAKLFGVSIGRK
RMRHDGGGDDDHAATVKAEPMDGRPHGKDEQSAETQAWPIYRPRPVYQPIRACNGYEYDRAGSDQDECGAKPSNQDK
KDLLRRWVVLISESLQLHGRQVVGAVQEWRRDRRGHRRPPAGHVQLRTCVNNLLVTGAGTLDAGREYETNSLPRPRR

>LOC_Os08g03150.1|PACid:21889591
MAGFSLITCAAGLVTFFSVCYMQPPAPVAALSFNYPTFASSDNQNIDIQGQASVSVGYVDISANSVSGMGNSAGRVV
YAPPVQLWDAATGEVASFTTRFSFNIIAPSDRSKKGDGMAFFLTSYPSRLPVGHEGGENLGLTNQTVGNVSTGQNRF
VAVEFDTFVNPFDPNTTNDHIGIDVNSVVSVTNESLPNFSLIGNMTATVDYNNNSRILSIKLWINETTTPYTLSSMV
DLKRALPENVTVGFSASTGSAFEQHQLTSWYFKSSSSFEQKLAAKVASPPPPSSPSPPPPSLTPITSHSRRGGVVAG
ATLGAVMFVILLFAMVAVLVRRRQSKKRREAEDGGWHGSDDDDDGEPIVEIEMGMGPRRFPYHELVDATKSFAPEEK
LGQGGFGAVYRGYLRELGLAVAIKRFAKNSSKQGRKEYKSEIKVISRLRHRNLVQLIGWCHGRTELLLVYELFPNRS
LDVHLHGNGTFLTWPMRINIVHGLGSALLYLHEEWDQCVVHRDIKPSNVMLDESFNAKLGDFGLARLIDHAVGIQTM
THPSGTPGYLDPECVITGKASAESDVYSFGIVLLEVACGRRPISLQDTQNNCLFRLVEWVWDLYGQGAVLNAADERL
NNEYDTTSMECVMAVGLCRYPSPYRGPGNVGVNNRSQFAEYFGYTAAMLSFRQATSVTKIGELELLVQFRTGHLLQS
SLPRGRTKS

>LOC_Os08g07970.3|PACid:21889808
MTSAAPAAAQFAAPAPAAMGIYDRRHHPLAAGVWGDHPFIRPDTTASTSNAAAAAMVVAPPPLTEPKFESQLALPLQ
HGDDQDNAAALQESPRHASDSFEQEASKPRDKIQRRLAQNREAARKSRLRKKAYIQNLETSRMKLAHLEQEITRARQ
QSAYINRSSNPATLPAPIDSGVVTFEVEYAQWVEEQGRQTAELRASLQAAAEGPELRAVVEAALAHYDRLFAAKREA

ARRDVFFVMSGVWRTGAERFFLWIAGFRPSEVIRVLAPQLEPMTERQAADVQGLQQKARHLEDALSQGMDKLKQTLA
DSLLAEAVVVSTSCDASPPPPPPEEEEPSSSAAGDGGCYMAQMGSAMGRLSNLVAFVDHVRHRRSPPPTSHLHVRRR
AELG

>LOC_Os08g37270.1|PACid:21889829
MDPAAARRKAERWIGVAEKLLMARDLEGCKQFVSQARADDPTVPGADDLAAAADILLSAQRRRLATGAPNPYAVLGL
DCADPASRDPDVVHSAYRRLSLLLNRSHPDRPCLHAFADAARLVAEAWAFLFDPVRKASLDSSLDAAAAAAPRPPP
APSPQKQQPQPQPRSPRPASPPPVPAAPEVASAVSTPPARPKRGRPPRAAKPQPTPERQQEAEVEAAATFWTACPSC
CNLHEYTRSYEARTLLCPSCRKPFFAAAMATPPPIVPGTDMYYCSWGFFPMGFPGGPAFARPTSSSSSSSSSSSSS
SSPTKQAPAALGFYPMGPYSLPLPAQGDAAEGNAAVGSGDGTVTAPSPPPPPPAAAPLPVKPKLVKLGARKRGRPKS
SKNKHVVIEIN

>LOC_Os08g29650.1|PACid:21890357
MSQSKEVRYTARSITPPADRNGTSKSKSPTPVRRSTSRSPPPKKSDSRSPPPRRRSTSRSPRPRRHGRSRSRSRDRS
RSRSRDDDLRNPGNNLYVTGLSTRVTEEDLEKFFSKEGKVQSCHVVLDPRTKESRGFAFVTMDSVDDARRCIKYLHR
TVLEGRLVTVEKAKRTRERTPTPGKYCGRRGSQRSSRSPSPYRSRRRERSRSRDRQRDRSRSRDRRRDRSRSRDRRR
DRSRSRDRRRDRSRSRDRRGSSPHDRDSHRRRGDRSRSPATNGNHKKD

>LOC_Os08g05260.1|PACid:21890509
MPPLLGVYVFTDRLAFAAADWCFRLHGWPIMPPLLGVYVFTDRLAFAATDWCFRLHGWPLCRRCWASTSSPTGLPSP
PPTGVSACTAGLLCRRCWASTSSPTGLPSPPPTGVSACTVGLYAAVVGRLRLHRPACLRRHRLVFPPARLAFMPPLL
GVYVFTDRPPRPPPTGASACTADVLCRRCWASTSSPTGRLVRRRLVLPPARLTYYAAVVGRLRLHRPAASTAADWCF
RLHGWPIMPPLLGVYVFTDRLAFAAADWCFRLHGWPLCRRCWASTSSPTGRLVRRRLVLPPARLTYYAAVVGRLRLH
RPAASTAADWCFRLHSLPIMPPLLGVYVFTDRPPRPPPTGASACTADLLCRRCWASTSSPTGRLDRRRLVFPPARLA
YYAAVVGRLRLHRPACLRRRRLVFPPARLAFMPPLLGVYVFTDRPPRPPPTGASACTADLLCRRCWASTSSPTGRLD
RRRLVFPPARWPIMPPLMGVCVFTDRLAFAAADWCLRLHGWPIMLSLLGVYDSTTVLPPSPPTGVSVCTAGLLCRRC
WASTSSSTGRLVRRRLVSPPTRLAYYAAVVGHLYLHRRSSRLLLTSALTSTSGYTATTTDGHHRLPSPTGYAAADWC
FYLHNCASSPPDFYLDIFNTYLQSSNDYSTTRSYSSRLYVQLFPN

>LOC_Os08g19374.1|PACid:21891121
MKRTMDARWAHIVYALLVPEAFFRDPDGRDGVASRSLRRPLAISAATLLSLPGEGRRYEAHDERAVGAHRVRAAGAR
GVLPRPRRPRRRRLLELSRSLRCPLALSASVTATSSSPLSPPPSIPPALPISPPLLSPLVSPSPPPPLASCR

>LOC_Os04g20900.1|PACid:21891721
MSSPTGWPSSAADWCFRLHGWPIMLPLLGVYVFTDRLAFAAADWIQIQICIKGIHGFKLISYDPGMDQSEHHHRLAS
TAADWCFRLHGWPIMPPLLGVYVFTDRLAFAAADWCPRLHGWPIMPPLLGAYVFTDRLAFVAADRCPRLHGWPLCRR
CWASTSSPTGLPSPPPTGVSACTAGLLCRRCWASTSSPTGLPSSPPTGVPACTAGLYAAVVGRLRLHRPACLRRRRL
VFPLARWPIMPPLLGVYVFTDRLAFAAADWCFRLHGWPIMPPLLGVYVFTDRLAFAAADWCFRLHGWPIMPPLLGVY
VFTDRLAFAAADWCFRLHGWPIMPPLLGVYVFTDRLAFAAADWCFRLHGWPLCRRCWASTSSPTGLPSPPPTGVSAC
TAGLYAAVVGRLRLHRPASLRRYRLVLLPARQTYYAAVVGRLRLHRPAAASAADWCHRLHGWPVITPTDATFFTAGY
IAVTTNRHQYRQHKLPTFAMAADWFLHFHGCMILPVLIGLIFFTAGRLVCC

>LOC_Os04g13400.1|PACid:21892249
MALGLSLTLQEWISQNITDWLAFAAADWCFRLHGWPLCRHCWASTSSPTGLPSPPPTGVSACTAGLYAAAVGRLRLH
RPACLRRRRLVFPPARLAFMPPLLGVYVFTDRPPRPPPTGASACTAGLLCRLCWASTSSPTGLPSPPPTGGSACTAG
LYAAVVGRLRLHRPAASSAADWCFRLHGWPIMPPLLGVYVFTDRLAFAAADWCFRLHGWPLCRRCWASTSSPTGRLV
RRRLVLPPARLAFMPPLLGVYVFTDRPPRPPPTGASACTAGLLCRRCWASTSSPTGLPSPPPTGVSACTAGLYAAVV
GRLRLHRPAASSAADWCFRLHGWPLCRRCWASTSSPTGRLVRRRLVLPPARLAFMPPLLGVYVFTDWLAFAAADWCF
RLHGWPLCRHCWASTSSPTGLPLPPPTGVSACTAGLYAAVVGRLRLHRPACLRRRRLVFPPARLAFMPPLLGVYVFT
DRPPRPPPTGASACTAGLLCRRCWASTSSPTGRLVRRRLVLPPARLAYYAAVVGRLRLHRPACLRRRRLVFPPARLA
FMPPLLGVYVFTDRLAFAAADWCFRLHGWPLCRRSWASTSSPTGLPSSPPTDVLACTAGLLCRRCWAPTSSPTGLPL
SPTTDVLACTAGLYAAVVGRLRLHRPACLRRRRLVFPPARLAFMPPLLGVYVFTDRPPRPPPTGASACTAGLYTAVV
GRLRLHRPAASSAADWCHRLHGWPVITPTDAIFFTAGYIAVTTNRHQYLQHKLPTFAMAADWFLHFHGCMILLVLIG
LTIFIAGRLVCC

>LOC_Os04g26540.1|PACid:21892576
MGVYDFTAGLPPSPPTGVLAYTVGWSHHRSWASTTSPPACLRRRRLVFLPTRLAYYATAVGRLRLHRRPASVAADWC

PRLYGRLVTPPFMGVYDFTAGLPPSPPTGASAYTGGLLCHRCWASTTSPAACLRRRRLVSRLYGWLVTPPFMGVYDF
TAGLPPSPADWCSRLYGWLVTPPFMGVYDFTAGLPPSPPTGVSACTVGLLCRRCWASTSSPTGLPSPPPTGVSACTA
GLYAAVVGRLRLHRLACLRRCRLVFPPARVAFMPPLLGVYVFTDRLAFAAADWCFRLHGWPLCRRCWASTSSPTGLP
SPPPTGVSACTAGLYAAVVGRLRLHRPACLRRRRLVFPPARLAFMPPLLGVYVVTDRLAFAAADWCFRLHGWPIMLP
LLGVYVFTGRPPHPPLTDVTASTAGLL

>LOC_Os04g58780.1|PACid:21894250
MQFLSLSAAAASSPAAAVLPPKPFKPLSSAAPFRRPSPPPPPPSPVPSPAPPPPPHRPSPSPPPNPLSSKLWLSSKL
SPPPPETLEQPEPSTTTTTTTPPPPEPEAEAEARRQEEFRHKGKVFVGNLPLWARKAEIAEFFRQFGPLEKVELVRG
HDDPERNVGFCFLYYGGDDADAAAERAVEVDGVDFRGRSLTVRLDDGRKGRARAEQRARWVNDGRRREPRSPWHEGR
DEACREFRRVVESRPDNWQAVVSAFERIPKPSRREFGLMIVYYAKRGDKHHARATFENMRARGIEPNAFVFTSLVHA
YAVARDMRGALSCVEEMKSEGLELTIVTYSILISGFAKINDSQSADNLFKEAKTKLSSLNGIIYSNIIHAHCQSGNM
ERAEELVREMEEDGIDAPIDVYHSMMHGYTIIQNENKCLVVFERLKECGFKPSIISYGCLLNLYVKIGKVAKALSIS
KEMESCGIKHNNKTYSMLINGFIHLHDFANAFAIFEEMLRSGLQPDRAIYNLLIEAFCKMGNMDRAICILEKMQKER
MQPSNRAFRPIIEGYAVAGDMKSALDTLDLMRRSGCVPTVMTYNALIHGLVRKHKVQRAVSVLDKMSIAGITPNEHT
YTIIMRGYAASGDIGKAFEYFTKIKESGLKLDVYIYETLLRACCKSGRMQSALAVTREMSFQKIPRNTFIYNILIDG
WARRGDVWEAEDLMKQMKEDGVPPNIHTYTSYINACCKAGDMQRAEKVIEEMVDVGLKPNVKTYTTLIKGWARVSLP
DRALKCFEEMKLAGLKPDEASYHCLVTSLLSRATVMEGSTYTGIISVCREMSENDLTVDLRTAVHWSRWLHKIERTG
GALTEALQRIFPPDWNSLEFLGEPSSSISMGESDDYSDSDFSGDEDEDHNIDDS

>LOC_Os04g20424.1|PACid:21894701
MNQIPLLPPAPTRASSPRNSRPPRSPQISRLSRKKKTRHSRSSRRQVATAAEAAPPILAPPVVRSPPGRTSTLPRFA
PRLLRRTLPSSPAPSPSPPPSPPSPPPPPPAPLPFRPPPLALVAGEAAQIPSPFAGEVFDGLLLLLPSARPPRVTRA
HARPATSRLGGRDAVPGLRPSRLCPPSQFESNRRARLID

>LOC_Os04g32090.1|PACid:21894737
MRRRNLIITSDSDSDDGGGGGGAATASTPASASASASFPSVSGGGCGDGWPSPQNPRSVPVQFPSPSSPPPSPPIEI
SDEEEAEAEVVVEEEEVVVVEDEEEEYEEVEEIEDPDGDSPFVDAPEHISPPPPPPPPARTPMPTPTPTPTPTPTRP
PVPVWAAPLPARTPTPTPSAPPRAAAPSPAGTPSPSPIPPSSTPVSALSGPLRQVDEFLRGLGLRLRPEWLESCAAG
VPGFYGLGGVEAMARRCFEQFLFADMNACGAGVLPEGVGSMHNAVLDGPLVLQVDEIVNLSAPLRERYRDAHAGPKR
CLKLSMTDGIQRIYGMEYRPIKDLEVLAPAGFKIVIRNVHIRRGLFMLVPEVIEILGGVDDELDEARNRLVSEVNKP
PRGKRKQGGLPLSSRATLAAWPTNANATNDAEQGASVPRTVNTPHPTRLGNASHASQVGRTTQPMVDNLIPHVVVSN
AQEQSRHIQEITMQGQPTSLNRHNKEASASYRYNAQCSISGTTRAMADEHVLVSNAQEQSPHIQEITMQDQSTSLNG
RNKEASASTSYRYNAQCSISGTTRAMADERVDPSFVGNNVHEQMQRVQGITMQDHISASSESKRELSVTTPSGYDSR
LAPHGVGNTGTRSGEATRSSNVDDGINNIGHPISLCGENEKPFTYIFNMLADWGVQQDTVPYIQGKIKGLITSVKRF
QYKQSMQYDLYVYIDDGSFITEAFVDRDIVQNMIGLSAEELAALSSGGPAQANIRKTMKAFEHFLVNFEGTILIEL
NRDSSVPIVREMNKGCSSSDAWQLLRRVKTFSGQGYMRSLDFMDTTP

>LOC_Os04g23220.1|PACid:21894957
MRAGAYTIHQSLTAEAAAVLKLALGIARRRGHAQVTPLHVAFALLSPACSPPQQQPAPPPYGLLKRACLRSHPSAAA
AVAAHPLQCRALELCFNVALNRLPTSAPHSPPPSSSAPSGAVAPPFASSLIQPNPTLSNALVAALKRAQANQRRGCV
ELQQQPPPPPPPPPPPPVAATAQQQQPLLAIKVELDQLIISILDDPSVSRVMREAGFSSSTVKSNLEGESALMMSTSS
SPPPPAIPPHFFLDPSIGVGGNGGGGGGGFMLWPAPFLSSPGMAVPSCKEDVRAVLEVMVRKQGRRTNPVVVGDSVS
MAEAVAGELLRRLEGGDVPDELAGAHLLKLQLSYVHVRLMSRADVDAKAAELRRSVDAVKRGGLVVYVGDLRWALDE
DHHHHHHPGADHHNTASSYSPVEHMVAELGRLLGDLRASAPPRGRVWLVATASYQTYMRCRRRRPSLESAWALQAVV
VPTGAGTGLALNNLHAVATTTSNGEPVQQAMVATNHQQQQQQLASPFVAMAAEPAARNELDDKLLVLCTECSHNYER
EASAVKAEAAADEEGPRAAGNLPGWLVPEPPKENYLIELKRKWSRLCRKLHLCGGGDPCSGQSFGAGAYGNGPSSLL
PWWSASCLLPNGGGKPSIAGFLGMEALRWSPPAAAALPSLSSLREPECQDVTTALALGSLPLSDSASSSGGGGGDGA
AARELERRLRKNVPWQRAAVAEIADAVAAGARSGNGTKGAGVWLLLKGSDHAAVRRVAAVIAETHCGSADRVVVVSA
DPNKFGCADDFRSDVVARASMAAAAGGNKLVLVVDDVERAPQHVVECLVAASRSGALKDKFGGQELDLSGSVVVMTT
SKLADAAVSGVISLRLYTSEQSPPSGDLKRKTPTSSPPTSDRKRARARRSAGNGHSLDLNLNLFAHDDDDNDAGDVD
DDDDGVPSDITHEGGGDDSGEHGHSHHRLLLESIATRVVTLDGDHHGAAAAVRERLSGRLDGGGRELRVDGEAAAAL
AAASGHFVDEVMERWVAEVFEPAAATVKNGGKAVVLGVGPSGGGAHESVGFMGSVLPSRVHVD

>LOC_Os04g45940.1|PACid:21895356
MASSSPTNAAAAARRVPPPCWTPDETLALARAYTARRLAVGRAHLTSADWAAVADAATPTKTARQCRHKVEKLRRRL

RSKRRRPCPLLDAIDLLDGPSPSASPSQSPSPPPPASPPPLPPAPSSPPPKKRRLPDGDADADAEDDGESDVVKALR
AIGEGFLRAEQRRMEAARKTQQMRMEMALRHLDSQRRLMEALVDRIIDSLE

>LOC_Os04g48830.1|PACid:21896027
MSPSAAAKMRLGGGGGGGGFMLGCGCRDAKAVAVAVSATSPCSAATETSTATTATWRRARTHPSASASASTGTLTVP
SASSSFLWDDADAEADGEEVDFKRESSATTPSFSGLLRQLNELEQSVMTWGWKSPRRGNHFSPPPPPPPPPPLPLRP
VVLHRAVDAGGKRSNKEDDAKFSSPPPSSHCPTTQLHRKVKSVDQRNREDDEAHFAPPAPPPLPLPPQQLRNVKGVD
KGGSKEDSKHCPPPPQAPKHRKTKSCDNNDGFTAGKLDGSLAVVKQSEDPRGDFRRSMLNMIVENRIVTGDELRELL
HRFLELNAPHHHDAILRAFAEIWDEVFAGPDEPRHGPPRPPPRQRTPPRRRHPLPAWRL

>LOC_Os04g58890.1|PACid:21896714
MGELASSPPPAAVLPVVFVDGDQTVDLGTVTVQPSLGVRKLQAVVADRVGLAPQQILASLARPRRARRVPLEEGTDL
AAAVAREGSGCYVLAALRRSRRERRGGRSRREKKAAAAAAAAGAAPAAPTQERTILKRLPAMDLASLASTPSPAAAA
MFGVWDYEAQLQELQRQREWYLMHTAAADPYFPLAPEREDPPFWSAQPLLRQGTPSSCPECEAAAAMMREPGFHWCV
RDAVTVGFRSLVGPIERPAKKSPSPSPSPPPLPPTPGRLPPSFVGMMPVY

>LOC_Os04g59140.1|PACid:21897157
MPATDSSSAAAPLTSFGRSFLSHRRDQIPPPPPDHHSHSHTQHPSSSDLEIDAFHRHAADLLHDLLSDSNSDPSAPD
LLSLAWTRRLLDSFLICLEEFRAILFALADSQPLSRPPLDRLLLDFLDRAVKALDLCNALRDGLDLIRQWRKHLAIA
AAALATAPAAQRGEAQIRRARKALTDLTILMLDDKDAGGVVGQRNRSFGSASTTRTPFPTATATTAGAAAAGAPGPA
LAPISGHSPGASPAHGPPRASSRPSGEASRCPARTTSPPPEASPPPSTPWAPCCSSSPGRSWQQSHARTAASRRTSQ
RCPGPSPGLAPSSHSSTASSTSPRRRTASTHAACSRRSTRLSAARGSSWRSLMLRSSPWPTTRIPRCRRPRRSWCKF
AGHSRMGWTLLSARCGRCSTGLCAPGQKSSTTSVGRTTPGEDSLLDPTLCEMQPLHRGSFSVKVSIYIYMSLHEEGL
ALVMIHLSYFFHFFSLLL

>LOC_Os04g19710.1|PACid:21897203
MDQSEHHHRLASTAADWCFRLHGWPIMLPLLGVYVFTDRLAFAAADWCFRLHGWPLCRRCWASTSSPTGLPSPLPTG
VSACTAGLYAAVVGRLRLHRPAASSAADWCPRLHGWPIMPPLLGAYVFTDWLAFVAADWCPRLYGWPIMAPLLGAYV
FTDRLAFVAADWCPRLHGWPIMPPLLGAYVFTDRLAFVAADWCPRLHGWPIMPPLLGAYVFTDRLAFAAADWCPRLH
GWPIMPPLLGAYVFTDRLAFAAADWCPRLHGWPIMPPLLGAYVFTDRLAFAAADWCPRLHGWPIMPPLLGAYVFTDR
LAFVAADWCPRLHGWPIMPPLLGAYVFTDRLAFAAADWCPRLHGWPIMPPLLGAYVFTDRLAFAAADWCPRLHGWPI
MPPLLGAYVFTDRLAFVAADWCPRLHGWPIMPPLLGAYVFTDRLAFVAADWCPRLHGWPIMPPLLGAYVFTDRLAFA
AADWCFRLHGWPLCRRCWAPTSSPTGSPSPPPTGVLACTAGLLCRRCWAPTSSPTGLPSPPPTGVLACTAGLLCRRC
WAPTSSPTGSPSSPPTGVLACTAGLLCRRCWAPTSSPTGSPSPPPTGVLACTAGLLCRRCWAPTSSPTGLPSPPPTG
VLACTAGLLCRRCCAPTSSPTGLPSLPPTGVLACTAGLLCRRCWAPTSSPTGLPSPPPTGVSACTAGLYAAVVGRLR
LHRPACLRRYRLVLPPARQTYYAAVVGRLRLHRPAAASAADWCHRLHGWPVITPTDATFFTAGYIAVTTNRHQYRQH
KLPTFAMAADWFLHFHGCMILPVLIGLIFFTAGRLVCC

>LOC_Os04g42540.1|PACid:21897381
MNSAAETETEGGGGAAAVEPQVVVVERVVTVEYLEPSMSRGLLGMFPDSSAFDFDYSQSGIWSPLNKVPRASSSPPP
PPRSGGGGAEGSRDFLIANPKRRARAAIGGRSSRSRRRLRLRREDGSFLNLHETGCARLDFSPPPPSSPLPAKEEG
WRRVLKAAIRKFKSRQRRSRPAPLLQMMLPTL

>LOC_Os04g42100.1|PACid:21897389
MDQMRRQRRRECIGVLCSALFFSVLAVAVAAGSGSSSQPPSSSPAPAASSATPAARAPSQRTPPTPATPAKAASPPA
TNSSSSPRTPAAPAPRPPQPPPATSPAPTKPSSPPAPKPPSPPAPSPSTTPSSPPAPKPSSPPPAATPTTKPSPPPS
SPPAPRPSPPLPPRTPPPPPPAAAPKPSPSPPPAPTNSTTNSSSPSTSTPVQLSPNFYAQSCPSVELAVRDVVRSAS
TLDSTIPGKLLRMLFHDCFVEGCDASVMIEGSGTERTDPANLSLGGFNVIDAAKRLLEAVCPVTVSCSDILVLAARD
AVTFTGGPLVPVSLGRLDGLVSLASNVRANIIDTGFSVDAMARSFSAKGLTLDDLVTLSGGHTIGSAHCTTFGERFR
VDANGSTVPADAAMNADYAGGLIRACSAVNNTVSSTAAVDCDEGSASRFDNAYFANLLAGRGLLRTDAVLVQNATTR
ATVEAFARSEGSFFASWAASFARLTSLGVRTGADGEVRRTCSRVNG

>LOC_Os07g01690.1|PACid:21897760
MARSWRRGGGGGRRGGGSKWRSSRASAAATSPPPDPAGGEATAAVEPSPPPDPAAATISFACGNDGGGLPTGDVIQM

>LOC_Os07g37045.1|PACid:21898456
MSFYQLLNTVIFTIQCRGVAPALTPARGYASRPAAARRFLGEEVCDAPQLAAEVVGACMENVPDRPCCRAIAAVVDF

GCFCPVAESSVIFSNGITPPVILTLYVECRGTKNRNIYSLWTDIKYLSVASPPPPPSPPPSPTPPPPPTTPPPSRSD
TR

>LOC_Os07g04730.1|PACid:21899118
MATGGGGATRATSEGEIEGEGGMLTKLAQRRGDNGNAAAMDSSGRHGNGGTPATSGEGGAAGAQHSLGERIEGLGRE
EEARYRGELRPEAAMAEVARVRGEGVVSVVARRKEAVAGLGIAAVKPVVTVTQCGGGSSHGERRPELAKAMAARVDR
GEGGSVGDWRNEAVAGARLGAVKPMAQAERRGDGQGSGGAQPESVEAVERGGARGGGASETGRGNGADAGVRKNSTL
FSGGKSTPKVPQLVIELQNRPAKPVIQRPSSIKTGYNRSFAAGGRMRVSAAGEGRRGGSDDGDERRGGDGGETASGR
RGGAARGRRGSARGLPRQPPPTAHEAHEAEEEAASGTAATTRQITEDITKGSYGHSCSSALRQPARRSSRCATTAPT
PSGRPPCPETQRSGAIGGGDFKLSPGANVSFPAPDGWSDRLWARTDCAPSGTASLACATGYFGGAVSCSLGGAPPVT
LAEFTLGGADGKDFYDGRLNLASAPASSVSTATSASSATTTAAADFAPSSRDIPLLPIGQFPQDHVNLGSASAAGDA
MINSAGAPAGAHQYMTNPYAGDAMINSSALAGAHPHMVNPPPYAGASSSRLPTASAEWNMDWLHDAMAEQQQPPPPP
QWQEQSVFSPPPPTASSPLHHSIWFTLHTHPSPPPPSPRSPPSDGSSCRCRVKFQPHTRRHSPQPISIPALFPPPPP
TP

>LOC_Os07g46180.1|PACid:21899504
MPPARKKRGAAAAAAAAAAAAAQWKVGDLVLAKMKGFPAWPAMISEPEKWGLSSAKKKLLVYFYGTKQIAFCNYTDLE
AFTEEKRKSLLAKRHGKGADFVRAVKEIVEIYDSLKNENNNKSDTSGTDVKPDVEDAAHNSNSDRGGRGEGSDLVND
NKQDSRPTSSMDHNVTKSPGSNIPAVESGRCVVNSAPDEPSSSFSKKSQNDVQQNISCALGNLTSPRKLRSSLGADL
RPTEDSCGPMNSINQPCVNVISDNKQVHSFQHTCIGDNKSNSDSLPAREDSSQGTCSKPEDSTVVVDDESLNSIDDV
QDIHHIEASKTEVKQNGTIDSMSTTVTFKRKRKPHTNHISNPIISVAPKTDEELQPKSSGNLADSPNSGNEVNKSDG
DEHLPLVKRARVRMGRALLEDTIVDECVISDNKTELATHENRCDKHDLYAGLGKDHSADMPPSMDPSSKIDLIMPSG
DAQTACKNKEYHPKVLSLDGEAALPPSKRLHRALEAMSANATETICSSLEVQKSESILKVKGCAASGASSPSNNSLD
AIAKVSRSVMTKSPTISSSGHSLDTPDGEKHILLKDIPSTIPLDLKNACSQNSLKEKVVEELHMDDKNITLTVCSRA
DNNVCGKAPTYSMESKASGNESMEPNGDPAHDFVKNVNGSAEPVSQANVVPSSNGNYNSVPHDDIRLAKPTVIVSDR
TSASSLVTKISCIQSDASSQTFEPHGSSAIALKEHNHRMYPKGKSLSPDMMPMKELIAAAHTRRFSHSSSFIDSFLC
SNGVPEPSVNVPSLKEGSSGQCSPSNHTIRFAADRIHTQQNSGAIPFDNMQQKGLNKLSGHDEASSARRAFEAFLGS
LTRTKESIGRATRLALECDKQGIAGEVMDIIIEHLEKESNLYKRVDLFFLVDSIIQCCRNQKGGVGNAFPSLIQAVL
PRILYASAPPGNSAWENRRQCLKVLKLWLDRKTLSEYIIRHHIKELEALNEASFGTSRRPSGTERALNDPLRDNEGM
LVDEYGSNTGFHLPNLIGTKLLEDEEGSSSEERSFEAVTPEHEATGANEQEASQMHVAKHRLVLEEVDGDHEMEDLA
PSSEAEGEAISSCQPDLTVARCATTKQNVDSVPPLPDDKPPSPPPLPSSPPPLPRPPCPVFQDSQVQGALAADRVQP
DPPRTTYNIQEQHPHSVANNRSNMDPCVVSSHPPAPYNCGYAGHANQMPLPPPPPPPLPPPPPVAPFHPPGPHFSGP
SVPPHHGNNYHQPPSVPPPNNAYHLQPPPHPPFPNQYPYPPEPQQNTQPWNCNPSYPERHQYGEHDRGPHAYDSRHH
FHHRGHHFDDGGHYFDDGAHHFDDRGHPFDDRGNYFDDRGHHFDERAIRGQMHHEVDRGRFPPHFPPGPPFPDHFDG
PSTPFHCRQPSNPPTGPGPGWSMPPRRFKYPPGPRHSMDHPVPHEGGWRRNGRHNHDKHPR

>LOC_Os07g01214.1|PACid:21899525
MSRRMARGGRARCVWPSSASASSTATSAPPHSTSTPPPSTAASATPTTSSASSPSSSTASSSSPSSSTSTSPSAPTT
TETAALSPSTPSSPATPRSASSPTSRPRTSSTSTSASPPRSAAPPSSASPPPPRSAPSGSRTSSRTAAPSASPSSSS
PSSPPPWSSATPASRPPSPSSPPSVASRTRRHTSTLVVWVTVGILVMLFAVQRFGTDKVGYLFAPVVLLWLLLIGGV
GVYNLAAHDVGVLRAFNPKYILDYFRRNGRHGWVSLGGVLLCFTGTEALFADLGCFSIRSIQLSFAFGLVPAVLLAY
AGQAAYLRVYPDHVGDAFYASTPQVLFWPTLVLALAASVVGSQAMISCAFATISHSQAMGCFPRVKVVHTSRQYQGQ
VYIPEINLLLGAAACVVTVAARDTVVIGEAHGICVVLVMLITTLLLTVVMVLVWRVNIGWVLVFACVFASTESVYLT
SVLYKFAHGGYIPVAMSAVLMGVMGVWHYVHVRRYKYEMERTVSTERVRELVSRRELQRVPGVGLFYTDLVQGIPPV
FPHLIDKIPSIHTVLLFVSVKHLPVPHVDPSERFLFRQVEPQEHKLFRCVARYGYRDRLEDARDFVANLVERLQYYV
RDVNLYGAAANNKVSYPSSRCDSMGIPKSASYAERLQLQRARSVAMLHSHSQHQQQPLPQQLGQLLQYSASTGEQQR
RSVYAEEMLTPAESFSEMGTMAASGRQLMAVAVKMSLEEMARIEEEQRFIQREMEKGVVFILGESEVVARPHSSLLK
KLVVNYAYSFLRRNCRQGDKMLAIPRSQLLKVGMSYEI

>LOC_Os07g48420.1|PACid:21899549
MAAPADDYYGDDDDEYDDYNPHPYGGGYDIFATYGSPIPPSPTTCYPVSSAAPTAPPPKPSPTPPPASPPPAPTPPQ
TRPPSPPPQQQQPRPVSPPPVAEPYYWPKPYDYGDAPREQPAYATPEVFRGWPFFAGARCHSRCGGRDYWRQFMRGL
DYLFGHADGYGERRIGVDYHGVPVYANRKGGVEEAVVIQVEPPATGTVEWHHAADDPEYNYNNGNRLSWDDNAKDET
YAYVQHNYSSYDRSYDQSYSLDAVSDETTWFPNQNYQHVYKEEESQYQEILSSSCAESKISAQPIYCYNQQFSEQPL
HVLVEPPETVYSQKLEYYESFSTYNHHNSNDDSDMLGHSYDIQLPDEHVPDEPFEPIKPSWAMHSGYYQSCTDGASA
EFENHTLSSSEFSGIASLFATSFYPQQTQIYECHGDENVSLQQNWQCNWNVVSENDFQSGYDSNHMNGSFWPFGDHS
A

>LOC_Os07g48420.2|PACid:21899550
MAAPADDYYGDDDDEYDDYNPHPYGGGYDIFATYGSPIPPSPTTCYPVSSAAPTAPPPKPSPTPPPASPPPAPTPPQ
TRPPSPPPQQQQPRPVSPPPVAEPYYWPKPYDYGDAPREQPAYATPEVFRGWPFFAGARCHSRCGGRDYWRQFMRGL
DYLFGHADGYGERRIGVDYHGVPVYANRKGGVEEAVVIQVEPPATGTVEWHHAADDPEYNYNNGNRLSWDDNAKDET
YAYVQHNYSSYDRSYDQSYSLDAVSDETTWFPNQNYQHVYKEEESQYQEILSSSCAESKISAQPIYCYNQQFSEQPL
HVLVEPPETVYSQKLEYYESFSTYNHHNSNDDSDMLGHSYDIQLPDEHVPDEPFEPIKPSWAMHSGYYQSCTDGASA
EFENHTLSSSEFSGIASLFATSFYPQQTQIYECHGDENVSLQQNWQCNWNVVSENDFQSVSVTVSMSVNVLF

>LOC_Os07g48420.3|PACid:21899551
MAAPADDYYGDDDDEYDDYNPHPYGGGYDIFATYGSPIPPSPTTCYPVSSAAPTAPPPKPSPTPPPASPPPAPTPPQ
TRPPSPPPQQQQPRPVSPPPVAEPYYWPKPYDYGDAPREQPAYATPEVFRGWPFFAGARCHSRCGGRDYWRQFMRGL
DYLFGHADGYGERRIGVDYHGVPVYANRKGGVEEAVVIQVEPPATGTVEWHHAADDPEYNYNNGNRLSWDDNAKDET
YAYVQHNYSSYDRSYDQSYSLDAVSDETTWFPNQNYQHVYKEEESQYQEILSSSCAESKISAQPIYCYNQQFSEQPL
HVLVEPPETVYSQKLEYYEVCLSSTNAVYSVFIW

>LOC_Os07g43950.1|PACid:21900023
MGRGYSYSPSPPPRSYRRRASSPIPRDRYGGRGRDLPTSLLVRNLRRDCRPEDLRRPFGQFGRLKDIYIPRDYYSGE
PRGFGFVQYYDPDDAADAKYYMDGQVILGREVAVVFAEENRKKPSEMRSRDRISGSRGRSYDQRYSRSPRYSPPPRG
RSPYRSPSYSRSPSPRYARRRMRERSYSPVESRSRSRSPVEEGYGGGSTRRERSLSVSE

>LOC_Os07g28614.1|PACid:21900145
MILVAIVAELLEEYTAAVARAMELLLSRAPPRIFPRRVRFLVLRSLPFASPPPSPLSPPPPFTVAAGTR

>LOC_Os07g46420.1|PACid:21900770
MEAAASPSPPPSRASPLRLNPAAFLLRTTTTTTVQPTTSADAPPPPPPTRQTAGVDRLISFLSSLIPRRGQRAKQP
TSPPPTAAAAAAMRRAAEREAEAERQLVGCAVPLFRPYVAQLPWHGGARAWLSRMFPRYGHYCGPNWSSGKEAGSVL
WDRRPADHLDFCCYCHDMAYDTHDQAQLLRADLAFLRCLQSSRQTPARDGIAAAAIYRSMCIFGLKTILIPYRTNLV
RLQTGPNYADAFADFVKRVASSSGRPTGGNKQRM

>LOC_Os07g10180.1|PACid:21901199
MERRRAPLPSPPPPPPLTSPPPPPSSDGEEEGAAAFSTTTTIAAVGSRRRHLPGCLTTAASPPAAKPPPPPPARSGG
GEGTVNRPSDHRRIPSYRRAATATFPPARSGGGEGAAATSRAVGGGKEEKGTTERGRRRRAVERGGGGGQWRGEEGD
GGEGREARTLGGGEKERIRSVWLPVGLISFSGPCYLGSAVGSA

>LOC_Os07g47400.1|PACid:21901265
MAYRVLEVTLHSARDLKNVNFISRMEVYAVATISGDPLTRQCTPPDPYGGRHPAWNATLRFTVPPTAASAAGCLHVL
LRAERSLGDRDIGEVIIPLADVLSGPYDLGARPPQFASYQVRKLHRSETRGVLHLSYRLGPVVAPQSVFAYPAPPPP
PPQLFETAPPSPPYVPPPPDAYLRKPSPPSPPPAKLSPPPPPQTQTQPLAKPPAPATPSRAGGHVAALAPPAVAKAD
RHVSTPSPAKADWQMVGTPTATKGASKHGSLEFERGLNAGLVGGAIGGMLVGTEMVSDAAFYHAGYRAGLADRDGWA
VY

>LOC_Os07g35979.1|PACid:21901976
MLHHHTSFADMWDPCTGVPRRPAFMRRRPRHTHRQLLLTPRSPPPSPAAPLCAKPASSTGRASRQSGLPPPAAPLAP
SRPPSPPPAPLAPSRPPLPSAALLCAKPVSDPSTARCHAGQMQPSPTPSASQARPPCLR

>LOC_Os07g06760.1|PACid:21902315
MSSTSSSSPPPSSKGKRRPGGIIRGPRPQPLIVSPPPPPPPASRPPKKPRVVASGGGGGGDAGPVIVYELTPRVVHV
EPEEFMAVVQKLTGNRKLSTATAAAAAPVDSTVRSADDQMTAGGGAEITDTAAATVADDQLALAFGQQQHWPAPPPE
IDDDNSANLPSPGSFFLSPTTMQALQELASNLF

>LOC_Os07g29380.1|PACid:21902782
MGEEDEGGGLEQRSEESPQPPGWHSIRRISPPPSLSAAAHRSEELPLPLPPIDVAAATAEAPLPMPPLLWRTPDADG
DAMAVAADDINVDLGILGDSQAPAPPPLAGGAAHGGRPPLPAATATPPRMVASPPPPAAIAARRPFSPPVTALLSAA
TAGSRLPALLRGTYRRPGQSVPPTAVADPLLSPRLPSPARCSRRVMRRNCPHRRLEQAGSCLVPVAVAQLPPGAQLA
VVVASVGGRRSAVLCCEGAAAPEPYHRGGWSAAAMEVALGGLVVARPAMKRLRRSPRPSAAAVARAPRLAVRRRRVL

KRQELLVSQLHELPPHLPVQPADLAARAAAAADGAYRAVRRVGQALAPGATLDLEVGGVEEHKSGNEDARWSTARSS
MVELTRRKPRAKALARAIPSYDPTFQRK

>LOC_Os01g57690.1|PACid:21904270
MVDMKAAICIAAAVSLIHVVSAADYTIGSAAGGWGGEYKAWVASQTFSPGDTLTFKYSSYHNVVEVTKDDYEACSAT
SPVSADSSGSTTIVLTTPGKRYFICGAPGHCQSGMKLVVDVADRPAPATPSPPPLLPPSPRHKRRTAPAPMPLPPAQ
APVWSPAPAPAATQRRHSGHKKHRSRHLPPKPAPAMAPTVQSVEADFPAAAFAPMSSPPPPPPMSSDASAVVRQKWS
DVIVGLVALGLVVLGV

>LOC_Os01g18770.1|PACid:21904705
MPLLLGVYDFTASLPPSPPTGVLAYTVGWSHHRSWASTSSPTGLPSPPPTGVSAYTAGLFMPPLLGVYDFTAGLPPS
PPTGVLAYTVGWSHHRSWASTSSPTGLPSPPPTGVSAYTASLLCRCCWASTTSPPACLRRRRLVSSPIRLVGHTTVH
GRPCLRLHGWPIMPPLLGVYDFTTGLPPSPPTGVPAYTVGWSHHR

>LOC_Os01g74130.1|PACid:21905196
MRLVGHTTVHGRPRLHRPAGLRRRRLVFPPARLAYYAAAIGHLRLHRRPTSVAADWCLRLHGWPIMPPLLGVYDFTA
GLPPSPPSGASAYTAGLLCRRCWASTTSPPACLRCRRVVPPPTRLAYYAAAVGRLRLHRRPASVAADWCPRLYGWLV
TPPFMGVHVFADRLAFAAADWCFRLHGWPIMPPLLDVYDFTAGLPPSPPTGVLAYTVGWSHHRSWASTSSPTGWPSP
PPTGVSAYTASLLCRCCWASTTSPLACLCRRRLVFPPTRLAYYAAAVGRLRLHRRPASVAADWCPRLYGWLVTPPFM
GVHVFADRLAFAAADWIQIQICIKGIHGFKLISYAPGMDQSEHHHRLALTAADWCFRLHGWPLCRRCWASTSSPTGL
PSPPPTGVSACTAGLYAAVVGRLRLYRPACLRRCRLVFPPARLAFMPPLLGVYVFTDRLAFAAADWCFRLHGWPLCR
RCWAPTSSPTGLPSPPPTGVSACTAGLYAAVVGRLRLYRPACLRRCRLVFPPARLAFMPPLLGVYVFTDRLAFAAAD
WCFRLHGWPLCRRCWASTSSPTGLPSPPPTGVSACTAGLYAAVVGRLRLHRPACLRRRRLVFPPARLAFMPPLLGVY
VFTDRLAFAAADWCFRLHGWPLCRRCWASTSSPTGRLDRRRLVLPPARLAFMPPLLGVYVFTDRPPHPPLTGVTACT
AGLL

>LOC_Os01g47200.1|PACid:21905707
MASPIHSPPPEPATDGDDAQSHPPPPTPHPATDPPPISPQNPTPPPPPLPASAAAPTTPSPNHSGDPSRPIPSQAPA
PPPPPTADPSPPLPHDNRTPQPRAAPPPAPAPDQPAPPPSPPPSLPPSPPAPGSPESMLEREASEADGEPENMTLALA
LAETETEKAMPPTPPKAAEAAESPTGSPQKESALTIAKLLSGEDHAGTETKPVPEKAAPAATTTTAAVASVGGGGGG
GGGGVGSKRWLLGGVPEKVRRSELRRAELGFRVLAAVFCLVSLSVMAADTTPGWSGDSFRRYNEYRYTLSASVLAFT
YSGFQLVVEVHYFVTGRRIIRDPSGKYFNLAMDQILAYLLLSASSAALSRNDVWVSRFGVDQFAKLINASASMAFLA
FVALGFSSIISAYHVFSSVF

>LOC_Os01g60090.1|PACid:21906415
MAGLAAGGWCRAAVGPWWWLAGWRRPAGSQGGESVGDENERRGGDEIGRQARSGIGEWGIGDRLGSGALGVRPSGST
RVFGRRQAGNRVGLLGWASILICPGSLRLASGSRASSSWLVISNELKCKLGSLGKPNEPSRAKPSFSRAERANEPRA
SWPALVSACTAGLLCRRCWASTSSPTGLPSPPPTGVSACTAGLYTAIVGRLRLHRPACLRRRRLVFPPARLAFMPPL
LGVYVFTDRPPRPPPTGASACTADLLCRRCWASTSSPTGRLDRRRLVFPPARLAFMPPLLGVYVFTDRLPRPPPTGV
SACTAGLLCRRCWASTSSPTGLPSPPPTGVSACTAGLYAAVVGRLRLHRPACLRRRRLVFPPARLAFMPPLLGVYVF
TDRLAFAAADWCFRLHGWPLCRRCWASTSSPTGRLVRRRLVLPPARLAFMPPLLGVYVFTDRPPRPPPTGVSACTAG
LYAAVVGRLRLHRPAASSAADWCFRLHGWPLCRRCWASTSSPTGRLVRRRLVLPPTRLTYYAAVVGRLRLHRPAAST
AADWCFRLHGWPLCRRCWASTSSPTGCLVRRRLVLPPARLTYYADVVGRLRLHRPAASTAADWCFRLHGWPIMPLLL
GVYVFADRPPRPPPTGVSACTAGLLCCRCWASTLSPTGRLVRRRLVSPPARLACYDAN

>LOC_Os01g42990.1|PACid:21906561
MATAAVFSASARRLLSTVAGGAGAGSAAELPVLIGHLRSLARAGRLADIDAALAPHLASHSVAAVSALSSVGLPDRA
SALLATIRNPTAAHLNALLAPLLRRHRLVGLIPTLLAAHPSVPRDDATEGIHAKALCIATGADSALHLLQRESPPPS
LQLFTSVIDSYYKQRKPHRAEQLWREMVEDHGIVPDAAAHNVRITYKAATGTVEEVKELIRAMREDAGLQPDIVSHN
GLMRAMARHGRVDEMMEVYKRLEKGSASAAAEGKSAPDCATYTCVVAALCKAGRWSEADDVFYEAVKRRKLADLGTA
RVLVRGLKEAGKGRAARRVVIGLRKKFPDRFDGPWKDLEELAGIAGEDEESDVEGEDDEQPPATTTTTAALNTSPIL
ELGTTPPPPTTRLLSGGGGARGPLPLGADDALGGRAAGSEAETGERGGRVRRSVREAAEEKKATAARSKAKTAAKAK
AEQSSGRDREQDDHGAKPLAPPSPPPLLAARLAVFRAVARRQPRLPLDWEINGIDRSGLDAQVAEAYSANLVQIASP
RTLRPVLVLTPVTADEVRAYVVCCRDHGLTVRARSGGHDYEGLSYCSLRPSGDGEGAARFAVVDVAALQAVRVDAAR
GVARTKAGATRAVCRIGAAGLPPPPPVSSGSRS

>LOC_Os01g40710.1|PACid:21906595
MSLAPSIPSIKVKVGGVAAVAVSPPRHRACRSSFAVIRSSKAEGAPRRPAAPPLSPPPKTPTLSTPPTLSQPPTPVK
PAAPSSSPPPSQDPEPKQAAAPVAVAAPAAAGAVTLEYQRKVAKDLQDYFKQKKLDEADQGPFFGFLGKNEISNGRW
AMFGFAVGMLTEYATGSDFVQQVKILLSNFGIVDLD

>LOC_Os01g56000.1|PACid:21907160
MASSAEPPPKKRRLVEVHVPSPAPSPSSSSAPAPASPRSPVPPPPGVPPPPPPPPQTLAAAASPRPEEAVRKRRNRE
ELRGLFECYRRIRLCVERKDARLLPELEQVYLSLIASSRGCTSVQRILADLIPQYASYCPTALEAATKVSINMYKWN
LAIVTRGEDVDGVAYQTARACILGLTDICSTASCEAPKSSVLTGICSAVYITVLTFFISTFDGKDIYHIGSRKLLNL
QDPVELLDILKQDSGGDNQQADDCLVELRALSLLCIFLLFPENILDACFALIASAEDVNGEGLYFLKQLTCHLNSGI
ITDALDVQGDGISQCTGMDVDHPSTKESVDSNLSSHSVGVSGSSMLQPNECYMTMAISRHPSLKGWILLRYKKLCDS
CRTDVVSEVSSCLNVLGSLTQLEDNKSHMGSESSVLDKIDENSGGYLQPNKLACSYEQRMLLKTDIADRYGGKTVQN
KNAQMVHTDEVKTEKLADVKLDVCKQGNVISDAGHQAARLDMHTPKSTHDSAGGAATLTSPGQNFGKAKHSYSEPSD
LYGAHFSRDVISVSKELWIGSLGNSAAESLVRSKFEEFGPLANFLFYPPKNFALVEYGNIMHAVHAYGCMQGSSVWG
GGLQIRYLDRLIGSKGFASGIAVGESRHIYVAKVKNKKDKDEVFDELKAAGLKRPCSFTDISSENALLLEFETATDA
AAAKAHIRRQLHPDVCSQDKNTSGHELFVRNIDNSVPDMEFINAFSRFGEVIRWQFNKLDGNCLIDYRSPNAAATAK
SHLHGARFGLKSISVESRSVHDKTLSPSVPMLAPSVSDSSHHEIRNPRVSGYNAGFTVPTERPIYGPSAPRAPQGIL
PCPPVSAHRGPVIPPPPIQTSFVRPVYPGPGSPWENTTPNPSFSHASPRMMPGSSFRMNPPAPLPFIPSSVTPLTQF
PGGSAQQSEKRPPPPPPPPNVAPPPFTRQDIPPPPPSPPPLPITQPPSVPPPPNSPPPLQPATDPSDSQKQRSHPRW
QGSLSKSGLHYCSIYASRIELDACRYEHAVSEPTGWPSRLDVTKRTDYQHVKTTFSNTPPSKREVCRLLPCSNGDQK
GFRDFISYLKQRECAGVIKIPSVKPLWSRLLFILPPTPEACGMLELPPHPTECLIALILPKETTAEAT

>LOC_Os01g65790.1|PACid:21908307
MSPPPLRLLLFLLLLHLPSSLSSRHHHHAPSPSKPAPASYAAPLAVLLACNATRFQPACVSTLSNASADASTPDLLA
ATLSALRARIPPAVSTARSVLAASSNVNLTNAATNCLTFLSFSTHRLSPPPSTSSPSLLSASTALLHLYDCWSAYKY
VNFSRTISDAMAYLDDTIAVNSNYISMLAALQRYGDDTFRWAPPQTERDGYWPPAAAGSAADEDALGVPKGLPPNVT
VCGAGCHYKTVGEAVAAAPDYGDEMFVVHVKEGVYKETVNVPLEKTNVVVVGDGMGKTVITGDLNADTPGVSTFNTA
TVGVLADGFMARDLTISNTAGPDAHQAVAFRSTGDRTVLDTVELLGHQDTLYAHAMRQFYTRCRVSGTVDFVFGNSA
TVLRDTALIVLPRQLRPEKGENDAVTAQGRTDPAQPTGIVLRGCVVNGSDDYMALYREKPDVHHVYLGRPWKEYSRT
VYVGCTLSEIVQPRGWMAWNGDFALKTLYYGEYESAGPGGDGASGSRIGWSSQVPRDHVDVYSVASFIQGDKWIPKI
H

>LOC_Os01g63470.1|PACid:21909721
MASPSPSSSPLHPQHQHQHPLPPHPQPQYQSPPPPMPPPPPAAPLKAIDLEVTVVSGKHLKNVNWRRGDLRAYVVAY
LDPSRRAATRPDDVGGCKPAWNERVVLPLPPHLSPHDPSLLLSLDVFHSKPSDSPKPLVGSARSPLRDLLFSTNPNP
SPDSPASALITLPLLRPSGRPQGKLRIRLALRERSPPPPEPQYPPPSSSPYYFPPPPPPAYSAPPPPQYGSEQYYRS
GGYYSAPPPPPQYEYTAGPSAPVEYNRQYDPRAGSGSGNGRYGVGTGIAVGTVTGALGGLAIDEGVKYKEEKSADRV
EDKVVPAGRDDDSRGYREY

>LOC_Os01g70060.1|PACid:21910585
MNAGFSPQILAQKLLKLNNSRQSIETLSHWCVFHYRHCRQVVETWESDFHSAPRERRVSLLYLANDIVQNSKKDSGR
YVNEFWRVIPAALNDVFVNGDDFGRNVVQRLVDIWEERNIFGSHGQSLKEDYSRRFKEFKSKSRNSSGELLEKVISC
YKHMLNAHVDDDTLMRKCQNALSFVDNLSNEYENNSILDCSNGSGFVEELQEQHNILRDSIEQFKTSELLRGNLISC
LKEALHEQEFKMERARSQIKEVQSRYKKADDLCQKLGIHVERQEQPNHGPKNSGSEMHGSFGPDSANASSFEKGQSS
AVMYSQENGGEHEIPNGVFSSRATKDNIEQKLEEHSTNKRQKLQNDVYVSRPQSPPPPLPSDAFEQPPPPPEHPPPP
ESTSPPPPPTSDPPPVPPPPPTTGSFMPIPSAPFAGLPVPAGPMTAVPYNSYPVFPPMNYPMVNIPPPFPSAPNTPP
GFQGLAGPFYGPPYPAPPPPPPPPMNRK

>LOC_Os03g12570.1|PACid:21911308
MVPEPAPAAATEPRRSTRRRLMTAAAMEAEAEAVADLDEIDREMSRAESRKRQRRTAKEKPGARKGATEWKPEDVEK
AAAAEGVAELDEIDREMPRPELRKRQRRTAKEKPSAHEGATEWKPEDVEKAAAQEPEGTELDSGLSPAESRGKRQRG
VEKVKRRTRKKTAKEKTKETTEKSAAQAPEKMKVNDAGGALAEDVCADEPDAEQMAMEEEEEAADVLEAEERMGKCV
GEGSAEKAATRKRVARPSTARRVEDSDDHFVGDPVPDDEARQRWPVRYSRKGSDSLLKQEPDEDEEMKARCHYLAAN
VDDEIYHLDDDVYVKAGPDEENYIGRITEFFEGVDRGSYFSCQWFFRTADTVISSKLLKVHDHRHNHKRVFLSKEKN
DNLIECIVSKVKIAHVDPNMTPQARAHAISDCDLYYDMSYSVAYSTFANLPADNDGALGSEATSNISCDDADNSSKG
KLSADIVAPYSEQTETASLLDLYSGCGAMSTGLCLGFAFSGINLETRWAVDINKYACACLKHNHPYSQVRNEKTEDF
LALIQQWDALCRKYVVHKNDTLEPSIDMPLNDADDVNEPLPEDIFDVEELLEICYGDPSNTGKNGLWFKVRWKGYDP

SYDTWEPIDGLSDCPERIKEFVEKGHKENILPLPGAVDVICGGPPCQGISGFNRFRKHNDPLEDEKNKQLVVFMDIV
KYLRPKYVLMENVVDILKFADGFLGRYAMSCLVAMNYQARLGMMAAGYYGLPQFRMRAFLWGALPSMVLPKFPLPTH
DAVVRGIVPTTFSQSVVAYNEVDTRCLRKALLLADAISDLPKVGNDQPKDVIEYSVAPKTEFQRYIRNNRKDIQDYS
FRGDDPSEEGKLFDHQPLKLNKDDYERVQRIPVKKGANFRDLKGVIVGPDNTVRLDPNISRERLSSGKPLVPDYAIS
FVKGKSTKPFGRLWWDETVPTVVTRAEPHNQIILHPSQDRVLTIRENARLQGFPDYYRLIGPLKEKYIQVGNAVAIP
VARALGYALGLAYRGESDGDRAVLKLPESFIYADQETVVKSSAGTPGSEIADSEQLFEYFINPNFFCMMFAFLKVNV
VIPLILDSKMGLSRRGAEEGEARRAVPFQNILSWEGWNRLDHRRGEIRLDVRREMDDSPLDNLFDGNGLDSSAGADS
SSSTGSPPSSTSSSPPSSQSPPPGSSPPPASPPPSTPSAPPTNSSGSAPSPPSPSQSAPPANTGGGGSPPPSHGSPP
APKAVQSQPAPKRSGDGGSSSDSGSSKEGGSSSDRGKSESNGNRPGPEAAIIAGMVIGFFTFALLLAIVACVCCSKK
KKRPPHMHMPYYTDENGKVYYANSMPRWQNSVDQGGGWHAQYSPGQAPPSSEMSGSHGAGPLPPPSPGMALGFSKSS
FSYDELALATGGFSSANLLGQGGFGYVYRGVLAGSGKEVAVKQLKAGSGQGEREFQAEVEIISRVHHRHLVSLVGYC
IAGSSQRLLVYEFVPNDTLEHHLHGLHIFPALFPAIVTRHSEGRAGDGLDDEARHRARLSQGPRVPSRRFLQGQILS
VRPSNDLICCIVVVTYEGHPRIIHRDIKAANILLDENFDAKVADFGLAKLTTDTNTHVSTRVMGTFGYLAPEYASSG
KLTDKSDVFSFGVMLLELITGRRPVDPTNYMEDSLVDWARPLLARALSEDGSFDELIDQRLENKFDRLEMERMAACA
AAAVRHSAKRRPKMKQIVRALEGDASLDDLNEGVKPGQSMMFSTGSEYDSGNYASDINRLRKVAFESSIEDSSEYGT
HSSADSGEPPRRQQHR

>LOC_Os03g10850.1|PACid:21911424
MPPPAWGWGSNPLEPVVHTVAAFSRRLLIAPDAAPDEARLRPLLSLSLSPPPTPPSPPPPPPEVLKKDSKAAPLTKE
EVGRATWMLLHTIAAQFPDEPTRQQRRDARELMAIISRLYPCKECAEHFKEVLKANPVQAGSQAEFSQWLCYVHNVV
NRSLGKPIFPCQRVNARWGKLDCPERSCDLEGSNDIIPNR

>LOC_Os03g10850.2|PACid:21911425
MPPPAWGWGSNPLEPVVHTVAAFSRRLLIAPDAAPDEARLRPLLSLSLSPPPTPPSPPPPPPEVLKKDSKAAPLTKE
EVGRATWMLLHTIAAQFPDEPTRQQRRDARELVSIKQDKQLVISL

>LOC_Os03g11580.1|PACid:21912026
MYGQGGNFNPQYRHAAPPPPPQQAGVTGGFPQQPLPPPPPRMAQYPQPPAMAAPPPGPYQHRMPLVQNQAYPFAQMH
QMPMLPQQRGYAQMPMPGPPSQPPPPQAMYQAHPQYPMPGSLPPPPPRPPSFAPENALPPSSPPPPSPPPPPPSSPP
PVPPSPTAAPTTGQSWNSEPERKEGATATDVGHDVKTEKVTNQLIVSDDSDMDMDADEDSPSREHVSPINSSLATAE
CTGNVNTRKPACDVSNLVKDSGGKAKTTNVTDEGRSTFQLIQGYASDDSEDEDGACAVSNLVPLTENNEPVHSIGTN
PDIGHQLLTEAAPCTERSLEDREHQLMSKSNPVKHDSDELGHPVKEDLSGNDSDRGQQTRRHGRSQWKRSRGQSPQG
RRSCSPLSQSLSSGRQSNSPLAKRANLLESKSPDGVGQTFRAQPGVKLGISKDGFYNDKHDSPVKVATPFDIHPAGG
HISGDRISEQDGLMGTKKFNGSPDDIDCNEKTNDASVGSFGPHGHGAVLTCGPSQSVASSANGSDPHKMQRSGRASI
PQSDMDKSSLGAHQSLSSQPPGISFATVHATEKNMMCDVLQPHSQNLCPPGQMPSGLRPAHIPSSNITPLPGQQLLS
TPEFPQMHFQPNVMAPANEFLQSQMQTYPAPDLPHPRPLDFHPHTLQPVVPPHQQPAAMVHSSFQRFTPNLPGSTEF
GAISDTDLPKSSIKPHYNPFASTFEQTDPTLNIGCDVIPNPVESASTKAAEHANALSPFGLSVPGSGTHVRENSAEV
VSSRQKQPHREFTSSAPYDPLLDSIEPSSSSINKMDLGREANLSASNHNASKIVNIEVESKNMHGLGLVAESEVEEF
GEVAADTEAGVVDNLSPEPLGAKDWSSDMPGDIDNDESVDKNKRTKDSRSMKLFKVAIADFVKEVLKPSWRQGNISR
EAFKTIVKKTVDKVSSSVPNNHIPKTPAKIKQYVQSSQKKVTKLVMGYVDKYVKL

>LOC_Os03g09045.1|PACid:21912301
MVMDKSSLPPPPPPPASSASAAAASSALKALNKSSYKISKQTSSSSLASMKASQSPPPPPPHPRRPSPPPLPPPPAP
PSSAPAAVDHPPPQPPVYNIDKSDFRDVVQKLTGSPCHLLPPQAQPPAPAAAVSMLPPQPPPTIMAPPPPPPPSAI
PTRLHRIRPPPLAPPRPPPILQPAPPALSPLPPLPAVCMTAESPISAYMRRLRGMPSPIHVPTSPLGFGCLPSPRTP
PSPGVPMPATSPRVRDP

>LOC_Os03g04910.1|PACid:21913189
MPPLLGVYVFTDRLAFAAADWCFRLHGWPLCRRCWASTSSPTGLPSPPPTGVSACTAGLYAAVVGRLRLHRPACLRR
CRLVFPPARLAFMPPLLGVYVFTDQLAFAAADWCFRLHGWPIMPPLLGVYVFTDRLAFAAADWCFRLHGWPLCRRCW
ASTSSPTGLPSPLPTGVSACTAGLYAAVVGRLRLHRPACLRRRLVFPPARLAYYAAVVGRLRLHRPACLRRRLVF
PPARLAFMPPLLGVYVFTDRLAFAAADWCFRLHGWPLCRRCWASTSSPTGLPSPPPTSVSACTAGLLCCRCWASTSS
PTGLPSPPPTGVSACTAGLYAAVVGRLRLHRPACLRRRLVFPPARLAFMPPLLGVYVFTDRLAFAAADWCFRLHGW
PLCRRCWASTSSPTGLPSPPPTGVSA

>LOC_Os03g41810.1|PACid:21913383
MGDKQETESTKTHFVDPTHNPTTDATLQQDEHESDLPEEFGVTSPPPLSPPPYPLSPSMEDDGMIYAEDLGYMSTLC
PSPPSDVDDLNPPEDPNNKIILHPAFIDDGGDLDIIQEDIYNFRYDQTPPRDAQSPVTRFKRHKRD

>LOC_Os03g63650.1|PACid:21913514
MQLKDMVMKLSGTSRHHGQQRRGGSPPPRGRTTSVYRSGYYRPGMVQDDMAVPPATYLGGGGTSMSSASSTPAWDFA
RPAEGEAREWVAQVEPGVQITFVSLAGGGGNDLKRIRFSREMYDKWQAQKWWGENNERIMELYNVRRFSRQVLPTPP
RSDDGERESFYSQVGSTRGSPAATPSPAPLTPDRVTSWSAFVRPPSASRQQQHSFRPLSPPPPSSSNPSERAWQQQ
QQPQRAGKSPAAASDAMDAARTTSCSSRDEVSISNASELEVTEWVIQDEPGVYITVRELADGTRELRRVRFSRERFA
ELNAKLWWEENKERIQAQYL

>LOC_Os03g62120.1|PACid:21913741
MATTHGEEAAGKAYKLAEDRFLVKDIAGALRAAREARRLFRSLPGLANAITAYEVHAPAATTRAGGRNWYAVLAVGD
RSAKTSSGGGGVTHESLKRQYHRLCLVVHPDKNRSAAAAGAFRLLQKAWDELSLRHPPRAAGAAAAPGQARPSALIA
THQIIRAPGLFGNSQQCNSRLVRVYLATSSSPMATGRDAEEAYELAENRFLANDIAGALRVAREAQRLIYPAALPAG
LANAVAAYEVHHAASRSDGGRWYAVLAVGDPSAPTTSSGINGAVITHKSLKQQYRRLCLVLHPDKNSSAAADGAFKL
LQEAWGELSLLHPPGSGATPVSWSSPPPPPAAAEAPEWKAPRQAKPRRRAMRCPHCGCSFVAVVSDAVSGVNCLDCN
RWVSTSSQSGPAPPPPPQPPPPPPPPPHQRETPSPSPPPQPQFPCPGNCSRCGAKFTATVSIGTRFVPCGVCKAFSM
VSVKSPSVATTWILG

>LOC_Os03g24339.1|PACid:21913837
MAPARKKRAAAAAAAAAAAAAAAAAAAQWKVGDLVLAKMKGFPAWPAMISEPEQWGQTSVKKKILVYFYGTKQIAFC
NYADLEAFTEEKRRSLLAKRQGKGSDFLRAVDEIIDVYDSLKEVKPGVEKLANNNSSLDTEGLVSSSNMGSDKKQED
HSIVASGHNTADSDEPSVTAVGSERCVVNSAPDDPTENVSILDEMRNIPLSTSSISKKLRDAQPQNCYTRSRVSSLR
RSRSSLNTDTRKAQDSGKLSGGTCLASVDLAADGTKEGSSHHVYVEDVKGNSGSPSTQDDVWLHSSAGIDNQPGTPG
TSNNNKKLNYTTKVDSTCDSETSQNGALETQFKSHDASSIPMKKSVIFKRKRKPSRNIFSHSTDKDDEFQAELSEKT
ADSPNPKTEVNKSDGDEHLPLVKRARVRMGRPQLEDSPVDGIDVTNNRPELAMLADLCNMHNTVALPGDDCSVDQSA
VVNSVSLTGRVANTVSNQSSKLYMPGPSGEGQSAWKNKEYQPKVLTLDVEAALPPSKRLHRALEAMSANVAETNNLP
EVTGSKQLIPNGFVASENSHSNKSADAVVTATNGSAIAESPRPSLCTESMHSPKCKTHSSESILQNNSVPASASVPS
EAKDDSHVTEGNICEETHVDSKTTDCLLVSSEVGNDDCGKGLALSMKLNESALGGTQTVAVPDRLSSSLGTASVSEV
AKPINFNEGPKPVDRPAYDTDRSVQRCDEPIYRPKLLSSNNNAISDSVLHNETVVAETVVNVADTASTSSLATKSSS
IQSDADTRTFEVHTFSALALKELNHRNLKDKSTSPDSMPMKELIAVAQARRFSRSTSFPDNFLNAKYIPETVVDTPA
FKEGSQKQLSPLNRIIRSTSTNDNIHSRSPFDSQQQKNLSKLTGHDEANAARKAFGSFLGTLTRTKENIARATRLAI
ECAKHGVAGEAIDIIVERLEKESNLYKRVDLFFLVDSITQYSRNQKGGAGDVYPSLIQAVLPRLLYAAAPPGNSAWE
NRRQCLKVLKLWFERKTLPEYIIRHHIRELEVINEASFGSSRRPSRTERALNDPLRDNEGMLVDEYGSNAGFQLPNL
ISTKVLEDEDGSSSEDRSFEAVTPEHDAPCTDEKEESQMPAEKHHRILEEVDGELEMEDVAPPSEVEASTRCRPEQI
DTKCRTSDRHTLGPPLPDDRPPSPPPLPSSPPPVPPPPPAPITQTGQLQRTLPMASDPVGPHPTRATNNIQTQQPNS
VVERPGSMNPSVAQLQPPPFCNSGYGGHPNQMPPPPPIAPLNPPGPHGNFPAPPAPYHGNNYHQPPMASVPNEGYHM
QPPPPPPPINQCPYRPPEPQQRPRPWSNNSSSYPERYRYDGHDRDHHRHDRRHHGHDRRHHFDDRGYHYDDRGYHYD
DRGHYFDDRGHHFDDRGRSFDERAIRGPMHHEVADRGRFPFPPGPPGPDHFEASSAAPVHYGRPSDPPPGPCSGWSM
PPRSSNYSPSRHSMEPPVPHVGAHGSWRPR

>LOC_Os03g06420.1|PACid:21914160
MAALVISKQRSHQQQSSDRRRKPSSHFASPQSMRGFNAVNCRAFHSSVSIGILPSPPPPPARTYSSPEPKTPKSQPH
HGKKRSRAISISPSTSPPSRPELWAGPAFSNSPPPSSLPIPKFSLHQKRSVSLELPPAGRSDDVVVPLHAKSAPSSP
TAGSGVSFFSDSDTAIATENLRRILHLKIADH

>LOC_Os03g59480.1|PACid:21915806
METEPFDEAELLALPASPVASPPRRLKRLKKSSQIATAAHPVVGSPPPPSPPPPPPLDEETLAQFPSPPTNPSPPPP
PPLDADAAAEAAPSPALTSPPPNPSSSPLPPTDTTEEEEEEEEDDGLDPLFSETFGAAGWDPLGMPRMEGEGDEEEM
LGGGLIEELRREKSAKKRLDMDEGEEGGGEIAMDAEVKGKRSKRRKKDEAPKDSARGKKRSEKERRVQLDSIHAESQ
RLLRETRSVSFKPSAQPVYKPISSVLEKIRLRKLEILKKSVSLLFLSATPNDDDDEEEDDVSSDPVSGTAGDLGAPQ
VKEVDAEGKDPKIDDIENEGGMNSGDVNQCDSVPENKDALNCDKDLDNCGSKDLDKELLENSQDNLEDKAQSSDNPN
NAADEIQSPPSSSPTESTDDISSEDEEYNDKENIAPSTPKDDVNVHEPLQRALAGDSCPDDAILKDFLDVEAEEEDD
SDDDMMRFKDNEEDDGSDENEVFNDLIEAGYEEGEIDHEKRNALHQKWLQQQDAAETNKFMQKLKFGHQEQKKVMDQ
DEDDAEDCEDESENEMSYDLTPTNVVRQNSEKAKQMIARMFTDDNDTYEHSDDEEIEEHLARQRISKREVHNSSFIS
PLENDSSREVFSLIKKLNIAPQPKRRGKQVTSNHELLTVGRDNSASSKSSFLGRTASGSLASSHRSAYRTYVFGRDD

SNSSNKSCLSTSESNADTYQTNSSQPKKAKFSSSQSKQAATKTNSKGDNSSGVSLIEVLRRSSSTSDKQEYTRQESC
AVITESQAAHQFSAFKLSRRFSRVGARN

>LOC_Os03g07040.1|PACid:21915891
MALSLSLTLQEWISQNITTGLPSPPPTGVSACTAGLLCRRCWASTSSPTGLPPPPPTGVSACTAGLYAAVVGRLRLH
RPACLRRRRLVFPPARLAFMPPLLGVYVFTDRLAFAAADWCFRLHGWPLCRRCWASMSSPTGLPSPPPTGVSACTAG
LYAAVVGRLRLHRPACLRRCRLVFPPARLAFMPPLLGVYVFTDRLAFAAADWCFRLHGWPLCRRCWASMSSPTGLPS
PPPTGVSACTAGLYAAVVGRLRLHRPACLRRCRLVFPPARLAFMPPLLGVYVFTDRLAFVAADWCPRLHGWPIMPPL
LGAYVFTDRLAFVAADRCPRLHGWPLCRRCWASTSSPIGLPSPPPTGVSACTAGLYAAVVGRLRLHRPAAASAADWC
HRLHGWPVITPTDAIFFTAGYIAVTTVGDMGPGVLRPEA

>LOC_Os03g02160.3|PACid:21916696
MPQTSSSAHSNVESHHDHYPSWSQVQHDPKNSLQAGSGKDLNSRYDIAQKLPSQRITRDVPSPVFAWSPSESRTASS
QHEGSCLSSTTNLCTHDELHSSIASAKAKSFAPATPVEDRGSSSPSGMLSLSERAPICSPQSAPSASASDTCKMEEN
MNQQKTLEADISNTSVNQSPQSKILPESSPDNQDAEHEYRSPPPISESKELSPQSRTTPGSSPDNQDTEREYPSPPP
ISGSKEISPQSRTILESSPDNQDNGHEYPSPPPIPESIELSPHSKALPESSPDNQDIEPECPSPPQIPESKELSRQS
KILPESSPGNQDIEPECPSPPQIPESKELSQQSKILPESSPDNHDIKCEYSSPTPIPESKELSLQSKILPESSSDNQ
DIKCEDPSPTPISKSKEVSPQSKILSESYLDNQDVERECPSSILITESKELAVDLPGSISLAPEKTASTDVGENSSL
AFIFPKSTLAGDDALKSVFDMAKAHLECEDSKVKEELYVESTVVIRDDMVVNPASGVESIDMSENLLESLMEQSCGT
FYMDGTTALEGFLSGSTKEEPQCSSPIALSTCSSPIALSPWGEHGYYQGDSVGSSLWGVQDDDPIGNIWPLSSQAPA
LQYSSAGSTAHFIDEATVTHGNNGVVLSSTPGEEVGLPNSGVCTDWGLVEQVNPETNDASVSMIDKNSGLVDSQPSA
NDGSDVGTARNTNHNTNLSLNHETAVPLSRSSGEASRKHGFITDLNVATSEEALGNTKNWNPYAGNANRGSQRNHHR
DRYSQISESWLLSSNYSRSRSDGFGTGGSSRSTPRGQTQRGICKFHENGYCRKGASCNYLHP

>LOC_Os03g02160.1|PACid:21916697
MPQTSSSAHSNVESHHDHYPSWSQVQHDPKNSLQAGSGKDLNSRYDIAQKLPSQRITRDVPSPVFAWSPSESRTASS
QHEGSCLSSTTNLCTHDELHSSIASAKAKSFAPATPVEDRGSSSPSGMLSLSERAPICSPQSAPSASASDTCKMEEN
MNQQKTLEADISNTSVNQSPQSKILPESSPDNQDAEHEYRSPPPISESKELSPQSRTTPGSSPDNQDTEREYPSPPP
ISGSKEISPQSRTILESSPDNQDNGHEYPSPPPIPESIELSPHSKALPESSPDNQDIEPECPSPPQIPESKELSRQS
KILPESSPGNQDIEPECPSPPQIPESKELSQQSKILPESSPDNHDIKCEYSSPTPIPESKELSLQSKILPESSSDNQ
DIKCEDPSPTPISKSKEVSPQSKILSESYLDNQDVERECPSSILITESKELAVDLPGSISLAPEKTASTDVGENSSL
AFIFPKSTLAGDDALKSVFDMAKAHLECEDSKVKEELYVESTVVIRDDMVVNPASGVESIDMSENLLESLMEQSCGT
FYMDGTTALEGFLSGSTKEEPQCSSPIALSTCSSPIALSPWGEHGYYQGDSVGSSLWGVQDDDPIGNIWPLSSQAPA
LQYSSGSTAHFIDEATVTHGNNGVVLSSTPGEEVGLPNSGVCTDWGLVEQVNPETNDASVSMIDKNSGLVDSQPSAN
DGSDVGTARNTNHNTNLSLNHETAVPLSRSSGEASRKHGFITDLNVATSEEALGNTKNWNPYAGNANRGSQRNHHRD
RYSQISESWLLSSNYSRSRSDGFGTGGSSRSTPRGQTQRGICKFHENGYCRKGASCNYLHP

>LOC_Os03g02160.2|PACid:21916698
MPQTSSSAHSNVESHHDHYPSWSQVQHDPKNSLQAGSGKDLNSRYDIAQKLPSQRITRDVPSPVFAWSPSESRTASS
QHEGSCLSSTTNLCTHDELHSSIASAKAKSFAPATPVEDRGSSSPSGMLSLSERAPICSPQSAPSASASDTCKMEEN
MNQQKTLEADISNTSVNQSPQSKILPESSPDNQDAEHEYRSPPPISESKELSPQSRTTPGSSPDNQDTEREYPSPPP
ISGSKEISPQSRTILESSPDNQDNGHEYPSPPPIPESIELSPHSKALPESSPDNQDIEPECPSPPQIPESKELSRQS
KILPESSPGNQDIEPECPSPPQIPESKELSQQSKILPESSPDNHDIKCEYSSPTPIPESKELSLQSKILPESSSDNQ
DIKCEDPSPTPISKSKEVSPQSKILSESYLDNQDVERECPSSILITESKELAVDLPGSISLAPEKTASTDVGENSSL
AFIFPKSTLAGDDALKSVFDMAKAHLECEDSKVKEELYVESTVVIRDDMVVNPASGVESIDMSENLLESLMEQSCGT
FYMDGTTALEGFLSGSTKEEPQCSSPIALSTCSSPIALSPWGEHGYYQGDSVGSSLWGVQDDDPIGNIWPLSSQAPA
LQYSSAGSTAHFIDEATVTHGNNGVVLSSTPGEEVNPETNDASVSMIDKNSGLVDSQPSANDGSDVGTARNTNHNTN
LSLNHETAVPLSRSSGEASRKHGFITDLNVATSEEALGNTKNWNPYAGNANRGSQRNHHRDRYSQISESWLLSSNYS
RSRSDGFGTGGSSRSTPRGQTQRGICKFHENGYCRKGASCNYLHP

>LOC_Os03g17960.1|PACid:21916810
MDPHPTPFAGKRRSVAAAPAKIAAPKPKSIASTRTKTTRKSPPAPPPPRPRRAFGTVRSSNAHDAPEKPPPLQKAPK
VSPPPPQKPDKVSPPPAQKPSKVSPPPPPPQKSAKVSPPPAAKPPKLSPPNLAKATKPSRLAAKPPKKAAPGPELDP
KPRKKAQRVSFQEDAAMSVAPGSGEKVKVSTDDAAGHTPMVAVRALEKKVSVVASAETPFFSAQNCSNCSLDPLEES
TYWLAHIHLAESVGKHRVAAAFFQLAFECQAQPIHRIQSELRNYTVRHESASTLTTLFDELLLAHGGIPVNQPKFET
DGFEVVDTPLTTDSDDKRLDSTTTQVDERCSECDCGGDIVDVAVPSIVKPLEEGMDQPSFERKLNDGFEFDDCEAVI
VDKLVGGHSDLEKIVDVNGPSDSETMQSACRSSIDRLSLKGSPVVSGLSQRQLSSDSPLDKLSPSARSLSAKRLSSV

SPLDKKSPFGSSSSKRLSSSCPSSKKSFSSKALSSKRMSSGNASAGVGDLNEVIADMEFDCPASDDQLELKEHGDSE
VCLRVLFTLWSSAEMI

>LOC_Os03g19310.1|PACid:21917124
MSKLQDRHGGEAAADVGRRARHQRLLLSFPVFPIVLLLLAPCTIFFFTSGDVPLPRIRIEYARRDAPTITAVAADTS
PPPPSPPSSSPPPLSFPPPPPPPSSPPPPALPVVDDHSDTQRSLRRLQLTDSPYTLGPAVTGYDARRAEWLRDHTE
FPASVGRGRPRVLMVTGSAPRRCKDPEGDHLLLRALKNKVDYCRVHGFDIFYSNTVLDAEMSGFWTKLPLLRALMLA
HPETELLWWVDSDVVFTDMLFEPPWGRYRRHNLVIHGWDGAVYGAKTWLGLNAGSFIIRNCQWSLDLLDAWAPMGPP
GPVRDMYGKIFAETLTNRPPYEADDQSALVFLLVTQRHRWGAKVFLENSYNLHGFWADIVDRYEEMRRQWRHPGLGD
DRWPLITHFVGCKPCGGDDASYDGERCRRGMDRAFNFADDQILELYGFAHESLDTMAVRRVRNDTGRPLDADNQELG
RLLHPTFKARKKKTSRAARPM

>LOC_Os02g05610.1|PACid:21919771
MADVLLPELRSPPPPPPPPPPTNDDRARAVDLPSPTPECDDRSPRRELGRSPEDHGVPLPPPPPLGSSRPERLASDR
PEEGASAAAQPCGGRSESPTARSMWPRRLSPASLPPRGGRRSESPTPRSIWRRLSPSPPPPLPPLPPKRPRLDGRRS
PPRGGRFGFEHERGRERSMNTSRRAPDCLDSGCDAPYNGQSNTRRKGLMTYKQFTQKLEDDVSPGEAESRYQEYKTS
YITSQKQDYFDHHKNEDRLKDMYHPTNLLSVIERRNELCKAAAKNLILDLRSGTLDLGPGMTAGTASKSGNDSDGIP
ADDEDYHNKRRRHHRGPLEETELVSVAPKAHPVSSHYRRIQTDIHQTLALVKKLDEEKGIVGNILTTGDHTKSNGDK
SYAGSTGPLVIVRGLSTVKGLDGFELLDTLLTYLWHVHGIDYYGMSESTNAKGLRHVRADTKNANMDKSSAADWEKK
LDYFWQERLTNGKDPLVALTAKDKIDASADKVLESYVTKVKDDNYGWTYGCGAKGCIKVFHAPDFVLKHLNLKHPDL
VSKLTSRVQEDIYFQNYMNDPNAPGGTPVMQQQSPEQQGPTPSELTPGAFGGQGSFVEMPTPPVLIPVPGAGPLGPF
VPAPPEVVMQMMRPVMPMYPPRPPNPRRLRSYKDLDAPDDEVTLVDYRSL

>LOC_Os02g10110.1|PACid:21919776
MAAAALPRLLLAAAVLCAAFAPVSGFTDPSDALGLWELYRTLDSPWQLSGWTSQGGDPCGRGGEQRPWHGVLCRDSS
IVALNISGLGVGGWLGLELLKFYSLKILPVFNFFASLNHDDRRDVSFNNIAGEIPRNLPPSVEYLNFAANQFEGSIP
PSLPWLHTLKYLNLSHNKLSGIIGDVFVNMESLGTMDLSFNSFSGDLPTSFSSLKNLHHLYLQHNEFTGSVILLADL
PLSSLNIENNSFSGYVPGTFESIPELRIDGNQFQPGFKRASPSFTRSAHSPPTPHPPPSSPPPPMSPPPPAVKENLK
HKPEPLKPSLSHSSMYNHNQHRKSHSRVTAAAIATVTGTAFVLLIVGLVLKSCTYSPKSTANNAKSPPANVEKVPKA
NEVLYSWNSLMNDCEASSSDVIKPERAMKTKVWAKTSKNFLTAKQFQAVDILAATRNFSKECFIGEGFTGQVYRGDF
PGGQLLAIKKINMVDLSLSEQDELIDMLGKMSNLKHPNISALVGYCVEFGHCALLYEYAENGSLDDILFSAATRSRA
LSWKARMKIALGVAYALEFMHSTCSPPVVHGNIKATNILLDAQLMPYLSHCGLARLSQFVSAIRTDSEALNSGKGYV
APELTDPATDSIKADIYSFGVILLVLLTGQKAFDSSRRQNEQFLVDWASPHLHNLDSLERITDPRIHASMPPQAIST
LGNIILLCIKKSPELRPPMTVITDKLLKLVQSTGLQKTSTTTQHLEVDAQEPSFKTTRPYFEPSFTVSQSATGGCIS
QR

>LOC_Os02g19180.1|PACid:21919893
MAQQHSPPPPPAPPSPPPALPCDPMPPPPRHDDTTLTLSLAPPAAARPLQAMVARAKCSSPTGDAPPCTECGRQFLS
WKALFGHMRCHPERHWRGITPPGGGGAGAASSTAASQFTLREREVAASLLMLSGAHPARSGAGKGKGKKRLLAPAAA
AAPHHSPATCADHKCAVCHRGFATGQALGGHKRCHWPDRSCADQAISMLAVSTAGSSSTTTTSASPPPAPATAATAL
DLNLNLPPPLARKCILLLLDLCLLNSIRGISGSLEWKSLQVLSFGYVCLVQIRAFLIVLNTEIGVLIDQKSAF

>LOC_Os02g46420.1|PACid:21919998
MGMRCKKHPYQAGGGVCATCLNDRLLALAAAQNGASSSPPPPAAPPAAAAPAFPRSVSPYVSRRKSDSSGGGALKHH
PSLLFFRTPQVGPAYGSSGGLEEGDIGYEKRRAGKFSVIATLFGHHHHHHRSEEKDKGGDNRESRNRSWLAGFMPRR
RKKQPPAPAAAAAASSPPPRRSCRVVSNRGLSPERDCDGSDEESSSPTDPPWQPSPSPMRRTPCRRRQTSTMPSGFA
VCLSPLVRPSPGRRHRHAHPPDPGTFSCELRPSPLHNLSSAASITRCRSRKLADGGRFR

>LOC_Os02g26370.1|PACid:21920449
MGSTSSPSPPPPPMIGRAGNLTVFITPPSPASTPRSSRPSESPRSGFSTPATAPRTAASPSPPSPAPSPQQQRVASP
PPTIPVKFSPPAAPVKVPPPPPVQVPPPQYEKASAGGKHDGSAFGFFWDAVARVQEAHASLDEYVANWFGLDQSKYQ
WALNDYYESTGKEVECGKAGKPKELTTSKVQKV

>LOC_Os02g26370.2|PACid:21920450
MGSTSSPSPPPPPMIGRAGNLTVFITPPSPASTPRSSRPSESPRSGFSTPATAPRTAASPSPPSPAPSPQQQRVASP
PPTIPVKFSPPAAPVKVPPPPPVQVPPPQYEKASAGGKHDGSAFGFFWDAVARVQEAHASLDEYVANWFGLDQSKYQ
WALNDYYESTGKVRSP

>LOC_Os02g39720.2|PACid:21920806
MARVYVGNLDPRVTAREIEDEFRVFGVLRSVWVARKPPGFAFIDFDDRRDAEDAIRDLDGKNGWRVELSTKAGSGRG
RDRSGGSDMKCYECGEPGHFARECRLRIGSGGLGSGRRRSRSRSRSPRYRGRSRSRSPRYRRSPSYGRRSYSPRDRS
PKRRSYSRSPPPARARSYSRSPPPPRERSYSRSPAQPANREESPYANNA

>LOC_Os02g39720.3|PACid:21920807
MARVYVGNLDPRVTAREIEDEFRVFGVLRSVWVARKPPGFAFIDFDDRRDAEDAIRDLDGKNGWRVELSTKAGSGRG
RDRSGGSDMKCYECGEPGHFARECRLRIGSGGLGSGRRRSRSRSRSPRYRGRSRSRSPRYRRSPSYGRSPRDRSPKR
RSYSRSPPPARARSYSRSPPPPRERSYSRSPAQPANREESPYANNA

>LOC_Os02g44450.1|PACid:21921026
MALSLSLTLQEWISQNITTGSPRPPPTGVSACTAGLLCRRCWASTSSPTGLPSPPPTGVSACTAGLYAAVVGRLRLH
RPAASTAADWCFRLHGWPIMPPLLGVYIFTDRLAFAAAGWCFRLHGWPLCRRCWASTSSPTGCLVRRRLVLPPARLT
YYAAVVGRLRLHRPAASTATDWCFRLHGWPIMPPLLGVYVFTDRLAFAAADWCFRLHGWPIMPPLLGVYVFTDRLAF
AAADWCFRLHGWPLCRRCWASTSSPTGRLDRRRLVFPPARLAFMPPLLGVYVFTDRLAFAAADWCFRLHGWPIMPPL
LGVYVFTDRLAFAAADWCFRLHGWPIMPPLLGVYVFTDRLAFAAADWCFRLHGWPLCRRCWASTSSPTGLPSPPPTG
VSACTAGLYAAVVGRLRLHRPAASSAADWCFRLHG

>LOC_Os02g49500.1|PACid:21921166
MPPLLGVYVFTDRLAFAAADWCFRLHGWPFMPPLLGVYVFTDRLAFAAADWCFRLHGWPLCRRCWASTSSSTGLPSP
PPTAFMPPLLGVYVFTDRPPRPPPTGASACTAGLYAAVVGRLRLHRPACLRRCRLVFPPARLAFMPPLLGVYVFTDR
PPRPPPTGASACMAGLYAAVVGRLRLHRPAASSAADWCFRLHGWPLCRRCWASTSSSTGLPSPLPTGVSACTADLYA
AVVGRLRLHRPAASSAADWCFRLHGWPLCRRCWASTSSPTGLPSPPPTGVSACTAGLLCRRCWASTSSPTGLPSPLP
TGVSACTAGLYAAVVGRLRLHRPAASTAADWCFRLHGWPIMPPLLGVYVFTDRLAFAAADWCFRLHGWPVMPPLLGV
YVFTDRPPHPPLTDVTASTAGLL

>LOC_Os02g38290.1|PACid:21921363
MAIASMAAAAAAAIVGAVREHVRASDLAVAGAVLFAFSAAVSAVRARRRGAPVLWPLVGIVPTLFVHRDDIYEWGSA
ALLRAGGVFPYRGTWGGGSSGVITSAPANVEHVLRANFGNYPKGPYYRERFVELLGGGIFNADGEAWRAQRRAATAE
MHSSRFVEFSVRSIEQLVYGRLVPLAERLSGGGAAVDLQEVLLRFTFDNICAVAFGVDAGCLADGLPDVPFARAFEL
ATELSLLRFVTPPFIWKAKRLLRAGSERRLVEATRAVREFAERAVADRRNEMRKVGSLRGRCDLLSRLMSSAPGADY
SNEFLRDFCISFILAGRDTSSVGLAWFFWLLAGHPDVESRVVGDVLAAGGDIKRMDYLHAALTEAMRLYPPVPVDFK
EALADDVLPDGTPVRARQRVIYYTYAIGRDPASWGDDAAAFRPERWMRGGAFAGGESPFKYAVFNAGPRLCIGKRFA
YTQMKTAAAAVLSRFAVEVVPGQEVKPKLTTTLYMKNGLMVRFRRRSPPPPSPPPRHVVADDDDDDDVAAGRHVAVG
SCNSNHL

>LOC_Os02g38290.2|PACid:21921364
MAIASMAAAAAAAIVGAVREHVRASDLAVAGAVLFAFSAAVSAVRARRRGAPVLWPLVGIVPTLFVHRDDIYEWGSA
ALLRAGGVFPYRGTWGGGSSGVITSAPANVEHVLRANFGNYPKGPYYRERFVELLGGGIFNADGEAWRAQRRAATAE
MHSSRFVEFSVRSIEQLVYGRLVPLAERLSGGGAAVDLQEVLLRFTFDNICAVAFGVDAGCLADGLPDVPFARAFEL
ATELSLLRFVTPPFIWKAKRLLRAGSERRLVEATRAVREFAERAVADRRNEMRKVGSLRGRCDLLSRLMSSAPGADY
SNEFLRDFCISFILAGRDTSSVGLAWFFWLLAGHPDVESRVVGDVLAAGGDIKRMDYLHAALTEAMRLYPPVPVDFK
EALADDVLPDGTPVRARQRVIYYTYAIGRDPASWGDDAAAFRPERWMRGGAFAGGESPFKYAVFNAGPRLCIGKRFA
YTQMKTAAAAVLSRFAVEVVPGQEVKPKLTTTLYMKNGLMVRFRRRSPPPPSPPPRHVVADDDDDDDVAAGRHVAVG
SCNTQE

>LOC_Os02g57790.1|PACid:21921777
MSVEDKHAGVVKRKRTKRPRHHAAPASSSESTTTEEEDMAHCLILLAQGAAVVDSKPSTPAPPPPPPAQPPVLAAPA
PAPPPPQPPVVVVKSERYTSRKYTEAATTADGVRAGFYVYECKTCNKCFPTFQALGGHRASHKKPRLPGADDDNVNN
VTNTNAIVVKSKPPLTTTTTPSAPSPPPPQADAVVVPDVTTVLSLNNVAAAGSIINKLRVHECSICGAEFGSGQALG
GHMRRHRPLHAPPERAATTAATTAATATAPDTKKEGSTSINLELDLNLPAPSDEESVSPPPPPPPPVLLALGGQFND
GKKPILQLTASAALVGCHY

>LOC_Os02g55430.1|PACid:21922264
MATAAVLGSHDALDNRMHLGAFAVSPASKPRRRRHSKAASPPPKVAAVSSPPVVKSAARVASPPPKAAAAAAVPQGR

RSPVARKQGSPTKELPKQRLVMGEIRILKRGEEPPVSSPSPAPVAAQAAAVDQRAPRVPAVAQAAPTDQKAPRPARS
KQQQHTAAVPTKIKSVADAPVYAGPAFSSASPEPSSLPFPAFIRRAEAEATRGLRCLLRIGELP

>LOC_Os02g33460.1|PACid:21923272
MLQAAGAAPAPGLLRAACLRSHSHPLQCKALELCFNVALNRLPASAGASPLLGHGHGVGVYYPPSLSNALVAAFKRA
QAHQRRGSVETQQQPVLAVKIELEQLVISILDDPSVSRVMREAGFSSTQVKANVEQAVSSSMEAATTKPQNPNPSSS
SPPPAAHQEAKPSRCIDQVVVREEDVAAILDCLATRSKKRVMVVAECAAAAEAAARAAVDRIRRGEARQHAQAQVVT
LAVSRFRGAPREEAERRLAELRCAVRGGGGRAVVLVVEDLAWAAEFWAGRRPPPSSCGAGAGGYYYCAVEHAVAEVR
ALACGGGGGGGGVWLVGHGTYQTNIRCRTGHPSLETLWGLHTLAVPAGSLALSLTCADADAAADDDDSGAMAAAAVN
HQSAKGANGSTSPSPCLSLLDAAAAGGACSSGQLAVMAAAVSGAYCGGDCAAATKALLPQSVVFMPPSATTTTTTIP
PWLHHCRDQEPAAHMKKWMSAHGGSPSRRTALNISSTAVSPCSSVSSYEQYTRLHQPYQPWLVADDDDEAEETKHPY
IAGDGGAGRLVPAAAKVVIKSDDSSASNGSVEVEWRRPRFKEVSAENLKVLCGALEKEVPWQKVIVPEIASTVLRCR
SGMAAPAMARRSSSCSSSKEHTWMLFLGGDADGKLRVARELASLVFGSSKSFVSIGGAANASPPPSSSSSSPARSSG
STEQPHRSKRPWAETTTTTTSGRDQDHLEALYDAVRDNPRRVILMERVDRADARCHDGIRDAIERGVVRSRGGGGEE
AFLGDAIVVLSCESLNPSSTTPAKKAKTEYSVEKLDQDGDDHHGKEAVAAAASPSCFDLNMSMDDDDEAAEERCTGE
EEEAGHHHHQLLLKAVDRVLFFRSIGE

>LOC_Os02g17220.1|PACid:21923325
MAARKARGSGWGEAVAAGAGGDVARRGRGGGRSRSCRPLRRWGRHVGEAETAVSDAGNTSPSTATATPPPPPGPSPS
PPPRPTSSPASSPSPPPSPPLSSLRVKPLPCRHLPDDDPDSDFAPDNLDLLRNLPVDRLLVVSVGVAANLLFAFLI
VYALARPPPPLSSPASSAADGEGGEKGGRERGREEEEGLEREDDMWAPHVSGSHNIYFFCVNDRWVP

>LOC_Os02g58770.1|PACid:21923848
MPPLLGVYVFTDRLAFAATDWCFRLHGWPFMPPLLGVYVFTDRLAFAAADWCFRLHGWPLCRCCWASTSSPTGLPSP
PPTGVSASTAVLLCRRCWASTSSPTGRLVRRRLVFSPARLAFMPLLLGVYVFTDRLAFAAADWCFRLHGWPIMPPLL
VFPPARLAFYAAVVGRLRLHRPACLRRRRLVFPPARLAFMPLLLGVHVFTDRLAFAAADWCFRQHGWPIMLPLLGVY
VFTDRPPRPPPTGVFACTAGLYAAVVGRLRLHRPACLRRRRLVFPPARLAFMPPLLGVYVFTDRLAFAATDWCFRLH
GWPLCRRCWASTSSPTGRLVRRRLVFPPARLAYYAAVVGRLRLHRPAASSAADWCFRLHGWPLCRCCWASTSSPTGL
PLPPPTGVSACTAGLYDRCCWASTSSPTGLPSPPPTGVSASTAGLLCRRCWASTSSPTGRLVRRRLVFPPARLAFYA
AVVGRLRLHRPACLRRRRLVFSPARLAFMPLLLGVHVFTDRLAFAAADWCFRQHGWPIMPPLLGVYVFTDRPPRPPP
TGVFACTAGLYAAVVGRLRLHRPACLRHRRLVFPPALLAFMPPLLGVYVFTDRLAFAATDWCFRLHGWPLCRRCWAS
TSSPTGRLVRRRLVFPPARLAYYAAVVGRLRLHRPAASSAADWCFRLHGWPLCRCCWASTSSPTGLPLPPPTGVSAC
TAGLYDRCCWASTSSPTGLPSPPPTGVSASTAGLLCRRCWASTSSPTGRLVRRRLVFPPARLAYYAAVVGRLRLHRP
ACLRRLRLVFPPARLAYYAAVGGRLRLHRPATASAADWCHRLHGWPVITPTDAIFFTAGYIAVTTNRHQYLQHKLPT
FAMAADWFLHFHGYMILPVLISLIFFTAGLVRYPLLLLPNSCFDKDNIIEGGDHNRPLYIEGNIGSAHLRRILIDPG
SAVNILPVRSLTRAGFTTKDLEPTDVVICGFDNQGKPTLGAITIKIQMSTFSFKVRFFVIEANTSYSALLGRPWIHK
YRVVPSTLHQCLKFLDGNGVQQCIIGNFSPYTIQESYHADAKYYFPVEENKQQLGRTAPAADIIIEPGTETTPKVRR
LIMPCSPIISESSSRNRKNSRRGNSSSATNLSTKLPFPAETSTSAPSTTPILLKARRSTSTPITLGATSRGSKKPPK
SNSDVPTTITLQARRSPSKEAALAQQGVVKKRVAEIEKVKAGAQCMQPPALYTLVSTPLEVWAIPDPSSSTSRTILY
KVPQVQECDSIIEFPKPYSDTEEACTTMKAEPRMARLLEKAGINLQRNNRLPPPPAVCEDWWAQAEEFIKRRCKEQP
KYGLGYINVDEPDDEDEVFEDDIFHCCTISTTTIGDALLQQHPFEVAAVGVEEELDVAGALKQLDDGGQPTIDELVE
MNLGTKDDPCPIFVSGMLTKEEREDYRSFLMEFRDCFAWTYKEMPGLDPRVATHKLAIDPQFRPVKQPPRRLRPEFQ
DQVIAKVDRLINVGFIKEIQYPRWLANIVPVEKKNGQVRVCVDFRDLNRACPKDDFPLPITEMVVYSTTGYGALSFM
DGSSGYNQIKMDPLDAFDTAFRTPKGNFYYTVMPFGLKNAGATYQRAMQFVLDDLIHHSVECYVDDMVVKTKDREHH
QKDLRIVFERLRRHQLKMNPLKCAFAVQSGVFLGFVVRHRGIEIEPKKIKVILNMPPPRELKDLRKLQGKLAYIRRF
ISNLSGRIQPFSKLMQKGTPFVWDEECQNGFDNIKRYLLNPPVLATPVKGSPLILYIATQPASIGALLAQHNDEGKE
VACYYLSRTMVGAEQNYSPIEKLCLALIFALKKLRHYMLAHQIQLIARADSIRYVLSQPVLTGRLGKLALLMMEYDI
TFVPQKAIKGQALAEFLAAHPVPDDSPLITNLPDEEIFTAELQEQWELYFDGASRKDVNPDGTPRRRAGAGLVFKTP
QRELVPYYTVARRLMDKFEHIEVIHVPRSKNAPADALAKLAAALVFQGDNPAQVVVEERWLLPAVLELIPEEVNIIT
TNSAKEEDWRQPFLDYFKHGSLPEDPVERRQLQRRLPSYIYKAGVLYKRSYGQEVLLRCVDRSEANQVLQEVHHGVC
GGHQSRPKMYHSIRLAGYYWPGIMADCLKTAKTCHGCQIHDNFKHQPPAPLHPTVPSWPFDAWGIDVIGLIDPPSPR
GHRFILAAIDYFSKWAEAVPLREVKSSDVINFLERHIIYRFGVPHRITSDNAKSFKSQKIYRFMEKYKIKWNYSTGY
YPQANGMAEAFNKTLGKILKKTELDAVEEERLGALQNLELYRQNMVRAYDKLVKQRVFRKGELVLVLRRPIVVTHKT
KGKFEPKWEGPYVIEQAYDGGAYQLIDHQGSRPMPPINGRFLKKYFV

>LOC_Os02g56730.1|PACid:21923915
MGVHVFADRLAFAAADWCFCLHGWPIMPPLLGVYDFTTGLPPSPPTGVLAYTVGWSHHRSWASTSSPTGWPSPPPTG

VSAYTVGLLCHRCWASTTSPPACLRRRRLVPPPTRVAYYAAAVGRLRLHRRPAFAAADWCFRLHGWPIMPPLLGVYD
FTAGLSPSPPSGASAYTAGLLCRRCWASTTSPPAYLRRRRVVPPPTRLAYYAAAVGRLRLHRRPASVAAEWCLRLHG
WPIMPPLLGVYDFTAGLPPSPPSGASAYTAGLLCRRCWMSTSSLLAHLRCRRLVFPLTRLTYYAAISGHLRFHRRPP
SSPPTGVFIVIDGRLCSHIGHFCHHQGEYYKAKNGSVRTSPPARLDRRRLVFPPARLAFMPPLLGVYVFTDRLAFAA
ADWCFRLHGWPLCRRCWASTSSPTGRLVRRRLVLPPARLAYYAAVVGRLRLHRPAASSAADWCFRLHGWPIMPPLLG
VYVFTDRLAFAAADWCFRLHGWPIMPPLLGVYVFTDRPPRPPPTGASACTAGLLCRRCWASTSSPTGLPSPPPTGVS
ACTAGLYATVVGRLRLHRLACLRRCRLVFPPARLAFMPPLLGVYVFTDRLAFAAADWCFRLHGWPLCRRCWASTSSP
TGLPSPPPTGVSACTAGLLCRRCWASTSSPTGRLVRRRLVLPPARLAYYAAVVGRIRLHRPACLRRCRLVFPPARLA
FMPPLLGVYVFTDRLAFAAADWCFRLHGWPLCHRCWASTSSPTGLPSPPPTGVSACTAGLYAAVVGRLRLHRPACLR
RRRLVFPPARLAFMPPLLGVYVFTDRLAFAAADWCFRLHGWPIMLPLLGVYVFTGRPPHPPLTDVTASTAGLL

>LOC_Os02g18870.1|PACid:21924546
MALPFLLLLAFALLFPLSAPPRCCSAAPASSPPPSPPPSPAAAAAAPRRTPLVPALFVIGDSTADVGTNNYLGTLAR
ADREPYGRDFDTRRPTGRFSNGRIPVDYIAEKLGLPFVPPYLEQNMRMGVGSVDLSNIDGMIQGVNYASAAAGILSS
SGSELGMHVSLSQQVQQVEDTYEQLSLALGEAATTDLFRKSVFFFSIGSNDFIHYYLRNVSGVQMRYLPWEFNQLLV
NAMRQEIKNLYNINVRKVVMMGLPPVGCAPHFLWEYGSQDGECIDYINNVVIQFNYALRYMSSEFIRQHPGSMISYC
DTFEGSVDILKNRDRYGFLTTTDACCGLGKYGGLFMCVLPQMACSDASSHVWWDEFHPTDAVNRILADNVWSGEHTK
MCYPVDLQQMVKLK

>LOC_Os09g24820.1|PACid:21925552
MEAMDVKYKPLVFPNGAIKKAAKPAAVAPAVGGGGGGETVYRECLKNHAASLGGHALDGCGEFMPSPAANPADPTSL
RCAACGCHRNFHRRLPEGSPPPPPPPALLPAPPMPPHRGEETPEVRLPGVDGDESDSDSDGSEYDDERSVSPPPPPL
AAAVAHQVYYPSAPHMLLSLGSSGQAQRLPPQVMSPAAAAAPPPGGGGGGMPRKRFRTKFTAEQKQRMQELSERLGW
RLQKRDEAIVDEWCRDIGVGKGVFKVWMHNNKHNYLGGHSARRSASSSSAAAAAAPPFNPPTSPPPPPPPPPHATDF
NINGTATAATAAAAATIAAGNHQENGASSPQSA

>LOC_Os09g07150.1|PACid:21925881
MPEADTAAAADSAAARVYLPSTAAAEDSSAAVRSASPPPPEASAAAADVSDDAAADSAARVYLPSTAAAEDSSAAVR
SASPPPPEASAAAAEVSDDAAADSAAARVYSPSPPPPPRTLPSPLRPNPSPARRRGRLRHSRGRLPRVRLRHRSRLP
PRRRRAAEVDSAATRVCLPRICLLLRRRARLPPPPRPTPRCRRHARHFFLSSFFPFFSDGDYHCRRDSSSNGSSAAA
VSPPYSPTSASPGGRRPMGTGCSSPRPAPRAGSHSPTSRRRRQAGLRATRSGLPPARSSEEHGAGHSRRHGQAGWHP
R

>LOC_Os09g29780.1|PACid:21925935
MARALVQVVGSPPPRPRSPRRVSRHRLPLFSPPPPPSSLSAAYDIGIGEGDEGDRRRRRPDGVGGGVPRRAADAGS
GGGRAVAGGRARPLRRRHRRALLRRLPPPLQARRVLATRCSSPPPQPPSE

>LOC_Os09g01800.1|PACid:21926093
MDQSEHHHRLASTAADWCFRLHGWPIMPPLLGVYVFTDRLAFAAADWCFRLHGWPLCRRCWASTSSPTGRLDRRRLV
FPPARLAYYAAVVGRLPLHRPACLRRRRLVFPPARLAFMPPLLGVYVFTDRPPRPPPTGVSACTAGLLCRRCWASTS
LPTGLPSPPPTGVSACTAGLLCRRCWASTSSPTGLPSPPPTGVSACTAGLYAAVVGRLRLHRPAASTAADWCFRLHG
WPLCRRCWASTSSPTGRLDRRRLVFPPARLAFMPPLLGVYVFTDRLPRLPPTGASACTADLLCRRCWASTSSPTGRL
DCRRLVFPPARLAYYAAVVGRLRLYRPACLRRRRLVFPPARLAYYAAVVGRLRLHRPACLRRRRLVFPPARLAFMPP
LLGVYVFTDRPPRPPPTGVSACTAGLLCRRCWASTSSPTGLPSPPPAGVSACTAGLYAAVVGRLRLHRPAASTAADW
CFRLHGWPIMPPLLGVYVFTDRLAFAAADWCFRLHGWPIMPPLLGVYVFTDRLAFAAADWCFRLHGWPLCRRCWAST
SSPTGRLDRRRLVFPPARLAFMPPLLGVYVFTNRLPRPPPTGASACTADLLCRRCWASTSSPTGRLDCRRLVFPPAR
LTYYAAVVGCLRLHRPAASTAADWCFRLHGWPIMLPLLGVYVFTDRLPRPPPTGASACTADLLCRRCWASTSSPTGL
PSPPPTGVSACTADLLCRRCWVSTSSPTGCLDRRRLLSLLMNIIVFRRRLVMLSPTSAFIFTTARLHYQFASVDADS
CSLHHGNATLSSW

>LOC_Os09g36490.1|PACid:21926097
MSSSLAVLLLVMRLAAAMAASPKSYISRTTEQQVIATVAPAVDVGQSAQPFLTSPSGSYAAYLRRAVDSSAGGPGAD
ACYVQIQQAGGGAGGGGSVWESECTLVGGADACDLAFSPVGLELFAGGHSLWDTGIDAEPGTLSLDDGGDMRIVSKD
GVSVWQASGEPWTGQQCGAAAPVSPSPTMDVLPPPSTTTTAKLLTPPASTLAGAGSSDLSFGDQLAPPVDTSLPASP
DQPPVDTMPDQPLLPPPPADATPATPDLPLPPPPPADTYPVSPDQPLYSSPPPAPTAFVPHTPLPPVDVPALSPPLP
HGKTPSGAPGGIALPPAAPAGGGMPQQHGSPHHLPLGASPPPEAAAVPDALAPSAGHGAAAGGLPIGGQGQQQGAFG
QHQVLNGAGQPLEDSSGERPRGAHAAVVVTSGLVSLLIALGFGF

>LOC_Os09g33610.1|PACid:21926116
MAARRCGSRRRRRRAAATRVGLISRLADTPCIKRGLESPSAWKSPWFVDMQFQGSYFVSPADRTYNALGFVKQINVQ
TAAALAEAREVLVSGGQSENINSGKENLESPNAKKEPGATTKLQAKIKIGKGLLPPLPMAATEHKPAGRKTAEKVEE
KPSPPPLLLLLLGVCFLWGESPRKQARLKSAIVVPETRKLSVFNRLSKGEPGAPSSIWQEVKPRFWWRQGRAPLPAT
ASRRPTTGVIPAWRPFNPATLGRCLRCLAQDHKIADCRDPPRCYICKRSGHISSGCPSKYKNKPSIFSCIYSTHPAP
PSKQEIPNPLAFPPLTGHKAPVRERLGPKMDYIPGLPENHPACGYAVVVADAAMEHEAMRLYNHGAVIKARLPNYDV
SAKEMAQALQQQVGTPNYDVRRCSKMSASSTNPNLLPRVSVFTLFPAGVGQVLETGGMPTPSQEVASAPLGRVRHLL
IHLDMVEDWRPLPARSSNSSGSTDSDDPPRRRVHRFDWHLGYEDGVSPPPLQPPQPPLPVRRLHEERGRPEQRGIDL
RNRLSQRGSPKAETTAGTAAAPPVAAIATSPAIGWVAPQPGAHPGRLLAAGALAGRARQARGVVRSRRVAVFAGALP
KVVGATREGEDPMFLELAVAAAFHAEKSANASTLEAGASVPIGSAAPLGPVAPIGPVSPGPVAPLGPDGSAASPGLI
ASPGLGLLELVGEQMEGTATQPMQGGVEPFFAPLPPPLVATPPVRRRGRPRKKVLLPSRHSKRLEARPSPVLISMRA
QVRIMKEMRVLKPGEPVGDEAVAEYIKRFQDPLPTVVVQGIRALTGLDSGKPLPPDVTEEADGATPEWCTSLGVTYG
SRMIEGLKILNWNVRGLNNPARREAICEVANNADASILCIQETKLANIDQFLGREIGGSRLRRFEFLPADGVRGGVA
IFWDDTKVQVDNTQIHTFSVSIRAQVIGGNVPFILTSVCGPSEDEHKSDFLAELESLVPQPLLPWVVLGDFNLICEA
SDKNNLNLDRRRMGQFRSTLNRCCLKEFKLQNRKFTWSNEQEAPTLERIDKVFCNIDGDILFSNHTLQALSSSCSDH
CPLLLAFTVSPPKRVVFRFENFWVWVPGFMEVVQQAWSSHTFGGHPFTKLSHKLTATAKALRKWSRSLFGEIKMQFH
VAQEVILRLDLAQEDRILTADEHQLRKDLKLRILGLAAVAKSKKRQRSRLTWLKEGDANTKFFHIHANSRRRKNHIH
QLHTTSAILTEHEEKSEALFNHFNSILGNKEARNCTLNWNAIDLPQVNPDELDEPFTEEEVWAALKQMQAEKAPGPD
GFNGGNFGVLNRAAITLLPKRNAPTLVTDFRPISLIHSVAKLISKVLAIRLARQMDGLISVAQSAFIKKRCIQDNFL
YVRNMLRNLNASKKPTLFFKLDIARAFDSVSWEYLLELMQNMGFGPRWRNWICLLFSTATSKVVLNGDQGECIAHRR
GLRQGDPISPYLFILAIDPLQRLLHLATEGGQLTNIGSRHARLRTSLYADDAAVFLNPVQSEVDVLITMLRDFGKAT
GLHVNLGKSLVAPIRCQEVDLDDVLCSFSGARASFPLTYLGLPLTPGRLRKVHFQPLQDKIKGRLSGWKPSLLYMGG
RRIMVNSVLSAIPTYTLTVLKPPKQSLQEIDKARRRFLWAGNENMHGGKCKVNWLKVCSPVRTPEKPWIGTETPCDE
LDKDLFAASTRVQIGDGQKASFWESNWIGGQRLKSQAPNLYRHSKRKTRTVQDALQQGRWINDILHSLTIPLLDEFF
HVWEILARNTPQLTEGVQDSIVWQWTTNGEYSAKSAYQFQFNGMVLLPSTKLSWEAWAPVKCKIFSWLVLQNRVWTA
DRLQIRGWPNNYFYQLCFRNLETIDHLLFECLVSKQIWFGILTWLHQGHIIPKWNTSRNLLDWWTQGTSDLEATKQK
GLRTLFLLIAWEIWKERNARIFRAKEATVHQMISRIQEELGWWSMAGAKHIGRMLP

>LOC_Os09g37700.1|PACid:21926316
MPAGDNPHSISEKKAALRESPKEPKNVGNQQPRTSPFPKDKAAGTVGIKRPQPNGPLNPTNPGTNGHLVYVRRRLET
DHSKVSSYASADSISSLSSKKTVVDRPQEQGLKHQNSSLQTPLAPAAAAATSPASPSGGSLPQNSLRKQSLGKVVVQ
PSIIVTANPPPRNVVSTTSVPQNSIAAKLACSSVAAASPPPRNLVSTTPVPRNSITANLASSSVAAASPPPRNLVST
TPVPHNSIAANLASSSVAAASPPPRNLVSTTPVPRNPIAANVASSSVAAASPPRNLASTTKVSQNSIAANLASSSVS
ATSTASRGAAPACYPVDPQRSSNQDWKERFIRLQAFLRNNEQSGQEEYIHMLRSLSSVGRSKLAIELENRAVKLLIE
EGKELQKMKVLNVLNKLSPTDALPLPTQPASVRHLAFPPR

>LOC_Os09g16950.1|PACid:21926343
MASPPSPAEYRSYRYVLDDNLSSPTPAPGPSQPPPSPPPPPLPSPPPPYPFSSPWSSPPSPQAPPPQILTAPPLPAQ
ALPLAQPKTNSSTKTIAMAVVVPTLAVCVVAAALLWLWRQRKRRRKNSPPPANNDSDQYSSDGQRQHGTADLERAVT
GGGPRRYQFHELAAATRDFAEEEKLGQGGFGNVYLGRLAVGTGGGEDHQEVAVKKFSMDSMSQGRREFEAEVRIISQ
LRHRNLVQLHGWCDSRKGLLLVYELVAGGSLDKHIYNTDRILTWPERYKIIMGLGAALRYLHQEWEQCILHGDIKPS
NIMVDSSYNTKLGDFGLARLVDHGKAWQATRSVLGTAGYIDPEFVNTRRPSTESDVYSFGVVLLEIVCAKPPVVLQE
NEPSFVLLRWVWNLYSQNAILDAVDERLRVVGVVRDERQMERVLVVGLWCAHPDLSERPSIARAMNVLQSDDARLPD
LSPQMYKSKASPPPRDVAVGVDYGGVSTGSTFSGSGVPTSATTTTTRSSGSFVG

>LOC_Os09g29900.1|PACid:21926494
MASSPETTVPPESTHMGSPPPSHSPSPPPPLQGDHSLPTDAPPEPSPQPHHATPISPPSQGQATKERPRVEEPQPPI
DGTPGAAGPPAQPSFFSLLELGTSAAPPAPAATRQPDSPSPHPSAEPSVEFYLGLAASSPSSSSYETAQDDWPAPPP
RAHSPTTGLLAGFTLHRVFPCTRSLRRGGGLARRTRAAAADTIGDKAKGYLRPTEPKNSLSSATSASQVERRRRGVH
GQISTIMGCLPPNVHFRGVPELNTYNCDVL

>LOC_Os06g49750.1|PACid:21928826
MMAAAKRPYCKPPPPTTTTTAITSSSGQNHKRLRPSLPAAAAMDGGMEEEAVAPPPPTTMTTTTPPQPLLPGLPDHL
AQLCLSPLPPRLLHAVCRPWRRLMYAPSFPPFLSLYALLDDADADAGVSFAAYDPLAGRWDALPSPPMPSPPPMLWH
PSFLARRLPLQSVAAAGRLVLVSGSTQSLHPALSRPLVFDPAAPTPRWQLGPRIPLSPRRWCAAGAARGRVFVAGGV
GAGYDPAVARSGATWDPAASPPPAAAWEPIPPLRDGRFSRDAAEAVCSGGKVCMVNLRTPGAKEGAVFDLRAGRWED

MPPGMLAGWKGPAAASPPDDGETIYVVDEERGALTAYDWGADRWRTVAESDRLKGAAEMTVAGGKACVVAPGGGKVL
IVDVTPPPPPPPAARRESWAAPPPPPRMWEVAAPGGKRVVSLHVLPRMTRPE
>LOC_Os06g15330.1|PACid:21928943
MSSTAKAAAAGAVGAKSARACDGCLRRRARWYCAADDAFLCQGCDTSVHSANPLARRHERLRLRPSSPPPLVPPSGS
GRRDEAVPAAWFKRKARTPRSHAAKSAAAFGQLLSRRLVVVPEAAAGSGGDSPEERKDEGEIVEEQEQLLYRVPIFD
PALSEFCSPPPLEDAAAAVSCCNEDGAVENPTKPSMTTTTATTPPLQFFPDGQANFGPTDAELREFAADMEALLGRG
LDDGNDEDSFCMETLGLIEPVDDDAGRVKVEADGDAGMTLAWCHELDTETSSGEMLDIDFDCGSPQAATTPDEKVGS
SGPAAADDDAQLQQSNLALSLNYEAIIESWGTSPWTDGERPHVKLDDSWPRDYSGVWMAAAGVFGHGGEEQALTPRL
GMDGGREARVSRYREKRRTRLFSKKIRYEVRKLNAEKRPRMKGRFVKRAAAAATAAVATACVA

>LOC_Os06g14430.1|PACid:21929083
MPPLLGVYVFTDRPPRPPPTGVSACTAGLYAAVVGRLRLHRPAASSAADWCFRLHGWPIMLPLLGVYVFTDRLAFAA
ADWCFRLHGWPLCRRCWASTSSPTGLPSPLPTGVSACTAGLYAAVVGRLRLHRPACLRRRRLVFPPARLAFMPPLLG
VYVFTDWLAFAAADWCFRLHGWPLCRRCWASTSSPTGRLVRRRLVFPPARLAYYAAVVGRLRLHRPAASSAADWCFR
LHGWPIMPPLLGVYVFTDRPPRQPPTGVSACTAGLYAAVVGRLRLHRPAASSAADWCFRLHGWPIMPPLLGVYVFTD
RLAFAAADWCFRLHGWPIMPPLLGVYVFTDRPPRPPPTGASACTAGLLCGRCWASTSSPTGLPSPPPTGVSACTAGL
LCRRCWASTSSPTGLPSPPPTGVSACTAGLLCRRCWASTSSPTGRLVRRRLVFPPARLAYYAAVVGRLRLHRPAASS
AADWCFRLHGWLLCRRCWASTSSPTGRLVRRRLVLPPARLAYYAAVVGRLRLHRPACLRRRRLVFLPARLAYYAAVV
GRLRLHRPAASSAADWCFRLHGWPIMPPLLGVYVFTDRPPRPPPTGVSACMAGLLCRRCWASTSSPTGRLVSRRLVF
PPARLAFMPPLLGVYVFTDRPPHPPLTGVTACTAGLL

>LOC_Os06g48490.1|PACid:21929664
MALGLSLTLQEWISQNITDWLAFAAADWCFRLHGWPLCRHCWASTSSPTGLPSPPPTGVSACTAGLYAAAVGRLRLH
RPACLRRRRLVFPPARLAFMPPLLGVYVFTDRPPRPPPTGASACTAGLLCRLCWASTSSPTGLPSPPPTGGSACTAG
LYAAVVGRLRLHRPAASSAADWCFRLHGWPIMPPLLGVYVFTDRLAFAAADWCFRLHGWPLCRRCWASTSSPTGRLV
RRRLVLPPARLAFMPPLLGVYVFTDRPPRPPPTGASACTAGLLCRRCWASTSSPTGLPSPPPTGVSACTAGLYAAVV
GRLRLHRPAASSAADWCFRLHGWPLCRRCWASTSSPTGRLVRRRLVLPPARLAFMPPLLGVYVFTDWLAFAAADWCF
RLHGWPLCRHCWASTSSPTGLPSPPPTGVSACTAGLYAAVVGRLRLHRPACLRRRRLVFPPARLAFMPPLLGVYVFT
DRPPRPPPTGASACTAGLLCRRCWASTSSPTGRLVRRRLVLPPARLAYYAAVVGRLRLHRPACLRRRRLVFPPARLA
FMPPLLGVYVFTDRLAFAAADWCFRLHGWPLCRRSWASTSSPTGLPSSPPTDVLACTAGLLCRRCWAPTSSPTGLPL
SPTTDVLACTAGLYAAVVGRLRLHRPACLRRRRLVFPPARLAFMPPLLGVYVFTDRPPRPPPTGASACTAGLYTAVV
GRLRLHRPAASSAADWCHRLHGWPVITPTDAIFFTAGYIAVTTNRHQYLQHKLPTFAMAADWFLHFHGCMILLVLIG
LTIFIAGRLVCC

>LOC_Os06g31480.1|PACid:21931631
MSWHRAGVLLLGAQSCLPVPGVPAVGGIGSVLLSMARMGWKLCHVFRPYTVVMAGSINRRGLDLTGFVLDLRGMCVV
LGYPHGVDYQGKQLPEQEGDDAEPHAAWEVTAVILTSSPERTSLAVTAGGDSFSAACQNDALLTIDTLHQRYPDELQ
HSPYRYNPRRGGARDYATFRDASSEDDTTIMHLARMVEAYDAARIDFHQMVRRGLVKNNMKILELRQENLQLKKDLD
ALEAQLCQLKVAQGEVCRPKCRRVCCSQKITARKSTSRPELVRQSLAWTCFVETPRAEPAPVVPQEGKPPALVARRM
RCCSPSALARRRSRRAVSRLARVFFLVCLPVGVAWGMVVPMVTTRRSANTGDGNQPEGSNHNNHGSPPPPPPPSPPP
PPDTNAILTQILAQQTNMMAAFLHHIQNLPQHNAPPPPPQHSKLAEFLRIRPPTFSSSNNPVDALDWLHAVGKKLDT
VQCSDEEKVIFAAHQLQGPASLWWDHFQATQPEGQPITWARFTAAFRRTHVPAGVMALKKREFRELKQGNRSVMEYL
LEFNNLARYAPEEVREDEEKQEKFLAGMDPELSVRLVSGDYPDFQRLVDKSIRLEAKHKELESHKRRLANFRNQQGA
NQRVRYTNPYPGGSSSQQQQQQQPRSAPRPQFVPGHFADKCPKPRRQQGQAPPRSSNGGKDVIRGRVNHVTAEDMLT
TPDVIVGMFLIHSIPATILFDSGASHSFISVPFVGRNQLGVERLRNPLLITTPGGVMTAKYYSPVVPIEIQGIPFPS
DLILLDTRNLDVILGMNWLAQFQGVVDCARRTVTLYRGPEQPVVFFAPPTSVGSSELHQIGLSEIPIVREFGDVFPE
ELPGMPPKREIEFRIDLAPGTTPQYKRPYRMASNELAEVKKQLEELKEKGYIRPSTSPWGAPVIFVEKKDKTKRMCV
DYRALNEVTIKNKYPLPRIDDLFDQLKGATVFSKIDLRSGYHQLRIREEDIPKTAFTTRYGLYEFTVMSFGLTNAPA
FFMNLMNKVFMEYLDKFVVVFIDDILVYSQSEEDHQKHLRLALGKLREHQLYAKLSKCEFWLSEVKFLGHVISAKGV
AVDPETVTAVTDWKQPKTVTQIRSFLGLAGYYRSPQCEKAFQTLKQKLVSSPVLILPNTRKDFMVYCDASRQGLGCV
LMQEGHVVAYASRQLRPHEGNYPTHDLELAAVVHALKIWRHYLIGNRSEIYTDHKSLKYIFTQSDLNLRQRRWLELI
KDYDVGIHYHPSKANVVADALSQKSHCNTLGVRGIPPELNQQMEALNLSIVGRGFLATLEAKPTLLDQIREAQKNDP
DMHGLLKNMKQGKAASFTEDEHGTLWNGNRVCVPDDRELKQLILQEAHESPYSIHPGSMKMYLDLKEKYWWVSMKRE
IAEFVALCDVCQRVKAEHQRPAGLLQPIQVPEWKWDEIGMDFITRLPKTQGGYDSIWVVVDRLTKVARFIPVKTTYG
GNKLAELYFARIILEDMLRACVLDFGKTWDKSLPYAEFSYNNSYQASIQMAPYEALYGHKCRTPLLWDQVGESQVFR
TDILREAEAKVRTIQDNLKVAQSQQKSYADNRRRNLEFAVDDFVYLRVTPLRGVHRFQTNGKLAPRFVGPFRIIARC

GEVAYQLELPASLGNVHDVFHVSQLKKCLRVPSEQADPEQIEVREDLTYMERPVKILDTMERRTRNRVIRFCKVQWS
NHAEEEATWEREDELKAAHPDLFASSSESRGRDSVYGG

>LOC_Os06g48530.1|PACid:21931828
MADVIDPASTEAPRARRPPPPPPDSPEGRSPPLPPPPPGGPPQPAATRKRSRSPPPPPPPPSLPPPPPLGSSRPERY
RDNHHRGGGGGRGGGSSSPPPYRSGRRHSPSRRSPSPPFKRSRRDDGYDRRGGRGSPPPRYGYGDRRYGYDHERGGG
RGGYDDDRYHGRYQNRAADWADSGFGASNDGPGITQREGLMTYKQFIQVLEDDISPAEAEKRYQEYRTEYITTQKRA
YFDLNKNDDRLKDKYHPTNLSSVIDRRNDSCKATAKDFFHDLQNGTLDLGPGITAAAASGSDGNSDDDGDSDKRRKH
GRGSSKETDPLSGAPVAHPVSSESRRVQVDIEQALALVRKLDTEKGIVGNILSSGDHDKSDVDKSHIGSMGPIIIIR
GLTTVKGLEGVELLDTLLTYLWRIHGVDYYGMSETNEAKGSRHVRADNKTSNTTNINAADWEKKVDTFWQERLRGQD
PMVILAAKDKIDAAAVEVLEPYVRKIRDEKYGWKYGCGAKGCTKLFHAPEFVHKHLRLKHPELVLELTSKVREDLYF
QNYMNDPNAPGGTPVMQQSAPDKSRQRPGMDNRLRYDRANRREYDRAERDGSRYGRGDRSPSLDGADDQMFDAFRGR
GPNAPFVPELPAPPILMPIPGAGPLGPFVPAPPEIAMHMLREQGPPPPFEPNGPPHANPGVLGPMMGGPAPIITMPP
SFRQDPRRLRSYNDLDAPDEEVTVLDYRSL

>LOC_Os06g33030.1|PACid:21932280
MADRDEEHILYDTIAEGSSQYWNEEEGNEDPNQYLNEEGNVERDAEGNDAEEASGSQPSAGQKRAREQRGAAKKIEG
RHIITEVDEDGRPSAPAEAAKNFVRHSGWVVRDNVPVSTVYWRRTRARGDDDSFVPDSEKEMLWTTMLETFTLPAGT
DNLVKQWTLKKMAEQFQSFKGDLYKKYILKGLTPNFDVFPKLRDHWDEFIAYKTGQQGQAMMARNKENAAKKKYHHH
LGSGGYSVAKPKWKEMEASLLERVVASSQGTFRPDREKDELSLALQTPEHPGRTRGKGVIPWKIGFKEDIHTYRSRM
RSKRDTEAKIADLEYRVSSYELSMQEEVARKVDERMAVHRSQDPQPYIHPAMVSPLGNRSSCASTGQVGSQSMDTMQ
TQDETSCPVDEITQRIPCELHIPFKNLSIKVASEMAVPTDPSGTYHCRPIPAGYSRVEVELVEALYEDLELDYPGGD
DETHLRDTSHAIILWRKRYIILPGRQAAFRAPSPPAPPSPPAPSPPPPPAAPSPSAPPPPPAAPSPPSPPPPPAPPC
PSAPPKTRSRQAPPPARTRARKKAKVDTTKNKEPPYDCSQEELDAYVAGEVKRQLKPRSPEKKIPIDPSVKKFFKGM
SKTNKEALQISDYDRTLQRAYHKKSKPVPQLGEQPNKEVEPLVTGEDFGITDFIADTGLSVDQLVGAALIPKAEVAY
KFELGKPLVKPEQLESQPTQMYKFHERYMEMSANGREMFGARIRNPDFLQGDDVLWIHFKDLFDLYQLDALDVSLLS
AWILMEIQRARRRRVYNTGFIDPQKINSEMINKVTVYDSMNKEEKIFDKVFKLIDRAWDRFRQLVRGTWKEKLGRRF
HFPCGKQEQGTNLCGYYVCEYAHCLSNQICTTRELDRIHMRDNLPHKDFITAVQEQLMGFINEEVLNPDGEFYYDGS
TIHNVGPSSSDITPASKSKS

>LOC_Os06g40540.1|PACid:21932369
MDQSEHHHRLASTAADWCFRLHGWPIMPPLLGVYVFTDRLAFAAADWCFRLHGWPIMPPLLGVCVFTDRLAFAAADW
CFRLHGWPLCRRCWASTSSPTGRLDHRRLVFPPARLAYYAADVGRLHLHRPAASSAADWCFRLHGWPLCRRCWAFTS
SPTGLPSPPPTGVSACTAGLLCRRCWASTSSPTGLPSPPPTGVSACTAGLYAAAVGRLRLHRPAASTAADWCFRLHG
WPLCRRCWASTSSPTGRLVRRRLVFPPARLAFMPPLLGVYVFTDRLAFAAADWCFRLHGWPLCRRCWAFTSLPTGLP
SPPPTGVSACTAGLLCRRCWASTSSPTGLPSPPPTGVSACTAGLYAAAVGRLRLHRPAASTAADWCFRLHGWPIMLP
LLGVYIFTDRPPRPPPTGVSACTAGLYAAAVGRLRLHRPAASSAADWCFRLHGWPLCRRCWASTSSPTGLPSPPPTG
VSACTAGLYAAAVGRLRLHRPACLRRRRLVFPPARLAYYAAAVGRLRPHRRPASVAAEWCLRLHGWPIMPLLLGVND
FTAGLPSPPPTGVSACTAGLYAAVVGRLRLHRPAASSAADWCFRLHGWPIMPPLLGVYVFTDRLAFAAADWCFRLHG
WPLCRRCWASTSSPTGLPSPPPTGVSACTAGLYAAVVGRLRLHRPAASSAADWCFRLHGWPIMSPLLGVYVFTDRLA
FAAADWCFRLHGWPLCRRCWASTSSPTGLPSSPPTGVLACTAGLYAAVVGRLRLHRPACLRRRRLVFPPARLAFMPP
LLGVYVFTDRPPHPPLTGVTACTAGLL

>LOC_Os06g26170.1|PACid:21933229
MGRRRCETSGRALDDGRLHAAAEASVHGDGARRGSGGRADGGMRGRAVGSGEGRGGGARRGGGRGRSAARDGGARPG
TQSWRAREMRRRRRHGTQSQRGVGCGSDGGQGRGRWLAWADEGGGRGGVATRREIVMGRNMHTTTFAVKELDTDDEE
TEEREAAMASILELYRRNLIERQPHLPPLTAASAPTRLRPRLLVYRTQPSLPPAARRPQPSPPPAARRRRLLASPPP
AAIRPIPAAIRPIPAAGRPSASTPRPPTAIHAAGRPSASAPRPPTARRRPPVGSRLLARPPPALFTEREGERGVKKG
VHGGVCGAEARLEIHRIDRNGDAIHGGAPGLLQISDGEVRVPERPLHEID

>LOC_Os12g42900.1|PACid:21934267
MPRVLEPKPSPPPPSPLPPPPEDYWSPTAVTPPEPTKPKPPPPSPPPPPQQPSQRYWTPPPAITPEPAKPAAGKPTS
SSPPSTKDSPRPPLPAPSPAANGGDPRLPATTPLSKQKRKEEKREKEAQEMGRAGAAAAEEERPPATATASGGGRRE
AEERRLMVNMAAAAAVLVGIIVSVWRTLSS

>LOC_Os12g12880.1|PACid:21934537
MWSSDSEADLTAAAAAMTTTTTTSSSSSSSSSPSSSPPPPRRRHRHHRRAARRTHPVEAEAEEVWRGAQWEAAWPRR

TKPVVVAEDEPPSGGVIVGACTAGGGEEGGGVGRARSLTDDDLEELKGCVDLGFGFSYDEIPELCGTLPALELCYSM
SQRFLDDHPQSHHSPPPPPPPMPSPSIPNWKISSPGDSPDEVKARLKYWAQAVACTVRLCS

>LOC_Os12g36380.1|PACid:21934783
MPPLLGVYDFTAGLPPSPPTGASAYTAGLLCRRCWASTTSPPACLRRRRLVPPPTRLAYYAAAVGRLRLHRRPASVA
ADWCLRLHGWPIMPPLLGVYDFTAGLPPLPPTGASAYTAGLLCRRCWASTTSPPASVAADWCLRLHGWPIMPPLLGV
YDFTAGLPPLPPTGASAYTAGLLCRRCWASTTSPPACLRRRRVVPPPTRLAYYAAAVGRLRLHRRPASVAAEWCLRL
HGWPIMPPLLGVYDFIAGLPPSPPTGVLAYTVGWIQIQICIKGIHGFKLISYAPGMDQSEHHHRLASTAADWCFRLH
GWPIMPPLLGVYVFTNRLAFAAADWCFRLHGWPLCRRCWASTSSPTGRLVRRRLVLPPARLAFMPPLLGVYVFTDRP
PSPPPTGVSACTAGLYAAVVGRLRLHRPACLRRCRLVFPPARLAFMPPLLGVYVFTDRPPRPPPTGASACTAGLYAT
VVGRLRLHRPAAFAAADWCFRLHGWPLYRRCWASTSSPTGLPSPPPTGVSACTAGLYAAVVGRLRLHRPACLRRCRL
VFPPARLAFMPPLLGVYVFTDRPPSPPPTGVSACTAGLLCRRCWASTSSPTGLPSPLPTGVSACTAGLYAAVVGCLR
LHRPAASSAADWCFRLHGWPIMPPLLGVYVFTDRLAFAAAY

>LOC_Os12g19285.1|PACid:21936199
MSPPPTAPPRPTAPRPLLLFPNPFPKRRHHVHHLSPPPSPTTAAPRHLSGRPRTASSPPLVLQGRGDPRPPPSSRPN
HWNPFPLAHRSRRCSAALPHLPVVVGASLALFFRGAPLPLLVVGRLPASPPRCFGAREYIAPFQKSAVVRKRVQKIE
VKKKGKSHIPLEHVDY

>LOC_Os12g19830.1|PACid:21936237
MPPLLGVYVFTDRLAFAAADWCFRLHGWPLCRRCWASTSLPTGLPSPLPTGASACTADLLCRRCWASTSSPTGLPSP
PPTGVSACTAGLYAAVIGRLRLYRPACLRRCRLVFPPARLAFMPPLLGVYVFTDRPPRPPPTGVSACTAGLYAAVVG
RLRLYRPACLRRCRLVFPPARLAFMPPLLGVYVFTDRPPRPPPTGASACTAGLLCCRCWASTSSPTGLPSPPPTGVS
ACTAGLYAAVVWRLRLHRPAAASAADWCHRLHGWPVITPTDAIFFTAGYIAVTTNRHQYLQHKLPTFAMAADWFLHF
HGCMILPVLIGLTIFIAGRLVCC

>LOC_Os12g36980.1|PACid:21936472
MGGGGRWSLPPSEPLRWRRSFKNLHLRELLDEEEEEEEAEADDAVEEDDDDRDADGAAAFATGAADGGGGGGGGGDS
HVRNGEWDRLRRRTDRHRRRPPRSSPAPTSSSSRGVSPPPTSGRSRIGRIWPLWKSSSAEDIAAGAGDGNLDEKSNS
NSYSSPSPPPSPPWWWWWWAGLAGALKKPPGKGASRAAGLDGALTNGVEHALSPPVARWWSSPETVSAAAADIAAAS
GGE

>LOC_Os12g02210.1|PACid:21936480
MLFLKELPIWQAFGFSLFTGALAGSILYAVCVLLLCCVDRRRRHAGAPPPDPKIWLPDHAHHRRRRDESSEMECSIC
LGELEEGERCCTLAACRHEFHKECIYRWLANRNTCPLCRHISLPPASSPPPAPPPHHASPPPSNVLAAAHV

>LOC_Os05g30830.1|PACid:21940088
MDQSEHHRPACLRRRRLVFPPARLAFMPPLLGVYVFTDRLAFAAADWCFRLHGWPLCRHCWASTSSPTGLPSPPPTG
VSACTAGLYAAVVGRLRLHRPACLRRRRLVFPPARLAFMPPLLGVYVFTDQPPRPPLTGASACTAGLLCRRCWASTS
SPTGLPSPPPTGVSACTAGLYAAVVGRLRLHRLACLRRCRLVFPPARLAFMPPLLGVYVFTDRLAFAAADWCFRLHG
WPLCRRCWASTSSPTGRLVRRRLVLPPARLAYYAAVVGRLRRHRPACLRRRRLVFPPARLAFMPPLLGVYVFTDWLA
FAAADWCFRLHGWPLCRHCWASTSSPTGLPSPPPTGVSACTAGLYAAVVGRLRLHRPACLRRRRLVFPPARLAFMPP
LLGVYVFTDRPPRPPPTGASACTAGLLCRRCWASTSSPTGRLVRRRLVLPPARLAYYADVVGRLRLHRPAASSAADW
CFRLHGWPIMPTLLGVYVFTDRPPRPPPTGVSACTAGLLCRRCWASTSSPTGLPSPPPTGVSACTAGLYAAAVGRLR
LHRPACLRRRRLVFPPARLAFMPPLLDVYVFTDRLAFAAADWCFRLHGWPLCRRCWASTSSPTGLPSPLPTGVSACT
AGLLCRRCWASTSSPTGRLVRRRLVLPPARLAYYAAVVGRLRLHRPACLRRRRLVFPPARLAFMPPLLGVYVFTDRP
PRPPPTGASACTAGLLCRRCWASTSSPTGLPSPPPTGVSACTAGLYAAAVGRLRLHRPAASTAADWCFRLHGWPIML
PLLGVYIFTDRPPRPPPTGVSVCTAGLYAAAVGRLRLHRPACLRRRRLVFPPARLAFMPPLLGVYVFTDRLAFAAAD
WCFRLHGWPIMPPVLGVYVFTDRLAFTAADWCFRLHGWPLCRRCWASTSSPTGRLDRRRLVFPPARLAYYAAVVGRL
RLHRPAAASAADWCHRLHGWPVITPTDTTFFTAGYIAVTTNRHQYLQHKLPTFAMAADWFLHFHGYMILPVLISLIF
FTAGHLVCC

>LOC_Os05g42340.1|PACid:21940800
MGCCVSKKSDELLPGSAAAAAGEEVEAPRRRVDQARDPPPTEEEKVKEVLSETPAPSARARPRQRRVAGAVAPSVER
GGGGGEKVARAKEGGGGGGRVRARRAVGAERATSEKSEAASESSVATTATGPERSPGKPARKRAVVSGELGRARRDR
GPAAAVHGAGRPGGGRASPSPPPPPRREPGERPTRRSPSPATKRPPDQRRTAASAGAAAGAASGPQRKPPVPPRPCG
RASPRRAQETNSPASSTTTTQSRGPPPHCSSPPPPQDAGASAAGAGEEVAGGRGEGKESLDNPSVAMECFIFL

>LOC_Os05g41730.1|PACid:21940841
MASPPEDAPAIPSNPSVPQEVGNQLPRRSPPPPPRLETAADEIGGVRCGVLCFHLPRRSKKKKKKKPPPPVMQLAGG
AAGIKSVQLETTATDDDDASSASPPPQRVTFLASASLSTWWPASPPAAAAAGGRGASSSSFSHWRRSLSSSRRRVTP
HGAAAAAPSAPTSFSFPSSPASASTSCTSTPKLVHGCHVD

>LOC_Os05g26550.1|PACid:21940852
MPPLLGVYVFTDRLAFVAADWCPRLHGWPLCRRCWASTSSPTGLPSPPPTGVSACTAGLYAAVVGRLRLHRPATASA
ADWCFRLHGWPIMPPLLVAYVFTDRLAFVAADRCPRLHGWPLCRRCWASTSSPTGLPSSPPTGVLACTAGLYAAVVG
RLRLHRPACLRRRRLVFPPARLAFMPPLLGVYVFTDRPPHPPPTGVSACTAGLLCRRCWAPTSSPTGLPSPPPTGVS
ACTAGLYAAVVGRLRLHRPAAASAADWCFRQHGWPIMPPLLGAYVFTDRLAFVAADRCPRLHGWPLCRRCWASTSSP
TGLPSPPPTGVSACTAGLYAAVVGRLRLHRPAAASAADWCFRLHGWPIMLPLLGVYVFTDRLAFAAADWCFRLHGWP
LCRRCWASTSSPTGRRIRR

>LOC_Os05g31680.1|PACid:21941250
MAASSLLPSTTTSPRRRLPFPPTRKPHSAAAAAAKPPTTLTTKPLRLARPLASSSSSPPPPPPPEEAEPKDPIALAF
ARAAAYKKERDSPPPPPPPPTPAPPPQPPAATEEDSRSSKESFARAVEYRNGNGGGLGSGGGSPLLGASPRFGQSTF
STEDGAFGKFTNKKEEYEYDETDFLGLDFFEKKSYKGPPPGLAPAADPFPDKDFPEVEIIIGDPSKFGKTHRSTEVQ
PADDSEPEETSRSTTEEKKEENKPDETPPSTVTEPEEDEDEYKPTVRSWGMFPRPQNISKAYGGGRNIRLGGETRSA
EEKAAKDKRTKELIAAYRNSQNMIVDAKTKAECTEALKEGDELMNTGRLKQALPYYEKVMQAVDFKVQGSHEHVQQT
QKSSKF

>LOC_Os05g03040.1|PACid:21941321
MELDLNNVAEGVVEKHETAARSDSGTSESSVLNGEASGAAIAPAEEGSSSTPPSPPPPPAAVLEFSILRSSASASGE
NDADDDEEEEATPSPPPHHQHQQLLVTRELFPSAAPSPQHWAELGFLRPDPPRPHPDIRILAHAPPPAPPPPPPQPQ
PQAAKKSRRGPRSRSSQYRGVTFYRRTGRWESHIWDCGKQVYLGGFDTAHAAARAYDRAAIKFRGVEADINFNLSDY
EEDMRQMKSLSKEEFVHVLRRQSTGFSRGSSKYRGVTLHKCGRWEARMGQFLGKKYIYLGLFDSEVEAARAYDKAAI
KCNGREAVTNFEPSTYDGELPTDAAAQGADVDLNLRISQPAASQQSPKRDSGSLGLQIHHGSFEGSEFKRAKNDAAP
SELASRPHRFPLLTEHPPIWTAQPHPLFPNNEDASRSSDQKRKPSEGVAVPSWAWKQVSHHHPAPPHTLPLPFFSSS
SSSPSSSSAAASSGFSKAATTAAAAQHTATLRFDPTAPSSSSSSRHHHHH

>LOC_Os05g03040.2|PACid:21941322
MELDLNNVAEGVVEKHETAARSDSGTSESSVLNGEASGAAIAPAEEGSSSTPPSPPPPPAAVLEFSILRSSASASGE
NDADDDEEEEATPSPPPHHQHQQLLVTRELFPSAAPSPQHWAELGFLRPDPPRPHPDIRILAHAPPPAPPPPPPQPQ
PQAAKKSRRGPRSRSSQYRGVTFYRRTGRWESHIWDCGKQVYLGGFDTAHAAARAYDRAAIKFRGVEADINFNLSDY
EEDMRQMKSLSKEEFVHVLRRQSTGFSRGSSKYRGVTLHKCGRWEARMGQFLGKKYIYLGLFDSEVEAARLSSAWIL
FSCTIDSNQKKVFSFLKFYFLVLLIFDGPCTDSNLLSFFFSSFFSSRFKHQGL

>LOC_Os05g13730.1|PACid:21941708
MSNEVNHVGKAPMFNGTNYSTWKIKMSTHLKAMSFHIWSIVDVGFAINGTPLTEIDHRNLQLNAQAMNALFNSLSQE
EFDRVSNLETAYEIWNKLAEIHEGTSEYKDAKLHFVKIQYETFSMLPHESVNDMYGRLNVIVNDLKGLGANYTDLEV
AQKMLRALPEKYETLVTMLINSDKSRMTPASLLGKINTNDMYKLKKKEMEEASPSKKCIALQAEVEDKGKGKVNEAN
EDLEEEIALLARRFNDLLGRRKERGRGSNSNRRRNRRPNKTLSNLRCFECGEKGHFASKCPSKDDDGDKSSKKKSGG
YKLMKKLKKEGKKIEAFIGEWDSNEESSASSGSEEEGGDDASSKKKKMAVVAIKEALSLFAPLCLMAKGSSKVTSLS
DSESNDNCNDVSYDELVSMFEELHAYSEKKIVKFMALKKDHASLEVLYEELKTSHERLTMWILWVPHTHTAWIQIQI
CIKGIHGFKLISYALGMDQSEHHHRPPRPPPTSVFACTVGLLCRRCWASTSSPTGLPSPPPTGVSACTAGLYAAVVG
RLRLHRPAASSAADWCFRLHGWPLCRRCWASTSSPTGRLVRRRLVLPPARLAFMPPLLGVYVFTDWLAFAAADWCFR
LHGWPLCRHCWASTSSSTGLPSPPPTGVSACTAGLYAAVVGRLRLHRPAASSAADWCFRLHGWPLCRRCWASTSSPT
GLPSPPPTGVSACTAGLYAAVVGRLRLHRPAASSAADWCFRLHGWPIMPPLLGVYVFTDRLAFAAADWCFRLHGWPL
CRRCWASASSPTGLPSPPPTGVSACTAGLYADVVGRLRLHRPAASSAADWCFRLHGWPIMPPLLGVYVFTDRLAFAA
ADWCFRLHGWPLCRRCWASTSSPTGLPSPPPTGVSACTAGLYAAVVGRLRLHRPAASSAADWCFRLHGWPIMPPLLG
VYVVTDRPPRPPPTGVSACTAGLLCRRCWASTSSPTGRRIRR

>LOC_Os05g11510.1|PACid:21942029
MSPPPPPYYHHLLLLRSSPTTTGGGARVLAAAELARMKLLCSACEAAEASVLCCADEAALCARCDRDIHAANRLAGK
HLRLPLLSPASSSSSSAAALAPPPPSPPKCDICQESHAYFFCLEDRALLCRSCDVAVHTANAFVSAHRRFLLTGVQV
GQEQDEHSPDPPEPSPPPPPPPPASKSDHPAPLYGEGGGGFSWDAADSPAAGGLPDWSAVVDQFGSPPPPRHTDTAT

VTTPPPTKRSPRAPAFGGQGGMMDWPLGEFFGGFTDFTGGFGFGFGDSGTSKADSGKLGGSTDGSPYYRSSSEDDRN
ADELFGQVPEIQWSVPELPSPPTASGLHWQRHPAATHGGGGGGPDTTAFVPDICSPDSCFPATTSKRRRQ

>LOC_Os05g01540.1|PACid:21943590
MRTAKKKKKKKKKKKKRKSKSKKKETKSKPKPIHHRISSSPSPSPAMATKPRRRSPSLSSSSSTSYSRSPASPSSSP
SRSRSPPRPAGGARSPSPPPPPPPPRKASPPPESTVLHVDHLSRNVNEDHLKEIFENYGEVVNVELSMDRVVNLPRG
YGYVEFKNRADAEKALLYLDGGQIDGNVVKVRFALPPQQQRAAASPKALHPSPKRDVVDHNKVGPSAEKATKQQPRK
PASPQRKPAPSPRRRSPPSRRVESPRCRPDSPPIRRRTAPSPIRHGGTPSRRPGSPIRRRSPSPPPRRLRSPRHLSP
RRDRGSPIRRRSPLPRRRLTPPRRMWSPPRRPQSLRHRSRSPIHRPIRSPSRSISPRRGRGPPVRRGRSSSSYSDSP
TPPRRGPRRVLRSRSPRRPLRGRSASSYSRSSSSSGSPIRN

>LOC_Os05g01540.2|PACid:21943591
MRTAKKKKKKKKKKKKRKSKSKKKETKSKPKPIHHRISSSPSPSPAMATKPRRRSPSLSSSSSTSYSRSPASPSSSP
SRSRSPPRPAGGARSPSPPPPPPPPRKASPPPESTVLHVDHLSRNVNEDHLKEIFENYGEVVNVELSMDRVVNLPRG
YGYVEFKNRADAEKALLYLDGGQIDGNVVKVRFALPPQQQRAAASPKALHPSPKRDVVDHNKVGPSAEKATKQQPRK
PSPQRKPAPSPRRRSPPSRRVESPRCRPDSPPIRRRTAPSPIRHGGTPSRRPGSPIRRRSPSPPPRRLRSPRHLSPR
RDRGSPIRRRSPLPRRRLTPPRRMWSPPRRPQSLRHRSRSPIHRPIRSPSRSISPRRGRGPPVRRGRSSSSYSDSPT
PPRRGPRRVLRSRSPRRPLRGRSASSYSRSSSSSGSPIRN

>LOC_Os11g17870.1|PACid:21944761
MEEGGAEEGDRAGRRGGAAEEAGGGADGVERRRRGLDGSGGSPPPSLPNLARGGGGKAGGGDDGVGQAAAAAPLPRS
LPNLTGGGREEAGGGADGMERRRRGLGGGGGSPPPSLPDLAGGGRGEAGGGNDGAGRAAATAARAERRRRLPSPAAS
QI

>LOC_Os11g34140.1|PACid:21946912
MPTSSTSKLDLKCGLGHWDQEASLCHSKGRKPPTFSQQPSSLSSQFSLPSRPPLHPVGDRAIHPPMPPASAAAGDGA
APDQEPPPSPPPPPPPPPPPRPAPSLASALPFWFYLTAAVSLLALLLPHLLPSSSPPPPLPSLLRSHASGGSVLKLH
PGPDLFAVTSKPTTTAAAHRPAVLVLPGLAAGSFSFRHALRSLSSRGLLAAAVDLPGQGMSPPCDAPPPPPPSKSAF
REIMDRGVFHAFEHLVETGEVPFQEPAAAPEPPSHARLYAPHEAAASVARAVDALGLGAAPVHLVLHDSALVAGAAF
VSANPAAVRSVTLVDATAVLPAFPVAVLGVPVLGSMVVRVPALFRGLLRLCCAREMGAEVAEAHRAAMRMEGKRDAV
FESWKALNQSFDLKEWRGSLEAVRKLPMMVLWSGSWTDRWIDEGKKVVAALPDAKFVYHSGGRWPQEDAYDELSELI
AEFVTSLPTSVRSQHIDQSSEQATAQE

>LOC_Os11g13930.1|PACid:21947502
MVSILQLQTRTEASPASSASAAATRIFAVRRQQQEQEGEEEEEEFEFQERMDLSGAQGELPIPMHASAAASPFAGMG
AHGGAGGGHVVELHRHEHVGNNGQAMAMASPPPTNVAVAAEQEGSPVAGKKRGGMAVVGGGGGVAVKYRECLKNHAA
AIGGNATDGCGEFMPSGEEGSLEALKCSACGCHRNFHRKEADDLDADSCAAALRAAAGRHHHLLGPALPHHHHKNGG
GLLVAGGDPYGAAYAAARALPPPPPPPPHGHHHHHQIIMPLNMIHTSESDEMDVSGGGGGVGRGGGSSSSSKKRFRT
KFTAEQKARMLEFAERVGWRLQKLDDAMVHHFCQEIGVKRRVLKVWMHNNKHNLAKKPLPSSPPPPPQIPPMSMPPS
PPPPQIPPMSMPPSPPPMPMPMPPSPPQLKLE

>LOC_Os11g02010.1|PACid:21948306
MSPVWIRRGASRATPAPHIAELAKECWREHLLGASIPFETFTFHVDADFSSSSASSADHRGSSVLPPGPGDIEVAHL
QSIASARLAAQGCEVLGKIKREQLVEFESVYGLHGYFDTTGTHDDASSSADHCVGPILPQLQSDRSAHFAAWAHSSW
ALAAAAAAAAVDDDDEGKWMKPCIGPVLACDCEPEYFDSDPAPPTPPTIARPPRPLPPASPPPPSIATPPPSPASPP
PPSTATPPPPSPTPTTTRASPTPPPIPTATVRPPPPLPRATVTPTPTTPPSTATTTPIPTWVWVGGVLVLSAGVAVL
AIKLAYDYLGGGRND

>LOC_Os11g19040.1|PACid:21948332
MGDKQETESTKTHFVVPTHNPTTDATPQQDEHESNLPEEFRVTSPPPLSPPPYPLSPSMEDDGMIYTEDLGHMSTPC
PSPLRTSRI

>LOC_Os03g46110.1|PACid:21914623
MERSHLAVLLGLLAFAAGVPAAAAATAVEGAQAATAEASCEPSILATQVSLFCAPDMPTAQCCEPVVASVDLGGGVP
CLCRVAAEPQLIISGLNATHLLTLYAACGGLRPGGARLAAACEGPAPPASIVTAPPPPVAFRRKPPAREAPPPPPAA
EKLSPPPQQHDDSDHNKRVGPLPRGSPPPYAQSVPVGPAAAPPPPRSGASSSLQAPLAATTTIVAITLIAAAQY

>LOC_Os06g40780.1|PACid:21929356
MHESDELLLLVAFLTLGISLSPSCPLLALCEGWKQGISACCGCMTQMHMSLLVGNDGVQVEVMIDCLIWGLRHSIQG
CPMPCPHIHSQLVELSPSPPLFAIFHLFANVLYQDALDYMQCETLTQLDQVWGVCLFLLQGSYLEAIINEDPTKGQN
MRWLETWEAAIASPDVGRARSPSLLNPPSSDAPSPPPPSPPPPPPSPAAHRRCSSSGGGGDDDGYGGGDGMLRSLH
SSSSSDTDNNSGGCKNNGGGGGEAAAAVEGGGDQRAVAAAAPSTRDLLLACADLLQRGDLPAARRAAEIVLAAAASP
RGDAADRLAYHFARALALRVDAKAGHGHVVVGGGAARPASSGAYLAFNQIAPFLRFAHLTANQAILEAVDGARRVHI
LDLDAVHGVQWPPLLQAIAERADPALGPPEVRVTGAGADRDTLLRTGNRLRAFARSIHLPFHFTPLLLSCATTAPHH
VAGTSTGAAAAASTAAAATGLEFHPDETLAVNCVMFLHNLAGHDELAAFLKWVKAMSPAVVTIAEREAGGGGGGGDH
IDDLPRRVGVAMDHYSAVFEALEATVPPGSRERLAVEQEVLGREIEAAVGPSGGRWWRGIERWGGAARAAGFAARPL
SAFAVSQARLLLRLHYPSEGYLVQEARGACFLGWQTRPLLSVSAWQPSSS

>LOC_Os06g35510.1|PACid:21933409
MPRRPDATVSHALGKPFEAAMPLMTATVTTTAAAAVEHSATSPSPSSPRTRLFPRCQRRHRLHSHGRHRRCHRREPP
PPQTAAAPSPSTTKQPATAAAVTNRRRQPTTQIRPRAAVDAADRGRRRQPPPPTAPPLEAPANGCCRRCRGPSTAVA
TATGAPSTSLPCCCRHVVASSAAPATSWLDPAAGWLDPPSGGRIRLQRGRIGASAAVAAVAIPAAVVPAFAVPAAVD
PAAPLPTPSPSPPPCPSPPPAATLPDPPEAARIWRSPSPLPWAAPPAARTRRGR

>LOC_Os03g45930.1|PACid:21913372
MPSPLKERPAGRLGRLLAALRPARAGPLPVQTGFPTSLADLVVKNHGRLKKPSASASRRKKRGGPEAPPSPSPSPSP
SPPPQPSSPPPPPPSPPPAAAVSVSPPTQPRPRPELPPVEAVLRRQPKGRVFGLGLGFVSLVGVVSLALLVIWSKKV
VAAVTVASFSLFLLESVRSSALSRRPRRPAANNKLDLDGRGYVSPIREVEPARASFSDSSRRSELSILTIEERSEVG
DDSIVAIEERIAAGGDDSSNAKVKTKKRSWRKLIPRKLQKGMKGKEAEDSSGSFRSSEGNRGDATATDSSDSRRGMR
TKAADAFVARSMDSSPSFRGNGGDTDADAYSNATRVEIDAPADVLAGDGDAVGGTRSSVALLVVAVVLVGLVAGKLP
AVVFTVLCGVFISSVQRLPAGGDGNGDRSFTWWFRIKPKDVN

>LOC_Os06g16700.1|PACid:21933009
MPGGFILAWLTRPDYTRGACVRGHPAACSGGLTNRLASVAAEWCLRLHGWPIMPPLLGVYDFTAGLPPSPPSGASAY
TAGLLRRRCWASTTSPPTGRLVSRRLMFLPARLAYYAAVVGRLRLHRRPASVAADWCPRLYGWLVTPPFMGVHVFAD
RLAFAAAEWCLRLHGWPIMPPLLGVYVFTVGLPPSPPIGASAYTAGLLCRRCWASTSSPSACLRRRRLVPPPTRLAY
YAAAVGRLRLHRRLASVAADWCLRLHGWPIMPPLLGVYDFTAGLPPSPPSGASAYTAGLLCRRCWASTTSPPACLRR
RRLVSSPIRLVGHTTVHGRPRLRDRLAFAAAEWCLRIHGWPIMPPLLGVYDFTAGLPLLPPIGAPAYTAGLLCRRCW
ASTTSPPACLRRRRLVSSPIRLVGHTTVHGRPRLHRRPTSVAADWCLRLHGWPIMPPLLGVYDFTAGLSPSPPTGVL
AYTVGWSHHRSWASTSSPTGWPSPPPSGASAYTAGLLCRRCWASTSSPSACLRRRRLVPPSTRLAYYAAAVGRLRLH
RRLASVAADWCLRLHGWPIMPPLLGVYDFTAGLPPSPPSGASAYTAGLLCRRCWASTTSPPACLRRRRLVSSPIRLV
GHTTIHGRPRLRRPAGLRRRRVVPPPTRLAYYAAVVGRLRLHRRPASVAADWCPRLYGWLVTPPFMGVHVFADRLAF
AAAEWCPRLYGWLVTPPFMGVHDFTAGLPPSPPIGASAYTAGLLCRRCWASTTSPPACLRRRRLVSSPIWLVGHTTV
HGRPRLHRRPASVAADWCPRLYGWLVTPPFMGVHVFTAGLPPSPPTGVLAYTLADFAAVNGLHHLHHWLGSTGADWC
LRLHGWPIMPPLSGVYDFTAGLPPSPPTGVFAYMLAFMPPLLGVYVFTDRLAFAAADWCFRLHGWPLCRHCWASTSS
PTGLPSPPPTGVSACTAGLYAAVVGRLRLHRPACLRRRRLVFPPARLAFMPPLLGVYVFTDRLAFVAADRCPRLHGW
PLCRRCWASTSSPTGLPSPPPTGVSACTAGLYAAVVGRLRLHRPAAASAADWCHRLHGWPVITPTDAIFFTAGYIAV
TTNRHQYLQHNLPTFAMAADWFLHFHGCMILPVLIGLTIFIAGRLVCC

>LOC_Os03g39040.1|PACid:21914482
MEIMQQLAAAATATATATATPLCLPEPPPTAMTTAQVEAAIATLHGKKQGLREAYDSLVLHSPIPLPFRWSDLDSHL
SSLQSSIHARFSQLQALQASRPAPPAAAAASHDEDVEMEDVQEQGEDEVMAPPSTVQVKEEPVEAAACASNTAGERA
GVGQDGFVRPGGMAAAKMASPSKVQVKEEPVEVSPSPPAGATGLTAAACASMDAPRLTDVCKRAGGGGGQGLCARNG
SGTAQSPPIPSCPVLQQQHTAGAPNGSHPVLRQHAANAMNAGHSAAFRPQQHMGKPRDTCDLRKAAAPNTGDRLPLQ
WRQQRVHANLTCPLPPPVVAGSSSSPPQQRVGVFPSPTPQIVGSSPPPPSQARVGEANVTNPSRQQFTASAPHAGDG
QLQKRPPWQRLQRVGVANPMNAGDLPPQKPHFTANARNAGEHPFQEQQQPPPVAKPTDAAAGDLLPQQKQLMANAPN
AGEHLLPEQQKQQPVTAAKPANAPPLQHAANATNPAVLRRQRQRQWVRLRPPTATNLPQTKQEQHHLFMADDGANAR
NPLSPPPPCGMAKPPNSGDPLTDQNNQQLMANTHSAPTPVSTPLVASNQSESSAMTTTTTTNSNQNSGGGRTGPQPV
AAGAAPNPAGNQQQGQRKGGANRRGGRGQGNKNNNVANTNISNMSKVIGKGNKGQGNGNPQLNKNGNPQFNKNVPGD
HHQKNKNAGQLCYRCGCRGHWSRICRTPEHLVKLYQQDRKAKLQAGANTNTVYVGAMTD

>LOC_Os05g45600.1|PACid:21940855
MPPLLGVYDFTAGLPPLPPSGASAYTAGLLCCRCWASTTSPPACLRCRRVVPPPTWLAYYAAAVGRLRLHRRPASVA
AEWCLRLHGWPIMPPLLGVYDFTAGLPPSPPTGVLAYTVGWSHHRSWASTSLPTGWPSSPPLLGVYDFTAGLPPLPP

SGASAYTAGLLCRRCWASTTSPPACLRSRRVVPPPTRLAYYAAAVGRLRLHRRPASVAAEWCLRLHGWPIMPPLLGV
YDFTAGLPPSPPTGVLAYTVGWSHHRSWASTSLPTGWPSSPPTGASACTAGLLCRRCWASTTSPPACLRRRRVVPPP
TRLAYYAAAVGRLRLHRRPASVAAEWCLRLHGWPIMPPLLGVYDFTAGLPPSPPTGVLAYTVGWSHHRSWASTSSPP
ACLRRRRVVPPPTRLAYHAAAVGRLRLHRRPASVAADWCLRLHGWPIMPPLLGVYDFTAGLPPSPPGGASAYTAGLL
CRRCWASTTSPPACLRRRRLVFLPIRLADFAAVNGLHHLHHWLGSTGADWIQIQICIKWIHGFKLISYAPGMDQSEH
HRRPASVAAEWCLRLHGWPIMPPLLGVYDFTAGLPPSPPSGASAYTAGLLCRRCWASTTSPPACLRRRRVVPPPTRL
AYYAAAVGRLRLHRRPASVAADWCLRLHGWPIMPPLLGVYDFTAGLPPSPPTGALAYMVGWSHHRSWASTSSPPACL
RRRRLVFPPARLAFMPPLLGVYVFTDRLAFAAADWCIRLHGWPLCRRCWASTSSPTGLPSPPPTGVSACTAGLYAAV
VGRLRFHRPACLRRHRLVYPPARLAFMPPLLGVYVFTDRLAFAAADWCFRLHGWPLCRRCWASTSSPTGLPSPPPTG
VSACTAGLYAAVVGRLRLHRPACLPPSDWCLRLHGWPLCRRCWASTSSPTGCLDRRRLVFPPARLAYYAAVVGRLRL
HRPAAASAADWCHRLHGWPVITPTDAIFFTAGYIAVTTNRHQYLQHKLPTFAMAAD

>LOC_Os01g72890.1|PACid:21907362
MAKPRRGRSASRSSSGSSSRSPSRSASSGSGSSRSRSRSRSFSSSSSPSRSRSPPAAKASSPGARRGRSPSSPAKRG
SPSKKGRSPSPPPPKKASPPRKASPAPESVVLHIDHLSRNVNEAHLKEIFGNFGEVVNVELSMDRMVNLPRGYGYIE
FKKRTDAEKALLYMDGGQIDGNVVKLRFTLAPRQRASSPMKAPPPPPKRDVPHNEKGAPSAEKDVQQRREPSPRRKP
ASPPRKRTPPNRRIESPRRQPDPSPRRRPDSPPIRRRADASPVRRGDTPPRRRPGSPVRRRSPSPPPRRRRSPMRPS
PRRLRGSPSPRRRSPGPIRRRSPPPPPRRPRSPPGRRLPPPRRHSRSPPPRRPPHSRSRSISPRTRRGPPLRRGRSD
SSYSRSPSPPRKGPRRVSRSRTPPRHRRGRSISSDSRSSSSPSPRRR

>LOC_Os08g14280.1|PACid:21888234
MPPLLGVYVFTDRLAFAAADWCFRLHSWPIMPPLLGAYVFTDRLAFVAAGWCPCLHGWPIMPPLLGAYVFTNRLAFV
AADWCPRLHGWPIMPPLLGAYVFTDRLAFAAADWCPRLHGWPIMPPLLGAYVFTDRLAFAAADWCPRLHGWLIMPPL
LGAYVFTDRLAFAAADWCFRLHGWPLCRRCWASTSSPTGSPSPPPTGVHACTAGLLCRRCWAPTSSPTGSPSSPPTG
VLACTAGLLCRRCWAPTSSPTGLPSPPPTGVLACTAGLLCRRCWAPTSSPTGLPSPPPTGVSACTAGLLCRRCWAST
SSPTGLPSPPPTGVSACTAGLLCRRCWASTSSPTGRFDRRRLVFPPARLAYYAAVVGRLRLHRPATASAADWCHRLH
GWPVITPTDAIFFTAGYIAVTTNRHQYLQHKLPTFAMAADWFLHFHGCYIAIDWITDIVIFINILSSLINIAYSAAV
DGKLRHHSRLSLLPLIIITVGCVCRRRLFSSSS

>LOC_Os04g08530.1|PACid:21891859
MPPLLGVYVFTDRLAFAAADWCFRLHGWPIMPPLLGVYVFTDRLAFAAADWCFRLHGWPIMLPLLGVYVFTDRPPRP
PPTGVTACTAGLLCRRCWASTSSPTGLPSPPPTGVSACTAGLLCRRCWASTSSPTGWPSPPPTGVSACTAGLLCCRC
WASTSSPTGRLVRRRLVSPPARLAYYAAVVGRLRLHRPACLRRRRLVFPPARLAYYAAVVGRLRLHRPAAFAAADWC
FRLHGWPIMPPLLGVYVFTDRLAFAAADWCFRLHGWPIMPPLLGVYVFTDRLAFAAADWCFRLHGWPIMPPLLGVYV
FTDRLAFAAADWCFRLHGWPIMPPLLGVYVFTDRPPRPPPTGVTACTAGLLCRRCWASTSSPTGLPSPPPTGVSACT
AGLLCHRCWASTSSPTGLPSPPPTGVSAYTAGLLCHRCWASTSLPTGLPSPPPTGVSACTAGLLCHRCWASTSSPTG
RLVRRRLVSPPARLAYYAAVVDRLRLHRPAASTAADWCFRLHGWPIMPPLLGVYIFTDRPPRPPPTGVSACTAGLLC
HRLWASTSSPTGLPPSPPTGVSVCTAGLLCRRCWASTTPPPACLHRRRLVFPSARLAYYAVVVGRLRLHHRPASIAA
DWCFRLHGWPIMPSVLGVYDSTTGLPPSPPTGVSVCTAGLLCRRCWASTS

>LOC_Os02g38830.1|PACid:21921389
MSSPARLAFMPPLLGVYVFTDRLAFAAADWCFRLHGWPIMPPLLGVYVFTDRLAFAAADWCPRLHGWPLCRRCWAST
SSPTGLPSPPPTGVSACTAGLLCRRCWASTSSPTGLPSPPPTGVPACTAGLYAAVVGRLRLHRPACLRRRRLVFPLA
RLAFMPPLLGVYVFTDRLAFAAADWCFRLHGWPLCRRCWASTSSPTGLPSPPPTGVSACTAGLYAAVVGRLRLHRPA
CLRRRRLVFPPARLPFMSPLLGVYVFTDRPPRPPPTGVSACTAGLVCCRCWASTSSPTGRLVRRRLVLPPARLAYYA
AVVGRLRLHRPAAASAADWCHRLHGWPVITPTDAIFFTAGYIAVTTYRHQYLQHKLPTFAMAADWFLHFHGCMILPV
LIGLTIFIAFRLVCC

>LOC_Os09g36010.1|PACid:21927192
MSASSFFPLFSPFLFSLSLFSGSGQAGTGARRERRSGGRRRGGGGGGEDDAGAGIHHPPTGELNPGAIKIGNSGGHH
NPKIQSIPIHIPELHIHGRRAAAAALGSLASWRGRLHRRRRAKKTCVFSMDSACSSSTSASKREDAAELTGMKEMDS
PGPTGRFPTSGVGHELGALEEEVEVKGNQRESEGGTERRPEVADGGGGGSSPSPPPMPSPAPALSSSPPPCSPDPAS
SPLPPPPRRRPPDLLSRLAPPDLLPRLAASPRRRLAQSQRREREEEGREEGKERGRHMGLTLSQQPRKTKPGSKPLR
NLL

>LOC_Os06g21660.1|PACid:21929857
MPPLLGVYVFTDRLAFAAADWCFRLHGWPFMPPLLGVYVFTDRLAFAAADWCFRLHGWPLCRCCWASTSSPTGLPSP

PPTGVSASTAGLLCRRCWASTSSPTGRLVRRRLVFSPARLAFMPLLLGVYVFTDRLAFAAADWCFRLHGWPLCRCCW
ASTSSPTGLPSPPPTGVSASTAGLLCRRCWASTSSPTGRLVRRRLVFSPARLAFMPLLLGVYVFTDRLAFAAADWCF
RLHGWPLCRRCWASTSSPTGLPSPPPTGVSACTAGLYAAVVGRLRLHRPAASSAADWCFRLHGWPIMPPLLGVYVFT
DRPPRPPPTGVFACTAGLYAAVVGRLRLHRPACLCRRRLVFPPARLAFMTAVVGRPRLHRPACLRRRRLVFPPARLA
YYAAVVGRLRLHRPAASSAADWCFRLHGWPIMPPLLGVYVFTDRLAFAASDWCFRLHGWPIMLPLVGVYVFTDRPPH
PPLTGVTACTAGLL

>LOC_Os09g38784.1|PACid:21927434
MEEGSSYLRPLHFFSIFVAFASFSQCGARVLRPDELLLDHYYYHSSSSDPYYSTPILPPYGDAFSPPNPPPPPPPMS
PSCLLPPIIPAPTFTYSSPPPPPLYYPPPPDISPSPPPSVTPLPPVVYPSPPEVTPSPPEIAPYPSPPEIVPSPPEI
TPYPSPPEIVPSPPEITPYPSPPEVVPGPPEINPYPSPPEIVPSPPEITPYPSPPEIVPSPPEITPYPSPPEIVPSP
PEIAPSPPIVTPMPPIIYPSPPEVTPSPPEITPYPSPPEVTPGPPEITPYPSPPEITPSPPEITPYPSPPEVVPSPP
KITPYPSPPEVTPSPPEITPYPSPPDIVPSPPSYEPSPPSYEPSPPEYAPEPPVYAPYPPGIFPSPPEYSPEPPSYV
PSPPQYAPQPPSYVPSPPEYAPEPPVYAPYPPGITPSPPEYAPEPPPGPPGGGGGYLPPVVFPPPYASRGPPGRRAV
WCVAKPSVPEGIIQPAMDYACGSGADCDSIQPSGPCFRPDTMIAHASYAFNSYWQRAKSNGATCDFGGTAMLITKDP
SYGGCHYSTM

Glycine max

>Glyma14g02170.1|PACid:26285174
MANENEPAKLLLPYLQRADELQKHEPLVAYYCRLYAMERGLKIPQSERTKTTNALLVSLMKQLEKDKKSIQLGPEDN
LYLEGFALNVFGKADKQDRAGRADLNTAKTFYAASIFFEILNQFGAVQPDLEQKQKYAVWKAAEIRKALKEGRKPTA
GPPDGDEDLSVPLSSSDRYDLGTTETTVSSPGPESDSSHSYHSPVNYQNLPSIQPTPKFHDTVNDQHSADIPPSMP
FHDRVDNNKHSSVVSPSSHSYTLGVYPSQDYHSPPPSQDYHPPPPSQDYHSPPPSQDYHHPPPSQDYHHPPPSQDYH
PPPPSQDYHPAPPSQDYHPPPARSESSYPEPYNHQQYSPENSQHLGPNYPSHETSSFSYPHFQSYPSFTESSLPSVP
SNYTYYQGSDASYSSQSAPLTTNHSSSAQHSSGSRNGTVVEPKPTTQTYQYDSSYQPAPEKIAEAHKAARFAVGALA
FDDVSIAVDYLKKSLELLTNPSAGQ

>Glyma14g09800.2|PACid:26285477
MCGKPLSVSDPTRWIPNCREGAVNPCTVSRVQSIRHSPMASAEQPLKKRKLYEPLPEPPPSSPPPESEATPPSPQTL
PTPSTPSLSQEDILAKRWNKDEIRSVYEGYKRIKRCLLRKDARSSMSELEQSYLALITSSRGCMRVQRIVADLIPRY
ACHCPTALEAAAKVVINMHNLSLTLISRGEDSSGIAFETARACICGLADVCCVASSVAPTSAVIRGICAAVFQNVLT
FFIALFEGKDVLQMVDKNFLNMQDTPEAFSELKQKVLDEDESSLTKLSKLRVLCLLWIFFSCPKDLLAACLDLLGSA
TKEGTNDEGQHFLSLVTSTFDDDKAVHLLERAIGGPKSCTDSIGSGIRDNEAGETIMTEDKHASGGDSSVGKSCLLI
QVLNKDPSLLKWMLCRCKKLLDLLSNASLEIASLVRGILGMFPQQTDLEDCQADSDEDKSDSSIYMNSNYIVPRISE
EHESIGESSVKGSSLRVHVGSSNDDFTDKVSDKYVMAHSSAVSLDHAPALKVGLLYDNGVSKPMSIGVGEDGNMPTP
RDSISHQMFSPAVRTPVNFRSNSFEGRNDFLNVEKNQVLNFNSPPLGSSSGSVSNSLASPNHHFMSPSASTKGQIVW
CCDGDPAAMGIVSASKQLWIGYVGPDVPESHIRFHIERFGPVEQFIFFPVKGFALVEYRRIVDAIKTRHCLPGCFPC
HVKFMDIGLGTRGAMNGVAVGSSSHIYVGNIPSQWAKDEIMHETRKVIHKGPLAFIDLSCEFALLMEFESPEEATTV
MLHLRQLRRERSNHNQHFCPGTVNVGIGHAYMDGARPIPAPPPPHLDLKVNNPAGSPHARTLSGSPADSSQTRISHL
STLLASLHTKYNINQNLGLNDNYMTGNNCPPMREEDMVPSSTLCITIPRSSSLFLTDDELMAICNLAIGNTGSIVQL
TQANMQMGCSWFVECSNVDGAVSVLKNLRGCPGLFFQIEFSKPGHQNAVPFSVKPENNSMELVSPRINSENHTSGIQ
GAPLLQSNWHFPGSTEMSEVGARKPDGYDNLSQDPHQGGNVPHSYSGAHGPSIPPPQQIQSFPFVHPVYVPPNGPWD
CQGINNHLPVGQFRTGVMPNHFHGNAVVSPFIPASVTPLAQIQGTPMHPYNQQVPPSIMPPPLSSLPPPQPEMPPPL
PPSPPPLPQVQPPLVPPLPSSPPPPPPPQLPVQEPVNMECSGQSLQYQWQGNLCKSGVNYCTIYASKADSNICRYSN
AIPEPAEWPSKLDMTKRTDLRHVKSTFAATPSHRREVCRLIPSSSSDHRRFQDFISYLKQRDCAGVIKIPASKSIWA
RLLFILPHSIETCSLLSIAHDPSDCLIALVLPKETNFDWI

>Glyma14g22930.2|PACid:26285581
MPVNRGQGQTTRWYQLCFIPTSSDNSDDSDIESVVLTPSNLTQTSSTWQELSPPIPPESEPEIDWSLPPPPPPPPPP
SPPPPLPNFLPLQRVVLTLGTPTSSTWQELFPPSSPLSSPPRPTRISSTWQELSPPSSPLSSRPPTNLEPEIDMSLS
LLSPPPLLPDFLQSRPLQRASARTFNIEVQQDRHQPPSPSHVFEGDQSDCSMNVKNGITRVRVSEIDKLFHCPICMD
EFKVGGDKACQLPCTHTYCSECILRWLDNNKTCPVCRLQLNGCSFDSINCNNGLDSQPQIPSLPSRSPIVHNDSPQR
WENFSQSPPYLLQGIENSINDNNGLDSQPEILLPPPPPPPSAHNDLHRPWEYYFESPYLIVQDIENSEGGNSDEADY
DSACDELGDPHEDGASQSVPAPLT

>Glyma14g36670.1|PACid:26286315
MRGRSYSPSPPPRHSRRGGGGRSPSPRGRYPPRPRQQDLPTSLLVRNLRHDCRPEDLRRPFGQFGPLKDIYLPKDYY
TGEPRGFGFVQYVDPADAADAKYHMDGQVLLGRELTVVFAEENRKKPTEMRTRERRGRFYDRRRSPPRYSRSPRYSR
SPPPRHRSRSRSRDYYSPPAKRREYSRSVSPEDRRYSRERSFSQHSRERSYSRSPPYNGGSRSRSQSPAKGPGQSRS
PSPNRDGREPARGRSPSQ

>Glyma14g28050.1|PACid:26286484
MASTQPNNYFPTFPPPSPSHPFNPPPPPSQSFYPPPPPSHFNPPPPPPHSFSPPPPPFRTSPPSPPRPFSPPPPKVR
PPPPPPRPIHPSPPPPPRPVRPPPLPPAPLPPSPSPNNPTVIIVVVVSLGGLLFLSMLAFALFCCIQRRKKKTQETE
VLHFDEHQTVKETIVPGPFGRNTVVVTVEDDVHIDEEIKTEKVGHGLHAKSSSPHEADRVTSGSIVEVATPLRALEH
HDAHHHHPENKP
>Glyma14g28090.1|PACid:26286701
MASTQPNNVYFPNFPPPSPSHPFNPPPPPPHSFSPPPPPFATPPPPLRPISPPPPKVLPPPPPPRPVLPSPPPPRPVR
PSPPPPRPIRPSPPPPPRPVRPPPLPPAPLPPSPSPNNPTIVIVVVASLGGLLLLSMLAFALFCCIQRRKKKTQETE
VIHFDEHKTVKETIVPGPFGRNTVVVTVEDDVHIDEEIKKKNEKGGHGLHAKSSSPEADQGTSSSIVEVAATPPPPE
NHDDHHHQPENKL

>Glyma14g07550.2|PACid:26286763
MQKVTFALAYYTCNKRKQAKSITPYFTYKLLFIYLASEGIKFQVPTTHTMVSTTKSWTTQKLTIVLLLFCITISSPV
EANTFSSKLDQSLVSQPTHTEIKCGSCPCGNPCDEQLLSPPPPPPASPPPLFLPEISSPTPPESCDDSSPLPPPPPP
SRPPPPPPPSRPPPTPPPPRFIYVTGVPGDGYAYYYSAAQNRVVGLLVFAGLGALSVTMLFG

>Glyma02g46460.1|PACid:26287469
MANENEPAKLLLPYLQRADELQKHEPLVAYYCRLYAMERGLKIPQSERTKTTNALLVSLMKQLEKDKKSIQLGPEDN
LYLEGFALNVFGKADKQDRAGRADLTTAKTFYAASIFFEILNQFGAVQPDLEQKQKYAVWKAAEIRKALKEGRKPTA
GPPDGDEDLSVPLSSSSDRYDLGTTENTVSSPGPESDSSRSYHNPANYQNLPSIHPAAPKFHDTVNDQHSANIPPSM
PFHDRVDNNKHSSVVSPSSHSFTPGVYPSQDYHSPPPSRDYHSPPPSQDYHSPPSSQDYHPPPPSQDYHPPPSQDYH
PPPARSEGSYSELYNHQQYSPENSQHLGPNYPSHETSSYSYPHFQSYPSFTESSLPSVPSNYTHYQGSDVSYSSQSA
PLTTNHSSSAQHSSRNETVEPKPTTTQAYQYDSNYQPAPEKIAEAHKAARFAVGALAFDDVSVAVDFLKKSLELLTN
PSAGQ

>Glyma02g46460.3|PACid:26287470
MLPVSFLRFLINLEQFSLMCVFHPLNLSFFLHISLHLETICSKNITSCKCMFDAFLNFFLQLEQKQKYAVWKAAEIR
KALKEGRKPTAGPPDGDEDLSVPLSSSSDRYDLGTTENTVSSPGPESDSSRSYHNPANYQNLPSIHPAAPKFHDTVN
DQHSANIPPSMPFHDRVDNNKHSSVVSPSSHSFTPGVYPSQDYHSPPPSRDYHSPPPSQDYHSPPSSQDYHPPPPSQ
DYHPPPSQDYHPPPARSEGSYSELYNHQQYSPENSQHLGPNYPSHETSSYSYPHFQSYPSFTESSLPSVPSNYTHYQ
GSDVSYSSQSAPLTTNHSSSAQHSSRNETVEPKPTTTQAYQYDSNYQPAPEKIAEAHKAARFAVGALAFDDVSVAVD
FLKKSLELLTNPSAGQ

>Glyma02g46460.2|PACid:26287471
MANENEPAKLLLPYLQRADELQKHEPLVAYYCRLYAMERGLKIPQSERTKTTNALLVSLMKQLEKDKKSIQLGPEDN
LYLEGFALNVFGKADKQDRAGRADLTTAKTFYAASIFFEILNQFGAVQPDLEQKQKYAVWKAAEIRKALKEGRKPTA
GPPDGDEDLSVPLSSSSDRYDLGTTENTVSSPGPESDSSRSYHNPANYQNLPSIHPAAPKFHDTVNDQHSANIPPSM
PFHDRVDNNKHSSVVSPSSHSFTPGVYPSQDYHSPPPSRDYHSPPPSQDYHSPPSSQDYHPPPPSQDYHPPPSQDYH
PPPARSEVLSKFYRKQPSISPIKLYSLSRIRCFIFFPVGSANYKPFIKCSTQ

>Glyma02g38540.1|PACid:26287895
MRGRSYSPSPPPPRYSRRGGGRSPSPRGRYPPRPRQQDLPTSLLVRNLRHDCRPEDLRRPFGQFGPLKDIYLPKDYY
TGEPRGFGFVQFVDPADAADAKYHMDGQVLLGRELTVVFAEENRKKPTEMRTRERRGRFSDRRRSPPRYSRSPRYSR
SPRYSRSPPPRHRSRSHSRDYYSPKRREYSRSVSPEGRRYSRERSYSQHNRERSFSRSPPYNGGSRSRSQSPAKGPG
RSRSPSSLNRDEREPARGRSPSQ

>Glyma02g38540.2|PACid:26287896
MRGRSYSPSPPPPRYSRRGGGRSPSPRGRYPPRPRQQDLPTSLLVRNLRHDCRPEDLRRPFGQFGPLKDIYLPKDYY
TGEPRGFGFVQFVDPADAADAKYHMDGQVLLGRELTVVFAEENRKKPTEMRTRERRGRFSDRRRSPPRYSRSPRYSR
SPRYSRSPPPRHRSRSHSRDYYSPKRREYSRYVACNNR

>Glyma02g03250.1|PACid:26290811
MKPYLRPIFLLFLILLHQSISASASNSRFIEFFSKLKNSFNPKSEELKSKDIKKLPSVEYLKKIHEGVKDLSQDLFI
PKIPFLNPKNPKLPRIPLFKSPPPPMYRLQLKSDPPPPMYRLQPKSPPPLPNAPPKQINIKGLSVVKDYLSDYGYIE
SSGPFNDSFDQEIISAIKTYQKFSNLQVTGGLNKQLIQQMLSIRCGVPDVNFDYNFTDDNISYPKAGHRWFPNRNLT
YGFLPENQIPDNMTKVFRDSFARWAQASGTLSLTETTYDNADIQVGFYNFTNSRIEVYGGSLIFLQPDSSKKGVVLL
NGNMGWLLPSENATLSKDDGVLDLETAAMHQIGHLLGLDHSHKEDSVMYPYILSSQQRKVQLSNSDKANIHLQFAKH
D

>Glyma02g43150.1|PACid:26290908
MAKNRKLTRFKDLSIAFRFVAVFFLLQKLGLCCSLNEEGNALLKLRQRIVSDPFGALSNWIDDEVSVDPCNWFGVEC
SDGRVVVALNLKDLCLGGTLGPELVKLVNIKSIILRNNSFSGIIPEGFVELEELEVLDLGYNYFSGHLPADLRSDIS
LAILLLDNNDFLVGFSPEINELRMLSECQVDENKLTNAAKMPACTKRVTTWNIDQGKSTRGLLQQKAKPRTNQGHFY
RVADPPVKSSPPPSASPSASAKPPGPKLAPHRKNGSDSPPPHSTPGSGTLSKTESNSPKVHTFPILPGVIGGAVFL
IFSSIGIYLCKTKVANVRPWATGLSGQLQKAFVTGAQKLRRSDLEAACEDFSNVIGTSPIGTLYKGTLSSGVEIAVA
FVPVTSSRNWSKTLEAQFRSKIDTLSKVNHKNFVNLIGYCEEEDPFTRVLVFEYAPNGTLFEHLHIKEAEHLDWGTR
LRVATGMAYCLQHMHQLDPPMTLIKLNSSAVYLTNDYAAKLSDLSFSNDITSAEARAIDMPIATPESNVYSFGVLLF

EMVTGRLPYSVEHRDSLENWASHYLEGDQPLIEMVDPILVSYQEDQLEQVAALITSCVHPDPQQRPTMKDVSERLRE
ITKITPESAVPKLSPLWWAELEIASAEAR

>Glyma12g02991.1|PACid:26292039
MEDVSGHPKDERPIFFDSYDEIDLQHQDSDRNLDPTSNISEEMSATPEGSPPMPLDSPPPPPPLPSSPPPPPPPLSP
SPPPPPPPPMLQPPFPPLPPSAPQVSLVPQSSGPARPLLSQSLMPPQSSHQSSPQLGYQQSVPHDFSGTTNGNQIVP
MVGNSFPGGHNNAVVKNEVFPQPTAYAPTAGCSSQEPSGFNPSRQLEYGQNDMYLNAQVPQPNHQFQQGNPPFAQRH
AHPAPPQNPPNLYSYSNPTVQQHLPHSFHPPFPLPSLPDGRRQFVADEQWRVSSSEFKTNNQHGVWRGRNPSSCPGP
PYGQEGHGVPQMMPCRPDIPAVNSWRPT

>Glyma11g36520.1|PACid:26294708
MKKKKQVPEWMNSALWSTPSDHGRFDPPSPPPPPPVSVKEDSRTIPTNQNSSPPPFSSSSVSSPTASADDISRQAQA
QAQAQVQLLAELSRKVIDMRELRRVASQGIPDAALRPTLWKLLLGYLPPDRALWFSELTKKRSQYKNFKDDLLMNPS
EITRRMYNSNSNSAAHDIDDAKSDTQTRLLLSRSQITHQDHPLSLGKTSIWNQFFQDTEIIEQIDRDVKRTHPDIDF
FSGDSHFAKSNQEALKTILIIFAKLNSGIRYVQGMNEVLAPLFYVFKNDPDEENAAFAEADTFFCFVELLSGFQDNF
CQQLDNSICGIRSTITRLSQLLKEHDEELWRHLEVTTEVNPQFYAFRWITLLLTQEFNFADILHIWDVILSDPEGPQ
ETLLRICCAMLILVRRRLLAGDFTSNLKLLQSYPYTNISHLLHVANKLRVQSV

>Glyma11g36520.2|PACid:26294709
MKKKKQVPEWMNSALWSTPSDHGRFDPPSPPPPPPVSVKEDSRTIPTNQNSSPPPFSSSSVSSPTASADDISRQAQA
QAQAQVQLLAELSRKVIDMRELRRVASQGIPDAALRPTLWKLLLGYLPPDRALWFSELTKKRSQYKNFKDDLLMNPS
EITRRMYNSNSNSAAHDIDDAKSDTQTRLLLSRSQITHQDHPLSLGKTSIWNQFFQDTEIIEQIDRDVKRTHPDIDF
FSGDSHFAKSNQEALKTILIIFAKLNSGIRYVQGMNEVLAPLFYVFKNDPDEENAAFAEADTFFCFVELLSGFQDNF
CQQLDNSICGIRSTITRLSQLLKEHDEELWRHLEVTTEVNPQFYAFRWITLLLTQEFNFADILHIWDVILSDPEGPQ
VQFYSYLSCTILDYP

>Glyma11g10671.1|PACid:26295043
MAPGRRRGANKAKANGHLSLGDLVLAKVKGFPAWPAKISRPEDWDKVPDPKKYFVQFFGTKEIAFVAPADIQAFTSE
AKNKLSARLQGKTKYFAQAVKEICAAFDEMQKQKASGLADDTDDSHIGSEAPSNDGVVGNLKDAADAVSNAEKDNID
MDNVCSNLEYCVPRIGENDSQDEKLSVSNHPNESSSVSSPVIKNKLAIGSETKKNANKSSFKGASNVNDFRQDANGH
SDLTNGTKTRKLDNGSRKKSEAASGSNRNGGSSTGKFMKEGNCTGRGDLSRSGETLKAVKKRKNAFSVKSDSPDTLK
PNDNGTTGEKDSNLMKVKTSHEVKNELQEISFDSEDADGKSSSMRKKTQLHAKHNVGGANESLHATKKLKLMDAKDD
STLGYTSKILKRASPVSTVIEDRPFKKLESKKSTPNLKTEKSLPSRGQIGGAGSDDSVHELLPGTKHHSQVQKIMPD
SAGIASDEKKERSFLRPKGDTNNVVIKQVERKRRAVCLFDDDDDDKPKTPVHGGAAKNMKSSSVSEVKKRNIVHSEK
SDVVQLAQRNSSELEDTHLKEPSSQLHDDRLSIQQPLKEKDDEVIPVHVPYSPEKLDLKQFPSNVTKLSSVSPLKSP
QLVPATKSNAERNKVSKVSLKVSSNATQKRAEHGSSKSSHNLSSSQNQVVTHKKKPAMSAEIFKTTSETLPQAVEVP
ATTVCSKDPDALHVDRLEVGTEEKNSIYTVSGTPESAKTMKHLIAAALAKRKQAHSQCLPSGFPNVQEGTPSPSTVQ
PFLPVSSNFVPADIQGVYEHTTLASPPTKELHSASHNQLDADDIEERRVGSVQRGLGGSLSGGTEAAVAREAFEGMI
ETLSRTKESIGRATRLAIDCAKYGIANEVVELLIRKLETETSFHRKVDLFFLVDSITQCSHNQKGIAGASYIPTVQA
ALPRLLGAAAPPGASARENRRQCLKVLRLWLERKIFPESVLRRYMDDIGVSNDDMTVSFSLRRPSRAERSVDDPIRE
MEGMLVDEYGSNATFQLPGFLSSHAFEEDEDEYEDAIPINLCKETCDASPADPPHTLGESETSTVTPNDKRHCILKD
VDGELEMEDVSGYPKDERPIFFNSSDEIDLQHQDSDRNLDPTSNISEETSVTPEGSPPMPLDSPPPPPPLPSSPPPP
PPPLSPSPPPPPPRPMLQPPPPPLPPSAPPVSLVPQSSGPARPLLSQSLMPSQSSHQSSPQLGYQQSVPHDFSGTTN
GNQIVPMTGNSFPGGHNNAVVKNEVFPQPSAYAPTAGCSSQEPSGFNPSRQLEYGQNDMYLNAQVPQPNHQFQQGNP
PFAQRHAHAAPPQNPPNPYSYSNPTVQQHLPHSFHPPFPLPSLPDGRRQFVADEQWRMSSSEFKTNSQHGVWRGRSP
SCPGPPYGQEGHFRPSLERPPVSTVGFQRPISGNLPVAPISGHVPQMMPCRPDIPAVNSWRPT

>Glyma11g17760.1|PACid:26295401
MPEMDGSSAKPPQISEMFQKFALAFKTKTFEFFSEEENNASPLLDDIDGFSLLDSTEEIITDQKVVVIKPDPDPSLK
SPPSPPPESPPPPPPPPPPPQITHPPPPPEFREPETPPPPPLTEAQIRETTHALISSVFAAVSAFEASYFQLQSAHVP
FVEEHVTSADKVLVSHLQRLSELKKFYCNPEPRGFPFGSRLGAEVEENQSKLRTLGTVSNRLQWELEQKHDEVVALR
AKLDEIHRGNVNLSKKLCARALNPSSDVLLTVKVFDSLLHDASRATHRFTKILIGLMRKAGWDLGLAANAVHPNVDY
AKKGHNQYALLSYVCLGIFHGFDSMNFGMEDGEELVVSNGHGSLDLEDRDGCLKQLLEHVSSNPMELLGIHPGCEFS
RFCEHKYERLIHPSMESSIFVNLEEKEAVLNSWRSLSMFYEAFVGMASAVWTLHKLSYTFDPTVEIFQVERGVEFSM
IYMEDVTKRLTWPNKGRAKVGFTVLPGFRIGRVVIQSQVYISNFKCTE

>Glyma11g16653.1|PACid:26296108
MNHLNKLVSKEPGKGLPKLKFVKEGRYNNQQKGIDFGETYVPLARVEVVRLLLAYAYVMDFKFEEAKLHGESKYCKE
LLKKFEMDKSKKVATPMATNCYLSADEKEKSIDQTKYTEISVLNRQFLSSSPPPHRPHGTQSPPPPTPMATLLLPNP
FSTKLPAQRYSHRRFTCSSITAQSRSAKEQLLALIADQDRGIKTQSDPAKHAAIVEAINAMAAAGEGSVTTGDALSA
TWRLLWTTEKEQLFIVEKAPLFGTQAGDVLQVIDVRNRTLNNVITFPPDGVFIVRSTIEVASPQRVNFSVHDLSIEG
GKGDKINRILILTTNDGEWVQVTRRRKTITETHGGPSRPENNHRLDRTVTWRDKADITTFYFCRFADWVNEKELWQI
FHRWGTVWEVFIPKTKNKEGHRYGFVRFKEVEDELRLERQLDNNIFFGKSKMFVNRPKFERGKRASRNQKNQFTPVN
GKPQGVTSYGRGAPRTNVTGARLRSYAEVVTMASPGQSILHNQRAACSQASQDVHRPPVVINTNMDQKAWLQKAWVG
RLKNRGMFKKVEDELKWVLDPEVTPCYWADDWIILLNLDDSKATQIMQEEKTHGSTPILDLQKWSQDIRPTHRLAWI
LLWGLPPTAWESESMGKMVEAIGDMVEVDELVEQRRRLDVARILIQTTMTPGIRTELKAVIDGIETTLHVVEDTTWL
GTRLDQKRMVSWMPPSPFSTEPNSPVVIGSDMYGADSMHETSEGSPVYDGDGFSTVGRVDRDLQTRRDQWVQAIGRC
SLDRSFTDIDDVDYPHEDNAILNLYPTVEAAVPSQHISSGQKVVKWDAINASSQGNRRESIHKQAPKRQVDSFAGQQ
RNPLEETFACTDCPAEGKQEQQLPQVVSLPTSSAQADNRDSISLDKNDIIKEKGNLGLRPSGPILGSAGTKYYVRRK
TVGGAREQAQYNQEMAVDPLTYPILPSPTKGTLKTAHTNIVSHLESECIKQDDEHGQINPHILEKQCLISRNGLDPW

>Glyma06g07380.1|PACid:26300496
MTKKKNPLVFMDVSIDGDPVERMVFELFYDVAPKTAENFRALCTGEKGISPNTGKSLHYKGSFFHQIIKGSIVQGGD
FVNRNGTAGESIYGSKFPDESPRLKHDATGLLSMAIADRDTLGSHFIITLKADHHLDRKHVVFGKLVQGHNVLKKIE
EFGDEEGHPTVTVKIINCGEYSEDGKKVNKSKMGKNKKSSKDRRKRRKYYSSESGSSSDSDTESLETDSDSESDMSS
SSYMSSSSDDRRKKRKRSKKDKYRRGKRKDKRRDKRRRKKDKRSKRKSKRESGSDSDSESNSDNSSEGENIGAQHKG
LKRKDNSQKNAETKSPLVAEKDLPPAHHEKEEMGMLENEEKLSKENGERRNNGIGGADYGSDRSEERQPDVMDDHSG
KTRSRSVSPKRPMSKSMSISPRRSASKSPSVTPKRRLSKSPSVRSPPPSQRSLSRSPARSISRSPSRSISRSPVRGR
NGRSVNRSPVRGRNGRSVSRSPVRGRNGRSVRRSPVRGRNGRSVRRSPVRGRNGRSVSRSPVRGRNGRNVNRTPVRG
GNGKSVSRSPVRTRSLKSVSRSPVRTRSLRSVSKSPVRARSQSHRSRSSPRAPSGKTISRSPVRVLRKSISRSPVRS
PARSLSRSSGWVPSKKSISRSPVRAPSRSIRRSYSRSPSPVRRTRTPRGRSVSRSVSPDASPKRIRRGRGFSERYSY
ARRYRTPSRSPVRSYRYNGRNDRDRYSSYRRFSPRRFRSPPPRGRTPPRYRSRRSRTPSVSRSPRYRARRYSRSRSP
VRSRSPVRSRSPVDRSRPTRVERRASSSRSRSPSKSRSRSRSRSRSSVESLPRASRDSRSRSSSRSPDGKKGLVSYGDG
SPY

>Glyma06g07380.3|PACid:26300497
MTKKKNPLVFMDVSIDGDPVERMVFELFYDVAPKTAENFRALCTGEKGISPNTGKSLHYKGSFFHQIIKGSIVQGGD
FVNRNGTAGESIYGSKFPDESPRLKHDATGLLSMAIADRDTLGSHFIITLKADHHLDRKHVVFGKLVQGHNVLKKIE
EFGDEEGHPTVTVKIINCGEYSEDGKKVNKSKMGKNKKSSKDRRKRRKYYSSESGSSSDSDTESLETDSDSESDMSS
SSYMSSSSDDRRKKRKRSKKDKYRRGKRKDKRRDKRRRKKDKRSKRKSKRESGSDSDSESNSDNSSEGENIGAQHKG
LKRKDNSQKNAETKSPLVAEKDLPPAHHEKEEMGMLENEEKLSKENGERRNNGIGGADYGSDRSEERQPDVMDDHSG
KTRSRSVSPKRPMSKSMSISPRRSASKSPSVTPKRRLSKSPSVRSPPPSQRSLSRSPARSISRSPSRSISRSPVRGR
NGRSVNRSPVRGRNGRSVSRSPVRGRNGRSVRRSPVRGRNGRSVSRSPVRGRNGRNVNRTPVRGGNGKSVSRSPVRT
RSLKSVSRSPVRTRSLRSVSKSPVRARSQSHRSRSSPRAPSGKTISRSPVRVLRKSISRSPVRSPARSLSRSSGWVP
SKKSISRSPVRAPSRSIRRSYSRSPSPVRRTRTPRGRSVSRSVSPDASPKRIRRGRGFSERYSYARRYRTPSRSPVR
SYRYNGRNDRDRYSSYRRFSPRRFRSPPPRGRTPPRYRSRRSRTPSVSRSPRYRARRYSRSRSPVRSRSPVRSRSPV
DRSRPTRVERRASSSRSRSPSKSRSRSRSRSRSSVESLPRASRDSRSRSSSRSPDGKKGLVSYGDGSPY

>Glyma06g02181.1|PACid:26300543
MGCNTSRLDRLPAVALCRDRCKFVDEALRQSYALADAHVAHMEALKTLGPALLCFFDGFDDSDETSVRIKETKVPVA
TKSPPPRSPSSLSSDSDDANVLLHSESETEDAEKEEFQLFTRARYDYAHSARYPPFSPPRGYSGSKPPSPPPPSGSA
WDFLNFFEPYEKYHVPYFPSGGDADGAEKDKGKVEKRDEFKSKGENEEVKKKKGDSKEKKVVSEKEEVVSVSEQCSD
SARVKSGKGFSEAMKEIQVLFEKASESGNPVLEMLDVGKLRYHRKFDLNPVSCKMMHVFTPSSPIGVKCIESSLLGR
RMGSGYEGDKGHSYGNLCSTLKKLCMWEKKLYHEVKAEEKLRMLHQKKCKQLRRMKQKDANAQKIDSVQTFVGILST
KMKISIQVVDKISITISKLREEELWPQINRFFLKFLGMWKDMQECYKCQYQQIVEAKTLDASSLNTKPGNAHIDATI
KLKSEVQKWNLSFLDWIHAQKSHVKALNGWLVRCLLYEPEEVPDDSTPFSPSKIGAPPVFVICHKWSQAVDNLSEKN
IIEAVNGFMLRVNELWEKYILDLQQKLTLDKEFERKVKMLEREEQKMHKVMRAHEKKMVTVGREESDALLRGDAVHH
ADVVDTTNLQSGLKQIFGAMEKFTDSTVRLYEELCQQIKQEEKPTKIN

>Glyma06g47265.1|PACid:26300594
MAEVINNMPPESLDQSPSSSAPPPPPPPPASSSAPADDLPPPPLPPPPRRRDRRDDRDFDRHPNRSRDYYDRDRDF
KRRRSPSPGYRDRRYSPPPPSRRSPPPYKRSRRGSPRGGGYGPDDRFGYDYSGGYERGAGGRTGYADEKSYGRLVHR
SAGGYHNGISDVDNSRGYADLSSGGAQREGLMSYKQFIQELEDDVLPAEAERRYQEYKSEYISTQKRAYFNAHKDEE

WLKDKYHPTNLLTVIERRNENARRLAKDFLLDLQSGTLDLNPGLNSTSSGKSGQASEPNSEEETDGKRRRHVRGPNK
DNDFSAAPKAHPISSEPRRIQADIQQAQAVVRKLDREKGIEDNILCTSDHNKNDDKAHSGSVGPIVIIRGLTSVKGL
EGVELLDTLITYLWRIHGVDYYGMVETNEAKGFRHVRPEGAGHEETSKSGSDWEKKLDSFWHGRLNGQDPLEVMTAK
EKIDAAATDVLDPHVRKIRDEKYGWKYGCGAKGCTKLFHAAEFVHKHLKLKHPEIVMELTSKMREDLYFQNYMNDPD
APGGMPVMQQPQKDRPLKRRLGGLEGRLKDDRGNRRDQDRSDRMNGDRPDGSPSHERQMGNHDEAMYDAYGGPGVPA
FTSDMPPPPVLMPVPGAGPLGPFVPAPPEVAMQMFREQGGPTSFDASGRKMRSGPHMGGPAPIIAVPPSFRPDPRRM
RSYEDLDAPEDEVTVIDYRSL

>Glyma06g34930.1|PACid:26301271
PQSAPPQAPHSRTYSTDPSSSSPAPSSQSPSHPAAARAPRTPSSPSRPPSPSPWPSPPPPPPPPPPSVPPSPPQSPP
PPPRSAPPSSPPSSPHPTSSTPPPPPTSSPPPRAPPAAP

>Glyma04g15990.2|PACid:26304513
MAEVINNMPPESLDHDQSPSASAPPPPAASSSAADDLPPPPLPLPPRRRDRRDDRDFDRHPNRSRDYYDRDRDFKRR
RSPSPGYRDRRYSPPPPSRRSPPPYKRSRRGSPRGGAYVPDDRFGYDYSGGYERGAGGRTGFADEKSYGRLAHRSAG
GYHNGISDVDNSRGYADLPSGGAQREGLMSYKQFIQELEDDVLPAEAERRYQEYKSEYISTQKRAYFNAHKDEEWLK
DKYHPTNLLTVIERRNENARRLAKDFLLDLQSGTLDLNPGLNSTSSGKSGQASEPNSEEETDGKRRRHGRGPNKDND
FSAAPKAHTISSEPRRIQTDIQQAQAVVRKLDREKGIDDNILCTSDHNKNDDKAHSGSVGPIVIVRGLTSVKGLEGV
ELLDTLITYLWRIHGVDYYGMIETNEAKGFRYVRPEGTGHEETRKSGSDWEKKLDSFWQGRLNGQDPLEVMTAKEKI
DAAATDVLDPHVRKIRDEKYGWKYGCGAKGCTKLFHAAEFVHKHLKLKHPELVMELTSKLREDLYFQNYMNDPDAPG
GTPVMQQPQKDRPLKRRLGGLEGRLKDDRGNRRDQDRSDRINGDRPDGSPSHERQMGNHDEAMYDAYGGLGVPQFTS
DMAPPPVLMPVPGAGPLGPFVPAPPEVAMQMFREQGGPSSYDASGRKIRSGPHMGGPAPIIAVPPSFRPDPRQMRSY
QDLDAPEDEVTVIDYRSL

>Glyma04g02080.2|PACid:26305119
MGCNTSRLDRLPAVALCRDRCKFLDDALRQSYALADAHVAHMEALKTLGHALLCFFDGFEDSDETNVPNKETKVPVA
TKSPPPHSPSSFSSDSDDAHVLLHSESETEDAEKEEFQLFTHARYDYAHSARYPPFSPPRGYSGSKPPSPPPPSGSA
WDFLNFFEPYEKYHVPYFPGGGDADVAENEKGKVEKRDGSKSKEENGEAKKKKGDSKEKKVVSEKAEVVLVSEQCSD
SAKGFSEAVKEIQILFEKASESGNPVLEMLDAGKLRYHRKFDLNPVSCKMMHVFTPSSPLGVRCMKSSVGRRMGSGY
EGDKDLTYANLCSTLKKLCMWEKKLYHEVKAEEKLRMLHQKKCKQLRRMKQKDADAQKIDSVQTFIGILSTKMKISI
QVVDKISITISKLREEELWPLIYRFILTFLGMWKDMQECYKCQYQQIVEAKTLDALSLNTKPGNAHIDATIKLKSEV
QKWNLSFLDWIHAQRSHVKALNGWLVRCLLYEPEEVPDDSTPFSPSKIGAPPVFVICHKWSRAVDNLSEKNVIEAVN
GFMLRVNELLEKHILDLQQKLTLDKEFERKVKMLEREEQKMHKVMRAHERKMVTVGREESDALLRGDAVHHADIVDS
TNLQSSLKQIFGAMEKFTDSTVRLYEELCQQIKQEDHVLGETNKIS

>Glyma04g07300.3|PACid:26305699
MTKKKNPLVFMDVSIDGDPVERMVFELFYDVAPKTAENFRALCTGEKGISPNTGKSLHYKGSFFHQIIKGSIVQGGD
FVNRNGTAGESIYGSKFPDESPKLKHDATGLLSMAIADRDTLGSHFIITLKADHHLDRKHVVFGKLVQGPNVLKKIE
EVGDEEGHPTVTVKIINCGEYSEDGKKVNKSKMGKNKKSSKDRRKRRKYYSSKSGSSSDSDTESSETDSDSESDMSS
SSYISSSSDDRRRKRKRSKKDKYRREKRRDKRRDKKRRKKDKRSKRKSKRESGSDSDSESNSDSSSEGEGIGAQHKG
LKLKDHSQKNAETRSALVAEKDLPPAHHEKEEVGMLENEEKFSKENGEQRSNGIGGADYGSDRSEERQPDVMDDHSG
KTRSRSVSPKRPISKSMSISPRRSARLSKSPSVTPKRRLSKSPSVTPKRRLSKSPSVRSPPPSQRSLSRSPARSISR
SPSRSISRSPVRGRNGRSVSRSPVRGRNGRSVSRSPVRVRNGRSVSRSSVRVRNGRSVSRSPVRVRNGRSVSRSPVR
GRNWRSVSRSPVRGRNGRSVSRSPARTRSLRSVSKSPVRSHSRSHRSRSSPRAPSGKIISKSPVRVLRKSISRSPVR
SPARSLSRSSGRVPSKKSISRSPVRAPSRSNRRSFSRSPSPVHRTRTPRGRSVSRSVSPDASPKRIRRGRGFSERYS
YARRYRTPSRSPVRSYRYNGRNDRDRYSTYRRFSPRRFRSPPPRGRTPPRYRSRRSRTPSVSRSPRYARRYSRSRS
PVRSRSLVDRSRPSRVERHASSSRSRSPSKSRSRSRSSVESPPRASRDSRSRSSSRSPDGKQGLVSYGDGSPY

>Glyma08g21490.1|PACid:26306871
MASYRPFPPQSSQIPNPIPGGNHQYNNNNNQNQNWGAYGDPSTSSFPQIPPNSNYHQNQHHVPYAPAPSNPHHPPHY
PYPPPPPPPPPPEASYQPPPPPPPPAPMYYPSSSQYSNQPPPPPPPLSPPPPPPPVSPPPPPPATQNNNEERRFKEP
SKSGRREYEHSNHGIAHKQHKQQQPPLPVKKMNNGPPGRAETDEEKRLRKKREFEKQRQEEKHRQQLKESQNTVLQK
THLLSSGKGHGMIAGSRMGERRSTPLLGAERVENRLKKPTTFLCKLKFRNELPDPSAQPKLMSFKKDKDQYAKYTIT
SLEKMYKPKLFVEPDLGIPLDLLDLSVYNPPRVRPPLAPEDEELLRDDEAATPIKKDGIKRKERPTDKGVAWLVKTQ
YISPLSMESTKQSLTEKQAKELREMKGRGILDNLNSRERQIREIQASFEAAKSDPVHATNKDLYPVEVMPLLPDFDR
YDDQFVVAAFDNAPTADSEMYAKMNKSVRDAFESKAVMKSYVATGLDPAPNPEKFLAYMAPAPGELSKDIYDENEDVS

YSWIREYHWDVRGDDADDPTTFLVAFDESEARYLPLPTKLVLRKKRAKEGRSGDEVEQCPVPARVTVRRRSSVAAIE
RKDSGVYTSSKGNSFKRVGLEMDDGLEDQHRGAPHQDNYQSSGAEDYMSD

>Glyma08g18310.1|PACid:26309162
MAKPGRGRRSHSGSVSGSSSRSSSPSDSRSSSRSRSVSRSRSPSRSRSSSPSSSSPSRSSRSRSPPPQRRRSPADAA
RRGRSPPPQSKRASPPPRKPSPVRESLVLHVEKLSRNVNEGHLKEIFSNFGEVISVELAMDRTVNLPKGYGYVQFKT
RGEAEKALLYMDGAQIDGNVIKARFTLPPRQKVSPPPKTSAVAPKREIPRTDNASADVDKDGPKRQRESSPRRKPPP
SPRRRSPVPRRAGSPRRPDSPRRRADSPVRRRVDSPYRRGGDTPPRRRPVSPGRGRSPSPPRRLRSPARLSPRRMRG
SPGRRRSPPPPPRRRSPPRRPRSPPRRSPIGRRRTRSPIRRSARSRSRSFSPRRGRPPVRRGRSSSYSDSPSPRKVS
RRSKSRSPRRPLRGRGSSNSSSSSPPPPPPPPPRKP

>Glyma08g10780.1|PACid:26310285
MESDSDSDGSHISATPPRKSTPSPPPPPPVVVSKKSRIRIKPSKSKPKPDPSGSSKTQSKSDPIQQATLLSLPTDLP
FQIRRPSDAPSISYSIETLPAGYFSSSHSASFSKIRRPLLNLEPSEVEQPVFTSGASDSRAEGADLRKRGKSQLPNL
IGSSAPMPSVKPRTSGAGEGNFVKLNMNGKRKKFLNKRGKFGGKRYKSWRFKSQREKEEEQEEAVGELKQRGGCKRR
EIDGDAVEEAATAARKEPSEENLVKLLRLVHGYDCFRDGQVEAIKMVLAGKSSVVVLPTGAGKSLCYQLPAVILPGV
TLVVSPLVALMIDQLRQLPHVIMGGLLSSTQTPEEASESLKQLRQGGIKVLFVSPERFLNEEFLSTISSLPAISLVV
IDEAHCISEWSHNFRPSFMRLRASLLHKTLNVRSVLAMTATATTTTLDAIMSALDIPSTNLIQKAQLRDNFHLSVSL
VRNRMKDLLNLIKSPPFVEVQSMIIYCKFQYETDQINRYLNDNNILAKSYHSGISAKERSYVQELFNSNKIRVVVAT
VAFGMGLDKRDVGAVIHYSLPESLEEYVQEIGRAGRDGRLSYCHLFYDDETYFKLRSLTHSEGVDEYAVNKFLREVF
PADKNSCGKICSLIKESASRRFDMKEEVMLTLLTRLELGDVQYLQLHPQINVTGTLNFHKSPPPLLAQKVSAIAVIL
KRSETKHGQYIFDIPTVANDMGVTAVELINQLYDLKLMGEITYEMKDLAYCYRIIEVPTDLLSLSADITRWLSEVEN
CKVRKMDAMFNAAYFALNLCDTMQGCSGANHTPCLQRKILDYFSGVDNADFCKKIGQSSPFLRADIKVFLQSNSQAR
FTPRAVARVMHGIASPAYPSTAWSKTHFWGRYTHIDFQVVMEAAKEELKNFVGKDTL

>Glyma08g28130.2|PACid:26310484
MTNSMGPPPPKNPNPPDTTPPSMPPPPCDSAEPSPPPSMPPPPCDSAEPSPPPPRDSSNAASQGAAVPYKIPPWSAA
PCHEFYLEVLKDGSIIGKFNVFEKGAYMFGRLDLCDFVLEHPTISRFHAVVQFKRSGDAYLYDLGSTHGTFLNKNQV
EKNTYVDLHVGDVIRFGRSSRLFIFQGPSDLMPPETNAKLMREVKMREAMLDKEASVQRARQEASLAEGISWGMGED
AIEEDEDDVEEVTWQSYKGQLTEKQEKTREKIIKRMEKIANMKKEINSIRVKDISQGGLTQGQQTQIARNEQRTMQI
LEELENLEETLNDSIRESMGARTGKLSHGKKKGAVEGEEEYLSDDDDEFYDRTKKKASHQKTGDNQSVETADTLLDK
KDVITKEMNEKKELLMIEKNKILSNPESATQDEVDDSLDAYMSGLSSQLVHDKSEQLEKELSTLQSELDRICYLLKI
ADPTGEAAKKRELKVHEPKPKKSEVTITIKKKPPAEAQKSSGPCAKADNKNPPVETLKIRETPVKEDGSIEGEKPGA
ATLGLDKSEPDSDRLKAENVVFAIPKPQWLGAVEDRVTDNTQQSMPSLLLHEIDESNQFVDYKDRNQILGSSDNAKT
SVGSTIESAAGLIIRKRKQVETTATNCNDASEQLTSSTSGEKMAEDAVALLLKHNKGLYTNDDEEKYEGQERRGPKR
VLGPEKPSFLNDEMDYDDSWVPPEGQSGDGRTSLNDRYGY

>Glyma17g35410.2|PACid:26311657
MRVVDTNFFEPVGSENHTIHARPDPTRPVVIPHFRRSAPNSWTACRLPSTVHSSLPMASAEQPLKKRKLYEPLPEPP
PFSPPQPPPPESEATPSSPQTLPTPSSTPPLSQEEILAKRRNKDEIRSVYEGYKRIKRCLLRKDAPSSMSELEQSYL
ALITSSRGCMSVQRIVADLIPRYACHCPTALEAAAKVVINMHNLSLALISRGEDSSGIAFETARACICGLADVCCDA
SSVAPTLAVIRGICEAVFQNVLTFFIALFEGKDVLQMVDKNFLNMQDTPEAFSELKQKILDEDESSLTKLSKLRVLC
LLRIFFSCPKDLLAACLDLFGSATKEATNAEGQRFLSLVTSTFDDDKAVHLFERAIGGSKSCTDSTGSGIRDNEAGE
AIMTEDNHVSGGDSSVGKSCLLMQVLDKDPLLRKWMLCRCKKLLDLLSDVSLEITSVLQGILGMFPRQTDLEDCQAD
SDEDKSDSSIYMNRNYMVPRISEEHESIGESSGKGSSLRVHVGSSDGFTDKYVMDHSSAVPLDHVPVLKVGSHYDNG
VSKPMSIGVGEEGNMPTPRDSVSHQMFSPAVRTPVDFRSNSFEGRNDFLNVEKNHVLNMNFNSPPLRSSSGSVSNSL
ASPNHHFMSPTASTKGQIVWCCDGDPAAMDIVSASKQLWIGYVGPDVPENHIRFHLERFGTIEQFIFFPVKGFALVE
YRRIIDAIKTRHCLPGCFPCRVKFMDIGLGTRGAMNGVAVGSSSHIYVGNIPSQWARDEIMHETRKVIHKGPLAFID
LSCEFALLMEFETPEEAATVMLHLRQLRRERSNYNQHFGPGTVNVGIGHAYMDGGRPIPAPPPPPPPPNLDLKVNNP
AGSPHARTLSGSPADSSRTRMSHLSTLLASLRTKYNINQNLGLSDNYTIGNNCPPMREEDMVPSSTLCITIPRSSSL
FLTDDELMAICNLAIGNSGSIVQLTQTNLQMGCSWFVECSNVDGAVSVLKNLRGCPGLFFQIEFSKPGNQIAVPFSV
KPENNSMELVSPRINSENHNLPQSNWHFPGSREMSELGARKPDGYDNLSQDPHQGGIVPHSHSGAHGPSIPPPQQIQ
SSPFVRPVYVPPNGPWDRRGINNHLPVSQFKTGVMPNNFHGNAVVSPFIPASVTPLAQIQGTPMHPYNQQVPPSIIP
PPLSSLPPPQPEMPPPLPPSPPPLPQVQPPLVPPLPSSPPPPPPPPLPVQEAVDMECSGQSLLYQWQGNLCKSGVNY
CTIYACKADSNICRYSNAIPEPAEWPSKLDMTKRTDLRHVKSTFAATPSHRREVCRLIPSSSSDHKRFQDFISYLKQ
RDCAGVIKIPASKSIWARLLFILPHSLETCSLLSIAHDPSDCLIALVLPKETNFEWI

>Glyma13g07240.2|PACid:26315351
MFKKLGKKIEREIKRPFRSGHRPRPSSPPPSQGPPSPPPPPLPPSTSPPPPPPPPPPPPPPPSPPPLPKQPNSPEYI
HPYLIKSSNSFLSLSYPSITFNSSFITQVFNPDITIASSECKTTPGSNTKHVISSFSDLSVTLDIPSSNLRFFLVRG
GPFVTGSYKWEKKGWGELLMLAHPLHLQLLNDSDYGVTVLHDLKYRSIDGELVGVVGDSWLLKADPVSVTWHSTRGI
KDEFHEEIFSALSEDVDGLNPLGVTTTSCYFYGKIIARAARLALIAEEVAFLDAMTEIKKFLKESIEPWLDGTFSGN
GFLYDGKWGGIVTKHGSKDSGADFGFGVYNDHHYNLGYFLYGIAVLAKIDPAWGRKYKPEAYSLVADFMNLGRRSNS
NYTRLRCFDLYKLHSWAGGLTEFADGRNQESTSEAVNAYYSAALMGLAYGDTQLIATGSTLAASEIHAAQMWWHVRE
GHKLYEEDFTKENKVVSVLWANKRDSGLWFAPAQWRECRLGIHVLPLSPITEALFSDVGYVKELVEWTLPNLNRKGV
GEGWKGFIYAMEGTYDKESALQKVRSLKVFDDGNSMTNLLWWIHTRGDVEEELVHGKQCWFGHYCH

>Glyma13g00300.1|PACid:26315725
MVSHNKPYTVATLFFLALFLISIFFFFSTRTTLHPAPSIHSDLYIHLQPLSPPPPLQPLSPPPPPFPSPHSSSATTE
TQPEPVGDIEDVADNATDAESDAQPVTENIAEDVALSLKVCDLFTGTWVKDESYPIYQPGSCPYVDEAYDCKVNGRT
DTLYSNWRWKPDACDLPRFNATDFLVRLKGKRLMLVGDSMNRNQFESILCLLREGLHNKSRMYEVHGHKITKGRGYF
VFKFEDYNCTVLFVRSHFLVREGVRLNGQGRSNPTLSIDRIDKTSGRWKKADILVFNTGHWWTHGKTARGINYYKEG
DYLYPKFDAVEAYRKAIKTWGKWIDDNINPRKQIVYYRGYSNAHFRGGDWDSGGSCYGETEPAFNGSILNNYPLKMK
VVEEVIRGMKVPVKLLNVTKLTNFRKDGHPSVFGKNTMGGRKVSTRKQDCSHWCLPGVPDAWNELIYATLVFQQTNS
RN

>Glyma13g24770.1|PACid:26317933
MSAPLGIGIRSGSYGSLDKQVHQNGTARKASKMLKEKEKERLFLWICKFAGRKKVGMLFLCLISAAVFIWVLYVGKA
EDSQDGNTVSSINVNESVSASDSTSENSTTNAMGLTKRSVLPPPPAGYFLGYRLPPGHPCNSFTLPPPPADKKRTGP
RPCPVCYLPVEEAIGLMPALPSPSPVLGNLTYVYEENLSRDGEFGGSDFGGYPTLKQRNDSFDIRESMSVHCGFVRG
IKPGRNTGFDIDEADHFEMEQCNGVVVASAIFGNFDEINEPNNISDYSRKTVCFLMFVDEETEKYLISSGKLGISKK
IGLWRIIVARNLPYPDARRTGKIPKLLLHRLVPNARYSIWLDGKLELVVDPYQILERFLWRKNATFAISKHYRRFDV
FIEAEANKAAGKYENASIDFQIEFYKNEGLTPYTEAKLPLISDVPEGCVIVREHVPISNLFTCLWFNEVDRFTSRDQ
ISFSTVRDKILSRVDFHFNMFLDCERRNFVVQKYHRDLLLRLAPPASPPPPSPPPPPPLPVLETSPEKGANSPIRRG
PGRRGRDRRAGSRHHRKVVAGSREMEPS

>Glyma13g09520.1|PACid:26319187
MKSLTTLWPMDILKTSSFVRITALSLFSLTLFLFLFTRFSPTSYSSLTFFSSTPEPPSREQAPPPQPNSPPPPPPPP
PPSPPPPGVKIFRVPPLPPVERMGVLDGNGVMTEDFKVGELDPGFEEDSLNDTVSSVSSKGGERVREKVEKYKTCDV
RTVDYVPCLDNVKAVKKYKESLRGEKYERHCKGMGLKCLVPRPKGYQRPIPWPKSRDEVWYSNVPHTRLVEDKGGQN
WILIKRDKFVFPGGGTQFIHGADKYLDQISEMVPEIAFGHNTRVALDVGCGVASFGAFLMQRNVTTLSVAPKDVHEN
QIQFALERGVPAMVAVFATHRLLFPSQAFDLIHCSRCRINWTRDDGILLLEANRLLRAGGYFVWAAQPVYKHEETLQ
EQWTEMENLTASICWELVRKEGYIAIWRKPLDNSCYLGRDIDAHPPLCESNDDPDNVWYVGLKACITPLPNNGYGAN
VTEWPLRLHQPPDRLHSIQLDAIISRDELLRADSKYWFEIIESYVRAFRWEDYNLRNVMDMRAGFGGVAAALHDLQI
DCWVMNVVPVSGFNTLPVIYDRGLTGVMHDWCEPFDTYPRTYDLLHAAGLFSVEKKRQKCNISTIMLEMDRMLRPGG
RVYIRDTTLVIGELQEIATALGWSTTINDVGEGPYSSWKILRSDKRF

>Glyma01g05810.1|PACid:26325040
MDLTKDTNTLDTNSQTPPQPFTTNGSLKDHHHHHPTTVSPPQQPPSTAVAYKECLKNHAASIGGHALDGCGEFMPSS
FSNPNEPRSLTCAACGCHRNFHRRRDTPENHHRSNSRPNFLSFYHSPPPSRHGAGPSSSPSPSPMSSPSPPPISHHF
PPSSHHFQGPIPAHGLLGLGNEHHHHMSFNFNSSSHWNPENSGGKKRHRTKFSHEQKEKMHNFAEKLGWRMQKGDEG
LVQDFCKEIGVSRGVFKVWMHNNKNTSGRKKSLEPGERINGTGSDSTDNNNNHPYNTNSTNDDTH

>Glyma01g17350.2|PACid:26325064
MPEMDGSSAKPPQISEMFQKFALAFKTKTFEFFSEEENNNASPLFDDIDGFSLLDSTEEIIPDQKVVVIKPDPDPSN
NSSPPPLLPQPQSPPPPPPPQIIPPPPPPPESREPETPPPPLTSAQIREMTHALVSSVFAAVSAFEASYFQLQSAHVP
FVEEHVTSADKVLVSHLQRLSELKRFYSNSEPCGFPLGLRLEAEVEENQSKLRTLGTVSNRLQWELEQKHDEVVALR
AKLDEIHRGNVNLSKKLCARALNPSSDVLLTVKVFDSLLLDASRATHRFTKILIGLMRKAGWDLGLAANAVHPNVDY
AKKGHNQYALLSYVCLGMFHGFDSLNFGMEEPVVLNGHGSDLEDRDGCLKQLLEHVSSNPMDLLGIHPGCKFSRFCE
HKYERLIHPSIESSIFVNLEEKEAVLNSWRSLSMFYETFVGMASAVWTLHKLSYAFNPAVEIFQVERGVEFSMIYME
DVTKRLTWPNKGRAKVGFSVLPGFKIGRVVIQSQVYISNFRCTE

>Glyma19g33636.1|PACid:26329042
MPPSRRKGGKKSAGGAAACRQFKLGDLVLAKVKGFPAWPATVSEPQKWGYSADRKKVFVCFFGAAPQIAFCNPADVE

VFTEEKKQSLAKRPGRGGEFARAVKEIIECYEKLRTENQDGDTGSKEQMDESYSPDPSANTGAKEQMDAPFTINSQM
KSSNCVIDRPEDAVALKDESYNIEASLEEATDNAIMTATVKSLFSTTQRNAPVQRSRSTLQVQNFVLPYSDGGNNGS
NSNDNISADAIEDTSIRRRKHIRKSPDLLGCDDTDSPAFAPNVSMEDNGSEIITIISDAFTLNEGSTIDSNLKLEQS
EPIECPEGEDDLNKGLDLEIKAVINKKKRKPNRKKETNDSGAQNASQSLQNMGGNSKERCPDQDGDEHLPLVKRARV
RMGKSSAEAELNSIAQVQVKCGEEDITDSPHQIITSSNCENGSLAEGGTSVLNSALVSVSPSNLIALCSENGSQICK
IKKDQMFGCSVDDEAALPPSKRIHRALEAMSANAAEEGEACMESSSIMTSSGRCCISTIKRCPCMTVNNQGGNDLEL
QRLDSCGIDSSHVSMYSFSTRSNTIISTENESSTEVDKHLAKFQCETGKDVIPGDRQQGGEDLSDSVVCHPAKIDSQ
IQSHGKISPDLDVKCCQVGNSKDSPCPSLLPNGDYNVRPSNHSDASDTLEHGGISLDPVAGDGESDKLVPQNSINVP
QNVVVACEDMGKQAVGGSSKINDTHEVVKEVKFKGQEEDMNSVSISNDYSGEKGNLVILSSPSLTDGRVFLPLGSPP
NTSVCNISTSDSSNILQNGSCSPDVHQKNTLSGPTDGWKDGIVENGRSRSVGKSTEAGDAALLYFEATLRTLKRTKE
SIGRATRIAIDCAKFGIATKVMEILVHDLEIESSLHRRVDLFFLVDSIAQCSRGLKGDIGGVYPSAMKAVLPRLLSA
AAPLGNAAKENRRQCLKVLRLWLERKILPEPIIRHHMQELDSYSSSVSAGVHSHRSLRRDRPFDDPVRDMEGMLDEY
GSNSSFQLPGFCMPRMLGDGGSDSDGGEFEAVTPEHDSETYEVQETTHAIEKRRHVLEDVDGELEMEDVAPSVDGEL
NSICNIDRGNAREFEKNLPVSFGPPLPQDLPPSSPPPPSSPPPPPPPPPPPPPPSLPLPPPPPPPTLHFKSATSDQYHV
AVDSKGFEDSLTVKANVLHPMAEPLAAPRNSQPISDAVQYTVPECRDMPMQMPESTCSFNTFPVQPTDNSRNTDGAT
MHNKGYLIPPPHHVPSNQFSFVHGEHRMKPQREVPPPPSYSNGHHFMPSMTREYGYDNHERLRPPYDYQERWNVPPC
SGPRYHDRGVPAPYGCHPSESVSFPGHGWRFPPPSMNYRDSLHFRPHFEDAIPVANRGPNFWQPR

>Glyma19g33636.2|PACid:26329043
MPPSRRKGGKKSAGGAAACRQFKLGDLVLAKVKGFPAWPATVSEPQKWGYSADRKKVFVCFFGAAPQIAFCNPADVE
VFTEEKKQSLAKRPGRGGEFARAVKEIIECYEKLRTENQDGDTGSKEQMDESYSPDPSANTGAKEQMDAPFTINSQM
KSSNCVIDRPEDAVALKDESYNIEASLEEATDNAIMTATVKSLFSTTQRNAPVQRSRSTLQVQNFVLPYSDGGNNGS
NSNDNISADAIEDTSIRRRKHIRKSPDLLGCDDTDSPAFAPNVSMEDNGSEIITIISDAFTLNEGSTIDSNLKLEQS
EPIECPEGEDDLNKGLDLEIKAVINKKKRKPNRKKETNDSGAQNASQSLQNMGGNSKERCPDQDGDEHLPLVKRARV
RMGKSSAEAELNSIAQVQVKCGEEDITDSPHQIITSSNCENGSLAEGGTSVLNSALVSVSPSNLIALCSENGSQICK
IKKDQMFGCSVDDEAALPPSKRIHRALEAMSANAAEEGEACMESSSIMTSSGRCCISTIKRCPCMTVNNQGENESST
EVDKHLAKFQCETGKDVIPGDRQQGGEDLSDSVVCHPAKIDSQIQSHGKISPDLDVKCCQVGNSKDSPCPSLLPNGD
YNVRPSNHSDASDTLEHGGISLDPVAGDGESDKLVPQNSINVPQNVVVACEDMGKQAVGGSSKINDTHEVVKEVKFK
GQEEDMNSVSISNDYSGEKGNLVILSSPSLTDGRVFLPLGSPPNTSVCNISTSDSSNILQNGSCSPDVHQKNTLSGP
TDGWKDGIVENGRSRSVGKSTEAGDAALLYFEATLRTLKRTKESIGRATRIAIDCAKFGIATKVMEILVHDLEIESS
LHRRVDLFFLVDSIAQCSRGLKGDIGGVYPSAMKAVLPRLLSAAAPLGNAAKENRRQCLKVLRLWLERKILPEPIIR
HHMQELDSYSSSVSAGVHSHRSLRRDRPFDDPVRDMEGMLDEYGSNSSFQLPGFCMPRMLGDGGSDSDGGEFEAVTP
EHDSETYEVQETTHAIEKRRHVLEDVDGELEMEDVAPSVDGELNSICNIDRGNAREFEKNLPVSFGPPLPQDLPPSS
PPPPSSPPPPPPPPPPPPPPSLPLPPPPPPPTLHFKSATSDQYHVAVDSKGFEDSLTVKANVLHPMAEPLAAPRNSQPI
SDAVQYTVPECRDMPMQMPESTCSFNTFPVQPTDNSRNTDGATMHNKGYLIPPPHHVPSNQFSFVHGEHRMKPQREV
PPPPSYSNGHHFMPSMTREYGYDNHERLRPPYDYQERWNVPPCSGPRYHDRGVPAPYGCHPSESVSFPGHGWRFPPP
SMNYRDSLHFRPHFEDAIPVANRGPNFWQPR

>Glyma15g37451.1|PACid:26329610
MGVVEQEMEEEEKEKHTNNHNHPSSLHPIKEECVSVRKPFSGDRVKRDEWSEGAVSTLLEAYEAKWVLRNRAKLKGH
DWEDVARHVSARANCTKSPKTSTQCKNKVESMKKRYRSESAATADHASSSWPLYSRLDLLLRGTGPVLSSPPPPPAM
MPAAMPVLSPVEPSQPPQLSPPPAAPSGPLANAQNSHGSNGIDEKLAKEDGLGTKSSDQVSSKNPLDTDSSTPALYS
KKDGLRCNKRKKKTIDNTGRRRKECVEIAESLRWLTEAMIRSEQARMDTMKEIERMRVEAEAKRGEMDLKRTEIIAN
TQLEIARIFASVNNKGVDSSLRIGRS

>Glyma15g18910.1|PACid:26330857
MPRRRSWHSSPPLLSPPLLIILFPIVILTLLFLAVPPILSAVTRLLRSASSDVKTSWDSLNILLVVFAILCGVFARR
NNDDEQTPNNHVHQHDDDAVSDRNAAFRRLHSEGQSQWFGFAEERKVYGNDTPLNQLQSLDTGGNRLRMRRNSSSYP
DLRQWETGDDRYKFRFYDDFEIDKPFRSPAREHFPAVEHRKRWPESPQPQQHQHQYQQQEDQVKDIPVDTFEIRPSP
PPVKSTSPPPPPPPPPPPPPESARHNTRRTHRKVERERESEITVELDDHEFTTIRSPPPAPPTPPQPPSVKTRSERKS
DRRKSTVKREMAMIWASVLSNQRKKKKKQKTKNNENQDPQYDDNADELTNNTTVPPPPPPPPPLPSVFHSLFRKGLG
KSKKIHSVSAPPPPPPPPPSKRKSQTPPPPEPPQRRNSGRPPLPNRTVTFNDETLNAGNQSPLIPVPPPPPPFKMKA
MKFVVRGDFVKIRSNQSSRCSSPEREDIINASETTINNDAVTDAVNDTVNDSVMDPNVFCPSPDVNAKAATFIARLR
GEWRLEKLNSLKEKDNNGSLPCSL

>Glyma15g40710.1|PACid:26331659
MAKPGRGRRSPSGSVSGSSSRSSSPSDSRSSSRSRSLSRSRSPSRSRSSSPSSSSSSRSSRSRSPPPQRRRSPADAT

RRGRSPPPQSKRASPPPRKPSPVRESLVLHVEKLSRNVNEGHLKEIFSNFGEVISVELAMDRTVNLPKGYGYVQFKT
RGDAEKALLYMDGAQIDGNVIKARFTLPPRQKVSPPPKASAVAPKRDTPRADNASADVDKDGPKRQRESSPRRKPPP
SPRRRSPVPRRAGSPRRPDSPRRRADSPVRRRLDSPYRRGGDTPPRRRPVSPGRGRSPSPPRRLRSPARLSPRRIRG
SPGRRRSPPPPPRRRSPPRRPRSPPRRSPIGRRRTRSPIRRSARSRSRSFSPRRGRPPVRRGRSSSYSDSPSPRKVS
RRSKSRSPRRPLRGRGSSNSSSSSSPPPPPPPPTRKP

>Glyma03g30790.1|PACid:26334026
MPPSRRKGGKKSGGGGAAASRQFKLGDLVLAKVKGFPAWPATVSEPQKWGYSADRKKVFVCFFGAPQIAFCNHADVE
AFTEEKKQSLAKRSGRGGEFACAVKEIIECYEKLRTENQDGDTSSGGEVAIANVSYSLDPSANTGLKDQTDAPFTIN
SQMKSSNCVIDRPEDAVALKDESYNIEASLEEATDNAIMTATVKSPFSITQRNAPVRRSRTRSTLQVQNFVVPCGDG
GNNVGNSDDNISADAIQDTSIRSKRIRKSPDLLRCDDTDSPAFAPNVSMEDNGSEIITINSDAFTLNEGSTIDSNLK
FEQSEPIVCPEGEGLDLEIKAVINKNKRKPNQKKETNDSGAQNASQSLQNMGGNSKERCPDQDGDEHLPLVKRARVR
MGKSSTEAELNSISQVQVKSGEEDITDSPHQIITCSNCENGLAEGGPSVLNSTLVNVSPSNLIASYSENGSQICKIK
KDQMFGCSVDDEAALPPSKRIHRALEAMSANAAEEGQACMESSSSIMTFSGRCCISAIKRCPCMTVNNQGVNELELQ
RLVSCGIDSSHVSVCSFSTRSNTIISTENELSTEVDKHLVKFQHESGKDVIPGASQQGGEDISDSVVCHPAKIDSLI
QSHGKISPNLDVKCCQVGNNKDSPGPSLLLNDDDNARTSNHSDASDTVEHVGISLDPVAGNSESDKLVPKNSINVTQ
NVVVACEDMMKHAVGDSSKPNDTHEVIKEVKFKGQEEDMNSVSISNDYSDEKGNLGILSSPSLTDVRVCLPLGSPPI
TSVCNISTSDSSNILQNGSCSPDVHQKNTLSGPTDGWKDGIVENEQSRSEGKSTEAGDAALLYFEATLRTLKRTKES
IGRATRIAIDCAKFGIATKVMEIVVHNLEIESSLHRRVDLFFLVDSIAQCSRGLKGDIGGVYPSTIKAVLPRLLSAA
APPGNAAKENRRQCLKVLRLWLERKILPEPIIQHHMRELDSYSSSVSAGVHARRSSRRERPFDDPVRDMEGMLDEYG
SNSSFQLPGFCMPRMLEDDGGSDSDEGEFEAVTPEHDSETYEVQETTHAIEKHRHVLEDVDGELEMEDVAPSVDGEL
NLICNIDRGNATEFEKNLPVSFGPPLPQDLPPSSPPPPSSPPPPPPAPPPPSLPLPPPPPPTLHFKSATSDQYHVAV
DSKGFEDSLTVEANVLHPMAQPLAAPRNSQSIGDAVQYTVPECRDMPIQMPESTCSFNTFPVQPTDNSRNTDGATMH
NKGYSIPPPHHVPSNQFSFVNGEHQMKSRREVPPPPSYSNGHHFMPSMMREYGYDSHERSRPPYDYQERWNVPPPCS
GPRYSDRGVPAPYGCHPSESVGFPGHGWRFPPPSMNYRDSLPFRPHFEDAIPVANRGPGFWQPR

>Glyma03g37030.1|PACid:26334248
MATLSACASPPWPNPDSPNSNPNPNFLPDQSQTLDFDSLMPPQPESPPPNHHHSPDASPPESSPSPPPPSILYLSFN
QDQACFAAAADNGFRIYNCDPFRELFRREFDGGGIGHVEMLFRCNILALVGGGPNPQYPPNKVMIWDDHQGRCIGEL
SFRAAVRGVRLRRDRIIVVVEQKIFVYNFADLKLVQQIETVPNPKGLCAVSQLSDSLVLACPGLHKGQIRVEHYAQK
KTKFISAHDSRIACFALTLDGQLIATASTKGTLIRIFDTDHGTLLQEVRRGANAAEIYSLAFSSTAQWLAVSSDKGT
VHVFSLKVNSSIPEQEKSQSSSNSDAAITPSSSSRSFIKLKGVLPKYFNSEWSVAQFHLQEGSHYTVAFGLQKNTVI
ILGMDGSFYRCQFDPVHGGEMTQLEYHNFLKPEPETAL

>Glyma03g37030.2|PACid:26334249
MATLSACASPPWPNPDSPNSNPNPNFLPDQSQTLDFDSLMPPQPESPPPNHHHSPDASPPESSPSPPPPSILYLSFN
QDQACFAAAADNGFRIYNCDPFRELFRREFDGGGIGHVEMLFRCNILALVGGGPNPQYPPNKVMIWDDHQGRCIGEL
SFRAAVRGVRLRRDRIIVVVEQKIFVYNFADLKLVQQIETVPNPKGLCAVSQLSDSLVLACPGLHKGQIRVEHYAQK
KTKFISAHDSRIACFALTLDGQLIATASTKGTLIRIFDTDHGTLLQEVRRGANAAEIYSLAFSSTAQWLAVSSDKGT
VHVFSLKVNSSIPEQEKSQSSSNSDAAITPSSSSRSFIKLKGVLPKYFNSEWSVAQFHLQEGSHYTVAFGLQKNTVI
ILASIDANLIQYMEGR

>Glyma03g42060.2|PACid:26334423
MVGPIATFGKGNRMKHLEKKENEVKEEGIIEHRSLTAPASKVTSKTVKMNVMVAVTVGYQAYFLKLKDMANPPPVRP
WFRLASIRSSPPPPPPPAPAPAQEPRLPAFRTASAPSSPQIQPTQPAPQPQEHPSSQHNRFSSSSLPTSPENKNITR
VPLPSPSHSPNTSPPPSPLALTPSQSRSETMIPDQPEPKTVLVQKTIDPRPKPWHNARHGQHDTVKESESKEKVVHR
KLSESEESGMRVITIAGENRGAYMELIQSPKKHDPKYLHKKGNSNINVDGVESEGSSAEEGTVNKKDKNQKGTSSSS
FPMSAYMNSNVQCVNNSLLYHASCSHHDPGVRLSLSKKPFGEGFHLKEHADSKHI

>Glyma03g42541.1|PACid:26336473
MAQESLSETPDISPPSSTAQSLPDPSLSRNVSFSRLNAQAPEFVPTRVSPRGDFQQPRLVVPSPSPSPPPPPPPPPP
ASGMVHVYSPPPTSQFHVPIQGHVVPVQNHHHHLAHQHHVPAHYRSHHHPHQYYVSSDSAVQQPQVDPDHAPSKSKM
SDEASQKILNQVEYYFSDLNLATTDHLMRFINKDPEGFVPISVVASFKKIKALIASHSQLATVLRNSSKLVVSEDGK
KIKRQYPLTESDIEELQSRIVVAENLPEDHCHQNLMKVFSAVGSVKTIRTCPPQTSNSGASATSRLGKVDGMPLSNK
LHAFVEYGSVELAERAVAELNDEGNWRSGLRIRLMLRRMSKPAQGRGKKGLDVEVGCDEDYTSVPEPQASEKQLEDA
SFPDTLLLEHAQGEEHGYDKESGQRKGRSRGRGKGRGRVHCHQNNRVGTPPSNNTIFTDQVIAKQPPGPRMPDGTRG
FSMGRGKPVAVNIA

>Glyma03g42541.2|PACid:26336474
MAQESLSETPDISPPSSTAQSLPDPSLSRNVSFSRLNAQAPEFVPTRVSPRGDFQQPRLVVPSPSPSPPPPPPPPPP
ASGMVHVYSPPPTSQFHVPIQGHVVPVQNHHHHLAHQHHVPAHYRSHHHPHQYYVSSDSAVQQPQVDPDHAPSKSKM
SDEASQKILNQVEYYFSDLNLATTDHLMRFINKDPEGFVPISVVASFKKIKALIASHSQLATVLRNSSKLVVSEDGK
KIKRQYPLTESDIEELQSRIVVAENLPEDHCHQNLMKVFSAVGSVKTIRTCPPQTSNSGASATSRLGKVDGMPLSNK
LHAFVEYGSVELAERAVAELNDEGNWRSGLRIRLMLRRMSKPAQGRGKKGLDVEVGCDEDYTSVPEPQASEKQLEDA
SFPDTLLLEHAGEEHGYDKESGQRKGRSRGRGKGRGRVHCHQNNRVGTPPSNNTIFTDQVIAKQPPGPRMPDGTRGF
SMGRGKPVAVNIA

>Glyma20g23550.1|PACid:26337174
MSEAPPPPTADASPPPYWCYHCEKRVSVETLANLPDVVCGECKNGFVESMPTPSRSRSPSASSDDPYFGSQFLQVLR
LIAQSARDDDASPPPPPSRSPENDFLRIELGGWDNDEENENDDDNGDEEFEEHEEAEDRSGNEDPHGGDDEDLRRRR
RELLRLRIRDLATRTRSMRNRILDWAEILMGLEDNSIEFRLQLPESDRYVGNPEDYVDAAEYEALLQTLAESDGGGR
RGAPPASKAALEALPTVKIASESEAVACAICKDLLGVGDAAKRLPCGHRYHGDCIVPWLSSRNSCPVCRFELPTDDK
EYEEERVVIAASSNSNGASSSGGGGSAIV

>Glyma09g32251.1|PACid:26340287
MKGTSKFIMGATLVMVVSLAIVLGLILVLLAELYCSLLLRRRQLRRKTNTIPISTTQTTTTISNHASSPSHTPQHSP
SPPPFCNDFYSQGVLQAPRSFLFPCKDEPNATPRGIGLVSAVSSSPSLASFLARAPPHSLLQQQNTLQVSASGINEI
PCADYNNKSCGGGVEHLVYISNPIYENEEGKASGANDTPFETPHTSPSRLARSGSSGEDDDHAGAQGSPPPPPSVTP
PLTPMKKLPAAEGSSVSLRDATSLGTSGSDSHSINGPSSSSSLDTPCTSPSW

>Glyma09g06230.1|PACid:26341451
MEGTLFPNRPVLPVPSHKPTQPPLKFKPTFLPPQSPPPSPPPSFQLDSLLQHLQHLSSVPITTHTLTIVPPSLDNTK
NFNKSVNSKHPTLGSDPIIDEDQFDDAKFRFLSDKGKLLLNSVVGSPLHELNDFFNSVKFELLEADFPSLLKALDLS
GNWERALLLFEWGWLHFGSDQNLRLDNQVVELMVRILGRESQHSIASKLFDLIPVEKYSLDVRAYTTILHAYARSGK
YKRAIDLFDKMEGIGLDPTLVTYNVMLDVYGKMGRSWGRILELLDEMRSKGLEFDEFTCSTVISACGREGMLDEARK
FLAELKLNGYKPGTVMYNSMLQVFGKAGIYTEALSILKEMEDNNCPPDSITYNELAATYVRAGFLDEGMAVIDTMTS
KGVMPNAITYTTVIDAYGKAGREDDALRLFSKMKDLGCAPNVYTYNSVLAMLGKKSRTEDVIKVLCEMKLNGCAPNR
ATWNTMLAVCSEEGKHNYVNKVLREMKNCGFEPDKDTFNTLISSYARCGSEVDSAKMYGEMVKSGFTPCVTTYNALL
NALAHRGDWKAAESVIQDMQTKGFKPNETSYSLLLHCYSKAGNVRGIEKVEKEIYDGQVFPSWILLRTLVLSNHKCR
HLRGMERAFDQLQKYGYKPDLVVINSMLSMFSRNKMFSKAREMLHFIHECGLQPNLFTYNCLMDLYVREDECWKAEE
VLKGIQNSVPEPDVVSYNTVIKGFCRKGLMQEAIRVLSEMTTKGIQPTIVTYNTFLSGYAGMELFDEANEVIRFMIE
HNCRPSELTYKILVDGYCKAGKHEEAMDFVTKIKEIDISFDDKSVKRLGSCIREKVGSTL

>Glyma09g05300.2|PACid:26341567
MAKEREKKLYVALLGNYAAELKLLLTTLLLLCAVATLLQFLPSRFTISASDLRVCISRVTQTTQTIPNSTNATSPPV
TATASASASASPLVASLSSTTSPSPPPPSPPPPSPPSSEQILPDGTIKRVFNPYGSAAYNFVTMGAYRGGLDTFAII
GIASKPLHVYAKPTYECAWFSLAGDKKKLSTTVGYKILPDWGYGRVYTVVIVNCTFPIPVNADNSGGKLVLFASTSG
GGDASFNTTDRIEALTEQPRTLDTTLFSSKPKYDYLYCGSSLYGNLNPQRVREWIAYHVKFFGPRSHFVIHDAGGVH
PQVLEVLKPWMDLGYVTLHDIRDQERFDGYYHNQFMVVNDCLHRYKFMAKWMFFFDVDEYIYVPPKSTIKSVLDSLS
EYSQFTIEQMPMSSKLCLTSDYGKTYRKWGFEKLVYKDSKKGIRRDRKYAVQPRSLYATGVHMSQNLAGKTTHKTEG
KIMYYHYHGTIAEKRESCKMLINSTEITYDKTPYVLDTTMRDIAGVIKKFELKMIGDRLQHKTRQ

>Glyma10g43280.1|PACid:26343936
MSEAPPSQPSPPTANASPPPYWCYHCEKRVSIETLANLPDVVCGECKNGFVESIHTPSRSRSPSASSDDPYFGSQFL
QVLRLIAQSARDDDASPPPPPSRSPENDFLRIELGGWDNDEENDNDNNDDEEFEEHEEAEDRSGNEDPHGRDDEDLR
RRRRELLRLRIRDLATRTRSMRNRILDWAEILMGLEDNSIEFRLQLPESDRYVGNPEDYVDAAEYEALLHTLAESDG
GGRRGAPPASKAAVEALPTVKIASESEAVACAICKDLLGVGDLAKRLPCGHGYHGDCIVPWLSSRNSCPVCRYELPT
DDKEYEEERVVNSASSSGGGGSAIV

>Glyma10g08280.1|PACid:26344147
MAEEHRCEPPEGHRLCVNNCGFFGSTATMNLCSKCYSAIRLKEQEEASTKSTIETALSSASSAKPSSSTSPPPSAVD
VLMESPPPSAAEVEVAVTVTVAVASSSISINSGSVAQPNRCATCRKRVGLTGFKCRCGVTFCGAHRYPEEHACGFDF
KTVGREEIARANPVIKAEKLRRI

>Glyma10g24391.1|PACid:26344584
MDDMDMDIPLPEELEFLESNSHFAEQQEEEDRHYYFPDLDPTAEIHESNSQQSPPPPDLAAPSAEPESSGHKRSCPP
SPSPPEEEKRAKVRVAVEEDSSAAAADEDWLRYSPPPVPEGEPAVEEMAFEKEKTLSRYASEIDGECMPITAPSGNR
VYAKLNRFQGEERVTKLDYNGYSTELSSEPVNVILERLEQEAFAKALEASSEGQSVLDVPEAQMVHERLWVDKYAPK
SFTELLSDEQTNREVLLWLKQWDSIVFGSEIRSTSDDVLSALKRHSSIVHNQKPLNSKFPRMSRGPRWSNGRRYKNS
RSMDESGSSKSIQDIWNAKSRNIGPPEPKILLLCGPPGLGKTTLAHVAARQCGYHVVEVNASDDRSTATIEAKILDV
VQMNSVLSDSRPKCLVVDEIDGALGDGKGAVEVLLKMISSERKPDAGKQSLGKGQQERKSSKKGSKTASLSRPVICI
CNDLYAPALRPLRQVAKVHIFVQPTVSRVVNRLTYICNKEGMKASAIALTALAEYTECDIRSCLNSLQFLFKKKQAL
NVFDIGSQVVGQKDMSKNVLDIWKEIFHKRRTKKIERKSHRGKSFEFDSLYSLVSNRGDSNLILDGIHENVLRLNYH
DPVMQKTVECFNNLGVYDLMHQYIMHTQQLPLYVYLPLVAITVHHIVSQVQKPTIEWPKSHQRYRTMMMEKMDILNT
WHHKIPPYIARNLSASSFVEDLISPLLHILSPPTIRPVAFQLLSDKEKNDLAQLVSTMVSYTITYKTVKSDMLPQTQ
RCEVADGLALSLVPPISDFINFKDYTSNHNVLSVAMKQVLVHEVEKHKILQVSNDKTGAFTNGGHEVIETGTNNIPL
ANTNHATAVDMKTNENQANVLSQQLNANPTAVSPNLNSDKSSIAADCGKLLNMGNVKKPSRTSSSFFDRFKKVNVKG
LQSNDRSLPEEATLDKDRYPILFKYNEGFTNAVKRPVRMREFLSL

>Glyma10g01910.2|PACid:26345844
MALNFSLVFTLYSIIFLLTTASSTTTTQTTTSTVGKDQVPRIMCNECELPPPPPQPKECPPPPSPPPPIPPSPPPPL
PASPPPPVIIKCPPPPKPLPPPPPVVIECPPPPKPPCGGGADCLLPTLPETPEEPQYLQPPPQLPSSPGTIDLDDII
SGANMINPLLRYFTLNSFTFLLLLCLPYYLFI

>Glyma07g01830.5|PACid:26348236
MASYRPFPPQSSQIPNPIPGGNHQYNNNQNQNWGAYGDPSTSSFPQIPPNSNYHQNQQHHVPYAPPNPHHPHYPYPP
PPPPPPPEASYQPPPPPPPAPMYYPSNNQYSNQPPPPPPPLSPPPPPPPVSPPPPPAVTQNNEERHFKEPSTSGRRE
YEHSNHGIAHKQHKQQPPVPVKKMNNGPPGRAETDEEKRLRKKREFEKQRQEEKHRQQLKESQNTVLQKTHMLSSGK
GHGMIAGSRMGERRSTPLLGAERVENRLKKPTTFLCKLKFRNELPDPSAQPKLMASKKDKDQYAKYTITSLEKMYKP
KLFVEPDLGIPLDLLDLSVYNPPSVRPPLAPEDKELLRDDEAVTPIKKDGIKRKERPTDKGVAWLVKTQYISPLSME
STKQSLTEKQAKELREMKGGRGILDNLNSRERQIREIEASFEAAKSDPVHATNKDLYPVEVMPLLPDFDRYDDQFVV
AAFDNAPTADSEMHAKMDKSVRDAFESKAVMKSYVATSSDPANPEKFLAYMVPAPGELSKDIYDENEDVSYSWIREY
HWDVRGDDADDPATFLVAFDESEARYLPLPTKLVLRKKRAKEGRSGDEVEQCPVPARVTVRRRSSVAAIERKDSGVY
TSSKGNSSKRGGLEMDDGLEDQHRGAPHQDNYQSSGAEDYMSD

>Glyma07g01830.4|PACid:26348238
MASYRPFPPQSSQIPNPIPGGNHQYNNNQNQNWGAYGDPSTSSFPQIPPNSNYHQNQQHHVPYAPPNPHHPHYPYPP
PPPPPPPEASYQPPPPPPPAPMYYPSNNQYSNQPPPPPPPLSPPPPPPPVSPPPPPAVTQNNEERHFKEPSTSGRRE
YEHSNHGIAHKQHKQQPPVPVKKMNNGPPGRAETDEEKRLRKKREFEKQRQEEKHRQQLKESQNTVLQKTHMLSSGK
GHGMIAGSRMGERRSTPLLGAERVENRLKKPTTFLCKLKFRNELPDPSAQPKLMASKKDKDQYAKYTITSLEKMYKP
KLFVEPDLGIPLDLLDLSVYNPPSVRPPLAPEDKELLRDDEAVTPIKKDGIKRKERPTDKGVAWLVKTQYISPLSME
STKQSLTEKQAKELREMKGGRGILDNLNSRERQIREIEASFEAAKSDPVHATNKDLYPVEVMPLLPDFDRYDDQFVV
AAFDNAPTADSEMHAKMDKSVRDAFESKAVMKSYVATSSDPANPEKFLAYMVPAPGELSKDIYDENEDVSYSWIREY
HWDVRGDDADDPATFLVAFDESEARYLPLPTKLVLRKKRAKEGRSGDEVEQCPVPARVTVRRRSSVAAIERKDSGVC
H

>Glyma07g31680.1|PACid:26348706
MSAPLGIGIRSGSYGSLDKQVQNGTARKASKMLKEKEKERLFLWICKFAGRKKVGMLFLCLISAAVFVWVLYVGKGE
DSQDGNTVTNINVNESVSTSDSTFENSMTNAMGLTKRLVLLPPPTGYFLGYHLPPGHPCNSFTLPPPPADKKRTGPR
PCPVCYLPVEEAIGLMPALPSPSPVLGNLTYVYEENLSRDGEFGGSDFGGYPTLKQRNDSFDIRESMTVHCGFVRGI
KPGRNTGFDIDGADLFEMEQCDGVVVASAIFGNFDVINEPNNISDYSRKTVCFLMFVDEQTEKYLISSGKLGISKKI
GLWRTIVARNLPYPDARRTGKIPKLLLHRLVPNARYSIWLDGKLELVVDPYQILERFLWRKNATFAISKHYRRFDVF
IEAEANKAAGKYDNASIDFQIEFYKKEGLTPYTEAKLPLISDVPEGCVIVREHVPISNLFACLWFNEVDRFTSRDQI
SFSTVRDKILSRVDFHFNMFLDCERRNFVVQKYHRDLLLRLAPPASPPPPSPPPPLPVLETSPEKGANSPIRRGPGR
RGKDRRAGSRRHRKVVAGGREMEPS

>Glyma07g09550.2|PACid:26349780
MKGTSKFIMGATLVMVVSLVIVLGLILVLLAELYCSLLLRRRQLRKKTNTIPISTTQTTTTIPNSSPSNTPQHSPSP
PPPPPPPFSNVYSQGVLQAPRSFLFPCKDEPNATPRGIGLVSVSSSPLASFLARAPPQSLLQVGASDNESPCVDNNK
YSCGGVEQHLVYISNPIYENGSSGEDDAQGSPPPSLTPPLTPMKKLPAQGSSVSLRGATSLGTSGSGGAPCTSSSW

>Glyma07g03950.1|PACid:26350549
MDQQEGNTWTMPPFWAVEQRNRRRLRRSYSLLLTSTSLLVLFLVLVLVFSLIVVPTLHSFASNIFKPRTVKNSWDSL
NLVLVLFAIFCGFLSRNNNTSETLTPRSRRTFSNPSTPPSAWYQNDYSQIHNRSFNRLRSFNSYPDLRQQIAADERF
RFYDDTYLLRHRNMRTETEEEEEKEIGIDNVATAPPQSPPQQGMEAVGRNVKRAYQVETVEEHKKEDLDAQISPPPP
ATRSKGVRRNVKRTYQAEAVEKHETNDFPVMDSRPPPVTITKGVRRNAKRTYQPETMEKRETNDFDEQNSQTLPSPP
PPPVTRRRGVRRNAKPEGNDSVVKSSPPPPPPPPLPAEEARTVSGKKKRGSATKEFLISLRGKKKKQRQRSVENFD
IILGSESLFSQPPPPPPLPPPKVFQNLFSLKKGKHKNRHIASVATTTTSVSNKRDHGSSSRLQDNVTMAGNESPLNP
IPPPPPLPPFKLPGWKFRVQGDFVRVDSMGSSRSGSPDDLDEVSVDTPTTSTHDETSQVNSPFAKDSEDSASVSAPS
LFCPSPDVDTKAHNFIESFRAGLRMAKMNSMKERQGIGRSNLAPSPNHEWN

>Glyma07g21210.1|PACid:26350617
MAYPFGAIPPTFEVQNDAPLVNERLKKAYVALQAWKEAIYSDPLNTTGNWVGEDVCSYNGVFCAPAIDDPTLNVVAG
VDLNNADIAGHLPEELGNLSDIALFHINSNRFCGIVPETLENLTLLHEFDISNNHFVGGFPSVVLKWPNLKYLDIRY
NDFEGPLPPELFEKDLDAIFLNNNRFTSIIPDTLGKSKVSVVTFANNKFTGCIPKSIVNMKNMNEIVFMGNDLGGCL
PQEIGLLESITVLDASHNGFVGTLPNLSGLKNVEVIDIEHNKLSGYVSNIVCQLPLLKNFTFSNNYFNGEAQTFNSP
PPPVSSPPPPVYSPPPSPINSPPPPTPVLSPSPLIHSPPPPVHSPPPPIYSPPPPVNSPPPPVSSPPPPVYSPPPPL
INSPPPPTLVLSPSPPIHSPPPPVHSPPPPVYSPPPPVYSPPPTWDDVVLPPHFGSSYQSPPPPTIAGY

>Glyma16g24850.1|PACid:26352340
MDEFLLKAEKANAIRRHNNDIFAVFSKALRILELCLVLVLLSWILTRLPFALALSAEFLRRLIAFAASPLVVFAVSN
AIIAALLAQSRRLSAPHSADSLYREFLHTRTAVTEPHAPPSPPPSPPPPEPEFHDKQVIVETVHDAPSAGAAAKYRR
SQSEKIKVDAAGKLPRRKQLRRSETEKRGECLPEILYPQDELSNEEFQRAIEAFIAKQLRFLREESSAIVVQNPS

>Glyma16g06320.2|PACid:26352426
MHSAMDFARLNTPKPPPIFTRPLTWVTSTALRLHRRQLSPPPPPPLPPPSPPPPPPHPSLSSIPSILTSKTLDSSKC
KSILPHLTPHHFDRLFLSLHRTVNPKTTHEFFRFATRHCNFRFTVRSYCLLLRSLLADSFVPRARFLLARLIDGHVP
TWSSKTTTSFHDRLREIASSMLELNQGSDEQRLGELDLLLHILCSQFKCLGSRCAFDIFVMFSKRGVFPCLKTCNLL
LSSLVKANELHKSYEVFDLACQGVAPDVFTFTTAINAFCKGGRVGDAVDLFCKMEGLGVFPNVVTYNNVIDGLFKSG
RFEEALRFKDRMVRSKVNPSVVTYGVLISGLMKLEMFEEANEVLVEMYSMGFAPNEVVFNALIDGYCRKGDMGEALR
VRDEMAMKGMKPNFVTFNTLLQGFCRSNQMEQAEQVLVYILSSGLSVNMDVCSYVIHRLMERSGFVSALKIVTKLLS
GNIRVSDSLLTPLVVGLCKCEGHSEAIELWFKLAAVKGLAANTVTSNALLHGLCERGNMEEVFEVLKQMLEKGLLLD
RISYNTLIFGCCKWGKIEEAFKLKEEMVQQEFQPDTYTYNFLMKGLADMGKIDDVHRLLHEAKEYGFVPNVYTYALL
LEGYCKADRIEDAVKFFKNLDYEKVELSSVVYNILIAAYCRIGNVTEAFKLRDAMKSRGILPTCATYSSLIHGMCCI
GRVDEAKEIFEEMRNEGLLPNVFCYTALIGGHCKLGQMDIVGSILLEMSSNGIRPNKITYTIMIDGYCKLGNMKEAR
ELLNEMIRNGIAPDTVTYNALQKGYCKERELTVTLQSDHKSNIGLPLEEEITYNTLIHKLHPHTAISNRE

>Glyma16g06320.3|PACid:26352427
MDFARLNTPKPPPIFTRPLTWVTSTALRLHRRQLSPPPPPPLPPPSPPPPPPHPSLSSIPSILTSKTLDSSKCKSIL
PHLTPHHFDRLFLSLHRTVNPKTTHEFFRFATRHCNFRFTVRSYCLLLRSLLADSFVPRARFLLARLIDGHVPTWSS
KTTTSFHDRLREIASSMLELNQGSDEQRLGELDLLLHILCSQFKCLGSRCAFDIFVMFSKRGVFPCLKTCNLLLSSL
VKANELHKSYEVFDLACQGVAPDVFTFTTAINAFCKGGRVGDAVDLFCKMEGLGVFPNVVTYNNVIDGLFKSGRFEE
ALRFKDRMVRSKVNPSVVTYGVLISGLMKLEMFEEANEVLVEMYSMGFAPNEVVFNALIDGYCRKGDMGEALRVRDE
MAMKGMKPNFVTFNTLLQGFCRSNQMEQAEQVLVYILSSGLSVNMDVCSYVIHRLMERSGFVSALKIVTKLLSGNIR
VSDSLLTPLVVGLCKCEGHSEAIELWFKLAAVKGLAANTVTSNALLHGLCERGNMEEVFEVLKQMLEKGLLLDRISY
NTLIFGCCKWGKIEEAFKLKEEMVQQEFQPDTYTYNFLMKGLADMGKIDDVHRLLHEAKEYGFVPNVYTYALLLEGY
CKADRIEDAVKFFKNLDYEKVELSSVVYNILIAAYCRIGNVTEAFKLRDAMKSRGILPTCATYSSLIHGMCCIGRVD
EAKEIFEEMRNEGLLPNVFCYTALIGGHCKLGQMDIVGSILLEMSSNGIRPNKITYTIMIDGYCKLGNMKEARELLN
EMIRNGIAPDTVTYNALQKGYCKERELTVTLQSDHKSNIGLPLEEEITYNTLIHKLHPHTAISNRE

>Glyma18g48685.1|PACid:26354571
MGCTASKLENEDTVRRCKERRRLMKEAVYARHHLAAAHSDYCRSLRLTGSALSTFSAGEPLSVSDHTPAVFLNNSQH
HPPPPQHQPSPSPSPPPPLFNPSPSPPPPKLATTNPRRRKPPPPKLPHILSDSSPSSTPRSHVTQQRTNPQIHDT
DSERSEYDFFDGKHETENEKTDSHHLPEEYTETEREEVQCSEWGDHYSTTSSSDDDGVEGDVESRSEIGTRSNFGSS
VRAESVVGVGGGGGAKRFDAASSVAAAEMKMVVRHRDLREIVEAIKENFDNAASAGDQVSEMLEISRAQLDRSFKQL
RKTVYHSSSILSNLSSSWTSKPPLAVKYRLDTGSLDEPGGPKSLCSTLERLLAWEKKLYEEVKAREGVKIEHEKKLS
ALQTQEYKGEDEAKIFKTKASINRLQSLIAVTSQAVSTTSTATIGLRDSDLVPQLVDLIHGFMYMWRSMHHYHEIQS
NIVQQVRGLVNRSSRGDSTSELHRQATRDLESAVSAWHSSFCRLIKFQRDFILSLHGWFKLSLVPVHNDNINSRETS

DTYQFFDEWKLALDRVPDTVASEAIKSFINVVHVISSKQVEELKIKKRTETASKELEKKASSLRNLERKFYSSYSMV
GISLPDSAPDNGQVLDARDPLAEKKLELATCQRRVEDEMLRHSKAVEVTRAMTLNNLQTGLPGVFQALTSFSSLFTE
ALESVCTRSYAIK

>Glyma18g12150.1|PACid:26355739
PGQPSPTPLLKSIHSPPPPPKNPYKYPSPPPPVYPHPYPHPHPHPHAHPHAHPPYVYHSPPLPPKKKPYYYHYPPPP
P

>Glyma02g14410.1|PACid:26289216
MAAIFWALAIISLCCKLDCNAQAPATVPVKLPPTTLPPTTPTATSPSSSTPLAVTQPPTVVASPPTTSTPPTTSQPP
ANVAPKPAPVKPSAPKVAPASSPKAPPPQIPIPQPPKPSPVSPPPLPPPPVASPPPLPPPKIAPTPAKTPPSPAPAK
ATPAPAPAPTKPAPTPSPVPPPPAATPAPAPVIEVPAPAPAPVIEVPAPAPAHHRHRRHRHRHRRHQAPAPAPTI
IRKSPPAPPDSTAESDTAPAPSPSLNLNAAPSNHQQGRNIWATAGLAIIVLLAVTGYSC

>Glyma02g01841.1|PACid:26291515
MMRPAIHNYTTQAYISLSLITKGLHQFSCSFLSPSTNHFQFKNMARNFSLVFTLSSIIFLVTTVLPSTVSSTSTLEK
HQVPRIMCDECKLPPPPPPPKPIECPPPPSPPPPIPPSPPPPLPPSPPPPVIKCPPPPKPLPLPPPPPVVVECPPPP
KPPCGGGGVDCLLPTFPETPDGPHYLLSPPGLPSPGTLDLDDIINGANMINPLLRYFSLHSFTFLLLLCLPYFLFV

>Glyma11g00280.1|PACid:26295241
METHLVFLLGLIFTLVAGVGAQAQGPSTSPAPPTPQASPPPVQSSPPPVPSSPPPSQSPPPASTPPPSPLSSPPPSS
PPPASPPPSSPPPASPPPSSPPPSSPPPFSPPPATPPPATPPPAVPPPSLTPTVTPLSSPPVSSPAPSPSKVFAPAL
SPSLAPGPSLSTISPSGDDSGAQNLWSFQKMIGSLVFGCALLSMLF

>Glyma04g05590.1|PACid:26304002
MNHTKFIKILFSWILKACFMGLALFATVTSSEEKVEDPSSGLFCISDCAKCPVICSPPPPMLITPPHPPTSPSVPHS
PPLSYLSPPPPKSQLPPPPPPPSRIAPPPPFKSYNTPPSGLTQPQPTVISAPHDFSYPYYYFYASGAASLSFHPPFFG
LVIVLNWVFCCW

>Glyma09g29566.1|PACid:26340796
MEKLRPAWAVKAIIVIVFTSILFRCVCGENHTVGGASGWDLRSNIQAWSSTTTFNIGDDLVFSYTPVHDVVEVNQLG
YNTCTIANALATYDNGETVIHLSDAKTRYFVCGRMRHCQQGLKLQVQILAQSNNGTSDDQNQSPGAGTGTVGSPPSP
SPPPPPPPPPPPPPPPPVSPPPHGDEQPPADEPVPCDCNRAEERHGVVPLITLVIVVALAHAPFFIAFPHLRFHLMR

>Glyma07g02955.1|PACid:26348412
MKKANTVTTTLFALLLLTHSLAAETRTTPLQDHKHEAELRGSRTSRRNRHSSGGRRTRGSNCDQFYLYLFGKCGLWP
FPTFTCPDNPFDQTPMPSSPFPPPITYTPRIPPPVQYSAPPPPPQSPPSPPPLPPSPPPPPPPLCPPWPLPCPSPPK
PACPSPPPPPPPPPVCSPPPVAPCCPLPPPVQPCCPLPSPSPPPLPPAPPLVPSPPPPAPTLLPPLVLPPPMSQMPT
FPWLSPPNPFMNNAPPLVPFLSSPPLLSTPTLPDISFPPPLEPTPFLSTPPVFTMPQTPNAFFPPPLVADVPPAFTV
PETPEMPEEPPTFNMPTTPDVFLAPPELPEDPPDVFLTPPELPEDPPDVFLAPPELPEEPPENLVPDPEEPPSPFTI
G

>Glyma16g00615.1|PACid:26352303
KNSCDSLNLVLVLFPIVCGFFSRSNTNTTSETQTPRSRRTFSNPSTPPSAWFQNDYSQIHNRSFNRLRSFDERFRFY
DNTYLLRHRHRHMRTEPEEEEKEIGIDNVPTAPPQQGTEAISPPPPATRTKGVRRNFKRTHQAETIEKHEANDLPVM
DSQPPPVTRTKGVRRNAKRTYQPETMEKRETNDFEAQNSQPPASPPCPPLPTTRTRGVRRNAKPEGNDSVVKSSPPP
PPPKVFQNLFSLKKGKHKNRHIVCVATITRASVSNKREHGSSSNVMMAGNESPLNPIPPPPPLPPFKLPGWKFRIGS
SRSGSPDDLDEVSVDTPTTSTHDETRSQVNSPFAKNGEDSASVSALSLFCPSPDVDTKAHHFIESFRAGLRMAKMNF
RAGDWEVQSCPFTKP
>Glyma15g13800.1|PACid:26330027
SSSSYEPPSPSPSISPTLSPFNSPLSLPSPSPISLEPSPSDSPSPSPSKSPLSSLPPPPASPAASPSDSPSPVSSPS
PPSNSPSPLSPPSPVSPAPAPSNSPSPSPPPPSISPAPSLSNTPPPPPPPPVSSPPPPLVSSPPPPPPPVSPPPPPL
VSSPPPPPPPVSPPPRNCPNLRFCTNILGGINIPGFPATTRECCTLISGLVDFDASFCLCTQIRAGIFGININPNIG
VNLVLNACGRRRNTTTNPCN

Medicago truncatula

>Medtr3g068990.1|PACid:23009112
MKSPINVDFLKTTNALKIVVFFFITFTFFYLGKHWSDGYQQLIFFTQNSDPDPNQNPNSAVSISPNFNKNFNISTLI
DQEKTLTQPPPPSPPPPSPPPSDSVEKFGIVNENGTMSEEFEIGSFDPAMVDDWVNETQVEKEGSESVTKFAIKKFG
LCSRGMSEYIPCLDNVEAIKKLPSTEKGERFERHCPEDGKKLNCLVPAPKGYRAPIPWPKSRDEVWFSNVPHTRLVE
DKGGQNWISRDKDKFKFPGGGTQFIHGADEYLDHISKMIPEITFGRHIRVALDVGCGVASFGAYLLQRNVITMSVAP
KDVHENQIQFALERGVPAMVAAFATRRLLYPSQAFDLIHCSRCRINWTRDDGILLLEVNRMLRAGGYFVWAAQPVYK
HEEALEEQWEEMLNLTTRLCWKFLKKDGYIAVWQKPFDNSCYLNREAGTKPPLCDPSDDPDNVWYVDLKACISELPK
NEYEANITDWPARLQTPPNRLQSIKVDAFISRKELFKAESKYWNEIIEAX
[truncated: 66,843 more chars]
