# Supplementary material for: Bioinformatic Identification and Analysis of Extensins in the Plant Kingdom
Source: PLoS One. 2016 Feb 26;11(2):e0150177. doi: 10.1371/journal.pone.0150177 (PMC4769139; doi:10.1371/journal.pone.0150177)

S4 Fig

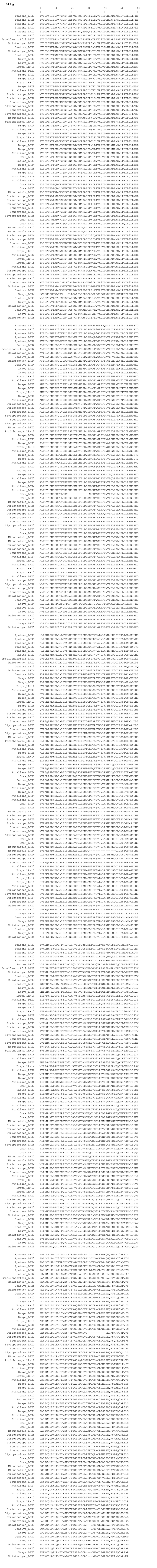

Supplement: S4 Fig — The analysis involved 78 amino acid sequences. There were a total of 294 positions in the final dataset. (PDF) [file pone.0150177.s004.pdf]
